# Supplementary material for: Testing the potential of a ribosomal 16S marker for DNA metabarcoding of insects
Source: PeerJ. 2016 Apr 19;4:e1966. doi: 10.7717/peerj.1966 (PMC4841222; doi:10.7717/peerj.1966)
Supplement: Figure S1 — 16S Fusion primers developed in this study. They include flow cell and sequencing primer binding regions for current Illumina sequencers. The amplified fragment has a size fo ∾157 bp and can be sequenced directly after purification (one step PCR). Up to 10 samples can be uniquely tagged from forward and reverse direction and pooled in one NextSeq run. The bases used for shifting on Ins_F and Ins_R can be used to uniquely tag samples (inline barcodes). It is recommended that all 10 primer pairs are used in the following combination to maximize sequence diversity and reduce effects of tag switching by uniquely tagging samples from both sides: P5_Ins_R0+P7_Ins_F4, P5_Ins_R1+P7_Ins_F3, P5_Ins_R2+P7_Ins_F2, P5_Ins_R3+P7_Ins_F1, P5_Ins_R4+P7_Ins_F0, P5_Ins_F0+P7_Ins_R4, P5_Ins_F1+P7_Ins_R3, P5_Ins_F2+P7_Ins_R2, P5_Ins_F3+P7_Ins_R1, P5_Ins_F4+P7_Ins_R0. [file peerj-04-1966-s001.pdf]

P5_Ins_F0 AATGATACGGCGACCACCGAGATCTACACTCTTTCCCTACACGACGCTCTTCCGATCTRGACGAGAAGACCCTATARA
P5_Ins_F1 AATGATACGGCGACCACCGAGATCTACACTCTTTCCCTACACGACGCTCTTCCGATCTTRGACGAGAAGACCCTATARA
P5_Ins_F2 AATGATACGGCGACCACCGAGATCTACACTCTTTCCCTACACGACGCTCTTCCGATCTCTRGACGAGAAGACCCTATARA
P5_Ins_F3 AATGATACGGCGACCACCGAGATCTACACTCTTTCCCTACACGACGCTCTTCCGATCTGTTRGACGAGAAGACCCTATARA
P5_Ins_F4 AATGATACGGCGACCACCGAGATCTACACTCTTTCCCTACACGACGCTCTTCCGATCTAACTRGACGAGAAGACCCTATARA
P5_Ins_R0 AATGATACGGCGACCACCGAGATCTACACTCTTTCCCTACACGACGCTCTTCCGATCTACGCTGTTATCCCTAARGTA
P5_Ins_R1 AATGATACGGCGACCACCGAGATCTACACTCTTTCCCTACACGACGCTCTTCCGATCTTACGCTGTTATCCCTAARGTA
P5_Ins_R2 AATGATACGGCGACCACCGAGATCTACACTCTTTCCCTACACGACGCTCTTCCGATCTTCACGCTGTTATCCCTAARGTA
P5_Ins_R3 AATGATACGGCGACCACCGAGATCTACACTCTTTCCCTACACGACGCTCTTCCGATCTGCTACGCTGTTATCCCTAARGTA
P5_Ins_R4 AATGATACGGCGACCACCGAGATCTACACTCTTTCCCTACACGACGCTCTTCCGATCTCGTTACGCTGTTATCCCTAARGTA

flow cell bind P5 sequ primer 1 bind Ins_F / Ins_R (Taberlet unpub.)
N = shift (Lundberg et al. 2013)

P7_Ins_R0 CAAGCAGAAGACGGCATACGAGATCGGTCTCGGCATTCCTGCTGAACCGCTCTTCCGATCTACGCTGTTATCCCTAARGTA
P7_Ins_R1 CAAGCAGAAGACGGCATACGAGATCGGTCTCGGCATTCCTGCTGAACCGCTCTTCCGATCTTACGCTGTTATCCCTAARGTA
P7_Ins_R2 CAAGCAGAAGACGGCATACGAGATCGGTCTCGGCATTCCTGCTGAACCGCTCTTCCGATCTTCACGCTGTTATCCCTAARGTA
P7_Ins_R3 CAAGCAGAAGACGGCATACGAGATCGGTCTCGGCATTCCTGCTGAACCGCTCTTCCGATCTGCTACGCTGTTATCCCTAARGTA
P7_Ins_R4 CAAGCAGAAGACGGCATACGAGATCGGTCTCGGCATTCCTGCTGAACCGCTCTTCCGATCTCGTTACGCTGTTATCCCTAARGTA
P7_Ins_F0 CAAGCAGAAGACGGCATACGAGATCGGTCTCGGCATTCCTGCTGAACCGCTCTTCCGATCTRGACGAGAAGACCCTATARA
P7_Ins_F1 CAAGCAGAAGACGGCATACGAGATCGGTCTCGGCATTCCTGCTGAACCGCTCTTCCGATCTTRGACGAGAAGACCCTATARA
P7_Ins_F2 CAAGCAGAAGACGGCATACGAGATCGGTCTCGGCATTCCTGCTGAACCGCTCTTCCGATCTCTRGACGAGAAGACCCTATARA
P7_Ins_F3 CAAGCAGAAGACGGCATACGAGATCGGTCTCGGCATTCCTGCTGAACCGCTCTTCCGATCTGTTRGACGAGAAGACCCTATARA
P7_Ins_F4 CAAGCAGAAGACGGCATACGAGATCGGTCTCGGCATTCCTGCTGAACCGCTCTTCCGATCTAACTRGACGAGAAGACCCTATARA

flow cell bind P7 sequ primer 2 bind Ins_F / Ins_R (Taberlet unpub.)
N = shift (Lundberg et al. 2013)

Figure S1. 16S Fusion primers developed in this study. They include flow cell and sequencing primer binding regions for current Illumina sequencers. The amplified fragment has a size fo ~157 bp and can be sequenced directly after purification (one step PCR). Up to 10 samples can be uniquely tagged from forward and reverse direction and pooled in one NextSeq run. The bases used for shifting on Ins_F and Ins_R can be used to uniquely tag samples (inline barcodes). It is recommended that all 10 primer pairs are used in the following combination to maximize sequence diversity and reduce effects of tag switching by uniquely tagging samples from both sides:

P5_Ins_R0+P7_Ins_F4, P5_Ins_R1+P7_Ins_F3, P5_Ins_R2+P7_Ins_F2, P5_Ins_R3+P7_Ins_F1, P5_Ins_R4+P7_Ins_F0,
P5_Ins_F0+P7_Ins_R4, P5_Ins_F1+P7_Ins_R3, P5_Ins_F2+P7_Ins_R2, P5_Ins_F3+P7_Ins_R1, P5_Ins_F4+P7_Ins_R0
