# Supplementary material for: Testing the potential of a ribosomal 16S marker for DNA metabarcoding of insects
Source: PeerJ. 2016 Apr 19;4:e1966. doi: 10.7717/peerj.1966 (PMC4841222; doi:10.7717/peerj.1966)
Supplement: Figure S2 — Number of sequences obtained per sample after library demultiplexing (A) and percentage of sequences excluded in different bioinformatic analysis steps (B). (A) Library demultiplexing; Numbers above bars indicate the relative contribution (in percent) to the total number of sequences obtained for each sample. Sequencing started with Ins_F (white) or Ins_R (black) is indicated by bar color. (B) Number of reads excluded in data processing steps. Mean percentage of sequence abundance in each processing step is written in brackets. Ins_F/Ins_R primer bias was tested with a t-test. [file peerj-04-1966-s002.pdf]

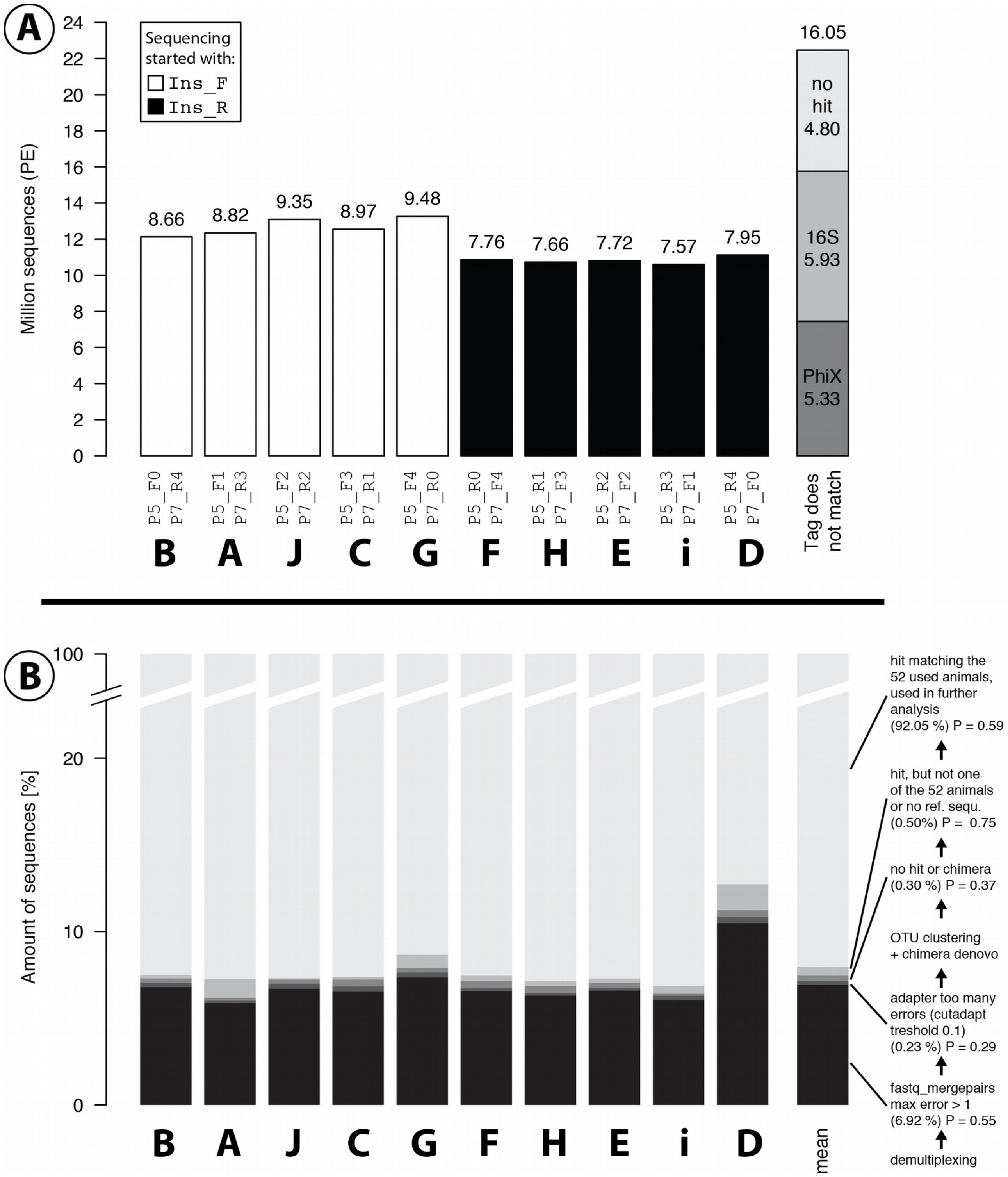

Figure S2. Number of sequences obtained per sample after library demultiplexing (A) and percentage of sequences excluded in different bioinformatic analysis steps (B). A: Library demultiplexing; Numbers above bars indicate the relative contribution (in percent) to the total number of sequences obtained for each sample. Sequencing started with Ins_F (white) or Ins_R (black) is indicated by bar color. **B:** Number of reads excluded in data processing steps. Mean percentage of sequence abundance in each processing step is written in brackets. Ins_F / Ins_R primer bias was tested with a t-test.
