# Supplementary material for: Testing the potential of a ribosomal 16S marker for DNA metabarcoding of insects
Source: PeerJ. 2016 Apr 19;4:e1966. doi: 10.7717/peerj.1966 (PMC4841222; doi:10.7717/peerj.1966)
Supplement: Supplemental Information 1 [file peerj-04-1966-s006.zip › scripts/5 match OTUs/blast_hits/OTU_1.csv_plot.pdf]

OTU_1.csv

Habroleptoides confusa

Ephemeroptera

100

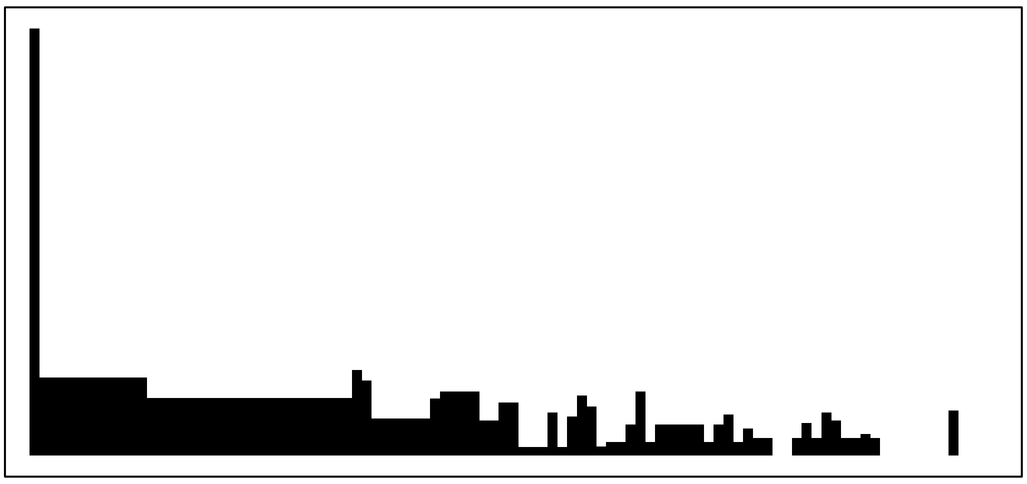

Habroleptoides confusa voucher BYU:IGCEP153 16S ri	GQ118289	100	100	5.22926e−70	Ephemeroptera		Habroleptoides confusa
Ephoron shigae mitochondrial gene for 16S ribosoma	AB874306	83.65	68.21	4.59521e−20	Ephemeroptera	Polymitarcyidae	Ephoron shigae
Ephoron shigae mitochondrial gene for 16S ribosoma	AB874293	83.65	68.21	4.59521e−20	Ephemeroptera	Polymitarcyidae	Ephoron shigae
Ephoron shigae mitochondrial gene for 16S ribosoma	AB874289	83.65	68.21	4.59521e−20	Ephemeroptera	Polymitarcyidae	Ephoron shigae
Ephoron shigae mitochondrial gene for 16S ribosoma	AB874283	83.65	68.21	4.59521e−20	Ephemeroptera	Polymitarcyidae	Ephoron shigae
Ephoron shigae mitochondrial gene for 16S ribosoma	AB874278	83.65	68.21	4.59521e−20	Ephemeroptera	Polymitarcyidae	Ephoron shigae
Ephoron shigae mitochondrial gene for 16S rRNA, pa	AB711533	83.65	68.21	4.59521e−20	Ephemeroptera	Polymitarcyidae	Ephoron shigae
Ephoron shigae mitochondrial gene for 16S rRNA, pa	AB711527	83.65	68.21	4.59521e−20	Ephemeroptera	Polymitarcyidae	Ephoron shigae
Ephoron shigae mitochondrial gene for 16S rRNA, pa	AB711523	83.65	68.21	4.59521e−20	Ephemeroptera	Polymitarcyidae	Ephoron shigae
Ephoron shigae mitochondrial gene for 16S rRNA, pa	AB711500	83.65	68.21	4.59521e−20	Ephemeroptera	Polymitarcyidae	Ephoron shigae
Ephoron shigae mitochondrial gene for 16S rRNA, pa	AB711493	83.65	68.21	4.59521e−20	Ephemeroptera	Polymitarcyidae	Ephoron shigae
Ephoron shigae mitochondrial gene for 16S rRNA, pa	AB711471	83.65	68.21	4.59521e−20	Ephemeroptera	Polymitarcyidae	Ephoron shigae
Ephoron shigae mitochondrial gene for 16S ribosoma	AB874305	82.69	68.21	5.59811e−19	Ephemeroptera	Polymitarcyidae	Ephoron shigae
Ephoron shigae mitochondrial gene for 16S ribosoma	AB874303	82.69	68.21	5.59811e−19	Ephemeroptera	Polymitarcyidae	Ephoron shigae
Ephoron shigae mitochondrial gene for 16S ribosoma	AB874302	82.69	68.21	5.59811e−19	Ephemeroptera	Polymitarcyidae	Ephoron shigae
Ephoron shigae mitochondrial gene for 16S ribosoma	AB874301	82.69	68.21	5.59811e−19	Ephemeroptera	Polymitarcyidae	Ephoron shigae
Ephoron shigae mitochondrial gene for 16S ribosoma	AB874299	82.69	68.21	5.59811e−19	Ephemeroptera	Polymitarcyidae	Ephoron shigae
Ephoron shigae mitochondrial gene for 16S ribosoma	AB874288	82.69	68.21	5.59811e−19	Ephemeroptera	Polymitarcyidae	Ephoron shigae
Ephoron shigae mitochondrial gene for 16S ribosoma	AB874284	82.69	68.21	5.59811e−19	Ephemeroptera	Polymitarcyidae	Ephoron shigae
Ephoron shigae mitochondrial gene for 16S ribosoma	AB874279	82.69	68.21	5.59811e−19	Ephemeroptera	Polymitarcyidae	Ephoron shigae
Ephoron shigae mitochondrial gene for 16S ribosoma	AB874277	82.69	68.21	5.59811e−19	Ephemeroptera	Polymitarcyidae	Ephoron shigae
Ephoron shigae mitochondrial gene for 16S rRNA, pa	AB711590	82.69	68.21	5.59811e−19	Ephemeroptera	Polymitarcyidae	Ephoron shigae
Ephoron shigae mitochondrial gene for 16S rRNA, pa	AB711582	82.69	68.21	5.59811e−19	Ephemeroptera	Polymitarcyidae	Ephoron shigae
Ephoron shigae mitochondrial gene for 16S rRNA, pa	AB711581	82.69	68.21	5.59811e−19	Ephemeroptera	Polymitarcyidae	Ephoron shigae
Ephoron shigae mitochondrial gene for 16S rRNA, pa	AB711575	82.69	68.21	5.59811e−19	Ephemeroptera	Polymitarcyidae	Ephoron shigae
Ephoron shigae mitochondrial gene for 16S rRNA, pa	AB711531	82.69	68.21	5.59811e−19	Ephemeroptera	Polymitarcyidae	Ephoron shigae
Ephoron shigae mitochondrial gene for 16S rRNA, pa	AB711530	82.69	68.21	5.59811e−19	Ephemeroptera	Polymitarcyidae	Ephoron shigae
Ephoron shigae mitochondrial gene for 16S rRNA, pa	AB711528	82.69	68.21	5.59811e−19	Ephemeroptera	Polymitarcyidae	Ephoron shigae
Ephoron shigae mitochondrial gene for 16S rRNA, pa	AB711521	82.69	68.21	5.59811e−19	Ephemeroptera	Polymitarcyidae	Ephoron shigae
Ephoron shigae mitochondrial gene for 16S rRNA, pa	AB711514	82.69	68.21	5.59811e−19	Ephemeroptera	Polymitarcyidae	Ephoron shigae
Ephoron shigae mitochondrial gene for 16S rRNA, pa	AB711501	82.69	68.21	5.59811e−19	Ephemeroptera	Polymitarcyidae	Ephoron shigae
Ephoron shigae mitochondrial gene for 16S rRNA, pa	AB711492	82.69	68.21	5.59811e−19	Ephemeroptera	Polymitarcyidae	Ephoron shigae
Ephoron shigae mitochondrial gene for 16S rRNA, pa	AB711482	82.69	68.21	5.59811e−19	Ephemeroptera	Polymitarcyidae	Ephoron shigae
Ephoron eophilum mitochondrial gene for 16S rRNA,	AB711631	84	64.24	6.8199e−18	Ephemeroptera	Polymitarcyidae	Ephoron eophilum
Penaphlebia sp. EP076 16S ribosomal RNA gene, part	AY749782	83.51	64.24	6.8199e−18	Ephemeroptera		Penaphlebia sp. EP076
Ephoron shigae mitochondrial gene for 16S ribosoma	AB874304	81.73	68.21	2.38038e−17	Ephemeroptera	Polymitarcyidae	Ephoron shigae
Ephoron shigae mitochondrial gene for 16S ribosoma	AB874300	81.73	68.21	2.38038e−17	Ephemeroptera	Polymitarcyidae	Ephoron shigae
Ephoron shigae mitochondrial gene for 16S ribosoma	AB874297	81.73	68.21	2.38038e−17	Ephemeroptera	Polymitarcyidae	Ephoron shigae
Ephoron shigae mitochondrial gene for 16S rRNA, pa	AB711598	81.73	68.21	2.38038e−17	Ephemeroptera	Polymitarcyidae	Ephoron shigae
Ephoron shigae mitochondrial gene for 16S rRNA, pa	AB711532	81.73	68.21	2.38038e−17	Ephemeroptera	Polymitarcyidae	Ephoron shigae
Ephoron shigae mitochondrial gene for 16S rRNA, pa	AB711522	81.73	68.21	2.38038e−17	Ephemeroptera	Polymitarcyidae	Ephoron shigae
Anthopotamus sp. EP022 16S ribosomal RNA gene, par	AY749766	82.55	64.24	8.30834e−17	Ephemeroptera	Potamanthidae	Anthopotamus sp. EP022
Ephoron eophilum mitochondrial gene for 16S rRNA,	AB711630	83	64.24	2.8999e−16	Ephemeroptera	Polymitarcyidae	Ephoron eophilum
Ephoron eophilum mitochondrial gene for 16S rRNA,	AB711621	83	64.24	2.8999e−16	Ephemeroptera	Polymitarcyidae	Ephoron eophilum
Ephoron eophilum mitochondrial gene for 16S rRNA,	AB711619	83	64.24	2.8999e−16	Ephemeroptera	Polymitarcyidae	Ephoron eophilum
Ephoron eophilum mitochondrial gene for 16S rRNA,	AB711615	83	64.24	2.8999e−16	Ephemeroptera	Polymitarcyidae	Ephoron eophilum
Ephemera orientalis voucher EPH01 tRNA−Leu, 16S ri	KF256907	81.63	64.24	1.01216e−15	Ephemeroptera	Ephemeridae	Ephemera orientalis
Ephemera orientalis mitochondrion, complete genome	EU591678	81.63	64.24	1.01216e−15	Ephemeroptera	Ephemeridae	Ephemera orientalis
Hypattalus sp. NLG−2013 voucher COL514 12S ribosom	KC524788	82.47	62.91	3.5328e−15	Coleoptera	Melyridae	Hypattalus sp. NLG−2013
Enochrus talamanca 16S ribosomal RNA gene, partial	KC992672	82.47	62.91	3.5328e−15	Coleoptera	Hydrophilidae	Enochrus talamanca
Ephoron leukon mitochondrial gene for 16S rRNA, pa	AB711641	80.39	66.89	3.5328e−15	Ephemeroptera	Polymitarcyidae	Ephoron leukon
Ephoron album mitochondrial gene for 16S rRNA, par	AB711636	80.39	66.89	3.5328e−15	Ephemeroptera	Polymitarcyidae	Ephoron album
Ephoron album mitochondrial gene for 16S rRNA, par	AB711635	80.39	66.89	3.5328e−15	Ephemeroptera	Polymitarcyidae	Ephoron album
Ephoron eophilum mitochondrial gene for 16S rRNA,	AB711633	82	64.24	3.5328e−15	Ephemeroptera	Polymitarcyidae	Ephoron eophilum
Hexagenia sp. BYU IGCEP003 16S ribosomal RNA gene,	GQ118286	80.39	66.89	3.5328e−15	Ephemeroptera	Ephemeridae	Hexagenia sp. BYU IGCEP003
Hercostomus brevidigitalis voucher RMBR:103445 16S	FJ808172	81.82	64.24	3.5328e−15	Diptera	Dolichopodidae	Hercostomus brevidigitalis
Neptis thisbe 16S ribosomal RNA gene, partial sequ	JX185871	82.8	60.26	1.23307e−14	Lepidoptera	Nymphalidae	Neptis thisbe
Phyllobaenus testaceus voucher 7228 16S ribosomal	KC524896	82.29	62.25	1.23307e−14	Coleoptera	Cleridae	Phyllobaenus testaceus
Paraleptophlebia vaciva 16S ribosomal RNA gene, pa	AY749759	80.41	64.24	1.23307e−14	Ephemeroptera		Paraleptophlebia vaciva
Protohermes yangi 16S ribosomal RNA gene, partial	KF944544	80.61	64.24	4.30383e−14	Megaloptera	Corydalidae	Protohermes yangi
Protohermes hunanensis 16S ribosomal RNA gene, par	KF944532	80.61	64.24	4.30383e−14	Megaloptera	Corydalidae	Protohermes hunanensis
Atrichiana placida voucher 842794 16S ribosomal RN	JX234487	81.44	62.91	4.30383e−14	Coleoptera	Scarabaeidae	Atrichiana placida
Neptis tshetverikovi 16S ribosomal RNA gene, parti	JX185894	82.98	60.26	4.30383e−14	Lepidoptera	Nymphalidae	Neptis tshetverikovi
Jellisonia amadoi mitochondrion, complete genome	KF322091	80.61	64.24	4.30383e−14	Siphonaptera	Ceratophyllidae	Jellisonia amadoi
Cafius velutinus isolate CNUIC−120 16S ribosomal R	JF796482	81.44	62.91	4.30383e−14	Coleoptera	Staphylinidae	Cafius velutinus
Afronurus sp. 213BoAf mitochondrial partial 16S rR	HE651422	81.44	62.91	4.30383e−14	Ephemeroptera	Heptageniidae	Afronurus sp. 213BoAf
Trox fascicularis rowei isolate 46A 16S ribosomal	EF570396	81.44	62.91	4.30383e−14	Coleoptera	Trogidae	Trox fascicularis rowei
Trox arcuatus isolate 69 16S ribosomal RNA gene, p	EF570395	81.44	62.91	4.30383e−14	Coleoptera	Trogidae	Trox arcuatus
Trox fascicularis rowei isolate 57A 16S ribosomal	EF570361	81.44	62.91	4.30383e−14	Coleoptera	Trogidae	Trox fascicularis rowei
Hymenoplia fulvipennis voucher DA0116 16S ribosoma	FJ956720	80.61	64.24	4.30383e−14	Coleoptera	Scarabaeidae	Hymenoplia fulvipennis
Pseudiron centralis 16S ribosomal RNA gene, partia	AY749799	81.44	62.91	4.30383e−14	Ephemeroptera	Pseudironidae	Pseudiron centralis
Epilachna tumida voucher KS135 16S ribosomal RNA g	KP143571	81.91	61.59	1.50218e−13	Coleoptera	Coccinellidae	Epilachna tumida
Anoplotrupes stercorosus voucher SK300 16S ribosom	KP250244	80.61	62.91	1.50218e−13	Coleoptera	Geotrupidae	Anoplotrupes stercorosus
Cephaloclerus sp. 1 NLG−2013 voucher 7183 16S ribo	KC524887	81.25	62.25	1.50218e−13	Coleoptera	Cleridae	Cephaloclerus sp. 1 NLG−2013
Myrmeleon bore isolate 74−boreD1 16S ribosomal RNA	KC195845	80.81	64.24	1.50218e−13	Neuroptera	Myrmeleontidae	Myrmeleon bore
Myrmeleon bore isolate 113−bore2 16S ribosomal RNA	KC195844	80.81	64.24	1.50218e−13	Neuroptera	Myrmeleontidae	Myrmeleon bore
Ephoron leukon mitochondrial gene for 16S rRNA, pa	AB711638	79.41	66.89	1.50218e−13	Ephemeroptera	Polymitarcyidae	Ephoron leukon
Ephoron leukon mitochondrial gene for 16S rRNA, pa	AB711637	79.41	66.89	1.50218e−13	Ephemeroptera	Polymitarcyidae	Ephoron leukon
Nemopterella longicornis isolate NLTS02 16S riboso	JX294150	80.81	64.24	1.50218e−13	Neuroptera	Nemopteridae	Nemopterella longicornis
Xylotrechus rusticus 16S ribosomal RNA gene, parti	JX177496	81.52	60.26	1.50218e−13	Coleoptera	Cerambycidae	Rusticoclytus rusticus
Tachytrechus notatus voucher Pollet 0279 (P) 16S r	EU864017	80.81	64.24	1.50218e−13	Diptera	Dolichopodidae	Tachytrechus notatus
Eatonica denysae voucher BYU:IGCEP225 16S ribosoma	GQ118284	82	64.24	1.50218e−13	Ephemeroptera	Ephemeridae	Eatonica denysae
Ephemerella atagosana 16S ribosomal RNA gene, part	FJ443039	81.63	64.24	1.50218e−13	Ephemeroptera	Ephemerellidae	Ephemerella atagosana
Leucochrysa robusta voucher CASC 00213 16S ribosom	DQ399281	80.81	64.24	1.50218e−13	Neuroptera	Chrysopidae	Leucochrysa robusta
Leucochrysa texana 16S ribosomal RNA gene, partial	DQ399279	80.81	64.24	1.50218e−13	Neuroptera	Chrysopidae	Leucochrysa texana
Italochrysa insignis 16S ribosomal RNA gene, parti	DQ399278	81	64.24	1.50218e−13	Neuroptera	Chrysopidae	Italochrysa insignis
Chaquihua sp. EP080 16S ribosomal RNA gene, partia	AY749784	80.81	64.24	1.50218e−13	Ephemeroptera	Ameletopsidae	Chaquihua sp. EP080
Leptophlebiidae sp. EP030 16S ribosomal RNA gene,	AY749770	79.38	64.24	1.50218e−13	Ephemeroptera		Leptophlebiidae sp. EP030
Neoneuromus tonkinensis mitochondrion, complete ge	KP126231	79.59	64.24	5.24313e−13	Megaloptera	Corydalidae	Neoneuromus tonkinensis
Protohermes sinuolatus 16S ribosomal RNA gene, par	KF944540	79.59	64.24	5.24313e−13	Megaloptera	Corydalidae	Protohermes sinuolatus
Protohermes gutianensis 16S ribosomal RNA gene, pa	KF944529	79.59	64.24	5.24313e−13	Megaloptera	Corydalidae	Protohermes gutianensis
Protohermes costalis 16S ribosomal RNA gene, parti	KF944522	79.59	64.24	5.24313e−13	Megaloptera	Corydalidae	Protohermes costalis
Platyneuromus sorror 16S ribosomal RNA gene, parti	KF944516	79.59	64.24	5.24313e−13	Megaloptera	Corydalidae	Platyneuromus sorror
Neoneuromus sp. 2 XL−2014 16S ribosomal RNA gene,	KF944513	79.59	64.24	5.24313e−13	Megaloptera	Corydalidae	Neoneuromus sp. 2 XL−2014
Epilachna cruciata voucher KS223 16S ribosomal RNA	KP143627	82.11	61.59	5.24313e−13	Coleoptera	Coccinellidae	Epilachna cruciata
Protohermes niger isolate H755 16S ribosomal RNA g	KF741319	79.59	64.24	5.24313e−13	Megaloptera	Corydalidae	Protohermes niger
Protohermes costalis isolate CAU00586 16S ribosoma	KF741309	79.59	64.24	5.24313e−13	Megaloptera	Corydalidae	Protohermes costalis
Protohermes costalis isolate CAU00211 16S ribosoma	KF741306	79.59	64.24	5.24313e−13	Megaloptera	Corydalidae	Protohermes costalis
Protohermes costalis isolate CAU00209 16S ribosoma	KF741305	79.59	64.24	5.24313e−13	Megaloptera	Corydalidae	Protohermes costalis
Protohermes gutianensis isolate CAU00196 16S ribos	KF741300	79.59	64.24	5.24313e−13	Megaloptera	Corydalidae	Protohermes gutianensis
