# Supplementary material for: Testing the potential of a ribosomal 16S marker for DNA metabarcoding of insects
Source: PeerJ. 2016 Apr 19;4:e1966. doi: 10.7717/peerj.1966 (PMC4841222; doi:10.7717/peerj.1966)
Supplement: Supplemental Information 1 [file peerj-04-1966-s006.zip › scripts/5 match OTUs/blast_hits/OTU_10.csv_plot.pdf]

OTU_10.csv

Ptychoptera sp. ATB−2011

Ptychopteridae

Diptera

91.14

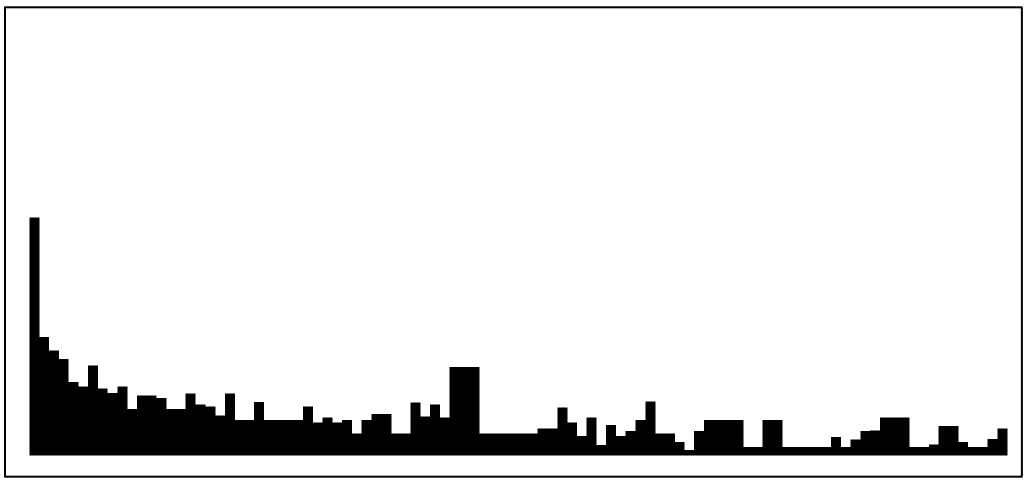

Ptychoptera sp. ATB−2011 mitochondrion, complete g	JN861744	91.14	100	1.07135e−53	Diptera	Ptychopteridae	Ptychoptera sp. ATB−2011
Trichocera bimacula mitochondrion, complete genome	JN861750	85.53	100	3.99607e−40	Diptera	Trichoceridae	Trichocera bimacula
Trichocera brevicornis 16S ribosomal RNA gene, par	KC177458	84.91	100	1.69917e−38	Diptera	Trichoceridae	Trichocera brevicornis
Urytalpa macrocera 16S ribosomal RNA gene, partial	FJ172025	84.52	97.47	2.52179e−36	Diptera	Keroplatidae	Urytalpa macrocera
Edwardsina gigantea 16S ribosomal RNA gene, partia	KC177460	83.44	99.37	8.80193e−36	Diptera	Blephariceridae	Edwardsina gigantea
Thaumatomyia notata 16S ribosomal RNA gene, partia	KC177449	83.23	99.37	3.07217e−35	Diptera	Chloropidae	Thaumatomyia notata
Leptomorphus walkeri 16S ribosomal RNA gene, parti	FJ172020	84.21	93.67	3.07217e−35	Diptera	Mycetophilidae	Leptomorphus walkeri
Limonia tahitiensis voucher O'Grady 202004 16S rib	EU005425	83.12	100	3.07217e−35	Diptera	Limoniidae	Dicranomyia tahitiensis
Parochlus steinenii isolate Sejong01 mitochondrion	KT003702	82.91	100	1.07229e−34	Diptera	Chironomidae	Parochlus steinenii
Bolitophila occlusa voucher JSB2 16S ribosomal RNA	KJ136713	83.23	97.47	1.07229e−34	Diptera	Mycetophilidae	Bolitophila occlusa
Dolichopodidae nr. Oedematopus sp. Pollet 0315 16S	HQ448979	82.17	99.37	3.74267e−34	Diptera	Dolichopodidae	Dolichopodidae nr. Oedematopus sp. Pollet 0315
Chrysops viduatus 16S ribosomal RNA gene, partial	HM132115	82.8	99.37	3.74267e−34	Diptera	Tabanidae	Chrysops viduatus
Chrysops carbonarius 16S ribosomal RNA gene, parti	AY123341	82.8	99.37	3.74267e−34	Diptera	Tabanidae	Chrysops carbonarius
Xylosciara betulae voucher 950 16S ribosomal RNA g	JQ613963	82.69	97.47	1.30632e−33	Diptera	Sciaridae	Xylosciara betulae
Chorisops tunisiae 16S ribosomal RNA gene, partial	GU947379	82.17	99.37	4.55951e−33	Diptera	Stratiomyidae	Chorisops tunisiae
Tabanus maculicornis 16S ribosomal RNA gene, parti	HM132112	82.17	99.37	4.55951e−33	Diptera	Tabanidae	Tabanus maculicornis
Speolepta leptogaster 16S ribosomal RNA gene, part	FJ172033	82.89	96.2	4.55951e−33	Diptera	Mycetophilidae	Speolepta leptogaster
Pedicia albivitta voucher O'Grady 200012 16S ribos	EU005435	82.39	100	4.55951e−33	Diptera	Pediciidae	Pedicia albivitta
Graphoderus liberus voucher KBMGrli69 16S ribosoma	KF979094	82.28	99.37	1.59143e−32	Coleoptera	Dytiscidae	Graphoderus liberus
Clusiodes ruficollis 16S ribosomal RNA gene, parti	EU268538	81.88	99.37	1.59143e−32	Diptera	Clusiidae	Clusiodes ruficollis
Boletina gripha isolate 033462 16S ribosomal RNA g	HQ230380	82.89	96.2	5.55462e−32	Diptera	Mycetophilidae	Boletina gripha
Cyphomyia notabilis 16S ribosomal RNA gene, partia	GU947403	81.65	100	5.55462e−32	Diptera	Stratiomyidae	Cyphomyia notabilis
Simulium nili 16S ribosomal RNA gene, partial sequ	AY944188	81.65	100	5.55462e−32	Diptera	Simuliidae	Simulium nili
Hydrotaea irritans 16S ribosomal RNA gene, partial	FJ025443	82.5	99.37	5.55462e−32	Diptera	Muscidae	Hydrotaea irritans
Simulium laterale mitochondrial gene for 16S rRNA,	AB093107	81.65	100	5.55462e−32	Diptera	Simuliidae	Simulium laterale
Simulium dumogaense mitochondrial gene for 16S rRN	AB093106	81.65	100	5.55462e−32	Diptera	Simuliidae	Simulium dumogaense
Simulium bishopi mitochondrial gene for 16S rRNA,	AB093105	81.65	100	5.55462e−32	Diptera	Simuliidae	Simulium bishopi
Graphoderus occidentalis voucher KBMGroc207 16S ri	KF979095	81.65	99.37	1.93875e−31	Coleoptera	Dytiscidae	Graphoderus occidentalis
Nodicornis nodicornis voucher Pollet 0072 (P) 16S	EU864014	82.28	99.37	1.93875e−31	Diptera	Dolichopodidae	Nodicornis nodicornis
Hercostomus verbekei voucher Pollet 0263 (P) 16S r	EU864004	81.53	99.37	1.93875e−31	Diptera	Dolichopodidae	Hercostomus verbekei
Libnotes orofenaae voucher O'Grady 202034 16S ribo	EU005431	81.76	100	1.93875e−31	Diptera	Limoniidae	Libnotes orofenaae
Tabanus rufofrater 16S ribosomal RNA gene, partial	AY140887	81.53	99.37	1.93875e−31	Diptera	Tabanidae	Tabanus rufofrater
Arganthomyza duplex isolate A50 16S ribosomal RNA	KJ418470	81.65	99.37	6.76691e−31	Diptera	Anthomyzidae	Arganthomyza duplex
Bertamyia notata isolate 1304 16S ribosomal RNA ge	KC699314	81.01	99.37	6.76691e−31	Diptera	Platypezidae	Bertamyia notata
Thermonectus nobilis voucher KBMThno256 16S riboso	KF979114	81.65	99.37	6.76691e−31	Coleoptera	Dytiscidae	Thermonectus nobilis
Boletina gripha isolate 057361 16S ribosomal RNA g	HQ230381	81.94	97.47	6.76691e−31	Diptera	Mycetophilidae	Boletina gripha
Boletina gripha isolate 015271 16S ribosomal RNA g	HQ230378	81.94	97.47	6.76691e−31	Diptera	Mycetophilidae	Boletina gripha
Simulium rufibasis 16S ribosomal RNA gene, partial	GQ866016	81.01	100	6.76691e−31	Diptera	Simuliidae	Simulium rufibasis
Simulium yuphae 16S ribosomal RNA gene, partial se	GQ866015	81.01	100	6.76691e−31	Diptera	Simuliidae	Simulium yuphae
Medina sp. Med_24022006 mitochondrial gene for 16S	AB465907	82.47	96.2	6.76691e−31	Diptera	Tachinidae	Medina sp. Med_24022006
Pyratula zonata 16S ribosomal RNA gene, partial se	FJ172004	81.82	94.94	6.76691e−31	Diptera	Keroplatidae	Pyratula zonata
Limnophora riparia 16S ribosomal RNA gene, partial	FJ025449	82.39	99.37	6.76691e−31	Diptera	Muscidae	Limnophora riparia
Limonia hawaiiensis voucher O'Grady 200106 16S rib	EU005403	81.76	100	6.76691e−31	Diptera	Limoniidae	Dicranomyia hawaiiensis
Ceriana sp. A 16S ribosomal RNA gene, partial sequ	AF154755	84.14	91.14	6.76691e−31	Diptera	Syrphidae	Ceriana sp. A
Ceriana sp. A 16S ribosomal RNA gene, partial sequ	AF154754	84.14	91.14	6.76691e−31	Diptera	Syrphidae	Ceriana sp. A
Ceriana sp. A 16S ribosomal RNA gene, partial sequ	AF154753	84.14	91.14	6.76691e−31	Diptera	Syrphidae	Ceriana sp. A
Simulium rufibasis mitochondrial gene for 16S rRNA	AB093121	81.01	100	6.76691e−31	Diptera	Simuliidae	Simulium rufibasis
Simulium nikkoense mitochondrial gene for 16S rRNA	AB093116	81.01	100	6.76691e−31	Diptera	Simuliidae	Simulium nikkoense
Simulium baltazarae mitochondrial gene for 16S rRN	AB093114	81.01	100	6.76691e−31	Diptera	Simuliidae	Simulium baltazarae
Simulium taalense mitochondrial gene for 16S rRNA,	AB093109	81.01	100	6.76691e−31	Diptera	Simuliidae	Simulium taalense
Simulium aokii mitochondrial gene for 16S ribosoma	AB056745	81.01	100	6.76691e−31	Diptera	Simuliidae	Simulium aokii
Simulium bivittatum mitochondrion 16S ribosomal RN	U17727	81.01	100	6.76691e−31	Diptera	Simuliidae	Simulium bivittatum
Cnephia dacotensis mitochondrial 16S ribosomal RNA	L13403	81.25	100	6.76691e−31	Diptera	Simuliidae	Cnephia dacotensis
Simulium suyoense mitochondrial gene for 16S ribos	LC035000	81.25	100	2.36188e−30	Diptera	Simuliidae	Simulium suyoense
Boletina griphoides isolate 009627 16S ribosomal R	HQ230370	82.24	96.2	2.36188e−30	Diptera	Mycetophilidae	Boletina griphoides
Clitellaria ephippium 16S ribosomal RNA gene, part	GU947399	81.53	99.37	2.36188e−30	Diptera	Stratiomyidae	Clitellaria ephippium
Actina chalybea 16S ribosomal RNA gene, partial se	GU947372	80.89	99.37	2.36188e−30	Diptera	Acroceridae	Actina chalybea
Simulium oblongum mitochondrial gene for 16S ribos	AB334089	81.76	100	2.36188e−30	Diptera	Simuliidae	Simulium oblongum
Derodontus emaculatus voucher BMNH:679374 16S ribo	DQ202609	80.49	100	2.36188e−30	Coleoptera		Derodontus emaculatus
Arachnocampa otwayiensis biotype 5 16S large subun	AY576348	81.41	97.47	2.36188e−30	Diptera	Keroplatidae	Arachnocampa otwayiensis
Gluma musgravei 16S ribosomal RNA gene, partial se	AF403467	80.89	99.37	2.36188e−30	Diptera	Coelopidae	Gluma musgravei
Simulium grossifilum mitochondrial gene for 16S rR	AB093102	81.13	100	2.36188e−30	Diptera	Simuliidae	Simulium grossifilum
Lispe nivalis voucher RMBR:116207 16S ribosomal RN	KJ476315	81.65	99.37	8.24379e−30	Diptera	Muscidae	Lispe nivalis
Diadocidia bruneicola voucher JSSJ16 16S ribosomal	KJ136744	82.52	90.51	8.24379e−30	Diptera	Diadocidiidae	Diadocidia bruneicola
Graphoderus zonatus voucher KBMGrzo66 16S ribosoma	KF979097	81.01	99.37	8.24379e−30	Coleoptera	Dytiscidae	Graphoderus zonatus
Pherbellia cinerella voucher MU_NUS_NSCU_3 16S rib	JN828223	81.01	99.37	8.24379e−30	Diptera	Sciomyzidae	Pherbellia cinerella
Carabus reitterianus reitterianus voucher CC00497	JQ647086	80.62	99.37	8.24379e−30	Coleoptera	Carabidae	Carabus reitterianus reitterianus
Cramptonomyia spenceri mitochondrion, complete gen	JN861747	80.25	99.37	8.24379e−30	Diptera	Pachyneuridae	Cramptonomyia spenceri
Poecilobothrus principalis voucher Pollet 0198 (P)	EU864010	81.13	99.37	8.24379e−30	Diptera	Dolichopodidae	Poecilobothrus principalis
Hercostomus praeceps voucher Pollet 0141 (P) 16S r	EU864003	81.65	99.37	8.24379e−30	Diptera	Dolichopodidae	Hercostomus praeceps
Hercostomus fulvicaudis voucher Pollet 0154 (P) 16	EU863992	81.65	99.37	8.24379e−30	Diptera	Dolichopodidae	Hercostomus fulvicaudis
Calozenillia tamara mitochondrial gene for 16S rRN	AB465934	81.65	99.37	8.24379e−30	Diptera	Tachinidae	Calozenillia tamara
Paraclius digitatus voucher RMBR:103462 16S riboso	FJ808190	81.65	99.37	8.24379e−30	Diptera	Dolichopodidae	Paraclius digitatus
Anthomyza pleuralis 16S ribosomal RNA gene, partia	EU268543	80.38	99.37	8.24379e−30	Diptera	Anthomyzidae	Anthomyza pleuralis
Anthomyza macra 16S ribosomal RNA gene, partial se	EU268526	80.38	99.37	8.24379e−30	Diptera	Anthomyzidae	Anthomyza macra
Melanostoma apicale 16S ribosomal RNA gene, partia	AF154791	81.65	99.37	8.24379e−30	Diptera	Syrphidae	Melanostoma apicale
Melanostoma apicale 16S ribosomal RNA gene, partia	AF154789	81.65	99.37	8.24379e−30	Diptera	Syrphidae	Melanostoma apicale
Simulium nobile isolate N5 16S ribosomal RNA gene,	KP661530	80.38	100	2.87736e−29	Diptera	Simuliidae	Simulium nobile
Simulium nobile isolate N4 16S ribosomal RNA gene,	KP661529	80.38	100	2.87736e−29	Diptera	Simuliidae	Simulium nobile
Simulium nobile isolate N3 16S ribosomal RNA gene,	KP661528	80.38	100	2.87736e−29	Diptera	Simuliidae	Simulium nobile
Simulium nobile isolate N2 16S ribosomal RNA gene,	KP661527	80.38	100	2.87736e−29	Diptera	Simuliidae	Simulium nobile
Simulium nobile isolate N1 16S ribosomal RNA gene,	KP661526	80.38	100	2.87736e−29	Diptera	Simuliidae	Simulium nobile
Ischnomyia spinosa isolate A48 16S ribosomal RNA g	KJ418468	80.86	99.37	2.87736e−29	Diptera	Anthomyzidae	Ischnomyia spinosa
Bertamyia notata isolate BN39 16S ribosomal RNA ge	KC699324	80.38	99.37	2.87736e−29	Diptera	Platypezidae	Bertamyia notata
Eretes griseus voucher UNM KBMErgr105 16S ribosoma	KJ548325	80.75	99.37	2.87736e−29	Coleoptera	Dytiscidae	Eretes griseus
Chiasmoneura anthracina voucher JSB6 16S ribosomal	KJ136717	81.13	97.47	2.87736e−29	Diptera	Keroplatidae	Chiasmoneura anthracina
Boletina sp. 014967 16S ribosomal RNA gene, partia	HQ230375	81.17	97.47	2.87736e−29	Diptera	Mycetophilidae	Boletina sp. 014967
Gymnopternus celer voucher Pollet 0018 (P) 16S rib	EU863977	81.76	99.37	2.87736e−29	Diptera	Dolichopodidae	Gymnopternus celer
Gymnopternus celer voucher Pollet 0051 (P) 16S rib	EU863978	81.76	99.37	2.87736e−29	Diptera	Dolichopodidae	Gymnopternus celer
Gymnopternus brevicornis voucher Pollet 0036 (P) 1	EU863974	81.76	99.37	2.87736e−29	Diptera	Dolichopodidae	Gymnopternus brevicornis
Simulium chamlongi 16S ribosomal RNA gene, partial	GQ866020	80.38	100	2.87736e−29	Diptera	Simuliidae	Simulium chamlongi
Simulium nobile 16S ribosomal RNA gene, partial se	GQ866008	80.38	100	2.87736e−29	Diptera	Simuliidae	Simulium nobile
Simulium siripoomense 16S ribosomal RNA gene, part	GQ866003	80.5	100	2.87736e−29	Diptera	Simuliidae	Simulium siripoomense
Musca domestica isolate CSU0907186180 16S ribosoma	GQ396696	81.37	99.37	2.87736e−29	Diptera	Muscidae	Musca domestica
Musca domestica isolate CSU0901283180 16S ribosoma	FJ763711	81.37	99.37	2.87736e−29	Diptera	Muscidae	Musca domestica
Hydrotaea dentipes 16S ribosomal RNA gene, partial	FJ025442	80.62	99.37	2.87736e−29	Diptera	Muscidae	Hydrotaea dentipes
Papuadytes perfectus mitochondrial 16S rRNA gene (	AM404029	80.38	99.37	2.87736e−29	Coleoptera	Dytiscidae	Papuadytes perfectus
Papuadytes sp. MB133 mitochondrial 16S rRNA gene (	AM404023	80.38	99.37	2.87736e−29	Coleoptera	Dytiscidae	Papuadytes sp. MB133
Arachnocampa otwayiensis biotype 1 16S large subun	AY576347	80.77	97.47	2.87736e−29	Diptera	Keroplatidae	Arachnocampa otwayiensis
Agabus labiatus 16S ribosomal RNA gene, partial se	AY138618	81.25	99.37	2.87736e−29	Coleoptera	Dytiscidae	Agabus labiatus
