# Supplementary material for: Testing the potential of a ribosomal 16S marker for DNA metabarcoding of insects
Source: PeerJ. 2016 Apr 19;4:e1966. doi: 10.7717/peerj.1966 (PMC4841222; doi:10.7717/peerj.1966)
Supplement: Supplemental Information 1 [file peerj-04-1966-s006.zip › scripts/5 match OTUs/blast_hits/OTU_100.csv_plot.pdf]

OTU_100.csv

Nemurella pictetii

Nemouridae

Plecoptera

100

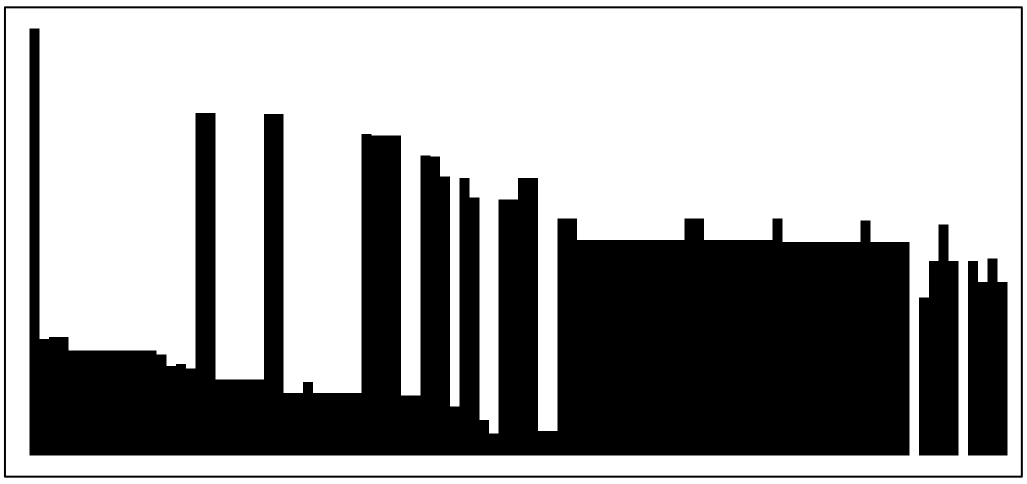

Nemurella pictetii 16S ribosomal RNA gene, partial	EF623192	100	64.74	7.58879e−43	Plecoptera	Nemouridae	Nemurella pictetii
Rhithrogena corcontica mitochondrial partial 16S r	LN868662	85.44	100	3.22684e−41	Ephemeroptera	Heptageniidae	Rhithrogena corcontica
Rhithrogena semicolorata mitochondrial partial 16S	LN868673	85.53	100	1.12628e−40	Ephemeroptera	Heptageniidae	Rhithrogena semicolorata
Rhithrogena sp. 4 LV−2015 mitochondrial partial 16	LN868652	85.53	100	1.12628e−40	Ephemeroptera	Heptageniidae	Rhithrogena sp. 4 LV−2015
Rhithrogena sp. 13 LV−2015 mitochondrial partial 1	LN868672	84.91	100	4.78905e−39	Ephemeroptera	Heptageniidae	Rhithrogena sp. 13 LV−2015
Rhithrogena sp. 12 LV−2015 mitochondrial partial 1	LN868671	84.91	100	4.78905e−39	Ephemeroptera	Heptageniidae	Rhithrogena sp. 12 LV−2015
Rhithrogena picteti mitochondrial partial 16S rRNA	LN868663	84.91	100	4.78905e−39	Ephemeroptera	Heptageniidae	Rhithrogena picteti
Rhithrogena sp. 3 LV−2015 mitochondrial partial 16	LN868659	84.91	100	4.78905e−39	Ephemeroptera	Heptageniidae	Rhithrogena sp. 3 LV−2015
Rhithrogena fonticola mitochondrial partial 16S rR	LN868656	84.91	100	4.78905e−39	Ephemeroptera	Heptageniidae	Rhithrogena fonticola
Rhithrogena sp. 2 LV−2015 mitochondrial partial 16	LN868654	84.91	100	4.78905e−39	Ephemeroptera	Heptageniidae	Rhithrogena sp. 2 LV−2015
Rhithrogena puytoraci mitochondrial partial 16S rR	LN868651	84.91	100	4.78905e−39	Ephemeroptera	Heptageniidae	Rhithrogena puytoraci
Rhithrogena iridina mitochondrial partial 16S rRNA	LN868646	84.91	100	4.78905e−39	Ephemeroptera	Heptageniidae	Rhithrogena iridina
Rhithrogena taurisca mitochondrial partial 16S rRN	LN868638	84.91	100	4.78905e−39	Ephemeroptera	Heptageniidae	Rhithrogena taurisca
Rhithrogena eatoni mitochondrial partial 16S rRNA	LN868666	84.71	100	1.67154e−38	Ephemeroptera	Heptageniidae	Rhithrogena eatoni
Rhithrogena gratianopolitana mitochondrial partial	LN868647	84.18	100	1.67154e−38	Ephemeroptera	Heptageniidae	Rhithrogena gratianopolitana
Rhithrogena sp. 5 LV−2015 mitochondrial partial 16	LN868653	84.28	100	5.83426e−38	Ephemeroptera	Heptageniidae	Rhithrogena sp. 5 LV−2015
Rhithrogena allobrogica mitochondrial partial 16S	LN868640	84.08	100	2.03636e−37	Ephemeroptera	Heptageniidae	Rhithrogena allobrogica
Ostrocerca foersteri 16S ribosomal RNA gene, parti	EF623191	96.04	64.74	2.03636e−37	Plecoptera	Nemouridae	Ostrocerca foersteri
Visoka cataractae 16S ribosomal RNA gene, partial	EF623189	96.04	64.74	2.03636e−37	Plecoptera	Nemouridae	Visoka cataractae
Rhithrogena degrangei mitochondrial partial 16S rR	LN868674	83.54	100	7.10758e−37	Ephemeroptera	Heptageniidae	Rhithrogena degrangei
Rhithrogena gratianopolitana mitochondrial partial	LN868648	83.54	100	7.10758e−37	Ephemeroptera	Heptageniidae	Rhithrogena gratianopolitana
Rhithrogena landai mitochondrial partial 16S rRNA	LN868644	83.54	100	7.10758e−37	Ephemeroptera	Heptageniidae	Rhithrogena landai
Rhithrogena hybrida mitochondrial partial 16S rRNA	LN868642	83.54	100	7.10758e−37	Ephemeroptera	Heptageniidae	Rhithrogena hybrida
Rhithrogena circumtatrica mitochondrial partial 16	LN868641	83.54	100	7.10758e−37	Ephemeroptera	Heptageniidae	Rhithrogena circumtatrica
Nemoura cinerea 16S ribosomal RNA gene, partial se	EF623194	96	64.1	7.10758e−37	Plecoptera	Nemouridae	Nemoura cinerea
Nemoura trispinosa 16S ribosomal RNA gene, partial	EF623187	96	64.1	7.10758e−37	Plecoptera	Nemouridae	Nemoura trispinosa
Rhithrogena sp. 7 LV−2015 mitochondrial partial 16	LN868669	82.91	100	8.65881e−36	Ephemeroptera	Heptageniidae	Rhithrogena sp. 7 LV−2015
Rhithrogena sp. 11 LV−2015 mitochondrial partial 1	LN868668	82.91	100	8.65881e−36	Ephemeroptera	Heptageniidae	Rhithrogena sp. 11 LV−2015
Rhithrogena insularis mitochondrial partial 16S rR	LN868665	83.44	100	8.65881e−36	Ephemeroptera	Heptageniidae	Rhithrogena insularis
Rhithrogena alpestris mitochondrial partial 16S rR	LN868657	82.91	100	8.65881e−36	Ephemeroptera	Heptageniidae	Rhithrogena alpestris
Rhithrogena sp. 6 LV−2015 mitochondrial partial 16	LN868655	82.91	100	8.65881e−36	Ephemeroptera	Heptageniidae	Rhithrogena sp. 6 LV−2015
Rhithrogena sp. 9 LV−2015 mitochondrial partial 16	LN868650	82.91	100	8.65881e−36	Ephemeroptera	Heptageniidae	Rhithrogena sp. 9 LV−2015
Rhithrogena nivata mitochondrial partial 16S rRNA	LN868643	82.91	100	8.65881e−36	Ephemeroptera	Heptageniidae	Rhithrogena nivata
Rhithrogena diensis mitochondrial partial 16S rRNA	LN868637	82.91	100	8.65881e−36	Ephemeroptera	Heptageniidae	Rhithrogena diensis
Malenka californica 16S ribosomal RNA gene, partia	EF623182	95.05	64.74	8.65881e−36	Plecoptera	Nemouridae	Malenka californica
Prostoia besametsa 16S ribosomal RNA gene, partial	EF623188	95	64.1	3.02222e−35	Plecoptera	Nemouridae	Prostoia besametsa
Paranemoura claasseni 16S ribosomal RNA gene, part	EF623185	95	64.1	3.02222e−35	Plecoptera	Nemouridae	Paranemoura claasseni
Shipsa rotunda 16S ribosomal RNA gene, partial seq	EF623184	95	64.1	3.02222e−35	Plecoptera	Nemouridae	Shipsa rotunda
Rhithrogena nuragica mitochondrial partial 16S rRN	LN868667	82.8	100	1.05486e−34	Ephemeroptera	Heptageniidae	Rhithrogena nuragica
Rhithrogena sp. 8 LV−2015 mitochondrial partial 16	LN868661	82.8	100	1.05486e−34	Ephemeroptera	Heptageniidae	Rhithrogena sp. 8 LV−2015
Nanonemoura wahkeena 16S ribosomal RNA gene, parti	EF623198	94.06	64.74	1.05486e−34	Plecoptera	Nemouridae	Nanonemoura wahkeena
Lednia tumana 16S ribosomal RNA gene, partial sequ	EF623197	94	64.1	3.68182e−34	Plecoptera	Nemouridae	Lednia tumana
Scopura montana 16S ribosomal RNA gene, partial se	EF623265	93.07	64.74	4.48537e−33	Plecoptera		Scopura montana
Rhithrogena beskidensis mitochondrial partial 16S	LN868645	82.28	100	1.56555e−32	Ephemeroptera	Heptageniidae	Rhithrogena beskidensis
Protonemura meyeri 16S ribosomal RNA gene, partial	EF623193	93	64.1	1.56555e−32	Plecoptera	Nemouridae	Protonemura meyeri
Zapada cinctipes 16S ribosomal RNA gene, partial s	EF623183	92.08	64.74	5.4643e−32	Plecoptera	Nemouridae	Zapada cinctipes
Rhithrogena sp. 10 LV−2015 mitochondrial partial 1	LN868664	81.65	100	1.90723e−31	Ephemeroptera	Heptageniidae	Rhithrogena sp. 10 LV−2015
Rhithrogena grischuna mitochondrial partial 16S rR	LN868639	81.01	100	1.90723e−31	Ephemeroptera	Heptageniidae	Rhithrogena grischuna
Doddsia occidentalis 16S ribosomal RNA gene, parti	EF623270	92	64.1	1.90723e−31	Plecoptera	Taeniopterygidae	Doddsia occidentalis
Brachyptera seticornis 16S ribosomal RNA gene, par	EF623268	92	64.1	1.90723e−31	Plecoptera	Taeniopterygidae	Brachyptera seticornis
Pteronarcys californica 16S ribosomal RNA gene, pa	EF623261	93	64.1	1.90723e−31	Plecoptera	Pteronarcyidae	Pteronarcys californica
Pteronarcys princeps mitochondrion, complete genom	AY687866	93	64.1	1.90723e−31	Plecoptera	Pteronarcyidae	Pteronarcys princeps
Rhithrogena germanica mitochondrial partial 16S rR	LN868660	81.13	100	6.65688e−31	Ephemeroptera	Heptageniidae	Rhithrogena germanica
Rhithrogena savoiensis mitochondrial partial 16S r	LN868658	81.13	100	6.65688e−31	Ephemeroptera	Heptageniidae	Rhithrogena savoiensis
Apteroperla tikumana mitochondrion, complete genom	KR604721	91.09	64.74	2.32348e−30	Plecoptera	Capniidae	Apteroperla tikumana
Oemopteryx vanduzeea 16S ribosomal RNA gene, parti	EF623266	91.09	64.74	2.32348e−30	Plecoptera	Taeniopterygidae	Oemopteryx vanduzeea
Mesocapnia arizonensis mitochondrion, partial geno	KP642637	90.1	64.74	2.83058e−29	Plecoptera	Capniidae	Mesocapnia arizonensis
Allocapnia recta isolate H19 16S ribosomal RNA gen	KC881054	90.1	64.74	2.83058e−29	Plecoptera	Capniidae	Allocapnia recta
Allocapnia recta isolate H16 16S ribosomal RNA gen	KC881051	90.1	64.74	2.83058e−29	Plecoptera	Capniidae	Allocapnia recta
Allocapnia recta isolate H15 16S ribosomal RNA gen	KC881050	90.1	64.74	2.83058e−29	Plecoptera	Capniidae	Allocapnia recta
Allocapnia recta isolate H14 16S ribosomal RNA gen	KC881049	90.1	64.74	2.83058e−29	Plecoptera	Capniidae	Allocapnia recta
Allocapnia recta isolate H13 16S ribosomal RNA gen	KC881048	90.1	64.74	2.83058e−29	Plecoptera	Capniidae	Allocapnia recta
Allocapnia recta isolate H11 16S ribosomal RNA gen	KC881046	90.1	64.74	2.83058e−29	Plecoptera	Capniidae	Allocapnia recta
Allocapnia recta isolate H10 16S ribosomal RNA gen	KC881045	90.1	64.74	2.83058e−29	Plecoptera	Capniidae	Allocapnia recta
Allocapnia recta isolate H7 16S ribosomal RNA gene	KC881042	90.1	64.74	2.83058e−29	Plecoptera	Capniidae	Allocapnia recta
Allocapnia recta isolate H6 16S ribosomal RNA gene	KC881041	90.1	64.74	2.83058e−29	Plecoptera	Capniidae	Allocapnia recta
Allocapnia recta isolate H3 16S ribosomal RNA gene	KC881038	90.1	64.74	2.83058e−29	Plecoptera	Capniidae	Allocapnia recta
Pteronarcys sachalina 16S ribosomal RNA gene, part	EF623264	91.09	64.74	2.83058e−29	Plecoptera	Pteronarcyidae	Pteronarcys sachalina
Moselia infuscata 16S ribosomal RNA gene, partial	EF623175	91.09	64.74	2.83058e−29	Plecoptera	Leuctridae	Moselia infuscata
Allocapnia minima 16S ribosomal RNA gene, partial	EF623130	90.1	64.74	2.83058e−29	Plecoptera	Capniidae	Allocapnia minima
Mesocapnia sp. BYU_PL010 16S ribosomal RNA gene, p	EF623127	90.1	64.74	2.83058e−29	Plecoptera	Capniidae	Mesocapnia sp. BYU_PL010
Allocapnia recta haplotype 12ar: H12 16S ribosomal	EU306875	90.1	64.74	2.83058e−29	Plecoptera	Capniidae	Allocapnia recta
Allocapnia recta haplotype 11ar: H011 16S ribosoma	EU306874	90.1	64.74	2.83058e−29	Plecoptera	Capniidae	Allocapnia recta
Allocapnia recta haplotype 7ar: H07 16S ribosomal	EU306870	90.1	64.74	2.83058e−29	Plecoptera	Capniidae	Allocapnia recta
Allocapnia recta haplotype 3ar: H03 16S ribosomal	DQ915181	90.1	64.74	2.83058e−29	Plecoptera	Capniidae	Allocapnia recta
Allocapnia recta haplotype 2ar: H02 16S ribosomal	DQ915180	90.1	64.74	2.83058e−29	Plecoptera	Capniidae	Allocapnia recta
Osmylus sp. EH−2004 16S ribosomal RNA gene, partia	AY620146	91.09	64.74	2.83058e−29	Neuroptera	Osmylidae	Osmylus sp. EH−2004
Allocapnia recta isolate H18 16S ribosomal RNA gen	KC881053	90	64.1	9.87969e−29	Plecoptera	Capniidae	Allocapnia recta
Allocapnia recta isolate H17 16S ribosomal RNA gen	KC881052	90	64.1	9.87969e−29	Plecoptera	Capniidae	Allocapnia recta
Allocapnia recta isolate H12 16S ribosomal RNA gen	KC881047	90	64.1	9.87969e−29	Plecoptera	Capniidae	Allocapnia recta
Allocapnia recta isolate H9 16S ribosomal RNA gene	KC881044	90	64.1	9.87969e−29	Plecoptera	Capniidae	Allocapnia recta
Allocapnia recta isolate H8 16S ribosomal RNA gene	KC881043	90	64.1	9.87969e−29	Plecoptera	Capniidae	Allocapnia recta
Allocapnia recta isolate H5 16S ribosomal RNA gene	KC881040	90	64.1	9.87969e−29	Plecoptera	Capniidae	Allocapnia recta
Allocapnia recta isolate H4 16S ribosomal RNA gene	KC881039	90	64.1	9.87969e−29	Plecoptera	Capniidae	Allocapnia recta
Strophopteryx appalachia 16S ribosomal RNA gene, p	EF623271	90	64.1	9.87969e−29	Plecoptera	Taeniopterygidae	Strophopteryx appalachia
Soyedina producta 16S ribosomal RNA gene, partial	EF623196	91	64.1	9.87969e−29	Plecoptera	Nemouridae	Soyedina producta
Allocapnia recta haplotype 10ar: H010 16S ribosoma	EU306873	90	64.1	9.87969e−29	Plecoptera	Capniidae	Allocapnia recta
Allocapnia recta haplotype 9ar: H09 16S ribosomal	EU306872	90	64.1	9.87969e−29	Plecoptera	Capniidae	Allocapnia recta
Allocapnia recta haplotype 8ar: H08 16S ribosomal	EU306871	90	64.1	9.87969e−29	Plecoptera	Capniidae	Allocapnia recta
Allocapnia recta haplotype 1ar: H01 16S ribosomal	DQ915179	90	64.1	9.87969e−29	Plecoptera	Capniidae	Allocapnia recta
Rhithrogena dorieri mitochondrial partial 16S rRNA	LN868649	79.87	100	3.44835e−28	Ephemeroptera	Heptageniidae	Rhithrogena dorieri
Bolotoperla rossi 16S ribosomal RNA gene, partial	EF623269	87.39	70.51	3.44835e−28	Plecoptera	Taeniopterygidae	Bolotoperla rossi
Allocapnia recta isolate H1 16S ribosomal RNA gene	KC881036	89.11	64.74	1.20359e−27	Plecoptera	Capniidae	Allocapnia recta
Pteronarcys scotti 16S ribosomal RNA gene, partial	EF623263	90.82	62.82	1.20359e−27	Plecoptera	Pteronarcyidae	Pteronarcys scotti
Udamocercia sp. BYU_PL150 16S ribosomal RNA gene,	EF623201	89.11	64.74	1.20359e−27	Plecoptera	Notonemouridae	Udamocercia sp. BYU_PL150
Rhithrogena sartorii mitochondrial partial 16S rRN	LN868670	79.25	100	4.20095e−27	Ephemeroptera	Heptageniidae	Rhithrogena sartorii
Cosumnoperla hypocrena 16S ribosomal RNA gene, par	EF623226	89.11	64.74	1.46628e−26	Plecoptera	Perlodidae	Cosumnoperla hypocrena
Amphinemura sulcicollis 16S ribosomal RNA gene, pa	EF623195	88.12	64.74	1.46628e−26	Plecoptera	Nemouridae	Amphinemura sulcicollis
Leuctra inermis 16S ribosomal RNA gene, partial se	EF623176	89.22	64.1	1.46628e−26	Plecoptera	Leuctridae	Leuctra inermis
Isocapnia hyalita 16S ribosomal RNA gene, partial	EF623131	88.12	64.74	1.46628e−26	Plecoptera	Capniidae	Isocapnia hyalita
