# Supplementary material for: Testing the potential of a ribosomal 16S marker for DNA metabarcoding of insects
Source: PeerJ. 2016 Apr 19;4:e1966. doi: 10.7717/peerj.1966 (PMC4841222; doi:10.7717/peerj.1966)
Supplement: Supplemental Information 1 [file peerj-04-1966-s006.zip › scripts/5 match OTUs/blast_hits/OTU_101.csv_plot.pdf]

OTU_101.csv

Boletina gripha

Mycetophilidae

Diptera

76.53

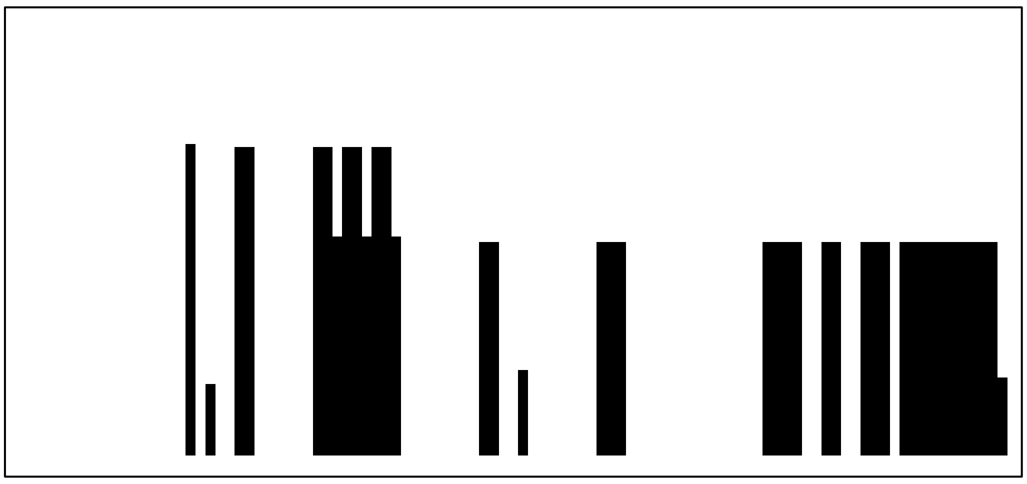

Boletina gripha isolate 057362 16S ribosomal RNA g	HQ230382	76.53	64.38	3.85717e−08	Diptera	Mycetophilidae	Boletina gripha
Paracantha forficula 16S ribosomal RNA gene, parti	DQ471385	76.84	63.7	3.85717e−08	Diptera	Tephritidae	Paracantha forficula
Seira sp. 1 FZ−2013 16S ribosomal RNA gene, partia	KC236214	76.47	65.07	1.34628e−07		Entomobryidae	Seira sp. 1 FZ−2013
Olisthaerus megacephalus voucher ZMUN:10008447 16S	JX536489	76.47	58.22	4.699e−07	Coleoptera	Staphylinidae	Olisthaerus megacephalus
Nalata nr. spinicollis 00052188 16S ribosomal RNA	JQ897812	76.27	73.97	5.72455e−06	Hemiptera	Reduviidae	Nalata nr. spinicollis 00052188
Dolichopus tanythrix voucher Pollet 0043 (P) 16S r	EU863951	79.22	51.37	5.72455e−06	Diptera	Dolichopodidae	Dolichopus tanythrix
Dolichopus nigricornis voucher Pollet 0023 (P) 16S	EU863922	77.92	51.37	5.72455e−06	Diptera	Dolichopodidae	Dolichopus nigricornis
Dolichopus migrans voucher Pollet 0114 (P) 16S rib	EU863921	79.22	51.37	5.72455e−06	Diptera	Dolichopodidae	Dolichopus migrans
Dolichopus bigeniculatus voucher RMBR:103435 16S r	FJ808164	79.22	51.37	5.72455e−06	Diptera	Dolichopodidae	Dolichopus bigeniculatus
Docosia gilvipes 16S ribosomal RNA gene, partial s	DQ787925	74.26	64.38	5.72455e−06	Diptera	Mycetophilidae	Docosia gilvipes
Chaetostomella vibrissata 16S ribosomal RNA gene,	DQ471415	74.53	69.86	5.72455e−06	Diptera	Tephritidae	Chaetostomella vibrissata
Ceratitis lentigera voucher ENT_000011690 16S ribo	KM023511	75.79	62.33	5.72455e−06	Diptera	Tephritidae	Ceratitis lentigera
Argiolestes roon voucher RMNH.INS.500848 16S ribos	KF369631	75	65.07	1.99806e−05	Odonata	Argiolestidae	Argiolestes roon
Neoclastobasis draskovitsae voucher JSL11 16S ribo	KC435583	76.74	57.53	1.99806e−05	Diptera	Mycetophilidae	Neoclastobasis draskovitsae
Boletina gripha isolate 033462 16S ribosomal RNA g	HQ230380	74.49	64.38	1.99806e−05	Diptera	Mycetophilidae	Boletina gripha
Isoneuromyia semirufa 16S ribosomal RNA gene, part	FJ172014	74.76	66.44	1.99806e−05	Diptera	Keroplatidae	Isoneuromyia semirufa
Human DNA sequence from clone RP3−431C21 on chromo	Z82210	94.59	25.34	1.99806e−05	Primates	Hominidae	Homo sapiens
Prosimulium fuscum mitochondrial 16S ribosomal RNA	L13399	76.47	65.07	1.99806e−05	Diptera	Simuliidae	Prosimulium fuscum
Folsomia quadrioculata 16S ribosomal RNA gene, par	KC236199	83.33	36.99	6.97393e−05		Isotomidae	Folsomia quadrioculata
Isotoma viridis 16S ribosomal RNA gene, partial se	JN970946	80	43.15	6.97393e−05		Isotomidae	Isotoma viridis
Hydraena arenicola voucher MNCN−AI504 large subuni	HM588479	77	66.44	6.97393e−05	Coleoptera	Hydraenidae	Hydraena arenicola
Homo sapiens FOSMID clone ABC24−1223N14 from chrom	AC240156	94.44	24.66	6.97393e−05	Primates	Hominidae	Homo sapiens
Homo sapiens FOSMID clone ABC27−417D24 from chromo	AC240257	94.44	24.66	6.97393e−05	Primates	Hominidae	Homo sapiens
Orellia falcata 16S ribosomal RNA gene, partial se	GQ175844	74.53	69.18	6.97393e−05	Diptera	Tephritidae	Orellia falcata
Rhagoletis fausta 16S ribosomal RNA gene, partial	U39433	73.79	66.44	6.97393e−05	Diptera	Tephritidae	Rhagoletis fausta
Docosia gilvipes 16S ribosomal RNA gene, partial s	FJ172027	73.27	64.38	6.97393e−05	Diptera	Mycetophilidae	Docosia gilvipes
Boletina plana 16S ribosomal RNA gene, partial seq	FJ172012	73.27	64.38	6.97393e−05	Diptera	Mycetophilidae	Boletina plana
Chaetogonopteron sp. 2 SNK−2009 voucher RMBR:10341	FJ808157	76.09	60.27	6.97393e−05	Diptera	Dolichopodidae	Chaetogonopteron sp. 2 SNK−2009
Ceratitis cosyra haplotype ClaI_B_DraI_B1_PsiI_B1	AY792079	77.11	55.48	6.97393e−05	Diptera	Tephritidae	Ceratitis cosyra
Homo sapiens chromosome 11, clone RP11−957I23, com	AC131288	94.44	24.66	6.97393e−05	Primates	Hominidae	Homo sapiens
Homo sapiens chromosome 11, clone RP11−280E5, comp	AC116901	94.44	24.66	6.97393e−05	Primates	Hominidae	Homo sapiens
Homo sapiens chromosome 17, clone CTD−2338C18, com	AC084809	90.24	28.08	6.97393e−05	Primates	Hominidae	Homo sapiens
Homo sapiens chromosome 18, clone RP11−465I4, comp	AC119868	94.44	24.66	6.97393e−05	Primates	Hominidae	Homo sapiens
Homo sapiens chromosome 11, clone RP11−227P3, comp	AC025787	94.44	24.66	6.97393e−05	Primates	Hominidae	Homo sapiens
Human DNA sequence from clone RP11−470P21 on chrom	AL353693	90.24	28.08	6.97393e−05	Primates	Hominidae	Homo sapiens
Homo sapiens chromosome 18, clone RP11−543H23, com	AC068254	94.44	24.66	6.97393e−05	Primates	Hominidae	Homo sapiens
Homo sapiens chromosome 11, clone RP11−574M7, comp	AC024405	94.44	24.66	6.97393e−05	Primates	Hominidae	Homo sapiens
Human DNA sequence from clone RP11−236O12 on chrom	AL589167	90.24	28.08	6.97393e−05	Primates	Hominidae	Homo sapiens
Prosimulium kiotoense mitochondrial gene for 16S r	LC035009	75.49	65.07	0.000243414	Diptera	Simuliidae	Prosimulium kiotoense
Simulium celebesense mitochondrial gene for 16S ri	LC034986	75.25	65.07	0.000243414	Diptera	Simuliidae	Simulium celebesense
Propebrevitrichia canuta 16S ribosomal RNA gene, p	KM878945	77.66	60.96	0.000243414	Diptera	Scenopinidae	Propebrevitrichia canuta
Ceratitis silvestrii voucher ENT_000010507 16S rib	KM023521	74	65.75	0.000243414	Diptera	Tephritidae	Ceratitis silvestrii
Ceratitis quinaria voucher AB31515648 16S ribosoma	KM023518	74	65.75	0.000243414	Diptera	Tephritidae	Ceratitis quinaria
Ceratitis quinaria voucher ENT_000010508 16S ribos	KM023517	74	65.75	0.000243414	Diptera	Tephritidae	Ceratitis quinaria
Ceratitis discussa voucher AB35136458 16S ribosoma	KM023508	74	65.75	0.000243414	Diptera	Tephritidae	Ceratitis discussa
Argiolestes roon voucher RMNH:INS:502073 16S ribos	KJ493085	74	65.07	0.000243414	Odonata	Argiolestidae	Argiolestes roon
Homo sapiens sodium channel and clathrin linker 1	NG_034202	90	27.4	0.000243414	Primates	Hominidae	Homo sapiens
Homo sapiens chromosome 15 clone VMRC53−276M3, com	AC254984	90	27.4	0.000243414	Primates	Hominidae	Homo sapiens
Eudicrana sp. DK−2013 voucher JSS12 16S ribosomal	KC435571	73.47	64.38	0.000243414	Diptera	Mycetophilidae	Eudicrana sp. DK−2013
Nalata setulosa strain 00003121 16S ribosomal RNA	JQ897813	76.4	58.9	0.000243414	Hemiptera	Reduviidae	Nalata setulosa
Leptosciarella (Leptosciarella) sp. 2 SS−2012 vouc	JQ613944	84	34.25	0.000243414	Diptera	Sciaridae	Leptosciarella (Leptosciarella) sp. 2 SS−2012
Hydraena circulata mitochondrial 16S rRNA gene (pa	HE971022	76.77	66.44	0.000243414	Coleoptera	Hydraenidae	Hydraena circulata
Choristoneura longicellana mitochondrion, complete	HQ452340	72.73	68.49	0.000243414	Lepidoptera	Tortricidae	Choristoneura longicellana
Boletina gripha isolate 057361 16S ribosomal RNA g	HQ230381	73.47	64.38	0.000243414	Diptera	Mycetophilidae	Boletina gripha
Boletina subtrivittata isolate 032921 16S ribosoma	HQ230379	74.49	64.38	0.000243414	Diptera	Mycetophilidae	Boletina subtrivittata
Boletina gripha isolate 015271 16S ribosomal RNA g	HQ230378	73.47	64.38	0.000243414	Diptera	Mycetophilidae	Boletina gripha
Gnoriste longirostris isolate 014972 16S ribosomal	HQ230376	73.47	64.38	0.000243414	Diptera	Mycetophilidae	Gnoriste longirostris
Boletina griphoides isolate 009627 16S ribosomal R	HQ230370	73.47	64.38	0.000243414	Diptera	Mycetophilidae	Boletina griphoides
Homo sapiens SATB homeobox 1 (SATB1), RefSeqGene o	NG_027814	90	27.4	0.000243414	Primates	Hominidae	Homo sapiens
Homo sapiens FOSMID clone ABC24−1239B9 from chromo	AC241516	90	27.4	0.000243414	Primates	Hominidae	Homo sapiens
Homo sapiens BAC clone RP11−785D17 from chromosome	AC240443	90	27.4	0.000243414	Primates	Hominidae	Homo sapiens
Dolichopus ungulatus voucher Pollet 0273 (P) 16S r	EU863960	77.92	51.37	0.000243414	Diptera	Dolichopodidae	Dolichopus ungulatus
Dolichopus ungulatus voucher Pollet 0024 (P) 16S r	EU863954	77.92	51.37	0.000243414	Diptera	Dolichopodidae	Dolichopus ungulatus
Dolichopus ungulatus voucher Pollet 0017 (P) 16S r	EU863953	77.92	51.37	0.000243414	Diptera	Dolichopodidae	Dolichopus ungulatus
Dolichopus subpennatus voucher Pollet 0118 (P) 16S	EU863949	77.92	51.37	0.000243414	Diptera	Dolichopodidae	Dolichopus subpennatus
Dolichopus signatus voucher Pollet 0046 (P) 16S ri	EU863945	77.92	51.37	0.000243414	Diptera	Dolichopodidae	Dolichopus signatus
Dolichopus popularis voucher Pollet 0002 (P) 16S r	EU863942	77.92	51.37	0.000243414	Diptera	Dolichopodidae	Dolichopus popularis
Dolichopus pennatus voucher Pollet 0013 (P) 16S ri	EU863926	77.92	51.37	0.000243414	Diptera	Dolichopodidae	Dolichopus pennatus
Dolichopus nubilus voucher Pollet 0180 (P) 16S rib	EU863925	77.92	51.37	0.000243414	Diptera	Dolichopodidae	Dolichopus nubilus
Dolichopus nitidus voucher Pollet 0218 (P) 16S rib	EU863924	77.92	51.37	0.000243414	Diptera	Dolichopodidae	Dolichopus nitidus
Dolichopus clavipes voucher Pollet 0193 (P) 16S ri	EU863907	77.92	51.37	0.000243414	Diptera	Dolichopodidae	Dolichopus clavipes
Dolichopus clavipes voucher Pollet 0113 (P) 16S ri	EU863906	77.92	51.37	0.000243414	Diptera	Dolichopodidae	Dolichopus clavipes
Dolichopus argyrotarsis voucher Pollet 0111 (P) 16	EU863899	77.92	51.37	0.000243414	Diptera	Dolichopodidae	Dolichopus argyrotarsis
Chaetorellia jaceae 16S ribosomal RNA gene, partia	GQ175842	74.07	71.23	0.000243414	Diptera	Tephritidae	Chaetorellia jaceae
Chaetostomella cylindrica 16S ribosomal RNA gene,	GQ175841	73.58	69.86	0.000243414	Diptera	Tephritidae	Chaetostomella cylindrica
Homo sapiens protocadherin 11 Y−linked (PCDH11Y),	NG_011652	90	27.4	0.000243414	Primates	Hominidae	Homo sapiens
Pan troglodytes chromosome X clone CH251−550L6 map	AC217568	90	27.4	0.000243414	Primates	Hominidae	Pan troglodytes
Pan troglodytes chromosome X clone PTB−104H11 map	AC232642	90	27.4	0.000243414	Primates	Hominidae	Pan troglodytes
Homo sapiens FOSMID clone ABC11−47394800A8 from ch	AC217022	90	27.4	0.000243414	Primates	Hominidae	Homo sapiens
Simulium celebesense mitochondrial gene for 16S ri	AB334095	75.25	65.07	0.000243414	Diptera	Simuliidae	Simulium celebesense
Picea sitchensis clone WS0279_O24 unknown mRNA	EF082104	73	61.64	0.000243414	Pinales	Pinaceae	Picea sitchensis
Pongo abelii BAC clone CH276−481J4 from chromosome	AC206035	90	27.4	0.000243414	Primates	Hominidae	Pongo abelii
Homo sapiens FOSMID clone ABC12−49095300M9 from ch	AC208004	90	27.4	0.000243414	Primates	Hominidae	Homo sapiens
Pseudobrachypeza helvetica 16S ribosomal RNA gene,	DQ787945	73.47	64.38	0.000243414	Diptera	Mycetophilidae	Pseudobrachypeza helvetica
Pseudorymosia fovea 16S ribosomal RNA gene, partia	DQ787935	74.26	66.44	0.000243414	Diptera	Mycetophilidae	Pseudorymosia fovea
Pan troglodytes BAC clone CH251−352J10 from chromo	AC188422	90	27.4	0.000243414	Primates	Hominidae	Pan troglodytes
Pan troglodytes BAC clone CH251−450L2 from chromos	AC192009	90	27.4	0.000243414	Primates	Hominidae	Pan troglodytes
Pan troglodytes chromosome X clone CH251−87H2 map	AC190129	90	27.4	0.000243414	Primates	Hominidae	Pan troglodytes
Chaetostomella cylindrica 16S ribosomal RNA gene,	DQ471414	73.58	69.86	0.000243414	Diptera	Tephritidae	Chaetostomella cylindrica
Homo sapiens chromosome 5 clone CTD−2322H6, comple	AC126752	90	27.4	0.000243414	Primates	Hominidae	Homo sapiens
Homo sapiens chromosome 8, clone CTD−3023L14, comp	AC114550	90	27.4	0.000243414	Primates	Hominidae	Homo sapiens
Homo sapiens chromosome 3 clone RP11−384L8, comple	AC097639	90	27.4	0.000243414	Primates	Hominidae	Homo sapiens
Homo sapiens chromosome 5 clone CTB−77B1, complete	AC021077	90	27.4	0.000243414	Primates	Hominidae	Homo sapiens
Homo sapiens chromosome 3 clone RP11−46B13, comple	AC103558	90	27.4	0.000243414	Primates	Hominidae	Homo sapiens
Homo sapiens chromosome 5 clone RP11−510I6, comple	AC106806	90	27.4	0.000243414	Primates	Hominidae	Homo sapiens
Homo sapiens BAC clone RP11−209D20 from 4, complet	AC093783	90	27.4	0.000243414	Primates	Hominidae	Homo sapiens
Human DNA sequence from clone RP11−393P6 on chromo	AL513317	90	27.4	0.000243414	Primates	Hominidae	Homo sapiens
Homo sapiens chromosome 8 clone CTA−398F10 map p22	AF186997	90	27.4	0.000243414	Primates	Hominidae	Homo sapiens
Homo sapiens chromosome 8, clone CTA−398F10, compl	AC103957	90	27.4	0.000243414	Primates	Hominidae	Homo sapiens
Homo sapiens chromosome 16 clone CTA−345G4, comple	AC130454	83.64	36.99	0.000243414	Primates	Hominidae	Homo sapiens
