# Supplementary material for: Testing the potential of a ribosomal 16S marker for DNA metabarcoding of insects
Source: PeerJ. 2016 Apr 19;4:e1966. doi: 10.7717/peerj.1966 (PMC4841222; doi:10.7717/peerj.1966)
Supplement: Supplemental Information 1 [file peerj-04-1966-s006.zip › scripts/5 match OTUs/blast_hits/OTU_103.csv_plot.pdf]

OTU_103.csv

Exorista cantans

Tachinidae

Diptera

79.65

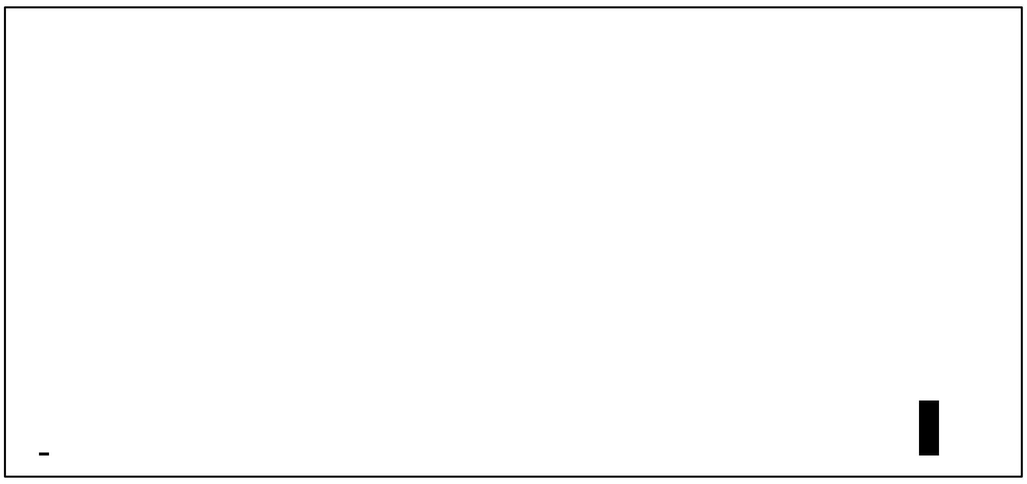

Exorista cantans mitochondrial gene for 16S rRNA,	AB257230	79.65	100	1.77256e−32	Diptera	Tachinidae	Exorista cantans
Cardiocladius sp. MNK−2008 16S ribosomal RNA gene,	EU669992	80.12	99.42	6.18685e−32	Diptera	Chironomidae	Cardiocladius sp. MNK−2008
Helina evecta 16S ribosomal RNA gene, partial sequ	FJ025439	79.43	100	9.18211e−30	Diptera	Muscidae	Helina evecta
Aedes flavopictus isolate A47 16S ribosomal RNA ge	DQ397928	79.07	100	9.18211e−30	Diptera	Culicidae	Aedes flavopictus
Sphyracephala munroi 16S ribosomal RNA gene, parti	AF304727	78.86	100	9.18211e−30	Diptera	Diopsidae	Sphyracephala munroi
Aedes albopictus mitochondrion, complete genome	KR068634	78.16	100	3.20487e−29	Diptera	Culicidae	Aedes albopictus
Culex mimeticus 16S ribosomal RNA gene, partial se	EF593021	77.59	100	3.20487e−29	Diptera	Culicidae	Culex mimeticus
Aedes albopictus isolate A21 16S ribosomal RNA gen	DQ397921	78.16	100	3.20487e−29	Diptera	Culicidae	Aedes albopictus
Aedes albopictus mitochondrion, complete genome	AY072044	78.16	100	3.20487e−29	Diptera	Culicidae	Aedes albopictus
Aedes albopictus mit−gene for large ribosomal subu	X01078	78.16	100	3.20487e−29	Diptera	Culicidae	Aedes albopictus
Teleopsis nr. dalmanni 2 MS−2015 voucher RMNH.INS.	KP978638	78.44	96.51	1.11861e−28	Diptera	Diopsidae	Teleopsis nr. dalmanni 2 MS−2015
Teleopsis dalmanni 16S ribosomal RNA gene, partial	KC177441	78.44	96.51	1.11861e−28	Diptera	Diopsidae	Teleopsis dalmanni
Cyrtodiopsis dalmanni 16S ribosomal RNA gene, part	AY123348	78.44	96.51	1.11861e−28	Diptera	Diopsidae	Teleopsis dalmanni
Diasemopsis sp. W 16S ribosomal RNA gene, partial	AF304726	79.19	100	1.11861e−28	Diptera	Diopsidae	Diasemopsis sp. W
Dideopsis aegrota 16S ribosomal RNA gene, partial	AF154774	79.19	100	1.11861e−28	Diptera	Syrphidae	Dideopsis aegrota
Dideopsis aegrota 16S ribosomal RNA gene, partial	AF154773	79.19	100	1.11861e−28	Diptera	Syrphidae	Dideopsis aegrota
Cyrtodiopsis dalmanni isolate CdGombak7 16S riboso	AY876567	78.44	96.51	1.11861e−28	Diptera	Diopsidae	Teleopsis dalmanni
Cyrtodiopsis dalmanni isolate CdGombak6 16S riboso	AY876566	78.44	96.51	1.11861e−28	Diptera	Diopsidae	Teleopsis dalmanni
Cyrtodiopsis dalmanni isolate CdGombak5 16S riboso	AY876565	78.44	96.51	1.11861e−28	Diptera	Diopsidae	Teleopsis dalmanni
Cyrtodiopsis dalmanni isolate CdGombak3 16S riboso	AY876563	78.44	96.51	1.11861e−28	Diptera	Diopsidae	Teleopsis dalmanni
Cyrtodiopsis dalmanni isolate CdGombak2 16S riboso	AY876562	78.44	96.51	1.11861e−28	Diptera	Diopsidae	Teleopsis dalmanni
Cyrtodiopsis dalmanni isolate CdGombak1 16S riboso	AY876561	78.44	96.51	1.11861e−28	Diptera	Diopsidae	Teleopsis dalmanni
Cyrtodiopsis dalmanni isolate CdBukitLawang3 16S r	AY876558	77.84	96.51	1.11861e−28	Diptera	Diopsidae	Teleopsis dalmanni
Prorates frommeri 16S ribosomal RNA gene, partial	KM878949	79.19	100	3.90433e−28	Diptera	Scenopinidae	Prorates frommeri
Helina fratercula voucher RMBR:116199 16S ribosoma	KJ476309	76.88	100	3.90433e−28	Diptera	Muscidae	Helina fratercula
Meigenia majuscula mitochondrial gene for 16S rRNA	AB465909	78.03	100	3.90433e−28	Diptera	Tachinidae	Meigenia majuscula
Neophryxe psychidis mitochondrial gene for 16S rRN	AB257232	78.03	100	3.90433e−28	Diptera	Tachinidae	Neophryxe psychidis
Aedes maehleri isolate A31 16S ribosomal RNA gene,	DQ397930	78.49	100	3.90433e−28	Diptera	Culicidae	Aedes maehleri
Aedes flavopictus isolate A49 16S ribosomal RNA ge	DQ397929	78.49	100	3.90433e−28	Diptera	Culicidae	Aedes flavopictus
Diasemopsis albifacies 16S ribosomal RNA gene, par	KT186652	78.03	100	1.36275e−27	Diptera	Diopsidae	Diasemopsis albifacies
Culex quinquefasciatus isolate A−270 16S ribosomal	EU711097	77.01	100	1.36275e−27	Diptera	Culicidae	Culex quinquefasciatus
Culex quinquefasciatus isolate A−267 16S ribosomal	EU711096	77.01	100	1.36275e−27	Diptera	Culicidae	Culex quinquefasciatus
Culex quinquefasciatus isolate A123 16S ribosomal	EU339145	77.01	100	1.36275e−27	Diptera	Culicidae	Culex quinquefasciatus
Culex tritaeniorhynchus 16S ribosomal RNA gene, pa	EF593020	77.01	100	1.36275e−27	Diptera	Culicidae	Culex tritaeniorhynchus
Culex bitaeniorhynchus 16S ribosomal RNA gene, par	EF593019	77.01	100	1.36275e−27	Diptera	Culicidae	Culex bitaeniorhynchus
Culex vishnui 16S ribosomal RNA gene, partial sequ	EF593018	77.01	100	1.36275e−27	Diptera	Culicidae	Culex vishnui
Culex tritaeniorhynchus 16S ribosomal RNA gene, mi	AF034469	77.01	100	1.36275e−27	Diptera	Culicidae	Culex tritaeniorhynchus
Teleopsis dalmanni 16S ribosomal RNA gene, partial	KT186654	77.84	96.51	4.75645e−27	Diptera	Diopsidae	Teleopsis dalmanni
Teleopsis nr. dalmanni 1 MS−2015 voucher FF−IIX3 1	KP978636	77.84	96.51	4.75645e−27	Diptera	Diopsidae	Teleopsis nr. dalmanni 1 MS−2015
Teleopsis nr. dalmanni 1 MS−2015 voucher RMNH.INS.	KP978617	77.84	96.51	4.75645e−27	Diptera	Diopsidae	Teleopsis nr. dalmanni 1 MS−2015
Aedes vigilax isolate Mi139 mitochondrion, complet	KP995260	77.33	100	4.75645e−27	Diptera	Culicidae	Ochlerotatus vigilax
Aedes notoscriptus isolate MC04 mitochondrion, com	KM676218	77.33	100	4.75645e−27	Diptera	Culicidae	Aedes notoscriptus
Lispe pygmaea 16S ribosomal RNA gene, partial sequ	JN226653	77.46	100	4.75645e−27	Diptera	Muscidae	Lispe pygmaea
Lispe leucospila 16S ribosomal RNA gene, partial s	JN226652	77.46	100	4.75645e−27	Diptera	Muscidae	Lispe leucospila
Exorista sorbillans mitochondrion, complete genome	HQ322500	77.33	100	4.75645e−27	Diptera	Tachinidae	Exorista sorbillans
Exorista sorbillans mitochondrial gene for 16S rRN	AB257227	77.33	100	4.75645e−27	Diptera	Tachinidae	Exorista sorbillans
Cyrtodiopsis dalmanni isolate CdLangat6−9a 16S rib	DQ098022	77.84	96.51	4.75645e−27	Diptera	Diopsidae	Teleopsis dalmanni
Cyrtodiopsis dalmanni isolate CdLangat6−6a 16S rib	DQ098019	77.84	96.51	4.75645e−27	Diptera	Diopsidae	Teleopsis dalmanni
Cyrtodiopsis dalmanni isolate CdBogor5 16S ribosom	AY876580	77.84	96.51	4.75645e−27	Diptera	Diopsidae	Teleopsis dalmanni
Cyrtodiopsis dalmanni isolate CdBogor4 16S ribosom	AY876579	77.84	96.51	4.75645e−27	Diptera	Diopsidae	Teleopsis dalmanni
Cyrtodiopsis dalmanni isolate CdBogor3 16S ribosom	AY876578	77.84	96.51	4.75645e−27	Diptera	Diopsidae	Teleopsis dalmanni
Cyrtodiopsis dalmanni isolate CdBogor2 16S ribosom	AY876577	77.84	96.51	4.75645e−27	Diptera	Diopsidae	Teleopsis dalmanni
Cyrtodiopsis dalmanni isolate CdBogor1 16S ribosom	AY876576	77.84	96.51	4.75645e−27	Diptera	Diopsidae	Teleopsis dalmanni
Cyrtodiopsis dalmanni isolate CdCameron4 16S ribos	AY876571	77.84	96.51	4.75645e−27	Diptera	Diopsidae	Teleopsis dalmanni
Cyrtodiopsis dalmanni isolate CdGombak4 16S riboso	AY876564	77.84	96.51	4.75645e−27	Diptera	Diopsidae	Teleopsis dalmanni
Cyrtodiopsis dalmanni isolate CdBukitLawang5 16S r	AY876560	77.25	96.51	4.75645e−27	Diptera	Diopsidae	Teleopsis dalmanni
Cyrtodiopsis dalmanni isolate CdBukitLawang4 16S r	AY876559	77.25	96.51	4.75645e−27	Diptera	Diopsidae	Teleopsis dalmanni
Cyrtodiopsis dalmanni isolate CdBukitLawang2 16S r	AY876557	77.25	96.51	4.75645e−27	Diptera	Diopsidae	Teleopsis dalmanni
Cyrtodiopsis dalmanni isolate CdBukitLawang1 16S r	AY876556	77.25	96.51	4.75645e−27	Diptera	Diopsidae	Teleopsis dalmanni
Cyrtodiopsis dalmanni isolate CdSoraya5 16S riboso	AY876555	77.25	96.51	4.75645e−27	Diptera	Diopsidae	Teleopsis dalmanni
Cyrtodiopsis dalmanni isolate CdSoraya4 16S riboso	AY876554	77.25	96.51	4.75645e−27	Diptera	Diopsidae	Teleopsis dalmanni
Cyrtodiopsis dalmanni isolate CdSoraya3 16S riboso	AY876553	77.25	96.51	4.75645e−27	Diptera	Diopsidae	Teleopsis dalmanni
Cyrtodiopsis dalmanni isolate CdSoraya2 16S riboso	AY876552	77.25	96.51	4.75645e−27	Diptera	Diopsidae	Teleopsis dalmanni
Cyrtodiopsis dalmanni isolate CdSoraya1 16S riboso	AY876551	77.25	96.51	4.75645e−27	Diptera	Diopsidae	Teleopsis dalmanni
Cyrtodiopsis dalmanni isolate CdBrunei5 16S riboso	AY876550	77.84	96.51	4.75645e−27	Diptera	Diopsidae	Teleopsis dalmanni
Cyrtodiopsis dalmanni isolate CdBrunei4 16S riboso	AY876549	77.84	96.51	4.75645e−27	Diptera	Diopsidae	Teleopsis dalmanni
Cyrtodiopsis dalmanni isolate CdBrunei3 16S riboso	AY876548	77.84	96.51	4.75645e−27	Diptera	Diopsidae	Teleopsis dalmanni
Cyrtodiopsis dalmanni isolate CdBrunei2 16S riboso	AY876547	77.84	96.51	4.75645e−27	Diptera	Diopsidae	Teleopsis dalmanni
Cyrtodiopsis dalmanni isolate CdBrunei1 16S riboso	AY876546	77.84	96.51	4.75645e−27	Diptera	Diopsidae	Teleopsis dalmanni
Aedes w−albus 16S ribosomal RNA gene, mitochondria	AF034472	78.49	100	4.75645e−27	Diptera	Culicidae	Aedes w−albus
Cyrtodiopsis nr. truncata KCADI049−14 16S ribosoma	KT186648	77.01	100	1.66016e−26	Diptera	Diopsidae	Cyrtodiopsis nr. truncata KCADI049−14
Chetogena sp. 16022006 mitochondrial gene for 16S	AB699939	77.46	100	1.66016e−26	Diptera	Tachinidae	Chetogena sp. 16022006
Aedes dybasi isolate A28 16S ribosomal RNA gene, p	DQ397925	78.61	100	1.66016e−26	Diptera	Culicidae	Aedes dybasi
Aedes palauensis isolate A26 16S ribosomal RNA gen	DQ397924	78.61	100	1.66016e−26	Diptera	Culicidae	Aedes palauensis
Culex bitaeniorhynchus 16S ribosomal RNA gene, mit	AF034474	77.01	100	1.66016e−26	Diptera	Culicidae	Culex bitaeniorhynchus
Teleopsis sp. n. 'gracilifemur' voucher RMNH.INS.5	KP978620	77.71	96.51	5.79454e−26	Diptera	Diopsidae	Teleopsis sp. n. 'gracilifemur'
Ceratitis connexa voucher ENT_000010509 16S riboso	KM023505	77.33	100	5.79454e−26	Diptera	Tephritidae	Ceratitis connexa
Suensonomyia nudinerva mitochondrial gene for 16S	AB465944	76.74	100	5.79454e−26	Diptera	Tachinidae	Suensonomyia nudinerva
Blondelia nigripes mitochondrial gene for 16S rRNA	AB465902	76.4	100	5.79454e−26	Diptera	Tachinidae	Blondelia nigripes
Trichodiopsis minuta 16S ribosomal RNA gene, parti	AF304724	77.46	100	5.79454e−26	Diptera	Diopsidae	Diasemopsis minuta
Diasemopsis albifacies 16S ribosomal RNA gene, par	AF304723	77.46	100	5.79454e−26	Diptera	Diopsidae	Diasemopsis albifacies
Diasemopsis obstans 16S ribosomal RNA gene, partia	AF304718	78.03	100	5.79454e−26	Diptera	Diopsidae	Diasemopsis obstans
Megalabops sp. BOLD:ACP5917 16S ribosomal RNA gene	KT186670	76.79	96.51	2.02249e−25	Diptera	Diopsidae	Megalabops sp. BOLD:ACP5917
Megalabops quadriguttata 16S ribosomal RNA gene, p	KT186655	76.79	96.51	2.02249e−25	Diptera	Diopsidae	Megalabops quadriguttata
Teleopsis pallifacies voucher RMNH.INS.555128 16S	KP978631	77.78	96.51	2.02249e−25	Diptera	Diopsidae	Teleopsis pallifacies
Ochlerotatus vigilax isolate Mi140 mitochondrion,	KP721463	76.74	100	2.02249e−25	Diptera	Culicidae	Ochlerotatus vigilax
Aedes notoscriptus isolate MC10 mitochondrion, com	KM676219	76.74	100	2.02249e−25	Diptera	Culicidae	Aedes notoscriptus
Exorista globosa mitochondrial gene for 16S rRNA,	AB699945	76.88	100	2.02249e−25	Diptera	Tachinidae	Exorista globosa
Chetogena sp. 1 MATM−2012 small subunit ribosomal	JQ246749	76.88	100	2.02249e−25	Diptera	Tachinidae	Chetogena sp. 1 MATM−2012
Dinera takanoi mitochondrial gene for 16S rRNA, pa	AB465958	76.88	100	2.02249e−25	Diptera	Tachinidae	Dinera takanoi
Admontia sp. Admon_23072006 mitochondrial gene for	AB465900	77.84	100	2.02249e−25	Diptera	Tachinidae	Admontia sp. Admon_23072006
Aedes riversi isolate A23 16S ribosomal RNA gene,	DQ397927	82.58	75	2.02249e−25	Diptera	Culicidae	Aedes riversi
Aedes riversi isolate A24 16S ribosomal RNA gene,	DQ397926	82.58	75	2.02249e−25	Diptera	Culicidae	Aedes riversi
Ochlerotatus japonicus isolate A81 16S ribosomal R	DQ397915	76.74	100	2.02249e−25	Diptera	Culicidae	Aedes japonicus
Somatia sp. YSUW04038 16S ribosomal RNA gene, part	AY573134	77.33	100	2.02249e−25	Diptera	Somatiidae	Somatia sp. YSUW04038
Armigeres subalbatus ASAP ID: 43761 large ribosoma	AY439688	76.74	100	2.02249e−25	Diptera	Culicidae	Armigeres subalbatus
Gitona bivisualis 16S ribosomal RNA gene, partial	AF479793	76.74	100	2.02249e−25	Diptera	Drosophilidae	Gitona bivisualis
Teloglabrus entabenensis 16S ribosomal RNA gene, p	AF304731	78.03	100	2.02249e−25	Diptera	Diopsidae	Teloglabrus entabenensis
Nephrocerus daeckei 16S ribosomal RNA gene, partia	AF154796	77.46	100	2.02249e−25	Diptera	Pipunculidae	Nephrocerus daeckei
Nephrocerus daeckei 16S ribosomal RNA gene, partia	AF154795	77.46	100	2.02249e−25	Diptera	Pipunculidae	Nephrocerus daeckei
