# Supplementary material for: Testing the potential of a ribosomal 16S marker for DNA metabarcoding of insects
Source: PeerJ. 2016 Apr 19;4:e1966. doi: 10.7717/peerj.1966 (PMC4841222; doi:10.7717/peerj.1966)
Supplement: Supplemental Information 1 [file peerj-04-1966-s006.zip › scripts/5 match OTUs/blast_hits/OTU_105.csv_plot.pdf]

OTU_105.csv

Sericostoma clypeatum

Sericostomatidae

Trichoptera

97.24

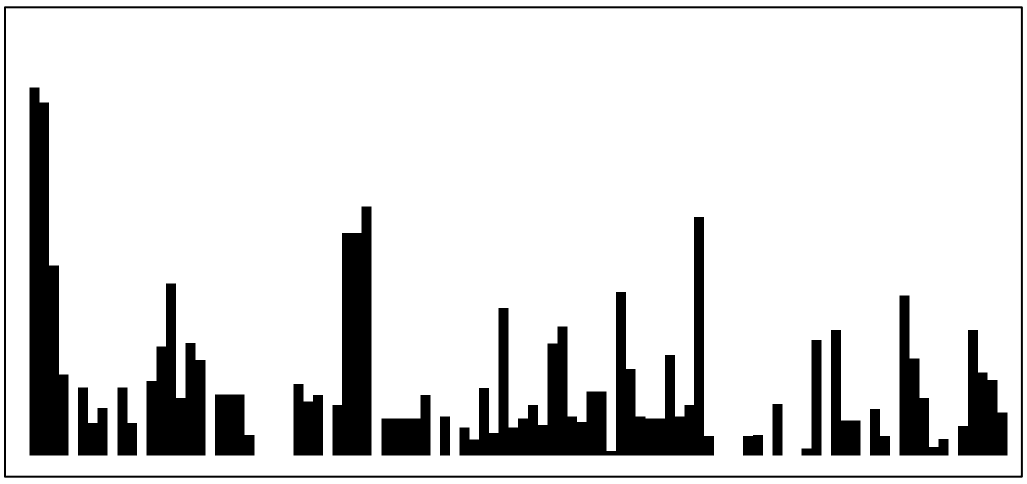

Sericostoma clypeatum voucher NHRS−EN CC4 16S ribo	FJ263207	97.24	100	2.90425e−60	Trichoptera	Sericostomatidae	Sericostoma clypeatum
Sericostoma personatum mitochondrion, partial geno	KP455290	96.53	100	3.5381e−59	Trichoptera	Sericostomatidae	Sericostoma personatum
Notidobiella chacayana isolate CL5 16S ribosomal R	EF394993	88.89	73.61	3.10637e−28	Trichoptera	Sericostomatidae	Notidobiella chacayana
Hypocera mordellaria isolate 200864 16S ribosomal	GU362000	83.78	70.14	4.31791e−20	Diptera	Phoridae	Hypocera mordellaria
Pelopidas agna voucher He013 16S ribosomal RNA gen	JX971182	79.39	85.42	5.2603e−19	Lepidoptera	Hesperiidae	Pelopidas agna
Polietina sp. SNK−2014 voucher RMBR:116219 16S rib	KJ476326	83.17	68.75	5.2603e−19	Diptera	Muscidae	Polietina sp. SNK−2014
Carterocephalus silvicola mitochondrion, complete	KJ629163	81.51	78.47	5.2603e−19	Lepidoptera	Hesperiidae	Carterocephalus silvicola
Sciodrepoides watsoni mitochondrial 16S rRNA gene	HE576705	82.2	78.47	5.2603e−19	Coleoptera	Leiodidae	Sciodrepoides watsoni
Attalus heydeni voucher UPOL001106 16S ribosomal R	HQ619658	78.62	92.36	5.2603e−19	Coleoptera	Melyridae	Attalus heydeni
Achalcus flavicollis voucher Pollet 0171 (P) 16S r	HQ448956	83.18	70.14	5.2603e−19	Diptera	Dolichopodidae	Achalcus flavicollis
Carterocephalus silvicola 16S ribosomal RNA gene,	GU372501	81.51	78.47	5.2603e−19	Lepidoptera	Hesperiidae	Carterocephalus silvicola
Pelopidas agna 16S ribosomal RNA gene, partial seq	KJ786312	79.84	85.42	1.83602e−18	Lepidoptera	Hesperiidae	Pelopidas agna
Lordithon thoracicus voucher ZMUN:10029203 16S rib	JX536486	83.47	78.47	1.83602e−18	Coleoptera	Staphylinidae	Lordithon thoracicus
Diplonevra florea isolate 16.xi.06−387 16S ribosom	HM367001	85.11	63.19	1.83602e−18	Diptera	Phoridae	Diplonevra florea
Limonia tahitiensis voucher O'Grady 202004 16S rib	EU005425	88.04	62.5	1.83602e−18	Diptera	Limoniidae	Dicranomyia tahitiensis
Graptomyza plumifer 16S ribosomal RNA gene, partia	AF154785	82.69	69.44	1.83602e−18	Diptera	Syrphidae	Graptomyza plumifer
Stenocallimerus prasinatus voucher UPOL001132 16S	KC758783	85.26	62.5	6.40835e−18	Coleoptera	Cleridae	Stenocallimerus prasinatus
Chaetoria stylata mitochondrial gene for 16S rRNA,	AB699938	84.47	65.97	6.40835e−18	Diptera	Tachinidae	Chaetoria stylata
Pelopidas mathias isolate F440 16S ribosomal RNA g	JQ421815	79.23	85.42	6.40835e−18	Lepidoptera	Hesperiidae	Pelopidas mathias
Costora seposita voucher NHRS−EN EN5 16S ribosomal	FJ263218	82.86	69.44	6.40835e−18	Trichoptera	Conoesucidae	Costora seposita
Paroligoneurus sp. JCB−2006 16S ribosomal RNA gene	DQ538568	82.86	71.53	6.40835e−18	Hymenoptera	Braconidae	Paroligoneurus sp. JCB−2006
Paroligoneurus sp. 16S ribosomal RNA gene, partial	AF003519	82.86	71.53	6.40835e−18	Hymenoptera	Braconidae	Paroligoneurus sp.
Pseudoborbo bevani voucher He018 16S ribosomal RNA	JX971180	80.95	79.86	2.23674e−17	Lepidoptera	Hesperiidae	Pseudoborbo bevani
Pelopidas mathias isolate F439 16S ribosomal RNA g	JQ421818	78.63	85.42	2.23674e−17	Lepidoptera	Hesperiidae	Pelopidas mathias
Pelopidas mathias isolate N273 16S ribosomal RNA g	JQ421813	78.63	85.42	2.23674e−17	Lepidoptera	Hesperiidae	Pelopidas mathias
Pelopidas mathias isolate F58 16S ribosomal RNA ge	JQ421808	78.63	85.42	2.23674e−17	Lepidoptera	Hesperiidae	Pelopidas mathias
Pelopidas mathias isolate F447 16S ribosomal RNA g	JQ421805	78.63	85.42	2.23674e−17	Lepidoptera	Hesperiidae	Pelopidas mathias
Conicera kempi isolate 200852 16S ribosomal RNA ge	GU361992	83.33	63.89	2.23674e−17	Diptera	Phoridae	Conicera kempi
Polietes lardarius 16S ribosomal RNA gene, partial	FJ025471	82.52	70.14	2.23674e−17	Diptera	Muscidae	Polietes lardarius
Muscina stabulans 16S ribosomal RNA gene, partial	EF531117	82.83	68.06	2.23674e−17	Diptera	Muscidae	Muscina stabulans
Notomma n. sp. 1000 16S ribosomal RNA gene, partia	AY792108	78.72	92.36	2.23674e−17	Diptera	Tephritidae	Notomma n. sp. 1000
Chylizosoma vittatum 16S ribosomal RNA gene, parti	DQ648654	82.35	64.58	2.23674e−17	Diptera	Scathophagidae	Chylizosoma vittatum
Protochauliodes biconicus incertus 16S ribosomal R	KF944494	90.41	50	7.80697e−17	Megaloptera	Corydalidae	Protochauliodes biconicus incertus
Protochauliodes incertus isolate H842 16S ribosoma	KF741322	90.41	50	7.80697e−17	Megaloptera	Corydalidae	Protochauliodes biconicus incertus
Diplonevra freyi isolate 13.viii.08−475 16S riboso	HM366989	91.67	48.61	7.80697e−17	Diptera	Phoridae	Diplonevra freyi
Attalus viridescens voucher UPOL001113 16S ribosom	HQ619662	77.3	90.28	7.80697e−17	Coleoptera	Melyridae	Attalus viridescens
Atherigona orientalis 16S ribosomal RNA gene, part	JN226640	81.73	71.53	7.80697e−17	Diptera	Muscidae	Atherigona orientalis
Neomyia bristocercus 16S ribosomal RNA gene, parti	JN226636	81.73	70.83	7.80697e−17	Diptera	Muscidae	Neomyia bristocercus
Neomyia gavisa 16S ribosomal RNA gene, partial seq	JN226635	81.73	70.83	7.80697e−17	Diptera	Muscidae	Neomyia gavisa
Neomyia timorensis 16S ribosomal RNA gene, partial	JN226634	81.73	70.83	7.80697e−17	Diptera	Muscidae	Neomyia timorensis
Hydrotaea irritans 16S ribosomal RNA gene, partial	FJ025443	82.83	68.06	7.80697e−17	Diptera	Muscidae	Hydrotaea irritans
Limonia sp. PMO 200984 16S ribosomal RNA gene, par	EU005416	80	83.33	7.80697e−17	Diptera	Limoniidae	Limonia sp. PMO 200984
Calliphora vomitoria isolate nk56 16S ribosomal RN	KP862541	81.82	68.06	2.7249e−16	Diptera	Calliphoridae	Calliphora vomitoria
Pelopidas mathias voucher He194 16S ribosomal RNA	JX971181	78.23	80.56	2.7249e−16	Lepidoptera	Hesperiidae	Pelopidas mathias
Ditomyia fasciata voucher JSSJ3 16S ribosomal RNA	KJ136734	81.31	66.67	2.7249e−16	Diptera	Ditomyiidae	Ditomyia fasciata
Rutylapa ruficornis voucher JSB8 16S ribosomal RNA	KJ136719	80.73	71.53	2.7249e−16	Diptera	Keroplatidae	Rutylapa ruficornis
Himalusa thailandensis voucher ZMUN:10051228 16S r	JX536406	83.16	65.28	2.7249e−16	Coleoptera	Staphylinidae	Himalusa thailandensis
Catops sp. IBE AF30 mitochondrial 16S rRNA gene (p	HE576691	81.03	76.39	2.7249e−16	Coleoptera	Leiodidae	Catops sp. IBE AF30
Stichillus sp. JMH−2012 isolate 322 16S ribosomal	JN196445	86.9	56.94	2.7249e−16	Diptera	Phoridae	Stichillus sp. JMH−2012
Achalcus vaillanti voucher Pollet 0182 (P) 16S rib	HQ448958	81.31	70.14	2.7249e−16	Diptera	Dolichopodidae	Achalcus vaillanti
Musca conducens 16S ribosomal RNA gene, partial se	JN226629	81.73	71.53	2.7249e−16	Diptera	Muscidae	Musca conducens
Cinara longipennis 12S small subunit ribosomal RNA	GU457835	82.35	69.44	2.7249e−16	Hemiptera	Lachnidae	Cinara longipennis
Culcua kolibaci 16S ribosomal RNA gene, partial se	GU947391	81.42	70.14	2.7249e−16	Diptera	Stratiomyidae	Culcua kolibaci
Melaloncha triangularis 16S ribosomal RNA gene, pa	GU550219	85.23	59.03	2.7249e−16	Diptera	Phoridae	Melaloncha triangularis
Toxotrypana curvicauda 16S ribosomal RNA gene, par	U39381	86.05	58.33	2.7249e−16	Diptera	Tephritidae	Toxotrypana curvicauda
Musca domestica isolate C2 16S ribosomal RNA gene,	EU021214	81.82	68.06	2.7249e−16	Diptera	Muscidae	Musca domestica
Paykullia maculata 16S ribosomal RNA gene, partial	FJ025466	81.55	70.14	2.7249e−16	Diptera	Rhinophoridae	Paykullia maculata
Mesembrina mystacea 16S ribosomal RNA gene, partia	FJ025453	82.98	64.58	2.7249e−16	Diptera	Muscidae	Mesembrina mystacea
Mesembrina meridiana 16S ribosomal RNA gene, parti	FJ025452	82.98	64.58	2.7249e−16	Diptera	Muscidae	Mesembrina meridiana
Hydrotaea dentipes 16S ribosomal RNA gene, partial	FJ025442	80.19	68.06	2.7249e−16	Diptera	Muscidae	Hydrotaea dentipes
Scatella hawaiiensis voucher 105726 NADH dehydroge	EU494299	87.65	54.86	2.7249e−16	Diptera	Ephydridae	Scatella hawaiiensis
Paramycodrosophila sp. POG−2002 16S ribosomal RNA	AF479810	84.04	64.58	2.7249e−16	Diptera	Drosophilidae	Paramycodrosophila n. sp. POG−2002
Musca sp. VH−2001 16S ribosomal RNA gene, partial	AF322436	81.82	68.06	2.7249e−16	Diptera	Muscidae	Musca sp. VH−2001
Lucilia sericata clone SCUBW05007 16S ribosomal RN	AY962646	81.73	71.53	2.7249e−16	Diptera	Calliphoridae	Lucilia sericata
Lucilia sericata clone SCUBW05006 16S ribosomal RN	AY962645	81.73	71.53	2.7249e−16	Diptera	Calliphoridae	Lucilia sericata
Trichobius longipes 16S ribosomal RNA gene, partia	DQ133052	84.71	59.03	2.7249e−16	Diptera	Streblidae	Trichobius longipes
Musca domestica 16S ribosomal RNA gene, partial se	DQ133039	81.82	68.06	2.7249e−16	Diptera	Muscidae	Musca domestica
Conicera similis 16S ribosomal RNA gene, partial s	AF126337	82.35	63.89	2.7249e−16	Diptera	Phoridae	Conicera similis
Atelestus pulicarius isolate AP47 16S ribosomal RN	KC699323	91.18	46.53	9.51084e−16	Diptera	Atelestidae	Atelestus pulicarius
Trirhithrum nigerrimum voucher ENT_000010570 16S r	KM023529	80.91	71.53	9.51084e−16	Diptera	Tephritidae	Trirhithrum nigerrimum
Caltoris bromus voucher He032 16S ribosomal RNA ge	JX971186	79.23	85.42	9.51084e−16	Lepidoptera	Hesperiidae	Caltoris bromus
Caltoris bromus voucher He024 16S ribosomal RNA ge	JX971184	79.23	85.42	9.51084e−16	Lepidoptera	Hesperiidae	Caltoris bromus
Caltoris bromus voucher He002 16S ribosomal RNA ge	JX971183	79.23	85.42	9.51084e−16	Lepidoptera	Hesperiidae	Caltoris bromus
Ampittia dioscorides voucher YXW007, complete geno	KM102732	80.91	72.22	9.51084e−16	Lepidoptera	Hesperiidae	Ampittia dioscorides
Lophosceles cinereiventris voucher RMBR:116208 16S	KJ476316	80.95	71.53	9.51084e−16	Diptera	Muscidae	Lophosceles cinereiventris
Caltoris bromus 16S ribosomal RNA gene, partial se	KJ786315	79.23	85.42	9.51084e−16	Lepidoptera	Hesperiidae	Caltoris bromus
Styracotechys dicelysma voucher AE167 16S ribosoma	KF318510	82.41	70.14	9.51084e−16	Hymenoptera	Pergidae	Styracotechys dicelysma
Pronomaea korgei voucher ZMUN:10008438 16S ribosom	JX536480	76.06	97.22	9.51084e−16	Coleoptera	Staphylinidae	Pronomaea korgei
Hoplicnema sp. CO968 16S ribosomal RNA gene, parti	JX844969	78.74	84.72	9.51084e−16	Coleoptera	Corylophidae	Hoplicnema sp. CO968
Cyclocephala sanguinicollis 16S ribosomal RNA gene	JX982879	80.33	79.86	9.51084e−16	Coleoptera	Scarabaeidae	Cyclocephala sanguinicollis
Dohrniphora fuliginata isolate 14.ix.08−508 16S ri	HM366995	85.39	59.03	9.51084e−16	Diptera	Phoridae	Dohrniphora fuliginata
Phloiotrya planiuscula voucher UPOL ZL0130 16S rib	FJ903741	79.82	78.47	9.51084e−16	Coleoptera	Melandryidae	Phloiotrya planiuscula
Philipomyia graeca 16S ribosomal RNA gene, partial	HM132110	85.88	57.64	9.51084e−16	Diptera	Tabanidae	Philipomyia graeca
Sphenometopa claripennis voucher RMBR:103685 16S r	GQ409159	81.63	63.89	9.51084e−16	Diptera	Sarcophagidae	Sphenometopa claripennis
Duckemyia latifrons voucher RMBR:103703 16S riboso	GQ409127	81.63	67.36	9.51084e−16	Diptera	Sarcophagidae	Duckemyia latifrons
Acrotaeniostola sexvittata voucher YSUW98071108 16	EU926919	77.54	92.36	9.51084e−16	Diptera	Tephritidae	Acrotaeniostola sexvittata
Drymeia hamata 16S ribosomal RNA gene, partial seq	FJ025431	82.18	69.44	9.51084e−16	Diptera	Muscidae	Drymeia hamata
Trirhithrum nigerrimum 16S ribosomal RNA gene, par	AY792109	80.91	71.53	9.51084e−16	Diptera	Tephritidae	Trirhithrum nigerrimum
Italochrysa neurodes isolate Itaneu1 16S ribosomal	AY620153	75.91	88.89	9.51084e−16	Neuroptera	Chrysopidae	Italochrysa neurodes
Megastrebla nigriceps '2' Di139 16S ribosomal RNA	DQ133032	87.5	54.86	9.51084e−16	Diptera	Streblidae	Megastrebla nigriceps '2' Di139
Trichobius hirsutulus 16S ribosomal RNA gene, part	DQ133023	84.52	58.33	9.51084e−16	Diptera	Streblidae	Trichobius hirsutulus
Toxotrypana littoralis 16S ribosomal RNA gene, par	AF152091	82.69	69.44	9.51084e−16	Diptera	Tephritidae	Toxotrypana littoralis
Ravinia pernix mitochondrion, complete genome	KM676414	80.39	70.14	3.31961e−15	Diptera	Sarcophagidae	Ravinia pernix
Gegenes nostrodamus voucher He240 16S ribosomal RN	JX971193	80.77	85.42	3.31961e−15	Lepidoptera	Hesperiidae	Gegenes nostrodamus
Huckettomyia watanabei voucher RMBR:116202 16S rib	KJ476311	79.63	68.75	3.31961e−15	Diptera	Muscidae	Huckettomyia watanabei
Coenosia agromyzina voucher RMBR:116185 16S riboso	KJ476299	81.37	70.14	3.31961e−15	Diptera	Muscidae	Coenosia agromyzina
Faronus parallelus isolate JPDG19 16S ribosomal RN	KM350426	85.88	55.56	3.31961e−15	Coleoptera	Staphylinidae	Faronus parallelus
Habrocerus capillaricornis voucher ZMUN:10008441 1	JX536488	83.87	62.5	3.31961e−15	Coleoptera	Staphylinidae	Habrocerus capillaricornis
Diplonevra gaudialis isolate 27.vi.02−56 16S ribos	HM366998	83.52	61.11	3.31961e−15	Diptera	Phoridae	Diplonevra gaudialis
Dohrniphora sp. Thai 1 isolate 6.ix.05−260 16S rib	HM366987	82	65.97	3.31961e−15	Diptera	Phoridae	Dohrniphora sp. Thai 1
