# Supplementary material for: Testing the potential of a ribosomal 16S marker for DNA metabarcoding of insects
Source: PeerJ. 2016 Apr 19;4:e1966. doi: 10.7717/peerj.1966 (PMC4841222; doi:10.7717/peerj.1966)
Supplement: Supplemental Information 1 [file peerj-04-1966-s006.zip › scripts/5 match OTUs/blast_hits/OTU_106.csv_plot.pdf]

OTU_106.csv

Gymnopternus aerosus

Dolichopodidae

Diptera

98.73

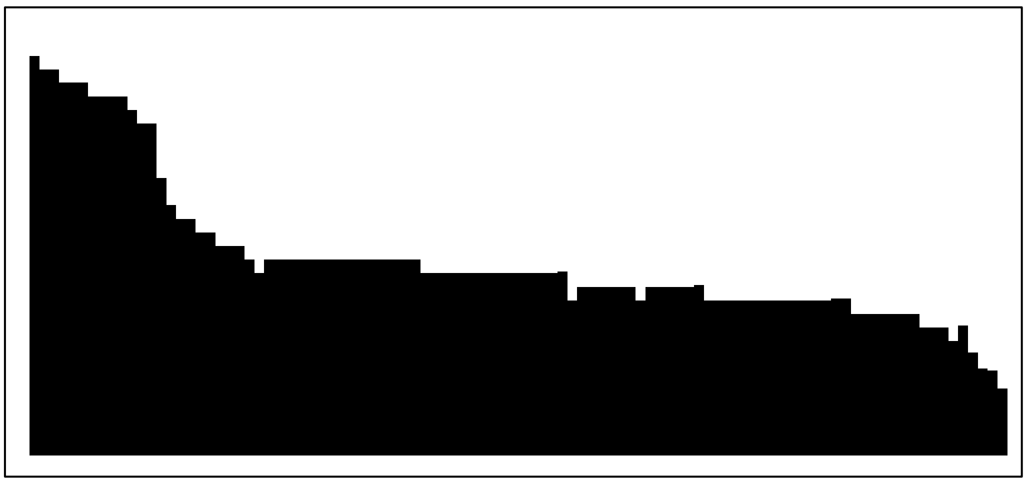

Gymnopternus aerosus voucher Pollet 0025 (P) 16S r	EU863970	98.73	100	1.9196e−69	Diptera	Dolichopodidae	Gymnopternus aerosus
Gymnopternus cupreus voucher Pollet 0021 (P) 16S r	EU863984	98.09	100	6.70005e−69	Diptera	Dolichopodidae	Gymnopternus cupreus
Gymnopternus angustifrons voucher Pollet 0052 (P)	EU863971	98.09	100	8.16233e−68	Diptera	Dolichopodidae	Gymnopternus angustifrons
Gymnopternus metallicus voucher Pollet 0030 (P) 16	EU863987	97.47	100	2.84893e−67	Diptera	Dolichopodidae	Gymnopternus metallicus
Gymnopternus helveticus voucher Pollet 0291 (P) 16	EU863986	97.47	100	2.84893e−67	Diptera	Dolichopodidae	Gymnopternus helveticus
Gymnopternus helveticus voucher Pollet 0290 (P) 16	EU863985	97.47	100	2.84893e−67	Diptera	Dolichopodidae	Gymnopternus helveticus
Gymnopternus celer voucher Pollet 0018 (P) 16S rib	EU863977	96.82	100	3.47071e−66	Diptera	Dolichopodidae	Gymnopternus celer
Gymnopternus celer voucher Pollet 0051 (P) 16S rib	EU863978	96.82	100	3.47071e−66	Diptera	Dolichopodidae	Gymnopternus celer
Gymnopternus brevicornis voucher Pollet 0036 (P) 1	EU863974	96.82	100	3.47071e−66	Diptera	Dolichopodidae	Gymnopternus brevicornis
Gymnopternus assimilis voucher Pollet 0088 (P) 16S	EU863972	96.82	100	4.22819e−65	Diptera	Dolichopodidae	Gymnopternus assimilis
Gymnopternus blankaartensis voucher Pollet 0090 (P	EU863973	96.18	100	5.15099e−64	Diptera	Dolichopodidae	Gymnopternus blankaartensis
Gymnopternus silvestris voucher Pollet 0082 (P) 16	EU863988	95.54	100	1.79787e−63	Diptera	Dolichopodidae	Gymnopternus silvestris
Gymnopternus celer voucher Pollet 0268 (P) 16S rib	EU863982	95.54	100	1.79787e−63	Diptera	Dolichopodidae	Gymnopternus celer
Dolichopus sp. HYH−2002 16S ribosomal RNA gene, pa	AY123343	92.99	100	5.87728e−57	Diptera	Dolichopodidae	Dolichopus sp. HYH−2002
Poecilobothrus principalis voucher Pollet 0198 (P)	EU864010	91.72	100	3.04451e−54	Diptera	Dolichopodidae	Poecilobothrus principalis
Hercostomus nigriplantis voucher Pollet 0210 (P) 1	EU863998	91.08	100	3.70897e−53	Diptera	Dolichopodidae	Hercostomus nigriplantis
Dolichopus sabinus voucher Pollet 0117 (P) 16S rib	EU863944	91.08	100	3.70897e−53	Diptera	Dolichopodidae	Dolichopus sabinus
Poecilobothrus nobilitatus voucher Pollet 0130 (P)	EU864008	90.45	100	1.57709e−51	Diptera	Dolichopodidae	Poecilobothrus nobilitatus
Hercostomus vockerothi voucher Pollet 0295 (P) 16S	EU864006	90.45	100	1.57709e−51	Diptera	Dolichopodidae	Hercostomus vockerothi
Hercostomus nigripennis voucher Pollet 0059 (P) 16	EU863997	89.81	100	1.92129e−50	Diptera	Dolichopodidae	Hercostomus nigripennis
Dolichopus lepidus voucher Pollet 0048 (P) 16S rib	EU863917	89.81	100	1.92129e−50	Diptera	Dolichopodidae	Dolichopus lepidus
Dolichopus brevipennis voucher Pollet 0014 (P) 16S	EU863901	89.81	100	1.92129e−50	Diptera	Dolichopodidae	Dolichopus brevipennis
Hercostomus praeceps voucher Pollet 0141 (P) 16S r	EU864003	89.17	100	8.16955e−49	Diptera	Dolichopodidae	Hercostomus praeceps
Hercostomus pilifer voucher Pollet 0176 (P) 16S ri	EU864001	88.54	100	8.16955e−49	Diptera	Dolichopodidae	Hercostomus pilifer
Hercostomus germanus voucher Pollet 0094 (P) 16S r	EU863993	89.17	100	8.16955e−49	Diptera	Dolichopodidae	Hercostomus germanus
Hercostomus fulvicaudis voucher Pollet 0154 (P) 16	EU863992	89.17	100	8.16955e−49	Diptera	Dolichopodidae	Hercostomus fulvicaudis
Dolichopus ungulatus voucher Pollet 0273 (P) 16S r	EU863960	89.17	100	8.16955e−49	Diptera	Dolichopodidae	Dolichopus ungulatus
Dolichopus trivialis voucher Pollet 0064 (P) 16S r	EU863952	89.17	100	8.16955e−49	Diptera	Dolichopodidae	Dolichopus trivialis
Dolichopus tanythrix voucher Pollet 0043 (P) 16S r	EU863951	89.17	100	8.16955e−49	Diptera	Dolichopodidae	Dolichopus tanythrix
Dolichopus popularis voucher Pollet 0002 (P) 16S r	EU863942	89.17	100	8.16955e−49	Diptera	Dolichopodidae	Dolichopus popularis
Dolichopus planitarsis voucher Pollet 0110 (P) 16S	EU863929	89.17	100	8.16955e−49	Diptera	Dolichopodidae	Dolichopus planitarsis
Dolichopus nitidus voucher Pollet 0218 (P) 16S rib	EU863924	89.17	100	8.16955e−49	Diptera	Dolichopodidae	Dolichopus nitidus
Dolichopus excisus voucher Pollet 0181 (P) 16S rib	EU863909	89.17	100	8.16955e−49	Diptera	Dolichopodidae	Dolichopus excisus
Dolichopus laticola voucher Pollet 0109 (P) 16S ri	EU863915	89.17	100	8.16955e−49	Diptera	Dolichopodidae	Dolichopus laticola
Dolichopus genicupallidus voucher Pollet 0100 (P)	EU863911	89.17	100	8.16955e−49	Diptera	Dolichopodidae	Dolichopus genicupallidus
Dolichopus clavipes voucher Pollet 0193 (P) 16S ri	EU863907	89.17	100	8.16955e−49	Diptera	Dolichopodidae	Dolichopus clavipes
Dolichopus clavipes voucher Pollet 0113 (P) 16S ri	EU863906	89.17	100	8.16955e−49	Diptera	Dolichopodidae	Dolichopus clavipes
Dolichopus arbustorum voucher Pollet 0108 (P) 16S	EU863898	89.17	100	8.16955e−49	Diptera	Dolichopodidae	Dolichopus arbustorum
Dolichopus acuticornis voucher Pollet 0222 (P) 16S	EU863897	89.17	100	8.16955e−49	Diptera	Dolichopodidae	Dolichopus acuticornis
Dolichopus bigeniculatus voucher RMBR:103435 16S r	FJ808164	89.17	100	8.16955e−49	Diptera	Dolichopodidae	Dolichopus bigeniculatus
Poecilobothrus chrysozygos voucher Pollet 0184 (P)	EU864007	88.54	100	9.95255e−48	Diptera	Dolichopodidae	Poecilobothrus chrysozygos
Hercostomus fugax voucher Pollet 0293 (P) 16S ribo	EU863991	88.54	100	9.95255e−48	Diptera	Dolichopodidae	Hercostomus fugax
Hercostomus chetifer voucher Pollet 0206 (P) 16S r	EU863990	88.54	100	9.95255e−48	Diptera	Dolichopodidae	Hercostomus chetifer
Hercostomus chaerophylli voucher Pollet 0105 (P) 1	EU863989	88.54	100	9.95255e−48	Diptera	Dolichopodidae	Hercostomus chaerophylli
Dolichopus ungulatus voucher Pollet 0024 (P) 16S r	EU863954	88.54	100	9.95255e−48	Diptera	Dolichopodidae	Dolichopus ungulatus
Dolichopus ungulatus voucher Pollet 0017 (P) 16S r	EU863953	88.54	100	9.95255e−48	Diptera	Dolichopodidae	Dolichopus ungulatus
Dolichopus subpennatus voucher Pollet 0118 (P) 16S	EU863949	88.54	100	9.95255e−48	Diptera	Dolichopodidae	Dolichopus subpennatus
Dolichopus nubilus voucher Pollet 0180 (P) 16S rib	EU863925	88.54	100	9.95255e−48	Diptera	Dolichopodidae	Dolichopus nubilus
Dolichopus longicornis voucher Pollet 0158 (P) 16S	EU863919	88.54	100	9.95255e−48	Diptera	Dolichopodidae	Dolichopus longicornis
Dolichopus griseipennis voucher Pollet 0194 (P) 16	EU863914	88.54	100	9.95255e−48	Diptera	Dolichopodidae	Dolichopus griseipennis
Dolichopus griseipennis voucher Pollet 0150 (P) 16	EU863912	88.54	100	9.95255e−48	Diptera	Dolichopodidae	Dolichopus griseipennis
Dolichopus festivus voucher Pollet 0142 (P) 16S ri	EU863910	88.54	100	9.95255e−48	Diptera	Dolichopodidae	Dolichopus festivus
Dolichopus claviger voucher Pollet 0015 (P) 16S ri	EU863904	88.54	100	9.95255e−48	Diptera	Dolichopodidae	Dolichopus claviger
Dolichopus cilifemoratus voucher Pollet 0177 (P) 1	EU863903	88.54	100	9.95255e−48	Diptera	Dolichopodidae	Dolichopus cilifemoratus
Tachytrechus tessellatus voucher RMBR:103478 16S r	FJ808199	88.61	100	3.47378e−47	Diptera	Dolichopodidae	Tachytrechus tessellatus
Pelastoneurus sp. Pollet 0309 16S ribosomal RNA ge	HQ448968	87.26	100	4.23193e−46	Diptera	Dolichopodidae	Pelastoneurus sp. Pollet 0309
Nodicornis nodicornis voucher Pollet 0072 (P) 16S	EU864014	87.9	100	4.23193e−46	Diptera	Dolichopodidae	Nodicornis nodicornis
Hercostomus nigrilamellatus voucher Pollet 0294 (P	EU863996	87.9	100	4.23193e−46	Diptera	Dolichopodidae	Hercostomus nigrilamellatus
Dolichopus wahlbergi voucher Pollet 0076 (P) 16S r	EU863965	87.9	100	4.23193e−46	Diptera	Dolichopodidae	Dolichopus wahlbergi
Dolichopus urbanus voucher Pollet 0001 (P) 16S rib	EU863963	87.9	100	4.23193e−46	Diptera	Dolichopodidae	Dolichopus urbanus
Dolichopus signatus voucher Pollet 0046 (P) 16S ri	EU863945	87.9	100	4.23193e−46	Diptera	Dolichopodidae	Dolichopus signatus
Dolichopus rupestris voucher Pollet 0116 (P) 16S r	EU863943	87.9	100	4.23193e−46	Diptera	Dolichopodidae	Dolichopus rupestris
Dolichopus polleti voucher Pollet 0213 (P) 16S rib	EU863941	87.26	100	4.23193e−46	Diptera	Dolichopodidae	Dolichopus polleti
Dolichopus plumipes voucher Pollet 0257 (P) 16S ri	EU863934	87.9	100	4.23193e−46	Diptera	Dolichopodidae	Dolichopus plumipes
Dolichopus pennatus voucher Pollet 0013 (P) 16S ri	EU863926	87.9	100	4.23193e−46	Diptera	Dolichopodidae	Dolichopus pennatus
Dolichopus migrans voucher Pollet 0114 (P) 16S rib	EU863921	87.9	100	4.23193e−46	Diptera	Dolichopodidae	Dolichopus migrans
Dolichopus atripes voucher Pollet 0060 (P) 16S rib	EU863900	87.9	100	4.23193e−46	Diptera	Dolichopodidae	Dolichopus atripes
Dolichopus argyrotarsis voucher Pollet 0111 (P) 16	EU863899	87.9	100	4.23193e−46	Diptera	Dolichopodidae	Dolichopus argyrotarsis
Ethiromyia chalybea voucher Pollet 0081 (P) 16S ri	EU863969	87.97	100	1.47709e−45	Diptera	Dolichopodidae	Ethiromyia chalybea
Sybistroma crinipes voucher Pollet 0278 (P) 16S ri	EU864012	87.26	100	5.15555e−45	Diptera	Dolichopodidae	Sybistroma crinipes
Poecilobothrus regalis voucher Pollet 0292 (P) 16S	EU864011	87.26	100	5.15555e−45	Diptera	Dolichopodidae	Poecilobothrus regalis
Hercostomus verbekei voucher Pollet 0263 (P) 16S r	EU864004	87.26	100	5.15555e−45	Diptera	Dolichopodidae	Hercostomus verbekei
Hercostomus longiventris voucher Pollet 0274 (P) 1	EU863994	87.26	100	5.15555e−45	Diptera	Dolichopodidae	Hercostomus longiventris
Dolichopus vitripennis voucher Pollet 0029 (P) 16S	EU863964	87.26	100	5.15555e−45	Diptera	Dolichopodidae	Dolichopus vitripennis
Dolichopus simplex voucher Pollet 0050 (P) 16S rib	EU863947	87.26	100	5.15555e−45	Diptera	Dolichopodidae	Dolichopus simplex
Dolichopus plumipes voucher Pollet 0272 (P) 16S ri	EU863940	87.26	100	5.15555e−45	Diptera	Dolichopodidae	Dolichopus plumipes
Dolichopus plumipes voucher Pollet 0212 (P) 16S ri	EU863932	87.26	100	5.15555e−45	Diptera	Dolichopodidae	Dolichopus plumipes
Dolichopus plumipes voucher Pollet 0003 (P) 16S ri	EU863930	87.26	100	5.15555e−45	Diptera	Dolichopodidae	Dolichopus plumipes
Dolichopus longitarsis voucher Pollet 0095 (P) 16S	EU863920	87.26	100	5.15555e−45	Diptera	Dolichopodidae	Dolichopus longitarsis
Dolichopus diadema voucher Pollet 0197 (P) 16S rib	EU863908	87.26	100	5.15555e−45	Diptera	Dolichopodidae	Dolichopus diadema
Dolichopus campestris voucher Pollet 0071 (P) 16S	EU863902	87.26	100	5.15555e−45	Diptera	Dolichopodidae	Dolichopus campestris
Paraclius digitatus voucher RMBR:103462 16S riboso	FJ808190	87.26	100	5.15555e−45	Diptera	Dolichopodidae	Paraclius digitatus
Tachytrechus transitorius voucher Pollet 0101 (P)	EU864018	87.34	100	1.79946e−44	Diptera	Dolichopodidae	Tachytrechus transitorius
Tachytrechus notatus voucher Pollet 0279 (P) 16S r	EU864017	87.34	100	2.19219e−43	Diptera	Dolichopodidae	Tachytrechus notatus
Sybistroma discipes voucher Pollet 0097 (P) 16S ri	EU864013	86.62	100	2.19219e−43	Diptera	Dolichopodidae	Sybistroma discipes
Hercostomus parvilamellatus voucher Pollet 0004 (P	EU864000	86.62	100	2.19219e−43	Diptera	Dolichopodidae	Hercostomus parvilamellatus
Hercostomus nanus voucher Pollet 0087 (P) 16S ribo	EU863995	86.62	100	2.19219e−43	Diptera	Dolichopodidae	Hercostomus nanus
Dolichopus plumipes voucher Pollet 0271 (P) 16S ri	EU863939	86.62	100	2.19219e−43	Diptera	Dolichopodidae	Dolichopus plumipes
Dolichopus picipes voucher Pollet 0065 (P) 16S rib	EU863928	86.62	100	2.19219e−43	Diptera	Dolichopodidae	Dolichopus picipes
Dolichopus linearis voucher Pollet 0157 (P) 16S ri	EU863918	86.62	100	2.19219e−43	Diptera	Dolichopodidae	Dolichopus linearis
Hercostomus meieri voucher RMBR:103442 16S ribosom	FJ808173	86.62	100	2.19219e−43	Diptera	Dolichopodidae	Hercostomus meieri
Sybistroma obscurellum voucher Pollet 0083 (P) 16S	EU864015	85.99	100	2.67064e−42	Diptera	Dolichopodidae	Sybistroma obscurellum
Dolichopus nigricornis voucher Pollet 0023 (P) 16S	EU863922	85.99	100	2.67064e−42	Diptera	Dolichopodidae	Dolichopus nigricornis
Lichtwardtia ziczac voucher RMBR:103446 16S riboso	FJ808174	85.99	100	2.67064e−42	Diptera	Dolichopodidae	Lichtwardtia ziczac
Tachytrechus sp. Pollet 0299 16S ribosomal RNA gen	HQ448969	85.35	100	9.32145e−42	Diptera	Dolichopodidae	Tachytrechus sp. Pollet 0299
Hercostomus plagiatus voucher Pollet 0185 (P) 16S	EU864002	86.08	100	1.13558e−40	Diptera	Dolichopodidae	Hercostomus plagiatus
Rhaphium consobrinum voucher Pollet 0201 (P) 16S r	HQ449003	84.81	100	3.96358e−40	Diptera	Dolichopodidae	Rhaphium consobrinum
Argyrochlamys impudicus voucher RMBR:103415 16S ri	FJ808154	84.08	100	5.88247e−38	Diptera	Dolichopodidae	Argyrochlamys impudicus
Limonia hawaiiensis voucher O'Grady 200106 16S rib	EU005403	83.97	99.36	5.88247e−38	Diptera	Limoniidae	Dicranomyia hawaiiensis
Paramonova sp. B SLW−2015 16S ribosomal RNA gene,	KM878938	83.12	100	2.50129e−36	Diptera	Scenopinidae	Paramonova sp. B SLW−2015
