# Supplementary material for: Testing the potential of a ribosomal 16S marker for DNA metabarcoding of insects
Source: PeerJ. 2016 Apr 19;4:e1966. doi: 10.7717/peerj.1966 (PMC4841222; doi:10.7717/peerj.1966)
Supplement: Supplemental Information 1 [file peerj-04-1966-s006.zip › scripts/5 match OTUs/blast_hits/OTU_107.csv_plot.pdf]

OTU_107.csv

Copelatus caelatipennis

Dytiscidae

Coleoptera

75.31

Copelatus caelatipennis mitochondrial partial 16S	AM946004	75.31	99.38	4.95174e−20	Coleoptera	Dytiscidae	Copelatus caelatipennis
Copelatus caelatipennis mitochondrial partial 16S	AM945976	75.31	99.38	4.95174e−20	Coleoptera	Dytiscidae	Copelatus caelatipennis
Copelatus sp. MB224 16S ribosomal RNA gene, partia	AY334122	75.31	99.38	4.95174e−20	Coleoptera	Dytiscidae	Copelatus sp. MB224
Copelatus caelatipennis mitochondrial partial 16S	AM945999	74.69	99.38	2.10553e−18	Coleoptera	Dytiscidae	Copelatus caelatipennis
Copelatus sp. MB188 16S ribosomal RNA gene, partia	AY334116	74.69	99.38	2.10553e−18	Coleoptera	Dytiscidae	Copelatus sp. MB188
Copelatus oblitus 16S ribosomal RNA gene, partial	AY334106	75.76	99.38	7.34903e−18	Coleoptera	Dytiscidae	Copelatus oblitus
Nepaletricha sp. DK−2014 voucher JSOUT10 16S ribos	KJ136733	76.54	97.5	2.56505e−17	Diptera	Rangomaramidae	Nepaletricha sp. DK−2014
Copelatus caelatipennis mitochondrial partial 16S	AM946013	74.07	99.38	2.56505e−17	Coleoptera	Dytiscidae	Copelatus caelatipennis
Copelatus sp. MB 311 mitochondrial partial 16S rRN	AM945980	75.46	99.38	8.95295e−17	Coleoptera	Dytiscidae	Copelatus sp. MB 311
Copelatus sp. MB 327 mitochondrial partial 16S rRN	AM945985	74.55	99.38	3.12489e−16	Coleoptera	Dytiscidae	Copelatus sp. MB 327
Papuadytes sp. MB165 mitochondrial 16S rRNA gene (	AM404028	74.55	99.38	3.12489e−16	Coleoptera	Dytiscidae	Papuadytes sp. MB165
Diaprysius fagniezi mitochondrial 16S rRNA gene (p	HG915665	74.53	97.5	3.80689e−15	Coleoptera	Leiodidae	Diaprysius fagniezi
Copelatus sp. MB110 16S ribosomal RNA gene, partia	AY334112	73.78	99.38	3.80689e−15	Coleoptera	Dytiscidae	Copelatus sp. MB110
Diaprysius serullazi muelleri mitochondrial 16S rR	HG915666	75.31	97.5	1.32874e−14	Coleoptera	Leiodidae	Diaprysius serullazi muelleri
Anastrepha flavipennis 16S ribosomal RNA gene, par	AF152065	75	99.38	4.63774e−14	Diptera	Tephritidae	Anastrepha flavipennis
Bidessonotus inconspicuus voucher UNM KBMBiin82 16	KJ548310	74.07	99.38	1.61873e−13	Coleoptera	Dytiscidae	Bidessonotus inconspicuus
Acalymma fairmairei 16S ribosomal RNA gene, partia	AY533638	74.38	97.5	5.64993e−13	Coleoptera	Chrysomelidae	Acalymma fairmairei
Nolima pinal 16S ribosomal RNA gene, partial seque	KM051514	73.41	99.38	1.97202e−12	Neuroptera	Mantispidae	Nolima pinal
Diaprysius sicardi mitochondrial 16S rRNA gene (pa	HG915667	73.75	97.5	1.97202e−12	Coleoptera	Leiodidae	Diaprysius sicardi
Aphelocheirus aestivalis isolate 465 16S ribosomal	AY585777	73.21	99.38	1.97202e−12	Hemiptera	Aphelocheiridae	Aphelocheirus aestivalis
Chalarus sp. A 16S ribosomal RNA gene, partial seq	AF154758	74.55	99.38	1.97202e−12	Diptera	Pipunculidae	Chalarus sp. A
Longitarsus niger voucher BMNH:851917 16S large su	KP763206	78.63	70.62	6.88302e−12	Coleoptera	Chrysomelidae	Longitarsus niger
Phyllobaenus testaceus voucher 7228 16S ribosomal	KC524896	74.05	96.88	6.88302e−12	Coleoptera	Cleridae	Phyllobaenus testaceus
Heza aurantia isolate RCW 637 16S ribosomal RNA ge	JQ888448	72.12	99.38	6.88302e−12	Hemiptera	Reduviidae	Heza aurantia
Agabus basalis mitochondrial 16S rRNA gene (partia	HF931443	73.33	99.38	6.88302e−12	Coleoptera	Dytiscidae	Agabus basalis
Forcipomyia bipunctata 16S ribosomal RNA gene, par	EF472584	74.1	99.38	6.88302e−12	Diptera	Ceratopogonidae	Forcipomyia bipunctata
Libnotes perkinsi voucher O'Grady 200981 16S ribos	EU005433	73.78	100	6.88302e−12	Diptera	Limoniidae	Libnotes perkinsi
Libnotes perkinsi voucher O'Grady 200947 16S ribos	EU005432	73.78	100	6.88302e−12	Diptera	Limoniidae	Libnotes perkinsi
Papuadytes boulevardi mitochondrial 16S rRNA gene	AM404048	72.84	99.38	6.88302e−12	Coleoptera	Dytiscidae	Papuadytes boulevardi
Agabus cf. nitidus IR−2001 16S ribosomal RNA gene,	AY039264	73.33	99.38	6.88302e−12	Coleoptera	Dytiscidae	Agabus cf. nitidus IR−2001
Pida apicalis voucher H205 large subunit ribosomal	KP081969	74.47	86.25	2.40241e−11	Lepidoptera	Erebidae	Pida apicalis
Placusa sp. prope tachyporoides HE−2010 voucher ZM	GQ980990	73.65	97.5	2.40241e−11	Coleoptera	Staphylinidae	Placusa sp. prope tachyporoides HE−2010
Fontecilla graphicus 16S ribosomal RNA gene, parti	EU734863	73.33	85	2.40241e−11	Neuroptera	Polystoechotidae	Fontecilla graphicus
Australysmus lacustris 16S ribosomal RNA gene, par	EU734849	72.29	99.38	2.40241e−11	Neuroptera	Osmylidae	Australysmus lacustris
Trichophya pilicornis voucher ZMUN:10008478 16S ri	JX536491	75	79.38	8.38524e−11	Coleoptera	Staphylinidae	Trichophya pilicornis
Oxypodini gen. sp. 2 ZMUN 10029209 16S ribosomal R	JX536464	75.63	72.5	8.38524e−11	Coleoptera	Staphylinidae	Oxypodini gen. sp. 2 ZMUN 10029209
Atrachya menetriesi voucher BMNH 835073 16S riboso	FJ973804	74	91.88	8.38524e−11	Coleoptera	Chrysomelidae	Atrachya menetriesi
Dysaules longicollis isolate MN202 16S ribosomal R	FJ806232	73.65	87.5	8.38524e−11	Mantodea	Tarachodidae	Dysaules longicollis
Bidessonotus longovalis partial 16S rRNA gene	AJ850329	72.84	99.38	8.38524e−11	Coleoptera	Dytiscidae	Bidessonotus longovalis
Rhantus calidus voucher UNM KBMRhca262 16S ribosom	KJ548368	71.51	99.38	2.92674e−10	Coleoptera	Dytiscidae	Rhantus calidus
Eucinetus haemorrhoidalis voucher UPOL 001319 16S	KF625806	72.19	100	2.92674e−10	Coleoptera	Eucinetidae	Eucinetus haemorrhoidalis
Orius minutus 16S ribosomal RNA gene, partial sequ	GQ258372	75	94.38	2.92674e−10	Hemiptera	Anthocoridae	Orius minutus
Hydrochus sp. BMNH 840193 mitochondrial sequence	HQ232801	77.12	70	2.92674e−10	Coleoptera	Hydrochidae	Hydrochus sp. BMNH 840193
Melaloncha stoneorum 16S ribosomal RNA gene, parti	GU550207	71.69	99.38	2.92674e−10	Diptera	Phoridae	Melaloncha stoneorum
Papuadytes sp. MB483 mitochondrial 16S rRNA gene (	AM404050	72.22	99.38	2.92674e−10	Coleoptera	Dytiscidae	Papuadytes sp. MB483
Papuadytes sp. MB481 mitochondrial 16S rRNA gene (	AM404049	72.22	99.38	2.92674e−10	Coleoptera	Dytiscidae	Papuadytes sp. MB481
Liopterus haemorrhoidalis 16S ribosomal RNA gene,	AY334125	71.6	99.38	2.92674e−10	Coleoptera	Dytiscidae	Copelatus aruensis
Liopterus atriceps 16S ribosomal RNA gene, partial	AY334124	71.6	99.38	2.92674e−10	Coleoptera	Dytiscidae	Liopterus atriceps
Liopterus haemorrhoidalis 16S ribosomal RNA gene,	AY071774	71.6	99.38	2.92674e−10	Coleoptera	Dytiscidae	Copelatus aruensis
Derovatellus lentus 16S ribosomal RNA gene, partia	KF575536	71.6	99.38	1.02153e−09	Coleoptera	Dytiscidae	Derovatellus lentus
Montina sp. 00002725 isolate RCW 1401 16S ribosoma	JQ888461	72.12	99.38	1.02153e−09	Hemiptera	Reduviidae	Montina sp. 00002725
Canthesancus sp. 00052211 16S ribosomal RNA gene,	JQ897784	76.99	68.75	1.02153e−09	Hemiptera	Reduviidae	Canthesancus sp. 00052211
Anthrax proconcisus partial mitochondrial 16S rRNA	AJ271846	72.89	99.38	1.02153e−09	Diptera	Bombyliidae	Anthrax proconcisus
Platynectes sp. UNM KBMPysp457 16S ribosomal RNA g	KJ548365	71.6	99.38	3.56549e−09	Coleoptera	Dytiscidae	Platynectes sp. UNM KBMPysp457
Periclitena vigorsi voucher BMNH 831740 16S riboso	FJ973858	74.48	88.75	3.56549e−09	Coleoptera	Chrysomelidae	Periclitena vigorsi
Agetocera mirablis voucher BMNH 831709 16S ribosom	FJ973793	72.61	93.75	3.56549e−09	Coleoptera	Chrysomelidae	Agetocera mirablis
Tetraphleps aterrimus 16S ribosomal RNA gene, part	GQ258378	73.33	99.38	3.56549e−09	Hemiptera	Anthocoridae	Tetraphleps aterrimus
Platynectes sp. MB362 16S ribosomal RNA (rrnL) gen	EF670011	71.6	99.38	3.56549e−09	Coleoptera	Dytiscidae	Platynectes sp. MB362
Anthocoris montanus 16S ribosomal RNA gene, partia	EF487284	73.33	99.38	3.56549e−09	Hemiptera	Anthocoridae	Anthocoris montanus
Chaetopsis fulvifrons 16S ribosomal RNA gene, part	AY573111	77.36	63.75	3.56549e−09	Diptera	Ulidiidae	Chaetopsis fulvifrons
Bathysciola finismillennii mitochondrial 16S rRNA	HG915647	73.25	96.25	1.24448e−08	Coleoptera	Leiodidae	Bathysciola finismillennii
Longitarsus sp. IBE−JGZ−01018 16S ribosomal RNA ge	FJ973839	74.15	89.38	1.24448e−08	Coleoptera	Chrysomelidae	Longitarsus sp. IBE−JGZ−01018
Aphelocheirus ellipsoideus mitochondrion, partial	FJ456939	72.02	99.38	1.24448e−08	Hemiptera	Aphelocheiridae	Aphelocheirus ellipsoideus
Osmylus fulvicephalus 16S ribosomal RNA gene, part	EU734887	71.78	99.38	1.24448e−08	Neuroptera	Osmylidae	Osmylus fulvicephalus
Diptera sp. BOLD:ACO2095 voucher BAH_201312_P02_A9	KP253902	71.69	99.38	4.34366e−08	Diptera		Diptera sp. BOLD:ACO2095
Labramachaerota korupa voucher 08−12−01−37 16S rib	KF525474	72.46	100	4.34366e−08	Hemiptera	Machaerotidae	Labramachaerota korupa
Paracymus limbatus 16S ribosomal RNA gene, partial	KC992701	72.97	88.12	4.34366e−08	Coleoptera	Hydrophilidae	Paracymus limbatus
Hygrotus nigrescens mitochondrial 16S rRNA gene (p	HF931386	70.35	99.38	4.34366e−08	Coleoptera	Dytiscidae	Hygrotus nigrescens
Chrysotus miripalpus voucher Pollet 0313 (P) 16S r	HQ448960	71.43	99.38	4.34366e−08	Diptera	Dolichopodidae	Chrysotus miripalpus
Clitea fulva voucher BMNH 831386 16S ribosomal RNA	FJ973814	71.92	88.75	4.34366e−08	Coleoptera	Chrysomelidae	Clitea fulva
Dysaules sp. MN067 16S ribosomal RNA gene, partial	FJ806176	72.3	87.5	4.34366e−08	Mantodea	Tarachodidae	Dysaules sp. MN067
Revelieria californica 16S ribosomal RNA gene, par	EU164598	71.97	94.38	4.34366e−08	Coleoptera	Latridiidae	Revelieria californica
Agaporomorphus tambopatensis mitochondrial partial	AM945997	70.24	99.38	4.34366e−08	Coleoptera	Dytiscidae	Agaporomorphus tambopatensis
Papuadytes simplex 2 note−SubSrc cryptic species 2	AY334154	71.52	99.38	4.34366e−08	Coleoptera	Dytiscidae	Papuadytes aff. simplex MB107
Gynandrobrotica lepida 16S ribosomal RNA gene, par	AY533643	74.03	91.88	4.34366e−08	Coleoptera	Chrysomelidae	Gynandrobrotica lepida
Philaethria wernickei voucher LMCI110−5 16S riboso	KF277295	72.52	80	1.51609e−07	Lepidoptera	Nymphalidae	Philaethria wernickei
Lipromela sp. BMNH 831429 16S ribosomal RNA gene,	FJ973837	70.89	95.62	1.51609e−07	Coleoptera	Chrysomelidae	Lipromela sp. BMNH 831429
Nanothinophilus hoplites voucher RMBR:103451 16S r	FJ808181	73.64	73.75	1.51609e−07	Diptera	Dolichopodidae	Nanothinophilus hoplites
Ostoma ferrugineum voucher BMNH:679285 16S ribosom	DQ202553	73.03	91.25	1.51609e−07	Coleoptera	Trogossitidae	Peltis ferruginea
Aphelocheirus venus isolate 506 16S ribosomal RNA	AY585778	74.4	75.62	1.51609e−07	Hemiptera	Aphelocheiridae	Aphelocheirus venus
Philaethria dido voucher 8700 ; mitochondrial	KP073342	74.4	75	5.29166e−07	Lepidoptera	Nymphalidae	Philaethria dido
Drilonius sp. UPOL 001274 16S ribosomal RNA gene,	KF625831	75	65.62	5.29166e−07	Coleoptera	Omethidae	Drilonius sp. UPOL 001274
Afzeliada afzelii voucher MHV0468 16S ribosomal RN	FJ168845	72.92	83.75	5.29166e−07	Hemiptera	Cicadidae	Afzeliada afzelii
Prosthiochaeta sp. YSUW04031 16S ribosomal RNA gen	AY573130	75.23	66.25	5.29166e−07	Diptera	Platystomatidae	Prosthiochaeta sp. YSUW04031
Acilius japonicus voucher KBMAcja84 16S ribosomal	KF979082	70.37	99.38	1.84697e−06	Coleoptera	Dytiscidae	Acilius japonicus
Phlogistus leucocosmus voucher COL1386 12S ribosom	KC524799	75.93	66.25	1.84697e−06	Coleoptera	Cleridae	Phlogistus leucocosmus
Neoplea striola isolate 605 16S ribosomal RNA gene	AY585785	72.02	99.38	1.84697e−06	Hemiptera	Pleidae	Neoplea striola
Bathysciola asperula mitochondrial 16S rRNA gene (	HG915642	70.86	93.12	6.44656e−06	Coleoptera	Leiodidae	Bathysciola asperula
Madaglymbus sp. BI−2011 voucher BMNH792905 16S rib	HQ381438	70.83	99.38	6.44656e−06	Coleoptera	Dytiscidae	Madaglymbus sp. BI−2011
Drilonius sp. UPOL 000M26 16S ribosomal RNA gene,	DQ198659	72.5	70.62	6.44656e−06	Coleoptera	Omethidae	Drilonius sp. UPOL 000M26
Plagiodera sp. BMNH#704394 mitochondrial partial 1	AJ841325	71.43	97.5	6.44656e−06	Coleoptera	Chrysomelidae	Plagiodera sp. BMNH#704394
Ramadra calligramma voucher H4 large subunit ribos	KP081967	72.55	91.25	2.25007e−05	Lepidoptera	Lymantriidae	Ramadra calligramma
Rhantus sinuatus voucher UNM KBMRhsi17 16S ribosom	KJ548369	70.3	99.38	2.25007e−05	Coleoptera	Dytiscidae	Rhantus sinuatus
Calliclopius nigripes voucher RCW_1246 UCR_ENT 000	JQ942235	71.9	73.75	2.25007e−05	Hemiptera	Reduviidae	Calliclopius nigripes
Schematiza flavofasciata 16S ribosomal RNA gene, p	EF197976	75.24	63.75	2.25007e−05	Coleoptera	Chrysomelidae	Schematiza flavofasciata
Scotomedes sp. WCW−2003 16S ribosomal RNA gene, pa	AY252771	75.24	63.12	2.25007e−05	Hemiptera	Velocipedidae	Scotomedes sp. WCW−2003
Leucania dorsalis 16S ribosomal RNA gene, partial	AF173078	73.33	71.25	2.25007e−05	Lepidoptera	Noctuidae	Leucania dorsalis
Galathea ohshimai voucher UF:25218 16S ribosomal R	KP203547	70.3	98.75	7.85352e−05	Decapoda	Galatheidae	Galathea ohshimai
Galathea nuda voucher MNHN:IU:2013−13918 16S ribos	KP203516	69.57	98.75	7.85352e−05	Decapoda	Galatheidae	Galathea nuda
Galathea bracteosa voucher MNHN:IU:2010−9904 16S r	KP203508	69.57	98.75	7.85352e−05	Decapoda	Galatheidae	Galathea bracteosa
