# Supplementary material for: Testing the potential of a ribosomal 16S marker for DNA metabarcoding of insects
Source: PeerJ. 2016 Apr 19;4:e1966. doi: 10.7717/peerj.1966 (PMC4841222; doi:10.7717/peerj.1966)
Supplement: Supplemental Information 1 [file peerj-04-1966-s006.zip › scripts/5 match OTUs/blast_hits/OTU_109.csv_plot.pdf]

OTU_109.csv

Oreodytes sanmarkii

Dytiscidae

Coleoptera

97.28

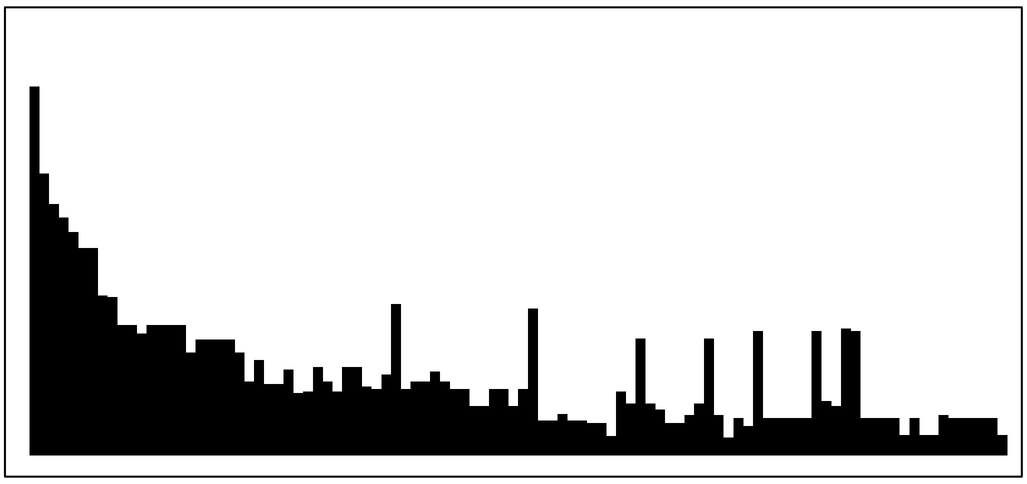

Oreodytes sanmarkii mitochondrial 16S rRNA gene (p	HF931524	97.28	82.12	2.58522e−62	Coleoptera	Dytiscidae	Oreodytes sanmarkii
Oreodytes obesus cordillerensis mitochondrial part	HF931516	93.2	82.12	3.59352e−54	Coleoptera	Dytiscidae	Oreodytes obesus cordillerensis
Oreodytes crassulus partial 16S rRNA gene	AJ850348	91.78	81.56	6.49723e−51	Coleoptera	Dytiscidae	Oreodytes crassulus
Oreodytes congruus partial 16S rRNA gene	AJ850347	91.16	82.12	7.91525e−50	Coleoptera	Dytiscidae	Oreodytes congruus
Oreodytes congruus 16S ribosomal RNA gene, partial	KF575550	90.48	82.12	9.64274e−49	Coleoptera	Dytiscidae	Oreodytes congruus
Oreodytes abbreviatus mitochondrial 16S rRNA gene	HF931477	89.73	81.56	1.43111e−46	Coleoptera	Dytiscidae	Oreodytes abbreviatus
Oreodytes rhyacophilus partial 16S rRNA gene	AJ850352	89.73	81.56	1.43111e−46	Coleoptera	Dytiscidae	Oreodytes rhyacophilus
Oreodytes mongolicus mitochondrial 16S rRNA gene (	HF931491	87.5	80.45	1.10024e−41	Coleoptera	Dytiscidae	Oreodytes mongolicus
Oreodytes snoqualmie mitochondrial partial 16S rRN	HF931519	87.41	79.89	3.8402e−41	Coleoptera	Dytiscidae	Oreodytes snoqualmie
Oreodytes septentrionalis mitochondrial 16S rRNA g	HF931492	86.11	80.45	5.69937e−39	Coleoptera	Dytiscidae	Oreodytes septentrionalis
Oreodytes shorti mitochondrial 16S rRNA gene (part	HF931488	86.11	80.45	5.69937e−39	Coleoptera	Dytiscidae	Oreodytes shorti
Deronectes parvicollis mitochondrial 16S rRNA gene	HF931454	85.71	82.12	5.69937e−39	Coleoptera	Dytiscidae	Deronectes parvicollis
Boreonectes striatellus mitochondrial 16S rRNA gen	HF931511	86.11	80.45	6.94325e−38	Coleoptera	Dytiscidae	Boreonectes striatellus
Boreonectes riberae mitochondrial 16S rRNA gene (p	HF931461	86.11	80.45	6.94325e−38	Coleoptera	Dytiscidae	Boreonectes riberae
Boreonectes griseostriatus mitochondrial partial 1	HF931387	86.11	80.45	6.94325e−38	Coleoptera	Dytiscidae	Boreonectes griseostriatus
Boreonectes multilineatus mitochondrial partial 16	HF931384	86.11	80.45	6.94325e−38	Coleoptera	Dytiscidae	Boreonectes multilineatus
Laccornis difformis 16S ribosomal RNA gene, partia	KF575544	84.83	81.01	8.45861e−37	Coleoptera	Dytiscidae	Laccornis difformis
Boreonectes griseostriatus mitochondrial 16S rRNA	HF931541	85.42	80.45	2.95234e−36	Coleoptera	Dytiscidae	Boreonectes griseostriatus
Boreonectes alpestris mitochondrial 16S rRNA gene	HF931512	85.42	80.45	2.95234e−36	Coleoptera	Dytiscidae	Boreonectes alpestris
Boreonectes macedonicus mitochondrial 16S rRNA gen	HF931359	85.42	80.45	2.95234e−36	Coleoptera	Dytiscidae	Boreonectes macedonicus
Stictotarsus ibericus 16S ribosomal RNA (rrnL) gen	EF670030	85.42	80.45	2.95234e−36	Coleoptera	Dytiscidae	Stictotarsus ibericus
Deronectes moestus mitochondrial 16S rRNA gene (pa	HE613726	84.83	80.45	1.25537e−34	Coleoptera	Dytiscidae	Deronectes moestus
Laccornis kocae partial 16S rRNA gene	AJ850419	83.45	81.01	4.38167e−34	Coleoptera	Dytiscidae	Laccornis kocae
Hydroporus glabriusculus 16S ribosomal RNA gene, p	AY365270	84.46	82.12	4.38167e−34	Coleoptera	Dytiscidae	Hydroporus glabriusculus
Oreodytes scitulus 16S ribosomal RNA gene, partial	KF575551	83.33	80.45	1.52935e−33	Coleoptera	Dytiscidae	Oreodytes scitulus
Oreodytes scitulus mitochondrial partial 16S rRNA	HF931515	83.33	80.45	1.52935e−33	Coleoptera	Dytiscidae	Oreodytes scitulus
Boreonectes griseostriatus mitochondrial 16S rRNA	HF931385	84.03	80.45	1.52935e−33	Coleoptera	Dytiscidae	Boreonectes griseostriatus
Hovahydrus sp. BI−2011 voucher BMNH831333 16S ribo	HQ381524	82.91	87.71	1.52935e−33	Coleoptera	Dytiscidae	Hovahydrus sp. BI−2011
Suphrodytes dorsalis 16S ribosomal RNA gene, parti	KF575577	82.99	82.12	5.33796e−33	Coleoptera	Dytiscidae	Suphrodytes dorsalis
Deronectes moestus 16S ribosomal RNA gene, partial	KF575561	84.14	80.45	5.33796e−33	Coleoptera	Dytiscidae	Deronectes moestus
Nebrioporus formaster mitochondrial partial 16S rR	HF931377	83.45	80.45	5.33796e−33	Coleoptera	Dytiscidae	Nebrioporus formaster
Suphrodytes dorsalis voucher BMNH:725966 16S ribos	JX221574	82.99	82.12	5.33796e−33	Coleoptera	Dytiscidae	Suphrodytes dorsalis
Deronectes brannani mitochondrial 16S rRNA gene (p	HE613695	84.14	80.45	5.33796e−33	Coleoptera	Dytiscidae	Deronectes brannanii
Deronectes moestus mitochondrial 16S rRNA gene (pa	HE613683	84.14	80.45	5.33796e−33	Coleoptera	Dytiscidae	Deronectes moestus
Scarodytes sp. IR−2003 16S ribosomal RNA gene, par	AY250939	83.22	82.12	5.33796e−33	Coleoptera	Dytiscidae	Scarodytes sp. IR−2003
Oreodytes quadrimaculatus 16S ribosomal RNA gene,	KF575567	83.1	79.33	1.86313e−32	Coleoptera	Dytiscidae	Oreodytes quadrimaculatus
Hydroporus foveolatus mitochondrial 16S rRNA gene	HE613724	83.78	82.12	1.86313e−32	Coleoptera	Dytiscidae	Hydroporus foveolatus
Hydroporus marginatus mitochondrial 16S rRNA gene	HE613699	87.1	69.27	1.86313e−32	Coleoptera	Dytiscidae	Hydroporus marginatus
Oreodytes quadrimaculatus partial 16S rRNA gene	AJ850351	83.1	79.33	1.86313e−32	Coleoptera	Dytiscidae	Oreodytes quadrimaculatus
Stictotarsus procerus mitochondrial 16S rRNA gene	HF931379	83.45	80.45	6.50297e−32	Coleoptera	Dytiscidae	Stictotarsus procerus
Deronectes doriae mitochondrial 16S rRNA gene (par	HE613710	83.45	80.45	6.50297e−32	Coleoptera	Dytiscidae	Deronectes doriae
Deronectes opatrinus mitochondrial 16S rRNA gene (	HE613701	83.92	79.33	6.50297e−32	Coleoptera	Dytiscidae	Deronectes opatrinus
Deronectes sahlbergi mitochondrial 16S rRNA gene (	HE613680	83.45	80.45	6.50297e−32	Coleoptera	Dytiscidae	Deronectes sahlbergi
Stictotarsus maghrebinus 16S ribosomal RNA gene, p	AY250947	83.11	82.12	6.50297e−32	Coleoptera	Dytiscidae	Stictotarsus maghrebinus
Hydroporus tessellatus 16S ribosomal RNA gene, par	JX434770	83.11	82.12	2.26976e−31	Coleoptera	Dytiscidae	Hydroporus tessellatus
Hygrotus novemlineatus mitochondrial partial 16S r	HF931523	82.31	82.12	2.26976e−31	Coleoptera	Dytiscidae	Hygrotus novemlineatus
Hygrotus marklini mitochondrial 16S rRNA gene (par	HF931467	82.31	82.12	2.26976e−31	Coleoptera	Dytiscidae	Hygrotus marklini
Hydroporus zimmermanni mitochondrial 16S rRNA gene	HE613679	83.11	82.12	2.26976e−31	Coleoptera	Dytiscidae	Hydroporus zimmermanni
Hydroporus basinotatus mitochondrial 16S rRNA gene	HE613678	83.11	82.12	2.26976e−31	Coleoptera	Dytiscidae	Hydroporus basinotatus
Hygrotus turbidus partial 16S rRNA gene	AJ850390	82.31	82.12	2.26976e−31	Coleoptera	Dytiscidae	Hygrotus turbidus
Hydroporus basinotatus 16S ribosomal RNA gene, par	AF518258	83.11	82.12	2.26976e−31	Coleoptera	Dytiscidae	Hydroporus basinotatus
Hydroporus marginatus 16S ribosomal RNA gene, part	AF518267	86.89	68.16	2.26976e−31	Coleoptera	Dytiscidae	Hydroporus marginatus
Aethionectes fulvonotatus voucher KBMAeop406 16S r	KF979088	81.63	82.12	7.92224e−31	Coleoptera	Dytiscidae	Aethionectes fulvonotatus
Deronectes bicostatus mitochondrial 16S rRNA gene	HE613705	81.63	82.12	7.92224e−31	Coleoptera	Dytiscidae	Deronectes bicostatus
Oreodytes natrix partial 16S rRNA gene	AJ850349	81.94	80.45	7.92224e−31	Coleoptera	Dytiscidae	Oreodytes natrix
Hygrotus patruelis mitochondrial 16S rRNA gene (pa	HF931388	81.63	82.12	2.76513e−30	Coleoptera	Dytiscidae	Hygrotus patruelis
Suphrodytes figuratus voucher BMNH:722148 16S ribo	JX221571	81.63	82.12	2.76513e−30	Coleoptera	Dytiscidae	Suphrodytes figuratus
Antiporus hollingsworthi mitochondrial partial 16S	FR727247	81.51	81.56	2.76513e−30	Coleoptera	Dytiscidae	Antiporus hollingsworthi
Antiporus hollingsworthi mitochondrial partial 16S	FR727246	81.51	81.56	2.76513e−30	Coleoptera	Dytiscidae	Antiporus hollingsworthi
Hovahydrus sp. IR−2007 16S ribosomal RNA gene, par	EF056677	80.89	87.71	2.76513e−30	Coleoptera	Dytiscidae	Hovahydrus sp. IR−2007
Coelhydrus brevicollis voucher MB1224 16S ribosoma	EF056670	82.99	81.56	2.76513e−30	Coleoptera	Dytiscidae	Coelhydrus brevicollis
Hydroporus analis mitochondrial partial 16S rRNA g	HE613731	82.43	82.12	9.65126e−30	Coleoptera	Dytiscidae	Hydroporus analis
Hydroporus fuscipennis mitochondrial 16S rRNA gene	HE613730	85.48	69.27	9.65126e−30	Coleoptera	Dytiscidae	Hydroporus fuscipennis
Hydroporus compunctus mitochondrial 16S rRNA gene	HE599693	82.43	82.12	9.65126e−30	Coleoptera	Dytiscidae	Hydroporus compunctus
Copelatus sp. BI−2011 voucher BMNH792945 16S ribos	HQ381441	82.14	78.21	9.65126e−30	Coleoptera	Dytiscidae	Copelatus sp. BI−2011
Hygrotus impressopunctatus partial 16S rRNA gene	AJ850405	81.51	81.56	9.65126e−30	Coleoptera	Dytiscidae	Hygrotus impressopunctatus
Hygrotus corpulentus partial 16S rRNA gene	AJ850387	81.51	81.56	9.65126e−30	Coleoptera	Dytiscidae	Hygrotus corpulentus
Hydroporus rufifrons 16S ribosomal RNA gene, parti	AF518277	81.88	82.12	9.65126e−30	Coleoptera	Dytiscidae	Hydroporus rufifrons
Hydroporus compunctus 16S ribosomal RNA gene, part	AF518259	82.43	82.12	9.65126e−30	Coleoptera	Dytiscidae	Hydroporus compunctus
Hydroporus fuscipennis 16S ribosomal RNA gene, par	AF518262	85.48	69.27	9.65126e−30	Coleoptera	Dytiscidae	Hydroporus fuscipennis
Hygrotus impressopunctatus voucher UNM KBMHtim80 1	KJ548334	81.88	81.56	3.36862e−29	Coleoptera	Dytiscidae	Hygrotus impressopunctatus
Cybister marginicollis voucher UNM KBMCyma416 16S	KJ548319	80.82	81.56	3.36862e−29	Coleoptera	Dytiscidae	Cybister marginicollis
Deronectes aubei voucher UNM KBMDeau758 16S riboso	KJ548292	81.76	82.12	3.36862e−29	Coleoptera	Dytiscidae	Deronectes aubei
Hygrotus sanfilippoi mitochondrial 16S rRNA gene (	HF931342	81.38	81.01	3.36862e−29	Coleoptera	Dytiscidae	Hygrotus sanfilippoi
Hydroporus brancoi gredensis mitochondrial partial	HF931329	85.83	67.04	3.36862e−29	Coleoptera	Dytiscidae	Hydroporus brancoi gredensis
Deronectes costipennis mitochondrial 16S rRNA gene	HE613697	81.76	82.12	3.36862e−29	Coleoptera	Dytiscidae	Deronectes costipennis
Deronectes platynotus mitochondrial 16S rRNA gene	HE613682	81.76	82.12	3.36862e−29	Coleoptera	Dytiscidae	Deronectes platynotus
Deronectes depressicollis mitochondrial 16S rRNA g	HE613681	81.76	82.12	3.36862e−29	Coleoptera	Dytiscidae	Deronectes depressicollis
Deronectes semirufus mitochondrial 16S rRNA gene (	HE613676	81.76	82.12	3.36862e−29	Coleoptera	Dytiscidae	Deronectes semirufus
Deronectes semirufus mitochondrial 16S rRNA gene (	HE613674	81.76	82.12	3.36862e−29	Coleoptera	Dytiscidae	Deronectes semirufus
Hydroporus necopinatus mitochondrial partial 16S r	HE599692	85.83	67.04	3.36862e−29	Coleoptera	Dytiscidae	Hydroporus necopinatus
Nirripirti megamacrocephalus isolate ABTC78781 16S	EU616935	82.55	82.12	3.36862e−29	Coleoptera	Dytiscidae	Paroster megamacrocephalus
Darwinhydrus solidus voucher MB354 16S ribosomal R	EF056671	82.31	81.56	3.36862e−29	Coleoptera	Dytiscidae	Darwinhydrus solidus
Hydroporus pubescens voucher NHM−IR39 16S ribosoma	EF419327	85.94	70.95	3.36862e−29	Coleoptera	Dytiscidae	Hydroporus pubescens
Hydroporus necopinatus 16S ribosomal RNA gene, par	AY365275	85.83	67.04	3.36862e−29	Coleoptera	Dytiscidae	Hydroporus necopinatus
Deronectes costipennis 16S ribosomal RNA gene, par	AY250951	81.76	82.12	3.36862e−29	Coleoptera	Dytiscidae	Deronectes costipennis
Deronectes aubei sanfilippoi haplotype 2 16S ribos	AY250908	81.76	82.12	3.36862e−29	Coleoptera	Dytiscidae	Deronectes aubei sanfilippoi
Deronectes aubei sanfilippoi haplotype 1 16S ribos	AY250907	81.76	82.12	3.36862e−29	Coleoptera	Dytiscidae	Deronectes aubei sanfilippoi
Deronectes aubei aubei haplotype 2 16S ribosomal R	AY250906	81.76	82.12	3.36862e−29	Coleoptera	Dytiscidae	Deronectes aubei aubei
Hygrotus lutescens mitochondrial partial 16S rRNA	HF931518	80.95	82.12	1.17576e−28	Coleoptera	Dytiscidae	Hygrotus lutescens
Hydroporus foveolatus mitochondrial 16S rRNA gene	HF931502	81.76	82.12	1.17576e−28	Coleoptera	Dytiscidae	Hydroporus foveolatus
Hygrotus fontinalis mitochondrial 16S rRNA gene (p	HF931494	80.95	82.12	1.17576e−28	Coleoptera	Dytiscidae	Hygrotus fontinalis
Suphrodytes figuratus voucher BMNH:722150 16S ribo	JX221572	80.95	82.12	1.17576e−28	Coleoptera	Dytiscidae	Suphrodytes figuratus
Deronectes hispanicus mitochondrial 16S rRNA gene	HE613727	81.88	82.12	1.17576e−28	Coleoptera	Dytiscidae	Deronectes hispanicus
Hydroporus discretus mitochondrial 16S rRNA gene (	HE613723	81.76	82.12	1.17576e−28	Coleoptera	Dytiscidae	Hydroporus discretus
Hydroporus decipiens mitochondrial 16S rRNA gene (	HE613722	81.76	82.12	1.17576e−28	Coleoptera	Dytiscidae	Hydroporus decipiens
Hydroporus pubescens mitochondrial 16S rRNA gene (	HE613707	81.76	82.12	1.17576e−28	Coleoptera	Dytiscidae	Hydroporus pubescens
Hydroporus limbatus mitochondrial 16S rRNA gene (p	HE613706	81.76	82.12	1.17576e−28	Coleoptera	Dytiscidae	Hydroporus limbatus
Hydroporus errans mitochondrial 16S rRNA gene (par	HE613704	81.76	82.12	1.17576e−28	Coleoptera	Dytiscidae	Hydroporus errans
Suphrodytes dorsalis mitochondrial 16S rRNA gene (	HE599700	80.95	82.12	1.17576e−28	Coleoptera	Dytiscidae	Suphrodytes dorsalis
