# Supplementary material for: Testing the potential of a ribosomal 16S marker for DNA metabarcoding of insects
Source: PeerJ. 2016 Apr 19;4:e1966. doi: 10.7717/peerj.1966 (PMC4841222; doi:10.7717/peerj.1966)
Supplement: Supplemental Information 1 [file peerj-04-1966-s006.zip › scripts/5 match OTUs/blast_hits/OTU_11.csv_plot.pdf]

OTU_11.csv

Protonemura meyeri

Nemouridae

Plecoptera

95.54

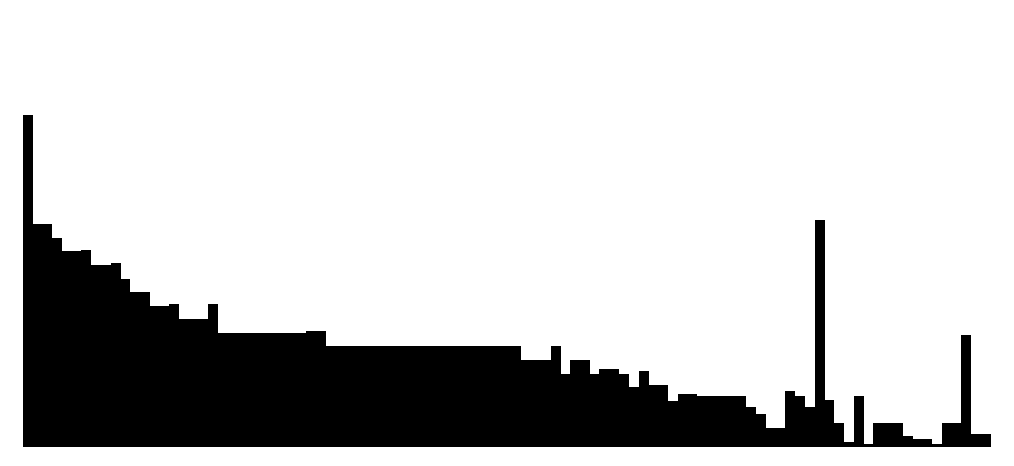

Protonemura meyeri 16S ribosomal RNA gene, partial	EF623193	95.54	100	1.79787e−63	Plecoptera	Nemouridae	Protonemura meyeri
Nanonemoura wahkeena 16S ribosomal RNA gene, parti	EF623198	90.45	100	1.29456e−52	Plecoptera	Nemouridae	Nanonemoura wahkeena
Ostrocerca foersteri 16S ribosomal RNA gene, parti	EF623191	90.45	100	1.29456e−52	Plecoptera	Nemouridae	Ostrocerca foersteri
Nemoura trispinosa 16S ribosomal RNA gene, partial	EF623187	89.81	100	1.57709e−51	Plecoptera	Nemouridae	Nemoura trispinosa
Nemurella pictetii 16S ribosomal RNA gene, partial	EF623192	89.17	100	6.70597e−50	Plecoptera	Nemouridae	Nemurella pictetii
Shipsa rotunda 16S ribosomal RNA gene, partial seq	EF623184	89.17	100	6.70597e−50	Plecoptera	Nemouridae	Shipsa rotunda
Nemoura cinerea 16S ribosomal RNA gene, partial se	EF623194	89.24	100	2.34061e−49	Plecoptera	Nemouridae	Nemoura cinerea
Visoka cataractae 16S ribosomal RNA gene, partial	EF623189	88.54	100	8.16955e−49	Plecoptera	Nemouridae	Visoka cataractae
Paranemoura claasseni 16S ribosomal RNA gene, part	EF623185	88.54	100	8.16955e−49	Plecoptera	Nemouridae	Paranemoura claasseni
Scopura montana 16S ribosomal RNA gene, partial se	EF623265	88.61	100	2.85145e−48	Plecoptera		Scopura montana
Prostoia besametsa 16S ribosomal RNA gene, partial	EF623188	87.9	100	3.47378e−47	Plecoptera	Nemouridae	Prostoia besametsa
Zapada cinctipes 16S ribosomal RNA gene, partial s	EF623183	87.26	100	4.23193e−46	Plecoptera	Nemouridae	Zapada cinctipes
Soyedina producta 16S ribosomal RNA gene, partial	EF623196	87.26	100	5.15555e−45	Plecoptera	Nemouridae	Soyedina producta
Lednia tumana 16S ribosomal RNA gene, partial sequ	EF623197	86.62	100	1.79946e−44	Plecoptera	Nemouridae	Lednia tumana
Malenka californica 16S ribosomal RNA gene, partia	EF623182	86.62	100	1.79946e−44	Plecoptera	Nemouridae	Malenka californica
Megaleuctra kincaidi 16S ribosomal RNA gene, parti	EF623174	86.71	100	6.28074e−44	Plecoptera	Leuctridae	Megaleuctra kincaidi
Apteroperla tikumana mitochondrion, complete genom	KR604721	85.99	100	2.19219e−43	Plecoptera	Capniidae	Apteroperla tikumana
Allocapnia minima 16S ribosomal RNA gene, partial	EF623130	85.99	100	2.19219e−43	Plecoptera	Capniidae	Allocapnia minima
Pteronarcys sachalina 16S ribosomal RNA gene, part	EF623264	85.99	100	2.67064e−42	Plecoptera	Pteronarcyidae	Pteronarcys sachalina
Moselia infuscata 16S ribosomal RNA gene, partial	EF623175	86.71	99.36	2.67064e−42	Plecoptera	Leuctridae	Moselia infuscata
Allocapnia recta isolate H19 16S ribosomal RNA gen	KC881054	85.35	100	9.32145e−42	Plecoptera	Capniidae	Allocapnia recta
Allocapnia recta isolate H16 16S ribosomal RNA gen	KC881051	85.35	100	9.32145e−42	Plecoptera	Capniidae	Allocapnia recta
Allocapnia recta isolate H15 16S ribosomal RNA gen	KC881050	85.35	100	9.32145e−42	Plecoptera	Capniidae	Allocapnia recta
Allocapnia recta isolate H14 16S ribosomal RNA gen	KC881049	85.35	100	9.32145e−42	Plecoptera	Capniidae	Allocapnia recta
Allocapnia recta isolate H10 16S ribosomal RNA gen	KC881045	85.35	100	9.32145e−42	Plecoptera	Capniidae	Allocapnia recta
Allocapnia recta isolate H6 16S ribosomal RNA gene	KC881041	85.35	100	9.32145e−42	Plecoptera	Capniidae	Allocapnia recta
Allocapnia recta haplotype 11ar: H011 16S ribosoma	EU306874	85.35	100	9.32145e−42	Plecoptera	Capniidae	Allocapnia recta
Allocapnia recta haplotype 7ar: H07 16S ribosomal	EU306870	85.35	100	9.32145e−42	Plecoptera	Capniidae	Allocapnia recta
Allocapnia recta haplotype 2ar: H02 16S ribosomal	DQ915180	85.35	100	9.32145e−42	Plecoptera	Capniidae	Allocapnia recta
Doddsia occidentalis 16S ribosomal RNA gene, parti	EF623270	85.44	100	3.2535e−41	Plecoptera	Taeniopterygidae	Doddsia occidentalis
Oemopteryx vanduzeea 16S ribosomal RNA gene, parti	EF623266	85.44	100	3.2535e−41	Plecoptera	Taeniopterygidae	Oemopteryx vanduzeea
Mesocapnia arizonensis mitochondrion, partial geno	KP642637	84.71	100	1.13558e−40	Plecoptera	Capniidae	Mesocapnia arizonensis
Allocapnia recta isolate H18 16S ribosomal RNA gen	KC881053	84.71	100	1.13558e−40	Plecoptera	Capniidae	Allocapnia recta
Allocapnia recta isolate H17 16S ribosomal RNA gen	KC881052	84.71	100	1.13558e−40	Plecoptera	Capniidae	Allocapnia recta
Allocapnia recta isolate H13 16S ribosomal RNA gen	KC881048	84.71	100	1.13558e−40	Plecoptera	Capniidae	Allocapnia recta
Allocapnia recta isolate H12 16S ribosomal RNA gen	KC881047	84.71	100	1.13558e−40	Plecoptera	Capniidae	Allocapnia recta
Allocapnia recta isolate H11 16S ribosomal RNA gen	KC881046	84.71	100	1.13558e−40	Plecoptera	Capniidae	Allocapnia recta
Allocapnia recta isolate H9 16S ribosomal RNA gene	KC881044	84.71	100	1.13558e−40	Plecoptera	Capniidae	Allocapnia recta
Allocapnia recta isolate H8 16S ribosomal RNA gene	KC881043	84.71	100	1.13558e−40	Plecoptera	Capniidae	Allocapnia recta
Allocapnia recta isolate H7 16S ribosomal RNA gene	KC881042	84.71	100	1.13558e−40	Plecoptera	Capniidae	Allocapnia recta
Allocapnia recta isolate H5 16S ribosomal RNA gene	KC881040	84.71	100	1.13558e−40	Plecoptera	Capniidae	Allocapnia recta
Allocapnia recta isolate H4 16S ribosomal RNA gene	KC881039	84.71	100	1.13558e−40	Plecoptera	Capniidae	Allocapnia recta
Allocapnia recta isolate H3 16S ribosomal RNA gene	KC881038	84.71	100	1.13558e−40	Plecoptera	Capniidae	Allocapnia recta
Allocapnia recta isolate H1 16S ribosomal RNA gene	KC881036	84.71	100	1.13558e−40	Plecoptera	Capniidae	Allocapnia recta
Mesocapnia sp. BYU_PL010 16S ribosomal RNA gene, p	EF623127	84.71	100	1.13558e−40	Plecoptera	Capniidae	Mesocapnia sp. BYU_PL010
Allocapnia recta haplotype 10ar: H010 16S ribosoma	EU306873	84.71	100	1.13558e−40	Plecoptera	Capniidae	Allocapnia recta
Allocapnia recta haplotype 9ar: H09 16S ribosomal	EU306872	84.71	100	1.13558e−40	Plecoptera	Capniidae	Allocapnia recta
Allocapnia recta haplotype 8ar: H08 16S ribosomal	EU306871	84.71	100	1.13558e−40	Plecoptera	Capniidae	Allocapnia recta
Allocapnia recta haplotype 3ar: H03 16S ribosomal	DQ915181	84.71	100	1.13558e−40	Plecoptera	Capniidae	Allocapnia recta
Allocapnia recta haplotype 1ar: H01 16S ribosomal	DQ915179	84.71	100	1.13558e−40	Plecoptera	Capniidae	Allocapnia recta
Pteronarcys princeps mitochondrion, complete genom	AY687866	84.71	100	1.38343e−39	Plecoptera	Pteronarcyidae	Pteronarcys princeps
Austrocerca rieka 16S ribosomal RNA gene, partial	EF623205	84.08	100	4.82863e−39	Plecoptera	Notonemouridae	Austrocerca rieka
Austrocercoides zwicki 16S ribosomal RNA gene, par	EF623199	84.08	100	4.82863e−39	Plecoptera	Notonemouridae	Austrocercoides zwicki
Allocapnia recta haplotype 12ar: H12 16S ribosomal	EU306875	84.08	100	4.82863e−39	Plecoptera	Capniidae	Allocapnia recta
Osmylus sp. EH−2004 16S ribosomal RNA gene, partia	AY620146	84.71	100	1.68536e−38	Neuroptera	Osmylidae	Osmylus sp. EH−2004
Notonemouridae sp. MD−2011 voucher ZMUC:JD−1507 16	JN615257	83.44	100	5.88247e−38	Plecoptera	Notonemouridae	Notonemouridae sp. MD−2011
Pteronarcys scotti 16S ribosomal RNA gene, partial	EF623263	84.08	100	5.88247e−38	Plecoptera	Pteronarcyidae	Pteronarcys scotti
Pteronarcys californica 16S ribosomal RNA gene, pa	EF623261	84.08	100	5.88247e−38	Plecoptera	Pteronarcyidae	Pteronarcys californica
Amphinemura sulcicollis 16S ribosomal RNA gene, pa	EF623195	83.44	100	5.88247e−38	Plecoptera	Nemouridae	Amphinemura sulcicollis
Paraleuctra occidentalis 16S ribosomal RNA gene, p	EF623178	83.65	100	2.05319e−37	Plecoptera	Leuctridae	Paraleuctra occidentalis
Brachyptera seticornis 16S ribosomal RNA gene, par	EF623268	83.65	100	7.16632e−37	Plecoptera	Taeniopterygidae	Brachyptera seticornis
Capnura wanica 16S ribosomal RNA gene, partial seq	EF623128	83.44	100	7.16632e−37	Plecoptera	Capniidae	Capnura wanica
Udamocercia sp. BYU_PL150 16S ribosomal RNA gene,	EF623201	82.8	100	2.50129e−36	Plecoptera	Notonemouridae	Udamocercia sp. BYU_PL150
Thaumatoperla sp. BYU_PL109 16S ribosomal RNA gene	EF623149	83.54	100	2.50129e−36	Plecoptera	Eustheniidae	Thaumatoperla sp. BYU_PL109
Strophopteryx appalachia 16S ribosomal RNA gene, p	EF623271	82.91	100	8.73037e−36	Plecoptera	Taeniopterygidae	Strophopteryx appalachia
Bolotoperla rossi 16S ribosomal RNA gene, partial	EF623269	82.91	100	8.73037e−36	Plecoptera	Taeniopterygidae	Bolotoperla rossi
Neonemura sp. BYU_PL153 16S ribosomal RNA gene, pa	EF623204	82.17	100	3.71225e−34	Plecoptera	Notonemouridae	Neonemura sp. BYU_PL153
Leptoperla sp. BYU_PL107 16S ribosomal RNA gene, p	EF623161	82.5	100	3.71225e−34	Plecoptera	Gripopterygidae	Leptoperla sp. BYU_PL107
Taenionema palladium 16S ribosomal RNA gene, parti	EF623267	82.5	100	1.2957e−33	Plecoptera	Taeniopterygidae	Taenionema palladium
Neopanorpa sp. M24899 16S ribosomal RNA gene, part	AF180059	82.39	100	1.2957e−33	Mecoptera	Panorpidae	Neopanorpa sp. M24899
Cryptoperla japonica mitochondrial gene for 16S rR	AB711202	82.39	100	4.52244e−33	Plecoptera	Peltoperlidae	Cryptoperla japonica
Cryptoperla japonica mitochondrial gene for 16S rR	AB711184	82.39	100	4.52244e−33	Plecoptera	Peltoperlidae	Cryptoperla japonica
Cryptoperla japonica mitochondrial gene for 16S rR	AB711183	82.39	100	4.52244e−33	Plecoptera	Peltoperlidae	Cryptoperla japonica
Cryptoperla japonica mitochondrial gene for 16S rR	AB711182	82.39	100	4.52244e−33	Plecoptera	Peltoperlidae	Cryptoperla japonica
Triznaka pintada 16S ribosomal RNA gene, partial s	EF623134	81.88	100	4.52244e−33	Plecoptera	Chloroperlidae	Triznaka pintada
Cascadoperla trictura 16S ribosomal RNA gene, part	EF623225	81.53	100	1.57849e−32	Plecoptera	Perlodidae	Cascadoperla trictura
Diamphipnoa virescentipennis 16S ribosomal RNA gen	EF623144	80.89	100	1.57849e−32	Plecoptera	Diamphipnoidae	Diamphipnoa virescentipennis
Isocapnia hyalita 16S ribosomal RNA gene, partial	EF623131	80.89	100	1.57849e−32	Plecoptera	Capniidae	Isocapnia hyalita
Leuctra inermis 16S ribosomal RNA gene, partial se	EF623176	82.61	100	5.50946e−32	Plecoptera	Leuctridae	Leuctra inermis
Eusthenia costalis 16S ribosomal RNA gene, partial	EF623150	82.39	100	5.50946e−32	Plecoptera	Eustheniidae	Eusthenia costalis
Cryptoperla sp. YK−2012−1 mitochondrial gene for 1	AB711243	81.88	100	1.92299e−31	Plecoptera	Peltoperlidae	Cryptoperla sp. YK−2012−1
Cosumnoperla hypocrena 16S ribosomal RNA gene, par	EF623226	90.65	68.15	1.92299e−31	Plecoptera	Perlodidae	Cosumnoperla hypocrena
Dinotoperla sp. BYU_PL105 16S ribosomal RNA gene,	EF623160	82.21	100	1.92299e−31	Plecoptera	Gripopterygidae	Dinotoperla sp. BYU_PL105
Neopanorpa pulchra mitochondrion, complete genome	JX569848	81.13	100	6.7119e−31	Mecoptera	Panorpidae	Neopanorpa pulchra
Amphinemura nigritta 16S ribosomal RNA gene, parti	EF623186	80.25	100	6.7119e−31	Plecoptera	Nemouridae	Amphinemura nigritta
Paraleuctra vershina 16S ribosomal RNA gene, parti	EF623173	82.39	90.45	6.7119e−31	Plecoptera	Leuctridae	Paraleuctra vershina
Dicranomyia iniquispina voucher 205222 16S ribosom	KC692800	80.12	100	2.34268e−30	Diptera	Limoniidae	Dicranomyia iniquispina
Cryptoperla japonica mitochondrial gene for 16S rR	AB711189	81.13	100	2.34268e−30	Plecoptera	Peltoperlidae	Cryptoperla japonica
Aeschnophlebia anisoptera mitochondrial gene for 1	AB707619	81.13	100	2.34268e−30	Odonata	Aeshnidae	Aeschnophlebia anisoptera
Caliaeschna microstigma 12S ribosomal RNA gene, pa	EU477639	81.13	100	2.34268e−30	Odonata	Aeshnidae	Caliaeschna microstigma
Peltoperla arcuata 16S ribosomal RNA gene, partial	EF623258	80.5	100	2.34268e−30	Plecoptera	Peltoperlidae	Peltoperla arcuata
Newmanoperla exigua 16S ribosomal RNA gene, partia	EF623162	80.38	100	2.34268e−30	Plecoptera	Gripopterygidae	Newmanoperla exigua
Klapopteryx armillata 16S ribosomal RNA gene, part	EF623123	80.38	100	2.34268e−30	Plecoptera	Austroperlidae	Klapopteryx armillata
Limonia iniquispina voucher O'Grady 201869 16S rib	EU005407	80.12	100	2.34268e−30	Diptera	Limoniidae	Dicranomyia iniquispina
Aeschnophlebia anisoptera 16S ribosomal RNA gene,	EF032712	81.13	100	2.34268e−30	Odonata	Aeshnidae	Aeschnophlebia anisoptera
Caliaeschna microstigma large subunit ribosomal RN	AF266092	81.13	100	2.34268e−30	Odonata	Aeshnidae	Caliaeschna microstigma
Frisonia picticeps 16S ribosomal RNA gene, partial	EF623227	85.25	77.71	8.17677e−30	Plecoptera	Perlodidae	Frisonia picticeps
Antarctoperla sp. BYU_PL142 16S ribosomal RNA gene	EF623163	80.62	100	8.17677e−30	Plecoptera	Gripopterygidae	Antarctoperla sp. BYU_PL142
Nesoperla fulvescens 16S ribosomal RNA gene, parti	EF623156	80.62	100	8.17677e−30	Plecoptera	Gripopterygidae	Nesoperla fulvescens
Diamphipnopsis sp. BYU_PL138 16S ribosomal RNA gen	EF623145	79.87	100	8.17677e−30	Plecoptera	Diamphipnoidae	Diamphipnopsis sp. BYU_PL138
