# Supplementary material for: Testing the potential of a ribosomal 16S marker for DNA metabarcoding of insects
Source: PeerJ. 2016 Apr 19;4:e1966. doi: 10.7717/peerj.1966 (PMC4841222; doi:10.7717/peerj.1966)
Supplement: Supplemental Information 1 [file peerj-04-1966-s006.zip › scripts/5 match OTUs/blast_hits/OTU_112.csv_plot.pdf]

OTU_112.csv

Oreodytes sanmarkii

Dytiscidae

Coleoptera

96.82

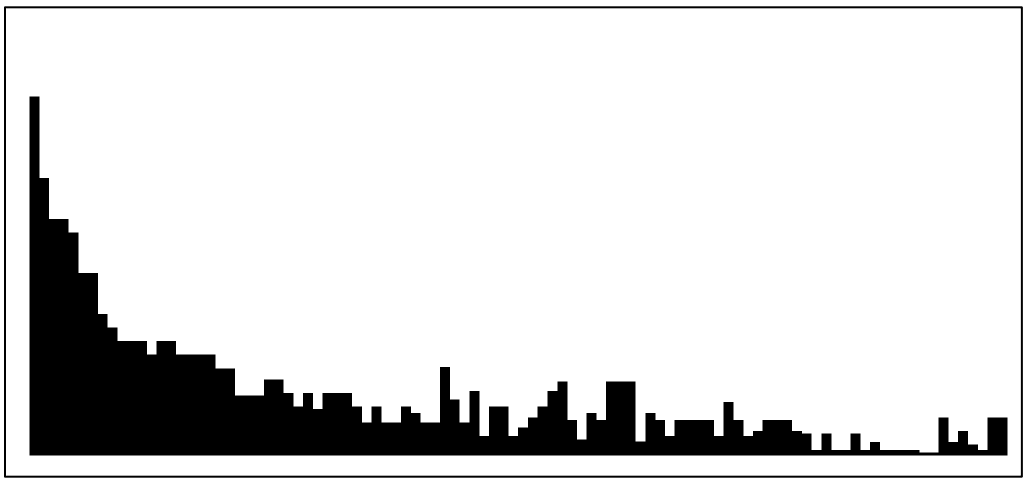

Oreodytes sanmarkii mitochondrial 16S rRNA gene (p	HF931524	96.82	100	3.47071e−66	Coleoptera	Dytiscidae	Oreodytes sanmarkii
Oreodytes obesus cordillerensis mitochondrial part	HF931516	92.99	100	4.82437e−58	Coleoptera	Dytiscidae	Oreodytes obesus cordillerensis
Oreodytes crassulus partial 16S rRNA gene	AJ850348	91.08	100	3.04451e−54	Coleoptera	Dytiscidae	Oreodytes crassulus
Oreodytes congruus partial 16S rRNA gene	AJ850347	91.08	100	3.04451e−54	Coleoptera	Dytiscidae	Oreodytes congruus
Oreodytes congruus 16S ribosomal RNA gene, partial	KF575550	90.45	100	1.29456e−52	Coleoptera	Dytiscidae	Oreodytes congruus
Oreodytes abbreviatus mitochondrial 16S rRNA gene	HF931477	88.54	100	8.16955e−49	Coleoptera	Dytiscidae	Oreodytes abbreviatus
Oreodytes rhyacophilus partial 16S rRNA gene	AJ850352	88.54	100	8.16955e−49	Coleoptera	Dytiscidae	Oreodytes rhyacophilus
Oreodytes mongolicus mitochondrial 16S rRNA gene (	HF931491	86.62	100	1.79946e−44	Coleoptera	Dytiscidae	Oreodytes mongolicus
Oreodytes snoqualmie mitochondrial partial 16S rRN	HF931519	85.99	100	2.19219e−43	Coleoptera	Dytiscidae	Oreodytes snoqualmie
Oreodytes septentrionalis mitochondrial 16S rRNA g	HF931492	85.35	100	9.32145e−42	Coleoptera	Dytiscidae	Oreodytes septentrionalis
Oreodytes shorti mitochondrial 16S rRNA gene (part	HF931488	85.35	100	9.32145e−42	Coleoptera	Dytiscidae	Oreodytes shorti
Boreonectes riberae mitochondrial 16S rRNA gene (p	HF931461	85.35	100	1.13558e−40	Coleoptera	Dytiscidae	Boreonectes riberae
Deronectes parvicollis mitochondrial 16S rRNA gene	HF931454	84.71	100	1.13558e−40	Coleoptera	Dytiscidae	Deronectes parvicollis
Boreonectes griseostriatus mitochondrial partial 1	HF931387	85.35	100	1.13558e−40	Coleoptera	Dytiscidae	Boreonectes griseostriatus
Boreonectes multilineatus mitochondrial partial 16	HF931384	85.35	100	1.13558e−40	Coleoptera	Dytiscidae	Boreonectes multilineatus
Boreonectes alpestris mitochondrial 16S rRNA gene	HF931512	84.71	100	1.38343e−39	Coleoptera	Dytiscidae	Boreonectes alpestris
Boreonectes striatellus mitochondrial 16S rRNA gen	HF931511	84.71	100	1.38343e−39	Coleoptera	Dytiscidae	Boreonectes striatellus
Boreonectes macedonicus mitochondrial 16S rRNA gen	HF931359	84.71	100	1.38343e−39	Coleoptera	Dytiscidae	Boreonectes macedonicus
Stictotarsus ibericus 16S ribosomal RNA (rrnL) gen	EF670030	84.71	100	1.38343e−39	Coleoptera	Dytiscidae	Stictotarsus ibericus
Laccornis difformis 16S ribosomal RNA gene, partia	KF575544	84.08	100	4.82863e−39	Coleoptera	Dytiscidae	Laccornis difformis
Boreonectes griseostriatus mitochondrial 16S rRNA	HF931541	84.08	100	5.88247e−38	Coleoptera	Dytiscidae	Boreonectes griseostriatus
Oreodytes scitulus 16S ribosomal RNA gene, partial	KF575551	82.8	100	2.50129e−36	Coleoptera	Dytiscidae	Oreodytes scitulus
Oreodytes scitulus mitochondrial partial 16S rRNA	HF931515	82.8	100	2.50129e−36	Coleoptera	Dytiscidae	Oreodytes scitulus
Laccornis kocae partial 16S rRNA gene	AJ850419	82.8	100	2.50129e−36	Coleoptera	Dytiscidae	Laccornis kocae
Hydroporus foveolatus mitochondrial 16S rRNA gene	HE613724	83.54	100	3.0472e−35	Coleoptera	Dytiscidae	Hydroporus foveolatus
Hydroporus glabriusculus 16S ribosomal RNA gene, p	AY365270	83.54	100	3.0472e−35	Coleoptera	Dytiscidae	Hydroporus glabriusculus
Aethionectes fulvonotatus voucher KBMAeop406 16S r	KF979088	82.91	100	1.06358e−34	Coleoptera	Dytiscidae	Aethionectes fulvonotatus
Nebrioporus formaster mitochondrial partial 16S rR	HF931377	82.28	100	1.06358e−34	Coleoptera	Dytiscidae	Nebrioporus formaster
Hydroporus tessellatus 16S ribosomal RNA gene, par	JX434770	82.91	100	3.71225e−34	Coleoptera	Dytiscidae	Hydroporus tessellatus
Boreonectes griseostriatus mitochondrial 16S rRNA	HF931385	82.17	100	3.71225e−34	Coleoptera	Dytiscidae	Boreonectes griseostriatus
Hydroporus zimmermanni mitochondrial 16S rRNA gene	HE613679	82.91	100	3.71225e−34	Coleoptera	Dytiscidae	Hydroporus zimmermanni
Hydroporus basinotatus mitochondrial 16S rRNA gene	HE613678	82.91	100	3.71225e−34	Coleoptera	Dytiscidae	Hydroporus basinotatus
Hydroporus basinotatus 16S ribosomal RNA gene, par	AF518258	82.91	100	3.71225e−34	Coleoptera	Dytiscidae	Hydroporus basinotatus
Suphrodytes dorsalis 16S ribosomal RNA gene, parti	KF575577	82.28	100	1.2957e−33	Coleoptera	Dytiscidae	Suphrodytes dorsalis
Oreodytes quadrimaculatus 16S ribosomal RNA gene,	KF575567	81.53	100	1.2957e−33	Coleoptera	Dytiscidae	Oreodytes quadrimaculatus
Suphrodytes dorsalis voucher BMNH:725966 16S ribos	JX221574	82.28	100	1.2957e−33	Coleoptera	Dytiscidae	Suphrodytes dorsalis
Oreodytes quadrimaculatus partial 16S rRNA gene	AJ850351	81.53	100	1.2957e−33	Coleoptera	Dytiscidae	Oreodytes quadrimaculatus
Oreodytes natrix partial 16S rRNA gene	AJ850349	81.53	100	1.2957e−33	Coleoptera	Dytiscidae	Oreodytes natrix
Stictotarsus maghrebinus 16S ribosomal RNA gene, p	AY250947	82.28	100	1.2957e−33	Coleoptera	Dytiscidae	Stictotarsus maghrebinus
Scarodytes sp. IR−2003 16S ribosomal RNA gene, par	AY250939	81.99	100	1.2957e−33	Coleoptera	Dytiscidae	Scarodytes sp. IR−2003
Hygrotus novemlineatus mitochondrial partial 16S r	HF931523	81.53	100	4.52244e−33	Coleoptera	Dytiscidae	Hygrotus novemlineatus
Hygrotus marklini mitochondrial 16S rRNA gene (par	HF931467	81.53	100	4.52244e−33	Coleoptera	Dytiscidae	Hygrotus marklini
Deronectes moestus mitochondrial 16S rRNA gene (pa	HE613726	84.14	91.72	4.52244e−33	Coleoptera	Dytiscidae	Deronectes moestus
Hydroporus marginatus mitochondrial 16S rRNA gene	HE613699	82.61	100	4.52244e−33	Coleoptera	Dytiscidae	Hydroporus marginatus
Hygrotus turbidus partial 16S rRNA gene	AJ850390	81.53	100	4.52244e−33	Coleoptera	Dytiscidae	Hygrotus turbidus
Nirripirti megamacrocephalus isolate ABTC78781 16S	EU616935	83.02	100	4.52244e−33	Coleoptera	Dytiscidae	Paroster megamacrocephalus
Cybister marginicollis voucher UNM KBMCyma416 16S	KJ548319	80.89	100	1.57849e−32	Coleoptera	Dytiscidae	Cybister marginicollis
Hydroporus analis mitochondrial partial 16S rRNA g	HE613731	82.28	100	1.57849e−32	Coleoptera	Dytiscidae	Hydroporus analis
Hydroporus compunctus mitochondrial 16S rRNA gene	HE599693	82.28	100	1.57849e−32	Coleoptera	Dytiscidae	Hydroporus compunctus
Copelatus sp. BI−2011 voucher BMNH792945 16S ribos	HQ381441	80.89	100	1.57849e−32	Coleoptera	Dytiscidae	Copelatus sp. BI−2011
Antiporus hollingsworthi mitochondrial partial 16S	FR727246	81.29	98.73	1.57849e−32	Coleoptera	Dytiscidae	Antiporus hollingsworthi
Hydroporus rufifrons 16S ribosomal RNA gene, parti	AF518277	81.76	100	1.57849e−32	Coleoptera	Dytiscidae	Hydroporus rufifrons
Hydroporus compunctus 16S ribosomal RNA gene, part	AF518259	82.28	100	1.57849e−32	Coleoptera	Dytiscidae	Hydroporus compunctus
Hydroporus marginatus 16S ribosomal RNA gene, part	AF518267	83.02	100	1.57849e−32	Coleoptera	Dytiscidae	Hydroporus marginatus
Deronectes moestus 16S ribosomal RNA gene, partial	KF575561	83.45	91.72	5.50946e−32	Coleoptera	Dytiscidae	Deronectes moestus
Stictotarsus procerus mitochondrial 16S rRNA gene	HF931379	81.65	100	5.50946e−32	Coleoptera	Dytiscidae	Stictotarsus procerus
Suphrodytes figuratus voucher BMNH:722148 16S ribo	JX221571	80.75	100	5.50946e−32	Coleoptera	Dytiscidae	Suphrodytes figuratus
Hydroporus fuscipennis mitochondrial 16S rRNA gene	HE613730	81.99	100	5.50946e−32	Coleoptera	Dytiscidae	Hydroporus fuscipennis
Deronectes opatrinus mitochondrial 16S rRNA gene (	HE613701	81.65	100	5.50946e−32	Coleoptera	Dytiscidae	Deronectes opatrinus
Deronectes brannani mitochondrial 16S rRNA gene (p	HE613695	83.45	91.72	5.50946e−32	Coleoptera	Dytiscidae	Deronectes brannanii
Deronectes moestus mitochondrial 16S rRNA gene (pa	HE613683	83.45	91.72	5.50946e−32	Coleoptera	Dytiscidae	Deronectes moestus
Hovahydrus sp. BI−2011 voucher BMNH831333 16S ribo	HQ381524	83.45	91.72	5.50946e−32	Coleoptera	Dytiscidae	Hovahydrus sp. BI−2011
Antiporus hollingsworthi mitochondrial partial 16S	FR727247	80.65	98.73	5.50946e−32	Coleoptera	Dytiscidae	Antiporus hollingsworthi
Hydroporus fuscipennis 16S ribosomal RNA gene, par	AF518262	81.99	100	5.50946e−32	Coleoptera	Dytiscidae	Hydroporus fuscipennis
Hydroporus foveolatus mitochondrial 16S rRNA gene	HF931502	81.65	100	1.92299e−31	Coleoptera	Dytiscidae	Hydroporus foveolatus
Hygrotus patruelis mitochondrial 16S rRNA gene (pa	HF931388	80.89	100	1.92299e−31	Coleoptera	Dytiscidae	Hygrotus patruelis
Hydroporus discretus mitochondrial 16S rRNA gene (	HE613723	81.65	100	1.92299e−31	Coleoptera	Dytiscidae	Hydroporus discretus
Hydroporus decipiens mitochondrial 16S rRNA gene (	HE613722	81.65	100	1.92299e−31	Coleoptera	Dytiscidae	Hydroporus decipiens
Hydroporus limbatus mitochondrial 16S rRNA gene (p	HE613706	81.65	100	1.92299e−31	Coleoptera	Dytiscidae	Hydroporus limbatus
Hydroporus errans mitochondrial 16S rRNA gene (par	HE613704	81.65	100	1.92299e−31	Coleoptera	Dytiscidae	Hydroporus errans
Nirripirti newhavenensis 16S ribosomal RNA gene, p	AY353844	80.89	100	1.92299e−31	Coleoptera	Dytiscidae	Paroster newhavenensis
Nirripirti napperbyensis 16S ribosomal RNA gene, p	AY353843	82.5	100	1.92299e−31	Coleoptera	Dytiscidae	Paroster napperbyensis
Hydroporus pubescens voucher NHM−IR39 16S ribosoma	EF419327	81.65	100	1.92299e−31	Coleoptera	Dytiscidae	Hydroporus pubescens
Hydroporus sp. IR33.4 16S ribosomal RNA gene, part	AY365261	80.89	100	1.92299e−31	Coleoptera	Dytiscidae	Hydroporus sp. IR33.4
Scarodytes halensis haplotype 3 16S ribosomal RNA	AY250937	81.13	100	1.92299e−31	Coleoptera	Dytiscidae	Scarodytes halensis
Hydroporus limbatus 16S ribosomal RNA gene, partia	AF518264	81.65	100	1.92299e−31	Coleoptera	Dytiscidae	Hydroporus limbatus
Hydroporus discretus 16S ribosomal RNA gene, parti	AF518260	81.65	100	1.92299e−31	Coleoptera	Dytiscidae	Hydroporus discretus
Hydroporus analis 16S ribosomal RNA gene, partial	AF518257	81.65	100	1.92299e−31	Coleoptera	Dytiscidae	Hydroporus analis
Hydroporus rufifrons 16S ribosomal RNA gene, parti	JX434758	81.13	100	6.7119e−31	Coleoptera	Dytiscidae	Hydroporus rufifrons
Deronectes doriae mitochondrial 16S rRNA gene (par	HE613710	81.01	100	6.7119e−31	Coleoptera	Dytiscidae	Deronectes doriae
Deronectes bicostatus mitochondrial 16S rRNA gene	HE613705	80.25	100	6.7119e−31	Coleoptera	Dytiscidae	Deronectes bicostatus
Deronectes sahlbergi mitochondrial 16S rRNA gene (	HE613680	81.01	100	6.7119e−31	Coleoptera	Dytiscidae	Deronectes sahlbergi
Cybister marginicollis voucher BMNH741827 16S ribo	HQ381392	80.25	100	6.7119e−31	Coleoptera	Dytiscidae	Cybister marginicollis
Copelatus sp. BI−2011 voucher BMNH671215 16S ribos	HQ381377	80.25	100	6.7119e−31	Coleoptera	Dytiscidae	Copelatus sp. BI−2011
Stictotarsus falli 16S ribosomal RNA (rrnL) gene,	EF670029	81.01	100	6.7119e−31	Coleoptera	Dytiscidae	Stictotarsus falli
Oreodytes picturatus partial 16S rRNA gene	AJ850350	80.25	100	6.7119e−31	Coleoptera	Dytiscidae	Oreodytes picturatus
Aethionectes oberthueri voucher 393H5/BMNH829993 1	KF979087	80.62	100	2.34268e−30	Coleoptera	Dytiscidae	Aethionectes oberthueri
Hygrotus lutescens mitochondrial partial 16S rRNA	HF931518	80.25	100	2.34268e−30	Coleoptera	Dytiscidae	Hygrotus lutescens
Hygrotus fontinalis mitochondrial 16S rRNA gene (p	HF931494	80.25	100	2.34268e−30	Coleoptera	Dytiscidae	Hygrotus fontinalis
Hygrotus sanfilippoi mitochondrial 16S rRNA gene (	HF931342	80.25	100	2.34268e−30	Coleoptera	Dytiscidae	Hygrotus sanfilippoi
Hydroporus brancoi gredensis mitochondrial partial	HF931329	80.25	100	2.34268e−30	Coleoptera	Dytiscidae	Hydroporus brancoi gredensis
Suphrodytes figuratus voucher BMNH:722150 16S ribo	JX221572	80.12	100	2.34268e−30	Coleoptera	Dytiscidae	Suphrodytes figuratus
Suphrodytes dorsalis mitochondrial 16S rRNA gene (	HE599700	80.12	100	2.34268e−30	Coleoptera	Dytiscidae	Suphrodytes dorsalis
Paroster sp. R401 Newhaven voucher ABTC:78974 16S	JQ745754	81.76	100	2.34268e−30	Coleoptera	Dytiscidae	Paroster sp. R401 Newhaven
Aethionectes oberthueri voucher BMNH829993 16S rib	HQ381496	80.62	100	2.34268e−30	Coleoptera	Dytiscidae	Aethionectes oberthueri
Pseudomigadops sp. PSM1 mitochondrial 16S rRNA gen	FM994738	81.13	100	2.34268e−30	Coleoptera	Carabidae	Pseudomigadops sp. PSM1
Megadytes sp. IR57 16S ribosomal RNA (rrnL) gene,	EF670018	80.5	100	2.34268e−30	Coleoptera	Dytiscidae	Megadytes sp. IR57
Hygrotus corpulentus partial 16S rRNA gene	AJ850387	80.25	100	2.34268e−30	Coleoptera	Dytiscidae	Hygrotus corpulentus
Paroster sp. 2 RL−2008 16S ribosomal RNA gene, par	EU616958	81.76	100	2.34268e−30	Coleoptera	Dytiscidae	Paroster sp. 2 RL−2008
Paroster sp. spnMtWedge 16S ribosomal RNA gene, pa	EU616954	81.76	100	2.34268e−30	Coleoptera	Dytiscidae	Paroster sp. spnMtWedge
