# Supplementary material for: Testing the potential of a ribosomal 16S marker for DNA metabarcoding of insects
Source: PeerJ. 2016 Apr 19;4:e1966. doi: 10.7717/peerj.1966 (PMC4841222; doi:10.7717/peerj.1966)
Supplement: Supplemental Information 1 [file peerj-04-1966-s006.zip › scripts/5 match OTUs/blast_hits/OTU_113.csv_plot.pdf]

OTU_113.csv

Agabus picotae

Dytiscidae

Coleoptera

90.53

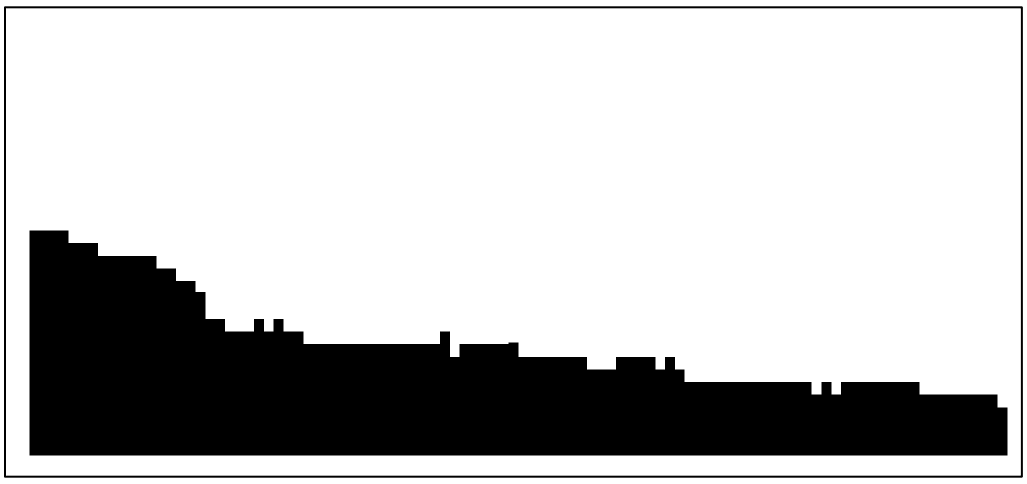

Agabus picotae mitochondrial 16S rRNA gene (partia	HF931356	90.53	100	1.24618e−59	Coleoptera	Dytiscidae	Agabus picotae
Agabus heydeni 16S ribosomal RNA gene, partial seq	AY039263	90.53	100	1.24618e−59	Coleoptera	Dytiscidae	Agabus heydeni
Agabus binotatus 16S ribosomal RNA gene, partial s	AY039260	90.53	100	1.24618e−59	Coleoptera	Dytiscidae	Agabus binotatus
Agabus biguttatus haplotype 1 16S ribosomal RNA ge	AY039258	90.53	100	1.24618e−59	Coleoptera	Dytiscidae	Agabus biguttatus
Agabus basalis mitochondrial 16S rRNA gene (partia	HF931443	89.94	100	5.29889e−58	Coleoptera	Dytiscidae	Agabus basalis
Agabus cf. nitidus IR−2001 16S ribosomal RNA gene,	AY039264	89.94	100	5.29889e−58	Coleoptera	Dytiscidae	Agabus cf. nitidus IR−2001
Agabus biguttatus haplotype 2 16S ribosomal RNA ge	AY039259	89.94	100	5.29889e−58	Coleoptera	Dytiscidae	Agabus biguttatus
Agabus guttatus mitochondrial 16S rRNA gene (parti	LM654777	89.35	100	6.45537e−57	Coleoptera	Dytiscidae	Agabus guttatus
Agabus caraboides mitochondrial partial 16S rRNA g	HF931451	89.35	100	6.45537e−57	Coleoptera	Dytiscidae	Agabus caraboides
Agabus guttatus 16S ribosomal RNA gene, partial se	AY039262	89.35	100	6.45537e−57	Coleoptera	Dytiscidae	Agabus guttatus
Agabus alexandrae 16S ribosomal RNA gene, partial	AY039257	89.35	100	6.45537e−57	Coleoptera	Dytiscidae	Agabus alexandrae
Agabus alexandrae 16S ribosomal RNA gene, partial	AF428199	89.35	100	6.45537e−57	Coleoptera	Dytiscidae	Agabus alexandrae
Agabus cephalotes mitochondrial 16S rRNA gene (par	HF931380	89.35	100	7.86426e−56	Coleoptera	Dytiscidae	Agabus cephalotes
Agabus faldermanni mitochondrial 16S rRNA gene (pa	LM654774	88.76	100	2.74489e−55	Coleoptera	Dytiscidae	Agabus faldermanni
Agabus faldermanni 16S ribosomal RNA gene, partial	AY138614	88.76	100	2.74489e−55	Coleoptera	Dytiscidae	Agabus faldermanni
Agabus glacialis mitochondrial 16S rRNA gene (part	LM654776	88.17	100	4.07379e−53	Coleoptera	Dytiscidae	Agabus glacialis
Agabus dilatatus 16S ribosomal RNA gene, partial s	AY039261	88.17	100	4.07379e−53	Coleoptera	Dytiscidae	Agabus dilatatus
Agabus maderensis 16S ribosomal RNA gene, partial	AY138621	87.65	100	4.96289e−52	Coleoptera	Dytiscidae	Agabus maderensis
Agabus undulatus mitochondrial 16S rRNA gene (part	HF931411	86.39	100	7.36558e−50	Coleoptera	Dytiscidae	Agabus undulatus
Agabus paludosus 16S ribosomal RNA gene, partial s	AY138625	86.39	100	8.97311e−49	Coleoptera	Dytiscidae	Agabus paludosus
Agabus nebulosus mitochondrial 16S rRNA gene (part	LM654779	85.8	100	1.09315e−47	Coleoptera	Dytiscidae	Agabus nebulosus
Agabus uliginosus mitochondrial 16S rRNA gene (par	HF931463	85.8	100	1.09315e−47	Coleoptera	Dytiscidae	Agabus uliginosus
Agabus dichrous mitochondrial partial 16S rRNA gen	HF931378	85.8	100	1.09315e−47	Coleoptera	Dytiscidae	Agabus dichrous
Agabus bipustulatus 16S ribosomal RNA gene, partia	AF428198	86.39	100	1.09315e−47	Coleoptera	Dytiscidae	Agabus bipustulatus
Agabus nebulosus 16S ribosomal RNA gene, partial s	AY138624	85.8	100	1.09315e−47	Coleoptera	Dytiscidae	Agabus nebulosus
Agabus melanarius 16S ribosomal RNA gene, partial	AY138623	86.39	100	1.09315e−47	Coleoptera	Dytiscidae	Agabus melanarius
Agabus lineatus 16S ribosomal RNA gene, partial se	AY138611	85.8	100	1.09315e−47	Coleoptera	Dytiscidae	Agabus lineatus
Agabus antennatus 16S ribosomal RNA gene, partial	AY138601	85.8	100	1.09315e−47	Coleoptera	Dytiscidae	Agabus antennatus
Agabus tristis voucher UNM KBMAgtr60 16S ribosomal	KJ548305	85.21	100	3.81546e−47	Coleoptera	Dytiscidae	Agabus tristis
Agabus rufulus mitochondrial 16S rRNA gene (partia	LM654818	85.21	100	4.64819e−46	Coleoptera	Dytiscidae	Agabus rufulus
Agabus rufulus mitochondrial 16S rRNA gene (partia	LM654817	85.21	100	4.64819e−46	Coleoptera	Dytiscidae	Agabus rufulus
Agabus ramblae mitochondrial 16S rRNA gene (partia	LM654816	85.21	100	4.64819e−46	Coleoptera	Dytiscidae	Agabus ramblae
Agabus brunneus mitochondrial 16S rRNA gene (parti	LM654791	85.21	100	4.64819e−46	Coleoptera	Dytiscidae	Agabus brunneus
Agabus brunneus mitochondrial 16S rRNA gene (parti	LM654790	85.21	100	4.64819e−46	Coleoptera	Dytiscidae	Agabus brunneus
Agabus brunneus mitochondrial 16S rRNA gene (parti	LM654789	85.21	100	4.64819e−46	Coleoptera	Dytiscidae	Agabus brunneus
Agabus brunneus mitochondrial 16S rRNA gene (parti	LM654788	85.21	100	4.64819e−46	Coleoptera	Dytiscidae	Agabus brunneus
Agabus brunneus mitochondrial 16S rRNA gene (parti	LM654787	85.21	100	4.64819e−46	Coleoptera	Dytiscidae	Agabus brunneus
Agabus rufulus mitochondrial partial 16S rRNA gene	LM654771	85.21	100	4.64819e−46	Coleoptera	Dytiscidae	Agabus rufulus
Agabus ramblae mitochondrial partial 16S rRNA gene	LM654770	85.21	100	4.64819e−46	Coleoptera	Dytiscidae	Agabus ramblae
Agabus brunneus mitochondrial partial 16S rRNA gen	LM654769	85.21	100	4.64819e−46	Coleoptera	Dytiscidae	Agabus brunneus
Agabus brunneus mitochondrial partial 16S rRNA gen	LM654768	85.21	100	4.64819e−46	Coleoptera	Dytiscidae	Agabus brunneus
Agabus brunneus mitochondrial partial 16S rRNA gen	LM654767	85.21	100	4.64819e−46	Coleoptera	Dytiscidae	Agabus brunneus
Agabus wollastoni 16S ribosomal RNA gene, partial	AY138636	85.8	100	4.64819e−46	Coleoptera	Dytiscidae	Agabus wollastoni
Agabus tristis 16S ribosomal RNA gene, partial seq	AY138634	84.62	100	4.64819e−46	Coleoptera	Dytiscidae	Agabus tristis
Agabus serricornis 16S ribosomal RNA gene, partial	AY138629	85.21	100	4.64819e−46	Coleoptera	Dytiscidae	Agabus serricornis
Agabus ramblae 16S ribosomal RNA gene, partial seq	AY138626	85.21	100	4.64819e−46	Coleoptera	Dytiscidae	Agabus ramblae
Agabus conspersus 16S ribosomal RNA gene, partial	AY138610	85.21	100	4.64819e−46	Coleoptera	Dytiscidae	Agabus conspersus
Agabus brunneus 16S ribosomal RNA gene, partial se	AY138607	85.21	100	4.64819e−46	Coleoptera	Dytiscidae	Agabus brunneus
Agabus bifarius 16S ribosomal RNA gene, partial se	AY138605	85.21	100	4.64819e−46	Coleoptera	Dytiscidae	Agabus bifarius
Agabus aubei 16S ribosomal RNA gene, partial seque	AY039265	85.29	100	1.62238e−45	Coleoptera	Dytiscidae	Agabus aubei
Agabus didymus mitochondrial 16S rRNA gene (partia	LM654773	84.62	100	5.66265e−45	Coleoptera	Dytiscidae	Agabus didymus
Agabus didymus voucher UNM KBMPmsp730 16S ribosoma	KJ548392	84.62	100	5.66265e−45	Coleoptera	Dytiscidae	Agabus didymus
Agabus godmanni mitochondrial 16S rRNA gene (parti	HF931348	84.62	100	5.66265e−45	Coleoptera	Dytiscidae	Agabus godmanni
Ilybius vandykei 16S ribosomal RNA gene, partial s	AY138671	84.62	100	5.66265e−45	Coleoptera	Dytiscidae	Ilybius vandykei
Agabus sturmii 16S ribosomal RNA gene, partial seq	AY138633	84.62	100	5.66265e−45	Coleoptera	Dytiscidae	Agabus sturmii
Agabus arcticus 16S ribosomal RNA gene, partial se	AY138603	84.62	100	5.66265e−45	Coleoptera	Dytiscidae	Agabus arcticus
Agabus affinis 16S ribosomal RNA gene, partial seq	AY138598	84.62	100	5.66265e−45	Coleoptera	Dytiscidae	Agabus affinis
Agabus disintegratus voucher UNM KBMAgdi412 16S ri	KJ548302	84.02	100	6.89852e−44	Coleoptera	Dytiscidae	Agabus disintegratus
Agabus disintegratus 16S ribosomal RNA gene, parti	AY071769	84.02	100	6.89852e−44	Coleoptera	Dytiscidae	Agabus disintegratus
Agabus striolatus mitochondrial 16S rRNA gene (par	LM654785	84.02	100	2.40782e−43	Coleoptera	Dytiscidae	Agabus striolatus
Agabus semipunctatus mitochondrial 16S rRNA gene (	LM654784	84.62	100	2.40782e−43	Coleoptera	Dytiscidae	Agabus semipunctatus
Agabus strigulosus 16S ribosomal RNA gene, partial	AY138632	84.62	100	2.40782e−43	Coleoptera	Dytiscidae	Agabus strigulosus
Agabus semipunctatus 16S ribosomal RNA gene, parti	AY138628	84.62	100	2.40782e−43	Coleoptera	Dytiscidae	Agabus semipunctatus
Agabus lutosus 16S ribosomal RNA gene, partial seq	AY138620	84.62	100	2.40782e−43	Coleoptera	Dytiscidae	Agabus lutosus
Agabus japonicus 16S ribosomal RNA gene, partial s	AY138617	84.02	100	2.40782e−43	Coleoptera	Dytiscidae	Agabus japonicus
Agabus sp. IR−sp2 16S ribosomal RNA gene, partial	AY138597	84.62	100	2.40782e−43	Coleoptera	Dytiscidae	Agabus sp. IR−sp2
Agabus morosus isolate Aga18.2R 16S ribosomal RNA	AY138592	84.02	100	2.40782e−43	Coleoptera	Dytiscidae	Agabus morosus
Agabus regimbarti mitochondrial 16S rRNA gene (par	LM654782	83.43	100	2.93332e−42	Coleoptera	Dytiscidae	Agabus regimbarti
Agabus ragazzii mitochondrial 16S rRNA gene (parti	LM654781	83.43	100	2.93332e−42	Coleoptera	Dytiscidae	Agabus ragazzii
Agabus pallidus mitochondrial 16S rRNA gene (parti	LM654780	83.43	100	2.93332e−42	Coleoptera	Dytiscidae	Agabus pallidus
Agabus labiatus mitochondrial 16S rRNA gene (parti	LM654778	83.43	100	2.93332e−42	Coleoptera	Dytiscidae	Agabus labiatus
Platambus stygius 16S ribosomal RNA gene, partial	AY138679	83.43	100	2.93332e−42	Coleoptera	Dytiscidae	Platambus stygius
Ilybius wasastjernae 16S ribosomal RNA gene, parti	AY138672	83.43	100	2.93332e−42	Coleoptera	Dytiscidae	Ilybius wasastjernae
Ilybius opacus 16S ribosomal RNA gene, partial seq	AY138668	83.43	100	2.93332e−42	Coleoptera	Dytiscidae	Ilybius opacus
Agabus erythropterus 16S ribosomal RNA gene, parti	AY138613	83.43	100	2.93332e−42	Coleoptera	Dytiscidae	Agabus erythropterus
Agabus elongatus 16S ribosomal RNA gene, partial s	AY138612	83.43	100	2.93332e−42	Coleoptera	Dytiscidae	Agabus elongatus
Agabus austinii 16S ribosomal RNA gene, partial se	AY138604	83.43	100	2.93332e−42	Coleoptera	Dytiscidae	Agabus austinii
Agabus thomsoni 16S ribosomal RNA gene, partial se	AY138595	83.43	100	2.93332e−42	Coleoptera	Dytiscidae	Agabus thomsoni
Agabus kootenai 16S ribosomal RNA gene, partial se	AY138594	83.43	100	2.93332e−42	Coleoptera	Dytiscidae	Agabus kootenai
Agabus morosus isolate Aga16.3 16S ribosomal RNA g	AY138591	83.43	100	2.93332e−42	Coleoptera	Dytiscidae	Agabus morosus
Agabus labiatus 16S ribosomal RNA gene, partial se	AY138618	82.84	100	3.57352e−41	Coleoptera	Dytiscidae	Agabus labiatus
Ilybius lineellus mitochondrial 16S rRNA gene (par	LM654802	83.43	100	1.24728e−40	Coleoptera	Dytiscidae	Ilybius lineellus
Agabus amoenus mitochondrial 16S rRNA gene (partia	LM654772	82.84	100	1.24728e−40	Coleoptera	Dytiscidae	Agabus amoenus
Ilybiosoma cordatum voucher UNM KBMAgco57 16S ribo	KJ548301	83.43	100	1.24728e−40	Coleoptera	Dytiscidae	Ilybiosoma cordatum
Ilybius walsinghami isolate Ilydisr 16S ribosomal	AY138660	83.43	100	1.24728e−40	Coleoptera	Dytiscidae	Ilybius walsinghami
Ilybiosoma seriatum isolate Ilbseri2R 16S ribosoma	AY138655	83.43	100	1.24728e−40	Coleoptera	Dytiscidae	Ilybiosoma seriatum
Ilybiosoma seriatum isolate IlbC2.1 16S ribosomal	AY138653	83.43	100	1.24728e−40	Coleoptera	Dytiscidae	Ilybiosoma seriatum
Ilybiosoma seriatum isolate Ilb7.1R 16S ribosomal	AY138651	83.43	100	1.24728e−40	Coleoptera	Dytiscidae	Ilybiosoma seriatum
Ilybiosoma perplexus 16S ribosomal RNA gene, parti	AY138650	83.43	100	1.24728e−40	Coleoptera	Dytiscidae	Ilybiosoma perplexus
Ilybiosoma seriatum isolate Ilb27.3 16S ribosomal	AY138649	83.43	100	1.24728e−40	Coleoptera	Dytiscidae	Ilybiosoma seriatum
Platambus sp. IR−sp1 16S ribosomal RNA gene, parti	AY138631	83.43	100	1.24728e−40	Coleoptera	Dytiscidae	Platambus sp. IR−sp1
Agabus matsumotoi 16S ribosomal RNA gene, partial	AY138622	82.84	100	1.24728e−40	Coleoptera	Dytiscidae	Agabus matsumotoi
Agabus hummeli 16S ribosomal RNA gene, partial seq	AY138616	82.84	100	1.24728e−40	Coleoptera	Dytiscidae	Agabus hummeli
Agabus congener 16S ribosomal RNA gene, partial se	AY138609	82.84	100	1.24728e−40	Coleoptera	Dytiscidae	Agabus congener
Agabus confinis 16S ribosomal RNA gene, partial se	AY138608	82.84	100	1.24728e−40	Coleoptera	Dytiscidae	Agabus confinis
Agabus anthracinus 16S ribosomal RNA gene, partial	AY138602	82.84	100	1.24728e−40	Coleoptera	Dytiscidae	Agabus anthracinus
Agabus amoenus 16S ribosomal RNA gene, partial seq	AY138600	82.84	100	1.24728e−40	Coleoptera	Dytiscidae	Agabus amoenus
Agabus ambiguus isolate Agaambi 16S ribosomal RNA	AY138599	82.84	100	1.24728e−40	Coleoptera	Dytiscidae	Agabus ambiguus
Agabus ambiguus isolate Aga32.1 16S ribosomal RNA	AY138596	82.84	100	1.24728e−40	Coleoptera	Dytiscidae	Agabus ambiguus
Agabus ruwenzoricus mitochondrial 16S rRNA gene (p	LM654783	82.25	100	1.5195e−39	Coleoptera	Dytiscidae	Agabus ruwenzoricus
