# Supplementary material for: Testing the potential of a ribosomal 16S marker for DNA metabarcoding of insects
Source: PeerJ. 2016 Apr 19;4:e1966. doi: 10.7717/peerj.1966 (PMC4841222; doi:10.7717/peerj.1966)
Supplement: Supplemental Information 1 [file peerj-04-1966-s006.zip › scripts/5 match OTUs/blast_hits/OTU_114.csv_plot.pdf]

OTU_114.csv

Asellus aquaticus

Asellidae

Isopoda

96.32

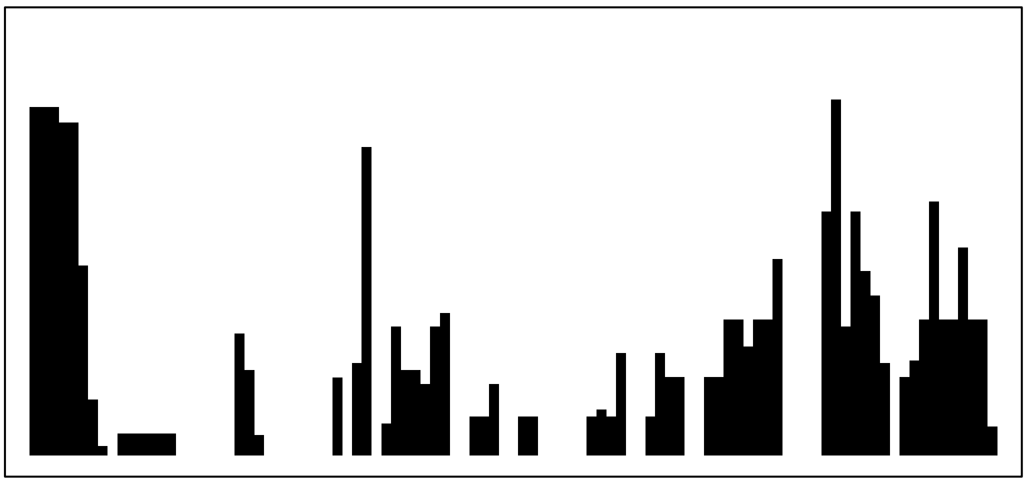

Asellus aquaticus isolate s180 16S ribosomal RNA g	KC610270	96.32	100	7.22122e−55	Isopoda	Asellidae	Asellus aquaticus
Asellus aquaticus 16S ribosomal RNA gene, partial	DQ305105	96.32	100	7.22122e−55	Isopoda	Asellidae	Asellus aquaticus
Asellus aquaticus 16S ribosomal RNA gene, partial	AF532161	96.32	100	7.22122e−55	Isopoda	Asellidae	Asellus aquaticus
Asellus aquaticus 16S ribosomal RNA gene, partial	DQ305106	95.59	100	8.79724e−54	Isopoda	Asellidae	Asellus aquaticus
Asellus aquaticus partial mitochondrial 16S rRNA g	AJ388077	95.59	100	8.79724e−54	Isopoda	Asellidae	Asellus aquaticus
Asellus levanidovorum 16S ribosomal RNA gene, part	AF532160	88.89	65.44	3.28422e−21	Isopoda	Asellidae	Asellus levanidovorum
Mesoasellus dybowskii 16S ribosomal RNA gene, part	AF532158	82.61	65.44	3.7473e−14	Isopoda	Asellidae	Mesoasellus dybowskii
Asellid sp. BH−2002 16S ribosomal RNA gene, partia	AF532159	80.43	65.44	1.94115e−11	Isopoda	Asellidae	Asellid sp. BH−2002
Proasellus coxalis isolate s194 16S ribosomal RNA	KC610284	77.17	66.91	4.27566e−07	Isopoda	Asellidae	Proasellus coxalis
Proasellus slavus isolate s118 16S ribosomal RNA g	KC610208	81.01	55.88	1.49235e−06	Isopoda	Asellidae	Proasellus slavus
Proasellus slavus isolate s117 16S ribosomal RNA g	KC610207	81.01	55.88	1.49235e−06	Isopoda	Asellidae	Proasellus slavus
Proasellus slavus isolate s105 16S ribosomal RNA g	KC610195	81.01	55.88	1.49235e−06	Isopoda	Asellidae	Proasellus slavus
Proasellus slavus isolate s104 16S ribosomal RNA g	KC610194	81.01	55.88	1.49235e−06	Isopoda	Asellidae	Proasellus slavus
Proasellus slavus isolate s103 16S ribosomal RNA g	KC610193	81.01	55.88	1.49235e−06	Isopoda	Asellidae	Proasellus slavus
Proasellus slavus isolate PRVOVERZ_201009_1ID_YCD5	JQ921809	81.01	55.88	1.49235e−06	Isopoda	Asellidae	Proasellus slavus
Proasellus beroni isolate BERNACCI_200810_3_YAS453	JQ921713	79.49	56.62	1.49235e−06	Isopoda	Asellidae	Proasellus beroni
Proasellus slavus isolate s113 16S ribosomal RNA g	KC610203	79.75	56.62	5.20882e−06	Isopoda	Asellidae	Proasellus slavus
Bragasellus cortesi isolate POUCA_201010_1ID_YCD83	JQ921676	76.6	66.18	5.20882e−06	Isopoda	Asellidae	Bragasellus cortesi
Proasellus beroni isolate s202 16S ribosomal RNA g	KC610292	76.67	65.44	1.81808e−05	Isopoda	Asellidae	Proasellus beroni
Proasellus beroni isolate s200 16S ribosomal RNA g	KC610290	78.21	56.62	6.34565e−05	Isopoda	Asellidae	Proasellus beroni
Stenasellus virei isolate ORFES_200906_2_YLK394 16	JQ921839	76.14	63.97	6.34565e−05	Isopoda	Stenasellidae	Stenasellus virei
Brachystelma minor clone 052_B141 photosystem II p	KP245158	85.71	36.03	0.000221485	Gentianales	Apocynaceae	Brachystelma minor
Dracunculus medinensis genome assembly D_medinensi	LK978197	84	36.76	0.000221485	Spirurida	Dracunculidae	Dracunculus medinensis
Megaphyllum montivagum voucher ZMUC:00101492 small	KF701067	80.95	44.85	0.000221485	Julida	Julidae	Megaphyllum montivagum
Proasellus ebrensis isolate s207 16S ribosomal RNA	KC610297	79.1	49.26	0.000221485	Isopoda	Asellidae	Proasellus ebrensis
Proasellus ebrensis isolate s206 16S ribosomal RNA	KC610296	79.1	49.26	0.000221485	Isopoda	Asellidae	Proasellus ebrensis
Proasellus sp. type locality of P. gourbaultae iso	KC610099	77.78	52.94	0.000221485	Isopoda	Asellidae	Proasellus sp. type locality of P. gourbaultae
Fusarium fujikuroi IMI 58289 draft genome, chromos	HF679030	77.94	50	0.000221485	Hypocreales	Nectriaceae	Fusarium fujikuroi IMI 58289
Proasellus spelaeus isolate ETCHEVE_200811_1ex_YAS	JQ921811	79.1	49.26	0.000221485	Isopoda	Asellidae	Proasellus spelaeus
Proasellus sp. n. Maglia isolate MAGLIA_200908_1ID	JQ921791	79.1	49.26	0.000221485	Isopoda	Asellidae	Proasellus sp. n. Maglia
Proasellus ebrensis isolate CERECEDA_200906_1_YLK1	JQ921747	79.1	49.26	0.000221485	Isopoda	Asellidae	Proasellus ebrensis
Proasellus coxalis isolate BUNAR_200402_1ID_YLK392	JQ921740	83.64	40.44	0.000221485	Isopoda	Asellidae	Proasellus coxalis
Proasellus beroni isolate ORTAMIN_200810_1ex_YAS42	JQ921715	76.47	61.76	0.000221485	Isopoda	Asellidae	Proasellus beroni
Dracunculus medinensis genome assembly D_medinensi	LK978210	84.31	36.03	0.000773058	Spirurida	Dracunculidae	Dracunculus medinensis
Cyprinus carpio genome assembly common carp genome	LN594801	94.44	25.74	0.000773058	Cypriniformes	Cyprinidae	Cyprinus carpio
Proasellus stocki isolate SAJA_201010_1ID_YCD838 1	JQ921730	72.41	83.82	0.000773058	Isopoda	Asellidae	Proasellus stocki
Julus scandinavius voucher ZMUC200018 16S ribosoma	JF320957	81.48	39.71	0.000773058	Julida	Julidae	Julus scandinavius
Ceropegia stenoloba var. stenoloba clone 142_B61 p	KP245247	86.05	31.62	0.00269824	Gentianales	Apocynaceae	Ceropegia stenoloba var. stenoloba
Brachystelma plocamoides clone 060_B4 photosystem	KP245166	84	36.03	0.00269824	Gentianales	Apocynaceae	Brachystelma plocamoides
Brachystelma nutans clone 056_B16 photosystem II p	KP245162	84	36.03	0.00269824	Gentianales	Apocynaceae	Brachystelma nutans
Candidatus Pantoea carbekii strain US, complete ge	CP010907	83.33	38.24	0.00269824	Enterobacteriales	Enterobacteriaceae	Candidatus Pantoea carbekii
Cyprinus carpio genome assembly common carp genome	LN596409	86.05	31.62	0.00269824	Cypriniformes	Cyprinidae	Cyprinus carpio
Cyprinus carpio genome assembly common carp genome	LN590709	86.67	33.09	0.00269824	Cypriniformes	Cyprinidae	Cyprinus carpio
Cyprinus carpio genome assembly common carp genome	LN590675	78.69	44.85	0.00269824	Cypriniformes	Cyprinidae	Cyprinus carpio
Proasellus sp. n. Verclause isolate s230 16S ribos	KC610320	77.94	50	0.00269824	Isopoda	Asellidae	Proasellus sp. n. Verclause
Proasellus coxalis isolate s193 16S ribosomal RNA	KC610283	81.82	40.44	0.00269824	Isopoda	Asellidae	Proasellus coxalis
Proasellus coxalis isolate s006 16S ribosomal RNA	KC610096	81.82	40.44	0.00269824	Isopoda	Asellidae	Proasellus coxalis
Halyomorpha halys symbiont DNA, complete genome	AP012554	83.33	38.24	0.00269824	Enterobacteriales	Enterobacteriaceae	Candidatus Pantoea carbekii
Proasellus sp. n. Verclause isolate LAPENNE_201002	JQ921797	77.94	50	0.00269824	Isopoda	Asellidae	Proasellus sp. n. Verclause
Proasellus karamani isolate ZALOMSKA_200305_1_YLK3	JQ921766	77.94	50	0.00269824	Isopoda	Asellidae	Proasellus karamani
Proasellus coxalis isolate STENSAN_200811_2ID_YAS4	JQ921743	81.82	40.44	0.00269824	Isopoda	Asellidae	Proasellus coxalis
Proasellus coxalis isolate CASAMOZA_200810_2_YAS39	JQ921739	81.82	40.44	0.00269824	Isopoda	Asellidae	Proasellus coxalis
Proasellus cantabricus isolate RESCANO_200906_1ID_	JQ921719	77.94	50	0.00269824	Isopoda	Asellidae	Proasellus cantabricus
Proasellus aquaecalidae isolate PEAUXCH_200811_1_Y	JQ921704	74.44	65.44	0.00269824	Isopoda	Asellidae	Proasellus aquaecalidae
Proasellus aff. lagari Henry & Magniez, 1982 isola	JQ921700	77.94	50	0.00269824	Isopoda	Asellidae	Proasellus aff. lagari Henry & Magniez, 1982
Bragasellus comasi isolate COBALLE2_200906_1_YLK19	JQ921672	73.4	65.44	0.00269824	Isopoda	Asellidae	Bragasellus comasi
Gossypium raimondii clone GR__Ba0033O07−hvk, compl	AC243111	79.45	50	0.00269824	Malvales	Malvaceae	Gossypium raimondii
Asellus aquaticus mitochondrion, partial genome	GU130252	81.82	40.44	0.00269824	Isopoda	Asellidae	Asellus aquaticus
Vitis vinifera contig VV78X250326.6, whole genome	AM488379	82.14	40.44	0.00269824	Vitales	Vitaceae	Vitis vinifera
Vitis vinifera contig VV78X219905.5, whole genome	AM438122	81.82	39.71	0.00269824	Vitales	Vitaceae	Vitis vinifera
Mus musculus chromosome 13, clone RP23−262I3, comp	AC120347	84.78	33.82	0.00269824	Rodentia	Muridae	Mus musculus
Lirceolus sp. JK−2004 isolate RS19 16S ribosomal R	AY570133	75.82	64.71	0.00269824	Isopoda	Asellidae	Lirceolus sp. Slaughter Bend
Proasellus sp. Netherlands−BH−2002 16S ribosomal R	AF532163	77.94	50	0.00269824	Isopoda	Asellidae	Proasellus sp. Netherlands−BH−2002
Proasellus coxalis 16S ribosomal RNA gene, partial	AF532162	81.82	40.44	0.00269824	Isopoda	Asellidae	Proasellus coxalis
Mus musculus BAC clone RP24−339I11 from chromosome	AC154231	84.78	33.82	0.00269824	Rodentia	Muridae	Mus musculus
Ceropegia stenoloba var. australis clone 141_B84 p	KP245246	83.67	36.03	0.00941777	Gentianales	Apocynaceae	Ceropegia stenoloba var. australis
Brachystelma swazicum clone 069_B138a photosystem	KP245174	83.67	36.03	0.00941777	Gentianales	Apocynaceae	Brachystelma swazicum
Cucumis melo genomic chromosome, chr_12	LN713266	78.57	47.79	0.00941777	Cucurbitales	Cucurbitaceae	Cucumis melo
Cucumis melo genomic scaffold, anchoredscaffold000	LN681931	78.57	47.79	0.00941777	Cucurbitales	Cucurbitaceae	Cucumis melo
Spiranthes ovalis var. erostellata isolate 19c tRN	KM283707	83.67	36.03	0.00941777	Asparagales	Orchidaceae	Spiranthes ovalis var. erostellata
Spiranthes ovalis var. ovalis isolate 18b tRNA−Leu	KM283704	83.67	36.03	0.00941777	Asparagales	Orchidaceae	Spiranthes ovalis var. ovalis
Dracunculus medinensis genome assembly D_medinensi	LK978211	86.36	32.35	0.00941777	Spirurida	Dracunculidae	Dracunculus medinensis
Brugia pahangi genome assembly B_pahangi_Glasgow ,	LK965589	86.36	31.62	0.00941777	Spirurida	Onchocercidae	Brugia pahangi
Soboliphyme baturini genome assembly S_baturini_Da	LM034464	85.11	33.82	0.00941777		Soboliphymatidae	Soboliphyme baturini
Cyprinus carpio genome assembly common carp genome	LN590718	86.36	31.62	0.00941777	Cypriniformes	Cyprinidae	Cyprinus carpio
Cyprinus carpio genome assembly common carp genome	LN590685	86.36	31.62	0.00941777	Cypriniformes	Cyprinidae	Cyprinus carpio
Solanum pennellii chromosome ch01, complete genome	HG975440	89.19	27.21	0.00941777	Solanales	Solanaceae	Solanum pennellii
Proasellus aquaecalidae isolate s198 16S ribosomal	KC610288	74.44	65.44	0.00941777	Isopoda	Asellidae	Proasellus aquaecalidae
Proasellus ebrensis isolate RUDRON_201010_1IDex_YC	JQ921720	77.61	49.26	0.00941777	Isopoda	Asellidae	Proasellus ebrensis
Proasellus aquaecalidae isolate OUEILNEE_200811_2_	JQ921703	74.44	65.44	0.00941777	Isopoda	Asellidae	Proasellus aquaecalidae
Paracymoriza prodigalis mitochondrion, complete ge	JX144892	76.81	50	0.00941777	Lepidoptera	Crambidae	Paracymoriza prodigalis
Acyrthosiphon pisum protein NPC2 homolog (LOC10016	NM_001162654	91.43	25.74	0.00941777	Hemiptera	Aphididae	Acyrthosiphon pisum
Karlodinium veneficum chloroplast, partial genome	JN039300	96.67	22.06	0.00941777	Gymnodiniales	Kareniaceae	Karlodinium veneficum
Ceropegia radicans subsp. smithii PsbA (psbA) gene	HM475456	86.05	31.62	0.00941777	Gentianales	Apocynaceae	Ceropegia radicans subsp. smithii
Acyrthosiphon pisum ACYPI002949 mRNA, clone: 408O7	AK339787	91.43	25.74	0.00941777	Hemiptera	Aphididae	Acyrthosiphon pisum
Zebrafish DNA sequence from clone CH73−23B23 in li	CU914775	88.64	31.62	0.00941777	Cypriniformes	Cyprinidae	Danio rerio
Glycine max strain Williams 82 clone GM_WBb0099N18	AC235362	87.5	29.41	0.00941777	Fabales	Fabaceae	Glycine max
Plasmodium falciparum 3D7 chromosome 6	AL844505	84.31	36.76	0.00941777	Haemosporida	Plasmodiidae	Plasmodium falciparum 3D7
Candidatus Phytoplasma mali strain AT complete chr	CU469464	80	47.06	0.00941777	Acholeplasmatales	Acholeplasmataceae	Candidatus Phytoplasma mali
Vitis vinifera contig VV78X264876.10, whole genome	AM449904	83.67	36.03	0.00941777	Vitales	Vitaceae	Vitis vinifera
Zebrafish DNA sequence from clone CH73−373E20 in l	CU062504	84.44	33.09	0.00941777	Cypriniformes	Cyprinidae	Danio rerio
Pan troglodytes BAC clone CH251−575K15 from chromo	AC191730	86.36	31.62	0.00941777	Primates	Hominidae	Pan troglodytes
Populus trichocarpa clone Pop1−26A1, complete sequ	AC182673	91.89	26.47	0.00941777	Malpighiales	Salicaceae	Populus trichocarpa
CH251−575K15, complete sequence	CT806410	86.36	31.62	0.00941777	Primates	Hominidae	Pan troglodytes
Homo sapiens X BAC RP13−106B4 (Roswell Park Cancer	AC117518	86.36	31.62	0.00941777	Primates	Hominidae	Homo sapiens
Homo sapiens chromosome 10 clone RP11−295O23, comp	AC011328	89.74	28.68	0.00941777	Primates	Hominidae	Homo sapiens
Homo sapiens Xp22−166−169 GSHB−523A23 (Genome Syst	AC005145	86.36	31.62	0.00941777	Primates	Hominidae	Homo sapiens
RP43−058A24, complete sequence	CT573089	86.36	31.62	0.00941777	Primates	Hominidae	Pan troglodytes
Homo sapiens chromosome 19 clone CTC−461H2, comple	AC008521	81.36	42.65	0.00941777	Primates	Hominidae	Homo sapiens
Proasellus ebrensis isolate MARFIL_200906_1ID_YAS8	JQ921748	73.13	49.26	0.400454	Isopoda	Asellidae	Proasellus ebrensis
