# Supplementary material for: Testing the potential of a ribosomal 16S marker for DNA metabarcoding of insects
Source: PeerJ. 2016 Apr 19;4:e1966. doi: 10.7717/peerj.1966 (PMC4841222; doi:10.7717/peerj.1966)
Supplement: Supplemental Information 1 [file peerj-04-1966-s006.zip › scripts/5 match OTUs/blast_hits/OTU_115.csv_plot.pdf]

OTU_115.csv

Aporrectodea rosea

Lumbricidae

Haplotaxida

100

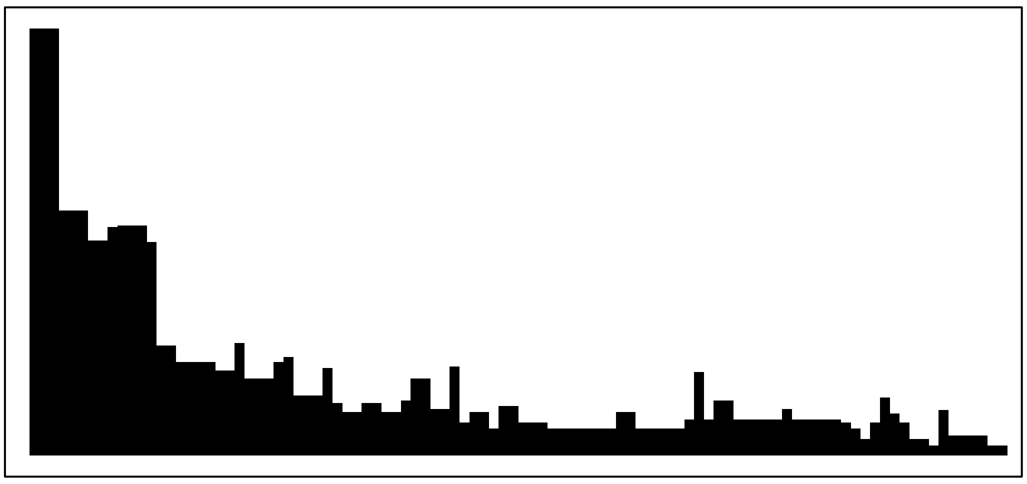

Aporrectodea rosea voucher 0301 16S ribosomal RNA	JN869773	100	100	1.28361e−57	Haplotaxida	Lumbricidae	Aporrectodea rosea
Aporrectodea rosea mitochondrial partial 16S rRNA	HE611648	100	100	1.28361e−57	Haplotaxida	Lumbricidae	Aporrectodea rosea
Aporrectodea rosea isolate MPL55 16S ribosomal RNA	FJ967642	100	100	1.28361e−57	Haplotaxida	Lumbricidae	Aporrectodea rosea
Aporrectodea rosea isolate MPL245 16S ribosomal RN	KJ912516	91.47	100	7.10569e−42	Haplotaxida	Lumbricidae	Aporrectodea rosea
Aporrectodea rosea voucher 0306 16S ribosomal RNA	JN869777	91.47	100	7.10569e−42	Haplotaxida	Lumbricidae	Aporrectodea rosea
Aporrectodea rosea mitochondrial partial 16S rRNA	HE611649	91.47	100	7.10569e−42	Haplotaxida	Lumbricidae	Aporrectodea rosea
Aporrectodea rosea isolate MPL428 16S ribosomal RN	KJ912517	90.08	100	8.65651e−41	Haplotaxida	Lumbricidae	Aporrectodea rosea
Aporrectodea rosea voucher 0302 16S ribosomal RNA	JN869774	90.08	100	8.65651e−41	Haplotaxida	Lumbricidae	Aporrectodea rosea
Aporrectodea rosea mitochondrial partial 16S rRNA	HE611653	90.7	100	8.65651e−41	Haplotaxida	Lumbricidae	Aporrectodea rosea
Aporrectodea rosea voucher 0304 16S ribosomal RNA	JN869775	90.77	100	3.02142e−40	Haplotaxida	Lumbricidae	Aporrectodea rosea
Aporrectodea rosea mitochondrial partial 16S rRNA	HE611651	90.77	100	3.02142e−40	Haplotaxida	Lumbricidae	Aporrectodea rosea
Aporrectodea rosea mitochondrial partial 16S rRNA	HE611650	90.77	100	3.02142e−40	Haplotaxida	Lumbricidae	Aporrectodea rosea
Aporrectodea rosea mitochondrial partial 16S rRNA	HE611652	90	100	1.28474e−38	Haplotaxida	Lumbricidae	Aporrectodea rosea
Allolobophora leoni isolate MPL410 16S ribosomal R	KJ912584	85.16	100	1.46589e−31	Haplotaxida	Lumbricidae	Allolobophora leoni
Allolobophora leoni isolate MPL330 16S ribosomal R	KJ912583	85.16	100	1.46589e−31	Haplotaxida	Lumbricidae	Allolobophora leoni
Allolobophora chlorotica voucher 0102 16S ribosoma	JN869756	84.38	100	1.78582e−30	Haplotaxida	Lumbricidae	Allolobophora chlorotica
Allolobophora chlorotica mitochondrial partial 16S	AM774389	84.38	100	1.78582e−30	Haplotaxida	Lumbricidae	Allolobophora chlorotica
Allolobophora chlorotica mitochondrial partial 16S	AM774375	84.38	100	1.78582e−30	Haplotaxida	Lumbricidae	Allolobophora chlorotica
Allolobophora chlorotica mitochondrial partial 16S	AM774374	84.38	100	1.78582e−30	Haplotaxida	Lumbricidae	Allolobophora chlorotica
Aporrectodea rosea isolate MPL447 16S ribosomal RN	KJ912518	83.97	100	6.23311e−30	Haplotaxida	Lumbricidae	Aporrectodea rosea
Aporrectodea rosea 16S ribosomal RNA gene, partial	JN794591	83.97	100	6.23311e−30	Haplotaxida	Lumbricidae	Aporrectodea rosea
Allolobophora chlorotica mitochondrial partial 16S	AM774369	85.27	100	6.23311e−30	Haplotaxida	Lumbricidae	Allolobophora chlorotica
Allolobophora chlorotica mitochondrial partial 16S	AM774391	83.59	100	7.59348e−29	Haplotaxida	Lumbricidae	Allolobophora chlorotica
Allolobophora chlorotica mitochondrial partial 16S	AM774388	83.59	100	7.59348e−29	Haplotaxida	Lumbricidae	Allolobophora chlorotica
Allolobophora chlorotica mitochondrial partial 16S	AM774387	83.59	100	7.59348e−29	Haplotaxida	Lumbricidae	Allolobophora chlorotica
Eisenia fetida isolate H33 16S ribosomal RNA gene,	JQ739934	84.38	100	2.65039e−28	Haplotaxida	Lumbricidae	Eisenia fetida
Allolobophora chlorotica mitochondrial partial 16S	AM774370	84.62	100	2.65039e−28	Haplotaxida	Lumbricidae	Allolobophora chlorotica
Allolobophora chlorotica isolate MPL257 16S riboso	KJ912502	82.81	100	9.25075e−28	Haplotaxida	Lumbricidae	Allolobophora chlorotica
Aporrectodea icterica mitochondrial partial 16S rR	HE611656	82.81	100	9.25075e−28	Haplotaxida	Lumbricidae	Aporrectodea icterica
Allolobophora chlorotica mitochondrial partial 16S	AM774360	82.81	100	9.25075e−28	Haplotaxida	Lumbricidae	Allolobophora chlorotica
Aporrectodea cupulifera mitochondrial partial 16S	HE611658	84.09	100	3.22883e−27	Haplotaxida	Lumbricidae	Aporrectodea cupulifera
Aporrectodea tuberculata voucher 0203 16S ribosoma	JN869761	82.44	100	3.93352e−26	Haplotaxida	Lumbricidae	Aporrectodea tuberculata
Aporrectodea icterica mitochondrial partial 16S rR	HE611655	82.03	100	3.93352e−26	Haplotaxida	Lumbricidae	Aporrectodea icterica
Allolobophora chlorotica mitochondrial partial 16S	HE611654	82.03	100	3.93352e−26	Haplotaxida	Lumbricidae	Allolobophora chlorotica
Aporrectodea tuberculata isolate MPL74 16S ribosom	FJ967653	82.44	100	3.93352e−26	Haplotaxida	Lumbricidae	Aporrectodea tuberculata
Aporrectodea tuberculata isolate MPL73 16S ribosom	FJ967652	82.44	100	3.93352e−26	Haplotaxida	Lumbricidae	Aporrectodea tuberculata
Allolobophora chlorotica mitochondrial partial 16S	AM774381	82.03	100	3.93352e−26	Haplotaxida	Lumbricidae	Allolobophora chlorotica
Allolobophora chlorotica mitochondrial partial 16S	AM774371	82.03	100	3.93352e−26	Haplotaxida	Lumbricidae	Allolobophora chlorotica
Proctodrilus antipai isolate MPL333 16S ribosomal	KJ912585	82.58	100	1.37293e−25	Haplotaxida	Lumbricidae	Proctodrilus antipai
Eisenia fetida isolate H38 16S ribosomal RNA gene,	JQ739939	83.59	100	1.37293e−25	Haplotaxida	Lumbricidae	Eisenia fetida
Eisenia fetida isolate H35 16S ribosomal RNA gene,	JQ739936	83.59	100	1.37293e−25	Haplotaxida	Lumbricidae	Eisenia fetida
Aporrectodea longa isolate MPL93 16S ribosomal RNA	FJ967663	82.17	100	1.37293e−25	Haplotaxida	Lumbricidae	Aporrectodea longa
Aporrectodea longa isolate MPL60 16S ribosomal RNA	FJ967646	82.17	100	1.37293e−25	Haplotaxida	Lumbricidae	Aporrectodea longa
Aporrectodea tuberculata isolate MPL56 16S ribosom	FJ967643	84.17	91.41	1.37293e−25	Haplotaxida	Lumbricidae	Aporrectodea tuberculata
Aporrectodea jassyensis isolate MPL404 16S ribosom	KJ912510	81.54	100	4.79201e−25	Haplotaxida	Lumbricidae	Aporrectodea jassyensis
Allolobophora chlorotica isolate MPL418 16S riboso	KJ912505	82.03	100	4.79201e−25	Haplotaxida	Lumbricidae	Allolobophora chlorotica
Allolobophora chlorotica isolate MPL258 16S riboso	KJ912503	82.03	100	4.79201e−25	Haplotaxida	Lumbricidae	Allolobophora chlorotica
Allolobophora chlorotica isolate MPL388 16S riboso	KJ912501	81.25	100	4.79201e−25	Haplotaxida	Lumbricidae	Allolobophora chlorotica
Aporrectodea trapezoides isolate MPL92 16S ribosom	FJ967662	82.31	100	4.79201e−25	Haplotaxida	Lumbricidae	Aporrectodea trapezoides
Aporrectodea trapezoides isolate MPL89 16S ribosom	FJ967661	82.31	100	4.79201e−25	Haplotaxida	Lumbricidae	Aporrectodea trapezoides
Aporrectodea caliginosa isolate MPL154 16S ribosom	FJ967626	81.54	100	4.79201e−25	Haplotaxida	Lumbricidae	Aporrectodea caliginosa
Aporrectodea caliginosa isolate MPL153 16S ribosom	FJ967625	81.54	100	4.79201e−25	Haplotaxida	Lumbricidae	Aporrectodea caliginosa
Aporrectodea caliginosa isolate MPL141 16S ribosom	FJ967619	81.54	100	4.79201e−25	Haplotaxida	Lumbricidae	Aporrectodea caliginosa
Allolobophora chlorotica mitochondrial partial 16S	AM774392	81.25	100	4.79201e−25	Haplotaxida	Lumbricidae	Allolobophora chlorotica
Allolobophora chlorotica mitochondrial partial 16S	AM774390	81.25	100	4.79201e−25	Haplotaxida	Lumbricidae	Allolobophora chlorotica
Allolobophora chlorotica mitochondrial partial 16S	AM774385	81.25	100	4.79201e−25	Haplotaxida	Lumbricidae	Allolobophora chlorotica
Allolobophora chlorotica mitochondrial partial 16S	AM774384	81.25	100	4.79201e−25	Haplotaxida	Lumbricidae	Allolobophora chlorotica
Allolobophora chlorotica mitochondrial partial 16S	AM774383	81.25	100	4.79201e−25	Haplotaxida	Lumbricidae	Allolobophora chlorotica
Allolobophora chlorotica mitochondrial partial 16S	AM774378	81.25	100	4.79201e−25	Haplotaxida	Lumbricidae	Allolobophora chlorotica
Allolobophora chlorotica mitochondrial partial 16S	AM774372	81.25	100	4.79201e−25	Haplotaxida	Lumbricidae	Allolobophora chlorotica
Allolobophora chlorotica mitochondrial partial 16S	AM774368	82.03	100	4.79201e−25	Haplotaxida	Lumbricidae	Allolobophora chlorotica
Allolobophora chlorotica mitochondrial partial 16S	AM774367	82.03	100	4.79201e−25	Haplotaxida	Lumbricidae	Allolobophora chlorotica
Allolobophora chlorotica mitochondrial partial 16S	AM774366	81.25	100	4.79201e−25	Haplotaxida	Lumbricidae	Allolobophora chlorotica
Allolobophora chlorotica mitochondrial partial 16S	AM774364	81.25	100	4.79201e−25	Haplotaxida	Lumbricidae	Allolobophora chlorotica
Allolobophora chlorotica mitochondrial partial 16S	AM774363	81.25	100	4.79201e−25	Haplotaxida	Lumbricidae	Allolobophora chlorotica
Allolobophora chlorotica mitochondrial partial 16S	AM774361	81.25	100	4.79201e−25	Haplotaxida	Lumbricidae	Allolobophora chlorotica
Allolobophora chlorotica mitochondrial partial 16S	AM774359	81.25	100	4.79201e−25	Haplotaxida	Lumbricidae	Allolobophora chlorotica
Octolasion lacteum isolate MPL260 16S ribosomal RN	KJ912579	81.68	100	1.67258e−24	Haplotaxida	Lumbricidae	Octolasion lacteum
Healyella jordanis isolate MPL316 16S ribosomal RN	KJ912560	83.9	90.62	1.67258e−24	Haplotaxida	Lumbricidae	Healyella jordanis
Aporrectodea georgii isolate MPL413 16S ribosomal	KJ912511	81.68	100	1.67258e−24	Haplotaxida	Lumbricidae	Aporrectodea georgii
Octolasion lacteum voucher 1721 16S ribosomal RNA	JN869843	82.58	100	1.67258e−24	Haplotaxida	Lumbricidae	Octolasion lacteum
Octolasion lacteum voucher 1706 16S ribosomal RNA	JN869836	82.58	100	1.67258e−24	Haplotaxida	Lumbricidae	Octolasion lacteum
Aporrectodea tuberculata voucher 0211 16S ribosoma	JN869769	81.68	100	1.67258e−24	Haplotaxida	Lumbricidae	Aporrectodea tuberculata
Aporrectodea tuberculata voucher 0210 16S ribosoma	JN869768	81.68	100	1.67258e−24	Haplotaxida	Lumbricidae	Aporrectodea tuberculata
Aporrectodea tuberculata voucher 0209 16S ribosoma	JN869767	81.68	100	1.67258e−24	Haplotaxida	Lumbricidae	Aporrectodea tuberculata
Aporrectodea tuberculata voucher 0205 16S ribosoma	JN869763	81.68	100	1.67258e−24	Haplotaxida	Lumbricidae	Aporrectodea tuberculata
Aporrectodea tuberculata voucher 0202 16S ribosoma	JN869760	81.68	100	1.67258e−24	Haplotaxida	Lumbricidae	Aporrectodea tuberculata
Lumbricus meliboeus mitochondrial partial 16S rRNA	HE611672	82.17	100	1.67258e−24	Haplotaxida	Lumbricidae	Lumbricus meliboeus
Aporrectodea tuberculata isolate MPL96 16S ribosom	FJ967665	81.68	100	1.67258e−24	Haplotaxida	Lumbricidae	Aporrectodea tuberculata
Aporrectodea tuberculata isolate MPL68 16S ribosom	FJ967651	81.68	100	1.67258e−24	Haplotaxida	Lumbricidae	Aporrectodea tuberculata
Aporrectodea tuberculata isolate MPL62 16S ribosom	FJ967648	81.68	100	1.67258e−24	Haplotaxida	Lumbricidae	Aporrectodea tuberculata
Octolasion tyrtaeum isolate 3C 16S ribosomal RNA g	DQ257301	81.68	100	1.67258e−24	Haplotaxida	Lumbricidae	Octolasion tyrtaeum
Octolasion tyrtaeum isolate 2C 16S ribosomal RNA g	DQ257300	81.68	100	1.67258e−24	Haplotaxida	Lumbricidae	Octolasion tyrtaeum
Octolasion lacteum isolate MPL411 16S ribosomal RN	KJ912581	81.54	100	5.83786e−24	Haplotaxida	Lumbricidae	Octolasion lacteum
Allolobophora chlorotica isolate MPL387 16S riboso	KJ912500	81.25	100	5.83786e−24	Haplotaxida	Lumbricidae	Allolobophora chlorotica
Aporrectodea trapezoides isolate D1EL1 16S ribosom	KC557022	80.77	100	5.83786e−24	Haplotaxida	Lumbricidae	Aporrectodea trapezoides
Octolasion lacteum voucher 1725 16S ribosomal RNA	JN869847	81.54	100	5.83786e−24	Haplotaxida	Lumbricidae	Octolasion lacteum
Octolasion lacteum voucher 1722 16S ribosomal RNA	JN869844	82.71	100	5.83786e−24	Haplotaxida	Lumbricidae	Octolasion lacteum
Octolasion lacteum voucher 1717 16S ribosomal RNA	JN869839	81.95	100	5.83786e−24	Haplotaxida	Lumbricidae	Octolasion lacteum
Octolasion lacteum voucher 1707 16S ribosomal RNA	JN869837	81.54	100	5.83786e−24	Haplotaxida	Lumbricidae	Octolasion lacteum
Aporrectodea nocturna isolate MPL306 16S ribosomal	FJ967629	80.77	100	5.83786e−24	Haplotaxida	Lumbricidae	Aporrectodea nocturna
Aporrectodea caliginosa isolate MPL143 16S ribosom	FJ967620	80.77	100	5.83786e−24	Haplotaxida	Lumbricidae	Aporrectodea caliginosa
Aporrectodea moebii isolate MPL349 16S ribosomal R	KJ912513	80.47	100	2.03761e−23	Haplotaxida	Lumbricidae	Aporrectodea moebii
Aporrectodea smaragdina voucher 4201 16S ribosomal	JN869757	82.11	92.19	2.03761e−23	Haplotaxida	Lumbricidae	Aporrectodea smaragdina
Aporrectodea longa mitochondrial partial 16S rRNA	HE611664	80.92	100	2.03761e−23	Haplotaxida	Lumbricidae	Aporrectodea longa
Aporrectodea longa isolate MPL308 16S ribosomal RN	FJ967631	80.92	100	2.03761e−23	Haplotaxida	Lumbricidae	Aporrectodea longa
Aporrectodea caliginosa isolate MPL150 16S ribosom	FJ967624	80.92	100	2.03761e−23	Haplotaxida	Lumbricidae	Aporrectodea caliginosa
Aporrectodea caliginosa isolate MPL149 16S ribosom	FJ967623	80.92	100	2.03761e−23	Haplotaxida	Lumbricidae	Aporrectodea caliginosa
Allolobophora chlorotica mitochondrial partial 16S	AM774393	80.47	100	2.03761e−23	Haplotaxida	Lumbricidae	Allolobophora chlorotica
Allolobophora chlorotica mitochondrial partial 16S	AM774380	80.47	100	2.03761e−23	Haplotaxida	Lumbricidae	Allolobophora chlorotica
