# Supplementary material for: Testing the potential of a ribosomal 16S marker for DNA metabarcoding of insects
Source: PeerJ. 2016 Apr 19;4:e1966. doi: 10.7717/peerj.1966 (PMC4841222; doi:10.7717/peerj.1966)
Supplement: Supplemental Information 1 [file peerj-04-1966-s006.zip › scripts/5 match OTUs/blast_hits/OTU_116.csv_plot.pdf]

# OTU_116.csv

## Strongyloides stercoralis

## Strongyloididae

## Rhabditida

## 71.01

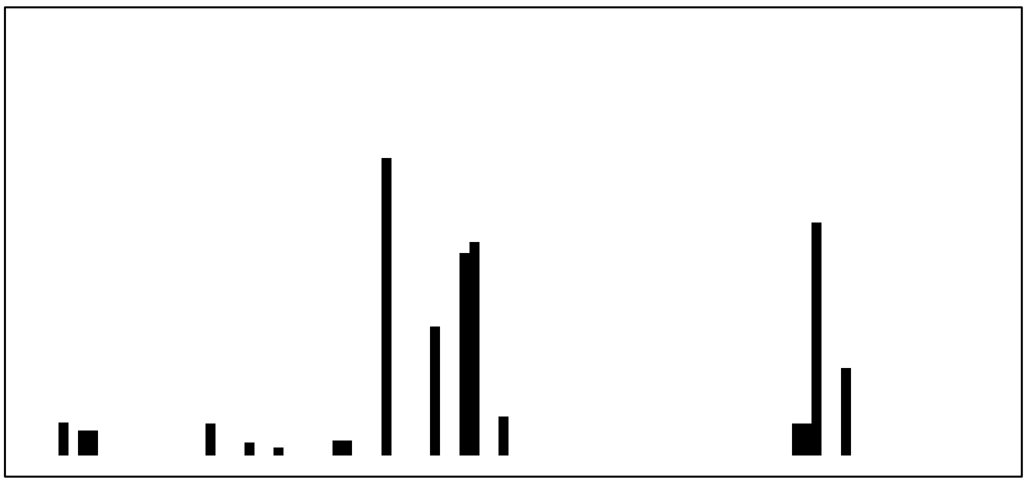

| | | | | | | | |
|---|---|---|---|---|---|---|---|
| Strongyloides stercoralis genome assembly S_sterco | LL999055 | 71.01 | 87.59 | 1.62533e−06 | Rhabditida | Strongyloididae | Strongyloides stercoralis |
| Cyprinus carpio genome assembly common carp genome | LN591245 | 73.61 | 93.1 | 1.98008e−05 | Cypriniformes | Cyprinidae | Cyprinus carpio |
| Candidatus Zinderia insecticola CARI, complete gen | CP002161 | 71.67 | 82.07 | 6.9111e−05 | Burkholderiales | Oxalobacteraceae | Candidatus Zinderia insecticola CARI |
| Strongyloides stercoralis genome assembly S_sterco | LL999054 | 81.54 | 43.45 | 0.000241221 | Rhabditida | Strongyloididae | Strongyloides stercoralis |
| Vitis vinifera contig VV78X250204.3, whole genome | AM435003 | 70.86 | 96.55 | 0.000241221 | Vitales | Vitaceae | Vitis vinifera |
| Cucumis melo genomic chromosome, chr_1 | LN713255 | 81.16 | 44.83 | 0.000841944 | Cucurbitales | Cucurbitaceae | Cucumis melo |
| Cucumis melo genomic scaffold, anchoredscaffold000 | LN681792 | 81.16 | 44.83 | 0.000841944 | Cucurbitales | Cucurbitaceae | Cucumis melo |
| Strongyloides ratti genome assembly S_ratti_ED321 | LN609396 | 76.14 | 58.62 | 0.000841944 | Rhabditida | Strongyloididae | Strongyloides ratti |
| Barnettozyma californica strain CBS 252 mitochondr | KC993183 | 71.43 | 96.55 | 0.000841944 | Saccharomycetales | Phaffomycetaceae | Barnettozyma californica |
| Dracunculus medinensis genome assembly D_medinensi | LK978196 | 69.86 | 94.48 | 0.00293867 | Spirurida | Dracunculidae | Dracunculus medinensis |
| Phytophthora alticola isolate P16053 SecY (secY) g | JF770686 | 79.1 | 46.21 | 0.00293867 | Peronosporales | | Phytophthora alticola |
| Phytophthora frigida isolate P16059 SecY (secY) ge | JF770489 | 79.1 | 46.21 | 0.00293867 | Peronosporales | | Phytophthora frigida |
| Zebrafish DNA sequence from clone CH73−348D8 in li | FQ311917 | 70.14 | 97.24 | 0.00293867 | Cypriniformes | Cyprinidae | Danio rerio |
| Chrysozephyrus smaragdinus 16S ribosomal RNA gene, | GU372446 | 70.07 | 97.24 | 0.00293867 | Lepidoptera | Lycaenidae | Chrysozephyrus smaragdinus |
| Zebrafish DNA sequence from clone CH73−151L10 in l | FP102802 | 70.14 | 97.24 | 0.00293867 | Cypriniformes | Cyprinidae | Danio rerio |
| Candida castellii complete mitochondrial genome, s | FM995165 | 72.58 | 84.14 | 0.00293867 | Saccharomycetales | Saccharomycetaceae | Candida castellii |
| Plasmodium falciparum 3D7 chromosome 6 | AL844505 | 77.03 | 51.03 | 0.00293867 | Haemosporida | Plasmodiidae | Plasmodium falciparum 3D7 |
| Plasmodium falciparum strain 3D7, chromosome 1 | AL844501 | 71.63 | 94.48 | 0.00293867 | Haemosporida | Plasmodiidae | Plasmodium falciparum 3D7 |
| Spiroplasma litorale strain TN−1, complete genome | CP012357 | 81.48 | 36.55 | 0.010257 | Entomoplasmatales | Spiroplasmataceae | Spiroplasma litorale |
| Botrytis cinerea B05.10 chromosome 15, complete se | CP009819 | 74.68 | 53.79 | 0.010257 | Helotiales | Sclerotiniaceae | Botrytis cinerea B05.10 |
| Ichneumonidae sp. MT−2014 isolate CL118 mitochondr | KM244711 | 77.46 | 43.45 | 0.010257 | Hymenoptera | Ichneumonidae | Ichneumonidae sp. MT−2014 |
| Dracunculus medinensis genome assembly D_medinensi | LK978189 | 70.37 | 89.66 | 0.010257 | Spirurida | Dracunculidae | Dracunculus medinensis |
| Strongyloides ratti genome assembly S_ratti_ED321 | LN609530 | 80.6 | 46.21 | 0.010257 | Rhabditida | Strongyloididae | Strongyloides ratti |
| Cyprinus carpio genome assembly common carp genome | LN593469 | 78.79 | 45.52 | 0.010257 | Cypriniformes | Cyprinidae | Cyprinus carpio |
| Brassica rapa subsp. pekinensis clone KBrB022F09, | AC240998 | 76.39 | 48.28 | 0.010257 | Brassicales | Brassicaceae | Brassica rapa subsp. pekinensis |
| Candidatus Sulcia muelleri DMIN, complete genome | CP001981 | 80.36 | 38.62 | 0.010257 | Flavobacteriales | | Candidatus Sulcia muelleri DMIN |
| Brassica rapa subsp. pekinensis clone KBrH006C14, | AC189554 | 76.39 | 48.28 | 0.010257 | Brassicales | Brassicaceae | Brassica rapa subsp. pekinensis |
| Zebrafish DNA sequence from clone CH211−206N3 in l | CT009597 | 69.92 | 88.28 | 0.010257 | Cypriniformes | Cyprinidae | Danio rerio |
| Zebrafish DNA sequence from clone DKEY−197I8 in li | CR388181 | 68.09 | 97.24 | 0.010257 | Cypriniformes | Cyprinidae | Danio rerio |
| F.caudatus 16S rRNA gene, partial | Z93695 | 70.47 | 93.1 | 0.010257 | Hymenoptera | Braconidae | Fopius caudatus |
| Epipremnum aureum chloroplast, complete genome | KR872391 | 72.39 | 88.28 | 0.0358004 | Alismatales | Araceae | Epipremnum aureum |
| Cucumis melo genomic chromosome, chr_6 | LN713260 | 80.7 | 37.24 | 0.0358004 | Cucurbitales | Cucurbitaceae | Cucumis melo |
| Cucumis melo genomic scaffold, anchoredscaffold000 | LN681851 | 80.7 | 37.24 | 0.0358004 | Cucurbitales | Cucurbitaceae | Cucumis melo |
| Fusarium graminearum chromosome 1, complete genome | HG970332 | 73.08 | 70.34 | 0.0358004 | Hypocreales | Nectriaceae | Fusarium graminearum |
| Fusarium graminearum chromosome 2, complete genome | HG970333 | 69.23 | 96.55 | 0.0358004 | Hypocreales | Nectriaceae | Fusarium graminearum |
| Protopolystoma xenopodis genome assembly P_xenopod | LM884722 | 76.62 | 50.34 | 0.0358004 | Polyopisthocotylea | Polystomatidae | Protopolystoma xenopodis |
| Schistosoma curassoni genome assembly S_curassoni_ | LM070743 | 93.94 | 22.07 | 0.0358004 | Strigeidida | Schistosomatidae | Schistosoma curassoni |
| Heligmosomoides polygyrus genome assembly H_bakeri | LL216568 | 69.5 | 92.41 | 0.0358004 | Rhabditida | Heligmosomatidae | Heligmosomoides polygyrus |
| Dracunculus medinensis genome assembly D_medinensi | LK978218 | 69.06 | 91.72 | 0.0358004 | Spirurida | Dracunculidae | Dracunculus medinensis |
| Strongyloides ratti genome assembly S_ratti_ED321 | LN609529 | 70.54 | 83.45 | 0.0358004 | Rhabditida | Strongyloididae | Strongyloides ratti |
| Cameraria ohridella mitochondrion, partial genome | KJ508042 | 75.71 | 46.9 | 0.0358004 | Lepidoptera | Gracillariidae | Cameraria ohridella |
| Solanum lycopersicum chromosome ch03, complete gen | HG975515 | 86.05 | 29.66 | 0.0358004 | Solanales | Solanaceae | Solanum lycopersicum |
| Solanum pennellii chromosome ch09, complete genome | HG975448 | 72.18 | 86.21 | 0.0358004 | Solanales | Solanaceae | Solanum pennellii |
| Candidatus Sulcia muelleri PSPU DNA, complete geno | AP013293 | 73.56 | 57.93 | 0.0358004 | Flavobacteriales | | Candidatus Sulcia muelleri PSPU |
| Emticicia oligotrophica DSM 17448, complete genome | CP002961 | 89.47 | 26.21 | 0.0358004 | Cytophagales | Cytophagaceae | Emticicia oligotrophica DSM 17448 |
| Flavobacterium branchiophilum FL−15, complete geno | FQ859183 | 90 | 27.59 | 0.0358004 | Flavobacteriales | Flavobacteriaceae | Flavobacterium branchiophilum FL−15 |
| Plasmodium falciparum 3D7 chromosome 9 | AL844508 | 75.34 | 48.28 | 0.0358004 | Haemosporida | Plasmodiidae | Plasmodium falciparum 3D7 |
| Plasmodium falciparum 3D7 chromosome 5 | AL844504 | 77.63 | 51.72 | 0.0358004 | Haemosporida | Plasmodiidae | Plasmodium falciparum 3D7 |
| M.truncatula DNA sequence from clone MTH2−67A13 on | CT962508 | 81.82 | 36.55 | 0.0358004 | Fabales | Fabaceae | Medicago truncatula |
| Homo sapiens 3 BAC RP11−392H18 (Roswell Park Cance | AC009520 | 79.41 | 46.21 | 0.0358004 | Primates | Hominidae | Homo sapiens |
| Zebrafish DNA sequence from clone DKEY−34F16 in li | BX248390 | 77.27 | 45.52 | 0.0358004 | Cypriniformes | Cyprinidae | Danio rerio |
| Cloning vector pDXA−GST | AJ510166 | 70.08 | 83.45 | 0.0358004 | | | Cloning vector pDXA−GST |
| Cloning vector pDXA−3FLAG | AJ510165 | 70.08 | 83.45 | 0.0358004 | | | Cloning vector pDXA−3FLAG |
| Cloning vector pDXA−3strep | AJ510164 | 70.08 | 83.45 | 0.0358004 | | | Cloning vector pDXA−3strep |
| Cloning vector pDXA−MCS−YFP | AJ510163 | 70.08 | 83.45 | 0.0358004 | | | Cloning vector pDXA−MCS−YFP |
| Cloning vector pDXA−YFP−NotI | AJ510162 | 70.08 | 83.45 | 0.0358004 | | | Cloning vector pDXA−YFP−NotI |
| Cloning vector pDXA−YFP−MCS | AJ510161 | 70.08 | 83.45 | 0.0358004 | | | Cloning vector pDXA−YFP−MCS |
| Cloning vector pDXA−CFP−MCS | AJ510160 | 70.08 | 83.45 | 0.0358004 | | | Cloning vector pDXA−CFP−MCS |
| Cloning vector pDXA−yFP | AJ510159 | 70.08 | 83.45 | 0.0358004 | | | Cloning vector pDXA−yFP |
| Cloning vector pDXA−CFP | AJ510158 | 70.08 | 83.45 | 0.0358004 | | | Cloning vector pDXA−CFP |
| Cloning vector pDXA−GFP2, complete sequence | AF269235 | 70.08 | 83.45 | 0.0358004 | | | Cloning vector pDXA−GFP2 |
| Homo sapiens, clone RP11−99A1, complete sequence | AC007673 | 78.38 | 48.28 | 0.0358004 | Primates | Hominidae | Homo sapiens |
| Cloning vector pDXA−FLAG, complete sequence | AF269236 | 70.08 | 83.45 | 0.0358004 | | | Cloning vector pDXA−FLAG |
| Dictyostelium discoideum actin A−6 gene, 5' flank | M29116 | 70.08 | 83.45 | 0.0358004 | Dictyosteliida | | Dictyostelium discoideum |
| Artificial sequences cloning vector DNA pDXD−3H | X85120 | 70.08 | 83.45 | 0.0358004 | | | synthetic construct |
| Artificial sequences cloning vector DNA pDXD−3C | X85121 | 70.08 | 83.45 | 0.0358004 | | | synthetic construct |
| Artificial sequences cloning vector DNA pDXA−HY | X85122 | 70.08 | 83.45 | 0.0358004 | | | synthetic construct |
| Artificial sequences cloning vector DNA pDXA−HC | X85123 | 70.08 | 83.45 | 0.0358004 | | | synthetic construct |
| Artificial sequences cloning vector DNA pDXA−3H | X85119 | 70.08 | 83.45 | 0.0358004 | | | synthetic construct |
| Artificial sequences cloning vector DNA pDXA−3C | X85118 | 70.08 | 83.45 | 0.0358004 | | | synthetic construct |
| Ovis canadensis canadensis isolate 43U chromosome | CP011893 | 75 | 49.66 | 0.124956 | | Bovidae | Ovis canadensis canadensis |
| Strongyloides venezuelensis genome assembly S_vene | LM524998 | 70.77 | 87.59 | 0.124956 | Rhabditida | Strongyloididae | Strongyloides venezuelensis |
| Francisella guangzhouensis strain 08HL01032, compl | CP010427 | 76.67 | 41.38 | 0.124956 | Thiotrichales | Francisellaceae | Francisella guangzhouensis |
| Sigalphus bicolor mitochondrion, partial genome | KF385878 | 75.68 | 50.34 | 0.124956 | Hymenoptera | Braconidae | Sigalphus bicolor |
| Pambolus sp. QL−2013 mitochondrion, partial genome | KF385875 | 69.3 | 75.86 | 0.124956 | Hymenoptera | Braconidae | Pambolus sp. QL−2013 |
| PREDICTED: Camelina sativa ATP−dependent DNA helic | XM_010481052 | 78.12 | 43.45 | 0.124956 | Brassicales | Brassicaceae | Camelina sativa |
| Strongyloides stercoralis genome assembly S_sterco | LL999073 | 78.46 | 44.14 | 0.124956 | Rhabditida | Strongyloididae | Strongyloides stercoralis |
| Strongyloides stercoralis genome assembly S_sterco | LL999051 | 70.83 | 80 | 0.124956 | Rhabditida | Strongyloididae | Strongyloides stercoralis |
| Brugia pahangi genome assembly B_pahangi_Glasgow , | LK964287 | 81.48 | 37.24 | 0.124956 | Spirurida | Onchocercidae | Brugia pahangi |
| Brugia timori genome assembly B_timori_Indonesia_v | LK919421 | 81.48 | 37.24 | 0.124956 | Spirurida | Onchocercidae | Brugia timori |
| Strongyloides ratti genome assembly S_ratti_ED321 | LN609528 | 90.91 | 22.76 | 0.124956 | Rhabditida | Strongyloididae | Strongyloides ratti |
| Strongyloides ratti genome assembly S_ratti_ED321 | LN609402 | 72.09 | 86.21 | 0.124956 | Rhabditida | Strongyloididae | Strongyloides ratti |
| Cyprinus carpio genome assembly common carp genome | LN594801 | 75.68 | 50.34 | 0.124956 | Cypriniformes | Cyprinidae | Cyprinus carpio |
| Cyprinus carpio genome assembly common carp genome | LN591896 | 84.09 | 28.97 | 0.124956 | Cypriniformes | Cyprinidae | Cyprinus carpio |
| Cyprinus carpio genome assembly common carp genome | LN590692 | 68.84 | 91.72 | 0.124956 | Cypriniformes | Cyprinidae | Cyprinus carpio |
| Solanum lycopersicum chromosome ch12, complete gen | HG975524 | 76.47 | 45.52 | 0.124956 | Solanales | Solanaceae | Solanum lycopersicum |
| Solanum pennellii chromosome ch12, complete genome | HG975451 | 76.47 | 44.83 | 0.124956 | Solanales | Solanaceae | Solanum pennellii |
| Solanum pennellii chromosome ch08, complete genome | HG975447 | 70.87 | 82.76 | 0.124956 | Solanales | Solanaceae | Solanum pennellii |
| Homo sapiens glutamate receptor, ionotropic, delta | NG_034113 | 75 | 51.03 | 0.124956 | Primates | Hominidae | Homo sapiens |
| Rapisma zayuanum mitochondrion, complete genome | KF626447 | 75.34 | 50.34 | 0.124956 | Neuroptera | Rapismatidae | Rapisma zayuanum |
| Rapisma xizangense mitochondrion, complete genome | KF626446 | 75.34 | 50.34 | 0.124956 | Neuroptera | Rapismatidae | Rapisma xizangense |
| Fusarium fujikuroi IMI 58289 draft genome, chromos | HF679032 | 77.78 | 48.28 | 0.124956 | Hypocreales | Nectriaceae | Fusarium fujikuroi IMI 58289 |
| Phytophthora plurivora isolate P1805 SecY (secY) g | JQ439396 | 70.29 | 90.34 | 0.124956 | Peronosporales | | Phytophthora plurivora |
| Phytophthora plurivora isolate P10627 SecY (secY) | JQ439395 | 70.29 | 90.34 | 0.124956 | Peronosporales | | Phytophthora plurivora |
| Phytophthora aff. citricola 1 FM−2012 isolate P107 | JQ439304 | 70.29 | 90.34 | 0.124956 | Peronosporales | | Phytophthora aff. citricola 1 FM−2012 |
| Phytophthora aff. citricola 1 FM−2012 isolate P107 | JQ439303 | 70.29 | 90.34 | 0.124956 | Peronosporales | | Phytophthora aff. citricola 1 FM−2012 |
| Phytophthora aff. citricola 1 FM−2012 isolate P107 | JQ439302 | 70.29 | 90.34 | 0.124956 | Peronosporales | | Phytophthora aff. citricola 1 FM−2012 |
| Vitis vinifera contig VV78X177641.5, whole genome | AM440566 | 70 | 69.66 | 0.124956 | Vitales | Vitaceae | Vitis vinifera |
| Ovis canadensis canadensis isolate 43U chromosome | CP011895 | 76.19 | 42.07 | 1.52227 | | Bovidae | Ovis canadensis canadensis |
