# Supplementary material for: Testing the potential of a ribosomal 16S marker for DNA metabarcoding of insects
Source: PeerJ. 2016 Apr 19;4:e1966. doi: 10.7717/peerj.1966 (PMC4841222; doi:10.7717/peerj.1966)
Supplement: Supplemental Information 1 [file peerj-04-1966-s006.zip › scripts/5 match OTUs/blast_hits/OTU_117.csv_plot.pdf]

OTU_117.csv

Ephemera danica

Ephemeridae

Ephemeroptera

86.18

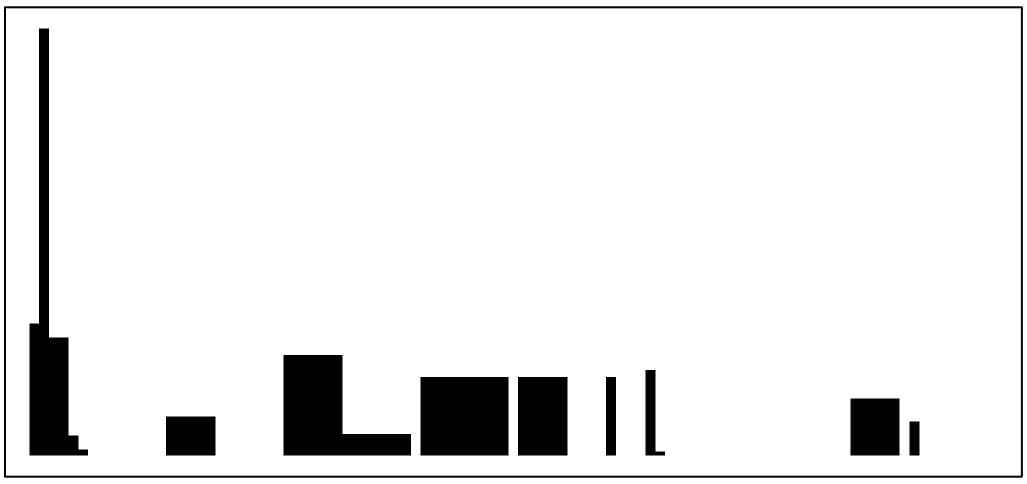

Ephemera danica 16S ribosomal RNA gene, partial se	KF855860	86.18	100	1.07974e−40	Ephemeroptera	Ephemeridae	Ephemera danica
Habroleptoides confusa voucher BYU:IGCEP153 16S ri	GQ118289	100	64.24	1.07974e−40	Ephemeroptera		Habroleptoides confusa
Ephemera orientalis voucher EPH01 tRNA−Leu, 16S ri	KF256907	85.53	100	1.31539e−39	Ephemeroptera	Ephemeridae	Ephemera orientalis
Ephemera orientalis mitochondrion, complete genome	EU591678	85.53	100	1.31539e−39	Ephemeroptera	Ephemeridae	Ephemera orientalis
Siphlonurus immanis voucher NKMT032 mitochondrion,	FJ606783	80.92	100	7.77463e−30	Ephemeroptera	Siphlonuridae	Siphlonurus immanis
Ephemera simulans voucher BYU:IGCEP038 16S ribosom	GQ118285	80.26	100	9.47144e−29	Ephemeroptera	Ephemeridae	Ephemera simulans
Protohermes sinuolatus 16S ribosomal RNA gene, par	KF944540	78.29	98.01	8.87085e−23	Megaloptera	Corydalidae	Protohermes sinuolatus
Ephemera sp. 1 BM−2008 12S ribosomal RNA gene, par	EU477611	77.78	100	8.87085e−23	Ephemeroptera	Ephemeridae	Ephemera sp. 1 BM−2008
Leucochrysa texana 16S ribosomal RNA gene, partial	DQ399279	78.85	99.34	8.87085e−23	Neuroptera	Chrysopidae	Leucochrysa texana
Ephemera sp. 'Bonn' large subunit ribosomal RNA ge	AF266047	77.78	100	8.87085e−23	Ephemeroptera	Ephemeridae	Ephemera sp. 'Bonn'
Anthopotamus sp. EP022 16S ribosomal RNA gene, par	AY749766	78.57	100	3.09623e−22	Ephemeroptera	Potamanthidae	Anthopotamus sp. EP022
Protohermes yangi 16S ribosomal RNA gene, partial	KF944544	78.48	99.34	1.31655e−20	Megaloptera	Corydalidae	Protohermes yangi
Protohermes hunanensis 16S ribosomal RNA gene, par	KF944532	78.48	99.34	1.31655e−20	Megaloptera	Corydalidae	Protohermes hunanensis
Protohermes arunachalensis 16S ribosomal RNA gene,	KF944519	78.48	99.34	1.31655e−20	Megaloptera	Corydalidae	Protohermes arunachalensis
Ephoron shigae mitochondrial gene for 16S ribosoma	AB874293	81.82	79.47	1.31655e−20	Ephemeroptera	Polymitarcyidae	Ephoron shigae
Ephoron shigae mitochondrial gene for 16S ribosoma	AB874289	81.82	79.47	1.31655e−20	Ephemeroptera	Polymitarcyidae	Ephoron shigae
Ephoron shigae mitochondrial gene for 16S rRNA, pa	AB711533	81.82	79.47	1.31655e−20	Ephemeroptera	Polymitarcyidae	Ephoron shigae
Ephoron shigae mitochondrial gene for 16S rRNA, pa	AB711527	81.82	79.47	1.31655e−20	Ephemeroptera	Polymitarcyidae	Ephoron shigae
Ephoron shigae mitochondrial gene for 16S rRNA, pa	AB711493	81.82	79.47	1.31655e−20	Ephemeroptera	Polymitarcyidae	Ephoron shigae
Protohermes gutianensis 16S ribosomal RNA gene, pa	KF944529	77.85	99.34	1.60389e−19	Megaloptera	Corydalidae	Protohermes gutianensis
Protohermes costalis 16S ribosomal RNA gene, parti	KF944522	77.07	99.34	1.60389e−19	Megaloptera	Corydalidae	Protohermes costalis
Protohermes costalis isolate CAU00586 16S ribosoma	KF741309	77.07	99.34	1.60389e−19	Megaloptera	Corydalidae	Protohermes costalis
Protohermes costalis isolate CAU00211 16S ribosoma	KF741306	77.07	99.34	1.60389e−19	Megaloptera	Corydalidae	Protohermes costalis
Protohermes costalis isolate CAU00209 16S ribosoma	KF741305	77.07	99.34	1.60389e−19	Megaloptera	Corydalidae	Protohermes costalis
Protohermes gutianensis isolate CAU00196 16S ribos	KF741300	77.85	99.34	1.60389e−19	Megaloptera	Corydalidae	Protohermes gutianensis
Atrichiana placida voucher 842794 16S ribosomal RN	JX234487	78	94.7	1.60389e−19	Coleoptera	Scarabaeidae	Atrichiana placida
Ephoron shigae mitochondrial gene for 16S ribosoma	AB874306	84.69	64.24	1.60389e−19	Ephemeroptera	Polymitarcyidae	Ephoron shigae
Ephoron shigae mitochondrial gene for 16S ribosoma	AB874283	84.69	64.24	1.60389e−19	Ephemeroptera	Polymitarcyidae	Ephoron shigae
Ephoron shigae mitochondrial gene for 16S ribosoma	AB874278	84.69	64.24	1.60389e−19	Ephemeroptera	Polymitarcyidae	Ephoron shigae
Ephoron shigae mitochondrial gene for 16S rRNA, pa	AB711523	84.69	64.24	1.60389e−19	Ephemeroptera	Polymitarcyidae	Ephoron shigae
Ephoron shigae mitochondrial gene for 16S rRNA, pa	AB711500	84.69	64.24	1.60389e−19	Ephemeroptera	Polymitarcyidae	Ephoron shigae
Ephoron shigae mitochondrial gene for 16S rRNA, pa	AB711471	84.69	64.24	1.60389e−19	Ephemeroptera	Polymitarcyidae	Ephoron shigae
Ephoron shigae mitochondrial gene for 16S rRNA, pa	AB711590	80.99	79.47	5.59811e−19	Ephemeroptera	Polymitarcyidae	Ephoron shigae
Ephoron shigae mitochondrial gene for 16S rRNA, pa	AB711582	80.99	79.47	5.59811e−19	Ephemeroptera	Polymitarcyidae	Ephoron shigae
Ephoron shigae mitochondrial gene for 16S rRNA, pa	AB711581	80.99	79.47	5.59811e−19	Ephemeroptera	Polymitarcyidae	Ephoron shigae
Ephoron shigae mitochondrial gene for 16S rRNA, pa	AB711575	80.99	79.47	5.59811e−19	Ephemeroptera	Polymitarcyidae	Ephoron shigae
Ephoron shigae mitochondrial gene for 16S rRNA, pa	AB711531	80.99	79.47	5.59811e−19	Ephemeroptera	Polymitarcyidae	Ephoron shigae
Ephoron shigae mitochondrial gene for 16S rRNA, pa	AB711530	80.99	79.47	5.59811e−19	Ephemeroptera	Polymitarcyidae	Ephoron shigae
Ephoron shigae mitochondrial gene for 16S rRNA, pa	AB711528	80.99	79.47	5.59811e−19	Ephemeroptera	Polymitarcyidae	Ephoron shigae
Leucochrysa robusta voucher CASC 00213 16S ribosom	DQ399281	76.92	99.34	5.59811e−19	Neuroptera	Chrysopidae	Leucochrysa robusta
Ephoron shigae mitochondrial gene for 16S ribosoma	AB874305	83.67	64.24	1.95393e−18	Ephemeroptera	Polymitarcyidae	Ephoron shigae
Ephoron shigae mitochondrial gene for 16S ribosoma	AB874303	83.67	64.24	1.95393e−18	Ephemeroptera	Polymitarcyidae	Ephoron shigae
Ephoron shigae mitochondrial gene for 16S ribosoma	AB874302	83.67	64.24	1.95393e−18	Ephemeroptera	Polymitarcyidae	Ephoron shigae
Ephoron shigae mitochondrial gene for 16S ribosoma	AB874301	83.67	64.24	1.95393e−18	Ephemeroptera	Polymitarcyidae	Ephoron shigae
Ephoron shigae mitochondrial gene for 16S ribosoma	AB874299	83.67	64.24	1.95393e−18	Ephemeroptera	Polymitarcyidae	Ephoron shigae
Ephoron shigae mitochondrial gene for 16S ribosoma	AB874288	83.67	64.24	1.95393e−18	Ephemeroptera	Polymitarcyidae	Ephoron shigae
Ephoron shigae mitochondrial gene for 16S ribosoma	AB874284	83.67	64.24	1.95393e−18	Ephemeroptera	Polymitarcyidae	Ephoron shigae
Ephoron shigae mitochondrial gene for 16S ribosoma	AB874279	83.67	64.24	1.95393e−18	Ephemeroptera	Polymitarcyidae	Ephoron shigae
Ephoron shigae mitochondrial gene for 16S ribosoma	AB874277	83.67	64.24	1.95393e−18	Ephemeroptera	Polymitarcyidae	Ephoron shigae
Phyllobaenus testaceus voucher 7228 16S ribosomal	KC524896	77.14	91.39	1.95393e−18	Coleoptera	Cleridae	Phyllobaenus testaceus
Ephoron shigae mitochondrial gene for 16S rRNA, pa	AB711521	83.67	64.24	1.95393e−18	Ephemeroptera	Polymitarcyidae	Ephoron shigae
Ephoron shigae mitochondrial gene for 16S rRNA, pa	AB711514	83.67	64.24	1.95393e−18	Ephemeroptera	Polymitarcyidae	Ephoron shigae
Ephoron shigae mitochondrial gene for 16S rRNA, pa	AB711501	83.67	64.24	1.95393e−18	Ephemeroptera	Polymitarcyidae	Ephoron shigae
Ephoron shigae mitochondrial gene for 16S rRNA, pa	AB711492	83.67	64.24	1.95393e−18	Ephemeroptera	Polymitarcyidae	Ephoron shigae
Ephoron shigae mitochondrial gene for 16S rRNA, pa	AB711482	83.67	64.24	1.95393e−18	Ephemeroptera	Polymitarcyidae	Ephoron shigae
Hoplandria lateralis voucher ZMUN:10002550 16S rib	GQ980980	77.62	90.07	1.95393e−18	Coleoptera	Staphylinidae	Hoplandria lateralis
Tachytrechus notatus voucher Pollet 0279 (P) 16S r	EU864017	79.84	78.81	1.95393e−18	Diptera	Dolichopodidae	Tachytrechus notatus
Hymenoplia fulvipennis voucher DA0116 16S ribosoma	FJ956720	76.97	98.68	1.95393e−18	Coleoptera	Scarabaeidae	Hymenoplia fulvipennis
Hydrochus carinatus mitochondrial partial 16S rRNA	AM287062	77.33	96.69	1.95393e−18	Coleoptera	Hydrochidae	Hydrochus carinatus
Penaphlebia sp. EP076 16S ribosomal RNA gene, part	AY749782	83.67	64.9	1.95393e−18	Ephemeroptera		Penaphlebia sp. EP076
Protohermes niger isolate H755 16S ribosomal RNA g	KF741319	77.12	98.68	6.8199e−18	Megaloptera	Corydalidae	Protohermes niger
Cephaloclerus sp. 1 NLG−2013 voucher 7183 16S ribo	KC524887	79.53	82.78	6.8199e−18	Coleoptera	Cleridae	Cephaloclerus sp. 1 NLG−2013
Enochrus talamanca 16S ribosomal RNA gene, partial	KC992672	78.23	96.03	6.8199e−18	Coleoptera	Hydrophilidae	Enochrus talamanca
Ephoron eophilum mitochondrial gene for 16S rRNA,	AB711631	84	64.24	6.8199e−18	Ephemeroptera	Polymitarcyidae	Ephoron eophilum
Ephoron shigae mitochondrial gene for 16S rRNA, pa	AB711598	80.17	79.47	6.8199e−18	Ephemeroptera	Polymitarcyidae	Ephoron shigae
Tachytrechus transitorius voucher Pollet 0101 (P)	EU864018	79.2	78.81	6.8199e−18	Diptera	Dolichopodidae	Tachytrechus transitorius
Hymenoplia fulvipennis voucher DA0214 16S ribosoma	FJ956723	76.16	98.68	6.8199e−18	Coleoptera	Scarabaeidae	Hymenoplia fulvipennis
Protohermes orientalis 16S ribosomal RNA gene, par	KF944538	77.37	84.77	2.38038e−17	Megaloptera	Corydalidae	Protohermes orientalis
Platyneuromus sorror 16S ribosomal RNA gene, parti	KF944516	75.64	99.34	2.38038e−17	Megaloptera	Corydalidae	Platyneuromus sorror
Asiagomphus melaenops mitochondrial gene for 16S r	AB707724	79.07	83.44	2.38038e−17	Odonata	Aeshnidae	Asiagomphus melaenops
Asiagomphus melaenops mitochondrial gene for 16S r	AB707720	79.07	83.44	2.38038e−17	Odonata	Aeshnidae	Asiagomphus melaenops
Asiagomphus melaenops mitochondrial gene for 16S r	AB707719	79.07	83.44	2.38038e−17	Odonata	Aeshnidae	Asiagomphus melaenops
Hercostomus parvilamellatus voucher Pollet 0004 (P	EU864000	76.67	96.03	2.38038e−17	Diptera	Dolichopodidae	Hercostomus parvilamellatus
Asiagomphus melaenops 12S ribosomal RNA gene, part	EU477664	79.07	83.44	2.38038e−17	Odonata	Aeshnidae	Asiagomphus melaenops
Libnotes orofenaae voucher O'Grady 202034 16S ribo	EU005431	75.95	100	2.38038e−17	Diptera	Limoniidae	Libnotes orofenaae
Ankylopteryx alluaudi 16S ribosomal RNA gene, part	DQ399264	76.47	98.01	2.38038e−17	Neuroptera	Chrysopidae	Ankylopteryx alluaudi
Dichochrysa tacta isolate Dictac1 16S ribosomal RN	AY620154	75.64	99.34	2.38038e−17	Neuroptera	Chrysopidae	Pseudomallada tactus
Dymasia dymas voucher NW27−7 16S ribosomal RNA gen	AF186876	77.14	90.73	2.38038e−17	Lepidoptera	Nymphalidae	Dymasia dymas
Protohermes weelei 16S ribosomal RNA gene, partial	KF944543	78.2	84.77	8.30834e−17	Megaloptera	Corydalidae	Protohermes weelei
Protohermes disjunctus 16S ribosomal RNA gene, par	KF944525	75.93	99.34	8.30834e−17	Megaloptera	Corydalidae	Protohermes disjunctus
Protohermes niger isolate Pro006 16S ribosomal RNA	KF741314	75.8	98.68	8.30834e−17	Megaloptera	Corydalidae	Protohermes niger
Protaetia marginicollis voucher 842877 16S ribosom	JX234546	76.67	97.35	8.30834e−17	Coleoptera	Scarabaeidae	Protaetia marginicollis
Protaetia angustata voucher 842871 16S ribosomal R	JX234542	80	80.79	8.30834e−17	Coleoptera	Scarabaeidae	Protaetia angustata
Protaetia angustata voucher 842767 16S ribosomal R	JX234468	80	80.79	8.30834e−17	Coleoptera	Scarabaeidae	Protaetia angustata
Ephoron shigae mitochondrial gene for 16S ribosoma	AB874304	82.65	64.24	8.30834e−17	Ephemeroptera	Polymitarcyidae	Ephoron shigae
Ephoron shigae mitochondrial gene for 16S ribosoma	AB874300	82.65	64.24	8.30834e−17	Ephemeroptera	Polymitarcyidae	Ephoron shigae
Ephoron shigae mitochondrial gene for 16S ribosoma	AB874297	82.65	64.24	8.30834e−17	Ephemeroptera	Polymitarcyidae	Ephoron shigae
Ephoron shigae mitochondrial gene for 16S rRNA, pa	AB711532	82.65	64.24	8.30834e−17	Ephemeroptera	Polymitarcyidae	Ephoron shigae
Ephoron shigae mitochondrial gene for 16S rRNA, pa	AB711522	82.65	64.24	8.30834e−17	Ephemeroptera	Polymitarcyidae	Ephoron shigae
Potamanthus luteus voucher BYU:IGCEP219 16S riboso	GQ118293	76.47	98.68	8.30834e−17	Ephemeroptera	Potamanthidae	Potamanthus luteus
Hercostomus brevidigitalis voucher RMBR:103445 16S	FJ808172	81.58	73.51	8.30834e−17	Diptera	Dolichopodidae	Hercostomus brevidigitalis
Neoserica sp. BMNH 671348 16S large subunit riboso	EF487856	78.03	83.44	8.30834e−17	Coleoptera	Scarabaeidae	Neoserica sp. BMNH 671348
Helictopleurus splendidicollis voucher Hel.splcol.	EF187966	78.79	84.11	8.30834e−17	Coleoptera	Scarabaeidae	Helictopleurus splendidicollis
Helictopleurus sp. 10 MTM−2009 voucher BMNH:669899	EF656657	78.79	84.11	8.30834e−17	Coleoptera	Scarabaeidae	Helictopleurus sp. 10 MTM−2009
Helictopleurus splendidicollis 16S ribosomal RNA g	DQ369538	78.79	84.11	8.30834e−17	Coleoptera	Scarabaeidae	Helictopleurus splendidicollis
Melitaea punica voucher NW34−11 16S ribosomal RNA	AF186914	77.3	90.73	8.30834e−17	Lepidoptera	Nymphalidae	Melitaea punica
Melitaea phoebe isolate 15−14 16S ribosomal RNA ge	AF186910	77.3	90.73	8.30834e−17	Lepidoptera	Nymphalidae	Melitaea phoebe
Protohermes sp. XL−2014 16S ribosomal RNA gene, pa	KF944548	75.82	98.68	2.8999e−16	Megaloptera	Corydalidae	Protohermes sp. XL−2014
Protohermes lii 16S ribosomal RNA gene, partial se	KF944537	75.16	96.03	2.8999e−16	Megaloptera	Corydalidae	Protohermes lii
Chloronia mexicana 16S ribosomal RNA gene, partial	KF944500	75.66	98.01	2.8999e−16	Megaloptera	Corydalidae	Chloronia mexicana
