# Supplementary material for: Testing the potential of a ribosomal 16S marker for DNA metabarcoding of insects
Source: PeerJ. 2016 Apr 19;4:e1966. doi: 10.7717/peerj.1966 (PMC4841222; doi:10.7717/peerj.1966)
Supplement: Supplemental Information 1 [file peerj-04-1966-s006.zip › scripts/5 match OTUs/blast_hits/OTU_118.csv_plot.pdf]

OTU_118.csv

Torleya major

Ephemerellidae

Ephemeroptera

100

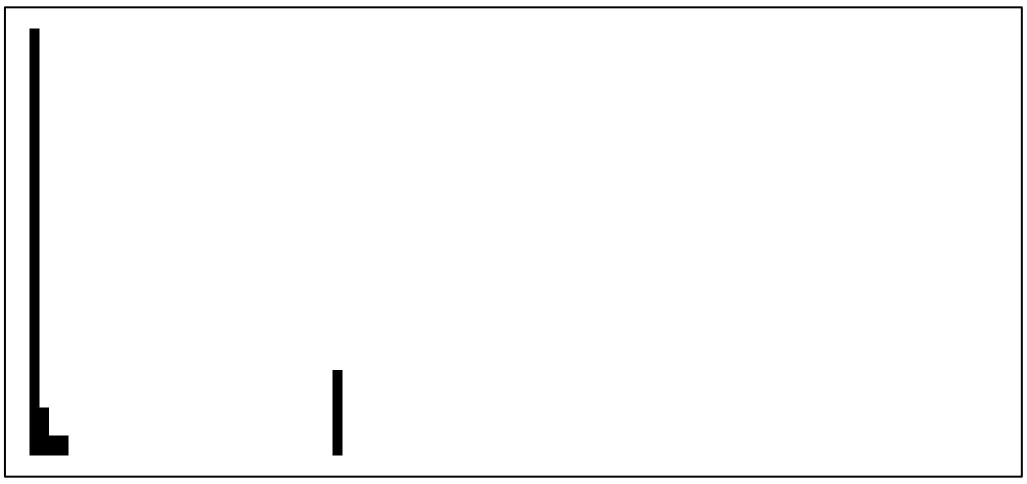

Torleya major 16S ribosomal RNA gene, partial sequ	FJ443047	100	64.9	3.0935e−41	Ephemeroptera	Ephemerellidae	Torleya major
Ephemera danica 16S ribosomal RNA gene, partial se	KF855860	82.24	100	1.50086e−32	Ephemeroptera	Ephemeridae	Ephemera danica
Ephemera orientalis voucher EPH01 tRNA−Leu, 16S ri	KF256907	80.92	100	7.77463e−30	Ephemeroptera	Ephemeridae	Ephemera orientalis
Ephemera orientalis mitochondrion, complete genome	EU591678	80.92	100	7.77463e−30	Ephemeroptera	Ephemeridae	Ephemera orientalis
Siphlonurus immanis voucher NKMT032 mitochondrion,	FJ606783	78.32	92.72	3.09623e−22	Ephemeroptera	Siphlonuridae	Siphlonurus immanis
Ephemera simulans voucher BYU:IGCEP038 16S ribosom	GQ118285	76.32	100	1.31655e−20	Ephemeroptera	Ephemeridae	Ephemera simulans
Ephemera sp. 1 BM−2008 12S ribosomal RNA gene, par	EU477611	74.51	100	2.8999e−16	Ephemeroptera	Ephemeridae	Ephemera sp. 1 BM−2008
Ephemera sp. 'Bonn' large subunit ribosomal RNA ge	AF266047	74.51	100	2.8999e−16	Ephemeroptera	Ephemeridae	Ephemera sp. 'Bonn'
Gymnopternus angustifrons voucher Pollet 0052 (P)	EU863971	73.55	99.34	1.50218e−13	Diptera	Dolichopodidae	Gymnopternus angustifrons
Gymnopternus celer voucher Pollet 0268 (P) 16S rib	EU863982	75.37	83.44	5.24313e−13	Diptera	Dolichopodidae	Gymnopternus celer
Melitaea arcesia isolate 10−9 16S ribosomal RNA ge	AF186855	76.06	89.4	5.24313e−13	Lepidoptera	Nymphalidae	Melitaea arcesia
Gymnopternus cupreus voucher Pollet 0021 (P) 16S r	EU863984	72.78	99.34	1.83003e−12	Diptera	Dolichopodidae	Gymnopternus cupreus
Gymnopternus celer voucher Pollet 0018 (P) 16S rib	EU863977	72.78	99.34	1.83003e−12	Diptera	Dolichopodidae	Gymnopternus celer
Gymnopternus celer voucher Pollet 0051 (P) 16S rib	EU863978	72.78	99.34	1.83003e−12	Diptera	Dolichopodidae	Gymnopternus celer
Gymnopternus brevicornis voucher Pollet 0036 (P) 1	EU863974	72.78	99.34	1.83003e−12	Diptera	Dolichopodidae	Gymnopternus brevicornis
Gymnopternus assimilis voucher Pollet 0088 (P) 16S	EU863972	72.9	99.34	1.83003e−12	Diptera	Dolichopodidae	Gymnopternus assimilis
Gymnopternus aerosus voucher Pollet 0025 (P) 16S r	EU863970	72.9	99.34	1.83003e−12	Diptera	Dolichopodidae	Gymnopternus aerosus
Chrysopodes spinella voucher CASC 00223 16S riboso	DQ399274	73.42	99.34	1.83003e−12	Neuroptera	Chrysopidae	Chrysopodes spinella
Rhabdotis aulica voucher 842782 16S ribosomal RNA	JX234477	76.34	82.78	6.38744e−12	Coleoptera	Scarabaeidae	Rhabdotis aulica
Comythovalgus sp. DA−2012 voucher 842778 16S ribos	JX234473	72.48	97.35	6.38744e−12	Coleoptera	Scarabaeidae	Comythovalgus sp. DA−2012
Dasytes coriaceipennis voucher UPOL001197 16S ribo	HQ619684	77.39	74.83	6.38744e−12	Coleoptera	Melyridae	Dasytes coriaceipennis
Dasytes emarginatus voucher UPOL001068 16S ribosom	HQ619637	74.03	97.35	6.38744e−12	Coleoptera	Melyridae	Dasytes emarginatus
Leucochrysa texana 16S ribosomal RNA gene, partial	DQ399279	73.72	99.34	6.38744e−12	Neuroptera	Chrysopidae	Leucochrysa texana
Gymnopternus silvestris voucher Pollet 0082 (P) 16	EU863988	72.15	99.34	2.22944e−11	Diptera	Dolichopodidae	Gymnopternus silvestris
Gymnopternus helveticus voucher Pollet 0291 (P) 16	EU863986	71.97	99.34	2.22944e−11	Diptera	Dolichopodidae	Gymnopternus helveticus
Gymnopternus helveticus voucher Pollet 0290 (P) 16	EU863985	71.97	99.34	2.22944e−11	Diptera	Dolichopodidae	Gymnopternus helveticus
Ephemerella atagosana 16S ribosomal RNA gene, part	FJ443039	77.5	78.81	2.22944e−11	Ephemeroptera	Ephemerellidae	Ephemerella atagosana
Chrysopodes divisa voucher CASC 00221 16S ribosoma	DQ399272	72.78	99.34	2.22944e−11	Neuroptera	Chrysopidae	Chrysopodes divisa
Protaetia angustata voucher 842871 16S ribosomal R	JX234542	76.98	80.79	7.7815e−11	Coleoptera	Scarabaeidae	Protaetia angustata
Protaetia angustata voucher 842767 16S ribosomal R	JX234468	76.98	80.79	7.7815e−11	Coleoptera	Scarabaeidae	Protaetia angustata
Ephoron shigae mitochondrial gene for 16S rRNA, pa	AB711581	76.23	79.47	7.7815e−11	Ephemeroptera	Polymitarcyidae	Ephoron shigae
Pelidnota parallela 16S ribosomal RNA gene, partia	JX982943	84	47.02	7.7815e−11	Coleoptera	Scarabaeidae	Pelidnota parallela
Danacea nigritarsis ingauna voucher UPOL001204 16S	HQ619688	73.68	97.35	7.7815e−11	Coleoptera	Melyridae	Danacea nigritarsis ingauna
Gymnopternus metallicus voucher Pollet 0030 (P) 16	EU863987	72.15	99.34	7.7815e−11	Diptera	Dolichopodidae	Gymnopternus metallicus
Gymnopternus blankaartensis voucher Pollet 0090 (P	EU863973	72.26	99.34	7.7815e−11	Diptera	Dolichopodidae	Gymnopternus blankaartensis
Argyreus hyperbius voucher jshao2005025 16S riboso	DQ784692	73.97	92.72	7.7815e−11	Lepidoptera	Nymphalidae	Argynnis hyperbius
Argyreus hyperbius 16S ribosomal RNA gene, partial	DQ445107	73.97	92.72	7.7815e−11	Lepidoptera	Nymphalidae	Argynnis hyperbius
Anthopotamus sp. EP022 16S ribosomal RNA gene, par	AY749766	73.25	100	7.7815e−11	Ephemeroptera	Potamanthidae	Anthopotamus sp. EP022
Megalabops sp. KCADI071−14 16S ribosomal RNA gene,	KT186671	72.5	99.34	2.71601e−10	Diptera	Diopsidae	Megalabops sp. KCADI071−14
Megalabops quadriguttata 16S ribosomal RNA gene, p	KT186655	72.5	99.34	2.71601e−10	Diptera	Diopsidae	Megalabops quadriguttata
Oxythyrea funesta voucher 678443 16S ribosomal RNA	JX234432	76.47	77.48	2.71601e−10	Coleoptera	Scarabaeidae	Oxythyrea funesta
Sphaerosoma sp. 1 16S ribosomal RNA gene, partial	GQ302155	75.41	77.48	2.71601e−10	Coleoptera	Alexiidae	Sphaerosoma sp. 1 FWS−2010
Pyratula zonata 16S ribosomal RNA gene, partial se	FJ172004	72.55	97.35	2.71601e−10	Diptera	Keroplatidae	Pyratula zonata
Potamanthus luteus voucher BYU:IGCEP219 16S riboso	GQ118293	73.72	98.68	2.71601e−10	Ephemeroptera	Potamanthidae	Potamanthus luteus
Danacea nigritarsis 16S ribosomal RNA gene, partia	EF508035	74.03	97.35	2.71601e−10	Coleoptera	Melyridae	Danacea nigritarsis
Chlosyne cyneas voucher NW38−17 16S ribosomal RNA	AF186870	74.26	87.42	2.71601e−10	Lepidoptera	Nymphalidae	Chlosyne cyneas
Porhydrus sp. UNM KBMPosp754 16S ribosomal RNA gen	KJ548289	72.19	98.01	9.47981e−10	Coleoptera	Dytiscidae	Porhydrus sp. UNM KBMPosp754
Protaetia marginicollis voucher 842877 16S ribosom	JX234546	73.33	97.35	9.47981e−10	Coleoptera	Scarabaeidae	Protaetia marginicollis
Neopanorpa pulchra mitochondrion, complete genome	JX569848	73.86	98.68	9.47981e−10	Mecoptera	Panorpidae	Neopanorpa pulchra
Ephoron shigae mitochondrial gene for 16S rRNA, pa	AB711582	75.21	79.47	9.47981e−10	Ephemeroptera	Polymitarcyidae	Ephoron shigae
Ephoron shigae mitochondrial gene for 16S rRNA, pa	AB711575	75.21	79.47	9.47981e−10	Ephemeroptera	Polymitarcyidae	Ephoron shigae
Cafius lithocharinus isolate CNUIC−106 16S ribosom	JF796468	74.22	82.12	9.47981e−10	Coleoptera	Staphylinidae	Cafius lithocharinus
Danacea pallipes voucher UPOL001203 16S ribosomal	HQ619687	73.03	97.35	9.47981e−10	Coleoptera	Melyridae	Danacea pallipes
Argynnis hyperbius mitochondrion, complete genome	JF439070	73.29	92.72	9.47981e−10	Lepidoptera	Nymphalidae	Argynnis hyperbius
Argynnis hyperbius 16S ribosomal RNA gene, partial	GU372435	73.29	92.72	9.47981e−10	Lepidoptera	Nymphalidae	Argynnis hyperbius
Netocia cuprea voucher BMNH 678460 16S large subun	EF487943	75.18	90.07	9.47981e−10	Coleoptera	Scarabaeidae	Protaetia cuprea
Protohermes sinuolatus 16S ribosomal RNA gene, par	KF944540	71.9	98.01	3.30878e−09	Megaloptera	Corydalidae	Protohermes sinuolatus
Ulomoides dermestoides mitochondrion, complete gen	KM046492	76.07	75.5	3.30878e−09	Coleoptera	Tenebrionidae	Ulomoides dermestoides
Ephoron eophilum mitochondrial gene for 16S rRNA,	AB711631	78.22	64.24	3.30878e−09	Ephemeroptera	Polymitarcyidae	Ephoron eophilum
Ephoron eophilum mitochondrial gene for 16S rRNA,	AB711630	79.41	64.24	3.30878e−09	Ephemeroptera	Polymitarcyidae	Ephoron eophilum
Ephoron eophilum mitochondrial gene for 16S rRNA,	AB711621	79.41	64.24	3.30878e−09	Ephemeroptera	Polymitarcyidae	Ephoron eophilum
Ephoron eophilum mitochondrial gene for 16S rRNA,	AB711619	79.41	64.24	3.30878e−09	Ephemeroptera	Polymitarcyidae	Ephoron eophilum
Ephoron eophilum mitochondrial gene for 16S rRNA,	AB711615	79.41	64.24	3.30878e−09	Ephemeroptera	Polymitarcyidae	Ephoron eophilum
Ephoron shigae mitochondrial gene for 16S rRNA, pa	AB711590	75.41	79.47	3.30878e−09	Ephemeroptera	Polymitarcyidae	Ephoron shigae
Palingenia longicauda mitochondrial partial 16S rR	HE650502	76.77	64.9	3.30878e−09	Ephemeroptera	Palingeniidae	Palingenia longicauda
Palingenia longicauda mitochondrial partial 16S rR	HE650484	76.77	64.9	3.30878e−09	Ephemeroptera	Palingeniidae	Palingenia longicauda
Lamproblatta albipalpus voucher ZMUC:JD−1555 16S r	JN615295	74.79	74.83	3.30878e−09	Blattodea	Lamproblattidae	Lamproblatta albipalpus
Papilio alexanor voucher FS−b−40 16S ribosomal RNA	GQ268343	73.61	93.38	3.30878e−09	Lepidoptera	Papilionidae	Papilio alexanor
Teloganopsis media 16S ribosomal RNA gene, partial	FJ443046	75.23	72.19	3.30878e−09	Ephemeroptera	Ephemerellidae	Teloganopsis media
Ephemerella cornutus 16S ribosomal RNA gene, parti	FJ443041	77	64.9	3.30878e−09	Ephemeroptera	Ephemerellidae	Ephemerella cornutus
Tetraserica sp. BMNH 671350 16S large subunit ribo	EF487874	74.05	83.44	3.30878e−09	Coleoptera	Scarabaeidae	Tetraserica sp. BMNH 671350
Australammoecius occidentalis 16S ribosomal RNA ge	EF487822	72.85	96.69	3.30878e−09	Coleoptera	Scarabaeidae	Australammoecius occidentalis
Helictopleurus fulgens purpuricollis voucher Hel.f	EF187934	75.41	78.81	3.30878e−09	Coleoptera	Scarabaeidae	Helictopleurus fulgens purpuricollis
Lobonyx aeneus 16S ribosomal RNA gene, partial seq	EF508039	72.19	96.69	3.30878e−09	Coleoptera	Prionoceridae	Lobonyx aeneus
Neofulla sp. BYU_PL152 16S ribosomal RNA gene, par	EF623203	73.88	85.43	3.30878e−09	Plecoptera	Notonemouridae	Neofulla sp. BYU_PL152
Libnotes orofenaae voucher O'Grady 202034 16S ribo	EU005431	71.52	100	3.30878e−09	Diptera	Limoniidae	Libnotes orofenaae
Limonia sp. PMO 200984 16S ribosomal RNA gene, par	EU005416	75	72.19	3.30878e−09	Diptera	Limoniidae	Limonia sp. PMO 200984
Helictopleurus purpuricollis 16S ribosomal RNA gen	DQ369534	75.41	78.81	3.30878e−09	Coleoptera	Scarabaeidae	Helictopleurus fulgens purpuricollis
Cerdistus sp. LRC−2002 16S ribosomal RNA gene, par	AF456850	75.21	74.83	3.30878e−09	Diptera	Asilidae	Cerdistus sp. LRC−2002
Neopanorpa sp. M24899 16S ribosomal RNA gene, part	AF180059	73.68	98.01	3.30878e−09	Mecoptera	Panorpidae	Neopanorpa sp. M24899
Thereva flavicincta voucher MEI 119977 16S ribosom	DQ250862	74.44	84.11	3.30878e−09	Diptera	Therevidae	Thereva flavicincta
Irwiniella oldroydi voucher MEI 146419 16S ribosom	DQ250843	74.81	84.77	3.30878e−09	Diptera	Therevidae	Irwiniella oldroydi
Phyciodes picta voucher NW34−7 16S ribosomal RNA g	AF186911	73.13	86.09	3.30878e−09	Lepidoptera	Nymphalidae	Phyciodes picta
Papilio alexanor mitochondrion, large subunit ribo	AJ224052	73.61	93.38	3.30878e−09	Lepidoptera	Papilionidae	Papilio alexanor
Megalabops sp. BOLD:ACP5917 16S ribosomal RNA gene	KT186670	71.88	99.34	1.15488e−08	Diptera	Diopsidae	Megalabops sp. BOLD:ACP5917
Tropha zambiensis voucher KS240 16S ribosomal RNA	KP143640	74.1	88.08	1.15488e−08	Coleoptera	Coccinellidae	Tropha zambiensis
Protaetia vidua voucher 842872 16S ribosomal RNA g	JX234543	74.4	80.79	1.15488e−08	Coleoptera	Scarabaeidae	Protaetia vidua
Myrmeleon mariaemathildae isolate 159−mmgam 16S ri	KF680151	73.68	84.11	1.15488e−08	Neuroptera	Myrmeleontidae	Myrmeleon mariaemathildae
Myrmeleon mariaemathildae isolate 26−mmgan2 16S ri	KC195852	73.68	84.11	1.15488e−08	Neuroptera	Myrmeleontidae	Myrmeleon mariaemathildae
Myrmeleon mariaemathildae isolate 30−mmsic2 16S ri	KC195848	73.68	84.11	1.15488e−08	Neuroptera	Myrmeleontidae	Myrmeleon mariaemathildae
Myrmeleon mariaemathildae isolate 62−gamch 16S rib	KC195847	73.68	84.11	1.15488e−08	Neuroptera	Myrmeleontidae	Myrmeleon mariaemathildae
Myrmeleon mariaemathildae isolate 63−gamch2 16S ri	KC195846	73.28	84.11	1.15488e−08	Neuroptera	Myrmeleontidae	Myrmeleon mariaemathildae
Myrmeleon inconspicuus isolate 40−inctu1 16S ribos	KC195839	75.41	76.82	1.15488e−08	Neuroptera	Myrmeleontidae	Myrmeleon inconspicuus
Pseudolycoriella sp. 1 SS−2012 voucher 586 16S rib	JQ613916	73.85	84.11	1.15488e−08	Diptera	Sciaridae	Pseudolycoriella sp. 1 SS−2012
Polyphylla decemlineata 16S ribosomal RNA gene, pa	JX994048	77.89	61.59	1.15488e−08	Coleoptera	Scarabaeidae	Polyphylla decemlineata
Notonemouridae sp. MD−2011 voucher ZMUC:JD−1507 16	JN615257	72.59	85.43	1.15488e−08	Plecoptera	Notonemouridae	Notonemouridae sp. MD−2011
Tachytrechus notatus voucher Pollet 0279 (P) 16S r	EU864017	74.4	78.81	1.15488e−08	Diptera	Dolichopodidae	Tachytrechus notatus
Hexagenia sp. BYU IGCEP003 16S ribosomal RNA gene,	GQ118286	77.14	66.89	1.15488e−08	Ephemeroptera	Ephemeridae	Hexagenia sp. BYU IGCEP003
Ephemerella sp. THO−2009 16S ribosomal RNA gene, p	FJ443044	76.53	64.9	1.15488e−08	Ephemeroptera	Ephemerellidae	Ephemerella sp. THO−2009
Serica tukucheana voucher BMNH 670899 16S large su	EF487905	73.48	83.44	1.15488e−08	Coleoptera	Scarabaeidae	Serica tukucheana
