# Supplementary material for: Testing the potential of a ribosomal 16S marker for DNA metabarcoding of insects
Source: PeerJ. 2016 Apr 19;4:e1966. doi: 10.7717/peerj.1966 (PMC4841222; doi:10.7717/peerj.1966)
Supplement: Supplemental Information 1 [file peerj-04-1966-s006.zip › scripts/5 match OTUs/blast_hits/OTU_119.csv_plot.pdf]

OTU_119.csv

Picea sitchensis

Pinaceae

Pinales

74.36

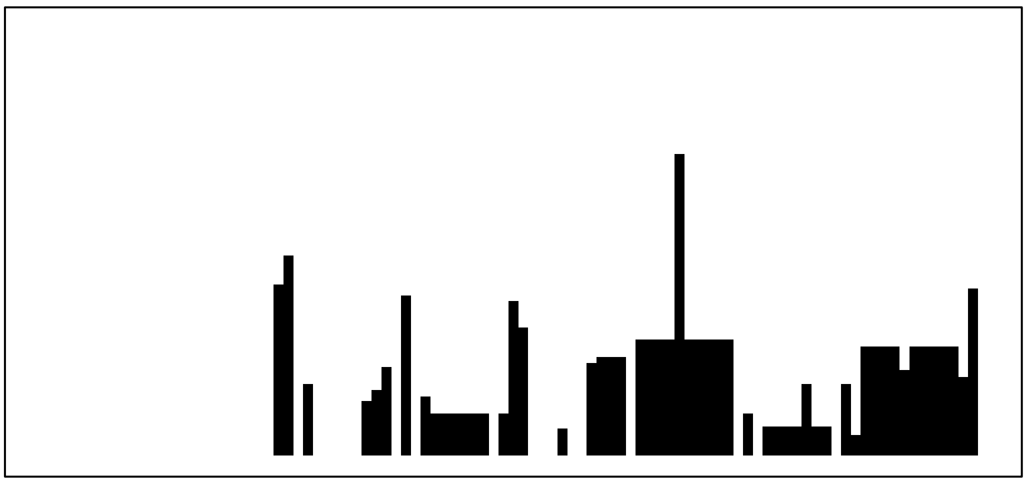

Picea sitchensis clone WS02745_D18 unknown mRNA	EF087003	74.36	97.47	6.45707e−20	Pinales	Pinaceae	Picea sitchensis
Picea sitchensis clone WS0279_O24 unknown mRNA	EF082104	75	86.36	2.74561e−18	Pinales	Pinaceae	Picea sitchensis
Plutella xylostella mitochondrion, complete genome	KM023645	73.98	92.93	9.58313e−18	Lepidoptera	Plutellidae	Plutella xylostella
Plutella xylostella mitochondrion, partial genome	JF911819	73.58	92.93	1.42226e−15	Lepidoptera	Plutellidae	Plutella xylostella
Grapholita dimorpha mitochondrion, complete genome	KJ671625	72.83	85.86	1.09343e−10	Lepidoptera	Tortricidae	Grapholita dimorpha
Grapholita molesta mitochondrion, complete genome	HQ392511	70.83	92.93	3.81646e−10	Lepidoptera	Tortricidae	Grapholita molesta
Grapholita molesta NADH dehydrogenase subunit 1 (N	EU661086	70.83	92.93	3.81646e−10	Lepidoptera	Tortricidae	Grapholita molesta
Toxopoda sp. 1 RM−2008 voucher su73 16S ribosomal	EU435619	67.6	88.38	1.33208e−09	Diptera	Sepsidae	Toxopoda sp. 1 RM−2008
Alucita montana mitochondrion, partial genome	KJ508059	71.2	89.9	4.6494e−09	Lepidoptera	Alucitidae	Alucita montana
Rhyacionia leptotubula mitochondrion, complete gen	JX028270	70.3	79.8	4.6494e−09	Lepidoptera	Tortricidae	Rhyacionia leptotubula
Grapholita molesta mitochondrion, complete genome	HQ116416	70.31	92.93	4.6494e−09	Lepidoptera	Tortricidae	Grapholita molesta
Vitis vinifera contig VV78X074153.3, whole genome	AM461859	72.07	83.33	4.6494e−09	Vitales	Vitaceae	Vitis vinifera
Vitis vinifera contig VV78X093339.2, whole genome	AM437270	71.82	83.33	1.6228e−08	Vitales	Vitaceae	Vitis vinifera
Sepsis thoracica voucher Lei35 16S ribosomal RNA g	KJ766344	67.2	93.43	5.66413e−08	Diptera	Sepsidae	Sepsis thoracica
Sepsis thoracica voucher su59 16S ribosomal RNA ge	EU435616	67.2	93.43	5.66413e−08	Diptera	Sepsidae	Sepsis thoracica
Vitis vinifera contig VV78X156159.7, whole genome	AM426150	71.86	80.3	5.66413e−08	Vitales	Vitaceae	Vitis vinifera
Pyrgus communis isolate FS−b−901 16S ribosomal RNA	DQ351078	66.32	95.45	5.66413e−08	Lepidoptera	Hesperiidae	Pyrgus communis
Toxopoda soror voucher Lei31 16S ribosomal RNA gen	KF199532	66.67	88.38	1.97698e−07	Diptera	Sepsidae	Toxopoda soror
Trichosia splendens voucher 970 16S ribosomal RNA	JQ613969	66.3	91.92	6.90033e−07	Diptera	Sciaridae	Trichosia splendens
Sepsis neocynipsea voucher su54 16S ribosomal RNA	EU435611	67.2	93.43	6.90033e−07	Diptera	Sepsidae	Sepsis neocynipsea
Perochaeta dikowi 16S ribosomal RNA gene, complete	EU375720	70.41	80.3	6.90033e−07	Diptera	Sepsidae	Perochaeta dikowi
Platytoxopoda sp. RM−2008 voucher su38 16S ribosom	EU435598	66.48	88.38	2.40845e−06	Diptera	Sepsidae	Platytoxopoda sp. RM−2008
Decachaetophora aeneipes voucher su14 16S ribosoma	EU435581	66.33	99.49	2.40845e−06	Diptera	Sepsidae	Decachaetophora aeneipes
Zemeros flegyas 16S ribosomal RNA gene, partial se	DQ445106	65.97	95.96	2.40845e−06	Lepidoptera	Riodinidae	Zemeros flegyas
Teleopsis nr. breviscopium BOLD:ACP5739 voucher RM	KP978632	65.15	99.49	8.40632e−06	Diptera	Diopsidae	Teleopsis nr. breviscopium BOLD:ACP5739
Plocopsylla wolffsohni isolate F264 16S ribosomal	KM891312	88	25.25	8.40632e−06	Siphonaptera	Stephanocircidae	Plocopsylla wolffsohni
Chaetopsylla appropinquans isolate F223 16S riboso	KM891283	89.36	23.74	8.40632e−06	Siphonaptera	Vermipsyllidae	Chaetopsylla appropinquans
Samia canningi mitochondrion, complete genome	KJ159909	71.75	76.26	8.40632e−06	Lepidoptera	Saturniidae	Samia canningi
Lonchoptera uniseta 16S ribosomal RNA gene, partia	KC177456	83.33	29.8	8.40632e−06	Diptera	Lonchopteridae	Lonchoptera uniseta
Samia cynthia ricini mitochondrion, complete genom	JN215366	71.75	76.26	8.40632e−06	Lepidoptera	Saturniidae	Samia ricini
Agelastica coerulea 16S ribosomal RNA gene, partia	EF421504	66.13	93.43	8.40632e−06	Coleoptera	Chrysomelidae	Agelastica coerulea
Themira minor mitochondrial partial 16S rRNA gene	AJ843106	71.43	80.3	8.40632e−06	Diptera	Sepsidae	Themira minor
Ochthebius sanabrensis mitochondrial 16S rRNA gene	HF931357	79.27	40.4	2.93409e−05	Coleoptera	Hydraenidae	Ochthebius sanabrensis
Macrocentrus camphoraphilus mitochondrion, partial	GU097656	70.11	81.82	2.93409e−05	Hymenoptera	Braconidae	Macrocentrus camphoraphilus
Pulex irritans 16S ribosomal RNA gene, partial seq	GQ387497	82.54	30.3	2.93409e−05	Siphonaptera	Pulicidae	Pulex irritans
Bothrideres geminatus 16S ribosomal RNA gene, part	EU164600	83.05	29.29	2.93409e−05	Coleoptera	Bothrideridae	Bothrideres geminatus
Saltella nigripes voucher su40 16S ribosomal RNA g	EU435600	84.13	30.3	2.93409e−05	Diptera	Sepsidae	Saltella nigripes
Tunga trimamillata isolate ecuMBOS1 16S ribosomal	AY425835	65.83	100	2.93409e−05	Siphonaptera	Hectopsyllidae	Tunga trimamillata
Stenoponia americana isolate FXG825 16S ribosomal	KM891401	87.5	23.23	0.00010241	Siphonaptera	Ctenophthalmidae	Stenoponia americana
Vitis vinifera contig VV78X157473.16, whole genome	AM484077	71.52	80.3	0.00010241	Vitales	Vitaceae	Vitis vinifera
Helictopleurus fungicola 16S ribosomal RNA gene, p	DQ369525	82.76	28.79	0.00010241	Coleoptera	Scarabaeidae	Helictopleurus fungicola
Tunga trimamillata isolate ecuMBOS6 16S ribosomal	AY425837	81.97	29.29	0.00010241	Siphonaptera	Hectopsyllidae	Tunga trimamillata
Tunga trimamillata isolate ecuMBOS3 16S ribosomal	AY425836	81.97	29.29	0.00010241	Siphonaptera	Hectopsyllidae	Tunga trimamillata
Tunga trimamillata isolate ecuCFEM7 16S ribosomal	AY425834	81.97	29.29	0.00010241	Siphonaptera	Hectopsyllidae	Tunga trimamillata
Tunga trimamillata isolate ecuCFEM5 16S ribosomal	AY425833	81.97	29.29	0.00010241	Siphonaptera	Hectopsyllidae	Tunga trimamillata
Tunga trimamillata isolate ecuCFEM4 16S ribosomal	AY425832	81.97	29.29	0.00010241	Siphonaptera	Hectopsyllidae	Tunga trimamillata
Tunga trimamillata isolate ecuAFEM4 16S ribosomal	AY425831	81.97	29.29	0.00010241	Siphonaptera	Hectopsyllidae	Tunga trimamillata
Italochrysa neurodes isolate Itaneu1 16S ribosomal	AY620153	65.76	92.42	0.00010241	Neuroptera	Chrysopidae	Italochrysa neurodes
Tunga trimamillata isolate EcaCAP1 16S ribosomal R	AF551755	81.97	29.29	0.00010241	Siphonaptera	Hectopsyllidae	Tunga trimamillata
Echidnophaga oschanini isolate F320 16S ribosomal	KM891362	87.23	23.74	0.000357446	Siphonaptera	Pulicidae	Echidnophaga oschanini
Barreropsylla excelsa isolate F142 16S ribosomal R	KM891272	86	25.25	0.000357446	Siphonaptera	Stephanocircidae	Barreropsylla excelsa
Brugia pahangi genome assembly B_pahangi_Glasgow ,	LK964856	74.74	45.45	0.000357446	Spirurida	Onchocercidae	Brugia pahangi
Ochthebius minimus mitochondrial 16S rRNA gene (pa	HE971074	69.74	73.74	0.000357446	Coleoptera	Hydraenidae	Ochthebius minimus
Samia cynthia ricini mitochondrion, complete genom	JF961379	71.19	76.26	0.000357446	Lepidoptera	Saturniidae	Samia ricini
Nycteridopsylla iae 16S ribosomal RNA gene, partia	FJ422790	81.25	30.81	0.000357446	Siphonaptera	Ischnopsyllidae	Nycteridopsylla iae
Sepsis fulgens voucher su49 16S ribosomal RNA gene	EU435606	65.41	93.43	0.000357446	Diptera	Sepsidae	Sepsis fulgens
Ochthebius minimus voucher BMNH:679120 16S ribosom	DQ202575	69.74	73.74	0.000357446	Coleoptera	Hydraenidae	Ochthebius minimus
Rhadinopsylla dahurica 16S ribosomal RNA gene, par	AY072637	84.31	25.76	0.000357446	Siphonaptera	Ctenophthalmidae	Rhadinopsylla dahurica
Themira pusilla mitochondrial partial 16S rRNA gen	AJ843108	84.62	25.76	0.000357446	Diptera	Sepsidae	Themira pusilla
Themira lucida mitochondrial partial 16S rRNA gene	AJ843105	84.62	25.76	0.000357446	Diptera	Sepsidae	Themira lucida
Themira flavicoxa mitochondrial partial 16S rRNA g	AJ843102	84.62	25.76	0.000357446	Diptera	Sepsidae	Themira flavicoxa
Irwiniella velutina voucher MEI 011319 16S ribosom	DQ250819	75.31	40.91	0.000357446	Diptera	Therevidae	Irwiniella velutina
Ctenocephalides felis felis isolate MerA1 16S ribo	KM891403	85.42	23.23	0.00124761	Siphonaptera	Pulicidae	Ctenocephalides felis felis
Hectopsylla pulex isolate F461 16S ribosomal RNA g	KM891392	85.42	23.23	0.00124761	Siphonaptera	Hectopsyllidae	Hectopsylla pulex
Hystrichopsylla dippiei isolate F428 16S ribosomal	KM891390	85.42	23.23	0.00124761	Siphonaptera	Hystrichopsyllidae	Hystrichopsylla dippiei
Hystrichopsylla sp. F404 16S ribosomal RNA gene, p	KM891382	85.42	23.23	0.00124761	Siphonaptera	Hystrichopsyllidae	Hystrichopsylla sp. F404
Xenopsylla conformis conformis isolate F324 16S ri	KM891366	94.12	17.17	0.00124761	Siphonaptera	Pulicidae	Xenopsylla conformis conformis
Paramonopsyllus scalonae isolate F318 16S ribosoma	KM891360	85.42	23.23	0.00124761	Siphonaptera	Ceratophyllidae	Paramonopsyllus scalonae
Ectinorus lareschiae isolate F283 16S ribosomal RN	KM891326	85.42	23.23	0.00124761	Siphonaptera	Rhopalopsyllidae	Ectinorus lareschiae
Listronius fortis isolate F273 16S ribosomal RNA g	KM891318	85.42	23.23	0.00124761	Siphonaptera	Rhopalopsyllidae	Listronius fortis
Ctenophthalmus cryptotis isolate F268 16S ribosoma	KM891315	85.42	23.23	0.00124761	Siphonaptera	Ctenophthalmidae	Ctenophthalmus cryptotis
Hystrichopsylla orophila isolate F191 16S ribosoma	KM891275	85.42	23.23	0.00124761	Siphonaptera	Hystrichopsyllidae	Hystrichopsylla orophila
Chloroniella peringueyi 16S ribosomal RNA gene, pa	KF944502	70.19	78.28	0.00124761	Megaloptera	Corydalidae	Chloroniella peringueyi
Asteia concinna isolate A63 16S ribosomal RNA gene	KJ418481	81.97	29.8	0.00124761	Diptera	Asteiidae	Asteia concinna
Trichosia sp. 1 nr. borealis SS−2012 voucher 858 1	JQ613945	78.87	32.83	0.00124761	Diptera	Sciaridae	Trichosia sp. 1 nr. borealis SS−2012
Listrocerus sp. 1186 voucher UPOL001186 16S riboso	HQ619681	81.36	29.29	0.00124761	Coleoptera	Melyridae	Listrocerus sp. 1186
Helictopleurus quadripunctatus voucher Hel.quapun.	EF187959	81.36	29.29	0.00124761	Coleoptera	Scarabaeidae	Helictopleurus quadripunctatus
Helictopleurus quadripunctatus voucher Hel.quapun.	EF187958	81.36	29.29	0.00124761	Coleoptera	Scarabaeidae	Helictopleurus quadripunctatus
Helictopleurus quadripunctatus voucher Hel.quapun.	EF187957	81.36	29.29	0.00124761	Coleoptera	Scarabaeidae	Helictopleurus quadripunctatus
Helictopleurus corruscus voucher Hel.cor.13.2 16S	EF187919	83.33	29.29	0.00124761	Coleoptera	Scarabaeidae	Helictopleurus corruscus
Helictopleurus quadripunctatus voucher BMNH:669892	EF656656	81.36	29.29	0.00124761	Coleoptera	Scarabaeidae	Helictopleurus quadripunctatus
Helictopleurus quadripunctatus 16S ribosomal RNA g	AY131551	81.36	29.29	0.00124761	Coleoptera	Scarabaeidae	Helictopleurus quadripunctatus
Helictopleurus sinuaticornis 16S ribosomal RNA gen	DQ369537	79.69	29.8	0.00124761	Coleoptera	Scarabaeidae	Helictopleurus sinuaticornis
Helictopleurus corruscus 16S ribosomal RNA gene, p	DQ369523	83.33	29.29	0.00124761	Coleoptera	Scarabaeidae	Helictopleurus corruscus
Nemopoda nitidula mitochondrial partial 16S rRNA g	AJ843096	80.95	30.3	0.00124761	Diptera	Sepsidae	Nemopoda nitidula
Pulex sp. F464 16S ribosomal RNA gene, partial seq	KM891395	85.11	23.74	0.00435458	Siphonaptera	Pulicidae	Pulex sp. F464
Ctenophthalmus sp. F462 16S ribosomal RNA gene, pa	KM891393	85.11	23.74	0.00435458	Siphonaptera	Ctenophthalmidae	Ctenophthalmus sp. F462
Ctenophthalmus sp. F423 16S ribosomal RNA gene, pa	KM891388	85.11	23.74	0.00435458	Siphonaptera	Ctenophthalmidae	Ctenophthalmus sp. F423
Ctenophthalmus sp. F405 16S ribosomal RNA gene, pa	KM891383	85.11	23.74	0.00435458	Siphonaptera	Ctenophthalmidae	Ctenophthalmus sp. F405
Craneopsylla minerva wolffheuglia isolate F336 16S	KM891378	84	25.25	0.00435458	Siphonaptera	Stephanocircidae	Craneopsylla minerva wolffheuglia
Tetrapsyllus maulinus isolate F333 16S ribosomal R	KM891375	85.11	23.74	0.00435458	Siphonaptera	Rhopalopsyllidae	Tetrapsyllus maulinus
Polygenis roberti roberti isolate F294 16S ribosom	KM891336	85.11	23.74	0.00435458	Siphonaptera	Rhopalopsyllidae	Polygenis roberti roberti
Tetrapsyllus maulinus isolate F267 16S ribosomal R	KM891314	85.11	23.74	0.00435458	Siphonaptera	Rhopalopsyllidae	Tetrapsyllus maulinus
Pygiopsylla hoplia isolate F244 16S ribosomal RNA	KM891300	85.11	23.74	0.00435458	Siphonaptera	Pygiopsyllidae	Pygiopsylla hoplia
Lentistivalius occidentayunnanus isolate F236 16S	KM891295	85.11	23.74	0.00435458	Siphonaptera	Stivaliidae	Lentistivalius occidentayunnanus
Xenopsylla ramesis isolate F206 16S ribosomal RNA	KM891278	83.67	23.23	0.00435458	Siphonaptera	Pulicidae	Xenopsylla ramesis
Protopolystoma xenopodis genome assembly P_xenopod	LM725368	87.8	20.71	0.00435458	Polyopisthocotylea	Polystomatidae	Protopolystoma xenopodis
Plasmodium yoelii genome assembly PY17X01, chromos	LM993668	79.03	30.3	0.00435458	Haemosporida	Plasmodiidae	Plasmodium yoelii
Plasmodium yoelii genome assembly PYYM01, chromoso	LK934642	79.03	30.3	0.00435458	Haemosporida	Plasmodiidae	Plasmodium yoelii
Plasmodium berghei ANKA genome assembly PBANKA01,	LK023129	79.03	30.3	0.00435458	Haemosporida	Plasmodiidae	Plasmodium berghei ANKA
