# Supplementary material for: Testing the potential of a ribosomal 16S marker for DNA metabarcoding of insects
Source: PeerJ. 2016 Apr 19;4:e1966. doi: 10.7717/peerj.1966 (PMC4841222; doi:10.7717/peerj.1966)
Supplement: Supplemental Information 1 [file peerj-04-1966-s006.zip › scripts/5 match OTUs/blast_hits/OTU_12.csv_plot.pdf]

OTU_12.csv

Ecdyonurus dispar

Heptageniidae

Ephemeroptera

89.68

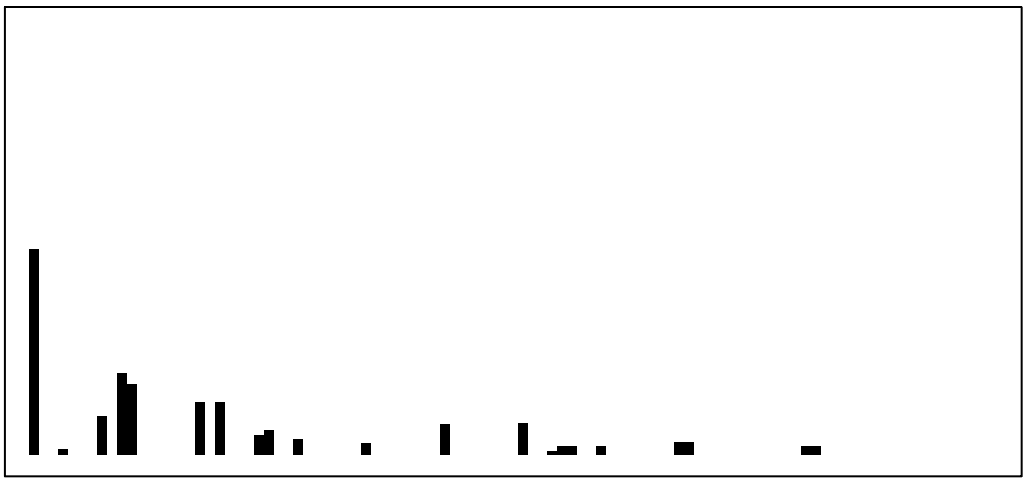

Ecdyonurus dispar 16S ribosomal RNA gene, partial	AY749775	89.68	100	1.8898e−50	Ephemeroptera	Heptageniidae	Ecdyonurus dispar
Atopopus tarsalis mitochondrial partial 16S rRNA g	HE651420	80	90.32	5.07551e−26	Ephemeroptera	Heptageniidae	Atopopus tarsalis
Atopopus tarsalis mitochondrial partial 16S rRNA g	HE651419	80	90.32	5.07551e−26	Ephemeroptera	Heptageniidae	Atopopus tarsalis
Thalerosphyrus lamuriensis mitochondrial partial 1	HE651425	80.29	85.16	3.203e−22	Ephemeroptera	Heptageniidae	Thalerosphyrus lamuriensis
Parafronurus youi mitochondrion, complete genome	EU349015	76.62	99.35	1.11796e−21	Ephemeroptera	Heptageniidae	Parafronurus youi
Thalerosphyrus sinuosus mitochondrial partial 16S	HE651430	76.92	92.26	1.36195e−20	Ephemeroptera	Heptageniidae	Thalerosphyrus sinuosus
Thalerosphyrus lamuriensis mitochondrial partial 1	HE651429	79.56	85.16	1.36195e−20	Ephemeroptera	Heptageniidae	Thalerosphyrus lamuriensis
Clioperla clio 16S ribosomal RNA gene, partial seq	EF623237	81.82	76.77	1.36195e−20	Plecoptera	Perlodidae	Clioperla clio
Heptagenia sulphurea mitochondrial partial 16S rRN	HE651431	77.7	89.03	4.75367e−20	Ephemeroptera	Heptageniidae	Heptagenia sulphurea
Afronurus sp. 212BoAf mitochondrial partial 16S rR	HE651421	83.84	63.87	4.75367e−20	Ephemeroptera	Heptageniidae	Afronurus sp. 212BoAf
Siphlonurus binotatus mitochondrial gene for 16S r	AB110269	83.33	65.81	4.75367e−20	Ephemeroptera	Siphlonuridae	Siphlonurus binotatus
Afronurus sp. 213BoAf mitochondrial partial 16S rR	HE651422	76.81	89.03	1.65919e−19	Ephemeroptera	Heptageniidae	Afronurus sp. 213BoAf
Siphlonurus sp. EP084 16S ribosomal RNA gene, part	AY749786	75.64	100	5.79115e−19	Ephemeroptera	Siphlonuridae	Siphlonurus sp. EP084
Isoperla fulva 16S ribosomal RNA gene, partial seq	EF623207	75	100	2.02131e−18	Plecoptera	Perlodidae	Isoperla fulva
Diamphipnopsis sp. BYU_PL138 16S ribosomal RNA gen	EF623145	77.54	87.74	2.02131e−18	Plecoptera	Diamphipnoidae	Diamphipnopsis sp. BYU_PL138
Isoperla oxylepis 16S ribosomal RNA gene, partial	EF623221	77.69	83.23	7.05507e−18	Plecoptera	Perlodidae	Isoperla oxylepis
Stenonema sp. EP026 16S ribosomal RNA gene, partia	AY749769	76.3	87.1	7.05507e−18	Ephemeroptera	Heptageniidae	Stenonema sp. EP026
Siphlonurus sp. MT−2014 isolate CL142 mitochondrio	KM244684	82.47	62.58	2.46246e−17	Ephemeroptera	Siphlonuridae	Siphlonurus sp. MT−2014
Afronurus peringueyi 16S ribosomal RNA gene, parti	AY749790	76.43	89.03	2.46246e−17	Ephemeroptera	Heptageniidae	Afronurus peringueyi
Parameletus columbiae 16S ribosomal RNA gene, part	AY749768	82.47	62.58	2.46246e−17	Ephemeroptera	Siphlonuridae	Parameletus columbiae
Paegniodes cupulatus mitochondrion, complete genom	HM004123	78.45	74.84	8.59483e−17	Ephemeroptera	Heptageniidae	Paegniodes cupulatus
Afronurus sp. 297SAAf mitochondrial partial 16S rR	HE651424	74.83	91.61	8.59483e−17	Ephemeroptera	Heptageniidae	Afronurus sp. 297SAAf
Pteronarcys sachalina 16S ribosomal RNA gene, part	EF623264	76.92	83.23	8.59483e−17	Plecoptera	Pteronarcyidae	Pteronarcys sachalina
Osobenus yakimae 16S ribosomal RNA gene, partial s	EF623232	80.95	67.1	8.59483e−17	Plecoptera	Perlodidae	Osobenus yakimae
Chernokrilus misnomus 16S ribosomal RNA gene, part	EF623209	81.19	65.16	8.59483e−17	Plecoptera	Perlodidae	Chernokrilus misnomus
Isoperla davisi 16S ribosomal RNA gene, partial se	EF623206	78.33	76.77	8.59483e−17	Plecoptera	Perlodidae	Isoperla davisi
Pseudiron centralis 16S ribosomal RNA gene, partia	AY749799	75.36	89.03	8.59483e−17	Ephemeroptera	Pseudironidae	Pseudiron centralis
Notonemouridae sp. MD−2011 voucher ZMUC:JD−1507 16	JN615257	80.77	66.45	2.99989e−16	Plecoptera	Notonemouridae	Notonemouridae sp. MD−2011
Kogotus nonus 16S ribosomal RNA gene, partial sequ	EF623213	80	67.74	2.99989e−16	Plecoptera	Perlodidae	Kogotus nonus
Eupholidoptera chabrieri voucher 173 16S ribosomal	EU120792	76.35	94.19	2.99989e−16	Orthoptera	Tettigoniidae	Eupholidoptera chabrieri
Cinygmina sp. EP133 16S ribosomal RNA gene, partia	AY749811	76.74	83.23	2.99989e−16	Ephemeroptera	Heptageniidae	Cinygmina sp. EP133
Cryptoperla japonica mitochondrial gene for 16S rR	AB711202	77.24	78.71	1.04707e−15	Plecoptera	Peltoperlidae	Cryptoperla japonica
Cryptoperla japonica mitochondrial gene for 16S rR	AB711184	77.24	78.71	1.04707e−15	Plecoptera	Peltoperlidae	Cryptoperla japonica
Nemoura cinerea 16S ribosomal RNA gene, partial se	EF623194	76.6	88.39	1.04707e−15	Plecoptera	Nemouridae	Nemoura cinerea
Amphinemura nigritta 16S ribosomal RNA gene, parti	EF623186	80.58	65.81	1.04707e−15	Plecoptera	Nemouridae	Amphinemura nigritta
Suwallia teleckojensis 16S ribosomal RNA gene, par	EF623142	75.52	90.97	1.04707e−15	Plecoptera	Chloroperlidae	Suwallia teleckojensis
Neaviperla forcipata 16S ribosomal RNA gene, parti	EF623139	76.09	87.74	1.04707e−15	Plecoptera	Chloroperlidae	Neaviperla forcipata
Ameletoides sp. EP159 16S ribosomal RNA gene, part	AY749826	75.71	89.68	1.04707e−15	Ephemeroptera	Nesameletidae	Ameletoides sp. EP159
Oniscigaster distans 16S ribosomal RNA gene, parti	AY749818	74.52	100	1.04707e−15	Ephemeroptera	Oniscigastridae	Oniscigaster distans
Macdunnoa hipawinia 16S ribosomal RNA gene, partia	AY749796	75.36	88.39	1.04707e−15	Ephemeroptera	Heptageniidae	Macdunnoa hipawinia
Rhithrogena nuragica mitochondrial partial 16S rRN	LN868667	74.83	90.97	3.65462e−15	Ephemeroptera	Heptageniidae	Rhithrogena nuragica
Cryptoperla sp. YK−2012−1 mitochondrial gene for 1	AB711243	78.57	71.61	3.65462e−15	Plecoptera	Peltoperlidae	Cryptoperla sp. YK−2012−1
Afronurus sp. 338SuAf mitochondrial partial 16S rR	HE651428	81.44	62.58	3.65462e−15	Ephemeroptera	Heptageniidae	Afronurus sp. 338SuAf
Ephemera simulans voucher BYU:IGCEP038 16S ribosom	GQ118285	78.05	78.71	3.65462e−15	Ephemeroptera	Ephemeridae	Ephemera simulans
Rickera sorpta 16S ribosomal RNA gene, partial seq	EF623235	79.05	67.74	3.65462e−15	Plecoptera	Perlodidae	Rickera sorpta
Perlinodes aurea 16S ribosomal RNA gene, partial s	EF623234	77.87	77.42	3.65462e−15	Plecoptera	Perlodidae	Perlinodes aurea
Frisonia picticeps 16S ribosomal RNA gene, partial	EF623227	77.87	77.42	3.65462e−15	Plecoptera	Perlodidae	Frisonia picticeps
Neofulla sp. BYU_PL152 16S ribosomal RNA gene, par	EF623203	76.34	83.23	3.65462e−15	Plecoptera	Notonemouridae	Neofulla sp. BYU_PL152
Isogenoides varians isolate BYU_PL079 16S ribosoma	EF623445	79.05	67.74	3.65462e−15	Plecoptera	Perlodidae	Isogenoides varians
Tasmanophlebia sp. EP158 16S ribosomal RNA gene, p	AY749825	75.54	89.03	3.65462e−15	Ephemeroptera	Oniscigastridae	Tasmanophlebia sp. EP158
Talitropsis sedilloti voucher CW160 16S ribosomal	HM594511	81.52	59.35	1.27559e−14	Orthoptera	Rhaphidophoridae	Talitropsis sedilloti
Udenus wnigrum voucher MAY 16S ribosomal RNA gene,	HM594504	78.26	72.9	1.27559e−14	Orthoptera	Rhaphidophoridae	Udenus wnigrum
Heteromallus cavicola voucher MIL 16S ribosomal RN	HM594499	78.07	72.9	1.27559e−14	Orthoptera	Rhaphidophoridae	Heteromallus cavicola
Helopicus bogaloosa 16S ribosomal RNA gene, partia	EF623228	80.2	64.52	1.27559e−14	Plecoptera	Perlodidae	Helopicus bogaloosa
Cosumnoperla hypocrena 16S ribosomal RNA gene, par	EF623226	80.41	62.58	1.27559e−14	Plecoptera	Perlodidae	Cosumnoperla hypocrena
Cascadoperla trictura 16S ribosomal RNA gene, part	EF623225	80.41	62.58	1.27559e−14	Plecoptera	Perlodidae	Cascadoperla trictura
Isogenoides olivaceus 16S ribosomal RNA gene, part	EF623224	79.25	67.74	1.27559e−14	Plecoptera	Perlodidae	Isogenoides olivaceus
Skwala americana 16S ribosomal RNA gene, partial s	EF623217	76.34	83.23	1.27559e−14	Plecoptera	Perlodidae	Skwala americana
Isoperla phalerata 16S ribosomal RNA gene, partial	EF623208	80.41	62.58	1.27559e−14	Plecoptera	Perlodidae	Isoperla phalerata
Plumiperla diversa 16S ribosomal RNA gene, partial	EF623132	74.21	100	1.27559e−14	Plecoptera	Chloroperlidae	Plumiperla diversa
Eupholidoptera megastyla isolate ALE1 16S ribosoma	KC852308	74.15	94.19	4.45223e−14	Orthoptera	Tettigoniidae	Eupholidoptera megastyla
Yoraperla uenoi mitochondrial gene for 16S rRNA, p	AB712375	75.56	83.23	4.45223e−14	Plecoptera	Peltoperlidae	Yoraperla uenoi
Cryptoperla japonica mitochondrial gene for 16S rR	AB711183	76.42	78.71	4.45223e−14	Plecoptera	Peltoperlidae	Cryptoperla japonica
Cryptoperla japonica mitochondrial gene for 16S rR	AB711182	76.42	78.71	4.45223e−14	Plecoptera	Peltoperlidae	Cryptoperla japonica
Dolichopoda sp. MR−2008 16S ribosomal RNA gene, pa	EU887863	74.5	92.26	4.45223e−14	Orthoptera	Rhaphidophoridae	Dolichopoda lustriae
Pteronarcys scotti 16S ribosomal RNA gene, partial	EF623263	78.3	68.39	4.45223e−14	Plecoptera	Pteronarcyidae	Pteronarcys scotti
Isogenoides hansoni 16S ribosomal RNA gene, partia	EF623223	80.61	62.58	4.45223e−14	Plecoptera	Perlodidae	Isogenoides hansoni
Yugus bulbosus 16S ribosomal RNA gene, partial seq	EF623214	80.61	62.58	4.45223e−14	Plecoptera	Perlodidae	Yugus bulbosus
Neuroperlopsis patris 16S ribosomal RNA gene, part	EF623152	74.13	91.61	4.45223e−14	Plecoptera	Eustheniidae	Neuroperlopsis patris
Osmylus sp. EH−2004 16S ribosomal RNA gene, partia	AY620146	78.3	68.39	4.45223e−14	Neuroptera	Osmylidae	Osmylus sp. EH−2004
Xiphydria mellipes 16S ribosomal RNA gene, partial	AF379857	76.09	87.1	4.45223e−14	Hymenoptera	Xiphydriidae	Xiphydria mellipes
Pteronarcys princeps mitochondrion, complete genom	AY687866	75.38	83.23	4.45223e−14	Plecoptera	Pteronarcyidae	Pteronarcys princeps
Panorpa nebulosa 16S ribosomal RNA gene, partial s	AF180058	76.67	76.77	4.45223e−14	Mecoptera	Panorpidae	Panorpa nebulosa
Ametropus neavei 16S ribosomal RNA gene, partial s	AY749800	74.65	90.97	4.45223e−14	Ephemeroptera	Ametropodidae	Ametropus neavei
Heptagenia sp. EP018 16S ribosomal RNA gene, parti	AY749762	75.38	83.23	4.45223e−14	Ephemeroptera	Heptageniidae	Heptagenia sp. EP018
Eupholidoptera smyrnensis isolate KOY1 16S ribosom	KC852309	75	94.19	1.55398e−13	Orthoptera	Tettigoniidae	Eupholidoptera smyrnensis
Eupholidoptera megastyla isolate MTO1 16S ribosoma	KC852307	75	94.19	1.55398e−13	Orthoptera	Tettigoniidae	Eupholidoptera megastyla
Eupholidoptera chabrieri isolate IMP1 16S ribosoma	KC852273	75	94.19	1.55398e−13	Orthoptera	Tettigoniidae	Eupholidoptera chabrieri
Cryptoperla ishigakiensis mitochondrial gene for 1	AB711242	74.66	92.26	1.55398e−13	Plecoptera	Peltoperlidae	Cryptoperla ishigakiensis
Afronurus sp. 336SuAf mitochondrial partial 16S rR	HE651427	80.41	62.58	1.55398e−13	Ephemeroptera	Heptageniidae	Afronurus sp. 336SuAf
Parudenus falklandicus voucher PAR 16S ribosomal R	HM594502	80.43	59.35	1.55398e−13	Orthoptera	Rhaphidophoridae	Parudenus falklandicus
Dolichopoda remyi voucher EDE 16S ribosomal RNA ge	EF216969	77.19	72.9	1.55398e−13	Orthoptera	Rhaphidophoridae	Dolichopoda remyi
Dolichopoda thasosensis voucher DRA 16S ribosomal	EF216956	74.67	92.26	1.55398e−13	Orthoptera	Rhaphidophoridae	Dolichopoda thasosensis
Dolichopoda pavesii voucher SPI 16S ribosomal RNA	EF216951	78.85	66.45	1.55398e−13	Orthoptera	Rhaphidophoridae	Dolichopoda pavesii
Peltoperla arcuata 16S ribosomal RNA gene, partial	EF623258	74.63	85.81	1.55398e−13	Plecoptera	Peltoperlidae	Peltoperla arcuata
Soyedina producta 16S ribosomal RNA gene, partial	EF623196	78.43	65.81	1.55398e−13	Plecoptera	Nemouridae	Soyedina producta
Protonemura meyeri 16S ribosomal RNA gene, partial	EF623193	75	86.45	1.55398e−13	Plecoptera	Nemouridae	Protonemura meyeri
Triznaka pintada 16S ribosomal RNA gene, partial s	EF623134	74.05	100	1.55398e−13	Plecoptera	Chloroperlidae	Triznaka pintada
Psorodonotus fieberi voucher 198 16S ribosomal RNA	EU120806	73.97	93.55	1.55398e−13	Orthoptera	Tettigoniidae	Psorodonotus fieberi
Dolichopoda remyi isolate EDE 16S ribosomal RNA ge	AY793589	77.19	72.9	1.55398e−13	Orthoptera	Rhaphidophoridae	Dolichopoda remyi
Siphlonella sp. EP083 16S ribosomal RNA gene, part	AY749785	78.43	65.81	1.55398e−13	Ephemeroptera	Oniscigastridae	Siphlonella sp. EP083
Chiloporter sp. EP051 16S ribosomal RNA gene, part	AY749776	79.38	62.58	1.55398e−13	Ephemeroptera	Ameletopsidae	Chiloporter sp. EP051
Phloiophilus edwardsi voucher UPOL001206 16S ribos	KC758772	78.5	68.39	5.42393e−13	Coleoptera	Phloiophilidae	Phloiophilus edwardsi
Eupholidoptera garganica isolate LEF1 16S ribosoma	KC852301	73.79	92.9	5.42393e−13	Orthoptera	Tettigoniidae	Eupholidoptera garganica
Cryptoperla kawasawai mitochondrial gene for 16S r	AB711226	76.32	73.55	5.42393e−13	Plecoptera	Peltoperlidae	Cryptoperla kawasawai
Cryptoperla kawasawai mitochondrial gene for 16S r	AB711225	76.32	73.55	5.42393e−13	Plecoptera	Peltoperlidae	Cryptoperla kawasawai
Cryptoperla kawasawai mitochondrial gene for 16S r	AB711224	76.58	71.61	5.42393e−13	Plecoptera	Peltoperlidae	Cryptoperla kawasawai
Cryptoperla kawasawai mitochondrial gene for 16S r	AB711220	76.32	73.55	5.42393e−13	Plecoptera	Peltoperlidae	Cryptoperla kawasawai
Cryptoperla kawasawai mitochondrial gene for 16S r	AB711219	76.32	73.55	5.42393e−13	Plecoptera	Peltoperlidae	Cryptoperla kawasawai
Compsoneuria sp. 10 LV−2012 mitochondrial partial	HE651412	72.78	100	5.42393e−13	Ephemeroptera	Heptageniidae	Compsoneuria sp. 10 LV−2012
