# Supplementary material for: Testing the potential of a ribosomal 16S marker for DNA metabarcoding of insects
Source: PeerJ. 2016 Apr 19;4:e1966. doi: 10.7717/peerj.1966 (PMC4841222; doi:10.7717/peerj.1966)
Supplement: Supplemental Information 1 [file peerj-04-1966-s006.zip › scripts/5 match OTUs/blast_hits/OTU_120.csv_plot.pdf]

OTU_120.csv

Pachygaster piriventris

Stratiomyidae

Diptera

82.69

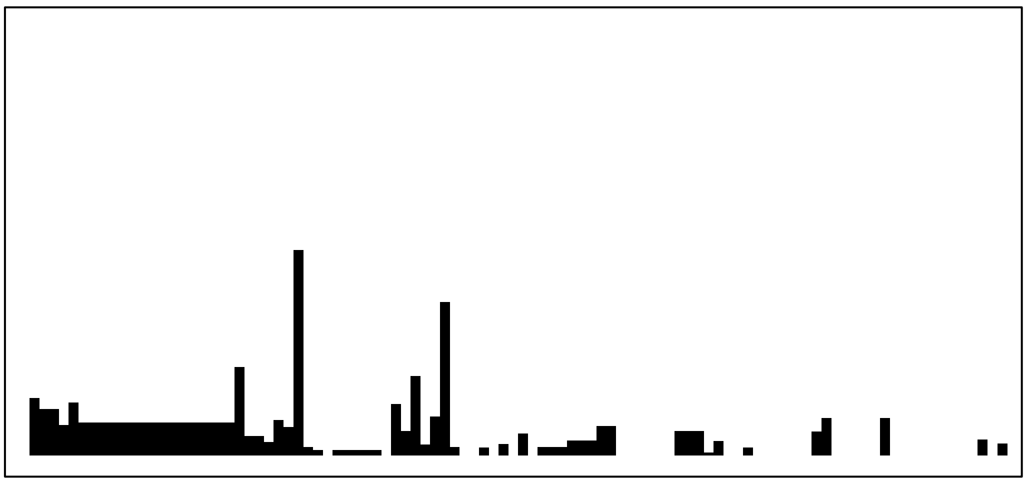

Pachygaster piriventris 16S ribosomal RNA gene, pa	GU947390	82.69	100	1.05486e−34	Diptera	Stratiomyidae	Pachygaster piriventris
Drosophila cardini voucher 103963 NADH dehydrogena	EU494310	82.17	100	4.48537e−33	Diptera	Drosophilidae	Drosophila cardini
Drosophila cardini 16S ribosomal RNA gene, partial	AF479800	82.17	100	4.48537e−33	Diptera	Drosophilidae	Drosophila cardini
Pedicia albivitta voucher O'Grady 200012 16S ribos	EU005435	81.41	99.36	5.4643e−32	Diptera	Pediciidae	Pedicia albivitta
Dichopygina aculeata voucher 947 16S ribosomal RNA	JQ613961	82.47	97.44	1.90723e−31	Diptera	Sciaridae	Dichopygina aculeata
Chorisops tunisiae 16S ribosomal RNA gene, partial	GU947379	81.53	100	1.90723e−31	Diptera	Stratiomyidae	Chorisops tunisiae
Drosophila dunni voucher 103969 NADH dehydrogenase	EU494311	81.53	100	1.90723e−31	Diptera	Drosophilidae	Drosophila dunni
Drosophila similis grenadensis NADH dehydrogenase	AF246519	81.53	100	1.90723e−31	Diptera	Drosophilidae	Drosophila similis grenadensis
Drosophila arawakana dominicensis NADH dehydrogena	AF246516	81.53	100	1.90723e−31	Diptera	Drosophilidae	Drosophila arawakana dominicensis
Drosophila dunni dunni strain Barranquitas, line 1	AF246512	81.53	100	1.90723e−31	Diptera	Drosophilidae	Drosophila dunni dunni
Drosophila cardini strain Constanza, line 4 NADH d	AF246511	81.53	100	1.90723e−31	Diptera	Drosophilidae	Drosophila cardini
Drosophila acutilabella strain 15181−2171.9 NADH d	AF246509	81.53	100	1.90723e−31	Diptera	Drosophilidae	Drosophila acutilabella
Drosophila arawakana arawakana strain 15182−2261.0	AF246499	81.53	100	1.90723e−31	Diptera	Drosophilidae	Drosophila arawakana arawakana
Drosophila dunni thomasensis NADH dehydrogenase su	AF246501	81.53	100	1.90723e−31	Diptera	Drosophilidae	Drosophila dunni thomasensis
Drosophila arawakana arawakana strain 15182−2261.1	AF246500	81.53	100	1.90723e−31	Diptera	Drosophilidae	Drosophila arawakana arawakana
Drosophila cardini strain 15181−2181.9 NADH dehydr	AF246498	81.53	100	1.90723e−31	Diptera	Drosophilidae	Drosophila cardini
Drosophila dunni dunni strain 15182−2291.0 NADH de	AF246497	81.53	100	1.90723e−31	Diptera	Drosophilidae	Drosophila dunni dunni
Drosophila acutilabella strain 15181−2171.2 NADH d	AF246496	81.53	100	1.90723e−31	Diptera	Drosophilidae	Drosophila acutilabella
Drosophila similis similis NADH dehydrogenase subu	AF246495	81.53	100	1.90723e−31	Diptera	Drosophilidae	Drosophila similis similis
Drosophila nigrodunni NADH dehydrogenase subunit 1	AF246494	81.53	100	1.90723e−31	Diptera	Drosophilidae	Drosophila nigrodunni
Drosophila caribiana NADH dehydrogenase subunit 1	AF246493	81.53	100	1.90723e−31	Diptera	Drosophilidae	Drosophila caribiana
Pseudolycoriella sp. SS−2012 voucher 84 16S riboso	JQ613888	84.14	89.1	2.32348e−30	Diptera	Sciaridae	Pseudolycoriella sp. SS−2012
Drosophila belladunni NADH dehydrogenase subunit 1	AF246506	80.89	100	2.32348e−30	Diptera	Drosophilidae	Drosophila belladunni
Drosophila parthenogenetica strain 15181−2221.0 NA	AF246503	80.89	100	2.32348e−30	Diptera	Drosophilidae	Drosophila parthenogenetica
Empis barbatoides 16S ribosomal RNA gene, partial	KC177443	80.62	100	8.10974e−30	Diptera	Empididae	Empis barbatoides
Stenopomyia angulata voucher MEI 146440 16S riboso	DQ250827	81.65	100	8.10974e−30	Diptera	Therevidae	Stenopomyia angulata
Cramptonomyia spenceri mitochondrion, complete gen	JN861747	81.33	94.87	2.83058e−29	Diptera	Pachyneuridae	Cramptonomyia spenceri
Cyrtodiopsis curranii 16S ribosomal RNA gene, parti	AF304746	89.62	67.31	2.83058e−29	Diptera	Diopsidae	Teleopsis currani
Eupsilocephala albodorsalis 16S ribosomal RNA gene	KM878912	80.38	100	9.87969e−29	Diptera	Therevidae	Eupsilocephala albodorsalis
Hermetia illucens 16S ribosomal RNA gene, partial	GU947384	80.25	100	9.87969e−29	Diptera	Stratiomyidae	Hermetia illucens
Tephritidae sp. HYH−2008 16S ribosomal RNA gene, p	EU926952	79.62	100	9.87969e−29	Diptera	Tephritidae	Tephritidae sp. HYH−2008
Drosophila neomorpha NADH dehydrogenase subunit 1	AF246517	80.25	100	9.87969e−29	Diptera	Drosophilidae	Drosophila neomorpha
Drosophila procardinoides NADH dehydrogenase subun	AF246508	80.25	100	9.87969e−29	Diptera	Drosophilidae	Drosophila procardinoides
Drosophila polymorpha strain 15181−2231.1 NADH deh	AF246507	80.25	100	9.87969e−29	Diptera	Drosophilidae	Drosophila polymorpha
Drosophila neocardini NADH dehydrogenase subunit 1	AF246505	80.25	100	9.87969e−29	Diptera	Drosophilidae	Drosophila neocardini
Drosophila polymorpha strain 15181−2231.2 NADH deh	AF246504	80.25	100	9.87969e−29	Diptera	Drosophilidae	Drosophila polymorpha
Sapromyza bentejui mitochondrion partial 16S rRNA	AJ439186	79.62	100	9.87969e−29	Diptera	Lauxaniidae	Sapromyza bentejui
Teleopsis sp. n. 'gracilifemur' voucher FF−V2 16S	KP978625	82.39	88.46	3.44835e−28	Diptera	Diopsidae	Teleopsis sp. n. 'gracilifemur'
Agathomyia unicolor isolate AU2 16S ribosomal RNA	KC699318	81.13	100	3.44835e−28	Diptera	Platypezidae	Agathomyia unicolor
Dixella sp. ZK−2014 mitochondrion, complete genome	KM245574	83.7	85.26	3.44835e−28	Diptera	Dixidae	Dixella sp. ZK−2014
Minettia flaveola 16S ribosomal RNA gene, partial	KC177448	80.5	100	3.44835e−28	Diptera	Lauxaniidae	Minettia flaveola
Beris morrisii 16S ribosomal RNA gene, partial seq	GU947373	81.82	91.03	3.44835e−28	Diptera	Stratiomyidae	Beris morrisii
Nemoraea takanoi mitochondrial gene for 16S rRNA,	AB465951	87.18	72.44	3.44835e−28	Diptera	Tachinidae	Nemoraea takanoi
Pexopsis pilosa mitochondrial gene for 16S rRNA, p	AB465940	80.38	100	3.44835e−28	Diptera	Tachinidae	Pexopsis pilosa
Parafreutreta regalis 16S ribosomal RNA gene, part	DQ471409	79.75	100	3.44835e−28	Diptera	Tephritidae	Parafreutreta regalis
Euaresta bella 16S ribosomal RNA gene, partial seq	DQ471398	79.75	100	3.44835e−28	Diptera	Tephritidae	Euaresta bella
Manestella ocellaris 16S ribosomal RNA gene, parti	KM878928	80.37	100	1.20359e−27	Diptera	Therevidae	Manestella ocellaris
Ectinorhynchus micans voucher MEI30034 16S ribosom	KM212982	79.62	100	1.20359e−27	Diptera	Therevidae	Ectinorhynchus micans
Brugia timori genome assembly B_timori_Indonesia_v	LK908083	80.52	94.87	1.20359e−27	Spirurida	Onchocercidae	Brugia timori
Hermetia illucens 16S ribosomal RNA gene, partial	KC177455	79.62	100	1.20359e−27	Diptera	Stratiomyidae	Hermetia illucens
Actina chalybea 16S ribosomal RNA gene, partial se	GU947372	81.01	100	1.20359e−27	Diptera	Acroceridae	Actina chalybea
Drosophila bifasciata voucher 109384 NADH dehydrog	EU494362	79.38	100	1.20359e−27	Diptera	Drosophilidae	Drosophila bifasciata
Limonia swezeyi voucher O'Grady 201882 16S ribosom	EU005424	80.38	99.36	1.20359e−27	Diptera	Limoniidae	Dicranomyia swezeyi
Limonia swezeyi voucher O'Grady 200931 16S ribosom	EU005421	80.38	99.36	1.20359e−27	Diptera	Limoniidae	Dicranomyia swezeyi
Limonia swezeyi voucher O'Grady 200126 16S ribosom	EU005420	80.38	99.36	1.20359e−27	Diptera	Limoniidae	Dicranomyia swezeyi
Melanostoma apicale 16S ribosomal RNA gene, partia	AF154791	80.69	92.31	1.20359e−27	Diptera	Syrphidae	Melanostoma apicale
Melanostoma apicale 16S ribosomal RNA gene, partia	AF154790	80.69	92.31	1.20359e−27	Diptera	Syrphidae	Melanostoma apicale
Melanostoma apicale 16S ribosomal RNA gene, partia	AF154789	80.69	92.31	1.20359e−27	Diptera	Syrphidae	Melanostoma apicale
Dideopsis aegrota 16S ribosomal RNA gene, partial	AF154774	81.38	92.31	1.20359e−27	Diptera	Syrphidae	Dideopsis aegrota
Dideopsis aegrota 16S ribosomal RNA gene, partial	AF154773	81.38	92.31	1.20359e−27	Diptera	Syrphidae	Dideopsis aegrota
Nanexila manni voucher MEI 120167 16S ribosomal RN	DQ250875	79.62	100	1.20359e−27	Diptera	Therevidae	Nanexila manni
Anabarhynchus tristis voucher MEI 119905 16S ribos	DQ250834	79.62	100	1.20359e−27	Diptera	Therevidae	Anabarhynchus tristis
Callomyia admirabilis isolate CA42 16S ribosomal R	KC699326	78.98	100	4.20095e−27	Diptera	Platypezidae	Callomyia admirabilis
Tipula abdominalis 16S ribosomal RNA gene, partial	KC177457	79.87	94.87	4.20095e−27	Diptera	Tipulidae	Tipula abdominalis
Tipula abdominalis mitochondrion, partial genome	JN861743	79.87	94.87	4.20095e−27	Diptera	Tipulidae	Tipula abdominalis
Euaresta festiva 16S ribosomal RNA gene, partial s	DQ471399	78.98	100	4.20095e−27	Diptera	Tephritidae	Euaresta festiva
Chalcosyrphus elongatus 16S ribosomal RNA gene, pa	AF154761	81.13	100	4.20095e−27	Diptera	Syrphidae	Chalcosyrphus elongatus
Chalcosyrphus elongatus 16S ribosomal RNA gene, pa	AF154760	81.13	100	4.20095e−27	Diptera	Syrphidae	Chalcosyrphus elongatus
Chalcosyrphus elongatus 16S ribosomal RNA gene, pa	AF154759	81.13	100	4.20095e−27	Diptera	Syrphidae	Chalcosyrphus elongatus
Hilara maura 16S ribosomal RNA gene, partial seque	AF126346	80.12	100	4.20095e−27	Diptera	Empididae	Hilara maura
Parochlus steinenii isolate Sejong01 mitochondrion	KT003702	80.67	94.87	1.46628e−26	Diptera	Chironomidae	Parochlus steinenii
Pamelamyia sp. PS33 16S ribosomal RNA gene, partia	KC699355	79.11	100	1.46628e−26	Diptera	Platypezidae	Pamelamyia sp. PS33
Drosophila simulans strain simw501 mitochondrion,	KC244284	78.75	100	1.46628e−26	Diptera	Drosophilidae	Drosophila simulans
Sciadocera rufomaculata 16S ribosomal RNA gene, pa	JN196447	80.37	100	1.46628e−26	Diptera	Sciadoceridae	Sciadocera rufomaculata
Dinera takanoi mitochondrial gene for 16S rRNA, pa	AB465958	79.38	100	1.46628e−26	Diptera	Tachinidae	Dinera takanoi
Drosophila simulans isolate KY215 mitochondrion, c	AY518672	78.75	100	1.46628e−26	Diptera	Drosophilidae	Drosophila simulans
Drosophila simulans isolate KY007 mitochondrion, c	AY518670	78.75	100	1.46628e−26	Diptera	Drosophilidae	Drosophila simulans
Drosophila simulans isolate RU35 mitochondrion, co	AF200845	78.75	100	1.46628e−26	Diptera	Drosophilidae	Drosophila simulans
Drosophila simulans isolate MD106 mitochondrion, c	AF200842	78.75	100	1.46628e−26	Diptera	Drosophilidae	Drosophila simulans
Drosophila simulans cytochrome b (cytb) gene, part	AF164588	78.75	100	1.46628e−26	Diptera	Drosophilidae	Drosophila simulans
Antissa sp. SLW−2002 16S ribosomal RNA gene, parti	AY140891	81.12	91.03	1.46628e−26	Diptera	Stratiomyidae	Antissa sp. SLW−2002
Melanostoma scalare 16S ribosomal RNA gene, partia	AF126336	81.76	92.31	1.46628e−26	Diptera	Syrphidae	Melanostoma scalare
Medomega bailmeup 16S ribosomal RNA gene, partial	KM878929	79.5	100	5.11781e−26	Diptera	Therevidae	Medomega bailmeup
Hemigephyra atra 16S ribosomal RNA gene, partial s	KM878915	78.98	100	5.11781e−26	Diptera	Therevidae	Hemigephyra atra
Microsania pectipennis isolate MP27 16S ribosomal	KC699344	79.63	100	5.11781e−26	Diptera	Platypezidae	Microsania pectipennis
Microsania pectipennis isolate MP12 16S ribosomal	KC699343	79.63	100	5.11781e−26	Diptera	Platypezidae	Microsania pectipennis
Agathomyia falleni isolate AF03 16S ribosomal RNA	KC699319	79.14	100	5.11781e−26	Diptera	Platypezidae	Agathomyia falleni
Agathomyia viduella isolate AV01 16S ribosomal RNA	KC699317	81.76	91.67	5.11781e−26	Diptera	Platypezidae	Agathomyia viduella
Therevidae gen. P sp. 'barakula' voucher QMT183200	KM212977	79.75	100	5.11781e−26	Diptera	Therevidae	Therevidae gen. P sp. 'barakula'
Ectinorhynchus brunneus voucher QMT183204 16S ribo	KM212973	79.11	100	5.11781e−26	Diptera	Therevidae	Ectinorhynchus brunneus
Johnmannia kosciuszkoensis voucher QMT183205 16S r	KM212971	79.11	100	5.11781e−26	Diptera	Therevidae	Johnmannia kosciuszkoensis
Rutilia goerlingiana mitochondrion, complete genom	JX913762	78.88	97.44	5.11781e−26	Diptera	Tachinidae	Rutilia goerlingiana
Cymatopus sp. Pollet 0321 16S ribosomal RNA gene,	HQ448972	78.34	100	5.11781e−26	Diptera	Dolichopodidae	Cymatopus sp. Pollet 0321
Limonia tahitiensis voucher O'Grady 202004 16S rib	EU005425	78.98	99.36	5.11781e−26	Diptera	Limoniidae	Dicranomyia tahitiensis
Limonia swezeyi voucher O'Grady 200982 16S ribosom	EU005423	79.75	99.36	5.11781e−26	Diptera	Limoniidae	Dicranomyia swezeyi
Limonia swezeyi voucher O'Grady 200949 16S ribosom	EU005422	79.75	99.36	5.11781e−26	Diptera	Limoniidae	Dicranomyia swezeyi
Limonia swezeyi voucher O'Grady 200105 16S ribosom	EU005419	79.75	99.36	5.11781e−26	Diptera	Limoniidae	Dicranomyia swezeyi
Melaloncha muricata isolate 2916S 16S ribosomal RN	EU068628	80.75	100	5.11781e−26	Diptera	Phoridae	Melaloncha muricata
Procecidochares atra 16S ribosomal RNA gene, parti	DQ471376	80	100	5.11781e−26	Diptera	Tephritidae	Procecidochares atra
Sphaerocera curvipes 16S ribosomal RNA gene, parti	AY573135	80.56	91.03	5.11781e−26	Diptera	Sphaeroceridae	Sphaerocera curvipes
