# Supplementary material for: Testing the potential of a ribosomal 16S marker for DNA metabarcoding of insects
Source: PeerJ. 2016 Apr 19;4:e1966. doi: 10.7717/peerj.1966 (PMC4841222; doi:10.7717/peerj.1966)
Supplement: Supplemental Information 1 [file peerj-04-1966-s006.zip › scripts/5 match OTUs/blast_hits/OTU_121.csv_plot.pdf]

OTU_121.csv

Pedicia albivitta

Pediciidae

Diptera

83.02

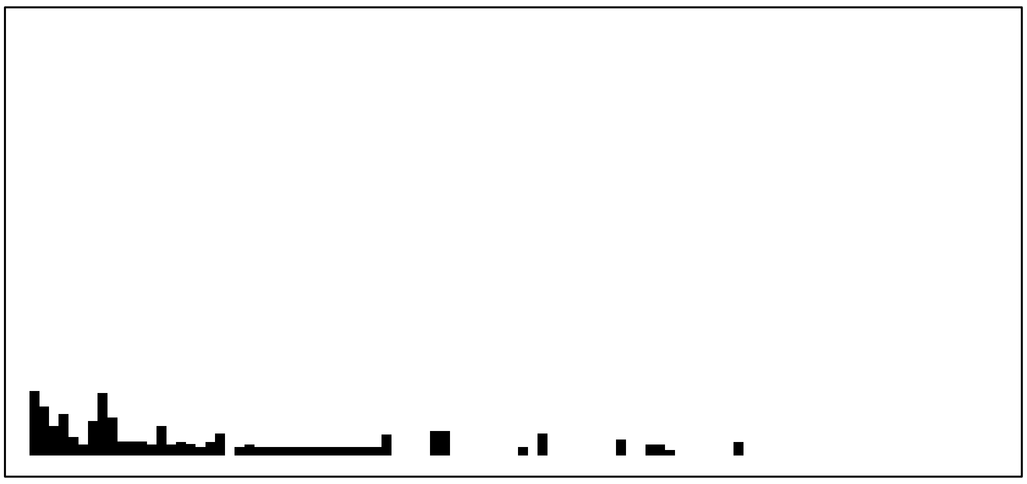

Pedicia albivitta voucher O'Grady 200012 16S ribos	EU005435	83.02	100	3.74267e−34	Diptera	Pediciidae	Pedicia albivitta
Parochlus steinenii isolate Sejong01 mitochondrion	KT003702	82.28	100	1.30632e−33	Diptera	Chironomidae	Parochlus steinenii
Libnotes perkinsi voucher O'Grady 202000 16S ribos	EU005434	81.37	100	1.59143e−32	Diptera	Limoniidae	Libnotes perkinsi
Scatopsciara atomaria voucher 981 16S ribosomal RN	JQ613973	81.94	97.47	5.55462e−32	Diptera	Sciaridae	Scatopsciara atomaria
Empis barbatoides 16S ribosomal RNA gene, partial	KC177443	80.86	99.37	6.76691e−31	Diptera	Empididae	Empis barbatoides
Somatia sp. YSUW04038 16S ribosomal RNA gene, part	AY573134	80.5	99.37	6.76691e−31	Diptera	Somatiidae	Somatia sp. YSUW04038
Libnotes orofenaae voucher O'Grady 202034 16S ribo	EU005431	81.6	100	2.36188e−30	Diptera	Limoniidae	Libnotes orofenaae
Bolitophila cinerea voucher JSB1 16S ribosomal RNA	KJ136712	82.91	97.47	8.24379e−30	Diptera	Mycetophilidae	Bolitophila cinerea
Drosophila procardinoides NADH dehydrogenase subun	AF246508	81.76	99.37	8.24379e−30	Diptera	Drosophilidae	Drosophila procardinoides
Dichopygina aculeata voucher 947 16S ribosomal RNA	JQ613961	80.65	97.47	2.87736e−29	Diptera	Sciaridae	Dichopygina aculeata
Lycoriella gerbatshevskayae voucher 601 16S riboso	JQ613919	80.65	97.47	2.87736e−29	Diptera	Sciaridae	Lycoriella gerbatshevskayae
Scatopsciara atomaria voucher 90 16S ribosomal RNA	JQ613890	80.65	97.47	2.87736e−29	Diptera	Sciaridae	Scatopsciara atomaria
Liriomyza trifolii mitochondrion, complete genome	JN570506	80.5	99.37	2.87736e−29	Diptera	Agromyzidae	Liriomyza trifolii
Exodontha dubia 16S ribosomal RNA gene, partial se	GU947396	81.37	99.37	2.87736e−29	Diptera	Stratiomyidae	Exodontha dubia
Liriomyza trifolii mitochondrion, complete genome	GU327644	80.5	99.37	2.87736e−29	Diptera	Agromyzidae	Liriomyza trifolii
Trichosia edwardsi voucher T39 16S ribosomal RNA g	JQ613980	80.62	98.73	1.0043e−28	Diptera	Sciaridae	Trichosia edwardsi
Camptochaeta tenuipalpalis voucher 566 16S ribosom	JQ613915	80.52	97.47	1.0043e−28	Diptera	Sciaridae	Camptochaeta tenuipalpalis
Haematopota pluvialis 16S ribosomal RNA gene, part	KC177454	80.38	99.37	1.0043e−28	Diptera	Tabanidae	Haematopota pluvialis
Sciadocera rufomaculata 16S ribosomal RNA gene, pa	JN196447	80.62	99.37	1.0043e−28	Diptera	Sciadoceridae	Sciadocera rufomaculata
Pachygaster piriventris 16S ribosomal RNA gene, pa	GU947390	81.01	99.37	1.0043e−28	Diptera	Stratiomyidae	Pachygaster piriventris
Tabanus bromius 16S ribosomal RNA gene, partial se	HM132111	79.75	99.37	1.0043e−28	Diptera	Tabanidae	Tabanus bromius
Drosophila dunni voucher 103969 NADH dehydrogenase	EU494311	80.38	99.37	1.0043e−28	Diptera	Drosophilidae	Drosophila dunni
Tipula sp. PMO 200011 16S ribosomal RNA gene, part	EU005437	80.5	100	1.0043e−28	Diptera	Tipulidae	Tipula sp. PMO 200011
Drosophila similis grenadensis NADH dehydrogenase	AF246519	80.38	99.37	1.0043e−28	Diptera	Drosophilidae	Drosophila similis grenadensis
Drosophila arawakana dominicensis NADH dehydrogena	AF246516	80.38	99.37	1.0043e−28	Diptera	Drosophilidae	Drosophila arawakana dominicensis
Drosophila dunni dunni strain Barranquitas, line 1	AF246512	80.38	99.37	1.0043e−28	Diptera	Drosophilidae	Drosophila dunni dunni
Drosophila acutilabella strain 15181−2171.9 NADH d	AF246509	80.38	99.37	1.0043e−28	Diptera	Drosophilidae	Drosophila acutilabella
Drosophila belladunni NADH dehydrogenase subunit 1	AF246506	80.38	99.37	1.0043e−28	Diptera	Drosophilidae	Drosophila belladunni
Drosophila arawakana arawakana strain 15182−2261.0	AF246499	80.38	99.37	1.0043e−28	Diptera	Drosophilidae	Drosophila arawakana arawakana
Drosophila dunni thomasensis NADH dehydrogenase su	AF246501	80.38	99.37	1.0043e−28	Diptera	Drosophilidae	Drosophila dunni thomasensis
Drosophila arawakana arawakana strain 15182−2261.1	AF246500	80.38	99.37	1.0043e−28	Diptera	Drosophilidae	Drosophila arawakana arawakana
Drosophila dunni dunni strain 15182−2291.0 NADH de	AF246497	80.38	99.37	1.0043e−28	Diptera	Drosophilidae	Drosophila dunni dunni
Drosophila acutilabella strain 15181−2171.2 NADH d	AF246496	80.38	99.37	1.0043e−28	Diptera	Drosophilidae	Drosophila acutilabella
Drosophila similis similis NADH dehydrogenase subu	AF246495	80.38	99.37	1.0043e−28	Diptera	Drosophilidae	Drosophila similis similis
Drosophila nigrodunni NADH dehydrogenase subunit 1	AF246494	80.38	99.37	1.0043e−28	Diptera	Drosophilidae	Drosophila nigrodunni
Drosophila caribiana NADH dehydrogenase subunit 1	AF246493	80.38	99.37	1.0043e−28	Diptera	Drosophilidae	Drosophila caribiana
Belosta albipilosa 16S ribosomal RNA gene, partial	KM878903	80.98	99.37	3.50535e−28	Diptera	Scenopinidae	Belosta albipilosa
Corynoptera blanda voucher 955 16S ribosomal RNA g	JQ613965	80	97.47	3.50535e−28	Diptera	Sciaridae	Corynoptera blanda
Scatopsciara necopinata voucher 623 16S ribosomal	JQ613921	80	97.47	3.50535e−28	Diptera	Sciaridae	Scatopsciara necopinata
Lycoriella flavicornis voucher 62 16S ribosomal RN	JQ613885	80	97.47	3.50535e−28	Diptera	Sciaridae	Lycoriella flavicornis
Drosophila bifasciata voucher 109384 NADH dehydrog	EU494362	79.87	99.37	3.50535e−28	Diptera	Drosophilidae	Drosophila bifasciata
Drosophila neocardini NADH dehydrogenase subunit 1	AF246505	81.13	99.37	3.50535e−28	Diptera	Drosophilidae	Drosophila neocardini
Drosophila parthenogenetica strain 15181−2221.0 NA	AF246503	81.13	99.37	3.50535e−28	Diptera	Drosophilidae	Drosophila parthenogenetica
Bradysiopsis vittigera voucher 948 16S ribosomal R	JQ613962	78.79	97.47	1.22349e−27	Diptera	Sciaridae	Bradysiopsis vittigera
Culcua kolibaci 16S ribosomal RNA gene, partial se	GU947391	79.62	99.37	1.22349e−27	Diptera	Stratiomyidae	Culcua kolibaci
Hermetia illucens 16S ribosomal RNA gene, partial	GU947384	79.62	99.37	1.22349e−27	Diptera	Stratiomyidae	Hermetia illucens
Platypezina diversa isolate PD29 16S ribosomal RNA	KC699349	79.25	99.37	4.27039e−27	Diptera	Platypezidae	Platypezina diversa
Helina fratercula voucher RMBR:116199 16S ribosoma	KJ476309	79.25	99.37	4.27039e−27	Diptera	Muscidae	Helina fratercula
Coboldia sp. SS−2012 voucher 905 16S ribosomal RNA	JQ613954	79.87	97.47	4.27039e−27	Diptera	Scatopsidae	Coboldia sp. SS−2012
Ptychoptera sp. ATB−2011 mitochondrion, complete g	JN861744	80	100	4.27039e−27	Diptera	Ptychopteridae	Ptychoptera sp. ATB−2011
Sybistroma discipes voucher Pollet 0097 (P) 16S ri	EU864013	80.38	99.37	4.27039e−27	Diptera	Dolichopodidae	Sybistroma discipes
Hercostomus nigriplantis voucher Pollet 0210 (P) 1	EU863998	79.75	99.37	4.27039e−27	Diptera	Dolichopodidae	Hercostomus nigriplantis
Bolitophila sp. ER−2009 16S ribosomal RNA gene, pa	FJ172021	81.01	97.47	4.27039e−27	Diptera	Mycetophilidae	Bolitophila sp. ER−2009
Empis producta voucher RMBR:103430 16S ribosomal R	FJ808167	79.75	99.37	4.27039e−27	Diptera	Empididae	Empis producta
Drosophila nebulosa voucher 107549 NADH dehydrogen	EU494370	79.87	99.37	4.27039e−27	Diptera	Drosophilidae	Drosophila nebulosa
Drosophila putrida voucher 103964 NADH dehydrogena	EU494348	79.25	99.37	4.27039e−27	Diptera	Drosophilidae	Drosophila putrida
Drosophila funebris voucher 103952 NADH dehydrogen	EU494313	79.75	99.37	4.27039e−27	Diptera	Drosophilidae	Drosophila funebris
Drosophila cardini voucher 103963 NADH dehydrogena	EU494310	79.75	99.37	4.27039e−27	Diptera	Drosophilidae	Drosophila cardini
Limonia kauaiensis voucher O'Grady 201868 16S ribo	EU005414	80	100	4.27039e−27	Diptera	Limoniidae	Dicranomyia kauaiensis
Limonia kauaiensis voucher O'Grady 200971 16S ribo	EU005412	80	100	4.27039e−27	Diptera	Limoniidae	Dicranomyia kauaiensis
Limonia jacobus voucher O'Grady 200974 16S ribosom	EU005408	80.75	100	4.27039e−27	Diptera	Limoniidae	Dicranomyia jacobus
Drosophila cardini 16S ribosomal RNA gene, partial	AF479800	79.75	99.37	4.27039e−27	Diptera	Drosophilidae	Drosophila cardini
Drosophila busckii 16S ribosomal RNA gene, partial	AF479796	79.25	99.37	4.27039e−27	Diptera	Drosophilidae	Drosophila busckii
Toxomerus marginatus 16S ribosomal RNA gene, parti	AF154820	80.5	99.37	4.27039e−27	Diptera	Syrphidae	Toxomerus marginatus
Toxomerus geminatus 16S ribosomal RNA gene, partia	AF154819	80.5	99.37	4.27039e−27	Diptera	Syrphidae	Toxomerus geminatus
Corynoptera boletiphaga voucher 995 16S ribosomal	JQ613974	80.25	97.47	1.49051e−26	Diptera	Sciaridae	Corynoptera boletiphaga
Scatopsciara atomaria gr. sp. SS−2012 voucher 978	JQ613972	80	97.47	1.49051e−26	Diptera	Sciaridae	Scatopsciara atomaria gr. sp. SS−2012
Lonchoptera uniseta 16S ribosomal RNA gene, partia	KC177456	79.5	99.37	1.49051e−26	Diptera	Lonchopteridae	Lonchoptera uniseta
Salticella fasciata voucher MU_NUS_NSCU_4 16S ribo	JN828238	78.75	99.37	1.49051e−26	Diptera	Sciomyzidae	Salticella fasciata
Trichopeza longicornis voucher Pollet 0243 (P) 16S	HQ449035	80	99.37	1.49051e−26	Diptera	Empididae	Trichopeza longicornis
Cymatopus sp. Pollet 0321 16S ribosomal RNA gene,	HQ448972	78.12	99.37	1.49051e−26	Diptera	Dolichopodidae	Cymatopus sp. Pollet 0321
Eudmeta marginata 16S ribosomal RNA gene, partial	GU947400	79.62	99.37	1.49051e−26	Diptera	Stratiomyidae	Eudmeta marginata
Hercostomus longiventris voucher Pollet 0274 (P) 1	EU863994	80.62	99.37	1.49051e−26	Diptera	Dolichopodidae	Hercostomus longiventris
Drosophila tripunctata voucher 107541 NADH dehydro	EU494351	80	99.37	1.49051e−26	Diptera	Drosophilidae	Drosophila tripunctata
Drosophila tripunctata 16S ribosomal RNA gene, par	AF479806	80	99.37	1.49051e−26	Diptera	Drosophilidae	Drosophila tripunctata
Anastrepha bezzii 16S ribosomal RNA gene, partial	AF152057	78.62	99.37	1.49051e−26	Diptera	Tephritidae	Anastrepha bezzii
Simulium suyoense mitochondrial gene for 16S ribos	LC035000	79.35	97.47	5.2024e−26	Diptera	Simuliidae	Simulium suyoense
Simulium suyoense mitochondrial gene for 16S ribos	LC034999	79.35	97.47	5.2024e−26	Diptera	Simuliidae	Simulium suyoense
Simulium suyoense mitochondrial gene for 16S ribos	LC034998	79.35	97.47	5.2024e−26	Diptera	Simuliidae	Simulium suyoense
Simulium suyoense mitochondrial gene for 16S ribos	LC034997	79.35	97.47	5.2024e−26	Diptera	Simuliidae	Simulium suyoense
Pseudatrichia bupennis 16S ribosomal RNA gene, par	KM878952	80	99.37	5.2024e−26	Diptera	Scenopinidae	Pseudatrichia bupennis
Dixella sp. ZK−2014 mitochondrion, complete genome	KM245574	79.11	99.37	5.2024e−26	Diptera	Dixidae	Dixella sp. ZK−2014
Diadocidia spinosula voucher JSSJ7 16S ribosomal R	KJ136737	79.74	96.2	5.2024e−26	Diptera	Diadocidiidae	Diadocidia spinosula
Trichosia confusa voucher 838 16S ribosomal RNA ge	JQ613942	78.75	98.73	5.2024e−26	Diptera	Sciaridae	Trichosia confusa
Leptosciarella (Leptospina) sp. 1 nr. dentata SS−2	JQ613937	79.63	97.47	5.2024e−26	Diptera	Sciaridae	Leptosciarella (Leptospina) sp. 1 nr. dentata SS−2012
Pseudovidalia rohdendorfi isolate PsvRoh1 16S ribo	JQ412062	79.38	99.37	5.2024e−26	Diptera	Tephritidae	Pseudovidalia rohdendorfi
Hermetia illucens 16S ribosomal RNA gene, partial	KC177455	78.98	99.37	5.2024e−26	Diptera	Stratiomyidae	Hermetia illucens
Clinocera sp. Pollet 0249 16S ribosomal RNA gene,	HQ449032	79.38	99.37	5.2024e−26	Diptera	Empididae	Clinocera sp. Pollet 0249
Clitellaria ephippium 16S ribosomal RNA gene, part	GU947399	79.75	99.37	5.2024e−26	Diptera	Stratiomyidae	Clitellaria ephippium
Oplodontha viridula 16S ribosomal RNA gene, partia	GU947377	78.98	99.37	5.2024e−26	Diptera	Stratiomyidae	Oplodontha viridula
Dolichopus ungulatus voucher Pollet 0273 (P) 16S r	EU863960	79.11	99.37	5.2024e−26	Diptera	Dolichopodidae	Dolichopus ungulatus
Diadocidia spinosula 16S ribosomal RNA gene, parti	FJ172010	79.74	96.2	5.2024e−26	Diptera	Diadocidiidae	Diadocidia spinosula
Hercostomus meieri voucher RMBR:103442 16S ribosom	FJ808173	79.11	99.37	5.2024e−26	Diptera	Dolichopodidae	Hercostomus meieri
Drosophila neocordata voucher 107545 NADH dehydrog	EU494366	79.25	99.37	5.2024e−26	Diptera	Drosophilidae	Drosophila neocordata
Simulium suyoense mitochondrial gene for 16S ribos	AB334099	79.35	97.47	5.2024e−26	Diptera	Simuliidae	Simulium suyoense
Libnotes perkinsi voucher O'Grady 200981 16S ribos	EU005433	79.25	100	5.2024e−26	Diptera	Limoniidae	Libnotes perkinsi
Libnotes perkinsi voucher O'Grady 200947 16S ribos	EU005432	79.25	100	5.2024e−26	Diptera	Limoniidae	Libnotes perkinsi
Limonia swezeyi voucher O'Grady 200105 16S ribosom	EU005419	79.38	100	5.2024e−26	Diptera	Limoniidae	Dicranomyia swezeyi
Tabanus rufofrater 16S ribosomal RNA gene, partial	AY140887	78.48	99.37	5.2024e−26	Diptera	Tabanidae	Tabanus rufofrater
Drosophila putrida 16S ribosomal RNA gene, partial	AF479805	79.25	99.37	5.2024e−26	Diptera	Drosophilidae	Drosophila putrida
