# Supplementary material for: Testing the potential of a ribosomal 16S marker for DNA metabarcoding of insects
Source: PeerJ. 2016 Apr 19;4:e1966. doi: 10.7717/peerj.1966 (PMC4841222; doi:10.7717/peerj.1966)
Supplement: Supplemental Information 1 [file peerj-04-1966-s006.zip › scripts/5 match OTUs/blast_hits/OTU_122.csv_plot.pdf]

OTU_122.csv

Sericostoma personatum

Sericostomatidae

Trichoptera

90.14

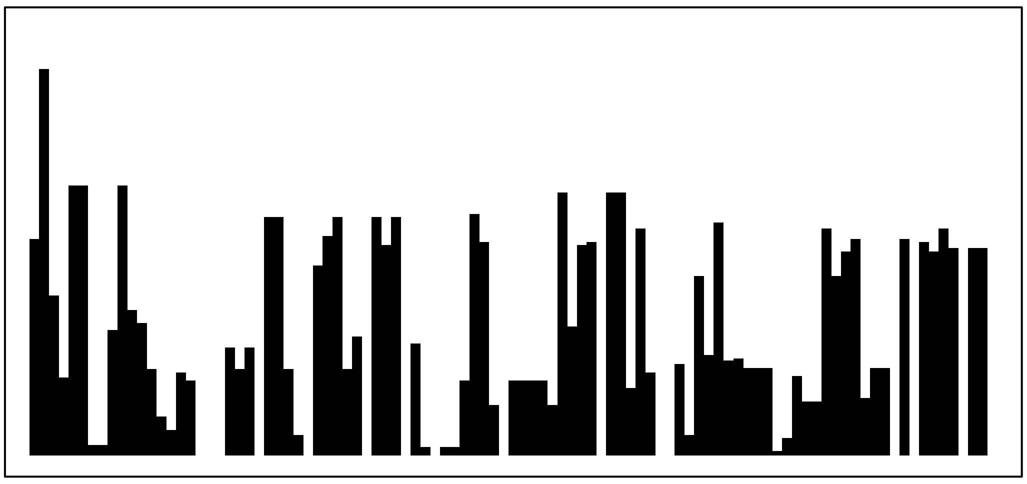

Sericostoma personatum mitochondrion, partial geno	KP455290	90.14	98.61	3.78099e−46	Trichoptera	Sericostomatidae	Sericostoma personatum
Sericostoma clypeatum voucher NHRS−EN CC4 16S ribo	FJ263207	98.11	73.61	6.83618e−43	Trichoptera	Sericostomatidae	Sericostoma clypeatum
Notidobiella chacayana isolate CL5 16S ribosomal R	EF394993	87.5	81.94	7.30547e−30	Trichoptera	Sericostomatidae	Notidobiella chacayana
Lordithon thoracicus voucher ZMUN:10029203 16S rib	JX536486	83.65	67.36	2.23674e−17	Coleoptera	Staphylinidae	Lordithon thoracicus
Protochauliodes biconicus incertus 16S ribosomal R	KF944494	92.65	46.53	7.80697e−17	Megaloptera	Corydalidae	Protochauliodes biconicus incertus
Protochauliodes incertus isolate H842 16S ribosoma	KF741322	92.65	46.53	7.80697e−17	Megaloptera	Corydalidae	Protochauliodes biconicus incertus
Nycteribia pygmaea mitochondrial genes for 16S rib	AB632579	80.49	84.72	7.80697e−17	Diptera	Nycteribiidae	Nycteribia pygmaea
Nycteribia pygmaea mitochondrial genes for 16S rib	AB632578	80.49	84.72	7.80697e−17	Diptera	Nycteribiidae	Nycteribia pygmaea
Hypocera mordellaria isolate 200864 16S ribosomal	GU362000	85.87	59.72	7.80697e−17	Diptera	Phoridae	Hypocera mordellaria
Hydrotaea irritans 16S ribosomal RNA gene, partial	FJ025443	92.65	46.53	7.80697e−17	Diptera	Muscidae	Hydrotaea irritans
Limonia tahitiensis voucher O'Grady 202004 16S rib	EU005425	86.81	61.81	7.80697e−17	Diptera	Limoniidae	Dicranomyia tahitiensis
Toxotrypana littoralis 16S ribosomal RNA gene, par	AF152091	86.21	59.03	7.80697e−17	Diptera	Tephritidae	Toxotrypana littoralis
Diopsis nr. gnu KCADI063−14 16S ribosomal RNA gene	KT186676	84.04	61.11	2.7249e−16	Diptera	Diopsidae	Diopsis nr. gnu KCADI063−14
Calliphora vomitoria isolate nk56 16S ribosomal RN	KP862541	81.82	68.06	2.7249e−16	Diptera	Calliphoridae	Calliphora vomitoria
Polietina sp. SNK−2014 voucher RMBR:116219 16S rib	KJ476326	81.19	68.75	2.7249e−16	Diptera	Muscidae	Polietina sp. SNK−2014
Arhopalus rusticus voucher HNAU.PP 006 16S ribosom	KF737722	83.87	59.72	2.7249e−16	Coleoptera	Cerambycidae	Arhopalus rusticus
Attalus heydeni voucher UPOL001106 16S ribosomal R	HQ619658	83.5	67.36	2.7249e−16	Coleoptera	Melyridae	Attalus heydeni
Trox terrestris isolate 7B 16S ribosomal RNA gene,	EF570394	78.42	91.67	2.7249e−16	Coleoptera	Trogidae	Trox terrestris
Trox terrestris isolate 7A 16S ribosomal RNA gene,	EF570383	78.42	91.67	2.7249e−16	Coleoptera	Trogidae	Trox terrestris
Ortholfersia minuta 16S ribosomal RNA gene, partia	EF531123	79.03	85.42	2.7249e−16	Diptera	Hippoboscidae	Ortholfersia minuta
Paroligoneurus sp. JCB−2006 16S ribosomal RNA gene	DQ538568	85.06	59.72	2.7249e−16	Hymenoptera	Braconidae	Paroligoneurus sp. JCB−2006
Diopsis gnu 16S ribosomal RNA gene, partial sequen	AF304744	84.04	61.11	2.7249e−16	Diptera	Diopsidae	Diopsis gnu
Paroligoneurus sp. 16S ribosomal RNA gene, partial	AF003519	85.06	59.72	2.7249e−16	Hymenoptera	Braconidae	Paroligoneurus sp.
Corydalus peruvianus 16S ribosomal RNA gene, parti	KF944507	80	79.17	9.51084e−16	Megaloptera	Corydalidae	Corydalus peruvianus
Atelestus pulicarius isolate AP47 16S ribosomal RN	KC699323	91.18	46.53	9.51084e−16	Diptera	Atelestidae	Atelestus pulicarius
Pseudoborbo bevani voucher He018 16S ribosomal RNA	JX971180	91.18	46.53	9.51084e−16	Lepidoptera	Hesperiidae	Pseudoborbo bevani
Carterocephalus silvicola mitochondrion, complete	KJ629163	84.04	60.42	9.51084e−16	Lepidoptera	Hesperiidae	Carterocephalus silvicola
Stenocrates laevicollis 16S ribosomal RNA gene, pa	JX982897	80.95	81.94	9.51084e−16	Coleoptera	Scarabaeidae	Stenocrates laevicollis
Sciodrepoides watsoni mitochondrial 16S rRNA gene	HE576705	78.12	81.94	9.51084e−16	Coleoptera	Leiodidae	Sciodrepoides watsoni
Diplonevra florea isolate 16.xi.06−387 16S ribosom	HM367001	88.89	48.61	9.51084e−16	Diptera	Phoridae	Diplonevra florea
Diplonevra freyi isolate 13.viii.08−475 16S riboso	HM366989	90.28	48.61	9.51084e−16	Diptera	Phoridae	Diplonevra freyi
Musca sorbens 16S ribosomal RNA gene, partial sequ	JN226628	91.18	46.53	9.51084e−16	Diptera	Muscidae	Musca sorbens
Carterocephalus silvicola 16S ribosomal RNA gene,	GU372501	84.04	60.42	9.51084e−16	Lepidoptera	Hesperiidae	Carterocephalus silvicola
Atheta vestita voucher ZMUN:10002613 16S ribosomal	GQ981033	85.56	61.11	9.51084e−16	Coleoptera	Staphylinidae	Atheta vestita
Corydalus armatus 16S ribosomal RNA gene, partial	EU734858	80	79.17	9.51084e−16	Megaloptera	Corydalidae	Corydalus armatus
Morellia aenescens 16S ribosomal RNA gene, partial	FJ025454	91.18	46.53	9.51084e−16	Diptera	Muscidae	Morellia aenescens
Drosophila nebulosa voucher 107549 NADH dehydrogen	EU494370	89.86	47.92	9.51084e−16	Diptera	Drosophilidae	Drosophila nebulosa
Chylizosoma vittatum 16S ribosomal RNA gene, parti	DQ648654	91.18	46.53	9.51084e−16	Diptera	Scathophagidae	Chylizosoma vittatum
Corydalus flavicornis 16S ribosomal RNA gene, part	KF944504	77.61	88.89	3.31961e−15	Megaloptera	Corydalidae	Corydalus flavicornis
Polyporivora boletina isolate 812 16S ribosomal RN	KC699311	85.23	59.72	3.31961e−15	Diptera	Platypezidae	Polyporivora boletina
Coenosia agromyzina voucher RMBR:116185 16S riboso	KJ476299	80.39	70.14	3.31961e−15	Diptera	Muscidae	Coenosia agromyzina
Diaulota densissima voucher ZMUN:10008421 16S ribo	JX536415	79.67	79.17	3.31961e−15	Coleoptera	Staphylinidae	Diaulota densissima
Chrysomya bezziana mitochondrion, complete genome	JX913737	80.39	70.14	3.31961e−15	Diptera	Calliphoridae	Chrysomya bezziana
Chrysomya bezziana isolate D3 small subunit riboso	JQ246710	80.39	70.14	3.31961e−15	Diptera	Calliphoridae	Chrysomya bezziana
Silpha carinata isolate 8 16S ribosomal RNA gene,	JF794603	83.5	67.36	3.31961e−15	Coleoptera	Silphidae	Silpha carinata
Conicera kempi isolate 200852 16S ribosomal RNA ge	GU361992	91.3	46.53	3.31961e−15	Diptera	Phoridae	Conicera kempi
Drosophila eurypeza voucher O201043 16S ribosomal	HQ170966	90	47.92	3.31961e−15	Diptera	Drosophilidae	Drosophila eurypeza
Dinaraea aequata voucher ZMUN:10002546 16S ribosom	GQ981040	82.35	67.36	3.31961e−15	Coleoptera	Staphylinidae	Dinaraea aequata
Atheta vaga voucher ZMUN:10002655 16S ribosomal RN	GQ981034	79.67	80.56	3.31961e−15	Coleoptera	Staphylinidae	Atheta vaga
Silpha carinata voucher CSU10020220B51 16S ribosom	HM051220	83.5	67.36	3.31961e−15	Coleoptera	Silphidae	Silpha carinata
Silpha carinata voucher CSU1002022059 16S ribosoma	HM051217	83.5	67.36	3.31961e−15	Coleoptera	Silphidae	Silpha carinata
Silpha carinata isolate CSU09100410N5 16S ribosoma	GU145268	83.5	67.36	3.31961e−15	Coleoptera	Silphidae	Silpha carinata
Silpha carinata isolate CSU0904046029 16S ribosoma	GQ118353	83.5	67.36	3.31961e−15	Coleoptera	Silphidae	Silpha carinata
Acrotaeniostola sexvittata voucher YSUW98071108 16	EU926919	82.35	67.36	3.31961e−15	Diptera	Tephritidae	Acrotaeniostola sexvittata
Musca domestica isolate C2 16S ribosomal RNA gene,	EU021214	92.31	44.44	3.31961e−15	Diptera	Muscidae	Musca domestica
Thricops cunctans 16S ribosomal RNA gene, partial	FJ025478	86.05	57.64	3.31961e−15	Diptera	Muscidae	Thricops cunctans
Hydrotaea dentipes 16S ribosomal RNA gene, partial	FJ025442	89.86	46.53	3.31961e−15	Diptera	Muscidae	Hydrotaea dentipes
Drosophila biseriata voucher 109453 NADH dehydroge	EU494377	90	47.92	3.31961e−15	Diptera	Drosophilidae	Drosophila biseriata
Corydalus sp. EH−2004 16S ribosomal RNA gene, part	AY620139	77.61	88.89	3.31961e−15	Megaloptera	Corydalidae	Corydalus sp. EH−2004
Musca sp. VH−2001 16S ribosomal RNA gene, partial	AF322436	92.31	44.44	3.31961e−15	Diptera	Muscidae	Musca sp. VH−2001
Musca domestica 16S ribosomal RNA gene, partial se	DQ133039	92.31	44.44	3.31961e−15	Diptera	Muscidae	Musca domestica
Pelopidas agna voucher He013 16S ribosomal RNA gen	JX971182	83.16	60.42	1.15866e−14	Lepidoptera	Hesperiidae	Pelopidas agna
Sonoma sp. JP−2014 isolate JPDG40 16S ribosomal RN	KM350447	90.62	44.44	1.15866e−14	Coleoptera	Staphylinidae	Sonoma sp. JP−2014
Pelopidas agna 16S ribosomal RNA gene, partial seq	KJ786312	83.87	60.42	1.15866e−14	Lepidoptera	Hesperiidae	Pelopidas agna
Thiasophila angulata voucher ZMUN:10030934 16S rib	JX536475	79.2	81.94	1.15866e−14	Coleoptera	Staphylinidae	Thiasophila angulata
Silusa opaca voucher ZMUN:10051225 16S ribosomal R	JX536409	78.74	81.94	1.15866e−14	Coleoptera	Staphylinidae	Silusa opaca
Achalcus flavicollis voucher Pollet 0171 (P) 16S r	HQ448956	84.27	59.72	1.15866e−14	Diptera	Dolichopodidae	Achalcus flavicollis
Phloiotrya planiuscula voucher UPOL ZL0130 16S rib	FJ903741	80.95	72.22	1.15866e−14	Coleoptera	Melandryidae	Phloiotrya planiuscula
Holostrophus orientalis voucher UPOL ZL0082 16S ri	FJ903723	88.41	47.92	1.15866e−14	Coleoptera	Tetratomidae	Holostrophus orientalis
Chondria armipes 16S ribosomal RNA gene, partial s	GQ302111	84.71	58.33	1.15866e−14	Coleoptera	Endomychidae	Chondria armipes
Hydraena unca voucher MNCN−AI276 large subunit rib	HM588431	90.91	45.14	1.15866e−14	Coleoptera	Hydraenidae	Hydraena unca
Chorisops tunisiae 16S ribosomal RNA gene, partial	GU947379	84.44	61.11	1.15866e−14	Diptera	Stratiomyidae	Chorisops tunisiae
Liogluta microptera voucher ZMUN:10002600 16S ribo	GQ981041	84.52	57.64	1.15866e−14	Coleoptera	Staphylinidae	Liogluta microptera
Dasylepida ishigakiensis mitochondrial gene for 16	AB499110	84.09	60.42	1.15866e−14	Coleoptera	Scarabaeidae	Dasylepida ishigakiensis
Dasylepida ishigakiensis mitochondrial gene for 16	AB499109	84.09	60.42	1.15866e−14	Coleoptera	Scarabaeidae	Dasylepida ishigakiensis
Dasylepida ishigakiensis mitochondrial gene for 16	AB499108	84.09	60.42	1.15866e−14	Coleoptera	Scarabaeidae	Dasylepida ishigakiensis
Stevenia atramentaria voucher RMBR:103670 16S ribo	GQ409161	80.2	69.44	1.15866e−14	Diptera	Rhinophoridae	Stevenia atramentaria
Sarcophaga australis voucher RMBR:103727 16S ribos	GQ409151	80.81	68.06	1.15866e−14	Diptera	Sarcophagidae	Sarcophaga australis
Drymeia hamata 16S ribosomal RNA gene, partial seq	FJ025431	83.72	59.03	1.15866e−14	Diptera	Muscidae	Drymeia hamata
Oxyporus niger mitochondrial gene for 16S ribosoma	AB285563	82.52	67.36	1.15866e−14	Coleoptera	Staphylinidae	Oxyporus niger
Eusilpha jakowlewi mitochondrial gene for 16S ribo	AB285547	82.52	67.36	1.15866e−14	Coleoptera	Silphidae	Eusilpha jakowlewi
Drosophila fumipennis voucher 108511 NADH dehydrog	EU494369	90.62	44.44	1.15866e−14	Diptera	Drosophilidae	Drosophila fumipennis
Drosophila equinoxialis voucher 107548 NADH dehydr	EU494368	88.41	47.92	1.15866e−14	Diptera	Drosophilidae	Drosophila equinoxialis
Drosophila austrosaltans voucher 106314 NADH dehyd	EU494364	89.55	46.53	1.15866e−14	Diptera	Drosophilidae	Drosophila austrosaltans
Delevea bertrandi 16S ribosomal RNA gene, partial	EF517584	90.14	48.61	1.15866e−14	Coleoptera	Torridincolidae	Delevea bertrandi
Chelonus sp. 4 NM−2007 16S ribosomal RNA gene, par	EU107070	82.69	67.36	1.15866e−14	Hymenoptera	Braconidae	Chelonus sp. 4 NM−2007
Dasylepida ishigakiensis mitochondrial genes for 1	AB332111	84.09	60.42	1.15866e−14	Coleoptera	Scarabaeidae	Dasylepida ishigakiensis
Dasylepida ishigakiensis mitochondrial genes for 1	AB332110	84.09	60.42	1.15866e−14	Coleoptera	Scarabaeidae	Dasylepida ishigakiensis
Onthophagus clypeatus 16S ribosomal RNA gene, part	EU162538	79.2	81.94	1.15866e−14	Coleoptera	Scarabaeidae	Onthophagus clypeatus
Delevea sp. TJH−2005b voucher BMNH:679387 16S ribo	DQ202611	90.14	48.61	1.15866e−14	Coleoptera	Torridincolidae	Delevea sp. TJH−2005b
Helicopsyche albescens isolate BR5 16S ribosomal R	EF394986	77.78	87.5	1.15866e−14	Trichoptera	Helicopsychidae	Helicopsyche albescens
Rivellia cestoventris 16S ribosomal RNA gene, part	DQ534711	90	47.22	1.15866e−14	Diptera	Platystomatidae	Rivellia cestoventris
Drosophila austrosaltans 16S ribosomal RNA gene, p	AF479808	89.55	46.53	1.15866e−14	Diptera	Drosophilidae	Drosophila austrosaltans
Graptomyza plumifer 16S ribosomal RNA gene, partia	AF154785	90.62	44.44	1.15866e−14	Diptera	Syrphidae	Graptomyza plumifer
Delia platura mitochondrion, partial genome	KP901268	89.71	46.53	4.04411e−14	Diptera	Anthomyiidae	Delia platura
Corydalus cornutus 16S ribosomal RNA gene, partial	KF944505	76.87	88.89	4.04411e−14	Megaloptera	Corydalidae	Corydalus cornutus
Drosophila paracracens voucher 202556 16S ribosoma	KM252237	89.71	46.53	4.04411e−14	Diptera	Drosophilidae	Drosophila paracracens
Drosophila orthoptera voucher 202540 16S ribosomal	KM252228	89.71	46.53	4.04411e−14	Diptera	Drosophilidae	Drosophila orthoptera
Caltoris bromus voucher He032 16S ribosomal RNA ge	JX971186	77.86	85.42	4.04411e−14	Lepidoptera	Hesperiidae	Caltoris bromus
Caltoris bromus 16S ribosomal RNA gene, partial se	KJ786315	77.86	85.42	4.04411e−14	Lepidoptera	Hesperiidae	Caltoris bromus
