# Supplementary material for: Testing the potential of a ribosomal 16S marker for DNA metabarcoding of insects
Source: PeerJ. 2016 Apr 19;4:e1966. doi: 10.7717/peerj.1966 (PMC4841222; doi:10.7717/peerj.1966)
Supplement: Supplemental Information 1 [file peerj-04-1966-s006.zip › scripts/5 match OTUs/blast_hits/OTU_123.csv_plot.pdf]

OTU_123.csv

Ephemera danica

Ephemeridae

Ephemeroptera

100

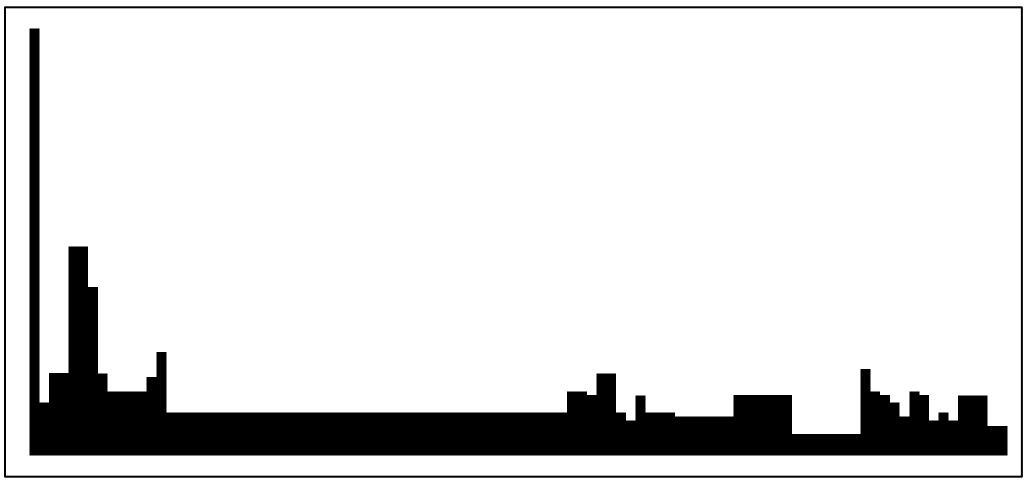

Ephemera danica 16S ribosomal RNA gene, partial se	KF855860	100	64.29	9.09223e−42	Ephemeroptera	Ephemeridae	Ephemera danica
Torleya major 16S ribosomal RNA gene, partial sequ	FJ443047	82.47	100	1.26384e−33	Ephemeroptera	Ephemerellidae	Torleya major
Ephemera orientalis voucher EPH01 tRNA−Leu, 16S ri	KF256907	83.85	84.42	2.78379e−29	Ephemeroptera	Ephemeridae	Ephemera orientalis
Ephemera orientalis mitochondrion, complete genome	EU591678	83.85	84.42	2.78379e−29	Ephemeroptera	Ephemeridae	Ephemera orientalis
Ephemera sp. 1 BM−2008 12S ribosomal RNA gene, par	EU477611	89.8	63.64	1.44204e−26	Ephemeroptera	Ephemeridae	Ephemera sp. 1 BM−2008
Ephemera sp. 'Bonn' large subunit ribosomal RNA ge	AF266047	89.8	63.64	1.44204e−26	Ephemeroptera	Ephemeridae	Ephemera sp. 'Bonn'
Ephemera simulans voucher BYU:IGCEP038 16S ribosom	GQ118285	87.88	64.29	1.75676e−25	Ephemeroptera	Ephemeridae	Ephemera simulans
Siphlonurus immanis voucher NKMT032 mitochondrion,	FJ606783	83.84	64.29	4.71405e−20	Ephemeroptera	Siphlonuridae	Siphlonurus immanis
Palingenia longicauda mitochondrial partial 16S rR	HE650662	83	64.94	5.74289e−19	Ephemeroptera	Palingeniidae	Palingenia longicauda
Palingenia longicauda mitochondrial partial 16S rR	HE650475	83	64.94	5.74289e−19	Ephemeroptera	Palingeniidae	Palingenia longicauda
Palingenia longicauda mitochondrial partial 16S rR	HE650547	83	64.94	5.74289e−19	Ephemeroptera	Palingeniidae	Palingenia longicauda
Palingenia longicauda mitochondrial partial 16S rR	HE650502	83	64.94	5.74289e−19	Ephemeroptera	Palingeniidae	Palingenia longicauda
Eresia plaginota isolate 38−4 16S ribosomal RNA ge	AF186912	83.67	62.99	2.00447e−18	Lepidoptera	Nymphalidae	Eresia plaginota
Ctenoplusia limbirena mitochondrion, complete geno	KM244665	84.85	62.99	6.99628e−18	Lepidoptera	Noctuidae	Ctenoplusia limbirena
Palingenia longicauda mitochondrial partial 16S rR	HE650589	82	64.94	6.99628e−18	Ephemeroptera	Palingeniidae	Palingenia longicauda
Palingenia longicauda mitochondrial partial 16S rR	HE650657	82	64.94	6.99628e−18	Ephemeroptera	Palingeniidae	Palingenia longicauda
Palingenia longicauda mitochondrial partial 16S rR	HE650650	82	64.94	6.99628e−18	Ephemeroptera	Palingeniidae	Palingenia longicauda
Palingenia longicauda mitochondrial partial 16S rR	HE650647	82	64.94	6.99628e−18	Ephemeroptera	Palingeniidae	Palingenia longicauda
Palingenia longicauda mitochondrial partial 16S rR	HE650637	82	64.94	6.99628e−18	Ephemeroptera	Palingeniidae	Palingenia longicauda
Palingenia longicauda mitochondrial partial 16S rR	HE650635	82	64.94	6.99628e−18	Ephemeroptera	Palingeniidae	Palingenia longicauda
Palingenia longicauda mitochondrial partial 16S rR	HE650631	82	64.94	6.99628e−18	Ephemeroptera	Palingeniidae	Palingenia longicauda
Palingenia longicauda mitochondrial partial 16S rR	HE650625	82	64.94	6.99628e−18	Ephemeroptera	Palingeniidae	Palingenia longicauda
Palingenia longicauda mitochondrial partial 16S rR	HE650623	82	64.94	6.99628e−18	Ephemeroptera	Palingeniidae	Palingenia longicauda
Palingenia longicauda mitochondrial partial 16S rR	HE650622	82	64.94	6.99628e−18	Ephemeroptera	Palingeniidae	Palingenia longicauda
Palingenia longicauda mitochondrial partial 16S rR	HE650618	82	64.94	6.99628e−18	Ephemeroptera	Palingeniidae	Palingenia longicauda
Palingenia longicauda mitochondrial partial 16S rR	HE650617	82	64.94	6.99628e−18	Ephemeroptera	Palingeniidae	Palingenia longicauda
Palingenia longicauda mitochondrial partial 16S rR	HE650604	82	64.94	6.99628e−18	Ephemeroptera	Palingeniidae	Palingenia longicauda
Palingenia longicauda mitochondrial partial 16S rR	HE650594	82	64.94	6.99628e−18	Ephemeroptera	Palingeniidae	Palingenia longicauda
Palingenia longicauda mitochondrial partial 16S rR	HE650588	82	64.94	6.99628e−18	Ephemeroptera	Palingeniidae	Palingenia longicauda
Palingenia longicauda mitochondrial partial 16S rR	HE650575	82	64.94	6.99628e−18	Ephemeroptera	Palingeniidae	Palingenia longicauda
Palingenia longicauda mitochondrial partial 16S rR	HE650569	82	64.94	6.99628e−18	Ephemeroptera	Palingeniidae	Palingenia longicauda
Palingenia longicauda mitochondrial partial 16S rR	HE650550	82	64.94	6.99628e−18	Ephemeroptera	Palingeniidae	Palingenia longicauda
Palingenia longicauda mitochondrial partial 16S rR	HE650542	82	64.94	6.99628e−18	Ephemeroptera	Palingeniidae	Palingenia longicauda
Palingenia longicauda mitochondrial partial 16S rR	HE650533	82	64.94	6.99628e−18	Ephemeroptera	Palingeniidae	Palingenia longicauda
Palingenia longicauda mitochondrial partial 16S rR	HE650503	82	64.94	6.99628e−18	Ephemeroptera	Palingeniidae	Palingenia longicauda
Palingenia longicauda mitochondrial partial 16S rR	HE650520	82	64.94	6.99628e−18	Ephemeroptera	Palingeniidae	Palingenia longicauda
Palingenia longicauda mitochondrial partial 16S rR	HE650519	82	64.94	6.99628e−18	Ephemeroptera	Palingeniidae	Palingenia longicauda
Palingenia longicauda mitochondrial partial 16S rR	HE650515	82	64.94	6.99628e−18	Ephemeroptera	Palingeniidae	Palingenia longicauda
Palingenia longicauda mitochondrial partial 16S rR	HE650514	82	64.94	6.99628e−18	Ephemeroptera	Palingeniidae	Palingenia longicauda
Palingenia longicauda mitochondrial partial 16S rR	HE650509	82	64.94	6.99628e−18	Ephemeroptera	Palingeniidae	Palingenia longicauda
Palingenia longicauda mitochondrial partial 16S rR	HE650507	82	64.94	6.99628e−18	Ephemeroptera	Palingeniidae	Palingenia longicauda
Palingenia longicauda mitochondrial partial 16S rR	HE650505	82	64.94	6.99628e−18	Ephemeroptera	Palingeniidae	Palingenia longicauda
Palingenia longicauda mitochondrial partial 16S rR	HE650499	82	64.94	6.99628e−18	Ephemeroptera	Palingeniidae	Palingenia longicauda
Palingenia longicauda mitochondrial partial 16S rR	HE650446	82	64.94	6.99628e−18	Ephemeroptera	Palingeniidae	Palingenia longicauda
Palingenia longicauda mitochondrial partial 16S rR	HE650435	82	64.94	6.99628e−18	Ephemeroptera	Palingeniidae	Palingenia longicauda
Palingenia longicauda mitochondrial partial 16S rR	HE650461	82	64.94	6.99628e−18	Ephemeroptera	Palingeniidae	Palingenia longicauda
Palingenia longicauda mitochondrial partial 16S rR	HE650449	82	64.94	6.99628e−18	Ephemeroptera	Palingeniidae	Palingenia longicauda
Palingenia longicauda mitochondrial partial 16S rR	HE650420	82	64.94	6.99628e−18	Ephemeroptera	Palingeniidae	Palingenia longicauda
Palingenia longicauda mitochondrial partial 16S rR	HE650440	82	64.94	6.99628e−18	Ephemeroptera	Palingeniidae	Palingenia longicauda
Palingenia longicauda mitochondrial partial 16S rR	HE650434	82	64.94	6.99628e−18	Ephemeroptera	Palingeniidae	Palingenia longicauda
Palingenia longicauda mitochondrial partial 16S rR	HE650430	82	64.94	6.99628e−18	Ephemeroptera	Palingeniidae	Palingenia longicauda
Palingenia longicauda mitochondrial partial 16S rR	HE650421	82	64.94	6.99628e−18	Ephemeroptera	Palingeniidae	Palingenia longicauda
Palingenia longicauda mitochondrial partial 16S rR	HE650424	82	64.94	6.99628e−18	Ephemeroptera	Palingeniidae	Palingenia longicauda
Palingenia longicauda mitochondrial partial 16S rR	HE650422	82	64.94	6.99628e−18	Ephemeroptera	Palingeniidae	Palingenia longicauda
Palingenia longicauda voucher BYU:IGCEP216 16S rib	GQ118292	82	64.94	6.99628e−18	Ephemeroptera	Palingeniidae	Palingenia longicauda
Euryades corethrus voucher FS−b−3716 16S ribosomal	GQ268348	83	62.99	2.44194e−17	Lepidoptera	Papilionidae	Euryades corethrus
Euryades corethrus mitochondrial partial 16S rRNA	AM283053	83	62.99	2.44194e−17	Lepidoptera	Papilionidae	Euryades corethrus
Ichthybotus hudsoni 16S ribosomal RNA gene, partia	AY749816	82.83	64.29	2.44194e−17	Ephemeroptera	Ichthybotidae	Ichthybotus hudsoni
Hygrostola dallolmoi voucher HYTAG7234 16S ribosom	KP682574	83.84	62.99	8.52321e−17	Lepidoptera	Noctuidae	Hygrostola dallolmoi
Hygrostola dallolmoi voucher HYTAG6454 16S ribosom	KP682573	83.84	62.99	8.52321e−17	Lepidoptera	Noctuidae	Hygrostola dallolmoi
Palingenia longicauda mitochondrial partial 16S rR	HE650642	82	64.94	8.52321e−17	Ephemeroptera	Palingeniidae	Palingenia longicauda
Palingenia longicauda mitochondrial partial 16S rR	HE650616	81.63	63.64	8.52321e−17	Ephemeroptera	Palingeniidae	Palingenia longicauda
Palingenia longicauda mitochondrial partial 16S rR	HE650443	82.8	60.39	8.52321e−17	Ephemeroptera	Palingeniidae	Palingenia longicauda
Neurosymploca sp. 3 (morphotype 'magnifica') mitoc	AJ831619	82	64.29	8.52321e−17	Lepidoptera	Zygaenidae	Neurosymploca sp. 3 (morphotype 'magnifica')
Neurosymploca sp. 2 (morphotype 'geertsemai') mito	AJ831618	82	64.29	8.52321e−17	Lepidoptera	Zygaenidae	Neurosymploca sp. 2 (morphotype 'geertsemai')
G.rothi mitochondrial 16S rRNA gene (partial)	Z93321	82	64.29	8.52321e−17	Grylloblattodea	Grylloblattidae	Grylloblatta rothi
Heliconius erato petiverana voucher 618 ; mitochon	KP073283	81.82	62.99	2.97489e−16	Lepidoptera	Nymphalidae	Heliconius erato petiverana
Heliconius erato favorinus voucher GS012 ; mitocho	KP073279	81.82	62.99	2.97489e−16	Lepidoptera	Nymphalidae	Heliconius erato favorinus
Heliconius erato erato voucher NCS2556 ; mitochond	KP073278	81.82	62.99	2.97489e−16	Lepidoptera	Nymphalidae	Heliconius erato erato
Heliconius erato emma voucher NCS1673 ; mitochondr	KP073277	81.82	62.99	2.97489e−16	Lepidoptera	Nymphalidae	Heliconius erato emma
Heliconius erato chestertonii voucher WOM0059 ; mi	KP073276	81.82	62.99	2.97489e−16	Lepidoptera	Nymphalidae	Heliconius erato chestertonii
Heliconius erato voucher LMCI159−1 16S ribosomal R	KF277292	81.82	62.99	2.97489e−16	Lepidoptera	Nymphalidae	Heliconius erato
Pachliopta aristolochiae isolate N296 16S ribosoma	JQ421944	82.83	62.99	2.97489e−16	Lepidoptera	Papilionidae	Pachliopta aristolochiae
Pachliopta aristolochiae isolate F566 16S ribosoma	JQ421940	82.83	62.99	2.97489e−16	Lepidoptera	Papilionidae	Pachliopta aristolochiae
Pachliopta aristolochiae isolate F177 16S ribosoma	JQ421938	82.83	62.99	2.97489e−16	Lepidoptera	Papilionidae	Pachliopta aristolochiae
Pachliopta aristolochiae isolate F317 16S ribosoma	JQ421934	82.83	62.99	2.97489e−16	Lepidoptera	Papilionidae	Pachliopta aristolochiae
Pachliopta aristolochiae isolate F520 16S ribosoma	JQ421931	82.83	62.99	2.97489e−16	Lepidoptera	Papilionidae	Pachliopta aristolochiae
Pachliopta aristolochiae isolate T75 16S ribosomal	JQ421926	82.83	62.99	2.97489e−16	Lepidoptera	Papilionidae	Pachliopta aristolochiae
Palingenia longicauda mitochondrial partial 16S rR	HE650658	81	64.94	2.97489e−16	Ephemeroptera	Palingeniidae	Palingenia longicauda
Palingenia longicauda mitochondrial partial 16S rR	HE650655	81	64.94	2.97489e−16	Ephemeroptera	Palingeniidae	Palingenia longicauda
Palingenia longicauda mitochondrial partial 16S rR	HE650524	81	64.94	2.97489e−16	Ephemeroptera	Palingeniidae	Palingenia longicauda
Palingenia longicauda mitochondrial partial 16S rR	HE650510	81	64.94	2.97489e−16	Ephemeroptera	Palingeniidae	Palingenia longicauda
Palingenia longicauda mitochondrial partial 16S rR	HE650498	81	64.94	2.97489e−16	Ephemeroptera	Palingeniidae	Palingenia longicauda
Palingenia longicauda mitochondrial partial 16S rR	HE650490	81	64.94	2.97489e−16	Ephemeroptera	Palingeniidae	Palingenia longicauda
Palingenia longicauda mitochondrial partial 16S rR	HE650484	81	64.94	2.97489e−16	Ephemeroptera	Palingeniidae	Palingenia longicauda
Trox niponensis isolate 100 16S ribosomal RNA gene	EF570413	84.04	59.74	2.97489e−16	Coleoptera	Trogidae	Trox niponensis
Trichopteryx ustata gene for 16S rRNA, partial seq	AB265432	83	62.99	2.97489e−16	Lepidoptera	Geometridae	Trichopteryx ustata
Pachliopta aristolochiae 16S ribosomal RNA gene, p	DQ673334	82.83	62.99	2.97489e−16	Lepidoptera	Papilionidae	Pachliopta aristolochiae
Atrophaneura dasarada 16S ribosomal RNA gene, part	DQ673329	82.47	62.34	2.97489e−16	Lepidoptera	Papilionidae	Atrophaneura dasarada
Heliconius erato hydara isolate STRI−B−440 16S rib	AY747877	81.82	62.99	2.97489e−16	Lepidoptera	Nymphalidae	Heliconius erato hydara
Papilio delalandei mitochondrion, large subunit ri	AJ224060	83	62.99	2.97489e−16	Lepidoptera	Papilionidae	Papilio delalandei
Hipparchia semele mitochondrion, partial genome	KM592976	82.83	62.34	1.03834e−15	Lepidoptera	Nymphalidae	Hipparchia semele
Operophtera brumata mitochondrion, complete genome	KP027400	81.63	62.99	1.03834e−15	Lepidoptera	Geometridae	Operophtera brumata
Heliconius erato lativitta voucher WOM03 ; mitocho	KP073281	82	62.99	1.03834e−15	Lepidoptera	Nymphalidae	Heliconius erato lativitta
Atrophaneura alcinous mitochondrion, complete geno	KJ540880	81.63	62.99	1.03834e−15	Lepidoptera	Papilionidae	Atrophaneura alcinous
Oxythyrea pantherina voucher 842859 16S ribosomal	JX234531	82.8	59.74	1.03834e−15	Coleoptera	Scarabaeidae	Oxythyrea pantherina
Leucocelis rubra voucher 842824 16S ribosomal RNA	JX234507	82.8	59.74	1.03834e−15	Coleoptera	Scarabaeidae	Leucocelis rubra
Oxythyrea funesta voucher 678443 16S ribosomal RNA	JX234432	82.8	59.74	1.03834e−15	Coleoptera	Scarabaeidae	Oxythyrea funesta
Semirhynchia sp. n. SV01 16S ribosomal RNA gene, p	JX294172	81.37	63.64	1.03834e−15	Neuroptera	Nemopteridae	Semirhynchia sp. n. SV01
Sicyoptera cuspidata isolate SCBB01 16S ribosomal	JX294167	81.37	63.64	1.03834e−15	Neuroptera	Nemopteridae	Sicyoptera cuspidata
