# Supplementary material for: Testing the potential of a ribosomal 16S marker for DNA metabarcoding of insects
Source: PeerJ. 2016 Apr 19;4:e1966. doi: 10.7717/peerj.1966 (PMC4841222; doi:10.7717/peerj.1966)
Supplement: Supplemental Information 1 [file peerj-04-1966-s006.zip › scripts/5 match OTUs/blast_hits/OTU_125.csv_plot.pdf]

OTU_125.csv

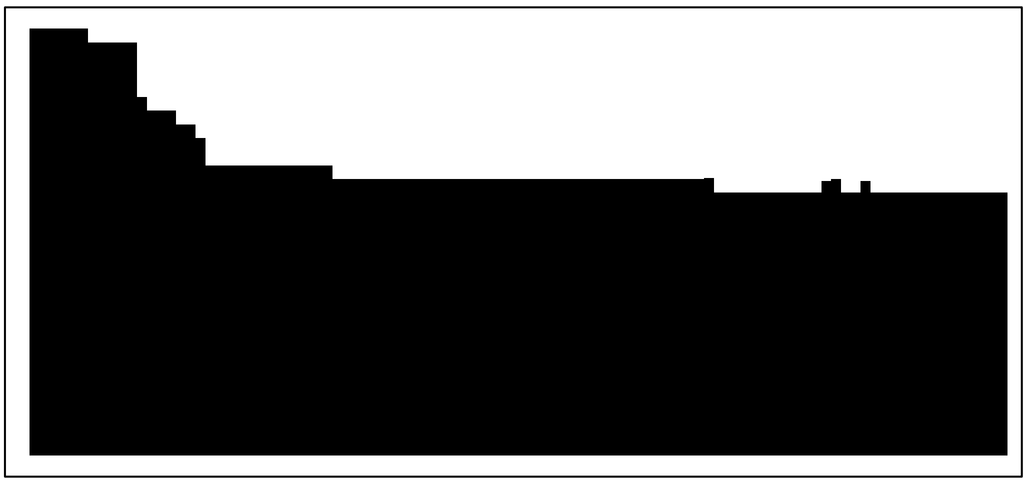

Simulium vernum

Simuliidae

Diptera

100

Simulium vernum voucher ACBSimFN1c 16S ribosomal R	GU073059	100	100	1.053e−72	Diptera	Simuliidae	Simulium vernum
Simulium silvestre voucher ACBSimMN11c1 16S riboso	GU073058	100	100	1.053e−72	Diptera	Simuliidae	Simulium silvestre
Simulium silvestre voucher ACBSimMN5c1 16S ribosom	GU073057	100	100	1.053e−72	Diptera	Simuliidae	Simulium silvestre
Simulium silvestre voucher ACBSimNu178 16S ribosom	GU073052	100	100	1.053e−72	Diptera	Simuliidae	Simulium silvestre
Simulium juxtacrenobium voucher ACBBsimEN1C 16S ri	GU073050	100	100	1.053e−72	Diptera	Simuliidae	Simulium juxtacrenobium
Simulium juxtacrenobium voucher ACBBsimFN100D 16S	GU073048	100	98.72	1.28281e−71	Diptera	Simuliidae	Simulium juxtacrenobium
Simulium juxtacrenobium voucher ACBBsimFN100I 16S	GU073042	99.36	100	1.28281e−71	Diptera	Simuliidae	Simulium juxtacrenobium
Simulium crenobium voucher ACBBsimAU1A 16S ribosom	GU073040	99.36	100	1.28281e−71	Diptera	Simuliidae	Simulium crenobium
Simulium silvestre voucher ACBBsimMN45b1 16S riboso	GU073056	99.36	100	4.47746e−71	Diptera	Simuliidae	Simulium silvestre
Simulium costatum voucher ACBBsimSW102H 16S riboso	GU073032	99.36	100	4.47746e−71	Diptera	Simuliidae	Simulium costatum
Simulium juxtacrenobium voucher ACBBsimFN100B 16S	GU073046	99.35	98.72	1.56279e−70	Diptera	Simuliidae	Simulium juxtacrenobium
Simulium uchidai mitochondrial gene for 16S riboso	AB056740	96.79	100	1.20147e−65	Diptera	Simuliidae	Simulium uchidai
Simulium khunklangense mitochondrial gene for 16S	AB820367	96.15	100	1.46369e−64	Diptera	Simuliidae	Simulium khunklangense
Simulium chomthongense mitochondrial gene for 16S	AB699899	96.15	100	1.46369e−64	Diptera	Simuliidae	Simulium chomthongense
Simulium caudisclerum 16S ribosomal RNA gene, part	GQ865999	96.15	100	1.46369e−64	Diptera	Simuliidae	Simulium caudisclerum
Simulium subcostatum mitochondrial gene for 16S ri	AB056739	95.51	100	6.22376e−63	Diptera	Simuliidae	Simulium subcostatum
Simulium bonninense mitochondrial gene for 16S rib	AB056738	95.51	100	6.22376e−63	Diptera	Simuliidae	Simulium bonninense
Simulium caudisclerum mitochondrial gene for 16S r	AB699900	94.87	100	7.58209e−62	Diptera	Simuliidae	Simulium caudisclerum
Simulium rufibasis 16S ribosomal RNA gene, partial	GQ866016	93.59	100	3.92762e−59	Diptera	Simuliidae	Simulium rufibasis
Simulium decuplum 16S ribosomal RNA gene, partial	GQ865987	93.59	100	3.92762e−59	Diptera	Simuliidae	Simulium decuplum
Simulium angulistylum 16S ribosomal RNA gene, part	GQ865985	93.59	100	3.92762e−59	Diptera	Simuliidae	Simulium angulistylum
Simulium angulistylum 16S ribosomal RNA gene, part	GQ865984	93.59	100	3.92762e−59	Diptera	Simuliidae	Simulium angulistylum
Simulium nili 16S ribosomal RNA gene, partial sequ	AY944188	93.59	100	3.92762e−59	Diptera	Simuliidae	Simulium nili
Simulium squamosum voucher P.Mansiangi Pm3(BNI) 16	DQ655644	93.59	100	3.92762e−59	Diptera	Simuliidae	Simulium squamosum
Simulium latipollex isolate SA 4 16S ribosomal RNA	AF416962	93.59	100	3.92762e−59	Diptera	Simuliidae	Simulium latipollex
Simulium damnosum s.l. Kulfo isolate Ku 3 16S ribo	AF416978	93.59	100	3.92762e−59	Diptera	Simuliidae	Simulium damnosum s.l. Kulfo
Simulium damnosum s.l. Mal4 16S ribosomal RNA gene	AF416964	93.59	100	3.92762e−59	Diptera	Simuliidae	Simulium damnosum s.l. Mal4
Simulium damnosum 16S ribosomal RNA gene, partial	AF127463	93.59	100	3.92762e−59	Diptera	Simuliidae	Simulium damnosum
Simulium sabahense mitochondrial gene for 16S rRNA	AB093122	93.59	100	3.92762e−59	Diptera	Simuliidae	Simulium sabahense
Simulium rufibasis mitochondrial gene for 16S rRNA	AB093121	93.59	100	3.92762e−59	Diptera	Simuliidae	Simulium rufibasis
Simulium aeneifacies mitochondrial gene for 16S rR	AB093119	93.59	100	3.92762e−59	Diptera	Simuliidae	Simulium aeneifacies
Simulium tani isolate ST1B 16S ribosomal RNA gene,	KM410188	92.95	100	1.67007e−57	Diptera	Simuliidae	Simulium tani
Simulium atipornae mitochondrial gene for 16S ribo	AB893948	92.95	100	1.67007e−57	Diptera	Simuliidae	Simulium atipornae
Simulium chamlongi 16S ribosomal RNA gene, partial	GQ866020	92.95	100	1.67007e−57	Diptera	Simuliidae	Simulium chamlongi
Simulium tani 16S ribosomal RNA gene, partial sequ	GQ866019	92.95	100	1.67007e−57	Diptera	Simuliidae	Simulium tani
Simulium tani 16S ribosomal RNA gene, partial sequ	GQ866018	92.95	100	1.67007e−57	Diptera	Simuliidae	Simulium tani
Simulium yuphae 16S ribosomal RNA gene, partial se	GQ866015	92.95	100	1.67007e−57	Diptera	Simuliidae	Simulium yuphae
Simulium chumpornense 16S ribosomal RNA gene, part	GQ865998	92.95	100	1.67007e−57	Diptera	Simuliidae	Simulium chumpornense
Simulium ruficorne 16S ribosomal RNA gene, partial	AY944189	92.95	100	1.67007e−57	Diptera	Simuliidae	Simulium ruficorne
Simulium arabicum 16S ribosomal RNA gene, partial	AY944186	92.95	100	1.67007e−57	Diptera	Simuliidae	Simulium arabicum
Simulium pandanophilum isolate KK1 16S ribosomal R	AF466184	92.95	100	1.67007e−57	Diptera	Simuliidae	Simulium pandanophilum
Simulium damnosum s.l. GH−D1 16S ribosomal RNA, pa	AY077709	92.95	100	1.67007e−57	Diptera	Simuliidae	Simulium damnosum s.l. GH−D1
Simulium damnosum s.l. Kulfo isolate Ku 1 16S ribo	AF416977	92.95	100	1.67007e−57	Diptera	Simuliidae	Simulium damnosum s.l. Kulfo
Simulium soubrense isolate Wa 52 16S ribosomal RNA	AF416976	92.95	100	1.67007e−57	Diptera	Simuliidae	Simulium soubrense
Simulium soubrense isolate Wa 47 16S ribosomal RNA	AF416975	92.95	100	1.67007e−57	Diptera	Simuliidae	Simulium soubrense
Simulium rasyani isolate rasy 19 16S ribosomal RNA	AF416974	92.95	100	1.67007e−57	Diptera	Simuliidae	Simulium rasyani
Simulium damnosum s.l. Me2 16S ribosomal RNA gene,	AF416972	92.95	100	1.67007e−57	Diptera	Simuliidae	Simulium damnosum s.l. Me2
Simulium damnosum s.l. GH−D2 16S ribosomal RNA, pa	AY077710	92.95	100	1.67007e−57	Diptera	Simuliidae	Simulium damnosum s.l. GH−D2
Simulium kilibanum isolate Ny 14 16S ribosomal RNA	AF416961	92.95	100	1.67007e−57	Diptera	Simuliidae	Simulium kilibanum
Simulium damnosum cytoform Kagera isolate Ka 6 16S	AF416955	92.95	100	1.67007e−57	Diptera	Simuliidae	Simulium damnosum cytoform Kagera
Simulium vorax 16S ribosomal RNA gene, partial seq	AF127462	92.95	100	1.67007e−57	Diptera	Simuliidae	Simulium vorax
Simulium pandanophilum 16S ribosomal RNA gene, par	AF081909	92.95	100	1.67007e−57	Diptera	Simuliidae	Simulium pandanophilum
Simulium damnosum country Uganda 16S ribosomal RNA	AF081904	92.95	100	1.67007e−57	Diptera	Simuliidae	Simulium damnosum
Simulium kilibanum isolate NY19 16S ribosomal RNA	AY885648	92.95	100	1.67007e−57	Diptera	Simuliidae	Simulium kilibanum
Simulium cabrerai mitochondrial gene for 16S rRNA,	AB093128	92.95	100	1.67007e−57	Diptera	Simuliidae	Simulium cabrerai
Simulium iridescens mitochondrial gene for 16S rRN	AB093127	92.95	100	1.67007e−57	Diptera	Simuliidae	Simulium iridescens
Simulium chamlongi mitochondrial gene for 16S rRNA	AB093124	92.95	100	1.67007e−57	Diptera	Simuliidae	Simulium chamlongi
Simulium tani mitochondrial gene for 16S rRNA, par	AB093123	92.95	100	1.67007e−57	Diptera	Simuliidae	Simulium tani
Simulium brevipar mitochondrial gene for 16S rRNA,	AB093120	92.95	100	1.67007e−57	Diptera	Simuliidae	Simulium brevipar
Simulium nikkoense mitochondrial gene for 16S rRNA	AB093116	92.95	100	1.67007e−57	Diptera	Simuliidae	Simulium nikkoense
Simulium dumogaense mitochondrial gene for 16S rRN	AB093106	92.95	100	1.67007e−57	Diptera	Simuliidae	Simulium dumogaense
Simulium maenoi mitochondrial gene for 16S rRNA, p	AB093104	92.95	100	1.67007e−57	Diptera	Simuliidae	Simulium maenoi
Simulium aokii mitochondrial gene for 16S ribosoma	AB056745	92.95	100	1.67007e−57	Diptera	Simuliidae	Simulium aokii
Simulium sasai mitochondrial gene for 16S ribosoma	AB056734	92.95	100	1.67007e−57	Diptera	Simuliidae	Simulium sasai
Simulium feuerborni mitochondrial gene for 16S rib	AB056728	92.95	100	1.67007e−57	Diptera	Simuliidae	Simulium feuerborni
Simulium leonense mitochondrion 16S ribosomal RNA	U17731	92.95	100	1.67007e−57	Diptera	Simuliidae	Simulium leonense
Simulium damnosum mitochondrion 16S ribosomal RNA	U17729	92.95	100	1.67007e−57	Diptera	Simuliidae	Simulium damnosum
Simulium venustum (individual CC3) mitochondrial 1	L13410	92.95	100	1.67007e−57	Diptera	Simuliidae	Simulium venustum
Simulium venustum (individual CC) mitochondrial 16	L13409	92.95	100	1.67007e−57	Diptera	Simuliidae	Simulium venustum
Stegopterna mutata mitochondrial 16S ribosomal RNA	L13404	92.99	100	5.82911e−57	Diptera	Simuliidae	Stegopterna mutata
Simulium ogonukii mitochondrial gene for 16S ribos	LC034993	92.31	100	2.03456e−56	Diptera	Simuliidae	Simulium ogonukii
Simulium izuae isolate I3B 16S ribosomal RNA gene,	KM410191	92.31	100	2.03456e−56	Diptera	Simuliidae	Simulium izuae
Simulium izuae isolate I2B 16S ribosomal RNA gene,	KM410190	92.31	100	2.03456e−56	Diptera	Simuliidae	Simulium izuae
Simulium capillatum mitochondrial gene for 16S rib	AB862986	92.31	100	2.03456e−56	Diptera	Simuliidae	Simulium capillatum
Cyphomyia notabilis 16S ribosomal RNA gene, partia	GU947403	92.31	100	2.03456e−56	Diptera	Stratiomyidae	Cyphomyia notabilis
Simulium charlesi mitochondrial gene for 16S ribos	AB535134	92.31	100	2.03456e−56	Diptera	Simuliidae	Simulium charlesi
Simulium baimaii 16S ribosomal RNA gene, partial s	GQ866002	92.31	100	2.03456e−56	Diptera	Simuliidae	Simulium baimaii
Simulium recurvum mitochondrial gene for 16S ribos	AB334097	92.31	100	2.03456e−56	Diptera	Simuliidae	Simulium recurvum
Simulium mengense isolate Me12 16S ribosomal RNA g	DQ022372	92.31	100	2.03456e−56	Diptera	Simuliidae	Simulium mengense
Simulium damnosum s.l. Kulfo isolate Ku 4 16S ribo	AF416979	92.31	100	2.03456e−56	Diptera	Simuliidae	Simulium damnosum s.l. Kulfo
Simulium damnosum isolate Ni 4 16S ribosomal RNA g	AF416973	92.31	100	2.03456e−56	Diptera	Simuliidae	Simulium damnosum
Simulium kilibanum isolate Nk 82 16S ribosomal RNA	AF416960	92.86	98.72	2.03456e−56	Diptera	Simuliidae	Simulium kilibanum
Simulium cf. damnosum 'Abu Hamed' 16S ribosomal RN	AF213336	92.95	100	2.03456e−56	Diptera	Simuliidae	Simulium damnosum cytoform Abu Hamed
Simulium damnosum strain Se−19 16S ribosomal RNA g	AF081908	92.31	100	2.03456e−56	Diptera	Simuliidae	Simulium damnosum
Simulium damnosum strain Se−12 16S ribosomal RNA g	AF081907	92.31	100	2.03456e−56	Diptera	Simuliidae	Simulium damnosum
Simulium kilibanum strain Ny−18 16S ribosomal RNA	AF081905	92.86	98.72	2.03456e−56	Diptera	Simuliidae	Simulium kilibanum
Simulium arakawae mitochondrial gene for 16S rRNA,	AB093125	92.31	100	2.03456e−56	Diptera	Simuliidae	Simulium arakawae
Simulium tumpaense mitochondrial gene for 16S rRNA	AB093110	92.31	100	2.03456e−56	Diptera	Simuliidae	Simulium tumpaense
Simulium taalense mitochondrial gene for 16S rRNA,	AB093109	92.31	100	2.03456e−56	Diptera	Simuliidae	Simulium taalense
Simulium laterale mitochondrial gene for 16S rRNA,	AB093107	92.31	100	2.03456e−56	Diptera	Simuliidae	Simulium laterale
Simulium bishopi mitochondrial gene for 16S rRNA,	AB093105	92.31	100	2.03456e−56	Diptera	Simuliidae	Simulium bishopi
Simulium japonicum mitochondrial gene for 16S rRNA	AB093103	92.31	100	2.03456e−56	Diptera	Simuliidae	Simulium japonicum
Simulium nigrogilvum mitochondrial gene for 16S rR	AB093099	92.31	100	2.03456e−56	Diptera	Simuliidae	Simulium nigrogilvum
Simulium whartoni mitochondrial gene for 16S ribos	AB056743	92.31	100	2.03456e−56	Diptera	Simuliidae	Simulium whartoni
Simulium konoi mitochondrial gene for 16S ribosoma	AB056741	92.31	100	2.03456e−56	Diptera	Simuliidae	Simulium konoi
Simulium aureohirtum mitochondrial gene for 16S ri	AB056736	92.31	100	2.03456e−56	Diptera	Simuliidae	Simulium aureohirtum
Simulium aureohirtum mitochondrial gene for 16S ri	AB056735	92.31	100	2.03456e−56	Diptera	Simuliidae	Simulium aureohirtum
Simulium saitoi mitochondrial gene for 16S ribosom	AB056733	92.31	100	2.03456e−56	Diptera	Simuliidae	Simulium saitoi
Simulium feuerborni mitochondrial gene for 16S rib	AB056729	92.31	100	2.03456e−56	Diptera	Simuliidae	Simulium feuerborni
Simulium meridionale mitochondrion 16S ribosomal R	U17732	92.31	100	2.03456e−56	Diptera	Simuliidae	Simulium meridionale
