# Supplementary material for: Testing the potential of a ribosomal 16S marker for DNA metabarcoding of insects
Source: PeerJ. 2016 Apr 19;4:e1966. doi: 10.7717/peerj.1966 (PMC4841222; doi:10.7717/peerj.1966)
Supplement: Supplemental Information 1 [file peerj-04-1966-s006.zip › scripts/5 match OTUs/blast_hits/OTU_127.csv_plot.pdf]

OTU_127.csv

Sericostoma personatum

Sericostomatidae

Trichoptera

90.41

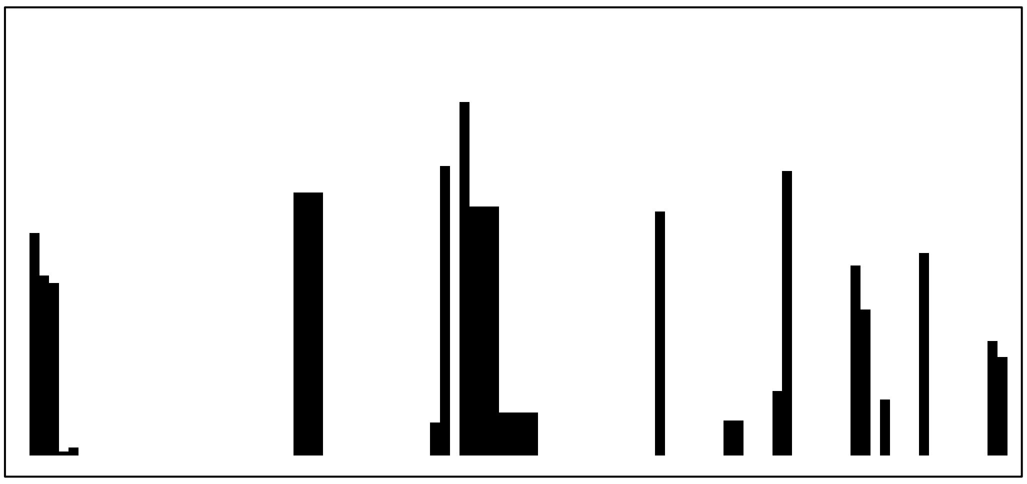

Sericostoma personatum mitochondrion, partial geno	KP455290	90.41	100	3.85036e−46	Trichoptera	Sericostomatidae	Sericostoma personatum
Sericostoma clypeatum voucher NHRS−EN CC4 16S ribo	FJ263207	88.44	100	2.96016e−41	Trichoptera	Sericostomatidae	Sericostoma clypeatum
Notidobiella chacayana isolate CL5 16S ribosomal R	EF394993	88.07	73.97	1.10412e−27	Trichoptera	Sericostomatidae	Notidobiella chacayana
Pliocaloca sp. CQ3 16S ribosomal RNA gene, partial	EF394999	80.18	73.97	4.11832e−14	Trichoptera	Calocidae	Pliocaloca sp. CQ3
Tamasia variegata mitochondrial 16S rRNA gene, spe	FN257669	80.37	72.6	1.75115e−12	Trichoptera	Calocidae	Tamasia variegata
Eosericostoma inaequispinum isolate CK9 16S riboso	EF394992	78.18	73.97	6.11212e−12	Trichoptera	Helicophidae	Eosericostoma inaequispinum
Eosericostoma aequispinum isolate CK7 16S ribosoma	EF394990	78.18	73.97	6.11212e−12	Trichoptera	Helicophidae	Eosericostoma aequispinum
Eosericostoma sp. CK5 16S ribosomal RNA gene, part	EF394989	78.18	73.97	6.11212e−12	Trichoptera	Helicophidae	Eosericostoma sp. CK5
Helicopsyche albescens isolate BR5 16S ribosomal R	EF394986	72.97	97.95	3.16616e−09	Trichoptera	Helicopsychidae	Helicopsyche albescens
Alloecentrella sp. BP8 16S ribosomal RNA gene, par	EF394983	76.64	73.29	3.16616e−09	Trichoptera	Calocidae	Alloecentrella sp. BP8
Zizina otis isolate N291 16S ribosomal RNA gene, p	JQ421901	73.28	77.4	1.1051e−08	Lepidoptera	Lycaenidae	Zizina otis
Zizina otis isolate F396 16S ribosomal RNA gene, p	JQ421860	73.28	77.4	1.1051e−08	Lepidoptera	Lycaenidae	Zizina otis
Zizina otis isolate N288 16S ribosomal RNA gene, p	JQ421854	73.28	77.4	1.1051e−08	Lepidoptera	Lycaenidae	Zizina otis
Pycnocentrella eruensis isolate BR4 16S ribosomal	EF394985	74.45	91.1	3.85717e−08	Trichoptera	Calocidae	Pycnocentrella eruensis
Chathamia integripennis voucher NHRS−EN DP2 16S ri	FJ263213	75.89	72.6	1.34628e−07	Trichoptera	Chathamiidae	Chathamia integripennis
Helicopsyche murrumba isolate CP6 16S ribosomal RN	EF394997	72.46	93.84	1.34628e−07	Trichoptera	Helicopsychidae	Helicopsyche murrumba
Zizina otis isolate T72 16S ribosomal RNA gene, pa	JQ421825	72.41	77.4	4.699e−07	Lepidoptera	Lycaenidae	Zizina otis
Gyrophaena conicientris 16S ribosomal RNA gene, p	EU477782	70.94	78.77	4.699e−07	Coleoptera	Staphylinidae	Gyrophaena conicientris
Lycocerus sp. UPOL RK0176 16S ribosomal RNA gene,	KF625886	72.22	72.6	1.64011e−06	Coleoptera	Cantharidae	Lycocerus sp. UPOL RK0176
Limbodessus gumwellensis voucher ABTC:78840_1 16S	JQ745739	72.9	73.29	1.64011e−06	Coleoptera	Dytiscidae	Limbodessus gumwellensis
Philanisus plebeius voucher NHRS−EN BY4 16S riboso	FJ263205	73.45	72.6	5.72455e−06	Trichoptera	Chathamiidae	Philanisus plebeius
Helicopsyche cochleaetesta isolate CP5 16S ribosom	EF394996	72.41	97.26	5.72455e−06	Trichoptera	Helicopsychidae	Helicopsyche cochleaetesta
Sejanus brittoni 16S ribosomal RNA gene, partial s	HQ667614	72.48	74.66	1.99806e−05	Hemiptera	Miridae	Sejanus brittoni
Trifolium pratense genome assembly redclover, chro	LN846350	70.59	91.1	0.000243414	Fabales	Fabaceae	Trifolium pratense
Oreodytes septentrionalis mitochondrial 16S rRNA g	HF931492	72.48	73.29	0.000243414	Coleoptera	Dytiscidae	Oreodytes septentrionalis
Austrocroce attenuata 16S ribosomal RNA gene, part	EU734859	72.48	73.29	0.000243414	Neuroptera	Nemopteridae	Austrocroce attenuata
Gergithus yayeyamensis 16S ribosomal RNA gene, par	AF158068	71.56	73.97	0.000243414	Hemiptera	Issidae	Gergithus yayeyamensis
PREDICTED: Gossypium raimondii uncharacterized LOC	XR_001134684	92.31	26.71	0.000849598	Malvales	Malvaceae	Gossypium raimondii
PREDICTED: Gossypium raimondii uncharacterized LOC	XR_001134683	92.31	26.71	0.000849598	Malvales	Malvaceae	Gossypium raimondii
PREDICTED: Gossypium raimondii uncharacterized LOC	XR_001134682	92.31	26.71	0.000849598	Malvales	Malvaceae	Gossypium raimondii
Dracunculus medinensis genome assembly D_medinensi	LK978195	73.33	61.64	0.000849598	Spirurida	Dracunculidae	Dracunculus medinensis
Gergithus sp. 1 NS−2013 16S ribosomal RNA gene, pa	JX556704	72.22	72.6	0.000849598	Hemiptera	Issidae	Gergithus sp. 1 NS−2013
Gergithus sp. 5 NS−2013 16S ribosomal RNA gene, pa	JX556703	72.22	72.6	0.000849598	Hemiptera	Issidae	Gergithus sp. 5 NS−2013
Nirripirti hinzeae 16S ribosomal RNA gene, partial	AF485940	70.4	84.25	0.000849598	Coleoptera	Dytiscidae	Paroster hinzeae
Strongyloides ratti genome assembly S_ratti_ED321	LN609400	70.77	84.93	0.00296539	Rhabditida	Strongyloididae	Strongyloides ratti
Elasmostethus humeralis mitochondrial gene for 16S	AB240571	71.03	73.29	0.00296539	Hemiptera	Acanthosomatidae	Elasmostethus humeralis
Human DNA sequence from clone RP11−542F9 on chromo	AL646090	76.19	49.32	0.00296539	Primates	Hominidae	Homo sapiens
Schizophyllum commune mitochondrial DNA, complete	AF402141	78.21	52.74	0.00296539	Agaricales	Schizophyllaceae	Schizophyllum commune
Hymenolepis nana genome assembly H_nana_Japan ,sca	LM400847	76.71	47.95	0.0103502	Cyclophyllidea	Hymenolepididae	Hymenolepis nana
Tanymecus palliatus partial mitochondrial 16S rRNA	AJ495507	69.75	81.51	0.0103502	Coleoptera	Curculionidae	Tanymecus palliatus
Athalia sp. Sus6 16S ribosomal RNA gene, partial s	AY206777	71.43	71.92	0.0103502	Hymenoptera	Tenthredinidae	Athalia sp. Sus6
Guillardia theta nucleomorph chromosome 1, complet	AF165818	81.54	43.15	0.0103502	Pyrenomonadales	Geminigeraceae	Guillardia theta
Ovis canadensis canadensis isolate 43U chromosome	CP011889	93.55	21.23	0.0361258		Bovidae	Ovis canadensis canadensis
Dracunculus medinensis genome assembly D_medinensi	LK978293	78.79	44.52	0.0361258	Spirurida	Dracunculidae	Dracunculus medinensis
Plasmodium chabaudi chabaudi genome assembly PCHAS	LK022891	96.55	19.86	0.0361258	Haemosporida	Plasmodiidae	Plasmodium chabaudi chabaudi
PREDICTED: Stegastes partitus vitamin K epoxide re	XM_008306889	91.67	24.66	0.0361258		Pomacentridae	Stegastes partitus
PREDICTED: Neolamprologus brichardi vitamin K epox	XM_006799567	91.67	24.66	0.0361258	Cichliformes	Cichlidae	Neolamprologus brichardi
PREDICTED: Haplochromis burtoni vitamin K epoxide	XM_005928446	91.67	24.66	0.0361258	Cichliformes	Cichlidae	Haplochromis burtoni
Papilio krishna thawgawa voucher CBGPFLC_00376 16S	JQ982213	82	32.88	0.0361258	Lepidoptera	Papilionidae	Papilio krishna thawgawa
Papilio krishna thawgawa voucher CBGPFLC_00058 16S	JQ982212	82	32.88	0.0361258	Lepidoptera	Papilionidae	Papilio krishna thawgawa
Papilio krishna mayumiae voucher CBGPFLC_00057 16S	JQ982211	82	32.88	0.0361258	Lepidoptera	Papilionidae	Papilio krishna mayumiae
Papilio krishna ssp. CBGPFLC_00055 16S ribosomal R	JQ982210	82	32.88	0.0361258	Lepidoptera	Papilionidae	Papilio krishna ssp. CBGPFLC_00055
Tuxedo drakei 16S ribosomal RNA gene, partial sequ	HQ667634	69.72	74.66	0.0361258	Hemiptera	Miridae	Tuxedo drakei
Pseudophylus stundjuki 16S ribosomal RNA gene, par	GU194601	69.72	74.66	0.0361258	Hemiptera	Miridae	Pseudophylus stundjuki
Delphacinae gen. 4 sp. 1 XHH−2010 isolate 229 16S	HM233799	68.63	99.32	0.0361258	Hemiptera	Delphacidae	Delphacinae gen. 4 sp. 1 XHH−2010
Lebinthus bitaeniatus voucher MNHN:ENSIF 2740 16S	JF972524	71.82	75.34	0.0361258	Orthoptera	Gryllidae	Lebinthus bitaeniatus
Prunus umbellata clone 908B sorbitol−6−phosphate d	EU168701	79.66	40.41	0.0361258	Rosales	Rosaceae	Prunus umbellata
Prunus maritima clone 002C sorbitol−6−phosphate de	EU168532	79.66	40.41	0.0361258	Rosales	Rosaceae	Prunus maritima
Prunus maritima clone 882C sorbitol−6−phosphate de	EU168578	79.66	40.41	0.0361258	Rosales	Rosaceae	Prunus maritima
Prunus maritima clone 882A sorbitol−6−phosphate de	EU168576	79.66	40.41	0.0361258	Rosales	Rosaceae	Prunus maritima
Prunus maritima clone 002D sorbitol−6−phosphate de	EU168533	79.66	40.41	0.0361258	Rosales	Rosaceae	Prunus maritima
Prunus maritima clone 002B sorbitol−6−phosphate de	EU168531	79.66	40.41	0.0361258	Rosales	Rosaceae	Prunus maritima
Prunus maritima clone 002A sorbitol−6−phosphate de	EU168530	79.66	40.41	0.0361258	Rosales	Rosaceae	Prunus maritima
Protopolystoma xenopodis genome assembly P_xenopod	LM721039	74.68	53.42	0.126092	Polyopisthocotylea	Polystomatidae	Protopolystoma xenopodis
Schistosoma margrebowiei genome assembly S_margreb	LL883732	91.43	23.29	0.126092	Strigeidida	Schistosomatidae	Schistosoma margrebowiei
Dracunculus medinensis genome assembly D_medinensi	LK978223	76.25	54.79	0.126092	Spirurida	Dracunculidae	Dracunculus medinensis
Brugia pahangi genome assembly B_pahangi_Glasgow ,	LK964181	75.36	47.26	0.126092	Spirurida	Onchocercidae	Brugia pahangi
Parastrongyloides trichosuri genome assembly P_tri	LM523159	70.37	88.36	0.126092	Rhabditida	Strongyloididae	Parastrongyloides trichosuri
Drosophila melanogaster chromosome 2L	AE014134	80	34.25	0.126092	Diptera	Drosophilidae	Drosophila melanogaster
Solanum pennellii chromosome ch03, complete genome	HG975442	69.77	88.36	0.126092	Solanales	Solanaceae	Solanum pennellii
Ichthyophthirius multifiliis hypothetical protein	XM_004025117	71.7	71.23	0.126092	Hymenostomatida		Ichthyophthirius multifiliis
Papilio hermeli voucher CBGPFLC_00045 16S ribosoma	JQ982201	81.63	32.19	0.126092	Lepidoptera	Papilionidae	Papilio hermeli
Papilio hermeli voucher CBGPFLC_00044 16S ribosoma	JQ982200	81.63	32.19	0.126092	Lepidoptera	Papilionidae	Papilio hermeli
Aspergillus niger clone AXAS101−N02, complete sequ	AC253892	70.07	95.89	0.126092	Eurotiales	Aspergillaceae	Aspergillus niger
Plasmodium falciparum 3D7 chromosome 2, complete s	AE001362	73.26	56.85	0.126092	Haemosporida	Plasmodiidae	Plasmodium falciparum 3D7
Drosophila melanogaster DNA, chromosome 2L, 31D1,	AB317178	80	34.25	0.126092	Diptera	Drosophilidae	Drosophila melanogaster
Vitis vinifera contig VV78X002396.11, whole genome	AM435740	83.02	34.25	0.126092	Vitales	Vitaceae	Vitis vinifera
Medicago truncatula clone mth2−175f4, complete seq	AC161863	93.33	20.55	0.126092	Fabales	Fabaceae	Medicago truncatula
Elasmostethus sp. WCW−2003 16S ribosomal RNA gene,	AY252797	69.81	72.6	0.126092	Hemiptera	Acanthosomatidae	Elasmostethus sp. WCW−2003
Stauralia compuncta 16S ribosomal RNA gene, partia	AY252741	67.39	94.52	0.126092	Hemiptera	Acanthosomatidae	Stauralia compuncta
Drosophila melanogaster, chromosome 2L, region 31C	AC092237	80	34.25	0.126092	Diptera	Drosophilidae	Drosophila melanogaster
Drosophila melanogaster, chromosome 2L, region 31B	AC009849	80	34.25	0.126092	Diptera	Drosophilidae	Drosophila melanogaster
Drosophila melanogaster, chromosome 2R, region 31C	AC005454	80	34.25	0.126092	Diptera	Drosophilidae	Drosophila melanogaster
Drosophila busckii chromosome 2R sequence	CP012524	71.95	53.42	0.440103	Diptera	Drosophilidae	Drosophila busckii
PREDICTED: Maylandia zebra vitamin K epoxide reduc	XM_004557439	88.89	24.66	0.440103	Cichliformes	Cichlidae	Maylandia zebra
Strongyloides venezuelensis genome assembly S_vene	LM524994	86.84	24.66	0.440103	Rhabditida	Strongyloididae	Strongyloides venezuelensis
Lactobacillus acetotolerans DNA, complete genome,	AP014808	78.33	39.73	0.440103	Lactobacillales	Lactobacillaceae	Lactobacillus acetotolerans
Strongyloides stercoralis genome assembly S_sterco	LL999121	82.61	30.82	0.440103	Rhabditida	Strongyloididae	Strongyloides stercoralis
Strongyloides stercoralis genome assembly S_sterco	LL999048	69.44	91.78	0.440103	Rhabditida	Strongyloididae	Strongyloides stercoralis
Schistosoma curassoni genome assembly S_curassoni_	LM068723	72.94	56.85	0.440103	Strigeidida	Schistosomatidae	Schistosoma curassoni
Soboliphyme baturini genome assembly S_baturini_Da	LM014852	77.97	39.73	0.440103		Soboliphymatidae	Soboliphyme baturini
Cyprinus carpio genome assembly common carp genome	LN594104	89.47	25.34	0.440103	Cypriniformes	Cyprinidae	Cyprinus carpio
Cyprinus carpio genome assembly common carp genome	LN592350	75.68	50.68	0.440103	Cypriniformes	Cyprinidae	Cyprinus carpio
Cyprinus carpio genome assembly common carp genome	LN590821	75.81	42.47	0.440103	Cypriniformes	Cyprinidae	Cyprinus carpio
Cyprinus carpio genome assembly common carp genome	LN590698	77.05	40.41	0.440103	Cypriniformes	Cyprinidae	Cyprinus carpio
Cyprinus carpio genome assembly common carp genome	LN590688	80	36.3	0.440103	Cypriniformes	Cyprinidae	Cyprinus carpio
Cyprinus carpio genome assembly common carp genome	LN590672	79.25	34.93	0.440103	Cypriniformes	Cyprinidae	Cyprinus carpio
Basilea psittacipulmonis DSM 24701, complete genom	CP009238	77.78	43.15	0.440103	Burkholderiales	Alcaligenaceae	Basilea psittacipulmonis DSM 24701
Ovis canadensis canadensis isolate 43U chromosome	CP011894	85.37	28.08	1.53611		Bovidae	Ovis canadensis canadensis
Ovis canadensis canadensis isolate 43U chromosome	CP011887	84.62	26.71	5.36155		Bovidae	Ovis canadensis canadensis
