# Supplementary material for: Testing the potential of a ribosomal 16S marker for DNA metabarcoding of insects
Source: PeerJ. 2016 Apr 19;4:e1966. doi: 10.7717/peerj.1966 (PMC4841222; doi:10.7717/peerj.1966)
Supplement: Supplemental Information 1 [file peerj-04-1966-s006.zip › scripts/5 match OTUs/blast_hits/OTU_128.csv_plot.pdf]

OTU_128.csv

Sericostoma personatum

Sericostomatidae

Trichoptera

80.42

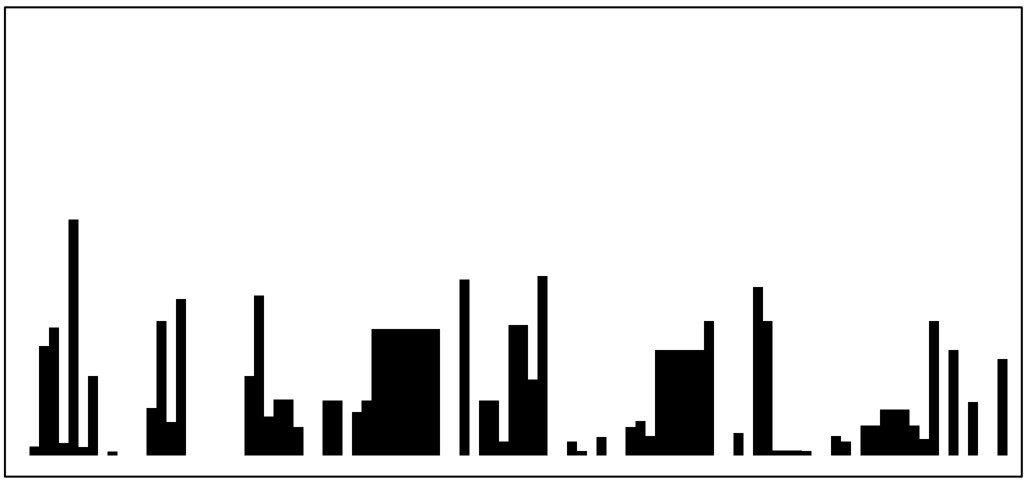

Sericostoma personatum mitochondrion, partial geno	KP455290	80.42	98.62	1.33298e−26	Trichoptera	Sericostomatidae	Sericostoma personatum
Notidobiella chacayana isolate CL5 16S ribosomal R	EF394993	85.12	82.07	1.6239e−25	Trichoptera	Sericostomatidae	Notidobiella chacayana
Sericostoma clypeatum voucher NHRS−EN CC4 16S ribo	FJ263207	85.98	73.79	1.97832e−24	Trichoptera	Sericostomatidae	Sericostoma clypeatum
Corydalus sp. EH−2004 16S ribosomal RNA gene, part	AY620139	80.58	70.34	9.5981e−16	Megaloptera	Corydalidae	Corydalus sp. EH−2004
Cardiocladius sp. MNK−2008 16S ribosomal RNA gene,	EU669992	91.04	45.52	3.35006e−15	Diptera	Chironomidae	Cardiocladius sp. MNK−2008
Acordulecera sp. SS2 voucher AE142 16S ribosomal R	KF318505	80.39	68.97	1.16929e−14	Hymenoptera	Pergidae	Acordulecera sp. SS2
Limnebius hispanicus mitochondrial 16S rRNA gene (	HF931402	83.72	59.31	1.16929e−14	Coleoptera	Hydraenidae	Limnebius hispanicus
Hydraena brevis mitochondrial 16S rRNA gene (parti	HE971007	77.86	83.45	1.16929e−14	Coleoptera	Hydraenidae	Hydraena brevis
Caenota plicata isolate CR9 16S ribosomal RNA gene	EF395003	80.18	73.79	1.16929e−14	Trichoptera	Calocidae	Caenota plicata
Pliocaloca sp. CQ3 16S ribosomal RNA gene, partial	EF394999	80	73.79	1.16929e−14	Trichoptera	Calocidae	Pliocaloca sp. CQ3
Corydalus cornutus 16S ribosomal RNA gene, partial	KF944505	79.61	70.34	4.08121e−14	Megaloptera	Corydalidae	Corydalus cornutus
Corydalus flavicornis 16S ribosomal RNA gene, part	KF944504	79.61	70.34	4.08121e−14	Megaloptera	Corydalidae	Corydalus flavicornis
Corydalus luteus 16S ribosomal RNA gene, partial s	KF944503	82.22	61.38	4.08121e−14	Megaloptera	Corydalidae	Corydalus luteus
Limnebius simplex mitochondrial 16S rRNA gene (par	HF931327	86.3	50.34	4.08121e−14	Coleoptera	Hydraenidae	Limnebius simplex
Xylosciara inornata voucher 230 16S ribosomal RNA	JQ613902	81.55	67.59	4.08121e−14	Diptera	Sciaridae	Xylosciara inornata
Limnebius nitidus mitochondrial 16S rRNA gene (par	HE613698	87.32	48.97	4.08121e−14	Coleoptera	Hydraenidae	Limnebius nitidus
Trox hamatus isolate 83A 16S ribosomal RNA gene, p	EF570388	78.99	77.24	4.08121e−14	Coleoptera	Trogidae	Trox hamatus
Trox hamatus isolate 83B 16S ribosomal RNA gene, p	EF570381	78.99	77.24	4.08121e−14	Coleoptera	Trogidae	Trox hamatus
Trox spinulosis isolate 96B 16S ribosomal RNA gene	EF570376	78.99	77.24	4.08121e−14	Coleoptera	Trogidae	Trox spinulosis
Trox spinulosis isolate 96A 16S ribosomal RNA gene	EF570362	78.99	77.24	4.08121e−14	Coleoptera	Trogidae	Trox spinulosis
Corydalus cornutus 16S ribosomal RNA gene, partial	EU734853	79.61	70.34	4.08121e−14	Megaloptera	Corydalidae	Corydalus cornutus
Corydalus cornutus mitochondrion, complete genome	FJ171323	79.61	70.34	4.08121e−14	Megaloptera	Corydalidae	Corydalus cornutus
Zygaena loti macedonica mitochondrial tRNA−Leu (CU	AJ831729	83.72	57.24	4.08121e−14	Lepidoptera	Zygaenidae	Zygaena loti macedonica
Hydraena sp. IBE AF202 mitochondrial 16S rRNA gene	HF971010	87.5	48.97	1.42448e−13	Coleoptera	Hydraenidae	Hydraena sp. IBE AF202
Mycetophagus atomarius voucher UPOL ZL0150 16S rib	FJ903753	81.82	58.62	1.42448e−13	Coleoptera	Mycetophagidae	Mycetophagus atomarius
Bactrocera minax mitochondrion, complete genome	HM776033	82.61	60	1.42448e−13	Diptera	Tephritidae	Bactrocera minax
Bactrocera minax 16S ribosomal RNA gene, partial s	FJ866821	82.61	60	1.42448e−13	Diptera	Tephritidae	Bactrocera minax
Diopsis nr. circularis KCADI065−14 16S ribosomal R	KT186651	81.32	58.62	4.97194e−13	Diptera	Diopsidae	Diopsis nr. circularis KCADI065−14
Corydalus ignotus 16S ribosomal RNA gene, partial	KF944506	78.64	70.34	4.97194e−13	Megaloptera	Corydalidae	Corydalus ignotus
Polytremis lubricans voucher He160 16S ribosomal R	JX971171	79.09	69.66	4.97194e−13	Lepidoptera	Hesperiidae	Polytremis lubricans
Limnebius theryi mitochondrial 16S rRNA gene (part	HF931484	82.56	59.31	4.97194e−13	Coleoptera	Hydraenidae	Limnebius theryi
Limnebius mucronatus mitochondrial 16S rRNA gene (	HF931452	82.56	59.31	4.97194e−13	Coleoptera	Hydraenidae	Limnebius mucronatus
Ochthebius salinator mitochondrial partial 16S rRN	HF931436	79.81	67.59	4.97194e−13	Coleoptera	Hydraenidae	Ochthebius salinator
Ochthebius heydeni mitochondrial 16S rRNA gene (pa	HF931419	82.02	60.69	4.97194e−13	Coleoptera	Hydraenidae	Ochthebius heydeni
Limnebius fretalis mitochondrial 16S rRNA gene (pa	HF931401	82.56	59.31	4.97194e−13	Coleoptera	Hydraenidae	Limnebius fretalis
Limnebius montanus mitochondrial 16S rRNA gene (pa	HE613718	85.92	48.97	4.97194e−13	Coleoptera	Hydraenidae	Limnebius montanus
Limnebius millani mitochondrial 16S rRNA gene (par	HE613717	85.92	48.97	4.97194e−13	Coleoptera	Hydraenidae	Limnebius millani
Limnebius aguilerai mitochondrial 16S rRNA gene (p	HE613715	85.92	48.97	4.97194e−13	Coleoptera	Hydraenidae	Limnebius aguilerai
Limnebius ordunyai mitochondrial 16S rRNA gene (pa	HE613712	85.92	48.97	4.97194e−13	Coleoptera	Hydraenidae	Limnebius ordunyai
Limnebius maurus mitochondrial 16S rRNA gene (part	HE613703	85.92	48.97	4.97194e−13	Coleoptera	Hydraenidae	Limnebius maurus
Limnebius gerhardti mitochondrial 16S rRNA gene (p	HE613700	85.92	48.97	4.97194e−13	Coleoptera	Hydraenidae	Limnebius gerhardti
Limnebius monfortei mitochondrial 16S rRNA gene (p	HE613672	85.92	48.97	4.97194e−13	Coleoptera	Hydraenidae	Limnebius monfortei
Trox terrestris isolate 7B 16S ribosomal RNA gene,	EF570394	76.98	91.72	4.97194e−13	Coleoptera	Trogidae	Trox terrestris
Trox terrestris isolate 7A 16S ribosomal RNA gene,	EF570383	76.98	91.72	4.97194e−13	Coleoptera	Trogidae	Trox terrestris
Aphodius fimetarius voucher BMNH 703033 16S large	EF487991	88.24	46.21	4.97194e−13	Coleoptera	Scarabaeidae	Aphodius fimetarius
Gracilipsodes koghiensis voucher NRM AF7 16S ribos	EF428481	79.09	71.72	4.97194e−13	Trichoptera	Leptoceridae	Gracilipsodes koghiensis
Zygaena ecki ecki mitochondrial tRNA−Leu (CUN) gen	AJ831721	82.56	57.24	4.97194e−13	Lepidoptera	Zygaenidae	Zygaena ecki ecki
Zygaena armena armena mitochondrial tRNA−Leu (CUN)	AJ831719	82.56	57.24	4.97194e−13	Lepidoptera	Zygaenidae	Zygaena armena armena
Zygaena algira algira mitochondrial tRNA−Leu (CUN)	AJ831626	80.65	63.45	4.97194e−13	Lepidoptera	Zygaenidae	Zygaena algira algira
Protochauliodes biconicus incertus 16S ribosomal R	KF944494	86.11	48.97	1.73538e−12	Megaloptera	Corydalidae	Protochauliodes biconicus incertus
Protochauliodes incertus isolate H842 16S ribosoma	KF741322	86.11	48.97	1.73538e−12	Megaloptera	Corydalidae	Protochauliodes biconicus incertus
Zethopsus sp. JP−2014 isolate JPDG46 16S ribosomal	KM350453	83.54	53.1	1.73538e−12	Coleoptera	Staphylinidae	Zethopsus sp. JP−2014
Pennisetia hylaeiformis mitochondrion, partial gen	KJ508049	88.41	46.9	1.73538e−12	Lepidoptera	Sesiidae	Pennisetia hylaeiformis
Ochthebius meridionalis mitochondrial 16S rRNA gen	HF931538	77.78	76.55	1.73538e−12	Coleoptera	Hydraenidae	Ochthebius meridionalis
Ochthebius lobicollis mitochondrial 16S rRNA gene	HF931534	76	82.07	1.73538e−12	Coleoptera	Hydraenidae	Ochthebius lobicollis
Ochthebius striatus mitochondrial 16S rRNA gene (p	HF931455	80.65	63.45	1.73538e−12	Coleoptera	Hydraenidae	Ochthebius striatus
Ochthebius rugulosus mitochondrial 16S rRNA gene (	HF931447	80.19	68.97	1.73538e−12	Coleoptera	Hydraenidae	Ochthebius rugulosus
Hypulus quercinus voucher UPOL ZL0158 16S ribosoma	FJ903757	78.64	67.59	1.73538e−12	Coleoptera	Melandryidae	Hypulus quercinus
Cordalia obscura voucher ZMUN:10002651 16S ribosom	GQ980973	80.85	63.45	1.73538e−12	Coleoptera	Staphylinidae	Cordalia obscura
Agulla bicolor 16S ribosomal RNA gene, partial seq	EU734847	74.31	95.17	1.73538e−12	Raphidioptera	Raphidiidae	Agulla bicolor
Gracilipsodes aureus voucher NRM AG9 16S ribosomal	EF428485	77.52	83.45	1.73538e−12	Trichoptera	Leptoceridae	Gracilipsodes aureus
Zygaena contaminei contaminei mitochondrial tRNA−L	AJ831673	81.32	60.69	1.73538e−12	Lepidoptera	Zygaenidae	Zygaena contaminei contaminei
Zygaena hilaris escorialensis mitochondrial tRNA−L	AJ831643	81.61	59.31	1.73538e−12	Lepidoptera	Zygaenidae	Zygaena hilaris escorialensis
Corydalus peruvianus 16S ribosomal RNA gene, parti	KF944507	80.9	60.69	6.05706e−12	Megaloptera	Corydalidae	Corydalus peruvianus
Heliconius sapho voucher 8126 ; mitochondrial	KP073323	84.93	48.97	6.05706e−12	Lepidoptera	Nymphalidae	Heliconius sapho
Heliconius sapho voucher 2739 ; mitochondrial	KP073322	84.93	48.97	6.05706e−12	Lepidoptera	Nymphalidae	Heliconius sapho
Heliconius sapho sapho voucher 260 ; mitochondrial	KP073321	84.93	48.97	6.05706e−12	Lepidoptera	Nymphalidae	Heliconius sapho sapho
Heliconius eleuchia eleuchia voucher 190 ; mitocho	KP073274	84.93	48.97	6.05706e−12	Lepidoptera	Nymphalidae	Heliconius eleuchia eleuchia
Heliconius antiochus voucher antiochus_S ; mitocho	KP073253	84.93	48.97	6.05706e−12	Lepidoptera	Nymphalidae	Heliconius antiochus
Atherigona varia voucher RMBR:116181 16S ribosomal	KJ476297	86.3	48.97	6.05706e−12	Diptera	Muscidae	Atherigona varia
Clambidae sp. UPOL 001320 16S ribosomal RNA gene,	KF625804	79.09	69.66	6.05706e−12	Coleoptera	Clambidae	Clambidae sp. UPOL 001320
Opitzia chiapas voucher 7167 16S ribosomal RNA gen	KC524881	80	59.31	6.05706e−12	Coleoptera	Cleridae	Opitzia chiapas
Laccobius serratus 16S ribosomal RNA gene, partial	KC992690	81.05	63.45	6.05706e−12	Coleoptera	Hydrophilidae	Laccobius serratus
Aulacochthebius exaratus mitochondrial 16S rRNA ge	HF931427	76.61	80.69	6.05706e−12	Coleoptera	Hydraenidae	Aulacochthebius exaratus
Hydraena cf. concinna IBE AF194 mitochondrial 16S	HE971009	87.88	44.83	6.05706e−12	Coleoptera	Hydraenidae	Hydraena cf. concinna IBE AF194
Chaetoria stylata mitochondrial gene for 16S rRNA,	AB699938	86.3	48.97	6.05706e−12	Diptera	Tachinidae	Chaetoria stylata
Tarucus nara isolate N308 16S ribosomal RNA gene,	JQ421899	80.22	59.31	6.05706e−12	Lepidoptera	Lycaenidae	Tarucus nara
Tarucus nara isolate F112 16S ribosomal RNA gene,	JQ421848	80.22	59.31	6.05706e−12	Lepidoptera	Lycaenidae	Tarucus nara
Tarucus nara isolate F124 16S ribosomal RNA gene,	JQ421841	80.22	59.31	6.05706e−12	Lepidoptera	Lycaenidae	Tarucus nara
Hypocera mordellaria isolate 200864 16S ribosomal	GU362000	80.21	62.07	6.05706e−12	Diptera	Phoridae	Hypocera mordellaria
Apatura nycteis 16S ribosomal RNA gene, partial se	GU372432	77.88	73.79	6.05706e−12	Lepidoptera	Nymphalidae	Apatura nycteis
Mongoloraphidia harmandi mitochondrion, complete g	FJ859902	78.5	71.72	6.05706e−12	Raphidioptera	Raphidiidae	Mongoloraphidia harmandi
Corydalus armatus 16S ribosomal RNA gene, partial	EU734858	80.9	60.69	6.05706e−12	Megaloptera	Corydalidae	Corydalus armatus
Nepaloserica sp. BMNH 670901 16S large subunit rib	EF487902	80.65	59.31	6.05706e−12	Coleoptera	Scarabaeidae	Nepaloserica sp. BMNH 670901
Clambus pubescens partial 16S rRNA gene, specimen	AM884186	79.09	69.66	6.05706e−12	Coleoptera	Clambidae	Clambus pubescens
Aphodius isaburoi 16S ribosomal RNA gene, partial	EF487814	81.4	55.17	6.05706e−12	Coleoptera	Scarabaeidae	Aphodius isaburoi
Zygaena lavandulae consobrina mitochondrial tRNA−L	AJ831726	81.4	58.62	6.05706e−12	Lepidoptera	Zygaenidae	Zygaena lavandulae consobrina
Zygaena transpamirina transpamirina mitochondrial	AJ831660	82.14	57.24	6.05706e−12	Lepidoptera	Zygaenidae	Zygaena transpamirina transpamirina
Zygaena transpamirina andarabensis mitochondrial t	AJ831659	82.14	57.24	6.05706e−12	Lepidoptera	Zygaenidae	Zygaena transpamirina andarabensis
Zygaena pamira pamira mitochondrial tRNA−Leu (CUN)	AJ831652	82.14	57.24	6.05706e−12	Lepidoptera	Zygaenidae	Zygaena pamira pamira
Zygaena occitanica huescacola mitochondrial tRNA−L	AJ831649	81.4	58.62	6.05706e−12	Lepidoptera	Zygaenidae	Zygaena occitanica huescacola
Cryptoscenea sp. N800014 16S ribosomal RNA gene, p	EF632340	80.77	66.9	6.05706e−12	Neuroptera	Coniopterygidae	Cryptoscenea sp. N800014
Somatia sp. YSUW04038 16S ribosomal RNA gene, part	AY573134	86.3	48.97	6.05706e−12	Diptera	Somatiidae	Somatia sp. YSUW04038
Tabanus rufofrater 16S ribosomal RNA gene, partial	AY140887	79.38	61.38	6.05706e−12	Diptera	Tabanidae	Tabanus rufofrater
Heliconius eleuchia isolate STRI−B−842 16S ribosom	AY747879	84.93	48.97	6.05706e−12	Lepidoptera	Nymphalidae	Heliconius eleuchia
Polytremis lubricans 16S ribosomal RNA gene, parti	KJ786310	78.18	69.66	2.11412e−11	Lepidoptera	Hesperiidae	Polytremis lubricans
Athalia japonica mitochondrial gene for 16S riboso	AB859181	82.5	53.79	2.11412e−11	Hymenoptera	Tenthredinidae	Athalia japonica
Conocoxa chalicipoda voucher AE088 16S ribosomal R	KF318484	77.98	73.1	2.11412e−11	Hymenoptera	Pergidae	Conocoxa chalicipoda
Arhopalus rusticus voucher HNAU.PP 006 16S ribosom	KF737722	79.79	60	2.11412e−11	Coleoptera	Cerambycidae	Arhopalus rusticus
Limnebius kocheri mitochondrial 16S rRNA gene (par	HF931546	84.51	48.97	2.11412e−11	Coleoptera	Hydraenidae	Limnebius kocheri
