# Supplementary material for: Testing the potential of a ribosomal 16S marker for DNA metabarcoding of insects
Source: PeerJ. 2016 Apr 19;4:e1966. doi: 10.7717/peerj.1966 (PMC4841222; doi:10.7717/peerj.1966)
Supplement: Supplemental Information 1 [file peerj-04-1966-s006.zip › scripts/5 match OTUs/blast_hits/OTU_129.csv_plot.pdf]

OTU_129.csv

Hansonoperla appalachia

Perlidae

Plecoptera

88.35

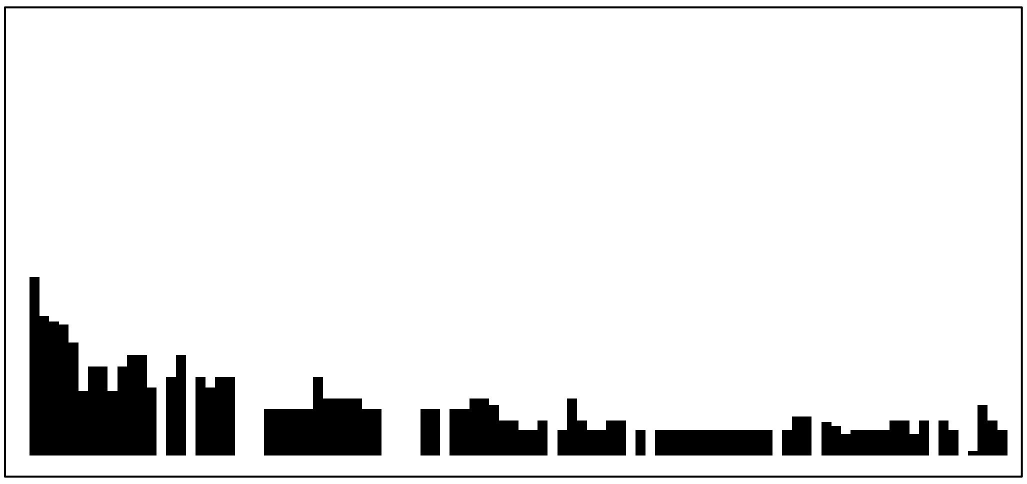

Hansonoperla appalachia 16S ribosomal RNA gene, pa	EF623249	88.35	62.35	1.87487e−25	Plecoptera	Perlidae	Hansonoperla appalachia
Acroneuria lycorias 16S ribosomal RNA gene, partia	EF623251	86.54	62.35	6.54393e−25	Plecoptera	Perlidae	Acroneuria lycorias
Eccoptura xanthenes 16S ribosomal RNA gene, partia	EF623252	86.27	62.35	2.78255e−23	Plecoptera	Perlidae	Eccoptura xanthenes
Calineuria californica 16S ribosomal RNA gene, par	EF623239	86.14	62.35	9.71205e−23	Plecoptera	Perlidae	Calineuria californica
Perlinella drymo 16S ribosomal RNA gene, partial s	EF623240	85.29	62.35	1.18317e−21	Plecoptera	Perlidae	Perlinella drymo
Dinocras cephalotes mitochondrion, complete genome	KF484757	83.02	62.35	4.12967e−21	Plecoptera	Perlidae	Dinocras cephalotes
Dolichopoda pavesii voucher SPI 16S ribosomal RNA	EF216951	84.16	62.35	4.12967e−21	Orthoptera	Rhaphidophoridae	Dolichopoda pavesii
Pictetoperla sp. BYU_PL156 16S ribosomal RNA gene,	EF623247	84.16	62.35	4.12967e−21	Plecoptera	Perlidae	Pictetoperla sp. BYU_PL156
Dinocras cephalotes 16S ribosomal RNA gene, partia	EF623244	83.02	62.35	4.12967e−21	Plecoptera	Perlidae	Dinocras cephalotes
Megaleuctra kincaidi 16S ribosomal RNA gene, parti	EF623174	84.16	62.35	4.12967e−21	Plecoptera	Leuctridae	Megaleuctra kincaidi
Cryptoperla japonica mitochondrial gene for 16S rR	AB711202	84.69	60.49	1.75598e−19	Plecoptera	Peltoperlidae	Cryptoperla japonica
Cryptoperla japonica mitochondrial gene for 16S rR	AB711184	84.69	60.49	1.75598e−19	Plecoptera	Peltoperlidae	Cryptoperla japonica
Notonemouridae sp. MD−2011 voucher ZMUC:JD−1507 16	JN615257	83.17	62.35	1.75598e−19	Plecoptera	Notonemouridae	Notonemouridae sp. MD−2011
Dolichopoda sp. MR−2008 16S ribosomal RNA gene, pa	EU887863	77.21	83.95	1.75598e−19	Orthoptera	Rhaphidophoridae	Dolichopoda lustriae
Scopura montana 16S ribosomal RNA gene, partial se	EF623265	83.67	60.49	1.75598e−19	Plecoptera		Scopura montana
Pteronarcys sachalina 16S ribosomal RNA gene, part	EF623264	84.69	60.49	1.75598e−19	Plecoptera	Pteronarcyidae	Pteronarcys sachalina
Agnetina capitata 16S ribosomal RNA gene, partial	EF623241	74.85	100	1.75598e−19	Plecoptera	Perlidae	Agnetina capitata
Hesperoperla pacifica 16S ribosomal RNA gene, part	EF623238	83.67	60.49	1.75598e−19	Plecoptera	Perlidae	Hesperoperla pacifica
Nemoura cinerea 16S ribosomal RNA gene, partial se	EF623194	83.17	62.35	1.75598e−19	Plecoptera	Nemouridae	Nemoura cinerea
Cryptoperla japonica mitochondrial gene for 16S rR	AB711183	83.67	60.49	2.13922e−18	Plecoptera	Peltoperlidae	Cryptoperla japonica
Cryptoperla japonica mitochondrial gene for 16S rR	AB711182	83.67	60.49	2.13922e−18	Plecoptera	Peltoperlidae	Cryptoperla japonica
Dolichopoda sp. VAR 16S ribosomal RNA gene, partia	EU887865	76.47	83.95	2.13922e−18	Orthoptera	Rhaphidophoridae	Dolichopoda sp. VAR
Dolichopoda sp. TAY 16S ribosomal RNA gene, partia	EU887864	76.47	83.95	2.13922e−18	Orthoptera	Rhaphidophoridae	Dolichopoda sp. TAY
Brachyptera seticornis 16S ribosomal RNA gene, par	EF623268	76.6	87.04	2.13922e−18	Plecoptera	Taeniopterygidae	Brachyptera seticornis
Pteronarcella badia 16S ribosomal RNA gene, partia	EF623262	82.18	62.35	2.13922e−18	Plecoptera	Pteronarcyidae	Pteronarcella badia
Prostoia besametsa 16S ribosomal RNA gene, partial	EF623188	82.18	62.35	2.13922e−18	Plecoptera	Nemouridae	Prostoia besametsa
Nemoura trispinosa 16S ribosomal RNA gene, partial	EF623187	82.18	62.35	2.13922e−18	Plecoptera	Nemouridae	Nemoura trispinosa
Paranemoura claasseni 16S ribosomal RNA gene, part	EF623185	82.18	62.35	2.13922e−18	Plecoptera	Nemouridae	Paranemoura claasseni
Shipsa rotunda 16S ribosomal RNA gene, partial seq	EF623184	82.18	62.35	2.13922e−18	Plecoptera	Nemouridae	Shipsa rotunda
Osmylus sp. EH−2004 16S ribosomal RNA gene, partia	AY620146	83.67	60.49	2.13922e−18	Neuroptera	Osmylidae	Osmylus sp. EH−2004
Apteroperla tikumana mitochondrion, complete genom	KR604721	82.65	60.49	7.46661e−18	Plecoptera	Capniidae	Apteroperla tikumana
Dolichopoda steriotisi voucher ANT 16S ribosomal R	EF216955	82.65	60.49	7.46661e−18	Orthoptera	Rhaphidophoridae	Dolichopoda steriotisi
Nemurella pictetii 16S ribosomal RNA gene, partial	EF623192	82.65	60.49	7.46661e−18	Plecoptera	Nemouridae	Nemurella pictetii
Malenka californica 16S ribosomal RNA gene, partia	EF623182	82.65	60.49	7.46661e−18	Plecoptera	Nemouridae	Malenka californica
Cryptoperla sp. YK−2012−1 mitochondrial gene for 1	AB711243	82.18	62.35	2.6061e−17	Plecoptera	Peltoperlidae	Cryptoperla sp. YK−2012−1
Cryptoperla ishigakiensis mitochondrial gene for 1	AB711242	82.18	62.35	2.6061e−17	Plecoptera	Peltoperlidae	Cryptoperla ishigakiensis
Micropathus tasmaniensis voucher JNC 16S ribosomal	HM594507	77.46	87.04	2.6061e−17	Orthoptera	Rhaphidophoridae	Micropathus tasmaniensis
Tessellana veyseli voucher 245 16S ribosomal RNA g	EU203889	74.84	98.15	2.6061e−17	Orthoptera	Tettigoniidae	Tessellana veyseli
Doddsia occidentalis 16S ribosomal RNA gene, parti	EF623270	75.17	91.98	2.6061e−17	Plecoptera	Taeniopterygidae	Doddsia occidentalis
Taenionema palladium 16S ribosomal RNA gene, parti	EF623267	74.83	92.59	2.6061e−17	Plecoptera	Taeniopterygidae	Taenionema palladium
Pteronarcys scotti 16S ribosomal RNA gene, partial	EF623263	82.18	62.35	2.6061e−17	Plecoptera	Pteronarcyidae	Pteronarcys scotti
Pteronarcys californica 16S ribosomal RNA gene, pa	EF623261	82.18	62.35	2.6061e−17	Plecoptera	Pteronarcyidae	Pteronarcys californica
Moselia infuscata 16S ribosomal RNA gene, partial	EF623175	74.23	100	2.6061e−17	Plecoptera	Leuctridae	Moselia infuscata
Eupholidoptera chabrieri voucher 173 16S ribosomal	EU120792	82.18	62.35	2.6061e−17	Orthoptera	Tettigoniidae	Eupholidoptera chabrieri
Pteronarcys princeps mitochondrion, complete genom	AY687866	82.18	62.35	2.6061e−17	Plecoptera	Pteronarcyidae	Pteronarcys princeps
Pleioplectron simplex voucher CW306 16S ribosomal	HM594512	82.65	60.49	9.0962e−17	Orthoptera	Rhaphidophoridae	Pleioplectron simplex
Pachyrhamma edwardsii voucher CW122 16S ribosomal	HM594508	82.65	60.49	9.0962e−17	Orthoptera	Rhaphidophoridae	Pachyrhamma edwardsii
Micropathus tasmaniensis voucher IDB 16S ribosomal	HM594505	82.35	62.35	9.0962e−17	Orthoptera	Rhaphidophoridae	Micropathus tasmaniensis
Dolichopoda geniculata geniculata isolate PRA20 16	AY507590	81.63	60.49	9.0962e−17	Orthoptera	Rhaphidophoridae	Dolichopoda geniculata geniculata
Dolichopoda annae voucher OLI 16S ribosomal RNA ge	EU887860	81.63	60.49	9.0962e−17	Orthoptera	Rhaphidophoridae	Dolichopoda annae
Dolichopoda hussoni voucher IZB 16S ribosomal RNA	EF216973	81.19	62.35	9.0962e−17	Orthoptera	Rhaphidophoridae	Dolichopoda hussoni
Dolichopoda thasosensis voucher DRA 16S ribosomal	EF216956	81.19	62.35	9.0962e−17	Orthoptera	Rhaphidophoridae	Dolichopoda thasosensis
Dolichopoda kiriakii voucher AGH 16S ribosomal RNA	EF216954	81.63	60.49	9.0962e−17	Orthoptera	Rhaphidophoridae	Dolichopoda kiriakii
Dolichopoda ithakii voucher ITA 16S ribosomal RNA	EF216949	76.06	87.04	9.0962e−17	Orthoptera	Rhaphidophoridae	Dolichopoda ithakii
Kempnyella sp. BYU_PL155 16S ribosomal RNA gene, p	EF623246	81.19	62.35	9.0962e−17	Plecoptera	Perlidae	Kempnyella sp. BYU_PL155
Cosumnoperla hypocrena 16S ribosomal RNA gene, par	EF623226	82.65	60.49	9.0962e−17	Plecoptera	Perlodidae	Cosumnoperla hypocrena
Nanonemoura wahkeena 16S ribosomal RNA gene, parti	EF623198	81.63	60.49	9.0962e−17	Plecoptera	Nemouridae	Nanonemoura wahkeena
Lednia tumana 16S ribosomal RNA gene, partial sequ	EF623197	81.19	62.35	9.0962e−17	Plecoptera	Nemouridae	Lednia tumana
Protonemura meyeri 16S ribosomal RNA gene, partial	EF623193	81.19	62.35	9.0962e−17	Plecoptera	Nemouridae	Protonemura meyeri
Ostrocerca foersteri 16S ribosomal RNA gene, parti	EF623191	81.63	60.49	9.0962e−17	Plecoptera	Nemouridae	Ostrocerca foersteri
Visoka cataractae 16S ribosomal RNA gene, partial	EF623189	81.63	60.49	9.0962e−17	Plecoptera	Nemouridae	Visoka cataractae
Tessellana veyseli voucher 260 16S ribosomal RNA g	EU120811	74.21	98.15	9.0962e−17	Orthoptera	Tettigoniidae	Tessellana veyseli
Eupholidoptera uvarovi isolate KAS1 16S ribosomal	KC852312	81.19	62.35	3.17489e−16	Orthoptera	Tettigoniidae	Eupholidoptera uvarovi
Eupholidoptera smyrnensis isolate KOY1 16S ribosom	KC852309	76.56	79.01	3.17489e−16	Orthoptera	Tettigoniidae	Eupholidoptera smyrnensis
Eupholidoptera megastyla isolate ALE1 16S ribosoma	KC852308	81.19	62.35	3.17489e−16	Orthoptera	Tettigoniidae	Eupholidoptera megastyla
Eupholidoptera hesperica isolate MAR3 16S ribosoma	KC852304	81.19	62.35	3.17489e−16	Orthoptera	Tettigoniidae	Eupholidoptera hesperica
Eupholidoptera hesperica isolate MAR1 16S ribosoma	KC852302	81.19	62.35	3.17489e−16	Orthoptera	Tettigoniidae	Eupholidoptera hesperica
Eupholidoptera danconai isolate ROG1 16S ribosomal	KC852297	81.19	62.35	3.17489e−16	Orthoptera	Tettigoniidae	Eupholidoptera danconai
Eupholidoptera danconai isolate TRL2 16S ribosomal	KC852296	81.19	62.35	3.17489e−16	Orthoptera	Tettigoniidae	Eupholidoptera danconai
Eupholidoptera danconai isolate JEN1 16S ribosomal	KC852294	81.19	62.35	3.17489e−16	Orthoptera	Tettigoniidae	Eupholidoptera danconai
Eupholidoptera danconai isolate PIE1 16S ribosomal	KC852292	81.19	62.35	3.17489e−16	Orthoptera	Tettigoniidae	Eupholidoptera danconai
Eupholidoptera bimucronata isolate MAD1 16S riboso	KC852289	81.19	62.35	3.17489e−16	Orthoptera	Tettigoniidae	Eupholidoptera bimucronata
Eupholidoptera bimucronata isolate PTR1 16S riboso	KC852287	81.19	62.35	3.17489e−16	Orthoptera	Tettigoniidae	Eupholidoptera bimucronata
Eupholidoptera bimucronata isolate GIB3 16S riboso	KC852285	81.19	62.35	3.17489e−16	Orthoptera	Tettigoniidae	Eupholidoptera bimucronata
Eupholidoptera bimucronata isolate GIB2 16S riboso	KC852284	81.19	62.35	3.17489e−16	Orthoptera	Tettigoniidae	Eupholidoptera bimucronata
Eupholidoptera bimucronata isolate GIB1 16S riboso	KC852283	81.19	62.35	3.17489e−16	Orthoptera	Tettigoniidae	Eupholidoptera bimucronata
Eupholidoptera magnifica isolate ARB1 16S ribosoma	KC852277	75.35	86.42	3.17489e−16	Orthoptera	Tettigoniidae	Eupholidoptera magnifica
Eupholidoptera magnifica isolate BIG1 16S ribosoma	KC852275	81.19	62.35	3.17489e−16	Orthoptera	Tettigoniidae	Eupholidoptera magnifica
Dolichopoda patrizii voucher PET 16S ribosomal RNA	EU887862	81.82	60.49	3.17489e−16	Orthoptera	Rhaphidophoridae	Dolichopoda patrizii
Dolichopoda graeca voucher PER 16S ribosomal RNA g	EF216953	81.82	60.49	3.17489e−16	Orthoptera	Rhaphidophoridae	Dolichopoda graeca
Dolichopoda giachinoi voucher ORO 16S ribosomal RN	EF216952	75.89	87.04	3.17489e−16	Orthoptera	Rhaphidophoridae	Dolichopoda giachinoi
Perlesta decipines 16S ribosomal RNA gene, partial	EF623242	81.55	62.35	3.17489e−16	Plecoptera	Perlidae	Perlesta decipines
Osobenus yakimae 16S ribosomal RNA gene, partial s	EF623232	81.37	62.96	3.17489e−16	Plecoptera	Perlodidae	Osobenus yakimae
Udamocercia sp. BYU_PL150 16S ribosomal RNA gene,	EF623201	81	61.73	3.17489e−16	Plecoptera	Notonemouridae	Udamocercia sp. BYU_PL150
Anabrus simplex voucher OR034 mitochondrion, compl	EF373911	81.19	62.35	3.17489e−16	Orthoptera	Tettigoniidae	Anabrus simplex
Eupholidoptera karatolosi voucher 421 16S ribosoma	EU120826	81.19	62.35	3.17489e−16	Orthoptera	Tettigoniidae	Eupholidoptera karatolosi
Anabrus simplex voucher 165 16S ribosomal RNA gene	EU120786	81.19	62.35	3.17489e−16	Orthoptera	Tettigoniidae	Anabrus simplex
Pholidoptera fallax voucher 160 16S ribosomal RNA	EU120781	81.19	62.35	3.17489e−16	Orthoptera	Tettigoniidae	Pholidoptera fallax
Cryptoperla sp. YK−2012−2 mitochondrial gene for 1	AB711244	81.63	60.49	1.10814e−15	Plecoptera	Peltoperlidae	Cryptoperla sp. YK−2012−2
Cryptoperla japonica mitochondrial gene for 16S rR	AB711189	81.63	60.49	1.10814e−15	Plecoptera	Peltoperlidae	Cryptoperla japonica
Hesperoperla pacifica strain PL008 16S ribosomal R	JN800541	81	60.49	1.10814e−15	Plecoptera	Perlidae	Hesperoperla pacifica
Pachyrhamma edwardsii voucher WEL 16S ribosomal RN	HM594509	81.63	60.49	1.10814e−15	Orthoptera	Rhaphidophoridae	Pachyrhamma edwardsii
Dolichopoda sp. KOR 16S ribosomal RNA gene, partia	EU887866	75.74	83.95	1.10814e−15	Orthoptera	Rhaphidophoridae	Dolichopoda sp. KOR
Dolichopoda unicolor voucher DIR 16S ribosomal RNA	EF216970	81.63	60.49	1.10814e−15	Orthoptera	Rhaphidophoridae	Dolichopoda unicolor
Dolichopoda remyi voucher EDE 16S ribosomal RNA ge	EF216969	81.19	62.35	1.10814e−15	Orthoptera	Rhaphidophoridae	Dolichopoda remyi
Dolichopoda dalensi voucher KEF 16S ribosomal RNA	EF216959	75.18	87.04	1.10814e−15	Orthoptera	Rhaphidophoridae	Dolichopoda dalensi
Strophopteryx appalachia 16S ribosomal RNA gene, p	EF623271	80.2	62.35	1.10814e−15	Plecoptera	Taeniopterygidae	Strophopteryx appalachia
Rickera sorpta 16S ribosomal RNA gene, partial seq	EF623235	82.35	62.35	1.10814e−15	Plecoptera	Perlodidae	Rickera sorpta
Neonemura sp. BYU_PL153 16S ribosomal RNA gene, pa	EF623204	81.63	60.49	1.10814e−15	Plecoptera	Notonemouridae	Neonemura sp. BYU_PL153
Soyedina producta 16S ribosomal RNA gene, partial	EF623196	81.19	62.35	1.10814e−15	Plecoptera	Nemouridae	Soyedina producta
