# Supplementary material for: Testing the potential of a ribosomal 16S marker for DNA metabarcoding of insects
Source: PeerJ. 2016 Apr 19;4:e1966. doi: 10.7717/peerj.1966 (PMC4841222; doi:10.7717/peerj.1966)
Supplement: Supplemental Information 1 [file peerj-04-1966-s006.zip › scripts/5 match OTUs/blast_hits/OTU_13.csv_plot.pdf]

OTU_13.csv

Velia caprai

Veliidae

Hemiptera

100

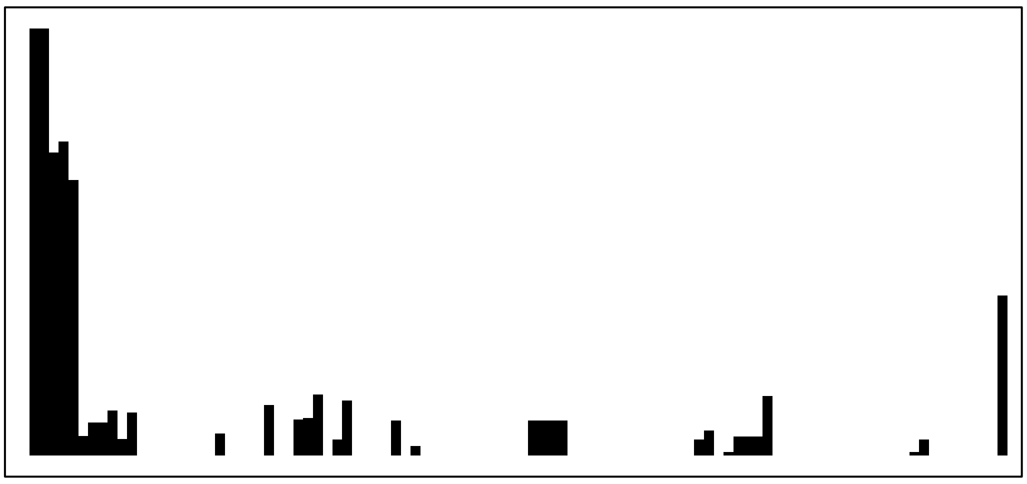

Velia caprai isolate 943 16S ribosomal RNA gene, p	EU871185	100	100	3.64495e−72	Hemiptera	Veliidae	Velia caprai
Velia caprai 16S ribosomal RNA gene, partial seque	AY648139	100	100	3.64495e−72	Hemiptera	Veliidae	Velia caprai
Velia affinis isolate 373 16S ribosomal RNA gene,	EU871189	94.19	100	1.11598e−59	Hemiptera	Veliidae	Velia affinis
Velia hoberlandti isolate 941 16S ribosomal RNA ge	EU871188	94.7	97.42	3.89516e−59	Hemiptera	Veliidae	Velia hoberlandti
Velia maderensis isolate 1862 16S ribosomal RNA ge	EU871187	92.9	100	5.78093e−57	Hemiptera	Veliidae	Velia maderensis
Rhagovelia tenuipes voucher MC36a 16S ribosomal RN	KC594837	80.89	100	6.60187e−31	Hemiptera	Veliidae	Rhagovelia tenuipes
Rhagovelia uncinata isolate 666 16S ribosomal RNA	EU871181	81.53	100	6.60187e−31	Hemiptera	Veliidae	Rhagovelia uncinata
Rhagovelia sp. 1 JD−2008 isolate 1602 16S ribosoma	EU871178	81.53	100	6.60187e−31	Hemiptera	Veliidae	Rhagovelia sp. 1 JD−2008
Perittopus asiaticus isolate 846 16S ribosomal RNA	EU871165	82.1	100	8.04272e−30	Hemiptera	Veliidae	Perittopus asiaticus
Ventidius usingeri isolate 1059 16S ribosomal RNA	EU871207	80.77	97.42	9.79804e−29	Hemiptera	Gerridae	Ventidius usingeri
Xenobates argentatus isolate 973 16S ribosomal RNA	EU871177	82.01	88.39	9.79804e−29	Hemiptera	Veliidae	Xenobates argentatus
Limnoporus genitalis 16S ribosomal RNA gene, parti	AY425217	79.87	97.42	9.79804e−29	Hemiptera	Gerridae	Limnoporus genitalis
Limnoporus canaliculatus 16S ribosomal RNA gene, p	AY425208	79.87	97.42	9.79804e−29	Hemiptera	Gerridae	Limnoporus canaliculatus
Gerris argenticollis 16S large subunit ribosomal R	AY951329	79.87	97.42	1.19365e−27	Hemiptera	Gerridae	Gerris argenticollis
Limnoporus dissortis 16S ribosomal RNA gene, parti	AY425228	79.22	97.42	1.19365e−27	Hemiptera	Gerridae	Limnoporus dissortis
Limnoporus esakii 16S ribosomal RNA gene, partial	AY425216	79.22	97.42	1.19365e−27	Hemiptera	Gerridae	Limnoporus esakii
Limnoporus notabilis 16S ribosomal RNA gene, parti	AY425210	79.22	97.42	1.19365e−27	Hemiptera	Gerridae	Limnoporus notabilis
Limnoporus esakii mitochondrial gene for 16S ribos	AB026592	79.22	97.42	1.19365e−27	Hemiptera	Gerridae	Limnoporus esakii
Limnoporus genitalis mitochondrial gene for 16S ri	AB026591	79.22	97.42	1.19365e−27	Hemiptera	Gerridae	Limnoporus genitalis
Rhagovelia robusta voucher MC35a 16S ribosomal RNA	KC594836	81.01	99.35	4.16623e−27	Hemiptera	Veliidae	Rhagovelia robusta
Amemboa sp. JD−2014 isolate 2440 16S large subunit	KC880875	79.75	97.42	1.45416e−26	Hemiptera	Gerridae	Amemboa sp. JD−2014
Gerris gracilicornis mitochondrial genes for 12S r	AB679162	79.22	97.42	1.45416e−26	Hemiptera	Gerridae	Gerris gracilicornis
Gerris gracilicornis mitochondrial genes for 12S r	AB679161	79.22	97.42	1.45416e−26	Hemiptera	Gerridae	Gerris gracilicornis
Gerris gracilicornis mitochondrial genes for 12S r	AB679160	79.22	97.42	1.45416e−26	Hemiptera	Gerridae	Gerris gracilicornis
Limnogonus hungerfordi isolate 1431 16S large subu	GU645089	82.35	85.16	1.45416e−26	Hemiptera	Gerridae	Limnogonus hungerfordi
Rhagovelia zela voucher MC37a 16S ribosomal RNA ge	KC594838	79.11	100	5.07551e−26	Hemiptera	Veliidae	Rhagovelia zela
Gerris gracilicornis mitochondrial genes for 12S r	AB679153	78.57	97.42	5.07551e−26	Hemiptera	Gerridae	Gerris gracilicornis
Neurigona lineata voucher Pollet 0183 (P) 16S ribo	HQ448992	81.68	82.58	5.07551e−26	Diptera	Dolichopodidae	Neurigona lineata
Limnogonus luctuosus isolate 2294 16S large subuni	GU645092	81.75	85.16	5.07551e−26	Hemiptera	Gerridae	Limnogonus luctuosus
Halovelia sp. JD−2008 isolate 1950 16S ribosomal R	EU871176	82.84	85.16	5.07551e−26	Hemiptera	Veliidae	Halovelia sp. JD−2008
Gerris sphagnetorum 16S ribosomal RNA gene, partia	AY425206	79.38	97.42	5.07551e−26	Hemiptera	Gerridae	Gerris sphagnetorum
Limnometra matsudai isolate 256 16S large subunit	KC880900	80.74	85.16	1.77153e−25	Hemiptera	Gerridae	Limnometra matsudai
Limnogonus pectoralis isolate 141 16S large subuni	GU645072	82.58	82.58	1.77153e−25	Hemiptera	Gerridae	Limnogonus pectoralis
Limnogonus recens isolate 1437 16S large subunit r	GU645063	78.98	97.42	1.77153e−25	Hemiptera	Gerridae	Limnogonus recens
Microvelia reticulata isolate 292 16S ribosomal RN	EU871172	79.49	97.42	1.77153e−25	Hemiptera	Veliidae	Microvelia reticulata
Amemboa sexualis 16S ribosomal RNA gene, partial s	AY648138	78.98	97.42	1.77153e−25	Hemiptera	Gerridae	Amemboa sexualis
Aquarius ventralis 16S ribosomal RNA gene, partial	AY425196	79.08	97.42	1.77153e−25	Hemiptera	Gerridae	Aquarius ventralis
Limnogonus aduncus voucher MC32a 16S ribosomal RNA	KC594827	81.62	85.16	6.18324e−25	Hemiptera	Gerridae	Limnogonus aduncus
Cylindrostethus palmaris voucher MC30a 16S ribosom	KC594823	78.26	100	6.18324e−25	Hemiptera	Gerridae	Cylindrostethus palmaris
Hypselonotus fulvus 16S ribosomal RNA gene, partia	KC537015	80.43	85.16	6.18324e−25	Hemiptera	Coreidae	Hypselonotus fulvus
Gerris gracilicornis mitochondrial genes for 12S r	AB679164	77.92	97.42	6.18324e−25	Hemiptera	Gerridae	Gerris gracilicornis
Gerris gracilicornis mitochondrial genes for 12S r	AB679163	77.92	97.42	6.18324e−25	Hemiptera	Gerridae	Gerris gracilicornis
Gerris gracilicornis mitochondrial genes for 12S r	AB679157	78.57	97.42	6.18324e−25	Hemiptera	Gerridae	Gerris gracilicornis
Gerris gracilicornis mitochondrial genes for 12S r	AB679158	78.57	97.42	6.18324e−25	Hemiptera	Gerridae	Gerris gracilicornis
Gerris gracilicornis mitochondrial genes for 12S r	AB679156	77.92	97.42	6.18324e−25	Hemiptera	Gerridae	Gerris gracilicornis
Gerris gracilicornis mitochondrial genes for 12S r	AB679154	77.92	97.42	6.18324e−25	Hemiptera	Gerridae	Gerris gracilicornis
Gerris gracilicornis mitochondrial genes for 12S r	AB679152	78.57	97.42	6.18324e−25	Hemiptera	Gerridae	Gerris gracilicornis
Gerris gracilicornis mitochondrial genes for 12S r	AB679151	78.57	97.42	6.18324e−25	Hemiptera	Gerridae	Gerris gracilicornis
Gerris gracilicornis mitochondrial genes for 12S r	AB679150	78.57	97.42	6.18324e−25	Hemiptera	Gerridae	Gerris gracilicornis
Gerris gracilicornis mitochondrial genes for 12S r	AB679149	78.57	97.42	6.18324e−25	Hemiptera	Gerridae	Gerris gracilicornis
Gerris sp. YT−2009 16S ribosomal RNA gene, partial	FJ372633	77.92	97.42	6.18324e−25	Hemiptera	Gerridae	Gerris sp. YT−2009
Limnogonus hungerfordi isolate 207 16S large subun	GU645085	81.62	85.16	6.18324e−25	Hemiptera	Gerridae	Limnogonus hungerfordi
Limnogonus nitidus isolate 2141 16S large subunit	GU645067	81.62	85.16	6.18324e−25	Hemiptera	Gerridae	Limnogonus nitidus
Limnogonus nitidus isolate 1983 16S large subunit	GU645066	81.62	85.16	6.18324e−25	Hemiptera	Gerridae	Limnogonus nitidus
Limnogonus ignotus isolate 2343 16S large subunit	GU645059	81.62	85.16	6.18324e−25	Hemiptera	Gerridae	Limnogonus ignotus
Cylindrostethus palmaris isolate 937 16S ribosomal	EU871198	78.26	100	6.18324e−25	Hemiptera	Gerridae	Cylindrostethus palmaris
Potamometropsis sumaldei 16S ribosomal RNA gene, p	AY743237	78.85	97.42	6.18324e−25	Hemiptera	Gerridae	Potamometropsis sumaldei
Riptortus sp. WCW−2003 16S ribosomal RNA gene, par	AY252665	79.62	97.42	6.18324e−25	Hemiptera	Alydidae	Riptortus sp. WCW−2003
Gerris caucasicus 16S large subunit ribosomal RNA	AY951328	78.57	97.42	6.18324e−25	Hemiptera	Gerridae	Gerris caucasicus
Gerris lateralis 16S ribosomal RNA gene, partial s	AY425231	77.92	97.42	6.18324e−25	Hemiptera	Gerridae	Gerris lateralis
Gerris odontogaster 16S ribosomal RNA gene, partia	AY425219	78.57	97.42	6.18324e−25	Hemiptera	Gerridae	Gerris odontogaster
Gerris yezoensis 16S ribosomal RNA gene, partial s	AY425203	78.57	97.42	6.18324e−25	Hemiptera	Gerridae	Gerris yezoensis
Gerris gracilicornis 16S ribosomal RNA gene, parti	AY425201	78.57	97.42	6.18324e−25	Hemiptera	Gerridae	Gerris gracilicornis
Gerris argentatus 16S ribosomal RNA gene, partial	AY425194	78.57	97.42	6.18324e−25	Hemiptera	Gerridae	Gerris argentatus
Gerris buenoi 16S ribosomal RNA gene, partial sequ	AY425191	78.57	97.42	6.18324e−25	Hemiptera	Gerridae	Gerris buenoi
Gerris asper 16S ribosomal RNA gene, partial seque	AY425184	77.92	97.42	6.18324e−25	Hemiptera	Gerridae	Gerris asper
Rhagovelia esakii mitochondrial gene for 16S ribos	AB026611	78.06	100	6.18324e−25	Hemiptera	Veliidae	Rhagovelia esakii
Gerris babai mitochondrial gene for 16S ribosomal	AB026585	78.57	97.42	6.18324e−25	Hemiptera	Gerridae	Gerris babai
Tenagogonus australiensis isolate 1482 16S large s	KC880888	80.74	85.16	2.15816e−24	Hemiptera	Gerridae	Tenagogonus australiensis
Limnogonus recens isolate 1434 16S large subunit r	GU645057	81.16	85.16	2.15816e−24	Hemiptera	Gerridae	Limnogonus recens
Rhagovelia sumatrensis 16S ribosomal RNA gene, par	AY648126	78.98	100	2.15816e−24	Hemiptera	Veliidae	Rhagovelia sumatrensis
Tenagogonus australiensis isolate 1479 16S large s	KC880887	80.15	85.16	7.53272e−24	Hemiptera	Gerridae	Tenagogonus australiensis
Limnogonus skusei isolate 149 16S large subunit ri	GU645094	80.88	85.16	7.53272e−24	Hemiptera	Gerridae	Limnogonus luctuosus
Limnogonus hungerfordi isolate 148 16S large subun	GU645084	80.88	85.16	7.53272e−24	Hemiptera	Gerridae	Limnogonus hungerfordi
Limnogonus intermedius isolate 140 16S large subun	GU645053	80.88	85.16	7.53272e−24	Hemiptera	Gerridae	Limnogonus intermedius
Bolitophila sp. ER−2009 16S ribosomal RNA gene, pa	FJ172021	82.79	76.13	7.53272e−24	Diptera	Mycetophilidae	Bolitophila sp. ER−2009
Halobates mjobergi 16S ribosomal RNA gene, partial	AY648134	79.29	85.16	7.53272e−24	Hemiptera	Gerridae	Halobates mjobergi
Aquarius cinereus 16S ribosomal RNA gene, partial	AY425234	77.92	97.42	7.53272e−24	Hemiptera	Gerridae	Aquarius cinereus
Gerris brasili 16S ribosomal RNA gene, partial seq	AY425229	77.92	97.42	7.53272e−24	Hemiptera	Gerridae	Gerris brasili
Gerris swakopensis 16S ribosomal RNA gene, partial	AY425223	77.92	97.42	7.53272e−24	Hemiptera	Gerridae	Gerris swakopensis
Aquarius elongatus 16S ribosomal RNA gene, partial	AY425200	77.92	97.42	7.53272e−24	Hemiptera	Gerridae	Aquarius elongatus
Aquarius najas 16S ribosomal RNA gene, partial seq	AY425199	77.92	97.42	7.53272e−24	Hemiptera	Gerridae	Aquarius najas
Gerris nepalensis 16S ribosomal RNA gene, partial	AY425192	77.92	97.42	7.53272e−24	Hemiptera	Gerridae	Gerris nepalensis
Aquarius chilensis 16S ribosomal RNA gene, partial	AY425189	77.92	97.42	7.53272e−24	Hemiptera	Gerridae	Aquarius chilensis
Microvelia horvathi mitochondrial gene for 16S rib	AB026612	77.71	97.42	7.53272e−24	Hemiptera	Veliidae	Microvelia horvathi
Rheumatogonus luzonicus mitochondrial gene for 16S	AB026609	78.98	97.42	7.53272e−24	Hemiptera	Gerridae	Rheumatogonus luzonicus
Aquarius elongatus mitochondrial gene for 16S ribo	AB026593	77.92	97.42	7.53272e−24	Hemiptera	Gerridae	Aquarius elongatus
Tachygerris opacus isolate 217 16S large subunit r	KC880879	78.71	97.42	2.62918e−23	Hemiptera	Gerridae	Tachygerris opacus
Gerris insularis mitochondrial genes for 12S rRNA,	AB679166	77.27	97.42	2.62918e−23	Hemiptera	Gerridae	Gerris insularis
Gerris cf. insularis 'type B' mitochondrial genes	AB679165	77.27	97.42	2.62918e−23	Hemiptera	Gerridae	Gerris cf. insularis 'type B'
Limnogonus hungerfordi isolate 1014 16S large subu	GU645086	80.15	85.16	2.62918e−23	Hemiptera	Gerridae	Limnogonus hungerfordi
Limnogonus cereiventris isolate 2390 16S large sub	GU645064	80.74	84.52	2.62918e−23	Hemiptera	Gerridae	Limnogonus cereiventris
Rheumatogonus luzonicus isolate 1062 16S ribosomal	EU871202	78.21	97.42	2.62918e−23	Hemiptera	Gerridae	Rheumatogonus luzonicus
Microvelia torquata isolate 435 16S ribosomal RNA	EU871174	78.71	97.42	2.62918e−23	Hemiptera	Veliidae	Microvelia torquata
Gerris insularis 16S large subunit ribosomal RNA g	AY951327	77.27	97.42	2.62918e−23	Hemiptera	Gerridae	Gerris insularis
Aquarius philippinensis 16S large subunit ribosoma	AY951324	78.71	97.42	2.62918e−23	Hemiptera	Gerridae	Aquarius philippinensis
Aquarius distanti 16S ribosomal RNA gene, partial	AY425198	77.42	97.42	2.62918e−23	Hemiptera	Gerridae	Aquarius distanti
Aquarius remigis 16S ribosomal RNA gene, partial s	AY425190	77.42	97.42	2.62918e−23	Hemiptera	Gerridae	Aquarius remigis
Leptinopterus femoratus voucher SK231 16S ribosoma	KP250254	77.85	98.71	9.17674e−23	Coleoptera	Lucanidae	Leptinopterus femoratus
Rhagovelia sp. Los Banos mitochondrial gene for 16	AB026610	87.5	60.65	9.17674e−23	Hemiptera	Veliidae	Rhagovelia sp. Los Banos
