# Supplementary material for: Testing the potential of a ribosomal 16S marker for DNA metabarcoding of insects
Source: PeerJ. 2016 Apr 19;4:e1966. doi: 10.7717/peerj.1966 (PMC4841222; doi:10.7717/peerj.1966)

OTU_131.csv

Asellus aquaticus

Asellidae

Isopoda

94.44

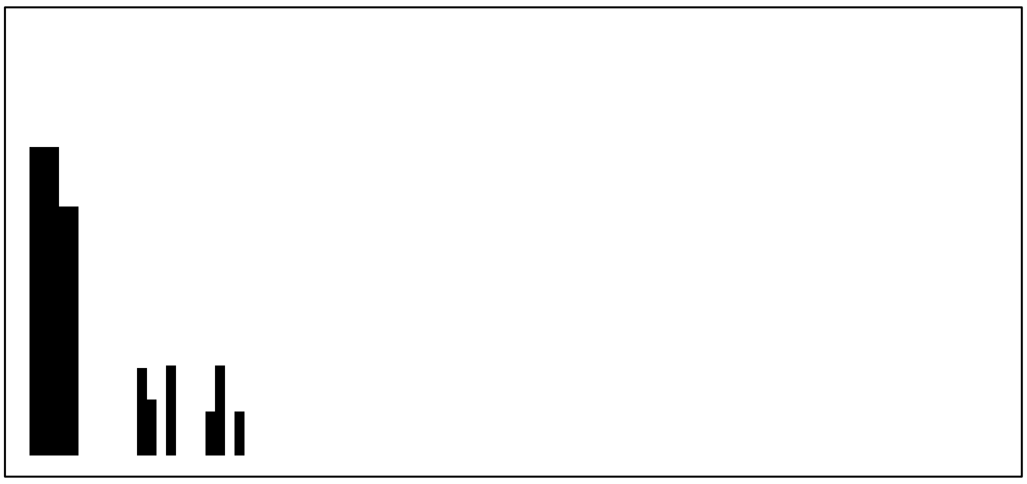

Asellus aquaticus isolate s180 16S ribosomal RNA g	KC610270	94.44	42.35	3.20525e−05	Isopoda	Asellidae	Asellus aquaticus
Asellus aquaticus 16S ribosomal RNA gene, partial	DQ305105	94.44	42.35	3.20525e−05	Isopoda	Asellidae	Asellus aquaticus
Asellus aquaticus 16S ribosomal RNA gene, partial	AF532161	94.44	42.35	3.20525e−05	Isopoda	Asellidae	Asellus aquaticus
Asellus aquaticus 16S ribosomal RNA gene, partial	DQ305106	91.67	42.35	0.00136291	Isopoda	Asellidae	Asellus aquaticus
Asellus aquaticus partial mitochondrial 16S rRNA g	AJ388077	91.67	42.35	0.00136291	Isopoda	Asellidae	Asellus aquaticus
Protopolystoma xenopodis genome assembly P_xenopod	LM693397	79.31	68.24	0.202274	Polyopisthocotylea	Polystomatidae	Protopolystoma xenopodis
Solanum lycopersicum chromosome ch05, complete gen	HG975517	78.18	64.71	0.202274	Solanales	Solanaceae	Solanum lycopersicum
Cucumis melo genomic chromosome, chr_7	LN713261	79.25	61.18	0.706004	Cucurbitales	Cucurbitaceae	Cucumis melo
Cucumis melo genomic scaffold, anchoredscaffold000	LN681864	79.25	61.18	0.706004	Cucurbitales	Cucurbitaceae	Cucumis melo
Zostera marina clone XTP02−B06, complete sequence	AC256214	78.85	60	2.4642	Alismatales	Zosteraceae	Zostera marina
Strongyloides ratti genome assembly S_ratti_ED321	LN609528	75.41	69.41	2.4642	Rhabditida	Strongyloididae	Strongyloides ratti
Cyprinus carpio genome assembly common carp genome	LN590675	84.09	51.76	2.4642	Cypriniformes	Cyprinidae	Cyprinus carpio
Cyprinus carpio genome assembly common carp genome	LN590671	82.61	51.76	2.4642	Cypriniformes	Cyprinidae	Cyprinus carpio
Brachyspira hyodysenteriae WA1, complete genome	CP001357	78.18	64.71	2.4642	Brachyspirales	Brachyspiraceae	Brachyspira hyodysenteriae WA1
Strongyloides stercoralis genome assembly S_sterco	LL999048	84.21	44.71	8.60089	Rhabditida	Strongyloididae	Strongyloides stercoralis
Cyprinus carpio genome assembly common carp genome	LN591031	79.17	55.29	8.60089	Cypriniformes	Cyprinidae	Cyprinus carpio
Cyprinus carpio clone 49961 microsatellite sequenc	JN745719	78.18	60	8.60089	Cypriniformes	Cyprinidae	Cyprinus carpio
Parachlorella kessleri culture−collection SAG:211−	FJ968741	76.79	64.71	8.60089	Chlorellales		Parachlorella kessleri
Mus musculus BAC clone RP24−468F14 from chromosome	AC122057	82.05	45.88	8.60089	Rodentia	Muridae	Mus musculus
Ehrlichia canis str. Jake, complete genome	CP000107	84.21	43.53	8.60089	Rickettsiales	Anaplasmataceae	Ehrlichia canis str. Jake
Lotus japonicus genomic DNA, chromosome 5, clone:	AP006659	75.86	68.24	8.60089	Fabales	Fabaceae	Lotus japonicus
Mouse DNA sequence from clone RP23−63E8 on chromos	CT010509	82.05	45.88	8.60089	Rodentia	Muridae	Mus musculus

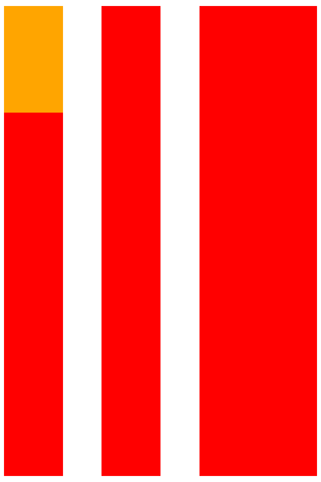

Supplement: Supplemental Information 1 [file peerj-04-1966-s006.zip › scripts/5 match OTUs/blast_hits/OTU_131.csv_plot.pdf]
