# Supplementary material for: Testing the potential of a ribosomal 16S marker for DNA metabarcoding of insects
Source: PeerJ. 2016 Apr 19;4:e1966. doi: 10.7717/peerj.1966 (PMC4841222; doi:10.7717/peerj.1966)
Supplement: Supplemental Information 1 [file peerj-04-1966-s006.zip › scripts/5 match OTUs/blast_hits/OTU_133.csv_plot.pdf]

OTU_133.csv

Folsomia candida

Isotomidae

82.29

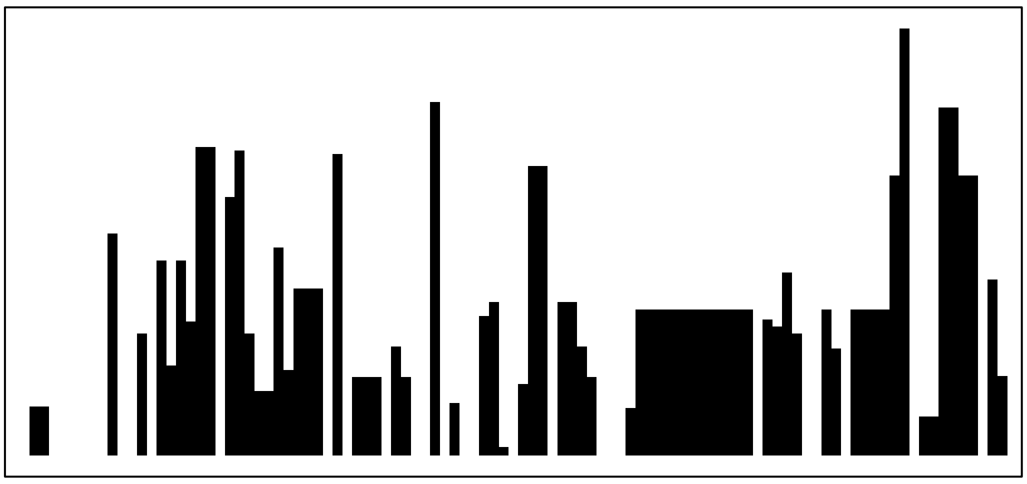

Folsomia candida voucher Folsomia_Candida 16S ribo	KF591920	82.29	63.7	1.43743e−13		Isotomidae	Folsomia candida
Folsomia candida 16S ribosomal RNA gene, partial s	KC236200	82.29	63.7	1.43743e−13		Isotomidae	Folsomia candida
Folsomia candida 16S ribosomal RNA gene, partial s	JN970944	75.2	84.25	6.11212e−12		Isotomidae	Folsomia candida
Folsomia candida 16S ribosomal RNA gene, partial s	AY555551	75.2	84.25	6.11212e−12		Isotomidae	Folsomia candida
Folsomia quadrioculata 16S ribosomal RNA gene, par	KC236199	79.12	61.64	2.59894e−10		Isotomidae	Folsomia quadrioculata
Cryptopygus sverdrupi voucher MNHN:cd468_065 16S r	KJ418952	77.66	63.7	3.16616e−09		Isotomidae	Cryptopygus sverdrupi
Cryptopygus sverdrupi voucher MNHN:EA010326 16S ri	KJ418902	77.66	63.7	3.16616e−09		Isotomidae	Cryptopygus sverdrupi
Cryptopygus sverdrupi voucher MNHN:cd325_065 16S r	KJ418901	77.66	63.7	3.16616e−09		Isotomidae	Cryptopygus sverdrupi
Salina sp. BXW−2014 16S ribosomal RNA gene, partia	KM978386	90.38	33.56	1.1051e−08		Paronellidae	Salina sp. BXW−2014
Isotoma viridis 16S ribosomal RNA gene, partial se	JN970946	78.26	62.33	1.1051e−08		Isotomidae	Isotoma viridis
Chrysochus auratus partial 16S rRNA gene	AJ781546	76.77	65.75	1.1051e−08	Coleoptera	Chrysomelidae	Chrysochus auratus
Callyntrura guangdongensis 16S ribosomal RNA gene,	KM978382	85.71	38.36	1.34628e−07		Paronellidae	Callyntrura guangdongensis
Pogonognathellus sp. HB07163 16S ribosomal RNA gen	KF591923	76.6	62.33	1.64011e−06		Tomoceridae	Pogonognathellus sp. HB07163
Pseudoparonella tanimbarensis 16S ribosomal RNA ge	KM978384	89.13	31.51	5.72455e−06		Paronellidae	Pseudoparonella tanimbarensis
Sinella curviseta 16S ribosomal RNA gene, partial	KC236219	84.21	39.04	5.72455e−06		Entomobryidae	Sinella curviseta
Lepidocyrtus (Ascocyrtus) sp. 2 FZ−2013 16S riboso	KC236190	89.13	31.51	5.72455e−06		Entomobryidae	Lepidocyrtus (Ascocyrtus) sp. 2 FZ−2013
Colotois pennaria gene for 16S rRNA, partial seque	AB265386	86.27	34.93	1.99806e−05	Lepidoptera	Geometridae	Colotois pennaria
Cyphoderus javanus 16S ribosomal RNA gene, partial	KM978390	94.44	24.66	6.97393e−05		Cyphoderidae	Cyphoderus javanus
Callyntrura sp. BXW−2014 16S ribosomal RNA gene, p	KM978383	94.44	24.66	6.97393e−05		Paronellidae	Callyntrura sp. BXW−2014
Copelatus sp. BI−2011 voucher BMNH792988 16S ribos	HQ381444	78.31	53.42	6.97393e−05	Coleoptera	Dytiscidae	Copelatus sp. BI−2011
Occiperipatoides sp. DNA104661 16S ribosomal RNA g	KC754543	92.11	26.03	0.000243414		Peripatopsidae	Occiperipatoides sp. DNA104661
Occiperipatoides gilesii voucher DNA103886 16S rib	KC754542	94.29	23.97	0.000243414		Peripatopsidae	Occiperipatoides gilesii
Seira sp. 2 FZ−2013 16S ribosomal RNA gene, partia	KC236215	85.71	33.56	0.000243414		Entomobryidae	Seira sp. 2 FZ−2013
Macronychini sp. FC_B03 16S ribosomal RNA (rrnL) g	EF209457	83.02	36.3	0.000243414	Coleoptera	Elmidae	Macronychini sp. FC_B03
Rhopalonychus levatorponderis 16S ribosomal RNA ge	DQ266477	83.02	36.3	0.000243414	Coleoptera	Elmidae	Rhopalonychus levatorponderis
Descoreba simplex gene for 16S rRNA, partial seque	AB265388	89.74	26.71	0.000849598	Lepidoptera	Geometridae	Descoreba simplex
Orchesellides sp. 9688−2 FZ−2013 16S ribosomal RNA	KC236217	84	34.25	0.00296539		Entomobryidae	Orchesellides sp. 9688−2 FZ−2013
Himalanura sp. FZ−2013 16S ribosomal RNA gene, par	KC236202	87.8	28.08	0.00296539		Entomobryidae	Himalanura sp. FZ−2013
Dosinia biscocta isolate Dosininabiscota0403 16S r	HM124691	87.8	28.08	0.00296539	Veneroida	Veneridae	Dosinia biscocta
Dosinia biscocta isolate Dosiniabiscota0323 16S ri	HM124685	87.8	28.08	0.00296539	Veneroida	Veneridae	Dosinia biscocta
Homoeogryllus sp. MCJ−2002 16S ribosomal RNA gene,	AF514594	75.27	63.01	0.00296539	Orthoptera	Gryllidae	Homoeogryllus sp. MCJ−2002
Apteryx australis mantelli genome assembly AptMant	LK065177	94.12	23.29	0.0103502	Apterygiformes	Apterygidae	Apteryx australis mantelli
Peripatopsidae gen. sp. DNA103744 16S ribosomal RN	KC754534	78.12	42.47	0.0103502		Peripatopsidae	Peripatopsidae gen. sp. DNA103744
Bugula neritina voucher BUGNE−H−FL 08 16S ribosoma	KC130072	83.67	33.56	0.0103502	Cheilostomatida	Bugulidae	Bugula neritina
Bugula neritina isolate D_H5 16S ribosomal RNA gen	JX183895	83.67	33.56	0.0103502	Cheilostomatida	Bugulidae	Bugula neritina
Bugula neritina isolate D_H4 16S ribosomal RNA gen	JX183894	83.67	33.56	0.0103502	Cheilostomatida	Bugulidae	Bugula neritina
Heteromurus nitidus 16S ribosomal RNA gene, partia	KC291493	73.63	62.33	0.0103502		Entomobryidae	Heteromurus nitidus
Eumunida funambulus isolate KC3100 16S ribosomal R	EU920922	85.11	31.51	0.0103502	Decapoda	Chirostylidae	Eumunida funambulus
Bugula neritina isolate TP large subunit ribosomal	AY633935	83.67	33.56	0.0103502	Cheilostomatida	Bugulidae	Bugula neritina
PREDICTED: Papilio polytes EH domain−binding prote	XM_013278397	79.71	46.58	0.0361258	Lepidoptera	Papilionidae	Papilio polytes
Euurobracon breviterebrae mitochondrion, partial g	KF385871	78.95	39.04	0.0361258	Hymenoptera	Braconidae	Euurobracon breviterebrae
Hymenolepis nana genome assembly H_nana_Japan ,sca	LM407417	96.55	19.86	0.0361258	Cyclophyllidea	Hymenolepididae	Hymenolepis nana
Cyprinus carpio genome assembly common carp genome	LN590706	75.61	54.11	0.0361258	Cypriniformes	Cyprinidae	Cyprinus carpio
Microhedyle sp. AB34500083 16S ribosomal RNA gene,	KF709268	82.46	36.99	0.0361258	Acochlidiacea	Microhedylidae	Microhedyle sp. AB34500083
Megalothorax perspicillum voucher EA042005 16S rib	KC900181	75.29	58.22	0.0361258		Neelidae	Megalothorax perspicillum
Willowsia sp. 1 FZ−2013 16S ribosomal RNA gene, pa	KC236205	77.05	41.78	0.0361258		Entomobryidae	Willowsia sp. 1 FZ−2013
Megalothorax sp. EA040001−cs1 16S ribosomal RNA ge	JN970967	86.54	33.56	0.0361258		Neelidae	Megalothorax sp. EA040001−cs1
Dosinia japonica isolate DoDja 16S ribosomal RNA g	JN969953	87.18	26.71	0.0361258	Veneroida	Veneridae	Dosinia japonica
Beania plurispinosa isolate BpluNH01 16S ribosomal	JF950316	80.39	31.51	0.0361258	Cheilostomatida	Beaniidae	Beania plurispinosa
Phigaliohybernia fulvinfula gene for 16S rRNA, par	AB265402	78.46	43.84	0.0361258	Lepidoptera	Geometridae	Phigaliohybernia fulvinfula
Copidozoum sp. Colony NA−10 16S ribosomal RNA gene	AY582970	83.33	33.56	0.0361258	Cheilostomatida	Calloporidae	Copidozoum sp. Colony NA−10
Homo sapiens 3 BAC RP11−36I16 (Roswell Park Cancer	AC119729	93.55	21.23	0.0361258	Primates	Hominidae	Homo sapiens
Homo sapiens 3 BAC RP11−341J3 (Roswell Park Cancer	AC117452	93.55	21.23	0.0361258	Primates	Hominidae	Homo sapiens
Marchantia paleacea subsp. diptera chloroplast DNA	LC035012	75.68	50.68	0.126092	Marchantiales	Marchantiaceae	Marchantia paleacea subsp. diptera
Fulvia mutica voucher FMNH 328595 16S ribosomal RN	KR422943	87.18	25.34	0.126092	Veneroida	Cardiidae	Fulvia mutica
Apteryx australis mantelli genome assembly AptMant	LK065216	87.18	25.34	0.126092	Apterygiformes	Apterygidae	Apteryx australis mantelli
Caulibugula pearsei voucher BRBRY−Cau2 16S ribosom	KF714807	85.11	32.19	0.126092	Hemiptera	Reduviidae	Caulibugula pearsei
Bugula microoecia voucher BRBRY−MZUSP 488 16S ribo	KF714801	83.67	33.56	0.126092	Cheilostomatida	Bugulidae	Bugula microoecia
Colophon primosi isolate C80 16S ribosomal RNA gen	KC763287	77.19	39.04	0.126092	Coleoptera	Lucanidae	Colophon primosi
Colophon primosi isolate C79 16S ribosomal RNA gen	KC763286	77.19	39.04	0.126092	Coleoptera	Lucanidae	Colophon primosi
Colophon primosi isolate C78 16S ribosomal RNA gen	KC763285	77.19	39.04	0.126092	Coleoptera	Lucanidae	Colophon primosi
Elaeophora elaphi genome assembly E_elaphi ,scaffo	LL710658	82.22	30.82	0.126092	Spirurida	Onchocercidae	Elaeophora elaphi
Bugula neritina voucher BUGNE−BF5 16S ribosomal RN	KC130074	86.84	26.03	0.126092	Cheilostomatida	Bugulidae	Bugula neritina
Bugula neritina voucher BUGNE−OY33 16S ribosomal R	KC130043	86.84	26.03	0.126092	Cheilostomatida	Bugulidae	Bugula neritina
Bugula neritina voucher BUGNE−N16 16S ribosomal RN	KC130020	86.84	26.03	0.126092	Cheilostomatida	Bugulidae	Bugula neritina
Bugula neritina voucher BUGNE−AS101 16S ribosomal	KC130001	86.84	26.03	0.126092	Cheilostomatida	Bugulidae	Bugula neritina
Bugula neritina voucher BUGNE−N42 16S ribosomal RN	KC129968	86.84	26.03	0.126092	Cheilostomatida	Bugulidae	Bugula neritina
Bugula neritina voucher BUGNE−BF11 16S ribosomal R	KC129967	86.84	26.03	0.126092	Cheilostomatida	Bugulidae	Bugula neritina
Bugula neritina voucher BUGNE−BF9 16S ribosomal RN	KC129964	86.84	26.03	0.126092	Cheilostomatida	Bugulidae	Bugula neritina
Bugula neritina isolate N_H7 16S ribosomal RNA gen	JX183897	86.84	26.03	0.126092	Cheilostomatida	Bugulidae	Bugula neritina
Bugula neritina isolate N_H6 16S ribosomal RNA gen	JX183896	86.84	26.03	0.126092	Cheilostomatida	Bugulidae	Bugula neritina
Bugula neritina isolate S_H3 16S ribosomal RNA gen	JX183893	86.84	26.03	0.126092	Cheilostomatida	Bugulidae	Bugula neritina
Bugula neritina isolate S_H2 16S ribosomal RNA gen	JX183892	86.84	26.03	0.126092	Cheilostomatida	Bugulidae	Bugula neritina
Bugula neritina isolate S_H1 16S ribosomal RNA gen	JX183891	86.84	26.03	0.126092	Cheilostomatida	Bugulidae	Bugula neritina
Cratilla lineata mitochondrial gene for 16S rRNA,	AB708007	78.18	37.67	0.126092	Odonata	Libellulidae	Cratilla lineata
Pseudogarypus banksi mitochondrion, complete genom	JQ040544	86.36	29.45	0.126092	Pseudoscorpiones	Pseudogarypidae	Pseudogarypus banksi
Megalothorax sp. EA040001−cs2 16S ribosomal RNA ge	JN970968	86.05	28.08	0.126092		Neelidae	Megalothorax sp. EA040001−cs2
Dosinia troscheli isolate Dosiniatroscheii1120 16S	HM124690	88.57	23.97	0.126092	Veneroida	Veneridae	Dosinia troscheli
Pig DNA sequence from clone CH242−324O16 on chromo	CU582856	85.71	28.77	0.126092		Suidae	Sus scrofa
Calocaris caribbaeus voucher ULLZ 7877 16S ribosom	EU882902	75.38	41.1	0.126092	Decapoda	Calocarididae	Calocaris caribbaeus
Calocaris caribbaeus voucher ULLZ 8285 16S ribosom	EU874929	75.38	41.1	0.126092	Decapoda	Calocarididae	Calocaris caribbaeus
Bugula neritina mitochondrion, complete genome	AY690838	86.84	26.03	0.126092	Cheilostomatida	Bugulidae	Bugula neritina
Pan troglodytes BAC clone CH251−267D14 from chromo	AC159743	85	27.4	0.126092	Primates	Hominidae	Pan troglodytes
Marchantia polymorpha chloroplast genome DNA	X04465	75.68	50.68	0.126092	Marchantiales	Marchantiaceae	Marchantia polymorpha
Bugula neritina 16S ribosomal RNA gene, partial se	DQ305338	86.84	26.03	0.126092	Cheilostomatida	Bugulidae	Bugula neritina
Bugula neritina isolate LH large subunit ribosomal	AY633937	86.84	26.03	0.126092	Cheilostomatida	Bugulidae	Bugula neritina
Bugula neritina isolate RI large subunit ribosomal	AY633936	86.84	26.03	0.126092	Cheilostomatida	Bugulidae	Bugula neritina
Bugula neritina isolate SP large subunit ribosomal	AY633934	86.84	26.03	0.126092	Cheilostomatida	Bugulidae	Bugula neritina
Ovis canadensis canadensis isolate 43U chromosome	CP011902	93.1	19.86	0.440103		Bovidae	Ovis canadensis canadensis
Apteryx australis mantelli genome assembly AptMant	LK064804	100	16.44	0.440103	Apterygiformes	Apterygidae	Apteryx australis mantelli
TPA_asm: Oryzias latipes strain Hd−rR, complete ge	HG313980	77.78	42.47	0.440103	Beloniformes	Adrianichthyidae	Oryzias latipes
PREDICTED: Sesamum indicum uncharacterized LOC1051	XR_849458	81.82	36.99	0.440103	Lamiales	Pedaliaceae	Sesamum indicum
PREDICTED: Sesamum indicum uncharacterized LOC1051	XR_849457	81.82	36.99	0.440103	Lamiales	Pedaliaceae	Sesamum indicum
Cucumis melo genomic chromosome, chr_4	LN713258	96.3	18.49	0.440103	Cucurbitales	Cucurbitaceae	Cucumis melo
Cucumis melo genomic scaffold, anchoredscaffold000	LN681827	96.3	18.49	0.440103	Cucurbitales	Cucurbitaceae	Cucumis melo
PREDICTED: Fukomys damarensis tetraspanin 8 (Tspan	XM_010617489	93.1	19.86	0.440103	Rodentia	Bathyergidae	Fukomys damarensis
PREDICTED: Fukomys damarensis tetraspanin 8 (Tspan	XM_010617488	93.1	19.86	0.440103	Rodentia	Bathyergidae	Fukomys damarensis
Schistosoma rodhaini genome assembly S_rodhaini_Bu	LL957229	77.59	39.73	0.440103	Strigeidida	Schistosomatidae	Schistosoma rodhaini
Trichobilharzia regenti genome assembly T_regenti_	LL013109	88.24	23.29	0.440103	Strigeidida	Schistosomatidae	Trichobilharzia regenti
Syphacia muris genome assembly S_muris_Valencia ,s	LK997924	83.72	29.45	0.440103	Oxyurida	Oxyuridae	Syphacia muris
