# Supplementary material for: Testing the potential of a ribosomal 16S marker for DNA metabarcoding of insects
Source: PeerJ. 2016 Apr 19;4:e1966. doi: 10.7717/peerj.1966 (PMC4841222; doi:10.7717/peerj.1966)
Supplement: Supplemental Information 1 [file peerj-04-1966-s006.zip › scripts/5 match OTUs/blast_hits/OTU_134.csv_plot.pdf]

OTU_134.csv

Anotogaster sieboldii

Cordulegastridae

Odonata

89.31

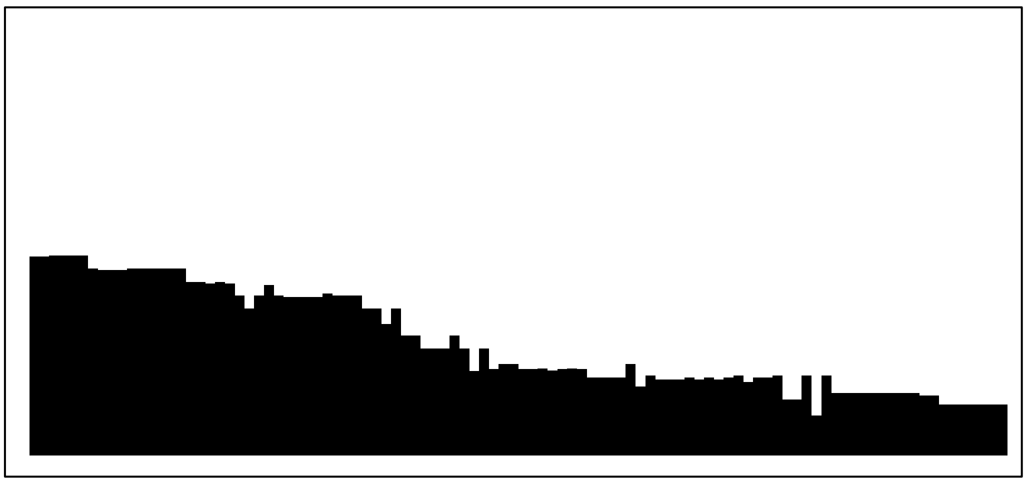

Anotogaster sieboldii mitochondrial gene for 16S r	AB707886	89.31	100	9.87097e−48	Odonata	Cordulegastridae	Anotogaster sieboldii
Anotogaster kuchenbeiseri mitochondrial gene for 1	AB707878	89.31	100	9.87097e−48	Odonata	Cordulegastridae	Anotogaster kuchenbeiseri
Anotogaster chaoi mitochondrial gene for 16S rRNA,	AB707858	89.38	100	9.87097e−48	Odonata	Cordulegastridae	Anotogaster chaoi
Cordulegaster boltonii 12S ribosomal RNA gene, par	EU477688	89.38	100	9.87097e−48	Odonata	Cordulegastridae	Cordulegaster boltonii
Cordulegaster dorsalis 16S ribosomal RNA gene, par	EU055084	89.38	100	9.87097e−48	Odonata	Cordulegastridae	Cordulegaster dorsalis
Cordulegaster boltoni large subunit ribosomal RNA	AF266056	89.38	100	9.87097e−48	Odonata	Cordulegastridae	Cordulegaster boltonii
Anotogaster sp. RF1283 mitochondrial gene for 16S	AB707895	88.75	100	3.44531e−47	Odonata	Cordulegastridae	Anotogaster sp. RF1283
Anotogaster sieboldii mitochondrial gene for 16S r	AB707885	88.68	100	1.20253e−46	Odonata	Cordulegastridae	Anotogaster sieboldii
Anotogaster sieboldii mitochondrial gene for 16S r	AB707882	88.68	100	1.20253e−46	Odonata	Cordulegastridae	Anotogaster sieboldii
Anotogaster sieboldii mitochondrial gene for 16S r	AB707880	88.68	100	1.20253e−46	Odonata	Cordulegastridae	Anotogaster sieboldii
Neallogaster pekinensis mitochondrial gene for 16S	AB707904	88.75	100	4.19724e−46	Odonata	Cordulegastridae	Neallogaster pekinensis
Anotogaster sieboldii mitochondrial gene for 16S r	AB707892	88.75	100	4.19724e−46	Odonata	Cordulegastridae	Anotogaster sieboldii
Anotogaster sieboldii mitochondrial gene for 16S r	AB707881	88.75	100	4.19724e−46	Odonata	Cordulegastridae	Anotogaster sieboldii
Anotogaster sieboldii 12S ribosomal RNA gene, part	EU477686	88.75	100	4.19724e−46	Odonata	Cordulegastridae	Anotogaster sieboldii
Anotogaster sieboldii mitochondrial gene for 16S r	AB127061	88.75	100	4.19724e−46	Odonata	Cordulegastridae	Anotogaster sieboldii
Anotogaster sieboldi large subunit ribosomal RNA g	AF266087	88.75	100	4.19724e−46	Odonata	Cordulegastridae	Anotogaster sieboldii
Neallogaster pekinensis mitochondrial gene for 16S	AB707903	88.12	100	5.11329e−45	Odonata	Cordulegastridae	Neallogaster pekinensis
Cordulegaster picta mitochondrial gene for 16S rRN	AB707902	88.12	100	5.11329e−45	Odonata	Cordulegastridae	Cordulegaster picta
Anotogaster sp. RF1898 mitochondrial gene for 16S	AB707899	88.05	100	5.11329e−45	Odonata	Cordulegastridae	Anotogaster sp. RF1898
Anotogaster sp. RF1284 mitochondrial gene for 16S	AB707896	88.12	100	5.11329e−45	Odonata	Cordulegastridae	Anotogaster sp. RF1284
Anotogaster klossi mitochondrial gene for 16S rRNA	AB707872	88.05	100	5.11329e−45	Odonata	Cordulegastridae	Anotogaster klossi
Kalyptogaster erronea 12S ribosomal RNA and 16S ri	EU477690	87.5	100	5.11329e−45	Odonata	Cordulegastridae	Kalyptogaster erronea
Cordulegaster maculata 12S ribosomal RNA and 16S r	EU477689	86.88	100	5.11329e−45	Odonata	Cordulegastridae	Cordulegaster maculata
Kalyptogaster erronea voucher JW_DNA_40 16S riboso	EF631561	87.5	100	5.11329e−45	Odonata	Cordulegastridae	Kalyptogaster erronea
Nemurella pictetii 16S ribosomal RNA gene, partial	EF623192	87.97	100	1.78471e−44	Plecoptera	Nemouridae	Nemurella pictetii
Sonjagaster insignis mitochondrial gene for 16S rR	AB707905	87.5	100	6.22926e−44	Odonata	Cordulegastridae	Sonjagaster insignis
Anotogaster klossi mitochondrial gene for 16S rRNA	AB707866	87.42	100	6.22926e−44	Odonata	Cordulegastridae	Anotogaster klossi
Anotogaster klossi mitochondrial gene for 16S rRNA	AB707864	87.42	100	6.22926e−44	Odonata	Cordulegastridae	Anotogaster klossi
Anotogaster klossi mitochondrial gene for 16S rRNA	AB707862	87.42	100	6.22926e−44	Odonata	Cordulegastridae	Anotogaster klossi
Anotogaster klossi mitochondrial gene for 16S rRNA	AB707860	87.42	100	6.22926e−44	Odonata	Cordulegastridae	Anotogaster klossi
Anotogaster sp. RF1894 mitochondrial gene for 16S	AB707898	87.58	100	7.58879e−43	Odonata	Cordulegastridae	Anotogaster sp. RF1894
Ostrocerca foersteri 16S ribosomal RNA gene, parti	EF623191	87.5	100	7.58879e−43	Plecoptera	Nemouridae	Ostrocerca foersteri
Anotogaster klossi mitochondrial gene for 16S rRNA	AB711440	87.5	100	2.64875e−42	Odonata	Cordulegastridae	Anotogaster klossi
Anotogaster klossi mitochondrial gene for 16S rRNA	AB707876	87.5	100	2.64875e−42	Odonata	Cordulegastridae	Anotogaster klossi
Cordulegaster picta 12S ribosomal RNA gene, partia	EU477685	86.88	100	2.64875e−42	Odonata	Cordulegastridae	Cordulegaster picta
Cordulegaster picta large subunit ribosomal RNA ge	AF266086	86.88	100	2.64875e−42	Odonata	Cordulegastridae	Cordulegaster picta
Cordulegaster sp. 1 BM−2008 12S ribosomal RNA gene	EU477687	86.16	100	3.22684e−41	Odonata	Cordulegastridae	Cordulegaster sp. 1 BM−2008
Visoka cataractae 16S ribosomal RNA gene, partial	EF623189	86.88	100	3.22684e−41	Plecoptera	Nemouridae	Visoka cataractae
Taeniogaster obliqua voucher JW_DNA_OTO 16S riboso	EF631533	85.62	100	3.22684e−41	Odonata	Cordulegastridae	Taeniogaster obliqua
Gynacantha japonica mitochondrial gene for 16S rRN	AB707679	85.62	100	1.67154e−38	Odonata	Aeshnidae	Gynacantha japonica
Phenes raptor isolate 2 12S ribosomal RNA gene, pa	EU477632	85	100	1.67154e−38	Odonata	Petaluridae	Phenes raptor
Phenes raptor 12S ribosomal RNA gene, partial sequ	EU477622	85	100	1.67154e−38	Odonata	Petaluridae	Phenes raptor
Phenes raptor 16S ribosomal RNA gene, partial sequ	EU055038	85	100	1.67154e−38	Odonata	Petaluridae	Phenes raptor
Nanonemoura wahkeena 16S ribosomal RNA gene, parti	EF623198	85.62	100	1.67154e−38	Plecoptera	Nemouridae	Nanonemoura wahkeena
Phenes raptor large subunit ribosomal RNA gene, pa	AF266083	85	100	1.67154e−38	Odonata	Petaluridae	Phenes raptor
Gynacantha japonica mitochondrial gene for 16S rRN	AB707678	83.95	100	2.03636e−37	Odonata	Aeshnidae	Gynacantha japonica
Shipsa rotunda 16S ribosomal RNA gene, partial seq	EF623184	85	100	2.03636e−37	Plecoptera	Nemouridae	Shipsa rotunda
Gynacantha japonica tRNA−Leu, 16S ribosomal RNA, a	KF256872	84.05	100	7.10758e−37	Odonata	Aeshnidae	Gynacantha japonica
Macromia manchurica voucher ANI29 tRNA−Leu, 16S ri	KF256863	84.28	100	7.10758e−37	Odonata	Macromiidae	Macromia manchurica
Macromia amphigena voucher ANI06 tRNA−Leu, 16S rib	KF256840	84.28	100	7.10758e−37	Odonata	Macromiidae	Macromia amphigena
Gynacantha japonica mitochondrial gene for 16S rRN	AB707677	84.05	100	7.10758e−37	Odonata	Aeshnidae	Gynacantha japonica
Gynacantha japonica mitochondrial gene for 16S rRN	AB707675	84.05	100	7.10758e−37	Odonata	Aeshnidae	Gynacantha japonica
Sympetrum costiferum isolate mtRNA03 12S ribosomal	JQ772596	84.08	99.36	7.10758e−37	Odonata	Libellulidae	Sympetrum costiferum
Synthemis eustalacta 12S ribosomal RNA gene, parti	EU477693	83.97	100	7.10758e−37	Odonata	Synthemistidae	Synthemis eustalacta
Gynacantha japonica isolate 2 12S ribosomal RNA ge	EU477644	84.05	100	7.10758e−37	Odonata	Aeshnidae	Gynacantha japonica
Sympetrum semicinctum 12S ribosomal RNA gene, part	EF640446	84.08	99.36	7.10758e−37	Odonata	Libellulidae	Sympetrum semicinctum
Gynacantha japonica 16S ribosomal RNA gene, partia	EF032710	84.05	100	7.10758e−37	Odonata	Aeshnidae	Gynacantha japonica
Shaogomphus postocularis voucher ANI03 tRNA−Leu, 1	KF256838	83.65	100	2.48079e−36	Odonata	Gomphidae	Shaogomphus postocularis
Gomphus pulchellus 12S ribosomal RNA gene, partial	EU477663	83.65	100	2.48079e−36	Odonata	Gomphidae	Gomphus pulchellus
Macromia clio mitochondrial gene for 16S rRNA, par	AB707941	83.65	100	8.65881e−36	Odonata	Macromiidae	Macromia clio
Macromia clio mitochondrial gene for 16S rRNA, par	AB707939	83.65	100	8.65881e−36	Odonata	Macromiidae	Macromia clio
Anax nigrofasciatus mitochondrial gene for 16S rRN	AB707659	84.28	100	8.65881e−36	Odonata	Aeshnidae	Anax nigrofasciatus
Petalura gigantea 16S ribosomal RNA gene, partial	EU055061	83.23	100	8.65881e−36	Odonata	Petaluridae	Petalura gigantea
Chlorolestes fasciatus voucher RMNH.INS.229156 16S	KF369666	83.75	100	3.02222e−35	Odonata	Synlestidae	Chlorolestes fasciatus
Sympetrum frequens mitochondrial gene for 16S rRNA	AB708215	83.54	99.36	3.02222e−35	Odonata	Libellulidae	Sympetrum frequens
Sympetrum depressiusculum mitochondrial gene for 1	AB708197	83.54	99.36	3.02222e−35	Odonata	Libellulidae	Sympetrum depressiusculum
Caliaeschna microstigma 12S ribosomal RNA gene, pa	EU477639	83.54	100	3.02222e−35	Odonata	Aeshnidae	Caliaeschna microstigma
Soyedina producta 16S ribosomal RNA gene, partial	EF623196	83.65	100	3.02222e−35	Plecoptera	Nemouridae	Soyedina producta
Caliaeschna microstigma large subunit ribosomal RN	AF266092	83.54	100	3.02222e−35	Odonata	Aeshnidae	Caliaeschna microstigma
Anax nigrofasciatus voucher ANI02 tRNA−Leu, 16S ri	KF256837	83.65	100	1.05486e−34	Odonata	Aeshnidae	Anax nigrofasciatus
Macromia callisto mitochondrial gene for 16S rRNA,	AB707937	83.54	100	1.05486e−34	Odonata	Macromiidae	Macromia callisto
Uropetala carovei 12S ribosomal RNA gene, partial	EU477633	83.65	100	1.05486e−34	Odonata	Petaluridae	Uropetala carovei
Paranemoura claasseni 16S ribosomal RNA gene, part	EF623185	83.75	100	1.05486e−34	Plecoptera	Nemouridae	Paranemoura claasseni
Macromia magnifica 12S ribosomal RNA gene, partial	EF640463	83.44	100	1.05486e−34	Odonata	Macromiidae	Macromia magnifica
Anax nigrofasciatus 16S ribosomal RNA gene, partia	EF032708	83.65	100	1.05486e−34	Odonata	Aeshnidae	Anax nigrofasciatus
Uropetala carovei large subunit ribosomal RNA gene	AY274506	83.65	100	1.05486e−34	Odonata	Petaluridae	Uropetala carovei
Polycanthagyna melanictera voucher ANI49 tRNA−Leu	KF256882	83.75	100	3.68182e−34	Odonata	Aeshnidae	Polycanthagyna melanictera
Stylurus nagoyanus mitochondrial gene for 16S rRNA	AB707824	82.61	100	3.68182e−34	Odonata	Gomphidae	Stylurus nagoyanus
Stylurus nagoyanus mitochondrial gene for 16S rRNA	AB707822	82.61	100	3.68182e−34	Odonata	Gomphidae	Stylurus nagoyanus
Polycanthagyna melanictera mitochondrial gene for	AB707698	83.75	100	3.68182e−34	Odonata	Aeshnidae	Polycanthagyna melanictera
Austrogomphus cf. guerini BM−2008 12S ribosomal RN	EU477682	81.88	100	3.68182e−34	Odonata	Gomphidae	Austrogomphus cf. guerini BM−2008
Polycanthagyna melanictera 16S ribosomal RNA gene,	EF032711	83.75	100	3.68182e−34	Odonata	Aeshnidae	Polycanthagyna melanictera
Sympetrum frequens voucher ANI23 tRNA−Leu, 16S rib	KF256857	82.91	99.36	1.28508e−33	Odonata	Libellulidae	Sympetrum frequens
Sympetrum frequens mitochondrial gene for 16S rRNA	AB708220	82.91	99.36	1.28508e−33	Odonata	Libellulidae	Sympetrum frequens
Sympetrum frequens mitochondrial gene for 16S rRNA	AB708214	82.91	99.36	1.28508e−33	Odonata	Libellulidae	Sympetrum frequens
Sympetrum depressiusculum mitochondrial gene for 1	AB708196	82.91	99.36	1.28508e−33	Odonata	Libellulidae	Sympetrum depressiusculum
Sympetrum depressiusculum mitochondrial gene for 1	AB708193	82.91	99.36	1.28508e−33	Odonata	Libellulidae	Sympetrum depressiusculum
Sympetrum depressiusculum mitochondrial gene for 1	AB708188	82.91	99.36	1.28508e−33	Odonata	Libellulidae	Sympetrum depressiusculum
Sympetrum depressiusculum mitochondrial gene for 1	AB708187	82.91	99.36	1.28508e−33	Odonata	Libellulidae	Sympetrum depressiusculum
Sympetrum depressiusculum mitochondrial gene for 1	AB708182	82.91	99.36	1.28508e−33	Odonata	Libellulidae	Sympetrum depressiusculum
Sympetrum depressiusculum mitochondrial gene for 1	AB708181	82.91	99.36	1.28508e−33	Odonata	Libellulidae	Sympetrum depressiusculum
Macromia kubokaiya mitochondrial gene for 16S rRNA	AB707948	82.8	100	1.28508e−33	Odonata	Macromiidae	Macromia kubokaiya
Macromia kubokaiya mitochondrial gene for 16S rRNA	AB707947	82.8	100	1.28508e−33	Odonata	Macromiidae	Macromia kubokaiya
Shaogomphus postocularis mitochondrial gene for 16	AB707782	82.39	100	1.28508e−33	Odonata	Gomphidae	Shaogomphus postocularis
Shaogomphus postocularis mitochondrial gene for 16	AB707778	82.39	100	1.28508e−33	Odonata	Gomphidae	Shaogomphus postocularis
Shaogomphus postocularis mitochondrial gene for 16	AB707774	82.39	100	1.28508e−33	Odonata	Gomphidae	Shaogomphus postocularis
Shaogomphus postocularis mitochondrial gene for 16	AB707775	82.39	100	1.28508e−33	Odonata	Gomphidae	Shaogomphus postocularis
Asiagomphus yayeyamensis mitochondrial gene for 16	AB707730	82.39	100	1.28508e−33	Odonata	Aeshnidae	Asiagomphus yayeyamensis
Asiagomphus yayeyamensis mitochondrial gene for 16	AB707728	82.39	100	1.28508e−33	Odonata	Aeshnidae	Asiagomphus yayeyamensis
Asiagomphus melaenops mitochondrial gene for 16S r	AB707724	82.39	100	1.28508e−33	Odonata	Aeshnidae	Asiagomphus melaenops
