# Supplementary material for: Testing the potential of a ribosomal 16S marker for DNA metabarcoding of insects
Source: PeerJ. 2016 Apr 19;4:e1966. doi: 10.7717/peerj.1966 (PMC4841222; doi:10.7717/peerj.1966)
Supplement: Supplemental Information 1 [file peerj-04-1966-s006.zip › scripts/5 match OTUs/blast_hits/OTU_135.csv_plot.pdf]

OTU_135.csv

Rhynchaenus fagi

Curculionidae

Coleoptera

100

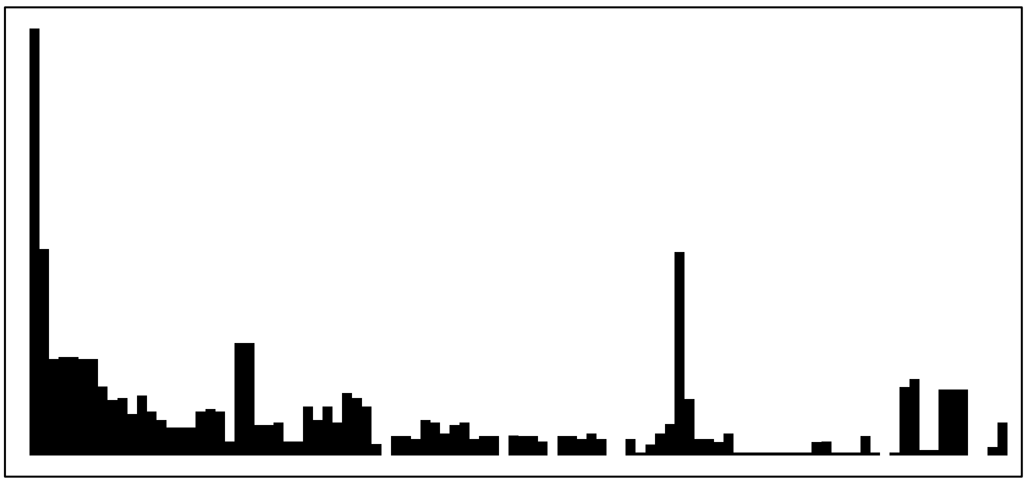

Rhynchaenus fagi partial mitochondrial 16S rRNA ge	AJ495548	100	100	3.64495e−72	Coleoptera	Curculionidae	Rhynchaenus fagi
Rhynchaenus quercus partial mitochondrial 16S rRNA	AJ495549	89.68	100	5.41436e−51	Coleoptera	Curculionidae	Rhynchaenus quercus
Cionus olens partial mitochondrial 16S rRNA gene	AJ495551	84.52	100	1.36075e−39	Coleoptera	Curculionidae	Cionus olens
Conotrachelus sp. SPN−002 mitochondrial partial 16	FN549255	84.62	100	4.74947e−39	Coleoptera	Curculionidae	Conotrachelus sp. SPN−002
Conotrachelus sp. SPN−047 mitochondrial partial 16	FN549222	84.62	100	4.74947e−39	Coleoptera	Curculionidae	Conotrachelus sp. SPN−047
Cionus sp. JJA−2010 16S ribosomal RNA gene, partia	GU988184	84.52	100	1.65773e−38	Coleoptera	Curculionidae	Cionus sp. JJA−2010
Adexius scrobipennis isolate ZFMK−DNA JJ0043 16S r	EU286289	84.52	100	1.65773e−38	Coleoptera	Curculionidae	Adexius scrobipennis
Conotrachelus punctiventris mitochondrial partial	FN549239	83.23	100	8.58725e−36	Coleoptera	Curculionidae	Conotrachelus punctiventris
Rhinusa canescens voucher 423i 16S ribosomal RNA g	JX091107	82.58	100	2.99724e−35	Coleoptera	Curculionidae	Rhinusa canescens
Pseudomeira obscurella mitochondrial partial 16S r	HE818405	82.69	100	1.04614e−34	Coleoptera	Curculionidae	Pseudomeira obscurella
Rhinusa canescens voucher 283t1 16S ribosomal RNA	JX091096	81.94	100	1.04614e−34	Coleoptera	Curculionidae	Rhinusa canescens
Conotrachelus punctiventris mitochondrial partial	FN549243	82.8	100	3.65139e−34	Coleoptera	Curculionidae	Conotrachelus punctiventris
Pseudomeira obscura mitochondrial partial 16S rRNA	HE818406	82.05	100	1.27446e−33	Coleoptera	Curculionidae	Pseudomeira obscura
Diaulota densissima voucher ZMUN:10008421 16S ribo	JX536415	81.65	100	1.55261e−32	Coleoptera	Staphylinidae	Diaulota densissima
Rhinusa collina voucher 303i 16S ribosomal RNA gen	JX091105	81.29	100	1.55261e−32	Coleoptera	Curculionidae	Rhinusa collina
Rhinusa neta voucher 350t1 16S ribosomal RNA gene,	JX091099	81.29	100	1.55261e−32	Coleoptera	Curculionidae	Rhinusa neta
Rhinusa eversmanni isolate Gy949 16S ribosomal RNA	JX455786	81.29	100	1.55261e−32	Coleoptera	Curculionidae	Rhinusa eversmanni
Bruchidius holosericeus voucher BRU−H11 16S riboso	HQ178224	82.05	100	1.55261e−32	Coleoptera	Chrysomelidae	Bruchidius holosericeus
Cortinicara gibbosa voucher BMNH:679214 16S riboso	DQ202543	82.17	100	1.55261e−32	Coleoptera	Latridiidae	Cortinicara gibbosa
Larinus turbinatus partial mitochondrial 16S rRNA	AJ495521	82.05	100	1.55261e−32	Coleoptera	Curculionidae	Larinus turbinatus
Rhinusa neta voucher 442t2 16S ribosomal RNA gene,	JX091104	80.65	100	5.41914e−32	Coleoptera	Curculionidae	Rhinusa neta
Acalles alcarazensis bio−material ZFMK−DNA JJ0353	GU988302	85.27	83.23	5.41914e−32	Coleoptera	Curculionidae	Acalles alcarazensis
Acalles alcarazensis bio−material ZFMK−DNA JJ0352	GU988301	85.27	83.23	5.41914e−32	Coleoptera	Curculionidae	Acalles alcarazensis
Conotrachelus sp. SPN−037 mitochondrial partial 16	FN549249	81.41	100	5.41914e−32	Coleoptera	Curculionidae	Conotrachelus sp. SPN−037
Laparocerus tibialis voucher Antonio Machado:AMC 8	EF583390	81.41	100	5.41914e−32	Coleoptera	Curculionidae	Laparocerus tibialis
Ericmodes sylvaticus voucher BMNH:697044 16S ribos	DQ202617	81.53	100	5.41914e−32	Coleoptera	Protocucujidae	Ericmodes sylvaticus
Cionus alauda partial mitochondrial 16S rRNA gene	AJ495550	80.65	100	5.41914e−32	Coleoptera	Curculionidae	Cionus alauda
Rhinusa collina isolate Gy872 16S ribosomal RNA ge	JX455774	80.65	100	1.89147e−31	Coleoptera	Curculionidae	Rhinusa collina
Lamproserica sp. BMNH 670867 16S large subunit rib	EF487887	82.28	100	1.89147e−31	Coleoptera	Scarabaeidae	Lamproserica sp. BMNH 670867
Sisyphus faciculatus 16S ribosomal RNA gene, parti	AY131625	81.65	99.35	1.89147e−31	Coleoptera	Scarabaeidae	Sisyphus faciculatus
Oxyops sp. JR−2002 partial mitochondrial 16S rRNA	AJ495591	82.28	100	1.89147e−31	Coleoptera	Curculionidae	Oxyops sp. JR−2002
Otiorhynchus raucus partial mitochondrial 16S rRNA	AJ495574	81.53	100	1.89147e−31	Coleoptera	Curculionidae	Otiorhynchus raucus
Dorytomus longimanus partial mitochondrial 16S rRN	AJ495536	82.91	100	1.89147e−31	Coleoptera	Brachyceridae	Dorytomus longimanus
Simo hirticornis partial mitochondrial 16S rRNA ge	AJ495486	82.69	100	1.89147e−31	Coleoptera	Brachyceridae	Simo hirticornis
Otiorhynchus porcatus partial mitochondrial 16S rR	AJ495481	82.28	100	1.89147e−31	Coleoptera	Curculionidae	Otiorhynchus porcatus
Orobitis apicalis mitochondrial gene for 12S ribos	AB232983	80.52	94.84	1.89147e−31	Coleoptera	Curculionidae	Orobitis apicalis
Rhinusa pilosa voucher Gy871 16S ribosomal RNA gen	JX091090	80	100	6.60187e−31	Coleoptera	Curculionidae	Rhinusa pilosa
Cafius subtilis isolate CNUIC−118 16S ribosomal RN	JF796480	80.89	99.35	6.60187e−31	Coleoptera	Staphylinidae	Cafius subtilis
Cafius bistriatus isolate CNUIC−100 16S ribosomal	JF796462	80.89	99.35	6.60187e−31	Coleoptera	Staphylinidae	Cafius bistriatus
Conotrachelus sp. SPN−035 mitochondrial partial 16	FN549246	80.77	100	6.60187e−31	Coleoptera	Curculionidae	Conotrachelus sp. SPN−035
Dendroxena sexcarinata mitochondrial gene for 16S	AB285535	81.65	100	6.60187e−31	Coleoptera	Silphidae	Dendroxena sexcarinata
Onthophagus clypeatus 16S ribosomal RNA gene, part	EU162538	81.53	98.71	6.60187e−31	Coleoptera	Scarabaeidae	Onthophagus clypeatus
Peritelus sphaeroides partial mitochondrial 16S rR	AJ495485	81.01	100	6.60187e−31	Coleoptera	Curculionidae	Peritelus sphaeroides
Cleonus piger partial mitochondrial 16S rRNA gene	AJ495524	81.41	100	6.60187e−31	Coleoptera	Curculionidae	Cleonus piger
Rhinotia haemoptera partial mitochondrial 16S rRNA	AJ495455	81.53	99.35	6.60187e−31	Coleoptera	Belidae	Rhinotia haemoptera
Hypera postica Budapest, Hungary mitochondrion 16S	U16967	80.77	100	6.60187e−31	Coleoptera	Curculionidae	Hypera postica
Eucryptorrhynchus chinensis voucher ECHIN20150110	KP455510	80.89	100	2.30428e−30	Coleoptera	Curculionidae	Eucryptorrhynchus chinensis
Eucryptorrhynchus chinensis mitochondrion, complet	KP410324	80.89	100	2.30428e−30	Coleoptera	Curculionidae	Eucryptorrhynchus chinensis
Rhinusa pilosa voucher Gy897 16S ribosomal RNA gen	JX091092	80	100	2.30428e−30	Coleoptera	Curculionidae	Rhinusa pilosa
Acalles asniensis bio−material ZFMK−DNA JJ0839 16S	GU988569	80.92	96.77	2.30428e−30	Coleoptera	Curculionidae	Acalles asniensis
Bruchidius watamuensis voucher BRU−GK193 16S ribos	HQ178171	80.89	100	2.30428e−30	Coleoptera	Chrysomelidae	Bruchidius watamuensis
Bruchidius watamuensis voucher BRU−GK166 16S ribos	HQ178170	80.89	100	2.30428e−30	Coleoptera	Chrysomelidae	Bruchidius watamuensis
Cortinicara sp. NPL−2007 16S ribosomal RNA gene, p	EU164611	80.65	100	2.30428e−30	Coleoptera	Latridiidae	Cortinicara sp. NPL−2007
Eutrichapion punctigerum partial mitochondrial 16S	AJ495469	79.87	100	2.30428e−30	Coleoptera	Brentidae	Eutrichapion punctigerum
Peritelus leucogrammus partial mitochondrial 16S r	AJ495484	80.89	100	2.30428e−30	Coleoptera	Curculionidae	Centricnemus leucogrammus
Curculio villosus partial mitochondrial 16S rRNA g	AJ495532	80.89	100	2.30428e−30	Coleoptera	Curculionidae	Curculio villosus
Bruchidius marginalis 16S ribosomal RNA gene, part	KP682970	80.77	100	8.04272e−30	Coleoptera	Chrysomelidae	Bruchidius marginalis
Trox cadaverinus cadaverinus isolate Tcad01 16S ri	KC801108	81.01	100	8.04272e−30	Coleoptera	Trogidae	Trox cadaverinus cadaverinus
Tomarus sallaei 16S ribosomal RNA gene, partial se	JX982898	80.77	99.35	8.04272e−30	Coleoptera	Scarabaeidae	Tomarus sallaei
Rhinusa neta isolate Gy935 16S ribosomal RNA gene,	JX455783	80	100	8.04272e−30	Coleoptera	Curculionidae	Rhinusa neta
Rhinusa neta isolate Gy640 16S ribosomal RNA gene,	JX455770	80	100	8.04272e−30	Coleoptera	Curculionidae	Rhinusa neta
Cafius sulcicollis isolate CNUIC−119 16S ribosomal	JF796481	80.77	99.35	8.04272e−30	Coleoptera	Staphylinidae	Cafius sulcicollis
Pentarthrum carmichaeli voucher 673223 16S ribosom	EF214122	80.13	99.35	8.04272e−30	Coleoptera	Curculionidae	Pentarthrum carmichaeli
Conotrachelus sp. SPN−009 mitochondrial partial 16	FN549244	80.5	100	8.04272e−30	Coleoptera	Curculionidae	Conotrachelus sp. SPN−009
Neuroserica mashona voucher BMNH 671442 16S large	EF487869	81.01	100	8.04272e−30	Coleoptera	Scarabaeidae	Neuroserica mashona
Rhyncogonus excavatus voucher Antonio Machado:AMC	EF583435	81.46	95.48	8.04272e−30	Coleoptera	Curculionidae	Rhyncogonus excavatus
Drouetius sp. AMC 3989 16S ribosomal RNA gene, par	EF583418	89.52	67.74	8.04272e−30	Coleoptera	Curculionidae	Drouetius sp. AMC 3989
Onthophagus praecellens 16S ribosomal RNA gene, pa	EU162560	82.54	90.32	8.04272e−30	Coleoptera	Scarabaeidae	Onthophagus praecellens
Larinus sturnus partial mitochondrial 16S rRNA gen	AJ495520	80.77	100	8.04272e−30	Coleoptera	Curculionidae	Larinus sturnus
Lixus angustatus partial mitochondrial 16S rRNA ge	AJ495517	80.77	100	8.04272e−30	Coleoptera	Curculionidae	Lixus angustatus
Pleophylla sp. BNHM834904 16S ribosomal RNA gene,	KC964529	80.62	100	2.80719e−29	Coleoptera	Scarabaeidae	Pleophylla sp. BNHM834904
Pleophylla sp. BNHM834734 16S ribosomal RNA gene,	KC964472	81.01	99.35	2.80719e−29	Coleoptera	Scarabaeidae	Pleophylla sp. BNHM834734
Rhinusa brondelii haplotype 16S−bron2 16S ribosoma	KJ420523	80.13	100	2.80719e−29	Coleoptera	Curculionidae	Rhinusa brondelii
Rhinusa brondelii haplotype 16S−bron1 16S ribosoma	KJ420522	80.13	100	2.80719e−29	Coleoptera	Curculionidae	Rhinusa brondelii
Rhinusa pilosa haplotype 16S−B5 16S ribosomal RNA	KJ420518	80.13	100	2.80719e−29	Coleoptera	Curculionidae	Rhinusa pilosa
Rhinusa pilosa haplotype 16S−B4 16S ribosomal RNA	KJ420517	80.13	100	2.80719e−29	Coleoptera	Curculionidae	Rhinusa pilosa
Rhinusa pilosa haplotype 16S−B3 16S ribosomal RNA	KJ420516	80.13	100	2.80719e−29	Coleoptera	Curculionidae	Rhinusa pilosa
Rhinusa pilosa haplotype 16S−B2 16S ribosomal RNA	KJ420515	80.13	100	2.80719e−29	Coleoptera	Curculionidae	Rhinusa pilosa
Rhinusa pilosa haplotype 16S−B1 16S ribosomal RNA	KJ420514	80.13	100	2.80719e−29	Coleoptera	Curculionidae	Rhinusa pilosa
Rhinusa pilosa haplotype 16S−A1 16S ribosomal RNA	KJ420513	80.13	100	2.80719e−29	Coleoptera	Curculionidae	Rhinusa pilosa
Trichius fasciatus voucher 842840 16S ribosomal RN	JX234518	80.62	100	2.80719e−29	Coleoptera	Scarabaeidae	Trichius fasciatus
Hylobitelus xiaoi mitochondrion, complete genome	JX847496	80.65	99.35	2.80719e−29	Coleoptera	Curculionidae	Hylobitelus xiaoi
Falklandiellus suffodens voucher 668315 16S riboso	EF214020	80.13	100	2.80719e−29	Coleoptera	Curculionidae	Falklandiellus suffodens
Falklandiellus suffodens voucher 668283 16S riboso	EF213994	80.13	100	2.80719e−29	Coleoptera	Curculionidae	Falklandiellus suffodens
Laparocerus oromii isolate ZFMK−DNA JJ0665 16S rib	FJ716536	80.13	100	2.80719e−29	Coleoptera	Curculionidae	Laparocerus oromii
Conotrachelus sp. SPN−010 mitochondrial partial 16	FN549226	80.89	100	2.80719e−29	Coleoptera	Curculionidae	Conotrachelus sp. SPN−010
Laparocerus excavatus voucher Antonio Machado:AMC	EF583431	80.13	100	2.80719e−29	Coleoptera	Curculionidae	Laparocerus excavatus
Drouetius azoricus voucher Antonio Machado:AMC 308	EF583417	80	100	2.80719e−29	Coleoptera	Curculionidae	Drouetius azoricus
Onthophagus vermiculatus 16S ribosomal RNA gene, p	EU162575	80.12	100	2.80719e−29	Coleoptera	Scarabaeidae	Onthophagus vermiculatus
Acalles sierrae voucher ZFMK−TIS−4108 16S ribosoma	KJ720557	83.21	79.35	9.79804e−29	Coleoptera	Curculionidae	Acalles sierrae
Artipus sp. BOLD:ACO0661 voucher BAH_201312_P01_G1	KP253881	83.58	85.16	9.79804e−29	Coleoptera	Curculionidae	Artipus sp. BOLD:ACO0661
Plaesiorrhinella trivittata voucher 842788 16S rib	JX234482	80.25	100	9.79804e−29	Coleoptera	Scarabaeidae	Plaesiorrhinella trivittata
Rentonellum sp. NLG−2013 voucher COL548 12S riboso	KC524797	80.25	100	9.79804e−29	Coleoptera	Trogossitidae	Rentonellum sp. NLG−2013
Rhinusa florum voucher 644i 16S ribosomal RNA gene	JX091111	83.08	83.87	9.79804e−29	Coleoptera	Curculionidae	Rhinusa florum
Rhinusa florum voucher 387t1 16S ribosomal RNA gen	JX091100	83.08	83.87	9.79804e−29	Coleoptera	Curculionidae	Rhinusa florum
Rhinusa griseohirta isolate Gy831 16S ribosomal RN	JX455773	83.08	83.87	9.79804e−29	Coleoptera	Curculionidae	Rhinusa griseohirta
Acalles sintraniensis bio−material ZFMK−DNA JJ0814	GU988550	79.62	100	9.79804e−29	Coleoptera	Curculionidae	Acalles sintraniensis
Acalles sintraniensis bio−material ZFMK−DNA JJ0811	GU988549	79.62	100	9.79804e−29	Coleoptera	Curculionidae	Acalles sintraniensis
Acalles pilula bio−material ZFMK−DNA JJ0651 16S ri	GU988458	80.38	100	9.79804e−29	Coleoptera	Curculionidae	Acalles pilula
Rhinusa griseohirta voucher Gy832 16S ribosomal RN	JX091087	81.54	83.87	4.16623e−27	Coleoptera	Curculionidae	Rhinusa griseohirta
