# Supplementary material for: Testing the potential of a ribosomal 16S marker for DNA metabarcoding of insects
Source: PeerJ. 2016 Apr 19;4:e1966. doi: 10.7717/peerj.1966 (PMC4841222; doi:10.7717/peerj.1966)
Supplement: Supplemental Information 1 [file peerj-04-1966-s006.zip › scripts/5 match OTUs/blast_hits/OTU_136.csv_plot.pdf]

# OTU_136.csv

## Hypogastrura vernalis

## Hypogastruridae

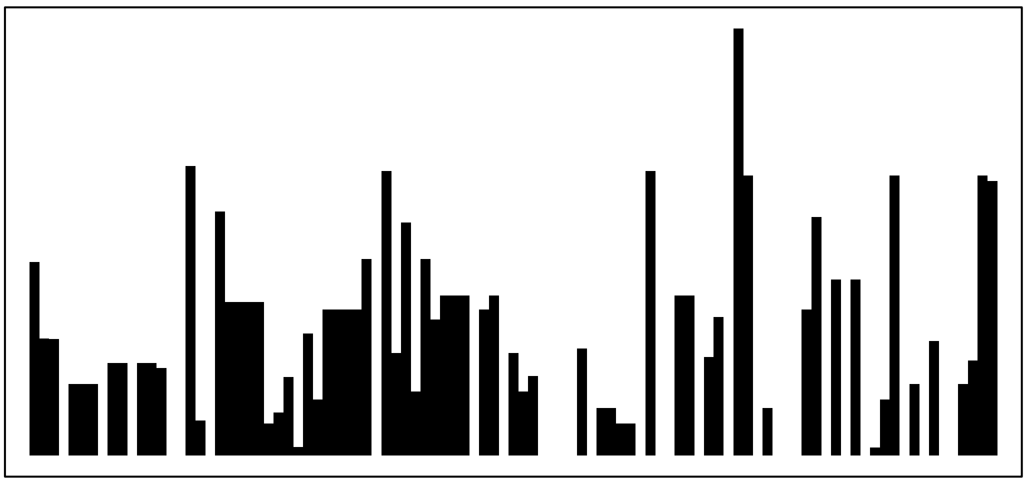

# 89.06

| | | | | | | | |
|---|---|---|---|---|---|---|---|
| Hypogastrura vernalis 16S ribosomal RNA gene, part | JN970945 | 89.06 | 42.28 | 7.64734e−11 | | Hypogastruridae | Hypogastrura vernalis |
| Pogonognathellus cf. flavescens FZ−2014 voucher Po | KF591937 | 85.48 | 40.94 | 3.96142e−08 | | Tomoceridae | Pogonognathellus cf. flavescens FZ−2014 |
| Podura aquatica mitochondrion, partial genome | AY639939 | 85.45 | 36.24 | 5.87927e−06 | | Poduridae | Podura aquatica |
| Isotoma viridis 16S ribosomal RNA gene, partial se | JN970946 | 78.79 | 44.3 | 7.16241e−05 | | Isotomidae | Isotoma viridis |
| Coecobrya communis 16S ribosomal RNA gene, partial | KC236218 | 83.33 | 32.21 | 0.00304554 | | Entomobryidae | Coecobrya communis |
| Coecobrya tenebricosa 16S ribosomal RNA gene, part | KC236191 | 83.33 | 32.21 | 0.00304554 | | Entomobryidae | Coecobrya tenebricosa |
| Buettikeria echinosa 16S ribosomal RNA gene, parti | EF487789 | 83.33 | 32.21 | 0.00304554 | Coleoptera | Scarabaeidae | Buettikeria echinosa |
| Schistosoma mattheei genome assembly S_mattheei_De | LM158611 | 79.69 | 42.95 | 0.01063 | Strigeidida | Schistosomatidae | Schistosoma mattheei |
| Congeria kusceri haplotype B 16S ribosomal RNA gen | JQ348915 | 84.31 | 32.89 | 0.01063 | Veneroida | Dreissenidae | Congeria kusceri |
| Congeria kusceri isolate D10 16S ribosomal RNA gen | JX099439 | 84.31 | 32.89 | 0.01063 | Veneroida | Dreissenidae | Congeria kusceri |
| Lepiserica sp. BMNH 671451 16S large subunit ribos | EF487924 | 78.46 | 42.28 | 0.01063 | Coleoptera | Scarabaeidae | Lepiserica sp. BMNH 671451 |
| Congeria kusceri 16S ribosomal RNA gene, partial s | AF320601 | 84.31 | 32.89 | 0.01063 | Veneroida | Dreissenidae | Congeria kusceri |
| Lotus japonicus genomic DNA, chromosome 4, clone: | AP006091 | 84.31 | 33.56 | 0.01063 | Fabales | Fabaceae | Lotus japonicus |
| Ovis canadensis canadensis isolate 43U chromosome | CP011891 | 84.09 | 29.53 | 0.0371022 | | Bovidae | Ovis canadensis canadensis |
| Strongyloides venezuelensis genome assembly S_vene | LM525031 | 76.81 | 44.97 | 0.0371022 | Rhabditida | Strongyloididae | Strongyloides venezuelensis |
| Strongyloides venezuelensis genome assembly S_vene | LM524996 | 76.81 | 44.97 | 0.0371022 | Rhabditida | Strongyloididae | Strongyloides venezuelensis |
| Clostridium scatologenes strain ATCC 25775, comple | CP009933 | 93.55 | 20.81 | 0.0371022 | Clostridiales | Clostridiaceae | Clostridium scatologenes |
| Strongyloides stercoralis genome assembly S_sterco | LL999054 | 81.63 | 32.89 | 0.0371022 | Rhabditida | Strongyloididae | Strongyloides stercoralis |
| Schistosoma curassoni genome assembly S_curassoni_ | LM068723 | 80 | 42.95 | 0.0371022 | Strigeidida | Schistosomatidae | Schistosoma curassoni |
| Wuchereria bancrofti genome assembly W_bancrofti_J | LM000688 | 91.43 | 22.15 | 0.0371022 | Spirurida | Onchocercidae | Wuchereria bancrofti |
| Anomala varicolor isolate BM677826 16S ribosomal R | DQ680967 | 87.18 | 26.17 | 0.0371022 | Coleoptera | Scarabaeidae | Anomala varicolor |
| Anomala varicolor isolate BM671388 16S ribosomal R | DQ680892 | 87.18 | 26.17 | 0.0371022 | Coleoptera | Scarabaeidae | Anomala varicolor |
| Vitis vinifera contig VV78X008195.12, whole genome | AM446568 | 87.18 | 26.17 | 0.0371022 | Vitales | Vitaceae | Vitis vinifera |
| Vitis vinifera contig VV78X008195.18, whole genome | AM438115 | 87.18 | 26.17 | 0.0371022 | Vitales | Vitaceae | Vitis vinifera |
| Vitis vinifera contig VV78X134311.9, whole genome | AM459239 | 81.48 | 36.24 | 0.0371022 | Vitales | Vitaceae | Vitis vinifera |
| Onychiurus orientalis mitochondrion, partial genom | AY639938 | 82 | 33.56 | 0.0371022 | | Onychiuridae | Onychiurus orientalis |
| Medicago truncatula clone mth2−34b13, complete seq | AC130799 | 83.67 | 30.87 | 0.0371022 | Fabales | Fabaceae | Medicago truncatula |
| Arabidopsis thaliana srp30 gene, exons 1−12 | AJ131214 | 80.39 | 34.23 | 0.0371022 | Brassicales | Brassicaceae | Arabidopsis thaliana |
| Ovis canadensis canadensis isolate 43U chromosome | CP011888 | 85.71 | 30.87 | 0.129499 | | Bovidae | Ovis canadensis canadensis |
| Strongyloides venezuelensis genome assembly S_vene | LM525123 | 82.61 | 30.87 | 0.129499 | Rhabditida | Strongyloididae | Strongyloides venezuelensis |
| PREDICTED: Linepithema humile neuroparsin−A−like ( | XM_012361527 | 86.84 | 25.5 | 0.129499 | Hymenoptera | Formicidae | Linepithema humile |
| PREDICTED: Linepithema humile neuroparsin−A−like ( | XM_012361526 | 86.84 | 25.5 | 0.129499 | Hymenoptera | Formicidae | Linepithema humile |
| PREDICTED: Linepithema humile neuroparsin−A−like ( | XM_012361525 | 86.84 | 25.5 | 0.129499 | Hymenoptera | Formicidae | Linepithema humile |
| PREDICTED: Linepithema humile neuroparsin−A−like ( | XM_012361524 | 86.84 | 25.5 | 0.129499 | Hymenoptera | Formicidae | Linepithema humile |
| PREDICTED: Linepithema humile pro−corazonin−like ( | XM_012359878 | 89.19 | 24.16 | 0.129499 | Hymenoptera | Formicidae | Linepithema humile |
| Strongyloides stercoralis genome assembly S_sterco | LL999050 | 79.25 | 35.57 | 0.129499 | Rhabditida | Strongyloididae | Strongyloides stercoralis |
| Strongyloides stercoralis genome assembly S_sterco | LL999048 | 93.33 | 20.13 | 0.129499 | Rhabditida | Strongyloididae | Strongyloides stercoralis |
| Schistosoma rodhaini genome assembly S_rodhaini_Bu | LL958232 | 84.78 | 28.86 | 0.129499 | Strigeidida | Schistosomatidae | Schistosoma rodhaini |
| Syphacia muris genome assembly S_muris_Valencia ,s | LK998325 | 90.91 | 22.15 | 0.129499 | Oxyurida | Oxyuridae | Syphacia muris |
| Syphacia muris genome assembly S_muris_Valencia ,s | LK996050 | 82.98 | 31.54 | 0.129499 | Oxyurida | Oxyuridae | Syphacia muris |
| Strongyloides papillosus genome assembly S_papillo | LM525569 | 89.19 | 24.16 | 0.129499 | Rhabditida | Strongyloididae | Strongyloides papillosus |
| Cyprinus carpio genome assembly common carp genome | LN590968 | 86.36 | 28.86 | 0.129499 | Cypriniformes | Cyprinidae | Cyprinus carpio |
| Plasmodium yoelii genome assembly PY17X01, chromos | LM993663 | 87.5 | 26.85 | 0.129499 | Haemosporida | Plasmodiidae | Plasmodium yoelii |
| Plasmodium yoelii genome assembly PYYM01, chromoso | LK934637 | 87.5 | 26.85 | 0.129499 | Haemosporida | Plasmodiidae | Plasmodium yoelii |
| Plasmodium berghei ANKA genome assembly PBANKA01, | LK023124 | 87.5 | 26.85 | 0.129499 | Haemosporida | Plasmodiidae | Plasmodium berghei ANKA |
| Eurys sp. SS2 voucher AE065 16S ribosomal RNA gene | KF318477 | 78.33 | 39.6 | 0.129499 | Hymenoptera | Pergidae | Eurys sp. SS2 |
| Callistethus sp. 6 VF−2014 voucher CEUA00105818 16 | KF738015 | 86.84 | 25.5 | 0.129499 | Coleoptera | Scarabaeidae | Callistethus sp. 6 VF−2014 |
| PREDICTED: Apis mellifera zinc finger protein 516− | XM_006567475 | 87.5 | 26.85 | 0.129499 | Hymenoptera | Apidae | Apis mellifera |
| Folsomia quadrioculata 16S ribosomal RNA gene, par | KC236199 | 78.18 | 36.91 | 0.129499 | | Isotomidae | Folsomia quadrioculata |
| Clostridium saccharoperbutylacetonicum N1−4(HMT), | CP004121 | 84.78 | 28.86 | 0.129499 | Clostridiales | Clostridiaceae | Clostridium saccharoperbutylacetonicum N1−4(HMT) |
| Podura aquatica 16S ribosomal RNA gene, partial se | JN970977 | 82.98 | 30.87 | 0.129499 | | Poduridae | Podura aquatica |
| Megalothorax sp. EA040003−cs17 16S ribosomal RNA g | JN970951 | 83.72 | 28.86 | 0.129499 | | Neelidae | Megalothorax sp. EA040003−cs17 |
| Myrmecolax sp. 5 DPM−2011 16S large subunit riboso | JN082867 | 74.36 | 49.66 | 0.129499 | Strepsiptera | Myrmecolacidae | Myrmecolax sp. 5 DPM−2011 |
| Myrmecolax sp. 4 DPM−2011 16S large subunit riboso | JN082866 | 74.36 | 49.66 | 0.129499 | Strepsiptera | Myrmecolacidae | Myrmecolax sp. 4 DPM−2011 |
| Homo sapiens T−box 20 (TBX20) gene, complete cds | HM015599 | 77.61 | 44.3 | 0.129499 | Primates | Hominidae | Homo sapiens |
| Homo sapiens T−box 20 (TBX20), RefSeqGene on chrom | NG_015805 | 77.61 | 44.3 | 0.129499 | Primates | Hominidae | Homo sapiens |
| Psychotria acuminata voucher STRI:BCI 711071 photo | GQ982336 | 85 | 26.85 | 0.129499 | Gentianales | Rubiaceae | Psychotria acuminata |
| Zebrafish DNA sequence from clone CH73−15N15 in li | FP102191 | 79.03 | 40.27 | 0.129499 | Cypriniformes | Cyprinidae | Danio rerio |
| Maladera sp. BMNH 671449 16S large subunit ribosom | EF487929 | 82.22 | 30.2 | 0.129499 | Coleoptera | Scarabaeidae | Maladera sp. BMNH 671449 |
| Lepiserica sp. BMNH 671452 16S large subunit ribos | EF487925 | 82.22 | 30.2 | 0.129499 | Coleoptera | Scarabaeidae | Lepiserica sp. BMNH 671452 |
| Phytophthora ramorum strain CBS 101553 mitochondri | EU427470 | 81.48 | 36.24 | 0.129499 | Peronosporales | | Phytophthora ramorum |
| Phytophthora ramorum mitochondrion, complete genom | DQ832718 | 81.48 | 36.24 | 0.129499 | Peronosporales | | Phytophthora ramorum |
| Vitis vinifera, whole genome shotgun sequence, con | AM480418 | 76.81 | 45.64 | 0.129499 | Vitales | Vitaceae | Vitis vinifera |
| Taeniopygia guttata chromosome UNK clone TGMCBa−9H | AC188314 | 93.33 | 20.13 | 0.129499 | Passeriformes | Estrildidae | Taeniopygia guttata |
| Homo sapiens chromosome 7 clone RP11−178P2, comple | AC009531 | 77.61 | 44.3 | 0.129499 | Primates | Hominidae | Homo sapiens |
| Homo sapiens clone SCb−41A3 from 7p14−15, complete | AC005826 | 77.61 | 44.3 | 0.129499 | Primates | Hominidae | Homo sapiens |
| Plasmodium yoelii yoelii str. 17XNL hypothetical p | XM_724929 | 87.5 | 26.85 | 0.129499 | Haemosporida | Plasmodiidae | Plasmodium yoelii yoelii 17XNL |
| Plasmodium berghei strain ANKA hypothetical protei | XM_663995 | 87.5 | 26.85 | 0.129499 | Haemosporida | Plasmodiidae | Plasmodium berghei ANKA |
| Homo sapiens BAC clone RP11−456N16 from 7p15.1−p13 | AC006379 | 77.61 | 44.3 | 0.129499 | Primates | Hominidae | Homo sapiens |
| Fusobacterium nucleatum subsp. vincentii ChDC F8 s | CP012714 | 84.62 | 26.17 | 0.451997 | Fusobacteriales | Fusobacteriaceae | Fusobacterium nucleatum subsp. vincentii ChDC F8 |
| Drosophila busckii chromosome 3R sequence | CP012526 | 86.49 | 24.83 | 0.451997 | Diptera | Drosophilidae | Drosophila busckii |
| Spiroplasma litorale strain TN−1, complete genome | CP012357 | 75.71 | 46.31 | 0.451997 | Entomoplasmatales | Spiroplasmataceae | Spiroplasma litorale |
| Pomacea maculata mitochondrion, complete genome | KR350466 | 100 | 16.11 | 0.451997 | | Ampullariidae | Pomacea maculata |
| Ovis canadensis canadensis isolate 43U chromosome | CP011886 | 93.1 | 19.46 | 0.451997 | | Bovidae | Ovis canadensis canadensis |
| Strongyloides venezuelensis genome assembly S_vene | LM525114 | 76.12 | 43.62 | 0.451997 | Rhabditida | Strongyloididae | Strongyloides venezuelensis |
| Strongyloides venezuelensis genome assembly S_vene | LM525008 | 82.22 | 30.2 | 0.451997 | Rhabditida | Strongyloididae | Strongyloides venezuelensis |
| Sinothela sp. XUX−2011−217 16S ribosomal RNA gene, | KP230154 | 80 | 36.24 | 0.451997 | Araneae | Liphistiidae | Sinothela sp. XUX−2011−217 |
| Vinathela abca voucher XUX−2013−047 16S ribosomal | KP230086 | 78.57 | 37.58 | 0.451997 | Araneae | Liphistiidae | Vinathela abca |
| Heptathela higoensis voucher XUX−2013−468 16S ribo | KP230064 | 78.57 | 35.57 | 0.451997 | Araneae | Heptathelidae | Heptathela higoensis |
| Elizabethkingia sp. BM10, complete genome | CP011059 | 86.84 | 25.5 | 0.451997 | Flavobacteriales | Flavobacteriaceae | Elizabethkingia sp. BM10 |
| Campylobacter volucris LMG 24379, complete genome | CP007774 | 91.18 | 22.15 | 0.451997 | Campylobacterales | Campylobacteraceae | Campylobacter volucris LMG 24379 |
| Cucumis melo genomic chromosome, chr_11 | LN713265 | 78.95 | 36.24 | 0.451997 | Cucurbitales | Cucurbitaceae | Cucumis melo |
| Cucumis melo genomic chromosome, chr_6 | LN713260 | 88.24 | 22.82 | 0.451997 | Cucurbitales | Cucurbitaceae | Cucumis melo |
| Cucumis melo genomic scaffold, anchoredscaffold000 | LN681921 | 78.95 | 36.24 | 0.451997 | Cucurbitales | Cucurbitaceae | Cucumis melo |
| Cucumis melo genomic scaffold, anchoredscaffold000 | LN681860 | 88.24 | 22.82 | 0.451997 | Cucurbitales | Cucurbitaceae | Cucumis melo |
| Parastrongyloides trichosuri genome assembly P_tri | LM523525 | 79.59 | 32.89 | 0.451997 | Rhabditida | Strongyloididae | Parastrongyloides trichosuri |
| Toxocara canis genome assembly T_canis_Equador ,sc | LM038155 | 80.36 | 36.24 | 0.451997 | Ascaridida | Toxocaridae | Toxocara canis |
| Strongyloides stercoralis genome assembly S_sterco | LL999108 | 82.61 | 30.87 | 0.451997 | Rhabditida | Strongyloididae | Strongyloides stercoralis |
| Strongyloides stercoralis genome assembly S_sterco | LL999053 | 93.1 | 19.46 | 0.451997 | Rhabditida | Strongyloididae | Strongyloides stercoralis |
| Onchocerca ochengi genome assembly O_ochengi_Ngaou | LL518239 | 75.71 | 46.98 | 0.451997 | Spirurida | Onchocercidae | Onchocerca ochengi |
| Heligmosomoides polygyrus genome assembly H_bakeri | LL195820 | 83.33 | 28.19 | 0.451997 | Rhabditida | Heligmosomatidae | Heligmosomoides polygyrus |
| Dracunculus medinensis genome assembly D_medinensi | LK978194 | 76.56 | 42.95 | 0.451997 | Spirurida | Dracunculidae | Dracunculus medinensis |
| Brugia pahangi genome assembly B_pahangi_Glasgow , | LK973504 | 85.37 | 26.85 | 0.451997 | Spirurida | Onchocercidae | Brugia pahangi |
| Brugia pahangi genome assembly B_pahangi_Glasgow , | LK968273 | 76.06 | 46.98 | 0.451997 | Spirurida | Onchocercidae | Brugia pahangi |
| Brugia pahangi genome assembly B_pahangi_Glasgow , | LK966974 | 77.27 | 43.62 | 0.451997 | Spirurida | Onchocercidae | Brugia pahangi |
| Brugia pahangi genome assembly B_pahangi_Glasgow , | LK965037 | 83.33 | 28.19 | 0.451997 | Spirurida | Onchocercidae | Brugia pahangi |
| Protopolystoma xenopodis genome assembly P_xenopod | LM990169 | 84.44 | 29.53 | 0.451997 | Polyopisthocotylea | Polystomatidae | Protopolystoma xenopodis |
| Toxocara canis genome assembly T_canis_Equador ,sc | LM041102 | 93.1 | 19.46 | 0.451997 | Ascaridida | Toxocaridae | Toxocara canis |
| Ovis canadensis canadensis isolate 43U chromosome | CP011887 | 92.86 | 18.79 | 1.57763 | | Bovidae | Ovis canadensis canadensis |
| Ovis canadensis canadensis isolate 43U chromosome | CP011905 | 72.31 | 41.61 | 1.57763 | | Bovidae | Ovis canadensis canadensis |
