# Supplementary material for: Testing the potential of a ribosomal 16S marker for DNA metabarcoding of insects
Source: PeerJ. 2016 Apr 19;4:e1966. doi: 10.7717/peerj.1966 (PMC4841222; doi:10.7717/peerj.1966)
Supplement: Supplemental Information 1 [file peerj-04-1966-s006.zip › scripts/5 match OTUs/blast_hits/OTU_139.csv_plot.pdf]

OTU_139.csv

Pedicia albivitta

Pediciidae

Diptera

88.54

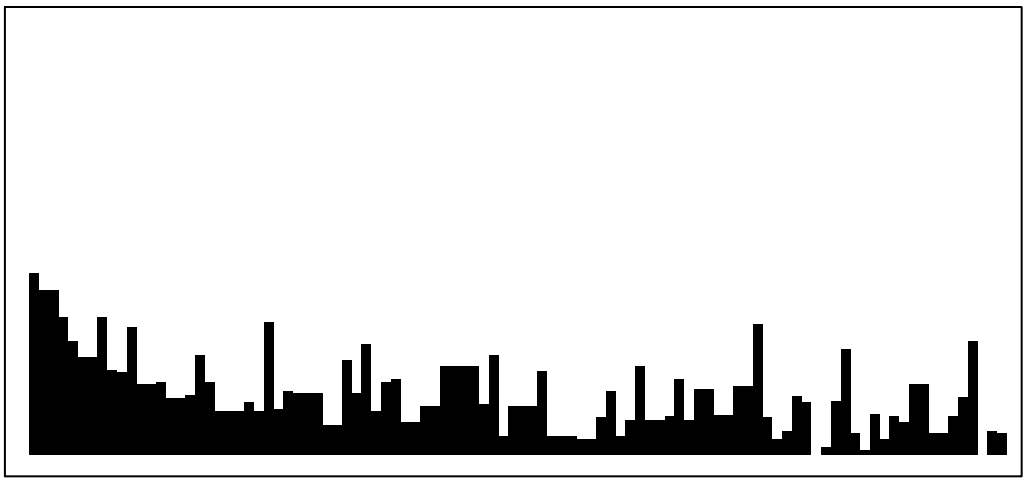

Pedicia albivitta voucher O'Grady 200012 16S ribos	EU005435	88.54	100	9.87097e−48	Diptera	Pediciidae	Pedicia albivitta
Bolitophila sp. ER−2009 16S ribosomal RNA gene, pa	FJ172021	87.74	97.44	2.17423e−43	Diptera	Mycetophilidae	Bolitophila sp. ER−2009
Bolitophila occlusa voucher JSB2 16S ribosomal RNA	KJ136713	87.76	93.59	3.22684e−41	Diptera	Mycetophilidae	Bolitophila occlusa
Bolitophila cinerea voucher JSB1 16S ribosomal RNA	KJ136712	86.45	97.44	1.12628e−40	Diptera	Mycetophilidae	Bolitophila cinerea
Tipula sp. PMO 200011 16S ribosomal RNA gene, part	EU005437	85.35	100	1.12628e−40	Diptera	Tipulidae	Tipula sp. PMO 200011
Tipula abdominalis 16S ribosomal RNA gene, partial	KC177457	84.62	100	3.93109e−40	Diptera	Tipulidae	Tipula abdominalis
Tipula abdominalis mitochondrion, partial genome	JN861743	84.62	100	3.93109e−40	Diptera	Tipulidae	Tipula abdominalis
Bolitophila hybrida 16S ribosomal RNA gene, partia	FJ172002	86.45	97.44	4.78905e−39	Diptera	Mycetophilidae	Bolitophila hybrida
Parochlus steinenii isolate Sejong01 mitochondrion	KT003702	83.97	100	1.67154e−38	Diptera	Chironomidae	Parochlus steinenii
Edwardsina gigantea 16S ribosomal RNA gene, partia	KC177460	83.87	99.36	5.83426e−38	Diptera	Blephariceridae	Edwardsina gigantea
Arachnocampa luminosa biotype 1 16S large subunit	AY576329	86	94.23	5.83426e−38	Diptera	Keroplatidae	Arachnocampa luminosa
Dicranomyia iniquispina voucher 205222 16S ribosom	KC692800	83.33	100	2.03636e−37	Diptera	Limoniidae	Dicranomyia iniquispina
Limonia iniquispina voucher O'Grady 201869 16S rib	EU005407	83.33	100	2.03636e−37	Diptera	Limoniidae	Dicranomyia iniquispina
Limonia swezeyi voucher O'Grady 200949 16S ribosom	EU005422	83.44	100	7.10758e−37	Diptera	Limoniidae	Dicranomyia swezeyi
Limonia hawaiiensis voucher O'Grady 201598 16S rib	EU005405	82.69	100	8.65881e−36	Diptera	Limoniidae	Dicranomyia hawaiiensis
Limonia hawaiiensis voucher O'Grady 200922 16S rib	EU005404	82.69	100	8.65881e−36	Diptera	Limoniidae	Dicranomyia hawaiiensis
Limonia swezeyi voucher O'Grady 200126 16S ribosom	EU005420	82.8	100	3.02222e−35	Diptera	Limoniidae	Dicranomyia swezeyi
Arachnocampa luminosa biotype 2 16S large subunit	AY576330	84.67	94.23	3.02222e−35	Diptera	Keroplatidae	Arachnocampa luminosa
Empis producta voucher RMBR:103430 16S ribosomal R	FJ808167	83.44	99.36	1.05486e−34	Diptera	Empididae	Empis producta
Limonia variabilis voucher O'Grady 201512 16S ribo	EU005428	82.05	100	1.05486e−34	Diptera	Limoniidae	Dicranomyia variabilis
Limonia kraussi voucher O'Grady 200013 16S ribosom	EU005415	82.05	100	1.05486e−34	Diptera	Limoniidae	Dicranomyia kraussi
Limonia variabilis voucher O'Grady 200973 16S ribo	EU005426	82.05	100	1.05486e−34	Diptera	Limoniidae	Dicranomyia variabilis
Limonia sp. PMO 200984 16S ribosomal RNA gene, par	EU005416	82.47	98.72	1.05486e−34	Diptera	Limoniidae	Limonia sp. PMO 200984
Limonia hawaiiensis voucher O'Grady 200106 16S rib	EU005403	82.05	100	1.05486e−34	Diptera	Limoniidae	Dicranomyia hawaiiensis
Arachnocampa luminosa biotype 3 16S large subunit	AY576331	86.23	87.82	1.05486e−34	Diptera	Keroplatidae	Arachnocampa luminosa
Limonia swezeyi voucher O'Grady 200105 16S ribosom	EU005419	82.17	100	3.68182e−34	Diptera	Limoniidae	Dicranomyia swezeyi
Limonia kauaiensis voucher O'Grady 201519 16S ribo	EU005413	83.02	100	1.28508e−33	Diptera	Limoniidae	Dicranomyia kauaiensis
Ectinorhynchus sp. 'Bribie' voucher UQIC41178 16S	KM212983	82.91	99.36	4.48537e−33	Diptera	Therevidae	Ectinorhynchus sp. 'Bribie'
Ectinorhynchus sp. 'Moffatt' voucher QMT183203 16S	KM212976	82.91	99.36	4.48537e−33	Diptera	Therevidae	Ectinorhynchus sp. 'Moffatt'
Ectinorhynchus sp. 'warrumbungles' voucher QMT1832	KM212974	82.91	99.36	4.48537e−33	Diptera	Therevidae	Ectinorhynchus sp. 'warrumbungles'
Limonia stygipennis voucher O'Grady 200980 16S rib	EU005418	81.41	100	4.48537e−33	Diptera	Limoniidae	Dicranomyia stygipennis
Limonia stygipennis voucher O'Grady 200948 16S rib	EU005417	81.41	100	4.48537e−33	Diptera	Limoniidae	Dicranomyia stygipennis
Caenophthalmus capensis voucher MEI 120162 16S rib	DQ250838	84.46	92.31	4.48537e−33	Diptera	Therevidae	Caenophthalmus capensis
Anabarhynchus tristis voucher MEI 119905 16S ribos	DQ250834	82.91	99.36	4.48537e−33	Diptera	Therevidae	Anabarhynchus tristis
Eudicrana sp. DK−2013 voucher JSS12 16S ribosomal	KC435571	85.19	85.26	1.56555e−32	Diptera	Mycetophilidae	Eudicrana sp. DK−2013
Bittacomorphella fenderiana mitochondrion, partial	JN861745	82.05	99.36	1.56555e−32	Diptera	Ptychopteridae	Bittacomorphella fenderiana
Beris morrisii 16S ribosomal RNA gene, partial seq	GU947373	83.44	99.36	1.56555e−32	Diptera	Stratiomyidae	Beris morrisii
Phthinia humilis 16S ribosomal RNA gene, partial s	FJ172009	83.56	92.95	1.56555e−32	Diptera	Mycetophilidae	Phthinia humilis
Limonia swezeyi voucher O'Grady 200982 16S ribosom	EU005423	81.53	100	1.56555e−32	Diptera	Limoniidae	Dicranomyia swezeyi
Limonia swezeyi voucher O'Grady 200931 16S ribosom	EU005421	81.53	100	1.56555e−32	Diptera	Limoniidae	Dicranomyia swezeyi
Somatia sp. YSUW04038 16S ribosomal RNA gene, part	AY573134	82.31	94.23	1.56555e−32	Diptera	Somatiidae	Somatia sp. YSUW04038
Ectinorhynchus levis voucher MEI29709 16S ribosoma	KM212980	82.28	99.36	5.4643e−32	Diptera	Therevidae	Ectinorhynchus levis
Boletina gripha isolate 057361 16S ribosomal RNA g	HQ230381	84.17	87.82	5.4643e−32	Diptera	Mycetophilidae	Boletina gripha
Boletina gripha isolate 033462 16S ribosomal RNA g	HQ230380	84.17	87.82	5.4643e−32	Diptera	Mycetophilidae	Boletina gripha
Boletina gripha isolate 015271 16S ribosomal RNA g	HQ230378	84.17	87.82	5.4643e−32	Diptera	Mycetophilidae	Boletina gripha
Boletina griphoides isolate 009627 16S ribosomal R	HQ230370	84.17	87.82	5.4643e−32	Diptera	Mycetophilidae	Boletina griphoides
Limonia jacobus voucher O'Grady 200974 16S ribosom	EU005408	82.39	100	5.4643e−32	Diptera	Limoniidae	Dicranomyia jacobus
Eupsilocephala albodorsalis 16S ribosomal RNA gene	KM878912	84.67	87.18	1.90723e−31	Diptera	Therevidae	Eupsilocephala albodorsalis
Dicranomyia grimshawi voucher 205223 16S ribosomal	KC692801	80.89	100	1.90723e−31	Diptera	Limoniidae	Dicranomyia grimshawi
Boletina sahlbergi isolate 057401 16S ribosomal RN	HQ230385	82.31	94.23	1.90723e−31	Diptera	Mycetophilidae	Boletina sahlbergi
Boletina sahlbergi isolate 057369 16S ribosomal RN	HQ230384	82.31	94.23	1.90723e−31	Diptera	Mycetophilidae	Boletina sahlbergi
Chrysops viduatus 16S ribosomal RNA gene, partial	HM132115	82.31	94.23	1.90723e−31	Diptera	Tabanidae	Chrysops viduatus
Allocotocera pulchella 16S ribosomal RNA gene, par	FJ172034	83.94	87.82	1.90723e−31	Diptera	Mycetophilidae	Allocotocera pulchella
Limonia swezeyi voucher O'Grady 201882 16S ribosom	EU005424	80.89	100	1.90723e−31	Diptera	Limoniidae	Dicranomyia swezeyi
Limonia kauaiensis voucher O'Grady 201868 16S ribo	EU005414	80.89	100	1.90723e−31	Diptera	Limoniidae	Dicranomyia kauaiensis
Limonia kauaiensis voucher O'Grady 200971 16S ribo	EU005412	80.89	100	1.90723e−31	Diptera	Limoniidae	Dicranomyia kauaiensis
Sylvicola fenestralis 16S ribosomal RNA gene, part	KC177461	80.77	100	6.65688e−31	Diptera	Anisopodidae	Sylvicola fenestralis
Sylvicola fenestralis mitochondrion, complete geno	JN861752	80.77	100	6.65688e−31	Diptera	Anisopodidae	Sylvicola fenestralis
Libnotes orofenaae voucher O'Grady 202034 16S ribo	EU005431	81.76	100	6.65688e−31	Diptera	Limoniidae	Libnotes orofenaae
Thereva cf. turneri 'knyssa' voucher MEI 123850 16	DQ250854	82.99	92.31	6.65688e−31	Diptera	Therevidae	Thereva cf. turneri 'knyssa'
Nanexila argentiquadris 16S ribosomal RNA gene, pa	KM878934	80.89	99.36	2.32348e−30	Diptera	Therevidae	Nanexila argentiquadris
Evansomyia sp. SLW−2015 16S ribosomal RNA gene, pa	KM878913	81.65	99.36	2.32348e−30	Diptera	Therevidae	Evansomyia sp. SLW−2015
Bolopus furcatus isolate 1305 16S ribosomal RNA ge	KC699315	84.17	87.82	2.32348e−30	Diptera	Platypezidae	Bolopus furcatus
Evansomyia sp. 'scuTas' voucher MEI137414 16S ribo	KM212984	81.65	99.36	2.32348e−30	Diptera	Therevidae	Evansomyia sp. 'scuTas'
Ectinorhynchus micans voucher MEI30034 16S ribosom	KM212982	81.65	99.36	2.32348e−30	Diptera	Therevidae	Ectinorhynchus micans
Dichopygina aculeata voucher 947 16S ribosomal RNA	JQ613961	81.82	97.44	2.32348e−30	Diptera	Sciaridae	Dichopygina aculeata
Zariquieya troglodytes partial mitochondrial 16S r	FR846366	83.57	89.1	2.32348e−30	Coleoptera	Carabidae	Zariquieya troglodytes
Boletina erythropyga isolate 057363 16S ribosomal	HQ230383	81.63	94.23	2.32348e−30	Diptera	Mycetophilidae	Boletina erythropyga
Syntemna stylata 16S ribosomal RNA gene, partial s	FJ172013	83.09	87.18	2.32348e−30	Diptera	Mycetophilidae	Syntemna stylata
Pyratula zonata 16S ribosomal RNA gene, partial se	FJ172004	83.09	87.18	2.32348e−30	Diptera	Keroplatidae	Pyratula zonata
Libnotes perkinsi voucher O'Grady 200981 16S ribos	EU005433	81.88	100	2.32348e−30	Diptera	Limoniidae	Libnotes perkinsi
Libnotes perkinsi voucher O'Grady 200947 16S ribos	EU005432	81.88	100	2.32348e−30	Diptera	Limoniidae	Libnotes perkinsi
Panorpa takenouchii isolate 26tak 16S ribosomal RN	EF041520	83.21	87.82	2.32348e−30	Mecoptera	Panorpidae	Panorpa takenouchii
Panorpa takenouchii 16S ribosomal RNA gene, partia	AF180064	83.21	87.82	2.32348e−30	Mecoptera	Panorpidae	Panorpa takenouchii
Manestella ocellaris 16S ribosomal RNA gene, parti	KM878928	86.15	81.41	8.10974e−30	Diptera	Therevidae	Manestella ocellaris
Belosta albipilosa 16S ribosomal RNA gene, partial	KM878903	81.76	99.36	8.10974e−30	Diptera	Scenopinidae	Belosta albipilosa
Agathomyia viduella isolate AV01 16S ribosomal RNA	KC699317	80.77	99.36	8.10974e−30	Diptera	Platypezidae	Agathomyia viduella
Simulium tribulatum isolate LAC 17−5 16S ribosomal	KP233212	81.13	100	8.10974e−30	Diptera	Simuliidae	Simulium tribulatum
Evansomyia sp. 'CannR' voucher QMT183206 16S ribos	KM212978	82.76	91.03	8.10974e−30	Diptera	Therevidae	Evansomyia sp. 'CannR'
Mycetophila alea voucher JSB11 16S ribosomal RNA g	KJ136722	82.48	87.82	8.10974e−30	Diptera	Mycetophilidae	Mycetophila alea
Tabanus bromius 16S ribosomal RNA gene, partial se	HM132111	80	99.36	8.10974e−30	Diptera	Tabanidae	Tabanus bromius
Neopanorpa pulchra mitochondrion, complete genome	FJ169955	80.38	100	8.10974e−30	Mecoptera	Panorpidae	Neopanorpa pulchra
Blondelia nigripes mitochondrial gene for 16S rRNA	AB465902	82.55	94.23	8.10974e−30	Diptera	Tachinidae	Blondelia nigripes
Urytalpa macrocera 16S ribosomal RNA gene, partial	FJ172025	84.96	84.62	8.10974e−30	Diptera	Keroplatidae	Urytalpa macrocera
Paraperla frontalis 16S ribosomal RNA gene, partia	EF623136	81.01	100	8.10974e−30	Plecoptera	Chloroperlidae	Paraperla frontalis
Limonia jacobus voucher O'Grady 201508 16S ribosom	EU005410	80.25	100	8.10974e−30	Diptera	Limoniidae	Dicranomyia jacobus
Phorinia flava mitochondrial gene for 16S rRNA, pa	AB257236	81.94	98.72	8.10974e−30	Diptera	Tachinidae	Phorinia flava
Penniverpa sp. MEI 090271 16S ribosomal RNA gene,	DQ250826	80.77	99.36	8.10974e−30	Diptera	Therevidae	Penniverpa sp. MEI 090271
Scenopinus fenestralis 16S ribosomal RNA gene, par	KM878964	81.82	97.44	2.83058e−29	Diptera	Scenopinidae	Scenopinus fenestralis
Patanothrix wilsoni 16S ribosomal RNA gene, partia	KM878941	81.53	99.36	2.83058e−29	Diptera	Therevidae	Patanothrix wilsoni
Callomyia admirabilis isolate CA42 16S ribosomal R	KC699326	83.33	87.82	2.83058e−29	Diptera	Platypezidae	Callomyia admirabilis
Callomyia amoena isolate CA06 16S ribosomal RNA ge	KC699325	83.33	87.82	2.83058e−29	Diptera	Platypezidae	Callomyia amoena
Therevidae gen. P sp. 'barakula' voucher QMT183200	KM212977	81.01	99.36	2.83058e−29	Diptera	Therevidae	Therevidae gen. P sp. 'barakula'
Ectinorhynchus brunneus voucher QMT183204 16S ribo	KM212973	81.01	99.36	2.83058e−29	Diptera	Therevidae	Ectinorhynchus brunneus
Leptomorphus sp. DK−2013 voucher JSS1 16S ribosoma	KC435575	81.82	91.67	2.83058e−29	Diptera	Mycetophilidae	Leptomorphus sp. DK−2013
Boletina gripha isolate 057362 16S ribosomal RNA g	HQ230382	82.73	87.82	2.83058e−29	Diptera	Mycetophilidae	Boletina gripha
Leptomorphus walkeri 16S ribosomal RNA gene, parti	FJ172020	85.37	78.21	2.83058e−29	Diptera	Mycetophilidae	Leptomorphus walkeri
Pteronarcys californica 16S ribosomal RNA gene, pa	EF623261	79.49	100	2.83058e−29	Plecoptera	Pteronarcyidae	Pteronarcys californica
Limonia jacobus voucher O'Grady 201886 16S ribosom	EU005411	81.13	100	2.83058e−29	Diptera	Limoniidae	Dicranomyia jacobus
Simulium sheilae isolate SH2B 16S ribosomal RNA ge	KM410214	81.01	100	9.87969e−29	Diptera	Simuliidae	Simulium sheilae
