# Supplementary material for: Testing the potential of a ribosomal 16S marker for DNA metabarcoding of insects
Source: PeerJ. 2016 Apr 19;4:e1966. doi: 10.7717/peerj.1966 (PMC4841222; doi:10.7717/peerj.1966)
Supplement: Supplemental Information 1 [file peerj-04-1966-s006.zip › scripts/5 match OTUs/blast_hits/OTU_14.csv_plot.pdf]

OTU_14.csv

Sericostoma personatum

Sericostomatidae

Trichoptera

81.94

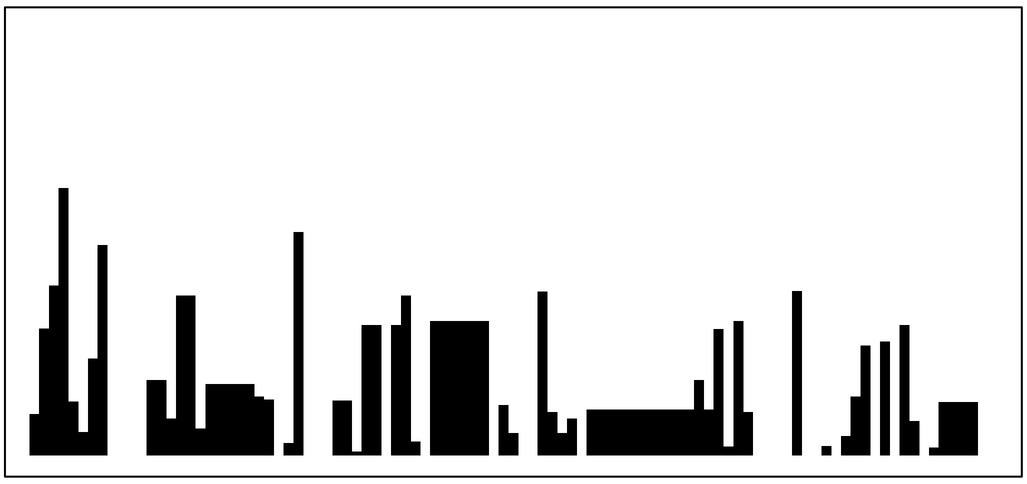

Sericostoma personatum mitochondrion, partial geno	KP455290	81.94	98.63	9.06319e−29	Trichoptera	Sericostomatidae	Sericostoma personatum
Notidobiella chacayana isolate CL5 16S ribosomal R	EF394993	85.95	82.19	1.10412e−27	Trichoptera	Sericostomatidae	Notidobiella chacayana
Sericostoma clypeatum voucher NHRS−EN CC4 16S ribo	FJ263207	87.96	73.97	1.3451e−26	Trichoptera	Sericostomatidae	Sericostoma clypeatum
Cardiocladius sp. MNK−2008 16S ribosomal RNA gene,	EU669992	92.54	45.89	2.27778e−17	Diptera	Chironomidae	Cardiocladius sp. MNK−2008
Xylosciara inornata voucher 230 16S ribosomal RNA	JQ613902	82.52	67.81	2.7749e−16	Diptera	Sciaridae	Xylosciara inornata
Caenota plicata isolate CR9 16S ribosomal RNA gene	EF395003	81.08	73.97	9.68535e−16	Trichoptera	Calocidae	Caenota plicata
Zygaena loti macedonica mitochondrial tRNA−Leu (CU	AJ831729	84.52	57.53	9.68535e−16	Lepidoptera	Zygaenidae	Zygaena loti macedonica
Pennisetia hylaeiformis mitochondrion, partial gen	KJ508049	89.86	47.26	3.38052e−15	Lepidoptera	Sesiidae	Pennisetia hylaeiformis
Trox hamatus isolate 83A 16S ribosomal RNA gene, p	EF570388	79.83	77.4	3.38052e−15	Coleoptera	Trogidae	Trox hamatus
Trox hamatus isolate 83B 16S ribosomal RNA gene, p	EF570381	79.83	77.4	3.38052e−15	Coleoptera	Trogidae	Trox hamatus
Trox spinulosis isolate 96B 16S ribosomal RNA gene	EF570376	79.83	77.4	3.38052e−15	Coleoptera	Trogidae	Trox spinulosis
Trox spinulosis isolate 96A 16S ribosomal RNA gene	EF570362	79.83	77.4	3.38052e−15	Coleoptera	Trogidae	Trox spinulosis
Bactrocera minax mitochondrion, complete genome	HM776033	83.52	60.27	3.38052e−15	Diptera	Tephritidae	Bactrocera minax
Bactrocera minax 16S ribosomal RNA gene, partial s	FJ866821	83.52	60.27	3.38052e−15	Diptera	Tephritidae	Bactrocera minax
Zygaena algira algira mitochondrial tRNA−Leu (CUN)	AJ831626	81.72	63.7	3.38052e−15	Lepidoptera	Zygaenidae	Zygaena algira algira
Protochauliodes biconicus incertus 16S ribosomal R	KF944494	87.5	49.32	1.17992e−14	Megaloptera	Corydalidae	Protochauliodes biconicus incertus
Protochauliodes incertus isolate H842 16S ribosoma	KF741322	87.5	49.32	1.17992e−14	Megaloptera	Corydalidae	Protochauliodes biconicus incertus
Hypocera mordellaria isolate 200864 16S ribosomal	GU362000	81.25	62.33	1.17992e−14	Diptera	Phoridae	Hypocera mordellaria
Zygaena ecki ecki mitochondrial tRNA−Leu (CUN) gen	AJ831721	83.33	57.53	1.17992e−14	Lepidoptera	Zygaenidae	Zygaena ecki ecki
Zygaena armena armena mitochondrial tRNA−Leu (CUN)	AJ831719	83.33	57.53	1.17992e−14	Lepidoptera	Zygaenidae	Zygaena armena armena
Zygaena transpamirina transpamirina mitochondrial	AJ831660	83.33	57.53	1.17992e−14	Lepidoptera	Zygaenidae	Zygaena transpamirina transpamirina
Zygaena transpamirina andarabensis mitochondrial t	AJ831659	83.33	57.53	1.17992e−14	Lepidoptera	Zygaenidae	Zygaena transpamirina andarabensis
Zygaena pamira pamira mitochondrial tRNA−Leu (CUN)	AJ831652	83.33	57.53	1.17992e−14	Lepidoptera	Zygaenidae	Zygaena pamira pamira
Zygaena hilaris escorialensis mitochondrial tRNA−L	AJ831643	82.76	59.59	1.17992e−14	Lepidoptera	Zygaenidae	Zygaena hilaris escorialensis
Diopsis nr. circularis KCADI065−14 16S ribosomal R	KT186651	82.61	58.9	4.11832e−14	Diptera	Diopsidae	Diopsis nr. circularis KCADI065−14
Polytremis lubricans voucher He160 16S ribosomal R	JX971171	80	69.86	4.11832e−14	Lepidoptera	Hesperiidae	Polytremis lubricans
Acordulecera sp. SS2 voucher AE142 16S ribosomal R	KF318505	80.58	69.18	4.11832e−14	Hymenoptera	Pergidae	Acordulecera sp. SS2
Bradysia atracornea voucher 133 16S ribosomal RNA	JQ613895	90.48	43.15	4.11832e−14	Diptera	Sciaridae	Bradysia atracornea
Chorisops tunisiae 16S ribosomal RNA gene, partial	GU947379	78.63	76.03	4.11832e−14	Diptera	Stratiomyidae	Chorisops tunisiae
Trox terrestris isolate 7B 16S ribosomal RNA gene,	EF570394	77.7	91.78	4.11832e−14	Coleoptera	Trogidae	Trox terrestris
Trox terrestris isolate 7A 16S ribosomal RNA gene,	EF570383	77.7	91.78	4.11832e−14	Coleoptera	Trogidae	Trox terrestris
Zygaena lavandulae consobrina mitochondrial tRNA−L	AJ831726	82.56	58.9	4.11832e−14	Lepidoptera	Zygaenidae	Zygaena lavandulae consobrina
Zygaena occitanica huescacola mitochondrial tRNA−L	AJ831649	82.56	58.9	4.11832e−14	Lepidoptera	Zygaenidae	Zygaena occitanica huescacola
Pliocaloca sp. CQ3 16S ribosomal RNA gene, partial	EF394999	80.18	73.97	4.11832e−14	Trichoptera	Calocidae	Pliocaloca sp. CQ3
Atelestus pulicarius isolate AP47 16S ribosomal RN	KC699323	86.11	49.32	1.43743e−13	Diptera	Atelestidae	Atelestus pulicarius
Keilbachia subacumina voucher 890 16S ribosomal RN	JQ613951	86.11	49.32	1.43743e−13	Diptera	Sciaridae	Keilbachia subacumina
Corynoptera micula voucher 792 16S ribosomal RNA g	JQ613934	78.4	81.51	1.43743e−13	Diptera	Sciaridae	Corynoptera micula
Pachygaster atra 16S ribosomal RNA gene, partial s	GU947397	86.11	49.32	1.43743e−13	Diptera	Stratiomyidae	Pachygaster atra
Drosophila neocordata voucher 107545 NADH dehydrog	EU494366	87.5	49.32	1.43743e−13	Diptera	Drosophilidae	Drosophila neocordata
Onthophagus pennsylvannicus 16S ribosomal RNA gene	EU162558	80.65	63.7	1.43743e−13	Coleoptera	Scarabaeidae	Onthophagus pennsylvanicus
Corydalus sp. EH−2004 16S ribosomal RNA gene, part	AY620139	79.81	70.55	1.43743e−13	Megaloptera	Corydalidae	Corydalus sp. EH−2004
Asteia concinna isolate A63 16S ribosomal RNA gene	KJ418481	86.3	49.32	5.01714e−13	Diptera	Asteiidae	Asteia concinna
Heliconius sapho voucher 8126 ; mitochondrial	KP073323	86.3	49.32	5.01714e−13	Lepidoptera	Nymphalidae	Heliconius sapho
Heliconius sapho voucher 2739 ; mitochondrial	KP073322	86.3	49.32	5.01714e−13	Lepidoptera	Nymphalidae	Heliconius sapho
Heliconius sapho sapho voucher 260 ; mitochondrial	KP073321	86.3	49.32	5.01714e−13	Lepidoptera	Nymphalidae	Heliconius sapho sapho
Heliconius eleuchia eleuchia voucher 190 ; mitocho	KP073274	86.3	49.32	5.01714e−13	Lepidoptera	Nymphalidae	Heliconius eleuchia eleuchia
Heliconius antiochus voucher antiochus_S ; mitocho	KP073253	86.3	49.32	5.01714e−13	Lepidoptera	Nymphalidae	Heliconius antiochus
Lestremiinae sp. DK−2014 voucher JSOUT6 16S riboso	KJ136728	75.97	86.3	5.01714e−13	Diptera	Cecidomyiidae	Lestremiinae sp. DK−2014
Carphurus sp. 2 NLG−2013 voucher COL358 12S riboso	KC524784	82.35	58.22	5.01714e−13	Coleoptera	Melyridae	Carphurus sp. 2 NLG−2013
Laccobius serratus 16S ribosomal RNA gene, partial	KC992690	81.05	63.7	5.01714e−13	Coleoptera	Hydrophilidae	Laccobius serratus
Eumanota humeralis voucher JSL5 16S ribosomal RNA	KC435572	76.8	81.51	5.01714e−13	Diptera	Mycetophilidae	Eumanota humeralis
Hydraena brevis mitochondrial 16S rRNA gene (parti	HE971007	77.27	83.56	5.01714e−13	Coleoptera	Hydraenidae	Hydraena brevis
Chaetoria stylata mitochondrial gene for 16S rRNA,	AB699938	87.67	49.32	5.01714e−13	Diptera	Tachinidae	Chaetoria stylata
Mycetophagus atomarius voucher UPOL ZL0150 16S rib	FJ903753	82.02	58.9	5.01714e−13	Coleoptera	Mycetophagidae	Mycetophagus atomarius
Echinocladius martini isolate M5 16S ribosomal RNA	EU669989	81.05	63.7	5.01714e−13	Diptera	Chironomidae	Echinocladius martini
Nepaloserica sp. BMNH 670901 16S large subunit rib	EF487902	81.72	59.59	5.01714e−13	Coleoptera	Scarabaeidae	Nepaloserica sp. BMNH 670901
Rivulophilus sakaii mitochondrial gene for 16S rib	AB355449	78.9	73.97	5.01714e−13	Trichoptera	Limnephilidae	Rivulophilus sakaii
Zygaena osterodensis validior mitochondrial tRNA−L	AJ831733	82.14	57.53	5.01714e−13	Lepidoptera	Zygaenidae	Zygaena osterodensis validior
Zygaena niphona niphona mitochondrial tRNA−Leu (CU	AJ831732	82.14	57.53	5.01714e−13	Lepidoptera	Zygaenidae	Zygaena niphona niphona
Zygaena tamara fahima mitochondrial tRNA−Leu (CUN)	AJ831715	82.14	57.53	5.01714e−13	Lepidoptera	Zygaenidae	Zygaena tamara fahima
Zygaena rubricollis ginnereissi mitochondrial tRNA	AJ831709	82.14	57.53	5.01714e−13	Lepidoptera	Zygaenidae	Zygaena rubricollis ginnereissi
Zygaena rubricollis flavicola mitochondrial tRNA−L	AJ831707	82.14	57.53	5.01714e−13	Lepidoptera	Zygaenidae	Zygaena rubricollis flavicola
Zygaena loyselis ungemachi mitochondrial tRNA−Leu	AJ831688	82.14	57.53	5.01714e−13	Lepidoptera	Zygaenidae	Zygaena loyselis ungemachi
Zygaena loyselis loyselis mitochondrial tRNA−Leu (	AJ831687	82.14	57.53	5.01714e−13	Lepidoptera	Zygaenidae	Zygaena loyselis loyselis
Zygaena hindukuschi cishindukuschi mitochondrial t	AJ831684	82.14	57.53	5.01714e−13	Lepidoptera	Zygaenidae	Zygaena hindukuschi cishindukuschi
Zygaena aisha mitochondrial tRNA−Leu (CUN) gene, 1	AJ831666	82.14	57.53	5.01714e−13	Lepidoptera	Zygaenidae	Zygaena aisha
Zygaena truchmena esseni mitochondrial tRNA−Leu (C	AJ831661	82.14	57.53	5.01714e−13	Lepidoptera	Zygaenidae	Zygaena truchmena esseni
Zygaena storaiae storaiae mitochondrial tRNA−Leu (	AJ831658	82.14	57.53	5.01714e−13	Lepidoptera	Zygaenidae	Zygaena storaiae storaiae
Zygaena johannae johannae mitochondrial partial 16	AJ831645	83.53	57.53	5.01714e−13	Lepidoptera	Zygaenidae	Zygaena johannae johannae
Zygaena cocandica minor mitochondrial tRNA−Leu (CU	AJ831632	82.14	57.53	5.01714e−13	Lepidoptera	Zygaenidae	Zygaena cocandica minor
Leptinotarsa juncta mitochondrial partial 16S rRNA	AJ841314	85.92	48.63	5.01714e−13	Coleoptera	Chrysomelidae	Leptinotarsa juncta
Tabanus rufofrater 16S ribosomal RNA gene, partial	AY140887	80.41	61.64	5.01714e−13	Diptera	Tabanidae	Tabanus rufofrater
Heliconius eleuchia isolate STRI−B−842 16S ribosom	AY747879	86.3	49.32	5.01714e−13	Lepidoptera	Nymphalidae	Heliconius eleuchia
Euproctis staudingeri voucher H192 large subunit r	KP082058	82.02	60.27	1.75115e−12	Lepidoptera	Lymantriidae	Euproctis staudingeri
Corydalus cornutus 16S ribosomal RNA gene, partial	KF944505	78.85	70.55	1.75115e−12	Megaloptera	Corydalidae	Corydalus cornutus
Corydalus flavicornis 16S ribosomal RNA gene, part	KF944504	78.85	70.55	1.75115e−12	Megaloptera	Corydalidae	Corydalus flavicornis
Polytremis lubricans voucher He095 16S ribosomal R	JX971170	79.09	69.86	1.75115e−12	Lepidoptera	Hesperiidae	Polytremis lubricans
Polytremis lubricans 16S ribosomal RNA gene, parti	KJ786310	79.09	69.86	1.75115e−12	Lepidoptera	Hesperiidae	Polytremis lubricans
Ditomyia fasciata voucher JSSJ3 16S ribosomal RNA	KJ136734	87.69	44.52	1.75115e−12	Diptera	Ditomyiidae	Ditomyia fasciata
Busalus sp. SS1 voucher AE003 16S ribosomal RNA ge	KF318463	79.81	71.23	1.75115e−12	Hymenoptera	Pergidae	Busalus sp. SS1
Arhopalus rusticus voucher HNAU.PP 006 16S ribosom	KF737722	79.79	60.27	1.75115e−12	Coleoptera	Cerambycidae	Arhopalus rusticus
Opitzia chiapas voucher 7167 16S ribosomal RNA gen	KC524881	80.43	59.59	1.75115e−12	Coleoptera	Cleridae	Opitzia chiapas
Ochthebius salinator mitochondrial partial 16S rRN	HF931436	78.85	67.81	1.75115e−12	Coleoptera	Hydraenidae	Ochthebius salinator
Ochthebius heydeni mitochondrial 16S rRNA gene (pa	HF931419	80.9	60.96	1.75115e−12	Coleoptera	Hydraenidae	Ochthebius heydeni
Limnebius hispanicus mitochondrial 16S rRNA gene (	HF931402	82.76	59.59	1.75115e−12	Coleoptera	Hydraenidae	Limnebius hispanicus
Cupido argiades mitochondrion, complete genome	KC310728	85.14	49.32	1.75115e−12	Lepidoptera	Lycaenidae	Cupido argiades
Bradysiopsis vittigera voucher 948 16S ribosomal R	JQ613962	79.38	62.33	1.75115e−12	Diptera	Sciaridae	Bradysiopsis vittigera
Trichosia confusa voucher 838 16S ribosomal RNA ge	JQ613942	85.33	49.32	1.75115e−12	Diptera	Sciaridae	Trichosia confusa
Chaetosciara sp. 3 SS−2012 voucher 216 16S ribosom	JQ613899	78.85	67.81	1.75115e−12	Diptera	Sciaridae	Chaetosciara sp. 3 SS−2012
Limnebius nitidus mitochondrial 16S rRNA gene (par	HE613698	86.11	49.32	1.75115e−12	Coleoptera	Hydraenidae	Limnebius nitidus
Spindasis sp. SSG−2012 isolate F562 16S ribosomal	JQ421910	81.61	58.9	1.75115e−12	Lepidoptera	Lycaenidae	Spindasis sp. SSG−2012
Tabanus maculicornis 16S ribosomal RNA gene, parti	HM132112	76.61	83.56	1.75115e−12	Diptera	Tabanidae	Tabanus maculicornis
Tamasia variegata mitochondrial 16S rRNA gene, spe	FN257669	80.37	72.6	1.75115e−12	Trichoptera	Calocidae	Tamasia variegata
Dasylepida ishigakiensis mitochondrial gene for 16	AB499119	82.5	54.79	1.75115e−12	Coleoptera	Scarabaeidae	Dasylepida ishigakiensis
Dasylepida ishigakiensis mitochondrial gene for 16	AB499118	82.5	54.79	1.75115e−12	Coleoptera	Scarabaeidae	Dasylepida ishigakiensis
Dasylepida ishigakiensis mitochondrial gene for 16	AB499117	82.5	54.79	1.75115e−12	Coleoptera	Scarabaeidae	Dasylepida ishigakiensis
Dasylepida ishigakiensis mitochondrial gene for 16	AB499116	82.5	54.79	1.75115e−12	Coleoptera	Scarabaeidae	Dasylepida ishigakiensis
Mongoloraphidia harmandi mitochondrion, complete g	FJ859902	78.18	71.92	1.75115e−12	Raphidioptera	Raphidiidae	Mongoloraphidia harmandi
Corydalus cornutus 16S ribosomal RNA gene, partial	EU734853	78.85	70.55	1.75115e−12	Megaloptera	Corydalidae	Corydalus cornutus
Corydalus cornutus mitochondrion, complete genome	FJ171323	78.85	70.55	1.75115e−12	Megaloptera	Corydalidae	Corydalus cornutus
