# Supplementary material for: Testing the potential of a ribosomal 16S marker for DNA metabarcoding of insects
Source: PeerJ. 2016 Apr 19;4:e1966. doi: 10.7717/peerj.1966 (PMC4841222; doi:10.7717/peerj.1966)
Supplement: Supplemental Information 1 [file peerj-04-1966-s006.zip › scripts/5 match OTUs/blast_hits/OTU_140.csv_plot.pdf]

OTU_140.csv

Epitonium ancillotoi

Epitoniidae

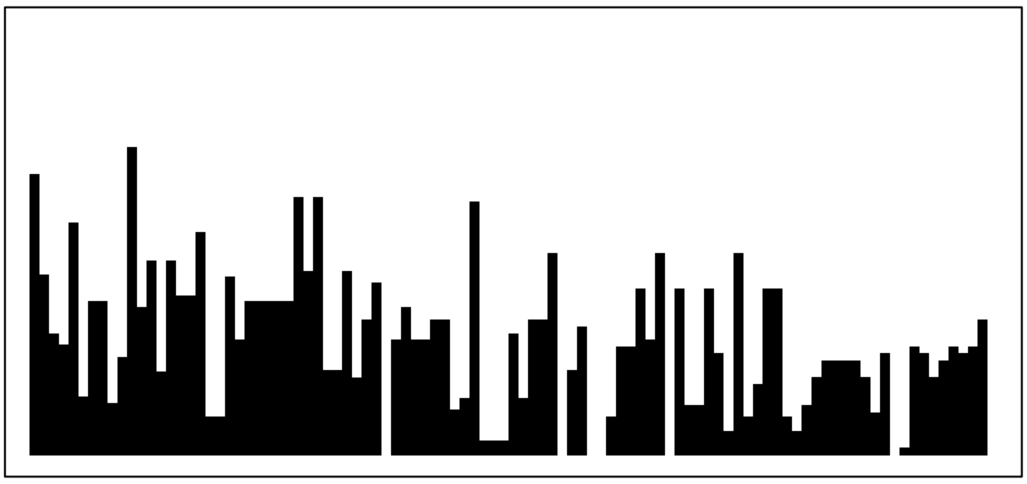

93.18

Epitonium ancillotoi voucher RMNH88484 16S ribosom	JF750949	93.18	29.33	1.3948e−07		Epitoniidae	Epitonium ancillotoi
Salina celebensis 16S ribosomal RNA gene, partial	KM978388	88.46	34.67	4.86833e−07		Paronellidae	Salina celebensis
Bugula bowiei voucher BRBRY−T5 16S ribosomal RNA g	KF714796	85.71	37.33	1.69921e−06	Cheilostomatida	Bugulidae	Bugula bowiei
Allorhogas ingavera strain CNIN1076 16S ribosomal	KJ586729	85.19	36	1.69921e−06	Hymenoptera	Braconidae	Allorhogas ingavera
Epitonium tinctum voucher UMMZ302904 16S ribosomal	JF750948	90.91	29.33	1.69921e−06		Epitoniidae	Epitonium tinctum
Apotolamprus quadrinotatus 16S ribosomal RNA gene,	DQ369612	82.76	38.67	5.93084e−06	Coleoptera	Scarabaeidae	Apotolamprus quadrinotatus
Eatoniella atropurpurea 16S ribosomal RNA gene, pa	KC109950	87.23	31.33	2.07007e−05		Eatoniellidae	Eatoniella atropurpurea
Eatoniella atropurpurea isolate HPL001 16S large s	JX970523	87.23	31.33	2.07007e−05		Eatoniellidae	Eatoniella atropurpurea
Apotolamprus quadrinotatus voucher Apo.quanot.10.1	EU247964	82.46	38	2.07007e−05	Coleoptera	Scarabaeidae	Apotolamprus quadrinotatus
Gomphiocephalus hodgsoni mitochondrion, complete g	AY191995	84.62	34.67	2.07007e−05		Hypogastruridae	Gomphiocephalus hodgsoni
Pseudosinella tumula 16S ribosomal RNA gene, parti	KC236210	94.44	24	7.22524e−05		Entomobryidae	Pseudosinella tumula
Drepanura sp. 1 FZ−2013 16S ribosomal RNA gene, pa	KC236193	86.96	30.67	7.22524e−05		Entomobryidae	Drepanura sp. 1 FZ−2013
Doryctes sp. JBW−2009 16S ribosomal RNA gene, part	GU135647	89.13	30.67	7.22524e−05	Hymenoptera	Braconidae	Doryctes sp. JBW−2009
Friesea grisea mitochondrion, complete genome	EU124719	83.93	36.67	7.22524e−05		Neanuridae	Friesea grisea
Podura aquatica mitochondrion, partial genome	AY639939	89.13	30.67	7.22524e−05		Poduridae	Podura aquatica
Sepia elegans 16S ribosomal RNA gene, partial sequ	AY293657	87.5	30.67	7.22524e−05	Sepiida	Sepiidae	Sepia elegans
Sepia elegans mitochondria lrRNA gene	X79591	87.5	30.67	7.22524e−05	Sepiida	Sepiidae	Sepia elegans
Laevicardium lobulatum voucher IM 200730151 16S ri	KR422958	90.48	28	0.000252186	Veneroida	Cardiidae	Laevicardium lobulatum
Gemmula periscelida isolate Gperisc0117025 large s	KM218647	81.82	36.67	0.000252186		Turridae	Gemmula periscelida
Gemmula periscelida isolate 2012−2 16S ribosomal R	KF915094	81.82	36.67	0.000252186		Turridae	Gemmula periscelida
Epitonium replicatum mitochondrial gene for 16S rR	AB930406	88.37	28.67	0.000252186		Epitoniidae	Epitonium replicatum
Tomocerus similis voucher C9674_1 16S ribosomal RN	KF591903	85.42	32	0.000252186		Tomoceridae	Tomocerus similis
Psyttalia fletcheri isolate UKM000141_57.3.4 16S r	KC594959	87.23	31.33	0.000252186	Hymenoptera	Braconidae	Psyttalia fletcheri
Psyttalia fletcheri isolate UKM000140_57.3.3 16S r	KC594958	87.23	31.33	0.000252186	Hymenoptera	Braconidae	Psyttalia fletcheri
Psyttalia fletcheri isolate UKM000139_57.3.2 16S r	KC594957	87.23	31.33	0.000252186	Hymenoptera	Braconidae	Psyttalia fletcheri
Psyttalia fletcheri isolate UKM000138_57.3.1 16S r	KC594956	87.23	31.33	0.000252186	Hymenoptera	Braconidae	Psyttalia fletcheri
Psyttalia fletcheri isolate UKM000136_57.1 16S rib	KC594954	87.23	31.33	0.000252186	Hymenoptera	Braconidae	Psyttalia fletcheri
Sinella cf. colorata FZ−2013 16S ribosomal RNA gen	KC236216	92.11	25.33	0.000252186		Entomobryidae	Sinella cf. colorata FZ−2013
Entomobrya sp. 9644 FZ−2013 16S ribosomal RNA gene	KC236194	88.64	29.33	0.000252186		Entomobryidae	Entomobrya sp. 9644 FZ−2013
Epidendrium sordidum voucher RMNH95286 16S ribosom	JF750945	92.11	25.33	0.000252186		Epitoniidae	Epidendrium sordidum
Anceya giraudi voucher 220.049a 16S ribosomal RNA	AY456584	84	33.33	0.000252186		Thiaridae	Anceya giraudi
Anceya giraudi voucher 220.000b 16S ribosomal RNA	AY456583	84	33.33	0.000252186		Thiaridae	Anceya giraudi
Fulvia lineonotata voucher UF 305206 16S ribosomal	KR422942	88.64	29.33	0.000880214	Veneroida	Cardiidae	Fulvia lineonotata
Alexania inazawai mitochondrial gene for 16S rRNA,	AB930407	83.64	35.33	0.000880214		Epitoniidae	Alexania inazawai
Allorhogas sp. CNIN786 16S ribosomal RNA gene, par	KJ586739	86.36	29.33	0.000880214	Hymenoptera	Braconidae	Allorhogas sp. CNIN786
Orchesellides sp. 9688−2 FZ−2013 16S ribosomal RNA	KC236217	88.1	28	0.000880214		Entomobryidae	Orchesellides sp. 9688−2 FZ−2013
Seira barnardi 16S ribosomal RNA gene, partial seq	KC236212	78.08	48	0.000880214		Entomobryidae	Seira barnardi
Heteromurus major 16S ribosomal RNA gene, partial	KC236201	85.42	32	0.000880214		Entomobryidae	Heteromurus major
Podura aquatica 16S ribosomal RNA gene, partial se	JN970977	86.96	30	0.000880214		Poduridae	Podura aquatica
Parisotoma notabilis 16S ribosomal RNA gene, parti	JN970975	85.42	32	0.000880214		Isotomidae	Parisotoma notabilis
Entomobrya lanuginosa 16S ribosomal RNA gene, part	JN970943	85.42	32	0.000880214		Entomobryidae	Entomobrya lanuginosa
Murex pecten mitochondrial partial 16S rRNA gene,	FN651903	86.36	29.33	0.000880214		Muricidae	Murex pecten
Brotia costula voucher ZMB:Moll 112783 16S ribosom	FJ377036	86.36	29.33	0.000880214		Pachychilidae	Brotia costula
Tylodinus sp. JJA−2007 isolate ZFMK−DNA JJ0220 16S	EU286362	82.14	37.33	0.000880214	Coleoptera	Curculionidae	Tylodinus sp. JJA−2007
Apotolamprus quadrinotatus voucher Apo.quanot.10.1	EU030538	82.69	34.67	0.000880214	Coleoptera	Scarabaeidae	Apotolamprus quadrinotatus
Lithobius variegatus rubriceps 16S ribosomal RNA g	AY084071	91.89	24.67	0.000880214	Lithobiomorpha	Lithobiidae	Lithobius variegatus rubriceps
Syrnolopsis minuta voucher 220.002x 16S ribosomal	AY456630	80.7	38	0.000880214		Thiaridae	Syrnolopsis minuta
Syrnolopsis lacustris voucher 220.046a 16S ribosom	AY456628	80.7	38	0.000880214		Thiaridae	Syrnolopsis lacustris
Syrnolopsis lacustris voucher 220.045a 16S ribosom	AY456627	80.7	38	0.000880214		Thiaridae	Syrnolopsis lacustris
Megalohelcon sp. 16S ribosomal RNA gene, partial s	AF003510	85.71	32	0.000880214	Hymenoptera	Braconidae	Megalohelcon sp.
Histeromerus mystacinus 16S ribosomal RNA gene, pa	AF003489	82.69	34.67	0.000880214	Hymenoptera	Braconidae	Histeromerus mystacinus
Metalabella curvicauda mitochondrial gene for 16S	AB052809	86.36	29.33	0.000880214	Dermaptera	Labiidae	Metalabella curvicauda
Brotia costula 16S ribosomal RNA gene, partial seq	DQ284986	86.36	29.33	0.000880214		Pachychilidae	Brotia costula
Acrilla acuminata 16S ribosomal RNA gene, partial	KP252993	89.47	25.33	0.00307225		Epitoniidae	Acrilla acuminata
Bursa rana 16S ribosomal RNA gene, partial sequenc	KP252985	79.31	38.67	0.00307225		Bursidae	Bursa rana
Caulibugula armata voucher BRBRY−Cau1 16S ribosoma	KF714806	84	33.33	0.00307225	Hemiptera	Reduviidae	Caulibugula armata
Abyssochrysos melanioides mitochondrial gene for 1	AB930403	86.05	28.67	0.00307225		Abyssochrysidae	Abyssochrysos melanioides
Haemaphysalis mageshimaensis mitochondrial gene fo	AB819212	79.69	40	0.00307225	Ixodida	Ixodidae	Haemaphysalis mageshimaensis
Haemaphysalis mageshimaensis mitochondrial gene fo	AB819211	79.69	40	0.00307225	Ixodida	Ixodidae	Haemaphysalis mageshimaensis
Xylobanus sp. UPOL A00076 voucher UPOLA00076 16S r	KC538752	81.82	36	0.00307225	Coleoptera	Lycidae	Xylobanus sp. UPOL A00076
Coecobrya communis 16S ribosomal RNA gene, partial	KC236218	85.11	29.33	0.00307225		Entomobryidae	Coecobrya communis
Coecobrya tenebricosa 16S ribosomal RNA gene, part	KC236191	85.11	29.33	0.00307225		Entomobryidae	Coecobrya tenebricosa
Epitonium jukesianum 16S ribosomal RNA gene, parti	KC109955	87.8	27.33	0.00307225		Epitoniidae	Epitonium jukesianum
Bowerbankia imbricata 16S ribosomal RNA (rrnL) gen	JN681072	85.42	32	0.00307225	Ctenostomatida	Vesiculariidae	Bowerbankia imbricata
Surrepifungium costulatum voucher RMNH95226 16S ri	JF750946	89.47	25.33	0.00307225		Epitoniidae	Surrepifungium costulatum
Bursa rana isolate LSGB22701 16S ribosomal RNA gen	HQ833983	79.31	38.67	0.00307225		Bursidae	Bursa rana
Arachnodes biimpressus voucher Ara.biim.5.1 16S ri	GQ341884	87.8	27.33	0.00307225	Coleoptera	Scarabaeidae	Arachnodes biimpressus
Apotolamprus sp. 1 HKW−2010 voucher Apo.VOHI4.52.1	GQ341878	82.35	34	0.00307225	Coleoptera	Scarabaeidae	Apotolamprus sp. 1 HKW−2010
Apotolamprus quadrimaculatus voucher Apo.quamac.5.	GQ341877	82.35	34	0.00307225	Coleoptera	Scarabaeidae	Apotolamprus quadrimaculatus
Brotia sumatrensis voucher ZMB:Moll 191693 16S rib	FJ377096	87.8	27.33	0.00307225		Pachychilidae	Brotia sumatrensis
Amblyomma geayi isolate 117373 16S ribosomal RNA g	EU805566	84.78	30.67	0.00307225	Ixodida	Ixodidae	Amblyomma geayi
Nanos bicoloratus voucher Nan.parpun.33.1 16S ribo	EU247994	81.13	35.33	0.00307225	Coleoptera	Scarabaeidae	Nanos bicoloratus
Euspira fusca 16S ribosomal RNA gene, partial sequ	EU332573	89.47	25.33	0.00307225		Naticidae	Euspira fusca
Episcopomantis chalybea isolate MN034 16S ribosoma	EF383329	81.82	36	0.00307225	Mantodea	Tarachodidae	Episcopomantis chalybea
Gromphas aeruginosa 16S ribosomal RNA gene, partia	AY131517	83.33	32	0.00307225	Coleoptera	Scarabaeidae	Gromphas aeruginosa
Brotia episcopalis voucher ZMB 200.119 16S ribosom	AY330785	87.8	27.33	0.00307225		Pachychilidae	Brotia episcopalis
Brotia episcopalis voucher ZMB 200.116 16S ribosom	AY330784	87.8	27.33	0.00307225		Pachychilidae	Brotia episcopalis
Episcopomantis chalybea isolate MN034 16S ribosoma	AY491123	81.82	36	0.00307225	Mantodea	Tarachodidae	Episcopomantis chalybea
Texola elada isolate 27−16 16S ribosomal RNA gene,	AF186878	81.13	35.33	0.00307225	Lepidoptera	Nymphalidae	Texola elada
Pleurocera acuta acuta 16S ribosomal RNA gene, par	AF100994	82.35	34	0.00307225		Pleuroceridae	Pleurocera acuta acuta
S.orbignyana mitochondria lrRNA gene	X79578	83.67	30.67	0.00307225	Sepiida	Sepiidae	Sepia orbignyana
Amblyomma longirostre isolate H23 16S ribosomal RN	KP835789	84.44	30	0.0107232	Ixodida	Ixodidae	Amblyomma longirostre
Amblyomma longirostre isolate H15 16S ribosomal RN	KP835788	84.44	30	0.0107232	Ixodida	Ixodidae	Amblyomma longirostre
Amblyomma longirostre isolate H24 16S ribosomal RN	KP835786	84.44	30	0.0107232	Ixodida	Ixodidae	Amblyomma longirostre
Amblyomma longirostre isolate H46 16S ribosomal RN	KP835785	84.44	30	0.0107232	Ixodida	Ixodidae	Amblyomma longirostre
Amblyomma mixtum isolate H08 16S ribosomal RNA gen	KP835781	83.67	32.67	0.0107232	Ixodida	Ixodidae	Amblyomma mixtum
Haemaphysalis asiatica 16S ribosomal RNA gene, par	KC170734	82	33.33	0.0107232	Ixodida	Ixodidae	Haemaphysalis asiatica
Haemaphysalis hystricis 16S ribosomal RNA gene, pa	KC170733	84.78	30.67	0.0107232	Ixodida	Ixodidae	Haemaphysalis hystricis
Conus nobilis skinneri 16S ribosomal RNA gene, par	KJ550692	80	36.67	0.0107232		Conidae	Conus nobilis skinneri
Conus melvilli voucher UF:367900 16S ribosomal RNA	KJ550669	80.36	35.33	0.0107232		Conidae	Conus melvilli
Allorhogas parvus strain ASDOR470 16S ribosomal RN	KJ586731	85.11	31.33	0.0107232	Hymenoptera	Braconidae	Allorhogas parvus
Haemaphysalis hystricis mitochondrial gene for 16S	AB819198	84.78	30.67	0.0107232	Ixodida	Ixodidae	Haemaphysalis hystricis
Haemaphysalis cornigera mitochondrial gene for 16S	AB819174	83.67	32	0.0107232	Ixodida	Ixodidae	Haemaphysalis cornigera
Amblyomma longirostre strain A25 16S ribosomal RNA	KF702347	84.44	30	0.0107232	Ixodida	Ixodidae	Amblyomma longirostre
Psyttalia incisi isolate UKM000090_34.3 16S riboso	JX233530	85.11	31.33	0.0107232	Hymenoptera	Braconidae	Psyttalia incisi
Haemaphysalis hystricis mitochondrion, partial gen	JX573137	84.78	30.67	0.0107232	Ixodida	Ixodidae	Haemaphysalis hystricis
Phaedrotoma sp. XYL−2012 isolate CVA4260 16S ribos	JQ736267	85.11	31.33	0.0107232	Hymenoptera	Braconidae	Phaedrotoma sp. XYL−2012
Phaedrotoma rudis isolate CVA4259 16S ribosomal RN	JQ736266	86.36	29.33	0.0107232	Hymenoptera	Braconidae	Phaedrotoma rudis
Bursa granularis isolate IM_2007_43069 16S ribosom	JX241128	78.95	38	0.0107232		Bursidae	Bursa granularis
Bursa granularis isolate IM_2007_43059 16S ribosom	JX241120	78.95	38	0.0107232		Bursidae	Bursa granularis
