# Supplementary material for: Testing the potential of a ribosomal 16S marker for DNA metabarcoding of insects
Source: PeerJ. 2016 Apr 19;4:e1966. doi: 10.7717/peerj.1966 (PMC4841222; doi:10.7717/peerj.1966)
Supplement: Supplemental Information 1 [file peerj-04-1966-s006.zip › scripts/5 match OTUs/blast_hits/OTU_141.csv_plot.pdf]

OTU_141.csv

uncultured organism

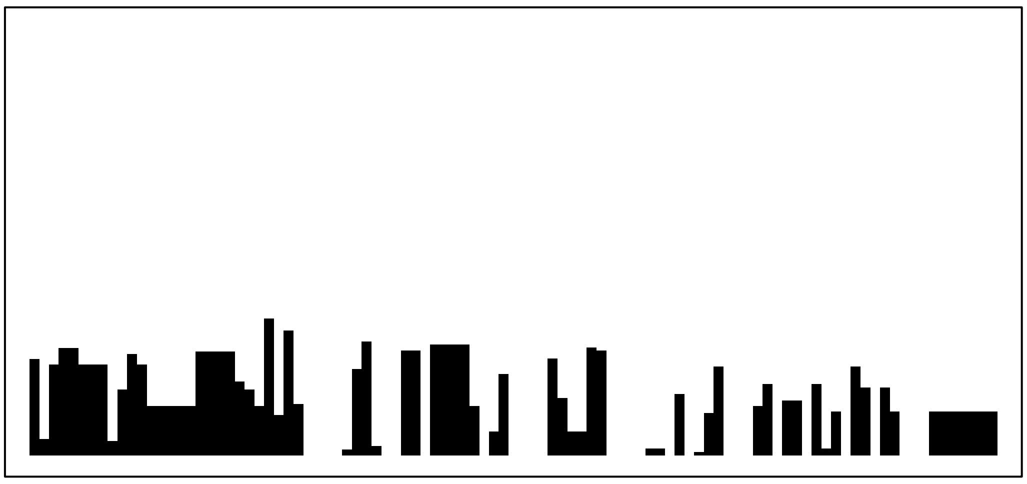

84.51

Uncultured organism isolate NR11 genomic sequence	EU548227	84.51	73.54	2.95716e−30			uncultured organism
Cardiocladius sp. MNK−2008 16S ribosomal RNA gene,	EU669992	80.77	77.25	1.25742e−28	Diptera	Chironomidae	Cardiocladius sp. MNK−2008
Aedes albopictus mitochondrion, complete genome	KR068634	84.25	66.14	5.34666e−27	Diptera	Culicidae	Aedes albopictus
Aedes flavopictus isolate A49 16S ribosomal RNA ge	DQ397929	85.04	66.14	5.34666e−27	Diptera	Culicidae	Aedes flavopictus
Aedes flavopictus isolate A47 16S ribosomal RNA ge	DQ397928	85.04	66.14	5.34666e−27	Diptera	Culicidae	Aedes flavopictus
Aedes albopictus isolate A21 16S ribosomal RNA gen	DQ397921	84.25	66.14	5.34666e−27	Diptera	Culicidae	Aedes albopictus
Aedes albopictus mitochondrion, complete genome	AY072044	84.25	66.14	5.34666e−27	Diptera	Culicidae	Aedes albopictus
Aedes albopictus mit−gene for large ribosomal subu	X01078	84.25	66.14	5.34666e−27	Diptera	Culicidae	Aedes albopictus
Culex bitaeniorhynchus 16S ribosomal RNA gene, par	EF593019	80.67	73.54	6.51357e−26	Diptera	Culicidae	Culex bitaeniorhynchus
Culex mimeticus 16S ribosomal RNA gene, partial se	EF593021	83.08	68.25	2.27346e−25	Diptera	Culicidae	Culex mimeticus
Aedes maehleri isolate A31 16S ribosomal RNA gene,	DQ397930	84.75	61.9	7.93515e−25	Diptera	Culicidae	Aedes maehleri
Aedes w−albus 16S ribosomal RNA gene, mitochondria	AF034472	84.25	66.14	7.93515e−25	Diptera	Culicidae	Aedes w−albus
Culex quinquefasciatus isolate A−270 16S ribosomal	EU711097	82.31	68.25	2.76964e−24	Diptera	Culicidae	Culex quinquefasciatus
Culex quinquefasciatus isolate A−267 16S ribosomal	EU711096	82.31	68.25	2.76964e−24	Diptera	Culicidae	Culex quinquefasciatus
Culex quinquefasciatus isolate A123 16S ribosomal	EU339145	82.31	68.25	2.76964e−24	Diptera	Culicidae	Culex quinquefasciatus
Culex tritaeniorhynchus 16S ribosomal RNA gene, pa	EF593020	82.31	68.25	2.76964e−24	Diptera	Culicidae	Culex tritaeniorhynchus
Culex vishnui 16S ribosomal RNA gene, partial sequ	EF593018	82.31	68.25	2.76964e−24	Diptera	Culicidae	Culex vishnui
Aedes riversi isolate A23 16S ribosomal RNA gene,	DQ397927	84.87	61.9	2.76964e−24	Diptera	Culicidae	Aedes riversi
Aedes riversi isolate A24 16S ribosomal RNA gene,	DQ397926	84.87	61.9	2.76964e−24	Diptera	Culicidae	Aedes riversi
Aedes dybasi isolate A28 16S ribosomal RNA gene, p	DQ397925	84.87	61.9	2.76964e−24	Diptera	Culicidae	Aedes dybasi
Aedes palauensis isolate A26 16S ribosomal RNA gen	DQ397924	84.87	61.9	2.76964e−24	Diptera	Culicidae	Aedes palauensis
Ochlerotatus japonicus isolate A81 16S ribosomal R	DQ397915	83.46	66.14	2.76964e−24	Diptera	Culicidae	Aedes japonicus
Culex bitaeniorhynchus 16S ribosomal RNA gene, mit	AF034474	83.08	68.25	2.76964e−24	Diptera	Culicidae	Culex bitaeniorhynchus
Culex tritaeniorhynchus 16S ribosomal RNA gene, mi	AF034469	82.31	68.25	2.76964e−24	Diptera	Culicidae	Culex tritaeniorhynchus
Promanota malaisei voucher JSL1 16S ribosomal RNA	KC435593	86.41	53.44	9.667e−24	Diptera	Mycetophilidae	Promanota malaisei
Aedes notoscriptus isolate MC04 mitochondrion, com	KM676218	81.89	66.14	3.37411e−23	Diptera	Culicidae	Aedes notoscriptus
Phytosciara ussuriensis voucher 883 16S ribosomal	JQ613948	85.85	56.08	3.37411e−23	Diptera	Sciaridae	Phytosciara ussuriensis
Armigeres subalbatus ASAP ID: 43761 large ribosoma	AY439688	82.4	65.61	3.37411e−23	Diptera	Culicidae	Armigeres subalbatus
Dideopsis aegrota 16S ribosomal RNA gene, partial	AF154774	79.47	77.78	3.37411e−23	Diptera	Syrphidae	Dideopsis aegrota
Dideopsis aegrota 16S ribosomal RNA gene, partial	AF154773	79.47	77.78	3.37411e−23	Diptera	Syrphidae	Dideopsis aegrota
Camptochaeta camptochaeta voucher 972 16S ribosoma	JQ613970	79.35	79.89	1.17768e−22	Diptera	Sciaridae	Camptochaeta camptochaeta
Megaselia scalaris 16S ribosomal RNA gene, partial	KC177442	79.35	79.89	1.17768e−22	Diptera	Phoridae	Megaselia scalaris
Neophryxe psychidis mitochondrial gene for 16S rRN	AB257232	80.27	76.72	1.17768e−22	Diptera	Tachinidae	Neophryxe psychidis
Aedes hensilli isolate A25 16S ribosomal RNA gene,	DQ397923	84.03	61.9	1.17768e−22	Diptera	Culicidae	Aedes hensilli
Aedes scutellaris isolate A29 16S ribosomal RNA ge	DQ397922	85.32	57.14	1.17768e−22	Diptera	Culicidae	Aedes scutellaris
Aedes esoensis isolate A83 16S ribosomal RNA gene,	DQ397916	80.43	71.43	1.17768e−22	Diptera	Culicidae	Aedes esoensis
Ochlerotatus nipponicus isolate A75 16S ribosomal	DQ397914	78.06	77.25	1.17768e−22	Diptera	Culicidae	Aedes nipponicus
Diasemopsis sp. W 16S ribosomal RNA gene, partial	AF304726	78.67	77.25	1.17768e−22	Diptera	Diopsidae	Diasemopsis sp. W
Lispe pygmaea 16S ribosomal RNA gene, partial sequ	JN226653	84.91	55.56	4.11051e−22	Diptera	Muscidae	Lispe pygmaea
Lispe leucospila 16S ribosomal RNA gene, partial s	JN226652	84.91	55.56	4.11051e−22	Diptera	Muscidae	Lispe leucospila
Sphyracephala munroi 16S ribosomal RNA gene, parti	AF304727	78.06	81.48	4.11051e−22	Diptera	Diopsidae	Sphyracephala munroi
Chalcosyrphus elongatus 16S ribosomal RNA gene, pa	AF154762	85.19	57.14	4.11051e−22	Diptera	Syrphidae	Chalcosyrphus elongatus
Chalcosyrphus elongatus 16S ribosomal RNA gene, pa	AF154761	85.19	57.14	4.11051e−22	Diptera	Syrphidae	Chalcosyrphus elongatus
Chalcosyrphus elongatus 16S ribosomal RNA gene, pa	AF154760	85.19	57.14	4.11051e−22	Diptera	Syrphidae	Chalcosyrphus elongatus
Chalcosyrphus elongatus 16S ribosomal RNA gene, pa	AF154759	85.19	57.14	4.11051e−22	Diptera	Syrphidae	Chalcosyrphus elongatus
Haemagogus janthinomys mitochondrion, complete gen	KT372555	82.31	68.25	1.43471e−21	Diptera	Culicidae	Haemagogus janthinomys
Diasemopsis aethiopica 16S ribosomal RNA gene, par	KT186657	78.12	80.95	1.43471e−21	Diptera	Diopsidae	Diasemopsis aethiopica
Aedes notoscriptus isolate MC10 mitochondrion, com	KM676219	81.1	66.14	1.43471e−21	Diptera	Culicidae	Aedes notoscriptus
Helina fratercula voucher RMBR:116199 16S ribosoma	KJ476309	83.81	55.56	1.43471e−21	Diptera	Muscidae	Helina fratercula
Diadocidia ferruginosa voucher SJ1 16S ribosomal R	KC435562	77.85	82.01	1.43471e−21	Diptera	Diadocidiidae	Diadocidia ferruginosa
Keilbachia subacumina voucher 890 16S ribosomal RN	JQ613951	74.36	97.88	1.43471e−21	Diptera	Sciaridae	Keilbachia subacumina
Corynoptera saetistyla voucher 876 16S ribosomal R	JQ613947	77.78	78.84	1.43471e−21	Diptera	Sciaridae	Corynoptera saetistyla
Cratyna ambigua voucher 777 16S ribosomal RNA gene	JQ613929	79.07	89.95	1.43471e−21	Diptera	Sciaridae	Cratyna ambigua
Bradysia chlorocornea voucher 736 16S ribosomal RN	JQ613928	84.55	57.67	1.43471e−21	Diptera	Sciaridae	Bradysia chlorocornea
Megaselia spiracularis isolate 200850 16S ribosoma	GU361991	82.68	66.14	1.43471e−21	Diptera	Phoridae	Megaselia spiracularis
Aedes galloisi isolate A87 16S ribosomal RNA gene,	DQ397920	81.1	66.14	1.43471e−21	Diptera	Culicidae	Aedes galloisi
Aedes galloisi isolate A77 16S ribosomal RNA gene,	DQ397919	81.1	66.14	1.43471e−21	Diptera	Culicidae	Aedes galloisi
Teleopsis pallifacies voucher RMNH.INS.555128 16S	KP978631	85.05	56.08	5.00763e−21	Diptera	Diopsidae	Teleopsis pallifacies
Pygophora unicolor voucher RMBR:116221 16S ribosom	KJ476328	84.91	55.56	5.00763e−21	Pygophora		Pygophora unicolor
Lestremiinae sp. DK−2014 voucher JSOUT6 16S riboso	KJ136728	77.12	76.72	5.00763e−21	Diptera	Cecidomyiidae	Lestremiinae sp. DK−2014
Simosyrphus grandicornis mitochondrion, complete g	DQ866050	78.71	77.78	5.00763e−21	Diptera	Syrphidae	Simosyrphus grandicornis
Diopsis sp. KCADI044−14 16S ribosomal RNA gene, pa	KT186663	77.63	77.25	1.74783e−20	Diptera	Diopsidae	Diopsis sp. KCADI044−14
Diopsis sp. BOLD:ACH1788 16S ribosomal RNA gene, p	KT186650	77.71	79.37	1.74783e−20	Diptera	Diopsidae	Diopsis sp. BOLD:ACH1788
Aedes vigilax isolate Mi139 mitochondrion, complet	KP995260	80.31	66.14	1.74783e−20	Diptera	Culicidae	Ochlerotatus vigilax
Ochlerotatus vigilax isolate Mi140 mitochondrion,	KP721463	80.31	66.14	1.74783e−20	Diptera	Culicidae	Ochlerotatus vigilax
Eumanota humeralis voucher JSL5 16S ribosomal RNA	KC435572	78.91	76.72	1.74783e−20	Diptera	Mycetophilidae	Eumanota humeralis
Spilogona dispar 16S ribosomal RNA gene, partial s	FJ025475	82.88	58.73	1.74783e−20	Diptera	Muscidae	Spilogona dispar
Culicoides arakawae mitochondrial DNA, complete ge	AB361004	77.07	81.48	1.74783e−20	Diptera	Ceratopogonidae	Culicoides arakawae
Aedes wadai isolate A85 16S ribosomal RNA gene, pa	DQ397918	80.15	69.31	1.74783e−20	Diptera	Culicidae	Aedes wadai
Somatia sp. YSUW04038 16S ribosomal RNA gene, part	AY573134	81.98	58.73	1.74783e−20	Diptera	Somatiidae	Somatia sp. YSUW04038
Arachnocampa luminosa biotype 3 16S large subunit	AY576331	84.16	53.44	1.74783e−20	Diptera	Keroplatidae	Arachnocampa luminosa
Diopsis longicornis 16S ribosomal RNA gene, partia	AF304745	77.63	77.25	1.74783e−20	Diptera	Diopsidae	Diopsis longicornis
Diopsis fumipennis 16S ribosomal RNA gene, partial	AF304743	77.71	79.37	1.74783e−20	Diptera	Diopsidae	Diopsis fumipennis
Dohrniphora trigonae 16S ribosomal RNA gene, parti	AF126343	78.71	79.89	1.74783e−20	Diptera	Phoridae	Dohrniphora trigonae
Megalabops sp. BOLD:ACP5917 16S ribosomal RNA gene	KT186670	82.3	59.26	6.10054e−20	Diptera	Diopsidae	Megalabops sp. BOLD:ACP5917
Teleopsis nr. sexguttata BOLD:ACP6074 voucher RMNH	KP978619	83.33	53.97	6.10054e−20	Diptera	Diopsidae	Teleopsis nr. sexguttata BOLD:ACP6074
Dinera takanoi mitochondrial gene for 16S rRNA, pa	AB465958	77.5	83.07	6.10054e−20	Diptera	Tachinidae	Dinera takanoi
Diasemopsis silvatica 16S ribosomal RNA gene, part	AF304716	82.57	56.61	6.10054e−20	Diptera	Diopsidae	Diasemopsis silvatica
Diasemopsis dubia 16S ribosomal RNA gene, partial	AF304715	82.57	56.61	6.10054e−20	Diptera	Diopsidae	Diasemopsis dubia
Diasemopsis meigenii 16S ribosomal RNA gene, parti	KT186666	77.78	79.37	2.1293e−19	Diptera	Diopsidae	Diasemopsis meigenii
Megalabops quadriguttata 16S ribosomal RNA gene, p	KT186655	83.33	56.08	2.1293e−19	Diptera	Diopsidae	Megalabops quadriguttata
Phytosciara semiferruginea voucher 220 16S ribosom	JQ613900	80.33	64.55	2.1293e−19	Diptera	Sciaridae	Phytosciara semiferruginea
Teleopsis dalmanni 16S ribosomal RNA gene, partial	KC177441	82.05	60.85	2.1293e−19	Diptera	Diopsidae	Teleopsis dalmanni
Fergusonina sp. LAN−2011 mitochondrion, complete g	HQ872008	76.32	79.37	2.1293e−19	Diptera	Fergusoninidae	Fergusonina taylori
Urytalpa macrocera 16S ribosomal RNA gene, partial	FJ172025	84.16	53.44	2.1293e−19	Diptera	Keroplatidae	Urytalpa macrocera
Coenosia tigrina 16S ribosomal RNA gene, partial s	FJ025427	83.18	55.56	2.1293e−19	Diptera	Muscidae	Coenosia tigrina
Leucophenga albofasciata voucher 109359 NADH dehyd	EU494303	77.93	74.6	2.1293e−19	Diptera	Drosophilidae	Leucophenga albofasciata
Arachnocampa luminosa biotype 2 16S large subunit	AY576330	83.17	53.44	2.1293e−19	Diptera	Keroplatidae	Arachnocampa luminosa
Cyrtodiopsis dalmanni 16S ribosomal RNA gene, part	AY123348	82.05	60.85	2.1293e−19	Diptera	Diopsidae	Teleopsis dalmanni
Diasemopsis elongata 16S ribosomal RNA gene, parti	AF304740	79.02	74.6	2.1293e−19	Diptera	Diopsidae	Diasemopsis elongata
Diasemopsis obstans 16S ribosomal RNA gene, partia	AF304718	76.82	77.25	2.1293e−19	Diptera	Diopsidae	Diasemopsis obstans
Chaetodiopsis meigenii 16S ribosomal RNA gene, par	AF304717	77.78	79.37	2.1293e−19	Diptera	Diopsidae	Diasemopsis meigenii
Cyrtodiopsis dalmanni isolate CdGombak7 16S riboso	AY876567	82.05	60.85	2.1293e−19	Diptera	Diopsidae	Teleopsis dalmanni
Cyrtodiopsis dalmanni isolate CdGombak6 16S riboso	AY876566	82.05	60.85	2.1293e−19	Diptera	Diopsidae	Teleopsis dalmanni
Cyrtodiopsis dalmanni isolate CdGombak5 16S riboso	AY876565	82.05	60.85	2.1293e−19	Diptera	Diopsidae	Teleopsis dalmanni
Cyrtodiopsis dalmanni isolate CdGombak4 16S riboso	AY876564	82.05	60.85	2.1293e−19	Diptera	Diopsidae	Teleopsis dalmanni
Cyrtodiopsis dalmanni isolate CdGombak3 16S riboso	AY876563	82.05	60.85	2.1293e−19	Diptera	Diopsidae	Teleopsis dalmanni
Cyrtodiopsis dalmanni isolate CdGombak2 16S riboso	AY876562	82.05	60.85	2.1293e−19	Diptera	Diopsidae	Teleopsis dalmanni
Cyrtodiopsis dalmanni isolate CdGombak1 16S riboso	AY876561	82.05	60.85	2.1293e−19	Diptera	Diopsidae	Teleopsis dalmanni
Diasemopsis comoroensis 16S ribosomal RNA gene, pa	AY910526	77.78	79.37	2.1293e−19	Diptera	Diopsidae	Diasemopsis comoroensis
