# Supplementary material for: Testing the potential of a ribosomal 16S marker for DNA metabarcoding of insects
Source: PeerJ. 2016 Apr 19;4:e1966. doi: 10.7717/peerj.1966 (PMC4841222; doi:10.7717/peerj.1966)
Supplement: Supplemental Information 1 [file peerj-04-1966-s006.zip › scripts/5 match OTUs/blast_hits/OTU_143.csv_plot.pdf]

OTU_143.csv

Rhithrogena sp. 13 LV−2015

Heptageniidae

Ephemeroptera

100

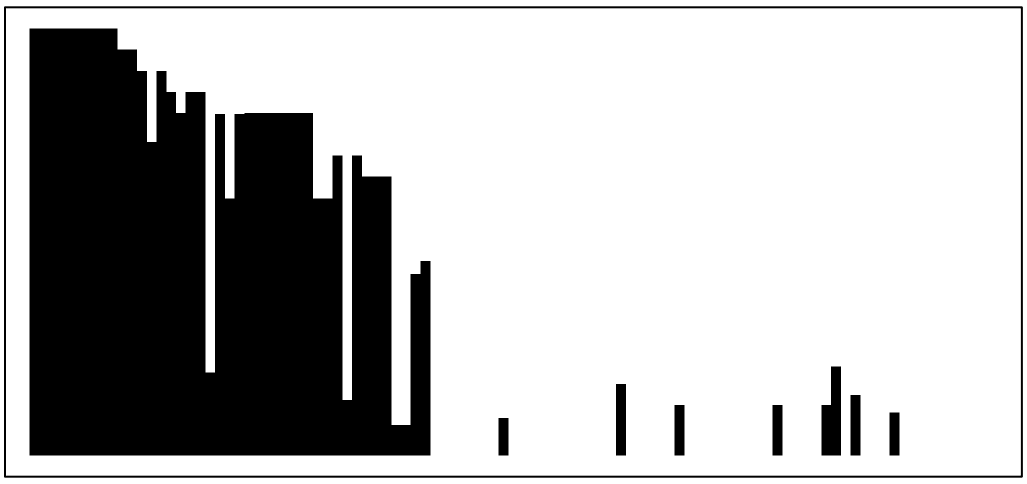

Rhithrogena sp. 13 LV−2015 mitochondrial partial 1	LN868672	100	64.74	7.58879e−43	Ephemeroptera	Heptageniidae	Rhithrogena sp. 13 LV−2015
Rhithrogena sp. 12 LV−2015 mitochondrial partial 1	LN868671	100	64.74	7.58879e−43	Ephemeroptera	Heptageniidae	Rhithrogena sp. 12 LV−2015
Rhithrogena picteti mitochondrial partial 16S rRNA	LN868663	100	64.74	7.58879e−43	Ephemeroptera	Heptageniidae	Rhithrogena picteti
Rhithrogena sp. 3 LV−2015 mitochondrial partial 16	LN868659	100	64.74	7.58879e−43	Ephemeroptera	Heptageniidae	Rhithrogena sp. 3 LV−2015
Rhithrogena sp. 2 LV−2015 mitochondrial partial 16	LN868654	100	64.74	7.58879e−43	Ephemeroptera	Heptageniidae	Rhithrogena sp. 2 LV−2015
Rhithrogena puytoraci mitochondrial partial 16S rR	LN868651	100	64.74	7.58879e−43	Ephemeroptera	Heptageniidae	Rhithrogena puytoraci
Rhithrogena iridina mitochondrial partial 16S rRNA	LN868646	100	64.74	7.58879e−43	Ephemeroptera	Heptageniidae	Rhithrogena iridina
Rhithrogena taurisca mitochondrial partial 16S rRN	LN868638	100	64.74	7.58879e−43	Ephemeroptera	Heptageniidae	Rhithrogena taurisca
Rhithrogena sp. 5 LV−2015 mitochondrial partial 16	LN868653	100	64.1	2.64875e−42	Ephemeroptera	Heptageniidae	Rhithrogena sp. 5 LV−2015
Rhithrogena semicolorata mitochondrial partial 16S	LN868673	99.01	64.74	3.22684e−41	Ephemeroptera	Heptageniidae	Rhithrogena semicolorata
Rhithrogena sp. 4 LV−2015 mitochondrial partial 16	LN868652	99.01	64.74	3.22684e−41	Ephemeroptera	Heptageniidae	Rhithrogena sp. 4 LV−2015
Rhithrogena fonticola mitochondrial partial 16S rR	LN868656	98.02	64.74	3.93109e−40	Ephemeroptera	Heptageniidae	Rhithrogena fonticola
Rhithrogena nivata mitochondrial partial 16S rRNA	LN868643	94.69	72.44	3.93109e−40	Ephemeroptera	Heptageniidae	Rhithrogena nivata
Rhithrogena sp. 7 LV−2015 mitochondrial partial 16	LN868669	98.02	64.74	4.78905e−39	Ephemeroptera	Heptageniidae	Rhithrogena sp. 7 LV−2015
Rhithrogena sp. 8 LV−2015 mitochondrial partial 16	LN868661	97.03	64.74	2.03636e−37	Ephemeroptera	Heptageniidae	Rhithrogena sp. 8 LV−2015
Rhithrogena savoiensis mitochondrial partial 16S r	LN868658	96.04	64.74	2.03636e−37	Ephemeroptera	Heptageniidae	Rhithrogena savoiensis
Rhithrogena sp. 9 LV−2015 mitochondrial partial 16	LN868650	97.03	64.74	2.03636e−37	Ephemeroptera	Heptageniidae	Rhithrogena sp. 9 LV−2015
Rhithrogena grischuna mitochondrial partial 16S rR	LN868639	97.03	64.74	2.03636e−37	Ephemeroptera	Heptageniidae	Rhithrogena grischuna
Rhithrogena insularis mitochondrial partial 16S rR	LN868665	83.87	99.36	7.10758e−37	Ephemeroptera	Heptageniidae	Rhithrogena insularis
Rhithrogena sp. 10 LV−2015 mitochondrial partial 1	LN868664	96	64.1	7.10758e−37	Ephemeroptera	Heptageniidae	Rhithrogena sp. 10 LV−2015
Rhithrogena germanica mitochondrial partial 16S rR	LN868660	92.04	72.44	7.10758e−37	Ephemeroptera	Heptageniidae	Rhithrogena germanica
Rhithrogena beskidensis mitochondrial partial 16S	LN868645	96	64.1	7.10758e−37	Ephemeroptera	Heptageniidae	Rhithrogena beskidensis
Rhithrogena degrangei mitochondrial partial 16S rR	LN868674	96.04	64.74	2.48079e−36	Ephemeroptera	Heptageniidae	Rhithrogena degrangei
Rhithrogena sp. 11 LV−2015 mitochondrial partial 1	LN868668	96.04	64.74	2.48079e−36	Ephemeroptera	Heptageniidae	Rhithrogena sp. 11 LV−2015
Rhithrogena alpestris mitochondrial partial 16S rR	LN868657	96.04	64.74	2.48079e−36	Ephemeroptera	Heptageniidae	Rhithrogena alpestris
Rhithrogena sp. 6 LV−2015 mitochondrial partial 16	LN868655	96.04	64.74	2.48079e−36	Ephemeroptera	Heptageniidae	Rhithrogena sp. 6 LV−2015
Rhithrogena hybrida mitochondrial partial 16S rRNA	LN868642	96.04	64.74	2.48079e−36	Ephemeroptera	Heptageniidae	Rhithrogena hybrida
Rhithrogena circumtatrica mitochondrial partial 16	LN868641	96.04	64.74	2.48079e−36	Ephemeroptera	Heptageniidae	Rhithrogena circumtatrica
Rhithrogena diensis mitochondrial partial 16S rRNA	LN868637	96.04	64.74	2.48079e−36	Ephemeroptera	Heptageniidae	Rhithrogena diensis
Rhithrogena gratianopolitana mitochondrial partial	LN868648	92.04	72.44	8.65881e−36	Ephemeroptera	Heptageniidae	Rhithrogena gratianopolitana
Rhithrogena gratianopolitana mitochondrial partial	LN868647	92.04	72.44	8.65881e−36	Ephemeroptera	Heptageniidae	Rhithrogena gratianopolitana
Rhithrogena sartorii mitochondrial partial 16S rRN	LN868670	94.06	64.74	1.05486e−34	Ephemeroptera	Heptageniidae	Rhithrogena sartorii
Rhithrogena nuragica mitochondrial partial 16S rRN	LN868667	82.58	99.36	3.68182e−34	Ephemeroptera	Heptageniidae	Rhithrogena nuragica
Rhithrogena eatoni mitochondrial partial 16S rRNA	LN868666	94.06	64.74	1.28508e−33	Ephemeroptera	Heptageniidae	Rhithrogena eatoni
Rhithrogena dorieri mitochondrial partial 16S rRNA	LN868649	93.07	64.74	4.48537e−33	Ephemeroptera	Heptageniidae	Rhithrogena dorieri
Rhithrogena corcontica mitochondrial partial 16S r	LN868662	93.07	64.74	5.4643e−32	Ephemeroptera	Heptageniidae	Rhithrogena corcontica
Rhithrogena landai mitochondrial partial 16S rRNA	LN868644	93.07	64.74	5.4643e−32	Ephemeroptera	Heptageniidae	Rhithrogena landai
Cinygmula sp. m185 mitochondrial partial 16S rRNA	LN868675	81.41	99.36	6.65688e−31	Ephemeroptera	Heptageniidae	Cinygmula sp. m185
Cinygmula sp. EP013 16S ribosomal RNA gene, partia	AY749760	81.41	99.36	6.65688e−31	Ephemeroptera	Heptageniidae	Cinygmula sp. EP013
Rhithrogena allobrogica mitochondrial partial 16S	LN868640	88.5	72.44	2.32348e−30	Ephemeroptera	Heptageniidae	Rhithrogena allobrogica
Rhithrogena sp. EP021 16S ribosomal RNA gene, part	AY749765	89.11	64.74	1.20359e−27	Ephemeroptera	Heptageniidae	Rhithrogena sp. EP021
Cinygma sp. EP123 16S ribosomal RNA gene, partial	AY749804	78.98	100	7.5955e−24	Ephemeroptera	Heptageniidae	Cinygma sp. EP123
Epeorus longimanus 16S ribosomal RNA gene, partial	AY749757	78.34	100	7.5955e−24	Ephemeroptera	Heptageniidae	Epeorus longimanus
Cloeon dipterum voucher EPH02 tRNA−Leu, 16S riboso	KF256908	78.48	100	2.65109e−23	Ephemeroptera	Baetidae	Cloeon dipterum
Tasmanophlebia sp. EP158 16S ribosomal RNA gene, p	AY749825	77.71	99.36	9.25321e−23	Ephemeroptera	Oniscigastridae	Tasmanophlebia sp. EP158
Epeorus sp. MT−2014 mitochondrion, complete genome	KM244708	78.48	100	3.22969e−22	Ephemeroptera	Heptageniidae	Epeorus sp. MT−2014
Siphlonurus sp. EP084 16S ribosomal RNA gene, part	AY749786	76.77	99.36	3.22969e−22	Ephemeroptera	Siphlonuridae	Siphlonurus sp. EP084
Capnura wanica 16S ribosomal RNA gene, partial seq	EF623128	77.22	99.36	3.93457e−21	Plecoptera	Capniidae	Capnura wanica
Paegniodes cupulatus mitochondrion, complete genom	HM004123	81.74	73.72	1.3733e−20	Ephemeroptera	Heptageniidae	Paegniodes cupulatus
Parameletus columbiae 16S ribosomal RNA gene, part	AY749768	76.92	99.36	1.3733e−20	Ephemeroptera	Siphlonuridae	Parameletus columbiae
Allocapnia recta haplotype 12ar: H12 16S ribosomal	EU306875	77.5	99.36	4.79328e−20	Plecoptera	Capniidae	Allocapnia recta
Siphlonella sp. EP083 16S ribosomal RNA gene, part	AY749785	76.43	99.36	4.79328e−20	Ephemeroptera	Oniscigastridae	Siphlonella sp. EP083
Austronemoura sp. BYU_PL149 16S ribosomal RNA gene	EF623200	76.43	99.36	1.67302e−19	Plecoptera	Notonemouridae	Austronemoura sp. BYU_PL149
Chiloporter sp. EP051 16S ribosomal RNA gene, part	AY749776	76.25	99.36	1.67302e−19	Ephemeroptera	Ameletopsidae	Chiloporter sp. EP051
Apteroperla tikumana mitochondrion, complete genom	KR604721	76.1	99.36	5.83941e−19	Plecoptera	Capniidae	Apteroperla tikumana
Mesocapnia arizonensis mitochondrion, partial geno	KP642637	76.1	99.36	5.83941e−19	Plecoptera	Capniidae	Mesocapnia arizonensis
Allocapnia recta isolate H13 16S ribosomal RNA gen	KC881048	76.1	99.36	5.83941e−19	Plecoptera	Capniidae	Allocapnia recta
Allocapnia recta isolate H11 16S ribosomal RNA gen	KC881046	76.1	99.36	5.83941e−19	Plecoptera	Capniidae	Allocapnia recta
Allocapnia recta isolate H7 16S ribosomal RNA gene	KC881042	76.1	99.36	5.83941e−19	Plecoptera	Capniidae	Allocapnia recta
Allocapnia recta isolate H3 16S ribosomal RNA gene	KC881038	76.1	99.36	5.83941e−19	Plecoptera	Capniidae	Allocapnia recta
Cultus pilatus 16S ribosomal RNA gene, partial seq	EF623212	83.33	65.38	5.83941e−19	Plecoptera	Perlodidae	Cultus pilatus
Mesocapnia sp. BYU_PL010 16S ribosomal RNA gene, p	EF623127	76.1	99.36	5.83941e−19	Plecoptera	Capniidae	Mesocapnia sp. BYU_PL010
Allocapnia recta haplotype 3ar: H03 16S ribosomal	DQ915181	76.1	99.36	5.83941e−19	Plecoptera	Capniidae	Allocapnia recta
Heptagenia sp. EP018 16S ribosomal RNA gene, parti	AY749762	77.36	99.36	5.83941e−19	Ephemeroptera	Heptageniidae	Heptagenia sp. EP018
Neopanorpa pulchra mitochondrion, complete genome	FJ169955	76.43	99.36	2.03816e−18	Mecoptera	Panorpidae	Neopanorpa pulchra
Siphlonurus sp. MT−2014 isolate CL142 mitochondrio	KM244684	74.84	99.36	7.11386e−18	Ephemeroptera	Siphlonuridae	Siphlonurus sp. MT−2014
Neopanorpa pulchra mitochondrion, complete genome	JX569848	82.35	65.38	7.11386e−18	Mecoptera	Panorpidae	Neopanorpa pulchra
Allocapnia recta isolate H15 16S ribosomal RNA gen	KC881050	75.47	99.36	7.11386e−18	Plecoptera	Capniidae	Allocapnia recta
Allocapnia recta isolate H14 16S ribosomal RNA gen	KC881049	75.47	99.36	7.11386e−18	Plecoptera	Capniidae	Allocapnia recta
Allocapnia recta isolate H10 16S ribosomal RNA gen	KC881045	75.47	99.36	7.11386e−18	Plecoptera	Capniidae	Allocapnia recta
Allocapnia recta isolate H6 16S ribosomal RNA gene	KC881041	75.47	99.36	7.11386e−18	Plecoptera	Capniidae	Allocapnia recta
Allocapnia recta isolate H1 16S ribosomal RNA gene	KC881036	75.47	99.36	7.11386e−18	Plecoptera	Capniidae	Allocapnia recta
Sicyoptera dilatata isolate SDGG01 16S ribosomal R	JX294163	75.78	100	7.11386e−18	Neuroptera	Nemopteridae	Sicyoptera dilatata
Cryptoperla ishigakiensis mitochondrial gene for 1	AB711242	75.95	99.36	7.11386e−18	Plecoptera	Peltoperlidae	Cryptoperla ishigakiensis
Cryptoperla japonica mitochondrial gene for 16S rR	AB711184	75.95	99.36	7.11386e−18	Plecoptera	Peltoperlidae	Cryptoperla japonica
Pelurgoperla personata 16S ribosomal RNA gene, par	EF623168	75.95	99.36	7.11386e−18	Plecoptera	Gripopterygidae	Pelurgoperla personata
Thaumatoperla sp. BYU_PL109 16S ribosomal RNA gene	EF623149	82.35	65.38	7.11386e−18	Plecoptera	Eustheniidae	Thaumatoperla sp. BYU_PL109
Allocapnia minima 16S ribosomal RNA gene, partial	EF623130	75.47	99.36	7.11386e−18	Plecoptera	Capniidae	Allocapnia minima
Penturoperla barbata 16S ribosomal RNA gene, parti	EF623124	76.71	93.59	7.11386e−18	Plecoptera	Austroperlidae	Penturoperla barbata
Allocapnia recta haplotype 11ar: H011 16S ribosoma	EU306874	75.47	99.36	7.11386e−18	Plecoptera	Capniidae	Allocapnia recta
Allocapnia recta haplotype 7ar: H07 16S ribosomal	EU306870	75.47	99.36	7.11386e−18	Plecoptera	Capniidae	Allocapnia recta
Neopanorpa sp. M24899 16S ribosomal RNA gene, part	AF180059	82.35	65.38	7.11386e−18	Mecoptera	Panorpidae	Neopanorpa sp. M24899
Simocephalus sp. EIZ−2011 isolate Brb1−Sim1 16S ri	JN874613	84.16	63.46	2.48298e−17	Diplostraca	Daphniidae	Simocephalus sp. EIZ−2011
Oniscigaster distans 16S ribosomal RNA gene, parti	AY749818	75	99.36	2.48298e−17	Ephemeroptera	Oniscigastridae	Oniscigaster distans
Arthroplea bipunctata 16S ribosomal RNA gene, part	AY749817	82.83	63.46	2.48298e−17	Ephemeroptera	Arthropleidae	Arthroplea bipunctata
Bidessodes sp. MB−2009 mitochondrial DNA sequence	FN546264	75.93	100	8.66646e−17	Coleoptera	Dytiscidae	Bidessodes sp. MB−2009
Bidessodes sp. MB−2009 mitochondrial DNA sequence	FN546263	75.93	100	8.66646e−17	Coleoptera	Dytiscidae	Bidessodes sp. MB−2009
Pteronarcys californica 16S ribosomal RNA gene, pa	EF623261	74.84	99.36	8.66646e−17	Plecoptera	Pteronarcyidae	Pteronarcys californica
Remenus bilobatus 16S ribosomal RNA gene, partial	EF623233	82	64.1	8.66646e−17	Plecoptera	Perlodidae	Remenus bilobatus
Kimminsoperla albomacula 16S ribosomal RNA gene, p	EF623202	75.32	99.36	8.66646e−17	Plecoptera	Notonemouridae	Kimminsoperla albomacula
Claudioperla sp. BYU_PL158 16S ribosomal RNA gene,	EF623169	75.95	99.36	8.66646e−17	Plecoptera	Gripopterygidae	Claudioperla sp. BYU_PL158
Neuroperlopsis patris 16S ribosomal RNA gene, part	EF623152	75.16	99.36	8.66646e−17	Plecoptera	Eustheniidae	Neuroperlopsis patris
Paraperla frontalis 16S ribosomal RNA gene, partia	EF623136	75.32	99.36	8.66646e−17	Plecoptera	Chloroperlidae	Paraperla frontalis
Allocapnia recta isolate H19 16S ribosomal RNA gen	KC881054	74.84	99.36	3.02489e−16	Plecoptera	Capniidae	Allocapnia recta
Allocapnia recta isolate H17 16S ribosomal RNA gen	KC881052	74.84	99.36	3.02489e−16	Plecoptera	Capniidae	Allocapnia recta
Allocapnia recta isolate H16 16S ribosomal RNA gen	KC881051	74.84	99.36	3.02489e−16	Plecoptera	Capniidae	Allocapnia recta
Allocapnia recta isolate H12 16S ribosomal RNA gen	KC881047	74.84	99.36	3.02489e−16	Plecoptera	Capniidae	Allocapnia recta
Allocapnia recta isolate H9 16S ribosomal RNA gene	KC881044	74.84	99.36	3.02489e−16	Plecoptera	Capniidae	Allocapnia recta
Allocapnia recta isolate H8 16S ribosomal RNA gene	KC881043	74.84	99.36	3.02489e−16	Plecoptera	Capniidae	Allocapnia recta
Allocapnia recta isolate H5 16S ribosomal RNA gene	KC881040	74.84	99.36	3.02489e−16	Plecoptera	Capniidae	Allocapnia recta
