# Supplementary material for: Testing the potential of a ribosomal 16S marker for DNA metabarcoding of insects
Source: PeerJ. 2016 Apr 19;4:e1966. doi: 10.7717/peerj.1966 (PMC4841222; doi:10.7717/peerj.1966)
Supplement: Supplemental Information 1 [file peerj-04-1966-s006.zip › scripts/5 match OTUs/blast_hits/OTU_144.csv_plot.pdf]

OTU_144.csv

Plutella xylostella

Plutellidae

Lepidoptera

66.84

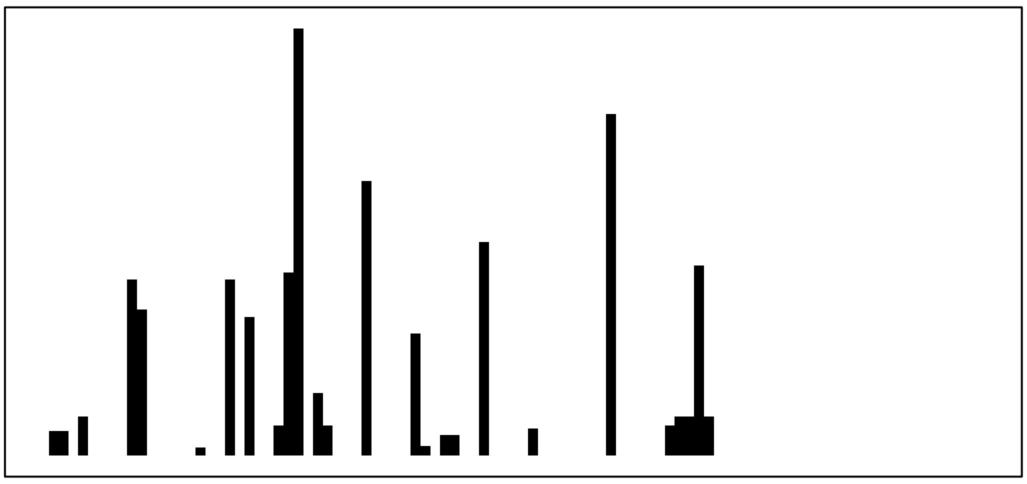

Plutella xylostella mitochondrion, partial genome	JF911819	66.84	90.4	0.00108687	Lepidoptera	Plutellidae	Plutella xylostella
Apteryx australis mantelli genome assembly AptMant	LK065141	76.62	42.94	0.0132408	Apterygiformes	Apterygidae	Apteryx australis mantelli
Brugia pahangi genome assembly B_pahangi_Glasgow ,	LK964334	81.13	28.25	0.0132408	Spirurida	Onchocercidae	Brugia pahangi
Brugia timori genome assembly B_timori_Indonesia_v	LK914412	81.13	28.25	0.0132408	Spirurida	Onchocercidae	Brugia timori
Schizaphis graminum mitochondrion, complete genome	AY531391	69.36	93.22	0.0132408	Hemiptera	Aphididae	Schizaphis graminum
Ascaris lumbricoides genome assembly A_lumbricoide	LK874853	81.82	30.51	0.046215	Ascaridida	Ascarididae	Ascaris lumbricoides
Trifolium pratense genome assembly redclover, chro	LN846352	70.23	70.62	0.161306	Fabales	Fabaceae	Trifolium pratense
Ascaris lumbricoides genome assembly A_lumbricoide	LK876664	70.42	74.58	0.161306	Ascaridida	Ascarididae	Ascaris lumbricoides
Cyprinus carpio genome assembly common carp genome	LN595271	76.92	42.37	0.161306	Cypriniformes	Cyprinidae	Cyprinus carpio
Methanobacterium lacus strain AL−21, complete geno	CP002551	74.12	46.33	0.161306	Methanobacteriales	Methanobacteriaceae	Methanobacterium lacus
PREDICTED: Mus musculus predicted gene, 34476 (Gm3	XR_396989	88.24	19.21	0.563014	Rodentia	Muridae	Mus musculus
Frischella perrara strain PEB0191, complete genome	CP009056	86.84	21.47	0.563014	Orbales	Orbaceae	Frischella perrara
Mycoplasma mycoides subsp. mycoides strain izsam_m	CP010267	79.66	31.07	0.563014	Mycoplasmatales	Mycoplasmataceae	Mycoplasma mycoides subsp. mycoides
Ascaris lumbricoides genome assembly A_lumbricoide	LK878731	70.45	68.36	0.563014	Ascaridida	Ascarididae	Ascaris lumbricoides
Mycoplasma leachii 99/014/6 complete genome	FR668087	79.66	31.07	0.563014	Mycoplasmatales	Mycoplasmataceae	Mycoplasma leachii 99/014/6
Mycoplasma leachii PG50 clone MU clone A8, complet	CP002108	79.66	31.07	0.563014	Mycoplasmatales	Mycoplasmataceae	Mycoplasma leachii PG50
Mycoplasma mycoides subsp. mycoides SC str. Gladys	CP002107	79.66	31.07	0.563014	Mycoplasmatales	Mycoplasmataceae	Mycoplasma mycoides subsp. mycoides SC str. Gladysdale
Xenorhabdus bovienii SS−2004 chromosome, complete	FN667741	80.36	28.81	0.563014	Enterobacteriales	Enterobacteriaceae	Xenorhabdus bovienii SS−2004
Mycoplasma mycoides subsp. mycoides SC str. PG1, c	BX293980	79.66	31.07	0.563014	Mycoplasmatales	Mycoplasmataceae	Mycoplasma mycoides subsp. mycoides SC str. PG1
Vitis vinifera contig VV78X072460.8, whole genome	AM478890	75.95	44.07	0.563014	Vitales	Vitaceae	Vitis vinifera
Mus musculus BAC clone RP24−261H7 from chromosome	AC124681	88.24	19.21	0.563014	Rodentia	Muridae	Mus musculus
Mycoplasma capricolum subsp. capricolum ATCC 27343	CP000123	79.66	31.07	0.563014	Mycoplasmatales	Mycoplasmataceae	Mycoplasma capricolum subsp. capricolum ATCC 27343
Zebrafish DNA sequence from clone CH211−207O21 in	AL831789	86.49	20.9	0.563014	Cypriniformes	Cyprinidae	Danio rerio
Cucumis melo genomic chromosome, chr_5	LN713259	74.39	45.76	1.96511	Cucurbitales	Cucurbitaceae	Cucumis melo
Cucumis melo genomic scaffold, anchoredscaffold000	LN681846	74.39	45.76	1.96511	Cucurbitales	Cucurbitaceae	Cucumis melo
Schistosoma curassoni genome assembly S_curassoni_	LM072165	81.4	24.29	1.96511	Strigeidida	Schistosomatidae	Schistosoma curassoni
Soboliphyme baturini genome assembly S_baturini_Da	LM019164	88.57	19.21	1.96511		Soboliphymatidae	Soboliphyme baturini
Strongyloides stercoralis genome assembly S_sterco	LL999118	100	12.99	1.96511	Rhabditida	Strongyloididae	Strongyloides stercoralis
Dracunculus medinensis genome assembly D_medinensi	LK978249	71.43	55.37	1.96511	Spirurida	Dracunculidae	Dracunculus medinensis
Dracunculus medinensis genome assembly D_medinensi	LK978198	82.93	23.16	1.96511	Spirurida	Dracunculidae	Dracunculus medinensis
Schistosoma mattheei genome assembly S_mattheei_De	LM164032	81.4	24.29	1.96511	Strigeidida	Schistosomatidae	Schistosoma mattheei
Elaeophora elaphi genome assembly E_elaphi ,scaffo	LL716714	78.18	30.51	1.96511	Spirurida	Onchocercidae	Elaeophora elaphi
Solanum pennellii chromosome ch05, complete genome	HG975444	74.29	39.55	1.96511	Solanales	Solanaceae	Solanum pennellii
Zebrafish DNA sequence from clone DKEYP−30F1 in li	BX321882	74.03	42.37	1.96511	Cypriniformes	Cyprinidae	Danio rerio
Brassica rapa subsp. pekinensis clone KBrH006E24,	AC189555	92.86	15.82	1.96511	Brassicales	Brassicaceae	Brassica rapa subsp. pekinensis
Vitis vinifera contig VV78X245481.12, whole genome	AM456182	78.57	26.55	1.96511	Vitales	Vitaceae	Vitis vinifera
Human DNA sequence from clone RP11−33J1 on chromos	AL645488	75.34	39.55	1.96511	Primates	Hominidae	Homo sapiens
Trifolium pratense genome assembly redclover, chro	LN846350	79.59	24.86	6.85892	Fabales	Fabaceae	Trifolium pratense
Mycoplasma capricolum subsp. capripneumoniae 87001	CP006959	77.59	31.07	6.85892	Mycoplasmatales	Mycoplasmataceae	Mycoplasma capricolum subsp. capripneumoniae 87001
PREDICTED: Corvus cornix cornix uncharacterized LO	XR_752022	85.71	19.77	6.85892	Passeriformes	Corvidae	Corvus cornix cornix
Protopolystoma xenopodis genome assembly P_xenopod	LM810099	80.43	24.86	6.85892	Polyopisthocotylea	Polystomatidae	Protopolystoma xenopodis
Protopolystoma xenopodis genome assembly P_xenopod	LM773081	80	25.42	6.85892	Polyopisthocotylea	Polystomatidae	Protopolystoma xenopodis
Schistosoma mattheei genome assembly S_mattheei_De	LM206825	80.95	23.73	6.85892	Strigeidida	Schistosomatidae	Schistosoma mattheei
Schistosoma curassoni genome assembly S_curassoni_	LM065117	80.95	23.73	6.85892	Strigeidida	Schistosomatidae	Schistosoma curassoni
Wuchereria bancrofti genome assembly W_bancrofti_J	LM005922	79.59	27.68	6.85892	Spirurida	Onchocercidae	Wuchereria bancrofti
Onchocerca ochengi genome assembly O_ochengi_Ngaou	LL517968	71.57	54.8	6.85892	Spirurida	Onchocercidae	Onchocerca ochengi
Syphacia muris genome assembly S_muris_Valencia ,s	LK996248	90	16.95	6.85892	Oxyurida	Oxyuridae	Syphacia muris
Dracunculus medinensis genome assembly D_medinensi	LK978205	78.69	31.64	6.85892	Spirurida	Dracunculidae	Dracunculus medinensis
Dicrocoelium dendriticum genome assembly D_dendrit	LK466331	80	25.42	6.85892	Plagiorchiida	Dicrocoeliidae	Dicrocoelium dendriticum
Dicrocoelium dendriticum genome assembly D_dendrit	LK405146	80	25.42	6.85892	Plagiorchiida	Dicrocoeliidae	Dicrocoelium dendriticum
Protopolystoma xenopodis genome assembly P_xenopod	LM732776	70.63	68.93	6.85892	Polyopisthocotylea	Polystomatidae	Protopolystoma xenopodis
Parastrongyloides trichosuri genome assembly P_tri	LM523168	81.25	27.12	6.85892	Rhabditida	Strongyloididae	Parastrongyloides trichosuri
Strongyloides ratti genome assembly S_ratti_ED321	LN609529	72.55	55.93	6.85892	Rhabditida	Strongyloididae	Strongyloides ratti
Mycoplasma capricolum subsp. capripneumoniae genom	LN515399	77.59	31.07	6.85892	Mycoplasmatales	Mycoplasmataceae	Mycoplasma capricolum subsp. capripneumoniae
Mycoplasma capricolum subsp. capripneumoniae genom	LN515398	77.59	31.07	6.85892	Mycoplasmatales	Mycoplasmataceae	Mycoplasma capricolum subsp. capripneumoniae
Mycoplasma capricolum subsp. capripneumoniae genom	LM995445	77.59	31.07	6.85892	Mycoplasmatales	Mycoplasmataceae	Mycoplasma capricolum subsp. capripneumoniae
Elaeophora elaphi genome assembly E_elaphi ,scaffo	LL711924	80	23.16	6.85892	Spirurida	Onchocercidae	Elaeophora elaphi
Solanum pennellii chromosome ch07, complete genome	HG975446	71.26	49.15	6.85892	Solanales	Solanaceae	Solanum pennellii
Solanum pennellii chromosome ch01, complete genome	HG975440	68.8	67.8	6.85892	Solanales	Solanaceae	Solanum pennellii
Schistosoma mansoni strain Puerto Rico chromosome	HE601626	96	14.12	6.85892	Strigeidida	Schistosomatidae	Schistosoma mansoni
Glycine max strain Williams 82 clone GM_WBb0115I18	AC235386	78.72	26.55	6.85892	Fabales	Fabaceae	Glycine max
Glycine max strain Williams 82 clone GM_WBb0083K09	AC235335	78.72	26.55	6.85892	Fabales	Fabaceae	Glycine max
Vitis vinifera contig VV78X037436.6, whole genome	AM475186	69.92	75.14	6.85892	Vitales	Vitaceae	Vitis vinifera
Vitis vinifera contig VV78X057287.4, whole genome	AM445017	70.09	55.93	6.85892	Vitales	Vitaceae	Vitis vinifera
Paramecium tetraurelia hypothetical protein (GSPAT	XM_001461834	73.85	32.77	6.85892	Peniculida	Parameciidae	Paramecium tetraurelia strain d4−2
Paramecium tetraurelia hypothetical protein (GSPAT	XM_001441888	81.4	22.03	6.85892	Peniculida	Parameciidae	Paramecium tetraurelia strain d4−2
Mus musculus BAC clone RP23−122B3 from 1, complete	AC098736	81.82	23.73	6.85892	Rodentia	Muridae	Mus musculus
Homo sapiens chromosome 5 clone RP11−395N10, compl	AC092363	81.82	24.86	6.85892	Primates	Hominidae	Homo sapiens
Medicago truncatula chromosome 2 BAC clone mth2−65	AC158498	88.89	19.77	6.85892	Fabales	Fabaceae	Medicago truncatula
Homo sapiens chromosome 5 clone CTD−2071A4, comple	AC125786	81.82	24.86	6.85892	Primates	Hominidae	Homo sapiens
Heterogynis penella partial mitochondrial 12S rRNA	AJ785617	70.71	55.37	6.85892	Lepidoptera	Heterogynidae	Heterogynis penella
Zebrafish DNA sequence from clone CH211−256J23 in	BX005280	72.09	45.76	6.85892	Cypriniformes	Cyprinidae	Danio rerio
