# Supplementary material for: Testing the potential of a ribosomal 16S marker for DNA metabarcoding of insects
Source: PeerJ. 2016 Apr 19;4:e1966. doi: 10.7717/peerj.1966 (PMC4841222; doi:10.7717/peerj.1966)
Supplement: Supplemental Information 1 [file peerj-04-1966-s006.zip › scripts/5 match OTUs/blast_hits/OTU_148.csv_plot.pdf]

# OTU_148.csv

## Allocoris sp. WCW−2003

## Thyreocoridae

## Hemiptera

# 80.77

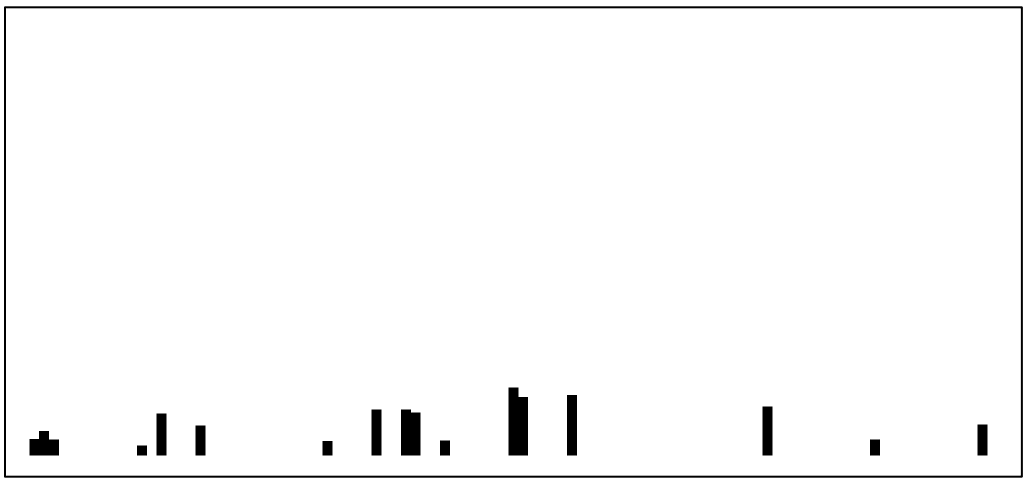

| | | | | | | | |
|---|---|---|---|---|---|---|---|
| Allocoris sp. WCW−2003 16S ribosomal RNA gene, par | AY252798 | 80.77 | 100 | 1.56555e−32 | Hemiptera | Thyreocoridae | Allocoris sp. WCW−2003 |
| Sehirus luctuosus voucher 3P1 16S ribosomal RNA ge | JQ234973 | 81.13 | 100 | 1.90723e−31 | Hemiptera | Cydnidae | Sehirus luctuosus |
| Stauralia chloracantha 16S ribosomal RNA gene, par | AY252740 | 80.75 | 100 | 3.44835e−28 | Hemiptera | Acanthosomatidae | Stauralia chloracantha |
| Elasmostethus sp. WCW−2003 16S ribosomal RNA gene, | AY252797 | 78.75 | 100 | 5.11781e−26 | Hemiptera | Acanthosomatidae | Elasmostethus sp. WCW−2003 |
| Stauralia compuncta 16S ribosomal RNA gene, partia | AY252741 | 78.98 | 100 | 1.78629e−25 | Hemiptera | Acanthosomatidae | Stauralia compuncta |
| Scutelleridae sp. BAHAR115−14 voucher BAH_201312_P | KP253967 | 77.78 | 100 | 6.23476e−25 | Hemiptera | Scutelleridae | Scutelleridae sp. BAHAR115−14 |
| Elasmostethus interstinctus 16S ribosomal RNA gene | JQ029152 | 78.12 | 100 | 6.23476e−25 | Hemiptera | Acanthosomatidae | Elasmostethus interstinctus |
| Cydninae sp. 3 WCW−2007 16S ribosomal RNA gene, pa | EF641130 | 79.01 | 100 | 6.23476e−25 | Hemiptera | Cydnidae | Cydninae sp. 3 WCW−2007 |
| Galgupha difficilis 16S ribosomal RNA gene, partia | JQ029145 | 78.21 | 98.72 | 2.65109e−23 | Hemiptera | Thyreocoridae | Galgupha difficilis |
| Amphaces sp. WCW−2003 16S ribosomal RNA gene, part | AY252743 | 78.26 | 100 | 2.65109e−23 | Hemiptera | Acanthosomatidae | Amphaces sp. WCW−2003 |
| Megymenum sp. WCW−2003 16S ribosomal RNA gene, par | AY252697 | 78.4 | 100 | 2.65109e−23 | Hemiptera | Dinidoridae | Megymenum sp. WCW−2003 |
| Elasmucha grisea 16S ribosomal RNA gene, partial s | JQ029151 | 80.45 | 84.62 | 9.25321e−23 | Hemiptera | Acanthosomatidae | Elasmucha grisea |
| Macroscytus gibbulus mitochondrion, complete genom | EU427338 | 78.43 | 96.15 | 9.25321e−23 | Hemiptera | Cydnidae | Macroscytus gibbulus |
| Elasmostethus humeralis mitochondrial gene for 16S | AB240571 | 81.97 | 75.64 | 9.25321e−23 | Hemiptera | Acanthosomatidae | Elasmostethus humeralis |
| Eumenotes obscura 16S ribosomal RNA gene, partial | JQ029142 | 77.78 | 100 | 3.22969e−22 | Hemiptera | Dinidoridae | Eumenotes obscura |
| Elasmostethus scotti 16S ribosomal RNA gene, parti | AY986804 | 76.88 | 100 | 3.22969e−22 | Hemiptera | Acanthosomatidae | Elasmostethus scotti |
| Neoneides muticus 16S ribosomal RNA gene, partial | AY252892 | 76.88 | 100 | 3.22969e−22 | Hemiptera | Berytidae | Neoneides muticus |
| Scutelleridae sp. BOLD:ACO1974 voucher BAH_201312_ | KP253969 | 81.4 | 80.13 | 3.93457e−21 | Hemiptera | Scutelleridae | Scutelleridae sp. BOLD:ACO1974 |
| Pachycoris torridus isolate Pt06 16S ribosomal RNA | KM676319 | 77.33 | 94.87 | 1.3733e−20 | Hemiptera | Scutelleridae | Pachycoris torridus |
| Pachycoris torridus isolate Pt48 16S ribosomal RNA | KM676314 | 77.33 | 94.87 | 1.3733e−20 | Hemiptera | Scutelleridae | Pachycoris torridus |
| Pachycoris torridus isolate Pt46 16S ribosomal RNA | KM676312 | 77.33 | 94.87 | 1.3733e−20 | Hemiptera | Scutelleridae | Pachycoris torridus |
| Pachycoris torridus isolate Pt43 16S ribosomal RNA | KM676309 | 77.33 | 94.87 | 1.3733e−20 | Hemiptera | Scutelleridae | Pachycoris torridus |
| Eurygaster maura 16S ribosomal RNA gene, partial s | JQ029150 | 77.64 | 100 | 1.3733e−20 | Hemiptera | Scutelleridae | Eurygaster maura |
| Macroscytus brunneus 16S ribosomal RNA gene, parti | JQ029138 | 78.72 | 88.46 | 1.3733e−20 | Hemiptera | Cydnidae | Macroscytus brunneus |
| Eurygaster testudinaria voucher 86ET 16S ribosomal | JQ234974 | 77.64 | 100 | 1.3733e−20 | Hemiptera | Scutelleridae | Eurygaster testudinaria |
| Pachycoris torridus isolate Pt33 16S ribosomal RNA | KM676302 | 77.18 | 94.23 | 4.79328e−20 | Hemiptera | Scutelleridae | Pachycoris torridus |
| Pachycoris torridus isolate Pt32 16S ribosomal RNA | KM676301 | 77.18 | 94.23 | 4.79328e−20 | Hemiptera | Scutelleridae | Pachycoris torridus |
| Choerocoris variegatus 16S ribosomal RNA gene, par | AY252748 | 78.57 | 89.1 | 4.79328e−20 | Hemiptera | Scutelleridae | Choerocoris variegatus |
| Austrotechus rugosus 16S ribosomal RNA gene, parti | AY252745 | 76.07 | 100 | 4.79328e−20 | Hemiptera | Pentatomidae | Austrotechus rugosus |
| Anischys sp. WCW−2003 16S ribosomal RNA gene, part | AY252742 | 77.64 | 100 | 4.79328e−20 | Hemiptera | Acanthosomatidae | Anischys sp. WCW−2003 |
| Tuxedo sp. WCW−2003g 16S ribosomal RNA gene, parti | AY252803 | 80.67 | 76.28 | 1.67302e−19 | Hemiptera | Miridae | Tuxedo sp. WCW−2003g |
| Riptortus sp. WCW−2003 16S ribosomal RNA gene, par | AY252665 | 76.83 | 100 | 1.67302e−19 | Hemiptera | Alydidae | Riptortus sp. WCW−2003 |
| Pachycoris torridus isolate Pt01 16S ribosomal RNA | KM676295 | 76.51 | 94.23 | 5.83941e−19 | Hemiptera | Scutelleridae | Pachycoris torridus |
| Pentatomidae sp. SJ−2010 16S ribosomal RNA gene, p | GU194578 | 74.07 | 100 | 5.83941e−19 | Hemiptera | Pentatomidae | Pentatomidae sp. SJ−2010 |
| Pachycoris torridus isolate Pt34 16S ribosomal RNA | KM676303 | 77.3 | 89.1 | 2.03816e−18 | Hemiptera | Scutelleridae | Pachycoris torridus |
| Parastrachia japonensis 16S ribosomal RNA gene, pa | JQ029136 | 82.14 | 70.51 | 2.03816e−18 | Hemiptera | Parastrachiidae | Parastrachia japonensis |
| Ausejanus minutus 16S ribosomal RNA gene, partial | HQ667608 | 76.13 | 94.87 | 2.03816e−18 | Hemiptera | Miridae | Ausejanus minutus |
| Ausejanus vividus 16S ribosomal RNA gene, partial | HQ667606 | 79.41 | 83.33 | 2.03816e−18 | Hemiptera | Miridae | Ausejanus vividus |
| Parastrachia japonensis 16S ribosomal RNA gene, pa | EF641132 | 82.14 | 70.51 | 2.03816e−18 | Hemiptera | Parastrachiidae | Parastrachia japonensis |
| Thyreocoris scarabaeoides 16S ribosomal RNA gene, | JQ029135 | 82 | 64.1 | 7.11386e−18 | Hemiptera | Thyreocoridae | Thyreocoris scarabaeoides |
| Ausejanus tasmaniae 16S ribosomal RNA gene, partia | HQ667605 | 77.22 | 97.44 | 7.11386e−18 | Hemiptera | Miridae | Ausejanus tasmaniae |
| Ausejanus mcdonaldi 16S ribosomal RNA gene, partia | HQ667604 | 77.22 | 97.44 | 7.11386e−18 | Hemiptera | Miridae | Ausejanus mcdonaldi |
| Hydaropsis longirostris mitochondrion, complete ge | EU427337 | 80.7 | 71.79 | 7.11386e−18 | Hemiptera | Coreidae | Hydaropsis longirostris |
| Poecilocoris lewisi mitochondrial gene for 16S rRN | AB240574 | 75.8 | 96.15 | 7.11386e−18 | Hemiptera | Scutelleridae | Poecilocoris lewisi |
| Phyllopidea picta 16S ribosomal RNA gene, partial | AY252822 | 76.13 | 94.87 | 7.11386e−18 | Hemiptera | Miridae | Phyllopidea picta |
| Peltocopta crassiventris 16S ribosomal RNA gene, p | AY252693 | 78.63 | 83.33 | 7.11386e−18 | Hemiptera | Tessaratomidae | Peltocopta crassiventris |
| Urochela quadrinotata mitochondrial gene for 16S r | AB858383 | 78.79 | 83.97 | 2.48298e−17 | Hemiptera | Urostylididae | Urochela quadrinotata |
| Urochela quadrinotata mitochondrial gene for 16S r | AB858382 | 78.79 | 83.97 | 2.48298e−17 | Hemiptera | Urostylididae | Urochela quadrinotata |
| Odontoscelis fuliginosa 16S ribosomal RNA gene, pa | JQ029149 | 75.78 | 100 | 2.48298e−17 | Hemiptera | Scutelleridae | Odontoscelis fuliginosa |
| Cyclopelta obscura 16S ribosomal RNA gene, partial | JQ029146 | 83.17 | 63.46 | 2.48298e−17 | Hemiptera | Dinidoridae | Cyclopelta obscura |
| Megymenum parallelum 16S ribosomal RNA gene, parti | JQ029140 | 82.73 | 69.23 | 2.48298e−17 | Hemiptera | Dinidoridae | Megymenum parallelum |
| Campylomma sp. KM−2011 16S ribosomal RNA gene, par | HQ667652 | 76.43 | 97.44 | 2.48298e−17 | Hemiptera | Miridae | Campylomma sp. KM−2011 |
| Urostylis westwoodi 16S ribosomal RNA gene, partia | AY252689 | 78.79 | 83.97 | 2.48298e−17 | Hemiptera | Urostylididae | Urostylis westwoodii |
| Campylomma sp. WCW−2003 16S ribosomal RNA gene, pa | AY252674 | 76.43 | 97.44 | 2.48298e−17 | Hemiptera | Miridae | Campylomma sp. WCW−2003 |
| Phylinae gen. sp. South African SA07 711 16S ribos | JX194137 | 75.32 | 94.87 | 8.66646e−17 | Hemiptera | Miridae | Phylinae gen. sp. South African SA07 711 |
| Heterocapillus sp. KLM−2013 16S ribosomal RNA gene | JX194136 | 82.83 | 62.82 | 8.66646e−17 | Hemiptera | Miridae | Heterocapillus sp. KLM−2013 |
| Malaysiamiris rufobadius 16S ribosomal RNA, partia | KF273182 | 77.69 | 81.41 | 8.66646e−17 | Hemiptera | Miridae | Malaysiamiris rufobadius |
| Atractotomus agrifoliae 16S ribosomal RNA, partial | KF273167 | 78.68 | 84.62 | 8.66646e−17 | Hemiptera | Miridae | Atractotomus agrifoliae |
| Hypseloecus sp. 6 KM−2011 16S ribosomal RNA gene, | JF414069 | 78.52 | 82.05 | 8.66646e−17 | Hemiptera | Miridae | Hypseloecus sp. 6 KM−2011 |
| Leucophoroptera quadrimaculata 16S ribosomal RNA g | HQ667624 | 76.73 | 97.44 | 8.66646e−17 | Hemiptera | Miridae | Leucophoroptera quadrimaculata |
| Schuhistes lekkersingia 16S ribosomal RNA gene, pa | HQ667620 | 74.68 | 94.87 | 8.66646e−17 | Hemiptera | Miridae | Schuhistes lekkersingia |
| Wallabicoris rutidosi 16S ribosomal RNA gene, part | HM142160 | 76.43 | 94.87 | 8.66646e−17 | Hemiptera | Miridae | Wallabicoris rutidosi |
| Wallabicoris helichrysi 16S ribosomal RNA gene, pa | HM142154 | 76.43 | 94.87 | 8.66646e−17 | Hemiptera | Miridae | Wallabicoris helichrysi |
| Mcateella sp. WCW−2003e 16S ribosomal RNA gene, pa | AY252733 | 77.08 | 91.03 | 8.66646e−17 | Hemiptera | Piesmatidae | Mcateella sp. WCW−2003e |
| Urochela quadrinotata mitochondrial gene for 16S r | AB858381 | 78.03 | 83.97 | 3.02489e−16 | Hemiptera | Urostylididae | Urochela quadrinotata |
| Tuponia elegans from China 16S ribosomal RNA gene, | KJ000472 | 75.51 | 89.1 | 3.02489e−16 | Hemiptera | Miridae | Tuponia elegans |
| Tuxedo susansolomonae 16S ribosomal RNA, partial s | KF273173 | 76.98 | 83.33 | 3.02489e−16 | Hemiptera | Miridae | Tuxedo susansolomonae |
| Ausejanus luteoelytratus 16S ribosomal RNA gene, p | HQ667611 | 76.58 | 97.44 | 3.02489e−16 | Hemiptera | Miridae | Ausejanus luteoelytratus |
| Tropidosteptes sp. WCW−2003 16S ribosomal RNA gene | AY252873 | 75 | 99.36 | 3.02489e−16 | Hemiptera | Miridae | Tropidosteptes sp. WCW−2003 |
| Plautia stali mitochondrial gene for 16S ribosomal | LC007442 | 74.21 | 100 | 1.05579e−15 | Hemiptera | Pentatomidae | Plautia stali |
| Plautia stali mitochondrial gene for 16S ribosomal | LC007388 | 74.21 | 100 | 1.05579e−15 | Hemiptera | Pentatomidae | Plautia stali |
| Plautia stali mitochondrial gene for 16S ribosomal | LC007193 | 74.21 | 100 | 1.05579e−15 | Hemiptera | Pentatomidae | Plautia stali |
| Plautia stali mitochondrial gene for 16S ribosomal | LC007174 | 74.21 | 100 | 1.05579e−15 | Hemiptera | Pentatomidae | Plautia stali |
| Coatonocapsus sp. n. 698 16S ribosomal RNA, partia | KF273192 | 79.34 | 75.92 | 1.05579e−15 | Hemiptera | Miridae | Coatonocapsus sp. n. 698 |
| Miridae gen. n. sp. South Africa 273 16S ribosomal | KF273190 | 74.52 | 94.87 | 1.05579e−15 | Hemiptera | Miridae | Miridae gen. n. sp. South Africa 273 |
| Tessaratoma quadrata 16S ribosomal RNA gene, parti | JQ029147 | 82.29 | 60.9 | 1.05579e−15 | Hemiptera | Tessaratomidae | Tessaratoma quadrata |
| Dilatops fici 16S ribosomal RNA gene, partial sequ | HQ667615 | 75.94 | 80.13 | 1.05579e−15 | Hemiptera | Miridae | Dilatops fici |
| Ausejanus femoralis 16S ribosomal RNA gene, partia | HQ667609 | 75.48 | 94.87 | 1.05579e−15 | Hemiptera | Miridae | Ausejanus femoralis |
| Ausejanus arvensus 16S ribosomal RNA gene, partial | HQ667607 | 76.73 | 97.44 | 1.05579e−15 | Hemiptera | Miridae | Ausejanus arvensus |
| Xiphoides sp. 'pallidus' 16S ribosomal RNA gene, p | HM142165 | 78.15 | 76.28 | 1.05579e−15 | Hemiptera | Miridae | Xiphoides sp. 'pallidus' |
| Wallabicoris chrysocephali 16S ribosomal RNA gene, | HM142149 | 74.52 | 94.87 | 1.05579e−15 | Hemiptera | Miridae | Wallabicoris chrysocephali |
| Stictopleurus subviridis mitochondrion, complete g | EU826088 | 75.66 | 94.87 | 1.05579e−15 | Hemiptera | Rhopalidae | Stictopleurus subviridis |
| Malcus inconspicuus mitochondrion, complete genome | EU427339 | 74.21 | 100 | 1.05579e−15 | Hemiptera | Malcidae | Malcus inconspicuus |
| Phloea subquadrata 16S ribosomal RNA gene, partial | EF641140 | 75.51 | 90.38 | 1.05579e−15 | Hemiptera | Phloeidae | Phloea subquadrata |
| Physopelta quadriguttata 16S ribosomal RNA gene, p | AY986817 | 74.07 | 100 | 1.05579e−15 | Hemiptera | Largidae | Physopelta quadriguttata |
| Poecilocoris latus 16S ribosomal RNA gene, partial | AY986800 | 75.91 | 86.54 | 1.05579e−15 | Hemiptera | Scutelleridae | Poecilocoris latus |
| Tuxedo sp. WCW−2003h 16S ribosomal RNA gene, parti | AY252823 | 80.73 | 69.23 | 1.05579e−15 | Hemiptera | Miridae | Tuxedo sp. WCW−2003h |
| Phytocoris vanduzeei 16S ribosomal RNA gene, parti | AY252814 | 76.98 | 83.33 | 1.05579e−15 | Hemiptera | Miridae | Phytocoris vanduzeei |
| Mcateella sp. WCW−2003d 16S ribosomal RNA gene, pa | AY252667 | 77.37 | 86.54 | 1.05579e−15 | Hemiptera | Piesmatidae | Mcateella sp. WCW−2003d |
| Plautia stali mitochondrial gene for 16S ribosomal | LC007155 | 73.75 | 100 | 3.68507e−15 | Hemiptera | Pentatomidae | Plautia stali |
| Plautia stali mitochondrial gene for 16S ribosomal | LC007154 | 73.75 | 100 | 3.68507e−15 | Hemiptera | Pentatomidae | Plautia stali |
| Plautia stali mitochondrial gene for 16S ribosomal | LC007152 | 73.78 | 100 | 3.68507e−15 | Hemiptera | Pentatomidae | Plautia stali |
| Plautia stali mitochondrial gene for 16S ribosomal | LC007148 | 73.75 | 100 | 3.68507e−15 | Hemiptera | Pentatomidae | Plautia stali |
| Reuteroscopus sp. KLM−2013 16S ribosomal RNA gene, | JX194134 | 76.22 | 88.46 | 3.68507e−15 | Hemiptera | Miridae | Reuteroscopus sp. KLM−2013 |
| Mormidea v−luteum 16S ribosomal RNA gene, partial | KC537026 | 75.16 | 100 | 3.68507e−15 | Hemiptera | Pentatomidae | Mormidea v−luteum |
| Miridae gen. n. sp. Bolivia 16S ribosomal RNA, par | KF273200 | 75.37 | 84.62 | 3.68507e−15 | Hemiptera | Miridae | Miridae gen. n. sp. Bolivia |
| Austrovelia caledonica voucher 2567 16S ribosomal | JQ362806 | 74.68 | 98.72 | 3.68507e−15 | Hemiptera | Mesoveliidae | Austrovelia caledonica |
| Eminoculus rugosus 16S ribosomal RNA gene, partial | HQ667637 | 81.44 | 62.18 | 3.68507e−15 | Hemiptera | Miridae | Eminoculus rugosus |
| Collessicoris bellissimus 16S ribosomal RNA gene, | HQ667623 | 75 | 97.44 | 3.68507e−15 | Hemiptera | Miridae | Collessicoris bellissimus |
| Phylus coryloides 16S ribosomal RNA gene, partial | GU194580 | 77.69 | 77.56 | 3.68507e−15 | Hemiptera | Miridae | Phylus coryloides |
