# Supplementary material for: Testing the potential of a ribosomal 16S marker for DNA metabarcoding of insects
Source: PeerJ. 2016 Apr 19;4:e1966. doi: 10.7717/peerj.1966 (PMC4841222; doi:10.7717/peerj.1966)
Supplement: Supplemental Information 1 [file peerj-04-1966-s006.zip › scripts/5 match OTUs/blast_hits/OTU_149.csv_plot.pdf]

OTU_149.csv

Isotoma viridis

Isotomidae

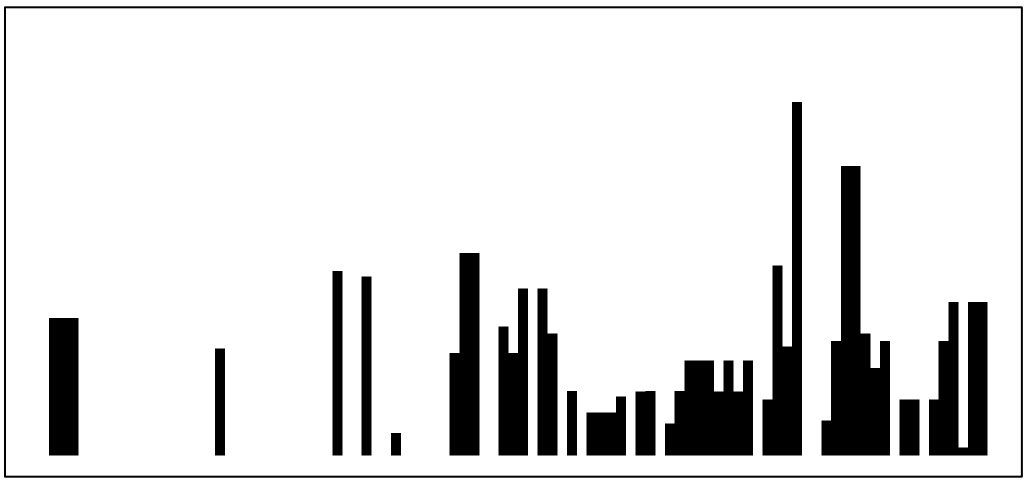

73.47

Isotoma viridis 16S ribosomal RNA gene, partial se	JN970946	73.47	100	1.75115e−12		Isotomidae	Isotoma viridis
Cryptopygus sverdrupi voucher MNHN:cd461_057 16S r	KJ418950	78.02	62.33	2.59894e−10		Isotomidae	Cryptopygus sverdrupi
Cryptopygus sverdrupi voucher MNHN:cd468_065 16S r	KJ418952	86.44	40.41	3.16616e−09		Isotomidae	Cryptopygus sverdrupi
Cryptopygus sverdrupi voucher MNHN:EA010326 16S ri	KJ418902	86.44	40.41	3.16616e−09		Isotomidae	Cryptopygus sverdrupi
Cryptopygus sverdrupi voucher MNHN:cd325_065 16S r	KJ418901	86.44	40.41	3.16616e−09		Isotomidae	Cryptopygus sverdrupi
Cryptopygus sverdrupi voucher MNHN:cd477_070 16S r	KJ418963	76.92	62.33	1.1051e−08		Isotomidae	Cryptopygus sverdrupi
Cryptopygus sverdrupi voucher MNHN:cd451_051 16S r	KJ418961	76.92	62.33	1.1051e−08		Isotomidae	Cryptopygus sverdrupi
Cryptopygus sverdrupi voucher MNHN:cd460_057 16S r	KJ418949	76.92	62.33	1.1051e−08		Isotomidae	Cryptopygus sverdrupi
Cryptopygus sverdrupi voucher MNHN:cd459_057 16S r	KJ418948	76.92	62.33	1.1051e−08		Isotomidae	Cryptopygus sverdrupi
Cryptopygus sverdrupi voucher MNHN:cd453_056 16S r	KJ418946	76.92	62.33	1.1051e−08		Isotomidae	Cryptopygus sverdrupi
Cryptopygus sverdrupi voucher MNHN:cd443_047 16S r	KJ418941	76.92	62.33	1.1051e−08		Isotomidae	Cryptopygus sverdrupi
Cryptopygus sverdrupi voucher MNHN:cd429_044 16S r	KJ418934	76.92	62.33	1.1051e−08		Isotomidae	Cryptopygus sverdrupi
Cryptopygus sverdrupi voucher MNHN:cd421_040 16S r	KJ418928	76.92	62.33	1.1051e−08		Isotomidae	Cryptopygus sverdrupi
Cryptopygus sverdrupi voucher MNHN:cd405_033 16S r	KJ418919	76.92	62.33	1.1051e−08		Isotomidae	Cryptopygus sverdrupi
Cryptopygus sverdrupi voucher MNHN:EA010324 16S ri	KJ418900	76.92	62.33	1.1051e−08		Isotomidae	Cryptopygus sverdrupi
Cryptopygus sverdrupi voucher MNHN:cd323_047 16S r	KJ418899	76.92	62.33	1.1051e−08		Isotomidae	Cryptopygus sverdrupi
Cryptopygus sverdrupi voucher MNHN:cd317_038 16S r	KJ418893	76.92	62.33	1.1051e−08		Isotomidae	Cryptopygus sverdrupi
Cryptopygus sverdrupi voucher MNHN:cd313_033 16S r	KJ418889	76.92	62.33	1.1051e−08		Isotomidae	Cryptopygus sverdrupi
Cryptopygus sverdrupi voucher MNHN:cd310_032 16S r	KJ418888	76.92	62.33	1.1051e−08		Isotomidae	Cryptopygus sverdrupi
Cryptopygus sverdrupi voucher MNHN:cd428_043 16S r	KJ418933	85	41.1	3.85717e−08		Isotomidae	Cryptopygus sverdrupi
Cryptopygus sverdrupi voucher MNHN:cd455_056 16S r	KJ418947	75.82	62.33	1.34628e−07		Isotomidae	Cryptopygus sverdrupi
Cryptopygus sverdrupi voucher MNHN:cd449_049 16S r	KJ418943	75.82	62.33	1.34628e−07		Isotomidae	Cryptopygus sverdrupi
Cryptopygus sverdrupi voucher MNHN:cd418_039 16S r	KJ418927	77.17	62.33	4.699e−07		Isotomidae	Cryptopygus sverdrupi
Cryptopygus sverdrupi voucher MNHN:cd412_038 16S r	KJ418925	77.17	62.33	4.699e−07		Isotomidae	Cryptopygus sverdrupi
Cryptopygus sverdrupi voucher MNHN:cd440_047 16S r	KJ418940	74.73	62.33	1.64011e−06		Isotomidae	Cryptopygus sverdrupi
Cryptopygus sverdrupi voucher MNHN:cd430_044 16S r	KJ418935	74.73	62.33	5.72455e−06		Isotomidae	Cryptopygus sverdrupi
Folsomia candida voucher Folsomia_Candida 16S ribo	KF591920	77.08	63.7	5.72455e−06		Isotomidae	Folsomia candida
Folsomia candida 16S ribosomal RNA gene, partial s	KC236200	77.08	63.7	5.72455e−06		Isotomidae	Folsomia candida
Folsomotoma octooculata mitochondrion, complete ge	KC862316	72.18	87.67	1.99806e−05		Isotomidae	Folsomotoma octooculata
Folsomia candida 16S ribosomal RNA gene, partial s	JN970944	73.12	63.7	1.99806e−05		Isotomidae	Folsomia candida
Folsomia candida 16S ribosomal RNA gene, partial s	AY555551	73.12	63.7	1.99806e−05		Isotomidae	Folsomia candida
Seira sp. 2 FZ−2013 16S ribosomal RNA gene, partia	KC236215	88.64	30.14	6.97393e−05		Entomobryidae	Seira sp. 2 FZ−2013
Folsomia quadrioculata 16S ribosomal RNA gene, par	KC236199	73.4	64.38	6.97393e−05		Isotomidae	Folsomia quadrioculata
Phauda mimica mitochondrial tRNA−Leu (CUN) gene, 1	AJ831600	74.26	65.75	6.97393e−05	Lepidoptera	Zygaenidae	Phauda mimica
Himalanura sp. FZ−2013 16S ribosomal RNA gene, par	KC236202	88.37	29.45	0.000243414		Entomobryidae	Himalanura sp. FZ−2013
Spodoptera dolichos voucher SPO−LSU27 16S ribosoma	HQ178278	79.71	46.58	0.000243414	Lepidoptera	Noctuidae	Spodoptera dolichos
Homeogryllus sp. MCJ−2002 16S ribosomal RNA gene,	AF514594	71.93	73.97	0.000243414	Orthoptera	Gryllidae	Homoeogryllus sp. MCJ−2002
Callyntrura guangdongensis 16S ribosomal RNA gene,	KM978382	81.03	39.73	0.000849598		Paronellidae	Callyntrura guangdongensis
Geometridae sp. BOLD:ACO2329 voucher BAH_201312_P0	KP253914	77.22	53.42	0.000849598	Lepidoptera	Geometridae	Geometridae sp. BOLD:ACO2329
Pogonognathellus cf. flavescens FZ−2014 voucher Po	KF591937	75	62.33	0.000849598		Tomoceridae	Pogonognathellus cf. flavescens FZ−2014
Antheraea yamamai mitochondrion, complete genome	EU726630	71.7	67.81	0.000849598	Lepidoptera	Saturniidae	Antheraea yamamai
Mogoplistinae sp. 'Botswana Cave' 16S ribosomal RN	AF514585	75	54.11	0.000849598	Orthoptera	Gryllidae	Mogoplistinae sp. 'Botswana Cave'
Epilachna patricia voucher KS133 16S ribosomal RNA	KP143570	73.68	63.7	0.00296539	Coleoptera	Coccinellidae	Epilachna patricia
Willowsia guangdongensis 16S ribosomal RNA gene, p	KC236223	84.78	31.51	0.00296539		Entomobryidae	Willowsia guangdongensis
Seira delamarei 16S ribosomal RNA gene, partial se	KC236213	89.47	26.03	0.00296539		Entomobryidae	Seira delamarei
Agnotecous azurensis isolate Aaz−RB 16S ribosomal	JX897358	89.47	26.03	0.00296539	Orthoptera	Gryllidae	Agnotecous azurensis
Spodoptera dolichos voucher SPO−B2 16S ribosomal R	HQ178271	78.26	46.58	0.00296539	Lepidoptera	Noctuidae	Spodoptera dolichos
Trichodes apiarius voucher UPOL001127 16S ribosoma	HQ619665	72.45	65.75	0.00296539	Coleoptera	Cleridae	Trichodes apiarius
Gempylodes lewisi 16S ribosomal RNA gene, partial	EF490156	86.05	29.45	0.00296539	Coleoptera	Zopheridae	Gempylodes lewisii
Sitaris muralis mitochondrial partial 16S rRNA gen	AM712132	84.78	31.51	0.00296539	Coleoptera	Meloidae	Sitaris muralis
Grylloblatta sp. BYU_gb29.1 16S ribosomal RNA gene	DQ457261	87.8	28.08	0.00296539	Grylloblattodea	Grylloblattidae	Grylloblatta sp. BYU_gb29.1
Spodoptera dolichos 16S ribosomal RNA gene, partia	AF173053	78.26	46.58	0.00296539	Lepidoptera	Noctuidae	Spodoptera dolichos
Croscherichia paykulli mitochondrial partial 16S r	AJ633667	87.8	28.08	0.00296539	Coleoptera	Meloidae	Croscherichia paykulli
Salina sp. BXW−2014 16S ribosomal RNA gene, partia	KM978386	85.71	28.77	0.0103502		Paronellidae	Salina sp. BXW−2014
Eremochelis bilobatus voucher DMNS:ZA.21949 16S ri	KT276610	75	54.79	0.0103502	Solifugae	Eremobatidae	Eremochelis bilobatus
Placamen calophylla 16S ribosomal RNA gene, partia	KP252975	83.02	36.3	0.0103502	Veneroida	Veneridae	Placamen calophylla
Opilo pallidus voucher UPOL001126 16S ribosomal RN	KC758780	72.55	65.07	0.0103502	Coleoptera	Cleridae	Opilo pallidus
Antheraea yamamai superba mitochondrial gene for 1	AB841459	82	34.25	0.0103502	Lepidoptera	Saturniidae	Antheraea yamamai superba
Antheraea yamamai superba mitochondrial gene for 1	AB841454	82	34.25	0.0103502	Lepidoptera	Saturniidae	Antheraea yamamai superba
Antheraea yamamai superba mitochondrial gene for 1	AB841453	82	34.25	0.0103502	Lepidoptera	Saturniidae	Antheraea yamamai superba
Nipponoserica koltzei 16S ribosomal RNA gene, part	JX112791	82.76	38.36	0.0103502	Coleoptera	Scarabaeidae	Nipponoserica koltzei
Savignium crenatum partial 16S rRNA gene, specimen	HG970425	74.49	63.01	0.0103502	Sessilia	Pyrgomatidae	Savignium crenatum
Berberomeloe majalis voucher MAB107 16S ribosomal	KC853076	82.98	32.19	0.0103502	Coleoptera	Meloidae	Berberomeloe majalis
Placamen calophylla isolate Placamencalophylla0805	HM124723	83.02	36.3	0.0103502	Veneroida	Veneridae	Placamen calophylla
Savignium crenatum clone NSB604 16S ribosomal RNA	FJ620744	74.49	63.01	0.0103502	Sessilia	Pyrgomatidae	Savignium crenatum
Orchesella villosa mitochondrion, complete genome	EU016195	81.48	36.99	0.0103502		Entomobryidae	Orchesella villosa
Placamen calophylla voucher FMNH 305977 large subu	DQ184734	83.02	36.3	0.0103502	Veneroida	Veneridae	Placamen calophylla
Grylloblatta rothi voucher BYU_gb06.4 16S ribosoma	DQ457233	84.44	30.82	0.0103502	Grylloblattodea	Grylloblattidae	Grylloblatta rothi
Grylloblatta gurneyi voucher BYU_gb05.2 16S riboso	DQ457232	84.44	30.82	0.0103502	Grylloblattodea	Grylloblattidae	Grylloblatta gurneyi
Grylloblatta gurneyi voucher BYU_gb05.1 16S riboso	DQ457231	84.44	30.82	0.0103502	Grylloblattodea	Grylloblattidae	Grylloblatta gurneyi
Medicago truncatula clone mth2−95k24, complete seq	AC153001	82.98	32.19	0.0103502	Fabales	Fabaceae	Medicago truncatula
Orthoptera sp. DJC−2002 16S ribosomal RNA gene, pa	AY144645	84.44	30.82	0.0103502	Orthoptera		Orthoptera sp. AMEBU8569
Medicago truncatula clone mth2−18l7, complete sequ	AC124961	82.98	32.19	0.0103502	Fabales	Fabaceae	Medicago truncatula
Grylloblatta sculleni mitochondrion, partial genom	DQ241796	84.44	30.82	0.0103502	Grylloblattodea	Grylloblattidae	Grylloblatta sculleni
Antheraea pernyi 16S ribosomal RNA gene, mitochond	AF027949	70.75	67.81	0.0103502	Lepidoptera	Saturniidae	Antheraea pernyi
Bartonella henselae genome assembly Bartonella hen	LN879429	82.61	31.51	0.0361258	Rhizobiales	Bartonellaceae	Bartonella henselae
Callyntrura sp. BXW−2014 16S ribosomal RNA gene, p	KM978383	88.89	24.66	0.0361258		Paronellidae	Callyntrura sp. BXW−2014
Massilia sp. NR 4−1, complete genome	CP012201	85.11	30.14	0.0361258	Burkholderiales	Oxalobacteraceae	Massilia sp. NR 4−1
Apteryx australis mantelli genome assembly AptMant	LK064922	96.55	19.86	0.0361258	Apterygiformes	Apterygidae	Apteryx australis mantelli
Epilachna insignis voucher KS166 16S ribosomal RNA	KP143593	71.13	64.38	0.0361258	Coleoptera	Coccinellidae	Epilachna insignis
Prays oleae isolate P38 mitochondrion, complete ge	KM874804	78.57	38.36	0.0361258	Lepidoptera	Yponomeutidae	Prays oleae
Glyphodes pyloalis mitochondrion, complete genome	KM576860	81.63	33.56	0.0361258	Lepidoptera	Crambidae	Glyphodes pyloalis
Platydema sp. 1 GJK−2014 16S ribosomal RNA gene, p	KJ002970	85.37	28.08	0.0361258	Coleoptera	Tenebrionidae	Platydema sp. 1 GJK−2014
Strongyloides papillosus genome assembly S_papillo	LM525836	93.55	21.23	0.0361258	Rhabditida	Strongyloididae	Strongyloides papillosus
Prochlorococcus sp. MIT 0801, complete genome	CP007754	93.55	21.23	0.0361258	Prochlorales	Prochlorococcaceae	Prochlorococcus sp. MIT 0801
Dracunculus medinensis genome assembly D_medinensi	LK978239	85.71	32.88	0.0361258	Spirurida	Dracunculidae	Dracunculus medinensis
Caenorhabditis elegans genome assembly C_elegans_B	LK927580	84.09	30.14	0.0361258	Rhabditida	Rhabditidae	Caenorhabditis elegans
Polyphaes balteata voucher 842807 16S ribosomal R	JX234495	85.37	28.08	0.0361258	Coleoptera	Scarabaeidae	Polyphaes balteata
Tetraclita sp. MM−2014 partial 16S rRNA gene, spec	HG970399	70.83	91.1	0.0361258	Sessilia	Tetraclitidae	Tetraclita sp. MM−2014
Bartonella henselae complete genome, strain BM1374	HG969191	82.61	31.51	0.0361258	Rhizobiales	Bartonellaceae	Bartonella henselae
Bartonella henselae, strain BM1374163 complete gen	HG965802	82.61	31.51	0.0361258	Rhizobiales	Bartonellaceae	Bartonella henselae
Pseudoperga guerinii voucher AE114 16S ribosomal R	KF318495	71.43	75.34	0.0361258	Hymenoptera	Pergidae	Pseudoperga guerinii
Clerus gilberti voucher COL1400 12S ribosomal RNA	KC524830	82.61	31.51	0.0361258	Coleoptera	Cleridae	Clerus gilberti
Platydema sp. NC FLC−2013 voucher OGBL_00001 16S r	KC160422	85.37	28.08	0.0361258	Coleoptera	Tenebrionidae	Platydema sp. NC FLC−2013
Sinella curviseta 16S ribosomal RNA gene, partial	KC236219	87.18	26.71	0.0361258		Entomobryidae	Sinella curviseta
Seira sp. 1 FZ−2013 16S ribosomal RNA gene, partia	KC236214	80.36	37.67	0.0361258		Entomobryidae	Seira sp. 1 FZ−2013
Lepidocyrtus sp. 2 FZ−2013 16S ribosomal RNA gene,	KC236207	87.18	26.71	0.0361258		Entomobryidae	Lepidocyrtus sp. 2 FZ−2013
Orchesellides sinensis 16S ribosomal RNA gene, par	KC236203	87.18	26.71	0.0361258		Entomobryidae	Homidia sinensis
Entomobrya multifasciata 16S ribosomal RNA gene, p	KC236196	79.66	36.99	0.0361258		Entomobryidae	Entomobrya multifasciata
Spodoptera marima voucher SPO−LSU19 16S ribosomal	HQ178341	78.69	41.1	0.0361258	Lepidoptera	Noctuidae	Spodoptera marima
