# Supplementary material for: Testing the potential of a ribosomal 16S marker for DNA metabarcoding of insects
Source: PeerJ. 2016 Apr 19;4:e1966. doi: 10.7717/peerj.1966 (PMC4841222; doi:10.7717/peerj.1966)
Supplement: Supplemental Information 1 [file peerj-04-1966-s006.zip › scripts/5 match OTUs/blast_hits/OTU_15.csv_plot.pdf]

OTU_15.csv

Rhithrogena sp. 13 LV−2015

Heptageniidae

Ephemeroptera

100

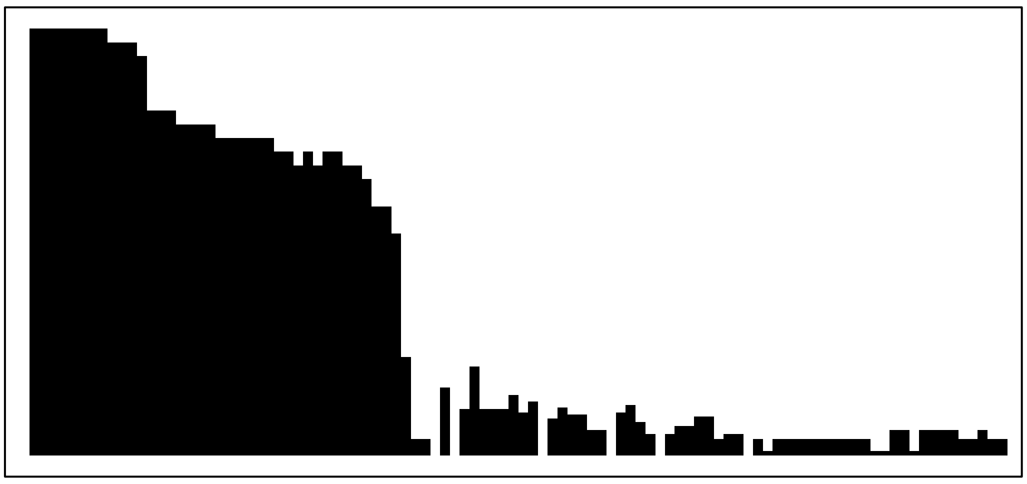

Rhithrogena sp. 13 LV−2015 mitochondrial partial 1	LN868672	100	100	1.053e−72	Ephemeroptera	Heptageniidae	Rhithrogena sp. 13 LV−2015
Rhithrogena sp. 12 LV−2015 mitochondrial partial 1	LN868671	100	100	1.053e−72	Ephemeroptera	Heptageniidae	Rhithrogena sp. 12 LV−2015
Rhithrogena picteti mitochondrial partial 16S rRNA	LN868663	100	100	1.053e−72	Ephemeroptera	Heptageniidae	Rhithrogena picteti
Rhithrogena sp. 3 LV−2015 mitochondrial partial 16	LN868659	100	100	1.053e−72	Ephemeroptera	Heptageniidae	Rhithrogena sp. 3 LV−2015
Rhithrogena sp. 2 LV−2015 mitochondrial partial 16	LN868654	100	100	1.053e−72	Ephemeroptera	Heptageniidae	Rhithrogena sp. 2 LV−2015
Rhithrogena puytoraci mitochondrial partial 16S rR	LN868651	100	100	1.053e−72	Ephemeroptera	Heptageniidae	Rhithrogena puytoraci
Rhithrogena iridina mitochondrial partial 16S rRNA	LN868646	100	100	1.053e−72	Ephemeroptera	Heptageniidae	Rhithrogena iridina
Rhithrogena taurisca mitochondrial partial 16S rRN	LN868638	100	100	1.053e−72	Ephemeroptera	Heptageniidae	Rhithrogena taurisca
Rhithrogena semicolorata mitochondrial partial 16S	LN868673	99.36	100	4.47746e−71	Ephemeroptera	Heptageniidae	Rhithrogena semicolorata
Rhithrogena sp. 5 LV−2015 mitochondrial partial 16	LN868653	99.36	100	4.47746e−71	Ephemeroptera	Heptageniidae	Rhithrogena sp. 5 LV−2015
Rhithrogena sp. 4 LV−2015 mitochondrial partial 16	LN868652	99.36	100	4.47746e−71	Ephemeroptera	Heptageniidae	Rhithrogena sp. 4 LV−2015
Rhithrogena fonticola mitochondrial partial 16S rR	LN868656	98.72	100	5.45466e−70	Ephemeroptera	Heptageniidae	Rhithrogena fonticola
Rhithrogena sp. 7 LV−2015 mitochondrial partial 16	LN868669	96.15	100	1.78314e−63	Ephemeroptera	Heptageniidae	Rhithrogena sp. 7 LV−2015
Rhithrogena alpestris mitochondrial partial 16S rR	LN868657	96.15	100	1.78314e−63	Ephemeroptera	Heptageniidae	Rhithrogena alpestris
Rhithrogena nivata mitochondrial partial 16S rRNA	LN868643	96.15	100	1.78314e−63	Ephemeroptera	Heptageniidae	Rhithrogena nivata
Rhithrogena degrangei mitochondrial partial 16S rR	LN868674	95.51	100	7.58209e−62	Ephemeroptera	Heptageniidae	Rhithrogena degrangei
Rhithrogena sp. 9 LV−2015 mitochondrial partial 16	LN868650	95.51	100	7.58209e−62	Ephemeroptera	Heptageniidae	Rhithrogena sp. 9 LV−2015
Rhithrogena hybrida mitochondrial partial 16S rRNA	LN868642	95.51	100	7.58209e−62	Ephemeroptera	Heptageniidae	Rhithrogena hybrida
Rhithrogena circumtatrica mitochondrial partial 16	LN868641	95.51	100	7.58209e−62	Ephemeroptera	Heptageniidae	Rhithrogena circumtatrica
Rhithrogena sp. 11 LV−2015 mitochondrial partial 1	LN868668	94.87	100	9.23688e−61	Ephemeroptera	Heptageniidae	Rhithrogena sp. 11 LV−2015
Rhithrogena eatoni mitochondrial partial 16S rRNA	LN868666	94.87	100	9.23688e−61	Ephemeroptera	Heptageniidae	Rhithrogena eatoni
Rhithrogena sp. 8 LV−2015 mitochondrial partial 16	LN868661	94.87	100	9.23688e−61	Ephemeroptera	Heptageniidae	Rhithrogena sp. 8 LV−2015
Rhithrogena sp. 6 LV−2015 mitochondrial partial 16	LN868655	94.87	100	9.23688e−61	Ephemeroptera	Heptageniidae	Rhithrogena sp. 6 LV−2015
Rhithrogena gratianopolitana mitochondrial partial	LN868647	94.87	100	9.23688e−61	Ephemeroptera	Heptageniidae	Rhithrogena gratianopolitana
Rhithrogena diensis mitochondrial partial 16S rRNA	LN868637	94.87	100	9.23688e−61	Ephemeroptera	Heptageniidae	Rhithrogena diensis
Rhithrogena germanica mitochondrial partial 16S rR	LN868660	94.23	100	3.22399e−60	Ephemeroptera	Heptageniidae	Rhithrogena germanica
Rhithrogena beskidensis mitochondrial partial 16S	LN868645	94.23	100	3.22399e−60	Ephemeroptera	Heptageniidae	Rhithrogena beskidensis
Rhithrogena sp. 10 LV−2015 mitochondrial partial 1	LN868664	93.59	100	3.92762e−59	Ephemeroptera	Heptageniidae	Rhithrogena sp. 10 LV−2015
Rhithrogena corcontica mitochondrial partial 16S r	LN868662	94.23	100	3.92762e−59	Ephemeroptera	Heptageniidae	Rhithrogena corcontica
Rhithrogena savoiensis mitochondrial partial 16S r	LN868658	93.59	100	3.92762e−59	Ephemeroptera	Heptageniidae	Rhithrogena savoiensis
Rhithrogena gratianopolitana mitochondrial partial	LN868648	94.23	100	3.92762e−59	Ephemeroptera	Heptageniidae	Rhithrogena gratianopolitana
Rhithrogena landai mitochondrial partial 16S rRNA	LN868644	94.23	100	3.92762e−59	Ephemeroptera	Heptageniidae	Rhithrogena landai
Rhithrogena insularis mitochondrial partial 16S rR	LN868665	93.59	100	4.78482e−58	Ephemeroptera	Heptageniidae	Rhithrogena insularis
Rhithrogena grischuna mitochondrial partial 16S rR	LN868639	93.59	100	4.78482e−58	Ephemeroptera	Heptageniidae	Rhithrogena grischuna
Rhithrogena nuragica mitochondrial partial 16S rRN	LN868667	92.95	100	2.03456e−56	Ephemeroptera	Heptageniidae	Rhithrogena nuragica
Rhithrogena dorieri mitochondrial partial 16S rRNA	LN868649	91.67	100	8.65116e−55	Ephemeroptera	Heptageniidae	Rhithrogena dorieri
Rhithrogena allobrogica mitochondrial partial 16S	LN868640	91.67	100	1.05393e−53	Ephemeroptera	Heptageniidae	Rhithrogena allobrogica
Rhithrogena sartorii mitochondrial partial 16S rRN	LN868670	90.38	100	4.48141e−52	Ephemeroptera	Heptageniidae	Rhithrogena sartorii
Rhithrogena sp. EP021 16S ribosomal RNA gene, part	AY749765	84.62	100	3.93109e−40	Ephemeroptera	Heptageniidae	Rhithrogena sp. EP021
Cinygmula sp. m185 mitochondrial partial 16S rRNA	LN868675	80.77	100	5.4643e−32	Ephemeroptera	Heptageniidae	Cinygmula sp. m185
Cinygmula sp. EP013 16S ribosomal RNA gene, partia	AY749760	80.77	100	5.4643e−32	Ephemeroptera	Heptageniidae	Cinygmula sp. EP013
Paegniodes cupulatus mitochondrion, complete genom	HM004123	77.07	100	3.93457e−21	Ephemeroptera	Heptageniidae	Paegniodes cupulatus
Cultus pilatus 16S ribosomal RNA gene, partial seq	EF623212	83.17	64.74	2.03816e−18	Plecoptera	Perlodidae	Cultus pilatus
Heptagenia sp. EP018 16S ribosomal RNA gene, parti	AY749762	77.54	87.82	2.03816e−18	Ephemeroptera	Heptageniidae	Heptagenia sp. EP018
Neopanorpa pulchra mitochondrion, complete genome	JX569848	82.18	64.74	2.48298e−17	Mecoptera	Panorpidae	Neopanorpa pulchra
Simocephalus sp. EIZ−2011 isolate Brb1−Sim1 16S ri	JN874613	84.16	63.46	2.48298e−17	Diplostraca	Daphniidae	Simocephalus sp. EIZ−2011
Neopanorpa pulchra mitochondrion, complete genome	FJ169955	82.18	64.74	2.48298e−17	Mecoptera	Panorpidae	Neopanorpa pulchra
Thaumatoperla sp. BYU_PL109 16S ribosomal RNA gene	EF623149	82.18	64.74	2.48298e−17	Plecoptera	Eustheniidae	Thaumatoperla sp. BYU_PL109
Neopanorpa sp. M24899 16S ribosomal RNA gene, part	AF180059	82.18	64.74	2.48298e−17	Mecoptera	Panorpidae	Neopanorpa sp. M24899
Arthroplea bipunctata 16S ribosomal RNA gene, part	AY749817	82.83	63.46	2.48298e−17	Ephemeroptera	Arthropleidae	Arthroplea bipunctata
Remenus bilobatus 16S ribosomal RNA gene, partial	EF623233	82	64.1	8.66646e−17	Plecoptera	Perlodidae	Remenus bilobatus
Capnura wanica 16S ribosomal RNA gene, partial seq	EF623128	82.52	64.74	8.66646e−17	Plecoptera	Capniidae	Capnura wanica
Chiloporter sp. EP051 16S ribosomal RNA gene, part	AY749776	75.16	100	8.66646e−17	Ephemeroptera	Ameletopsidae	Chiloporter sp. EP051
Apteroperla tikumana mitochondrion, complete genom	KR604721	81.73	64.74	3.02489e−16	Plecoptera	Capniidae	Apteroperla tikumana
Simocephalus vetulus 16S ribosomal RNA gene, parti	DQ470576	82.24	67.31	3.02489e−16	Diplostraca	Daphniidae	Simocephalus vetulus
Isoperla oxylepis 16S ribosomal RNA gene, partial	EF623221	81.9	66.67	1.05579e−15	Plecoptera	Perlodidae	Isoperla oxylepis
Isoperla davisi 16S ribosomal RNA gene, partial se	EF623206	81.9	66.67	1.05579e−15	Plecoptera	Perlodidae	Isoperla davisi
Eusthenia costalis 16S ribosomal RNA gene, partial	EF623150	81.19	64.74	1.05579e−15	Plecoptera	Eustheniidae	Eusthenia costalis
Penturoperla barbata 16S ribosomal RNA gene, parti	EF623124	81.19	64.74	1.05579e−15	Plecoptera	Austroperlidae	Penturoperla barbata
Austroperla cyrene 16S ribosomal RNA gene, partial	EF623120	77.08	89.1	1.05579e−15	Plecoptera	Austroperlidae	Austroperla cyrene
Cinygma sp. EP123 16S ribosomal RNA gene, partial	AY749804	82	64.1	1.05579e−15	Ephemeroptera	Heptageniidae	Cinygma sp. EP123
Analetris eximia 16S ribosomal RNA gene, partial s	AY749797	82.35	64.74	1.05579e−15	Ephemeroptera	Acanthametropodidae	Analetris eximia
Pachyrhamma edwardsii voucher CW122 16S ribosomal	HM594508	81.55	64.74	3.68507e−15	Orthoptera	Rhaphidophoridae	Pachyrhamma edwardsii
Heteromallus cavicola voucher MIL 16S ribosomal RN	HM594499	81	64.1	3.68507e−15	Orthoptera	Rhaphidophoridae	Heteromallus cavicola
Bolotoperla rossi 16S ribosomal RNA gene, partial	EF623269	79.82	70.51	3.68507e−15	Plecoptera	Taeniopterygidae	Bolotoperla rossi
Pteronarcys californica 16S ribosomal RNA gene, pa	EF623261	81	64.1	3.68507e−15	Plecoptera	Pteronarcyidae	Pteronarcys californica
Perlinodes aurea 16S ribosomal RNA gene, partial s	EF623234	81.37	64.74	3.68507e−15	Plecoptera	Perlodidae	Perlinodes aurea
Austronemoura sp. BYU_PL149 16S ribosomal RNA gene	EF623200	81.37	64.74	3.68507e−15	Plecoptera	Notonemouridae	Austronemoura sp. BYU_PL149
Calileuctra ephemera 16S ribosomal RNA gene, parti	EF623181	81.82	62.82	3.68507e−15	Plecoptera	Leuctridae	Calileuctra ephemera
Perlomyia utahensis 16S ribosomal RNA gene, partia	EF623179	81.82	62.82	3.68507e−15	Plecoptera	Leuctridae	Perlomyia utahensis
Chloroperla tripunctata 16S ribosomal RNA gene, pa	EF623137	80.77	66.03	3.68507e−15	Plecoptera	Chloroperlidae	Chloroperla tripunctata
Pteronarcys princeps mitochondrion, complete genom	AY687866	81	64.1	3.68507e−15	Plecoptera	Pteronarcyidae	Pteronarcys princeps
Dipteromimus sp. Ofunato−1 mitochondrial gene for	AB110236	81	64.1	3.68507e−15	Ephemeroptera	Dipteromimidae	Dipteromimus sp. Ofunato−1
Epeorus longimanus 16S ribosomal RNA gene, partial	AY749757	74.36	99.36	3.68507e−15	Ephemeroptera	Heptageniidae	Epeorus longimanus
Mesocapnia arizonensis mitochondrion, partial geno	KP642637	80.77	64.74	1.28622e−14	Plecoptera	Capniidae	Mesocapnia arizonensis
Apterodorcus bacchus voucher SK121 16S ribosomal R	KP250211	80.2	64.74	1.28622e−14	Coleoptera	Lucanidae	Apterodorcus bacchus
Allocapnia recta isolate H19 16S ribosomal RNA gen	KC881054	80.77	64.74	1.28622e−14	Plecoptera	Capniidae	Allocapnia recta
Allocapnia recta isolate H16 16S ribosomal RNA gen	KC881051	80.77	64.74	1.28622e−14	Plecoptera	Capniidae	Allocapnia recta
Allocapnia recta isolate H15 16S ribosomal RNA gen	KC881050	80.77	64.74	1.28622e−14	Plecoptera	Capniidae	Allocapnia recta
Allocapnia recta isolate H14 16S ribosomal RNA gen	KC881049	80.77	64.74	1.28622e−14	Plecoptera	Capniidae	Allocapnia recta
Allocapnia recta isolate H13 16S ribosomal RNA gen	KC881048	80.77	64.74	1.28622e−14	Plecoptera	Capniidae	Allocapnia recta
Allocapnia recta isolate H11 16S ribosomal RNA gen	KC881046	80.77	64.74	1.28622e−14	Plecoptera	Capniidae	Allocapnia recta
Allocapnia recta isolate H10 16S ribosomal RNA gen	KC881045	80.77	64.74	1.28622e−14	Plecoptera	Capniidae	Allocapnia recta
Allocapnia recta isolate H7 16S ribosomal RNA gene	KC881042	80.77	64.74	1.28622e−14	Plecoptera	Capniidae	Allocapnia recta
Allocapnia recta isolate H6 16S ribosomal RNA gene	KC881041	80.77	64.74	1.28622e−14	Plecoptera	Capniidae	Allocapnia recta
Allocapnia recta isolate H3 16S ribosomal RNA gene	KC881038	80.77	64.74	1.28622e−14	Plecoptera	Capniidae	Allocapnia recta
Dolichopoda hyrcana voucher LEN 16S ribosomal RNA	EU887871	80.2	64.74	1.28622e−14	Orthoptera	Rhaphidophoridae	Dolichopoda hyrcana
Pteronarcys sachalina 16S ribosomal RNA gene, part	EF623264	80.2	64.74	1.28622e−14	Plecoptera	Pteronarcyidae	Pteronarcys sachalina
Salmoperla sylvanica 16S ribosomal RNA gene, parti	EF623222	81.19	64.1	1.28622e−14	Plecoptera	Perlodidae	Salmoperla sylvanica
Megarcys signata 16S ribosomal RNA gene, partial s	EF623215	81.19	64.1	1.28622e−14	Plecoptera	Perlodidae	Megarcys signata
Kimminsoperla albomacula 16S ribosomal RNA gene, p	EF623202	80.2	64.74	1.28622e−14	Plecoptera	Notonemouridae	Kimminsoperla albomacula
Claudioperla sp. BYU_PL158 16S ribosomal RNA gene,	EF623169	81.19	64.1	1.28622e−14	Plecoptera	Gripopterygidae	Claudioperla sp. BYU_PL158
Pelurgoperla personata 16S ribosomal RNA gene, par	EF623168	81.19	64.1	1.28622e−14	Plecoptera	Gripopterygidae	Pelurgoperla personata
Antarctoperla sp. BYU_PL142 16S ribosomal RNA gene	EF623163	81.19	64.1	1.28622e−14	Plecoptera	Gripopterygidae	Antarctoperla sp. BYU_PL142
Diamphipnopsis sp. BYU_PL138 16S ribosomal RNA gen	EF623145	81.19	64.1	1.28622e−14	Plecoptera	Diamphipnoidae	Diamphipnopsis sp. BYU_PL138
Allocapnia minima 16S ribosomal RNA gene, partial	EF623130	80.77	64.74	1.28622e−14	Plecoptera	Capniidae	Allocapnia minima
Mesocapnia sp. BYU_PL010 16S ribosomal RNA gene, p	EF623127	80.77	64.74	1.28622e−14	Plecoptera	Capniidae	Mesocapnia sp. BYU_PL010
Tasmanoperla larvalis 16S ribosomal RNA gene, part	EF623125	81.19	64.1	1.28622e−14	Plecoptera	Austroperlidae	Tasmanoperla larvalis
Allocapnia recta haplotype 12ar: H12 16S ribosomal	EU306875	80.77	64.74	1.28622e−14	Plecoptera	Capniidae	Allocapnia recta
Allocapnia recta haplotype 11ar: H011 16S ribosoma	EU306874	80.77	64.74	1.28622e−14	Plecoptera	Capniidae	Allocapnia recta
