# Supplementary material for: Testing the potential of a ribosomal 16S marker for DNA metabarcoding of insects
Source: PeerJ. 2016 Apr 19;4:e1966. doi: 10.7717/peerj.1966 (PMC4841222; doi:10.7717/peerj.1966)
Supplement: Supplemental Information 1 [file peerj-04-1966-s006.zip › scripts/5 match OTUs/blast_hits/OTU_150.csv_plot.pdf]

OTU_150.csv

Isotoma viridis

Isotomidae

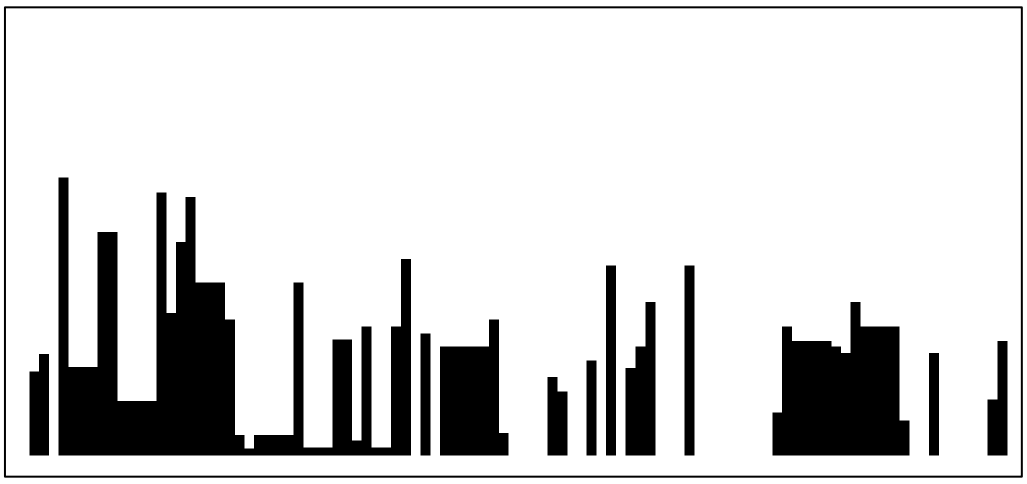

83.92

Isotoma viridis 16S ribosomal RNA gene, partial se	JN970946	83.92	98.62	3.34711e−34		Isotomidae	Isotoma viridis
Folsomia quadrioculata 16S ribosomal RNA gene, par	KC236199	84.75	40.69	1.33416e−07		Isotomidae	Folsomia quadrioculata
Salina sp. BXW−2014 16S ribosomal RNA gene, partia	KM978386	76.09	63.45	4.65666e−07		Paronellidae	Salina sp. BXW−2014
Himalanura sp. FZ−2013 16S ribosomal RNA gene, par	KC236202	93.02	29.66	4.65666e−07		Entomobryidae	Himalanura sp. FZ−2013
Chelarctus cultrifer voucher NTOU M00980 16S ribos	JN701712	84.13	42.76	1.62533e−06	Decapoda	Scyllaridae	Chelarctus cultrifer
Scyllarus pygmaeus isolate M03L043 16S ribosomal R	GQ922072	84.13	42.76	1.62533e−06	Decapoda	Scyllaridae	Scyllarus pygmaeus
Scyllarus pygmaeus 16S ribosomal RNA gene, partial	DQ377973	84.13	42.76	1.62533e−06	Decapoda	Scyllaridae	Scyllarus pygmaeus
Pseudoparonella tanimbarensis 16S ribosomal RNA ge	KM978384	90.48	28.97	1.98006e−05		Paronellidae	Pseudoparonella tanimbarensis
Lepidocyrtus (Ascocyrtus) sp. 2 FZ−2013 16S riboso	KC236190	90.48	28.97	1.98006e−05		Entomobryidae	Lepidocyrtus (Ascocyrtus) sp. 2 FZ−2013
Petrarctus veliger voucher NTOU M00739 16S ribosom	JN701728	82.54	42.76	1.98006e−05	Decapoda	Scyllaridae	Petrarctus veliger
Galearctus kitanoviriosus voucher NTOU M00981 16S	JN701718	82.54	42.76	1.98006e−05	Decapoda	Scyllaridae	Galearctus kitanoviriosus
Crenarctus bicuspidatus voucher MNHN−Pa1028 16S ri	JN701713	82.54	42.76	1.98006e−05	Decapoda	Scyllaridae	Crenarctus bicuspidatus
Scyllarus pygmaeus 16S ribosomal RNA gene, partial	FJ174908	82.54	42.76	1.98006e−05	Decapoda	Scyllaridae	Scyllarus pygmaeus
Sinella curviseta 16S ribosomal RNA gene, partial	KC236219	92.31	26.9	6.9111e−05		Entomobryidae	Sinella curviseta
Palinurellus wieneckii mitochondrion, complete gen	KC847078	86.67	31.03	0.000241221	Decapoda	Synaxidae	Palinurellus wieneckii
Willowsia japonica 16S ribosomal RNA gene, partial	KC236224	90	27.59	0.000241221		Entomobryidae	Willowsia japonica
Entomobrya aino 16S ribosomal RNA gene, partial se	KC236195	92.11	26.21	0.000241221		Entomobryidae	Entomobrya aino
Cryptopygus sverdrupi voucher MNHN:cd468_065 16S r	KJ418952	88.1	28.97	0.000841944		Isotomidae	Cryptopygus sverdrupi
Cryptopygus sverdrupi voucher MNHN:EA010326 16S ri	KJ418902	88.1	28.97	0.000841944		Isotomidae	Cryptopygus sverdrupi
Cryptopygus sverdrupi voucher MNHN:cd325_065 16S r	KJ418901	88.1	28.97	0.000841944		Isotomidae	Cryptopygus sverdrupi
Willowsia sp. 1 FZ−2013 16S ribosomal RNA gene, pa	KC236205	86.36	30.34	0.000841944		Entomobryidae	Willowsia sp. 1 FZ−2013
Scammarctus batei voucher NTOU M00984 16S ribosoma	JN701731	80.95	42.76	0.000841944	Decapoda	Scyllaridae	Scammarctus batei
Gibbularctus gibberosus voucher MNHN−Pa1857 16S ri	JN701722	80.33	42.07	0.000841944	Decapoda	Scyllaridae	Gibbularctus gibberosus
Galearctus timidus voucher NTOU M00982 16S ribosom	JN701721	80.95	42.76	0.000841944	Decapoda	Scyllaridae	Galearctus timidus
Galearctus avulsus voucher MNHN−IU−2010−1910 16S r	JN701719	80.95	42.76	0.000841944	Decapoda	Scyllaridae	Galearctus avulsus
Galearctus aurora voucher NTOU M00863 16S ribosoma	JN701717	80.95	42.76	0.000841944	Decapoda	Scyllaridae	Galearctus aurora
Antipodarctus aoteanus voucher NIWA 57499 16S ribo	JN701705	80.95	42.76	0.000841944	Decapoda	Scyllaridae	Antipodarctus aoteanus
Sminthurinus bimaculatus 16S ribosomal RNA gene, p	AY555555	88.1	28.97	0.000841944		Katiannidae	Sminthurinus bimaculatus
Spodoptera eridania voucher SPO−B70 16S ribosomal	HQ178282	80.36	38.62	0.00293867	Lepidoptera	Noctuidae	Spodoptera eridania
Spodoptera eridania voucher SPO−B16 16S ribosomal	HQ178280	80.36	38.62	0.00293867	Lepidoptera	Noctuidae	Spodoptera eridania
Spodoptera evanida voucher SPO−B155 16S ribosomal	HQ178269	80.36	38.62	0.00293867	Lepidoptera	Noctuidae	Spodoptera evanida
Galearctus rapanus voucher MNHN−IU−2008−16596 16S	JN701720	85.42	32.41	0.00293867	Decapoda	Scyllaridae	Galearctus rapanus
Bathyarctus formosanus voucher NTOU M00976 16S rib	JN701707	85.42	32.41	0.00293867	Decapoda	Scyllaridae	Bathyarctus formosanus
Tylodina fungina voucher EED−Phy−851 16S ribosomal	GU213046	80.7	39.31	0.00293867		Tylodinidae	Tylodina fungina
Piromis sp. KJO−2011 voucher LACM−AHF POLY 2264 16	HQ326961	86.05	29.66	0.00293867	Flabelligerida	Flabelligeridae	Piromis sp. KJO−2011
Spodoptera evanida 16S ribosomal RNA gene, partial	AF173062	80.36	38.62	0.00293867	Lepidoptera	Noctuidae	Spodoptera evanida
Spodoptera eridania 16S ribosomal RNA gene, partia	AF173054	80.36	38.62	0.00293867	Lepidoptera	Noctuidae	Spodoptera eridania
Orchesella villosa 16S ribosomal RNA gene, partial	AY555550	86.05	29.66	0.00293867		Entomobryidae	Orchesella villosa
Callyntrura guangdongensis 16S ribosomal RNA gene,	KM978382	89.19	25.52	0.010257		Paronellidae	Callyntrura guangdongensis
Procordulia smithii isolate MLn187 16S ribosomal R	KM973579	79.31	40	0.010257	Odonata	Corduliidae	Procordulia smithii
Antipodochlora braueri isolate MLn126 16S ribosoma	KM973575	85.71	28.97	0.010257	Odonata	Corduliidae	Antipodochlora braueri
Parribacus antarcticus voucher ICMD13120205 16S ri	KJ150686	79.37	42.76	0.010257	Decapoda	Scyllaridae	Parribacus antarcticus
Acantharctus delfini voucher RCL15 16S ribosomal R	JN566207	85.11	31.72	0.010257	Decapoda	Scyllaridae	Acantharctus delfini
Acantharctus delfini voucher RCL16 16S ribosomal R	JN566208	85.11	31.72	0.010257	Decapoda	Scyllaridae	Acantharctus delfini
Acantharctus delfini voucher RCL10 16S ribosomal R	JN566202	85.11	31.72	0.010257	Decapoda	Scyllaridae	Acantharctus delfini
Acantharctus delfini voucher RCL2 16S ribosomal RN	JN566194	85.11	31.72	0.010257	Decapoda	Scyllaridae	Acantharctus delfini
Acantharctus delfini voucher RCL8 16S ribosomal RN	JN566200	85.11	31.72	0.010257	Decapoda	Scyllaridae	Acantharctus delfini
Aspidosiphon parvulus voucher MCZ DNA100375 16S ri	JN864967	86.36	29.66	0.010257	Aspidosiphonidormes	Aspidosiphonidae	Aspidosiphon parvulus
Eduarctus reticulatus voucher MNHN−Pa1335 16S ribo	JN701716	81.03	39.31	0.010257	Decapoda	Scyllaridae	Eduarctus reticulatus
Parribacus antarcticus voucher KC6087 16S ribosoma	JN701704	79.37	42.76	0.010257	Decapoda	Scyllaridae	Parribacus antarcticus
Parribacus antarcticus voucher KC6016 16S ribosoma	JN701703	79.37	42.76	0.010257	Decapoda	Scyllaridae	Parribacus antarcticus
Parribacus antarcticus voucher KC6015 16S ribosoma	JN701702	79.37	42.76	0.010257	Decapoda	Scyllaridae	Parribacus antarcticus
Laurentaeglyphea neocaledonica 16S ribosomal RNA g	HQ241517	76.54	55.17	0.010257	Decapoda	Glypheidae	Laurentaeglyphea neocaledonica
Ibacus peronii clone PD066 16S ribosomal RNA gene,	HM015419	83.67	33.1	0.010257	Decapoda	Scyllaridae	Ibacus peronii
Progoniada regularis 16S ribosomal RNA gene, parti	GQ426615	82.98	32.41	0.010257	Phyllodocida	Goniadidae	Progoniada regularis
Parribacus antarcticus 16S ribosomal RNA gene, par	FJ174913	79.37	42.76	0.010257	Decapoda	Scyllaridae	Parribacus antarcticus
Scyllarus caparti 16S ribosomal RNA gene, partial	FJ174909	79.37	42.07	0.010257	Decapoda	Scyllaridae	Scyllarus caparti
Chaetopleura apiculata 16S ribosomal RNA gene, par	AY377590	84.44	31.03	0.010257	Neoloricata	Chaetopleuridae	Chaetopleura apiculata
Arctornis sp. H148 large subunit ribosomal RNA gen	KP081943	78.57	38.62	0.0358004	Lepidoptera	Lymantriidae	Arctornis sp. H148
Callyntrura sp. BXW−2014 16S ribosomal RNA gene, p	KM978383	88.89	24.83	0.0358004		Paronellidae	Callyntrura sp. BXW−2014
Spodoptera littoralis voucher SPCAM0144 16S riboso	KP682579	78.57	38.62	0.0358004	Lepidoptera	Noctuidae	Spodoptera littoralis
Epilachna convergens voucher KS185 16S ribosomal R	KP143607	84.09	30.34	0.0358004	Coleoptera	Coccinellidae	Epilachna convergens
Osedax sp. 'MB16' 16S ribosomal RNA gene, partial	KP119581	85.11	31.03	0.0358004	Sabellida	Siboglinidae	Osedax sp. 'MB16'
Eunice gracilicirrata voucher CAS187532 16S riboso	JX559748	87.18	26.9	0.0358004	Eunicida	Eunicidae	Eunice gracilicirrata
Mononeuron duguetiae strain CNIN754 16S ribosomal	KJ586745	72.45	63.45	0.0358004	Hymenoptera	Braconidae	Mononeuron duguetiae
Omorgus monachus isolate 93C 16S ribosomal RNA gen	KC801115	79.31	39.31	0.0358004	Coleoptera	Trogidae	Omorgus monachus
Endochus sp. 00019754 isolate RCW 2661 16S ribosom	JQ888422	76.06	48.97	0.0358004	Hemiptera	Reduviidae	Endochus sp. 00019754
Heteromurus nitidus 16S ribosomal RNA gene, partia	KC291493	88.89	24.83	0.0358004		Entomobryidae	Heteromurus nitidus
Spodoptera ochrea voucher SPO−B110 16S ribosomal R	HQ178349	78.57	38.62	0.0358004	Lepidoptera	Noctuidae	Spodoptera ochrea
Spodoptera ochrea voucher SPO−B86 16S ribosomal RN	HQ178348	78.57	38.62	0.0358004	Lepidoptera	Noctuidae	Spodoptera ochrea
Spodoptera ochrea voucher SPO−B84 16S ribosomal RN	HQ178346	78.57	38.62	0.0358004	Lepidoptera	Noctuidae	Spodoptera ochrea
Spodoptera litura voucher SPO−B15 16S ribosomal RN	HQ178334	78.57	38.62	0.0358004	Lepidoptera	Noctuidae	Spodoptera litura
Spodoptera littoralis voucher SPO−B14 16S ribosoma	HQ178324	78.57	38.62	0.0358004	Lepidoptera	Noctuidae	Spodoptera littoralis
Spodoptera latifascia voucher SPO−B3 16S ribosomal	HQ178314	78.57	38.62	0.0358004	Lepidoptera	Noctuidae	Spodoptera latifascia
Spodoptera dolichos voucher SPO−B2 16S ribosomal R	HQ178271	78.57	38.62	0.0358004	Lepidoptera	Noctuidae	Spodoptera dolichos
Spodoptera cosmiodes voucher SPO−B19 16S ribosomal	HQ178256	78.57	38.62	0.0358004	Lepidoptera	Noctuidae	Spodoptera cosmioides
Electra sp. Eom 1026 partial 16S rRNA gene, specim	FR754522	82	34.48	0.0358004	Cheilostomatida	Electridae	Electra sp. Eom 1026
Panulirus interruptus voucher KC6006 16S ribosomal	JN701738	86.05	28.97	0.0358004	Decapoda	Palinuridae	Panulirus interruptus
Onuphis iridescens 16S ribosomal RNA gene, partial	HM746715	85.37	28.28	0.0358004	Eunicida	Onuphidae	Onuphis iridescens
Onuphis cf. iridescens JZ−2010 voucher USNM1121744	GQ478127	85.37	28.28	0.0358004	Eunicida	Onuphidae	Onuphis cf. iridescens JZ−2010
Progoniada regularis 16S ribosomal RNA gene, parti	GQ426616	85.37	28.28	0.0358004	Phyllodocida	Goniadidae	Progoniada regularis
Goniadella bobrezkii 16S ribosomal RNA gene, parti	GQ426614	85.37	28.28	0.0358004	Phyllodocida	Goniadidae	Goniadella bobrezkii
Osedax sp. 'white−collar' isolate V2874−7 16S ribo	FJ347659	85.11	31.03	0.0358004	Sabellida	Siboglinidae	Osedax sp. 'white−collar'
Phyllodoce groenlandica voucher SMNH<SWE>:F. Pleij	EU431118	84.78	31.03	0.0358004	Phyllodocida	Phyllodocidae	Phyllodoce groenlandica
Cryptopygus antarcticus mitochondrion, complete ge	EU016194	87.18	26.9	0.0358004		Isotomidae	Cryptopygus antarcticus
Panulirus interruptus isolate RBM2 16S ribosomal R	EF546600	86.05	28.97	0.0358004	Decapoda	Palinuridae	Panulirus interruptus
Panulirus interruptus isolate RBM1 16S ribosomal R	EF546599	86.05	28.97	0.0358004	Decapoda	Palinuridae	Panulirus interruptus
Panulirus interruptus isolate RGU2 16S ribosomal R	EF546598	86.05	28.97	0.0358004	Decapoda	Palinuridae	Panulirus interruptus
Panulirus interruptus isolate RES2 16S ribosomal R	EF546597	86.05	28.97	0.0358004	Decapoda	Palinuridae	Panulirus interruptus
Nannophlebia risi voucher JW_DNA_11 16S ribosomal	EF631569	81.63	33.79	0.0358004	Odonata	Libellulidae	Nannophlebia risi
Trichopteryx ustata gene for 16S rRNA, partial seq	AB265432	78.57	38.62	0.0358004	Lepidoptera	Geometridae	Trichopteryx ustata
Trichopteryx miracula gene for 16S rRNA, partial s	AB265431	78.57	38.62	0.0358004	Lepidoptera	Geometridae	Trichopteryx miracula
Phyllodoce groenlandica 16S ribosomal RNA gene, pa	AY996057	84.78	31.03	0.0358004	Phyllodocida	Phyllodocidae	Phyllodoce groenlandica
Spodoptera pectinicornis 16S ribosomal RNA gene, p	AF173072	78.57	38.62	0.0358004	Lepidoptera	Noctuidae	Spodoptera pectinicornis
Spodoptera ochrea 16S ribosomal RNA gene, partial	AF173070	78.57	38.62	0.0358004	Lepidoptera	Noctuidae	Spodoptera ochrea
Spodoptera picta 16S ribosomal RNA gene, partial s	AF173066	78.57	38.62	0.0358004	Lepidoptera	Noctuidae	Spodoptera picta
Spodoptera littoralis 16S ribosomal RNA gene, part	AF173064	78.57	38.62	0.0358004	Lepidoptera	Noctuidae	Spodoptera littoralis
Spodoptera descoinsi 16S ribosomal RNA gene, parti	AF173063	78.57	38.62	0.0358004	Lepidoptera	Noctuidae	Spodoptera descoinsi
Lasaea sp. DOF−02−2000 isolate P2 16S ribosomal RN	AF215750	82.61	31.72	0.0358004	Veneroida	Lasaeidae	Lasaea sp. DOF−02−2000
Morulina verrucosa 16S ribosomal RNA gene, partial	AY555554	85.37	28.28	0.0358004		Neanuridae	Morulina verrucosa
