# Supplementary material for: Testing the potential of a ribosomal 16S marker for DNA metabarcoding of insects
Source: PeerJ. 2016 Apr 19;4:e1966. doi: 10.7717/peerj.1966 (PMC4841222; doi:10.7717/peerj.1966)
Supplement: Supplemental Information 1 [file peerj-04-1966-s006.zip › scripts/5 match OTUs/blast_hits/OTU_151.csv_plot.pdf]

OTU_151.csv

Hydraena nigrita

Hydraenidae

Coleoptera

100

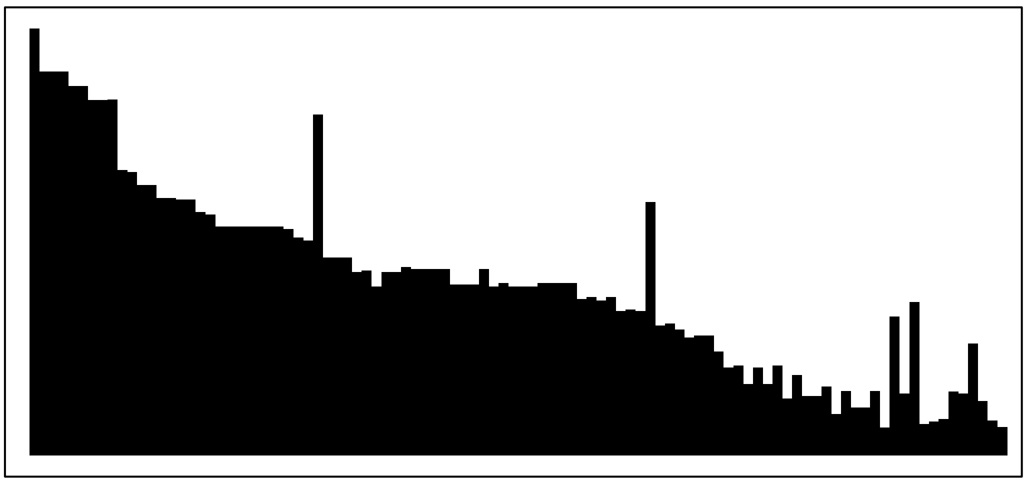

Hydraena nigrita voucher MNCN−AI345 large subunit	HM588450	100	100	6.2607e−69	Coleoptera	Hydraenidae	Hydraena nigrita
Hydraena subsequens mitochondrial partial 16S rRNA	HE971060	97.99	100	1.37901e−64	Coleoptera	Hydraenidae	Hydraena subsequens
Hydraena subimpressa mitochondrial 16S rRNA gene (	HE971059	97.99	100	1.37901e−64	Coleoptera	Hydraenidae	Hydraena subimpressa
Hydraena sicula mitochondrial 16S rRNA gene (parti	HE971032	97.99	100	1.37901e−64	Coleoptera	Hydraenidae	Hydraena sicula
Hydraena helena mitochondrial 16S rRNA gene (parti	HE971091	97.32	100	1.67998e−63	Coleoptera	Hydraenidae	Hydraena helena
Hydraena speciosa mitochondrial 16S rRNA gene (par	HE971054	97.32	100	1.67998e−63	Coleoptera	Hydraenidae	Hydraena speciosa
Hydraena subjuncta mitochondrial 16S rRNA gene (pa	HE971050	96.64	100	7.14346e−62	Coleoptera	Hydraenidae	Hydraena subjuncta
Hydraena similis mitochondrial 16S rRNA gene (part	HE971041	96.64	100	7.14346e−62	Coleoptera	Hydraenidae	Hydraena similis
Hydraena quilisi voucher MNCN−AI1085 large subunit	HM588416	96.67	100	2.49331e−61	Coleoptera	Hydraenidae	Hydraena quilisi
Hydraena carbonaria mitochondrial 16S rRNA gene (p	HE971096	93.38	100	2.33521e−55	Coleoptera	Hydraenidae	Hydraena carbonaria
Hydraena servilia voucher MNCN−AI274 large subunit	HM588430	93.29	100	2.33521e−55	Coleoptera	Hydraenidae	Hydraena servilia
Hydraena schubertorum mitochondrial 16S rRNA gene	HE971093	92.67	100	3.46576e−53	Coleoptera	Hydraenidae	Hydraena schubertorum
Hydraena sardoa mitochondrial 16S rRNA gene (parti	HE971011	92.67	100	3.46576e−53	Coleoptera	Hydraenidae	Hydraena sardoa
Hydraena riberai voucher MNCN−AI568 large subunit	HM588486	92.05	100	1.20967e−52	Coleoptera	Hydraenidae	Hydraena riberai
Hydraena reyi voucher MNCN−AI320 large subunit rib	HM588443	92.05	100	1.20967e−52	Coleoptera	Hydraenidae	Hydraena reyi
Hydraena dentipalpis voucher MNCN−AI492 large subu	HM588478	92	100	4.22216e−52	Coleoptera	Hydraenidae	Hydraena dentipalpis
Hydraena subacuminata voucher MNCN−AI305 large sub	HM588439	92	100	4.22216e−52	Coleoptera	Hydraenidae	Hydraena subacuminata
Hydraena melas mitochondrial 16S rRNA gene (partia	HE971066	91.39	100	1.47368e−51	Coleoptera	Hydraenidae	Hydraena melas
Hydraena pachyptera mitochondrial 16S rRNA gene (p	HE971028	91.28	98.66	1.79531e−50	Coleoptera	Hydraenidae	Hydraena pachyptera
Hydraena brachymera mitochondrial 16S rRNA gene (p	HE971064	90.73	100	6.26624e−50	Coleoptera	Hydraenidae	Hydraena brachymera
Hydraena allomorpha mitochondrial 16S rRNA gene (p	HE971063	90.73	100	6.26624e−50	Coleoptera	Hydraenidae	Hydraena allomorpha
Hydraena riparia mitochondrial 16S rRNA gene (part	HE971062	90.73	100	6.26624e−50	Coleoptera	Hydraenidae	Hydraena riparia
Hydraena britteni mitochondrial 16S rRNA gene (par	HE971046	90.73	100	6.26624e−50	Coleoptera	Hydraenidae	Hydraena britteni
Hydraena assimilis mitochondrial 16S rRNA gene (pa	HE971044	90.73	100	6.26624e−50	Coleoptera	Hydraenidae	Hydraena assimilis
Hydraena assimilis isolate 29_1 large subunit ribo	HQ425872	90.73	100	6.26624e−50	Coleoptera	Hydraenidae	Hydraena assimilis
Hydraena capta voucher MNCN−AI167 large subunit ri	HM588423	90.73	100	6.26624e−50	Coleoptera	Hydraenidae	Hydraena capta
Hydraena sharpi mitochondrial 16S rRNA gene (parti	HE971075	90.6	100	7.63384e−49	Coleoptera	Hydraenidae	Hydraena sharpi
Hydraena pygmaea voucher MNCN−AI346 large subunit	HM588451	90.2	100	7.63384e−49	Coleoptera	Hydraenidae	Hydraena pygmaea
Hydraena morio voucher MNCN−AI1203 large subunit r	HM588418	90.07	100	7.63384e−49	Coleoptera	Hydraenidae	Hydraena morio
Hydraena holdhausi voucher MNCN−AI1025 large subun	HM588410	95.97	82.55	3.24599e−47	Coleoptera	Hydraenidae	Hydraena holdhausi
Hydraena dochula voucher MNCN−AI518 large subunit	HM588483	89.26	100	3.95443e−46	Coleoptera	Hydraenidae	Hydraena dochula
Hydraena kasyi voucher MNCN−AI1026 large subunit r	HM588411	89.26	100	3.95443e−46	Coleoptera	Hydraenidae	Hydraena kasyi
Hydraena sp. MNCN AI518/23864 mitochondrial partia	FM946171	89.26	100	3.95443e−46	Coleoptera	Hydraenidae	Hydraena sp. MNCN AI518/23864
Hydraena gavarrensis voucher MNCN−AI1288 large sub	HM588419	88.59	100	1.38023e−45	Coleoptera	Hydraenidae	Hydraena gavarrensis
Hydraena fontiscarsavii isolate 134_1 large subuni	HQ425871	88.67	100	4.81748e−45	Coleoptera	Hydraenidae	Hydraena fontiscarsavii
Hydraena intermedia mitochondrial 16S rRNA gene (p	HE971073	87.92	100	1.68146e−44	Coleoptera	Hydraenidae	Hydraena intermedia
Hydraena sp. IBE RA97 mitochondrial 16S rRNA gene	HE971030	88.59	100	1.68146e−44	Coleoptera	Hydraenidae	Hydraena sp. IBE RA97
Hydraena pulchella mitochondrial 16S rRNA gene (pa	HE971024	88.59	100	1.68146e−44	Coleoptera	Hydraenidae	Hydraena pulchella
Hydraena sp. MNCN AI691 large subunit ribosomal RN	HM588487	88.82	100	1.68146e−44	Coleoptera	Hydraenidae	Hydraena sp. MNCN AI691
Hydraena coryleti mitochondrial 16S rRNA gene (par	HE971090	88.74	100	5.86889e−44	Coleoptera	Hydraenidae	Hydraena coryleti
Hydraena angulosa mitochondrial 16S rRNA gene (par	HE971053	88.74	100	5.86889e−44	Coleoptera	Hydraenidae	Hydraena angulosa
Hydraena subirregularis mitochondrial 16S rRNA gen	HE971042	88.74	100	5.86889e−44	Coleoptera	Hydraenidae	Hydraena subirregularis
Hydraena armipes mitochondrial 16S rRNA gene (part	HE971025	88.74	100	5.86889e−44	Coleoptera	Hydraenidae	Hydraena armipes
Hydraena sinope mitochondrial 16S rRNA gene (parti	FR773522	88	100	5.86889e−44	Coleoptera	Hydraenidae	Hydraena sinope
Hydraena septemlacuum isolate 141_1 large subunit	HQ425869	88	100	5.86889e−44	Coleoptera	Hydraenidae	Hydraena septemlacuum
Hydraena sinope voucher MNCN−AI788 large subunit r	HM588491	88	100	5.86889e−44	Coleoptera	Hydraenidae	Hydraena sinope
Hydraena delia voucher MNCN−AI1061 large subunit r	HM588415	88.74	100	5.86889e−44	Coleoptera	Hydraenidae	Hydraena delia
Hydraena andreini mitochondrial 16S rRNA gene (par	HE971095	87.92	100	2.04844e−43	Coleoptera	Hydraenidae	Hydraena andreini
Hydraena cordata mitochondrial 16S rRNA gene (part	HE971068	88.08	100	2.04844e−43	Coleoptera	Hydraenidae	Hydraena cordata
Hydraena affusa mitochondrial 16S rRNA gene (parti	HE971043	87.92	100	2.04844e−43	Coleoptera	Hydraenidae	Hydraena affusa
Hydraena chiesai mitochondrial 16S rRNA gene (part	HE971023	87.92	100	2.04844e−43	Coleoptera	Hydraenidae	Hydraena chiesai
Hydraena persica mitochondrial 16S rRNA gene (part	HE971017	87.92	100	2.04844e−43	Coleoptera	Hydraenidae	Hydraena persica
Hydraena rigua mitochondrial 16S rRNA gene (partia	HE971086	88.08	100	7.14977e−43	Coleoptera	Hydraenidae	Hydraena rigua
Hydraena attaleiae mitochondrial 16S rRNA gene (pa	HE971078	88.08	100	7.14977e−43	Coleoptera	Hydraenidae	Hydraena attaleiae
Hydraena stussineri mitochondrial partial 16S rRNA	HE971058	88.08	100	7.14977e−43	Coleoptera	Hydraenidae	Hydraena stussineri
Hydraena graphica mitochondrial 16S rRNA gene (par	HE971051	88.08	100	2.49552e−42	Coleoptera	Hydraenidae	Hydraena graphica
Hydraena scitula isolate 142_1 large subunit ribos	HQ425870	87.33	100	2.49552e−42	Coleoptera	Hydraenidae	Hydraena scitula
Hydraena barrosi voucher MNCN−AI954 large subunit	HM588496	87.42	100	8.7102e−42	Coleoptera	Hydraenidae	Hydraena barrosi
Hydraena balearica voucher MNCN−AI175 large subuni	HM588427	87.25	100	8.7102e−42	Coleoptera	Hydraenidae	Hydraena balearica
Hydraena bolivari voucher MNCN−AI171 large subunit	HM588425	87.42	100	8.7102e−42	Coleoptera	Hydraenidae	Hydraena bolivari
Hydraena albai mitochondrial partial 16S rRNA gene	HE971097	86.75	100	1.06112e−40	Coleoptera	Hydraenidae	Hydraena albai
Hydraena palustris voucher MNCN−AI309 large subuni	HM588440	86.84	100	1.06112e−40	Coleoptera	Hydraenidae	Hydraena palustris
Hydraena corinna voucher MNCN−AI284 large subunit	HM588432	86.75	100	1.06112e−40	Coleoptera	Hydraenidae	Hydraena corinna
Hydraena platycnemis mitochondrial 16S rRNA gene (	HE971026	91.87	82.55	3.70367e−40	Coleoptera	Hydraenidae	Hydraena platycnemis
Hydraena croatica mitochondrial 16S rRNA gene (par	HE971027	86.09	100	4.512e−39	Coleoptera	Hydraenidae	Hydraena croatica
Hydraena circulata mitochondrial 16S rRNA gene (pa	HE971022	86.18	100	4.512e−39	Coleoptera	Hydraenidae	Hydraena circulata
Hydraena antiatlantica voucher MNCN−AI567 large su	HM588485	85.91	100	4.512e−39	Coleoptera	Hydraenidae	Hydraena antiatlantica
Hydraena sp. MNCN AI610 mitochondrial 16S rRNA gen	HE971085	85.53	100	1.57484e−38	Coleoptera	Hydraenidae	Hydraena sp. MNCN AI610
Hydraena vandykei mitochondrial 16S rRNA gene (par	HE971052	85.62	100	6.6964e−37	Coleoptera	Hydraenidae	Hydraena vandykei
Hydraena arenicola voucher MNCN−AI504 large subuni	HM588479	85.62	100	6.6964e−37	Coleoptera	Hydraenidae	Hydraena arenicola
Hydraena marcosae voucher MNCN−AI904 large subunit	HM588495	84.87	100	2.33727e−36	Coleoptera	Hydraenidae	Hydraena marcosae
Hydraena curta mitochondrial 16S rRNA gene (partia	HE971067	84.11	100	2.84738e−35	Coleoptera	Hydraenidae	Hydraena curta
Hydraena cf. concinna IBE AF194 mitochondrial 16S	HE971009	84.21	100	2.84738e−35	Coleoptera	Hydraenidae	Hydraena cf. concinna IBE AF194
Hydraena sp. MNCN AI541 mitochondrial 16S rRNA gen	HE971081	83.33	100	3.46882e−34	Coleoptera	Hydraenidae	Hydraena sp. MNCN AI541
Hydraena hosiwergi mitochondrial 16S rRNA gene (pa	HE971037	84.11	100	3.46882e−34	Coleoptera	Hydraenidae	Hydraena hosiwergi
Hydraena petila voucher MNCN−AI465 large subunit r	HM588475	83.33	100	3.46882e−34	Coleoptera	Hydraenidae	Hydraena petila
Hydraena exarata voucher MNCN−AI169 large subunit	HM588424	84.21	100	1.21074e−33	Coleoptera	Hydraenidae	Hydraena exarata
Hydraena marinae mitochondrial partial 16S rRNA ge	HE971057	82.67	100	4.22589e−33	Coleoptera	Hydraenidae	Hydraena marinae
Hydraena sp. IBE RA322 mitochondrial 16S rRNA gene	HE971020	83.77	100	4.22589e−33	Coleoptera	Hydraenidae	Hydraena sp. IBE RA322
Hydraena pacifica mitochondrial 16S rRNA gene (par	HE971048	82.78	100	1.47498e−32	Coleoptera	Hydraenidae	Hydraena pacifica
Hydraena bisulcata voucher MNCN−AI172 large subuni	HM588426	82.78	100	1.47498e−32	Coleoptera	Hydraenidae	Hydraena bisulcata
Hydraena manguao mitochondrial 16S rRNA gene (part	HE971036	83.22	99.33	1.79689e−31	Coleoptera	Hydraenidae	Hydraena manguao
Chondria armipes 16S ribosomal RNA gene, partial s	GQ302111	81.94	100	1.79689e−31	Coleoptera	Endomychidae	Chondria armipes
Hydraena cf. rhinoceros IBE RA319 mitochondrial 16	HE971018	83.01	100	6.27177e−31	Coleoptera	Hydraenidae	Hydraena cf. rhinoceros IBE RA319
Hydraena miyatakei voucher MNCN−AI828 large subuni	HM588494	82.24	100	6.27177e−31	Coleoptera	Hydraenidae	Hydraena miyatakei
Hydraena monikae voucher MNCN−AI455 large subunit	HM588473	82.24	100	6.27177e−31	Coleoptera	Hydraenidae	Hydraena monikae
Hydraena sp. MNCN AI429 mitochondrial 16S rRNA gen	HE971072	83.01	100	2.18906e−30	Coleoptera	Hydraenidae	Hydraena sp. MNCN AI429
Chondria nigra 16S ribosomal RNA gene, partial seq	GQ302112	81.29	100	2.18906e−30	Coleoptera	Endomychidae	Chondria nigra
Hydraena fontana voucher MNCN−AI708 large subunit	HM588488	86.51	82.55	2.18906e−30	Coleoptera	Hydraenidae	Hydraena fontana
Hydraena sp. IBE AF202 mitochondrial 16S rRNA gene	HE971010	82.89	100	7.64058e−30	Coleoptera	Hydraenidae	Hydraena sp. IBE AF202
Endomychus biguttatus 16S ribosomal RNA gene, part	GQ302115	87.18	78.52	7.64058e−30	Coleoptera	Endomychidae	Endomychus biguttatus
Hydraena (Hydraenopsis) sp. MNCN AI423 large subun	HM588467	81.46	100	7.64058e−30	Coleoptera	Hydraenidae	Hydraena (Hydraenopsis) sp. MNCN AI423
Hydraena rugosa voucher MNCN−AI392 large subunit r	HM588464	81.58	100	7.64058e−30	Coleoptera	Hydraenidae	Hydraena rugosa
Hydraena cooperi voucher MNCN−AI456 large subunit	HM588474	81.7	100	2.66683e−29	Coleoptera	Hydraenidae	Hydraena cooperi
Euphydryas aurinia 16S ribosomal RNA gene, partial	JX185896	82.99	97.32	9.30814e−29	Lepidoptera	Nymphalidae	Euphydryas aurinia
Hydraena sp. MNCN AI687 mitochondrial 16S rRNA gen	HE971088	82.89	100	9.30814e−29	Coleoptera	Hydraenidae	Hydraena sp. MNCN AI687
Hydraena sp. IBE RA323 mitochondrial 16S rRNA gene	HE971021	85.25	81.21	9.30814e−29	Coleoptera	Hydraenidae	Hydraena sp. IBE RA323
Phyciodes tharos voucher NW34−2 16S ribosomal RNA	AF186916	82.55	95.97	3.24886e−28	Lepidoptera	Nymphalidae	Phyciodes tharos
Anthanassa ptolyca isolate 34−8 16S ribosomal RNA	AF186913	81.63	95.97	3.24886e−28	Lepidoptera	Nymphalidae	Anthanassa ptolyca
Papilio anactus mitochondrion, large subunit ribos	AJ224053	81.33	100	3.24886e−28	Lepidoptera	Papilionidae	Papilio anactus
