# Supplementary material for: Testing the potential of a ribosomal 16S marker for DNA metabarcoding of insects
Source: PeerJ. 2016 Apr 19;4:e1966. doi: 10.7717/peerj.1966 (PMC4841222; doi:10.7717/peerj.1966)
Supplement: Supplemental Information 1 [file peerj-04-1966-s006.zip › scripts/5 match OTUs/blast_hits/OTU_157.csv_plot.pdf]

OTU_157.csv

Ephemera danica

Ephemeridae

Ephemeroptera

100

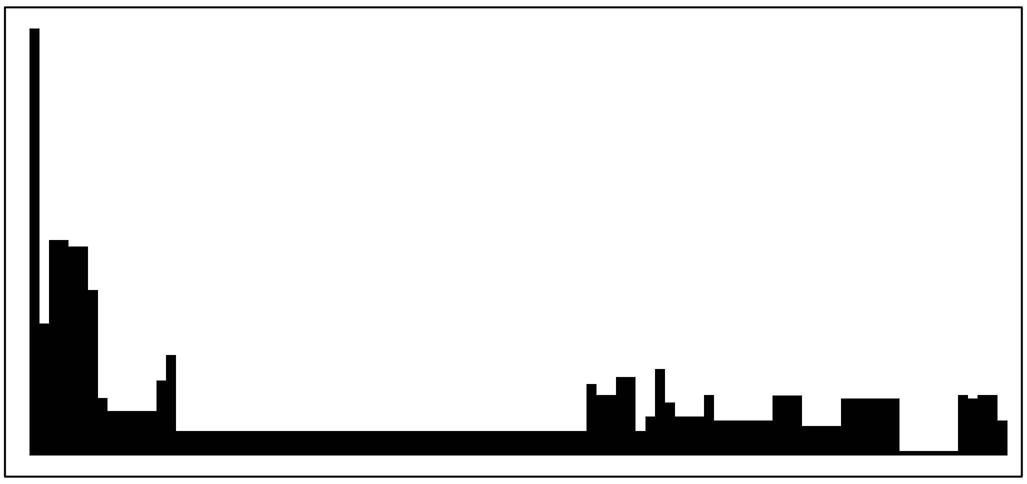

Ephemera danica 16S ribosomal RNA gene, partial se	KF855860	100	64.47	3.12016e−41	Ephemeroptera	Ephemeridae	Ephemera danica
Habroleptoides confusa voucher BYU:IGCEP153 16S ri	GQ118289	86.18	100	1.08904e−40	Ephemeroptera		Habroleptoides confusa
Ephemera sp. 1 BM−2008 12S ribosomal RNA gene, par	EU477611	90.1	66.45	3.33436e−28	Ephemeroptera	Ephemeridae	Ephemera sp. 1 BM−2008
Ephemera sp. 'Bonn' large subunit ribosomal RNA ge	AF266047	90.1	66.45	3.33436e−28	Ephemeroptera	Ephemeridae	Ephemera sp. 'Bonn'
Ephemera orientalis voucher EPH01 tRNA−Leu, 16S ri	KF256907	89.8	64.47	1.1638e−27	Ephemeroptera	Ephemeridae	Ephemera orientalis
Ephemera orientalis mitochondrion, complete genome	EU591678	89.8	64.47	1.1638e−27	Ephemeroptera	Ephemeridae	Ephemera orientalis
Ephemera simulans voucher BYU:IGCEP038 16S ribosom	GQ118285	87.76	64.47	6.02866e−25	Ephemeroptera	Ephemeridae	Ephemera simulans
Siphlonurus immanis voucher NKMT032 mitochondrion,	FJ606783	82.69	68.42	4.63483e−20	Ephemeroptera	Siphlonuridae	Siphlonurus immanis
Palingenia longicauda mitochondrial partial 16S rR	HE650662	82.08	69.08	1.97078e−18	Ephemeroptera	Palingeniidae	Palingenia longicauda
Palingenia longicauda mitochondrial partial 16S rR	HE650616	82.08	69.08	1.97078e−18	Ephemeroptera	Palingeniidae	Palingenia longicauda
Palingenia longicauda mitochondrial partial 16S rR	HE650475	82.08	69.08	1.97078e−18	Ephemeroptera	Palingeniidae	Palingenia longicauda
Palingenia longicauda mitochondrial partial 16S rR	HE650547	82.08	69.08	1.97078e−18	Ephemeroptera	Palingeniidae	Palingenia longicauda
Palingenia longicauda mitochondrial partial 16S rR	HE650502	82.08	69.08	1.97078e−18	Ephemeroptera	Palingeniidae	Palingenia longicauda
Eresia plaginota isolate 38−4 16S ribosomal RNA ge	AF186912	83.51	63.16	6.87869e−18	Lepidoptera	Nymphalidae	Eresia plaginota
Ctenoplusia limbirena mitochondrion, complete geno	KM244665	84.69	63.16	2.4009e−17	Lepidoptera	Noctuidae	Ctenoplusia limbirena
Palingenia longicauda mitochondrial partial 16S rR	HE650589	81.13	69.08	2.4009e−17	Ephemeroptera	Palingeniidae	Palingenia longicauda
Palingenia longicauda mitochondrial partial 16S rR	HE650657	81.13	69.08	2.4009e−17	Ephemeroptera	Palingeniidae	Palingenia longicauda
Palingenia longicauda mitochondrial partial 16S rR	HE650650	81.13	69.08	2.4009e−17	Ephemeroptera	Palingeniidae	Palingenia longicauda
Palingenia longicauda mitochondrial partial 16S rR	HE650647	81.13	69.08	2.4009e−17	Ephemeroptera	Palingeniidae	Palingenia longicauda
Palingenia longicauda mitochondrial partial 16S rR	HE650637	81.13	69.08	2.4009e−17	Ephemeroptera	Palingeniidae	Palingenia longicauda
Palingenia longicauda mitochondrial partial 16S rR	HE650635	81.13	69.08	2.4009e−17	Ephemeroptera	Palingeniidae	Palingenia longicauda
Palingenia longicauda mitochondrial partial 16S rR	HE650631	81.13	69.08	2.4009e−17	Ephemeroptera	Palingeniidae	Palingenia longicauda
Palingenia longicauda mitochondrial partial 16S rR	HE650625	81.13	69.08	2.4009e−17	Ephemeroptera	Palingeniidae	Palingenia longicauda
Palingenia longicauda mitochondrial partial 16S rR	HE650623	81.13	69.08	2.4009e−17	Ephemeroptera	Palingeniidae	Palingenia longicauda
Palingenia longicauda mitochondrial partial 16S rR	HE650622	81.13	69.08	2.4009e−17	Ephemeroptera	Palingeniidae	Palingenia longicauda
Palingenia longicauda mitochondrial partial 16S rR	HE650618	81.13	69.08	2.4009e−17	Ephemeroptera	Palingeniidae	Palingenia longicauda
Palingenia longicauda mitochondrial partial 16S rR	HE650617	81.13	69.08	2.4009e−17	Ephemeroptera	Palingeniidae	Palingenia longicauda
Palingenia longicauda mitochondrial partial 16S rR	HE650604	81.13	69.08	2.4009e−17	Ephemeroptera	Palingeniidae	Palingenia longicauda
Palingenia longicauda mitochondrial partial 16S rR	HE650594	81.13	69.08	2.4009e−17	Ephemeroptera	Palingeniidae	Palingenia longicauda
Palingenia longicauda mitochondrial partial 16S rR	HE650588	81.13	69.08	2.4009e−17	Ephemeroptera	Palingeniidae	Palingenia longicauda
Palingenia longicauda mitochondrial partial 16S rR	HE650575	81.13	69.08	2.4009e−17	Ephemeroptera	Palingeniidae	Palingenia longicauda
Palingenia longicauda mitochondrial partial 16S rR	HE650569	81.13	69.08	2.4009e−17	Ephemeroptera	Palingeniidae	Palingenia longicauda
Palingenia longicauda mitochondrial partial 16S rR	HE650550	81.13	69.08	2.4009e−17	Ephemeroptera	Palingeniidae	Palingenia longicauda
Palingenia longicauda mitochondrial partial 16S rR	HE650542	81.13	69.08	2.4009e−17	Ephemeroptera	Palingeniidae	Palingenia longicauda
Palingenia longicauda mitochondrial partial 16S rR	HE650533	81.13	69.08	2.4009e−17	Ephemeroptera	Palingeniidae	Palingenia longicauda
Palingenia longicauda mitochondrial partial 16S rR	HE650503	81.13	69.08	2.4009e−17	Ephemeroptera	Palingeniidae	Palingenia longicauda
Palingenia longicauda mitochondrial partial 16S rR	HE650520	81.13	69.08	2.4009e−17	Ephemeroptera	Palingeniidae	Palingenia longicauda
Palingenia longicauda mitochondrial partial 16S rR	HE650519	81.13	69.08	2.4009e−17	Ephemeroptera	Palingeniidae	Palingenia longicauda
Palingenia longicauda mitochondrial partial 16S rR	HE650515	81.13	69.08	2.4009e−17	Ephemeroptera	Palingeniidae	Palingenia longicauda
Palingenia longicauda mitochondrial partial 16S rR	HE650514	81.13	69.08	2.4009e−17	Ephemeroptera	Palingeniidae	Palingenia longicauda
Palingenia longicauda mitochondrial partial 16S rR	HE650510	81.13	69.08	2.4009e−17	Ephemeroptera	Palingeniidae	Palingenia longicauda
Palingenia longicauda mitochondrial partial 16S rR	HE650509	81.13	69.08	2.4009e−17	Ephemeroptera	Palingeniidae	Palingenia longicauda
Palingenia longicauda mitochondrial partial 16S rR	HE650507	81.13	69.08	2.4009e−17	Ephemeroptera	Palingeniidae	Palingenia longicauda
Palingenia longicauda mitochondrial partial 16S rR	HE650505	81.13	69.08	2.4009e−17	Ephemeroptera	Palingeniidae	Palingenia longicauda
Palingenia longicauda mitochondrial partial 16S rR	HE650499	81.13	69.08	2.4009e−17	Ephemeroptera	Palingeniidae	Palingenia longicauda
Palingenia longicauda mitochondrial partial 16S rR	HE650446	81.13	69.08	2.4009e−17	Ephemeroptera	Palingeniidae	Palingenia longicauda
Palingenia longicauda mitochondrial partial 16S rR	HE650435	81.13	69.08	2.4009e−17	Ephemeroptera	Palingeniidae	Palingenia longicauda
Palingenia longicauda mitochondrial partial 16S rR	HE650461	81.13	69.08	2.4009e−17	Ephemeroptera	Palingeniidae	Palingenia longicauda
Palingenia longicauda mitochondrial partial 16S rR	HE650449	81.13	69.08	2.4009e−17	Ephemeroptera	Palingeniidae	Palingenia longicauda
Palingenia longicauda mitochondrial partial 16S rR	HE650420	81.13	69.08	2.4009e−17	Ephemeroptera	Palingeniidae	Palingenia longicauda
Palingenia longicauda mitochondrial partial 16S rR	HE650440	81.13	69.08	2.4009e−17	Ephemeroptera	Palingeniidae	Palingenia longicauda
Palingenia longicauda mitochondrial partial 16S rR	HE650434	81.13	69.08	2.4009e−17	Ephemeroptera	Palingeniidae	Palingenia longicauda
Palingenia longicauda mitochondrial partial 16S rR	HE650430	81.13	69.08	2.4009e−17	Ephemeroptera	Palingeniidae	Palingenia longicauda
Palingenia longicauda mitochondrial partial 16S rR	HE650421	81.13	69.08	2.4009e−17	Ephemeroptera	Palingeniidae	Palingenia longicauda
Palingenia longicauda mitochondrial partial 16S rR	HE650424	81.13	69.08	2.4009e−17	Ephemeroptera	Palingeniidae	Palingenia longicauda
Palingenia longicauda mitochondrial partial 16S rR	HE650422	81.13	69.08	2.4009e−17	Ephemeroptera	Palingeniidae	Palingenia longicauda
Palingenia longicauda voucher BYU:IGCEP216 16S rib	GQ118292	81.13	69.08	2.4009e−17	Ephemeroptera	Palingeniidae	Palingenia longicauda
Ichthybotus hudsoni 16S ribosomal RNA gene, partia	AY749816	83.33	63.16	2.4009e−17	Ephemeroptera	Ichthybotidae	Ichthybotus hudsoni
Euryades corethrus voucher FS−b−3716 16S ribosomal	GQ268348	82.83	63.16	8.37996e−17	Lepidoptera	Papilionidae	Euryades corethrus
Euryades corethrus mitochondrial partial 16S rRNA	AM283053	82.83	63.16	8.37996e−17	Lepidoptera	Papilionidae	Euryades corethrus
Hygrostola dallolmoi voucher HYTAG7234 16S ribosom	KP682574	83.67	63.16	2.92489e−16	Lepidoptera	Noctuidae	Hygrostola dallolmoi
Hygrostola dallolmoi voucher HYTAG6454 16S ribosom	KP682573	83.67	63.16	2.92489e−16	Lepidoptera	Noctuidae	Hygrostola dallolmoi
Palingenia longicauda mitochondrial partial 16S rR	HE650642	81.13	69.08	2.92489e−16	Ephemeroptera	Palingeniidae	Palingenia longicauda
Palingenia longicauda mitochondrial partial 16S rR	HE650443	81.82	64.47	2.92489e−16	Ephemeroptera	Palingeniidae	Palingenia longicauda
Trox niponensis isolate 100 16S ribosomal RNA gene	EF570413	84.04	60.53	2.92489e−16	Coleoptera	Trogidae	Trox niponensis
Atrophaneura dasarada 16S ribosomal RNA gene, part	DQ673329	82.47	63.16	2.92489e−16	Lepidoptera	Papilionidae	Atrophaneura dasarada
Neurosymploca sp. 3 (morphotype 'magnifica') mitoc	AJ831619	81.82	64.47	2.92489e−16	Lepidoptera	Zygaenidae	Neurosymploca sp. 3 (morphotype 'magnifica')
Neurosymploca sp. 2 (morphotype 'geertsemai') mito	AJ831618	81.82	64.47	2.92489e−16	Lepidoptera	Zygaenidae	Neurosymploca sp. 2 (morphotype 'geertsemai')
G.rothi mitochondrial 16S rRNA gene (partial)	Z93321	81.82	64.47	2.92489e−16	Grylloblattodea	Grylloblattidae	Grylloblatta rothi
Hipparchia semele mitochondrion, partial genome	KM592976	82.83	63.16	1.02089e−15	Lepidoptera	Nymphalidae	Hipparchia semele
Heliconius erato petiverana voucher 618 ; mitochon	KP073283	81.63	63.16	1.02089e−15	Lepidoptera	Nymphalidae	Heliconius erato petiverana
Heliconius erato favorinus voucher GS012 ; mitocho	KP073279	81.63	63.16	1.02089e−15	Lepidoptera	Nymphalidae	Heliconius erato favorinus
Heliconius erato erato voucher NCS2556 ; mitochond	KP073278	81.63	63.16	1.02089e−15	Lepidoptera	Nymphalidae	Heliconius erato erato
Heliconius erato emma voucher NCS1673 ; mitochondr	KP073277	81.63	63.16	1.02089e−15	Lepidoptera	Nymphalidae	Heliconius erato emma
Heliconius erato chestertonii voucher WOM0059 ; mi	KP073276	81.63	63.16	1.02089e−15	Lepidoptera	Nymphalidae	Heliconius erato chestertonii
Heliconius erato voucher LMCI159−1 16S ribosomal R	KF277292	81.63	63.16	1.02089e−15	Lepidoptera	Nymphalidae	Heliconius erato
Oxythyrea pantherina voucher 842859 16S ribosomal	JX234531	82.8	60.53	1.02089e−15	Coleoptera	Scarabaeidae	Oxythyrea pantherina
Leucocelis rubra voucher 842824 16S ribosomal RNA	JX234507	82.8	60.53	1.02089e−15	Coleoptera	Scarabaeidae	Leucocelis rubra
Oxythyrea funesta voucher 678443 16S ribosomal RNA	JX234432	82.8	60.53	1.02089e−15	Coleoptera	Scarabaeidae	Oxythyrea funesta
Semirhynchia sp. n. SV01 16S ribosomal RNA gene, p	JX294172	81.37	64.47	1.02089e−15	Neuroptera	Nemopteridae	Semirhynchia sp. n. SV01
Sicyoptera cuspidata isolate SCBB01 16S ribosomal	JX294167	81.37	64.47	1.02089e−15	Neuroptera	Nemopteridae	Sicyoptera cuspidata
Sicyoptera dilatata isolate SDGG01 16S ribosomal R	JX294163	81.37	64.47	1.02089e−15	Neuroptera	Nemopteridae	Sicyoptera dilatata
Nemeura gracilis isolate NGW01 16S ribosomal RNA g	JX294132	81.37	64.47	1.02089e−15	Neuroptera	Nemopteridae	Nemeura gracilis
Pachliopta aristolochiae isolate N296 16S ribosoma	JQ421944	82.65	63.16	1.02089e−15	Lepidoptera	Papilionidae	Pachliopta aristolochiae
Pachliopta aristolochiae isolate F566 16S ribosoma	JQ421940	82.65	63.16	1.02089e−15	Lepidoptera	Papilionidae	Pachliopta aristolochiae
Pachliopta aristolochiae isolate F177 16S ribosoma	JQ421938	82.65	63.16	1.02089e−15	Lepidoptera	Papilionidae	Pachliopta aristolochiae
Pachliopta aristolochiae isolate F317 16S ribosoma	JQ421934	82.65	63.16	1.02089e−15	Lepidoptera	Papilionidae	Pachliopta aristolochiae
Pachliopta aristolochiae isolate F520 16S ribosoma	JQ421931	82.65	63.16	1.02089e−15	Lepidoptera	Papilionidae	Pachliopta aristolochiae
Pachliopta aristolochiae isolate T75 16S ribosomal	JQ421926	82.65	63.16	1.02089e−15	Lepidoptera	Papilionidae	Pachliopta aristolochiae
Palingenia longicauda mitochondrial partial 16S rR	HE650658	80.19	69.08	1.02089e−15	Ephemeroptera	Palingeniidae	Palingenia longicauda
Palingenia longicauda mitochondrial partial 16S rR	HE650655	80.19	69.08	1.02089e−15	Ephemeroptera	Palingeniidae	Palingenia longicauda
Palingenia longicauda mitochondrial partial 16S rR	HE650524	80.19	69.08	1.02089e−15	Ephemeroptera	Palingeniidae	Palingenia longicauda
Palingenia longicauda mitochondrial partial 16S rR	HE650498	80.19	69.08	1.02089e−15	Ephemeroptera	Palingeniidae	Palingenia longicauda
Palingenia longicauda mitochondrial partial 16S rR	HE650490	80.19	69.08	1.02089e−15	Ephemeroptera	Palingeniidae	Palingenia longicauda
Palingenia longicauda mitochondrial partial 16S rR	HE650484	80.19	69.08	1.02089e−15	Ephemeroptera	Palingeniidae	Palingenia longicauda
Trichopteryx ustata gene for 16S rRNA, partial seq	AB265432	82.83	63.16	1.02089e−15	Lepidoptera	Geometridae	Trichopteryx ustata
Pachliopta aristolochiae 16S ribosomal RNA gene, p	DQ673334	82.65	63.16	1.02089e−15	Lepidoptera	Papilionidae	Pachliopta aristolochiae
Hipparchia aristeus 16S ribosomal RNA gene, partia	AF214604	82.83	63.16	1.02089e−15	Lepidoptera	Nymphalidae	Hipparchia aristeus
Hipparchia semele 16S ribosomal RNA gene, partial	AF214600	82.83	63.16	1.02089e−15	Lepidoptera	Nymphalidae	Hipparchia semele
Heliconius erato hydara isolate STRI−B−440 16S rib	AY747877	81.63	63.16	1.02089e−15	Lepidoptera	Nymphalidae	Heliconius erato hydara
