# Supplementary material for: Testing the potential of a ribosomal 16S marker for DNA metabarcoding of insects
Source: PeerJ. 2016 Apr 19;4:e1966. doi: 10.7717/peerj.1966 (PMC4841222; doi:10.7717/peerj.1966)
Supplement: Supplemental Information 1 [file peerj-04-1966-s006.zip › scripts/5 match OTUs/blast_hits/OTU_16.csv_plot.pdf]

OTU_16.csv

Torleya major

Ephemerellidae

Ephemeroptera

100

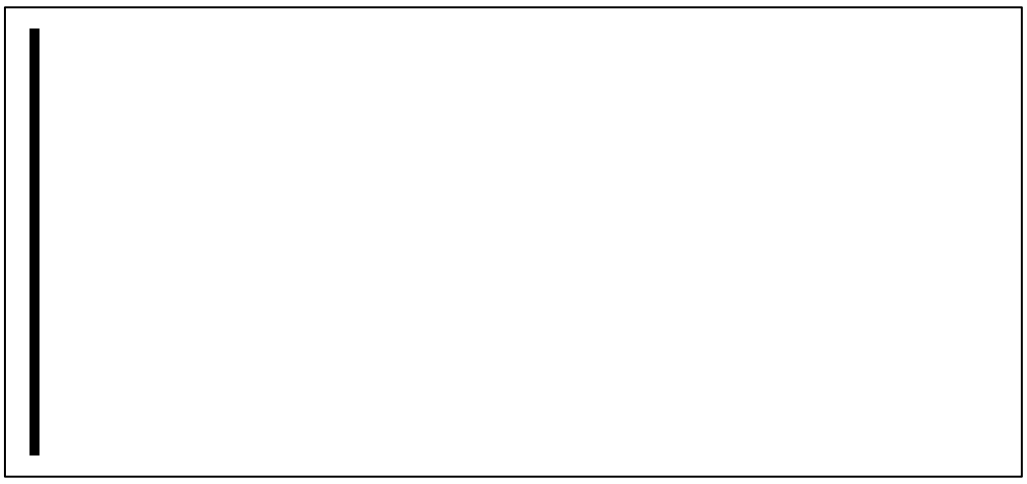

Torleya major 16S ribosomal RNA gene, partial sequ	FJ443047	100	100	4.36644e−71	Ephemeroptera	Ephemerellidae	Torleya major
Teloganopsis media 16S ribosomal RNA gene, partial	FJ443046	72.73	100	1.86159e−12	Ephemeroptera	Ephemerellidae	Teloganopsis media
Ephemerella atagosana 16S ribosomal RNA gene, part	FJ443039	79.8	64.05	2.26788e−11	Ephemeroptera	Ephemerellidae	Ephemerella atagosana
Ephemerella cornutus 16S ribosomal RNA gene, parti	FJ443041	72.66	89.54	2.76284e−10	Ephemeroptera	Ephemerellidae	Ephemerella cornutus
Ephoron shigae mitochondrial gene for 16S rRNA, pa	AB711581	77.78	63.4	9.64325e−10	Ephemeroptera	Polymitarcyidae	Ephoron shigae
Palingenia longicauda mitochondrial partial 16S rR	HE650502	77	64.71	9.64325e−10	Ephemeroptera	Palingeniidae	Palingenia longicauda
Palingenia longicauda mitochondrial partial 16S rR	HE650484	77	64.71	9.64325e−10	Ephemeroptera	Palingeniidae	Palingenia longicauda
Rhabdotis aulica voucher 842782 16S ribosomal RNA	JX234477	77.55	62.75	3.36583e−09	Coleoptera	Scarabaeidae	Rhabdotis aulica
Ephoron eophilum mitochondrial gene for 16S rRNA,	AB711631	78.22	63.4	3.36583e−09	Ephemeroptera	Polymitarcyidae	Ephoron eophilum
Ephoron eophilum mitochondrial gene for 16S rRNA,	AB711630	79.41	63.4	3.36583e−09	Ephemeroptera	Polymitarcyidae	Ephoron eophilum
Ephoron eophilum mitochondrial gene for 16S rRNA,	AB711621	79.41	63.4	3.36583e−09	Ephemeroptera	Polymitarcyidae	Ephoron eophilum
Ephoron eophilum mitochondrial gene for 16S rRNA,	AB711619	79.41	63.4	3.36583e−09	Ephemeroptera	Polymitarcyidae	Ephoron eophilum
Ephoron eophilum mitochondrial gene for 16S rRNA,	AB711615	79.41	63.4	3.36583e−09	Ephemeroptera	Polymitarcyidae	Ephoron eophilum
Hexagenia sp. BYU IGCEP003 16S ribosomal RNA gene,	GQ118286	77.67	64.71	3.36583e−09	Ephemeroptera	Ephemeridae	Hexagenia sp. BYU IGCEP003
Ephoron shigae mitochondrial gene for 16S rRNA, pa	AB711590	76.77	63.4	1.17479e−08	Ephemeroptera	Polymitarcyidae	Ephoron shigae
Ephoron shigae mitochondrial gene for 16S rRNA, pa	AB711582	76.53	63.4	1.17479e−08	Ephemeroptera	Polymitarcyidae	Ephoron shigae
Ephoron shigae mitochondrial gene for 16S rRNA, pa	AB711575	76.53	63.4	1.17479e−08	Ephemeroptera	Polymitarcyidae	Ephoron shigae
Polyphylla decemlineata 16S ribosomal RNA gene, pa	JX994048	77.89	60.78	1.17479e−08	Coleoptera	Scarabaeidae	Polyphylla decemlineata
Palingenia longicauda mitochondrial partial 16S rR	HE650589	76	64.71	1.17479e−08	Ephemeroptera	Palingeniidae	Palingenia longicauda
Palingenia longicauda mitochondrial partial 16S rR	HE650647	76	64.71	1.17479e−08	Ephemeroptera	Palingeniidae	Palingenia longicauda
Palingenia longicauda mitochondrial partial 16S rR	HE650637	76	64.71	1.17479e−08	Ephemeroptera	Palingeniidae	Palingenia longicauda
Palingenia longicauda mitochondrial partial 16S rR	HE650625	76	64.71	1.17479e−08	Ephemeroptera	Palingeniidae	Palingenia longicauda
Palingenia longicauda mitochondrial partial 16S rR	HE650604	76	64.71	1.17479e−08	Ephemeroptera	Palingeniidae	Palingenia longicauda
Palingenia longicauda mitochondrial partial 16S rR	HE650588	76	64.71	1.17479e−08	Ephemeroptera	Palingeniidae	Palingenia longicauda
Palingenia longicauda mitochondrial partial 16S rR	HE650575	76	64.71	1.17479e−08	Ephemeroptera	Palingeniidae	Palingenia longicauda
Palingenia longicauda mitochondrial partial 16S rR	HE650547	76	64.71	1.17479e−08	Ephemeroptera	Palingeniidae	Palingenia longicauda
Palingenia longicauda mitochondrial partial 16S rR	HE650550	76	64.71	1.17479e−08	Ephemeroptera	Palingeniidae	Palingenia longicauda
Palingenia longicauda mitochondrial partial 16S rR	HE650542	76	64.71	1.17479e−08	Ephemeroptera	Palingeniidae	Palingenia longicauda
Palingenia longicauda mitochondrial partial 16S rR	HE650503	76	64.71	1.17479e−08	Ephemeroptera	Palingeniidae	Palingenia longicauda
Palingenia longicauda mitochondrial partial 16S rR	HE650520	76	64.71	1.17479e−08	Ephemeroptera	Palingeniidae	Palingenia longicauda
Palingenia longicauda mitochondrial partial 16S rR	HE650519	76	64.71	1.17479e−08	Ephemeroptera	Palingeniidae	Palingenia longicauda
Palingenia longicauda mitochondrial partial 16S rR	HE650515	76	64.71	1.17479e−08	Ephemeroptera	Palingeniidae	Palingenia longicauda
Palingenia longicauda mitochondrial partial 16S rR	HE650507	76	64.71	1.17479e−08	Ephemeroptera	Palingeniidae	Palingenia longicauda
Palingenia longicauda mitochondrial partial 16S rR	HE650505	76	64.71	1.17479e−08	Ephemeroptera	Palingeniidae	Palingenia longicauda
Palingenia longicauda mitochondrial partial 16S rR	HE650499	76	64.71	1.17479e−08	Ephemeroptera	Palingeniidae	Palingenia longicauda
Palingenia longicauda mitochondrial partial 16S rR	HE650498	76	64.71	1.17479e−08	Ephemeroptera	Palingeniidae	Palingenia longicauda
Palingenia longicauda mitochondrial partial 16S rR	HE650446	76	64.71	1.17479e−08	Ephemeroptera	Palingeniidae	Palingenia longicauda
Palingenia longicauda mitochondrial partial 16S rR	HE650435	76	64.71	1.17479e−08	Ephemeroptera	Palingeniidae	Palingenia longicauda
Palingenia longicauda mitochondrial partial 16S rR	HE650420	76	64.71	1.17479e−08	Ephemeroptera	Palingeniidae	Palingenia longicauda
Palingenia longicauda mitochondrial partial 16S rR	HE650424	76	64.71	1.17479e−08	Ephemeroptera	Palingeniidae	Palingenia longicauda
Palingenia longicauda mitochondrial partial 16S rR	HE650422	76	64.71	1.17479e−08	Ephemeroptera	Palingeniidae	Palingenia longicauda
Palingenia longicauda voucher BYU:IGCEP216 16S rib	GQ118292	76	64.71	1.17479e−08	Ephemeroptera	Palingeniidae	Palingenia longicauda
Ephemerella sp. THO−2009 16S ribosomal RNA gene, p	FJ443044	76.53	64.05	1.17479e−08	Ephemeroptera	Ephemerellidae	Ephemerella sp. THO−2009
Argyreus hyperbius voucher jshao2005025 16S riboso	DQ784692	78.26	58.82	1.17479e−08	Lepidoptera	Nymphalidae	Argynnis hyperbius
Argyreus hyperbius 16S ribosomal RNA gene, partial	DQ445107	78.26	58.82	1.17479e−08	Lepidoptera	Nymphalidae	Argynnis hyperbius
Pantoporia hordonia isolate N629 mitochondrion, co	KF590534	78.02	58.17	4.10042e−08	Lepidoptera	Nymphalidae	Pantoporia hordonia
Ephemera orientalis voucher EPH01 tRNA−Leu, 16S ri	KF256907	72.31	84.31	4.10042e−08	Ephemeroptera	Ephemeridae	Ephemera orientalis
Sphaerosoma sp. 1 16S ribosomal RNA gene, partial	GQ302155	78.02	58.17	4.10042e−08	Coleoptera	Alexiidae	Sphaerosoma sp. 1 FWS−2010
Ephemera orientalis mitochondrion, complete genome	EU591678	72.31	84.31	4.10042e−08	Ephemeroptera	Ephemeridae	Ephemera orientalis
Phaedyma columella voucher jshao2005036 16S riboso	DQ784713	78.02	58.17	4.10042e−08	Lepidoptera	Nymphalidae	Phaedyma columella
Tropha zambiensis voucher KS240 16S ribosomal RNA	KP143640	77.89	60.78	1.43119e−07	Coleoptera	Coccinellidae	Tropha zambiensis
Euroleon nostras isolate 60−nosmar1 16S ribosomal	KF321986	76	64.05	1.43119e−07	Neuroptera	Myrmeleontidae	Euroleon nostras
Euroleon nostras isolate 34−nostur2 16S ribosomal	KF321983	76	64.05	1.43119e−07	Neuroptera	Myrmeleontidae	Euroleon nostras
Ephoron eophilum mitochondrial gene for 16S rRNA,	AB711633	78.43	63.4	1.43119e−07	Ephemeroptera	Polymitarcyidae	Ephoron eophilum
Ephoron shigae mitochondrial gene for 16S rRNA, pa	AB711598	75.51	63.4	1.43119e−07	Ephemeroptera	Polymitarcyidae	Ephoron shigae
Pantoporia hordonia isolate F97 16S ribosomal RNA	JQ416138	77.17	58.17	1.43119e−07	Lepidoptera	Nymphalidae	Pantoporia hordonia
Dasytes coriaceipennis voucher UPOL001197 16S ribo	HQ619684	76.53	62.75	1.43119e−07	Coleoptera	Melyridae	Dasytes coriaceipennis
Palingenia longicauda mitochondrial partial 16S rR	HE650616	75.51	63.4	1.43119e−07	Ephemeroptera	Palingeniidae	Palingenia longicauda
Lamproblatta albipalpus voucher ZMUC:JD−1555 16S r	JN615295	76.53	62.75	1.43119e−07	Blattodea	Lamproblattidae	Lamproblatta albipalpus
Gymnopternus celer voucher Pollet 0268 (P) 16S rib	EU863982	75.73	64.05	1.43119e−07	Diptera	Dolichopodidae	Gymnopternus celer
Gymnopternus angustifrons voucher Pollet 0052 (P)	EU863971	76	64.05	1.43119e−07	Diptera	Dolichopodidae	Gymnopternus angustifrons
Simulium parahiyangum 16S ribosomal RNA gene, part	GQ865991	76	64.05	1.43119e−07	Diptera	Simuliidae	Simulium parahiyangum
Drunella tuberculata 16S ribosomal RNA gene, parti	FJ443037	75.25	66.01	1.43119e−07	Ephemeroptera	Ephemerellidae	Drunella tuberculata
Limonia variabilis voucher O'Grady 201512 16S ribo	EU005428	76	64.05	1.43119e−07	Diptera	Limoniidae	Dicranomyia variabilis
Limonia kraussi voucher O'Grady 200013 16S ribosom	EU005415	76	64.05	1.43119e−07	Diptera	Limoniidae	Dicranomyia kraussi
Limonia variabilis voucher O'Grady 200973 16S ribo	EU005426	76	64.05	1.43119e−07	Diptera	Limoniidae	Dicranomyia variabilis
Limonia hawaiiensis voucher O'Grady 201598 16S rib	EU005405	76	64.05	1.43119e−07	Diptera	Limoniidae	Dicranomyia hawaiiensis
Limonia hawaiiensis voucher O'Grady 200922 16S rib	EU005404	76	64.05	1.43119e−07	Diptera	Limoniidae	Dicranomyia hawaiiensis
Uloma sp. 5 GJK−2014 16S ribosomal RNA gene, parti	KJ003074	73.6	78.43	4.99533e−07	Coleoptera	Tenebrionidae	Uloma sp. 5 GJK−2014
Atrichelaphinis tigrina voucher 842817 16S ribosom	JX234502	76.29	62.09	4.99533e−07	Coleoptera	Scarabaeidae	Atrichelaphinis tigrina
Comythovalgus sp. DA−2012 voucher 842778 16S ribos	JX234473	75.26	62.09	4.99533e−07	Coleoptera	Scarabaeidae	Comythovalgus sp. DA−2012
Oxythyrea funesta voucher 678443 16S ribosomal RNA	JX234432	76.29	62.09	4.99533e−07	Coleoptera	Scarabaeidae	Oxythyrea funesta
Myrmeleon inconspicuus isolate 9−incfo2 16S riboso	KC195842	74.76	63.4	4.99533e−07	Neuroptera	Myrmeleontidae	Myrmeleon inconspicuus
Ephoron leukon mitochondrial gene for 16S rRNA, pa	AB711641	75	64.71	4.99533e−07	Ephemeroptera	Polymitarcyidae	Ephoron leukon
Ephoron album mitochondrial gene for 16S rRNA, par	AB711636	75	64.71	4.99533e−07	Ephemeroptera	Polymitarcyidae	Ephoron album
Ephoron album mitochondrial gene for 16S rRNA, par	AB711635	75	64.71	4.99533e−07	Ephemeroptera	Polymitarcyidae	Ephoron album
Cafius lithocharinus isolate CNUIC−106 16S ribosom	JF796468	75.76	63.4	4.99533e−07	Coleoptera	Staphylinidae	Cafius lithocharinus
Palingenia longicauda mitochondrial partial 16S rR	HE650662	75	64.71	4.99533e−07	Ephemeroptera	Palingeniidae	Palingenia longicauda
Palingenia longicauda mitochondrial partial 16S rR	HE650658	75	64.71	4.99533e−07	Ephemeroptera	Palingeniidae	Palingenia longicauda
Palingenia longicauda mitochondrial partial 16S rR	HE650657	75	64.71	4.99533e−07	Ephemeroptera	Palingeniidae	Palingenia longicauda
Palingenia longicauda mitochondrial partial 16S rR	HE650650	75	64.71	4.99533e−07	Ephemeroptera	Palingeniidae	Palingenia longicauda
Palingenia longicauda mitochondrial partial 16S rR	HE650635	75	64.71	4.99533e−07	Ephemeroptera	Palingeniidae	Palingenia longicauda
Palingenia longicauda mitochondrial partial 16S rR	HE650631	75	64.71	4.99533e−07	Ephemeroptera	Palingeniidae	Palingenia longicauda
Palingenia longicauda mitochondrial partial 16S rR	HE650623	75	64.71	4.99533e−07	Ephemeroptera	Palingeniidae	Palingenia longicauda
Palingenia longicauda mitochondrial partial 16S rR	HE650622	75	64.71	4.99533e−07	Ephemeroptera	Palingeniidae	Palingenia longicauda
Palingenia longicauda mitochondrial partial 16S rR	HE650618	75	64.71	4.99533e−07	Ephemeroptera	Palingeniidae	Palingenia longicauda
Palingenia longicauda mitochondrial partial 16S rR	HE650617	75	64.71	4.99533e−07	Ephemeroptera	Palingeniidae	Palingenia longicauda
Palingenia longicauda mitochondrial partial 16S rR	HE650594	75	64.71	4.99533e−07	Ephemeroptera	Palingeniidae	Palingenia longicauda
Palingenia longicauda mitochondrial partial 16S rR	HE650475	75	64.71	4.99533e−07	Ephemeroptera	Palingeniidae	Palingenia longicauda
Palingenia longicauda mitochondrial partial 16S rR	HE650569	75	64.71	4.99533e−07	Ephemeroptera	Palingeniidae	Palingenia longicauda
Palingenia longicauda mitochondrial partial 16S rR	HE650533	75	64.71	4.99533e−07	Ephemeroptera	Palingeniidae	Palingenia longicauda
Palingenia longicauda mitochondrial partial 16S rR	HE650524	75	64.71	4.99533e−07	Ephemeroptera	Palingeniidae	Palingenia longicauda
Palingenia longicauda mitochondrial partial 16S rR	HE650514	75	64.71	4.99533e−07	Ephemeroptera	Palingeniidae	Palingenia longicauda
Palingenia longicauda mitochondrial partial 16S rR	HE650509	75	64.71	4.99533e−07	Ephemeroptera	Palingeniidae	Palingenia longicauda
Palingenia longicauda mitochondrial partial 16S rR	HE650490	75	64.71	4.99533e−07	Ephemeroptera	Palingeniidae	Palingenia longicauda
Palingenia longicauda mitochondrial partial 16S rR	HE650461	75	64.71	4.99533e−07	Ephemeroptera	Palingeniidae	Palingenia longicauda
Palingenia longicauda mitochondrial partial 16S rR	HE650443	75	64.71	4.99533e−07	Ephemeroptera	Palingeniidae	Palingenia longicauda
Palingenia longicauda mitochondrial partial 16S rR	HE650440	75	64.71	4.99533e−07	Ephemeroptera	Palingeniidae	Palingenia longicauda
Palingenia longicauda mitochondrial partial 16S rR	HE650434	75	64.71	4.99533e−07	Ephemeroptera	Palingeniidae	Palingenia longicauda
Palingenia longicauda mitochondrial partial 16S rR	HE650430	75	64.71	4.99533e−07	Ephemeroptera	Palingeniidae	Palingenia longicauda
