# Supplementary material for: Testing the potential of a ribosomal 16S marker for DNA metabarcoding of insects
Source: PeerJ. 2016 Apr 19;4:e1966. doi: 10.7717/peerj.1966 (PMC4841222; doi:10.7717/peerj.1966)
Supplement: Supplemental Information 1 [file peerj-04-1966-s006.zip › scripts/5 match OTUs/blast_hits/OTU_163.csv_plot.pdf]

OTU_163.csv

Scaptodrosophila lebanonensis

Drosophilidae

Diptera

80.75

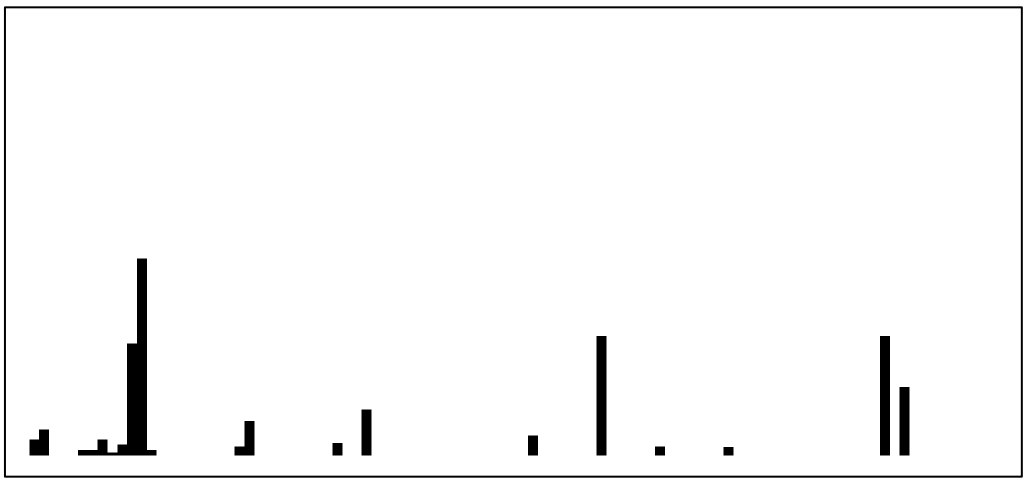

Scaptodrosophila lebanonensis voucher 105639 NADH	EU494411	80.75	100	2.80719e−29	Diptera	Drosophilidae	Scaptodrosophila lebanonensis
Culicoides actoni mitochondrial DNA, large subunit	AB360971	81.21	94.19	9.79804e−29	Diptera	Ceratopogonidae	Culicoides actoni
Megalabops sp. KCADI071−14 16S ribosomal RNA gene,	KT186671	79.38	100	1.19365e−27	Diptera	Diopsidae	Megalabops sp. KCADI071−14
Megalabops sp. BOLD:ACP5917 16S ribosomal RNA gene	KT186670	79.38	100	1.19365e−27	Diptera	Diopsidae	Megalabops sp. BOLD:ACP5917
Palaeosepsis bucki voucher Lei19 16S ribosomal RNA	KF199519	79.62	100	1.19365e−27	Diptera	Sepsidae	Palaeosepsis bucki
Drosophila camargoi voucher 103971 NADH dehydrogen	EU494312	80.25	100	1.19365e−27	Diptera	Drosophilidae	Drosophila camargoi
Drosophila camargoi 16S ribosomal RNA gene, partia	AF479801	80.25	100	1.19365e−27	Diptera	Drosophilidae	Drosophila camargoi
Spiniphora punctipennis 16S ribosomal RNA gene, pa	AF126342	80.75	100	1.19365e−27	Diptera	Phoridae	Spiniphora punctipennis
Diasemopsis longipedunculata 16S ribosomal RNA gen	KT186667	80.12	100	4.16623e−27	Diptera	Diopsidae	Diasemopsis longipedunculata
Asteia concinna isolate A63 16S ribosomal RNA gene	KJ418481	80.5	100	4.16623e−27	Diptera	Asteiidae	Asteia concinna
Cardiocladius sp. MNK−2008 16S ribosomal RNA gene,	EU669992	85.25	75.48	4.16623e−27	Diptera	Chironomidae	Cardiocladius sp. MNK−2008
Culicoides oxystoma mitochondrial DNA, large subun	AB360979	89.22	65.81	4.16623e−27	Diptera	Ceratopogonidae	Culicoides oxystoma
Cladodiopsis seyrigi 16S ribosomal RNA gene, parti	DQ317406	80.25	100	4.16623e−27	Diptera	Diopsidae	Cladodiopsis seyrigi
Sepsis luteipes voucher Lei33 16S ribosomal RNA ge	KJ766342	79.62	100	1.45416e−26	Diptera	Sepsidae	Sepsis luteipes
Archisepsis sp. LZ−2013 16S ribosomal RNA gene, pa	KF199498	80	96.13	1.45416e−26	Diptera	Sepsidae	Archisepsis sp. LZ−2013
Archisepsis armata voucher 106676 16S ribosomal RN	KF199495	80	96.13	1.45416e−26	Diptera	Sepsidae	Archisepsis armata
Lonchoptera uniseta 16S ribosomal RNA gene, partia	KC177456	79.62	100	1.45416e−26	Diptera	Lonchopteridae	Lonchoptera uniseta
Sepsis fulgens voucher su49 16S ribosomal RNA gene	EU435606	79.62	100	1.45416e−26	Diptera	Sepsidae	Sepsis fulgens
Sepsis neglecta voucher su53 16S ribosomal RNA gen	EU435610	79.62	100	1.45416e−26	Diptera	Sepsidae	Sepsis neglecta
Megalabops quadriguttata 16S ribosomal RNA gene, p	KT186655	78.75	100	5.07551e−26	Diptera	Diopsidae	Megalabops quadriguttata
Atelestus pulicarius isolate AP47 16S ribosomal RN	KC699323	79.08	96.13	5.07551e−26	Diptera	Atelestidae	Atelestus pulicarius
Scaptomyza lobifera voucher 202596 16S ribosomal R	KC609604	80.42	89.03	5.07551e−26	Diptera	Drosophilidae	Scaptomyza lobifera
Oestroidea sp. 'McAlpine's fly' voucher RMBR:10442	GQ409133	81.6	100	5.07551e−26	Diptera		Oestroidea sp. 'McAlpine's fly'
Drosophila austrosaltans voucher 106314 NADH dehyd	EU494364	79.25	100	5.07551e−26	Diptera	Drosophilidae	Drosophila austrosaltans
Bezzia pygmaea 16S ribosomal RNA gene, partial seq	EF472588	79.75	100	5.07551e−26	Diptera	Ceratopogonidae	Bezzia pygmaea
Drosophila austrosaltans 16S ribosomal RNA gene, p	AF479808	79.25	100	5.07551e−26	Diptera	Drosophilidae	Drosophila austrosaltans
Diasemopsis elongata 16S ribosomal RNA gene, parti	AF304740	79.38	100	5.07551e−26	Diptera	Diopsidae	Diasemopsis elongata
Trupanea pantosticta isolate Tpanh1 16S ribosomal	KM079210	78.48	100	1.77153e−25	Diptera	Tephritidae	Trupanea pantosticta
Meroplius fasciculata voucher 106683 16S ribosomal	KF199514	79.87	100	1.77153e−25	Diptera	Sepsidae	Meroplius fasciculatus
Colobaea bifasciella voucher MU_NUS_SG3 16S riboso	JN828202	78.21	100	1.77153e−25	Diptera	Sciomyzidae	Colobaea bifasciella
Scaptomyza tumidula voucher O201059a 16S ribosomal	HQ171050	79.86	89.03	1.77153e−25	Diptera	Drosophilidae	Scaptomyza tumidula
Scaptomyza sp. 'akalae' voucher O201528 16S riboso	HQ171045	80.58	89.03	1.77153e−25	Diptera	Drosophilidae	Scaptomyza sp. 'akalae'
Drosophila micromyia voucher O201044 16S ribosomal	HQ170981	78.12	100	1.77153e−25	Diptera	Drosophilidae	Drosophila micromyia
Limnophora riparia 16S ribosomal RNA gene, partial	FJ025449	79.35	98.71	1.77153e−25	Diptera	Muscidae	Limnophora riparia
Zaprionus lineosus voucher 103961 NADH dehydrogena	EU494414	82.14	89.03	1.77153e−25	Diptera	Drosophilidae	Zaprionus lineosus
Drosophila micromyia voucher 109454 NADH dehydroge	EU494392	78.12	100	1.77153e−25	Diptera	Drosophilidae	Drosophila micromyia
Aciura afghana 16S ribosomal RNA gene, partial seq	DQ471406	78.62	100	1.77153e−25	Diptera	Tephritidae	Aciura afghana
Culicoides sp. LJH−2002 16S ribosomal RNA gene, pa	AY140904	78.75	100	1.77153e−25	Diptera	Ceratopogonidae	Culicoides sp. LJH−2002
Diasemopsis longipedunculata 16S ribosomal RNA gen	AF304741	78.88	100	1.77153e−25	Diptera	Diopsidae	Diasemopsis longipedunculata
Cyrtodiopsis dalmanni isolate CdGombak4 16S riboso	AY876564	79.63	100	1.77153e−25	Diptera	Diopsidae	Teleopsis dalmanni
Diopsis nr. circularis KCADI065−14 16S ribosomal R	KT186651	78.75	100	6.18324e−25	Diptera	Diopsidae	Diopsis nr. circularis KCADI065−14
Teleopsis pallifacies voucher RMNH.INS.555138 16S	KP978628	79.38	100	6.18324e−25	Diptera	Diopsidae	Teleopsis pallifacies
Evocoa chilensis 16S ribosomal RNA gene, partial s	KM878914	78.75	100	6.18324e−25	Diptera	Evocoidae	Evocoa chilensis
Phorocerosoma vicarium mitochondrial gene for 16S	AB465926	79.75	100	6.18324e−25	Diptera	Tachinidae	Phorocerosoma vicarium
Drosophila proceriseta voucher O201880 16S ribosom	HQ170994	78.4	100	6.18324e−25	Diptera	Drosophilidae	Drosophila proceriseta
Mikia patellipalpis mitochondrial gene for 16S rRN	AB465956	79.25	100	6.18324e−25	Diptera	Tachinidae	Mikia patellipalpis
Sepsis neocynipsea voucher su54 16S ribosomal RNA	EU435611	78.98	100	6.18324e−25	Diptera	Sepsidae	Sepsis neocynipsea
Sepsis cynipsea voucher su44 16S ribosomal RNA gen	EU435602	78.98	100	6.18324e−25	Diptera	Sepsidae	Sepsis cynipsea
Drosophila funebris voucher 103952 NADH dehydrogen	EU494313	79.08	98.71	6.18324e−25	Diptera	Drosophilidae	Drosophila funebris
Perochaeta dikowi 16S ribosomal RNA gene, complete	EU375720	78.34	100	6.18324e−25	Diptera	Sepsidae	Perochaeta dikowi
Sphyracephala europaea 16S ribosomal RNA gene, par	DQ452962	78.62	100	6.18324e−25	Diptera	Diopsidae	Sphyracephala europaea
Themira biloba mitochondrial partial 16S rRNA gene	AJ843101	80.92	94.19	6.18324e−25	Diptera	Sepsidae	Themira biloba
Cyrtodiopsis currani 16S ribosomal RNA gene, parti	AF304746	77.99	100	6.18324e−25	Diptera	Diopsidae	Teleopsis currani
Diasemopsis sp. W 16S ribosomal RNA gene, partial	AF304726	78.57	98.06	6.18324e−25	Diptera	Diopsidae	Diasemopsis sp. W
Diopsis nr. gnu KCADI063−14 16S ribosomal RNA gene	KT186676	78.26	100	2.15816e−24	Diptera	Diopsidae	Diopsis nr. gnu KCADI063−14
Trupanea pantosticta isolate Tpanh2 16S ribosomal	KM079211	77.85	100	2.15816e−24	Diptera	Tephritidae	Trupanea pantosticta
Trupanea joycei isolate TjoyMo 16S ribosomal RNA g	KM079209	77.85	100	2.15816e−24	Diptera	Tephritidae	Trupanea joycei
Trupanea crassipes isolate Tcash1 16S ribosomal RN	KM079199	77.85	100	2.15816e−24	Diptera	Tephritidae	Trupanea crassipes
Stiphrosoma humerale isolate A58 16S ribosomal RNA	KJ418477	85.59	70.32	2.15816e−24	Diptera	Anthomyzidae	Stiphrosoma humerale
Drosophila paracracens voucher 202556 16S ribosoma	KM252237	79.86	89.03	2.15816e−24	Diptera	Drosophilidae	Drosophila paracracens
Drosophila orthoptera voucher 202540 16S ribosomal	KM252228	79.86	89.03	2.15816e−24	Diptera	Drosophilidae	Drosophila orthoptera
Brachythoracosepsis saothomensis voucher Lei9 16S	KF199503	78.85	99.35	2.15816e−24	Diptera	Sepsidae	Brachythoracosepsis saothomensis
Brachythoracosepsis pseudonotosa voucher Lei7 16S	KF199501	78.85	99.35	2.15816e−24	Diptera	Sepsidae	Brachythoracosepsis pseudonotosa
Brachythoracosepsis butikensis voucher Lei4 16S ri	KF199499	78.85	99.35	2.15816e−24	Diptera	Sepsidae	Brachythoracosepsis butikensis
Scaptomyza nigrita voucher 10004 16S ribosomal RNA	KC609624	80.42	89.03	2.15816e−24	Diptera	Drosophilidae	Scaptomyza nigrita
Scaptomyza apicata voucher 10003 16S ribosomal RNA	KC609623	79.72	89.03	2.15816e−24	Diptera	Drosophilidae	Scaptomyza apicata
Scaptomyza crassifemur voucher 202688 16S ribosoma	KC609614	79.86	89.03	2.15816e−24	Diptera	Drosophilidae	Scaptomyza crassifemur
Scaptomyza cuspidata voucher 202656 16S ribosomal	KC609610	79.72	89.03	2.15816e−24	Diptera	Drosophilidae	Scaptomyza cuspidata
Scaptomyza dentata voucher 202625 16S ribosomal RN	KC609607	79.86	89.03	2.15816e−24	Diptera	Drosophilidae	Scaptomyza dentata
Scaptomyza protensa voucher 202622 16S ribosomal R	KC609606	79.86	89.03	2.15816e−24	Diptera	Drosophilidae	Scaptomyza protensa
Scaptomyza buccata voucher 202560 16S ribosomal RN	KC609600	77.99	100	2.15816e−24	Diptera	Drosophilidae	Scaptomyza buccata
Scythropochroa nr. quercicola SS−2012 voucher 778	JQ613930	80.38	96.13	2.15816e−24	Diptera	Sciaridae	Scythropochroa nr. quercicola SS−2012
Teleopsis dalmanni 16S ribosomal RNA gene, partial	KC177441	79.5	100	2.15816e−24	Diptera	Diopsidae	Teleopsis dalmanni
Drosophila dasycnemia voucher 202331a 16S ribosoma	HQ703698	77.5	100	2.15816e−24	Diptera	Drosophilidae	Drosophila dasycnemia
Drosophila neutralis voucher 202329b 16S ribosomal	HQ703696	77.5	100	2.15816e−24	Diptera	Drosophilidae	Drosophila neutralis
Drosophila sordidapex voucher O202321a 16S ribosom	HQ171025	77.5	100	2.15816e−24	Diptera	Drosophilidae	Drosophila sordidapex
Drosophila sordidapex voucher 202318a 16S ribosoma	HQ703693	77.5	100	2.15816e−24	Diptera	Drosophilidae	Drosophila sordidapex
Drosophila percnosoma voucher O200125a 16S ribosom	HQ171022	77.5	100	2.15816e−24	Diptera	Drosophilidae	Drosophila percnosoma
Drosophila sodomae voucher O34.2 16S ribosomal RNA	HQ171041	77.5	100	2.15816e−24	Diptera	Drosophilidae	Drosophila sodomae
Drosophila conformis voucher O201312a 16S ribosoma	HQ171023	77.5	100	2.15816e−24	Diptera	Drosophilidae	Drosophila conformis
Drosophila contorta voucher O200120a 16S ribosomal	HQ171021	79.72	89.03	2.15816e−24	Diptera	Drosophilidae	Drosophila contorta
Drosophila tetraspilota voucher O200995 16S riboso	HQ171015	77.5	100	2.15816e−24	Diptera	Drosophilidae	Drosophila tetraspilota
Drosophila nr. hirtitarsus RL−2010 voucher O200998	HQ171010	77.5	100	2.15816e−24	Diptera	Drosophilidae	Drosophila nr. hirtitarsus RL−2010
Drosophila desallei voucher O200989 16S ribosomal	HQ170963	78.12	100	2.15816e−24	Diptera	Drosophilidae	Drosophila desallei
Drosophila antecedens voucher O205061 16S ribosoma	HQ170953	77.5	100	2.15816e−24	Diptera	Drosophilidae	Drosophila antecedens
Tephritis divisa 16S ribosomal RNA gene, partial s	GQ175827	78.26	100	2.15816e−24	Diptera	Tephritidae	Tephritis divisa
Senometopia cariniforceps mitochondrial gene for 1	AB465923	79.14	100	2.15816e−24	Diptera	Tachinidae	Senometopia cariniforceps
Stiphrosoma laetum 16S ribosomal RNA gene, partial	EU268534	85.59	70.32	2.15816e−24	Diptera	Anthomyzidae	Stiphrosoma laetum
Parapalaeosepsis apicalis voucher su34 16S ribosom	EU435595	78.66	100	2.15816e−24	Diptera	Sepsidae	Parapalaeosepsis apicalis
Scaptomyza undulata voucher 109452 NADH dehydrogen	EU494407	83.19	76.13	2.15816e−24	Diptera	Drosophilidae	Scaptomyza undulata
Scaptomyza frustulifera voucher 108518 NADH dehydr	EU494405	77.5	100	2.15816e−24	Diptera	Drosophilidae	Scaptomyza frustulifera
Drosophila percnosoma voucher 105685 NADH dehydrog	EU494396	77.5	100	2.15816e−24	Diptera	Drosophilidae	Drosophila percnosoma
Drosophila nigra voucher 105821 NADH dehydrogenase	EU494394	78.12	100	2.15816e−24	Diptera	Drosophilidae	Drosophila nigra
Drosophila adunca voucher 105818 NADH dehydrogenas	EU494374	79.86	89.03	2.15816e−24	Diptera	Drosophilidae	Drosophila adunca
Drosophila neoperkinsi 16S ribosomal RNA gene, par	AY006398	78.12	100	2.15816e−24	Diptera	Drosophilidae	Drosophila neoperkinsi
Drosophila hemipeza 16S ribosomal RNA gene, partia	AY006391	78.12	100	2.15816e−24	Diptera	Drosophilidae	Drosophila hemipeza
Cyrtodiopsis dalmanni 16S ribosomal RNA gene, part	AY123348	79.5	100	2.15816e−24	Diptera	Diopsidae	Teleopsis dalmanni
Diopsis gnu 16S ribosomal RNA gene, partial sequen	AF304744	78.26	100	2.15816e−24	Diptera	Diopsidae	Diopsis gnu
Cyrtodiopsis dalmanni isolate CdGombak7 16S riboso	AY876567	79.5	100	2.15816e−24	Diptera	Diopsidae	Teleopsis dalmanni
Afraciura zernyi 16S ribosomal RNA gene, partial s	DQ471395	77.71	100	7.53272e−24	Diptera	Tephritidae	Afraciura zernyi
