# Supplementary material for: Testing the potential of a ribosomal 16S marker for DNA metabarcoding of insects
Source: PeerJ. 2016 Apr 19;4:e1966. doi: 10.7717/peerj.1966 (PMC4841222; doi:10.7717/peerj.1966)
Supplement: Supplemental Information 1 [file peerj-04-1966-s006.zip › scripts/5 match OTUs/blast_hits/OTU_18.csv_plot.pdf]

OTU_18.csv

Dinocras cephalotes

Perlidae

Plecoptera

100

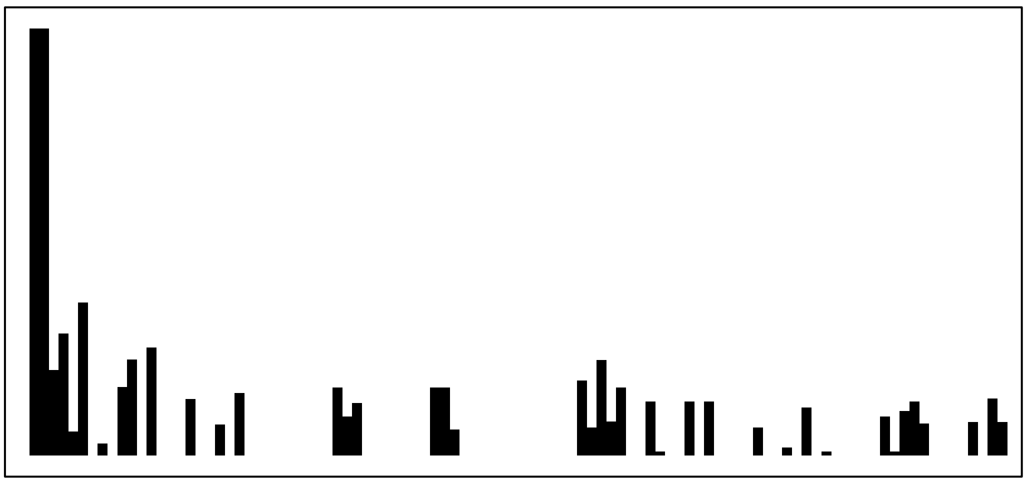

Dinocras cephalotes mitochondrion, complete genome	KF484757	100	100	5.09667e−77	Plecoptera	Perlidae	Dinocras cephalotes
Dinocras cephalotes 16S ribosomal RNA gene, partia	EF623244	100	100	5.09667e−77	Plecoptera	Perlidae	Dinocras cephalotes
Calineuria californica 16S ribosomal RNA gene, par	EF623239	84	90.24	3.92524e−34	Plecoptera	Perlidae	Calineuria californica
Agnetina capitata 16S ribosomal RNA gene, partial	EF623241	85.71	72.56	1.05329e−28	Plecoptera	Perlidae	Agnetina capitata
Perlinella drymo 16S ribosomal RNA gene, partial s	EF623240	81.12	87.2	1.28317e−27	Plecoptera	Perlidae	Perlinella drymo
Hansonoperla appalachia 16S ribosomal RNA gene, pa	EF623249	87.16	65.85	1.56322e−26	Plecoptera	Perlidae	Hansonoperla appalachia
Taenionema palladium 16S ribosomal RNA gene, parti	EF623267	78.05	99.39	5.45617e−26	Plecoptera	Taeniopterygidae	Taenionema palladium
Pteronarcys princeps mitochondrion, complete genom	AY687866	80.56	87.2	6.64698e−25	Plecoptera	Pteronarcyidae	Pteronarcys princeps
Claassenia sabulosa 16S ribosomal RNA gene, partia	EF623250	79.47	92.07	2.32002e−24	Plecoptera	Perlidae	Claassenia sabulosa
Perlesta decipines 16S ribosomal RNA gene, partial	EF623242	83.2	76.22	2.32002e−24	Plecoptera	Perlidae	Perlesta decipines
Udamocercia sp. BYU_PL150 16S ribosomal RNA gene,	EF623201	84.48	70.73	2.32002e−24	Plecoptera	Notonemouridae	Udamocercia sp. BYU_PL150
Moselia infuscata 16S ribosomal RNA gene, partial	EF623175	78.18	100	2.32002e−24	Plecoptera	Leuctridae	Mosella infuscata
Kathroperla perdita 16S ribosomal RNA gene, partia	EF623135	85.05	65.24	2.32002e−24	Plecoptera	Chloroperlidae	Kathroperla perdita
Plumiperla diversa 16S ribosomal RNA gene, partial	EF623132	79.73	89.02	2.32002e−24	Plecoptera	Chloroperlidae	Plumiperla diversa
Pteronarcys californica 16S ribosomal RNA gene, pa	EF623261	79.86	87.2	8.09768e−24	Plecoptera	Pteronarcyidae	Pteronarcys californica
Cosumnoperla hypocrena 16S ribosomal RNA gene, par	EF623226	79.02	87.2	8.09768e−24	Plecoptera	Perlodidae	Cosumnoperla hypocrena
Leuctra inermis 16S ribosomal RNA gene, partial se	EF623176	82.64	73.17	8.09768e−24	Plecoptera	Leuctridae	Leuctra inermis
Neaviperla forcipata 16S ribosomal RNA gene, parti	EF623139	77.92	93.9	8.09768e−24	Plecoptera	Chloroperlidae	Neaviperla forcipata
Doddsia occidentalis 16S ribosomal RNA gene, parti	EF623270	76.83	99.39	2.82637e−23	Plecoptera	Taeniopterygidae	Doddsia occidentalis
Scopura montana 16S ribosomal RNA gene, partial se	EF623265	81.45	74.39	2.82637e−23	Plecoptera		Scopura montana
Pteronarcys sachalina 16S ribosomal RNA gene, part	EF623264	79.31	87.2	2.82637e−23	Plecoptera	Pteronarcyidae	Pteronarcys sachalina
Triznaka pintada 16S ribosomal RNA gene, partial s	EF623134	82.93	74.39	2.82637e−23	Plecoptera	Chloroperlidae	Triznaka pintada
Leuctra tenuis haplotype 10lt: HO10 16S ribosomal	EU327682	76.83	99.39	2.82637e−23	Plecoptera	Leuctridae	Leuctra tenuis
Leuctra tenuis haplotype 9lt: HO9 16S ribosomal RN	EU327681	76.83	99.39	2.82637e−23	Plecoptera	Leuctridae	Leuctra tenuis
Leuctra tenuis haplotype 8lt: HO8 16S ribosomal RN	EU327680	76.83	99.39	2.82637e−23	Plecoptera	Leuctridae	Leuctra tenuis
Leuctra tenuis haplotype 7lt: HO7 16S ribosomal RN	EU327679	76.83	99.39	2.82637e−23	Plecoptera	Leuctridae	Leuctra tenuis
Leuctra tenuis haplotype 6lt: HO6 16S ribosomal RN	EU327678	76.83	99.39	2.82637e−23	Plecoptera	Leuctridae	Leuctra tenuis
Leuctra tenuis haplotype 3lt: H03 16S ribosomal RN	DQ915184	76.83	99.39	2.82637e−23	Plecoptera	Leuctridae	Leuctra tenuis
Leuctra tenuis haplotype 2lt: H02 16S ribosomal RN	DQ915183	76.83	99.39	2.82637e−23	Plecoptera	Leuctridae	Leuctra tenuis
Leuctra tenuis haplotype 1lt: H01 16S ribosomal RN	DQ915182	76.83	99.39	2.82637e−23	Plecoptera	Leuctridae	Leuctra tenuis
Pteronarcys scotti 16S ribosomal RNA gene, partial	EF623263	79.02	87.2	9.86499e−23	Plecoptera	Pteronarcyidae	Pteronarcys scotti
Acroneuria lycorias 16S ribosomal RNA gene, partia	EF623251	83.19	67.07	9.86499e−23	Plecoptera	Perlidae	Acroneuria lycorias
Despaxia augusta 16S ribosomal RNA gene, partial s	EF623177	81.82	73.17	9.86499e−23	Plecoptera	Leuctridae	Despaxia augusta
Suwallia teleckojensis 16S ribosomal RNA gene, par	EF623142	82.46	69.51	9.86499e−23	Plecoptera	Chloroperlidae	Suwallia teleckojensis
Styloperla sp. mitochondrion, partial genome	KR088971	76.28	93.29	3.44322e−22	Plecoptera	Styloperlidae	Styloperla sp. YZD−2015
Cascadoperla trictura 16S ribosomal RNA gene, part	EF623225	79.17	85.98	3.44322e−22	Plecoptera	Perlodidae	Cascadoperla trictura
Skwala americana 16S ribosomal RNA gene, partial s	EF623217	78.47	87.2	3.44322e−22	Plecoptera	Perlodidae	Skwala americana
Prostoia besametsa 16S ribosomal RNA gene, partial	EF623188	76.88	96.95	3.44322e−22	Plecoptera	Nemouridae	Prostoia besametsa
Leuctra tenuis haplotype 5lt: HO5 16S ribosomal RN	EU327677	76.22	99.39	3.44322e−22	Plecoptera	Leuctridae	Leuctra tenuis
Cryptoperla sp. YK−2012−1 mitochondrial gene for 1	AB711243	79.43	84.76	1.2018e−21	Plecoptera	Peltoperlidae	Cryptoperla sp. YK−2012−1
Dolichopoda bormansi isolate BRA3 16S ribosomal RN	AY507605	76.33	99.39	1.2018e−21	Orthoptera	Rhaphidophoridae	Dolichopoda bormansi
Pictetoperla sp. BYU_PL156 16S ribosomal RNA gene,	EF623247	83.18	65.24	1.2018e−21	Plecoptera	Perlidae	Pictetoperla sp. BYU_PL156
Nemoura cinerea 16S ribosomal RNA gene, partial se	EF623194	83.18	65.24	1.2018e−21	Plecoptera	Nemouridae	Nemoura cinerea
Leuctra tenuis haplotype 4lt: HO4 16S ribosomal RN	EU327676	81.2	71.34	1.2018e−21	Plecoptera	Leuctridae	Leuctra tenuis
Dolichopoda bormansi isolate BRA 16S ribosomal RNA	AY793578	76.33	99.39	1.2018e−21	Orthoptera	Rhaphidophoridae	Dolichopoda bormansi
Cerconychia sp. BYU_PL114 16S ribosomal RNA gene,	EF623260	77.03	90.24	4.1947e−21	Plecoptera	Styloperlidae	Cerconychia sp. BYU_PL114
Siphonoperla torrentium 16S ribosomal RNA gene, pa	EF623138	77.62	87.2	4.1947e−21	Plecoptera	Chloroperlidae	Siphonoperla torrentium
D.geniculata mitochondrial 16S rRNA gene	Y07551	75	99.39	4.1947e−21	Orthoptera	Rhaphidophoridae	Dolichopoda geniculata
Dolichopoda bormansi isolate BRA5 16S ribosomal RN	AY507606	75.74	99.39	1.46409e−20	Orthoptera	Rhaphidophoridae	Dolichopoda bormansi
Strophopteryx appalachia 16S ribosomal RNA gene, p	EF623271	80	75.61	1.46409e−20	Plecoptera	Taeniopterygidae	Strophopteryx appalachia
Salmoperla sylvanica 16S ribosomal RNA gene, parti	EF623222	75.97	93.29	1.46409e−20	Plecoptera	Perlodidae	Salmoperla sylvanica
Lednia tumana 16S ribosomal RNA gene, partial sequ	EF623197	80	75.61	1.46409e−20	Plecoptera	Nemouridae	Lednia tumana
Nemoura trispinosa 16S ribosomal RNA gene, partial	EF623187	80	75.61	1.46409e−20	Plecoptera	Nemouridae	Nemoura trispinosa
Paranemoura claasseni 16S ribosomal RNA gene, part	EF623185	80	75.61	1.46409e−20	Plecoptera	Nemouridae	Paranemoura claasseni
Shipsa rotunda 16S ribosomal RNA gene, partial seq	EF623184	80	75.61	1.46409e−20	Plecoptera	Nemouridae	Shipsa rotunda
Cosmioperla australis 16S ribosomal RNA gene, part	EF623148	75.64	91.46	1.46409e−20	Plecoptera	Eustheniidae	Cosmioperla australis
Sasquaperla hoopa 16S ribosomal RNA gene, partial	EF623143	83.5	62.8	1.46409e−20	Plecoptera	Chloroperlidae	Sasquaperla hoopa
Chloroperla tripunctata 16S ribosomal RNA gene, pa	EF623137	81.3	74.39	1.46409e−20	Plecoptera	Chloroperlidae	Chloroperla tripunctata
Paraperla frontalis 16S ribosomal RNA gene, partia	EF623136	84.47	62.8	1.46409e−20	Plecoptera	Chloroperlidae	Paraperla frontalis
Isoperla fulva 16S ribosomal RNA gene, partial seq	EF623207	81.58	69.51	5.11019e−20	Plecoptera	Perlodidae	Isoperla fulva
Megaleuctra kincaidi 16S ribosomal RNA gene, parti	EF623174	83.18	65.24	5.11019e−20	Plecoptera	Leuctridae	Megaleuctra kincaidi
Dolichopoda pavesii voucher SPI 16S ribosomal RNA	EF216951	79.69	75	1.78363e−19	Orthoptera	Rhaphidophoridae	Dolichopoda pavesii
Brachyptera seticornis 16S ribosomal RNA gene, par	EF623268	74.85	99.39	1.78363e−19	Plecoptera	Taeniopterygidae	Brachyptera seticornis
Perlinodes aurea 16S ribosomal RNA gene, partial s	EF623234	82.52	62.8	1.78363e−19	Plecoptera	Perlodidae	Perlinodes aurea
Frisonia picticeps 16S ribosomal RNA gene, partial	EF623227	80.17	70.73	1.78363e−19	Plecoptera	Perlodidae	Frisonia picticeps
Protonemura meyeri 16S ribosomal RNA gene, partial	EF623193	76.62	92.68	1.78363e−19	Plecoptera	Nemouridae	Protonemura meyeri
Nemurella pictetii 16S ribosomal RNA gene, partial	EF623192	77.63	90.24	1.78363e−19	Plecoptera	Nemouridae	Nemurella pictetii
Malenka californica 16S ribosomal RNA gene, partia	EF623182	82.52	62.8	1.78363e−19	Plecoptera	Nemouridae	Malenka californica
Alloperla severa 16S ribosomal RNA gene, partial s	EF623141	76.82	89.02	1.78363e−19	Plecoptera	Chloroperlidae	Alloperla severa
Sweltsa coloradensis 16S ribosomal RNA gene, parti	EF623133	82.52	62.8	1.78363e−19	Plecoptera	Chloroperlidae	Sweltsa coloradensis
Ceraeochrysa cincta voucher CASC 00217 16S ribosom	DQ399268	79.66	71.95	1.78363e−19	Neuroptera	Chrysopidae	Ceraeochrysa cincta
Osmylus sp. EH−2004 16S ribosomal RNA gene, partia	AY620146	77.93	87.2	1.78363e−19	Neuroptera	Osmylidae	Osmylus sp. EH−2004
Blattella vaga mitochondrion 16S ribosomal RNA, pa	U17775	77.33	91.46	1.78363e−19	Blattodea	Ectobiidae	Blattella vaga
Eupholidoptera krueperi voucher 408 16S ribosomal	EU203912	76.51	88.41	6.22549e−19	Orthoptera	Tettigoniidae	Eupholidoptera krueperi
Pteronarcella badia 16S ribosomal RNA gene, partia	EF623262	81.31	65.24	6.22549e−19	Plecoptera	Pteronarcyidae	Pteronarcella badia
Eccoptura xanthenes 16S ribosomal RNA gene, partia	EF623252	79.2	75.61	6.22549e−19	Plecoptera	Perlidae	Eccoptura xanthenes
Osobenus yakimae 16S ribosomal RNA gene, partial s	EF623232	75.97	90.85	6.22549e−19	Plecoptera	Perlodidae	Osobenus yakimae
Isogenoides olivaceus 16S ribosomal RNA gene, part	EF623224	80.36	68.29	6.22549e−19	Plecoptera	Perlodidae	Isogenoides olivaceus
Neonemura sp. BYU_PL153 16S ribosomal RNA gene, pa	EF623204	75.8	94.51	6.22549e−19	Plecoptera	Notonemouridae	Neonemura sp. BYU_PL153
Paraleuctra vershina 16S ribosomal RNA gene, parti	EF623173	82.24	65.24	6.22549e−19	Plecoptera	Leuctridae	Paraleuctra vershina
Macroxyela ferruginea 16S ribosomal RNA gene, part	AY206773	79.51	74.39	6.22549e−19	Hymenoptera	Xyelidae	Macroxyela ferruginea
Cryptoperla ishigakiensis mitochondrial gene for 1	AB711242	80.17	70.73	2.17291e−18	Plecoptera	Peltoperlidae	Cryptoperla ishigakiensis
Udenus wnigrum voucher MAY 16S ribosomal RNA gene,	HM594504	80	68.9	2.17291e−18	Orthoptera	Rhaphidophoridae	Udenus wnigrum
Dolichopoda geniculata geniculata isolate VAL1 16S	AY507600	73.81	99.39	2.17291e−18	Orthoptera	Rhaphidophoridae	Dolichopoda geniculata geniculata
Dolichopoda geniculata geniculata isolate PRA20 16	AY507590	73.81	99.39	2.17291e−18	Orthoptera	Rhaphidophoridae	Dolichopoda geniculata geniculata
Dolichopoda geniculata geniculata isolate AUS1 16S	AY507566	73.81	99.39	2.17291e−18	Orthoptera	Rhaphidophoridae	Dolichopoda geniculata geniculata
Paragnetina media 16S ribosomal RNA gene, partial	EF623253	74.55	100	2.17291e−18	Plecoptera	Perlidae	Paragnetina media
Kempnyella sp. BYU_PL155 16S ribosomal RNA gene, p	EF623246	81.82	66.46	2.17291e−18	Plecoptera	Perlidae	Kempnyella sp. BYU_PL155
Clioperla clio 16S ribosomal RNA gene, partial seq	EF623237	80.17	70.73	2.17291e−18	Plecoptera	Perlodidae	Clioperla clio
Malirekus iroquois 16S ribosomal RNA gene, partial	EF623231	82.08	64.63	2.17291e−18	Plecoptera	Perlodidae	Malirekus iroquois
Isoperla oxylepis 16S ribosomal RNA gene, partial	EF623221	82.52	62.8	2.17291e−18	Plecoptera	Perlodidae	Isoperla oxylepis
Chernokrilus misnomus 16S ribosomal RNA gene, part	EF623209	81.48	65.85	2.17291e−18	Plecoptera	Perlodidae	Chernokrilus misnomus
Austrocerca rieka 16S ribosomal RNA gene, partial	EF623205	75.61	99.39	2.17291e−18	Plecoptera	Notonemouridae	Austrocerca rieka
Austrocercoides zwicki 16S ribosomal RNA gene, par	EF623199	75.61	99.39	2.17291e−18	Plecoptera	Notonemouridae	Austrocercoides zwicki
Dolichopoda geniculata isolate VAL 16S ribosomal R	AY793583	73.81	99.39	2.17291e−18	Orthoptera	Rhaphidophoridae	Dolichopoda geniculata
Apteroperla tikumana mitochondrion, complete genom	KR604721	76.39	87.2	7.5842e−18	Plecoptera	Capniidae	Apteroperla tikumana
Bolotoperla rossi 16S ribosomal RNA gene, partial	EF623269	81.55	62.8	7.5842e−18	Plecoptera	Taeniopterygidae	Bolotoperla rossi
Oconoperla innubila 16S ribosomal RNA gene, partia	EF623230	74.7	99.39	7.5842e−18	Plecoptera	Perlodidae	Oconoperla innubila
Isogenoides hansoni 16S ribosomal RNA gene, partia	EF623223	82.65	59.76	7.5842e−18	Plecoptera	Perlodidae	Isogenoides hansoni
Nanonemoura wahkeena 16S ribosomal RNA gene, parti	EF623198	81.55	62.8	7.5842e−18	Plecoptera	Nemouridae	Nanonemoura wahkeena
