# Supplementary material for: Testing the potential of a ribosomal 16S marker for DNA metabarcoding of insects
Source: PeerJ. 2016 Apr 19;4:e1966. doi: 10.7717/peerj.1966 (PMC4841222; doi:10.7717/peerj.1966)
Supplement: Supplemental Information 1 [file peerj-04-1966-s006.zip › scripts/5 match OTUs/blast_hits/OTU_185.csv_plot.pdf]

OTU_185.csv

Ceratophysella sp. FZ−2014

Hypogastruridae

74.23

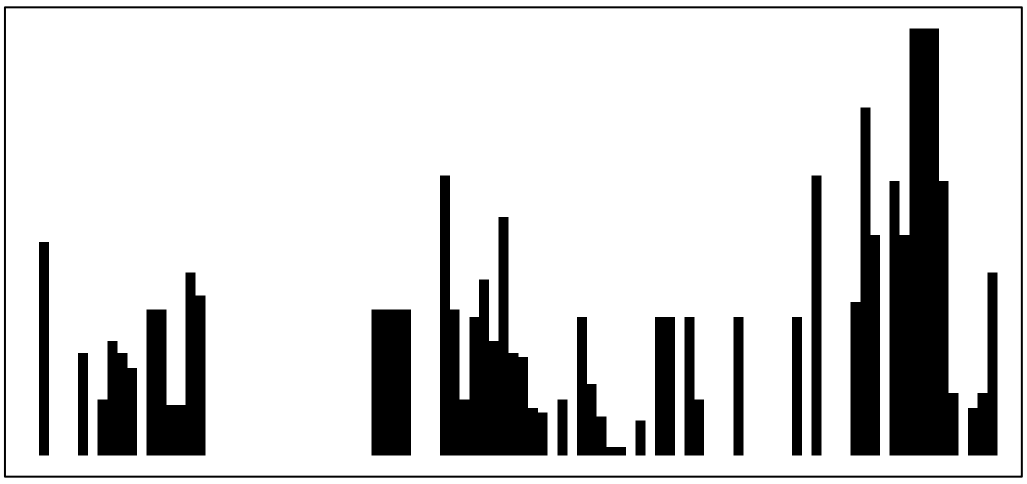

Ceratophysella sp. FZ−2014 voucher Ceratophysella_	KF591912	74.23	75.61	0.000192977		Hypogastruridae	Ceratophysella sp. FZ−2014
Solanum lycopersicum chromosome ch12, complete gen	HG975524	90	31.71	0.00235094	Solanales	Solanaceae	Solanum lycopersicum
Typhlocharis sp. MNHN AF119 genomic DNA containing	FR729594	76.39	58.54	0.00820558	Coleoptera	Carabidae	Typhlocharis sp. MNHN AF119
Epitheca canis 12S ribosomal RNA gene, partial seq	EU477712	77.94	52.85	0.00820558	Odonata	Corduliidae	Tetragoneuria canis
Solanum lycopersicum chromosome ch04, complete gen	HG975516	77.78	58.54	0.0286403	Solanales	Solanaceae	Solanum lycopersicum
Arabidopsis thaliana chromosome 3, complete sequence	CP002686	84.78	36.59	0.0286403	Brassicales	Brassicaceae	Arabidopsis thaliana
S.lycopersicum DNA sequence from clone LE_HBa−188D	CU915746	77.78	58.54	0.0286403	Solanales	Solanaceae	Solanum lycopersicum
Mycoplasma agalactiae PG2 chromosome, complete seq	CU179680	82.61	37.4	0.0286403	Mycoplasmatales	Mycoplasmataceae	Mycoplasma agalactiae PG2
Morulina verrucosa 16S ribosomal RNA gene, partial	AY555554	85.37	33.33	0.0286403		Neanuridae	Morulina verrucosa
Arabidopsis thaliana DNA chromosome 3, BAC clone T	AL137079	84.78	36.59	0.0286403	Brassicales	Brassicaceae	Arabidopsis thaliana
Ovis canadensis canadensis isolate 43U chromosome	CP011888	84.09	35.77	0.0999645		Bovidae	Ovis canadensis canadensis
Spodoptera littoralis voucher SPCAM0144 16S riboso	KP682579	78.12	49.59	0.0999645	Lepidoptera	Noctuidae	Spodoptera littoralis
Cucumis melo genomic chromosome, chr_11	LN713265	86.84	30.89	0.0999645	Cucurbitales	Cucurbitaceae	Cucumis melo
Cucumis melo genomic scaffold, anchoredscaffold000	LN681913	86.84	30.89	0.0999645	Cucurbitales	Cucurbitaceae	Cucumis melo
Schistosoma curassoni genome assembly S_curassoni_	LM065287	82.35	39.84	0.0999645	Strigeidida	Schistosomatidae	Schistosoma curassoni
Schistosoma mattheei genome assembly S_mattheei_De	LM151378	82.35	39.84	0.0999645	Strigeidida	Schistosomatidae	Schistosoma mattheei
Borrelia valaisiana Tom4006, complete genome	CP009117	88.57	28.46	0.0999645	Spirochaetales	Borreliaceae	Borrelia valaisiana Tom4006
Solanum pennellii chromosome ch12, complete genome	HG975451	87.5	31.71	0.0999645	Solanales	Solanaceae	Solanum pennellii
Solanum pennellii chromosome ch10, complete genome	HG975449	77.46	57.72	0.0999645	Solanales	Solanaceae	Solanum pennellii
Spodoptera litura mitochondrion, complete genome	KF543065	78.12	49.59	0.0999645	Lepidoptera	Noctuidae	Spodoptera litura
Spodoptera litura isolate Delhi15_S_I 16S ribosoma	KC352618	78.12	49.59	0.0999645	Lepidoptera	Noctuidae	Spodoptera litura
Spodoptera litura mitochondrion, complete genome	KF701043	78.12	49.59	0.0999645	Lepidoptera	Noctuidae	Spodoptera litura
Fusarium fujikuroi IMI 58289 draft genome, chromos	HF679027	77.19	40.65	0.0999645	Hypocreales	Nectriaceae	Fusarium fujikuroi IMI 58289
Spodoptera litura voucher SPO−B113 16S ribosomal R	HQ178335	78.12	49.59	0.0999645	Lepidoptera	Noctuidae	Spodoptera litura
Spodoptera litura voucher SPO−B15 16S ribosomal RN	HQ178334	78.12	49.59	0.0999645	Lepidoptera	Noctuidae	Spodoptera litura
Spodoptera littoralis voucher SPO−B14 16S ribosoma	HQ178324	78.12	49.59	0.0999645	Lepidoptera	Noctuidae	Spodoptera littoralis
Thermoanaerobacterium xylanolyticum LX−11, complet	CP002739	80	40.65	0.0999645	Thermoanaerobacterales	Thermoanaerobacterales Family III. Incertae Sedis	Thermoanaerobacterium xylanolyticum LX−11
Anomala xanthoptera isolate BM677788 16S ribosomal	DQ681060	76.39	56.91	0.0999645	Coleoptera	Scarabaeidae	Anomala xanthoptera
Anomala xanthoptera isolate BM677779 16S ribosomal	DQ681055	76.39	56.91	0.0999645	Coleoptera	Scarabaeidae	Anomala xanthoptera
Anomala xanthoptera isolate BM677728 16S ribosomal	DQ681049	76.39	56.91	0.0999645	Coleoptera	Scarabaeidae	Anomala xanthoptera
Anomala xanthoptera isolate BM677806 16S ribosomal	DQ680973	76.39	56.91	0.0999645	Coleoptera	Scarabaeidae	Anomala xanthoptera
Anomala xanthoptera isolate BM670906 16S ribosomal	DQ680917	76.39	56.91	0.0999645	Coleoptera	Scarabaeidae	Anomala xanthoptera
Anomala xanthoptera isolate BM677776 16S ribosomal	DQ680871	76.39	56.91	0.0999645	Coleoptera	Scarabaeidae	Anomala xanthoptera
Anomala sp. BM670922 16S ribosomal RNA gene, parti	DQ680862	76.39	56.91	0.0999645	Coleoptera	Scarabaeidae	Anomala sp. BM670922
Anomala xanthoptera isolate BM677744 16S ribosomal	DQ680858	76.39	56.91	0.0999645	Coleoptera	Scarabaeidae	Anomala xanthoptera
Canis Familiaris chromosome 11, clone XX−2553E14,	AC191160	86.84	30.89	0.0999645	Carnivora	Canidae	Canis lupus familiaris
Canis Familiaris chromosome 11, clone XX−334N9, co	AC188522	86.84	30.89	0.0999645	Carnivora	Canidae	Canis lupus familiaris
Canis Familiaris chromosome 11, clone XX−402C16, c	AC186998	86.84	30.89	0.0999645	Carnivora	Canidae	Canis lupus familiaris
Canis Familiaris chromosome 11, clone XX−80M13, co	AC187313	86.84	30.89	0.0999645	Carnivora	Canidae	Canis lupus familiaris
Spodoptera litura 16S ribosomal RNA gene, partial	AF173065	78.12	49.59	0.0999645	Lepidoptera	Noctuidae	Spodoptera litura
Spodoptera littoralis 16S ribosomal RNA gene, part	AF173064	78.12	49.59	0.0999645	Lepidoptera	Noctuidae	Spodoptera littoralis
Ovis canadensis canadensis isolate 43U chromosome	CP011892	79.25	42.28	0.34891		Bovidae	Ovis canadensis canadensis
Tetrahymena thermophila SB210 transmembrane protei	XM_012798190	93.1	23.58	0.34891	Hymenostomatida	Tetrahymenidae	Tetrahymena thermophila SB210
Trifolium pratense genome assembly redclover, chro	LN846354	86.84	28.46	0.34891	Fabales	Fabaceae	Trifolium pratense
Esox lucius high choriolytic enzyme 1−like (LOC105	NM_001303719	82.61	36.59	0.34891	Esociformes	Esocidae	Esox lucius
Clostridium botulinum DNA, complete genome, strain	AP014696	86.49	30.08	0.34891	Clostridiales	Clostridiaceae	Clostridium botulinum
Strongyloides stercoralis genome assembly S_sterco	LL999055	88.24	27.64	0.34891	Rhabditida	Strongyloididae	Strongyloides stercoralis
Brugia pahangi genome assembly B_pahangi_Glasgow ,	LK967806	85.37	32.52	0.34891	Spirurida	Onchocercidae	Brugia pahangi
Angiostrongylus cantonensis genome assembly A_cant	LK945875	91.18	27.64	0.34891	Rhabditida	Angiostrongylidae	Angiostrongylus cantonensis
Strongyloides papillosus genome assembly S_papillo	LM525561	84.78	35.77	0.34891	Rhabditida	Strongyloididae	Strongyloides papillosus
Strongyloides ratti genome assembly S_ratti_ED321	LN609529	84.62	31.71	0.34891	Rhabditida	Strongyloididae	Strongyloides ratti
Cyprinus carpio genome assembly common carp genome	LN590687	82.22	36.59	0.34891	Cypriniformes	Cyprinidae	Cyprinus carpio
Methanocaldococcus sp. JH146, complete genome	CP009149	82	40.65	0.34891	Methanococcales	Methanocaldococcaceae	Methanocaldococcus sp. JH146
Solanum pennellii chromosome ch05, complete genome	HG975444	74.36	63.41	0.34891	Solanales	Solanaceae	Solanum pennellii
Lotharella sp. CCMP622 chromosome 1 nucleomorph se	CP006627	82.61	37.4	0.34891			Lotharella oceanica
Aeschnophlebia longistigma voucher ANI01 tRNA−Leu,	KF256836	75	58.54	0.34891	Odonata	Aeshnidae	Aeschnophlebia longistigma
Cenchrus americanus plastid, complete genome	KJ490012	86.49	30.08	0.34891	Poales	Poaceae	Cenchrus americanus
Pogonognathellus cf. flavescens FZ−2014 voucher Po	KF591937	83.33	34.15	0.34891		Tomoceridae	Pogonognathellus cf. flavescens FZ−2014
Xylobanus sp. UPOL A00076 voucher UPOLA00076 16S r	KC538752	81.82	35.77	0.34891	Coleoptera	Lycidae	Xylobanus sp. UPOL A00076
Epichloe sp. E4305 elymoclavine monooxygenase (clo	KC989575	80.39	40.65	0.34891	Hypocreales	Clavicipitaceae	Epichloe sp. E4305
Neotyphodium coenophialum strain e4163 isolate EAS	KC989569	80.39	40.65	0.34891	Hypocreales	Clavicipitaceae	Epichloe coenophiala
Sminthurus viridis 16S ribosomal RNA gene, partial	JN970982	76.06	54.47	0.34891		Sminthuridae	Sminthurus viridis
Colotis dissociatus voucher TBL−009 16S ribosomal	HQ682529	81.63	39.02	0.34891	Lepidoptera	Pieridae	Colotis dissociatus
Monosynamma bohemanni 16S ribosomal RNA gene, part	GU194568	74.39	65.04	0.34891	Hemiptera	Miridae	Monosynamma bohemanni
Clostridium botulinum H04402 065, complete genome	FR773526	86.49	30.08	0.34891	Clostridiales	Clostridiaceae	Clostridium botulinum H04402 065
Clostridium botulinum F str. 230613, complete geno	CP002011	86.49	30.08	0.34891	Clostridiales	Clostridiaceae	Clostridium botulinum F str. 230613
Caridina sp. Malili 1 mitochondrial partial 16S rR	FM201963	78.85	39.02	0.34891	Decapoda	Atyidae	Caridina sp. Malili 1
Clostridium botulinum A2 str. Kyoto, complete geno	CP001581	86.49	30.08	0.34891	Clostridiales	Clostridiaceae	Clostridium botulinum A2 str. Kyoto
Esox lucius clone eluc−evq−530−313 High choriolyti	BT079568	82.61	36.59	0.34891	Esociformes	Esocidae	Esox lucius
Caridina lanceolata mitochondrial partial 16S rRNA	AM747665	78.85	39.02	0.34891	Decapoda	Atyidae	Caridina lanceolata
Epitheca costalis 12S ribosomal RNA gene, partial	EU477713	76.47	52.85	0.34891	Odonata	Corduliidae	Epitheca costalis
Sminthurus viridis mitochondrion, complete genome	EU016192	76.06	54.47	0.34891		Sminthuridae	Sminthurus viridis
Clostridium botulinum B1 str. Okra, complete genom	CP000939	86.49	30.08	0.34891	Clostridiales	Clostridiaceae	Clostridium botulinum B1 str. Okra
Anomala albopilosa albopilosa mitochondrial genes	AB330391	75	55.28	0.34891	Coleoptera	Scarabaeidae	Anomala albopilosa albopilosa
Anomala albopilosa albopilosa mitochondrial genes	AB330390	75	55.28	0.34891	Coleoptera	Scarabaeidae	Anomala albopilosa albopilosa
Anomala albopilosa gracilis mitochondrial genes fo	AB330389	76.47	55.28	0.34891	Coleoptera	Scarabaeidae	Anomala albopilosa gracilis
Anomala albopilosa sakishimana mitochondrial genes	AB330387	75	55.28	0.34891	Coleoptera	Scarabaeidae	Anomala albopilosa sakishimana
Caridina lanceolata voucher ZMB Crust. 29093 16S r	EF432583	78.85	39.02	0.34891	Decapoda	Atyidae	Caridina lanceolata
Clostridium botulinum F str. Langeland, complete g	CP000728	86.49	30.08	0.34891	Clostridiales	Clostridiaceae	Clostridium botulinum F str. Langeland
Anomala sp. BM671387 16S ribosomal RNA gene, parti	DQ680885	75	55.28	0.34891	Coleoptera	Scarabaeidae	Anomala sp. BM671387
Vitis vinifera contig VV78X247621.8, whole genome	AM436068	93.1	23.58	0.34891	Vitales	Vitaceae	Vitis vinifera
Ochlerotatus japonicus isolate A81 16S ribosomal R	DQ397915	78.33	48.78	0.34891	Diptera	Culicidae	Aedes japonicus
Spodoptera picta 16S ribosomal RNA gene, partial s	AF173066	76.92	49.59	0.34891	Lepidoptera	Noctuidae	Spodoptera picta
Tetragoneuria williamsoni 16S ribosomal RNA gene,	AF037193	76.47	52.85	0.34891	Odonata	Corduliidae	Tetragoneuria williamsoni
Frankliniella sp. KY−2004 16S ribosomal RNA gene,	DQ104743	87.18	31.71	0.34891	Thysanoptera	Thripidae	Frankliniella sp. KY−2004
Mus musculus BAC clone RP23−236A6 from 10, complet	AC142273	96.3	21.95	0.34891	Rodentia	Muridae	Mus musculus
Bacillus sp. FJAT−4402 genome	CP012600	90.32	25.2	1.21782	Bacillales	Bacillaceae	Bacillus sp. FJAT−4402
Ovis canadensis canadensis isolate 43U chromosome	CP011903	78.95	43.9	1.21782		Bovidae	Ovis canadensis canadensis
Acinetobacter baumannii genome assembly CIP70.10,	LN865143	92.86	22.76	1.21782	Pseudomonadales	Moraxellaceae	Acinetobacter baumannii
Spiroplasma eriocheiris strain DSM 21848, complete	CP011856	90.32	25.2	1.21782	Entomoplasmatales	Spiroplasmataceae	Spiroplasma eriocheiris
PREDICTED: Hydra vulgaris uncharacterized LOC10020	XM_012703853	100	18.7	1.21782	Anthoathecata	Hydridae	Hydra vulgaris
PREDICTED: Hydra vulgaris uncharacterized LOC10584	XM_012698612	100	18.7	1.21782	Anthoathecata	Hydridae	Hydra vulgaris
PREDICTED: Hydra vulgaris uncharacterized LOC10584	XM_012702315	100	18.7	1.21782	Anthoathecata	Hydridae	Hydra vulgaris
Trifolium pratense genome assembly redclover, chro	LN846355	92.86	22.76	1.21782	Fabales	Fabaceae	Trifolium pratense
PREDICTED: Mandrillus leucophaeus cysteine/tyrosin	XM_011998314	82.93	33.33	1.21782	Primates	Cercopithecidae	Mandrillus leucophaeus
PREDICTED: Felis catus eukaryotic translation init	XM_004000333	79.17	39.02	1.21782	Carnivora	Felidae	Felis catus
Bacillus thuringiensis strain 97−27, complete geno	CP010088	82.22	35.77	1.21782	Bacillales	Bacillaceae	Bacillus thuringiensis
Vitis vinifera contig VV78X222883.24, whole genome	AM430323	82.93	33.33	1.21782	Vitales	Vitaceae	Vitis vinifera
Vitis vinifera contig VV78X215330.6, whole genome	AM449349	88.57	28.46	1.21782	Vitales	Vitaceae	Vitis vinifera
Ovis canadensis canadensis isolate 43U chromosome	CP011912	80	36.59	4.2506		Bovidae	Ovis canadensis canadensis
