# Supplementary material for: Testing the potential of a ribosomal 16S marker for DNA metabarcoding of insects
Source: PeerJ. 2016 Apr 19;4:e1966. doi: 10.7717/peerj.1966 (PMC4841222; doi:10.7717/peerj.1966)
Supplement: Supplemental Information 1 [file peerj-04-1966-s006.zip › scripts/5 match OTUs/blast_hits/OTU_187.csv_plot.pdf]

OTU_187.csv

Notidobiella chacayana

Sericostomatidae

Trichoptera

78.85

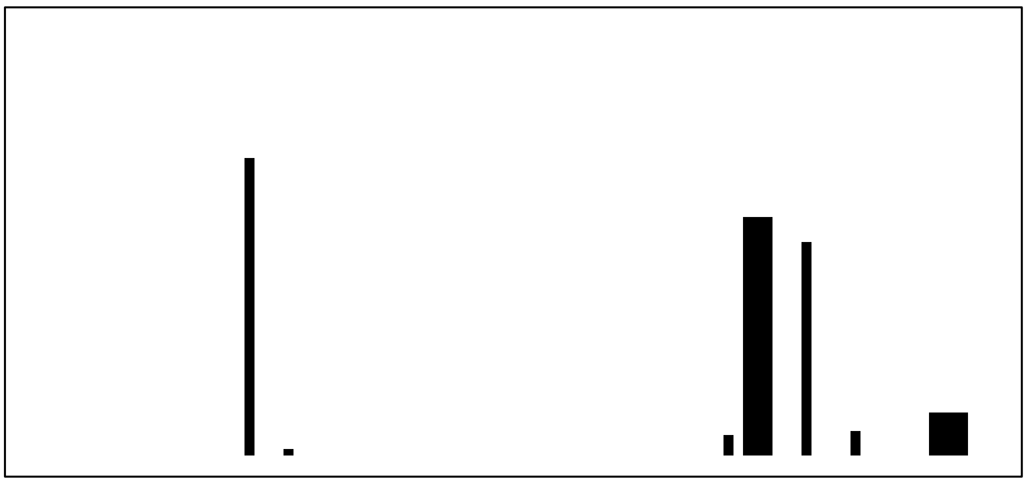

Notidobiella chacayana isolate CL5 16S ribosomal R	EF394993	78.85	74.64	1.62494e−12	Trichoptera	Sericostomatidae	Notidobiella chacayana
Sericostoma clypeatum voucher NHRS−EN CC4 16S ribo	FJ263207	76.47	85.51	1.97959e−11	Trichoptera	Sericostomatidae	Sericostoma clypeatum
Sericostoma personatum mitochondrion, partial geno	KP455290	76.7	74.64	2.93797e−09	Trichoptera	Sericostomatidae	Sericostoma personatum
Caenota plicata isolate CR9 16S ribosomal RNA gene	EF395003	75.7	74.64	5.31197e−06	Trichoptera	Calocidae	Caenota plicata
Acordulecera sp. SS2 voucher AE142 16S ribosomal R	KF318505	70.99	93.48	6.4713e−05	Hymenoptera	Pergidae	Acordulecera sp. SS2
Mucroberotha vesicaria 16S ribosomal RNA gene, par	EU734872	79.71	48.55	6.4713e−05	Neuroptera	Berothidae	Mucroberotha vesicaria
Episyrphus balteatus 16S ribosomal RNA gene, parti	AY573115	70.83	79.71	0.000225871	Diptera	Syrphidae	Episyrphus balteatus
Melanostoma apicale 16S ribosomal RNA gene, partia	AF154791	72.28	71.01	0.000225871	Diptera	Syrphidae	Melanostoma apicale
Melanostoma apicale 16S ribosomal RNA gene, partia	AF154790	72.28	71.01	0.000225871	Diptera	Syrphidae	Melanostoma apicale
Melanostoma apicale 16S ribosomal RNA gene, partia	AF154789	72.28	71.01	0.000225871	Diptera	Syrphidae	Melanostoma apicale
Argestina inconstans 16S ribosomal RNA gene, parti	KM200168	79.69	44.2	0.000788366	Lepidoptera	Nymphalidae	Argestina inconstans
Plasmodium yoelii genome assembly PY17X01, chromos	LM993663	70.92	98.55	0.000788366	Haemosporida	Plasmodiidae	Plasmodium yoelii
Plasmodium yoelii genome assembly PYYM01, chromoso	LK934637	70.92	98.55	0.000788366	Haemosporida	Plasmodiidae	Plasmodium yoelii
Xylosciara inornata voucher 230 16S ribosomal RNA	JQ613902	74.75	68.12	0.000788366	Diptera	Sciaridae	Xylosciara inornata
Eosericostoma inaequispinum isolate CK9 16S riboso	EF394992	73.53	73.91	0.000788366	Trichoptera	Helicophidae	Eosericostoma inaequispinum
Eosericostoma aequispinum isolate CK7 16S ribosoma	EF394990	73.53	73.91	0.000788366	Trichoptera	Helicophidae	Eosericostoma aequispinum
Eosericostoma sp. CK5 16S ribosomal RNA gene, part	EF394989	73.53	73.91	0.000788366	Trichoptera	Helicophidae	Eosericostoma sp. CK5
Onycholyda amplecta 16S ribosomal RNA gene, partia	AY206785	78.26	49.28	0.000788366	Hymenoptera	Pamphiliidae	Onycholyda amplecta
Toxomerus marginatus 16S ribosomal RNA gene, parti	AF154820	72.38	72.46	0.000788366	Diptera	Syrphidae	Toxomerus marginatus
Toxomerus geminatus 16S ribosomal RNA gene, partia	AF154819	72.38	72.46	0.000788366	Diptera	Syrphidae	Toxomerus geminatus
Sthenaridea piceonigra 16S ribosomal RNA gene, par	JX194130	78.46	47.1	0.00275167	Hemiptera	Miridae	Sthenaridea piceonigra
Sthenaridea vulgaris 16S ribosomal RNA gene, parti	JX194129	76	54.35	0.00275167	Hemiptera	Miridae	Sthenaridea vulgaris
Strongyloides stercoralis genome assembly S_sterco	LL999123	93.94	23.91	0.00275167	Rhabditida	Strongyloididae	Strongyloides stercoralis
Ichthyophthirius multifiliis hypothetical protein	XM_004024101	75.28	64.49	0.00275167	Hymenostomatida		Ichthyophthirius multifiliis
Sthenaridea piceonigra 16S ribosomal RNA gene, par	JF414076	78.46	47.1	0.00275167	Hemiptera	Miridae	Sthenaridea piceonigra
Sthenaridea vulgaris 16S ribosomal RNA gene, parti	JF414075	76	54.35	0.00275167	Hemiptera	Miridae	Sthenaridea vulgaris
Dictyostelium purpureum hypothetical protein, mRNA	XM_003284470	80.3	47.1	0.00275167	Dictyosteliida		Dictyostelium purpureum
Drosophila bifasciata voucher 109384 NADH dehydrog	EU494362	72.97	73.19	0.00275167	Diptera	Drosophilidae	Drosophila bifasciata
Simosyrphus grandicornis mitochondrion, complete g	DQ866050	71.17	73.19	0.00275167	Diptera	Syrphidae	Simosyrphus grandicornis
Cordilura ontario 16S ribosomal RNA gene, partial	DQ648662	73	68.84	0.00275167	Diptera	Scathophagidae	Cordilura ontario
Mucroberotha vesicaria isolate Mucves4 16S ribosom	AY620166	78.26	48.55	0.00275167	Neuroptera	Berothidae	Mucroberotha vesicaria
Drosophila mauritiana isolate G52 mitochondrion, c	AF200830	74.47	59.42	0.00275167	Diptera	Drosophilidae	Drosophila mauritiana
Ips knausi 16S ribosomal RNA gene, partial sequenc	AF397485	78.26	47.83	0.00275167	Coleoptera	Curculionidae	Ips knausi
Dideopsis aegrota 16S ribosomal RNA gene, partial	AF154775	71.55	78.26	0.00275167	Diptera	Syrphidae	Dideopsis aegrota
Plasmodium yoelii genome assembly PY17X01, chromos	LM993655	70.71	95.65	0.00960426	Haemosporida	Plasmodiidae	Plasmodium yoelii
Plasmodium yoelii genome assembly PYYM01, chromoso	LK934629	70.71	95.65	0.00960426	Haemosporida	Plasmodiidae	Plasmodium yoelii
Cupido argiades mitochondrion, complete genome	KC310728	77.14	48.55	0.00960426	Lepidoptera	Lycaenidae	Cupido argiades
Ceratovacuna orientalis mitochondrial genes for sm	AB710259	79.1	47.1	0.00960426	Hemiptera	Hormaphididae	Ceratovacuna orientalis
Ceratovacuna orientalis mitochondrial genes for sm	AB710252	79.1	47.1	0.00960426	Hemiptera	Hormaphididae	Ceratovacuna orientalis
Ceratovacuna orientalis mitochondrial genes for sm	AB710253	79.1	47.1	0.00960426	Hemiptera	Hormaphididae	Ceratovacuna orientalis
Ceratovacuna oplismeni mitochondrial genes for sma	AB710251	79.1	47.1	0.00960426	Hemiptera	Hormaphididae	Ceratovacuna oplismeni
Ceratovacuna oplismeni mitochondrial genes for sma	AB710249	79.1	47.1	0.00960426	Hemiptera	Hormaphididae	Ceratovacuna oplismeni
Ceratovacuna oplismeni mitochondrial genes for sma	AB710245	79.1	47.1	0.00960426	Hemiptera	Hormaphididae	Ceratovacuna oplismeni
Ceratovacuna oplismeni mitochondrial genes for sma	AB710246	79.1	47.1	0.00960426	Hemiptera	Hormaphididae	Ceratovacuna oplismeni
Ceratovacuna oplismeni mitochondrial genes for sma	AB710244	79.1	47.1	0.00960426	Hemiptera	Hormaphididae	Ceratovacuna oplismeni
Ceratovacuna oplismeni mitochondrial genes for sma	AB710243	79.1	47.1	0.00960426	Hemiptera	Hormaphididae	Ceratovacuna oplismeni
Ceratovacuna oplismeni mitochondrial genes for sma	AB710242	79.1	47.1	0.00960426	Hemiptera	Hormaphididae	Ceratovacuna oplismeni
Ceratovacuna oplismeni mitochondrial genes for sma	AB710241	79.1	47.1	0.00960426	Hemiptera	Hormaphididae	Ceratovacuna oplismeni
Ceratovacuna nekoashi mitochondrial genes for smal	AB710240	79.1	47.1	0.00960426	Hemiptera	Hormaphididae	Ceratovacuna nekoashi
Ceratovacuna nekoashi mitochondrial genes for smal	AB710239	79.1	47.1	0.00960426	Hemiptera	Hormaphididae	Ceratovacuna nekoashi
Ceratovacuna nekoashi mitochondrial genes for smal	AB710238	79.1	47.1	0.00960426	Hemiptera	Hormaphididae	Ceratovacuna nekoashi
Ceratovacuna nekoashi mitochondrial genes for smal	AB710226	79.1	47.1	0.00960426	Hemiptera	Hormaphididae	Ceratovacuna nekoashi
Ceratovacuna nekoashi mitochondrial genes for smal	AB710235	79.1	47.1	0.00960426	Hemiptera	Hormaphididae	Ceratovacuna nekoashi
Ceratovacuna nekoashi mitochondrial genes for smal	AB710234	79.1	47.1	0.00960426	Hemiptera	Hormaphididae	Ceratovacuna nekoashi
Ceratovacuna nekoashi mitochondrial genes for smal	AB710229	79.1	47.1	0.00960426	Hemiptera	Hormaphididae	Ceratovacuna nekoashi
Ceratovacuna nekoashi mitochondrial genes for smal	AB710232	79.1	47.1	0.00960426	Hemiptera	Hormaphididae	Ceratovacuna nekoashi
Ceratovacuna nekoashi mitochondrial genes for smal	AB710231	79.1	47.1	0.00960426	Hemiptera	Hormaphididae	Ceratovacuna nekoashi
Ceratovacuna nekoashi mitochondrial genes for smal	AB710230	79.1	47.1	0.00960426	Hemiptera	Hormaphididae	Ceratovacuna nekoashi
Ceratovacuna nekoashi mitochondrial genes for smal	AB710228	79.1	47.1	0.00960426	Hemiptera	Hormaphididae	Ceratovacuna nekoashi
Ceratovacuna nekoashi mitochondrial genes for smal	AB710225	79.1	47.1	0.00960426	Hemiptera	Hormaphididae	Ceratovacuna nekoashi
Ceratovacuna nekoashi mitochondrial genes for smal	AB710224	79.1	47.1	0.00960426	Hemiptera	Hormaphididae	Ceratovacuna nekoashi
Platypleura signifera voucher MHV0888 16S ribosoma	FJ168939	71.3	72.46	0.00960426	Hemiptera	Cicadidae	Platypleura signifera
Tamasia variegata mitochondrial 16S rRNA gene, spe	FN257669	73.79	73.19	0.00960426	Trichoptera	Calocidae	Tamasia variegata
Pliocaloca sp. CQ3 16S ribosomal RNA gene, partial	EF394999	73.39	73.91	0.00960426	Trichoptera	Calocidae	Pliocaloca sp. CQ3
Helicopha paniensis isolate K4 16S ribosomal RNA g	EF394946	73.96	68.12	0.00960426	Trichoptera	Helicophidae	Helicopha paniensis
Pamphilius cf. hortorum Sus39 voucher Sus39 16S ri	EF032172	76.81	49.28	0.00960426	Hymenoptera	Pamphiliidae	Pamphilius cf. hortorum Sus39
Procecidochares atra 16S ribosomal RNA gene, parti	DQ471376	75.28	61.59	0.00960426	Diptera	Tephritidae	Procecidochares atra
Ips emarginatus 16S ribosomal RNA gene, partial se	AF397480	77.27	46.38	0.00960426	Coleoptera	Curculionidae	Ips emarginatus
Coelopella popeae 16S ribosomal RNA gene, partial	AF403464	72	71.01	0.00960426	Diptera	Coelopidae	Coelopella popeae
Ceratovacuna nekoashi mitochondrial genes for smal	AB035879	79.1	47.1	0.00960426	Hemiptera	Hormaphididae	Ceratovacuna nekoashi
Melanostoma scalare 16S ribosomal RNA gene, partia	AF126336	71.29	71.01	0.00960426	Diptera	Syrphidae	Melanostoma scalare
Drosophila busckii chromosome X sequence	CP012528	80.95	43.48	0.0335222	Diptera	Drosophilidae	Drosophila busckii
Stichophthalma howqua 16S ribosomal RNA gene, part	KJ777745	74.7	58.7	0.0335222	Lepidoptera	Nymphalidae	Stichophthalma howqua
PREDICTED: Bombus terrestris uncharacterized LOC10	XM_012312432	91.18	24.64	0.0335222	Hymenoptera	Apidae	Bombus terrestris
PREDICTED: Bombus terrestris uncharacterized LOC10	XM_012312431	91.18	24.64	0.0335222	Hymenoptera	Apidae	Bombus terrestris
PREDICTED: Bombus terrestris uncharacterized LOC10	XM_003398136	91.18	24.64	0.0335222	Hymenoptera	Apidae	Bombus terrestris
Stichophthalma louisa voucher JH001 mitochondrion,	KP247523	74.7	58.7	0.0335222	Lepidoptera	Nymphalidae	Stichophthalma louisa
Muscina stabulans mitochondrion, complete genome	KM676394	72.28	71.01	0.0335222	Diptera	Muscidae	Muscina stabulans
Stichophthalma howqua mitochondrion, partial genom	KF990129	74.7	58.7	0.0335222	Lepidoptera	Nymphalidae	Stichophthalma howqua
PREDICTED: Meleagris gallopavo uncharacterized LOC	XR_794697	90	28.99	0.0335222	Galliformes	Phasianidae	Meleagris gallopavo
Huckettomyia watanabei voucher RMBR:116202 16S rib	KJ476311	76.81	48.55	0.0335222	Diptera	Muscidae	Huckettomyia watanabei
Atherigona varia voucher RMBR:116181 16S ribosomal	KJ476297	76.81	48.55	0.0335222	Diptera	Muscidae	Atherigona varia
Brugia pahangi genome assembly B_pahangi_Glasgow ,	LK970232	80	41.3	0.0335222	Spirurida	Onchocercidae	Brugia pahangi
Strongyloides ratti genome assembly S_ratti_ED321	LN609529	74.12	61.59	0.0335222	Rhabditida	Strongyloididae	Strongyloides ratti
Strongyloides ratti genome assembly S_ratti_ED321	LN609396	81.13	37.68	0.0335222	Rhabditida	Strongyloididae	Strongyloides ratti
Polyura arja isolate N1696 mitochondrion, complete	KF590540	75.32	53.62	0.0335222	Lepidoptera	Nymphalidae	Polyura arja
Graphium timur mitochondrion, complete genome	KJ472924	77.94	46.38	0.0335222	Lepidoptera	Papilionidae	Graphium timur
Ochthebius rugulosus mitochondrial 16S rRNA gene (	HF931447	75.31	55.07	0.0335222	Coleoptera	Hydraenidae	Ochthebius rugulosus
Procecidochares utilis mitochondrion, complete gen	KC355248	72.12	68.84	0.0335222	Diptera	Tephritidae	Procecidochares utilis
Bradysia longimentula voucher 232 16S ribosomal RN	JQ613903	73.86	63.04	0.0335222	Diptera	Sciaridae	Bradysia longimentula
Drosophila simulans strain simw501 mitochondrion,	KC244284	73.4	59.42	0.0335222	Diptera	Drosophilidae	Drosophila simulans
Drosophila simulans strain sm21 mitochondrion, com	KC244283	73.4	59.42	0.0335222	Diptera	Drosophilidae	Drosophila simulans
Papilio krishna thawgawa voucher CBGPFLC_00376 16S	JQ982213	82	34.78	0.0335222	Lepidoptera	Papilionidae	Papilio krishna thawgawa
Papilio krishna thawgawa voucher CBGPFLC_00058 16S	JQ982212	82	34.78	0.0335222	Lepidoptera	Papilionidae	Papilio krishna thawgawa
Papilio krishna mayumiae voucher CBGPFLC_00057 16S	JQ982211	82	34.78	0.0335222	Lepidoptera	Papilionidae	Papilio krishna mayumiae
Papilio krishna ssp. CBGPFLC_00055 16S ribosomal R	JQ982210	82	34.78	0.0335222	Lepidoptera	Papilionidae	Papilio krishna ssp. CBGPFLC_00055
Canualna liberiana voucher MHV1553 16S large subun	GU344094	73.33	59.42	0.0335222	Hemiptera	Cicadidae	Canualna liberiana
Sciapus wiedemanni voucher Pollet 0192 (P) 16S rib	HQ449008	75	55.07	0.0335222	Diptera	Dolichopodidae	Sciapus wiedemanni
Muscina stabulans 16S ribosomal RNA gene, partial	JN226646	72.28	71.01	0.0335222	Diptera	Muscidae	Muscina stabulans
Cupido argiades hellotia 16S ribosomal RNA gene, p	GU372447	76.06	48.55	0.0335222	Lepidoptera	Lycaenidae	Cupido argiades hellotia
