# Supplementary material for: Testing the potential of a ribosomal 16S marker for DNA metabarcoding of insects
Source: PeerJ. 2016 Apr 19;4:e1966. doi: 10.7717/peerj.1966 (PMC4841222; doi:10.7717/peerj.1966)
Supplement: Supplemental Information 1 [file peerj-04-1966-s006.zip › scripts/5 match OTUs/blast_hits/OTU_19.csv_plot.pdf]

OTU_19.csv

Cordulegaster boltonii

Cordulegastridae

Odonata

99.37

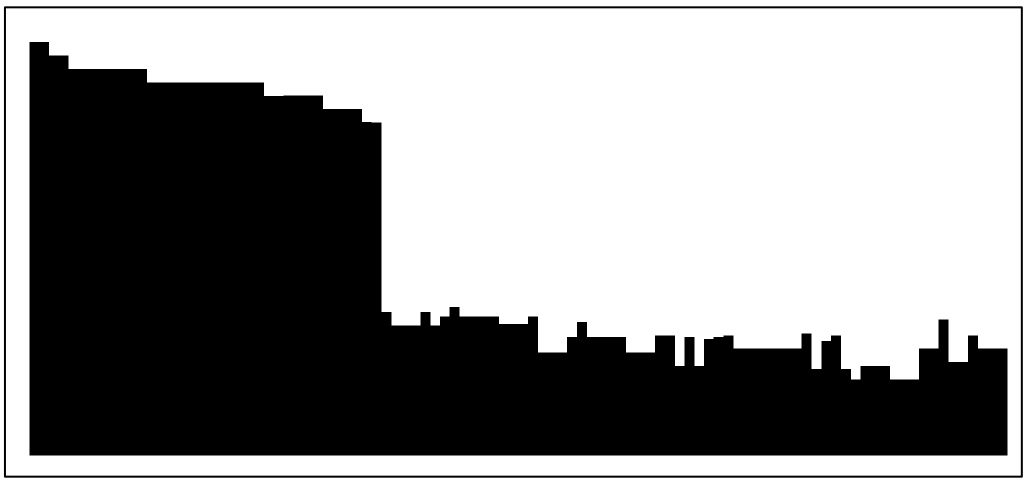

Cordulegaster boltonii 12S ribosomal RNA gene, par	EU477688	99.37	100	3.73607e−72	Odonata	Cordulegastridae	Cordulegaster boltonii
Cordulegaster boltoni large subunit ribosomal RNA	AF266056	99.37	100	3.73607e−72	Odonata	Cordulegastridae	Cordulegaster boltonii
Neallogaster pekinensis mitochondrial gene for 16S	AB707904	98.74	100	1.58862e−70	Odonata	Cordulegastridae	Neallogaster pekinensis
Anotogaster kuchenbeiseri mitochondrial gene for 1	AB707878	98.74	100	1.58862e−70	Odonata	Cordulegastridae	Anotogaster kuchenbeiseri
Neallogaster pekinensis mitochondrial gene for 16S	AB707903	98.11	100	6.75497e−69	Odonata	Cordulegastridae	Neallogaster pekinensis
Anotogaster sp. RF1898 mitochondrial gene for 16S	AB707899	98.11	100	6.75497e−69	Odonata	Cordulegastridae	Anotogaster sp. RF1898
Anotogaster sieboldii mitochondrial gene for 16S r	AB707886	98.11	100	6.75497e−69	Odonata	Cordulegastridae	Anotogaster sieboldii
Anotogaster klossi mitochondrial gene for 16S rRNA	AB707872	98.11	100	6.75497e−69	Odonata	Cordulegastridae	Anotogaster klossi
Kalyptogaster erronea 12S ribosomal RNA and 16S ri	EU477690	98.11	100	6.75497e−69	Odonata	Cordulegastridae	Kalyptogaster erronea
Cordulegaster maculata 12S ribosomal RNA and 16S r	EU477689	98.11	100	6.75497e−69	Odonata	Cordulegastridae	Cordulegaster maculata
Cordulegaster dorsalis 16S ribosomal RNA gene, par	EU055084	98.11	100	6.75497e−69	Odonata	Cordulegastridae	Cordulegaster dorsalis
Kalyptogaster erronea voucher JW_DNA_40 16S riboso	EF631561	98.11	100	6.75497e−69	Odonata	Cordulegastridae	Kalyptogaster erronea
Sonjagaster insignis mitochondrial gene for 16S rR	AB707905	97.47	100	2.35772e−68	Odonata	Cordulegastridae	Sonjagaster insignis
Anotogaster sp. RF1284 mitochondrial gene for 16S	AB707896	97.48	100	8.22924e−68	Odonata	Cordulegastridae	Anotogaster sp. RF1284
Anotogaster sieboldii mitochondrial gene for 16S r	AB707892	97.48	100	8.22924e−68	Odonata	Cordulegastridae	Anotogaster sieboldii
Anotogaster sieboldii mitochondrial gene for 16S r	AB707881	97.48	100	8.22924e−68	Odonata	Cordulegastridae	Anotogaster sieboldii
Anotogaster sieboldii mitochondrial gene for 16S r	AB707882	97.48	100	8.22924e−68	Odonata	Cordulegastridae	Anotogaster sieboldii
Anotogaster klossi mitochondrial gene for 16S rRNA	AB707866	97.48	100	8.22924e−68	Odonata	Cordulegastridae	Anotogaster klossi
Anotogaster klossi mitochondrial gene for 16S rRNA	AB707864	97.48	100	8.22924e−68	Odonata	Cordulegastridae	Anotogaster klossi
Anotogaster klossi mitochondrial gene for 16S rRNA	AB707862	97.48	100	8.22924e−68	Odonata	Cordulegastridae	Anotogaster klossi
Anotogaster klossi mitochondrial gene for 16S rRNA	AB707860	97.48	100	8.22924e−68	Odonata	Cordulegastridae	Anotogaster klossi
Anotogaster sieboldii 12S ribosomal RNA gene, part	EU477686	97.48	100	8.22924e−68	Odonata	Cordulegastridae	Anotogaster sieboldii
Anotogaster sieboldii mitochondrial gene for 16S r	AB127061	97.48	100	8.22924e−68	Odonata	Cordulegastridae	Anotogaster sieboldii
Anotogaster sieboldi large subunit ribosomal RNA g	AF266087	97.48	100	8.22924e−68	Odonata	Cordulegastridae	Anotogaster sieboldii
Cordulegaster picta 12S ribosomal RNA gene, partia	EU477685	96.84	100	1.00253e−66	Odonata	Cordulegastridae	Cordulegaster picta
Cordulegaster picta large subunit ribosomal RNA ge	AF266086	96.84	100	1.00253e−66	Odonata	Cordulegastridae	Cordulegaster picta
Anotogaster sieboldii mitochondrial gene for 16S r	AB707885	96.86	100	3.49916e−66	Odonata	Cordulegastridae	Anotogaster sieboldii
Anotogaster sieboldii mitochondrial gene for 16S r	AB707880	96.86	100	3.49916e−66	Odonata	Cordulegastridae	Anotogaster sieboldii
Anotogaster chaoi mitochondrial gene for 16S rRNA,	AB707858	96.86	100	3.49916e−66	Odonata	Cordulegastridae	Anotogaster chaoi
Anotogaster sp. RF1894 mitochondrial gene for 16S	AB707898	96.88	100	1.22133e−65	Odonata	Cordulegastridae	Anotogaster sp. RF1894
Anotogaster klossi mitochondrial gene for 16S rRNA	AB711440	96.23	100	4.26285e−65	Odonata	Cordulegastridae	Anotogaster klossi
Cordulegaster picta mitochondrial gene for 16S rRN	AB707902	96.23	100	4.26285e−65	Odonata	Cordulegastridae	Cordulegaster picta
Anotogaster klossi mitochondrial gene for 16S rRNA	AB707876	96.23	100	4.26285e−65	Odonata	Cordulegastridae	Anotogaster klossi
Cordulegaster sp. 1 BM−2008 12S ribosomal RNA gene	EU477687	96.23	100	4.26285e−65	Odonata	Cordulegastridae	Cordulegaster sp. 1 BM−2008
Anotogaster sp. RF1283 mitochondrial gene for 16S	AB707895	95.62	100	6.32663e−63	Odonata	Cordulegastridae	Anotogaster sp. RF1283
Taeniogaster obliqua voucher JW_DNA_OTO 16S riboso	EF631533	95.6	100	2.20821e−62	Odonata	Cordulegastridae	Taeniogaster obliqua
Uropetala carovei 12S ribosomal RNA gene, partial	EU477633	86.71	100	6.33222e−44	Odonata	Petaluridae	Uropetala carovei
Phenes raptor isolate 2 12S ribosomal RNA gene, pa	EU477632	86.08	100	6.33222e−44	Odonata	Petaluridae	Phenes raptor
Phenes raptor 12S ribosomal RNA gene, partial sequ	EU477622	86.08	100	6.33222e−44	Odonata	Petaluridae	Phenes raptor
Phenes raptor 16S ribosomal RNA gene, partial sequ	EU055038	86.08	100	6.33222e−44	Odonata	Petaluridae	Phenes raptor
Uropetala carovei large subunit ribosomal RNA gene	AY274506	86.71	100	6.33222e−44	Odonata	Petaluridae	Uropetala carovei
Phenes raptor large subunit ribosomal RNA gene, pa	AF266083	86.08	100	6.33222e−44	Odonata	Petaluridae	Phenes raptor
Gynacantha japonica tRNA−Leu, 16S ribosomal RNA, a	KF256872	86.5	100	7.71423e−43	Odonata	Aeshnidae	Gynacantha japonica
Gynacantha japonica mitochondrial gene for 16S rRN	AB707679	86.96	100	7.71423e−43	Odonata	Aeshnidae	Gynacantha japonica
Gynacantha japonica mitochondrial gene for 16S rRN	AB707677	86.5	100	7.71423e−43	Odonata	Aeshnidae	Gynacantha japonica
Gynacantha japonica mitochondrial gene for 16S rRN	AB707675	86.5	100	7.71423e−43	Odonata	Aeshnidae	Gynacantha japonica
Gynacantha japonica isolate 2 12S ribosomal RNA ge	EU477644	86.5	100	7.71423e−43	Odonata	Aeshnidae	Gynacantha japonica
Gynacantha japonica 16S ribosomal RNA gene, partia	EF032710	86.5	100	7.71423e−43	Odonata	Aeshnidae	Gynacantha japonica
Epiophlebia superstes mitochondrial gene for 16S r	AB707607	86.16	100	2.69253e−42	Odonata	Epiophlebiidae	Epiophlebia superstes
Epiophlebia superstes mitochondrial gene for 16S r	AB707604	86.16	100	2.69253e−42	Odonata	Epiophlebiidae	Epiophlebia superstes
Epiophlebia superstes mitochondrial gene for 16S r	AB127062	86.16	100	2.69253e−42	Odonata	Epiophlebiidae	Epiophlebia superstes
Gynacantha japonica mitochondrial gene for 16S rRN	AB707678	86.5	100	9.39785e−42	Odonata	Aeshnidae	Gynacantha japonica
Chlorogomphus brunneus mitochondrial gene for 16S	AB707908	84.81	100	3.28017e−41	Odonata	Chlorogomphidae	Chlorogomphus brunneus
Chlorogomphus brunneus 12S ribosomal RNA gene, par	EU477691	84.81	100	3.28017e−41	Odonata	Chlorogomphidae	Chlorogomphus brunneus
Chlorogomphus brunneus large subunit ribosomal RNA	AF266088	84.81	100	3.28017e−41	Odonata	Chlorogomphidae	Chlorogomphus brunneus
Epiophlebia superstes voucher ANISO01 tRNA−Leu, 16	KF256906	85.53	100	1.14489e−40	Odonata	Epiophlebiidae	Epiophlebia superstes
Anax nigrofasciatus mitochondrial gene for 16S rRN	AB707659	86.25	100	1.14489e−40	Odonata	Aeshnidae	Anax nigrofasciatus
Epiophlebia superstes mitochondrial gene for 16S r	AB707615	85.53	100	1.14489e−40	Odonata	Epiophlebiidae	Epiophlebia superstes
Epiophlebia superstes mitochondrial gene for 16S r	AB707613	85.53	100	1.14489e−40	Odonata	Epiophlebiidae	Epiophlebia superstes
Epiophlebia superstes mitochondrial gene for 16S r	AB707602	85.53	100	1.14489e−40	Odonata	Epiophlebiidae	Epiophlebia superstes
Epiophlebia superstes 16S ribosomal RNA gene, part	EU055132	85.53	100	1.14489e−40	Odonata	Epiophlebiidae	Epiophlebia superstes
Shaogomphus postocularis voucher ANI03 tRNA−Leu, 1	KF256838	84.81	100	3.99607e−40	Odonata	Gomphidae	Shaogomphus postocularis
Stylurus annulatus mitochondrial gene for 16S rRNA	AB707821	84.81	100	3.99607e−40	Odonata	Gomphidae	Stylurus annulatus
Stylurus annulatus mitochondrial gene for 16S rRNA	AB707820	84.81	100	3.99607e−40	Odonata	Gomphidae	Stylurus annulatus
Epiophlebia superstes 12S ribosomal RNA gene, part	EU477631	85.62	100	3.99607e−40	Odonata	Epiophlebiidae	Epiophlebia superstes
Epiophlebia superstes large subunit ribosomal RNA	AF266094	85.62	100	3.99607e−40	Odonata	Epiophlebiidae	Epiophlebia superstes
Chlorogomphus iriomotensis mitochondrial gene for	AB707919	84.18	100	1.39476e−39	Odonata	Chlorogomphidae	Chlorogomphus iriomotensis
Caliaeschna microstigma 12S ribosomal RNA gene, pa	EU477639	85.53	100	1.39476e−39	Odonata	Aeshnidae	Caliaeschna microstigma
Tachopteryx thoreyi isolate 2 12S ribosomal RNA ge	EU477636	84.18	100	1.39476e−39	Odonata	Petaluridae	Tachopteryx thoreyi
Eusynthemis nigra 16S ribosomal RNA gene, partial	EU055069	85.44	100	1.39476e−39	Odonata	Synthemistidae	Eusynthemis nigra
Caliaeschna microstigma large subunit ribosomal RN	AF266092	85.53	100	1.39476e−39	Odonata	Aeshnidae	Caliaeschna microstigma
Anax nigrofasciatus voucher ANI02 tRNA−Leu, 16S ri	KF256837	85.62	100	4.86821e−39	Odonata	Aeshnidae	Anax nigrofasciatus
Trigomphus ogumai mitochondrial gene for 16S rRNA,	AB707850	85	100	4.86821e−39	Odonata	Gomphidae	Trigomphus ogumai
Trigomphus ogumai mitochondrial gene for 16S rRNA,	AB707849	85	100	4.86821e−39	Odonata	Gomphidae	Trigomphus ogumai
Trigomphus ogumai mitochondrial gene for 16S rRNA,	AB707848	85	100	4.86821e−39	Odonata	Gomphidae	Trigomphus ogumai
Trigomphus melampus mitochondrial gene for 16S rRN	AB707836	85	100	4.86821e−39	Odonata	Gomphidae	Trigomphus melampus
Trigomphus melampus mitochondrial gene for 16S rRN	AB707840	85	100	4.86821e−39	Odonata	Gomphidae	Trigomphus melampus
Trigomphus melampus mitochondrial gene for 16S rRN	AB707837	85	100	4.86821e−39	Odonata	Gomphidae	Trigomphus melampus
Trigomphus interruptus mitochondrial gene for 16S	AB707833	85	100	4.86821e−39	Odonata	Gomphidae	Trigomphus interruptus
Anax junius mitochondrial gene for 16S rRNA, parti	AB707656	85.71	100	4.86821e−39	Odonata	Aeshnidae	Anax junius
Onychogomphus uncatus 12S ribosomal RNA gene, part	EU477671	84.05	100	4.86821e−39	Odonata	Gomphidae	Onychogomphus uncatus
Uracis fastigiata 12S ribosomal RNA gene, partial	EF640390	85.35	99.37	4.86821e−39	Odonata	Libellulidae	Uracis fastigiata
Anax nigrofasciatus 16S ribosomal RNA gene, partia	EF032708	85.62	100	4.86821e−39	Odonata	Aeshnidae	Anax nigrofasciatus
Onychogomphus uncatus large subunit ribosomal RNA	AF266054	84.05	100	4.86821e−39	Odonata	Gomphidae	Onychogomphus uncatus
Chlorogomphus brunneus mitochondrial gene for 16S	AB707906	83.54	100	1.69917e−38	Odonata	Chlorogomphidae	Chlorogomphus brunneus
Stylurus nagoyanus mitochondrial gene for 16S rRNA	AB707824	84.18	100	1.69917e−38	Odonata	Gomphidae	Stylurus nagoyanus
Stylurus nagoyanus mitochondrial gene for 16S rRNA	AB707822	84.18	100	1.69917e−38	Odonata	Gomphidae	Stylurus nagoyanus
Gomphus pulchellus 12S ribosomal RNA gene, partial	EU477663	84.18	100	1.69917e−38	Odonata	Gomphidae	Gomphus pulchellus
Chlorogomphus brunneus 16S ribosomal RNA gene, par	EU055091	83.54	100	1.69917e−38	Odonata	Chlorogomphidae	Chlorogomphus brunneus
Antipodogomphus acolythus 16S ribosomal RNA gene,	EU055068	83.54	100	1.69917e−38	Odonata	Gomphidae	Antipodogomphus acolythus
Arigomphus cornutus large subunit ribosomal RNA ge	AF266072	83.54	100	1.69917e−38	Odonata	Gomphidae	Arigomphus cornutus
Anax parthenope voucher ANI19 tRNA−Leu, 16S riboso	KF256853	85	100	5.93069e−38	Odonata	Aeshnidae	Anax parthenope
Aeschnophlebia longistigma voucher ANI01 tRNA−Leu,	KF256836	85	100	5.93069e−38	Odonata	Aeshnidae	Aeschnophlebia longistigma
Epiophlebia superstes mitochondrion, complete geno	JX050223	86.36	96.84	5.93069e−38	Odonata	Epiophlebiidae	Epiophlebia superstes
Trigomphus melampus mitochondrial gene for 16S rRN	AB707843	84.38	100	5.93069e−38	Odonata	Gomphidae	Trigomphus melampus
Trigomphus interruptus mitochondrial gene for 16S	AB707834	84.38	100	5.93069e−38	Odonata	Gomphidae	Trigomphus interruptus
Tetracanthagyna plagiata mitochondrial gene for 16	AB707709	85.62	100	5.93069e−38	Odonata	Aeshnidae	Tetracanthagyna plagiata
Anax parthenope mitochondrial gene for 16S rRNA, p	AB707664	85	100	5.93069e−38	Odonata	Aeshnidae	Anax parthenope
Anax parthenope mitochondrial gene for 16S rRNA, p	AB707666	85	100	5.93069e−38	Odonata	Aeshnidae	Anax parthenope
Anax panybeus mitochondrial gene for 16S rRNA, par	AB707660	85	100	5.93069e−38	Odonata	Aeshnidae	Anax panybeus
