# Supplementary material for: Testing the potential of a ribosomal 16S marker for DNA metabarcoding of insects
Source: PeerJ. 2016 Apr 19;4:e1966. doi: 10.7717/peerj.1966 (PMC4841222; doi:10.7717/peerj.1966)
Supplement: Supplemental Information 1 [file peerj-04-1966-s006.zip › scripts/5 match OTUs/blast_hits/OTU_190.csv_plot.pdf]

# OTU_190.csv

## Oreodytes sanmarkii

## Dytiscidae

## Coleoptera

# 98.09

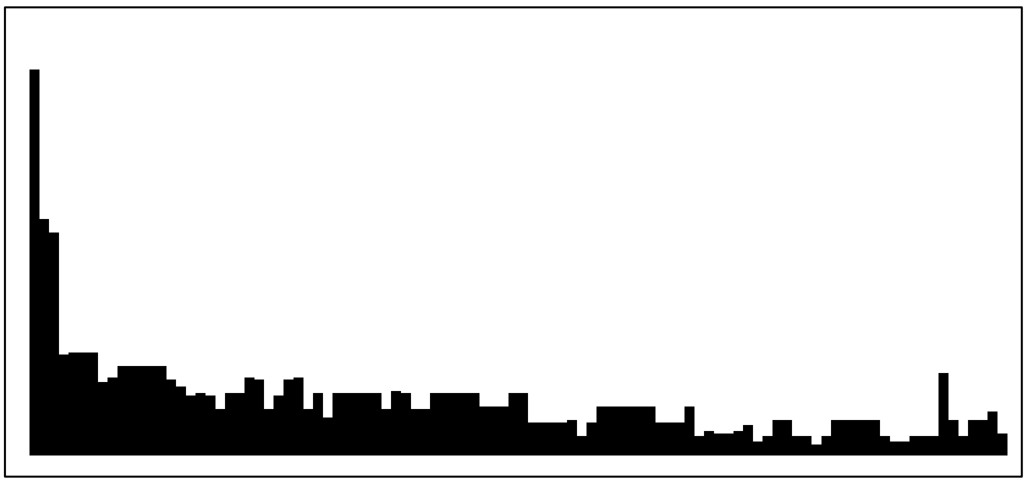

| | | | | | | | |
|---|---|---|---|---|---|---|---|
| Oreodytes sanmarkii mitochondrial 16S rRNA gene (p | HF931524 | 98.09 | 100 | 6.70005e−69 | Coleoptera | Dytiscidae | Oreodytes sanmarkii |
| Oreodytes congruus partial 16S rRNA gene | AJ850347 | 91.08 | 100 | 3.04451e−54 | Coleoptera | Dytiscidae | Oreodytes congruus |
| Oreodytes congruus 16S ribosomal RNA gene, partial | KF575550 | 90.45 | 100 | 1.29456e−52 | Coleoptera | Dytiscidae | Oreodytes congruus |
| Laccornis difformis 16S ribosomal RNA gene, partia | KF575544 | 84.71 | 100 | 1.13558e−40 | Coleoptera | Dytiscidae | Laccornis difformis |
| Nebrioporus formaster mitochondrial partial 16S rR | HF931377 | 84.81 | 100 | 3.96358e−40 | Coleoptera | Dytiscidae | Nebrioporus formaster |
| Stictotarsus maghrebinus 16S ribosomal RNA gene, p | AY250947 | 84.81 | 100 | 4.82863e−39 | Coleoptera | Dytiscidae | Stictotarsus maghrebinus |
| Hydroporus foveolatus mitochondrial 16S rRNA gene | HE613724 | 84.81 | 100 | 5.88247e−38 | Coleoptera | Dytiscidae | Hydroporus foveolatus |
| Copelatus sp. BI−2011 voucher BMNH671215 16S ribos | HQ381377 | 83.44 | 100 | 5.88247e−38 | Coleoptera | Dytiscidae | Copelatus sp. BI−2011 |
| Scarodytes sp. IR−2003 16S ribosomal RNA gene, par | AY250939 | 83.65 | 100 | 5.88247e−38 | Coleoptera | Dytiscidae | Scarodytes sp. IR−2003 |
| Stictotarsus procerus mitochondrial 16S rRNA gene | HF931379 | 84.18 | 100 | 2.05319e−37 | Coleoptera | Dytiscidae | Stictotarsus procerus |
| Hydroporus tessellatus 16S ribosomal RNA gene, par | JX434770 | 84.18 | 100 | 7.16632e−37 | Coleoptera | Dytiscidae | Hydroporus tessellatus |
| Hydroporus zimmermanni mitochondrial 16S rRNA gene | HE613679 | 84.18 | 100 | 7.16632e−37 | Coleoptera | Dytiscidae | Hydroporus zimmermanni |
| Hydroporus basinotatus mitochondrial 16S rRNA gene | HE613678 | 84.18 | 100 | 7.16632e−37 | Coleoptera | Dytiscidae | Hydroporus basinotatus |
| Hydroporus basinotatus 16S ribosomal RNA gene, par | AF518258 | 84.18 | 100 | 7.16632e−37 | Coleoptera | Dytiscidae | Hydroporus basinotatus |
| Nirripirti megamacrocephalus isolate ABTC78781 16S | EU616935 | 83.54 | 100 | 2.50129e−36 | Coleoptera | Dytiscidae | Paroster megamacrocephalus |
| Hydroporus marginatus mitochondrial 16S rRNA gene | HE613699 | 83.23 | 100 | 8.73037e−36 | Coleoptera | Dytiscidae | Hydroporus marginatus |
| Hydroporus sp. IR33.4 16S ribosomal RNA gene, part | AY365261 | 82.8 | 100 | 8.73037e−36 | Coleoptera | Dytiscidae | Hydroporus sp. IR33.4 |
| Scarodytes halensis haplotype 3 16S ribosomal RNA | AY250937 | 82.91 | 100 | 8.73037e−36 | Coleoptera | Dytiscidae | Scarodytes halensis |
| Hydroporus marginatus 16S ribosomal RNA gene, part | AF518267 | 82.8 | 100 | 8.73037e−36 | Coleoptera | Dytiscidae | Hydroporus marginatus |
| Hydaticus cinctipennis voucher UNM KBMHyci20 16S r | KJ548338 | 82.17 | 100 | 3.0472e−35 | Coleoptera | Dytiscidae | Hydaticus cinctipennis |
| Suphrodytes dorsalis 16S ribosomal RNA gene, parti | KF575581 | 82.91 | 100 | 3.0472e−35 | Coleoptera | Dytiscidae | Suphrodytes dorsalis |
| Suphrodytes dorsalis voucher BMNH:725966 16S ribos | JX221574 | 82.91 | 100 | 3.0472e−35 | Coleoptera | Dytiscidae | Suphrodytes dorsalis |
| Hydroporus fuscipennis mitochondrial 16S rRNA gene | HE613730 | 83.65 | 100 | 3.0472e−35 | Coleoptera | Dytiscidae | Hydroporus fuscipennis |
| Hydroporus compunctus mitochondrial 16S rRNA gene | HE599693 | 83.54 | 100 | 3.0472e−35 | Coleoptera | Dytiscidae | Hydroporus compunctus |
| Nirripirti byroensis isolate ABTC78677−8 16S ribos | EU616910 | 82.17 | 100 | 3.0472e−35 | Coleoptera | Dytiscidae | Paroster byroensis |
| Nirripirti newhavenensis 16S ribosomal RNA gene, p | AY353844 | 82.8 | 100 | 3.0472e−35 | Coleoptera | Dytiscidae | Paroster newhavenensis |
| Hydroporus compunctus 16S ribosomal RNA gene, part | AF518259 | 83.54 | 100 | 3.0472e−35 | Coleoptera | Dytiscidae | Hydroporus compunctus |
| Hydroporus fuscipennis 16S ribosomal RNA gene, par | AF518262 | 83.65 | 100 | 3.0472e−35 | Coleoptera | Dytiscidae | Hydroporus fuscipennis |
| Hydroporus appalachius 16S ribosomal RNA gene, par | JX434765 | 82.17 | 100 | 3.71225e−34 | Coleoptera | Dytiscidae | Hydroporus appalachius |
| Hydroporus foveolatus mitochondrial 16S rRNA gene | HF931502 | 82.91 | 100 | 3.71225e−34 | Coleoptera | Dytiscidae | Hydroporus foveolatus |
| Scarodytes halensis mitochondrial 16S rRNA gene (p | HF931324 | 81.76 | 100 | 3.71225e−34 | Coleoptera | Dytiscidae | Scarodytes halensis |
| Hydroporus discretus mitochondrial 16S rRNA gene ( | HE613723 | 82.91 | 100 | 3.71225e−34 | Coleoptera | Dytiscidae | Hydroporus discretus |
| Hydroporus decipiens mitochondrial 16S rRNA gene ( | HE613722 | 82.91 | 100 | 3.71225e−34 | Coleoptera | Dytiscidae | Hydroporus decipiens |
| Hydroporus pubescens mitochondrial 16S rRNA gene ( | HE613707 | 82.91 | 100 | 3.71225e−34 | Coleoptera | Dytiscidae | Hydroporus pubescens |
| Hydroporus limbatus mitochondrial 16S rRNA gene (p | HE613706 | 82.91 | 100 | 3.71225e−34 | Coleoptera | Dytiscidae | Hydroporus limbatus |
| Hydroporus errans mitochondrial 16S rRNA gene (par | HE613704 | 82.91 | 100 | 3.71225e−34 | Coleoptera | Dytiscidae | Hydroporus errans |
| Hydroporus pervicinus mitochondrial 16S rRNA gene | HE599695 | 82.17 | 100 | 3.71225e−34 | Coleoptera | Dytiscidae | Hydroporus pervicinus |
| Nirripirti napperbyensis 16S ribosomal RNA gene, p | AY353843 | 83.02 | 100 | 3.71225e−34 | Coleoptera | Dytiscidae | Paroster napperbyensis |
| Hydroporus pubescens voucher NHM−IR39 16S ribosoma | EF419327 | 82.91 | 100 | 3.71225e−34 | Coleoptera | Dytiscidae | Hydroporus pubescens |
| Hydroporus occidentalis 16S ribosomal RNA gene, pa | AY365280 | 82.17 | 100 | 3.71225e−34 | Coleoptera | Dytiscidae | Hydroporus occidentalis |
| Hydroporus hirtellus 16S ribosomal RNA gene, parti | AY365271 | 82.17 | 100 | 3.71225e−34 | Coleoptera | Dytiscidae | Hydroporus hirtellus |
| Hydroporus rufifrons 16S ribosomal RNA gene, parti | AF518277 | 82.91 | 100 | 3.71225e−34 | Coleoptera | Dytiscidae | Hydroporus rufifrons |
| Hydroporus pubescens 16S ribosomal RNA gene, parti | AF518276 | 82.91 | 100 | 3.71225e−34 | Coleoptera | Dytiscidae | Hydroporus pubescens |
| Hydroporus limbatus 16S ribosomal RNA gene, partia | AF518264 | 82.91 | 100 | 3.71225e−34 | Coleoptera | Dytiscidae | Hydroporus limbatus |
| Hydroporus discretus 16S ribosomal RNA gene, parti | AF518260 | 82.91 | 100 | 3.71225e−34 | Coleoptera | Dytiscidae | Hydroporus discretus |
| Hydroporus analis 16S ribosomal RNA gene, partial | AF518257 | 82.91 | 100 | 3.71225e−34 | Coleoptera | Dytiscidae | Hydroporus analis |
| Paroster sp. R401 Newhaven voucher ABTC:78974 16S | JQ745754 | 82.28 | 100 | 1.2957e−33 | Coleoptera | Dytiscidae | Paroster sp. R401 Newhaven |
| Paroster sp. 2 RL−2008 16S ribosomal RNA gene, par | EU616958 | 82.28 | 100 | 1.2957e−33 | Coleoptera | Dytiscidae | Paroster sp. 2 RL−2008 |
| Paroster sp. spnMtWedge 16S ribosomal RNA gene, pa | EU616954 | 82.28 | 100 | 1.2957e−33 | Coleoptera | Dytiscidae | Paroster sp. spnMtWedge |
| Nirripirti bulbus isolate ABTC78658−9 16S ribosoma | EU616918 | 82.91 | 100 | 1.2957e−33 | Coleoptera | Dytiscidae | Paroster bulbus |
| Nirripirti skaphites isolate 2 ABTC78694−6 16S rib | EU616916 | 82.91 | 100 | 1.2957e−33 | Coleoptera | Dytiscidae | Paroster skaphites |
| Hygrotus novemlineatus mitochondrial partial 16S r | HF931523 | 81.53 | 100 | 4.52244e−33 | Coleoptera | Dytiscidae | Hygrotus novemlineatus |
| Suphrodytes figuratus voucher BMNH:722148 16S ribo | JX221571 | 81.53 | 100 | 4.52244e−33 | Coleoptera | Dytiscidae | Suphrodytes figuratus |
| Hygrotus turbidus partial 16S rRNA gene | AJ850390 | 81.53 | 100 | 4.52244e−33 | Coleoptera | Dytiscidae | Hygrotus turbidus |
| Nirripirti wedgeensis isolate ABTC78773−4 16S ribo | EU616938 | 81.53 | 100 | 4.52244e−33 | Coleoptera | Dytiscidae | Paroster wedgeensis |
| Scarodytes antoni voucher MNCN−AC6 16S ribosomal R | EU156492 | 81.65 | 100 | 4.52244e−33 | Coleoptera | Dytiscidae | Scarodytes antoni |
| Hydaticus aruspex voucher UNM KBMHyar68 16S riboso | KJ548337 | 80.89 | 100 | 1.57849e−32 | Coleoptera | Dytiscidae | Hydaticus aruspex |
| Hydaticus aruspex voucher NHRS/JLKB000000298 16S r | KF979119 | 81.53 | 100 | 1.57849e−32 | Coleoptera | Dytiscidae | Hydaticus aruspex |
| Hydroporus rufifrons 16S ribosomal RNA gene, parti | JX434758 | 82.28 | 100 | 1.57849e−32 | Coleoptera | Dytiscidae | Hydroporus rufifrons |
| Hydroporus discretus mitochondrial 16S rRNA gene ( | HF931449 | 82.28 | 100 | 1.57849e−32 | Coleoptera | Dytiscidae | Hydroporus discretus |
| Hydroporus thracicus mitochondrial 16S rRNA gene ( | HF931413 | 82.28 | 100 | 1.57849e−32 | Coleoptera | Dytiscidae | Hydroporus thracicus |
| Hygrotus patruelis mitochondrial 16S rRNA gene (pa | HF931388 | 82.28 | 100 | 1.57849e−32 | Coleoptera | Dytiscidae | Hygrotus patruelis |
| Hydroporus analis mitochondrial partial 16S rRNA g | HE613731 | 82.28 | 100 | 1.57849e−32 | Coleoptera | Dytiscidae | Hydroporus analis |
| Hydroporus macedonicus mitochondrial 16S rRNA gene | HE613690 | 82.28 | 100 | 1.57849e−32 | Coleoptera | Dytiscidae | Hydroporus macedonicus |
| Nirripirti arachnoides isolate ABTC78660−1 16S rib | EU616917 | 81.53 | 100 | 1.57849e−32 | Coleoptera | Dytiscidae | Paroster arachnoides |
| Nirripirti innouendyensis isolate ABTC78672−3 16S | EU616913 | 81.53 | 100 | 1.57849e−32 | Coleoptera | Dytiscidae | Paroster innouendyensis |
| Nirripirti eurypleuron isolate ABTC78681 16S ribos | EU616909 | 81.53 | 100 | 1.57849e−32 | Coleoptera | Dytiscidae | Paroster eurypleuron |
| Hydroporus macedonicus 16S ribosomal RNA gene, par | DQ195529 | 82.28 | 100 | 1.57849e−32 | Coleoptera | Dytiscidae | Hydroporus macedonicus |
| Aglymbus gestroi 16S ribosomal RNA gene, partial s | AY138639 | 80.89 | 100 | 1.57849e−32 | Coleoptera | Dytiscidae | Aglymbus gestroi |
| Paroster nigroadumbratus mitochondrial partial 16S | HG940414 | 81.13 | 100 | 5.50946e−32 | Coleoptera | Dytiscidae | Paroster nigroadumbratus |
| Scarodytes malickyi mitochondrial 16S rRNA gene (p | HF931334 | 81.01 | 100 | 5.50946e−32 | Coleoptera | Dytiscidae | Scarodytes malickyi |
| Scarodytes savinensis voucher MNCN−AI135 16S ribos | EU156491 | 81.01 | 100 | 5.50946e−32 | Coleoptera | Dytiscidae | Scarodytes savinensis |
| Paroster nigroadumbratus 16S ribosomal RNA gene, p | AY353824 | 81.13 | 100 | 5.50946e−32 | Coleoptera | Dytiscidae | Paroster nigroadumbratus |
| Scarodytes nigriventris 16S ribosomal RNA gene, pa | AY250938 | 81.41 | 98.73 | 5.50946e−32 | Coleoptera | Dytiscidae | Scarodytes nigriventris |
| Chostonectes sharpi mitochondrial partial 16S rRNA | HG940420 | 80.65 | 98.73 | 1.92299e−31 | Coleoptera | Dytiscidae | Chostonectes sharpi |
| Hydroporus submuticus 16S ribosomal RNA gene, part | JX434777 | 80.89 | 100 | 1.92299e−31 | Coleoptera | Dytiscidae | Hydroporus submuticus |
| Hydroporus erythrocephalus 16S ribosomal RNA gene, | JX434775 | 81.65 | 100 | 1.92299e−31 | Coleoptera | Dytiscidae | Hydroporus erythrocephalus |
| Hydroporus nigrita 16S ribosomal RNA gene, partial | JX434769 | 81.65 | 100 | 1.92299e−31 | Coleoptera | Dytiscidae | Hydroporus nigrita |
| Hygrotus lutescens mitochondrial partial 16S rRNA | HF931518 | 80.89 | 100 | 1.92299e−31 | Coleoptera | Dytiscidae | Hygrotus lutescens |
| Hygrotus fontinalis mitochondrial 16S rRNA gene (p | HF931494 | 80.89 | 100 | 1.92299e−31 | Coleoptera | Dytiscidae | Hygrotus fontinalis |
| Scarodytes halensis mitochondrial 16S rRNA gene (p | HF931408 | 80.5 | 100 | 1.92299e−31 | Coleoptera | Dytiscidae | Scarodytes halensis |
| Suphrodytes figuratus voucher BMNH:722150 16S ribo | JX221572 | 80.89 | 100 | 1.92299e−31 | Coleoptera | Dytiscidae | Suphrodytes figuratus |
| Hydroporus nigrita mitochondrial 16S rRNA gene (pa | HE613725 | 81.65 | 100 | 1.92299e−31 | Coleoptera | Dytiscidae | Hydroporus nigrita |
| Hydroporus sabaudus mitochondrial 16S rRNA gene (p | HE613720 | 81.65 | 100 | 1.92299e−31 | Coleoptera | Dytiscidae | Hydroporus sabaudus |
| Hydroporus discretus mitochondrial 16S rRNA gene ( | HE613709 | 81.65 | 100 | 1.92299e−31 | Coleoptera | Dytiscidae | Hydroporus discretus |
| Hydroporus brancuccii mitochondrial 16S rRNA gene | HE613696 | 81.65 | 100 | 1.92299e−31 | Coleoptera | Dytiscidae | Hydroporus brancuccii |
| Hydroporus kozlovskii mitochondrial 16S rRNA gene | HE613689 | 81.65 | 100 | 1.92299e−31 | Coleoptera | Dytiscidae | Hydroporus kozlovskii |
| Suphrodytes dorsalis mitochondrial 16S rRNA gene ( | HE599700 | 80.89 | 100 | 1.92299e−31 | Coleoptera | Dytiscidae | Suphrodytes dorsalis |
| Chostonectes sharpi partial 16S rRNA gene | AJ850313 | 80.65 | 98.73 | 1.92299e−31 | Coleoptera | Dytiscidae | Chostonectes sharpi |
| Chostonectes sharpi isolate ABTC78964 16S ribosoma | EU616947 | 80.65 | 98.73 | 1.92299e−31 | Coleoptera | Dytiscidae | Chostonectes sharpi |
| Nirripirti tetrameres isolate ABTC78764−5 16S ribo | EU616930 | 80.89 | 100 | 1.92299e−31 | Coleoptera | Dytiscidae | Paroster tetrameres |
| Nirripirti verrucosus isolate ABTC78682 16S riboso | EU616908 | 80.89 | 100 | 1.92299e−31 | Coleoptera | Dytiscidae | Paroster verrucosus |
| Paroster peelensis voucher ABTC79000−1 16S ribosom | EU371112 | 80.89 | 100 | 1.92299e−31 | Coleoptera | Dytiscidae | Paroster peelensis |
| Darwinhydrus solidus voucher MB354 16S ribosomal R | EF056671 | 83.85 | 100 | 1.92299e−31 | Coleoptera | Dytiscidae | Darwinhydrus solidus |
| Hydroporus vespertinus 16S ribosomal RNA gene, par | AY365291 | 81.65 | 100 | 1.92299e−31 | Coleoptera | Dytiscidae | Hydroporus vespertinus |
| Hydroporus sp. IR23.2 16S ribosomal RNA gene, part | AY365258 | 80.89 | 100 | 1.92299e−31 | Coleoptera | Dytiscidae | Hydroporus sp. IR23.2 |
| Hydroporus nigrita 16S ribosomal RNA gene, partial | AF518271 | 81.65 | 100 | 1.92299e−31 | Coleoptera | Dytiscidae | Hydroporus nigrita |
| Hydroporus erythrocephalus 16S ribosomal RNA gene, | AF518261 | 81.65 | 100 | 1.92299e−31 | Coleoptera | Dytiscidae | Hydroporus erythrocephalus |
| Hygrotus impressopunctatus voucher UNM KBMHtim80 1 | KJ548334 | 82.05 | 97.45 | 6.7119e−31 | Coleoptera | Dytiscidae | Hygrotus impressopunctatus |
| Paroster gibbi mitochondrial partial 16S rRNA gene | HG940398 | 81.01 | 100 | 2.34268e−30 | Coleoptera | Dytiscidae | Paroster gibbi |
