# Supplementary material for: Testing the potential of a ribosomal 16S marker for DNA metabarcoding of insects
Source: PeerJ. 2016 Apr 19;4:e1966. doi: 10.7717/peerj.1966 (PMC4841222; doi:10.7717/peerj.1966)
Supplement: Supplemental Information 1 [file peerj-04-1966-s006.zip › scripts/5 match OTUs/blast_hits/OTU_191.csv_plot.pdf]

OTU_191.csv

Parochlus steinenii

Chironomidae

Diptera

88.46

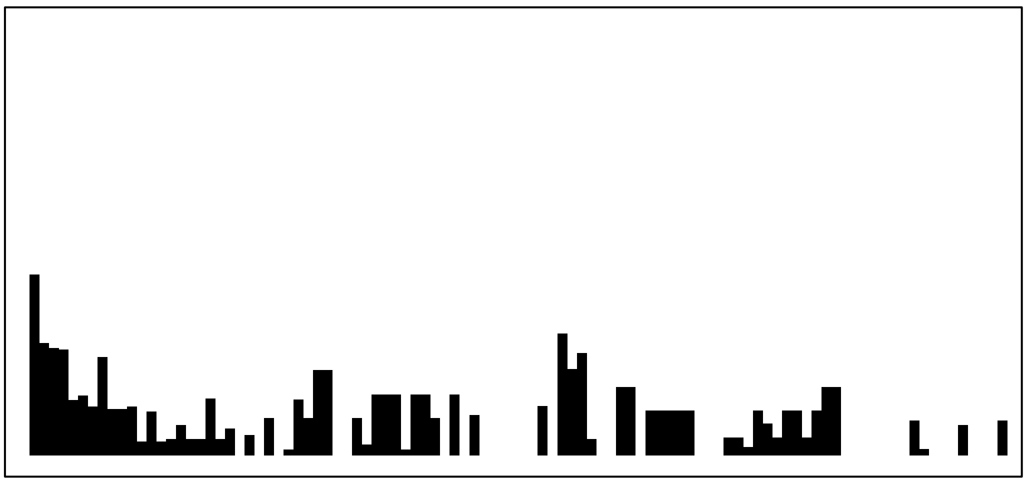

Parochlus steinenii isolate Sejong01 mitochondrion	KT003702	88.46	100	3.41683e−47	Diptera	Chironomidae	Parochlus steinenii
Bolitophila sp. ER−2009 16S ribosomal RNA gene, pa	FJ172021	85.26	97.42	2.99724e−35	Diptera	Mycetophilidae	Bolitophila sp. ER−2009
Bolitophila occlusa voucher JSB2 16S ribosomal RNA	KJ136713	85.03	93.55	1.04614e−34	Diptera	Mycetophilidae	Bolitophila occlusa
Bolitophila cinerea voucher JSB1 16S ribosomal RNA	KJ136712	84.97	96.13	1.04614e−34	Diptera	Mycetophilidae	Bolitophila cinerea
Edwardsina gigantea 16S ribosomal RNA gene, partia	KC177460	82.58	99.35	3.65139e−34	Diptera	Blephariceridae	Edwardsina gigantea
Pedicia albivitta voucher O'Grady 200012 16S ribos	EU005435	82.8	100	3.65139e−34	Diptera	Pediciidae	Pedicia albivitta
Clinocera sp. Pollet 0249 16S ribosomal RNA gene,	HQ449032	82.28	99.35	1.55261e−32	Diptera	Empididae	Clinocera sp. Pollet 0249
Bolitophila hybrida 16S ribosomal RNA gene, partia	FJ172002	84.62	97.42	1.55261e−32	Diptera	Mycetophilidae	Bolitophila hybrida
Tipula abdominalis 16S ribosomal RNA gene, partial	KC177457	82.17	100	5.41914e−32	Diptera	Tipulidae	Tipula abdominalis
Tipula abdominalis mitochondrion, partial genome	JN861743	82.17	100	5.41914e−32	Diptera	Tipulidae	Tipula abdominalis
Tipula sp. PMO 200011 16S ribosomal RNA gene, part	EU005437	82.28	100	1.89147e−31	Diptera	Tipulidae	Tipula sp. PMO 200011
Agathomyia elegantula isolate AE04 16S ribosomal R	KC699320	80.65	99.35	2.30428e−30	Diptera	Platypezidae	Agathomyia elegantula
Beris morrisii 16S ribosomal RNA gene, partial seq	GU947373	82.05	99.35	2.30428e−30	Diptera	Stratiomyidae	Beris morrisii
Geomyza tripunctata 16S ribosomal RNA gene, partia	EU268537	80.65	99.35	2.30428e−30	Diptera	Opomyzidae	Geomyza tripunctata
Agathomyia sexmaculata isolate 816 16S ribosomal R	KC699313	80.77	99.35	8.04272e−30	Diptera	Platypezidae	Agathomyia sexmaculata
Agathomyia zetterstedti isolate 814 16S ribosomal	KC699312	81.41	99.35	8.04272e−30	Diptera	Platypezidae	Agathomyia zetterstedti
Sylvicola fenestralis 16S ribosomal RNA gene, part	KC177461	80.77	100	8.04272e−30	Diptera	Anisopodidae	Sylvicola fenestralis
Sylvicola fenestralis mitochondrion, complete geno	JN861752	80.77	100	8.04272e−30	Diptera	Anisopodidae	Sylvicola fenestralis
Arachnocampa luminosa biotype 1 16S large subunit	AY576329	82.67	94.19	8.04272e−30	Diptera	Keroplatidae	Arachnocampa luminosa
Agathomyia lundbecki isolate 233 16S ribosomal RNA	KC699306	80.77	99.35	9.79804e−29	Diptera	Platypezidae	Agathomyia lundbecki
Tongamya stuckenbergi 16S ribosomal RNA gene, part	KM878969	81.25	99.35	3.41985e−28	Diptera	Mydidae	Tongamya stuckenbergi
Agathomyia viduella isolate AV01 16S ribosomal RNA	KC699317	79.35	99.35	1.19365e−27	Diptera	Platypezidae	Agathomyia viduella
Chrysops viduatus 16S ribosomal RNA gene, partial	HM132115	80.95	94.19	1.19365e−27	Diptera	Tabanidae	Chrysops viduatus
Tabanus bromius 16S ribosomal RNA gene, partial se	HM132111	79.35	99.35	1.19365e−27	Diptera	Tabanidae	Tabanus bromius
Callomyia admirabilis isolate CA42 16S ribosomal R	KC699326	81.75	87.74	4.16623e−27	Diptera	Platypezidae	Callomyia admirabilis
Empis producta voucher RMBR:103430 16S ribosomal R	FJ808167	79.49	99.35	4.16623e−27	Diptera	Empididae	Empis producta
Somatia sp. YSUW04038 16S ribosomal RNA gene, part	AY573134	80.27	94.19	4.16623e−27	Diptera	Somatiidae	Somatia sp. YSUW04038
Arachnocampa luminosa biotype 3 16S large subunit	AY576331	82.61	87.74	4.16623e−27	Diptera	Keroplatidae	Arachnocampa luminosa
Coelopa alluaudi 16S ribosomal RNA gene, partial s	AF403455	81.75	87.74	4.16623e−27	Diptera	Coelopidae	Coelopa alluaudi
Simulium tenederoi mitochondrial gene for 16S ribo	LC035002	84	79.35	1.45416e−26	Diptera	Simuliidae	Simulium tenederoi
Simulium tenederoi mitochondrial gene for 16S ribo	LC035001	84	79.35	1.45416e−26	Diptera	Simuliidae	Simulium tenederoi
Simulium sheilae isolate SH1B 16S ribosomal RNA ge	KM410213	79.62	100	1.45416e−26	Diptera	Simuliidae	Simulium sheilae
Dichopygina aculeata voucher 947 16S ribosomal RNA	JQ613961	79.74	97.42	1.45416e−26	Diptera	Sciaridae	Dichopygina aculeata
Caenophthalmus capensis voucher MEI 120162 16S rib	DQ250838	81.76	92.26	1.45416e−26	Diptera	Therevidae	Caenophthalmus capensis
Simulium suyoense mitochondrial gene for 16S ribos	LC035000	80.5	100	5.07551e−26	Diptera	Simuliidae	Simulium suyoense
Simulium roslihashimi isolate R1B 16S ribosomal RN	KM410192	82.84	85.16	5.07551e−26	Diptera	Simuliidae	Simulium roslihashimi
Simulium izuae isolate I3B 16S ribosomal RNA gene,	KM410191	82.84	85.16	5.07551e−26	Diptera	Simuliidae	Simulium izuae
Simulium izuae isolate I2B 16S ribosomal RNA gene,	KM410190	82.84	85.16	5.07551e−26	Diptera	Simuliidae	Simulium izuae
Haematopota pluvialis 16S ribosomal RNA gene, part	KC177454	80.27	94.19	5.07551e−26	Diptera	Tabanidae	Haematopota pluvialis
Simulium tuenense mitochondrial gene for 16S ribos	AB591244	82.84	85.16	5.07551e−26	Diptera	Simuliidae	Simulium tuenense
Simulium inthanonense 16S ribosomal RNA gene, part	GQ865995	82.84	85.16	5.07551e−26	Diptera	Simuliidae	Simulium inthanonense
Allocotocera pulchella 16S ribosomal RNA gene, par	FJ172034	81.75	87.74	5.07551e−26	Diptera	Mycetophilidae	Allocotocera pulchella
Dialysis sp. SLW−2002 16S ribosomal RNA gene, part	AY140888	78.71	99.35	5.07551e−26	Diptera	Xylophagidae	Dialysis sp. SLW−2002
Simulium cabrerai mitochondrial gene for 16S rRNA,	AB093128	82.84	85.16	5.07551e−26	Diptera	Simuliidae	Simulium cabrerai
Microsania collarti isolate CAN8 16S ribosomal RNA	KC699334	78.85	99.35	1.77153e−25	Diptera	Platypezidae	Microsania collarti
Callomyia amoena isolate CA06 16S ribosomal RNA ge	KC699325	81.88	87.74	1.77153e−25	Diptera	Platypezidae	Callomyia amoena
Agathomyia woodella isolate 448 16S ribosomal RNA	KC699307	79.45	93.55	1.77153e−25	Diptera	Platypezidae	Agathomyia woodella
Ectinorhynchus sp. 'Bribie' voucher UQIC41178 16S	KM212983	79.75	99.35	1.77153e−25	Diptera	Therevidae	Ectinorhynchus sp. 'Bribie'
Ectinorhynchus sp. 'Moffatt' voucher QMT183203 16S	KM212976	79.75	99.35	1.77153e−25	Diptera	Therevidae	Ectinorhynchus sp. 'Moffatt'
Ectinorhynchus sp. 'warrumbungles' voucher QMT1832	KM212974	79.75	99.35	1.77153e−25	Diptera	Therevidae	Ectinorhynchus sp. 'warrumbungles'
Diadocidia stanfordensis voucher JSSJ14 16S riboso	KJ136742	79.73	90.32	1.77153e−25	Diptera	Diadocidiidae	Diadocidia stanfordensis
Coboldia sp. SS−2012 voucher 905 16S ribosomal RNA	JQ613954	79.22	97.42	1.77153e−25	Diptera	Scatopsidae	Coboldia sp. SS−2012
Keilbachia subacumina voucher 890 16S ribosomal RN	JQ613951	82.31	81.94	1.77153e−25	Diptera	Sciaridae	Keilbachia subacumina
Zygoneura bidens voucher 312 16S ribosomal RNA gen	JQ613910	79.08	96.13	1.77153e−25	Diptera	Sciaridae	Zygoneura bidens
Paracladura trichoptera mitochondrion, complete ge	JN861751	85.71	75.48	1.77153e−25	Diptera	Trichoceridae	Paracladura trichoptera
Leptomorphus walkeri 16S ribosomal RNA gene, parti	FJ172020	84.03	76.13	1.77153e−25	Diptera	Mycetophilidae	Leptomorphus walkeri
Hercostomus meieri voucher RMBR:103442 16S ribosom	FJ808173	84.8	79.35	1.77153e−25	Diptera	Dolichopodidae	Hercostomus meieri
Phorinia flava mitochondrial gene for 16S rRNA, pa	AB257236	80.77	98.71	1.77153e−25	Diptera	Tachinidae	Phorinia flava
Prosimulium kiotoense mitochondrial gene for 16S r	LC035009	78.98	100	6.18324e−25	Diptera	Simuliidae	Prosimulium kiotoense
Simulium tuyense mitochondrial gene for 16S riboso	LC035004	79.74	97.42	6.18324e−25	Diptera	Simuliidae	Simulium tuyense
Simulium claveriaense mitochondrial gene for 16S r	LC034988	83.2	79.35	6.18324e−25	Diptera	Simuliidae	Simulium claveriaense
Simulium claveriaense mitochondrial gene for 16S r	LC034987	83.2	79.35	6.18324e−25	Diptera	Simuliidae	Simulium claveriaense
Simulium sheilae isolate SH2B 16S ribosomal RNA ge	KM410214	78.98	100	6.18324e−25	Diptera	Simuliidae	Simulium sheilae
Simulium brinchangense isolate B2B 16S ribosomal R	KM410202	82.09	85.16	6.18324e−25	Diptera	Simuliidae	Simulium brinchangense
Simulium brinchangense isolate B1B 16S ribosomal R	KM410201	82.09	85.16	6.18324e−25	Diptera	Simuliidae	Simulium brinchangense
Simulium tanahrataense isolate TR2B 16S ribosomal	KM410196	82.09	85.16	6.18324e−25	Diptera	Simuliidae	Simulium tanahrataense
Simulium tanahrataense isolate TR1B 16S ribosomal	KM410195	82.09	85.16	6.18324e−25	Diptera	Simuliidae	Simulium tanahrataense
Simulium izuae isolate I1B 16S ribosomal RNA gene,	KM410189	82.09	85.16	6.18324e−25	Diptera	Simuliidae	Simulium izuae
Riekiella bolgarti 16S ribosomal RNA gene, partial	KM878954	79.5	99.35	6.18324e−25	Diptera	Scenopinidae	Riekiella bolgarti
Paramonova sp. A SLW−2015 16S ribosomal RNA gene,	KM878937	79.5	99.35	6.18324e−25	Diptera	Scenopinidae	Paramonova sp. A SLW−2015
Chydaeopeza tibialis isolate CT44 16S ribosomal RN	KC699336	78.06	99.35	6.18324e−25	Diptera	Platypezidae	Chydaeopeza tibialis
Simulium khunklangense mitochondrial gene for 16S	AB820367	80.82	92.26	6.18324e−25	Diptera	Simuliidae	Simulium khunklangense
Simulium chomthongense mitochondrial gene for 16S	AB699899	80.82	92.26	6.18324e−25	Diptera	Simuliidae	Simulium chomthongense
Trichopeza longicornis voucher Pollet 0243 (P) 16S	HQ449035	80.38	99.35	6.18324e−25	Diptera	Empididae	Trichopeza longicornis
Cyphomyia notabilis 16S ribosomal RNA gene, partia	GU947403	82.09	85.16	6.18324e−25	Diptera	Stratiomyidae	Cyphomyia notabilis
Pachygaster atra 16S ribosomal RNA gene, partial s	GU947397	81.48	86.45	6.18324e−25	Diptera	Stratiomyidae	Pachygaster atra
Simulium caudisclerum 16S ribosomal RNA gene, part	GQ865999	80.82	92.26	6.18324e−25	Diptera	Simuliidae	Simulium caudisclerum
Simulium chumpornense 16S ribosomal RNA gene, part	GQ865998	82.09	85.16	6.18324e−25	Diptera	Simuliidae	Simulium chumpornense
Simulium decuplum 16S ribosomal RNA gene, partial	GQ865986	82.09	85.16	6.18324e−25	Diptera	Simuliidae	Simulium decuplum
Simulium arabicum 16S ribosomal RNA gene, partial	AY944186	80.82	92.26	6.18324e−25	Diptera	Simuliidae	Simulium arabicum
Simulium recurvum mitochondrial gene for 16S ribos	AB334097	82.09	85.16	6.18324e−25	Diptera	Simuliidae	Simulium recurvum
Simulium claveriaense mitochondrial gene for 16S r	AB334096	83.2	79.35	6.18324e−25	Diptera	Simuliidae	Simulium claveriaense
Simulium kobayashii mitochondrial gene for 16S rib	AB334093	83.2	79.35	6.18324e−25	Diptera	Simuliidae	Simulium kobayashii
Euaresta festiva 16S ribosomal RNA gene, partial s	DQ471399	79.59	94.19	6.18324e−25	Diptera	Tephritidae	Euaresta festiva
Eutreta novaeboracensis 16S ribosomal RNA gene, pa	DQ471384	78.34	99.35	6.18324e−25	Diptera	Tephritidae	Eutreta novaeboracensis
Panorpa annexa 16S ribosomal RNA gene, partial seq	AF180048	79.73	93.55	6.18324e−25	Mecoptera	Panorpidae	Panorpa annexa
Cnephia dacotensis mitochondrial 16S ribosomal RNA	L13403	80	97.42	6.18324e−25	Diptera	Simuliidae	Cnephia dacotensis
Simulium suyoense mitochondrial gene for 16S ribos	LC034999	79.87	100	2.15816e−24	Diptera	Simuliidae	Simulium suyoense
Simulium suyoense mitochondrial gene for 16S ribos	LC034998	79.87	100	2.15816e−24	Diptera	Simuliidae	Simulium suyoense
Simulium suyoense mitochondrial gene for 16S ribos	LC034997	79.87	100	2.15816e−24	Diptera	Simuliidae	Simulium suyoense
Simulium spinosibranchium mitochondrial gene for 1	LC034996	81.62	85.16	2.15816e−24	Diptera	Simuliidae	Simulium spinosibranchium
Platypezina diversa isolate PD29 16S ribosomal RNA	KC699349	80.29	87.74	2.15816e−24	Diptera	Platypezidae	Platypezina diversa
Agathomyia unicolor isolate AU2 16S ribosomal RNA	KC699318	78.85	99.35	2.15816e−24	Diptera	Platypezidae	Agathomyia unicolor
Leptomorphus sp. DK−2013 voucher JSS1 16S ribosoma	KC435575	79.72	91.61	2.15816e−24	Diptera	Mycetophilidae	Leptomorphus sp. DK−2013
Musca ventrosa 16S ribosomal RNA gene, partial seq	JN226633	78.85	99.35	2.15816e−24	Diptera	Muscidae	Musca ventrosa
Voria ruralis mitochondrial gene for 16S rRNA, par	AB465959	81.43	88.39	2.15816e−24	Diptera	Tachinidae	Voria ruralis
Admontia sp. Admon_23072006 mitochondrial gene for	AB465900	79.49	94.84	2.15816e−24	Diptera	Tachinidae	Admontia sp. Admon_23072006
Neoplatyura flava 16S ribosomal RNA gene, partial	FJ172019	79.47	96.13	2.15816e−24	Diptera	Keroplatidae	Neoplatyura flava
Simulium suyoense mitochondrial gene for 16S ribos	AB334099	79.87	100	2.15816e−24	Diptera	Simuliidae	Simulium suyoense
Simulium spinosibranchium mitochondrial gene for 1	AB334098	81.62	85.16	2.15816e−24	Diptera	Simuliidae	Simulium spinosibranchium
