# Supplementary material for: Testing the potential of a ribosomal 16S marker for DNA metabarcoding of insects
Source: PeerJ. 2016 Apr 19;4:e1966. doi: 10.7717/peerj.1966 (PMC4841222; doi:10.7717/peerj.1966)
Supplement: Supplemental Information 1 [file peerj-04-1966-s006.zip › scripts/5 match OTUs/blast_hits/OTU_2.csv_plot.pdf]

OTU_2.csv

Nemurella pictetii

Nemouridae

Plecoptera

100

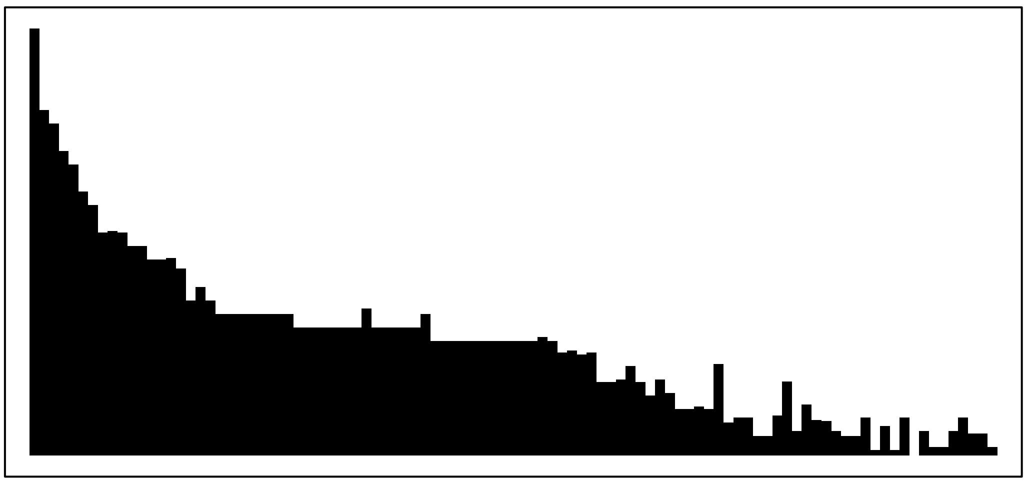

Nemurella pictetii 16S ribosomal RNA gene, partial	EF623192	100	100	3.04182e−73	Plecoptera	Nemouridae	Nemurella pictetii
Ostrocerca foersteri 16S ribosomal RNA gene, parti	EF623191	96.18	100	4.22819e−65	Plecoptera	Nemouridae	Ostrocerca foersteri
Visoka cataractae 16S ribosomal RNA gene, partial	EF623189	95.54	100	1.79787e−63	Plecoptera	Nemouridae	Visoka cataractae
Nanonemoura wahkeena 16S ribosomal RNA gene, parti	EF623198	94.27	100	9.31322e−61	Plecoptera	Nemouridae	Nanonemoura wahkeena
Shipsa rotunda 16S ribosomal RNA gene, partial seq	EF623184	93.63	100	1.13458e−59	Plecoptera	Nemouridae	Shipsa rotunda
Paranemoura claasseni 16S ribosomal RNA gene, part	EF623185	92.36	100	5.87728e−57	Plecoptera	Nemouridae	Paranemoura claasseni
Nemoura trispinosa 16S ribosomal RNA gene, partial	EF623187	91.72	100	2.49908e−55	Plecoptera	Nemouridae	Nemoura trispinosa
Lednia tumana 16S ribosomal RNA gene, partial sequ	EF623197	90.45	100	1.29456e−52	Plecoptera	Nemouridae	Lednia tumana
Nemoura cinerea 16S ribosomal RNA gene, partial se	EF623194	90.51	100	4.51845e−52	Plecoptera	Nemouridae	Nemoura cinerea
Soyedina producta 16S ribosomal RNA gene, partial	EF623196	90.45	100	1.57709e−51	Plecoptera	Nemouridae	Soyedina producta
Protonemura meyeri 16S ribosomal RNA gene, partial	EF623193	89.81	100	1.57709e−51	Plecoptera	Nemouridae	Protonemura meyeri
Malenka californica 16S ribosomal RNA gene, partia	EF623182	89.81	100	1.57709e−51	Plecoptera	Nemouridae	Malenka californica
Prostoia besametsa 16S ribosomal RNA gene, partial	EF623188	89.17	100	6.70597e−50	Plecoptera	Nemouridae	Prostoia besametsa
Zapada cinctipes 16S ribosomal RNA gene, partial s	EF623183	89.17	100	6.70597e−50	Plecoptera	Nemouridae	Zapada cinctipes
Scopura montana 16S ribosomal RNA gene, partial se	EF623265	89.24	100	2.34061e−49	Plecoptera		Scopura montana
Oemopteryx vanduzeea 16S ribosomal RNA gene, parti	EF623266	88.75	100	8.16955e−49	Plecoptera	Taeniopterygidae	Oemopteryx vanduzeea
Apteroperla tikumana mitochondrion, complete genom	KR604721	87.26	100	4.23193e−46	Plecoptera	Capniidae	Apteroperla tikumana
Pteronarcys californica 16S ribosomal RNA gene, pa	EF623261	87.9	100	4.23193e−46	Plecoptera	Pteronarcyidae	Pteronarcys californica
Pteronarcys princeps mitochondrion, complete genom	AY687866	87.26	100	5.15555e−45	Plecoptera	Pteronarcyidae	Pteronarcys princeps
Mesocapnia arizonensis mitochondrion, partial geno	KP642637	86.62	100	1.79946e−44	Plecoptera	Capniidae	Mesocapnia arizonensis
Allocapnia recta isolate H13 16S ribosomal RNA gen	KC881048	86.62	100	1.79946e−44	Plecoptera	Capniidae	Allocapnia recta
Allocapnia recta isolate H11 16S ribosomal RNA gen	KC881046	86.62	100	1.79946e−44	Plecoptera	Capniidae	Allocapnia recta
Allocapnia recta isolate H7 16S ribosomal RNA gene	KC881042	86.62	100	1.79946e−44	Plecoptera	Capniidae	Allocapnia recta
Allocapnia recta isolate H3 16S ribosomal RNA gene	KC881038	86.62	100	1.79946e−44	Plecoptera	Capniidae	Allocapnia recta
Allocapnia minima 16S ribosomal RNA gene, partial	EF623130	86.62	100	1.79946e−44	Plecoptera	Capniidae	Allocapnia minima
Mesocapnia sp. BYU_PL010 16S ribosomal RNA gene, p	EF623127	86.62	100	1.79946e−44	Plecoptera	Capniidae	Mesocapnia sp. BYU_PL010
Allocapnia recta haplotype 3ar: H03 16S ribosomal	DQ915181	86.62	100	1.79946e−44	Plecoptera	Capniidae	Allocapnia recta
Allocapnia recta isolate H19 16S ribosomal RNA gen	KC881054	85.99	100	2.19219e−43	Plecoptera	Capniidae	Allocapnia recta
Allocapnia recta isolate H16 16S ribosomal RNA gen	KC881051	85.99	100	2.19219e−43	Plecoptera	Capniidae	Allocapnia recta
Allocapnia recta isolate H15 16S ribosomal RNA gen	KC881050	85.99	100	2.19219e−43	Plecoptera	Capniidae	Allocapnia recta
Allocapnia recta isolate H14 16S ribosomal RNA gen	KC881049	85.99	100	2.19219e−43	Plecoptera	Capniidae	Allocapnia recta
Allocapnia recta isolate H10 16S ribosomal RNA gen	KC881045	85.99	100	2.19219e−43	Plecoptera	Capniidae	Allocapnia recta
Allocapnia recta isolate H6 16S ribosomal RNA gene	KC881041	85.99	100	2.19219e−43	Plecoptera	Capniidae	Allocapnia recta
Allocapnia recta isolate H1 16S ribosomal RNA gene	KC881036	85.99	100	2.19219e−43	Plecoptera	Capniidae	Allocapnia recta
Doddsia occidentalis 16S ribosomal RNA gene, parti	EF623270	86.88	100	2.19219e−43	Plecoptera	Taeniopterygidae	Doddsia occidentalis
Allocapnia recta haplotype 12ar: H12 16S ribosomal	EU306875	85.99	100	2.19219e−43	Plecoptera	Capniidae	Allocapnia recta
Allocapnia recta haplotype 11ar: H011 16S ribosoma	EU306874	85.99	100	2.19219e−43	Plecoptera	Capniidae	Allocapnia recta
Allocapnia recta haplotype 7ar: H07 16S ribosomal	EU306870	85.99	100	2.19219e−43	Plecoptera	Capniidae	Allocapnia recta
Allocapnia recta haplotype 2ar: H02 16S ribosomal	DQ915180	85.99	100	2.19219e−43	Plecoptera	Capniidae	Allocapnia recta
Pteronarcys sachalina 16S ribosomal RNA gene, part	EF623264	85.99	100	2.67064e−42	Plecoptera	Pteronarcyidae	Pteronarcys sachalina
Osmylus sp. EH−2004 16S ribosomal RNA gene, partia	AY620146	86.62	100	2.67064e−42	Neuroptera	Osmylidae	Osmylus sp. EH−2004
Allocapnia recta isolate H18 16S ribosomal RNA gen	KC881053	85.35	100	9.32145e−42	Plecoptera	Capniidae	Allocapnia recta
Allocapnia recta isolate H17 16S ribosomal RNA gen	KC881052	85.35	100	9.32145e−42	Plecoptera	Capniidae	Allocapnia recta
Allocapnia recta isolate H12 16S ribosomal RNA gen	KC881047	85.35	100	9.32145e−42	Plecoptera	Capniidae	Allocapnia recta
Allocapnia recta isolate H9 16S ribosomal RNA gene	KC881044	85.35	100	9.32145e−42	Plecoptera	Capniidae	Allocapnia recta
Allocapnia recta isolate H8 16S ribosomal RNA gene	KC881043	85.35	100	9.32145e−42	Plecoptera	Capniidae	Allocapnia recta
Allocapnia recta isolate H5 16S ribosomal RNA gene	KC881040	85.35	100	9.32145e−42	Plecoptera	Capniidae	Allocapnia recta
Allocapnia recta isolate H4 16S ribosomal RNA gene	KC881039	85.35	100	9.32145e−42	Plecoptera	Capniidae	Allocapnia recta
Allocapnia recta haplotype 10ar: H010 16S ribosoma	EU306873	85.35	100	9.32145e−42	Plecoptera	Capniidae	Allocapnia recta
Allocapnia recta haplotype 9ar: H09 16S ribosomal	EU306872	85.35	100	9.32145e−42	Plecoptera	Capniidae	Allocapnia recta
Allocapnia recta haplotype 8ar: H08 16S ribosomal	EU306871	85.35	100	9.32145e−42	Plecoptera	Capniidae	Allocapnia recta
Allocapnia recta haplotype 1ar: H01 16S ribosomal	DQ915179	85.35	100	9.32145e−42	Plecoptera	Capniidae	Allocapnia recta
Brachyptera seticornis 16S ribosomal RNA gene, par	EF623268	85.53	100	3.2535e−41	Plecoptera	Taeniopterygidae	Brachyptera seticornis
Capnura wanica 16S ribosomal RNA gene, partial seq	EF623128	85.35	100	1.13558e−40	Plecoptera	Capniidae	Capnura wanica
Bolotoperla rossi 16S ribosomal RNA gene, partial	EF623269	84.81	100	3.96358e−40	Plecoptera	Taeniopterygidae	Bolotoperla rossi
Isocapnia hyalita 16S ribosomal RNA gene, partial	EF623131	84.91	100	3.96358e−40	Plecoptera	Capniidae	Isocapnia hyalita
Pteronarcys scotti 16S ribosomal RNA gene, partial	EF623263	84.71	100	1.38343e−39	Plecoptera	Pteronarcyidae	Pteronarcys scotti
Moselia infuscata 16S ribosomal RNA gene, partial	EF623175	84.81	99.36	1.68536e−38	Plecoptera	Leuctridae	Moselia infuscata
Austrocerca rieka 16S ribosomal RNA gene, partial	EF623205	83.44	100	5.88247e−38	Plecoptera	Notonemouridae	Austrocerca rieka
Austrocercoides zwicki 16S ribosomal RNA gene, par	EF623199	83.44	100	5.88247e−38	Plecoptera	Notonemouridae	Austrocercoides zwicki
Megaleuctra kincaidi 16S ribosomal RNA gene, parti	EF623174	83.54	100	2.05319e−37	Plecoptera	Leuctridae	Megaleuctra kincaidi
Triznaka pintada 16S ribosomal RNA gene, partial s	EF623134	84.18	100	2.05319e−37	Plecoptera	Chloroperlidae	Triznaka pintada
Neonemura sp. BYU_PL153 16S ribosomal RNA gene, pa	EF623204	83.44	100	7.16632e−37	Plecoptera	Notonemouridae	Neonemura sp. BYU_PL153
Udamocercia sp. BYU_PL150 16S ribosomal RNA gene,	EF623201	82.8	100	2.50129e−36	Plecoptera	Notonemouridae	Udamocercia sp. BYU_PL150
Thaumatoperla sp. BYU_PL109 16S ribosomal RNA gene	EF623149	83.54	100	2.50129e−36	Plecoptera	Eustheniidae	Thaumatoperla sp. BYU_PL109
Strophopteryx appalachia 16S ribosomal RNA gene, p	EF623271	82.91	100	8.73037e−36	Plecoptera	Taeniopterygidae	Strophopteryx appalachia
Amphinemura sulcicollis 16S ribosomal RNA gene, pa	EF623195	82.17	100	3.0472e−35	Plecoptera	Nemouridae	Amphinemura sulcicollis
Amphinemura nigritta 16S ribosomal RNA gene, parti	EF623186	82.17	100	3.0472e−35	Plecoptera	Nemouridae	Amphinemura nigritta
Alloperla severa 16S ribosomal RNA gene, partial s	EF623141	82.28	100	1.06358e−34	Plecoptera	Chloroperlidae	Alloperla severa
Sierraperla cora 16S ribosomal RNA gene, partial s	EF623255	82.17	100	3.71225e−34	Plecoptera	Peltoperlidae	Sierraperla cora
Kathroperla perdita 16S ribosomal RNA gene, partia	EF623135	84.29	89.17	3.71225e−34	Plecoptera	Chloroperlidae	Kathroperla perdita
Paraperla frontalis 16S ribosomal RNA gene, partia	EF623136	81.53	100	1.2957e−33	Plecoptera	Chloroperlidae	Paraperla frontalis
Notonemouridae sp. MD−2011 voucher ZMUC:JD−1507 16	JN615257	81.76	100	4.52244e−33	Plecoptera	Notonemouridae	Notonemouridae sp. MD−2011
Paraleuctra occidentalis 16S ribosomal RNA gene, p	EF623178	81.76	100	4.52244e−33	Plecoptera	Leuctridae	Paraleuctra occidentalis
Perlinodes aurea 16S ribosomal RNA gene, partial s	EF623234	80.89	100	1.57849e−32	Plecoptera	Perlodidae	Perlinodes aurea
Salmoperla sylvanica 16S ribosomal RNA gene, parti	EF623222	80.89	100	1.57849e−32	Plecoptera	Perlodidae	Salmoperla sylvanica
Utaperla sopladora 16S ribosomal RNA gene, partial	EF623140	81.88	100	1.57849e−32	Plecoptera	Chloroperlidae	Utaperla sopladora
Yoraperla uenoi mitochondrial gene for 16S rRNA, p	AB712375	83.45	92.36	5.50946e−32	Plecoptera	Peltoperlidae	Yoraperla uenoi
Megarcys signata 16S ribosomal RNA gene, partial s	EF623215	81.13	100	5.50946e−32	Plecoptera	Perlodidae	Megarcys signata
Eusthenia costalis 16S ribosomal RNA gene, partial	EF623150	82.39	100	5.50946e−32	Plecoptera	Eustheniidae	Eusthenia costalis
Plumiperla diversa 16S ribosomal RNA gene, partial	EF623132	81.65	100	5.50946e−32	Plecoptera	Chloroperlidae	Plumiperla diversa
Taenionema palladium 16S ribosomal RNA gene, parti	EF623267	81.6	100	1.92299e−31	Plecoptera	Taeniopterygidae	Taenionema palladium
Peltoperla arcuata 16S ribosomal RNA gene, partial	EF623258	81.13	100	1.92299e−31	Plecoptera	Peltoperlidae	Peltoperla arcuata
Cascadoperla trictura 16S ribosomal RNA gene, part	EF623225	80.89	100	1.92299e−31	Plecoptera	Perlodidae	Cascadoperla trictura
Limnoperla sp. BYU_PL145 16S ribosomal RNA gene, p	EF623166	80.89	100	1.92299e−31	Plecoptera	Gripopterygidae	Limnoperla sp. BYU_PL145
Soliperla campanula 16S ribosomal RNA gene, partia	EF623256	81.76	99.36	6.7119e−31	Plecoptera	Peltoperlidae	Soliperla campanula
Skwala americana 16S ribosomal RNA gene, partial s	EF623217	80.25	100	6.7119e−31	Plecoptera	Perlodidae	Skwala americana
Diamphipnopsis sp. BYU_PL138 16S ribosomal RNA gen	EF623145	81.37	100	6.7119e−31	Plecoptera	Diamphipnoidae	Diamphipnopsis sp. BYU_PL138
Diamphipnoa virescentipennis 16S ribosomal RNA gen	EF623144	80.25	100	6.7119e−31	Plecoptera	Diamphipnoidae	Diamphipnoa virescentipennis
Caliaeschna microstigma 12S ribosomal RNA gene, pa	EU477639	81.76	100	2.34268e−30	Odonata	Aeshnidae	Caliaeschna microstigma
Pteronarcella badia 16S ribosomal RNA gene, partia	EF623262	79.5	100	2.34268e−30	Plecoptera	Pteronarcyidae	Pteronarcella badia
Cosumnoperla hypocrena 16S ribosomal RNA gene, par	EF623226	81.13	100	2.34268e−30	Plecoptera	Perlodidae	Cosumnoperla hypocrena
Suwallia teleckojensis 16S ribosomal RNA gene, par	EF623142	80.38	100	2.34268e−30	Plecoptera	Chloroperlidae	Suwallia teleckojensis
Neaviperla forcipata 16S ribosomal RNA gene, parti	EF623139	80.38	100	2.34268e−30	Plecoptera	Chloroperlidae	Neaviperla forcipata
Klapopteryx armillata 16S ribosomal RNA gene, part	EF623123	81.13	100	2.34268e−30	Plecoptera	Austroperlidae	Klapopteryx armillata
Caliaeschna microstigma large subunit ribosomal RN	AF266092	81.76	100	2.34268e−30	Odonata	Aeshnidae	Caliaeschna microstigma
Microchorista philpotti 16S ribosomal RNA gene, pa	KC177459	81.01	100	8.17677e−30	Mecoptera	Nannochoristidae	Nannochorista philpotti
Microchorista philpotti mitochondrion, partial gen	HQ696580	81.01	100	8.17677e−30	Mecoptera	Nannochoristidae	Nannochorista philpotti
Isoperla oxylepis 16S ribosomal RNA gene, partial	EF623221	80.38	100	8.17677e−30	Plecoptera	Perlodidae	Isoperla oxylepis
Chloroperla tripunctata 16S ribosomal RNA gene, pa	EF623137	79.62	100	8.17677e−30	Plecoptera	Chloroperlidae	Chloroperla tripunctata
