# Supplementary material for: Testing the potential of a ribosomal 16S marker for DNA metabarcoding of insects
Source: PeerJ. 2016 Apr 19;4:e1966. doi: 10.7717/peerj.1966 (PMC4841222; doi:10.7717/peerj.1966)
Supplement: Supplemental Information 1 [file peerj-04-1966-s006.zip › scripts/5 match OTUs/blast_hits/OTU_20.csv_plot.pdf]

OTU_20.csv

Parochlus steinenii

Chironomidae

Diptera

88.46

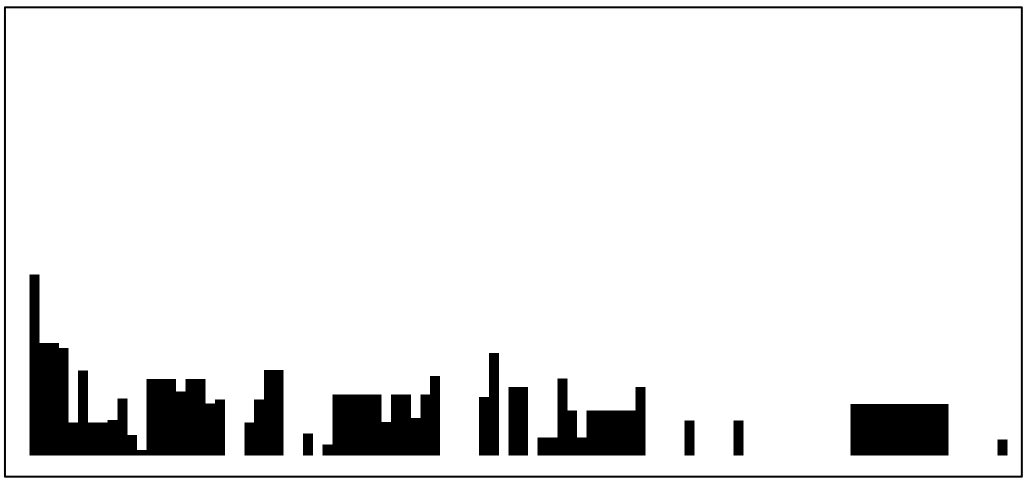

Parochlus steinenii isolate Sejong01 mitochondrion	KT003702	88.46	100	2.82808e−48	Diptera	Chironomidae	Parochlus steinenii
Bolitophila cinerea voucher JSB1 16S ribosomal RNA	KJ136712	85.26	97.44	2.48079e−36	Diptera	Mycetophilidae	Bolitophila cinerea
Bolitophila sp. ER−2009 16S ribosomal RNA gene, pa	FJ172021	85.26	97.44	2.48079e−36	Diptera	Mycetophilidae	Bolitophila sp. ER−2009
Bolitophila occlusa voucher JSB2 16S ribosomal RNA	KJ136713	85.03	93.59	8.65881e−36	Diptera	Mycetophilidae	Bolitophila occlusa
Pedicia albivitta voucher O'Grady 200012 16S ribos	EU005435	81.53	100	1.56555e−32	Diptera	Pediciidae	Pedicia albivitta
Bolitophila hybrida 16S ribosomal RNA gene, partia	FJ172002	83.97	97.44	5.4643e−32	Diptera	Mycetophilidae	Bolitophila hybrida
Tipula abdominalis 16S ribosomal RNA gene, partial	KC177457	81.53	100	1.90723e−31	Diptera	Tipulidae	Tipula abdominalis
Tipula abdominalis mitochondrion, partial genome	JN861743	81.53	100	1.90723e−31	Diptera	Tipulidae	Tipula abdominalis
Tipula sp. PMO 200011 16S ribosomal RNA gene, part	EU005437	81.65	100	6.65688e−31	Diptera	Tipulidae	Tipula sp. PMO 200011
Arachnocampa luminosa biotype 1 16S large subunit	AY576329	82.67	94.23	6.65688e−31	Diptera	Keroplatidae	Arachnocampa luminosa
Somatia sp. YSUW04038 16S ribosomal RNA gene, part	AY573134	80.95	94.23	8.10974e−30	Diptera	Somatiidae	Somatia sp. YSUW04038
Simulium sheilae isolate SH1B 16S ribosomal RNA ge	KM410213	80.25	100	9.87969e−29	Diptera	Simuliidae	Simulium sheilae
Simulium roslihashimi isolate R1B 16S ribosomal RN	KM410192	83.58	85.26	9.87969e−29	Diptera	Simuliidae	Simulium roslihashimi
Simulium izuae isolate I3B 16S ribosomal RNA gene,	KM410191	83.58	85.26	9.87969e−29	Diptera	Simuliidae	Simulium izuae
Simulium izuae isolate I2B 16S ribosomal RNA gene,	KM410190	83.58	85.26	9.87969e−29	Diptera	Simuliidae	Simulium izuae
Paracladura trichoptera mitochondrion, complete ge	JN861751	82.98	86.54	9.87969e−29	Diptera	Trichoceridae	Paracladura trichoptera
Simulium tuenense mitochondrial gene for 16S ribos	AB591244	83.58	85.26	9.87969e−29	Diptera	Simuliidae	Simulium tuenense
Simulium inthanonense 16S ribosomal RNA gene, part	GQ865995	83.58	85.26	9.87969e−29	Diptera	Simuliidae	Simulium inthanonense
Caenophthalmus capensis voucher MEI 120162 16S rib	DQ250838	82.43	92.31	9.87969e−29	Diptera	Therevidae	Caenophthalmus capensis
Callomyia admirabilis isolate CA42 16S ribosomal R	KC699326	82.61	87.82	3.44835e−28	Diptera	Platypezidae	Callomyia admirabilis
Sylvicola fenestralis 16S ribosomal RNA gene, part	KC177461	79.49	100	3.44835e−28	Diptera	Anisopodidae	Sylvicola fenestralis
Sylvicola fenestralis mitochondrion, complete geno	JN861752	79.49	100	3.44835e−28	Diptera	Anisopodidae	Sylvicola fenestralis
Beris morrisii 16S ribosomal RNA gene, partial seq	GU947373	81.53	99.36	3.44835e−28	Diptera	Stratiomyidae	Beris morrisii
Arachnocampa luminosa biotype 3 16S large subunit	AY576331	82.61	87.82	3.44835e−28	Diptera	Keroplatidae	Arachnocampa luminosa
Simulium tenederoi mitochondrial gene for 16S ribo	LC035002	84	79.49	1.20359e−27	Diptera	Simuliidae	Simulium tenederoi
Simulium tenederoi mitochondrial gene for 16S ribo	LC035001	84	79.49	1.20359e−27	Diptera	Simuliidae	Simulium tenederoi
Simulium sheilae isolate SH2B 16S ribosomal RNA ge	KM410214	79.62	100	1.20359e−27	Diptera	Simuliidae	Simulium sheilae
Liriomyza sativae mitochondrion, complete genome	JQ862475	78.98	99.36	1.20359e−27	Diptera	Agromyzidae	Liriomyza sativae
Trichopeza longicornis voucher Pollet 0243 (P) 16S	HQ449035	81.01	99.36	1.20359e−27	Diptera	Empididae	Trichopeza longicornis
Liriomyza sativae mitochondrion, complete genome	HQ333260	78.98	99.36	1.20359e−27	Diptera	Agromyzidae	Liriomyza sativae
Simulium suyoense mitochondrial gene for 16S ribos	LC035000	80.5	100	4.20095e−27	Diptera	Simuliidae	Simulium suyoense
Simulium brinchangense isolate B2B 16S ribosomal R	KM410202	82.84	85.26	4.20095e−27	Diptera	Simuliidae	Simulium brinchangense
Simulium brinchangense isolate B1B 16S ribosomal R	KM410201	82.84	85.26	4.20095e−27	Diptera	Simuliidae	Simulium brinchangense
Simulium tanahrataense isolate TR2B 16S ribosomal	KM410196	82.84	85.26	4.20095e−27	Diptera	Simuliidae	Simulium tanahrataense
Simulium tanahrataense isolate TR1B 16S ribosomal	KM410195	82.84	85.26	4.20095e−27	Diptera	Simuliidae	Simulium tanahrataense
Simulium izuae isolate I1B 16S ribosomal RNA gene,	KM410189	82.84	85.26	4.20095e−27	Diptera	Simuliidae	Simulium izuae
Ptychoptera sp. ATB−2011 mitochondrion, complete g	JN861744	81.56	89.1	4.20095e−27	Diptera	Ptychopteridae	Ptychoptera sp. ATB−2011
Simulium chumpornense 16S ribosomal RNA gene, part	GQ865998	82.84	85.26	4.20095e−27	Diptera	Simuliidae	Simulium chumpornense
Simulium decuplum 16S ribosomal RNA gene, partial	GQ865986	82.84	85.26	4.20095e−27	Diptera	Simuliidae	Simulium decuplum
Allocotocera pulchella 16S ribosomal RNA gene, par	FJ172034	81.75	87.82	4.20095e−27	Diptera	Mycetophilidae	Allocotocera pulchella
Simulium cabrerai mitochondrial gene for 16S rRNA,	AB093128	82.84	85.26	4.20095e−27	Diptera	Simuliidae	Simulium cabrerai
Simulium palauense mitochondrial gene for 16S ribo	AB056742	83.7	85.26	4.20095e−27	Diptera	Simuliidae	Simulium palauense
Simulium mie mitochondrial gene for 16S ribosomal	AB056732	79.87	100	4.20095e−27	Diptera	Simuliidae	Simulium mie
Simulium mie mitochondrial gene for 16S ribosomal	AB056731	79.87	100	4.20095e−27	Diptera	Simuliidae	Simulium mie
Simulium mie mitochondrial gene for 16S ribosomal	AB056730	79.87	100	4.20095e−27	Diptera	Simuliidae	Simulium mie
Tongamya stuckenbergi 16S ribosomal RNA gene, part	KM878969	80	99.36	1.46628e−26	Diptera	Mydidae	Tongamya stuckenbergi
Callomyia amoena isolate CA06 16S ribosomal RNA ge	KC699325	82.73	87.82	1.46628e−26	Diptera	Platypezidae	Callomyia amoena
Hercostomus meieri voucher RMBR:103442 16S ribosom	FJ808173	84.8	79.49	1.46628e−26	Diptera	Dolichopodidae	Hercostomus meieri
Simulium tuyense mitochondrial gene for 16S riboso	LC035004	79.74	97.44	5.11781e−26	Diptera	Simuliidae	Simulium tuyense
Simulium claveriaense mitochondrial gene for 16S r	LC034988	83.2	79.49	5.11781e−26	Diptera	Simuliidae	Simulium claveriaense
Simulium claveriaense mitochondrial gene for 16S r	LC034987	83.2	79.49	5.11781e−26	Diptera	Simuliidae	Simulium claveriaense
Diadocidia stanfordensis voucher JSSJ14 16S riboso	KJ136742	79.47	91.67	5.11781e−26	Diptera	Diadocidiidae	Diadocidia stanfordensis
Simulium khunklangense mitochondrial gene for 16S	AB820367	80.82	92.31	5.11781e−26	Diptera	Simuliidae	Simulium khunklangense
Simulium chomthongense mitochondrial gene for 16S	AB699899	80.82	92.31	5.11781e−26	Diptera	Simuliidae	Simulium chomthongense
Keilbachia subacumina voucher 890 16S ribosomal RN	JQ613951	83.61	77.56	5.11781e−26	Diptera	Sciaridae	Keilbachia subacumina
Cyphomyia notabilis 16S ribosomal RNA gene, partia	GU947403	82.09	85.26	5.11781e−26	Diptera	Stratiomyidae	Cyphomyia notabilis
Simulium caudisclerum 16S ribosomal RNA gene, part	GQ865999	80.82	92.31	5.11781e−26	Diptera	Simuliidae	Simulium caudisclerum
Simulium sheilae 16S ribosomal RNA gene, partial s	GQ865996	82.09	85.26	5.11781e−26	Diptera	Simuliidae	Simulium sheilae
Simulium siamense 16S ribosomal RNA gene, partial	GQ865994	82.09	85.26	5.11781e−26	Diptera	Simuliidae	Simulium siamense
Simulium siamense 16S ribosomal RNA gene, partial	GQ865993	82.09	85.26	5.11781e−26	Diptera	Simuliidae	Simulium siamense
Simulium siamense 16S ribosomal RNA gene, partial	GQ865992	82.09	85.26	5.11781e−26	Diptera	Simuliidae	Simulium siamense
Simulium recurvum mitochondrial gene for 16S ribos	AB334097	82.09	85.26	5.11781e−26	Diptera	Simuliidae	Simulium recurvum
Simulium claveriaense mitochondrial gene for 16S r	AB334096	83.2	79.49	5.11781e−26	Diptera	Simuliidae	Simulium claveriaense
Cnephia dacotensis mitochondrial 16S ribosomal RNA	L13403	80	97.44	5.11781e−26	Diptera	Simuliidae	Cnephia dacotensis
Simulium suyoense mitochondrial gene for 16S ribos	LC034999	79.87	100	1.78629e−25	Diptera	Simuliidae	Simulium suyoense
Simulium suyoense mitochondrial gene for 16S ribos	LC034998	79.87	100	1.78629e−25	Diptera	Simuliidae	Simulium suyoense
Simulium suyoense mitochondrial gene for 16S ribos	LC034997	79.87	100	1.78629e−25	Diptera	Simuliidae	Simulium suyoense
Simulium spinosibranchium mitochondrial gene for 1	LC034996	81.62	85.26	1.78629e−25	Diptera	Simuliidae	Simulium spinosibranchium
Microsania collarti isolate CAN8 16S ribosomal RNA	KC699334	78.21	99.36	1.78629e−25	Diptera	Platypezidae	Microsania collarti
Simulium tribulatum isolate LAC 17−5 16S ribosomal	KP233212	79.25	100	1.78629e−25	Diptera	Simuliidae	Simulium tribulatum
Leptomorphus sp. DK−2013 voucher JSS1 16S ribosoma	KC435575	79.72	91.67	1.78629e−25	Diptera	Mycetophilidae	Leptomorphus sp. DK−2013
Simulium suyoense mitochondrial gene for 16S ribos	AB334099	79.87	100	1.78629e−25	Diptera	Simuliidae	Simulium suyoense
Simulium spinosibranchium mitochondrial gene for 1	AB334098	81.62	85.26	1.78629e−25	Diptera	Simuliidae	Simulium spinosibranchium
Simulium chiharuae mitochondrial gene for 16S ribo	AB334092	80	91.67	1.78629e−25	Diptera	Simuliidae	Simulium chiharuae
Simulium squamosum voucher P.Mansiangi Pm3(BNI) 16	DQ655644	79.19	92.31	1.78629e−25	Diptera	Simuliidae	Simulium squamosum
Arachnocampa luminosa biotype 2 16S large subunit	AY576330	80	94.23	1.78629e−25	Diptera	Keroplatidae	Arachnocampa luminosa
Simulium damnosum s.l. GH−D1 16S ribosomal RNA, pa	AY077709	79.19	92.31	1.78629e−25	Diptera	Simuliidae	Simulium damnosum s.l. GH−D1
Simulium damnosum s.l. Kulfo isolate Ku 3 16S ribo	AF416978	79.19	92.31	1.78629e−25	Diptera	Simuliidae	Simulium damnosum s.l. Kulfo
Simulium soubrense isolate Wa 52 16S ribosomal RNA	AF416976	79.19	92.31	1.78629e−25	Diptera	Simuliidae	Simulium soubrense
Simulium soubrense isolate Wa 47 16S ribosomal RNA	AF416975	79.19	92.31	1.78629e−25	Diptera	Simuliidae	Simulium soubrense
Simulium damnosum strain Se−12 16S ribosomal RNA g	AF081907	79.19	92.31	1.78629e−25	Diptera	Simuliidae	Simulium damnosum
Simulium sasai mitochondrial gene for 16S ribosoma	AB056734	80	91.67	1.78629e−25	Diptera	Simuliidae	Simulium sasai
Simulium feuerborni mitochondrial gene for 16S rib	AB056728	80	91.67	1.78629e−25	Diptera	Simuliidae	Simulium feuerborni
Simulium leonense mitochondrion 16S ribosomal RNA	U17731	79.19	92.31	1.78629e−25	Diptera	Simuliidae	Simulium leonense
Simulium nobile isolate N5 16S ribosomal RNA gene,	KP661530	82.4	79.49	6.23476e−25	Diptera	Simuliidae	Simulium nobile
Simulium nobile isolate N4 16S ribosomal RNA gene,	KP661529	82.4	79.49	6.23476e−25	Diptera	Simuliidae	Simulium nobile
Simulium nobile isolate N3 16S ribosomal RNA gene,	KP661528	82.4	79.49	6.23476e−25	Diptera	Simuliidae	Simulium nobile
Simulium nobile isolate N2 16S ribosomal RNA gene,	KP661527	82.4	79.49	6.23476e−25	Diptera	Simuliidae	Simulium nobile
Simulium nobile isolate N1 16S ribosomal RNA gene,	KP661526	82.4	79.49	6.23476e−25	Diptera	Simuliidae	Simulium nobile
Simulium timorense isolate TM5 16S ribosomal RNA g	KP661525	82.4	79.49	6.23476e−25	Diptera	Simuliidae	Simulium timorense
Simulium timorense isolate TM4 16S ribosomal RNA g	KP661524	82.4	79.49	6.23476e−25	Diptera	Simuliidae	Simulium timorense
Simulium timorense isolate TM3 16S ribosomal RNA g	KP661523	82.4	79.49	6.23476e−25	Diptera	Simuliidae	Simulium timorense
Simulium timorense isolate TM2 16S ribosomal RNA g	KP661522	82.4	79.49	6.23476e−25	Diptera	Simuliidae	Simulium timorense
Simulium timorense isolate TM1 16S ribosomal RNA g	KP661521	82.4	79.49	6.23476e−25	Diptera	Simuliidae	Simulium timorense
Nanexila argentiquadris 16S ribosomal RNA gene, pa	KM878934	78.34	99.36	6.23476e−25	Diptera	Therevidae	Nanexila argentiquadris
Ectinorhynchus sp. 'Bribie' voucher UQIC41178 16S	KM212983	79.11	99.36	6.23476e−25	Diptera	Therevidae	Ectinorhynchus sp. 'Bribie'
Ectinorhynchus sp. 'Moffatt' voucher QMT183203 16S	KM212976	79.11	99.36	6.23476e−25	Diptera	Therevidae	Ectinorhynchus sp. 'Moffatt'
Ectinorhynchus sp. 'warrumbungles' voucher QMT1832	KM212974	79.11	99.36	6.23476e−25	Diptera	Therevidae	Ectinorhynchus sp. 'warrumbungles'
Coboldia sp. SS−2012 voucher 905 16S ribosomal RNA	JQ613954	78.57	97.44	6.23476e−25	Diptera	Scatopsidae	Coboldia sp. SS−2012
Pachygaster atra 16S ribosomal RNA gene, partial s	GU947397	80.74	86.54	6.23476e−25	Diptera	Stratiomyidae	Pachygaster atra
