# Supplementary material for: Testing the potential of a ribosomal 16S marker for DNA metabarcoding of insects
Source: PeerJ. 2016 Apr 19;4:e1966. doi: 10.7717/peerj.1966 (PMC4841222; doi:10.7717/peerj.1966)
Supplement: Supplemental Information 1 [file peerj-04-1966-s006.zip › scripts/5 match OTUs/blast_hits/OTU_203.csv_plot.pdf]

OTU_203.csv

Parochlus steinenii

Chironomidae

Diptera

87.58

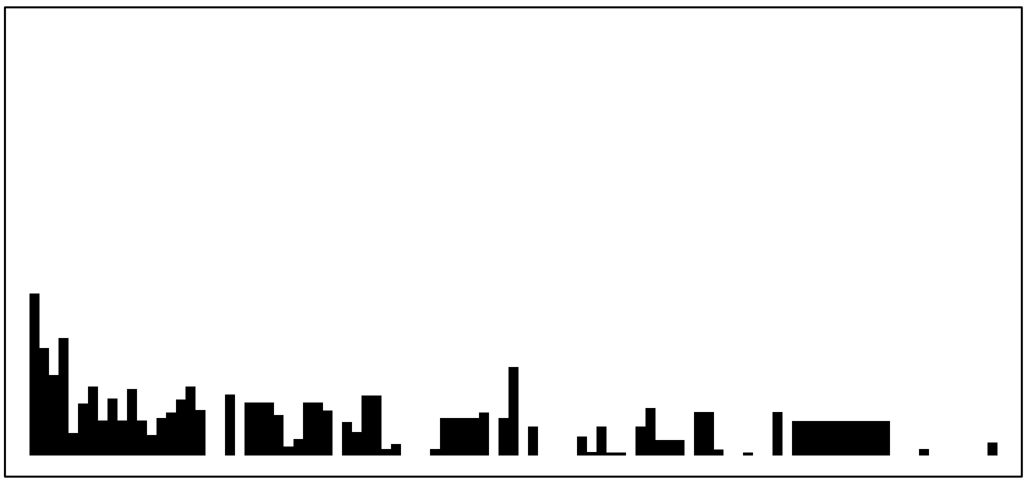

Parochlus steinenii isolate Sejong01 mitochondrion	KT003702	87.58	98.71	5.07103e−45	Diptera	Chironomidae	Parochlus steinenii
Bolitophila occlusa voucher JSB2 16S ribosomal RNA	KJ136713	85.03	94.19	8.58725e−36	Diptera	Mycetophilidae	Bolitophila occlusa
Bolitophila cinerea voucher JSB1 16S ribosomal RNA	KJ136712	83.77	97.42	1.04614e−34	Diptera	Mycetophilidae	Bolitophila cinerea
Bolitophila sp. ER−2009 16S ribosomal RNA gene, pa	FJ172021	85.51	88.39	1.04614e−34	Diptera	Mycetophilidae	Bolitophila sp. ER−2009
Edwardsina gigantea 16S ribosomal RNA gene, partia	KC177460	81.05	98.71	1.89147e−31	Diptera	Blephariceridae	Edwardsina gigantea
Agathomyia zetterstedti isolate 814 16S ribosomal	KC699312	82.43	94.84	6.60187e−31	Diptera	Platypezidae	Agathomyia zetterstedti
Bolitophila hybrida 16S ribosomal RNA gene, partia	FJ172002	83.22	91.61	6.60187e−31	Diptera	Mycetophilidae	Bolitophila hybrida
Pedicia albivitta voucher O'Grady 200012 16S ribos	EU005435	81.63	94.84	6.60187e−31	Diptera	Pediciidae	Pedicia albivitta
Arachnocampa luminosa biotype 1 16S large subunit	AY576329	82.67	94.84	6.60187e−31	Diptera	Keroplatidae	Arachnocampa luminosa
Tipula abdominalis 16S ribosomal RNA gene, partial	KC177457	81.63	94.84	2.30428e−30	Diptera	Tipulidae	Tipula abdominalis
Clinocera sp. Pollet 0249 16S ribosomal RNA gene,	HQ449032	83.1	89.68	2.30428e−30	Diptera	Empididae	Clinocera sp. Pollet 0249
Tipula abdominalis mitochondrion, partial genome	JN861743	81.63	94.84	2.30428e−30	Diptera	Tipulidae	Tipula abdominalis
Agathomyia elegantula isolate AE04 16S ribosomal R	KC699320	80.95	94.84	8.04272e−30	Diptera	Platypezidae	Agathomyia elegantula
Agathomyia lundbecki isolate 233 16S ribosomal RNA	KC699306	81.76	94.84	2.80719e−29	Diptera	Platypezidae	Agathomyia lundbecki
Tipula sp. PMO 200011 16S ribosomal RNA gene, part	EU005437	82.01	89.68	2.80719e−29	Diptera	Tipulidae	Tipula sp. PMO 200011
Arachnocampa luminosa biotype 3 16S large subunit	AY576331	82.61	88.39	3.41985e−28	Diptera	Keroplatidae	Arachnocampa luminosa
Simulium izuae isolate I1B 16S ribosomal RNA gene,	KM410189	83.21	85.81	1.19365e−27	Diptera	Simuliidae	Simulium izuae
Beris morrisii 16S ribosomal RNA gene, partial seq	GU947373	82.12	96.13	1.19365e−27	Diptera	Stratiomyidae	Beris morrisii
Geomyza tripunctata 16S ribosomal RNA gene, partia	EU268537	79.08	98.71	1.19365e−27	Diptera	Opomyzidae	Geomyza tripunctata
Agathomyia sexmaculata isolate 816 16S ribosomal R	KC699313	79.22	98.71	4.16623e−27	Diptera	Platypezidae	Agathomyia sexmaculata
Empis producta voucher RMBR:103430 16S ribosomal R	FJ808167	82.86	88.39	4.16623e−27	Diptera	Empididae	Empis producta
Somatia sp. YSUW04038 16S ribosomal RNA gene, part	AY573134	79.59	94.84	4.16623e−27	Diptera	Somatiidae	Somatia sp. YSUW04038
Simulium roslihashimi isolate R1B 16S ribosomal RN	KM410192	82.48	85.81	1.45416e−26	Diptera	Simuliidae	Simulium roslihashimi
Simulium izuae isolate I3B 16S ribosomal RNA gene,	KM410191	82.48	85.81	1.45416e−26	Diptera	Simuliidae	Simulium izuae
Simulium izuae isolate I2B 16S ribosomal RNA gene,	KM410190	82.48	85.81	1.45416e−26	Diptera	Simuliidae	Simulium izuae
Callomyia admirabilis isolate CA42 16S ribosomal R	KC699326	81.88	88.39	1.45416e−26	Diptera	Platypezidae	Callomyia admirabilis
Agathomyia viduella isolate AV01 16S ribosomal RNA	KC699317	80.41	94.84	1.45416e−26	Diptera	Platypezidae	Agathomyia viduella
Trichopeza longicornis voucher Pollet 0243 (P) 16S	HQ449035	80.77	98.71	1.45416e−26	Diptera	Empididae	Trichopeza longicornis
Simulium tuenense mitochondrial gene for 16S ribos	AB591244	82.48	85.81	1.45416e−26	Diptera	Simuliidae	Simulium tuenense
Simulium inthanonense 16S ribosomal RNA gene, part	GQ865995	82.48	85.81	1.45416e−26	Diptera	Simuliidae	Simulium inthanonense
Simulium sheilae isolate SH1B 16S ribosomal RNA ge	KM410213	82.09	85.81	5.07551e−26	Diptera	Simuliidae	Simulium sheilae
Diadocidia stanfordensis voucher JSSJ14 16S riboso	KJ136742	79.47	92.26	5.07551e−26	Diptera	Diadocidiidae	Diadocidia stanfordensis
Paracladura trichoptera mitochondrion, complete ge	JN861751	81.56	87.1	5.07551e−26	Diptera	Trichoceridae	Paracladura trichoptera
Caenophthalmus capensis voucher MEI 120162 16S rib	DQ250838	81.08	92.9	5.07551e−26	Diptera	Therevidae	Caenophthalmus capensis
Simulium tenederoi mitochondrial gene for 16S ribo	LC035002	82.81	80	1.77153e−25	Diptera	Simuliidae	Simulium tenederoi
Simulium tenederoi mitochondrial gene for 16S ribo	LC035001	82.81	80	1.77153e−25	Diptera	Simuliidae	Simulium tenederoi
Platypezina diversa isolate PD29 16S ribosomal RNA	KC699349	80.29	88.39	1.77153e−25	Diptera	Platypezidae	Platypezina diversa
Agathomyia woodella isolate 448 16S ribosomal RNA	KC699307	80.54	94.19	1.77153e−25	Diptera	Platypezidae	Agathomyia woodella
Sylvicola fenestralis 16S ribosomal RNA gene, part	KC177461	77.71	98.71	1.77153e−25	Diptera	Anisopodidae	Sylvicola fenestralis
Sylvicola fenestralis mitochondrion, complete geno	JN861752	77.71	98.71	1.77153e−25	Diptera	Anisopodidae	Sylvicola fenestralis
Arachnocampa luminosa biotype 2 16S large subunit	AY576330	80	94.84	1.77153e−25	Diptera	Keroplatidae	Arachnocampa luminosa
Coelopa alluaudi 16S ribosomal RNA gene, partial s	AF403455	80.29	88.39	1.77153e−25	Diptera	Coelopidae	Coelopa alluaudi
Simulium brinchangense isolate B2B 16S ribosomal R	KM410202	81.75	85.81	6.18324e−25	Diptera	Simuliidae	Simulium brinchangense
Simulium brinchangense isolate B1B 16S ribosomal R	KM410201	81.75	85.81	6.18324e−25	Diptera	Simuliidae	Simulium brinchangense
Simulium tanahrataense isolate TR2B 16S ribosomal	KM410196	81.75	85.81	6.18324e−25	Diptera	Simuliidae	Simulium tanahrataense
Simulium tanahrataense isolate TR1B 16S ribosomal	KM410195	81.75	85.81	6.18324e−25	Diptera	Simuliidae	Simulium tanahrataense
Callomyia amoena isolate CA06 16S ribosomal RNA ge	KC699325	82.01	88.39	6.18324e−25	Diptera	Platypezidae	Callomyia amoena
Zygoneura bidens voucher 312 16S ribosomal RNA gen	JQ613910	78.71	97.42	6.18324e−25	Diptera	Sciaridae	Zygoneura bidens
Simulium chumpornense 16S ribosomal RNA gene, part	GQ865998	81.75	85.81	6.18324e−25	Diptera	Simuliidae	Simulium chumpornense
Hercostomus meieri voucher RMBR:103442 16S ribosom	FJ808173	84.13	80	6.18324e−25	Diptera	Dolichopodidae	Hercostomus meieri
Simulium suyoense mitochondrial gene for 16S ribos	LC035000	79.87	95.48	2.15816e−24	Diptera	Simuliidae	Simulium suyoense
Simulium sheilae isolate SH2B 16S ribosomal RNA ge	KM410214	81.34	85.81	2.15816e−24	Diptera	Simuliidae	Simulium sheilae
Grossoseta pacifica isolate 1384 16S ribosomal RNA	KC699316	79.56	88.39	2.15816e−24	Diptera	Platypezidae	Grossoseta pacifica
Ectinorhynchus sp. 'Bribie' voucher UQIC41178 16S	KM212983	80	91.61	2.15816e−24	Diptera	Therevidae	Ectinorhynchus sp. 'Bribie'
Ectinorhynchus sp. 'Moffatt' voucher QMT183203 16S	KM212976	80	91.61	2.15816e−24	Diptera	Therevidae	Ectinorhynchus sp. 'Moffatt'
Ectinorhynchus sp. 'warrumbungles' voucher QMT1832	KM212974	80	91.61	2.15816e−24	Diptera	Therevidae	Ectinorhynchus sp. 'warrumbungles'
Dichopygina aculeata voucher 947 16S ribosomal RNA	JQ613961	80.88	87.1	2.15816e−24	Diptera	Sciaridae	Dichopygina aculeata
Ptychoptera sp. ATB−2011 mitochondrion, complete g	JN861744	80.14	89.68	2.15816e−24	Diptera	Ptychopteridae	Ptychoptera sp. ATB−2011
Simulium decuplum 16S ribosomal RNA gene, partial	GQ865986	81.34	85.81	2.15816e−24	Diptera	Simuliidae	Simulium decuplum
Drosophila rubida voucher 109400 NADH dehydrogenas	EU494327	80.13	92.9	2.15816e−24	Diptera	Drosophilidae	Drosophila rubida
Drosophila hypocausta voucher 103972 NADH dehydrog	EU494319	80.13	92.9	2.15816e−24	Diptera	Drosophilidae	Drosophila hypocausta
Dialysis sp. SLW−2002 16S ribosomal RNA gene, part	AY140888	78.87	91.61	2.15816e−24	Diptera	Xylophagidae	Dialysis sp. SLW−2002
Simulium cabrerai mitochondrial gene for 16S rRNA,	AB093128	81.34	85.81	2.15816e−24	Diptera	Simuliidae	Simulium cabrerai
Simulium palauense mitochondrial gene for 16S ribo	AB056742	82.22	85.81	2.15816e−24	Diptera	Simuliidae	Simulium palauense
Simulium mie mitochondrial gene for 16S ribosomal	AB056732	80.71	87.1	2.15816e−24	Diptera	Simuliidae	Simulium mie
Simulium mie mitochondrial gene for 16S ribosomal	AB056731	80.71	87.1	2.15816e−24	Diptera	Simuliidae	Simulium mie
Simulium mie mitochondrial gene for 16S ribosomal	AB056730	80.71	87.1	2.15816e−24	Diptera	Simuliidae	Simulium mie
Simulium tuyense mitochondrial gene for 16S riboso	LC035004	78.85	98.06	7.53272e−24	Diptera	Simuliidae	Simulium tuyense
Simulium claveriaense mitochondrial gene for 16S r	LC034988	82.03	80	7.53272e−24	Diptera	Simuliidae	Simulium claveriaense
Simulium claveriaense mitochondrial gene for 16S r	LC034987	82.03	80	7.53272e−24	Diptera	Simuliidae	Simulium claveriaense
Tongamya stuckenbergi 16S ribosomal RNA gene, part	KM878969	80.27	93.55	7.53272e−24	Diptera	Mydidae	Tongamya stuckenbergi
Protoclythia rufa isolate PR22 16S ribosomal RNA g	KC699354	78.52	94.84	7.53272e−24	Diptera	Platypezidae	Protoclythia rufa
Protoclythia modesta isolate PM21 16S ribosomal RN	KC699350	78.52	94.84	7.53272e−24	Diptera	Platypezidae	Protoclythia modesta
Simulium tribulatum isolate LAC 17−5 16S ribosomal	KP233212	80.14	92.26	7.53272e−24	Diptera	Simuliidae	Simulium tribulatum
Liriomyza sativae mitochondrion, complete genome	JQ862475	77.42	98.71	7.53272e−24	Diptera	Agromyzidae	Liriomyza sativae
Liriomyza sativae mitochondrion, complete genome	HQ333260	77.42	98.71	7.53272e−24	Diptera	Agromyzidae	Liriomyza sativae
Simulium claveriaense mitochondrial gene for 16S r	AB334096	82.03	80	7.53272e−24	Diptera	Simuliidae	Simulium claveriaense
Protoclythia modesta 16S ribosomal RNA gene, parti	AF126348	78.52	94.84	7.53272e−24	Diptera	Platypezidae	Protoclythia modesta
Simulium nobile isolate N5 16S ribosomal RNA gene,	KP661530	81.6	80	2.62918e−23	Diptera	Simuliidae	Simulium nobile
Simulium nobile isolate N4 16S ribosomal RNA gene,	KP661529	81.6	80	2.62918e−23	Diptera	Simuliidae	Simulium nobile
Simulium nobile isolate N3 16S ribosomal RNA gene,	KP661528	81.6	80	2.62918e−23	Diptera	Simuliidae	Simulium nobile
Simulium nobile isolate N2 16S ribosomal RNA gene,	KP661527	81.6	80	2.62918e−23	Diptera	Simuliidae	Simulium nobile
Simulium nobile isolate N1 16S ribosomal RNA gene,	KP661526	81.6	80	2.62918e−23	Diptera	Simuliidae	Simulium nobile
Simulium timorense isolate TM5 16S ribosomal RNA g	KP661525	81.6	80	2.62918e−23	Diptera	Simuliidae	Simulium timorense
Simulium timorense isolate TM4 16S ribosomal RNA g	KP661524	81.6	80	2.62918e−23	Diptera	Simuliidae	Simulium timorense
Simulium timorense isolate TM3 16S ribosomal RNA g	KP661523	81.6	80	2.62918e−23	Diptera	Simuliidae	Simulium timorense
Simulium timorense isolate TM2 16S ribosomal RNA g	KP661522	81.6	80	2.62918e−23	Diptera	Simuliidae	Simulium timorense
Simulium timorense isolate TM1 16S ribosomal RNA g	KP661521	81.6	80	2.62918e−23	Diptera	Simuliidae	Simulium timorense
Chydaeopeza tibialis isolate CT44 16S ribosomal RN	KC699336	77.92	98.71	2.62918e−23	Diptera	Platypezidae	Chydaeopeza tibialis
Agathomyia falleni isolate AF03 16S ribosomal RNA	KC699319	77.12	98.71	2.62918e−23	Diptera	Platypezidae	Agathomyia falleni
Haematobia irritans mitochondrion, partial genome	KM669714	77.92	98.71	2.62918e−23	Diptera	Muscidae	Haematobia irritans
Ectinorhynchus brunneus voucher QMT183204 16S ribo	KM212973	80.29	87.74	2.62918e−23	Diptera	Therevidae	Ectinorhynchus brunneus
Simulium khunklangense mitochondrial gene for 16S	AB820367	79.45	92.9	2.62918e−23	Diptera	Simuliidae	Simulium khunklangense
Simulium chomthongense mitochondrial gene for 16S	AB699899	79.45	92.9	2.62918e−23	Diptera	Simuliidae	Simulium chomthongense
Coboldia sp. SS−2012 voucher 905 16S ribosomal RNA	JQ613954	77.78	97.42	2.62918e−23	Diptera	Scatopsidae	Coboldia sp. SS−2012
Haematopota pluvialis 16S ribosomal RNA gene, part	KC177454	78.23	94.84	2.62918e−23	Diptera	Tabanidae	Haematopota pluvialis
Haematobia irritans isolate D50 small subunit ribo	JQ246755	77.92	98.71	2.62918e−23	Diptera	Muscidae	Haematobia irritans
Cramptonomyia spenceri mitochondrion, complete gen	JN861747	79.61	95.48	2.62918e−23	Diptera	Pachyneuridae	Cramptonomyia spenceri
Cyphomyia notabilis 16S ribosomal RNA gene, partia	GU947403	80.6	85.81	2.62918e−23	Diptera	Stratiomyidae	Cyphomyia notabilis
Pachygaster atra 16S ribosomal RNA gene, partial s	GU947397	80	87.1	2.62918e−23	Diptera	Stratiomyidae	Pachygaster atra
