# Supplementary material for: Testing the potential of a ribosomal 16S marker for DNA metabarcoding of insects
Source: PeerJ. 2016 Apr 19;4:e1966. doi: 10.7717/peerj.1966 (PMC4841222; doi:10.7717/peerj.1966)
Supplement: Supplemental Information 1 [file peerj-04-1966-s006.zip › scripts/5 match OTUs/blast_hits/OTU_21.csv_plot.pdf]

OTU_21.csv

Protonemura meyeri

Nemouridae

Plecoptera

91.72

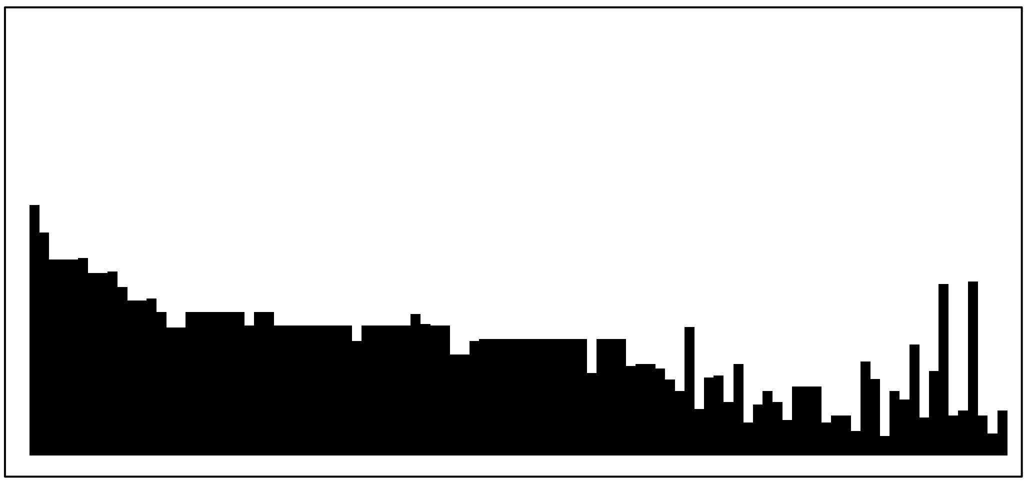

Protonemura meyeri 16S ribosomal RNA gene, partial	EF623193	91.72	100	2.49908e−55	Plecoptera	Nemouridae	Protonemura meyeri
Nanonemoura wahkeena 16S ribosomal RNA gene, parti	EF623198	90.45	100	1.29456e−52	Plecoptera	Nemouridae	Nanonemoura wahkeena
Ostrocerca foersteri 16S ribosomal RNA gene, parti	EF623191	89.17	100	6.70597e−50	Plecoptera	Nemouridae	Ostrocerca foersteri
Visoka cataractae 16S ribosomal RNA gene, partial	EF623189	89.17	100	6.70597e−50	Plecoptera	Nemouridae	Visoka cataractae
Shipsa rotunda 16S ribosomal RNA gene, partial seq	EF623184	89.17	100	6.70597e−50	Plecoptera	Nemouridae	Shipsa rotunda
Scopura montana 16S ribosomal RNA gene, partial se	EF623265	89.24	100	2.34061e−49	Plecoptera		Scopura montana
Nemoura trispinosa 16S ribosomal RNA gene, partial	EF623187	88.54	100	8.16955e−49	Plecoptera	Nemouridae	Nemoura trispinosa
Paranemoura claasseni 16S ribosomal RNA gene, part	EF623185	88.54	100	8.16955e−49	Plecoptera	Nemouridae	Paranemoura claasseni
Nemoura cinerea 16S ribosomal RNA gene, partial se	EF623194	88.61	100	2.85145e−48	Plecoptera	Nemouridae	Nemoura cinerea
Nemurella pictetii 16S ribosomal RNA gene, partial	EF623192	87.9	100	3.47378e−47	Plecoptera	Nemouridae	Nemurella pictetii
Lednia tumana 16S ribosomal RNA gene, partial sequ	EF623197	87.26	100	4.23193e−46	Plecoptera	Nemouridae	Lednia tumana
Prostoia besametsa 16S ribosomal RNA gene, partial	EF623188	87.26	100	4.23193e−46	Plecoptera	Nemouridae	Prostoia besametsa
Oemopteryx vanduzeea 16S ribosomal RNA gene, parti	EF623266	87.34	100	1.47709e−45	Plecoptera	Taeniopterygidae	Oemopteryx vanduzeea
Doddsia occidentalis 16S ribosomal RNA gene, parti	EF623270	86.71	100	6.28074e−44	Plecoptera	Taeniopterygidae	Doddsia occidentalis
Zapada cinctipes 16S ribosomal RNA gene, partial s	EF623183	85.99	100	2.19219e−43	Plecoptera	Nemouridae	Zapada cinctipes
Malenka californica 16S ribosomal RNA gene, partia	EF623182	85.99	100	2.19219e−43	Plecoptera	Nemouridae	Malenka californica
Mesocapnia arizonensis mitochondrion, partial geno	KP642637	86.71	100	7.65151e−43	Plecoptera	Capniidae	Mesocapnia arizonensis
Allocapnia recta isolate H13 16S ribosomal RNA gen	KC881048	86.71	100	7.65151e−43	Plecoptera	Capniidae	Allocapnia recta
Allocapnia recta isolate H11 16S ribosomal RNA gen	KC881046	86.71	100	7.65151e−43	Plecoptera	Capniidae	Allocapnia recta
Allocapnia recta isolate H7 16S ribosomal RNA gene	KC881042	86.71	100	7.65151e−43	Plecoptera	Capniidae	Allocapnia recta
Allocapnia recta isolate H3 16S ribosomal RNA gene	KC881038	86.71	100	7.65151e−43	Plecoptera	Capniidae	Allocapnia recta
Allocapnia recta isolate H1 16S ribosomal RNA gene	KC881036	86.71	100	7.65151e−43	Plecoptera	Capniidae	Allocapnia recta
Megaleuctra kincaidi 16S ribosomal RNA gene, parti	EF623174	86.08	100	7.65151e−43	Plecoptera	Leuctridae	Megaleuctra kincaidi
Mesocapnia sp. BYU_PL010 16S ribosomal RNA gene, p	EF623127	86.71	100	7.65151e−43	Plecoptera	Capniidae	Mesocapnia sp. BYU_PL010
Allocapnia recta haplotype 3ar: H03 16S ribosomal	DQ915181	86.71	100	7.65151e−43	Plecoptera	Capniidae	Allocapnia recta
Apteroperla tikumana mitochondrion, complete genom	KR604721	86.08	100	9.32145e−42	Plecoptera	Capniidae	Apteroperla tikumana
Allocapnia recta isolate H19 16S ribosomal RNA gen	KC881054	86.08	100	9.32145e−42	Plecoptera	Capniidae	Allocapnia recta
Allocapnia recta isolate H16 16S ribosomal RNA gen	KC881051	86.08	100	9.32145e−42	Plecoptera	Capniidae	Allocapnia recta
Allocapnia recta isolate H15 16S ribosomal RNA gen	KC881050	86.08	100	9.32145e−42	Plecoptera	Capniidae	Allocapnia recta
Allocapnia recta isolate H14 16S ribosomal RNA gen	KC881049	86.08	100	9.32145e−42	Plecoptera	Capniidae	Allocapnia recta
Allocapnia recta isolate H10 16S ribosomal RNA gen	KC881045	86.08	100	9.32145e−42	Plecoptera	Capniidae	Allocapnia recta
Allocapnia recta isolate H6 16S ribosomal RNA gene	KC881041	86.08	100	9.32145e−42	Plecoptera	Capniidae	Allocapnia recta
Notonemouridae sp. MD−2011 voucher ZMUC:JD−1507 16	JN615257	86.08	100	9.32145e−42	Plecoptera	Notonemouridae	Notonemouridae sp. MD−2011
Amphinemura sulcicollis 16S ribosomal RNA gene, pa	EF623195	85.35	100	9.32145e−42	Plecoptera	Nemouridae	Amphinemura sulcicollis
Allocapnia minima 16S ribosomal RNA gene, partial	EF623130	86.08	100	9.32145e−42	Plecoptera	Capniidae	Allocapnia minima
Allocapnia recta haplotype 12ar: H12 16S ribosomal	EU306875	86.08	100	9.32145e−42	Plecoptera	Capniidae	Allocapnia recta
Allocapnia recta haplotype 11ar: H011 16S ribosoma	EU306874	86.08	100	9.32145e−42	Plecoptera	Capniidae	Allocapnia recta
Allocapnia recta haplotype 7ar: H07 16S ribosomal	EU306870	86.08	100	9.32145e−42	Plecoptera	Capniidae	Allocapnia recta
Allocapnia recta haplotype 2ar: H02 16S ribosomal	DQ915180	86.08	100	9.32145e−42	Plecoptera	Capniidae	Allocapnia recta
Moselia infuscata 16S ribosomal RNA gene, partial	EF623175	86.62	99.36	3.2535e−41	Plecoptera	Leuctridae	Moselia infuscata
Thaumatoperla sp. BYU_PL109 16S ribosomal RNA gene	EF623149	86.16	100	3.2535e−41	Plecoptera	Eustheniidae	Thaumatoperla sp. BYU_PL109
Pteronarcys sachalina 16S ribosomal RNA gene, part	EF623264	86.08	100	1.13558e−40	Plecoptera	Pteronarcyidae	Pteronarcys sachalina
Pteronarcys californica 16S ribosomal RNA gene, pa	EF623261	86.08	100	1.13558e−40	Plecoptera	Pteronarcyidae	Pteronarcys californica
Austrocerca rieka 16S ribosomal RNA gene, partial	EF623205	84.71	100	1.13558e−40	Plecoptera	Notonemouridae	Austrocerca rieka
Austrocercoides zwicki 16S ribosomal RNA gene, par	EF623199	84.71	100	1.13558e−40	Plecoptera	Notonemouridae	Austrocercoides zwicki
Soyedina producta 16S ribosomal RNA gene, partial	EF623196	85.35	100	1.13558e−40	Plecoptera	Nemouridae	Soyedina producta
Allocapnia recta isolate H18 16S ribosomal RNA gen	KC881053	85.44	100	3.96358e−40	Plecoptera	Capniidae	Allocapnia recta
Allocapnia recta isolate H17 16S ribosomal RNA gen	KC881052	85.44	100	3.96358e−40	Plecoptera	Capniidae	Allocapnia recta
Allocapnia recta isolate H12 16S ribosomal RNA gen	KC881047	85.44	100	3.96358e−40	Plecoptera	Capniidae	Allocapnia recta
Allocapnia recta isolate H9 16S ribosomal RNA gene	KC881044	85.44	100	3.96358e−40	Plecoptera	Capniidae	Allocapnia recta
Allocapnia recta isolate H8 16S ribosomal RNA gene	KC881043	85.44	100	3.96358e−40	Plecoptera	Capniidae	Allocapnia recta
Allocapnia recta isolate H5 16S ribosomal RNA gene	KC881040	85.44	100	3.96358e−40	Plecoptera	Capniidae	Allocapnia recta
Allocapnia recta isolate H4 16S ribosomal RNA gene	KC881039	85.44	100	3.96358e−40	Plecoptera	Capniidae	Allocapnia recta
Allocapnia recta haplotype 10ar: H010 16S ribosoma	EU306873	85.44	100	3.96358e−40	Plecoptera	Capniidae	Allocapnia recta
Allocapnia recta haplotype 9ar: H09 16S ribosomal	EU306872	85.44	100	3.96358e−40	Plecoptera	Capniidae	Allocapnia recta
Allocapnia recta haplotype 8ar: H08 16S ribosomal	EU306871	85.44	100	3.96358e−40	Plecoptera	Capniidae	Allocapnia recta
Allocapnia recta haplotype 1ar: H01 16S ribosomal	DQ915179	85.44	100	3.96358e−40	Plecoptera	Capniidae	Allocapnia recta
Strophopteryx appalachia 16S ribosomal RNA gene, p	EF623271	83.85	100	4.82863e−39	Plecoptera	Taeniopterygidae	Strophopteryx appalachia
Pteronarcys scotti 16S ribosomal RNA gene, partial	EF623263	85.44	100	4.82863e−39	Plecoptera	Pteronarcyidae	Pteronarcys scotti
Capnura wanica 16S ribosomal RNA gene, partial seq	EF623128	85.44	100	4.82863e−39	Plecoptera	Capniidae	Capnura wanica
Pteronarcys princeps mitochondrion, complete genom	AY687866	85.44	100	4.82863e−39	Plecoptera	Pteronarcyidae	Pteronarcys princeps
Bolotoperla rossi 16S ribosomal RNA gene, partial	EF623269	84.18	100	1.68536e−38	Plecoptera	Taeniopterygidae	Bolotoperla rossi
Brachyptera seticornis 16S ribosomal RNA gene, par	EF623268	84.28	100	1.68536e−38	Plecoptera	Taeniopterygidae	Brachyptera seticornis
Taenionema palladium 16S ribosomal RNA gene, parti	EF623267	84.28	100	5.88247e−38	Plecoptera	Taeniopterygidae	Taenionema palladium
Osmylus sp. EH−2004 16S ribosomal RNA gene, partia	AY620146	84.08	100	7.16632e−37	Neuroptera	Osmylidae	Osmylus sp. EH−2004
Eusthenia costalis 16S ribosomal RNA gene, partial	EF623150	83.54	100	2.50129e−36	Plecoptera	Eustheniidae	Eusthenia costalis
Paraleuctra occidentalis 16S ribosomal RNA gene, p	EF623178	83.02	100	8.73037e−36	Plecoptera	Leuctridae	Paraleuctra occidentalis
Triznaka pintada 16S ribosomal RNA gene, partial s	EF623134	86.01	89.17	8.73037e−36	Plecoptera	Chloroperlidae	Triznaka pintada
Amphinemura nigritta 16S ribosomal RNA gene, parti	EF623186	82.17	100	3.0472e−35	Plecoptera	Nemouridae	Amphinemura nigritta
Frisonia picticeps 16S ribosomal RNA gene, partial	EF623227	83.65	100	1.06358e−34	Plecoptera	Perlodidae	Frisonia picticeps
Cosumnoperla hypocrena 16S ribosomal RNA gene, par	EF623226	83.75	100	3.71225e−34	Plecoptera	Perlodidae	Cosumnoperla hypocrena
Antarctoperla sp. BYU_PL142 16S ribosomal RNA gene	EF623163	82.5	100	3.71225e−34	Plecoptera	Gripopterygidae	Antarctoperla sp. BYU_PL142
Kathroperla perdita 16S ribosomal RNA gene, partia	EF623135	84.29	89.17	3.71225e−34	Plecoptera	Chloroperlidae	Kathroperla perdita
Udamocercia sp. BYU_PL150 16S ribosomal RNA gene,	EF623201	81.53	100	1.2957e−33	Plecoptera	Notonemouridae	Udamocercia sp. BYU_PL150
Neopanorpa sp. M24899 16S ribosomal RNA gene, part	AF180059	82.39	100	1.2957e−33	Mecoptera	Panorpidae	Neopanorpa sp. M24899
Salmoperla sylvanica 16S ribosomal RNA gene, parti	EF623222	83.02	100	4.52244e−33	Plecoptera	Perlodidae	Salmoperla sylvanica
Claudioperla sp. BYU_PL158 16S ribosomal RNA gene,	EF623169	82.5	100	4.52244e−33	Plecoptera	Gripopterygidae	Claudioperla sp. BYU_PL158
Suwallia teleckojensis 16S ribosomal RNA gene, par	EF623142	81.65	100	4.52244e−33	Plecoptera	Chloroperlidae	Suwallia teleckojensis
Cryptoperla japonica mitochondrial gene for 16S rR	AB711184	83.23	100	1.57849e−32	Plecoptera	Peltoperlidae	Cryptoperla japonica
Cryptoperla japonica mitochondrial gene for 16S rR	AB711183	83.23	100	1.57849e−32	Plecoptera	Peltoperlidae	Cryptoperla japonica
Cryptoperla japonica mitochondrial gene for 16S rR	AB711182	83.23	100	1.57849e−32	Plecoptera	Peltoperlidae	Cryptoperla japonica
Neonemura sp. BYU_PL153 16S ribosomal RNA gene, pa	EF623204	81.53	100	1.57849e−32	Plecoptera	Notonemouridae	Neonemura sp. BYU_PL153
Pelurgoperla personata 16S ribosomal RNA gene, par	EF623168	81.88	100	1.57849e−32	Plecoptera	Gripopterygidae	Pelurgoperla personata
Leptoperla sp. BYU_PL107 16S ribosomal RNA gene, p	EF623161	81.88	100	1.57849e−32	Plecoptera	Gripopterygidae	Leptoperla sp. BYU_PL107
Diamphipnopsis sp. BYU_PL138 16S ribosomal RNA gen	EF623145	81.13	100	1.57849e−32	Plecoptera	Diamphipnoidae	Diamphipnopsis sp. BYU_PL138
Neaviperla forcipata 16S ribosomal RNA gene, parti	EF623139	84.4	89.17	1.57849e−32	Plecoptera	Chloroperlidae	Neaviperla forcipata
Plumiperla diversa 16S ribosomal RNA gene, partial	EF623132	83.57	89.17	1.57849e−32	Plecoptera	Chloroperlidae	Plumiperla diversa
Isocapnia hyalita 16S ribosomal RNA gene, partial	EF623131	80.89	100	1.57849e−32	Plecoptera	Capniidae	Isocapnia hyalita
Cascadoperla trictura 16S ribosomal RNA gene, part	EF623225	83.02	100	5.50946e−32	Plecoptera	Perlodidae	Cascadoperla trictura
Leuctra inermis 16S ribosomal RNA gene, partial se	EF623176	82.61	100	5.50946e−32	Plecoptera	Leuctridae	Leuctra inermis
Notoperla sp. BYU_PL146 16S ribosomal RNA gene, pa	EF623167	85.19	85.35	5.50946e−32	Plecoptera	Gripopterygidae	Notoperla sp. BYU_PL146
Neuroperlopsis patris 16S ribosomal RNA gene, part	EF623152	81.76	100	5.50946e−32	Plecoptera	Eustheniidae	Neuroperlopsis patris
Yoraperla uenoi mitochondrial gene for 16S rRNA, p	AB712375	83.94	87.26	1.92299e−31	Plecoptera	Peltoperlidae	Yoraperla uenoi
Cryptoperla japonica mitochondrial gene for 16S rR	AB711202	88.03	74.52	1.92299e−31	Plecoptera	Peltoperlidae	Cryptoperla japonica
Perlinodes aurea 16S ribosomal RNA gene, partial s	EF623234	81.88	100	1.92299e−31	Plecoptera	Perlodidae	Perlinodes aurea
Cryptoperla sp. YK−2012−1 mitochondrial gene for 1	AB711243	82.1	100	6.7119e−31	Plecoptera	Peltoperlidae	Cryptoperla sp. YK−2012−1
Cerconychia sp. BYU_PL114 16S ribosomal RNA gene,	EF623260	88.14	71.97	6.7119e−31	Plecoptera	Styloperlidae	Cerconychia sp. BYU_PL114
Soliperla campanula 16S ribosomal RNA gene, partia	EF623256	81.88	99.36	6.7119e−31	Plecoptera	Peltoperlidae	Soliperla campanula
Baumanella alameda 16S ribosomal RNA gene, partial	EF623236	81.01	100	6.7119e−31	Plecoptera	Perlodidae	Baumanella alameda
Skwala americana 16S ribosomal RNA gene, partial s	EF623217	82.1	100	6.7119e−31	Plecoptera	Perlodidae	Skwala americana
