# Supplementary material for: Testing the potential of a ribosomal 16S marker for DNA metabarcoding of insects
Source: PeerJ. 2016 Apr 19;4:e1966. doi: 10.7717/peerj.1966 (PMC4841222; doi:10.7717/peerj.1966)
Supplement: Supplemental Information 1 [file peerj-04-1966-s006.zip › scripts/5 match OTUs/blast_hits/OTU_22.csv_plot.pdf]

OTU_22.csv

Hansonoperla appalachia

Perlidae

Plecoptera

88.35

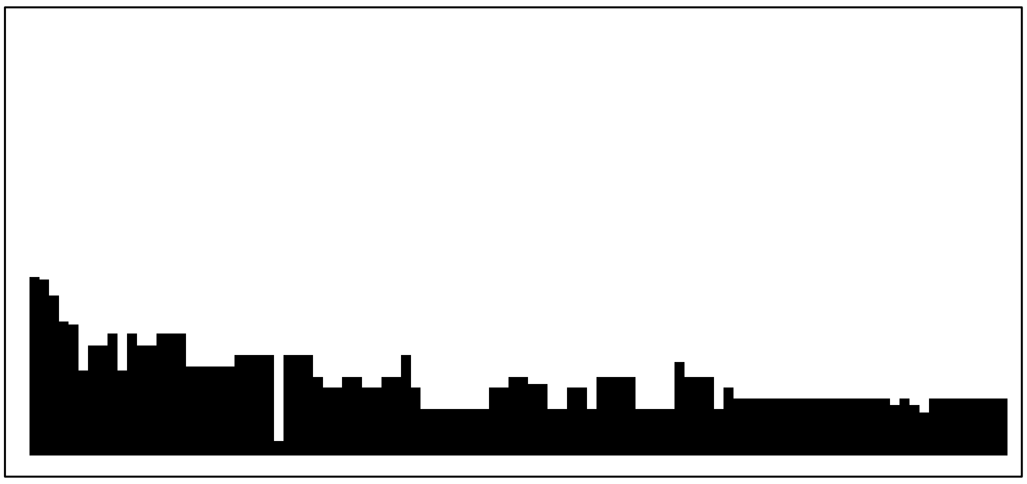

Hansonoperla appalachia 16S ribosomal RNA gene, pa	EF623249	88.35	62.35	1.53898e−26	Plecoptera	Perlidae	Hansonoperla appalachia
Eccoptura xanthenes 16S ribosomal RNA gene, partia	EF623252	88.24	62.35	5.37158e−26	Plecoptera	Perlidae	Eccoptura xanthenes
Acroneuria lycorias 16S ribosomal RNA gene, partia	EF623251	87.5	62.35	5.37158e−26	Plecoptera	Perlidae	Acroneuria lycorias
Perlinella drymo 16S ribosomal RNA gene, partial s	EF623240	86.27	62.35	2.78255e−23	Plecoptera	Perlidae	Perlinella drymo
Calineuria californica 16S ribosomal RNA gene, par	EF623239	86.14	62.35	9.71205e−23	Plecoptera	Perlidae	Calineuria californica
Dinocras cephalotes mitochondrion, complete genome	KF484757	83.96	62.35	3.38984e−22	Plecoptera	Perlidae	Dinocras cephalotes
Notonemouridae sp. MD−2011 voucher ZMUC:JD−1507 16	JN615257	85.15	62.35	3.38984e−22	Plecoptera	Notonemouridae	Notonemouridae sp. MD−2011
Dolichopoda pavesii voucher SPI 16S ribosomal RNA	EF216951	85.15	62.35	3.38984e−22	Orthoptera	Rhaphidophoridae	Dolichopoda pavesii
Scopura montana 16S ribosomal RNA gene, partial se	EF623265	85.71	60.49	3.38984e−22	Plecoptera		Scopura montana
Dinocras cephalotes 16S ribosomal RNA gene, partia	EF623244	83.96	62.35	3.38984e−22	Plecoptera	Perlidae	Dinocras cephalotes
Agnetina capitata 16S ribosomal RNA gene, partial	EF623241	85.71	60.49	3.38984e−22	Plecoptera	Perlidae	Agnetina capitata
Nemoura cinerea 16S ribosomal RNA gene, partial se	EF623194	85.15	62.35	3.38984e−22	Plecoptera	Nemouridae	Nemoura cinerea
Megaleuctra kincaidi 16S ribosomal RNA gene, parti	EF623174	85.15	62.35	3.38984e−22	Plecoptera	Leuctridae	Megaleuctra kincaidi
Cryptoperla japonica mitochondrial gene for 16S rR	AB711202	85.71	60.49	4.12967e−21	Plecoptera	Peltoperlidae	Cryptoperla japonica
Cryptoperla japonica mitochondrial gene for 16S rR	AB711184	85.71	60.49	4.12967e−21	Plecoptera	Peltoperlidae	Cryptoperla japonica
Pteronarcys sachalina 16S ribosomal RNA gene, part	EF623264	85.71	60.49	4.12967e−21	Plecoptera	Pteronarcyidae	Pteronarcys sachalina
Pictetoperla sp. BYU_PL156 16S ribosomal RNA gene,	EF623247	84.16	62.35	4.12967e−21	Plecoptera	Perlidae	Pictetoperla sp. BYU_PL156
Prostoia besametsa 16S ribosomal RNA gene, partial	EF623188	84.16	62.35	4.12967e−21	Plecoptera	Nemouridae	Prostoia besametsa
Nemoura trispinosa 16S ribosomal RNA gene, partial	EF623187	84.16	62.35	4.12967e−21	Plecoptera	Nemouridae	Nemoura trispinosa
Paranemoura claasseni 16S ribosomal RNA gene, part	EF623185	84.16	62.35	4.12967e−21	Plecoptera	Nemouridae	Paranemoura claasseni
Shipsa rotunda 16S ribosomal RNA gene, partial seq	EF623184	84.16	62.35	4.12967e−21	Plecoptera	Nemouridae	Shipsa rotunda
Apteroperla tikumana mitochondrion, complete genom	KR604721	84.69	60.49	1.4414e−20	Plecoptera	Capniidae	Apteroperla tikumana
Dolichopoda sp. MR−2008 16S ribosomal RNA gene, pa	EU887863	84.69	60.49	1.4414e−20	Orthoptera	Rhaphidophoridae	Dolichopoda lustriae
Nemurella pictetii 16S ribosomal RNA gene, partial	EF623192	84.69	60.49	1.4414e−20	Plecoptera	Nemouridae	Nemurella pictetii
Malenka californica 16S ribosomal RNA gene, partia	EF623182	84.69	60.49	1.4414e−20	Plecoptera	Nemouridae	Malenka californica
Brachyptera seticornis 16S ribosomal RNA gene, par	EF623268	80.67	73.46	5.03097e−20	Plecoptera	Taeniopterygidae	Brachyptera seticornis
Cryptoperla japonica mitochondrial gene for 16S rR	AB711183	84.69	60.49	1.75598e−19	Plecoptera	Peltoperlidae	Cryptoperla japonica
Cryptoperla japonica mitochondrial gene for 16S rR	AB711182	84.69	60.49	1.75598e−19	Plecoptera	Peltoperlidae	Cryptoperla japonica
Pachyrhamma edwardsii voucher CW122 16S ribosomal	HM594508	84.69	60.49	1.75598e−19	Orthoptera	Rhaphidophoridae	Pachyrhamma edwardsii
Dolichopoda steriotisi voucher ANT 16S ribosomal R	EF216955	83.67	60.49	1.75598e−19	Orthoptera	Rhaphidophoridae	Dolichopoda steriotisi
Doddsia occidentalis 16S ribosomal RNA gene, parti	EF623270	83.17	62.35	1.75598e−19	Plecoptera	Taeniopterygidae	Doddsia occidentalis
Pteronarcella badia 16S ribosomal RNA gene, partia	EF623262	83.17	62.35	1.75598e−19	Plecoptera	Pteronarcyidae	Pteronarcella badia
Hesperoperla pacifica 16S ribosomal RNA gene, part	EF623238	83.67	60.49	1.75598e−19	Plecoptera	Perlidae	Hesperoperla pacifica
Nanonemoura wahkeena 16S ribosomal RNA gene, parti	EF623198	83.67	60.49	1.75598e−19	Plecoptera	Nemouridae	Nanonemoura wahkeena
Lednia tumana 16S ribosomal RNA gene, partial sequ	EF623197	83.17	62.35	1.75598e−19	Plecoptera	Nemouridae	Lednia tumana
Protonemura meyeri 16S ribosomal RNA gene, partial	EF623193	83.17	62.35	1.75598e−19	Plecoptera	Nemouridae	Protonemura meyeri
Ostrocerca foersteri 16S ribosomal RNA gene, parti	EF623191	83.67	60.49	1.75598e−19	Plecoptera	Nemouridae	Ostrocerca foersteri
Visoka cataractae 16S ribosomal RNA gene, partial	EF623189	83.67	60.49	1.75598e−19	Plecoptera	Nemouridae	Visoka cataractae
Osmylus sp. EH−2004 16S ribosomal RNA gene, partia	AY620146	84.69	60.49	1.75598e−19	Neuroptera	Osmylidae	Osmylus sp. EH−2004
Eupholidoptera chabrieri voucher 173 16S ribosomal	EU120792	83.17	62.35	6.12897e−19	Orthoptera	Tettigoniidae	Eupholidoptera chabrieri
Allocapnia recta isolate H18 16S ribosomal RNA gen	KC881053	82.18	62.35	2.13922e−18	Plecoptera	Capniidae	Allocapnia recta
Allocapnia recta isolate H17 16S ribosomal RNA gen	KC881052	82.18	62.35	2.13922e−18	Plecoptera	Capniidae	Allocapnia recta
Allocapnia recta isolate H12 16S ribosomal RNA gen	KC881047	82.18	62.35	2.13922e−18	Plecoptera	Capniidae	Allocapnia recta
Allocapnia recta isolate H9 16S ribosomal RNA gene	KC881044	82.18	62.35	2.13922e−18	Plecoptera	Capniidae	Allocapnia recta
Allocapnia recta isolate H8 16S ribosomal RNA gene	KC881043	82.18	62.35	2.13922e−18	Plecoptera	Capniidae	Allocapnia recta
Allocapnia recta isolate H5 16S ribosomal RNA gene	KC881040	82.18	62.35	2.13922e−18	Plecoptera	Capniidae	Allocapnia recta
Allocapnia recta isolate H4 16S ribosomal RNA gene	KC881039	82.18	62.35	2.13922e−18	Plecoptera	Capniidae	Allocapnia recta
Cryptoperla sp. YK−2012−1 mitochondrial gene for 1	AB711243	83.17	62.35	2.13922e−18	Plecoptera	Peltoperlidae	Cryptoperla sp. YK−2012−1
Cryptoperla ishigakiensis mitochondrial gene for 1	AB711242	83.17	62.35	2.13922e−18	Plecoptera	Peltoperlidae	Cryptoperla ishigakiensis
Pleioplectron simplex voucher CW306 16S ribosomal	HM594512	83.67	60.49	2.13922e−18	Orthoptera	Rhaphidophoridae	Pleioplectron simplex
Pachyrhamma edwardsii voucher WEL 16S ribosomal RN	HM594509	83.67	60.49	2.13922e−18	Orthoptera	Rhaphidophoridae	Pachyrhamma edwardsii
Micropathus tasmaniensis voucher JNC 16S ribosomal	HM594507	83.33	62.35	2.13922e−18	Orthoptera	Rhaphidophoridae	Micropathus tasmaniensis
Micropathus tasmaniensis voucher IDB 16S ribosomal	HM594505	83.33	62.35	2.13922e−18	Orthoptera	Rhaphidophoridae	Micropathus tasmaniensis
Dolichopoda hussoni voucher IZB 16S ribosomal RNA	EF216973	82.18	62.35	2.13922e−18	Orthoptera	Rhaphidophoridae	Dolichopoda hussoni
Dolichopoda thasosensis voucher DRA 16S ribosomal	EF216956	82.18	62.35	2.13922e−18	Orthoptera	Rhaphidophoridae	Dolichopoda thasosensis
Pteronarcys scotti 16S ribosomal RNA gene, partial	EF623263	83.17	62.35	2.13922e−18	Plecoptera	Pteronarcyidae	Pteronarcys scotti
Pteronarcys californica 16S ribosomal RNA gene, pa	EF623261	83.17	62.35	2.13922e−18	Plecoptera	Pteronarcyidae	Pteronarcys californica
Kempnyella sp. BYU_PL155 16S ribosomal RNA gene, p	EF623246	82.18	62.35	2.13922e−18	Plecoptera	Perlidae	Kempnyella sp. BYU_PL155
Cosumnoperla hypocrena 16S ribosomal RNA gene, par	EF623226	83.67	60.49	2.13922e−18	Plecoptera	Perlodidae	Cosumnoperla hypocrena
Neonemura sp. BYU_PL153 16S ribosomal RNA gene, pa	EF623204	83.67	60.49	2.13922e−18	Plecoptera	Notonemouridae	Neonemura sp. BYU_PL153
Moselia infuscata 16S ribosomal RNA gene, partial	EF623175	83.67	60.49	2.13922e−18	Plecoptera	Leuctridae	Moselia infuscata
Galloisiana nipponensis 16S ribosomal RNA gene, pa	EF623153	83.67	60.49	2.13922e−18	Grylloblattodea	Grylloblattidae	Galloisiana nipponensis
Kathroperla perdita 16S ribosomal RNA gene, partia	EF623135	82.18	62.35	2.13922e−18	Plecoptera	Chloroperlidae	Kathroperla perdita
Allocapnia recta haplotype 10ar: H010 16S ribosoma	EU306873	82.18	62.35	2.13922e−18	Plecoptera	Capniidae	Allocapnia recta
Allocapnia recta haplotype 9ar: H09 16S ribosomal	EU306872	82.18	62.35	2.13922e−18	Plecoptera	Capniidae	Allocapnia recta
Allocapnia recta haplotype 8ar: H08 16S ribosomal	EU306871	82.18	62.35	2.13922e−18	Plecoptera	Capniidae	Allocapnia recta
Grylloblatta sp. BYU_gb29.1 16S ribosomal RNA gene	DQ457261	84.38	59.26	2.13922e−18	Grylloblattodea	Grylloblattidae	Grylloblatta sp. BYU_gb29.1
Galloisiana yuasai voucher BYU_gb13.2 16S ribosoma	DQ457244	83.67	60.49	2.13922e−18	Grylloblattodea	Grylloblattidae	Galloisiana yuasai
Galloisiana yuasai voucher BYU_gb13.1 16S ribosoma	DQ457243	83.67	60.49	2.13922e−18	Grylloblattodea	Grylloblattidae	Galloisiana yuasai
Galloisiana yuasai voucher BYU_gb12.1 16S ribosoma	DQ457242	83.67	60.49	2.13922e−18	Grylloblattodea	Grylloblattidae	Galloisiana yuasai
Allocapnia recta haplotype 1ar: H01 16S ribosomal	DQ915179	82.18	62.35	2.13922e−18	Plecoptera	Capniidae	Allocapnia recta
Pteronarcys princeps mitochondrion, complete genom	AY687866	83.17	62.35	2.13922e−18	Plecoptera	Pteronarcyidae	Pteronarcys princeps
Mesocapnia arizonensis mitochondrion, partial geno	KP642637	82.65	60.49	7.46661e−18	Plecoptera	Capniidae	Mesocapnia arizonensis
Allocapnia recta isolate H19 16S ribosomal RNA gen	KC881054	82.65	60.49	7.46661e−18	Plecoptera	Capniidae	Allocapnia recta
Allocapnia recta isolate H16 16S ribosomal RNA gen	KC881051	82.65	60.49	7.46661e−18	Plecoptera	Capniidae	Allocapnia recta
Allocapnia recta isolate H15 16S ribosomal RNA gen	KC881050	82.65	60.49	7.46661e−18	Plecoptera	Capniidae	Allocapnia recta
Allocapnia recta isolate H14 16S ribosomal RNA gen	KC881049	82.65	60.49	7.46661e−18	Plecoptera	Capniidae	Allocapnia recta
Allocapnia recta isolate H13 16S ribosomal RNA gen	KC881048	82.65	60.49	7.46661e−18	Plecoptera	Capniidae	Allocapnia recta
Allocapnia recta isolate H11 16S ribosomal RNA gen	KC881046	82.65	60.49	7.46661e−18	Plecoptera	Capniidae	Allocapnia recta
Allocapnia recta isolate H10 16S ribosomal RNA gen	KC881045	82.65	60.49	7.46661e−18	Plecoptera	Capniidae	Allocapnia recta
Allocapnia recta isolate H7 16S ribosomal RNA gene	KC881042	82.65	60.49	7.46661e−18	Plecoptera	Capniidae	Allocapnia recta
Allocapnia recta isolate H6 16S ribosomal RNA gene	KC881041	82.65	60.49	7.46661e−18	Plecoptera	Capniidae	Allocapnia recta
Allocapnia recta isolate H3 16S ribosomal RNA gene	KC881038	82.65	60.49	7.46661e−18	Plecoptera	Capniidae	Allocapnia recta
Allocapnia recta isolate H1 16S ribosomal RNA gene	KC881036	82.65	60.49	7.46661e−18	Plecoptera	Capniidae	Allocapnia recta
Dolichopoda geniculata geniculata isolate PRA20 16	AY507590	82.65	60.49	7.46661e−18	Orthoptera	Rhaphidophoridae	Dolichopoda geniculata geniculata
Dolichopoda sp. VAR 16S ribosomal RNA gene, partia	EU887865	82.65	60.49	7.46661e−18	Orthoptera	Rhaphidophoridae	Dolichopoda sp. VAR
Dolichopoda sp. TAY 16S ribosomal RNA gene, partia	EU887864	82.65	60.49	7.46661e−18	Orthoptera	Rhaphidophoridae	Dolichopoda sp. TAY
Dolichopoda kiriakii voucher AGH 16S ribosomal RNA	EF216954	82.65	60.49	7.46661e−18	Orthoptera	Rhaphidophoridae	Dolichopoda kiriakii
Taenionema palladium 16S ribosomal RNA gene, parti	EF623267	82.35	62.35	7.46661e−18	Plecoptera	Taeniopterygidae	Taenionema palladium
Oemopteryx vanduzeea 16S ribosomal RNA gene, parti	EF623266	82.65	60.49	7.46661e−18	Plecoptera	Taeniopterygidae	Oemopteryx vanduzeea
Osobenus yakimae 16S ribosomal RNA gene, partial s	EF623232	82.35	62.96	7.46661e−18	Plecoptera	Perlodidae	Osobenus yakimae
Udamocercia sp. BYU_PL150 16S ribosomal RNA gene,	EF623201	82	61.73	7.46661e−18	Plecoptera	Notonemouridae	Udamocercia sp. BYU_PL150
Zapada cinctipes 16S ribosomal RNA gene, partial s	EF623183	82.65	60.49	7.46661e−18	Plecoptera	Nemouridae	Zapada cinctipes
Allocapnia minima 16S ribosomal RNA gene, partial	EF623130	82.65	60.49	7.46661e−18	Plecoptera	Capniidae	Allocapnia minima
Mesocapnia sp. BYU_PL010 16S ribosomal RNA gene, p	EF623127	82.65	60.49	7.46661e−18	Plecoptera	Capniidae	Mesocapnia sp. BYU_PL010
Allocapnia recta haplotype 12ar: H12 16S ribosomal	EU306875	82.65	60.49	7.46661e−18	Plecoptera	Capniidae	Allocapnia recta
Allocapnia recta haplotype 11ar: H011 16S ribosoma	EU306874	82.65	60.49	7.46661e−18	Plecoptera	Capniidae	Allocapnia recta
Allocapnia recta haplotype 7ar: H07 16S ribosomal	EU306870	82.65	60.49	7.46661e−18	Plecoptera	Capniidae	Allocapnia recta
Allocapnia recta haplotype 3ar: H03 16S ribosomal	DQ915181	82.65	60.49	7.46661e−18	Plecoptera	Capniidae	Allocapnia recta
Allocapnia recta haplotype 2ar: H02 16S ribosomal	DQ915180	82.65	60.49	7.46661e−18	Plecoptera	Capniidae	Allocapnia recta
