# Supplementary material for: Testing the potential of a ribosomal 16S marker for DNA metabarcoding of insects
Source: PeerJ. 2016 Apr 19;4:e1966. doi: 10.7717/peerj.1966 (PMC4841222; doi:10.7717/peerj.1966)
Supplement: Supplemental Information 1 [file peerj-04-1966-s006.zip › scripts/5 match OTUs/blast_hits/OTU_228.csv_plot.pdf]

OTU_228.csv

Nemoura cinerea

Nemouridae

Plecoptera

90.1

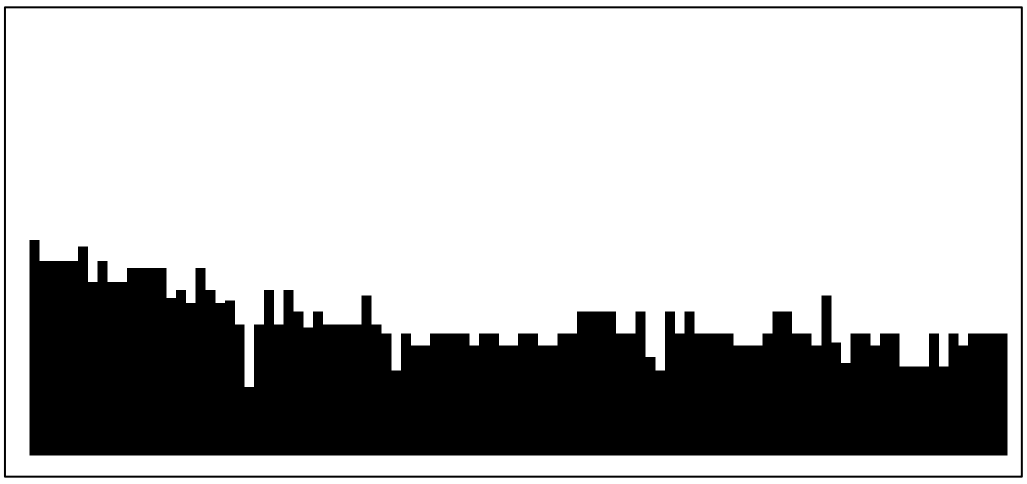

Nemoura cinerea 16S ribosomal RNA gene, partial se	EF623194	90.1	62.35	2.97094e−29	Plecoptera	Nemouridae	Nemoura cinerea
Prostoia besametsa 16S ribosomal RNA gene, partial	EF623188	89.11	62.35	1.26327e−27	Plecoptera	Nemouridae	Prostoia besametsa
Nemoura trispinosa 16S ribosomal RNA gene, partial	EF623187	89.11	62.35	1.26327e−27	Plecoptera	Nemouridae	Nemoura trispinosa
Paranemoura claasseni 16S ribosomal RNA gene, part	EF623185	89.11	62.35	1.26327e−27	Plecoptera	Nemouridae	Paranemoura claasseni
Shipsa rotunda 16S ribosomal RNA gene, partial seq	EF623184	89.11	62.35	1.26327e−27	Plecoptera	Nemouridae	Shipsa rotunda
Malenka californica 16S ribosomal RNA gene, partia	EF623182	89.8	60.49	1.26327e−27	Plecoptera	Nemouridae	Malenka californica
Notonemouridae sp. MD−2011 voucher ZMUC:JD−1507 16	JN615257	88.12	62.35	1.53898e−26	Plecoptera	Notonemouridae	Notonemouridae sp. MD−2011
Calineuria californica 16S ribosomal RNA gene, par	EF623239	89.11	62.35	1.53898e−26	Plecoptera	Perlidae	Calineuria californica
Lednia tumana 16S ribosomal RNA gene, partial sequ	EF623197	88.12	62.35	1.53898e−26	Plecoptera	Nemouridae	Lednia tumana
Protonemura meyeri 16S ribosomal RNA gene, partial	EF623193	88.12	62.35	1.53898e−26	Plecoptera	Nemouridae	Protonemura meyeri
Scopura montana 16S ribosomal RNA gene, partial se	EF623265	88.78	60.49	5.37158e−26	Plecoptera		Scopura montana
Nanonemoura wahkeena 16S ribosomal RNA gene, parti	EF623198	88.78	60.49	5.37158e−26	Plecoptera	Nemouridae	Nanonemoura wahkeena
Ostrocerca foersteri 16S ribosomal RNA gene, parti	EF623191	88.78	60.49	5.37158e−26	Plecoptera	Nemouridae	Ostrocerca foersteri
Visoka cataractae 16S ribosomal RNA gene, partial	EF623189	88.78	60.49	5.37158e−26	Plecoptera	Nemouridae	Visoka cataractae
Hansonoperla appalachia 16S ribosomal RNA gene, pa	EF623249	87.38	62.35	1.87487e−25	Plecoptera	Perlidae	Hansonoperla appalachia
Apteroperla tikumana mitochondrion, complete genom	KR604721	87.76	60.49	6.54393e−25	Plecoptera	Capniidae	Apteroperla tikumana
Dolichopoda pavesii voucher SPI 16S ribosomal RNA	EF216951	87.13	62.35	6.54393e−25	Orthoptera	Rhaphidophoridae	Dolichopoda pavesii
Pteronarcys sachalina 16S ribosomal RNA gene, part	EF623264	88.78	60.49	6.54393e−25	Plecoptera	Pteronarcyidae	Pteronarcys sachalina
Zapada cinctipes 16S ribosomal RNA gene, partial s	EF623183	87.76	60.49	6.54393e−25	Plecoptera	Nemouridae	Zapada cinctipes
Megaleuctra kincaidi 16S ribosomal RNA gene, parti	EF623174	87.13	62.35	6.54393e−25	Plecoptera	Leuctridae	Megaleuctra kincaidi
Perlinella drymo 16S ribosomal RNA gene, partial s	EF623240	87.25	62.35	2.28405e−24	Plecoptera	Perlidae	Perlinella drymo
Doddsia occidentalis 16S ribosomal RNA gene, parti	EF623270	86.14	62.35	7.97213e−24	Plecoptera	Taeniopterygidae	Doddsia occidentalis
Brachyptera seticornis 16S ribosomal RNA gene, par	EF623268	83.19	73.46	7.97213e−24	Plecoptera	Taeniopterygidae	Brachyptera seticornis
Pteronarcella badia 16S ribosomal RNA gene, partia	EF623262	86.14	62.35	7.97213e−24	Plecoptera	Pteronarcyidae	Pteronarcella badia
Moselia infuscata 16S ribosomal RNA gene, partial	EF623175	87.76	60.49	7.97213e−24	Plecoptera	Leuctridae	Moselia infuscata
Kathroperla perdita 16S ribosomal RNA gene, partia	EF623135	86.14	62.35	7.97213e−24	Plecoptera	Chloroperlidae	Kathroperla perdita
Osmylus sp. EH−2004 16S ribosomal RNA gene, partia	AY620146	87.76	60.49	7.97213e−24	Neuroptera	Osmylidae	Osmylus sp. EH−2004
Dolichopoda sp. MR−2008 16S ribosomal RNA gene, pa	EU887863	86.73	60.49	2.78255e−23	Orthoptera	Rhaphidophoridae	Dolichopoda lustriae
Udamocercia sp. BYU_PL150 16S ribosomal RNA gene,	EF623201	86	61.73	2.78255e−23	Plecoptera	Notonemouridae	Udamocercia sp. BYU_PL150
Nemurella pictetii 16S ribosomal RNA gene, partial	EF623192	86.73	60.49	2.78255e−23	Plecoptera	Nemouridae	Nemurella pictetii
Cryptoperla sp. YK−2012−1 mitochondrial gene for 1	AB711243	86.14	62.35	9.71205e−23	Plecoptera	Peltoperlidae	Cryptoperla sp. YK−2012−1
Cryptoperla ishigakiensis mitochondrial gene for 1	AB711242	86.14	62.35	9.71205e−23	Plecoptera	Peltoperlidae	Cryptoperla ishigakiensis
Pteronarcys scotti 16S ribosomal RNA gene, partial	EF623263	86.14	62.35	9.71205e−23	Plecoptera	Pteronarcyidae	Pteronarcys scotti
Pteronarcys californica 16S ribosomal RNA gene, pa	EF623261	86.14	62.35	9.71205e−23	Plecoptera	Pteronarcyidae	Pteronarcys californica
Grylloblatta sp. BYU_gb29.1 16S ribosomal RNA gene	DQ457261	87.5	59.26	9.71205e−23	Grylloblattodea	Grylloblattidae	Grylloblatta sp. BYU_gb29.1
Pteronarcys princeps mitochondrion, complete genom	AY687866	86.14	62.35	9.71205e−23	Plecoptera	Pteronarcyidae	Pteronarcys princeps
Mesocapnia arizonensis mitochondrion, partial geno	KP642637	85.71	60.49	3.38984e−22	Plecoptera	Capniidae	Mesocapnia arizonensis
Dinocras cephalotes mitochondrion, complete genome	KF484757	83.96	62.35	3.38984e−22	Plecoptera	Perlidae	Dinocras cephalotes
Allocapnia recta isolate H19 16S ribosomal RNA gen	KC881054	85.71	60.49	3.38984e−22	Plecoptera	Capniidae	Allocapnia recta
Allocapnia recta isolate H18 16S ribosomal RNA gen	KC881053	85.15	62.35	3.38984e−22	Plecoptera	Capniidae	Allocapnia recta
Allocapnia recta isolate H17 16S ribosomal RNA gen	KC881052	85.15	62.35	3.38984e−22	Plecoptera	Capniidae	Allocapnia recta
Allocapnia recta isolate H16 16S ribosomal RNA gen	KC881051	85.71	60.49	3.38984e−22	Plecoptera	Capniidae	Allocapnia recta
Allocapnia recta isolate H15 16S ribosomal RNA gen	KC881050	85.71	60.49	3.38984e−22	Plecoptera	Capniidae	Allocapnia recta
Allocapnia recta isolate H14 16S ribosomal RNA gen	KC881049	85.71	60.49	3.38984e−22	Plecoptera	Capniidae	Allocapnia recta
Allocapnia recta isolate H13 16S ribosomal RNA gen	KC881048	85.71	60.49	3.38984e−22	Plecoptera	Capniidae	Allocapnia recta
Allocapnia recta isolate H12 16S ribosomal RNA gen	KC881047	85.15	62.35	3.38984e−22	Plecoptera	Capniidae	Allocapnia recta
Allocapnia recta isolate H11 16S ribosomal RNA gen	KC881046	85.71	60.49	3.38984e−22	Plecoptera	Capniidae	Allocapnia recta
Allocapnia recta isolate H10 16S ribosomal RNA gen	KC881045	85.71	60.49	3.38984e−22	Plecoptera	Capniidae	Allocapnia recta
Allocapnia recta isolate H9 16S ribosomal RNA gene	KC881044	85.15	62.35	3.38984e−22	Plecoptera	Capniidae	Allocapnia recta
Allocapnia recta isolate H8 16S ribosomal RNA gene	KC881043	85.15	62.35	3.38984e−22	Plecoptera	Capniidae	Allocapnia recta
Allocapnia recta isolate H7 16S ribosomal RNA gene	KC881042	85.71	60.49	3.38984e−22	Plecoptera	Capniidae	Allocapnia recta
Allocapnia recta isolate H6 16S ribosomal RNA gene	KC881041	85.71	60.49	3.38984e−22	Plecoptera	Capniidae	Allocapnia recta
Allocapnia recta isolate H5 16S ribosomal RNA gene	KC881040	85.15	62.35	3.38984e−22	Plecoptera	Capniidae	Allocapnia recta
Allocapnia recta isolate H4 16S ribosomal RNA gene	KC881039	85.15	62.35	3.38984e−22	Plecoptera	Capniidae	Allocapnia recta
Allocapnia recta isolate H3 16S ribosomal RNA gene	KC881038	85.71	60.49	3.38984e−22	Plecoptera	Capniidae	Allocapnia recta
Allocapnia recta isolate H1 16S ribosomal RNA gene	KC881036	85.71	60.49	3.38984e−22	Plecoptera	Capniidae	Allocapnia recta
Cryptoperla japonica mitochondrial gene for 16S rR	AB711202	86.73	60.49	3.38984e−22	Plecoptera	Peltoperlidae	Cryptoperla japonica
Cryptoperla japonica mitochondrial gene for 16S rR	AB711184	86.73	60.49	3.38984e−22	Plecoptera	Peltoperlidae	Cryptoperla japonica
Cryptoperla japonica mitochondrial gene for 16S rR	AB711183	86.73	60.49	3.38984e−22	Plecoptera	Peltoperlidae	Cryptoperla japonica
Cryptoperla japonica mitochondrial gene for 16S rR	AB711182	86.73	60.49	3.38984e−22	Plecoptera	Peltoperlidae	Cryptoperla japonica
Dolichopoda steriotisi voucher ANT 16S ribosomal R	EF216955	85.71	60.49	3.38984e−22	Orthoptera	Rhaphidophoridae	Dolichopoda steriotisi
Oemopteryx vanduzeea 16S ribosomal RNA gene, parti	EF623266	85.71	60.49	3.38984e−22	Plecoptera	Taeniopterygidae	Oemopteryx vanduzeea
Cerconychia sp. BYU_PL114 16S ribosomal RNA gene,	EF623260	86.73	60.49	3.38984e−22	Plecoptera	Styloperlidae	Cerconychia sp. BYU_PL114
Acroneuria lycorias 16S ribosomal RNA gene, partia	EF623251	84.62	62.35	3.38984e−22	Plecoptera	Perlidae	Acroneuria lycorias
Dinocras cephalotes 16S ribosomal RNA gene, partia	EF623244	83.96	62.35	3.38984e−22	Plecoptera	Perlidae	Dinocras cephalotes
Cosumnoperla hypocrena 16S ribosomal RNA gene, par	EF623226	86.73	60.49	3.38984e−22	Plecoptera	Perlodidae	Cosumnoperla hypocrena
Amphinemura sulcicollis 16S ribosomal RNA gene, pa	EF623195	85.71	60.49	3.38984e−22	Plecoptera	Nemouridae	Amphinemura sulcicollis
Galloisiana nipponensis 16S ribosomal RNA gene, pa	EF623153	86.73	60.49	3.38984e−22	Grylloblattodea	Grylloblattidae	Galloisiana nipponensis
Allocapnia minima 16S ribosomal RNA gene, partial	EF623130	85.71	60.49	3.38984e−22	Plecoptera	Capniidae	Allocapnia minima
Mesocapnia sp. BYU_PL010 16S ribosomal RNA gene, p	EF623127	85.71	60.49	3.38984e−22	Plecoptera	Capniidae	Mesocapnia sp. BYU_PL010
Allocapnia recta haplotype 12ar: H12 16S ribosomal	EU306875	85.71	60.49	3.38984e−22	Plecoptera	Capniidae	Allocapnia recta
Allocapnia recta haplotype 11ar: H011 16S ribosoma	EU306874	85.71	60.49	3.38984e−22	Plecoptera	Capniidae	Allocapnia recta
Allocapnia recta haplotype 10ar: H010 16S ribosoma	EU306873	85.15	62.35	3.38984e−22	Plecoptera	Capniidae	Allocapnia recta
Allocapnia recta haplotype 9ar: H09 16S ribosomal	EU306872	85.15	62.35	3.38984e−22	Plecoptera	Capniidae	Allocapnia recta
Allocapnia recta haplotype 8ar: H08 16S ribosomal	EU306871	85.15	62.35	3.38984e−22	Plecoptera	Capniidae	Allocapnia recta
Allocapnia recta haplotype 7ar: H07 16S ribosomal	EU306870	85.71	60.49	3.38984e−22	Plecoptera	Capniidae	Allocapnia recta
Galloisiana yuasai voucher BYU_gb13.2 16S ribosoma	DQ457244	86.73	60.49	3.38984e−22	Grylloblattodea	Grylloblattidae	Galloisiana yuasai
Galloisiana yuasai voucher BYU_gb13.1 16S ribosoma	DQ457243	86.73	60.49	3.38984e−22	Grylloblattodea	Grylloblattidae	Galloisiana yuasai
Allocapnia recta haplotype 3ar: H03 16S ribosomal	DQ915181	85.71	60.49	3.38984e−22	Plecoptera	Capniidae	Allocapnia recta
Allocapnia recta haplotype 2ar: H02 16S ribosomal	DQ915180	85.71	60.49	3.38984e−22	Plecoptera	Capniidae	Allocapnia recta
Allocapnia recta haplotype 1ar: H01 16S ribosomal	DQ915179	85.15	62.35	3.38984e−22	Plecoptera	Capniidae	Allocapnia recta
Thanatophyllum akinetum voucher BYU IGC CR001 16S	KF372495	87.5	59.26	1.18317e−21	Blattodea	Blaberidae	Thanatophyllum akinetum
Eccoptura xanthenes 16S ribosomal RNA gene, partia	EF623252	85.29	62.35	1.18317e−21	Plecoptera	Perlidae	Eccoptura xanthenes
Helopicus bogaloosa 16S ribosomal RNA gene, partia	EF623228	84.31	62.96	1.18317e−21	Plecoptera	Perlodidae	Helopicus bogaloosa
Gerstaeckerella chilensis 16S ribosomal RNA gene,	KM051513	85.71	60.49	4.12967e−21	Neuroptera	Mantispidae	Gerstaeckerella chilensis
Cryptoperla sp. YK−2012−2 mitochondrial gene for 1	AB711244	85.71	60.49	4.12967e−21	Plecoptera	Peltoperlidae	Cryptoperla sp. YK−2012−2
Cryptoperla kawasawai mitochondrial gene for 16S r	AB711236	85.15	62.35	4.12967e−21	Plecoptera	Peltoperlidae	Cryptoperla kawasawai
Galloisiana yuasai voucher ZMUC:JD−650 16S ribosom	JN615261	85.71	60.49	4.12967e−21	Grylloblattodea	Grylloblattidae	Galloisiana yuasai
Pachyrhamma edwardsii voucher CW122 16S ribosomal	HM594508	85.71	60.49	4.12967e−21	Orthoptera	Rhaphidophoridae	Pachyrhamma edwardsii
Dolichopoda hussoni voucher IZB 16S ribosomal RNA	EF216973	84.16	62.35	4.12967e−21	Orthoptera	Rhaphidophoridae	Dolichopoda hussoni
Dolichopoda thasosensis voucher DRA 16S ribosomal	EF216956	84.16	62.35	4.12967e−21	Orthoptera	Rhaphidophoridae	Dolichopoda thasosensis
Strophopteryx appalachia 16S ribosomal RNA gene, p	EF623271	84.16	62.35	4.12967e−21	Plecoptera	Taeniopterygidae	Strophopteryx appalachia
Soliperla campanula 16S ribosomal RNA gene, partia	EF623256	85.71	60.49	4.12967e−21	Plecoptera	Peltoperlidae	Soliperla campanula
Pictetoperla sp. BYU_PL156 16S ribosomal RNA gene,	EF623247	84.16	62.35	4.12967e−21	Plecoptera	Perlidae	Pictetoperla sp. BYU_PL156
Isoperla fulva 16S ribosomal RNA gene, partial seq	EF623207	85.71	60.49	4.12967e−21	Plecoptera	Perlodidae	Isoperla fulva
Soyedina producta 16S ribosomal RNA gene, partial	EF623196	85.15	62.35	4.12967e−21	Plecoptera	Nemouridae	Soyedina producta
Galloisiana yuasai 16S large subunit ribosomal RNA	DQ890284	85.71	60.49	4.12967e−21	Grylloblattodea	Grylloblattidae	Galloisiana yuasai
Galloisiana yuasai voucher BYU_gb12.1 16S ribosoma	DQ457242	85.71	60.49	4.12967e−21	Grylloblattodea	Grylloblattidae	Galloisiana yuasai
Macroxyela ferruginea 16S ribosomal RNA gene, part	AY206773	85.71	60.49	4.12967e−21	Hymenoptera	Xyelidae	Macroxyela ferruginea
Xyela sp. Sus1 16S ribosomal RNA gene, partial seq	AY206772	85.71	60.49	4.12967e−21	Hymenoptera	Xyelidae	Xyela sp. Sus1
