# Supplementary material for: Testing the potential of a ribosomal 16S marker for DNA metabarcoding of insects
Source: PeerJ. 2016 Apr 19;4:e1966. doi: 10.7717/peerj.1966 (PMC4841222; doi:10.7717/peerj.1966)
Supplement: Supplemental Information 1 [file peerj-04-1966-s006.zip › scripts/5 match OTUs/blast_hits/OTU_23.csv_plot.pdf]

OTU_23.csv

Sialis californica

Sialidae

Megaloptera

94.3

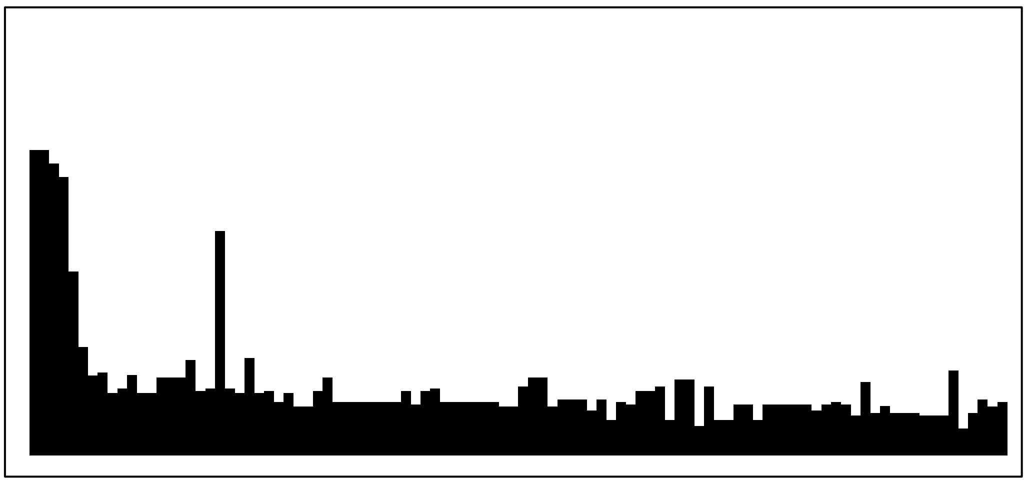

Sialis californica 16S ribosomal RNA gene, partial	EU734902	94.3	100	2.69015e−61	Megaloptera	Sialidae	Sialis californica
Sialis lutaria isolate Sialut1 16S ribosomal RNA g	AY620141	94.3	100	2.69015e−61	Megaloptera	Sialidae	Sialis lutaria
Sialis hamata mitochondrion, complete genome	FJ859905	93.67	100	3.27728e−60	Megaloptera	Sialidae	Sialis hamata
Nipponosialis kumejimae isolate H841 16S ribosomal	KF741321	93.04	100	1.39353e−58	Megaloptera	Sialidae	Nipponosialis kumejimae
Sialis nr. mohri SLW−2008 16S ribosomal RNA gene,	EU734903	88.61	100	2.3598e−49	Megaloptera	Sialidae	Sialis nr. mohri SLW−2008
Dasytes depexus voucher UPOL001173 16S ribosomal R	HQ619676	85.06	97.47	1.39476e−39	Coleoptera	Melyridae	Dasytes depexus
Exorista patelliforceps mitochondrial gene for 16S	AB257231	83.75	100	7.22506e−37	Diptera	Tachinidae	Exorista patelliforceps
Dasytes otini voucher UPOL001084 16S ribosomal RNA	HQ619645	83.87	97.47	2.52179e−36	Coleoptera	Melyridae	Dasytes otini
Sternhydrus scutellaris voucher UNM KBMOnsc107 16S	KJ548362	82.91	100	8.80193e−36	Coleoptera	Dytiscidae	Sternhydrus scutellaris
Exorista lepis mitochondrial gene for 16S rRNA, pa	AB699948	83.12	100	8.80193e−36	Diptera	Tachinidae	Exorista lepis
Dolichosoma lineare voucher UPOL001063 16S ribosom	HQ619634	83.77	97.47	8.80193e−36	Coleoptera	Melyridae	Dolichosoma lineare
Onychohydrus larva partial 16S rRNA gene	AJ850371	82.91	100	8.80193e−36	Coleoptera	Dytiscidae	Onychohydrus larva
Ochlerotatus nipponicus isolate A75 16S ribosomal	DQ397914	82.91	100	8.80193e−36	Diptera	Culicidae	Aedes nipponicus
Semisuturia sp. LRC−2002 16S ribosomal RNA gene, p	AF456848	83.65	100	8.80193e−36	Diptera	Tachinidae	Semisuturia sp. LRC−2002
Teloglabrus entabenensis 16S ribosomal RNA gene, p	AF304731	83.65	100	8.80193e−36	Diptera	Diopsidae	Teloglabrus entabenensis
Anabarhynchus tristis voucher MEI 119905 16S ribos	DQ250834	83.65	100	8.80193e−36	Diptera	Therevidae	Anabarhynchus tristis
Limonia tahitiensis voucher O'Grady 202004 16S rib	EU005425	84.47	99.37	3.07217e−35	Diptera	Limoniidae	Dicranomyia tahitiensis
Irwiniella natalensis voucher MEI 119909 16S ribos	DQ250845	83.02	100	3.07217e−35	Diptera	Therevidae	Irwiniella natalensis
Agabus paludosus 16S ribosomal RNA gene, partial s	AY138625	83.12	100	3.07217e−35	Coleoptera	Dytiscidae	Agabus paludosus
Teleopsis trichophora 16S ribosomal RNA gene, part	KT186661	90.52	72.78	1.07229e−34	Diptera	Diopsidae	Teleopsis trichophora
Acilius abbreviatus voucher KBMAcab72 16S ribosoma	KF979078	83.12	100	1.07229e−34	Coleoptera	Dytiscidae	Acilius abbreviatus
Exorista rustica mitochondrial gene for 16S rRNA,	AB699940	82.91	100	1.07229e−34	Diptera	Tachinidae	Exorista rustica
Dasytes emarginatus voucher UPOL001068 16S ribosom	HQ619637	84.56	94.3	1.07229e−34	Coleoptera	Melyridae	Dasytes emarginatus
Exorista mimula mitochondrial gene for 16S rRNA, p	AB465929	82.91	100	1.07229e−34	Diptera	Tachinidae	Exorista mimula
Isosturmia japonica mitochondrial gene for 16S rRN	AB465919	83.02	100	1.07229e−34	Diptera	Tachinidae	Isosturmia japonica
Blondelia nigripes mitochondrial gene for 16S rRNA	AB465902	82.5	100	1.07229e−34	Diptera	Tachinidae	Blondelia nigripes
Exorista mimula mitochondrial gene for 16S rRNA, p	AB257223	82.91	100	1.07229e−34	Diptera	Tachinidae	Exorista mimula
Culex bitaeniorhynchus 16S ribosomal RNA gene, par	EF593019	82.28	100	1.07229e−34	Diptera	Culicidae	Culex bitaeniorhynchus
Mallada luctuosus 16S ribosomal RNA gene, partial	DQ399288	82.28	100	1.07229e−34	Neuroptera	Chrysopidae	Pseudomallada luctuosus
Cionophora sp. MEI 124032 16S ribosomal RNA gene,	DQ250835	83.02	100	1.07229e−34	Diptera	Therevidae	Cionophora sp. MEI 124032
Chymomyza amoena mitochondrial 16S ribosomal RNA,	M93986	83.65	100	1.07229e−34	Diptera	Drosophilidae	Chymomyza amoena
Agabus nebulosus mitochondrial 16S rRNA gene (part	LM654779	82.5	100	3.74267e−34	Coleoptera	Dytiscidae	Agabus nebulosus
Agabus glacialis mitochondrial 16S rRNA gene (part	LM654776	82.5	100	3.74267e−34	Coleoptera	Dytiscidae	Agabus glacialis
Agabus fuscipennis mitochondrial 16S rRNA gene (pa	LM654775	82.5	100	3.74267e−34	Coleoptera	Dytiscidae	Agabus fuscipennis
Agabus uliginosus mitochondrial 16S rRNA gene (par	HF931463	82.5	100	3.74267e−34	Coleoptera	Dytiscidae	Agabus uliginosus
Agabus cephalotes mitochondrial 16S rRNA gene (par	HF931380	82.5	100	3.74267e−34	Coleoptera	Dytiscidae	Agabus cephalotes
Agabus dichrous mitochondrial partial 16S rRNA gen	HF931378	82.5	100	3.74267e−34	Coleoptera	Dytiscidae	Agabus dichrous
Agabus godmanni mitochondrial 16S rRNA gene (parti	HF931348	82.5	100	3.74267e−34	Coleoptera	Dytiscidae	Agabus godmanni
Zeugomantispa minuta 16S ribosomal RNA gene, parti	EU734871	83.02	100	3.74267e−34	Neuroptera	Mantispidae	Zeugomantispa minuta
Notomma n. sp. 1000 16S ribosomal RNA gene, partia	AY792108	82.39	100	3.74267e−34	Diptera	Tephritidae	Notomma n. sp. 1000
Gitona bivisualis 16S ribosomal RNA gene, partial	AF479793	83.02	100	3.74267e−34	Diptera	Drosophilidae	Gitona bivisualis
Litolinga tergisa voucher MEI 119911 16S ribosomal	DQ250846	83.12	100	3.74267e−34	Diptera	Therevidae	Litolinga tergisa
Agabus nebulosus 16S ribosomal RNA gene, partial s	AY138624	82.5	100	3.74267e−34	Coleoptera	Dytiscidae	Agabus nebulosus
Agabus japonicus 16S ribosomal RNA gene, partial s	AY138617	82.5	100	3.74267e−34	Coleoptera	Dytiscidae	Agabus japonicus
Agabus dilatatus 16S ribosomal RNA gene, partial s	AY039261	82.5	100	3.74267e−34	Coleoptera	Dytiscidae	Agabus dilatatus
Agabus lineatus 16S ribosomal RNA gene, partial se	AY138611	82.5	100	3.74267e−34	Coleoptera	Dytiscidae	Agabus lineatus
Agabus congener 16S ribosomal RNA gene, partial se	AY138609	82.5	100	3.74267e−34	Coleoptera	Dytiscidae	Agabus congener
Agabus morosus isolate Aga18.2R 16S ribosomal RNA	AY138592	82.5	100	3.74267e−34	Coleoptera	Dytiscidae	Agabus morosus
Sarcophagidae sp. BAHAR107−14 voucher BAH_201312_P	KP253964	82.28	100	1.30632e−33	Diptera	Sarcophagidae	Sarcophagidae sp. BAHAR107−14
Lispe bivittata 16S ribosomal RNA gene, partial se	JN226651	82.28	100	1.30632e−33	Diptera	Muscidae	Lispe bivittata
Leistus spinibarbis 16S ribosomal RNA (rrnl) gene,	JF778797	83.23	100	1.30632e−33	Coleoptera	Carabidae	Leistus spinibarbis
Hymenoplia rugulosa voucher DA0142 16S ribosomal R	FJ956741	83.65	99.37	1.30632e−33	Coleoptera	Scarabaeidae	Hymenoplia rugulosa
Hymenoplia rugulosa voucher DA0140 16S ribosomal R	FJ956739	83.65	99.37	1.30632e−33	Coleoptera	Scarabaeidae	Hymenoplia rugulosa
Exorista cantans mitochondrial gene for 16S rRNA,	AB257230	82.28	100	1.30632e−33	Diptera	Tachinidae	Exorista cantans
Agabus aubei 16S ribosomal RNA gene, partial seque	AY039265	82.61	100	1.30632e−33	Coleoptera	Dytiscidae	Agabus aubei
Agabus biguttatus haplotype 1 16S ribosomal RNA ge	AY039258	82.61	100	1.30632e−33	Coleoptera	Dytiscidae	Agabus biguttatus
Agabus alexandrae 16S ribosomal RNA gene, partial	AY039257	82.61	100	1.30632e−33	Coleoptera	Dytiscidae	Agabus alexandrae
Teloglabrus milleri 16S ribosomal RNA gene, partia	AF304732	82.1	100	1.30632e−33	Diptera	Diopsidae	Teloglabrus milleri
Agabus alexandrae 16S ribosomal RNA gene, partial	AF428199	82.61	100	1.30632e−33	Coleoptera	Dytiscidae	Agabus alexandrae
Megadytes robustus voucher UNM KBMMero254 16S ribo	KJ548356	81.65	100	4.55951e−33	Coleoptera	Dytiscidae	Megadytes robustus
Acilius semisulcatus voucher KBMAcse7 16S ribosoma	KF979085	82.5	100	4.55951e−33	Coleoptera	Dytiscidae	Acilius semisulcatus
Exorista globosa mitochondrial gene for 16S rRNA,	AB699945	82.39	100	4.55951e−33	Diptera	Tachinidae	Exorista globosa
Lispe pygmaea 16S ribosomal RNA gene, partial sequ	JN226653	83.02	100	4.55951e−33	Diptera	Muscidae	Lispe pygmaea
Lispe leucospila 16S ribosomal RNA gene, partial s	JN226652	83.02	100	4.55951e−33	Diptera	Muscidae	Lispe leucospila
Omaliomimus venator voucher 668293 16S ribosomal R	EF214004	83.22	94.3	4.55951e−33	Coleoptera	Staphylinidae	Omaliomimus venator
Sphenella marginata 16S ribosomal RNA gene, partia	GQ175836	81.65	100	4.55951e−33	Diptera	Tephritidae	Sphenella marginata
Hymenoplia fulvipennis voucher DA0214 16S ribosoma	FJ956723	83.54	99.37	4.55951e−33	Coleoptera	Scarabaeidae	Hymenoplia fulvipennis
Hymenoplia clypealis voucher DA0164 16S ribosomal	FJ956712	83.54	98.73	4.55951e−33	Coleoptera	Scarabaeidae	Hymenoplia clypealis
Medina sp. Med_24022006 mitochondrial gene for 16S	AB465907	81.37	100	4.55951e−33	Diptera	Tachinidae	Medina sp. Med_24022006
Omaliomimus venator mitochondrial 16S rRNA gene (p	FM994733	83.22	94.3	4.55951e−33	Coleoptera	Staphylinidae	Omaliomimus venator
Megadytes sp. IR98 partial 16S rRNA gene	AJ850368	81.65	100	4.55951e−33	Coleoptera	Dytiscidae	Megadytes sp. IR98
Megadytes australis partial 16S rRNA gene	AJ850367	81.65	100	4.55951e−33	Coleoptera	Dytiscidae	Megadytes australis
Scatella hawaiiensis voucher 105726 NADH dehydroge	EU494299	82.39	100	4.55951e−33	Diptera	Ephydridae	Scatella hawaiiensis
Exorista tubigera mitochondrial gene for 16S rRNA,	AB257224	82.39	100	4.55951e−33	Diptera	Tachinidae	Exorista tubigera
Culex mimeticus 16S ribosomal RNA gene, partial se	EF593021	81.65	100	4.55951e−33	Diptera	Culicidae	Culex mimeticus
Thereva cf. turneri 'knyssa' voucher MEI 123850 16	DQ250854	82.39	100	4.55951e−33	Diptera	Therevidae	Thereva cf. turneri 'knyssa'
Ozodiceromyia sp. MEI 089435 16S ribosomal RNA gen	DQ250823	82.39	100	4.55951e−33	Diptera	Therevidae	Ozodiceromyia sp. MEI 089435
Lysilinga aurentiaca voucher MEI 089531 16S riboso	DQ250821	82.39	100	4.55951e−33	Diptera	Therevidae	Lysilinga aurentiaca
Platambus princeps 16S ribosomal RNA gene, partial	AY138677	82.39	100	4.55951e−33	Coleoptera	Dytiscidae	Platambus princeps
Agabus sturmii 16S ribosomal RNA gene, partial seq	AY138633	82.39	100	4.55951e−33	Coleoptera	Dytiscidae	Agabus sturmii
Agabus maderensis 16S ribosomal RNA gene, partial	AY138621	82.1	100	4.55951e−33	Coleoptera	Dytiscidae	Agabus maderensis
Agabus arcticus 16S ribosomal RNA gene, partial se	AY138603	82.39	100	4.55951e−33	Coleoptera	Dytiscidae	Agabus arcticus
Sapromyza sexpunctata mitochondrion partial 16S rR	AJ439185	82.5	100	4.55951e−33	Diptera	Lauxaniidae	Sapromyza sexpunctata
Toxotrypana littoralis 16S ribosomal RNA gene, par	AF152091	82.39	100	4.55951e−33	Diptera	Tephritidae	Toxotrypana littoralis
Agabus ragazzii mitochondrial 16S rRNA gene (parti	LM654781	81.88	100	1.59143e−32	Coleoptera	Dytiscidae	Agabus ragazzii
Ochthebius nr. difficilis IBE RA126 mitochondrial	HF931532	83.44	92.41	1.59143e−32	Coleoptera	Hydraenidae	Ochthebius nr. difficilis IBE RA126
Carabus cancellatus dolens voucher CC00816 16S rib	JQ647183	81.99	99.37	1.59143e−32	Coleoptera	Carabidae	Carabus cancellatus dolens
Carabus yuae guixiensis voucher CC00805 16S riboso	JQ647172	82.32	99.37	1.59143e−32	Coleoptera	Carabidae	Carabus yuae guixiensis
Carabus cancellatus dolens voucher CC00801 16S rib	JQ647168	81.99	99.37	1.59143e−32	Coleoptera	Carabidae	Carabus cancellatus dolens
Carabus cancellatus isolate TACHcc816 large subuni	JQ689798	81.99	99.37	1.59143e−32	Coleoptera	Carabidae	Carabus cancellatus
Carabus cancellatus isolate ROMA94106 large subuni	JQ689792	81.99	99.37	1.59143e−32	Coleoptera	Carabidae	Carabus cancellatus
Nebria bellorum voucher DHK0509 16S ribosomal RNA	JN847627	81.88	100	1.59143e−32	Coleoptera	Carabidae	Nebria bellorum
Nebria lacustris voucher DHK0004 16S ribosomal RNA	JN847615	81.88	100	1.59143e−32	Coleoptera	Carabidae	Nebria lacustris
Nebria gyllenhali voucher DHK0010 16S ribosomal RN	JN847607	81.88	100	1.59143e−32	Coleoptera	Carabidae	Nebria gyllenhali
Stenotarsus lemniscatus−CO322 16S ribosomal RNA ge	GQ302098	83.97	97.47	1.59143e−32	Coleoptera	Endomychidae	Stenotarsus lemniscatus
Ceromya silacea mitochondrial gene for 16S rRNA, p	AB465952	81.25	100	1.59143e−32	Diptera	Tachinidae	Ceromya silacea
Rhantus suturellus mitochondrial DNA sequence cont	FN298840	81.99	100	1.59143e−32	Coleoptera	Dytiscidae	Rhantus suturellus
Anthomyza socculata 16S ribosomal RNA gene, partia	EU268546	82.61	100	1.59143e−32	Diptera	Anthomyzidae	Anthomyza socculata
Lispe tentaculata 16S ribosomal RNA gene, partial	FJ025450	82.28	100	1.59143e−32	Diptera	Muscidae	Lispe tentaculata
Drymeia alpicola 16S ribosomal RNA gene, partial s	FJ025430	82.5	100	1.59143e−32	Diptera	Muscidae	Drymeia alpicola
