# Supplementary material for: Testing the potential of a ribosomal 16S marker for DNA metabarcoding of insects
Source: PeerJ. 2016 Apr 19;4:e1966. doi: 10.7717/peerj.1966 (PMC4841222; doi:10.7717/peerj.1966)
Supplement: Supplemental Information 1 [file peerj-04-1966-s006.zip › scripts/5 match OTUs/blast_hits/OTU_238.csv_plot.pdf]

OTU_238.csv

Pedicia albivitta

Pediciidae

Diptera

86.36

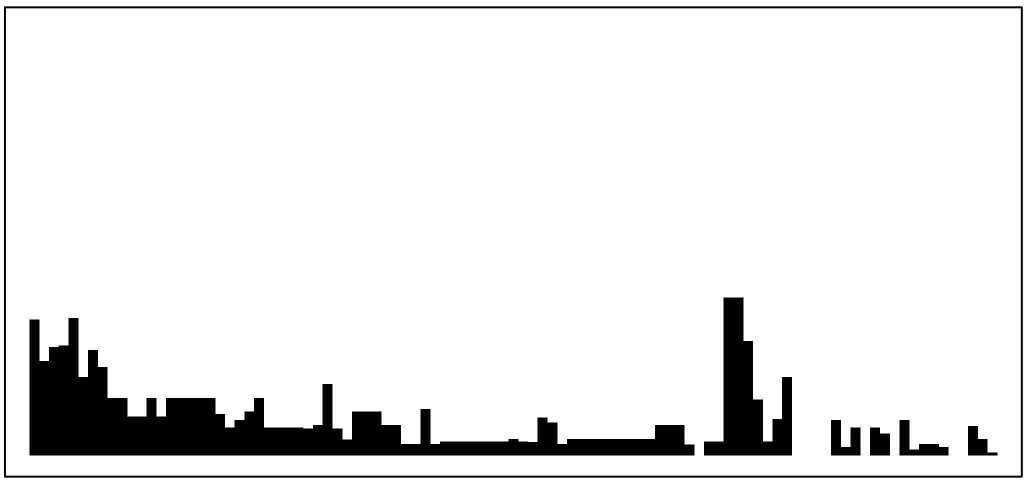

Pedicia albivitta voucher O'Grady 200012 16S ribos	EU005435	86.36	98.08	9.24504e−42	Diptera	Pediciidae	Pedicia albivitta
Edwardsina gigantea 16S ribosomal RNA gene, partia	KC177460	84.42	98.72	4.78905e−39	Diptera	Blephariceridae	Edwardsina gigantea
Bolitophila cinerea voucher JSB1 16S ribosomal RNA	KJ136712	85.06	97.44	5.83426e−38	Diptera	Mycetophilidae	Bolitophila cinerea
Eudicrana sp. DK−2013 voucher JSS12 16S ribosomal	KC435571	85.14	93.59	7.10758e−37	Diptera	Mycetophilidae	Eudicrana sp. DK−2013
Bolitophila sp. ER−2009 16S ribosomal RNA gene, pa	FJ172021	86.43	89.1	2.48079e−36	Diptera	Mycetophilidae	Bolitophila sp. ER−2009
Corynoptera blanda voucher 955 16S ribosomal RNA g	JQ613965	83.66	97.44	8.65881e−36	Diptera	Sciaridae	Corynoptera blanda
Tipula sp. PMO 200011 16S ribosomal RNA gene, part	EU005437	84.93	92.95	3.02222e−35	Diptera	Tipulidae	Tipula sp. PMO 200011
Parochlus steinenii isolate Sejong01 mitochondrion	KT003702	84.14	92.95	3.68182e−34	Diptera	Chironomidae	Parochlus steinenii
Tipula abdominalis 16S ribosomal RNA gene, partial	KC177457	82.69	98.08	1.28508e−33	Diptera	Tipulidae	Tipula abdominalis
Tipula abdominalis mitochondrion, partial genome	JN861743	82.69	98.08	1.28508e−33	Diptera	Tipulidae	Tipula abdominalis
Drosophila takahashii cytochrome b (cytb) gene, pa	AF164592	81.82	98.72	1.28508e−33	Diptera	Drosophilidae	Drosophila takahashii
Tinda javana 16S ribosomal RNA gene, partial seque	GU947393	81.82	98.72	1.56555e−32	Diptera	Stratiomyidae	Tinda javana
Chrysoperla nipponensis mitochondrial DNA, complet	AP011623	82.69	98.72	1.56555e−32	Neuroptera	Chrysopidae	Chrysoperla nipponensis
Derodontus emaculatus voucher BMNH:679374 16S ribo	DQ202609	81.82	98.08	1.56555e−32	Coleoptera		Derodontus emaculatus
Chrysoperla agilis isolate A−083 16S large subunit	AY743751	82.69	98.72	1.56555e−32	Neuroptera	Chrysopidae	Chrysoperla agilis
Chrysoperla lucasina isolate L−079 16S large subun	AY743746	82.69	98.72	1.56555e−32	Neuroptera	Chrysopidae	Chrysoperla lucasina
Chrysoperla lucasina isolate L−070 16S large subun	AY743744	82.69	98.72	1.56555e−32	Neuroptera	Chrysopidae	Chrysoperla lucasina
Chrysoperla lucasina isolate L−029 16S large subun	AY743737	82.69	98.72	1.56555e−32	Neuroptera	Chrysopidae	Chrysoperla lucasina
Chrysoperla lucasina isolate L−099 16S large subun	AY743732	82.69	98.72	1.56555e−32	Neuroptera	Chrysopidae	Chrysoperla lucasina
Culcua kolibaci 16S ribosomal RNA gene, partial se	GU947391	81.94	98.72	5.4643e−32	Diptera	Stratiomyidae	Culcua kolibaci
Limonia tahitiensis voucher O'Grady 202004 16S rib	EU005425	81.29	98.08	5.4643e−32	Diptera	Limoniidae	Dicranomyia tahitiensis
Chrysoperla carnea isolate Chrcar3 16S ribosomal R	AY620150	81.65	98.72	5.4643e−32	Neuroptera	Chrysopidae	Chrysoperla carnea
Chrysoperla nipponensis mitochondrion, complete ge	KP027407	82.05	98.72	1.90723e−31	Neuroptera	Chrysopidae	Chrysoperla nipponensis
Cychrus caraboides caraboides voucher CC00817 16S	JQ647184	82.69	98.08	1.90723e−31	Coleoptera	Carabidae	Cychrus caraboides caraboides
Nebria bellorum voucher DHK0509 16S ribosomal RNA	JN847627	81.29	98.72	1.90723e−31	Coleoptera	Carabidae	Nebria bellorum
Nebria lacustris voucher DHK0004 16S ribosomal RNA	JN847615	81.29	98.72	1.90723e−31	Coleoptera	Carabidae	Nebria lacustris
Nebria hudsonica voucher DHK0381a 16S ribosomal RN	JN847614	81.29	98.72	1.90723e−31	Coleoptera	Carabidae	Nebria hudsonica
Nebria gyllenhali voucher DHK0010 16S ribosomal RN	JN847607	81.29	98.72	1.90723e−31	Coleoptera	Carabidae	Nebria gyllenhali
Calosoma sp. BYU−CO241 mitochondrion, complete gen	GU176340	81.25	98.08	1.90723e−31	Coleoptera	Carabidae	Calosoma sp. BYU−CO241
Pterostichus mucronatus mitochondrial gene for 16S	AB253849	81.41	98.08	1.90723e−31	Coleoptera	Carabidae	Pterostichus mucronatus
Bolitophila hybrida 16S ribosomal RNA gene, partia	FJ172002	83.33	91.67	1.90723e−31	Diptera	Mycetophilidae	Bolitophila hybrida
Calosoma scrutator 16S ribosomal RNA gene, partial	EU734850	81.25	98.08	1.90723e−31	Coleoptera	Carabidae	Calosoma scrutator
Mallada luctuosus 16S ribosomal RNA gene, partial	DQ399288	80.75	98.72	1.90723e−31	Neuroptera	Chrysopidae	Pseudomallada luctuosus
Chrysoperla carnea isolate Chrcar2 16S ribosomal R	AY620152	82.05	98.72	1.90723e−31	Neuroptera	Chrysopidae	Chrysoperla carnea
Chrysoperla sp. isolate 82 16S large subunit ribos	AY743750	82.05	98.72	1.90723e−31	Neuroptera	Chrysopidae	Chrysoperla sp. isolate 82
Chrysoperla carnea isolate C−097 16S large subunit	AY743731	82.05	98.72	1.90723e−31	Neuroptera	Chrysopidae	Chrysoperla carnea
Abax parallelus isolate CAar7 16S ribosomal RNA ge	EU694429	81.41	98.72	6.65688e−31	Coleoptera	Carabidae	Abax parallelus
Abax parallelus isolate CAar29 16S ribosomal RNA g	EU694420	81.41	98.72	6.65688e−31	Coleoptera	Carabidae	Abax parallelus
Drosophila malerkotliana voucher 105504 NADH dehyd	EU494357	80.52	98.72	6.65688e−31	Diptera	Drosophilidae	Drosophila malerkotliana
Drosophila malerkotliana cytochrome b (cytb) gene,	AF164579	80.52	98.72	6.65688e−31	Diptera	Drosophilidae	Drosophila malerkotliana
Chrysoperla lucasina isolate L−078 16S large subun	AY743776	82.17	98.72	6.65688e−31	Neuroptera	Chrysopidae	Chrysoperla lucasina
Minettia longipennis mitochondrion partial 16S rRN	AJ439184	80.52	98.72	6.65688e−31	Diptera	Lauxaniidae	Minettia longipennis
Nebria lacustris voucher DHK0066 16S ribosomal RNA	JN847616	80.65	98.72	2.32348e−30	Coleoptera	Carabidae	Nebria lacustris
Nebria gouleti voucher DHK0006 16S ribosomal RNA g	JN847612	80.65	98.72	2.32348e−30	Coleoptera	Carabidae	Nebria gouleti
Nebria subdilatata voucher DHK0012 16S ribosomal R	JN847611	80.65	98.72	2.32348e−30	Coleoptera	Carabidae	Nebria subdilatata
Nebria baicalica voucher DHK0386a 16S ribosomal RN	JN847610	80.65	98.72	2.32348e−30	Coleoptera	Carabidae	Nebria baicalica
Nebria nivalis voucher DHK0388a 16S ribosomal RNA	JN847609	80.65	98.72	2.32348e−30	Coleoptera	Carabidae	Nebria nivalis
Nebria nivalis voucher DHK0387a 16S ribosomal RNA	JN847608	80.65	98.72	2.32348e−30	Coleoptera	Carabidae	Nebria nivalis
Nebria crassicornis voucher DHK0021 16S ribosomal	JN847606	80.65	98.72	2.32348e−30	Coleoptera	Carabidae	Nebria crassicornis
Drosophila funebris voucher 103952 NADH dehydrogen	EU494313	80.77	98.72	2.32348e−30	Diptera	Drosophilidae	Drosophila funebris
Amphizoa lecontei 16S ribosomal RNA gene, partial	AY071771	80.65	98.72	2.32348e−30	Coleoptera	Amphizoidae	Amphizoa lecontei
Chrysoperla rufilabris isolate R−065 16S large sub	AY743741	80.62	98.72	2.32348e−30	Neuroptera	Chrysopidae	Chrysoperla rufilabris
Toxotrypana littoralis 16S ribosomal RNA gene, par	AF152091	81.76	98.72	2.32348e−30	Diptera	Tephritidae	Toxotrypana littoralis
Dysmicohermes ingens mitochondrion, complete genom	KJ806318	81.53	98.72	8.10974e−30	Megaloptera	Corydalidae	Dysmicohermes ingens
Cramptonomyia spenceri mitochondrion, complete gen	JN861747	80.52	98.08	8.10974e−30	Diptera	Pachyneuridae	Cramptonomyia spenceri
Pseudomigadops n. sp. MSL2007 voucher 668381 16S r	EF214073	80.77	98.72	8.10974e−30	Coleoptera	Carabidae	Pseudomigadops n. sp. MSL2007
Pseudomigadops n. sp. MSL2007 voucher 668379 16S r	EF214072	80.77	98.72	8.10974e−30	Coleoptera	Carabidae	Pseudomigadops n. sp. MSL2007
Pseudomigadops n. sp. MSL2007 voucher 668338 16S r	EF214034	80.77	98.72	8.10974e−30	Coleoptera	Carabidae	Pseudomigadops n. sp. MSL2007
Pseudomigadops n. sp. MSL2007 voucher 668317 16S r	EF214022	80.77	98.72	8.10974e−30	Coleoptera	Carabidae	Pseudomigadops n. sp. MSL2007
Pseudomigadops n. sp. MSL2007 voucher 668302 16S r	EF214012	80.77	98.72	8.10974e−30	Coleoptera	Carabidae	Pseudomigadops n. sp. MSL2007
Pseudomigadops n. sp. MSL2007 voucher 668300 16S r	EF214010	80.77	98.72	8.10974e−30	Coleoptera	Carabidae	Pseudomigadops n. sp. MSL2007
Pseudomigadops n. sp. MSL2007 voucher 668299 16S r	EF214009	80.77	98.72	8.10974e−30	Coleoptera	Carabidae	Pseudomigadops n. sp. MSL2007
Pseudomigadops sp. PSM1 mitochondrial 16S rRNA gen	FM994738	80.77	98.72	8.10974e−30	Coleoptera	Carabidae	Pseudomigadops sp. PSM1
Pseudomigadops n. sp. MSL2007 voucher 668369 16S r	EF214062	80.77	98.72	8.10974e−30	Coleoptera	Carabidae	Pseudomigadops n. sp. MSL2007
Chrysoperla carnea isolate C−116 16S large subunit	AY743760	81.41	98.72	8.10974e−30	Neuroptera	Chrysopidae	Chrysoperla carnea
Chrysoperla carnea isolate C−102 16S large subunit	AY743755	81.41	98.72	8.10974e−30	Neuroptera	Chrysopidae	Chrysoperla carnea
Chrysoperla carnea isolate C−089 16S large subunit	AY743729	81.41	98.72	8.10974e−30	Neuroptera	Chrysopidae	Chrysoperla carnea
Aedes vigilax isolate Mi139 mitochondrion, complet	KP995260	80.5	98.72	2.83058e−29	Diptera	Culicidae	Ochlerotatus vigilax
Drosophila ananassae isolate Delhi1 16S ribosomal	JX896435	79.87	98.72	2.83058e−29	Diptera	Drosophilidae	Drosophila ananassae
Pterostichus fasciatopunctatus isolate CAar143 16S	EU694417	80.65	98.08	2.83058e−29	Coleoptera	Carabidae	Pterostichus fasciatopunctatus
Pterostichus fasciatopunctatus isolate CAar142 16S	EU694416	80.65	98.08	2.83058e−29	Coleoptera	Carabidae	Pterostichus fasciatopunctatus
Spelaeiacris tabulae voucher KAL 16S ribosomal RNA	HM594496	87.39	71.15	2.83058e−29	Orthoptera	Rhaphidophoridae	Spelaeiacris tabulae
Spelaeiacris tabulae voucher BOO 16S ribosomal RNA	HM594494	87.39	71.15	2.83058e−29	Orthoptera	Rhaphidophoridae	Spelaeiacris tabulae
Leptomorphus walkeri 16S ribosomal RNA gene, parti	FJ172020	85.37	78.21	2.83058e−29	Diptera	Mycetophilidae	Leptomorphus walkeri
Pyratula zonata 16S ribosomal RNA gene, partial se	FJ172004	82.61	88.46	2.83058e−29	Diptera	Keroplatidae	Pyratula zonata
Conops sp. JFC−2009 isolate CSU0904046119 16S ribo	GQ118390	80.65	98.72	2.83058e−29	Diptera	Conopidae	Conops sp. JFC−2009
Tephritidae sp. HYH−2008 16S ribosomal RNA gene, p	EU926952	81.7	97.44	2.83058e−29	Diptera	Tephritidae	Tephritidae sp. HYH−2008
Dendroxena sexcarinata mitochondrial gene for 16S	AB285535	83.67	92.31	2.83058e−29	Coleoptera	Silphidae	Dendroxena sexcarinata
Drosophila signata voucher 109405 NADH dehydrogena	EU494328	79.87	98.72	2.83058e−29	Diptera	Drosophilidae	Drosophila signata
Drosophila rubida voucher 109400 NADH dehydrogenas	EU494327	79.87	98.72	2.83058e−29	Diptera	Drosophilidae	Drosophila rubida
Drosophila kepulauana voucher 109407 NADH dehydrog	EU494321	79.87	98.72	2.83058e−29	Diptera	Drosophilidae	Drosophila kepulauana
Drosophila hypocausta voucher 103972 NADH dehydrog	EU494319	79.87	98.72	2.83058e−29	Diptera	Drosophilidae	Drosophila hypocausta
Lipoptena cervi 16S ribosomal RNA gene, partial se	EF531114	81.65	98.72	2.83058e−29	Diptera	Hippoboscidae	Lipoptena cervi
Brachypanorpa sp. CP−2006 16S ribosomal RNA gene,	EF041521	80.38	98.08	2.83058e−29	Mecoptera	Panorpodidae	Brachypanorpa sp. CP−2006
Panorpa takenouchii isolate 26tak 16S ribosomal RN	EF041520	81.29	98.08	2.83058e−29	Mecoptera	Panorpidae	Panorpa takenouchii
Drosophila kepulauana 16S ribosomal RNA gene, part	AF387334	79.87	98.72	2.83058e−29	Diptera	Drosophilidae	Drosophila kepulauana
Panorpa takenouchii 16S ribosomal RNA gene, partia	AF180064	81.29	98.08	2.83058e−29	Mecoptera	Panorpidae	Panorpa takenouchii
Chrysoperla carnea isolate C−101 16S large subunit	AY743754	81.01	98.72	2.83058e−29	Neuroptera	Chrysopidae	Chrysoperla carnea
Sarcophagidae sp. BAHAR107−14 voucher BAH_201312_P	KP253964	79.62	98.72	9.87969e−29	Diptera	Sarcophagidae	Sarcophagidae sp. BAHAR107−14
Acilius athabascae voucher 305G09/BMNH799009 16S r	KF979079	81.65	98.72	9.87969e−29	Coleoptera	Dytiscidae	Acilius athabascae
Phthinia winnertzi voucher JSS2 16S ribosomal RNA	KC435591	80.26	97.44	9.87969e−29	Diptera	Mycetophilidae	Phthinia winnertzi
Trichocera brevicornis 16S ribosomal RNA gene, par	KC177458	80.52	98.08	9.87969e−29	Diptera	Trichoceridae	Trichocera brevicornis
Trichocera bimacula mitochondrion, complete genome	JN861750	80.52	98.08	9.87969e−29	Diptera	Trichoceridae	Trichocera bimacula
Drepanepteryx phalaenoides 16S ribosomal RNA gene,	EU734861	80.38	97.44	9.87969e−29	Neuroptera	Hemerobiidae	Drepanepteryx phalaenoides
Papuadytes aubei mitochondrial 16S rRNA gene (part	AM404022	80	98.72	9.87969e−29	Coleoptera	Dytiscidae	Papuadytes aubei
Papuadytes aubei mitochondrial 16S rRNA gene (part	AM404074	80	98.72	9.87969e−29	Coleoptera	Dytiscidae	Papuadytes aubei
Notomma n. sp. 1000 16S ribosomal RNA gene, partia	AY792108	81.38	92.31	9.87969e−29	Diptera	Tephritidae	Notomma n. sp. 1000
Ochlerotatus nipponicus isolate A75 16S ribosomal	DQ397914	80.77	98.72	9.87969e−29	Diptera	Culicidae	Aedes nipponicus
Leucochrysa marquezi voucher CASC 00212 16S riboso	DQ399280	80.13	98.72	9.87969e−29	Neuroptera	Chrysopidae	Leucochrysa marquezi
Papuadytes simplex 2 note−SubSrc cryptic species 2	AY334154	80	98.72	9.87969e−29	Coleoptera	Dytiscidae	Papuadytes aff. simplex MB107
