# Supplementary material for: Testing the potential of a ribosomal 16S marker for DNA metabarcoding of insects
Source: PeerJ. 2016 Apr 19;4:e1966. doi: 10.7717/peerj.1966 (PMC4841222; doi:10.7717/peerj.1966)
Supplement: Supplemental Information 1 [file peerj-04-1966-s006.zip › scripts/5 match OTUs/blast_hits/OTU_250.csv_plot.pdf]

OTU_250.csv

Oreodytes sanmarkii

Dytiscidae

Coleoptera

99.36

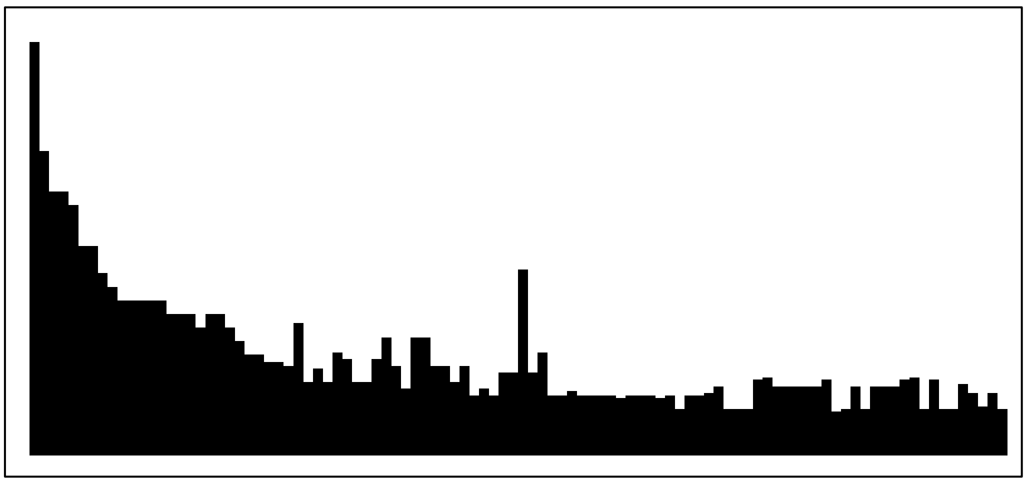

Oreodytes sanmarkii mitochondrial 16S rRNA gene (p	HF931524	99.36	99.37	1.58862e−70	Coleoptera	Dytiscidae	Oreodytes sanmarkii
Oreodytes obesus cordillerensis mitochondrial part	HF931516	94.27	99.37	1.14388e−59	Coleoptera	Dytiscidae	Oreodytes obesus cordillerensis
Oreodytes crassulus partial 16S rRNA gene	AJ850348	92.36	99.37	7.21868e−56	Coleoptera	Dytiscidae	Oreodytes crassulus
Oreodytes congruus partial 16S rRNA gene	AJ850347	92.36	99.37	7.21868e−56	Coleoptera	Dytiscidae	Oreodytes congruus
Oreodytes congruus 16S ribosomal RNA gene, partial	KF575550	91.72	99.37	3.06946e−54	Coleoptera	Dytiscidae	Oreodytes congruus
Oreodytes abbreviatus mitochondrial 16S rRNA gene	HF931477	89.81	99.37	1.93704e−50	Coleoptera	Dytiscidae	Oreodytes abbreviatus
Oreodytes rhyacophilus partial 16S rRNA gene	AJ850352	89.81	99.37	1.93704e−50	Coleoptera	Dytiscidae	Oreodytes rhyacophilus
Oreodytes mongolicus mitochondrial 16S rRNA gene (	HF931491	88.54	99.37	1.00341e−47	Coleoptera	Dytiscidae	Oreodytes mongolicus
Oreodytes snoqualmie mitochondrial partial 16S rRN	HF931519	87.9	99.37	4.26662e−46	Coleoptera	Dytiscidae	Oreodytes snoqualmie
Oreodytes septentrionalis mitochondrial 16S rRNA g	HF931492	87.26	99.37	5.1978e−45	Coleoptera	Dytiscidae	Oreodytes septentrionalis
Oreodytes shorti mitochondrial 16S rRNA gene (part	HF931488	87.26	99.37	5.1978e−45	Coleoptera	Dytiscidae	Oreodytes shorti
Boreonectes riberae mitochondrial 16S rRNA gene (p	HF931461	87.26	99.37	6.33222e−44	Coleoptera	Dytiscidae	Boreonectes riberae
Boreonectes griseostriatus mitochondrial partial 1	HF931387	87.26	99.37	6.33222e−44	Coleoptera	Dytiscidae	Boreonectes griseostriatus
Boreonectes multilineatus mitochondrial partial 16	HF931384	87.26	99.37	6.33222e−44	Coleoptera	Dytiscidae	Boreonectes multilineatus
Laccornis difformis 16S ribosomal RNA gene, partia	KF575544	86.62	99.37	2.21016e−43	Coleoptera	Dytiscidae	Laccornis difformis
Boreonectes alpestris mitochondrial 16S rRNA gene	HF931512	86.62	99.37	2.69253e−42	Coleoptera	Dytiscidae	Boreonectes alpestris
Boreonectes striatellus mitochondrial 16S rRNA gen	HF931511	86.62	99.37	2.69253e−42	Coleoptera	Dytiscidae	Boreonectes striatellus
Deronectes parvicollis mitochondrial 16S rRNA gene	HF931454	85.99	99.37	2.69253e−42	Coleoptera	Dytiscidae	Deronectes parvicollis
Boreonectes macedonicus mitochondrial 16S rRNA gen	HF931359	86.62	99.37	2.69253e−42	Coleoptera	Dytiscidae	Boreonectes macedonicus
Stictotarsus ibericus 16S ribosomal RNA (rrnL) gen	EF670030	86.62	99.37	2.69253e−42	Coleoptera	Dytiscidae	Stictotarsus ibericus
Boreonectes griseostriatus mitochondrial 16S rRNA	HF931541	85.99	99.37	3.28017e−41	Coleoptera	Dytiscidae	Boreonectes griseostriatus
Laccornis kocae partial 16S rRNA gene	AJ850419	85.35	99.37	1.14489e−40	Coleoptera	Dytiscidae	Laccornis kocae
Oreodytes scitulus 16S ribosomal RNA gene, partial	KF575551	84.71	99.37	1.39476e−39	Coleoptera	Dytiscidae	Oreodytes scitulus
Oreodytes scitulus mitochondrial partial 16S rRNA	HF931515	84.71	99.37	1.39476e−39	Coleoptera	Dytiscidae	Oreodytes scitulus
Aethionectes fulvonotatus voucher KBMAeop406 16S r	KF979088	84.38	99.37	1.69917e−38	Coleoptera	Dytiscidae	Aethionectes fulvonotatus
Scarodytes sp. IR−2003 16S ribosomal RNA gene, par	AY250939	84.38	99.37	5.93069e−38	Coleoptera	Dytiscidae	Scarodytes sp. IR−2003
Nebrioporus formaster mitochondrial partial 16S rR	HF931377	84.18	99.37	2.07001e−37	Coleoptera	Dytiscidae	Nebrioporus formaster
Deronectes moestus mitochondrial 16S rRNA gene (pa	HE613726	86.21	91.14	2.07001e−37	Coleoptera	Dytiscidae	Deronectes moestus
Suphrodytes dorsalis 16S ribosomal RNA gene, parti	KF575577	83.44	99.37	7.22506e−37	Coleoptera	Dytiscidae	Suphrodytes dorsalis
Boreonectes griseostriatus mitochondrial 16S rRNA	HF931385	84.08	99.37	7.22506e−37	Coleoptera	Dytiscidae	Boreonectes griseostriatus
Suphrodytes dorsalis voucher BMNH:725966 16S ribos	JX221574	83.44	99.37	7.22506e−37	Coleoptera	Dytiscidae	Suphrodytes dorsalis
Hydroporus foveolatus mitochondrial 16S rRNA gene	HE613724	84.81	99.37	7.22506e−37	Coleoptera	Dytiscidae	Hydroporus foveolatus
Deronectes opatrinus mitochondrial 16S rRNA gene (	HE613701	84.52	97.47	7.22506e−37	Coleoptera	Dytiscidae	Deronectes opatrinus
Copelatus sp. BI−2011 voucher BMNH792945 16S ribos	HQ381441	83.44	99.37	7.22506e−37	Coleoptera	Dytiscidae	Copelatus sp. BI−2011
Oreodytes natrix partial 16S rRNA gene	AJ850349	83.44	99.37	7.22506e−37	Coleoptera	Dytiscidae	Oreodytes natrix
Stictotarsus maghrebinus 16S ribosomal RNA gene, p	AY250947	84.52	97.47	7.22506e−37	Coleoptera	Dytiscidae	Stictotarsus maghrebinus
Deronectes moestus 16S ribosomal RNA gene, partial	KF575561	85.52	91.14	8.80193e−36	Coleoptera	Dytiscidae	Deronectes moestus
Hydroporus tessellatus 16S ribosomal RNA gene, par	JX434770	84.18	99.37	8.80193e−36	Coleoptera	Dytiscidae	Hydroporus tessellatus
Deronectes bicostatus mitochondrial 16S rRNA gene	HE613705	83.12	97.47	8.80193e−36	Coleoptera	Dytiscidae	Deronectes bicostatus
Deronectes brannani mitochondrial 16S rRNA gene (p	HE613695	85.52	91.14	8.80193e−36	Coleoptera	Dytiscidae	Deronectes brannanii
Deronectes moestus mitochondrial 16S rRNA gene (pa	HE613683	85.52	91.14	8.80193e−36	Coleoptera	Dytiscidae	Deronectes moestus
Hydroporus zimmermanni mitochondrial 16S rRNA gene	HE613679	84.18	99.37	8.80193e−36	Coleoptera	Dytiscidae	Hydroporus zimmermanni
Hydroporus basinotatus mitochondrial 16S rRNA gene	HE613678	84.18	99.37	8.80193e−36	Coleoptera	Dytiscidae	Hydroporus basinotatus
Hydroporus glabriusculus 16S ribosomal RNA gene, p	AY365270	83.44	99.37	8.80193e−36	Coleoptera	Dytiscidae	Hydroporus glabriusculus
Hydroporus basinotatus 16S ribosomal RNA gene, par	AF518258	84.18	99.37	8.80193e−36	Coleoptera	Dytiscidae	Hydroporus basinotatus
Hydaticus cinctipennis voucher UNM KBMHyci20 16S r	KJ548338	82.8	99.37	3.07217e−35	Coleoptera	Dytiscidae	Hydaticus cinctipennis
Hygrotus impressopunctatus voucher UNM KBMHtim80 1	KJ548334	83.12	99.37	3.07217e−35	Coleoptera	Dytiscidae	Hygrotus impressopunctatus
Oreodytes quadrimaculatus 16S ribosomal RNA gene,	KF575567	82.8	99.37	3.07217e−35	Coleoptera	Dytiscidae	Oreodytes quadrimaculatus
Stictotarsus procerus mitochondrial 16S rRNA gene	HF931379	83.87	97.47	3.07217e−35	Coleoptera	Dytiscidae	Stictotarsus procerus
Deronectes doriae mitochondrial 16S rRNA gene (par	HE613710	83.87	97.47	3.07217e−35	Coleoptera	Dytiscidae	Deronectes doriae
Hydroporus marginatus mitochondrial 16S rRNA gene	HE613699	88.71	78.48	3.07217e−35	Coleoptera	Dytiscidae	Hydroporus marginatus
Deronectes sahlbergi mitochondrial 16S rRNA gene (	HE613680	83.87	97.47	3.07217e−35	Coleoptera	Dytiscidae	Deronectes sahlbergi
Hovahydrus sp. BI−2011 voucher BMNH831333 16S ribo	HQ381524	84.81	98.73	3.07217e−35	Coleoptera	Dytiscidae	Hovahydrus sp. BI−2011
Copelatus sp. BI−2011 voucher BMNH671215 16S ribos	HQ381377	82.8	99.37	3.07217e−35	Coleoptera	Dytiscidae	Copelatus sp. BI−2011
Stictotarsus falli 16S ribosomal RNA (rrnL) gene,	EF670029	82.8	99.37	3.07217e−35	Coleoptera	Dytiscidae	Stictotarsus falli
Hygrotus impressopunctatus partial 16S rRNA gene	AJ850405	83.02	99.37	3.07217e−35	Coleoptera	Dytiscidae	Hygrotus impressopunctatus
Oreodytes quadrimaculatus partial 16S rRNA gene	AJ850351	82.8	99.37	3.07217e−35	Coleoptera	Dytiscidae	Oreodytes quadrimaculatus
Hygrotus novemlineatus mitochondrial partial 16S r	HF931523	82.8	99.37	1.07229e−34	Coleoptera	Dytiscidae	Hygrotus novemlineatus
Hygrotus marklini mitochondrial 16S rRNA gene (par	HF931467	82.8	99.37	1.07229e−34	Coleoptera	Dytiscidae	Hygrotus marklini
Suphrodytes figuratus voucher BMNH:722148 16S ribo	JX221571	82.8	99.37	1.07229e−34	Coleoptera	Dytiscidae	Suphrodytes figuratus
Antiporus hollingsworthi mitochondrial partial 16S	FR727246	82.69	98.73	1.07229e−34	Coleoptera	Dytiscidae	Antiporus hollingsworthi
Hygrotus turbidus partial 16S rRNA gene	AJ850390	82.8	99.37	1.07229e−34	Coleoptera	Dytiscidae	Hygrotus turbidus
Hygrotus corpulentus partial 16S rRNA gene	AJ850387	82.8	99.37	1.07229e−34	Coleoptera	Dytiscidae	Hygrotus corpulentus
Hydroporus sp. IR33.4 16S ribosomal RNA gene, part	AY365261	82.8	99.37	1.07229e−34	Coleoptera	Dytiscidae	Hydroporus sp. IR33.4
Nebrioporus assimilis 16S ribosomal RNA gene, part	AY250921	82.69	98.73	1.07229e−34	Coleoptera	Dytiscidae	Nebrioporus assimilis
Hydroporus marginatus 16S ribosomal RNA gene, part	AF518267	82.8	99.37	1.07229e−34	Coleoptera	Dytiscidae	Hydroporus marginatus
Agabus glacialis mitochondrial 16S rRNA gene (part	LM654776	82.17	99.37	3.74267e−34	Coleoptera	Dytiscidae	Agabus glacialis
Agabus didymus mitochondrial 16S rRNA gene (partia	LM654773	82.8	99.37	3.74267e−34	Coleoptera	Dytiscidae	Agabus didymus
Agabus didymus voucher UNM KBMPmsp730 16S ribosoma	KJ548392	82.8	99.37	3.74267e−34	Coleoptera	Dytiscidae	Agabus didymus
Herophydrus rohani voucher UNM KBMHpsp574 16S ribo	KJ548382	82.91	99.37	3.74267e−34	Coleoptera	Dytiscidae	Herophydrus rohani
Deronectes aubei voucher UNM KBMDeau758 16S riboso	KJ548292	83.23	97.47	3.74267e−34	Coleoptera	Dytiscidae	Deronectes aubei
Ilybius neglectus mitochondrial 16S rRNA gene (par	HF931450	82.17	99.37	3.74267e−34	Coleoptera	Dytiscidae	Ilybius neglectus
Agabus dichrous mitochondrial partial 16S rRNA gen	HF931378	82.17	99.37	3.74267e−34	Coleoptera	Dytiscidae	Agabus dichrous
Agabus godmanni mitochondrial 16S rRNA gene (parti	HF931348	82.17	99.37	3.74267e−34	Coleoptera	Dytiscidae	Agabus godmanni
Hydroporus analis mitochondrial partial 16S rRNA g	HE613731	83.54	99.37	3.74267e−34	Coleoptera	Dytiscidae	Hydroporus analis
Hydroporus fuscipennis mitochondrial 16S rRNA gene	HE613730	83.65	99.37	3.74267e−34	Coleoptera	Dytiscidae	Hydroporus fuscipennis
Deronectes costipennis mitochondrial 16S rRNA gene	HE613697	83.23	97.47	3.74267e−34	Coleoptera	Dytiscidae	Deronectes costipennis
Deronectes platynotus mitochondrial 16S rRNA gene	HE613682	83.23	97.47	3.74267e−34	Coleoptera	Dytiscidae	Deronectes platynotus
Deronectes depressicollis mitochondrial 16S rRNA g	HE613681	83.23	97.47	3.74267e−34	Coleoptera	Dytiscidae	Deronectes depressicollis
Deronectes semirufus mitochondrial 16S rRNA gene (	HE613676	83.23	97.47	3.74267e−34	Coleoptera	Dytiscidae	Deronectes semirufus
Deronectes semirufus mitochondrial 16S rRNA gene (	HE613674	83.23	97.47	3.74267e−34	Coleoptera	Dytiscidae	Deronectes semirufus
Hydroporus compunctus mitochondrial 16S rRNA gene	HE599693	83.54	99.37	3.74267e−34	Coleoptera	Dytiscidae	Hydroporus compunctus
Antiporus hollingsworthi mitochondrial partial 16S	FR727247	82.05	98.73	3.74267e−34	Coleoptera	Dytiscidae	Antiporus hollingsworthi
Nirripirti byroensis isolate ABTC78677−8 16S ribos	EU616910	82.17	99.37	3.74267e−34	Coleoptera	Dytiscidae	Paroster byroensis
Deronectes costipennis 16S ribosomal RNA gene, par	AY250951	83.23	97.47	3.74267e−34	Coleoptera	Dytiscidae	Deronectes costipennis
Nebrioporus airumlus 16S ribosomal RNA gene, parti	AY250920	82.17	99.37	3.74267e−34	Coleoptera	Dytiscidae	Nebrioporus airumlus
Deronectes aubei sanfilippoi haplotype 2 16S ribos	AY250908	83.23	97.47	3.74267e−34	Coleoptera	Dytiscidae	Deronectes aubei sanfilippoi
Deronectes aubei sanfilippoi haplotype 1 16S ribos	AY250907	83.23	97.47	3.74267e−34	Coleoptera	Dytiscidae	Deronectes aubei sanfilippoi
Deronectes aubei aubei haplotype 2 16S ribosomal R	AY250906	83.23	97.47	3.74267e−34	Coleoptera	Dytiscidae	Deronectes aubei aubei
Hydroporus compunctus 16S ribosomal RNA gene, part	AF518259	83.54	99.37	3.74267e−34	Coleoptera	Dytiscidae	Hydroporus compunctus
Hydroporus fuscipennis 16S ribosomal RNA gene, par	AF518262	83.65	99.37	3.74267e−34	Coleoptera	Dytiscidae	Hydroporus fuscipennis
Ilybius satuni 16S ribosomal RNA gene, partial seq	AY138670	82.17	99.37	3.74267e−34	Coleoptera	Dytiscidae	Ilybius satunini
Ilybius erichsoni 16S ribosomal RNA gene, partial	AY138661	83.54	99.37	3.74267e−34	Coleoptera	Dytiscidae	Ilybius erichsoni
Agabus dilatatus 16S ribosomal RNA gene, partial s	AY039261	82.17	99.37	3.74267e−34	Coleoptera	Dytiscidae	Agabus dilatatus
Agabus bifarius 16S ribosomal RNA gene, partial se	AY138605	82.17	99.37	3.74267e−34	Coleoptera	Dytiscidae	Agabus bifarius
Deronectes hispanicus mitochondrial 16S rRNA gene	HE613727	83.33	97.47	1.30632e−33	Coleoptera	Dytiscidae	Deronectes hispanicus
Nirripirti megamacrocephalus isolate ABTC78781 16S	EU616935	82.91	99.37	1.30632e−33	Coleoptera	Dytiscidae	Paroster megamacrocephalus
Scarodytes halensis haplotype 3 16S ribosomal RNA	AY250937	82.28	99.37	1.30632e−33	Coleoptera	Dytiscidae	Scarodytes halensis
Herophydrus inquinatus 16S ribosomal RNA gene, par	KF575572	82.91	99.37	4.55951e−33	Coleoptera	Dytiscidae	Herophydrus inquinatus
Hydaticus aruspex voucher NHRS/JLKB000000298 16S r	KF979119	82.17	99.37	4.55951e−33	Coleoptera	Dytiscidae	Hydaticus aruspex
