# Supplementary material for: Testing the potential of a ribosomal 16S marker for DNA metabarcoding of insects
Source: PeerJ. 2016 Apr 19;4:e1966. doi: 10.7717/peerj.1966 (PMC4841222; doi:10.7717/peerj.1966)
Supplement: Supplemental Information 1 [file peerj-04-1966-s006.zip › scripts/5 match OTUs/blast_hits/OTU_260.csv_plot.pdf]

OTU_260.csv

Oreodytes sanmarkii

Dytiscidae

Coleoptera

98.09

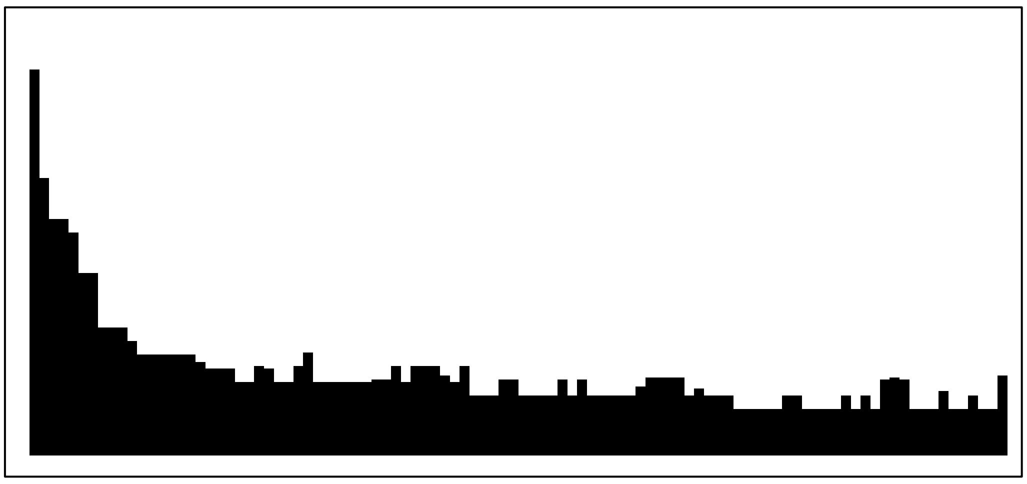

Oreodytes sanmarkii mitochondrial 16S rRNA gene (p	HF931524	98.09	100	6.70005e−69	Coleoptera	Dytiscidae	Oreodytes sanmarkii
Oreodytes obesus cordillerensis mitochondrial part	HF931516	92.99	100	4.82437e−58	Coleoptera	Dytiscidae	Oreodytes obesus cordillerensis
Oreodytes crassulus partial 16S rRNA gene	AJ850348	91.08	100	3.04451e−54	Coleoptera	Dytiscidae	Oreodytes crassulus
Oreodytes congruus partial 16S rRNA gene	AJ850347	91.08	100	3.04451e−54	Coleoptera	Dytiscidae	Oreodytes congruus
Oreodytes congruus 16S ribosomal RNA gene, partial	KF575550	90.45	100	1.29456e−52	Coleoptera	Dytiscidae	Oreodytes congruus
Oreodytes abbreviatus mitochondrial 16S rRNA gene	HF931477	88.54	100	8.16955e−49	Coleoptera	Dytiscidae	Oreodytes abbreviatus
Oreodytes rhyacophilus partial 16S rRNA gene	AJ850352	88.54	100	8.16955e−49	Coleoptera	Dytiscidae	Oreodytes rhyacophilus
Laccornis difformis 16S ribosomal RNA gene, partia	KF575544	85.99	100	2.19219e−43	Coleoptera	Dytiscidae	Laccornis difformis
Lancetes nigriceps partial 16S rRNA gene	AJ850418	85.99	100	2.19219e−43	Coleoptera	Dytiscidae	Lancetes nigriceps
Lancetes nigriceps 16S ribosomal RNA gene, partial	AY334132	85.99	100	2.19219e−43	Coleoptera	Dytiscidae	Lancetes nigriceps
Lancetes nigriceps voucher UNM KBMLcni390 16S ribo	KJ548350	85.35	100	9.32145e−42	Coleoptera	Dytiscidae	Lancetes nigriceps
Ilybius fraterculus mitochondrial 16S rRNA gene (p	LM654800	84.71	100	1.13558e−40	Coleoptera	Dytiscidae	Ilybius fraterculus
Ilybius ater mitochondrial 16S rRNA gene (partial)	LM654794	84.71	100	1.13558e−40	Coleoptera	Dytiscidae	Ilybius ater
Lancetes varius voucher UNM KBMLcva196 16S ribosom	KJ548351	84.71	100	1.13558e−40	Coleoptera	Dytiscidae	Lancetes varius
Lancetes angusticollis voucher UNM KBMLcan301 16S	KJ548347	84.71	100	1.13558e−40	Coleoptera	Dytiscidae	Lancetes angusticollis
Ilybius fraterculus voucher UNM KBMIlfr409 16S rib	KJ548342	84.71	100	1.13558e−40	Coleoptera	Dytiscidae	Ilybius fraterculus
Hydaticus cinctipennis voucher UNM KBMHyci20 16S r	KJ548338	84.71	100	1.13558e−40	Coleoptera	Dytiscidae	Hydaticus cinctipennis
Aethionectes fulvonotatus voucher KBMAeop406 16S r	KF979088	84.38	100	1.38343e−39	Coleoptera	Dytiscidae	Aethionectes fulvonotatus
Lancetes lanceolatus voucher UNM KBMLcla91 16S rib	KJ548348	84.08	100	4.82863e−39	Coleoptera	Dytiscidae	Lancetes lanceolatus
Lancetes varius 16S ribosomal RNA gene, partial se	AY071784	84.08	100	4.82863e−39	Coleoptera	Dytiscidae	Lancetes varius
Lancetes lanceolatus 16S ribosomal RNA gene, parti	AY138673	84.08	100	4.82863e−39	Coleoptera	Dytiscidae	Lancetes lanceolatus
Agabus glacialis mitochondrial 16S rRNA gene (part	LM654776	83.44	100	5.88247e−38	Coleoptera	Dytiscidae	Agabus glacialis
Hydaticus aruspex voucher UNM KBMHyar68 16S riboso	KJ548337	83.44	100	5.88247e−38	Coleoptera	Dytiscidae	Hydaticus aruspex
Suphrodytes dorsalis 16S ribosomal RNA gene, parti	KF575577	84.18	100	5.88247e−38	Coleoptera	Dytiscidae	Suphrodytes dorsalis
Hydaticus aruspex voucher NHRS/JLKB000000298 16S r	KF979119	84.08	100	5.88247e−38	Coleoptera	Dytiscidae	Hydaticus aruspex
Agabus dichrous mitochondrial partial 16S rRNA gen	HF931378	83.44	100	5.88247e−38	Coleoptera	Dytiscidae	Agabus dichrous
Agabus godmanni mitochondrial 16S rRNA gene (parti	HF931348	83.44	100	5.88247e−38	Coleoptera	Dytiscidae	Agabus godmanni
Suphrodytes dorsalis voucher BMNH:725966 16S ribos	JX221574	84.18	100	5.88247e−38	Coleoptera	Dytiscidae	Suphrodytes dorsalis
Hydroporus foveolatus mitochondrial 16S rRNA gene	HE613724	84.81	100	5.88247e−38	Coleoptera	Dytiscidae	Hydroporus foveolatus
Hydaticus transversalis partial 16S rRNA gene	AJ850365	83.44	100	5.88247e−38	Coleoptera	Dytiscidae	Prodaticus transversalis
Hydaticus transversalis 16S ribosomal RNA gene, pa	AY334126	83.44	100	5.88247e−38	Coleoptera	Dytiscidae	Prodaticus transversalis
Ilybius gagates 16S ribosomal RNA gene, partial se	AY138663	83.44	100	5.88247e−38	Coleoptera	Dytiscidae	Ilybius gagates
Aglymbus gestroi 16S ribosomal RNA gene, partial s	AY138639	83.44	100	5.88247e−38	Coleoptera	Dytiscidae	Aglymbus gestroi
Agabus dilatatus 16S ribosomal RNA gene, partial s	AY039261	83.44	100	5.88247e−38	Coleoptera	Dytiscidae	Agabus dilatatus
Agabus bifarius 16S ribosomal RNA gene, partial se	AY138605	83.44	100	5.88247e−38	Coleoptera	Dytiscidae	Agabus bifarius
Lancetes waterhousei voucher UNM KBMLcwa303 16S ri	KJ548352	83.54	100	2.05319e−37	Coleoptera	Dytiscidae	Lancetes waterhousei
Lancetes marginatus voucher UNM KBMLcma302 16S rib	KJ548349	83.54	100	2.05319e−37	Coleoptera	Dytiscidae	Lancetes marginatus
Ilybius pseudoneglectus mitochondrial 16S rRNA gen	HF931542	84.18	100	2.05319e−37	Coleoptera	Dytiscidae	Ilybius pseudoneglectus
Hydroporus umbrosus voucher UNM KBMHyum551 16S rib	KJ548394	83.44	100	7.16632e−37	Coleoptera	Dytiscidae	Hydroporus umbrosus
Hydroporus tessellatus 16S ribosomal RNA gene, par	JX434770	84.18	100	7.16632e−37	Coleoptera	Dytiscidae	Hydroporus tessellatus
Hydroporus zimmermanni mitochondrial 16S rRNA gene	HE613679	84.18	100	7.16632e−37	Coleoptera	Dytiscidae	Hydroporus zimmermanni
Hydroporus basinotatus mitochondrial 16S rRNA gene	HE613678	84.18	100	7.16632e−37	Coleoptera	Dytiscidae	Hydroporus basinotatus
Acilius duvergeri partial 16S rRNA gene	AJ850357	83.75	100	7.16632e−37	Coleoptera	Dytiscidae	Acilius duvergeri
Hydroporus umbrosus 16S ribosomal RNA gene, partia	AF518280	83.44	100	7.16632e−37	Coleoptera	Dytiscidae	Hydroporus umbrosus
Hydroporus basinotatus 16S ribosomal RNA gene, par	AF518258	84.18	100	7.16632e−37	Coleoptera	Dytiscidae	Hydroporus basinotatus
Ilybius similis mitochondrial 16S rRNA gene (parti	LM654807	82.8	100	2.50129e−36	Coleoptera	Dytiscidae	Ilybius similis
Ilybius lineellus mitochondrial 16S rRNA gene (par	LM654802	82.8	100	2.50129e−36	Coleoptera	Dytiscidae	Ilybius lineellus
Prodaticus fabricii voucher UNM KBMHyfa119 16S rib	KJ548340	82.8	100	2.50129e−36	Coleoptera	Dytiscidae	Prodaticus fabricii
Thermonectus margineguttatus voucher KBMThmg199 16	KF979117	83.54	100	2.50129e−36	Coleoptera	Dytiscidae	Thermonectus margineguttatus
Ilybius pederzanii mitochondrial 16S rRNA gene (pa	HF931460	83.54	100	2.50129e−36	Coleoptera	Dytiscidae	Ilybius pederzanii
Agabus cephalotes mitochondrial 16S rRNA gene (par	HF931380	82.8	100	2.50129e−36	Coleoptera	Dytiscidae	Agabus cephalotes
Graphoderus cinereus voucher BMNH829956 16S riboso	HQ381495	82.8	100	2.50129e−36	Coleoptera	Dytiscidae	Graphoderus cinereus
Rhantus bistriatus mitochondrial DNA sequence cont	FN298834	82.8	100	2.50129e−36	Coleoptera	Dytiscidae	Rhantus bistriatus
Prodaticus pictus partial 16S rRNA gene	AJ850372	82.8	100	2.50129e−36	Coleoptera	Dytiscidae	Prodaticus pictus
Ilybius chalconatus haplotype 3 16S ribosomal RNA	AF428200	83.54	100	2.50129e−36	Coleoptera	Dytiscidae	Ilybius chalconatus
Ilybius walsinghami isolate Ilydisr 16S ribosomal	AY138660	82.8	100	2.50129e−36	Coleoptera	Dytiscidae	Ilybius walsinghami
Ilybiosoma perplexus 16S ribosomal RNA gene, parti	AY138650	83.54	100	2.50129e−36	Coleoptera	Dytiscidae	Ilybiosoma perplexus
Agabus paludosus 16S ribosomal RNA gene, partial s	AY138625	82.8	100	2.50129e−36	Coleoptera	Dytiscidae	Agabus paludosus
Agabus conspersus 16S ribosomal RNA gene, partial	AY138610	82.8	100	2.50129e−36	Coleoptera	Dytiscidae	Agabus conspersus
Hydroporus puberulus 16S ribosomal RNA gene, parti	JX434766	82.8	100	8.73037e−36	Coleoptera	Dytiscidae	Hydroporus puberulus
Hygrotus novemlineatus mitochondrial partial 16S r	HF931523	82.8	100	8.73037e−36	Coleoptera	Dytiscidae	Hygrotus novemlineatus
Suphrodytes figuratus voucher BMNH:722148 16S ribo	JX221571	82.8	100	8.73037e−36	Coleoptera	Dytiscidae	Suphrodytes figuratus
Hydroporus marginatus mitochondrial 16S rRNA gene	HE613699	83.23	100	8.73037e−36	Coleoptera	Dytiscidae	Hydroporus marginatus
Pseudomigadops n. sp. MSL2007 voucher 668381 16S r	EF214073	83.65	100	8.73037e−36	Coleoptera	Carabidae	Pseudomigadops n. sp. MSL2007
Pseudomigadops n. sp. MSL2007 voucher 668379 16S r	EF214072	83.65	100	8.73037e−36	Coleoptera	Carabidae	Pseudomigadops n. sp. MSL2007
Pseudomigadops n. sp. MSL2007 voucher 668300 16S r	EF214010	83.65	100	8.73037e−36	Coleoptera	Carabidae	Pseudomigadops n. sp. MSL2007
Pseudomigadops sp. PSM1 mitochondrial 16S rRNA gen	FM994738	83.65	100	8.73037e−36	Coleoptera	Carabidae	Pseudomigadops sp. PSM1
Hygrotus turbidus partial 16S rRNA gene	AJ850390	82.8	100	8.73037e−36	Coleoptera	Dytiscidae	Hygrotus turbidus
Acilius sulcatus partial 16S rRNA gene	AJ850358	83.12	100	8.73037e−36	Coleoptera	Dytiscidae	Acilius sulcatus
Hydroporus puberulus 16S ribosomal RNA gene, parti	AY365283	82.8	100	8.73037e−36	Coleoptera	Dytiscidae	Hydroporus puberulus
Hydroporus sp. IR33.4 16S ribosomal RNA gene, part	AY365261	82.8	100	8.73037e−36	Coleoptera	Dytiscidae	Hydroporus sp. IR33.4
Hydroporus marginatus 16S ribosomal RNA gene, part	AF518267	82.8	100	8.73037e−36	Coleoptera	Dytiscidae	Hydroporus marginatus
Prodaticus luczonicus voucher DBAU−BT−30_B94_Dytis	KM064623	82.17	100	3.0472e−35	Coleoptera	Dytiscidae	Prodaticus luczonicus
Agabus striolatus mitochondrial 16S rRNA gene (par	LM654785	82.17	100	3.0472e−35	Coleoptera	Dytiscidae	Agabus striolatus
Agabus pallidus mitochondrial 16S rRNA gene (parti	LM654780	82.17	100	3.0472e−35	Coleoptera	Dytiscidae	Agabus pallidus
Agabus nebulosus mitochondrial 16S rRNA gene (part	LM654779	82.17	100	3.0472e−35	Coleoptera	Dytiscidae	Agabus nebulosus
Agabus labiatus mitochondrial 16S rRNA gene (parti	LM654778	82.17	100	3.0472e−35	Coleoptera	Dytiscidae	Agabus labiatus
Agabus didymus mitochondrial 16S rRNA gene (partia	LM654773	82.8	100	3.0472e−35	Coleoptera	Dytiscidae	Agabus didymus
Agabus didymus voucher UNM KBMPmsp730 16S ribosoma	KJ548392	82.8	100	3.0472e−35	Coleoptera	Dytiscidae	Agabus didymus
Prodaticus xanthomelas voucher UNM KBMHyxa67 16S r	KJ548341	82.17	100	3.0472e−35	Coleoptera	Dytiscidae	Prodaticus xanthomelas
Cybister marginicollis voucher UNM KBMCyma416 16S	KJ548319	82.17	100	3.0472e−35	Coleoptera	Dytiscidae	Cybister marginicollis
Ilybius sp. UNM KBMAgsp411 16S ribosomal RNA gene,	KJ548304	82.17	100	3.0472e−35	Coleoptera	Dytiscidae	Ilybius sp. UNM KBMAgsp411
Ilybiosoma cordatum voucher UNM KBMAgco57 16S ribo	KJ548301	82.17	100	3.0472e−35	Coleoptera	Dytiscidae	Ilybiosoma cordatum
Stictotarsus roffii 16S ribosomal RNA gene, partia	KF575555	82.8	100	3.0472e−35	Coleoptera	Dytiscidae	Stictotarsus roffii
Thermonectus alfredi voucher KBMThal264 16S riboso	KF979107	82.17	100	3.0472e−35	Coleoptera	Dytiscidae	Thermonectus alfredi
Eretes sticticus voucher 411G2/BMNH832075 16S ribo	KF979076	82.8	100	3.0472e−35	Coleoptera	Dytiscidae	Eretes sticticus
Agabus uliginosus mitochondrial 16S rRNA gene (par	HF931463	82.17	100	3.0472e−35	Coleoptera	Dytiscidae	Agabus uliginosus
Hydroporus analis mitochondrial partial 16S rRNA g	HE613731	83.54	100	3.0472e−35	Coleoptera	Dytiscidae	Hydroporus analis
Hydroporus fuscipennis mitochondrial 16S rRNA gene	HE613730	83.65	100	3.0472e−35	Coleoptera	Dytiscidae	Hydroporus fuscipennis
Hydroporus compunctus mitochondrial 16S rRNA gene	HE599693	83.54	100	3.0472e−35	Coleoptera	Dytiscidae	Hydroporus compunctus
Copelatus sp. BI−2011 voucher BMNH792956 16S ribos	HQ381443	82.17	100	3.0472e−35	Coleoptera	Dytiscidae	Copelatus sp. BI−2011
Copelatus sp. BI−2011 voucher BMNH792915 16S ribos	HQ381439	82.17	100	3.0472e−35	Coleoptera	Dytiscidae	Copelatus sp. BI−2011
Copelatus sp. BI−2011 voucher BMNH729890 16S ribos	HQ381391	82.17	100	3.0472e−35	Coleoptera	Dytiscidae	Copelatus sp. BI−2011
Penetretus temporalis genomic DNA containing mitoc	FR729595	83.02	100	3.0472e−35	Coleoptera	Carabidae	Penetretus temporalis
Rhantus wallisi mitochondrial DNA sequence contain	FN298838	82.17	100	3.0472e−35	Coleoptera	Dytiscidae	Rhantus wallisi
Rhantus suturalis mitochondrial DNA sequence conta	FN298835	82.17	100	3.0472e−35	Coleoptera	Dytiscidae	Rhantus suturalis
Stictotarsus roffi partial 16S rRNA gene	AJ850355	82.8	100	3.0472e−35	Coleoptera	Dytiscidae	Stictotarsus roffii
Papuadytes simplex 2 note−SubSrc cryptic species 2	AY334154	82.17	100	3.0472e−35	Coleoptera	Dytiscidae	Papuadytes aff. simplex MB107
Ilybius vandykei 16S ribosomal RNA gene, partial s	AY138671	82.17	100	3.0472e−35	Coleoptera	Dytiscidae	Ilybius vandykei
Ilybius montanus 16S ribosomal RNA gene, partial s	AY138666	83.75	100	3.0472e−35	Coleoptera	Dytiscidae	Ilybius montanus
