# Supplementary material for: Testing the potential of a ribosomal 16S marker for DNA metabarcoding of insects
Source: PeerJ. 2016 Apr 19;4:e1966. doi: 10.7717/peerj.1966 (PMC4841222; doi:10.7717/peerj.1966)
Supplement: Supplemental Information 1 [file peerj-04-1966-s006.zip › scripts/5 match OTUs/blast_hits/OTU_263.csv_plot.pdf]

OTU_263.csv

Nemurella pictetii

Nemouridae

Plecoptera

98.73

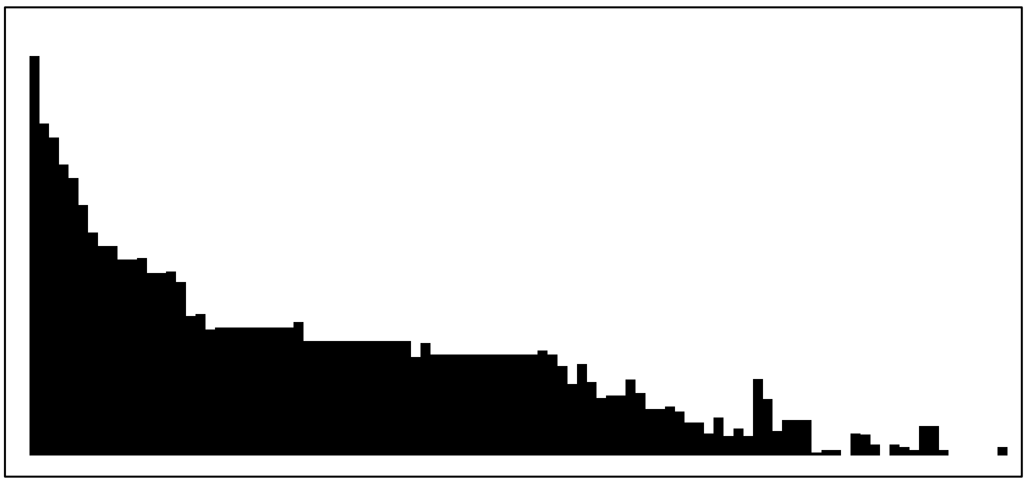

Nemurella pictetii 16S ribosomal RNA gene, partial	EF623192	98.73	100	2.30021e−68	Plecoptera	Nemouridae	Nemurella pictetii
Ostrocerca foersteri 16S ribosomal RNA gene, parti	EF623191	95.54	100	2.62454e−61	Plecoptera	Nemouridae	Ostrocerca foersteri
Visoka cataractae 16S ribosomal RNA gene, partial	EF623189	94.9	100	3.19734e−60	Plecoptera	Nemouridae	Visoka cataractae
Nanonemoura wahkeena 16S ribosomal RNA gene, parti	EF623198	93.63	100	1.65626e−57	Plecoptera	Nemouridae	Nanonemoura wahkeena
Shipsa rotunda 16S ribosomal RNA gene, partial seq	EF623184	92.99	100	7.04262e−56	Plecoptera	Nemouridae	Shipsa rotunda
Paranemoura claasseni 16S ribosomal RNA gene, part	EF623185	91.72	100	3.64817e−53	Plecoptera	Nemouridae	Paranemoura claasseni
Nemoura trispinosa 16S ribosomal RNA gene, partial	EF623187	90.45	100	1.8898e−50	Plecoptera	Nemouridae	Nemoura trispinosa
Lednia tumana 16S ribosomal RNA gene, partial sequ	EF623197	89.81	100	2.30224e−49	Plecoptera	Nemouridae	Lednia tumana
Soyedina producta 16S ribosomal RNA gene, partial	EF623196	89.81	100	2.80471e−48	Plecoptera	Nemouridae	Soyedina producta
Protonemura meyeri 16S ribosomal RNA gene, partial	EF623193	89.17	100	9.78939e−48	Plecoptera	Nemouridae	Protonemura meyeri
Malenka californica 16S ribosomal RNA gene, partia	EF623182	89.17	100	9.78939e−48	Plecoptera	Nemouridae	Malenka californica
Nemoura cinerea 16S ribosomal RNA gene, partial se	EF623194	89.24	100	3.41683e−47	Plecoptera	Nemouridae	Nemoura cinerea
Prostoia besametsa 16S ribosomal RNA gene, partial	EF623188	88.54	100	1.19259e−46	Plecoptera	Nemouridae	Prostoia besametsa
Zapada cinctipes 16S ribosomal RNA gene, partial s	EF623183	88.54	100	1.19259e−46	Plecoptera	Nemouridae	Zapada cinctipes
Scopura montana 16S ribosomal RNA gene, partial se	EF623265	88.61	100	4.16255e−46	Plecoptera		Scopura montana
Oemopteryx vanduzeea 16S ribosomal RNA gene, parti	EF623266	88.12	100	1.45287e−45	Plecoptera	Taeniopterygidae	Oemopteryx vanduzeea
Pteronarcys californica 16S ribosomal RNA gene, pa	EF623261	86.54	100	7.52607e−43	Plecoptera	Pteronarcyidae	Pteronarcys californica
Apteroperla tikumana mitochondrion, complete genom	KR604721	86.62	100	2.62686e−42	Plecoptera	Capniidae	Apteroperla tikumana
Pteronarcys princeps mitochondrion, complete genom	AY687866	85.9	100	9.16864e−42	Plecoptera	Pteronarcyidae	Pteronarcys princeps
Mesocapnia arizonensis mitochondrion, partial geno	KP642637	85.99	100	3.20017e−41	Plecoptera	Capniidae	Mesocapnia arizonensis
Allocapnia recta isolate H13 16S ribosomal RNA gen	KC881048	85.99	100	3.20017e−41	Plecoptera	Capniidae	Allocapnia recta
Allocapnia recta isolate H11 16S ribosomal RNA gen	KC881046	85.99	100	3.20017e−41	Plecoptera	Capniidae	Allocapnia recta
Allocapnia recta isolate H7 16S ribosomal RNA gene	KC881042	85.99	100	3.20017e−41	Plecoptera	Capniidae	Allocapnia recta
Allocapnia recta isolate H3 16S ribosomal RNA gene	KC881038	85.99	100	3.20017e−41	Plecoptera	Capniidae	Allocapnia recta
Allocapnia minima 16S ribosomal RNA gene, partial	EF623130	85.99	100	3.20017e−41	Plecoptera	Capniidae	Allocapnia minima
Mesocapnia sp. BYU_PL010 16S ribosomal RNA gene, p	EF623127	85.99	100	3.20017e−41	Plecoptera	Capniidae	Mesocapnia sp. BYU_PL010
Allocapnia recta haplotype 3ar: H03 16S ribosomal	DQ915181	85.99	100	3.20017e−41	Plecoptera	Capniidae	Allocapnia recta
Doddsia occidentalis 16S ribosomal RNA gene, parti	EF623270	86.25	100	3.8986e−40	Plecoptera	Taeniopterygidae	Doddsia occidentalis
Allocapnia recta isolate H19 16S ribosomal RNA gen	KC881054	85.35	100	1.36075e−39	Plecoptera	Capniidae	Allocapnia recta
Allocapnia recta isolate H16 16S ribosomal RNA gen	KC881051	85.35	100	1.36075e−39	Plecoptera	Capniidae	Allocapnia recta
Allocapnia recta isolate H15 16S ribosomal RNA gen	KC881050	85.35	100	1.36075e−39	Plecoptera	Capniidae	Allocapnia recta
Allocapnia recta isolate H14 16S ribosomal RNA gen	KC881049	85.35	100	1.36075e−39	Plecoptera	Capniidae	Allocapnia recta
Allocapnia recta isolate H10 16S ribosomal RNA gen	KC881045	85.35	100	1.36075e−39	Plecoptera	Capniidae	Allocapnia recta
Allocapnia recta isolate H6 16S ribosomal RNA gene	KC881041	85.35	100	1.36075e−39	Plecoptera	Capniidae	Allocapnia recta
Allocapnia recta isolate H1 16S ribosomal RNA gene	KC881036	85.35	100	1.36075e−39	Plecoptera	Capniidae	Allocapnia recta
Allocapnia recta haplotype 12ar: H12 16S ribosomal	EU306875	85.35	100	1.36075e−39	Plecoptera	Capniidae	Allocapnia recta
Allocapnia recta haplotype 11ar: H011 16S ribosoma	EU306874	85.35	100	1.36075e−39	Plecoptera	Capniidae	Allocapnia recta
Allocapnia recta haplotype 7ar: H07 16S ribosomal	EU306870	85.35	100	1.36075e−39	Plecoptera	Capniidae	Allocapnia recta
Allocapnia recta haplotype 2ar: H02 16S ribosomal	DQ915180	85.35	100	1.36075e−39	Plecoptera	Capniidae	Allocapnia recta
Pteronarcys sachalina 16S ribosomal RNA gene, part	EF623264	84.62	100	4.74947e−39	Plecoptera	Pteronarcyidae	Pteronarcys sachalina
Osmylus sp. EH−2004 16S ribosomal RNA gene, partia	AY620146	85.26	100	4.74947e−39	Neuroptera	Osmylidae	Osmylus sp. EH−2004
Allocapnia recta isolate H18 16S ribosomal RNA gen	KC881053	84.71	100	1.65773e−38	Plecoptera	Capniidae	Allocapnia recta
Allocapnia recta isolate H17 16S ribosomal RNA gen	KC881052	84.71	100	1.65773e−38	Plecoptera	Capniidae	Allocapnia recta
Allocapnia recta isolate H12 16S ribosomal RNA gen	KC881047	84.71	100	1.65773e−38	Plecoptera	Capniidae	Allocapnia recta
Allocapnia recta isolate H9 16S ribosomal RNA gene	KC881044	84.71	100	1.65773e−38	Plecoptera	Capniidae	Allocapnia recta
Allocapnia recta isolate H8 16S ribosomal RNA gene	KC881043	84.71	100	1.65773e−38	Plecoptera	Capniidae	Allocapnia recta
Allocapnia recta isolate H5 16S ribosomal RNA gene	KC881040	84.71	100	1.65773e−38	Plecoptera	Capniidae	Allocapnia recta
Allocapnia recta isolate H4 16S ribosomal RNA gene	KC881039	84.71	100	1.65773e−38	Plecoptera	Capniidae	Allocapnia recta
Allocapnia recta haplotype 10ar: H010 16S ribosoma	EU306873	84.71	100	1.65773e−38	Plecoptera	Capniidae	Allocapnia recta
Allocapnia recta haplotype 9ar: H09 16S ribosomal	EU306872	84.71	100	1.65773e−38	Plecoptera	Capniidae	Allocapnia recta
Allocapnia recta haplotype 8ar: H08 16S ribosomal	EU306871	84.71	100	1.65773e−38	Plecoptera	Capniidae	Allocapnia recta
Allocapnia recta haplotype 1ar: H01 16S ribosomal	DQ915179	84.71	100	1.65773e−38	Plecoptera	Capniidae	Allocapnia recta
Brachyptera seticornis 16S ribosomal RNA gene, par	EF623268	84.91	100	2.01953e−37	Plecoptera	Taeniopterygidae	Brachyptera seticornis
Capnura wanica 16S ribosomal RNA gene, partial seq	EF623128	84.71	100	2.01953e−37	Plecoptera	Capniidae	Capnura wanica
Bolotoperla rossi 16S ribosomal RNA gene, partial	EF623269	84.18	100	2.46029e−36	Plecoptera	Taeniopterygidae	Bolotoperla rossi
Pteronarcys scotti 16S ribosomal RNA gene, partial	EF623263	83.33	100	2.46029e−36	Plecoptera	Pteronarcyidae	Pteronarcys scotti
Isocapnia hyalita 16S ribosomal RNA gene, partial	EF623131	84.28	100	2.46029e−36	Plecoptera	Capniidae	Isocapnia hyalita
Moselia infuscata 16S ribosomal RNA gene, partial	EF623175	83.44	99.35	2.99724e−35	Plecoptera	Leuctridae	Moselia infuscata
Neonemura sp. BYU_PL153 16S ribosomal RNA gene, pa	EF623204	82.69	100	1.04614e−34	Plecoptera	Notonemouridae	Neonemura sp. BYU_PL153
Austrocerca rieka 16S ribosomal RNA gene, partial	EF623205	82.8	100	3.65139e−34	Plecoptera	Notonemouridae	Austrocerca rieka
Austrocercoides zwicki 16S ribosomal RNA gene, par	EF623199	82.8	100	3.65139e−34	Plecoptera	Notonemouridae	Austrocercoides zwicki
Triznaka pintada 16S ribosomal RNA gene, partial s	EF623134	83.54	100	3.65139e−34	Plecoptera	Chloroperlidae	Triznaka pintada
Megaleuctra kincaidi 16S ribosomal RNA gene, parti	EF623174	82.91	100	1.27446e−33	Plecoptera	Leuctridae	Megaleuctra kincaidi
Udamocercia sp. BYU_PL150 16S ribosomal RNA gene,	EF623201	82.17	100	4.4483e−33	Plecoptera	Notonemouridae	Udamocercia sp. BYU_PL150
Thaumatoperla sp. BYU_PL109 16S ribosomal RNA gene	EF623149	82.17	100	4.4483e−33	Plecoptera	Eustheniidae	Thaumatoperla sp. BYU_PL109
Strophopteryx appalachia 16S ribosomal RNA gene, p	EF623271	82.28	100	1.55261e−32	Plecoptera	Taeniopterygidae	Strophopteryx appalachia
Sierraperla cora 16S ribosomal RNA gene, partial s	EF623255	82.05	100	1.55261e−32	Plecoptera	Peltoperlidae	Sierraperla cora
Amphinemura sulcicollis 16S ribosomal RNA gene, pa	EF623195	81.53	100	1.89147e−31	Plecoptera	Nemouridae	Amphinemura sulcicollis
Amphinemura nigritta 16S ribosomal RNA gene, parti	EF623186	81.53	100	1.89147e−31	Plecoptera	Nemouridae	Amphinemura nigritta
Alloperla severa 16S ribosomal RNA gene, partial s	EF623141	81.01	100	6.60187e−31	Plecoptera	Chloroperlidae	Alloperla severa
Soliperla campanula 16S ribosomal RNA gene, partia	EF623256	81.76	99.35	2.30428e−30	Plecoptera	Peltoperlidae	Soliperla campanula
Salmoperla sylvanica 16S ribosomal RNA gene, parti	EF623222	80.89	100	2.30428e−30	Plecoptera	Perlodidae	Salmoperla sylvanica
Utaperla sopladora 16S ribosomal RNA gene, partial	EF623140	81.25	100	2.30428e−30	Plecoptera	Chloroperlidae	Utaperla sopladora
Paraperla frontalis 16S ribosomal RNA gene, partia	EF623136	80.89	100	2.30428e−30	Plecoptera	Chloroperlidae	Paraperla frontalis
Kathroperla perdita 16S ribosomal RNA gene, partia	EF623135	83.57	89.03	2.30428e−30	Plecoptera	Chloroperlidae	Kathroperla perdita
Yoraperla uenoi mitochondrial gene for 16S rRNA, p	AB712375	82.64	92.26	8.04272e−30	Plecoptera	Peltoperlidae	Yoraperla uenoi
Notonemouridae sp. MD−2011 voucher ZMUC:JD−1507 16	JN615257	81.13	100	8.04272e−30	Plecoptera	Notonemouridae	Notonemouridae sp. MD−2011
Caliaeschna microstigma 12S ribosomal RNA gene, pa	EU477639	81.65	100	8.04272e−30	Odonata	Aeshnidae	Caliaeschna microstigma
Eusthenia costalis 16S ribosomal RNA gene, partial	EF623150	81.65	100	8.04272e−30	Plecoptera	Eustheniidae	Eusthenia costalis
Caliaeschna microstigma large subunit ribosomal RN	AF266092	81.65	100	8.04272e−30	Odonata	Aeshnidae	Caliaeschna microstigma
Pteronarcella badia 16S ribosomal RNA gene, partia	EF623262	80.12	100	2.80719e−29	Plecoptera	Pteronarcyidae	Pteronarcella badia
Perlinodes aurea 16S ribosomal RNA gene, partial s	EF623234	80.25	100	9.79804e−29	Plecoptera	Perlodidae	Perlinodes aurea
Megarcys signata 16S ribosomal RNA gene, partial s	EF623215	80.25	100	9.79804e−29	Plecoptera	Perlodidae	Megarcys signata
Limnoperla sp. BYU_PL145 16S ribosomal RNA gene, p	EF623166	79.87	100	9.79804e−29	Plecoptera	Gripopterygidae	Limnoperla sp. BYU_PL145
Plumiperla diversa 16S ribosomal RNA gene, partial	EF623132	81.01	100	9.79804e−29	Plecoptera	Chloroperlidae	Plumiperla diversa
Taenionema palladium 16S ribosomal RNA gene, parti	EF623267	80.98	100	3.41985e−28	Plecoptera	Taeniopterygidae	Taenionema palladium
Peltoperla arcuata 16S ribosomal RNA gene, partial	EF623258	80.5	100	3.41985e−28	Plecoptera	Peltoperlidae	Peltoperla arcuata
Cascadoperla trictura 16S ribosomal RNA gene, part	EF623225	79.49	100	3.41985e−28	Plecoptera	Perlodidae	Cascadoperla trictura
Paraleuctra occidentalis 16S ribosomal RNA gene, p	EF623178	80.5	100	3.41985e−28	Plecoptera	Leuctridae	Paraleuctra occidentalis
Klapopteryx armillata 16S ribosomal RNA gene, part	EF623123	80.38	100	3.41985e−28	Plecoptera	Austroperlidae	Klapopteryx armillata
Microchorista philpotti 16S ribosomal RNA gene, pa	KC177459	80.25	100	1.19365e−27	Mecoptera	Nannochoristidae	Nannochorista philpotti
Boyeria maclachlani mitochondrial gene for 16S rRN	AB707674	81.37	100	1.19365e−27	Odonata	Aeshnidae	Boyeria maclachlani
Boyeria maclachlani mitochondrial gene for 16S rRN	AB707672	81.37	100	1.19365e−27	Odonata	Aeshnidae	Boyeria maclachlani
Microchorista philpotti mitochondrion, partial gen	HQ696580	80.25	100	1.19365e−27	Mecoptera	Nannochoristidae	Nannochorista philpotti
Isoperla oxylepis 16S ribosomal RNA gene, partial	EF623221	79.75	100	1.19365e−27	Plecoptera	Perlodidae	Isoperla oxylepis
Skwala americana 16S ribosomal RNA gene, partial s	EF623217	79.62	100	1.19365e−27	Plecoptera	Perlodidae	Skwala americana
Diamphipnoa virescentipennis 16S ribosomal RNA gen	EF623144	79.62	100	1.19365e−27	Plecoptera	Diamphipnoidae	Diamphipnoa virescentipennis
Chloroperla tripunctata 16S ribosomal RNA gene, pa	EF623137	79.62	100	1.19365e−27	Plecoptera	Chloroperlidae	Chloroperla tripunctata
Cryptoperla sp. YK−2012−1 mitochondrial gene for 1	AB711243	79.75	100	4.16623e−27	Plecoptera	Peltoperlidae	Cryptoperla sp. YK−2012−1
Aeschnophlebia longistigma mitochondrial gene for	AB707621	80.38	100	4.16623e−27	Odonata	Aeshnidae	Aeschnophlebia longistigma
