# Supplementary material for: Testing the potential of a ribosomal 16S marker for DNA metabarcoding of insects
Source: PeerJ. 2016 Apr 19;4:e1966. doi: 10.7717/peerj.1966 (PMC4841222; doi:10.7717/peerj.1966)
Supplement: Supplemental Information 1 [file peerj-04-1966-s006.zip › scripts/5 match OTUs/blast_hits/OTU_271.csv_plot.pdf]

OTU_271.csv

Ephemera danica

Ephemeridae

Ephemeroptera

96.71

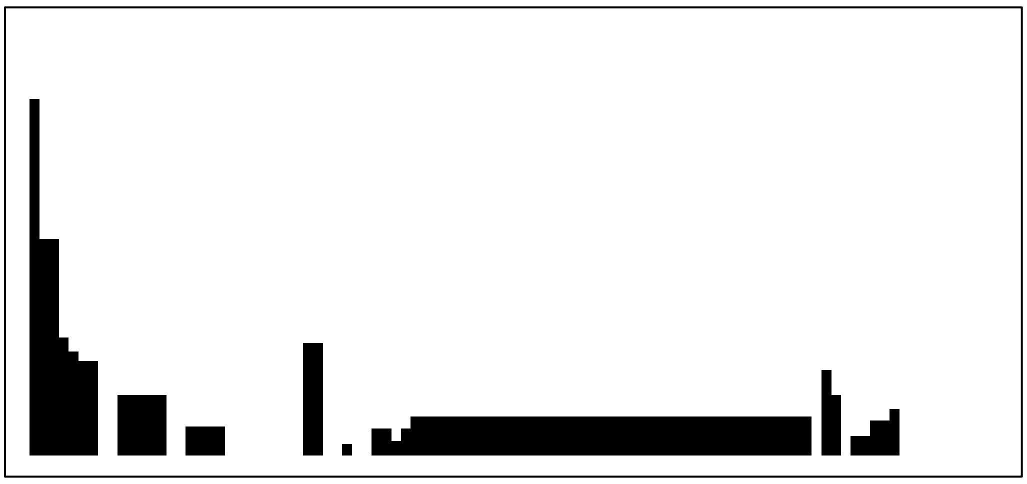

Ephemera danica 16S ribosomal RNA gene, partial se	KF855860	96.71	100	1.72419e−63	Ephemeroptera	Ephemeridae	Ephemera danica
Ephemera orientalis voucher EPH01 tRNA−Leu, 16S ri	KF256907	90.13	100	6.43114e−50	Ephemeroptera	Ephemeridae	Ephemera orientalis
Ephemera orientalis mitochondrion, complete genome	EU591678	90.13	100	6.43114e−50	Ephemeroptera	Ephemeridae	Ephemera orientalis
Ephemera simulans voucher BYU:IGCEP038 16S ribosom	GQ118285	85.53	100	1.08904e−40	Ephemeroptera	Ephemeridae	Ephemera simulans
Siphlonurus immanis voucher NKMT032 mitochondrion,	FJ606783	84.87	100	4.63073e−39	Ephemeroptera	Siphlonuridae	Siphlonurus immanis
Ephemera sp. 1 BM−2008 12S ribosomal RNA gene, par	EU477611	84.42	100	8.37256e−36	Ephemeroptera	Ephemeridae	Ephemera sp. 1 BM−2008
Ephemera sp. 'Bonn' large subunit ribosomal RNA ge	AF266047	84.42	100	8.37256e−36	Ephemeroptera	Ephemeridae	Ephemera sp. 'Bonn'
Potamanthus luteus voucher BYU:IGCEP219 16S riboso	GQ118293	77.78	98.68	1.3279e−20	Ephemeroptera	Potamanthidae	Potamanthus luteus
Leptoperla sp. BYU_PL107 16S ribosomal RNA gene, p	EF623161	79.7	83.55	5.64637e−19	Plecoptera	Gripopterygidae	Leptoperla sp. BYU_PL107
Palingenia longicauda mitochondrial partial 16S rR	HE650662	82.83	65.13	1.97078e−18	Ephemeroptera	Palingeniidae	Palingenia longicauda
Palingenia longicauda mitochondrial partial 16S rR	HE650658	82.83	65.13	1.97078e−18	Ephemeroptera	Palingeniidae	Palingenia longicauda
Palingenia longicauda mitochondrial partial 16S rR	HE650475	82.83	65.13	1.97078e−18	Ephemeroptera	Palingeniidae	Palingenia longicauda
Palingenia longicauda mitochondrial partial 16S rR	HE650547	82.83	65.13	1.97078e−18	Ephemeroptera	Palingeniidae	Palingenia longicauda
Palingenia longicauda mitochondrial partial 16S rR	HE650502	82.83	65.13	1.97078e−18	Ephemeroptera	Palingeniidae	Palingenia longicauda
Pterostichus biexcisus mitochondrial gene for 16S	AB253862	75.95	100	1.97078e−18	Coleoptera	Carabidae	Pterostichus biexcisus
Pterostichus fujimurai mitochondrial gene for 16S	AB253834	75.95	100	1.97078e−18	Coleoptera	Carabidae	Pterostichus fujimurai
Galloisiana nipponensis 16S ribosomal RNA gene, pa	EF623153	81.36	75.66	1.97078e−18	Grylloblattodea	Grylloblattidae	Galloisiana nipponensis
Galloisiana yuasai voucher BYU_gb13.2 16S ribosoma	DQ457244	81.36	75.66	1.97078e−18	Grylloblattodea	Grylloblattidae	Galloisiana yuasai
Galloisiana yuasai voucher BYU_gb13.1 16S ribosoma	DQ457243	81.36	75.66	1.97078e−18	Grylloblattodea	Grylloblattidae	Galloisiana yuasai
Galloisiana sp. ONGP001 mitochondrial gene for 16S	AB119537	81.36	75.66	1.97078e−18	Grylloblattodea	Grylloblattidae	Galloisiana sp. ONGP001
Anthopotamus sp. EP022 16S ribosomal RNA gene, par	AY749766	76.58	100	1.97078e−18	Ephemeroptera	Potamanthidae	Anthopotamus sp. EP022
Indolestes peregrinus voucher ZYG08 tRNA−Leu, 16S	KF256891	75.97	98.03	6.87869e−18	Odonata	Lestidae	Indolestes peregrinus
Sympecma paedisca voucher ZYG07 tRNA−Leu, 16S ribo	KF256890	75.97	98.03	6.87869e−18	Odonata	Lestidae	Sympecma paedisca
Indolestes peregrinus mitochondrial gene for 16S r	AB707356	75.97	98.03	6.87869e−18	Odonata	Lestidae	Indolestes peregrinus
Pterostichus asymmetricus mitochondrial gene for 1	AB253866	75.8	99.34	6.87869e−18	Coleoptera	Carabidae	Pterostichus asymmetricus
Eunotoperla sp. BYU_PL102 16S ribosomal RNA gene,	EF623159	78.79	83.55	6.87869e−18	Plecoptera	Gripopterygidae	Eunotoperla sp. BYU_PL102
Klapopteryx armillata 16S ribosomal RNA gene, part	EF623123	79.55	83.55	6.87869e−18	Plecoptera	Austroperlidae	Klapopteryx armillata
Indolestes gracilis 16S ribosomal RNA gene, partia	EF032700	75.97	98.03	6.87869e−18	Odonata	Lestidae	Indolestes gracilis
Hygrostola dallolmoi voucher HYTAG7234 16S ribosom	KP682574	85.26	61.18	2.4009e−17	Lepidoptera	Noctuidae	Hygrostola dallolmoi
Hygrostola dallolmoi voucher HYTAG6454 16S ribosom	KP682573	85.26	61.18	2.4009e−17	Lepidoptera	Noctuidae	Hygrostola dallolmoi
Ephoron shigae mitochondrial gene for 16S ribosoma	AB874293	79.34	79.61	2.4009e−17	Ephemeroptera	Polymitarcyidae	Ephoron shigae
Ephoron shigae mitochondrial gene for 16S ribosoma	AB874289	79.34	79.61	2.4009e−17	Ephemeroptera	Polymitarcyidae	Ephoron shigae
Ephoron album mitochondrial gene for 16S rRNA, par	AB711636	80.53	73.68	2.4009e−17	Ephemeroptera	Polymitarcyidae	Ephoron album
Ephoron shigae mitochondrial gene for 16S rRNA, pa	AB711533	79.34	79.61	2.4009e−17	Ephemeroptera	Polymitarcyidae	Ephoron shigae
Ephoron shigae mitochondrial gene for 16S rRNA, pa	AB711527	79.34	79.61	2.4009e−17	Ephemeroptera	Polymitarcyidae	Ephoron shigae
Semirhynchia sp. n. SV01 16S ribosomal RNA gene, p	JX294172	81.25	71.05	2.4009e−17	Neuroptera	Nemopteridae	Semirhynchia sp. n. SV01
Sicyoptera cuspidata isolate SCBB01 16S ribosomal	JX294167	81.25	71.05	2.4009e−17	Neuroptera	Nemopteridae	Sicyoptera cuspidata
Sicyoptera dilatata isolate SDGG01 16S ribosomal R	JX294163	80.67	75.66	2.4009e−17	Neuroptera	Nemopteridae	Sicyoptera dilatata
Nemeura gracilis isolate NGW01 16S ribosomal RNA g	JX294132	81.25	71.05	2.4009e−17	Neuroptera	Nemopteridae	Nemeura gracilis
Palingenia longicauda mitochondrial partial 16S rR	HE650589	81.82	65.13	2.4009e−17	Ephemeroptera	Palingeniidae	Palingenia longicauda
Palingenia longicauda mitochondrial partial 16S rR	HE650657	81.82	65.13	2.4009e−17	Ephemeroptera	Palingeniidae	Palingenia longicauda
Palingenia longicauda mitochondrial partial 16S rR	HE650650	81.82	65.13	2.4009e−17	Ephemeroptera	Palingeniidae	Palingenia longicauda
Palingenia longicauda mitochondrial partial 16S rR	HE650647	81.82	65.13	2.4009e−17	Ephemeroptera	Palingeniidae	Palingenia longicauda
Palingenia longicauda mitochondrial partial 16S rR	HE650637	81.82	65.13	2.4009e−17	Ephemeroptera	Palingeniidae	Palingenia longicauda
Palingenia longicauda mitochondrial partial 16S rR	HE650635	81.82	65.13	2.4009e−17	Ephemeroptera	Palingeniidae	Palingenia longicauda
Palingenia longicauda mitochondrial partial 16S rR	HE650631	81.82	65.13	2.4009e−17	Ephemeroptera	Palingeniidae	Palingenia longicauda
Palingenia longicauda mitochondrial partial 16S rR	HE650625	81.82	65.13	2.4009e−17	Ephemeroptera	Palingeniidae	Palingenia longicauda
Palingenia longicauda mitochondrial partial 16S rR	HE650623	81.82	65.13	2.4009e−17	Ephemeroptera	Palingeniidae	Palingenia longicauda
Palingenia longicauda mitochondrial partial 16S rR	HE650622	81.82	65.13	2.4009e−17	Ephemeroptera	Palingeniidae	Palingenia longicauda
Palingenia longicauda mitochondrial partial 16S rR	HE650618	81.82	65.13	2.4009e−17	Ephemeroptera	Palingeniidae	Palingenia longicauda
Palingenia longicauda mitochondrial partial 16S rR	HE650617	81.82	65.13	2.4009e−17	Ephemeroptera	Palingeniidae	Palingenia longicauda
Palingenia longicauda mitochondrial partial 16S rR	HE650604	81.82	65.13	2.4009e−17	Ephemeroptera	Palingeniidae	Palingenia longicauda
Palingenia longicauda mitochondrial partial 16S rR	HE650594	81.82	65.13	2.4009e−17	Ephemeroptera	Palingeniidae	Palingenia longicauda
Palingenia longicauda mitochondrial partial 16S rR	HE650588	81.82	65.13	2.4009e−17	Ephemeroptera	Palingeniidae	Palingenia longicauda
Palingenia longicauda mitochondrial partial 16S rR	HE650575	81.82	65.13	2.4009e−17	Ephemeroptera	Palingeniidae	Palingenia longicauda
Palingenia longicauda mitochondrial partial 16S rR	HE650569	81.82	65.13	2.4009e−17	Ephemeroptera	Palingeniidae	Palingenia longicauda
Palingenia longicauda mitochondrial partial 16S rR	HE650550	81.82	65.13	2.4009e−17	Ephemeroptera	Palingeniidae	Palingenia longicauda
Palingenia longicauda mitochondrial partial 16S rR	HE650542	81.82	65.13	2.4009e−17	Ephemeroptera	Palingeniidae	Palingenia longicauda
Palingenia longicauda mitochondrial partial 16S rR	HE650533	81.82	65.13	2.4009e−17	Ephemeroptera	Palingeniidae	Palingenia longicauda
Palingenia longicauda mitochondrial partial 16S rR	HE650503	81.82	65.13	2.4009e−17	Ephemeroptera	Palingeniidae	Palingenia longicauda
Palingenia longicauda mitochondrial partial 16S rR	HE650520	81.82	65.13	2.4009e−17	Ephemeroptera	Palingeniidae	Palingenia longicauda
Palingenia longicauda mitochondrial partial 16S rR	HE650519	81.82	65.13	2.4009e−17	Ephemeroptera	Palingeniidae	Palingenia longicauda
Palingenia longicauda mitochondrial partial 16S rR	HE650515	81.82	65.13	2.4009e−17	Ephemeroptera	Palingeniidae	Palingenia longicauda
Palingenia longicauda mitochondrial partial 16S rR	HE650514	81.82	65.13	2.4009e−17	Ephemeroptera	Palingeniidae	Palingenia longicauda
Palingenia longicauda mitochondrial partial 16S rR	HE650509	81.82	65.13	2.4009e−17	Ephemeroptera	Palingeniidae	Palingenia longicauda
Palingenia longicauda mitochondrial partial 16S rR	HE650507	81.82	65.13	2.4009e−17	Ephemeroptera	Palingeniidae	Palingenia longicauda
Palingenia longicauda mitochondrial partial 16S rR	HE650505	81.82	65.13	2.4009e−17	Ephemeroptera	Palingeniidae	Palingenia longicauda
Palingenia longicauda mitochondrial partial 16S rR	HE650499	81.82	65.13	2.4009e−17	Ephemeroptera	Palingeniidae	Palingenia longicauda
Palingenia longicauda mitochondrial partial 16S rR	HE650446	81.82	65.13	2.4009e−17	Ephemeroptera	Palingeniidae	Palingenia longicauda
Palingenia longicauda mitochondrial partial 16S rR	HE650435	81.82	65.13	2.4009e−17	Ephemeroptera	Palingeniidae	Palingenia longicauda
Palingenia longicauda mitochondrial partial 16S rR	HE650461	81.82	65.13	2.4009e−17	Ephemeroptera	Palingeniidae	Palingenia longicauda
Palingenia longicauda mitochondrial partial 16S rR	HE650449	81.82	65.13	2.4009e−17	Ephemeroptera	Palingeniidae	Palingenia longicauda
Palingenia longicauda mitochondrial partial 16S rR	HE650420	81.82	65.13	2.4009e−17	Ephemeroptera	Palingeniidae	Palingenia longicauda
Palingenia longicauda mitochondrial partial 16S rR	HE650440	81.82	65.13	2.4009e−17	Ephemeroptera	Palingeniidae	Palingenia longicauda
Palingenia longicauda mitochondrial partial 16S rR	HE650434	81.82	65.13	2.4009e−17	Ephemeroptera	Palingeniidae	Palingenia longicauda
Palingenia longicauda mitochondrial partial 16S rR	HE650430	81.82	65.13	2.4009e−17	Ephemeroptera	Palingeniidae	Palingenia longicauda
Palingenia longicauda mitochondrial partial 16S rR	HE650421	81.82	65.13	2.4009e−17	Ephemeroptera	Palingeniidae	Palingenia longicauda
Palingenia longicauda mitochondrial partial 16S rR	HE650424	81.82	65.13	2.4009e−17	Ephemeroptera	Palingeniidae	Palingenia longicauda
Palingenia longicauda mitochondrial partial 16S rR	HE650422	81.82	65.13	2.4009e−17	Ephemeroptera	Palingeniidae	Palingenia longicauda
Palingenia longicauda voucher BYU:IGCEP216 16S rib	GQ118292	81.82	65.13	2.4009e−17	Ephemeroptera	Palingeniidae	Palingenia longicauda
Megarcys signata 16S ribosomal RNA gene, partial s	EF623215	79.73	92.11	2.4009e−17	Plecoptera	Perlodidae	Megarcys signata
Grylloblatta n. sp. 'Trout Lake' voucher BYU_gb23.	DQ457256	84	64.47	2.4009e−17	Grylloblattodea	Grylloblattidae	Grylloblatta sp. n. 'Trout Lake'
Ichthybotus hudsoni 16S ribosomal RNA gene, partia	AY749816	82.83	65.13	2.4009e−17	Ephemeroptera	Ichthybotidae	Ichthybotus hudsoni
Neope pulaha 16S ribosomal RNA gene, partial seque	KJ777753	75.82	94.74	8.37996e−17	Lepidoptera	Nymphalidae	Neope pulaha
Ephoron leukon mitochondrial gene for 16S rRNA, pa	AB711641	80.91	71.71	8.37996e−17	Ephemeroptera	Polymitarcyidae	Ephoron leukon
Ephoron album mitochondrial gene for 16S rRNA, par	AB711635	80.91	71.71	8.37996e−17	Ephemeroptera	Polymitarcyidae	Ephoron album
Ephoron shigae mitochondrial gene for 16S rRNA, pa	AB711523	81.63	64.47	8.37996e−17	Ephemeroptera	Polymitarcyidae	Ephoron shigae
Palingenia longicauda mitochondrial partial 16S rR	HE650616	81.63	64.47	8.37996e−17	Ephemeroptera	Palingeniidae	Palingenia longicauda
Palingenia longicauda mitochondrial partial 16S rR	HE650490	82.18	65.13	8.37996e−17	Ephemeroptera	Palingeniidae	Palingenia longicauda
Galloisiana yuasai voucher ZMUC:JD−650 16S ribosom	JN615261	79.84	77.63	8.37996e−17	Grylloblattodea	Grylloblattidae	Galloisiana yuasai
Papilio alexanor voucher FS−b−40 16S ribosomal RNA	GQ268343	76.71	94.74	8.37996e−17	Lepidoptera	Papilionidae	Papilio alexanor
Pterostichus dandonis mitochondrial gene for 16S r	AB253857	75.32	100	8.37996e−17	Coleoptera	Carabidae	Pterostichus dandonis
Pterostichus spiculifer mitochondrial gene for 16S	AB253856	75.32	100	8.37996e−17	Coleoptera	Carabidae	Pterostichus spiculifer
Pterostichus seunglaki mitochondrial gene for 16S	AB253846	75.32	100	8.37996e−17	Coleoptera	Carabidae	Pterostichus seunglaki
Acruroperla atra 16S ribosomal RNA gene, partial s	EF623121	78.36	84.21	8.37996e−17	Plecoptera	Austroperlidae	Acruroperla atra
Doleschallia bisaltide voucher jshao2005048 16S ri	DQ784697	76.43	90.79	8.37996e−17	Lepidoptera	Nymphalidae	Doleschallia bisaltide
Neurocordulia obsoleta voucher JW_DNA_ONO 16S ribo	EF631509	77.95	80.26	8.37996e−17	Odonata	Corduliidae	Neurocordulia obsoleta
Galloisiana yuasai 16S large subunit ribosomal RNA	DQ890284	79.84	77.63	8.37996e−17	Grylloblattodea	Grylloblattidae	Galloisiana yuasai
Tegosa anieta isolate 22−2 16S ribosomal RNA gene,	AF186853	75.17	94.74	8.37996e−17	Lepidoptera	Nymphalidae	Tegosa anieta
Papilio alexanor mitochondrion, large subunit ribo	AJ224052	76.71	94.74	8.37996e−17	Lepidoptera	Papilionidae	Papilio alexanor
