# Supplementary material for: Testing the potential of a ribosomal 16S marker for DNA metabarcoding of insects
Source: PeerJ. 2016 Apr 19;4:e1966. doi: 10.7717/peerj.1966 (PMC4841222; doi:10.7717/peerj.1966)
Supplement: Supplemental Information 1 [file peerj-04-1966-s006.zip › scripts/5 match OTUs/blast_hits/OTU_28.csv_plot.pdf]

OTU_28.csv

Limnius volckmari

Elmidae

Coleoptera

100

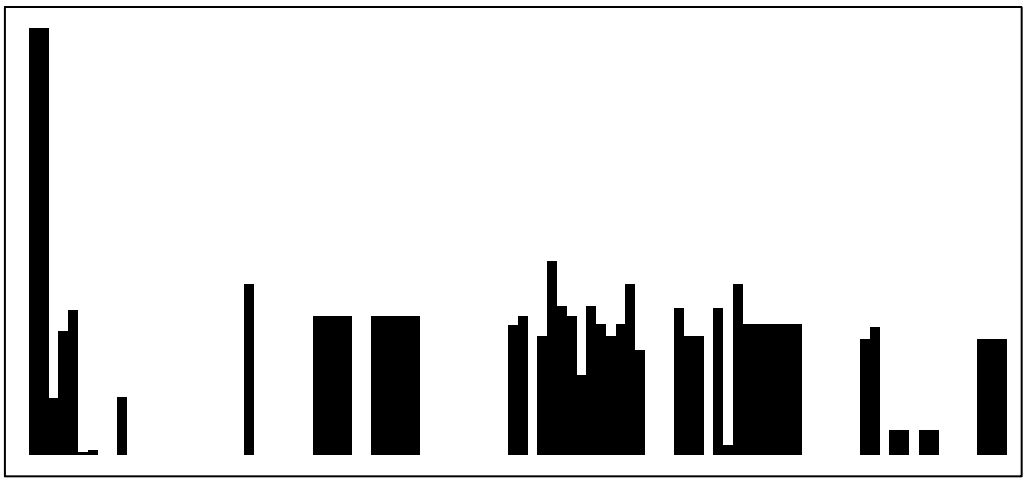

Limnius volckmari 16S ribosomal RNA gene, partial	DQ198627	100	100	1.053e−72	Coleoptera	Elmidae	Limnius volckmari
Limnius intermedius 16S ribosomal RNA gene, partia	DQ266475	100	100	1.053e−72	Coleoptera	Elmidae	Limnius intermedius
Elmis latreillei 16S ribosomal RNA (rrnL) gene, pa	EF209449	82.69	100	8.65881e−36	Coleoptera	Elmidae	Elmis latreillei
Lucholelmis sp. FC_D19 16S ribosomal RNA (rrnL) ge	EF209455	85.82	83.97	5.4643e−32	Coleoptera	Elmidae	Lucholelmis sp. FC_D19
Elminae sp. FC_B22 16S ribosomal RNA (rrnL) gene,	EF209442	86.78	77.56	5.4643e−32	Coleoptera	Elmidae	Elminae sp. FC_B22
Oulimnius echinatus 16S ribosomal RNA gene, partia	GU935679	80.13	100	2.32348e−30	Coleoptera	Elmidae	Oulimnius echinatus
Phloiotrya flavitarsis voucher UPOL ZL0138 16S rib	FJ903746	80.25	100	9.87969e−29	Coleoptera	Melandryidae	Phloiotrya flavitarsis
Esolus angustatus 16S ribosomal RNA (rrnL) gene, p	EF209450	79.62	100	9.87969e−29	Coleoptera	Elmidae	Esolus angustatus
Riolus subviolaceus 16S ribosomal RNA (rrnL) gene,	EF209465	79.62	100	1.20359e−27	Coleoptera	Elmidae	Riolus subviolaceus
Dupophilus sp. FC_D04 16S ribosomal RNA (rrnL) gen	EF209446	82.71	84.62	1.20359e−27	Coleoptera	Elmidae	Dupophilus sp. FC_D04
Oulimnius bertrandi isolate SP01 16S ribosomal RNA	GU935697	78.21	100	1.46628e−26	Coleoptera	Elmidae	Oulimnius bertrandi
Oulimnius tuberculatus isolate SK2 16S ribosomal R	GU935696	78.21	100	1.46628e−26	Coleoptera	Elmidae	Oulimnius tuberculatus
Oulimnius perezi isolate 318 16S ribosomal RNA gen	GU935691	78.21	100	1.46628e−26	Coleoptera	Elmidae	Oulimnius perezi
Oulimnius perezi isolate 317 16S ribosomal RNA gen	GU935690	78.21	100	1.46628e−26	Coleoptera	Elmidae	Oulimnius perezi
Oulimnius perezi isolate 316 16S ribosomal RNA gen	GU935689	78.21	100	1.46628e−26	Coleoptera	Elmidae	Oulimnius perezi
Oulimnius perezi isolate PT3 16S ribosomal RNA gen	GU935688	78.21	100	1.46628e−26	Coleoptera	Elmidae	Oulimnius perezi
Oulimnius perezi isolate PT2 16S ribosomal RNA gen	GU935687	78.21	100	1.46628e−26	Coleoptera	Elmidae	Oulimnius perezi
Oulimnius perezi isolate PT1 16S ribosomal RNA gen	GU935686	78.21	100	1.46628e−26	Coleoptera	Elmidae	Oulimnius perezi
Oulimnius perezi isolate SP1 16S ribosomal RNA gen	GU935685	78.21	100	1.46628e−26	Coleoptera	Elmidae	Oulimnius perezi
Oulimnius bertrandi isolate 2 16S ribosomal RNA ge	GU935678	78.21	100	1.46628e−26	Coleoptera	Elmidae	Oulimnius bertrandi
Oulimnius bertrandi isolate 1 16S ribosomal RNA ge	GU935677	78.21	100	1.46628e−26	Coleoptera	Elmidae	Oulimnius bertrandi
Omaloplia ruricola 16S ribosomal RNA gene, partial	EF487790	79.11	100	1.46628e−26	Coleoptera	Scarabaeidae	Omaloplia ruricola
Placusa pinearum voucher NJFU 619584−30 16S riboso	JX569194	88	64.1	5.11781e−26	Coleoptera	Staphylinidae	Placusa pinearum
Stenelmis grossa 16S ribosomal RNA gene, partial s	HQ629798	79.11	100	5.11781e−26	Coleoptera	Elmidae	Stenelmis grossa
Normandia sp. FC_B08 16S ribosomal RNA (rrnL) gene	EF209460	78.98	100	5.11781e−26	Coleoptera	Elmidae	Normandia sp. FC_B08
Larainae sp. FC_E15 16S ribosomal RNA (rrnL) gene,	EF209452	78.98	100	5.11781e−26	Coleoptera	Elmidae	Larainae sp. FC_E15
Elminae sp. FC_E03 16S ribosomal RNA (rrnL) gene,	EF209447	78.34	100	5.11781e−26	Coleoptera	Elmidae	Elminae sp. FC_E03
Dubiraphia sp. MNCN−FC−E10 16S ribosomal RNA gene,	DQ267445	79.11	100	5.11781e−26	Coleoptera	Elmidae	Dubiraphia sp. MNCN−FC−E10
Oulimnius tuberculatus 16S ribosomal RNA gene, par	DQ266476	78.98	100	5.11781e−26	Coleoptera	Elmidae	Oulimnius tuberculatus
Rhantus binotatus voucher UNM KBMRhbi3 16S ribosom	KJ548367	86.54	66.67	1.78629e−25	Coleoptera	Dytiscidae	Rhantus binotatus
Rhantus atricolor voucher UNM KBMRhat10 16S riboso	KJ548366	86.54	66.67	1.78629e−25	Coleoptera	Dytiscidae	Rhantus atricolor
Rhantus souzannae 16S ribosomal RNA gene, partial	KJ637890	86.54	66.67	1.78629e−25	Coleoptera	Dytiscidae	Rhantus souzannae
Rhantus gutticollis 16S ribosomal RNA gene, partia	KJ637884	86.54	66.67	1.78629e−25	Coleoptera	Dytiscidae	Rhantus gutticollis
Placusa pinearum voucher NJFU 619584−32 16S riboso	JX661721	77.85	100	1.78629e−25	Coleoptera	Staphylinidae	Placusa pinearum
Placusa pinearum voucher NJFU 619584−33 16S riboso	JX661720	77.85	100	1.78629e−25	Coleoptera	Staphylinidae	Placusa pinearum
Rhantus bula mitochondrial DNA sequence containing	FN298851	86.54	66.67	1.78629e−25	Coleoptera	Dytiscidae	Rhantus bula
Rhantus kini mitochondrial DNA sequence containing	FN298850	86.54	66.67	1.78629e−25	Coleoptera	Dytiscidae	Rhantus kini
Rhantus vitiensis mitochondrial DNA sequence conta	FN298849	86.54	66.67	1.78629e−25	Coleoptera	Dytiscidae	Rhantus vitiensis
Rhantus bistriatus mitochondrial DNA sequence cont	FN298834	86.54	66.67	1.78629e−25	Coleoptera	Dytiscidae	Rhantus bistriatus
Rhantus pederzanii mitochondrial DNA sequence cont	FN298816	86.54	66.67	1.78629e−25	Coleoptera	Dytiscidae	Rhantus pederzanii
Hoperius planatus partial 16S rRNA gene	AJ850337	78.81	96.15	1.78629e−25	Coleoptera	Dytiscidae	Hoperius planatus
Agabus strigulosus 16S ribosomal RNA gene, partial	AY138632	79.08	97.44	1.78629e−25	Coleoptera	Dytiscidae	Agabus strigulosus
Agabus sp. IR−sp2 16S ribosomal RNA gene, partial	AY138597	79.08	97.44	1.78629e−25	Coleoptera	Dytiscidae	Agabus sp. IR−sp2
Hoperius planatus voucher UNM KBMHopl453 16S ribos	KJ548330	78.15	96.15	6.23476e−25	Coleoptera	Dytiscidae	Hoperius planatus
Habroscelimorpha boops voucher BMNH 697593 16S rib	KC963513	80	94.87	6.23476e−25	Coleoptera	Carabidae	Habroscelimorpha boops
Oulimnius tuberculatus isolate CH 16S ribosomal RN	GU935694	77.56	100	6.23476e−25	Coleoptera	Elmidae	Oulimnius tuberculatus
Oulimnius tuberculatus isolate GB 16S ribosomal RN	GU935693	77.56	100	6.23476e−25	Coleoptera	Elmidae	Oulimnius tuberculatus
Oulimnius tuberculatus isolate AU 16S ribosomal RN	GU935692	77.56	100	6.23476e−25	Coleoptera	Elmidae	Oulimnius tuberculatus
Malvapion malvae partial mitochondrial 16S rRNA ge	AJ495464	79.75	100	6.23476e−25	Coleoptera	Brentidae	Malvapion malvae
Hebascus sp. MJM−2013 voucher BYU CO624 16S riboso	KC966531	86.11	67.95	2.17615e−24	Coleoptera	Nitidulidae	Hebascus sp. MJM−2013
Neoscutopterus angustus voucher UNM KBMNean25 16S	KJ548357	86.54	66.67	2.17615e−24	Coleoptera	Dytiscidae	Neoscutopterus angustus
Hoperius planatus 16S ribosomal RNA gene, partial	KJ637889	78.15	96.15	2.17615e−24	Coleoptera	Dytiscidae	Hoperius planatus
Neoscutopterus hornii 16S ribosomal RNA gene, part	KJ637886	85.58	66.67	2.17615e−24	Coleoptera	Dytiscidae	Neoscutopterus hornii
Psephenidae sp. UPOL RK0168 16S ribosomal RNA gene	KF625820	89.11	63.46	2.17615e−24	Coleoptera	Psephenidae	Psephenidae sp. UPOL RK0168
Placusa pinearum voucher NJFU 619584−35 16S riboso	JX661719	87	64.1	2.17615e−24	Coleoptera	Staphylinidae	Placusa pinearum
Neoscutopterus horni partial 16S rRNA gene	AJ850340	86.54	66.67	2.17615e−24	Coleoptera	Dytiscidae	Neoscutopterus hornii
Papuadytes sp. MB659 mitochondrial 16S rRNA gene (	AM404058	83.74	78.21	2.17615e−24	Coleoptera	Dytiscidae	Papuadytes sp. MB659
Agabus serricornis 16S ribosomal RNA gene, partial	AY138629	87	64.1	2.17615e−24	Coleoptera	Dytiscidae	Agabus serricornis
Ilybiosoma sp. IBE−RA301 mitochondrial 16S rRNA ge	LM654793	86.14	64.74	7.5955e−24	Coleoptera	Dytiscidae	Ilybiosoma sp. IBE−RA301
Crepidodera aurata voucher BMNH:853107 16S large s	KP763226	85.58	66.67	7.5955e−24	Coleoptera	Chrysomelidae	Crepidodera aurata
Ilybiosoma cordatum voucher UNM KBMAgco57 16S ribo	KJ548301	86.14	64.74	7.5955e−24	Coleoptera	Dytiscidae	Ilybiosoma cordatum
Psephenidae sp. UPOL RK0169 16S ribosomal RNA gene	KF625821	88	63.46	7.5955e−24	Coleoptera	Psephenidae	Psephenidae sp. UPOL RK0169
Speonomus sp. IR−2014 mitochondrial 16S rRNA gene	HG915701	84.91	67.95	7.5955e−24	Coleoptera	Leiodidae	Speonomus sp. IR−2014
Graphoderus occidentalis voucher KBMGroc207 16S ri	KF979095	78.43	97.44	7.5955e−24	Coleoptera	Dytiscidae	Graphoderus occidentalis
Quaestus pasensis mitochondrial 16S rRNA gene (par	HE663533	78.75	99.36	7.5955e−24	Coleoptera	Leiodidae	Quaestus pasensis
Oulimnius fuscipes 16S ribosomal RNA gene, partial	GU935680	77.56	100	7.5955e−24	Coleoptera	Elmidae	Oulimnius fuscipes
Dytiscus dimidiatus mitochondrial DNA sequence con	FN298837	86.87	63.46	7.5955e−24	Coleoptera	Dytiscidae	Dytiscus dimidiatus
Rhantus consputus mitochondrial DNA sequence conta	FN298833	85.58	66.67	7.5955e−24	Coleoptera	Dytiscidae	Rhantus consputus
Rhantus latitans mitochondrial DNA sequence contai	FN298832	85.58	66.67	7.5955e−24	Coleoptera	Dytiscidae	Rhantus latitans
Lepadoretus ovatoides voucher BMNH 671466 16S larg	EF487914	77.85	100	7.5955e−24	Coleoptera	Scarabaeidae	Lepadoretus ovatoides
Elminae sp. FC_D20 16S ribosomal RNA (rrnL) gene,	EF209454	86.87	63.46	7.5955e−24	Coleoptera	Elmidae	Elminae sp. FC_D20
Oulimnius rivularis 16S ribosomal RNA gene, partia	DQ198628	80.45	85.26	7.5955e−24	Coleoptera	Elmidae	Oulimnius rivularis
Dytiscus circumcinctus contains 16S ribosomal RNA	AY368226	88	63.46	7.5955e−24	Coleoptera	Dytiscidae	Dytiscus circumcinctus
Ilybiosoma seriatum isolate IIbseri2R 16S ribosoma	AY138655	86.14	64.74	7.5955e−24	Coleoptera	Dytiscidae	Ilybiosoma seriatum
Ilybiosoma seriatum isolate IIbC2.1 16S ribosomal	AY138653	86.14	64.74	7.5955e−24	Coleoptera	Dytiscidae	Ilybiosoma seriatum
Ilybiosoma seriatum isolate IIb7.1R 16S ribosomal	AY138651	86.14	64.74	7.5955e−24	Coleoptera	Dytiscidae	Ilybiosoma seriatum
Ilybiosoma perplexus 16S ribosomal RNA gene, parti	AY138650	86.14	64.74	7.5955e−24	Coleoptera	Dytiscidae	Ilybiosoma perplexus
Ilybiosoma seriatum isolate IIb27.3 16S ribosomal	AY138649	86.14	64.74	7.5955e−24	Coleoptera	Dytiscidae	Ilybiosoma seriatum
Ilybiosoma pandurus 16S ribosomal RNA gene, partia	AY138648	86.14	64.74	7.5955e−24	Coleoptera	Dytiscidae	Ilybiosoma pandurus
Agabus matsumotoi 16S ribosomal RNA gene, partial	AY138622	78.43	97.44	7.5955e−24	Coleoptera	Dytiscidae	Agabus matsumotoi
Agabus japonicus 16S ribosomal RNA gene, partial s	AY138617	78.43	97.44	7.5955e−24	Coleoptera	Dytiscidae	Agabus japonicus
Agabus morosus isolate Aga18.2R 16S ribosomal RNA	AY138592	78.43	97.44	7.5955e−24	Coleoptera	Dytiscidae	Agabus morosus
Cylindera debilis 16S large subunit ribosomal RNA	AF133002	78.57	97.44	7.5955e−24	Coleoptera	Carabidae	Cylindera debilis
Cicindela longilabris large subunit ribosomal RNA	AF438883	78.57	97.44	7.5955e−24	Coleoptera	Carabidae	Cicindela longilabris
Psylliodes chrysocephalus voucher BMNH:847996 16S	KP763113	79.25	100	2.65109e−23	Coleoptera	Chrysomelidae	Psylliodes chrysocephalus
Meladema coriacea voucher UNM KBMMdco475 16S ribos	KJ548354	85.44	66.03	2.65109e−23	Coleoptera	Dytiscidae	Meladema coriacea
Placusa pinearum voucher NJFU 619584−31 16S riboso	JX661722	86	64.1	2.65109e−23	Coleoptera	Staphylinidae	Placusa pinearum
Agabus dichrous mitochondrial partial 16S rRNA gen	HF931378	78.57	97.44	2.65109e−23	Coleoptera	Dytiscidae	Agabus dichrous
Carabus cancellatus dolens voucher CC00816 16S rib	JQ647183	81.16	83.33	2.65109e−23	Coleoptera	Carabidae	Carabus cancellatus dolens
Carabus cancellatus dolens voucher CC00801 16S rib	JQ647168	81.16	83.33	2.65109e−23	Coleoptera	Carabidae	Carabus cancellatus dolens
Carabus semiopacus semiopacus voucher CC00780 16S	JQ647147	79.5	98.08	2.65109e−23	Coleoptera	Carabidae	Carabus semiopacus semiopacus
Carabus cancellatus isolate TACHcc816 large subuni	JQ689798	81.16	83.33	2.65109e−23	Coleoptera	Carabidae	Carabus cancellatus
Carabus cancellatus isolate ROMA94106 large subuni	JQ689792	81.16	83.33	2.65109e−23	Coleoptera	Carabidae	Carabus cancellatus
Omaloplia nigromarginata 16S ribosomal RNA gene, p	EF487791	77.07	100	2.65109e−23	Coleoptera	Scarabaeidae	Omaloplia nigromarginata
Dryopomorphus sp. FC_C16 16S ribosomal RNA (rrnL)	EF209445	78.21	99.36	2.65109e−23	Coleoptera	Elmidae	Dryopomorphus sp. FC_C16
Hellaserica elongata voucher BMNH 678381 16S large	EF487885	78.26	100	2.65109e−23	Coleoptera	Scarabaeidae	Hellaserica elongata
Copris sinicus 16S ribosomal RNA gene, partial seq	AY131495	77.07	100	2.65109e−23	Coleoptera	Scarabaeidae	Copris sinicus
Meladema lanio haplotype 6 16S ribosomal RNA gene,	AF428194	85.44	66.03	2.65109e−23	Coleoptera	Dytiscidae	Meladema lanio
Meladema coriacea haplotype 2 16S ribosomal RNA ge	AF428188	85.44	66.03	2.65109e−23	Coleoptera	Dytiscidae	Meladema coriacea
Meladema coriacea haplotype 1 16S ribosomal RNA ge	AF428187	85.44	66.03	2.65109e−23	Coleoptera	Dytiscidae	Meladema coriacea
