# Supplementary material for: Testing the potential of a ribosomal 16S marker for DNA metabarcoding of insects
Source: PeerJ. 2016 Apr 19;4:e1966. doi: 10.7717/peerj.1966 (PMC4841222; doi:10.7717/peerj.1966)
Supplement: Supplemental Information 1 [file peerj-04-1966-s006.zip › scripts/5 match OTUs/blast_hits/OTU_292.csv_plot.pdf]

OTU_292.csv

Annitella apfelbecki

Limnephilidae

Trichoptera

93.9

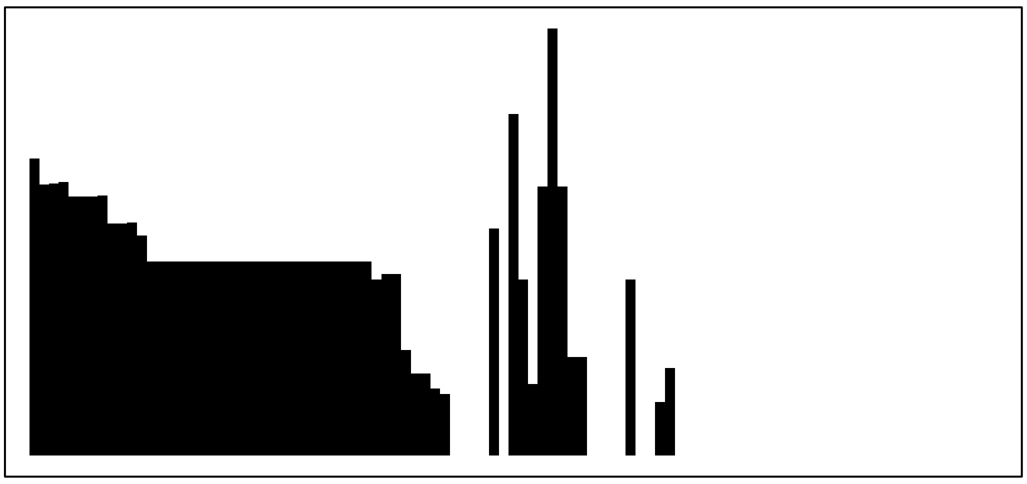

Annitella apfelbecki voucher AaIM1 16S ribosomal R	FJ002819	93.9	98.8	1.9305e−63	Trichoptera	Limnephilidae	Annitella apfelbecki
Chaetopterygopsis maclachlani voucher CM001 16S ri	EU215176	92.68	98.8	1.00003e−60	Trichoptera	Limnephilidae	Chaetopterygopsis maclachlani
Anomalopterygella chauviniana voucher AC001 16S ri	EU215174	92.73	98.8	3.49043e−60	Trichoptera	Limnephilidae	Anomalopterygella chauviniana
Cryptothrix nebulicola voucher CN006 16S ribosomal	EU215177	92.81	98.8	1.21828e−59	Trichoptera	Limnephilidae	Cryptothrix nebulicola
Chaetopteryx fusca voucher ChfIM1 16S ribosomal RN	FJ002820	92.12	98.8	1.48417e−58	Trichoptera	Limnephilidae	Chaetopteryx fusca
Drusus chrysotus voucher DCh004 16S ribosomal RNA	EU215189	92.12	98.8	1.48417e−58	Trichoptera	Limnephilidae	Drusus chrysotus
Drusus chrysotus voucher DCh001 16S ribosomal RNA	EU215188	92.12	98.8	1.48417e−58	Trichoptera	Limnephilidae	Drusus chrysotus
Ecclisocosmoecus spinosus mitochondrial gene for 1	AB355447	92.17	98.8	5.18026e−58	Trichoptera	Limnephilidae	Ecclisocosmoecus spinosus
Chaetopteryx rugulosa voucher CRu002 16S ribosomal	EU215178	90.85	98.8	6.31085e−57	Trichoptera	Limnephilidae	Chaetopteryx rugulosa
Allogamus uncatus voucher AUn003 16S ribosomal RNA	KM001840	90.85	98.8	2.2027e−56	Trichoptera	Limnephilidae	Allogamus uncatus
Asynarchus sachalinensis mitochondrial gene for 16	AB355450	90.91	98.8	9.36613e−55	Trichoptera	Limnephilidae	Asynarchus sachalinensis
Rivulophilus sakaii mitochondrial gene for 16S rib	AB355449	90.3	98.8	1.14103e−53	Trichoptera	Limnephilidae	Rivulophilus sakaii
Ecclisopteryx keroveci voucher E2JAL2 16S ribosoma	KM001845	89.09	98.8	4.85178e−52	Trichoptera	Limnephilidae	Ecclisopteryx keroveci
Ecclisopteryx keroveci voucher E2JAL1 16S ribosoma	KM001844	89.09	98.8	4.85178e−52	Trichoptera	Limnephilidae	Ecclisopteryx keroveci
Ecclisopteryx keroveci voucher E1JAL2 16S ribosoma	KM001843	89.09	98.8	4.85178e−52	Trichoptera	Limnephilidae	Ecclisopteryx keroveci
Ecclisopteryx keroveci voucher E1JAL1 16S ribosoma	KM001842	89.09	98.8	4.85178e−52	Trichoptera	Limnephilidae	Ecclisopteryx keroveci
Ecclisopteryx ivkae voucher fEda1202L 16S ribosoma	KM001810	89.09	98.8	4.85178e−52	Trichoptera	Limnephilidae	Ecclisopteryx ivkae
Ecclisopteryx ivkae voucher fEda1201L 16S ribosoma	KM001809	89.09	98.8	4.85178e−52	Trichoptera	Limnephilidae	Ecclisopteryx ivkae
Ecclisopteryx ivkae voucher fEda0102M 16S ribosoma	KM001808	89.09	98.8	4.85178e−52	Trichoptera	Limnephilidae	Ecclisopteryx ivkae
Ecclisopteryx ivkae voucher fEda0102F 16S ribosoma	KM001807	89.09	98.8	4.85178e−52	Trichoptera	Limnephilidae	Ecclisopteryx ivkae
Ecclisopteryx ivkae voucher fEda0101M 16S ribosoma	KM001806	89.09	98.8	4.85178e−52	Trichoptera	Limnephilidae	Ecclisopteryx ivkae
Ecclisopteryx ivkae voucher fDsp3301L 16S ribosoma	KM001804	89.09	98.8	4.85178e−52	Trichoptera	Limnephilidae	Ecclisopteryx ivkae
Ecclisopteryx keroveci voucher fEns0101M 16S ribos	KM001784	89.09	98.8	4.85178e−52	Trichoptera	Limnephilidae	Ecclisopteryx keroveci
Ecclisopteryx keroveci voucher fEda1101F 16S ribos	KM001783	89.09	98.8	4.85178e−52	Trichoptera	Limnephilidae	Ecclisopteryx keroveci
Ecclisopteryx keroveci voucher fEda1001M 16S ribos	KM001782	89.09	98.8	4.85178e−52	Trichoptera	Limnephilidae	Ecclisopteryx keroveci
Ecclisopteryx keroveci voucher fEda0301M 16S ribos	KM001781	89.09	98.8	4.85178e−52	Trichoptera	Limnephilidae	Ecclisopteryx keroveci
Ecclisopteryx keroveci voucher fEda0202F 16S ribos	KM001780	89.09	98.8	4.85178e−52	Trichoptera	Limnephilidae	Ecclisopteryx keroveci
Ecclisopteryx keroveci voucher fEda0201M 16S ribos	KM001779	89.09	98.8	4.85178e−52	Trichoptera	Limnephilidae	Ecclisopteryx keroveci
Ecclisopteryx keroveci voucher fDs30109F 16S ribos	KM001778	89.09	98.8	4.85178e−52	Trichoptera	Limnephilidae	Ecclisopteryx keroveci
Ecclisopteryx keroveci voucher fDs30106F 16S ribos	KM001777	89.09	98.8	4.85178e−52	Trichoptera	Limnephilidae	Ecclisopteryx keroveci
Ecclisopteryx keroveci voucher fDs30105M 16S ribos	KM001776	89.09	98.8	4.85178e−52	Trichoptera	Limnephilidae	Ecclisopteryx keroveci
Ecclisopteryx keroveci voucher fDs30103M 16S ribos	KM001775	89.09	98.8	4.85178e−52	Trichoptera	Limnephilidae	Ecclisopteryx keroveci
Ecclisopteryx keroveci voucher fDs30102M 16S ribos	KM001774	89.09	98.8	4.85178e−52	Trichoptera	Limnephilidae	Ecclisopteryx keroveci
Ecclisopteryx keroveci voucher fDs30101M 16S ribos	KM001773	89.09	98.8	4.85178e−52	Trichoptera	Limnephilidae	Ecclisopteryx keroveci
Ecclisopteryx dalecarlica voucher EdIM1 16S riboso	FJ002818	89.09	98.8	4.85178e−52	Trichoptera	Limnephilidae	Ecclisopteryx dalecarlica
Consorophylax consors voucher CCon001 16S ribosoma	EU215175	88.24	98.8	1.69344e−51	Trichoptera	Limnephilidae	Consorophylax consors
Ecclisopteryx ivkae voucher fEda0101F 16S ribosoma	KM001805	88.48	98.8	2.06303e−50	Trichoptera	Limnephilidae	Ecclisopteryx ivkae
Hydatophylax festivus mitochondrial gene for 16S r	AB355448	88.48	98.8	2.51328e−49	Trichoptera	Limnephilidae	Hydatophylax festivus
Ironoquia punctatissima mitochondrial gene for 16S	AB355446	84.94	98.8	8.21596e−43	Trichoptera	Limnephilidae	Ironoquia punctatissima
Nothopsyche montivaga mitochondrial gene for 16S r	AB355431	83.83	98.8	7.69498e−37	Trichoptera	Limnephilidae	Nothopsyche montivaga
Nothopsyche montivaga mitochondrial gene for 16S r	AB355432	83.83	98.8	7.69498e−37	Trichoptera	Limnephilidae	Nothopsyche montivaga
Dicosmoecus jozankeanus mitochondrial gene for 16S	AB355445	83.12	94.58	3.9861e−34	Trichoptera	Limnephilidae	Dicosmoecus jozankeanus
Taskiropsyche lacustris voucher NHRS−EN CS5 16S ri	FJ263209	82.88	84.34	3.06451e−29	Trichoptera	Kokiriidae	Taskiropsyche lacustris
Eubasilissa regina mitochondrion, complete genome	KF756943	76.97	98.8	1.81129e−19	Trichoptera		Eubasilissa regina
Myiodactylus osmyloides 16S ribosomal RNA gene, pa	EU734873	77.7	83.13	1.14305e−15	Neuroptera	Nymphidae	Myiodactylus osmyloides
Bambusiphaga fascia isolate 181 16S ribosomal RNA	HM233787	71.34	95.18	2.35808e−05	Hemiptera	Delphacidae	Bambusiphaga fascia
Mycoplasma canadense DNA, complete genome, strain:	AP014631	77.78	34.94	0.042635	Mycoplasmatales	Mycoplasmataceae	Mycoplasma canadense
Ovis canadensis canadensis isolate 43U chromosome	CP011888	90.62	19.28	0.519401		Bovidae	Ovis canadensis canadensis
Cyprinus carpio genome assembly common carp genome	LN594807	76.81	40.36	0.519401	Cypriniformes	Cyprinidae	Cyprinus carpio
Apteryx australis mantelli genome assembly AptMant	LK391415	96	15.06	6.32759	Apterygiformes	Apterygidae	Apteryx australis mantelli
Tetrahymena thermophila SB210 transmembrane protei	XM_012796639	88.24	20.48	6.32759	Hymenostomatida	Tetrahymenidae	Tetrahymena thermophila SB210
Onchocerca flexuosa genome assembly O_flexuosa_Cor	LM539701	83.33	25.3	6.32759	Spirurida	Onchocercidae	Onchocerca flexuosa
Trichobilharzia regenti genome assembly T_regenti_	LL009804	92.59	16.27	6.32759	Strigeidida	Schistosomatidae	Trichobilharzia regenti
Rhabditophanes sp. KR3021 genome assembly Rhabdito	LK995574	100	13.25	6.32759	Rhabditida	Alloionematidae	Rhabditophanes sp. KR3021
Protopolystoma xenopodis genome assembly P_xenopod	LM682778	92.59	16.27	6.32759	Polyopisthocotylea	Polystomatidae	Protopolystoma xenopodis
Strongyloides papillosus genome assembly S_papillo	LM525635	84.62	23.49	6.32759	Rhabditida	Strongyloididae	Strongyloides papillosus
Cyprinus carpio genome assembly common carp genome	LN590687	84.62	22.89	6.32759	Cypriniformes	Cyprinidae	Cyprinus carpio
Solanum lycopersicum chromosome ch03, complete gen	HG975515	80	27.11	6.32759	Solanales	Solanaceae	Solanum lycopersicum
Wakarumbia nepeensis (nomen nudum) voucher UPOLMD0	KC538618	77.42	37.35	6.32759	Coleoptera	Lycidae	Wakarumbia nepeensis (nomen nudum)
Wakarumbia linearis voucher UPOLMD0134 NADH dehydr	KC538617	77.42	37.35	6.32759	Coleoptera	Lycidae	Wakarumbia linearis
Wakarumbia nepeensis (nomen nudum) voucher UPOLMD0	KC538613	77.42	37.35	6.32759	Coleoptera	Lycidae	Wakarumbia nepeensis (nomen nudum)
Fluviicola taffensis DSM 16823, complete genome	CP002542	88.24	19.88	6.32759	Flavobacteriales	Cryomorphaceae	Fluviicola taffensis DSM 16823
Hemiselmis andersenii hypothetical protein (HAN_1g	XM_001712135	69.9	62.05	6.32759	Cryptomonadales	Hemiselmidaceae	Hemiselmis andersenii
Hemiselmis andersenii chromosome 1, complete seque	CP000881	69.9	62.05	6.32759	Cryptomonadales	Hemiselmidaceae	Hemiselmis andersenii
Human DNA sequence from clone RP11−289M23 on chrom	AL157370	82.5	24.1	6.32759	Primates	Hominidae	Homo sapiens
Zebrafish DNA sequence from clone CH211−215M13 in	CR533575	84.09	25.9	6.32759	Cypriniformes	Cyprinidae	Danio rerio
