# Supplementary material for: Testing the potential of a ribosomal 16S marker for DNA metabarcoding of insects
Source: PeerJ. 2016 Apr 19;4:e1966. doi: 10.7717/peerj.1966 (PMC4841222; doi:10.7717/peerj.1966)
Supplement: Supplemental Information 1 [file peerj-04-1966-s006.zip › scripts/5 match OTUs/blast_hits/OTU_3.csv_plot.pdf]

OTU_3.csv

Isoperla oxylepis

Perlodidae

Plecoptera

96.79

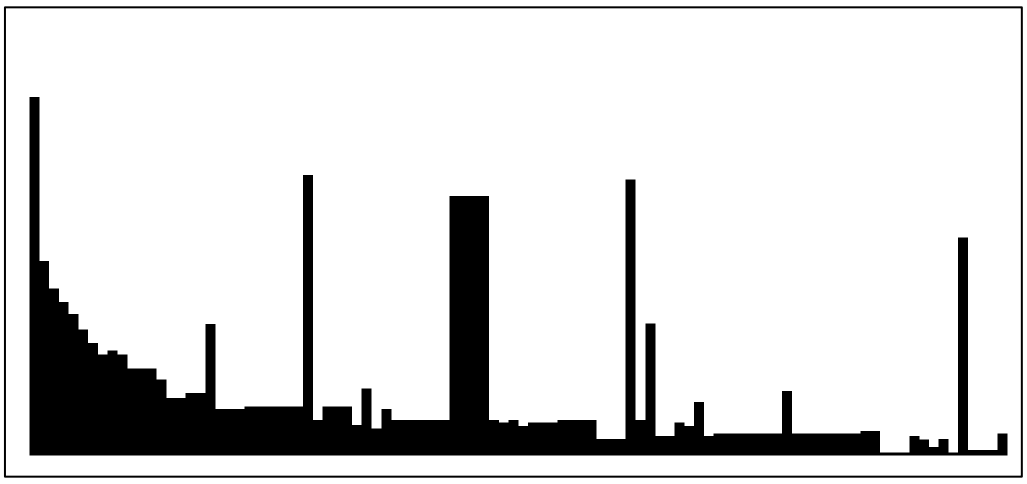

Isoperla oxylepis 16S ribosomal RNA gene, partial	EF623221	96.79	100	1.20147e−65	Plecoptera	Perlodidae	Isoperla oxylepis
Isoperla davisi 16S ribosomal RNA gene, partial se	EF623206	89.1	100	2.32143e−49	Plecoptera	Perlodidae	Isoperla davisi
Isoperla fulva 16S ribosomal RNA gene, partial seq	EF623207	87.82	100	1.20253e−46	Plecoptera	Perlodidae	Isoperla fulva
Isoperla phalerata 16S ribosomal RNA gene, partial	EF623208	87.18	100	1.46498e−45	Plecoptera	Perlodidae	Isoperla phalerata
Perlinodes aurea 16S ribosomal RNA gene, partial s	EF623234	86.62	100	2.17423e−43	Plecoptera	Perlodidae	Perlinodes aurea
Cascadoperla trictura 16S ribosomal RNA gene, part	EF623225	85.9	100	7.58879e−43	Plecoptera	Perlodidae	Cascadoperla trictura
Clioperla clio 16S ribosomal RNA gene, partial seq	EF623237	85.26	100	3.22684e−41	Plecoptera	Perlodidae	Clioperla clio
Frisonia picticeps 16S ribosomal RNA gene, partial	EF623227	84.71	100	1.37209e−39	Plecoptera	Perlodidae	Frisonia picticeps
Brachyptera seticornis 16S ribosomal RNA gene, par	EF623268	84.91	100	1.67154e−38	Plecoptera	Taeniopterygidae	Brachyptera seticornis
Rickera sorpta 16S ribosomal RNA gene, partial seq	EF623235	84.71	100	1.67154e−38	Plecoptera	Perlodidae	Rickera sorpta
Cosumnoperla hypocrena 16S ribosomal RNA gene, par	EF623226	84.08	100	5.83426e−38	Plecoptera	Perlodidae	Cosumnoperla hypocrena
Megarcys signata 16S ribosomal RNA gene, partial s	EF623215	84.08	100	5.83426e−38	Plecoptera	Perlodidae	Megarcys signata
Kogotus nonus 16S ribosomal RNA gene, partial sequ	EF623213	84.08	100	7.10758e−37	Plecoptera	Perlodidae	Kogotus nonus
Perlodes dispar 16S ribosomal RNA gene, partial se	EF623220	83.54	100	2.48079e−36	Plecoptera	Perlodidae	Perlodes dispar
Pictetiella expansa 16S ribosomal RNA gene, partia	EF623216	82.69	100	8.65881e−36	Plecoptera	Perlodidae	Pictetiella expansa
Sweltsa coloradensis 16S ribosomal RNA gene, parti	EF623133	82.69	100	8.65881e−36	Plecoptera	Chloroperlidae	Sweltsa coloradensis
Zapada cinctipes 16S ribosomal RNA gene, partial s	EF623183	82.91	100	3.02222e−35	Plecoptera	Nemouridae	Zapada cinctipes
Suwallia teleckojensis 16S ribosomal RNA gene, par	EF623142	82.91	100	1.05486e−34	Plecoptera	Chloroperlidae	Suwallia teleckojensis
Peltoperla arcuata 16S ribosomal RNA gene, partial	EF623258	86.15	83.33	3.68182e−34	Plecoptera	Peltoperlidae	Peltoperla arcuata
Salmoperla sylvanica 16S ribosomal RNA gene, parti	EF623222	82.17	100	3.68182e−34	Plecoptera	Perlodidae	Salmoperla sylvanica
Skwala americana 16S ribosomal RNA gene, partial s	EF623217	82.17	100	3.68182e−34	Plecoptera	Perlodidae	Skwala americana
Paraperla frontalis 16S ribosomal RNA gene, partia	EF623136	82.17	100	3.68182e−34	Plecoptera	Chloroperlidae	Paraperla frontalis
Mesocapnia arizonensis mitochondrion, partial geno	KP642637	82.28	100	1.28508e−33	Plecoptera	Capniidae	Mesocapnia arizonensis
Allocapnia recta isolate H13 16S ribosomal RNA gen	KC881048	82.28	100	1.28508e−33	Plecoptera	Capniidae	Allocapnia recta
Allocapnia recta isolate H11 16S ribosomal RNA gen	KC881046	82.28	100	1.28508e−33	Plecoptera	Capniidae	Allocapnia recta
Allocapnia recta isolate H7 16S ribosomal RNA gene	KC881042	82.28	100	1.28508e−33	Plecoptera	Capniidae	Allocapnia recta
Allocapnia recta isolate H3 16S ribosomal RNA gene	KC881038	82.28	100	1.28508e−33	Plecoptera	Capniidae	Allocapnia recta
Allocapnia recta isolate H1 16S ribosomal RNA gene	KC881036	82.28	100	1.28508e−33	Plecoptera	Capniidae	Allocapnia recta
Cryptoperla sp. YK−2012−1 mitochondrial gene for 1	AB711243	93.14	65.38	1.28508e−33	Plecoptera	Peltoperlidae	Cryptoperla sp. YK−2012−1
Setvena wahkeena 16S ribosomal RNA gene, partial s	EF623211	81.65	100	1.28508e−33	Plecoptera	Perlodidae	Setvena wahkeena
Mesocapnia sp. BYU_PL010 16S ribosomal RNA gene, p	EF623127	82.28	100	1.28508e−33	Plecoptera	Capniidae	Mesocapnia sp. BYU_PL010
Allocapnia recta haplotype 12ar: H12 16S ribosomal	EU306875	82.28	100	1.28508e−33	Plecoptera	Capniidae	Allocapnia recta
Allocapnia recta haplotype 3ar: H03 16S ribosomal	DQ915181	82.28	100	1.28508e−33	Plecoptera	Capniidae	Allocapnia recta
Cultus pilatus 16S ribosomal RNA gene, partial seq	EF623212	81.41	100	4.48537e−33	Plecoptera	Perlodidae	Cultus pilatus
Nemoura cinerea 16S ribosomal RNA gene, partial se	EF623194	83.12	100	4.48537e−33	Plecoptera	Nemouridae	Nemoura cinerea
Thaumatoperla sp. BYU_PL109 16S ribosomal RNA gene	EF623149	81.25	100	4.48537e−33	Plecoptera	Eustheniidae	Thaumatoperla sp. BYU_PL109
Capnura wanica 16S ribosomal RNA gene, partial seq	EF623128	82.17	100	4.48537e−33	Plecoptera	Capniidae	Capnura wanica
Allocapnia recta isolate H19 16S ribosomal RNA gen	KC881054	81.65	100	1.56555e−32	Plecoptera	Capniidae	Allocapnia recta
Allocapnia recta isolate H16 16S ribosomal RNA gen	KC881051	81.65	100	1.56555e−32	Plecoptera	Capniidae	Allocapnia recta
Allocapnia recta isolate H15 16S ribosomal RNA gen	KC881050	81.65	100	1.56555e−32	Plecoptera	Capniidae	Allocapnia recta
Allocapnia recta isolate H14 16S ribosomal RNA gen	KC881049	81.65	100	1.56555e−32	Plecoptera	Capniidae	Allocapnia recta
Allocapnia recta isolate H10 16S ribosomal RNA gen	KC881045	81.65	100	1.56555e−32	Plecoptera	Capniidae	Allocapnia recta
Allocapnia recta isolate H6 16S ribosomal RNA gene	KC881041	81.65	100	1.56555e−32	Plecoptera	Capniidae	Allocapnia recta
Cryptoperla japonica mitochondrial gene for 16S rR	AB711202	92.16	65.38	1.56555e−32	Plecoptera	Peltoperlidae	Cryptoperla japonica
Cryptoperla japonica mitochondrial gene for 16S rR	AB711184	92.16	65.38	1.56555e−32	Plecoptera	Peltoperlidae	Cryptoperla japonica
Cryptoperla japonica mitochondrial gene for 16S rR	AB711183	92.16	65.38	1.56555e−32	Plecoptera	Peltoperlidae	Cryptoperla japonica
Cryptoperla japonica mitochondrial gene for 16S rR	AB711182	92.16	65.38	1.56555e−32	Plecoptera	Peltoperlidae	Cryptoperla japonica
Austrocerca rieka 16S ribosomal RNA gene, partial	EF623205	81.65	100	1.56555e−32	Plecoptera	Notonemouridae	Austrocerca rieka
Austronemoura sp. BYU_PL149 16S ribosomal RNA gene	EF623200	81.53	100	1.56555e−32	Plecoptera	Notonemouridae	Austronemoura sp. BYU_PL149
Austrocercoides zwicki 16S ribosomal RNA gene, par	EF623199	81.65	100	1.56555e−32	Plecoptera	Notonemouridae	Austrocercoides zwicki
Cosmioperla australis 16S ribosomal RNA gene, part	EF623148	81.37	100	1.56555e−32	Plecoptera	Eustheniidae	Cosmioperla australis
Triznaka pintada 16S ribosomal RNA gene, partial s	EF623134	81.53	100	1.56555e−32	Plecoptera	Chloroperlidae	Triznaka pintada
Plumiperla diversa 16S ribosomal RNA gene, partial	EF623132	81.53	100	1.56555e−32	Plecoptera	Chloroperlidae	Plumiperla diversa
Isogenoides frontalis isolate BYU_PL080 16S riboso	EF623446	81.53	100	1.56555e−32	Plecoptera	Perlodidae	Isogenoides frontalis
Allocapnia recta haplotype 11ar: H011 16S ribosoma	EU306874	81.65	100	1.56555e−32	Plecoptera	Capniidae	Allocapnia recta
Allocapnia recta haplotype 7ar: H07 16S ribosomal	EU306870	81.65	100	1.56555e−32	Plecoptera	Capniidae	Allocapnia recta
Allocapnia recta haplotype 2ar: H02 16S ribosomal	DQ915180	81.65	100	1.56555e−32	Plecoptera	Capniidae	Allocapnia recta
Perlodes mortoni 16S ribosomal RNA gene, partial s	KF855870	81.65	100	5.4643e−32	Plecoptera	Perlodidae	Perlodes mortoni
Osobenus yakimae 16S ribosomal RNA gene, partial s	EF623232	80.77	100	5.4643e−32	Plecoptera	Perlodidae	Osobenus yakimae
Malirekus iroquois 16S ribosomal RNA gene, partial	EF623231	80.77	100	5.4643e−32	Plecoptera	Perlodidae	Malirekus iroquois
Hydroperla crosbyi 16S ribosomal RNA gene, partial	EF623219	80.77	100	5.4643e−32	Plecoptera	Perlodidae	Hydroperla crosbyi
Chernokrilus misnomus 16S ribosomal RNA gene, part	EF623209	92.93	63.46	5.4643e−32	Plecoptera	Perlodidae	Chernokrilus misnomus
Neaviperla forcipata 16S ribosomal RNA gene, parti	EF623139	81.65	100	5.4643e−32	Plecoptera	Chloroperlidae	Neaviperla forcipata
Cryptoperla sp. YK−2012−2 mitochondrial gene for 1	AB711244	86.18	78.85	1.90723e−31	Plecoptera	Peltoperlidae	Cryptoperla sp. YK−2012−2
Oconoperla innubila 16S ribosomal RNA gene, partia	EF623230	80.89	100	1.90723e−31	Plecoptera	Perlodidae	Oconoperla innubila
Yugus bulbosus 16S ribosomal RNA gene, partial seq	EF623214	80.89	100	1.90723e−31	Plecoptera	Perlodidae	Yugus bulbosus
Neonemura sp. BYU_PL153 16S ribosomal RNA gene, pa	EF623204	81.53	100	1.90723e−31	Plecoptera	Notonemouridae	Neonemura sp. BYU_PL153
Malenka californica 16S ribosomal RNA gene, partia	EF623182	81.37	100	1.90723e−31	Plecoptera	Nemouridae	Malenka californica
Megaleuctra kincaidi 16S ribosomal RNA gene, parti	EF623174	82.5	100	1.90723e−31	Plecoptera	Leuctridae	Megaleuctra kincaidi
Isogenoides colubrinus isolate BYU_PL130 16S ribos	EF623448	80.89	100	1.90723e−31	Plecoptera	Perlodidae	Isogenoides colubrinus
Allocapnia recta isolate H18 16S ribosomal RNA gen	KC881053	81.01	100	6.65688e−31	Plecoptera	Capniidae	Allocapnia recta
Allocapnia recta isolate H17 16S ribosomal RNA gen	KC881052	81.01	100	6.65688e−31	Plecoptera	Capniidae	Allocapnia recta
Allocapnia recta isolate H12 16S ribosomal RNA gen	KC881047	81.01	100	6.65688e−31	Plecoptera	Capniidae	Allocapnia recta
Allocapnia recta isolate H9 16S ribosomal RNA gene	KC881044	81.01	100	6.65688e−31	Plecoptera	Capniidae	Allocapnia recta
Allocapnia recta isolate H8 16S ribosomal RNA gene	KC881043	81.01	100	6.65688e−31	Plecoptera	Capniidae	Allocapnia recta
Allocapnia recta isolate H5 16S ribosomal RNA gene	KC881040	81.01	100	6.65688e−31	Plecoptera	Capniidae	Allocapnia recta
Allocapnia recta isolate H4 16S ribosomal RNA gene	KC881039	81.01	100	6.65688e−31	Plecoptera	Capniidae	Allocapnia recta
Kimminsoperla albomacula 16S ribosomal RNA gene, p	EF623202	83.02	100	6.65688e−31	Plecoptera	Notonemouridae	Kimminsoperla albomacula
Nanonemoura wahkeena 16S ribosomal RNA gene, parti	EF623198	81.01	100	6.65688e−31	Plecoptera	Nemouridae	Nanonemoura wahkeena
Nemoura trispinosa 16S ribosomal RNA gene, partial	EF623187	81.01	100	6.65688e−31	Plecoptera	Nemouridae	Nemoura trispinosa
Allocapnia minima 16S ribosomal RNA gene, partial	EF623130	81.01	100	6.65688e−31	Plecoptera	Capniidae	Allocapnia minima
Allocapnia recta haplotype 10ar: H010 16S ribosoma	EU306873	81.01	100	6.65688e−31	Plecoptera	Capniidae	Allocapnia recta
Allocapnia recta haplotype 9ar: H09 16S ribosomal	EU306872	81.01	100	6.65688e−31	Plecoptera	Capniidae	Allocapnia recta
Allocapnia recta haplotype 8ar: H08 16S ribosomal	EU306871	81.01	100	6.65688e−31	Plecoptera	Capniidae	Allocapnia recta
Allocapnia recta haplotype 1ar: H01 16S ribosomal	DQ915179	81.01	100	6.65688e−31	Plecoptera	Capniidae	Allocapnia recta
Doddsia occidentalis 16S ribosomal RNA gene, parti	EF623270	81.13	100	2.32348e−30	Plecoptera	Taeniopterygidae	Doddsia occidentalis
Scopura montana 16S ribosomal RNA gene, partial se	EF623265	81.13	100	2.32348e−30	Plecoptera		Scopura montana
Pteronarcella badia 16S ribosomal RNA gene, partia	EF623262	80.12	100	2.32348e−30	Plecoptera	Pteronarcyidae	Pteronarcella badia
Pteronarcys californica 16S ribosomal RNA gene, pa	EF623261	80.13	100	2.32348e−30	Plecoptera	Pteronarcyidae	Pteronarcys californica
Soyedina producta 16S ribosomal RNA gene, partial	EF623196	80.13	100	2.32348e−30	Plecoptera	Nemouridae	Soyedina producta
Siphonoperla torrentium 16S ribosomal RNA gene, pa	EF623138	80.89	100	2.32348e−30	Plecoptera	Chloroperlidae	Siphonoperla torrentium
Neopanorpa sp. M24899 16S ribosomal RNA gene, part	AF180059	80.75	100	2.32348e−30	Mecoptera	Panorpidae	Neopanorpa sp. M24899
Apteroperla tikumana mitochondrion, complete genom	KR604721	80.38	100	8.10974e−30	Plecoptera	Capniidae	Apteroperla tikumana
Chloronia mexicana 16S ribosomal RNA gene, partial	KF944500	80.77	99.36	8.10974e−30	Megaloptera	Corydalidae	Chloronia mexicana
Cryptoperla ishigakiensis mitochondrial gene for 1	AB711242	80.12	100	8.10974e−30	Plecoptera	Peltoperlidae	Cryptoperla ishigakiensis
Cryptoperla japonica mitochondrial gene for 16S rR	AB711189	90.2	65.38	8.10974e−30	Plecoptera	Peltoperlidae	Cryptoperla japonica
Baumanella alameda 16S ribosomal RNA gene, partial	EF623236	80.25	100	8.10974e−30	Plecoptera	Perlodidae	Baumanella alameda
Susulus venastus 16S ribosomal RNA gene, partial s	EF623229	80.25	100	8.10974e−30	Plecoptera	Perlodidae	Susulus venastus
Isogenoides olivaceus 16S ribosomal RNA gene, part	EF623224	80.25	100	8.10974e−30	Plecoptera	Perlodidae	Isogenoides olivaceus
Isogenoides doratus isolate BYU_PL131 16S ribosoma	EF623449	81.01	100	8.10974e−30	Plecoptera	Perlodidae	Isogenoides doratus
