# Supplementary material for: Testing the potential of a ribosomal 16S marker for DNA metabarcoding of insects
Source: PeerJ. 2016 Apr 19;4:e1966. doi: 10.7717/peerj.1966 (PMC4841222; doi:10.7717/peerj.1966)
Supplement: Supplemental Information 1 [file peerj-04-1966-s006.zip › scripts/5 match OTUs/blast_hits/OTU_30.csv_plot.pdf]

OTU_30.csv

Sericostoma personatum

Sericostomatidae

Trichoptera

98.61

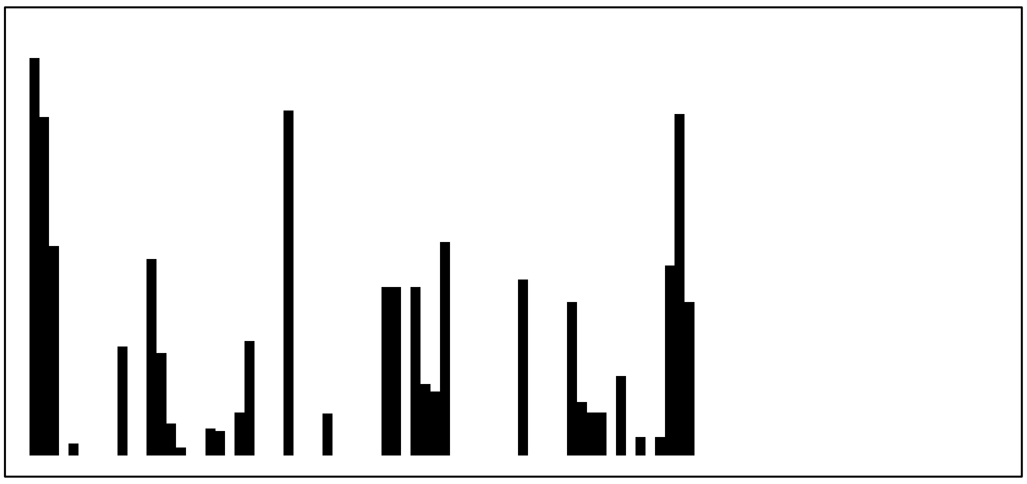

Sericostoma personatum mitochondrion, partial geno	KP455290	98.61	100	1.6063e−63	Trichoptera	Sericostomatidae	Sericostoma personatum
Sericostoma clypeatum voucher NHRS−EN CC4 16S ribo	FJ263207	95.86	100	1.50444e−57	Trichoptera	Sericostomatidae	Sericostoma clypeatum
Notidobiella chacayana isolate CL5 16S ribosomal R	EF394993	89.81	73.61	7.30547e−30	Trichoptera	Sericostomatidae	Notidobiella chacayana
Helicopsyche cochleaetesta isolate CP5 16S ribosom	EF394996	75.52	97.22	1.41153e−13	Trichoptera	Helicopsychidae	Helicopsyche cochleaetesta
Pliocaloca sp. CQ3 16S ribosomal RNA gene, partial	EF394999	80.56	73.61	1.7196e−12	Trichoptera	Calocidae	Pliocaloca sp. CQ3
Caloca saneva mitochondrial 16S rRNA gene, specime	FN257670	79.05	72.22	7.31193e−11	Trichoptera	Calocidae	Caloca saneva
Pycnocentrella eruensis isolate BR4 16S ribosomal	EF394985	75.74	90.97	2.55211e−10	Trichoptera	Calocidae	Pycnocentrella eruensis
Alloecentrella sp. BP8 16S ribosomal RNA gene, par	EF394983	76.19	72.92	3.10911e−09	Trichoptera	Calocidae	Alloecentrella sp. BP8
Chrysis fasciata mitochondrial partial 16S rRNA ge	AJ514346	72.09	88.19	4.61433e−07	Hymenoptera	Chrysididae	Chrysis fasciata
Sesamum indicum clone TilSSR_29963 microsatellite	KJ058762	85.11	32.64	0.0101637	Lamiales	Pedaliaceae	Sesamum indicum
Daltonia sp. 16S ribosomal RNA gene, partial seque	L12999	71.53	96.53	0.0101637	Hemiptera	Cicadellidae	Daltonia sp.
Glycine max DNA, BAC clone: WBb35C13, complete seq	AP011822	78.67	50.69	0.0354749	Fabales	Fabaceae	Glycine max
Trichobilharzia regenti genome assembly T_regenti_	LL000277	89.19	25	0.12382	Strigeidida	Schistosomatidae	Trichobilharzia regenti
PREDICTED: Heterocephalus glaber guanylate−binding	XM_004841310	84.78	29.86	0.12382	Rodentia	Bathyergidae	Heterocephalus glaber
Brassica rapa subsp. pekinensis clone KBrB060J18,	AC232507	81.48	36.81	0.12382	Brassicales	Brassicaceae	Brassica rapa subsp. pekinensis
Mus musculus chromosome 1, clone RP24−289K8, compl	AC154144	80.36	38.89	0.12382	Rodentia	Muridae	Mus musculus
Mus musculus BAC clone RP24−476G13 from chromosome	AC158389	74.07	55.56	0.12382	Rodentia	Muridae	Mus musculus
Strongyloides venezuelensis genome assembly S_vene	LM524971	73.68	52.78	0.432173	Rhabditida	Strongyloididae	Strongyloides venezuelensis
Trifolium pratense genome assembly redclover, chro	LN846353	81.25	33.33	0.432173	Fabales	Fabaceae	Trifolium pratense
PREDICTED: Plutella xylostella uncharacterized LOC	XM_011564277	81.13	36.11	0.432173	Lepidoptera	Plutellidae	Plutella xylostella
Strongyloides papillosus genome assembly S_papillo	LM527306	76	51.39	0.432173	Rhabditida	Strongyloididae	Strongyloides papillosus
Schistosoma curassoni genome assembly S_curassoni_	LM086212	82	34.72	0.432173	Strigeidida	Schistosomatidae	Schistosoma curassoni
Cyprinus carpio genome assembly common carp genome	LN595784	85.37	28.47	0.432173	Cypriniformes	Cyprinidae	Cyprinus carpio
Solanum lycopersicum chromosome ch12, complete gen	HG975524	77.65	58.33	0.432173	Solanales	Solanaceae	Solanum lycopersicum
Solanum lycopersicum chromosome ch01, complete gen	HG975513	78.21	53.47	0.432173	Solanales	Solanaceae	Solanum lycopersicum
Vitis vinifera contig VV78X188519.2, whole genome	AM439753	75.34	50.69	0.432173	Vitales	Vitaceae	Vitis vinifera
Protopolystoma xenopodis genome assembly P_xenopod	LM743394	96.15	18.06	1.50843	Polyopisthocotylea	Polystomatidae	Protopolystoma xenopodis
Wuchereria bancrofti genome assembly W_bancrofti_J	LM005816	76.12	45.14	1.50843	Spirurida	Onchocercidae	Wuchereria bancrofti
Wuchereria bancrofti genome assembly W_bancrofti_J	LM001050	78.43	31.94	1.50843	Spirurida	Onchocercidae	Wuchereria bancrofti
Strongyloides stercoralis genome assembly S_sterco	LL999057	73.49	57.64	1.50843	Rhabditida	Strongyloididae	Strongyloides stercoralis
Strongyloides papillosus genome assembly S_papillo	LM525585	81.97	42.36	1.50843	Rhabditida	Strongyloididae	Strongyloides papillosus
Parastrongyloides trichosuri genome assembly P_tri	LM523301	79.25	36.81	1.50843	Rhabditida	Strongyloididae	Parastrongyloides trichosuri
PREDICTED: Microplitis demolitor histone−lysine N−	XM_008545570	75.36	47.92	1.50843	Hymenoptera	Braconidae	Microplitis demolitor
PREDICTED: Microplitis demolitor histone−lysine N−	XM_008545562	75.36	47.92	1.50843	Hymenoptera	Braconidae	Microplitis demolitor
PREDICTED: Microplitis demolitor histone−lysine N−	XM_008545553	75.36	47.92	1.50843	Hymenoptera	Braconidae	Microplitis demolitor
PREDICTED: Microplitis demolitor histone−lysine N−	XM_008545547	75.36	47.92	1.50843	Hymenoptera	Braconidae	Microplitis demolitor
Solanum lycopersicum chromosome ch03, complete gen	HG975515	87.88	22.92	1.50843	Solanales	Solanaceae	Solanum lycopersicum
Solanum lycopersicum chromosome 3 clone C03SLm0111	AC238478	87.88	22.92	1.50843	Solanales	Solanaceae	Solanum lycopersicum
Brassica rapa subsp. pekinensis clone KBrB084M06,	AC189491	77.42	40.97	1.50843	Brassicales	Brassicaceae	Brassica rapa subsp. pekinensis
Vitis vinifera contig VV78X043279.8, whole genome	AM429387	87.88	22.92	1.50843	Vitales	Vitaceae	Vitis vinifera
Zebrafish DNA sequence from clone CH211−272H9 in l	CR847899	83.33	29.17	1.50843	Cypriniformes	Cyprinidae	Danio rerio
M.truncatula DNA sequence from clone MTH2−36C8 on	CT967304	82.98	31.94	1.50843	Fabales	Fabaceae	Medicago truncatula
Anabaena sp. wa102 strain WA102, complete genome	CP011456	90	20.83	5.26495	Nostocales	Nostocaceae	Anabaena sp. wa102
PREDICTED: Elaeis guineensis uncharacterized LOC10	XM_010943985	75.32	51.39	5.26495	Arecales	Arecaceae	Elaeis guineensis
Kluyveromyces marxianus mitochondrial DNA, complet	AP014607	68.84	93.75	5.26495	Saccharomycetales	Saccharomycetaceae	Kluyveromyces marxianus
Strongyloides stercoralis genome assembly S_sterco	LL999476	72.5	55.56	5.26495	Rhabditida	Strongyloididae	Strongyloides stercoralis
Strongyloides stercoralis genome assembly S_sterco	LL999081	75.58	59.03	5.26495	Rhabditida	Strongyloididae	Strongyloides stercoralis
Dracunculus medinensis genome assembly D_medinensi	LK978207	70.79	57.64	5.26495	Spirurida	Dracunculidae	Dracunculus medinensis
Protopolystoma xenopodis genome assembly P_xenopod	LM677454	76.81	44.44	5.26495	Polyopisthocotylea	Polystomatidae	Protopolystoma xenopodis
Strongyloides ratti genome assembly S_ratti_ED321	LN609530	77.59	40.28	5.26495	Rhabditida	Strongyloididae	Strongyloides ratti
Cyprinus carpio genome assembly common carp genome	LN590681	88.24	23.61	5.26495	Cypriniformes	Cyprinidae	Cyprinus carpio
Solanum pennellii chromosome ch12, complete genome	HG975451	74.36	53.47	5.26495	Solanales	Solanaceae	Solanum pennellii
Solanum pennellii chromosome ch10, complete genome	HG975449	76.47	45.14	5.26495	Solanales	Solanaceae	Solanum pennellii
Telamonia vlijmi mitochondrion, complete genome	KJ598073	69.44	94.44	5.26495	Araneae	Salticidae	Telamonia vlijmi
Kluyveromyces marxianus DMKU3−1042 mitochondrial D	AP012221	68.84	93.75	5.26495	Saccharomycetales	Saccharomycetaceae	Kluyveromyces marxianus DMKU3−1042
Homo sapiens dihydropyrimidine dehydrogenase (DPYD	NG_008807	87.18	26.39	5.26495	Primates	Hominidae	Homo sapiens
Ichthyophthirius multifiliis hypothetical protein	XM_004030935	82.5	27.78	5.26495	Hymenostomatida		Ichthyophthirius multifiliis
Phialocephala fortinii strain 7_6_7v ribosomal pro	JN091519	82	34.72	5.26495	Helotiales		Phialocephala fortinii
Phialocephala subalpina voucher UAMH:11012 mitocho	JN031566	82	34.72	5.26495	Helotiales		Phialocephala subalpina
Jatropha curcas DNA, clone: JHL25H03, complete seq	AP011975	73.49	56.25	5.26495	Malpighiales	Euphorbiaceae	Jatropha curcas
Zebrafish DNA sequence from clone CH73−322F21 in l	CU855801	83.72	29.86	5.26495	Cypriniformes	Cyprinidae	Danio rerio
Vitis vinifera contig VV78X013557.19, whole genome	AM480535	74.47	64.58	5.26495	Vitales	Vitaceae	Vitis vinifera
Candida albicans SC5314 hypothetical protein (CaO1	XM_705673	80.85	32.64	5.26495	Saccharomycetales	Debaryomycetaceae	Candida albicans SC5314
Oryza sativa Japonica Group chromosome 5 clone OJ1	AC108873	78.72	32.64	5.26495	Poales	Poaceae	Oryza sativa Japonica Group
Candida albicans genomic DNA, chromosome 7, comple	AP006852	80.85	32.64	5.26495	Saccharomycetales	Debaryomycetaceae	Candida albicans
Human DNA sequence from clone RP11−94H3 on chromos	AL731550	88.89	24.31	5.26495	Primates	Hominidae	Homo sapiens
Oryza sativa Japonica Group genomic DNA, chromosom	AP005681	96	17.36	5.26495	Poales	Poaceae	Oryza sativa Japonica Group
Homo sapiens chromosome 1 clone RP11−359C24, compl	AC093576	87.18	26.39	5.26495	Primates	Hominidae	Homo sapiens
