# Supplementary material for: Testing the potential of a ribosomal 16S marker for DNA metabarcoding of insects
Source: PeerJ. 2016 Apr 19;4:e1966. doi: 10.7717/peerj.1966 (PMC4841222; doi:10.7717/peerj.1966)
Supplement: Supplemental Information 1 [file peerj-04-1966-s006.zip › scripts/5 match OTUs/blast_hits/OTU_304.csv_plot.pdf]

OTU_304.csv

Oreodytes sanmarkii

Dytiscidae

Coleoptera

96.82

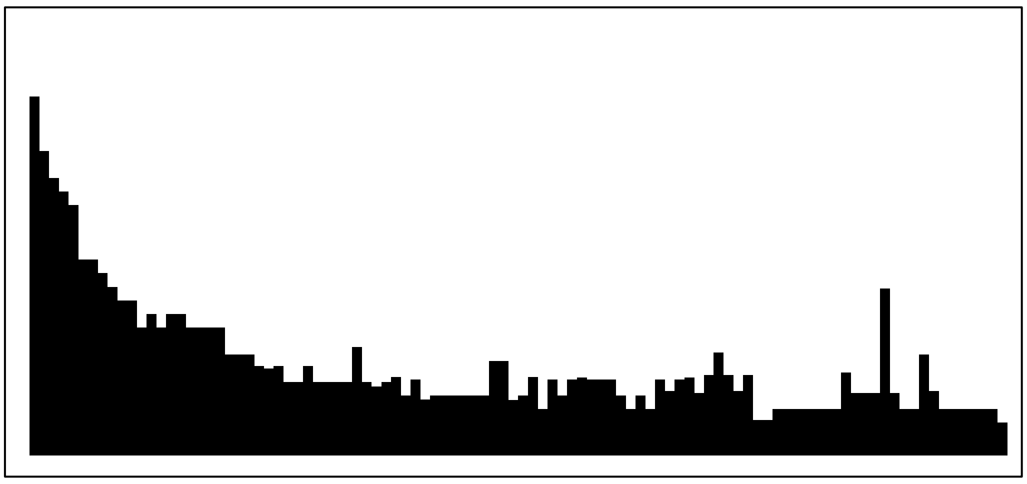

Oreodytes sanmarkii mitochondrial 16S rRNA gene (p	HF931524	96.82	100	4.19354e−65	Coleoptera	Dytiscidae	Oreodytes sanmarkii
Oreodytes obesus cordillerensis mitochondrial part	HF931516	94.27	100	1.12528e−59	Coleoptera	Dytiscidae	Oreodytes obesus cordillerensis
Oreodytes congruus partial 16S rRNA gene	AJ850347	92.99	100	5.82911e−57	Coleoptera	Dytiscidae	Oreodytes congruus
Oreodytes congruus 16S ribosomal RNA gene, partial	KF575550	92.36	100	7.10131e−56	Coleoptera	Dytiscidae	Oreodytes congruus
Oreodytes crassulus partial 16S rRNA gene	AJ850348	91.72	100	3.01955e−54	Coleoptera	Dytiscidae	Oreodytes crassulus
Oreodytes abbreviatus mitochondrial 16S rRNA gene	HF931477	89.17	100	8.10258e−49	Coleoptera	Dytiscidae	Oreodytes abbreviatus
Oreodytes rhyacophilus partial 16S rRNA gene	AJ850352	89.17	100	8.10258e−49	Coleoptera	Dytiscidae	Oreodytes rhyacophilus
Oreodytes mongolicus mitochondrial 16S rRNA gene (	HF931491	88.54	100	9.87097e−48	Coleoptera	Dytiscidae	Oreodytes mongolicus
Oreodytes snoqualmie mitochondrial partial 16S rRN	HF931519	87.9	100	4.19724e−46	Coleoptera	Dytiscidae	Oreodytes snoqualmie
Oreodytes septentrionalis mitochondrial 16S rRNA g	HF931492	87.26	100	5.11329e−45	Coleoptera	Dytiscidae	Oreodytes septentrionalis
Oreodytes shorti mitochondrial 16S rRNA gene (part	HF931488	87.26	100	5.11329e−45	Coleoptera	Dytiscidae	Oreodytes shorti
Laccornis difformis 16S ribosomal RNA gene, partia	KF575544	85.99	100	2.64875e−42	Coleoptera	Dytiscidae	Laccornis difformis
Boreonectes alpestris mitochondrial 16S rRNA gene	HF931512	86.62	100	2.64875e−42	Coleoptera	Dytiscidae	Boreonectes alpestris
Deronectes parvicollis mitochondrial 16S rRNA gene	HF931454	85.99	100	2.64875e−42	Coleoptera	Dytiscidae	Deronectes parvicollis
Boreonectes macedonicus mitochondrial 16S rRNA gen	HF931359	86.62	100	2.64875e−42	Coleoptera	Dytiscidae	Boreonectes macedonicus
Stictotarsus ibericus 16S ribosomal RNA (rrnL) gen	EF670030	86.62	100	2.64875e−42	Coleoptera	Dytiscidae	Stictotarsus ibericus
Boreonectes striatellus mitochondrial 16S rRNA gen	HF931511	85.99	100	3.22684e−41	Coleoptera	Dytiscidae	Boreonectes striatellus
Boreonectes riberae mitochondrial 16S rRNA gene (p	HF931461	85.99	100	3.22684e−41	Coleoptera	Dytiscidae	Boreonectes riberae
Boreonectes griseostriatus mitochondrial partial 1	HF931387	85.99	100	3.22684e−41	Coleoptera	Dytiscidae	Boreonectes griseostriatus
Boreonectes multilineatus mitochondrial partial 16	HF931384	85.99	100	3.22684e−41	Coleoptera	Dytiscidae	Boreonectes multilineatus
Oreodytes scitulus 16S ribosomal RNA gene, partial	KF575551	84.71	100	1.37209e−39	Coleoptera	Dytiscidae	Oreodytes scitulus
Oreodytes scitulus mitochondrial partial 16S rRNA	HF931515	84.71	100	1.37209e−39	Coleoptera	Dytiscidae	Oreodytes scitulus
Boreonectes griseostriatus mitochondrial 16S rRNA	HF931541	84.71	100	1.67154e−38	Coleoptera	Dytiscidae	Boreonectes griseostriatus
Nebrioporus formaster mitochondrial partial 16S rR	HF931377	84.18	100	2.03636e−37	Coleoptera	Dytiscidae	Nebrioporus formaster
Hydroporus glabriusculus 16S ribosomal RNA gene, p	AY365270	84.08	100	2.03636e−37	Coleoptera	Dytiscidae	Hydroporus glabriusculus
Suphrodytes dorsalis 16S ribosomal RNA gene, parti	KF575577	84.18	100	7.10758e−37	Coleoptera	Dytiscidae	Suphrodytes dorsalis
Oreodytes quadrimaculatus 16S ribosomal RNA gene,	KF575567	83.44	100	7.10758e−37	Coleoptera	Dytiscidae	Oreodytes quadrimaculatus
Aethionectes fulvonotatus voucher KBMAeop406 16S r	KF979088	83.44	100	7.10758e−37	Coleoptera	Dytiscidae	Aethionectes fulvonotatus
Suphrodytes dorsalis voucher BMNH:725966 16S ribos	JX221574	84.18	100	7.10758e−37	Coleoptera	Dytiscidae	Suphrodytes dorsalis
Copelatus sp. BI−2011 voucher BMNH792945 16S ribos	HQ381441	83.44	100	7.10758e−37	Coleoptera	Dytiscidae	Copelatus sp. BI−2011
Laccornis kocae partial 16S rRNA gene	AJ850419	83.44	100	7.10758e−37	Coleoptera	Dytiscidae	Laccornis kocae
Oreodytes quadrimaculatus partial 16S rRNA gene	AJ850351	83.44	100	7.10758e−37	Coleoptera	Dytiscidae	Oreodytes quadrimaculatus
Oreodytes natrix partial 16S rRNA gene	AJ850349	83.44	100	7.10758e−37	Coleoptera	Dytiscidae	Oreodytes natrix
Deronectes opatrinus mitochondrial 16S rRNA gene (	HE613701	85.06	97.44	2.48079e−36	Coleoptera	Dytiscidae	Deronectes opatrinus
Hygrotus marklini mitochondrial 16S rRNA gene (par	HF931467	83.44	100	8.65881e−36	Coleoptera	Dytiscidae	Hygrotus marklini
Antiporus hollingsworthi mitochondrial partial 16S	FR727246	83.23	98.72	8.65881e−36	Coleoptera	Dytiscidae	Antiporus hollingsworthi
Hygrotus turbidus partial 16S rRNA gene	AJ850390	83.44	100	8.65881e−36	Coleoptera	Dytiscidae	Hygrotus turbidus
Deronectes bicostatus mitochondrial 16S rRNA gene	HE613705	83.66	97.44	3.02222e−35	Coleoptera	Dytiscidae	Deronectes bicostatus
Copelatus sp. BI−2011 voucher BMNH671215 16S ribos	HQ381377	82.8	100	3.02222e−35	Coleoptera	Dytiscidae	Copelatus sp. BI−2011
Stictotarsus maghrebinus 16S ribosomal RNA gene, p	AY250947	83.54	100	3.02222e−35	Coleoptera	Dytiscidae	Stictotarsus maghrebinus
Scarodytes sp. IR−2003 16S ribosomal RNA gene, par	AY250939	82.61	100	3.02222e−35	Coleoptera	Dytiscidae	Scarodytes sp. IR−2003
Hygrotus lutescens mitochondrial partial 16S rRNA	HF931518	82.8	100	1.05486e−34	Coleoptera	Dytiscidae	Hygrotus lutescens
Hygrotus fontinalis mitochondrial 16S rRNA gene (p	HF931494	82.8	100	1.05486e−34	Coleoptera	Dytiscidae	Hygrotus fontinalis
Hygrotus unguicularis mitochondrial partial 16S rR	HF931489	82.8	100	1.05486e−34	Coleoptera	Dytiscidae	Hygrotus unguicularis
Hygrotus patruelis mitochondrial 16S rRNA gene (pa	HF931388	82.8	100	1.05486e−34	Coleoptera	Dytiscidae	Hygrotus patruelis
Hygrotus sanfilippoi mitochondrial 16S rRNA gene (	HF931342	82.8	100	1.05486e−34	Coleoptera	Dytiscidae	Hygrotus sanfilippoi
Suphrodytes figuratus voucher BMNH:722148 16S ribo	JX221571	82.8	100	1.05486e−34	Coleoptera	Dytiscidae	Suphrodytes figuratus
Deronectes doriae mitochondrial 16S rRNA gene (par	HE613710	84.42	97.44	1.05486e−34	Coleoptera	Dytiscidae	Deronectes doriae
Deronectes sahlbergi mitochondrial 16S rRNA gene (	HE613680	84.42	97.44	1.05486e−34	Coleoptera	Dytiscidae	Deronectes sahlbergi
Antiporus hollingsworthi mitochondrial partial 16S	FR727247	82.58	98.72	1.05486e−34	Coleoptera	Dytiscidae	Antiporus hollingsworthi
Hydroporus sp. IR33.4 16S ribosomal RNA gene, part	AY365261	82.8	100	1.05486e−34	Coleoptera	Dytiscidae	Hydroporus sp. IR33.4
Herophydrus rohani voucher UNM KBMHpsp574 16S ribo	KJ548382	83.66	97.44	3.68182e−34	Coleoptera	Dytiscidae	Herophydrus rohani
Cybister marginicollis voucher UNM KBMCyma416 16S	KJ548319	82.17	100	3.68182e−34	Coleoptera	Dytiscidae	Cybister marginicollis
Hydroporus tessellatus 16S ribosomal RNA gene, par	JX434770	83.54	100	3.68182e−34	Coleoptera	Dytiscidae	Hydroporus tessellatus
Boreonectes griseostriatus mitochondrial 16S rRNA	HF931385	82.8	100	3.68182e−34	Coleoptera	Dytiscidae	Boreonectes griseostriatus
Hydroporus analis mitochondrial partial 16S rRNA g	HE613731	83.54	100	3.68182e−34	Coleoptera	Dytiscidae	Hydroporus analis
Hydroporus fuscipennis mitochondrial 16S rRNA gene	HE613730	83.65	100	3.68182e−34	Coleoptera	Dytiscidae	Hydroporus fuscipennis
Hydroporus foveolatus mitochondrial 16S rRNA gene	HE613724	83.54	100	3.68182e−34	Coleoptera	Dytiscidae	Hydroporus foveolatus
Hydroporus basinotatus mitochondrial 16S rRNA gene	HE613678	83.54	100	3.68182e−34	Coleoptera	Dytiscidae	Hydroporus basinotatus
Hydroporus compunctus mitochondrial 16S rRNA gene	HE599693	83.54	100	3.68182e−34	Coleoptera	Dytiscidae	Hydroporus compunctus
Nirripirti tetrameres isolate ABTC78764−5 16S ribo	EU616930	82.8	100	3.68182e−34	Coleoptera	Dytiscidae	Paroster tetrameres
Nirripirti byroensis isolate ABTC78677−8 16S ribos	EU616910	82.17	100	3.68182e−34	Coleoptera	Dytiscidae	Paroster byroensis
Nirripirti newhavenensis 16S ribosomal RNA gene, p	AY353844	82.8	100	3.68182e−34	Coleoptera	Dytiscidae	Paroster newhavenensis
Nebrioporus airumlus 16S ribosomal RNA gene, parti	AY250920	82.17	100	3.68182e−34	Coleoptera	Dytiscidae	Nebrioporus airumlus
Hydroporus basinotatus 16S ribosomal RNA gene, par	AF518258	83.54	100	3.68182e−34	Coleoptera	Dytiscidae	Hydroporus basinotatus
Hydroporus rufifrons 16S ribosomal RNA gene, parti	AF518277	83.02	100	3.68182e−34	Coleoptera	Dytiscidae	Hydroporus rufifrons
Hydroporus compunctus 16S ribosomal RNA gene, part	AF518259	83.54	100	3.68182e−34	Coleoptera	Dytiscidae	Hydroporus compunctus
Hydroporus fuscipennis 16S ribosomal RNA gene, par	AF518262	83.65	100	3.68182e−34	Coleoptera	Dytiscidae	Hydroporus fuscipennis
Stictotarsus procerus mitochondrial 16S rRNA gene	HF931379	82.91	100	1.28508e−33	Coleoptera	Dytiscidae	Stictotarsus procerus
Deronectes costipennis mitochondrial 16S rRNA gene	HE613697	83.77	97.44	1.28508e−33	Coleoptera	Dytiscidae	Deronectes costipennis
Deronectes brannani mitochondrial 16S rRNA gene (p	HE613695	84.83	91.67	1.28508e−33	Coleoptera	Dytiscidae	Deronectes brannanii
Deronectes platynotus mitochondrial 16S rRNA gene	HE613682	83.77	97.44	1.28508e−33	Coleoptera	Dytiscidae	Deronectes platynotus
Deronectes depressicollis mitochondrial 16S rRNA g	HE613681	83.01	97.44	1.28508e−33	Coleoptera	Dytiscidae	Deronectes depressicollis
Deronectes costipennis 16S ribosomal RNA gene, par	AY250951	83.77	97.44	1.28508e−33	Coleoptera	Dytiscidae	Deronectes costipennis
Scarodytes halensis haplotype 3 16S ribosomal RNA	AY250937	81.65	100	1.28508e−33	Coleoptera	Dytiscidae	Scarodytes halensis
Ilybius hozgargantae 16S ribosomal RNA gene, parti	AY138664	81.65	100	1.28508e−33	Coleoptera	Dytiscidae	Ilybius hozgargantae
Agabus didymus mitochondrial 16S rRNA gene (partia	LM654773	82.17	100	4.48537e−33	Coleoptera	Dytiscidae	Agabus didymus
Agabus didymus voucher UNM KBMPmsp730 16S ribosoma	KJ548392	82.17	100	4.48537e−33	Coleoptera	Dytiscidae	Agabus didymus
Herophydrus inquinatus 16S ribosomal RNA gene, par	KF575572	82.17	100	4.48537e−33	Coleoptera	Dytiscidae	Herophydrus inquinatus
Hydroporus appalachius 16S ribosomal RNA gene, par	JX434765	82.17	100	4.48537e−33	Coleoptera	Dytiscidae	Hydroporus appalachius
Hygrotus novemlineatus mitochondrial partial 16S r	HF931523	82.17	100	4.48537e−33	Coleoptera	Dytiscidae	Hygrotus novemlineatus
Boreonectes funereus mitochondrial 16S rRNA gene (	HF931393	82.17	100	4.48537e−33	Coleoptera	Dytiscidae	Boreonectes funereus
Suphrodytes figuratus voucher BMNH:722150 16S ribo	JX221572	82.17	100	4.48537e−33	Coleoptera	Dytiscidae	Suphrodytes figuratus
Deronectes hispanicus mitochondrial 16S rRNA gene	HE613727	83.87	97.44	4.48537e−33	Coleoptera	Dytiscidae	Deronectes hispanicus
Hydroporus decipiens mitochondrial 16S rRNA gene (	HE613722	82.91	100	4.48537e−33	Coleoptera	Dytiscidae	Hydroporus decipiens
Hydroporus pubescens mitochondrial 16S rRNA gene (	HE613707	82.91	100	4.48537e−33	Coleoptera	Dytiscidae	Hydroporus pubescens
Hydroporus limbatus mitochondrial 16S rRNA gene (p	HE613706	82.91	100	4.48537e−33	Coleoptera	Dytiscidae	Hydroporus limbatus
Hydroporus marginatus mitochondrial 16S rRNA gene	HE613699	87.8	78.85	4.48537e−33	Coleoptera	Dytiscidae	Hydroporus marginatus
Hydroporus zimmermanni mitochondrial 16S rRNA gene	HE613679	82.91	100	4.48537e−33	Coleoptera	Dytiscidae	Hydroporus zimmermanni
Suphrodytes dorsalis mitochondrial 16S rRNA gene (	HE599700	82.17	100	4.48537e−33	Coleoptera	Dytiscidae	Suphrodytes dorsalis
Hydroporus pervicinus mitochondrial 16S rRNA gene	HE599695	82.17	100	4.48537e−33	Coleoptera	Dytiscidae	Hydroporus pervicinus
Hovahydrus sp. BI−2011 voucher BMNH831333 16S ribo	HQ381524	84.71	98.72	4.48537e−33	Coleoptera	Dytiscidae	Hovahydrus sp. BI−2011
Pseudomigadops sp. PSM1 mitochondrial 16S rRNA gen	FM994738	83.02	100	4.48537e−33	Coleoptera	Carabidae	Pseudomigadops sp. PSM1
Hygrotus corpulentus partial 16S rRNA gene	AJ850387	82.17	100	4.48537e−33	Coleoptera	Dytiscidae	Hygrotus corpulentus
Herophydrus cf. gigas BMNH 681835 partial 16S rRNA	AJ850383	82.17	100	4.48537e−33	Coleoptera	Dytiscidae	Herophydrus cf. gigas BMNH 681835
Nirripirti bulbus isolate ABTC78658−9 16S ribosoma	EU616918	82.17	100	4.48537e−33	Coleoptera	Dytiscidae	Paroster bulbus
Nirripirti arachnoides isolate ABTC78660−1 16S rib	EU616917	82.17	100	4.48537e−33	Coleoptera	Dytiscidae	Paroster arachnoides
Nirripirti skaphites isolate 2 ABTC78694−6 16S rib	EU616916	82.17	100	4.48537e−33	Coleoptera	Dytiscidae	Paroster skaphites
Nirripirti innouendyensis isolate ABTC78672−3 16S	EU616913	82.17	100	4.48537e−33	Coleoptera	Dytiscidae	Paroster innouendyensis
Nirripirti wedgeensis isolate ABTC78773−4 16S ribo	EU616938	81.53	100	5.4643e−32	Coleoptera	Dytiscidae	Paroster wedgeensis
