# Supplementary material for: Testing the potential of a ribosomal 16S marker for DNA metabarcoding of insects
Source: PeerJ. 2016 Apr 19;4:e1966. doi: 10.7717/peerj.1966 (PMC4841222; doi:10.7717/peerj.1966)
Supplement: Supplemental Information 1 [file peerj-04-1966-s006.zip › scripts/5 match OTUs/blast_hits/OTU_310.csv_plot.pdf]

OTU_310.csv

Parochlus steinenii

Chironomidae

Diptera

88.46

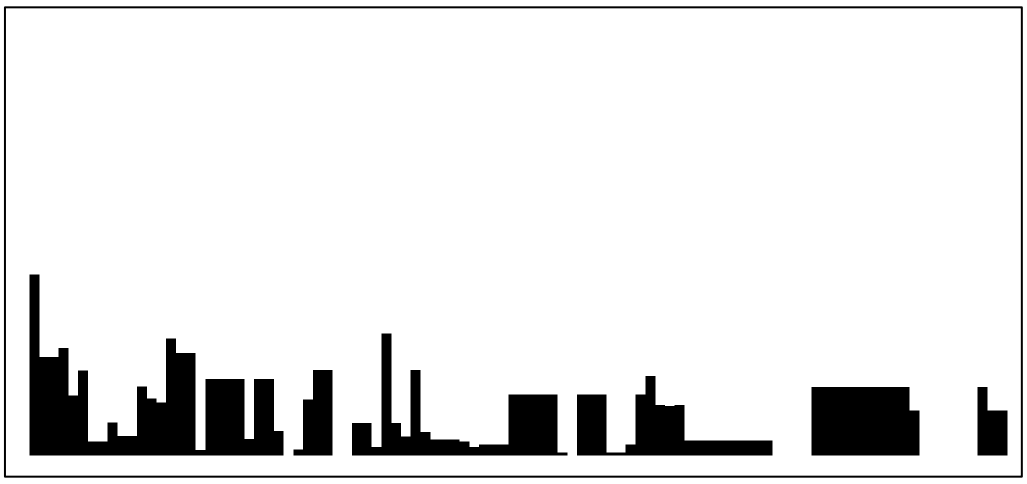

Parochlus steinenii isolate Sejong01 mitochondrion	KT003702	88.46	100	3.41683e−47	Diptera	Chironomidae	Parochlus steinenii
Bolitophila cinerea voucher JSB1 16S ribosomal RNA	KJ136712	84.62	97.42	2.99724e−35	Diptera	Mycetophilidae	Bolitophila cinerea
Bolitophila sp. ER−2009 16S ribosomal RNA gene, pa	FJ172021	84.62	97.42	2.99724e−35	Diptera	Mycetophilidae	Bolitophila sp. ER−2009
Bolitophila occlusa voucher JSB2 16S ribosomal RNA	KJ136713	85.03	93.55	1.04614e−34	Diptera	Mycetophilidae	Bolitophila occlusa
Pedicia albivitta voucher O'Grady 200012 16S ribos	EU005435	82.8	100	3.65139e−34	Diptera	Pediciidae	Pedicia albivitta
Bolitophila hybrida 16S ribosomal RNA gene, partia	FJ172002	83.97	97.42	1.55261e−32	Diptera	Mycetophilidae	Bolitophila hybrida
Sylvicola fenestralis 16S ribosomal RNA gene, part	KC177461	80.65	100	1.89147e−31	Diptera	Anisopodidae	Sylvicola fenestralis
Sylvicola fenestralis mitochondrion, complete geno	JN861752	80.65	100	1.89147e−31	Diptera	Anisopodidae	Sylvicola fenestralis
Tipula sp. PMO 200011 16S ribosomal RNA gene, part	EU005437	81.53	100	1.89147e−31	Diptera	Tipulidae	Tipula sp. PMO 200011
Tipula abdominalis 16S ribosomal RNA gene, partial	KC177457	80.89	100	2.30428e−30	Diptera	Tipulidae	Tipula abdominalis
Tipula abdominalis mitochondrion, partial genome	JN861743	80.89	100	2.30428e−30	Diptera	Tipulidae	Tipula abdominalis
Callomyia admirabilis isolate CA42 16S ribosomal R	KC699326	83.21	87.74	8.04272e−30	Diptera	Platypezidae	Callomyia admirabilis
Arachnocampa luminosa biotype 1 16S large subunit	AY576329	82.67	94.19	8.04272e−30	Diptera	Keroplatidae	Arachnocampa luminosa
Callomyia amoena isolate CA06 16S ribosomal RNA ge	KC699325	82.48	87.74	9.79804e−29	Diptera	Platypezidae	Callomyia amoena
Hercostomus meieri voucher RMBR:103442 16S ribosom	FJ808173	85.48	79.35	9.79804e−29	Diptera	Dolichopodidae	Hercostomus meieri
Simulium tenederoi mitochondrial gene for 16S ribo	LC035002	84.8	79.35	1.19365e−27	Diptera	Simuliidae	Simulium tenederoi
Simulium tenederoi mitochondrial gene for 16S ribo	LC035001	84.8	79.35	1.19365e−27	Diptera	Simuliidae	Simulium tenederoi
Simulium sheilae isolate SH1B 16S ribosomal RNA ge	KM410213	80.25	100	1.19365e−27	Diptera	Simuliidae	Simulium sheilae
Simulium roslihashimi isolate R1B 16S ribosomal RN	KM410192	83.58	85.16	1.19365e−27	Diptera	Simuliidae	Simulium roslihashimi
Simulium izuae isolate I3B 16S ribosomal RNA gene,	KM410191	83.58	85.16	1.19365e−27	Diptera	Simuliidae	Simulium izuae
Simulium izuae isolate I2B 16S ribosomal RNA gene,	KM410190	83.58	85.16	1.19365e−27	Diptera	Simuliidae	Simulium izuae
Simulium tuenense mitochondrial gene for 16S ribos	AB591244	83.58	85.16	1.19365e−27	Diptera	Simuliidae	Simulium tuenense
Beris morrisii 16S ribosomal RNA gene, partial seq	GU947373	80.77	99.35	1.19365e−27	Diptera	Stratiomyidae	Beris morrisii
Simulium inthanonense 16S ribosomal RNA gene, part	GQ865995	83.58	85.16	1.19365e−27	Diptera	Simuliidae	Simulium inthanonense
Simulium cabrerai mitochondrial gene for 16S rRNA,	AB093128	83.58	85.16	1.19365e−27	Diptera	Simuliidae	Simulium cabrerai
Simulium suyoense mitochondrial gene for 16S ribos	LC035000	81.13	100	4.16623e−27	Diptera	Simuliidae	Simulium suyoense
Microsania collarti isolate CAN8 16S ribosomal RNA	KC699334	79.49	99.35	4.16623e−27	Diptera	Platypezidae	Microsania collarti
Somatia sp. YSUW04038 16S ribosomal RNA gene, part	AY573134	80.27	94.19	4.16623e−27	Diptera	Somatiidae	Somatia sp. YSUW04038
Arachnocampa luminosa biotype 3 16S large subunit	AY576331	82.61	87.74	4.16623e−27	Diptera	Keroplatidae	Arachnocampa luminosa
Simulium claveriaense mitochondrial gene for 16S r	LC034988	84	79.35	1.45416e−26	Diptera	Simuliidae	Simulium claveriaense
Simulium claveriaense mitochondrial gene for 16S r	LC034987	84	79.35	1.45416e−26	Diptera	Simuliidae	Simulium claveriaense
Simulium sheilae isolate SH2B 16S ribosomal RNA ge	KM410214	79.62	100	1.45416e−26	Diptera	Simuliidae	Simulium sheilae
Tongamya stuckenbergi 16S ribosomal RNA gene, part	KM878969	80	99.35	1.45416e−26	Diptera	Mydidae	Tongamya stuckenbergi
Simulium khunklangense mitochondrial gene for 16S	AB820367	81.51	92.26	1.45416e−26	Diptera	Simuliidae	Simulium khunklangense
Simulium chomthongense mitochondrial gene for 16S	AB699899	81.51	92.26	1.45416e−26	Diptera	Simuliidae	Simulium chomthongense
Trichopeza longicornis voucher Pollet 0243 (P) 16S	HQ449035	80.38	99.35	1.45416e−26	Diptera	Empididae	Trichopeza longicornis
Paracladura trichoptera mitochondrion, complete ge	JN861751	85.71	75.48	1.45416e−26	Diptera	Trichoceridae	Paracladura trichoptera
Simulium caudisclerum 16S ribosomal RNA gene, part	GQ865999	81.51	92.26	1.45416e−26	Diptera	Simuliidae	Simulium caudisclerum
Allocotocera pulchella 16S ribosomal RNA gene, par	FJ172034	80.88	87.74	1.45416e−26	Diptera	Mycetophilidae	Allocotocera pulchella
Simulium claveriaense mitochondrial gene for 16S r	AB334096	84	79.35	1.45416e−26	Diptera	Simuliidae	Simulium claveriaense
Caenophthalmus capensis voucher MEI 120162 16S rib	DQ250838	81.08	92.26	1.45416e−26	Diptera	Therevidae	Caenophthalmus capensis
Simulium mie mitochondrial gene for 16S ribosomal	AB056732	80.75	100	1.45416e−26	Diptera	Simuliidae	Simulium mie
Simulium mie mitochondrial gene for 16S ribosomal	AB056731	80.75	100	1.45416e−26	Diptera	Simuliidae	Simulium mie
Simulium mie mitochondrial gene for 16S ribosomal	AB056730	80.75	100	1.45416e−26	Diptera	Simuliidae	Simulium mie
Cnephia dacotensis mitochondrial 16S ribosomal RNA	L13403	80.65	97.42	1.45416e−26	Diptera	Simuliidae	Cnephia dacotensis
Simulium tuyense mitochondrial gene for 16S riboso	LC035004	80.39	97.42	5.07551e−26	Diptera	Simuliidae	Simulium tuyense
Simulium suyoense mitochondrial gene for 16S ribos	LC034999	80.5	100	5.07551e−26	Diptera	Simuliidae	Simulium suyoense
Simulium suyoense mitochondrial gene for 16S ribos	LC034998	80.5	100	5.07551e−26	Diptera	Simuliidae	Simulium suyoense
Simulium suyoense mitochondrial gene for 16S ribos	LC034997	80.5	100	5.07551e−26	Diptera	Simuliidae	Simulium suyoense
Simulium brinchangense isolate B2B 16S ribosomal R	KM410202	82.84	85.16	5.07551e−26	Diptera	Simuliidae	Simulium brinchangense
Simulium brinchangense isolate B1B 16S ribosomal R	KM410201	82.84	85.16	5.07551e−26	Diptera	Simuliidae	Simulium brinchangense
Simulium tanahrataense isolate TR2B 16S ribosomal	KM410196	82.84	85.16	5.07551e−26	Diptera	Simuliidae	Simulium tanahrataense
Simulium tanahrataense isolate TR1B 16S ribosomal	KM410195	82.84	85.16	5.07551e−26	Diptera	Simuliidae	Simulium tanahrataense
Simulium izuae isolate I1B 16S ribosomal RNA gene,	KM410189	82.84	85.16	5.07551e−26	Diptera	Simuliidae	Simulium izuae
Diadocidia stanfordensis voucher JSSJ14 16S riboso	KJ136742	80.13	91.61	5.07551e−26	Diptera	Diadocidiidae	Diadocidia stanfordensis
Musca ventrosa 16S ribosomal RNA gene, partial seq	JN226633	79.49	99.35	5.07551e−26	Diptera	Muscidae	Musca ventrosa
Cyphomyia notabilis 16S ribosomal RNA gene, partia	GU947403	82.84	85.16	5.07551e−26	Diptera	Stratiomyidae	Cyphomyia notabilis
Simulium chumpornense 16S ribosomal RNA gene, part	GQ865998	82.84	85.16	5.07551e−26	Diptera	Simuliidae	Simulium chumpornense
Simulium decuplum 16S ribosomal RNA gene, partial	GQ865986	82.84	85.16	5.07551e−26	Diptera	Simuliidae	Simulium decuplum
Drosophila rubida voucher 109400 NADH dehydrogenas	EU494327	80.14	92.26	5.07551e−26	Diptera	Drosophilidae	Drosophila rubida
Drosophila hypocausta voucher 103972 NADH dehydrog	EU494319	80.14	92.26	5.07551e−26	Diptera	Drosophilidae	Drosophila hypocausta
Simulium suyoense mitochondrial gene for 16S ribos	AB334099	80.5	100	5.07551e−26	Diptera	Simuliidae	Simulium suyoense
Simulium recurvum mitochondrial gene for 16S ribos	AB334097	82.84	85.16	5.07551e−26	Diptera	Simuliidae	Simulium recurvum
Simulium palauense mitochondrial gene for 16S ribo	AB056742	83.7	85.16	5.07551e−26	Diptera	Simuliidae	Simulium palauense
Simulium spinosibranchium mitochondrial gene for 1	LC034996	82.35	85.16	1.77153e−25	Diptera	Simuliidae	Simulium spinosibranchium
Keilbachia subacumina voucher 890 16S ribosomal RN	JQ613951	82.31	81.94	1.77153e−25	Diptera	Sciaridae	Keilbachia subacumina
Simulium spinosibranchium mitochondrial gene for 1	AB334098	82.35	85.16	1.77153e−25	Diptera	Simuliidae	Simulium spinosibranchium
Simulium chiharuae mitochondrial gene for 16S ribo	AB334092	80.69	91.61	1.77153e−25	Diptera	Simuliidae	Simulium chiharuae
Simulium squamosum voucher P.Mansiangi Pm3(BNI) 16	DQ655644	80.69	92.26	1.77153e−25	Diptera	Simuliidae	Simulium squamosum
Simulium damnosum s.l. GH−D1 16S ribosomal RNA, pa	AY077709	80.69	92.26	1.77153e−25	Diptera	Simuliidae	Simulium damnosum s.l. GH−D1
Simulium damnosum s.l. Kulfo isolate Ku 3 16S ribo	AF416978	80.69	92.26	1.77153e−25	Diptera	Simuliidae	Simulium damnosum s.l. Kulfo
Simulium soubrense isolate Wa 52 16S ribosomal RNA	AF416976	80.69	92.26	1.77153e−25	Diptera	Simuliidae	Simulium soubrense
Simulium soubrense isolate Wa 47 16S ribosomal RNA	AF416975	80.69	92.26	1.77153e−25	Diptera	Simuliidae	Simulium soubrense
Simulium damnosum strain Se−12 16S ribosomal RNA g	AF081907	80.69	92.26	1.77153e−25	Diptera	Simuliidae	Simulium damnosum
Simulium uchidai mitochondrial gene for 16S riboso	AB056740	80.69	92.26	1.77153e−25	Diptera	Simuliidae	Simulium uchidai
Simulium leonense mitochondrion 16S ribosomal RNA	U17731	80.69	92.26	1.77153e−25	Diptera	Simuliidae	Simulium leonense
Simulium tuyense mitochondrial gene for 16S riboso	LC035008	79.74	97.42	6.18324e−25	Diptera	Simuliidae	Simulium tuyense
Simulium tuyense mitochondrial gene for 16S riboso	LC035007	79.74	97.42	6.18324e−25	Diptera	Simuliidae	Simulium tuyense
Simulium tuyense mitochondrial gene for 16S riboso	LC035005	79.74	97.42	6.18324e−25	Diptera	Simuliidae	Simulium tuyense
Simulium tuyense mitochondrial gene for 16S riboso	LC035003	79.74	97.42	6.18324e−25	Diptera	Simuliidae	Simulium tuyense
Simulium nobile isolate N5 16S ribosomal RNA gene,	KP661530	83.2	79.35	6.18324e−25	Diptera	Simuliidae	Simulium nobile
Simulium nobile isolate N4 16S ribosomal RNA gene,	KP661529	83.2	79.35	6.18324e−25	Diptera	Simuliidae	Simulium nobile
Simulium nobile isolate N3 16S ribosomal RNA gene,	KP661528	83.2	79.35	6.18324e−25	Diptera	Simuliidae	Simulium nobile
Simulium nobile isolate N2 16S ribosomal RNA gene,	KP661527	83.2	79.35	6.18324e−25	Diptera	Simuliidae	Simulium nobile
Simulium nobile isolate N1 16S ribosomal RNA gene,	KP661526	83.2	79.35	6.18324e−25	Diptera	Simuliidae	Simulium nobile
Simulium timorense isolate TM5 16S ribosomal RNA g	KP661525	83.2	79.35	6.18324e−25	Diptera	Simuliidae	Simulium timorense
Simulium timorense isolate TM4 16S ribosomal RNA g	KP661524	83.2	79.35	6.18324e−25	Diptera	Simuliidae	Simulium timorense
Simulium timorense isolate TM3 16S ribosomal RNA g	KP661523	83.2	79.35	6.18324e−25	Diptera	Simuliidae	Simulium timorense
Simulium timorense isolate TM2 16S ribosomal RNA g	KP661522	83.2	79.35	6.18324e−25	Diptera	Simuliidae	Simulium timorense
Simulium timorense isolate TM1 16S ribosomal RNA g	KP661521	83.2	79.35	6.18324e−25	Diptera	Simuliidae	Simulium timorense
Simulium leparense isolate LPR1B 16S ribosomal RNA	KM502576	82.09	85.16	6.18324e−25	Diptera	Simuliidae	Simulium leparense
Liriomyza sativae mitochondrion, complete genome	JQ862475	77.71	99.35	6.18324e−25	Diptera	Agromyzidae	Liriomyza sativae
Haematopota pluvialis 16S ribosomal RNA gene, part	KC177454	79.59	94.19	6.18324e−25	Diptera	Tabanidae	Haematopota pluvialis
Bittacomorphella fenderiana mitochondrion, partial	JN861745	78.06	99.35	6.18324e−25	Diptera	Ptychopteridae	Bittacomorphella fenderiana
Liriomyza sativae mitochondrion, complete genome	HQ333260	77.71	99.35	6.18324e−25	Diptera	Agromyzidae	Liriomyza sativae
Chrysops viduatus 16S ribosomal RNA gene, partial	HM132115	79.59	94.19	6.18324e−25	Diptera	Tabanidae	Chrysops viduatus
Tabanus bromius 16S ribosomal RNA gene, partial se	HM132111	78.06	99.35	6.18324e−25	Diptera	Tabanidae	Tabanus bromius
Simulium nobile 16S ribosomal RNA gene, partial se	GQ866008	83.2	79.35	6.18324e−25	Diptera	Simuliidae	Simulium nobile
Simulium sheilae 16S ribosomal RNA gene, partial s	GQ865996	82.09	85.16	6.18324e−25	Diptera	Simuliidae	Simulium sheilae
Simulium siamense 16S ribosomal RNA gene, partial	GQ865994	82.09	85.16	6.18324e−25	Diptera	Simuliidae	Simulium siamense
