# Supplementary material for: Testing the potential of a ribosomal 16S marker for DNA metabarcoding of insects
Source: PeerJ. 2016 Apr 19;4:e1966. doi: 10.7717/peerj.1966 (PMC4841222; doi:10.7717/peerj.1966)
Supplement: Supplemental Information 1 [file peerj-04-1966-s006.zip › scripts/5 match OTUs/blast_hits/OTU_312.csv_plot.pdf]

OTU_312.csv

Oreodytes sanmarkii

Dytiscidae

Coleoptera

99.36

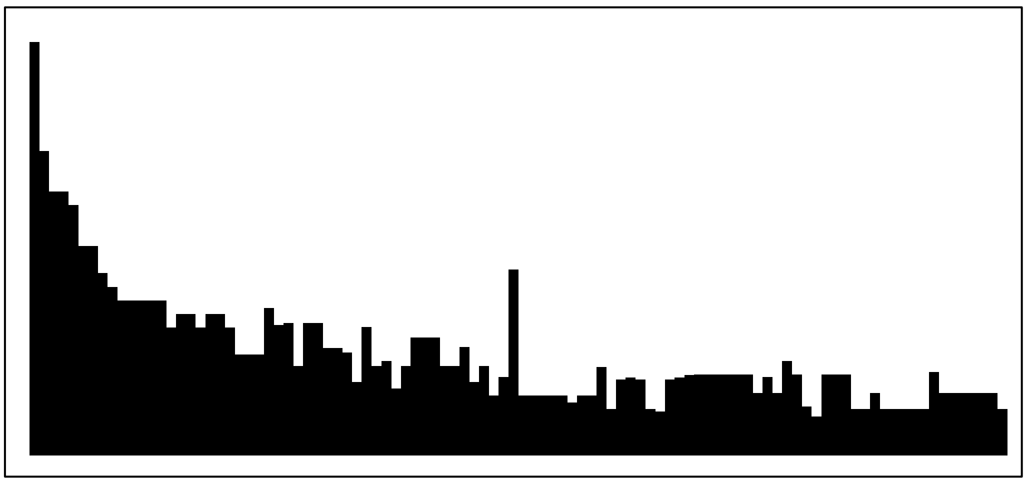

Oreodytes sanmarkii mitochondrial 16S rRNA gene (p	HF931524	99.36	100	1.56279e−70	Coleoptera	Dytiscidae	Oreodytes sanmarkii
Oreodytes obesus cordillerensis mitochondrial part	HF931516	94.27	100	1.12528e−59	Coleoptera	Dytiscidae	Oreodytes obesus cordillerensis
Oreodytes crassulus partial 16S rRNA gene	AJ850348	92.36	100	7.10131e−56	Coleoptera	Dytiscidae	Oreodytes crassulus
Oreodytes congruus partial 16S rRNA gene	AJ850347	92.36	100	7.10131e−56	Coleoptera	Dytiscidae	Oreodytes congruus
Oreodytes congruus 16S ribosomal RNA gene, partial	KF575550	91.72	100	3.01955e−54	Coleoptera	Dytiscidae	Oreodytes congruus
Oreodytes abbreviatus mitochondrial 16S rRNA gene	HF931477	89.81	100	1.90555e−50	Coleoptera	Dytiscidae	Oreodytes abbreviatus
Oreodytes rhyacophilus partial 16S rRNA gene	AJ850352	89.81	100	1.90555e−50	Coleoptera	Dytiscidae	Oreodytes rhyacophilus
Oreodytes mongolicus mitochondrial 16S rRNA gene (	HF931491	88.54	100	9.87097e−48	Coleoptera	Dytiscidae	Oreodytes mongolicus
Oreodytes snoqualmie mitochondrial partial 16S rRN	HF931519	87.9	100	4.19724e−46	Coleoptera	Dytiscidae	Oreodytes snoqualmie
Oreodytes septentrionalis mitochondrial 16S rRNA g	HF931492	87.26	100	5.11329e−45	Coleoptera	Dytiscidae	Oreodytes septentrionalis
Oreodytes shorti mitochondrial 16S rRNA gene (part	HF931488	87.26	100	5.11329e−45	Coleoptera	Dytiscidae	Oreodytes shorti
Boreonectes riberae mitochondrial 16S rRNA gene (p	HF931461	87.26	100	6.22926e−44	Coleoptera	Dytiscidae	Boreonectes riberae
Boreonectes griseostriatus mitochondrial partial 1	HF931387	87.26	100	6.22926e−44	Coleoptera	Dytiscidae	Boreonectes griseostriatus
Boreonectes multilineatus mitochondrial partial 16	HF931384	87.26	100	6.22926e−44	Coleoptera	Dytiscidae	Boreonectes multilineatus
Laccornis difformis 16S ribosomal RNA gene, partia	KF575544	85.99	100	2.64875e−42	Coleoptera	Dytiscidae	Laccornis difformis
Boreonectes alpestris mitochondrial 16S rRNA gene	HF931512	86.62	100	2.64875e−42	Coleoptera	Dytiscidae	Boreonectes alpestris
Boreonectes striatellus mitochondrial 16S rRNA gen	HF931511	86.62	100	2.64875e−42	Coleoptera	Dytiscidae	Boreonectes striatellus
Deronectes parvicollis mitochondrial 16S rRNA gene	HF931454	85.99	100	2.64875e−42	Coleoptera	Dytiscidae	Deronectes parvicollis
Boreonectes macedonicus mitochondrial 16S rRNA gen	HF931359	86.62	100	2.64875e−42	Coleoptera	Dytiscidae	Boreonectes macedonicus
Stictotarsus ibericus 16S ribosomal RNA (rrnL) gen	EF670030	86.62	100	2.64875e−42	Coleoptera	Dytiscidae	Stictotarsus ibericus
Boreonectes griseostriatus mitochondrial 16S rRNA	HF931541	85.99	100	3.22684e−41	Coleoptera	Dytiscidae	Boreonectes griseostriatus
Oreodytes scitulus 16S ribosomal RNA gene, partial	KF575551	84.71	100	1.37209e−39	Coleoptera	Dytiscidae	Oreodytes scitulus
Oreodytes scitulus mitochondrial partial 16S rRNA	HF931515	84.71	100	1.37209e−39	Coleoptera	Dytiscidae	Oreodytes scitulus
Laccornis kocae partial 16S rRNA gene	AJ850419	84.71	100	1.37209e−39	Coleoptera	Dytiscidae	Laccornis kocae
Deronectes moestus mitochondrial 16S rRNA gene (pa	HE613726	86.9	92.31	1.67154e−38	Coleoptera	Dytiscidae	Deronectes moestus
Boreonectes griseostriatus mitochondrial 16S rRNA	HF931385	86.11	92.31	5.83426e−38	Coleoptera	Dytiscidae	Boreonectes griseostriatus
Deronectes moestus 16S ribosomal RNA gene, partial	KF575561	86.21	92.31	2.03636e−37	Coleoptera	Dytiscidae	Deronectes moestus
Nebrioporus formaster mitochondrial partial 16S rR	HF931377	84.18	100	2.03636e−37	Coleoptera	Dytiscidae	Nebrioporus formaster
Deronectes brannani mitochondrial 16S rRNA gene (p	HE613695	86.21	92.31	2.03636e−37	Coleoptera	Dytiscidae	Deronectes brannanii
Deronectes moestus mitochondrial 16S rRNA gene (pa	HE613683	86.21	92.31	2.03636e−37	Coleoptera	Dytiscidae	Deronectes moestus
Suphrodytes dorsalis 16S ribosomal RNA gene, parti	KF575577	85.03	94.23	7.10758e−37	Coleoptera	Dytiscidae	Suphrodytes dorsalis
Suphrodytes dorsalis voucher BMNH:725966 16S ribos	JX221574	85.03	94.23	7.10758e−37	Coleoptera	Dytiscidae	Suphrodytes dorsalis
Hydroporus foveolatus mitochondrial 16S rRNA gene	HE613724	84.81	100	7.10758e−37	Coleoptera	Dytiscidae	Hydroporus foveolatus
Oreodytes natrix partial 16S rRNA gene	AJ850349	83.44	100	7.10758e−37	Coleoptera	Dytiscidae	Oreodytes natrix
Deronectes opatrinus mitochondrial 16S rRNA gene (	HE613701	86.01	91.03	2.48079e−36	Coleoptera	Dytiscidae	Deronectes opatrinus
Stictotarsus maghrebinus 16S ribosomal RNA gene, p	AY250947	84.18	100	2.48079e−36	Coleoptera	Dytiscidae	Stictotarsus maghrebinus
Scarodytes sp. IR−2003 16S ribosomal RNA gene, par	AY250939	84.42	97.44	2.48079e−36	Coleoptera	Dytiscidae	Scarodytes sp. IR−2003
Aethionectes fulvonotatus voucher KBMAeop406 16S r	KF979088	83.12	100	8.65881e−36	Coleoptera	Dytiscidae	Aethionectes fulvonotatus
Hydroporus tessellatus 16S ribosomal RNA gene, par	JX434770	84.18	100	8.65881e−36	Coleoptera	Dytiscidae	Hydroporus tessellatus
Stictotarsus procerus mitochondrial 16S rRNA gene	HF931379	85.52	92.31	8.65881e−36	Coleoptera	Dytiscidae	Stictotarsus procerus
Deronectes doriae mitochondrial 16S rRNA gene (par	HE613710	85.52	92.31	8.65881e−36	Coleoptera	Dytiscidae	Deronectes doriae
Deronectes sahlbergi mitochondrial 16S rRNA gene (	HE613680	85.52	92.31	8.65881e−36	Coleoptera	Dytiscidae	Deronectes sahlbergi
Hydroporus zimmermanni mitochondrial 16S rRNA gene	HE613679	84.18	100	8.65881e−36	Coleoptera	Dytiscidae	Hydroporus zimmermanni
Hydroporus basinotatus mitochondrial 16S rRNA gene	HE613678	84.18	100	8.65881e−36	Coleoptera	Dytiscidae	Hydroporus basinotatus
Hovahydrus sp. BI−2011 voucher BMNH831333 16S ribo	HQ381524	85.06	97.44	8.65881e−36	Coleoptera	Dytiscidae	Hovahydrus sp. BI−2011
Hydroporus glabriusculus 16S ribosomal RNA gene, p	AY365270	83.44	100	8.65881e−36	Coleoptera	Dytiscidae	Hydroporus glabriusculus
Hydroporus basinotatus 16S ribosomal RNA gene, par	AF518258	84.18	100	8.65881e−36	Coleoptera	Dytiscidae	Hydroporus basinotatus
Oreodytes quadrimaculatus 16S ribosomal RNA gene,	KF575567	82.8	100	3.02222e−35	Coleoptera	Dytiscidae	Oreodytes quadrimaculatus
Deronectes bicostatus mitochondrial 16S rRNA gene	HE613705	83.67	94.23	3.02222e−35	Coleoptera	Dytiscidae	Deronectes bicostatus
Hydroporus marginatus mitochondrial 16S rRNA gene	HE613699	88.71	79.49	3.02222e−35	Coleoptera	Dytiscidae	Hydroporus marginatus
Copelatus sp. BI−2011 voucher BMNH792945 16S ribos	HQ381441	82.8	100	3.02222e−35	Coleoptera	Dytiscidae	Copelatus sp. BI−2011
Oreodytes quadrimaculatus partial 16S rRNA gene	AJ850351	82.8	100	3.02222e−35	Coleoptera	Dytiscidae	Oreodytes quadrimaculatus
Hygrotus novemlineatus mitochondrial partial 16S r	HF931523	82.8	100	1.05486e−34	Coleoptera	Dytiscidae	Hygrotus novemlineatus
Hygrotus marklini mitochondrial 16S rRNA gene (par	HF931467	82.8	100	1.05486e−34	Coleoptera	Dytiscidae	Hygrotus marklini
Suphrodytes figuratus voucher BMNH:722148 16S ribo	JX221571	82.8	100	1.05486e−34	Coleoptera	Dytiscidae	Suphrodytes figuratus
Antiporus hollingsworthi mitochondrial partial 16S	FR727246	82.47	98.72	1.05486e−34	Coleoptera	Dytiscidae	Antiporus hollingsworthi
Hygrotus turbidus partial 16S rRNA gene	AJ850390	82.8	100	1.05486e−34	Coleoptera	Dytiscidae	Hygrotus turbidus
Hydroporus sp. IR33.4 16S ribosomal RNA gene, part	AY365261	82.8	100	1.05486e−34	Coleoptera	Dytiscidae	Hydroporus sp. IR33.4
Hydroporus marginatus 16S ribosomal RNA gene, part	AF518267	84.14	92.95	1.05486e−34	Coleoptera	Dytiscidae	Hydroporus marginatus
Hydaticus cinctipennis voucher UNM KBMHyci20 16S r	KJ548338	82.17	100	3.68182e−34	Coleoptera	Dytiscidae	Hydaticus cinctipennis
Hydroporus analis mitochondrial partial 16S rRNA g	HE613731	83.54	100	3.68182e−34	Coleoptera	Dytiscidae	Hydroporus analis
Hydroporus fuscipennis mitochondrial 16S rRNA gene	HE613730	83.65	100	3.68182e−34	Coleoptera	Dytiscidae	Hydroporus fuscipennis
Hydroporus compunctus mitochondrial 16S rRNA gene	HE599693	83.54	100	3.68182e−34	Coleoptera	Dytiscidae	Hydroporus compunctus
Copelatus sp. BI−2011 voucher BMNH671215 16S ribos	HQ381377	82.17	100	3.68182e−34	Coleoptera	Dytiscidae	Copelatus sp. BI−2011
Antiporus hollingsworthi mitochondrial partial 16S	FR727247	82.05	98.72	3.68182e−34	Coleoptera	Dytiscidae	Antiporus hollingsworthi
Hydroporus compunctus 16S ribosomal RNA gene, part	AF518259	83.54	100	3.68182e−34	Coleoptera	Dytiscidae	Hydroporus compunctus
Hydroporus fuscipennis 16S ribosomal RNA gene, par	AF518262	83.65	100	3.68182e−34	Coleoptera	Dytiscidae	Hydroporus fuscipennis
Hygrotus impressopunctatus voucher UNM KBMHtim80 1	KJ548334	83.77	96.79	1.28508e−33	Coleoptera	Dytiscidae	Hygrotus impressopunctatus
Deronectes aubei voucher UNM KBMDeau758 16S riboso	KJ548292	83.78	94.23	1.28508e−33	Coleoptera	Dytiscidae	Deronectes aubei
Deronectes costipennis mitochondrial 16S rRNA gene	HE613697	83.78	94.23	1.28508e−33	Coleoptera	Dytiscidae	Deronectes costipennis
Deronectes platynotus mitochondrial 16S rRNA gene	HE613682	83.78	94.23	1.28508e−33	Coleoptera	Dytiscidae	Deronectes platynotus
Deronectes depressicollis mitochondrial 16S rRNA g	HE613681	83.78	94.23	1.28508e−33	Coleoptera	Dytiscidae	Deronectes depressicollis
Deronectes semirufus mitochondrial 16S rRNA gene (	HE613676	83.78	94.23	1.28508e−33	Coleoptera	Dytiscidae	Deronectes semirufus
Deronectes semirufus mitochondrial 16S rRNA gene (	HE613674	83.78	94.23	1.28508e−33	Coleoptera	Dytiscidae	Deronectes semirufus
Stictotarsus falli 16S ribosomal RNA (rrnL) gene,	EF670029	82.91	100	1.28508e−33	Coleoptera	Dytiscidae	Stictotarsus falli
Hygrotus impressopunctatus partial 16S rRNA gene	AJ850405	83.66	96.79	1.28508e−33	Coleoptera	Dytiscidae	Hygrotus impressopunctatus
Nirripirti megamacrocephalus isolate ABTC78781 16S	EU616935	82.91	100	1.28508e−33	Coleoptera	Dytiscidae	Paroster megamacrocephalus
Darwinhydrus solidus voucher MB354 16S ribosomal R	EF056671	84.42	97.44	1.28508e−33	Coleoptera	Dytiscidae	Darwinhydrus solidus
Deronectes costipennis 16S ribosomal RNA gene, par	AY250951	83.78	94.23	1.28508e−33	Coleoptera	Dytiscidae	Deronectes costipennis
Scarodytes halensis haplotype 3 16S ribosomal RNA	AY250937	82.28	100	1.28508e−33	Coleoptera	Dytiscidae	Scarodytes halensis
Nebrioporus assimilis 16S ribosomal RNA gene, part	AY250921	81.82	98.72	1.28508e−33	Coleoptera	Dytiscidae	Nebrioporus assimilis
Deronectes aubei sanfilippoi haplotype 2 16S ribos	AY250908	83.78	94.23	1.28508e−33	Coleoptera	Dytiscidae	Deronectes aubei sanfilippoi
Deronectes aubei sanfilippoi haplotype 1 16S ribos	AY250907	83.78	94.23	1.28508e−33	Coleoptera	Dytiscidae	Deronectes aubei sanfilippoi
Deronectes aubei aubei haplotype 2 16S ribosomal R	AY250906	83.78	94.23	1.28508e−33	Coleoptera	Dytiscidae	Deronectes aubei aubei
Hydroporus appalachius 16S ribosomal RNA gene, par	JX434765	82.17	100	4.48537e−33	Coleoptera	Dytiscidae	Hydroporus appalachius
Hygrotus lutescens mitochondrial partial 16S rRNA	HF931518	82.17	100	4.48537e−33	Coleoptera	Dytiscidae	Hygrotus lutescens
Hydroporus foveolatus mitochondrial 16S rRNA gene	HF931502	82.91	100	4.48537e−33	Coleoptera	Dytiscidae	Hydroporus foveolatus
Hygrotus fontinalis mitochondrial 16S rRNA gene (p	HF931494	82.17	100	4.48537e−33	Coleoptera	Dytiscidae	Hygrotus fontinalis
Boreonectes funereus mitochondrial 16S rRNA gene (	HF931393	82.17	100	4.48537e−33	Coleoptera	Dytiscidae	Boreonectes funereus
Hygrotus patruelis mitochondrial 16S rRNA gene (pa	HF931388	82.17	100	4.48537e−33	Coleoptera	Dytiscidae	Hygrotus patruelis
Hydroporus brancoi gredensis mitochondrial partial	HF931329	82.17	100	4.48537e−33	Coleoptera	Dytiscidae	Hydroporus brancoi gredensis
Suphrodytes figuratus voucher BMNH:722150 16S ribo	JX221572	82.17	100	4.48537e−33	Coleoptera	Dytiscidae	Suphrodytes figuratus
Deronectes hispanicus mitochondrial 16S rRNA gene	HE613727	83.89	94.23	4.48537e−33	Coleoptera	Dytiscidae	Deronectes hispanicus
Hydroporus discretus mitochondrial 16S rRNA gene (	HE613723	82.91	100	4.48537e−33	Coleoptera	Dytiscidae	Hydroporus discretus
Hydroporus decipiens mitochondrial 16S rRNA gene (	HE613722	82.91	100	4.48537e−33	Coleoptera	Dytiscidae	Hydroporus decipiens
Hydroporus pubescens mitochondrial 16S rRNA gene (	HE613707	82.91	100	4.48537e−33	Coleoptera	Dytiscidae	Hydroporus pubescens
Hydroporus limbatus mitochondrial 16S rRNA gene (p	HE613706	82.91	100	4.48537e−33	Coleoptera	Dytiscidae	Hydroporus limbatus
Hydroporus errans mitochondrial 16S rRNA gene (par	HE613704	82.91	100	4.48537e−33	Coleoptera	Dytiscidae	Hydroporus errans
Hydroporus macedonicus mitochondrial 16S rRNA gene	HE613690	82.91	100	4.48537e−33	Coleoptera	Dytiscidae	Hydroporus macedonicus
Suphrodytes dorsalis mitochondrial 16S rRNA gene (	HE599700	82.17	100	4.48537e−33	Coleoptera	Dytiscidae	Suphrodytes dorsalis
