# Supplementary material for: Testing the potential of a ribosomal 16S marker for DNA metabarcoding of insects
Source: PeerJ. 2016 Apr 19;4:e1966. doi: 10.7717/peerj.1966 (PMC4841222; doi:10.7717/peerj.1966)
Supplement: Supplemental Information 1 [file peerj-04-1966-s006.zip › scripts/5 match OTUs/blast_hits/OTU_316.csv_plot.pdf]

OTU_316.csv

Neallogaster pekinensis

Cordulegastridae

Odonata

96.86

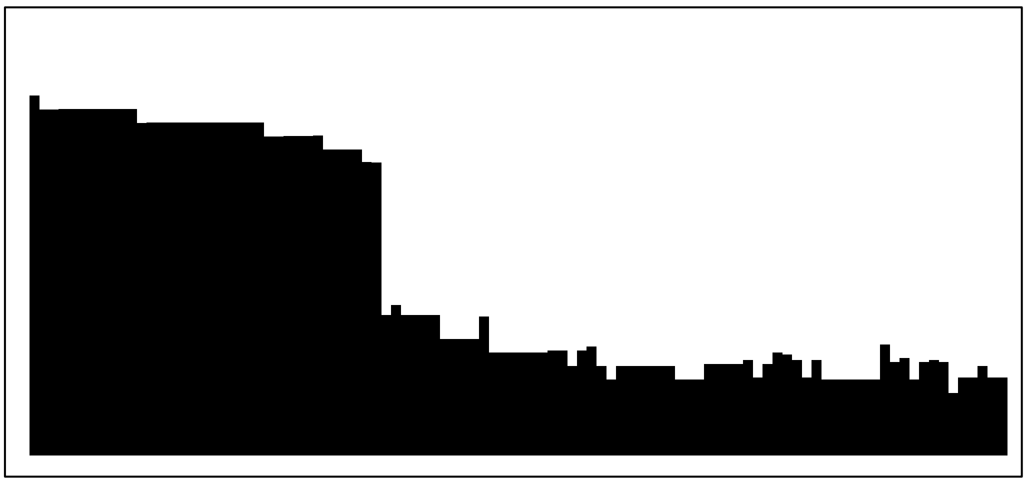

Neallogaster pekinensis mitochondrial gene for 16S	AB707904	96.86	100	3.49916e−66	Odonata	Cordulegastridae	Neallogaster pekinensis
Cordulegaster boltonii 12S ribosomal RNA gene, par	EU477688	96.2	100	1.22133e−65	Odonata	Cordulegastridae	Cordulegaster boltonii
Cordulegaster boltoni large subunit ribosomal RNA	AF266056	96.2	100	1.22133e−65	Odonata	Cordulegastridae	Cordulegaster boltonii
Neallogaster pekinensis mitochondrial gene for 16S	AB707903	96.23	100	4.26285e−65	Odonata	Cordulegastridae	Neallogaster pekinensis
Anotogaster sp. RF1898 mitochondrial gene for 16S	AB707899	96.23	100	4.26285e−65	Odonata	Cordulegastridae	Anotogaster sp. RF1898
Anotogaster sieboldii mitochondrial gene for 16S r	AB707886	96.23	100	4.26285e−65	Odonata	Cordulegastridae	Anotogaster sieboldii
Anotogaster klossi mitochondrial gene for 16S rRNA	AB707872	96.23	100	4.26285e−65	Odonata	Cordulegastridae	Anotogaster klossi
Kalyptogaster erronea 12S ribosomal RNA and 16S ri	EU477690	96.23	100	4.26285e−65	Odonata	Cordulegastridae	Kalyptogaster erronea
Cordulegaster maculata 12S ribosomal RNA and 16S r	EU477689	96.23	100	4.26285e−65	Odonata	Cordulegastridae	Cordulegaster maculata
Cordulegaster dorsalis 16S ribosomal RNA gene, par	EU055084	96.23	100	4.26285e−65	Odonata	Cordulegastridae	Cordulegaster dorsalis
Kalyptogaster erronea voucher JW_DNA_40 16S riboso	EF631561	96.23	100	4.26285e−65	Odonata	Cordulegastridae	Kalyptogaster erronea
Sonjagaster insignis mitochondrial gene for 16S rR	AB707905	95.57	100	5.19322e−64	Odonata	Cordulegastridae	Sonjagaster insignis
Anotogaster sp. RF1284 mitochondrial gene for 16S	AB707896	95.6	100	1.81261e−63	Odonata	Cordulegastridae	Anotogaster sp. RF1284
Anotogaster sieboldii mitochondrial gene for 16S r	AB707892	95.6	100	1.81261e−63	Odonata	Cordulegastridae	Anotogaster sieboldii
Anotogaster sieboldii mitochondrial gene for 16S r	AB707881	95.6	100	1.81261e−63	Odonata	Cordulegastridae	Anotogaster sieboldii
Anotogaster sieboldii mitochondrial gene for 16S r	AB707882	95.6	100	1.81261e−63	Odonata	Cordulegastridae	Anotogaster sieboldii
Anotogaster kuchenbeiseri mitochondrial gene for 1	AB707878	95.6	100	1.81261e−63	Odonata	Cordulegastridae	Anotogaster kuchenbeiseri
Anotogaster klossi mitochondrial gene for 16S rRNA	AB707866	95.6	100	1.81261e−63	Odonata	Cordulegastridae	Anotogaster klossi
Anotogaster klossi mitochondrial gene for 16S rRNA	AB707864	95.6	100	1.81261e−63	Odonata	Cordulegastridae	Anotogaster klossi
Anotogaster klossi mitochondrial gene for 16S rRNA	AB707862	95.6	100	1.81261e−63	Odonata	Cordulegastridae	Anotogaster klossi
Anotogaster klossi mitochondrial gene for 16S rRNA	AB707860	95.6	100	1.81261e−63	Odonata	Cordulegastridae	Anotogaster klossi
Anotogaster sieboldii 12S ribosomal RNA gene, part	EU477686	95.6	100	1.81261e−63	Odonata	Cordulegastridae	Anotogaster sieboldii
Anotogaster sieboldii mitochondrial gene for 16S r	AB127061	95.6	100	1.81261e−63	Odonata	Cordulegastridae	Anotogaster sieboldii
Anotogaster sieboldi large subunit ribosomal RNA g	AF266087	95.6	100	1.81261e−63	Odonata	Cordulegastridae	Anotogaster sieboldii
Cordulegaster picta 12S ribosomal RNA gene, partia	EU477685	94.94	100	6.32663e−63	Odonata	Cordulegastridae	Cordulegaster picta
Cordulegaster picta large subunit ribosomal RNA ge	AF266086	94.94	100	6.32663e−63	Odonata	Cordulegastridae	Cordulegaster picta
Anotogaster sieboldii mitochondrial gene for 16S r	AB707885	94.97	100	2.20821e−62	Odonata	Cordulegastridae	Anotogaster sieboldii
Anotogaster sieboldii mitochondrial gene for 16S r	AB707880	94.97	100	2.20821e−62	Odonata	Cordulegastridae	Anotogaster sieboldii
Anotogaster chaoi mitochondrial gene for 16S rRNA,	AB707858	94.97	100	2.20821e−62	Odonata	Cordulegastridae	Anotogaster chaoi
Anotogaster sp. RF1894 mitochondrial gene for 16S	AB707898	95	100	7.70742e−62	Odonata	Cordulegastridae	Anotogaster sp. RF1894
Anotogaster klossi mitochondrial gene for 16S rRNA	AB711440	94.34	100	9.38956e−61	Odonata	Cordulegastridae	Anotogaster klossi
Cordulegaster picta mitochondrial gene for 16S rRN	AB707902	94.34	100	9.38956e−61	Odonata	Cordulegastridae	Cordulegaster picta
Anotogaster klossi mitochondrial gene for 16S rRNA	AB707876	94.34	100	9.38956e−61	Odonata	Cordulegastridae	Anotogaster klossi
Cordulegaster sp. 1 BM−2008 12S ribosomal RNA gene	EU477687	94.34	100	9.38956e−61	Odonata	Cordulegastridae	Cordulegaster sp. 1 BM−2008
Anotogaster sp. RF1283 mitochondrial gene for 16S	AB707895	93.75	100	3.99254e−59	Odonata	Cordulegastridae	Anotogaster sp. RF1283
Taeniogaster obliqua voucher JW_DNA_OTO 16S riboso	EF631533	93.71	100	1.39353e−58	Odonata	Cordulegastridae	Taeniogaster obliqua
Gynacantha japonica tRNA−Leu, 16S ribosomal RNA, a	KF256872	86.59	100	7.71423e−43	Odonata	Aeshnidae	Gynacantha japonica
Gynacantha japonica mitochondrial gene for 16S rRN	AB707679	87.04	100	7.71423e−43	Odonata	Aeshnidae	Gynacantha japonica
Gynacantha japonica mitochondrial gene for 16S rRN	AB707677	86.59	100	7.71423e−43	Odonata	Aeshnidae	Gynacantha japonica
Gynacantha japonica mitochondrial gene for 16S rRN	AB707675	86.59	100	7.71423e−43	Odonata	Aeshnidae	Gynacantha japonica
Gynacantha japonica isolate 2 12S ribosomal RNA ge	EU477644	86.59	100	7.71423e−43	Odonata	Aeshnidae	Gynacantha japonica
Gynacantha japonica 16S ribosomal RNA gene, partia	EF032710	86.59	100	7.71423e−43	Odonata	Aeshnidae	Gynacantha japonica
Phenes raptor isolate 2 12S ribosomal RNA gene, pa	EU477632	85.44	100	2.69253e−42	Odonata	Petaluridae	Phenes raptor
Phenes raptor 12S ribosomal RNA gene, partial sequ	EU477622	85.44	100	2.69253e−42	Odonata	Petaluridae	Phenes raptor
Phenes raptor 16S ribosomal RNA gene, partial sequ	EU055038	85.44	100	2.69253e−42	Odonata	Petaluridae	Phenes raptor
Phenes raptor large subunit ribosomal RNA gene, pa	AF266083	85.44	100	2.69253e−42	Odonata	Petaluridae	Phenes raptor
Gynacantha japonica mitochondrial gene for 16S rRN	AB707678	86.5	100	9.39785e−42	Odonata	Aeshnidae	Gynacantha japonica
Shaogomphus postocularis mitochondrial gene for 16	AB707782	84.81	100	3.99607e−40	Odonata	Gomphidae	Shaogomphus postocularis
Shaogomphus postocularis mitochondrial gene for 16	AB707778	84.81	100	3.99607e−40	Odonata	Gomphidae	Shaogomphus postocularis
Shaogomphus postocularis mitochondrial gene for 16	AB707774	84.81	100	3.99607e−40	Odonata	Gomphidae	Shaogomphus postocularis
Shaogomphus postocularis mitochondrial gene for 16	AB707775	84.81	100	3.99607e−40	Odonata	Gomphidae	Shaogomphus postocularis
Uropetala carovei 12S ribosomal RNA gene, partial	EU477633	84.81	100	3.99607e−40	Odonata	Petaluridae	Uropetala carovei
Uropetala carovei large subunit ribosomal RNA gene	AY274506	84.81	100	3.99607e−40	Odonata	Petaluridae	Uropetala carovei
Epiophlebia superstes mitochondrial gene for 16S r	AB707607	84.91	100	1.39476e−39	Odonata	Epiophlebiidae	Epiophlebia superstes
Epiophlebia superstes mitochondrial gene for 16S r	AB707604	84.91	100	1.39476e−39	Odonata	Epiophlebiidae	Epiophlebia superstes
Tachopteryx thoreyi isolate 2 12S ribosomal RNA ge	EU477636	84.18	100	1.39476e−39	Odonata	Petaluridae	Tachopteryx thoreyi
Epiophlebia superstes mitochondrial gene for 16S r	AB127062	84.91	100	1.39476e−39	Odonata	Epiophlebiidae	Epiophlebia superstes
Anax nigrofasciatus mitochondrial gene for 16S rRN	AB707659	85.09	100	4.86821e−39	Odonata	Aeshnidae	Anax nigrofasciatus
Shaogomphus postocularis voucher ANI03 tRNA−Leu, 1	KF256838	84.18	100	1.69917e−38	Odonata	Gomphidae	Shaogomphus postocularis
Chlorogomphus brunneus mitochondrial gene for 16S	AB707908	83.54	100	1.69917e−38	Odonata	Chlorogomphidae	Chlorogomphus brunneus
Tanypteryx pryeri mitochondrial gene for 16S rRNA,	AB707856	84.18	100	1.69917e−38	Odonata	Petaluridae	Tanypteryx pryeri
Tanypteryx pryeri mitochondrial gene for 16S rRNA,	AB707853	84.18	100	1.69917e−38	Odonata	Petaluridae	Tanypteryx pryeri
Stylurus annulatus mitochondrial gene for 16S rRNA	AB707821	84.18	100	1.69917e−38	Odonata	Gomphidae	Stylurus annulatus
Stylurus annulatus mitochondrial gene for 16S rRNA	AB707820	84.18	100	1.69917e−38	Odonata	Gomphidae	Stylurus annulatus
Shaogomphus postocularis mitochondrial gene for 16	AB707780	84.18	100	1.69917e−38	Odonata	Gomphidae	Shaogomphus postocularis
Shaogomphus postocularis mitochondrial gene for 16	AB707773	84.18	100	1.69917e−38	Odonata	Gomphidae	Shaogomphus postocularis
Chlorogomphus brunneus 12S ribosomal RNA gene, par	EU477691	83.54	100	1.69917e−38	Odonata	Chlorogomphidae	Chlorogomphus brunneus
Chlorogomphus brunneus large subunit ribosomal RNA	AF266088	83.54	100	1.69917e−38	Odonata	Chlorogomphidae	Chlorogomphus brunneus
Arigomphus cornutus large subunit ribosomal RNA ge	AF266072	83.54	100	1.69917e−38	Odonata	Gomphidae	Arigomphus cornutus
Epiophlebia superstes voucher ANISO01 tRNA−Leu, 16	KF256906	84.28	100	5.93069e−38	Odonata	Epiophlebiidae	Epiophlebia superstes
Epiophlebia superstes mitochondrial gene for 16S r	AB707615	84.28	100	5.93069e−38	Odonata	Epiophlebiidae	Epiophlebia superstes
Epiophlebia superstes mitochondrial gene for 16S r	AB707613	84.28	100	5.93069e−38	Odonata	Epiophlebiidae	Epiophlebia superstes
Epiophlebia superstes mitochondrial gene for 16S r	AB707602	84.28	100	5.93069e−38	Odonata	Epiophlebiidae	Epiophlebia superstes
Caliaeschna microstigma 12S ribosomal RNA gene, pa	EU477639	84.47	100	5.93069e−38	Odonata	Aeshnidae	Caliaeschna microstigma
Tachopteryx thoreyi 12S ribosomal RNA gene, partia	EU477634	83.65	100	5.93069e−38	Odonata	Petaluridae	Tachopteryx thoreyi
Epiophlebia superstes 16S ribosomal RNA gene, part	EU055132	84.28	100	5.93069e−38	Odonata	Epiophlebiidae	Epiophlebia superstes
Eusynthemis nigra 16S ribosomal RNA gene, partial	EU055069	84.81	100	5.93069e−38	Odonata	Synthemistidae	Eusynthemis nigra
Uracis fastigiata 12S ribosomal RNA gene, partial	EF640390	84.71	99.37	5.93069e−38	Odonata	Libellulidae	Uracis fastigiata
Caliaeschna microstigma large subunit ribosomal RN	AF266092	84.47	100	5.93069e−38	Odonata	Aeshnidae	Caliaeschna microstigma
Tachopteryx thoreyi large subunit ribosomal RNA ge	AF266081	83.65	100	5.93069e−38	Odonata	Petaluridae	Tachopteryx thoreyi
Anax nigrofasciatus voucher ANI02 tRNA−Leu, 16S ri	KF256837	84.47	100	2.07001e−37	Odonata	Aeshnidae	Anax nigrofasciatus
Tanypteryx pryeri mitochondrial gene for 16S rRNA,	AB707854	83.54	100	2.07001e−37	Odonata	Petaluridae	Tanypteryx pryeri
Tanypteryx pryeri mitochondrial gene for 16S rRNA,	AB707851	83.54	100	2.07001e−37	Odonata	Petaluridae	Tanypteryx pryeri
Stylurus nagoyanus mitochondrial gene for 16S rRNA	AB707824	83.54	100	2.07001e−37	Odonata	Gomphidae	Stylurus nagoyanus
Stylurus nagoyanus mitochondrial gene for 16S rRNA	AB707822	83.54	100	2.07001e−37	Odonata	Gomphidae	Stylurus nagoyanus
Asiagomphus yayeyamensis mitochondrial gene for 16	AB707730	83.54	100	2.07001e−37	Odonata	Aeshnidae	Asiagomphus yayeyamensis
Asiagomphus yayeyamensis mitochondrial gene for 16	AB707728	83.54	100	2.07001e−37	Odonata	Aeshnidae	Asiagomphus yayeyamensis
Tetracanthagyna plagiata mitochondrial gene for 16	AB707709	85.19	100	2.07001e−37	Odonata	Aeshnidae	Tetracanthagyna plagiata
Sarasaeschna lieni mitochondrial gene for 16S rRNA	AB707705	84.38	100	2.07001e−37	Odonata	Aeshnidae	Sarasaeschna lieni
Anax junius mitochondrial gene for 16S rRNA, parti	AB707656	84.57	100	2.07001e−37	Odonata	Aeshnidae	Anax junius
Gomphus pulchellus 12S ribosomal RNA gene, partial	EU477663	83.54	100	2.07001e−37	Odonata	Gomphidae	Gomphus pulchellus
Epiophlebia superstes 12S ribosomal RNA gene, part	EU477631	84.38	100	2.07001e−37	Odonata	Epiophlebiidae	Epiophlebia superstes
Anax nigrofasciatus 16S ribosomal RNA gene, partia	EF032708	84.47	100	2.07001e−37	Odonata	Aeshnidae	Anax nigrofasciatus
Epiophlebia superstes large subunit ribosomal RNA	AF266094	84.38	100	2.07001e−37	Odonata	Epiophlebiidae	Epiophlebia superstes
Chlorogomphus iriomotensis mitochondrial gene for	AB707919	82.91	100	7.22506e−37	Odonata	Chlorogomphidae	Chlorogomphus iriomotensis
Tanypteryx pryeri mitochondrial gene for 16S rRNA,	AB707852	83.65	100	7.22506e−37	Odonata	Petaluridae	Tanypteryx pryeri
Tanypteryx pryeri 12S ribosomal RNA gene, partial	EU477635	83.65	100	7.22506e−37	Odonata	Petaluridae	Tanypteryx pryeri
Choristhemis flavoterminata 16S ribosomal RNA gene	EU055070	84.18	100	7.22506e−37	Odonata	Synthemistidae	Choristhemis flavoterminata
Tanypteryx pryeri mitochondrial gene for 16S rRNA,	AB127060	83.65	100	7.22506e−37	Odonata	Petaluridae	Tanypteryx pryeri
Tanypteryx pryeri large subunit ribosomal RNA gene	AF266082	83.65	100	7.22506e−37	Odonata	Petaluridae	Tanypteryx pryeri
