# Supplementary material for: Testing the potential of a ribosomal 16S marker for DNA metabarcoding of insects
Source: PeerJ. 2016 Apr 19;4:e1966. doi: 10.7717/peerj.1966 (PMC4841222; doi:10.7717/peerj.1966)
Supplement: Supplemental Information 1 [file peerj-04-1966-s006.zip › scripts/5 match OTUs/blast_hits/OTU_321.csv_plot.pdf]

OTU_321.csv

Diasemopsis longipedunculata

Diopsidae

Diptera

80.25

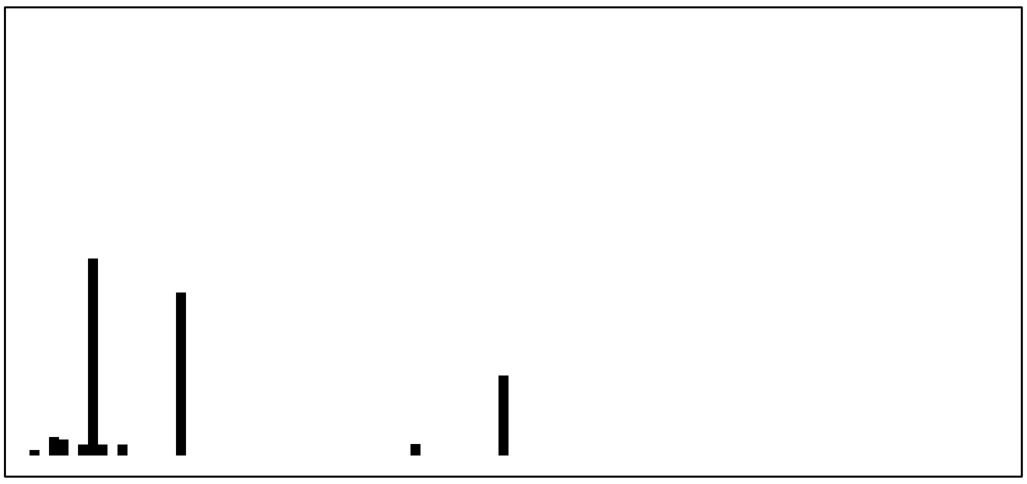

Diasemopsis longipedunculata 16S ribosomal RNA gen	KT186667	80.25	100	3.41985e−28	Diptera	Diopsidae	Diasemopsis longipedunculata
Scaptodrosophila lebanonensis voucher 105639 NADH	EU494411	79.63	100	1.19365e−27	Diptera	Drosophilidae	Scaptodrosophila lebanonensis
Spiniphora punctipennis 16S ribosomal RNA gene, pa	AF126342	80.86	100	1.19365e−27	Diptera	Phoridae	Spiniphora punctipennis
Asteia concinna isolate A63 16S ribosomal RNA gene	KJ418481	80.75	100	1.45416e−26	Diptera	Asteiidae	Asteia concinna
Diasemopsis longipedunculata 16S ribosomal RNA gen	AF304741	79.01	100	1.45416e−26	Diptera	Diopsidae	Diasemopsis longipedunculata
Drosophila camargoi voucher 103971 NADH dehydrogen	EU494312	80.5	100	5.07551e−26	Diptera	Drosophilidae	Drosophila camargoi
Culicoides oxystoma mitochondrial DNA, large subun	AB360979	89.22	65.16	5.07551e−26	Diptera	Ceratopogonidae	Culicoides oxystoma
Drosophila camargoi 16S ribosomal RNA gene, partia	AF479801	80.5	100	5.07551e−26	Diptera	Drosophilidae	Drosophila camargoi
Lonchoptera uniseta 16S ribosomal RNA gene, partia	KC177456	79.75	100	6.18324e−25	Diptera	Lonchopteridae	Lonchoptera uniseta
Zaprionus lineosus voucher 103961 NADH dehydrogena	EU494414	80.5	100	6.18324e−25	Diptera	Drosophilidae	Zaprionus lineosus
Evocoa chilensis 16S ribosomal RNA gene, partial s	KM878914	79.01	100	2.15816e−24	Diptera	Evocoidae	Evocoa chilensis
Palaeosepsis bucki voucher Lei19 16S ribosomal RNA	KF199519	79.25	100	2.15816e−24	Diptera	Sepsidae	Palaeosepsis bucki
Oestroidea sp. 'McAlpine's fly' voucher RMBR:10442	GQ409133	80	100	2.15816e−24	Diptera		Oestroidea sp. 'McAlpine's fly'
Senometopia cariniforceps mitochondrial gene for 1	AB465923	79.63	100	2.15816e−24	Diptera	Tachinidae	Senometopia cariniforceps
Drosophila austrosaltans voucher 106314 NADH dehyd	EU494364	78.88	100	2.15816e−24	Diptera	Drosophilidae	Drosophila austrosaltans
Culicoides actoni mitochondrial DNA, large subunit	AB360971	87.62	65.81	2.15816e−24	Diptera	Ceratopogonidae	Culicoides actoni
Drosophila austrosaltans 16S ribosomal RNA gene, p	AF479808	78.88	100	2.15816e−24	Diptera	Drosophilidae	Drosophila austrosaltans
Megalabops sp. KCADI071−14 16S ribosomal RNA gene,	KT186671	78.05	100	7.53272e−24	Diptera	Diopsidae	Megalabops sp. KCADI071−14
Megalabops sp. BOLD:ACP5917 16S ribosomal RNA gene	KT186670	78.05	100	7.53272e−24	Diptera	Diopsidae	Megalabops sp. BOLD:ACP5917
Dohrniphora segregata 16S ribosomal RNA gene, part	JQ707933	79.5	100	7.53272e−24	Diptera	Phoridae	Dohrniphora segregata
Drosophila micromyia voucher O201044 16S ribosomal	HQ170981	78.4	100	7.53272e−24	Diptera	Drosophilidae	Drosophila micromyia
Drosophila micromyia voucher 109454 NADH dehydroge	EU494392	78.4	100	7.53272e−24	Diptera	Drosophilidae	Drosophila micromyia
Diasemopsis elongata 16S ribosomal RNA gene, parti	AF304740	78.4	100	7.53272e−24	Diptera	Diopsidae	Diasemopsis elongata
Sepsis luteipes voucher Lei33 16S ribosomal RNA ge	KJ766342	79.25	100	2.62918e−23	Diptera	Sepsidae	Sepsis luteipes
Archisepsis sp. LZ−2013 16S ribosomal RNA gene, pa	KF199498	79.49	96.13	2.62918e−23	Diptera	Sepsidae	Archisepsis sp. LZ−2013
Archisepsis armata voucher 106676 16S ribosomal RN	KF199495	79.49	96.13	2.62918e−23	Diptera	Sepsidae	Archisepsis armata
Scaptomyza crassifemur voucher 202688 16S ribosoma	KC609614	78.48	100	2.62918e−23	Diptera	Drosophilidae	Scaptomyza crassifemur
Scaptomyza lobifera voucher 202596 16S ribosomal R	KC609604	78.48	100	2.62918e−23	Diptera	Drosophilidae	Scaptomyza lobifera
Colobaea bifasciella voucher MU_NUS_SG3 16S riboso	JN828202	78.48	100	2.62918e−23	Diptera	Sciomyzidae	Colobaea bifasciella
Phorocerosoma vicarium mitochondrial gene for 16S	AB465926	80	100	2.62918e−23	Diptera	Tachinidae	Phorocerosoma vicarium
Liriomyza trifolii mitochondrion, complete genome	JN570506	78.48	98.71	2.62918e−23	Diptera	Agromyzidae	Liriomyza trifolii
Scaptomyza sp. 'akalae' voucher O201528 16S riboso	HQ171045	78.48	100	2.62918e−23	Diptera	Drosophilidae	Scaptomyza sp. 'akalae'
Drosophila proceriseta voucher O201880 16S ribosom	HQ170994	77.91	100	2.62918e−23	Diptera	Drosophilidae	Drosophila proceriseta
Liriomyza trifolii mitochondrion, complete genome	GU327644	78.48	98.71	2.62918e−23	Diptera	Agromyzidae	Liriomyza trifolii
Parapalaeosepsis apicalis voucher su34 16S ribosom	EU435595	79.14	100	2.62918e−23	Diptera	Sepsidae	Parapalaeosepsis apicalis
Scaptomyza undulata voucher 109452 NADH dehydrogen	EU494407	78.48	100	2.62918e−23	Diptera	Drosophilidae	Scaptomyza undulata
Bezzia pygmaea 16S ribosomal RNA gene, partial seq	EF472588	79.25	100	2.62918e−23	Diptera	Ceratopogonidae	Bezzia pygmaea
Phorinia flava mitochondrial gene for 16S rRNA, pa	AB257236	79.27	100	2.62918e−23	Diptera	Tachinidae	Phorinia flava
Afraciura zernyi 16S ribosomal RNA gene, partial s	DQ471395	77.99	100	2.62918e−23	Diptera	Tephritidae	Afraciura zernyi
Themira nigricornis mitochondrial partial 16S rRNA	AJ843107	80.52	94.19	2.62918e−23	Diptera	Sepsidae	Themira nigricornis
Themira biloba mitochondrial partial 16S rRNA gene	AJ843101	79.74	94.19	2.62918e−23	Diptera	Sepsidae	Themira biloba
Diopsis nr. gnu KCADI063−14 16S ribosomal RNA gene	KT186676	78.4	100	9.17674e−23	Diptera	Diopsidae	Diopsis nr. gnu KCADI063−14
Diopsis nr. circularis KCADI065−14 16S ribosomal R	KT186651	77.91	100	9.17674e−23	Diptera	Diopsidae	Diopsis nr. circularis KCADI065−14
Atelestus pulicarius isolate AP47 16S ribosomal RN	KC699323	79.22	96.13	9.17674e−23	Diptera	Atelestidae	Atelestus pulicarius
Dichaetomyia luteiventris voucher RMBR:116193 16S	KJ476304	78.79	100	9.17674e−23	Diptera	Muscidae	Dichaetomyia luteiventris
Scythropochroa nr. quercicola SS−2012 voucher 778	JQ613930	79.25	96.13	9.17674e−23	Diptera	Sciaridae	Scythropochroa nr. quercicola SS−2012
Scaptomyza tumidula voucher O201059a 16S ribosomal	HQ171050	78.12	100	9.17674e−23	Diptera	Drosophilidae	Scaptomyza tumidula
Scaptomyza sp. 'neocyrtandrae' voucher O201368 16S	HQ171049	78.12	100	9.17674e−23	Diptera	Drosophilidae	Scaptomyza sp. 'neocyrtandrae'
Cardiocladius sp. MNK−2008 16S ribosomal RNA gene,	EU669992	83.74	74.84	9.17674e−23	Diptera	Chironomidae	Cardiocladius sp. MNK−2008
Willistoniella pleuropunctata voucher su6 16S ribo	EU435573	78.88	100	9.17674e−23	Diptera	Sepsidae	Willistoniella pleuropunctata
Aciura afghana 16S ribosomal RNA gene, partial seq	DQ471406	77.5	100	9.17674e−23	Diptera	Tephritidae	Aciura afghana
Sphaerocera curvipes 16S ribosomal RNA gene, parti	AY573135	77.58	100	9.17674e−23	Diptera	Sphaeroceridae	Sphaerocera curvipes
Culicoides sp. LJH−2002 16S ribosomal RNA gene, pa	AY140904	78.12	100	9.17674e−23	Diptera	Ceratopogonidae	Culicoides sp. LJH−2002
Cladodiopsis seyrigi 16S ribosomal RNA gene, parti	DQ317406	78.53	100	9.17674e−23	Diptera	Diopsidae	Cladodiopsis seyrigi
Diopsis gnu 16S ribosomal RNA gene, partial sequen	AF304744	78.4	100	9.17674e−23	Diptera	Diopsidae	Diopsis gnu
Trichodiopsis minuta 16S ribosomal RNA gene, parti	AF304724	79.01	100	9.17674e−23	Diptera	Diopsidae	Diasemopsis minuta
Cyrtodiopsis dalmanni isolate CdGombak4 16S riboso	AY876564	78.26	100	9.17674e−23	Diptera	Diopsidae	Teleopsis dalmanni
Megalabops quadriguttata 16S ribosomal RNA gene, p	KT186655	77.44	100	3.203e−22	Diptera	Diopsidae	Megalabops quadriguttata
Drosophila paracracens voucher 202556 16S ribosoma	KM252237	77.85	100	3.203e−22	Diptera	Drosophilidae	Drosophila paracracens
Drosophila orthoptera voucher 202540 16S ribosomal	KM252228	77.85	100	3.203e−22	Diptera	Drosophilidae	Drosophila orthoptera
Drosophila kokeensis voucher 201055 16S ribosomal	KM252222	77.85	100	3.203e−22	Diptera	Drosophilidae	Drosophila kokeensis
Lophosceles cinereiventris voucher RMBR:116208 16S	KJ476316	77.85	100	3.203e−22	Diptera	Muscidae	Lophosceles cinereiventris
Brachythoracosepsis saothomensis voucher Lei9 16S	KF199503	78.88	100	3.203e−22	Diptera	Sepsidae	Brachythoracosepsis saothomensis
Brachythoracosepsis pseudonotosa voucher Lei7 16S	KF199501	78.88	100	3.203e−22	Diptera	Sepsidae	Brachythoracosepsis pseudonotosa
Brachythoracosepsis butikensis voucher Lei4 16S ri	KF199499	78.88	100	3.203e−22	Diptera	Sepsidae	Brachythoracosepsis butikensis
Scaptomyza apicata voucher 10003 16S ribosomal RNA	KC609623	77.85	100	3.203e−22	Diptera	Drosophilidae	Scaptomyza apicata
Scaptomyza nasalis voucher 202692 16S ribosomal RN	KC609617	77.85	100	3.203e−22	Diptera	Drosophilidae	Scaptomyza nasalis
Scaptomyza ampliloba voucher 202690 16S ribosomal	KC609616	77.85	100	3.203e−22	Diptera	Drosophilidae	Scaptomyza amplilobus
Scaptomyza reducta voucher 202686 16S ribosomal RN	KC609613	77.85	100	3.203e−22	Diptera	Drosophilidae	Scaptomyza reducta
Scaptomyza cuspidata voucher 202656 16S ribosomal	KC609610	77.85	100	3.203e−22	Diptera	Drosophilidae	Scaptomyza cuspidata
Scaptomyza dentata voucher 202625 16S ribosomal RN	KC609607	77.85	100	3.203e−22	Diptera	Drosophilidae	Scaptomyza dentata
Teleopsis dalmanni 16S ribosomal RNA gene, partial	KC177441	78.12	100	3.203e−22	Diptera	Diopsidae	Teleopsis dalmanni
Drosophila conformis voucher 202493a 16S ribosomal	HQ703701	77.85	100	3.203e−22	Diptera	Drosophilidae	Drosophila conformis
Drosophila incognita voucher O202333a 16S ribosoma	HQ171027	77.85	100	3.203e−22	Diptera	Drosophilidae	Drosophila incognita
Drosophila dasycnemia voucher 202331a 16S ribosoma	HQ703698	77.78	100	3.203e−22	Diptera	Drosophilidae	Drosophila dasycnemia
Drosophila neutralis voucher 202329b 16S ribosomal	HQ703696	77.78	100	3.203e−22	Diptera	Drosophilidae	Drosophila neutralis
Drosophila sordidapex voucher O202321a 16S ribosom	HQ171025	77.78	100	3.203e−22	Diptera	Drosophilidae	Drosophila sordidapex
Drosophila sordidapex voucher 202318a 16S ribosoma	HQ703693	77.78	100	3.203e−22	Diptera	Drosophilidae	Drosophila sordidapex
Drosophila percnosoma voucher O200125a 16S ribosom	HQ171022	77.78	100	3.203e−22	Diptera	Drosophilidae	Drosophila percnosoma
Drosophila sodomae voucher O34.2 16S ribosomal RNA	HQ171041	77.78	100	3.203e−22	Diptera	Drosophilidae	Drosophila sodomae
Drosophila conformis voucher O201312a 16S ribosoma	HQ171023	77.78	100	3.203e−22	Diptera	Drosophilidae	Drosophila conformis
Drosophila contorta voucher O200120a 16S ribosomal	HQ171021	77.78	100	3.203e−22	Diptera	Drosophilidae	Drosophila contorta
Drosophila tetraspilota voucher O200995 16S riboso	HQ171015	77.78	100	3.203e−22	Diptera	Drosophilidae	Drosophila tetraspilota
Drosophila nr. hirtitarsus RL−2010 voucher O200998	HQ171010	77.78	100	3.203e−22	Diptera	Drosophilidae	Drosophila nr. hirtitarsus RL−2010
Drosophila eurypeza voucher O201043 16S ribosomal	HQ170966	77.85	100	3.203e−22	Diptera	Drosophilidae	Drosophila eurypeza
Drosophila desallei voucher O200989 16S ribosomal	HQ170963	77.78	100	3.203e−22	Diptera	Drosophilidae	Drosophila desallei
Drosophila antecedens voucher O205061 16S ribosoma	HQ170953	77.78	100	3.203e−22	Diptera	Drosophilidae	Drosophila antecedens
Diopsina draconigena partial 16S rRNA gene, isolat	FN813724	78.12	100	3.203e−22	Diptera	Diopsidae	Diopsina draconigena
Atylostoma towadensis mitochondrial gene for 16S r	AB465949	78.4	100	3.203e−22	Diptera	Tachinidae	Atylostoma towadensis
Limnophora riparia 16S ribosomal RNA gene, partial	FJ025449	78.98	98.71	3.203e−22	Diptera	Muscidae	Limnophora riparia
Archisepsis diversiformis voucher su7 16S ribosoma	EU435574	78.85	96.13	3.203e−22	Diptera	Sepsidae	Archisepsis diversiformis
Drosophila percnosoma voucher 105685 NADH dehydrog	EU494396	77.78	100	3.203e−22	Diptera	Drosophilidae	Drosophila percnosoma
Drosophila biseriata voucher 109453 NADH dehydroge	EU494377	77.85	100	3.203e−22	Diptera	Drosophilidae	Drosophila biseriata
Drosophila adunca voucher 105818 NADH dehydrogenas	EU494374	77.85	100	3.203e−22	Diptera	Drosophilidae	Drosophila adunca
Drosophila paramelanica voucher 109372 NADH dehydr	EU494346	77.85	100	3.203e−22	Diptera	Drosophilidae	Drosophila paramelanica
Drosophila melanica voucher 105499 NADH dehydrogen	EU494344	77.85	100	3.203e−22	Diptera	Drosophilidae	Drosophila melanica
Perochaeta dikowi 16S ribosomal RNA gene, complete	EU375720	78.26	100	3.203e−22	Diptera	Sepsidae	Perochaeta dikowi
Cyrtodiopsis dalmanni isolate CdBrastagi3a 16S rib	DQ098014	76.88	100	3.203e−22	Diptera	Diopsidae	Teleopsis dalmanni
Paramycodrosophila sp. POG−2002 16S ribosomal RNA	AF479810	77.85	100	3.203e−22	Diptera	Drosophilidae	Paramycodrosophila n. sp. POG−2002
Scaptomyza buccata voucher 202560 16S ribosomal RN	KC609600	77.36	100	1.11796e−21	Diptera	Drosophilidae	Scaptomyza buccata
