# Supplementary material for: Testing the potential of a ribosomal 16S marker for DNA metabarcoding of insects
Source: PeerJ. 2016 Apr 19;4:e1966. doi: 10.7717/peerj.1966 (PMC4841222; doi:10.7717/peerj.1966)
Supplement: Supplemental Information 1 [file peerj-04-1966-s006.zip › scripts/5 match OTUs/blast_hits/OTU_33.csv_plot.pdf]

OTU_33.csv

Anomalopterygella chauviniana

Limnephilidae

Trichoptera

100

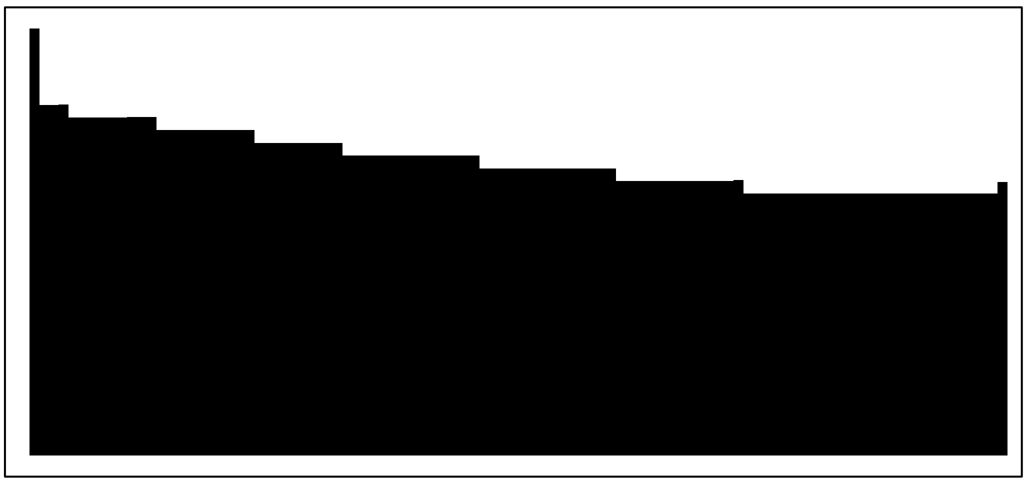

Anomalopterygella chauviniana voucher AC001 16S ri	EU215174	100	100	3.54059e−79	Trichoptera	Limnephilidae	Anomalopterygella chauviniana
Drusus melanchaetes voucher DMel001 16S ribosomal	EU215199	96.43	100	4.9215e−71	Trichoptera	Limnephilidae	Drusus melanchaetes
Drusus annulatus voucher DAn001 16S ribosomal RNA	EU215181	96.43	100	4.9215e−71	Trichoptera	Limnephilidae	Drusus annulatus
Drusus brunneus voucher DBr002 16S ribosomal RNA g	EU215187	96.45	100	1.71777e−70	Trichoptera	Limnephilidae	Drusus brunneus
Metanoea flavipennis voucher MF001 16S ribosomal R	EU215224	95.83	100	2.09268e−69	Trichoptera	Limnephilidae	Metanoea flavipennis
Drusus rectus voucher DRec005 16S ribosomal RNA ge	EU215211	95.83	100	2.09268e−69	Trichoptera	Limnephilidae	Drusus rectus
Drusus nigrescens voucher DNig001 16S ribosomal RN	EU215206	95.83	100	2.09268e−69	Trichoptera	Limnephilidae	Drusus nigrescens
Drusus monticola voucher DMon001 16S ribosomal RNA	EU215201	95.83	100	2.09268e−69	Trichoptera	Limnephilidae	Drusus monticola
Drusus biguttatus voucher DBig003 16S ribosomal RN	EU215184	95.83	100	2.09268e−69	Trichoptera	Limnephilidae	Drusus biguttatus
Drusus annulatus voucher DAn005 16S ribosomal RNA	EU215182	95.83	100	2.09268e−69	Trichoptera	Limnephilidae	Drusus annulatus
Drusus trifidus voucher DTri007 16S ribosomal RNA	EU215215	95.86	100	7.30416e−69	Trichoptera	Limnephilidae	Drusus trifidus
Drusus trifidus voucher DTri006 16S ribosomal RNA	EU215214	95.86	100	7.30416e−69	Trichoptera	Limnephilidae	Drusus trifidus
Drusus brunneus voucher DBr001 16S ribosomal RNA g	EU215186	95.86	100	7.30416e−69	Trichoptera	Limnephilidae	Drusus brunneus
Drusus improvisus voucher RIS DImp004 16S ribosoma	EU999189	95.24	100	2.5494e−68	Trichoptera	Limnephilidae	Drusus improvisus
Drusus improvisus voucher RIS DImp001 16S ribosoma	EU999187	95.24	100	2.5494e−68	Trichoptera	Limnephilidae	Drusus improvisus
Drusus croaticus voucher DcVRL17 16S ribosomal RNA	FJ002846	95.24	100	2.5494e−68	Trichoptera	Limnephilidae	Drusus croaticus
Metanoea rhaetica voucher MR005 16S ribosomal RNA	EU215226	95.24	100	2.5494e−68	Trichoptera	Limnephilidae	Metanoea rhaetica
Metanoea rhaetica voucher MR001 16S ribosomal RNA	EU215225	95.24	100	2.5494e−68	Trichoptera	Limnephilidae	Metanoea rhaetica
Drusus muelleri voucher DMue005 16S ribosomal RNA	EU215205	95.24	100	2.5494e−68	Trichoptera	Limnephilidae	Drusus muelleri
Drusus muelleri voucher DMue004 16S ribosomal RNA	EU215204	95.24	100	2.5494e−68	Trichoptera	Limnephilidae	Drusus muelleri
Drusus muelleri voucher DMue003 16S ribosomal RNA	EU215203	95.24	100	2.5494e−68	Trichoptera	Limnephilidae	Drusus muelleri
Drusus mixtus voucher DMix001 16S ribosomal RNA ge	EU215200	95.24	100	2.5494e−68	Trichoptera	Limnephilidae	Drusus mixtus
Cryptothrix nebulicola voucher CN006 16S ribosomal	EU215177	95.24	100	2.5494e−68	Trichoptera	Limnephilidae	Cryptothrix nebulicola
Drusus croaticus voucher DcVRL24 16S ribosomal RNA	FJ002853	94.64	100	1.08403e−66	Trichoptera	Limnephilidae	Drusus croaticus
Drusus croaticus voucher DcGTVL12 16S ribosomal RN	FJ002783	94.64	100	1.08403e−66	Trichoptera	Limnephilidae	Drusus croaticus
Drusus croaticus voucher DcDTL8 16S ribosomal RNA	FJ002762	94.64	100	1.08403e−66	Trichoptera	Limnephilidae	Drusus croaticus
Drusus croaticus voucher DCCAIM1 16S ribosomal RNA	FJ002739	94.64	100	1.08403e−66	Trichoptera	Limnephilidae	Drusus croaticus
Drusus romanicus romanicus voucher DR003 16S ribos	EU215209	94.64	100	1.08403e−66	Trichoptera	Limnephilidae	Drusus romanicus romanicus
Drusus romanicus romanicus voucher DR001 16S ribos	EU215208	94.64	100	1.08403e−66	Trichoptera	Limnephilidae	Drusus romanicus romanicus
Drusus destitutus voucher DDes001 16S ribosomal RN	EU215193	94.64	100	1.08403e−66	Trichoptera	Limnephilidae	Drusus destitutus
Drusus chrysotus voucher DCh004 16S ribosomal RNA	EU215189	94.64	100	1.08403e−66	Trichoptera	Limnephilidae	Drusus chrysotus
Drusus chrysotus voucher DCh001 16S ribosomal RNA	EU215188	94.64	100	1.08403e−66	Trichoptera	Limnephilidae	Drusus chrysotus
Drusus improvisus voucher RIS DImp003 16S ribosoma	EU999188	94.05	100	1.32062e−65	Trichoptera	Limnephilidae	Drusus improvisus
Drusus croaticus voucher DcVIL6 16S ribosomal RNA	FJ002845	94.05	100	1.32062e−65	Trichoptera	Limnephilidae	Drusus croaticus
Drusus croaticus voucher DcVIL3 16S ribosomal RNA	FJ002844	94.05	100	1.32062e−65	Trichoptera	Limnephilidae	Drusus croaticus
Drusus croaticus voucher DcPLL1 16S ribosomal RNA	FJ002823	94.05	100	1.32062e−65	Trichoptera	Limnephilidae	Drusus croaticus
Drusus croaticus voucher DcGMVIM3 16S ribosomal RN	FJ002766	94.05	100	1.32062e−65	Trichoptera	Limnephilidae	Drusus croaticus
Drusus croaticus voucher DCCAL3 16S ribosomal RNA	FJ002744	94.05	100	1.32062e−65	Trichoptera	Limnephilidae	Drusus croaticus
Drusus croaticus voucher DcBUL5 16S ribosomal RNA	FJ002737	94.05	100	1.32062e−65	Trichoptera	Limnephilidae	Drusus croaticus
Drusus croaticus voucher DcBUL1 16S ribosomal RNA	FJ002734	94.05	100	1.32062e−65	Trichoptera	Limnephilidae	Drusus croaticus
Drusus croaticus voucher DcBRL9 16S ribosomal RNA	FJ002733	94.05	100	1.32062e−65	Trichoptera	Limnephilidae	Drusus croaticus
Drusus croaticus voucher DcBRL10 16S ribosomal RNA	FJ002722	94.05	100	1.32062e−65	Trichoptera	Limnephilidae	Drusus croaticus
Ecclisopteryx asterix voucher EAst003 16S ribosoma	EU215216	94.05	100	1.32062e−65	Trichoptera	Limnephilidae	Ecclisopteryx asterix
Drusus destitutus voucher DDes005 16S ribosomal RN	EU215194	94.05	100	1.32062e−65	Trichoptera	Limnephilidae	Drusus destitutus
Drusus alpinus voucher DAlp003 16S ribosomal RNA g	EU215180	94.05	100	1.32062e−65	Trichoptera	Limnephilidae	Drusus alpinus
Drusus alpinus voucher DAlp002 16S ribosomal RNA g	EU215179	94.05	100	1.32062e−65	Trichoptera	Limnephilidae	Drusus alpinus
Ecclisopteryx asterix voucher EAstDDes002 16S ribo	EU215217	93.45	100	1.60885e−64	Trichoptera	Limnephilidae	Ecclisopteryx asterix
Drusus discolor voucher DdCAIM1 16S ribosomal RNA	KM001841	93.45	100	5.61543e−64	Trichoptera	Limnephilidae	Drusus discolor
Ecclisopteryx asterix voucher fEas0103M 16S riboso	KM001756	93.45	100	5.61543e−64	Trichoptera	Limnephilidae	Ecclisopteryx asterix
Ecclisopteryx asterix voucher fEas0102M 16S riboso	KM001755	93.45	100	5.61543e−64	Trichoptera	Limnephilidae	Ecclisopteryx asterix
Ecclisopteryx asterix voucher fEas0101M 16S riboso	KM001754	93.45	100	5.61543e−64	Trichoptera	Limnephilidae	Ecclisopteryx asterix
Drusus vespertinus voucher DvRL1 16S ribosomal RNA	FJ002817	93.45	100	5.61543e−64	Trichoptera	Limnephilidae	Drusus vespertinus
Drusus ramae voucher DraIM1 16S ribosomal RNA gene	FJ002809	93.45	100	5.61543e−64	Trichoptera	Limnephilidae	Drusus ramae
Drusus medianus voucher DmIM1 16S ribosomal RNA ge	FJ002807	93.45	100	5.61543e−64	Trichoptera	Limnephilidae	Drusus medianus
Drusus discolor voucher DdIM2 16S ribosomal RNA ge	FJ002806	93.45	100	5.61543e−64	Trichoptera	Limnephilidae	Drusus discolor
Ecclisopteryx guttulata voucher EGut009 16S riboso	EU215220	93.45	100	5.61543e−64	Trichoptera	Limnephilidae	Ecclisopteryx guttulata
Drusus romanicus meridionalis voucher DR007 16S ri	EU215210	93.45	100	5.61543e−64	Trichoptera	Limnephilidae	Drusus romanicus meridionalis
Drusus franzi voucher DFrII 16S ribosomal RNA gene	EU215198	93.45	100	5.61543e−64	Trichoptera	Limnephilidae	Drusus franzi
Drusus discophorus pallidus voucher DDis001 16S ri	EU215195	93.45	100	5.61543e−64	Trichoptera	Limnephilidae	Drusus discophorus pallidus
Drusus discolor voucher DD373 16S ribosomal RNA ge	EU215190	93.45	100	5.61543e−64	Trichoptera	Limnephilidae	Drusus discolor
Ecclisopteryx madida voucher fEma0102M 16S ribosom	KM001738	92.86	100	6.84099e−63	Trichoptera	Limnephilidae	Ecclisopteryx madida
Ecclisopteryx madida voucher fEma0101M 16S ribosom	KM001737	92.86	100	6.84099e−63	Trichoptera	Limnephilidae	Ecclisopteryx madida
Ecclisopteryx madida voucher EM006 16S ribosomal R	KM001736	92.86	100	6.84099e−63	Trichoptera	Limnephilidae	Ecclisopteryx madida
Ecclisopteryx madida voucher EM005 16S ribosomal R	KM001735	92.86	100	6.84099e−63	Trichoptera	Limnephilidae	Ecclisopteryx madida
Ecclisopteryx madida voucher EM003 16S ribosomal R	KM001734	92.86	100	6.84099e−63	Trichoptera	Limnephilidae	Ecclisopteryx madida
Drusus bosnicus voucher DbsVML2 16S ribosomal RNA	GQ470605	92.86	100	6.84099e−63	Trichoptera	Limnephilidae	Drusus bosnicus
Drusus radovanovici radovanovici voucher DRRIM1 16	GQ470601	92.86	100	6.84099e−63	Trichoptera	Limnephilidae	Drusus radovanovici radovanovici
Drusus bosnicus voucher DbsIM1 16S ribosomal RNA g	FJ002821	92.86	100	6.84099e−63	Trichoptera	Limnephilidae	Drusus bosnicus
Drusus schmidi voucher DsIM4 16S ribosomal RNA gen	FJ002816	92.86	100	6.84099e−63	Trichoptera	Limnephilidae	Drusus schmidi
Ecclisopteryx madida voucher EM001 16S ribosomal R	EU215221	92.86	100	6.84099e−63	Trichoptera	Limnephilidae	Ecclisopteryx madida
Drusus franzi voucher DFr007 16S ribosomal RNA gen	EU215197	92.86	100	6.84099e−63	Trichoptera	Limnephilidae	Drusus franzi
Drusus balcanicus voucher DBal001 16S ribosomal RN	EU215183	92.86	100	6.84099e−63	Trichoptera	Limnephilidae	Drusus balcanicus
Ecclisopteryx malickyi voucher EMal002 16S ribosom	KM001725	92.9	100	2.38774e−62	Trichoptera	Limnephilidae	Ecclisopteryx malickyi
Ecclisopteryx keroveci voucher E2JAL2 16S ribosoma	KM001845	92.26	100	2.90886e−61	Trichoptera	Limnephilidae	Ecclisopteryx keroveci
Ecclisopteryx keroveci voucher E2JAL1 16S ribosoma	KM001844	92.26	100	2.90886e−61	Trichoptera	Limnephilidae	Ecclisopteryx keroveci
Ecclisopteryx keroveci voucher E1JAL2 16S ribosoma	KM001843	92.26	100	2.90886e−61	Trichoptera	Limnephilidae	Ecclisopteryx keroveci
Ecclisopteryx keroveci voucher E1JAL1 16S ribosoma	KM001842	92.26	100	2.90886e−61	Trichoptera	Limnephilidae	Ecclisopteryx keroveci
Ecclisopteryx ivkae voucher fEda1202L 16S ribosoma	KM001810	92.26	100	2.90886e−61	Trichoptera	Limnephilidae	Ecclisopteryx ivkae
Ecclisopteryx ivkae voucher fEda1201L 16S ribosoma	KM001809	92.26	100	2.90886e−61	Trichoptera	Limnephilidae	Ecclisopteryx ivkae
Ecclisopteryx ivkae voucher fEda0102M 16S ribosoma	KM001808	92.26	100	2.90886e−61	Trichoptera	Limnephilidae	Ecclisopteryx ivkae
Ecclisopteryx ivkae voucher fEda0102F 16S ribosoma	KM001807	92.26	100	2.90886e−61	Trichoptera	Limnephilidae	Ecclisopteryx ivkae
Ecclisopteryx ivkae voucher fEda0101M 16S ribosoma	KM001806	92.26	100	2.90886e−61	Trichoptera	Limnephilidae	Ecclisopteryx ivkae
Ecclisopteryx ivkae voucher fDsp3301L 16S ribosoma	KM001804	92.26	100	2.90886e−61	Trichoptera	Limnephilidae	Ecclisopteryx ivkae
Ecclisopteryx keroveci voucher fEns0101M 16S ribos	KM001784	92.26	100	2.90886e−61	Trichoptera	Limnephilidae	Ecclisopteryx keroveci
Ecclisopteryx keroveci voucher fEda1101F 16S ribos	KM001783	92.26	100	2.90886e−61	Trichoptera	Limnephilidae	Ecclisopteryx keroveci
Ecclisopteryx keroveci voucher fEda1001M 16S ribos	KM001782	92.26	100	2.90886e−61	Trichoptera	Limnephilidae	Ecclisopteryx keroveci
Ecclisopteryx keroveci voucher fEda0301M 16S ribos	KM001781	92.26	100	2.90886e−61	Trichoptera	Limnephilidae	Ecclisopteryx keroveci
Ecclisopteryx keroveci voucher fEda0202F 16S ribos	KM001780	92.26	100	2.90886e−61	Trichoptera	Limnephilidae	Ecclisopteryx keroveci
Ecclisopteryx keroveci voucher fEda0201M 16S ribos	KM001779	92.26	100	2.90886e−61	Trichoptera	Limnephilidae	Ecclisopteryx keroveci
Ecclisopteryx keroveci voucher fDs30109F 16S ribos	KM001778	92.26	100	2.90886e−61	Trichoptera	Limnephilidae	Ecclisopteryx keroveci
Ecclisopteryx keroveci voucher fDs30106F 16S ribos	KM001777	92.26	100	2.90886e−61	Trichoptera	Limnephilidae	Ecclisopteryx keroveci
Ecclisopteryx keroveci voucher fDs30105M 16S ribos	KM001776	92.26	100	2.90886e−61	Trichoptera	Limnephilidae	Ecclisopteryx keroveci
Ecclisopteryx keroveci voucher fDs30103M 16S ribos	KM001775	92.26	100	2.90886e−61	Trichoptera	Limnephilidae	Ecclisopteryx keroveci
Ecclisopteryx keroveci voucher fDs30102M 16S ribos	KM001774	92.26	100	2.90886e−61	Trichoptera	Limnephilidae	Ecclisopteryx keroveci
Ecclisopteryx keroveci voucher fDs30101M 16S ribos	KM001773	92.26	100	2.90886e−61	Trichoptera	Limnephilidae	Ecclisopteryx keroveci
Ecclisopteryx guttulata voucher fEgu0104M 16S ribo	KM001749	92.26	100	2.90886e−61	Trichoptera	Limnephilidae	Ecclisopteryx guttulata
Ecclisopteryx guttulata voucher fEgu0103M 16S ribo	KM001748	92.26	100	2.90886e−61	Trichoptera	Limnephilidae	Ecclisopteryx guttulata
Ecclisopteryx guttulata voucher fEgu0101M 16S ribo	KM001746	92.26	100	2.90886e−61	Trichoptera	Limnephilidae	Ecclisopteryx guttulata
Ecclisopteryx madida voucher EM002 16S ribosomal R	KM001733	92.26	100	2.90886e−61	Trichoptera	Limnephilidae	Ecclisopteryx madida
Ecclisopteryx malickyi voucher EMal001 16S ribosom	EU215223	92.81	98.81	2.90886e−61	Trichoptera	Limnephilidae	Ecclisopteryx malickyi
