# Supplementary material for: Testing the potential of a ribosomal 16S marker for DNA metabarcoding of insects
Source: PeerJ. 2016 Apr 19;4:e1966. doi: 10.7717/peerj.1966 (PMC4841222; doi:10.7717/peerj.1966)
Supplement: Supplemental Information 1 [file peerj-04-1966-s006.zip › scripts/5 match OTUs/blast_hits/OTU_34.csv_plot.pdf]

OTU_34.csv

Asellus aquaticus

Asellidae

Isopoda

100

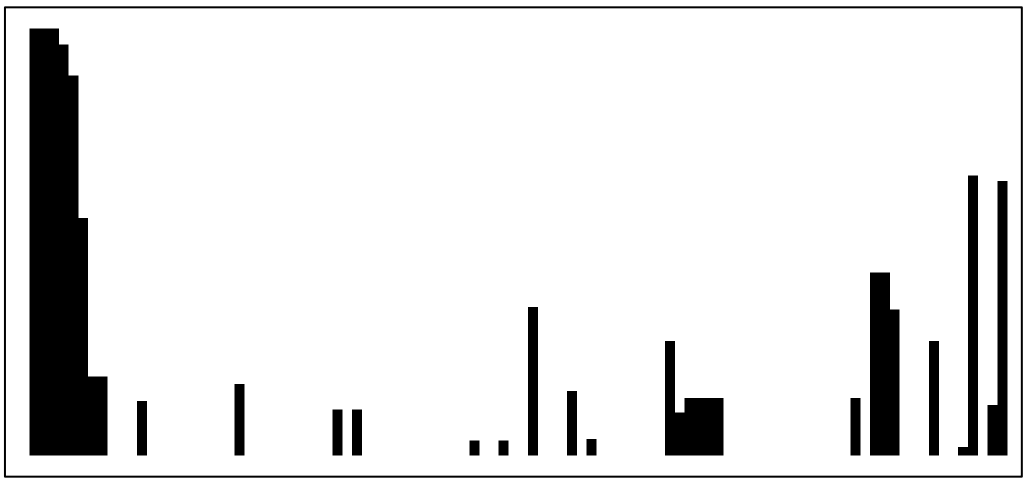

Asellus aquaticus isolate s180 16S ribosomal RNA g	KC610270	100	100	6.32885e−62	Isopoda	Asellidae	Asellus aquaticus
Asellus aquaticus 16S ribosomal RNA gene, partial	DQ305105	100	100	6.32885e−62	Isopoda	Asellidae	Asellus aquaticus
Asellus aquaticus 16S ribosomal RNA gene, partial	AF532161	100	100	6.32885e−62	Isopoda	Asellidae	Asellus aquaticus
Asellus aquaticus partial mitochondrial 16S rRNA g	AJ388077	99.26	100	2.6911e−60	Isopoda	Asellidae	Asellus aquaticus
Asellus aquaticus 16S ribosomal RNA gene, partial	DQ305106	97.79	100	1.39402e−57	Isopoda	Asellidae	Asellus aquaticus
Asellus levanidovorum 16S ribosomal RNA gene, part	AF532160	91.11	65.44	6.34004e−24	Isopoda	Asellidae	Asellus levanidovorum
Mesoasellus dybowskii 16S ribosomal RNA gene, part	AF532158	83.7	65.44	8.8128e−16	Isopoda	Asellidae	Mesoasellus dybowskii
Asellid sp. BH−2002 16S ribosomal RNA gene, partia	AF532159	83.7	65.44	3.7473e−14	Isopoda	Asellidae	Asellid sp. BH−2002
Stenasellus virei isolate ORFES_200906_2_YLK394 16	JQ921839	78.41	63.97	1.225e−07	Isopoda	Stenasellidae	Stenasellus virei
Bragasellus cortesi isolate POUCA_201010_1ID_YCD83	JQ921676	77.66	66.18	4.27566e−07	Isopoda	Asellidae	Bragasellus cortesi
Proasellus beroni isolate s202 16S ribosomal RNA g	KC610292	76.4	65.44	5.20882e−06	Isopoda	Asellidae	Proasellus beroni
Megaphyllum montivagum voucher ZMUC:00101492 small	KF701067	82.54	44.85	1.81805e−05	Julida	Julidae	Megaphyllum montivagum
Proasellus slavus isolate s118 16S ribosomal RNA g	KC610208	79.75	55.88	6.34565e−05	Isopoda	Asellidae	Proasellus slavus
Proasellus slavus isolate s117 16S ribosomal RNA g	KC610207	79.75	55.88	6.34565e−05	Isopoda	Asellidae	Proasellus slavus
Proasellus slavus isolate s105 16S ribosomal RNA g	KC610195	79.75	55.88	6.34565e−05	Isopoda	Asellidae	Proasellus slavus
Proasellus slavus isolate s104 16S ribosomal RNA g	KC610194	79.75	55.88	6.34565e−05	Isopoda	Asellidae	Proasellus slavus
Proasellus slavus isolate s103 16S ribosomal RNA g	KC610193	79.75	55.88	6.34565e−05	Isopoda	Asellidae	Proasellus slavus
Proasellus slavus isolate PRVOVERZ_201009_1ID_YCD5	JQ921809	79.75	55.88	6.34565e−05	Isopoda	Asellidae	Proasellus slavus
Proasellus karamani isolate ZALOMSKA_200305_1_YLK3	JQ921766	79.41	50	6.34565e−05	Isopoda	Asellidae	Proasellus karamani
Proasellus coxalis isolate BUNAR_200402_1ID_YLK392	JQ921740	76.34	66.18	6.34565e−05	Isopoda	Asellidae	Proasellus coxalis
Proasellus aff. lagari Henry & Magniez, 1982 isola	JQ921700	79.41	50	6.34565e−05	Isopoda	Asellidae	Proasellus aff. lagari Henry & Magniez, 1982
Julus scandinavius voucher ZMUC200018 16S ribosoma	JF320957	83.33	39.71	6.34565e−05	Julida	Julidae	Julus scandinavius
Proasellus ebrensis isolate s207 16S ribosomal RNA	KC610297	79.1	49.26	0.000221485	Isopoda	Asellidae	Proasellus ebrensis
Proasellus ebrensis isolate s206 16S ribosomal RNA	KC610296	79.1	49.26	0.000221485	Isopoda	Asellidae	Proasellus ebrensis
Proasellus sp. type locality of P. gourbaultae iso	KC610099	75.28	65.44	0.000221485	Isopoda	Asellidae	Proasellus sp. type locality of P. gourbaultae
Proasellus spelaeus isolate ETCHEVE_200811_1ex_YAS	JQ921811	75.28	65.44	0.000221485	Isopoda	Asellidae	Proasellus spelaeus
Proasellus sp. n. Maglia isolate MAGLIA_200908_1ID	JQ921791	75.28	65.44	0.000221485	Isopoda	Asellidae	Proasellus sp. n. Maglia
Proasellus ebrensis isolate CERECEDA_200906_1_YLK1	JQ921747	79.1	49.26	0.000221485	Isopoda	Asellidae	Proasellus ebrensis
Proasellus beroni isolate ORTAMIN_200810_1ex_YAS42	JQ921715	76.19	61.76	0.000221485	Isopoda	Asellidae	Proasellus beroni
Proasellus sp. Netherlands−BH−2002 16S ribosomal R	AF532163	76.34	66.18	0.000221485	Isopoda	Asellidae	Proasellus sp. Netherlands−BH−2002
Proasellus sp. n. Verclause isolate s230 16S ribos	KC610320	74.44	66.18	0.000773058	Isopoda	Asellidae	Proasellus sp. n. Verclause
Proasellus meijersae isolate s223 16S ribosomal RN	KC610313	82.14	41.18	0.000773058	Isopoda	Asellidae	Proasellus meijersae
Proasellus sp. n. Verclause isolate LAPENNE_201002	JQ921797	74.44	66.18	0.000773058	Isopoda	Asellidae	Proasellus sp. n. Verclause
Proasellus meijersae isolate MUNDO_200910_3_YDE001	JQ921777	82.14	41.18	0.000773058	Isopoda	Asellidae	Proasellus meijersae
Proasellus coxalis isolate STENSAN_200811_2ID_YAS4	JQ921743	76.84	66.18	0.000773058	Isopoda	Asellidae	Proasellus coxalis
Proasellus stocki isolate SAJA_201010_1ID_YCD838 1	JQ921730	76.32	55.88	0.000773058	Isopoda	Asellidae	Proasellus stocki
Asellus aquaticus mitochondrion, partial genome	GU130252	76.84	66.18	0.000773058	Isopoda	Asellidae	Asellus aquaticus
Proasellus aff. escolai s209 16S ribosomal RNA gen	KC610299	75.28	65.44	0.00269824	Isopoda	Asellidae	Proasellus aff. escolai s209
Proasellus cantabricus isolate RESCANO_200906_1ID_	JQ921719	77.94	50	0.00269824	Isopoda	Asellidae	Proasellus cantabricus
Proasellus aquaecalidae isolate PEAUXCH_200811_1_Y	JQ921704	74.44	65.44	0.00269824	Isopoda	Asellidae	Proasellus aquaecalidae
Proasellus aff. escolai Henry & Magniez, 1982 isol	JQ921699	75.28	65.44	0.00269824	Isopoda	Asellidae	Proasellus aff. escolai Henry & Magniez, 1982
Bragasellus comasi isolate COBALLE2_200906_1_YLK19	JQ921672	72.04	65.44	0.00269824	Isopoda	Asellidae	Bragasellus comasi
Monolistra pretneri spinulosa 16S ribosomal RNA ge	FJ842042	74.71	63.24	0.00269824	Isopoda	Sphaeromatidae	Monolistra pretneri spinulosa
Monolistra pretneri 16S ribosomal RNA gene, partia	FJ842016	74.71	63.24	0.00269824	Isopoda	Sphaeromatidae	Monolistra pretneri
Tityus pachyurus isolate 24 16S ribosomal RNA gene	AY586753	76.19	58.82	0.00269824	Scorpiones	Buthidae	Tityus pachyurus
Stenasellus breuili isolate s237 16S ribosomal RNA	KC610327	80.7	41.91	0.00941777	Isopoda	Stenasellidae	Stenasellus breuili
Proasellus aquaecalidae isolate s198 16S ribosomal	KC610288	74.44	65.44	0.00941777	Isopoda	Asellidae	Proasellus aquaecalidae
Synasellus sp. n. Aboim isolate ABOIM_201010_1ID_Y	JQ921860	73.33	65.44	0.00941777	Isopoda	Asellidae	Synasellus sp. n. Aboim
Stenasellus breuili isolate ALLI_200906_1ID_YLK130	JQ921830	80.7	41.91	0.00941777	Isopoda	Stenasellidae	Stenasellus breuili
Proasellus ebrensis isolate RUDRON_201010_1IDex_YC	JQ921720	77.61	49.26	0.00941777	Isopoda	Asellidae	Proasellus ebrensis
Proasellus aquaecalidae isolate OUEILNEE_200811_2_	JQ921703	74.44	65.44	0.00941777	Isopoda	Asellidae	Proasellus aquaecalidae
Ophyiulus pilosus voucher ZMUC200176 16S ribosomal	JF320969	86.96	33.09	0.00941777	Julida	Julidae	Ophyiulus pilosus
Megaphyllum bosniense voucher ZMUC:00021391 small	KF701073	72.53	63.97	0.0328713	Julida	Julidae	Megaphyllum bosniense
Proasellus slovenicus isolate JAMAPOD_200409_1_YLK	JQ921810	75	65.44	0.0328713	Isopoda	Asellidae	Proasellus slovenicus
Megaphyllum projectum voucher ZMUC200023 16S ribos	JF320963	72.73	61.76	0.0328713	Julida	Julidae	Megaphyllum projectum
Phonognatha graeffei voucher FAPMCZ02 16S ribosoma	EU003275	83.02	38.24	0.0328713	Araneae	Tetragnathidae	Phonognatha graeffei
Synalpheus williamsi 16S ribosomal RNA gene, parti	AF230264	79.22	50	0.0328713	Decapoda	Alpheidae	Synalpheus williamsi
Metaiulus pratensis voucher ZMUC:00101657 small su	KF701064	80.77	36.76	0.114732	Julida	Julidae	Metaiulus pratensis
Proasellus aff. escolai s208 16S ribosomal RNA gen	KC610298	74.16	65.44	0.114732	Isopoda	Asellidae	Proasellus aff. escolai s208
Proasellus slavus isolate s110 16S ribosomal RNA g	KC610200	75	65.44	0.114732	Isopoda	Asellidae	Proasellus slavus
Proasellus slavus isolate s108 16S ribosomal RNA g	KC610198	75	65.44	0.114732	Isopoda	Asellidae	Proasellus slavus
Carapoia genitalis bio−material ZFMK:DNA100446401	JX023897	75.64	56.62	0.114732	Araneae	Pholcidae	Carapoia genitalis
Proasellus escolai isolate SALAR_200910_2_YCD140 1	JQ921749	78.12	47.06	0.114732	Isopoda	Asellidae	Proasellus escolai
Proasellus aragonensis isolate BURGUI_200906_2_YLK	JQ921706	76.12	49.26	0.114732	Isopoda	Asellidae	Proasellus aragonensis
Proasellus aragonensis isolate BURGUI_200906_1ID_Y	JQ921705	76.12	49.26	0.114732	Isopoda	Asellidae	Proasellus aragonensis
Parachtes limbarae voucher crba001231 16S ribosoma	JN705772	85.37	27.94	0.114732	Araneae	Dysderidae	Parachtes limbarae
Syedra oii voucher IZCL108 16S ribosomal RNA gene,	GU338615	82	36.76	0.114732	Araneae	Linyphiidae	Syedra oii
Mysmeninae sp. MYSM−033−MAD 16S ribosomal RNA gene	GU456754	82.69	38.24	0.114732	Araneae	Mysmenidae	Mysmeninae sp. MYSM−033−MAD
Mysmeninae sp. MYSM−032−MAD 16S ribosomal RNA gene	GU456753	82.69	38.24	0.114732	Araneae	Mysmenidae	Mysmeninae sp. MYSM−032−MAD
Mysmeninae sp. MYSM−031−MAD 16S ribosomal RNA gene	GU456752	82.69	38.24	0.114732	Araneae	Mysmenidae	Mysmeninae sp. MYSM−031−MAD
Mysmeninae sp. MYSM−026−MAD 16S ribosomal RNA gene	GU456751	82.69	38.24	0.114732	Araneae	Mysmenidae	Mysmeninae sp. MYSM−026−MAD
Modisimus vittatus voucher DNA07−NF41 16S ribosoma	FJ227991	79.66	38.97	0.114732	Araneae	Pholcidae	Modisimus vittatus
Monolistra schotlanderi 16S ribosomal RNA gene, pa	FJ842063	73.56	63.24	0.114732	Isopoda	Sphaeromatidae	Monolistra schotlanderi
Monolistra caeca 16S ribosomal RNA gene, partial s	FJ842054	73.56	63.24	0.114732	Isopoda	Sphaeromatidae	Monolistra caeca
Monolistra sp. RV−2009 isolate Rupecica 16S riboso	FJ842049	73.56	63.24	0.114732	Isopoda	Sphaeromatidae	Monolistra sp. RV−2009
Monolistra sketi 16S ribosomal RNA gene, partial s	FJ842046	73.56	63.24	0.114732	Isopoda	Sphaeromatidae	Monolistra sketi
Monolistra pretneri isolate Obrovac 16S ribosomal	FJ842044	73.56	63.24	0.114732	Isopoda	Sphaeromatidae	Monolistra pretneri
Monolistra spinosissima isolate Vrhnika & Logatec	FJ842027	73.56	63.24	0.114732	Isopoda	Sphaeromatidae	Monolistra spinosissima
Monolistra sp. 1 RV−2009 16S ribosomal RNA gene, p	FJ842033	73.56	63.24	0.114732	Isopoda	Sphaeromatidae	Monolistra sp. 1 RV−2009
Monolistra spinosa 16S ribosomal RNA gene, partial	FJ842007	73.56	63.24	0.114732	Isopoda	Sphaeromatidae	Monolistra spinosa
Monolistra racovitzai isolate Postojna 16S ribosom	FJ842024	74.42	62.5	0.114732	Isopoda	Sphaeromatidae	Monolistra racovitzai
Monolistra velkovrhi 16S ribosomal RNA gene, parti	FJ842019	73.86	63.24	0.114732	Isopoda	Sphaeromatidae	Monolistra velkovrhi
Monolistra bolei 16S ribosomal RNA gene, partial s	FJ842013	73.56	63.24	0.114732	Isopoda	Sphaeromatidae	Monolistra bolei
Monolistra sp. 2 RV−2009 16S ribosomal RNA gene, p	FJ842010	73.56	63.24	0.114732	Isopoda	Sphaeromatidae	Monolistra sp. 2 RV−2009
Leucauge argyra voucher FAPDNA041 16S ribosomal RN	EU003264	82.69	38.24	0.114732	Araneae	Tetragnathidae	Leucauge argyra
Monolistra bolei isolate b−brevispinosa−T 16S ribo	FJ851102	73.56	63.24	0.114732	Isopoda	Sphaeromatidae	Monolistra bolei
Betamorpha fusiformis isolate BF138 16S ribosomal	EF116535	88.57	25.74	0.114732	Isopoda	Munnopsidae	Betamorpha fusiformis
Betamorpha fusiformis isolate BF91 16S ribosomal R	EF116533	88.57	25.74	0.114732	Isopoda	Munnopsidae	Betamorpha fusiformis
Betamorpha fusiformis isolate BF57 16S ribosomal R	EF116529	86.84	27.94	0.114732	Isopoda	Munnopsidae	Betamorpha fusiformis
Stylodactylus sp. CARID161−14 voucher MNHN−IU−2011	KP725653	79.37	41.18	0.400454	Decapoda	Stylodactylidae	Stylodactylus sp. CARID161−14
Megaphyllum transsylvanicum voucher ZMUC:00021392	KF701074	77.19	38.97	0.400454	Julida	Julidae	Megaphyllum transsylvanicum
Proasellus chauvini isolate s204 16S ribosomal RNA	KC610294	79.63	39.71	0.400454	Isopoda	Asellidae	Proasellus chauvini
Heptischius lactuca voucher CUMZ−D00007 16S riboso	KC519547	85.37	30.15	0.400454	Spirostreptida	Harpagophoridae	Heptischius lactuca
Proasellus ebrensis isolate MARFIL_200906_1ID_YAS8	JQ921748	73.13	49.26	0.400454	Isopoda	Asellidae	Proasellus ebrensis
Proasellus chauvini isolate HAYTZA_200811_2_YAS534	JQ921733	79.63	39.71	0.400454	Isopoda	Asellidae	Proasellus chauvini
Proasellus sp. n. Aquamalas Pontones isolate AQUAL	JQ921727	80.39	36.76	0.400454	Isopoda	Asellidae	Proasellus sp. n. Aquamalas Pontones
Brachyiulus bagnalli voucher ZMUC100790 16S riboso	JF320984	93.1	21.32	0.400454	Julida	Julidae	Brachyiulus bagnalli
Dolichoiulus tongiorgii voucher ZMUC200178 16S rib	JF320971	77.78	39.71	0.400454	Julida	Julidae	Dolichoiulus tongiorgii
Microdipoena guttata 16S ribosomal RNA gene, parti	GU456747	82.35	37.5	0.400454	Araneae	Mysmenidae	Microdipoena guttata
Apteryx australis mantelli genome assembly AptMant	LK064785	92.86	20.59	1.39772	Apterygiformes	Apterygidae	Apteryx australis mantelli
