# Supplementary material for: Testing the potential of a ribosomal 16S marker for DNA metabarcoding of insects
Source: PeerJ. 2016 Apr 19;4:e1966. doi: 10.7717/peerj.1966 (PMC4841222; doi:10.7717/peerj.1966)
Supplement: Supplemental Information 1 [file peerj-04-1966-s006.zip › scripts/5 match OTUs/blast_hits/OTU_341.csv_plot.pdf]

OTU_341.csv

Torleya major

Ephemerellidae

Ephemeroptera

96.73

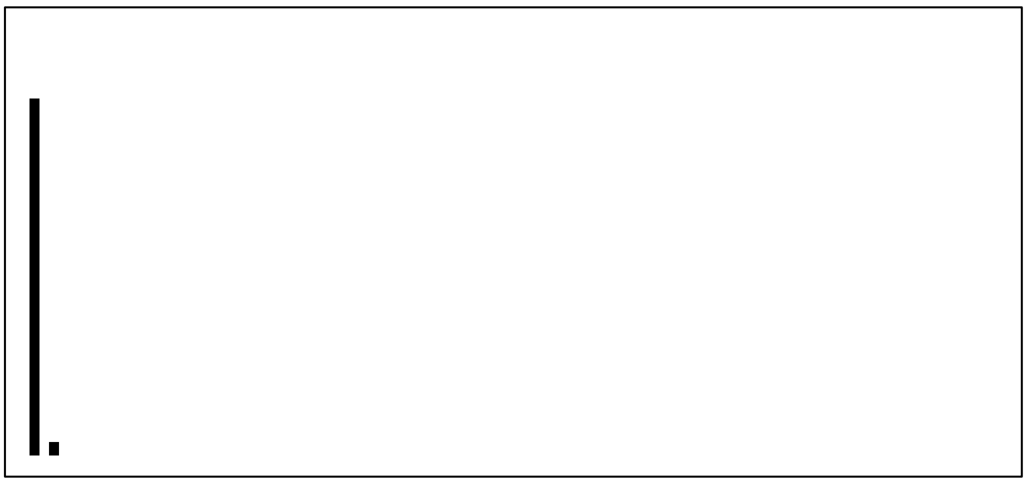

Torleya major 16S ribosomal RNA gene, partial sequ	FJ443047	96.73	100	4.98211e−64	Ephemeroptera	Ephemerellidae	Torleya major
Teloganopsis media 16S ribosomal RNA gene, partial	FJ443046	75.32	100	6.93748e−18	Ephemeroptera	Ephemerellidae	Teloganopsis media
Ephemerella atagosana 16S ribosomal RNA gene, part	FJ443039	80.61	64.05	4.37803e−14	Ephemeroptera	Ephemerellidae	Ephemerella atagosana
Ephemerella sp. THO−2009 16S ribosomal RNA gene, p	FJ443044	79.59	64.05	5.33353e−13	Ephemeroptera	Ephemerellidae	Ephemerella sp. THO−2009
Ephemerella cornutus 16S ribosomal RNA gene, parti	FJ443041	74.64	89.54	5.33353e−13	Ephemeroptera	Ephemerellidae	Ephemerella cornutus
Drunella pelosa 16S ribosomal RNA gene, partial se	FJ443033	77.55	64.05	2.76284e−10	Ephemeroptera	Ephemerellidae	Drunella pelosa
Ephoron shigae mitochondrial gene for 16S rRNA, pa	AB711581	77.78	63.4	9.64325e−10	Ephemeroptera	Polymitarcyidae	Ephoron shigae
Drunella doddsi 16S ribosomal RNA gene, partial se	AY749756	73.28	85.62	9.64325e−10	Ephemeroptera	Ephemerellidae	Drunella doddsi
Ephoron eophilum mitochondrial gene for 16S rRNA,	AB711631	77.23	63.4	3.36583e−09	Ephemeroptera	Polymitarcyidae	Ephoron eophilum
Ephoron eophilum mitochondrial gene for 16S rRNA,	AB711630	78.43	63.4	3.36583e−09	Ephemeroptera	Polymitarcyidae	Ephoron eophilum
Ephoron eophilum mitochondrial gene for 16S rRNA,	AB711621	78.43	63.4	3.36583e−09	Ephemeroptera	Polymitarcyidae	Ephoron eophilum
Ephoron eophilum mitochondrial gene for 16S rRNA,	AB711619	78.43	63.4	3.36583e−09	Ephemeroptera	Polymitarcyidae	Ephoron eophilum
Ephoron eophilum mitochondrial gene for 16S rRNA,	AB711615	78.43	63.4	3.36583e−09	Ephemeroptera	Polymitarcyidae	Ephoron eophilum
Ephoron shigae mitochondrial gene for 16S rRNA, pa	AB711590	76.77	63.4	1.17479e−08	Ephemeroptera	Polymitarcyidae	Ephoron shigae
Drunella ishiyamana 16S ribosomal RNA gene, partia	FJ443035	72.44	100	1.17479e−08	Ephemeroptera	Ephemerellidae	Drunella ishiyamana
Ephacerella longicaudata 16S ribosomal RNA gene, p	FJ443038	75.76	64.71	4.10042e−08	Ephemeroptera	Ephemerellidae	Ephacerella longicaudata
Drunella spinifera 16S ribosomal RNA gene, partial	FJ443034	75.76	64.71	4.10042e−08	Ephemeroptera	Ephemerellidae	Drunella spinifera
Caudatella hystrix 16S ribosomal RNA gene, partial	AY749787	75	65.36	4.10042e−08	Ephemeroptera	Ephemerellidae	Caudatella hystrix
Rhabdotis aulica voucher 842782 16S ribosomal RNA	JX234477	76.53	62.75	1.43119e−07	Coleoptera	Scarabaeidae	Rhabdotis aulica
Ephoron eophilum mitochondrial gene for 16S rRNA,	AB711633	77.45	63.4	1.43119e−07	Ephemeroptera	Polymitarcyidae	Ephoron eophilum
Ephoron shigae mitochondrial gene for 16S rRNA, pa	AB711582	75.51	63.4	1.43119e−07	Ephemeroptera	Polymitarcyidae	Ephoron shigae
Ephoron shigae mitochondrial gene for 16S rRNA, pa	AB711575	75.51	63.4	1.43119e−07	Ephemeroptera	Polymitarcyidae	Ephoron shigae
Polyphylla decemlineata 16S ribosomal RNA gene, pa	JX994048	76.84	60.78	1.43119e−07	Coleoptera	Scarabaeidae	Polyphylla decemlineata
Hexagenia sp. BYU IGCEP003 16S ribosomal RNA gene,	GQ118286	76.7	64.71	1.43119e−07	Ephemeroptera	Ephemeridae	Hexagenia sp. BYU IGCEP003
Drunella tuberculata 16S ribosomal RNA gene, parti	FJ443037	75.25	66.01	1.43119e−07	Ephemeroptera	Ephemerellidae	Drunella tuberculata
Drunella trispina 16S ribosomal RNA gene, partial	FJ443036	75.25	66.01	1.43119e−07	Ephemeroptera	Ephemerellidae	Drunella trispina
Sphaerosoma sp. 1 16S ribosomal RNA gene, partial	GQ302155	76.92	58.17	4.99533e−07	Coleoptera	Alexiidae	Sphaerosoma sp. 1 FWS−2010
Tropha zambiensis voucher KS240 16S ribosomal RNA	KP143640	76.84	60.78	1.74354e−06	Coleoptera	Coccinellidae	Tropha zambiensis
Dasytes coriaceipennis voucher UPOL001197 16S ribo	HQ619684	75.51	62.75	1.74354e−06	Coleoptera	Melyridae	Dasytes coriaceipennis
Lamproblatta albipalpus voucher ZMUC:JD−1555 16S r	JN615295	75.51	62.75	1.74354e−06	Blattodea	Lamproblattidae	Lamproblatta albipalpus
Gymnopternus celer voucher Pollet 0268 (P) 16S rib	EU863982	74.76	64.05	1.74354e−06	Diptera	Dolichopodidae	Gymnopternus celer
Serratella serrata 16S ribosomal RNA gene, partial	FJ443045	73.53	66.67	1.74354e−06	Ephemeroptera	Ephemerellidae	Serratella serrata
Phaedyma columella voucher jshao2005036 16S riboso	DQ784713	76.92	58.17	1.74354e−06	Lepidoptera	Nymphalidae	Phaedyma columella
Cerdistus sp. LRC−2002 16S ribosomal RNA gene, par	AF456850	75.49	64.05	1.74354e−06	Diptera	Asilidae	Cerdistus sp. LRC−2002
Euploea mulciber 16S ribosomal RNA gene, partial s	KJ777733	76.09	58.82	6.08556e−06	Lepidoptera	Nymphalidae	Euploea mulciber
Ephemerella sp. MT−2014 isolate CL54 mitochondrion	KM244691	74.49	64.05	6.08556e−06	Ephemeroptera	Ephemerellidae	Ephemerella sp. MT−2014
Atrichelaphinis tigrina voucher 842817 16S ribosom	JX234502	75.26	62.09	6.08556e−06	Coleoptera	Scarabaeidae	Atrichelaphinis tigrina
Comythovalgus sp. DA−2012 voucher 842778 16S ribos	JX234473	74.23	62.09	6.08556e−06	Coleoptera	Scarabaeidae	Comythovalgus sp. DA−2012
Ephoron shigae mitochondrial gene for 16S ribosoma	AB874293	74.75	63.4	6.08556e−06	Ephemeroptera	Polymitarcyidae	Ephoron shigae
Ephoron shigae mitochondrial gene for 16S ribosoma	AB874289	74.75	63.4	6.08556e−06	Ephemeroptera	Polymitarcyidae	Ephoron shigae
Ephoron shigae mitochondrial gene for 16S ribosoma	AB874283	74.75	63.4	6.08556e−06	Ephemeroptera	Polymitarcyidae	Ephoron shigae
Ephoron shigae mitochondrial gene for 16S ribosoma	AB874278	74.75	63.4	6.08556e−06	Ephemeroptera	Polymitarcyidae	Ephoron shigae
Euroleon nostras isolate 60−nosmar1 16S ribosomal	KF321986	75	64.05	6.08556e−06	Neuroptera	Myrmeleontidae	Euroleon nostras
Euroleon nostras isolate 34−nostur2 16S ribosomal	KF321983	75	64.05	6.08556e−06	Neuroptera	Myrmeleontidae	Euroleon nostras
Ephoron leukon mitochondrial gene for 16S rRNA, pa	AB711641	74	64.71	6.08556e−06	Ephemeroptera	Polymitarcyidae	Ephoron leukon
Ephoron album mitochondrial gene for 16S rRNA, par	AB711636	74	64.71	6.08556e−06	Ephemeroptera	Polymitarcyidae	Ephoron album
Ephoron album mitochondrial gene for 16S rRNA, par	AB711635	74	64.71	6.08556e−06	Ephemeroptera	Polymitarcyidae	Ephoron album
Ephoron shigae mitochondrial gene for 16S rRNA, pa	AB711598	74.49	63.4	6.08556e−06	Ephemeroptera	Polymitarcyidae	Ephoron shigae
Ephoron shigae mitochondrial gene for 16S rRNA, pa	AB711533	74.75	63.4	6.08556e−06	Ephemeroptera	Polymitarcyidae	Ephoron shigae
Ephoron shigae mitochondrial gene for 16S rRNA, pa	AB711527	74.75	63.4	6.08556e−06	Ephemeroptera	Polymitarcyidae	Ephoron shigae
Ephoron shigae mitochondrial gene for 16S rRNA, pa	AB711523	74.75	63.4	6.08556e−06	Ephemeroptera	Polymitarcyidae	Ephoron shigae
Ephoron shigae mitochondrial gene for 16S rRNA, pa	AB711500	74.75	63.4	6.08556e−06	Ephemeroptera	Polymitarcyidae	Ephoron shigae
Ephoron shigae mitochondrial gene for 16S rRNA, pa	AB711493	74.75	63.4	6.08556e−06	Ephemeroptera	Polymitarcyidae	Ephoron shigae
Ephoron shigae mitochondrial gene for 16S rRNA, pa	AB711492	74.75	63.4	6.08556e−06	Ephemeroptera	Polymitarcyidae	Ephoron shigae
Ephoron shigae mitochondrial gene for 16S rRNA, pa	AB711471	74.75	63.4	6.08556e−06	Ephemeroptera	Polymitarcyidae	Ephoron shigae
Pantoporia hordonia isolate F97 16S ribosomal RNA	JQ416138	76.09	58.17	6.08556e−06	Lepidoptera	Nymphalidae	Pantoporia hordonia
Euploea mulciber mitochondrion, complete genome	HQ378507	76.09	58.82	6.08556e−06	Lepidoptera	Nymphalidae	Euploea mulciber
Gymnopternus angustifrons voucher Pollet 0052 (P)	EU863971	75	64.05	6.08556e−06	Diptera	Dolichopodidae	Gymnopternus angustifrons
Simulium parahiyangum 16S ribosomal RNA gene, part	GQ865991	75	64.05	6.08556e−06	Diptera	Simuliidae	Simulium parahiyangum
Proboscidoplocia sp. BYU IGCEP162 16S ribosomal RN	GQ118287	75.76	64.05	6.08556e−06	Ephemeroptera	Euthyplociidae	Proboscidoplocia sp. BYU IGCEP162
Attenella margarita 16S ribosomal RNA gene, partia	FJ443027	73.47	64.05	6.08556e−06	Ephemeroptera	Ephemerellidae	Attenella margarita
Limonia hawaiiensis voucher O'Grady 201598 16S rib	EU005405	75	64.05	6.08556e−06	Diptera	Limoniidae	Dicranomyia hawaiiensis
Limonia hawaiiensis voucher O'Grady 200922 16S rib	EU005404	75	64.05	6.08556e−06	Diptera	Limoniidae	Dicranomyia hawaiiensis
Argyreus hyperbius voucher jshao2005025 16S riboso	DQ784692	76.09	58.82	6.08556e−06	Lepidoptera	Nymphalidae	Argynnis hyperbius
Aethriamanta rezia voucher JW_DNA_69 16S ribosomal	EF631501	75.76	63.4	6.08556e−06	Odonata	Libellulidae	Aethriamanta rezia
Argyreus hyperbius 16S ribosomal RNA gene, partial	DQ445107	76.09	58.82	6.08556e−06	Lepidoptera	Nymphalidae	Argynnis hyperbius
Attenella margarita 16S ribosomal RNA gene, partia	AY749805	73.47	64.05	6.08556e−06	Ephemeroptera	Ephemerellidae	Attenella margarita
Leptophlebiidae sp. EP030 16S ribosomal RNA gene,	AY749770	73.47	64.05	6.08556e−06	Ephemeroptera		Leptophlebiidae sp. EP030
Pantoporia hordonia isolate N629 mitochondrion, co	KF590534	75.82	58.17	2.12407e−05	Lepidoptera	Nymphalidae	Pantoporia hordonia
Neptis soma isolate N546 mitochondrion, complete g	KF590533	75.82	58.17	2.12407e−05	Lepidoptera	Nymphalidae	Neptis soma
Ephoron shigae mitochondrial gene for 16S ribosoma	AB874305	74.26	63.4	2.12407e−05	Ephemeroptera	Polymitarcyidae	Ephoron shigae
Ephoron shigae mitochondrial gene for 16S ribosoma	AB874303	74.26	63.4	2.12407e−05	Ephemeroptera	Polymitarcyidae	Ephoron shigae
Ephoron shigae mitochondrial gene for 16S ribosoma	AB874302	74.26	63.4	2.12407e−05	Ephemeroptera	Polymitarcyidae	Ephoron shigae
Ephoron shigae mitochondrial gene for 16S ribosoma	AB874301	74.26	63.4	2.12407e−05	Ephemeroptera	Polymitarcyidae	Ephoron shigae
Ephoron shigae mitochondrial gene for 16S ribosoma	AB874300	74.26	63.4	2.12407e−05	Ephemeroptera	Polymitarcyidae	Ephoron shigae
Ephoron shigae mitochondrial gene for 16S ribosoma	AB874299	74.26	63.4	2.12407e−05	Ephemeroptera	Polymitarcyidae	Ephoron shigae
Ephoron shigae mitochondrial gene for 16S ribosoma	AB874288	74.26	63.4	2.12407e−05	Ephemeroptera	Polymitarcyidae	Ephoron shigae
Ephoron shigae mitochondrial gene for 16S ribosoma	AB874279	74.26	63.4	2.12407e−05	Ephemeroptera	Polymitarcyidae	Ephoron shigae
Ephoron shigae mitochondrial gene for 16S rRNA, pa	AB711531	74.26	63.4	2.12407e−05	Ephemeroptera	Polymitarcyidae	Ephoron shigae
Ephoron shigae mitochondrial gene for 16S rRNA, pa	AB711530	74.26	63.4	2.12407e−05	Ephemeroptera	Polymitarcyidae	Ephoron shigae
Ephoron shigae mitochondrial gene for 16S rRNA, pa	AB711528	74.26	63.4	2.12407e−05	Ephemeroptera	Polymitarcyidae	Ephoron shigae
Ephoron shigae mitochondrial gene for 16S rRNA, pa	AB711521	74.26	63.4	2.12407e−05	Ephemeroptera	Polymitarcyidae	Ephoron shigae
Ephoron shigae mitochondrial gene for 16S rRNA, pa	AB711514	74.26	63.4	2.12407e−05	Ephemeroptera	Polymitarcyidae	Ephoron shigae
Ephoron shigae mitochondrial gene for 16S rRNA, pa	AB711501	74.26	63.4	2.12407e−05	Ephemeroptera	Polymitarcyidae	Ephoron shigae
Ephoron shigae mitochondrial gene for 16S rRNA, pa	AB711482	74.26	63.4	2.12407e−05	Ephemeroptera	Polymitarcyidae	Ephoron shigae
Cafius lithocharinus isolate CNUIC−106 16S ribosom	JF796468	74.75	63.4	2.12407e−05	Coleoptera	Staphylinidae	Cafius lithocharinus
Ephemerella berneri 16S ribosomal RNA gene, partia	FJ443040	73.74	64.71	2.12407e−05	Ephemeroptera	Ephemerellidae	Ephemerella berneri
Caurinella idahoensis 16S ribosomal RNA gene, part	FJ443028	74.23	63.4	2.12407e−05	Ephemeroptera	Ephemerellidae	Caurinella idahoensis
Caenophthalmus capensis voucher MEI 120162 16S rib	DQ250838	74.51	64.05	2.12407e−05	Diptera	Therevidae	Caenophthalmus capensis
Iranotrichia nigra 16S ribosomal RNA gene, partial	KM878921	74	64.05	7.41372e−05	Diptera	Scenopinidae	Iranotrichia nigra
Afidenta godarti voucher KS177 16S ribosomal RNA g	KP143601	75.79	60.78	7.41372e−05	Coleoptera	Coccinellidae	Afidenta godarti
Chileistomus cucullatus voucher SK188 16S ribosoma	KP250278	74.49	62.75	7.41372e−05	Coleoptera	Lucanidae	Chileistomus cucullatus
Mausoleopsis amabilis voucher 842820 16S ribosomal	JX234504	74.75	62.09	7.41372e−05	Coleoptera	Scarabaeidae	Mausoleopsis amabilis
Ephemera orientalis voucher EPH01 tRNA−Leu, 16S ri	KF256907	71.76	84.31	7.41372e−05	Ephemeroptera	Ephemeridae	Ephemera orientalis
Ephoron shigae mitochondrial gene for 16S ribosoma	AB874304	73.47	63.4	7.41372e−05	Ephemeroptera	Polymitarcyidae	Ephoron shigae
Neptis rivularis 16S ribosomal RNA gene, partial s	JX185895	75	58.17	7.41372e−05	Lepidoptera	Nymphalidae	Neptis rivularis
Euroleon nostras isolate 59−nostor1 16S ribosomal	KF321985	74	64.05	7.41372e−05	Neuroptera	Myrmeleontidae	Euroleon nostras
Euroleon nostras isolate 33−nostur1 16S ribosomal	KF321982	74	64.05	7.41372e−05	Neuroptera	Myrmeleontidae	Euroleon nostras
Euroleon nostras isolate 31−nosfe1 16S ribosomal R	KF321980	74	64.05	7.41372e−05	Neuroptera	Myrmeleontidae	Euroleon nostras
Trichosia morio voucher 945 16S ribosomal RNA gene	JQ613960	73.74	62.75	7.41372e−05	Diptera	Sciaridae	Trichosia morio
