# Supplementary material for: Testing the potential of a ribosomal 16S marker for DNA metabarcoding of insects
Source: PeerJ. 2016 Apr 19;4:e1966. doi: 10.7717/peerj.1966 (PMC4841222; doi:10.7717/peerj.1966)

OTU_35.csv

Annitella apfelbecki

Limnephilidae

Trichoptera

93.41

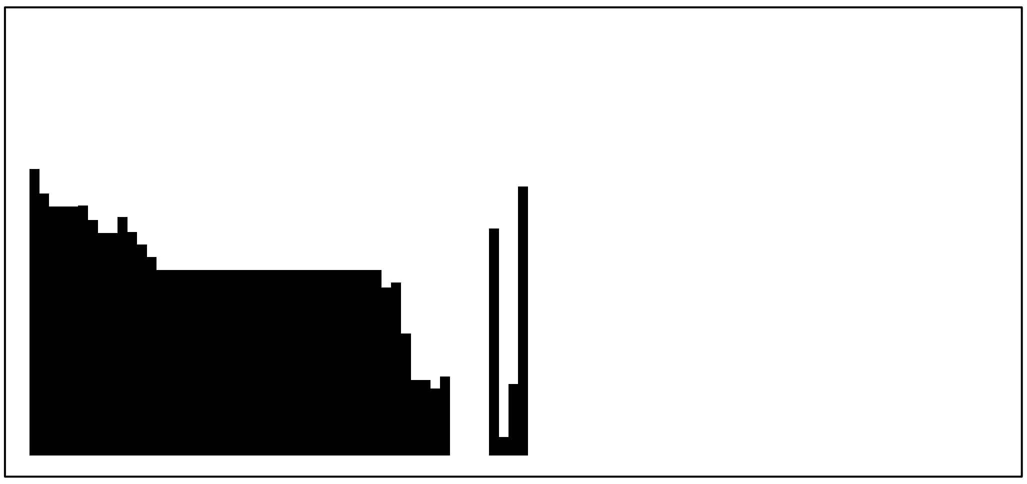

Annitella apfelbecki voucher AaIM1 16S ribosomal R	FJ002819	93.41	100	1.94524e−63	Trichoptera	Limnephilidae	Annitella apfelbecki
Anomalopterygella chauviniana voucher AC001 16S ri	EU215174	92.26	100	3.51708e−60	Trichoptera	Limnephilidae	Anomalopterygella chauviniana
Chaetopteryx fusca voucher ChfIM1 16S ribosomal RN	FJ002820	91.67	100	4.28468e−59	Trichoptera	Limnephilidae	Chaetopteryx fusca
Drusus chrysotus voucher DCh004 16S ribosomal RNA	EU215189	91.67	100	4.28468e−59	Trichoptera	Limnephilidae	Drusus chrysotus
Drusus chrysotus voucher DCh001 16S ribosomal RNA	EU215188	91.67	100	4.28468e−59	Trichoptera	Limnephilidae	Drusus chrysotus
Ecclisocosmoecus spinosus mitochondrial gene for 1	AB355447	91.72	100	1.4955e−58	Trichoptera	Limnephilidae	Ecclisocosmoecus spinosus
Chaetopterygopsis maclachlani voucher CM001 16S ri	EU215176	91.02	100	5.21981e−58	Trichoptera	Limnephilidae	Chaetopterygopsis maclachlani
Chaetopteryx rugulosa voucher CRu002 16S ribosomal	EU215178	90.42	100	1.82189e−57	Trichoptera	Limnephilidae	Chaetopteryx rugulosa
Allogamus uncatus voucher AUn003 16S ribosomal RNA	KM001840	90.42	100	6.35903e−57	Trichoptera	Limnephilidae	Allogamus uncatus
Cryptothrix nebulicola voucher CN006 16S ribosomal	EU215177	91.18	100	6.35903e−57	Trichoptera	Limnephilidae	Cryptothrix nebulicola
Asynarchus sachalinensis mitochondrial gene for 16	AB355450	90.48	100	2.70393e−55	Trichoptera	Limnephilidae	Asynarchus sachalinensis
Rivulophilus sakaii mitochondrial gene for 16S rib	AB355449	89.88	100	1.14974e−53	Trichoptera	Limnephilidae	Rivulophilus sakaii
Hydatophylax festivus mitochondrial gene for 16S r	AB355448	89.29	100	1.40067e−52	Trichoptera	Limnephilidae	Hydatophylax festivus
Ecclisopteryx keroveci voucher E2JAL2 16S ribosoma	KM001845	88.69	100	4.88881e−52	Trichoptera	Limnephilidae	Ecclisopteryx keroveci
Ecclisopteryx keroveci voucher E2JAL1 16S ribosoma	KM001844	88.69	100	4.88881e−52	Trichoptera	Limnephilidae	Ecclisopteryx keroveci
Ecclisopteryx keroveci voucher E1JAL2 16S ribosoma	KM001843	88.69	100	4.88881e−52	Trichoptera	Limnephilidae	Ecclisopteryx keroveci
Ecclisopteryx keroveci voucher E1JAL1 16S ribosoma	KM001842	88.69	100	4.88881e−52	Trichoptera	Limnephilidae	Ecclisopteryx keroveci
Ecclisopteryx ivkae voucher fEda1202L 16S ribosoma	KM001810	88.69	100	4.88881e−52	Trichoptera	Limnephilidae	Ecclisopteryx ivkae
Ecclisopteryx ivkae voucher fEda1201L 16S ribosoma	KM001809	88.69	100	4.88881e−52	Trichoptera	Limnephilidae	Ecclisopteryx ivkae
Ecclisopteryx ivkae voucher fEda0102M 16S ribosoma	KM001808	88.69	100	4.88881e−52	Trichoptera	Limnephilidae	Ecclisopteryx ivkae
Ecclisopteryx ivkae voucher fEda0102F 16S ribosoma	KM001807	88.69	100	4.88881e−52	Trichoptera	Limnephilidae	Ecclisopteryx ivkae
Ecclisopteryx ivkae voucher fEda0101M 16S ribosoma	KM001806	88.69	100	4.88881e−52	Trichoptera	Limnephilidae	Ecclisopteryx ivkae
Ecclisopteryx ivkae voucher fDsp3301L 16S ribosoma	KM001804	88.69	100	4.88881e−52	Trichoptera	Limnephilidae	Ecclisopteryx ivkae
Ecclisopteryx keroveci voucher fEns0101M 16S ribos	KM001784	88.69	100	4.88881e−52	Trichoptera	Limnephilidae	Ecclisopteryx keroveci
Ecclisopteryx keroveci voucher fEda1101F 16S ribos	KM001783	88.69	100	4.88881e−52	Trichoptera	Limnephilidae	Ecclisopteryx keroveci
Ecclisopteryx keroveci voucher fEda1001M 16S ribos	KM001782	88.69	100	4.88881e−52	Trichoptera	Limnephilidae	Ecclisopteryx keroveci
Ecclisopteryx keroveci voucher fEda0301M 16S ribos	KM001781	88.69	100	4.88881e−52	Trichoptera	Limnephilidae	Ecclisopteryx keroveci
Ecclisopteryx keroveci voucher fEda0202F 16S ribos	KM001780	88.69	100	4.88881e−52	Trichoptera	Limnephilidae	Ecclisopteryx keroveci
Ecclisopteryx keroveci voucher fEda0201M 16S ribos	KM001779	88.69	100	4.88881e−52	Trichoptera	Limnephilidae	Ecclisopteryx keroveci
Ecclisopteryx keroveci voucher fDs30109F 16S ribos	KM001778	88.69	100	4.88881e−52	Trichoptera	Limnephilidae	Ecclisopteryx keroveci
Ecclisopteryx keroveci voucher fDs30106F 16S ribos	KM001777	88.69	100	4.88881e−52	Trichoptera	Limnephilidae	Ecclisopteryx keroveci
Ecclisopteryx keroveci voucher fDs30105M 16S ribos	KM001776	88.69	100	4.88881e−52	Trichoptera	Limnephilidae	Ecclisopteryx keroveci
Ecclisopteryx keroveci voucher fDs30103M 16S ribos	KM001775	88.69	100	4.88881e−52	Trichoptera	Limnephilidae	Ecclisopteryx keroveci
Ecclisopteryx keroveci voucher fDs30102M 16S ribos	KM001774	88.69	100	4.88881e−52	Trichoptera	Limnephilidae	Ecclisopteryx keroveci
Ecclisopteryx keroveci voucher fDs30101M 16S ribos	KM001773	88.69	100	4.88881e−52	Trichoptera	Limnephilidae	Ecclisopteryx keroveci
Ecclisopteryx dalecarlica voucher EdIM1 16S riboso	FJ002818	88.69	100	4.88881e−52	Trichoptera	Limnephilidae	Ecclisopteryx dalecarlica
Consorophylax consors voucher CCon001 16S ribosoma	EU215175	87.86	100	4.88881e−52	Trichoptera	Limnephilidae	Consorophylax consors
Ecclisopteryx ivkae voucher fEda0101F 16S ribosoma	KM001805	88.1	100	5.95579e−51	Trichoptera	Limnephilidae	Ecclisopteryx ivkae
Ironoquia punctatissima mitochondrial gene for 16S	AB355446	85.71	100	2.37188e−43	Trichoptera	Limnephilidae	Ironoquia punctatissima
Nothopsyche montivaga mitochondrial gene for 16S r	AB355431	83.53	100	2.22148e−37	Trichoptera	Limnephilidae	Nothopsyche montivaga
Nothopsyche montivaga mitochondrial gene for 16S r	AB355432	83.53	100	2.22148e−37	Trichoptera	Limnephilidae	Nothopsyche montivaga
Dicosmoecus jozankeanus mitochondrial gene for 16S	AB355445	83.12	94.01	4.01653e−34	Trichoptera	Limnephilidae	Dicosmoecus jozankeanus
Taskiropsyche lacustris voucher NHRS−EN CS5 16S ri	FJ263209	83.69	80.84	3.0879e−29	Trichoptera	Kokiriidae	Taskiropsyche lacustris
Eubasilissa regina mitochondrion, complete genome	KF756943	76.19	100	2.22344e−18	Trichoptera		Eubasilissa regina
Myiodactylus osmyloides 16S ribosomal RNA gene, pa	EU734873	75	100	4.89746e−14	Neuroptera	Nymphidae	Myiodactylus osmyloides
Bambusiphaga fascia isolate 181 16S ribosomal RNA	HM233787	70.73	94.61	0.000289465	Hemiptera	Delphacidae	Bambusiphaga fascia
Mycoplasma canadense DNA, complete genome, strain:	AP014631	79.37	34.73	0.00352641	Mycoplasmatales	Mycoplasmataceae	Mycoplasma canadense
Ovis canadensis canadensis isolate 43U chromosome	CP011888	90.62	19.16	0.523366		Bovidae	Ovis canadensis canadensis
Solanum lycopersicum chromosome ch10, complete gen	HG975522	80.85	28.14	1.82673	Solanales	Solanaceae	Solanum lycopersicum
Onchocerca flexuosa genome assembly O_flexuosa_Cor	LM539701	83.33	25.15	6.3759	Spirurida	Onchocercidae	Onchocerca flexuosa
Protopolystoma xenopodis genome assembly P_xenopod	LM682778	92.59	16.17	6.3759	Polyopisthocotylea	Polystomatidae	Protopolystoma xenopodis
Wakarumbia nepeensis (nomen nudum) voucher UPOLMD0	KC538618	77.42	37.13	6.3759	Coleoptera	Lycidae	Wakarumbia nepeensis (nomen nudum)
Wakarumbia linearis voucher UPOLMD0134 NADH dehydr	KC538617	77.42	37.13	6.3759	Coleoptera	Lycidae	Wakarumbia linearis
Wakarumbia nepeensis (nomen nudum) voucher UPOLMD0	KC538613	77.42	37.13	6.3759	Coleoptera	Lycidae	Wakarumbia nepeensis (nomen nudum)

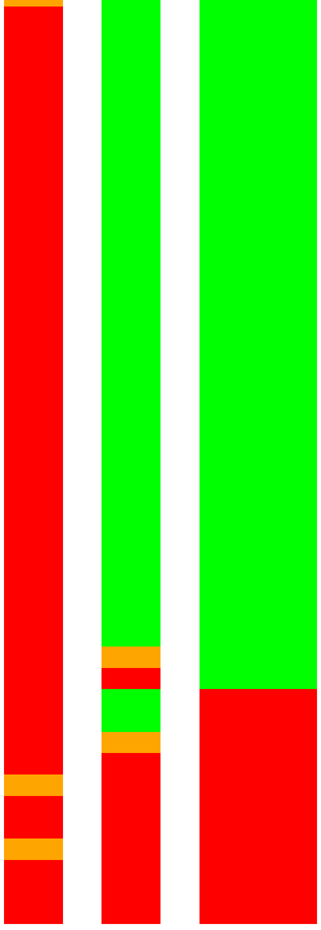

Supplement: Supplemental Information 1 [file peerj-04-1966-s006.zip › scripts/5 match OTUs/blast_hits/OTU_35.csv_plot.pdf]
