# Supplementary material for: Testing the potential of a ribosomal 16S marker for DNA metabarcoding of insects
Source: PeerJ. 2016 Apr 19;4:e1966. doi: 10.7717/peerj.1966 (PMC4841222; doi:10.7717/peerj.1966)
Supplement: Supplemental Information 1 [file peerj-04-1966-s006.zip › scripts/5 match OTUs/blast_hits/OTU_36.csv_plot.pdf]

OTU_36.csv

Allogamus uncatus

Limnephilidae

Trichoptera

89.82

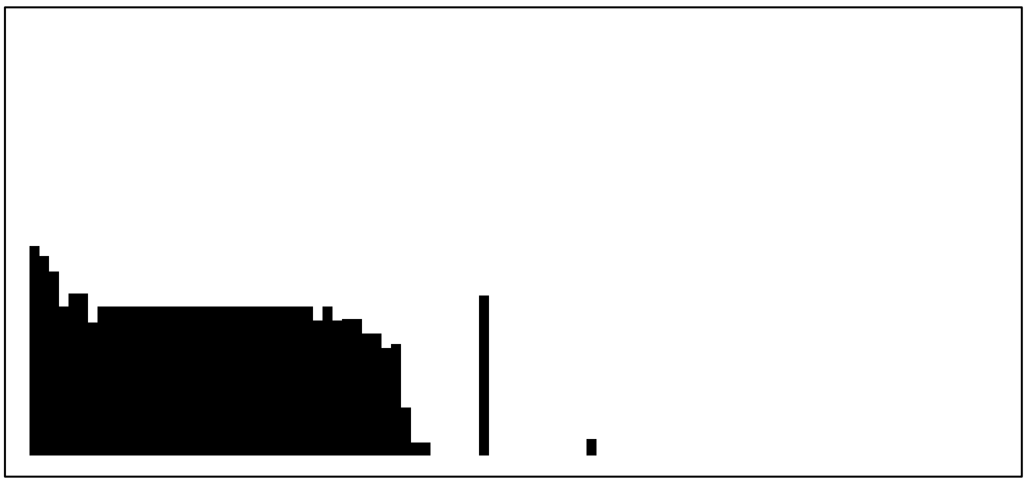

Allogamus uncatus voucher AUn003 16S ribosomal RNA	KM001840	89.82	100	2.70393e−55	Trichoptera	Limnephilidae	Allogamus uncatus
Consorophylax consors voucher CCon001 16S ribosoma	EU215175	89.35	100	4.01298e−53	Trichoptera	Limnephilidae	Consorophylax consors
Annitella apfelbecki voucher AaIM1 16S ribosomal R	FJ002819	88.62	100	1.40067e−52	Trichoptera	Limnephilidae	Annitella apfelbecki
Ecclisocosmoecus spinosus mitochondrial gene for 1	AB355447	86.98	100	1.07683e−47	Trichoptera	Limnephilidae	Ecclisocosmoecus spinosus
Drusus chrysotus voucher DCh004 16S ribosomal RNA	EU215189	87.57	100	1.07683e−47	Trichoptera	Limnephilidae	Drusus chrysotus
Drusus chrysotus voucher DCh001 16S ribosomal RNA	EU215188	87.57	100	1.07683e−47	Trichoptera	Limnephilidae	Drusus chrysotus
Chaetopteryx rugulosa voucher CRu002 16S ribosomal	EU215178	86.23	100	1.07683e−47	Trichoptera	Limnephilidae	Chaetopteryx rugulosa
Ecclisopteryx keroveci voucher E2JAL2 16S ribosoma	KM001845	86.98	100	1.31185e−46	Trichoptera	Limnephilidae	Ecclisopteryx keroveci
Ecclisopteryx keroveci voucher E2JAL1 16S ribosoma	KM001844	86.98	100	1.31185e−46	Trichoptera	Limnephilidae	Ecclisopteryx keroveci
Ecclisopteryx keroveci voucher E1JAL2 16S ribosoma	KM001843	86.98	100	1.31185e−46	Trichoptera	Limnephilidae	Ecclisopteryx keroveci
Ecclisopteryx keroveci voucher E1JAL1 16S ribosoma	KM001842	86.98	100	1.31185e−46	Trichoptera	Limnephilidae	Ecclisopteryx keroveci
Ecclisopteryx ivkae voucher fEda1202L 16S ribosoma	KM001810	86.98	100	1.31185e−46	Trichoptera	Limnephilidae	Ecclisopteryx ivkae
Ecclisopteryx ivkae voucher fEda1201L 16S ribosoma	KM001809	86.98	100	1.31185e−46	Trichoptera	Limnephilidae	Ecclisopteryx ivkae
Ecclisopteryx ivkae voucher fEda0102M 16S ribosoma	KM001808	86.98	100	1.31185e−46	Trichoptera	Limnephilidae	Ecclisopteryx ivkae
Ecclisopteryx ivkae voucher fEda0102F 16S ribosoma	KM001807	86.98	100	1.31185e−46	Trichoptera	Limnephilidae	Ecclisopteryx ivkae
Ecclisopteryx ivkae voucher fEda0101M 16S ribosoma	KM001806	86.98	100	1.31185e−46	Trichoptera	Limnephilidae	Ecclisopteryx ivkae
Ecclisopteryx ivkae voucher fDsp3301L 16S ribosoma	KM001804	86.98	100	1.31185e−46	Trichoptera	Limnephilidae	Ecclisopteryx ivkae
Ecclisopteryx keroveci voucher fEns0101M 16S ribos	KM001784	86.98	100	1.31185e−46	Trichoptera	Limnephilidae	Ecclisopteryx keroveci
Ecclisopteryx keroveci voucher fEda1101F 16S ribos	KM001783	86.98	100	1.31185e−46	Trichoptera	Limnephilidae	Ecclisopteryx keroveci
Ecclisopteryx keroveci voucher fEda1001M 16S ribos	KM001782	86.98	100	1.31185e−46	Trichoptera	Limnephilidae	Ecclisopteryx keroveci
Ecclisopteryx keroveci voucher fEda0301M 16S ribos	KM001781	86.98	100	1.31185e−46	Trichoptera	Limnephilidae	Ecclisopteryx keroveci
Ecclisopteryx keroveci voucher fEda0202F 16S ribos	KM001780	86.98	100	1.31185e−46	Trichoptera	Limnephilidae	Ecclisopteryx keroveci
Ecclisopteryx keroveci voucher fEda0201M 16S ribos	KM001779	86.98	100	1.31185e−46	Trichoptera	Limnephilidae	Ecclisopteryx keroveci
Ecclisopteryx keroveci voucher fDs30109F 16S ribos	KM001778	86.98	100	1.31185e−46	Trichoptera	Limnephilidae	Ecclisopteryx keroveci
Ecclisopteryx keroveci voucher fDs30106F 16S ribos	KM001777	86.98	100	1.31185e−46	Trichoptera	Limnephilidae	Ecclisopteryx keroveci
Ecclisopteryx keroveci voucher fDs30105M 16S ribos	KM001776	86.98	100	1.31185e−46	Trichoptera	Limnephilidae	Ecclisopteryx keroveci
Ecclisopteryx keroveci voucher fDs30103M 16S ribos	KM001775	86.98	100	1.31185e−46	Trichoptera	Limnephilidae	Ecclisopteryx keroveci
Ecclisopteryx keroveci voucher fDs30102M 16S ribos	KM001774	86.98	100	1.31185e−46	Trichoptera	Limnephilidae	Ecclisopteryx keroveci
Ecclisopteryx keroveci voucher fDs30101M 16S ribos	KM001773	86.98	100	1.31185e−46	Trichoptera	Limnephilidae	Ecclisopteryx keroveci
Chaetopteryx fusca voucher ChfIM1 16S ribosomal RN	FJ002820	86.31	100	1.31185e−46	Trichoptera	Limnephilidae	Chaetopteryx fusca
Ecclisopteryx dalecarlica voucher EdIM1 16S riboso	FJ002818	86.98	100	1.31185e−46	Trichoptera	Limnephilidae	Ecclisopteryx dalecarlica
Anomalopterygella chauviniana voucher AC001 16S ri	EU215174	86.31	100	1.31185e−46	Trichoptera	Limnephilidae	Anomalopterygella chauviniana
Ecclisopteryx ivkae voucher fEda0101F 16S ribosoma	KM001805	86.39	100	5.57813e−45	Trichoptera	Limnephilidae	Ecclisopteryx ivkae
Cryptothrix nebulicola voucher CN006 16S ribosomal	EU215177	86.39	100	5.57813e−45	Trichoptera	Limnephilidae	Cryptothrix nebulicola
Hydatophylax festivus mitochondrial gene for 16S r	AB355448	85.71	100	1.94696e−44	Trichoptera	Limnephilidae	Hydatophylax festivus
Asynarchus sachalinensis mitochondrial gene for 16	AB355450	85.71	100	1.94696e−44	Trichoptera	Limnephilidae	Asynarchus sachalinensis
Chaetopterygopsis maclachlani voucher CM001 16S ri	EU215176	85.03	100	1.94696e−44	Trichoptera	Limnephilidae	Chaetopterygopsis maclachlani
Rivulophilus sakaii mitochondrial gene for 16S rib	AB355449	85.21	100	3.52019e−41	Trichoptera	Limnephilidae	Rivulophilus sakaii
Ironoquia punctatissima mitochondrial gene for 16S	AB355446	82.25	100	1.15075e−34	Trichoptera	Limnephilidae	Ironoquia punctatissima
Nothopsyche montivaga mitochondrial gene for 16S r	AB355431	80.59	100	5.96106e−32	Trichoptera	Limnephilidae	Nothopsyche montivaga
Nothopsyche montivaga mitochondrial gene for 16S r	AB355432	80.59	100	5.96106e−32	Trichoptera	Limnephilidae	Nothopsyche montivaga
Dicosmoecus jozankeanus mitochondrial gene for 16S	AB355445	79.5	94.01	1.94868e−25	Trichoptera	Limnephilidae	Dicosmoecus jozankeanus
Taskiropsyche lacustris voucher NHRS−EN CS5 16S ri	FJ263209	78.91	83.83	3.5233e−22	Trichoptera	Kokiriidae	Taskiropsyche lacustris
Myiodactylus osmyloides 16S ribosomal RNA gene, pa	EU734873	75.15	98.8	7.76058e−18	Neuroptera	Nymphidae	Myiodactylus osmyloides
Eubasilissa regina mitochondrion, complete genome	KF756943	75.58	100	1.15177e−15	Trichoptera		Eubasilissa regina
Bambusiphaga fascia isolate 181 16S ribosomal RNA	HM233787	70.34	83.83	1.82673	Hemiptera	Delphacidae	Bambusiphaga fascia
Ovis canadensis canadensis isolate 43U chromosome	CP011888	87.5	19.16	6.3759		Bovidae	Ovis canadensis canadensis
Apis mellifera mellifera isolate 13.4.15 mitochond	KJ396190	69.63	76.65	6.3759	Hymenoptera	Apidae	Apis mellifera mellifera
Apis mellifera mellifera isolate 4.2.15 mitochondr	KJ396189	69.63	76.65	6.3759	Hymenoptera	Apidae	Apis mellifera mellifera
Apis mellifera mellifera isolate 1.4.15 mitochondr	KJ396188	69.63	76.65	6.3759	Hymenoptera	Apidae	Apis mellifera mellifera
Apis mellifera mellifera isolate 2.2.15 mitochondr	KJ396187	69.63	76.65	6.3759	Hymenoptera	Apidae	Apis mellifera mellifera
Apis mellifera mellifera isolate 16.1.5 mitochondr	KJ396186	69.63	76.65	6.3759	Hymenoptera	Apidae	Apis mellifera mellifera
Apis mellifera mellifera isolate 15.4.25 mitochond	KJ396184	69.63	76.65	6.3759	Hymenoptera	Apidae	Apis mellifera mellifera
Apis mellifera mellifera isolate 22.2.5 mitochondr	KJ396182	69.63	76.65	6.3759	Hymenoptera	Apidae	Apis mellifera mellifera
Apis mellifera scutellata mitochondrion, complete	KJ601784	69.63	76.65	6.3759	Hymenoptera	Apidae	Apis mellifera scutellata
Gracilaria salicornia chloroplast, complete genome	KF861575	74.24	35.93	6.3759	Gracilariales	Gracilariaceae	Gracilaria salicornia
Cyberlindnera suaveolens strain CBS 1670 mitochond	KC993193	76.12	40.12	6.3759	Saccharomycetales	Phaffomycetaceae	Cyberlindnera suaveolens
Naegleria gruberi predicted protein, mRNA	XM_002675263	80.77	28.14	6.3759	Schizopyrenida	Vahlkampfiidae	Naegleria gruberi strain NEG−M
Apis mellifera scutellata isolate WSS5455 cytochro	EF184029	69.63	76.65	6.3759	Hymenoptera	Apidae	Apis mellifera scutellata
Apis mellifera cytochrome b (cytb) gene, partial c	EF184030	69.63	76.65	6.3759	Hymenoptera	Apidae	Apis mellifera
Apis mellifera scutellata isolate WSS8554 cytochro	EF184027	69.63	76.65	6.3759	Hymenoptera	Apidae	Apis mellifera scutellata
Apis mellifera scutellata isolate WSS5449 cytochro	EF184025	69.63	76.65	6.3759	Hymenoptera	Apidae	Apis mellifera scutellata
