# Supplementary material for: Testing the potential of a ribosomal 16S marker for DNA metabarcoding of insects
Source: PeerJ. 2016 Apr 19;4:e1966. doi: 10.7717/peerj.1966 (PMC4841222; doi:10.7717/peerj.1966)
Supplement: Supplemental Information 1 [file peerj-04-1966-s006.zip › scripts/5 match OTUs/blast_hits/OTU_366.csv_plot.pdf]

OTU_366.csv

Rhithrogena semicolorata

Heptageniidae

Ephemeroptera

99.33

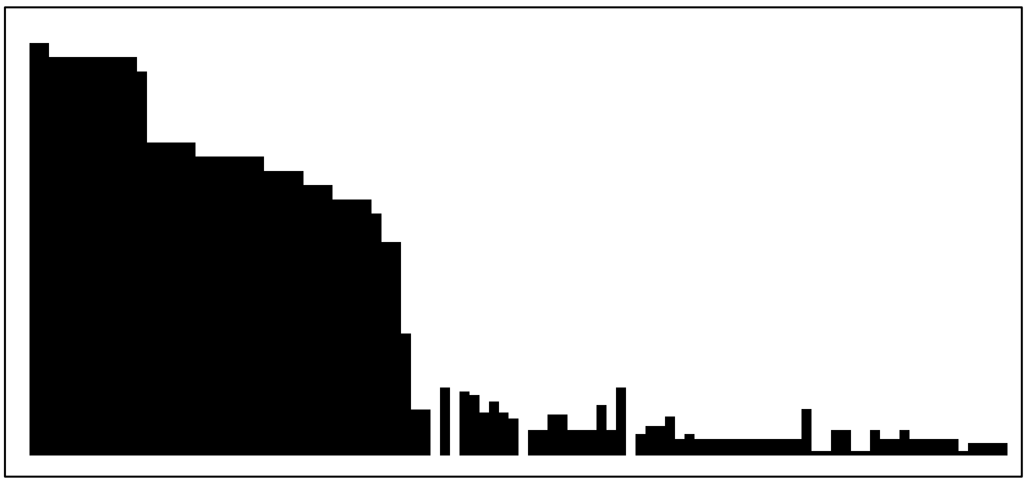

Rhithrogena semicolorata mitochondrial partial 16S	LN868673	99.33	98.04	7.89472e−68	Ephemeroptera	Heptageniidae	Rhithrogena semicolorata
Rhithrogena sp. 4 LV−2015 mitochondrial partial 16	LN868652	99.33	98.04	7.89472e−68	Ephemeroptera	Heptageniidae	Rhithrogena sp. 4 LV−2015
Rhithrogena sp. 13 LV−2015 mitochondrial partial 1	LN868672	98.67	98.04	9.61773e−67	Ephemeroptera	Heptageniidae	Rhithrogena sp. 13 LV−2015
Rhithrogena sp. 12 LV−2015 mitochondrial partial 1	LN868671	98.67	98.04	9.61773e−67	Ephemeroptera	Heptageniidae	Rhithrogena sp. 12 LV−2015
Rhithrogena picteti mitochondrial partial 16S rRNA	LN868663	98.67	98.04	9.61773e−67	Ephemeroptera	Heptageniidae	Rhithrogena picteti
Rhithrogena sp. 3 LV−2015 mitochondrial partial 16	LN868659	98.67	98.04	9.61773e−67	Ephemeroptera	Heptageniidae	Rhithrogena sp. 3 LV−2015
Rhithrogena fonticola mitochondrial partial 16S rR	LN868656	98.67	98.04	9.61773e−67	Ephemeroptera	Heptageniidae	Rhithrogena fonticola
Rhithrogena sp. 2 LV−2015 mitochondrial partial 16	LN868654	98.67	98.04	9.61773e−67	Ephemeroptera	Heptageniidae	Rhithrogena sp. 2 LV−2015
Rhithrogena puytoraci mitochondrial partial 16S rR	LN868651	98.67	98.04	9.61773e−67	Ephemeroptera	Heptageniidae	Rhithrogena puytoraci
Rhithrogena iridina mitochondrial partial 16S rRNA	LN868646	98.67	98.04	9.61773e−67	Ephemeroptera	Heptageniidae	Rhithrogena iridina
Rhithrogena taurisca mitochondrial partial 16S rRN	LN868638	98.67	98.04	9.61773e−67	Ephemeroptera	Heptageniidae	Rhithrogena taurisca
Rhithrogena sp. 5 LV−2015 mitochondrial partial 16	LN868653	98	98.04	4.08956e−65	Ephemeroptera	Heptageniidae	Rhithrogena sp. 5 LV−2015
Rhithrogena sp. 7 LV−2015 mitochondrial partial 16	LN868669	94.67	98.04	1.62866e−57	Ephemeroptera	Heptageniidae	Rhithrogena sp. 7 LV−2015
Rhithrogena eatoni mitochondrial partial 16S rRNA	LN868666	94.67	98.04	1.62866e−57	Ephemeroptera	Heptageniidae	Rhithrogena eatoni
Rhithrogena alpestris mitochondrial partial 16S rR	LN868657	94.67	98.04	1.62866e−57	Ephemeroptera	Heptageniidae	Rhithrogena alpestris
Rhithrogena gratianopolitana mitochondrial partial	LN868647	94.67	98.04	1.62866e−57	Ephemeroptera	Heptageniidae	Rhithrogena gratianopolitana
Rhithrogena nivata mitochondrial partial 16S rRNA	LN868643	94.67	98.04	1.62866e−57	Ephemeroptera	Heptageniidae	Rhithrogena nivata
Rhithrogena degrangei mitochondrial partial 16S rR	LN868674	94	98.04	6.92524e−56	Ephemeroptera	Heptageniidae	Rhithrogena degrangei
Rhithrogena corcontica mitochondrial partial 16S r	LN868662	94	98.04	6.92524e−56	Ephemeroptera	Heptageniidae	Rhithrogena corcontica
Rhithrogena sp. 9 LV−2015 mitochondrial partial 16	LN868650	94	98.04	6.92524e−56	Ephemeroptera	Heptageniidae	Rhithrogena sp. 9 LV−2015
Rhithrogena gratianopolitana mitochondrial partial	LN868648	94	98.04	6.92524e−56	Ephemeroptera	Heptageniidae	Rhithrogena gratianopolitana
Rhithrogena landai mitochondrial partial 16S rRNA	LN868644	94	98.04	6.92524e−56	Ephemeroptera	Heptageniidae	Rhithrogena landai
Rhithrogena hybrida mitochondrial partial 16S rRNA	LN868642	94	98.04	6.92524e−56	Ephemeroptera	Heptageniidae	Rhithrogena hybrida
Rhithrogena circumtatrica mitochondrial partial 16	LN868641	94	98.04	6.92524e−56	Ephemeroptera	Heptageniidae	Rhithrogena circumtatrica
Rhithrogena sp. 11 LV−2015 mitochondrial partial 1	LN868668	93.33	98.04	8.43667e−55	Ephemeroptera	Heptageniidae	Rhithrogena sp. 11 LV−2015
Rhithrogena sp. 8 LV−2015 mitochondrial partial 16	LN868661	93.33	98.04	8.43667e−55	Ephemeroptera	Heptageniidae	Rhithrogena sp. 8 LV−2015
Rhithrogena sp. 6 LV−2015 mitochondrial partial 16	LN868655	93.33	98.04	8.43667e−55	Ephemeroptera	Heptageniidae	Rhithrogena sp. 6 LV−2015
Rhithrogena diensis mitochondrial partial 16S rRNA	LN868637	93.33	98.04	8.43667e−55	Ephemeroptera	Heptageniidae	Rhithrogena diensis
Rhithrogena germanica mitochondrial partial 16S rR	LN868660	92.67	98.04	2.94469e−54	Ephemeroptera	Heptageniidae	Rhithrogena germanica
Rhithrogena beskidensis mitochondrial partial 16S	LN868645	92.67	98.04	2.94469e−54	Ephemeroptera	Heptageniidae	Rhithrogena beskidensis
Rhithrogena nuragica mitochondrial partial 16S rRN	LN868667	92.67	98.04	3.58736e−53	Ephemeroptera	Heptageniidae	Rhithrogena nuragica
Rhithrogena sp. 10 LV−2015 mitochondrial partial 1	LN868664	92	98.04	3.58736e−53	Ephemeroptera	Heptageniidae	Rhithrogena sp. 10 LV−2015
Rhithrogena savoiensis mitochondrial partial 16S r	LN868658	92	98.04	3.58736e−53	Ephemeroptera	Heptageniidae	Rhithrogena savoiensis
Rhithrogena insularis mitochondrial partial 16S rR	LN868665	92	98.04	4.3703e−52	Ephemeroptera	Heptageniidae	Rhithrogena insularis
Rhithrogena grischuna mitochondrial partial 16S rR	LN868639	92	98.04	4.3703e−52	Ephemeroptera	Heptageniidae	Rhithrogena grischuna
Rhithrogena allobrogica mitochondrial partial 16S	LN868640	91.33	98.04	1.8583e−50	Ephemeroptera	Heptageniidae	Rhithrogena allobrogica
Rhithrogena sartorii mitochondrial partial 16S rRN	LN868670	90	98.04	7.90169e−49	Ephemeroptera	Heptageniidae	Rhithrogena sartorii
Rhithrogena dorieri mitochondrial partial 16S rRNA	LN868649	90	98.04	7.90169e−49	Ephemeroptera	Heptageniidae	Rhithrogena dorieri
Rhithrogena sp. EP021 16S ribosomal RNA gene, part	AY749765	85.71	91.5	6.93136e−37	Ephemeroptera	Heptageniidae	Rhithrogena sp. EP021
Cinygmula sp. m185 mitochondrial partial 16S rRNA	LN868675	82.14	91.5	7.90868e−30	Ephemeroptera	Heptageniidae	Cinygmula sp. m185
Cinygmula sp. EP013 16S ribosomal RNA gene, partia	AY749760	82.14	91.5	7.90868e−30	Ephemeroptera	Heptageniidae	Cinygmula sp. EP013
Paegniodes cupulatus mitochondrion, complete genom	HM004123	77.3	92.16	4.67444e−20	Ephemeroptera	Heptageniidae	Paegniodes cupulatus
Cultus pilatus 16S ribosomal RNA gene, partial seq	EF623212	83.17	66.01	1.98762e−18	Plecoptera	Perlodidae	Cultus pilatus
Heptagenia sp. EP018 16S ribosomal RNA gene, parti	AY749762	77.54	89.54	1.98762e−18	Ephemeroptera	Heptageniidae	Heptagenia sp. EP018
Dipteromimus sp. Ofunato−1 mitochondrial gene for	AB110236	83	65.36	6.93748e−18	Ephemeroptera	Dipteromimidae	Dipteromimus sp. Ofunato−1
Arthroplea bipunctata 16S ribosomal RNA gene, part	AY749817	82.83	64.71	2.42142e−17	Ephemeroptera	Arthropleidae	Arthroplea bipunctata
Remenus bilobatus 16S ribosomal RNA gene, partial	EF623233	82	65.36	8.45159e−17	Plecoptera	Perlodidae	Remenus bilobatus
Capnura wanica 16S ribosomal RNA gene, partial seq	EF623128	82.52	66.01	8.45159e−17	Plecoptera	Capniidae	Capnura wanica
Tasmanophlebia sp. EP158 16S ribosomal RNA gene, p	AY749825	82	65.36	8.45159e−17	Ephemeroptera	Oniscigastridae	Tasmanophlebia sp. EP158
Apteroperla tikumana mitochondrion, complete genom	KR604721	81.73	66.01	2.94989e−16	Plecoptera	Capniidae	Apteroperla tikumana
Chiloporter sp. EP051 16S ribosomal RNA gene, part	AY749776	77.86	84.97	2.94989e−16	Ephemeroptera	Ameletopsidae	Chiloporter sp. EP051
Neopanorpa pulchra mitochondrion, complete genome	JX569848	81.19	66.01	1.02961e−15	Mecoptera	Panorpidae	Neopanorpa pulchra
Neopanorpa pulchra mitochondrion, complete genome	FJ169955	81.19	66.01	1.02961e−15	Mecoptera	Panorpidae	Neopanorpa pulchra
Isoperla oxylepis 16S ribosomal RNA gene, partial	EF623221	81.9	67.97	1.02961e−15	Plecoptera	Perlodidae	Isoperla oxylepis
Isoperla davisi 16S ribosomal RNA gene, partial se	EF623206	81.9	67.97	1.02961e−15	Plecoptera	Perlodidae	Isoperla davisi
Thaumatoperla sp. BYU_PL109 16S ribosomal RNA gene	EF623149	81.19	66.01	1.02961e−15	Plecoptera	Eustheniidae	Thaumatoperla sp. BYU_PL109
Penturoperla barbata 16S ribosomal RNA gene, parti	EF623124	81.19	66.01	1.02961e−15	Plecoptera	Austroperlidae	Penturoperla barbata
Neopanorpa sp. M24899 16S ribosomal RNA gene, part	AF180059	81.19	66.01	1.02961e−15	Mecoptera	Panorpidae	Neopanorpa sp. M24899
Analetris eximia 16S ribosomal RNA gene, partial s	AY749797	82.35	66.01	1.02961e−15	Ephemeroptera	Acanthametropodidae	Analetris eximia
Siphlonurus sp. EP084 16S ribosomal RNA gene, part	AY749786	81.19	66.01	1.02961e−15	Ephemeroptera	Siphlonuridae	Siphlonurus sp. EP084
Ephoron eophilum mitochondrial gene for 16S rRNA,	AB711631	83.17	65.36	3.59371e−15	Ephemeroptera	Polymitarcyidae	Ephoron eophilum
Bolotoperla rossi 16S ribosomal RNA gene, partial	EF623269	79.82	71.9	3.59371e−15	Plecoptera	Taeniopterygidae	Bolotoperla rossi
Pteronarcys californica 16S ribosomal RNA gene, pa	EF623261	81	65.36	3.59371e−15	Plecoptera	Pteronarcyidae	Pteronarcys californica
Perlinodes aurea 16S ribosomal RNA gene, partial s	EF623234	81.37	66.01	3.59371e−15	Plecoptera	Perlodidae	Perlinodes aurea
Austronemoura sp. BYU_PL149 16S ribosomal RNA gene	EF623200	81.37	66.01	3.59371e−15	Plecoptera	Notonemouridae	Austronemoura sp. BYU_PL149
Perlomyia utahensis 16S ribosomal RNA gene, partia	EF623179	81.82	64.05	3.59371e−15	Plecoptera	Leuctridae	Perlomyia utahensis
Chloroperla tripunctata 16S ribosomal RNA gene, pa	EF623137	80.77	67.32	3.59371e−15	Plecoptera	Chloroperlidae	Chloroperla tripunctata
Pteronarcys princeps mitochondrion, complete genom	AY687866	81	65.36	3.59371e−15	Plecoptera	Pteronarcyidae	Pteronarcys princeps
Mesocapnia arizonensis mitochondrion, partial geno	KP642637	80.77	66.01	1.25433e−14	Plecoptera	Capniidae	Mesocapnia arizonensis
Allocapnia recta isolate H19 16S ribosomal RNA gen	KC881054	80.77	66.01	1.25433e−14	Plecoptera	Capniidae	Allocapnia recta
Allocapnia recta isolate H16 16S ribosomal RNA gen	KC881051	80.77	66.01	1.25433e−14	Plecoptera	Capniidae	Allocapnia recta
Allocapnia recta isolate H15 16S ribosomal RNA gen	KC881050	80.77	66.01	1.25433e−14	Plecoptera	Capniidae	Allocapnia recta
Allocapnia recta isolate H14 16S ribosomal RNA gen	KC881049	80.77	66.01	1.25433e−14	Plecoptera	Capniidae	Allocapnia recta
Allocapnia recta isolate H13 16S ribosomal RNA gen	KC881048	80.77	66.01	1.25433e−14	Plecoptera	Capniidae	Allocapnia recta
Allocapnia recta isolate H11 16S ribosomal RNA gen	KC881046	80.77	66.01	1.25433e−14	Plecoptera	Capniidae	Allocapnia recta
Allocapnia recta isolate H10 16S ribosomal RNA gen	KC881045	80.77	66.01	1.25433e−14	Plecoptera	Capniidae	Allocapnia recta
Allocapnia recta isolate H7 16S ribosomal RNA gene	KC881042	80.77	66.01	1.25433e−14	Plecoptera	Capniidae	Allocapnia recta
Allocapnia recta isolate H6 16S ribosomal RNA gene	KC881041	80.77	66.01	1.25433e−14	Plecoptera	Capniidae	Allocapnia recta
Allocapnia recta isolate H3 16S ribosomal RNA gene	KC881038	80.77	66.01	1.25433e−14	Plecoptera	Capniidae	Allocapnia recta
Simocephalus sp. EIZ−2011 isolate Brb1−Sim1 16S ri	JN874613	82.18	64.71	1.25433e−14	Diplostraca	Daphniidae	Simocephalus sp. EIZ−2011
Dolichopoda hyrcana voucher LEN 16S ribosomal RNA	EU887871	80.2	66.01	1.25433e−14	Orthoptera	Rhaphidophoridae	Dolichopoda hyrcana
Pteronarcys sachalina 16S ribosomal RNA gene, part	EF623264	80.2	66.01	1.25433e−14	Plecoptera	Pteronarcyidae	Pteronarcys sachalina
Salmoperla sylvanica 16S ribosomal RNA gene, parti	EF623222	81.19	65.36	1.25433e−14	Plecoptera	Perlodidae	Salmoperla sylvanica
Megarcys signata 16S ribosomal RNA gene, partial s	EF623215	81.19	65.36	1.25433e−14	Plecoptera	Perlodidae	Megarcys signata
Kimminsoperla albomacula 16S ribosomal RNA gene, p	EF623202	80.2	66.01	1.25433e−14	Plecoptera	Notonemouridae	Kimminsoperla albomacula
Eusthenia costalis 16S ribosomal RNA gene, partial	EF623150	80.2	66.01	1.25433e−14	Plecoptera	Eustheniidae	Eusthenia costalis
Diamphipnopsis sp. BYU_PL138 16S ribosomal RNA gen	EF623145	81.19	65.36	1.25433e−14	Plecoptera	Diamphipnoidae	Diamphipnopsis sp. BYU_PL138
Allocapnia minima 16S ribosomal RNA gene, partial	EF623130	80.77	66.01	1.25433e−14	Plecoptera	Capniidae	Allocapnia minima
Mesocapnia sp. BYU_PL010 16S ribosomal RNA gene, p	EF623127	80.77	66.01	1.25433e−14	Plecoptera	Capniidae	Mesocapnia sp. BYU_PL010
Tasmanoperla larvalis 16S ribosomal RNA gene, part	EF623125	81.19	65.36	1.25433e−14	Plecoptera	Austroperlidae	Tasmanoperla larvalis
Allocapnia recta haplotype 12ar: H12 16S ribosomal	EU306875	80.77	66.01	1.25433e−14	Plecoptera	Capniidae	Allocapnia recta
Allocapnia recta haplotype 11ar: H011 16S ribosoma	EU306874	80.77	66.01	1.25433e−14	Plecoptera	Capniidae	Allocapnia recta
Allocapnia recta haplotype 7ar: H07 16S ribosomal	EU306870	80.77	66.01	1.25433e−14	Plecoptera	Capniidae	Allocapnia recta
Allocapnia recta haplotype 3ar: H03 16S ribosomal	DQ915181	80.77	66.01	1.25433e−14	Plecoptera	Capniidae	Allocapnia recta
Allocapnia recta haplotype 2ar: H02 16S ribosomal	DQ915180	80.77	66.01	1.25433e−14	Plecoptera	Capniidae	Allocapnia recta
Osmylus sp. EH−2004 16S ribosomal RNA gene, partia	AY620146	80.2	66.01	1.25433e−14	Neuroptera	Osmylidae	Osmylus sp. EH−2004
Allocapnia recta isolate H18 16S ribosomal RNA gen	KC881053	80.58	65.36	4.37803e−14	Plecoptera	Capniidae	Allocapnia recta
Allocapnia recta isolate H17 16S ribosomal RNA gen	KC881052	80.58	65.36	4.37803e−14	Plecoptera	Capniidae	Allocapnia recta
Allocapnia recta isolate H12 16S ribosomal RNA gen	KC881047	80.58	65.36	4.37803e−14	Plecoptera	Capniidae	Allocapnia recta
Allocapnia recta isolate H9 16S ribosomal RNA gene	KC881044	80.58	65.36	4.37803e−14	Plecoptera	Capniidae	Allocapnia recta
