# Supplementary material for: Testing the potential of a ribosomal 16S marker for DNA metabarcoding of insects
Source: PeerJ. 2016 Apr 19;4:e1966. doi: 10.7717/peerj.1966 (PMC4841222; doi:10.7717/peerj.1966)
Supplement: Supplemental Information 1 [file peerj-04-1966-s006.zip › scripts/5 match OTUs/blast_hits/OTU_369.csv_plot.pdf]

OTU_369.csv

Dinocras cephalotes

Perlidae

Plecoptera

96.95

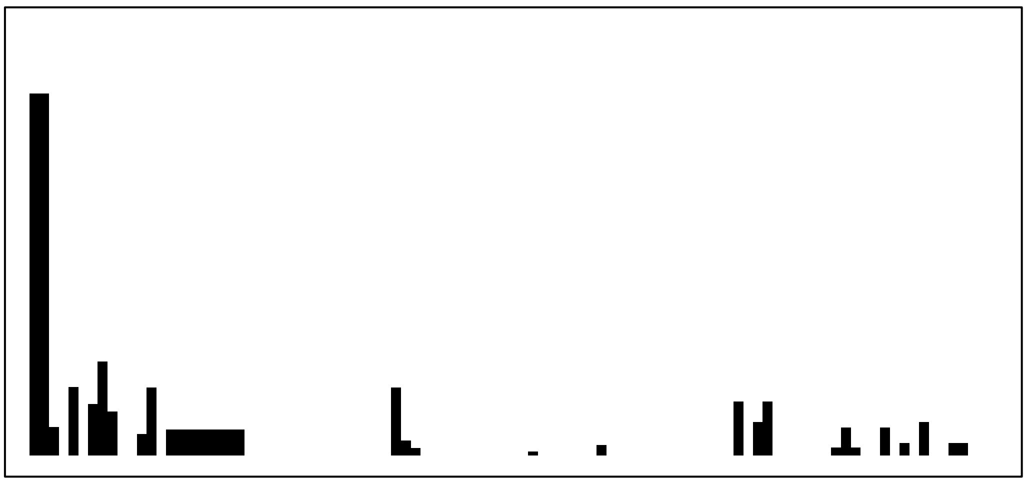

Dinocras cephalotes mitochondrion, complete genome	KF484757	96.95	100	7.02956e−69	Plecoptera	Perlidae	Dinocras cephalotes
Dinocras cephalotes 16S ribosomal RNA gene, partia	EF623244	96.95	100	7.02956e−69	Plecoptera	Perlidae	Dinocras cephalotes
Calineuria californica 16S ribosomal RNA gene, par	EF623239	81.33	90.8	1.04512e−28	Plecoptera	Perlidae	Calineuria californica
Perlinella drymo 16S ribosomal RNA gene, partial s	EF623240	79.72	87.73	6.59545e−25	Plecoptera	Perlidae	Perlinella drymo
Agnetina capitata 16S ribosomal RNA gene, partial	EF623241	83.19	73.01	2.30204e−24	Plecoptera	Perlidae	Agnetina capitata
Taenionema palladium 16S ribosomal RNA gene, parti	EF623267	77.44	99.39	2.80446e−23	Plecoptera	Taeniopterygidae	Taenionema palladium
Perlesta decipiens 16S ribosomal RNA gene, partial	EF623242	82.4	76.69	2.80446e−23	Plecoptera	Perlidae	Perlesta decipiens
Hansonoperla appalachia 16S ribosomal RNA gene, pa	EF623249	84.4	66.26	9.78852e−23	Plecoptera	Perlidae	Hansonoperla appalachia
Leuctra tenuis haplotype 5lt: HO5 16S ribosomal RN	EU327677	82.05	71.78	9.78852e−23	Plecoptera	Leuctridae	Leuctra tenuis
Moselia infuscata 16S ribosomal RNA gene, partial	EF623175	76.83	100	3.41653e−22	Plecoptera	Leuctridae	Moselia infuscata
Claassenia sabulosa 16S ribosomal RNA gene, partia	EF623250	78.15	92.64	1.19249e−21	Plecoptera	Perlidae	Claassenia sabulosa
Despaxia augusta 16S ribosomal RNA gene, partial s	EF623177	80.99	73.62	1.19249e−21	Plecoptera	Leuctridae	Despaxia augusta
Kathroperla perdita 16S ribosomal RNA gene, partia	EF623135	83.18	65.64	1.19249e−21	Plecoptera	Chloroperlidae	Kathroperla perdita
Plumiperla diversa 16S ribosomal RNA gene, partial	EF623132	78.38	89.57	1.19249e−21	Plecoptera	Chloroperlidae	Plumiperla diversa
Leuctra tenuis haplotype 10lt: HO10 16S ribosomal	EU327682	81.2	71.78	1.19249e−21	Plecoptera	Leuctridae	Leuctra tenuis
Leuctra tenuis haplotype 9lt: HO9 16S ribosomal RN	EU327681	81.2	71.78	1.19249e−21	Plecoptera	Leuctridae	Leuctra tenuis
Leuctra tenuis haplotype 8lt: HO8 16S ribosomal RN	EU327680	81.2	71.78	1.19249e−21	Plecoptera	Leuctridae	Leuctra tenuis
Leuctra tenuis haplotype 7lt: HO7 16S ribosomal RN	EU327679	81.2	71.78	1.19249e−21	Plecoptera	Leuctridae	Leuctra tenuis
Leuctra tenuis haplotype 6lt: HO6 16S ribosomal RN	EU327678	81.2	71.78	1.19249e−21	Plecoptera	Leuctridae	Leuctra tenuis
Leuctra tenuis haplotype 3lt: H03 16S ribosomal RN	DQ915184	81.2	71.78	1.19249e−21	Plecoptera	Leuctridae	Leuctra tenuis
Leuctra tenuis haplotype 2lt: H02 16S ribosomal RN	DQ915183	81.2	71.78	1.19249e−21	Plecoptera	Leuctridae	Leuctra tenuis
Leuctra tenuis haplotype 1lt: H01 16S ribosomal RN	DQ915182	81.2	71.78	1.19249e−21	Plecoptera	Leuctridae	Leuctra tenuis
Prostoia besametsa 16S ribosomal RNA gene, partial	EF623188	77.36	96.93	4.16218e−21	Plecoptera	Nemouridae	Prostoia besametsa
Allocapnia recta isolate H18 16S ribosomal RNA gen	KC881053	77.78	87.73	1.45275e−20	Plecoptera	Capniidae	Allocapnia recta
Allocapnia recta isolate H17 16S ribosomal RNA gen	KC881052	77.78	87.73	1.45275e−20	Plecoptera	Capniidae	Allocapnia recta
Allocapnia recta isolate H12 16S ribosomal RNA gen	KC881047	77.78	87.73	1.45275e−20	Plecoptera	Capniidae	Allocapnia recta
Allocapnia recta isolate H9 16S ribosomal RNA gene	KC881044	77.78	87.73	1.45275e−20	Plecoptera	Capniidae	Allocapnia recta
Allocapnia recta isolate H8 16S ribosomal RNA gene	KC881043	77.78	87.73	1.45275e−20	Plecoptera	Capniidae	Allocapnia recta
Allocapnia recta isolate H5 16S ribosomal RNA gene	KC881040	77.78	87.73	1.45275e−20	Plecoptera	Capniidae	Allocapnia recta
Allocapnia recta isolate H4 16S ribosomal RNA gene	KC881039	77.78	87.73	1.45275e−20	Plecoptera	Capniidae	Allocapnia recta
Pteronarcys sachalina 16S ribosomal RNA gene, part	EF623264	77.93	87.73	1.45275e−20	Plecoptera	Pteronarcyidae	Pteronarcys sachalina
Cosumnoperla hypocrena 16S ribosomal RNA gene, par	EF623226	76.13	95.09	1.45275e−20	Plecoptera	Perlodidae	Cosumnoperla hypocrena
Neaviperla forcipata 16S ribosomal RNA gene, parti	EF623139	77.62	87.73	1.45275e−20	Plecoptera	Chloroperlidae	Neaviperla forcipata
Allocapnia recta haplotype 10ar: H010 16S ribosoma	EU306873	77.78	87.73	1.45275e−20	Plecoptera	Capniidae	Allocapnia recta
Allocapnia recta haplotype 9ar: H09 16S ribosomal	EU306872	77.78	87.73	1.45275e−20	Plecoptera	Capniidae	Allocapnia recta
Allocapnia recta haplotype 8ar: H08 16S ribosomal	EU306871	77.78	87.73	1.45275e−20	Plecoptera	Capniidae	Allocapnia recta
Allocapnia recta haplotype 1ar: H01 16S ribosomal	DQ915179	77.78	87.73	1.45275e−20	Plecoptera	Capniidae	Allocapnia recta
Nemoura cinerea 16S ribosomal RNA gene, partial se	EF623194	83.18	65.64	5.07058e−20	Plecoptera	Nemouridae	Nemoura cinerea
Suwallia teleckojensis 16S ribosomal RNA gene, par	EF623142	80.7	69.94	5.07058e−20	Plecoptera	Chloroperlidae	Suwallia teleckojensis
Leuctra tenuis haplotype 4lt: HO4 16S ribosomal RN	EU327676	80.34	71.78	5.07058e−20	Plecoptera	Leuctridae	Leuctra tenuis
Allocapnia recta isolate H19 16S ribosomal RNA gen	KC881054	77.08	87.73	1.76981e−19	Plecoptera	Capniidae	Allocapnia recta
Allocapnia recta isolate H16 16S ribosomal RNA gen	KC881051	77.08	87.73	1.76981e−19	Plecoptera	Capniidae	Allocapnia recta
Allocapnia recta isolate H15 16S ribosomal RNA gen	KC881050	77.08	87.73	1.76981e−19	Plecoptera	Capniidae	Allocapnia recta
Allocapnia recta isolate H14 16S ribosomal RNA gen	KC881049	77.08	87.73	1.76981e−19	Plecoptera	Capniidae	Allocapnia recta
Allocapnia recta isolate H10 16S ribosomal RNA gen	KC881045	77.08	87.73	1.76981e−19	Plecoptera	Capniidae	Allocapnia recta
Allocapnia recta isolate H6 16S ribosomal RNA gene	KC881041	77.08	87.73	1.76981e−19	Plecoptera	Capniidae	Allocapnia recta
Dolichopoda bormansi isolate BRA3 16S ribosomal RN	AY507605	76.33	99.39	1.76981e−19	Orthoptera	Rhaphidophoridae	Dolichopoda bormansi
Acroneuria lycorias 16S ribosomal RNA gene, partia	EF623251	75.45	99.39	1.76981e−19	Plecoptera	Perlidae	Acroneuria lycorias
Skwala americana 16S ribosomal RNA gene, partial s	EF623217	77.08	87.73	1.76981e−19	Plecoptera	Perlodidae	Skwala americana
Paranemoura claasseni 16S ribosomal RNA gene, part	EF623185	76.92	95.09	1.76981e−19	Plecoptera	Nemouridae	Paranemoura claasseni
Shipsa rotunda 16S ribosomal RNA gene, partial seq	EF623184	76.92	95.09	1.76981e−19	Plecoptera	Nemouridae	Shipsa rotunda
Leuctra inermis 16S ribosomal RNA gene, partial se	EF623176	80.17	73.62	1.76981e−19	Plecoptera	Leuctridae	Leuctra inermis
Allocapnia recta haplotype 11ar: H011 16S ribosoma	EU306874	77.08	87.73	1.76981e−19	Plecoptera	Capniidae	Allocapnia recta
Allocapnia recta haplotype 7ar: H07 16S ribosomal	EU306870	77.08	87.73	1.76981e−19	Plecoptera	Capniidae	Allocapnia recta
Allocapnia recta haplotype 2ar: H02 16S ribosomal	DQ915180	77.08	87.73	1.76981e−19	Plecoptera	Capniidae	Allocapnia recta
Dolichopoda bormansi isolate BRA 16S ribosomal RNA	AY793578	76.33	99.39	1.76981e−19	Orthoptera	Rhaphidophoridae	Dolichopoda bormansi
Styloperla sp. mitochondrion, partial genome	KR088971	75.51	90.18	6.17723e−19	Plecoptera	Styloperlidae	Styloperla sp. YZD−2015
Cryptoperla sp. YK−2012−1 mitochondrial gene for 1	AB711243	75.8	95.09	6.17723e−19	Plecoptera	Peltoperlidae	Cryptoperla sp. YK−2012−1
Chloroperla tripunctata 16S ribosomal RNA gene, pa	EF623137	80.49	74.85	6.17723e−19	Plecoptera	Chloroperlidae	Chloroperla tripunctata
Triznaka pintada 16S ribosomal RNA gene, partial s	EF623134	79.67	74.85	6.17723e−19	Plecoptera	Chloroperlidae	Triznaka pintada
Cosmioperla australis 16S ribosomal RNA gene, part	EF623148	74.38	94.48	2.15607e−18	Plecoptera	Eustheniidae	Cosmioperla australis
Capnura wanica 16S ribosomal RNA gene, partial seq	EF623128	77.08	87.73	2.15607e−18	Plecoptera	Capniidae	Capnura wanica
D.geniculata mitochondrial 16S rRNA gene	Y07551	75	99.39	2.15607e−18	Orthoptera	Rhaphidophoridae	Dolichopoda geniculata
Mesocapnia arizonensis mitochondrion, partial geno	KP642637	76.39	87.73	7.52541e−18	Plecoptera	Capniidae	Mesocapnia arizonensis
Allocapnia recta isolate H13 16S ribosomal RNA gen	KC881048	76.39	87.73	7.52541e−18	Plecoptera	Capniidae	Allocapnia recta
Allocapnia recta isolate H11 16S ribosomal RNA gen	KC881046	76.39	87.73	7.52541e−18	Plecoptera	Capniidae	Allocapnia recta
Allocapnia recta isolate H7 16S ribosomal RNA gene	KC881042	76.39	87.73	7.52541e−18	Plecoptera	Capniidae	Allocapnia recta
Allocapnia recta isolate H3 16S ribosomal RNA gene	KC881038	76.39	87.73	7.52541e−18	Plecoptera	Capniidae	Allocapnia recta
Allocapnia recta isolate H1 16S ribosomal RNA gene	KC881036	76.39	87.73	7.52541e−18	Plecoptera	Capniidae	Allocapnia recta
Dolichopoda bormansi isolate BRA5 16S ribosomal RN	AY507606	75.74	99.39	7.52541e−18	Orthoptera	Rhaphidophoridae	Dolichopoda bormansi
Cascadoperla trictura 16S ribosomal RNA gene, part	EF623225	76.39	86.5	7.52541e−18	Plecoptera	Perlodidae	Cascadoperla trictura
Lednia tumana 16S ribosomal RNA gene, partial sequ	EF623197	78.4	76.07	7.52541e−18	Plecoptera	Nemouridae	Lednia tumana
Malenka californica 16S ribosomal RNA gene, partia	EF623182	82.52	63.19	7.52541e−18	Plecoptera	Nemouridae	Malenka californica
Dinotoperla sp. BYU_PL105 16S ribosomal RNA gene,	EF623160	75.61	99.39	7.52541e−18	Plecoptera	Gripopterygidae	Dinotoperla sp. BYU_PL105
Sasquaperla hoopa 16S ribosomal RNA gene, partial	EF623143	81.55	63.19	7.52541e−18	Plecoptera	Chloroperlidae	Sasquaperla hoopa
Paraperla frontalis 16S ribosomal RNA gene, partia	EF623136	82.52	63.19	7.52541e−18	Plecoptera	Chloroperlidae	Paraperla frontalis
Allocapnia minima 16S ribosomal RNA gene, partial	EF623130	76.39	87.73	7.52541e−18	Plecoptera	Capniidae	Allocapnia minima
Mesocapnia sp. BYU_PL010 16S ribosomal RNA gene, p	EF623127	76.39	87.73	7.52541e−18	Plecoptera	Capniidae	Mesocapnia sp. BYU_PL010
Allocapnia recta haplotype 3ar: H03 16S ribosomal	DQ915181	76.39	87.73	7.52541e−18	Plecoptera	Capniidae	Allocapnia recta
Udenus wnigrum voucher MAY 16S ribosomal RNA gene,	HM594504	79.13	69.33	2.62662e−17	Orthoptera	Rhaphidophoridae	Udenus wnigrum
Eupholidoptera krueperi voucher 408 16S ribosomal	EU203912	76.51	88.96	2.62662e−17	Orthoptera	Tettigoniidae	Eupholidoptera krueperi
Dolichopoda pavesii voucher SPI 16S ribosomal RNA	EF216951	75.45	99.39	2.62662e−17	Orthoptera	Rhaphidophoridae	Dolichopoda pavesii
Pteronarcella badia 16S ribosomal RNA gene, partia	EF623262	80.37	65.64	2.62662e−17	Plecoptera	Pteronarcyidae	Pteronarcella badia
Pictetoperla sp. BYU_PL156 16S ribosomal RNA gene,	EF623247	81.31	65.64	2.62662e−17	Plecoptera	Perlidae	Pictetoperla sp. BYU_PL156
Salmoperla sylvanica 16S ribosomal RNA gene, parti	EF623222	80.37	65.64	2.62662e−17	Plecoptera	Perlodidae	Salmoperla sylvanica
Austrocerca rieka 16S ribosomal RNA gene, partial	EF623205	74.85	99.39	2.62662e−17	Plecoptera	Notonemouridae	Austrocerca rieka
Austrocercoides zwicki 16S ribosomal RNA gene, par	EF623199	74.85	99.39	2.62662e−17	Plecoptera	Notonemouridae	Austrocercoides zwicki
Megaleuctra kincaidi 16S ribosomal RNA gene, parti	EF623174	81.31	65.64	2.62662e−17	Plecoptera	Leuctridae	Megaleuctra kincaidi
Cerconychia sp. BYU_PL114 16S ribosomal RNA gene,	EF623260	74.32	90.8	9.16782e−17	Plecoptera	Styloperlidae	Cerconychia sp. BYU_PL114
Perlinodes aurea 16S ribosomal RNA gene, partial s	EF623234	80.58	63.19	9.16782e−17	Plecoptera	Perlodidae	Perlinodes aurea
Frisonia picticeps 16S ribosomal RNA gene, partial	EF623227	78.45	71.17	9.16782e−17	Plecoptera	Perlodidae	Frisonia picticeps
Nanonemoura wahkeena 16S ribosomal RNA gene, parti	EF623198	81.55	63.19	9.16782e−17	Plecoptera	Nemouridae	Nanonemoura wahkeena
Riekoperla triloba 16S ribosomal RNA gene, partial	EF623171	74.52	95.71	9.16782e−17	Plecoptera	Gripopterygidae	Riekoperla triloba
Acroperla trivacauta 16S ribosomal RNA gene, parti	EF623155	74.39	99.39	9.16782e−17	Plecoptera	Gripopterygidae	Acroperla trivacauta
Sweltsa coloradensis 16S ribosomal RNA gene, parti	EF623133	80.58	63.19	9.16782e−17	Plecoptera	Chloroperlidae	Sweltsa coloradensis
Allocapnia recta haplotype 12ar: H12 16S ribosomal	EU306875	80.58	63.19	9.16782e−17	Plecoptera	Capniidae	Allocapnia recta
Ceraeochrysa cincta voucher CASC 00217 16S ribosom	DQ399268	77.97	72.39	9.16782e−17	Neuroptera	Chrysopidae	Ceraeochrysa cincta
Pollenia rudis mitochondrion, partial genome	JX913761	78.86	74.85	3.19988e−16	Diptera	Calliphoridae	Pollenia rudis
Prodegeeria japonica mitochondrial gene for 16S rR	AB465910	75.32	93.87	3.19988e−16	Diptera	Tachinidae	Prodegeeria japonica
Osobenus yakimae 16S ribosomal RNA gene, partial s	EF623232	74.68	91.41	3.19988e−16	Plecoptera	Perlodidae	Osobenus yakimae
