# Supplementary material for: Testing the potential of a ribosomal 16S marker for DNA metabarcoding of insects
Source: PeerJ. 2016 Apr 19;4:e1966. doi: 10.7717/peerj.1966 (PMC4841222; doi:10.7717/peerj.1966)
Supplement: Supplemental Information 1 [file peerj-04-1966-s006.zip › scripts/5 match OTUs/blast_hits/OTU_37.csv_plot.pdf]

OTU_37.csv

Agabus guttatus

Dytiscidae

Coleoptera

100

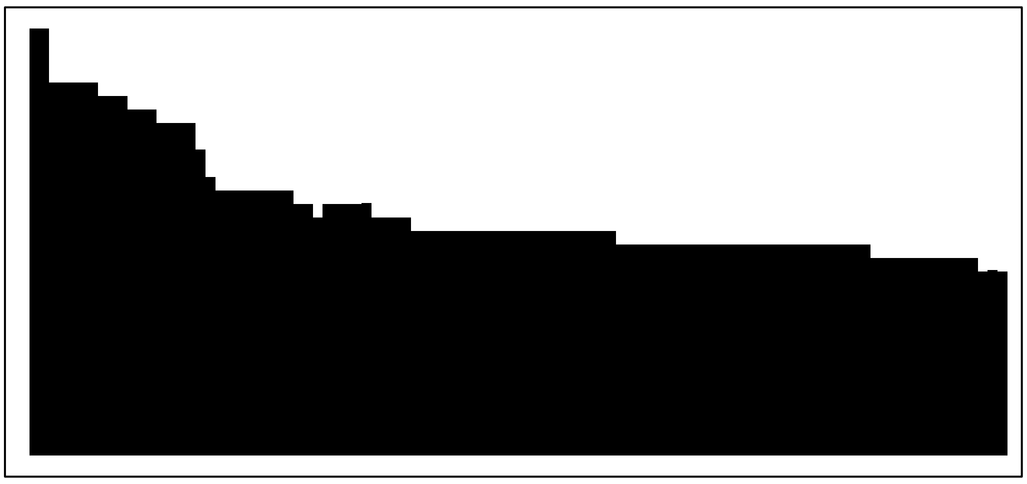

Agabus guttatus mitochondrial 16S rRNA gene (parti	LM654777	100	100	8.78639e−74	Coleoptera	Dytiscidae	Agabus guttatus
Agabus guttatus 16S ribosomal RNA gene, partial se	AY039262	100	100	8.78639e−74	Coleoptera	Dytiscidae	Agabus guttatus
Agabus caraboides mitochondrial partial 16S rRNA g	HF931451	97.47	100	2.35772e−68	Coleoptera	Dytiscidae	Agabus caraboides
Agabus picotae mitochondrial 16S rRNA gene (partia	HF931356	97.47	100	2.35772e−68	Coleoptera	Dytiscidae	Agabus picotae
Agabus heydeni 16S ribosomal RNA gene, partial seq	AY039263	97.47	100	2.35772e−68	Coleoptera	Dytiscidae	Agabus heydeni
Agabus binotatus 16S ribosomal RNA gene, partial s	AY039260	97.47	100	2.35772e−68	Coleoptera	Dytiscidae	Agabus binotatus
Agabus biguttatus haplotype 1 16S ribosomal RNA ge	AY039258	97.47	100	2.35772e−68	Coleoptera	Dytiscidae	Agabus biguttatus
Agabus basalis mitochondrial 16S rRNA gene (partia	HF931443	96.84	100	1.00253e−66	Coleoptera	Dytiscidae	Agabus basalis
Agabus cf. nitidus IR−2001 16S ribosomal RNA gene,	AY039264	96.84	100	1.00253e−66	Coleoptera	Dytiscidae	Agabus cf. nitidus IR−2001
Agabus biguttatus haplotype 2 16S ribosomal RNA ge	AY039259	96.84	100	1.00253e−66	Coleoptera	Dytiscidae	Agabus biguttatus
Agabus alexandrae 16S ribosomal RNA gene, partial	AY039257	96.2	100	1.22133e−65	Coleoptera	Dytiscidae	Agabus alexandrae
Agabus alexandrae 16S ribosomal RNA gene, partial	AF428199	96.2	100	1.22133e−65	Coleoptera	Dytiscidae	Agabus alexandrae
Agabus cephalotes mitochondrial 16S rRNA gene (par	HF931380	96.2	100	1.48788e−64	Coleoptera	Dytiscidae	Agabus cephalotes
Agabus faldermanni mitochondrial 16S rRNA gene (pa	LM654774	95.57	100	5.19322e−64	Coleoptera	Dytiscidae	Agabus faldermanni
Agabus faldermanni 16S ribosomal RNA gene, partial	AY138614	95.57	100	5.19322e−64	Coleoptera	Dytiscidae	Agabus faldermanni
Agabus glacialis mitochondrial 16S rRNA gene (part	LM654776	95.57	100	6.32663e−63	Coleoptera	Dytiscidae	Agabus glacialis
Agabus dilatatus 16S ribosomal RNA gene, partial s	AY039261	95.57	100	6.32663e−63	Coleoptera	Dytiscidae	Agabus dilatatus
Agabus maderensis 16S ribosomal RNA gene, partial	AY138621	94.34	100	9.38956e−61	Coleoptera	Dytiscidae	Agabus maderensis
Agabus paludosus 16S ribosomal RNA gene, partial s	AY138625	93.04	100	1.69767e−57	Coleoptera	Dytiscidae	Agabus paludosus
Agabus pallidus mitochondrial 16S rRNA gene (parti	LM654780	92.41	100	2.06819e−56	Coleoptera	Dytiscidae	Agabus pallidus
Agabus nebulosus mitochondrial 16S rRNA gene (part	LM654779	92.41	100	2.06819e−56	Coleoptera	Dytiscidae	Agabus nebulosus
Agabus labiatus mitochondrial 16S rRNA gene (parti	LM654778	92.41	100	2.06819e−56	Coleoptera	Dytiscidae	Agabus labiatus
Agabus uliginosus mitochondrial 16S rRNA gene (par	HF931463	92.41	100	2.06819e−56	Coleoptera	Dytiscidae	Agabus uliginosus
Agabus dichrous mitochondrial partial 16S rRNA gen	HF931378	92.41	100	2.06819e−56	Coleoptera	Dytiscidae	Agabus dichrous
Agabus nebulosus 16S ribosomal RNA gene, partial s	AY138624	92.41	100	2.06819e−56	Coleoptera	Dytiscidae	Agabus nebulosus
Agabus lineatus 16S ribosomal RNA gene, partial se	AY138611	92.41	100	2.06819e−56	Coleoptera	Dytiscidae	Agabus lineatus
Agabus bifarius 16S ribosomal RNA gene, partial se	AY138605	92.41	100	2.06819e−56	Coleoptera	Dytiscidae	Agabus bifarius
Agabus undulatus mitochondrial 16S rRNA gene (part	HF931411	91.77	100	7.21868e−56	Coleoptera	Dytiscidae	Agabus undulatus
Agabus labiatus 16S ribosomal RNA gene, partial se	AY138618	91.77	100	2.51957e−55	Coleoptera	Dytiscidae	Agabus labiatus
Agabus tristis voucher UNM KBMAgtr60 16S ribosomal	KJ548305	91.14	100	8.79416e−55	Coleoptera	Dytiscidae	Agabus tristis
Agabus serricornis 16S ribosomal RNA gene, partial	AY138629	91.77	100	8.79416e−55	Coleoptera	Dytiscidae	Agabus serricornis
Agabus conspersus 16S ribosomal RNA gene, partial	AY138610	91.77	100	8.79416e−55	Coleoptera	Dytiscidae	Agabus conspersus
Agabus antennatus 16S ribosomal RNA gene, partial	AY138601	91.77	100	8.79416e−55	Coleoptera	Dytiscidae	Agabus antennatus
Agabus affinis 16S ribosomal RNA gene, partial seq	AY138598	91.77	100	8.79416e−55	Coleoptera	Dytiscidae	Agabus affinis
Agabus aubei 16S ribosomal RNA gene, partial seque	AY039265	91.82	100	3.06946e−54	Coleoptera	Dytiscidae	Agabus aubei
Agabus godmanni mitochondrial 16S rRNA gene (parti	HF931348	91.14	100	1.07135e−53	Coleoptera	Dytiscidae	Agabus godmanni
Agabus wollastoni 16S ribosomal RNA gene, partial	AY138636	91.14	100	1.07135e−53	Coleoptera	Dytiscidae	Agabus wollastoni
Agabus melanarius 16S ribosomal RNA gene, partial	AY138623	91.14	100	1.07135e−53	Coleoptera	Dytiscidae	Agabus melanarius
Agabus morosus isolate Aga18.2R 16S ribosomal RNA	AY138592	91.14	100	1.07135e−53	Coleoptera	Dytiscidae	Agabus morosus
Agabus tristis 16S ribosomal RNA gene, partial seq	AY138634	90.51	100	3.73937e−53	Coleoptera	Dytiscidae	Agabus tristis
Agabus didymus mitochondrial 16S rRNA gene (partia	LM654773	90.51	100	1.30517e−52	Coleoptera	Dytiscidae	Agabus didymus
Agabus didymus voucher UNM KBMPmsp730 16S ribosoma	KJ548392	90.51	100	1.30517e−52	Coleoptera	Dytiscidae	Agabus didymus
Agabus striolatus mitochondrial 16S rRNA gene (par	LM654785	90.51	100	4.55549e−52	Coleoptera	Dytiscidae	Agabus striolatus
Agabus semipunctatus mitochondrial 16S rRNA gene (	LM654784	90.51	100	4.55549e−52	Coleoptera	Dytiscidae	Agabus semipunctatus
Agabus regimbarti mitochondrial 16S rRNA gene (par	LM654782	90.51	100	4.55549e−52	Coleoptera	Dytiscidae	Agabus regimbarti
Agabus ragazzii mitochondrial 16S rRNA gene (parti	LM654781	90.51	100	4.55549e−52	Coleoptera	Dytiscidae	Agabus ragazzii
Agabus bipustulatus 16S ribosomal RNA gene, partia	AF428198	90.51	100	4.55549e−52	Coleoptera	Dytiscidae	Agabus bipustulatus
Ilybius vandykei 16S ribosomal RNA gene, partial s	AY138671	90.51	100	4.55549e−52	Coleoptera	Dytiscidae	Ilybius vandykei
Agabus sturmii 16S ribosomal RNA gene, partial seq	AY138633	90.51	100	4.55549e−52	Coleoptera	Dytiscidae	Agabus sturmii
Agabus strigulosus 16S ribosomal RNA gene, partial	AY138632	90.51	100	4.55549e−52	Coleoptera	Dytiscidae	Agabus strigulosus
Agabus semipunctatus 16S ribosomal RNA gene, parti	AY138628	90.51	100	4.55549e−52	Coleoptera	Dytiscidae	Agabus semipunctatus
Agabus japonicus 16S ribosomal RNA gene, partial s	AY138617	90.51	100	4.55549e−52	Coleoptera	Dytiscidae	Agabus japonicus
Agabus erythropterus 16S ribosomal RNA gene, parti	AY138613	90.51	100	4.55549e−52	Coleoptera	Dytiscidae	Agabus erythropterus
Agabus elongatus 16S ribosomal RNA gene, partial s	AY138612	90.51	100	4.55549e−52	Coleoptera	Dytiscidae	Agabus elongatus
Agabus austinii 16S ribosomal RNA gene, partial se	AY138604	90.51	100	4.55549e−52	Coleoptera	Dytiscidae	Agabus austinii
Agabus arcticus 16S ribosomal RNA gene, partial se	AY138603	90.51	100	4.55549e−52	Coleoptera	Dytiscidae	Agabus arcticus
Agabus sp. IR−sp2 16S ribosomal RNA gene, partial	AY138597	90.51	100	4.55549e−52	Coleoptera	Dytiscidae	Agabus sp. IR−sp2
Agabus thomsoni 16S ribosomal RNA gene, partial se	AY138595	90.51	100	4.55549e−52	Coleoptera	Dytiscidae	Agabus thomsoni
Agabus kootenai 16S ribosomal RNA gene, partial se	AY138594	90.51	100	4.55549e−52	Coleoptera	Dytiscidae	Agabus kootenai
Agabus morosus isolate Aga16.3 16S ribosomal RNA g	AY138591	90.51	100	4.55549e−52	Coleoptera	Dytiscidae	Agabus morosus
Agabus rufulus mitochondrial 16S rRNA gene (partia	LM654818	89.87	100	5.54972e−51	Coleoptera	Dytiscidae	Agabus rufulus
Agabus rufulus mitochondrial 16S rRNA gene (partia	LM654817	89.87	100	5.54972e−51	Coleoptera	Dytiscidae	Agabus rufulus
Agabus ramblae mitochondrial 16S rRNA gene (partia	LM654816	89.87	100	5.54972e−51	Coleoptera	Dytiscidae	Agabus ramblae
Agabus brunneus mitochondrial 16S rRNA gene (parti	LM654791	89.87	100	5.54972e−51	Coleoptera	Dytiscidae	Agabus brunneus
Agabus brunneus mitochondrial 16S rRNA gene (parti	LM654790	89.87	100	5.54972e−51	Coleoptera	Dytiscidae	Agabus brunneus
Agabus brunneus mitochondrial 16S rRNA gene (parti	LM654789	89.87	100	5.54972e−51	Coleoptera	Dytiscidae	Agabus brunneus
Agabus brunneus mitochondrial 16S rRNA gene (parti	LM654788	89.87	100	5.54972e−51	Coleoptera	Dytiscidae	Agabus brunneus
Agabus brunneus mitochondrial 16S rRNA gene (parti	LM654787	89.87	100	5.54972e−51	Coleoptera	Dytiscidae	Agabus brunneus
Agabus unguicularis mitochondrial 16S rRNA gene (p	LM654786	89.87	100	5.54972e−51	Coleoptera	Dytiscidae	Agabus unguicularis
Agabus fuscipennis mitochondrial 16S rRNA gene (pa	LM654775	89.87	100	5.54972e−51	Coleoptera	Dytiscidae	Agabus fuscipennis
Agabus rufulus mitochondrial partial 16S rRNA gene	LM654771	89.87	100	5.54972e−51	Coleoptera	Dytiscidae	Agabus rufulus
Agabus ramblae mitochondrial partial 16S rRNA gene	LM654770	89.87	100	5.54972e−51	Coleoptera	Dytiscidae	Agabus ramblae
Agabus brunneus mitochondrial partial 16S rRNA gen	LM654769	89.87	100	5.54972e−51	Coleoptera	Dytiscidae	Agabus brunneus
Agabus brunneus mitochondrial partial 16S rRNA gen	LM654768	89.87	100	5.54972e−51	Coleoptera	Dytiscidae	Agabus brunneus
Agabus brunneus mitochondrial partial 16S rRNA gen	LM654767	89.87	100	5.54972e−51	Coleoptera	Dytiscidae	Agabus brunneus
Platambus stygius 16S ribosomal RNA gene, partial	AY138679	89.87	100	5.54972e−51	Coleoptera	Dytiscidae	Platambus stygius
Agabus ramblae 16S ribosomal RNA gene, partial seq	AY138626	89.87	100	5.54972e−51	Coleoptera	Dytiscidae	Agabus ramblae
Agabus matsumotoi 16S ribosomal RNA gene, partial	AY138622	89.87	100	5.54972e−51	Coleoptera	Dytiscidae	Agabus matsumotoi
Agabus lutosus 16S ribosomal RNA gene, partial seq	AY138620	89.87	100	5.54972e−51	Coleoptera	Dytiscidae	Agabus lutosus
Agabus congener 16S ribosomal RNA gene, partial se	AY138609	89.87	100	5.54972e−51	Coleoptera	Dytiscidae	Agabus congener
Agabus confinis 16S ribosomal RNA gene, partial se	AY138608	89.87	100	5.54972e−51	Coleoptera	Dytiscidae	Agabus confinis
Agabus brunneus 16S ribosomal RNA gene, partial se	AY138607	89.87	100	5.54972e−51	Coleoptera	Dytiscidae	Agabus brunneus
Agabus anthracinus 16S ribosomal RNA gene, partial	AY138602	89.87	100	5.54972e−51	Coleoptera	Dytiscidae	Agabus anthracinus
Agabus ambiguus isolate Agaambi 16S ribosomal RNA	AY138599	89.87	100	5.54972e−51	Coleoptera	Dytiscidae	Agabus ambiguus
Agabus ambiguus isolate Aga32.1 16S ribosomal RNA	AY138596	89.87	100	5.54972e−51	Coleoptera	Dytiscidae	Agabus ambiguus
Agabus cf. lutosus/griseipennis IR−2002 16S riboso	AY138593	89.87	100	5.54972e−51	Coleoptera	Dytiscidae	Agabus cf. lutosus/griseipennis IR−2002
Agabus ruwenzoricus mitochondrial 16S rRNA gene (p	LM654783	89.24	100	6.76094e−50	Coleoptera	Dytiscidae	Agabus ruwenzoricus
Agabus disintegratus voucher UNM KBMAgdi412 16S ri	KJ548302	89.24	100	6.76094e−50	Coleoptera	Dytiscidae	Agabus disintegratus
Agabus disintegratus 16S ribosomal RNA gene, parti	AY071769	89.24	100	6.76094e−50	Coleoptera	Dytiscidae	Agabus disintegratus
Ilybius lineellus mitochondrial 16S rRNA gene (par	LM654802	89.24	100	2.3598e−49	Coleoptera	Dytiscidae	Ilybius lineellus
Agabus amoenus mitochondrial 16S rRNA gene (partia	LM654772	89.24	100	2.3598e−49	Coleoptera	Dytiscidae	Agabus amoenus
Platambus semivittatus 16S ribosomal RNA gene, par	AY138678	89.24	100	2.3598e−49	Coleoptera	Dytiscidae	Platambus semivittatus
Ilybius walsinghami isolate Ilydisr 16S ribosomal	AY138660	89.24	100	2.3598e−49	Coleoptera	Dytiscidae	Ilybius walsinghami
Agabus unguicularis 16S ribosomal RNA gene, partia	AY138635	89.24	100	2.3598e−49	Coleoptera	Dytiscidae	Agabus unguicularis
Agabus setulosus 16S ribosomal RNA gene, partial s	AY138630	89.24	100	2.3598e−49	Coleoptera	Dytiscidae	Agabus setulosus
Agabus hummeli 16S ribosomal RNA gene, partial seq	AY138616	89.24	100	2.3598e−49	Coleoptera	Dytiscidae	Agabus hummeli
Agabus amoenus 16S ribosomal RNA gene, partial seq	AY138600	89.24	100	2.3598e−49	Coleoptera	Dytiscidae	Agabus amoenus
Acilius athabascae voucher 305G09/BMNH799009 16S r	KF979079	88.61	100	8.23651e−49	Coleoptera	Dytiscidae	Acilius athabascae
Ilybius walsinghami isolate Ily16.4 16S ribosomal	AY138657	88.68	100	8.23651e−49	Coleoptera	Dytiscidae	Ilybius walsinghami
Lancetes varius voucher UNM KBMLcva196 16S ribosom	KJ548351	88.61	100	2.87482e−48	Coleoptera	Dytiscidae	Lancetes varius
