# Supplementary material for: Testing the potential of a ribosomal 16S marker for DNA metabarcoding of insects
Source: PeerJ. 2016 Apr 19;4:e1966. doi: 10.7717/peerj.1966 (PMC4841222; doi:10.7717/peerj.1966)
Supplement: Supplemental Information 1 [file peerj-04-1966-s006.zip › scripts/5 match OTUs/blast_hits/OTU_383.csv_plot.pdf]

# OTU_383.csv

## Oreodytes sanmarkii

## Dytiscidae

## Coleoptera

# 98.72

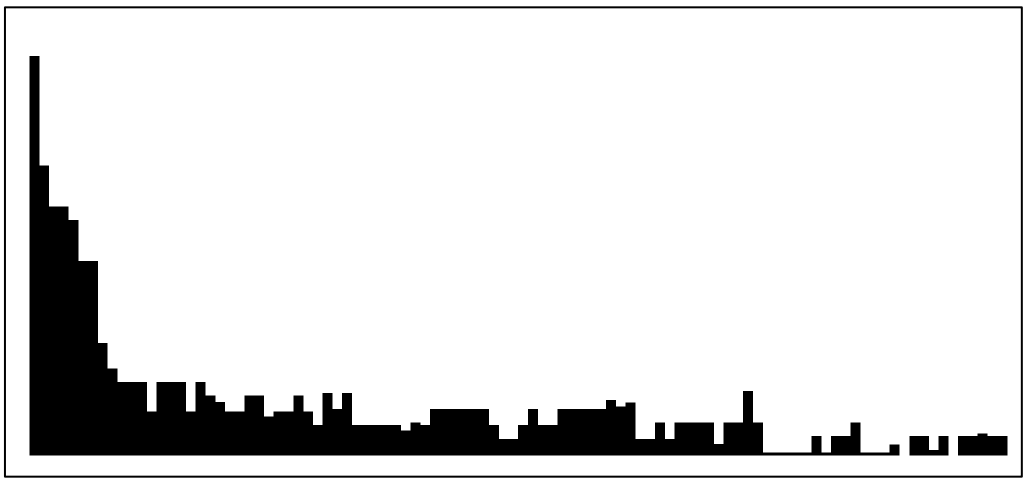

| | | | | | | | |
|---|---|---|---|---|---|---|---|
| Oreodytes sanmarkii mitochondrial 16S rRNA gene (p | HF931524 | 98.72 | 99.36 | 5.49974e−70 | Coleoptera | Dytiscidae | Oreodytes sanmarkii |
| Oreodytes obesus cordillerensis mitochondrial part | HF931516 | 93.59 | 99.36 | 3.96008e−59 | Coleoptera | Dytiscidae | Oreodytes obesus cordillerensis |
| Oreodytes crassulus partial 16S rRNA gene | AJ850348 | 91.67 | 99.36 | 8.72266e−55 | Coleoptera | Dytiscidae | Oreodytes crassulus |
| Oreodytes congruus partial 16S rRNA gene | AJ850347 | 91.67 | 99.36 | 8.72266e−55 | Coleoptera | Dytiscidae | Oreodytes congruus |
| Oreodytes congruus 16S ribosomal RNA gene, partial | KF575550 | 91.03 | 99.36 | 1.06264e−53 | Coleoptera | Dytiscidae | Oreodytes congruus |
| Oreodytes abbreviatus mitochondrial 16S rRNA gene | HF931477 | 89.1 | 99.36 | 2.34061e−49 | Coleoptera | Dytiscidae | Oreodytes abbreviatus |
| Oreodytes rhyacophilus partial 16S rRNA gene | AJ850352 | 89.1 | 99.36 | 2.34061e−49 | Coleoptera | Dytiscidae | Oreodytes rhyacophilus |
| Laccornis difformis 16S ribosomal RNA gene, partia | KF575544 | 85.26 | 99.36 | 3.2535e−41 | Coleoptera | Dytiscidae | Laccornis difformis |
| Hydroporus foveolatus mitochondrial 16S rRNA gene | HE613724 | 84.08 | 99.36 | 2.50129e−36 | Coleoptera | Dytiscidae | Hydroporus foveolatus |
| Suphrodytes dorsalis 16S ribosomal RNA gene, parti | KF575577 | 83.44 | 99.36 | 8.73037e−36 | Coleoptera | Dytiscidae | Suphrodytes dorsalis |
| Suphrodytes dorsalis voucher BMNH:725966 16S ribos | JX221574 | 83.44 | 99.36 | 8.73037e−36 | Coleoptera | Dytiscidae | Suphrodytes dorsalis |
| Stictotarsus maghrebinus 16S ribosomal RNA gene, p | AY250947 | 83.44 | 99.36 | 8.73037e−36 | Coleoptera | Dytiscidae | Stictotarsus maghrebinus |
| Oreodytes quadrimaculatus 16S ribosomal RNA gene, | KF575567 | 82.05 | 99.36 | 1.06358e−34 | Coleoptera | Dytiscidae | Oreodytes quadrimaculatus |
| Hydroporus tessellatus 16S ribosomal RNA gene, par | JX434770 | 83.44 | 99.36 | 1.06358e−34 | Coleoptera | Dytiscidae | Hydroporus tessellatus |
| Hydroporus zimmermanni mitochondrial 16S rRNA gene | HE613679 | 83.44 | 99.36 | 1.06358e−34 | Coleoptera | Dytiscidae | Hydroporus zimmermanni |
| Hydroporus basinotatus mitochondrial 16S rRNA gene | HE613678 | 83.44 | 99.36 | 1.06358e−34 | Coleoptera | Dytiscidae | Hydroporus basinotatus |
| Oreodytes quadrimaculatus partial 16S rRNA gene | AJ850351 | 82.05 | 99.36 | 1.06358e−34 | Coleoptera | Dytiscidae | Oreodytes quadrimaculatus |
| Hydroporus basinotatus 16S ribosomal RNA gene, par | AF518258 | 83.44 | 99.36 | 1.06358e−34 | Coleoptera | Dytiscidae | Hydroporus basinotatus |
| Stictotarsus procerus mitochondrial 16S rRNA gene | HF931379 | 82.8 | 99.36 | 3.71225e−34 | Coleoptera | Dytiscidae | Stictotarsus procerus |
| Hydroporus marginatus mitochondrial 16S rRNA gene | HE613699 | 82.5 | 99.36 | 3.71225e−34 | Coleoptera | Dytiscidae | Hydroporus marginatus |
| Hygrotus novemlineatus mitochondrial partial 16S r | HF931523 | 82.05 | 99.36 | 1.2957e−33 | Coleoptera | Dytiscidae | Hygrotus novemlineatus |
| Suphrodytes figuratus voucher BMNH:722148 16S ribo | JX221571 | 82.05 | 99.36 | 1.2957e−33 | Coleoptera | Dytiscidae | Suphrodytes figuratus |
| Hydroporus analis mitochondrial partial 16S rRNA g | HE613731 | 82.8 | 99.36 | 1.2957e−33 | Coleoptera | Dytiscidae | Hydroporus analis |
| Hydroporus compunctus mitochondrial 16S rRNA gene | HE599693 | 82.8 | 99.36 | 1.2957e−33 | Coleoptera | Dytiscidae | Hydroporus compunctus |
| Antiporus hollingsworthi mitochondrial partial 16S | FR727246 | 81.82 | 98.09 | 1.2957e−33 | Coleoptera | Dytiscidae | Antiporus hollingsworthi |
| Hygrotus turbidus partial 16S rRNA gene | AJ850390 | 82.05 | 99.36 | 1.2957e−33 | Coleoptera | Dytiscidae | Hygrotus turbidus |
| Hydroporus sp. IR33.4 16S ribosomal RNA gene, part | AY365261 | 82.05 | 99.36 | 1.2957e−33 | Coleoptera | Dytiscidae | Hydroporus sp. IR33.4 |
| Hydroporus compunctus 16S ribosomal RNA gene, part | AF518259 | 82.8 | 99.36 | 1.2957e−33 | Coleoptera | Dytiscidae | Hydroporus compunctus |
| Hydroporus marginatus 16S ribosomal RNA gene, part | AF518267 | 82.05 | 99.36 | 1.2957e−33 | Coleoptera | Dytiscidae | Hydroporus marginatus |
| Hydaticus cinctipennis voucher UNM KBMHyci20 16S r | KJ548338 | 81.41 | 99.36 | 4.52244e−33 | Coleoptera | Dytiscidae | Hydaticus cinctipennis |
| Hydroporus fuscipennis mitochondrial 16S rRNA gene | HE613730 | 82.91 | 99.36 | 4.52244e−33 | Coleoptera | Dytiscidae | Hydroporus fuscipennis |
| Nirripirti megamacrocephalus isolate ABTC78781 16S | EU616935 | 82.17 | 99.36 | 4.52244e−33 | Coleoptera | Dytiscidae | Paroster megamacrocephalus |
| Hydroporus fuscipennis 16S ribosomal RNA gene, par | AF518262 | 82.91 | 99.36 | 4.52244e−33 | Coleoptera | Dytiscidae | Hydroporus fuscipennis |
| Hygrotus lutescens mitochondrial partial 16S rRNA | HF931518 | 81.41 | 99.36 | 1.57849e−32 | Coleoptera | Dytiscidae | Hygrotus lutescens |
| Hygrotus fontinalis mitochondrial 16S rRNA gene (p | HF931494 | 81.41 | 99.36 | 1.57849e−32 | Coleoptera | Dytiscidae | Hygrotus fontinalis |
| Hygrotus patruelis mitochondrial 16S rRNA gene (pa | HF931388 | 81.41 | 99.36 | 1.57849e−32 | Coleoptera | Dytiscidae | Hygrotus patruelis |
| Suphrodytes figuratus voucher BMNH:722150 16S ribo | JX221572 | 81.41 | 99.36 | 1.57849e−32 | Coleoptera | Dytiscidae | Suphrodytes figuratus |
| Suphrodytes dorsalis mitochondrial 16S rRNA gene ( | HE599700 | 81.41 | 99.36 | 1.57849e−32 | Coleoptera | Dytiscidae | Suphrodytes dorsalis |
| Antiporus hollingsworthi mitochondrial partial 16S | FR727247 | 81.17 | 98.09 | 1.57849e−32 | Coleoptera | Dytiscidae | Antiporus hollingsworthi |
| Herophydrus rohani voucher UNM KBMHpsp574 16S ribo | KJ548382 | 81.53 | 99.36 | 5.50946e−32 | Coleoptera | Dytiscidae | Herophydrus rohani |
| Hydroporus appalachius 16S ribosomal RNA gene, par | JX434765 | 81.41 | 99.36 | 5.50946e−32 | Coleoptera | Dytiscidae | Hydroporus appalachius |
| Hydroporus foveolatus mitochondrial 16S rRNA gene | HF931502 | 82.17 | 99.36 | 5.50946e−32 | Coleoptera | Dytiscidae | Hydroporus foveolatus |
| Hydroporus discretus mitochondrial 16S rRNA gene ( | HE613723 | 82.17 | 99.36 | 5.50946e−32 | Coleoptera | Dytiscidae | Hydroporus discretus |
| Hydroporus decipiens mitochondrial 16S rRNA gene ( | HE613722 | 82.17 | 99.36 | 5.50946e−32 | Coleoptera | Dytiscidae | Hydroporus decipiens |
| Hydroporus pubescens mitochondrial 16S rRNA gene ( | HE613707 | 82.17 | 99.36 | 5.50946e−32 | Coleoptera | Dytiscidae | Hydroporus pubescens |
| Hydroporus limbatus mitochondrial 16S rRNA gene (p | HE613706 | 82.17 | 99.36 | 5.50946e−32 | Coleoptera | Dytiscidae | Hydroporus limbatus |
| Hydroporus errans mitochondrial 16S rRNA gene (par | HE613704 | 82.17 | 99.36 | 5.50946e−32 | Coleoptera | Dytiscidae | Hydroporus errans |
| Hydroporus pervicinus mitochondrial 16S rRNA gene | HE599695 | 81.41 | 99.36 | 5.50946e−32 | Coleoptera | Dytiscidae | Hydroporus pervicinus |
| Antiporus interrogationis mitochondrial partial 16 | FR727252 | 80.77 | 99.36 | 5.50946e−32 | Coleoptera | Dytiscidae | Antiporus interrogationis |
| Antiporus sp. MB1421 mitochondrial partial 16S rRN | FR727245 | 80.77 | 99.36 | 5.50946e−32 | Coleoptera | Dytiscidae | Antiporus sp. MB1421 |
| Nirripirti newhavenensis 16S ribosomal RNA gene, p | AY353844 | 81.41 | 99.36 | 5.50946e−32 | Coleoptera | Dytiscidae | Paroster newhavenensis |
| Hydroporus pubescens voucher NHM−IR39 16S ribosoma | EF419327 | 82.17 | 99.36 | 5.50946e−32 | Coleoptera | Dytiscidae | Hydroporus pubescens |
| Hydroporus occidentalis 16S ribosomal RNA gene, pa | AY365280 | 81.41 | 99.36 | 5.50946e−32 | Coleoptera | Dytiscidae | Hydroporus occidentalis |
| Hydroporus hirtellus 16S ribosomal RNA gene, parti | AY365271 | 81.41 | 99.36 | 5.50946e−32 | Coleoptera | Dytiscidae | Hydroporus hirtellus |
| Hydroporus rufifrons 16S ribosomal RNA gene, parti | AF518277 | 82.17 | 99.36 | 5.50946e−32 | Coleoptera | Dytiscidae | Hydroporus rufifrons |
| Hydroporus pubescens 16S ribosomal RNA gene, parti | AF518276 | 82.17 | 99.36 | 5.50946e−32 | Coleoptera | Dytiscidae | Hydroporus pubescens |
| Hydroporus limbatus 16S ribosomal RNA gene, partia | AF518264 | 82.17 | 99.36 | 5.50946e−32 | Coleoptera | Dytiscidae | Hydroporus limbatus |
| Hydroporus discretus 16S ribosomal RNA gene, parti | AF518260 | 82.17 | 99.36 | 5.50946e−32 | Coleoptera | Dytiscidae | Hydroporus discretus |
| Hydroporus analis 16S ribosomal RNA gene, partial | AF518257 | 82.17 | 99.36 | 5.50946e−32 | Coleoptera | Dytiscidae | Hydroporus analis |
| Hygrotus impressopunctatus voucher UNM KBMHtim80 1 | KJ548334 | 82.58 | 96.82 | 1.92299e−31 | Coleoptera | Dytiscidae | Hygrotus impressopunctatus |
| Hygrotus armeniacus mitochondrial partial 16S rRNA | HF931520 | 82.28 | 99.36 | 1.92299e−31 | Coleoptera | Dytiscidae | Hygrotus armeniacus |
| Hygrotus impressopunctatus partial 16S rRNA gene | AJ850405 | 82.47 | 96.82 | 1.92299e−31 | Coleoptera | Dytiscidae | Hygrotus impressopunctatus |
| Agabus didymus mitochondrial 16S rRNA gene (partia | LM654773 | 80.77 | 99.36 | 6.7119e−31 | Coleoptera | Dytiscidae | Agabus didymus |
| Agabus didymus voucher UNM KBMPmsp730 16S ribosoma | KJ548392 | 80.77 | 99.36 | 6.7119e−31 | Coleoptera | Dytiscidae | Agabus didymus |
| Herophydrus inquinatus 16S ribosomal RNA gene, par | KF575572 | 81.53 | 99.36 | 6.7119e−31 | Coleoptera | Dytiscidae | Herophydrus inquinatus |
| Hydaticus aruspex voucher NHRS/JLKB000000298 16S r | KF979119 | 80.77 | 99.36 | 6.7119e−31 | Coleoptera | Dytiscidae | Hydaticus aruspex |
| Hydroporus rufifrons 16S ribosomal RNA gene, parti | JX434758 | 81.53 | 99.36 | 6.7119e−31 | Coleoptera | Dytiscidae | Hydroporus rufifrons |
| Hydroporus discretus mitochondrial 16S rRNA gene ( | HF931449 | 81.53 | 99.36 | 6.7119e−31 | Coleoptera | Dytiscidae | Hydroporus discretus |
| Hydroporus thracicus mitochondrial 16S rRNA gene ( | HF931413 | 81.53 | 99.36 | 6.7119e−31 | Coleoptera | Dytiscidae | Hydroporus thracicus |
| Hydroporus macedonicus mitochondrial 16S rRNA gene | HE613690 | 81.53 | 99.36 | 6.7119e−31 | Coleoptera | Dytiscidae | Hydroporus macedonicus |
| Antiporus wilsoni mitochondrial partial 16S rRNA g | FR727249 | 80.52 | 98.09 | 6.7119e−31 | Coleoptera | Dytiscidae | Antiporus wilsoni |
| Herophydrus cf. gigas BMNH 681835 partial 16S rRNA | AJ850383 | 81.53 | 99.36 | 6.7119e−31 | Coleoptera | Dytiscidae | Herophydrus cf. gigas BMNH 681835 |
| Herophydrus inquinatus voucher MB1227 16S ribosoma | EF056676 | 81.53 | 99.36 | 6.7119e−31 | Coleoptera | Dytiscidae | Herophydrus inquinatus |
| Darwinhydrus solidus voucher MB354 16S ribosomal R | EF056671 | 83.02 | 99.36 | 6.7119e−31 | Coleoptera | Dytiscidae | Darwinhydrus solidus |
| Hydroporus macedonicus 16S ribosomal RNA gene, par | DQ195529 | 81.53 | 99.36 | 6.7119e−31 | Coleoptera | Dytiscidae | Hydroporus macedonicus |
| Agabus glacialis mitochondrial 16S rRNA gene (part | LM654776 | 80.13 | 99.36 | 2.34268e−30 | Coleoptera | Dytiscidae | Agabus glacialis |
| Hydaticus aruspex voucher UNM KBMHyar68 16S riboso | KJ548337 | 80.13 | 99.36 | 2.34268e−30 | Coleoptera | Dytiscidae | Hydaticus aruspex |
| Cybister marginicollis voucher UNM KBMCyma416 16S | KJ548319 | 80.13 | 99.36 | 2.34268e−30 | Coleoptera | Dytiscidae | Cybister marginicollis |
| Agabus dichrous mitochondrial partial 16S rRNA gen | HF931378 | 80.13 | 99.36 | 2.34268e−30 | Coleoptera | Dytiscidae | Agabus dichrous |
| Agabus godmanni mitochondrial 16S rRNA gene (parti | HF931348 | 80.13 | 99.36 | 2.34268e−30 | Coleoptera | Dytiscidae | Agabus godmanni |
| Paroster sp. R401 Newhaven voucher ABTC:78974 16S | JQ745754 | 80.89 | 99.36 | 2.34268e−30 | Coleoptera | Dytiscidae | Paroster sp. R401 Newhaven |
| Antiporus interrogationis mitochondrial partial 16 | FR727250 | 80.13 | 99.36 | 2.34268e−30 | Coleoptera | Dytiscidae | Antiporus interrogationis |
| Paroster sp. 2 RL−2008 16S ribosomal RNA gene, par | EU616958 | 80.89 | 99.36 | 2.34268e−30 | Coleoptera | Dytiscidae | Paroster sp. 2 RL−2008 |
| Paroster sp. spnMtWedge 16S ribosomal RNA gene, pa | EU616954 | 80.89 | 99.36 | 2.34268e−30 | Coleoptera | Dytiscidae | Paroster sp. spnMtWedge |
| Nirripirti bulbus isolate ABTC78658−9 16S ribosoma | EU616918 | 81.53 | 99.36 | 2.34268e−30 | Coleoptera | Dytiscidae | Paroster bulbus |
| Aglymbus gestroi 16S ribosomal RNA gene, partial s | AY138639 | 80.13 | 99.36 | 2.34268e−30 | Coleoptera | Dytiscidae | Aglymbus gestroi |
| Agabus dilatatus 16S ribosomal RNA gene, partial s | AY039261 | 80.13 | 99.36 | 2.34268e−30 | Coleoptera | Dytiscidae | Agabus dilatatus |
| Hydroporus submuticus 16S ribosomal RNA gene, part | JX434777 | 80.13 | 99.36 | 8.17677e−30 | Coleoptera | Dytiscidae | Hydroporus submuticus |
| Acilius duvergeri partial 16S rRNA gene | AJ850357 | 80.5 | 99.36 | 8.17677e−30 | Coleoptera | Dytiscidae | Acilius duvergeri |
| Agabus nebulosus mitochondrial 16S rRNA gene (part | LM654779 | 79.49 | 99.36 | 2.85397e−29 | Coleoptera | Dytiscidae | Agabus nebulosus |
| Hydroporus erythrocephalus 16S ribosomal RNA gene, | JX434775 | 80.89 | 99.36 | 2.85397e−29 | Coleoptera | Dytiscidae | Hydroporus erythrocephalus |
| Hydroporus nigrita 16S ribosomal RNA gene, partial | JX434769 | 80.89 | 99.36 | 2.85397e−29 | Coleoptera | Dytiscidae | Hydroporus nigrita |
| Hygrotus picatus mitochondrial partial 16S rRNA ge | HF931517 | 80.25 | 99.36 | 2.85397e−29 | Coleoptera | Dytiscidae | Hygrotus picatus |
| Hygrotus urgensis mitochondrial 16S rRNA gene (par | HF931486 | 80.89 | 99.36 | 2.85397e−29 | Coleoptera | Dytiscidae | Hygrotus urgensis |
| Agabus cephalotes mitochondrial 16S rRNA gene (par | HF931380 | 79.49 | 99.36 | 2.85397e−29 | Coleoptera | Dytiscidae | Agabus cephalotes |
| Hydroporus nigrita mitochondrial 16S rRNA gene (pa | HE613725 | 80.89 | 99.36 | 2.85397e−29 | Coleoptera | Dytiscidae | Hydroporus nigrita |
| Hydroporus sabaudus mitochondrial 16S rRNA gene (p | HE613720 | 80.89 | 99.36 | 2.85397e−29 | Coleoptera | Dytiscidae | Hydroporus sabaudus |
| Hydroporus kabakovi mitochondrial 16S rRNA gene (p | HE613714 | 81.01 | 99.36 | 2.85397e−29 | Coleoptera | Dytiscidae | Hydroporus kabakovi |
| Hydroporus discretus mitochondrial 16S rRNA gene ( | HE613709 | 80.89 | 99.36 | 2.85397e−29 | Coleoptera | Dytiscidae | Hydroporus discretus |
| Hydroporus brancuccii mitochondrial 16S rRNA gene | HE613696 | 80.89 | 99.36 | 2.85397e−29 | Coleoptera | Dytiscidae | Hydroporus brancuccii |
