# Supplementary material for: Testing the potential of a ribosomal 16S marker for DNA metabarcoding of insects
Source: PeerJ. 2016 Apr 19;4:e1966. doi: 10.7717/peerj.1966 (PMC4841222; doi:10.7717/peerj.1966)
Supplement: Supplemental Information 1 [file peerj-04-1966-s006.zip › scripts/5 match OTUs/blast_hits/OTU_389.csv_plot.pdf]

OTU_389.csv

Oreodytes sanmarkii

Dytiscidae

Coleoptera

97.42

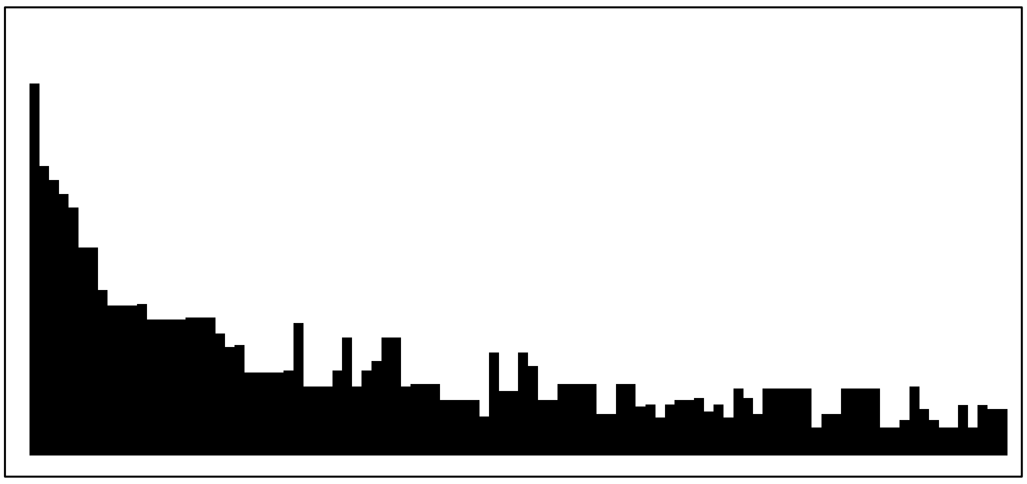

Oreodytes sanmarkii mitochondrial 16S rRNA gene (p	HF931524	97.42	98.72	1.20147e−65	Coleoptera	Dytiscidae	Oreodytes sanmarkii
Oreodytes obesus cordillerensis mitochondrial part	HF931516	93.55	98.72	1.67007e−57	Coleoptera	Dytiscidae	Oreodytes obesus cordillerensis
Oreodytes crassulus partial 16S rRNA gene	AJ850348	92.9	98.72	7.10131e−56	Coleoptera	Dytiscidae	Oreodytes crassulus
Oreodytes congruus partial 16S rRNA gene	AJ850347	92.26	98.72	8.65116e−55	Coleoptera	Dytiscidae	Oreodytes congruus
Oreodytes congruus 16S ribosomal RNA gene, partial	KF575550	91.61	98.72	3.67857e−53	Coleoptera	Dytiscidae	Oreodytes congruus
Oreodytes abbreviatus mitochondrial 16S rRNA gene	HF931477	89.74	98.72	9.87097e−48	Coleoptera	Dytiscidae	Oreodytes abbreviatus
Oreodytes rhyacophilus partial 16S rRNA gene	AJ850352	89.74	98.72	9.87097e−48	Coleoptera	Dytiscidae	Oreodytes rhyacophilus
Oreodytes mongolicus mitochondrial 16S rRNA gene (	HF931491	87.74	98.72	5.11329e−45	Coleoptera	Dytiscidae	Oreodytes mongolicus
Boreonectes riberae mitochondrial 16S rRNA gene (p	HF931461	87.01	98.72	1.78471e−44	Coleoptera	Dytiscidae	Boreonectes riberae
Boreonectes griseostriatus mitochondrial partial 1	HF931387	87.01	98.72	1.78471e−44	Coleoptera	Dytiscidae	Boreonectes griseostriatus
Boreonectes multilineatus mitochondrial partial 16	HF931384	87.01	98.72	1.78471e−44	Coleoptera	Dytiscidae	Boreonectes multilineatus
Oreodytes snoqualmie mitochondrial partial 16S rRN	HF931519	87.1	98.72	6.22926e−44	Coleoptera	Dytiscidae	Oreodytes snoqualmie
Boreonectes alpestris mitochondrial 16S rRNA gene	HF931512	86.36	98.72	7.58879e−43	Coleoptera	Dytiscidae	Boreonectes alpestris
Boreonectes striatellus mitochondrial 16S rRNA gen	HF931511	86.36	98.72	7.58879e−43	Coleoptera	Dytiscidae	Boreonectes striatellus
Boreonectes macedonicus mitochondrial 16S rRNA gen	HF931359	86.36	98.72	7.58879e−43	Coleoptera	Dytiscidae	Boreonectes macedonicus
Stictotarsus ibericus 16S ribosomal RNA (rrnL) gen	EF670030	86.36	98.72	7.58879e−43	Coleoptera	Dytiscidae	Stictotarsus ibericus
Oreodytes septentrionalis mitochondrial 16S rRNA g	HF931492	86.45	98.72	2.64875e−42	Coleoptera	Dytiscidae	Oreodytes septentrionalis
Oreodytes shorti mitochondrial 16S rRNA gene (part	HF931488	86.45	98.72	2.64875e−42	Coleoptera	Dytiscidae	Oreodytes shorti
Deronectes parvicollis mitochondrial 16S rRNA gene	HF931454	86.45	98.72	2.64875e−42	Coleoptera	Dytiscidae	Deronectes parvicollis
Boreonectes griseostriatus mitochondrial 16S rRNA	HF931541	85.71	98.72	9.24504e−42	Coleoptera	Dytiscidae	Boreonectes griseostriatus
Boreonectes griseostriatus mitochondrial 16S rRNA	HF931385	85.06	98.72	3.93109e−40	Coleoptera	Dytiscidae	Boreonectes griseostriatus
Laccornis difformis 16S ribosomal RNA gene, partia	KF575544	85.16	98.72	1.37209e−39	Coleoptera	Dytiscidae	Laccornis difformis
Oreodytes scitulus 16S ribosomal RNA gene, partial	KF575551	83.87	98.72	7.10758e−37	Coleoptera	Dytiscidae	Oreodytes scitulus
Oreodytes scitulus mitochondrial partial 16S rRNA	HF931515	83.87	98.72	7.10758e−37	Coleoptera	Dytiscidae	Oreodytes scitulus
Laccornis kocae partial 16S rRNA gene	AJ850419	83.87	98.72	7.10758e−37	Coleoptera	Dytiscidae	Laccornis kocae
Oreodytes natrix partial 16S rRNA gene	AJ850349	83.87	98.72	7.10758e−37	Coleoptera	Dytiscidae	Oreodytes natrix
Nebrioporus formaster mitochondrial partial 16S rR	HF931377	83.97	98.72	2.48079e−36	Coleoptera	Dytiscidae	Nebrioporus formaster
Deronectes moestus mitochondrial 16S rRNA gene (pa	HE613726	86.21	91.67	2.48079e−36	Coleoptera	Dytiscidae	Deronectes moestus
Oreodytes quadrimaculatus 16S ribosomal RNA gene,	KF575567	83.23	98.72	8.65881e−36	Coleoptera	Dytiscidae	Oreodytes quadrimaculatus
Aethionectes fulvonotatus voucher KBMAeop406 16S r	KF979088	83.23	98.72	8.65881e−36	Coleoptera	Dytiscidae	Aethionectes fulvonotatus
Oreodytes quadrimaculatus partial 16S rRNA gene	AJ850351	83.23	98.72	8.65881e−36	Coleoptera	Dytiscidae	Oreodytes quadrimaculatus
Stictotarsus maghrebinus 16S ribosomal RNA gene, p	AY250947	83.97	98.72	3.02222e−35	Coleoptera	Dytiscidae	Stictotarsus maghrebinus
Deronectes moestus 16S ribosomal RNA gene, partial	KF575561	85.52	91.67	1.05486e−34	Coleoptera	Dytiscidae	Deronectes moestus
Hygrotus patruelis mitochondrial 16S rRNA gene (pa	HF931388	83.23	98.72	1.05486e−34	Coleoptera	Dytiscidae	Hygrotus patruelis
Hydroporus foveolatus mitochondrial 16S rRNA gene	HE613724	83.97	98.72	1.05486e−34	Coleoptera	Dytiscidae	Hydroporus foveolatus
Deronectes opatrinus mitochondrial 16S rRNA gene (	HE613701	84.42	97.44	1.05486e−34	Coleoptera	Dytiscidae	Deronectes opatrinus
Deronectes brannani mitochondrial 16S rRNA gene (p	HE613695	85.52	91.67	1.05486e−34	Coleoptera	Dytiscidae	Deronectes brannanii
Deronectes moestus mitochondrial 16S rRNA gene (pa	HE613683	85.52	91.67	1.05486e−34	Coleoptera	Dytiscidae	Deronectes moestus
Hydroporus glabriusculus 16S ribosomal RNA gene, p	AY365270	83.23	98.72	1.05486e−34	Coleoptera	Dytiscidae	Hydroporus glabriusculus
Suphrodytes dorsalis 16S ribosomal RNA gene, parti	KF575577	83.33	98.72	3.68182e−34	Coleoptera	Dytiscidae	Suphrodytes dorsalis
Stictotarsus procerus mitochondrial 16S rRNA gene	HF931379	83.33	98.72	3.68182e−34	Coleoptera	Dytiscidae	Stictotarsus procerus
Suphrodytes dorsalis voucher BMNH:725966 16S ribos	JX221574	83.33	98.72	3.68182e−34	Coleoptera	Dytiscidae	Suphrodytes dorsalis
Copelatus sp. BI−2011 voucher BMNH792945 16S ribos	HQ381441	82.58	98.72	3.68182e−34	Coleoptera	Dytiscidae	Copelatus sp. BI−2011
Stictotarsus falli 16S ribosomal RNA (rrnL) gene,	EF670029	82.58	98.72	3.68182e−34	Coleoptera	Dytiscidae	Stictotarsus falli
Hygrotus novemlineatus mitochondrial partial 16S r	HF931523	82.58	98.72	1.28508e−33	Coleoptera	Dytiscidae	Hygrotus novemlineatus
Hygrotus marklini mitochondrial 16S rRNA gene (par	HF931467	82.58	98.72	1.28508e−33	Coleoptera	Dytiscidae	Hygrotus marklini
Boreonectes funereus mitochondrial 16S rRNA gene (	HF931393	81.82	98.72	1.28508e−33	Coleoptera	Dytiscidae	Boreonectes funereus
Deronectes doriae mitochondrial 16S rRNA gene (par	HE613710	84.83	91.67	1.28508e−33	Coleoptera	Dytiscidae	Deronectes doriae
Deronectes bicostatus mitochondrial 16S rRNA gene	HE613705	83.01	97.44	1.28508e−33	Coleoptera	Dytiscidae	Deronectes bicostatus
Hydroporus marginatus mitochondrial 16S rRNA gene	HE613699	83.02	98.72	1.28508e−33	Coleoptera	Dytiscidae	Hydroporus marginatus
Deronectes sahlbergi mitochondrial 16S rRNA gene (	HE613680	84.83	91.67	1.28508e−33	Coleoptera	Dytiscidae	Deronectes sahlbergi
Hovahydrus sp. BI−2011 voucher BMNH831333 16S ribo	HQ381524	84.18	99.36	1.28508e−33	Coleoptera	Dytiscidae	Hovahydrus sp. BI−2011
Hygrotus turbidus partial 16S rRNA gene	AJ850390	82.58	98.72	1.28508e−33	Coleoptera	Dytiscidae	Hygrotus turbidus
Hydroporus marginatus 16S ribosomal RNA gene, part	AF518267	82.58	98.72	1.28508e−33	Coleoptera	Dytiscidae	Hydroporus marginatus
Hydroporus tessellatus 16S ribosomal RNA gene, par	JX434770	83.33	98.72	4.48537e−33	Coleoptera	Dytiscidae	Hydroporus tessellatus
Hydroporus limbatus mitochondrial 16S rRNA gene (p	HE613706	83.33	98.72	4.48537e−33	Coleoptera	Dytiscidae	Hydroporus limbatus
Hydroporus zimmermanni mitochondrial 16S rRNA gene	HE613679	83.33	98.72	4.48537e−33	Coleoptera	Dytiscidae	Hydroporus zimmermanni
Hydroporus basinotatus mitochondrial 16S rRNA gene	HE613678	83.33	98.72	4.48537e−33	Coleoptera	Dytiscidae	Hydroporus basinotatus
Copelatus sp. BI−2011 voucher BMNH671215 16S ribos	HQ381377	81.94	98.72	4.48537e−33	Coleoptera	Dytiscidae	Copelatus sp. BI−2011
Antiporus hollingsworthi mitochondrial partial 16S	FR727246	81.94	98.72	4.48537e−33	Coleoptera	Dytiscidae	Antiporus hollingsworthi
Hydroporus basinotatus 16S ribosomal RNA gene, par	AF518258	83.33	98.72	4.48537e−33	Coleoptera	Dytiscidae	Hydroporus basinotatus
Hydroporus limbatus 16S ribosomal RNA gene, partia	AF518264	83.33	98.72	4.48537e−33	Coleoptera	Dytiscidae	Hydroporus limbatus
Suphrodytes figuratus voucher BMNH:722148 16S ribo	JX221571	82.28	98.72	1.56555e−32	Coleoptera	Dytiscidae	Suphrodytes figuratus
Hydroporus analis mitochondrial partial 16S rRNA g	HE613731	82.39	98.72	1.56555e−32	Coleoptera	Dytiscidae	Hydroporus analis
Hydroporus fuscipennis mitochondrial 16S rRNA gene	HE613730	81.76	98.72	1.56555e−32	Coleoptera	Dytiscidae	Hydroporus fuscipennis
Hydroporus compunctus mitochondrial 16S rRNA gene	HE599693	82.39	98.72	1.56555e−32	Coleoptera	Dytiscidae	Hydroporus compunctus
Hydrocolus sahlbergi mitochondrial 16S rRNA gene (	HE599691	82.58	98.72	1.56555e−32	Coleoptera	Dytiscidae	Hydrocolus sahlbergi
Hydrocolus picicornis partial 16S rRNA gene	AJ850379	82.58	98.72	1.56555e−32	Coleoptera	Dytiscidae	Hydrocolus picicornis
Nirripirti megamacrocephalus isolate ABTC78781 16S	EU616935	82.69	98.72	1.56555e−32	Coleoptera	Dytiscidae	Paroster megamacrocephalus
Scarodytes halensis haplotype 3 16S ribosomal RNA	AY250937	82.05	98.72	1.56555e−32	Coleoptera	Dytiscidae	Scarodytes halensis
Hydroporus compunctus 16S ribosomal RNA gene, part	AF518259	82.39	98.72	1.56555e−32	Coleoptera	Dytiscidae	Hydroporus compunctus
Hydroporus fuscipennis 16S ribosomal RNA gene, par	AF518262	81.76	98.72	1.56555e−32	Coleoptera	Dytiscidae	Hydroporus fuscipennis
Deronectes aubei voucher UNM KBMDeau758 16S riboso	KJ548292	83.12	97.44	5.4643e−32	Coleoptera	Dytiscidae	Deronectes aubei
Hydroporus thracicus mitochondrial 16S rRNA gene (	HF931413	82.69	98.72	5.4643e−32	Coleoptera	Dytiscidae	Hydroporus thracicus
Hygrotus sanfilippoi mitochondrial 16S rRNA gene (	HF931342	81.94	98.72	5.4643e−32	Coleoptera	Dytiscidae	Hygrotus sanfilippoi
Deronectes costipennis mitochondrial 16S rRNA gene	HE613697	83.12	97.44	5.4643e−32	Coleoptera	Dytiscidae	Deronectes costipennis
Deronectes platynotus mitochondrial 16S rRNA gene	HE613682	83.12	97.44	5.4643e−32	Coleoptera	Dytiscidae	Deronectes platynotus
Deronectes depressicollis mitochondrial 16S rRNA g	HE613681	83.12	97.44	5.4643e−32	Coleoptera	Dytiscidae	Deronectes depressicollis
Deronectes semirufus mitochondrial 16S rRNA gene (	HE613676	83.12	97.44	5.4643e−32	Coleoptera	Dytiscidae	Deronectes semirufus
Deronectes semirufus mitochondrial 16S rRNA gene (	HE613674	83.12	97.44	5.4643e−32	Coleoptera	Dytiscidae	Deronectes semirufus
Antiporus hollingsworthi mitochondrial partial 16S	FR727247	81.29	98.72	5.4643e−32	Coleoptera	Dytiscidae	Antiporus hollingsworthi
Nirripirti newhavenensis 16S ribosomal RNA gene, p	AY353844	81.94	98.72	5.4643e−32	Coleoptera	Dytiscidae	Paroster newhavenensis
Hydroporus sp. IR33.4 16S ribosomal RNA gene, part	AY365261	81.94	98.72	5.4643e−32	Coleoptera	Dytiscidae	Hydroporus sp. IR33.4
Deronectes costipennis 16S ribosomal RNA gene, par	AY250951	83.12	97.44	5.4643e−32	Coleoptera	Dytiscidae	Deronectes costipennis
Deronectes aubei sanfilippoi haplotype 2 16S ribos	AY250908	83.12	97.44	5.4643e−32	Coleoptera	Dytiscidae	Deronectes aubei sanfilippoi
Deronectes aubei sanfilippoi haplotype 1 16S ribos	AY250907	83.12	97.44	5.4643e−32	Coleoptera	Dytiscidae	Deronectes aubei sanfilippoi
Deronectes aubei aubei haplotype 2 16S ribosomal R	AY250906	83.12	97.44	5.4643e−32	Coleoptera	Dytiscidae	Deronectes aubei aubei
Hydaticus cinctipennis voucher UNM KBMHyci20 16S r	KJ548338	81.29	98.72	1.90723e−31	Coleoptera	Dytiscidae	Hydaticus cinctipennis
Cybister marginicollis voucher UNM KBMCyma416 16S	KJ548319	81.29	98.72	1.90723e−31	Coleoptera	Dytiscidae	Cybister marginicollis
Suphrodytes figuratus voucher BMNH:722150 16S ribo	JX221572	81.65	98.72	1.90723e−31	Coleoptera	Dytiscidae	Suphrodytes figuratus
Deronectes hispanicus mitochondrial 16S rRNA gene	HE613727	83.23	97.44	1.90723e−31	Coleoptera	Dytiscidae	Deronectes hispanicus
Hydroporus pubescens mitochondrial 16S rRNA gene (	HE613707	82.17	98.72	1.90723e−31	Coleoptera	Dytiscidae	Hydroporus pubescens
Suphrodytes dorsalis mitochondrial 16S rRNA gene (	HE599700	81.65	98.72	1.90723e−31	Coleoptera	Dytiscidae	Suphrodytes dorsalis
Antiporus wilsoni mitochondrial partial 16S rRNA g	FR727249	81.29	98.72	1.90723e−31	Coleoptera	Dytiscidae	Antiporus wilsoni
Antiporus sp. MB1421 mitochondrial partial 16S rRN	FR727245	81.29	98.72	1.90723e−31	Coleoptera	Dytiscidae	Antiporus sp. MB1421
Hygrotus impressopunctatus partial 16S rRNA gene	AJ850405	82.35	97.44	1.90723e−31	Coleoptera	Dytiscidae	Hygrotus impressopunctatus
Nirripirti byroensis isolate ABTC78677−8 16S ribos	EU616910	81.29	98.72	1.90723e−31	Coleoptera	Dytiscidae	Paroster byroensis
Deronectes delarouzei haplotype 2 16S ribosomal RN	AY250909	82.35	97.44	1.90723e−31	Coleoptera	Dytiscidae	Deronectes delarouzei
Hydroporus rufifrons 16S ribosomal RNA gene, parti	AF518277	82.17	98.72	1.90723e−31	Coleoptera	Dytiscidae	Hydroporus rufifrons
Hydroporus pubescens 16S ribosomal RNA gene, parti	AF518276	82.17	98.72	1.90723e−31	Coleoptera	Dytiscidae	Hydroporus pubescens
