# Supplementary material for: Testing the potential of a ribosomal 16S marker for DNA metabarcoding of insects
Source: PeerJ. 2016 Apr 19;4:e1966. doi: 10.7717/peerj.1966 (PMC4841222; doi:10.7717/peerj.1966)
Supplement: Supplemental Information 1 [file peerj-04-1966-s006.zip › scripts/5 match OTUs/blast_hits/OTU_39.csv_plot.pdf]

OTU_39.csv

Rhyacophila balcanica

Rhyacophilidae

Trichoptera

90.58

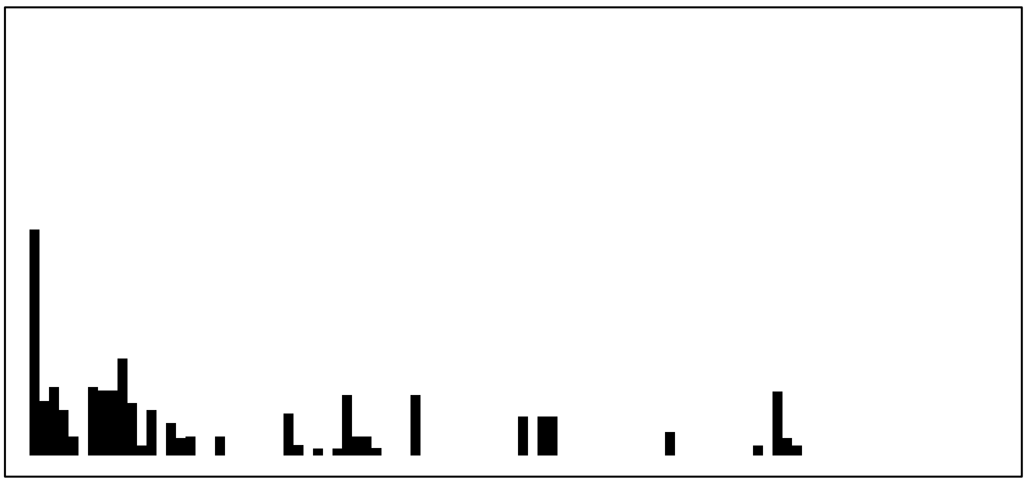

Rhyacophila balcanica voucher RbalL1 16S ribosomal	FJ002822	90.58	100	1.51922e−44	Trichoptera	Rhyacophilidae	Rhyacophila balcanica
Camptomyia heterobia voucher 12 16S ribosomal RNA	JQ613882	82.54	90.58	1.85242e−24	Diptera	Cecidomyiidae	Camptomyia heterobia
Scatella hawaiiensis voucher 105726 NADH dehydroge	EU494299	83.2	88.41	1.85242e−24	Diptera	Ephydridae	Scatella hawaiiensis
Sphindus americanus voucher BYU CO611 16S ribosoma	KC966534	82.11	89.13	6.46559e−24	Coleoptera	Sphindidae	Sphindus americanus
Aneugmenus kiotonis mitochondrial gene for 16S rib	AB859178	80.88	97.1	2.25671e−23	Hymenoptera	Tenthredinidae	Aneugmenus kiotonis
Ditomyia fasciata voucher JSSJ3 16S ribosomal RNA	KJ136734	79.55	95.65	7.8767e−23	Diptera	Ditomyiidae	Ditomyia fasciata
Catogenus rufus voucher BYU CO613 16S ribosomal RN	KC966532	83.19	86.23	2.74924e−22	Coleoptera	Passandridae	Catogenus rufus
Mycetophagus quadripustulatus voucher BMNH840485 m	HQ232824	83.04	81.16	9.59578e−22	Coleoptera	Mycetophagidae	Mycetophagus quadripustulatus
Mycetophagus quadripustulatus 16S ribosomal RNA ge	EF490159	83.04	81.16	9.59578e−22	Coleoptera	Mycetophagidae	Mycetophagus quadripustulatus
Phytobaenus amabilis voucher BMNH:679279 16S ribos	DQ202594	84.55	77.54	9.59578e−22	Coleoptera	Aderidae	Phytobaenus amabilis
Pyxidicerina sp. JP−2014 isolate JPDG37 16S riboso	KM350444	82.46	82.61	1.16901e−20	Coleoptera	Staphylinidae	Pyxidicerina sp. JP−2014
Conocoxa chalicipoda voucher AE088 16S ribosomal R	KF318484	80.45	93.48	1.16901e−20	Hymenoptera	Pergidae	Conocoxa chalicipoda
Alticini sp. BMNH 835476 16S ribosomal RNA gene, p	FJ973809	82.11	88.41	1.16901e−20	Coleoptera	Chrysomelidae	Alticini sp. BMNH 835476
Brachygluta simplex voucher BMNH:667999 16S riboso	DQ202574	78.17	97.1	1.16901e−20	Coleoptera	Staphylinidae	Brachygluta simplex
Semidalis aleyrodiformis 16S ribosomal RNA gene, p	EF632331	81.51	86.23	1.16901e−20	Neuroptera	Coniopterygidae	Semidalis aleyrodiformis
Calicnemia sinensis voucher RMNH.INS.229127 16S ri	KF369654	80.8	89.86	4.08023e−20	Odonata	Platycnemididae	Calicnemia sinensis
Silvacalles pedestris bio−material ZFMK−DNA JJ0155	GU988220	80.87	83.33	4.08023e−20	Coleoptera	Curculionidae	Silvacalles pedestris
Blepharhymenus sp. ZMUN 10008487 16S ribosomal RNA	JX536427	79.55	94.2	1.42414e−19	Coleoptera	Staphylinidae	Blepharhymenus sp. ZMUN 10008487
Lamprochromus strobli voucher Pollet 0152 (P) 16S	HQ449016	78.57	97.83	1.42414e−19	Diptera	Dolichopodidae	Lamprochromus strobli
Silvacalles pedestris bio−material ZFMK−DNA JJ0494	GU988365	80.87	83.33	1.42414e−19	Coleoptera	Curculionidae	Silvacalles pedestris
Falklandius antarcticus voucher 673222 16S ribosom	EF214121	78.99	96.38	1.42414e−19	Coleoptera	Curculionidae	Falklandius antarcticus
Falklandius antarcticus voucher 673236 16S ribosom	EF214129	78.99	96.38	1.42414e−19	Coleoptera	Curculionidae	Falklandius antarcticus
Falklandius antarcticus voucher 668282 16S ribosom	EF213993	78.99	96.38	1.42414e−19	Coleoptera	Curculionidae	Falklandius antarcticus
Falklandius antarcticus mitochondrial 16S rRNA gen	FM994722	78.99	96.38	1.42414e−19	Coleoptera	Curculionidae	Falklandius antarcticus
Lasinus mikado isolate JPDG26 16S ribosomal RNA ge	KM350433	78.36	97.1	4.97074e−19	Coleoptera	Staphylinidae	Lasinus mikado
Coleoptera sp. BI−2011 voucher BMNH831308 16S ribo	HQ381521	79.07	93.48	4.97074e−19	Coleoptera		Coleoptera sp. BI−2011
Aphanocephalus sp. 1 NPL−2007 16S ribosomal RNA ge	EU164591	81.97	86.96	4.97074e−19	Coleoptera	Discolomatidae	Aphanocephalus sp. 1 NPL−2007
Xyela sp. Sus1 16S ribosomal RNA gene, partial seq	AY206772	80.49	88.41	4.97074e−19	Hymenoptera	Xyelidae	Xyela sp. Sus1
Cyrtodiopsis quinqueguttata 16S ribosomal RNA gene	AF304748	79.37	89.86	4.97074e−19	Diptera	Diopsidae	Teleopsis quinqueguttata
Cyrtodiopsis quinqueguttata isolate Cq3 16S riboso	AY876593	80.31	89.86	4.97074e−19	Diptera	Diopsidae	Teleopsis quinqueguttata
Cyrtodiopsis quinqueguttata isolate Cq2 16S riboso	AY876592	79.37	89.86	4.97074e−19	Diptera	Diopsidae	Teleopsis quinqueguttata
Cyrtodiopsis quinqueguttata isolate Cq1 16S riboso	AY876591	80.31	89.86	4.97074e−19	Diptera	Diopsidae	Teleopsis quinqueguttata
Epitheca bimaculata mitochondrial gene for 16S rRN	AB707952	82.83	71.74	1.73496e−18	Odonata	Corduliidae	Epitheca bimaculata
Silvacalles pedestris bio−material ZFMK−DNA JJ0775	GU988523	80.87	83.33	1.73496e−18	Coleoptera	Curculionidae	Silvacalles pedestris
Silvacalles pedestris bio−material ZFMK−DNA JJ0156	GU988221	80.87	83.33	1.73496e−18	Coleoptera	Curculionidae	Silvacalles pedestris
Melandrya sp. UPOL ZL0120 16S ribosomal RNA gene,	FJ903739	80.34	83.33	1.73496e−18	Coleoptera	Melandryidae	Melandrya sp. UPOL ZL0120
Orectogyrus ornaticollis voucher BMNH831252 16S ri	HQ381518	79.84	86.96	1.73496e−18	Coleoptera	Gyrinidae	Orectogyrus ornaticollis
Lesticus magnus mitochondrial gene for 16S ribosom	AB620066	79.37	88.41	1.73496e−18	Coleoptera	Carabidae	Lesticus magnus
Darwinella sp. DAM2 mitochondrial 16S rRNA gene (p	FM994752	79.07	90.58	1.73496e−18	Coleoptera	Perimylopidae	Darwinella sp. DAM2
Epitheca canis 12S ribosomal RNA gene, partial seq	EU477712	82.83	71.74	1.73496e−18	Odonata	Corduliidae	Tetragoneuria canis
Darwinella amaroides voucher 673161 16S ribosomal	EF214088	79.23	90.58	1.73496e−18	Coleoptera	Perimylopidae	Darwinella amaroides
Brachypterolus sp. BMNH 697022 16S ribosomal RNA g	EF512351	79.53	91.3	1.73496e−18	Coleoptera	Kateretidae	Brachypterolus sp. BMNH 697022
Trichiosoma sp. KNS mitochondrial gene for 16S rib	AB859198	78.23	84.78	6.0556e−18	Hymenoptera	Cimbicidae	Trichiosoma sp. KNS
Calicnemia chaseni voucher RMNH.INS.500057 16S rib	KF369653	80	81.88	6.0556e−18	Odonata	Platycnemididae	Calicnemia chaseni
Dziedzickia marginata voucher JSM7 16S ribosomal R	KC435569	78.95	94.2	6.0556e−18	Diptera	Mycetophilidae	Dziedzickia marginata
Ruteria hypocrita bio−material ZFMK−DNA JJ0324 16S	GU213538	77.94	96.38	6.0556e−18	Coleoptera	Curculionidae	Echinodera hypocrita
Ruteria hypocrita isolate ZFMK−DNA JJ0205 16S ribo	EU286356	77.94	96.38	6.0556e−18	Coleoptera	Curculionidae	Echinodera hypocrita
Laricobius erichsoni voucher BMNH:679165 16S ribos	DQ202530	78.57	89.86	6.0556e−18	Coleoptera		Laricobius erichsoni
Philomastix xanthophylax voucher AE104 16S ribosom	KF318487	78.52	95.65	2.11361e−17	Hymenoptera	Pergidae	Philomastix xanthophylax
Chaetarthria incisa 16S ribosomal RNA gene, partia	KC992655	76.76	96.38	2.11361e−17	Coleoptera	Hydrophilidae	Chaetarthria incisa
Epitheca bimaculata mitochondrial gene for 16S rRN	AB707954	81.82	71.74	2.11361e−17	Odonata	Corduliidae	Epitheca bimaculata
Neurigona pectinata voucher RMBR:103457 16S riboso	FJ808185	78.29	92.03	2.11361e−17	Diptera	Dolichopodidae	Neurigona pectinata
Epitheca costalis 12S ribosomal RNA gene, partial	EU477713	81.82	71.74	2.11361e−17	Odonata	Corduliidae	Epitheca costalis
Epitheca bimaculata 12S ribosomal RNA gene, partia	EU477708	81.82	71.74	2.11361e−17	Odonata	Corduliidae	Epitheca bimaculata
Meru phyllisae mitochondrial partial 16S rRNA gene	FM163591	77.94	94.93	2.11361e−17	Coleoptera	Meruidae	Meru phyllisae
Glypta fumiferanae 16S ribosomal RNA gene, partial	DQ538563	79.03	86.23	2.11361e−17	Hymenoptera	Ichneumonidae	Glypta fumiferanae
Lophyrotoma analis voucher Sus37 16S ribosomal RNA	EF032171	78.01	98.55	2.11361e−17	Hymenoptera	Pergidae	Lophyrotoma analis
Xiphydria mellipes 16S ribosomal RNA gene, partial	AF379857	77.87	88.41	2.11361e−17	Hymenoptera	Xiphydriidae	Xiphydria mellipes
Hydrellia modesta 16S ribosomal RNA gene, partial	AF126334	78.86	88.41	2.11361e−17	Diptera	Ephydridae	Hydrellia modesta
Philomastix macleaii voucher AE090 16S ribosomal R	KF318486	78.03	94.2	7.37723e−17	Hymenoptera	Pergidae	Philomastix macleaii
Coeliccia ryukyuensis mitochondrial gene for 16S r	AB707500	79.53	89.86	7.37723e−17	Odonata	Platycnemididae	Coeliccia ryukyuensis
Dolichopodidae nr. Oedematopus sp. Pollet 0315 16S	HQ448979	78.69	86.96	7.37723e−17	Diptera	Dolichopodidae	Dolichopodidae nr. Oedematopus sp. Pollet 0315
Melandrya dubia voucher UPOL ZL0155 16S ribosomal	FJ903755	79.49	83.33	7.37723e−17	Coleoptera	Melandryidae	Melandrya dubia
Andogyrus sedilloti voucher BMNH831248 16S ribosom	HQ381517	79.03	86.96	7.37723e−17	Coleoptera	Gyrinidae	Andogyrus sedilloti
Acalles pedestris isolate ZFMK−DNA JJ0650 16S ribo	FJ716528	80	83.33	7.37723e−17	Coleoptera	Curculionidae	Silvacalles pedestris
Tychius quinquepunctatus partial mitochondrial 16S	AJ495547	81.08	79.71	7.37723e−17	Coleoptera	Curculionidae	Tychius quinquepunctatus
Aphanarthrum glabrum isolate 59 La Palma 16S ribos	AY514262	77.21	95.65	7.37723e−17	Coleoptera	Curculionidae	Aphanarthrum glabrum
Akis spinosa 16S ribosomal RNA gene, partial seque	KJ003010	77.7	96.38	2.57491e−16	Coleoptera	Tenebrionidae	Akis spinosa
Semiclaviger sikorae isolate JPDG39 16S ribosomal	KM350446	79.2	89.86	2.57491e−16	Coleoptera	Staphylinidae	Semiclaviger sikorae
Thrinax japonica mitochondrial gene for 16S riboso	AB859194	75.18	96.38	2.57491e−16	Hymenoptera	Tenthredinidae	Thrinax japonica
Antiperga antiopa voucher AE187 16S ribosomal RNA	KF318513	78.51	86.23	2.57491e−16	Hymenoptera	Pergidae	Antiperga antiopa
Pterygophorus nr. turneri SS1 voucher AE051 16S ri	KF318475	75.89	96.38	2.57491e−16	Hymenoptera	Pergidae	Pterygophorus nr. turneri SS1
Neodiprion sertifer voucher AE004 16S ribosomal RN	KF318464	77.95	89.86	2.57491e−16	Hymenoptera	Diprionidae	Neodiprion sertifer
Coeliccia poungyi voucher RMNH.INS.229162 16S ribo	KF369674	79.66	84.06	2.57491e−16	Odonata	Platycnemididae	Coeliccia poungyi
Coeliccia cyanomelas voucher RMNH.INS.228208 16S r	KF369670	80.47	89.86	2.57491e−16	Odonata	Platycnemididae	Coeliccia cyanomelas
Micromorphus albipes voucher Pollet 0156 (P) 16S r	HQ448998	77.87	88.41	2.57491e−16	Diptera	Dolichopodidae	Micromorphus albipes
Mycetophagus atomarius voucher UPOL ZL0150 16S rib	FJ903753	82.98	68.12	2.57491e−16	Coleoptera	Mycetophagidae	Mycetophagus atomarius
Tetragoneuria cynosura 12S ribosomal RNA gene, par	EU477709	80.81	71.74	2.57491e−16	Odonata	Corduliidae	Tetragoneuria cynosura
Coeliccia cyanomelas 16S ribosomal RNA gene, parti	EU055081	80.47	89.86	2.57491e−16	Odonata	Platycnemididae	Coeliccia cyanomelas
Nannothemis bella 12S ribosomal RNA gene, partial	EF640388	77.17	91.3	2.57491e−16	Odonata	Libellulidae	Nannothemis bella
Somatochlora clavata 16S ribosomal RNA gene, parti	EF032714	78.51	87.68	2.57491e−16	Odonata	Corduliidae	Somatochlora clavata
Selandria serva voucher Sus32 16S ribosomal RNA ge	EF032166	76.69	96.38	2.57491e−16	Hymenoptera	Tenthredinidae	Selandria serva
Aphanarthrum glabrum glabrum isolate 154 Tenerife	DQ460296	76.47	95.65	2.57491e−16	Coleoptera	Curculionidae	Aphanarthrum glabrum glabrum
Daphnephila sp. SYY−2001−12 16S ribosomal RNA gene	AF334197	78.4	89.86	2.57491e−16	Diptera	Cecidomyiidae	Daphnephila sp. SYY−2001−12
Agathemera crassa 16S ribosomal RNA gene, partial	KJ024421	78.33	86.23	8.98731e−16	Phasmatodea	Agathemeridae	Agathemera crassa
Haplostegus epimelas voucher AE174 16S ribosomal R	KF318511	76.43	98.55	8.98731e−16	Hymenoptera	Pergidae	Haplostegus epimelas
Indocnemis ambigua voucher RMNH.INS.228684 16S rib	KF369742	79.37	89.86	8.98731e−16	Odonata	Platycnemididae	Indocnemis ambigua
Argia insipida 16S ribosomal RNA gene, partial seq	JX121161	77.24	89.13	8.98731e−16	Odonata	Coenagrionidae	Argia insipida
Agathemera crassa voucher MNHNCHPHA 0000009 16S ri	JN228848	78.33	86.23	8.98731e−16	Phasmatodea	Agathemeridae	Agathemera crassa
Agathemera crassa voucher MNHNCHPHA 0000008 16S ri	JN228847	78.33	86.23	8.98731e−16	Phasmatodea	Agathemeridae	Agathemera crassa
Aphrosylus celtiber voucher Pollet 0195 (P) 16S ri	HQ448971	79.46	79.71	8.98731e−16	Diptera	Dolichopodidae	Aphrosylus celtiber
Lygistorrhina sp. ER−2009 16S ribosomal RNA gene,	FJ172007	78.38	80.43	8.98731e−16	Diptera	Lygistorrhinidae	Lygistorrhina sp. ER−2009
Aphanarthrum subglabrum isolate 121 La Palma 16S r	DQ460271	76.47	95.65	8.98731e−16	Coleoptera	Curculionidae	Aphanarthrum subglabrum
Aphanarthrum subglabrum isolate 122 La Palma 16S r	DQ460272	76.47	95.65	8.98731e−16	Coleoptera	Curculionidae	Aphanarthrum subglabrum
Daphnephila sp. SYY−2001−13 16S ribosomal RNA gene	AF334198	78.57	89.86	8.98731e−16	Diptera	Cecidomyiidae	Daphnephila sp. SYY−2001−13
Aphanarthrum subglabrum isolate 51 La Palma 16S ri	AY514256	76.47	95.65	8.98731e−16	Coleoptera	Curculionidae	Aphanarthrum subglabrum
Aphanarthrum subglabrum isolate 42 La Palma 16S ri	AY514248	76.47	95.65	8.98731e−16	Coleoptera	Curculionidae	Aphanarthrum subglabrum
Plecia sp. DK−2014 voucher JSOUT2 16S ribosomal RN	KJ136725	79.66	83.33	3.13688e−15	Diptera	Bibionidae	Plecia sp. DK−2014
Nematus sp. 1_KMKC mitochondrial gene for 16S ribo	AB859199	77.69	86.96	3.13688e−15	Hymenoptera	Tenthredinidae	Nematus sp. 1_KMKC
Nematinus luteus voucher Sus124 16S ribosomal RNA	JF505486	76.52	94.2	3.13688e−15	Hymenoptera	Tenthredinidae	Nematinus luteus
