# Supplementary material for: Testing the potential of a ribosomal 16S marker for DNA metabarcoding of insects
Source: PeerJ. 2016 Apr 19;4:e1966. doi: 10.7717/peerj.1966 (PMC4841222; doi:10.7717/peerj.1966)
Supplement: Supplemental Information 1 [file peerj-04-1966-s006.zip › scripts/5 match OTUs/blast_hits/OTU_394.csv_plot.pdf]

OTU_394.csv

Oreodytes sanmarkii

Dytiscidae

Coleoptera

96.82

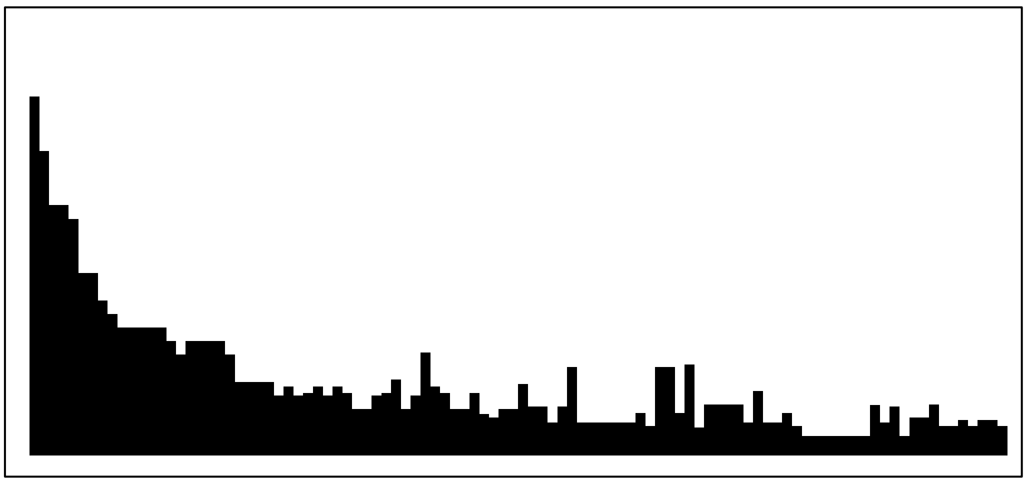

Oreodytes sanmarkii mitochondrial 16S rRNA gene (p	HF931524	96.82	100	4.19354e−65	Coleoptera	Dytiscidae	Oreodytes sanmarkii
Oreodytes obesus cordillerensis mitochondrial part	HF931516	94.27	100	1.12528e−59	Coleoptera	Dytiscidae	Oreodytes obesus cordillerensis
Oreodytes crassulus partial 16S rRNA gene	AJ850348	91.72	100	3.01955e−54	Coleoptera	Dytiscidae	Oreodytes crassulus
Oreodytes congruus partial 16S rRNA gene	AJ850347	91.72	100	3.01955e−54	Coleoptera	Dytiscidae	Oreodytes congruus
Oreodytes congruus 16S ribosomal RNA gene, partial	KF575550	91.08	100	3.67857e−53	Coleoptera	Dytiscidae	Oreodytes congruus
Oreodytes abbreviatus mitochondrial 16S rRNA gene	HF931477	88.54	100	9.87097e−48	Coleoptera	Dytiscidae	Oreodytes abbreviatus
Oreodytes rhyacophilus partial 16S rRNA gene	AJ850352	88.54	100	9.87097e−48	Coleoptera	Dytiscidae	Oreodytes rhyacophilus
Oreodytes mongolicus mitochondrial 16S rRNA gene (	HF931491	87.26	100	5.11329e−45	Coleoptera	Dytiscidae	Oreodytes mongolicus
Oreodytes snoqualmie mitochondrial partial 16S rRN	HF931519	86.62	100	2.17423e−43	Coleoptera	Dytiscidae	Oreodytes snoqualmie
Oreodytes septentrionalis mitochondrial 16S rRNA g	HF931492	85.99	100	2.64875e−42	Coleoptera	Dytiscidae	Oreodytes septentrionalis
Oreodytes shorti mitochondrial 16S rRNA gene (part	HF931488	85.99	100	2.64875e−42	Coleoptera	Dytiscidae	Oreodytes shorti
Boreonectes riberae mitochondrial 16S rRNA gene (p	HF931461	85.99	100	3.22684e−41	Coleoptera	Dytiscidae	Boreonectes riberae
Boreonectes griseostriatus mitochondrial partial 1	HF931387	85.99	100	3.22684e−41	Coleoptera	Dytiscidae	Boreonectes griseostriatus
Boreonectes multilineatus mitochondrial partial 16	HF931384	85.99	100	3.22684e−41	Coleoptera	Dytiscidae	Boreonectes multilineatus
Deronectes parvicollis mitochondrial 16S rRNA gene	HF931454	85.35	100	1.12628e−40	Coleoptera	Dytiscidae	Deronectes parvicollis
Laccornis difformis 16S ribosomal RNA gene, partia	KF575544	84.71	100	1.37209e−39	Coleoptera	Dytiscidae	Laccornis difformis
Boreonectes alpestris mitochondrial 16S rRNA gene	HF931512	85.35	100	1.37209e−39	Coleoptera	Dytiscidae	Boreonectes alpestris
Boreonectes striatellus mitochondrial 16S rRNA gen	HF931511	85.35	100	1.37209e−39	Coleoptera	Dytiscidae	Boreonectes striatellus
Boreonectes macedonicus mitochondrial 16S rRNA gen	HF931359	85.35	100	1.37209e−39	Coleoptera	Dytiscidae	Boreonectes macedonicus
Stictotarsus ibericus 16S ribosomal RNA (rrnL) gen	EF670030	85.35	100	1.37209e−39	Coleoptera	Dytiscidae	Stictotarsus ibericus
Boreonectes griseostriatus mitochondrial 16S rRNA	HF931541	84.71	100	1.67154e−38	Coleoptera	Dytiscidae	Boreonectes griseostriatus
Oreodytes scitulus 16S ribosomal RNA gene, partial	KF575551	83.44	100	7.10758e−37	Coleoptera	Dytiscidae	Oreodytes scitulus
Oreodytes scitulus mitochondrial partial 16S rRNA	HF931515	83.44	100	7.10758e−37	Coleoptera	Dytiscidae	Oreodytes scitulus
Laccornis kocae partial 16S rRNA gene	AJ850419	83.44	100	7.10758e−37	Coleoptera	Dytiscidae	Laccornis kocae
Hydroporus glabriusculus 16S ribosomal RNA gene, p	AY365270	83.44	100	8.65881e−36	Coleoptera	Dytiscidae	Hydroporus glabriusculus
Oreodytes natrix partial 16S rRNA gene	AJ850349	82.8	100	3.02222e−35	Coleoptera	Dytiscidae	Oreodytes natrix
Hydroporus tessellatus 16S ribosomal RNA gene, par	JX434770	83.23	100	1.05486e−34	Coleoptera	Dytiscidae	Hydroporus tessellatus
Hygrotus novemlineatus mitochondrial partial 16S r	HF931523	82.8	100	1.05486e−34	Coleoptera	Dytiscidae	Hygrotus novemlineatus
Nebrioporus formaster mitochondrial partial 16S rR	HF931377	82.91	100	1.05486e−34	Coleoptera	Dytiscidae	Nebrioporus formaster
Hydroporus basinotatus mitochondrial 16S rRNA gene	HE613678	83.23	100	1.05486e−34	Coleoptera	Dytiscidae	Hydroporus basinotatus
Hygrotus turbidus partial 16S rRNA gene	AJ850390	82.8	100	1.05486e−34	Coleoptera	Dytiscidae	Hygrotus turbidus
Hydroporus basinotatus 16S ribosomal RNA gene, par	AF518258	83.23	100	1.05486e−34	Coleoptera	Dytiscidae	Hydroporus basinotatus
Suphrodytes dorsalis 16S ribosomal RNA gene, parti	KF575577	82.91	100	3.68182e−34	Coleoptera	Dytiscidae	Suphrodytes dorsalis
Oreodytes quadrimaculatus 16S ribosomal RNA gene,	KF575567	82.17	100	3.68182e−34	Coleoptera	Dytiscidae	Oreodytes quadrimaculatus
Aethionectes fulvonotatus voucher KBMAeop406 16S r	KF979088	82.17	100	3.68182e−34	Coleoptera	Dytiscidae	Aethionectes fulvonotatus
Boreonectes griseostriatus mitochondrial 16S rRNA	HF931385	82.8	100	3.68182e−34	Coleoptera	Dytiscidae	Boreonectes griseostriatus
Suphrodytes dorsalis voucher BMNH:725966 16S ribos	JX221574	82.91	100	3.68182e−34	Coleoptera	Dytiscidae	Suphrodytes dorsalis
Hydroporus foveolatus mitochondrial 16S rRNA gene	HE613724	83.54	100	3.68182e−34	Coleoptera	Dytiscidae	Hydroporus foveolatus
Oreodytes quadrimaculatus partial 16S rRNA gene	AJ850351	82.17	100	3.68182e−34	Coleoptera	Dytiscidae	Oreodytes quadrimaculatus
Hydrocolus paugus 16S ribosomal RNA gene, partial	KF575539	82.8	100	1.28508e−33	Coleoptera	Dytiscidae	Hydrocolus paugus
Deronectes moestus mitochondrial 16S rRNA gene (pa	HE613726	84.83	91.67	1.28508e−33	Coleoptera	Dytiscidae	Deronectes moestus
Hydroporus marginatus mitochondrial 16S rRNA gene	HE613699	83.23	97.44	1.28508e−33	Coleoptera	Dytiscidae	Hydroporus marginatus
Stictotarsus maghrebinus 16S ribosomal RNA gene, p	AY250947	82.91	100	1.28508e−33	Coleoptera	Dytiscidae	Stictotarsus maghrebinus
Hydroporus umbrosus voucher UNM KBMHyum551 16S rib	KJ548394	82.17	100	4.48537e−33	Coleoptera	Dytiscidae	Hydroporus umbrosus
Hygrotus patruelis mitochondrial 16S rRNA gene (pa	HF931388	82.17	100	4.48537e−33	Coleoptera	Dytiscidae	Hygrotus patruelis
Hydroporus zimmermanni mitochondrial 16S rRNA gene	HE613679	82.91	100	4.48537e−33	Coleoptera	Dytiscidae	Hydroporus zimmermanni
Antiporus hollingsworthi mitochondrial partial 16S	FR727246	81.94	98.72	4.48537e−33	Coleoptera	Dytiscidae	Antiporus hollingsworthi
Scarodytes sp. IR−2003 16S ribosomal RNA gene, par	AY250939	81.76	100	4.48537e−33	Coleoptera	Dytiscidae	Scarodytes sp. IR−2003
Hydroporus umbrosus 16S ribosomal RNA gene, partia	AF518280	82.17	100	4.48537e−33	Coleoptera	Dytiscidae	Hydroporus umbrosus
Hydroporus marginatus 16S ribosomal RNA gene, part	AF518267	82.17	100	4.48537e−33	Coleoptera	Dytiscidae	Hydroporus marginatus
Hygrotus impressopunctatus voucher UNM KBMHtim80 1	KJ548334	83.33	97.44	1.56555e−32	Coleoptera	Dytiscidae	Hygrotus impressopunctatus
Stictotarsus procerus mitochondrial 16S rRNA gene	HF931379	82.28	100	1.56555e−32	Coleoptera	Dytiscidae	Stictotarsus procerus
Deronectes opatrinus mitochondrial 16S rRNA gene (	HE613701	82.28	100	1.56555e−32	Coleoptera	Dytiscidae	Deronectes opatrinus
Copelatus sp. BI−2011 voucher BMNH792945 16S ribos	HQ381441	81.53	100	1.56555e−32	Coleoptera	Dytiscidae	Copelatus sp. BI−2011
Nirripirti megamacrocephalus isolate ABTC78781 16S	EU616935	82.28	100	1.56555e−32	Coleoptera	Dytiscidae	Paroster megamacrocephalus
Deronectes moestus 16S ribosomal RNA gene, partial	KF575561	84.14	91.67	5.4643e−32	Coleoptera	Dytiscidae	Deronectes moestus
Hydroporus puberulus 16S ribosomal RNA gene, parti	JX434766	81.53	100	5.4643e−32	Coleoptera	Dytiscidae	Hydroporus puberulus
Hygrotus lutescens mitochondrial partial 16S rRNA	HF931518	81.53	100	5.4643e−32	Coleoptera	Dytiscidae	Hygrotus lutescens
Hygrotus fontinalis mitochondrial 16S rRNA gene (p	HF931494	81.53	100	5.4643e−32	Coleoptera	Dytiscidae	Hygrotus fontinalis
Hygrotus marklini mitochondrial 16S rRNA gene (par	HF931467	81.53	100	5.4643e−32	Coleoptera	Dytiscidae	Hygrotus marklini
Hygrotus sanfilippoi mitochondrial 16S rRNA gene (	HF931342	81.53	100	5.4643e−32	Coleoptera	Dytiscidae	Hygrotus sanfilippoi
Suphrodytes figuratus voucher BMNH:722148 16S ribo	JX221571	81.53	100	5.4643e−32	Coleoptera	Dytiscidae	Suphrodytes figuratus
Hydroporus analis mitochondrial partial 16S rRNA g	HE613731	81.99	100	5.4643e−32	Coleoptera	Dytiscidae	Hydroporus analis
Hydroporus fuscipennis mitochondrial 16S rRNA gene	HE613730	81.37	100	5.4643e−32	Coleoptera	Dytiscidae	Hydroporus fuscipennis
Deronectes brannani mitochondrial 16S rRNA gene (p	HE613695	84.14	91.67	5.4643e−32	Coleoptera	Dytiscidae	Deronectes brannanii
Deronectes moestus mitochondrial 16S rRNA gene (pa	HE613683	84.14	91.67	5.4643e−32	Coleoptera	Dytiscidae	Deronectes moestus
Hydroporus compunctus mitochondrial 16S rRNA gene	HE599693	81.99	100	5.4643e−32	Coleoptera	Dytiscidae	Hydroporus compunctus
Hovahydrus sp. BI−2011 voucher BMNH831333 16S ribo	HQ381524	84.25	91.67	5.4643e−32	Coleoptera	Dytiscidae	Hovahydrus sp. BI−2011
Antiporus hollingsworthi mitochondrial partial 16S	FR727247	81.29	98.72	5.4643e−32	Coleoptera	Dytiscidae	Antiporus hollingsworthi
Pseudomigadops n. sp. MSL2007 voucher 668381 16S r	EF214073	82.39	100	5.4643e−32	Coleoptera	Carabidae	Pseudomigadops n. sp. MSL2007
Pseudomigadops n. sp. MSL2007 voucher 668379 16S r	EF214072	82.39	100	5.4643e−32	Coleoptera	Carabidae	Pseudomigadops n. sp. MSL2007
Pseudomigadops n. sp. MSL2007 voucher 668300 16S r	EF214010	82.39	100	5.4643e−32	Coleoptera	Carabidae	Pseudomigadops n. sp. MSL2007
Pseudomigadops sp. PSM1 mitochondrial 16S rRNA gen	FM994738	82.39	100	5.4643e−32	Coleoptera	Carabidae	Pseudomigadops sp. PSM1
Hygrotus corpulentus partial 16S rRNA gene	AJ850387	81.53	100	5.4643e−32	Coleoptera	Dytiscidae	Hygrotus corpulentus
Darwinhydrus solidus voucher MB354 16S ribosomal R	EF056671	83.02	100	5.4643e−32	Coleoptera	Dytiscidae	Darwinhydrus solidus
Hydroporus puberulus 16S ribosomal RNA gene, parti	AY365283	81.53	100	5.4643e−32	Coleoptera	Dytiscidae	Hydroporus puberulus
Hydroporus sp. IR33.4 16S ribosomal RNA gene, part	AY365261	81.53	100	5.4643e−32	Coleoptera	Dytiscidae	Hydroporus sp. IR33.4
Hydroporus compunctus 16S ribosomal RNA gene, part	AF518259	81.99	100	5.4643e−32	Coleoptera	Dytiscidae	Hydroporus compunctus
Hydroporus fuscipennis 16S ribosomal RNA gene, par	AF518262	81.37	100	5.4643e−32	Coleoptera	Dytiscidae	Hydroporus fuscipennis
Ilybius fraterculus mitochondrial 16S rRNA gene (p	LM654800	80.89	100	1.90723e−31	Coleoptera	Dytiscidae	Ilybius fraterculus
Ilybius ater mitochondrial 16S rRNA gene (partial)	LM654794	80.89	100	1.90723e−31	Coleoptera	Dytiscidae	Ilybius ater
Ilybius fraterculus voucher UNM KBMIlfr409 16S rib	KJ548342	80.89	100	1.90723e−31	Coleoptera	Dytiscidae	Ilybius fraterculus
Hydaticus cinctipennis voucher UNM KBMHyci20 16S r	KJ548338	80.89	100	1.90723e−31	Coleoptera	Dytiscidae	Hydaticus cinctipennis
Cybister marginicollis voucher UNM KBMCyma416 16S	KJ548319	80.89	100	1.90723e−31	Coleoptera	Dytiscidae	Cybister marginicollis
Deronectes bicostatus mitochondrial 16S rRNA gene	HE613705	80.89	100	1.90723e−31	Coleoptera	Dytiscidae	Deronectes bicostatus
Copelatus sp. BI−2011 voucher BMNH671215 16S ribos	HQ381377	80.89	100	1.90723e−31	Coleoptera	Dytiscidae	Copelatus sp. BI−2011
Hygrotus impressopunctatus partial 16S rRNA gene	AJ850405	82.35	97.44	1.90723e−31	Coleoptera	Dytiscidae	Hygrotus impressopunctatus
Nirripirti newhavenensis 16S ribosomal RNA gene, p	AY353844	81.53	100	1.90723e−31	Coleoptera	Dytiscidae	Paroster newhavenensis
Coelhydrus brevicollis voucher MB1224 16S ribosoma	EF056670	82.28	100	1.90723e−31	Coleoptera	Dytiscidae	Coelhydrus brevicollis
Nebrioporus airumlus 16S ribosomal RNA gene, parti	AY250920	80.89	100	1.90723e−31	Coleoptera	Dytiscidae	Nebrioporus airumlus
Hydroporus rufifrons 16S ribosomal RNA gene, parti	AF518277	81.76	100	1.90723e−31	Coleoptera	Dytiscidae	Hydroporus rufifrons
Herophydrus rohani voucher UNM KBMHpsp574 16S ribo	KJ548382	81.76	100	6.65688e−31	Coleoptera	Dytiscidae	Herophydrus rohani
Hygrotus armeniacus mitochondrial partial 16S rRNA	HF931520	82.39	100	6.65688e−31	Coleoptera	Dytiscidae	Hygrotus armeniacus
Hydroporus discretus mitochondrial 16S rRNA gene (	HE613723	81.37	100	6.65688e−31	Coleoptera	Dytiscidae	Hydroporus discretus
Hydroporus decipiens mitochondrial 16S rRNA gene (	HE613722	81.37	100	6.65688e−31	Coleoptera	Dytiscidae	Hydroporus decipiens
Deronectes doriae mitochondrial 16S rRNA gene (par	HE613710	81.65	100	6.65688e−31	Coleoptera	Dytiscidae	Deronectes doriae
Hydroporus errans mitochondrial 16S rRNA gene (par	HE613704	81.37	100	6.65688e−31	Coleoptera	Dytiscidae	Hydroporus errans
Deronectes sahlbergi mitochondrial 16S rRNA gene (	HE613680	81.65	100	6.65688e−31	Coleoptera	Dytiscidae	Deronectes sahlbergi
Stictotarsus falli 16S ribosomal RNA (rrnL) gene,	EF670029	81.65	100	6.65688e−31	Coleoptera	Dytiscidae	Stictotarsus falli
Hydroporus pubescens voucher NHM−IR39 16S ribosoma	EF419327	81.37	100	6.65688e−31	Coleoptera	Dytiscidae	Hydroporus pubescens
