# Supplementary material for: Testing the potential of a ribosomal 16S marker for DNA metabarcoding of insects
Source: PeerJ. 2016 Apr 19;4:e1966. doi: 10.7717/peerj.1966 (PMC4841222; doi:10.7717/peerj.1966)
Supplement: Supplemental Information 1 [file peerj-04-1966-s006.zip › scripts/5 match OTUs/blast_hits/OTU_4.csv_plot.pdf]

OTU_4.csv

Ephemera danica

Ephemeridae

Ephemeroptera

100

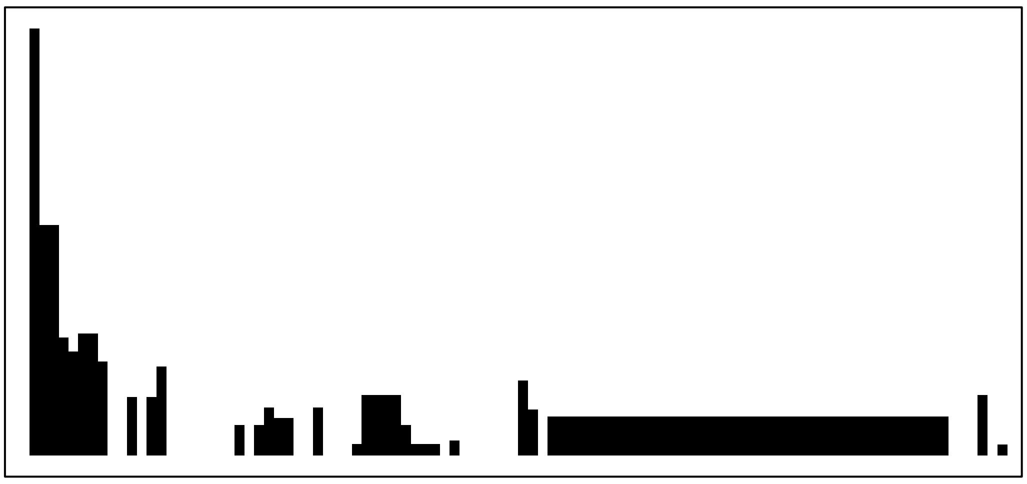

Ephemera danica 16S ribosomal RNA gene, partial se	KF855860	100	100	1.51112e−70	Ephemeroptera	Ephemeridae	Ephemera danica
Ephemera orientalis voucher EPH01 tRNA−Leu, 16S ri	KF256907	90.79	100	1.51246e−51	Ephemeroptera	Ephemeridae	Ephemera orientalis
Ephemera orientalis mitochondrion, complete genome	EU591678	90.79	100	1.51246e−51	Ephemeroptera	Ephemeridae	Ephemera orientalis
Ephemera simulans voucher BYU:IGCEP038 16S ribosom	GQ118285	85.53	100	1.08904e−40	Ephemeroptera	Ephemeridae	Ephemera simulans
Siphlonurus immanis voucher NKMT032 mitochondrion,	FJ606783	84.87	100	4.63073e−39	Ephemeroptera	Siphlonuridae	Siphlonurus immanis
Ephemera sp. 1 BM−2008 12S ribosomal RNA gene, par	EU477611	85.71	100	1.61629e−38	Ephemeroptera	Ephemeridae	Ephemera sp. 1 BM−2008
Ephemera sp. 'Bonn' large subunit ribosomal RNA ge	AF266047	85.71	100	1.61629e−38	Ephemeroptera	Ephemeridae	Ephemera sp. 'Bonn'
Ctenoplusia limbirena mitochondrion, complete geno	KM244665	84.4	70.39	1.3279e−20	Lepidoptera	Noctuidae	Ctenoplusia limbirena
Doleschallia bisaltide voucher jshao2005048 16S ri	DQ784697	77.78	93.42	1.3279e−20	Lepidoptera	Nymphalidae	Doleschallia bisaltide
Neope pulaha 16S ribosomal RNA gene, partial seque	KJ777753	77.07	97.37	4.63483e−20	Lepidoptera	Nymphalidae	Neope pulaha
Euryades corethrus voucher FS−b−3716 16S ribosomal	GQ268348	82.73	70.39	4.63483e−20	Lepidoptera	Papilionidae	Euryades corethrus
Papilio alexanor voucher FS−b−40 16S ribosomal RNA	GQ268343	78	97.37	4.63483e−20	Lepidoptera	Papilionidae	Papilio alexanor
Euryades corethrus mitochondrial partial 16S rRNA	AM283053	82.73	70.39	4.63483e−20	Lepidoptera	Papilionidae	Euryades corethrus
Eresia plaginota isolate 38−4 16S ribosomal RNA ge	AF186912	84.16	65.79	4.63483e−20	Lepidoptera	Nymphalidae	Eresia plaginota
Papilio alexanor mitochondrion, large subunit ribo	AJ224052	78	97.37	4.63483e−20	Lepidoptera	Papilionidae	Papilio alexanor
Pachliopta aristolochiae isolate N296 16S ribosoma	JQ421944	78.32	91.45	1.61771e−19	Lepidoptera	Papilionidae	Pachliopta aristolochiae
Pachliopta aristolochiae isolate F566 16S ribosoma	JQ421940	78.32	91.45	1.61771e−19	Lepidoptera	Papilionidae	Pachliopta aristolochiae
Pachliopta aristolochiae isolate F177 16S ribosoma	JQ421938	78.32	91.45	1.61771e−19	Lepidoptera	Papilionidae	Pachliopta aristolochiae
Pachliopta aristolochiae isolate F317 16S ribosoma	JQ421934	78.32	91.45	1.61771e−19	Lepidoptera	Papilionidae	Pachliopta aristolochiae
Pachliopta aristolochiae isolate F520 16S ribosoma	JQ421931	78.32	91.45	1.61771e−19	Lepidoptera	Papilionidae	Pachliopta aristolochiae
Pachliopta aristolochiae isolate T75 16S ribosomal	JQ421926	78.32	91.45	1.61771e−19	Lepidoptera	Papilionidae	Pachliopta aristolochiae
Atrophaneura alcinous mitochondrial partial 16S rR	AM283055	81.42	73.68	1.61771e−19	Lepidoptera	Papilionidae	Atrophaneura alcinous
Pachliopta aristolochiae 16S ribosomal RNA gene, p	DQ673334	78.32	91.45	1.61771e−19	Lepidoptera	Papilionidae	Pachliopta aristolochiae
Atrophaneura dasarada 16S ribosomal RNA gene, part	DQ673329	81.42	73.68	1.61771e−19	Lepidoptera	Papilionidae	Atrophaneura dasarada
Atrophaneura alcinous mitochondrion, complete geno	KJ540880	82.24	69.74	5.64637e−19	Lepidoptera	Papilionidae	Atrophaneura alcinous
Oxythyrea pantherina voucher 842859 16S ribosomal	JX234531	81.74	75	5.64637e−19	Coleoptera	Scarabaeidae	Oxythyrea pantherina
Oxythyrea funesta voucher 678443 16S ribosomal RNA	JX234432	81.74	75	5.64637e−19	Coleoptera	Scarabaeidae	Oxythyrea funesta
Pterostichus katashinensis mitochondrial gene for	AB253865	76.43	99.34	5.64637e−19	Coleoptera	Carabidae	Pterostichus katashinensis
Potamanthus luteus voucher BYU:IGCEP219 16S riboso	GQ118293	77.12	98.68	5.64637e−19	Ephemeroptera	Potamanthidae	Potamanthus luteus
Atrophaneura mencius 16S ribosomal RNA gene, parti	DQ673330	82.24	69.74	5.64637e−19	Lepidoptera	Papilionidae	Atrophaneura mencius
Dymasia dymas voucher NW27−7 16S ribosomal RNA gen	AF186876	77.08	93.42	5.64637e−19	Lepidoptera	Nymphalidae	Dymasia dymas
Chlosyne cyneas voucher NW38−17 16S ribosomal RNA	AF186870	77.3	91.45	5.64637e−19	Lepidoptera	Nymphalidae	Chlosyne cyneas
Kuromondokuga niphonis voucher H167 large subunit	KP081963	78.43	96.71	1.97078e−18	Lepidoptera	Lymantriidae	Kuromondokuga niphonis
Papilio demoleus mitochondrion, complete genome	KR024009	80.53	73.68	1.97078e−18	Lepidoptera	Papilionidae	Papilio demoleus
Palingenia longicauda mitochondrial partial 16S rR	HE650662	82.83	65.13	1.97078e−18	Ephemeroptera	Palingeniidae	Palingenia longicauda
Palingenia longicauda mitochondrial partial 16S rR	HE650475	82.83	65.13	1.97078e−18	Ephemeroptera	Palingeniidae	Palingenia longicauda
Palingenia longicauda mitochondrial partial 16S rR	HE650547	82.83	65.13	1.97078e−18	Ephemeroptera	Palingeniidae	Palingenia longicauda
Palingenia longicauda mitochondrial partial 16S rR	HE650502	82.83	65.13	1.97078e−18	Ephemeroptera	Palingeniidae	Palingenia longicauda
Trox niponensis isolate 100 16S ribosomal RNA gene	EF570413	81.42	73.03	1.97078e−18	Coleoptera	Trogidae	Trox niponensis
Papilio demoleus 16S ribosomal RNA gene, partial s	DQ673336	80.53	73.68	1.97078e−18	Lepidoptera	Papilionidae	Papilio demoleus
Papilio demoleus isolate FS−a−68 16S ribosomal RNA	DQ351090	80.53	73.68	1.97078e−18	Lepidoptera	Papilionidae	Papilio demoleus
Papilio demoleus mitochondrion, large subunit ribo	AJ224061	80.53	73.68	1.97078e−18	Lepidoptera	Papilionidae	Papilio demoleus
Neope muirheadii 16S ribosomal RNA gene, partial s	KM200160	77.21	84.21	6.87869e−18	Lepidoptera	Nymphalidae	Neope muirheadii
Atrophaneura alcinous 16S ribosomal RNA gene, part	GU372456	80.7	73.68	6.87869e−18	Lepidoptera	Papilionidae	Atrophaneura alcinous
Pterostichus asymmetricus mitochondrial gene for 1	AB253866	75.8	99.34	6.87869e−18	Coleoptera	Carabidae	Pterostichus asymmetricus
Pterostichus koheii mitochondrial gene for 16S rRN	AB253841	76.4	100	6.87869e−18	Coleoptera	Carabidae	Pterostichus koheii
Argyreus hyperbius voucher jshao2005025 16S riboso	DQ784692	76.47	96.71	6.87869e−18	Lepidoptera	Nymphalidae	Argynnis hyperbius
Trichopteryx ustata gene for 16S rRNA, partial seq	AB265432	76.28	97.37	6.87869e−18	Lepidoptera	Geometridae	Trichopteryx ustata
Argyreus hyperbius 16S ribosomal RNA gene, partial	DQ445107	76.47	96.71	6.87869e−18	Lepidoptera	Nymphalidae	Argynnis hyperbius
Neope muirheadii 16S ribosomal RNA gene, partial s	DQ445102	77.21	84.21	6.87869e−18	Lepidoptera	Nymphalidae	Neope muirheadii
Papilio delalandei mitochondrion, large subunit ri	AJ224060	83.5	65.79	6.87869e−18	Lepidoptera	Papilionidae	Papilio delalandei
Papilio dardanus mitochondrion, complete genome	JX313686	82.14	70.39	2.4009e−17	Lepidoptera	Papilionidae	Papilio dardanus
Papilio montrouzieri voucher CBGPFLC_00106 16S rib	JQ982226	76.43	97.37	2.4009e−17	Lepidoptera	Papilionidae	Papilio montrouzieri
Palingenia longicauda mitochondrial partial 16S rR	HE650589	81.82	65.13	2.4009e−17	Ephemeroptera	Palingeniidae	Palingenia longicauda
Palingenia longicauda mitochondrial partial 16S rR	HE650657	81.82	65.13	2.4009e−17	Ephemeroptera	Palingeniidae	Palingenia longicauda
Palingenia longicauda mitochondrial partial 16S rR	HE650650	81.82	65.13	2.4009e−17	Ephemeroptera	Palingeniidae	Palingenia longicauda
Palingenia longicauda mitochondrial partial 16S rR	HE650647	81.82	65.13	2.4009e−17	Ephemeroptera	Palingeniidae	Palingenia longicauda
Palingenia longicauda mitochondrial partial 16S rR	HE650637	81.82	65.13	2.4009e−17	Ephemeroptera	Palingeniidae	Palingenia longicauda
Palingenia longicauda mitochondrial partial 16S rR	HE650635	81.82	65.13	2.4009e−17	Ephemeroptera	Palingeniidae	Palingenia longicauda
Palingenia longicauda mitochondrial partial 16S rR	HE650631	81.82	65.13	2.4009e−17	Ephemeroptera	Palingeniidae	Palingenia longicauda
Palingenia longicauda mitochondrial partial 16S rR	HE650625	81.82	65.13	2.4009e−17	Ephemeroptera	Palingeniidae	Palingenia longicauda
Palingenia longicauda mitochondrial partial 16S rR	HE650623	81.82	65.13	2.4009e−17	Ephemeroptera	Palingeniidae	Palingenia longicauda
Palingenia longicauda mitochondrial partial 16S rR	HE650622	81.82	65.13	2.4009e−17	Ephemeroptera	Palingeniidae	Palingenia longicauda
Palingenia longicauda mitochondrial partial 16S rR	HE650618	81.82	65.13	2.4009e−17	Ephemeroptera	Palingeniidae	Palingenia longicauda
Palingenia longicauda mitochondrial partial 16S rR	HE650617	81.82	65.13	2.4009e−17	Ephemeroptera	Palingeniidae	Palingenia longicauda
Palingenia longicauda mitochondrial partial 16S rR	HE650604	81.82	65.13	2.4009e−17	Ephemeroptera	Palingeniidae	Palingenia longicauda
Palingenia longicauda mitochondrial partial 16S rR	HE650594	81.82	65.13	2.4009e−17	Ephemeroptera	Palingeniidae	Palingenia longicauda
Palingenia longicauda mitochondrial partial 16S rR	HE650588	81.82	65.13	2.4009e−17	Ephemeroptera	Palingeniidae	Palingenia longicauda
Palingenia longicauda mitochondrial partial 16S rR	HE650575	81.82	65.13	2.4009e−17	Ephemeroptera	Palingeniidae	Palingenia longicauda
Palingenia longicauda mitochondrial partial 16S rR	HE650569	81.82	65.13	2.4009e−17	Ephemeroptera	Palingeniidae	Palingenia longicauda
Palingenia longicauda mitochondrial partial 16S rR	HE650550	81.82	65.13	2.4009e−17	Ephemeroptera	Palingeniidae	Palingenia longicauda
Palingenia longicauda mitochondrial partial 16S rR	HE650542	81.82	65.13	2.4009e−17	Ephemeroptera	Palingeniidae	Palingenia longicauda
Palingenia longicauda mitochondrial partial 16S rR	HE650533	81.82	65.13	2.4009e−17	Ephemeroptera	Palingeniidae	Palingenia longicauda
Palingenia longicauda mitochondrial partial 16S rR	HE650503	81.82	65.13	2.4009e−17	Ephemeroptera	Palingeniidae	Palingenia longicauda
Palingenia longicauda mitochondrial partial 16S rR	HE650520	81.82	65.13	2.4009e−17	Ephemeroptera	Palingeniidae	Palingenia longicauda
Palingenia longicauda mitochondrial partial 16S rR	HE650519	81.82	65.13	2.4009e−17	Ephemeroptera	Palingeniidae	Palingenia longicauda
Palingenia longicauda mitochondrial partial 16S rR	HE650515	81.82	65.13	2.4009e−17	Ephemeroptera	Palingeniidae	Palingenia longicauda
Palingenia longicauda mitochondrial partial 16S rR	HE650514	81.82	65.13	2.4009e−17	Ephemeroptera	Palingeniidae	Palingenia longicauda
Palingenia longicauda mitochondrial partial 16S rR	HE650509	81.82	65.13	2.4009e−17	Ephemeroptera	Palingeniidae	Palingenia longicauda
Palingenia longicauda mitochondrial partial 16S rR	HE650507	81.82	65.13	2.4009e−17	Ephemeroptera	Palingeniidae	Palingenia longicauda
Palingenia longicauda mitochondrial partial 16S rR	HE650505	81.82	65.13	2.4009e−17	Ephemeroptera	Palingeniidae	Palingenia longicauda
Palingenia longicauda mitochondrial partial 16S rR	HE650499	81.82	65.13	2.4009e−17	Ephemeroptera	Palingeniidae	Palingenia longicauda
Palingenia longicauda mitochondrial partial 16S rR	HE650446	81.82	65.13	2.4009e−17	Ephemeroptera	Palingeniidae	Palingenia longicauda
Palingenia longicauda mitochondrial partial 16S rR	HE650435	81.82	65.13	2.4009e−17	Ephemeroptera	Palingeniidae	Palingenia longicauda
Palingenia longicauda mitochondrial partial 16S rR	HE650461	81.82	65.13	2.4009e−17	Ephemeroptera	Palingeniidae	Palingenia longicauda
Palingenia longicauda mitochondrial partial 16S rR	HE650449	81.82	65.13	2.4009e−17	Ephemeroptera	Palingeniidae	Palingenia longicauda
Palingenia longicauda mitochondrial partial 16S rR	HE650420	81.82	65.13	2.4009e−17	Ephemeroptera	Palingeniidae	Palingenia longicauda
Palingenia longicauda mitochondrial partial 16S rR	HE650440	81.82	65.13	2.4009e−17	Ephemeroptera	Palingeniidae	Palingenia longicauda
Palingenia longicauda mitochondrial partial 16S rR	HE650434	81.82	65.13	2.4009e−17	Ephemeroptera	Palingeniidae	Palingenia longicauda
Palingenia longicauda mitochondrial partial 16S rR	HE650430	81.82	65.13	2.4009e−17	Ephemeroptera	Palingeniidae	Palingenia longicauda
Palingenia longicauda mitochondrial partial 16S rR	HE650421	81.82	65.13	2.4009e−17	Ephemeroptera	Palingeniidae	Palingenia longicauda
Palingenia longicauda mitochondrial partial 16S rR	HE650424	81.82	65.13	2.4009e−17	Ephemeroptera	Palingeniidae	Palingenia longicauda
Palingenia longicauda mitochondrial partial 16S rR	HE650422	81.82	65.13	2.4009e−17	Ephemeroptera	Palingeniidae	Palingenia longicauda
Palingenia longicauda voucher BYU:IGCEP216 16S rib	GQ118292	81.82	65.13	2.4009e−17	Ephemeroptera	Palingeniidae	Palingenia longicauda
Archon apollinaris mitochondrial partial 16S rRNA	AJ972036	76.13	97.37	2.4009e−17	Lepidoptera	Papilionidae	Archon apollinaris
Archon apollinaris mitochondrial partial 16S rRNA	AM284149	77.12	96.05	2.4009e−17	Lepidoptera	Papilionidae	Archon apollinaris
Archon apollinaris isolate FS−b−2025 16S ribosomal	DQ351095	76.13	97.37	2.4009e−17	Lepidoptera	Papilionidae	Archon apollinaris
Ichthybotus hudsoni mitochondrial 16S ribosomal RN	AY749816	82.83	65.13	2.4009e−17	Ephemeroptera	Ichthybotidae	Ichthybotus hudsoni
Anthopotamus sp. EP022 16S ribosomal RNA gene, par	AY749766	75.8	100	2.4009e−17	Ephemeroptera	Potamanthidae	Anthopotamus sp. EP022
Heliconius demeter isolate STRI−B−8562 16S ribosom	AY747871	80.51	75.66	2.4009e−17	Lepidoptera	Nymphalidae	Heliconius demeter
