# Supplementary material for: Testing the potential of a ribosomal 16S marker for DNA metabarcoding of insects
Source: PeerJ. 2016 Apr 19;4:e1966. doi: 10.7717/peerj.1966 (PMC4841222; doi:10.7717/peerj.1966)
Supplement: Supplemental Information 1 [file peerj-04-1966-s006.zip › scripts/5 match OTUs/blast_hits/OTU_405.csv_plot.pdf]

OTU_405.csv

Protonemura meyeri

Nemouridae

Plecoptera

94.27

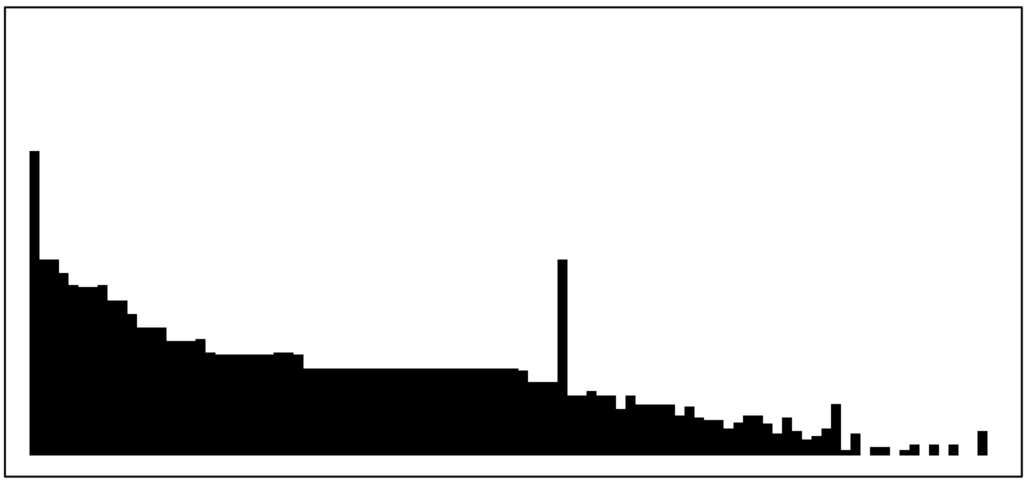

Protonemura meyeri 16S ribosomal RNA gene, partial	EF623193	94.27	100	1.12528e−59	Plecoptera	Nemouridae	Protonemura meyeri
Nanonemoura wahkeena 16S ribosomal RNA gene, parti	EF623198	89.17	100	8.10258e−49	Plecoptera	Nemouridae	Nanonemoura wahkeena
Ostrocerca foersteri 16S ribosomal RNA gene, parti	EF623191	89.17	100	8.10258e−49	Plecoptera	Nemouridae	Ostrocerca foersteri
Nemoura trispinosa 16S ribosomal RNA gene, partial	EF623187	88.54	100	9.87097e−48	Plecoptera	Nemouridae	Nemoura trispinosa
Nemoura cinerea 16S ribosomal RNA gene, partial se	EF623194	87.97	100	3.44531e−47	Plecoptera	Nemouridae	Nemoura cinerea
Nemurella pictetii 16S ribosomal RNA gene, partial	EF623192	87.9	100	4.19724e−46	Plecoptera	Nemouridae	Nemurella pictetii
Shipsa rotunda 16S ribosomal RNA gene, partial seq	EF623184	87.9	100	4.19724e−46	Plecoptera	Nemouridae	Shipsa rotunda
Scopura montana 16S ribosomal RNA gene, partial se	EF623265	87.97	100	1.46498e−45	Plecoptera		Scopura montana
Visoka cataractae 16S ribosomal RNA gene, partial	EF623189	87.26	100	5.11329e−45	Plecoptera	Nemouridae	Visoka cataractae
Paranemoura claasseni 16S ribosomal RNA gene, part	EF623185	87.26	100	5.11329e−45	Plecoptera	Nemouridae	Paranemoura claasseni
Prostoia besametsa 16S ribosomal RNA gene, partial	EF623188	86.62	100	2.17423e−43	Plecoptera	Nemouridae	Prostoia besametsa
Lednia tumana 16S ribosomal RNA gene, partial sequ	EF623197	85.99	100	2.64875e−42	Plecoptera	Nemouridae	Lednia tumana
Zapada cinctipes 16S ribosomal RNA gene, partial s	EF623183	85.99	100	2.64875e−42	Plecoptera	Nemouridae	Zapada cinctipes
Soyedina producta 16S ribosomal RNA gene, partial	EF623196	85.99	100	3.22684e−41	Plecoptera	Nemouridae	Soyedina producta
Apteroperla tikumana mitochondrion, complete genom	KR604721	85.35	100	1.12628e−40	Plecoptera	Capniidae	Apteroperla tikumana
Malenka californica 16S ribosomal RNA gene, partia	EF623182	85.35	100	1.12628e−40	Plecoptera	Nemouridae	Malenka californica
Allocapnia minima 16S ribosomal RNA gene, partial	EF623130	85.35	100	1.12628e−40	Plecoptera	Capniidae	Allocapnia minima
Moselia infuscata 16S ribosomal RNA gene, partial	EF623175	85.44	99.36	3.93109e−40	Plecoptera	Leuctridae	Moselia infuscata
Megaleuctra kincaidi 16S ribosomal RNA gene, parti	EF623174	84.81	100	3.93109e−40	Plecoptera	Leuctridae	Megaleuctra kincaidi
Allocapnia recta isolate H15 16S ribosomal RNA gen	KC881050	84.71	100	1.37209e−39	Plecoptera	Capniidae	Allocapnia recta
Allocapnia recta isolate H14 16S ribosomal RNA gen	KC881049	84.71	100	1.37209e−39	Plecoptera	Capniidae	Allocapnia recta
Allocapnia recta isolate H10 16S ribosomal RNA gen	KC881045	84.71	100	1.37209e−39	Plecoptera	Capniidae	Allocapnia recta
Allocapnia recta isolate H6 16S ribosomal RNA gene	KC881041	84.71	100	1.37209e−39	Plecoptera	Capniidae	Allocapnia recta
Allocapnia recta haplotype 11ar: H011 16S ribosoma	EU306874	84.71	100	1.37209e−39	Plecoptera	Capniidae	Allocapnia recta
Allocapnia recta haplotype 7ar: H07 16S ribosomal	EU306870	84.71	100	1.37209e−39	Plecoptera	Capniidae	Allocapnia recta
Doddsia occidentalis 16S ribosomal RNA gene, parti	EF623270	84.81	100	4.78905e−39	Plecoptera	Taeniopterygidae	Doddsia occidentalis
Oemopteryx vanduzeea 16S ribosomal RNA gene, parti	EF623266	84.81	100	4.78905e−39	Plecoptera	Taeniopterygidae	Oemopteryx vanduzeea
Pteronarcys sachalina 16S ribosomal RNA gene, part	EF623264	84.71	100	1.67154e−38	Plecoptera	Pteronarcyidae	Pteronarcys sachalina
Mesocapnia arizonensis mitochondrion, partial geno	KP642637	84.08	100	5.83426e−38	Plecoptera	Capniidae	Mesocapnia arizonensis
Allocapnia recta isolate H19 16S ribosomal RNA gen	KC881054	84.08	100	5.83426e−38	Plecoptera	Capniidae	Allocapnia recta
Allocapnia recta isolate H17 16S ribosomal RNA gen	KC881052	84.08	100	5.83426e−38	Plecoptera	Capniidae	Allocapnia recta
Allocapnia recta isolate H16 16S ribosomal RNA gen	KC881051	84.08	100	5.83426e−38	Plecoptera	Capniidae	Allocapnia recta
Allocapnia recta isolate H13 16S ribosomal RNA gen	KC881048	84.08	100	5.83426e−38	Plecoptera	Capniidae	Allocapnia recta
Allocapnia recta isolate H12 16S ribosomal RNA gen	KC881047	84.08	100	5.83426e−38	Plecoptera	Capniidae	Allocapnia recta
Allocapnia recta isolate H11 16S ribosomal RNA gen	KC881046	84.08	100	5.83426e−38	Plecoptera	Capniidae	Allocapnia recta
Allocapnia recta isolate H9 16S ribosomal RNA gene	KC881044	84.08	100	5.83426e−38	Plecoptera	Capniidae	Allocapnia recta
Allocapnia recta isolate H8 16S ribosomal RNA gene	KC881043	84.08	100	5.83426e−38	Plecoptera	Capniidae	Allocapnia recta
Allocapnia recta isolate H7 16S ribosomal RNA gene	KC881042	84.08	100	5.83426e−38	Plecoptera	Capniidae	Allocapnia recta
Allocapnia recta isolate H5 16S ribosomal RNA gene	KC881040	84.08	100	5.83426e−38	Plecoptera	Capniidae	Allocapnia recta
Allocapnia recta isolate H4 16S ribosomal RNA gene	KC881039	84.08	100	5.83426e−38	Plecoptera	Capniidae	Allocapnia recta
Allocapnia recta isolate H3 16S ribosomal RNA gene	KC881038	84.08	100	5.83426e−38	Plecoptera	Capniidae	Allocapnia recta
Allocapnia recta isolate H1 16S ribosomal RNA gene	KC881036	84.08	100	5.83426e−38	Plecoptera	Capniidae	Allocapnia recta
Paraleuctra occidentalis 16S ribosomal RNA gene, p	EF623178	84.08	100	5.83426e−38	Plecoptera	Leuctridae	Paraleuctra occidentalis
Mesocapnia sp. BYU_PL010 16S ribosomal RNA gene, p	EF623127	84.08	100	5.83426e−38	Plecoptera	Capniidae	Mesocapnia sp. BYU_PL010
Allocapnia recta haplotype 10ar: H010 16S ribosoma	EU306873	84.08	100	5.83426e−38	Plecoptera	Capniidae	Allocapnia recta
Allocapnia recta haplotype 9ar: H09 16S ribosomal	EU306872	84.08	100	5.83426e−38	Plecoptera	Capniidae	Allocapnia recta
Allocapnia recta haplotype 8ar: H08 16S ribosomal	EU306871	84.08	100	5.83426e−38	Plecoptera	Capniidae	Allocapnia recta
Allocapnia recta haplotype 3ar: H03 16S ribosomal	DQ915181	84.08	100	5.83426e−38	Plecoptera	Capniidae	Allocapnia recta
Allocapnia recta haplotype 2ar: H02 16S ribosomal	DQ915180	84.08	100	5.83426e−38	Plecoptera	Capniidae	Allocapnia recta
Allocapnia recta haplotype 1ar: H01 16S ribosomal	DQ915179	84.08	100	5.83426e−38	Plecoptera	Capniidae	Allocapnia recta
Osmylus sp. EH−2004 16S ribosomal RNA gene, partia	AY620146	83.97	100	2.03636e−37	Neuroptera	Osmylidae	Osmylus sp. EH−2004
Allocapnia recta isolate H18 16S ribosomal RNA gen	KC881053	83.44	100	7.10758e−37	Plecoptera	Capniidae	Allocapnia recta
Allocapnia recta haplotype 12ar: H12 16S ribosomal	EU306875	83.44	100	7.10758e−37	Plecoptera	Capniidae	Allocapnia recta
Pteronarcys princeps mitochondrion, complete genom	AY687866	83.44	100	8.65881e−36	Plecoptera	Pteronarcyidae	Pteronarcys princeps
Notonemouridae sp. MD−2011 voucher ZMUC:JD−1507 16	JN615257	89.17	76.92	3.02222e−35	Plecoptera	Notonemouridae	Notonemouridae sp. MD−2011
Austrocerca rieka 16S ribosomal RNA gene, partial	EF623205	82.8	100	3.02222e−35	Plecoptera	Notonemouridae	Austrocerca rieka
Austrocercoides zwicki 16S ribosomal RNA gene, par	EF623199	82.8	100	3.02222e−35	Plecoptera	Notonemouridae	Austrocercoides zwicki
Brachyptera seticornis 16S ribosomal RNA gene, par	EF623268	83.02	100	1.05486e−34	Plecoptera	Taeniopterygidae	Brachyptera seticornis
Pteronarcys scotti 16S ribosomal RNA gene, partial	EF623263	82.8	100	3.68182e−34	Plecoptera	Pteronarcyidae	Pteronarcys scotti
Pteronarcys californica 16S ribosomal RNA gene, pa	EF623261	82.8	100	3.68182e−34	Plecoptera	Pteronarcyidae	Pteronarcys californica
Amphinemura sulcicollis 16S ribosomal RNA gene, pa	EF623195	82.17	100	3.68182e−34	Plecoptera	Nemouridae	Amphinemura sulcicollis
Capnura wanica 16S ribosomal RNA gene, partial seq	EF623128	82.8	100	3.68182e−34	Plecoptera	Capniidae	Capnura wanica
Cryptoperla japonica mitochondrial gene for 16S rR	AB711202	82.39	100	4.48537e−33	Plecoptera	Peltoperlidae	Cryptoperla japonica
Cryptoperla japonica mitochondrial gene for 16S rR	AB711184	82.39	100	4.48537e−33	Plecoptera	Peltoperlidae	Cryptoperla japonica
Cryptoperla japonica mitochondrial gene for 16S rR	AB711183	82.39	100	4.48537e−33	Plecoptera	Peltoperlidae	Cryptoperla japonica
Cryptoperla japonica mitochondrial gene for 16S rR	AB711182	82.39	100	4.48537e−33	Plecoptera	Peltoperlidae	Cryptoperla japonica
Udamocercia sp. BYU_PL150 16S ribosomal RNA gene,	EF623201	81.88	100	4.48537e−33	Plecoptera	Notonemouridae	Udamocercia sp. BYU_PL150
Thaumatoperla sp. BYU_PL109 16S ribosomal RNA gene	EF623149	82.28	100	1.56555e−32	Plecoptera	Eustheniidae	Thaumatoperla sp. BYU_PL109
Cryptoperla sp. YK−2012−1 mitochondrial gene for 1	AB711243	81.76	100	5.4643e−32	Plecoptera	Peltoperlidae	Cryptoperla sp. YK−2012−1
Strophopteryx appalachia 16S ribosomal RNA gene, p	EF623271	81.65	100	5.4643e−32	Plecoptera	Taeniopterygidae	Strophopteryx appalachia
Bolotoperla rossi 16S ribosomal RNA gene, partial	EF623269	81.65	100	5.4643e−32	Plecoptera	Taeniopterygidae	Bolotoperla rossi
Triznaka pintada 16S ribosomal RNA gene, partial s	EF623134	81.25	100	5.4643e−32	Plecoptera	Chloroperlidae	Triznaka pintada
Cascadoperla trictura 16S ribosomal RNA gene, part	EF623225	81.53	100	1.90723e−31	Plecoptera	Perlodidae	Cascadoperla trictura
Leuctra inermis 16S ribosomal RNA gene, partial se	EF623176	81.88	100	1.90723e−31	Plecoptera	Leuctridae	Leuctra inermis
Leptoperla sp. BYU_PL107 16S ribosomal RNA gene, p	EF623161	81.88	100	1.90723e−31	Plecoptera	Gripopterygidae	Leptoperla sp. BYU_PL107
Dinotoperla sp. BYU_PL105 16S ribosomal RNA gene,	EF623160	81.48	100	6.65688e−31	Plecoptera	Gripopterygidae	Dinotoperla sp. BYU_PL105
Diamphipnoa virescentipennis 16S ribosomal RNA gen	EF623144	81.01	100	6.65688e−31	Plecoptera	Diamphipnoidae	Diamphipnoa virescentipennis
Neopanorpa sp. M24899 16S ribosomal RNA gene, part	AF180059	81.76	100	6.65688e−31	Mecoptera	Panorpidae	Neopanorpa sp. M24899
Cryptoperla japonica mitochondrial gene for 16S rR	AB711189	81.13	100	2.32348e−30	Plecoptera	Peltoperlidae	Cryptoperla japonica
Cosumnoperla hypocrena 16S ribosomal RNA gene, par	EF623226	80.75	100	2.32348e−30	Plecoptera	Perlodidae	Cosumnoperla hypocrena
Neonemura sp. BYU_PL153 16S ribosomal RNA gene, pa	EF623204	80.89	100	2.32348e−30	Plecoptera	Notonemouridae	Neonemura sp. BYU_PL153
Taenionema palladium 16S ribosomal RNA gene, parti	EF623267	81.25	100	8.10974e−30	Plecoptera	Taeniopterygidae	Taenionema palladium
Paraleuctra vershina 16S ribosomal RNA gene, parti	EF623173	82.39	90.38	8.10974e−30	Plecoptera	Leuctridae	Paraleuctra vershina
Isocapnia hyalita 16S ribosomal RNA gene, partial	EF623131	80.25	100	8.10974e−30	Plecoptera	Capniidae	Isocapnia hyalita
Panorpodes colei isolate 117Pcol 16S ribosomal RNA	EF041524	81.01	100	8.10974e−30	Mecoptera	Panorpodidae	Panorpodes colei
Cerconychia sp. BYU_PL114 16S ribosomal RNA gene,	EF623260	80	100	2.83058e−29	Plecoptera	Styloperlidae	Cerconychia sp. BYU_PL114
Alloperla severa 16S ribosomal RNA gene, partial s	EF623141	80.38	100	2.83058e−29	Plecoptera	Chloroperlidae	Alloperla severa
Klapopteryx armillata 16S ribosomal RNA gene, part	EF623123	80.38	100	2.83058e−29	Plecoptera	Austroperlidae	Klapopteryx armillata
Kathroperla perdita 16S ribosomal RNA gene, partia	EF623135	79.62	100	9.87969e−29	Plecoptera	Chloroperlidae	Kathroperla perdita
Sweltsa coloradensis 16S ribosomal RNA gene, parti	EF623133	80.25	100	9.87969e−29	Plecoptera	Chloroperlidae	Sweltsa coloradensis
Neopanorpa pulchra mitochondrion, complete genome	JX569848	80.5	100	3.44835e−28	Mecoptera	Panorpidae	Neopanorpa pulchra
Dicranomyia iniquispina voucher 205222 16S ribosom	KC692800	79.38	100	3.44835e−28	Diptera	Limoniidae	Dicranomyia iniquispina
Microchorista philpotti 16S ribosomal RNA gene, pa	KC177459	80.5	100	3.44835e−28	Mecoptera	Nannochoristidae	Nannochorista philpotti
Aeschnophlebia anisoptera mitochondrial gene for 1	AB707619	79.87	100	3.44835e−28	Odonata	Aeshnidae	Aeschnophlebia anisoptera
Microchorista philpotti mitochondrion, partial gen	HQ696580	80.5	100	3.44835e−28	Mecoptera	Nannochoristidae	Nannochorista philpotti
Caliaeschna microstigma 12S ribosomal RNA gene, pa	EU477639	79.87	100	3.44835e−28	Odonata	Aeshnidae	Caliaeschna microstigma
Newmanoperla exigua 16S ribosomal RNA gene, partia	EF623162	79.75	100	3.44835e−28	Plecoptera	Gripopterygidae	Newmanoperla exigua
Eusthenia costalis 16S ribosomal RNA gene, partial	EF623150	81.13	100	3.44835e−28	Plecoptera	Eustheniidae	Eusthenia costalis
Suwallia teleckojensis 16S ribosomal RNA gene, par	EF623142	79.75	100	3.44835e−28	Plecoptera	Chloroperlidae	Suwallia teleckojensis
Limonia iniquispina voucher O'Grady 201869 16S rib	EU005407	79.38	100	3.44835e−28	Diptera	Limoniidae	Dicranomyia iniquispina
