# Supplementary material for: Testing the potential of a ribosomal 16S marker for DNA metabarcoding of insects
Source: PeerJ. 2016 Apr 19;4:e1966. doi: 10.7717/peerj.1966 (PMC4841222; doi:10.7717/peerj.1966)
Supplement: Supplemental Information 1 [file peerj-04-1966-s006.zip › scripts/5 match OTUs/blast_hits/OTU_407.csv_plot.pdf]

OTU_407.csv

Oreodytes sanmarkii

Dytiscidae

Coleoptera

97.45

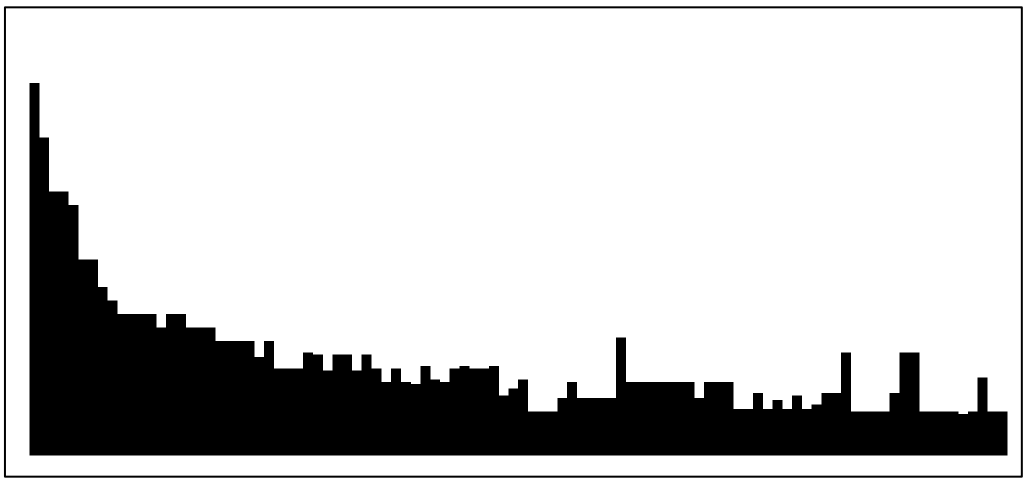

Oreodytes sanmarkii mitochondrial 16S rRNA gene (p	HF931524	97.45	100	9.86225e−67	Coleoptera	Dytiscidae	Oreodytes sanmarkii
Oreodytes obesus cordillerensis mitochondrial part	HF931516	94.9	100	2.64641e−61	Coleoptera	Dytiscidae	Oreodytes obesus cordillerensis
Oreodytes crassulus partial 16S rRNA gene	AJ850348	92.36	100	7.10131e−56	Coleoptera	Dytiscidae	Oreodytes crassulus
Oreodytes congruus partial 16S rRNA gene	AJ850347	92.36	100	7.10131e−56	Coleoptera	Dytiscidae	Oreodytes congruus
Oreodytes congruus 16S ribosomal RNA gene, partial	KF575550	91.72	100	3.01955e−54	Coleoptera	Dytiscidae	Oreodytes congruus
Oreodytes abbreviatus mitochondrial 16S rRNA gene	HF931477	89.17	100	8.10258e−49	Coleoptera	Dytiscidae	Oreodytes abbreviatus
Oreodytes rhyacophilus partial 16S rRNA gene	AJ850352	89.17	100	8.10258e−49	Coleoptera	Dytiscidae	Oreodytes rhyacophilus
Oreodytes mongolicus mitochondrial 16S rRNA gene (	HF931491	87.9	100	4.19724e−46	Coleoptera	Dytiscidae	Oreodytes mongolicus
Oreodytes snoqualmie mitochondrial 16S rRNA gene	HF931519	87.26	100	5.11329e−45	Coleoptera	Dytiscidae	Oreodytes snoqualmie
Oreodytes septentrionalis mitochondrial 16S rRNA g	HF931492	86.62	100	2.17423e−43	Coleoptera	Dytiscidae	Oreodytes septentrionalis
Oreodytes shorti mitochondrial 16S rRNA gene (part	HF931488	86.62	100	2.17423e−43	Coleoptera	Dytiscidae	Oreodytes shorti
Boreonectes striatellus mitochondrial 16S rRNA gen	HF931511	86.62	100	2.64875e−42	Coleoptera	Dytiscidae	Boreonectes striatellus
Boreonectes riberae mitochondrial 16S rRNA gene (p	HF931461	86.62	100	2.64875e−42	Coleoptera	Dytiscidae	Boreonectes riberae
Deronectes parvicollis mitochondrial 16S rRNA gene	HF931454	85.99	100	2.64875e−42	Coleoptera	Dytiscidae	Deronectes parvicollis
Boreonectes griseostriatus mitochondrial partial 1	HF931387	86.62	100	2.64875e−42	Coleoptera	Dytiscidae	Boreonectes griseostriatus
Boreonectes multilineatus mitochondrial partial 16	HF931384	86.62	100	2.64875e−42	Coleoptera	Dytiscidae	Boreonectes multilineatus
Boreonectes alpestris mitochondrial 16S rRNA gene	HF931512	85.99	100	3.22684e−41	Coleoptera	Dytiscidae	Boreonectes alpestris
Boreonectes macedonicus mitochondrial 16S rRNA gen	HF931359	85.99	100	3.22684e−41	Coleoptera	Dytiscidae	Boreonectes macedonicus
Stictotarsus ibericus 16S ribosomal RNA (rrnL) gen	EF670030	85.99	100	3.22684e−41	Coleoptera	Dytiscidae	Stictotarsus ibericus
Oreodytes scitulus 16S ribosomal RNA gene, partial	KF575551	85.35	100	1.12628e−40	Coleoptera	Dytiscidae	Oreodytes scitulus
Laccornis difformis 16S ribosomal RNA gene, partia	KF575544	85.35	100	1.12628e−40	Coleoptera	Dytiscidae	Laccornis difformis
Oreodytes scitulus mitochondrial partial 16S rRNA	HF931515	85.35	100	1.12628e−40	Coleoptera	Dytiscidae	Oreodytes scitulus
Boreonectes griseostriatus mitochondrial 16S rRNA	HF931541	85.35	100	1.37209e−39	Coleoptera	Dytiscidae	Boreonectes griseostriatus
Hydroporus glabriusculus 16S ribosomal RNA gene, p	AY365270	84.62	100	4.78905e−39	Coleoptera	Dytiscidae	Hydroporus glabriusculus
Hydroporus foveolatus mitochondrial 16S rRNA gene	HE613724	85.35	100	1.67154e−38	Coleoptera	Dytiscidae	Hydroporus foveolatus
Oreodytes quadrimaculatus 16S ribosomal RNA gene,	KF575567	84.08	100	5.83426e−38	Coleoptera	Dytiscidae	Oreodytes quadrimaculatus
Laccornis kocae partial 16S rRNA gene	AJ850419	84.08	100	5.83426e−38	Coleoptera	Dytiscidae	Laccornis kocae
Oreodytes quadrimaculatus partial 16S rRNA gene	AJ850351	84.08	100	5.83426e−38	Coleoptera	Dytiscidae	Oreodytes quadrimaculatus
Stictotarsus maghrebinus 16S ribosomal RNA gene, p	AY250947	84.81	100	5.83426e−38	Coleoptera	Dytiscidae	Stictotarsus maghrebinus
Hydroporus tessellatus 16S ribosomal RNA gene, par	JX434770	84.71	100	2.03636e−37	Coleoptera	Dytiscidae	Hydroporus tessellatus
Hygrotus novemlineatus mitochondrial partial 16S r	HF931523	83.97	100	2.03636e−37	Coleoptera	Dytiscidae	Hygrotus novemlineatus
Hydroporus zimmermanni mitochondrial 16S rRNA gene	HE613679	84.71	100	2.03636e−37	Coleoptera	Dytiscidae	Hydroporus zimmermanni
Hydroporus basinotatus mitochondrial 16S rRNA gene	HE613678	84.71	100	2.03636e−37	Coleoptera	Dytiscidae	Hydroporus basinotatus
Hygrotus turbidus partial 16S rRNA gene	AJ850390	83.97	100	2.03636e−37	Coleoptera	Dytiscidae	Hygrotus turbidus
Hydroporus basinotatus 16S ribosomal RNA gene, par	AF518258	84.71	100	2.03636e−37	Coleoptera	Dytiscidae	Hydroporus basinotatus
Suphrodytes dorsalis 16S ribosomal RNA gene, parti	KF575577	84.08	100	7.10758e−37	Coleoptera	Dytiscidae	Suphrodytes dorsalis
Aethionectes fulvonotatus voucher KBMAeop406 16S r	KF979088	83.44	100	7.10758e−37	Coleoptera	Dytiscidae	Aethionectes fulvonotatus
Suphrodytes dorsalis voucher BMNH:725966 16S ribos	JX221574	84.08	100	7.10758e−37	Coleoptera	Dytiscidae	Suphrodytes dorsalis
Oreodytes natrix partial 16S rRNA gene	AJ850349	83.44	100	7.10758e−37	Coleoptera	Dytiscidae	Oreodytes natrix
Hygrotus patruelis mitochondrial 16S rRNA gene (pa	HF931388	83.33	100	2.48079e−36	Coleoptera	Dytiscidae	Hygrotus patruelis
Stictotarsus procerus mitochondrial 16S rRNA gene	HF931379	84.18	100	2.48079e−36	Coleoptera	Dytiscidae	Stictotarsus procerus
Nebrioporus formaster mitochondrial partial 16S rR	HF931377	83.54	100	2.48079e−36	Coleoptera	Dytiscidae	Nebrioporus formaster
Boreonectes griseostriatus mitochondrial 16S rRNA	HF931385	83.44	100	8.65881e−36	Coleoptera	Dytiscidae	Boreonectes griseostriatus
Hydroporus analis mitochondrial partial 16S rRNA g	HE613731	84.08	100	8.65881e−36	Coleoptera	Dytiscidae	Hydroporus analis
Hydroporus fuscipennis mitochondrial 16S rRNA gene	HE613730	84.18	100	8.65881e−36	Coleoptera	Dytiscidae	Hydroporus fuscipennis
Hydroporus compunctus mitochondrial 16S rRNA gene	HE599693	84.08	100	8.65881e−36	Coleoptera	Dytiscidae	Hydroporus compunctus
Hydroporus compunctus 16S ribosomal RNA gene, part	AF518259	84.08	100	8.65881e−36	Coleoptera	Dytiscidae	Hydroporus compunctus
Hydroporus fuscipennis 16S ribosomal RNA gene, par	AF518262	84.18	100	8.65881e−36	Coleoptera	Dytiscidae	Hydroporus fuscipennis
Hydaticus cinctipennis voucher UNM KBMHyci20 16S r	KJ548338	82.8	100	3.02222e−35	Coleoptera	Dytiscidae	Hydaticus cinctipennis
Hydroporus marginatus mitochondrial 16S rRNA gene	HE613699	83.12	100	3.02222e−35	Coleoptera	Dytiscidae	Hydroporus marginatus
Nirripirti megamacrocephalus isolate ABTC78781 16S	EU616935	83.54	100	3.02222e−35	Coleoptera	Dytiscidae	Paroster megamacrocephalus
Agabus didymus mitochondrial 16S rRNA gene (partia	LM654773	82.05	100	1.05486e−34	Coleoptera	Dytiscidae	Agabus didymus
Agabus didymus voucher UNM KBMPmsp730 16S ribosoma	KJ548392	82.05	100	1.05486e−34	Coleoptera	Dytiscidae	Agabus didymus
Hydaticus aruspex voucher NHRS/JLKB000000298 16S r	KF979119	82.05	100	1.05486e−34	Coleoptera	Dytiscidae	Hydaticus aruspex
Hygrotus lutescens mitochondrial partial 16S rRNA	HF931518	82.69	100	1.05486e−34	Coleoptera	Dytiscidae	Hygrotus lutescens
Hydroporus foveolatus mitochondrial 16S rRNA gene	HF931502	83.44	100	1.05486e−34	Coleoptera	Dytiscidae	Hydroporus foveolatus
Hygrotus fontinalis mitochondrial 16S rRNA gene (p	HF931494	82.69	100	1.05486e−34	Coleoptera	Dytiscidae	Hygrotus fontinalis
Hygrotus marklini mitochondrial 16S rRNA gene (par	HF931467	82.69	100	1.05486e−34	Coleoptera	Dytiscidae	Hygrotus marklini
Hydroporus brancoi gredensis mitochondrial partial	HF931329	82.69	100	1.05486e−34	Coleoptera	Dytiscidae	Hydroporus brancoi gredensis
Suphrodytes figuratus voucher BMNH:722148 16S ribo	JX221571	82.69	100	1.05486e−34	Coleoptera	Dytiscidae	Suphrodytes figuratus
Deronectes moestus mitochondrial 16S rRNA gene (pa	HE613726	85.52	91.67	1.05486e−34	Coleoptera	Dytiscidae	Deronectes moestus
Hydroporus discretus mitochondrial 16S rRNA gene (	HE613723	83.44	100	1.05486e−34	Coleoptera	Dytiscidae	Hydroporus discretus
Hydroporus decipiens mitochondrial 16S rRNA gene (	HE613722	83.44	100	1.05486e−34	Coleoptera	Dytiscidae	Hydroporus decipiens
Hydroporus pubescens mitochondrial 16S rRNA gene (	HE613707	83.44	100	1.05486e−34	Coleoptera	Dytiscidae	Hydroporus pubescens
Hydroporus limbatus mitochondrial 16S rRNA gene (p	HE613706	83.44	100	1.05486e−34	Coleoptera	Dytiscidae	Hydroporus limbatus
Hydroporus errans mitochondrial 16S rRNA gene (par	HE613704	83.44	100	1.05486e−34	Coleoptera	Dytiscidae	Hydroporus errans
Hydroporus pubescens voucher NHM−IR39 16S ribosoma	EF419327	83.44	100	1.05486e−34	Coleoptera	Dytiscidae	Hydroporus pubescens
Hydroporus pubescens 16S ribosomal RNA gene, parti	AF518276	83.44	100	1.05486e−34	Coleoptera	Dytiscidae	Hydroporus pubescens
Hydroporus marginatus 16S ribosomal RNA gene, part	AF518267	82.69	100	1.05486e−34	Coleoptera	Dytiscidae	Hydroporus marginatus
Hydroporus limbatus 16S ribosomal RNA gene, partia	AF518264	83.44	100	1.05486e−34	Coleoptera	Dytiscidae	Hydroporus limbatus
Hydroporus discretus 16S ribosomal RNA gene, parti	AF518260	83.44	100	1.05486e−34	Coleoptera	Dytiscidae	Hydroporus discretus
Hydroporus analis 16S ribosomal RNA gene, partial	AF518257	83.44	100	1.05486e−34	Coleoptera	Dytiscidae	Hydroporus analis
Hydaticus aruspex voucher UNM KBMHyar68 16S riboso	KJ548337	82.17	100	3.68182e−34	Coleoptera	Dytiscidae	Hydaticus aruspex
Cybister marginicollis voucher UNM KBMCyma416 16S	KJ548319	82.17	100	3.68182e−34	Coleoptera	Dytiscidae	Cybister marginicollis
Hydroporus planus mitochondrial 16S rRNA gene (par	HE613708	82.91	100	3.68182e−34	Coleoptera	Dytiscidae	Hydroporus planus
Copelatus sp. BI−2011 voucher BMNH792945 16S ribos	HQ381441	82.17	100	3.68182e−34	Coleoptera	Dytiscidae	Copelatus sp. BI−2011
Antiporus hollingsworthi mitochondrial partial 16S	FR727246	82.58	98.72	3.68182e−34	Coleoptera	Dytiscidae	Antiporus hollingsworthi
Acilius duvergeri partial 16S rRNA gene	AJ850357	82.17	100	3.68182e−34	Coleoptera	Dytiscidae	Acilius duvergeri
Nirripirti newhavenensis 16S ribosomal RNA gene, p	AY353844	82.8	100	3.68182e−34	Coleoptera	Dytiscidae	Paroster newhavenensis
Amphizoa lecontei 16S ribosomal RNA gene, partial	AY071771	82.17	100	3.68182e−34	Coleoptera	Amphizoidae	Amphizoa lecontei
Scarodytes sp. IR−2003 16S ribosomal RNA gene, par	AY250939	82.39	100	3.68182e−34	Coleoptera	Dytiscidae	Scarodytes sp. IR−2003
Hydroporus rufifrons 16S ribosomal RNA gene, parti	AF518277	82.91	100	3.68182e−34	Coleoptera	Dytiscidae	Hydroporus rufifrons
Hydroporus planus 16S ribosomal RNA gene, partial	AF518275	82.91	100	3.68182e−34	Coleoptera	Dytiscidae	Hydroporus planus
Deronectes moestus 16S ribosomal RNA gene, partial	KF575561	84.83	91.67	1.28508e−33	Coleoptera	Dytiscidae	Deronectes moestus
Hygrotus tumidiventris mitochondrial 16S rRNA gene	HF931496	82.05	100	1.28508e−33	Coleoptera	Dytiscidae	Hygrotus tumidiventris
Hygrotus unguicularis mitochondrial partial 16S rR	HF931489	82.05	100	1.28508e−33	Coleoptera	Dytiscidae	Hygrotus unguicularis
Hygrotus sanfilippoi mitochondrial 16S rRNA gene (	HF931342	82.05	100	1.28508e−33	Coleoptera	Dytiscidae	Hygrotus sanfilippoi
Suphrodytes figuratus voucher BMNH:722150 16S ribo	JX221572	82.05	100	1.28508e−33	Coleoptera	Dytiscidae	Suphrodytes figuratus
Deronectes opatrinus mitochondrial 16S rRNA gene (	HE613701	82.91	100	1.28508e−33	Coleoptera	Dytiscidae	Deronectes opatrinus
Deronectes brannani mitochondrial 16S rRNA gene (p	HE613695	84.83	91.67	1.28508e−33	Coleoptera	Dytiscidae	Deronectes brannanii
Deronectes moestus mitochondrial 16S rRNA gene (pa	HE613683	84.83	91.67	1.28508e−33	Coleoptera	Dytiscidae	Deronectes moestus
Suphrodytes dorsalis mitochondrial 16S rRNA gene (	HE599700	82.05	100	1.28508e−33	Coleoptera	Dytiscidae	Suphrodytes dorsalis
Hydroporus necopinatus mitochondrial partial 16S r	HE599692	82.05	100	1.28508e−33	Coleoptera	Dytiscidae	Hydroporus necopinatus
Hydroporus brancoi mitochondrial 16S rRNA gene (pa	HE599678	82.05	100	1.28508e−33	Coleoptera	Dytiscidae	Hydroporus brancoi
Hydroporus cantabricus mitochondrial partial 16S r	HE599676	82.05	100	1.28508e−33	Coleoptera	Dytiscidae	Hydroporus cantabricus
Antiporus hollingsworthi mitochondrial partial 16S	FR727247	81.94	98.72	1.28508e−33	Coleoptera	Dytiscidae	Antiporus hollingsworthi
Hygrotus corpulentus partial 16S rRNA gene	AJ850387	82.05	100	1.28508e−33	Coleoptera	Dytiscidae	Hygrotus corpulentus
Darwinhydrus solidus voucher MB354 16S ribosomal R	EF056671	83.65	100	1.28508e−33	Coleoptera	Dytiscidae	Darwinhydrus solidus
Hydroporus necopinatus 16S ribosomal RNA gene, par	AY365275	82.05	100	1.28508e−33	Coleoptera	Dytiscidae	Hydroporus necopinatus
Hydroporus sp. IR33.4 16S ribosomal RNA gene, part	AY365261	82.05	100	1.28508e−33	Coleoptera	Dytiscidae	Hydroporus sp. IR33.4
