# Supplementary material for: Testing the potential of a ribosomal 16S marker for DNA metabarcoding of insects
Source: PeerJ. 2016 Apr 19;4:e1966. doi: 10.7717/peerj.1966 (PMC4841222; doi:10.7717/peerj.1966)
Supplement: Supplemental Information 1 [file peerj-04-1966-s006.zip › scripts/5 match OTUs/blast_hits/OTU_41.csv_plot.pdf]

OTU_41.csv

Leuctra inermis

Leuctridae

Plecoptera

88.68

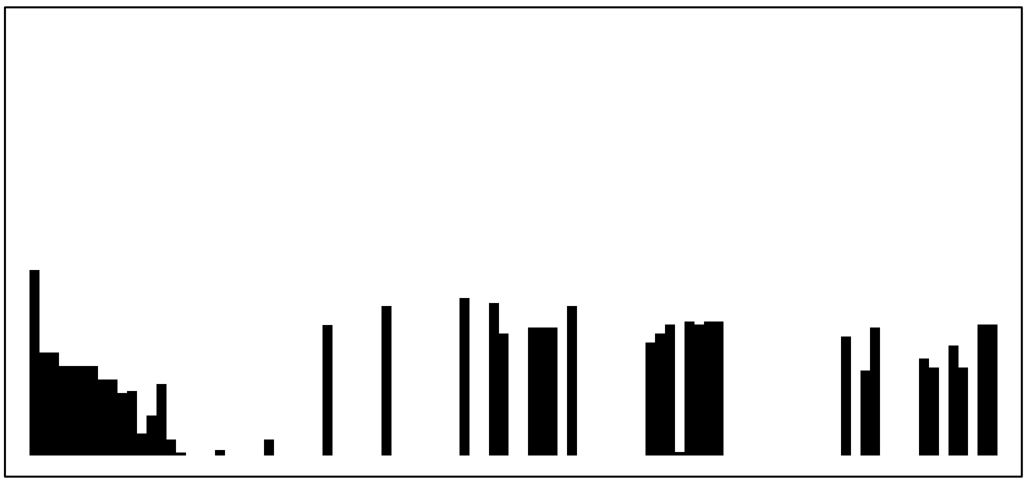

Leuctra inermis 16S ribosomal RNA gene, partial se	EF623176	88.68	100	8.23651e−49	Plecoptera	Leuctridae	Leuctra inermis
Leuctra tenuis haplotype 8lt: HO8 16S ribosomal RN	EU327680	84.81	100	3.28017e−41	Plecoptera	Leuctridae	Leuctra tenuis
Leuctra tenuis haplotype 7lt: HO7 16S ribosomal RN	EU327679	84.81	100	3.28017e−41	Plecoptera	Leuctridae	Leuctra tenuis
Leuctra tenuis haplotype 10lt: HO10 16S ribosomal	EU327682	84.18	100	1.39476e−39	Plecoptera	Leuctridae	Leuctra tenuis
Leuctra tenuis haplotype 9lt: HO9 16S ribosomal RN	EU327681	84.18	100	1.39476e−39	Plecoptera	Leuctridae	Leuctra tenuis
Leuctra tenuis haplotype 3lt: H03 16S ribosomal RN	DQ915184	84.18	100	1.39476e−39	Plecoptera	Leuctridae	Leuctra tenuis
Leuctra tenuis haplotype 2lt: H02 16S ribosomal RN	DQ915183	84.18	100	1.39476e−39	Plecoptera	Leuctridae	Leuctra tenuis
Leuctra tenuis haplotype 6lt: HO6 16S ribosomal RN	EU327678	83.54	100	1.69917e−38	Plecoptera	Leuctridae	Leuctra tenuis
Leuctra tenuis haplotype 1lt: H01 16S ribosomal RN	DQ915182	83.54	100	1.69917e−38	Plecoptera	Leuctridae	Leuctra tenuis
Leuctra tenuis haplotype 5lt: HO5 16S ribosomal RN	EU327677	82.91	100	7.22506e−37	Plecoptera	Leuctridae	Leuctra tenuis
Leuctra tenuis haplotype 4lt: HO4 16S ribosomal RN	EU327676	83.02	100	3.07217e−35	Plecoptera	Leuctridae	Leuctra tenuis
Despaxia augusta 16S ribosomal RNA gene, partial s	EF623177	81.01	100	5.55462e−32	Plecoptera	Leuctridae	Despaxia augusta
Soyedina producta 16S ribosomal RNA gene, partial	EF623196	81.88	100	1.93875e−31	Plecoptera	Nemouridae	Soyedina producta
Moselia infuscata 16S ribosomal RNA gene, partial	EF623175	83.33	87.34	2.36188e−30	Plecoptera	Leuctridae	Moselia infuscata
Pteronarcys californica 16S ribosomal RNA gene, pa	EF623261	80.75	100	8.24379e−30	Plecoptera	Pteronarcyidae	Pteronarcys californica
Pteronarcys princeps mitochondrion, complete genom	AY687866	80.12	100	1.0043e−28	Plecoptera	Pteronarcyidae	Pteronarcys princeps
Nemurella pictetii 16S ribosomal RNA gene, partial	EF623192	79.88	100	1.22349e−27	Plecoptera	Nemouridae	Nemurella pictetii
Kathroperla perdita 16S ribosomal RNA gene, partia	EF623135	79.11	100	1.22349e−27	Plecoptera	Chloroperlidae	Kathroperla perdita
Pteronarcys scotti 16S ribosomal RNA gene, partial	EF623263	79.5	100	4.27039e−27	Plecoptera	Pteronarcyidae	Pteronarcys scotti
Doddsia occidentalis 16S ribosomal RNA gene, parti	EF623270	80.25	100	1.49051e−26	Plecoptera	Taeniopterygidae	Doddsia occidentalis
Nemoura trispinosa 16S ribosomal RNA gene, partial	EF623187	79.14	100	1.49051e−26	Plecoptera	Nemouridae	Nemoura trispinosa
Pteronarcys sachalina 16S ribosomal RNA gene, part	EF623264	78.88	100	5.2024e−26	Plecoptera	Pteronarcyidae	Pteronarcys sachalina
Shipsa rotunda 16S ribosomal RNA gene, partial seq	EF623184	79.27	100	5.2024e−26	Plecoptera	Nemouridae	Shipsa rotunda
Malenka californica 16S ribosomal RNA gene, partia	EF623182	79.39	100	5.2024e−26	Plecoptera	Nemouridae	Malenka californica
Klapopteryx armillata 16S ribosomal RNA gene, part	EF623123	80.74	85.44	5.2024e−26	Plecoptera	Austroperlidae	Klapopteryx armillata
Protonemura meyeri 16S ribosomal RNA gene, partial	EF623193	79.5	100	1.81582e−25	Plecoptera	Nemouridae	Protonemura meyeri
Ameletoides sp. EP159 16S ribosomal RNA gene, part	AY749826	79.11	100	1.81582e−25	Ephemeroptera	Nesameletidae	Ameletoides sp. EP159
Oemopteryx vanduzeea 16S ribosomal RNA gene, parti	EF623266	78.53	100	6.33782e−25	Plecoptera	Taeniopterygidae	Oemopteryx vanduzeea
Scopura montana 16S ribosomal RNA gene, partial se	EF623265	79.14	100	6.33782e−25	Plecoptera		Scopura montana
Cerconychia sp. BYU_PL114 16S ribosomal RNA gene,	EF623260	79.27	100	6.33782e−25	Plecoptera	Styloperlidae	Cerconychia sp. BYU_PL114
Nemoura cinerea 16S ribosomal RNA gene, partial se	EF623194	86.11	66.46	6.33782e−25	Plecoptera	Nemouridae	Nemoura cinerea
Triznaka pintada 16S ribosomal RNA gene, partial s	EF623134	78.62	100	6.33782e−25	Plecoptera	Chloroperlidae	Triznaka pintada
Ostrocerca foersteri 16S ribosomal RNA gene, parti	EF623191	78.31	100	2.21212e−24	Plecoptera	Nemouridae	Ostrocerca foersteri
Visoka cataractae 16S ribosomal RNA gene, partial	EF623189	78.18	100	2.21212e−24	Plecoptera	Nemouridae	Visoka cataractae
Paranemoura claasseni 16S ribosomal RNA gene, part	EF623185	78.31	100	2.21212e−24	Plecoptera	Nemouridae	Paranemoura claasseni
Capnura wanica 16S ribosomal RNA gene, partial seq	EF623128	78.4	100	2.21212e−24	Plecoptera	Capniidae	Capnura wanica
Tasmanoperla larvalis 16S ribosomal RNA gene, part	EF623125	87	63.29	2.21212e−24	Plecoptera	Austroperlidae	Tasmanoperla larvalis
Apteroperla tikumana mitochondrion, complete genom	KR604721	77.91	100	7.72104e−24	Plecoptera	Capniidae	Apteroperla tikumana
Mesocapnia arizonensis mitochondrion, partial geno	KP642637	77.91	100	7.72104e−24	Plecoptera	Capniidae	Mesocapnia arizonensis
Allocapnia recta isolate H13 16S ribosomal RNA gen	KC881048	77.91	100	7.72104e−24	Plecoptera	Capniidae	Allocapnia recta
Allocapnia recta isolate H11 16S ribosomal RNA gen	KC881046	77.91	100	7.72104e−24	Plecoptera	Capniidae	Allocapnia recta
Allocapnia recta isolate H7 16S ribosomal RNA gene	KC881042	77.91	100	7.72104e−24	Plecoptera	Capniidae	Allocapnia recta
Allocapnia recta isolate H3 16S ribosomal RNA gene	KC881038	77.91	100	7.72104e−24	Plecoptera	Capniidae	Allocapnia recta
Susulus venastus 16S ribosomal RNA gene, partial s	EF623229	78.53	100	7.72104e−24	Plecoptera	Perlodidae	Susulus venastus
Lednia tumana 16S ribosomal RNA gene, partial sequ	EF623197	87.38	64.56	7.72104e−24	Plecoptera	Nemouridae	Lednia tumana
Mesocapnia sp. BYU_PL010 16S ribosomal RNA gene, p	EF623127	77.91	100	7.72104e−24	Plecoptera	Capniidae	Mesocapnia sp. BYU_PL010
Allocapnia recta haplotype 3ar: H03 16S ribosomal	DQ915181	77.91	100	7.72104e−24	Plecoptera	Capniidae	Allocapnia recta
Isoperla davisi 16S ribosomal RNA gene, partial se	EF623206	87.13	63.29	2.69491e−23	Plecoptera	Perlodidae	Isoperla davisi
Prostoia besametsa 16S ribosomal RNA gene, partial	EF623188	85.71	64.56	2.69491e−23	Plecoptera	Nemouridae	Prostoia besametsa
Amphinemura nigritta 16S ribosomal RNA gene, parti	EF623186	77.36	100	2.69491e−23	Plecoptera	Nemouridae	Amphinemura nigritta
Paraleuctra occidentalis 16S ribosomal RNA gene, p	EF623178	78.12	100	2.69491e−23	Plecoptera	Leuctridae	Paraleuctra occidentalis
Riekoperla triloba 16S ribosomal RNA gene, partial	EF623171	86	63.29	2.69491e−23	Plecoptera	Gripopterygidae	Riekoperla triloba
Sasquaperla hoopa 16S ribosomal RNA gene, partial	EF623143	86	63.29	2.69491e−23	Plecoptera	Chloroperlidae	Sasquaperla hoopa
Chloroperla tripunctata 16S ribosomal RNA gene, pa	EF623137	86	63.29	2.69491e−23	Plecoptera	Chloroperlidae	Chloroperla tripunctata
Paraperla frontalis 16S ribosomal RNA gene, partia	EF623136	77.99	100	2.69491e−23	Plecoptera	Chloroperlidae	Paraperla frontalis
Penturoperla barbata 16S ribosomal RNA gene, parti	EF623124	87	63.29	2.69491e−23	Plecoptera	Austroperlidae	Penturoperla barbata
Osmylus sp. EH−2004 16S ribosomal RNA gene, partia	AY620146	78.4	100	2.69491e−23	Neuroptera	Osmylidae	Osmylus sp. EH−2004
Allocapnia recta isolate H17 16S ribosomal RNA gen	KC881052	78.26	100	9.40616e−23	Plecoptera	Capniidae	Allocapnia recta
Allocapnia recta isolate H12 16S ribosomal RNA gen	KC881047	78.26	100	9.40616e−23	Plecoptera	Capniidae	Allocapnia recta
Allocapnia recta isolate H9 16S ribosomal RNA gene	KC881044	78.26	100	9.40616e−23	Plecoptera	Capniidae	Allocapnia recta
Allocapnia recta isolate H8 16S ribosomal RNA gene	KC881043	78.26	100	9.40616e−23	Plecoptera	Capniidae	Allocapnia recta
Allocapnia recta isolate H5 16S ribosomal RNA gene	KC881040	78.26	100	9.40616e−23	Plecoptera	Capniidae	Allocapnia recta
Allocapnia recta isolate H4 16S ribosomal RNA gene	KC881039	78.26	100	9.40616e−23	Plecoptera	Capniidae	Allocapnia recta
Pteronarcella badia 16S ribosomal RNA gene, partia	EF623262	85.29	64.56	9.40616e−23	Plecoptera	Pteronarcyidae	Pteronarcella badia
Acroneuria lycorias 16S ribosomal RNA gene, partia	EF623251	85.71	64.56	9.40616e−23	Plecoptera	Perlidae	Acroneuria lycorias
Udamocercia sp. BYU_PL150 16S ribosomal RNA gene,	EF623201	86.14	62.03	9.40616e−23	Plecoptera	Notonemouridae	Udamocercia sp. BYU_PL150
Zapada cinctipes 16S ribosomal RNA gene, partial s	EF623183	80.14	87.34	9.40616e−23	Plecoptera	Nemouridae	Zapada cinctipes
Suwallia teleckojensis 16S ribosomal RNA gene, par	EF623142	86.27	63.29	9.40616e−23	Plecoptera	Chloroperlidae	Suwallia teleckojensis
Alloperla severa 16S ribosomal RNA gene, partial s	EF623141	86.14	63.29	9.40616e−23	Plecoptera	Chloroperlidae	Alloperla severa
Neaviperla forcipata 16S ribosomal RNA gene, parti	EF623139	86.27	63.29	9.40616e−23	Plecoptera	Chloroperlidae	Neaviperla forcipata
Plumiperla diversa 16S ribosomal RNA gene, partial	EF623132	86.27	63.29	9.40616e−23	Plecoptera	Chloroperlidae	Plumiperla diversa
Isocapnia hyalita 16S ribosomal RNA gene, partial	EF623131	77.02	100	9.40616e−23	Plecoptera	Capniidae	Isocapnia hyalita
Allocapnia recta haplotype 10ar: H010 16S ribosoma	EU306873	78.26	100	9.40616e−23	Plecoptera	Capniidae	Allocapnia recta
Allocapnia recta haplotype 9ar: H09 16S ribosomal	EU306872	78.26	100	9.40616e−23	Plecoptera	Capniidae	Allocapnia recta
Allocapnia recta haplotype 8ar: H08 16S ribosomal	EU306871	78.26	100	9.40616e−23	Plecoptera	Capniidae	Allocapnia recta
Allocapnia recta haplotype 1ar: H01 16S ribosomal	DQ915179	78.26	100	9.40616e−23	Plecoptera	Capniidae	Allocapnia recta
Allocapnia recta isolate H15 16S ribosomal RNA gen	KC881050	77.3	100	3.28307e−22	Plecoptera	Capniidae	Allocapnia recta
Allocapnia recta isolate H14 16S ribosomal RNA gen	KC881049	77.3	100	3.28307e−22	Plecoptera	Capniidae	Allocapnia recta
Allocapnia recta isolate H10 16S ribosomal RNA gen	KC881045	77.3	100	3.28307e−22	Plecoptera	Capniidae	Allocapnia recta
Allocapnia recta isolate H6 16S ribosomal RNA gene	KC881041	77.3	100	3.28307e−22	Plecoptera	Capniidae	Allocapnia recta
Allocapnia recta isolate H1 16S ribosomal RNA gene	KC881036	77.3	100	3.28307e−22	Plecoptera	Capniidae	Allocapnia recta
Anterastes serbicus haplotype 38BUL 16S ribosomal	HM856177	77.78	99.37	3.28307e−22	Orthoptera	Tettigoniidae	Anterastes serbicus
Strophopteryx appalachia 16S ribosomal RNA gene, p	EF623271	77.16	100	3.28307e−22	Plecoptera	Taeniopterygidae	Strophopteryx appalachia
Brachyptera seticornis 16S ribosomal RNA gene, par	EF623268	85.58	64.56	3.28307e−22	Plecoptera	Taeniopterygidae	Brachyptera seticornis
Nanonemoura wahkeena 16S ribosomal RNA gene, parti	EF623198	77.44	100	3.28307e−22	Plecoptera	Nemouridae	Nanonemoura wahkeena
Amphinemura sulcicollis 16S ribosomal RNA gene, pa	EF623195	83.96	67.09	3.28307e−22	Plecoptera	Nemouridae	Amphinemura sulcicollis
Sweltsa coloradensis 16S ribosomal RNA gene, parti	EF623133	86	63.29	3.28307e−22	Plecoptera	Chloroperlidae	Sweltsa coloradensis
Allocapnia minima 16S ribosomal RNA gene, partial	EF623130	77.3	100	3.28307e−22	Plecoptera	Capniidae	Allocapnia minima
Allocapnia recta haplotype 12ar: H12 16S ribosomal	EU306875	77.3	100	3.28307e−22	Plecoptera	Capniidae	Allocapnia recta
Allocapnia recta haplotype 11ar: H011 16S ribosoma	EU306874	77.3	100	3.28307e−22	Plecoptera	Capniidae	Allocapnia recta
Allocapnia recta haplotype 7ar: H07 16S ribosomal	EU306870	77.3	100	3.28307e−22	Plecoptera	Capniidae	Allocapnia recta
Siphlonurus sp. EP084 16S ribosomal RNA gene, part	AY749786	84.55	69.62	3.28307e−22	Ephemeroptera	Siphlonuridae	Siphlonurus sp. EP084
Dinocras cephalotes mitochondrion, complete genome	KF484757	84.11	64.56	1.1459e−21	Plecoptera	Perlidae	Dinocras cephalotes
Allocapnia recta isolate H18 16S ribosomal RNA gen	KC881053	77.64	100	1.1459e−21	Plecoptera	Capniidae	Allocapnia recta
Sierraperla cora 16S ribosomal RNA gene, partial s	EF623255	85.15	63.92	1.1459e−21	Plecoptera	Peltoperlidae	Sierraperla cora
Dinocras cephalotes 16S ribosomal RNA gene, partia	EF623244	84.11	64.56	1.1459e−21	Plecoptera	Perlidae	Dinocras cephalotes
Rickera sorpta 16S ribosomal RNA gene, partial seq	EF623235	77.64	100	1.1459e−21	Plecoptera	Perlodidae	Rickera sorpta
Cosumnoperla hypocrena 16S ribosomal RNA gene, par	EF623226	86.14	63.29	1.1459e−21	Plecoptera	Perlodidae	Cosumnoperla hypocrena
Isoperla oxylepis 16S ribosomal RNA gene, partial	EF623221	86.14	63.29	1.1459e−21	Plecoptera	Perlodidae	Isoperla oxylepis
Megarcys signata 16S ribosomal RNA gene, partial s	EF623215	76.88	100	1.1459e−21	Plecoptera	Perlodidae	Megarcys signata
