# Supplementary material for: Testing the potential of a ribosomal 16S marker for DNA metabarcoding of insects
Source: PeerJ. 2016 Apr 19;4:e1966. doi: 10.7717/peerj.1966 (PMC4841222; doi:10.7717/peerj.1966)
Supplement: Supplemental Information 1 [file peerj-04-1966-s006.zip › scripts/5 match OTUs/blast_hits/OTU_42.csv_plot.pdf]

OTU_42.csv

Pedicia albivitta

Pediciidae

Diptera

94.9

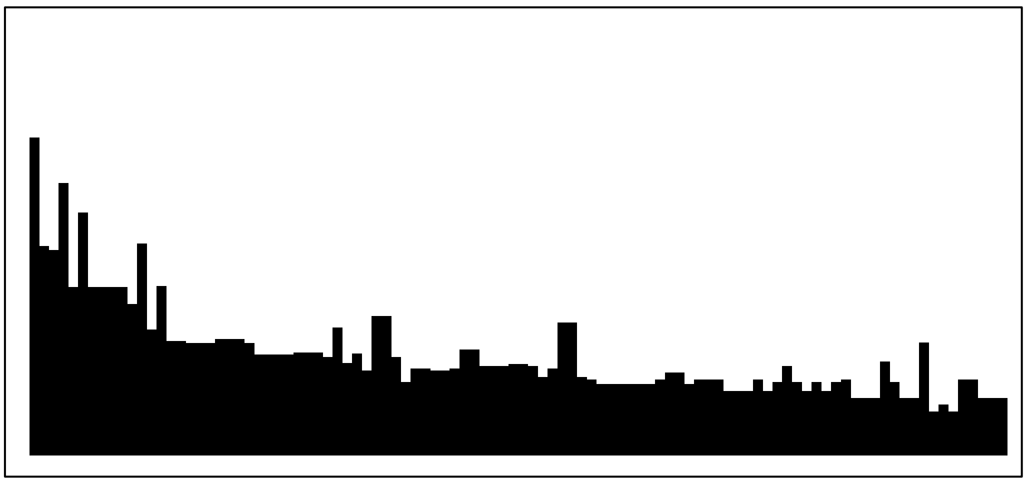

Pedicia albivitta voucher O'Grady 200012 16S ribos	EU005435	94.9	100	2.19026e−62	Diptera	Pediciidae	Pedicia albivitta
Tipula sp. PMO 200011 16S ribosomal RNA gene, part	EU005437	89.81	100	1.57709e−51	Diptera	Tipulidae	Tipula sp. PMO 200011
Bolitophila cinerea voucher JSB1 16S ribosomal RNA	KJ136712	89.61	97.45	8.16955e−49	Diptera	Mycetophilidae	Bolitophila cinerea
Bolitophila sp. ER−2009 16S ribosomal RNA gene, pa	FJ172021	92.75	87.26	2.85145e−48	Diptera	Mycetophilidae	Bolitophila sp. ER−2009
Parochlus steinenii isolate Sejong01 mitochondrion	KT003702	87.9	100	4.23193e−46	Diptera	Chironomidae	Parochlus steinenii
Bolitophila occlusa voucher JSB2 16S ribosomal RNA	KJ136713	91.37	87.9	4.23193e−46	Diptera	Mycetophilidae	Bolitophila occlusa
Dicranomyia iniquispina voucher 205222 16S ribosom	KC692800	87.9	100	4.23193e−46	Diptera	Limoniidae	Dicranomyia iniquispina
Tipula abdominalis 16S ribosomal RNA gene, partial	KC177457	87.9	100	4.23193e−46	Diptera	Tipulidae	Tipula abdominalis
Tipula abdominalis mitochondrion, partial genome	JN861743	87.9	100	4.23193e−46	Diptera	Tipulidae	Tipula abdominalis
Limonia iniquispina voucher O'Grady 201869 16S rib	EU005407	87.9	100	4.23193e−46	Diptera	Limoniidae	Dicranomyia iniquispina
Limonia sp. PMO 200984 16S ribosomal RNA gene, par	EU005416	87.1	98.73	6.28074e−44	Diptera	Limoniidae	Limonia sp. PMO 200984
Bolitophila hybrida 16S ribosomal RNA gene, partia	FJ172002	89.93	87.9	2.19219e−43	Diptera	Mycetophilidae	Bolitophila hybrida
Edwardsina gigantea 16S ribosomal RNA gene, partia	KC177460	85.9	99.36	9.32145e−42	Diptera	Blephariceridae	Edwardsina gigantea
Arachnocampa luminosa biotype 1 16S large subunit	AY576329	87.94	89.81	3.2535e−41	Diptera	Keroplatidae	Arachnocampa luminosa
Limonia hawaiiensis voucher O'Grady 201598 16S rib	EU005405	85.35	100	1.13558e−40	Diptera	Limoniidae	Dicranomyia hawaiiensis
Limonia hawaiiensis voucher O'Grady 200922 16S rib	EU005404	85.35	100	1.13558e−40	Diptera	Limoniidae	Dicranomyia hawaiiensis
Ectinorhynchus sp. 'Bribie' voucher UQIC41178 16S	KM212983	85.26	99.36	3.96358e−40	Diptera	Therevidae	Ectinorhynchus sp. 'Bribie'
Ectinorhynchus sp. 'Moffatt' voucher QMT183203 16S	KM212976	85.26	99.36	3.96358e−40	Diptera	Therevidae	Ectinorhynchus sp. 'Moffatt'
Ectinorhynchus sp. 'warrumbungles' voucher QMT1832	KM212974	85.26	99.36	3.96358e−40	Diptera	Therevidae	Ectinorhynchus sp. 'warrumbungles'
Limonia swezeyi voucher O'Grady 201882 16S ribosom	EU005424	85.44	100	3.96358e−40	Diptera	Limoniidae	Dicranomyia swezeyi
Limonia swezeyi voucher O'Grady 200982 16S ribosom	EU005423	85.44	100	3.96358e−40	Diptera	Limoniidae	Dicranomyia swezeyi
Limonia swezeyi voucher O'Grady 200126 16S ribosom	EU005420	85.44	100	3.96358e−40	Diptera	Limoniidae	Dicranomyia swezeyi
Anabarhynchus tristis voucher MEI 119905 16S ribos	DQ250834	85.26	99.36	3.96358e−40	Diptera	Therevidae	Anabarhynchus tristis
Limonia variabilis voucher O'Grady 201512 16S ribo	EU005428	84.71	100	1.38343e−39	Diptera	Limoniidae	Dicranomyia variabilis
Limonia kraussi voucher O'Grady 200013 16S ribosom	EU005415	84.71	100	1.38343e−39	Diptera	Limoniidae	Dicranomyia kraussi
Limonia variabilis voucher O'Grady 200973 16S ribo	EU005426	84.71	100	1.38343e−39	Diptera	Limoniidae	Dicranomyia variabilis
Limonia hawaiiensis voucher O'Grady 200106 16S rib	EU005403	84.71	100	1.38343e−39	Diptera	Limoniidae	Dicranomyia hawaiiensis
Limonia swezeyi voucher O'Grady 200949 16S ribosom	EU005422	84.81	100	4.82863e−39	Diptera	Limoniidae	Dicranomyia swezeyi
Limonia swezeyi voucher O'Grady 200931 16S ribosom	EU005421	84.81	100	4.82863e−39	Diptera	Limoniidae	Dicranomyia swezeyi
Limonia swezeyi voucher O'Grady 200105 16S ribosom	EU005419	84.81	100	4.82863e−39	Diptera	Limoniidae	Dicranomyia swezeyi
Penniverpa sp. MEI 090271 16S ribosomal RNA gene,	DQ250826	84.62	99.36	4.82863e−39	Diptera	Therevidae	Penniverpa sp. MEI 090271
Eudicrana sp. DK−2013 voucher JSS12 16S ribosomal	KC435571	85.99	97.45	1.68536e−38	Diptera	Mycetophilidae	Eudicrana sp. DK−2013
Docosia muranica voucher JSM10 16S ribosomal RNA g	KC435567	84.31	97.45	1.68536e−38	Diptera	Mycetophilidae	Docosia muranica
Syntemna stylata 16S ribosomal RNA gene, partial s	FJ172013	84.77	96.18	1.68536e−38	Diptera	Mycetophilidae	Syntemna stylata
Somatia sp. YSUW04038 16S ribosomal RNA gene, part	AY573134	83.97	99.36	1.68536e−38	Diptera	Somatiidae	Somatia sp. YSUW04038
Arachnocampa luminosa biotype 3 16S large subunit	AY576331	86.52	89.81	1.68536e−38	Diptera	Keroplatidae	Arachnocampa luminosa
Arachnocampa luminosa biotype 2 16S large subunit	AY576330	86.52	89.81	1.68536e−38	Diptera	Keroplatidae	Arachnocampa luminosa
Clitellaria ephippium 16S ribosomal RNA gene, part	GU947399	84.62	99.36	5.88247e−38	Diptera	Stratiomyidae	Clitellaria ephippium
Libnotes orofenaae voucher O'Grady 202034 16S ribo	EU005431	83.44	100	5.88247e−38	Diptera	Limoniidae	Libnotes orofenaae
Limonia stygipennis voucher O'Grady 200980 16S rib	EU005418	84.08	100	5.88247e−38	Diptera	Limoniidae	Dicranomyia stygipennis
Limonia stygipennis voucher O'Grady 200948 16S rib	EU005417	84.08	100	5.88247e−38	Diptera	Limoniidae	Dicranomyia stygipennis
Eupsilocephala albodorsalis 16S ribosomal RNA gene	KM878912	83.97	99.36	2.05319e−37	Diptera	Therevidae	Eupsilocephala albodorsalis
Ectinorhynchus levis voucher MEI29709 16S ribosoma	KM212980	83.97	99.36	2.05319e−37	Diptera	Therevidae	Ectinorhynchus levis
Dixella sp. ZK−2014 mitochondrion, complete genome	KM245574	84.08	99.36	2.05319e−37	Diptera	Dixidae	Dixella sp. ZK−2014
Boletina gripha isolate 033462 16S ribosomal RNA g	HQ230380	84.97	96.18	2.05319e−37	Diptera	Mycetophilidae	Boletina gripha
Boletina griphoides isolate 009627 16S ribosomal R	HQ230370	84.97	96.18	2.05319e−37	Diptera	Mycetophilidae	Boletina griphoides
Libnotes perkinsi voucher O'Grady 200981 16S ribos	EU005433	84.18	100	2.05319e−37	Diptera	Limoniidae	Libnotes perkinsi
Libnotes perkinsi voucher O'Grady 200947 16S ribos	EU005432	84.18	100	2.05319e−37	Diptera	Limoniidae	Libnotes perkinsi
Limonia kauaiensis voucher O'Grady 201519 16S ribo	EU005413	84.18	100	2.05319e−37	Diptera	Limoniidae	Dicranomyia kauaiensis
Panorpa takenouchii isolate 26tak 16S ribosomal RN	EF041520	84.28	100	2.05319e−37	Mecoptera	Panorpidae	Panorpa takenouchii
Panorpa takenouchii 16S ribosomal RNA gene, partia	AF180064	84.28	100	2.05319e−37	Mecoptera	Panorpidae	Panorpa takenouchii
Nanexila argentiquadris 16S ribosomal RNA gene, pa	KM878934	84.18	99.36	7.16632e−37	Diptera	Therevidae	Nanexila argentiquadris
Dichopygina aculeata voucher 947 16S ribosomal RNA	JQ613961	83.66	97.45	7.16632e−37	Diptera	Sciaridae	Dichopygina aculeata
Laemostenus terricola 16S ribosomal RNA (rrnl) gen	JF778796	84.08	99.36	7.16632e−37	Coleoptera	Carabidae	Laemostenus terricola
Boletina sahlbergi isolate 057401 16S ribosomal RN	HQ230385	86.23	87.9	7.16632e−37	Diptera	Mycetophilidae	Boletina sahlbergi
Boletina sahlbergi isolate 057369 16S ribosomal RN	HQ230384	86.23	87.9	7.16632e−37	Diptera	Mycetophilidae	Boletina sahlbergi
Grzegorzekia collaris 16S ribosomal RNA gene, part	FJ172016	83.66	97.45	7.16632e−37	Diptera	Mycetophilidae	Grzegorzekia collaris
Manestella ocellaris 16S ribosomal RNA gene, parti	KM878928	83.54	99.36	2.50129e−36	Diptera	Therevidae	Manestella ocellaris
Evansomyia sp. SLW−2015 16S ribosomal RNA gene, pa	KM878913	83.33	99.36	2.50129e−36	Diptera	Therevidae	Evansomyia sp. SLW−2015
Evansomyia sp. 'scuTas' voucher MEI137414 16S ribo	KM212984	83.33	99.36	2.50129e−36	Diptera	Therevidae	Evansomyia sp. 'scuTas'
Ectinorhynchus micans voucher MEI30034 16S ribosom	KM212982	83.33	99.36	2.50129e−36	Diptera	Therevidae	Ectinorhynchus micans
Ectinorhynchus sp. 9 FT−2014 voucher MEI29882 16S	KM212981	83.33	99.36	2.50129e−36	Diptera	Therevidae	Ectinorhynchus sp. 9 FT−2014
Ectinorhynchus terminalis voucher QMT183231 16S ri	KM212979	83.33	99.36	2.50129e−36	Diptera	Therevidae	Ectinorhynchus terminalis
Ectinorhynchus brunneus voucher QMT183204 16S ribo	KM212973	83.33	99.36	2.50129e−36	Diptera	Therevidae	Ectinorhynchus brunneus
Ptychoptera sp. ATB−2011 mitochondrion, complete g	JN861744	83.54	100	2.50129e−36	Diptera	Ptychopteridae	Ptychoptera sp. ATB−2011
Boletina gripha isolate 057361 16S ribosomal RNA g	HQ230381	83.87	97.45	2.50129e−36	Diptera	Mycetophilidae	Boletina gripha
Boletina gripha isolate 015271 16S ribosomal RNA g	HQ230378	83.87	97.45	2.50129e−36	Diptera	Mycetophilidae	Boletina gripha
Tabanus bromius 16S ribosomal RNA gene, partial se	HM132111	83.33	99.36	2.50129e−36	Diptera	Tabanidae	Tabanus bromius
Limonia kauaiensis voucher O'Grady 201868 16S ribo	EU005414	83.54	100	2.50129e−36	Diptera	Limoniidae	Dicranomyia kauaiensis
Limonia kauaiensis voucher O'Grady 200971 16S ribo	EU005412	83.54	100	2.50129e−36	Diptera	Limoniidae	Dicranomyia kauaiensis
Limonia jacobus voucher O'Grady 200974 16S ribosom	EU005408	83.54	100	2.50129e−36	Diptera	Limoniidae	Dicranomyia jacobus
Scatopsciara atomaria voucher 981 16S ribosomal RN	JQ613973	83.01	97.45	8.73037e−36	Diptera	Sciaridae	Scatopsciara atomaria
Scatopsciara atomaria voucher 90 16S ribosomal RNA	JQ613890	83.01	97.45	8.73037e−36	Diptera	Sciaridae	Scatopsciara atomaria
Docosia sp. 010122 16S ribosomal RNA gene, partial	HQ230372	83.01	97.45	8.73037e−36	Diptera	Mycetophilidae	Docosia sp. 010122
Beris morrisii 16S ribosomal RNA gene, partial seq	GU947373	83.54	99.36	8.73037e−36	Diptera	Stratiomyidae	Beris morrisii
Leptomorphus walkeri 16S ribosomal RNA gene, parti	FJ172020	83.01	97.45	8.73037e−36	Diptera	Mycetophilidae	Leptomorphus walkeri
Ochlerotatus japonicus isolate A81 16S ribosomal R	DQ397915	83.44	99.36	8.73037e−36	Diptera	Culicidae	Aedes japonicus
Bolopus furcatus isolate 1305 16S ribosomal RNA ge	KC699315	84.18	99.36	3.0472e−35	Diptera	Platypezidae	Bolopus furcatus
Protoplasa fitchii 16S ribosomal RNA gene, partial	KC177462	83.44	97.45	3.0472e−35	Diptera	Tanyderidae	Protoplasa fitchii
Liriomyza trifolii mitochondrion, complete genome	JN570506	83.02	99.36	3.0472e−35	Diptera	Agromyzidae	Liriomyza trifolii
Protoplasa fitchii mitochondrion, complete genome	JN861746	83.44	97.45	3.0472e−35	Diptera	Tanyderidae	Protoplasa fitchii
Liriomyza trifolii mitochondrion, complete genome	GU327644	83.02	99.36	3.0472e−35	Diptera	Agromyzidae	Liriomyza trifolii
Agapophytus bicolor 16S ribosomal RNA gene, partia	KM878897	83.44	99.36	1.06358e−34	Diptera	Therevidae	Agapophytus bicolor
Aedes notoscriptus isolate MC04 mitochondrion, com	KM676218	83.55	94.9	1.06358e−34	Diptera	Culicidae	Aedes notoscriptus
Evansomyia sp. 'CannR' voucher QMT183206 16S ribos	KM212978	82.69	99.36	1.06358e−34	Diptera	Therevidae	Evansomyia sp. 'CannR'
Therevidae gen. P sp. 'barakula' voucher QMT183200	KM212977	82.69	99.36	1.06358e−34	Diptera	Therevidae	Therevidae gen. P sp. 'barakula'
Ectinorhynchus sp. A FT−2014 voucher QMT183201 16S	KM212975	82.69	99.36	1.06358e−34	Diptera	Therevidae	Ectinorhynchus sp. A FT−2014
Mycetophila alea voucher JSB11 16S ribosomal RNA g	KJ136722	84.4	89.81	1.06358e−34	Diptera	Mycetophilidae	Mycetophila alea
Bittacomorphella fenderiana mitochondrion, partial	JN861745	83.44	99.36	1.06358e−34	Diptera	Ptychopteridae	Bittacomorphella fenderiana
Chorisops tunisiae 16S ribosomal RNA gene, partial	GU947379	82.69	99.36	1.06358e−34	Diptera	Stratiomyidae	Chorisops tunisiae
Chrysops viduatus 16S ribosomal RNA gene, partial	HM132115	82.69	99.36	1.06358e−34	Diptera	Tabanidae	Chrysops viduatus
Pyratula zonata 16S ribosomal RNA gene, partial se	FJ172004	85.29	86.62	1.06358e−34	Diptera	Keroplatidae	Pyratula zonata
Drosophila bifasciata voucher 109384 NADH dehydrog	EU494362	82.05	99.36	1.06358e−34	Diptera	Drosophilidae	Drosophila bifasciata
Derodontus emaculatus voucher BMNH:679374 16S ribo	DQ202609	82.39	100	1.06358e−34	Coleoptera		Derodontus emaculatus
Euaresta festiva 16S ribosomal RNA gene, partial s	DQ471399	82.05	99.36	1.06358e−34	Diptera	Tephritidae	Euaresta festiva
Aedes galloisi isolate A87 16S ribosomal RNA gene,	DQ397920	83.55	94.9	1.06358e−34	Diptera	Culicidae	Aedes galloisi
Aedes galloisi isolate A77 16S ribosomal RNA gene,	DQ397919	83.55	94.9	1.06358e−34	Diptera	Culicidae	Aedes galloisi
Chrysops carbonarius 16S ribosomal RNA gene, parti	AY123341	82.69	99.36	1.06358e−34	Diptera	Tabanidae	Chrysops carbonarius
Cionophora sp. MEI 124032 16S ribosomal RNA gene,	DQ250835	82.69	99.36	1.06358e−34	Diptera	Therevidae	Cionophora sp. MEI 124032
Arenigena marcida voucher MEI 120161 16S ribosomal	DQ250832	82.69	99.36	1.06358e−34	Diptera	Therevidae	Arenigena marcida
