# Supplementary material for: Testing the potential of a ribosomal 16S marker for DNA metabarcoding of insects
Source: PeerJ. 2016 Apr 19;4:e1966. doi: 10.7717/peerj.1966 (PMC4841222; doi:10.7717/peerj.1966)
Supplement: Supplemental Information 1 [file peerj-04-1966-s006.zip › scripts/5 match OTUs/blast_hits/OTU_420.csv_plot.pdf]

OTU_420.csv

Rhithrogena semicolorata

Heptageniidae

Ephemeroptera

98.68

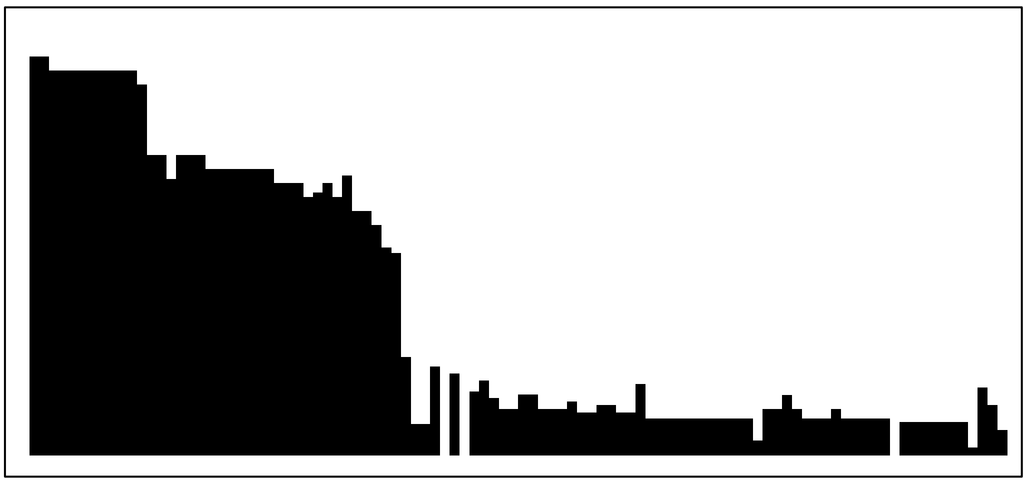

Rhithrogena semicolorata mitochondrial partial 16S	LN868673	98.68	98.7	7.96162e−68	Ephemeroptera	Heptageniidae	Rhithrogena semicolorata
Rhithrogena sp. 4 LV−2015 mitochondrial partial 16	LN868652	98.68	98.7	7.96162e−68	Ephemeroptera	Heptageniidae	Rhithrogena sp. 4 LV−2015
Rhithrogena sp. 13 LV−2015 mitochondrial partial 1	LN868672	98.03	98.7	3.38537e−66	Ephemeroptera	Heptageniidae	Rhithrogena sp. 13 LV−2015
Rhithrogena sp. 12 LV−2015 mitochondrial partial 1	LN868671	98.03	98.7	3.38537e−66	Ephemeroptera	Heptageniidae	Rhithrogena sp. 12 LV−2015
Rhithrogena picteti mitochondrial partial 16S rRNA	LN868663	98.03	98.7	3.38537e−66	Ephemeroptera	Heptageniidae	Rhithrogena picteti
Rhithrogena sp. 3 LV−2015 mitochondrial partial 16	LN868659	98.03	98.7	3.38537e−66	Ephemeroptera	Heptageniidae	Rhithrogena sp. 3 LV−2015
Rhithrogena fonticola mitochondrial partial 16S rR	LN868656	98.03	98.7	3.38537e−66	Ephemeroptera	Heptageniidae	Rhithrogena fonticola
Rhithrogena sp. 2 LV−2015 mitochondrial partial 16	LN868654	98.03	98.7	3.38537e−66	Ephemeroptera	Heptageniidae	Rhithrogena sp. 2 LV−2015
Rhithrogena puytoraci mitochondrial partial 16S rR	LN868651	98.03	98.7	3.38537e−66	Ephemeroptera	Heptageniidae	Rhithrogena puytoraci
Rhithrogena iridina mitochondrial partial 16S rRNA	LN868646	98.03	98.7	3.38537e−66	Ephemeroptera	Heptageniidae	Rhithrogena iridina
Rhithrogena taurisca mitochondrial partial 16S rRN	LN868638	98.03	98.7	3.38537e−66	Ephemeroptera	Heptageniidae	Rhithrogena taurisca
Rhithrogena sp. 5 LV−2015 mitochondrial partial 16	LN868653	97.37	98.7	4.12422e−65	Ephemeroptera	Heptageniidae	Rhithrogena sp. 5 LV−2015
Rhithrogena sp. 7 LV−2015 mitochondrial partial 16	LN868669	94.08	98.7	5.73276e−57	Ephemeroptera	Heptageniidae	Rhithrogena sp. 7 LV−2015
Rhithrogena eatoni mitochondrial partial 16S rRNA	LN868666	94.08	98.7	5.73276e−57	Ephemeroptera	Heptageniidae	Rhithrogena eatoni
Rhithrogena germanica mitochondrial partial 16S rR	LN868660	92.95	100	5.73276e−57	Ephemeroptera	Heptageniidae	Rhithrogena germanica
Rhithrogena alpestris mitochondrial partial 16S rR	LN868657	94.08	98.7	5.73276e−57	Ephemeroptera	Heptageniidae	Rhithrogena alpestris
Rhithrogena gratianopolitana mitochondrial partial	LN868647	94.08	98.7	5.73276e−57	Ephemeroptera	Heptageniidae	Rhithrogena gratianopolitana
Rhithrogena nivata mitochondrial partial 16S rRNA	LN868643	94.08	98.7	5.73276e−57	Ephemeroptera	Heptageniidae	Rhithrogena nivata
Rhithrogena degrangei mitochondrial partial 16S rR	LN868674	93.42	98.7	6.98393e−56	Ephemeroptera	Heptageniidae	Rhithrogena degrangei
Rhithrogena corcontica mitochondrial partial 16S r	LN868662	93.42	98.7	6.98393e−56	Ephemeroptera	Heptageniidae	Rhithrogena corcontica
Rhithrogena sp. 9 LV−2015 mitochondrial partial 16	LN868650	93.42	98.7	6.98393e−56	Ephemeroptera	Heptageniidae	Rhithrogena sp. 9 LV−2015
Rhithrogena gratianopolitana mitochondrial partial	LN868648	93.42	98.7	6.98393e−56	Ephemeroptera	Heptageniidae	Rhithrogena gratianopolitana
Rhithrogena landai mitochondrial partial 16S rRNA	LN868644	93.42	98.7	6.98393e−56	Ephemeroptera	Heptageniidae	Rhithrogena landai
Rhithrogena hybrida mitochondrial partial 16S rRNA	LN868642	93.42	98.7	6.98393e−56	Ephemeroptera	Heptageniidae	Rhithrogena hybrida
Rhithrogena circumtatrica mitochondrial partial 16	LN868641	93.42	98.7	6.98393e−56	Ephemeroptera	Heptageniidae	Rhithrogena circumtatrica
Rhithrogena sp. 11 LV−2015 mitochondrial partial 1	LN868668	92.76	98.7	2.96964e−54	Ephemeroptera	Heptageniidae	Rhithrogena sp. 11 LV−2015
Rhithrogena sp. 8 LV−2015 mitochondrial partial 16	LN868661	92.76	98.7	2.96964e−54	Ephemeroptera	Heptageniidae	Rhithrogena sp. 8 LV−2015
Rhithrogena sp. 6 LV−2015 mitochondrial partial 16	LN868655	92.76	98.7	2.96964e−54	Ephemeroptera	Heptageniidae	Rhithrogena sp. 6 LV−2015
Rhithrogena beskidensis mitochondrial partial 16S	LN868645	92.11	98.7	2.96964e−54	Ephemeroptera	Heptageniidae	Rhithrogena beskidensis
Rhithrogena grischuna mitochondrial partial 16S rR	LN868639	92.31	100	2.96964e−54	Ephemeroptera	Heptageniidae	Rhithrogena grischuna
Rhithrogena diensis mitochondrial partial 16S rRNA	LN868637	92.76	98.7	2.96964e−54	Ephemeroptera	Heptageniidae	Rhithrogena diensis
Rhithrogena nuragica mitochondrial partial 16S rRN	LN868667	92.11	98.7	3.61776e−53	Ephemeroptera	Heptageniidae	Rhithrogena nuragica
Rhithrogena sp. 10 LV−2015 mitochondrial partial 1	LN868664	93.1	94.16	3.61776e−53	Ephemeroptera	Heptageniidae	Rhithrogena sp. 10 LV−2015
Rhithrogena savoiensis mitochondrial partial 16S r	LN868658	91.45	98.7	1.26272e−52	Ephemeroptera	Heptageniidae	Rhithrogena savoiensis
Rhithrogena insularis mitochondrial partial 16S rR	LN868665	91.45	98.7	1.53831e−51	Ephemeroptera	Heptageniidae	Rhithrogena insularis
Rhithrogena allobrogica mitochondrial partial 16S	LN868640	90.79	98.7	1.87405e−50	Ephemeroptera	Heptageniidae	Rhithrogena allobrogica
Rhithrogena sartorii mitochondrial partial 16S rRN	LN868670	89.74	100	6.54107e−50	Ephemeroptera	Heptageniidae	Rhithrogena sartorii
Rhithrogena dorieri mitochondrial partial 16S rRNA	LN868649	89.47	98.7	7.96866e−49	Ephemeroptera	Heptageniidae	Rhithrogena dorieri
Rhithrogena sp. EP021 16S ribosomal RNA gene, part	AY749765	84.62	100	4.70989e−39	Ephemeroptera	Heptageniidae	Rhithrogena sp. EP021
Cinygmula sp. m185 mitochondrial partial 16S rRNA	LN868675	81.46	98.05	5.37398e−32	Ephemeroptera	Heptageniidae	Cinygmula sp. m185
Cinygmula sp. EP013 16S ribosomal RNA gene, partia	AY749760	81.46	98.05	5.37398e−32	Ephemeroptera	Heptageniidae	Cinygmula sp. EP013
Cultus pilatus 16S ribosomal RNA gene, partial seq	EF623212	84.16	65.58	4.71405e−20	Plecoptera	Perlodidae	Cultus pilatus
Heptagenia sp. EP018 16S ribosomal RNA gene, parti	AY749762	78.26	88.96	4.71405e−20	Ephemeroptera	Heptageniidae	Heptagenia sp. EP018
Arthroplea bipunctata 16S ribosomal RNA gene, part	AY749817	83.84	64.29	5.74289e−19	Ephemeroptera	Arthropleidae	Arthroplea bipunctata
Paegniodes cupulatus mitochondrion, complete genom	HM004123	76.6	91.56	2.00447e−18	Ephemeroptera	Heptageniidae	Paegniodes cupulatus
Remenus bilobatus 16S ribosomal RNA gene, partial	EF623233	83	64.94	6.99628e−18	Plecoptera	Perlodidae	Remenus bilobatus
Capnura wanica 16S ribosomal RNA gene, partial seq	EF623128	83.5	65.58	6.99628e−18	Plecoptera	Capniidae	Capnura wanica
Apteroperla tikumana mitochondrion, complete genom	KR604721	82.69	65.58	2.44194e−17	Plecoptera	Capniidae	Apteroperla tikumana
Neopanorpa pulchra mitochondrion, complete genome	JX569848	82.18	65.58	2.44194e−17	Mecoptera	Panorpidae	Neopanorpa pulchra
Neopanorpa pulchra mitochondrion, complete genome	FJ169955	82.18	65.58	2.44194e−17	Mecoptera	Panorpidae	Neopanorpa pulchra
Isoperla oxylepis 16S ribosomal RNA gene, partial	EF623221	82.86	67.53	2.44194e−17	Plecoptera	Perlodidae	Isoperla oxylepis
Isoperla davisi 16S ribosomal RNA gene, partial se	EF623206	82.86	67.53	2.44194e−17	Plecoptera	Perlodidae	Isoperla davisi
Thaumatoperla sp. BYU_PL109 16S ribosomal RNA gene	EF623149	82.18	65.58	2.44194e−17	Plecoptera	Eustheniidae	Thaumatoperla sp. BYU_PL109
Penturoperla barbata 16S ribosomal RNA gene, parti	EF623124	82.18	65.58	2.44194e−17	Plecoptera	Austroperlidae	Penturoperla barbata
Neopanorpa sp. M24899 16S ribosomal RNA gene, part	AF180059	82.18	65.58	2.44194e−17	Mecoptera	Panorpidae	Neopanorpa sp. M24899
Pachyrhamma edwardsii voucher CW122 16S ribosomal	HM594508	82.52	65.58	8.52321e−17	Orthoptera	Rhaphidophoridae	Pachyrhamma edwardsii
Heteromallus cavicola voucher MIL 16S ribosomal RN	HM594499	82	64.94	8.52321e−17	Orthoptera	Rhaphidophoridae	Heteromallus cavicola
Pteronarcys californica 16S ribosomal RNA gene, pa	EF623261	82	64.94	8.52321e−17	Plecoptera	Pteronarcyidae	Pteronarcys californica
Perlinodes aurea 16S ribosomal RNA gene, partial s	EF623234	82.35	65.58	8.52321e−17	Plecoptera	Perlodidae	Perlinodes aurea
Austronemoura sp. BYU_PL149 16S ribosomal RNA gene	EF623200	82.35	65.58	8.52321e−17	Plecoptera	Notonemouridae	Austronemoura sp. BYU_PL149
Pteronarcys princeps mitochondrion, complete genom	AY687866	82	64.94	8.52321e−17	Plecoptera	Pteronarcyidae	Pteronarcys princeps
Dipteromimus sp. Ofunato−1 mitochondrial gene for	AB110236	82	64.94	8.52321e−17	Ephemeroptera	Dipteromimidae	Dipteromimus sp. Ofunato−1
Analetris eximia 16S ribosomal RNA gene, partial s	AY749797	83.33	65.58	8.52321e−17	Ephemeroptera	Acanthametropodidae	Analetris eximia
Mesocapnia arizonensis mitochondrion, partial geno	KP642637	81.73	65.58	2.97489e−16	Plecoptera	Capniidae	Mesocapnia arizonensis
Allocapnia recta isolate H19 16S ribosomal RNA gen	KC881054	81.73	65.58	2.97489e−16	Plecoptera	Capniidae	Allocapnia recta
Allocapnia recta isolate H16 16S ribosomal RNA gen	KC881051	81.73	65.58	2.97489e−16	Plecoptera	Capniidae	Allocapnia recta
Allocapnia recta isolate H15 16S ribosomal RNA gen	KC881050	81.73	65.58	2.97489e−16	Plecoptera	Capniidae	Allocapnia recta
Allocapnia recta isolate H14 16S ribosomal RNA gen	KC881049	81.73	65.58	2.97489e−16	Plecoptera	Capniidae	Allocapnia recta
Allocapnia recta isolate H13 16S ribosomal RNA gen	KC881048	81.73	65.58	2.97489e−16	Plecoptera	Capniidae	Allocapnia recta
Allocapnia recta isolate H11 16S ribosomal RNA gen	KC881046	81.73	65.58	2.97489e−16	Plecoptera	Capniidae	Allocapnia recta
Allocapnia recta isolate H10 16S ribosomal RNA gen	KC881045	81.73	65.58	2.97489e−16	Plecoptera	Capniidae	Allocapnia recta
Allocapnia recta isolate H7 16S ribosomal RNA gene	KC881042	81.73	65.58	2.97489e−16	Plecoptera	Capniidae	Allocapnia recta
Allocapnia recta isolate H6 16S ribosomal RNA gene	KC881041	81.73	65.58	2.97489e−16	Plecoptera	Capniidae	Allocapnia recta
Allocapnia recta isolate H3 16S ribosomal RNA gene	KC881038	81.73	65.58	2.97489e−16	Plecoptera	Capniidae	Allocapnia recta
Bolotoperla rossi 16S ribosomal RNA gene, partial	EF623269	80.7	71.43	2.97489e−16	Plecoptera	Taeniopterygidae	Bolotoperla rossi
Salmoperla sylvanica 16S ribosomal RNA gene, parti	EF623222	82.18	64.94	2.97489e−16	Plecoptera	Perlodidae	Salmoperla sylvanica
Megarcys signata 16S ribosomal RNA gene, partial s	EF623215	82.18	64.94	2.97489e−16	Plecoptera	Perlodidae	Megarcys signata
Perlomyia utahensis 16S ribosomal RNA gene, partia	EF623179	82.83	63.64	2.97489e−16	Plecoptera	Leuctridae	Perlomyia utahensis
Diamphipnopsis sp. BYU_PL138 16S ribosomal RNA gen	EF623145	82.18	64.94	2.97489e−16	Plecoptera	Diamphipnoidae	Diamphipnopsis sp. BYU_PL138
Chloroperla tripunctata 16S ribosomal RNA gene, pa	EF623137	81.73	66.88	2.97489e−16	Plecoptera	Chloroperlidae	Chloroperla tripunctata
Allocapnia minima 16S ribosomal RNA gene, partial	EF623130	81.73	65.58	2.97489e−16	Plecoptera	Capniidae	Allocapnia minima
Mesocapnia sp. BYU_PL010 16S ribosomal RNA gene, p	EF623127	81.73	65.58	2.97489e−16	Plecoptera	Capniidae	Mesocapnia sp. BYU_PL010
Tasmanoperla larvalis 16S ribosomal RNA gene, part	EF623125	82.18	64.94	2.97489e−16	Plecoptera	Austroperlidae	Tasmanoperla larvalis
Allocapnia recta haplotype 12ar: H12 16S ribosomal	EU306875	81.73	65.58	2.97489e−16	Plecoptera	Capniidae	Allocapnia recta
Allocapnia recta haplotype 11ar: H011 16S ribosoma	EU306874	81.73	65.58	2.97489e−16	Plecoptera	Capniidae	Allocapnia recta
Allocapnia recta haplotype 7ar: H07 16S ribosomal	EU306870	81.73	65.58	2.97489e−16	Plecoptera	Capniidae	Allocapnia recta
Allocapnia recta haplotype 3ar: H03 16S ribosomal	DQ915181	81.73	65.58	2.97489e−16	Plecoptera	Capniidae	Allocapnia recta
Allocapnia recta haplotype 2ar: H02 16S ribosomal	DQ915180	81.73	65.58	2.97489e−16	Plecoptera	Capniidae	Allocapnia recta
Chiloporter sp. EP051 16S ribosomal RNA gene, part	AY749776	75.16	98.7	2.97489e−16	Ephemeroptera	Ameletopsidae	Chiloporter sp. EP051
Allocapnia recta isolate H18 16S ribosomal RNA gen	KC881053	81.55	64.94	1.03834e−15	Plecoptera	Capniidae	Allocapnia recta
Allocapnia recta isolate H17 16S ribosomal RNA gen	KC881052	81.55	64.94	1.03834e−15	Plecoptera	Capniidae	Allocapnia recta
Allocapnia recta isolate H12 16S ribosomal RNA gen	KC881047	81.55	64.94	1.03834e−15	Plecoptera	Capniidae	Allocapnia recta
Allocapnia recta isolate H9 16S ribosomal RNA gene	KC881044	81.55	64.94	1.03834e−15	Plecoptera	Capniidae	Allocapnia recta
Allocapnia recta isolate H8 16S ribosomal RNA gene	KC881043	81.55	64.94	1.03834e−15	Plecoptera	Capniidae	Allocapnia recta
Allocapnia recta isolate H5 16S ribosomal RNA gene	KC881040	81.55	64.94	1.03834e−15	Plecoptera	Capniidae	Allocapnia recta
Allocapnia recta isolate H4 16S ribosomal RNA gene	KC881039	81.55	64.94	1.03834e−15	Plecoptera	Capniidae	Allocapnia recta
Enicopus ater voucher UPOL001164 16S ribosomal RNA	HQ619670	80.36	72.08	1.03834e−15	Coleoptera	Melyridae	Enicopus ater
Simocephalus sp. EIZ−2011 isolate Brb1−Sim1 16S ri	JN874613	83.17	64.29	1.03834e−15	Diplostraca	Daphniidae	Simocephalus sp. EIZ−2011
Pachyrhamma edwardsii voucher WEL 16S ribosomal RN	HM594509	82.35	65.58	1.03834e−15	Orthoptera	Rhaphidophoridae	Pachyrhamma edwardsii
Dolichopoda hyrcana voucher LEN 16S ribosomal RNA	EU887871	81.19	65.58	1.03834e−15	Orthoptera	Rhaphidophoridae	Dolichopoda hyrcana
