# Supplementary material for: Testing the potential of a ribosomal 16S marker for DNA metabarcoding of insects
Source: PeerJ. 2016 Apr 19;4:e1966. doi: 10.7717/peerj.1966 (PMC4841222; doi:10.7717/peerj.1966)
Supplement: Supplemental Information 1 [file peerj-04-1966-s006.zip › scripts/5 match OTUs/blast_hits/OTU_422.csv_plot.pdf]

OTU_422.csv

Habroleptoides confusa

Ephemeroptera

96.69

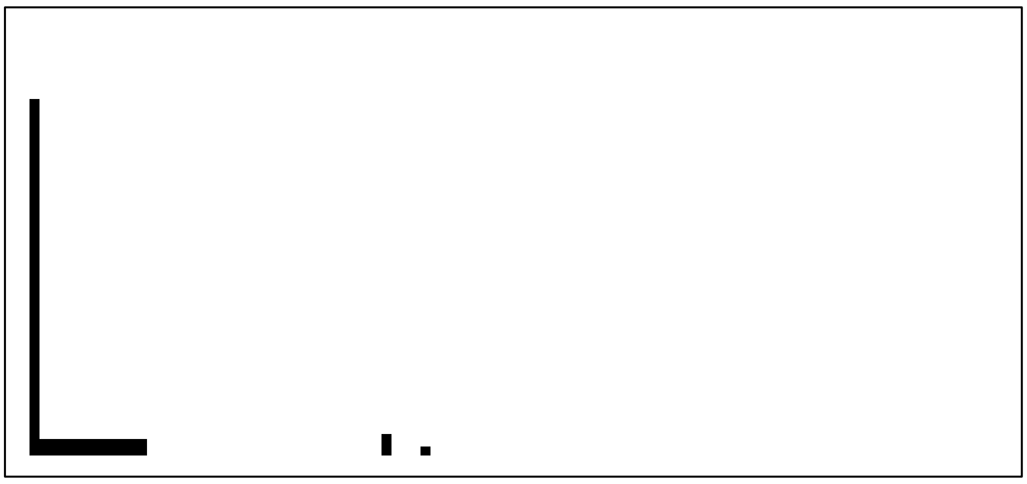

Habroleptoides confusa voucher BYU:IGCEP153 16S ri	GQ118289	96.69	100	5.96658e−63	Ephemeroptera		Habroleptoides confusa
Ephoron shigae mitochondrial gene for 16S ribosoma	AB874306	80.77	68.21	2.8999e−16	Ephemeroptera	Polymitarcyidae	Ephoron shigae
Ephoron shigae mitochondrial gene for 16S ribosoma	AB874293	80.77	68.21	2.8999e−16	Ephemeroptera	Polymitarcyidae	Ephoron shigae
Ephoron shigae mitochondrial gene for 16S ribosoma	AB874289	80.77	68.21	2.8999e−16	Ephemeroptera	Polymitarcyidae	Ephoron shigae
Ephoron shigae mitochondrial gene for 16S ribosoma	AB874283	80.77	68.21	2.8999e−16	Ephemeroptera	Polymitarcyidae	Ephoron shigae
Ephoron shigae mitochondrial gene for 16S ribosoma	AB874278	80.77	68.21	2.8999e−16	Ephemeroptera	Polymitarcyidae	Ephoron shigae
Ephoron shigae mitochondrial gene for 16S rRNA, pa	AB711533	80.77	68.21	2.8999e−16	Ephemeroptera	Polymitarcyidae	Ephoron shigae
Ephoron shigae mitochondrial gene for 16S rRNA, pa	AB711527	80.77	68.21	2.8999e−16	Ephemeroptera	Polymitarcyidae	Ephoron shigae
Ephoron shigae mitochondrial gene for 16S rRNA, pa	AB711523	80.77	68.21	2.8999e−16	Ephemeroptera	Polymitarcyidae	Ephoron shigae
Ephoron shigae mitochondrial gene for 16S rRNA, pa	AB711500	80.77	68.21	2.8999e−16	Ephemeroptera	Polymitarcyidae	Ephoron shigae
Ephoron shigae mitochondrial gene for 16S rRNA, pa	AB711493	80.77	68.21	2.8999e−16	Ephemeroptera	Polymitarcyidae	Ephoron shigae
Ephoron shigae mitochondrial gene for 16S rRNA, pa	AB711471	80.77	68.21	2.8999e−16	Ephemeroptera	Polymitarcyidae	Ephoron shigae
Ephoron shigae mitochondrial gene for 16S ribosoma	AB874305	79.81	68.21	1.23307e−14	Ephemeroptera	Polymitarcyidae	Ephoron shigae
Ephoron shigae mitochondrial gene for 16S ribosoma	AB874303	79.81	68.21	1.23307e−14	Ephemeroptera	Polymitarcyidae	Ephoron shigae
Ephoron shigae mitochondrial gene for 16S ribosoma	AB874302	79.81	68.21	1.23307e−14	Ephemeroptera	Polymitarcyidae	Ephoron shigae
Ephoron shigae mitochondrial gene for 16S ribosoma	AB874301	79.81	68.21	1.23307e−14	Ephemeroptera	Polymitarcyidae	Ephoron shigae
Ephoron shigae mitochondrial gene for 16S ribosoma	AB874299	79.81	68.21	1.23307e−14	Ephemeroptera	Polymitarcyidae	Ephoron shigae
Ephoron shigae mitochondrial gene for 16S ribosoma	AB874288	79.81	68.21	1.23307e−14	Ephemeroptera	Polymitarcyidae	Ephoron shigae
Ephoron shigae mitochondrial gene for 16S ribosoma	AB874284	79.81	68.21	1.23307e−14	Ephemeroptera	Polymitarcyidae	Ephoron shigae
Ephoron shigae mitochondrial gene for 16S ribosoma	AB874279	79.81	68.21	1.23307e−14	Ephemeroptera	Polymitarcyidae	Ephoron shigae
Ephoron shigae mitochondrial gene for 16S ribosoma	AB874277	79.81	68.21	1.23307e−14	Ephemeroptera	Polymitarcyidae	Ephoron shigae
Ephoron shigae mitochondrial gene for 16S rRNA, pa	AB711590	79.81	68.21	1.23307e−14	Ephemeroptera	Polymitarcyidae	Ephoron shigae
Ephoron shigae mitochondrial gene for 16S rRNA, pa	AB711582	79.81	68.21	1.23307e−14	Ephemeroptera	Polymitarcyidae	Ephoron shigae
Ephoron shigae mitochondrial gene for 16S rRNA, pa	AB711581	79.81	68.21	1.23307e−14	Ephemeroptera	Polymitarcyidae	Ephoron shigae
Ephoron shigae mitochondrial gene for 16S rRNA, pa	AB711575	79.81	68.21	1.23307e−14	Ephemeroptera	Polymitarcyidae	Ephoron shigae
Ephoron shigae mitochondrial gene for 16S rRNA, pa	AB711531	79.81	68.21	1.23307e−14	Ephemeroptera	Polymitarcyidae	Ephoron shigae
Ephoron shigae mitochondrial gene for 16S rRNA, pa	AB711530	79.81	68.21	1.23307e−14	Ephemeroptera	Polymitarcyidae	Ephoron shigae
Ephoron shigae mitochondrial gene for 16S rRNA, pa	AB711528	79.81	68.21	1.23307e−14	Ephemeroptera	Polymitarcyidae	Ephoron shigae
Ephoron shigae mitochondrial gene for 16S rRNA, pa	AB711521	79.81	68.21	1.23307e−14	Ephemeroptera	Polymitarcyidae	Ephoron shigae
Ephoron shigae mitochondrial gene for 16S rRNA, pa	AB711514	79.81	68.21	1.23307e−14	Ephemeroptera	Polymitarcyidae	Ephoron shigae
Ephoron shigae mitochondrial gene for 16S rRNA, pa	AB711501	79.81	68.21	1.23307e−14	Ephemeroptera	Polymitarcyidae	Ephoron shigae
Ephoron shigae mitochondrial gene for 16S rRNA, pa	AB711492	79.81	68.21	1.23307e−14	Ephemeroptera	Polymitarcyidae	Ephoron shigae
Ephoron shigae mitochondrial gene for 16S rRNA, pa	AB711482	79.81	68.21	1.23307e−14	Ephemeroptera	Polymitarcyidae	Ephoron shigae
Ephoron shigae mitochondrial gene for 16S ribosoma	AB874304	78.85	68.21	1.50218e−13	Ephemeroptera	Polymitarcyidae	Ephoron shigae
Ephoron shigae mitochondrial gene for 16S ribosoma	AB874300	78.85	68.21	1.50218e−13	Ephemeroptera	Polymitarcyidae	Ephoron shigae
Ephoron shigae mitochondrial gene for 16S ribosoma	AB874297	78.85	68.21	1.50218e−13	Ephemeroptera	Polymitarcyidae	Ephoron shigae
Ephoron eophilum mitochondrial gene for 16S rRNA,	AB711631	81	64.24	1.50218e−13	Ephemeroptera	Polymitarcyidae	Ephoron eophilum
Ephoron shigae mitochondrial gene for 16S rRNA, pa	AB711598	78.85	68.21	1.50218e−13	Ephemeroptera	Polymitarcyidae	Ephoron shigae
Ephoron shigae mitochondrial gene for 16S rRNA, pa	AB711532	78.85	68.21	1.50218e−13	Ephemeroptera	Polymitarcyidae	Ephoron shigae
Ephoron shigae mitochondrial gene for 16S rRNA, pa	AB711522	78.85	68.21	1.50218e−13	Ephemeroptera	Polymitarcyidae	Ephoron shigae
Penaphlebia sp. EP076 16S ribosomal RNA gene, part	AY749782	80.41	64.24	1.50218e−13	Ephemeroptera		Penaphlebia sp. EP076
Anthopotamus sp. EP022 16S ribosomal RNA gene, par	AY749766	79.59	64.24	5.24313e−13	Ephemeroptera	Potamanthidae	Anthopotamus sp. EP022
Ephoron eophilum mitochondrial gene for 16S rRNA,	AB711630	80	64.24	1.83003e−12	Ephemeroptera	Polymitarcyidae	Ephoron eophilum
Ephoron eophilum mitochondrial gene for 16S rRNA,	AB711621	80	64.24	1.83003e−12	Ephemeroptera	Polymitarcyidae	Ephoron eophilum
Ephoron eophilum mitochondrial gene for 16S rRNA,	AB711619	80	64.24	1.83003e−12	Ephemeroptera	Polymitarcyidae	Ephoron eophilum
Ephoron eophilum mitochondrial gene for 16S rRNA,	AB711615	80	64.24	1.83003e−12	Ephemeroptera	Polymitarcyidae	Ephoron eophilum
Atrichiana placida voucher 842794 16S ribosomal RN	JX234487	78.35	62.91	2.22944e−11	Coleoptera	Scarabaeidae	Atrichiana placida
Niphetophora sp. DA−2012 voucher 842732 16S riboso	JX234445	79.38	62.91	2.22944e−11	Coleoptera	Scarabaeidae	Niphetophora sp. DA−2012
Ephemera orientalis voucher EPH01 tRNA−Leu, 16S ri	KF256907	78.57	64.24	2.22944e−11	Ephemeroptera	Ephemeridae	Ephemera orientalis
Hypattalus sp. NLG−2013 voucher COL514 12S ribosom	KC524788	79.38	62.91	2.22944e−11	Coleoptera	Melyridae	Hypattalus sp. NLG−2013
Enochrus talamanca 16S ribosomal RNA gene, partial	KC992672	79.38	62.91	2.22944e−11	Coleoptera	Hydrophilidae	Enochrus talamanca
Ephemera orientalis mitochondrion, complete genome	EU591678	78.57	64.24	2.22944e−11	Ephemeroptera	Ephemeridae	Ephemera orientalis
Pseudiron centralis 16S ribosomal RNA gene, partia	AY749799	79.38	62.91	2.22944e−11	Ephemeroptera	Pseudironidae	Pseudiron centralis
Phyllobaenus testaceus voucher 7228 16S ribosomal	KC524896	79.17	62.25	7.7815e−11	Coleoptera	Cleridae	Phyllobaenus testaceus
Ephoron leukon mitochondrial gene for 16S rRNA, pa	AB711641	77.45	66.89	7.7815e−11	Ephemeroptera	Polymitarcyidae	Ephoron leukon
Ephoron album mitochondrial gene for 16S rRNA, par	AB711636	77.45	66.89	7.7815e−11	Ephemeroptera	Polymitarcyidae	Ephoron album
Ephoron album mitochondrial gene for 16S rRNA, par	AB711635	77.45	66.89	7.7815e−11	Ephemeroptera	Polymitarcyidae	Ephoron album
Ephoron eophilum mitochondrial gene for 16S rRNA,	AB711633	79	64.24	7.7815e−11	Ephemeroptera	Polymitarcyidae	Ephoron eophilum
Hercostomus brevidigitalis voucher RMBR:103445 16S	FJ808172	78.79	64.24	7.7815e−11	Diptera	Dolichopodidae	Hercostomus brevidigitalis
Paraleptophlebia vaciva 16S ribosomal RNA gene, pa	AY749759	77.32	64.24	7.7815e−11	Ephemeroptera		Paraleptophlebia vaciva
Protohermes yangi 16S ribosomal RNA gene, partial	KF944544	77.55	64.24	2.71601e−10	Megaloptera	Corydalidae	Protohermes yangi
Protohermes hunanensis 16S ribosomal RNA gene, par	KF944532	77.55	64.24	2.71601e−10	Megaloptera	Corydalidae	Protohermes hunanensis
Neptis thisbe 16S ribosomal RNA gene, partial sequ	JX185871	79.57	60.26	2.71601e−10	Lepidoptera	Nymphalidae	Neptis thisbe
Jellisonia amadoi mitochondrion, complete genome	KF322091	77.55	64.24	2.71601e−10	Siphonaptera	Ceratophyllidae	Jellisonia amadoi
Hymenoplia fulvipennis voucher DA0116 16S ribosoma	FJ956720	77.55	64.24	2.71601e−10	Coleoptera	Scarabaeidae	Hymenoplia fulvipennis
Limonia kauaiensis voucher O'Grady 201868 16S ribo	EU005414	77	64.24	2.71601e−10	Diptera	Limoniidae	Dicranomyia kauaiensis
Limonia kauaiensis voucher O'Grady 200971 16S ribo	EU005412	77	64.24	2.71601e−10	Diptera	Limoniidae	Dicranomyia kauaiensis
Limonia jacobus voucher O'Grady 200974 16S ribosom	EU005408	77	64.24	2.71601e−10	Diptera	Limoniidae	Dicranomyia jacobus
Epilachna tumida voucher KS135 16S ribosomal RNA g	KP143571	78.72	61.59	9.47981e−10	Coleoptera	Coccinellidae	Epilachna tumida
Myrmeleon fasciatus group sp. 195−MyfaPant 16S rib	KP063912	76.77	64.24	9.47981e−10	Neuroptera	Myrmeleontidae	Myrmeleon fasciatus group sp. 195−MyfaPant
Myrmeleon fasciatus group sp. 194−MypsSb1 16S ribo	KP063911	76.77	64.24	9.47981e−10	Neuroptera	Myrmeleontidae	Myrmeleon fasciatus group sp. 194−MypsSb1
Myrmeleon fasciatus group sp. 193−MyspSb2 16S ribo	KP063910	76.77	64.24	9.47981e−10	Neuroptera	Myrmeleontidae	Myrmeleon fasciatus group sp. 193−MyspSb2
Neptis tshetverikovi 16S ribosomal RNA gene, parti	JX185894	79.79	60.26	9.47981e−10	Lepidoptera	Nymphalidae	Neptis tshetverikovi
Myrmeleon bore isolate 74−boreD1 16S ribosomal RNA	KC195845	77.78	64.24	9.47981e−10	Neuroptera	Myrmeleontidae	Myrmeleon bore
Myrmeleon bore isolate 113−bore2 16S ribosomal RNA	KC195844	77.78	64.24	9.47981e−10	Neuroptera	Myrmeleontidae	Myrmeleon bore
Ephoron leukon mitochondrial gene for 16S rRNA, pa	AB711638	76.47	66.89	9.47981e−10	Ephemeroptera	Polymitarcyidae	Ephoron leukon
Ephoron leukon mitochondrial gene for 16S rRNA, pa	AB711637	76.47	66.89	9.47981e−10	Ephemeroptera	Polymitarcyidae	Ephoron leukon
Semirhynchia sp. n. SV01 16S ribosomal RNA gene, p	JX294172	77.78	64.24	9.47981e−10	Neuroptera	Nemopteridae	Semirhynchia sp. n. SV01
Sicyoptera cuspidata isolate SCBB01 16S ribosomal	JX294167	77.78	64.24	9.47981e−10	Neuroptera	Nemopteridae	Sicyoptera cuspidata
Sicyoptera dilatata isolate SDGG01 16S ribosomal R	JX294163	77.78	64.24	9.47981e−10	Neuroptera	Nemopteridae	Sicyoptera dilatata
Nemopterella longicornis isolate NLTS02 16S riboso	JX294150	77.78	64.24	9.47981e−10	Neuroptera	Nemopteridae	Nemopterella longicornis
Nemeura gracilis isolate NGW01 16S ribosomal RNA g	JX294132	77.78	64.24	9.47981e−10	Neuroptera	Nemopteridae	Nemeura gracilis
Xylotrechus rusticus 16S ribosomal RNA gene, parti	JX177496	78.26	60.26	9.47981e−10	Coleoptera	Cerambycidae	Rusticoclytus rusticus
Afronurus sp. 213BoAf mitochondrial partial 16S rR	HE651422	78.35	62.91	9.47981e−10	Ephemeroptera	Heptageniidae	Afronurus sp. 213BoAf
Trox fascicularis rowei isolate 46A 16S ribosomal	EF570396	78.35	62.91	9.47981e−10	Coleoptera	Trogidae	Trox fascicularis rowei
Trox arcuatus isolate 69 16S ribosomal RNA gene, p	EF570395	78.35	62.91	9.47981e−10	Coleoptera	Trogidae	Trox arcuatus
Trox fascicularis rowei isolate 57A 16S ribosomal	EF570361	78.35	62.91	9.47981e−10	Coleoptera	Trogidae	Trox fascicularis rowei
Tachytrechus notatus voucher Pollet 0279 (P) 16S r	EU864017	77.78	64.24	9.47981e−10	Diptera	Dolichopodidae	Tachytrechus notatus
Eatonica denysae voucher BYU:IGCEP225 16S ribosoma	GQ118284	79	64.24	9.47981e−10	Ephemeroptera	Ephemeridae	Eatonica denysae
Leucochrysa robusta voucher CASC 00213 16S ribosom	DQ399281	76.77	64.24	9.47981e−10	Neuroptera	Chrysopidae	Leucochrysa robusta
Leucochrysa texana 16S ribosomal RNA gene, partial	DQ399279	77.78	64.24	9.47981e−10	Neuroptera	Chrysopidae	Leucochrysa texana
Chaquihua sp. EP080 16S ribosomal RNA gene, partia	AY749784	77.78	64.24	9.47981e−10	Ephemeroptera	Ameletopsidae	Chaquihua sp. EP080
Epilachna cruciata voucher KS223 16S ribosomal RNA	KP143627	78.95	61.59	3.30878e−09	Coleoptera	Coccinellidae	Epilachna cruciata
Anoplotrupes stercorosus voucher SK300 16S ribosom	KP250244	77.55	62.91	3.30878e−09	Coleoptera	Geotrupidae	Anoplotrupes stercorosus
Abrota ganga isolate N793 mitochondrion, complete	KF590536	78.49	60.26	3.30878e−09	Lepidoptera	Nymphalidae	Abrota ganga
Dophla evelina isolate N529 mitochondrion, complet	KF590532	78.49	60.26	3.30878e−09	Lepidoptera	Nymphalidae	Dophla evelina
Cephaloclerus sp. 1 NLG−2013 voucher 7183 16S ribo	KC524887	78.12	62.25	3.30878e−09	Coleoptera	Cleridae	Cephaloclerus sp. 1 NLG−2013
Myrmeleon inconspicuus isolate 6−incgre1 16S ribos	KC195843	77	64.24	3.30878e−09	Neuroptera	Myrmeleontidae	Myrmeleon inconspicuus
Myrmeleon inconspicuus isolate 49−inclaz 16S ribos	KC195841	77	64.24	3.30878e−09	Neuroptera	Myrmeleontidae	Myrmeleon inconspicuus
Myrmeleon inconspicuus isolate 40−inctu1 16S ribos	KC195839	77	64.24	3.30878e−09	Neuroptera	Myrmeleontidae	Myrmeleon inconspicuus
