# Supplementary material for: Testing the potential of a ribosomal 16S marker for DNA metabarcoding of insects
Source: PeerJ. 2016 Apr 19;4:e1966. doi: 10.7717/peerj.1966 (PMC4841222; doi:10.7717/peerj.1966)
Supplement: Supplemental Information 1 [file peerj-04-1966-s006.zip › scripts/5 match OTUs/blast_hits/OTU_424.csv_plot.pdf]

OTU_424.csv

Hansonoperla appalachia

Perlidae

Plecoptera

88.35

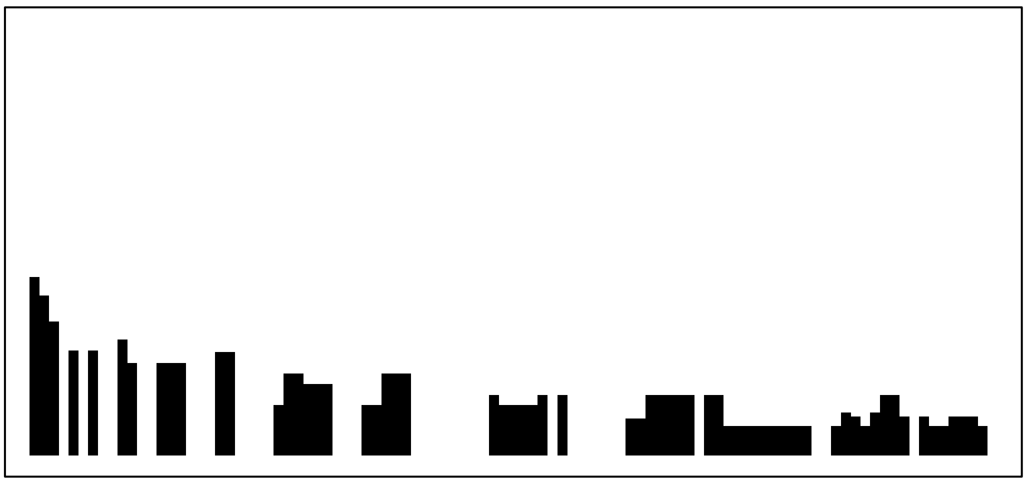

Hansonoperla appalachia 16S ribosomal RNA gene, pa	EF623249	88.35	62.58	1.5511e−26	Plecoptera	Perlidae	Hansonoperla appalachia
Acroneuria lycorias 16S ribosomal RNA gene, partia	EF623251	87.5	62.58	5.41388e−26	Plecoptera	Perlidae	Acroneuria lycorias
Eccoptura xanthenes 16S ribosomal RNA gene, partia	EF623252	86.27	62.58	2.30204e−24	Plecoptera	Perlidae	Eccoptura xanthenes
Agnetina capitata 16S ribosomal RNA gene, partial	EF623241	77.85	91.41	9.78852e−23	Plecoptera	Perlidae	Agnetina capitata
Dinocras cephalotes mitochondrion, complete genome	KF484757	84.91	62.58	3.41653e−22	Plecoptera	Perlidae	Dinocras cephalotes
Dolichopoda pavesii voucher SPI 16S ribosomal RNA	EF216951	79.26	82.82	3.41653e−22	Orthoptera	Rhaphidophoridae	Dolichopoda pavesii
Dinocras cephalotes 16S ribosomal RNA gene, partia	EF623244	84.91	62.58	3.41653e−22	Plecoptera	Perlidae	Dinocras cephalotes
Dolichopoda sp. MR−2008 16S ribosomal RNA gene, pa	EU887863	78.83	84.05	1.19249e−21	Orthoptera	Rhaphidophoridae	Dolichopoda lustriae
Dolichopoda ithakii voucher ITA 16S ribosomal RNA	EF216949	78.17	87.12	1.19249e−21	Orthoptera	Rhaphidophoridae	Dolichopoda ithakii
Perlinella drymo 16S ribosomal RNA gene, partial s	EF623240	85.44	62.58	4.16218e−21	Plecoptera	Perlidae	Perlinella drymo
Notonemouridae sp. MD−2011 voucher ZMUC:JD−1507 16	JN615257	84.31	62.58	1.45275e−20	Plecoptera	Notonemouridae	Notonemouridae sp. MD−2011
Dolichopoda patrizii voucher PET 16S ribosomal RNA	EU887862	77.46	87.12	1.45275e−20	Orthoptera	Rhaphidophoridae	Dolichopoda patrizii
Dolichopoda graeca voucher PER 16S ribosomal RNA g	EF216953	77.78	82.82	1.45275e−20	Orthoptera	Rhaphidophoridae	Dolichopoda graeca
Pteronarcella badia 16S ribosomal RNA gene, partia	EF623262	84.31	62.58	1.45275e−20	Plecoptera	Pteronarcyidae	Pteronarcella badia
Calineuria californica 16S ribosomal RNA gene, par	EF623239	84.31	62.58	1.45275e−20	Plecoptera	Perlidae	Calineuria californica
Megaleuctra kincaidi 16S ribosomal RNA gene, parti	EF623174	84.31	62.58	1.45275e−20	Plecoptera	Leuctridae	Megaleuctra kincaidi
Dolichopoda remyi voucher EDE 16S ribosomal RNA ge	EF216969	77.37	84.05	1.76981e−19	Orthoptera	Rhaphidophoridae	Dolichopoda remyi
Dolichopoda steriotisi voucher ANT 16S ribosomal R	EF216955	77.78	82.82	1.76981e−19	Orthoptera	Rhaphidophoridae	Dolichopoda steriotisi
Dolichopoda remyi isolate EDE 16S ribosomal RNA ge	AY793589	77.37	84.05	1.76981e−19	Orthoptera	Rhaphidophoridae	Dolichopoda remyi
Cryptoperla japonica mitochondrial gene for 16S rR	AB711202	84.85	60.74	6.17723e−19	Plecoptera	Peltoperlidae	Cryptoperla japonica
Cryptoperla japonica mitochondrial gene for 16S rR	AB711184	84.85	60.74	6.17723e−19	Plecoptera	Peltoperlidae	Cryptoperla japonica
Dolichopoda geniculata geniculata isolate PRA20 16	AY507590	77.37	84.05	6.17723e−19	Orthoptera	Rhaphidophoridae	Dolichopoda geniculata geniculata
Dolichopoda hussoni voucher IZB 16S ribosomal RNA	EF216973	77.37	84.05	6.17723e−19	Orthoptera	Rhaphidophoridae	Dolichopoda hussoni
Dolichopoda matsakisi voucher KASA 16S ribosomal R	EF216957	76.55	87.12	6.17723e−19	Orthoptera	Rhaphidophoridae	Dolichopoda matsakisi
Dolichopoda thasosensis voucher DRA 16S ribosomal	EF216956	77.37	84.05	6.17723e−19	Orthoptera	Rhaphidophoridae	Dolichopoda thasosensis
Taenionema palladium 16S ribosomal RNA gene, parti	EF623267	82.35	62.58	6.17723e−19	Plecoptera	Taeniopterygidae	Taenionema palladium
Scopura montana 16S ribosomal RNA gene, partial se	EF623265	83.84	60.74	6.17723e−19	Plecoptera		Scopura montana
Pteronarcys sachalina 16S ribosomal RNA gene, part	EF623264	83.84	60.74	6.17723e−19	Plecoptera	Pteronarcyidae	Pteronarcys sachalina
Pictetoperla sp. BYU_PL156 16S ribosomal RNA gene,	EF623247	83.33	62.58	6.17723e−19	Plecoptera	Perlidae	Pictetoperla sp. BYU_PL156
Kempnyella sp. BYU_PL155 16S ribosomal RNA gene, p	EF623246	83.33	62.58	6.17723e−19	Plecoptera	Perlidae	Kempnyella sp. BYU_PL155
Nemoura cinerea 16S ribosomal RNA gene, partial se	EF623194	83.33	62.58	6.17723e−19	Plecoptera	Nemouridae	Nemoura cinerea
Eupholidoptera smyrnensis isolate KOY1 16S ribosom	KC852309	77.52	79.14	2.15607e−18	Orthoptera	Tettigoniidae	Eupholidoptera smyrnensis
Eupholidoptera magnifica isolate ARB1 16S ribosoma	KC852277	76.22	86.5	2.15607e−18	Orthoptera	Tettigoniidae	Eupholidoptera magnifica
Eupholidoptera chabrieri voucher 173 16S ribosomal	EU120792	76.22	86.5	2.15607e−18	Orthoptera	Tettigoniidae	Eupholidoptera chabrieri
Cryptoperla sp. YK−2012−1 mitochondrial gene for 1	AB711243	82.35	62.58	7.52541e−18	Plecoptera	Peltoperlidae	Cryptoperla sp. YK−2012−1
Cryptoperla ishigakiensis mitochondrial gene for 1	AB711242	82.35	62.58	7.52541e−18	Plecoptera	Peltoperlidae	Cryptoperla ishigakiensis
Cryptoperla japonica mitochondrial gene for 16S rR	AB711183	83.84	60.74	7.52541e−18	Plecoptera	Peltoperlidae	Cryptoperla japonica
Cryptoperla japonica mitochondrial gene for 16S rR	AB711182	83.84	60.74	7.52541e−18	Plecoptera	Peltoperlidae	Cryptoperla japonica
Pachyrhamma edwardsii voucher CW122 16S ribosomal	HM594508	83.84	60.74	7.52541e−18	Orthoptera	Rhaphidophoridae	Pachyrhamma edwardsii
Dolichopoda sp. VAR 16S ribosomal RNA gene, partia	EU887865	76.64	84.05	7.52541e−18	Orthoptera	Rhaphidophoridae	Dolichopoda sp. VAR
Dolichopoda sp. TAY 16S ribosomal RNA gene, partia	EU887864	76.64	84.05	7.52541e−18	Orthoptera	Rhaphidophoridae	Dolichopoda sp. TAY
Dolichopoda annae voucher OLI 16S ribosomal RNA ge	EU887860	76.64	84.05	7.52541e−18	Orthoptera	Rhaphidophoridae	Dolichopoda annae
Dolichopoda dalensi voucher KEF 16S ribosomal RNA	EF216959	76.76	87.12	7.52541e−18	Orthoptera	Rhaphidophoridae	Dolichopoda dalensi
Dolichopoda kiriakii voucher AGH 16S ribosomal RNA	EF216954	77.04	82.82	7.52541e−18	Orthoptera	Rhaphidophoridae	Dolichopoda kiriakii
Dolichopoda gasparoi voucher CHI 16S ribosomal RNA	EF216950	77.04	82.82	7.52541e−18	Orthoptera	Rhaphidophoridae	Dolichopoda gasparoi
Brachyptera seticornis 16S ribosomal RNA gene, par	EF623268	76.76	87.12	7.52541e−18	Plecoptera	Taeniopterygidae	Brachyptera seticornis
Pteronarcys scotti 16S ribosomal RNA gene, partial	EF623263	75.97	93.87	7.52541e−18	Plecoptera	Pteronarcyidae	Pteronarcys scotti
Cosumnoperla hypocrena 16S ribosomal RNA gene, par	EF623226	82.83	60.74	7.52541e−18	Plecoptera	Perlodidae	Cosumnoperla hypocrena
Prostoia besametsa 16S ribosomal RNA gene, partial	EF623188	82.35	62.58	7.52541e−18	Plecoptera	Nemouridae	Prostoia besametsa
Nemoura trispinosa 16S ribosomal RNA gene, partial	EF623187	82.35	62.58	7.52541e−18	Plecoptera	Nemouridae	Nemoura trispinosa
Paranemoura claasseni 16S ribosomal RNA gene, part	EF623185	82.35	62.58	7.52541e−18	Plecoptera	Nemouridae	Paranemoura claasseni
Shipsa rotunda 16S ribosomal RNA gene, partial seq	EF623184	82.35	62.58	7.52541e−18	Plecoptera	Nemouridae	Shipsa rotunda
Osmylus sp. EH−2004 16S ribosomal RNA gene, partia	AY620146	82.83	60.74	7.52541e−18	Neuroptera	Osmylidae	Osmylus sp. EH−2004
Dolichopoda cyrnensis isolate SAB 16S ribosomal RN	AY793576	76.64	84.05	7.52541e−18	Orthoptera	Rhaphidophoridae	Dolichopoda cyrnensis
Apteroperla tikumana mitochondrion, complete genom	KR604721	82.83	60.74	2.62662e−17	Plecoptera	Capniidae	Apteroperla tikumana
Eupholidoptera smyrnensis isolate MRM1 16S ribosom	KC852310	76.74	79.14	2.62662e−17	Orthoptera	Tettigoniidae	Eupholidoptera smyrnensis
Eupholidoptera megastyla isolate ALE1 16S ribosoma	KC852308	76.74	79.14	2.62662e−17	Orthoptera	Tettigoniidae	Eupholidoptera megastyla
Eupholidoptera hesperica isolate MAR3 16S ribosoma	KC852304	75.52	86.5	2.62662e−17	Orthoptera	Tettigoniidae	Eupholidoptera hesperica
Eupholidoptera hesperica isolate MAR1 16S ribosoma	KC852302	75.52	86.5	2.62662e−17	Orthoptera	Tettigoniidae	Eupholidoptera hesperica
Eupholidoptera danconai isolate ROG1 16S ribosomal	KC852297	75.52	86.5	2.62662e−17	Orthoptera	Tettigoniidae	Eupholidoptera danconai
Eupholidoptera bimucronata isolate GIB2 16S riboso	KC852284	75.52	86.5	2.62662e−17	Orthoptera	Tettigoniidae	Eupholidoptera bimucronata
Micropathus tasmaniensis voucher JNC 16S ribosomal	HM594507	81.73	62.58	2.62662e−17	Orthoptera	Rhaphidophoridae	Micropathus tasmaniensis
Micropathus tasmaniensis voucher IDB 16S ribosomal	HM594505	81.73	62.58	2.62662e−17	Orthoptera	Rhaphidophoridae	Micropathus tasmaniensis
Dolichopoda bormansi isolate BRA5 16S ribosomal RN	AY507606	82.83	60.74	2.62662e−17	Orthoptera	Rhaphidophoridae	Dolichopoda bormansi
Dolichopoda bormansi isolate BRA3 16S ribosomal RN	AY507605	82.83	60.74	2.62662e−17	Orthoptera	Rhaphidophoridae	Dolichopoda bormansi
Hesperoperla pacifica 16S ribosomal RNA gene, part	EF623238	82.83	60.74	2.62662e−17	Plecoptera	Perlidae	Hesperoperla pacifica
Nemurella pictetii 16S ribosomal RNA gene, partial	EF623192	82.83	60.74	2.62662e−17	Plecoptera	Nemouridae	Nemurella pictetii
Malenka californica 16S ribosomal RNA gene, partia	EF623182	82.83	60.74	2.62662e−17	Plecoptera	Nemouridae	Malenka californica
Eupholidoptera karatolosi voucher 421 16S ribosoma	EU120826	75.52	86.5	2.62662e−17	Orthoptera	Tettigoniidae	Eupholidoptera karatolosi
Dolichopoda bormansi isolate BRA 16S ribosomal RNA	AY793578	82.83	60.74	2.62662e−17	Orthoptera	Rhaphidophoridae	Dolichopoda bormansi
Dolichopoda muceddai isolate SAR1 16S ribosomal RN	AY793575	82.83	60.74	2.62662e−17	Orthoptera	Rhaphidophoridae	Dolichopoda muceddai
Eupholidoptera uvarovi isolate KAS1 16S ribosomal	KC852312	81.37	62.58	9.16782e−17	Orthoptera	Tettigoniidae	Eupholidoptera uvarovi
Eupholidoptera danconai isolate TRL2 16S ribosomal	KC852296	81.37	62.58	9.16782e−17	Orthoptera	Tettigoniidae	Eupholidoptera danconai
Eupholidoptera danconai isolate JEN1 16S ribosomal	KC852294	81.37	62.58	9.16782e−17	Orthoptera	Tettigoniidae	Eupholidoptera danconai
Eupholidoptera danconai isolate PIE1 16S ribosomal	KC852292	81.37	62.58	9.16782e−17	Orthoptera	Tettigoniidae	Eupholidoptera danconai
Eupholidoptera bimucronata isolate MAD1 16S riboso	KC852289	81.37	62.58	9.16782e−17	Orthoptera	Tettigoniidae	Eupholidoptera bimucronata
Eupholidoptera bimucronata isolate PTR1 16S riboso	KC852287	81.37	62.58	9.16782e−17	Orthoptera	Tettigoniidae	Eupholidoptera bimucronata
Eupholidoptera bimucronata isolate GIB3 16S riboso	KC852285	81.37	62.58	9.16782e−17	Orthoptera	Tettigoniidae	Eupholidoptera bimucronata
Eupholidoptera bimucronata isolate GIB1 16S riboso	KC852283	81.37	62.58	9.16782e−17	Orthoptera	Tettigoniidae	Eupholidoptera bimucronata
Eupholidoptera magnifica isolate BIG1 16S ribosoma	KC852275	81.37	62.58	9.16782e−17	Orthoptera	Tettigoniidae	Eupholidoptera magnifica
Dolichopoda unicolor voucher DIR 16S ribosomal RNA	EF216970	76.64	84.05	9.16782e−17	Orthoptera	Rhaphidophoridae	Dolichopoda unicolor
Doddsia occidentalis 16S ribosomal RNA gene, parti	EF623270	75.33	92.02	9.16782e−17	Plecoptera	Taeniopterygidae	Doddsia occidentalis
Pteronarcys californica 16S ribosomal RNA gene, pa	EF623261	81.37	62.58	9.16782e−17	Plecoptera	Pteronarcyidae	Pteronarcys californica
Claassenia sabulosa 16S ribosomal RNA gene, partia	EF623250	82	60.74	9.16782e−17	Plecoptera	Perlidae	Claassenia sabulosa
Macroxyela ferruginea 16S ribosomal RNA gene, part	AY206773	81.82	60.74	9.16782e−17	Hymenoptera	Xyelidae	Macroxyela ferruginea
Pteronarcys princeps mitochondrion, complete genom	AY687866	81.37	62.58	9.16782e−17	Plecoptera	Pteronarcyidae	Pteronarcys princeps
Cryptoperla sp. YK−2012−2 mitochondrial gene for 1	AB711244	82	60.74	3.19988e−16	Plecoptera	Peltoperlidae	Cryptoperla sp. YK−2012−2
Pleioplectron simplex voucher CW306 16S ribosomal	HM594512	82.83	60.74	3.19988e−16	Orthoptera	Rhaphidophoridae	Pleioplectron simplex
Pachyrhamma edwardsii voucher WEL 16S ribosomal RN	HM594509	82.83	60.74	3.19988e−16	Orthoptera	Rhaphidophoridae	Pachyrhamma edwardsii
Dolichopoda baccettii isolate PST17 16S ribosomal	AY507593	81.82	60.74	3.19988e−16	Orthoptera	Rhaphidophoridae	Dolichopoda baccettii
Perlesta decipines 16S ribosomal RNA gene, partial	EF623242	78.51	73.62	3.19988e−16	Plecoptera	Perlidae	Perlesta decipines
Nanonemoura wahkeena 16S ribosomal RNA gene, parti	EF623198	81.82	60.74	3.19988e−16	Plecoptera	Nemouridae	Nanonemoura wahkeena
Lednia tumana 16S ribosomal RNA gene, partial sequ	EF623197	81.37	62.58	3.19988e−16	Plecoptera	Nemouridae	Lednia tumana
Protonemura meyeri 16S ribosomal RNA gene, partial	EF623193	81.37	62.58	3.19988e−16	Plecoptera	Nemouridae	Protonemura meyeri
Ostrocerca foersteri 16S ribosomal RNA gene, parti	EF623191	81.82	60.74	3.19988e−16	Plecoptera	Nemouridae	Ostrocerca foersteri
Visoka cataractae 16S ribosomal RNA gene, partial	EF623189	81.82	60.74	3.19988e−16	Plecoptera	Nemouridae	Visoka cataractae
Paraperla frontalis 16S ribosomal RNA gene, partia	EF623136	81.82	60.74	3.19988e−16	Plecoptera	Chloroperlidae	Paraperla frontalis
Kathroperla perdita 16S ribosomal RNA gene, partia	EF623135	81.37	62.58	3.19988e−16	Plecoptera	Chloroperlidae	Kathroperla perdita
Tessellana veyseli voucher 260 16S ribosomal RNA g	EU120811	73.75	98.16	3.19988e−16	Orthoptera	Tettigoniidae	Tessellana veyseli
Dolichopoda geniculata pontiana isolate PNZ 16S ri	AY793586	75.91	84.05	3.19988e−16	Orthoptera	Rhaphidophoridae	Dolichopoda geniculata pontiana
