# Supplementary material for: Testing the potential of a ribosomal 16S marker for DNA metabarcoding of insects
Source: PeerJ. 2016 Apr 19;4:e1966. doi: 10.7717/peerj.1966 (PMC4841222; doi:10.7717/peerj.1966)
Supplement: Supplemental Information 1 [file peerj-04-1966-s006.zip › scripts/5 match OTUs/blast_hits/OTU_425.csv_plot.pdf]

OTU_425.csv

Oreodytes sanmarkii

Dytiscidae

Coleoptera

98.09

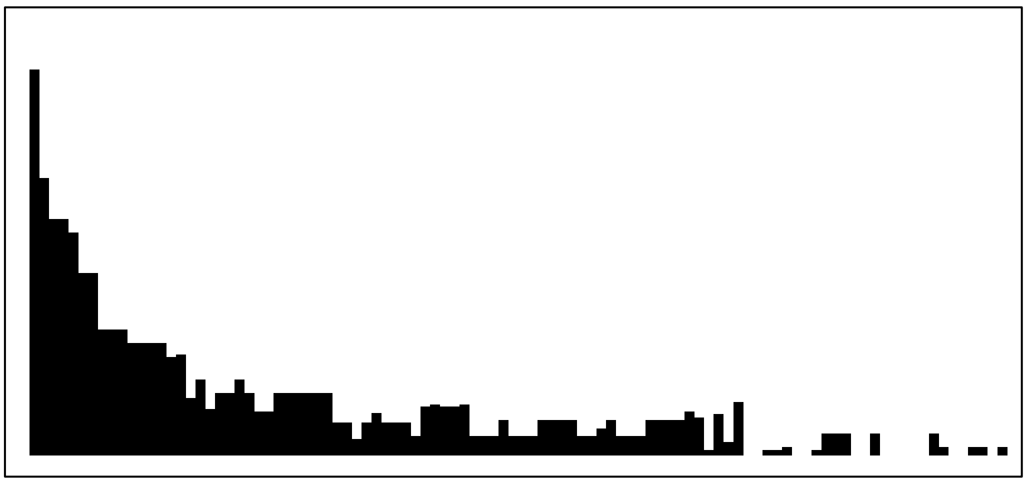

Oreodytes sanmarkii mitochondrial 16S rRNA gene (p	HF931524	98.09	100	8.09543e−68	Coleoptera	Dytiscidae	Oreodytes sanmarkii
Oreodytes obesus cordillerensis mitochondrial part	HF931516	92.99	100	5.82911e−57	Coleoptera	Dytiscidae	Oreodytes obesus cordillerensis
Oreodytes crassulus partial 16S rRNA gene	AJ850348	91.08	100	3.67857e−53	Coleoptera	Dytiscidae	Oreodytes crassulus
Oreodytes congruus partial 16S rRNA gene	AJ850347	91.08	100	3.67857e−53	Coleoptera	Dytiscidae	Oreodytes congruus
Oreodytes congruus 16S ribosomal RNA gene, partial	KF575550	90.45	100	1.56417e−51	Coleoptera	Dytiscidae	Oreodytes congruus
Oreodytes abbreviatus mitochondrial 16S rRNA gene	HF931477	88.54	100	9.87097e−48	Coleoptera	Dytiscidae	Oreodytes abbreviatus
Oreodytes rhyacophilus partial 16S rRNA gene	AJ850352	88.54	100	9.87097e−48	Coleoptera	Dytiscidae	Oreodytes rhyacophilus
Boreonectes riberae mitochondrial 16S rRNA gene (p	HF931461	85.9	100	7.58879e−43	Coleoptera	Dytiscidae	Boreonectes riberae
Boreonectes griseostriatus mitochondrial partial 1	HF931387	85.9	100	7.58879e−43	Coleoptera	Dytiscidae	Boreonectes griseostriatus
Boreonectes multilineatus mitochondrial partial 16	HF931384	85.9	100	7.58879e−43	Coleoptera	Dytiscidae	Boreonectes multilineatus
Boreonectes alpestris mitochondrial 16S rRNA gene	HF931512	85.26	100	3.22684e−41	Coleoptera	Dytiscidae	Boreonectes alpestris
Boreonectes striatellus mitochondrial 16S rRNA gen	HF931511	85.26	100	3.22684e−41	Coleoptera	Dytiscidae	Boreonectes striatellus
Boreonectes macedonicus mitochondrial 16S rRNA gen	HF931359	85.26	100	3.22684e−41	Coleoptera	Dytiscidae	Boreonectes macedonicus
Stictotarsus ibericus 16S ribosomal RNA (rrnL) gen	EF670030	85.26	100	3.22684e−41	Coleoptera	Dytiscidae	Stictotarsus ibericus
Boreonectes griseostriatus mitochondrial 16S rRNA	HF931541	84.62	100	3.93109e−40	Coleoptera	Dytiscidae	Boreonectes griseostriatus
Laccornis difformis 16S ribosomal RNA gene, partia	KF575544	84.71	100	1.37209e−39	Coleoptera	Dytiscidae	Laccornis difformis
Boreonectes griseostriatus mitochondrial 16S rRNA	HF931385	82.69	100	8.65881e−36	Coleoptera	Dytiscidae	Boreonectes griseostriatus
Stictotarsus maghrebinus 16S ribosomal RNA gene, p	AY250947	83.54	100	3.02222e−35	Coleoptera	Dytiscidae	Stictotarsus maghrebinus
Hydaticus cinctipennis voucher UNM KBMHyci20 16S r	KJ548338	82.17	100	3.68182e−34	Coleoptera	Dytiscidae	Hydaticus cinctipennis
Suphrodytes dorsalis 16S ribosomal RNA gene, parti	KF575577	82.91	100	3.68182e−34	Coleoptera	Dytiscidae	Suphrodytes dorsalis
Suphrodytes dorsalis voucher BMNH:725966 16S ribos	JX221574	82.91	100	3.68182e−34	Coleoptera	Dytiscidae	Suphrodytes dorsalis
Hydroporus foveolatus mitochondrial 16S rRNA gene	HE613724	83.54	100	3.68182e−34	Coleoptera	Dytiscidae	Hydroporus foveolatus
Stictotarsus procerus mitochondrial 16S rRNA gene	HF931379	82.91	100	1.28508e−33	Coleoptera	Dytiscidae	Stictotarsus procerus
Hydrocolus sahlbergi mitochondrial 16S rRNA gene (	HE599691	82.05	100	1.28508e−33	Coleoptera	Dytiscidae	Hydrocolus sahlbergi
Hydrocolus picicornis partial 16S rRNA gene	AJ850379	82.05	100	1.28508e−33	Coleoptera	Dytiscidae	Hydrocolus picicornis
Hydroporus tessellatus 16S ribosomal RNA gene, par	JX434770	82.91	100	4.48537e−33	Coleoptera	Dytiscidae	Hydroporus tessellatus
Hydroporus limbatus mitochondrial 16S rRNA gene (p	HE613706	82.91	100	4.48537e−33	Coleoptera	Dytiscidae	Hydroporus limbatus
Hydroporus zimmermanni mitochondrial 16S rRNA gene	HE613679	82.91	100	4.48537e−33	Coleoptera	Dytiscidae	Hydroporus zimmermanni
Hydroporus basinotatus mitochondrial 16S rRNA gene	HE613678	82.91	100	4.48537e−33	Coleoptera	Dytiscidae	Hydroporus basinotatus
Hydroporus basinotatus 16S ribosomal RNA gene, par	AF518258	82.91	100	4.48537e−33	Coleoptera	Dytiscidae	Hydroporus basinotatus
Hydroporus limbatus 16S ribosomal RNA gene, partia	AF518264	82.91	100	4.48537e−33	Coleoptera	Dytiscidae	Hydroporus limbatus
Stictotarsus falli 16S ribosomal RNA (rrnL) gene,	EF670029	81.53	100	1.56555e−32	Coleoptera	Dytiscidae	Stictotarsus falli
Hygrotus novemlineatus mitochondrial partial 16S r	HF931523	81.53	100	5.4643e−32	Coleoptera	Dytiscidae	Hygrotus novemlineatus
Boreonectes funereus mitochondrial 16S rRNA gene (	HF931393	80.77	100	5.4643e−32	Coleoptera	Dytiscidae	Boreonectes funereus
Suphrodytes figuratus voucher BMNH:722148 16S ribo	JX221571	81.53	100	5.4643e−32	Coleoptera	Dytiscidae	Suphrodytes figuratus
Hydroporus marginatus mitochondrial 16S rRNA gene	HE613699	81.99	100	5.4643e−32	Coleoptera	Dytiscidae	Hydroporus marginatus
Hygrotus turbidus partial 16S rRNA gene	AJ850390	81.53	100	5.4643e−32	Coleoptera	Dytiscidae	Hygrotus turbidus
Hydroporus sp. IR33.4 16S ribosomal RNA gene, part	AY365261	81.53	100	5.4643e−32	Coleoptera	Dytiscidae	Hydroporus sp. IR33.4
Hydroporus marginatus 16S ribosomal RNA gene, part	AF518267	81.53	100	5.4643e−32	Coleoptera	Dytiscidae	Hydroporus marginatus
Hydaticus aruspex voucher UNM KBMHyar68 16S riboso	KJ548337	80.89	100	1.90723e−31	Coleoptera	Dytiscidae	Hydaticus aruspex
Hydroporus analis mitochondrial partial 16S rRNA g	HE613731	82.28	100	1.90723e−31	Coleoptera	Dytiscidae	Hydroporus analis
Hydroporus fuscipennis mitochondrial 16S rRNA gene	HE613730	82.39	100	1.90723e−31	Coleoptera	Dytiscidae	Hydroporus fuscipennis
Hydroporus compunctus mitochondrial 16S rRNA gene	HE599693	82.28	100	1.90723e−31	Coleoptera	Dytiscidae	Hydroporus compunctus
Hydroporus compunctus 16S ribosomal RNA gene, part	AF518259	82.28	100	1.90723e−31	Coleoptera	Dytiscidae	Hydroporus compunctus
Hydroporus fuscipennis 16S ribosomal RNA gene, par	AF518262	82.39	100	1.90723e−31	Coleoptera	Dytiscidae	Hydroporus fuscipennis
Aglymbus gestroi 16S ribosomal RNA gene, partial s	AY138639	80.89	100	1.90723e−31	Coleoptera	Dytiscidae	Aglymbus gestroi
Hydroporus appalachius 16S ribosomal RNA gene, par	JX434765	80.89	100	2.32348e−30	Coleoptera	Dytiscidae	Hydroporus appalachius
Hygrotus lutescens mitochondrial partial 16S rRNA	HF931518	80.89	100	2.32348e−30	Coleoptera	Dytiscidae	Hygrotus lutescens
Hydroporus foveolatus mitochondrial 16S rRNA gene	HF931502	81.65	100	2.32348e−30	Coleoptera	Dytiscidae	Hydroporus foveolatus
Hygrotus fontinalis mitochondrial 16S rRNA gene (p	HF931494	80.89	100	2.32348e−30	Coleoptera	Dytiscidae	Hygrotus fontinalis
Hygrotus patruelis mitochondrial 16S rRNA gene (pa	HF931388	80.89	100	2.32348e−30	Coleoptera	Dytiscidae	Hygrotus patruelis
Suphrodytes figuratus voucher BMNH:722150 16S ribo	JX221572	80.89	100	2.32348e−30	Coleoptera	Dytiscidae	Suphrodytes figuratus
Hydroporus discretus mitochondrial 16S rRNA gene (	HE613723	81.65	100	2.32348e−30	Coleoptera	Dytiscidae	Hydroporus discretus
Hydroporus decipiens mitochondrial 16S rRNA gene (	HE613722	81.65	100	2.32348e−30	Coleoptera	Dytiscidae	Hydroporus decipiens
Hydroporus pubescens mitochondrial 16S rRNA gene (	HE613707	81.65	100	2.32348e−30	Coleoptera	Dytiscidae	Hydroporus pubescens
Hydroporus errans mitochondrial 16S rRNA gene (par	HE613704	81.65	100	2.32348e−30	Coleoptera	Dytiscidae	Hydroporus errans
Suphrodytes dorsalis mitochondrial 16S rRNA gene (	HE599700	80.89	100	2.32348e−30	Coleoptera	Dytiscidae	Suphrodytes dorsalis
Hydroporus pervicinus mitochondrial 16S rRNA gene	HE599695	80.89	100	2.32348e−30	Coleoptera	Dytiscidae	Hydroporus pervicinus
Acilius duvergeri partial 16S rRNA gene	AJ850357	81.25	100	2.32348e−30	Coleoptera	Dytiscidae	Acilius duvergeri
Hydroporus pubescens voucher NHM−IR39 16S ribosoma	EF419327	81.65	100	2.32348e−30	Coleoptera	Dytiscidae	Hydroporus pubescens
Hydroporus occidentalis 16S ribosomal RNA gene, pa	AY365280	80.89	100	2.32348e−30	Coleoptera	Dytiscidae	Hydroporus occidentalis
Hydroporus hirtellus 16S ribosomal RNA gene, parti	AY365271	80.89	100	2.32348e−30	Coleoptera	Dytiscidae	Hydroporus hirtellus
Deronectes delarouzei haplotype 2 16S ribosomal RN	AY250909	80.89	100	2.32348e−30	Coleoptera	Dytiscidae	Deronectes delarouzei
Hydroporus rufifrons 16S ribosomal RNA gene, parti	AF518277	81.65	100	2.32348e−30	Coleoptera	Dytiscidae	Hydroporus rufifrons
Hydroporus pubescens 16S ribosomal RNA gene, parti	AF518276	81.65	100	2.32348e−30	Coleoptera	Dytiscidae	Hydroporus pubescens
Hydroporus discretus 16S ribosomal RNA gene, parti	AF518260	81.65	100	2.32348e−30	Coleoptera	Dytiscidae	Hydroporus discretus
Hydroporus analis 16S ribosomal RNA gene, partial	AF518257	81.65	100	2.32348e−30	Coleoptera	Dytiscidae	Hydroporus analis
Hygrotus impressopunctatus voucher UNM KBMHtim80 1	KJ548334	82.05	97.44	8.10974e−30	Coleoptera	Dytiscidae	Hygrotus impressopunctatus
Hygrotus armeniacus mitochondrial partial 16S rRNA	HF931520	81.76	100	8.10974e−30	Coleoptera	Dytiscidae	Hygrotus armeniacus
Prodaticus pictus partial 16S rRNA gene	AJ850372	80.25	100	8.10974e−30	Coleoptera	Dytiscidae	Prodaticus pictus
Hygrotus impressopunctatus partial 16S rRNA gene	AJ850405	81.94	97.44	2.83058e−29	Coleoptera	Dytiscidae	Hygrotus impressopunctatus
Acilius sulcatus partial 16S rRNA gene	AJ850358	80.62	100	2.83058e−29	Coleoptera	Dytiscidae	Acilius sulcatus
Darwinhydrus solidus voucher MB354 16S ribosomal R	EF056671	82.5	100	2.83058e−29	Coleoptera	Dytiscidae	Darwinhydrus solidus
Prodaticus luczonicus voucher DBAU−BT−30_B94_Dytis	KM064623	79.62	100	9.87969e−29	Coleoptera	Dytiscidae	Prodaticus luczonicus
Agabus glacialis mitochondrial 16S rRNA gene (part	LM654776	79.62	100	9.87969e−29	Coleoptera	Dytiscidae	Agabus glacialis
Agabus didymus mitochondrial 16S rRNA gene (partia	LM654773	80.25	100	9.87969e−29	Coleoptera	Dytiscidae	Agabus didymus
Agabus didymus voucher UNM KBMPmsp730 16S ribosoma	KJ548392	80.25	100	9.87969e−29	Coleoptera	Dytiscidae	Agabus didymus
Herophydrus rohani voucher UNM KBMHpsp574 16S ribo	KJ548382	80.38	100	9.87969e−29	Coleoptera	Dytiscidae	Herophydrus rohani
Prodaticus xanthomelas voucher UNM KBMHyxa67 16S r	KJ548341	79.62	100	9.87969e−29	Coleoptera	Dytiscidae	Prodaticus xanthomelas
Cybister marginicollis voucher UNM KBMCyma416 16S	KJ548319	79.62	100	9.87969e−29	Coleoptera	Dytiscidae	Cybister marginicollis
Eretes sticticus voucher 411G2/BMNH832075 16S ribo	KF979076	80.25	100	9.87969e−29	Coleoptera	Dytiscidae	Eretes sticticus
Hydroporus rufifrons 16S ribosomal RNA gene, parti	JX434758	81.01	100	9.87969e−29	Coleoptera	Dytiscidae	Hydroporus rufifrons
Hydroporus discretus mitochondrial 16S rRNA gene (	HF931449	81.01	100	9.87969e−29	Coleoptera	Dytiscidae	Hydroporus discretus
Hydroporus thracicus mitochondrial 16S rRNA gene (	HF931413	81.01	100	9.87969e−29	Coleoptera	Dytiscidae	Hydroporus thracicus
Agabus dichrous mitochondrial partial 16S rRNA gen	HF931378	79.62	100	9.87969e−29	Coleoptera	Dytiscidae	Agabus dichrous
Agabus godmanni mitochondrial 16S rRNA gene (parti	HF931348	79.62	100	9.87969e−29	Coleoptera	Dytiscidae	Agabus godmanni
Hydroporus macedonicus mitochondrial 16S rRNA gene	HE613690	81.01	100	9.87969e−29	Coleoptera	Dytiscidae	Hydroporus macedonicus
Prodaticus bivittatus voucher BMNH829907 16S ribos	HQ381491	79.62	100	9.87969e−29	Coleoptera	Dytiscidae	Prodaticus bivittatus
Copelatus sp. BI−2011 voucher BMNH792956 16S ribos	HQ381443	79.62	100	9.87969e−29	Coleoptera	Dytiscidae	Copelatus sp. BI−2011
Copelatus sp. BI−2011 voucher BMNH792915 16S ribos	HQ381439	79.62	100	9.87969e−29	Coleoptera	Dytiscidae	Copelatus sp. BI−2011
Copelatus sp. BI−2011 voucher BMNH729890 16S ribos	HQ381391	79.62	100	9.87969e−29	Coleoptera	Dytiscidae	Copelatus sp. BI−2011
Oreodytes picturatus partial 16S rRNA gene	AJ850350	79.62	100	9.87969e−29	Coleoptera	Dytiscidae	Oreodytes picturatus
Hydroporus macedonicus 16S ribosomal RNA gene, par	DQ195529	81.01	100	9.87969e−29	Coleoptera	Dytiscidae	Hydroporus macedonicus
Iberoporus cermenius 16S ribosomal RNA gene, parti	AY250918	80.38	100	9.87969e−29	Coleoptera	Dytiscidae	Iberoporus cermenius
Agabus paludosus 16S ribosomal RNA gene, partial s	AY138625	79.62	100	9.87969e−29	Coleoptera	Dytiscidae	Agabus paludosus
Agabus dilatatus 16S ribosomal RNA gene, partial s	AY039261	79.62	100	9.87969e−29	Coleoptera	Dytiscidae	Agabus dilatatus
Prodaticus servillianus voucher BMNH742590 16S rib	HQ381394	80.38	100	3.44835e−28	Coleoptera	Dytiscidae	Prodaticus servillianus
Hydaticus leander partial 16S rRNA gene	AJ850364	80.38	100	3.44835e−28	Coleoptera	Dytiscidae	Prodaticus leander
Nirripirti plutonicensis 16S ribosomal RNA gene, p	AY353839	79.49	100	3.44835e−28	Coleoptera	Dytiscidae	Paroster plutonicensis
Herophydrus inquinatus 16S ribosomal RNA gene, par	KF575572	80.38	100	1.20359e−27	Coleoptera	Dytiscidae	Herophydrus inquinatus
