# Supplementary material for: Testing the potential of a ribosomal 16S marker for DNA metabarcoding of insects
Source: PeerJ. 2016 Apr 19;4:e1966. doi: 10.7717/peerj.1966 (PMC4841222; doi:10.7717/peerj.1966)
Supplement: Supplemental Information 1 [file peerj-04-1966-s006.zip › scripts/5 match OTUs/blast_hits/OTU_433.csv_plot.pdf]

OTU_433.csv

Ecdyonurus dispar

Heptageniidae

Ephemeroptera

89.68

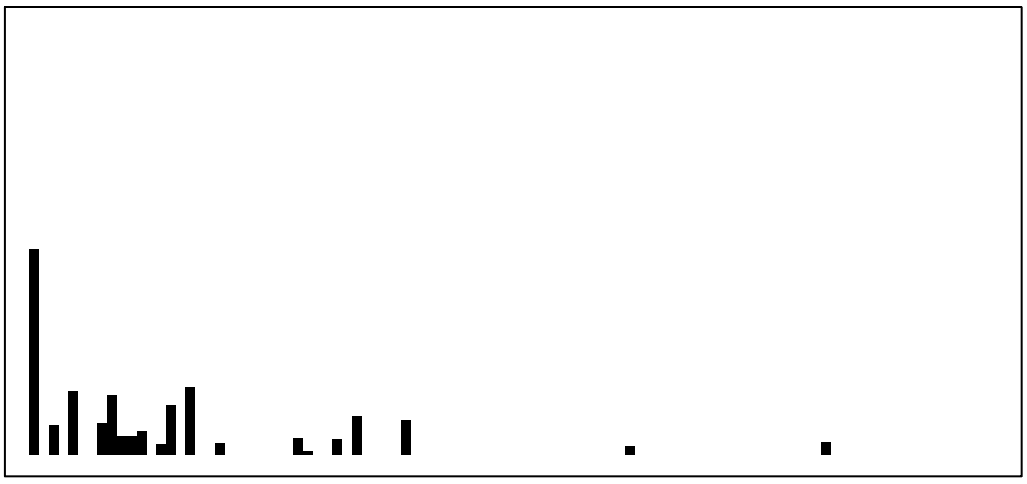

Ecdyonurus dispar 16S ribosomal RNA gene, partial	AY749775	89.68	100	1.8898e−50	Ephemeroptera	Heptageniidae	Ecdyonurus dispar
Parafronurus youi mitochondrion, complete genome	EU349015	78.57	99.35	1.77153e−25	Ephemeroptera	Heptageniidae	Parafronurus youi
Pseudiron centralis 16S ribosomal RNA gene, partia	AY749799	81.42	72.9	1.36195e−20	Ephemeroptera	Pseudironidae	Pseudiron centralis
Clioperla clio 16S ribosomal RNA gene, partial seq	EF623237	75.64	100	4.75367e−20	Plecoptera	Perlodidae	Clioperla clio
Afronurus sp. 212BoAf mitochondrial partial 16S rR	HE651421	83	64.52	5.79115e−19	Ephemeroptera	Heptageniidae	Afronurus sp. 212BoAf
Atopopus tarsalis mitochondrial partial 16S rRNA g	HE651420	74.84	100	5.79115e−19	Ephemeroptera	Heptageniidae	Atopopus tarsalis
Atopopus tarsalis mitochondrial partial 16S rRNA g	HE651419	74.84	100	5.79115e−19	Ephemeroptera	Heptageniidae	Atopopus tarsalis
Siphlonurus sp. MT−2014 isolate CL142 mitochondrio	KM244684	81.48	69.68	2.02131e−18	Ephemeroptera	Siphlonuridae	Siphlonurus sp. MT−2014
Afronurus sp. 213BoAf mitochondrial partial 16S rR	HE651422	82.83	63.87	2.02131e−18	Ephemeroptera	Heptageniidae	Afronurus sp. 213BoAf
Heteromallus cavicola voucher MIL 16S ribosomal RN	HM594499	80.87	72.9	2.02131e−18	Orthoptera	Rhaphidophoridae	Heteromallus cavicola
Dolichopoda sp. MR−2008 16S ribosomal RNA gene, pa	EU887863	80.87	72.9	2.02131e−18	Orthoptera	Rhaphidophoridae	Dolichopoda lustriae
Pteronarcys sachalina 16S ribosomal RNA gene, part	EF623264	81.13	68.39	2.02131e−18	Plecoptera	Pteronarcyidae	Pteronarcys sachalina
Oniscigaster distans 16S ribosomal RNA gene, parti	AY749818	75.8	100	2.02131e−18	Ephemeroptera	Oniscigastridae	Oniscigaster distans
Siphlonurus sp. EP084 16S ribosomal RNA gene, part	AY749786	80.51	75.48	2.02131e−18	Ephemeroptera	Siphlonuridae	Siphlonurus sp. EP084
Heptagenia sulphurea mitochondrial partial 16S rRN	HE651431	82.35	65.16	7.05507e−18	Ephemeroptera	Heptageniidae	Heptagenia sulphurea
Cinygmina sp. EP133 16S ribosomal RNA gene, partia	AY749811	74.84	100	7.05507e−18	Ephemeroptera	Heptageniidae	Cinygmina sp. EP133
Afronurus peringueyi 16S ribosomal RNA gene, parti	AY749790	83.17	63.87	7.05507e−18	Ephemeroptera	Heptageniidae	Afronurus peringueyi
Osmylus sp. EH−2004 16S ribosomal RNA gene, partia	AY620146	75	100	2.46246e−17	Neuroptera	Osmylidae	Osmylus sp. EH−2004
Rallidens mcfarlanei 16S ribosomal RNA gene, parti	AY749794	75.16	100	2.46246e−17	Ephemeroptera	Rallidentidae	Rallidens mcfarlanei
Paegniodes cupulatus mitochondrion, complete genom	HM004123	80.58	66.45	8.59483e−17	Ephemeroptera	Heptageniidae	Paegniodes cupulatus
Panorpa alpina 16S ribosomal RNA gene, partial seq	AF180046	78.95	72.9	2.99989e−16	Mecoptera	Panorpidae	Panorpa alpina
Hexagenia sp. BYU IGCEP003 16S ribosomal RNA gene,	GQ118286	78.76	72.9	1.04707e−15	Ephemeroptera	Ephemeridae	Hexagenia sp. BYU IGCEP003
Dolichopoda sp. VAR 16S ribosomal RNA gene, partia	EU887865	79.13	72.9	1.04707e−15	Orthoptera	Rhaphidophoridae	Dolichopoda sp. VAR
Dolichopoda sp. TAY 16S ribosomal RNA gene, partia	EU887864	79.13	72.9	1.04707e−15	Orthoptera	Rhaphidophoridae	Dolichopoda sp. TAY
Dolichopoda annae voucher OLI 16S ribosomal RNA ge	EU887860	79.13	72.9	1.04707e−15	Orthoptera	Rhaphidophoridae	Dolichopoda annae
Siphlonurus binotatus mitochondrial gene for 16S r	AB110269	75.57	84.52	1.04707e−15	Ephemeroptera	Siphlonuridae	Siphlonurus binotatus
Australostoma sp. MCJ−2002 16S ribosomal RNA gene,	AF514481	74.21	100	1.04707e−15	Orthoptera	Stenopelmatidae	Australostoma sp. MCJ−2002
Stenonema sp. EP026 16S ribosomal RNA gene, partia	AY749769	80.81	63.87	1.04707e−15	Ephemeroptera	Heptageniidae	Stenonema sp. EP026
Parameletus columbiae 16S ribosomal RNA gene, part	AY749768	80.2	65.16	1.04707e−15	Ephemeroptera	Siphlonuridae	Parameletus columbiae
Thalerosphyrus lamuriensis mitochondrial partial 1	HE651425	77.78	75.48	3.65462e−15	Ephemeroptera	Heptageniidae	Thalerosphyrus lamuriensis
Dolichopoda pavesii voucher SPI 16S ribosomal RNA	EF216951	80	67.1	3.65462e−15	Orthoptera	Rhaphidophoridae	Dolichopoda pavesii
Yugus bulbosus 16S ribosomal RNA gene, partial seq	EF623214	80.77	65.81	3.65462e−15	Plecoptera	Perlodidae	Yugus bulbosus
Isoperla davisi 16S ribosomal RNA gene, partial se	EF623206	79.44	68.39	3.65462e−15	Plecoptera	Perlodidae	Isoperla davisi
Ichthybotus hudsoni 16S ribosomal RNA gene, partia	AY749816	81.82	63.87	3.65462e−15	Ephemeroptera	Ichthybotidae	Ichthybotus hudsoni
Udenus wnigrum voucher MAY 16S ribosomal RNA gene,	HM594504	77.39	72.26	1.27559e−14	Orthoptera	Rhaphidophoridae	Udenus wnigrum
Udenus wnigrum voucher CER 16S ribosomal RNA gene,	HM594503	77.39	72.26	1.27559e−14	Orthoptera	Rhaphidophoridae	Udenus wnigrum
Dolichopoda remyi voucher EDE 16S ribosomal RNA ge	EF216969	78.07	72.9	1.27559e−14	Orthoptera	Rhaphidophoridae	Dolichopoda remyi
Dolichopoda giachinoi voucher ORO 16S ribosomal RN	EF216952	78.85	67.1	1.27559e−14	Orthoptera	Rhaphidophoridae	Dolichopoda giachinoi
Osobenus yakimae 16S ribosomal RNA gene, partial s	EF623232	81.63	62.58	1.27559e−14	Plecoptera	Perlodidae	Osobenus yakimae
Cosumnoperla hypocrena 16S ribosomal RNA gene, par	EF623226	77.59	74.19	1.27559e−14	Plecoptera	Perlodidae	Cosumnoperla hypocrena
Cascadoperla trictura 16S ribosomal RNA gene, part	EF623225	78.38	70.97	1.27559e−14	Plecoptera	Perlodidae	Cascadoperla trictura
Isoperla oxylepis 16S ribosomal RNA gene, partial	EF623221	79.41	65.81	1.27559e−14	Plecoptera	Perlodidae	Isoperla oxylepis
Isoperla fulva 16S ribosomal RNA gene, partial seq	EF623207	73.08	100	1.27559e−14	Plecoptera	Perlodidae	Isoperla fulva
Dolichopoda remyi isolate EDE 16S ribosomal RNA ge	AY793589	78.07	72.9	1.27559e−14	Orthoptera	Rhaphidophoridae	Dolichopoda remyi
Panorpa acuta 16S ribosomal RNA gene, partial sequ	AF180047	78.07	72.9	1.27559e−14	Mecoptera	Panorpidae	Panorpa acuta
Lachlania saskatchewanensis 16S ribosomal RNA gene	AY749801	79.8	63.87	1.27559e−14	Ephemeroptera	Oligoneuriidae	Lachlania saskatchewanensis
Chaquihua sp. EP080 16S ribosomal RNA gene, partia	AY749784	79.41	65.81	1.27559e−14	Ephemeroptera	Ameletopsidae	Chaquihua sp. EP080
Parudenus falklandicus voucher PAR 16S ribosomal R	HM594502	78.07	72.26	4.45223e−14	Orthoptera	Rhaphidophoridae	Parudenus falklandicus
Dolichopoda hussoni voucher IZB 16S ribosomal RNA	EF216973	78.26	72.9	4.45223e−14	Orthoptera	Rhaphidophoridae	Dolichopoda hussoni
Dolichopoda thasosensis voucher DRA 16S ribosomal	EF216956	78.26	72.9	4.45223e−14	Orthoptera	Rhaphidophoridae	Dolichopoda thasosensis
Dolichopoda steriotisi voucher ANT 16S ribosomal R	EF216955	79.05	67.1	4.45223e−14	Orthoptera	Rhaphidophoridae	Dolichopoda steriotisi
Pteronarcys scotti 16S ribosomal RNA gene, partial	EF623263	78.3	68.39	4.45223e−14	Plecoptera	Pteronarcyidae	Pteronarcys scotti
Zealeuctra arnoldi 16S ribosomal RNA gene, partial	EF623180	78.5	67.74	4.45223e−14	Plecoptera	Leuctridae	Zealeuctra arnoldi
Neuroperlopsis patris 16S ribosomal RNA gene, part	EF623152	73.65	94.84	4.45223e−14	Plecoptera	Eustheniidae	Neuroperlopsis patris
Acruroperla atra 16S ribosomal RNA gene, partial s	EF623121	74	95.48	4.45223e−14	Plecoptera	Austroperlidae	Acruroperla atra
Isogenoides varians isolate BYU_PL079 16S ribosoma	EF623445	79.21	65.16	4.45223e−14	Plecoptera	Perlodidae	Isogenoides varians
Perlodes mortoni 16S ribosomal RNA gene, partial s	KF855870	79.41	65.16	1.55398e−13	Plecoptera	Perlodidae	Perlodes mortoni
Thalerosphyrus lamuriensis mitochondrial partial 1	HE651429	76.92	75.48	1.55398e−13	Ephemeroptera	Heptageniidae	Thalerosphyrus lamuriensis
Afronurus sp. 297SAAf mitochondrial partial 16S rR	HE651424	78.43	65.81	1.55398e−13	Ephemeroptera	Heptageniidae	Afronurus sp. 297SAAf
Compsoneuria sp. 4 LV−2012 mitochondrial partial 1	HE651398	77.59	74.19	1.55398e−13	Ephemeroptera	Heptageniidae	Compsoneuria sp. 4 LV−2012
Talitropsis sedilloti voucher CW160 16S ribosomal	HM594511	79	64.52	1.55398e−13	Orthoptera	Rhaphidophoridae	Talitropsis sedilloti
Psorodonotus caucasicus voucher 397 16S ribosomal	EU203908	80.41	62.58	1.55398e−13	Orthoptera	Tettigoniidae	Psorodonotus caucasicus
Dolichopoda graeca voucher PER 16S ribosomal RNA g	EF216953	78.3	67.1	1.55398e−13	Orthoptera	Rhaphidophoridae	Dolichopoda graeca
Isogenoides olivaceus 16S ribosomal RNA gene, part	EF623224	79.41	65.16	1.55398e−13	Plecoptera	Perlodidae	Isogenoides olivaceus
Hydroperla crosbyi 16S ribosomal RNA gene, partial	EF623219	79.38	62.58	1.55398e−13	Plecoptera	Perlodidae	Hydroperla crosbyi
Neofulla sp. BYU_PL152 16S ribosomal RNA gene, par	EF623203	77.68	71.61	1.55398e−13	Plecoptera	Notonemouridae	Neofulla sp. BYU_PL152
Kimminsoperla albomacula 16S ribosomal RNA gene, p	EF623202	76.72	72.9	1.55398e−13	Plecoptera	Notonemouridae	Kimminsoperla albomacula
Despaxia augusta 16S ribosomal RNA gene, partial s	EF623177	79.38	62.58	1.55398e−13	Plecoptera	Leuctridae	Despaxia augusta
Suwallia teleckojensis 16S ribosomal RNA gene, par	EF623142	73.46	100	1.55398e−13	Plecoptera	Chloroperlidae	Suwallia teleckojensis
Neopanorpa sp. M24899 16S ribosomal RNA gene, part	AF180059	77.19	72.9	1.55398e−13	Mecoptera	Panorpidae	Neopanorpa sp. M24899
Panorpa nebulosa 16S ribosomal RNA gene, partial s	AF180058	77.19	72.9	1.55398e−13	Mecoptera	Panorpidae	Panorpa nebulosa
Tasmanophlebia sp. EP158 16S ribosomal RNA gene, p	AY749825	79.38	62.58	1.55398e−13	Ephemeroptera	Oniscigastridae	Tasmanophlebia sp. EP158
Siphlonella sp. EP083 16S ribosomal RNA gene, part	AY749785	79.38	62.58	1.55398e−13	Ephemeroptera	Oniscigastridae	Siphlonella sp. EP083
Chiloporter sp. EP051 16S ribosomal RNA gene, part	AY749776	79	64.52	1.55398e−13	Ephemeroptera	Ameletopsidae	Chiloporter sp. EP051
Heptagenia sp. EP018 16S ribosomal RNA gene, parti	AY749762	74.1	89.03	1.55398e−13	Ephemeroptera	Heptageniidae	Heptagenia sp. EP018
Rhithrogena nuragica mitochondrial partial 16S rRN	LN868667	72.61	100	5.42393e−13	Ephemeroptera	Heptageniidae	Rhithrogena nuragica
Tettigonia ussuriana isolate CLN008 16S large subu	EF198430	78.64	66.45	5.42393e−13	Orthoptera	Tettigoniidae	Tettigonia ussuriana
Dolichopoda dalensi voucher KEF 16S ribosomal RNA	EF216959	77.88	71.61	5.42393e−13	Orthoptera	Rhaphidophoridae	Dolichopoda dalensi
Pteronarcys californica 16S ribosomal RNA gene, pa	EF623261	77.36	68.39	5.42393e−13	Plecoptera	Pteronarcyidae	Pteronarcys californica
Baumanella alameda 16S ribosomal RNA gene, partial	EF623236	78.64	65.81	5.42393e−13	Plecoptera	Perlodidae	Baumanella alameda
Perlinodes aurea 16S ribosomal RNA gene, partial s	EF623234	78.64	65.81	5.42393e−13	Plecoptera	Perlodidae	Perlinodes aurea
Kogotus nonus 16S ribosomal RNA gene, partial sequ	EF623213	80.61	62.58	5.42393e−13	Plecoptera	Perlodidae	Kogotus nonus
Udamocercia sp. BYU_PL150 16S ribosomal RNA gene,	EF623201	78.18	70.32	5.42393e−13	Plecoptera	Notonemouridae	Udamocercia sp. BYU_PL150
Nanonemoura wahkeena 16S ribosomal RNA gene, parti	EF623198	73.2	91.61	5.42393e−13	Plecoptera	Nemouridae	Nanonemoura wahkeena
Amphinemura nigritta 16S ribosomal RNA gene, parti	EF623186	78.3	67.74	5.42393e−13	Plecoptera	Nemouridae	Amphinemura nigritta
Zapada cinctipes 16S ribosomal RNA gene, partial s	EF623183	78.64	65.81	5.42393e−13	Plecoptera	Nemouridae	Zapada cinctipes
Dinotoperla sp. BYU_PL105 16S ribosomal RNA gene,	EF623160	73.17	100	5.42393e−13	Plecoptera	Gripopterygidae	Dinotoperla sp. BYU_PL105
Diamphipnopsis sp. BYU_PL138 16S ribosomal RNA gen	EF623145	74.64	87.74	5.42393e−13	Plecoptera	Diamphipnoidae	Diamphipnopsis sp. BYU_PL138
Plumiperla diversa 16S ribosomal RNA gene, partial	EF623132	73.75	100	5.42393e−13	Plecoptera	Chloroperlidae	Plumiperla diversa
Penturoperla barbata 16S ribosomal RNA gene, parti	EF623124	78.22	65.16	5.42393e−13	Plecoptera	Austroperlidae	Penturoperla barbata
Pteronarcys princeps mitochondrion, complete genom	AY687866	77.36	68.39	5.42393e−13	Plecoptera	Pteronarcyidae	Pteronarcys princeps
Tettigonia viridissima mitochondrial 16S rRNA gene	Z97622	78.64	66.45	5.42393e−13	Orthoptera	Tettigoniidae	Tettigonia viridissima
Cryptoperla japonica mitochondrial gene for 16S rR	AB711202	78.85	66.45	1.89314e−12	Plecoptera	Peltoperlidae	Cryptoperla japonica
Cryptoperla japonica mitochondrial gene for 16S rR	AB711184	78.85	66.45	1.89314e−12	Plecoptera	Peltoperlidae	Cryptoperla japonica
Afronurus sp. 338SuAf mitochondrial partial 16S rR	HE651428	79.38	62.58	1.89314e−12	Ephemeroptera	Heptageniidae	Afronurus sp. 338SuAf
Notonurus josettae mitochondrial partial 16S rRNA	HE651408	76.52	74.19	1.89314e−12	Ephemeroptera	Heptageniidae	Notonurus josettae
Notonurus josettae mitochondrial partial 16S rRNA	HE651399	76.52	74.19	1.89314e−12	Ephemeroptera	Heptageniidae	Notonurus josettae
Notonemouridae sp. MD−2011 voucher ZMUC:JD−1507 16	JN615257	78.1	67.1	1.89314e−12	Plecoptera	Notonemouridae	Notonemouridae sp. MD−2011
Bittacus pilicornis mitochondrion, complete genome	HQ696578	78.85	65.81	1.89314e−12	Mecoptera	Bittacidae	Bittacus pilicornis
Dolichopoda matsakisi voucher KASA 16S ribosomal R	EF216957	76.07	71.61	1.89314e−12	Orthoptera	Rhaphidophoridae	Dolichopoda matsakisi
