# Supplementary material for: Testing the potential of a ribosomal 16S marker for DNA metabarcoding of insects
Source: PeerJ. 2016 Apr 19;4:e1966. doi: 10.7717/peerj.1966 (PMC4841222; doi:10.7717/peerj.1966)
Supplement: Supplemental Information 1 [file peerj-04-1966-s006.zip › scripts/5 match OTUs/blast_hits/OTU_437.csv_plot.pdf]

OTU_437.csv

Leuctra inermis

Leuctridae

Plecoptera

87.42

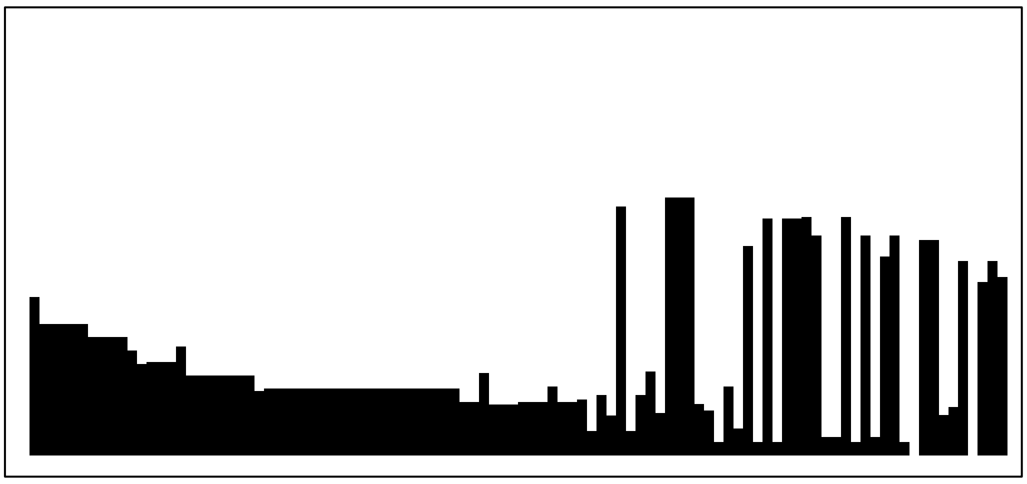

Leuctra inermis 16S ribosomal RNA gene, partial se	EF623176	87.42	100	3.53073e−47	Plecoptera	Leuctridae	Leuctra inermis
Leuctra tenuis haplotype 10lt: HO10 16S ribosomal	EU327682	86.16	100	2.22813e−43	Plecoptera	Leuctridae	Leuctra tenuis
Leuctra tenuis haplotype 9lt: HO9 16S ribosomal RN	EU327681	86.16	100	2.22813e−43	Plecoptera	Leuctridae	Leuctra tenuis
Leuctra tenuis haplotype 3lt: H03 16S ribosomal RN	DQ915184	86.16	100	2.22813e−43	Plecoptera	Leuctridae	Leuctra tenuis
Leuctra tenuis haplotype 2lt: H02 16S ribosomal RN	DQ915183	86.16	100	2.22813e−43	Plecoptera	Leuctridae	Leuctra tenuis
Moselia infuscata 16S ribosomal RNA gene, partial	EF623175	86.16	99.37	9.47426e−42	Plecoptera	Leuctridae	Moselia infuscata
Leuctra tenuis haplotype 8lt: HO8 16S ribosomal RN	EU327680	85.53	100	9.47426e−42	Plecoptera	Leuctridae	Leuctra tenuis
Leuctra tenuis haplotype 7lt: HO7 16S ribosomal RN	EU327679	85.53	100	9.47426e−42	Plecoptera	Leuctridae	Leuctra tenuis
Leuctra tenuis haplotype 6lt: HO6 16S ribosomal RN	EU327678	85.53	100	9.47426e−42	Plecoptera	Leuctridae	Leuctra tenuis
Leuctra tenuis haplotype 1lt: H01 16S ribosomal RN	DQ915182	85.53	100	9.47426e−42	Plecoptera	Leuctridae	Leuctra tenuis
Leuctra tenuis haplotype 5lt: HO5 16S ribosomal RN	EU327677	84.91	100	1.1542e−40	Plecoptera	Leuctridae	Leuctra tenuis
Leuctra tenuis haplotype 4lt: HO4 16S ribosomal RN	EU327676	84.28	100	4.90779e−39	Plecoptera	Leuctridae	Leuctra tenuis
Apteroperla tikumana mitochondrion, complete genom	KR604721	84.38	100	5.97891e−38	Plecoptera	Capniidae	Apteroperla tikumana
Nemurella pictetii 16S ribosomal RNA gene, partial	EF623192	84.38	100	5.97891e−38	Plecoptera	Nemouridae	Nemurella pictetii
Allocapnia minima 16S ribosomal RNA gene, partial	EF623130	84.38	100	5.97891e−38	Plecoptera	Capniidae	Allocapnia minima
Protonemura meyeri 16S ribosomal RNA gene, partial	EF623193	85.09	100	2.08684e−37	Plecoptera	Nemouridae	Protonemura meyeri
Allocapnia recta isolate H15 16S ribosomal RNA gen	KC881050	83.75	100	2.5423e−36	Plecoptera	Capniidae	Allocapnia recta
Allocapnia recta isolate H14 16S ribosomal RNA gen	KC881049	83.75	100	2.5423e−36	Plecoptera	Capniidae	Allocapnia recta
Allocapnia recta isolate H10 16S ribosomal RNA gen	KC881045	83.75	100	2.5423e−36	Plecoptera	Capniidae	Allocapnia recta
Allocapnia recta isolate H6 16S ribosomal RNA gene	KC881041	83.75	100	2.5423e−36	Plecoptera	Capniidae	Allocapnia recta
Ostrocerca foersteri 16S ribosomal RNA gene, parti	EF623191	83.75	100	2.5423e−36	Plecoptera	Nemouridae	Ostrocerca foersteri
Allocapnia recta haplotype 11ar: H011 16S ribosoma	EU306874	83.75	100	2.5423e−36	Plecoptera	Capniidae	Allocapnia recta
Allocapnia recta haplotype 7ar: H07 16S ribosomal	EU306870	83.75	100	2.5423e−36	Plecoptera	Capniidae	Allocapnia recta
Capnura wanica 16S ribosomal RNA gene, partial seq	EF623128	83.02	100	8.87349e−36	Plecoptera	Capniidae	Capnura wanica
Mesocapnia arizonensis mitochondrion, partial geno	KP642637	83.12	100	3.09715e−35	Plecoptera	Capniidae	Mesocapnia arizonensis
Allocapnia recta isolate H19 16S ribosomal RNA gen	KC881054	83.12	100	3.09715e−35	Plecoptera	Capniidae	Allocapnia recta
Allocapnia recta isolate H17 16S ribosomal RNA gen	KC881052	83.12	100	3.09715e−35	Plecoptera	Capniidae	Allocapnia recta
Allocapnia recta isolate H16 16S ribosomal RNA gen	KC881051	83.12	100	3.09715e−35	Plecoptera	Capniidae	Allocapnia recta
Allocapnia recta isolate H13 16S ribosomal RNA gen	KC881048	83.12	100	3.09715e−35	Plecoptera	Capniidae	Allocapnia recta
Allocapnia recta isolate H12 16S ribosomal RNA gen	KC881047	83.12	100	3.09715e−35	Plecoptera	Capniidae	Allocapnia recta
Allocapnia recta isolate H11 16S ribosomal RNA gen	KC881046	83.12	100	3.09715e−35	Plecoptera	Capniidae	Allocapnia recta
Allocapnia recta isolate H9 16S ribosomal RNA gene	KC881044	83.12	100	3.09715e−35	Plecoptera	Capniidae	Allocapnia recta
Allocapnia recta isolate H8 16S ribosomal RNA gene	KC881043	83.12	100	3.09715e−35	Plecoptera	Capniidae	Allocapnia recta
Allocapnia recta isolate H7 16S ribosomal RNA gene	KC881042	83.12	100	3.09715e−35	Plecoptera	Capniidae	Allocapnia recta
Allocapnia recta isolate H5 16S ribosomal RNA gene	KC881040	83.12	100	3.09715e−35	Plecoptera	Capniidae	Allocapnia recta
Allocapnia recta isolate H4 16S ribosomal RNA gene	KC881039	83.12	100	3.09715e−35	Plecoptera	Capniidae	Allocapnia recta
Allocapnia recta isolate H3 16S ribosomal RNA gene	KC881038	83.12	100	3.09715e−35	Plecoptera	Capniidae	Allocapnia recta
Mesocapnia sp. BYU_PL010 16S ribosomal RNA gene, p	EF623127	83.12	100	3.09715e−35	Plecoptera	Capniidae	Mesocapnia sp. BYU_PL010
Allocapnia recta haplotype 10ar: H010 16S ribosoma	EU306873	83.12	100	3.09715e−35	Plecoptera	Capniidae	Allocapnia recta
Allocapnia recta haplotype 9ar: H09 16S ribosomal	EU306872	83.12	100	3.09715e−35	Plecoptera	Capniidae	Allocapnia recta
Allocapnia recta haplotype 8ar: H08 16S ribosomal	EU306871	83.12	100	3.09715e−35	Plecoptera	Capniidae	Allocapnia recta
Allocapnia recta haplotype 3ar: H03 16S ribosomal	DQ915181	83.12	100	3.09715e−35	Plecoptera	Capniidae	Allocapnia recta
Allocapnia recta haplotype 2ar: H02 16S ribosomal	DQ915180	83.12	100	3.09715e−35	Plecoptera	Capniidae	Allocapnia recta
Allocapnia recta haplotype 1ar: H01 16S ribosomal	DQ915179	83.12	100	3.09715e−35	Plecoptera	Capniidae	Allocapnia recta
Doddsia occidentalis 16S ribosomal RNA gene, parti	EF623270	82.5	100	1.08101e−34	Plecoptera	Taeniopterygidae	Doddsia occidentalis
Oemopteryx vanduzeea 16S ribosomal RNA gene, parti	EF623266	82.5	100	1.08101e−34	Plecoptera	Taeniopterygidae	Oemopteryx vanduzeea
Visoka cataractae 16S ribosomal RNA gene, partial	EF623189	83.85	100	1.08101e−34	Plecoptera	Nemouridae	Visoka cataractae
Cascadoperla trictura 16S ribosomal RNA gene, part	EF623225	82.39	100	3.7731e−34	Plecoptera	Perlodidae	Cascadoperla trictura
Soyedina producta 16S ribosomal RNA gene, partial	EF623196	82.39	100	3.7731e−34	Plecoptera	Nemouridae	Soyedina producta
Paraleuctra occidentalis 16S ribosomal RNA gene, p	EF623178	82.39	100	3.7731e−34	Plecoptera	Leuctridae	Paraleuctra occidentalis
Allocapnia recta isolate H18 16S ribosomal RNA gen	KC881053	82.5	100	1.31694e−33	Plecoptera	Capniidae	Allocapnia recta
Allocapnia recta isolate H1 16S ribosomal RNA gene	KC881036	82.5	100	1.31694e−33	Plecoptera	Capniidae	Allocapnia recta
Nemoura trispinosa 16S ribosomal RNA gene, partial	EF623187	82.5	100	1.31694e−33	Plecoptera	Nemouridae	Nemoura trispinosa
Shipsa rotunda 16S ribosomal RNA gene, partial seq	EF623184	83.23	100	1.31694e−33	Plecoptera	Nemouridae	Shipsa rotunda
Isocapnia hyalita 16S ribosomal RNA gene, partial	EF623131	82.5	100	1.31694e−33	Plecoptera	Capniidae	Isocapnia hyalita
Allocapnia recta haplotype 12ar: H12 16S ribosomal	EU306875	82.5	100	1.31694e−33	Plecoptera	Capniidae	Allocapnia recta
Nemoura cinerea 16S ribosomal RNA gene, partial se	EF623194	82.61	100	4.59658e−33	Plecoptera	Nemouridae	Nemoura cinerea
Bolotoperla rossi 16S ribosomal RNA gene, partial	EF623269	81.13	100	1.60436e−32	Plecoptera	Taeniopterygidae	Bolotoperla rossi
Scopura montana 16S ribosomal RNA gene, partial se	EF623265	82.82	100	1.60436e−32	Plecoptera		Scopura montana
Nanonemoura wahkeena 16S ribosomal RNA gene, parti	EF623198	81.88	100	1.60436e−32	Plecoptera	Nemouridae	Nanonemoura wahkeena
Zapada cinctipes 16S ribosomal RNA gene, partial s	EF623183	91.67	67.3	1.60436e−32	Plecoptera	Nemouridae	Zapada cinctipes
Despaxia augusta 16S ribosomal RNA gene, partial s	EF623177	81.13	100	1.60436e−32	Plecoptera	Leuctridae	Despaxia augusta
Paranemoura claasseni 16S ribosomal RNA gene, part	EF623185	82.82	100	1.95452e−32	Plecoptera	Nemouridae	Paranemoura claasseni
Pteronarcys princeps mitochondrion, complete genom	AY687866	83.92	89.31	1.95452e−31	Plecoptera	Pteronarcyidae	Pteronarcys princeps
Malenka californica 16S ribosomal RNA gene, partia	EF623182	81.99	100	6.82193e−31	Plecoptera	Nemouridae	Malenka californica
Suwallia teleckojensis 16S ribosomal RNA gene, par	EF623142	92.08	63.52	6.82193e−31	Plecoptera	Chloroperlidae	Suwallia teleckojensis
Neaviperla forcipata 16S ribosomal RNA gene, parti	EF623139	92.08	63.52	6.82193e−31	Plecoptera	Chloroperlidae	Neaviperla forcipata
Plumiperla diversa 16S ribosomal RNA gene, partial	EF623132	92.08	63.52	6.82193e−31	Plecoptera	Chloroperlidae	Plumiperla diversa
Pteronarcys sachalina 16S ribosomal RNA gene, part	EF623264	82.39	89.31	2.38109e−30	Plecoptera	Pteronarcyidae	Pteronarcys sachalina
Triznaka pintada 16S ribosomal RNA gene, partial s	EF623134	82.1	100	2.38109e−30	Plecoptera	Chloroperlidae	Triznaka pintada
Notonemouridae sp. MD−2011 voucher ZMUC:JD−1507 16	JN615257	80.62	100	8.31081e−30	Plecoptera	Notonemouridae	Notonemouridae sp. MD−2011
Pteronarcys californica 16S ribosomal RNA gene, pa	EF623261	83.22	89.31	8.31081e−30	Plecoptera	Pteronarcyidae	Pteronarcys californica
Perlinodes aurea 16S ribosomal RNA gene, partial s	EF623234	81.25	100	8.31081e−30	Plecoptera	Perlodidae	Perlinodes aurea
Udamocercia sp. BYU_PL150 16S ribosomal RNA gene,	EF623201	89.81	67.3	8.31081e−30	Plecoptera	Notonemouridae	Udamocercia sp. BYU_PL150
Thaumatoperla sp. BYU_PL109 16S ribosomal RNA gene	EF623149	80.62	100	8.31081e−30	Plecoptera	Eustheniidae	Thaumatoperla sp. BYU_PL109
Siphonoperla torrentium 16S ribosomal RNA gene, pa	EF623138	91.09	63.52	8.31081e−30	Plecoptera	Chloroperlidae	Siphonoperla torrentium
Kathroperla perdita 16S ribosomal RNA gene, partia	EF623135	80.62	100	8.31081e−30	Plecoptera	Chloroperlidae	Kathroperla perdita
Sweltsa coloradensis 16S ribosomal RNA gene, parti	EF623133	91.09	63.52	8.31081e−30	Plecoptera	Chloroperlidae	Sweltsa coloradensis
Strophopteryx appalachia 16S ribosomal RNA gene, p	EF623271	91.09	63.52	2.90076e−29	Plecoptera	Taeniopterygidae	Strophopteryx appalachia
Brachyptera seticornis 16S ribosomal RNA gene, par	EF623268	91.18	63.52	2.90076e−29	Plecoptera	Taeniopterygidae	Brachyptera seticornis
Frisonia picticeps 16S ribosomal RNA gene, partial	EF623227	90.29	64.78	2.90076e−29	Plecoptera	Perlodidae	Frisonia picticeps
Austrocerca rieka 16S ribosomal RNA gene, partial	EF623205	80.86	100	2.90076e−29	Plecoptera	Notonemouridae	Austrocerca rieka
Austrocercoides zwicki 16S ribosomal RNA gene, par	EF623199	80.86	100	2.90076e−29	Plecoptera	Notonemouridae	Austrocercoides zwicki
Prostoia besametsa 16S ribosomal RNA gene, partial	EF623188	91.18	63.52	2.90076e−29	Plecoptera	Nemouridae	Prostoia besametsa
Alloperla severa 16S ribosomal RNA gene, partial s	EF623141	80.62	100	2.90076e−29	Plecoptera	Chloroperlidae	Alloperla severa
Osmylus sp. EH−2004 16S ribosomal RNA gene, partia	AY620146	90.29	64.78	2.90076e−29	Neuroptera	Osmylidae	Osmylus sp. EH−2004
Megaleuctra kincaidi 16S ribosomal RNA gene, parti	EF623174	80.86	100	1.01246e−28	Plecoptera	Leuctridae	Megaleuctra kincaidi
Cosumnoperla hypocrena 16S ribosomal RNA gene, par	EF623226	89.32	64.78	3.53385e−28	Plecoptera	Perlodidae	Cosumnoperla hypocrena
Isoperla davisi 16S ribosomal RNA gene, partial se	EF623206	90.29	64.78	3.53385e−28	Plecoptera	Perlodidae	Isoperla davisi
Lednia tumana 16S ribosomal RNA gene, partial sequ	EF623197	80.62	100	3.53385e−28	Plecoptera	Nemouridae	Lednia tumana
Perlomyia utahensis 16S ribosomal RNA gene, partia	EF623179	79.25	100	3.53385e−28	Plecoptera	Leuctridae	Perlomyia utahensis
Sasquaperla hoopa 16S ribosomal RNA gene, partial	EF623143	90.1	63.52	3.53385e−28	Plecoptera	Chloroperlidae	Sasquaperla hoopa
Chloroperla tripunctata 16S ribosomal RNA gene, pa	EF623137	90.1	63.52	3.53385e−28	Plecoptera	Chloroperlidae	Chloroperla tripunctata
Pteronarcys scotti 16S ribosomal RNA gene, partial	EF623263	81.88	93.71	1.23343e−27	Plecoptera	Pteronarcyidae	Pteronarcys scotti
Paraleuctra vershina 16S ribosomal RNA gene, parti	EF623173	82.27	87.42	1.23343e−27	Plecoptera	Leuctridae	Paraleuctra vershina
Limnoperla sp. BYU_PL145 16S ribosomal RNA gene, p	EF623166	89.11	63.52	4.30511e−27	Plecoptera	Gripopterygidae	Limnoperla sp. BYU_PL145
Diamphipnoa virescentipennis 16S ribosomal RNA gen	EF623144	79.25	100	4.30511e−27	Plecoptera	Diamphipnoidae	Diamphipnoa virescentipennis
Taenionema palladium 16S ribosomal RNA gene, parti	EF623267	88.12	63.52	1.50263e−26	Plecoptera	Taeniopterygidae	Taenionema palladium
Pteronarcella badia 16S ribosomal RNA gene, partia	EF623262	89.11	63.52	1.50263e−26	Plecoptera	Pteronarcyidae	Pteronarcella badia
Cerconychia sp. BYU_PL114 16S ribosomal RNA gene,	EF623260	88.35	64.78	1.50263e−26	Plecoptera	Styloperlidae	Cerconychia sp. BYU_PL114
