# Supplementary material for: Testing the potential of a ribosomal 16S marker for DNA metabarcoding of insects
Source: PeerJ. 2016 Apr 19;4:e1966. doi: 10.7717/peerj.1966 (PMC4841222; doi:10.7717/peerj.1966)
Supplement: Supplemental Information 1 [file peerj-04-1966-s006.zip › scripts/5 match OTUs/blast_hits/OTU_44.csv_plot.pdf]

OTU_44.csv

Bolitophila occlusa

Mycetophilidae

Diptera

88.96

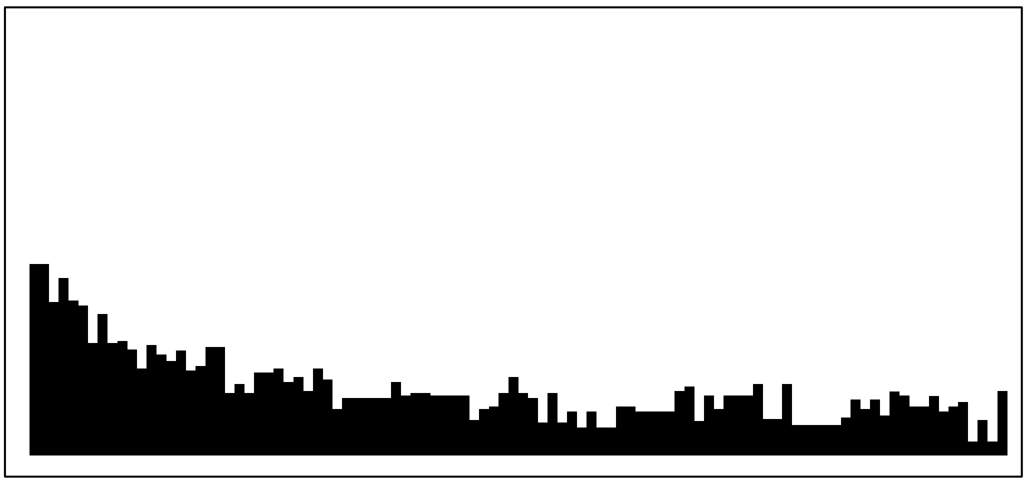

Bolitophila occlusa voucher JSB2 16S ribosomal RNA	KJ136713	88.96	97.44	4.19724e−46	Diptera	Mycetophilidae	Bolitophila occlusa
Bolitophila cinerea voucher JSB1 16S ribosomal RNA	KJ136712	88.96	97.44	4.19724e−46	Diptera	Mycetophilidae	Bolitophila cinerea
Parochlus steinenii isolate Sejong01 mitochondrion	KT003702	87.18	100	1.46498e−45	Diptera	Chironomidae	Parochlus steinenii
Bolitophila sp. ER−2009 16S ribosomal RNA gene, pa	FJ172021	88.31	97.44	5.11329e−45	Diptera	Mycetophilidae	Bolitophila sp. ER−2009
Pedicia albivitta voucher O'Grady 200012 16S ribos	EU005435	87.26	100	5.11329e−45	Diptera	Pediciidae	Pedicia albivitta
Bolitophila hybrida 16S ribosomal RNA gene, partia	FJ172002	87.01	97.44	2.64875e−42	Diptera	Mycetophilidae	Bolitophila hybrida
Tipula abdominalis 16S ribosomal RNA gene, partial	KC177457	85.26	100	3.22684e−41	Diptera	Tipulidae	Tipula abdominalis
Trichopeza longicornis voucher Pollet 0243 (P) 16S	HQ449035	86.62	99.36	3.22684e−41	Diptera	Empididae	Trichopeza longicornis
Tipula abdominalis mitochondrion, partial genome	JN861743	85.26	100	3.22684e−41	Diptera	Tipulidae	Tipula abdominalis
Edwardsina gigantea 16S ribosomal RNA gene, partia	KC177460	85.35	99.36	3.93109e−40	Diptera	Blephariceridae	Edwardsina gigantea
Pyratula zonata 16S ribosomal RNA gene, partial se	FJ172004	84.97	97.44	1.67154e−38	Diptera	Keroplatidae	Pyratula zonata
Tipula sp. PMO 200011 16S ribosomal RNA gene, part	EU005437	84.08	100	5.83426e−38	Diptera	Tipulidae	Tipula sp. PMO 200011
Arachnocampa luminosa biotype 1 16S large subunit	AY576329	85.16	97.44	5.83426e−38	Diptera	Keroplatidae	Arachnocampa luminosa
Empis producta voucher RMBR:103430 16S ribosomal R	FJ808167	84.71	99.36	2.03636e−37	Diptera	Empididae	Empis producta
Urytalpa macrocera 16S ribosomal RNA gene, partial	FJ172025	84.42	97.44	7.10758e−37	Diptera	Keroplatidae	Urytalpa macrocera
Belosta albipilosa 16S ribosomal RNA gene, partial	KM878903	84.91	99.36	2.48079e−36	Diptera	Scenopinidae	Belosta albipilosa
Hercostomus verbekei voucher Pollet 0263 (P) 16S r	EU864004	83.97	99.36	2.48079e−36	Diptera	Dolichopodidae	Hercostomus verbekei
Libnotes orofenaae voucher O'Grady 202034 16S ribo	EU005431	84.18	100	2.48079e−36	Diptera	Limoniidae	Libnotes orofenaae
Coboldia fuscipes voucher JSOUT4 16S ribosomal RNA	KJ136727	85.06	97.44	8.65881e−36	Diptera	Scatopsidae	Coboldia fuscipes
Camptochaeta tenuipalpalis voucher 566 16S ribosom	JQ613915	85.06	97.44	8.65881e−36	Diptera	Sciaridae	Camptochaeta tenuipalpalis
Dicranomyia iniquispina voucher 205222 16S ribosom	KC692800	82.91	100	3.02222e−35	Diptera	Limoniidae	Dicranomyia iniquispina
Gymnopternus aerosus voucher Pollet 0025 (P) 16S r	EU863970	83.33	99.36	3.02222e−35	Diptera	Dolichopodidae	Gymnopternus aerosus
Limonia iniquispina voucher O'Grady 201869 16S rib	EU005407	82.91	100	3.02222e−35	Diptera	Limoniidae	Dicranomyia iniquispina
Arachnocampa luminosa biotype 3 16S large subunit	AY576331	83.87	97.44	3.02222e−35	Diptera	Keroplatidae	Arachnocampa luminosa
Arachnocampa luminosa biotype 2 16S large subunit	AY576330	83.87	97.44	3.02222e−35	Diptera	Keroplatidae	Arachnocampa luminosa
Eupsilocephala albodorsalis 16S ribosomal RNA gene	KM878912	84.08	99.36	1.05486e−34	Diptera	Therevidae	Eupsilocephala albodorsalis
Bolopus furcatus isolate 1305 16S ribosomal RNA ge	KC699315	83.44	99.36	1.05486e−34	Diptera	Platypezidae	Bolopus furcatus
Allocotocera pulchella 16S ribosomal RNA gene, par	FJ172034	83.66	97.44	1.05486e−34	Diptera	Mycetophilidae	Allocotocera pulchella
Neoplatyura flava 16S ribosomal RNA gene, partial	FJ172019	83.01	97.44	1.05486e−34	Diptera	Keroplatidae	Neoplatyura flava
Anabarhynchus tristis voucher MEI 119905 16S ribos	DQ250834	84.08	99.36	1.05486e−34	Diptera	Therevidae	Anabarhynchus tristis
Simulium asakoae isolate A1B 16S ribosomal RNA gen	KM410198	83.54	100	3.68182e−34	Diptera	Simuliidae	Simulium asakoae
Libnotes perkinsi voucher O'Grady 202000 16S ribos	EU005434	82.17	100	3.68182e−34	Diptera	Limoniidae	Libnotes perkinsi
Poecilobothrus nobilitatus voucher Pollet 0130 (P)	EU864008	82.69	99.36	1.28508e−33	Diptera	Dolichopodidae	Poecilobothrus nobilitatus
Hercostomus nigriplantis voucher Pollet 0210 (P) 1	EU863998	82.69	99.36	1.28508e−33	Diptera	Dolichopodidae	Hercostomus nigriplantis
Gymnopternus blankaartensis voucher Pollet 0090 (P	EU863973	82.69	99.36	1.28508e−33	Diptera	Dolichopodidae	Gymnopternus blankaartensis
Gymnopternus angustifrons voucher Pollet 0052 (P)	EU863971	82.69	99.36	1.28508e−33	Diptera	Dolichopodidae	Gymnopternus angustifrons
Dolichopus sp. HYH−2002 16S ribosomal RNA gene, pa	AY123343	82.69	99.36	1.28508e−33	Diptera	Dolichopodidae	Dolichopus sp. HYH−2002
Callomyia amoena isolate CA06 16S ribosomal RNA ge	KC699325	83.44	94.87	4.48537e−33	Diptera	Platypezidae	Callomyia amoena
Cramptonomyia spenceri mitochondrion, complete gen	JN861747	82.8	99.36	4.48537e−33	Diptera	Pachyneuridae	Cramptonomyia spenceri
Poecilobothrus regalis voucher Pollet 0292 (P) 16S	EU864011	82.91	99.36	4.48537e−33	Diptera	Dolichopodidae	Poecilobothrus regalis
Poecilobothrus principalis voucher Pollet 0198 (P)	EU864010	82.91	99.36	4.48537e−33	Diptera	Dolichopodidae	Poecilobothrus principalis
Gymnopternus cupreus voucher Pollet 0021 (P) 16S r	EU863984	82.8	99.36	4.48537e−33	Diptera	Dolichopodidae	Gymnopternus cupreus
Gymnopternus celer voucher Pollet 0018 (P) 16S rib	EU863977	82.8	99.36	4.48537e−33	Diptera	Dolichopodidae	Gymnopternus celer
Gymnopternus celer voucher Pollet 0051 (P) 16S rib	EU863978	82.8	99.36	4.48537e−33	Diptera	Dolichopodidae	Gymnopternus celer
Gymnopternus brevicornis voucher Pollet 0036 (P) 1	EU863974	82.8	99.36	4.48537e−33	Diptera	Dolichopodidae	Gymnopternus brevicornis
Limonia hawaiiensis voucher O'Grady 200106 16S rib	EU005403	81.65	100	4.48537e−33	Diptera	Limoniidae	Dicranomyia hawaiiensis
Somatia sp. YSUW04038 16S ribosomal RNA gene, part	AY573134	82.17	99.36	4.48537e−33	Diptera	Somatiidae	Somatia sp. YSUW04038
Evocoa chilensis 16S ribosomal RNA gene, partial s	KM878914	82.28	99.36	1.56555e−32	Diptera	Evocoidae	Evocoa chilensis
Lindneromyia sp. CAN4 16S ribosomal RNA gene, part	KC699330	82.91	99.36	1.56555e−32	Diptera	Platypezidae	Lindneromyia sp. CAN4
Sternhydrus atratus voucher UNM KBMStat165 16S rib	KJ548361	83.66	96.15	1.56555e−32	Coleoptera	Dytiscidae	Sternhydrus atratus
Simulium capillatum mitochondrial gene for 16S rib	AB862986	82.91	100	1.56555e−32	Diptera	Simuliidae	Simulium capillatum
Thaumatomyia notata 16S ribosomal RNA gene, partia	KC177449	82.69	97.44	1.56555e−32	Diptera	Chloropidae	Thaumatomyia notata
Ptychoptera sp. ATB−2011 mitochondrion, complete g	JN861744	81.53	100	1.56555e−32	Diptera	Ptychopteridae	Ptychoptera sp. ATB−2011
Simulium metatarsale mitochondrial gene for 16S ri	AB591242	82.91	100	1.56555e−32	Diptera	Simuliidae	Simulium metatarsale
Licinus punctatulus granulatus 16S ribosomal RNA (	JF778802	81.53	100	1.56555e−32	Coleoptera	Carabidae	Licinus punctatulus granulatus
Poecilobothrus chrysozygos voucher Pollet 0184 (P)	EU864007	82.05	99.36	1.56555e−32	Diptera	Dolichopodidae	Poecilobothrus chrysozygos
Hercostomus vockerothi voucher Pollet 0295 (P) 16S	EU864006	81.29	99.36	1.56555e−32	Diptera	Dolichopodidae	Hercostomus vockerothi
Hercostomus longiventris voucher Pollet 0274 (P) 1	EU863994	82.05	99.36	1.56555e−32	Diptera	Dolichopodidae	Hercostomus longiventris
Hercostomus germanus voucher Pollet 0094 (P) 16S r	EU863993	81.29	99.36	1.56555e−32	Diptera	Dolichopodidae	Hercostomus germanus
Hercostomus chaerophylli voucher Pollet 0105 (P) 1	EU863989	81.29	99.36	1.56555e−32	Diptera	Dolichopodidae	Hercostomus chaerophylli
Gymnopternus helveticus voucher Pollet 0291 (P) 16	EU863986	82.28	99.36	1.56555e−32	Diptera	Dolichopodidae	Gymnopternus helveticus
Gymnopternus helveticus voucher Pollet 0290 (P) 16	EU863985	82.28	99.36	1.56555e−32	Diptera	Dolichopodidae	Gymnopternus helveticus
Gymnopternus assimilis voucher Pollet 0088 (P) 16S	EU863972	82.05	99.36	1.56555e−32	Diptera	Dolichopodidae	Gymnopternus assimilis
Dolichopus sabinus voucher Pollet 0117 (P) 16S rib	EU863944	82.05	99.36	1.56555e−32	Diptera	Dolichopodidae	Dolichopus sabinus
Dolichopus picipes voucher Pollet 0065 (P) 16S rib	EU863928	82.05	99.36	1.56555e−32	Diptera	Dolichopodidae	Dolichopus picipes
Dolichopus lepidus voucher Pollet 0048 (P) 16S rib	EU863917	82.05	99.36	1.56555e−32	Diptera	Dolichopodidae	Dolichopus lepidus
Voria ruralis mitochondrial gene for 16S rRNA, par	AB465959	83.02	100	1.56555e−32	Diptera	Tachinidae	Voria ruralis
Clusiodes ruficollis 16S ribosomal RNA gene, parti	EU268538	83.23	97.44	1.56555e−32	Diptera	Clusiidae	Clusiodes ruficollis
Derodontus emaculatus voucher BMNH:679374 16S ribo	DQ202609	81.6	100	1.56555e−32	Coleoptera		Derodontus emaculatus
Agapophytus bicolor 16S ribosomal RNA gene, partia	KM878897	82.8	99.36	5.4643e−32	Diptera	Therevidae	Agapophytus bicolor
Microsania collarti isolate CAN8 16S ribosomal RNA	KC699334	82.17	99.36	5.4643e−32	Diptera	Platypezidae	Microsania collarti
Ectinorhynchus sp. 'Bribie' voucher UQIC41178 16S	KM212983	82.8	99.36	5.4643e−32	Diptera	Therevidae	Ectinorhynchus sp. 'Bribie'
Ectinorhynchus sp. 'Moffatt' voucher QMT183203 16S	KM212976	82.8	99.36	5.4643e−32	Diptera	Therevidae	Ectinorhynchus sp. 'Moffatt'
Ectinorhynchus sp. 'warrumbungles' voucher QMT1832	KM212974	82.8	99.36	5.4643e−32	Diptera	Therevidae	Ectinorhynchus sp. 'warrumbungles'
Phytosciara flavipes voucher JSOUT8 16S ribosomal	KJ136731	83.33	97.44	5.4643e−32	Diptera	Sciaridae	Phytosciara flavipes
Eudicrana sp. DK−2013 16S ribosomal RNA gene, part	KC435571	81.7	97.44	5.4643e−32	Diptera	Mycetophilidae	Eudicrana sp. DK−2013
Zygoneura bidens voucher 312 16S ribosomal RNA gen	JQ613910	81.7	97.44	5.4643e−32	Diptera	Sciaridae	Zygoneura bidens
Phytosciara flavipes voucher 88 16S ribosomal RNA	JQ613889	83.33	97.44	5.4643e−32	Diptera	Sciaridae	Phytosciara flavipes
Nebria bellorum voucher DHK0509 16S ribosomal RNA	JN847627	81.41	99.36	5.4643e−32	Coleoptera	Carabidae	Nebria bellorum
Nebria lacustris voucher DHK0004 16S ribosomal RNA	JN847615	81.41	99.36	5.4643e−32	Coleoptera	Carabidae	Nebria lacustris
Nebria hudsonica voucher DHK0381a 16S ribosomal RN	JN847614	81.41	99.36	5.4643e−32	Coleoptera	Carabidae	Nebria hudsonica
Nebria gouleti voucher DHK0006 16S ribosomal RNA g	JN847612	81.41	99.36	5.4643e−32	Coleoptera	Carabidae	Nebria gouleti
Nebria gyllenhali voucher DHK0010 16S ribosomal RN	JN847607	81.41	99.36	5.4643e−32	Coleoptera	Carabidae	Nebria gyllenhali
Zariquieya troglodytes partial mitochondrial 16S r	FR846366	81.76	100	5.4643e−32	Coleoptera	Carabidae	Zariquieya troglodytes
Hybos femoratus voucher Pollet 0247 (P) 16S riboso	HQ449036	82.61	99.36	5.4643e−32	Diptera	Hybotidae	Hybos femoratus
Gymnopternus celer voucher Pollet 0268 (P) 16S rib	EU863982	82.17	99.36	5.4643e−32	Diptera	Dolichopodidae	Gymnopternus celer
Senometopia cariniforceps mitochondrial gene for 1	AB465923	82.61	99.36	5.4643e−32	Diptera	Tachinidae	Senometopia cariniforceps
Blondelia nigripes mitochondrial gene for 16S rRNA	AB465902	81.88	99.36	5.4643e−32	Diptera	Tachinidae	Blondelia nigripes
Phthinia humilis 16S ribosomal RNA gene, partial s	FJ172009	82.99	92.95	5.4643e−32	Diptera	Mycetophilidae	Phthinia humilis
Dialysis sp. SLW−2002 16S ribosomal RNA gene, part	AY140888	82.8	99.36	5.4643e−32	Diptera	Xylophagidae	Dialysis sp. SLW−2002
Lindneromyia argentifascia isolate CAN5 16S riboso	KC699331	82.28	99.36	1.90723e−31	Diptera	Platypezidae	Lindneromyia argentifascia
Lindneromyia denticulata isolate CAN1 16S ribosoma	KC699327	82.28	99.36	1.90723e−31	Diptera	Platypezidae	Lindneromyia denticulata
Callomyia admirabilis isolate CA42 16S ribosomal R	KC699326	82.78	94.87	1.90723e−31	Diptera	Platypezidae	Callomyia admirabilis
Lispe nana voucher RMBR:116206 16S ribosomal RNA g	KJ476314	82.05	99.36	1.90723e−31	Diptera	Muscidae	Lispe nana
Achalcus flavicollis voucher Pollet 0171 (P) 16S r	HQ448956	82.28	99.36	1.90723e−31	Diptera	Dolichopodidae	Achalcus flavicollis
Paracladura trichoptera mitochondrion, complete ge	JN861751	82.5	100	1.90723e−31	Diptera	Trichoceridae	Paracladura trichoptera
Hercostomus nigripennis voucher Pollet 0059 (P) 16	EU863997	80.65	99.36	1.90723e−31	Diptera	Dolichopodidae	Hercostomus nigripennis
Gymnopternus metallicus voucher Pollet 0030 (P) 16	EU863987	81.65	99.36	1.90723e−31	Diptera	Dolichopodidae	Gymnopternus metallicus
Dolichopus ungulatus voucher Pollet 0273 (P) 16S r	EU863960	80.65	99.36	1.90723e−31	Diptera	Dolichopodidae	Dolichopus ungulatus
Simulium duolongum 16S ribosomal RNA gene, partial	GQ865988	83.01	97.44	1.90723e−31	Diptera	Simuliidae	Simulium duolongum
