# Supplementary material for: Testing the potential of a ribosomal 16S marker for DNA metabarcoding of insects
Source: PeerJ. 2016 Apr 19;4:e1966. doi: 10.7717/peerj.1966 (PMC4841222; doi:10.7717/peerj.1966)
Supplement: Supplemental Information 1 [file peerj-04-1966-s006.zip › scripts/5 match OTUs/blast_hits/OTU_443.csv_plot.pdf]

OTU_443.csv

Pedicia albivitta

Pediciidae

Diptera

88.68

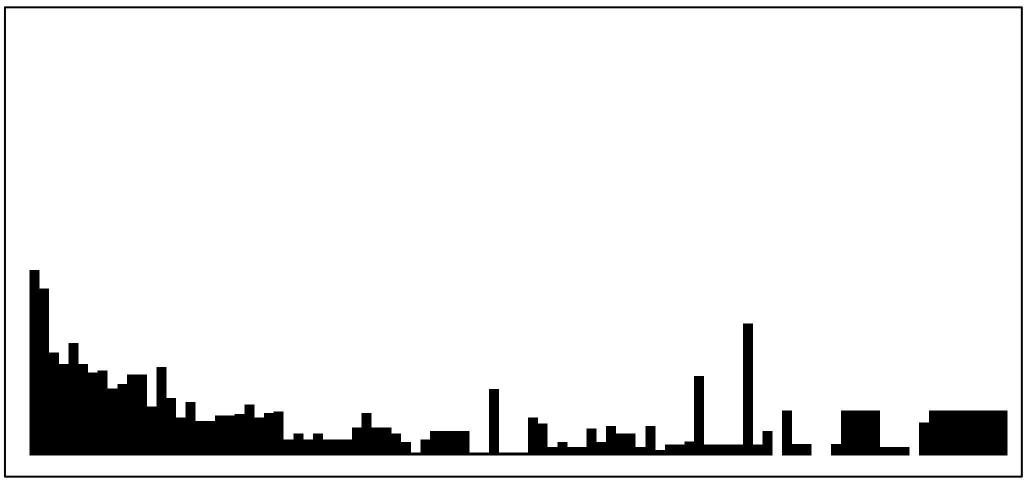

Pedicia albivitta voucher O'Grady 200012 16S ribos	EU005435	88.68	100	8.30347e−49	Diptera	Pediciidae	Pedicia albivitta
Bolitophila cinerea voucher JSB1 16S ribosomal RNA	KJ136712	87.82	97.48	5.24006e−45	Diptera	Mycetophilidae	Bolitophila cinerea
Edwardsina gigantea 16S ribosomal RNA gene, partia	KC177460	84.81	99.37	1.1542e−40	Diptera	Blephariceridae	Edwardsina gigantea
Parochlus steinenii isolate Sejong01 mitochondrion	KT003702	84.28	100	1.4061e−39	Diptera	Chironomidae	Parochlus steinenii
Bolitophila sp. ER−2009 16S ribosomal RNA gene, pa	FJ172021	85.26	97.48	1.4061e−39	Diptera	Mycetophilidae	Bolitophila sp. ER−2009
Tipula sp. PMO 200011 16S ribosomal RNA gene, part	EU005437	84.28	100	1.4061e−39	Diptera	Tipulidae	Tipula sp. PMO 200011
Corynoptera blanda voucher 955 16S ribosomal RNA g	JQ613965	83.87	97.48	2.08684e−37	Diptera	Sciaridae	Corynoptera blanda
Bolitophila hybrida 16S ribosomal RNA gene, partia	FJ172002	83.97	97.48	7.2838e−37	Diptera	Mycetophilidae	Bolitophila hybrida
Derodontus emaculatus voucher BMNH:679374 16S ribo	DQ202609	83.12	100	2.5423e−36	Coleoptera		Derodontus emaculatus
Phytosciara flavipes voucher JSOUT8 16S ribosomal	KJ136731	83.33	97.48	3.09715e−35	Diptera	Sciaridae	Phytosciara flavipes
Tipula abdominalis 16S ribosomal RNA gene, partial	KC177457	83.78	91.19	3.09715e−35	Diptera	Tipulidae	Tipula abdominalis
Tipula abdominalis mitochondrion, partial genome	JN861743	83.78	91.19	3.09715e−35	Diptera	Tipulidae	Tipula abdominalis
Minettia longipennis mitochondrion partial 16S rRN	AJ439184	82.28	99.37	3.09715e−35	Diptera	Lauxaniidae	Minettia longipennis
Eudicrana sp. DK−2013 voucher JSS12 16S ribosomal	KC435571	84.14	89.94	1.08101e−34	Diptera	Mycetophilidae	Eudicrana sp. DK−2013
Bolitophila occlusa voucher JSB2 16S ribosomal RNA	KJ136713	82.69	97.48	3.7731e−34	Diptera	Mycetophilidae	Bolitophila occlusa
Libnotes orofenae voucher O'Grady 202034 16S ribo	EU005431	81.76	100	3.7731e−34	Diptera	Limoniidae	Libnotes orofenae
Ptychoptera sp. ATB−2011 mitochondrion, complete g	JN861744	82.5	100	1.31694e−33	Diptera	Ptychopteridae	Ptychoptera sp. ATB−2011
Calosoma sp. BYU−CO241 mitochondrion, complete gen	GU176340	81.6	100	1.31694e−33	Coleoptera	Carabidae	Calosoma sp. BYU−CO241
Calosoma scrutator 16S ribosomal RNA gene, partial	EU734850	81.6	100	1.31694e−33	Coleoptera	Carabidae	Calosoma scrutator
Libnotes perkinsi voucher O'Grady 200981 16S ribos	EU005433	81.88	100	1.31694e−33	Diptera	Limoniidae	Libnotes perkinsi
Libnotes perkinsi voucher O'Grady 200947 16S ribos	EU005432	81.88	100	1.31694e−33	Diptera	Limoniidae	Libnotes perkinsi
Lycoriella flavicornis voucher 62 16S ribosomal RN	JQ613885	81.94	97.48	4.59658e−33	Diptera	Sciaridae	Lycoriella flavicornis
Cramptonomyia spenceri mitochondrion, complete gen	JN861747	82.39	99.37	4.59658e−33	Diptera	Pachyneuridae	Cramptonomyia spenceri
Gonocephalum pusillum isolate CSU0904046030 16S ri	GQ118381	81.76	99.37	4.59658e−33	Coleoptera	Tenebrionidae	Gonocephalum pusillum
Limonia tahitiensis voucher O'Grady 202004 16S rib	EU005425	81.99	100	4.59658e−33	Diptera	Limoniidae	Dicranomyia tahitiensis
Phytosciara flavipes voucher 88 16S ribosomal RNA	JQ613889	82.05	97.48	1.60436e−32	Diptera	Sciaridae	Phytosciara flavipes
Chrysoperla nipponensis mitochondrial DNA, complet	AP011623	80.75	99.37	1.60436e−32	Neuroptera	Chrysopidae	Chrysoperla nipponensis
Drosophila funebris voucher 103952 NADH dehydrogen	EU494313	81.01	99.37	1.60436e−32	Diptera	Drosophilidae	Drosophila funebris
Mallada luctuosus 16S ribosomal RNA gene, partial	DQ399288	80.75	99.37	1.60436e−32	Neuroptera	Chrysopidae	Pseudomallada luctuosus
Drosophila takahashii cytochrome b (cytb) gene, pa	AF164592	81.01	99.37	1.60436e−32	Diptera	Drosophilidae	Drosophila takahashii
Chrysoperla agilis isolate A−083 16S large subunit	AY743751	80.75	99.37	1.60436e−32	Neuroptera	Chrysopidae	Chrysoperla agilis
Chrysoperla lucasina isolate L−029 16S large subun	AY743737	80.75	99.37	1.60436e−32	Neuroptera	Chrysopidae	Chrysoperla lucasina
Chrysoperla lucasina isolate L−099 16S large subun	AY743732	80.75	99.37	1.60436e−32	Neuroptera	Chrysopidae	Chrysoperla lucasina
Lycoriella gerbatshevskayae voucher 601 16S riboso	JQ613919	81.29	97.48	5.59978e−32	Diptera	Sciaridae	Lycoriella gerbatshevskayae
Cychrus caraboides caraboides voucher CC00817 16S	JQ647184	81.99	100	5.59978e−32	Coleoptera	Carabidae	Cychrus caraboides caraboides
Grzegorzekia collaris 16S ribosomal RNA gene, part	FJ172016	81.29	97.48	5.59978e−32	Diptera	Mycetophilidae	Grzegorzekia collaris
Pyratula zonata 16S ribosomal RNA gene, partial se	FJ172004	81.29	97.48	5.59978e−32	Diptera	Keroplatidae	Pyratula zonata
Amphizoa lecontei 16S ribosomal RNA gene, partial	AY071771	81.01	99.37	5.59978e−32	Coleoptera	Amphizoidae	Amphizoa lecontei
Chrysoperla lucasina isolate L−070 16S large subun	AY743744	80.62	98.74	5.59978e−32	Neuroptera	Chrysopidae	Chrysoperla lucasina
Chrysoperla nipponensis mitochondrion, complete ge	KP027407	80.12	99.37	1.95452e−31	Neuroptera	Chrysopidae	Chrysoperla nipponensis
Sarcophagidae sp. BAHAR107−14 voucher BAH_201312_P	KP253964	80.75	99.37	1.95452e−31	Diptera	Sarcophagidae	Sarcophagidae sp. BAHAR107−14
Pseudomigadops n. sp. MSL2007 voucher 668381 16S r	EF214073	81.13	99.37	1.95452e−31	Coleoptera	Carabidae	Pseudomigadops n. sp. MSL2007
Pseudomigadops n. sp. MSL2007 voucher 668379 16S r	EF214072	81.13	99.37	1.95452e−31	Coleoptera	Carabidae	Pseudomigadops n. sp. MSL2007
Pseudomigadops n. sp. MSL2007 voucher 668300 16S r	EF214010	81.13	99.37	1.95452e−31	Coleoptera	Carabidae	Pseudomigadops n. sp. MSL2007
Pseudomigadops sp. PSM1 mitochondrial 16S rRNA gen	FM994738	81.13	99.37	1.95452e−31	Coleoptera	Carabidae	Pseudomigadops sp. PSM1
Chrysoperla carnea isolate Chrcar2 16S ribosomal R	AY620152	80.12	99.37	1.95452e−31	Neuroptera	Chrysopidae	Chrysoperla carnea
Chrysoperla carnea isolate Chrcar3 16S ribosomal R	AY620150	80.12	99.37	1.95452e−31	Neuroptera	Chrysopidae	Chrysoperla carnea
Arachnocampa luminosa biotype 1 16S large subunit	AY576329	83.1	89.31	1.95452e−31	Diptera	Keroplatidae	Arachnocampa luminosa
Chrysoperla sp. isolate 82 16S large subunit ribos	AY743750	80.12	99.37	1.95452e−31	Neuroptera	Chrysopidae	Chrysoperla sp. isolate 82
Chrysoperla lucasina isolate L−079 16S large subun	AY743746	80.12	99.37	1.95452e−31	Neuroptera	Chrysopidae	Chrysoperla lucasina
Chrysoperla carnea isolate C−097 16S large subunit	AY743731	80.12	99.37	1.95452e−31	Neuroptera	Chrysopidae	Chrysoperla carnea
Acilius athabascae voucher 305G09/BMNH799009 16S r	KF979079	81.76	99.37	6.82193e−31	Coleoptera	Dytiscidae	Acilius athabascae
Bradysiopsis vittigera voucher 948 16S ribosomal R	JQ613962	81.48	97.48	6.82193e−31	Diptera	Sciaridae	Bradysiopsis vittigera
Minettia flaveola 16S ribosomal RNA gene, partial	KC177448	80.38	99.37	6.82193e−31	Diptera	Lauxaniidae	Minettia flaveola
Lispe tentaculata 16S ribosomal RNA gene, partial	FJ025450	80.62	99.37	6.82193e−31	Diptera	Muscidae	Lispe tentaculata
Drosophila tripunctata voucher 107541 NADH dehydro	EU494351	80.38	99.37	6.82193e−31	Diptera	Drosophilidae	Drosophila tripunctata
Drosophila putrida voucher 103964 NADH dehydrogena	EU494348	80.38	99.37	6.82193e−31	Diptera	Drosophilidae	Drosophila putrida
Thaumatoperla sp. BYU_PL109 16S ribosomal RNA gene	EF623149	81.25	100	6.82193e−31	Plecoptera	Eustheniidae	Thaumatoperla sp. BYU_PL109
Libnotes perkinsi voucher O'Grady 202000 16S ribos	EU005434	80.62	100	6.82193e−31	Diptera	Limoniidae	Libnotes perkinsi
Panorpa takenouchii isolate 26tak 16S ribosomal RN	EF041520	81.37	100	6.82193e−31	Mecoptera	Panorpidae	Panorpa takenouchii
Leucochrysa marquezi voucher CASC 00212 16S riboso	DQ399280	81.01	99.37	6.82193e−31	Neuroptera	Chrysopidae	Leucochrysa marquezi
Leucochrysa lancala voucher CASC 00211 16S ribosom	DQ399277	81.01	99.37	6.82193e−31	Neuroptera	Chrysopidae	Leucochrysa lancala
Drosophila tripunctata 16S ribosomal RNA gene, par	AF479806	80.38	99.37	6.82193e−31	Diptera	Drosophilidae	Drosophila tripunctata
Panorpa takenouchii 16S ribosomal RNA gene, partia	AF180064	81.37	100	6.82193e−31	Mecoptera	Panorpidae	Panorpa takenouchii
Chrysoperla lucasina isolate L−078 16S large subun	AY743776	80.25	99.37	6.82193e−31	Neuroptera	Chrysopidae	Chrysoperla lucasina
Polyporivora picta isolate PP45m 16S ribosomal RNA	KC699353	80.5	99.37	2.38109e−30	Diptera	Platypezidae	Polyporivora picta
Polyporivora picta isolate PP45f 16S ribosomal RNA	KC699352	80.5	99.37	2.38109e−30	Diptera	Platypezidae	Polyporivora picta
Zygoneura sciarina voucher 311 16S ribosomal RNA g	JQ613909	80.65	97.48	2.38109e−30	Diptera	Sciaridae	Zygoneura sciarina
Eudmeta marginata 16S ribosomal RNA gene, partial	GU947400	83.7	84.91	2.38109e−30	Diptera	Stratiomyidae	Eudmeta marginata
Pseudomigadops n. sp. MSL2007 voucher 668338 16S r	EF214034	80.5	99.37	2.38109e−30	Coleoptera	Carabidae	Pseudomigadops n. sp. MSL2007
Pseudomigadops n. sp. MSL2007 voucher 668317 16S r	EF214022	80.5	99.37	2.38109e−30	Coleoptera	Carabidae	Pseudomigadops n. sp. MSL2007
Pseudomigadops n. sp. MSL2007 voucher 668302 16S r	EF214012	80.5	99.37	2.38109e−30	Coleoptera	Carabidae	Pseudomigadops n. sp. MSL2007
Pseudomigadops n. sp. MSL2007 voucher 668298 16S r	EF214009	80.5	99.37	2.38109e−30	Coleoptera	Carabidae	Pseudomigadops n. sp. MSL2007
Leptomorphus walkeri 16S ribosomal RNA gene, parti	FJ172020	86.18	76.73	2.38109e−30	Diptera	Mycetophilidae	Leptomorphus walkeri
Pseudomigadops n. sp. MSL2007 voucher 668369 16S r	EF214062	80.5	99.37	2.38109e−30	Coleoptera	Carabidae	Pseudomigadops n. sp. MSL2007
Ochlerotatus nipponicus isolate A75 16S ribosomal	DQ397914	81.13	99.37	2.38109e−30	Diptera	Culicidae	Aedes nipponicus
Polyporivora ornata isolate PO25 16S ribosomal RNA	KC699351	79.75	99.37	8.31081e−30	Diptera	Platypezidae	Polyporivora ornata
Wuchereria bancrofti genome assembly W_bancrofti_J	LM000940	82.1	99.37	8.31081e−30	Spirurida	Onchocercidae	Wuchereria bancrofti
Rhantaticus congestus voucher NHRS/JLK000000091 16	KF979103	80.52	96.23	8.31081e−30	Coleoptera	Dytiscidae	Rhantaticus congestus
Rhantaticus congestus voucher 412B12_BMNH832121 16	KF979100	80.52	96.23	8.31081e−30	Coleoptera	Dytiscidae	Rhantaticus congestus
Colobaea bifasciella voucher MU_NUS_SG3 16S riboso	JN828202	79.75	99.37	8.31081e−30	Diptera	Sciomyzidae	Colobaea bifasciella
Hybos femoratus voucher Pollet 0247 (P) 16S riboso	HQ449036	80	99.37	8.31081e−30	Diptera	Hybotidae	Hybos femoratus
Rhantaticus congestus voucher BMNH829999 16S ribos	HQ381497	80.52	96.23	8.31081e−30	Coleoptera	Dytiscidae	Rhantaticus congestus
Culex quinquefasciatus from USA mitochondrion, com	HQ724617	82.1	99.37	8.31081e−30	Diptera	Culicidae	Culex quinquefasciatus
Culex pipiens pipiens from Turkey mitochondrion, c	HQ724616	82.1	99.37	8.31081e−30	Diptera	Culicidae	Culex pipiens pipiens
Culex pipiens pipiens from Tunisia mitochondrion,	HQ724614	82.1	99.37	8.31081e−30	Diptera	Culicidae	Culex pipiens pipiens
Culex quinquefasciatus mitochondrion, complete gen	GU188856	82.1	99.37	8.31081e−30	Diptera	Culicidae	Culex quinquefasciatus
Hercostomus praeceps voucher Pollet 0141 (P) 16S r	EU864003	80.38	99.37	8.31081e−30	Diptera	Dolichopodidae	Hercostomus praeceps
Hercostomus longiventris voucher Pollet 0274 (P) 1	EU863994	80.38	99.37	8.31081e−30	Diptera	Dolichopodidae	Hercostomus longiventris
Hercostomus fulvicaudis voucher Pollet 0154 (P) 16	EU863992	80.38	99.37	8.31081e−30	Diptera	Dolichopodidae	Hercostomus fulvicaudis
Senometopia cariniforceps mitochondrial gene for 1	AB465923	80	99.37	8.31081e−30	Diptera	Tachinidae	Senometopia cariniforceps
Phthinia humilis 16S ribosomal RNA gene, partial s	FJ172009	81.53	97.48	8.31081e−30	Diptera	Mycetophilidae	Phthinia humilis
Culex quinquefasciatus isolate A−373 16S ribosomal	EU711101	82.1	99.37	8.31081e−30	Diptera	Culicidae	Culex quinquefasciatus
Culex quinquefasciatus isolate A−365 16S ribosomal	EU711099	82.1	99.37	8.31081e−30	Diptera	Culicidae	Culex quinquefasciatus
Culex quinquefasciatus isolate A−274 16S ribosomal	EU711098	82.1	99.37	8.31081e−30	Diptera	Culicidae	Culex quinquefasciatus
Culex quinquefasciatus isolate A−266 16S ribosomal	EU711095	82.1	99.37	8.31081e−30	Diptera	Culicidae	Culex quinquefasciatus
Culex quinquefasciatus isolate A−263 16S ribosomal	EU711094	82.1	99.37	8.31081e−30	Diptera	Culicidae	Culex quinquefasciatus
Culex quinquefasciatus isolate A−128 16S ribosomal	EU711088	82.1	99.37	8.31081e−30	Diptera	Culicidae	Culex quinquefasciatus
Culex quinquefasciatus isolate A−173 16S ribosomal	EU711092	82.1	99.37	8.31081e−30	Diptera	Culicidae	Culex quinquefasciatus
Culex quinquefasciatus isolate A−169 16S ribosomal	EU711091	82.1	99.37	8.31081e−30	Diptera	Culicidae	Culex quinquefasciatus
