# Supplementary material for: Testing the potential of a ribosomal 16S marker for DNA metabarcoding of insects
Source: PeerJ. 2016 Apr 19;4:e1966. doi: 10.7717/peerj.1966 (PMC4841222; doi:10.7717/peerj.1966)
Supplement: Supplemental Information 1 [file peerj-04-1966-s006.zip › scripts/5 match OTUs/blast_hits/OTU_444.csv_plot.pdf]

OTU_444.csv

Oreodytes sanmarkii

Dytiscidae

Coleoptera

98.09

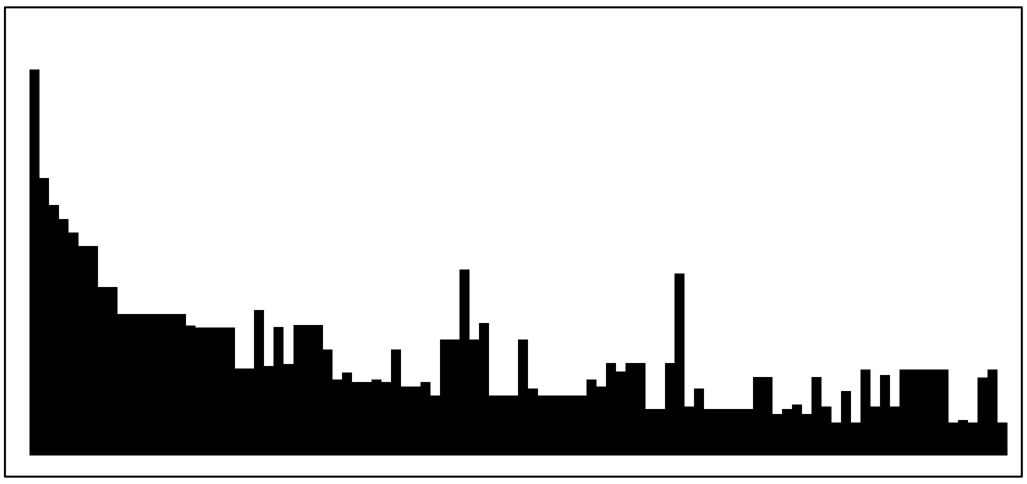

Oreodytes sanmarkii mitochondrial 16S rRNA gene (p	HF931524	98.09	100	8.09543e−68	Coleoptera	Dytiscidae	Oreodytes sanmarkii
Oreodytes obesus cordillerensis mitochondrial part	HF931516	92.99	100	5.82911e−57	Coleoptera	Dytiscidae	Oreodytes obesus cordillerensis
Oreodytes crassulus partial 16S rRNA gene	AJ850348	91.72	100	3.01955e−54	Coleoptera	Dytiscidae	Oreodytes crassulus
Oreodytes congruus partial 16S rRNA gene	AJ850347	91.08	100	3.67857e−53	Coleoptera	Dytiscidae	Oreodytes congruus
Oreodytes congruus 16S ribosomal RNA gene, partial	KF575550	90.45	100	1.56417e−51	Coleoptera	Dytiscidae	Oreodytes congruus
Oreodytes abbreviatus mitochondrial 16S rRNA gene	HF931477	89.81	100	1.90555e−50	Coleoptera	Dytiscidae	Oreodytes abbreviatus
Oreodytes rhyacophilus partial 16S rRNA gene	AJ850352	89.81	100	1.90555e−50	Coleoptera	Dytiscidae	Oreodytes rhyacophilus
Oreodytes snoqualmie mitochondrial partial 16S rRN	HF931519	87.9	100	4.19724e−46	Coleoptera	Dytiscidae	Oreodytes snoqualmie
Oreodytes mongolicus mitochondrial 16S rRNA gene (	HF931491	87.9	100	4.19724e−46	Coleoptera	Dytiscidae	Oreodytes mongolicus
Oreodytes septentrionalis mitochondrial 16S rRNA g	HF931492	86.62	100	2.17423e−43	Coleoptera	Dytiscidae	Oreodytes septentrionalis
Oreodytes shorti mitochondrial 16S rRNA gene (part	HF931488	86.62	100	2.17423e−43	Coleoptera	Dytiscidae	Oreodytes shorti
Deronectes parvicollis mitochondrial 16S rRNA gene	HF931454	86.62	100	2.17423e−43	Coleoptera	Dytiscidae	Deronectes parvicollis
Boreonectes striatellus mitochondrial 16S rRNA gen	HF931511	86.62	100	2.64875e−42	Coleoptera	Dytiscidae	Boreonectes striatellus
Boreonectes riberae mitochondrial 16S rRNA gene (p	HF931461	86.62	100	2.64875e−42	Coleoptera	Dytiscidae	Boreonectes riberae
Boreonectes griseostriatus mitochondrial partial 1	HF931387	86.62	100	2.64875e−42	Coleoptera	Dytiscidae	Boreonectes griseostriatus
Boreonectes multilineatus mitochondrial partial 16	HF931384	86.62	100	2.64875e−42	Coleoptera	Dytiscidae	Boreonectes multilineatus
Laccornis difformis 16S ribosomal RNA gene, partia	KF575544	86.08	100	9.24504e−42	Coleoptera	Dytiscidae	Laccornis difformis
Boreonectes griseostriatus mitochondrial 16S rRNA	HF931541	85.99	100	3.22684e−41	Coleoptera	Dytiscidae	Boreonectes griseostriatus
Boreonectes alpestris mitochondrial 16S rRNA gene	HF931512	85.99	100	3.22684e−41	Coleoptera	Dytiscidae	Boreonectes alpestris
Boreonectes macedonicus mitochondrial 16S rRNA gen	HF931359	85.99	100	3.22684e−41	Coleoptera	Dytiscidae	Boreonectes macedonicus
Stictotarsus ibericus 16S ribosomal RNA (rrnL) gen	EF670030	85.99	100	3.22684e−41	Coleoptera	Dytiscidae	Stictotarsus ibericus
Oreodytes scitulus 16S ribosomal RNA gene, partial	KF575551	84.08	100	5.83426e−38	Coleoptera	Dytiscidae	Oreodytes scitulus
Oreodytes scitulus mitochondrial partial 16S rRNA	HF931515	84.08	100	5.83426e−38	Coleoptera	Dytiscidae	Oreodytes scitulus
Deronectes moestus mitochondrial 16S rRNA gene (pa	HE613726	86.81	91.67	5.83426e−38	Coleoptera	Dytiscidae	Deronectes moestus
Laccornis kocae partial 16S rRNA gene	AJ850419	84.18	100	5.83426e−38	Coleoptera	Dytiscidae	Laccornis kocae
Boreonectes griseostriatus mitochondrial 16S rRNA	HF931385	86.01	91.67	2.03636e−37	Coleoptera	Dytiscidae	Boreonectes griseostriatus
Nebrioporus formaster mitochondrial partial 16S rR	HF931377	84.28	100	2.03636e−37	Coleoptera	Dytiscidae	Nebrioporus formaster
Deronectes moestus 16S ribosomal RNA gene, partial	KF575561	86.11	91.67	7.10758e−37	Coleoptera	Dytiscidae	Deronectes moestus
Deronectes brannani mitochondrial 16S rRNA gene (p	HE613695	86.11	91.67	7.10758e−37	Coleoptera	Dytiscidae	Deronectes brannanii
Deronectes moestus mitochondrial 16S rRNA gene (pa	HE613683	86.11	91.67	7.10758e−37	Coleoptera	Dytiscidae	Deronectes moestus
Deronectes opatrinus mitochondrial 16S rRNA gene (	HE613701	84.97	97.44	2.48079e−36	Coleoptera	Dytiscidae	Deronectes opatrinus
Suphrodytes dorsalis 16S ribosomal RNA gene, parti	KF575577	83.55	97.44	8.65881e−36	Coleoptera	Dytiscidae	Suphrodytes dorsalis
Aethionectes fulvonotatus voucher KBMAeop406 16S r	KF979088	83.87	97.44	8.65881e−36	Coleoptera	Dytiscidae	Aethionectes fulvonotatus
Hygrotus novemlineatus mitochondrial partial 16S r	HF931523	83.44	100	8.65881e−36	Coleoptera	Dytiscidae	Hygrotus novemlineatus
Hygrotus marklini mitochondrial 16S rRNA gene (par	HF931467	83.44	100	8.65881e−36	Coleoptera	Dytiscidae	Hygrotus marklini
Suphrodytes dorsalis voucher BMNH:725966 16S ribos	JX221574	83.55	97.44	8.65881e−36	Coleoptera	Dytiscidae	Suphrodytes dorsalis
Suphrodytes figuratus voucher BMNH:722148 16S ribo	JX221571	83.44	100	8.65881e−36	Coleoptera	Dytiscidae	Suphrodytes figuratus
Hydroporus foveolatus mitochondrial 16S rRNA gene	HE613724	84.97	97.44	8.65881e−36	Coleoptera	Dytiscidae	Hydroporus foveolatus
Antiporus hollingsworthi mitochondrial partial 16S	FR727247	83.23	98.72	8.65881e−36	Coleoptera	Dytiscidae	Antiporus hollingsworthi
Antiporus hollingsworthi mitochondrial partial 16S	FR727246	83.23	98.72	8.65881e−36	Coleoptera	Dytiscidae	Antiporus hollingsworthi
Hygrotus turbidus partial 16S rRNA gene	AJ850390	83.44	100	8.65881e−36	Coleoptera	Dytiscidae	Hygrotus turbidus
Oreodytes quadrimaculatus 16S ribosomal RNA gene,	KF575567	82.8	100	3.02222e−35	Coleoptera	Dytiscidae	Oreodytes quadrimaculatus
Stictotarsus procerus mitochondrial 16S rRNA gene	HF931379	85.42	91.67	3.02222e−35	Coleoptera	Dytiscidae	Stictotarsus procerus
Deronectes doriae mitochondrial 16S rRNA gene (par	HE613710	85.42	91.67	3.02222e−35	Coleoptera	Dytiscidae	Deronectes doriae
Hydroporus marginatus mitochondrial 16S rRNA gene	HE613699	88.71	79.49	3.02222e−35	Coleoptera	Dytiscidae	Hydroporus marginatus
Deronectes sahlbergi mitochondrial 16S rRNA gene (	HE613680	85.42	91.67	3.02222e−35	Coleoptera	Dytiscidae	Deronectes sahlbergi
Hovahydrus sp. BI−2011 voucher BMNH831333 16S ribo	HQ381524	86.21	91.67	3.02222e−35	Coleoptera	Dytiscidae	Hovahydrus sp. BI−2011
Copelatus sp. BI−2011 voucher BMNH792945 16S ribos	HQ381441	82.8	100	3.02222e−35	Coleoptera	Dytiscidae	Copelatus sp. BI−2011
Oreodytes quadrimaculatus partial 16S rRNA gene	AJ850351	82.8	100	3.02222e−35	Coleoptera	Dytiscidae	Oreodytes quadrimaculatus
Oreodytes natrix partial 16S rRNA gene	AJ850349	82.8	100	3.02222e−35	Coleoptera	Dytiscidae	Oreodytes natrix
Stictotarsus maghrebinus 16S ribosomal RNA gene, p	AY250947	85.42	91.67	3.02222e−35	Coleoptera	Dytiscidae	Stictotarsus maghrebinus
Scarodytes sp. IR−2003 16S ribosomal RNA gene, par	AY250939	83.12	100	3.02222e−35	Coleoptera	Dytiscidae	Scarodytes sp. IR−2003
Hygrotus lutescens mitochondrial partial 16S rRNA	HF931518	82.8	100	1.05486e−34	Coleoptera	Dytiscidae	Hygrotus lutescens
Hygrotus fontinalis mitochondrial 16S rRNA gene (p	HF931494	82.8	100	1.05486e−34	Coleoptera	Dytiscidae	Hygrotus fontinalis
Hygrotus patruelis mitochondrial 16S rRNA gene (pa	HF931388	82.8	100	1.05486e−34	Coleoptera	Dytiscidae	Hygrotus patruelis
Suphrodytes figuratus voucher BMNH:722150 16S ribo	JX221572	82.8	100	1.05486e−34	Coleoptera	Dytiscidae	Suphrodytes figuratus
Suphrodytes dorsalis mitochondrial 16S rRNA gene (	HE599700	82.8	100	1.05486e−34	Coleoptera	Dytiscidae	Suphrodytes dorsalis
Hydroporus glabriusculus 16S ribosomal RNA gene, p	AY365270	83.55	97.44	1.05486e−34	Coleoptera	Dytiscidae	Hydroporus glabriusculus
Hygrotus impressopunctatus voucher UNM KBMHtim80 1	KJ548334	83.23	97.44	3.68182e−34	Coleoptera	Dytiscidae	Hygrotus impressopunctatus
Hydroporus tessellatus 16S ribosomal RNA gene, par	JX434770	84.31	97.44	3.68182e−34	Coleoptera	Dytiscidae	Hydroporus tessellatus
Deronectes bicostatus mitochondrial 16S rRNA gene	HE613705	83.92	91.67	3.68182e−34	Coleoptera	Dytiscidae	Deronectes bicostatus
Hydroporus zimmermanni mitochondrial 16S rRNA gene	HE613679	84.31	97.44	3.68182e−34	Coleoptera	Dytiscidae	Hydroporus zimmermanni
Hydroporus basinotatus mitochondrial 16S rRNA gene	HE613678	84.31	97.44	3.68182e−34	Coleoptera	Dytiscidae	Hydroporus basinotatus
Copelatus sp. BI−2011 voucher BMNH671215 16S ribos	HQ381377	82.17	100	3.68182e−34	Coleoptera	Dytiscidae	Copelatus sp. BI−2011
Antiporus sp. MB1421 mitochondrial partial 16S rRN	FR727245	82.17	100	3.68182e−34	Coleoptera	Dytiscidae	Antiporus sp. MB1421
Hydroporus basinotatus 16S ribosomal RNA gene, par	AF518258	84.31	97.44	3.68182e−34	Coleoptera	Dytiscidae	Hydroporus basinotatus
Hydroporus marginatus 16S ribosomal RNA gene, part	AF518267	88.52	78.21	3.68182e−34	Coleoptera	Dytiscidae	Hydroporus marginatus
Hydaticus cinctipennis voucher UNM KBMHyci20 16S r	KJ548338	82.28	100	1.28508e−33	Coleoptera	Dytiscidae	Hydaticus cinctipennis
Hygrotus impressopunctatus partial 16S rRNA gene	AJ850405	83.12	97.44	1.28508e−33	Coleoptera	Dytiscidae	Hygrotus impressopunctatus
Agabus didymus mitochondrial 16S rRNA gene (partia	LM654773	82.17	100	4.48537e−33	Coleoptera	Dytiscidae	Agabus didymus
Agabus didymus voucher UNM KBMPmsp730 16S ribosoma	KJ548392	82.17	100	4.48537e−33	Coleoptera	Dytiscidae	Agabus didymus
Hygrotus tumidiventris mitochondrial 16S rRNA gene	HF931496	82.17	100	4.48537e−33	Coleoptera	Dytiscidae	Hygrotus tumidiventris
Hygrotus unguicularis mitochondrial partial 16S rR	HF931489	82.17	100	4.48537e−33	Coleoptera	Dytiscidae	Hygrotus unguicularis
Hygrotus urgensis mitochondrial 16S rRNA gene (par	HF931486	82.17	100	4.48537e−33	Coleoptera	Dytiscidae	Hygrotus urgensis
Hydroporus analis mitochondrial partial 16S rRNA g	HE613731	83.66	97.44	4.48537e−33	Coleoptera	Dytiscidae	Hydroporus analis
Hydroporus compunctus mitochondrial 16S rRNA gene	HE599693	83.66	97.44	4.48537e−33	Coleoptera	Dytiscidae	Hydroporus compunctus
Antiporus wilsoni mitochondrial partial 16S rRNA g	FR727249	81.94	98.72	4.48537e−33	Coleoptera	Dytiscidae	Antiporus wilsoni
Hygrotus corpulentus partial 16S rRNA gene	AJ850387	82.17	100	4.48537e−33	Coleoptera	Dytiscidae	Hygrotus corpulentus
Scarodytes halensis haplotype 3 16S ribosomal RNA	AY250937	82.39	100	4.48537e−33	Coleoptera	Dytiscidae	Scarodytes halensis
Nebrioporus assimilis 16S ribosomal RNA gene, part	AY250921	81.94	98.72	4.48537e−33	Coleoptera	Dytiscidae	Nebrioporus assimilis
Hydroporus compunctus 16S ribosomal RNA gene, part	AF518259	83.66	97.44	4.48537e−33	Coleoptera	Dytiscidae	Hydroporus compunctus
Prodaticus luczonicus voucher DBAU−BT−30_B94_Dytis	KM064623	82.28	100	1.56555e−32	Coleoptera	Dytiscidae	Prodaticus luczonicus
Agabus glacialis mitochondrial 16S rRNA gene (part	LM654776	81.53	100	1.56555e−32	Coleoptera	Dytiscidae	Agabus glacialis
Herophydrus rohani voucher UNM KBMHpsp574 16S ribo	KJ548382	83.01	97.44	1.56555e−32	Coleoptera	Dytiscidae	Herophydrus rohani
Cybister marginicollis voucher UNM KBMCyma416 16S	KJ548319	81.53	100	1.56555e−32	Coleoptera	Dytiscidae	Cybister marginicollis
Deronectes aubei voucher UNM KBMDeau758 16S riboso	KJ548292	84.03	91.67	1.56555e−32	Coleoptera	Dytiscidae	Deronectes aubei
Boreonectes funereus mitochondrial 16S rRNA gene (	HF931393	82.28	100	1.56555e−32	Coleoptera	Dytiscidae	Boreonectes funereus
Hydroporus fuscipennis mitochondrial 16S rRNA gene	HE613730	83.77	97.44	1.56555e−32	Coleoptera	Dytiscidae	Hydroporus fuscipennis
Ilybius apicalis mitochondrial 16S rRNA gene (part	HE613729	82.28	100	1.56555e−32	Coleoptera	Dytiscidae	Ilybius apicalis
Deronectes costipennis mitochondrial 16S rRNA gene	HE613697	84.03	91.67	1.56555e−32	Coleoptera	Dytiscidae	Deronectes costipennis
Deronectes platynotus mitochondrial 16S rRNA gene	HE613682	84.03	91.67	1.56555e−32	Coleoptera	Dytiscidae	Deronectes platynotus
Deronectes depressicollis mitochondrial 16S rRNA g	HE613681	84.03	91.67	1.56555e−32	Coleoptera	Dytiscidae	Deronectes depressicollis
Deronectes semirufus mitochondrial 16S rRNA gene (	HE613676	84.03	91.67	1.56555e−32	Coleoptera	Dytiscidae	Deronectes semirufus
Deronectes semirufus mitochondrial 16S rRNA gene (	HE613674	84.03	91.67	1.56555e−32	Coleoptera	Dytiscidae	Deronectes semirufus
Antiporus interrogationis mitochondrial partial 16	FR727252	81.53	100	1.56555e−32	Coleoptera	Dytiscidae	Antiporus interrogationis
Stictotarsus falli 16S ribosomal RNA (rrnL) gene,	EF670029	81.65	100	1.56555e−32	Coleoptera	Dytiscidae	Stictotarsus falli
Oreodytes picturatus partial 16S rRNA gene	AJ850350	81.53	100	1.56555e−32	Coleoptera	Dytiscidae	Oreodytes picturatus
Coelhydrus brevicollis voucher MB1224 16S ribosoma	EF056670	83.65	100	1.56555e−32	Coleoptera	Dytiscidae	Coelhydrus brevicollis
Deronectes costipennis 16S ribosomal RNA gene, par	AY250951	84.03	91.67	1.56555e−32	Coleoptera	Dytiscidae	Deronectes costipennis
Nebrioporus airumlus 16S ribosomal RNA gene, parti	AY250920	81.53	100	1.56555e−32	Coleoptera	Dytiscidae	Nebrioporus airumlus
