# Supplementary material for: Testing the potential of a ribosomal 16S marker for DNA metabarcoding of insects
Source: PeerJ. 2016 Apr 19;4:e1966. doi: 10.7717/peerj.1966 (PMC4841222; doi:10.7717/peerj.1966)
Supplement: Supplemental Information 1 [file peerj-04-1966-s006.zip › scripts/5 match OTUs/blast_hits/OTU_445.csv_plot.pdf]

OTU_445.csv

Oreodytes sanmarkii

Dytiscidae

Coleoptera

97.45

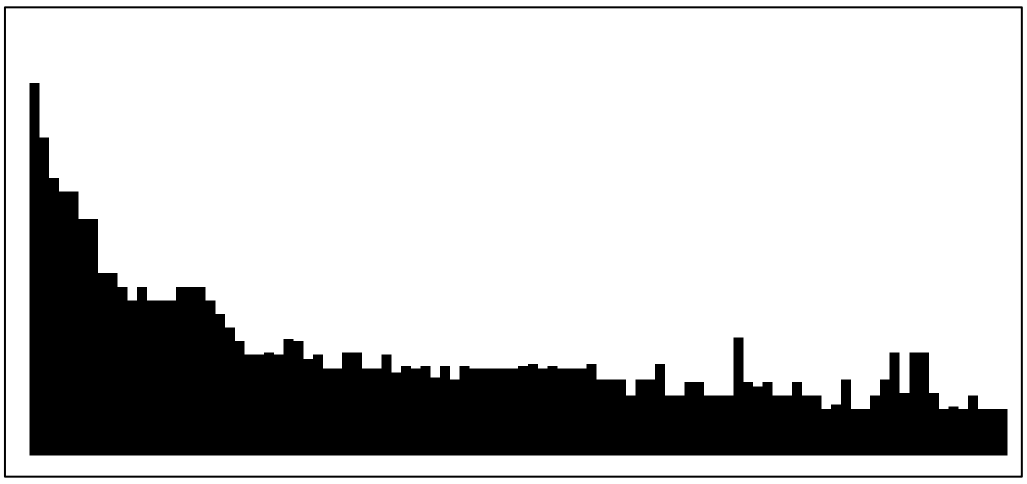

Oreodytes sanmarkii mitochondrial 16S rRNA gene (p	HF931524	97.45	100	1.19154e−65	Coleoptera	Dytiscidae	Oreodytes sanmarkii
Oreodytes obesus cordillerensis mitochondrial part	HF931516	94.9	100	3.19734e−60	Coleoptera	Dytiscidae	Oreodytes obesus cordillerensis
Oreodytes congruus 16S ribosomal RNA gene, partial	KF575550	92.99	100	7.04262e−56	Coleoptera	Dytiscidae	Oreodytes congruus
Oreodytes crassulus partial 16S rRNA gene	AJ850348	92.36	100	8.57966e−55	Coleoptera	Dytiscidae	Oreodytes crassulus
Oreodytes congruus partial 16S rRNA gene	AJ850347	92.36	100	8.57966e−55	Coleoptera	Dytiscidae	Oreodytes congruus
Oreodytes abbreviatus mitochondrial 16S rRNA gene	HF931477	91.08	100	4.44438e−52	Coleoptera	Dytiscidae	Oreodytes abbreviatus
Oreodytes rhyacophilus partial 16S rRNA gene	AJ850352	91.08	100	4.44438e−52	Coleoptera	Dytiscidae	Oreodytes rhyacophilus
Boreonectes riberae mitochondrial 16S rRNA gene (p	HF931461	88.54	100	1.45287e−45	Coleoptera	Dytiscidae	Boreonectes riberae
Boreonectes multilineatus mitochondrial partial 16	HF931384	88.54	100	1.45287e−45	Coleoptera	Dytiscidae	Boreonectes multilineatus
Oreodytes mongolicus mitochondrial 16S rRNA gene (	HF931491	87.9	100	5.07103e−45	Coleoptera	Dytiscidae	Oreodytes mongolicus
Oreodytes snoqualmie mitochondrial partial 16S rRN	HF931519	87.26	100	6.17778e−44	Coleoptera	Dytiscidae	Oreodytes snoqualmie
Boreonectes alpestris mitochondrial 16S rRNA gene	HF931512	87.9	100	6.17778e−44	Coleoptera	Dytiscidae	Boreonectes alpestris
Oreodytes septentrionalis mitochondrial 16S rRNA g	HF931492	87.26	100	6.17778e−44	Coleoptera	Dytiscidae	Oreodytes septentrionalis
Oreodytes shorti mitochondrial 16S rRNA gene (part	HF931488	87.26	100	6.17778e−44	Coleoptera	Dytiscidae	Oreodytes shorti
Deronectes parvicollis mitochondrial 16S rRNA gene	HF931454	87.26	100	6.17778e−44	Coleoptera	Dytiscidae	Deronectes parvicollis
Boreonectes griseostriatus mitochondrial partial 1	HF931387	87.9	100	6.17778e−44	Coleoptera	Dytiscidae	Boreonectes griseostriatus
Boreonectes macedonicus mitochondrial 16S rRNA gen	HF931359	87.9	100	6.17778e−44	Coleoptera	Dytiscidae	Boreonectes macedonicus
Stictotarsus ibericus 16S ribosomal RNA (rrnL) gen	EF670030	87.9	100	6.17778e−44	Coleoptera	Dytiscidae	Stictotarsus ibericus
Boreonectes griseostriatus mitochondrial 16S rRNA	HF931541	87.26	100	7.52607e−43	Coleoptera	Dytiscidae	Boreonectes griseostriatus
Boreonectes striatellus mitochondrial 16S rRNA gen	HF931511	86.62	100	3.20017e−41	Coleoptera	Dytiscidae	Boreonectes striatellus
Laccornis kocae partial 16S rRNA gene	AJ850419	85.99	100	3.20017e−41	Coleoptera	Dytiscidae	Laccornis kocae
Laccornis difformis 16S ribosomal RNA gene, partia	KF575544	85.35	100	1.36075e−39	Coleoptera	Dytiscidae	Laccornis difformis
Oreodytes scitulus 16S ribosomal RNA gene, partial	KF575551	84.71	100	1.65773e−38	Coleoptera	Dytiscidae	Oreodytes scitulus
Oreodytes scitulus mitochondrial partial 16S rRNA	HF931515	84.71	100	1.65773e−38	Coleoptera	Dytiscidae	Oreodytes scitulus
Nebrioporus formaster mitochondrial partial 16S rR	HF931377	84.81	100	5.78604e−38	Coleoptera	Dytiscidae	Nebrioporus formaster
Hygrotus marklini mitochondrial 16S rRNA gene (par	HF931467	84.71	100	2.01953e−37	Coleoptera	Dytiscidae	Hygrotus marklini
Hydroporus foveolatus mitochondrial 16S rRNA gene	HE613724	85.44	100	2.01953e−37	Coleoptera	Dytiscidae	Hydroporus foveolatus
Hydroporus zimmermanni mitochondrial 16S rRNA gene	HE613679	85.35	100	2.01953e−37	Coleoptera	Dytiscidae	Hydroporus zimmermanni
Antiporus hollingsworthi mitochondrial partial 16S	FR727246	84.52	98.71	2.01953e−37	Coleoptera	Dytiscidae	Antiporus hollingsworthi
Hygrotus turbidus partial 16S rRNA gene	AJ850390	84.71	100	2.01953e−37	Coleoptera	Dytiscidae	Hygrotus turbidus
Oreodytes quadrimaculatus 16S ribosomal RNA gene,	KF575567	84.08	100	7.04884e−37	Coleoptera	Dytiscidae	Oreodytes quadrimaculatus
Oreodytes quadrimaculatus partial 16S rRNA gene	AJ850351	84.08	100	7.04884e−37	Coleoptera	Dytiscidae	Oreodytes quadrimaculatus
Nirripirti megamacrocephalus isolate ABTC78781 16S	EU616935	84.81	100	7.04884e−37	Coleoptera	Dytiscidae	Paroster megamacrocephalus
Stictotarsus maghrebinus 16S ribosomal RNA gene, p	AY250947	84.81	100	7.04884e−37	Coleoptera	Dytiscidae	Stictotarsus maghrebinus
Hygrotus novemlineatus mitochondrial partial 16S r	HF931523	84.08	100	2.46029e−36	Coleoptera	Dytiscidae	Hygrotus novemlineatus
Hygrotus patruelis mitochondrial 16S rRNA gene (pa	HF931388	84.08	100	2.46029e−36	Coleoptera	Dytiscidae	Hygrotus patruelis
Hydroporus analis mitochondrial partial 16S rRNA g	HE613731	84.71	100	2.46029e−36	Coleoptera	Dytiscidae	Hydroporus analis
Antiporus hollingsworthi mitochondrial partial 16S	FR727247	83.87	98.71	2.46029e−36	Coleoptera	Dytiscidae	Antiporus hollingsworthi
Suphrodytes dorsalis 16S ribosomal RNA gene, parti	KF575577	84.18	100	8.58725e−36	Coleoptera	Dytiscidae	Suphrodytes dorsalis
Boreonectes griseostriatus mitochondrial 16S rRNA	HF931385	84.08	100	8.58725e−36	Coleoptera	Dytiscidae	Boreonectes griseostriatus
Suphrodytes dorsalis voucher BMNH:725966 16S ribos	JX221574	84.18	100	8.58725e−36	Coleoptera	Dytiscidae	Suphrodytes dorsalis
Scarodytes sp. IR−2003 16S ribosomal RNA gene, par	AY250939	83.65	100	8.58725e−36	Coleoptera	Dytiscidae	Scarodytes sp. IR−2003
Stictotarsus procerus mitochondrial 16S rRNA gene	HF931379	84.18	100	2.99724e−35	Coleoptera	Dytiscidae	Stictotarsus procerus
Scarodytes halensis haplotype 3 16S ribosomal RNA	AY250937	83.54	100	2.99724e−35	Coleoptera	Dytiscidae	Scarodytes halensis
Hydroporus tessellatus 16S ribosomal RNA gene, par	JX434770	84.18	100	1.04614e−34	Coleoptera	Dytiscidae	Hydroporus tessellatus
Hygrotus foveolatus mitochondrial 16S rRNA gene	HF931502	84.08	100	1.04614e−34	Coleoptera	Dytiscidae	Hygrotus foveolatus
Hydroporus discretus mitochondrial 16S rRNA gene (	HE613723	84.08	100	1.04614e−34	Coleoptera	Dytiscidae	Hydroporus discretus
Hydroporus decipiens mitochondrial 16S rRNA gene (	HE613722	84.08	100	1.04614e−34	Coleoptera	Dytiscidae	Hydroporus decipiens
Hydroporus pubescens mitochondrial 16S rRNA gene (	HE613707	84.08	100	1.04614e−34	Coleoptera	Dytiscidae	Hydroporus pubescens
Hydroporus errans mitochondrial 16S rRNA gene (par	HE613704	84.08	100	1.04614e−34	Coleoptera	Dytiscidae	Hydroporus errans
Hydroporus basinotatus mitochondrial 16S rRNA gene	HE613678	84.18	100	1.04614e−34	Coleoptera	Dytiscidae	Hydroporus basinotatus
Nirripirti napperbyensis 16S ribosomal RNA gene, p	AY353843	84.28	100	1.04614e−34	Coleoptera	Dytiscidae	Paroster napperbyensis
Hydroporus pubescens voucher NHM−IR39 16S ribosoma	EF419327	84.08	100	1.04614e−34	Coleoptera	Dytiscidae	Hydroporus pubescens
Hydroporus basinotatus 16S ribosomal RNA gene, par	AF518258	84.18	100	1.04614e−34	Coleoptera	Dytiscidae	Hydroporus basinotatus
Hydroporus pubescens 16S ribosomal RNA gene, parti	AF518276	84.08	100	1.04614e−34	Coleoptera	Dytiscidae	Hydroporus pubescens
Hydroporus discretus 16S ribosomal RNA gene, parti	AF518260	84.08	100	1.04614e−34	Coleoptera	Dytiscidae	Hydroporus discretus
Hydroporus analis 16S ribosomal RNA gene, partial	AF518257	84.08	100	1.04614e−34	Coleoptera	Dytiscidae	Hydroporus analis
Hydroporus fuscipennis mitochondrial 16S rRNA gene	HE613730	84.28	100	3.65139e−34	Coleoptera	Dytiscidae	Hydroporus fuscipennis
Deronectes doriae mitochondrial 16S rRNA gene (par	HE613710	83.54	100	3.65139e−34	Coleoptera	Dytiscidae	Deronectes doriae
Paroster sp. R401 Newhaven voucher ABTC:78974 16S	JQ745754	83.54	100	3.65139e−34	Coleoptera	Dytiscidae	Paroster sp. R401 Newhaven
Stictotarsus falli 16S ribosomal RNA (rrnL) gene,	EF670029	83.54	100	3.65139e−34	Coleoptera	Dytiscidae	Stictotarsus falli
Oreodytes natrix partial 16S rRNA gene	AJ850349	82.8	100	3.65139e−34	Coleoptera	Dytiscidae	Oreodytes natrix
Paroster sp. 2 RL−2008 16S ribosomal RNA gene, par	EU616958	83.54	100	3.65139e−34	Coleoptera	Dytiscidae	Paroster sp. 2 RL−2008
Paroster sp. spnMtWedge 16S ribosomal RNA gene, pa	EU616954	83.54	100	3.65139e−34	Coleoptera	Dytiscidae	Paroster sp. spnMtWedge
Hydroporus fuscipennis 16S ribosomal RNA gene, par	AF518262	84.28	100	3.65139e−34	Coleoptera	Dytiscidae	Hydroporus fuscipennis
Hygrotus tumidiventris mitochondrial 16S rRNA gene	HF931496	82.8	100	1.27446e−33	Coleoptera	Dytiscidae	Hygrotus tumidiventris
Hygrotus unguicularis mitochondrial partial 16S rR	HF931489	82.8	100	1.27446e−33	Coleoptera	Dytiscidae	Hygrotus unguicularis
Hydroporus discretus mitochondrial 16S rRNA gene (	HF931449	83.44	100	1.27446e−33	Coleoptera	Dytiscidae	Hydroporus discretus
Hydroporus thracicus mitochondrial 16S rRNA gene (	HF931413	83.44	100	1.27446e−33	Coleoptera	Dytiscidae	Hydroporus thracicus
Hydroporus brancoi gredensis mitochondrial partial	HF931329	82.8	100	1.27446e−33	Coleoptera	Dytiscidae	Hydroporus brancoi gredensis
Suphrodytes figuratus voucher BMNH:722150 16S ribo	JX221572	82.8	100	1.27446e−33	Coleoptera	Dytiscidae	Suphrodytes figuratus
Suphrodytes figuratus voucher BMNH:722148 16S ribo	JX221571	82.8	100	1.27446e−33	Coleoptera	Dytiscidae	Suphrodytes figuratus
Deronectes moestus mitochondrial 16S rRNA gene (pa	HE613726	85.52	91.61	1.27446e−33	Coleoptera	Dytiscidae	Deronectes moestus
Hydroporus discretus mitochondrial 16S rRNA gene (	HE613709	83.44	100	1.27446e−33	Coleoptera	Dytiscidae	Hydroporus discretus
Hydroporus marginatus mitochondrial 16S rRNA gene	HE613699	83.23	100	1.27446e−33	Coleoptera	Dytiscidae	Hydroporus marginatus
Hydroporus macedonicus mitochondrial 16S rRNA gene	HE613690	83.44	100	1.27446e−33	Coleoptera	Dytiscidae	Hydroporus macedonicus
Suphrodytes dorsalis mitochondrial 16S rRNA gene (	HE599700	82.8	100	1.27446e−33	Coleoptera	Dytiscidae	Suphrodytes dorsalis
Nirripirti wedgeensis isolate ABTC78773−4 16S ribo	EU616938	82.8	100	1.27446e−33	Coleoptera	Dytiscidae	Paroster wedgeensis
Hydroporus macedonicus 16S ribosomal RNA gene, par	DQ195529	83.44	100	1.27446e−33	Coleoptera	Dytiscidae	Hydroporus macedonicus
Hydroporus glabriusculus 16S ribosomal RNA gene, p	AY365270	82.8	100	1.27446e−33	Coleoptera	Dytiscidae	Hydroporus glabriusculus
Hydroporus marginatus 16S ribosomal RNA gene, part	AF518267	82.8	100	1.27446e−33	Coleoptera	Dytiscidae	Hydroporus marginatus
Aethionectes fulvonotatus voucher KBMAeop406 16S r	KF979088	82.17	100	4.4483e−33	Coleoptera	Dytiscidae	Aethionectes fulvonotatus
Scarodytes halensis mitochondrial 16S rRNA gene (p	HF931324	82.39	100	4.4483e−33	Coleoptera	Dytiscidae	Scarodytes halensis
Hydroporus compunctus mitochondrial 16S rRNA gene	HE599693	83.54	100	4.4483e−33	Coleoptera	Dytiscidae	Hydroporus compunctus
Antiporus sp. MB1421 mitochondrial partial 16S rRN	FR727245	82.17	100	4.4483e−33	Coleoptera	Dytiscidae	Antiporus sp. MB1421
Oreodytes picturatus partial 16S rRNA gene	AJ850350	82.17	100	4.4483e−33	Coleoptera	Dytiscidae	Oreodytes picturatus
Nirripirti newhavenensis 16S ribosomal RNA gene, p	AY353844	82.8	100	4.4483e−33	Coleoptera	Dytiscidae	Paroster newhavenensis
Hydroporus compunctus 16S ribosomal RNA gene, part	AF518259	83.54	100	4.4483e−33	Coleoptera	Dytiscidae	Hydroporus compunctus
Deronectes moestus 16S ribosomal RNA gene, partial	KF575561	84.83	91.61	1.55261e−32	Coleoptera	Dytiscidae	Deronectes moestus
Deronectes opatrinus mitochondrial 16S rRNA gene (	HE613701	82.91	100	1.55261e−32	Coleoptera	Dytiscidae	Deronectes opatrinus
Deronectes brannani mitochondrial 16S rRNA gene (p	HE613695	84.83	91.61	1.55261e−32	Coleoptera	Dytiscidae	Deronectes brannanii
Deronectes moestus mitochondrial 16S rRNA gene (pa	HE613683	84.83	91.61	1.55261e−32	Coleoptera	Dytiscidae	Deronectes moestus
Deronectes sahlbergi mitochondrial 16S rRNA gene (	HE613680	82.91	100	1.55261e−32	Coleoptera	Dytiscidae	Deronectes sahlbergi
Nirripirti byroensis isolate ABTC78677−8 16S ribos	EU616910	82.17	100	1.55261e−32	Coleoptera	Dytiscidae	Paroster byroensis
Scarodytes antoni voucher MNCN−AC6 16S ribosomal R	EU156492	82.28	100	1.55261e−32	Coleoptera	Dytiscidae	Scarodytes antoni
Hydroporus submuticus 16S ribosomal RNA gene, part	JX434777	82.17	100	5.41914e−32	Coleoptera	Dytiscidae	Hydroporus submuticus
Hydroporus nigrita 16S ribosomal RNA gene, partial	JX434769	82.8	100	5.41914e−32	Coleoptera	Dytiscidae	Hydroporus nigrita
Hygrotus lutescens mitochondrial partial 16S rRNA	HF931518	82.17	100	5.41914e−32	Coleoptera	Dytiscidae	Hygrotus lutescens
Hydroporus semenowi mitochondrial partial 16S rRNA	HF931509	82.17	100	5.41914e−32	Coleoptera	Dytiscidae	Hydroporus semenowi
Hygrotus fontinalis mitochondrial 16S rRNA gene (p	HF931494	82.17	100	5.41914e−32	Coleoptera	Dytiscidae	Hygrotus fontinalis
