# Supplementary material for: Testing the potential of a ribosomal 16S marker for DNA metabarcoding of insects
Source: PeerJ. 2016 Apr 19;4:e1966. doi: 10.7717/peerj.1966 (PMC4841222; doi:10.7717/peerj.1966)
Supplement: Supplemental Information 1 [file peerj-04-1966-s006.zip › scripts/5 match OTUs/blast_hits/OTU_449.csv_plot.pdf]

OTU_449.csv

Oreodytes sanmarkii

Dytiscidae

Coleoptera

98.09

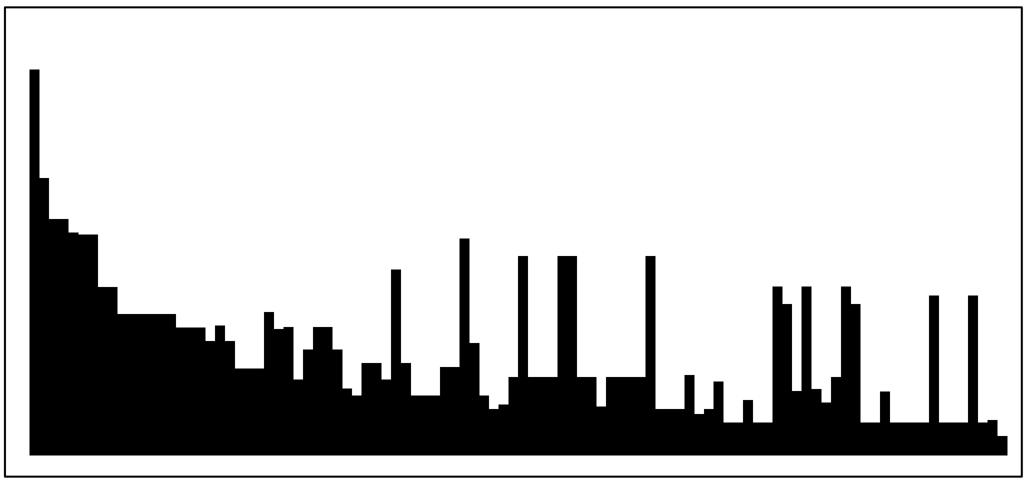

Oreodytes sanmarkii mitochondrial 16S rRNA gene (p	HF931524	98.09	100	8.09543e−68	Coleoptera	Dytiscidae	Oreodytes sanmarkii
Oreodytes obesus cordillerensis mitochondrial part	HF931516	92.99	100	5.82911e−57	Coleoptera	Dytiscidae	Oreodytes obesus cordillerensis
Oreodytes crassulus partial 16S rRNA gene	AJ850348	91.08	100	3.67857e−53	Coleoptera	Dytiscidae	Oreodytes crassulus
Oreodytes congruus partial 16S rRNA gene	AJ850347	91.08	100	3.67857e−53	Coleoptera	Dytiscidae	Oreodytes congruus
Oreodytes congruus 16S ribosomal RNA gene, partial	KF575550	90.45	100	1.56417e−51	Coleoptera	Dytiscidae	Oreodytes congruus
Oreodytes abbreviatus mitochondrial 16S rRNA gene	HF931477	90.34	92.95	9.87097e−48	Coleoptera	Dytiscidae	Oreodytes abbreviatus
Oreodytes rhyacophilus partial 16S rRNA gene	AJ850352	90.34	92.95	9.87097e−48	Coleoptera	Dytiscidae	Oreodytes rhyacophilus
Oreodytes snoqualmie mitochondrial partial 16S rRN	HF931519	87.9	100	4.19724e−46	Coleoptera	Dytiscidae	Oreodytes snoqualmie
Oreodytes mongolicus mitochondrial 16S rRNA gene (	HF931491	87.9	100	4.19724e−46	Coleoptera	Dytiscidae	Oreodytes mongolicus
Oreodytes septentrionalis mitochondrial 16S rRNA g	HF931492	86.62	100	2.17423e−43	Coleoptera	Dytiscidae	Oreodytes septentrionalis
Oreodytes shorti mitochondrial 16S rRNA gene (part	HF931488	86.62	100	2.17423e−43	Coleoptera	Dytiscidae	Oreodytes shorti
Boreonectes striatellus mitochondrial 16S rRNA gen	HF931511	86.62	100	2.64875e−42	Coleoptera	Dytiscidae	Boreonectes striatellus
Boreonectes riberae mitochondrial 16S rRNA gene (p	HF931461	86.62	100	2.64875e−42	Coleoptera	Dytiscidae	Boreonectes riberae
Boreonectes griseostriatus mitochondrial partial 1	HF931387	86.62	100	2.64875e−42	Coleoptera	Dytiscidae	Boreonectes griseostriatus
Boreonectes multilineatus mitochondrial partial 16	HF931384	86.62	100	2.64875e−42	Coleoptera	Dytiscidae	Boreonectes multilineatus
Boreonectes alpestris mitochondrial 16S rRNA gene	HF931512	85.99	100	3.22684e−41	Coleoptera	Dytiscidae	Boreonectes alpestris
Boreonectes macedonicus mitochondrial 16S rRNA gen	HF931359	85.99	100	3.22684e−41	Coleoptera	Dytiscidae	Boreonectes macedonicus
Stictotarsus ibericus 16S ribosomal RNA (rrnL) gen	EF670030	85.99	100	3.22684e−41	Coleoptera	Dytiscidae	Stictotarsus ibericus
Laccornis difformis 16S ribosomal RNA gene, partia	KF575544	85.35	100	1.12628e−40	Coleoptera	Dytiscidae	Laccornis difformis
Boreonectes griseostriatus mitochondrial 16S rRNA	HF931541	86.08	100	1.12628e−40	Coleoptera	Dytiscidae	Boreonectes griseostriatus
Deronectes parvicollis mitochondrial 16S rRNA gene	HF931454	85.35	100	1.12628e−40	Coleoptera	Dytiscidae	Deronectes parvicollis
Oreodytes scitulus 16S ribosomal RNA gene, partial	KF575551	84.08	100	5.83426e−38	Coleoptera	Dytiscidae	Oreodytes scitulus
Oreodytes scitulus mitochondrial partial 16S rRNA	HF931515	84.08	100	5.83426e−38	Coleoptera	Dytiscidae	Oreodytes scitulus
Laccornis kocae partial 16S rRNA gene	AJ850419	84.08	100	5.83426e−38	Coleoptera	Dytiscidae	Laccornis kocae
Deronectes moestus mitochondrial 16S rRNA gene (pa	HE613726	86.71	91.03	2.03636e−37	Coleoptera	Dytiscidae	Deronectes moestus
Boreonectes griseostriatus mitochondrial partial 1	HF931385	85.92	91.03	7.10758e−37	Coleoptera	Dytiscidae	Boreonectes griseostriatus
Deronectes moestus 16S ribosomal RNA gene, partial	KF575561	86.01	91.03	2.48079e−36	Coleoptera	Dytiscidae	Deronectes moestus
Nebrioporus formaster mitochondrial partial 16S rR	HF931377	83.54	100	2.48079e−36	Coleoptera	Dytiscidae	Nebrioporus formaster
Deronectes opatrinus mitochondrial 16S rRNA gene (	HE613701	84.97	97.44	2.48079e−36	Coleoptera	Dytiscidae	Deronectes opatrinus
Deronectes brannani mitochondrial 16S rRNA gene (p	HE613695	86.01	91.03	2.48079e−36	Coleoptera	Dytiscidae	Deronectes brannanii
Deronectes moestus mitochondrial 16S rRNA gene (pa	HE613683	86.01	91.03	2.48079e−36	Coleoptera	Dytiscidae	Deronectes moestus
Stictotarsus maghrebinus 16S ribosomal RNA gene, p	AY250947	84.97	97.44	2.48079e−36	Coleoptera	Dytiscidae	Stictotarsus maghrebinus
Aethionectes fulvonotatus voucher KBMAeop406 16S r	KF979088	83.12	100	8.65881e−36	Coleoptera	Dytiscidae	Aethionectes fulvonotatus
Oreodytes quadrimaculatus 16S ribosomal RNA gene,	KF575567	82.8	100	3.02222e−35	Coleoptera	Dytiscidae	Oreodytes quadrimaculatus
Stictotarsus procerus mitochondrial 16S rRNA gene	HF931379	84.31	97.44	3.02222e−35	Coleoptera	Dytiscidae	Stictotarsus procerus
Deronectes doriae mitochondrial 16S rRNA gene (par	HE613710	84.31	97.44	3.02222e−35	Coleoptera	Dytiscidae	Deronectes doriae
Deronectes bicostatus mitochondrial 16S rRNA gene	HE613705	83.55	97.44	3.02222e−35	Coleoptera	Dytiscidae	Deronectes bicostatus
Hydroporus marginatus mitochondrial 16S rRNA gene	HE613699	88.71	79.49	3.02222e−35	Coleoptera	Dytiscidae	Hydroporus marginatus
Deronectes sahlbergi mitochondrial 16S rRNA gene (	HE613680	84.31	97.44	3.02222e−35	Coleoptera	Dytiscidae	Deronectes sahlbergi
Copelatus sp. BI−2011 voucher BMNH792945 16S ribos	HQ381441	82.8	100	3.02222e−35	Coleoptera	Dytiscidae	Copelatus sp. BI−2011
Oreodytes quadrimaculatus partial 16S rRNA gene	AJ850351	82.8	100	3.02222e−35	Coleoptera	Dytiscidae	Oreodytes quadrimaculatus
Oreodytes natrix partial 16S rRNA gene	AJ850349	82.8	100	3.02222e−35	Coleoptera	Dytiscidae	Oreodytes natrix
Suphrodytes dorsalis 16S ribosomal RNA gene, parti	KF575577	84.14	92.95	1.05486e−34	Coleoptera	Dytiscidae	Suphrodytes dorsalis
Suphrodytes dorsalis voucher BMNH:725966 16S ribos	JX221574	84.14	92.95	1.05486e−34	Coleoptera	Dytiscidae	Suphrodytes dorsalis
Hydroporus foveolatus mitochondrial 16S rRNA gene	HE613724	90.16	77.56	1.05486e−34	Coleoptera	Dytiscidae	Hydroporus foveolatus
Hovahydrus sp. BI−2011 voucher BMNH831333 16S ribo	HQ381524	85.26	98.72	1.05486e−34	Coleoptera	Dytiscidae	Hovahydrus sp. BI−2011
Hydroporus marginatus 16S ribosomal RNA gene, part	AF518267	82.8	100	1.05486e−34	Coleoptera	Dytiscidae	Hydroporus marginatus
Copelatus sp. BI−2011 voucher BMNH671215 16S ribos	HQ381377	82.17	100	3.68182e−34	Coleoptera	Dytiscidae	Copelatus sp. BI−2011
Scarodytes sp. IR−2003 16S ribosomal RNA gene, par	AY250939	82.39	100	3.68182e−34	Coleoptera	Dytiscidae	Scarodytes sp. IR−2003
Deronectes aubei voucher UNM KBMDeau758 16S riboso	KJ548292	83.66	97.44	1.28508e−33	Coleoptera	Dytiscidae	Deronectes aubei
Hydroporus tessellatus 16S ribosomal RNA gene, par	JX434770	89.34	77.56	1.28508e−33	Coleoptera	Dytiscidae	Hydroporus tessellatus
Deronectes costipennis mitochondrial 16S rRNA gene	HE613697	83.66	97.44	1.28508e−33	Coleoptera	Dytiscidae	Deronectes costipennis
Deronectes platynotus mitochondrial 16S rRNA gene	HE613682	83.66	97.44	1.28508e−33	Coleoptera	Dytiscidae	Deronectes platynotus
Deronectes depressicollis mitochondrial 16S rRNA g	HE613681	83.66	97.44	1.28508e−33	Coleoptera	Dytiscidae	Deronectes depressicollis
Hydroporus zimmermanni mitochondrial 16S rRNA gene	HE613679	89.34	77.56	1.28508e−33	Coleoptera	Dytiscidae	Hydroporus zimmermanni
Hydroporus basinotatus mitochondrial 16S rRNA gene	HE613678	89.34	77.56	1.28508e−33	Coleoptera	Dytiscidae	Hydroporus basinotatus
Deronectes semirufus mitochondrial 16S rRNA gene (	HE613676	83.66	97.44	1.28508e−33	Coleoptera	Dytiscidae	Deronectes semirufus
Deronectes semirufus mitochondrial 16S rRNA gene (	HE613674	83.66	97.44	1.28508e−33	Coleoptera	Dytiscidae	Deronectes semirufus
Stictotarsus falli 16S ribosomal RNA (rrnL) gene,	EF670029	82.28	100	1.28508e−33	Coleoptera	Dytiscidae	Stictotarsus falli
Deronectes costipennis 16S ribosomal RNA gene, par	AY250951	83.66	97.44	1.28508e−33	Coleoptera	Dytiscidae	Deronectes costipennis
Deronectes aubei sanfilippoi haplotype 2 16S ribos	AY250908	83.66	97.44	1.28508e−33	Coleoptera	Dytiscidae	Deronectes aubei sanfilippoi
Deronectes aubei sanfilippoi haplotype 1 16S ribos	AY250907	83.66	97.44	1.28508e−33	Coleoptera	Dytiscidae	Deronectes aubei sanfilippoi
Deronectes aubei aubei haplotype 2 16S ribosomal R	AY250906	83.66	97.44	1.28508e−33	Coleoptera	Dytiscidae	Deronectes aubei aubei
Hydroporus basinotatus 16S ribosomal RNA gene, par	AF518258	89.34	77.56	1.28508e−33	Coleoptera	Dytiscidae	Hydroporus basinotatus
Hygrotus novemlineatus mitochondrial partial 16S r	HF931523	82.17	100	4.48537e−33	Coleoptera	Dytiscidae	Hygrotus novemlineatus
Hygrotus marklini mitochondrial 16S rRNA gene (par	HF931467	82.17	100	4.48537e−33	Coleoptera	Dytiscidae	Hygrotus marklini
Suphrodytes figuratus voucher BMNH:722148 16S ribo	JX221571	82.17	100	4.48537e−33	Coleoptera	Dytiscidae	Suphrodytes figuratus
Deronectes hispanicus mitochondrial 16S rRNA gene	HE613727	83.77	97.44	4.48537e−33	Coleoptera	Dytiscidae	Deronectes hispanicus
Antiporus hollingsworthi mitochondrial partial 16S	FR727246	81.94	98.72	4.48537e−33	Coleoptera	Dytiscidae	Antiporus hollingsworthi
Hygrotus turbidus partial 16S rRNA gene	AJ850390	82.17	100	4.48537e−33	Coleoptera	Dytiscidae	Hygrotus turbidus
Hydroporus glabriusculus 16S ribosomal RNA gene, p	AY365270	83.45	92.95	4.48537e−33	Coleoptera	Dytiscidae	Hydroporus glabriusculus
Agabus glacialis mitochondrial 16S rRNA gene (part	LM654776	81.53	100	1.56555e−32	Coleoptera	Dytiscidae	Agabus glacialis
Hydaticus cinctipennis voucher UNM KBMHyci20 16S r	KJ548338	81.53	100	1.56555e−32	Coleoptera	Dytiscidae	Hydaticus cinctipennis
Hygrotus impressopunctatus voucher UNM KBMHtim80 1	KJ548334	82.58	97.44	1.56555e−32	Coleoptera	Dytiscidae	Hygrotus impressopunctatus
Agabus dichrous mitochondrial partial 16S rRNA gen	HF931378	81.53	100	1.56555e−32	Coleoptera	Dytiscidae	Agabus dichrous
Agabus godmanni mitochondrial 16S rRNA gene (parti	HF931348	81.53	100	1.56555e−32	Coleoptera	Dytiscidae	Agabus godmanni
Hydroporus analis mitochondrial partial 16S rRNA g	HE613731	87.9	79.49	1.56555e−32	Coleoptera	Dytiscidae	Hydroporus analis
Hydroporus fuscipennis mitochondrial 16S rRNA gene	HE613730	87.1	79.49	1.56555e−32	Coleoptera	Dytiscidae	Hydroporus fuscipennis
Deronectes platynotus mitochondrial 16S rRNA gene	HE613692	83.01	97.44	1.56555e−32	Coleoptera	Dytiscidae	Deronectes platynotus
Hydroporus compunctus mitochondrial 16S rRNA gene	HE599693	87.9	79.49	1.56555e−32	Coleoptera	Dytiscidae	Hydroporus compunctus
Antiporus hollingsworthi mitochondrial partial 16S	FR727247	83.1	91.03	1.56555e−32	Coleoptera	Dytiscidae	Antiporus hollingsworthi
Hygrotus impressopunctatus partial 16S rRNA gene	AJ850405	82.47	97.44	1.56555e−32	Coleoptera	Dytiscidae	Hygrotus impressopunctatus
Deronectes delarouzei haplotype 2 16S ribosomal RN	AY250909	83.66	97.44	1.56555e−32	Coleoptera	Dytiscidae	Deronectes delarouzei
Hydroporus compunctus 16S ribosomal RNA gene, part	AF518259	87.9	79.49	1.56555e−32	Coleoptera	Dytiscidae	Hydroporus compunctus
Hydroporus fuscipennis 16S ribosomal RNA gene, par	AF518262	87.1	79.49	1.56555e−32	Coleoptera	Dytiscidae	Hydroporus fuscipennis
Agabus dilatatus 16S ribosomal RNA gene, partial s	AY039261	81.53	100	1.56555e−32	Coleoptera	Dytiscidae	Agabus dilatatus
Agabus bifarius 16S ribosomal RNA gene, partial se	AY138605	81.53	100	1.56555e−32	Coleoptera	Dytiscidae	Agabus bifarius
Deronectes latus voucher UNM KBMDnla560 16S riboso	KJ548380	82.98	90.38	5.4643e−32	Coleoptera	Dytiscidae	Deronectes latus
Hygrotus lutescens mitochondrial partial 16S rRNA	HF931518	81.53	100	5.4643e−32	Coleoptera	Dytiscidae	Hygrotus lutescens
Hygrotus fontinalis mitochondrial 16S rRNA gene (p	HF931494	81.53	100	5.4643e−32	Coleoptera	Dytiscidae	Hygrotus fontinalis
Hygrotus patruelis mitochondrial 16S rRNA gene (pa	HF931388	81.53	100	5.4643e−32	Coleoptera	Dytiscidae	Hygrotus patruelis
Hygrotus sanfilippoi mitochondrial 16S rRNA gene (	HF931342	81.53	100	5.4643e−32	Coleoptera	Dytiscidae	Hygrotus sanfilippoi
Hydroporus brancoi gredensis mitochondrial partial	HF931329	87.5	76.92	5.4643e−32	Coleoptera	Dytiscidae	Hydroporus brancoi gredensis
Suphrodytes figuratus voucher BMNH:722150 16S ribo	JX221572	81.53	100	5.4643e−32	Coleoptera	Dytiscidae	Suphrodytes figuratus
Suphrodytes dorsalis mitochondrial 16S rRNA gene (	HE599700	81.53	100	5.4643e−32	Coleoptera	Dytiscidae	Suphrodytes dorsalis
Hygrotus corpulentus partial 16S rRNA gene	AJ850387	81.53	100	5.4643e−32	Coleoptera	Dytiscidae	Hygrotus corpulentus
Hydroporus pubescens voucher NHM−IR39 16S ribosoma	EF419327	87.5	81.41	5.4643e−32	Coleoptera	Dytiscidae	Hydroporus pubescens
Hydroporus sp. IR33.4 16S ribosomal RNA gene, part	AY365261	81.53	100	5.4643e−32	Coleoptera	Dytiscidae	Hydroporus sp. IR33.4
Scarodytes halensis haplotype 3 16S ribosomal RNA	AY250937	81.65	100	5.4643e−32	Coleoptera	Dytiscidae	Scarodytes halensis
Agabus nebulosus mitochondrial 16S rRNA gene (part	LM654779	80.89	100	1.90723e−31	Coleoptera	Dytiscidae	Agabus nebulosus
