# Supplementary material for: Testing the potential of a ribosomal 16S marker for DNA metabarcoding of insects
Source: PeerJ. 2016 Apr 19;4:e1966. doi: 10.7717/peerj.1966 (PMC4841222; doi:10.7717/peerj.1966)
Supplement: Supplemental Information 1 [file peerj-04-1966-s006.zip › scripts/5 match OTUs/blast_hits/OTU_459.csv_plot.pdf]

OTU_459.csv

Parochlus steinenii

Chironomidae

Diptera

87.18

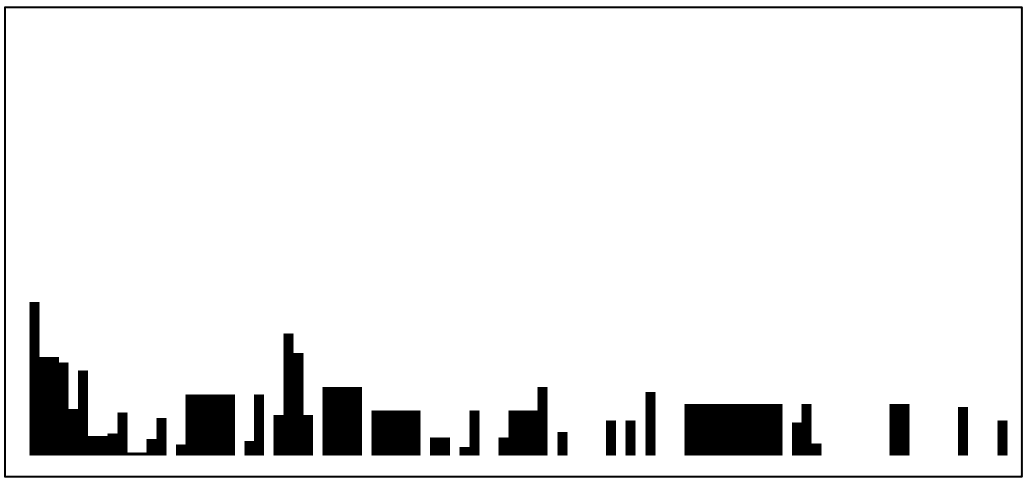

Parochlus steinenii isolate Sejong01 mitochondrion	KT003702	87.18	100	1.46498e−45	Diptera	Chironomidae	Parochlus steinenii
Bolitophila cinerea voucher JSB1 16S ribosomal RNA	KJ136712	84.62	97.44	1.05486e−34	Diptera	Mycetophilidae	Bolitophila cinerea
Bolitophila sp. ER−2009 16S ribosomal RNA gene, pa	FJ172021	84.62	97.44	1.05486e−34	Diptera	Mycetophilidae	Bolitophila sp. ER−2009
Bolitophila occlusa voucher JSB2 16S ribosomal RNA	KJ136713	84.35	93.59	3.68182e−34	Diptera	Mycetophilidae	Bolitophila occlusa
Pedicia albivitta voucher O'Grady 200012 16S ribos	EU005435	82.17	100	3.68182e−34	Diptera	Pediciidae	Pedicia albivitta
Bolitophila hybrida 16S ribosomal RNA gene, partia	FJ172002	83.97	97.44	5.4643e−32	Diptera	Mycetophilidae	Bolitophila hybrida
Tipula abdominalis 16S ribosomal RNA gene, partial	KC177457	80.89	100	2.32348e−30	Diptera	Tipulidae	Tipula abdominalis
Tipula abdominalis mitochondrion, partial genome	JN861743	80.89	100	2.32348e−30	Diptera	Tipulidae	Tipula abdominalis
Tipula sp. PMO 200011 16S ribosomal RNA gene, part	EU005437	81.01	100	8.10974e−30	Diptera	Tipulidae	Tipula sp. PMO 200011
Arachnocampa luminosa biotype 1 16S large subunit	AY576329	82	94.23	8.10974e−30	Diptera	Keroplatidae	Arachnocampa luminosa
Sylvicola fenestralis 16S ribosomal RNA gene, part	KC177461	80.13	100	2.83058e−29	Diptera	Anisopodidae	Sylvicola fenestralis
Sylvicola fenestralis mitochondrion, complete geno	JN861752	80.13	100	2.83058e−29	Diptera	Anisopodidae	Sylvicola fenestralis
Beris morrisii 16S ribosomal RNA gene, partial seq	GU947373	80.77	99.36	9.87969e−29	Diptera	Stratiomyidae	Beris morrisii
Callomyia admirabilis isolate CA42 16S ribosomal R	KC699326	81.75	87.82	3.44835e−28	Diptera	Platypezidae	Callomyia admirabilis
Simulium sheilae isolate SH1B 16S ribosomal RNA ge	KM410213	79.62	100	1.20359e−27	Diptera	Simuliidae	Simulium sheilae
Simulium suyoense mitochondrial gene for 16S ribos	LC035000	80.5	100	4.20095e−27	Diptera	Simuliidae	Simulium suyoense
Simulium roslihashimi isolate R1B 16S ribosomal RN	KM410192	82.84	85.26	4.20095e−27	Diptera	Simuliidae	Simulium roslihashimi
Simulium izuae isolate I3B 16S ribosomal RNA gene,	KM410191	82.84	85.26	4.20095e−27	Diptera	Simuliidae	Simulium izuae
Simulium izuae isolate I2B 16S ribosomal RNA gene,	KM410190	82.84	85.26	4.20095e−27	Diptera	Simuliidae	Simulium izuae
Simulium tuenense mitochondrial gene for 16S ribos	AB591244	82.84	85.26	4.20095e−27	Diptera	Simuliidae	Simulium tuenense
Simulium inthanonense 16S ribosomal RNA gene, part	GQ865995	82.84	85.26	4.20095e−27	Diptera	Simuliidae	Simulium inthanonense
Somatia sp. YSUW04038 16S ribosomal RNA gene, part	AY573134	79.59	94.23	4.20095e−27	Diptera	Somatiidae	Somatia sp. YSUW04038
Arachnocampa luminosa biotype 2 16S large subunit	AY576330	80.67	94.23	4.20095e−27	Diptera	Keroplatidae	Arachnocampa luminosa
Simulium cabrerai mitochondrial gene for 16S rRNA,	AB093131	82.84	85.26	4.20095e−27	Diptera	Simuliidae	Simulium cabrerai
Microsania collarti isolate CAN8 16S ribosomal RNA	KC699334	78.85	99.36	1.46628e−26	Diptera	Platypezidae	Microsania collarti
Callomyia amoena isolate CA06 16S ribosomal RNA ge	KC699325	81.88	87.82	1.46628e−26	Diptera	Platypezidae	Callomyia amoena
Paracladura trichoptera mitochondrion, complete ge	JN861751	85.71	75.64	1.46628e−26	Diptera	Trichoceridae	Paracladura trichoptera
Hercostomus meieri voucher RMBR:103442 16S ribosom	FJ808173	84.8	79.49	1.46628e−26	Diptera	Dolichopodidae	Hercostomus meieri
Arachnocampa luminosa biotype 3 16S large subunit	AY576331	81.88	87.82	1.46628e−26	Diptera	Keroplatidae	Arachnocampa luminosa
Simulium tuyense mitochondrial gene for 16S riboso	LC035004	79.74	97.44	5.11781e−26	Diptera	Simuliidae	Simulium tuyense
Simulium tenederoi mitochondrial gene for 16S ribo	LC035002	83.2	79.49	5.11781e−26	Diptera	Simuliidae	Simulium tenederoi
Simulium tenederoi mitochondrial gene for 16S ribo	LC035001	83.2	79.49	5.11781e−26	Diptera	Simuliidae	Simulium tenederoi
Simulium claveriaense mitochondrial gene for 16S r	LC034988	83.2	79.49	5.11781e−26	Diptera	Simuliidae	Simulium claveriaense
Simulium claveriaense mitochondrial gene for 16S r	LC034987	83.2	79.49	5.11781e−26	Diptera	Simuliidae	Simulium claveriaense
Simulium sheilae isolate SH2B 16S ribosomal RNA ge	KM410214	78.98	100	5.11781e−26	Diptera	Simuliidae	Simulium sheilae
Simulium brinchangense isolate B2B 16S ribosomal R	KM410202	82.09	85.26	5.11781e−26	Diptera	Simuliidae	Simulium brinchangense
Simulium brinchangense isolate B1B 16S ribosomal R	KM410201	82.09	85.26	5.11781e−26	Diptera	Simuliidae	Simulium brinchangense
Simulium tanahrataense isolate TR2B 16S ribosomal	KM410196	82.09	85.26	5.11781e−26	Diptera	Simuliidae	Simulium tanahrataense
Simulium tanahrataense isolate TR1B 16S ribosomal	KM410195	82.09	85.26	5.11781e−26	Diptera	Simuliidae	Simulium tanahrataense
Simulium izuae isolate I1B 16S ribosomal RNA gene,	KM410189	82.09	85.26	5.11781e−26	Diptera	Simuliidae	Simulium izuae
Diadocidia stanfordensis voucher JSSJ14 16S riboso	KJ136742	79.47	91.67	5.11781e−26	Diptera	Diadocidiidae	Diadocidia stanfordensis
Simulium khunklangense mitochondrial gene for 16S	AB820367	80.82	92.31	5.11781e−26	Diptera	Simuliidae	Simulium khunklangense
Simulium chomthongense mitochondrial gene for 16S	AB699899	80.82	92.31	5.11781e−26	Diptera	Simuliidae	Simulium chomthongense
Haematopota pluvialis 16S ribosomal RNA gene, part	KC177454	79.59	94.23	5.11781e−26	Diptera	Tabanidae	Haematopota pluvialis
Trichopeza longicornis voucher Pollet 0243 (P) 16S	HQ449035	80.38	99.36	5.11781e−26	Diptera	Empididae	Trichopeza longicornis
Cyphomyia notabilis 16S ribosomal RNA gene, partia	GU947403	82.09	85.26	5.11781e−26	Diptera	Stratiomyidae	Cyphomyia notabilis
Chrysops viduatus 16S ribosomal RNA gene, partial	HM132115	79.59	94.23	5.11781e−26	Diptera	Tabanidae	Chrysops viduatus
Tabanus bromius 16S ribosomal RNA gene, partial se	HM132111	78.06	99.36	5.11781e−26	Diptera	Tabanidae	Tabanus bromius
Simulium caudisclerum 16S ribosomal RNA gene, part	GQ865999	80.82	92.31	5.11781e−26	Diptera	Simuliidae	Simulium caudisclerum
Simulium chumpornense 16S ribosomal RNA gene, part	GQ865998	82.09	85.26	5.11781e−26	Diptera	Simuliidae	Simulium chumpornense
Simulium decuplum 16S ribosomal RNA gene, partial	GQ865986	82.09	85.26	5.11781e−26	Diptera	Simuliidae	Simulium decuplum
Simulium recurvum mitochondrial gene for 16S ribos	AB334097	82.09	85.26	5.11781e−26	Diptera	Simuliidae	Simulium recurvum
Simulium claveriaense mitochondrial gene for 16S r	AB334096	83.2	79.49	5.11781e−26	Diptera	Simuliidae	Simulium claveriaense
Eutreta novaeboracensis 16S ribosomal RNA gene, pa	DQ471384	79.11	99.36	5.11781e−26	Diptera	Tephritidae	Eutreta novaeboracensis
Caenophthalmus capensis voucher MEI 120162 16S rib	DQ250838	81.08	92.31	5.11781e−26	Diptera	Therevidae	Caenophthalmus capensis
Cnephia dacotensis mitochondrial 16S ribosomal RNA	L13403	80	97.44	5.11781e−26	Diptera	Simuliidae	Cnephia dacotensis
Simulium suyoense mitochondrial gene for 16S ribos	LC034999	79.87	100	1.78629e−25	Diptera	Simuliidae	Simulium suyoense
Simulium suyoense mitochondrial gene for 16S ribos	LC034998	79.87	100	1.78629e−25	Diptera	Simuliidae	Simulium suyoense
Simulium suyoense mitochondrial gene for 16S ribos	LC034997	79.87	100	1.78629e−25	Diptera	Simuliidae	Simulium suyoense
Simulium spinosibranchium mitochondrial gene for 1	LC034996	81.62	85.26	1.78629e−25	Diptera	Simuliidae	Simulium spinosibranchium
Simulium suyoense mitochondrial gene for 16S ribos	AB334099	79.87	100	1.78629e−25	Diptera	Simuliidae	Simulium suyoense
Simulium spinosibranchium mitochondrial gene for 1	AB334098	81.62	85.26	1.78629e−25	Diptera	Simuliidae	Simulium spinosibranchium
Simulium chiharuae mitochondrial gene for 16S ribo	AB334092	80	91.67	1.78629e−25	Diptera	Simuliidae	Simulium chiharuae
Simulium palauense mitochondrial gene for 16S ribo	AB056742	82.96	85.26	1.78629e−25	Diptera	Simuliidae	Simulium palauense
Simulium mie mitochondrial gene for 16S ribosomal	AB056732	79.38	100	1.78629e−25	Diptera	Simuliidae	Simulium mie
Simulium mie mitochondrial gene for 16S ribosomal	AB056731	79.38	100	1.78629e−25	Diptera	Simuliidae	Simulium mie
Simulium mie mitochondrial gene for 16S ribosomal	AB056730	79.38	100	1.78629e−25	Diptera	Simuliidae	Simulium mie
Simulium nobile isolate N5 16S ribosomal RNA gene,	KP661530	82.4	79.49	6.23476e−25	Diptera	Simuliidae	Simulium nobile
Simulium nobile isolate N4 16S ribosomal RNA gene,	KP661529	82.4	79.49	6.23476e−25	Diptera	Simuliidae	Simulium nobile
Simulium nobile isolate N3 16S ribosomal RNA gene,	KP661528	82.4	79.49	6.23476e−25	Diptera	Simuliidae	Simulium nobile
Simulium nobile isolate N2 16S ribosomal RNA gene,	KP661527	82.4	79.49	6.23476e−25	Diptera	Simuliidae	Simulium nobile
Simulium nobile isolate N1 16S ribosomal RNA gene,	KP661526	82.4	79.49	6.23476e−25	Diptera	Simuliidae	Simulium nobile
Simulium timorense isolate TM5 16S ribosomal RNA g	KP661525	82.4	79.49	6.23476e−25	Diptera	Simuliidae	Simulium timorense
Simulium timorense isolate TM4 16S ribosomal RNA g	KP661524	82.4	79.49	6.23476e−25	Diptera	Simuliidae	Simulium timorense
Simulium timorense isolate TM3 16S ribosomal RNA g	KP661523	82.4	79.49	6.23476e−25	Diptera	Simuliidae	Simulium timorense
Simulium timorense isolate TM2 16S ribosomal RNA g	KP661522	82.4	79.49	6.23476e−25	Diptera	Simuliidae	Simulium timorense
Simulium timorense isolate TM1 16S ribosomal RNA g	KP661521	82.4	79.49	6.23476e−25	Diptera	Simuliidae	Simulium timorense
Tongamya stuckenbergi 16S ribosomal RNA gene, part	KM878969	79.38	99.36	6.23476e−25	Diptera	Mydidae	Tongamya stuckenbergi
Keilbachia subacumina voucher 890 16S ribosomal RN	JQ613951	81.54	82.05	6.23476e−25	Diptera	Sciaridae	Keilbachia subacumina
Simulium nobile 16S ribosomal RNA gene, partial se	GQ866008	82.4	79.49	6.23476e−25	Diptera	Simuliidae	Simulium nobile
Clusiodes ruficollis 16S ribosomal RNA gene, parti	EU268538	80.56	91.03	6.23476e−25	Diptera	Clusiidae	Clusiodes ruficollis
Euaresta festiva 16S ribosomal RNA gene, partial s	DQ471399	78.91	94.23	6.23476e−25	Diptera	Tephritidae	Euaresta festiva
Simulium squamosum voucher P.Mansiangi Pm3(BNI) 16	DQ655644	80	92.31	6.23476e−25	Diptera	Simuliidae	Simulium squamosum
Simulium damnosum s.l. GH−D1 16S ribosomal RNA, pa	AY077709	80	92.31	6.23476e−25	Diptera	Simuliidae	Simulium damnosum s.l. GH−D1
Simulium damnosum s.l. Kulfo isolate Ku 3 16S ribo	AF416978	80	92.31	6.23476e−25	Diptera	Simuliidae	Simulium damnosum s.l. Kulfo
Simulium soubrense isolate Wa 52 16S ribosomal RNA	AF416976	80	92.31	6.23476e−25	Diptera	Simuliidae	Simulium soubrense
Simulium soubrense isolate Wa 47 16S ribosomal RNA	AF416975	80	92.31	6.23476e−25	Diptera	Simuliidae	Simulium soubrense
Simulium damnosum strain Se−12 16S ribosomal RNA g	AF081907	80	92.31	6.23476e−25	Diptera	Simuliidae	Simulium damnosum
Simulium nobile mitochondrial gene for 16S rRNA, p	AB093115	82.4	79.49	6.23476e−25	Diptera	Simuliidae	Simulium nobile
Simulium baltazarae mitochondrial gene for 16S rRN	AB093114	82.4	79.49	6.23476e−25	Diptera	Simuliidae	Simulium baltazarae
Simulium uchidai mitochondrial gene for 16S riboso	AB056740	80	92.31	6.23476e−25	Diptera	Simuliidae	Simulium uchidai
Simulium saitoi mitochondrial gene for 16S ribosom	AB056733	79.73	91.67	6.23476e−25	Diptera	Simuliidae	Simulium saitoi
Simulium leonense mitochondrion 16S ribosomal RNA	U17731	80	92.31	6.23476e−25	Diptera	Simuliidae	Simulium leonense
Simulium tuyense mitochondrial gene for 16S riboso	LC035008	79.08	97.44	2.17615e−24	Diptera	Simuliidae	Simulium tuyense
Simulium tuyense mitochondrial gene for 16S riboso	LC035007	79.08	97.44	2.17615e−24	Diptera	Simuliidae	Simulium tuyense
Simulium tuyense mitochondrial gene for 16S riboso	LC035006	82.26	78.85	2.17615e−24	Diptera	Simuliidae	Simulium tuyense
Simulium tuyense mitochondrial gene for 16S riboso	LC035005	79.08	97.44	2.17615e−24	Diptera	Simuliidae	Simulium tuyense
Simulium tuyense mitochondrial gene for 16S riboso	LC035003	79.08	97.44	2.17615e−24	Diptera	Simuliidae	Simulium tuyense
Artystona sp. 1 GJK−2014 16S ribosomal RNA gene, p	KJ003047	79.73	93.59	2.17615e−24	Coleoptera	Tenebrionidae	Artystona sp. 1 GJK−2014
Keroplatus testaceus voucher JSB7 16S ribosomal RN	KJ136718	81.62	86.54	2.17615e−24	Diptera	Keroplatidae	Keroplatus testaceus
