# Supplementary material for: Testing the potential of a ribosomal 16S marker for DNA metabarcoding of insects
Source: PeerJ. 2016 Apr 19;4:e1966. doi: 10.7717/peerj.1966 (PMC4841222; doi:10.7717/peerj.1966)
Supplement: Supplemental Information 1 [file peerj-04-1966-s006.zip › scripts/5 match OTUs/blast_hits/OTU_47.csv_plot.pdf]

OTU_47.csv

Bactrocera abdopallescens

Tephritidae

Diptera

74.36

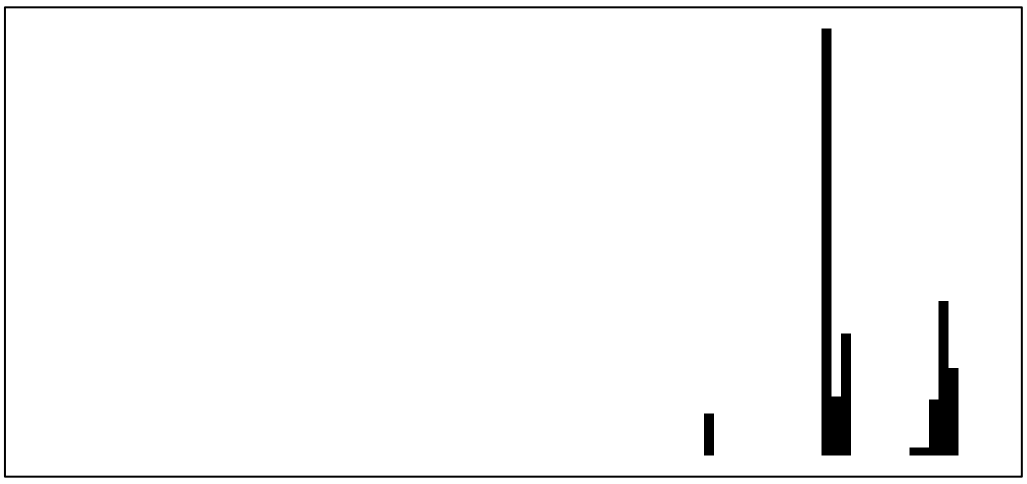

Bactrocera abdopallescens voucher Babdopa222 16S r	JQ671100	74.36	98.69	1.02961e−15	Diptera	Tephritidae	Bactrocera abdopallescens
Atheta hampshirensis voucher ZMUN:10002558 16S rib	GQ981020	74.34	98.69	3.59371e−15	Coleoptera	Staphylinidae	Atheta hampshirensis
Atheta hampshirensis voucher ZMUN:10002545 16S rib	GQ981019	74.34	98.69	3.59371e−15	Coleoptera	Staphylinidae	Atheta hampshirensis
Sphyracephala brevicornis 16S ribosomal RNA gene,	AF304730	73.12	98.69	4.37803e−14	Diptera	Diopsidae	Sphyracephala brevicornis
Cicindela laetescripta 16S ribosomal RNA gene, par	KC963598	76.92	98.04	6.49757e−12	Coleoptera	Carabidae	Cicindela laetescripta
Heniartes sp. 00004418 16S ribosomal RNA gene, par	JQ942249	71.61	98.69	6.49757e−12	Hemiptera	Reduviidae	Heniartes sp. 00004418
Ochthebius gibbosus voucher MNCN−AI365/23711 16S r	GU143727	74.17	98.04	6.49757e−12	Coleoptera	Hydraenidae	Ochthebius gibbosus
Ochthebius gibbosus voucher MNCN−AI794/24140 16S r	GU143726	74.17	98.04	6.49757e−12	Coleoptera	Hydraenidae	Ochthebius gibbosus
Semidalis prorecta 16S ribosomal RNA gene, partial	EF632332	73.89	98.69	6.49757e−12	Neuroptera	Coniopterygidae	Semidalis prorecta
Brachyaciura limbata 16S ribosomal RNA gene, parti	DQ471393	71.52	98.69	6.49757e−12	Diptera	Tephritidae	Brachyaciura limbata
Cicindela laetescripta mitochondrial gene for 16S	AB118719	76.92	98.04	6.49757e−12	Coleoptera	Carabidae	Cicindela laetescripta
Cicindela laetescripta mitochondrial gene for 16S	AB118715	76.92	98.04	6.49757e−12	Coleoptera	Carabidae	Cicindela laetescripta
Cicindela laetescripta mitochondrial gene for 16S	AB118713	76.92	98.04	6.49757e−12	Coleoptera	Carabidae	Cicindela laetescripta
Cicindela laetescripta mitochondrial gene for 16S	AB118712	76.92	98.04	6.49757e−12	Coleoptera	Carabidae	Cicindela laetescripta
Cicindela laetescripta mitochondrial gene for 16S	AB118711	76.92	98.04	6.49757e−12	Coleoptera	Carabidae	Cicindela laetescripta
Cicindela laetescripta mitochondrial gene for 16S	AB118709	76.92	98.04	6.49757e−12	Coleoptera	Carabidae	Cicindela laetescripta
Trichosia morio voucher 945 16S ribosomal RNA gene	JQ613960	72.26	98.04	2.26788e−11	Diptera	Sciaridae	Trichosia morio
Cicindela laetescripta mitochondrial gene for 16S	AB118718	76.28	98.04	7.91566e−11	Coleoptera	Carabidae	Cicindela laetescripta
Cicindela laetescripta mitochondrial gene for 16S	AB118714	75.64	98.04	7.91566e−11	Coleoptera	Carabidae	Cicindela laetescripta
Cicindela laetescripta mitochondrial gene for 16S	AB118710	76.28	98.04	7.91566e−11	Coleoptera	Carabidae	Cicindela laetescripta
Necrobia rufipes 16S ribosomal RNA gene, partial s	EF508044	72.26	98.69	2.76284e−10	Coleoptera	Cleridae	Necrobia rufipes
Amphizoa lecontei 16S ribosomal RNA gene, partial	AY071771	72.9	100	2.76284e−10	Coleoptera	Amphizoidae	Amphizoa lecontei
Phormia regina mitochondrion, complete genome	KC005712	70.78	100	9.64325e−10	Diptera	Calliphoridae	Phormia regina
Baoris farri voucher He049 16S ribosomal RNA gene,	JX971190	72	94.12	9.64325e−10	Lepidoptera	Hesperiidae	Baoris farri
Phormia regina voucher NICC0266 16S ribosomal RNA	KF908064	70.78	100	9.64325e−10	Diptera	Calliphoridae	Phormia regina
Phormia regina voucher NICC0261 16S ribosomal RNA	KF908060	70.78	100	9.64325e−10	Diptera	Calliphoridae	Phormia regina
Phormia regina voucher NICC0256 16S ribosomal RNA	KF908058	70.78	100	9.64325e−10	Diptera	Calliphoridae	Phormia regina
Phormia regina voucher NICC0038 16S ribosomal RNA	KF908054	70.78	100	9.64325e−10	Diptera	Calliphoridae	Phormia regina
Rhyncomya soyauxi isolate D39 small subunit riboso	JQ246744	70.78	100	9.64325e−10	Diptera	Calliphoridae	Rhyncomya soyauxi
Phormia regina isolate D12 small subunit ribosomal	JQ246718	70.78	100	9.64325e−10	Diptera	Calliphoridae	Phormia regina
Cynomya mortuorum voucher RMBR:103606 16S ribosoma	GQ409125	70.78	100	9.64325e−10	Diptera	Calliphoridae	Cynomya mortuorum
Delia platura large subunit ribosomal RNA gene, mi	AF086867	70.13	100	9.64325e−10	Diptera	Anthomyiidae	Delia platura
Baoris farri voucher He091 16S ribosomal RNA gene,	JX971189	71.52	94.12	3.36583e−09	Lepidoptera	Hesperiidae	Baoris farri
Paramanota orientalis voucher JSL2 16S ribosomal R	KC435587	71.43	98.04	3.36583e−09	Diptera	Mycetophilidae	Paramanota orientalis
Silpha carinata isolate 8 16S ribosomal RNA gene,	JF794603	71.07	98.04	3.36583e−09	Coleoptera	Silphidae	Silpha carinata
Silpha carinata voucher CSU10020220B51 16S ribosom	HM051220	71.07	98.04	3.36583e−09	Coleoptera	Silphidae	Silpha carinata
Silpha carinata voucher CSU1002022059 16S ribosoma	HM051217	71.07	98.04	3.36583e−09	Coleoptera	Silphidae	Silpha carinata
Necrobia rufipes voucher COL970 12S ribosomal RNA	KC524798	71.61	98.69	1.17479e−08	Coleoptera	Cleridae	Necrobia rufipes
Tetragoneura sylvatica 16S ribosomal RNA gene, par	FJ172028	70.97	98.04	1.17479e−08	Diptera	Mycetophilidae	Tetragoneura sylvatica
Drepanepteryx phalaenoides 16S ribosomal RNA gene,	EU734861	71.15	98.69	1.17479e−08	Neuroptera	Hemerobiidae	Drepanepteryx phalaenoides
Antiporus hollingsworthi mitochondrial partial 16S	FR727246	71.26	100	1.43119e−07	Coleoptera	Dytiscidae	Antiporus hollingsworthi
Aspidytes niobe completemitochondrial genome	AM493668	72.15	100	1.43119e−07	Coleoptera	Aspidytidae	Aspidytes niobe
Aspidytes niobe 16S ribosomal RNA gene, partial se	AY071782	72.15	100	1.43119e−07	Coleoptera	Aspidytidae	Aspidytes niobe
Elaphrus cupreus mitochondrial large subunit 16S r	L42917	70.78	100	1.43119e−07	Coleoptera	Carabidae	Elaphrus cupreus
Coenosia semifumosa voucher RMBR:116187 16S riboso	KJ476301	68.83	100	4.99533e−07	Diptera	Muscidae	Coenosia semifumosa
Atherigona seticauda voucher RMBR:116178 16S ribos	KJ476295	70.59	98.69	4.99533e−07	Diptera	Muscidae	Atherigona seticauda
Antiporus hollingsworthi mitochondrial partial 16S	FR727247	70.66	100	4.99533e−07	Coleoptera	Dytiscidae	Antiporus hollingsworthi
Argynnis anadyomene ella 16S ribosomal RNA gene, p	GU372438	71.05	94.12	4.99533e−07	Lepidoptera	Nymphalidae	Argynnis anadyomene ella
Sundaptinus sp. MSL2007−1 voucher 677493 16S ribos	FJ613834	70.7	98.69	4.99533e−07	Coleoptera	Ptinidae	Sundaptinus sp. MSL2007−1
Calyptoglossa frontalis mitochondrial large subuni	L42909	73.38	98.04	4.99533e−07	Coleoptera	Carabidae	Calyptoglossa frontalis
Neomuscina instabilis voucher RMBR:116213 16S ribo	KJ476320	69.28	99.35	1.74354e−06	Diptera	Muscidae	Neomuscina instabilis
Listrocerus kuscheli voucher UPOL001182 16S riboso	HQ619679	71.34	98.04	1.74354e−06	Coleoptera	Melyridae	Listrocerus kuscheli
Holops sp. SLW−2002 16S ribosomal RNA gene, partia	AY140861	70.7	97.39	1.74354e−06	Diptera	Acroceridae	Holops sp. SLW−2002
Pityokteines lasiocarpi isolate Pitlas1 16S riboso	KF862731	74.76	67.32	6.08556e−06	Coleoptera	Curculionidae	Pityokteines lasiocarpi
Stephanorrhina princeps voucher 842739 16S ribosom	JX234449	70.78	98.69	6.08556e−06	Coleoptera	Scarabaeidae	Stephanorrhina princeps
Boreonectes funereus mitochondrial 16S rRNA gene (	HF931393	70.78	99.35	6.08556e−06	Coleoptera	Dytiscidae	Boreonectes funereus
Drymeia alpicola 16S ribosomal RNA gene, partial s	FJ025430	69.87	100	6.08556e−06	Diptera	Muscidae	Drymeia alpicola
Oreodytes congruus partial 16S rRNA gene	AJ850347	70.51	100	6.08556e−06	Coleoptera	Dytiscidae	Oreodytes congruus
Rhaphium appendiculatum voucher Pollet 0008 (P) 16	EU864026	69.18	98.69	2.12407e−05	Diptera	Dolichopodidae	Rhaphium appendiculatum
Battus philenor isolate FS−a−3 16S ribosomal RNA g	DQ351083	69.54	94.12	2.12407e−05	Lepidoptera	Papilionidae	Battus philenor
Epilachna anhweiana voucher KS201 16S ribosomal RN	KP143614	69.54	88.89	7.41372e−05	Coleoptera	Coccinellidae	Epilachna anhweiana
Angiostrongylus cantonensis genome assembly A_cant	LK946873	75.86	55.56	7.41372e−05	Rhabditida	Angiostrongylidae	Angiostrongylus cantonensis
Zemeros flegyas 16S ribosomal RNA gene, partial se	DQ445106	68.99	98.04	7.41372e−05	Lepidoptera	Riodinidae	Zemeros flegyas
Aspidytes niobe 16S ribosomal RNA gene, partial se	AY071783	70.89	100	7.41372e−05	Coleoptera	Aspidytidae	Aspidytes niobe
Amphizoa insolens 16S ribosomal RNA gene, partial	AY071770	70.97	100	7.41372e−05	Coleoptera	Amphizoidae	Amphizoa insolens
Battus philenor mitochondrion, large subunit ribos	AJ224048	69.08	94.12	7.41372e−05	Lepidoptera	Papilionidae	Battus philenor
Charaxes bernardus 16S ribosomal RNA gene, partial	KJ777737	69.54	94.12	0.000258764	Lepidoptera	Nymphalidae	Charaxes bernardus
Perreyia flavipes voucher AE127 16S ribosomal RNA	KF318499	68.87	98.04	0.000258764	Hymenoptera	Pergidae	Perreyia flavipes
Pharmacophagus antenor voucher FS−b−1646 16S ribos	GQ268346	70.68	83.66	0.000258764	Lepidoptera	Papilionidae	Pharmacophagus antenor
Chlorocebus aethiops BAC clone CH252−473I8 from ch	AC239374	81.97	37.91	0.000258764	Primates	Cercopithecidae	Chlorocebus aethiops
Echinodera ariadnae isolate ZFMK−DNA JJ0124 16S ri	EU286314	72.39	82.35	0.000258764	Coleoptera	Curculionidae	Echinodera ariadnae
Pharmacophagus antenor mitochondrial partial 16S r	AM283054	70.9	83.66	0.000258764	Lepidoptera	Papilionidae	Pharmacophagus antenor
Oreodytes sanmarkii mitochondrial 16S rRNA gene (p	HF931524	70.25	100	0.000903177	Coleoptera	Dytiscidae	Oreodytes sanmarkii
Epilachna anhweiana isolate LBB242 16S ribosomal R	EU392398	68.21	88.89	0.000903177	Coleoptera	Coccinellidae	Epilachna anhweiana
Echinodera siciliensis bio−material ZFMK−DNA JJ012	GU213510	70.9	82.35	0.000903177	Coleoptera	Curculionidae	Echinodera siciliensis
Populus trichocarpa clone POP040−E22, complete seq	AC209002	72.31	79.74	0.000903177	Malpighiales	Salicaceae	Populus trichocarpa
Tetrigus cyprius voucher UPOL RK0228 16S ribosomal	KF626032	69.93	98.69	0.0031524	Coleoptera	Elateridae	Tetrigus cyprius
Atherigona falcata 16S ribosomal RNA gene, partial	JN226638	68.63	98.69	0.0031524	Diptera	Muscidae	Atherigona falcata
Antiporus wilsoni mitochondrial partial 16S rRNA g	FR727249	69.33	100	0.0031524	Coleoptera	Dytiscidae	Antiporus wilsoni
Ophyra spinigera isolate CSU0904046107 16S ribosom	GQ118352	68.63	98.69	0.0031524	Diptera	Muscidae	Ophyra spinigera
Oreodytes crassulus partial 16S rRNA gene	AJ850348	69.68	100	0.0031524	Coleoptera	Dytiscidae	Oreodytes crassulus
Soboliphyme baturini genome assembly S_baturini_Da	LM028046	100	17.65	0.0110029		Soboliphymatidae	Soboliphyme baturini
Strongyloides ratti genome assembly S_ratti_ED321	LN609408	82.76	37.25	0.0110029	Rhabditida	Strongyloididae	Strongyloides ratti
Cyprinus carpio genome assembly common carp genome	LN590707	85.71	31.37	0.0110029	Cypriniformes	Cyprinidae	Cyprinus carpio
Echinodera siciliensis bio−material ZFMK−DNA JJ078	GU988530	70.15	82.35	0.0110029	Coleoptera	Curculionidae	Echinodera siciliensis
Echinodera siciliensis bio−material ZFMK−DNA JJ002	GU988175	70.15	82.35	0.0110029	Coleoptera	Curculionidae	Echinodera siciliensis
Echinodera siciliensis bio−material ZFMK−DNA JJ088	GU213632	70.15	82.35	0.0110029	Coleoptera	Curculionidae	Echinodera siciliensis
Echinodera siciliensis isolate ZFMK−DNA JJ0126 16S	EU286315	70.15	82.35	0.0110029	Coleoptera	Curculionidae	Echinodera siciliensis
Papuadytes sp. MB166 16S ribosomal RNA gene, parti	AY334141	68.39	99.35	0.0110029	Coleoptera	Dytiscidae	Papuadytes sp. MB166
Monosiga brevicollis mitochondrion, complete genom	AF538053	72.22	78.43	0.0110029	Choanoflagellida	Codonosigidae	Monosiga brevicollis
Cucumis melo genomic chromosome, chr_2	LN713256	80.36	36.6	0.038404	Cucurbitales	Cucurbitaceae	Cucumis melo
Cucumis melo genomic scaffold, anchoredscaffold000	LN681807	80.36	36.6	0.038404	Cucurbitales	Cucurbitaceae	Cucumis melo
PREDICTED: Apaloderma vittatum forkhead box O1 (FO	XM_009872611	82.61	30.07	0.038404	Trogoniformes	Trogonidae	Apaloderma vittatum
Parastrongyloides trichosuri genome assembly P_tri	LM523160	87.23	29.41	0.038404	Rhabditida	Strongyloididae	Parastrongyloides trichosuri
Anopheles cruzii mitochondrion, complete genome	KJ701506	84.09	28.76	0.038404	Diptera	Culicidae	Anopheles cruzii
Hellula undalis mitochondrion, partial genome	KJ636057	70.13	94.12	0.038404	Lepidoptera	Crambidae	Hellula undalis
Allorhogas ingavera strain CNIN1076 16S ribosomal	KJ586729	68.83	98.69	0.038404	Hymenoptera	Braconidae	Allorhogas ingavera
Spiroplasma taiwanense CT−1 plasmid, complete sequ	CP005075	69.92	84.31	0.038404	Entomoplasmatales	Spiroplasmataceae	Spiroplasma taiwanense CT−1
Antiporus bakewellii mitochondrial partial 16S rRN	FR727259	68.71	100	0.038404	Coleoptera	Dytiscidae	Antiporus bakewellii
Antiporus wilsoni mitochondrial partial 16S rRNA g	FR727256	68.71	100	0.038404	Coleoptera	Dytiscidae	Antiporus wilsoni
