# Supplementary material for: Testing the potential of a ribosomal 16S marker for DNA metabarcoding of insects
Source: PeerJ. 2016 Apr 19;4:e1966. doi: 10.7717/peerj.1966 (PMC4841222; doi:10.7717/peerj.1966)
Supplement: Supplemental Information 1 [file peerj-04-1966-s006.zip › scripts/5 match OTUs/blast_hits/OTU_491.csv_plot.pdf]

OTU_491.csv

Oreodytes sanmarkii

Dytiscidae

Coleoptera

99.35

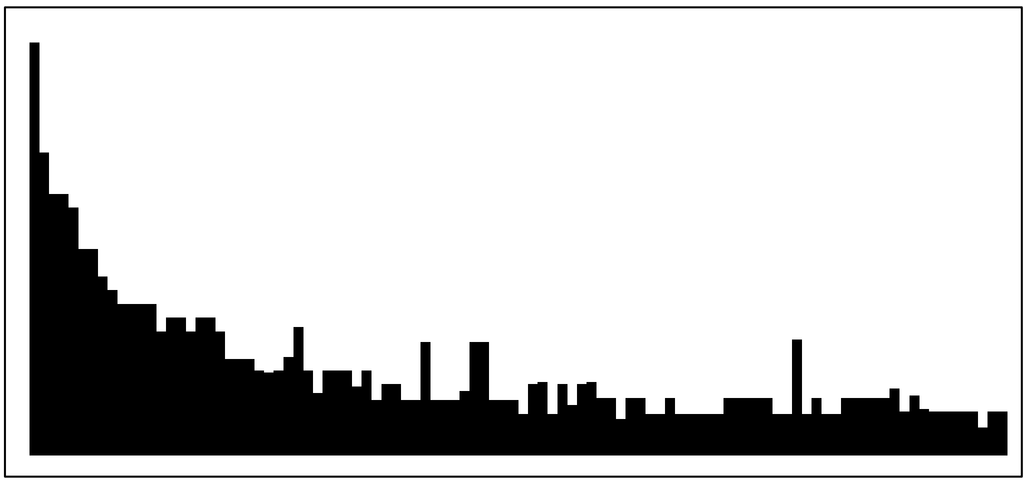

Oreodytes sanmarkii mitochondrial 16S rRNA gene (p	HF931524	99.35	98.73	1.5757e−70	Coleoptera	Dytiscidae	Oreodytes sanmarkii
Oreodytes obesus cordillerensis mitochondrial part	HF931516	94.19	98.73	1.13458e−59	Coleoptera	Dytiscidae	Oreodytes obesus cordillerensis
Oreodytes crassulus partial 16S rRNA gene	AJ850348	92.26	98.73	7.15999e−56	Coleoptera	Dytiscidae	Oreodytes crassulus
Oreodytes congruus partial 16S rRNA gene	AJ850347	92.26	98.73	7.15999e−56	Coleoptera	Dytiscidae	Oreodytes congruus
Oreodytes congruus 16S ribosomal RNA gene, partial	KF575550	91.61	98.73	3.04451e−54	Coleoptera	Dytiscidae	Oreodytes congruus
Oreodytes abbreviatus mitochondrial 16S rRNA gene	HF931477	89.68	98.73	1.92129e−50	Coleoptera	Dytiscidae	Oreodytes abbreviatus
Oreodytes rhyacophilus partial 16S rRNA gene	AJ850352	89.68	98.73	1.92129e−50	Coleoptera	Dytiscidae	Oreodytes rhyacophilus
Oreodytes mongolicus mitochondrial 16S rRNA gene (	HF931491	88.39	98.73	9.95255e−48	Coleoptera	Dytiscidae	Oreodytes mongolicus
Oreodytes snoqualmie mitochondrial partial 16S rRN	HF931519	87.74	98.73	4.23193e−46	Coleoptera	Dytiscidae	Oreodytes snoqualmie
Oreodytes shorti mitochondrial 16S rRNA gene (part	HF931488	87.1	98.73	5.15555e−45	Coleoptera	Dytiscidae	Oreodytes shorti
Boreonectes riberae mitochondrial 16S rRNA gene (p	HF931461	87.1	98.73	6.28074e−44	Coleoptera	Dytiscidae	Boreonectes riberae
Boreonectes griseostriatus mitochondrial partial 1	HF931387	87.1	98.73	6.28074e−44	Coleoptera	Dytiscidae	Boreonectes griseostriatus
Boreonectes multilineatus mitochondrial partial 16	HF931384	87.1	98.73	6.28074e−44	Coleoptera	Dytiscidae	Boreonectes multilineatus
Laccornis difformis 16S ribosomal RNA gene, partia	KF575544	85.81	98.73	2.67064e−42	Coleoptera	Dytiscidae	Laccornis difformis
Boreonectes alpestris mitochondrial 16S rRNA gene	HF931512	86.45	98.73	2.67064e−42	Coleoptera	Dytiscidae	Boreonectes alpestris
Boreonectes striatellus mitochondrial 16S rRNA gen	HF931511	86.45	98.73	2.67064e−42	Coleoptera	Dytiscidae	Boreonectes striatellus
Deronectes parvicollis mitochondrial 16S rRNA gene	HF931454	85.81	98.73	2.67064e−42	Coleoptera	Dytiscidae	Deronectes parvicollis
Boreonectes macedonicus mitochondrial 16S rRNA gen	HF931359	86.45	98.73	2.67064e−42	Coleoptera	Dytiscidae	Boreonectes macedonicus
Stictotarsus ibericus 16S ribosomal RNA (rrnL) gen	EF670030	86.45	98.73	2.67064e−42	Coleoptera	Dytiscidae	Stictotarsus ibericus
Boreonectes griseostriatus mitochondrial 16S rRNA	HF931541	85.81	98.73	3.2535e−41	Coleoptera	Dytiscidae	Boreonectes griseostriatus
Oreodytes scitulus 16S ribosomal RNA gene, partial	KF575551	84.52	98.73	1.38343e−39	Coleoptera	Dytiscidae	Oreodytes scitulus
Oreodytes scitulus mitochondrial partial 16S rRNA	HF931515	84.52	98.73	1.38343e−39	Coleoptera	Dytiscidae	Oreodytes scitulus
Laccornis kocae partial 16S rRNA gene	AJ850419	84.52	98.73	1.38343e−39	Coleoptera	Dytiscidae	Laccornis kocae
Suphrodytes dorsalis 16S ribosomal RNA gene, parti	KF575577	83.97	98.73	7.16632e−37	Coleoptera	Dytiscidae	Suphrodytes dorsalis
Boreonectes griseostriatus mitochondrial 16S rRNA	HF931385	83.87	98.73	7.16632e−37	Coleoptera	Dytiscidae	Boreonectes griseostriatus
Suphrodytes dorsalis voucher BMNH:725966 16S ribos	JX221574	83.97	98.73	7.16632e−37	Coleoptera	Dytiscidae	Suphrodytes dorsalis
Hydroporus foveolatus mitochondrial 16S rRNA gene	HE613724	84.62	98.73	7.16632e−37	Coleoptera	Dytiscidae	Hydroporus foveolatus
Deronectes moestus mitochondrial 16S rRNA gene (pa	HE613726	86.01	90.45	2.50129e−36	Coleoptera	Dytiscidae	Deronectes moestus
Stictotarsus maghrebinus 16S ribosomal RNA gene, p	AY250947	83.97	98.73	2.50129e−36	Coleoptera	Dytiscidae	Stictotarsus maghrebinus
Aethionectes fulvonotatus voucher KBMAeop406 16S r	KF979088	82.91	98.73	8.73037e−36	Coleoptera	Dytiscidae	Aethionectes fulvonotatus
Hydroporus tessellatus 16S ribosomal RNA gene, par	JX434770	83.97	98.73	8.73037e−36	Coleoptera	Dytiscidae	Hydroporus tessellatus
Hydroporus zimmermanni mitochondrial 16S rRNA gene	HE613679	83.97	98.73	8.73037e−36	Coleoptera	Dytiscidae	Hydroporus zimmermanni
Hydroporus basinotatus mitochondrial 16S rRNA gene	HE613678	83.97	98.73	8.73037e−36	Coleoptera	Dytiscidae	Hydroporus basinotatus
Hydroporus glabriusculus 16S ribosomal RNA gene, p	AY365270	83.23	98.73	8.73037e−36	Coleoptera	Dytiscidae	Hydroporus glabriusculus
Hydroporus basinotatus 16S ribosomal RNA gene, par	AF518258	83.97	98.73	8.73037e−36	Coleoptera	Dytiscidae	Hydroporus basinotatus
Oreodytes quadrimaculatus 16S ribosomal RNA gene,	KF575567	82.58	98.73	3.0472e−35	Coleoptera	Dytiscidae	Oreodytes quadrimaculatus
Stictotarsus procerus mitochondrial 16S rRNA gene	HF931379	83.33	98.73	3.0472e−35	Coleoptera	Dytiscidae	Stictotarsus procerus
Deronectes opatrinus mitochondrial 16S rRNA gene (	HE613701	83.33	98.73	3.0472e−35	Coleoptera	Dytiscidae	Deronectes opatrinus
Copelatus sp. BI−2011 voucher BMNH792945 16S ribos	HQ381441	82.58	98.73	3.0472e−35	Coleoptera	Dytiscidae	Copelatus sp. BI−2011
Oreodytes quadrimaculatus partial 16S rRNA gene	AJ850351	82.58	98.73	3.0472e−35	Coleoptera	Dytiscidae	Oreodytes quadrimaculatus
Deronectes moestus 16S ribosomal RNA gene, partial	KF575561	85.31	90.45	1.06358e−34	Coleoptera	Dytiscidae	Deronectes moestus
Hygrotus novemlineatus mitochondrial partial 16S r	HF931523	82.58	98.73	1.06358e−34	Coleoptera	Dytiscidae	Hygrotus novemlineatus
Hygrotus marklini mitochondrial 16S rRNA gene (par	HF931467	82.58	98.73	1.06358e−34	Coleoptera	Dytiscidae	Hygrotus marklini
Suphrodytes figuratus voucher BMNH:722148 16S ribo	JX221571	82.58	98.73	1.06358e−34	Coleoptera	Dytiscidae	Suphrodytes figuratus
Hydroporus marginatus mitochondrial 16S rRNA gene	HE613699	83.02	98.73	1.06358e−34	Coleoptera	Dytiscidae	Hydroporus marginatus
Deronectes brannani mitochondrial 16S rRNA gene (p	HE613695	85.31	90.45	1.06358e−34	Coleoptera	Dytiscidae	Deronectes brannanii
Deronectes moestus mitochondrial 16S rRNA gene (pa	HE613683	85.31	90.45	1.06358e−34	Coleoptera	Dytiscidae	Deronectes moestus
Hygrotus turbidus partial 16S rRNA gene	AJ850390	82.58	98.73	1.06358e−34	Coleoptera	Dytiscidae	Hygrotus turbidus
Hydroporus sp. IR33.4 16S ribosomal RNA gene, part	AY365261	82.58	98.73	1.06358e−34	Coleoptera	Dytiscidae	Hydroporus sp. IR33.4
Hydroporus marginatus 16S ribosomal RNA gene, part	AF518267	82.58	98.73	1.06358e−34	Coleoptera	Dytiscidae	Hydroporus marginatus
Hydaticus cinctipennis voucher UNM KBMHyci20 16S r	KJ548338	81.94	98.73	3.71225e−34	Coleoptera	Dytiscidae	Hydaticus cinctipennis
Hydroporus analis mitochondrial partial 16S rRNA g	HE613731	83.33	98.73	3.71225e−34	Coleoptera	Dytiscidae	Hydroporus analis
Hydroporus fuscipennis mitochondrial 16S rRNA gene	HE613730	83.44	98.73	3.71225e−34	Coleoptera	Dytiscidae	Hydroporus fuscipennis
Deronectes bicostatus mitochondrial 16S rRNA gene	HE613705	81.94	98.73	3.71225e−34	Coleoptera	Dytiscidae	Deronectes bicostatus
Hydroporus compunctus mitochondrial 16S rRNA gene	HE599693	83.33	98.73	3.71225e−34	Coleoptera	Dytiscidae	Hydroporus compunctus
Antiporus hollingsworthi mitochondrial partial 16S	FR727246	82.35	97.45	3.71225e−34	Coleoptera	Dytiscidae	Antiporus hollingsworthi
Hydroporus compunctus 16S ribosomal RNA gene, part	AF518259	83.33	98.73	3.71225e−34	Coleoptera	Dytiscidae	Hydroporus compunctus
Hydroporus fuscipennis 16S ribosomal RNA gene, par	AF518262	83.44	98.73	3.71225e−34	Coleoptera	Dytiscidae	Hydroporus fuscipennis
Deronectes doriae mitochondrial 16S rRNA gene (par	HE613710	82.69	98.73	1.2957e−33	Coleoptera	Dytiscidae	Deronectes doriae
Deronectes sahlbergi mitochondrial 16S rRNA gene (	HE613680	82.69	98.73	1.2957e−33	Coleoptera	Dytiscidae	Deronectes sahlbergi
Antiporus hollingsworthi mitochondrial partial 16S	FR727247	81.7	97.45	1.2957e−33	Coleoptera	Dytiscidae	Antiporus hollingsworthi
Stictotarsus falli 16S ribosomal RNA (rrnL) gene,	EF670029	82.69	98.73	1.2957e−33	Coleoptera	Dytiscidae	Stictotarsus falli
Nirripirti megamacrocephalus isolate ABTC78781 16S	EU616935	82.69	98.73	1.2957e−33	Coleoptera	Dytiscidae	Paroster megamacrocephalus
Hydroporus appalachius 16S ribosomal RNA gene, par	JX434765	81.94	98.73	4.52244e−33	Coleoptera	Dytiscidae	Hydroporus appalachius
Hygrotus lutescens mitochondrial partial 16S rRNA	HF931518	81.94	98.73	4.52244e−33	Coleoptera	Dytiscidae	Hygrotus lutescens
Hydroporus foveolatus mitochondrial 16S rRNA gene	HF931502	82.69	98.73	4.52244e−33	Coleoptera	Dytiscidae	Hydroporus foveolatus
Hygrotus fontinalis mitochondrial 16S rRNA gene (p	HF931494	81.94	98.73	4.52244e−33	Coleoptera	Dytiscidae	Hygrotus fontinalis
Boreonectes funereus mitochondrial 16S rRNA gene (	HF931393	81.94	98.73	4.52244e−33	Coleoptera	Dytiscidae	Boreonectes funereus
Hygrotus patruelis mitochondrial 16S rRNA gene (pa	HF931388	81.94	98.73	4.52244e−33	Coleoptera	Dytiscidae	Hygrotus patruelis
Hydroporus brancoi gredensis mitochondrial partial	HF931329	81.94	98.73	4.52244e−33	Coleoptera	Dytiscidae	Hydroporus brancoi gredensis
Suphrodytes figuratus voucher BMNH:722150 16S ribo	JX221572	81.94	98.73	4.52244e−33	Coleoptera	Dytiscidae	Suphrodytes figuratus
Hydroporus discretus mitochondrial 16S rRNA gene (	HE613723	82.69	98.73	4.52244e−33	Coleoptera	Dytiscidae	Hydroporus discretus
Hydroporus decipiens mitochondrial 16S rRNA gene (	HE613722	82.69	98.73	4.52244e−33	Coleoptera	Dytiscidae	Hydroporus decipiens
Hydroporus pubescens mitochondrial 16S rRNA gene (	HE613707	82.69	98.73	4.52244e−33	Coleoptera	Dytiscidae	Hydroporus pubescens
Hydroporus limbatus mitochondrial 16S rRNA gene (p	HE613706	82.69	98.73	4.52244e−33	Coleoptera	Dytiscidae	Hydroporus limbatus
Hydroporus errans mitochondrial 16S rRNA gene (par	HE613704	82.69	98.73	4.52244e−33	Coleoptera	Dytiscidae	Hydroporus errans
Suphrodytes dorsalis mitochondrial 16S rRNA gene (	HE599700	81.94	98.73	4.52244e−33	Coleoptera	Dytiscidae	Suphrodytes dorsalis
Hydroporus pervicinus mitochondrial 16S rRNA gene	HE599695	81.94	98.73	4.52244e−33	Coleoptera	Dytiscidae	Hydroporus pervicinus
Hovahydrus sp. BI−2011 voucher BMNH831333 16S ribo	HQ381524	85.42	90.45	4.52244e−33	Coleoptera	Dytiscidae	Hovahydrus sp. BI−2011
Nirripirti newhavenensis 16S ribosomal RNA gene, p	AY353844	81.94	98.73	4.52244e−33	Coleoptera	Dytiscidae	Paroster newhavenensis
Hydroporus pubescens voucher NHM−IR39 16S ribosoma	EF419327	82.69	98.73	4.52244e−33	Coleoptera	Dytiscidae	Hydroporus pubescens
Hydroporus occidentalis 16S ribosomal RNA gene, pa	AY365280	81.94	98.73	4.52244e−33	Coleoptera	Dytiscidae	Hydroporus occidentalis
Hydroporus hirtellus 16S ribosomal RNA gene, parti	AY365271	81.94	98.73	4.52244e−33	Coleoptera	Dytiscidae	Hydroporus hirtellus
Hydroporus rufifrons 16S ribosomal RNA gene, parti	AF518277	82.69	98.73	4.52244e−33	Coleoptera	Dytiscidae	Hydroporus rufifrons
Hydroporus pubescens 16S ribosomal RNA gene, parti	AF518276	82.69	98.73	4.52244e−33	Coleoptera	Dytiscidae	Hydroporus pubescens
Hydroporus limbatus 16S ribosomal RNA gene, partia	AF518264	82.69	98.73	4.52244e−33	Coleoptera	Dytiscidae	Hydroporus limbatus
Hydroporus discretus 16S ribosomal RNA gene, parti	AF518260	82.69	98.73	4.52244e−33	Coleoptera	Dytiscidae	Hydroporus discretus
Hydroporus analis 16S ribosomal RNA gene, partial	AF518257	82.69	98.73	4.52244e−33	Coleoptera	Dytiscidae	Hydroporus analis
Hygrotus impressopunctatus voucher UNM KBMHtim80 1	KJ548334	83.12	96.18	1.57849e−32	Coleoptera	Dytiscidae	Hygrotus impressopunctatus
Deronectes aubei voucher UNM KBMDeau758 16S riboso	KJ548292	82.05	98.73	1.57849e−32	Coleoptera	Dytiscidae	Deronectes aubei
Hygrotus armeniacus mitochondrial partial 16S rRNA	HF931520	82.8	98.73	1.57849e−32	Coleoptera	Dytiscidae	Hygrotus armeniacus
Hydroporus planus mitochondrial 16S rRNA gene (par	HE613708	82.17	98.73	1.57849e−32	Coleoptera	Dytiscidae	Hydroporus planus
Deronectes costipennis mitochondrial 16S rRNA gene	HE613697	82.05	98.73	1.57849e−32	Coleoptera	Dytiscidae	Deronectes costipennis
Deronectes platynotus mitochondrial 16S rRNA gene	HE613682	82.05	98.73	1.57849e−32	Coleoptera	Dytiscidae	Deronectes platynotus
Deronectes depressicollis mitochondrial 16S rRNA g	HE613681	82.05	98.73	1.57849e−32	Coleoptera	Dytiscidae	Deronectes depressicollis
Deronectes semirufus mitochondrial 16S rRNA gene (	HE613676	82.05	98.73	1.57849e−32	Coleoptera	Dytiscidae	Deronectes semirufus
Deronectes semirufus mitochondrial 16S rRNA gene (	HE613674	82.05	98.73	1.57849e−32	Coleoptera	Dytiscidae	Deronectes semirufus
Antiporus sp. MB1421 mitochondrial partial 16S rRN	FR727245	81.29	98.73	1.57849e−32	Coleoptera	Dytiscidae	Antiporus sp. MB1421
Deronectes costipennis 16S ribosomal RNA gene, par	AY250951	82.05	98.73	1.57849e−32	Coleoptera	Dytiscidae	Deronectes costipennis
Deronectes aubei sanfilippoi haplotype 2 16S ribos	AY250908	82.05	98.73	1.57849e−32	Coleoptera	Dytiscidae	Deronectes aubei sanfilippoi
