# Supplementary material for: Testing the potential of a ribosomal 16S marker for DNA metabarcoding of insects
Source: PeerJ. 2016 Apr 19;4:e1966. doi: 10.7717/peerj.1966 (PMC4841222; doi:10.7717/peerj.1966)
Supplement: Supplemental Information 1 [file peerj-04-1966-s006.zip › scripts/5 match OTUs/blast_hits/OTU_50.csv_plot.pdf]

OTU_50.csv

Simulium vernum

Simuliidae

Diptera

96.15

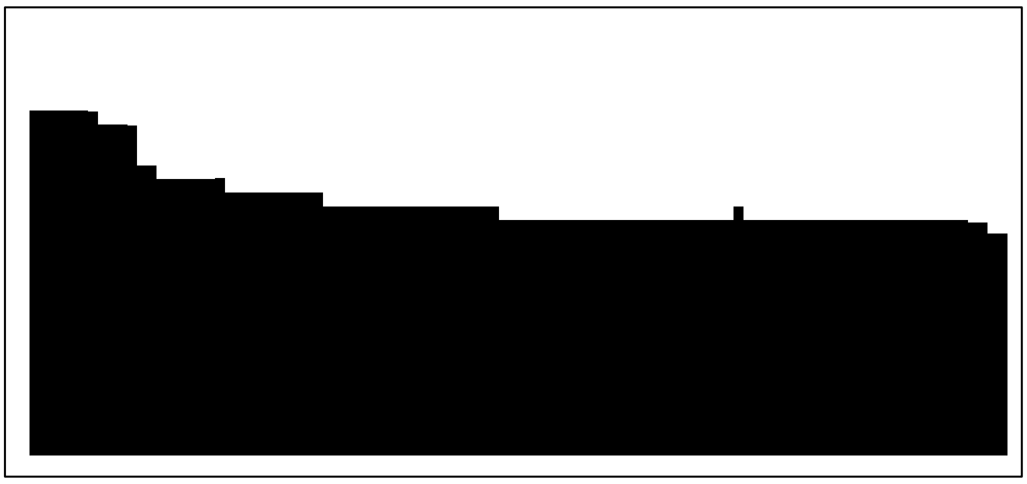

Simulium vernum voucher ACBSimFN1c 16S ribosomal R	GU073059	96.15	100	1.46369e−64	Diptera	Simuliidae	Simulium vernum
Simulium silvestre voucher ACBSimMN11c1 16S riboso	GU073058	96.15	100	1.46369e−64	Diptera	Simuliidae	Simulium silvestre
Simulium silvestre voucher ACBSimMN5c1 16S ribosom	GU073057	96.15	100	1.46369e−64	Diptera	Simuliidae	Simulium silvestre
Simulium silvestre voucher ACBSimMN45b1 16S riboso	GU073056	96.15	100	1.46369e−64	Diptera	Simuliidae	Simulium silvestre
Simulium silvestre voucher ACBSimNu178 16S ribosom	GU073052	96.15	100	1.46369e−64	Diptera	Simuliidae	Simulium silvestre
Simulium juxtacrenobium voucher ACBBsimEN1C 16S ri	GU073050	96.15	100	1.46369e−64	Diptera	Simuliidae	Simulium juxtacrenobium
Simulium juxtacrenobium voucher ACBBsimFN100D 16S	GU073048	96.1	98.72	1.78314e−63	Diptera	Simuliidae	Simulium juxtacrenobium
Simulium juxtacrenobium voucher ACBBsimFN100I 16S	GU073042	95.51	100	1.78314e−63	Diptera	Simuliidae	Simulium juxtacrenobium
Simulium crenobium voucher ACBBsimAU1A 16S ribosom	GU073040	95.51	100	1.78314e−63	Diptera	Simuliidae	Simulium crenobium
Simulium costatum voucher ACBBsimSW102H 16S riboso	GU073032	95.51	100	6.22376e−63	Diptera	Simuliidae	Simulium costatum
Simulium juxtacrenobium voucher ACBBsimFN100B 16S	GU073046	95.45	98.72	2.17231e−62	Diptera	Simuliidae	Simulium juxtacrenobium
Simulium sabahense mitochondrial gene for 16S rRNA	AB093122	93.59	100	3.92762e−59	Diptera	Simuliidae	Simulium sabahense
Simulium aeneifacies mitochondrial gene for 16S rR	AB093119	93.59	100	3.92762e−59	Diptera	Simuliidae	Simulium aeneifacies
Simulium tani isolate ST1B 16S ribosomal RNA gene,	KM410188	92.95	100	1.67007e−57	Diptera	Simuliidae	Simulium tani
Simulium tani 16S ribosomal RNA gene, partial sequ	GQ866019	92.95	100	1.67007e−57	Diptera	Simuliidae	Simulium tani
Simulium tani 16S ribosomal RNA gene, partial sequ	GQ866018	92.95	100	1.67007e−57	Diptera	Simuliidae	Simulium tani
Simulium tani mitochondrial gene for 16S rRNA, par	AB093123	92.95	100	1.67007e−57	Diptera	Simuliidae	Simulium tani
Simulium japonicum mitochondrial gene for 16S rRNA	AB093103	92.95	100	1.67007e−57	Diptera	Simuliidae	Simulium japonicum
Simulium uchidai mitochondrial gene for 16S riboso	AB056740	92.95	100	1.67007e−57	Diptera	Simuliidae	Simulium uchidai
Stegopterna mutata mitochondrial 16S ribosomal RNA	L13404	92.99	100	5.82911e−57	Diptera	Simuliidae	Stegopterna mutata
Simulium khunklangense mitochondrial gene for 16S	AB820367	92.31	100	2.03456e−56	Diptera	Simuliidae	Simulium khunklangense
Simulium caudisclerum mitochondrial gene for 16S r	AB699900	92.31	100	2.03456e−56	Diptera	Simuliidae	Simulium caudisclerum
Simulium chomthongense mitochondrial gene for 16S	AB699899	92.31	100	2.03456e−56	Diptera	Simuliidae	Simulium chomthongense
Simulium caudisclerum 16S ribosomal RNA gene, part	GQ865999	92.31	100	2.03456e−56	Diptera	Simuliidae	Simulium caudisclerum
Simulium nili 16S ribosomal RNA gene, partial sequ	AY944188	92.31	100	2.03456e−56	Diptera	Simuliidae	Simulium nili
Simulium latipollex isolate SA 4 16S ribosomal RNA	AF416962	92.31	100	2.03456e−56	Diptera	Simuliidae	Simulium latipollex
Simulium damnosum s.l. Mal4 16S ribosomal RNA gene	AF416964	92.31	100	2.03456e−56	Diptera	Simuliidae	Simulium damnosum s.l. Mal4
Simulium damnosum 16S ribosomal RNA gene, partial	AF127463	92.31	100	2.03456e−56	Diptera	Simuliidae	Simulium damnosum
Simulium nigrogilvum mitochondrial gene for 16S rR	AB093099	92.31	100	2.03456e−56	Diptera	Simuliidae	Simulium nigrogilvum
Simulium subcostatum mitochondrial gene for 16S ri	AB056739	92.31	100	2.03456e−56	Diptera	Simuliidae	Simulium subcostatum
Simulium ogonukii mitochondrial gene for 16S ribos	LC034993	91.67	100	8.65116e−55	Diptera	Simuliidae	Simulium ogonukii
Simulium tani 16S ribosomal RNA gene, partial sequ	GQ866017	91.67	100	8.65116e−55	Diptera	Simuliidae	Simulium tani
Simulium rufibasis 16S ribosomal RNA gene, partial	GQ866016	91.67	100	8.65116e−55	Diptera	Simuliidae	Simulium rufibasis
Simulium chiangmaiense 16S ribosomal RNA gene, par	GQ866009	91.67	100	8.65116e−55	Diptera	Simuliidae	Simulium chiangmaiense
Simulium siripoomense 16S ribosomal RNA gene, part	GQ866003	91.67	100	8.65116e−55	Diptera	Simuliidae	Simulium siripoomense
Simulium squamosum voucher P.Mansiangi Pm3(BNI) 16	DQ655644	91.67	100	8.65116e−55	Diptera	Simuliidae	Simulium squamosum
Simulium pandanophilum isolate KK1 16S ribosomal R	AF466184	91.67	100	8.65116e−55	Diptera	Simuliidae	Simulium pandanophilum
Simulium damnosum s.l. Kulfo isolate Ku 3 16S ribo	AF416978	91.67	100	8.65116e−55	Diptera	Simuliidae	Simulium damnosum s.l. Kulfo
Simulium pandanophilum 16S ribosomal RNA gene, par	AF081909	91.67	100	8.65116e−55	Diptera	Simuliidae	Simulium pandanophilum
Simulium bidentatum mitochondrial gene for 16S rRN	AB093126	91.67	100	8.65116e−55	Diptera	Simuliidae	Simulium bidentatum
Simulium arakawae mitochondrial gene for 16S rRNA,	AB093125	91.67	100	8.65116e−55	Diptera	Simuliidae	Simulium arakawae
Simulium rufibasis mitochondrial gene for 16S rRNA	AB093121	91.67	100	8.65116e−55	Diptera	Simuliidae	Simulium rufibasis
Simulium tumpaense mitochondrial gene for 16S rRNA	AB093110	91.67	100	8.65116e−55	Diptera	Simuliidae	Simulium tumpaense
Simulium konoi mitochondrial gene for 16S ribosoma	AB056741	91.67	100	8.65116e−55	Diptera	Simuliidae	Simulium konoi
Simulium bonninense mitochondrial gene for 16S rib	AB056738	91.67	100	8.65116e−55	Diptera	Simuliidae	Simulium bonninense
Simulium verecundum isolate AA 16S ribosomal RNA g	L13414	91.67	100	8.65116e−55	Diptera	Simuliidae	Simulium verecundum
Simulium venustum (individual CC3) mitochondrial 1	L13410	91.67	100	8.65116e−55	Diptera	Simuliidae	Simulium venustum
Simulium venustum (individual CC) mitochondrial 16	L13409	91.67	100	8.65116e−55	Diptera	Simuliidae	Simulium venustum
Simulium atipornae mitochondrial gene for 16S ribo	AB893948	91.03	100	1.05393e−53	Diptera	Simuliidae	Simulium atipornae
Simulium chamlongi 16S ribosomal RNA gene, partial	GQ866020	91.03	100	1.05393e−53	Diptera	Simuliidae	Simulium chamlongi
Simulium yuphae 16S ribosomal RNA gene, partial se	GQ866015	91.03	100	1.05393e−53	Diptera	Simuliidae	Simulium yuphae
Simulium nakhonense 16S ribosomal RNA gene, partia	GQ866011	91.03	100	1.05393e−53	Diptera	Simuliidae	Simulium nakhonense
Simulium quinquestriatum 16S ribosomal RNA gene, p	GQ866013	91.03	100	1.05393e−53	Diptera	Simuliidae	Simulium quinquestriatum
Simulium nakhonense 16S ribosomal RNA gene, partia	GQ866012	91.03	100	1.05393e−53	Diptera	Simuliidae	Simulium nakhonense
Simulium nakhonense 16S ribosomal RNA gene, partia	GQ866010	91.03	100	1.05393e−53	Diptera	Simuliidae	Simulium nakhonense
Simulium fenestratum 16S ribosomal RNA gene, parti	GQ866007	91.03	100	1.05393e−53	Diptera	Simuliidae	Simulium fenestratum
Simulium chainarongi 16S ribosomal RNA gene, parti	GQ866004	91.03	100	1.05393e−53	Diptera	Simuliidae	Simulium chainarongi
Simulium rudnicki 16S ribosomal RNA gene, partial	GQ866001	91.03	100	1.05393e−53	Diptera	Simuliidae	Simulium rudnicki
Simulium grossifilum 16S ribosomal RNA gene, parti	GQ866000	91.03	100	1.05393e−53	Diptera	Simuliidae	Simulium grossifilum
Simulium chumpornense 16S ribosomal RNA gene, part	GQ865998	91.03	100	1.05393e−53	Diptera	Simuliidae	Simulium chumpornense
Simulium yemenense 16S ribosomal RNA gene, partial	AY944190	91.03	100	1.05393e−53	Diptera	Simuliidae	Simulium yemenense
Simulium arabicum 16S ribosomal RNA gene, partial	AY944186	91.03	100	1.05393e−53	Diptera	Simuliidae	Simulium arabicum
Simulium oblongum mitochondrial gene for 16S ribos	AB334089	91.03	100	1.05393e−53	Diptera	Simuliidae	Simulium oblongum
Simulium damnosum s.l. GH−D1 16S ribosomal RNA, pa	AY077709	91.03	100	1.05393e−53	Diptera	Simuliidae	Simulium damnosum s.l. GH−D1
Simulium damnosum s.l. Kulfo isolate Ku 1 16S ribo	AF416977	91.03	100	1.05393e−53	Diptera	Simuliidae	Simulium damnosum s.l. Kulfo
Simulium soubrense isolate Wa 52 16S ribosomal RNA	AF416976	91.03	100	1.05393e−53	Diptera	Simuliidae	Simulium soubrense
Simulium soubrense isolate Wa 47 16S ribosomal RNA	AF416975	91.03	100	1.05393e−53	Diptera	Simuliidae	Simulium soubrense
Simulium rasyani isolate rasy 19 16S ribosomal RNA	AF416974	91.03	100	1.05393e−53	Diptera	Simuliidae	Simulium rasyani
Simulium damnosum s.l. Me2 16S ribosomal RNA gene,	AF416972	91.03	100	1.05393e−53	Diptera	Simuliidae	Simulium damnosum s.l. Me2
Simulium damnosum s.l. GH−D2 16S ribosomal RNA, pa	AY077710	91.03	100	1.05393e−53	Diptera	Simuliidae	Simulium damnosum s.l. GH−D2
Simulium kilibanum isolate Ny 14 16S ribosomal RNA	AF416961	91.03	100	1.05393e−53	Diptera	Simuliidae	Simulium kilibanum
Simulium damnosum cytoform Kagera isolate Ka 6 16S	AF416955	91.03	100	1.05393e−53	Diptera	Simuliidae	Simulium damnosum cytoform Kagera
Simulium cf. damnosum 'Abu Hamed' 16S ribosomal RN	AF213336	91.67	100	1.05393e−53	Diptera	Simuliidae	Simulium damnosum cytoform Abu Hamed
Simulium vorax 16S ribosomal RNA gene, partial seq	AF127462	91.03	100	1.05393e−53	Diptera	Simuliidae	Simulium vorax
Simulium hargreavesi 16S ribosomal RNA gene, parti	AF127461	91.03	100	1.05393e−53	Diptera	Simuliidae	Simulium hargreavesi
Simulium damnosum country Uganda 16S ribosomal RNA	AF081904	91.03	100	1.05393e−53	Diptera	Simuliidae	Simulium damnosum
Simulium kilibanum isolate NY19 16S ribosomal RNA	AY885648	91.03	100	1.05393e−53	Diptera	Simuliidae	Simulium kilibanum
Simulium iridescens mitochondrial gene for 16S rRN	AB093127	91.03	100	1.05393e−53	Diptera	Simuliidae	Simulium iridescens
Simulium chamlongi mitochondrial gene for 16S rRNA	AB093124	91.03	100	1.05393e−53	Diptera	Simuliidae	Simulium chamlongi
Simulium brevipar mitochondrial gene for 16S rRNA,	AB093120	91.03	100	1.05393e−53	Diptera	Simuliidae	Simulium brevipar
Simulium iwatense mitochondrial gene for 16S rRNA,	AB093117	91.03	100	1.05393e−53	Diptera	Simuliidae	Simulium iwatense
Simulium nikkoense mitochondrial gene for 16S rRNA	AB093116	91.03	100	1.05393e−53	Diptera	Simuliidae	Simulium nikkoense
Simulium sakishimaense mitochondrial gene for 16S	AB093113	91.03	100	1.05393e−53	Diptera	Simuliidae	Simulium sakishimaense
Simulium hirtinervis mitochondrial gene for 16S rR	AB093111	91.03	100	1.05393e−53	Diptera	Simuliidae	Simulium hirtinervis
Simulium taalense mitochondrial gene for 16S rRNA,	AB093109	91.03	100	1.05393e−53	Diptera	Simuliidae	Simulium taalense
Simulium laterale mitochondrial gene for 16S rRNA,	AB093107	91.03	100	1.05393e−53	Diptera	Simuliidae	Simulium laterale
Simulium dumogaense mitochondrial gene for 16S rRN	AB093106	91.03	100	1.05393e−53	Diptera	Simuliidae	Simulium dumogaense
Simulium bishopi mitochondrial gene for 16S rRNA,	AB093105	91.03	100	1.05393e−53	Diptera	Simuliidae	Simulium bishopi
Simulium maenoi mitochondrial gene for 16S rRNA, p	AB093104	91.03	100	1.05393e−53	Diptera	Simuliidae	Simulium maenoi
Simulium quinquestriatum mitochondrial gene for 16	AB056746	91.03	100	1.05393e−53	Diptera	Simuliidae	Simulium quinquestriatum
Simulium aokii mitochondrial gene for 16S ribosoma	AB056745	91.03	100	1.05393e−53	Diptera	Simuliidae	Simulium aokii
Simulium yahense mitochondrion 16S ribosomal RNA g	U17741	91.03	100	1.05393e−53	Diptera	Simuliidae	Simulium yahense
Simulium squamosum mitochondrion 16S ribosomal RNA	U17738	91.03	100	1.05393e−53	Diptera	Simuliidae	Simulium squamosum
Simulium leonense mitochondrion 16S ribosomal RNA	U17731	91.03	100	1.05393e−53	Diptera	Simuliidae	Simulium leonense
Simulium damnosum mitochondrion 16S ribosomal RNA	U17729	91.03	100	1.05393e−53	Diptera	Simuliidae	Simulium damnosum
Simulium venustum (individual CC4) mitochondrial 1	L13411	91.03	100	1.05393e−53	Diptera	Simuliidae	Simulium venustum
Simulium kilibanum isolate Nk 82 16S ribosomal RNA	AF416960	90.91	98.72	1.28395e−52	Diptera	Simuliidae	Simulium kilibanum
Simulium kilibanum strain Ny−18 16S ribosomal RNA	AF081905	90.91	98.72	1.28395e−52	Diptera	Simuliidae	Simulium kilibanum
Simulium tuyense mitochondrial gene for 16S riboso	LC035006	90.38	100	4.48141e−52	Diptera	Simuliidae	Simulium tuyense
Simulium marilogense mitochondrial gene for 16S ri	LC034991	90.38	100	4.48141e−52	Diptera	Simuliidae	Simulium marilogense
