# Supplementary material for: Testing the potential of a ribosomal 16S marker for DNA metabarcoding of insects
Source: PeerJ. 2016 Apr 19;4:e1966. doi: 10.7717/peerj.1966 (PMC4841222; doi:10.7717/peerj.1966)
Supplement: Supplemental Information 1 [file peerj-04-1966-s006.zip › scripts/5 match OTUs/blast_hits/OTU_505.csv_plot.pdf]

OTU_505.csv

Oreodytes sanmarkii

Dytiscidae

Coleoptera

98.09

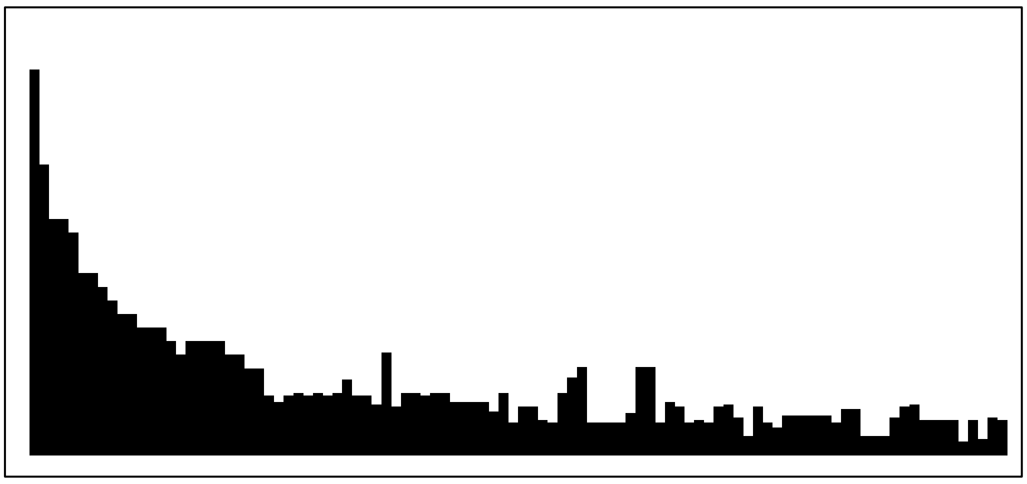

Oreodytes sanmarkii mitochondrial 16S rRNA gene (p	HF931524	98.09	100	2.28104e−68	Coleoptera	Dytiscidae	Oreodytes sanmarkii
Oreodytes obesus cordillerensis mitochondrial part	HF931516	93.63	100	3.8627e−59	Coleoptera	Dytiscidae	Oreodytes obesus cordillerensis
Oreodytes crassulus partial 16S rRNA gene	AJ850348	91.08	100	1.03651e−53	Coleoptera	Dytiscidae	Oreodytes crassulus
Oreodytes congruus partial 16S rRNA gene	AJ850347	91.08	100	1.03651e−53	Coleoptera	Dytiscidae	Oreodytes congruus
Oreodytes congruus 16S ribosomal RNA gene, partial	KF575550	90.45	100	4.40734e−52	Coleoptera	Dytiscidae	Oreodytes congruus
Oreodytes abbreviatus mitochondrial 16S rRNA gene	HF931477	88.54	100	2.78133e−48	Coleoptera	Dytiscidae	Oreodytes abbreviatus
Oreodytes rhyacophilus partial 16S rRNA gene	AJ850352	88.54	100	2.78133e−48	Coleoptera	Dytiscidae	Oreodytes rhyacophilus
Oreodytes mongolicus mitochondrial 16S rRNA gene (	HF931491	87.9	100	1.18265e−46	Coleoptera	Dytiscidae	Oreodytes mongolicus
Oreodytes snoqualmie mitochondrial partial 16S rRN	HF931519	87.26	100	1.44077e−45	Coleoptera	Dytiscidae	Oreodytes snoqualmie
Oreodytes septentrionalis mitochondrial 16S rRNA g	HF931492	86.62	100	6.1263e−44	Coleoptera	Dytiscidae	Oreodytes septentrionalis
Oreodytes shorti mitochondrial 16S rRNA gene (part	HF931488	86.62	100	6.1263e−44	Coleoptera	Dytiscidae	Oreodytes shorti
Boreonectes riberae mitochondrial 16S rRNA gene (p	HF931461	85.99	100	9.09223e−42	Coleoptera	Dytiscidae	Boreonectes riberae
Boreonectes griseostriatus mitochondrial partial 1	HF931387	85.99	100	9.09223e−42	Coleoptera	Dytiscidae	Boreonectes griseostriatus
Boreonectes multilineatus mitochondrial partial 16	HF931384	85.99	100	9.09223e−42	Coleoptera	Dytiscidae	Boreonectes multilineatus
Deronectes parvicollis mitochondrial 16S rRNA gene	HF931454	85.35	100	3.1735e−41	Coleoptera	Dytiscidae	Deronectes parvicollis
Laccornis difformis 16S ribosomal RNA gene, partia	KF575544	84.71	100	3.86611e−40	Coleoptera	Dytiscidae	Laccornis difformis
Boreonectes alpestris mitochondrial 16S rRNA gene	HF931512	85.35	100	3.86611e−40	Coleoptera	Dytiscidae	Boreonectes alpestris
Boreonectes striatellus mitochondrial 16S rRNA gen	HF931511	85.35	100	3.86611e−40	Coleoptera	Dytiscidae	Boreonectes striatellus
Boreonectes macedonicus mitochondrial 16S rRNA gen	HF931359	85.35	100	3.86611e−40	Coleoptera	Dytiscidae	Boreonectes macedonicus
Stictotarsus ibericus 16S ribosomal RNA (rrnL) gen	EF670030	85.35	100	3.86611e−40	Coleoptera	Dytiscidae	Stictotarsus ibericus
Laccornis kocae partial 16S rRNA gene	AJ850419	84.71	100	3.86611e−40	Coleoptera	Dytiscidae	Laccornis kocae
Boreonectes griseostriatus mitochondrial 16S rRNA	HF931541	84.71	100	4.70989e−39	Coleoptera	Dytiscidae	Boreonectes griseostriatus
Oreodytes scitulus 16S ribosomal RNA gene, partial	KF575551	84.08	100	1.64391e−38	Coleoptera	Dytiscidae	Oreodytes scitulus
Oreodytes scitulus mitochondrial partial 16S rRNA	HF931515	84.08	100	1.64391e−38	Coleoptera	Dytiscidae	Oreodytes scitulus
Oreodytes natrix partial 16S rRNA gene	AJ850349	82.8	100	8.51568e−36	Coleoptera	Dytiscidae	Oreodytes natrix
Aethionectes fulvonotatus voucher KBMAeop406 16S r	KF979088	82.5	100	2.97227e−35	Coleoptera	Dytiscidae	Aethionectes fulvonotatus
Hygrotus marklini mitochondrial 16S rRNA gene (par	HF931467	82.8	100	2.97227e−35	Coleoptera	Dytiscidae	Hygrotus marklini
Nebrioporus formaster mitochondrial partial 16S rR	HF931377	82.91	100	2.97227e−35	Coleoptera	Dytiscidae	Nebrioporus formaster
Hydroporus glabriusculus 16S ribosomal RNA gene, p	AY365270	82.8	100	2.97227e−35	Coleoptera	Dytiscidae	Hydroporus glabriusculus
Suphrodytes dorsalis 16S ribosomal RNA gene, parti	KF575577	82.91	100	1.03742e−34	Coleoptera	Dytiscidae	Suphrodytes dorsalis
Boreonectes griseostriatus mitochondrial 16S rRNA	HF931385	82.8	100	1.03742e−34	Coleoptera	Dytiscidae	Boreonectes griseostriatus
Suphrodytes dorsalis voucher BMNH:725966 16S ribos	JX221574	82.91	100	1.03742e−34	Coleoptera	Dytiscidae	Suphrodytes dorsalis
Hydroporus foveolatus mitochondrial 16S rRNA gene	HE613724	83.54	100	1.03742e−34	Coleoptera	Dytiscidae	Hydroporus foveolatus
Nirripirti megamacrocephalus isolate ABTC78781 16S	EU616935	82.8	100	1.03742e−34	Coleoptera	Dytiscidae	Paroster megamacrocephalus
Nirripirti byroensis isolate ABTC78677−8 16S ribos	EU616910	82.8	100	1.03742e−34	Coleoptera	Dytiscidae	Paroster byroensis
Scarodytes sp. IR−2003 16S ribosomal RNA gene, par	AY250939	82.39	100	1.03742e−34	Coleoptera	Dytiscidae	Scarodytes sp. IR−2003
Deronectes moestus mitochondrial 16S rRNA gene (pa	HE613726	84.83	91.56	3.62096e−34	Coleoptera	Dytiscidae	Deronectes moestus
Copelatus sp. BI−2011 voucher BMNH792945 16S ribos	HQ381441	82.28	100	3.62096e−34	Coleoptera	Dytiscidae	Copelatus sp. BI−2011
Stictotarsus maghrebinus 16S ribosomal RNA gene, p	AY250947	82.91	100	3.62096e−34	Coleoptera	Dytiscidae	Stictotarsus maghrebinus
Hydroporus tessellatus 16S ribosomal RNA gene, par	JX434770	82.91	100	1.26384e−33	Coleoptera	Dytiscidae	Hydroporus tessellatus
Boreonectes funereus mitochondrial 16S rRNA gene (	HF931393	82.8	100	1.26384e−33	Coleoptera	Dytiscidae	Boreonectes funereus
Hydroporus zimmermanni mitochondrial 16S rRNA gene	HE613679	82.91	100	1.26384e−33	Coleoptera	Dytiscidae	Hydroporus zimmermanni
Hydroporus basinotatus mitochondrial 16S rRNA gene	HE613678	82.91	100	1.26384e−33	Coleoptera	Dytiscidae	Hydroporus basinotatus
Pseudomigadops n. sp. MSL2007 voucher 668381 16S r	EF214073	82.5	100	1.26384e−33	Coleoptera	Carabidae	Pseudomigadops n. sp. MSL2007
Pseudomigadops n. sp. MSL2007 voucher 668379 16S r	EF214072	82.5	100	1.26384e−33	Coleoptera	Carabidae	Pseudomigadops n. sp. MSL2007
Pseudomigadops n. sp. MSL2007 voucher 668300 16S r	EF214010	82.5	100	1.26384e−33	Coleoptera	Carabidae	Pseudomigadops n. sp. MSL2007
Pseudomigadops sp. PSM1 mitochondrial 16S rRNA gen	FM994738	82.5	100	1.26384e−33	Coleoptera	Carabidae	Pseudomigadops sp. PSM1
Nirripirti newhavenensis 16S ribosomal RNA gene, p	AY353844	82.05	100	1.26384e−33	Coleoptera	Dytiscidae	Paroster newhavenensis
Hydroporus basinotatus 16S ribosomal RNA gene, par	AF518258	82.91	100	1.26384e−33	Coleoptera	Dytiscidae	Hydroporus basinotatus
Oreodytes quadrimaculatus 16S ribosomal RNA gene,	KF575567	81.53	100	4.41123e−33	Coleoptera	Dytiscidae	Oreodytes quadrimaculatus
Stictotarsus procerus mitochondrial 16S rRNA gene	HF931379	82.28	100	4.41123e−33	Coleoptera	Dytiscidae	Stictotarsus procerus
Deronectes opatrinus mitochondrial 16S rRNA gene (	HE613701	82.28	100	4.41123e−33	Coleoptera	Dytiscidae	Deronectes opatrinus
Copelatus sp. BI−2011 voucher BMNH671215 16S ribos	HQ381377	81.65	100	4.41123e−33	Coleoptera	Dytiscidae	Copelatus sp. BI−2011
Oreodytes quadrimaculatus partial 16S rRNA gene	AJ850351	81.53	100	4.41123e−33	Coleoptera	Dytiscidae	Oreodytes quadrimaculatus
Paroster sp. spnMtWedge 16S ribosomal RNA gene, pa	EU616954	82.91	100	4.41123e−33	Coleoptera	Dytiscidae	Paroster sp. spnMtWedge
Nirripirti bulbus isolate ABTC78658−9 16S ribosoma	EU616918	83.65	100	4.41123e−33	Coleoptera	Dytiscidae	Paroster bulbus
Deronectes moestus 16S ribosomal RNA gene, partial	KF575561	84.14	91.56	1.53967e−32	Coleoptera	Dytiscidae	Deronectes moestus
Hygrotus novemlineatus mitochondrial partial 16S r	HF931523	81.53	100	1.53967e−32	Coleoptera	Dytiscidae	Hygrotus novemlineatus
Hygrotus tumidiventris mitochondrial 16S rRNA gene	HF931496	81.53	100	1.53967e−32	Coleoptera	Dytiscidae	Hygrotus tumidiventris
Hydroporus brancoi gredensis mitochondrial partial	HF931329	81.53	100	1.53967e−32	Coleoptera	Dytiscidae	Hydroporus brancoi gredensis
Suphrodytes figuratus voucher BMNH:722148 16S ribo	JX221571	81.53	100	1.53967e−32	Coleoptera	Dytiscidae	Suphrodytes figuratus
Hydroporus marginatus mitochondrial 16S rRNA gene	HE613699	81.99	100	1.53967e−32	Coleoptera	Dytiscidae	Hydroporus marginatus
Deronectes brannani mitochondrial 16S rRNA gene (p	HE613695	84.14	91.56	1.53967e−32	Coleoptera	Dytiscidae	Deronectes brannanii
Deronectes moestus mitochondrial 16S rRNA gene (pa	HE613683	84.14	91.56	1.53967e−32	Coleoptera	Dytiscidae	Deronectes moestus
Hygrotus turbidus partial 16S rRNA gene	AJ850390	81.53	100	1.53967e−32	Coleoptera	Dytiscidae	Hygrotus turbidus
Trichonectes otini partial 16S rRNA gene	AJ850356	82.5	100	1.53967e−32	Coleoptera	Dytiscidae	Trichonectes otini
Nirripirti napperbyensis 16S ribosomal RNA gene, p	AY353843	82.28	100	1.53967e−32	Coleoptera	Dytiscidae	Paroster napperbyensis
Hydroporus sp. IR33.4 16S ribosomal RNA gene, part	AY365261	81.53	100	1.53967e−32	Coleoptera	Dytiscidae	Hydroporus sp. IR33.4
Scarodytes halensis haplotype 3 16S ribosomal RNA	AY250937	81.65	100	1.53967e−32	Coleoptera	Dytiscidae	Scarodytes halensis
Hydroporus marginatus 16S ribosomal RNA gene, part	AF518267	81.53	100	1.53967e−32	Coleoptera	Dytiscidae	Hydroporus marginatus
Hydroporus analis mitochondrial partial 16S rRNA g	HE613731	82.28	100	5.37398e−32	Coleoptera	Dytiscidae	Hydroporus analis
Hydroporus fuscipennis mitochondrial 16S rRNA gene	HE613730	82.39	100	5.37398e−32	Coleoptera	Dytiscidae	Hydroporus fuscipennis
Hydroporus planus mitochondrial 16S rRNA gene (par	HE613708	81.76	100	5.37398e−32	Coleoptera	Dytiscidae	Hydroporus planus
Deronectes bicostatus mitochondrial 16S rRNA gene	HE613705	80.89	100	5.37398e−32	Coleoptera	Dytiscidae	Deronectes bicostatus
Hydroporus compunctus mitochondrial 16S rRNA gene	HE599693	82.28	100	5.37398e−32	Coleoptera	Dytiscidae	Hydroporus compunctus
Paroster sp. R401 Newhaven voucher ABTC:78974 16S	JQ745754	81.53	100	5.37398e−32	Coleoptera	Dytiscidae	Paroster sp. R401 Newhaven
Antiporus hollingsworthi mitochondrial partial 16S	FR727246	81.29	98.7	5.37398e−32	Coleoptera	Dytiscidae	Antiporus hollingsworthi
Pseudomigadops n. sp. MSL2007 voucher 668338 16S r	EF214034	81.88	100	5.37398e−32	Coleoptera	Carabidae	Pseudomigadops n. sp. MSL2007
Pseudomigadops n. sp. MSL2007 voucher 668317 16S r	EF214022	81.88	100	5.37398e−32	Coleoptera	Carabidae	Pseudomigadops n. sp. MSL2007
Pseudomigadops n. sp. MSL2007 voucher 668302 16S r	EF214012	81.88	100	5.37398e−32	Coleoptera	Carabidae	Pseudomigadops n. sp. MSL2007
Pseudomigadops n. sp. MSL2007 voucher 668299 16S r	EF214009	81.88	100	5.37398e−32	Coleoptera	Carabidae	Pseudomigadops n. sp. MSL2007
Pseudomigadops n. sp. MSL2007 voucher 668369 16S r	EF214062	81.88	100	5.37398e−32	Coleoptera	Carabidae	Pseudomigadops n. sp. MSL2007
Paroster sp. 2 RL−2008 16S ribosomal RNA gene, par	EU616958	81.53	100	5.37398e−32	Coleoptera	Dytiscidae	Paroster sp. 2 RL−2008
Nirripirti arachnoides isolate ABTC78660−1 16S rib	EU616917	82.17	100	5.37398e−32	Coleoptera	Dytiscidae	Paroster arachnoides
Nirripirti skaphites isolate 2 ABTC78694−6 16S rib	EU616916	82.17	100	5.37398e−32	Coleoptera	Dytiscidae	Paroster skaphites
Papuadytes simplex 2 note−SubSrc cryptic species 2	AY334154	80.89	100	5.37398e−32	Coleoptera	Dytiscidae	Papuadytes aff. simplex MB107
Amphizoa lecontei 16S ribosomal RNA gene, partial	AY071771	80.89	100	5.37398e−32	Coleoptera	Amphizoidae	Amphizoa lecontei
Amphizoa insolens 16S ribosomal RNA gene, partial	AY071770	80.89	100	5.37398e−32	Coleoptera	Amphizoidae	Amphizoa insolens
Hydroporus planus 16S ribosomal RNA gene, partial	AF518275	81.76	100	5.37398e−32	Coleoptera	Dytiscidae	Hydroporus planus
Hydroporus compunctus 16S ribosomal RNA gene, part	AF518259	82.28	100	5.37398e−32	Coleoptera	Dytiscidae	Hydroporus compunctus
Hydroporus fuscipennis 16S ribosomal RNA gene, par	AF518262	82.39	100	5.37398e−32	Coleoptera	Dytiscidae	Hydroporus fuscipennis
Deronectes doriae mitochondrial 16S rRNA gene (par	HE613710	81.65	100	1.8757e−31	Coleoptera	Dytiscidae	Deronectes doriae
Deronectes costipennis mitochondrial 16S rRNA gene	HE613697	81.65	100	1.8757e−31	Coleoptera	Dytiscidae	Deronectes costipennis
Deronectes platynotus mitochondrial 16S rRNA gene	HE613682	81.65	100	1.8757e−31	Coleoptera	Dytiscidae	Deronectes platynotus
Deronectes sahlbergi mitochondrial 16S rRNA gene (	HE613680	81.65	100	1.8757e−31	Coleoptera	Dytiscidae	Deronectes sahlbergi
Antiporus hollingsworthi mitochondrial partial 16S	FR727247	80.65	98.7	1.8757e−31	Coleoptera	Dytiscidae	Antiporus hollingsworthi
Stictotarsus falli 16S ribosomal RNA (rrnL) gene,	EF670029	81.65	100	1.8757e−31	Coleoptera	Dytiscidae	Stictotarsus falli
Nirripirti wedgeensis isolate ABTC78773−4 16S ribo	EU616938	80.77	100	1.8757e−31	Coleoptera	Dytiscidae	Paroster wedgeensis
Nirripirti verrucosus isolate ABTC78682 16S riboso	EU616908	81.76	100	1.8757e−31	Coleoptera	Dytiscidae	Paroster verrucosus
Deronectes costipennis 16S ribosomal RNA gene, par	AY250951	81.65	100	1.8757e−31	Coleoptera	Dytiscidae	Deronectes costipennis
