# Supplementary material for: Testing the potential of a ribosomal 16S marker for DNA metabarcoding of insects
Source: PeerJ. 2016 Apr 19;4:e1966. doi: 10.7717/peerj.1966 (PMC4841222; doi:10.7717/peerj.1966)
Supplement: Supplemental Information 1 [file peerj-04-1966-s006.zip › scripts/5 match OTUs/blast_hits/OTU_508.csv_plot.pdf]

OTU_508.csv

Oreodytes sanmarkii

Dytiscidae

Coleoptera

98.73

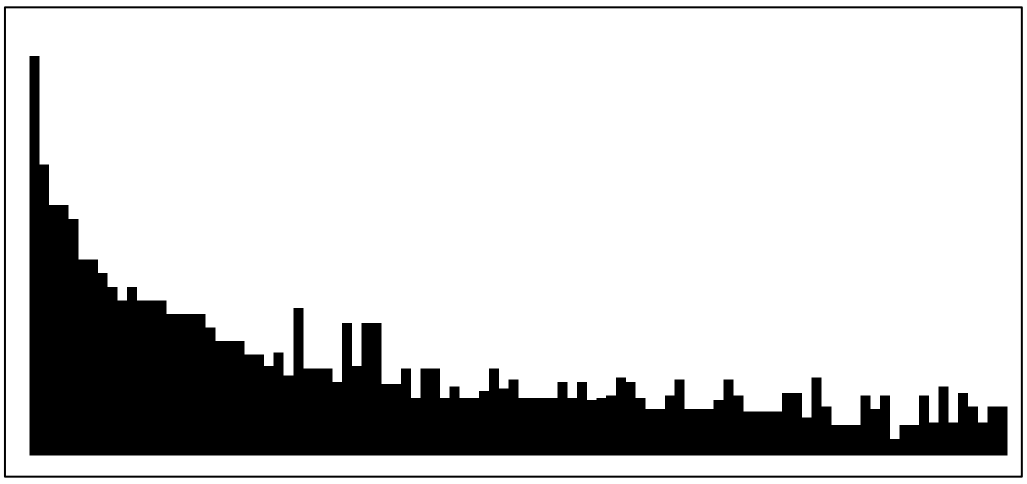

Oreodytes sanmarkii mitochondrial 16S rRNA gene (p	HF931524	98.73	100	2.30021e−68	Coleoptera	Dytiscidae	Oreodytes sanmarkii
Oreodytes obesus cordillerensis mitochondrial part	HF931516	93.63	100	1.65626e−57	Coleoptera	Dytiscidae	Oreodytes obesus cordillerensis
Oreodytes crassulus partial 16S rRNA gene	AJ850348	91.72	100	3.64817e−53	Coleoptera	Dytiscidae	Oreodytes crassulus
Oreodytes congruus partial 16S rRNA gene	AJ850347	91.72	100	3.64817e−53	Coleoptera	Dytiscidae	Oreodytes congruus
Oreodytes congruus 16S ribosomal RNA gene, partial	KF575550	91.08	100	4.44438e−52	Coleoptera	Dytiscidae	Oreodytes congruus
Oreodytes abbreviatus mitochondrial 16S rRNA gene	HF931477	89.17	100	9.78939e−48	Coleoptera	Dytiscidae	Oreodytes abbreviatus
Oreodytes rhyacophilus partial 16S rRNA gene	AJ850352	89.17	100	9.78939e−48	Coleoptera	Dytiscidae	Oreodytes rhyacophilus
Oreodytes mongolicus mitochondrial 16S rRNA gene (	HF931491	88.54	100	1.19259e−46	Coleoptera	Dytiscidae	Oreodytes mongolicus
Oreodytes snoqualmie mitochondrial partial 16S rRN	HF931519	87.9	100	5.07103e−45	Coleoptera	Dytiscidae	Oreodytes snoqualmie
Oreodytes shorti mitochondrial 16S rRNA gene (part	HF931488	87.26	100	6.17778e−44	Coleoptera	Dytiscidae	Oreodytes shorti
Boreonectes griseostriatus mitochondrial partial 1	HF931387	87.9	100	6.17778e−44	Coleoptera	Dytiscidae	Boreonectes griseostriatus
Boreonectes striatellus mitochondrial 16S rRNA gen	HF931511	87.26	100	7.52607e−43	Coleoptera	Dytiscidae	Boreonectes striatellus
Boreonectes riberae mitochondrial 16S rRNA gene (p	HF931461	87.26	100	7.52607e−43	Coleoptera	Dytiscidae	Boreonectes riberae
Boreonectes multilineatus mitochondrial partial 16	HF931384	87.26	100	7.52607e−43	Coleoptera	Dytiscidae	Boreonectes multilineatus
Oreodytes septentrionalis mitochondrial 16S rRNA g	HF931492	86.62	100	2.62686e−42	Coleoptera	Dytiscidae	Oreodytes septentrionalis
Boreonectes alpestris mitochondrial 16S rRNA gene	HF931512	86.62	100	3.20017e−41	Coleoptera	Dytiscidae	Boreonectes alpestris
Boreonectes macedonicus mitochondrial 16S rRNA gen	HF931359	86.62	100	3.20017e−41	Coleoptera	Dytiscidae	Boreonectes macedonicus
Stictotarsus ibericus 16S ribosomal RNA (rrnL) gen	EF670030	86.62	100	3.20017e−41	Coleoptera	Dytiscidae	Stictotarsus ibericus
Boreonectes griseostriatus mitochondrial 16S rRNA	HF931541	85.99	100	3.8986e−40	Coleoptera	Dytiscidae	Boreonectes griseostriatus
Laccornis difformis 16S ribosomal RNA gene, partia	KF575544	85.35	100	1.36075e−39	Coleoptera	Dytiscidae	Laccornis difformis
Deronectes parvicollis mitochondrial 16S rRNA gene	HF931454	85.35	100	1.36075e−39	Coleoptera	Dytiscidae	Deronectes parvicollis
Laccornis kocae partial 16S rRNA gene	AJ850419	85.35	100	1.36075e−39	Coleoptera	Dytiscidae	Laccornis kocae
Oreodytes scitulus 16S ribosomal RNA gene, partial	KF575551	84.71	100	1.65773e−38	Coleoptera	Dytiscidae	Oreodytes scitulus
Oreodytes scitulus mitochondrial partial 16S rRNA	HF931515	84.71	100	1.65773e−38	Coleoptera	Dytiscidae	Oreodytes scitulus
Nebrioporus formaster mitochondrial partial 16S rR	HF931377	84.18	100	5.78604e−38	Coleoptera	Dytiscidae	Nebrioporus formaster
Stictotarsus maghrebinus 16S ribosomal RNA gene, p	AY250947	84.81	100	7.04884e−37	Coleoptera	Dytiscidae	Stictotarsus maghrebinus
Aethionectes fulvonotatus voucher KBMAeop406 16S r	KF979088	83.75	100	2.46029e−36	Coleoptera	Dytiscidae	Aethionectes fulvonotatus
Deronectes moestus mitochondrial 16S rRNA gene (pa	HE613726	86.9	91.61	2.46029e−36	Coleoptera	Dytiscidae	Deronectes moestus
Suphrodytes dorsalis 16S ribosomal RNA gene, parti	KF575577	84.08	100	8.58725e−36	Coleoptera	Dytiscidae	Suphrodytes dorsalis
Boreonectes griseostriatus mitochondrial 16S rRNA	HF931385	84.08	100	8.58725e−36	Coleoptera	Dytiscidae	Boreonectes griseostriatus
Suphrodytes dorsalis voucher BMNH:725966 16S ribos	JX221574	84.08	100	8.58725e−36	Coleoptera	Dytiscidae	Suphrodytes dorsalis
Oreodytes natrix partial 16S rRNA gene	AJ850349	83.44	100	8.58725e−36	Coleoptera	Dytiscidae	Oreodytes natrix
Deronectes moestus 16S ribosomal RNA gene, partial	KF575561	86.21	91.61	2.99724e−35	Coleoptera	Dytiscidae	Deronectes moestus
Stictotarsus procerus mitochondrial 16S rRNA gene	HF931379	84.18	100	2.99724e−35	Coleoptera	Dytiscidae	Stictotarsus procerus
Deronectes brannani mitochondrial 16S rRNA gene (p	HE613695	86.21	91.61	2.99724e−35	Coleoptera	Dytiscidae	Deronectes brannanii
Deronectes moestus mitochondrial 16S rRNA gene (pa	HE613683	86.21	91.61	2.99724e−35	Coleoptera	Dytiscidae	Deronectes moestus
Hydroporus glabriusculus 16S ribosomal RNA gene, p	AY365270	83.33	100	2.99724e−35	Coleoptera	Dytiscidae	Hydroporus glabriusculus
Hydroporus sp. IR33.4 16S ribosomal RNA gene, part	AY365261	83.33	100	2.99724e−35	Coleoptera	Dytiscidae	Hydroporus sp. IR33.4
Hydroporus tessellatus 16S ribosomal RNA gene, par	JX434770	84.08	100	1.04614e−34	Coleoptera	Dytiscidae	Hydroporus tessellatus
Hydroporus appalachius 16S ribosomal RNA gene, par	JX434765	82.69	100	1.04614e−34	Coleoptera	Dytiscidae	Hydroporus appalachius
Hydroporus foveolatus mitochondrial 16S rRNA gene	HE613724	84.08	100	1.04614e−34	Coleoptera	Dytiscidae	Hydroporus foveolatus
Hydroporus basinotatus mitochondrial 16S rRNA gene	HE613678	84.08	100	1.04614e−34	Coleoptera	Dytiscidae	Hydroporus basinotatus
Hydroporus pervicinus mitochondrial 16S rRNA gene	HE599695	82.69	100	1.04614e−34	Coleoptera	Dytiscidae	Hydroporus pervicinus
Antiporus hollingsworthi mitochondrial partial 16S	FR727246	83.23	98.71	1.04614e−34	Coleoptera	Dytiscidae	Antiporus hollingsworthi
Hydroporus occidentalis 16S ribosomal RNA gene, pa	AY365280	82.69	100	1.04614e−34	Coleoptera	Dytiscidae	Hydroporus occidentalis
Hydroporus hirtellus 16S ribosomal RNA gene, parti	AY365271	82.69	100	1.04614e−34	Coleoptera	Dytiscidae	Hydroporus hirtellus
Scarodytes sp. IR−2003 16S ribosomal RNA gene, par	AY250939	83.02	100	1.04614e−34	Coleoptera	Dytiscidae	Scarodytes sp. IR−2003
Hydroporus basinotatus 16S ribosomal RNA gene, par	AF518258	84.08	100	1.04614e−34	Coleoptera	Dytiscidae	Hydroporus basinotatus
Hydroporus marginatus mitochondrial 16S rRNA gene	HE613699	83.12	100	3.65139e−34	Coleoptera	Dytiscidae	Hydroporus marginatus
Nirripirti megamacrocephalus isolate ABTC78781 16S	EU616935	83.54	100	3.65139e−34	Coleoptera	Dytiscidae	Paroster megamacrocephalus
Hygrotus novemlineatus mitochondrial partial 16S r	HF931523	82.69	100	1.27446e−33	Coleoptera	Dytiscidae	Hygrotus novemlineatus
Hygrotus marklini mitochondrial 16S rRNA gene (par	HF931467	82.69	100	1.27446e−33	Coleoptera	Dytiscidae	Hygrotus marklini
Suphrodytes figuratus voucher BMNH:722150 16S ribo	JX221572	82.69	100	1.27446e−33	Coleoptera	Dytiscidae	Suphrodytes figuratus
Suphrodytes figuratus voucher BMNH:722148 16S ribo	JX221571	82.69	100	1.27446e−33	Coleoptera	Dytiscidae	Suphrodytes figuratus
Hydroporus zimmermanni mitochondrial 16S rRNA gene	HE613679	83.44	100	1.27446e−33	Coleoptera	Dytiscidae	Hydroporus zimmermanni
Suphrodytes dorsalis mitochondrial 16S rRNA gene (	HE599700	82.69	100	1.27446e−33	Coleoptera	Dytiscidae	Suphrodytes dorsalis
Hydroporus compunctus mitochondrial 16S rRNA gene	HE599693	83.44	100	1.27446e−33	Coleoptera	Dytiscidae	Hydroporus compunctus
Antiporus hollingsworthi mitochondrial partial 16S	FR727247	82.58	98.71	1.27446e−33	Coleoptera	Dytiscidae	Antiporus hollingsworthi
Hygrotus turbidus partial 16S rRNA gene	AJ850390	82.69	100	1.27446e−33	Coleoptera	Dytiscidae	Hygrotus turbidus
Nirripirti wedgeensis isolate ABTC78773−4 16S ribo	EU616938	82.8	100	1.27446e−33	Coleoptera	Dytiscidae	Paroster wedgeensis
Nirripirti napperbyensis 16S ribosomal RNA gene, p	AY353843	83.65	100	1.27446e−33	Coleoptera	Dytiscidae	Paroster napperbyensis
Hydroporus compunctus 16S ribosomal RNA gene, part	AF518259	83.44	100	1.27446e−33	Coleoptera	Dytiscidae	Hydroporus compunctus
Hydroporus marginatus 16S ribosomal RNA gene, part	AF518267	82.69	100	1.27446e−33	Coleoptera	Dytiscidae	Hydroporus marginatus
Oreodytes quadrimaculatus 16S ribosomal RNA gene,	KF575567	82.17	100	4.4483e−33	Coleoptera	Dytiscidae	Oreodytes quadrimaculatus
Sanfilippodytes sp. KBM−2014 16S ribosomal RNA gen	KF575554	82.17	100	4.4483e−33	Coleoptera	Dytiscidae	Sanfilippodytes sp. KBM−2014
Boreonectes funereus mitochondrial 16S rRNA gene (	HF931393	82.8	100	4.4483e−33	Coleoptera	Dytiscidae	Boreonectes funereus
Hydroporus fuscipennis mitochondrial 16S rRNA gene	HE613730	83.54	100	4.4483e−33	Coleoptera	Dytiscidae	Hydroporus fuscipennis
Copelatus sp. BI−2011 voucher BMNH792945 16S ribos	HQ381441	82.17	100	4.4483e−33	Coleoptera	Dytiscidae	Copelatus sp. BI−2011
Oreodytes quadrimaculatus partial 16S rRNA gene	AJ850351	82.17	100	4.4483e−33	Coleoptera	Dytiscidae	Oreodytes quadrimaculatus
Amphizoa lecontei 16S ribosomal RNA gene, partial	AY071771	82.17	100	4.4483e−33	Coleoptera	Amphizoidae	Amphizoa lecontei
Nebrioporus assimilis 16S ribosomal RNA gene, part	AY250921	82.58	98.71	4.4483e−33	Coleoptera	Dytiscidae	Nebrioporus assimilis
Hydroporus fuscipennis 16S ribosomal RNA gene, par	AF518262	83.54	100	4.4483e−33	Coleoptera	Dytiscidae	Hydroporus fuscipennis
Ilybius erichsoni 16S ribosomal RNA gene, partial	AY138661	82.8	100	4.4483e−33	Coleoptera	Dytiscidae	Ilybius erichsoni
Hygrotus lutescens mitochondrial partial 16S rRNA	HF931518	82.05	100	1.55261e−32	Coleoptera	Dytiscidae	Hygrotus lutescens
Hygrotus tumidiventris mitochondrial 16S rRNA gene	HF931496	82.05	100	1.55261e−32	Coleoptera	Dytiscidae	Hygrotus tumidiventris
Hygrotus fontinalis mitochondrial 16S rRNA gene (p	HF931494	82.05	100	1.55261e−32	Coleoptera	Dytiscidae	Hygrotus fontinalis
Hygrotus patruelis mitochondrial 16S rRNA gene (pa	HF931388	82.05	100	1.55261e−32	Coleoptera	Dytiscidae	Hygrotus patruelis
Deronectes opatrinus mitochondrial 16S rRNA gene (	HE613701	82.91	100	1.55261e−32	Coleoptera	Dytiscidae	Deronectes opatrinus
Stictotarsus falli 16S ribosomal RNA (rrnL) gene,	EF670029	82.91	100	1.55261e−32	Coleoptera	Dytiscidae	Stictotarsus falli
Acilius duvergeri partial 16S rRNA gene	AJ850357	81.76	100	1.55261e−32	Coleoptera	Dytiscidae	Acilius duvergeri
Darwinhydrus solidus voucher MB354 16S ribosomal R	EF056671	83.65	100	1.55261e−32	Coleoptera	Dytiscidae	Darwinhydrus solidus
Scarodytes halensis haplotype 3 16S ribosomal RNA	AY250937	82.28	100	1.55261e−32	Coleoptera	Dytiscidae	Scarodytes halensis
Agabus didymus mitochondrial 16S rRNA gene (partia	LM654773	81.41	100	5.41914e−32	Coleoptera	Dytiscidae	Agabus didymus
Agabus didymus voucher UNM KBMPmsp730 16S ribosoma	KJ548392	81.41	100	5.41914e−32	Coleoptera	Dytiscidae	Agabus didymus
Hydaticus aruspex voucher NHRS/JLKB000000298 16S r	KF979119	81.41	100	5.41914e−32	Coleoptera	Dytiscidae	Hydaticus aruspex
Hydroporus foveolatus mitochondrial 16S rRNA gene	HF931502	82.8	100	5.41914e−32	Coleoptera	Dytiscidae	Hydroporus foveolatus
Hydroporus brancoi gredensis mitochondrial partial	HF931329	82.17	100	5.41914e−32	Coleoptera	Dytiscidae	Hydroporus brancoi gredensis
Hydroporus analis mitochondrial partial 16S rRNA g	HE613731	82.8	100	5.41914e−32	Coleoptera	Dytiscidae	Hydroporus analis
Nirripirti byroensis isolate ABTC78677−8 16S ribos	EU616910	80.77	100	5.41914e−32	Coleoptera	Dytiscidae	Paroster byroensis
Nirripirti newhavenensis 16S ribosomal RNA gene, p	AY353844	81.41	100	5.41914e−32	Coleoptera	Dytiscidae	Paroster newhavenensis
Hydroporus sp. IR23.2 16S ribosomal RNA gene, part	AY365258	81.41	100	5.41914e−32	Coleoptera	Dytiscidae	Hydroporus sp. IR23.2
Hydroporus rufifrons 16S ribosomal RNA gene, parti	AF518277	82.8	100	5.41914e−32	Coleoptera	Dytiscidae	Hydroporus rufifrons
Hydaticus cinctipennis voucher UNM KBMHyci20 16S r	KJ548338	81.53	100	1.89147e−31	Coleoptera	Dytiscidae	Hydaticus cinctipennis
Hygrotus impressopunctatus voucher UNM KBMHtim80 1	KJ548334	83.23	97.42	1.89147e−31	Coleoptera	Dytiscidae	Hygrotus impressopunctatus
Cybister marginicollis voucher UNM KBMCyma416 16S	KJ548319	81.53	100	1.89147e−31	Coleoptera	Dytiscidae	Cybister marginicollis
Hygrotus armeniacus mitochondrial partial 16S rRNA	HF931520	82.91	100	1.89147e−31	Coleoptera	Dytiscidae	Hygrotus armeniacus
Deronectes doriae mitochondrial 16S rRNA gene (par	HE613710	82.28	100	1.89147e−31	Coleoptera	Dytiscidae	Deronectes doriae
Deronectes bicostatus mitochondrial 16S rRNA gene	HE613705	81.53	100	1.89147e−31	Coleoptera	Dytiscidae	Deronectes bicostatus
Deronectes sahlbergi mitochondrial 16S rRNA gene (	HE613680	82.28	100	1.89147e−31	Coleoptera	Dytiscidae	Deronectes sahlbergi
Paroster sp. R401 Newhaven voucher ABTC:78974 16S	JQ745754	82.28	100	1.89147e−31	Coleoptera	Dytiscidae	Paroster sp. R401 Newhaven
