# Supplementary material for: Testing the potential of a ribosomal 16S marker for DNA metabarcoding of insects
Source: PeerJ. 2016 Apr 19;4:e1966. doi: 10.7717/peerj.1966 (PMC4841222; doi:10.7717/peerj.1966)
Supplement: Supplemental Information 1 [file peerj-04-1966-s006.zip › scripts/5 match OTUs/blast_hits/OTU_511.csv_plot.pdf]

OTU_511.csv

Oreodytes sanmarkii

Dytiscidae

Coleoptera

96.82

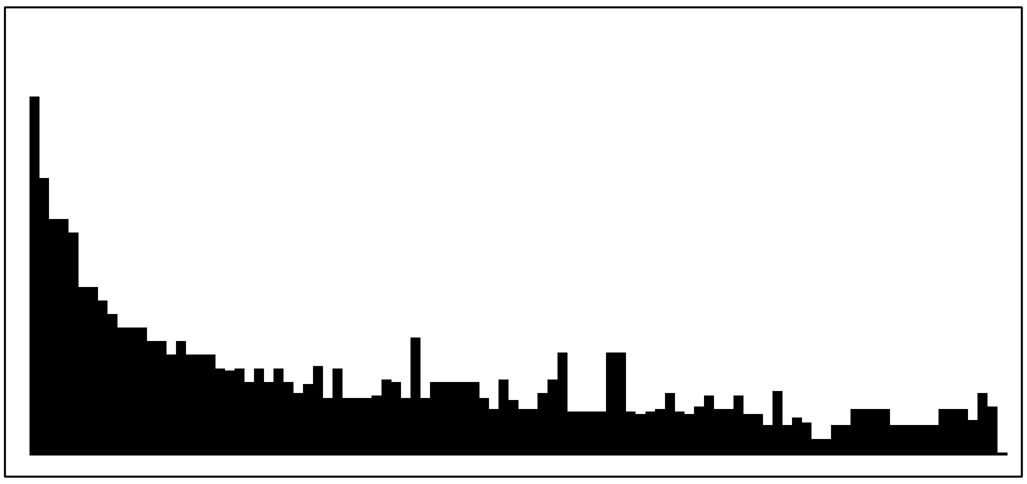

Oreodytes sanmarkii mitochondrial 16S rRNA gene (p	HF931524	96.82	100	4.19354e−65	Coleoptera	Dytiscidae	Oreodytes sanmarkii
Oreodytes obesus cordillerensis mitochondrial part	HF931516	92.99	100	5.82911e−57	Coleoptera	Dytiscidae	Oreodytes obesus cordillerensis
Oreodytes crassulus partial 16S rRNA gene	AJ850348	91.08	100	3.67857e−53	Coleoptera	Dytiscidae	Oreodytes crassulus
Oreodytes congruus partial 16S rRNA gene	AJ850347	91.08	100	3.67857e−53	Coleoptera	Dytiscidae	Oreodytes congruus
Oreodytes congruus 16S ribosomal RNA gene, partial	KF575550	90.45	100	1.56417e−51	Coleoptera	Dytiscidae	Oreodytes congruus
Oreodytes abbreviatus mitochondrial 16S rRNA gene	HF931477	87.9	100	4.19724e−46	Coleoptera	Dytiscidae	Oreodytes abbreviatus
Oreodytes rhyacophilus partial 16S rRNA gene	AJ850352	87.9	100	4.19724e−46	Coleoptera	Dytiscidae	Oreodytes rhyacophilus
Oreodytes mongolicus mitochondrial 16S rRNA gene (	HF931491	87.26	100	5.11329e−45	Coleoptera	Dytiscidae	Oreodytes mongolicus
Oreodytes snoqualmie mitochondrial partial 16S rRN	HF931519	86.62	100	2.17423e−43	Coleoptera	Dytiscidae	Oreodytes snoqualmie
Oreodytes shorti mitochondrial 16S rRNA gene (part	HF931488	85.99	100	2.64875e−42	Coleoptera	Dytiscidae	Oreodytes shorti
Boreonectes striatellus mitochondrial 16S rRNA gen	HF931511	85.99	100	3.22684e−41	Coleoptera	Dytiscidae	Boreonectes striatellus
Boreonectes griseostriatus mitochondrial partial 1	HF931387	85.99	100	3.22684e−41	Coleoptera	Dytiscidae	Boreonectes griseostriatus
Oreodytes septentrionalis mitochondrial 16S rRNA g	HF931492	85.35	100	1.12628e−40	Coleoptera	Dytiscidae	Oreodytes septentrionalis
Boreonectes riberae mitochondrial 16S rRNA gene (p	HF931461	85.35	100	1.37209e−39	Coleoptera	Dytiscidae	Boreonectes riberae
Deronectes parvicollis mitochondrial 16S rRNA gene	HF931454	84.71	100	1.37209e−39	Coleoptera	Dytiscidae	Deronectes parvicollis
Boreonectes multilineatus mitochondrial partial 16	HF931384	85.35	100	1.37209e−39	Coleoptera	Dytiscidae	Boreonectes multilineatus
Boreonectes alpestris mitochondrial 16S rRNA gene	HF931512	84.71	100	1.67154e−38	Coleoptera	Dytiscidae	Boreonectes alpestris
Boreonectes macedonicus mitochondrial 16S rRNA gen	HF931359	84.71	100	1.67154e−38	Coleoptera	Dytiscidae	Boreonectes macedonicus
Stictotarsus ibericus 16S ribosomal RNA (rrnL) gen	EF670030	84.71	100	1.67154e−38	Coleoptera	Dytiscidae	Stictotarsus ibericus
Laccornis difformis 16S ribosomal RNA gene, partia	KF575544	84.08	100	5.83426e−38	Coleoptera	Dytiscidae	Laccornis difformis
Hydroporus glabriusculus 16S ribosomal RNA gene, p	AY365270	83.97	100	2.03636e−37	Coleoptera	Dytiscidae	Hydroporus glabriusculus
Suphrodytes dorsalis 16S ribosomal RNA gene, parti	KF575577	84.08	100	7.10758e−37	Coleoptera	Dytiscidae	Suphrodytes dorsalis
Oreodytes scitulus 16S ribosomal RNA gene, partial	KF575551	83.44	100	7.10758e−37	Coleoptera	Dytiscidae	Oreodytes scitulus
Boreonectes griseostriatus mitochondrial 16S rRNA	HF931541	84.08	100	7.10758e−37	Coleoptera	Dytiscidae	Boreonectes griseostriatus
Oreodytes scitulus mitochondrial partial 16S rRNA	HF931515	83.44	100	7.10758e−37	Coleoptera	Dytiscidae	Oreodytes scitulus
Suphrodytes dorsalis voucher BMNH:725966 16S ribos	JX221574	84.08	100	7.10758e−37	Coleoptera	Dytiscidae	Suphrodytes dorsalis
Laccornis kocae partial 16S rRNA gene	AJ850419	83.44	100	7.10758e−37	Coleoptera	Dytiscidae	Laccornis kocae
Nebrioporus formaster mitochondrial partial 16S rR	HF931377	82.91	100	2.48079e−36	Coleoptera	Dytiscidae	Nebrioporus formaster
Hydroporus sp. IR33.4 16S ribosomal RNA gene, part	AY365261	83.33	100	2.48079e−36	Coleoptera	Dytiscidae	Hydroporus sp. IR33.4
Stictotarsus maghrebinus 16S ribosomal RNA gene, p	AY250947	84.18	100	2.48079e−36	Coleoptera	Dytiscidae	Stictotarsus maghrebinus
Hydroporus appalachius 16S ribosomal RNA gene, par	JX434765	82.69	100	8.65881e−36	Coleoptera	Dytiscidae	Hydroporus appalachius
Hydroporus foveolatus mitochondrial 16S rRNA gene	HE613724	84.08	100	8.65881e−36	Coleoptera	Dytiscidae	Hydroporus foveolatus
Hydroporus pervicinus mitochondrial 16S rRNA gene	HE599695	82.69	100	8.65881e−36	Coleoptera	Dytiscidae	Hydroporus pervicinus
Hydroporus occidentalis 16S ribosomal RNA gene, pa	AY365280	82.69	100	8.65881e−36	Coleoptera	Dytiscidae	Hydroporus occidentalis
Hydroporus hirtellus 16S ribosomal RNA gene, parti	AY365271	82.69	100	8.65881e−36	Coleoptera	Dytiscidae	Hydroporus hirtellus
Aethionectes fulvonotatus voucher KBMAeop406 16S r	KF979088	82.8	100	3.02222e−35	Coleoptera	Dytiscidae	Aethionectes fulvonotatus
Stictotarsus procerus mitochondrial 16S rRNA gene	HF931379	83.54	100	3.02222e−35	Coleoptera	Dytiscidae	Stictotarsus procerus
Hydroporus tessellatus 16S ribosomal RNA gene, par	JX434770	83.44	100	1.05486e−34	Coleoptera	Dytiscidae	Hydroporus tessellatus
Suphrodytes figuratus voucher BMNH:722148 16S ribo	JX221571	82.69	100	1.05486e−34	Coleoptera	Dytiscidae	Suphrodytes figuratus
Deronectes moestus mitochondrial 16S rRNA gene (pa	HE613726	85.52	91.67	1.05486e−34	Coleoptera	Dytiscidae	Deronectes moestus
Hydroporus marginatus mitochondrial 16S rRNA gene	HE613699	82.69	100	1.05486e−34	Coleoptera	Dytiscidae	Hydroporus marginatus
Hydroporus zimmermanni mitochondrial 16S rRNA gene	HE613679	83.44	100	1.05486e−34	Coleoptera	Dytiscidae	Hydroporus zimmermanni
Hydroporus basinotatus mitochondrial 16S rRNA gene	HE613678	83.44	100	1.05486e−34	Coleoptera	Dytiscidae	Hydroporus basinotatus
Hydroporus compunctus mitochondrial 16S rRNA gene	HE599693	83.44	100	1.05486e−34	Coleoptera	Dytiscidae	Hydroporus compunctus
Hydroporus basinotatus 16S ribosomal RNA gene, par	AF518258	83.44	100	1.05486e−34	Coleoptera	Dytiscidae	Hydroporus basinotatus
Hydroporus compunctus 16S ribosomal RNA gene, part	AF518259	83.44	100	1.05486e−34	Coleoptera	Dytiscidae	Hydroporus compunctus
Hydroporus marginatus 16S ribosomal RNA gene, part	AF518267	82.69	100	1.05486e−34	Coleoptera	Dytiscidae	Hydroporus marginatus
Oreodytes quadrimaculatus 16S ribosomal RNA gene,	KF575567	82.17	100	3.68182e−34	Coleoptera	Dytiscidae	Oreodytes quadrimaculatus
Hydroporus fuscipennis mitochondrial 16S rRNA gene	HE613730	83.54	100	3.68182e−34	Coleoptera	Dytiscidae	Hydroporus fuscipennis
Antiporus hollingsworthi mitochondrial partial 16S	FR727246	82.58	98.72	3.68182e−34	Coleoptera	Dytiscidae	Antiporus hollingsworthi
Oreodytes quadrimaculatus partial 16S rRNA gene	AJ850351	82.17	100	3.68182e−34	Coleoptera	Dytiscidae	Oreodytes quadrimaculatus
Oreodytes natrix partial 16S rRNA gene	AJ850349	82.17	100	3.68182e−34	Coleoptera	Dytiscidae	Oreodytes natrix
Hydroporus rufifrons 16S ribosomal RNA gene, parti	AF518277	82.91	100	3.68182e−34	Coleoptera	Dytiscidae	Hydroporus rufifrons
Hydroporus fuscipennis 16S ribosomal RNA gene, par	AF518262	83.54	100	3.68182e−34	Coleoptera	Dytiscidae	Hydroporus fuscipennis
Deronectes moestus 16S ribosomal RNA gene, partial	KF575561	84.83	91.67	1.28508e−33	Coleoptera	Dytiscidae	Deronectes moestus
Hygrotus novemlineatus mitochondrial partial 16S r	HF931523	82.05	100	1.28508e−33	Coleoptera	Dytiscidae	Hygrotus novemlineatus
Hygrotus semenowi mitochondrial partial 16S rRNA	HF931509	82.05	100	1.28508e−33	Coleoptera	Dytiscidae	Hygrotus semenowi
Hygrotus marklini mitochondrial 16S rRNA gene (par	HF931467	82.05	100	1.28508e−33	Coleoptera	Dytiscidae	Hygrotus marklini
Suphrodytes figuratus voucher BMNH:722150 16S ribo	JX221572	82.05	100	1.28508e−33	Coleoptera	Dytiscidae	Suphrodytes figuratus
Deronectes brannani mitochondrial 16S rRNA gene (p	HE613695	84.83	91.67	1.28508e−33	Coleoptera	Dytiscidae	Deronectes brannanii
Deronectes moestus mitochondrial 16S rRNA gene (pa	HE613683	84.83	91.67	1.28508e−33	Coleoptera	Dytiscidae	Deronectes moestus
Suphrodytes dorsalis mitochondrial 16S rRNA gene (	HE599700	82.05	100	1.28508e−33	Coleoptera	Dytiscidae	Suphrodytes dorsalis
Antiporus hollingsworthi mitochondrial partial 16S	FR727247	81.94	98.72	1.28508e−33	Coleoptera	Dytiscidae	Antiporus hollingsworthi
Hygrotus turbidus partial 16S rRNA gene	AJ850390	82.05	100	1.28508e−33	Coleoptera	Dytiscidae	Hygrotus turbidus
Nirripirti wedgeensis isolate ABTC78773−4 16S ribo	EU616938	82.17	100	1.28508e−33	Coleoptera	Dytiscidae	Paroster wedgeensis
Nirripirti megamacrocephalus isolate ABTC78781 16S	EU616935	82.91	100	1.28508e−33	Coleoptera	Dytiscidae	Paroster megamacrocephalus
Hydroporus morio 16S ribosomal RNA gene, partial s	AY365274	82.05	100	1.28508e−33	Coleoptera	Dytiscidae	Hydroporus morio
Chostonectes sharpi mitochondrial partial 16S rRNA	HG940420	81.94	98.72	4.48537e−33	Coleoptera	Dytiscidae	Chostonectes sharpi
Hydroporus rufifrons 16S ribosomal RNA gene, parti	JX434758	82.28	100	4.48537e−33	Coleoptera	Dytiscidae	Hydroporus rufifrons
Hydroporus foveolatus mitochondrial 16S rRNA gene	HF931502	82.8	100	4.48537e−33	Coleoptera	Dytiscidae	Hydroporus foveolatus
Boreonectes griseostriatus mitochondrial 16S rRNA	HF931385	82.17	100	4.48537e−33	Coleoptera	Dytiscidae	Boreonectes griseostriatus
Hydroporus brancoi gredensis mitochondrial partial	HF931329	82.17	100	4.48537e−33	Coleoptera	Dytiscidae	Hydroporus brancoi gredensis
Hydroporus analis mitochondrial 16S rRNA g	HE613731	82.8	100	4.48537e−33	Coleoptera	Dytiscidae	Hydroporus analis
Chostonectes sharpi partial 16S rRNA gene	AJ850313	81.94	98.72	4.48537e−33	Coleoptera	Dytiscidae	Chostonectes sharpi
Chostonectes sharpi isolate ABTC78964 16S ribosoma	EU616947	81.94	98.72	4.48537e−33	Coleoptera	Dytiscidae	Chostonectes sharpi
Nirripirti newhavenensis 16S ribosomal RNA gene, p	AY353844	81.41	100	4.48537e−33	Coleoptera	Dytiscidae	Paroster newhavenensis
Nirripirti napperbyensis 16S ribosomal RNA gene, p	AY353843	83.02	100	4.48537e−33	Coleoptera	Dytiscidae	Paroster napperbyensis
Hydroporus sp. IR23.2 16S ribosomal RNA gene, part	AY365258	81.41	100	4.48537e−33	Coleoptera	Dytiscidae	Hydroporus sp. IR23.2
Scarodytes sp. IR−2003 16S ribosomal RNA gene, par	AY250939	81.76	100	4.48537e−33	Coleoptera	Dytiscidae	Scarodytes sp. IR−2003
Copelatus sp. BI−2011 voucher BMNH671215 16S ribos	HQ381377	81.53	100	1.56555e−32	Coleoptera	Dytiscidae	Copelatus sp. BI−2011
Agabus didymus mitochondrial 16S rRNA gene (partia	LM654773	80.77	100	5.4643e−32	Coleoptera	Dytiscidae	Agabus didymus
Agabus didymus voucher UNM KBMPmsp730 16S ribosoma	KJ548392	80.77	100	5.4643e−32	Coleoptera	Dytiscidae	Agabus didymus
Hydroporus neglectus 16S ribosomal RNA gene, parti	JX434768	81.41	100	5.4643e−32	Coleoptera	Dytiscidae	Hydroporus neglectus
Hygrotus patruelis mitochondrial 16S rRNA gene (pa	HF931388	81.41	100	5.4643e−32	Coleoptera	Dytiscidae	Hygrotus patruelis
Hydroporus discretus mitochondrial 16S rRNA gene (	HE613723	82.17	100	5.4643e−32	Coleoptera	Dytiscidae	Hydroporus discretus
Hydroporus decipiens mitochondrial 16S rRNA gene (	HE613722	82.17	100	5.4643e−32	Coleoptera	Dytiscidae	Hydroporus decipiens
Hydroporus pubescens mitochondrial 16S rRNA gene (	HE613707	82.17	100	5.4643e−32	Coleoptera	Dytiscidae	Hydroporus pubescens
Hydroporus errans mitochondrial 16S rRNA gene (par	HE613704	82.17	100	5.4643e−32	Coleoptera	Dytiscidae	Hydroporus errans
Hydroporus necopinatus mitochondrial partial 16S r	HE599692	81.41	100	5.4643e−32	Coleoptera	Dytiscidae	Hydroporus necopinatus
Hydroporus brancoi mitochondrial 16S rRNA gene (pa	HE599678	81.41	100	5.4643e−32	Coleoptera	Dytiscidae	Hydroporus brancoi
Hydroporus cantabricus mitochondrial partial 16S r	HE599676	81.41	100	5.4643e−32	Coleoptera	Dytiscidae	Hydroporus cantabricus
Hydroporus neglectus 16S ribosomal RNA gene, parti	AY365276	81.41	100	5.4643e−32	Coleoptera	Dytiscidae	Hydroporus neglectus
Hydroporus necopinatus 16S ribosomal RNA gene, par	AY365275	81.41	100	5.4643e−32	Coleoptera	Dytiscidae	Hydroporus necopinatus
Hydroporus pubescens 16S ribosomal RNA gene, parti	AF518276	82.17	100	5.4643e−32	Coleoptera	Dytiscidae	Hydroporus pubescens
Hydroporus discretus 16S ribosomal RNA gene, parti	AF518260	82.17	100	5.4643e−32	Coleoptera	Dytiscidae	Hydroporus discretus
Hydroporus analis 16S ribosomal RNA gene, partial	AF518257	82.17	100	5.4643e−32	Coleoptera	Dytiscidae	Hydroporus analis
Hydroporus planus mitochondrial 16S rRNA gene (par	HE613708	81.65	100	1.90723e−31	Coleoptera	Dytiscidae	Hydroporus planus
Hydroporus limbatus mitochondrial 16S rRNA gene (p	HE613706	82.91	100	1.90723e−31	Coleoptera	Dytiscidae	Hydroporus limbatus
Hydroporus brancuccii mitochondrial 16S rRNA gene	HE613696	82.28	100	1.90723e−31	Coleoptera	Dytiscidae	Hydroporus brancuccii
Nirripirti byroensis isolate ABTC78677−8 16S ribos	EU616910	80.13	100	1.90723e−31	Coleoptera	Dytiscidae	Paroster byroensis
