# Supplementary material for: Testing the potential of a ribosomal 16S marker for DNA metabarcoding of insects
Source: PeerJ. 2016 Apr 19;4:e1966. doi: 10.7717/peerj.1966 (PMC4841222; doi:10.7717/peerj.1966)
Supplement: Supplemental Information 1 [file peerj-04-1966-s006.zip › scripts/5 match OTUs/blast_hits/OTU_513.csv_plot.pdf]

OTU_513.csv

Protonemura meyeri

Nemouridae

Plecoptera

92.36

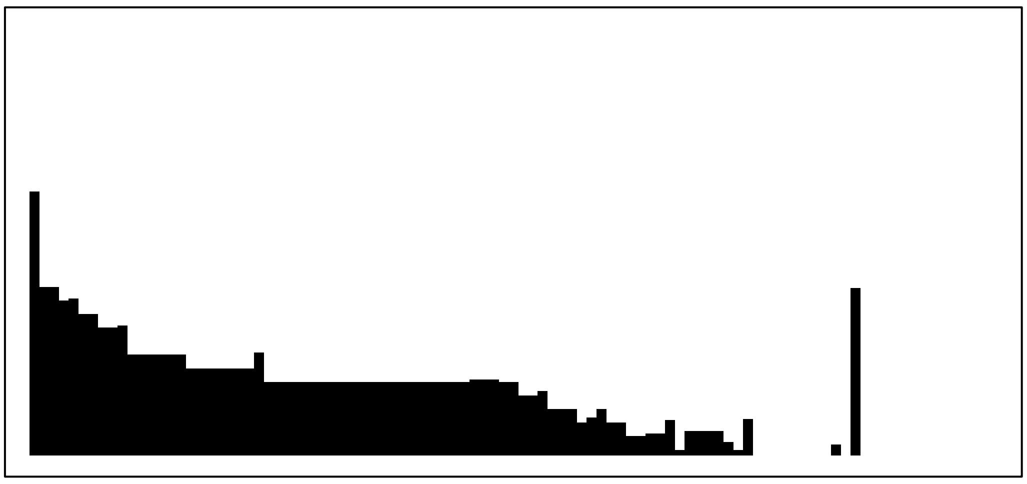

Protonemura meyeri 16S ribosomal RNA gene, partial	EF623193	92.36	100	5.87728e−57	Plecoptera	Nemouridae	Protonemura meyeri
Nanonemoura wahkeena 16S ribosomal RNA gene, parti	EF623198	87.9	100	3.47378e−47	Plecoptera	Nemouridae	Nanonemoura wahkeena
Ostrocerca foersteri 16S ribosomal RNA gene, parti	EF623191	87.9	100	3.47378e−47	Plecoptera	Nemouridae	Ostrocerca foersteri
Nemoura trispinosa 16S ribosomal RNA gene, partial	EF623187	87.26	100	4.23193e−46	Plecoptera	Nemouridae	Nemoura trispinosa
Scopura montana 16S ribosomal RNA gene, partial se	EF623265	87.34	100	1.47709e−45	Plecoptera		Scopura montana
Nemurella pictetii 16S ribosomal RNA gene, partial	EF623192	86.62	100	1.79946e−44	Plecoptera	Nemouridae	Nemurella pictetii
Shipsa rotunda 16S ribosomal RNA gene, partial seq	EF623184	86.62	100	1.79946e−44	Plecoptera	Nemouridae	Shipsa rotunda
Visoka cataractae 16S ribosomal RNA gene, partial	EF623189	85.99	100	2.19219e−43	Plecoptera	Nemouridae	Visoka cataractae
Paranemoura claasseni 16S ribosomal RNA gene, part	EF623185	85.99	100	2.19219e−43	Plecoptera	Nemouridae	Paranemoura claasseni
Nemoura cinerea 16S ribosomal RNA gene, partial se	EF623194	86.08	100	7.65151e−43	Plecoptera	Nemouridae	Nemoura cinerea
Apteroperla tikumana mitochondrion, complete genom	KR604721	84.71	100	1.13558e−40	Plecoptera	Capniidae	Apteroperla tikumana
Lednia tumana 16S ribosomal RNA gene, partial sequ	EF623197	84.71	100	1.13558e−40	Plecoptera	Nemouridae	Lednia tumana
Prostoia besametsa 16S ribosomal RNA gene, partial	EF623188	84.71	100	1.13558e−40	Plecoptera	Nemouridae	Prostoia besametsa
Zapada cinctipes 16S ribosomal RNA gene, partial s	EF623183	84.71	100	1.13558e−40	Plecoptera	Nemouridae	Zapada cinctipes
Allocapnia minima 16S ribosomal RNA gene, partial	EF623130	84.71	100	1.13558e−40	Plecoptera	Capniidae	Allocapnia minima
Soyedina producta 16S ribosomal RNA gene, partial	EF623196	84.71	100	1.38343e−39	Plecoptera	Nemouridae	Soyedina producta
Allocapnia recta isolate H15 16S ribosomal RNA gen	KC881050	84.08	100	4.82863e−39	Plecoptera	Capniidae	Allocapnia recta
Allocapnia recta isolate H14 16S ribosomal RNA gen	KC881049	84.08	100	4.82863e−39	Plecoptera	Capniidae	Allocapnia recta
Allocapnia recta isolate H10 16S ribosomal RNA gen	KC881045	84.08	100	4.82863e−39	Plecoptera	Capniidae	Allocapnia recta
Allocapnia recta isolate H6 16S ribosomal RNA gene	KC881041	84.08	100	4.82863e−39	Plecoptera	Capniidae	Allocapnia recta
Malenka californica 16S ribosomal RNA gene, partia	EF623182	84.08	100	4.82863e−39	Plecoptera	Nemouridae	Malenka californica
Allocapnia recta haplotype 11ar: H011 16S ribosoma	EU306874	84.08	100	4.82863e−39	Plecoptera	Capniidae	Allocapnia recta
Allocapnia recta haplotype 7ar: H07 16S ribosomal	EU306870	84.08	100	4.82863e−39	Plecoptera	Capniidae	Allocapnia recta
Moselia infuscata 16S ribosomal RNA gene, partial	EF623175	84.81	99.36	1.68536e−38	Plecoptera	Leuctridae	Moselia infuscata
Mesocapnia arizonensis mitochondrion, partial geno	KP642637	83.44	100	5.88247e−38	Plecoptera	Capniidae	Mesocapnia arizonensis
Allocapnia recta isolate H19 16S ribosomal RNA gen	KC881054	83.44	100	5.88247e−38	Plecoptera	Capniidae	Allocapnia recta
Allocapnia recta isolate H17 16S ribosomal RNA gen	KC881052	83.44	100	5.88247e−38	Plecoptera	Capniidae	Allocapnia recta
Allocapnia recta isolate H16 16S ribosomal RNA gen	KC881051	83.44	100	5.88247e−38	Plecoptera	Capniidae	Allocapnia recta
Allocapnia recta isolate H13 16S ribosomal RNA gen	KC881048	83.44	100	5.88247e−38	Plecoptera	Capniidae	Allocapnia recta
Allocapnia recta isolate H12 16S ribosomal RNA gen	KC881047	83.44	100	5.88247e−38	Plecoptera	Capniidae	Allocapnia recta
Allocapnia recta isolate H11 16S ribosomal RNA gen	KC881046	83.44	100	5.88247e−38	Plecoptera	Capniidae	Allocapnia recta
Allocapnia recta isolate H9 16S ribosomal RNA gene	KC881044	83.44	100	5.88247e−38	Plecoptera	Capniidae	Allocapnia recta
Allocapnia recta isolate H8 16S ribosomal RNA gene	KC881043	83.44	100	5.88247e−38	Plecoptera	Capniidae	Allocapnia recta
Allocapnia recta isolate H7 16S ribosomal RNA gene	KC881042	83.44	100	5.88247e−38	Plecoptera	Capniidae	Allocapnia recta
Allocapnia recta isolate H5 16S ribosomal RNA gene	KC881040	83.44	100	5.88247e−38	Plecoptera	Capniidae	Allocapnia recta
Allocapnia recta isolate H4 16S ribosomal RNA gene	KC881039	83.44	100	5.88247e−38	Plecoptera	Capniidae	Allocapnia recta
Allocapnia recta isolate H3 16S ribosomal RNA gene	KC881038	83.44	100	5.88247e−38	Plecoptera	Capniidae	Allocapnia recta
Allocapnia recta isolate H1 16S ribosomal RNA gene	KC881036	83.44	100	5.88247e−38	Plecoptera	Capniidae	Allocapnia recta
Mesocapnia sp. BYU_PL010 16S ribosomal RNA gene, p	EF623127	83.44	100	5.88247e−38	Plecoptera	Capniidae	Mesocapnia sp. BYU_PL010
Allocapnia recta haplotype 10ar: H010 16S ribosoma	EU306873	83.44	100	5.88247e−38	Plecoptera	Capniidae	Allocapnia recta
Allocapnia recta haplotype 9ar: H09 16S ribosomal	EU306872	83.44	100	5.88247e−38	Plecoptera	Capniidae	Allocapnia recta
Allocapnia recta haplotype 8ar: H08 16S ribosomal	EU306871	83.44	100	5.88247e−38	Plecoptera	Capniidae	Allocapnia recta
Allocapnia recta haplotype 3ar: H03 16S ribosomal	DQ915181	83.44	100	5.88247e−38	Plecoptera	Capniidae	Allocapnia recta
Allocapnia recta haplotype 2ar: H02 16S ribosomal	DQ915180	83.44	100	5.88247e−38	Plecoptera	Capniidae	Allocapnia recta
Allocapnia recta haplotype 1ar: H01 16S ribosomal	DQ915179	83.44	100	5.88247e−38	Plecoptera	Capniidae	Allocapnia recta
Doddsia occidentalis 16S ribosomal RNA gene, partial	EF623270	83.54	100	2.05319e−37	Plecoptera	Taeniopterygidae	Doddsia occidentalis
Oemopteryx vanduzeea 16S ribosomal RNA gene, parti	EF623266	83.54	100	2.05319e−37	Plecoptera	Taeniopterygidae	Oemopteryx vanduzeea
Megaleuctra kincaidi 16S ribosomal RNA gene, parti	EF623174	83.54	100	2.05319e−37	Plecoptera	Leuctridae	Megaleuctra kincaidi
Pteronarcys sachalina 16S ribosomal RNA gene, part	EF623264	83.44	100	7.16632e−37	Plecoptera	Pteronarcyidae	Pteronarcys sachalina
Capnura wanica 16S ribosomal RNA gene, partial seq	EF623128	83.44	100	7.16632e−37	Plecoptera	Capniidae	Capnura wanica
Allocapnia recta isolate H18 16S ribosomal RNA gen	KC881053	82.8	100	2.50129e−36	Plecoptera	Capniidae	Allocapnia recta
Allocapnia recta haplotype 12ar: H12 16S ribosomal	EU306875	82.8	100	2.50129e−36	Plecoptera	Capniidae	Allocapnia recta
Paraleuctra occidentalis 16S ribosomal RNA gene, p	EF623178	83.02	100	8.73037e−36	Plecoptera	Leuctridae	Paraleuctra occidentalis
Austrocerca rieka 16S ribosomal RNA gene, partial	EF623205	82.17	100	3.0472e−35	Plecoptera	Notonemouridae	Austrocerca rieka
Austrocercoides zwicki 16S ribosomal RNA gene, par	EF623199	82.17	100	3.0472e−35	Plecoptera	Notonemouridae	Austrocercoides zwicki
Pteronarcys princeps mitochondrion, complete genom	AY687866	82.17	100	3.71225e−34	Plecoptera	Pteronarcyidae	Pteronarcys princeps
Notonemouridae sp. MD−2011 voucher ZMUC:JD−1507 16	JN615257	81.53	100	1.2957e−33	Plecoptera	Notonemouridae	Notonemouridae sp. MD−2011
Brachyptera seticornis 16S ribosomal RNA gene, par	EF623268	81.76	100	4.52244e−33	Plecoptera	Taeniopterygidae	Brachyptera seticornis
Osmylus sp. EH−2004 16S ribosomal RNA gene, partia	AY620146	82.17	100	4.52244e−33	Neuroptera	Osmylidae	Osmylus sp. EH−2004
Pteronarcys scotti 16S ribosomal RNA gene, partial	EF623263	81.53	100	1.57849e−32	Plecoptera	Pteronarcyidae	Pteronarcys scotti
Pteronarcys californica 16S ribosomal RNA gene, pa	EF623261	81.53	100	1.57849e−32	Plecoptera	Pteronarcyidae	Pteronarcys californica
Udamocercia sp. BYU_PL150 16S ribosomal RNA gene,	EF623201	80.89	100	1.57849e−32	Plecoptera	Notonemouridae	Udamocercia sp. BYU_PL150
Amphinemura sulcicollis 16S ribosomal RNA gene, pa	EF623195	80.89	100	1.57849e−32	Plecoptera	Nemouridae	Amphinemura sulcicollis
Strophopteryx appalachia 16S ribosomal RNA gene, p	EF623271	81.01	100	5.50946e−32	Plecoptera	Taeniopterygidae	Strophopteryx appalachia
Bolotoperla rossi 16S ribosomal RNA gene, partial	EF623269	81.01	100	5.50946e−32	Plecoptera	Taeniopterygidae	Bolotoperla rossi
Thaumatoperla sp. BYU_PL109 16S ribosomal RNA gene	EF623149	81.65	100	5.50946e−32	Plecoptera	Eustheniidae	Thaumatoperla sp. BYU_PL109
Diamphipnoa virescentipennis 16S ribosomal RNA gen	EF623144	80.25	100	6.7119e−31	Plecoptera	Diamphipnoidae	Diamphipnoa virescentipennis
Cryptoperla japonica mitochondrial gene for 16S rR	AB711202	81.13	100	2.34268e−30	Plecoptera	Peltoperlidae	Cryptoperla japonica
Cryptoperla japonica mitochondrial gene for 16S rR	AB711184	81.13	100	2.34268e−30	Plecoptera	Peltoperlidae	Cryptoperla japonica
Cryptoperla japonica mitochondrial gene for 16S rR	AB711183	81.13	100	2.34268e−30	Plecoptera	Peltoperlidae	Cryptoperla japonica
Cryptoperla japonica mitochondrial gene for 16S rR	AB711182	81.13	100	2.34268e−30	Plecoptera	Peltoperlidae	Cryptoperla japonica
Taenionema palladium 16S ribosomal RNA gene, parti	EF623267	80.62	100	8.17677e−30	Plecoptera	Taeniopterygidae	Taenionema palladium
Neonemura sp. BYU_PL153 16S ribosomal RNA gene, pa	EF623204	80.25	100	8.17677e−30	Plecoptera	Notonemouridae	Neonemura sp. BYU_PL153
Paraleuctra vershina 16S ribosomal RNA gene, parti	EF623173	81.69	90.45	8.17677e−30	Plecoptera	Leuctridae	Paraleuctra vershina
Isocapnia hyalita 16S ribosomal RNA gene, partial	EF623131	79.62	100	8.17677e−30	Plecoptera	Capniidae	Isocapnia hyalita
Cascadoperla trictura 16S ribosomal RNA gene, part	EF623225	79.62	100	9.96134e−29	Plecoptera	Perlodidae	Cascadoperla trictura
Leptoperla sp. BYU_PL107 16S ribosomal RNA gene, p	EF623161	80	100	9.96134e−29	Plecoptera	Gripopterygidae	Leptoperla sp. BYU_PL107
Nesoperla fulvescens 16S ribosomal RNA gene, parti	EF623156	80	100	9.96134e−29	Plecoptera	Gripopterygidae	Nesoperla fulvescens
Triznaka pintada 16S ribosomal RNA gene, partial s	EF623134	79.75	100	3.47685e−28	Plecoptera	Chloroperlidae	Triznaka pintada
Neopanorpa sp. M24899 16S ribosomal RNA gene, part	AF180059	79.87	100	3.47685e−28	Mecoptera	Panorpidae	Neopanorpa sp. M24899
Cryptoperla sp. YK−2012−1 mitochondrial gene for 1	AB711243	79.87	100	1.21354e−27	Plecoptera	Peltoperlidae	Cryptoperla sp. YK−2012−1
Cryptoperla japonica mitochondrial gene for 16S rR	AB711189	79.87	100	1.21354e−27	Plecoptera	Peltoperlidae	Cryptoperla japonica
Eusthenia costalis 16S ribosomal RNA gene, partial	EF623150	80.5	100	1.21354e−27	Plecoptera	Eustheniidae	Eusthenia costalis
Frisonia picticeps 16S ribosomal RNA gene, partial	EF623227	78.34	100	4.23567e−27	Plecoptera	Perlodidae	Frisonia picticeps
Cosumnoperla hypocrena 16S ribosomal RNA gene, par	EF623226	87.85	68.15	4.23567e−27	Plecoptera	Perlodidae	Cosumnoperla hypocrena
Amphinemura nigritta 16S ribosomal RNA gene, parti	EF623186	78.34	100	4.23567e−27	Plecoptera	Nemouridae	Amphinemura nigritta
Diamphipnopsis sp. BYU_PL138 16S ribosomal RNA gen	EF623145	78.62	100	4.23567e−27	Plecoptera	Diamphipnoidae	Diamphipnopsis sp. BYU_PL138
Alloperla severa 16S ribosomal RNA gene, partial s	EF623141	79.5	100	4.23567e−27	Plecoptera	Chloroperlidae	Alloperla severa
Kathroperla perdita 16S ribosomal RNA gene, partia	EF623135	78.34	100	4.23567e−27	Plecoptera	Chloroperlidae	Kathroperla perdita
Aeschnophlebia anisoptera mitochondrial gene for 1	AB707619	79.25	100	1.47839e−26	Odonata	Aeshnidae	Aeschnophlebia anisoptera
Caliaeschna microstigma 12S ribosomal RNA gene, pa	EU477639	79.25	100	1.47839e−26	Odonata	Aeshnidae	Caliaeschna microstigma
Newmanoperla exigua 16S ribosomal RNA gene, partia	EF623162	78.48	100	1.47839e−26	Plecoptera	Gripopterygidae	Newmanoperla exigua
Sweltsa coloradensis 16S ribosomal RNA gene, parti	EF623133	80	100	1.47839e−26	Plecoptera	Chloroperlidae	Sweltsa coloradensis
Klapopteryx armillata 16S ribosomal RNA gene, part	EF623123	78.48	100	1.47839e−26	Plecoptera	Austroperlidae	Klapopteryx armillata
Aeschnophlebia anisoptera 16S ribosomal RNA gene,	EF032712	79.25	100	1.47839e−26	Odonata	Aeshnidae	Aeschnophlebia anisoptera
Caliaeschna microstigma large subunit ribosomal RN	AF266092	79.25	100	1.47839e−26	Odonata	Aeshnidae	Caliaeschna microstigma
Cerconychia sp. BYU_PL114 16S ribosomal RNA gene,	EF623260	78.92	100	5.1601e−26	Plecoptera	Styloperlidae	Cerconychia sp. BYU_PL114
Peltoperla arcuata 16S ribosomal RNA gene, partial	EF623258	78.62	100	5.1601e−26	Plecoptera	Peltoperlidae	Peltoperla arcuata
Neopanorpa pulchra mitochondrion, complete genome	JX569848	78.62	100	1.80105e−25	Mecoptera	Panorpidae	Neopanorpa pulchra
Pteronarcella badia 16S ribosomal RNA gene, partia	EF623262	77.91	100	1.80105e−25	Plecoptera	Pteronarcyidae	Pteronarcella badia
