# Supplementary material for: Testing the potential of a ribosomal 16S marker for DNA metabarcoding of insects
Source: PeerJ. 2016 Apr 19;4:e1966. doi: 10.7717/peerj.1966 (PMC4841222; doi:10.7717/peerj.1966)
Supplement: Supplemental Information 1 [file peerj-04-1966-s006.zip › scripts/5 match OTUs/blast_hits/OTU_52.csv_plot.pdf]

OTU_52.csv

Agabus binotatus

Dytiscidae

Coleoptera

99.37

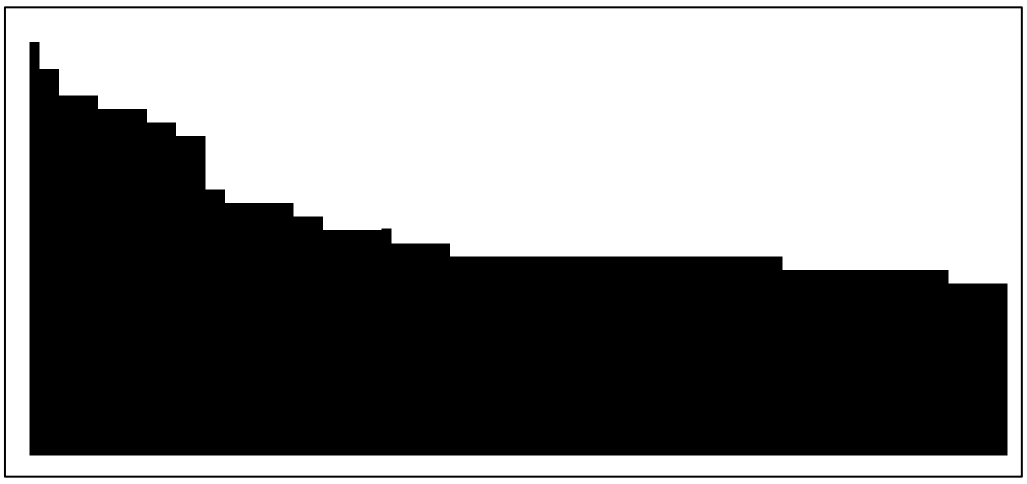

Agabus binotatus 16S ribosomal RNA gene, partial s	AY039260	99.37	100	1.31462e−71	Coleoptera	Dytiscidae	Agabus binotatus
Agabus picotae mitochondrial 16S rRNA gene (partia	HF931356	98.11	100	6.80989e−69	Coleoptera	Dytiscidae	Agabus picotae
Agabus heydeni 16S ribosomal RNA gene, partial seq	AY039263	98.11	100	6.80989e−69	Coleoptera	Dytiscidae	Agabus heydeni
Agabus guttatus mitochondrial 16S rRNA gene (parti	LM654777	96.86	100	3.52761e−66	Coleoptera	Dytiscidae	Agabus guttatus
Agabus caraboides mitochondrial partial 16S rRNA g	HF931451	96.86	100	3.52761e−66	Coleoptera	Dytiscidae	Agabus caraboides
Agabus guttatus 16S ribosomal RNA gene, partial se	AY039262	96.86	100	3.52761e−66	Coleoptera	Dytiscidae	Agabus guttatus
Agabus biguttatus haplotype 1 16S ribosomal RNA ge	AY039258	96.86	100	3.52761e−66	Coleoptera	Dytiscidae	Agabus biguttatus
Agabus faldermanni mitochondrial 16S rRNA gene (pa	LM654774	96.23	100	4.29751e−65	Coleoptera	Dytiscidae	Agabus faldermanni
Agabus basalis mitochondrial 16S rRNA gene (partia	HF931443	96.23	100	4.29751e−65	Coleoptera	Dytiscidae	Agabus basalis
Agabus cf. nitidus IR−2001 16S ribosomal RNA gene,	AY039264	96.23	100	4.29751e−65	Coleoptera	Dytiscidae	Agabus cf. nitidus IR−2001
Agabus biguttatus haplotype 2 16S ribosomal RNA ge	AY039259	96.23	100	4.29751e−65	Coleoptera	Dytiscidae	Agabus biguttatus
Agabus faldermanni 16S ribosomal RNA gene, partial	AY138614	96.23	100	4.29751e−65	Coleoptera	Dytiscidae	Agabus faldermanni
Agabus cephalotes mitochondrial 16S rRNA gene (par	HF931380	95.6	100	5.23544e−64	Coleoptera	Dytiscidae	Agabus cephalotes
Agabus alexandrae 16S ribosomal RNA gene, partial	AY039257	95.6	100	1.82735e−63	Coleoptera	Dytiscidae	Agabus alexandrae
Agabus alexandrae 16S ribosomal RNA gene, partial	AF428199	95.6	100	1.82735e−63	Coleoptera	Dytiscidae	Agabus alexandrae
Agabus maderensis 16S ribosomal RNA gene, partial	AY138621	94.97	100	1.82735e−63	Coleoptera	Dytiscidae	Agabus maderensis
Agabus glacialis mitochondrial 16S rRNA gene (part	LM654776	94.97	100	2.22616e−62	Coleoptera	Dytiscidae	Agabus glacialis
Agabus dilatatus 16S ribosomal RNA gene, partial s	AY039261	94.97	100	2.22616e−62	Coleoptera	Dytiscidae	Agabus dilatatus
Agabus paludosus 16S ribosomal RNA gene, partial s	AY138625	92.45	100	5.97363e−57	Coleoptera	Dytiscidae	Agabus paludosus
Agabus conspersus 16S ribosomal RNA gene, partial	AY138610	92.45	100	5.97363e−57	Coleoptera	Dytiscidae	Agabus conspersus
Agabus nebulosus mitochondrial 16S rRNA gene (part	LM654779	91.82	100	7.27737e−56	Coleoptera	Dytiscidae	Agabus nebulosus
Agabus uliginosus mitochondrial 16S rRNA gene (par	HF931463	91.82	100	7.27737e−56	Coleoptera	Dytiscidae	Agabus uliginosus
Agabus dichrous mitochondrial partial 16S rRNA gen	HF931378	91.82	100	7.27737e−56	Coleoptera	Dytiscidae	Agabus dichrous
Agabus godmanni mitochondrial 16S rRNA gene (parti	HF931348	91.82	100	7.27737e−56	Coleoptera	Dytiscidae	Agabus godmanni
Agabus nebulosus 16S ribosomal RNA gene, partial s	AY138624	91.82	100	7.27737e−56	Coleoptera	Dytiscidae	Agabus nebulosus
Agabus lineatus 16S ribosomal RNA gene, partial se	AY138611	91.82	100	7.27737e−56	Coleoptera	Dytiscidae	Agabus lineatus
Agabus bifarius 16S ribosomal RNA gene, partial se	AY138605	91.82	100	7.27737e−56	Coleoptera	Dytiscidae	Agabus bifarius
Agabus undulatus mitochondrial 16S rRNA gene (part	HF931411	91.19	100	3.09442e−54	Coleoptera	Dytiscidae	Agabus undulatus
Agabus serricornis 16S ribosomal RNA gene, partial	AY138629	91.19	100	3.09442e−54	Coleoptera	Dytiscidae	Agabus serricornis
Agabus antennatus 16S ribosomal RNA gene, partial	AY138601	91.19	100	3.09442e−54	Coleoptera	Dytiscidae	Agabus antennatus
Agabus pallidus mitochondrial 16S rRNA gene (parti	LM654780	90.57	100	3.76977e−53	Coleoptera	Dytiscidae	Agabus pallidus
Agabus labiatus mitochondrial 16S rRNA gene (parti	LM654778	90.57	100	3.76977e−53	Coleoptera	Dytiscidae	Agabus labiatus
Agabus bipustulatus 16S ribosomal RNA gene, partia	AF428198	90.57	100	3.76977e−53	Coleoptera	Dytiscidae	Agabus bipustulatus
Agabus melanarius 16S ribosomal RNA gene, partial	AY138623	90.57	100	3.76977e−53	Coleoptera	Dytiscidae	Agabus melanarius
Agabus affinis 16S ribosomal RNA gene, partial seq	AY138598	90.57	100	3.76977e−53	Coleoptera	Dytiscidae	Agabus affinis
Agabus tristis voucher UNM KBMAgtr60 16S ribosomal	KJ548305	90.57	100	1.31578e−52	Coleoptera	Dytiscidae	Agabus tristis
Agabus aubei 16S ribosomal RNA gene, partial seque	AY039265	90.62	100	1.31578e−52	Coleoptera	Dytiscidae	Agabus aubei
Agabus labiatus 16S ribosomal RNA gene, partial se	AY138618	89.94	100	4.59252e−52	Coleoptera	Dytiscidae	Agabus labiatus
Agabus striolatus mitochondrial 16S rRNA gene (par	LM654785	89.94	100	1.60295e−51	Coleoptera	Dytiscidae	Agabus striolatus
Agabus wollastoni 16S ribosomal RNA gene, partial	AY138636	89.94	100	1.60295e−51	Coleoptera	Dytiscidae	Agabus wollastoni
Agabus tristis 16S ribosomal RNA gene, partial seq	AY138634	89.94	100	1.60295e−51	Coleoptera	Dytiscidae	Agabus tristis
Agabus japonicus 16S ribosomal RNA gene, partial s	AY138617	89.94	100	1.60295e−51	Coleoptera	Dytiscidae	Agabus japonicus
Agabus morosus isolate Aga18.2R 16S ribosomal RNA	AY138592	89.94	100	1.60295e−51	Coleoptera	Dytiscidae	Agabus morosus
Agabus rufulus mitochondrial 16S rRNA gene (partia	LM654818	89.31	100	1.95279e−50	Coleoptera	Dytiscidae	Agabus rufulus
Agabus rufulus mitochondrial 16S rRNA gene (partia	LM654817	89.31	100	1.95279e−50	Coleoptera	Dytiscidae	Agabus rufulus
Agabus ramblae mitochondrial 16S rRNA gene (partia	LM654816	89.31	100	1.95279e−50	Coleoptera	Dytiscidae	Agabus ramblae
Agabus brunneus mitochondrial 16S rRNA gene (parti	LM654791	89.31	100	1.95279e−50	Coleoptera	Dytiscidae	Agabus brunneus
Agabus brunneus mitochondrial 16S rRNA gene (parti	LM654790	89.31	100	1.95279e−50	Coleoptera	Dytiscidae	Agabus brunneus
Agabus brunneus mitochondrial 16S rRNA gene (parti	LM654789	89.31	100	1.95279e−50	Coleoptera	Dytiscidae	Agabus brunneus
Agabus brunneus mitochondrial 16S rRNA gene (parti	LM654788	89.31	100	1.95279e−50	Coleoptera	Dytiscidae	Agabus brunneus
Agabus brunneus mitochondrial 16S rRNA gene (parti	LM654787	89.31	100	1.95279e−50	Coleoptera	Dytiscidae	Agabus brunneus
Agabus unguicularis mitochondrial 16S rRNA gene (p	LM654786	89.31	100	1.95279e−50	Coleoptera	Dytiscidae	Agabus unguicularis
Agabus semipunctatus mitochondrial 16S rRNA gene (	LM654784	89.31	100	1.95279e−50	Coleoptera	Dytiscidae	Agabus semipunctatus
Agabus regimbarti mitochondrial 16S rRNA gene (par	LM654782	89.31	100	1.95279e−50	Coleoptera	Dytiscidae	Agabus regimbarti
Agabus ragazzii mitochondrial 16S rRNA gene (parti	LM654781	89.31	100	1.95279e−50	Coleoptera	Dytiscidae	Agabus ragazzii
Agabus fuscipennis mitochondrial 16S rRNA gene (pa	LM654775	89.31	100	1.95279e−50	Coleoptera	Dytiscidae	Agabus fuscipennis
Agabus didymus mitochondrial 16S rRNA gene (partia	LM654773	89.31	100	1.95279e−50	Coleoptera	Dytiscidae	Agabus didymus
Agabus rufulus mitochondrial partial 16S rRNA gene	LM654771	89.31	100	1.95279e−50	Coleoptera	Dytiscidae	Agabus rufulus
Agabus ramblae mitochondrial partial 16S rRNA gene	LM654770	89.31	100	1.95279e−50	Coleoptera	Dytiscidae	Agabus ramblae
Agabus brunneus mitochondrial partial 16S rRNA gen	LM654769	89.31	100	1.95279e−50	Coleoptera	Dytiscidae	Agabus brunneus
Agabus brunneus mitochondrial partial 16S rRNA gen	LM654768	89.31	100	1.95279e−50	Coleoptera	Dytiscidae	Agabus brunneus
Agabus brunneus mitochondrial partial 16S rRNA gen	LM654767	89.31	100	1.95279e−50	Coleoptera	Dytiscidae	Agabus brunneus
Agabus didymus voucher UNM KBMPmsp730 16S ribosoma	KJ548392	89.31	100	1.95279e−50	Coleoptera	Dytiscidae	Agabus didymus
Ilybius vandykei 16S ribosomal RNA gene, partial s	AY138671	89.31	100	1.95279e−50	Coleoptera	Dytiscidae	Ilybius vandykei
Agabus sturmii 16S ribosomal RNA gene, partial seq	AY138633	89.31	100	1.95279e−50	Coleoptera	Dytiscidae	Agabus sturmii
Agabus strigulosus 16S ribosomal RNA gene, partial	AY138632	89.31	100	1.95279e−50	Coleoptera	Dytiscidae	Agabus strigulosus
Agabus semipunctatus 16S ribosomal RNA gene, parti	AY138628	89.31	100	1.95279e−50	Coleoptera	Dytiscidae	Agabus semipunctatus
Agabus ramblae 16S ribosomal RNA gene, partial seq	AY138626	89.31	100	1.95279e−50	Coleoptera	Dytiscidae	Agabus ramblae
Agabus erythropterus 16S ribosomal RNA gene, parti	AY138613	89.31	100	1.95279e−50	Coleoptera	Dytiscidae	Agabus erythropterus
Agabus elongatus 16S ribosomal RNA gene, partial s	AY138612	89.31	100	1.95279e−50	Coleoptera	Dytiscidae	Agabus elongatus
Agabus brunneus 16S ribosomal RNA gene, partial se	AY138607	89.31	100	1.95279e−50	Coleoptera	Dytiscidae	Agabus brunneus
Agabus austinii 16S ribosomal RNA gene, partial se	AY138604	89.31	100	1.95279e−50	Coleoptera	Dytiscidae	Agabus austinii
Agabus arcticus 16S ribosomal RNA gene, partial se	AY138603	89.31	100	1.95279e−50	Coleoptera	Dytiscidae	Agabus arcticus
Agabus sp. IR−sp2 16S ribosomal RNA gene, partial	AY138597	89.31	100	1.95279e−50	Coleoptera	Dytiscidae	Agabus sp. IR−sp2
Agabus thomsoni 16S ribosomal RNA gene, partial se	AY138595	89.31	100	1.95279e−50	Coleoptera	Dytiscidae	Agabus thomsoni
Agabus kootenai 16S ribosomal RNA gene, partial se	AY138594	89.31	100	1.95279e−50	Coleoptera	Dytiscidae	Agabus kootenai
Agabus morosus isolate Aga16.3 16S ribosomal RNA g	AY138591	89.31	100	1.95279e−50	Coleoptera	Dytiscidae	Agabus morosus
Agabus disintegratus voucher UNM KBMAgdi412 16S ri	KJ548302	88.68	100	2.37899e−49	Coleoptera	Dytiscidae	Agabus disintegratus
Acilius athabascae voucher 305G09/BMNH799009 16S r	KF979079	88.68	100	2.37899e−49	Coleoptera	Dytiscidae	Acilius athabascae
Agabus disintegratus 16S ribosomal RNA gene, parti	AY071769	88.68	100	2.37899e−49	Coleoptera	Dytiscidae	Agabus disintegratus
Platambus stygius 16S ribosomal RNA gene, partial	AY138679	88.68	100	2.37899e−49	Coleoptera	Dytiscidae	Platambus stygius
Agabus amoenus mitochondrial 16S rRNA gene (partia	LM654772	88.68	100	8.30347e−49	Coleoptera	Dytiscidae	Agabus amoenus
Platambus semivittatus 16S ribosomal RNA gene, par	AY138678	88.68	100	8.30347e−49	Coleoptera	Dytiscidae	Platambus semivittatus
Agabus unguicularis 16S ribosomal RNA gene, partia	AY138635	88.68	100	8.30347e−49	Coleoptera	Dytiscidae	Agabus unguicularis
Agabus matsumotoi 16S ribosomal RNA gene, partial	AY138622	88.68	100	8.30347e−49	Coleoptera	Dytiscidae	Agabus matsumotoi
Agabus lutosus 16S ribosomal RNA gene, partial seq	AY138620	88.68	100	8.30347e−49	Coleoptera	Dytiscidae	Agabus lutosus
Agabus hummeli 16S ribosomal RNA gene, partial seq	AY138616	88.68	100	8.30347e−49	Coleoptera	Dytiscidae	Agabus hummeli
Agabus congener 16S ribosomal RNA gene, partial se	AY138609	88.68	100	8.30347e−49	Coleoptera	Dytiscidae	Agabus congener
Agabus confinis 16S ribosomal RNA gene, partial se	AY138608	88.68	100	8.30347e−49	Coleoptera	Dytiscidae	Agabus confinis
Agabus anthracinus 16S ribosomal RNA gene, partial	AY138602	88.68	100	8.30347e−49	Coleoptera	Dytiscidae	Agabus anthracinus
Agabus amoenus 16S ribosomal RNA gene, partial seq	AY138600	88.68	100	8.30347e−49	Coleoptera	Dytiscidae	Agabus amoenus
Agabus ambiguus isolate Agaambi 16S ribosomal RNA	AY138599	88.68	100	8.30347e−49	Coleoptera	Dytiscidae	Agabus ambiguus
Agabus ambiguus isolate Aga32.1 16S ribosomal RNA	AY138596	88.68	100	8.30347e−49	Coleoptera	Dytiscidae	Agabus ambiguus
Agabus cf. lutosus/griseipennis IR−2002 16S riboso	AY138593	88.68	100	8.30347e−49	Coleoptera	Dytiscidae	Agabus cf. lutosus/griseipennis IR−2002
Ilybius euryonus 16S ribosomal RNA gene, partial s	AY138662	88.05	100	2.8982e−48	Coleoptera	Dytiscidae	Ilybius euryonus
Ilybius lineellus mitochondrial 16S rRNA gene (par	LM654802	88.05	100	1.01157e−47	Coleoptera	Dytiscidae	Ilybius lineellus
Agabus ruwenzoricus mitochondrial 16S rRNA gene (p	LM654783	88.05	100	1.01157e−47	Coleoptera	Dytiscidae	Agabus ruwenzoricus
Lancetes varius voucher UNM KBMLcva196 16S ribosom	KJ548351	88.05	100	1.01157e−47	Coleoptera	Dytiscidae	Lancetes varius
Ilybius walsinghami isolate Ilydisr 16S ribosomal	AY138660	88.05	100	1.01157e−47	Coleoptera	Dytiscidae	Ilybius walsinghami
Platambus sp. IR−sp1 16S ribosomal RNA gene, parti	AY138631	88.05	100	1.01157e−47	Coleoptera	Dytiscidae	Platambus sp. IR−sp1
