# Supplementary material for: Testing the potential of a ribosomal 16S marker for DNA metabarcoding of insects
Source: PeerJ. 2016 Apr 19;4:e1966. doi: 10.7717/peerj.1966 (PMC4841222; doi:10.7717/peerj.1966)
Supplement: Supplemental Information 1 [file peerj-04-1966-s006.zip › scripts/5 match OTUs/blast_hits/OTU_524.csv_plot.pdf]

OTU_524.csv

Leuctra inermis

Leuctridae

Plecoptera

87.42

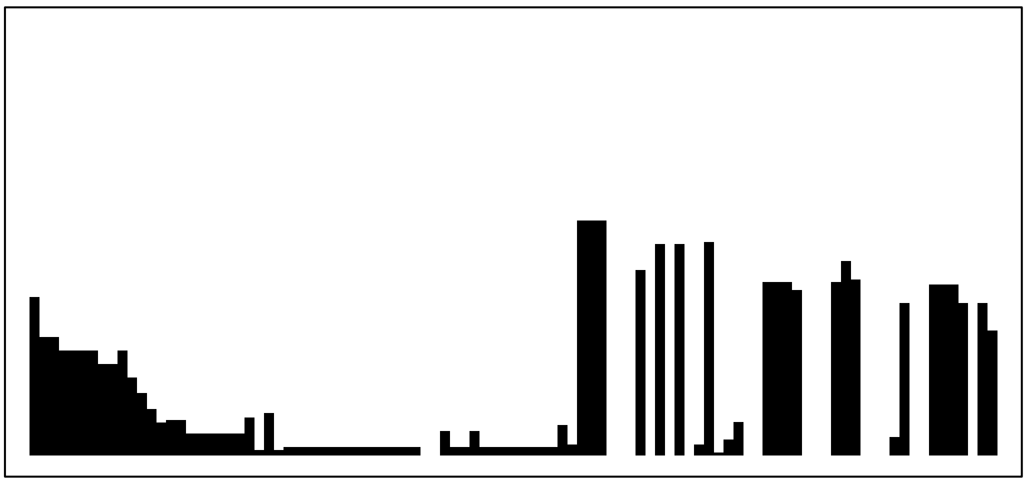

Leuctra inermis 16S ribosomal RNA gene, partial se	EF623176	87.42	100	5.11329e−45	Plecoptera	Leuctridae	Leuctra inermis
Leuctra tenuis haplotype 8lt: HO8 16S ribosomal RN	EU327680	85.53	100	3.93109e−40	Plecoptera	Leuctridae	Leuctra tenuis
Leuctra tenuis haplotype 7lt: HO7 16S ribosomal RN	EU327679	85.53	100	3.93109e−40	Plecoptera	Leuctridae	Leuctra tenuis
Leuctra tenuis haplotype 10lt: HO10 16S ribosomal	EU327682	84.91	100	1.67154e−38	Plecoptera	Leuctridae	Leuctra tenuis
Leuctra tenuis haplotype 9lt: HO9 16S ribosomal RN	EU327681	84.91	100	1.67154e−38	Plecoptera	Leuctridae	Leuctra tenuis
Leuctra tenuis haplotype 3lt: H03 16S ribosomal RN	DQ915184	84.91	100	1.67154e−38	Plecoptera	Leuctridae	Leuctra tenuis
Leuctra tenuis haplotype 2lt: H02 16S ribosomal RN	DQ915183	84.91	100	1.67154e−38	Plecoptera	Leuctridae	Leuctra tenuis
Leuctra tenuis haplotype 6lt: HO6 16S ribosomal RN	EU327678	84.28	100	2.03636e−37	Plecoptera	Leuctridae	Leuctra tenuis
Leuctra tenuis haplotype 1lt: H01 16S ribosomal RN	DQ915182	84.28	100	2.03636e−37	Plecoptera	Leuctridae	Leuctra tenuis
Moselia infuscata 16S ribosomal RNA gene, partial	EF623175	84.91	99.36	7.10758e−37	Plecoptera	Leuctridae	Moselia infuscata
Leuctra tenuis haplotype 5lt: HO5 16S ribosomal RN	EU327677	83.65	100	8.65881e−36	Plecoptera	Leuctridae	Leuctra tenuis
Leuctra tenuis haplotype 4lt: HO4 16S ribosomal RN	EU327676	82.91	100	1.05486e−34	Plecoptera	Leuctridae	Leuctra tenuis
Despaxia augusta 16S ribosomal RNA gene, partial s	EF623177	82.17	100	3.68182e−34	Plecoptera	Leuctridae	Despaxia augusta
Cascadoperla trictura 16S ribosomal RNA gene, part	EF623225	81.53	100	1.90723e−31	Plecoptera	Perlodidae	Cascadoperla trictura
Apteroperla tikumana mitochondrion, complete genom	KR604721	81.65	100	6.65688e−31	Plecoptera	Capniidae	Apteroperla tikumana
Allocapnia minima 16S ribosomal RNA gene, partial	EF623130	81.65	100	6.65688e−31	Plecoptera	Capniidae	Allocapnia minima
Allocapnia recta isolate H15 16S ribosomal RNA gen	KC881050	81.01	100	8.10974e−30	Plecoptera	Capniidae	Allocapnia recta
Allocapnia recta isolate H14 16S ribosomal RNA gen	KC881049	81.01	100	8.10974e−30	Plecoptera	Capniidae	Allocapnia recta
Allocapnia recta isolate H10 16S ribosomal RNA gen	KC881045	81.01	100	8.10974e−30	Plecoptera	Capniidae	Allocapnia recta
Allocapnia recta isolate H6 16S ribosomal RNA gene	KC881041	81.01	100	8.10974e−30	Plecoptera	Capniidae	Allocapnia recta
Allocapnia recta haplotype 11ar: H011 16S ribosoma	EU306874	81.01	100	8.10974e−30	Plecoptera	Capniidae	Allocapnia recta
Allocapnia recta haplotype 7ar: H07 16S ribosomal	EU306870	81.01	100	8.10974e−30	Plecoptera	Capniidae	Allocapnia recta
Protonemura meyeri 16S ribosomal RNA gene, partial	EF623193	81.76	100	2.83058e−29	Plecoptera	Nemouridae	Protonemura meyeri
Soyedina producta 16S ribosomal RNA gene, partial	EF623196	80.25	100	9.87969e−29	Plecoptera	Nemouridae	Soyedina producta
Triznaka pintada 16S ribosomal RNA gene, partial s	EF623134	81.99	100	9.87969e−29	Plecoptera	Chloroperlidae	Triznaka pintada
Capnura wanica 16S ribosomal RNA gene, partial seq	EF623128	80.25	100	9.87969e−29	Plecoptera	Capniidae	Capnura wanica
Mesocapnia arizonensis mitochondrion, partial geno	KP642637	80.38	100	3.44835e−28	Plecoptera	Capniidae	Mesocapnia arizonensis
Allocapnia recta isolate H19 16S ribosomal RNA gen	KC881054	80.38	100	3.44835e−28	Plecoptera	Capniidae	Allocapnia recta
Allocapnia recta isolate H17 16S ribosomal RNA gen	KC881052	80.38	100	3.44835e−28	Plecoptera	Capniidae	Allocapnia recta
Allocapnia recta isolate H16 16S ribosomal RNA gen	KC881051	80.38	100	3.44835e−28	Plecoptera	Capniidae	Allocapnia recta
Allocapnia recta isolate H13 16S ribosomal RNA gen	KC881048	80.38	100	3.44835e−28	Plecoptera	Capniidae	Allocapnia recta
Allocapnia recta isolate H12 16S ribosomal RNA gen	KC881047	80.38	100	3.44835e−28	Plecoptera	Capniidae	Allocapnia recta
Allocapnia recta isolate H11 16S ribosomal RNA gen	KC881046	80.38	100	3.44835e−28	Plecoptera	Capniidae	Allocapnia recta
Allocapnia recta isolate H9 16S ribosomal RNA gene	KC881044	80.38	100	3.44835e−28	Plecoptera	Capniidae	Allocapnia recta
Allocapnia recta isolate H8 16S ribosomal RNA gene	KC881043	80.38	100	3.44835e−28	Plecoptera	Capniidae	Allocapnia recta
Allocapnia recta isolate H7 16S ribosomal RNA gene	KC881042	80.38	100	3.44835e−28	Plecoptera	Capniidae	Allocapnia recta
Allocapnia recta isolate H5 16S ribosomal RNA gene	KC881040	80.38	100	3.44835e−28	Plecoptera	Capniidae	Allocapnia recta
Allocapnia recta isolate H4 16S ribosomal RNA gene	KC881039	80.38	100	3.44835e−28	Plecoptera	Capniidae	Allocapnia recta
Allocapnia recta isolate H3 16S ribosomal RNA gene	KC881038	80.38	100	3.44835e−28	Plecoptera	Capniidae	Allocapnia recta
Allocapnia recta isolate H1 16S ribosomal RNA gene	KC881036	80.38	100	3.44835e−28	Plecoptera	Capniidae	Allocapnia recta
Doddsia occidentalis 16S ribosomal RNA gene, parti	EF623270	79.75	100	3.44835e−28	Plecoptera	Taeniopterygidae	Doddsia occidentalis
Oemopteryx vanduzeea 16S ribosomal RNA gene, parti	EF623266	79.75	100	3.44835e−28	Plecoptera	Taeniopterygidae	Oemopteryx vanduzeea
Perlinodes aurea 16S ribosomal RNA gene, partial s	EF623234	81.13	100	3.44835e−28	Plecoptera	Perlodidae	Perlinodes aurea
Nemurella pictetii 16S ribosomal RNA gene, partial	EF623192	80.38	100	3.44835e−28	Plecoptera	Nemouridae	Nemurella pictetii
Ostrocerca foersteri 16S ribosomal RNA gene, parti	EF623191	80.38	100	3.44835e−28	Plecoptera	Nemouridae	Ostrocerca foersteri
Shipsa rotunda 16S ribosomal RNA gene, partial seq	EF623184	81.13	100	3.44835e−28	Plecoptera	Nemouridae	Shipsa rotunda
Isocapnia hyalita 16S ribosomal RNA gene, partial	EF623131	80.38	100	3.44835e−28	Plecoptera	Capniidae	Isocapnia hyalita
Mesocapnia sp. BYU_PL010 16S ribosomal RNA gene, p	EF623127	80.38	100	3.44835e−28	Plecoptera	Capniidae	Mesocapnia sp. BYU_PL010
Allocapnia recta haplotype 10ar: H010 16S ribosoma	EU306873	80.38	100	3.44835e−28	Plecoptera	Capniidae	Allocapnia recta
Allocapnia recta haplotype 9ar: H09 16S ribosomal	EU306872	80.38	100	3.44835e−28	Plecoptera	Capniidae	Allocapnia recta
Allocapnia recta haplotype 8ar: H08 16S ribosomal	EU306871	80.38	100	3.44835e−28	Plecoptera	Capniidae	Allocapnia recta
Allocapnia recta haplotype 3ar: H03 16S ribosomal	DQ915181	80.38	100	3.44835e−28	Plecoptera	Capniidae	Allocapnia recta
Allocapnia recta haplotype 2ar: H02 16S ribosomal	DQ915180	80.38	100	3.44835e−28	Plecoptera	Capniidae	Allocapnia recta
Allocapnia recta haplotype 1ar: H01 16S ribosomal	DQ915179	80.38	100	3.44835e−28	Plecoptera	Capniidae	Allocapnia recta
Pteronarcys sachalina 16S ribosomal RNA gene, part	EF623264	81.43	89.1	1.20359e−27	Plecoptera	Pteronarcyidae	Pteronarcys sachalina
Nemoura cinerea 16S ribosomal RNA gene, partial se	EF623194	80.5	100	1.20359e−27	Plecoptera	Nemouridae	Nemoura cinerea
Suwallia teleckojensis 16S ribosomal RNA gene, par	EF623142	91	62.82	1.20359e−27	Plecoptera	Chloroperlidae	Suwallia teleckojensis
Neaviperla forcipata 16S ribosomal RNA gene, parti	EF623139	91	62.82	1.20359e−27	Plecoptera	Chloroperlidae	Neaviperla forcipata
Plumiperla diversa 16S ribosomal RNA gene, partial	EF623132	91	62.82	1.20359e−27	Plecoptera	Chloroperlidae	Plumiperla diversa
Allocapnia recta isolate H18 16S ribosomal RNA gen	KC881053	79.75	100	4.20095e−27	Plecoptera	Capniidae	Allocapnia recta
Nanonemoura wahkeena 16S ribosomal RNA gene, parti	EF623198	79.75	100	4.20095e−27	Plecoptera	Nemouridae	Nanonemoura wahkeena
Nemoura trispinosa 16S ribosomal RNA gene, partial	EF623187	79.75	100	4.20095e−27	Plecoptera	Nemouridae	Nemoura trispinosa
Zapada cinctipes 16S ribosomal RNA gene, partial s	EF623183	88.68	66.67	4.20095e−27	Plecoptera	Nemouridae	Zapada cinctipes
Paraleuctra occidentalis 16S ribosomal RNA gene, p	EF623178	79.75	100	4.20095e−27	Plecoptera	Leuctridae	Paraleuctra occidentalis
Siphonoperla torrentium 16S ribosomal RNA gene, pa	EF623138	89.9	62.82	4.20095e−27	Plecoptera	Chloroperlidae	Siphonoperla torrentium
Kathroperla perdita 16S ribosomal RNA gene, partia	EF623135	79.75	100	4.20095e−27	Plecoptera	Chloroperlidae	Kathroperla perdita
Sweltsa coloradensis 16S ribosomal RNA gene, parti	EF623133	89.9	62.82	4.20095e−27	Plecoptera	Chloroperlidae	Sweltsa coloradensis
Allocapnia recta haplotype 12ar: H12 16S ribosomal	EU306875	79.75	100	4.20095e−27	Plecoptera	Capniidae	Allocapnia recta
Visoka cataractae 16S ribosomal RNA gene, partial	EF623189	80.5	100	1.46628e−26	Plecoptera	Nemouridae	Visoka cataractae
Chloroperla tripunctata 16S ribosomal RNA gene, pa	EF623137	90	62.82	1.46628e−26	Plecoptera	Chloroperlidae	Chloroperla tripunctata
Scopura montana 16S ribosomal RNA gene, partial se	EF623265	80.12	100	5.11781e−26	Plecoptera		Scopura montana
Paranemoura claasseni 16S ribosomal RNA gene, part	EF623185	80.75	100	5.11781e−26	Plecoptera	Nemouridae	Paranemoura claasseni
Pteronarcys princeps mitochondrion, complete genom	AY687866	81.56	89.1	5.11781e−26	Plecoptera	Pteronarcyidae	Pteronarcys princeps
Bolotoperla rossi 16S ribosomal RNA gene, partial	EF623269	78.48	100	1.78629e−25	Plecoptera	Taeniopterygidae	Bolotoperla rossi
Osobenus yakimae 16S ribosomal RNA gene, partial s	EF623232	79.38	100	1.78629e−25	Plecoptera	Perlodidae	Osobenus yakimae
Cosumnoperla hypocrena 16S ribosomal RNA gene, par	EF623226	88.12	64.1	1.78629e−25	Plecoptera	Perlodidae	Cosumnoperla hypocrena
Isoperla oxylepis 16S ribosomal RNA gene, partial	EF623221	88.12	64.1	1.78629e−25	Plecoptera	Perlodidae	Isoperla oxylepis
Isoperla davisi 16S ribosomal RNA gene, partial se	EF623206	88.12	64.1	1.78629e−25	Plecoptera	Perlodidae	Isoperla davisi
Udamocercia sp. BYU_PL150 16S ribosomal RNA gene,	EF623201	87.74	66.67	1.78629e−25	Plecoptera	Notonemouridae	Udamocercia sp. BYU_PL150
Malenka californica 16S ribosomal RNA gene, partia	EF623182	79.87	100	1.78629e−25	Plecoptera	Nemouridae	Malenka californica
Thaumatoperla sp. BYU_PL109 16S ribosomal RNA gene	EF623149	79.11	100	1.78629e−25	Plecoptera	Eustheniidae	Thaumatoperla sp. BYU_PL109
Paraperla frontalis 16S ribosomal RNA gene, partia	EF623136	79.87	100	1.78629e−25	Plecoptera	Chloroperlidae	Paraperla frontalis
Osmylus sp. EH−2004 16S ribosomal RNA gene, partia	AY620146	88.12	64.1	1.78629e−25	Neuroptera	Osmylidae	Osmylus sp. EH−2004
Pteronarcella badia 16S ribosomal RNA gene, partia	EF623262	89.11	62.82	6.23476e−25	Plecoptera	Pteronarcyidae	Pteronarcella badia
Frisonia picticeps 16S ribosomal RNA gene, partial	EF623227	88.24	64.1	6.23476e−25	Plecoptera	Perlodidae	Frisonia picticeps
Cultus pilatus 16S ribosomal RNA gene, partial seq	EF623212	79.01	100	6.23476e−25	Plecoptera	Perlodidae	Cultus pilatus
Megaleuctra kincaidi 16S ribosomal RNA gene, parti	EF623174	79.38	100	6.23476e−25	Plecoptera	Leuctridae	Megaleuctra kincaidi
Notonemouridae sp. MD−2011 voucher ZMUC:JD−1507 16	JN615257	78.48	100	2.17615e−24	Plecoptera	Notonemouridae	Notonemouridae sp. MD−2011
Pteronarcys californica 16S ribosomal RNA gene, pa	EF623261	80.85	89.1	2.17615e−24	Plecoptera	Pteronarcyidae	Pteronarcys californica
Cerconychia sp. BYU_PL114 16S ribosomal RNA gene,	EF623260	87.13	64.1	7.5955e−24	Plecoptera	Styloperlidae	Cerconychia sp. BYU_PL114
Austrocerca rieka 16S ribosomal RNA gene, partial	EF623205	78.75	100	7.5955e−24	Plecoptera	Notonemouridae	Austrocerca rieka
Austrocercoides zwicki 16S ribosomal RNA gene, par	EF623199	78.75	100	7.5955e−24	Plecoptera	Notonemouridae	Austrocercoides zwicki
Prostoia besametsa 16S ribosomal RNA gene, partial	EF623188	88	62.82	7.5955e−24	Plecoptera	Nemouridae	Prostoia besametsa
Sasquaperla hoopa 16S ribosomal RNA gene, partial	EF623143	88	62.82	7.5955e−24	Plecoptera	Chloroperlidae	Sasquaperla hoopa
Tasmanoperla larvalis 16S ribosomal RNA gene, part	EF623125	88	62.82	7.5955e−24	Plecoptera	Austroperlidae	Tasmanoperla larvalis
Penturoperla barbata 16S ribosomal RNA gene, parti	EF623124	87.13	64.1	7.5955e−24	Plecoptera	Austroperlidae	Penturoperla barbata
Klapopteryx armillata 16S ribosomal RNA gene, part	EF623123	78.62	100	7.5955e−24	Plecoptera	Austroperlidae	Klapopteryx armillata
Neopanorpa sp. M24899 16S ribosomal RNA gene, part	AF180059	87.13	64.1	7.5955e−24	Mecoptera	Panorpidae	Neopanorpa sp. M24899
Cryptoperla sp. YK−2012−1 mitochondrial gene for 1	AB711243	85.85	67.31	2.65109e−23	Plecoptera	Peltoperlidae	Cryptoperla sp. YK−2012−1
Yugus bulbosus 16S ribosomal RNA gene, partial seq	EF623214	79.01	100	2.65109e−23	Plecoptera	Perlodidae	Yugus bulbosus
