# Supplementary material for: Testing the potential of a ribosomal 16S marker for DNA metabarcoding of insects
Source: PeerJ. 2016 Apr 19;4:e1966. doi: 10.7717/peerj.1966 (PMC4841222; doi:10.7717/peerj.1966)
Supplement: Supplemental Information 1 [file peerj-04-1966-s006.zip › scripts/5 match OTUs/blast_hits/OTU_528.csv_plot.pdf]

OTU_528.csv

Parochlus steinenii

Chironomidae

Diptera

86.54

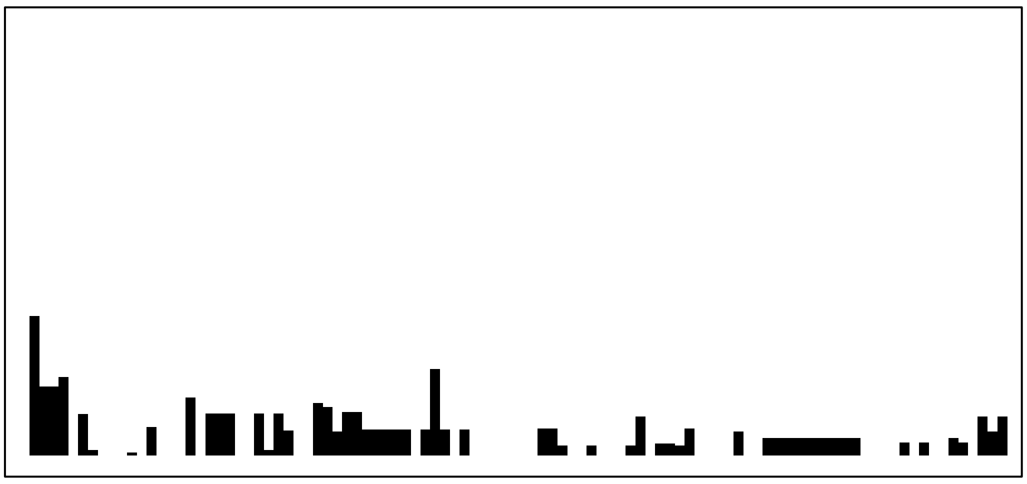

Parochlus steinenii isolate Sejong01 mitochondrion	KT003702	86.54	100	6.22926e−44	Diptera	Chironomidae	Parochlus steinenii
Bolitophila cinerea voucher JSB1 16S ribosomal RNA	KJ136712	83.23	97.44	1.28508e−33	Diptera	Mycetophilidae	Bolitophila cinerea
Bolitophila sp. ER−2009 16S ribosomal RNA gene, pa	FJ172021	83.23	97.44	1.28508e−33	Diptera	Mycetophilidae	Bolitophila sp. ER−2009
Bolitophila occlusa voucher JSB2 16S ribosomal RNA	KJ136713	83.67	93.59	4.48537e−33	Diptera	Mycetophilidae	Bolitophila occlusa
Edwardsina gigantea 16S ribosomal RNA gene, partia	KC177460	80	99.36	8.10974e−30	Diptera	Blephariceridae	Edwardsina gigantea
Bolitophila hybrida 16S ribosomal RNA gene, partia	FJ172002	81.94	97.44	8.10974e−30	Diptera	Mycetophilidae	Bolitophila hybrida
Pedicia albivitta voucher O'Grady 200012 16S ribos	EU005435	80.25	100	8.10974e−30	Diptera	Pediciidae	Pedicia albivitta
Tipula abdominalis 16S ribosomal RNA gene, partial	KC177457	79.49	100	2.83058e−29	Diptera	Tipulidae	Tipula abdominalis
Tipula abdominalis mitochondrion, partial genome	JN861743	79.49	100	2.83058e−29	Diptera	Tipulidae	Tipula abdominalis
Tipula sp. PMO 200011 16S ribosomal RNA gene, part	EU005437	79.62	100	9.87969e−29	Diptera	Tipulidae	Tipula sp. PMO 200011
Agathomyia zetterstedti isolate 814 16S ribosomal	KC699312	80.13	99.36	3.44835e−28	Diptera	Platypezidae	Agathomyia zetterstedti
Clinocera sp. Pollet 0249 16S ribosomal RNA gene,	HQ449032	79.75	99.36	3.44835e−28	Diptera	Empididae	Clinocera sp. Pollet 0249
Arachnocampa luminosa biotype 1 16S large subunit	AY576329	81.33	94.23	3.44835e−28	Diptera	Keroplatidae	Arachnocampa luminosa
Agathomyia elegantula isolate AE04 16S ribosomal R	KC699320	78.71	99.36	4.20095e−27	Diptera	Platypezidae	Agathomyia elegantula
Agathomyia lundbecki isolate 233 16S ribosomal RNA	KC699306	79.49	99.36	4.20095e−27	Diptera	Platypezidae	Agathomyia lundbecki
Geomyza tripunctata 16S ribosomal RNA gene, partia	EU268537	78.71	99.36	4.20095e−27	Diptera	Opomyzidae	Geomyza tripunctata
Simulium izuae isolate I1B 16S ribosomal RNA gene,	KM410189	82.71	84.62	1.46628e−26	Diptera	Simuliidae	Simulium izuae
Agathomyia sexmaculata isolate 816 16S ribosomal R	KC699313	78.85	99.36	1.46628e−26	Diptera	Platypezidae	Agathomyia sexmaculata
Simulium roslihashimi isolate R1B 16S ribosomal RN	KM410192	81.95	84.62	1.78629e−25	Diptera	Simuliidae	Simulium roslihashimi
Simulium izuae isolate I3B 16S ribosomal RNA gene,	KM410191	81.95	84.62	1.78629e−25	Diptera	Simuliidae	Simulium izuae
Simulium izuae isolate I2B 16S ribosomal RNA gene,	KM410190	81.95	84.62	1.78629e−25	Diptera	Simuliidae	Simulium izuae
Sylvicola fenestralis 16S ribosomal RNA gene, part	KC177461	78.21	100	1.78629e−25	Diptera	Anisopodidae	Sylvicola fenestralis
Sylvicola fenestralis mitochondrion, complete geno	JN861752	78.21	100	1.78629e−25	Diptera	Anisopodidae	Sylvicola fenestralis
Simulium tuenense mitochondrial gene for 16S ribos	AB591244	81.95	84.62	1.78629e−25	Diptera	Simuliidae	Simulium tuenense
Beris morrisii 16S ribosomal RNA gene, partial seq	GU947373	80.25	99.36	1.78629e−25	Diptera	Stratiomyidae	Beris morrisii
Simulium inthanonense 16S ribosomal RNA gene, part	GQ865995	81.95	84.62	1.78629e−25	Diptera	Simuliidae	Simulium inthanonense
Arachnocampa luminosa biotype 3 16S large subunit	AY576331	81.16	87.82	1.78629e−25	Diptera	Keroplatidae	Arachnocampa luminosa
Trichopeza longicornis voucher Pollet 0243 (P) 16S	HQ449035	79.75	99.36	6.23476e−25	Diptera	Empididae	Trichopeza longicornis
Agathomyia viduella isolate AV01 16S ribosomal RNA	KC699317	78.21	99.36	2.17615e−24	Diptera	Platypezidae	Agathomyia viduella
Diadocidia stanfordensis voucher JSSJ14 16S riboso	KJ136742	82.44	82.69	2.17615e−24	Diptera	Diadocidiidae	Diadocidia stanfordensis
Hercostomus meieri voucher RMBR:103442 16S ribosom	FJ808173	82.26	79.49	2.17615e−24	Diptera	Dolichopodidae	Hercostomus meieri
Somatia sp. YSUW04038 16S ribosomal RNA gene, part	AY573134	81.1	81.41	2.17615e−24	Diptera	Somatiidae	Somatia sp. YSUW04038
Simulium tenederoi mitochondrial gene for 16S ribo	LC035002	82.03	79.49	7.5955e−24	Diptera	Simuliidae	Simulium tenederoi
Simulium tenederoi mitochondrial gene for 16S ribo	LC035001	82.03	79.49	7.5955e−24	Diptera	Simuliidae	Simulium tenederoi
Simulium sheilae isolate SH1B 16S ribosomal RNA ge	KM410213	81.2	84.62	7.5955e−24	Diptera	Simuliidae	Simulium sheilae
Simulium brinchangense isolate B2B 16S ribosomal R	KM410202	81.2	84.62	7.5955e−24	Diptera	Simuliidae	Simulium brinchangense
Simulium brinchangense isolate B1B 16S ribosomal R	KM410201	81.2	84.62	7.5955e−24	Diptera	Simuliidae	Simulium brinchangense
Simulium tanahrataense isolate TR2B 16S ribosomal	KM410196	81.2	84.62	7.5955e−24	Diptera	Simuliidae	Simulium tanahrataense
Simulium tanahrataense isolate TR1B 16S ribosomal	KM410195	81.2	84.62	7.5955e−24	Diptera	Simuliidae	Simulium tanahrataense
Tongamya stuckenbergi 16S ribosomal RNA gene, part	KM878969	78.75	99.36	7.5955e−24	Diptera	Mydidae	Tongamya stuckenbergi
Callomyia admirabilis isolate CA42 16S ribosomal R	KC699326	81.2	84.62	7.5955e−24	Diptera	Platypezidae	Callomyia admirabilis
Paracladura trichoptera mitochondrion, complete ge	JN861751	84.03	75.64	7.5955e−24	Diptera	Trichoceridae	Paracladura trichoptera
Simulium chumpornense 16S ribosomal RNA gene, part	GQ865998	81.2	84.62	7.5955e−24	Diptera	Simuliidae	Simulium chumpornense
Empis producta voucher RMBR:103430 16S ribosomal R	FJ808167	78.34	99.36	7.5955e−24	Diptera	Empididae	Empis producta
Simulium cabrerai mitochondrial gene for 16S rRNA,	AB093128	81.2	84.62	7.5955e−24	Diptera	Simuliidae	Simulium cabrerai
Zygoneura bidens voucher 312 16S ribosomal RNA gen	JQ613910	78.57	97.44	2.65109e−23	Diptera	Sciaridae	Zygoneura bidens
Celyphidae sp. SNK−2008 16S ribosomal RNA gene, pa	FJ025463	77.85	99.36	2.65109e−23	Diptera	Celyphidae	Celyphidae sp. SNK−2008
Coelopa pilipes 16S ribosomal RNA gene, partial se	AF403458	79.25	99.36	2.65109e−23	Diptera	Coelopidae	Coelopa pilipes
Caenophthalmus capensis voucher MEI 120162 16S rib	DQ250838	79.73	92.31	2.65109e−23	Diptera	Therevidae	Caenophthalmus capensis
Simulium mie mitochondrial gene for 16S ribosomal	AB056732	78.4	100	2.65109e−23	Diptera	Simuliidae	Simulium mie
Simulium mie mitochondrial gene for 16S ribosomal	AB056731	78.4	100	2.65109e−23	Diptera	Simuliidae	Simulium mie
Simulium mie mitochondrial gene for 16S ribosomal	AB056730	78.4	100	2.65109e−23	Diptera	Simuliidae	Simulium mie
Simulium claveriaense mitochondrial gene for 16S r	LC034988	81.25	79.49	9.25321e−23	Diptera	Simuliidae	Simulium claveriaense
Simulium claveriaense mitochondrial gene for 16S r	LC034987	81.25	79.49	9.25321e−23	Diptera	Simuliidae	Simulium claveriaense
Simulium sheilae isolate SH2B 16S ribosomal RNA ge	KM410214	80.45	84.62	9.25321e−23	Diptera	Simuliidae	Simulium sheilae
Platypezina diversa isolate PD29 16S ribosomal RNA	KC699349	79.55	84.62	9.25321e−23	Diptera	Platypezidae	Platypezina diversa
Chydaeopeza tibialis isolate CT44 16S ribosomal RN	KC699336	77.56	99.36	9.25321e−23	Diptera	Platypezidae	Chydaeopeza tibialis
Callomyia amoena isolate CA06 16S ribosomal RNA ge	KC699325	80.45	84.62	9.25321e−23	Diptera	Platypezidae	Callomyia amoena
Agathomyia woodella isolate 448 16S ribosomal RNA	KC699307	79.19	93.59	9.25321e−23	Diptera	Platypezidae	Agathomyia woodella
Haematobia irritans mitochondrion, partial genome	KM669714	77.56	99.36	9.25321e−23	Diptera	Muscidae	Haematobia irritans
Haematobia irritans isolate D50 small subunit ribo	JQ246755	77.56	99.36	9.25321e−23	Diptera	Muscidae	Haematobia irritans
Simulium decuplum 16S ribosomal RNA gene, partial	GQ865986	80.45	84.62	9.25321e−23	Diptera	Simuliidae	Simulium decuplum
Allocotocera pulchella 16S ribosomal RNA gene, par	FJ172034	81.82	77.56	9.25321e−23	Diptera	Mycetophilidae	Allocotocera pulchella
Haematobia irritans 16S ribosomal RNA gene, partia	FJ025436	77.56	99.36	9.25321e−23	Diptera	Muscidae	Haematobia irritans
Drosophila rubida voucher 109400 NADH dehydrogenas	EU494327	80.56	89.1	9.25321e−23	Diptera	Drosophilidae	Drosophila rubida
Drosophila hypocausta voucher 103972 NADH dehydrog	EU494319	80.56	89.1	9.25321e−23	Diptera	Drosophilidae	Drosophila hypocausta
Simulium recurvum mitochondrial gene for 16S ribos	AB334097	80.45	84.62	9.25321e−23	Diptera	Simuliidae	Simulium recurvum
Simulium claveriaense mitochondrial gene for 16S r	AB334096	81.25	79.49	9.25321e−23	Diptera	Simuliidae	Simulium claveriaense
Arachnocampa luminosa biotype 2 16S large subunit	AY576330	78.67	94.23	9.25321e−23	Diptera	Keroplatidae	Arachnocampa luminosa
Haematobia irritans irritans mitochondrion, comple	DQ029097	77.56	99.36	9.25321e−23	Diptera	Muscidae	Haematobia irritans irritans
Coelopa alluaudi 16S ribosomal RNA gene, partial s	AF403455	78.83	87.82	9.25321e−23	Diptera	Coelopidae	Coelopa alluaudi
Chaetocoelopa littoralis 16S ribosomal RNA gene, p	AF403453	77.56	99.36	9.25321e−23	Diptera	Coelopidae	Chaetocoelopa littoralis
Simulium tuyense mitochondrial gene for 16S ribos	LC035004	81.1	78.85	3.22969e−22	Diptera	Simuliidae	Simulium tuyense
Simulium suyoense mitochondrial gene for 16S ribos	LC035000	78.4	100	3.22969e−22	Diptera	Simuliidae	Simulium suyoense
Simulium spinosibranchium mitochondrial gene for 1	LC034996	80	84.62	3.22969e−22	Diptera	Simuliidae	Simulium spinosibranchium
Simulium nobile isolate N5 16S ribosomal RNA gene,	KP661530	80.8	79.49	3.22969e−22	Diptera	Simuliidae	Simulium nobile
Simulium nobile isolate N4 16S ribosomal RNA gene,	KP661529	80.8	79.49	3.22969e−22	Diptera	Simuliidae	Simulium nobile
Simulium nobile isolate N3 16S ribosomal RNA gene,	KP661528	80.8	79.49	3.22969e−22	Diptera	Simuliidae	Simulium nobile
Simulium nobile isolate N2 16S ribosomal RNA gene,	KP661527	80.8	79.49	3.22969e−22	Diptera	Simuliidae	Simulium nobile
Simulium nobile isolate N1 16S ribosomal RNA gene,	KP661526	80.8	79.49	3.22969e−22	Diptera	Simuliidae	Simulium nobile
Simulium timorense isolate TM5 16S ribosomal RNA g	KP661525	80.8	79.49	3.22969e−22	Diptera	Simuliidae	Simulium timorense
Simulium timorense isolate TM4 16S ribosomal RNA g	KP661524	80.8	79.49	3.22969e−22	Diptera	Simuliidae	Simulium timorense
Simulium timorense isolate TM3 16S ribosomal RNA g	KP661523	80.8	79.49	3.22969e−22	Diptera	Simuliidae	Simulium timorense
Simulium timorense isolate TM2 16S ribosomal RNA g	KP661522	80.8	79.49	3.22969e−22	Diptera	Simuliidae	Simulium timorense
Simulium timorense isolate TM1 16S ribosomal RNA g	KP661521	80.8	79.49	3.22969e−22	Diptera	Simuliidae	Simulium timorense
Agathomyia unicolor isolate AU2 16S ribosomal RNA	KC699318	77.85	99.36	3.22969e−22	Diptera	Platypezidae	Agathomyia unicolor
Ectinorhynchus sp. 'Bribie' voucher UQIC41178 16S	KM212983	77.85	99.36	3.22969e−22	Diptera	Therevidae	Ectinorhynchus sp. 'Bribie'
Ectinorhynchus sp. 'Moffatt' voucher QMT183203 16S	KM212976	77.85	99.36	3.22969e−22	Diptera	Therevidae	Ectinorhynchus sp. 'Moffatt'
Ectinorhynchus sp. 'warrumbungles' voucher QMT1832	KM212974	77.85	99.36	3.22969e−22	Diptera	Therevidae	Ectinorhynchus sp. 'warrumbungles'
Simulium khunklangense mitochondrial gene for 16S	AB820367	80.6	84.62	3.22969e−22	Diptera	Simuliidae	Simulium khunklangense
Liriomyza sativae mitochondrion, complete genome	JQ862475	76.43	99.36	3.22969e−22	Diptera	Agromyzidae	Liriomyza sativae
Simulium chomthongense mitochondrial gene for 16S	AB699899	80.6	84.62	3.22969e−22	Diptera	Simuliidae	Simulium chomthongense
Dichopygina aculeata voucher 947 16S ribosomal RNA	JQ613961	77.92	97.44	3.22969e−22	Diptera	Sciaridae	Dichopygina aculeata
Liriomyza sativae mitochondrion, complete genome	HQ333260	76.43	99.36	3.22969e−22	Diptera	Agromyzidae	Liriomyza sativae
Simulium nobile 16S ribosomal RNA gene, partial se	GQ866008	80.8	79.49	3.22969e−22	Diptera	Simuliidae	Simulium nobile
Simulium caudisclerum 16S ribosomal RNA gene, part	GQ865999	80.6	84.62	3.22969e−22	Diptera	Simuliidae	Simulium caudisclerum
Simulium spinosibranchium mitochondrial gene for 1	AB334098	80	84.62	3.22969e−22	Diptera	Simuliidae	Simulium spinosibranchium
Simulium squamosum voucher P.Mansiangi Pm3(BNI) 16	DQ655644	81.82	77.56	3.22969e−22	Diptera	Simuliidae	Simulium squamosum
Arachnocampa tropicus biotype 6 16S large subunit	AY576335	81.1	80.77	3.22969e−22	Diptera	Keroplatidae	Arachnocampa tropicus
Simulium rasyani isolate rasy 19 16S ribosomal RNA	AF416974	81.82	77.56	3.22969e−22	Diptera	Simuliidae	Simulium rasyani
