# Supplementary material for: Testing the potential of a ribosomal 16S marker for DNA metabarcoding of insects
Source: PeerJ. 2016 Apr 19;4:e1966. doi: 10.7717/peerj.1966 (PMC4841222; doi:10.7717/peerj.1966)
Supplement: Supplemental Information 1 [file peerj-04-1966-s006.zip › scripts/5 match OTUs/blast_hits/OTU_53.csv_plot.pdf]

OTU_53.csv

Baetis pseudorhodani

Baetidae

Ephemeroptera

83.78

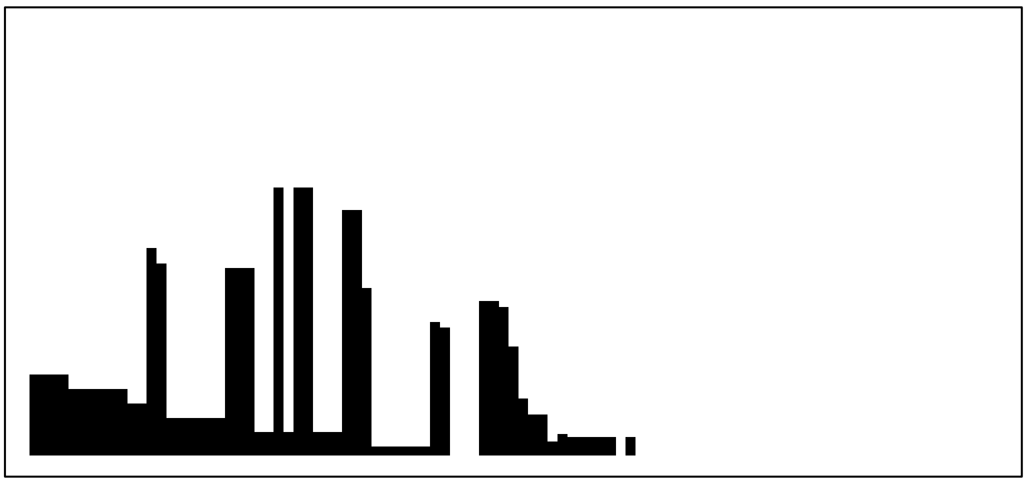

Baetis pseudorhodani isolate TF3_745823 16S riboso	KF438121	83.78	100	9.85116e−35	Ephemeroptera	Baetidae	Baetis pseudorhodani
Baetis pseudorhodani isolate LG1_830004 16S riboso	KF438113	83.78	100	9.85116e−35	Ephemeroptera	Baetidae	Baetis pseudorhodani
Baetis harrisoni voucher llp_032 16S ribosomal RNA	HM637001	83.78	100	9.85116e−35	Ephemeroptera	Baetidae	Baetis harrisoni
Baetis harrisoni voucher llp_022 16S ribosomal RNA	HM636993	83.78	100	9.85116e−35	Ephemeroptera	Baetidae	Baetis harrisoni
Baetis harrisoni voucher llp_046 16S ribosomal RNA	HM637014	83.11	100	4.18882e−33	Ephemeroptera	Baetidae	Baetis harrisoni
Baetis harrisoni voucher llp_045 16S ribosomal RNA	HM637013	83.11	100	4.18882e−33	Ephemeroptera	Baetidae	Baetis harrisoni
Baetis harrisoni voucher llp_042 16S ribosomal RNA	HM637010	83.11	100	4.18882e−33	Ephemeroptera	Baetidae	Baetis harrisoni
Baetis harrisoni voucher llp_039 16S ribosomal RNA	HM637007	83.11	100	4.18882e−33	Ephemeroptera	Baetidae	Baetis harrisoni
Baetis sp. PC−2010 mitochondrion, complete genome	GU936204	83.11	100	4.18882e−33	Ephemeroptera	Baetidae	Baetis sp. PC−2010
Baetis harrisoni partial mitochondrial 16S rRNA ge	AJ971743	83.11	100	4.18882e−33	Ephemeroptera	Baetidae	Baetis harrisoni
Baetis harrisoni voucher llp_044 16S ribosomal RNA	HM637012	82.43	100	5.10303e−32	Ephemeroptera	Baetidae	Baetis harrisoni
Baetis harrisoni voucher llp_041 16S ribosomal RNA	HM637009	82.43	100	5.10303e−32	Ephemeroptera	Baetidae	Baetis harrisoni
Baetis enigmaticus isolate MD2_745837 16S ribosoma	KF438116	89.72	72.3	6.21676e−31	Ephemeroptera	Baetidae	Baetis enigmaticus
Baetis canariensis isolate LG1_830001 16S ribosoma	KF438112	88.99	73.65	6.21676e−31	Ephemeroptera	Baetidae	Baetis canariensis
Baetis harrisoni voucher llp_055 16S ribosomal RNA	HM637022	81.76	100	2.16986e−30	Ephemeroptera	Baetidae	Baetis harrisoni
Baetis harrisoni voucher llp_050 16S ribosomal RNA	HM637018	81.76	100	2.16986e−30	Ephemeroptera	Baetidae	Baetis harrisoni
Baetis harrisoni voucher llp_038 16S ribosomal RNA	HM637006	81.76	100	2.16986e−30	Ephemeroptera	Baetidae	Baetis harrisoni
Baetis harrisoni voucher llp_037 16S ribosomal RNA	HM637005	81.76	100	2.16986e−30	Ephemeroptera	Baetidae	Baetis harrisoni
Baetis harrisoni voucher llp_020 16S ribosomal RNA	HM636991	81.76	100	2.16986e−30	Ephemeroptera	Baetidae	Baetis harrisoni
Baetis harrisoni voucher llp_004 16S ribosomal RNA	HM636980	81.76	100	2.16986e−30	Ephemeroptera	Baetidae	Baetis harrisoni
Baetis rhodani isolate MO_745832 16S ribosomal RNA	KF438119	88.79	72.3	7.57356e−30	Ephemeroptera	Baetidae	Baetis rhodani
Baetis rhodani isolate MO_745831 16S ribosomal RNA	KF438118	88.79	72.3	7.57356e−30	Ephemeroptera	Baetidae	Baetis rhodani
Baetis atlanticus isolate MD3_745567 16S ribosomal	KF438117	88.79	72.3	7.57356e−30	Ephemeroptera	Baetidae	Baetis atlanticus
Baetis rhodani isolate CH_745580_neo 16S ribosomal	KF438105	81.08	100	2.64343e−29	Ephemeroptera	Baetidae	Baetis rhodani
Baetis harrisoni voucher llp_058 16S ribosomal RNA	HM637025	81.08	100	2.64343e−29	Ephemeroptera	Baetidae	Baetis harrisoni
Baetis harrisoni voucher llp_054 16S ribosomal RNA	HM637021	92.55	63.51	2.64343e−29	Ephemeroptera	Baetidae	Baetis harrisoni
Baetis rhodani voucher llp_048 16S ribosomal RNA g	HM637016	81.08	100	2.64343e−29	Ephemeroptera	Baetidae	Baetis rhodani
Baetis harrisoni voucher llp_029 16S ribosomal RNA	HM636998	92.55	63.51	2.64343e−29	Ephemeroptera	Baetidae	Baetis harrisoni
Baetis harrisoni voucher llp_028 16S ribosomal RNA	HM636997	92.55	63.51	2.64343e−29	Ephemeroptera	Baetidae	Baetis harrisoni
Baetis harrisoni voucher llp_019 16S ribosomal RNA	HM636990	81.08	100	2.64343e−29	Ephemeroptera	Baetidae	Baetis harrisoni
Baetis harrisoni voucher llp_017 16S ribosomal RNA	HM636988	81.08	100	2.64343e−29	Ephemeroptera	Baetidae	Baetis harrisoni
Baetis harrisoni voucher llp_016 16S ribosomal RNA	HM636987	81.08	100	2.64343e−29	Ephemeroptera	Baetidae	Baetis harrisoni
Baetis harrisoni voucher llp_027 16S ribosomal RNA	HM636996	91.49	63.51	3.22036e−28	Ephemeroptera	Baetidae	Baetis harrisoni
Baetis harrisoni voucher llp_014 16S ribosomal RNA	HM636985	91.49	63.51	3.22036e−28	Ephemeroptera	Baetidae	Baetis harrisoni
Labiobaetis sp. BMNH 704637 mitochondrial 16S rRNA	AJ971775	87.85	72.3	3.22036e−28	Ephemeroptera	Baetidae	Labiobaetis sp. BMNH 704637
Baetis rhodani isolate ES_200043 16S ribosomal RNA	KF438110	80.41	100	1.12402e−27	Ephemeroptera	Baetidae	Baetis rhodani
Baetis rhodani isolate ES_200033 16S ribosomal RNA	KF438109	80.41	100	1.12402e−27	Ephemeroptera	Baetidae	Baetis rhodani
Baetis ingridae isolate CO_830458 16S ribosomal RN	KF438106	80.41	100	1.12402e−27	Ephemeroptera	Baetidae	Baetis ingridae
Baetis harrisoni voucher llp_051 16S ribosomal RNA	HM637019	80.41	100	1.12402e−27	Ephemeroptera	Baetidae	Baetis harrisoni
Baetis harrisoni voucher llp_040 16S ribosomal RNA	HM637008	80.41	100	1.12402e−27	Ephemeroptera	Baetidae	Baetis harrisoni
Baetis harrisoni voucher llp_018 16S ribosomal RNA	HM636989	80.41	100	1.12402e−27	Ephemeroptera	Baetidae	Baetis harrisoni
Baetis canariensis isolate TF3_745824 16S ribosoma	KF438122	86.24	73.65	1.36933e−26	Ephemeroptera	Baetidae	Baetis canariensis
Baetis ingridae isolate CO_830478 16S ribosomal RN	KF438108	85.98	72.3	1.66819e−25	Ephemeroptera	Baetidae	Baetis ingridae
Baetis tricaudatus voucher BYU:IGCEP011 16S riboso	GQ118283	79.05	100	5.82255e−25	Ephemeroptera	Baetidae	Baetis tricaudatus
Baetis cyrneus isolate CO_830461 16S ribosomal RNA	KF438107	79.19	100	2.03227e−24	Ephemeroptera	Baetidae	Baetis cyrneus
Baetis alpinus partial mitochondrial 16S rRNA gene	AJ971726	79.19	100	2.03227e−24	Ephemeroptera	Baetidae	Baetis alpinus
Baetis sp. SR−2014 isolate TN_745843 16S ribosomal	KF438123	87.23	62.84	1.05274e−21	Ephemeroptera	Baetidae	Baetis sp. SR−2014
Baetis canariensis isolate GC1_250012 16S ribosoma	KF438111	87.23	62.84	1.05274e−21	Ephemeroptera	Baetidae	Baetis canariensis
Labiobaetis sp. BMNH 704655 mitochondrial 16S rRNA	AJ971783	86.96	62.16	1.05274e−21	Ephemeroptera	Baetidae	Labiobaetis sp. BMNH 704655
Labiobaetis fabulosus partial mitochondrial 16S rR	AJ971734	85.11	62.84	5.45334e−19	Ephemeroptera	Baetidae	Labiobaetis fabulosus
Diphetor sp. EP127 16S ribosomal RNA gene, partial	AY749808	82.65	66.22	6.64352e−18	Ephemeroptera	Baetidae	Diphetor sp. EP127
Labiobaetis gilliesi partial mitochondrial 16S rRN	AJ971735	81.91	62.84	1.20118e−14	Ephemeroptera	Baetidae	Labiobaetis gilliesi
Labiobaetis vulgaris partial mitochondrial 16S rRN	AJ971725	81.91	62.84	1.20118e−14	Ephemeroptera	Baetidae	Labiobaetis vulgaris
Edmulmeatus grandis partial mitochondrial 16S rRNA	AJ971737	80.65	62.84	4.19252e−14	Ephemeroptera	Baetidae	Edmulmeatus grandis
Labiobaetis sp. BMNH 704646 mitochondrial 16S rRNA	AJ971780	81	66.22	1.46333e−13	Ephemeroptera	Baetidae	Labiobaetis sp. BMNH 704646
Pseudocloeon vinosum partial mitochondrial 16S rRN	AJ971763	80.85	62.84	1.46333e−13	Ephemeroptera	Baetidae	Pseudocloeon vinosum
Labiobaetis punctatus partial mitochondrial 16S rR	AJ971733	80.85	62.84	1.46333e−13	Ephemeroptera	Baetidae	Labiobaetis punctatus
Baetis tricaudatus 16S ribosomal RNA gene, partial	AY749771	80.85	62.84	1.46333e−13	Ephemeroptera	Baetidae	Baetis tricaudatus
Alainites yixiani mitochondrion, complete genome	GU479735	80.85	62.84	1.7827e−12	Ephemeroptera	Baetidae	Takobia yixiani
Demoreptus natalensis partial mitochondrial 16S rR	AJ971765	80.85	62.84	1.7827e−12	Ephemeroptera	Baetidae	Demoreptus natalensis
Pseudocloeon glaucum partial mitochondrial 16S rRN	AJ971762	78.57	66.22	1.7827e−12	Ephemeroptera	Baetidae	Pseudocloeon glaucum
Ophelmastoma camerunense partial mitochondrial 16S	AJ971747	80.85	62.84	1.7827e−12	Ephemeroptera	Baetidae	Ophelmastoma camerunense
Labiobaetis longicercus partial mitochondrial 16S	AJ971724	73.15	100	1.7827e−12	Ephemeroptera	Baetidae	Labiobaetis longicercus
Callibaetis ferrugineus 16S ribosomal RNA gene, pa	AF370873	79.44	70.95	6.22225e−12	Ephemeroptera	Baetidae	Callibaetis ferrugineus
Labiobaetis sp. BMNH 704656 mitochondrial 16S rRNA	AJ971784	76.99	72.97	6.22225e−12	Ephemeroptera	Baetidae	Labiobaetis sp. BMNH 704656
Pseudocloeon vitale partial mitochondrial 16S rRNA	AJ971781	79.79	62.84	6.22225e−12	Ephemeroptera	Baetidae	Pseudocloeon vitale
Pseudopannota maculosa partial mitochondrial 16S r	AJ971745	72.97	100	6.22225e−12	Ephemeroptera	Baetidae	Pseudopannota maculosa
Callibaetis ferrugineus hageni isolate I_38 16S ri	HM003946	79.79	62.84	7.58025e−11	Ephemeroptera	Baetidae	Callibaetis ferrugineus hageni
Pseudopannota vinckei partial mitochondrial 16S rR	AJ971754	78.72	62.84	7.58025e−11	Ephemeroptera	Baetidae	Pseudopannota vinckei
Callibaetis ferrugineus hageni isolate QQ_17 16S r	HM003975	79	66.22	2.64577e−10	Ephemeroptera	Baetidae	Callibaetis ferrugineus hageni
Callibaetis ferrugineus hageni isolate O_5 16S rib	HM003950	79	66.22	2.64577e−10	Ephemeroptera	Baetidae	Callibaetis ferrugineus hageni
Callibaetis ferrugineus hageni isolate K_13 16S ri	HM003949	79	66.22	2.64577e−10	Ephemeroptera	Baetidae	Callibaetis ferrugineus hageni
Callibaetis ferrugineus hageni isolate J_10 16S ri	HM003947	79	66.22	2.64577e−10	Ephemeroptera	Baetidae	Callibaetis ferrugineus hageni
Callibaetis ferrugineus hageni isolate W_38 16S ri	HM003945	79	66.22	2.64577e−10	Ephemeroptera	Baetidae	Callibaetis ferrugineus hageni
Callibaetis ferrugineus hageni isolate E_20 16S ri	HM003944	79	66.22	2.64577e−10	Ephemeroptera	Baetidae	Callibaetis ferrugineus hageni
Jubabaetis sp. EP139 16S ribosomal RNA gene, parti	AY749813	78	66.89	2.64577e−10	Ephemeroptera	Baetidae	Jubabaetis sp. EP139
Callibaetis ferrugineus hageni isolate V_37 16S ri	HM003943	78.72	62.84	9.23464e−10	Ephemeroptera	Baetidae	Callibaetis ferrugineus hageni
Callibaetis ferrugineus hageni isolate T_15 16S ri	HM003940	78.72	62.84	9.23464e−10	Ephemeroptera	Baetidae	Callibaetis ferrugineus hageni
Callibaetis ferrugineus hageni isolate A_3 16S rib	HM003937	78.72	62.84	9.23464e−10	Ephemeroptera	Baetidae	Callibaetis ferrugineus hageni
Callibaetis ferrugineus hageni isolate D_3 16S rib	HM003933	78.72	62.84	9.23464e−10	Ephemeroptera	Baetidae	Callibaetis ferrugineus hageni
Callibaetis ferrugineus hageni isolate L_17 16S ri	HM003974	78	66.22	3.22321e−09	Ephemeroptera	Baetidae	Callibaetis ferrugineus hageni
Callibaetis ferrugineus hageni isolate PP_13 16S r	HM003973	78	66.22	3.22321e−09	Ephemeroptera	Baetidae	Callibaetis ferrugineus hageni
Callibaetis ferrugineus hageni isolate OO_11 16S r	HM003972	78	66.22	3.22321e−09	Ephemeroptera	Baetidae	Callibaetis ferrugineus hageni
Callibaetis ferrugineus hageni isolate NN_11 16S r	HM003971	78	66.22	3.22321e−09	Ephemeroptera	Baetidae	Callibaetis ferrugineus hageni
Callibaetis ferrugineus hageni isolate FF_7 16S ri	HM003961	78	66.22	3.22321e−09	Ephemeroptera	Baetidae	Callibaetis ferrugineus hageni
Callibaetis ferrugineus hageni isolate EE_7 16S ri	HM003958	78	66.22	3.22321e−09	Ephemeroptera	Baetidae	Callibaetis ferrugineus hageni
Callibaetis ferrugineus hageni isolate DD_7 16S ri	HM003957	78	66.22	3.22321e−09	Ephemeroptera	Baetidae	Callibaetis ferrugineus hageni
Callibaetis ferrugineus hageni isolate F_7 16S rib	HM003955	78	66.22	3.22321e−09	Ephemeroptera	Baetidae	Callibaetis ferrugineus hageni
Callibaetis ferrugineus hageni isolate H_7 16S rib	HM003939	78	66.22	3.22321e−09	Ephemeroptera	Baetidae	Callibaetis ferrugineus hageni
Nigrobaetis bethunae partial mitochondrial 16S rRN	AJ971785	78.57	64.86	3.22321e−09	Ephemeroptera	Baetidae	Nigrobaetis bethunae
Callibaetis sp. EP020 16S ribosomal RNA gene, part	AY749764	78	66.22	3.22321e−09	Ephemeroptera	Baetidae	Callibaetis sp. EP020
Cloeon sp. MG−2011 isolate 300005 16S ribosomal RN	JN627431	77.66	62.16	3.92667e−08	Ephemeroptera	Baetidae	Cloeon sp. MG−2011
Callibaetis ferrugineus hageni isolate JJ_8 16S ri	HM003967	77.66	62.84	3.92667e−08	Ephemeroptera	Baetidae	Callibaetis ferrugineus hageni
Callibaetis ferrugineus hageni isolate M_7 16S rib	HM003963	77.66	62.84	3.92667e−08	Ephemeroptera	Baetidae	Callibaetis ferrugineus hageni
Callibaetis ferrugineus hageni isolate GG_7 16S ri	HM003962	77.66	62.84	3.92667e−08	Ephemeroptera	Baetidae	Callibaetis ferrugineus hageni
Callibaetis ferrugineus hageni isolate C_7 16S rib	HM003959	77.66	62.84	3.92667e−08	Ephemeroptera	Baetidae	Callibaetis ferrugineus hageni
Callibaetis ferrugineus hageni isolate CC_7 16S ri	HM003956	77.66	62.84	3.92667e−08	Ephemeroptera	Baetidae	Callibaetis ferrugineus hageni
Callibaetis ferrugineus hageni isolate AA_5 16S ri	HM003953	77.66	62.84	3.92667e−08	Ephemeroptera	Baetidae	Callibaetis ferrugineus hageni
Callibaetis ferrugineus hageni isolate Y_5 16S rib	HM003951	77.66	62.84	3.92667e−08	Ephemeroptera	Baetidae	Callibaetis ferrugineus hageni
Callibaetis ferrugineus hageni isolate X_21 16S ri	HM003948	76.6	62.84	3.92667e−08	Ephemeroptera	Baetidae	Callibaetis ferrugineus hageni
