# Supplementary material for: Testing the potential of a ribosomal 16S marker for DNA metabarcoding of insects
Source: PeerJ. 2016 Apr 19;4:e1966. doi: 10.7717/peerj.1966 (PMC4841222; doi:10.7717/peerj.1966)
Supplement: Supplemental Information 1 [file peerj-04-1966-s006.zip › scripts/5 match OTUs/blast_hits/OTU_531.csv_plot.pdf]

OTU_531.csv

Sialis californica

Sialidae

Megaloptera

93.08

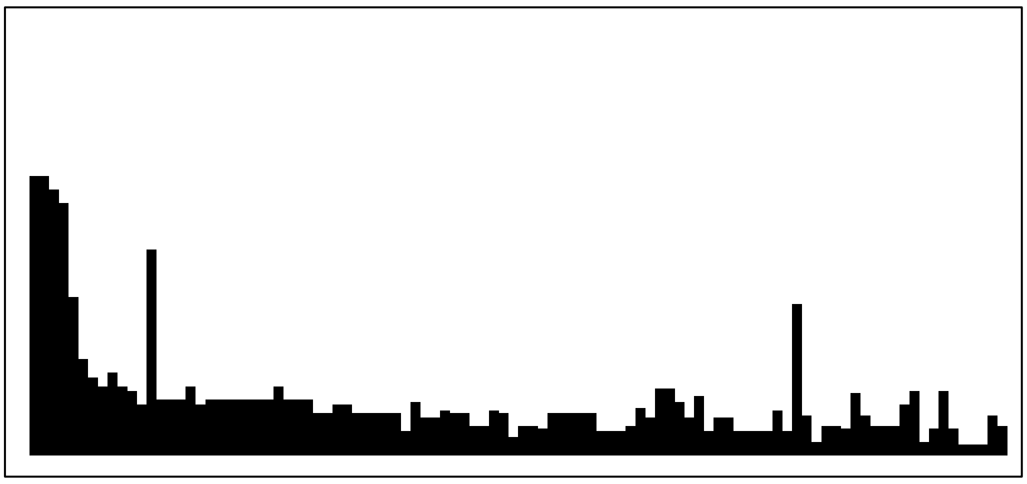

Sialis californica 16S ribosomal RNA gene, partial	EU734902	93.08	100	5.92546e−57	Megaloptera	Sialidae	Sialis californica
Sialis lutaria isolate Sialut1 16S ribosomal RNA g	AY620141	93.08	100	5.92546e−57	Megaloptera	Sialidae	Sialis lutaria
Sialis hamata mitochondrion, complete genome	FJ859905	92.45	100	7.21868e−56	Megaloptera	Sialidae	Sialis hamata
Nipponosialis kumejimae isolate H841 16S ribosomal	KF741321	91.82	100	3.06946e−54	Megaloptera	Sialidae	Nipponosialis kumejimae
Sialis nr. mohri SLW−2008 16S ribosomal RNA gene,	EU734903	87.42	100	5.1978e−45	Megaloptera	Sialidae	Sialis nr. mohri SLW−2008
Dasytes depexus voucher UPOL001173 16S ribosomal R	HQ619676	84.52	97.47	2.52179e−36	Coleoptera	Melyridae	Dasytes depexus
Ochlerotatus nipponicus isolate A75 16S ribosomal	DQ397914	83.65	100	8.80193e−36	Diptera	Culicidae	Aedes nipponicus
Dasytes otini voucher UPOL001084 16S ribosomal RNA	HQ619645	83.23	97.47	1.07229e−34	Coleoptera	Melyridae	Dasytes otini
Dolichosoma lineare voucher UPOL001063 16S ribosom	HQ619634	83.87	97.47	3.74267e−34	Coleoptera	Melyridae	Dolichosoma lineare
Exorista patelliforceps mitochondrial gene for 16S	AB257231	83.23	100	3.74267e−34	Diptera	Tachinidae	Exorista patelliforceps
Semisuturia sp. LRC−2002 16S ribosomal RNA gene, p	AF456848	83.02	100	3.74267e−34	Diptera	Tachinidae	Semisuturia sp. LRC−2002
Irwiniella natalensis voucher MEI 119909 16S ribos	DQ250845	82.39	100	3.74267e−34	Diptera	Therevidae	Irwiniella natalensis
Teleopsis trichophora 16S ribosomal RNA gene, part	KT186661	89.66	72.78	1.30632e−33	Diptera	Diopsidae	Teleopsis trichophora
Agabus biguttatus haplotype 1 16S ribosomal RNA ge	AY039258	82.61	100	1.30632e−33	Coleoptera	Dytiscidae	Agabus biguttatus
Agabus alexandrae 16S ribosomal RNA gene, partial	AY039257	82.61	100	1.30632e−33	Coleoptera	Dytiscidae	Agabus alexandrae
Agabus alexandrae 16S ribosomal RNA gene, partial	AF428199	82.61	100	1.30632e−33	Coleoptera	Dytiscidae	Agabus alexandrae
Agabus paludosus 16S ribosomal RNA gene, partial s	AY138625	83.23	100	1.30632e−33	Coleoptera	Dytiscidae	Agabus paludosus
Gitona bivisualis 16S ribosomal RNA gene, partial	AF479793	82.39	100	4.55951e−33	Diptera	Drosophilidae	Gitona bivisualis
Agabus nebulosus mitochondrial 16S rRNA gene (part	LM654779	82.61	100	1.59143e−32	Coleoptera	Dytiscidae	Agabus nebulosus
Agabus glacialis mitochondrial 16S rRNA gene (part	LM654776	82.61	100	1.59143e−32	Coleoptera	Dytiscidae	Agabus glacialis
Agabus uliginosus mitochondrial 16S rRNA gene (par	HF931463	82.61	100	1.59143e−32	Coleoptera	Dytiscidae	Agabus uliginosus
Agabus cephalotes mitochondrial 16S rRNA gene (par	HF931380	82.61	100	1.59143e−32	Coleoptera	Dytiscidae	Agabus cephalotes
Agabus dichrous mitochondrial partial 16S rRNA gen	HF931378	82.61	100	1.59143e−32	Coleoptera	Dytiscidae	Agabus dichrous
Agabus godmanni mitochondrial 16S rRNA gene (parti	HF931348	82.61	100	1.59143e−32	Coleoptera	Dytiscidae	Agabus godmanni
Exorista lepis mitochondrial gene for 16S rRNA, pa	AB699948	82.61	100	1.59143e−32	Diptera	Tachinidae	Exorista lepis
Limonia tahitiensis voucher O'Grady 202004 16S rib	EU005425	83.23	99.37	1.59143e−32	Diptera	Limoniidae	Dicranomyia tahitiensis
Agabus nebulosus 16S ribosomal RNA gene, partial s	AY138624	82.61	100	1.59143e−32	Coleoptera	Dytiscidae	Agabus nebulosus
Agabus dilatatus 16S ribosomal RNA gene, partial s	AY039261	82.61	100	1.59143e−32	Coleoptera	Dytiscidae	Agabus dilatatus
Agabus lineatus 16S ribosomal RNA gene, partial se	AY138611	82.61	100	1.59143e−32	Coleoptera	Dytiscidae	Agabus lineatus
Agabus guttatus mitochondrial 16S rRNA gene (parti	LM654777	81.99	100	5.55462e−32	Coleoptera	Dytiscidae	Agabus guttatus
Agabus basalis mitochondrial 16S rRNA gene (partia	HF931443	81.99	100	5.55462e−32	Coleoptera	Dytiscidae	Agabus basalis
Lispe pygmaea 16S ribosomal RNA gene, partial sequ	JN226653	82.39	100	5.55462e−32	Diptera	Muscidae	Lispe pygmaea
Lispe leucospila 16S ribosomal RNA gene, partial s	JN226652	82.39	100	5.55462e−32	Diptera	Muscidae	Lispe leucospila
Agabus cf. nitidus IR−2001 16S ribosomal RNA gene,	AY039264	81.99	100	5.55462e−32	Coleoptera	Dytiscidae	Agabus cf. nitidus IR−2001
Agabus guttatus 16S ribosomal RNA gene, partial se	AY039262	81.99	100	5.55462e−32	Coleoptera	Dytiscidae	Agabus guttatus
Agabus biguttatus haplotype 2 16S ribosomal RNA ge	AY039259	81.99	100	5.55462e−32	Coleoptera	Dytiscidae	Agabus biguttatus
Irwiniella oldroydi voucher MEI 146419 16S ribosom	DQ250843	81.99	100	5.55462e−32	Diptera	Therevidae	Irwiniella oldroydi
Acilius abbreviatus voucher KBMAcab72 16S ribosoma	KF979078	81.99	100	1.93875e−31	Coleoptera	Dytiscidae	Acilius abbreviatus
Agabus undulatus mitochondrial 16S rRNA gene (part	HF931411	81.13	100	1.93875e−31	Coleoptera	Dytiscidae	Agabus undulatus
Isosturmia japonica mitochondrial gene for 16S rRN	AB465919	82.5	100	1.93875e−31	Diptera	Tachinidae	Isosturmia japonica
Exorista tubigera mitochondrial gene for 16S rRNA,	AB257224	81.76	100	1.93875e−31	Diptera	Tachinidae	Exorista tubigera
Culex bitaeniorhynchus 16S ribosomal RNA gene, par	EF593019	81.76	100	1.93875e−31	Diptera	Culicidae	Culex bitaeniorhynchus
Agabus maderensis 16S ribosomal RNA gene, partial	AY138621	82.1	100	1.93875e−31	Coleoptera	Dytiscidae	Agabus maderensis
Agabus fuscipennis mitochondrial 16S rRNA gene (pa	LM654775	81.99	100	6.76691e−31	Coleoptera	Dytiscidae	Agabus fuscipennis
Brugia timori genome assembly B_timori_Indonesia_v	LK908083	81.99	100	6.76691e−31	Spirurida	Onchocercidae	Brugia timori
Agabus caraboides mitochondrial partial 16S rRNA g	HF931451	81.37	100	6.76691e−31	Coleoptera	Dytiscidae	Agabus caraboides
Agabus picotae mitochondrial 16S rRNA gene (partia	HF931356	81.37	100	6.76691e−31	Coleoptera	Dytiscidae	Agabus picotae
Leistus spinibarbis 16S ribosomal RNA (rrnl) gene,	JF778797	82.1	100	6.76691e−31	Coleoptera	Carabidae	Leistus spinibarbis
Anthomyza socculata 16S ribosomal RNA gene, partia	EU268546	81.99	100	6.76691e−31	Diptera	Anthomyzidae	Anthomyza socculata
Paranthomyza nitida 16S ribosomal RNA gene, partia	EU268531	80.86	100	6.76691e−31	Diptera	Anthomyzidae	Paranthomyza nitida
Agabus heydeni 16S ribosomal RNA gene, partial seq	AY039263	81.37	100	6.76691e−31	Coleoptera	Dytiscidae	Agabus heydeni
Agabus binotatus 16S ribosomal RNA gene, partial s	AY039260	81.37	100	6.76691e−31	Coleoptera	Dytiscidae	Agabus binotatus
Ilybius euryonus 16S ribosomal RNA gene, partial s	AY138662	81.25	100	6.76691e−31	Coleoptera	Dytiscidae	Ilybius euryonus
Agabus japonicus 16S ribosomal RNA gene, partial s	AY138617	81.99	100	6.76691e−31	Coleoptera	Dytiscidae	Agabus japonicus
Agabus conspersus 16S ribosomal RNA gene, partial	AY138610	81.99	100	6.76691e−31	Coleoptera	Dytiscidae	Agabus conspersus
Agabus congener 16S ribosomal RNA gene, partial se	AY138609	81.99	100	6.76691e−31	Coleoptera	Dytiscidae	Agabus congener
Agabus morosus isolate Aga18.2R 16S ribosomal RNA	AY138592	81.99	100	6.76691e−31	Coleoptera	Dytiscidae	Agabus morosus
Conicera similis 16S ribosomal RNA gene, partial s	AF126337	81.99	100	6.76691e−31	Diptera	Phoridae	Conicera similis
Agabus didymus mitochondrial 16S rRNA gene (partia	LM654773	81.13	100	2.36188e−30	Coleoptera	Dytiscidae	Agabus didymus
Aedes albopictus mitochondrion, complete genome	KR068634	81.13	100	2.36188e−30	Diptera	Culicidae	Aedes albopictus
Agabus didymus voucher UNM KBMPmsp730 16S ribosoma	KJ548392	81.13	100	2.36188e−30	Coleoptera	Dytiscidae	Agabus didymus
Acilius semisulcatus voucher KBMAcse7 16S ribosoma	KF979085	81.37	100	2.36188e−30	Coleoptera	Dytiscidae	Acilius semisulcatus
Exorista sabahensis mitochondrial gene for 16S rRN	AB699949	82.21	100	2.36188e−30	Diptera	Tachinidae	Exorista sabahensis
Exorista rustica mitochondrial gene for 16S rRNA,	AB699940	81.76	100	2.36188e−30	Diptera	Tachinidae	Exorista rustica
Hymenoplia rugulosa voucher DA0142 16S ribosomal R	FJ956741	83.12	99.37	2.36188e−30	Coleoptera	Scarabaeidae	Hymenoplia rugulosa
Hymenoplia rugulosa voucher DA0140 16S ribosomal R	FJ956739	83.12	99.37	2.36188e−30	Coleoptera	Scarabaeidae	Hymenoplia rugulosa
Hymenoplia lineolata voucher DA0186 16S ribosomal	FJ956734	82.5	99.37	2.36188e−30	Coleoptera	Scarabaeidae	Hymenoplia lineolata
Exorista mimula mitochondrial gene for 16S rRNA, p	AB465929	81.76	100	2.36188e−30	Diptera	Tachinidae	Exorista mimula
Falkocholeva falklandica mitochondrial partial 16S	FM994861	82.78	94.94	2.36188e−30	Coleoptera	Leiodidae	Falkocholeva falklandica
Scatella hawaiiensis voucher 105726 NADH dehydroge	EU494299	81.13	100	2.36188e−30	Diptera	Ephydridae	Scatella hawaiiensis
Exorista cantans mitochondrial gene for 16S rRNA,	AB257230	81.76	100	2.36188e−30	Diptera	Tachinidae	Exorista cantans
Exorista mimula mitochondrial gene for 16S rRNA, p	AB257223	81.76	100	2.36188e−30	Diptera	Tachinidae	Exorista mimula
Culex mimeticus 16S ribosomal RNA gene, partial se	EF593021	81.13	100	2.36188e−30	Diptera	Culicidae	Culex mimeticus
Aedes flavopictus isolate A47 16S ribosomal RNA ge	DQ397928	81.13	100	2.36188e−30	Diptera	Culicidae	Aedes flavopictus
Aedes albopictus isolate A21 16S ribosomal RNA gen	DQ397921	81.13	100	2.36188e−30	Diptera	Culicidae	Aedes albopictus
Aedes albopictus mitochondrion, complete genome	AY072044	81.13	100	2.36188e−30	Diptera	Culicidae	Aedes albopictus
Agabus aubei 16S ribosomal RNA gene, partial seque	AY039265	82.1	100	2.36188e−30	Coleoptera	Dytiscidae	Agabus aubei
Aedes albopictus mit−gene for large ribosomal subu	X01078	81.13	100	2.36188e−30	Diptera	Culicidae	Aedes albopictus
Lymantria sp. SM01 large subunit ribosomal RNA gen	KP081993	87.1	75.32	8.24379e−30	Lepidoptera	Lymantriidae	Lymantria sp. SM01
Aedes vigilax isolate Mi139 mitochondrion, complet	KP995260	81.88	100	8.24379e−30	Diptera	Culicidae	Ochlerotatus vigilax
Agabus ragazzii mitochondrial 16S rRNA gene (parti	LM654781	80.62	100	8.24379e−30	Coleoptera	Dytiscidae	Agabus ragazzii
Agabus pallidus mitochondrial 16S rRNA gene (parti	LM654780	81.37	100	8.24379e−30	Coleoptera	Dytiscidae	Agabus pallidus
Agabus labiatus mitochondrial 16S rRNA gene (parti	LM654778	81.37	100	8.24379e−30	Coleoptera	Dytiscidae	Agabus labiatus
Bolopus furcatus isolate 1305 16S ribosomal RNA ge	KC699315	81.25	100	8.24379e−30	Diptera	Platypezidae	Bolopus furcatus
Sciophila thoracica voucher JSS4 16S ribosomal RNA	KC435595	82.91	97.47	8.24379e−30	Diptera	Mycetophilidae	Sciophila thoracica
Stichillus sp. JMH−2012 isolate 322 16S ribosomal	JN196445	81.88	100	8.24379e−30	Diptera	Phoridae	Stichillus sp. JMH−2012
Nebria bellorum voucher DHK0509 16S ribosomal RNA	JN847627	81.37	100	8.24379e−30	Coleoptera	Carabidae	Nebria bellorum
Nebria lacustris voucher DHK0004 16S ribosomal RNA	JN847615	81.37	100	8.24379e−30	Coleoptera	Carabidae	Nebria lacustris
Nebria gyllenhali voucher DHK0010 16S ribosomal RN	JN847607	81.37	100	8.24379e−30	Coleoptera	Carabidae	Nebria gyllenhali
Hymenoplia fulvipennis voucher DA0214 16S ribosoma	FJ956723	82.39	99.37	8.24379e−30	Coleoptera	Scarabaeidae	Hymenoplia fulvipennis
Hymenoplia clypealis voucher DA0164 16S ribosomal	FJ956712	83.02	98.73	8.24379e−30	Coleoptera	Scarabaeidae	Hymenoplia clypealis
Ceromya silacea mitochondrial gene for 16S rRNA, p	AB465952	80.62	100	8.24379e−30	Diptera	Tachinidae	Ceromya silacea
Thelairoleskia sp. Thle_05022006 mitochondrial gen	AB465950	81.25	100	8.24379e−30	Diptera	Tachinidae	Thelairoleskia sp. Thle_05022006
Nanos minutus voucher Ara.MA22.11.3 16S ribosomal	EU247966	83.02	96.84	8.24379e−30	Coleoptera	Scarabaeidae	Nanos minutus
Drymeia alpicola 16S ribosomal RNA gene, partial s	FJ025430	81.25	100	8.24379e−30	Diptera	Muscidae	Drymeia alpicola
Aedes riversi isolate A23 16S ribosomal RNA gene,	DQ397927	80.5	100	8.24379e−30	Diptera	Culicidae	Aedes riversi
Aedes riversi isolate A24 16S ribosomal RNA gene,	DQ397926	80.5	100	8.24379e−30	Diptera	Culicidae	Aedes riversi
Aedes hensilli isolate A25 16S ribosomal RNA gene,	DQ397923	80.5	100	8.24379e−30	Diptera	Culicidae	Aedes hensilli
Platambus princeps 16S ribosomal RNA gene, partial	AY138677	81.88	100	8.24379e−30	Coleoptera	Dytiscidae	Platambus princeps
Platambus obtusatus 16S ribosomal RNA gene, partia	AY138675	81.37	100	8.24379e−30	Coleoptera	Dytiscidae	Platambus obtusatus
