# Supplementary material for: Testing the potential of a ribosomal 16S marker for DNA metabarcoding of insects
Source: PeerJ. 2016 Apr 19;4:e1966. doi: 10.7717/peerj.1966 (PMC4841222; doi:10.7717/peerj.1966)
Supplement: Supplemental Information 1 [file peerj-04-1966-s006.zip › scripts/5 match OTUs/blast_hits/OTU_533.csv_plot.pdf]

OTU_533.csv

Oreodytes sanmarkii

Dytiscidae

Coleoptera

98.09

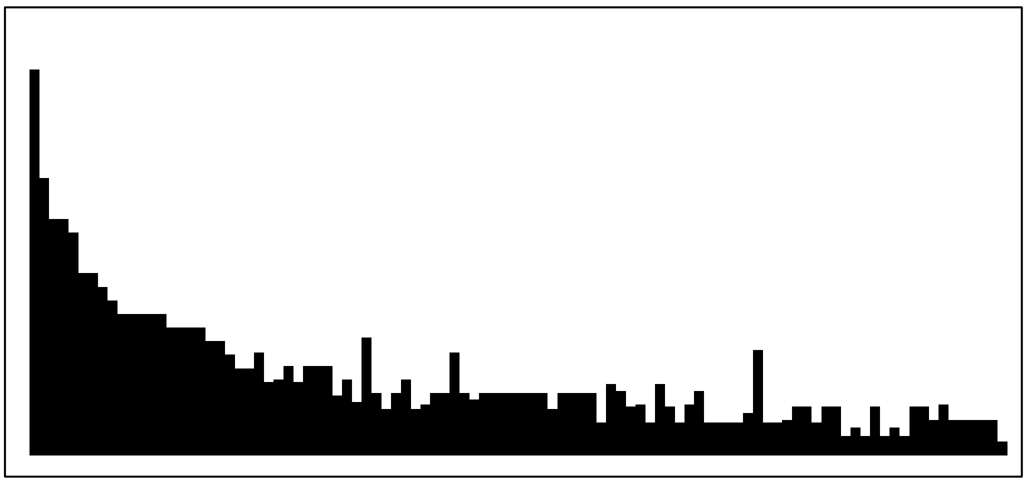

Oreodytes sanmarkii mitochondrial 16S rRNA gene (p	HF931524	98.09	100	6.70005e−69	Coleoptera	Dytiscidae	Oreodytes sanmarkii
Oreodytes obesus cordillerensis mitochondrial part	HF931516	92.99	100	4.82437e−58	Coleoptera	Dytiscidae	Oreodytes obesus cordillerensis
Oreodytes crassulus partial 16S rRNA gene	AJ850348	91.08	100	3.04451e−54	Coleoptera	Dytiscidae	Oreodytes crassulus
Oreodytes congruus partial 16S rRNA gene	AJ850347	91.08	100	3.04451e−54	Coleoptera	Dytiscidae	Oreodytes congruus
Oreodytes congruus 16S ribosomal RNA gene, partial	KF575550	90.45	100	1.29456e−52	Coleoptera	Dytiscidae	Oreodytes congruus
Oreodytes abbreviatus mitochondrial 16S rRNA gene	HF931477	88.54	100	8.16955e−49	Coleoptera	Dytiscidae	Oreodytes abbreviatus
Oreodytes rhyacophilus partial 16S rRNA gene	AJ850352	88.54	100	8.16955e−49	Coleoptera	Dytiscidae	Oreodytes rhyacophilus
Oreodytes mongolicus mitochondrial 16S rRNA gene (	HF931491	87.9	100	3.47378e−47	Coleoptera	Dytiscidae	Oreodytes mongolicus
Oreodytes snoqualmie mitochondrial partial 16S rRN	HF931519	87.26	100	4.23193e−46	Coleoptera	Dytiscidae	Oreodytes snoqualmie
Oreodytes septentrionalis mitochondrial 16S rRNA g	HF931492	86.62	100	1.79946e−44	Coleoptera	Dytiscidae	Oreodytes septentrionalis
Oreodytes shorti mitochondrial 16S rRNA gene (part	HF931488	86.62	100	1.79946e−44	Coleoptera	Dytiscidae	Oreodytes shorti
Boreonectes riberae mitochondrial 16S rRNA gene (p	HF931461	86.62	100	2.19219e−43	Coleoptera	Dytiscidae	Boreonectes riberae
Boreonectes griseostriatus mitochondrial partial 1	HF931387	86.62	100	2.19219e−43	Coleoptera	Dytiscidae	Boreonectes griseostriatus
Boreonectes multilineatus mitochondrial partial 16	HF931384	86.62	100	2.19219e−43	Coleoptera	Dytiscidae	Boreonectes multilineatus
Boreonectes alpestris mitochondrial 16S rRNA gene	HF931512	85.99	100	2.67064e−42	Coleoptera	Dytiscidae	Boreonectes alpestris
Boreonectes striatellus mitochondrial 16S rRNA gene	HF931511	85.99	100	2.67064e−42	Coleoptera	Dytiscidae	Boreonectes striatellus
Boreonectes macedonicus mitochondrial 16S rRNA gen	HF931359	85.99	100	2.67064e−42	Coleoptera	Dytiscidae	Boreonectes macedonicus
Stictotarsus ibericus 16S ribosomal RNA (rrnL) gen	EF670030	85.99	100	2.67064e−42	Coleoptera	Dytiscidae	Stictotarsus ibericus
Laccornis difformis 16S ribosomal RNA gene, partia	KF575544	85.35	100	9.32145e−42	Coleoptera	Dytiscidae	Laccornis difformis
Boreonectes griseostriatus mitochondrial 16S rRNA	HF931541	85.35	100	1.13558e−40	Coleoptera	Dytiscidae	Boreonectes griseostriatus
Deronectes parvicollis mitochondrial 16S rRNA gene	HF931454	84.71	100	1.13558e−40	Coleoptera	Dytiscidae	Deronectes parvicollis
Oreodytes scitulus 16S ribosomal RNA gene, partial	KF575551	84.08	100	4.82863e−39	Coleoptera	Dytiscidae	Oreodytes scitulus
Oreodytes scitulus mitochondrial partial 16S rRNA	HF931515	84.08	100	4.82863e−39	Coleoptera	Dytiscidae	Oreodytes scitulus
Hydroporus foveolatus mitochondrial 16S rRNA gene	HE613724	84.81	100	5.88247e−38	Coleoptera	Dytiscidae	Hydroporus foveolatus
Laccornis kocae partial 16S rRNA gene	AJ850419	83.44	100	5.88247e−38	Coleoptera	Dytiscidae	Laccornis kocae
Nebrioporus formaster mitochondrial partial 16S rR	HF931377	83.54	100	2.05319e−37	Coleoptera	Dytiscidae	Nebrioporus formaster
Hydroporus tessellatus 16S ribosomal RNA gene, par	JX434770	84.18	100	7.16632e−37	Coleoptera	Dytiscidae	Hydroporus tessellatus
Boreonectes griseostriatus mitochondrial 16S rRNA	HF931385	83.44	100	7.16632e−37	Coleoptera	Dytiscidae	Boreonectes griseostriatus
Hydroporus zimmermanni mitochondrial 16S rRNA gene	HE613679	84.18	100	7.16632e−37	Coleoptera	Dytiscidae	Hydroporus zimmermanni
Hydroporus basinotatus mitochondrial 16S rRNA gene	HE613678	84.18	100	7.16632e−37	Coleoptera	Dytiscidae	Hydroporus basinotatus
Hydroporus basinotatus 16S ribosomal RNA gene, par	AF518258	84.18	100	7.16632e−37	Coleoptera	Dytiscidae	Hydroporus basinotatus
Oreodytes natrix partial 16S rRNA gene	AJ850349	82.8	100	2.50129e−36	Coleoptera	Dytiscidae	Oreodytes natrix
Stictotarsus maghrebinus 16S ribosomal RNA gene, p	AY250947	83.54	100	2.50129e−36	Coleoptera	Dytiscidae	Stictotarsus maghrebinus
Aethionectes fulvonotatus voucher KBMAeop406 16S r	KF979088	82.5	100	8.73037e−36	Coleoptera	Dytiscidae	Aethionectes fulvonotatus
Deronectes moestus mitochondrial 16S rRNA gene (pa	HE613726	85.52	91.72	8.73037e−36	Coleoptera	Dytiscidae	Deronectes moestus
Suphrodytes dorsalis 16S ribosomal RNA gene, parti	KF575577	82.91	100	3.0472e−35	Coleoptera	Dytiscidae	Suphrodytes dorsalis
Oreodytes quadrimaculatus 16S ribosomal RNA gene,	KF575567	82.17	100	3.0472e−35	Coleoptera	Dytiscidae	Oreodytes quadrimaculatus
Suphrodytes dorsalis voucher BMNH:725966 16S ribos	JX221574	82.91	100	3.0472e−35	Coleoptera	Dytiscidae	Suphrodytes dorsalis
Hydroporus analis mitochondrial 16S rRNA g	HE613731	83.54	100	3.0472e−35	Coleoptera	Dytiscidae	Hydroporus analis
Oreodytes quadrimaculatus partial 16S rRNA gene	AJ850351	82.17	100	3.0472e−35	Coleoptera	Dytiscidae	Oreodytes quadrimaculatus
Scarodytes sp. IR−2003 16S ribosomal RNA gene, par	AY250939	82.39	100	3.0472e−35	Coleoptera	Dytiscidae	Scarodytes sp. IR−2003
Stictotarsus procerus mitochondrial 16S rRNA gene	HF931379	82.91	100	1.06358e−34	Coleoptera	Dytiscidae	Stictotarsus procerus
Deronectes opatrinus mitochondrial 16S rRNA gene (	HE613701	82.91	100	1.06358e−34	Coleoptera	Dytiscidae	Deronectes opatrinus
Deronectes brannani mitochondrial 16S rRNA gene (p	HE613695	84.83	91.72	1.06358e−34	Coleoptera	Dytiscidae	Deronectes brannanii
Stictotarsus falli 16S ribosomal RNA (rrnL) gene,	EF670029	82.91	100	1.06358e−34	Coleoptera	Dytiscidae	Stictotarsus falli
Hydroporus marginatus 16S ribosomal RNA gene, part	AF518267	82.61	100	1.06358e−34	Coleoptera	Dytiscidae	Hydroporus marginatus
Hydroporus foveolatus mitochondrial 16S rRNA gene	HF931502	82.91	100	3.71225e−34	Coleoptera	Dytiscidae	Hydroporus foveolatus
Hydroporus discretus mitochondrial 16S rRNA gene (	HE613723	82.91	100	3.71225e−34	Coleoptera	Dytiscidae	Hydroporus discretus
Hydroporus decipiens mitochondrial 16S rRNA gene (	HE613722	82.91	100	3.71225e−34	Coleoptera	Dytiscidae	Hydroporus decipiens
Hydroporus pubescens mitochondrial 16S rRNA gene (	HE613707	82.91	100	3.71225e−34	Coleoptera	Dytiscidae	Hydroporus pubescens
Hydroporus limbatus mitochondrial 16S rRNA gene (p	HE613706	82.91	100	3.71225e−34	Coleoptera	Dytiscidae	Hydroporus limbatus
Hydroporus errans mitochondrial 16S rRNA gene (par	HE613704	82.91	100	3.71225e−34	Coleoptera	Dytiscidae	Hydroporus errans
Hydroporus pubescens voucher NHM−IR39 16S ribosoma	EF419327	82.91	100	3.71225e−34	Coleoptera	Dytiscidae	Hydroporus pubescens
Hydroporus glabriusculus 16S ribosomal RNA gene, p	AY365270	82.17	100	3.71225e−34	Coleoptera	Dytiscidae	Hydroporus glabriusculus
Hydroporus pubescens 16S ribosomal RNA gene, parti	AF518276	82.91	100	3.71225e−34	Coleoptera	Dytiscidae	Hydroporus pubescens
Hydroporus limbatus 16S ribosomal RNA gene, partia	AF518264	82.91	100	3.71225e−34	Coleoptera	Dytiscidae	Hydroporus limbatus
Hydroporus discretus 16S ribosomal RNA gene, parti	AF518260	82.91	100	3.71225e−34	Coleoptera	Dytiscidae	Hydroporus discretus
Hydroporus analis 16S ribosomal RNA gene, partial	AF518257	82.91	100	3.71225e−34	Coleoptera	Dytiscidae	Hydroporus analis
Hydaticus cinctipennis voucher UNM KBMHyci20 16S r	KJ548338	81.53	100	1.2957e−33	Coleoptera	Dytiscidae	Hydaticus cinctipennis
Deronectes moestus 16S ribosomal RNA gene, partial	KF575561	83.33	91.72	1.2957e−33	Coleoptera	Dytiscidae	Deronectes moestus
Hydroporus fuscipennis mitochondrial 16S rRNA gene	HE613730	83.02	100	1.2957e−33	Coleoptera	Dytiscidae	Hydroporus fuscipennis
Deronectes doriae mitochondrial 16S rRNA gene (par	HE613710	82.28	100	1.2957e−33	Coleoptera	Dytiscidae	Deronectes doriae
Hydroporus planus mitochondrial 16S rRNA gene (par	HE613708	82.39	100	1.2957e−33	Coleoptera	Dytiscidae	Hydroporus planus
Deronectes bicostatus mitochondrial 16S rRNA gene	HE613705	81.53	100	1.2957e−33	Coleoptera	Dytiscidae	Deronectes bicostatus
Deronectes moestus mitochondrial 16S rRNA gene (pa	HE613683	83.33	91.72	1.2957e−33	Coleoptera	Dytiscidae	Deronectes moestus
Deronectes sahlbergi mitochondrial 16S rRNA gene (	HE613680	82.28	100	1.2957e−33	Coleoptera	Dytiscidae	Deronectes sahlbergi
Copelatus sp. BI−2011 voucher BMNH792945 16S ribos	HQ381441	81.53	100	1.2957e−33	Coleoptera	Dytiscidae	Copelatus sp. BI−2011
Hydroporus planus 16S ribosomal RNA gene, partial	AF518275	82.39	100	1.2957e−33	Coleoptera	Dytiscidae	Hydroporus planus
Hydroporus fuscipennis 16S ribosomal RNA gene, par	AF518262	83.02	100	1.2957e−33	Coleoptera	Dytiscidae	Hydroporus fuscipennis
Hygrotus novemlineatus mitochondrial partial 16S r	HF931523	81.53	100	4.52244e−33	Coleoptera	Dytiscidae	Hygrotus novemlineatus
Hygrotus marklini mitochondrial 16S rRNA gene (par	HF931467	81.53	100	4.52244e−33	Coleoptera	Dytiscidae	Hygrotus marklini
Hydroporus brancoi gredensis mitochondrial partial	HF931329	81.53	100	4.52244e−33	Coleoptera	Dytiscidae	Hydroporus brancoi gredensis
Suphrodytes figuratus voucher BMNH:722148 16S ribo	JX221571	81.53	100	4.52244e−33	Coleoptera	Dytiscidae	Suphrodytes figuratus
Hydroporus marginatus mitochondrial 16S rRNA gene	HE613699	81.99	100	4.52244e−33	Coleoptera	Dytiscidae	Hydroporus marginatus
Hovahydrus sp. BI−2011 voucher BMNH831333 16S ribo	HQ381524	84.93	91.72	4.52244e−33	Coleoptera	Dytiscidae	Hovahydrus sp. BI−2011
Hygrotus turbidus partial 16S rRNA gene	AJ850390	81.53	100	4.52244e−33	Coleoptera	Dytiscidae	Hygrotus turbidus
Hydroporus sp. IR33.4 16S ribosomal RNA gene, part	AY365261	81.53	100	4.52244e−33	Coleoptera	Dytiscidae	Hydroporus sp. IR33.4
Scarodytes halensis haplotype 3 16S ribosomal RNA	AY250937	81.65	100	4.52244e−33	Coleoptera	Dytiscidae	Scarodytes halensis
Hydroporus discretus mitochondrial 16S rRNA gene (	HF931449	82.28	100	1.57849e−32	Coleoptera	Dytiscidae	Hydroporus discretus
Hydroporus thracicus mitochondrial 16S rRNA gene (	HF931413	82.28	100	1.57849e−32	Coleoptera	Dytiscidae	Hydroporus thracicus
Boreonectes funereus mitochondrial 16S rRNA gene (	HF931393	81.53	100	1.57849e−32	Coleoptera	Dytiscidae	Boreonectes funereus
Hydroporus macedonicus mitochondrial 16S rRNA gene	HE613690	82.28	100	1.57849e−32	Coleoptera	Dytiscidae	Hydroporus macedonicus
Hydroporus compunctus mitochondrial 16S rRNA gene	HE599693	82.28	100	1.57849e−32	Coleoptera	Dytiscidae	Hydroporus compunctus
Copelatus sp. BI−2011 voucher BMNH671215 16S ribos	HQ381377	80.89	100	1.57849e−32	Coleoptera	Dytiscidae	Copelatus sp. BI−2011
Antiporus hollingsworthi mitochondrial partial 16S	FR727246	81.29	98.73	1.57849e−32	Coleoptera	Dytiscidae	Antiporus hollingsworthi
Antiporus sp. MB1421 mitochondrial partial 16S rRN	FR727245	80.89	100	1.57849e−32	Coleoptera	Dytiscidae	Antiporus sp. MB1421
Hydroporus macedonicus 16S ribosomal RNA gene, par	DQ195529	82.28	100	1.57849e−32	Coleoptera	Dytiscidae	Hydroporus macedonicus
Amphizoa insolens 16S ribosomal RNA gene, partial	AY071770	80.89	100	1.57849e−32	Coleoptera	Amphizoidae	Amphizoa insolens
Nebrioporus assimilis 16S ribosomal RNA gene, part	AY250921	81.29	98.73	1.57849e−32	Coleoptera	Dytiscidae	Nebrioporus assimilis
Nebrioporus airumlus 16S ribosomal RNA gene, parti	AY250920	80.89	100	1.57849e−32	Coleoptera	Dytiscidae	Nebrioporus airumlus
Hydroporus rufifrons 16S ribosomal RNA gene, parti	AF518277	82.28	100	1.57849e−32	Coleoptera	Dytiscidae	Hydroporus rufifrons
Hydroporus compunctus 16S ribosomal RNA gene, part	AF518259	82.28	100	1.57849e−32	Coleoptera	Dytiscidae	Hydroporus compunctus
Deronectes aubei voucher UNM KBMDeau758 16S riboso	KJ548292	81.65	100	5.50946e−32	Coleoptera	Dytiscidae	Deronectes aubei
Hygrotus armeniacus mitochondrial partial 16S rRNA	HF931520	82.39	100	5.50946e−32	Coleoptera	Dytiscidae	Hygrotus armeniacus
Deronectes costipennis mitochondrial 16S rRNA gene	HE613697	81.65	100	5.50946e−32	Coleoptera	Dytiscidae	Deronectes costipennis
Deronectes platynotus mitochondrial 16S rRNA gene	HE613682	81.65	100	5.50946e−32	Coleoptera	Dytiscidae	Deronectes platynotus
Deronectes depressicollis mitochondrial 16S rRNA g	HE613681	81.65	100	5.50946e−32	Coleoptera	Dytiscidae	Deronectes depressicollis
Deronectes semirufus mitochondrial 16S rRNA gene (	HE613676	81.65	100	5.50946e−32	Coleoptera	Dytiscidae	Deronectes semirufus
Deronectes semirufus mitochondrial 16S rRNA gene (	HE613674	81.65	100	5.50946e−32	Coleoptera	Dytiscidae	Deronectes semirufus
Antiporus hollingsworthi mitochondrial partial 16S	FR727247	80.65	98.73	5.50946e−32	Coleoptera	Dytiscidae	Antiporus hollingsworthi
