# Supplementary material for: Testing the potential of a ribosomal 16S marker for DNA metabarcoding of insects
Source: PeerJ. 2016 Apr 19;4:e1966. doi: 10.7717/peerj.1966 (PMC4841222; doi:10.7717/peerj.1966)
Supplement: Supplemental Information 1 [file peerj-04-1966-s006.zip › scripts/5 match OTUs/blast_hits/OTU_539.csv_plot.pdf]

OTU_539.csv

Oreodytes sanmarkii

Dytiscidae

Coleoptera

98.09

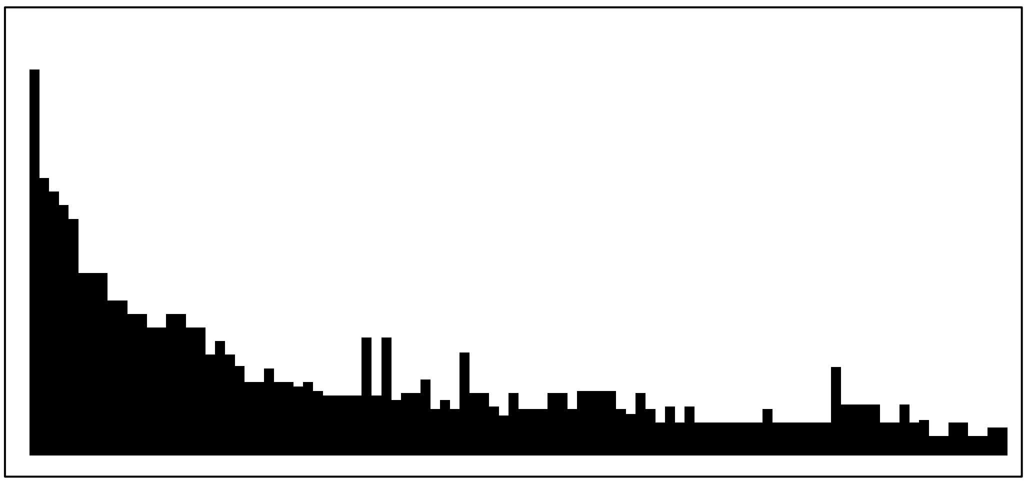

Oreodytes sanmarkii mitochondrial 16S rRNA gene (p	HF931524	98.09	100	6.70005e−69	Coleoptera	Dytiscidae	Oreodytes sanmarkii
Oreodytes obesus cordillerensis mitochondrial part	HF931516	92.99	100	4.82437e−58	Coleoptera	Dytiscidae	Oreodytes obesus cordillerensis
Oreodytes congruus partial 16S rRNA gene	AJ850347	92.36	100	5.87728e−57	Coleoptera	Dytiscidae	Oreodytes congruus
Oreodytes congruus 16S ribosomal RNA gene, partial	KF575550	91.72	100	2.49908e−55	Coleoptera	Dytiscidae	Oreodytes congruus
Oreodytes crassulus partial 16S rRNA gene	AJ850348	91.08	100	3.04451e−54	Coleoptera	Dytiscidae	Oreodytes crassulus
Oreodytes mongolicus mitochondrial 16S rRNA gene (	HF931491	88.54	100	8.16955e−49	Coleoptera	Dytiscidae	Oreodytes mongolicus
Oreodytes abbreviatus mitochondrial 16S rRNA gene	HF931477	88.54	100	8.16955e−49	Coleoptera	Dytiscidae	Oreodytes abbreviatus
Oreodytes rhyacophilus partial 16S rRNA gene	AJ850352	88.54	100	8.16955e−49	Coleoptera	Dytiscidae	Oreodytes rhyacophilus
Boreonectes riberae mitochondrial 16S rRNA gene (p	HF931461	87.26	100	5.15555e−45	Coleoptera	Dytiscidae	Boreonectes riberae
Boreonectes multilineatus mitochondrial partial 16	HF931384	87.26	100	5.15555e−45	Coleoptera	Dytiscidae	Boreonectes multilineatus
Oreodytes snoqualmie mitochondrial partial 16S rRN	HF931519	86.62	100	1.79946e−44	Coleoptera	Dytiscidae	Oreodytes snoqualmie
Boreonectes alpestris mitochondrial 16S rRNA gene	HF931512	86.62	100	2.19219e−43	Coleoptera	Dytiscidae	Boreonectes alpestris
Oreodytes septentrionalis mitochondrial 16S rRNA g	HF931492	85.99	100	2.19219e−43	Coleoptera	Dytiscidae	Oreodytes septentrionalis
Oreodytes shorti mitochondrial 16S rRNA gene (part	HF931488	85.99	100	2.19219e−43	Coleoptera	Dytiscidae	Oreodytes shorti
Boreonectes macedonicus mitochondrial 16S rRNA gen	HF931359	86.62	100	2.19219e−43	Coleoptera	Dytiscidae	Boreonectes macedonicus
Stictotarsus ibericus 16S ribosomal RNA (rrnL) gen	EF670030	86.62	100	2.19219e−43	Coleoptera	Dytiscidae	Stictotarsus ibericus
Boreonectes griseostriatus mitochondrial 16S rRNA	HF931541	85.99	100	2.67064e−42	Coleoptera	Dytiscidae	Boreonectes griseostriatus
Boreonectes griseostriatus mitochondrial partial 1	HF931387	85.99	100	2.67064e−42	Coleoptera	Dytiscidae	Boreonectes griseostriatus
Laccornis difformis 16S ribosomal RNA gene, partia	KF575544	84.71	100	1.13558e−40	Coleoptera	Dytiscidae	Laccornis difformis
Boreonectes striatellus mitochondrial 16S rRNA gen	HF931511	85.35	100	1.13558e−40	Coleoptera	Dytiscidae	Boreonectes striatellus
Deronectes parvicollis mitochondrial 16S rRNA gene	HF931454	84.71	100	1.13558e−40	Coleoptera	Dytiscidae	Deronectes parvicollis
Nebrioporus formaster mitochondrial partial 16S rR	HF931377	84.18	100	1.68536e−38	Coleoptera	Dytiscidae	Nebrioporus formaster
Oreodytes scitulus 16S ribosomal RNA gene, partial	KF575551	83.44	100	5.88247e−38	Coleoptera	Dytiscidae	Oreodytes scitulus
Oreodytes scitulus mitochondrial partial 16S rRNA	HF931515	83.44	100	5.88247e−38	Coleoptera	Dytiscidae	Oreodytes scitulus
Boreonectes griseostriatus mitochondrial 16S rRNA	HF931385	84.08	100	5.88247e−38	Coleoptera	Dytiscidae	Boreonectes griseostriatus
Laccornis kocae partial 16S rRNA gene	AJ850419	83.44	100	5.88247e−38	Coleoptera	Dytiscidae	Laccornis kocae
Stictotarsus roffii 16S ribosomal RNA gene, partia	KF575555	83.44	100	7.16632e−37	Coleoptera	Dytiscidae	Stictotarsus roffii
Antiporus hollingsworthi mitochondrial partial 16S	FR727246	83.23	98.73	7.16632e−37	Coleoptera	Dytiscidae	Antiporus hollingsworthi
Stictotarsus roffi partial 16S rRNA gene	AJ850355	83.44	100	7.16632e−37	Coleoptera	Dytiscidae	Stictotarsus roffii
Scarodytes sp. IR−2003 16S ribosomal RNA gene, par	AY250939	83.02	100	7.16632e−37	Coleoptera	Dytiscidae	Scarodytes sp. IR−2003
Oreodytes quadrimaculatus 16S ribosomal RNA gene,	KF575567	82.8	100	2.50129e−36	Coleoptera	Dytiscidae	Oreodytes quadrimaculatus
Antiporus sp. MB1421 mitochondrial partial 16S rRN	FR727245	82.8	100	2.50129e−36	Coleoptera	Dytiscidae	Antiporus sp. MB1421
Oreodytes quadrimaculatus partial 16S rRNA gene	AJ850351	82.8	100	2.50129e−36	Coleoptera	Dytiscidae	Oreodytes quadrimaculatus
Oreodytes natrix partial 16S rRNA gene	AJ850349	82.8	100	2.50129e−36	Coleoptera	Dytiscidae	Oreodytes natrix
Deronectes moestus 16S ribosomal RNA gene, partial	KF575561	85.52	91.72	8.73037e−36	Coleoptera	Dytiscidae	Deronectes moestus
Hygrotus novemlineatus mitochondrial partial 16S r	HF931523	82.8	100	8.73037e−36	Coleoptera	Dytiscidae	Hygrotus novemlineatus
Deronectes moestus mitochondrial 16S rRNA gene (pa	HE613683	85.52	91.72	8.73037e−36	Coleoptera	Dytiscidae	Deronectes moestus
Antiporus hollingsworthi mitochondrial partial 16S	FR727247	82.58	98.73	8.73037e−36	Coleoptera	Dytiscidae	Antiporus hollingsworthi
Suphrodytes dorsalis 16S ribosomal RNA gene, parti	KF575577	82.91	100	3.0472e−35	Coleoptera	Dytiscidae	Suphrodytes dorsalis
Suphrodytes dorsalis voucher BMNH:725966 16S ribos	JX221574	82.91	100	3.0472e−35	Coleoptera	Dytiscidae	Suphrodytes dorsalis
Hydroporus foveolatus mitochondrial 16S rRNA gene	HE613724	83.54	100	3.0472e−35	Coleoptera	Dytiscidae	Hydroporus foveolatus
Antiporus interrogationis mitochondrial partial 16	FR727252	82.17	100	3.0472e−35	Coleoptera	Dytiscidae	Antiporus interrogationis
Nebrioporus assimilis 16S ribosomal RNA gene, part	AY250921	82.58	98.73	3.0472e−35	Coleoptera	Dytiscidae	Nebrioporus assimilis
Nebrioporus airumlus 16S ribosomal RNA gene, parti	AY250920	82.17	100	3.0472e−35	Coleoptera	Dytiscidae	Nebrioporus airumlus
Deronectes moestus mitochondrial 16S rRNA gene (pa	HE613726	84.83	91.72	1.06358e−34	Coleoptera	Dytiscidae	Deronectes moestus
Stictotarsus falli 16S ribosomal RNA (rrnL) gene,	EF670029	82.91	100	1.06358e−34	Coleoptera	Dytiscidae	Stictotarsus falli
Stictotarsus maghrebinus 16S ribosomal RNA gene, p	AY250947	82.91	100	1.06358e−34	Coleoptera	Dytiscidae	Stictotarsus maghrebinus
Scarodytes halensis haplotype 3 16S ribosomal RNA	AY250937	82.28	100	1.06358e−34	Coleoptera	Dytiscidae	Scarodytes halensis
Aethionectes fulvonotatus voucher KBMAeop406 16S r	KF979088	81.88	100	3.71225e−34	Coleoptera	Dytiscidae	Aethionectes fulvonotatus
Hydroporus tessellatus 16S ribosomal RNA gene, par	JX434770	82.91	100	3.71225e−34	Coleoptera	Dytiscidae	Hydroporus tessellatus
Boreonectes funereus mitochondrial 16S rRNA gene (	HF931393	82.17	100	3.71225e−34	Coleoptera	Dytiscidae	Boreonectes funereus
Suphrodytes figuratus voucher BMNH:722150 16S ribo	JX221572	82.17	100	3.71225e−34	Coleoptera	Dytiscidae	Suphrodytes figuratus
Hydroporus marginatus mitochondrial 16S rRNA gene	HE613699	82.17	100	3.71225e−34	Coleoptera	Dytiscidae	Hydroporus marginatus
Hydroporus zimmermanni mitochondrial 16S rRNA gene	HE613679	82.91	100	3.71225e−34	Coleoptera	Dytiscidae	Hydroporus zimmermanni
Hydroporus basinotatus mitochondrial 16S rRNA gene	HE613678	82.91	100	3.71225e−34	Coleoptera	Dytiscidae	Hydroporus basinotatus
Suphrodytes dorsalis mitochondrial 16S rRNA gene (	HE599700	82.17	100	3.71225e−34	Coleoptera	Dytiscidae	Suphrodytes dorsalis
Pseudomigadops n. sp. MSL2007 voucher 668381 16S r	EF214073	83.02	100	3.71225e−34	Coleoptera	Carabidae	Pseudomigadops n. sp. MSL2007
Pseudomigadops n. sp. MSL2007 voucher 668379 16S r	EF214072	83.02	100	3.71225e−34	Coleoptera	Carabidae	Pseudomigadops n. sp. MSL2007
Pseudomigadops n. sp. MSL2007 voucher 668300 16S r	EF214010	83.02	100	3.71225e−34	Coleoptera	Carabidae	Pseudomigadops n. sp. MSL2007
Pseudomigadops sp. PSM1 mitochondrial 16S rRNA gen	FM994738	83.02	100	3.71225e−34	Coleoptera	Carabidae	Pseudomigadops sp. PSM1
Hydroporus glabriusculus 16S ribosomal RNA gene, p	AY365270	82.17	100	3.71225e−34	Coleoptera	Dytiscidae	Hydroporus glabriusculus
Nebrioporus croceus 16S ribosomal RNA gene, partia	AY250925	81.94	98.73	3.71225e−34	Coleoptera	Dytiscidae	Nebrioporus croceus
Hydroporus basinotatus 16S ribosomal RNA gene, par	AF518258	82.91	100	3.71225e−34	Coleoptera	Dytiscidae	Hydroporus basinotatus
Hydroporus marginatus 16S ribosomal RNA gene, part	AF518267	82.17	100	3.71225e−34	Coleoptera	Dytiscidae	Hydroporus marginatus
Nebrioporus rotundatus 16S ribosomal RNA gene, par	KF575570	81.53	100	1.2957e−33	Coleoptera	Dytiscidae	Nebrioporus rotundatus
Stictotarsus procerus mitochondrial 16S rRNA gene	HF931379	82.28	100	1.2957e−33	Coleoptera	Dytiscidae	Stictotarsus procerus
Nebrioporus rotundatus mitochondrial partial 16S r	HF931328	81.53	100	1.2957e−33	Coleoptera	Dytiscidae	Nebrioporus rotundatus
Deronectes opatrinus mitochondrial 16S rRNA gene (	HE613701	82.28	100	1.2957e−33	Coleoptera	Dytiscidae	Deronectes opatrinus
Copelatus sp. BI−2011 voucher BMNH792945 16S ribos	HQ381441	81.53	100	1.2957e−33	Coleoptera	Dytiscidae	Copelatus sp. BI−2011
Antiporus gilbertii mitochondrial partial 16S rRNA	FR727263	81.53	100	1.2957e−33	Coleoptera	Dytiscidae	Antiporus gilbertii
Antiporus gilbertii mitochondrial partial 16S rRNA	FR727262	81.53	100	1.2957e−33	Coleoptera	Dytiscidae	Antiporus gilbertii
Antiporus femoralis mitochondrial partial 16S rRNA	FR727260	81.53	100	1.2957e−33	Coleoptera	Dytiscidae	Antiporus femoralis
Antiporus interrogationis mitochondrial partial 16	FR727250	81.53	100	1.2957e−33	Coleoptera	Dytiscidae	Antiporus interrogationis
Antiporus femoralis partial 16S rRNA gene	AJ850309	81.53	100	1.2957e−33	Coleoptera	Dytiscidae	Antiporus femoralis
Antiporus gilbertii isolate ABTC78750−1 16S riboso	EU616925	81.53	100	1.2957e−33	Coleoptera	Dytiscidae	Antiporus gilbertii
Hydrocolus paugus 16S ribosomal RNA gene, partial	KF575539	82.17	100	4.52244e−33	Coleoptera	Dytiscidae	Hydrocolus paugus
Hygrotus lutescens mitochondrial partial 16S rRNA	HF931518	81.53	100	4.52244e−33	Coleoptera	Dytiscidae	Hygrotus lutescens
Hygrotus tumidiventris mitochondrial 16S rRNA gene	HF931496	81.53	100	4.52244e−33	Coleoptera	Dytiscidae	Hygrotus tumidiventris
Hygrotus fontinalis mitochondrial 16S rRNA gene (p	HF931494	81.53	100	4.52244e−33	Coleoptera	Dytiscidae	Hygrotus fontinalis
Hygrotus marklini mitochondrial 16S rRNA gene (par	HF931467	81.53	100	4.52244e−33	Coleoptera	Dytiscidae	Hygrotus marklini
Hygrotus fresnedai mitochondrial 16S rRNA gene (pa	HF931325	81.53	100	4.52244e−33	Coleoptera	Dytiscidae	Hygrotus fresnedai
Suphrodytes figuratus voucher BMNH:722148 16S ribo	JX221571	81.53	100	4.52244e−33	Coleoptera	Dytiscidae	Suphrodytes figuratus
Deronectes brannani mitochondrial 16S rRNA gene (p	HE613695	84.14	91.72	4.52244e−33	Coleoptera	Dytiscidae	Deronectes brannanii
Pseudomigadops n. sp. MSL2007 voucher 668338 16S r	EF214034	82.39	100	4.52244e−33	Coleoptera	Carabidae	Pseudomigadops n. sp. MSL2007
Pseudomigadops n. sp. MSL2007 voucher 668317 16S r	EF214022	82.39	100	4.52244e−33	Coleoptera	Carabidae	Pseudomigadops n. sp. MSL2007
Pseudomigadops n. sp. MSL2007 voucher 668302 16S r	EF214012	82.39	100	4.52244e−33	Coleoptera	Carabidae	Pseudomigadops n. sp. MSL2007
Pseudomigadops n. sp. MSL2007 voucher 668299 16S r	EF214009	82.39	100	4.52244e−33	Coleoptera	Carabidae	Pseudomigadops n. sp. MSL2007
Hygrotus turbidus partial 16S rRNA gene	AJ850390	81.53	100	4.52244e−33	Coleoptera	Dytiscidae	Hygrotus turbidus
Hygrotus saginatus partial 16S rRNA gene	AJ850389	81.53	100	4.52244e−33	Coleoptera	Dytiscidae	Hygrotus saginatus
Pseudomigadops n. sp. MSL2007 voucher 668369 16S r	EF214062	82.39	100	4.52244e−33	Coleoptera	Carabidae	Pseudomigadops n. sp. MSL2007
Hydroporus sp. IR33.4 16S ribosomal RNA gene, part	AY365261	81.53	100	4.52244e−33	Coleoptera	Dytiscidae	Hydroporus sp. IR33.4
Nebrioporus nipponicus 16S ribosomal RNA gene, par	AY250931	81.65	100	4.52244e−33	Coleoptera	Dytiscidae	Nebrioporus nipponicus
Ilybius fraterculus mitochondrial 16S rRNA gene (p	LM654800	80.89	100	1.57849e−32	Coleoptera	Dytiscidae	Ilybius fraterculus
Ilybius ater mitochondrial 16S rRNA gene (partial)	LM654794	80.89	100	1.57849e−32	Coleoptera	Dytiscidae	Ilybius ater
Agabus didymus mitochondrial 16S rRNA gene (partia	LM654773	81.53	100	1.57849e−32	Coleoptera	Dytiscidae	Agabus didymus
Agabus didymus voucher UNM KBMPmsp730 16S ribosoma	KJ548392	81.53	100	1.57849e−32	Coleoptera	Dytiscidae	Agabus didymus
Ilybius fraterculus voucher UNM KBMIlfr409 16S rib	KJ548342	80.89	100	1.57849e−32	Coleoptera	Dytiscidae	Ilybius fraterculus
Hydaticus cinctipennis voucher UNM KBMHyci20 16S r	KJ548338	80.89	100	1.57849e−32	Coleoptera	Dytiscidae	Hydaticus cinctipennis
Antiporus blakeii 16S ribosomal RNA gene, partial	KF575566	81.29	98.73	1.57849e−32	Coleoptera	Dytiscidae	Antiporus blakeii
Antiporus femoralis 16S ribosomal RNA gene, partia	KF575532	81.29	98.73	1.57849e−32	Coleoptera	Dytiscidae	Antiporus femoralis
