# Supplementary material for: Testing the potential of a ribosomal 16S marker for DNA metabarcoding of insects
Source: PeerJ. 2016 Apr 19;4:e1966. doi: 10.7717/peerj.1966 (PMC4841222; doi:10.7717/peerj.1966)
Supplement: Supplemental Information 1 [file peerj-04-1966-s006.zip › scripts/5 match OTUs/blast_hits/OTU_542.csv_plot.pdf]

OTU_542.csv

Nemurella pictetii

Nemouridae

Plecoptera

98.09

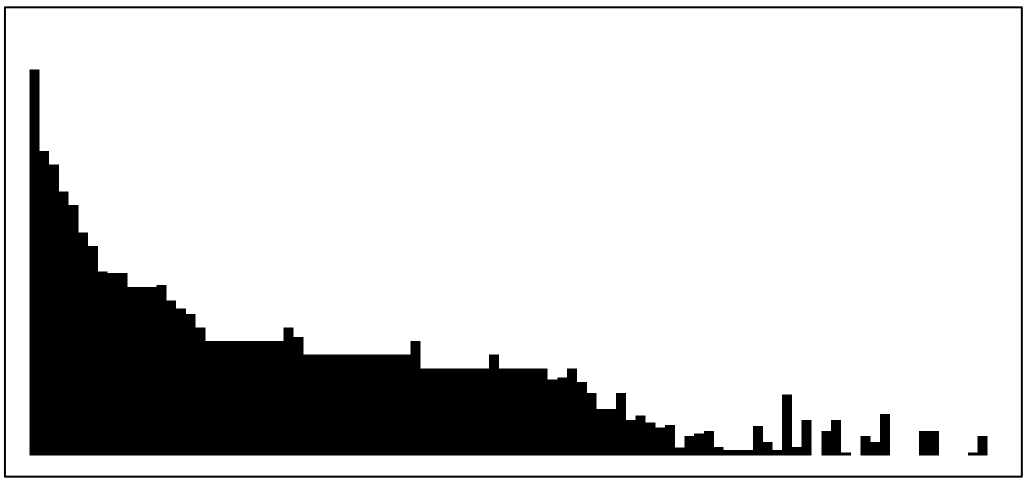

Nemurella pictetii 16S ribosomal RNA gene, partial	EF623192	98.09	100	2.77888e−67	Plecoptera	Nemouridae	Nemurella pictetii
Ostrocerca foersteri 16S ribosomal RNA gene, parti	EF623191	94.27	100	3.8627e−59	Plecoptera	Nemouridae	Ostrocerca foersteri
Visoka cataractae 16S ribosomal RNA gene, partial	EF623189	93.63	100	1.64246e−57	Plecoptera	Nemouridae	Visoka cataractae
Nanonemoura wahkeena 16S ribosomal RNA gene, parti	EF623198	92.36	100	8.50817e−55	Plecoptera	Nemouridae	Nanonemoura wahkeena
Shipsa rotunda 16S ribosomal RNA gene, partial seq	EF623184	91.72	100	1.03651e−53	Plecoptera	Nemouridae	Shipsa rotunda
Paranemoura claasseni 16S ribosomal RNA gene, part	EF623185	90.45	100	5.36924e−51	Plecoptera	Nemouridae	Paranemoura claasseni
Nemoura trispinosa 16S ribosomal RNA gene, partial	EF623187	89.81	100	2.28306e−49	Plecoptera	Nemouridae	Nemoura trispinosa
Nemoura cinerea 16S ribosomal RNA gene, partial se	EF623194	88.61	100	9.70781e−48	Plecoptera	Nemouridae	Nemoura cinerea
Lednia tumana 16S ribosomal RNA gene, partial sequ	EF623197	88.54	100	1.18265e−46	Plecoptera	Nemouridae	Lednia tumana
Soyedina producta 16S ribosomal RNA gene, partial	EF623196	88.54	100	1.44077e−45	Plecoptera	Nemouridae	Soyedina producta
Protonemura meyeri 16S ribosomal RNA gene, partial	EF623193	87.9	100	1.44077e−45	Plecoptera	Nemouridae	Protonemura meyeri
Prostoia besametsa 16S ribosomal RNA gene, partial	EF623188	87.9	100	1.44077e−45	Plecoptera	Nemouridae	Prostoia besametsa
Malenka californica 16S ribosomal RNA gene, partia	EF623182	87.9	100	1.44077e−45	Plecoptera	Nemouridae	Malenka californica
Scopura montana 16S ribosomal RNA gene, partial se	EF623265	87.97	100	5.02877e−45	Plecoptera		Scopura montana
Zapada cinctipes 16S ribosomal RNA gene, partial s	EF623183	87.26	100	6.1263e−44	Plecoptera	Nemouridae	Zapada cinctipes
Oemopteryx vanduzeea 16S ribosomal RNA gene, parti	EF623266	86.88	100	7.46336e−43	Plecoptera	Taeniopterygidae	Oemopteryx vanduzeea
Pteronarcys californica 16S ribosomal RNA gene, pa	EF623261	86.62	100	9.09223e−42	Plecoptera	Pteronarcyidae	Pteronarcys californica
Apteroperla tikumana mitochondrion, complete genom	KR604721	85.99	100	3.1735e−41	Plecoptera	Capniidae	Apteroperla tikumana
Mesocapnia arizonensis mitochondrion, partial geno	KP642637	85.35	100	3.86611e−40	Plecoptera	Capniidae	Mesocapnia arizonensis
Allocapnia recta isolate H13 16S ribosomal RNA gen	KC881048	85.35	100	3.86611e−40	Plecoptera	Capniidae	Allocapnia recta
Allocapnia recta isolate H11 16S ribosomal RNA gen	KC881046	85.35	100	3.86611e−40	Plecoptera	Capniidae	Allocapnia recta
Allocapnia recta isolate H7 16S ribosomal RNA gene	KC881042	85.35	100	3.86611e−40	Plecoptera	Capniidae	Allocapnia recta
Allocapnia recta isolate H3 16S ribosomal RNA gene	KC881038	85.35	100	3.86611e−40	Plecoptera	Capniidae	Allocapnia recta
Allocapnia minima 16S ribosomal RNA gene, partial	EF623130	85.35	100	3.86611e−40	Plecoptera	Capniidae	Allocapnia minima
Mesocapnia sp. BYU_PL010 16S ribosomal RNA gene, p	EF623127	85.35	100	3.86611e−40	Plecoptera	Capniidae	Mesocapnia sp. BYU_PL010
Allocapnia recta haplotype 3ar: H03 16S ribosomal	DQ915181	85.35	100	3.86611e−40	Plecoptera	Capniidae	Allocapnia recta
Pteronarcys princeps mitochondrion, complete genom	AY687866	85.99	100	3.86611e−40	Plecoptera	Pteronarcyidae	Pteronarcys princeps
Doddsia occidentalis 16S ribosomal RNA gene, parti	EF623270	85.53	100	4.70989e−39	Plecoptera	Taeniopterygidae	Doddsia occidentalis
Allocapnia recta isolate H19 16S ribosomal RNA gen	KC881054	84.71	100	1.64391e−38	Plecoptera	Capniidae	Allocapnia recta
Allocapnia recta isolate H16 16S ribosomal RNA gen	KC881051	84.71	100	1.64391e−38	Plecoptera	Capniidae	Allocapnia recta
Allocapnia recta isolate H15 16S ribosomal RNA gen	KC881050	84.71	100	1.64391e−38	Plecoptera	Capniidae	Allocapnia recta
Allocapnia recta isolate H14 16S ribosomal RNA gen	KC881049	84.71	100	1.64391e−38	Plecoptera	Capniidae	Allocapnia recta
Allocapnia recta isolate H10 16S ribosomal RNA gen	KC881045	84.71	100	1.64391e−38	Plecoptera	Capniidae	Allocapnia recta
Allocapnia recta isolate H6 16S ribosomal RNA gene	KC881041	84.71	100	1.64391e−38	Plecoptera	Capniidae	Allocapnia recta
Allocapnia recta isolate H1 16S ribosomal RNA gene	KC881036	84.71	100	1.64391e−38	Plecoptera	Capniidae	Allocapnia recta
Allocapnia recta haplotype 12ar: H12 16S ribosomal	EU306875	84.71	100	1.64391e−38	Plecoptera	Capniidae	Allocapnia recta
Allocapnia recta haplotype 11ar: H011 16S ribosoma	EU306874	84.71	100	1.64391e−38	Plecoptera	Capniidae	Allocapnia recta
Allocapnia recta haplotype 7ar: H07 16S ribosomal	EU306870	84.71	100	1.64391e−38	Plecoptera	Capniidae	Allocapnia recta
Allocapnia recta haplotype 2ar: H02 16S ribosomal	DQ915180	84.71	100	1.64391e−38	Plecoptera	Capniidae	Allocapnia recta
Osmylus sp. EH−2004 16S ribosomal RNA gene, partia	AY620146	85.35	100	5.73782e−38	Neuroptera	Osmylidae	Osmylus sp. EH−2004
Allocapnia recta isolate H18 16S ribosomal RNA gen	KC881053	84.08	100	2.0027e−37	Plecoptera	Capniidae	Allocapnia recta
Allocapnia recta isolate H17 16S ribosomal RNA gen	KC881052	84.08	100	2.0027e−37	Plecoptera	Capniidae	Allocapnia recta
Allocapnia recta isolate H12 16S ribosomal RNA gen	KC881047	84.08	100	2.0027e−37	Plecoptera	Capniidae	Allocapnia recta
Allocapnia recta isolate H9 16S ribosomal RNA gene	KC881044	84.08	100	2.0027e−37	Plecoptera	Capniidae	Allocapnia recta
Allocapnia recta isolate H8 16S ribosomal RNA gene	KC881043	84.08	100	2.0027e−37	Plecoptera	Capniidae	Allocapnia recta
Allocapnia recta isolate H5 16S ribosomal RNA gene	KC881040	84.08	100	2.0027e−37	Plecoptera	Capniidae	Allocapnia recta
Allocapnia recta isolate H4 16S ribosomal RNA gene	KC881039	84.08	100	2.0027e−37	Plecoptera	Capniidae	Allocapnia recta
Pteronarcys sachalina 16S ribosomal RNA gene, part	EF623264	84.71	100	2.0027e−37	Plecoptera	Pteronarcyidae	Pteronarcys sachalina
Allocapnia recta haplotype 10ar: H010 16S ribosoma	EU306873	84.08	100	2.0027e−37	Plecoptera	Capniidae	Allocapnia recta
Allocapnia recta haplotype 9ar: H09 16S ribosomal	EU306872	84.08	100	2.0027e−37	Plecoptera	Capniidae	Allocapnia recta
Allocapnia recta haplotype 8ar: H08 16S ribosomal	EU306871	84.08	100	2.0027e−37	Plecoptera	Capniidae	Allocapnia recta
Allocapnia recta haplotype 1ar: H01 16S ribosomal	DQ915179	84.08	100	2.0027e−37	Plecoptera	Capniidae	Allocapnia recta
Capnura wanica 16S ribosomal RNA gene, partial seq	EF623128	84.08	100	2.43978e−36	Plecoptera	Capniidae	Capnura wanica
Isocapnia hyalita 16S ribosomal RNA gene, partial	EF623131	83.54	100	8.51568e−36	Plecoptera	Capniidae	Isocapnia hyalita
Brachyptera seticornis 16S ribosomal RNA gene, par	EF623268	83.65	100	2.97227e−35	Plecoptera	Taeniopterygidae	Brachyptera seticornis
Moselia infuscata 16S ribosomal RNA gene, partial	EF623175	84.08	99.35	2.97227e−35	Plecoptera	Leuctridae	Moselia infuscata
Pteronarcys scotti 16S ribosomal RNA gene, partial	EF623263	83.44	100	1.03742e−34	Plecoptera	Pteronarcyidae	Pteronarcys scotti
Bolotoperla rossi 16S ribosomal RNA gene, partial	EF623269	82.91	100	3.62096e−34	Plecoptera	Taeniopterygidae	Bolotoperla rossi
Austrocerca rieka 16S ribosomal RNA gene, partial	EF623205	82.17	100	4.41123e−33	Plecoptera	Notonemouridae	Austrocerca rieka
Austrocercoides zwicki 16S ribosomal RNA gene, par	EF623199	82.17	100	4.41123e−33	Plecoptera	Notonemouridae	Austrocercoides zwicki
Triznaka pintada 16S ribosomal RNA gene, partial s	EF623134	82.91	100	4.41123e−33	Plecoptera	Chloroperlidae	Triznaka pintada
Megaleuctra kincaidi 16S ribosomal RNA gene, parti	EF623174	81.65	100	1.53967e−32	Plecoptera	Leuctridae	Megaleuctra kincaidi
Thaumatoperla sp. BYU_PL109 16S ribosomal RNA gene	EF623149	81.88	100	1.53967e−32	Plecoptera	Eustheniidae	Thaumatoperla sp. BYU_PL109
Udamocercia sp. BYU_PL150 16S ribosomal RNA gene,	EF623201	81.53	100	5.37398e−32	Plecoptera	Notonemouridae	Udamocercia sp. BYU_PL150
Sierraperla cora 16S ribosomal RNA gene, partial s	EF623255	81.29	100	1.8757e−31	Plecoptera	Peltoperlidae	Sierraperla cora
Neonemura sp. BYU_PL153 16S ribosomal RNA gene, pa	EF623204	81.41	100	6.54685e−31	Plecoptera	Notonemouridae	Neonemura sp. BYU_PL153
Pteronarcella badia 16S ribosomal RNA gene, partia	EF623262	80.37	100	2.28508e−30	Plecoptera	Pteronarcyidae	Pteronarcella badia
Paraleuctra occidentalis 16S ribosomal RNA gene, p	EF623178	80.89	100	2.28508e−30	Plecoptera	Leuctridae	Paraleuctra occidentalis
Strophopteryx appalachia 16S ribosomal RNA gene, p	EF623271	81.01	100	7.9757e−30	Plecoptera	Taeniopterygidae	Strophopteryx appalachia
Salmoperla sylvanica 16S ribosomal RNA gene, parti	EF623222	81.13	100	7.9757e−30	Plecoptera	Perlodidae	Salmoperla sylvanica
Alloperla severa 16S ribosomal RNA gene, partial s	EF623141	80.38	100	7.9757e−30	Plecoptera	Chloroperlidae	Alloperla severa
Megarcys signata 16S ribosomal RNA gene, partial s	EF623215	80.25	100	2.78379e−29	Plecoptera	Perlodidae	Megarcys signata
Amphinemura sulcicollis 16S ribosomal RNA gene, pa	EF623195	80.25	100	2.78379e−29	Plecoptera	Nemouridae	Amphinemura sulcicollis
Amphinemura nigritta 16S ribosomal RNA gene, parti	EF623186	80.25	100	2.78379e−29	Plecoptera	Nemouridae	Amphinemura nigritta
Eusthenia costalis 16S ribosomal RNA gene, partial	EF623150	81.37	100	2.78379e−29	Plecoptera	Eustheniidae	Eusthenia costalis
Utaperla sopladora 16S ribosomal RNA gene, partial	EF623140	80.62	100	2.78379e−29	Plecoptera	Chloroperlidae	Utaperla sopladora
Paraperla frontalis 16S ribosomal RNA gene, partia	EF623136	80.25	100	2.78379e−29	Plecoptera	Chloroperlidae	Paraperla frontalis
Kathroperla perdita 16S ribosomal RNA gene, partia	EF623135	82.86	88.96	2.78379e−29	Plecoptera	Chloroperlidae	Kathroperla perdita
Notonemouridae sp. MD−2011 voucher ZMUC:JD−1507 16	JN615257	80.38	100	9.71639e−29	Plecoptera	Notonemouridae	Notonemouridae sp. MD−2011
Caliaeschna microstigma 12S ribosomal RNA gene, pa	EU477639	81.65	100	9.71639e−29	Odonata	Aeshnidae	Caliaeschna microstigma
Perlinodes aurea 16S ribosomal RNA gene, partial s	EF623234	79.87	100	9.71639e−29	Plecoptera	Perlodidae	Perlinodes aurea
Limnoperla sp. BYU_PL145 16S ribosomal RNA gene, p	EF623166	81.13	100	9.71639e−29	Plecoptera	Gripopterygidae	Limnoperla sp. BYU_PL145
Caliaeschna microstigma large subunit ribosomal RN	AF266092	81.65	100	9.71639e−29	Odonata	Aeshnidae	Caliaeschna microstigma
Parochlus steinenii isolate Sejong01 mitochondrion	KT003702	80.13	100	3.39135e−28	Diptera	Chironomidae	Parochlus steinenii
Peltoperla arcuata 16S ribosomal RNA gene, partial	EF623258	80	100	3.39135e−28	Plecoptera	Peltoperlidae	Peltoperla arcuata
Soliperla campanula 16S ribosomal RNA gene, partia	EF623256	80.89	99.35	3.39135e−28	Plecoptera	Peltoperlidae	Soliperla campanula
Plumiperla diversa 16S ribosomal RNA gene, partial	EF623132	80.62	100	3.39135e−28	Plecoptera	Chloroperlidae	Plumiperla diversa
Yoraperla uenoi mitochondrial gene for 16S rRNA, p	AB712375	81.94	92.21	1.1837e−27	Plecoptera	Peltoperlidae	Yoraperla uenoi
Microchorista philpotti 16S ribosomal RNA gene, pa	KC177459	78.66	100	1.1837e−27	Mecoptera	Nannochoristidae	Nannochorista philpotti
Microchorista philpotti mitochondrion, partial gen	HQ696580	78.66	100	1.1837e−27	Mecoptera	Nannochoristidae	Nannochorista philpotti
Cascadoperla trictura 16S ribosomal RNA gene, part	EF623225	79.75	100	1.1837e−27	Plecoptera	Perlodidae	Cascadoperla trictura
Boyeria maclachlani mitochondrial gene for 16S rRN	AB707674	81.13	100	4.13151e−27	Odonata	Aeshnidae	Boyeria maclachlani
Boyeria maclachlani mitochondrial gene for 16S rRN	AB707672	81.13	100	4.13151e−27	Odonata	Aeshnidae	Boyeria maclachlani
Dolichopoda unicolor voucher DIR 16S ribosomal RNA	EF216970	79.38	100	4.13151e−27	Orthoptera	Rhaphidophoridae	Dolichopoda unicolor
Chloroperla tripunctata 16S ribosomal RNA gene, pa	EF623137	79.87	100	4.13151e−27	Plecoptera	Chloroperlidae	Chloroperla tripunctata
Klapopteryx armillata 16S ribosomal RNA gene, part	EF623123	79.87	100	4.13151e−27	Plecoptera	Austroperlidae	Klapopteryx armillata
Trithetrum navasi 12S ribosomal RNA gene, partial	EF640448	80.13	99.35	4.13151e−27	Odonata	Libellulidae	Trithetrum navasi
Hendrella caloptera 16S ribosomal RNA gene, partia	DQ471407	80.89	99.35	4.13151e−27	Diptera	Tephritidae	Hendrella caloptera
Xyela sp. Sus1 16S ribosomal RNA gene, partial seq	AY206772	79.49	100	4.13151e−27	Hymenoptera	Xyelidae	Xyela sp. Sus1
Dicranomyia iniquispina voucher 205222 16S ribosom	KC692800	78.4	100	1.44204e−26	Diptera	Limoniidae	Dicranomyia iniquispina
