# Supplementary material for: Testing the potential of a ribosomal 16S marker for DNA metabarcoding of insects
Source: PeerJ. 2016 Apr 19;4:e1966. doi: 10.7717/peerj.1966 (PMC4841222; doi:10.7717/peerj.1966)
Supplement: Supplemental Information 1 [file peerj-04-1966-s006.zip › scripts/5 match OTUs/blast_hits/OTU_55.csv_plot.pdf]

OTU_55.csv

Nemoura trispinosa

Nemouridae

Plecoptera

95.54

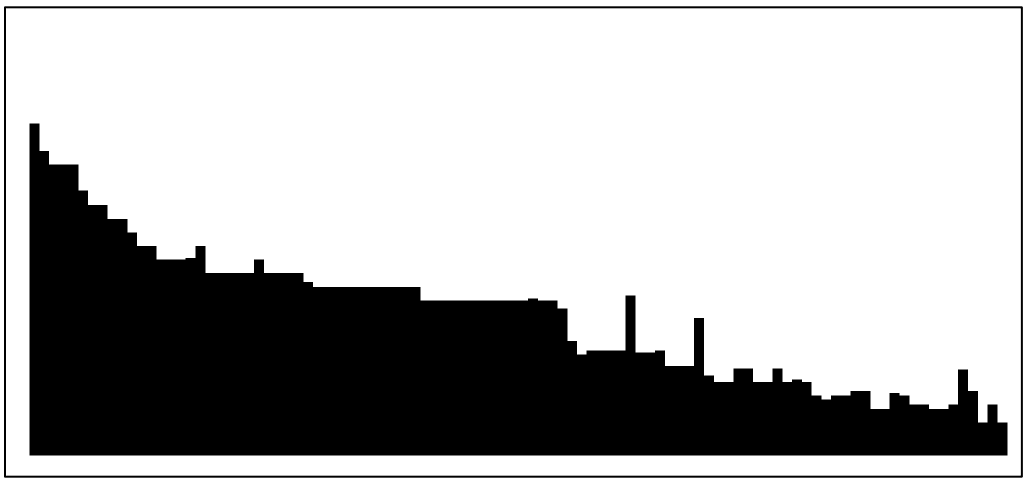

Nemoura trispinosa 16S ribosomal RNA gene, partial	EF623187	95.54	100	1.79787e−63	Plecoptera	Nemouridae	Nemoura trispinosa
Visoka cataractae 16S ribosomal RNA gene, partial	EF623189	94.27	100	9.31322e−61	Plecoptera	Nemouridae	Visoka cataractae
Nanonemoura wahkeena 16S ribosomal RNA gene, parti	EF623198	93.63	100	1.13458e−59	Plecoptera	Nemouridae	Nanonemoura wahkeena
Nemurella pictetii 16S ribosomal RNA gene, partial	EF623192	93.63	100	1.13458e−59	Plecoptera	Nemouridae	Nemurella pictetii
Ostrocerca foersteri 16S ribosomal RNA gene, parti	EF623191	93.63	100	1.13458e−59	Plecoptera	Nemouridae	Ostrocerca foersteri
Nemoura cinerea 16S ribosomal RNA gene, partial se	EF623194	92.41	100	2.05137e−56	Plecoptera	Nemouridae	Nemoura cinerea
Shipsa rotunda 16S ribosomal RNA gene, partial seq	EF623184	91.72	100	2.49908e−55	Plecoptera	Nemouridae	Shipsa rotunda
Malenka californica 16S ribosomal RNA gene, partia	EF623182	91.72	100	2.49908e−55	Plecoptera	Nemouridae	Malenka californica
Paranemoura claasseni 16S ribosomal RNA gene, part	EF623185	91.08	100	3.04451e−54	Plecoptera	Nemouridae	Paranemoura claasseni
Zapada cinctipes 16S ribosomal RNA gene, partial s	EF623183	91.08	100	3.04451e−54	Plecoptera	Nemouridae	Zapada cinctipes
Pteronarcys californica 16S ribosomal RNA gene, pa	EF623261	90.45	100	1.57709e−51	Plecoptera	Pteronarcyidae	Pteronarcys californica
Soyedina producta 16S ribosomal RNA gene, partial	EF623196	89.81	100	1.92129e−50	Plecoptera	Nemouridae	Soyedina producta
Pteronarcys princeps mitochondrion, complete genom	AY687866	89.81	100	1.92129e−50	Plecoptera	Pteronarcyidae	Pteronarcys princeps
Apteroperla tikumana mitochondrion, complete genom	KR604721	89.17	100	6.70597e−50	Plecoptera	Capniidae	Apteroperla tikumana
Allocapnia recta isolate H1 16S ribosomal RNA gene	KC881036	89.17	100	6.70597e−50	Plecoptera	Capniidae	Allocapnia recta
Lednia tumana 16S ribosomal RNA gene, partial sequ	EF623197	89.17	100	6.70597e−50	Plecoptera	Nemouridae	Lednia tumana
Scopura montana 16S ribosomal RNA gene, partial se	EF623265	89.24	100	2.34061e−49	Plecoptera		Scopura montana
Osmylus sp. EH−2004 16S ribosomal RNA gene, partia	AY620146	89.81	100	2.34061e−49	Neuroptera	Osmylidae	Osmylus sp. EH−2004
Mesocapnia arizonensis mitochondrion, partial geno	KP642637	88.54	100	8.16955e−49	Plecoptera	Capniidae	Mesocapnia arizonensis
Allocapnia recta isolate H13 16S ribosomal RNA gen	KC881048	88.54	100	8.16955e−49	Plecoptera	Capniidae	Allocapnia recta
Allocapnia recta isolate H11 16S ribosomal RNA gen	KC881046	88.54	100	8.16955e−49	Plecoptera	Capniidae	Allocapnia recta
Allocapnia recta isolate H7 16S ribosomal RNA gene	KC881042	88.54	100	8.16955e−49	Plecoptera	Capniidae	Allocapnia recta
Allocapnia recta isolate H3 16S ribosomal RNA gene	KC881038	88.54	100	8.16955e−49	Plecoptera	Capniidae	Allocapnia recta
Pteronarcys sachalina 16S ribosomal RNA gene, part	EF623264	89.17	100	8.16955e−49	Plecoptera	Pteronarcyidae	Pteronarcys sachalina
Protonemura meyeri 16S ribosomal RNA gene, partial	EF623193	88.54	100	8.16955e−49	Plecoptera	Nemouridae	Protonemura meyeri
Allocapnia minima 16S ribosomal RNA gene, partial	EF623130	88.54	100	8.16955e−49	Plecoptera	Capniidae	Allocapnia minima
Mesocapnia sp. BYU_PL010 16S ribosomal RNA gene, p	EF623127	88.54	100	8.16955e−49	Plecoptera	Capniidae	Mesocapnia sp. BYU_PL010
Allocapnia recta haplotype 3ar: H03 16S ribosomal	DQ915181	88.54	100	8.16955e−49	Plecoptera	Capniidae	Allocapnia recta
Brachyptera seticornis 16S ribosomal RNA gene, par	EF623268	88.12	100	9.95255e−48	Plecoptera	Taeniopterygidae	Brachyptera seticornis
Allocapnia recta isolate H19 16S ribosomal RNA gen	KC881054	87.9	100	3.47378e−47	Plecoptera	Capniidae	Allocapnia recta
Allocapnia recta isolate H16 16S ribosomal RNA gen	KC881051	87.9	100	3.47378e−47	Plecoptera	Capniidae	Allocapnia recta
Allocapnia recta isolate H15 16S ribosomal RNA gen	KC881050	87.9	100	3.47378e−47	Plecoptera	Capniidae	Allocapnia recta
Allocapnia recta isolate H14 16S ribosomal RNA gen	KC881049	87.9	100	3.47378e−47	Plecoptera	Capniidae	Allocapnia recta
Allocapnia recta isolate H10 16S ribosomal RNA gen	KC881045	87.9	100	3.47378e−47	Plecoptera	Capniidae	Allocapnia recta
Allocapnia recta isolate H6 16S ribosomal RNA gene	KC881041	87.9	100	3.47378e−47	Plecoptera	Capniidae	Allocapnia recta
Prostoia besametsa 16S ribosomal RNA gene, partial	EF623188	87.9	100	3.47378e−47	Plecoptera	Nemouridae	Prostoia besametsa
Allocapnia recta haplotype 12ar: H12 16S ribosomal	EU306875	87.9	100	3.47378e−47	Plecoptera	Capniidae	Allocapnia recta
Allocapnia recta haplotype 11ar: H011 16S ribosoma	EU306874	87.9	100	3.47378e−47	Plecoptera	Capniidae	Allocapnia recta
Allocapnia recta haplotype 7ar: H07 16S ribosomal	EU306870	87.9	100	3.47378e−47	Plecoptera	Capniidae	Allocapnia recta
Allocapnia recta haplotype 2ar: H02 16S ribosomal	DQ915180	87.9	100	3.47378e−47	Plecoptera	Capniidae	Allocapnia recta
Allocapnia recta isolate H18 16S ribosomal RNA gen	KC881053	87.26	100	4.23193e−46	Plecoptera	Capniidae	Allocapnia recta
Allocapnia recta isolate H17 16S ribosomal RNA gen	KC881052	87.26	100	4.23193e−46	Plecoptera	Capniidae	Allocapnia recta
Allocapnia recta isolate H12 16S ribosomal RNA gen	KC881047	87.26	100	4.23193e−46	Plecoptera	Capniidae	Allocapnia recta
Allocapnia recta isolate H9 16S ribosomal RNA gene	KC881044	87.26	100	4.23193e−46	Plecoptera	Capniidae	Allocapnia recta
Allocapnia recta isolate H8 16S ribosomal RNA gene	KC881043	87.26	100	4.23193e−46	Plecoptera	Capniidae	Allocapnia recta
Allocapnia recta isolate H5 16S ribosomal RNA gene	KC881040	87.26	100	4.23193e−46	Plecoptera	Capniidae	Allocapnia recta
Allocapnia recta isolate H4 16S ribosomal RNA gene	KC881039	87.26	100	4.23193e−46	Plecoptera	Capniidae	Allocapnia recta
Allocapnia recta haplotype 10ar: H010 16S ribosoma	EU306873	87.26	100	4.23193e−46	Plecoptera	Capniidae	Allocapnia recta
Allocapnia recta haplotype 9ar: H09 16S ribosomal	EU306872	87.26	100	4.23193e−46	Plecoptera	Capniidae	Allocapnia recta
Allocapnia recta haplotype 8ar: H08 16S ribosomal	EU306871	87.26	100	4.23193e−46	Plecoptera	Capniidae	Allocapnia recta
Allocapnia recta haplotype 1ar: H01 16S ribosomal	DQ915179	87.26	100	4.23193e−46	Plecoptera	Capniidae	Allocapnia recta
Oemopteryx vanduzeea 16S ribosomal RNA gene, parti	EF623266	87.34	100	1.47709e−45	Plecoptera	Taeniopterygidae	Oemopteryx vanduzeea
Pteronarcys scotti 16S ribosomal RNA gene, partial	EF623263	87.26	100	5.15555e−45	Plecoptera	Pteronarcyidae	Pteronarcys scotti
Capnura wanica 16S ribosomal RNA gene, partial seq	EF623128	87.26	100	5.15555e−45	Plecoptera	Capniidae	Capnura wanica
Doddsia occidentalis 16S ribosomal RNA gene, parti	EF623270	86.88	100	2.19219e−43	Plecoptera	Taeniopterygidae	Doddsia occidentalis
Amphinemura sulcicollis 16S ribosomal RNA gene, pa	EF623195	85.35	100	9.32145e−42	Plecoptera	Nemouridae	Amphinemura sulcicollis
Triznaka pintada 16S ribosomal RNA gene, partial s	EF623134	84.71	100	1.13558e−40	Plecoptera	Chloroperlidae	Triznaka pintada
Megarcys signata 16S ribosomal RNA gene, partial s	EF623215	84.91	100	3.96358e−40	Plecoptera	Perlodidae	Megarcys signata
Austrocerca rieka 16S ribosomal RNA gene, partial	EF623205	84.91	100	3.96358e−40	Plecoptera	Notonemouridae	Austrocerca rieka
Austrocercoides zwicki 16S ribosomal RNA gene, par	EF623199	84.91	100	3.96358e−40	Plecoptera	Notonemouridae	Austrocercoides zwicki
Isocapnia hyalita 16S ribosomal RNA gene, partial	EF623131	84.91	100	3.96358e−40	Plecoptera	Capniidae	Isocapnia hyalita
Moselia infuscata 16S ribosomal RNA gene, partial	EF623175	87.5	89.81	1.38343e−39	Plecoptera	Leuctridae	Moselia infuscata
Isoperla oxylepis 16S ribosomal RNA gene, partial	EF623221	84.81	100	4.82863e−39	Plecoptera	Perlodidae	Isoperla oxylepis
Thaumatoperla sp. BYU_PL109 16S ribosomal RNA gene	EF623149	84.81	100	4.82863e−39	Plecoptera	Eustheniidae	Thaumatoperla sp. BYU_PL109
Cosumnoperla hypocrena 16S ribosomal RNA gene, par	EF623226	84.91	100	1.68536e−38	Plecoptera	Perlodidae	Cosumnoperla hypocrena
Setvena wahkeena 16S ribosomal RNA gene, partial s	EF623211	84.18	100	1.68536e−38	Plecoptera	Perlodidae	Setvena wahkeena
Suwallia teleckojensis 16S ribosomal RNA gene, par	EF623142	84.18	100	1.68536e−38	Plecoptera	Chloroperlidae	Suwallia teleckojensis
Alloperla severa 16S ribosomal RNA gene, partial s	EF623141	84.18	100	1.68536e−38	Plecoptera	Chloroperlidae	Alloperla severa
Strophopteryx appalachia 16S ribosomal RNA gene, p	EF623271	86.43	89.17	5.88247e−38	Plecoptera	Taeniopterygidae	Strophopteryx appalachia
Bolotoperla rossi 16S ribosomal RNA gene, partial	EF623269	83.75	100	5.88247e−38	Plecoptera	Taeniopterygidae	Bolotoperla rossi
Perlinodes aurea 16S ribosomal RNA gene, partial s	EF623234	83.44	100	5.88247e−38	Plecoptera	Perlodidae	Perlinodes aurea
Skwala americana 16S ribosomal RNA gene, partial s	EF623217	83.44	100	5.88247e−38	Plecoptera	Perlodidae	Skwala americana
Isoperla fulva 16S ribosomal RNA gene, partial seq	EF623207	84.08	100	5.88247e−38	Plecoptera	Perlodidae	Isoperla fulva
Neonemura sp. BYU_PL153 16S ribosomal RNA gene, pa	EF623204	84.08	100	5.88247e−38	Plecoptera	Notonemouridae	Neonemura sp. BYU_PL153
Udamocercia sp. BYU_PL150 16S ribosomal RNA gene,	EF623201	83.44	100	5.88247e−38	Plecoptera	Notonemouridae	Udamocercia sp. BYU_PL150
Paraperla frontalis 16S ribosomal RNA gene, partia	EF623136	83.44	100	5.88247e−38	Plecoptera	Chloroperlidae	Paraperla frontalis
Sweltsa coloradensis 16S ribosomal RNA gene, parti	EF623133	84.08	100	5.88247e−38	Plecoptera	Chloroperlidae	Sweltsa coloradensis
Plumiperla diversa 16S ribosomal RNA gene, partial	EF623132	83.44	100	5.88247e−38	Plecoptera	Chloroperlidae	Plumiperla diversa
Neaviperla forcipata 16S ribosomal RNA gene, parti	EF623139	83.54	100	2.05319e−37	Plecoptera	Chloroperlidae	Neaviperla forcipata
Cascadoperla trictura 16S ribosomal RNA gene, part	EF623225	83.44	100	7.16632e−37	Plecoptera	Perlodidae	Cascadoperla trictura
Amphinemura nigritta 16S ribosomal RNA gene, parti	EF623186	82.8	100	2.50129e−36	Plecoptera	Nemouridae	Amphinemura nigritta
Megaleuctra kincaidi 16S ribosomal RNA gene, parti	EF623174	82.61	100	2.50129e−36	Plecoptera	Leuctridae	Megaleuctra kincaidi
Chloroperla tripunctata 16S ribosomal RNA gene, pa	EF623137	82.8	100	2.50129e−36	Plecoptera	Chloroperlidae	Chloroperla tripunctata
Kathroperla perdita 16S ribosomal RNA gene, partia	EF623135	82.8	100	2.50129e−36	Plecoptera	Chloroperlidae	Kathroperla perdita
Notonemouridae sp. MD−2011 voucher ZMUC:JD−1507 16	JN615257	83.02	100	8.73037e−36	Plecoptera	Notonemouridae	Notonemouridae sp. MD−2011
Taenionema palladium 16S ribosomal RNA gene, parti	EF623267	83.02	100	3.0472e−35	Plecoptera	Taeniopterygidae	Taenionema palladium
Frisonia picticeps 16S ribosomal RNA gene, partial	EF623227	82.17	100	3.0472e−35	Plecoptera	Perlodidae	Frisonia picticeps
Salmoperla sylvanica 16S ribosomal RNA gene, parti	EF623222	82.17	100	3.0472e−35	Plecoptera	Perlodidae	Salmoperla sylvanica
Isoperla davisi 16S ribosomal RNA gene, partial se	EF623206	82.91	100	3.0472e−35	Plecoptera	Perlodidae	Isoperla davisi
Siphonoperla torrentium 16S ribosomal RNA gene, pa	EF623138	82.8	100	3.0472e−35	Plecoptera	Chloroperlidae	Siphonoperla torrentium
Paraleuctra occidentalis 16S ribosomal RNA gene, p	EF623178	82.39	100	1.06358e−34	Plecoptera	Leuctridae	Paraleuctra occidentalis
Peltoperla arcuata 16S ribosomal RNA gene, partial	EF623258	82.39	100	3.71225e−34	Plecoptera	Peltoperlidae	Peltoperla arcuata
Sierraperla cora 16S ribosomal RNA gene, partial s	EF623255	82.17	100	3.71225e−34	Plecoptera	Peltoperlidae	Sierraperla cora
Isoperla phalerata 16S ribosomal RNA gene, partial	EF623208	82.17	100	3.71225e−34	Plecoptera	Perlodidae	Isoperla phalerata
Utaperla sopladora 16S ribosomal RNA gene, partial	EF623140	82.39	100	3.71225e−34	Plecoptera	Chloroperlidae	Utaperla sopladora
Yoraperla uenoi mitochondrial gene for 16S rRNA, p	AB712375	84.03	91.72	1.2957e−33	Plecoptera	Peltoperlidae	Yoraperla uenoi
Eusthenia costalis 16S ribosomal RNA gene, partial	EF623150	83.02	100	4.52244e−33	Plecoptera	Eustheniidae	Eusthenia costalis
Sasquaperla hoopa 16S ribosomal RNA gene, partial	EF623143	81.53	100	4.52244e−33	Plecoptera	Chloroperlidae	Sasquaperla hoopa
Soliperla campanula 16S ribosomal RNA gene, partia	EF623256	82.39	99.36	1.57849e−32	Plecoptera	Peltoperlidae	Soliperla campanula
Clioperla clio 16S ribosomal RNA gene, partial seq	EF623237	81.53	100	1.57849e−32	Plecoptera	Perlodidae	Clioperla clio
