# Supplementary material for: Testing the potential of a ribosomal 16S marker for DNA metabarcoding of insects
Source: PeerJ. 2016 Apr 19;4:e1966. doi: 10.7717/peerj.1966 (PMC4841222; doi:10.7717/peerj.1966)
Supplement: Supplemental Information 1 [file peerj-04-1966-s006.zip › scripts/5 match OTUs/blast_hits/OTU_558.csv_plot.pdf]

OTU_558.csv

Parochlus steinenii

Chironomidae

Diptera

87.18

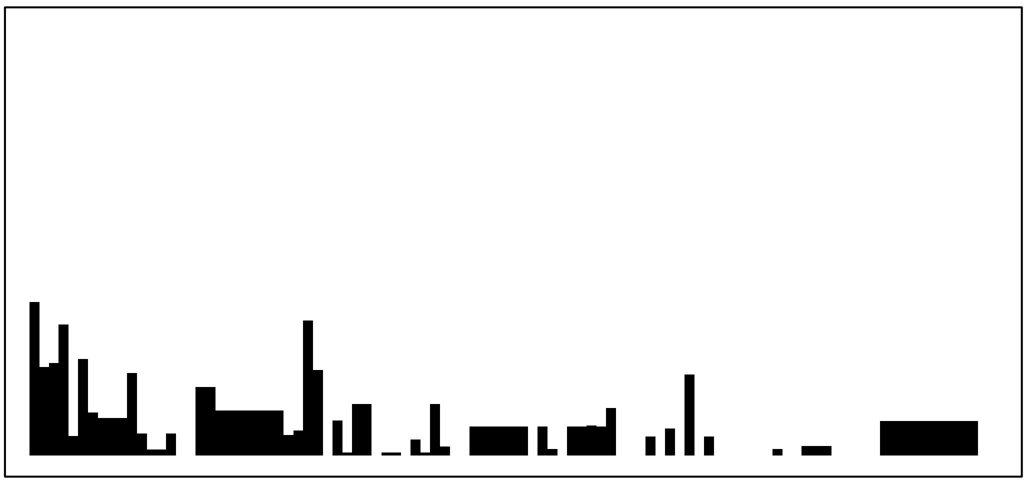

Parochlus steinenii isolate Sejong01 mitochondrion	KT003702	87.18	100	1.46498e−45	Diptera	Chironomidae	Parochlus steinenii
Bolitophila occlusa voucher JSB2 16S ribosomal RNA	KJ136713	84.14	92.31	4.48537e−33	Diptera	Mycetophilidae	Bolitophila occlusa
Bolitophila cinerea voucher JSB1 16S ribosomal RNA	KJ136712	84.31	95.51	4.48537e−33	Diptera	Mycetophilidae	Bolitophila cinerea
Bolitophila sp. ER−2009 16S ribosomal RNA gene, pa	FJ172021	86.13	86.54	4.48537e−33	Diptera	Mycetophilidae	Bolitophila sp. ER−2009
Pedicia albivitta voucher O'Grady 200012 16S ribos	EU005435	80.89	100	1.90723e−31	Diptera	Pediciidae	Pedicia albivitta
Bolitophila hybrida 16S ribosomal RNA gene, partia	FJ172002	84.51	89.74	6.65688e−31	Diptera	Mycetophilidae	Bolitophila hybrida
Beris morrisii 16S ribosomal RNA gene, partial seq	GU947373	82	95.51	2.83058e−29	Diptera	Stratiomyidae	Beris morrisii
Tipula abdominalis 16S ribosomal RNA gene, partial	KC177457	81.76	94.23	9.87969e−29	Diptera	Tipulidae	Tipula abdominalis
Tipula abdominalis mitochondrion, partial genome	JN861743	81.76	94.23	9.87969e−29	Diptera	Tipulidae	Tipula abdominalis
Arachnocampa luminosa biotype 1 16S large subunit	AY576329	81.76	92.95	9.87969e−29	Diptera	Keroplatidae	Arachnocampa luminosa
Tipula sp. PMO 200011 16S ribosomal RNA gene, part	EU005437	83.85	82.69	1.20359e−27	Diptera	Tipulidae	Tipula sp. PMO 200011
Callomyia admirabilis isolate CA42 16S ribosomal R	KC699326	81.02	87.82	4.20095e−27	Diptera	Platypezidae	Callomyia admirabilis
Haematopota pluvialis 16S ribosomal RNA gene, part	KC177454	80.27	94.23	4.20095e−27	Diptera	Tabanidae	Haematopota pluvialis
Chrysops viduatus 16S ribosomal RNA gene, partial	HM132115	80.27	94.23	4.20095e−27	Diptera	Tabanidae	Chrysops viduatus
Tabanus bromius 16S ribosomal RNA gene, partial se	HM132111	81.02	87.82	4.20095e−27	Diptera	Tabanidae	Tabanus bromius
Sylvicola fenestralis 16S ribosomal RNA gene, part	KC177461	78.85	100	1.46628e−26	Diptera	Anisopodidae	Sylvicola fenestralis
Sylvicola fenestralis mitochondrion, complete geno	JN861752	78.85	100	1.46628e−26	Diptera	Anisopodidae	Sylvicola fenestralis
Simulium tenederoi mitochondrial gene for 16S ribo	LC035002	83.2	79.49	5.11781e−26	Diptera	Simuliidae	Simulium tenederoi
Simulium tenederoi mitochondrial gene for 16S ribo	LC035001	83.2	79.49	5.11781e−26	Diptera	Simuliidae	Simulium tenederoi
Simulium sheilae isolate SH1B 16S ribosomal RNA ge	KM410213	82.09	85.26	5.11781e−26	Diptera	Simuliidae	Simulium sheilae
Simulium roslihashimi isolate R1B 16S ribosomal RN	KM410192	82.09	85.26	5.11781e−26	Diptera	Simuliidae	Simulium roslihashimi
Simulium izuae isolate I3B 16S ribosomal RNA gene,	KM410191	82.09	85.26	5.11781e−26	Diptera	Simuliidae	Simulium izuae
Simulium izuae isolate I2B 16S ribosomal RNA gene,	KM410190	82.09	85.26	5.11781e−26	Diptera	Simuliidae	Simulium izuae
Simulium tuenense mitochondrial gene for 16S ribos	AB591244	82.09	85.26	5.11781e−26	Diptera	Simuliidae	Simulium tuenense
Simulium inthanonense 16S ribosomal RNA gene, part	GQ865995	82.09	85.26	5.11781e−26	Diptera	Simuliidae	Simulium inthanonense
Simulium cabrerai mitochondrial gene for 16S rRNA,	AB093128	82.09	85.26	5.11781e−26	Diptera	Simuliidae	Simulium cabrerai
Tongamya stuckenbergi 16S ribosomal RNA gene, part	KM878969	80.95	92.95	1.78629e−25	Diptera	Mydidae	Tongamya stuckenbergi
Callomyia amoena isolate CA06 16S ribosomal RNA ge	KC699325	81.16	87.82	1.78629e−25	Diptera	Platypezidae	Callomyia amoena
Paracladura trichoptera mitochondrion, complete ge	JN861751	80.32	74.36	1.78629e−25	Diptera	Trichoceridae	Paracladura trichoptera
Hercostomus meieri voucher RMBR:103442 16S ribosom	FJ808173	84	79.49	1.78629e−25	Diptera	Dolichopodidae	Hercostomus meieri
Somatia sp. YSUW04038 16S ribosomal RNA gene, part	AY573134	78.91	94.23	1.78629e−25	Diptera	Somatiidae	Somatia sp. YSUW04038
Arachnocampa luminosa biotype 3 16S large subunit	AY576331	81.62	86.54	1.78629e−25	Diptera	Keroplatidae	Arachnocampa luminosa
Simulium suyoense mitochondrial gene for 16S ribos	LC035000	80.13	94.87	6.23476e−25	Diptera	Simuliidae	Simulium suyoense
Simulium claveriaense mitochondrial gene for 16S r	LC034988	82.4	79.49	6.23476e−25	Diptera	Simuliidae	Simulium claveriaense
Simulium claveriaense mitochondrial gene for 16S r	LC034987	82.4	79.49	6.23476e−25	Diptera	Simuliidae	Simulium claveriaense
Diadocidia stanfordensis voucher JSSJ14 16S riboso	KJ136742	79.19	90.38	6.23476e−25	Diptera	Diadocidiidae	Diadocidia stanfordensis
Simulium khunklangense mitochondrial gene for 16S	AB820367	80.14	92.31	6.23476e−25	Diptera	Simuliidae	Simulium khunklangense
Simulium chomthongense mitochondrial gene for 16S	AB699899	80.14	92.31	6.23476e−25	Diptera	Simuliidae	Simulium chomthongense
Coboldia sp. SS−2012 voucher 905 16S ribosomal RNA	JQ613954	78.81	95.51	6.23476e−25	Diptera	Scatopsidae	Coboldia sp. SS−2012
Pachygaster atra 16S ribosomal RNA gene, partial s	GU947397	80.74	86.54	6.23476e−25	Diptera	Stratiomyidae	Pachygaster atra
Simulium caudisclerum 16S ribosomal RNA gene, part	GQ865999	80.14	92.31	6.23476e−25	Diptera	Simuliidae	Simulium caudisclerum
Simulium claveriaense mitochondrial gene for 16S r	AB334096	82.4	79.49	6.23476e−25	Diptera	Simuliidae	Simulium claveriaense
Caenophthalmus capensis voucher MEI 120162 16S rib	DQ250838	80.41	92.31	6.23476e−25	Diptera	Therevidae	Caenophthalmus capensis
Cnephia dacotensis mitochondrial 16S ribosomal RNA	L13403	79.35	97.44	6.23476e−25	Diptera	Simuliidae	Cnephia dacotensis
Simulium tuyense mitochondrial gene for 16S riboso	LC035004	79.08	97.44	2.17615e−24	Diptera	Simuliidae	Simulium tuyense
Simulium sheilae isolate SH2B 16S ribosomal RNA ge	KM410214	81.34	85.26	2.17615e−24	Diptera	Simuliidae	Simulium sheilae
Simulium brinchangense isolate B2B 16S ribosomal R	KM410202	81.34	85.26	2.17615e−24	Diptera	Simuliidae	Simulium brinchangense
Simulium brinchangense isolate B1B 16S ribosomal R	KM410201	81.34	85.26	2.17615e−24	Diptera	Simuliidae	Simulium brinchangense
Simulium tanahrataense isolate TR2B 16S ribosomal	KM410196	81.34	85.26	2.17615e−24	Diptera	Simuliidae	Simulium tanahrataense
Simulium tanahrataense isolate TR1B 16S ribosomal	KM410195	81.34	85.26	2.17615e−24	Diptera	Simuliidae	Simulium tanahrataense
Simulium izuae isolate I1B 16S ribosomal RNA gene,	KM410189	81.34	85.26	2.17615e−24	Diptera	Simuliidae	Simulium izuae
Microsania collarti isolate CAN8 16S ribosomal RNA	KC699334	77.92	98.08	2.17615e−24	Diptera	Platypezidae	Microsania collarti
Cyphomyia notabilis 16S ribosomal RNA gene, partia	GU947403	81.34	85.26	2.17615e−24	Diptera	Stratiomyidae	Cyphomyia notabilis
Exodontha dubia 16S ribosomal RNA gene, partial se	GU947396	80.29	87.82	2.17615e−24	Diptera	Stratiomyidae	Exodontha dubia
Hermetia illucens 16S ribosomal RNA gene, partial	GU947384	79.17	91.03	2.17615e−24	Diptera	Stratiomyidae	Hermetia illucens
Simulium chumpornense 16S ribosomal RNA gene, part	GQ865989	81.34	85.26	2.17615e−24	Diptera	Simuliidae	Simulium chumpornense
Simulium decuplum 16S ribosomal RNA gene, partial	GQ865986	81.34	85.26	2.17615e−24	Diptera	Simuliidae	Simulium decuplum
Allocotocera pulchella 16S ribosomal RNA gene, par	FJ172034	81.4	82.69	2.17615e−24	Diptera	Mycetophilidae	Allocotocera pulchella
Simulium recurvum mitochondrial gene for 16S ribos	AB334097	81.34	85.26	2.17615e−24	Diptera	Simuliidae	Simulium recurvum
Simulium palauense mitochondrial gene for 16S ribo	AB056742	82.22	85.26	2.17615e−24	Diptera	Simuliidae	Simulium palauense
Simulium suyoense mitochondrial gene for 16S ribos	LC034999	79.47	94.87	7.5955e−24	Diptera	Simuliidae	Simulium suyoense
Simulium suyoense mitochondrial gene for 16S ribos	LC034998	79.47	94.87	7.5955e−24	Diptera	Simuliidae	Simulium suyoense
Simulium suyoense mitochondrial gene for 16S ribos	LC034997	79.47	94.87	7.5955e−24	Diptera	Simuliidae	Simulium suyoense
Simulium spinosibranchium mitochondrial gene for 1	LC034996	80.88	85.26	7.5955e−24	Diptera	Simuliidae	Simulium spinosibranchium
Coboldia fuscipes voucher JSOUT4 16S ribosomal RNA	KJ136727	77.85	95.51	7.5955e−24	Diptera	Scatopsidae	Coboldia fuscipes
Keilbachia subacumina voucher 890 16S ribosomal RN	JQ613951	81.25	80.77	7.5955e−24	Diptera	Sciaridae	Keilbachia subacumina
Trichopeza longicornis voucher Pollet 0243 (P) 16S	HQ449035	79.49	98.08	7.5955e−24	Diptera	Empididae	Trichopeza longicornis
Leptomorphus walkeri 16S ribosomal RNA gene, parti	FJ172020	83.78	71.15	7.5955e−24	Diptera	Mycetophilidae	Leptomorphus walkeri
Simulium suyoense mitochondrial gene for 16S ribos	AB334099	79.47	94.87	7.5955e−24	Diptera	Simuliidae	Simulium suyoense
Simulium spinosibranchium mitochondrial gene for 1	AB334098	80.88	85.26	7.5955e−24	Diptera	Simuliidae	Simulium spinosibranchium
Simulium chiharuae mitochondrial gene for 16S ribo	AB334092	79.31	91.67	7.5955e−24	Diptera	Simuliidae	Simulium chiharuae
Simulium squamosum voucher P.Mansiangi Pm3(BNI) 16	DQ655644	79.31	92.31	7.5955e−24	Diptera	Simuliidae	Simulium squamosum
Simulium damnosum s.l. GH−D1 16S ribosomal RNA, pa	AY077709	79.31	92.31	7.5955e−24	Diptera	Simuliidae	Simulium damnosum s.l. GH−D1
Simulium damnosum s.l. Kulfo isolate Ku 3 16S ribo	AF416978	79.31	92.31	7.5955e−24	Diptera	Simuliidae	Simulium damnosum s.l. Kulfo
Simulium soubrense isolate Wa 52 16S ribosomal RNA	AF416976	79.31	92.31	7.5955e−24	Diptera	Simuliidae	Simulium soubrense
Simulium soubrense isolate Wa 47 16S ribosomal RNA	AF416975	79.31	92.31	7.5955e−24	Diptera	Simuliidae	Simulium soubrense
Panorpa annexa 16S ribosomal RNA gene, partial seq	AF180048	80.29	86.54	7.5955e−24	Mecoptera	Panorpidae	Panorpa annexa
Simulium damnosum strain Se−12 16S ribosomal RNA g	AF081907	79.31	92.31	7.5955e−24	Diptera	Simuliidae	Simulium damnosum
Simulium uchidai mitochondrial gene for 16S riboso	AB056740	79.31	92.31	7.5955e−24	Diptera	Simuliidae	Simulium uchidai
Simulium mie mitochondrial gene for 16S ribosomal	AB056732	80.43	86.54	7.5955e−24	Diptera	Simuliidae	Simulium mie
Simulium mie mitochondrial gene for 16S ribosomal	AB056731	80.43	86.54	7.5955e−24	Diptera	Simuliidae	Simulium mie
Simulium mie mitochondrial gene for 16S ribosomal	AB056730	80.43	86.54	7.5955e−24	Diptera	Simuliidae	Simulium mie
Simulium leonense mitochondrion 16S ribosomal RNA	U17731	79.31	92.31	7.5955e−24	Diptera	Simuliidae	Simulium leonense
Simulium tuyense mitochondrial gene for 16S riboso	LC035008	78.43	97.44	2.65109e−23	Diptera	Simuliidae	Simulium tuyense
Simulium tuyense mitochondrial gene for 16S riboso	LC035007	78.43	97.44	2.65109e−23	Diptera	Simuliidae	Simulium tuyense
Simulium tuyense mitochondrial gene for 16S riboso	LC035005	78.43	97.44	2.65109e−23	Diptera	Simuliidae	Simulium tuyense
Simulium tuyense mitochondrial gene for 16S riboso	LC035003	78.43	97.44	2.65109e−23	Diptera	Simuliidae	Simulium tuyense
Simulium nobile isolate N5 16S ribosomal RNA gene,	KP661530	81.6	79.49	2.65109e−23	Diptera	Simuliidae	Simulium nobile
Simulium nobile isolate N4 16S ribosomal RNA gene,	KP661529	81.6	79.49	2.65109e−23	Diptera	Simuliidae	Simulium nobile
Simulium nobile isolate N3 16S ribosomal RNA gene,	KP661528	81.6	79.49	2.65109e−23	Diptera	Simuliidae	Simulium nobile
Simulium nobile isolate N2 16S ribosomal RNA gene,	KP661527	81.6	79.49	2.65109e−23	Diptera	Simuliidae	Simulium nobile
Simulium nobile isolate N1 16S ribosomal RNA gene,	KP661526	81.6	79.49	2.65109e−23	Diptera	Simuliidae	Simulium nobile
Simulium timorense isolate TM5 16S ribosomal RNA g	KP661525	81.6	79.49	2.65109e−23	Diptera	Simuliidae	Simulium timorense
Simulium timorense isolate TM4 16S ribosomal RNA g	KP661524	81.6	79.49	2.65109e−23	Diptera	Simuliidae	Simulium timorense
Simulium timorense isolate TM3 16S ribosomal RNA g	KP661523	81.6	79.49	2.65109e−23	Diptera	Simuliidae	Simulium timorense
Simulium timorense isolate TM2 16S ribosomal RNA g	KP661522	81.6	79.49	2.65109e−23	Diptera	Simuliidae	Simulium timorense
Simulium timorense isolate TM1 16S ribosomal RNA g	KP661521	81.6	79.49	2.65109e−23	Diptera	Simuliidae	Simulium timorense
Leptomorphus sp. DK−2013 voucher JSS1 16S ribosoma	KC435575	79.85	85.9	2.65109e−23	Diptera	Mycetophilidae	Leptomorphus sp. DK−2013
Hermetia illucens 16S ribosomal RNA gene, partial	KC177455	78.47	91.03	2.65109e−23	Diptera	Stratiomyidae	Hermetia illucens
Ptychoptera sp. ATB−2011 mitochondrion, complete g	JN861744	79.43	89.1	2.65109e−23	Diptera	Ptychopteridae	Ptychoptera sp. ATB−2011
