# Supplementary material for: Testing the potential of a ribosomal 16S marker for DNA metabarcoding of insects
Source: PeerJ. 2016 Apr 19;4:e1966. doi: 10.7717/peerj.1966 (PMC4841222; doi:10.7717/peerj.1966)
Supplement: Supplemental Information 1 [file peerj-04-1966-s006.zip › scripts/5 match OTUs/blast_hits/OTU_565.csv_plot.pdf]

OTU_565.csv

Ecdyonurus dispar

Heptageniidae

Ephemeroptera

88.39

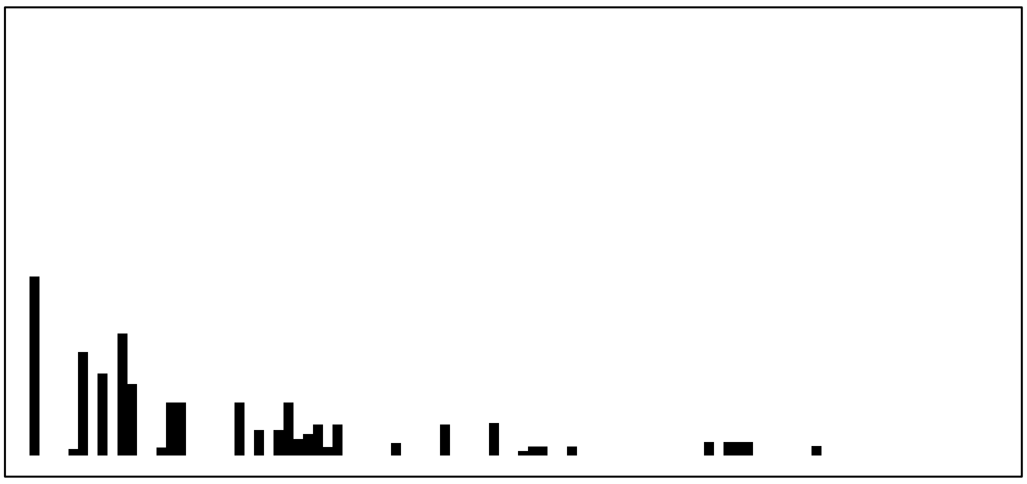

Ecdyonurus dispar 16S ribosomal RNA gene, partial	AY749775	88.39	100	1.17875e−46	Ephemeroptera	Heptageniidae	Ecdyonurus dispar
Atopopus tarsalis mitochondrial partial 16S rRNA g	HE651420	79.29	90.2	7.4453e−24	Ephemeroptera	Heptageniidae	Atopopus tarsalis
Atopopus tarsalis mitochondrial partial 16S rRNA g	HE651419	79.29	90.2	7.4453e−24	Ephemeroptera	Heptageniidae	Atopopus tarsalis
Thalerosphyrus sinuosus mitochondrial partial 16S	HE651430	77.86	90.2	3.85676e−21	Ephemeroptera	Heptageniidae	Thalerosphyrus sinuosus
Thalerosphyrus lamuriensis mitochondrial partial 1	HE651425	80.29	84.97	3.85676e−21	Ephemeroptera	Heptageniidae	Thalerosphyrus lamuriensis
Afronurus sp. 212BoAf mitochondrial partial 16S rR	HE651421	84.85	64.71	3.85676e−21	Ephemeroptera	Heptageniidae	Afronurus sp. 212BoAf
Thalerosphyrus lamuriensis mitochondrial partial 1	HE651429	79.56	84.97	4.6985e−20	Ephemeroptera	Heptageniidae	Thalerosphyrus lamuriensis
Afronurus sp. 213BoAf mitochondrial partial 16S rR	HE651422	83.84	64.71	4.6985e−20	Ephemeroptera	Heptageniidae	Afronurus sp. 213BoAf
Parafronurus youi mitochondrion, complete genome	EU349015	77.86	90.2	4.6985e−20	Ephemeroptera	Heptageniidae	Parafronurus youi
Clioperla clio 16S ribosomal RNA gene, partial seq	EF623237	85.71	63.4	4.6985e−20	Plecoptera	Perlodidae	Clioperla clio
Siphlonurus binotatus mitochondrial gene for 16S r	AB110269	83.33	65.67	4.6985e−20	Ephemeroptera	Siphlonuridae	Siphlonurus binotatus
Heptagenia sulphurea mitochondrial partial 16S rRN	HE651431	77.7	88.89	1.63994e−19	Ephemeroptera	Heptageniidae	Heptagenia sulphurea
Afronurus peringueyi 16S ribosomal RNA gene, parti	AY749790	77.86	88.89	1.63994e−19	Ephemeroptera	Heptageniidae	Afronurus peringueyi
Parameletus columbiae 16S ribosomal RNA gene, part	AY749768	80.36	71.9	6.97319e−18	Ephemeroptera	Siphlonuridae	Parameletus columbiae
Siphlonurus sp. MT−2014 isolate CL142 mitochondrio	KM244684	82.47	63.4	2.43388e−17	Ephemeroptera	Siphlonuridae	Siphlonurus sp. MT−2014
Paegniodes cupulatus mitochondrion, complete genom	HM004123	82.47	63.4	2.43388e−17	Ephemeroptera	Heptageniidae	Paegniodes cupulatus
Pteronarcys sachalina 16S ribosomal RNA gene, part	EF623264	76.92	83.01	2.43388e−17	Plecoptera	Pteronarcyidae	Pteronarcys sachalina
Neofulla sp. BYU_PL152 16S ribosomal RNA gene, par	EF623203	77.69	83.01	2.43388e−17	Plecoptera	Notonemouridae	Neofulla sp. BYU_PL152
Suwallia teleckojensis 16S ribosomal RNA gene, par	EF623142	75.86	90.85	2.43388e−17	Plecoptera	Chloroperlidae	Suwallia teleckojensis
Neaviperla forcipata 16S ribosomal RNA gene, parti	EF623139	76.43	87.58	2.43388e−17	Plecoptera	Chloroperlidae	Neaviperla forcipata
Pseudiron centralis 16S ribosomal RNA gene, partia	AY749799	76.09	88.89	2.43388e−17	Ephemeroptera	Pseudironidae	Pseudiron centralis
Siphlonurus sp. EP084 16S ribosomal RNA gene, part	AY749786	82.47	63.4	2.43388e−17	Ephemeroptera	Siphlonuridae	Siphlonurus sp. EP084
Yoraperla uenoi mitochondrial gene for 16S rRNA, p	AB712375	75.89	91.5	8.49508e−17	Plecoptera	Peltoperlidae	Yoraperla uenoi
Chernokrilus misnomus 16S ribosomal RNA gene, part	EF623209	81.19	66.01	8.49508e−17	Plecoptera	Perlodidae	Chernokrilus misnomus
Ameletoides sp. EP159 16S ribosomal RNA gene, part	AY749826	74.36	100	8.49508e−17	Ephemeroptera	Nesameletidae	Ameletoides sp. EP159
Stenonema sp. EP026 16S ribosomal RNA gene, partia	AY749769	81.19	66.01	8.49508e−17	Ephemeroptera	Heptageniidae	Stenonema sp. EP026
Afronurus sp. 338SuAf mitochondrial partial 16S rR	HE651428	82.47	63.4	2.96508e−16	Ephemeroptera	Heptageniidae	Afronurus sp. 338SuAf
Notonemouridae sp. MD−2011 voucher ZMUC:JD−1507 16	JN615257	80.77	67.32	2.96508e−16	Plecoptera	Notonemouridae	Notonemouridae sp. MD−2011
Osobenus yakimae 16S ribosomal RNA gene, partial s	EF623232	81	65.36	2.96508e−16	Plecoptera	Perlodidae	Osobenus yakimae
Isoperla oxylepis 16S ribosomal RNA gene, partial	EF623221	81.44	63.4	2.96508e−16	Plecoptera	Perlodidae	Isoperla oxylepis
Kogotus nonus 16S ribosomal RNA gene, partial sequ	EF623213	80.39	66.67	2.96508e−16	Plecoptera	Perlodidae	Kogotus nonus
Isoperla fulva 16S ribosomal RNA gene, partial seq	EF623207	81.44	63.4	2.96508e−16	Plecoptera	Perlodidae	Isoperla fulva
Isoperla davisi 16S ribosomal RNA gene, partial se	EF623206	80	68.63	2.96508e−16	Plecoptera	Perlodidae	Isoperla davisi
Nemoura cinerea 16S ribosomal RNA gene, partial se	EF623194	75.89	88.24	2.96508e−16	Plecoptera	Nemouridae	Nemoura cinerea
Diamphipnopsis sp. BYU_PL138 16S ribosomal RNA gen	EF623145	76.09	87.58	2.96508e−16	Plecoptera	Diamphipnoidae	Diamphipnopsis sp. BYU_PL138
Oniscigaster distans 16S ribosomal RNA gene, parti	AY749818	75.16	100	2.96508e−16	Ephemeroptera	Oniscigastridae	Oniscigaster distans
Cosumnoperla hypocrena 16S ribosomal RNA gene, par	EF623226	77.05	79.74	1.03491e−15	Plecoptera	Perlodidae	Cosumnoperla hypocrena
Amphinemura nigritta 16S ribosomal RNA gene, parti	EF623186	80.58	66.67	1.03491e−15	Plecoptera	Nemouridae	Amphinemura nigritta
Eupholidoptera chabrieri voucher 173 16S ribosomal	EU120792	76.19	94.12	1.03491e−15	Orthoptera	Tettigoniidae	Eupholidoptera chabrieri
Panorpa maculosa 16S ribosomal RNA gene, partial s	AF180055	77.05	79.74	1.03491e−15	Mecoptera	Panorpidae	Panorpa maculosa
Panorpa germanica isolate Eifel 16S ribosomal RNA	AF180052	77.05	79.74	1.03491e−15	Mecoptera	Panorpidae	Panorpa germanica
Panorpa acuta 16S ribosomal RNA gene, partial sequ	AF180047	77.87	79.74	1.03491e−15	Mecoptera	Panorpidae	Panorpa acuta
Afronurus sp. 336SuAf mitochondrial partial 16S rR	HE651427	81.44	63.4	3.6122e−15	Ephemeroptera	Heptageniidae	Afronurus sp. 336SuAf
Udenus wnigrum voucher MAY 16S ribosomal RNA gene,	HM594504	78.95	72.55	3.6122e−15	Orthoptera	Rhaphidophoridae	Udenus wnigrum
Ephemera simulans voucher BYU:IGCEP038 16S ribosom	GQ118285	77.5	78.43	3.6122e−15	Ephemeroptera	Ephemeridae	Ephemera simulans
Osmylus sp. EH−2004 16S ribosomal RNA gene, partia	AY620146	76.74	83.01	3.6122e−15	Neuroptera	Osmylidae	Osmylus sp. EH−2004
Afronurus sp. 297SAAf mitochondrial partial 16S rR	HE651424	79.41	66.67	1.26078e−14	Ephemeroptera	Heptageniidae	Afronurus sp. 297SAAf
Talitropsis sedilloti voucher CW160 16S ribosomal	HM594511	81.52	60.13	1.26078e−14	Orthoptera	Rhaphidophoridae	Talitropsis sedilloti
Pteronarcys scotti 16S ribosomal RNA gene, partial	EF623263	74.64	90.2	1.26078e−14	Plecoptera	Pteronarcyidae	Pteronarcys scotti
Rickera sorpta 16S ribosomal RNA gene, partial seq	EF623235	79.41	66.67	1.26078e−14	Plecoptera	Perlodidae	Rickera sorpta
Helopicus bogaloosa 16S ribosomal RNA gene, partia	EF623228	80.2	65.36	1.26078e−14	Plecoptera	Perlodidae	Helopicus bogaloosa
Cascadoperla trictura 16S ribosomal RNA gene, part	EF623225	80.41	63.4	1.26078e−14	Plecoptera	Perlodidae	Cascadoperla trictura
Isoperla phalerata 16S ribosomal RNA gene, partial	EF623208	80.41	63.4	1.26078e−14	Plecoptera	Perlodidae	Isoperla phalerata
Chloroperla tripunctata 16S ribosomal RNA gene, pa	EF623137	75	87.58	1.26078e−14	Plecoptera	Chloroperlidae	Chloroperla tripunctata
Plumiperla diversa 16S ribosomal RNA gene, partial	EF623132	78.9	69.28	1.26078e−14	Plecoptera	Chloroperlidae	Plumiperla diversa
Isogenoides varians isolate BYU_PL079 16S ribosoma	EF623445	80.41	63.4	1.26078e−14	Plecoptera	Perlodidae	Isogenoides varians
Dipteromimus tipuliformis mitochondrial gene for 1	AB110257	77.24	78.43	1.26078e−14	Ephemeroptera	Dipteromimidae	Dipteromimus tipuliformis
Tasmanophlebia sp. EP158 16S ribosomal RNA gene, p	AY749825	74.64	88.89	1.26078e−14	Ephemeroptera	Oniscigastridae	Tasmanophlebia sp. EP158
Ametropus neavei 16S ribosomal RNA gene, partial s	AY749800	75.35	90.85	1.26078e−14	Ephemeroptera	Ametropodidae	Ametropus neavei
Eutelia adulatricoides mitochondrion, complete gen	KJ185131	76.72	75.82	4.40056e−14	Lepidoptera	Noctuidae	Eutelia adulatricoides
Cryptoperla sp. YK−2012−1 mitochondrial gene for 1	AB711243	77.05	79.74	4.40056e−14	Plecoptera	Peltoperlidae	Cryptoperla sp. YK−2012−1
Cryptoperla japonica mitochondrial gene for 16S rR	AB711202	77.05	79.74	4.40056e−14	Plecoptera	Peltoperlidae	Cryptoperla japonica
Cryptoperla japonica mitochondrial gene for 16S rR	AB711184	77.05	79.74	4.40056e−14	Plecoptera	Peltoperlidae	Cryptoperla japonica
Compsoneuria sp. 10 LV−2012 mitochondrial partial	HE651412	73.08	100	4.40056e−14	Ephemeroptera	Heptageniidae	Compsoneuria sp. 10 LV−2012
Udenus wnigrum voucher CER 16S ribosomal RNA gene,	HM594503	78.07	72.55	4.40056e−14	Orthoptera	Rhaphidophoridae	Udenus wnigrum
Heteromallus cavicola voucher MIL 16S ribosomal RN	HM594499	78.07	72.55	4.40056e−14	Orthoptera	Rhaphidophoridae	Heteromallus cavicola
Dolichopoda sp. MR−2008 16S ribosomal RNA gene, pa	EU887863	74.32	91.5	4.40056e−14	Orthoptera	Rhaphidophoridae	Dolichopoda lustriae
Dolichopoda remyi voucher EDE 16S ribosomal RNA ge	EF216969	77.19	72.55	4.40056e−14	Orthoptera	Rhaphidophoridae	Dolichopoda remyi
Peltoperla arcuata 16S ribosomal RNA gene, partial	EF623258	74.63	85.62	4.40056e−14	Plecoptera	Peltoperlidae	Peltoperla arcuata
Perlinodes aurea 16S ribosomal RNA gene, partial s	EF623234	80.61	63.4	4.40056e−14	Plecoptera	Perlodidae	Perlinodes aurea
Oconoperla innubila 16S ribosomal RNA gene, partia	EF623230	77.78	70.59	4.40056e−14	Plecoptera	Perlodidae	Oconoperla innubila
Isogenoides olivaceus 16S ribosomal RNA gene, part	EF623224	80.61	63.4	4.40056e−14	Plecoptera	Perlodidae	Isogenoides olivaceus
Isogenoides hansoni 16S ribosomal RNA gene, partia	EF623223	80.61	63.4	4.40056e−14	Plecoptera	Perlodidae	Isogenoides hansoni
Yugus bulbosus 16S ribosomal RNA gene, partial seq	EF623214	80.61	63.4	4.40056e−14	Plecoptera	Perlodidae	Yugus bulbosus
Protonemura meyeri 16S ribosomal RNA gene, partial	EF623193	75	86.27	4.40056e−14	Plecoptera	Nemouridae	Protonemura meyeri
Triznaka pintada 16S ribosomal RNA gene, partial s	EF623134	74.83	90.2	4.40056e−14	Plecoptera	Chloroperlidae	Triznaka pintada
Dolichopoda remyi isolate EDE 16S ribosomal RNA ge	AY793589	77.19	72.55	4.40056e−14	Orthoptera	Rhaphidophoridae	Dolichopoda remyi
Pteronarcys princeps mitochondrion, complete genom	AY687866	74.82	90.2	4.40056e−14	Plecoptera	Pteronarcyidae	Pteronarcys princeps
Panorpa nebulosa 16S ribosomal RNA gene, partial s	AF180058	77.05	79.74	4.40056e−14	Mecoptera	Panorpidae	Panorpa nebulosa
Cinygmina sp. EP133 16S ribosomal RNA gene, partia	AY749811	75.97	83.01	4.40056e−14	Ephemeroptera	Heptageniidae	Cinygmina sp. EP133
Parudenus falklandicus voucher PAR 16S ribosomal R	HM594502	80.43	60.13	1.53595e−13	Orthoptera	Rhaphidophoridae	Parudenus falklandicus
Hexagenia sp. BYU IGCEP003 16S ribosomal RNA gene,	GQ118286	79.8	64.71	1.53595e−13	Ephemeroptera	Ephemeridae	Hexagenia sp. BYU IGCEP003
Dolichopoda thasosensis voucher DRA 16S ribosomal	EF216956	74.5	91.5	1.53595e−13	Orthoptera	Rhaphidophoridae	Dolichopoda thasosensis
Dolichopoda pavesii voucher SPI 16S ribosomal RNA	EF216951	78.85	67.32	1.53595e−13	Orthoptera	Rhaphidophoridae	Dolichopoda pavesii
Frisonia picticeps 16S ribosomal RNA gene, partial	EF623227	78.85	67.32	1.53595e−13	Plecoptera	Perlodidae	Frisonia picticeps
Skwala americana 16S ribosomal RNA gene, partial s	EF623217	80	66.67	1.53595e−13	Plecoptera	Perlodidae	Skwala americana
Austronemoura sp. BYU_PL149 16S ribosomal RNA gene	EF623200	78.9	70.59	1.53595e−13	Plecoptera	Notonemouridae	Austronemoura sp. BYU_PL149
Soyedina producta 16S ribosomal RNA gene, partial	EF623196	78.43	66.67	1.53595e−13	Plecoptera	Nemouridae	Soyedina producta
Neuroperlopsis patris 16S ribosomal RNA gene, part	EF623152	73.43	91.5	1.53595e−13	Plecoptera	Eustheniidae	Neuroperlopsis patris
Panorpa rufostigma isolate 33rufAl 16S ribosomal R	EF041517	76.03	77.12	1.53595e−13	Mecoptera	Panorpidae	Panorpa rufostigma
Xiphydria mellipes 16S ribosomal RNA gene, partial	AF379857	75.18	86.93	1.53595e−13	Hymenoptera	Xiphydriidae	Xiphydria mellipes
Dipteromimus tipuliformis mitochondrial gene for 1	AB110255	76.86	77.12	1.53595e−13	Ephemeroptera	Dipteromimidae	Dipteromimus tipuliformis
Panorpa communis 16S ribosomal RNA gene, partial s	AF180050	76.42	79.74	1.53595e−13	Mecoptera	Panorpidae	Panorpa communis
Macdunnoa hipawinia 16S ribosomal RNA gene, partia	AY749796	79.41	66.01	1.53595e−13	Ephemeroptera	Heptageniidae	Macdunnoa hipawinia
Rallidens mcfarlanei 16S ribosomal RNA gene, parti	AY749794	76.72	75.82	1.53595e−13	Ephemeroptera	Rallidentidae	Rallidens mcfarlanei
Siphlonella sp. EP083 16S ribosomal RNA gene, part	AY749785	78.43	66.67	1.53595e−13	Ephemeroptera	Oniscigastridae	Siphlonella sp. EP083
Chaquihua sp. EP080 16S ribosomal RNA gene, partia	AY749784	78.5	69.28	1.53595e−13	Ephemeroptera	Ameletopsidae	Chaquihua sp. EP080
Chiloporter sp. EP051 16S ribosomal RNA gene, part	AY749776	79.38	63.4	1.53595e−13	Ephemeroptera	Ameletopsidae	Chiloporter sp. EP051
Rhithrogena nuragica mitochondrial partial 16S rRN	LN868667	74.83	90.85	5.36098e−13	Ephemeroptera	Heptageniidae	Rhithrogena nuragica
Eupholidoptera smyrnensis isolate MRM1 16S ribosom	KC852310	74.83	94.12	5.36098e−13	Orthoptera	Tettigoniidae	Eupholidoptera smyrnensis
