# Supplementary material for: Testing the potential of a ribosomal 16S marker for DNA metabarcoding of insects
Source: PeerJ. 2016 Apr 19;4:e1966. doi: 10.7717/peerj.1966 (PMC4841222; doi:10.7717/peerj.1966)
Supplement: Supplemental Information 1 [file peerj-04-1966-s006.zip › scripts/5 match OTUs/blast_hits/OTU_57.csv_plot.pdf]

OTU_57.csv

Limnodrilus udekemianus

Naididae

Haplotaxida

93.13

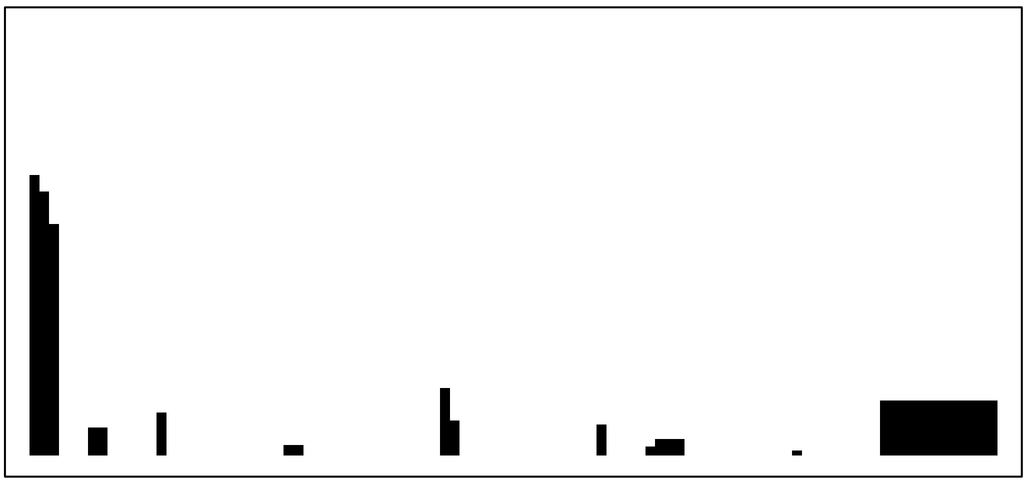

Limnodrilus udekemianus 16S ribosomal RNA gene, pa	AY885612	93.13	100	1.1623e−45	Haplotaxida	Naididae	Limnodrilus udekemianus
Limnodrilus udekemianus isolate 95BTub 16S ribosom	HM991165	92.37	100	1.41597e−44	Haplotaxida	Naididae	Limnodrilus udekemianus
Limnodrilus udekemianus 16S ribosomal RNA gene, pa	AF325986	90.84	100	6.02086e−43	Haplotaxida	Naididae	Limnodrilus udekemianus
Limnodrilus hoffmeisteri haplotype 24_III 16S ribo	EU160492	79.55	100	1.32735e−19	Haplotaxida	Naididae	Limnodrilus hoffmeisteri
Limnodrilus hoffmeisteri haplotype 23_III 16S ribo	EU160466	79.55	100	1.32735e−19	Haplotaxida	Naididae	Limnodrilus hoffmeisteri
Limnodrilus hoffmeisteri haplotype 22_II 16S ribos	EU160465	79.55	100	1.32735e−19	Haplotaxida	Naididae	Limnodrilus hoffmeisteri
Limnodrilus hoffmeisteri isolate WG5 16S ribosomal	AF325980	81.3	92.37	4.63292e−19	Haplotaxida	Naididae	Limnodrilus hoffmeisteri
Limnodrilus hoffmeisteri isolate WG4 16S ribosomal	AF325979	81.3	92.37	4.63292e−19	Haplotaxida	Naididae	Limnodrilus hoffmeisteri
Limnodrilus claparedeanus isolate 303BTub 16S ribo	JF783979	78.79	100	1.61705e−18	Haplotaxida	Naididae	Limnodrilus claparedeanus
Limnodrilus cervix isolate WG2 16S ribosomal RNA g	AF325984	78.79	100	1.61705e−18	Haplotaxida	Naididae	Limnodrilus cervix
Limnodrilus hoffmeisteri isolate 233BTub 16S ribos	JF783983	77.44	100	6.87587e−17	Haplotaxida	Naididae	Limnodrilus hoffmeisteri
Tubificoides kozloffi isolate CE2134 16S ribosomal	HM459963	77.27	100	6.87587e−17	Haplotaxida	Naididae	Tubificoides kozloffi
Tubificoides kozloffi isolate CE2062 16S ribosomal	HM459962	77.27	100	6.87587e−17	Haplotaxida	Naididae	Tubificoides kozloffi
Limnodrilus hoffmeisteri haplotype 31_VI 16S ribos	EU160493	82	76.34	6.87587e−17	Haplotaxida	Naididae	Limnodrilus hoffmeisteri
Limnodrilus hoffmeisteri haplotype 33_VII 16S ribo	EU160485	77.44	100	6.87587e−17	Haplotaxida	Naididae	Limnodrilus hoffmeisteri
Limnodrilus hoffmeisteri haplotype 13_I 16S riboso	EU160471	77.27	100	6.87587e−17	Haplotaxida	Naididae	Limnodrilus hoffmeisteri
Limnodrilus cervix isolate WG1 16S ribosomal RNA g	AF325983	78.03	100	6.87587e−17	Haplotaxida	Naididae	Limnodrilus cervix
Limnodrilus hoffmeisteri isolate 190BTub 16S ribos	JF783981	77.27	100	8.37652e−16	Haplotaxida	Naididae	Limnodrilus hoffmeisteri
Limnodrilus hoffmeisteri voucher Lh2 16S ribosomal	EU117547	77.27	100	8.37652e−16	Haplotaxida	Naididae	Limnodrilus hoffmeisteri
Limnodrilus hoffmeisteri haplotype 26_III 16S ribo	EU160491	77.27	100	8.37652e−16	Haplotaxida	Naididae	Limnodrilus hoffmeisteri
Limnodrilus hoffmeisteri haplotype 27_III 16S ribo	EU160490	77.27	100	8.37652e−16	Haplotaxida	Naididae	Limnodrilus hoffmeisteri
Limnodrilus hoffmeisteri haplotype 29_IV 16S ribos	EU160486	77.27	100	8.37652e−16	Haplotaxida	Naididae	Limnodrilus hoffmeisteri
Limnodrilus hoffmeisteri haplotype 25_III 16S ribo	EU160470	77.27	100	8.37652e−16	Haplotaxida	Naididae	Limnodrilus hoffmeisteri
Limnodrilus hoffmeisteri 16S ribosomal RNA gene, p	EF089340	77.27	100	8.37652e−16	Haplotaxida	Naididae	Limnodrilus hoffmeisteri
Tubifex sp. HRV−2001 16S ribosomal RNA gene, parti	AF315057	77.27	100	8.37652e−16	Haplotaxida	Naididae	Tubifex sp. HRV−2001
Limnodrilus hoffmeisteri isolate parvus 16S riboso	AF325985	77.27	100	8.37652e−16	Haplotaxida	Naididae	Limnodrilus hoffmeisteri
Limnodrilus hoffmeisteri isolate WG6 16S ribosomal	AF325981	80.49	91.6	8.37652e−16	Haplotaxida	Naididae	Limnodrilus hoffmeisteri
Limnodrilus hoffmeisteri isolate WG1 16S ribosomal	AF325976	80.49	91.6	8.37652e−16	Haplotaxida	Naididae	Limnodrilus hoffmeisteri
Limnodrilus hoffmeisteri 16S ribosomal RNA gene, p	AY885613	77.27	100	8.37652e−16	Haplotaxida	Naididae	Limnodrilus hoffmeisteri
Limnodrilus hoffmeisteri haplotype 07_I 16S riboso	EU160483	76.52	100	2.92369e−15	Haplotaxida	Naididae	Limnodrilus hoffmeisteri
Limnodrilus hoffmeisteri haplotype 06_I 16S riboso	EU160481	76.52	100	2.92369e−15	Haplotaxida	Naididae	Limnodrilus hoffmeisteri
Limnodrilus hoffmeisteri haplotype 11_I 16S riboso	EU160480	76.52	100	2.92369e−15	Haplotaxida	Naididae	Limnodrilus hoffmeisteri
Limnodrilus hoffmeisteri haplotype 08_I 16S riboso	EU160476	76.52	100	2.92369e−15	Haplotaxida	Naididae	Limnodrilus hoffmeisteri
Limnodrilus hoffmeisteri haplotype 09_I 16S riboso	EU160475	76.52	100	2.92369e−15	Haplotaxida	Naididae	Limnodrilus hoffmeisteri
Limnodrilus hoffmeisteri haplotype 04_I 16S riboso	EU160474	76.52	100	2.92369e−15	Haplotaxida	Naididae	Limnodrilus hoffmeisteri
Limnodrilus hoffmeisteri haplotype 05_I 16S riboso	EU160473	76.52	100	2.92369e−15	Haplotaxida	Naididae	Limnodrilus hoffmeisteri
Limnodrilus hoffmeisteri haplotype 10_I 16S riboso	EU160472	76.52	100	2.92369e−15	Haplotaxida	Naididae	Limnodrilus hoffmeisteri
Limnodrilus hoffmeisteri haplotype 12_I 16S riboso	EU160469	76.52	100	2.92369e−15	Haplotaxida	Naididae	Limnodrilus hoffmeisteri
Limnodrilus hoffmeisteri haplotype 03_I 16S riboso	EU160467	76.52	100	2.92369e−15	Haplotaxida	Naididae	Limnodrilus hoffmeisteri
Limnodrilus hoffmeisteri haplotype 01_I 16S riboso	EU160464	76.52	100	2.92369e−15	Haplotaxida	Naididae	Limnodrilus hoffmeisteri
Limnodrilus hoffmeisteri isolate LBR 16S ribosomal	AF325982	79.67	92.37	2.92369e−15	Haplotaxida	Naididae	Limnodrilus hoffmeisteri
Limnodrilus hoffmeisteri Mitochondrion 16S Ribosom	AJ225912	76.52	100	2.92369e−15	Haplotaxida	Naididae	Limnodrilus hoffmeisteri
Marionina southerni clone CE674 16S ribosomal RNA	JN799953	83.16	71.76	1.02047e−14	Haplotaxida	Enchytraeidae	Marionina southerni
Limnodrilus hoffmeisteri haplotype 28_III 16S ribo	EU160489	81.63	74.05	1.02047e−14	Haplotaxida	Naididae	Limnodrilus hoffmeisteri
Limnodrilus hoffmeisteri haplotype 19_II 16S ribos	EU160487	76.34	100	1.02047e−14	Haplotaxida	Naididae	Limnodrilus hoffmeisteri
Limnodrilus hoffmeisteri haplotype 16_II 16S ribos	EU160484	76.34	100	1.02047e−14	Haplotaxida	Naididae	Limnodrilus hoffmeisteri
Limnodrilus hoffmeisteri haplotype 21_II 16S ribos	EU160482	76.34	100	1.02047e−14	Haplotaxida	Naididae	Limnodrilus hoffmeisteri
Limnodrilus hoffmeisteri haplotype 15_II 16S ribos	EU160478	76.34	100	1.02047e−14	Haplotaxida	Naididae	Limnodrilus hoffmeisteri
Limnodrilus hoffmeisteri haplotype 17_II 16S ribos	EU160477	76.34	100	1.02047e−14	Haplotaxida	Naididae	Limnodrilus hoffmeisteri
Limnodrilus hoffmeisteri haplotype 20_II 16S ribos	EU160463	76.34	100	1.02047e−14	Haplotaxida	Naididae	Limnodrilus hoffmeisteri
Limnodrilus hoffmeisteri haplotype 14_II 16S ribos	EU160462	76.34	100	1.02047e−14	Haplotaxida	Naididae	Limnodrilus hoffmeisteri
Guestphalinus sp. PDW−2010 isolate CE865 16S ribos	GU592334	76.52	100	3.56179e−14	Lumbriculida	Lumbriculidae	Guestphalinus sp. PDW−2010
Tubifex tubifex voucher SpaTt3 16S ribosomal RNA g	EU117525	75.76	100	3.56179e−14	Haplotaxida	Naididae	Tubifex tubifex
Limnodrilus hoffmeisteri haplotype 02_I 16S riboso	EU160468	75.76	100	3.56179e−14	Haplotaxida	Naididae	Limnodrilus hoffmeisteri
Limnodrilus hoffmeisteri isolate 248BTub 16S ribos	JF783980	76.61	92.37	1.24319e−13	Haplotaxida	Naididae	Limnodrilus hoffmeisteri
Limnodrilus hoffmeisteri haplotype 18_II 16S ribos	EU160479	75.57	100	1.24319e−13	Haplotaxida	Naididae	Limnodrilus hoffmeisteri
Limnodrilus hoffmeisteri Mitochondrion 16S Ribosom	AJ225901	76.61	92.37	1.24319e−13	Haplotaxida	Naididae	Limnodrilus hoffmeisteri
Tubifex tubifex voucher Tt01 16S ribosomal RNA gen	HQ603822	75	100	4.33914e−13	Haplotaxida	Naididae	Tubifex tubifex
Parachilota sp. 570 16S ribosomal RNA gene, partia	HQ232624	81.44	72.52	4.33914e−13	Haplotaxida	Acanthodrilidae	Parachilota sp. 570
Tubifex tubifex voucher SpaTt4 16S ribosomal RNA g	EU117526	75	100	4.33914e−13	Haplotaxida	Naididae	Tubifex tubifex
Limnodrilus hoffmeisteri haplotype 30_V 16S riboso	EU160488	78.86	91.6	4.33914e−13	Haplotaxida	Naididae	Limnodrilus hoffmeisteri
Limnodrilus hoffmeisteri isolate 235BTub 16S ribos	JF783982	75	100	1.51451e−12	Haplotaxida	Naididae	Limnodrilus hoffmeisteri
Limnodrilus hoffmeisteri voucher Lh1 16S ribosomal	EU117546	75	100	1.51451e−12	Haplotaxida	Naididae	Limnodrilus hoffmeisteri
Parachilota sp. 561 16S ribosomal RNA gene, partia	HQ232623	80.41	72.52	5.28616e−12	Haplotaxida	Acanthodrilidae	Parachilota sp. 561
Rhynchelmis glandula isolate CE864 16S ribosomal R	GU592338	80.77	77.1	5.28616e−12	Lumbriculida	Lumbriculidae	Rhynchelmis glandula
Rhynchelmis elrodi isolate CE580 16S ribosomal RNA	GU592337	80.77	77.1	5.28616e−12	Lumbriculida	Lumbriculidae	Rhynchelmis elrodi
Rhynchelmis elrodi 16S ribosomal RNA gene, partial	AY764152	80.77	77.1	5.28616e−12	Lumbriculida	Lumbriculidae	Rhynchelmis elrodi
Enchytraeus sp. 16S ribosomal RNA gene, mitochondr	U55841	77.52	96.95	5.28616e−12	Haplotaxida	Enchytraeidae	Enchytraeus sp.
Tectidrilus bori 16S ribosomal RNA gene, partial s	AY885631	75.56	100	5.28616e−12	Haplotaxida	Naididae	Tectidrilus bori
Thalassodrilides bruneti 16S ribosomal RNA gene, p	AY885625	74.26	100	5.28616e−12	Haplotaxida	Naididae	Thalassodrilides bruneti
Fimoscolex sp. PG 16S ribosomal RNA gene, partial	JF267885	80	67.94	1.84505e−11	Haplotaxida	Glossoscolecidae	Fimoscolex sp. PG
Isochaetides gianii voucher Ig02 16S ribosomal RNA	HQ603817	77.98	80.92	1.84505e−11	Haplotaxida	Naididae	Isochaetides gianii
Tubifex tubifex isolate 207BTub 16S ribosomal RNA	JF783971	78	76.34	1.84505e−11	Haplotaxida	Naididae	Tubifex tubifex
Henlea nasuta isolate CE824 16S ribosomal RNA gene	GU901823	75.94	100	1.84505e−11	Haplotaxida	Enchytraeidae	Henlea nasuta
Tubificoides brownae isolate CE3387 16S ribosomal	HM459978	75	100	1.84505e−11	Haplotaxida	Naididae	Tubificoides brownae
Tubificoides diazi isolate CE3410 16S ribosomal RN	HM459944	74.1	100	1.84505e−11	Haplotaxida	Naididae	Tubificoides diazi
Tubificoides diazi isolate CE3409 16S ribosomal RN	HM459943	74.1	100	1.84505e−11	Haplotaxida	Naididae	Tubificoides diazi
Tubifex tubifex voucher Tt10 16S ribosomal RNA gen	EU311258	78	76.34	1.84505e−11	Haplotaxida	Naididae	Tubifex tubifex
Aporrectodea dubiosa isolate MPL429 16S ribosomal	KJ912509	80.22	68.7	6.43986e−11	Haplotaxida	Lumbricidae	Aporrectodea dubiosa
Chamaedrilus aff. sphagnetorum C SM−2014 isolate C	KF672460	78.95	72.52	6.43986e−11	Haplotaxida	Enchytraeidae	Chamaedrilus aff. sphagnetorum C SM−2014
Chamaedrilus aff. sphagnetorum C SM−2014 isolate C	KF672457	78.95	72.52	6.43986e−11	Haplotaxida	Enchytraeidae	Chamaedrilus aff. sphagnetorum C SM−2014
Glossoscolex paulistus 16S ribosomal RNA gene, par	JF267888	78.1	78.63	6.43986e−11	Haplotaxida	Glossoscolecidae	Glossoscolex paulistus
Psammoryctides moravicus isolate 222BTub 16S ribos	JF783973	77.98	80.15	6.43986e−11	Haplotaxida	Naididae	Psammoryctides moravicus
Tubificoides pseudogaster isolate CE199−3 16S ribo	HM459965	77.78	75.57	6.43986e−11	Haplotaxida	Naididae	Tubificoides pseudogaster
Tubifex ignotus isolate 2 16S ribosomal RNA gene,	AF325988	78.43	75.57	6.43986e−11	Haplotaxida	Naididae	Tubifex ignotus
Tubifex ignotus isolate 1 16S ribosomal RNA gene,	AF325987	78.43	75.57	6.43986e−11	Haplotaxida	Naididae	Tubifex ignotus
Tubifex ignotus 16S ribosomal RNA gene, partial se	AY885610	78.43	75.57	6.43986e−11	Haplotaxida	Naididae	Tubifex ignotus
Hormogaster samnitica voucher UCMLT 00046 16S ribo	KF975031	82.56	64.12	2.24773e−10	Haplotaxida	Hormogastridae	Hormogaster samnitica
Hormogaster samnitica voucher UCMLT 00045 16S ribo	KF975030	82.56	64.12	2.24773e−10	Haplotaxida	Hormogastridae	Hormogaster samnitica
Hormogaster samnitica voucher UCMLT 00044 16S ribo	KF975029	82.56	64.12	2.24773e−10	Haplotaxida	Hormogastridae	Hormogaster samnitica
Hormogaster samnitica voucher UCMLT 00043 16S ribo	KF975028	82.56	64.12	2.24773e−10	Haplotaxida	Hormogastridae	Hormogaster samnitica
Hormogaster samnitica voucher UCMLT 00042 16S ribo	KF975027	82.56	64.12	2.24773e−10	Haplotaxida	Hormogastridae	Hormogaster samnitica
Hormogaster samnitica voucher UCMLT 00041 16S ribo	KF975026	82.56	64.12	2.24773e−10	Haplotaxida	Hormogastridae	Hormogaster samnitica
Hormogaster samnitica voucher UCMLT 00040 16S ribo	KF975025	82.56	64.12	2.24773e−10	Haplotaxida	Hormogastridae	Hormogaster samnitica
Hormogaster samnitica voucher UCMLT 00039 16S ribo	KF975024	82.56	64.12	2.24773e−10	Haplotaxida	Hormogastridae	Hormogaster samnitica
Hormogaster samnitica voucher UCMLT 00038 16S ribo	KF975023	82.56	64.12	2.24773e−10	Haplotaxida	Hormogastridae	Hormogaster samnitica
Hormogaster samnitica voucher UCMLT 00049 16S ribo	KF975022	82.56	64.12	2.24773e−10	Haplotaxida	Hormogastridae	Hormogaster samnitica
Hormogaster samnitica voucher UCMLT 00048 16S ribo	KF975021	82.56	64.12	2.24773e−10	Haplotaxida	Hormogastridae	Hormogaster samnitica
Hormogaster samnitica voucher UCMLT 00047 16S ribo	KF975020	82.56	64.12	2.24773e−10	Haplotaxida	Hormogastridae	Hormogaster samnitica
Isochaetides gianii voucher Ig01 16S ribosomal RNA	HQ603816	77.06	80.92	2.24773e−10	Haplotaxida	Naididae	Isochaetides gianii
