# Supplementary material for: Testing the potential of a ribosomal 16S marker for DNA metabarcoding of insects
Source: PeerJ. 2016 Apr 19;4:e1966. doi: 10.7717/peerj.1966 (PMC4841222; doi:10.7717/peerj.1966)
Supplement: Supplemental Information 1 [file peerj-04-1966-s006.zip › scripts/5 match OTUs/blast_hits/OTU_58.csv_plot.pdf]

OTU_58.csv

Erpobdella obscura

Erpobdellidae

Arhynchobdellida

89.52

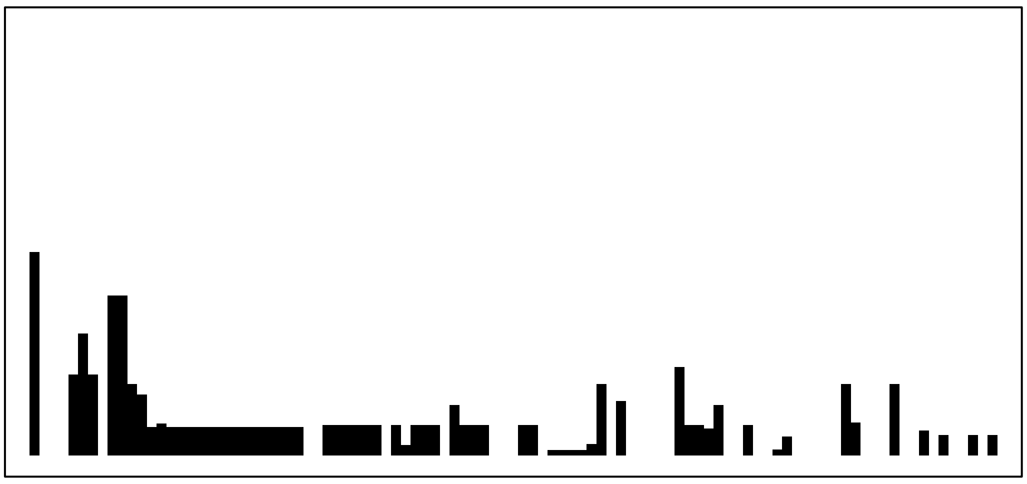

Erpobdella obscura isolate Eobs 16S ribosomal RNA	JQ821464	89.52	97.64	1.88622e−36	Arhynchobdellida	Erpobdellidae	Erpobdella obscura
Toutellus adecus isolate 894 16S ribosomal RNA gen	HQ232611	78.29	97.64	1.45141e−12	Haplotaxida	Megascolecidae	Toutellus adecus
Amynthas sp. FJ201109−01 isolate S44J 16S ribosoma	KF205704	79.8	77.17	1.76817e−11	Haplotaxida	Megascolecidae	Amynthas sp. FJ201109−01
Toutellus sp. 391 16S ribosomal RNA gene, partial	JF267896	77.68	85.83	7.51847e−10	Haplotaxida	Megascolecidae	Toutellus sp. 391
Amynthas glabrus voucher SC201008−02 16S ribosomal	KF179549	83.78	56.69	9.15937e−09	Haplotaxida	Megascolecidae	Amynthas glabrus
Amynthas hainanicus strain HN201115−01 16S ribosom	JX315376	85.71	49.61	9.15937e−09	Haplotaxida	Megascolecidae	Amynthas hainanicus
Amynthas glabrus mitochondrial gene for 16S riboso	AB474285	83.78	56.69	9.15937e−09	Haplotaxida	Megascolecidae	Amynthas glabrus
Amynthas sp. 2 QZ−2012 16S ribosomal RNA gene, par	JQ904531	77.89	73.23	3.19693e−08	Haplotaxida	Megascolecidae	Amynthas sp. 2 QZ−2012
Pirodrilus minutus 16S ribosomal RNA gene, partial	DQ459958	87.5	47.24	3.19693e−08	Haplotaxida	Naididae	Pirodrilus minutus
Pirodrilus minutus 16S ribosomal RNA gene, partial	AY885590	87.5	47.24	3.19693e−08	Haplotaxida	Naididae	Pirodrilus minutus
Thalassodrilus prostatus isolate CE970 16S ribosom	KJ753883	83.33	59.06	1.11584e−07	Haplotaxida	Naididae	Thalassodrilus prostatus
Duplodicodrilus schmardae isolate S63T 16S ribosom	KF205896	82.86	53.54	1.11584e−07	Haplotaxida	Megascolecidae	Duplodicodrilus schmardae
Amynthas hupeiensis isolate S2F 16S ribosomal RNA	KF205757	81.33	58.27	1.11584e−07	Haplotaxida	Megascolecidae	Amynthas hupeiensis
Amynthas sp. YN201101−04 isolate S4M 16S ribosomal	KF205725	81.48	62.2	1.11584e−07	Haplotaxida	Megascolecidae	Amynthas sp. YN201101−04
Amynthas hupeiensis isolate SR4 16S ribosomal RNA	JQ236795	81.33	58.27	1.11584e−07	Haplotaxida	Megascolecidae	Amynthas hupeiensis
Amynthas hupeiensis isolate SV4 16S ribosomal RNA	JQ236799	81.33	58.27	1.11584e−07	Haplotaxida	Megascolecidae	Amynthas hupeiensis
Amynthas hupeiensis isolate SL10−8 16S ribosomal R	JQ236793	81.33	58.27	1.11584e−07	Haplotaxida	Megascolecidae	Amynthas hupeiensis
Amynthas hupeiensis isolate SL10−6 16S ribosomal R	JQ236791	81.33	58.27	1.11584e−07	Haplotaxida	Megascolecidae	Amynthas hupeiensis
Amynthas hupeiensis isolate SL1−3 16S ribosomal RN	JQ236789	81.33	58.27	1.11584e−07	Haplotaxida	Megascolecidae	Amynthas hupeiensis
Amynthas hupeiensis isolate SL−1 16S ribosomal RNA	JQ236788	81.33	58.27	1.11584e−07	Haplotaxida	Megascolecidae	Amynthas hupeiensis
Amynthas hupeiensis isolate SB2−8 16S ribosomal RN	JQ236787	81.33	58.27	1.11584e−07	Haplotaxida	Megascolecidae	Amynthas hupeiensis
Amynthas hupeiensis isolate SB2−3 16S ribosomal RN	JQ236784	81.33	58.27	1.11584e−07	Haplotaxida	Megascolecidae	Amynthas hupeiensis
Amynthas hupeiensis isolate SB2−4 16S ribosomal RN	JQ236785	81.33	58.27	1.11584e−07	Haplotaxida	Megascolecidae	Amynthas hupeiensis
Amynthas hupeiensis isolate SB2−1 16S ribosomal RN	JQ236783	81.33	58.27	1.11584e−07	Haplotaxida	Megascolecidae	Amynthas hupeiensis
Amynthas hupeiensis isolate QZ5 16S ribosomal RNA	JQ236781	81.33	58.27	1.11584e−07	Haplotaxida	Megascolecidae	Amynthas hupeiensis
Amynthas hupeiensis isolate LG14 16S ribosomal RNA	JQ236778	81.33	58.27	1.11584e−07	Haplotaxida	Megascolecidae	Amynthas hupeiensis
Amynthas hupeiensis mitochondrial gene for 16S rib	AB474293	81.33	58.27	1.11584e−07	Haplotaxida	Megascolecidae	Amynthas hupeiensis
Amynthas hupeiensis mitochondrial gene for 16S rib	AB474294	81.33	58.27	1.11584e−07	Haplotaxida	Megascolecidae	Amynthas hupeiensis
Hormogaster samnitica voucher UCMLT 00182 16S ribo	KF975156	79.75	62.2	3.89466e−07	Haplotaxida	Hormogastridae	Hormogaster samnitica
Parachilota sp. 561 16S ribosomal RNA gene, partia	HQ232623	78.35	72.44	3.89466e−07	Haplotaxida	Acanthodrilidae	Parachilota sp. 561
Amynthas corticis mitochondrion, complete genome	KM199290	81.43	53.54	1.35937e−06	Haplotaxida	Megascolecidae	Amynthas corticis
Amynthas corticis haplotype 14 16S ribosomal RNA g	KP214595	81.43	53.54	1.35937e−06	Haplotaxida	Megascolecidae	Amynthas corticis
Amynthas corticis haplotype 10 16S ribosomal RNA g	KP214594	81.43	53.54	1.35937e−06	Haplotaxida	Megascolecidae	Amynthas corticis
Amynthas corticis haplotype 8 16S ribosomal RNA ge	KP214592	81.43	53.54	1.35937e−06	Haplotaxida	Megascolecidae	Amynthas corticis
Amynthas corticis haplotype 7 16S ribosomal RNA ge	KP214591	81.43	53.54	1.35937e−06	Haplotaxida	Megascolecidae	Amynthas corticis
Amynthas corticis haplotype 4 16S ribosomal RNA ge	KP214588	81.43	53.54	1.35937e−06	Haplotaxida	Megascolecidae	Amynthas corticis
Amynthas corticis haplotype 1 16S ribosomal RNA ge	KP214585	77.32	74.02	1.35937e−06	Haplotaxida	Megascolecidae	Amynthas corticis
Amynthas sp. HN201111−02 isolate S24I 16S ribosoma	KF205882	81.43	53.54	1.35937e−06	Haplotaxida	Megascolecidae	Amynthas sp. HN201111−02
Amynthas sp. ZJ201101−05 isolate S5U 16S ribosomal	KF205758	80.49	62.99	1.35937e−06	Haplotaxida	Megascolecidae	Amynthas sp. ZJ201101−05
Amynthas corticis isolate S1P 16S ribosomal RNA ge	KF205751	81.43	53.54	1.35937e−06	Haplotaxida	Megascolecidae	Amynthas corticis
Amynthas hawayanus 16S ribosomal RNA gene, partial	JX196393	81.43	53.54	1.35937e−06	Haplotaxida	Megascolecidae	Amynthas hawayanus
Plutellus sp. 2 PK−2012 16S ribosomal RNA gene, pa	JX046749	81.43	53.54	1.35937e−06	Haplotaxida	Megascolecidae	Plutellus sp. 2 PK−2012
Argilophilus sp. 900 16S ribosomal RNA gene, parti	HQ232613	75.96	78.74	1.35937e−06	Haplotaxida	Megascolecidae	Argilophilus sp. 900
Metaphire communissima mitochondrial gene for 16S	AB474338	82.35	51.97	1.35937e−06	Haplotaxida	Megascolecidae	Metaphire communissima
Amynthas corticis mitochondrial gene for 16S ribos	AB474291	81.43	53.54	1.35937e−06	Haplotaxida	Megascolecidae	Amynthas corticis
Diplocardia eiseni isolate 42 16S ribosomal RNA ge	EF126212	81.43	53.54	1.35937e−06	Haplotaxida	Megascolecidae	Diplocardia eiseni
Amynthas sp. AMVK−2006 16S ribosomal RNA gene, par	EF126187	81.43	53.54	1.35937e−06	Haplotaxida	Megascolecidae	Amynthas sp. AMVK−2006
Diplocardia komareki isolate 6.16S1 16S ribosomal	DQ533722	73.64	99.21	1.35937e−06	Haplotaxida	Megascolecidae	Diplocardia komareki
Amynthas diffringens isolate 5I 16S ribosomal RNA	DQ257309	77.32	74.02	1.35937e−06	Haplotaxida	Megascolecidae	Amynthas diffringens
Amynthas diffringens isolate 4I 16S ribosomal RNA	DQ257308	77.32	74.02	1.35937e−06	Haplotaxida	Megascolecidae	Amynthas diffringens
Amynthas diffringens isolate 2I 16S ribosomal RNA	DQ257306	81.43	53.54	1.35937e−06	Haplotaxida	Megascolecidae	Amynthas diffringens
Amynthas diffringens isolate 1I 16S ribosomal RNA	DQ257305	81.43	53.54	1.35937e−06	Haplotaxida	Megascolecidae	Amynthas diffringens
Hormogaster samnitica voucher UCMLT 00179 16S ribo	KF975153	76.7	80.31	4.74467e−06	Haplotaxida	Hormogastridae	Hormogaster samnitica
Hormogaster samnitica voucher UCMLT 00178 16S ribo	KF975152	80.25	62.99	4.74467e−06	Haplotaxida	Hormogastridae	Hormogaster samnitica
Hormogaster samnitica voucher UCMLT 00177 16S ribo	KF975151	80.25	62.99	4.74467e−06	Haplotaxida	Hormogastridae	Hormogaster samnitica
Hormogaster samnitica voucher UCMLT 00176 16S ribo	KF975150	80.25	62.99	4.74467e−06	Haplotaxida	Hormogastridae	Hormogaster samnitica
Amynthas sp. YN201109−02 isolate S64M 16S ribosoma	KF205738	80.25	62.2	4.74467e−06	Haplotaxida	Megascolecidae	Amynthas sp. YN201109−02
Parachilota sp. 570 16S ribosomal RNA gene, partia	HQ232624	80.52	59.06	4.74467e−06	Haplotaxida	Acanthodrilidae	Parachilota sp. 570
Diplocardia eiseni isolate 426 16S ribosomal RNA g	HQ232493	83.33	47.24	4.74467e−06	Haplotaxida	Megascolecidae	Diplocardia eiseni
Diplocardia farmvillensis isolate 420 16S ribosoma	HQ232491	74.8	96.85	4.74467e−06	Haplotaxida	Megascolecidae	Diplocardia farmvillensis
Metaphire acincta mitochondrial gene for 16S ribos	AB474313	82.54	49.61	4.74467e−06	Haplotaxida	Megascolecidae	Duplodicodrilus acinctus
Hormogaster samnitica voucher UCMLT 00186 16S ribo	KF975160	78.48	62.2	1.65605e−05	Haplotaxida	Hormogastridae	Hormogaster samnitica
Hormogaster samnitica voucher UCMLT 00185 16S ribo	KF975159	78.48	62.2	1.65605e−05	Haplotaxida	Hormogastridae	Hormogaster samnitica
Hormogaster samnitica voucher UCMLT 00181 16S ribo	KF975155	78.48	62.2	1.65605e−05	Haplotaxida	Hormogastridae	Hormogaster samnitica
Hormogaster samnitica voucher UCMLT 00180 16S ribo	KF975154	78.48	62.2	1.65605e−05	Haplotaxida	Hormogastridae	Hormogaster samnitica
Amynthas sp. FJ201002−02 isolate S6D 16S ribosomal	KF205887	78.82	65.35	1.65605e−05	Haplotaxida	Megascolecidae	Amynthas sp. FJ201002−02
Amynthas sp. HB201101−15 isolate S15P 16S ribosoma	KF205754	84.13	48.82	1.65605e−05	Haplotaxida	Megascolecidae	Amynthas sp. HB201101−15
Amynthas sp. FJ201110−16 isolate S62J 16S ribosoma	KF205715	81.43	53.54	1.65605e−05	Haplotaxida	Megascolecidae	Amynthas sp. FJ201110−16
Amynthas sp. FJ201110−09 isolate S55J 16S ribosoma	KF205711	81.43	53.54	1.65605e−05	Haplotaxida	Megascolecidae	Amynthas sp. FJ201110−09
Amynthas sp. FJ201106−03 isolate S11J 16S ribosoma	KF205696	81.25	50.39	1.65605e−05	Haplotaxida	Megascolecidae	Amynthas sp. FJ201106−03
Amynthas sp. GD201101−04 isolate S4T 16S ribosomal	KF205671	82.35	52.76	1.65605e−05	Haplotaxida	Megascolecidae	Amynthas sp. GD201101−04
Driloleirus sp. 917 16S ribosomal RNA gene, partia	JF267898	76.47	77.95	1.65605e−05	Haplotaxida	Megascolecidae	Driloleirus sp. 917
Megascolex sp. NewCal982 isolate 982 16S ribosomal	HQ232607	76.84	72.44	1.65605e−05	Haplotaxida	Megascolecidae	Megascolex sp. NewCal982
Deinodrilus n. sp. 3 TRB−2010 isolate WM95 16S rib	HQ232596	81.43	51.97	1.65605e−05	Haplotaxida	Acanthodrilidae	Deinodrilus n. sp. 3 TRB−2010
Pontodrilus sp. WM19 16S ribosomal RNA gene, parti	HQ232527	73.64	97.64	1.65605e−05	Haplotaxida	Megascolecidae	Pontodrilus sp. WM19
Megascolex novaezealandiae isolate WM103 16S ribos	HQ232508	78.41	67.72	1.65605e−05	Haplotaxida	Megascolecidae	Megascolex novaezealandiae
Metaphire sieboldi mitochondrial gene for 16S ribo	AB474353	80.28	55.91	1.65605e−05	Haplotaxida	Megascolecidae	Metaphire sieboldi
Metaphire communissima mitochondrial gene for 16S	AB474337	80.88	51.97	1.65605e−05	Haplotaxida	Megascolecidae	Metaphire communissima
Amynthas surcatus mitochondrial gene for 16S ribos	AB474320	77.65	65.35	1.65605e−05	Haplotaxida	Megascolecidae	Amynthas surcatus
Metaphire abdita 16S ribosomal RNA gene, partial s	EF490527	77.78	62.99	1.65605e−05	Haplotaxida	Megascolecidae	Metaphire abdita
Metaphire tschiliensis tschiliensis 16S ribosomal	EF490525	77.78	62.99	1.65605e−05	Haplotaxida	Megascolecidae	Metaphire tschiliensis tschiliensis
Terrisswalkerius windsori isolate oli116 16S ribos	AF406587	77	75.59	1.65605e−05	Haplotaxida	Megascolecidae	Terrisswalkerius windsori
Terrisswalkerius moritzi isolate oli85 16S ribosom	AF406560	76.84	72.44	1.65605e−05	Haplotaxida	Megascolecidae	Terrisswalkerius moritzi
Aporrectodea dubiosa isolate MPL429 16S ribosomal	KJ912509	83.33	47.24	5.78019e−05	Haplotaxida	Lumbricidae	Aporrectodea dubiosa
Amynthas sp. FJ201110−15 isolate S61J 16S ribosoma	KF205714	81.54	51.18	5.78019e−05	Haplotaxida	Megascolecidae	Amynthas sp. FJ201110−15
Amynthas sp. FJ201110−13 isolate S59J 16S ribosoma	KF205713	80	53.54	5.78019e−05	Haplotaxida	Megascolecidae	Amynthas sp. FJ201110−13
Amynthas sp. FJ201108−06 isolate S33J 16S ribosoma	KF205700	75.53	72.44	5.78019e−05	Haplotaxida	Megascolecidae	Amynthas sp. FJ201108−06
Amynthas sp. 13 JS−2012 16S ribosomal RNA gene, pa	JX081525	78.05	62.99	5.78019e−05	Haplotaxida	Megascolecidae	Amynthas sp. 13 JS−2012
Plutellus sp. 5 PK−2012 16S ribosomal RNA gene, pa	JX157597	83.33	47.24	5.78019e−05	Haplotaxida	Megascolecidae	Plutellus sp. 5 PK−2012
Arctiostrotus sp. 389 16S ribosomal RNA gene, part	JF267895	76.6	72.44	5.78019e−05	Haplotaxida	Acanthodrilidae	Arctiostrotus sp. 389
Glossodrilus sp. 862 16S ribosomal RNA gene, parti	JF267889	77.5	62.2	5.78019e−05	Haplotaxida	Glossoscolecidae	Glossodrilus sp. 862
Dichogaster sp. Ke 16S ribosomal RNA gene, partial	JF267875	81.16	52.76	5.78019e−05	Haplotaxida	Megascolecidae	Dichogaster sp. Ke
Maoridrilus n. sp. 8 TRB−2010 isolate WM44 16S rib	HQ232551	78.49	71.65	5.78019e−05	Haplotaxida	Acanthodrilidae	Maoridrilus n. sp. 8 TRB−2010
Metaphire sieboldi mitochondrial gene for 16S ribo	AB482154	80.95	49.61	5.78019e−05	Haplotaxida	Megascolecidae	Metaphire sieboldi
Metaphire sieboldi mitochondrial gene for 16S ribo	AB482150	77.5	62.2	5.78019e−05	Haplotaxida	Megascolecidae	Metaphire sieboldi
Metaphire sieboldi mitochondrial gene for 16S ribo	AB482143	77.5	62.2	5.78019e−05	Haplotaxida	Megascolecidae	Metaphire sieboldi
Metaphire sieboldi mitochondrial gene for 16S ribo	AB482121	80.95	49.61	5.78019e−05	Haplotaxida	Megascolecidae	Metaphire sieboldi
Metaphire sieboldi mitochondrial gene for 16S ribo	AB482119	77.5	62.2	5.78019e−05	Haplotaxida	Megascolecidae	Metaphire sieboldi
Metaphire sieboldi mitochondrial gene for 16S ribo	AB482113	80.95	49.61	5.78019e−05	Haplotaxida	Megascolecidae	Metaphire sieboldi
Metaphire servina mitochondrial gene for 16S ribos	AB474350	80	53.54	5.78019e−05	Haplotaxida	Megascolecidae	Metaphire servina
