# Supplementary material for: Testing the potential of a ribosomal 16S marker for DNA metabarcoding of insects
Source: PeerJ. 2016 Apr 19;4:e1966. doi: 10.7717/peerj.1966 (PMC4841222; doi:10.7717/peerj.1966)
Supplement: Supplemental Information 1 [file peerj-04-1966-s006.zip › scripts/5 match OTUs/blast_hits/OTU_583.csv_plot.pdf]

OTU_583.csv

Oreodytes sanmarkii

Dytiscidae

Coleoptera

98.09

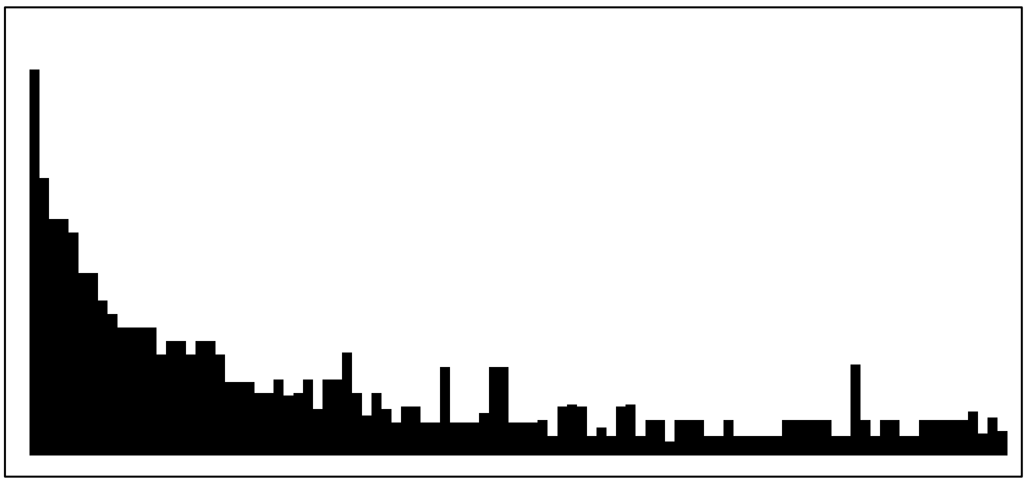

Oreodytes sanmarkii mitochondrial 16S rRNA gene (p	HF931524	98.09	100	6.73453e−69	Coleoptera	Dytiscidae	Oreodytes sanmarkii
Oreodytes obesus cordillerensis mitochondrial part	HF931516	92.99	100	4.8492e−58	Coleoptera	Dytiscidae	Oreodytes obesus cordillerensis
Oreodytes crassulus partial 16S rRNA gene	AJ850348	91.08	100	3.06018e−54	Coleoptera	Dytiscidae	Oreodytes crassulus
Oreodytes congruus partial 16S rRNA gene	AJ850347	91.08	100	3.06018e−54	Coleoptera	Dytiscidae	Oreodytes congruus
Oreodytes congruus 16S ribosomal RNA gene, partial	KF575550	90.45	100	1.30122e−52	Coleoptera	Dytiscidae	Oreodytes congruus
Oreodytes abbreviatus mitochondrial 16S rRNA gene	HF931477	88.54	100	8.21159e−49	Coleoptera	Dytiscidae	Oreodytes abbreviatus
Oreodytes rhyacophilus partial 16S rRNA gene	AJ850352	88.54	100	8.21159e−49	Coleoptera	Dytiscidae	Oreodytes rhyacophilus
Oreodytes mongolicus mitochondrial 16S rRNA gene (	HF931491	87.26	100	4.25371e−46	Coleoptera	Dytiscidae	Oreodytes mongolicus
Oreodytes snoqualmie mitochondrial partial 16S rRN	HF931519	86.62	100	1.80872e−44	Coleoptera	Dytiscidae	Oreodytes snoqualmie
Oreodytes shorti mitochondrial 16S rRNA gene (part	HF931488	85.99	100	2.20348e−43	Coleoptera	Dytiscidae	Oreodytes shorti
Boreonectes riberae mitochondrial 16S rRNA gene (p	HF931461	85.99	100	2.68438e−42	Coleoptera	Dytiscidae	Boreonectes riberae
Boreonectes griseostriatus mitochondrial partial 1	HF931387	85.99	100	2.68438e−42	Coleoptera	Dytiscidae	Boreonectes griseostriatus
Boreonectes multilineatus mitochondrial partial 16	HF931384	85.99	100	2.68438e−42	Coleoptera	Dytiscidae	Boreonectes multilineatus
Laccornis difformis 16S ribosomal RNA gene, partia	KF575544	84.71	100	1.14143e−40	Coleoptera	Dytiscidae	Laccornis difformis
Boreonectes alpestris mitochondrial 16S rRNA gene	HF931512	85.35	100	1.14143e−40	Coleoptera	Dytiscidae	Boreonectes alpestris
Boreonectes striatellus mitochondrial 16S rRNA gen	HF931511	85.35	100	1.14143e−40	Coleoptera	Dytiscidae	Boreonectes striatellus
Deronectes parvicollis mitochondrial 16S rRNA gene	HF931454	84.71	100	1.14143e−40	Coleoptera	Dytiscidae	Deronectes parvicollis
Boreonectes macedonicus mitochondrial 16S rRNA gen	HF931359	85.35	100	1.14143e−40	Coleoptera	Dytiscidae	Boreonectes macedonicus
Stictotarsus ibericus 16S ribosomal RNA (rrnL) gen	EF670030	85.35	100	1.14143e−40	Coleoptera	Dytiscidae	Stictotarsus ibericus
Boreonectes griseostriatus mitochondrial 16S rRNA	HF931541	84.71	100	1.39055e−39	Coleoptera	Dytiscidae	Boreonectes griseostriatus
Oreodytes scitulus 16S ribosomal RNA gene, partial	KF575551	83.44	100	5.91275e−38	Coleoptera	Dytiscidae	Oreodytes scitulus
Oreodytes scitulus mitochondrial partial 16S rRNA	HF931515	83.44	100	5.91275e−38	Coleoptera	Dytiscidae	Oreodytes scitulus
Laccornis kocae partial 16S rRNA gene	AJ850419	83.44	100	5.91275e−38	Coleoptera	Dytiscidae	Laccornis kocae
Nebrioporus formaster mitochondrial partial 16S rR	HF931377	82.91	100	8.7753e−36	Coleoptera	Dytiscidae	Nebrioporus formaster
Suphrodytes dorsalis 16S ribosomal RNA gene, parti	KF575577	82.91	100	3.06288e−35	Coleoptera	Dytiscidae	Suphrodytes dorsalis
Hydroporus tessellatus 16S ribosomal RNA gene, par	JX434770	83.54	100	3.06288e−35	Coleoptera	Dytiscidae	Hydroporus tessellatus
Boreonectes griseostriatus mitochondrial 16S rRNA	HF931385	82.8	100	3.06288e−35	Coleoptera	Dytiscidae	Boreonectes griseostriatus
Suphrodytes dorsalis voucher BMNH:725966 16S ribos	JX221574	82.91	100	3.06288e−35	Coleoptera	Dytiscidae	Suphrodytes dorsalis
Hydroporus foveolatus mitochondrial 16S rRNA gene	HE613724	83.54	100	3.06288e−35	Coleoptera	Dytiscidae	Hydroporus foveolatus
Deronectes bicostatus mitochondrial 16S rRNA gene	HE613705	82.17	100	3.06288e−35	Coleoptera	Dytiscidae	Deronectes bicostatus
Hydroporus basinotatus mitochondrial 16S rRNA gene	HE613678	83.54	100	3.06288e−35	Coleoptera	Dytiscidae	Hydroporus basinotatus
Hydroporus basinotatus 16S ribosomal RNA gene, par	AF518258	83.54	100	3.06288e−35	Coleoptera	Dytiscidae	Hydroporus basinotatus
Deronectes moestus mitochondrial 16S rRNA gene (pa	HE613726	84.83	91.72	1.06905e−34	Coleoptera	Dytiscidae	Deronectes moestus
Stictotarsus maghrebinus 16S ribosomal RNA gene, p	AY250947	82.91	100	1.06905e−34	Coleoptera	Dytiscidae	Stictotarsus maghrebinus
Aethionectes fulvonotatus voucher KBMAeop406 16S r	KF979088	81.88	100	3.73135e−34	Coleoptera	Dytiscidae	Aethionectes fulvonotatus
Hydroporus zimmermanni mitochondrial 16S rRNA gene	HE613679	82.91	100	3.73135e−34	Coleoptera	Dytiscidae	Hydroporus zimmermanni
Hydroporus glabriusculus 16S ribosomal RNA gene, p	AY365270	82.17	100	3.73135e−34	Coleoptera	Dytiscidae	Hydroporus glabriusculus
Oreodytes quadrimaculatus 16S ribosomal RNA gene,	KF575567	81.53	100	1.30237e−33	Coleoptera	Dytiscidae	Oreodytes quadrimaculatus
Stictotarsus procerus mitochondrial 16S rRNA gene	HF931379	82.28	100	1.30237e−33	Coleoptera	Dytiscidae	Stictotarsus procerus
Deronectes opatrinus mitochondrial 16S rRNA gene (	HE613701	82.28	100	1.30237e−33	Coleoptera	Dytiscidae	Deronectes opatrinus
Copelatus sp. BI−2011 voucher BMNH792945 16S ribos	HQ381441	81.53	100	1.30237e−33	Coleoptera	Dytiscidae	Copelatus sp. BI−2011
Oreodytes quadrimaculatus partial 16S rRNA gene	AJ850351	81.53	100	1.30237e−33	Coleoptera	Dytiscidae	Oreodytes quadrimaculatus
Deronectes moestus 16S ribosomal RNA gene, partial	KF575561	84.14	91.72	4.54572e−33	Coleoptera	Dytiscidae	Deronectes moestus
Hygrotus novemlineatus mitochondrial partial 16S r	HF931523	81.53	100	4.54572e−33	Coleoptera	Dytiscidae	Hygrotus novemlineatus
Hygrotus marklini mitochondrial 16S rRNA gene (par	HF931467	81.53	100	4.54572e−33	Coleoptera	Dytiscidae	Hygrotus marklini
Suphrodytes figuratus voucher BMNH:722148 16S ribo	JX221571	81.53	100	4.54572e−33	Coleoptera	Dytiscidae	Suphrodytes figuratus
Hydroporus marginatus mitochondrial 16S rRNA gene	HE613699	81.99	100	4.54572e−33	Coleoptera	Dytiscidae	Hydroporus marginatus
Deronectes brannani mitochondrial 16S rRNA gene (p	HE613695	84.14	91.72	4.54572e−33	Coleoptera	Dytiscidae	Deronectes brannanii
Deronectes moestus mitochondrial 16S rRNA gene (pa	HE613683	84.14	91.72	4.54572e−33	Coleoptera	Dytiscidae	Deronectes moestus
Hygrotus turbidus partial 16S rRNA gene	AJ850390	81.53	100	4.54572e−33	Coleoptera	Dytiscidae	Hygrotus turbidus
Hydroporus sp. IR33.4 16S ribosomal RNA gene, part	AY365261	81.53	100	4.54572e−33	Coleoptera	Dytiscidae	Hydroporus sp. IR33.4
Hydroporus marginatus 16S ribosomal RNA gene, part	AF518267	81.53	100	4.54572e−33	Coleoptera	Dytiscidae	Hydroporus marginatus
Herophydrus rohani voucher UNM KBMHpsp574 16S ribo	KJ548382	81.65	100	1.58661e−32	Coleoptera	Dytiscidae	Herophydrus rohani
Hydaticus cinctipennis voucher UNM KBMHyci20 16S r	KJ548338	80.89	100	1.58661e−32	Coleoptera	Dytiscidae	Hydaticus cinctipennis
Hydroporus analis mitochondrial partial 16S rRNA g	HE613731	82.28	100	1.58661e−32	Coleoptera	Dytiscidae	Hydroporus analis
Hydroporus fuscipennis mitochondrial 16S rRNA gene	HE613730	82.39	100	1.58661e−32	Coleoptera	Dytiscidae	Hydroporus fuscipennis
Hydroporus compunctus mitochondrial 16S rRNA gene	HE599693	82.28	100	1.58661e−32	Coleoptera	Dytiscidae	Hydroporus compunctus
Copelatus sp. BI−2011 voucher BMNH671215 16S ribos	HQ381377	80.89	100	1.58661e−32	Coleoptera	Dytiscidae	Copelatus sp. BI−2011
Antiporus hollingsworthi mitochondrial partial 16S	FR727246	81.29	98.73	1.58661e−32	Coleoptera	Dytiscidae	Antiporus hollingsworthi
Amphizoa insolens 16S ribosomal RNA gene, partial	AY071770	80.89	100	1.58661e−32	Coleoptera	Amphizoidae	Amphizoa insolens
Hydroporus compunctus 16S ribosomal RNA gene, part	AF518259	82.28	100	1.58661e−32	Coleoptera	Dytiscidae	Hydroporus compunctus
Hydroporus fuscipennis 16S ribosomal RNA gene, par	AF518262	82.39	100	1.58661e−32	Coleoptera	Dytiscidae	Hydroporus fuscipennis
Aglymbus gestroi 16S ribosomal RNA gene, partial s	AY138639	80.89	100	1.58661e−32	Coleoptera	Dytiscidae	Aglymbus gestroi
Deronectes doriae mitochondrial 16S rRNA gene (par	HE613710	81.65	100	5.53782e−32	Coleoptera	Dytiscidae	Deronectes doriae
Deronectes sahlbergi mitochondrial 16S rRNA gene (	HE613680	81.65	100	5.53782e−32	Coleoptera	Dytiscidae	Deronectes sahlbergi
Antiporus hollingsworthi mitochondrial partial 16S	FR727247	80.65	98.73	5.53782e−32	Coleoptera	Dytiscidae	Antiporus hollingsworthi
Stictotarsus falli 16S ribosomal RNA (rrnL) gene,	EF670029	81.65	100	5.53782e−32	Coleoptera	Dytiscidae	Stictotarsus falli
Nirripirti megamacrocephalus isolate ABTC78781 16S	EU616935	81.65	100	5.53782e−32	Coleoptera	Dytiscidae	Paroster megamacrocephalus
Herophydrus inquinatus 16S ribosomal RNA gene, par	KF575572	81.65	100	1.93289e−31	Coleoptera	Dytiscidae	Herophydrus inquinatus
Hydroporus appalachius 16S ribosomal RNA gene, par	JX434765	80.89	100	1.93289e−31	Coleoptera	Dytiscidae	Hydroporus appalachius
Hygrotus lutescens mitochondrial partial 16S rRNA	HF931518	80.89	100	1.93289e−31	Coleoptera	Dytiscidae	Hygrotus lutescens
Hydroporus foveolatus mitochondrial 16S rRNA gene	HF931502	81.65	100	1.93289e−31	Coleoptera	Dytiscidae	Hydroporus foveolatus
Hygrotus fontinalis mitochondrial 16S rRNA gene (p	HF931494	80.89	100	1.93289e−31	Coleoptera	Dytiscidae	Hygrotus fontinalis
Boreonectes funereus mitochondrial 16S rRNA gene (	HF931393	80.89	100	1.93289e−31	Coleoptera	Dytiscidae	Boreonectes funereus
Hygrotus patruelis mitochondrial 16S rRNA gene (pa	HF931388	80.89	100	1.93289e−31	Coleoptera	Dytiscidae	Hygrotus patruelis
Hydroporus brancoi gredensis mitochondrial partial	HF931329	80.89	100	1.93289e−31	Coleoptera	Dytiscidae	Hydroporus brancoi gredensis
Suphrodytes figuratus voucher BMNH:722150 16S ribo	JX221572	80.89	100	1.93289e−31	Coleoptera	Dytiscidae	Suphrodytes figuratus
Hydroporus discretus mitochondrial 16S rRNA gene (	HE613723	81.65	100	1.93289e−31	Coleoptera	Dytiscidae	Hydroporus discretus
Hydroporus decipiens mitochondrial 16S rRNA gene (	HE613722	81.65	100	1.93289e−31	Coleoptera	Dytiscidae	Hydroporus decipiens
Hydroporus pubescens mitochondrial 16S rRNA gene (	HE613707	81.65	100	1.93289e−31	Coleoptera	Dytiscidae	Hydroporus pubescens
Hydroporus limbatus mitochondrial 16S rRNA gene (p	HE613706	81.65	100	1.93289e−31	Coleoptera	Dytiscidae	Hydroporus limbatus
Hydroporus errans mitochondrial 16S rRNA gene (par	HE613704	81.65	100	1.93289e−31	Coleoptera	Dytiscidae	Hydroporus errans
Suphrodytes dorsalis mitochondrial 16S rRNA gene (	HE599700	80.89	100	1.93289e−31	Coleoptera	Dytiscidae	Suphrodytes dorsalis
Hydroporus pervicinus mitochondrial 16S rRNA gene	HE599695	80.89	100	1.93289e−31	Coleoptera	Dytiscidae	Hydroporus pervicinus
Hovahydrus sp. BI−2011 voucher BMNH831333 16S ribo	HQ381524	84.25	91.72	1.93289e−31	Coleoptera	Dytiscidae	Hovahydrus sp. BI−2011
Herophydrus cf. gigas BMNH 681835 partial 16S rRNA	AJ850383	81.65	100	1.93289e−31	Coleoptera	Dytiscidae	Herophydrus cf. gigas BMNH 681835
Nirripirti newhavenensis 16S ribosomal RNA gene, p	AY353844	80.89	100	1.93289e−31	Coleoptera	Dytiscidae	Paroster newhavenensis
Herophydrus inquinatus voucher MB1227 16S ribosoma	EF056676	81.65	100	1.93289e−31	Coleoptera	Dytiscidae	Herophydrus inquinatus
Hydroporus pubescens voucher NHM−IR39 16S ribosoma	EF419327	81.65	100	1.93289e−31	Coleoptera	Dytiscidae	Hydroporus pubescens
Hydroporus occidentalis 16S ribosomal RNA gene, pa	AY365280	80.89	100	1.93289e−31	Coleoptera	Dytiscidae	Hydroporus occidentalis
Hydroporus hirtellus 16S ribosomal RNA gene, parti	AY365271	80.89	100	1.93289e−31	Coleoptera	Dytiscidae	Hydroporus hirtellus
Hydroporus rufifrons 16S ribosomal RNA gene, parti	AF518277	81.65	100	1.93289e−31	Coleoptera	Dytiscidae	Hydroporus rufifrons
Hydroporus pubescens 16S ribosomal RNA gene, parti	AF518276	81.65	100	1.93289e−31	Coleoptera	Dytiscidae	Hydroporus pubescens
Hydroporus limbatus 16S ribosomal RNA gene, partia	AF518264	81.65	100	1.93289e−31	Coleoptera	Dytiscidae	Hydroporus limbatus
Hydroporus discretus 16S ribosomal RNA gene, parti	AF518260	81.65	100	1.93289e−31	Coleoptera	Dytiscidae	Hydroporus discretus
Hydroporus analis 16S ribosomal RNA gene, partial	AF518257	81.65	100	1.93289e−31	Coleoptera	Dytiscidae	Hydroporus analis
Hygrotus impressopunctatus voucher UNM KBMHtim80 1	KJ548334	82.05	97.45	6.74644e−31	Coleoptera	Dytiscidae	Hygrotus impressopunctatus
Deronectes aubei voucher UNM KBMDeau758 16S riboso	KJ548292	81.01	100	6.74644e−31	Coleoptera	Dytiscidae	Deronectes aubei
Hygrotus armeniacus mitochondrial partial 16S rRNA	HF931520	81.76	100	6.74644e−31	Coleoptera	Dytiscidae	Hygrotus armeniacus
Hydroporus planus mitochondrial 16S rRNA gene (par	HE613708	81.13	100	6.74644e−31	Coleoptera	Dytiscidae	Hydroporus planus
