# Supplementary material for: Testing the potential of a ribosomal 16S marker for DNA metabarcoding of insects
Source: PeerJ. 2016 Apr 19;4:e1966. doi: 10.7717/peerj.1966 (PMC4841222; doi:10.7717/peerj.1966)
Supplement: Supplemental Information 1 [file peerj-04-1966-s006.zip › scripts/5 match OTUs/blast_hits/OTU_585.csv_plot.pdf]

OTU_585.csv

Leuctra inermis

Leuctridae

Plecoptera

87.42

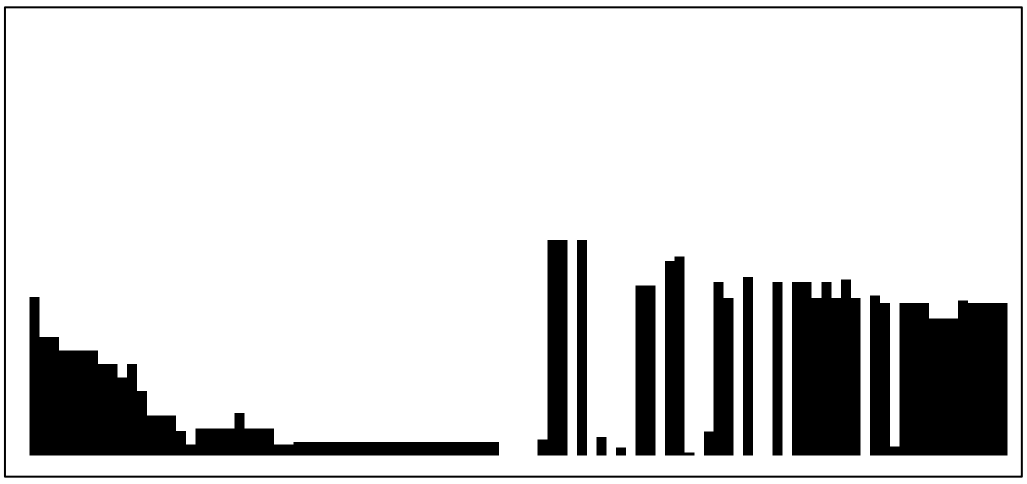

Leuctra inermis 16S ribosomal RNA gene, partial se	EF623176	87.42	100	4.28858e−46	Plecoptera	Leuctridae	Leuctra inermis
Leuctra tenuis haplotype 8lt: HO8 16S ribosomal RN	EU327680	85.53	100	1.15079e−40	Plecoptera	Leuctridae	Leuctra tenuis
Leuctra tenuis haplotype 7lt: HO7 16S ribosomal RN	EU327679	85.53	100	1.15079e−40	Plecoptera	Leuctridae	Leuctra tenuis
Leuctra tenuis haplotype 10lt: HO10 16S ribosomal	EU327682	84.91	100	1.40194e−39	Plecoptera	Leuctridae	Leuctra tenuis
Leuctra tenuis haplotype 9lt: HO9 16S ribosomal RN	EU327681	84.91	100	1.40194e−39	Plecoptera	Leuctridae	Leuctra tenuis
Leuctra tenuis haplotype 3lt: H03 16S ribosomal RN	DQ915184	84.91	100	1.40194e−39	Plecoptera	Leuctridae	Leuctra tenuis
Leuctra tenuis haplotype 2lt: H02 16S ribosomal RN	DQ915183	84.91	100	1.40194e−39	Plecoptera	Leuctridae	Leuctra tenuis
Leuctra tenuis haplotype 6lt: HO6 16S ribosomal RN	EU327678	84.28	100	5.96121e−38	Plecoptera	Leuctridae	Leuctra tenuis
Leuctra tenuis haplotype 1lt: H01 16S ribosomal RN	DQ915182	84.28	100	5.96121e−38	Plecoptera	Leuctridae	Leuctra tenuis
Leuctra tenuis haplotype 5lt: HO5 16S ribosomal RN	EU327677	83.65	100	7.26225e−37	Plecoptera	Leuctridae	Leuctra tenuis
Moselia infuscata 16S ribosomal RNA gene, partial	EF623175	84.28	99.37	2.53477e−36	Plecoptera	Leuctridae	Moselia infuscata
Leuctra tenuis haplotype 4lt: HO4 16S ribosomal RN	EU327676	83.02	100	3.08799e−35	Plecoptera	Leuctridae	Leuctra tenuis
Apteroperla tikumana mitochondrion, complete genom	KR604721	81.88	100	1.94873e−31	Plecoptera	Capniidae	Apteroperla tikumana
Isocapnia hyalita 16S ribosomal RNA gene, partial	EF623131	81.88	100	1.94873e−31	Plecoptera	Capniidae	Isocapnia hyalita
Allocapnia minima 16S ribosomal RNA gene, partial	EF623130	81.88	100	1.94873e−31	Plecoptera	Capniidae	Allocapnia minima
Cascadoperla trictura 16S ribosomal RNA gene, part	EF623225	81.13	100	2.37404e−30	Plecoptera	Perlodidae	Cascadoperla trictura
Despaxia augusta 16S ribosomal RNA gene, partial s	EF623177	80.5	100	2.37404e−30	Plecoptera	Leuctridae	Despaxia augusta
Allocapnia recta isolate H15 16S ribosomal RNA gen	KC881050	81.25	100	8.28622e−30	Plecoptera	Capniidae	Allocapnia recta
Allocapnia recta isolate H14 16S ribosomal RNA gen	KC881049	81.25	100	8.28622e−30	Plecoptera	Capniidae	Allocapnia recta
Allocapnia recta isolate H10 16S ribosomal RNA gen	KC881045	81.25	100	8.28622e−30	Plecoptera	Capniidae	Allocapnia recta
Allocapnia recta isolate H6 16S ribosomal RNA gene	KC881041	81.25	100	8.28622e−30	Plecoptera	Capniidae	Allocapnia recta
Protonemura meyeri 16S ribosomal RNA gene, partial	EF623193	81.99	100	8.28622e−30	Plecoptera	Nemouridae	Protonemura meyeri
Nemurella pictetii 16S ribosomal RNA gene, partial	EF623192	81.25	100	8.28622e−30	Plecoptera	Nemouridae	Nemurella pictetii
Allocapnia recta haplotype 11ar: H011 16S ribosoma	EU306874	81.25	100	8.28622e−30	Plecoptera	Capniidae	Allocapnia recta
Allocapnia recta haplotype 7ar: H07 16S ribosomal	EU306870	81.25	100	8.28622e−30	Plecoptera	Capniidae	Allocapnia recta
Soyedina producta 16S ribosomal RNA gene, partial	EF623196	80.5	100	2.89217e−29	Plecoptera	Nemouridae	Soyedina producta
Capnura wanica 16S ribosomal RNA gene, partial seq	EF623128	80.5	100	2.89217e−29	Plecoptera	Capniidae	Capnura wanica
Mesocapnia arizonensis mitochondrion, partial geno	KP642637	80.62	100	1.00947e−28	Plecoptera	Capniidae	Mesocapnia arizonensis
Allocapnia recta isolate H19 16S ribosomal RNA gen	KC881054	80.62	100	1.00947e−28	Plecoptera	Capniidae	Allocapnia recta
Allocapnia recta isolate H17 16S ribosomal RNA gen	KC881052	80.62	100	1.00947e−28	Plecoptera	Capniidae	Allocapnia recta
Allocapnia recta isolate H16 16S ribosomal RNA gen	KC881051	80.62	100	1.00947e−28	Plecoptera	Capniidae	Allocapnia recta
Allocapnia recta isolate H13 16S ribosomal RNA gen	KC881048	80.62	100	1.00947e−28	Plecoptera	Capniidae	Allocapnia recta
Allocapnia recta isolate H12 16S ribosomal RNA gen	KC881047	80.62	100	1.00947e−28	Plecoptera	Capniidae	Allocapnia recta
Allocapnia recta isolate H11 16S ribosomal RNA gen	KC881046	80.62	100	1.00947e−28	Plecoptera	Capniidae	Allocapnia recta
Allocapnia recta isolate H9 16S ribosomal RNA gene	KC881044	80.62	100	1.00947e−28	Plecoptera	Capniidae	Allocapnia recta
Allocapnia recta isolate H8 16S ribosomal RNA gene	KC881043	80.62	100	1.00947e−28	Plecoptera	Capniidae	Allocapnia recta
Allocapnia recta isolate H7 16S ribosomal RNA gene	KC881042	80.62	100	1.00947e−28	Plecoptera	Capniidae	Allocapnia recta
Allocapnia recta isolate H5 16S ribosomal RNA gene	KC881040	80.62	100	1.00947e−28	Plecoptera	Capniidae	Allocapnia recta
Allocapnia recta isolate H4 16S ribosomal RNA gene	KC881039	80.62	100	1.00947e−28	Plecoptera	Capniidae	Allocapnia recta
Allocapnia recta isolate H3 16S ribosomal RNA gene	KC881038	80.62	100	1.00947e−28	Plecoptera	Capniidae	Allocapnia recta
Ostrocerca foersteri 16S ribosomal RNA gene, parti	EF623191	80.62	100	1.00947e−28	Plecoptera	Nemouridae	Ostrocerca foersteri
Mesocapnia sp. BYU_PL010 16S ribosomal RNA gene, p	EF623127	80.62	100	1.00947e−28	Plecoptera	Capniidae	Mesocapnia sp. BYU_PL010
Allocapnia recta haplotype 10ar: H010 16S ribosoma	EU306873	80.62	100	1.00947e−28	Plecoptera	Capniidae	Allocapnia recta
Allocapnia recta haplotype 9ar: H09 16S ribosomal	EU306872	80.62	100	1.00947e−28	Plecoptera	Capniidae	Allocapnia recta
Allocapnia recta haplotype 8ar: H08 16S ribosomal	EU306871	80.62	100	1.00947e−28	Plecoptera	Capniidae	Allocapnia recta
Allocapnia recta haplotype 3ar: H03 16S ribosomal	DQ915181	80.62	100	1.00947e−28	Plecoptera	Capniidae	Allocapnia recta
Allocapnia recta haplotype 2ar: H02 16S ribosomal	DQ915180	80.62	100	1.00947e−28	Plecoptera	Capniidae	Allocapnia recta
Allocapnia recta haplotype 1ar: H01 16S ribosomal	DQ915179	80.62	100	1.00947e−28	Plecoptera	Capniidae	Allocapnia recta
Doddsia occidentalis 16S ribosomal RNA gene, parti	EF623270	80	100	3.52339e−28	Plecoptera	Taeniopterygidae	Doddsia occidentalis
Oemopteryx vanduzeea 16S ribosomal RNA gene, parti	EF623266	80	100	3.52339e−28	Plecoptera	Taeniopterygidae	Oemopteryx vanduzeea
Allocapnia recta isolate H18 16S ribosomal RNA gen	KC881053	80	100	4.29237e−27	Plecoptera	Capniidae	Allocapnia recta
Allocapnia recta isolate H1 16S ribosomal RNA gene	KC881036	80	100	4.29237e−27	Plecoptera	Capniidae	Allocapnia recta
Visoka cataractae 16S ribosomal RNA gene, partial	EF623189	80.75	100	4.29237e−27	Plecoptera	Nemouridae	Visoka cataractae
Suwallia teleckojensis 16S ribosomal RNA gene, par	EF623142	90.1	63.29	4.29237e−27	Plecoptera	Chloroperlidae	Suwallia teleckojensis
Neaviperla forcipata 16S ribosomal RNA gene, parti	EF623139	90.1	63.29	4.29237e−27	Plecoptera	Chloroperlidae	Neaviperla forcipata
Kathroperla perdita 16S ribosomal RNA gene, partia	EF623135	80	100	4.29237e−27	Plecoptera	Chloroperlidae	Kathroperla perdita
Plumiperla diversa 16S ribosomal RNA gene, partial	EF623132	90.1	63.29	4.29237e−27	Plecoptera	Chloroperlidae	Plumiperla diversa
Allocapnia recta haplotype 12ar: H12 16S ribosomal	EU306875	80	100	4.29237e−27	Plecoptera	Capniidae	Allocapnia recta
Triznaka pintada 16S ribosomal RNA gene, partial s	EF623134	80.86	100	1.49818e−26	Plecoptera	Chloroperlidae	Triznaka pintada
Bolotoperla rossi 16S ribosomal RNA gene, partial	EF623269	78.62	100	5.22917e−26	Plecoptera	Taeniopterygidae	Bolotoperla rossi
Scopura montana 16S ribosomal RNA gene, partial se	EF623265	80.37	100	5.22917e−26	Plecoptera		Scopura montana
Perlinodes aurea 16S ribosomal RNA gene, partial s	EF623234	80	100	5.22917e−26	Plecoptera	Perlodidae	Perlinodes aurea
Udamocercia sp. BYU_PL150 16S ribosomal RNA gene,	EF623201	87.96	67.09	5.22917e−26	Plecoptera	Notonemouridae	Udamocercia sp. BYU_PL150
Zapada cinctipes 16S ribosomal RNA gene, partial s	EF623183	87.96	67.09	5.22917e−26	Plecoptera	Nemouridae	Zapada cinctipes
Paraleuctra occidentalis 16S ribosomal RNA gene, p	EF623178	79.25	100	5.22917e−26	Plecoptera	Leuctridae	Paraleuctra occidentalis
Siphonoperla torrentium 16S ribosomal RNA gene, pa	EF623138	89.11	63.29	5.22917e−26	Plecoptera	Chloroperlidae	Siphonoperla torrentium
Isoperla davisi 16S ribosomal RNA gene, partial se	EF623206	89.32	64.56	1.82516e−25	Plecoptera	Perlodidae	Isoperla davisi
Shipsa rotunda 16S ribosomal RNA gene, partial seq	EF623184	80.12	100	1.82516e−25	Plecoptera	Nemouridae	Shipsa rotunda
Nemoura cinerea 16S ribosomal RNA gene, partial se	EF623194	79.14	100	6.37044e−25	Plecoptera	Nemouridae	Nemoura cinerea
Pteronarcys princeps mitochondrion, complete genom	AY687866	81.12	89.24	6.37044e−25	Plecoptera	Pteronarcyidae	Pteronarcys princeps
Strophopteryx appalachia 16S ribosomal RNA gene, p	EF623271	88.12	63.29	2.2235e−24	Plecoptera	Taeniopterygidae	Strophopteryx appalachia
Pteronarcys sachalina 16S ribosomal RNA gene, part	EF623264	87.38	64.56	2.2235e−24	Plecoptera	Pteronarcyidae	Pteronarcys sachalina
Osobenus yakimae 16S ribosomal RNA gene, partial s	EF623232	79.63	100	2.2235e−24	Plecoptera	Perlodidae	Osobenus yakimae
Isoperla oxylepis 16S ribosomal RNA gene, partial	EF623221	88.35	64.56	2.2235e−24	Plecoptera	Perlodidae	Isoperla oxylepis
Nanonemoura wahkeena 16S ribosomal RNA gene, parti	EF623198	78.75	100	2.2235e−24	Plecoptera	Nemouridae	Nanonemoura wahkeena
Nemoura trispinosa 16S ribosomal RNA gene, partial	EF623187	78.75	100	2.2235e−24	Plecoptera	Nemouridae	Nemoura trispinosa
Riekoperla triloba 16S ribosomal RNA gene, partial	EF623171	88.12	63.29	2.2235e−24	Plecoptera	Gripopterygidae	Riekoperla triloba
Thaumatoperla sp. BYU_PL109 16S ribosomal RNA gene	EF623149	78.75	100	2.2235e−24	Plecoptera	Eustheniidae	Thaumatoperla sp. BYU_PL109
Chloroperla tripunctata 16S ribosomal RNA gene, pa	EF623137	88.12	63.29	2.2235e−24	Plecoptera	Chloroperlidae	Chloroperla tripunctata
Sweltsa coloradensis 16S ribosomal RNA gene, parti	EF623133	88.12	63.29	2.2235e−24	Plecoptera	Chloroperlidae	Sweltsa coloradensis
Penturoperla barbata 16S ribosomal RNA gene, parti	EF623124	87.38	64.56	2.2235e−24	Plecoptera	Austroperlidae	Penturoperla barbata
Klapopteryx armillata 16S ribosomal RNA gene, part	EF623123	88.12	63.29	2.2235e−24	Plecoptera	Austroperlidae	Klapopteryx armillata
Osmylus sp. EH−2004 16S ribosomal RNA gene, partia	AY620146	87.38	64.56	2.2235e−24	Neuroptera	Osmylidae	Osmylus sp. EH−2004
Brachyptera seticornis 16S ribosomal RNA gene, par	EF623268	88.24	63.29	7.76078e−24	Plecoptera	Taeniopterygidae	Brachyptera seticornis
Frisonia picticeps 16S ribosomal RNA gene, partial	EF623227	87.38	64.56	7.76078e−24	Plecoptera	Perlodidae	Frisonia picticeps
Paranemoura claasseni 16S ribosomal RNA gene, part	EF623185	79.75	100	7.76078e−24	Plecoptera	Nemouridae	Paranemoura claasseni
Malenka californica 16S ribosomal RNA gene, partia	EF623182	87.5	64.56	7.76078e−24	Plecoptera	Nemouridae	Malenka californica
Alloperla severa 16S ribosomal RNA gene, partial s	EF623141	87.13	63.29	7.76078e−24	Plecoptera	Chloroperlidae	Alloperla severa
Pteronarcys californica 16S ribosomal RNA gene, pa	EF623261	80.42	89.24	2.70878e−23	Plecoptera	Pteronarcyidae	Pteronarcys californica
Peltoperla arcuata 16S ribosomal RNA gene, partial	EF623258	87.13	63.29	2.70878e−23	Plecoptera	Peltoperlidae	Peltoperla arcuata
Limnoperla sp. BYU_PL145 16S ribosomal RNA gene, p	EF623166	87.13	63.29	2.70878e−23	Plecoptera	Gripopterygidae	Limnoperla sp. BYU_PL145
Pteronarcella badia 16S ribosomal RNA gene, partia	EF623262	87.13	63.29	9.45457e−23	Plecoptera	Pteronarcyidae	Pteronarcella badia
Cerconychia sp. BYU_PL114 16S ribosomal RNA gene,	EF623260	86.41	64.56	9.45457e−23	Plecoptera	Styloperlidae	Cerconychia sp. BYU_PL114
Cosumnoperla hypocrena 16S ribosomal RNA gene, par	EF623226	86.41	64.56	9.45457e−23	Plecoptera	Perlodidae	Cosumnoperla hypocrena
Yugus bulbosus 16S ribosomal RNA gene, partial seq	EF623214	86.41	64.56	9.45457e−23	Plecoptera	Perlodidae	Yugus bulbosus
Prostoia besametsa 16S ribosomal RNA gene, partial	EF623188	87.25	63.29	9.45457e−23	Plecoptera	Nemouridae	Prostoia besametsa
Claudioperla sp. BYU_PL158 16S ribosomal RNA gene,	EF623169	87.13	63.29	9.45457e−23	Plecoptera	Gripopterygidae	Claudioperla sp. BYU_PL158
Pelurgoperla personata 16S ribosomal RNA gene, par	EF623168	87.13	63.29	9.45457e−23	Plecoptera	Gripopterygidae	Pelurgoperla personata
Antarctoperla sp. BYU_PL142 16S ribosomal RNA gene	EF623163	87.13	63.29	9.45457e−23	Plecoptera	Gripopterygidae	Antarctoperla sp. BYU_PL142
Acroperla trivacauta 16S ribosomal RNA gene, parti	EF623155	87.13	63.29	9.45457e−23	Plecoptera	Gripopterygidae	Acroperla trivacauta
