# Supplementary material for: Testing the potential of a ribosomal 16S marker for DNA metabarcoding of insects
Source: PeerJ. 2016 Apr 19;4:e1966. doi: 10.7717/peerj.1966 (PMC4841222; doi:10.7717/peerj.1966)
Supplement: Supplemental Information 1 [file peerj-04-1966-s006.zip › scripts/5 match OTUs/blast_hits/OTU_59.csv_plot.pdf]

OTU_59.csv

Cladodiopsis seyrigi

Diopsidae

Diptera

81.66

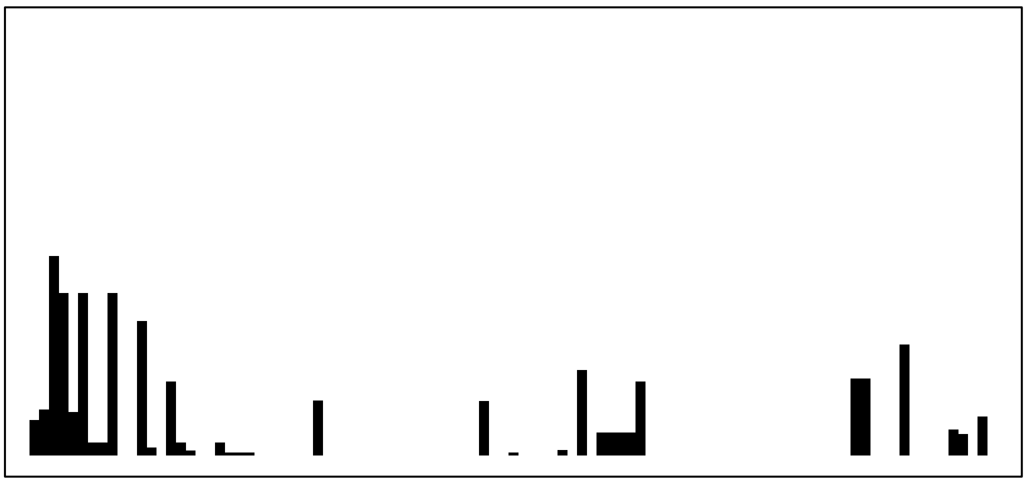

Cladodiopsis seyrigi 16S ribosomal RNA gene, parti	DQ317406	81.66	97.6	1.40191e−33	Diptera	Diopsidae	Cladodiopsis seyrigi
Sphyracephala detrahens 16S ribosomal RNA gene, pa	KT186668	82.14	100	4.89313e−33	Diptera	Diopsidae	Sphyracephala detrahens
Sphyracephala bipunctipennis 16S ribosomal RNA gen	AF304735	89.34	71.86	1.70787e−32	Diptera	Diopsidae	Sphyracephala bipunctipennis
Cladodiopsis leptophylla 16S ribosomal RNA gene, p	KT186664	87.6	71.86	5.96106e−32	Diptera	Diopsidae	Cladodiopsis leptophylla
Uromedina eumorphophaga mitochondrial gene for 16S	AB465914	82.04	97.6	7.26205e−31	Diptera	Tachinidae	Uromedina eumorphophaga
Sphyracephala beccarii 16S ribosomal RNA gene, par	KT186669	87.6	71.86	8.84699e−30	Diptera	Diopsidae	Sphyracephala beccarii
Musca confiscata voucher RMBR:116210 16S ribosomal	KJ476318	80.61	97.6	8.84699e−30	Diptera	Muscidae	Musca confiscata
Coenosia obscuricula voucher RMBR:116186 16S ribos	KJ476300	80.61	97.6	8.84699e−30	Diptera	Muscidae	Coenosia obscuricula
Sphyracephala beccarii 16S ribosomal RNA gene, par	AF304737	87.6	71.86	8.84699e−30	Diptera	Diopsidae	Sphyracephala beccarii
Microsania pectipennis isolate MP27 16S ribosomal	KC699344	79.76	100	3.0879e−29	Diptera	Platypezidae	Microsania pectipennis
Microsania pectipennis isolate MP12 16S ribosomal	KC699343	79.76	100	3.0879e−29	Diptera	Platypezidae	Microsania pectipennis
Nemoraea takanoi mitochondrial gene for 16S rRNA,	AB465951	86.29	73.05	1.07778e−28	Diptera	Tachinidae	Nemoraea takanoi
Coenosia testacea 16S ribosomal RNA gene, partial	FJ025426	80.36	97.6	1.07778e−28	Diptera	Muscidae	Coenosia testacea
Procecidochares atra 16S ribosomal RNA gene, parti	DQ471376	80	100	3.76184e−28	Diptera	Tephritidae	Procecidochares atra
Sphyracephala munroi 16S ribosomal RNA gene, parti	AF304727	83.46	79.04	3.76184e−28	Diptera	Diopsidae	Sphyracephala munroi
Lispe nana voucher RMBR:116206 16S ribosomal RNA g	KJ476314	80.61	97.6	1.31301e−27	Diptera	Muscidae	Lispe nana
Linnaemya picta voucher UBZ MU LP1 16S ribosomal R	FJ222719	80.23	97.6	1.31301e−27	Diptera	Tachinidae	Linnaemya picta
Hemigephyra atra 16S ribosomal RNA gene, partial s	KM878915	77.98	100	4.58286e−27	Diptera	Therevidae	Hemigephyra atra
Limnophora simulans voucher RMBR:116205 16S riboso	KJ476313	80	97.6	4.58286e−27	Diptera	Muscidae	Limnophora simulans
Helina cinerella voucher RMBR:116198 16S ribosomal	KJ476308	80.59	97.6	4.58286e−27	Diptera	Muscidae	Helina cinerella
Dinera ferina isolate FE2 16S ribosomal RNA gene,	HM770484	80.12	97.6	4.58286e−27	Diptera	Tachinidae	Dinera ferina
Dinera ferina isolate FE1 16S ribosomal RNA gene,	HM770483	80.12	97.6	4.58286e−27	Diptera	Tachinidae	Dinera ferina
Dinera ferina isolate MU_UBZ_16S_DF 16S ribosomal	FJ913863	80.12	97.6	4.58286e−27	Diptera	Tachinidae	Dinera ferina
Trupanea pantosticta isolate Tpanh2 16S ribosomal	KM079211	78.79	97.6	1.59957e−26	Diptera	Tephritidae	Trupanea pantosticta
Trupanea joycei isolate TjoyMo 16S ribosomal RNA g	KM079209	78.79	97.6	1.59957e−26	Diptera	Tephritidae	Trupanea joycei
Sciadocera rufomaculata 16S ribosomal RNA gene, pa	JN196447	78.57	100	1.59957e−26	Diptera	Sciadoceridae	Sciadocera rufomaculata
Cymatopus sp. Pollet 0321 16S ribosomal RNA gene,	HQ448972	79.17	100	1.59957e−26	Diptera	Dolichopodidae	Cymatopus sp. Pollet 0321
Dinera takanoi mitochondrial gene for 16S rRNA, pa	AB465958	78.44	97.6	1.59957e−26	Diptera	Tachinidae	Dinera takanoi
Diasemopsis aethiopica 16S ribosomal RNA gene, par	KT186657	78.57	100	5.58306e−26	Diptera	Diopsidae	Diasemopsis aethiopica
Trupanea sp. 'waikamoi' 16S ribosomal RNA gene, pa	KM079216	82.58	78.44	5.58306e−26	Diptera	Tephritidae	Trupanea sp. 'waikamoi'
Cephalispa sp. SNK−2014 voucher RMBR:116183 16S ri	KJ476298	80	97.6	5.58306e−26	Diptera	Muscidae	Cephalispa sp. SNK−2014
Dohrniphora membranea isolate 29.v.08−460 16S ribo	HM366969	78.24	100	5.58306e−26	Diptera	Phoridae	Dohrniphora membranea
Sphyracephala europaea 16S ribosomal RNA gene, par	DQ452962	78.11	100	5.58306e−26	Diptera	Diopsidae	Sphyracephala europaea
Trupanea sp. 'pohakuloa' 16S ribosomal RNA gene, p	KM079213	78.18	97.6	1.94868e−25	Diptera	Tephritidae	Trupanea sp. 'pohakuloa'
Trupanea perkinsi isolate Tper 16S ribosomal RNA g	KM079212	78.18	97.6	1.94868e−25	Diptera	Tephritidae	Trupanea perkinsi
Trupanea pantosticta isolate Tpanh1 16S ribosomal	KM079210	78.18	97.6	1.94868e−25	Diptera	Tephritidae	Trupanea pantosticta
Trupanea joycei isolate TjoyMa 16S ribosomal RNA g	KM079208	78.79	97.6	1.94868e−25	Diptera	Tephritidae	Trupanea joycei
Trupanea dubautiae isolate TdubKa 16S ribosomal RN	KM079204	78.18	97.6	1.94868e−25	Diptera	Tephritidae	Trupanea dubautiae
Trupanea cratericola isolate Tcra 16S ribosomal RN	KM079201	78.18	97.6	1.94868e−25	Diptera	Tephritidae	Trupanea cratericola
Trupanea crassipes isolate Tcash2 16S ribosomal RN	KM079200	78.18	97.6	1.94868e−25	Diptera	Tephritidae	Trupanea crassipes
Trupanea arboreae isolate Tarb 16S ribosomal RNA g	KM079196	78.18	97.6	1.94868e−25	Diptera	Tephritidae	Trupanea arboreae
Microsania collarti isolate CAN8 16S ribosomal RNA	KC699334	78.79	97.6	1.94868e−25	Diptera	Platypezidae	Microsania collarti
Minettia flaveola 16S ribosomal RNA gene, partial	KC177448	77.98	100	1.94868e−25	Diptera	Lauxaniidae	Minettia flaveola
Dinera carinifrons isolate L41 16S ribosomal RNA g	HM770476	79.52	97.6	1.94868e−25	Diptera	Tachinidae	Dinera carinifrons
Dinera carinifrons isolate SP1 16S ribosomal RNA g	HM770475	79.52	97.6	1.94868e−25	Diptera	Tachinidae	Dinera carinifrons
Dinera sp. AT−2009 16S ribosomal RNA gene, partial	FJ913861	79.52	97.6	1.94868e−25	Diptera	Tachinidae	Dinera sp. AT−2009
Trupanea stellata 16S ribosomal RNA gene, partial	GQ175834	82.54	74.85	1.94868e−25	Diptera	Tephritidae	Trupanea stellata
Hermya beelzebul mitochondrial gene for 16S rRNA,	AB465960	79.52	97.6	1.94868e−25	Diptera	Tachinidae	Hermya beelzebul
Dohrniphora trigonae 16S ribosomal RNA gene, parti	AF126343	78.57	100	1.94868e−25	Diptera	Phoridae	Dohrniphora trigonae
Antipoda sp. large subunit ribosomal RNA gene, mit	AF086863	80.12	97.6	1.94868e−25	Diptera	Muscidae	Antipoda sp.
Trupanea denotata isolate Tdenh2 16S ribosomal RNA	KM079203	76.79	100	6.80156e−25	Diptera	Tephritidae	Trupanea denotata
Trupanea denotata isolate Tdenh1 16S ribosomal RNA	KM079202	76.79	100	6.80156e−25	Diptera	Tephritidae	Trupanea denotata
Atherigona varia voucher RMBR:116181 16S ribosomal	KJ476297	78.57	97.6	6.80156e−25	Diptera	Muscidae	Atherigona varia
Hybos femoratus voucher Pollet 0247 (P) 16S riboso	HQ449036	77.38	100	6.80156e−25	Diptera	Hybotidae	Hybos femoratus
Panzeria melanopyga mitochondrial gene for 16S rRN	AB465948	80.24	97.6	6.80156e−25	Diptera	Tachinidae	Panzeria melanopyga
Nemoraea pellucida 16S ribosomal RNA gene, partial	AF332432	79.04	97.6	6.80156e−25	Diptera	Tachinidae	Nemoraea pellucida
Stenopomyia angulata voucher MEI 146440 16S riboso	DQ250827	84	71.26	6.80156e−25	Diptera	Therevidae	Stenopomyia angulata
Belvosia sp. Di68 16S ribosomal RNA gene, partial	DQ133027	79.04	97.6	6.80156e−25	Diptera	Tachinidae	Belvosia sp. Di68
Trupanea swezeyi isolate TsweMa 16S ribosomal RNA	KM079215	81.06	78.44	2.37398e−24	Diptera	Tephritidae	Trupanea swezeyi
Trupanea dubautiae isolate TdubOa 16S ribosomal RN	KM079206	81.06	78.44	2.37398e−24	Diptera	Tephritidae	Trupanea dubautiae
Trupanea dubautiae isolate TdubMa 16S ribosomal RN	KM079205	81.06	78.44	2.37398e−24	Diptera	Tephritidae	Trupanea dubautiae
Trupanea beardsleyi isolate Tbea 16S ribosomal RNA	KM079198	81.06	78.44	2.37398e−24	Diptera	Tephritidae	Trupanea beardsleyi
Pseudatrichia bupennis 16S ribosomal RNA gene, par	KM878952	83.46	72.46	2.37398e−24	Diptera	Scenopinidae	Pseudatrichia bupennis
Microsania capnophila isolate CAN7 16S ribosomal R	KC699333	78.18	97.6	2.37398e−24	Diptera	Platypezidae	Microsania capnophila
Helina fratercula voucher RMBR:116199 16S ribosoma	KJ476309	78.79	97.6	2.37398e−24	Diptera	Muscidae	Helina fratercula
Dinera fuscata isolate L51 16S ribosomal RNA gene,	HM770478	78.92	97.6	2.37398e−24	Diptera	Tachinidae	Dinera fuscata
Dinera carinifrons isolate CA3 16S ribosomal RNA g	HM770473	78.92	97.6	2.37398e−24	Diptera	Tachinidae	Dinera carinifrons
Dinera carinifrons isolate CA2 16S ribosomal RNA g	HM770472	78.92	97.6	2.37398e−24	Diptera	Tachinidae	Dinera carinifrons
Ophyra ignava 16S ribosomal RNA gene, partial sequ	JN226644	78.92	97.6	2.37398e−24	Diptera	Muscidae	Ophyra ignava
Dinera carinifrons isolate MU_UBZ_16S_DC 16S ribos	FJ913862	78.92	97.6	2.37398e−24	Diptera	Tachinidae	Dinera carinifrons
Ophyra chalcogaster isolate H109 16S ribosomal RNA	HM016627	78.92	97.6	2.37398e−24	Diptera	Muscidae	Ophyra chalcogaster
Ophyra chalcogaster isolate CSU09100410I5 16S ribo	GU145258	78.92	97.6	2.37398e−24	Diptera	Muscidae	Ophyra chalcogaster
Ophyra chalcogaster isolate H101 16S ribosomal RNA	HM016623	78.92	97.6	2.37398e−24	Diptera	Muscidae	Ophyra chalcogaster
Gymnosoma rotundatum mitochondrial gene for 16S rR	AB465962	78.79	97.6	2.37398e−24	Diptera	Tachinidae	Gymnosoma rotundatum
Phorinia spinulosa mitochondrial gene for 16S rRNA	AB465931	78.18	97.6	2.37398e−24	Diptera	Tachinidae	Phorinia spinulosa
Thricops genarum 16S ribosomal RNA gene, partial s	FJ025479	78.92	97.6	2.37398e−24	Diptera	Muscidae	Thricops genarum
Mydaea rufinervis 16S ribosomal RNA gene, partial	FJ025461	79.52	97.6	2.37398e−24	Diptera	Muscidae	Mydaea rufinervis
Phorinia spinulosa mitochondrial gene for 16S rRNA	AB257237	78.18	97.6	2.37398e−24	Diptera	Tachinidae	Phorinia spinulosa
Ophyra capensis voucher SCUBW 05039 16S ribosomal	AY965657	78.92	97.6	2.37398e−24	Diptera	Muscidae	Ophyra capensis
Ophyra capensis voucher SCUBW 05042 16S ribosomal	AY965635	78.92	97.6	2.37398e−24	Diptera	Muscidae	Ophyra capensis
Ophyra capensis voucher SCUBW 05041 16S ribosomal	AY965634	78.92	97.6	2.37398e−24	Diptera	Muscidae	Ophyra capensis
Ophyra capensis voucher SCUBW 05040 16S ribosomal	AY965633	78.92	97.6	2.37398e−24	Diptera	Muscidae	Ophyra capensis
Protidricerus japonicus isolate jap10 16S ribosoma	KM259924	79.75	92.22	8.286e−24	Neuroptera	Ascalaphidae	Protidricerus japonicus
Trupanea crassipes isolate Tcash1 16S ribosomal RN	KM079199	77.58	97.6	8.286e−24	Diptera	Tephritidae	Trupanea crassipes
Irwiniana irwini 16S ribosomal RNA gene, partial s	KM878923	83.61	71.86	8.286e−24	Diptera	Scenopinidae	Irwiniana irwini
Irwiniana graciliparamera 16S ribosomal RNA gene,	KM878922	83.61	71.86	8.286e−24	Diptera	Scenopinidae	Irwiniana graciliparamera
Mumetopia nigrimana group sp. AT−2015 isolate Mn2	KJ418483	76.19	100	8.286e−24	Diptera	Anthomyzidae	Mumetopia nigrimana group sp. AT−2015
Mumetopia nigrimana group sp. AT−2015 isolate Mn1	KJ418482	76.19	100	8.286e−24	Diptera	Anthomyzidae	Mumetopia nigrimana group sp. AT−2015
Xylosciara inornata voucher 230 16S ribosomal RNA	JQ613902	78.7	96.41	8.286e−24	Diptera	Sciaridae	Xylosciara inornata
Tetanura pallidiventris voucher MU_NUS_SG29 16S ri	JN828248	85.19	64.67	8.286e−24	Diptera	Sciomyzidae	Tetanura pallidiventris
Dinera xuei isolate L25 16S ribosomal RNA gene, pa	HM770487	79.64	97.6	8.286e−24	Diptera	Tachinidae	Dinera xuei
Dinera xuei isolate L24 16S ribosomal RNA gene, pa	HM770486	79.64	97.6	8.286e−24	Diptera	Tachinidae	Dinera xuei
Dinera xuei isolate L23 16S ribosomal RNA gene, pa	HM770485	79.64	97.6	8.286e−24	Diptera	Tachinidae	Dinera xuei
Billaea triangulifera isolate MU_UBZ_16S_ET2 16S r	FJ913860	78.44	97.6	8.286e−24	Diptera	Tachinidae	Billaea triangulifera
Trupanea amoena 16S ribosomal RNA gene, partial se	GQ175833	81.2	78.44	8.286e−24	Diptera	Tephritidae	Trupanea amoena
Musca domestica isolate CSU0907186180 16S ribosoma	GQ396696	80.99	78.44	8.286e−24	Diptera	Muscidae	Musca domestica
Hemerodromia sp. SNK−2009 voucher RMBR:103443 16S	FJ808170	76.61	100	8.286e−24	Diptera	Empididae	Hemerodromia sp. SNK−2009
Empis producta voucher RMBR:103430 16S ribosomal R	FJ808167	81.82	77.25	8.286e−24	Diptera	Empididae	Empis producta
Dohrniphora biseriata isolate 23.xii.03−141 16S ri	HM366964	77.06	100	2.8921e−23	Diptera	Phoridae	Dohrniphora biseriata
Dohrniphora longinoi isolate 11.x.05−281 16S ribos	HM366962	76.47	100	2.8921e−23	Diptera	Phoridae	Dohrniphora longinoi
