# Supplementary material for: Testing the potential of a ribosomal 16S marker for DNA metabarcoding of insects
Source: PeerJ. 2016 Apr 19;4:e1966. doi: 10.7717/peerj.1966 (PMC4841222; doi:10.7717/peerj.1966)
Supplement: Supplemental Information 1 [file peerj-04-1966-s006.zip › scripts/5 match OTUs/blast_hits/OTU_595.csv_plot.pdf]

OTU_595.csv

Pedicia albivitta

Pediciidae

Diptera

85.9

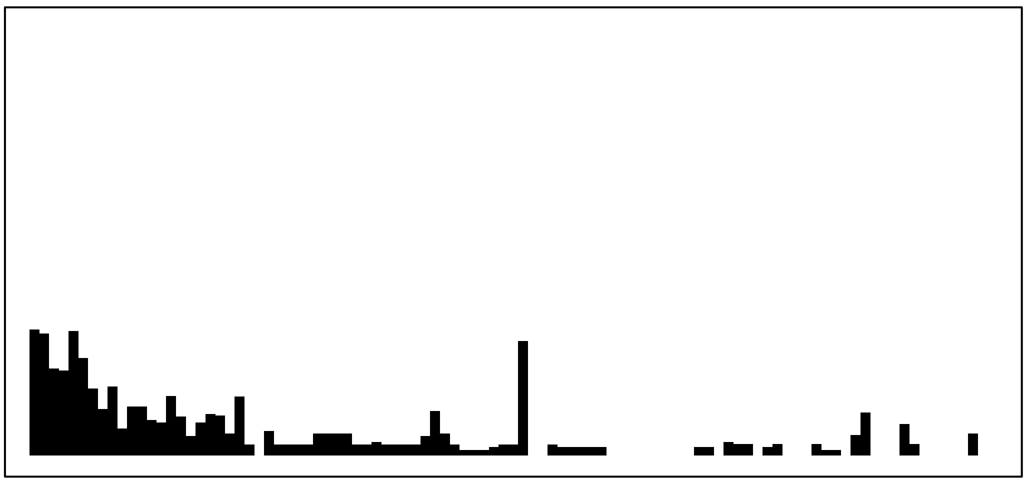

Pedicia albivitta voucher O'Grady 200012 16S ribos	EU005435	85.9	99.36	7.69089e−43	Diptera	Pediciidae	Pedicia albivitta
Bolitophila cinerea voucher JSB1 16S ribosomal RNA	KJ136712	85.71	97.45	1.14143e−40	Diptera	Mycetophilidae	Bolitophila cinerea
Edwardsina gigantea 16S ribosomal RNA gene, partia	KC177460	84.08	100	5.91275e−38	Diptera	Blephariceridae	Edwardsina gigantea
Parochlus steinenii isolate Sejong01 mitochondrion	KT003702	83.97	99.36	2.06375e−37	Diptera	Chironomidae	Parochlus steinenii
Bolitophila sp. ER−2009 16S ribosomal RNA gene, pa	FJ172021	85.82	89.17	8.7753e−36	Diptera	Mycetophilidae	Bolitophila sp. ER−2009
Tipula sp. PMO 200011 16S ribosomal RNA gene, part	EU005437	84.56	94.27	8.7753e−36	Diptera	Tipulidae	Tipula sp. PMO 200011
Corynoptera blanda voucher 955 16S ribosomal RNA g	JQ613965	83.12	97.45	3.73135e−34	Diptera	Sciaridae	Corynoptera blanda
Minettia longipennis mitochondrion partial 16S rRN	AJ439184	82.17	100	3.73135e−34	Diptera	Lauxaniidae	Minettia longipennis
Eudicrana sp. DK−2013 voucher JSS12 16S ribosomal	KC435571	83.22	93.63	1.30237e−33	Diptera	Mycetophilidae	Eudicrana sp. DK−2013
Derodontus emaculatus voucher BMNH:679374 16S ribo	DQ202609	81.25	99.36	1.30237e−33	Coleoptera		Derodontus emaculatus
Tipula abdominalis 16S ribosomal RNA gene, partial	KC177457	82.28	99.36	4.54572e−33	Diptera	Tipulidae	Tipula abdominalis
Tipula abdominalis mitochondrion, partial genome	JN861743	82.28	99.36	4.54572e−33	Diptera	Tipulidae	Tipula abdominalis
Gonocephalum pusillum isolate CSU0904046030 16S ri	GQ118381	81.65	100	4.54572e−33	Coleoptera	Tenebrionidae	Gonocephalum pusillum
Chorisops tunisiae 16S ribosomal RNA gene, partial	GU947379	81.53	100	1.58661e−32	Diptera	Stratiomyidae	Chorisops tunisiae
Ptychoptera sp. ATB−2011 mitochondrion, complete g	JN861744	82.78	94.9	5.53782e−32	Diptera	Ptychopteridae	Ptychoptera sp. ATB−2011
Corynoptera subtilis voucher T34 16S ribosomal RNA	JQ613978	81.82	97.45	1.93289e−31	Diptera	Sciaridae	Corynoptera subtilis
Minettia flaveola 16S ribosomal RNA gene, partial	KC177448	80.89	100	1.93289e−31	Diptera	Lauxaniidae	Minettia flaveola
Actina chalybea 16S ribosomal RNA gene, partial se	GU947372	81.53	100	1.93289e−31	Diptera	Acroceridae	Actina chalybea
Phytosciara flavipes voucher JSOUT8 16S ribosomal	KJ136731	81.94	97.45	6.74644e−31	Diptera	Sciaridae	Phytosciara flavipes
Acilius athabascae voucher 305G09/BMNH799009 16S r	KF979079	81.88	100	6.74644e−31	Coleoptera	Dytiscidae	Acilius athabascae
Chrysoperla nipponensis mitochondrial DNA, complet	AP011623	81.01	100	6.74644e−31	Neuroptera	Chrysopidae	Chrysoperla nipponensis
Bolitophila hybrida 16S ribosomal RNA gene, partia	FJ172002	82.76	91.72	6.74644e−31	Diptera	Mycetophilidae	Bolitophila hybrida
Drosophila funebris voucher 103952 NADH dehydrogen	EU494313	80.5	100	6.74644e−31	Diptera	Drosophilidae	Drosophila funebris
Mallada luctuosus 16S ribosomal RNA gene, partial	DQ399288	79.75	100	6.74644e−31	Neuroptera	Chrysopidae	Pseudomallada luctuosus
Leucochrysa marquezi voucher CASC 00212 16S riboso	DQ399280	81.13	100	6.74644e−31	Neuroptera	Chrysopidae	Leucochrysa marquezi
Drosophila arawakana dominicensis NADH dehydrogena	AF246516	80.5	100	6.74644e−31	Diptera	Drosophilidae	Drosophila arawakana dominicensis
Drosophila arawakana arawakana strain 15182−2261.0	AF246499	80.5	100	6.74644e−31	Diptera	Drosophilidae	Drosophila arawakana arawakana
Drosophila arawakana arawakana strain 15182−2261.1	AF246500	80.5	100	6.74644e−31	Diptera	Drosophilidae	Drosophila arawakana arawakana
Drosophila nigrodunni NADH dehydrogenase subunit 1	AF246494	80.5	100	6.74644e−31	Diptera	Drosophilidae	Drosophila nigrodunni
Chrysoperla agilis isolate A−083 16S large subunit	AY743751	81.01	100	6.74644e−31	Neuroptera	Chrysopidae	Chrysoperla agilis
Chrysoperla lucasina isolate L−079 16S large subun	AY743746	81.01	100	6.74644e−31	Neuroptera	Chrysopidae	Chrysoperla lucasina
Chrysoperla lucasina isolate L−029 16S large subun	AY743737	81.01	100	6.74644e−31	Neuroptera	Chrysopidae	Chrysoperla lucasina
Chrysoperla lucasina isolate L−099 16S large subun	AY743732	81.01	100	6.74644e−31	Neuroptera	Chrysopidae	Chrysoperla lucasina
Calosoma sp. BYU−CO241 mitochondrion, complete gen	GU176340	80.5	99.36	2.35474e−30	Coleoptera	Carabidae	Calosoma sp. BYU−CO241
Calosoma scrutator 16S ribosomal RNA gene, partial	EU734850	80.5	99.36	2.35474e−30	Coleoptera	Carabidae	Calosoma scrutator
Chrysoperla carnea isolate Chrcar3 16S ribosomal R	AY620150	80.62	100	2.35474e−30	Neuroptera	Chrysopidae	Chrysoperla carnea
Drosophila simulans isolate NC48 mitochondrion, co	AF200838	80.5	100	2.35474e−30	Diptera	Drosophilidae	Drosophila simulans
Drosophila simulans isolate NC37 mitochondrion, co	AF200837	80.5	100	2.35474e−30	Diptera	Drosophilidae	Drosophila simulans
Drosophila simulans isolate TT01 mitochondrion, co	AF200834	80.5	100	2.35474e−30	Diptera	Drosophilidae	Drosophila simulans
Drosophila simulans isolate TT00 mitochondrion, co	AF200833	80.5	100	2.35474e−30	Diptera	Drosophilidae	Drosophila simulans
Chrysoperla lucasina isolate L−070 16S large subun	AY743744	80.89	99.36	2.35474e−30	Neuroptera	Chrysopidae	Chrysoperla lucasina
Bolitophila occlusa voucher JSB2 16S ribosomal RNA	KJ136713	82.07	91.72	8.21885e−30	Diptera	Mycetophilidae	Bolitophila occlusa
Cychrus caraboides caraboides voucher CC00817 16S	JQ647184	81.01	99.36	8.21885e−30	Coleoptera	Carabidae	Cychrus caraboides caraboides
Cramptonomyia spenceri mitochondrion, complete gen	JN861747	80.5	99.36	8.21885e−30	Diptera	Pachyneuridae	Cramptonomyia spenceri
Dolichopus sabinus voucher Pollet 0117 (P) 16S rib	EU863944	80.25	100	8.21885e−30	Diptera	Dolichopodidae	Dolichopus sabinus
Drosophila robusta voucher 103967 NADH dehydrogena	EU494347	80.25	100	8.21885e−30	Diptera	Drosophilidae	Drosophila robusta
Drosophila takahashii cytochrome b (cytb) gene, pa	AF164592	80.25	100	8.21885e−30	Diptera	Drosophilidae	Drosophila takahashii
Chrysoperla nipponensis mitochondrion, complete ge	KP027407	80.38	100	2.86866e−29	Neuroptera	Chrysopidae	Chrysoperla nipponensis
Hercostomus praeceps voucher Pollet 0141 (P) 16S r	EU864003	80.5	100	2.86866e−29	Diptera	Dolichopodidae	Hercostomus praeceps
Hercostomus fulvicaudis voucher Pollet 0154 (P) 16	EU863992	80.5	100	2.86866e−29	Diptera	Dolichopodidae	Hercostomus fulvicaudis
Leptomorphus walkeri 16S ribosomal RNA gene, parti	FJ172020	85.37	77.71	2.86866e−29	Diptera	Mycetophilidae	Leptomorphus walkeri
Drosophila dunni voucher 103969 NADH dehydrogenase	EU494311	79.87	100	2.86866e−29	Diptera	Drosophilidae	Drosophila dunni
Limonia tahitiensis voucher O'Grady 202004 16S rib	EU005425	80	99.36	2.86866e−29	Diptera	Limoniidae	Dicranomyia tahitiensis
Leucochrysa lancala voucher CASC 00211 16S ribosom	DQ399277	80.5	100	2.86866e−29	Neuroptera	Chrysopidae	Leucochrysa lancala
Chrysoperla carnea isolate Chrcar2 16S ribosomal R	AY620152	80.38	100	2.86866e−29	Neuroptera	Chrysopidae	Chrysoperla carnea
Drosophila simulans isolate HW09 mitochondrion, co	AF200836	80.38	100	2.86866e−29	Diptera	Drosophilidae	Drosophila simulans
Drosophila simulans isolate HW00 mitochondrion, co	AF200835	80.38	100	2.86866e−29	Diptera	Drosophilidae	Drosophila simulans
Drosophila sechellia type line mitochondrion, comp	AF200832	80.38	100	2.86866e−29	Diptera	Drosophilidae	Drosophila sechellia
Drosophila sechellia cytochrome b (cytb) gene, par	AF164589	80.38	100	2.86866e−29	Diptera	Drosophilidae	Drosophila sechellia
Drosophila similis grenadensis NADH dehydrogenase	AF246519	79.87	100	2.86866e−29	Diptera	Drosophilidae	Drosophila similis grenadensis
Drosophila dunni dunni strain Barranquitas, line 1	AF246512	79.87	100	2.86866e−29	Diptera	Drosophilidae	Drosophila dunni dunni
Drosophila procardinoides NADH dehydrogenase subun	AF246508	79.87	100	2.86866e−29	Diptera	Drosophilidae	Drosophila procardinoides
Drosophila acutilabella strain 15181−2171.9 NADH d	AF246509	79.87	100	2.86866e−29	Diptera	Drosophilidae	Drosophila acutilabella
Drosophila dunni thomasensis NADH dehydrogenase su	AF246501	79.87	100	2.86866e−29	Diptera	Drosophilidae	Drosophila dunni thomasensis
Drosophila dunni dunni strain 15182−2291.0 NADH de	AF246497	79.87	100	2.86866e−29	Diptera	Drosophilidae	Drosophila dunni dunni
Drosophila acutilabella strain 15181−2171.2 NADH d	AF246496	79.87	100	2.86866e−29	Diptera	Drosophilidae	Drosophila acutilabella
Drosophila similis similis NADH dehydrogenase subu	AF246495	79.87	100	2.86866e−29	Diptera	Drosophilidae	Drosophila similis similis
Drosophila caribiana NADH dehydrogenase subunit 1	AF246493	79.87	100	2.86866e−29	Diptera	Drosophilidae	Drosophila caribiana
Chrysoperla sp. isolate 82 16S large subunit ribos	AY743750	80.38	100	2.86866e−29	Neuroptera	Chrysopidae	Chrysoperla sp. isolate 82
Chrysoperla carnea isolate C−097 16S large subunit	AY743731	80.38	100	2.86866e−29	Neuroptera	Chrysopidae	Chrysoperla carnea
Platambus princeps 16S ribosomal RNA gene, partial	AY138677	79.87	100	2.86866e−29	Coleoptera	Dytiscidae	Platambus princeps
Dysmicohermes ingens mitochondrion, complete genom	KJ806318	80.62	100	1.00126e−28	Megaloptera	Corydalidae	Dysmicohermes ingens
Rhantaticus congestus voucher NHRS/JLK000000091 16	KF979103	80.54	93.63	1.00126e−28	Coleoptera	Dytiscidae	Rhantaticus congestus
Rhantaticus congestus voucher 412B12_BMNH832121 16	KF979100	80.54	93.63	1.00126e−28	Coleoptera	Dytiscidae	Rhantaticus congestus
Zygoneura sciarina voucher 311 16S ribosomal RNA g	JQ613909	79.74	97.45	1.00126e−28	Diptera	Sciaridae	Zygoneura sciarina
Phytosciara semiferruginea voucher 220 16S ribosom	JQ613900	80.38	97.45	1.00126e−28	Diptera	Sciaridae	Phytosciara semiferruginea
Lycoriella flavicornis voucher 62 16S ribosomal RN	JQ613885	80.52	97.45	1.00126e−28	Diptera	Sciaridae	Lycoriella flavicornis
Drosophila simulans strain sm21 mitochondrion, com	KC244283	79.87	100	1.00126e−28	Diptera	Drosophilidae	Drosophila simulans
Colobaea bifasciella voucher MU_NUS_SG3 16S riboso	JN828202	79.62	100	1.00126e−28	Diptera	Sciomyzidae	Colobaea bifasciella
Hybos femoratus voucher Pollet 0247 (P) 16S riboso	HQ449036	79.87	100	1.00126e−28	Diptera	Hybotidae	Hybos femoratus
Rhantaticus congestus voucher BMNH829999 16S ribos	HQ381497	80.54	93.63	1.00126e−28	Coleoptera	Dytiscidae	Rhantaticus congestus
Prosopochrysa vitripennis 16S ribosomal RNA gene,	GU947402	80.25	100	1.00126e−28	Diptera	Stratiomyidae	Prosopochrysa vitripennis
Exodontha dubia 16S ribosomal RNA gene, partial se	GU947396	80.25	100	1.00126e−28	Diptera	Stratiomyidae	Exodontha dubia
Hermetia illucens 16S ribosomal RNA gene, partial	GU947384	79.62	100	1.00126e−28	Diptera	Stratiomyidae	Hermetia illucens
Lasiopa balius 16S ribosomal RNA gene, partial seq	GU947382	80.95	93.63	1.00126e−28	Diptera	Stratiomyidae	Lasiopa balius
Pyratula zonata 16S ribosomal RNA gene, partial se	FJ172004	82.01	88.54	1.00126e−28	Diptera	Keroplatidae	Pyratula zonata
Hercostomus meieri voucher RMBR:103442 16S ribosom	FJ808173	79.62	100	1.00126e−28	Diptera	Dolichopodidae	Hercostomus meieri
Elaphropeza neesoonensis voucher RMBR:103437 16S r	FJ808166	79.62	100	1.00126e−28	Diptera	Hybotidae	Elaphropeza neesoonensis
Conops sp. JFC−2009 16S ribosomal RNA gene, partia	FJ763680	79.62	100	1.00126e−28	Diptera	Conopidae	Conops sp. JFC−2009
Clusiodes ruficollis 16S ribosomal RNA gene, parti	EU268538	81.46	94.27	1.00126e−28	Diptera	Clusiidae	Clusiodes ruficollis
Rhantaticus congestus partial 16S rRNA gene	AJ850373	80.54	93.63	1.00126e−28	Coleoptera	Dytiscidae	Rhantaticus congestus
Zaprionus tuberculatus voucher 105498 NADH dehydro	EU494416	79.62	100	1.00126e−28	Diptera	Drosophilidae	Zaprionus tuberculatus
Drosophila signata voucher 109405 NADH dehydrogena	EU494328	79.62	100	1.00126e−28	Diptera	Drosophilidae	Drosophila signata
Drosophila rubida voucher 109400 NADH dehydrogenas	EU494327	79.62	100	1.00126e−28	Diptera	Drosophilidae	Drosophila rubida
Drosophila kepulauana voucher 109407 NADH dehydrog	EU494321	79.62	100	1.00126e−28	Diptera	Drosophilidae	Drosophila kepulauana
Drosophila hypocausta voucher 103972 NADH dehydrog	EU494319	79.62	100	1.00126e−28	Diptera	Drosophilidae	Drosophila hypocausta
Panorpa takenouchii isolate 26tak 16S ribosomal RN	EF041520	81.01	99.36	1.00126e−28	Mecoptera	Panorpidae	Panorpa takenouchii
Apertochrysa edwardsi 16S ribosomal RNA gene, part	DQ399265	80	100	1.00126e−28	Neuroptera	Chrysopidae	Apertochrysa edwardsi
Drosophila kepulauana 16S ribosomal RNA gene, part	AF387334	79.62	100	1.00126e−28	Diptera	Drosophilidae	Drosophila kepulauana
Drosophila simulans isolate AU023 mitochondrion, c	AY518674	79.87	100	1.00126e−28	Diptera	Drosophilidae	Drosophila simulans
