# Supplementary material for: Testing the potential of a ribosomal 16S marker for DNA metabarcoding of insects
Source: PeerJ. 2016 Apr 19;4:e1966. doi: 10.7717/peerj.1966 (PMC4841222; doi:10.7717/peerj.1966)
Supplement: Supplemental Information 1 [file peerj-04-1966-s006.zip › scripts/5 match OTUs/blast_hits/OTU_596.csv_plot.pdf]

OTU_596.csv

Parochlus steinenii

Chironomidae

Diptera

87.82

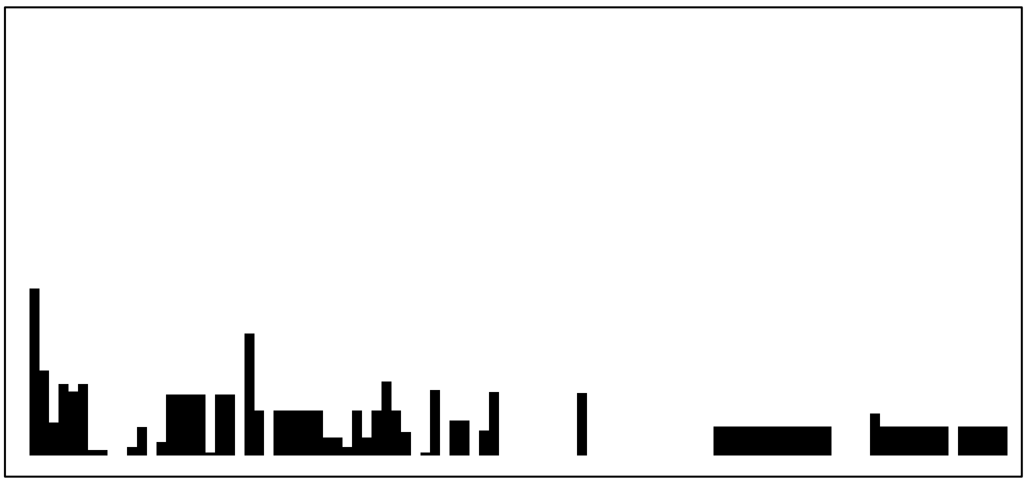

Parochlus steinenii isolate Sejong01 mitochondrion	KT003702	87.82	100	1.46035e−45	Diptera	Chironomidae	Parochlus steinenii
Bolitophila cinerea voucher JSB1 16S ribosomal RNA	KJ136712	83.97	97.42	1.5606e−32	Diptera	Mycetophilidae	Bolitophila cinerea
Pedicia albivitta voucher O'Grady 200012 16S ribos	EU005435	81.53	100	1.9012e−31	Diptera	Pediciidae	Pedicia albivitta
Bolitophila sp. ER−2009 16S ribosomal RNA gene, pa	FJ172021	83.33	97.42	6.63584e−31	Diptera	Mycetophilidae	Bolitophila sp. ER−2009
Bolitophila occlusa voucher JSB2 16S ribosomal RNA	KJ136713	82.99	93.55	2.31614e−30	Diptera	Mycetophilidae	Bolitophila occlusa
Bolitophila hybrida 16S ribosomal RNA gene, partia	FJ172002	83.33	97.42	8.08411e−30	Diptera	Mycetophilidae	Bolitophila hybrida
Tipula abdominalis 16S ribosomal RNA gene, partial	KC177457	80.25	100	1.19979e−27	Diptera	Tipulidae	Tipula abdominalis
Tipula abdominalis mitochondrion, partial genome	JN861743	80.25	100	1.19979e−27	Diptera	Tipulidae	Tipula abdominalis
Sylvicola fenestralis 16S ribosomal RNA gene, part	KC177461	79.49	100	4.18767e−27	Diptera	Anisopodidae	Sylvicola fenestralis
Sylvicola fenestralis mitochondrion, complete geno	JN861752	79.49	100	4.18767e−27	Diptera	Anisopodidae	Sylvicola fenestralis
Tipula sp. PMO 200011 16S ribosomal RNA gene, part	EU005437	80.38	100	4.18767e−27	Diptera	Tipulidae	Tipula sp. PMO 200011
Arachnocampa luminosa biotype 1 16S large subunit	AY576329	81.33	94.19	4.18767e−27	Diptera	Keroplatidae	Arachnocampa luminosa
Simulium sheilae isolate SH1B 16S ribosomal RNA ge	KM410213	79.62	100	1.46164e−26	Diptera	Simuliidae	Simulium sheilae
Tongamya stuckenbergi 16S ribosomal RNA gene, part	KM878969	80.62	99.35	1.46164e−26	Diptera	Mydidae	Tongamya stuckenbergi
Simulium roslihashimi isolate R1B 16S ribosomal RN	KM410192	82.84	85.16	5.10163e−26	Diptera	Simuliidae	Simulium roslihashimi
Simulium izuae isolate I3B 16S ribosomal RNA gene,	KM410191	82.84	85.16	5.10163e−26	Diptera	Simuliidae	Simulium izuae
Simulium izuae isolate I2B 16S ribosomal RNA gene,	KM410190	82.84	85.16	5.10163e−26	Diptera	Simuliidae	Simulium izuae
Simulium tuenense mitochondrial gene for 16S ribos	AB591244	82.84	85.16	5.10163e−26	Diptera	Simuliidae	Simulium tuenense
Beris morrisii 16S ribosomal RNA gene, partial seq	GU947373	80.13	99.35	5.10163e−26	Diptera	Stratiomyidae	Beris morrisii
Simulium inthanonense 16S ribosomal RNA gene, part	GQ865995	82.84	85.16	5.10163e−26	Diptera	Simuliidae	Simulium inthanonense
Simulium cabrerai mitochondrial gene for 16S rRNA,	AB093128	82.84	85.16	5.10163e−26	Diptera	Simuliidae	Simulium cabrerai
Simulium tuyense mitochondrial gene for 16S riboso	LC035004	79.49	97.42	1.78064e−25	Diptera	Simuliidae	Simulium tuyense
Paracladura trichoptera mitochondrion, complete ge	JN861751	85.71	75.48	1.78064e−25	Diptera	Trichoceridae	Paracladura trichoptera
Simulium tenederoi mitochondrial gene for 16S ribo	LC035001	82.09	85.16	6.21506e−25	Diptera	Simuliidae	Simulium tenederoi
Simulium sheilae isolate SH2B 16S ribosomal RNA ge	KM410214	78.98	100	6.21506e−25	Diptera	Simuliidae	Simulium sheilae
Simulium brinchangense isolate B2B 16S ribosomal R	KM410202	82.09	85.16	6.21506e−25	Diptera	Simuliidae	Simulium brinchangense
Simulium brinchangense isolate B1B 16S ribosomal R	KM410201	82.09	85.16	6.21506e−25	Diptera	Simuliidae	Simulium brinchangense
Simulium tanahrataense isolate TR2B 16S ribosomal	KM410196	82.09	85.16	6.21506e−25	Diptera	Simuliidae	Simulium tanahrataense
Simulium tanahrataense isolate TR1B 16S ribosomal	KM410195	82.09	85.16	6.21506e−25	Diptera	Simuliidae	Simulium tanahrataense
Simulium izuae isolate I1B 16S ribosomal RNA gene,	KM410189	82.09	85.16	6.21506e−25	Diptera	Simuliidae	Simulium izuae
Simulium khunklangense mitochondrial gene for 16S	AB820367	80.82	92.26	6.21506e−25	Diptera	Simuliidae	Simulium khunklangense
Simulium chomthongense mitochondrial gene for 16S	AB699899	80.82	92.26	6.21506e−25	Diptera	Simuliidae	Simulium chomthongense
Trichopeza longicornis voucher Pollet 0243 (P) 16S	HQ449035	80.38	99.35	6.21506e−25	Diptera	Empididae	Trichopeza longicornis
Cyphomyia notabilis 16S ribosomal RNA gene, partia	GU947403	82.09	85.16	6.21506e−25	Diptera	Stratiomyidae	Cyphomyia notabilis
Simulium caudisclerum 16S ribosomal RNA gene, part	GQ865999	80.82	92.26	6.21506e−25	Diptera	Simuliidae	Simulium caudisclerum
Simulium chumpornense 16S ribosomal RNA gene, part	GQ865998	82.09	85.16	6.21506e−25	Diptera	Simuliidae	Simulium chumpornense
Simulium decuplum 16S ribosomal RNA gene, partial	GQ865986	83.46	80	6.21506e−25	Diptera	Simuliidae	Simulium decuplum
Simulium recurvum mitochondrial gene for 16S ribos	AB334097	82.09	85.16	6.21506e−25	Diptera	Simuliidae	Simulium recurvum
Caenophthalmus capensis voucher MEI 120162 16S rib	DQ250838	81.08	92.26	6.21506e−25	Diptera	Therevidae	Caenophthalmus capensis
Cnephia dacotensis mitochondrial 16S ribosomal RNA	L13403	80	97.42	6.21506e−25	Diptera	Simuliidae	Cnephia dacotensis
Simulium tuyense mitochondrial gene for 16S riboso	LC035006	80.13	93.55	2.16927e−24	Diptera	Simuliidae	Simulium tuyense
Simulium tenederoi mitochondrial gene for 16S ribo	LC035002	83.06	78.71	2.16927e−24	Diptera	Simuliidae	Simulium tenederoi
Simulium suyoense mitochondrial gene for 16S ribos	LC035000	79.87	100	2.16927e−24	Diptera	Simuliidae	Simulium suyoense
Simulium spinosibranchium mitochondrial gene for 1	LC034996	81.62	85.16	2.16927e−24	Diptera	Simuliidae	Simulium spinosibranchium
Simulium spinosibranchium mitochondrial gene for 1	AB334098	81.62	85.16	2.16927e−24	Diptera	Simuliidae	Simulium spinosibranchium
Simulium chiharuae mitochondrial gene for 16S ribo	AB334092	80	91.61	2.16927e−24	Diptera	Simuliidae	Simulium chiharuae
Arachnocampa luminosa biotype 3 16S large subunit	AY576331	81.16	87.74	2.16927e−24	Diptera	Keroplatidae	Arachnocampa luminosa
Simulium palauense mitochondrial gene for 16S ribo	AB056742	82.96	85.16	2.16927e−24	Diptera	Simuliidae	Simulium palauense
Simulium mie mitochondrial gene for 16S ribosomal	AB056732	79.38	100	2.16927e−24	Diptera	Simuliidae	Simulium mie
Simulium mie mitochondrial gene for 16S ribosomal	AB056731	79.38	100	2.16927e−24	Diptera	Simuliidae	Simulium mie
Simulium mie mitochondrial gene for 16S ribosomal	AB056730	79.38	100	2.16927e−24	Diptera	Simuliidae	Simulium mie
Simulium tuyense mitochondrial gene for 16S riboso	LC035008	78.85	97.42	7.57149e−24	Diptera	Simuliidae	Simulium tuyense
Simulium tuyense mitochondrial gene for 16S riboso	LC035007	78.85	97.42	7.57149e−24	Diptera	Simuliidae	Simulium tuyense
Simulium tuyense mitochondrial gene for 16S riboso	LC035005	78.85	97.42	7.57149e−24	Diptera	Simuliidae	Simulium tuyense
Simulium tuyense mitochondrial gene for 16S riboso	LC035003	78.85	97.42	7.57149e−24	Diptera	Simuliidae	Simulium tuyense
Ptychoptera sp. ATB−2011 mitochondrion, complete g	JN861744	79.73	93.55	7.57149e−24	Diptera	Ptychopteridae	Ptychoptera sp. ATB−2011
Hercostomus meieri voucher RMBR:103442 16S ribosom	FJ808173	82.93	78.71	7.57149e−24	Diptera	Dolichopodidae	Hercostomus meieri
Simulium tuyense mitochondrial gene for 16S riboso	AB334100	78.85	97.42	7.57149e−24	Diptera	Simuliidae	Simulium tuyense
Simulium squamosum voucher P.Mansiangi Pm3(BNI) 16	DQ655644	80	92.26	7.57149e−24	Diptera	Simuliidae	Simulium squamosum
Simulium damnosum s.l. GH−D1 16S ribosomal RNA, pa	AY077709	80	92.26	7.57149e−24	Diptera	Simuliidae	Simulium damnosum s.l. GH−D1
Simulium damnosum s.l. Kulfo isolate Ku 3 16S ribo	AF416978	80	92.26	7.57149e−24	Diptera	Simuliidae	Simulium damnosum s.l. Kulfo
Simulium soubrense isolate Wa 52 16S ribosomal RNA	AF416976	80	92.26	7.57149e−24	Diptera	Simuliidae	Simulium soubrense
Simulium soubrense isolate Wa 47 16S ribosomal RNA	AF416975	80	92.26	7.57149e−24	Diptera	Simuliidae	Simulium soubrense
Simulium damnosum strain Se−12 16S ribosomal RNA g	AF081907	80	92.26	7.57149e−24	Diptera	Simuliidae	Simulium damnosum
Simulium uchidai mitochondrial gene for 16S riboso	AB056740	80	92.26	7.57149e−24	Diptera	Simuliidae	Simulium uchidai
Simulium saitoi mitochondrial gene for 16S ribosom	AB056733	79.73	91.61	7.57149e−24	Diptera	Simuliidae	Simulium saitoi
Simulium leonense mitochondrion 16S ribosomal RNA	U17731	80	92.26	7.57149e−24	Diptera	Simuliidae	Simulium leonense
Simulium suyoense mitochondrial gene for 16S ribos	LC034999	79.25	100	2.64271e−23	Diptera	Simuliidae	Simulium suyoense
Simulium suyoense mitochondrial gene for 16S ribos	LC034998	79.25	100	2.64271e−23	Diptera	Simuliidae	Simulium suyoense
Simulium suyoense mitochondrial gene for 16S ribos	LC034997	79.25	100	2.64271e−23	Diptera	Simuliidae	Simulium suyoense
Simulium claveriaense mitochondrial gene for 16S r	LC034988	81.34	85.16	2.64271e−23	Diptera	Simuliidae	Simulium claveriaense
Simulium claveriaense mitochondrial gene for 16S r	LC034987	81.34	85.16	2.64271e−23	Diptera	Simuliidae	Simulium claveriaense
Simulium nobile isolate N5 16S ribosomal RNA gene,	KP661530	81.34	85.16	2.64271e−23	Diptera	Simuliidae	Simulium nobile
Simulium nobile isolate N4 16S ribosomal RNA gene,	KP661529	81.34	85.16	2.64271e−23	Diptera	Simuliidae	Simulium nobile
Simulium nobile isolate N3 16S ribosomal RNA gene,	KP661528	81.34	85.16	2.64271e−23	Diptera	Simuliidae	Simulium nobile
Simulium nobile isolate N2 16S ribosomal RNA gene,	KP661527	81.34	85.16	2.64271e−23	Diptera	Simuliidae	Simulium nobile
Simulium nobile isolate N1 16S ribosomal RNA gene,	KP661526	81.34	85.16	2.64271e−23	Diptera	Simuliidae	Simulium nobile
Simulium timorense isolate TM5 16S ribosomal RNA g	KP661525	81.34	85.16	2.64271e−23	Diptera	Simuliidae	Simulium timorense
Simulium timorense isolate TM4 16S ribosomal RNA g	KP661524	81.34	85.16	2.64271e−23	Diptera	Simuliidae	Simulium timorense
Simulium timorense isolate TM3 16S ribosomal RNA g	KP661523	81.34	85.16	2.64271e−23	Diptera	Simuliidae	Simulium timorense
Simulium timorense isolate TM2 16S ribosomal RNA g	KP661522	81.34	85.16	2.64271e−23	Diptera	Simuliidae	Simulium timorense
Simulium timorense isolate TM1 16S ribosomal RNA g	KP661521	81.34	85.16	2.64271e−23	Diptera	Simuliidae	Simulium timorense
Diadocidia stanfordensis voucher JSSJ14 16S riboso	KJ136742	78.81	91.61	2.64271e−23	Diptera	Diadocidiidae	Diadocidia stanfordensis
Haematopota pluvialis 16S ribosomal RNA gene, part	KC177454	78.91	94.19	2.64271e−23	Diptera	Tabanidae	Haematopota pluvialis
Chrysops viduatus 16S ribosomal RNA gene, partial	HM132115	78.91	94.19	2.64271e−23	Diptera	Tabanidae	Chrysops viduatus
Tabanus bromius 16S ribosomal RNA gene, partial se	HM132111	77.42	99.35	2.64271e−23	Diptera	Tabanidae	Tabanus bromius
Gymnopternus blankaartensis voucher Pollet 0090 (P	EU863973	81.97	78.06	2.64271e−23	Diptera	Dolichopodidae	Gymnopternus blankaartensis
Simulium nobile 16S ribosomal RNA gene, partial se	GQ866008	81.34	85.16	2.64271e−23	Diptera	Simuliidae	Simulium nobile
Simulium sheilae 16S ribosomal RNA gene, partial s	GQ865996	81.34	85.16	2.64271e−23	Diptera	Simuliidae	Simulium sheilae
Simulium siamense 16S ribosomal RNA gene, partial	GQ865994	81.34	85.16	2.64271e−23	Diptera	Simuliidae	Simulium siamense
Simulium siamense 16S ribosomal RNA gene, partial	GQ865993	81.34	85.16	2.64271e−23	Diptera	Simuliidae	Simulium siamense
Simulium siamense 16S ribosomal RNA gene, partial	GQ865992	81.34	85.16	2.64271e−23	Diptera	Simuliidae	Simulium siamense
Simulium decuplum 16S ribosomal RNA gene, partial	GQ865987	81.34	85.16	2.64271e−23	Diptera	Simuliidae	Simulium decuplum
Simulium angulistylum 16S ribosomal RNA gene, part	GQ865985	81.34	85.16	2.64271e−23	Diptera	Simuliidae	Simulium angulistylum
Simulium suyoense mitochondrial gene for 16S ribos	AB334099	79.25	100	2.64271e−23	Diptera	Simuliidae	Simulium suyoense
Simulium claveriaense mitochondrial gene for 16S r	AB334096	81.34	85.16	2.64271e−23	Diptera	Simuliidae	Simulium claveriaense
Simulium damnosum cytoform Sanje isolate SJ02−1 16	AY722112	81.34	85.16	2.64271e−23	Diptera	Simuliidae	Simulium damnosum cytoform Sanje
Simulium damnosum sensu lato isolate SJ11 16S ribo	AF466187	81.34	85.16	2.64271e−23	Diptera	Simuliidae	Simulium damnosum s.l. SJ11
Simulium iridescens mitochondrial gene for 16S rRN	AB093127	81.34	85.16	2.64271e−23	Diptera	Simuliidae	Simulium iridescens
Simulium nobile mitochondrial gene for 16S rRNA, p	AB093115	81.34	85.16	2.64271e−23	Diptera	Simuliidae	Simulium nobile
