# Supplementary material for: Testing the potential of a ribosomal 16S marker for DNA metabarcoding of insects
Source: PeerJ. 2016 Apr 19;4:e1966. doi: 10.7717/peerj.1966 (PMC4841222; doi:10.7717/peerj.1966)
Supplement: Supplemental Information 1 [file peerj-04-1966-s006.zip › scripts/5 match OTUs/blast_hits/OTU_599.csv_plot.pdf]

OTU_599.csv

Parochlus steinenii

Chironomidae

Diptera

87.18

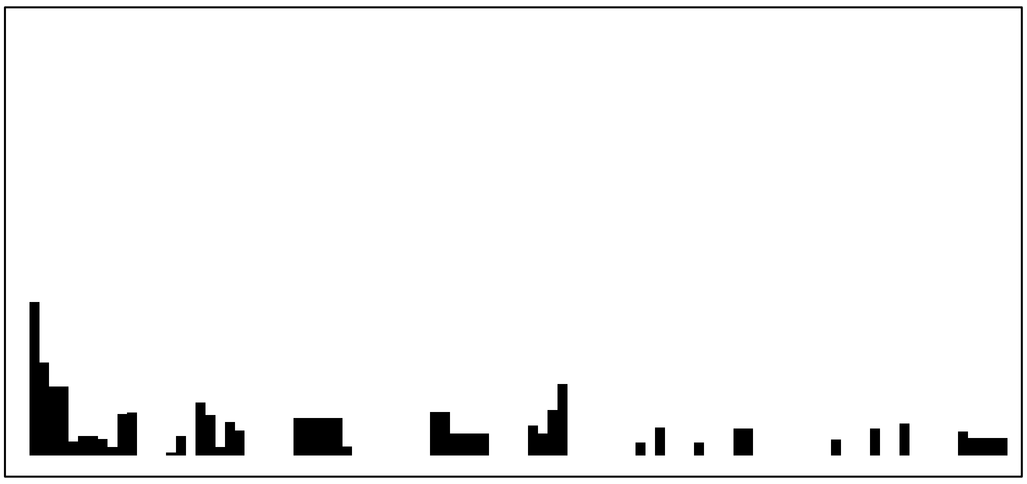

Parochlus steinenii isolate Sejong01 mitochondrion	KT003702	87.18	100	1.47252e−45	Diptera	Chironomidae	Parochlus steinenii
Bolitophila occlusa voucher JSB2 16S ribosomal RNA	KJ136713	84.35	93.59	3.70077e−34	Diptera	Mycetophilidae	Bolitophila occlusa
Bolitophila cinerea voucher JSB1 16S ribosomal RNA	KJ136712	83.23	97.44	1.29169e−33	Diptera	Mycetophilidae	Bolitophila cinerea
Bolitophila sp. ER−2009 16S ribosomal RNA gene, pa	FJ172021	83.23	97.44	1.29169e−33	Diptera	Mycetophilidae	Bolitophila sp. ER−2009
Edwardsina gigantea 16S ribosomal RNA gene, partia	KC177460	80.65	99.36	1.91704e−31	Diptera	Blephariceridae	Edwardsina gigantea
Tipula sp. PMO 200011 16S ribosomal RNA gene, part	EU005437	80.89	100	1.91704e−31	Diptera	Tipulidae	Tipula sp. PMO 200011
Pedicia albivitta voucher O'Grady 200012 16S ribos	EU005435	80.89	100	1.91704e−31	Diptera	Pediciidae	Pedicia albivitta
Agathomyia zetterstedti isolate 814 16S ribosomal	KC699312	80.77	99.36	8.15148e−30	Diptera	Platypezidae	Agathomyia zetterstedti
Clinocera sp. Pollet 0249 16S ribosomal RNA gene,	HQ449032	80.38	99.36	8.15148e−30	Diptera	Empididae	Clinocera sp. Pollet 0249
Bolitophila hybrida 16S ribosomal RNA gene, partia	FJ172002	81.94	97.44	8.15148e−30	Diptera	Mycetophilidae	Bolitophila hybrida
Arachnocampa luminosa biotype 1 16S large subunit	AY576329	82	94.23	8.15148e−30	Diptera	Keroplatidae	Arachnocampa luminosa
Tipula abdominalis 16S ribosomal RNA gene, partial	KC177457	79.49	100	2.84515e−29	Diptera	Tipulidae	Tipula abdominalis
Tipula abdominalis mitochondrion, partial genome	JN861743	79.49	100	2.84515e−29	Diptera	Tipulidae	Tipula abdominalis
Agathomyia elegantula isolate AE04 16S ribosomal R	KC699320	79.35	99.36	9.93054e−29	Diptera	Platypezidae	Agathomyia elegantula
Agathomyia lundbecki isolate 233 16S ribosomal RNA	KC699306	80.13	99.36	3.4661e−28	Diptera	Platypezidae	Agathomyia lundbecki
Beris morrisii 16S ribosomal RNA gene, partial seq	GU947373	80.89	99.36	4.22257e−27	Diptera	Stratiomyidae	Beris morrisii
Geomyza tripunctata 16S ribosomal RNA gene, partia	EU268537	78.71	99.36	4.22257e−27	Diptera	Opomyzidae	Geomyza tripunctata
Simulium izuae isolate I1B 16S ribosomal RNA gene,	KM410189	82.48	85.26	1.47382e−26	Diptera	Simuliidae	Simulium izuae
Arachnocampa luminosa biotype 3 16S large subunit	AY576331	81.88	87.82	1.47382e−26	Diptera	Keroplatidae	Arachnocampa luminosa
Trichopeza longicornis voucher Pollet 0243 (P) 16S	HQ449035	80.38	99.36	5.14415e−26	Diptera	Empididae	Trichopeza longicornis
Paracladura trichoptera mitochondrion, complete ge	JN861751	81.56	86.54	5.14415e−26	Diptera	Trichoceridae	Paracladura trichoptera
Callomyia admirabilis isolate CA42 16S ribosomal R	KC699326	81.16	87.82	1.79548e−25	Diptera	Platypezidae	Callomyia admirabilis
Agathomyia viduella isolate AV01 16S ribosomal RNA	KC699317	78.85	99.36	1.79548e−25	Diptera	Platypezidae	Agathomyia viduella
Sylvicola fenestralis 16S ribosomal RNA gene, part	KC177461	77.5	100	1.79548e−25	Diptera	Anisopodidae	Sylvicola fenestralis
Sylvicola fenestralis mitochondrion, complete geno	JN861752	77.5	100	1.79548e−25	Diptera	Anisopodidae	Sylvicola fenestralis
Somatia sp. YSUW04038 16S ribosomal RNA gene, part	AY573134	78.91	94.23	1.79548e−25	Diptera	Somatiidae	Somatia sp. YSUW04038
Simulium sheilae isolate SH1B 16S ribosomal RNA ge	KM410213	78.34	100	6.26685e−25	Diptera	Simuliidae	Simulium sheilae
Simulium roslihashimi isolate R1B 16S ribosomal RN	KM410192	81.75	85.26	6.26685e−25	Diptera	Simuliidae	Simulium roslihashimi
Simulium izuae isolate I3B 16S ribosomal RNA gene,	KM410191	81.75	85.26	6.26685e−25	Diptera	Simuliidae	Simulium izuae
Simulium izuae isolate I2B 16S ribosomal RNA gene,	KM410190	81.75	85.26	6.26685e−25	Diptera	Simuliidae	Simulium izuae
Simulium tuenense mitochondrial gene for 16S ribos	AB591244	81.75	85.26	6.26685e−25	Diptera	Simuliidae	Simulium tuenense
Simulium inthanonense 16S ribosomal RNA gene, part	GQ865995	81.75	85.26	6.26685e−25	Diptera	Simuliidae	Simulium inthanonense
Caenophthalmus capensis voucher MEI 120162 16S rib	DQ250838	80.41	92.31	6.26685e−25	Diptera	Therevidae	Caenophthalmus capensis
Platypezina diversa isolate PD29 16S ribosomal RNA	KC699349	79.56	87.82	2.18735e−24	Diptera	Platypezidae	Platypezina diversa
Agathomyia woodella isolate 448 16S ribosomal RNA	KC699307	79.87	93.59	2.18735e−24	Diptera	Platypezidae	Agathomyia woodella
Diadocidia stanfordensis voucher JSSJ14 16S riboso	KJ136742	78.81	91.67	2.18735e−24	Diptera	Diadocidiidae	Diadocidia stanfordensis
Zygoneura bidens voucher 312 16S ribosomal RNA gen	JQ613910	78.21	97.44	2.18735e−24	Diptera	Sciaridae	Zygoneura bidens
Chloromyia formosa 16S ribosomal RNA gene, partial	GU947385	80	95.51	2.18735e−24	Diptera	Stratiomyidae	Chloromyia formosa
Arachnocampa luminosa biotype 2 16S large subunit	AY576330	79.33	94.23	2.18735e−24	Diptera	Keroplatidae	Arachnocampa luminosa
Coelopa alluaudi 16S ribosomal RNA gene, partial s	AF403455	79.56	87.82	2.18735e−24	Diptera	Coelopidae	Coelopa alluaudi
Anthrax proconcisus partial mitochondrial 16S rRNA	AJ271846	76.88	99.36	2.18735e−24	Diptera	Bombyliidae	Anthrax proconcisus
Simulium tenederoi mitochondrial gene for 16S ribo	LC035002	82.03	79.49	7.63459e−24	Diptera	Simuliidae	Simulium tenederoi
Simulium tenederoi mitochondrial gene for 16S ribo	LC035001	82.03	79.49	7.63459e−24	Diptera	Simuliidae	Simulium tenederoi
Simulium brinchangense isolate B2B 16S ribosomal R	KM410202	81.02	85.26	7.63459e−24	Diptera	Simuliidae	Simulium brinchangense
Simulium brinchangense isolate B1B 16S ribosomal R	KM410201	81.02	85.26	7.63459e−24	Diptera	Simuliidae	Simulium brinchangense
Simulium tanahrataense isolate TR2B 16S ribosomal	KM410196	81.02	85.26	7.63459e−24	Diptera	Simuliidae	Simulium tanahrataense
Simulium tanahrataense isolate TR1B 16S ribosomal	KM410195	81.02	85.26	7.63459e−24	Diptera	Simuliidae	Simulium tanahrataense
Ectinorhynchus sp. 'Bribie' voucher UQIC41178 16S	KM212983	78.48	99.36	7.63459e−24	Diptera	Therevidae	Ectinorhynchus sp. 'Bribie'
Ectinorhynchus sp. 'Moffatt' voucher QMT183203 16S	KM212976	78.48	99.36	7.63459e−24	Diptera	Therevidae	Ectinorhynchus sp. 'Moffatt'
Ectinorhynchus sp. 'warrumbungles' voucher QMT1832	KM212974	78.48	99.36	7.63459e−24	Diptera	Therevidae	Ectinorhynchus sp. 'warrumbungles'
Leptomorphus sp. DK−2013 voucher JSS1 16S ribosoma	KC435575	79.02	91.67	7.63459e−24	Diptera	Mycetophilidae	Leptomorphus sp. DK−2013
Coboldia sp. SS−2012 voucher 905 16S ribosomal RNA	JQ613954	81.4	81.41	7.63459e−24	Diptera	Scatopsidae	Coboldia sp. SS−2012
Simulium chumpornense 16S ribosomal RNA gene, part	GQ865998	81.02	85.26	7.63459e−24	Diptera	Simuliidae	Simulium chumpornense
Leptomorphus walkeri 16S ribosomal RNA gene, parti	FJ172020	82.11	78.21	7.63459e−24	Diptera	Mycetophilidae	Leptomorphus walkeri
Hercostomus meieri voucher RMBR:103442 16S ribosom	FJ808173	83.33	79.49	7.63459e−24	Diptera	Dolichopodidae	Hercostomus meieri
Simulium suyoense mitochondrial gene for 16S ribos	LC035000	79.01	100	2.66473e−23	Diptera	Simuliidae	Simulium suyoense
Simulium sheilae isolate SH2B 16S ribosomal RNA ge	KM410214	77.71	100	2.66473e−23	Diptera	Simuliidae	Simulium sheilae
Agathomyia falleni isolate AF03 16S ribosomal RNA	KC699319	76.77	99.36	2.66473e−23	Diptera	Platypezidae	Agathomyia falleni
Liriomyza sativae mitochondrion, complete genome	JQ862475	77.07	99.36	2.66473e−23	Diptera	Agromyzidae	Liriomyza sativae
Dichopygina aculeata voucher 947 16S ribosomal RNA	JQ613961	78.57	97.44	2.66473e−23	Diptera	Sciaridae	Dichopygina aculeata
Ptychoptera sp. ATB−2011 mitochondrion, complete g	JN861744	79.43	89.1	2.66473e−23	Diptera	Ptychopteridae	Ptychoptera sp. ATB−2011
Liriomyza sativae mitochondrion, complete genome	HQ333260	77.07	99.36	2.66473e−23	Diptera	Agromyzidae	Liriomyza sativae
Simulium decuplum 16S ribosomal RNA gene, partial	GQ865986	80.6	85.26	2.66473e−23	Diptera	Simuliidae	Simulium decuplum
Panorpa takenouchii isolate 26tak 16S ribosomal RN	EF041520	79.56	87.82	2.66473e−23	Mecoptera	Panorpidae	Panorpa takenouchii
Neotephritis finalis 16S ribosomal RNA gene, parti	DQ471400	81.3	78.85	2.66473e−23	Diptera	Tephritidae	Neotephritis finalis
Eutreta novaeboracensis 16S ribosomal RNA gene, pa	DQ471384	77.85	99.36	2.66473e−23	Diptera	Tephritidae	Eutreta novaeboracensis
Dialysis sp. SLW−2002 16S ribosomal RNA gene, part	AY140888	76.77	99.36	2.66473e−23	Diptera	Xylophagidae	Dialysis sp. SLW−2002
Panorpa takenouchii 16S ribosomal RNA gene, partia	AF180064	79.56	87.82	2.66473e−23	Mecoptera	Panorpidae	Panorpa takenouchii
Simulium cabrerai mitochondrial gene for 16S rRNA,	AB093128	80.6	85.26	2.66473e−23	Diptera	Simuliidae	Simulium cabrerai
Simulium mie mitochondrial gene for 16S ribosomal	AB056732	78.4	100	2.66473e−23	Diptera	Simuliidae	Simulium mie
Simulium mie mitochondrial gene for 16S ribosomal	AB056731	78.4	100	2.66473e−23	Diptera	Simuliidae	Simulium mie
Simulium mie mitochondrial gene for 16S ribosomal	AB056730	78.4	100	2.66473e−23	Diptera	Simuliidae	Simulium mie
Simulium claveriaense mitochondrial gene for 16S r	LC034988	81.25	79.49	9.30083e−23	Diptera	Simuliidae	Simulium claveriaense
Simulium claveriaense mitochondrial gene for 16S r	LC034987	81.25	79.49	9.30083e−23	Diptera	Simuliidae	Simulium claveriaense
Protoclythia rufa isolate PR22 16S ribosomal RNA g	KC699354	77.07	99.36	9.30083e−23	Diptera	Platypezidae	Protoclythia rufa
Protoclythia modesta isolate PM21 16S ribosomal RN	KC699350	77.07	99.36	9.30083e−23	Diptera	Platypezidae	Protoclythia modesta
Chydaeopeza tibialis isolate CT44 16S ribosomal RN	KC699336	77.56	99.36	9.30083e−23	Diptera	Platypezidae	Chydaeopeza tibialis
Agathomyia unicolor isolate AU2 16S ribosomal RNA	KC699318	78.34	99.36	9.30083e−23	Diptera	Platypezidae	Agathomyia unicolor
Grossoseta pacifica isolate 1384 16S ribosomal RNA	KC699316	78.83	87.82	9.30083e−23	Diptera	Platypezidae	Grossoseta pacifica
Simulium tribulatum isolate LAC 17−5 16S ribosomal	KP233212	77.99	100	9.30083e−23	Diptera	Simuliidae	Simulium tribulatum
Haematobia irritans mitochondrion, partial genome	KM669714	77.56	99.36	9.30083e−23	Diptera	Muscidae	Haematobia irritans
Haematobia irritans isolate D50 small subunit ribo	JQ246755	77.56	99.36	9.30083e−23	Diptera	Muscidae	Haematobia irritans
Eudmeta marginata 16S ribosomal RNA gene, partial	GU947400	80.74	85.9	9.30083e−23	Diptera	Stratiomyidae	Eudmeta marginata
Haematobia irritans 16S ribosomal RNA gene, partia	FJ025436	77.56	99.36	9.30083e−23	Diptera	Muscidae	Haematobia irritans
Drosophila rubida voucher 109400 NADH dehydrogenas	EU494327	79.47	92.31	9.30083e−23	Diptera	Drosophilidae	Drosophila rubida
Drosophila hypocausta voucher 103972 NADH dehydrog	EU494319	79.47	92.31	9.30083e−23	Diptera	Drosophilidae	Drosophila hypocausta
Simulium claveriaense mitochondrial gene for 16S r	AB334096	81.25	79.49	9.30083e−23	Diptera	Simuliidae	Simulium claveriaense
Haematobia irritans irritans mitochondrion, comple	DQ029097	77.56	99.36	9.30083e−23	Diptera	Muscidae	Haematobia irritans irritans
Chaetocoelopa littoralis 16S ribosomal RNA gene, p	AF403453	77.56	99.36	9.30083e−23	Diptera	Coelopidae	Chaetocoelopa littoralis
Simulium palauense mitochondrial gene for 16S ribo	AB056742	81.48	85.26	9.30083e−23	Diptera	Simuliidae	Simulium palauense
Protoclythia modesta 16S ribosomal RNA gene, parti	AF126348	77.07	99.36	9.30083e−23	Diptera	Platypezidae	Protoclythia modesta
Simulium tuyense mitochondrial gene for 16S riboso	LC035004	78.21	97.44	3.24631e−22	Diptera	Simuliidae	Simulium tuyense
Simulium suyoense mitochondrial gene for 16S ribos	LC034999	78.4	100	3.24631e−22	Diptera	Simuliidae	Simulium suyoense
Simulium suyoense mitochondrial gene for 16S ribos	LC034998	78.4	100	3.24631e−22	Diptera	Simuliidae	Simulium suyoense
Simulium suyoense mitochondrial gene for 16S ribos	LC034997	78.4	100	3.24631e−22	Diptera	Simuliidae	Simulium suyoense
Simulium celebesense mitochondrial gene for 16S ri	LC034986	81.1	78.85	3.24631e−22	Diptera	Simuliidae	Simulium celebesense
Simulium nobile isolate N5 16S ribosomal RNA gene,	KP661530	80.8	79.49	3.24631e−22	Diptera	Simuliidae	Simulium nobile
Simulium nobile isolate N4 16S ribosomal RNA gene,	KP661529	80.8	79.49	3.24631e−22	Diptera	Simuliidae	Simulium nobile
Simulium nobile isolate N3 16S ribosomal RNA gene,	KP661528	80.8	79.49	3.24631e−22	Diptera	Simuliidae	Simulium nobile
Simulium nobile isolate N2 16S ribosomal RNA gene,	KP661527	80.8	79.49	3.24631e−22	Diptera	Simuliidae	Simulium nobile
