# Supplementary material for: Testing the potential of a ribosomal 16S marker for DNA metabarcoding of insects
Source: PeerJ. 2016 Apr 19;4:e1966. doi: 10.7717/peerj.1966 (PMC4841222; doi:10.7717/peerj.1966)
Supplement: Supplemental Information 1 [file peerj-04-1966-s006.zip › scripts/5 match OTUs/blast_hits/OTU_6.csv_plot.pdf]

# OTU_6.csv

## Leuctra inermis

## Leuctridae

## Plecoptera

## 89.31

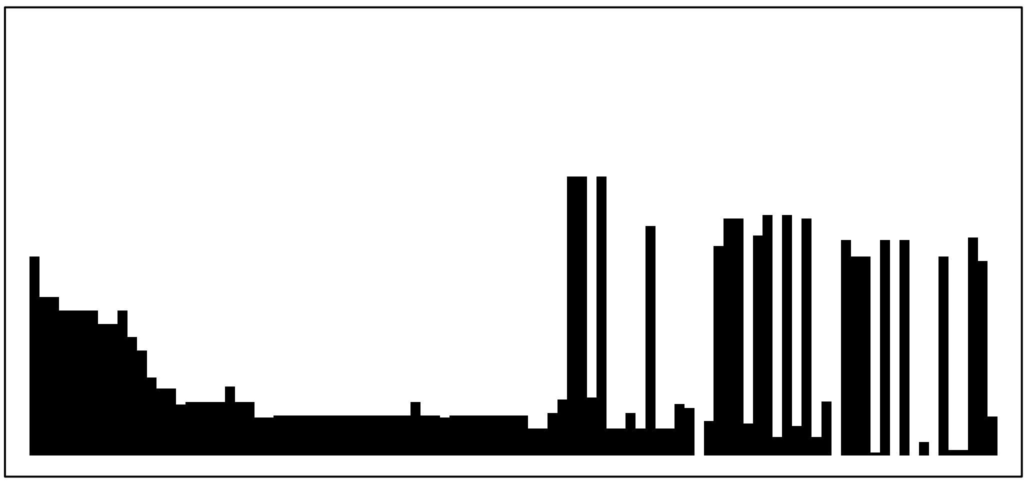

| | | | | | | | |
|---|---|---|---|---|---|---|---|
| Leuctra inermis 16S ribosomal RNA gene, partial se | EF623176 | 89.31 | 100 | 5.59484e−51 | Plecoptera | Leuctridae | Leuctra inermis |
| Leuctra tenuis haplotype 8lt: HO8 16S ribosomal RN | EU327680 | 87.42 | 100 | 4.30131e−46 | Plecoptera | Leuctridae | Leuctra tenuis |
| Leuctra tenuis haplotype 7lt: HO7 16S ribosomal RN | EU327679 | 87.42 | 100 | 4.30131e−46 | Plecoptera | Leuctridae | Leuctra tenuis |
| Leuctra tenuis haplotype 10lt: HO10 16S ribosomal | EU327682 | 86.79 | 100 | 1.82896e−44 | Plecoptera | Leuctridae | Leuctra tenuis |
| Leuctra tenuis haplotype 9lt: HO9 16S ribosomal RN | EU327681 | 86.79 | 100 | 1.82896e−44 | Plecoptera | Leuctridae | Leuctra tenuis |
| Leuctra tenuis haplotype 3lt: H03 16S ribosomal RN | DQ915184 | 86.79 | 100 | 1.82896e−44 | Plecoptera | Leuctridae | Leuctra tenuis |
| Leuctra tenuis haplotype 2lt: H02 16S ribosomal RN | DQ915183 | 86.79 | 100 | 1.82896e−44 | Plecoptera | Leuctridae | Leuctra tenuis |
| Leuctra tenuis haplotype 6lt: HO6 16S ribosomal RN | EU327678 | 86.16 | 100 | 2.22813e−43 | Plecoptera | Leuctridae | Leuctra tenuis |
| Leuctra tenuis haplotype 1lt: H01 16S ribosomal RN | DQ915182 | 86.16 | 100 | 2.22813e−43 | Plecoptera | Leuctridae | Leuctra tenuis |
| Moselia infuscata 16S ribosomal RNA gene, partial | EF623175 | 86.79 | 99.37 | 7.77694e−43 | Plecoptera | Leuctridae | Moselia infuscata |
| Leuctra tenuis haplotype 5lt: HO5 16S ribosomal RN | EU327677 | 85.53 | 100 | 9.47426e−42 | Plecoptera | Leuctridae | Leuctra tenuis |
| Leuctra tenuis haplotype 4lt: HO4 16S ribosomal RN | EU327676 | 84.91 | 100 | 1.1542e−40 | Plecoptera | Leuctridae | Leuctra tenuis |
| Despaxia augusta 16S ribosomal RNA gene, partial s | EF623177 | 83.65 | 100 | 5.97891e−38 | Plecoptera | Leuctridae | Despaxia augusta |
| Apteroperla tikumana mitochondrion, complete genom | KR604721 | 83.12 | 100 | 3.09715e−35 | Plecoptera | Capniidae | Apteroperla tikumana |
| Allocapnia minima 16S ribosomal RNA gene, partial | EF623130 | 83.12 | 100 | 3.09715e−35 | Plecoptera | Capniidae | Allocapnia minima |
| Cascadoperla trictura 16S ribosomal RNA gene, part | EF623225 | 82.39 | 100 | 3.7731e−34 | Plecoptera | Perlodidae | Cascadoperla trictura |
| Allocapnia recta isolate H15 16S ribosomal RNA gen | KC881050 | 82.5 | 100 | 1.31694e−33 | Plecoptera | Capniidae | Allocapnia recta |
| Allocapnia recta isolate H14 16S ribosomal RNA gen | KC881049 | 82.5 | 100 | 1.31694e−33 | Plecoptera | Capniidae | Allocapnia recta |
| Allocapnia recta isolate H10 16S ribosomal RNA gen | KC881045 | 82.5 | 100 | 1.31694e−33 | Plecoptera | Capniidae | Allocapnia recta |
| Allocapnia recta isolate H6 16S ribosomal RNA gene | KC881041 | 82.5 | 100 | 1.31694e−33 | Plecoptera | Capniidae | Allocapnia recta |
| Protonemura meyeri 16S ribosomal RNA gene, partial | EF623193 | 83.23 | 100 | 1.31694e−33 | Plecoptera | Nemouridae | Protonemura meyeri |
| Allocapnia recta haplotype 11ar: H011 16S ribosoma | EU306874 | 82.5 | 100 | 1.31694e−33 | Plecoptera | Capniidae | Allocapnia recta |
| Allocapnia recta haplotype 7ar: H07 16S ribosomal | EU306870 | 82.5 | 100 | 1.31694e−33 | Plecoptera | Capniidae | Allocapnia recta |
| Soyedina producta 16S ribosomal RNA gene, partial | EF623196 | 81.76 | 100 | 4.59658e−33 | Plecoptera | Nemouridae | Soyedina producta |
| Capnura wanica 16S ribosomal RNA gene, partial seq | EF623128 | 81.76 | 100 | 4.59658e−33 | Plecoptera | Capniidae | Capnura wanica |
| Mesocapnia arizonensis mitochondrion, partial geno | KP642637 | 81.88 | 100 | 1.60436e−32 | Plecoptera | Capniidae | Mesocapnia arizonensis |
| Allocapnia recta isolate H19 16S ribosomal RNA gen | KC881054 | 81.88 | 100 | 1.60436e−32 | Plecoptera | Capniidae | Allocapnia recta |
| Allocapnia recta isolate H17 16S ribosomal RNA gen | KC881052 | 81.88 | 100 | 1.60436e−32 | Plecoptera | Capniidae | Allocapnia recta |
| Allocapnia recta isolate H16 16S ribosomal RNA gen | KC881051 | 81.88 | 100 | 1.60436e−32 | Plecoptera | Capniidae | Allocapnia recta |
| Allocapnia recta isolate H13 16S ribosomal RNA gen | KC881048 | 81.88 | 100 | 1.60436e−32 | Plecoptera | Capniidae | Allocapnia recta |
| Allocapnia recta isolate H12 16S ribosomal RNA gen | KC881047 | 81.88 | 100 | 1.60436e−32 | Plecoptera | Capniidae | Allocapnia recta |
| Allocapnia recta isolate H11 16S ribosomal RNA gen | KC881046 | 81.88 | 100 | 1.60436e−32 | Plecoptera | Capniidae | Allocapnia recta |
| Allocapnia recta isolate H9 16S ribosomal RNA gene | KC881044 | 81.88 | 100 | 1.60436e−32 | Plecoptera | Capniidae | Allocapnia recta |
| Allocapnia recta isolate H8 16S ribosomal RNA gene | KC881043 | 81.88 | 100 | 1.60436e−32 | Plecoptera | Capniidae | Allocapnia recta |
| Allocapnia recta isolate H7 16S ribosomal RNA gene | KC881042 | 81.88 | 100 | 1.60436e−32 | Plecoptera | Capniidae | Allocapnia recta |
| Allocapnia recta isolate H5 16S ribosomal RNA gene | KC881040 | 81.88 | 100 | 1.60436e−32 | Plecoptera | Capniidae | Allocapnia recta |
| Allocapnia recta isolate H4 16S ribosomal RNA gene | KC881039 | 81.88 | 100 | 1.60436e−32 | Plecoptera | Capniidae | Allocapnia recta |
| Allocapnia recta isolate H3 16S ribosomal RNA gene | KC881038 | 81.88 | 100 | 1.60436e−32 | Plecoptera | Capniidae | Allocapnia recta |
| Allocapnia recta isolate H1 16S ribosomal RNA gene | KC881036 | 81.88 | 100 | 1.60436e−32 | Plecoptera | Capniidae | Allocapnia recta |
| Perlinodes aurea 16S ribosomal RNA gene, partial s | EF623234 | 82.5 | 100 | 1.60436e−32 | Plecoptera | Perlodidae | Perlinodes aurea |
| Nemurella pictetii 16S ribosomal RNA gene, partial | EF623192 | 81.88 | 100 | 1.60436e−32 | Plecoptera | Nemouridae | Nemurella pictetii |
| Ostrocerca foersteri 16S ribosomal RNA gene, parti | EF623191 | 81.88 | 100 | 1.60436e−32 | Plecoptera | Nemouridae | Ostrocerca foersteri |
| Paraleuctra occidentalis 16S ribosomal RNA gene, p | EF623178 | 81.76 | 100 | 1.60436e−32 | Plecoptera | Leuctridae | Paraleuctra occidentalis |
| Isocapnia hyalita 16S ribosomal RNA gene, partial | EF623131 | 81.88 | 100 | 1.60436e−32 | Plecoptera | Capniidae | Isocapnia hyalita |
| Mesocapnia sp. BYU_PL010 16S ribosomal RNA gene, p | EF623127 | 81.88 | 100 | 1.60436e−32 | Plecoptera | Capniidae | Mesocapnia sp. BYU_PL010 |
| Allocapnia recta haplotype 10ar: H010 16S ribosoma | EU306873 | 81.88 | 100 | 1.60436e−32 | Plecoptera | Capniidae | Allocapnia recta |
| Allocapnia recta haplotype 9ar: H09 16S ribosomal | EU306872 | 81.88 | 100 | 1.60436e−32 | Plecoptera | Capniidae | Allocapnia recta |
| Allocapnia recta haplotype 8ar: H08 16S ribosomal | EU306871 | 81.88 | 100 | 1.60436e−32 | Plecoptera | Capniidae | Allocapnia recta |
| Allocapnia recta haplotype 3ar: H03 16S ribosomal | DQ915181 | 81.88 | 100 | 1.60436e−32 | Plecoptera | Capniidae | Allocapnia recta |
| Allocapnia recta haplotype 2ar: H02 16S ribosomal | DQ915180 | 81.88 | 100 | 1.60436e−32 | Plecoptera | Capniidae | Allocapnia recta |
| Allocapnia recta haplotype 1ar: H01 16S ribosomal | DQ915179 | 81.88 | 100 | 1.60436e−32 | Plecoptera | Capniidae | Allocapnia recta |
| Doddsia occidentalis 16S ribosomal RNA gene, parti | EF623270 | 81.25 | 100 | 5.59978e−32 | Plecoptera | Taeniopterygidae | Doddsia occidentalis |
| Oemopteryx vanduzeea 16S ribosomal RNA gene, parti | EF623266 | 81.25 | 100 | 5.59978e−32 | Plecoptera | Taeniopterygidae | Oemopteryx vanduzeea |
| Nemoura cinerea 16S ribosomal RNA gene, partial se | EF623194 | 81.99 | 100 | 5.59978e−32 | Plecoptera | Nemouridae | Nemoura cinerea |
| Shipsa rotunda 16S ribosomal RNA gene, partial seq | EF623184 | 82.61 | 100 | 5.59978e−32 | Plecoptera | Nemouridae | Shipsa rotunda |
| Suwallia teleckojensis 16S ribosomal RNA gene, par | EF623142 | 93.07 | 63.52 | 5.59978e−32 | Plecoptera | Chloroperlidae | Suwallia teleckojensis |
| Neaviperla forcipata 16S ribosomal RNA gene, parti | EF623139 | 93.07 | 63.52 | 5.59978e−32 | Plecoptera | Chloroperlidae | Neaviperla forcipata |
| Triznaka pintada 16S ribosomal RNA gene, partial s | EF623134 | 82.72 | 100 | 5.59978e−32 | Plecoptera | Chloroperlidae | Triznaka pintada |
| Plumiperla diversa 16S ribosomal RNA gene, partial | EF623132 | 93.07 | 63.52 | 5.59978e−32 | Plecoptera | Chloroperlidae | Plumiperla diversa |
| Allocapnia recta isolate H18 16S ribosomal RNA gen | KC881053 | 81.25 | 100 | 6.82193e−31 | Plecoptera | Capniidae | Allocapnia recta |
| Nanonemoura wahkeena 16S ribosomal RNA gene, parti | EF623198 | 81.25 | 100 | 6.82193e−31 | Plecoptera | Nemouridae | Nanonemoura wahkeena |
| Visoka cataractae 16S ribosomal RNA gene, partial | EF623189 | 81.99 | 100 | 6.82193e−31 | Plecoptera | Nemouridae | Visoka cataractae |
| Nemoura trispinosa 16S ribosomal RNA gene, partial | EF623187 | 81.25 | 100 | 6.82193e−31 | Plecoptera | Nemouridae | Nemoura trispinosa |
| Zapada cinctipes 16S ribosomal RNA gene, partial s | EF623183 | 90.74 | 67.3 | 6.82193e−31 | Plecoptera | Nemouridae | Zapada cinctipes |
| Kathroperla perdita 16S ribosomal RNA gene, partia | EF623135 | 81.25 | 100 | 6.82193e−31 | Plecoptera | Chloroperlidae | Kathroperla perdita |
| Allocapnia recta haplotype 12ar: H12 16S ribosomal | EU306875 | 81.25 | 100 | 6.82193e−31 | Plecoptera | Capniidae | Allocapnia recta |
| Pteronarcys sachalina 16S ribosomal RNA gene, part | EF623264 | 82.39 | 89.31 | 2.38109e−30 | Plecoptera | Pteronarcyidae | Pteronarcys sachalina |
| Paranemoura claasseni 16S ribosomal RNA gene, part | EF623185 | 82.21 | 100 | 2.38109e−30 | Plecoptera | Nemouridae | Paranemoura claasseni |
| Bolotoperla rossi 16S ribosomal RNA gene, partial | EF623269 | 79.87 | 100 | 8.31081e−30 | Plecoptera | Taeniopterygidae | Bolotoperla rossi |
| Scopura montana 16S ribosomal RNA gene, partial se | EF623265 | 81.6 | 100 | 8.31081e−30 | Plecoptera | | Scopura montana |
| Udamocercia sp. BYU_PL150 16S ribosomal RNA gene, | EF623201 | 89.81 | 67.3 | 8.31081e−30 | Plecoptera | Notonemouridae | Udamocercia sp. BYU_PL150 |
| Siphonoperla torrentium 16S ribosomal RNA gene, pa | EF623138 | 91.09 | 63.52 | 8.31081e−30 | Plecoptera | Chloroperlidae | Siphonoperla torrentium |
| Sweltsa coloradensis 16S ribosomal RNA gene, parti | EF623133 | 91.09 | 63.52 | 8.31081e−30 | Plecoptera | Chloroperlidae | Sweltsa coloradensis |
| Osobenus yakimae 16S ribosomal RNA gene, partial s | EF623232 | 81.48 | 100 | 2.90076e−29 | Plecoptera | Perlodidae | Osobenus yakimae |
| Frisonia picticeps 16S ribosomal RNA gene, partial | EF623227 | 90.29 | 64.78 | 2.90076e−29 | Plecoptera | Perlodidae | Frisonia picticeps |
| Isoperla oxylepis 16S ribosomal RNA gene, partial | EF623221 | 91.26 | 64.78 | 2.90076e−29 | Plecoptera | Perlodidae | Isoperla oxylepis |
| Yugus bulbosus 16S ribosomal RNA gene, partial seq | EF623214 | 80.86 | 100 | 2.90076e−29 | Plecoptera | Perlodidae | Yugus bulbosus |
| Isoperla davisi 16S ribosomal RNA gene, partial se | EF623206 | 91.26 | 64.78 | 2.90076e−29 | Plecoptera | Perlodidae | Isoperla davisi |
| Malenka californica 16S ribosomal RNA gene, partia | EF623182 | 81.37 | 100 | 2.90076e−29 | Plecoptera | Nemouridae | Malenka californica |
| Chloroperla tripunctata 16S ribosomal RNA gene, pa | EF623137 | 91.09 | 63.52 | 2.90076e−29 | Plecoptera | Chloroperlidae | Chloroperla tripunctata |
| Megaleuctra kincaidi 16S ribosomal RNA gene, parti | EF623174 | 80.86 | 100 | 1.01246e−28 | Plecoptera | Leuctridae | Megaleuctra kincaidi |
| Pteronarcys princeps mitochondrion, complete genom | AY687866 | 82.52 | 89.31 | 1.01246e−28 | Plecoptera | Pteronarcyidae | Pteronarcys princeps |
| Notonemouridae sp. MD−2011 voucher ZMUC:JD−1507 16 | JN615257 | 80 | 100 | 3.53385e−28 | Plecoptera | Notonemouridae | Notonemouridae sp. MD−2011 |
| Pteronarcella badia 16S ribosomal RNA gene, partia | EF623262 | 90.1 | 63.52 | 3.53385e−28 | Plecoptera | Pteronarcyidae | Pteronarcella badia |
| Cerconychia sp. BYU_PL114 16S ribosomal RNA gene, | EF623260 | 89.32 | 64.78 | 3.53385e−28 | Plecoptera | Styloperlidae | Cerconychia sp. BYU_PL114 |
| Cosumnoperla hypocrena 16S ribosomal RNA gene, par | EF623226 | 89.32 | 64.78 | 3.53385e−28 | Plecoptera | Perlodidae | Cosumnoperla hypocrena |
| Cultus pilatus 16S ribosomal RNA gene, partial seq | EF623212 | 80.12 | 100 | 3.53385e−28 | Plecoptera | Perlodidae | Cultus pilatus |
| Acroperla trivacauta 16S ribosomal RNA gene, parti | EF623155 | 90.1 | 63.52 | 3.53385e−28 | Plecoptera | Gripopterygidae | Acroperla trivacauta |
| Thaumatoperla sp. BYU_PL109 16S ribosomal RNA gene | EF623149 | 80 | 100 | 3.53385e−28 | Plecoptera | Eustheniidae | Thaumatoperla sp. BYU_PL109 |
| Sasquaperla hoopa 16S ribosomal RNA gene, partial | EF623143 | 90.1 | 63.52 | 3.53385e−28 | Plecoptera | Chloroperlidae | Sasquaperla hoopa |
| Alloperla severa 16S ribosomal RNA gene, partial s | EF623141 | 80 | 100 | 3.53385e−28 | Plecoptera | Chloroperlidae | Alloperla severa |
| Paraperla frontalis 16S ribosomal RNA gene, partia | EF623136 | 80.62 | 100 | 3.53385e−28 | Plecoptera | Chloroperlidae | Paraperla frontalis |
| Klapopteryx armillata 16S ribosomal RNA gene, part | EF623123 | 80 | 100 | 3.53385e−28 | Plecoptera | Austroperlidae | Klapopteryx armillata |
| Osmylus sp. EH−2004 16S ribosomal RNA gene, partia | AY620146 | 89.32 | 64.78 | 3.53385e−28 | Neuroptera | Osmylidae | Osmylus sp. EH−2004 |
| Austrocerca rieka 16S ribosomal RNA gene, partial | EF623205 | 80.25 | 100 | 1.23343e−27 | Plecoptera | Notonemouridae | Austrocerca rieka |
| Austrocercoides zwicki 16S ribosomal RNA gene, par | EF623199 | 80.25 | 100 | 1.23343e−27 | Plecoptera | Notonemouridae | Austrocercoides zwicki |
| Prostoia besametsa 16S ribosomal RNA gene, partial | EF623188 | 90.2 | 63.52 | 1.23343e−27 | Plecoptera | Nemouridae | Prostoia besametsa |
| Utaperla sopladora 16S ribosomal RNA gene, partial | EF623140 | 89.11 | 63.52 | 1.23343e−27 | Plecoptera | Chloroperlidae | Utaperla sopladora |
| Pteronarcys californica 16S ribosomal RNA gene, pa | EF623261 | 81.82 | 89.31 | 4.30511e−27 | Plecoptera | Pteronarcyidae | Pteronarcys californica |
| Lednia tumana 16S ribosomal RNA gene, partial sequ | EF623197 | 80 | 100 | 4.30511e−27 | Plecoptera | Nemouridae | Lednia tumana |
