# Supplementary material for: Testing the potential of a ribosomal 16S marker for DNA metabarcoding of insects
Source: PeerJ. 2016 Apr 19;4:e1966. doi: 10.7717/peerj.1966 (PMC4841222; doi:10.7717/peerj.1966)
Supplement: Supplemental Information 1 [file peerj-04-1966-s006.zip › scripts/5 match OTUs/blast_hits/OTU_60.csv_plot.pdf]

OTU_60.csv

Pedicia albivitta

Pediciidae

Diptera

84.97

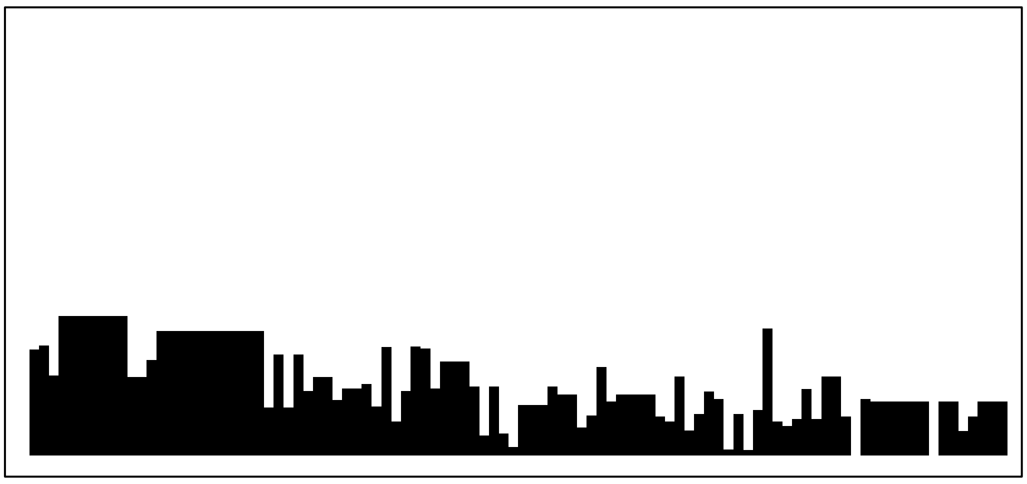

Pedicia albivitta voucher O'Grady 200012 16S ribos	EU005435	84.97	97.42	2.01953e−37	Diptera	Pediciidae	Pedicia albivitta
Chorisops tunisiae 16S ribosomal RNA gene, partial	GU947379	85.14	92.9	2.99724e−35	Diptera	Stratiomyidae	Chorisops tunisiae
Bolitophila occlusa voucher JSB2 16S ribosomal RNA	KJ136713	83.75	100	1.04614e−34	Diptera	Mycetophilidae	Bolitophila occlusa
Drosophila cardini voucher 103963 NADH dehydrogena	EU494310	86.52	89.68	3.65139e−34	Diptera	Drosophilidae	Drosophila cardini
Drosophila cardini 16S ribosomal RNA gene, partial	AF479800	86.52	89.68	3.65139e−34	Diptera	Drosophilidae	Drosophila cardini
Drosophila neomorpha NADH dehydrogenase subunit 1	AF246517	86.52	89.68	3.65139e−34	Diptera	Drosophilidae	Drosophila neomorpha
Drosophila arawakana dominicensis NADH dehydrogena	AF246516	86.52	89.68	3.65139e−34	Diptera	Drosophilidae	Drosophila arawakana dominicensis
Drosophila arawakana arawakana strain 15182−2261.0	AF246499	86.52	89.68	3.65139e−34	Diptera	Drosophilidae	Drosophila arawakana arawakana
Drosophila arawakana arawakana strain 15182−2261.1	AF246500	86.52	89.68	3.65139e−34	Diptera	Drosophilidae	Drosophila arawakana arawakana
Drosophila nigrodunni NADH dehydrogenase subunit 1	AF246494	86.52	89.68	3.65139e−34	Diptera	Drosophilidae	Drosophila nigrodunni
Melanostoma apicale 16S ribosomal RNA gene, partia	AF154791	83.66	97.42	1.27446e−33	Diptera	Syrphidae	Melanostoma apicale
Melanostoma apicale 16S ribosomal RNA gene, partia	AF154789	83.66	97.42	1.27446e−33	Diptera	Syrphidae	Melanostoma apicale
Actina chalybea 16S ribosomal RNA gene, partial se	GU947372	84.46	92.9	4.4483e−33	Diptera	Acroceridae	Actina chalybea
Drosophila dunni voucher 103969 NADH dehydrogenase	EU494311	85.82	89.68	4.4483e−33	Diptera	Drosophilidae	Drosophila dunni
Drosophila similis grenadensis NADH dehydrogenase	AF246519	85.82	89.68	4.4483e−33	Diptera	Drosophilidae	Drosophila similis grenadensis
Drosophila dunni dunni strain Barranquitas, line 1	AF246512	85.82	89.68	4.4483e−33	Diptera	Drosophilidae	Drosophila dunni dunni
Drosophila cardini strain Constanza, line 4 NADH d	AF246511	85.82	89.68	4.4483e−33	Diptera	Drosophilidae	Drosophila cardini
Drosophila acutilabella strain 15181−2171.9 NADH d	AF246509	85.82	89.68	4.4483e−33	Diptera	Drosophilidae	Drosophila acutilabella
Drosophila dunni thomasensis NADH dehydrogenase su	AF246501	85.82	89.68	4.4483e−33	Diptera	Drosophilidae	Drosophila dunni thomasensis
Drosophila cardini strain 15181−2181.9 NADH dehydr	AF246498	85.82	89.68	4.4483e−33	Diptera	Drosophilidae	Drosophila cardini
Drosophila dunni dunni strain 15182−2291.0 NADH de	AF246497	85.82	89.68	4.4483e−33	Diptera	Drosophilidae	Drosophila dunni dunni
Drosophila acutilabella strain 15181−2171.2 NADH d	AF246496	85.82	89.68	4.4483e−33	Diptera	Drosophilidae	Drosophila acutilabella
Drosophila similis similis NADH dehydrogenase subu	AF246495	85.82	89.68	4.4483e−33	Diptera	Drosophilidae	Drosophila similis similis
Drosophila caribiana NADH dehydrogenase subunit 1	AF246493	85.82	89.68	4.4483e−33	Diptera	Drosophilidae	Drosophila caribiana
Tipula abdominalis 16S ribosomal RNA gene, partial	KC177457	82.24	97.42	1.55261e−32	Diptera	Tipulidae	Tipula abdominalis
Hermetia illucens 16S ribosomal RNA gene, partial	KC177455	84.72	90.97	1.55261e−32	Diptera	Stratiomyidae	Hermetia illucens
Tipula abdominalis mitochondrion, partial genome	JN861743	82.24	97.42	1.55261e−32	Diptera	Tipulidae	Tipula abdominalis
Hermetia illucens 16S ribosomal RNA gene, partial	GU947384	84.72	90.97	1.55261e−32	Diptera	Stratiomyidae	Hermetia illucens
Melanostoma apicale 16S ribosomal RNA gene, partia	AF154790	83.01	97.42	1.55261e−32	Diptera	Syrphidae	Melanostoma apicale
Dideopsis aegrota 16S ribosomal RNA gene, partial	AF154774	83.66	97.42	1.55261e−32	Diptera	Syrphidae	Dideopsis aegrota
Dideopsis aegrota 16S ribosomal RNA gene, partial	AF154773	83.66	97.42	1.55261e−32	Diptera	Syrphidae	Dideopsis aegrota
Empis sp. SNK−2009 voucher RMBR:103438 16S ribosom	FJ808168	82.58	97.42	5.41914e−32	Diptera	Empididae	Empis sp. SNK−2009
Paragus politus 16S ribosomal RNA gene, partial se	AF154800	83.12	97.42	5.41914e−32	Diptera	Syrphidae	Paragus politus
Paragus politus 16S ribosomal RNA gene, partial se	AF154799	83.12	97.42	5.41914e−32	Diptera	Syrphidae	Paragus politus
Agathomyia unicolor isolate AU2 16S ribosomal RNA	KC699318	83.33	92.26	1.89147e−31	Diptera	Platypezidae	Agathomyia unicolor
Pseudolycoriella sp. SS−2012 voucher 84 16S riboso	JQ613888	82.28	100	1.89147e−31	Diptera	Sciaridae	Pseudolycoriella sp. SS−2012
Empis producta voucher RMBR:103430 16S ribosomal R	FJ808167	85.07	85.16	1.89147e−31	Diptera	Empididae	Empis producta
Limonia sp. PMO 200984 16S ribosomal RNA gene, par	EU005416	81.58	97.42	1.89147e−31	Diptera	Limoniidae	Limonia sp. PMO 200984
Dideopsis aegrota 16S ribosomal RNA gene, partial	AF154775	83.01	97.42	1.89147e−31	Diptera	Syrphidae	Dideopsis aegrota
Drosophila belladunni NADH dehydrogenase subunit 1	AF246506	85.11	89.68	1.89147e−31	Diptera	Drosophilidae	Drosophila belladunni
Clitellaria ephippium 16S ribosomal RNA gene, part	GU947399	85	89.03	6.60187e−31	Diptera	Stratiomyidae	Clitellaria ephippium
Gluma musgravei 16S ribosomal RNA gene, partial se	AF403467	83.12	97.42	6.60187e−31	Diptera	Coelopidae	Gluma musgravei
Drosophila procardinoides NADH dehydrogenase subun	AF246508	84.4	89.68	2.30428e−30	Diptera	Drosophilidae	Drosophila procardinoides
Drosophila polymorpha strain 15181−2231.1 NADH deh	AF246507	84.4	89.68	2.30428e−30	Diptera	Drosophilidae	Drosophila polymorpha
Drosophila polymorpha strain 15181−2231.2 NADH deh	AF246504	84.4	89.68	2.30428e−30	Diptera	Drosophilidae	Drosophila polymorpha
Scaptomyza recta voucher 202667 16S ribosomal RNA	KC609611	83.22	89.68	8.04272e−30	Diptera	Drosophilidae	Scaptomyza recta
Trichocera bimacula mitochondrion, complete genome	JN861750	80.92	97.42	8.04272e−30	Diptera	Trichoceridae	Trichocera bimacula
Drosophila latigena voucher O201015 16S ribosomal	HQ170977	83.22	89.68	8.04272e−30	Diptera	Drosophilidae	Drosophila latigena
Atheta pasadenae voucher ZMUN:10002642 16S ribosom	GQ981025	81.01	100	8.04272e−30	Coleoptera	Staphylinidae	Atheta pasadenae
Atheta aeneipennis voucher ZMUN:10002583 16S ribos	GQ981017	80.38	100	8.04272e−30	Coleoptera	Staphylinidae	Atheta aeneipennis
Episyrphus balteatus 16S ribosomal RNA gene, parti	AY573115	82.35	97.42	8.04272e−30	Diptera	Syrphidae	Episyrphus balteatus
Toxomerus marginatus 16S ribosomal RNA gene, parti	AF154820	82.35	97.42	8.04272e−30	Diptera	Syrphidae	Toxomerus marginatus
Toxomerus geminatus 16S ribosomal RNA gene, partia	AF154819	82.35	97.42	8.04272e−30	Diptera	Syrphidae	Toxomerus geminatus
Hemigephyra atra 16S ribosomal RNA gene, partial s	KM878915	83.23	97.42	2.80719e−29	Diptera	Therevidae	Hemigephyra atra
Drosophila unicula voucher O200997 16S ribosomal R	HQ171017	82.86	89.68	2.80719e−29	Diptera	Drosophilidae	Drosophila unicula
Drosophila nr. melanopedis RL−2010 voucher O201016	HQ171012	82.86	89.68	2.80719e−29	Diptera	Drosophilidae	Drosophila nr. melanopedis RL−2010
Isoneuromyia semirufa 16S ribosomal RNA gene, part	FJ172014	81.29	99.35	2.80719e−29	Diptera	Keroplatidae	Isoneuromyia semirufa
Aphodius nigrita 16S ribosomal RNA gene, partial s	EF487829	81.88	100	2.80719e−29	Coleoptera	Scarabaeidae	Aphodius nigrita
Dicranosepsis cf. sauteri RM−2008 voucher su16 16S	EU435582	84.14	91.61	2.80719e−29	Diptera	Sepsidae	Dicranosepsis cf. sauteri RM−2008
Drosophila bifasciata voucher 109384 NADH dehydrog	EU494362	82.52	91.61	2.80719e−29	Diptera	Drosophilidae	Drosophila bifasciata
Drosophila putrida voucher 103964 NADH dehydrogena	EU494348	82.86	89.68	2.80719e−29	Diptera	Drosophilidae	Drosophila putrida
Drosophila quinaria voucher 107542 NADH dehydrogen	EU494338	82.86	89.68	2.80719e−29	Diptera	Drosophilidae	Drosophila quinaria
Drosophila falleni 16S ribosomal RNA gene, partial	AF479802	82.86	89.68	2.80719e−29	Diptera	Drosophilidae	Drosophila falleni
Drosophila bizonata 16S ribosomal RNA gene, partia	AF479797	82.86	89.68	2.80719e−29	Diptera	Drosophilidae	Drosophila bizonata
Triglyphus fulvicornis 16S ribosomal RNA gene, par	AF154822	81.82	97.42	2.80719e−29	Diptera	Syrphidae	Triglyphus fulvicornis
Drosophila mediodiffusa NADH dehydrogenase subunit	AF246464	81.58	97.42	2.80719e−29	Diptera	Drosophilidae	Drosophila mediodiffusa
Drosophila unipunctata NADH dehydrogenase subunit	AF246515	83.69	89.68	2.80719e−29	Diptera	Drosophilidae	Drosophila unipunctata
Melanostoma scalare 16S ribosomal RNA gene, partia	AF126336	81.17	97.42	2.80719e−29	Diptera	Syrphidae	Melanostoma scalare
Chydaeopeza tibialis isolate CT44 16S ribosomal RN	KC699336	81.94	92.26	9.79804e−29	Diptera	Platypezidae	Chydaeopeza tibialis
Bertamyia notata isolate BN39 16S ribosomal RNA ge	KC699324	82.99	92.26	9.79804e−29	Diptera	Platypezidae	Bertamyia notata
Agathomyia antennata isolate AA27 16S ribosomal RN	KC699322	82.64	92.26	9.79804e−29	Diptera	Platypezidae	Agathomyia antennata
Trichocera brevicornis 16S ribosomal RNA gene, par	KC177458	80.26	97.42	9.79804e−29	Diptera	Trichoceridae	Trichocera brevicornis
Drosophila nr. humeralis RL−2010 voucher O201041 1	HQ171011	81.94	92.26	9.79804e−29	Diptera	Drosophilidae	Drosophila nr. humeralis RL−2010
Pella humeralis voucher ZMUN:10002569 16S ribosoma	GQ980988	80.25	100	9.79804e−29	Coleoptera	Staphylinidae	Pella humeralis
Billaea triangulifera isolate MU_UBZ_16S_ET2 16S r	FJ913860	82.12	94.19	9.79804e−29	Diptera	Tachinidae	Billaea triangulifera
Hypoderma lineatum mitochondrion, complete genome	GU584123	85.95	76.13	9.79804e−29	Diptera	Oestridae	Hypoderma lineatum
Helina celsa 16S ribosomal RNA gene, partial seque	FJ025438	81.58	94.84	9.79804e−29	Diptera	Muscidae	Helina celsa
Aphodius nigrivittis 16S ribosomal RNA gene, parti	EF487824	81.37	100	9.79804e−29	Coleoptera	Scarabaeidae	Aphodius nigrivittis
Simosyrphus grandicornis mitochondrion, complete g	DQ866050	81.7	97.42	9.79804e−29	Diptera	Syrphidae	Simosyrphus grandicornis
Drosophila putrida 16S ribosomal RNA gene, partial	AF479805	83.1	89.68	9.79804e−29	Diptera	Drosophilidae	Drosophila putrida
Toxomerus marginatus 16S ribosomal RNA gene, parti	AF154821	81.7	97.42	9.79804e−29	Diptera	Syrphidae	Toxomerus marginatus
Drosophila neocardini NADH dehydrogenase subunit 1	AF246505	83.69	89.68	9.79804e−29	Diptera	Drosophilidae	Drosophila neocardini
Drosophila parthenogenetica strain 15181−2221.0 NA	AF246503	83.69	89.68	9.79804e−29	Diptera	Drosophilidae	Drosophila parthenogenetica
Xestomyza lugubris 16S ribosomal RNA gene, partial	KM878970	81.82	97.42	3.41985e−28	Diptera	Therevidae	Xestomyza lugubris
Amphisternus malaccanus voucher UPOL002080 16S rib	KP143650	79.75	100	3.41985e−28	Coleoptera	Endomychidae	Amphisternus malaccanus
Calotarsa pallipes isolate CAN3 16S ribosomal RNA	KC699329	82.64	89.68	3.41985e−28	Diptera	Platypezidae	Calotarsa pallipes
Drosophila kraussi voucher 202553 16S ribosomal RN	KM252236	82.52	89.68	3.41985e−28	Diptera	Drosophilidae	Drosophila kraussi
Drosophila williamsi voucher 202551 16S ribosomal	KM252235	82.52	89.68	3.41985e−28	Diptera	Drosophilidae	Drosophila williamsi
Drosophila yooni voucher 202550 16S ribosomal RNA	KM252234	82.52	89.68	3.41985e−28	Diptera	Drosophilidae	Drosophila yooni
Drosophila cracens voucher 202538 16S ribosomal RN	KM252227	82.52	89.68	3.41985e−28	Diptera	Drosophilidae	Drosophila cracens
Drosophila nr. dorsigera RTL−2014 voucher 201052 1	KM252219	82.52	89.68	3.41985e−28	Diptera	Drosophilidae	Drosophila nr. dorsigera RTL−2014
Drosophila capitata voucher 201050 16S ribosomal R	KM252217	82.52	89.68	3.41985e−28	Diptera	Drosophilidae	Drosophila capitata
Mycetophila alea voucher JSB11 16S ribosomal RNA g	KJ136722	79.49	100	3.41985e−28	Diptera	Mycetophilidae	Mycetophila alea
Scaptomyza undulata voucher 202684 16S ribosomal R	KC609612	82.52	89.68	3.41985e−28	Diptera	Drosophilidae	Scaptomyza undulata
Scaptomyza pusilla voucher 202601 16S ribosomal RN	KC609605	82.52	89.68	3.41985e−28	Diptera	Drosophilidae	Scaptomyza pusilla
Dohrniphora irawanensis isolate 28.iii.05−228 16S	HM366953	81.13	97.42	3.41985e−28	Diptera	Phoridae	Dohrniphora irawanensis
Dohrniphora cornuta isolate 200862 16S ribosomal R	GU361998	81.82	97.42	3.41985e−28	Diptera	Phoridae	Dohrniphora cornuta
Drosophila dasycnemia voucher 202331a 16S ribosoma	HQ703698	82.52	89.68	3.41985e−28	Diptera	Drosophilidae	Drosophila dasycnemia
Drosophila neutralis voucher 202329b 16S ribosomal	HQ703696	82.52	89.68	3.41985e−28	Diptera	Drosophilidae	Drosophila neutralis
Drosophila sordidapex voucher O202321a 16S ribosom	HQ171025	82.52	89.68	3.41985e−28	Diptera	Drosophilidae	Drosophila sordidapex
