# Supplementary material for: Testing the potential of a ribosomal 16S marker for DNA metabarcoding of insects
Source: PeerJ. 2016 Apr 19;4:e1966. doi: 10.7717/peerj.1966 (PMC4841222; doi:10.7717/peerj.1966)
Supplement: Supplemental Information 1 [file peerj-04-1966-s006.zip › scripts/5 match OTUs/blast_hits/OTU_606.csv_plot.pdf]

OTU_606.csv

Rhithrogena semicolorata

Heptageniidae

Ephemeroptera

98.72

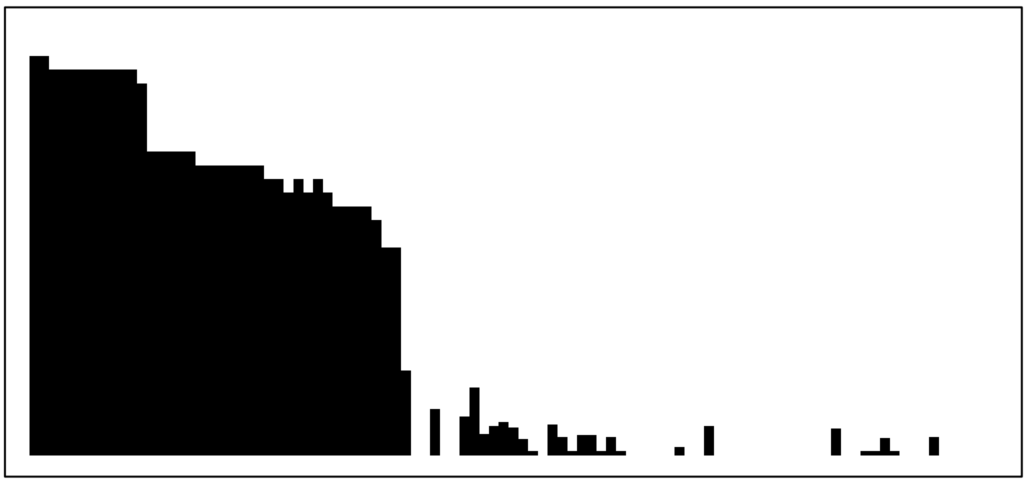

Rhithrogena semicolorata mitochondrial partial 16S	LN868673	98.72	100	6.62413e−69	Ephemeroptera	Heptageniidae	Rhithrogena semicolorata
Rhithrogena sp. 4 LV−2015 mitochondrial partial 16	LN868652	98.72	100	6.62413e−69	Ephemeroptera	Heptageniidae	Rhithrogena sp. 4 LV−2015
Rhithrogena sp. 13 LV−2015 mitochondrial partial 1	LN868672	98.08	100	2.81665e−67	Ephemeroptera	Heptageniidae	Rhithrogena sp. 13 LV−2015
Rhithrogena sp. 12 LV−2015 mitochondrial partial 1	LN868671	98.08	100	2.81665e−67	Ephemeroptera	Heptageniidae	Rhithrogena sp. 12 LV−2015
Rhithrogena picteti mitochondrial partial 16S rRNA	LN868663	98.08	100	2.81665e−67	Ephemeroptera	Heptageniidae	Rhithrogena picteti
Rhithrogena sp. 3 LV−2015 mitochondrial partial 16	LN868659	98.08	100	2.81665e−67	Ephemeroptera	Heptageniidae	Rhithrogena sp. 3 LV−2015
Rhithrogena fonticola mitochondrial partial 16S rR	LN868656	98.08	100	2.81665e−67	Ephemeroptera	Heptageniidae	Rhithrogena fonticola
Rhithrogena sp. 2 LV−2015 mitochondrial partial 16	LN868654	98.08	100	2.81665e−67	Ephemeroptera	Heptageniidae	Rhithrogena sp. 2 LV−2015
Rhithrogena puytoraci mitochondrial partial 16S rR	LN868651	98.08	100	2.81665e−67	Ephemeroptera	Heptageniidae	Rhithrogena puytoraci
Rhithrogena iridina mitochondrial partial 16S rRNA	LN868646	98.08	100	2.81665e−67	Ephemeroptera	Heptageniidae	Rhithrogena iridina
Rhithrogena taurisca mitochondrial partial 16S rRN	LN868638	98.08	100	2.81665e−67	Ephemeroptera	Heptageniidae	Rhithrogena taurisca
Rhithrogena sp. 5 LV−2015 mitochondrial partial 16	LN868653	97.44	100	3.43139e−66	Ephemeroptera	Heptageniidae	Rhithrogena sp. 5 LV−2015
Rhithrogena sp. 7 LV−2015 mitochondrial partial 16	LN868669	94.23	100	4.7697e−58	Ephemeroptera	Heptageniidae	Rhithrogena sp. 7 LV−2015
Rhithrogena eatoni mitochondrial partial 16S rRNA	LN868666	94.23	100	4.7697e−58	Ephemeroptera	Heptageniidae	Rhithrogena eatoni
Rhithrogena alpestris mitochondrial partial 16S rR	LN868657	94.23	100	4.7697e−58	Ephemeroptera	Heptageniidae	Rhithrogena alpestris
Rhithrogena gratianopolitana mitochondrial partial	LN868647	94.23	100	4.7697e−58	Ephemeroptera	Heptageniidae	Rhithrogena gratianopolitana
Rhithrogena nivata mitochondrial partial 16S rRNA	LN868643	94.23	100	4.7697e−58	Ephemeroptera	Heptageniidae	Rhithrogena nivata
Rhithrogena degrangei mitochondrial partial 16S rR	LN868674	93.59	100	5.81068e−57	Ephemeroptera	Heptageniidae	Rhithrogena degrangei
Rhithrogena corcontica mitochondrial partial 16S r	LN868662	93.59	100	5.81068e−57	Ephemeroptera	Heptageniidae	Rhithrogena corcontica
Rhithrogena sp. 9 LV−2015 mitochondrial partial 16	LN868650	93.59	100	5.81068e−57	Ephemeroptera	Heptageniidae	Rhithrogena sp. 9 LV−2015
Rhithrogena gratianopolitana mitochondrial partial	LN868648	93.59	100	5.81068e−57	Ephemeroptera	Heptageniidae	Rhithrogena gratianopolitana
Rhithrogena landai mitochondrial partial 16S rRNA	LN868644	93.59	100	5.81068e−57	Ephemeroptera	Heptageniidae	Rhithrogena landai
Rhithrogena hybrida mitochondrial partial 16S rRNA	LN868642	93.59	100	5.81068e−57	Ephemeroptera	Heptageniidae	Rhithrogena hybrida
Rhithrogena circumtatrica mitochondrial partial 16	LN868641	93.59	100	5.81068e−57	Ephemeroptera	Heptageniidae	Rhithrogena circumtatrica
Rhithrogena sp. 11 LV−2015 mitochondrial partial 1	LN868668	92.95	100	2.47077e−55	Ephemeroptera	Heptageniidae	Rhithrogena sp. 11 LV−2015
Rhithrogena sp. 8 LV−2015 mitochondrial partial 16	LN868661	92.95	100	2.47077e−55	Ephemeroptera	Heptageniidae	Rhithrogena sp. 8 LV−2015
Rhithrogena germanica mitochondrial partial 16S rR	LN868660	92.31	100	2.47077e−55	Ephemeroptera	Heptageniidae	Rhithrogena germanica
Rhithrogena sp. 6 LV−2015 mitochondrial partial 16	LN868655	92.95	100	2.47077e−55	Ephemeroptera	Heptageniidae	Rhithrogena sp. 6 LV−2015
Rhithrogena beskidensis mitochondrial partial 16S	LN868645	92.31	100	2.47077e−55	Ephemeroptera	Heptageniidae	Rhithrogena beskidensis
Rhithrogena diensis mitochondrial partial 16S rRNA	LN868637	92.95	100	2.47077e−55	Ephemeroptera	Heptageniidae	Rhithrogena diensis
Rhithrogena nuragica mitochondrial partial 16S rRN	LN868667	92.31	100	3.01001e−54	Ephemeroptera	Heptageniidae	Rhithrogena nuragica
Rhithrogena sp. 10 LV−2015 mitochondrial partial 1	LN868664	91.67	100	1.0506e−53	Ephemeroptera	Heptageniidae	Rhithrogena sp. 10 LV−2015
Rhithrogena savoiensis mitochondrial partial 16S r	LN868658	91.67	100	1.0506e−53	Ephemeroptera	Heptageniidae	Rhithrogena savoiensis
Rhithrogena insularis mitochondrial partial 16S rR	LN868665	91.67	100	1.27989e−52	Ephemeroptera	Heptageniidae	Rhithrogena insularis
Rhithrogena grischuna mitochondrial partial 16S rR	LN868639	91.67	100	1.27989e−52	Ephemeroptera	Heptageniidae	Rhithrogena grischuna
Rhithrogena allobrogica mitochondrial partial 16S	LN868640	91.03	100	1.55922e−51	Ephemeroptera	Heptageniidae	Rhithrogena allobrogica
Rhithrogena sartorii mitochondrial partial 16S rRN	LN868670	89.74	100	6.62999e−50	Ephemeroptera	Heptageniidae	Rhithrogena sartorii
Rhithrogena dorieri mitochondrial partial 16S rRNA	LN868649	89.74	100	6.62999e−50	Ephemeroptera	Heptageniidae	Rhithrogena dorieri
Rhithrogena sp. EP021 16S ribosomal RNA gene, part	AY749765	83.97	100	2.02992e−37	Ephemeroptera	Heptageniidae	Rhithrogena sp. EP021
Cinygmula sp. m185 mitochondrial partial 16S rRNA	LN868675	79.49	100	3.43745e−28	Ephemeroptera	Heptageniidae	Cinygmula sp. m185
Cinygmula sp. EP013 16S ribosomal RNA gene, partia	AY749760	79.49	100	3.43745e−28	Ephemeroptera	Heptageniidae	Cinygmula sp. EP013
Cultus pilatus 16S ribosomal RNA gene, partial seq	EF623212	82.18	64.52	3.01533e−16	Plecoptera	Perlodidae	Cultus pilatus
Heptagenia sp. EP018 16S ribosomal RNA gene, parti	AY749762	76.81	87.74	3.01533e−16	Ephemeroptera	Heptageniidae	Heptagenia sp. EP018
Paegniodes cupulatus mitochondrion, complete genom	HM004123	75.16	100	1.05245e−15	Ephemeroptera	Heptageniidae	Paegniodes cupulatus
Arthroplea bipunctata 16S ribosomal RNA gene, part	AY749817	81.82	63.23	3.67342e−15	Ephemeroptera	Arthropleidae	Arthroplea bipunctata
Simocephalus sp. EIZ−2011 isolate Brb1−Sim1 16S ri	JN874613	83.17	63.23	1.28215e−14	Diplostraca	Daphniidae	Simocephalus sp. EIZ−2011
Remenus bilobatus 16S ribosomal RNA gene, partial	EF623233	81	63.87	4.47515e−14	Plecoptera	Perlodidae	Remenus bilobatus
Austronemoura sp. BYU_PL149 16S ribosomal RNA gene	EF623200	81.37	64.52	4.47515e−14	Plecoptera	Notonemouridae	Austronemoura sp. BYU_PL149
Capnura wanica 16S ribosomal RNA gene, partial seq	EF623128	81.55	64.52	4.47515e−14	Plecoptera	Capniidae	Capnura wanica
Simocephalus vetulus 16S ribosomal RNA gene, parti	DQ470576	81.31	67.1	4.47515e−14	Diplostraca	Daphniidae	Simocephalus vetulus
Apteroperla tikumana mitochondrion, complete genom	KR604721	80.77	64.52	1.56198e−13	Plecoptera	Capniidae	Apteroperla tikumana
Neopanorpa pulchra mitochondrion, complete genome	JX569848	80.2	64.52	1.56198e−13	Mecoptera	Panorpidae	Neopanorpa pulchra
Enicopus ater voucher UPOL001164 16S ribosomal RNA	HQ619670	79.63	69.03	1.56198e−13	Coleoptera	Melyridae	Enicopus ater
Pachyrhamma edwardsii voucher CW122 16S ribosomal	HM594508	81.44	61.29	1.56198e−13	Orthoptera	Rhaphidophoridae	Pachyrhamma edwardsii
Heteromallus cavicola voucher MIL 16S ribosomal RN	HM594499	80.85	60.65	1.56198e−13	Orthoptera	Rhaphidophoridae	Heteromallus cavicola
Neopanorpa pulchra mitochondrion, complete genome	FJ169955	80.2	64.52	1.56198e−13	Mecoptera	Panorpidae	Neopanorpa pulchra
Isoperla oxylepis 16S ribosomal RNA gene, partial	EF623221	80.95	66.45	1.56198e−13	Plecoptera	Perlodidae	Isoperla oxylepis
Isoperla davisi 16S ribosomal RNA gene, partial se	EF623206	80.95	66.45	1.56198e−13	Plecoptera	Perlodidae	Isoperla davisi
Thaumatoperla sp. BYU_PL109 16S ribosomal RNA gene	EF623149	80.2	64.52	1.56198e−13	Plecoptera	Eustheniidae	Thaumatoperla sp. BYU_PL109
Penturoperla barbata 16S ribosomal RNA gene, parti	EF623124	80.85	60.65	1.56198e−13	Plecoptera	Austroperlidae	Penturoperla barbata
Neopanorpa sp. M24899 16S ribosomal RNA gene, part	AF180059	80.2	64.52	1.56198e−13	Mecoptera	Panorpidae	Neopanorpa sp. M24899
Apterodorcus bacchus voucher SK121 16S ribosomal R	KP250211	79.59	63.23	5.45185e−13	Coleoptera	Lucanidae	Apterodorcus bacchus
Actias sinensis subaurea mitochondrial gene for 16	AB841441	79.59	63.23	5.45185e−13	Lepidoptera	Saturniidae	Actias sinensis subaurea
Actias sinensis subaurea mitochondrial gene for 16	AB841440	79.59	63.23	5.45185e−13	Lepidoptera	Saturniidae	Actias sinensis subaurea
Actias sinensis subaurea mitochondrial gene for 16	AB841439	79.59	63.23	5.45185e−13	Lepidoptera	Saturniidae	Actias sinensis subaurea
Pteronarcys californica 16S ribosomal RNA gene, pa	EF623261	80	63.87	5.45185e−13	Plecoptera	Pteronarcyidae	Pteronarcys californica
Perlinodes aurea 16S ribosomal RNA gene, partial s	EF623234	80.39	64.52	5.45185e−13	Plecoptera	Perlodidae	Perlinodes aurea
Pteronarcys princeps mitochondrion, complete genom	AY687866	80	63.87	5.45185e−13	Plecoptera	Pteronarcyidae	Pteronarcys princeps
Dipteromimus sp. Ofunato−1 mitochondrial gene for	AB110236	80	63.87	5.45185e−13	Ephemeroptera	Dipteromimidae	Dipteromimus sp. Ofunato−1
Analetris eximia 16S ribosomal RNA gene, partial s	AY749797	81.37	64.52	5.45185e−13	Ephemeroptera	Acanthametropodidae	Analetris eximia
Mesocapnia arizonensis mitochondrion, partial geno	KP642637	79.81	64.52	1.90288e−12	Plecoptera	Capniidae	Mesocapnia arizonensis
Allocapnia recta isolate H19 16S ribosomal RNA gen	KC881054	79.81	64.52	1.90288e−12	Plecoptera	Capniidae	Allocapnia recta
Allocapnia recta isolate H16 16S ribosomal RNA gen	KC881051	79.81	64.52	1.90288e−12	Plecoptera	Capniidae	Allocapnia recta
Allocapnia recta isolate H15 16S ribosomal RNA gen	KC881050	79.81	64.52	1.90288e−12	Plecoptera	Capniidae	Allocapnia recta
Allocapnia recta isolate H14 16S ribosomal RNA gen	KC881049	79.81	64.52	1.90288e−12	Plecoptera	Capniidae	Allocapnia recta
Allocapnia recta isolate H13 16S ribosomal RNA gen	KC881048	79.81	64.52	1.90288e−12	Plecoptera	Capniidae	Allocapnia recta
Allocapnia recta isolate H11 16S ribosomal RNA gen	KC881046	79.81	64.52	1.90288e−12	Plecoptera	Capniidae	Allocapnia recta
Allocapnia recta isolate H10 16S ribosomal RNA gen	KC881045	79.81	64.52	1.90288e−12	Plecoptera	Capniidae	Allocapnia recta
Allocapnia recta isolate H7 16S ribosomal RNA gene	KC881042	79.81	64.52	1.90288e−12	Plecoptera	Capniidae	Allocapnia recta
Allocapnia recta isolate H6 16S ribosomal RNA gene	KC881041	79.81	64.52	1.90288e−12	Plecoptera	Capniidae	Allocapnia recta
Allocapnia recta isolate H3 16S ribosomal RNA gene	KC881038	79.81	64.52	1.90288e−12	Plecoptera	Capniidae	Allocapnia recta
Psilothrix viridicoeruleus voucher UPOL001076 16S	HQ619641	80	61.29	1.90288e−12	Coleoptera	Melyridae	Psilothrix viridicoeruleus
Pachyrhamma edwardsii voucher WEL 16S ribosomal RN	HM594509	81.25	61.29	1.90288e−12	Orthoptera	Rhaphidophoridae	Pachyrhamma edwardsii
Dolichopoda hyrcana voucher LEN 16S ribosomal RNA	EU887871	80	61.29	1.90288e−12	Orthoptera	Rhaphidophoridae	Dolichopoda hyrcana
Bolotoperla rossi 16S ribosomal RNA gene, partial	EF623269	78.95	70.32	1.90288e−12	Plecoptera	Taeniopterygidae	Bolotoperla rossi
Salmoperla sylvanica 16S ribosomal RNA gene, parti	EF623222	80.2	63.87	1.90288e−12	Plecoptera	Perlodidae	Salmoperla sylvanica
Megarcys signata 16S ribosomal RNA gene, partial s	EF623215	80.2	63.87	1.90288e−12	Plecoptera	Perlodidae	Megarcys signata
Perlomyia utahensis 16S ribosomal RNA gene, partia	EF623179	80.81	62.58	1.90288e−12	Plecoptera	Leuctridae	Perlomyia utahensis
Diamphipnopsis sp. BYU_PL138 16S ribosomal RNA gen	EF623145	80.2	63.87	1.90288e−12	Plecoptera	Diamphipnoidae	Diamphipnopsis sp. BYU_PL138
Chloroperla tripunctata 16S ribosomal RNA gene, pa	EF623137	79.81	65.81	1.90288e−12	Plecoptera	Chloroperlidae	Chloroperla tripunctata
Allocapnia minima 16S ribosomal RNA gene, partial	EF623130	79.81	64.52	1.90288e−12	Plecoptera	Capniidae	Allocapnia minima
Mesocapnia sp. BYU_PL010 16S ribosomal RNA gene, p	EF623127	79.81	64.52	1.90288e−12	Plecoptera	Capniidae	Mesocapnia sp. BYU_PL010
Tasmanoperla larvalis 16S ribosomal RNA gene, part	EF623125	80.85	60	1.90288e−12	Plecoptera	Austroperlidae	Tasmanoperla larvalis
Allocapnia recta haplotype 12ar: H12 16S ribosomal	EU306875	79.81	64.52	1.90288e−12	Plecoptera	Capniidae	Allocapnia recta
Allocapnia recta haplotype 11ar: H011 16S ribosoma	EU306874	79.81	64.52	1.90288e−12	Plecoptera	Capniidae	Allocapnia recta
Allocapnia recta haplotype 7ar: H07 16S ribosomal	EU306870	79.81	64.52	1.90288e−12	Plecoptera	Capniidae	Allocapnia recta
Psilothrix viridicoeruleus voucher BMNH:679198 16S	DQ202538	80	61.29	1.90288e−12	Coleoptera	Melyridae	Psilothrix viridicoeruleus
Allocapnia recta haplotype 3ar: H03 16S ribosomal	DQ915181	79.81	64.52	1.90288e−12	Plecoptera	Capniidae	Allocapnia recta
Allocapnia recta haplotype 2ar: H02 16S ribosomal	DQ915180	79.81	64.52	1.90288e−12	Plecoptera	Capniidae	Allocapnia recta
Lophocateres sp. UPOL001123 16S ribosomal RNA gene	KC758777	77.78	69.03	5.64171e−12	Coleoptera	Trogossitidae	Lophocateres sp. UPOL001123
