# Supplementary material for: Testing the potential of a ribosomal 16S marker for DNA metabarcoding of insects
Source: PeerJ. 2016 Apr 19;4:e1966. doi: 10.7717/peerj.1966 (PMC4841222; doi:10.7717/peerj.1966)
Supplement: Supplemental Information 1 [file peerj-04-1966-s006.zip › scripts/5 match OTUs/blast_hits/OTU_607.csv_plot.pdf]

OTU_607.csv

Oreodytes sanmarkii

Dytiscidae

Coleoptera

98.1

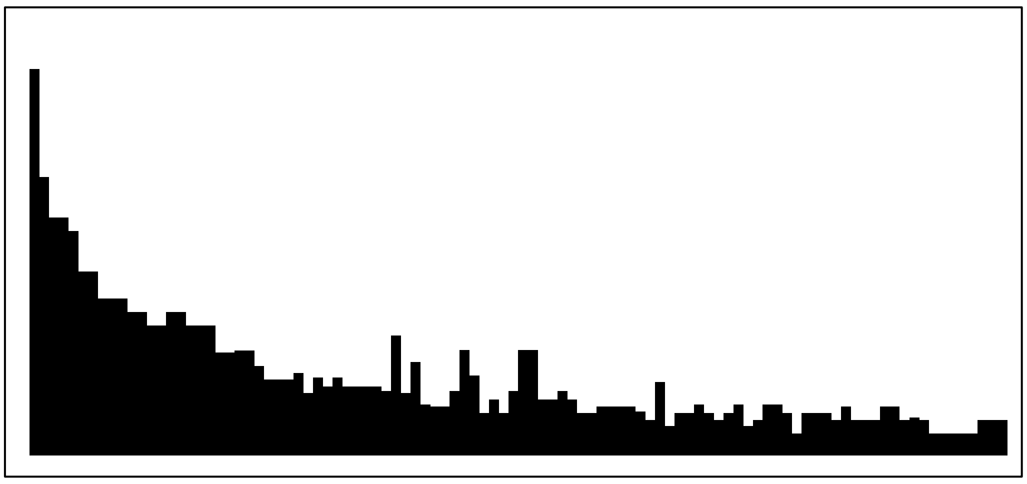

Oreodytes sanmarkii mitochondrial 16S rRNA gene (p	HF931524	98.1	100	2.36985e−68	Coleoptera	Dytiscidae	Oreodytes sanmarkii
Oreodytes obesus cordillerensis mitochondrial part	HF931516	93.04	100	1.70641e−57	Coleoptera	Dytiscidae	Oreodytes obesus cordillerensis
Oreodytes crassulus partial 16S rRNA gene	AJ850348	91.14	100	1.07686e−53	Coleoptera	Dytiscidae	Oreodytes crassulus
Oreodytes congruus partial 16S rRNA gene	AJ850347	91.14	100	1.07686e−53	Coleoptera	Dytiscidae	Oreodytes congruus
Oreodytes congruus 16S ribosomal RNA gene, partial	KF575550	90.51	100	4.57893e−52	Coleoptera	Dytiscidae	Oreodytes congruus
Oreodytes abbreviatus mitochondrial 16S rRNA gene	HF931477	88.61	100	2.88962e−48	Coleoptera	Dytiscidae	Oreodytes abbreviatus
Oreodytes rhyacophilus partial 16S rRNA gene	AJ850352	88.61	100	2.88962e−48	Coleoptera	Dytiscidae	Oreodytes rhyacophilus
Oreodytes mongolicus mitochondrial 16S rRNA gene (	HF931491	87.34	100	1.49686e−45	Coleoptera	Dytiscidae	Oreodytes mongolicus
Boreonectes riberae mitochondrial 16S rRNA gene (p	HF931461	87.34	100	1.82355e−44	Coleoptera	Dytiscidae	Boreonectes riberae
Boreonectes multilineatus mitochondrial partial 16	HF931384	87.34	100	1.82355e−44	Coleoptera	Dytiscidae	Boreonectes multilineatus
Oreodytes snoqualmie mitochondrial partial 16S rRN	HF931519	86.71	100	6.36481e−44	Coleoptera	Dytiscidae	Oreodytes snoqualmie
Boreonectes alpestris mitochondrial 16S rRNA gene	HF931512	86.71	100	7.75393e−43	Coleoptera	Dytiscidae	Boreonectes alpestris
Oreodytes septentrionalis mitochondrial 16S rRNA g	HF931492	86.08	100	7.75393e−43	Coleoptera	Dytiscidae	Oreodytes septentrionalis
Oreodytes shorti mitochondrial 16S rRNA gene (part	HF931488	86.08	100	7.75393e−43	Coleoptera	Dytiscidae	Oreodytes shorti
Boreonectes macedonicus mitochondrial 16S rRNA gen	HF931359	86.71	100	7.75393e−43	Coleoptera	Dytiscidae	Boreonectes macedonicus
Stictotarsus ibericus 16S ribosomal RNA (rrnL) gen	EF670030	86.71	100	7.75393e−43	Coleoptera	Dytiscidae	Stictotarsus ibericus
Boreonectes griseostriatus mitochondrial 16S rRNA	HF931541	86.08	100	9.44622e−42	Coleoptera	Dytiscidae	Boreonectes griseostriatus
Boreonectes striatellus mitochondrial 16S rRNA gen	HF931511	86.08	100	9.44622e−42	Coleoptera	Dytiscidae	Boreonectes striatellus
Boreonectes griseostriatus mitochondrial partial 1	HF931387	86.08	100	9.44622e−42	Coleoptera	Dytiscidae	Boreonectes griseostriatus
Laccornis difformis 16S ribosomal RNA gene, partia	KF575544	84.81	100	4.01663e−40	Coleoptera	Dytiscidae	Laccornis difformis
Deronectes parvicollis mitochondrial 16S rRNA gene	HF931454	84.81	100	4.01663e−40	Coleoptera	Dytiscidae	Deronectes parvicollis
Oreodytes scitulus 16S ribosomal RNA gene, partial	KF575551	84.91	100	1.70792e−38	Coleoptera	Dytiscidae	Oreodytes scitulus
Oreodytes scitulus mitochondrial partial 16S rRNA	HF931515	84.91	100	1.70792e−38	Coleoptera	Dytiscidae	Oreodytes scitulus
Boreonectes griseostriatus mitochondrial 16S rRNA	HF931385	84.18	100	2.08067e−37	Coleoptera	Dytiscidae	Boreonectes griseostriatus
Laccornis kocae partial 16S rRNA gene	AJ850419	83.54	100	2.08067e−37	Coleoptera	Dytiscidae	Laccornis kocae
Lancetes nigriceps partial 16S rRNA gene	AJ850418	83.54	100	2.08067e−37	Coleoptera	Dytiscidae	Lancetes nigriceps
Lancetes nigriceps 16S ribosomal RNA gene, partial	AY334132	83.54	100	2.08067e−37	Coleoptera	Dytiscidae	Lancetes nigriceps
Hydroporus marginatus mitochondrial 16S rRNA gene	HE613699	83.85	100	7.26225e−37	Coleoptera	Dytiscidae	Hydroporus marginatus
Lancetes nigriceps voucher UNM KBMLcni390 16S ribo	KJ548350	82.91	100	8.84723e−36	Coleoptera	Dytiscidae	Lancetes nigriceps
Suphrodytes dorsalis 16S ribosomal RNA gene, parti	KF575577	83.65	100	8.84723e−36	Coleoptera	Dytiscidae	Suphrodytes dorsalis
Hydroporus tessellatus 16S ribosomal RNA gene, par	JX434770	83.23	100	8.84723e−36	Coleoptera	Dytiscidae	Hydroporus tessellatus
Suphrodytes dorsalis voucher BMNH:725966 16S ribos	JX221574	83.65	100	8.84723e−36	Coleoptera	Dytiscidae	Suphrodytes dorsalis
Hydroporus foveolatus mitochondrial 16S rRNA gene	HE613724	83.23	100	8.84723e−36	Coleoptera	Dytiscidae	Hydroporus foveolatus
Hydroporus basinotatus mitochondrial 16S rRNA gene	HE613678	83.23	100	8.84723e−36	Coleoptera	Dytiscidae	Hydroporus basinotatus
Hydroporus basinotatus 16S ribosomal RNA gene, par	AF518258	83.23	100	8.84723e−36	Coleoptera	Dytiscidae	Hydroporus basinotatus
Hydroporus marginatus 16S ribosomal RNA gene, part	AF518267	83.23	100	8.84723e−36	Coleoptera	Dytiscidae	Hydroporus marginatus
Nebrioporus formaster mitochondrial partial 16S rR	HF931377	83.02	100	3.08799e−35	Coleoptera	Dytiscidae	Nebrioporus formaster
Deronectes moestus mitochondrial 16S rRNA gene (pa	HE613726	85.62	91.77	3.08799e−35	Coleoptera	Dytiscidae	Deronectes moestus
Hydroporus glabriusculus 16S ribosomal RNA gene, p	AY365270	82.91	100	3.08799e−35	Coleoptera	Dytiscidae	Hydroporus glabriusculus
Stictotarsus maghrebinus 16S ribosomal RNA gene, p	AY250947	84.38	100	3.08799e−35	Coleoptera	Dytiscidae	Stictotarsus maghrebinus
Scarodytes sp. IR−2003 16S ribosomal RNA gene, par	AY250939	82.39	100	3.08799e−35	Coleoptera	Dytiscidae	Scarodytes sp. IR−2003
Oreodytes natrix partial 16S rRNA gene	AJ850349	82.28	100	1.07781e−34	Coleoptera	Dytiscidae	Oreodytes natrix
Nebrioporus airumlus 16S ribosomal RNA gene, parti	AY250920	82.28	100	1.07781e−34	Coleoptera	Dytiscidae	Nebrioporus airumlus
Oreodytes quadrimaculatus 16S ribosomal RNA gene,	KF575567	83.02	100	3.76194e−34	Coleoptera	Dytiscidae	Oreodytes quadrimaculatus
Deronectes moestus 16S ribosomal RNA gene, partial	KF575561	84.93	91.77	3.76194e−34	Coleoptera	Dytiscidae	Deronectes moestus
Stictotarsus procerus mitochondrial 16S rRNA gene	HF931379	83.75	100	3.76194e−34	Coleoptera	Dytiscidae	Stictotarsus procerus
Suphrodytes figuratus voucher BMNH:722148 16S ribo	JX221571	81.99	100	3.76194e−34	Coleoptera	Dytiscidae	Suphrodytes figuratus
Hydroporus analis mitochondrial partial 16S rRNA g	HE613731	82.61	100	3.76194e−34	Coleoptera	Dytiscidae	Hydroporus analis
Hydroporus fuscipennis mitochondrial 16S rRNA gene	HE613730	81.99	100	3.76194e−34	Coleoptera	Dytiscidae	Hydroporus fuscipennis
Deronectes opatrinus mitochondrial 16S rRNA gene (	HE613701	83.02	100	3.76194e−34	Coleoptera	Dytiscidae	Deronectes opatrinus
Deronectes brannani mitochondrial 16S rRNA gene (p	HE613695	84.93	91.77	3.76194e−34	Coleoptera	Dytiscidae	Deronectes brannanii
Deronectes moestus mitochondrial 16S rRNA gene (pa	HE613683	84.93	91.77	3.76194e−34	Coleoptera	Dytiscidae	Deronectes moestus
Hydroporus zimmermanni mitochondrial 16S rRNA gene	HE613679	82.61	100	3.76194e−34	Coleoptera	Dytiscidae	Hydroporus zimmermanni
Hydroporus compunctus mitochondrial 16S rRNA gene	HE599693	82.61	100	3.76194e−34	Coleoptera	Dytiscidae	Hydroporus compunctus
Oreodytes quadrimaculatus partial 16S rRNA gene	AJ850351	83.02	100	3.76194e−34	Coleoptera	Dytiscidae	Oreodytes quadrimaculatus
Hydroporus compunctus 16S ribosomal RNA gene, part	AF518259	82.61	100	3.76194e−34	Coleoptera	Dytiscidae	Hydroporus compunctus
Hydroporus fuscipennis 16S ribosomal RNA gene, par	AF518262	81.99	100	3.76194e−34	Coleoptera	Dytiscidae	Hydroporus fuscipennis
Aethionectes fulvonotatus voucher KBMAeop406 16S r	KF979088	81.99	100	1.31304e−33	Coleoptera	Dytiscidae	Aethionectes fulvonotatus
Hygrotus novemlineatus mitochondrial partial 16S r	HF931523	82.28	100	1.31304e−33	Coleoptera	Dytiscidae	Hygrotus novemlineatus
Hygrotus marklini mitochondrial 16S rRNA gene (par	HF931467	82.28	100	1.31304e−33	Coleoptera	Dytiscidae	Hygrotus marklini
Hygrotus turbidus partial 16S rRNA gene	AJ850390	82.28	100	1.31304e−33	Coleoptera	Dytiscidae	Hygrotus turbidus
Nirripirti newhavenensis 16S ribosomal RNA gene, p	AY353844	82.28	100	1.31304e−33	Coleoptera	Dytiscidae	Paroster newhavenensis
Nebrioporus assimilis 16S ribosomal RNA gene, part	AY250921	82.05	98.73	1.31304e−33	Coleoptera	Dytiscidae	Nebrioporus assimilis
Lancetes angusticollis voucher UNM KBMLcan301 16S	KJ548347	81.65	100	4.58298e−33	Coleoptera	Dytiscidae	Lancetes angusticollis
Hygrotus impressopunctatus voucher UNM KBMHtim80 1	KJ548334	83.44	97.47	4.58298e−33	Coleoptera	Dytiscidae	Hygrotus impressopunctatus
Suphrodytes figuratus voucher BMNH:722150 16S ribo	JX221572	81.37	100	4.58298e−33	Coleoptera	Dytiscidae	Suphrodytes figuratus
Hydroporus discretus mitochondrial 16S rRNA gene (	HE613723	81.99	100	4.58298e−33	Coleoptera	Dytiscidae	Hydroporus discretus
Hydroporus decipiens mitochondrial 16S rRNA gene (	HE613722	81.99	100	4.58298e−33	Coleoptera	Dytiscidae	Hydroporus decipiens
Deronectes doriae mitochondrial 16S rRNA gene (par	HE613710	82.39	100	4.58298e−33	Coleoptera	Dytiscidae	Deronectes doriae
Hydroporus planus mitochondrial 16S rRNA gene (par	HE613708	81.99	100	4.58298e−33	Coleoptera	Dytiscidae	Hydroporus planus
Deronectes bicostatus mitochondrial 16S rRNA gene	HE613705	81.65	100	4.58298e−33	Coleoptera	Dytiscidae	Deronectes bicostatus
Hydroporus errans mitochondrial 16S rRNA gene (par	HE613704	81.99	100	4.58298e−33	Coleoptera	Dytiscidae	Hydroporus errans
Deronectes sahlbergi mitochondrial 16S rRNA gene (	HE613680	82.39	100	4.58298e−33	Coleoptera	Dytiscidae	Deronectes sahlbergi
Suphrodytes dorsalis mitochondrial 16S rRNA gene (	HE599700	81.37	100	4.58298e−33	Coleoptera	Dytiscidae	Suphrodytes dorsalis
Copelatus sp. BI−2011 voucher BMNH792945 16S ribos	HQ381441	81.65	100	4.58298e−33	Coleoptera	Dytiscidae	Copelatus sp. BI−2011
Stictotarsus falli 16S ribosomal RNA (rrnL) gene,	EF670029	82.39	100	4.58298e−33	Coleoptera	Dytiscidae	Stictotarsus falli
Nirripirti megamacrocephalus isolate ABTC78781 16S	EU616935	82.39	100	4.58298e−33	Coleoptera	Dytiscidae	Paroster megamacrocephalus
Hydroporus pubescens voucher NHM−IR39 16S ribosoma	EF419327	81.99	100	4.58298e−33	Coleoptera	Dytiscidae	Hydroporus pubescens
Scarodytes halensis haplotype 3 16S ribosomal RNA	AY250937	81.01	100	4.58298e−33	Coleoptera	Dytiscidae	Scarodytes halensis
Hydroporus planus 16S ribosomal RNA gene, partial	AF518275	81.99	100	4.58298e−33	Coleoptera	Dytiscidae	Hydroporus planus
Hydroporus discretus 16S ribosomal RNA gene, parti	AF518260	81.99	100	4.58298e−33	Coleoptera	Dytiscidae	Hydroporus discretus
Hydroporus analis 16S ribosomal RNA gene, partial	AF518257	81.99	100	4.58298e−33	Coleoptera	Dytiscidae	Hydroporus analis
Hydroporus umbrosus voucher UNM KBMHyum551 16S rib	KJ548394	81.65	100	1.59962e−32	Coleoptera	Dytiscidae	Hydroporus umbrosus
Hydrocolus paugus 16S ribosomal RNA gene, partial	KF575539	82.28	100	1.59962e−32	Coleoptera	Dytiscidae	Hydrocolus paugus
Hygrotus lutescens mitochondrial partial 16S rRNA	HF931518	81.65	100	1.59962e−32	Coleoptera	Dytiscidae	Hygrotus lutescens
Hygrotus fontinalis mitochondrial 16S rRNA gene (p	HF931494	81.65	100	1.59962e−32	Coleoptera	Dytiscidae	Hygrotus fontinalis
Hygrotus patruelis mitochondrial 16S rRNA gene (pa	HF931388	81.65	100	1.59962e−32	Coleoptera	Dytiscidae	Hygrotus patruelis
Hydrocolus sahlbergi mitochondrial 16S rRNA gene (	HE599691	82.28	100	1.59962e−32	Coleoptera	Dytiscidae	Hydrocolus sahlbergi
Hydrocolus picicornis partial 16S rRNA gene	AJ850379	82.28	100	1.59962e−32	Coleoptera	Dytiscidae	Hydrocolus picicornis
Hydroporus sp. IR33.4 16S ribosomal RNA gene, part	AY365261	81.65	100	1.59962e−32	Coleoptera	Dytiscidae	Hydroporus sp. IR33.4
Nebrioporus nipponicus 16S ribosomal RNA gene, par	AY250931	81.76	100	1.59962e−32	Coleoptera	Dytiscidae	Nebrioporus nipponicus
Hydroporus umbrosus 16S ribosomal RNA gene, partia	AF518280	81.65	100	1.59962e−32	Coleoptera	Dytiscidae	Hydroporus umbrosus
Ilybius fraterculus mitochondrial 16S rRNA gene (p	LM654800	81.01	100	5.58321e−32	Coleoptera	Dytiscidae	Ilybius fraterculus
Ilybius ater mitochondrial 16S rRNA gene (partial)	LM654794	81.01	100	5.58321e−32	Coleoptera	Dytiscidae	Ilybius ater
Lancetes varius voucher UNM KBMLcva196 16S ribosom	KJ548351	81.01	100	5.58321e−32	Coleoptera	Dytiscidae	Lancetes varius
Ilybius fraterculus voucher UNM KBMIlfr409 16S rib	KJ548342	81.01	100	5.58321e−32	Coleoptera	Dytiscidae	Ilybius fraterculus
Hydaticus cinctipennis voucher UNM KBMHyci20 16S r	KJ548338	81.01	100	5.58321e−32	Coleoptera	Dytiscidae	Hydaticus cinctipennis
Hydroporus appalachius 16S ribosomal RNA gene, par	JX434765	81.65	100	5.58321e−32	Coleoptera	Dytiscidae	Hydroporus appalachius
Boreonectes funereus mitochondrial 16S rRNA gene (	HF931393	81.65	100	5.58321e−32	Coleoptera	Dytiscidae	Boreonectes funereus
Hydroporus pervicinus mitochondrial 16S rRNA gene	HE599695	81.65	100	5.58321e−32	Coleoptera	Dytiscidae	Hydroporus pervicinus
