# Supplementary material for: Testing the potential of a ribosomal 16S marker for DNA metabarcoding of insects
Source: PeerJ. 2016 Apr 19;4:e1966. doi: 10.7717/peerj.1966 (PMC4841222; doi:10.7717/peerj.1966)
Supplement: Supplemental Information 1 [file peerj-04-1966-s006.zip › scripts/5 match OTUs/blast_hits/OTU_61.csv_plot.pdf]

OTU_61.csv

Notidobiella chacayana

Sericostomatidae

Trichoptera

77.78

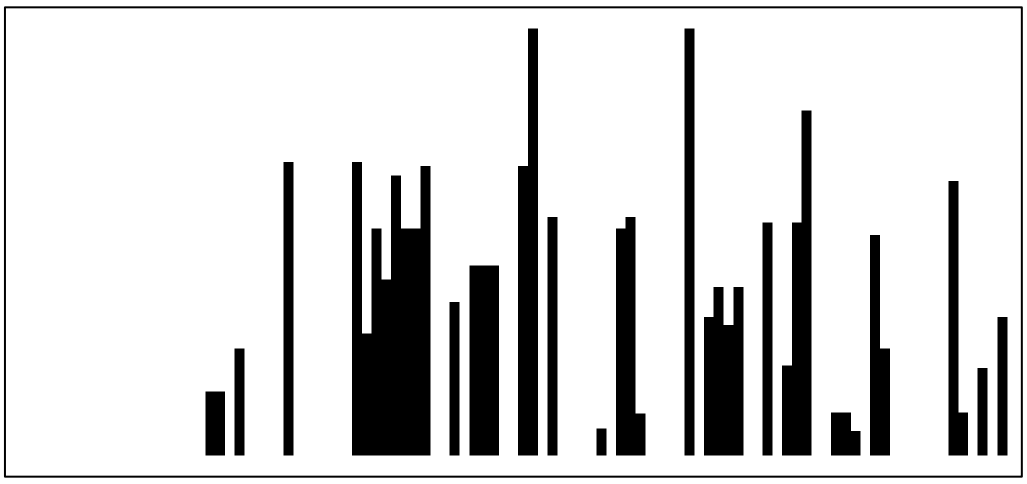

Notidobiella chacayana isolate CL5 16S ribosomal R	EF394993	77.78	98.53	3.07597e−15	Trichoptera	Sericostomatidae	Notidobiella chacayana
Sericostoma clypeatum voucher NHRS−EN CC4 16S ribo	FJ263207	75.61	86.76	5.56148e−12	Trichoptera	Sericostomatidae	Sericostoma clypeatum
Sericostoma personatum mitochondrion, partial geno	KP455290	76.7	75.74	2.88092e−09	Trichoptera	Sericostomatidae	Sericostoma personatum
Zebrafish DNA sequence from clone CH211−87J1 in li	FP102071	73.15	77.94	0.000221485	Cypriniformes	Cyprinidae	Danio rerio
Zebrafish DNA sequence from clone CH1073−416M20 in	CU651673	73.15	77.94	0.000221485	Cypriniformes	Cyprinidae	Danio rerio
Tamasia variegata mitochondrial 16S rRNA gene, spe	FN257669	75	74.26	0.000773058	Trichoptera	Calocidae	Tamasia variegata
Eosericostoma inaequispinum isolate CK9 16S riboso	EF394992	72.38	75	0.00269824	Trichoptera	Helicophidae	Eosericostoma inaequispinum
Eosericostoma aequispinum isolate CK7 16S ribosoma	EF394990	72.38	75	0.00269824	Trichoptera	Helicophidae	Eosericostoma aequispinum
Eosericostoma sp. CK5 16S ribosomal RNA gene, part	EF394989	72.38	75	0.00269824	Trichoptera	Helicophidae	Eosericostoma sp. CK5
Alloecentrella sp. BP8 16S ribosomal RNA gene, par	EF394983	73.27	74.26	0.00269824	Trichoptera	Calocidae	Alloecentrella sp. BP8
Tetrix tubercarina 16S ribosomal RNA gene, partial	AY590170	74.7	61.03	0.00269824	Orthoptera	Tetrigidae	Tetrix tubercarina
Dictyostelium purpureum hypothetical protein, mRNA	XM_003284470	79.69	46.32	0.00941777	Dictyosteliida		Dictyostelium purpureum
Elassogaster sp. HWB−2006 16S ribosomal RNA gene,	DQ534708	72.16	67.65	0.00941777	Diptera	Platystomatidae	Elassogaster sp. HWB−2006
Strongyloides ratti genome assembly S_ratti_ED321	LN609529	74.12	62.5	0.0328713	Rhabditida	Strongyloididae	Strongyloides ratti
Cyprinus carpio genome assembly common carp genome	LN590688	77.94	49.26	0.0328713	Cypriniformes	Cyprinidae	Cyprinus carpio
Vitis vinifera contig VV78X000758.6, whole genome	AM429030	71.3	70.59	0.0328713	Vitales	Vitaceae	Vitis vinifera
Mycoplasma yeatsii GM274B	CP007520	73.42	58.09	0.114732	Mycoplasmatales	Mycoplasmataceae	Mycoplasma yeatsii GM274B
Xenorhabdus bovienii str. CS03 chromosome, complet	FO818637	72.16	71.32	0.114732	Enterobacteriales	Enterobacteriaceae	Xenorhabdus bovienii
Cucumis melo genomic chromosome, chr_10	LN713264	82.98	34.56	0.114732	Cucurbitales	Cucurbitaceae	Cucumis melo
Cucumis melo genomic scaffold, anchoredscaffold000	LN681904	82.98	34.56	0.114732	Cucurbitales	Cucurbitaceae	Cucumis melo
Strongyloides stercoralis genome assembly S_sterco	LL999058	74.16	64.71	0.114732	Rhabditida	Strongyloididae	Strongyloides stercoralis
Gongylonema pulchrum genome assembly G_pulchrum_Ho	LL750493	85	29.41	0.114732	Spirurida	Gongylonematidae	Gongylonema pulchrum
Heligmosomoides polygyrus genome assembly H_bakeri	LL189433	76.81	50	0.114732	Rhabditida	Heligmosomatidae	Heligmosomoides polygyrus
Plasmodium yoelii genome assembly PY17X01, chromos	LM993663	70.21	98.53	0.114732	Haemosporida	Plasmodiidae	Plasmodium yoelii
Plasmodium yoelii genome assembly PYYM01, chromoso	LK934637	70.21	98.53	0.114732	Haemosporida	Plasmodiidae	Plasmodium yoelii
Aspergillus niger clone AXAS101−N02, complete sequ	AC253892	74.26	67.65	0.114732	Eurotiales	Aspergillaceae	Aspergillus niger
Arabidopsis thaliana chromosome 5 sequence	CP002688	93.75	22.79	0.114732	Brassicales	Brassicaceae	Arabidopsis thaliana
Pinus taeda clone PT_7Ba0044C12, complete sequence	AC241286	72.63	64.71	0.114732	Pinales	Pinaceae	Pinus taeda
Medicago truncatula clone mth2−5f17, complete sequ	AC144591	73.97	50.74	0.114732	Fabales	Fabaceae	Medicago truncatula
Pliocaloca sp. CQ3 16S ribosomal RNA gene, partial	EF394999	72.48	75	0.114732	Trichoptera	Calocidae	Pliocaloca sp. CQ3
Zebrafish DNA sequence from clone DKEYP−90G2 in li	BX511254	73.81	61.03	0.114732	Cypriniformes	Cyprinidae	Danio rerio
Zebrafish DNA sequence from clone DKEY−22L9 in lin	CR388032	75	60.29	0.114732	Cypriniformes	Cyprinidae	Danio rerio
Zebrafish DNA sequence from clone DKEY−238D17 in l	BX511246	75	60.29	0.114732	Cypriniformes	Cyprinidae	Danio rerio
Arabidopsis thaliana genomic DNA, chromosome 5, P1	AB005231	93.75	22.79	0.114732	Brassicales	Brassicaceae	Arabidopsis thaliana
Homo sapiens chromosome 4 clone C0190D22 map 4p16,	AC007103	85.71	30.88	0.114732	Primates	Hominidae	Homo sapiens
Drosophila busckii chromosome 2R sequence	CP012524	90.62	23.53	0.400454	Diptera	Drosophilidae	Drosophila busckii
Trifolium pratense genome assembly redclover, chro	LN846351	88.24	25	0.400454	Fabales	Fabaceae	Trifolium pratense
PREDICTED: Pogonomyrmex barbatus major royal jelly	XM_011640908	93.1	21.32	0.400454	Hymenoptera	Formicidae	Pogonomyrmex barbatus
PREDICTED: Esox lucius interleukin 20 receptor, al	XM_010882954	90.62	23.53	0.400454	Esociformes	Esocidae	Esox lucius
PREDICTED: Esox lucius interleukin 20 receptor, al	XM_010882953	90.62	23.53	0.400454	Esociformes	Esocidae	Esox lucius
Schistosoma rodhaini genome assembly S_rodhaini_Bu	LL959551	93.55	22.79	0.400454	Strigeidida	Schistosomatidae	Schistosoma rodhaini
Dracunculus medinensis genome assembly D_medinensi	LK978291	72.12	72.06	0.400454	Spirurida	Dracunculidae	Dracunculus medinensis
Schistosoma curassoni genome assembly S_curassoni_	LM078077	70.59	72.06	0.400454	Strigeidida	Schistosomatidae	Schistosoma curassoni
PREDICTED: Brassica rapa spermidine hydroxycinnamo	XM_009114326	87.18	27.94	0.400454	Brassicales	Brassicaceae	Brassica rapa
Solanum pennellii chromosome ch01, complete genome	HG975440	75.9	59.56	0.400454	Solanales	Solanaceae	Solanum pennellii
PREDICTED: Stegastes partitus vitamin K epoxide re	XM_008306889	88.89	26.47	0.400454		Pomacentridae	Stegastes partitus
PREDICTED: Neolamprologus brichardi vitamin K epox	XM_006799567	88.89	26.47	0.400454	Cichliformes	Cichlidae	Neolamprologus brichardi
PREDICTED: Haplochromis burtoni vitamin K epoxide	XM_005928446	88.89	26.47	0.400454	Cichliformes	Cichlidae	Haplochromis burtoni
Leucocytozoon caulleryi apicoplast DNA, complete s	AP013071	75.32	53.68	0.400454	Haemosporida	Leucocytozoidae	Leucocytozoon caulleryi
Candidatus Carsonella ruddii PC isolate NHV, compl	CP003545	70.83	65.44	0.400454	Oceanospirillales	Halomonadaceae	Candidatus Carsonella ruddii PC isolate NHV
Schistosoma mansoni strain Puerto Rico chromosome	HE601630	93.55	22.79	0.400454	Strigeidida	Schistosomatidae	Schistosoma mansoni
Arabidopsis thaliana chromosome 1 sequence	CP002684	100	17.65	0.400454	Brassicales	Brassicaceae	Arabidopsis thaliana
Pycnocentrella eruensis isolate BR4 16S ribosomal	EF394985	72.9	75	0.400454	Trichoptera	Calocidae	Pycnocentrella eruensis
Medicago truncatula clone mth2−30e7, complete sequ	AC137837	91.18	24.26	0.400454	Fabales	Fabaceae	Medicago truncatula
Plasmodium falciparum 3D7 chromosome 4	AL844503	72.97	54.41	0.400454	Haemosporida	Plasmodiidae	Plasmodium falciparum 3D7
Medicago truncatula clone mth2−14o4, complete sequ	AC171266	72.53	62.5	0.400454	Fabales	Fabaceae	Medicago truncatula
Vitis vinifera contig VV78X101012.2, whole genome	AM475927	71.13	69.12	0.400454	Vitales	Vitaceae	Vitis vinifera
Medicago truncatula chromosome 5 clone mth2−57p20,	CU459035	72.92	62.5	0.400454	Fabales	Fabaceae	Medicago truncatula
Ixora sp. Davis 2297 acetyl−CoA carboxylase beta s	DQ180516	81.25	33.09	0.400454	Gentianales	Rubiaceae	Ixora sp. Davis 2297
Vitis vinifera, whole genome shotgun sequence, con	AM435059	71.13	69.12	0.400454	Vitales	Vitaceae	Vitis vinifera
Oenococcus oeni PSU−1, complete genome	CP000411	90.62	23.53	0.400454	Lactobacillales	Leuconostocaceae	Oenococcus oeni PSU−1
Medicago truncatula clone mth2−76i7, complete sequ	AC153460	91.18	24.26	0.400454	Fabales	Fabaceae	Medicago truncatula
Gracilaria tenuistipitata var. liui chloroplast, c	AY673996	81.97	42.65	0.400454	Gracilariales	Gracilariaceae	Gracilaria tenuistipitata var. liui
Guillardia theta nucleomorph chromosome 1, complet	AF165818	76.39	51.47	0.400454	Pyrenomonadales	Geminigeraceae	Guillardia theta
Wigglesworthia glossinidia endosymbiont of Glossin	BA000021	72.92	69.12	0.400454	Enterobacteriales	Enterobacteriaceae	Wigglesworthia glossinidia endosymbiont of Glossina brevipalpis
Geisha distinetissima 16S ribosomal RNA gene, part	AF158052	73.49	60.29	0.400454	Hemiptera	Flatidae	Geisha distinctissima
Medicago truncatula chromosome 2 clone mth2−89f22,	AC174468	72.92	62.5	0.400454	Fabales	Fabaceae	Medicago truncatula
Sequence of BAC F7G19 from Arabidopsis thaliana ch	AC000106	100	17.65	0.400454	Brassicales	Brassicaceae	Arabidopsis thaliana
Drosophila busckii chromosome X sequence	CP012528	79.37	44.12	1.39772	Diptera	Drosophilidae	Drosophila busckii
Plasmodium reichenowi conserved Plasmodium protein	XM_012908979	86.49	25.74	1.39772	Haemosporida	Plasmodiidae	Plasmodium reichenowi
TPA_asm: Oryzias latipes strain Hd−rR, complete ge	HG313994	87.88	24.26	1.39772	Beloniformes	Adrianichthyidae	Oryzias latipes
PREDICTED: Strongylocentrotus purpuratus uncharact	XR_972630	86.11	22.79	1.39772	Echinoida	Strongylocentrotidae	Strongylocentrotus purpuratus
PREDICTED: Oryzias latipes metastasis associated i	XM_004077815	87.88	24.26	1.39772	Beloniformes	Adrianichthyidae	Oryzias latipes
Botrytis cinerea B05.10 chromosome 6, complete seq	CP009810	74.29	51.47	1.39772	Helotiales	Sclerotiniaceae	Botrytis cinerea B05.10
Cucumis melo genomic chromosome, chr_6	LN713260	71.91	63.24	1.39772	Cucurbitales	Cucurbitaceae	Cucumis melo
Cucumis melo genomic chromosome, chr_4	LN713258	90.91	23.53	1.39772	Cucurbitales	Cucurbitaceae	Cucumis melo
Cucumis melo genomic scaffold, anchoredscaffold000	LN681860	71.91	63.24	1.39772	Cucurbitales	Cucurbitaceae	Cucumis melo
Cucumis melo genomic scaffold, anchoredscaffold000	LN681841	84.21	27.94	1.39772	Cucurbitales	Cucurbitaceae	Cucumis melo
Cucumis melo genomic scaffold, anchoredscaffold001	LN681831	90.91	23.53	1.39772	Cucurbitales	Cucurbitaceae	Cucumis melo
Protopolystoma xenopodis genome assembly P_xenopod	LM911455	96.15	19.12	1.39772	Polyopisthocotylea	Polystomatidae	Protopolystoma xenopodis
Hymenolepis diminuta genome assembly H_diminuta_De	LM390132	73.49	61.03	1.39772	Cyclophyllidea	Hymenolepididae	Hymenolepis diminuta
Strongyloides ratti genome assembly S_ratti_ED321	LN609396	79.25	38.24	1.39772	Rhabditida	Strongyloididae	Strongyloides ratti
Cyprinus carpio genome assembly common carp genome	LN592105	82	36.03	1.39772	Cypriniformes	Cyprinidae	Cyprinus carpio
Cyprinus carpio genome assembly common carp genome	LN590712	82	36.03	1.39772	Cypriniformes	Cyprinidae	Cyprinus carpio
Plasmodium berghei ANKA genome assembly PBANKA01,	LK023121	81.13	38.24	1.39772	Haemosporida	Plasmodiidae	Plasmodium berghei ANKA
Aureococcus anophagefferens virus isolate BtV−01,	KJ645900	80	36.76	1.39772		Phycodnaviridae	Aureococcus anophagefferens virus
Elaeophora elaphi genome assembly E_elaphi ,scaffo	LL713316	90.32	22.79	1.39772	Spirurida	Onchocercidae	Elaeophora elaphi
Solanum pennellii chromosome ch05, complete genome	HG975444	85	29.41	1.39772	Solanales	Solanaceae	Solanum pennellii
Solanum pennellii chromosome ch04, complete genome	HG975443	77.42	45.59	1.39772	Solanales	Solanaceae	Solanum pennellii
Gossypium hirsutum clone NBRI_GE11951 microsatelli	JX579665	73.61	52.94	1.39772	Malvales	Malvaceae	Gossypium hirsutum
Papilio krishna thawgawa voucher CBGPFLC_00376 16S	JQ982213	80	35.29	1.39772	Lepidoptera	Papilionidae	Papilio krishna thawgawa
Papilio krishna thawgawa voucher CBGPFLC_00058 16S	JQ982212	80	35.29	1.39772	Lepidoptera	Papilionidae	Papilio krishna thawgawa
Papilio krishna mayumiae voucher CBGPFLC_00057 16S	JQ982211	80	35.29	1.39772	Lepidoptera	Papilionidae	Papilio krishna mayumiae
Papilio krishna ssp. CBGPFLC_00055 16S ribosomal R	JQ982210	80	35.29	1.39772	Lepidoptera	Papilionidae	Papilio krishna ssp. CBGPFLC_00055
Cyprinus carpio clone 646631 microsatellite sequen	JN757338	92.86	20.59	1.39772	Cypriniformes	Cyprinidae	Cyprinus carpio
Cyprinus carpio clone 621360 microsatellite sequen	JN754486	82	36.03	1.39772	Cypriniformes	Cyprinidae	Cyprinus carpio
Methanothermococcus okinawensis IH1, complete geno	CP002792	75	52.94	1.39772	Methanococcales	Methanococcaceae	Methanothermococcus okinawensis IH1
Arabidopsis thaliana chromosome 3, complete sequen	CP002686	84.09	31.62	1.39772	Brassicales	Brassicaceae	Arabidopsis thaliana
Dictyostelium purpureum hypothetical protein, mRNA	XM_003283811	76.56	46.32	1.39772	Dictyosteliida		Dictyostelium purpureum
Plasmodium falciparum 3D7 conserved Plasmodium pro	XM_002808951	86.49	25.74	1.39772	Haemosporida	Plasmodiidae	Plasmodium falciparum 3D7
