# Supplementary material for: Testing the potential of a ribosomal 16S marker for DNA metabarcoding of insects
Source: PeerJ. 2016 Apr 19;4:e1966. doi: 10.7717/peerj.1966 (PMC4841222; doi:10.7717/peerj.1966)
Supplement: Supplemental Information 1 [file peerj-04-1966-s006.zip › scripts/5 match OTUs/blast_hits/OTU_619.csv_plot.pdf]

OTU_619.csv

Rhithrogena semicolorata

Heptageniidae

Ephemeroptera

98.69

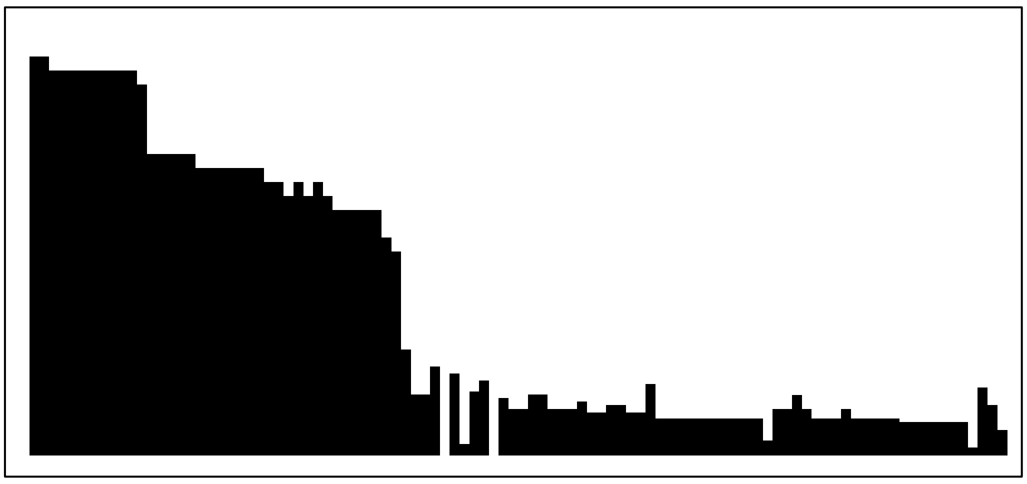

Rhithrogena semicolorata mitochondrial partial 16S	LN868673	98.69	98.71	2.31205e−68	Ephemeroptera	Heptageniidae	Rhithrogena semicolorata
Rhithrogena sp. 4 LV−2015 mitochondrial partial 16	LN868652	98.69	98.71	2.31205e−68	Ephemeroptera	Heptageniidae	Rhithrogena sp. 4 LV−2015
Rhithrogena sp. 13 LV−2015 mitochondrial partial 1	LN868672	98.04	98.71	9.83108e−67	Ephemeroptera	Heptageniidae	Rhithrogena sp. 13 LV−2015
Rhithrogena sp. 12 LV−2015 mitochondrial partial 1	LN868671	98.04	98.71	9.83108e−67	Ephemeroptera	Heptageniidae	Rhithrogena sp. 12 LV−2015
Rhithrogena picteti mitochondrial partial 16S rRNA	LN868663	98.04	98.71	9.83108e−67	Ephemeroptera	Heptageniidae	Rhithrogena picteti
Rhithrogena sp. 3 LV−2015 mitochondrial partial 16	LN868659	98.04	98.71	9.83108e−67	Ephemeroptera	Heptageniidae	Rhithrogena sp. 3 LV−2015
Rhithrogena fonticola mitochondrial partial 16S rR	LN868656	98.04	98.71	9.83108e−67	Ephemeroptera	Heptageniidae	Rhithrogena fonticola
Rhithrogena sp. 2 LV−2015 mitochondrial partial 16	LN868654	98.04	98.71	9.83108e−67	Ephemeroptera	Heptageniidae	Rhithrogena sp. 2 LV−2015
Rhithrogena puytoraci mitochondrial partial 16S rR	LN868651	98.04	98.71	9.83108e−67	Ephemeroptera	Heptageniidae	Rhithrogena puytoraci
Rhithrogena iridina mitochondrial partial 16S rRNA	LN868646	98.04	98.71	9.83108e−67	Ephemeroptera	Heptageniidae	Rhithrogena iridina
Rhithrogena taurisca mitochondrial partial 16S rRN	LN868638	98.04	98.71	9.83108e−67	Ephemeroptera	Heptageniidae	Rhithrogena taurisca
Rhithrogena sp. 5 LV−2015 mitochondrial partial 16	LN868653	97.39	98.71	1.19767e−65	Ephemeroptera	Heptageniidae	Rhithrogena sp. 5 LV−2015
Rhithrogena sp. 7 LV−2015 mitochondrial partial 16	LN868669	94.12	98.71	1.66479e−57	Ephemeroptera	Heptageniidae	Rhithrogena sp. 7 LV−2015
Rhithrogena eatoni mitochondrial partial 16S rRNA	LN868666	94.12	98.71	1.66479e−57	Ephemeroptera	Heptageniidae	Rhithrogena eatoni
Rhithrogena alpestris mitochondrial partial 16S rR	LN868657	94.12	98.71	1.66479e−57	Ephemeroptera	Heptageniidae	Rhithrogena alpestris
Rhithrogena gratianopolitana mitochondrial partial	LN868647	94.12	98.71	1.66479e−57	Ephemeroptera	Heptageniidae	Rhithrogena gratianopolitana
Rhithrogena nivata mitochondrial partial 16S rRNA	LN868643	94.12	98.71	1.66479e−57	Ephemeroptera	Heptageniidae	Rhithrogena nivata
Rhithrogena degrangei mitochondrial partial 16S rR	LN868674	93.46	98.71	2.02813e−56	Ephemeroptera	Heptageniidae	Rhithrogena degrangei
Rhithrogena corcontica mitochondrial partial 16S r	LN868662	93.46	98.71	2.02813e−56	Ephemeroptera	Heptageniidae	Rhithrogena corcontica
Rhithrogena sp. 9 LV−2015 mitochondrial partial 16	LN868650	93.46	98.71	2.02813e−56	Ephemeroptera	Heptageniidae	Rhithrogena sp. 9 LV−2015
Rhithrogena gratianopolitana mitochondrial partial	LN868648	93.46	98.71	2.02813e−56	Ephemeroptera	Heptageniidae	Rhithrogena gratianopolitana
Rhithrogena landai mitochondrial partial 16S rRNA	LN868644	93.46	98.71	2.02813e−56	Ephemeroptera	Heptageniidae	Rhithrogena landai
Rhithrogena hybrida mitochondrial partial 16S rRNA	LN868642	93.46	98.71	2.02813e−56	Ephemeroptera	Heptageniidae	Rhithrogena hybrida
Rhithrogena circumtatrica mitochondrial partial 16	LN868641	93.46	98.71	2.02813e−56	Ephemeroptera	Heptageniidae	Rhithrogena circumtatrica
Rhithrogena sp. 11 LV−2015 mitochondrial partial 1	LN868668	92.81	98.71	8.62382e−55	Ephemeroptera	Heptageniidae	Rhithrogena sp. 11 LV−2015
Rhithrogena sp. 8 LV−2015 mitochondrial partial 16	LN868661	92.81	98.71	8.62382e−55	Ephemeroptera	Heptageniidae	Rhithrogena sp. 8 LV−2015
Rhithrogena germanica mitochondrial partial 16S rR	LN868660	92.16	98.71	8.62382e−55	Ephemeroptera	Heptageniidae	Rhithrogena germanica
Rhithrogena sp. 6 LV−2015 mitochondrial partial 16	LN868655	92.81	98.71	8.62382e−55	Ephemeroptera	Heptageniidae	Rhithrogena sp. 6 LV−2015
Rhithrogena beskidensis mitochondrial partial 16S	LN868645	92.16	98.71	8.62382e−55	Ephemeroptera	Heptageniidae	Rhithrogena beskidensis
Rhithrogena diensis mitochondrial partial 16S rRNA	LN868637	92.81	98.71	8.62382e−55	Ephemeroptera	Heptageniidae	Rhithrogena diensis
Rhithrogena nuragica mitochondrial partial 16S rRN	LN868667	92.16	98.71	1.0506e−53	Ephemeroptera	Heptageniidae	Rhithrogena nuragica
Rhithrogena sp. 10 LV−2015 mitochondrial partial 1	LN868664	91.5	98.71	3.66694e−53	Ephemeroptera	Heptageniidae	Rhithrogena sp. 10 LV−2015
Rhithrogena savoiensis mitochondrial partial 16S r	LN868658	91.5	98.71	3.66694e−53	Ephemeroptera	Heptageniidae	Rhithrogena savoiensis
Rhithrogena insularis mitochondrial partial 16S rR	LN868665	91.5	98.71	4.46725e−52	Ephemeroptera	Heptageniidae	Rhithrogena insularis
Rhithrogena allobrogica mitochondrial partial 16S	LN868640	91.5	98.71	4.46725e−52	Ephemeroptera	Heptageniidae	Rhithrogena allobrogica
Rhithrogena grischuna mitochondrial partial 16S rR	LN868639	91.5	98.71	4.46725e−52	Ephemeroptera	Heptageniidae	Rhithrogena grischuna
Rhithrogena sartorii mitochondrial partial 16S rRN	LN868670	90.2	98.71	1.89952e−50	Ephemeroptera	Heptageniidae	Rhithrogena sartorii
Rhithrogena dorieri mitochondrial partial 16S rRNA	LN868649	89.54	98.71	2.31409e−49	Ephemeroptera	Heptageniidae	Rhithrogena dorieri
Rhithrogena sp. EP021 16S ribosomal RNA gene, part	AY749765	84.97	98.71	1.36775e−39	Ephemeroptera	Heptageniidae	Rhithrogena sp. EP021
Cinygmula sp. m185 mitochondrial partial 16S rRNA	LN868675	82.86	90.32	1.9012e−31	Ephemeroptera	Heptageniidae	Cinygmula sp. m185
Cinygmula sp. EP013 16S ribosomal RNA gene, partia	AY749760	82.86	90.32	1.9012e−31	Ephemeroptera	Heptageniidae	Cinygmula sp. EP013
Cultus pilatus 16S ribosomal RNA gene, partial seq	EF623212	84.16	65.16	4.77813e−20	Plecoptera	Perlodidae	Cultus pilatus
Heptagenia sp. EP018 16S ribosomal RNA gene, parti	AY749762	77.86	89.68	1.66773e−19	Ephemeroptera	Heptageniidae	Heptagenia sp. EP018
Arthroplea bipunctata 16S ribosomal RNA gene, part	AY749817	83.84	63.87	5.82096e−19	Ephemeroptera	Arthropleidae	Arthroplea bipunctata
Paegniodes cupulatus mitochondrion, complete genom	HM004123	80.53	72.9	2.03171e−18	Ephemeroptera	Heptageniidae	Paegniodes cupulatus
Remenus bilobatus 16S ribosomal RNA gene, partial	EF623233	83	64.52	7.09138e−18	Plecoptera	Perlodidae	Remenus bilobatus
Capnura wanica 16S ribosomal RNA gene, partial seq	EF623128	83.5	65.16	7.09138e−18	Plecoptera	Capniidae	Capnura wanica
Chiloporter sp. EP051 16S ribosomal RNA gene, part	AY749776	75.97	98.71	7.09138e−18	Ephemeroptera	Ameletopsidae	Chiloporter sp. EP051
Apteroperla tikumana mitochondrion, complete genom	KR604721	82.69	65.16	2.47513e−17	Plecoptera	Capniidae	Apteroperla tikumana
Neopanorpa pulchra mitochondrion, complete genome	JX569848	82.18	65.16	2.47513e−17	Mecoptera	Panorpidae	Neopanorpa pulchra
Neopanorpa pulchra mitochondrion, complete genome	FJ169955	82.18	65.16	2.47513e−17	Mecoptera	Panorpidae	Neopanorpa pulchra
Isoperla oxylepis 16S ribosomal RNA gene, partial	EF623221	82.86	67.1	2.47513e−17	Plecoptera	Perlodidae	Isoperla oxylepis
Isoperla davisi 16S ribosomal RNA gene, partial se	EF623206	82.86	67.1	2.47513e−17	Plecoptera	Perlodidae	Isoperla davisi
Thaumatoperla sp. BYU_PL109 16S ribosomal RNA gene	EF623149	82.18	65.16	2.47513e−17	Plecoptera	Eustheniidae	Thaumatoperla sp. BYU_PL109
Penturoperla barbata 16S ribosomal RNA gene, parti	EF623124	82.18	65.16	2.47513e−17	Plecoptera	Austroperlidae	Penturoperla barbata
Neopanorpa sp. M24899 16S ribosomal RNA gene, part	AF180059	82.18	65.16	2.47513e−17	Mecoptera	Panorpidae	Neopanorpa sp. M24899
Pachyrhamma edwardsii voucher CW122 16S ribosomal	HM594508	82.52	65.16	8.63907e−17	Orthoptera	Rhaphidophoridae	Pachyrhamma edwardsii
Heteromallus cavicola voucher MIL 16S ribosomal RN	HM594499	82	64.52	8.63907e−17	Orthoptera	Rhaphidophoridae	Heteromallus cavicola
Pteronarcys californica 16S ribosomal RNA gene, pa	EF623261	82	64.52	8.63907e−17	Plecoptera	Pteronarcyidae	Pteronarcys californica
Perlinodes aurea 16S ribosomal RNA gene, partial s	EF623234	82.35	65.16	8.63907e−17	Plecoptera	Perlodidae	Perlinodes aurea
Austronemoura sp. BYU_PL149 16S ribosomal RNA gene	EF623200	82.35	65.16	8.63907e−17	Plecoptera	Notonemouridae	Austronemoura sp. BYU_PL149
Pteronarcys princeps mitochondrion, complete genom	AY687866	82	64.52	8.63907e−17	Plecoptera	Pteronarcyidae	Pteronarcys princeps
Dipteromimus sp. Ofunato−1 mitochondrial gene for	AB110236	82	64.52	8.63907e−17	Ephemeroptera	Dipteromimidae	Dipteromimus sp. Ofunato−1
Analetris eximia 16S ribosomal RNA gene, partial s	AY749797	83.33	65.16	8.63907e−17	Ephemeroptera	Acanthametropodidae	Analetris eximia
Mesocapnia arizonensis mitochondrion, partial geno	KP642637	81.73	65.16	3.01533e−16	Plecoptera	Capniidae	Mesocapnia arizonensis
Allocapnia recta isolate H19 16S ribosomal RNA gen	KC881054	81.73	65.16	3.01533e−16	Plecoptera	Capniidae	Allocapnia recta
Allocapnia recta isolate H16 16S ribosomal RNA gen	KC881051	81.73	65.16	3.01533e−16	Plecoptera	Capniidae	Allocapnia recta
Allocapnia recta isolate H15 16S ribosomal RNA gen	KC881050	81.73	65.16	3.01533e−16	Plecoptera	Capniidae	Allocapnia recta
Allocapnia recta isolate H14 16S ribosomal RNA gen	KC881049	81.73	65.16	3.01533e−16	Plecoptera	Capniidae	Allocapnia recta
Allocapnia recta isolate H13 16S ribosomal RNA gen	KC881048	81.73	65.16	3.01533e−16	Plecoptera	Capniidae	Allocapnia recta
Allocapnia recta isolate H11 16S ribosomal RNA gen	KC881046	81.73	65.16	3.01533e−16	Plecoptera	Capniidae	Allocapnia recta
Allocapnia recta isolate H10 16S ribosomal RNA gen	KC881045	81.73	65.16	3.01533e−16	Plecoptera	Capniidae	Allocapnia recta
Allocapnia recta isolate H7 16S ribosomal RNA gene	KC881042	81.73	65.16	3.01533e−16	Plecoptera	Capniidae	Allocapnia recta
Allocapnia recta isolate H6 16S ribosomal RNA gene	KC881041	81.73	65.16	3.01533e−16	Plecoptera	Capniidae	Allocapnia recta
Allocapnia recta isolate H3 16S ribosomal RNA gene	KC881038	81.73	65.16	3.01533e−16	Plecoptera	Capniidae	Allocapnia recta
Bolotoperla rossi 16S ribosomal RNA gene, partial	EF623269	80.7	70.97	3.01533e−16	Plecoptera	Taeniopterygidae	Bolotoperla rossi
Salmoperla sylvanica 16S ribosomal RNA gene, parti	EF623222	82.18	64.52	3.01533e−16	Plecoptera	Perlodidae	Salmoperla sylvanica
Megarcys signata 16S ribosomal RNA gene, partial s	EF623215	82.18	64.52	3.01533e−16	Plecoptera	Perlodidae	Megarcys signata
Perlomyia utahensis 16S ribosomal RNA gene, partia	EF623179	82.83	63.23	3.01533e−16	Plecoptera	Leuctridae	Perlomyia utahensis
Diamphipnopsis sp. BYU_PL138 16S ribosomal RNA gen	EF623145	82.18	64.52	3.01533e−16	Plecoptera	Diamphipnoidae	Diamphipnopsis sp. BYU_PL138
Chloroperla tripunctata 16S ribosomal RNA gene, pa	EF623137	81.73	66.45	3.01533e−16	Plecoptera	Chloroperlidae	Chloroperla tripunctata
Allocapnia minima 16S ribosomal RNA gene, partial	EF623130	81.73	65.16	3.01533e−16	Plecoptera	Capniidae	Allocapnia minima
Mesocapnia sp. BYU_PL010 16S ribosomal RNA gene, p	EF623127	81.73	65.16	3.01533e−16	Plecoptera	Capniidae	Mesocapnia sp. BYU_PL010
Tasmanoperla larvalis 16S ribosomal RNA gene, part	EF623125	82.18	64.52	3.01533e−16	Plecoptera	Austroperlidae	Tasmanoperla larvalis
Allocapnia recta haplotype 12ar: H12 16S ribosomal	EU306875	81.73	65.16	3.01533e−16	Plecoptera	Capniidae	Allocapnia recta
Allocapnia recta haplotype 11ar: H011 16S ribosoma	EU306874	81.73	65.16	3.01533e−16	Plecoptera	Capniidae	Allocapnia recta
Allocapnia recta haplotype 7ar: H07 16S ribosomal	EU306870	81.73	65.16	3.01533e−16	Plecoptera	Capniidae	Allocapnia recta
Allocapnia recta haplotype 3ar: H03 16S ribosomal	DQ915181	81.73	65.16	3.01533e−16	Plecoptera	Capniidae	Allocapnia recta
Allocapnia recta haplotype 2ar: H02 16S ribosomal	DQ915180	81.73	65.16	3.01533e−16	Plecoptera	Capniidae	Allocapnia recta
Allocapnia recta isolate H18 16S ribosomal RNA gen	KC881053	81.55	64.52	1.05245e−15	Plecoptera	Capniidae	Allocapnia recta
Allocapnia recta isolate H17 16S ribosomal RNA gen	KC881052	81.55	64.52	1.05245e−15	Plecoptera	Capniidae	Allocapnia recta
Allocapnia recta isolate H12 16S ribosomal RNA gen	KC881047	81.55	64.52	1.05245e−15	Plecoptera	Capniidae	Allocapnia recta
Allocapnia recta isolate H9 16S ribosomal RNA gene	KC881044	81.55	64.52	1.05245e−15	Plecoptera	Capniidae	Allocapnia recta
Allocapnia recta isolate H8 16S ribosomal RNA gene	KC881043	81.55	64.52	1.05245e−15	Plecoptera	Capniidae	Allocapnia recta
Allocapnia recta isolate H5 16S ribosomal RNA gene	KC881040	81.55	64.52	1.05245e−15	Plecoptera	Capniidae	Allocapnia recta
Allocapnia recta isolate H4 16S ribosomal RNA gene	KC881039	81.55	64.52	1.05245e−15	Plecoptera	Capniidae	Allocapnia recta
Enicopus ater voucher UPOL001164 16S ribosomal RNA	HQ619670	80.36	71.61	1.05245e−15	Coleoptera	Melyridae	Enicopus ater
Simocephalus sp. EIZ−2011 isolate Brb1−Sim1 16S ri	JN874613	83.17	63.87	1.05245e−15	Diplostraca	Daphniidae	Simocephalus sp. EIZ−2011
Pachyrhamma edwardsii voucher WEL 16S ribosomal RN	HM594509	82.35	65.16	1.05245e−15	Orthoptera	Rhaphidophoridae	Pachyrhamma edwardsii
Dolichopoda hyrcana voucher LEN 16S ribosomal RNA	EU887871	81.19	65.16	1.05245e−15	Orthoptera	Rhaphidophoridae	Dolichopoda hyrcana
