# Supplementary figures and images for: Testing the potential of a ribosomal 16S marker for DNA metabarcoding of insects
Source: PeerJ. 2016 Apr 19;4:e1966. doi: 10.7717/peerj.1966 (PMC4841222; doi:10.7717/peerj.1966)

OTU\_124.csv

# Dinocras cephalotes

# Perlidae

# Plecoptera

96.95

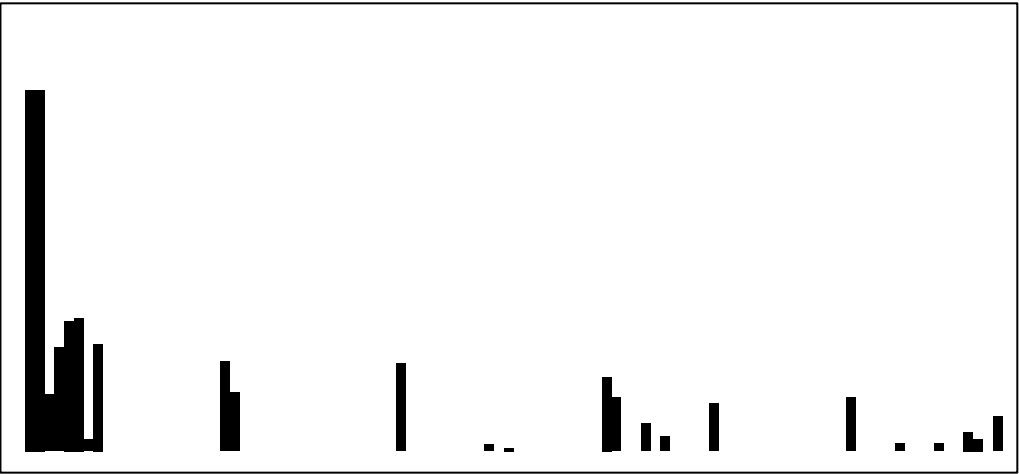

Supplement: Supplemental Information 1 [file peerj-04-1966-s006.zip › scripts/5 match OTUs/blast_hits/OTU_124.csv_plot.pdf]
