# Supplementary material for: Testing the potential of a ribosomal 16S marker for DNA metabarcoding of insects
Source: PeerJ. 2016 Apr 19;4:e1966. doi: 10.7717/peerj.1966 (PMC4841222; doi:10.7717/peerj.1966)
Supplement: Supplemental Information 1 [file OTU_108.csv_plot.pdf › scripts/5 match OTUs/blast_hits/OTU_108.csv_plot.pdf]

OTU_108.csv

Velia hoberlandti

Veliidae

Hemiptera

88.16

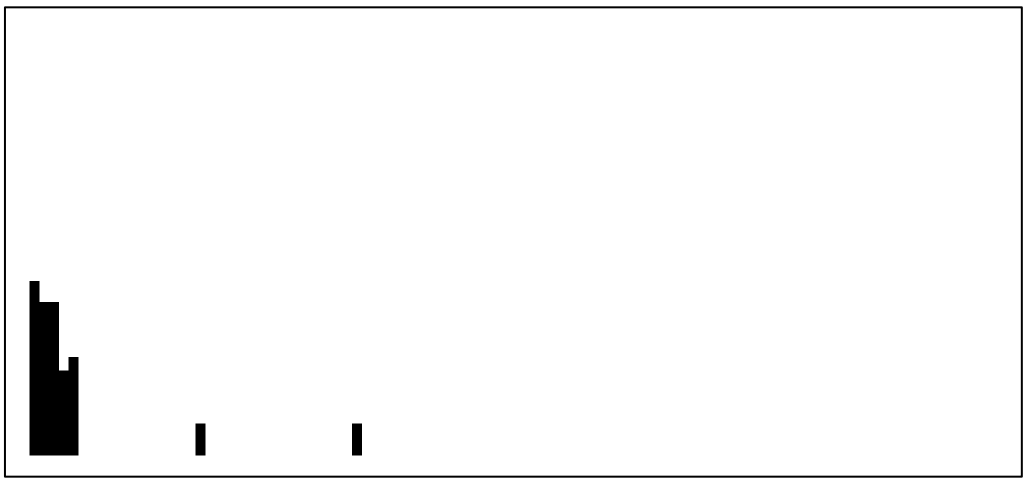

Velia hoberlandti isolate 941 16S ribosomal RNA ge	EU871188	88.16	97.2	3.07515e−47	Hemiptera	Veliidae	Velia hoberlandti
Velia caprai isolate 943 16S ribosomal RNA gene, p	EU871185	87.18	100	1.07333e−46	Hemiptera	Veliidae	Velia caprai
Velia caprai 16S ribosomal RNA gene, partial seque	AY648139	87.18	100	1.07333e−46	Hemiptera	Veliidae	Velia caprai
Velia affinis isolate 373 16S ribosomal RNA gene,	EU871189	83.97	100	1.22467e−39	Hemiptera	Veliidae	Velia affinis
Velia maderensis isolate 1862 16S ribosomal RNA ge	EU871187	84.62	100	1.22467e−39	Hemiptera	Veliidae	Velia maderensis
Perittopus asiaticus isolate 846 16S ribosomal RNA	EU871165	77.3	100	5.56491e−25	Hemiptera	Veliidae	Perittopus asiaticus
Rhagovelia sp. 1 JD−2008 isolate 1602 16S ribosoma	EU871178	76.43	100	2.36626e−23	Hemiptera	Veliidae	Rhagovelia sp. 1 JD−2008
Rhagovelia robusta voucher MC35a 16S ribosomal RNA	KC594836	76.13	99.3	1.22575e−20	Hemiptera	Veliidae	Rhagovelia robusta
Ventidius usingeri isolate 1059 16S ribosomal RNA	EU871207	75.64	97.2	1.49327e−19	Hemiptera	Gerridae	Ventidius usingeri
Rhagovelia uncinata isolate 666 16S ribosomal RNA	EU871181	75.68	93.71	1.49327e−19	Hemiptera	Veliidae	Rhagovelia uncinata
Tenagogonus madagascariensis isolate 1240 16S larg	KC880885	74.36	97.2	1.81918e−18	Hemiptera	Gerridae	Tenagogonus madagascariensis
Limnogonus hungerfordi isolate 1431 16S large subu	GU645089	74.84	97.2	1.81918e−18	Hemiptera	Gerridae	Limnogonus hungerfordi
Rheumatogonus luzonicus isolate 1062 16S ribosomal	EU871202	74.36	97.2	1.81918e−18	Hemiptera	Gerridae	Rheumatogonus luzonicus
Limnoporus esakii 16S ribosomal RNA gene, partial	AY425216	74.19	97.2	1.81918e−18	Hemiptera	Gerridae	Limnoporus esakii
Limnoporus esakii mitochondrial gene for 16S ribos	AB026592	74.19	97.2	1.81918e−18	Hemiptera	Gerridae	Limnoporus esakii
Limnogonus luctuosus isolate 2294 16S large subuni	GU645092	74.36	97.2	6.34956e−18	Hemiptera	Gerridae	Limnogonus luctuosus
Microvelia reticulata isolate 292 16S ribosomal RN	EU871172	73.72	97.2	6.34956e−18	Hemiptera	Veliidae	Microvelia reticulata
Rheumatogonus luzonicus mitochondrial gene for 16S	AB026609	81.48	74.13	6.34956e−18	Hemiptera	Gerridae	Rheumatogonus luzonicus
Rhagovelia tenuipes voucher MC36a 16S ribosomal RN	KC594837	76	93.71	2.21621e−17	Hemiptera	Veliidae	Rhagovelia tenuipes
Gerris gracilicornis mitochondrial genes for 12S r	AB679162	74.03	97.2	2.21621e−17	Hemiptera	Gerridae	Gerris gracilicornis
Gerris gracilicornis mitochondrial genes for 12S r	AB679161	74.03	97.2	2.21621e−17	Hemiptera	Gerridae	Gerris gracilicornis
Gerris gracilicornis mitochondrial genes for 12S r	AB679160	74.03	97.2	2.21621e−17	Hemiptera	Gerridae	Gerris gracilicornis
Gerris argenticollis 16S large subunit ribosomal R	AY951329	74.19	97.2	2.21621e−17	Hemiptera	Gerridae	Gerris argenticollis
Gerris caucasicus 16S large subunit ribosomal RNA	AY951328	74.84	97.2	2.21621e−17	Hemiptera	Gerridae	Gerris caucasicus
Gerris odontogaster 16S ribosomal RNA gene, partia	AY425219	74.84	97.2	2.21621e−17	Hemiptera	Gerridae	Gerris odontogaster
Gerris sphagnetorum 16S ribosomal RNA gene, partia	AY425206	74.05	97.2	2.21621e−17	Hemiptera	Gerridae	Gerris sphagnetorum
Gerris argentatus 16S ribosomal RNA gene, partial	AY425194	74.84	97.2	2.21621e−17	Hemiptera	Gerridae	Gerris argentatus
Gerris nepalensis 16S ribosomal RNA gene, partial	AY425192	74.84	97.2	2.21621e−17	Hemiptera	Gerridae	Gerris nepalensis
Gerris buenoi 16S ribosomal RNA gene, partial sequ	AY425191	74.84	97.2	2.21621e−17	Hemiptera	Gerridae	Gerris buenoi
Gerris babai mitochondrial gene for 16S ribosomal	AB026585	74.84	97.2	2.21621e−17	Hemiptera	Gerridae	Gerris babai
Limnogonus aduncus voucher MC32a 16S ribosomal RNA	KC594827	74.19	97.2	7.73535e−17	Hemiptera	Gerridae	Limnogonus aduncus
Limnogonus nitidus isolate 2141 16S large subunit	GU645067	74.19	97.2	7.73535e−17	Hemiptera	Gerridae	Limnogonus nitidus
Limnogonus ignotus isolate 2343 16S large subunit	GU645059	74.19	97.2	7.73535e−17	Hemiptera	Gerridae	Limnogonus ignotus
Asclepios annandalei isolate H169 16S ribosomal RN	EU871206	81.48	74.13	7.73535e−17	Hemiptera	Gerridae	Asclepios annandalei
Eurygerris fuscinervis 16S ribosomal RNA gene, par	AY648137	75	97.2	7.73535e−17	Hemiptera	Gerridae	Eurygerris fuscinervis
Limnoporus dissortis 16S ribosomal RNA gene, parti	AY425228	73.55	97.2	7.73535e−17	Hemiptera	Gerridae	Limnoporus dissortis
Limnoporus notabilis 16S ribosomal RNA gene, parti	AY425210	73.55	97.2	7.73535e−17	Hemiptera	Gerridae	Limnoporus notabilis
Aquarius ventralis 16S ribosomal RNA gene, partial	AY425196	73.86	97.2	7.73535e−17	Hemiptera	Gerridae	Aquarius ventralis
Limnoporus genitalis mitochondrial gene for 16S ri	AB026591	73.55	97.2	7.73535e−17	Hemiptera	Gerridae	Limnoporus genitalis
Leptinopterus femoratus voucher SK231 16S ribosoma	KP250254	73.58	98.6	2.6999e−16	Coleoptera	Lucanidae	Leptinopterus femoratus
Amemboa sp. JD−2014 isolate 2440 16S large subunit	KC880875	73.89	97.2	2.6999e−16	Hemiptera	Gerridae	Amemboa sp. JD−2014
Gerris gracilicornis mitochondrial genes for 12S r	AB679157	73.38	97.2	2.6999e−16	Hemiptera	Gerridae	Gerris gracilicornis
Gerris gracilicornis mitochondrial genes for 12S r	AB679158	73.38	97.2	2.6999e−16	Hemiptera	Gerridae	Gerris gracilicornis
Gerris gracilicornis mitochondrial genes for 12S r	AB679152	73.38	97.2	2.6999e−16	Hemiptera	Gerridae	Gerris gracilicornis
Gerris gracilicornis mitochondrial genes for 12S r	AB679151	73.38	97.2	2.6999e−16	Hemiptera	Gerridae	Gerris gracilicornis
Gerris gracilicornis mitochondrial genes for 12S r	AB679150	73.38	97.2	2.6999e−16	Hemiptera	Gerridae	Gerris gracilicornis
Gerris gracilicornis mitochondrial genes for 12S r	AB679149	73.38	97.2	2.6999e−16	Hemiptera	Gerridae	Gerris gracilicornis
Gerris brasili 16S ribosomal RNA gene, partial seq	AY425229	73.38	97.2	2.6999e−16	Hemiptera	Gerridae	Gerris brasili
Gerris swakopensis 16S ribosomal RNA gene, partial	AY425223	73.38	97.2	2.6999e−16	Hemiptera	Gerridae	Gerris swakopensis
Gerris yezoensis 16S ribosomal RNA gene, partial s	AY425203	73.38	97.2	2.6999e−16	Hemiptera	Gerridae	Gerris yezoensis
Gerris gracilicornis 16S ribosomal RNA gene, parti	AY425201	73.38	97.2	2.6999e−16	Hemiptera	Gerridae	Gerris gracilicornis
Limnometra matsudai isolate 256 16S large subunit	KC880900	72.9	97.2	9.42359e−16	Hemiptera	Gerridae	Limnometra matsudai
Gerris gracilicornis mitochondrial genes for 12S r	AB679153	72.9	97.2	9.42359e−16	Hemiptera	Gerridae	Gerris gracilicornis
Limnogonus windi isolate 2394 16S large subunit ri	GU645097	73.25	97.2	9.42359e−16	Hemiptera	Gerridae	Limnogonus windi
Limnogonus skusei isolate 149 16S large subunit ri	GU645094	73.55	97.2	9.42359e−16	Hemiptera	Gerridae	Limnogonus luctuosus
Limnogonus hungerfordi isolate 207 16S large subun	GU645085	73.55	97.2	9.42359e−16	Hemiptera	Gerridae	Limnogonus hungerfordi
Limnogonus intermedius isolate 140 16S large subun	GU645053	73.55	97.2	9.42359e−16	Hemiptera	Gerridae	Limnogonus intermedius
Rheumatometroides sp. JD−2008 isolate 971 16S ribo	EU871193	74.5	91.61	9.42359e−16	Hemiptera	Gerridae	Rheumatometroides sp. JD−2008
Potamometropsis sumaldei 16S ribosomal RNA gene, p	AY743237	73.25	97.2	9.42359e−16	Hemiptera	Gerridae	Potamometropsis sumaldei
Aquarius philippinensis 16S large subunit ribosoma	AY951324	74.36	97.2	9.42359e−16	Hemiptera	Gerridae	Aquarius philippinensis
Aquarius elongatus 16S ribosomal RNA gene, partial	AY425200	74.19	97.2	9.42359e−16	Hemiptera	Gerridae	Aquarius elongatus
Aquarius najas 16S ribosomal RNA gene, partial seq	AY425199	73.55	97.2	9.42359e−16	Hemiptera	Gerridae	Aquarius najas
Aquarius remigis 16S ribosomal RNA gene, partial s	AY425190	72.9	97.2	9.42359e−16	Hemiptera	Gerridae	Aquarius remigis
Rhagovelia esakii mitochondrial gene for 16S ribos	AB026611	73.55	100	9.42359e−16	Hemiptera	Veliidae	Rhagovelia esakii
Aquarius elongatus mitochondrial gene for 16S ribo	AB026593	74.19	97.2	9.42359e−16	Hemiptera	Gerridae	Aquarius elongatus
Limnogonus pectoralis isolate 141 16S large subuni	GU645072	75.76	81.12	3.28915e−15	Hemiptera	Gerridae	Limnogonus pectoralis
Gelastocoris oculatus isolate 498 16S ribosomal RN	AY585760	74.52	97.2	3.28915e−15	Hemiptera	Gelastocoridae	Gelastocoris oculatus
Rhagovelia sumatrensis 16S ribosomal RNA gene, par	AY648126	73.25	100	3.28915e−15	Hemiptera	Veliidae	Rhagovelia sumatrensis
Aquarius lili 16S ribosomal RNA gene, partial sequ	AY425232	73.89	97.2	3.28915e−15	Hemiptera	Gerridae	Aquarius lili
Aquarius adelaidis 16S ribosomal RNA gene, partial	AY425202	73.89	97.2	3.28915e−15	Hemiptera	Gerridae	Aquarius adelaidis
Gerris nepalensis mitochondrial gene for 16S ribos	AB026587	74.36	97.2	3.28915e−15	Hemiptera	Gerridae	Gerris nepalensis
Tenagogonus australiensis isolate 1479 16S large s	KC880887	75.18	83.92	1.14803e−14	Hemiptera	Gerridae	Tenagogonus australiensis
Limnogonus aduncus isolate 2344 16S large subunit	GU645058	72.61	97.2	1.14803e−14	Hemiptera	Gerridae	Limnogonus aduncus
Limnogonus aduncus isolate 1430 16S large subunit	GU645056	73.25	97.2	1.14803e−14	Hemiptera	Gerridae	Limnogonus aduncus
Halovelia sp. JD−2008 isolate 1950 16S ribosomal R	EU871176	73.91	87.41	1.14803e−14	Hemiptera	Veliidae	Halovelia sp. JD−2008
Microvelia torquata isolate 435 16S ribosomal RNA	EU871174	73.55	97.2	1.14803e−14	Hemiptera	Veliidae	Microvelia torquata
Gerris sahlbergi isolate 109 16S ribosomal RNA gen	DQ988688	73.55	97.2	1.14803e−14	Hemiptera	Gerridae	Gerris sahlbergi
Nerthra fuscipes isolate 499 16S ribosomal RNA gen	AY585759	72.85	93.71	1.14803e−14	Hemiptera	Gelastocoridae	Nerthra fuscipes
Aquarius cinereus 16S ribosomal RNA gene, partial	AY425234	72.73	97.2	1.14803e−14	Hemiptera	Gerridae	Aquarius cinereus
Gerris lacustris 16S ribosomal RNA gene, partial s	AY425227	73.55	97.2	1.14803e−14	Hemiptera	Gerridae	Gerris lacustris
Gerris costae costae 16S ribosomal RNA gene, parti	AY425226	73.55	97.2	1.14803e−14	Hemiptera	Gerridae	Gerris costae costae
Gerris latiabdominis 16S ribosomal RNA gene, parti	AY425215	73.55	97.2	1.14803e−14	Hemiptera	Gerridae	Gerris latiabdominis
Gerris gibbifer 16S ribosomal RNA gene, partial se	AY425195	73.55	97.2	1.14803e−14	Hemiptera	Gerridae	Gerris gibbifer
Gerris argenticollis 16S ribosomal RNA gene, parti	AY425193	73.55	97.2	1.14803e−14	Hemiptera	Gerridae	Gerris argenticollis
Gerris latiabdominis mitochondrial gene for 16S ri	AB026586	73.55	97.2	1.14803e−14	Hemiptera	Gerridae	Gerris latiabdominis
Lyctocoris sp. SJ−2013 16S ribosomal RNA gene, par	JQ782757	72.85	93.71	4.00701e−14	Hemiptera	Lyctocoridae	Lyctocoris sp. SJ−2013
Microvelia longipes voucher MC33a 16S ribosomal RN	KC594830	72.9	97.2	4.00701e−14	Hemiptera	Veliidae	Microvelia longipes
Gerris gracilicornis mitochondrial genes for 12S r	AB679164	72.26	97.2	4.00701e−14	Hemiptera	Gerridae	Gerris gracilicornis
Gerris gracilicornis mitochondrial genes for 12S r	AB679163	72.26	97.2	4.00701e−14	Hemiptera	Gerridae	Gerris gracilicornis
Gerris gracilicornis mitochondrial genes for 12S r	AB679156	72.26	97.2	4.00701e−14	Hemiptera	Gerridae	Gerris gracilicornis
Gerris gracilicornis mitochondrial genes for 12S r	AB679154	72.26	97.2	4.00701e−14	Hemiptera	Gerridae	Gerris gracilicornis
Gerris sp. YT−2009 16S ribosomal RNA gene, partial	FJ372633	72.26	97.2	4.00701e−14	Hemiptera	Gerridae	Gerris sp. YT−2009
Lyctocoris beneficus 16S ribosomal RNA gene, parti	GQ258369	72.85	93.71	4.00701e−14	Hemiptera	Lyctocoridae	Lyctocoris beneficus
Limnogonus hungerfordi isolate 148 16S large subun	GU645084	72.9	97.2	4.00701e−14	Hemiptera	Gerridae	Limnogonus hungerfordi
Limnogonus nitidus isolate 1983 16S large subunit	GU645066	72.9	97.2	4.00701e−14	Hemiptera	Gerridae	Limnogonus nitidus
Limnogonus capensis isolate 1806 16S large subunit	GU645047	73.58	97.2	4.00701e−14	Hemiptera	Gerridae	Limnogonus capensis
Xenobates argentatus isolate 973 16S ribosomal RNA	EU871177	73.57	87.41	4.00701e−14	Hemiptera	Veliidae	Xenobates argentatus
Lyctocoris beneficus 16S ribosomal RNA gene, parti	EF487282	72.85	93.71	4.00701e−14	Hemiptera	Lyctocoridae	Lyctocoris beneficus
Aquarius amplus 16S ribosomal RNA gene, partial se	AY425182	72.26	97.2	4.00701e−14	Hemiptera	Gerridae	Aquarius amplus
Rhagovelia sp. Los Banos mitochondrial gene for 16	AB026610	71.33	94.41	4.00701e−14	Hemiptera	Veliidae	Rhagovelia sp. Los Banos
