# Supplementary material for: Testing the potential of a ribosomal 16S marker for DNA metabarcoding of insects
Source: PeerJ. 2016 Apr 19;4:e1966. doi: 10.7717/peerj.1966 (PMC4841222; doi:10.7717/peerj.1966)
Supplement: Supplemental Information 1 [file OTU_111.csv_plot.pdf › scripts/5 match OTUs/blast_hits/OTU_111.csv_plot.pdf]

OTU_111.csv

Entomobrya aino

Entomobryidae

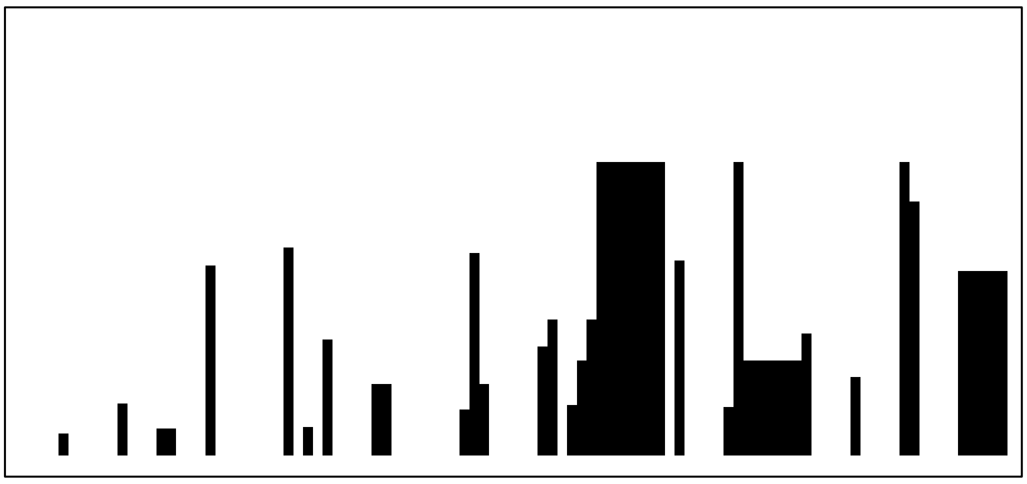

79.8

Entomobrya aino 16S ribosomal RNA gene, partial se	KC236195	79.8	70.9	5.45135e−12		Entomobryidae	Entomobrya aino
Himalanura sp. FZ−2013 16S ribosomal RNA gene, par	KC236202	79.78	64.18	3.44018e−08		Entomobryidae	Himalanura sp. FZ−2013
Orchesella villosa 16S ribosomal RNA gene, partial	AY555550	78.07	82.84	3.44018e−08		Entomobryidae	Orchesella villosa
Pseudoparonella sp. BXW−2014 16S ribosomal RNA gen	KM978385	81.01	57.46	4.191e−07		Paronellidae	Pseudoparonella sp. BXW−2014
Lepidocyrtus sp. 2 FZ−2013 16S ribosomal RNA gene,	KC236207	73.73	85.07	4.191e−07		Entomobryidae	Lepidocyrtus sp. 2 FZ−2013
Willowsia sp. 1 FZ−2013 16S ribosomal RNA gene, pa	KC236205	77.27	65.67	4.191e−07		Entomobryidae	Willowsia sp. 1 FZ−2013
Isotoma viridis 16S ribosomal RNA gene, partial se	JN970946	75.73	73.13	4.191e−07		Isotomidae	Isotoma viridis
Sinella curviseta 16S ribosomal RNA gene, partial	KC236219	75.24	76.87	5.10568e−06		Entomobryidae	Sinella curviseta
Entomobrya proxima 16S ribosomal RNA gene, partial	KC236197	75.7	78.36	5.10568e−06		Entomobryidae	Entomobrya proxima
Helicoradomenia sp. AO−2003 16S ribosomal RNA gene	AY377613	82.43	54.48	5.10568e−06	Cavibelonia	Simrothiellidae	Helicoradomenia sp. AO−2003
Willowsia japonica 16S ribosomal RNA gene, partial	KC236224	75.79	70.15	6.21999e−05		Entomobryidae	Willowsia japonica
Orchesella cincta 16S ribosomal RNA gene, partial	JN970973	72.27	79.1	6.21999e−05		Entomobryidae	Orchesella cincta
Bilobella aurantiaca mitochondrion, complete genom	EU084034	74.19	60.45	6.21999e−05		Neanuridae	Bilobella aurantiaca
Achatinella lila isolate 2 16S ribosomal RNA gene,	AF159473	81.25	45.52	6.21999e−05		Achatinellidae	Achatinella lila
Achatinella lila isolate 1 16S ribosomal RNA gene,	AF159472	81.25	45.52	6.21999e−05		Achatinellidae	Achatinella lila
Aivalykus sp. CNIN952 16S ribosomal RNA gene, part	KJ586753	75	57.46	0.000217099	Hymenoptera	Braconidae	Aivalykus sp. CNIN952
Orchesellides sinensis 16S ribosomal RNA gene, par	KC236209	77.91	57.46	0.000217099		Entomobryidae	Orchesellides sinensis
Teinobasis ariel isolate OD205 16S ribosomal RNA g	JQ966662	78.16	61.19	0.000217099	Odonata	Coenagrionidae	Teinobasis ariel
Callopora sp. QY−2004 16S ribosomal RNA gene, part	AY789089	88.89	33.58	0.000217099	Cheilostomatida	Calloporidae	Callopora sp. QY−2004
Daphnia similis from Israel 16S ribosomal RNA gene	AY921455	77.63	55.22	0.000217099	Diplostraca	Daphniidae	Daphnia similis
Sminthurinus bimaculatus 16S ribosomal RNA gene, p	AY555555	77.63	55.97	0.000217099		Katiannidae	Sminthurinus bimaculatus
Pseudoparonella tanimbarensis 16S ribosomal RNA ge	KM978384	73.96	68.66	0.00075775		Paronellidae	Pseudoparonella tanimbarensis
Lepidocyrtus (Ascocyrtus) sp. 2 FZ−2013 16S riboso	KC236190	73.96	68.66	0.00075775		Entomobryidae	Lepidocyrtus (Ascocyrtus) sp. 2 FZ−2013
Protoplasa fitchii 16S ribosomal RNA gene, partial	KC177462	76.06	48.51	0.00075775	Diptera	Tanyderidae	Protoplasa fitchii
Protoplasa fitchii mitochondrion, complete genome	JN861746	76.06	48.51	0.00075775	Diptera	Tanyderidae	Protoplasa fitchii
Cryptopygus antarcticus mitochondrion, complete ge	EU016194	73.33	60.45	0.00075775		Isotomidae	Cryptopygus antarcticus
Thalamoporella californica 16S ribosomal RNA gene,	AF156293	89.74	29.1	0.00075775	Cheilostomatida	Thalamoporellidae	Thalamoporella californica
Perdicella helena 16S ribosomal RNA gene, partial	AF159494	79.45	50	0.00075775		Achatinellidae	Perdicella helena
Partulina proxima isolate 2 16S ribosomal RNA gene	AF159492	81.33	55.22	0.00075775		Achatinellidae	Partulina proxima
Achatinella fulgens 16S ribosomal RNA gene, partia	AF159474	79.41	50	0.00075775		Achatinellidae	Achatinella fulgens
Olea hansineensis isolate Ol_han_10FHL01 16S ribos	KM204269	85.42	35.82	0.00264481		Limapontiidae	Olea hansineensis
Tomocerus nigrus voucher HB0851_2 16S ribosomal RN	KF591926	75.32	57.46	0.00264481		Tomoceridae	Tomocerus nigrus
Ethmostigmus rubripes rubripes voucher IZ130653 16	KF676475	75	62.69	0.00264481	Scolopendromorpha	Scolopendridae	Ethmostigmus rubripes rubripes
Willowsia guangdongensis 16S ribosomal RNA gene, p	KC236223	75	58.21	0.00264481		Entomobryidae	Willowsia guangdongensis
Orchesella cincta 16S ribosomal RNA gene, partial	KC236208	71.07	80.6	0.00264481		Entomobryidae	Orchesella cincta
Semirhynchia sp. n. SV01 16S ribosomal RNA gene, p	JX294172	83.33	38.06	0.00264481	Neuroptera	Nemopteridae	Semirhynchia sp. n. SV01
Sicyoptera dilatata isolate SDGG01 16S ribosomal R	JX294163	83.33	38.06	0.00264481	Neuroptera	Nemopteridae	Sicyoptera dilatata
Paramunida belone isolate PAR74 16S ribosomal RNA	GU814731	76.14	62.69	0.00264481	Decapoda	Munididae	Paramunida belone
Paramunida belone isolate PAR73 16S ribosomal RNA	GU814730	76.14	62.69	0.00264481	Decapoda	Munididae	Paramunida belone
Paramunida belone isolate PAR72 16S ribosomal RNA	GU814729	76.14	62.69	0.00264481	Decapoda	Munididae	Paramunida belone
Paramunida belone isolate PAR130 16S ribosomal RNA	GU814655	76.14	62.69	0.00264481	Decapoda	Munididae	Paramunida belone
Paramunida belone isolate PAR129 16S ribosomal RNA	GU814653	76.14	62.69	0.00264481	Decapoda	Munididae	Paramunida belone
Paramunida belone isolate PAR72 16S ribosomal RNA	HM173512	76.14	62.69	0.00264481	Decapoda	Munididae	Paramunida belone
Paramunida belone isolate PAR129 16S ribosomal RNA	HM173406	76.14	62.69	0.00264481	Decapoda	Munididae	Paramunida belone
Heteromenopon psittacum 16S ribosomal RNA gene, pa	GU569239	82.14	39.55	0.00264481	Phthiraptera	Menoponidae	Heteromenopon psittacum
Cf. Spionidae sp. DH−2009 isolate A7polych06 16S r	GU227035	89.47	28.36	0.00264481	Spionida	Spionidae	cf. Spionidae sp. DH−2009
Lertha barbara 16S ribosomal RNA gene, partial seq	EU734879	83.33	38.06	0.00264481	Neuroptera	Nemopteridae	Lertha barbara
Everettia jucunda voucher P970 16S ribosomal RNA g	FJ160636	77.14	52.24	0.00264481		Ariophantidae	Everettia jucunda
Paramunida belone voucher Fo154 16S ribosomal RNA	AY351199	76.14	62.69	0.00264481	Decapoda	Munididae	Paramunida belone
Paramunida belone voucher Fo196 16S ribosomal RNA	AY351198	76.14	62.69	0.00264481	Decapoda	Munididae	Paramunida belone
Spodoptera picta 16S ribosomal RNA gene, partial s	AF173066	74.16	64.18	0.00264481	Lepidoptera	Noctuidae	Spodoptera picta
Neanura muscorum 16S ribosomal RNA gene, partial s	AY555553	78.38	52.99	0.00264481		Neanuridae	Neanura muscorum
Gascoignella jabae isolate Ga_jab_09Tha01 16S ribo	KM204256	85.11	35.07	0.00923128		Platyhedylidae	Gascoignella jabae
Siphonaria belcheri isolate S2 16S ribosomal RNA g	KM492938	86.36	32.84	0.00923128		Siphonariidae	Siphonaria belcheri
Paramunida salai isolate PAR222 16S ribosomal RNA	KJ021652	76.92	56.72	0.00923128	Decapoda	Munididae	Paramunida salai
Valvata sp. II CC−2014 voucher UGSB 11149 16S larg	KJ186055	82.35	36.57	0.00923128		Valvatidae	Valvata sp. II CC−2014
Petaloconchus varians isolate Petvarians0315946 12	KC583381	84.44	33.58	0.00923128		Vermetidae	Petaloconchus varians
Siphonaria sp. UF 296586 clone 690−1 16S ribosomal	KF000900	86.36	32.84	0.00923128		Siphonariidae	Siphonaria sp. UF 296586
Arenaeus cribrarius isolate 11 voucher CCDB<BRA>:3	JX123468	93.75	23.88	0.00923128	Decapoda	Portunidae	Arenaeus cribrarius
Arenaeus cribrarius voucher CCDB<BRA>:3182 16S rib	JX123466	93.75	23.88	0.00923128	Decapoda	Portunidae	Arenaeus cribrarius
Arenaeus cribrarius voucher CCDB<BRA>:291 16S ribo	JX123463	93.75	23.88	0.00923128	Decapoda	Portunidae	Arenaeus cribrarius
Arenaeus cribrarius voucher CCDB<BRA>:1536 16S rib	JX123462	93.75	23.88	0.00923128	Decapoda	Portunidae	Arenaeus cribrarius
Arenaeus cribrarius voucher CCDB<BRA>:3391 16S rib	JX123460	93.75	23.88	0.00923128	Decapoda	Portunidae	Arenaeus cribrarius
Arenaeus cribrarius voucher CCDB<BRA>:3635 16S rib	JX123459	93.75	23.88	0.00923128	Decapoda	Portunidae	Arenaeus cribrarius
Arenaeus cribrarius voucher MZUCR:2470−10 16S ribo	JX123457	93.75	23.88	0.00923128	Decapoda	Portunidae	Arenaeus cribrarius
Lepidocyrtus sp. 1 FZ−2013 16S ribosomal RNA gene,	KC236206	71.43	80.6	0.00923128		Entomobryidae	Lepidocyrtus sp. 1 FZ−2013
Learchis poica 16S ribosomal RNA gene, partial seq	JQ699556	89.13	32.84	0.00923128	Nudibranchia	Facelinidae	Learchis poica
Spodoptera dolichos voucher SPO−B2 16S ribosomal R	HQ178271	77.46	51.49	0.00923128	Lepidoptera	Noctuidae	Spodoptera dolichos
Spodoptera evanida voucher SPO−B155 16S ribosomal	HQ178269	77.46	51.49	0.00923128	Lepidoptera	Noctuidae	Spodoptera evanida
Sternopriscus eikei mitochondrial partial 16S rRNA	HE818947	74.42	64.18	0.00923128	Coleoptera	Dytiscidae	Sternopriscus eikei
Paramunida salai isolate PAR177 16S ribosomal RNA	HM173450	76.92	56.72	0.00923128	Decapoda	Munididae	Paramunida salai
Boletina villosa isolate 014966 16S ribosomal RNA	HQ230374	82.26	45.52	0.00923128	Diptera	Mycetophilidae	Boletina villosa
Arenaeus cribrarius mitochondrial 16S rRNA gene (p	FM208749	93.75	23.88	0.00923128	Decapoda	Portunidae	Arenaeus cribrarius
Petaloconchus cf. varians AM−2010 voucher MNCN:Pet	GU290143	84.44	33.58	0.00923128		Vermetidae	Petaloconchus cf. varians AM−2010
Petaloconchus cf. varians AM−2010 voucher MNCN:Pet	GU290133	84.44	33.58	0.00923128		Vermetidae	Petaloconchus cf. varians AM−2010
Petaloconchus cf. varians AM−2010 voucher MNCN:Pet	GU290141	84.44	33.58	0.00923128		Vermetidae	Petaloconchus cf. varians AM−2010
Petaloconchus cf. varians AM−2010 voucher MNCN:Pet	GU290139	84.44	33.58	0.00923128		Vermetidae	Petaloconchus cf. varians AM−2010
Petaloconchus cf. varians AM−2010 voucher MNCN:Pet	GU290138	84.44	33.58	0.00923128		Vermetidae	Petaloconchus cf. varians AM−2010
Petaloconchus cf. varians AM−2010 voucher MNCN:Pet	GU290131	84.44	33.58	0.00923128		Vermetidae	Petaloconchus cf. varians AM−2010
Petaloconchus cf. varians AM−2010 voucher MNCN:Pet	GU290130	85.71	31.34	0.00923128		Vermetidae	Petaloconchus cf. varians AM−2010
Ornithoptera priamus voucher FS−b−3776 16S ribosom	GQ268350	77.27	47.76	0.00923128	Lepidoptera	Papilionidae	Ornithoptera priamus
Progoniada regularis 16S ribosomal RNA gene, parti	GQ426616	78.46	44.78	0.00923128	Phyllodocida	Goniadidae	Progoniada regularis
Paramunida salai isolate S29 16S ribosomal RNA gen	EU417985	76.92	56.72	0.00923128	Decapoda	Munididae	Paramunida salai
Paramunida salai isolate S26 16S ribosomal RNA gen	EU417982	76.92	56.72	0.00923128	Decapoda	Munididae	Paramunida salai
Munida oblongata isolate S22 16S ribosomal RNA gen	EU417978	83.67	36.57	0.00923128	Decapoda	Munididae	Munida oblongata
Paramunida salai isolate S7 16S ribosomal RNA gene	EU417970	76.92	56.72	0.00923128	Decapoda	Munididae	Paramunida salai
Plataspididae sp. 2 WCW−2007 16S ribosomal RNA gen	EF641142	76.71	48.51	0.00923128	Hemiptera	Plataspidae	Plataspididae sp. 2 WCW−2007
Ornithoptera priamus mitochondrial partial 16S rRN	AM283052	77.27	47.76	0.00923128	Lepidoptera	Papilionidae	Ornithoptera priamus
Eupithecia proterva gene for 16S rRNA, partial seq	AB265421	76.06	51.49	0.00923128	Lepidoptera	Geometridae	Eupithecia proterva
Arenaeus cribrarius voucher ULLZ−5173 16S ribosoma	DQ407667	93.75	23.88	0.00923128	Decapoda	Portunidae	Arenaeus cribrarius
Alderia modesta haplotype 1 16S ribosomal RNA gene	DQ364408	91.89	26.87	0.00923128		Limapontiidae	Alderia modesta
Spodoptera evanida 16S ribosomal RNA gene, partial	AF173062	77.46	51.49	0.00923128	Lepidoptera	Noctuidae	Spodoptera evanida
Spodoptera dolichos 16S ribosomal RNA gene, partia	AF173053	77.46	51.49	0.00923128	Lepidoptera	Noctuidae	Spodoptera dolichos
Epimenia australis 16S ribosomal RNA gene, partial	AY377614	77.78	58.21	0.00923128	Cavibelonia	Epimeniidae	Epimenia australis
Panorpa claripennis 16S ribosomal RNA gene, partia	AF180068	78.26	50.75	0.00923128	Mecoptera	Panorpidae	Panorpa claripennis
Achatinella sowerbyana isolate 5 16S ribosomal RNA	AF159468	88.64	32.09	0.00923128		Achatinellidae	Achatinella sowerbyana
Achatinella sowerbyana isolate 2 16S ribosomal RNA	AF159467	88.64	32.09	0.00923128		Achatinellidae	Achatinella sowerbyana
Achatinella sowerbyana isolate 1 16S ribosomal RNA	AF159466	88.64	32.09	0.00923128		Achatinellidae	Achatinella sowerbyana
Achatinella livida isolate 2 16S ribosomal RNA gen	AF159465	88.64	32.09	0.00923128		Achatinellidae	Achatinella livida
Achatinella livida isolate 1 16S ribosomal RNA gen	AF159464	88.64	32.09	0.00923128		Achatinellidae	Achatinella livida
