# Supplementary material for: Testing the potential of a ribosomal 16S marker for DNA metabarcoding of insects
Source: PeerJ. 2016 Apr 19;4:e1966. doi: 10.7717/peerj.1966 (PMC4841222; doi:10.7717/peerj.1966)
Supplement: Supplemental Information 1 [file OTU_126.csv_plot.pdf › scripts/5 match OTUs/blast_hits/OTU_126.csv_plot.pdf]

OTU_126.csv

Neanura muscorum

Neanuridae

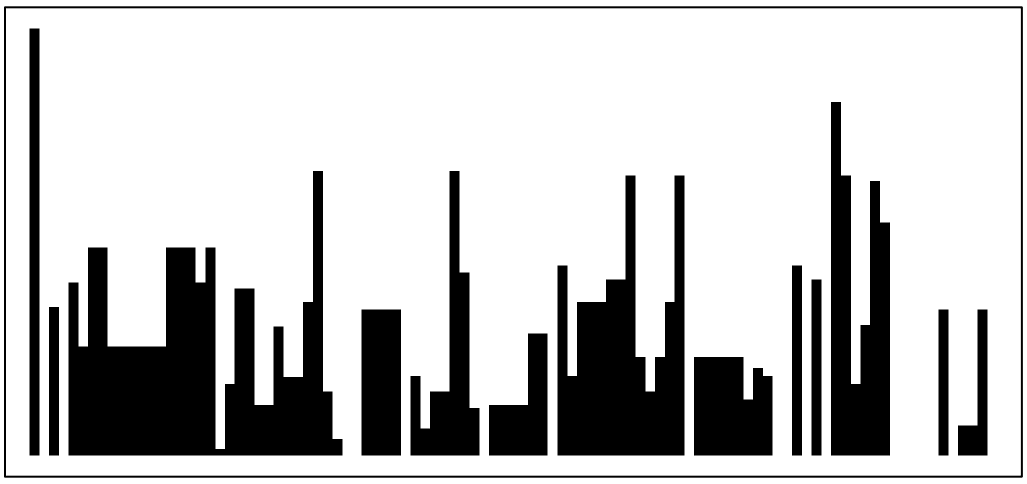

100

Neanura muscorum 16S ribosomal RNA gene, partial s	AY555553	100	100	3.10088e−66		Neanuridae	Neanura muscorum
Bilobella aurantiaca mitochondrion, complete genom	EU084034	76.92	61.81	1.96206e−05		Neanuridae	Bilobella aurantiaca
Heteromenopon psittacum 16S ribosomal RNA gene, pa	GU569239	86.96	30.56	0.00083429	Phthiraptera	Menoponidae	Heteromenopon psittacum
Leuctra inermis 16S ribosomal RNA gene, partial se	EF623176	75.27	61.81	0.00291196	Plecoptera	Leuctridae	Leuctra inermis
TPA_asm: Oryzias latipes strain Hd−rR, complete ge	HG314002	88.1	29.17	0.0101637	Beloniformes	Adrianichthyidae	Oryzias latipes
Petaloconchus varians isolate Petvarians0315946 12	KC583381	85.11	31.94	0.0101637		Vermetidae	Petaloconchus varians
Velarifictorus micado isolate TA 16S ribosomal RNA	KJ612521	89.74	27.08	0.0101637	Orthoptera	Gryllidae	Velarifictorus micado
Velarifictorus micado isolate BB 16S ribosomal RNA	KJ612520	89.74	27.08	0.0101637	Orthoptera	Gryllidae	Velarifictorus micado
Petaloconchus cf. varians AM−2010 voucher MNCN:Pet	GU290143	85.11	31.94	0.0101637		Vermetidae	Petaloconchus cf. varians AM−2010
Petaloconchus cf. varians AM−2010 voucher MNCN:Pet	GU290133	85.11	31.94	0.0101637		Vermetidae	Petaloconchus cf. varians AM−2010
Petaloconchus cf. varians AM−2010 voucher MNCN:Pet	GU290141	85.11	31.94	0.0101637		Vermetidae	Petaloconchus cf. varians AM−2010
Petaloconchus cf. varians AM−2010 voucher MNCN:Pet	GU290139	85.11	31.94	0.0101637		Vermetidae	Petaloconchus cf. varians AM−2010
Petaloconchus cf. varians AM−2010 voucher MNCN:Pet	GU290138	85.11	31.94	0.0101637		Vermetidae	Petaloconchus cf. varians AM−2010
Petaloconchus cf. varians AM−2010 voucher MNCN:Pet	GU290131	85.11	31.94	0.0101637		Vermetidae	Petaloconchus cf. varians AM−2010
Velarifictorus ornatus 16S ribosomal RNA gene, par	AY509569	89.74	27.08	0.0101637	Orthoptera	Gryllidae	Velarifictorus ornatus
Velarifictorus ornatus Velar−A2 16S ribosomal RNA	AY239104	89.74	27.08	0.0101637	Orthoptera	Gryllidae	Velarifictorus ornatus
Velarifictorus ornatus Velar−A1 16S ribosomal RNA	AY239103	89.74	27.08	0.0101637	Orthoptera	Gryllidae	Velarifictorus ornatus
Myara sordida 16S ribosomal RNA gene, partial sequ	AY905312	88.1	29.17	0.0101637	Orthoptera	Gryllidae	Myara sordida
Velarifictorus micado 16S ribosomal RNA gene, part	AF514613	89.74	27.08	0.0101637	Orthoptera	Gryllidae	Velarifictorus micado
Morulina verrucosa 16S ribosomal RNA gene, partial	AY555554	80.3	43.75	0.0101637		Neanuridae	Morulina verrucosa
Rhopaea magnicornis mitochondrion, complete genome	FJ859903	83.33	32.64	0.0354749	Coleoptera	Scarabaeidae	Rhopaea magnicornis
Salmanites wittilliko 16S ribosomal RNA gene, part	AY905318	87.8	28.47	0.0354749	Orthoptera	Gryllidae	Salmanites wittilliko
Salmanites taltantris 16S ribosomal RNA gene, part	AY905317	87.8	28.47	0.0354749	Orthoptera	Gryllidae	Salmanites taltantris
Eurepella mjobergi 16S ribosomal RNA gene, partial	AY905306	82.35	35.42	0.0354749	Orthoptera	Gryllidae	Eurepella mjobergi
Eurepa marginipennis 16S ribosomal RNA gene, parti	AY905303	82.35	35.42	0.0354749	Orthoptera	Gryllidae	Eurepa marginipennis
Mus musculus chromosome 8, clone RP24−421B13, comp	AC110740	86.05	29.86	0.0354749	Rodentia	Muridae	Mus musculus
Fusobacterium nucleatum subsp. nucleatum strain KC	CP012717	83.67	32.64	0.12382	Fusobacteriales	Fusobacteriaceae	Fusobacterium nucleatum subsp. nucleatum
Fusobacterium nucleatum subsp. nucleatum ChDC F316	CP012716	83.67	32.64	0.12382	Fusobacteriales	Fusobacteriaceae	Fusobacterium nucleatum subsp. nucleatum ChDC F316
Wuchereria bancrofti genome assembly W_bancrofti_J	LM003748	87.18	25	0.12382	Spirurida	Onchocercidae	Wuchereria bancrofti
Wuchereria bancrofti genome assembly W_bancrofti_J	LM002748	93.33	20.83	0.12382	Spirurida	Onchocercidae	Wuchereria bancrofti
Brugia timori genome assembly B_timori_Indonesia_v	LK908416	82.98	32.64	0.12382	Spirurida	Onchocercidae	Brugia timori
Aivalykus sp. CNIN952 16S ribosomal RNA gene, part	KJ586753	80.77	36.11	0.12382	Hymenoptera	Braconidae	Aivalykus sp. CNIN952
Macroperipatus torquatus voucher DNA104657 16S rib	KC754522	79.25	36.81	0.12382		Peripatidae	Macroperipatus torquatus
Entomobrya aino 16S ribosomal RNA gene, partial se	KC236195	76.12	45.14	0.12382		Entomobryidae	Entomobrya aino
Ephoron eophilum mitochondrial gene for 16S rRNA,	AB711630	86.84	26.39	0.12382	Ephemeroptera	Polymitarcyidae	Ephoron eophilum
Ephoron eophilum mitochondrial gene for 16S rRNA,	AB711621	86.84	26.39	0.12382	Ephemeroptera	Polymitarcyidae	Ephoron eophilum
Ephoron eophilum mitochondrial gene for 16S rRNA,	AB711619	86.84	26.39	0.12382	Ephemeroptera	Polymitarcyidae	Ephoron eophilum
Ephoron eophilum mitochondrial gene for 16S rRNA,	AB711615	86.84	26.39	0.12382	Ephemeroptera	Polymitarcyidae	Ephoron eophilum
Schoettella ununguiculata 16S ribosomal RNA gene,	JN970980	77.42	41.67	0.12382		Hypogastruridae	Schoettella ununguiculata
Anurida maritima 16S ribosomal RNA gene, partial s	JN970941	83.72	29.86	0.12382		Neanuridae	Anurida maritima
Anticheira sp. FrG1 16S large subunit ribosomal RN	HQ711600	81.25	33.33	0.12382	Coleoptera	Scarabaeidae	Anticheira sp. FrG1
Petaloconchus cf. varians AM−2010 voucher MNCN:Pet	GU290132	82.98	31.94	0.12382		Vermetidae	Petaloconchus cf. varians AM−2010
Petaloconchus cf. varians AM−2010 voucher MNCN:Pet	GU290130	82.98	31.94	0.12382		Vermetidae	Petaloconchus cf. varians AM−2010
Zunongwangia profunda SM−A87, complete genome	CP001650	93.33	20.83	0.12382	Flavobacteriales	Flavobacteriaceae	Zunongwangia profunda SM−A87
Hexagenia sp. BYU IGCEP003 16S ribosomal RNA gene,	GQ118286	88.57	24.31	0.12382	Ephemeroptera	Ephemeridae	Hexagenia sp. BYU IGCEP003
Eatonica denysae voucher BYU:IGCEP225 16S ribosoma	GQ118284	82.22	31.25	0.12382	Ephemeroptera	Ephemeridae	Eatonica denysae
Bhutanitis mansfieldi isolate FS−b−1589 16S riboso	DQ351097	74.12	57.64	0.12382	Lepidoptera	Papilionidae	Bhutanitis mansfieldi
Crosnierita urizae voucher Fo25 16S ribosomal RNA	AY351087	82.35	35.42	0.12382	Decapoda	Munididae	Crosnierita urizae
Crosnierita urizae voucher Fo41 16S ribosomal RNA	AY351090	82.35	35.42	0.12382	Decapoda	Munididae	Crosnierita urizae
Crosnierita urizae voucher Fo27 16S ribosomal RNA	AY351089	82.35	35.42	0.12382	Decapoda	Munididae	Crosnierita urizae
Crosnierita urizae voucher Fo24 16S ribosomal RNA	AY351086	82.35	35.42	0.12382	Decapoda	Munididae	Crosnierita urizae
Eurepella torowatta 16S ribosomal RNA gene, partia	AY905308	85.71	29.17	0.12382	Orthoptera	Gryllidae	Eurepella torowatta
Eurepella moojerra 16S ribosomal RNA gene, partial	AY905307	85.71	29.17	0.12382	Orthoptera	Gryllidae	Eurepella moojerra
Orchesella villosa 16S ribosomal RNA gene, partial	AY555550	73.4	63.89	0.12382		Entomobryidae	Orchesella villosa
Apteryx australis mantelli genome assembly AptMant	LK064790	88.89	25	0.432173	Apterygiformes	Apterygidae	Apteryx australis mantelli
Auxicerus platyceps voucher SK131 16S ribosomal RN	KP252212	83.72	28.47	0.432173	Coleoptera	Lucanidae	Auxicerus platyceps
Rangia cuneata voucher RC4 16S ribosomal RNA gene,	KP052753	87.18	27.08	0.432173	Veneroida	Mactridae	Rangia cuneata
Rangia cuneata voucher RC2 16S ribosomal RNA gene,	KP052752	87.18	27.08	0.432173	Veneroida	Mactridae	Rangia cuneata
Rangia cuneata voucher RC1 16S ribosomal RNA gene,	KP052751	87.18	27.08	0.432173	Veneroida	Mactridae	Rangia cuneata
Cucumis melo genomic chromosome, chr_9	LN713263	88.24	23.61	0.432173	Cucurbitales	Cucurbitaceae	Cucumis melo
Cucumis melo genomic scaffold, anchoredscaffold015	LN681892	88.24	23.61	0.432173	Cucurbitales	Cucurbitaceae	Cucumis melo
Strongyloides papillosus genome assembly S_papillo	LM526440	93.1	20.14	0.432173	Rhabditida	Strongyloididae	Strongyloides papillosus
Heligmosomoides polygyrus genome assembly H_bakeri	LL188374	84.62	27.08	0.432173	Rhabditida	Heligmosomatidae	Heligmosomoides polygyrus
Solanum pennellii chromosome ch12, complete genome	HG975451	82.98	29.86	0.432173	Solanales	Solanaceae	Solanum pennellii
Ephoron shigae mitochondrial gene for 16S ribosoma	AB874306	84.62	27.08	0.432173	Ephemeroptera	Polymitarcyidae	Ephoron shigae
Rangia cuneata voucher BivAToL−280 16S ribosomal R	KC429310	87.18	27.08	0.432173	Veneroida	Mactridae	Rangia cuneata
Clostridium botulinum B str. Osaka05 DNA, contig:	AP014512	93.1	20.14	0.432173	Clostridiales	Clostridiaceae	Clostridium botulinum B str. Osaka05
Oocyclus funestus 16S ribosomal RNA gene, partial	KC992693	73.86	58.33	0.432173	Coleoptera	Hydrophilidae	Oocyclus funestus
Ephoron leukon mitochondrial gene for 16S rRNA, pa	AB711641	84.62	27.08	0.432173	Ephemeroptera	Polymitarcyidae	Ephoron leukon
Ephoron leukon mitochondrial gene for 16S rRNA, pa	AB711638	84.62	27.08	0.432173	Ephemeroptera	Polymitarcyidae	Ephoron leukon
Ephoron leukon mitochondrial gene for 16S rRNA, pa	AB711637	84.62	27.08	0.432173	Ephemeroptera	Polymitarcyidae	Ephoron leukon
Ephoron album mitochondrial gene for 16S rRNA, par	AB711636	84.62	27.08	0.432173	Ephemeroptera	Polymitarcyidae	Ephoron album
Ephoron album mitochondrial gene for 16S rRNA, par	AB711635	84.62	27.08	0.432173	Ephemeroptera	Polymitarcyidae	Ephoron album
Meimuna gakokizana voucher 02.TW.NT.PLC.01 elongat	GQ527124	82.61	31.25	0.432173	Hemiptera	Cicadidae	Meimuna gakokizana
Ephemera simulans voucher BYU:IGCEP038 16S ribosom	GQ118285	84.09	30.56	0.432173	Ephemeroptera	Ephemeridae	Ephemera simulans
Zebrafish DNA sequence from clone DKEYP−51F12 in l	BX323071	83.72	28.47	0.432173	Cypriniformes	Cyprinidae	Danio rerio
Epitheca princeps 12S ribosomal RNA gene, partial	EU477710	73.56	59.03	0.432173	Odonata	Corduliidae	Epitheca princeps
Ancyrona sp. UPOL 001122 16S ribosomal RNA gene, p	EF508042	79.63	36.81	0.432173	Coleoptera	Trogossitidae	Ancyrona sp. UPOL 001122
Serratia proteamaculans 568, complete genome	CP000826	88.89	25	0.432173	Enterobacteriales	Enterobacteriaceae	Serratia proteamaculans 568
Mus musculus chromosome 15, clone RP24−189C9, comp	AC122774	78.33	40.28	0.432173	Rodentia	Muridae	Mus musculus
Dendrodoris albobrunnea 16S ribosomal RNA gene, pa	AF430349	88.24	23.61	0.432173	Nudibranchia	Dendrodorididae	Dendrodoris albobrunnea
Human DNA sequence from clone RP11−96K19 on chromo	AL360270	78.85	33.33	0.432173	Primates	Hominidae	Homo sapiens
Mus musculus BAC clone RP23−131F11 from chromosome	AC154705	96.55	20.14	0.432173	Rodentia	Muridae	Mus musculus
Oryza sativa Japonica Group genomic DNA, chromosom	AP003334	93.1	20.14	0.432173	Poales	Poaceae	Oryza sativa Japonica Group
Chaquihua sp. EP080 16S ribosomal RNA gene, partia	AY749784	83.33	32.64	0.432173	Ephemeroptera	Ameletopsidae	Chaquihua sp. EP080
Oryza nivara DNA, chromosome 7, BAC clone: IRGC105	LC060742	86.11	25	1.50843	Poales	Poaceae	Oryza nivara
Ovis canadensis canadensis isolate 43U chromosome	CP011899	92.86	19.44	1.50843		Bovidae	Ovis canadensis canadensis
Tetrahymena thermophila SB210 IQ calmodulin−bindin	XM_001011181	90.91	22.22	1.50843	Hymenostomatida	Tetrahymenidae	Tetrahymena thermophila SB210
PREDICTED: Linepithema humile poly(rC)−binding pro	XM_012360461	75	47.22	1.50843	Hymenoptera	Formicidae	Linepithema humile
PREDICTED: Linepithema humile poly(rC)−binding pro	XM_012360460	75	47.22	1.50843	Hymenoptera	Formicidae	Linepithema humile
PREDICTED: Linepithema humile poly(rC)−binding pro	XM_012360459	75	47.22	1.50843	Hymenoptera	Formicidae	Linepithema humile
PREDICTED: Linepithema humile poly(rC)−binding pro	XR_001100046	75	47.22	1.50843	Hymenoptera	Formicidae	Linepithema humile
PREDICTED: Linepithema humile poly(rC)−binding pro	XM_012360458	75	47.22	1.50843	Hymenoptera	Formicidae	Linepithema humile
TPA_asm: Oryzias latipes strain Hd−rR, complete ge	HG313987	86.84	26.39	1.50843	Beloniformes	Adrianichthyidae	Oryzias latipes
Cyphophthalmus solentiensis voucher MCZ:IZ:129787	KJ857532	73.75	55.56	1.50843	Opiliones	Sironidae	Cyphophthalmus solentiensis
Cucumis melo genomic chromosome, chr_1	LN713255	81.4	26.39	1.50843	Cucurbitales	Cucurbitaceae	Cucumis melo
Cucumis melo genomic scaffold, anchoredscaffold000	LN681798	81.4	26.39	1.50843	Cucurbitales	Cucurbitaceae	Cucumis melo
Hymenolepis nana genome assembly H_nana_Japan ,sca	LM401844	86.84	25.69	1.50843	Cyclophyllidea	Hymenolepididae	Hymenolepis nana
