# Supplementary material for: Testing the potential of a ribosomal 16S marker for DNA metabarcoding of insects
Source: PeerJ. 2016 Apr 19;4:e1966. doi: 10.7717/peerj.1966 (PMC4841222; doi:10.7717/peerj.1966)
Supplement: Supplemental Information 1 [file OTU_132.csv_plot.pdf › scripts/5 match OTUs/blast_hits/OTU_132.csv_plot.pdf]

OTU_132.csv

Dinocras cephalotes

Perlidae

Plecoptera

95.12

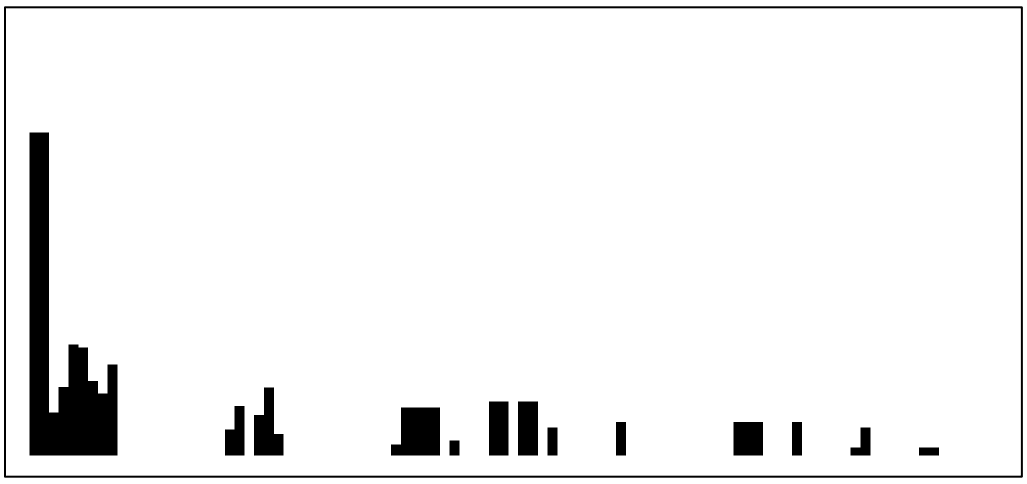

Dinocras cephalotes mitochondrion, complete genome	KF484757	95.12	100	3.66985e−66	Plecoptera	Perlidae	Dinocras cephalotes
Dinocras cephalotes 16S ribosomal RNA gene, partia	EF623244	95.12	100	3.66985e−66	Plecoptera	Perlidae	Dinocras cephalotes
Calineuria californica 16S ribosomal RNA gene, par	EF623239	82	90.24	1.05329e−28	Plecoptera	Perlidae	Calineuria californica
Perlesta decipines 16S ribosomal RNA gene, partial	EF623242	83.2	76.22	2.32002e−24	Plecoptera	Perlidae	Perlesta decipines
Perlinella drymo 16S ribosomal RNA gene, partial s	EF623240	85.19	65.85	2.32002e−24	Plecoptera	Perlidae	Perlinella drymo
Kathroperla perdita 16S ribosomal RNA gene, partia	EF623135	85.05	65.24	2.32002e−24	Plecoptera	Chloroperlidae	Kathroperla perdita
Leuctra inermis 16S ribosomal RNA gene, partial se	EF623176	83.47	73.17	8.09768e−24	Plecoptera	Leuctridae	Leuctra inermis
Agnetina capitata 16S ribosomal RNA gene, partial	EF623241	82.91	71.34	2.82637e−23	Plecoptera	Perlidae	Agnetina capitata
Hansonoperla appalachia 16S ribosomal RNA gene, pa	EF623249	84.26	65.85	9.86499e−23	Plecoptera	Perlidae	Hansonoperla appalachia
Pteronarcys princeps mitochondrion, complete genom	AY687866	79.02	87.2	9.86499e−23	Plecoptera	Pteronarcyidae	Pteronarcys princeps
Skwala americana 16S ribosomal RNA gene, partial s	EF623217	78.47	87.2	3.44322e−22	Plecoptera	Perlodidae	Skwala americana
Leuctra tenuis haplotype 10lt: HO10 16S ribosomal	EU327682	76.07	99.39	3.44322e−22	Plecoptera	Leuctridae	Leuctra tenuis
Leuctra tenuis haplotype 9lt: HO9 16S ribosomal RN	EU327681	76.07	99.39	3.44322e−22	Plecoptera	Leuctridae	Leuctra tenuis
Leuctra tenuis haplotype 8lt: HO8 16S ribosomal RN	EU327680	76.07	99.39	3.44322e−22	Plecoptera	Leuctridae	Leuctra tenuis
Leuctra tenuis haplotype 7lt: HO7 16S ribosomal RN	EU327679	76.07	99.39	3.44322e−22	Plecoptera	Leuctridae	Leuctra tenuis
Leuctra tenuis haplotype 6lt: HO6 16S ribosomal RN	EU327678	76.07	99.39	3.44322e−22	Plecoptera	Leuctridae	Leuctra tenuis
Leuctra tenuis haplotype 3lt: H03 16S ribosomal RN	DQ915184	76.07	99.39	3.44322e−22	Plecoptera	Leuctridae	Leuctra tenuis
Leuctra tenuis haplotype 2lt: H02 16S ribosomal RN	DQ915183	76.07	99.39	3.44322e−22	Plecoptera	Leuctridae	Leuctra tenuis
Leuctra tenuis haplotype 1lt: H01 16S ribosomal RN	DQ915182	76.07	99.39	3.44322e−22	Plecoptera	Leuctridae	Leuctra tenuis
Taenionema palladium 16S ribosomal RNA gene, parti	EF623267	76.22	99.39	1.2018e−21	Plecoptera	Taeniopterygidae	Taenionema palladium
Scopura montana 16S ribosomal RNA gene, partial se	EF623265	81.2	71.34	1.2018e−21	Plecoptera		Scopura montana
Acroneuria lycorias 16S ribosomal RNA gene, partia	EF623251	82.3	67.07	1.2018e−21	Plecoptera	Perlidae	Acroneuria lycorias
Claassenia sabulosa 16S ribosomal RNA gene, partia	EF623250	78.15	92.07	1.2018e−21	Plecoptera	Perlidae	Claassenia sabulosa
Udamocercia sp. BYU_PL150 16S ribosomal RNA gene,	EF623201	81.9	70.73	1.2018e−21	Plecoptera	Notonemouridae	Udamocercia sp. BYU_PL150
Nemoura cinerea 16S ribosomal RNA gene, partial se	EF623194	83.18	65.24	1.2018e−21	Plecoptera	Nemouridae	Nemoura cinerea
Despaxia augusta 16S ribosomal RNA gene, partial s	EF623177	80.99	73.17	1.2018e−21	Plecoptera	Leuctridae	Despaxia augusta
Moselia infuscata 16S ribosomal RNA gene, partial	EF623175	76.97	100	1.2018e−21	Plecoptera	Leuctridae	Moselia infuscata
Plumiperla diversa 16S ribosomal RNA gene, partial	EF623132	79.05	89.02	1.2018e−21	Plecoptera	Chloroperlidae	Plumiperla diversa
Pteronarcys sachalina 16S ribosomal RNA gene, part	EF623264	78.32	87.2	4.1947e−21	Plecoptera	Pteronarcyidae	Pteronarcys sachalina
Pteronarcys scotti 16S ribosomal RNA gene, partial	EF623263	78.32	87.2	4.1947e−21	Plecoptera	Pteronarcyidae	Pteronarcys scotti
Pteronarcys californica 16S ribosomal RNA gene, pa	EF623261	78.32	87.2	4.1947e−21	Plecoptera	Pteronarcyidae	Pteronarcys californica
Neaviperla forcipata 16S ribosomal RNA gene, parti	EF623139	76.62	93.9	4.1947e−21	Plecoptera	Chloroperlidae	Neaviperla forcipata
Leuctra tenuis haplotype 5lt: HO5 16S ribosomal RN	EU327677	75.46	99.39	4.1947e−21	Plecoptera	Leuctridae	Leuctra tenuis
Styloperla sp. mitochondrion, partial genome	KR088971	75.64	93.29	1.46409e−20	Plecoptera	Styloperlidae	Styloperla sp. YZD−2015
Doddsia occidentalis 16S ribosomal RNA gene, parti	EF623270	75.61	99.39	1.46409e−20	Plecoptera	Taeniopterygidae	Doddsia occidentalis
Prostoia besametsa 16S ribosomal RNA gene, partial	EF623188	76.25	96.95	1.46409e−20	Plecoptera	Nemouridae	Prostoia besametsa
Chloroperla tripunctata 16S ribosomal RNA gene, pa	EF623137	77.63	89.02	1.46409e−20	Plecoptera	Chloroperlidae	Chloroperla tripunctata
Leuctra tenuis haplotype 4lt: HO4 16S ribosomal RN	EU327676	80.51	71.95	1.46409e−20	Plecoptera	Leuctridae	Leuctra tenuis
Lednia tumana 16S ribosomal RNA gene, partial sequ	EF623197	82.24	65.24	5.11019e−20	Plecoptera	Nemouridae	Lednia tumana
Nemoura trispinosa 16S ribosomal RNA gene, partial	EF623187	82.24	65.24	5.11019e−20	Plecoptera	Nemouridae	Nemoura trispinosa
Paranemoura claasseni 16S ribosomal RNA gene, part	EF623185	82.24	65.24	5.11019e−20	Plecoptera	Nemouridae	Paranemoura claasseni
Shipsa rotunda 16S ribosomal RNA gene, partial seq	EF623184	82.24	65.24	5.11019e−20	Plecoptera	Nemouridae	Shipsa rotunda
Cardioperla lobata 16S ribosomal RNA gene, partial	EF623164	76.97	91.46	5.11019e−20	Plecoptera	Gripopterygidae	Cardioperla lobata
Suwallia teleckojensis 16S ribosomal RNA gene, par	EF623142	80.7	69.51	5.11019e−20	Plecoptera	Chloroperlidae	Suwallia teleckojensis
Notonemouridae sp. MD−2011 voucher ZMUC:JD−1507 16	JN615257	79.2	75.61	1.78363e−19	Plecoptera	Notonemouridae	Notonemouridae sp. MD−2011
Cosumnoperla hypocrena 16S ribosomal RNA gene, par	EF623226	76.92	87.2	1.78363e−19	Plecoptera	Perlodidae	Cosumnoperla hypocrena
Salmoperla sylvanica 16S ribosomal RNA gene, parti	EF623222	76.47	93.29	1.78363e−19	Plecoptera	Perlodidae	Salmoperla sylvanica
Nemurella pictetii 16S ribosomal RNA gene, partial	EF623192	82.52	62.8	1.78363e−19	Plecoptera	Nemouridae	Nemurella pictetii
Malenka californica 16S ribosomal RNA gene, partia	EF623182	82.52	62.8	1.78363e−19	Plecoptera	Nemouridae	Malenka californica
Riekoperla triloba 16S ribosomal RNA gene, partial	EF623171	75	99.39	1.78363e−19	Plecoptera	Gripopterygidae	Riekoperla triloba
Sasquaperla hoopa 16S ribosomal RNA gene, partial	EF623143	82.52	62.8	1.78363e−19	Plecoptera	Chloroperlidae	Sasquaperla hoopa
Triznaka pintada 16S ribosomal RNA gene, partial s	EF623134	82.52	62.8	1.78363e−19	Plecoptera	Chloroperlidae	Triznaka pintada
Dolichopoda bormansi isolate BRA3 16S ribosomal RN	AY507605	74.56	99.39	6.22549e−19	Orthoptera	Rhaphidophoridae	Dolichopoda bormansi
Paraleuctra vershina 16S ribosomal RNA gene, parti	EF623173	81.31	65.24	6.22549e−19	Plecoptera	Leuctridae	Paraleuctra vershina
Claudioperla sp. BYU_PL158 16S ribosomal RNA gene,	EF623169	76.32	91.46	6.22549e−19	Plecoptera	Gripopterygidae	Claudioperla sp. BYU_PL158
Dinotoperla sp. BYU_PL105 16S ribosomal RNA gene,	EF623160	75.61	99.39	6.22549e−19	Plecoptera	Gripopterygidae	Dinotoperla sp. BYU_PL105
Dolichopoda bormansi isolate BRA 16S ribosomal RNA	AY793578	74.56	99.39	6.22549e−19	Orthoptera	Rhaphidophoridae	Dolichopoda bormansi
Cryptoperla sp. YK−2012−1 mitochondrial gene for 1	AB711243	79.31	70.73	2.17291e−18	Plecoptera	Peltoperlidae	Cryptoperla sp. YK−2012−1
Cerconychia sp. BYU_PL114 16S ribosomal RNA gene,	EF623260	75	90.24	2.17291e−18	Plecoptera	Styloperlidae	Cerconychia sp. BYU_PL114
Siphonoperla torrentium 16S ribosomal RNA gene, pa	EF623138	76.92	87.2	2.17291e−18	Plecoptera	Chloroperlidae	Siphonoperla torrentium
Sweltsa coloradensis 16S ribosomal RNA gene, parti	EF623133	81.55	62.8	2.17291e−18	Plecoptera	Chloroperlidae	Sweltsa coloradensis
Tasmanoperla larvalis 16S ribosomal RNA gene, part	EF623125	76.92	93.9	2.17291e−18	Plecoptera	Austroperlidae	Tasmanoperla larvalis
Allocapnia recta isolate H18 16S ribosomal RNA gen	KC881053	76.39	87.2	7.5842e−18	Plecoptera	Capniidae	Allocapnia recta
Allocapnia recta isolate H17 16S ribosomal RNA gen	KC881052	76.39	87.2	7.5842e−18	Plecoptera	Capniidae	Allocapnia recta
Allocapnia recta isolate H12 16S ribosomal RNA gen	KC881047	76.39	87.2	7.5842e−18	Plecoptera	Capniidae	Allocapnia recta
Allocapnia recta isolate H9 16S ribosomal RNA gene	KC881044	76.39	87.2	7.5842e−18	Plecoptera	Capniidae	Allocapnia recta
Allocapnia recta isolate H8 16S ribosomal RNA gene	KC881043	76.39	87.2	7.5842e−18	Plecoptera	Capniidae	Allocapnia recta
Allocapnia recta isolate H5 16S ribosomal RNA gene	KC881040	76.39	87.2	7.5842e−18	Plecoptera	Capniidae	Allocapnia recta
Allocapnia recta isolate H4 16S ribosomal RNA gene	KC881039	76.39	87.2	7.5842e−18	Plecoptera	Capniidae	Allocapnia recta
Dolichopoda bormansi isolate BRA5 16S ribosomal RN	AY507606	73.96	99.39	7.5842e−18	Orthoptera	Rhaphidophoridae	Dolichopoda bormansi
Frisonia picticeps 16S ribosomal RNA gene, partial	EF623227	78.4	75.61	7.5842e−18	Plecoptera	Perlodidae	Frisonia picticeps
Cascadoperla trictura 16S ribosomal RNA gene, part	EF623225	76.39	85.98	7.5842e−18	Plecoptera	Perlodidae	Cascadoperla trictura
Nanonemoura wahkeena 16S ribosomal RNA gene, parti	EF623198	81.55	62.8	7.5842e−18	Plecoptera	Nemouridae	Nanonemoura wahkeena
Ostrocerca foersteri 16S ribosomal RNA gene, parti	EF623191	81.55	62.8	7.5842e−18	Plecoptera	Nemouridae	Ostrocerca foersteri
Visoka cataractae 16S ribosomal RNA gene, partial	EF623189	81.55	62.8	7.5842e−18	Plecoptera	Nemouridae	Visoka cataractae
Dinotoperla serricauda 16S ribosomal RNA gene, par	EF623165	79.2	75.61	7.5842e−18	Plecoptera	Gripopterygidae	Dinotoperla serricauda
Cosmioperla australis 16S ribosomal RNA gene, part	EF623148	74.36	91.46	7.5842e−18	Plecoptera	Eustheniidae	Cosmioperla australis
Alloperla severa 16S ribosomal RNA gene, partial s	EF623141	76.32	89.02	7.5842e−18	Plecoptera	Chloroperlidae	Alloperla severa
Paraperla frontalis 16S ribosomal RNA gene, partia	EF623136	81.55	62.8	7.5842e−18	Plecoptera	Chloroperlidae	Paraperla frontalis
Allocapnia recta haplotype 10ar: H010 16S ribosoma	EU306873	76.39	87.2	7.5842e−18	Plecoptera	Capniidae	Allocapnia recta
Allocapnia recta haplotype 9ar: H09 16S ribosomal	EU306872	76.39	87.2	7.5842e−18	Plecoptera	Capniidae	Allocapnia recta
Allocapnia recta haplotype 8ar: H08 16S ribosomal	EU306871	76.39	87.2	7.5842e−18	Plecoptera	Capniidae	Allocapnia recta
Allocapnia recta haplotype 1ar: H01 16S ribosomal	DQ915179	76.39	87.2	7.5842e−18	Plecoptera	Capniidae	Allocapnia recta
Eupholidoptera krueperi voucher 408 16S ribosomal	EU203912	75.17	88.41	2.64715e−17	Orthoptera	Tettigoniidae	Eupholidoptera krueperi
Pteronarcella badia 16S ribosomal RNA gene, partia	EF623262	80.37	65.24	2.64715e−17	Plecoptera	Pteronarcyidae	Pteronarcella badia
Pictetoperla sp. BYU_PL156 16S ribosomal RNA gene,	EF623247	81.31	65.24	2.64715e−17	Plecoptera	Perlidae	Pictetoperla sp. BYU_PL156
Kempnyella sp. BYU_PL155 16S ribosomal RNA gene, p	EF623246	79.82	66.46	2.64715e−17	Plecoptera	Perlidae	Kempnyella sp. BYU_PL155
Osobenus yakimae 16S ribosomal RNA gene, partial s	EF623232	75.32	90.85	2.64715e−17	Plecoptera	Perlodidae	Osobenus yakimae
Isoperla fulva 16S ribosomal RNA gene, partial seq	EF623207	79.82	69.51	2.64715e−17	Plecoptera	Perlodidae	Isoperla fulva
Austrocerca rieka 16S ribosomal RNA gene, partial	EF623205	74.23	99.39	2.64715e−17	Plecoptera	Notonemouridae	Austrocerca rieka
Austrocercoides zwicki 16S ribosomal RNA gene, par	EF623199	74.23	99.39	2.64715e−17	Plecoptera	Notonemouridae	Austrocercoides zwicki
Protonemura meyeri 16S ribosomal RNA gene, partial	EF623193	80.37	65.24	2.64715e−17	Plecoptera	Nemouridae	Protonemura meyeri
Megaleuctra kincaidi 16S ribosomal RNA gene, parti	EF623174	80.37	65.24	2.64715e−17	Plecoptera	Leuctridae	Megaleuctra kincaidi
Pelurgoperla personata 16S ribosomal RNA gene, par	EF623168	75.66	91.46	2.64715e−17	Plecoptera	Gripopterygidae	Pelurgoperla personata
Antarctoperla sp. BYU_PL142 16S ribosomal RNA gene	EF623163	75.66	91.46	2.64715e−17	Plecoptera	Gripopterygidae	Antarctoperla sp. BYU_PL142
Osmylus sp. EH−2004 16S ribosomal RNA gene, partia	AY620146	76.92	87.2	2.64715e−17	Neuroptera	Osmylidae	Osmylus sp. EH−2004
Allocapnia recta isolate H19 16S ribosomal RNA gen	KC881054	75.69	87.2	9.23945e−17	Plecoptera	Capniidae	Allocapnia recta
Allocapnia recta isolate H16 16S ribosomal RNA gen	KC881051	75.69	87.2	9.23945e−17	Plecoptera	Capniidae	Allocapnia recta
Allocapnia recta isolate H15 16S ribosomal RNA gen	KC881050	75.69	87.2	9.23945e−17	Plecoptera	Capniidae	Allocapnia recta
Allocapnia recta isolate H14 16S ribosomal RNA gen	KC881049	75.69	87.2	9.23945e−17	Plecoptera	Capniidae	Allocapnia recta
