# Supplementary material for: Testing the potential of a ribosomal 16S marker for DNA metabarcoding of insects
Source: PeerJ. 2016 Apr 19;4:e1966. doi: 10.7717/peerj.1966 (PMC4841222; doi:10.7717/peerj.1966)
Supplement: Supplemental Information 1 [file OTU_146.csv_plot.pdf › scripts/5 match OTUs/blast_hits/OTU_146.csv_plot.pdf]

OTU_146.csv

Velia caprai

Veliidae

Hemiptera

92.81

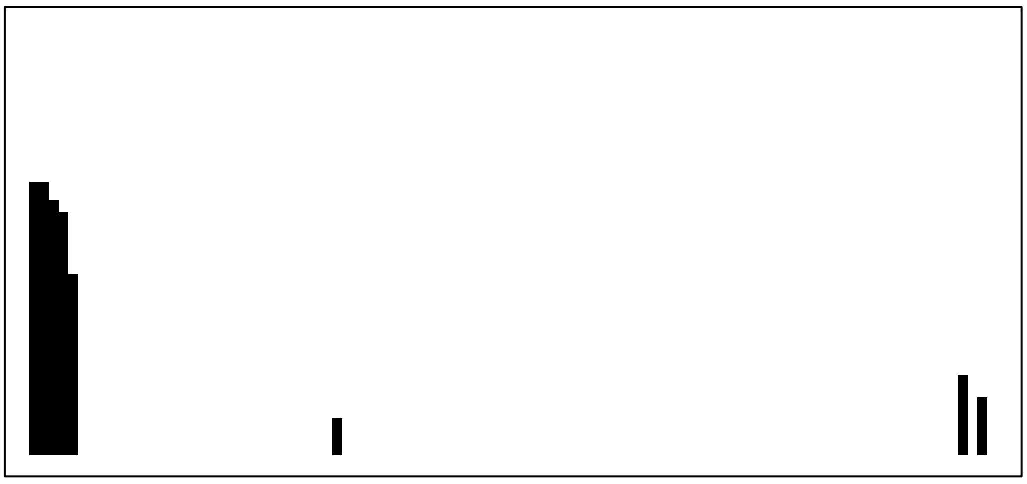

Velia caprai isolate 943 16S ribosomal RNA gene, p	EU871185	92.81	95.86	6.04637e−50	Hemiptera	Veliidae	Velia caprai
Velia caprai 16S ribosomal RNA gene, partial seque	AY648139	92.81	95.86	6.04637e−50	Hemiptera	Veliidae	Velia caprai
Velia hoberlandti isolate 941 16S ribosomal RNA ge	EU871188	91.97	94.48	3.1321e−47	Hemiptera	Veliidae	Velia hoberlandti
Velia maderensis isolate 1862 16S ribosomal RNA ge	EU871187	91.37	95.86	3.1321e−47	Hemiptera	Veliidae	Velia maderensis
Velia affinis isolate 373 16S ribosomal RNA gene,	EU871189	88.49	95.86	8.40458e−42	Hemiptera	Veliidae	Velia affinis
Xenobates argentatus isolate 973 16S ribosomal RNA	EU871177	79.39	88.97	3.57688e−21	Hemiptera	Veliidae	Xenobates argentatus
Rhagovelia sp. 1 JD−2008 isolate 1602 16S ribosoma	EU871178	76.98	95.86	5.30858e−19	Hemiptera	Veliidae	Rhagovelia sp. 1 JD−2008
Limnometra matsudai isolate 256 16S large subunit	KC880900	76.03	98.62	1.85287e−18	Hemiptera	Gerridae	Limnometra matsudai
Tachygerris opacus isolate 217 16S large subunit r	KC880879	78.38	98.62	1.85287e−18	Hemiptera	Gerridae	Tachygerris opacus
Ventidius usingeri isolate 1059 16S ribosomal RNA	EU871207	77.78	89.66	2.25726e−17	Hemiptera	Gerridae	Ventidius usingeri
Aquarius distanti 16S ribosomal RNA gene, partial	AY425198	79.03	84.83	7.8786e−17	Hemiptera	Gerridae	Aquarius distanti
Limnogonus windi isolate 2394 16S large subunit ri	GU645097	75.68	98.62	2.7499e−16	Hemiptera	Gerridae	Limnogonus windi
Teuchophorus ornatulus voucher RMBR:103493 16S rib	FJ808201	76.67	98.62	2.7499e−16	Diptera	Dolichopodidae	Teuchophorus ornatulus
Halobates mjobergi 16S ribosomal RNA gene, partial	AY648134	75	99.31	2.7499e−16	Hemiptera	Gerridae	Halobates mjobergi
Gerris argenticollis 16S large subunit ribosomal R	AY951329	77.61	89.66	2.7499e−16	Hemiptera	Gerridae	Gerris argenticollis
Limnoporus dissortis 16S ribosomal RNA gene, parti	AY425228	75.94	89.66	2.7499e−16	Hemiptera	Gerridae	Limnoporus dissortis
Limnoporus genitalis 16S ribosomal RNA gene, parti	AY425217	75.94	89.66	2.7499e−16	Hemiptera	Gerridae	Limnoporus genitalis
Limnoporus notabilis 16S ribosomal RNA gene, parti	AY425210	75.94	89.66	2.7499e−16	Hemiptera	Gerridae	Limnoporus notabilis
Limnoporus canaliculatus 16S ribosomal RNA gene, p	AY425208	75.94	89.66	2.7499e−16	Hemiptera	Gerridae	Limnoporus canaliculatus
Limnoporus genitalis mitochondrial gene for 16S ri	AB026591	75.94	89.66	2.7499e−16	Hemiptera	Gerridae	Limnoporus genitalis
Limnogonus aduncus voucher MC32a 16S ribosomal RNA	KC594827	76.03	98.62	9.5981e−16	Hemiptera	Gerridae	Limnogonus aduncus
Limnogonus hungerfordi isolate 1431 16S large subu	GU645089	76.03	98.62	9.5981e−16	Hemiptera	Gerridae	Limnogonus hungerfordi
Limnogonus nitidus isolate 2141 16S large subunit	GU645067	76.03	98.62	9.5981e−16	Hemiptera	Gerridae	Limnogonus nitidus
Limnogonus ignotus isolate 2343 16S large subunit	GU645059	76.03	98.62	9.5981e−16	Hemiptera	Gerridae	Limnogonus ignotus
Rhagovelia uncinata isolate 666 16S ribosomal RNA	EU871181	77.69	88.28	9.5981e−16	Hemiptera	Veliidae	Rhagovelia uncinata
Riptortus sp. WCW−2003 16S ribosomal RNA gene, par	AY252665	75.5	100	9.5981e−16	Hemiptera	Alydidae	Riptortus sp. WCW−2003
Rhagovelia tenuipes voucher MC36a 16S ribosomal RN	KC594837	77.1	88.28	3.35006e−15	Hemiptera	Veliidae	Rhagovelia tenuipes
Limnometra pulchra isolate 231 16S large subunit r	KC880904	79.28	75.17	3.35006e−15	Hemiptera	Gerridae	Limnometra pulchra
Amemboa sp. JD−2014 isolate 2440 16S large subunit	KC880875	75.52	94.48	3.35006e−15	Hemiptera	Gerridae	Amemboa sp. JD−2014
Limnogonus luctuosus isolate 2294 16S large subuni	GU645092	75.51	98.62	3.35006e−15	Hemiptera	Gerridae	Limnogonus luctuosus
Microvelia horvathi mitochondrial gene for 16S rib	AB026612	76.47	89.66	3.35006e−15	Hemiptera	Veliidae	Microvelia horvathi
Hypselonotus fulvus 16S ribosomal RNA gene, partia	KC537015	81.72	61.38	1.16929e−14	Hemiptera	Coreidae	Hypselonotus fulvus
Limnometra palauana isolate 230 16S large subunit	KC880902	79.81	71.72	1.16929e−14	Hemiptera	Gerridae	Limnometra palauana
Gerris gracilicornis mitochondrial genes for 12S r	AB679153	73.97	98.62	1.16929e−14	Hemiptera	Gerridae	Gerris gracilicornis
Limnogonus nitidus isolate 1983 16S large subunit	GU645066	75.34	98.62	1.16929e−14	Hemiptera	Gerridae	Limnogonus nitidus
Limnogonus intermedius isolate 140 16S large subun	GU645053	75.34	98.62	1.16929e−14	Hemiptera	Gerridae	Limnogonus intermedius
Halovelia sp. JD−2008 isolate 1950 16S ribosomal R	EU871176	78.81	80	1.16929e−14	Hemiptera	Veliidae	Halovelia sp. JD−2008
Perittopus asiaticus isolate 846 16S ribosomal RNA	EU871165	75.86	97.93	1.16929e−14	Hemiptera	Veliidae	Perittopus asiaticus
Limnoporus esakii 16S ribosomal RNA gene, partial	AY425216	75.19	89.66	1.16929e−14	Hemiptera	Gerridae	Limnoporus esakii
Gerris nepalensis 16S ribosomal RNA gene, partial	AY425192	75.51	98.62	1.16929e−14	Hemiptera	Gerridae	Gerris nepalensis
Rhagovelia esakii mitochondrial gene for 16S ribos	AB026611	74.82	95.86	1.16929e−14	Hemiptera	Veliidae	Rhagovelia esakii
Limnoporus esakii mitochondrial gene for 16S ribos	AB026592	75.19	89.66	1.16929e−14	Hemiptera	Gerridae	Limnoporus esakii
Limnogonus pectoralis isolate 141 16S large subuni	GU645072	78.05	82.76	4.08121e−14	Hemiptera	Gerridae	Limnogonus pectoralis
Brachymetra unca 16S ribosomal RNA gene, partial s	AY648136	76.61	84.83	4.08121e−14	Hemiptera	Gerridae	Brachymetra unca
Gerris nepalensis mitochondrial gene for 16S ribos	AB026587	77.42	84.83	4.08121e−14	Hemiptera	Gerridae	Gerris nepalensis
Leptinopterus femoratus voucher SK231 16S ribosoma	KP250254	75.18	90.34	1.42448e−13	Coleoptera	Lucanidae	Leptinopterus femoratus
Rhagovelia zela voucher MC37a 16S ribosomal RNA ge	KC594838	76.52	88.28	1.42448e−13	Hemiptera	Veliidae	Rhagovelia zela
Limnometra sp. JD−2014 isolate 1562 16S large subu	KC880899	79.81	71.72	1.42448e−13	Hemiptera	Gerridae	Limnometra sp. JD−2014
Limnometra lipovskyi isolate 1071 16S large subuni	KC880898	76.98	84.83	1.42448e−13	Hemiptera	Gerridae	Limnometra lipovskyi
Limnometra femorata isolate 1054 16S large subunit	KC880897	78.85	71.72	1.42448e−13	Hemiptera	Gerridae	Limnometra femorata
Gerris gracilicornis mitochondrial genes for 12S r	AB679162	75.19	89.66	1.42448e−13	Hemiptera	Gerridae	Gerris gracilicornis
Gerris gracilicornis mitochondrial genes for 12S r	AB679161	75.19	89.66	1.42448e−13	Hemiptera	Gerridae	Gerris gracilicornis
Gerris gracilicornis mitochondrial genes for 12S r	AB679160	75.19	89.66	1.42448e−13	Hemiptera	Gerridae	Gerris gracilicornis
Gerris gracilicornis mitochondrial genes for 12S r	AB679157	73.97	98.62	1.42448e−13	Hemiptera	Gerridae	Gerris gracilicornis
Gerris gracilicornis mitochondrial genes for 12S r	AB679158	73.97	98.62	1.42448e−13	Hemiptera	Gerridae	Gerris gracilicornis
Gerris gracilicornis mitochondrial genes for 12S r	AB679152	73.97	98.62	1.42448e−13	Hemiptera	Gerridae	Gerris gracilicornis
Gerris gracilicornis mitochondrial genes for 12S r	AB679151	73.97	98.62	1.42448e−13	Hemiptera	Gerridae	Gerris gracilicornis
Gerris gracilicornis mitochondrial genes for 12S r	AB679150	73.97	98.62	1.42448e−13	Hemiptera	Gerridae	Gerris gracilicornis
Gerris gracilicornis mitochondrial genes for 12S r	AB679149	73.97	98.62	1.42448e−13	Hemiptera	Gerridae	Gerris gracilicornis
Limnogonus fossarum gilguy isolate gilguy_2395 16S	GU645080	75.68	98.62	1.42448e−13	Hemiptera	Gerridae	Limnogonus fossarum gilguy
Limnogonus fossarum gilguy isolate gilguy_1435 16S	GU645078	75.68	98.62	1.42448e−13	Hemiptera	Gerridae	Limnogonus fossarum gilguy
Limnogonus aduncus isolate 2344 16S large subunit	GU645058	74.83	98.62	1.42448e−13	Hemiptera	Gerridae	Limnogonus aduncus
Hercostomus verbekei voucher Pollet 0263 (P) 16S r	EU864004	73.97	98.62	1.42448e−13	Diptera	Dolichopodidae	Hercostomus verbekei
Hercostomus plagiatus voucher Pollet 0185 (P) 16S	EU864002	74.83	98.62	1.42448e−13	Diptera	Dolichopodidae	Hercostomus plagiatus
Gymnopternus aerosus voucher Pollet 0025 (P) 16S r	EU863970	75.57	86.9	1.42448e−13	Diptera	Dolichopodidae	Gymnopternus aerosus
Bolitophila sp. ER−2009 16S ribosomal RNA gene, pa	FJ172021	78.9	71.72	1.42448e−13	Diptera	Mycetophilidae	Bolitophila sp. ER−2009
Microvelia reticulata isolate 292 16S ribosomal RN	EU871172	76.12	88.97	1.42448e−13	Hemiptera	Veliidae	Microvelia reticulata
Rhagovelia sumatrensis 16S ribosomal RNA gene, par	AY648126	74.1	95.86	1.42448e−13	Hemiptera	Veliidae	Rhagovelia sumatrensis
Gerris caucasicus 16S large subunit ribosomal RNA	AY951328	76.12	89.66	1.42448e−13	Hemiptera	Gerridae	Gerris caucasicus
Gerris odontogaster 16S ribosomal RNA gene, partia	AY425219	76.12	89.66	1.42448e−13	Hemiptera	Gerridae	Gerris odontogaster
Gerris gracilicornis 16S ribosomal RNA gene, parti	AY425201	73.97	98.62	1.42448e−13	Hemiptera	Gerridae	Gerris gracilicornis
Gerris argentatus 16S ribosomal RNA gene, partial	AY425194	76.12	89.66	1.42448e−13	Hemiptera	Gerridae	Gerris argentatus
Gerris buenoi 16S ribosomal RNA gene, partial sequ	AY425191	76.12	89.66	1.42448e−13	Hemiptera	Gerridae	Gerris buenoi
Limnogonus fossarum fossarum mitochondrial gene fo	AB026601	75.68	98.62	1.42448e−13	Hemiptera	Gerridae	Limnogonus fossarum fossarum
Gerris babai mitochondrial gene for 16S ribosomal	AB026585	76.12	89.66	1.42448e−13	Hemiptera	Gerridae	Gerris babai
Syntormon flexibile isolate Synt.flexibile104 16S	KM282992	74.48	95.17	4.97194e−13	Diptera	Dolichopodidae	Syntormon flexibile
Palaeodocosia vittata voucher JSM6 16S ribosomal R	KC435585	75.51	92.41	4.97194e−13	Diptera	Mycetophilidae	Palaeodocosia vittata
Gerris gracilicornis mitochondrial genes for 12S r	AB679164	73.29	98.62	4.97194e−13	Hemiptera	Gerridae	Gerris gracilicornis
Gerris gracilicornis mitochondrial genes for 12S r	AB679163	73.29	98.62	4.97194e−13	Hemiptera	Gerridae	Gerris gracilicornis
Gerris gracilicornis mitochondrial genes for 12S r	AB679156	73.29	98.62	4.97194e−13	Hemiptera	Gerridae	Gerris gracilicornis
Gerris gracilicornis mitochondrial genes for 12S r	AB679154	73.29	98.62	4.97194e−13	Hemiptera	Gerridae	Gerris gracilicornis
Gerris sp. YT−2009 16S ribosomal RNA gene, partial	FJ372633	73.29	98.62	4.97194e−13	Hemiptera	Gerridae	Gerris sp. YT−2009
Limnogonus hungerfordi isolate 1014 16S large subu	GU645086	73.97	98.62	4.97194e−13	Hemiptera	Gerridae	Limnogonus hungerfordi
Limnogonus hungerfordi isolate 1995 16S large subu	GU645087	73.97	98.62	4.97194e−13	Hemiptera	Gerridae	Limnogonus hungerfordi
Limnogonus hungerfordi isolate 207 16S large subun	GU645085	74.66	98.62	4.97194e−13	Hemiptera	Gerridae	Limnogonus hungerfordi
Nodicornis nodicornis voucher Pollet 0072 (P) 16S	EU864014	75.61	82.76	4.97194e−13	Diptera	Dolichopodidae	Nodicornis nodicornis
Hercostomus longiventris voucher Pollet 0274 (P) 1	EU863994	75.61	82.76	4.97194e−13	Diptera	Dolichopodidae	Hercostomus longiventris
Hercostomus chetifer voucher Pollet 0206 (P) 16S r	EU863990	74.81	89.66	4.97194e−13	Diptera	Dolichopodidae	Hercostomus chetifer
Syntormon flexibile voucher RMBR:103475 16S riboso	FJ808198	74.48	95.17	4.97194e−13	Diptera	Dolichopodidae	Syntormon flexibile
Syntormon flexibile voucher RMBR:103476 16S riboso	FJ808197	74.48	95.17	4.97194e−13	Diptera	Dolichopodidae	Syntormon flexibile
Amemboa sexualis 16S ribosomal RNA gene, partial s	AY648138	75	95.17	4.97194e−13	Hemiptera	Gerridae	Amemboa sexualis
Aquarius fabricii 16S ribosomal RNA gene, partial	AY425204	74.15	98.62	4.97194e−13	Hemiptera	Gerridae	Aquarius fabricii
Gerris yezoensis 16S ribosomal RNA gene, partial s	AY425203	75	88.97	4.97194e−13	Hemiptera	Gerridae	Gerris yezoensis
Aquarius ventralis 16S ribosomal RNA gene, partial	AY425196	75	89.66	4.97194e−13	Hemiptera	Gerridae	Aquarius ventralis
Aquarius remigis 16S ribosomal RNA gene, partial s	AY425190	75.61	84.83	4.97194e−13	Hemiptera	Gerridae	Aquarius remigis
Rhagovelia sp. Los Banos mitochondrial gene for 16	AB026610	83.75	53.79	4.97194e−13	Hemiptera	Veliidae	Rhagovelia sp. Los Banos
Limnogonus fossarum gilguy mitochondrial gene for	AB026602	75.17	98.62	4.97194e−13	Hemiptera	Gerridae	Limnogonus fossarum gilguy
Simulium molawinense mitochondrial gene for 16S ri	LC034992	82.72	53.79	1.73538e−12	Diptera	Simuliidae	Simulium molawinense
Limnometra pseudoinsularis isolate 1909 16S large	KC880903	78.85	71.72	1.73538e−12	Hemiptera	Gerridae	Limnometra pseudoinsularis
Limnometra fluviorum isolate 227 16S large subunit	KC880894	76.15	85.52	1.73538e−12	Hemiptera	Gerridae	Limnometra fluviorum
