# Supplementary material for: Testing the potential of a ribosomal 16S marker for DNA metabarcoding of insects
Source: PeerJ. 2016 Apr 19;4:e1966. doi: 10.7717/peerj.1966 (PMC4841222; doi:10.7717/peerj.1966)
Supplement: Supplemental Information 1 [file OTU_152.csv_plot.pdf › scripts/5 match OTUs/blast_hits/OTU_152.csv_plot.pdf]

OTU_152.csv

Dinocras cephalotes

Perlidae

Plecoptera

94.51

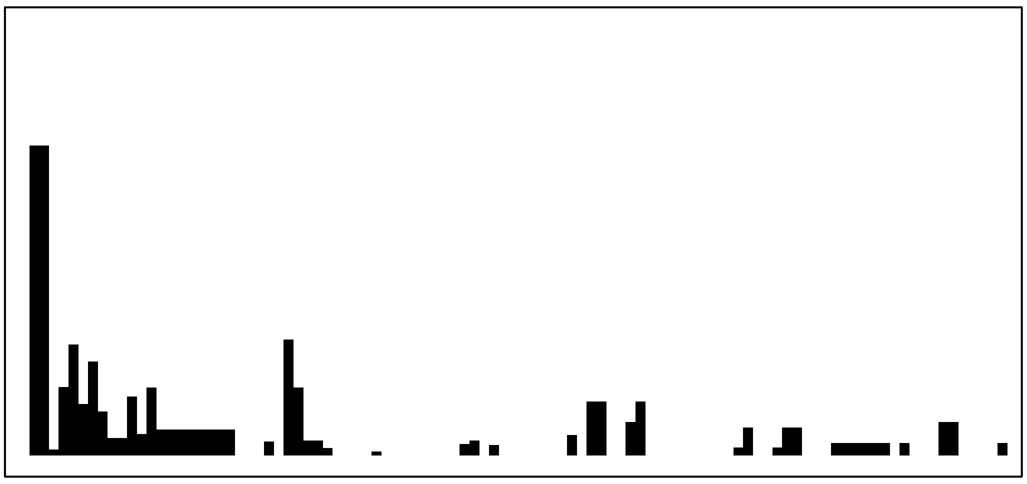

Dinocras cephalotes mitochondrion, complete genome	KF484757	94.51	100	1.51207e−64	Plecoptera	Perlidae	Dinocras cephalotes
Dinocras cephalotes 16S ribosomal RNA gene, partia	EF623244	94.51	100	1.51207e−64	Plecoptera	Perlidae	Dinocras cephalotes
Calineuria californica 16S ribosomal RNA gene, par	EF623239	80.26	93.75	4.33983e−27	Plecoptera	Perlidae	Calineuria californica
Agnetina capitata 16S ribosomal RNA gene, partial	EF623241	83.19	74.38	2.24809e−24	Plecoptera	Perlidae	Agnetina capitata
Perlinella drymo 16S ribosomal RNA gene, partial s	EF623240	85.19	67.5	2.24809e−24	Plecoptera	Perlidae	Perlinella drymo
Perlesta decipines 16S ribosomal RNA gene, partial	EF623242	82.4	78.12	2.73873e−23	Plecoptera	Perlidae	Perlesta decipines
Hansonoperla appalachia 16S ribosomal RNA gene, pa	EF623249	84.4	67.5	9.5591e−23	Plecoptera	Perlidae	Hansonoperla appalachia
Leuctra tenuis haplotype 5lt: HO5 16S ribosomal RN	EU327677	82.05	73.12	9.5591e−23	Plecoptera	Leuctridae	Leuctra tenuis
Notonemouridae sp. MD−2011 voucher ZMUC:JD−1507 16	JN615257	80.8	77.5	3.33645e−22	Plecoptera	Notonemouridae	Notonemouridae sp. MD−2011
Taenionema palladium 16S ribosomal RNA gene, parti	EF623267	80.8	77.5	1.16454e−21	Plecoptera	Taeniopterygidae	Taenionema palladium
Udamocercia sp. BYU_PL150 16S ribosomal RNA gene,	EF623201	82.76	72.5	1.16454e−21	Plecoptera	Notonemouridae	Udamocercia sp. BYU_PL150
Despaxia augusta 16S ribosomal RNA gene, partial s	EF623177	80.99	75	1.16454e−21	Plecoptera	Leuctridae	Despaxia augusta
Kathroperla perdita 16S ribosomal RNA gene, partia	EF623135	83.18	66.88	1.16454e−21	Plecoptera	Chloroperlidae	Kathroperla perdita
Leuctra tenuis haplotype 10lt: HO10 16S ribosomal	EU327682	81.2	73.12	1.16454e−21	Plecoptera	Leuctridae	Leuctra tenuis
Leuctra tenuis haplotype 9lt: HO9 16S ribosomal RN	EU327681	81.2	73.12	1.16454e−21	Plecoptera	Leuctridae	Leuctra tenuis
Leuctra tenuis haplotype 8lt: HO8 16S ribosomal RN	EU327680	81.2	73.12	1.16454e−21	Plecoptera	Leuctridae	Leuctra tenuis
Leuctra tenuis haplotype 7lt: HO7 16S ribosomal RN	EU327679	81.2	73.12	1.16454e−21	Plecoptera	Leuctridae	Leuctra tenuis
Leuctra tenuis haplotype 6lt: HO6 16S ribosomal RN	EU327678	81.2	73.12	1.16454e−21	Plecoptera	Leuctridae	Leuctra tenuis
Leuctra tenuis haplotype 3lt: H03 16S ribosomal RN	DQ915184	81.2	73.12	1.16454e−21	Plecoptera	Leuctridae	Leuctra tenuis
Leuctra tenuis haplotype 2lt: H02 16S ribosomal RN	DQ915183	81.2	73.12	1.16454e−21	Plecoptera	Leuctridae	Leuctra tenuis
Leuctra tenuis haplotype 1lt: H01 16S ribosomal RN	DQ915182	81.2	73.12	1.16454e−21	Plecoptera	Leuctridae	Leuctra tenuis
Prostoia besametsa 16S ribosomal RNA gene, partial	EF623188	77.71	96.88	4.06463e−21	Plecoptera	Nemouridae	Prostoia besametsa
Plumiperla diversa 16S ribosomal RNA gene, partial	EF623132	80	78.12	4.06463e−21	Plecoptera	Chloroperlidae	Plumiperla diversa
Pteronarcys princeps mitochondrion, complete genom	AY687866	76.07	99.38	4.06463e−21	Plecoptera	Pteronarcyidae	Pteronarcys princeps
Scopura montana 16S ribosomal RNA gene, partial se	EF623265	80.65	76.25	1.4187e−20	Plecoptera		Scopura montana
Pteronarcys californica 16S ribosomal RNA gene, pa	EF623261	75.46	99.38	4.95174e−20	Plecoptera	Pteronarcyidae	Pteronarcys californica
Claassenia sabulosa 16S ribosomal RNA gene, partia	EF623250	85.44	64.38	4.95174e−20	Plecoptera	Perlidae	Claassenia sabulosa
Nemoura cinerea 16S ribosomal RNA gene, partial se	EF623194	83.18	66.88	4.95174e−20	Plecoptera	Nemouridae	Nemoura cinerea
Suwallia teleckojensis 16S ribosomal RNA gene, par	EF623142	80.7	71.25	4.95174e−20	Plecoptera	Chloroperlidae	Suwallia teleckojensis
Neaviperla forcipata 16S ribosomal RNA gene, parti	EF623139	80.7	71.25	4.95174e−20	Plecoptera	Chloroperlidae	Neaviperla forcipata
Leuctra tenuis haplotype 4lt: HO4 16S ribosomal RN	EU327676	80.34	73.12	4.95174e−20	Plecoptera	Leuctridae	Leuctra tenuis
Pteronarcys sachalina 16S ribosomal RNA gene, part	EF623264	75.61	99.38	1.72833e−19	Plecoptera	Pteronarcyidae	Pteronarcys sachalina
Nemoura trispinosa 16S ribosomal RNA gene, partial	EF623187	80	77.5	1.72833e−19	Plecoptera	Nemouridae	Nemoura trispinosa
Paranemoura claasseni 16S ribosomal RNA gene, part	EF623185	77.27	95	1.72833e−19	Plecoptera	Nemouridae	Paranemoura claasseni
Shipsa rotunda 16S ribosomal RNA gene, partial seq	EF623184	77.27	95	1.72833e−19	Plecoptera	Nemouridae	Shipsa rotunda
Leuctra inermis 16S ribosomal RNA gene, partial se	EF623176	80.17	75	1.72833e−19	Plecoptera	Leuctridae	Leuctra inermis
Allocapnia recta isolate H18 16S ribosomal RNA gen	KC881053	76.35	91.88	6.03245e−19	Plecoptera	Capniidae	Allocapnia recta
Allocapnia recta isolate H17 16S ribosomal RNA gen	KC881052	76.35	91.88	6.03245e−19	Plecoptera	Capniidae	Allocapnia recta
Allocapnia recta isolate H12 16S ribosomal RNA gen	KC881047	76.35	91.88	6.03245e−19	Plecoptera	Capniidae	Allocapnia recta
Allocapnia recta isolate H9 16S ribosomal RNA gene	KC881044	76.35	91.88	6.03245e−19	Plecoptera	Capniidae	Allocapnia recta
Allocapnia recta isolate H8 16S ribosomal RNA gene	KC881043	76.35	91.88	6.03245e−19	Plecoptera	Capniidae	Allocapnia recta
Allocapnia recta isolate H5 16S ribosomal RNA gene	KC881040	76.35	91.88	6.03245e−19	Plecoptera	Capniidae	Allocapnia recta
Allocapnia recta isolate H4 16S ribosomal RNA gene	KC881039	76.35	91.88	6.03245e−19	Plecoptera	Capniidae	Allocapnia recta
Pteronarcys scotti 16S ribosomal RNA gene, partial	EF623263	74.69	99.38	6.03245e−19	Plecoptera	Pteronarcyidae	Pteronarcys scotti
Acroneuria lycorias 16S ribosomal RNA gene, partia	EF623251	80.53	68.75	6.03245e−19	Plecoptera	Perlidae	Acroneuria lycorias
Cosumnoperla hypocrena 16S ribosomal RNA gene, par	EF623226	80.7	71.25	6.03245e−19	Plecoptera	Perlodidae	Cosumnoperla hypocrena
Claudioperla sp. BYU_PL158 16S ribosomal RNA gene,	EF623169	75.16	99.38	6.03245e−19	Plecoptera	Gripopterygidae	Claudioperla sp. BYU_PL158
Chloroperla tripunctata 16S ribosomal RNA gene, pa	EF623137	80.49	76.25	6.03245e−19	Plecoptera	Chloroperlidae	Chloroperla tripunctata
Triznaka pintada 16S ribosomal RNA gene, partial s	EF623134	79.67	76.25	6.03245e−19	Plecoptera	Chloroperlidae	Triznaka pintada
Allocapnia recta haplotype 10ar: H010 16S ribosoma	EU306873	76.35	91.88	6.03245e−19	Plecoptera	Capniidae	Allocapnia recta
Allocapnia recta haplotype 9ar: H09 16S ribosomal	EU306872	76.35	91.88	6.03245e−19	Plecoptera	Capniidae	Allocapnia recta
Allocapnia recta haplotype 8ar: H08 16S ribosomal	EU306871	76.35	91.88	6.03245e−19	Plecoptera	Capniidae	Allocapnia recta
Allocapnia recta haplotype 1ar: H01 16S ribosomal	DQ915179	76.35	91.88	6.03245e−19	Plecoptera	Capniidae	Allocapnia recta
Styloperla sp. mitochondrion, partial genome	KR088971	75.91	85.62	7.34903e−18	Plecoptera	Styloperlidae	Styloperla sp. YZD−2015
Strophopteryx appalachia 16S ribosomal RNA gene, p	EF623271	78.4	77.5	7.34903e−18	Plecoptera	Taeniopterygidae	Strophopteryx appalachia
Skwala americana 16S ribosomal RNA gene, partial s	EF623217	80.95	65.62	7.34903e−18	Plecoptera	Perlodidae	Skwala americana
Lednia tumana 16S ribosomal RNA gene, partial sequ	EF623197	78.4	77.5	7.34903e−18	Plecoptera	Nemouridae	Lednia tumana
Nemurella pictetii 16S ribosomal RNA gene, partial	EF623192	82.52	64.38	7.34903e−18	Plecoptera	Nemouridae	Nemurella pictetii
Malenka californica 16S ribosomal RNA gene, partia	EF623182	82.52	64.38	7.34903e−18	Plecoptera	Nemouridae	Malenka californica
Moselia infuscata 16S ribosomal RNA gene, partial	EF623175	75	100	7.34903e−18	Plecoptera	Leuctridae	Moselia infuscata
Dinotoperla sp. BYU_PL105 16S ribosomal RNA gene,	EF623160	74.69	99.38	7.34903e−18	Plecoptera	Gripopterygidae	Dinotoperla sp. BYU_PL105
Sasquaperla hoopa 16S ribosomal RNA gene, partial	EF623143	81.55	64.38	7.34903e−18	Plecoptera	Chloroperlidae	Sasquaperla hoopa
Paraperla frontalis 16S ribosomal RNA gene, partia	EF623136	82.52	64.38	7.34903e−18	Plecoptera	Chloroperlidae	Paraperla frontalis
Allocapnia recta isolate H19 16S ribosomal RNA gen	KC881054	75.68	91.88	2.56506e−17	Plecoptera	Capniidae	Allocapnia recta
Allocapnia recta isolate H16 16S ribosomal RNA gen	KC881051	75.68	91.88	2.56506e−17	Plecoptera	Capniidae	Allocapnia recta
Allocapnia recta isolate H15 16S ribosomal RNA gen	KC881050	75.68	91.88	2.56506e−17	Plecoptera	Capniidae	Allocapnia recta
Allocapnia recta isolate H14 16S ribosomal RNA gen	KC881049	75.68	91.88	2.56506e−17	Plecoptera	Capniidae	Allocapnia recta
Allocapnia recta isolate H10 16S ribosomal RNA gen	KC881045	75.68	91.88	2.56506e−17	Plecoptera	Capniidae	Allocapnia recta
Allocapnia recta isolate H6 16S ribosomal RNA gene	KC881041	75.68	91.88	2.56506e−17	Plecoptera	Capniidae	Allocapnia recta
Cryptoperla sp. YK−2012−1 mitochondrial gene for 1	AB711243	78.45	72.5	2.56506e−17	Plecoptera	Peltoperlidae	Cryptoperla sp. YK−2012−1
Udenus wnigrum voucher MAY 16S ribosomal RNA gene,	HM594504	79.13	70.62	2.56506e−17	Orthoptera	Rhaphidophoridae	Udenus wnigrum
Doddsia occidentalis 16S ribosomal RNA gene, parti	EF623270	75.33	93.12	2.56506e−17	Plecoptera	Taeniopterygidae	Doddsia occidentalis
Pteronarcella badia 16S ribosomal RNA gene, partia	EF623262	80.37	66.88	2.56506e−17	Plecoptera	Pteronarcyidae	Pteronarcella badia
Pictetoperla sp. BYU_PL156 16S ribosomal RNA gene,	EF623247	81.31	66.88	2.56506e−17	Plecoptera	Perlidae	Pictetoperla sp. BYU_PL156
Osobenus yakimae 16S ribosomal RNA gene, partial s	EF623232	75.84	90	2.56506e−17	Plecoptera	Perlodidae	Osobenus yakimae
Cascadoperla trictura 16S ribosomal RNA gene, part	EF623225	75.33	91.88	2.56506e−17	Plecoptera	Perlodidae	Cascadoperla trictura
Salmoperla sylvanica 16S ribosomal RNA gene, parti	EF623222	80.37	66.88	2.56506e−17	Plecoptera	Perlodidae	Salmoperla sylvanica
Megaleuctra kincaidi 16S ribosomal RNA gene, parti	EF623174	81.31	66.88	2.56506e−17	Plecoptera	Leuctridae	Megaleuctra kincaidi
Paraleuctra vershina 16S ribosomal RNA gene, parti	EF623173	81.31	66.88	2.56506e−17	Plecoptera	Leuctridae	Paraleuctra vershina
Allocapnia recta haplotype 11ar: H011 16S ribosoma	EU306874	75.68	91.88	2.56506e−17	Plecoptera	Capniidae	Allocapnia recta
Allocapnia recta haplotype 7ar: H07 16S ribosomal	EU306870	75.68	91.88	2.56506e−17	Plecoptera	Capniidae	Allocapnia recta
Allocapnia recta haplotype 2ar: H02 16S ribosomal	DQ915180	75.68	91.88	2.56506e−17	Plecoptera	Capniidae	Allocapnia recta
Mesocapnia arizonensis mitochondrion, partial geno	KP642637	80.58	64.38	8.95295e−17	Plecoptera	Capniidae	Mesocapnia arizonensis
Allocapnia recta isolate H13 16S ribosomal RNA gen	KC881048	80.58	64.38	8.95295e−17	Plecoptera	Capniidae	Allocapnia recta
Allocapnia recta isolate H11 16S ribosomal RNA gen	KC881046	80.58	64.38	8.95295e−17	Plecoptera	Capniidae	Allocapnia recta
Allocapnia recta isolate H7 16S ribosomal RNA gene	KC881042	80.58	64.38	8.95295e−17	Plecoptera	Capniidae	Allocapnia recta
Allocapnia recta isolate H3 16S ribosomal RNA gene	KC881038	80.58	64.38	8.95295e−17	Plecoptera	Capniidae	Allocapnia recta
Allocapnia recta isolate H1 16S ribosomal RNA gene	KC881036	80.58	64.38	8.95295e−17	Plecoptera	Capniidae	Allocapnia recta
Dolichopoda pavesii voucher SPI 16S ribosomal RNA	EF216951	78.12	76.88	8.95295e−17	Orthoptera	Rhaphidophoridae	Dolichopoda pavesii
Perlinodes aurea 16S ribosomal RNA gene, partial s	EF623234	80.58	64.38	8.95295e−17	Plecoptera	Perlodidae	Perlinodes aurea
Frisonia picticeps 16S ribosomal RNA gene, partial	EF623227	78.45	72.5	8.95295e−17	Plecoptera	Perlodidae	Frisonia picticeps
Nanonemoura wahkeena 16S ribosomal RNA gene, parti	EF623198	75.16	95	8.95295e−17	Plecoptera	Nemouridae	Nanonemoura wahkeena
Protonemura meyeri 16S ribosomal RNA gene, partial	EF623193	77.6	77.5	8.95295e−17	Plecoptera	Nemouridae	Protonemura meyeri
Ostrocerca foersteri 16S ribosomal RNA gene, parti	EF623191	81.55	64.38	8.95295e−17	Plecoptera	Nemouridae	Ostrocerca foersteri
Visoka cataractae 16S ribosomal RNA gene, partial	EF623189	81.55	64.38	8.95295e−17	Plecoptera	Nemouridae	Visoka cataractae
Pelurgoperla personata 16S ribosomal RNA gene, par	EF623168	77.6	77.5	8.95295e−17	Plecoptera	Gripopterygidae	Pelurgoperla personata
Cardioperla lobata 16S ribosomal RNA gene, partial	EF623164	77.6	77.5	8.95295e−17	Plecoptera	Gripopterygidae	Cardioperla lobata
Antarctoperla sp. BYU_PL142 16S ribosomal RNA gene	EF623163	74.07	99.38	8.95295e−17	Plecoptera	Gripopterygidae	Antarctoperla sp. BYU_PL142
Siphonoperla torrentium 16S ribosomal RNA gene, pa	EF623138	77.87	76.25	8.95295e−17	Plecoptera	Chloroperlidae	Siphonoperla torrentium
Sweltsa coloradensis 16S ribosomal RNA gene, parti	EF623133	80.58	64.38	8.95295e−17	Plecoptera	Chloroperlidae	Sweltsa coloradensis
