# Supplementary material for: Testing the potential of a ribosomal 16S marker for DNA metabarcoding of insects
Source: PeerJ. 2016 Apr 19;4:e1966. doi: 10.7717/peerj.1966 (PMC4841222; doi:10.7717/peerj.1966)

OTU_159.csv

Chaetopteryx fusca

Limnephilidae

Trichoptera

94.08

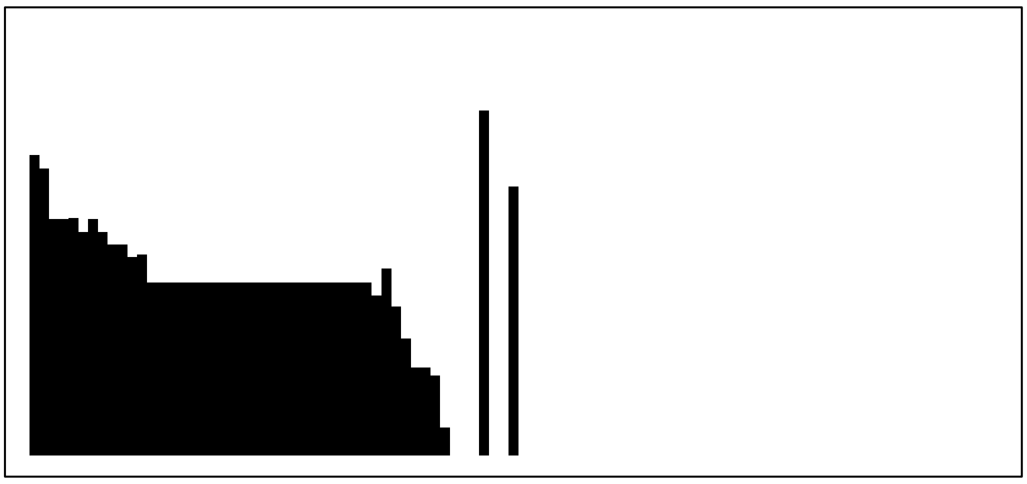

Chaetopteryx fusca voucher ChfIM1 16S ribosomal RN	FJ002820	94.08	100	5.61543e−64	Trichoptera	Limnephilidae	Chaetopteryx fusca
Annitella apfelbecki voucher AaIM1 16S ribosomal R	FJ002819	93.45	100	6.84099e−63	Trichoptera	Limnephilidae	Annitella apfelbecki
Drusus chrysotus voucher DCh004 16S ribosomal RNA	EU215189	91.07	100	1.50683e−58	Trichoptera	Limnephilidae	Drusus chrysotus
Drusus chrysotus voucher DCh001 16S ribosomal RNA	EU215188	91.07	100	1.50683e−58	Trichoptera	Limnephilidae	Drusus chrysotus
Ecclisocosmoecus spinosus mitochondrial gene for 1	AB355447	91.12	100	5.25935e−58	Trichoptera	Limnephilidae	Ecclisocosmoecus spinosus
Cryptothrix nebulicola voucher CN006 16S ribosomal	EU215177	90.48	100	1.83569e−57	Trichoptera	Limnephilidae	Cryptothrix nebulicola
Chaetopterygopsis maclachlani voucher CM001 16S ri	EU215176	91.07	100	1.83569e−57	Trichoptera	Limnephilidae	Chaetopterygopsis maclachlani
Chaetopteryx rugulosa voucher CRu002 16S ribosomal	EU215178	90.48	100	6.4072e−57	Trichoptera	Limnephilidae	Chaetopteryx rugulosa
Anomalopterygella chauviniana voucher AC001 16S ri	EU215174	89.88	100	7.80557e−56	Trichoptera	Limnephilidae	Anomalopterygella chauviniana
Allogamus uncatus voucher AUn003 16S ribosomal RNA	KM001840	89.88	100	9.50913e−55	Trichoptera	Limnephilidae	Allogamus uncatus
Asynarchus sachalinensis mitochondrial gene for 16	AB355450	89.29	100	1.15845e−53	Trichoptera	Limnephilidae	Asynarchus sachalinensis
Consorophylax consors voucher CCon001 16S ribosoma	EU215175	89.41	100	1.41128e−52	Trichoptera	Limnephilidae	Consorophylax consors
Ecclisopteryx keroveci voucher E2JAL2 16S ribosoma	KM001845	88.1	100	4.92585e−52	Trichoptera	Limnephilidae	Ecclisopteryx keroveci
Ecclisopteryx keroveci voucher E2JAL1 16S ribosoma	KM001844	88.1	100	4.92585e−52	Trichoptera	Limnephilidae	Ecclisopteryx keroveci
Ecclisopteryx keroveci voucher E1JAL2 16S ribosoma	KM001843	88.1	100	4.92585e−52	Trichoptera	Limnephilidae	Ecclisopteryx keroveci
Ecclisopteryx keroveci voucher E1JAL1 16S ribosoma	KM001842	88.1	100	4.92585e−52	Trichoptera	Limnephilidae	Ecclisopteryx keroveci
Ecclisopteryx ivkae voucher fEda1202L 16S ribosoma	KM001810	88.1	100	4.92585e−52	Trichoptera	Limnephilidae	Ecclisopteryx ivkae
Ecclisopteryx ivkae voucher fEda1201L 16S ribosoma	KM001809	88.1	100	4.92585e−52	Trichoptera	Limnephilidae	Ecclisopteryx ivkae
Ecclisopteryx ivkae voucher fEda0102M 16S ribosoma	KM001808	88.1	100	4.92585e−52	Trichoptera	Limnephilidae	Ecclisopteryx ivkae
Ecclisopteryx ivkae voucher fEda0102F 16S ribosoma	KM001807	88.1	100	4.92585e−52	Trichoptera	Limnephilidae	Ecclisopteryx ivkae
Ecclisopteryx ivkae voucher fEda0101M 16S ribosoma	KM001806	88.1	100	4.92585e−52	Trichoptera	Limnephilidae	Ecclisopteryx ivkae
Ecclisopteryx ivkae voucher fDsp3301L 16S ribosoma	KM001804	88.1	100	4.92585e−52	Trichoptera	Limnephilidae	Ecclisopteryx ivkae
Ecclisopteryx keroveci voucher fEns0101M 16S ribos	KM001784	88.1	100	4.92585e−52	Trichoptera	Limnephilidae	Ecclisopteryx keroveci
Ecclisopteryx keroveci voucher fEda1101F 16S ribos	KM001783	88.1	100	4.92585e−52	Trichoptera	Limnephilidae	Ecclisopteryx keroveci
Ecclisopteryx keroveci voucher fEda1001M 16S ribos	KM001782	88.1	100	4.92585e−52	Trichoptera	Limnephilidae	Ecclisopteryx keroveci
Ecclisopteryx keroveci voucher fEda0301M 16S ribos	KM001781	88.1	100	4.92585e−52	Trichoptera	Limnephilidae	Ecclisopteryx keroveci
Ecclisopteryx keroveci voucher fEda0202F 16S ribos	KM001780	88.1	100	4.92585e−52	Trichoptera	Limnephilidae	Ecclisopteryx keroveci
Ecclisopteryx keroveci voucher fEda0201M 16S ribos	KM001779	88.1	100	4.92585e−52	Trichoptera	Limnephilidae	Ecclisopteryx keroveci
Ecclisopteryx keroveci voucher fDs30109F 16S ribos	KM001778	88.1	100	4.92585e−52	Trichoptera	Limnephilidae	Ecclisopteryx keroveci
Ecclisopteryx keroveci voucher fDs30106F 16S ribos	KM001777	88.1	100	4.92585e−52	Trichoptera	Limnephilidae	Ecclisopteryx keroveci
Ecclisopteryx keroveci voucher fDs30105M 16S ribos	KM001776	88.1	100	4.92585e−52	Trichoptera	Limnephilidae	Ecclisopteryx keroveci
Ecclisopteryx keroveci voucher fDs30103M 16S ribos	KM001775	88.1	100	4.92585e−52	Trichoptera	Limnephilidae	Ecclisopteryx keroveci
Ecclisopteryx keroveci voucher fDs30102M 16S ribos	KM001774	88.1	100	4.92585e−52	Trichoptera	Limnephilidae	Ecclisopteryx keroveci
Ecclisopteryx keroveci voucher fDs30101M 16S ribos	KM001773	88.1	100	4.92585e−52	Trichoptera	Limnephilidae	Ecclisopteryx keroveci
Ecclisopteryx dalecarlica voucher EdIM1 16S riboso	FJ002818	88.1	100	4.92585e−52	Trichoptera	Limnephilidae	Ecclisopteryx dalecarlica
Ecclisopteryx ivkae voucher fEda0101F 16S ribosoma	KM001805	87.5	100	2.09452e−50	Trichoptera	Limnephilidae	Ecclisopteryx ivkae
Rivulophilus sakaii mitochondrial gene for 16S rib	AB355449	88.76	100	2.09452e−50	Trichoptera	Limnephilidae	Rivulophilus sakaii
Hydatophylax festivus mitochondrial gene for 16S r	AB355448	86.98	100	1.32179e−46	Trichoptera	Limnephilidae	Hydatophylax festivus
Ironoquia punctatissima mitochondrial gene for 16S	AB355446	85.47	100	1.01619e−41	Trichoptera	Limnephilidae	Ironoquia punctatissima
Nothopsyche montivaga mitochondrial gene for 16S r	AB355431	84.12	100	1.50816e−39	Trichoptera	Limnephilidae	Nothopsyche montivaga
Nothopsyche montivaga mitochondrial gene for 16S r	AB355432	84.12	100	1.50816e−39	Trichoptera	Limnephilidae	Nothopsyche montivaga
Dicosmoecus jozankeanus mitochondrial gene for 16S	AB355445	83.75	94.05	2.72682e−36	Trichoptera	Limnephilidae	Dicosmoecus jozankeanus
Taskiropsyche lacustris voucher NHRS−EN CS5 16S ri	FJ263209	81.29	80.95	8.34877e−24	Trichoptera	Kokiriidae	Taskiropsyche lacustris
Eubasilissa regina mitochondrion, complete genome	KF756943	77.51	100	1.23907e−21	Trichoptera		Eubasilissa regina
Myiodactylus osmyloides 16S ribosomal RNA gene, pa	EU734873	73.56	100	7.32353e−12	Neuroptera	Nymphidae	Myiodactylus osmyloides
Bambusiphaga fascia isolate 181 16S ribosomal RNA	HM233787	73.17	94.64	3.79368e−09	Hemiptera	Delphacidae	Bambusiphaga fascia
Ovis canadensis canadensis isolate 43U chromosome	CP011888	96.15	15.48	1.84056		Bovidae	Ovis canadensis canadensis
Strongyloides venezuelensis genome assembly S_vene	LM524980	75.86	34.52	1.84056	Rhabditida	Strongyloididae	Strongyloides venezuelensis
Mycoplasma canadense DNA, complete genome, strain:	AP014631	77.42	35.12	1.84056	Mycoplasmatales	Mycoplasmataceae	Mycoplasma canadense
Protopolystoma xenopodis genome assembly P_xenopod	LM682778	92.59	16.07	6.4242	Polyopisthocotylea	Polystomatidae	Protopolystoma xenopodis

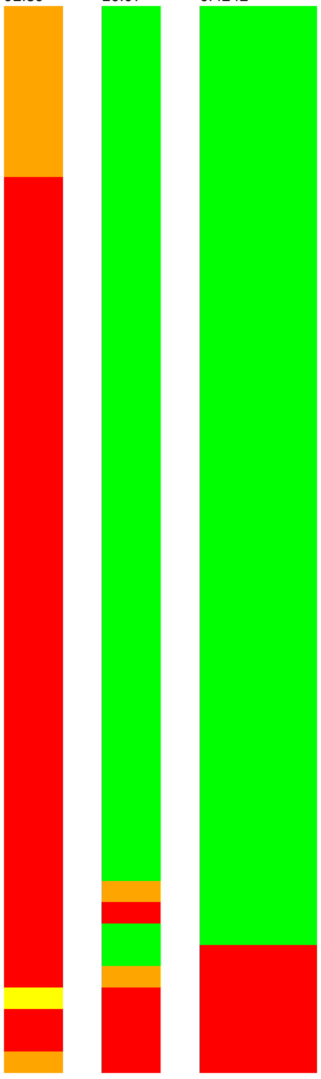

Supplement: Supplemental Information 1 [file OTU_159.csv_plot.pdf › scripts/5 match OTUs/blast_hits/OTU_159.csv_plot.pdf]
