# Supplementary material for: Testing the potential of a ribosomal 16S marker for DNA metabarcoding of insects
Source: PeerJ. 2016 Apr 19;4:e1966. doi: 10.7717/peerj.1966 (PMC4841222; doi:10.7717/peerj.1966)
Supplement: Supplemental Information 1 [file OTU_161.csv_plot.pdf › scripts/5 match OTUs/blast_hits/OTU_161.csv_plot.pdf]

OTU_161.csv

Dinocras cephalotes

Perlidae

Plecoptera

90.85

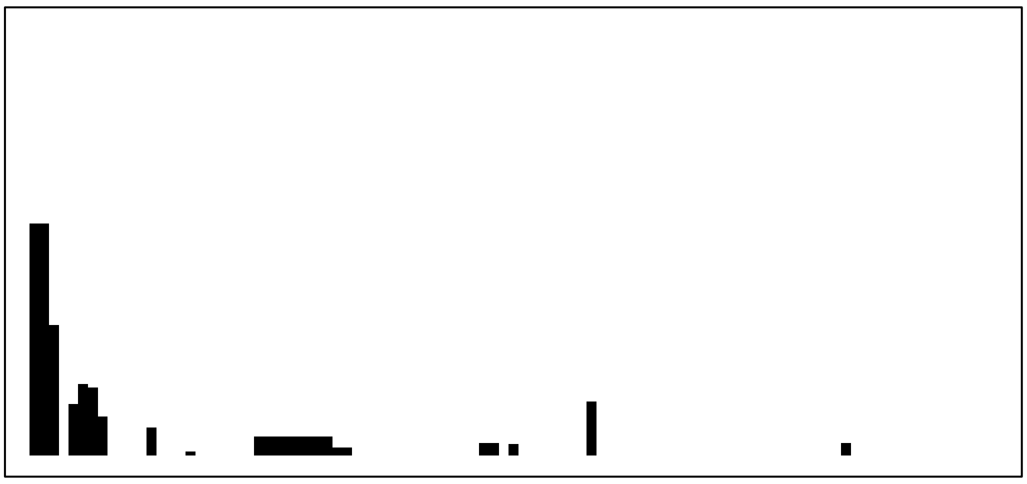

Dinocras cephalotes mitochondrion, complete genome	KF484757	90.85	100	7.45344e−56	Plecoptera	Perlidae	Dinocras cephalotes
Dinocras cephalotes 16S ribosomal RNA gene, partia	EF623244	90.85	100	7.45344e−56	Plecoptera	Perlidae	Dinocras cephalotes
Perlinella drymo 16S ribosomal RNA gene, partial s	EF623240	86.11	66.67	2.28405e−24	Plecoptera	Perlidae	Perlinella drymo
Calineuria californica 16S ribosomal RNA gene, par	EF623239	78.95	93.83	7.97213e−24	Plecoptera	Perlidae	Calineuria californica
Perlesta decipines 16S ribosomal RNA gene, partial	EF623242	82.4	77.16	2.78255e−23	Plecoptera	Perlidae	Perlesta decipines
Hansonoperla appalachia 16S ribosomal RNA gene, pa	EF623249	83.33	66.67	1.18317e−21	Plecoptera	Perlidae	Hansonoperla appalachia
Kathroperla perdita 16S ribosomal RNA gene, partia	EF623135	83.18	66.05	1.18317e−21	Plecoptera	Chloroperlidae	Kathroperla perdita
Agnetina capitata 16S ribosomal RNA gene, partial	EF623241	81.82	67.9	1.4414e−20	Plecoptera	Perlidae	Agnetina capitata
Notonemouridae sp. MD−2011 voucher ZMUC:JD−1507 16	JN615257	79.2	76.54	1.75598e−19	Plecoptera	Notonemouridae	Notonemouridae sp. MD−2011
Pteronarcys californica 16S ribosomal RNA gene, pa	EF623261	77.93	88.27	1.75598e−19	Plecoptera	Pteronarcyidae	Pteronarcys californica
Dinotoperla sp. BYU_PL105 16S ribosomal RNA gene,	EF623160	75.76	99.38	1.75598e−19	Plecoptera	Gripopterygidae	Dinotoperla sp. BYU_PL105
Pteronarcys princeps mitochondrion, complete genom	AY687866	77.93	88.27	1.75598e−19	Plecoptera	Pteronarcyidae	Pteronarcys princeps
Nemoura trispinosa 16S ribosomal RNA gene, partial	EF623187	81.31	66.05	6.12897e−19	Plecoptera	Nemouridae	Nemoura trispinosa
Dinotoperla serricauda 16S ribosomal RNA gene, par	EF623165	80	76.54	6.12897e−19	Plecoptera	Gripopterygidae	Dinotoperla serricauda
Pteronarcys scotti 16S ribosomal RNA gene, partial	EF623263	76.92	88.27	2.13922e−18	Plecoptera	Pteronarcyidae	Pteronarcys scotti
Udamocercia sp. BYU_PL150 16S ribosomal RNA gene,	EF623201	80	70.99	2.13922e−18	Plecoptera	Notonemouridae	Udamocercia sp. BYU_PL150
Leuctra inermis 16S ribosomal RNA gene, partial se	EF623176	80.17	74.07	2.13922e−18	Plecoptera	Leuctridae	Leuctra inermis
Moselia infuscata 16S ribosomal RNA gene, partial	EF623175	75.61	100	2.13922e−18	Plecoptera	Leuctridae	Moselia infuscata
Cardioperla lobata 16S ribosomal RNA gene, partial	EF623164	76.32	92.59	2.13922e−18	Plecoptera	Gripopterygidae	Cardioperla lobata
Scopura montana 16S ribosomal RNA gene, partial se	EF623265	79.13	70.99	7.46661e−18	Plecoptera		Scopura montana
Acroneuria lycorias 16S ribosomal RNA gene, partia	EF623251	75	99.38	7.46661e−18	Plecoptera	Perlidae	Acroneuria lycorias
Prostoia besametsa 16S ribosomal RNA gene, partial	EF623188	75.32	96.91	7.46661e−18	Plecoptera	Nemouridae	Prostoia besametsa
Plumiperla diversa 16S ribosomal RNA gene, partial	EF623132	77.03	90.12	7.46661e−18	Plecoptera	Chloroperlidae	Plumiperla diversa
Leuctra tenuis haplotype 10lt: HO10 16S ribosomal	EU327682	80.87	70.37	7.46661e−18	Plecoptera	Leuctridae	Leuctra tenuis
Leuctra tenuis haplotype 9lt: HO9 16S ribosomal RN	EU327681	80.87	70.37	7.46661e−18	Plecoptera	Leuctridae	Leuctra tenuis
Leuctra tenuis haplotype 8lt: HO8 16S ribosomal RN	EU327680	80.87	70.37	7.46661e−18	Plecoptera	Leuctridae	Leuctra tenuis
Leuctra tenuis haplotype 7lt: HO7 16S ribosomal RN	EU327679	80.87	70.37	7.46661e−18	Plecoptera	Leuctridae	Leuctra tenuis
Leuctra tenuis haplotype 6lt: HO6 16S ribosomal RN	EU327678	80.87	70.37	7.46661e−18	Plecoptera	Leuctridae	Leuctra tenuis
Leuctra tenuis haplotype 3lt: H03 16S ribosomal RN	DQ915184	80.87	70.37	7.46661e−18	Plecoptera	Leuctridae	Leuctra tenuis
Leuctra tenuis haplotype 2lt: H02 16S ribosomal RN	DQ915183	80.87	70.37	7.46661e−18	Plecoptera	Leuctridae	Leuctra tenuis
Leuctra tenuis haplotype 1lt: H01 16S ribosomal RN	DQ915182	80.87	70.37	7.46661e−18	Plecoptera	Leuctridae	Leuctra tenuis
Lednia tumana 16S ribosomal RNA gene, partial sequ	EF623197	80.37	66.05	2.6051e−17	Plecoptera	Nemouridae	Lednia tumana
Nemoura cinerea 16S ribosomal RNA gene, partial se	EF623194	80.37	66.05	2.6051e−17	Plecoptera	Nemouridae	Nemoura cinerea
Riekoperla triloba 16S ribosomal RNA gene, partial	EF623171	75.8	95.68	2.6051e−17	Plecoptera	Gripopterygidae	Riekoperla triloba
Styloperla sp. mitochondrion, partial genome	KR088971	74.84	91.98	9.0962e−17	Plecoptera	Styloperlidae	Styloperla sp. YZD−2015
Allocapnia recta isolate H18 16S ribosomal RNA gen	KC881053	75.69	88.27	9.0962e−17	Plecoptera	Capniidae	Allocapnia recta
Allocapnia recta isolate H17 16S ribosomal RNA gen	KC881052	75.69	88.27	9.0962e−17	Plecoptera	Capniidae	Allocapnia recta
Allocapnia recta isolate H12 16S ribosomal RNA gen	KC881047	75.69	88.27	9.0962e−17	Plecoptera	Capniidae	Allocapnia recta
Allocapnia recta isolate H9 16S ribosomal RNA gene	KC881044	75.69	88.27	9.0962e−17	Plecoptera	Capniidae	Allocapnia recta
Allocapnia recta isolate H8 16S ribosomal RNA gene	KC881043	75.69	88.27	9.0962e−17	Plecoptera	Capniidae	Allocapnia recta
Allocapnia recta isolate H5 16S ribosomal RNA gene	KC881040	75.69	88.27	9.0962e−17	Plecoptera	Capniidae	Allocapnia recta
Allocapnia recta isolate H4 16S ribosomal RNA gene	KC881039	75.69	88.27	9.0962e−17	Plecoptera	Capniidae	Allocapnia recta
Allocapnia recta isolate H1 16S ribosomal RNA gene	KC881036	75.69	88.27	9.0962e−17	Plecoptera	Capniidae	Allocapnia recta
Taenionema palladium 16S ribosomal RNA gene, parti	EF623267	75	99.38	9.0962e−17	Plecoptera	Taeniopterygidae	Taenionema palladium
Frisonia picticeps 16S ribosomal RNA gene, partial	EF623227	78.4	76.54	9.0962e−17	Plecoptera	Perlodidae	Frisonia picticeps
Cascadoperla trictura 16S ribosomal RNA gene, part	EF623225	76.67	88.89	9.0962e−17	Plecoptera	Perlodidae	Cascadoperla trictura
Nanonemoura wahkeena 16S ribosomal RNA gene, parti	EF623198	80.58	63.58	9.0962e−17	Plecoptera	Nemouridae	Nanonemoura wahkeena
Nemurella pictetii 16S ribosomal RNA gene, partial	EF623192	80.58	63.58	9.0962e−17	Plecoptera	Nemouridae	Nemurella pictetii
Diamphipnopsis sp. BYU_PL138 16S ribosomal RNA gen	EF623145	78.4	76.54	9.0962e−17	Plecoptera	Diamphipnoidae	Diamphipnopsis sp. BYU_PL138
Penturoperla barbata 16S ribosomal RNA gene, parti	EF623124	80.53	69.75	9.0962e−17	Plecoptera	Austroperlidae	Penturoperla barbata
Allocapnia recta haplotype 10ar: H010 16S ribosoma	EU306873	75.69	88.27	9.0962e−17	Plecoptera	Capniidae	Allocapnia recta
Allocapnia recta haplotype 9ar: H09 16S ribosomal	EU306872	75.69	88.27	9.0962e−17	Plecoptera	Capniidae	Allocapnia recta
Allocapnia recta haplotype 8ar: H08 16S ribosomal	EU306871	75.69	88.27	9.0962e−17	Plecoptera	Capniidae	Allocapnia recta
Leuctra tenuis haplotype 5lt: HO5 16S ribosomal RN	EU327677	80	70.37	9.0962e−17	Plecoptera	Leuctridae	Leuctra tenuis
Leuctra tenuis haplotype 4lt: HO4 16S ribosomal RN	EU327676	80	70.37	9.0962e−17	Plecoptera	Leuctridae	Leuctra tenuis
Allocapnia recta haplotype 1ar: H01 16S ribosomal	DQ915179	75.69	88.27	9.0962e−17	Plecoptera	Capniidae	Allocapnia recta
Udenus wnigrum voucher MAY 16S ribosomal RNA gene,	HM594504	77.97	71.6	3.17489e−16	Orthoptera	Rhaphidophoridae	Udenus wnigrum
Claassenia sabulosa 16S ribosomal RNA gene, partia	EF623250	82.52	63.58	3.17489e−16	Plecoptera	Perlidae	Claassenia sabulosa
Isoperla davisi 16S ribosomal RNA gene, partial se	EF623206	76.51	90.12	3.17489e−16	Plecoptera	Perlodidae	Isoperla davisi
Paranemoura claasseni 16S ribosomal RNA gene, part	EF623185	79.44	66.05	3.17489e−16	Plecoptera	Nemouridae	Paranemoura claasseni
Shipsa rotunda 16S ribosomal RNA gene, partial seq	EF623184	79.44	66.05	3.17489e−16	Plecoptera	Nemouridae	Shipsa rotunda
Despaxia augusta 16S ribosomal RNA gene, partial s	EF623177	77.69	74.07	3.17489e−16	Plecoptera	Leuctridae	Despaxia augusta
Claudioperla sp. BYU_PL158 16S ribosomal RNA gene,	EF623169	74.83	92.59	3.17489e−16	Plecoptera	Gripopterygidae	Claudioperla sp. BYU_PL158
Neaviperla forcipata 16S ribosomal RNA gene, parti	EF623139	75.5	90.74	3.17489e−16	Plecoptera	Chloroperlidae	Neaviperla forcipata
Chloroperla tripunctata 16S ribosomal RNA gene, pa	EF623137	77.95	75.93	3.17489e−16	Plecoptera	Chloroperlidae	Chloroperla tripunctata
Tasmanoperla larvalis 16S ribosomal RNA gene, part	EF623125	79.46	69.14	3.17489e−16	Plecoptera	Austroperlidae	Tasmanoperla larvalis
Pachyrhamma edwardsii voucher CW122 16S ribosomal	HM594508	79.61	63.58	1.10814e−15	Orthoptera	Rhaphidophoridae	Pachyrhamma edwardsii
Doddsia occidentalis 16S ribosomal RNA gene, parti	EF623270	74.39	99.38	1.10814e−15	Plecoptera	Taeniopterygidae	Doddsia occidentalis
Pteronarcys sachalina 16S ribosomal RNA gene, part	EF623264	75.52	88.27	1.10814e−15	Plecoptera	Pteronarcyidae	Pteronarcys sachalina
Suwallia teleckojensis 16S ribosomal RNA gene, par	EF623142	78.95	70.37	1.10814e−15	Plecoptera	Chloroperlidae	Suwallia teleckojensis
Mesocapnia arizonensis mitochondrion, partial geno	KP642637	75	88.27	3.8678e−15	Plecoptera	Capniidae	Mesocapnia arizonensis
Allocapnia recta isolate H19 16S ribosomal RNA gen	KC881054	75	88.27	3.8678e−15	Plecoptera	Capniidae	Allocapnia recta
Allocapnia recta isolate H16 16S ribosomal RNA gen	KC881051	75	88.27	3.8678e−15	Plecoptera	Capniidae	Allocapnia recta
Allocapnia recta isolate H15 16S ribosomal RNA gen	KC881050	75	88.27	3.8678e−15	Plecoptera	Capniidae	Allocapnia recta
Allocapnia recta isolate H14 16S ribosomal RNA gen	KC881049	75	88.27	3.8678e−15	Plecoptera	Capniidae	Allocapnia recta
Allocapnia recta isolate H13 16S ribosomal RNA gen	KC881048	75	88.27	3.8678e−15	Plecoptera	Capniidae	Allocapnia recta
Allocapnia recta isolate H11 16S ribosomal RNA gen	KC881046	75	88.27	3.8678e−15	Plecoptera	Capniidae	Allocapnia recta
Allocapnia recta isolate H10 16S ribosomal RNA gen	KC881045	75	88.27	3.8678e−15	Plecoptera	Capniidae	Allocapnia recta
Allocapnia recta isolate H7 16S ribosomal RNA gene	KC881042	75	88.27	3.8678e−15	Plecoptera	Capniidae	Allocapnia recta
Allocapnia recta isolate H6 16S ribosomal RNA gene	KC881041	75	88.27	3.8678e−15	Plecoptera	Capniidae	Allocapnia recta
Allocapnia recta isolate H3 16S ribosomal RNA gene	KC881038	75	88.27	3.8678e−15	Plecoptera	Capniidae	Allocapnia recta
Prodegeeria japonica mitochondrial gene for 16S rR	AB465910	73.72	95.68	3.8678e−15	Diptera	Tachinidae	Prodegeeria japonica
Dolichopoda pavesii voucher SPI 16S ribosomal RNA	EF216951	73.86	91.36	3.8678e−15	Orthoptera	Rhaphidophoridae	Dolichopoda pavesii
Perlinodes aurea 16S ribosomal RNA gene, partial s	EF623234	80.58	63.58	3.8678e−15	Plecoptera	Perlodidae	Perlinodes aurea
Skwala americana 16S ribosomal RNA gene, partial s	EF623217	75	88.27	3.8678e−15	Plecoptera	Perlodidae	Skwala americana
Austrocerca rieka 16S ribosomal RNA gene, partial	EF623205	73.46	99.38	3.8678e−15	Plecoptera	Notonemouridae	Austrocerca rieka
Austrocercoides zwicki 16S ribosomal RNA gene, par	EF623199	73.46	99.38	3.8678e−15	Plecoptera	Notonemouridae	Austrocercoides zwicki
Protonemura meyeri 16S ribosomal RNA gene, partial	EF623193	76.8	76.54	3.8678e−15	Plecoptera	Nemouridae	Protonemura meyeri
Ostrocerca foersteri 16S ribosomal RNA gene, parti	EF623191	79.61	63.58	3.8678e−15	Plecoptera	Nemouridae	Ostrocerca foersteri
Visoka cataractae 16S ribosomal RNA gene, partial	EF623189	79.61	63.58	3.8678e−15	Plecoptera	Nemouridae	Visoka cataractae
Zapada cinctipes 16S ribosomal RNA gene, partial s	EF623183	79.61	63.58	3.8678e−15	Plecoptera	Nemouridae	Zapada cinctipes
Malenka californica 16S ribosomal RNA gene, partia	EF623182	79.61	63.58	3.8678e−15	Plecoptera	Nemouridae	Malenka californica
Megaleuctra kincaidi 16S ribosomal RNA gene, parti	EF623174	76.8	76.54	3.8678e−15	Plecoptera	Leuctridae	Megaleuctra kincaidi
Limnoperla sp. BYU_PL145 16S ribosomal RNA gene, p	EF623166	74.69	95.68	3.8678e−15	Plecoptera	Gripopterygidae	Limnoperla sp. BYU_PL145
Triznaka pintada 16S ribosomal RNA gene, partial s	EF623134	77.24	75.31	3.8678e−15	Plecoptera	Chloroperlidae	Triznaka pintada
Isocapnia hyalita 16S ribosomal RNA gene, partial	EF623131	79.61	63.58	3.8678e−15	Plecoptera	Capniidae	Isocapnia hyalita
Mesocapnia sp. BYU_PL010 16S ribosomal RNA gene, p	EF623127	75	88.27	3.8678e−15	Plecoptera	Capniidae	Mesocapnia sp. BYU_PL010
Allocapnia recta haplotype 11ar: H011 16S ribosoma	EU306874	75	88.27	3.8678e−15	Plecoptera	Capniidae	Allocapnia recta
Allocapnia recta haplotype 7ar: H07 16S ribosomal	EU306870	75	88.27	3.8678e−15	Plecoptera	Capniidae	Allocapnia recta
Allocapnia recta haplotype 3ar: H03 16S ribosomal	DQ915181	75	88.27	3.8678e−15	Plecoptera	Capniidae	Allocapnia recta
