# Supplementary material for: Testing the potential of a ribosomal 16S marker for DNA metabarcoding of insects
Source: PeerJ. 2016 Apr 19;4:e1966. doi: 10.7717/peerj.1966 (PMC4841222; doi:10.7717/peerj.1966)
Supplement: Supplemental Information 1 [file OTU_17.csv_plot.pdf › scripts/5 match OTUs/blast_hits/OTU_17.csv_plot.pdf]

OTU_17.csv

Cincticostella insolta

Ephemerellidae

Ephemeroptera

80.65

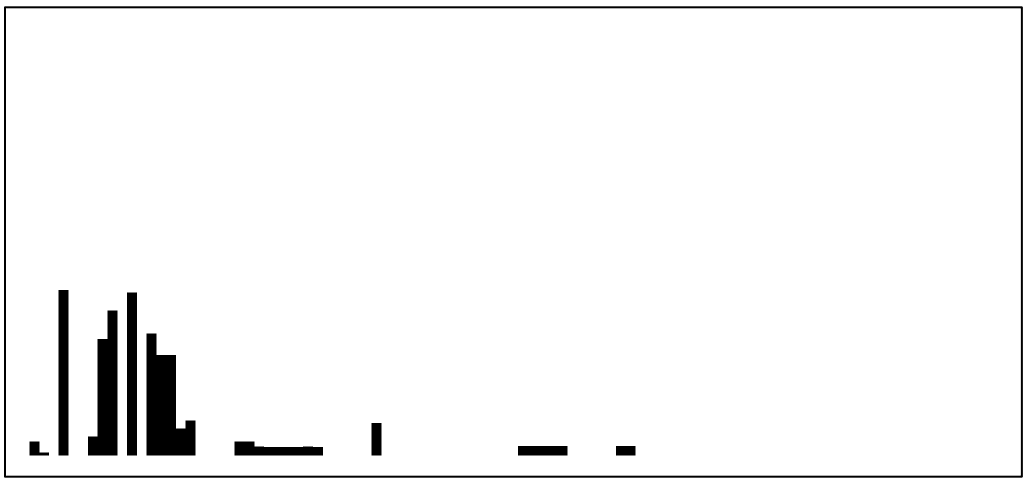

Cincticostella insolta 16S ribosomal RNA gene, par	FJ443029	80.65	100	7.77463e−30	Ephemeroptera	Ephemerellidae	Cincticostella insolta
Drunella pelosa 16S ribosomal RNA gene, partial se	FJ443033	80.13	100	2.71361e−29	Ephemeroptera	Ephemerellidae	Drunella pelosa
Drunella spinifera 16S ribosomal RNA gene, partial	FJ443034	79.47	100	1.15386e−27	Ephemeroptera	Ephemerellidae	Drunella spinifera
Drunella coloradensis 16S ribosomal RNA gene, part	FJ443032	87.74	70.2	1.15386e−27	Ephemeroptera	Ephemerellidae	Drunella coloradensis
Caurinella idahoensis 16S ribosomal RNA gene, part	FJ443028	79.47	100	1.15386e−27	Ephemeroptera	Ephemerellidae	Caurinella idahoensis
Ephemerella atagosana 16S ribosomal RNA gene, part	FJ443039	78.81	100	1.40569e−26	Ephemeroptera	Ephemerellidae	Ephemerella atagosana
Ephacerella longicaudata 16S ribosomal RNA gene, p	FJ443038	80.88	90.07	1.40569e−26	Ephemeroptera	Ephemerellidae	Ephacerella longicaudata
Ephemerella sp. MT−2014 isolate CL54 mitochondrion	KM244691	85.45	72.85	4.90633e−26	Ephemeroptera	Ephemerellidae	Ephemerella sp. MT−2014
Drunella ishiyamana 16S ribosomal RNA gene, partia	FJ443035	86.79	69.54	1.71248e−25	Ephemeroptera	Ephemerellidae	Drunella ishiyamana
Ephemerella needhami 16S ribosomal RNA gene, parti	FJ443042	78.15	100	5.97713e−25	Ephemeroptera	Ephemerellidae	Ephemerella needhami
Ephemerella berneri 16S ribosomal RNA gene, partia	FJ443040	87.63	64.24	2.08622e−24	Ephemeroptera	Ephemerellidae	Ephemerella berneri
Ephemerella sp. THO−2009 16S ribosomal RNA gene, p	FJ443044	78.29	100	2.54154e−23	Ephemeroptera	Ephemerellidae	Ephemerella sp. THO−2009
Drunella doddsi 16S ribosomal RNA gene, partial se	AY749756	85.71	64.9	3.09623e−22	Ephemeroptera	Ephemerellidae	Drunella doddsi
Drunella tuberculata 16S ribosomal RNA gene, parti	FJ443037	84.69	64.9	1.31655e−20	Ephemeroptera	Ephemerellidae	Drunella tuberculata
Drunella trispina 16S ribosomal RNA gene, partial	FJ443036	84.69	64.9	1.31655e−20	Ephemeroptera	Ephemerellidae	Drunella trispina
Teloganopsis media 16S ribosomal RNA gene, partial	FJ443046	81.25	72.85	8.30834e−17	Ephemeroptera	Ephemerellidae	Teloganopsis media
Serratella serrata 16S ribosomal RNA gene, partial	FJ443045	81.63	64.24	1.01216e−15	Ephemeroptera	Ephemerellidae	Serratella serrata
Kirinia epimenides 16S ribosomal RNA gene, partial	JX185909	78.95	71.52	1.50218e−13	Lepidoptera	Nymphalidae	Kirinia epimenides
Attenella margarita 16S ribosomal RNA gene, partia	FJ443027	78.64	67.55	5.24313e−13	Ephemeroptera	Ephemerellidae	Attenella margarita
Attenella margarita 16S ribosomal RNA gene, partia	AY749805	78.64	67.55	5.24313e−13	Ephemeroptera	Ephemerellidae	Attenella margarita
Davidina armandi 16S ribosomal RNA gene, partial s	KM200162	79.82	67.55	1.83003e−12	Lepidoptera	Nymphalidae	Davidina armandi
Parnara guttata voucher He001 16S ribosomal RNA ge	JX971164	80.65	58.94	1.83003e−12	Lepidoptera	Hesperiidae	Parnara guttatus
Parnara guttata guttata 16S ribosomal RNA gene, pa	KJ786305	80.65	58.94	1.83003e−12	Lepidoptera	Hesperiidae	Parnara guttata guttata
Miomantis sp. MD−2011 voucher ZMUC:JD−588 16S ribo	JN615263	80.41	62.91	1.83003e−12	Mantodea	Mantidae	Miomantis sp. MD−2011
Melanargia ines voucher DNAwthmel 224 from Spain 1	GQ201286	80.39	63.58	1.83003e−12	Lepidoptera	Nymphalidae	Melanargia ines
Melanargia ines voucher DNAwthmel 101 from Morocco	GQ201282	80.39	63.58	1.83003e−12	Lepidoptera	Nymphalidae	Melanargia ines
Melanargia ines voucher DNAwthmel 004 from Morocco	GQ201279	80.39	63.58	1.83003e−12	Lepidoptera	Nymphalidae	Melanargia ines
Melanargia ines voucher DNAwthmel 002 from Spain 1	GQ201278	80.39	63.58	1.83003e−12	Lepidoptera	Nymphalidae	Melanargia ines
Mantodea sp. JD−2003 16S ribosomal RNA gene, parti	AY318882	80.41	62.91	1.83003e−12	Mantodea		Mantodea sp. JD−2003
Hipparchia neomiris 16S ribosomal RNA gene, partia	AF214605	80.39	63.58	1.83003e−12	Lepidoptera	Nymphalidae	Hipparchia neomiris
Derops uenoi voucher ZMUN:10029217 16S ribosomal R	JX536485	78.1	66.89	6.38744e−12	Coleoptera	Staphylinidae	Derops uenoi
Miomantis aurea isolate MN228 16S ribosomal RNA ge	EF383435	79.59	63.58	6.38744e−12	Mantodea	Mantidae	Miomantis aurea
Oeneis urda 16S ribosomal RNA gene, partial sequen	JX185886	78.7	67.55	2.22944e−11	Lepidoptera	Nymphalidae	Oeneis urda
Ephoron eophilum mitochondrial gene for 16S rRNA,	AB711633	78.43	66.23	2.22944e−11	Ephemeroptera	Polymitarcyidae	Ephoron eophilum
Miomantis sp. MN191 16S ribosomal RNA gene, partia	FJ806227	79.38	62.91	2.22944e−11	Mantodea	Mantidae	Miomantis sp. MN191
Parnara guttata 16S ribosomal RNA gene, partial se	DQ445110	81.52	58.94	2.22944e−11	Lepidoptera	Hesperiidae	Parnara guttatus
Apimela sp. ZMUN 10029190 16S ribosomal RNA gene,	JX536422	78.1	67.55	7.7815e−11	Coleoptera	Staphylinidae	Apimela sp. ZMUN 10029190
Pharmacophagus antenor voucher FS−b−1646 16S ribos	GQ268346	79.41	63.58	7.7815e−11	Lepidoptera	Papilionidae	Pharmacophagus antenor
Melanargia ines voucher DNAwthmel 098 from Morocco	GQ201280	79.41	63.58	7.7815e−11	Lepidoptera	Nymphalidae	Melanargia ines
Melanargia arge voucher GCB−M 7 from Italy 16S rib	GQ201236	79.41	63.58	7.7815e−11	Lepidoptera	Nymphalidae	Melanargia arge
Gyrophaena flavicornis 16S ribosomal RNA gene, par	EU477783	78.64	66.23	7.7815e−11	Coleoptera	Staphylinidae	Gyrophaena flavicornis
Pharmacophagus antenor mitochondrial partial 16S r	AM283054	79.41	63.58	7.7815e−11	Lepidoptera	Papilionidae	Pharmacophagus antenor
Leptophlebiidae sp. EP030 16S ribosomal RNA gene,	AY749770	79.05	67.55	7.7815e−11	Ephemeroptera		Leptophlebiidae sp. EP030
Anthopotamus sp. EP022 16S ribosomal RNA gene, par	AY749766	78.79	64.24	7.7815e−11	Ephemeroptera	Potamanthidae	Anthopotamus sp. EP022
Pararge aegeria mitochondrion, partial genome	KM592968	77.88	70.2	2.71601e−10	Lepidoptera	Nymphalidae	Pararge aegeria
Pseudochazara hippolyte 16S ribosomal RNA gene, pa	KM200166	77.78	67.55	2.71601e−10	Lepidoptera	Nymphalidae	Pseudochazara hippolyte
Hyponephele sifanica 16S ribosomal RNA gene, parti	KM200147	77.48	69.54	2.71601e−10	Lepidoptera	Nymphalidae	Hyponephele sifanica
Danaus genutia 16S ribosomal RNA gene, partial seq	KJ777731	79.8	62.25	2.71601e−10	Lepidoptera	Nymphalidae	Danaus genutia
Libythea myrrha 16S ribosomal RNA gene, partial se	KJ777730	78.72	59.6	2.71601e−10	Lepidoptera	Nymphalidae	Libythea myrrha
Conicobruchus cicatricosus voucher GK448 16S ribos	KM378102	75.81	80.13	2.71601e−10	Coleoptera	Chrysomelidae	Conicobruchus cicatricosus
Parnara bada voucher He012 16S ribosomal RNA gene,	JX971169	80.43	58.94	2.71601e−10	Lepidoptera	Hesperiidae	Parnara bada
Parnara ganga voucher He029 16S ribosomal RNA gene	JX971167	80.43	58.94	2.71601e−10	Lepidoptera	Hesperiidae	Parnara ganga
Parnara ganga voucher He028 16S ribosomal RNA gene	JX971166	80.43	58.94	2.71601e−10	Lepidoptera	Hesperiidae	Parnara ganga
Parnara bada 16S ribosomal RNA gene, partial seque	KJ786307	80.43	58.94	2.71601e−10	Lepidoptera	Hesperiidae	Parnara bada
Parnara guttata mangala 16S ribosomal RNA gene, pa	KJ786306	80.43	58.94	2.71601e−10	Lepidoptera	Hesperiidae	Parnara guttata mangala
Pararge aegeria aegeria mitochondrion, complete ge	KJ547676	77.88	70.2	2.71601e−10	Lepidoptera	Nymphalidae	Pararge aegeria aegeria
Coenonympha hero 16S ribosomal RNA gene, partial s	JX185898	79	63.58	2.71601e−10	Lepidoptera	Nymphalidae	Coenonympha hero
Neothetalia canadiana voucher ZMUN:10008446 16S ri	JX536451	76.36	71.52	2.71601e−10	Coleoptera	Staphylinidae	Neothetalia canadiana
Danaus genutia isolate F450 16S ribosomal RNA gene	JQ416115	79.8	62.25	2.71601e−10	Lepidoptera	Nymphalidae	Danaus genutia
Danaus genutia isolate F386 16S ribosomal RNA gene	JQ416051	79.8	62.25	2.71601e−10	Lepidoptera	Nymphalidae	Danaus genutia
Parnara guttatus isolate F427 16S ribosomal RNA ge	JQ421806	80.43	58.94	2.71601e−10	Lepidoptera	Hesperiidae	Parnara guttatus
Parnara guttatus isolate F431 16S ribosomal RNA ge	JQ421802	80.43	58.94	2.71601e−10	Lepidoptera	Hesperiidae	Parnara guttatus
Melanargia parce voucher DNAwthmel 156 from Tajiki	GQ201338	77.78	67.55	2.71601e−10	Lepidoptera	Nymphalidae	Melanargia parce
Melanargia parce voucher DNAwthmel 157 from Tajiki	GQ201339	77.78	67.55	2.71601e−10	Lepidoptera	Nymphalidae	Melanargia parce
Melanargia occitanica voucher DNAwthmel 008 from M	GQ201332	78.1	66.89	2.71601e−10	Lepidoptera	Nymphalidae	Melanargia occitanica
Melanargia occitanica voucher DNAwthmel 161 from M	GQ201328	78.1	66.89	2.71601e−10	Lepidoptera	Nymphalidae	Melanargia occitanica
Ephemerella cornutus 16S ribosomal RNA gene, parti	FJ443041	76.7	67.55	2.71601e−10	Ephemeroptera	Ephemerellidae	Ephemerella cornutus
Lasioderma sp. MSL2007 voucher 677505 16S ribosoma	EF213843	77.67	66.89	2.71601e−10	Coleoptera	Anobiidae	Lasioderma serricorne
Leucochrysa robusta voucher CASC 00213 16S ribosom	DQ399281	76.38	81.46	2.71601e−10	Neuroptera	Chrysopidae	Leucochrysa robusta
Themira arctica mitochondrial partial 16S rRNA gen	AJ843100	75.86	72.85	2.71601e−10	Diptera	Sepsidae	Themira arctica
Hyrtanella sp. EP144 16S ribosomal RNA gene, parti	AY749815	76.7	67.55	2.71601e−10	Ephemeroptera	Ephemerellidae	Hyrtanella sp. EP144
Hipparchia semele mitochondrion, partial genome	KM592976	78.43	63.58	9.47981e−10	Lepidoptera	Nymphalidae	Hipparchia semele
Loxerebia saxicola 16S ribosomal RNA gene, partial	KM200173	76.86	76.16	9.47981e−10	Lepidoptera	Nymphalidae	Loxerebia saxicola
Colotis aurigineus voucher GVDP077 16S ribosomal R	HQ682523	78.57	62.25	9.47981e−10	Lepidoptera	Pieridae	Colotis aurigineus
Eumorphus drescheri 16S ribosomal RNA gene, partia	GQ302146	78.22	64.9	9.47981e−10	Coleoptera	Endomychidae	Eumorphus drescheri
Ahlbergia korea 16S ribosomal RNA gene, partial se	GU372488	77.27	70.86	9.47981e−10	Lepidoptera	Lycaenidae	Ahlbergia korea
Melanargia sp. VN−2009 voucher VNLEP00257 from Ira	GQ201276	78.43	63.58	9.47981e−10	Lepidoptera	Nymphalidae	Melanargia sp. VN−2009
Melanargia syriaca voucher DNAwthmel 088 from Turk	GQ201357	78.43	63.58	9.47981e−10	Lepidoptera	Nymphalidae	Melanargia syriaca
Melanargia russiae voucher DNAwthmel 032 from Iran	GQ201345	78.43	63.58	9.47981e−10	Lepidoptera	Nymphalidae	Melanargia russiae
Melanargia parce voucher DNAwthmel 153 from Kyrgyz	GQ201335	78.43	63.58	9.47981e−10	Lepidoptera	Nymphalidae	Melanargia parce
Melanargia parce voucher DNAwthmel 154 from Kyrgyz	GQ201336	78.43	63.58	9.47981e−10	Lepidoptera	Nymphalidae	Melanargia parce
Melanargia occitanica voucher DNAwthmel 005 from F	GQ201330	78.43	63.58	9.47981e−10	Lepidoptera	Nymphalidae	Melanargia occitanica
Melanargia leda voucher GCB−M 10 from China 16S ri	GQ201309	78.43	63.58	9.47981e−10	Lepidoptera	Nymphalidae	Melanargia leda
Melanargia leda voucher GCB−M 9 from China 16S rib	GQ201308	78.43	63.58	9.47981e−10	Lepidoptera	Nymphalidae	Melanargia leda
Melanargia leda voucher GCB−M 11 from China 16S ri	GQ201307	78.43	63.58	9.47981e−10	Lepidoptera	Nymphalidae	Melanargia leda
Melanargia larissa voucher ARSA−256l−001 from Iran	GQ201306	78.43	63.58	9.47981e−10	Lepidoptera	Nymphalidae	Melanargia larissa
Melanargia hylata voucher DNAwthmel 075 from Iran	GQ201269	78.43	63.58	9.47981e−10	Lepidoptera	Nymphalidae	Melanargia hylata
Melanargia evartianae voucher DNAwthmel 042 from I	GQ201246	78.43	63.58	9.47981e−10	Lepidoptera	Nymphalidae	Melanargia evartianae
Oliarces clara 16S ribosomal RNA gene, partial seq	EU734885	76.85	68.87	9.47981e−10	Neuroptera	Ithonidae	Oliarces clara
Onthophagus rorarius 16S ribosomal RNA gene, parti	AY131586	78.57	62.25	9.47981e−10	Coleoptera	Scarabaeidae	Onthophagus rorarius
Milichus apicalis 16S ribosomal RNA gene, partial	AY131566	78.57	62.25	9.47981e−10	Coleoptera	Scarabaeidae	Milichus apicalis
Canthon viridis 16S ribosomal RNA gene, partial se	AY131446	76.85	67.55	9.47981e−10	Coleoptera	Scarabaeidae	Canthon viridis
Notocrypta curvifascia 16S ribosomal RNA gene, par	DQ445109	78.43	62.91	9.47981e−10	Lepidoptera	Hesperiidae	Notocrypta curvifascia
Donacia japana isolate N264 16S ribosomal RNA gene	AY232555	77.45	66.23	9.47981e−10	Coleoptera	Chrysomelidae	Donacia japana
Donacia japana isolate N119 16S ribosomal RNA gene	AY232554	77.45	66.23	9.47981e−10	Coleoptera	Chrysomelidae	Donacia japana
Oeneis taygete 16S ribosomal RNA gene, partial seq	AF214628	78.43	63.58	9.47981e−10	Lepidoptera	Nymphalidae	Oeneis taygete
Erebia aethiopella 16S ribosomal RNA gene, partial	AF214619	77.27	67.55	9.47981e−10	Lepidoptera	Nymphalidae	Erebia aethiopella
Hipparchia aristeus 16S ribosomal RNA gene, partia	AF214604	78.43	63.58	9.47981e−10	Lepidoptera	Nymphalidae	Hipparchia aristeus
Hipparchia semele 16S ribosomal RNA gene, partial	AF214600	78.43	63.58	9.47981e−10	Lepidoptera	Nymphalidae	Hipparchia semele
Tasmanophlebia sp. EP158 16S ribosomal RNA gene, p	AY749825	79	63.58	9.47981e−10	Ephemeroptera	Oniscigastridae	Tasmanophlebia sp. EP158
