# Supplementary material for: Testing the potential of a ribosomal 16S marker for DNA metabarcoding of insects
Source: PeerJ. 2016 Apr 19;4:e1966. doi: 10.7717/peerj.1966 (PMC4841222; doi:10.7717/peerj.1966)
Supplement: Supplemental Information 1 [file OTU_307.csv_plot.pdf › scripts/5 match OTUs/blast_hits/OTU_307.csv_plot.pdf]

OTU_307.csv

Ecdyonurus dispar

Heptageniidae

Ephemeroptera

89.68

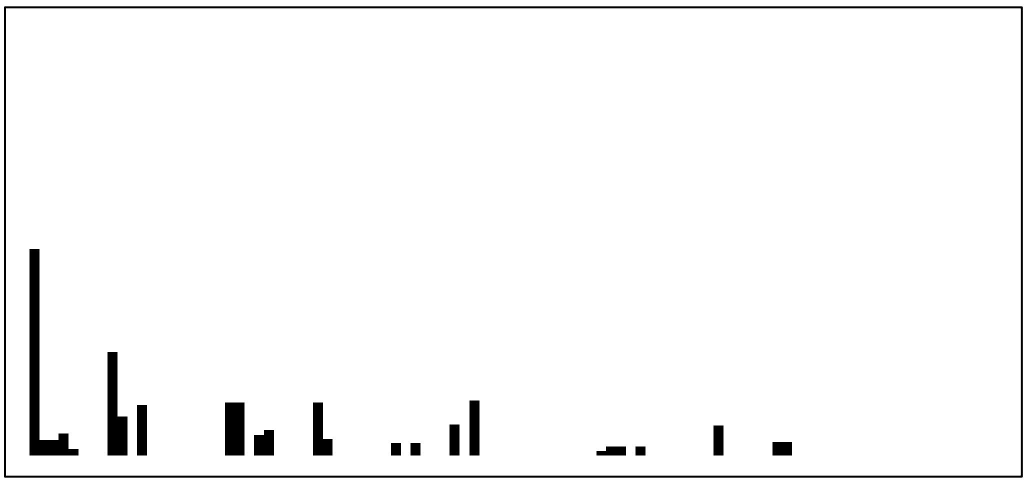

Ecdyonurus dispar 16S ribosomal RNA gene, partial	AY749775	89.68	100	2.28306e−49	Ephemeroptera	Heptageniidae	Ecdyonurus dispar
Atopopus tarsalis mitochondrial partial 16S rRNA g	HE651420	80.71	90.26	5.03321e−26	Ephemeroptera	Heptageniidae	Atopopus tarsalis
Atopopus tarsalis mitochondrial partial 16S rRNA g	HE651419	80.71	90.26	5.03321e−26	Ephemeroptera	Heptageniidae	Atopopus tarsalis
Thalerosphyrus lamuriensis mitochondrial partial 1	HE651425	81.02	85.06	3.1763e−22	Ephemeroptera	Heptageniidae	Thalerosphyrus lamuriensis
Thalerosphyrus lamuriensis mitochondrial partial 1	HE651429	80.29	85.06	3.86953e−21	Ephemeroptera	Heptageniidae	Thalerosphyrus lamuriensis
Parafronurus youi mitochondrion, complete genome	EU349015	78.57	90.26	3.86953e−21	Ephemeroptera	Heptageniidae	Parafronurus youi
Thalerosphyrus sinuosus mitochondrial partial 16S	HE651430	77.86	90.26	1.3506e−20	Ephemeroptera	Heptageniidae	Thalerosphyrus sinuosus
Afronurus sp. 213BoAf mitochondrial partial 16S rR	HE651422	78.03	85.71	4.71405e−20	Ephemeroptera	Heptageniidae	Afronurus sp. 213BoAf
Afronurus sp. 212BoAf mitochondrial partial 16S rR	HE651421	84.85	63.64	4.71405e−20	Ephemeroptera	Heptageniidae	Afronurus sp. 212BoAf
Clioperla clio 16S ribosomal RNA gene, partial seq	EF623237	81.82	76.62	1.64537e−19	Plecoptera	Perlodidae	Clioperla clio
Heptagenia sulphurea mitochondrial partial 16S rRN	HE651431	77.7	88.96	5.74289e−19	Ephemeroptera	Heptageniidae	Heptagenia sulphurea
Siphlonurus binotatus mitochondrial gene for 16S r	AB110269	82.35	65.58	6.99628e−18	Ephemeroptera	Siphlonuridae	Siphlonurus binotatus
Afronurus peringueyi 16S ribosomal RNA gene, parti	AY749790	77.14	88.96	6.99628e−18	Ephemeroptera	Heptageniidae	Afronurus peringueyi
Stenonema sp. EP026 16S ribosomal RNA gene, partia	AY749769	77.04	87.01	6.99628e−18	Ephemeroptera	Heptageniidae	Stenonema sp. EP026
Paegniodes cupulatus mitochondrion, complete genom	HM004123	79.31	74.68	2.44194e−17	Ephemeroptera	Heptageniidae	Paegniodes cupulatus
Diamphipnopsis sp. BYU_PL138 16S ribosomal RNA gen	EF623145	77.54	87.66	2.44194e−17	Plecoptera	Diamphipnoidae	Diamphipnopsis sp. BYU_PL138
Pseudiron centralis 16S ribosomal RNA gene, partia	AY749799	76.52	85.71	2.44194e−17	Ephemeroptera	Pseudironidae	Pseudiron centralis
Isoperla oxylepis 16S ribosomal RNA gene, partial	EF623221	77.69	83.12	8.52321e−17	Plecoptera	Perlodidae	Isoperla oxylepis
Isoperla fulva 16S ribosomal RNA gene, partial seq	EF623207	79.17	76.62	8.52321e−17	Plecoptera	Perlodidae	Isoperla fulva
Siphlonurus sp. EP084 16S ribosomal RNA gene, part	AY749786	76.98	88.96	8.52321e−17	Ephemeroptera	Siphlonuridae	Siphlonurus sp. EP084
Siphlonurus sp. MT−2014 isolate CL142 mitochondrio	KM244684	82.47	62.34	2.97489e−16	Ephemeroptera	Siphlonuridae	Siphlonurus sp. MT−2014
Parameletus columbiae 16S ribosomal RNA gene, part	AY749768	82.47	62.34	2.97489e−16	Ephemeroptera	Siphlonuridae	Parameletus columbiae
Pteronarcys sachalina 16S ribosomal RNA gene, part	EF623264	76.92	83.12	1.03834e−15	Plecoptera	Pteronarcyidae	Pteronarcys sachalina
Osobenus yakimae 16S ribosomal RNA gene, partial s	EF623232	80.95	66.88	1.03834e−15	Plecoptera	Perlodidae	Osobenus yakimae
Chernokrilus misnomus 16S ribosomal RNA gene, part	EF623209	81.19	64.94	1.03834e−15	Plecoptera	Perlodidae	Chernokrilus misnomus
Isoperla davisi 16S ribosomal RNA gene, partial se	EF623206	78.33	76.62	1.03834e−15	Plecoptera	Perlodidae	Isoperla davisi
Suwallia teleckojensis 16S ribosomal RNA gene, par	EF623142	75.86	90.91	1.03834e−15	Plecoptera	Chloroperlidae	Suwallia teleckojensis
Neaviperla forcipata 16S ribosomal RNA gene, parti	EF623139	76.43	87.66	1.03834e−15	Plecoptera	Chloroperlidae	Neaviperla forcipata
Cinygmina sp. EP133 16S ribosomal RNA gene, partia	AY749811	77.24	79.87	1.03834e−15	Ephemeroptera	Heptageniidae	Cinygmina sp. EP133
Afronurus sp. 338SuAf mitochondrial partial 16S rR	HE651428	82.47	62.34	3.62416e−15	Ephemeroptera	Heptageniidae	Afronurus sp. 338SuAf
Notonemouridae sp. MD−2011 voucher ZMUC:JD−1507 16	JN615257	80.77	66.23	3.62416e−15	Plecoptera	Notonemouridae	Notonemouridae sp. MD−2011
Kogotus nonus 16S ribosomal RNA gene, partial sequ	EF623213	80	67.53	3.62416e−15	Plecoptera	Perlodidae	Kogotus nonus
Ameletoides sp. EP159 16S ribosomal RNA gene, part	AY749826	74.36	100	3.62416e−15	Ephemeroptera	Nesameletidae	Ameletoides sp. EP159
Cryptoperla japonica mitochondrial gene for 16S rR	AB711202	77.24	78.57	1.26496e−14	Plecoptera	Peltoperlidae	Cryptoperla japonica
Cryptoperla japonica mitochondrial gene for 16S rR	AB711184	77.24	78.57	1.26496e−14	Plecoptera	Peltoperlidae	Cryptoperla japonica
Skwala americana 16S ribosomal RNA gene, partial s	EF623217	77.44	83.12	1.26496e−14	Plecoptera	Perlodidae	Skwala americana
Nemoura cinerea 16S ribosomal RNA gene, partial se	EF623194	76.6	88.31	1.26496e−14	Plecoptera	Nemouridae	Nemoura cinerea
Amphinemura nigritta 16S ribosomal RNA gene, parti	EF623186	80.58	65.58	1.26496e−14	Plecoptera	Nemouridae	Amphinemura nigritta
Eupholidoptera chabrieri voucher 173 16S ribosomal	EU120792	75.86	92.86	1.26496e−14	Orthoptera	Tettigoniidae	Eupholidoptera chabrieri
Oniscigaster distans 16S ribosomal RNA gene, parti	AY749818	80.58	65.58	1.26496e−14	Ephemeroptera	Oniscigastridae	Oniscigaster distans
Macdunnoa hipawinia 16S ribosomal RNA gene, partia	AY749796	75.36	88.31	1.26496e−14	Ephemeroptera	Heptageniidae	Macdunnoa hipawinia
Rhithrogena nuragica mitochondrial partial 16S rRN	LN868667	74.83	90.91	4.41513e−14	Ephemeroptera	Heptageniidae	Rhithrogena nuragica
Cryptoperla sp. YK−2012−1 mitochondrial gene for 1	AB711243	78.57	71.43	4.41513e−14	Plecoptera	Peltoperlidae	Cryptoperla sp. YK−2012−1
Afronurus sp. 336SuAf mitochondrial partial 16S rR	HE651427	81.44	62.34	4.41513e−14	Ephemeroptera	Heptageniidae	Afronurus sp. 336SuAf
Afronurus sp. 297SAAf mitochondrial partial 16S rR	HE651424	74.17	91.56	4.41513e−14	Ephemeroptera	Heptageniidae	Afronurus sp. 297SAAf
Talitropsis sedilloti voucher CW160 16S ribosomal	HM594511	82.56	55.84	4.41513e−14	Orthoptera	Rhaphidophoridae	Talitropsis sedilloti
Udenus wnigrum voucher MAY 16S ribosomal RNA gene,	HM594504	78.9	69.48	4.41513e−14	Orthoptera	Rhaphidophoridae	Udenus wnigrum
Heteromallus cavicola voucher MIL 16S ribosomal RN	HM594499	78.7	69.48	4.41513e−14	Orthoptera	Rhaphidophoridae	Heteromallus cavicola
Hexagenia sp. BYU IGCEP003 16S ribosomal RNA gene,	GQ118286	79.05	68.18	4.41513e−14	Ephemeroptera	Ephemeridae	Hexagenia sp. BYU IGCEP003
Ephemera simulans voucher BYU:IGCEP038 16S ribosom	GQ118285	78.05	78.57	4.41513e−14	Ephemeroptera	Ephemeridae	Ephemera simulans
Rickera sorpta 16S ribosomal RNA gene, partial seq	EF623235	79.05	67.53	4.41513e−14	Plecoptera	Perlodidae	Rickera sorpta
Perlinodes aurea 16S ribosomal RNA gene, partial s	EF623234	77.87	77.27	4.41513e−14	Plecoptera	Perlodidae	Perlinodes aurea
Frisonia picticeps 16S ribosomal RNA gene, partial	EF623227	77.87	77.27	4.41513e−14	Plecoptera	Perlodidae	Frisonia picticeps
Neofulla sp. BYU_PL152 16S ribosomal RNA gene, par	EF623203	76.34	83.12	4.41513e−14	Plecoptera	Notonemouridae	Neofulla sp. BYU_PL152
Isogenoides varians isolate BYU_PL079 16S ribosoma	EF623445	79.05	67.53	4.41513e−14	Plecoptera	Perlodidae	Isogenoides varians
Xiphydria mellipes 16S ribosomal RNA gene, partial	AF379857	76.81	87.01	4.41513e−14	Hymenoptera	Xiphydriidae	Xiphydria mellipes
Panorpa nebulosa 16S ribosomal RNA gene, partial s	AF180058	77.5	76.62	4.41513e−14	Mecoptera	Panorpidae	Panorpa nebulosa
Tasmanophlebia sp. EP158 16S ribosomal RNA gene, p	AY749825	75.54	88.96	4.41513e−14	Ephemeroptera	Oniscigastridae	Tasmanophlebia sp. EP158
Helopicus bogaloosa 16S ribosomal RNA gene, partia	EF623228	80.2	64.29	1.54103e−13	Plecoptera	Perlodidae	Helopicus bogaloosa
Cosumnoperla hypocrena 16S ribosomal RNA gene, par	EF623226	80.41	62.34	1.54103e−13	Plecoptera	Perlodidae	Cosumnoperla hypocrena
Cascadoperla trictura 16S ribosomal RNA gene, part	EF623225	80.41	62.34	1.54103e−13	Plecoptera	Perlodidae	Cascadoperla trictura
Isogenoides olivaceus 16S ribosomal RNA gene, part	EF623224	79.25	67.53	1.54103e−13	Plecoptera	Perlodidae	Isogenoides olivaceus
Isoperla phalerata 16S ribosomal RNA gene, partial	EF623208	80.41	62.34	1.54103e−13	Plecoptera	Perlodidae	Isoperla phalerata
Plumiperla diversa 16S ribosomal RNA gene, partial	EF623132	76.06	87.66	1.54103e−13	Plecoptera	Chloroperlidae	Plumiperla diversa
Phloiophilus edwardsi voucher UPOL001206 16S ribos	KC758772	78.18	70.78	5.37873e−13	Coleoptera	Phloiophilidae	Phloiophilus edwardsi
Mecopoda elongata mitochondrion, complete genome	JQ917910	74.15	94.16	5.37873e−13	Orthoptera	Tettigoniidae	Mecopoda elongata
Yoraperla uenoi mitochondrial gene for 16S rRNA, p	AB712375	75.56	83.12	5.37873e−13	Plecoptera	Peltoperlidae	Yoraperla uenoi
Cryptoperla japonica mitochondrial gene for 16S rR	AB711183	76.42	78.57	5.37873e−13	Plecoptera	Peltoperlidae	Cryptoperla japonica
Cryptoperla japonica mitochondrial gene for 16S rR	AB711182	76.42	78.57	5.37873e−13	Plecoptera	Peltoperlidae	Cryptoperla japonica
Compsoneuria sp. 10 LV−2012 mitochondrial partial	HE651412	73.42	100	5.37873e−13	Ephemeroptera	Heptageniidae	Compsoneuria sp. 10 LV−2012
Parudenus falklandicus voucher PAR 16S ribosomal R	HM594502	81.4	55.84	5.37873e−13	Orthoptera	Rhaphidophoridae	Parudenus falklandicus
Phloiophilus edwardsi partial 16S rRNA gene, speci	AM884187	78.18	70.78	5.37873e−13	Coleoptera	Phloiophilidae	Phloiophilus edwardsi
Dolichopoda sp. MR−2008 16S ribosomal RNA gene, pa	EU887863	74.65	88.31	5.37873e−13	Orthoptera	Rhaphidophoridae	Dolichopoda lustriae
Dolichopoda remyi voucher EDE 16S ribosomal RNA ge	EF216969	77.78	69.48	5.37873e−13	Orthoptera	Rhaphidophoridae	Dolichopoda remyi
Dolichopoda pavesii voucher SPI 16S ribosomal RNA	EF216951	79.59	62.99	5.37873e−13	Orthoptera	Rhaphidophoridae	Dolichopoda pavesii
Pteronarcys scotti 16S ribosomal RNA gene, partial	EF623263	78.3	68.18	5.37873e−13	Plecoptera	Pteronarcyidae	Pteronarcys scotti
Isogenoides hansoni 16S ribosomal RNA gene, partia	EF623223	80.61	62.34	5.37873e−13	Plecoptera	Perlodidae	Isogenoides hansoni
Yugus bulbosus 16S ribosomal RNA gene, partial seq	EF623214	80.61	62.34	5.37873e−13	Plecoptera	Perlodidae	Yugus bulbosus
Neuroperlopsis patris 16S ribosomal RNA gene, part	EF623152	74.13	91.56	5.37873e−13	Plecoptera	Eustheniidae	Neuroperlopsis patris
Chloroperla tripunctata 16S ribosomal RNA gene, pa	EF623137	75	87.66	5.37873e−13	Plecoptera	Chloroperlidae	Chloroperla tripunctata
Osmylus sp. EH−2004 16S ribosomal RNA gene, partia	AY620146	78.3	68.18	5.37873e−13	Neuroptera	Osmylidae	Osmylus sp. EH−2004
Dolichopoda remyi isolate EDE 16S ribosomal RNA ge	AY793589	77.78	69.48	5.37873e−13	Orthoptera	Rhaphidophoridae	Dolichopoda remyi
Pteronarcys princeps mitochondrion, complete genom	AY687866	75.38	83.12	5.37873e−13	Plecoptera	Pteronarcyidae	Pteronarcys princeps
Dipteromimus tipuliformis mitochondrial gene for 1	AB110257	76.42	78.57	5.37873e−13	Ephemeroptera	Dipteromimidae	Dipteromimus tipuliformis
Dipteromimus tipuliformis mitochondrial gene for 1	AB110255	76.27	75.97	5.37873e−13	Ephemeroptera	Dipteromimidae	Dipteromimus tipuliformis
Panorpa maculosa 16S ribosomal RNA gene, partial s	AF180055	76.67	76.62	5.37873e−13	Mecoptera	Panorpidae	Panorpa maculosa
Panorpa germanica isolate Eifel 16S ribosomal RNA	AF180052	76.67	76.62	5.37873e−13	Mecoptera	Panorpidae	Panorpa germanica
Panorpa acuta 16S ribosomal RNA gene, partial sequ	AF180047	76.67	76.62	5.37873e−13	Mecoptera	Panorpidae	Panorpa acuta
Ametropus neavei 16S ribosomal RNA gene, partial s	AY749800	74.65	90.91	5.37873e−13	Ephemeroptera	Ametropodidae	Ametropus neavei
Heptagenia sp. EP018 16S ribosomal RNA gene, parti	AY749762	75.38	83.12	5.37873e−13	Ephemeroptera	Heptageniidae	Heptagenia sp. EP018
Eutelia adulatricoides mitochondrion, complete gen	KJ185131	78	64.94	1.87736e−12	Lepidoptera	Noctuidae	Eutelia adulatricoides
Eupholidoptera megastyla isolate ALE1 16S ribosoma	KC852308	73.61	92.86	1.87736e−12	Orthoptera	Tettigoniidae	Eupholidoptera megastyla
Macropathus filifer voucher CW301 16S ribosomal RN	HM594510	77.27	71.43	1.87736e−12	Orthoptera	Rhaphidophoridae	Macropathus filifer
Udenus wnigrum voucher CER 16S ribosomal RNA gene,	HM594503	77.98	69.48	1.87736e−12	Orthoptera	Rhaphidophoridae	Udenus wnigrum
Dolichopoda thasosensis voucher DRA 16S ribosomal	EF216956	74.83	88.31	1.87736e−12	Orthoptera	Rhaphidophoridae	Dolichopoda thasosensis
Peltoperla arcuata 16S ribosomal RNA gene, partial	EF623258	74.63	85.71	1.87736e−12	Plecoptera	Peltoperlidae	Peltoperla arcuata
Soyedina producta 16S ribosomal RNA gene, partial	EF623196	78.43	65.58	1.87736e−12	Plecoptera	Nemouridae	Soyedina producta
Protonemura meyeri 16S ribosomal RNA gene, partial	EF623193	75	86.36	1.87736e−12	Plecoptera	Nemouridae	Protonemura meyeri
Triznaka pintada 16S ribosomal RNA gene, partial s	EF623134	74.83	90.26	1.87736e−12	Plecoptera	Chloroperlidae	Triznaka pintada
Namaquaphasma ookiepense isolate 813 16S large sub	DQ890292	75	79.87	1.87736e−12	Mantophasmatodea	Austrophasmatidae	Namaquaphasma ookiepense
