# Supplementary material for: Testing the potential of a ribosomal 16S marker for DNA metabarcoding of insects
Source: PeerJ. 2016 Apr 19;4:e1966. doi: 10.7717/peerj.1966 (PMC4841222; doi:10.7717/peerj.1966)

OTU_31.csv

Chaetopteryx fusca

Limnephilidae

Trichoptera

97.63

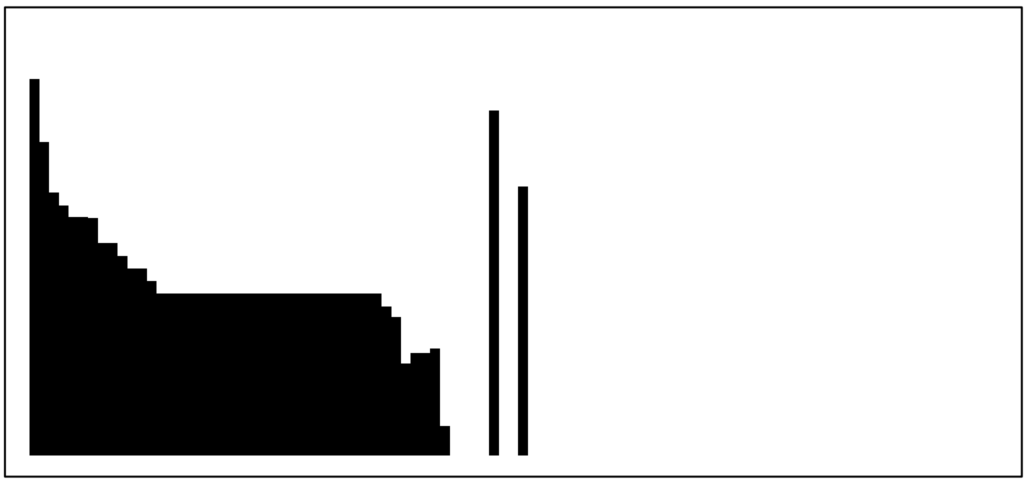

Chaetopteryx fusca voucher ChfIM1 16S ribosomal RN	FJ002820	97.63	100	3.34101e−73	Trichoptera	Limnephilidae	Chaetopteryx fusca
Annitella apfelbecki voucher AaIM1 16S ribosomal R	FJ002819	94.67	100	4.64408e−65	Trichoptera	Limnephilidae	Annitella apfelbecki
Chaetopterygopsis maclachlani voucher CM001 16S ri	EU215176	92.31	100	1.24618e−59	Trichoptera	Limnephilidae	Chaetopterygopsis maclachlani
Chaetopteryx rugulosa voucher CRu002 16S ribosomal	EU215178	91.72	100	4.3496e−59	Trichoptera	Limnephilidae	Chaetopteryx rugulosa
Ecclisocosmoecus spinosus mitochondrial gene for 1	AB355447	91.18	100	1.8495e−57	Trichoptera	Limnephilidae	Ecclisocosmoecus spinosus
Consorophylax consors voucher CCon001 16S ribosoma	EU215175	91.18	100	1.8495e−57	Trichoptera	Limnephilidae	Consorophylax consors
Allogamus uncatus voucher AUn003 16S ribosomal RNA	KM001840	91.12	100	6.45537e−57	Trichoptera	Limnephilidae	Allogamus uncatus
Drusus chrysotus voucher DCh004 16S ribosomal RNA	EU215189	89.94	100	2.74489e−55	Trichoptera	Limnephilidae	Drusus chrysotus
Drusus chrysotus voucher DCh001 16S ribosomal RNA	EU215188	89.94	100	2.74489e−55	Trichoptera	Limnephilidae	Drusus chrysotus
Cryptothrix nebulicola voucher CN006 16S ribosomal	EU215177	89.35	100	3.34397e−54	Trichoptera	Limnephilidae	Cryptothrix nebulicola
Anomalopterygella chauviniana voucher AC001 16S ri	EU215174	88.76	100	1.42189e−52	Trichoptera	Limnephilidae	Anomalopterygella chauviniana
Asynarchus sachalinensis mitochondrial gene for 16	AB355450	88.76	100	1.73222e−51	Trichoptera	Limnephilidae	Asynarchus sachalinensis
Rivulophilus sakaii mitochondrial gene for 16S rib	AB355449	88.17	100	2.11027e−50	Trichoptera	Limnephilidae	Rivulophilus sakaii
Ecclisopteryx keroveci voucher E2JAL2 16S ribosoma	KM001845	87.57	100	7.36558e−50	Trichoptera	Limnephilidae	Ecclisopteryx keroveci
Ecclisopteryx keroveci voucher E2JAL1 16S ribosoma	KM001844	87.57	100	7.36558e−50	Trichoptera	Limnephilidae	Ecclisopteryx keroveci
Ecclisopteryx keroveci voucher E1JAL2 16S ribosoma	KM001843	87.57	100	7.36558e−50	Trichoptera	Limnephilidae	Ecclisopteryx keroveci
Ecclisopteryx keroveci voucher E1JAL1 16S ribosoma	KM001842	87.57	100	7.36558e−50	Trichoptera	Limnephilidae	Ecclisopteryx keroveci
Ecclisopteryx ivkae voucher fEda1202L 16S ribosoma	KM001810	87.57	100	7.36558e−50	Trichoptera	Limnephilidae	Ecclisopteryx ivkae
Ecclisopteryx ivkae voucher fEda1201L 16S ribosoma	KM001809	87.57	100	7.36558e−50	Trichoptera	Limnephilidae	Ecclisopteryx ivkae
Ecclisopteryx ivkae voucher fEda0102M 16S ribosoma	KM001808	87.57	100	7.36558e−50	Trichoptera	Limnephilidae	Ecclisopteryx ivkae
Ecclisopteryx ivkae voucher fEda0102F 16S ribosoma	KM001807	87.57	100	7.36558e−50	Trichoptera	Limnephilidae	Ecclisopteryx ivkae
Ecclisopteryx ivkae voucher fEda0101M 16S ribosoma	KM001806	87.57	100	7.36558e−50	Trichoptera	Limnephilidae	Ecclisopteryx ivkae
Ecclisopteryx ivkae voucher fDsp3301L 16S ribosoma	KM001804	87.57	100	7.36558e−50	Trichoptera	Limnephilidae	Ecclisopteryx ivkae
Ecclisopteryx keroveci voucher fEns0101M 16S ribos	KM001784	87.57	100	7.36558e−50	Trichoptera	Limnephilidae	Ecclisopteryx keroveci
Ecclisopteryx keroveci voucher fEda1101F 16S ribos	KM001783	87.57	100	7.36558e−50	Trichoptera	Limnephilidae	Ecclisopteryx keroveci
Ecclisopteryx keroveci voucher fEda1001M 16S ribos	KM001782	87.57	100	7.36558e−50	Trichoptera	Limnephilidae	Ecclisopteryx keroveci
Ecclisopteryx keroveci voucher fEda0301M 16S ribos	KM001781	87.57	100	7.36558e−50	Trichoptera	Limnephilidae	Ecclisopteryx keroveci
Ecclisopteryx keroveci voucher fEda0202F 16S ribos	KM001780	87.57	100	7.36558e−50	Trichoptera	Limnephilidae	Ecclisopteryx keroveci
Ecclisopteryx keroveci voucher fEda0201M 16S ribos	KM001779	87.57	100	7.36558e−50	Trichoptera	Limnephilidae	Ecclisopteryx keroveci
Ecclisopteryx keroveci voucher fDs30109F 16S ribos	KM001778	87.57	100	7.36558e−50	Trichoptera	Limnephilidae	Ecclisopteryx keroveci
Ecclisopteryx keroveci voucher fDs30106F 16S ribos	KM001777	87.57	100	7.36558e−50	Trichoptera	Limnephilidae	Ecclisopteryx keroveci
Ecclisopteryx keroveci voucher fDs30105M 16S ribos	KM001776	87.57	100	7.36558e−50	Trichoptera	Limnephilidae	Ecclisopteryx keroveci
Ecclisopteryx keroveci voucher fDs30103M 16S ribos	KM001775	87.57	100	7.36558e−50	Trichoptera	Limnephilidae	Ecclisopteryx keroveci
Ecclisopteryx keroveci voucher fDs30102M 16S ribos	KM001774	87.57	100	7.36558e−50	Trichoptera	Limnephilidae	Ecclisopteryx keroveci
Ecclisopteryx keroveci voucher fDs30101M 16S ribos	KM001773	87.57	100	7.36558e−50	Trichoptera	Limnephilidae	Ecclisopteryx keroveci
Ecclisopteryx dalecarlica voucher EdIM1 16S riboso	FJ002818	87.57	100	7.36558e−50	Trichoptera	Limnephilidae	Ecclisopteryx dalecarlica
Ecclisopteryx ivkae voucher fEda0101F 16S ribosoma	KM001805	86.98	100	8.97311e−49	Trichoptera	Limnephilidae	Ecclisopteryx ivkae
Hydatophylax festivus mitochondrial gene for 16S r	AB355448	86.47	100	1.97646e−44	Trichoptera	Limnephilidae	Hydatophylax festivus
Ironoquia punctatissima mitochondrial gene for 16S	AB355446	84.3	100	1.02383e−41	Trichoptera	Limnephilidae	Ironoquia punctatissima
Nothopsyche montivaga mitochondrial gene for 16S r	AB355431	84.8	100	1.24728e−40	Trichoptera	Limnephilidae	Nothopsyche montivaga
Nothopsyche montivaga mitochondrial gene for 16S r	AB355432	84.8	100	1.24728e−40	Trichoptera	Limnephilidae	Nothopsyche montivaga
Dicosmoecus jozankeanus mitochondrial gene for 16S	AB355445	85	94.08	4.35344e−40	Trichoptera	Limnephilidae	Dicosmoecus jozankeanus
Taskiropsyche lacustris voucher NHRS−EN CS5 16S ri	FJ263209	81.38	84.02	6.90461e−25	Trichoptera	Kokiriidae	Taskiropsyche lacustris
Eubasilissa regina mitochondrion, complete genome	KF756943	77.06	100	4.35729e−21	Trichoptera		Eubasilissa regina
Myiodactylus osmyloides 16S ribosomal RNA gene, pa	EU734873	75.14	100	4.08099e−15	Neuroptera	Nymphidae	Myiodactylus osmyloides
Bambusiphaga fascia isolate 181 16S ribosomal RNA	HM233787	71.95	94.67	1.62524e−07	Hemiptera	Delphacidae	Bambusiphaga fascia
Ichthyophthirius multifiliis leucine rich repeat p	XM_004039058	69.92	71.6	0.531295	Hymenostomatida		Ichthyophthirius multifiliis
Ovis canadensis canadensis isolate 43U chromosome	CP011888	96.15	15.38	1.8544		Bovidae	Ovis canadensis canadensis
Strongyloides venezuelensis genome assembly S_vene	LM524980	75.86	34.32	1.8544	Rhabditida	Strongyloididae	Strongyloides venezuelensis
Mycoplasma canadense DNA, complete genome, strain:	AP014631	77.42	34.91	1.8544	Mycoplasmatales	Mycoplasmataceae	Mycoplasma canadense
Protopolystoma xenopodis genome assembly P_xenopod	LM682778	92.59	15.98	6.4725	Polyopisthocotylea	Polystomatidae	Protopolystoma xenopodis

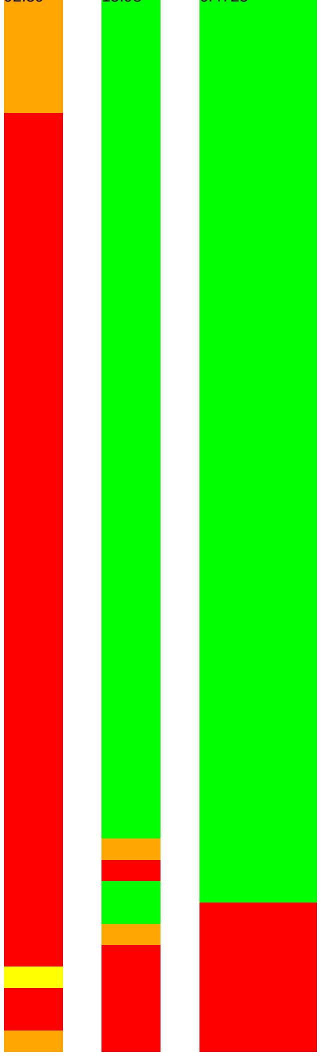

Supplement: Supplemental Information 1 [file OTU_31.csv_plot.pdf › scripts/5 match OTUs/blast_hits/OTU_31.csv_plot.pdf]
