# Supplementary material for: Testing the potential of a ribosomal 16S marker for DNA metabarcoding of insects
Source: PeerJ. 2016 Apr 19;4:e1966. doi: 10.7717/peerj.1966 (PMC4841222; doi:10.7717/peerj.1966)
Supplement: Supplemental Information 1 [file OTU_345.csv_plot.pdf › scripts/5 match OTUs/blast_hits/OTU_345.csv_plot.pdf]

OTU_345.csv

Dinocras cephalotes

Perlidae

Plecoptera

96.95

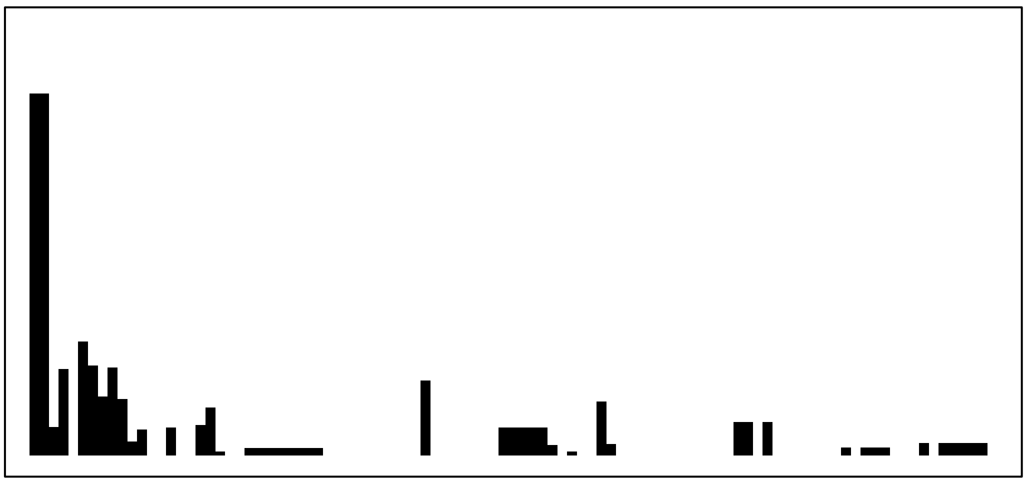

Dinocras cephalotes mitochondrion, complete genome	KF484757	96.95	100	5.8153e−70	Plecoptera	Perlidae	Dinocras cephalotes
Dinocras cephalotes 16S ribosomal RNA gene, partia	EF623244	96.95	100	5.8153e−70	Plecoptera	Perlidae	Dinocras cephalotes
Calineuria californica 16S ribosomal RNA gene, par	EF623239	81.33	90.24	1.05329e−28	Plecoptera	Perlidae	Calineuria californica
Agnetina capitata 16S ribosomal RNA gene, partial	EF623241	84.03	72.56	5.45617e−26	Plecoptera	Perlidae	Agnetina capitata
Perlinella drymo 16S ribosomal RNA gene, partial s	EF623240	79.72	87.2	6.64698e−25	Plecoptera	Perlidae	Perlinella drymo
Hansonoperla appalachia 16S ribosomal RNA gene, pa	EF623249	85.32	65.85	8.09768e−24	Plecoptera	Perlidae	Hansonoperla appalachia
Perlesta decipines 16S ribosomal RNA gene, partial	EF623242	84.21	69.51	9.86499e−23	Plecoptera	Perlidae	Perlesta decipines
Udamocercia sp. BYU_PL150 16S ribosomal RNA gene,	EF623201	82.76	70.73	9.86499e−23	Plecoptera	Notonemouridae	Udamocercia sp. BYU_PL150
Kathroperla perdita 16S ribosomal RNA gene, partia	EF623135	84.11	65.24	9.86499e−23	Plecoptera	Chloroperlidae	Kathroperla perdita
Leuctra inermis 16S ribosomal RNA gene, partial se	EF623176	82.64	73.17	3.44322e−22	Plecoptera	Leuctridae	Leuctra inermis
Scopura montana 16S ribosomal RNA gene, partial se	EF623265	80.65	74.39	1.2018e−21	Plecoptera		Scopura montana
Leuctra tenuis haplotype 5lt: HO5 16S ribosomal RN	EU327677	81.2	71.34	1.2018e−21	Plecoptera	Leuctridae	Leuctra tenuis
Pteronarcys princeps mitochondrion, complete genom	AY687866	76.22	99.39	4.1947e−21	Plecoptera	Pteronarcyidae	Pteronarcys princeps
Taenionema palladium 16S ribosomal RNA gene, parti	EF623267	75.61	99.39	1.46409e−20	Plecoptera	Taeniopterygidae	Taenionema palladium
Triznaka pintada 16S ribosomal RNA gene, partial s	EF623134	81.3	74.39	1.46409e−20	Plecoptera	Chloroperlidae	Triznaka pintada
Notonemouridae sp. MD−2011 voucher ZMUC:JD−1507 16	JN615257	79.84	75	5.11019e−20	Plecoptera	Notonemouridae	Notonemouridae sp. MD−2011
Pteronarcys californica 16S ribosomal RNA gene, pa	EF623261	75.61	99.39	5.11019e−20	Plecoptera	Pteronarcyidae	Pteronarcys californica
Acroneuria lycorias 16S ribosomal RNA gene, partia	EF623251	81.42	67.07	5.11019e−20	Plecoptera	Perlidae	Acroneuria lycorias
Nemoura cinerea 16S ribosomal RNA gene, partial se	EF623194	82.24	65.24	5.11019e−20	Plecoptera	Nemouridae	Nemoura cinerea
Despaxia augusta 16S ribosomal RNA gene, partial s	EF623177	80.17	73.17	5.11019e−20	Plecoptera	Leuctridae	Despaxia augusta
Moselia infuscata 16S ribosomal RNA gene, partial	EF623175	75.9	100	5.11019e−20	Plecoptera	Leuctridae	Moselia infuscata
Neaviperla forcipata 16S ribosomal RNA gene, parti	EF623139	75.97	93.9	5.11019e−20	Plecoptera	Chloroperlidae	Neaviperla forcipata
Leuctra tenuis haplotype 10lt: HO10 16S ribosomal	EU327682	80.34	71.34	5.11019e−20	Plecoptera	Leuctridae	Leuctra tenuis
Leuctra tenuis haplotype 9lt: HO9 16S ribosomal RN	EU327681	80.34	71.34	5.11019e−20	Plecoptera	Leuctridae	Leuctra tenuis
Leuctra tenuis haplotype 8lt: HO8 16S ribosomal RN	EU327680	80.34	71.34	5.11019e−20	Plecoptera	Leuctridae	Leuctra tenuis
Leuctra tenuis haplotype 7lt: HO7 16S ribosomal RN	EU327679	80.34	71.34	5.11019e−20	Plecoptera	Leuctridae	Leuctra tenuis
Leuctra tenuis haplotype 6lt: HO6 16S ribosomal RN	EU327678	80.34	71.34	5.11019e−20	Plecoptera	Leuctridae	Leuctra tenuis
Leuctra tenuis haplotype 3lt: H03 16S ribosomal RN	DQ915184	80.34	71.34	5.11019e−20	Plecoptera	Leuctridae	Leuctra tenuis
Leuctra tenuis haplotype 2lt: H02 16S ribosomal RN	DQ915183	80.34	71.34	5.11019e−20	Plecoptera	Leuctridae	Leuctra tenuis
Leuctra tenuis haplotype 1lt: H01 16S ribosomal RN	DQ915182	80.34	71.34	5.11019e−20	Plecoptera	Leuctridae	Leuctra tenuis
Allocapnia recta isolate H18 16S ribosomal RNA gen	KC881053	77.08	87.2	1.78363e−19	Plecoptera	Capniidae	Allocapnia recta
Allocapnia recta isolate H17 16S ribosomal RNA gen	KC881052	77.08	87.2	1.78363e−19	Plecoptera	Capniidae	Allocapnia recta
Allocapnia recta isolate H12 16S ribosomal RNA gen	KC881047	77.08	87.2	1.78363e−19	Plecoptera	Capniidae	Allocapnia recta
Allocapnia recta isolate H9 16S ribosomal RNA gene	KC881044	77.08	87.2	1.78363e−19	Plecoptera	Capniidae	Allocapnia recta
Allocapnia recta isolate H8 16S ribosomal RNA gene	KC881043	77.08	87.2	1.78363e−19	Plecoptera	Capniidae	Allocapnia recta
Allocapnia recta isolate H5 16S ribosomal RNA gene	KC881040	77.08	87.2	1.78363e−19	Plecoptera	Capniidae	Allocapnia recta
Allocapnia recta isolate H4 16S ribosomal RNA gene	KC881039	77.08	87.2	1.78363e−19	Plecoptera	Capniidae	Allocapnia recta
Pteronarcys sachalina 16S ribosomal RNA gene, part	EF623264	75.76	99.39	1.78363e−19	Plecoptera	Pteronarcyidae	Pteronarcys sachalina
Cosumnoperla hypocrena 16S ribosomal RNA gene, par	EF623226	76.92	87.2	1.78363e−19	Plecoptera	Perlodidae	Cosumnoperla hypocrena
Utaperla sopladora 16S ribosomal RNA gene, partial	EF623140	76.4	95.73	1.78363e−19	Plecoptera	Chloroperlidae	Utaperla sopladora
Paraperla frontalis 16S ribosomal RNA gene, partia	EF623136	83.5	62.8	1.78363e−19	Plecoptera	Chloroperlidae	Paraperla frontalis
Plumiperla diversa 16S ribosomal RNA gene, partial	EF623132	79.51	74.39	1.78363e−19	Plecoptera	Chloroperlidae	Plumiperla diversa
Allocapnia recta haplotype 10ar: H010 16S ribosoma	EU306873	77.08	87.2	1.78363e−19	Plecoptera	Capniidae	Allocapnia recta
Allocapnia recta haplotype 9ar: H09 16S ribosomal	EU306872	77.08	87.2	1.78363e−19	Plecoptera	Capniidae	Allocapnia recta
Allocapnia recta haplotype 8ar: H08 16S ribosomal	EU306871	77.08	87.2	1.78363e−19	Plecoptera	Capniidae	Allocapnia recta
Allocapnia recta haplotype 1ar: H01 16S ribosomal	DQ915179	77.08	87.2	1.78363e−19	Plecoptera	Capniidae	Allocapnia recta
Pteronarcys scotti 16S ribosomal RNA gene, partial	EF623263	74.85	99.39	6.22549e−19	Plecoptera	Pteronarcyidae	Pteronarcys scotti
Claassenia sabulosa 16S ribosomal RNA gene, partia	EF623250	76.16	92.07	6.22549e−19	Plecoptera	Perlidae	Claassenia sabulosa
Lednia tumana 16S ribosomal RNA gene, partial sequ	EF623197	81.31	65.24	6.22549e−19	Plecoptera	Nemouridae	Lednia tumana
Prostoia besametsa 16S ribosomal RNA gene, partial	EF623188	81.31	65.24	6.22549e−19	Plecoptera	Nemouridae	Prostoia besametsa
Nemoura trispinosa 16S ribosomal RNA gene, partial	EF623187	81.31	65.24	6.22549e−19	Plecoptera	Nemouridae	Nemoura trispinosa
Paranemoura claasseni 16S ribosomal RNA gene, part	EF623185	81.31	65.24	6.22549e−19	Plecoptera	Nemouridae	Paranemoura claasseni
Shipsa rotunda 16S ribosomal RNA gene, partial seq	EF623184	81.31	65.24	6.22549e−19	Plecoptera	Nemouridae	Shipsa rotunda
Chloroperla tripunctata 16S ribosomal RNA gene, pa	EF623137	80.49	74.39	6.22549e−19	Plecoptera	Chloroperlidae	Chloroperla tripunctata
Leuctra tenuis haplotype 4lt: HO4 16S ribosomal RN	EU327676	79.49	71.34	6.22549e−19	Plecoptera	Leuctridae	Leuctra tenuis
Cryptoperla sp. YK−2012−1 mitochondrial gene for 1	AB711243	80.17	70.73	2.17291e−18	Plecoptera	Peltoperlidae	Cryptoperla sp. YK−2012−1
Claudioperla sp. BYU_PL158 16S ribosomal RNA gene,	EF623169	75.8	94.51	2.17291e−18	Plecoptera	Gripopterygidae	Claudioperla sp. BYU_PL158
Suwallia teleckojensis 16S ribosomal RNA gene, par	EF623142	79.82	69.51	2.17291e−18	Plecoptera	Chloroperlidae	Suwallia teleckojensis
Capnura wanica 16S ribosomal RNA gene, partial seq	EF623128	82.52	62.8	2.17291e−18	Plecoptera	Capniidae	Capnura wanica
Penturoperla barbata 16S ribosomal RNA gene, parti	EF623124	80.53	68.9	2.17291e−18	Plecoptera	Austroperlidae	Penturoperla barbata
Osmylus sp. EH−2004 16S ribosomal RNA gene, partia	AY620146	75.15	99.39	2.17291e−18	Neuroptera	Osmylidae	Osmylus sp. EH−2004
D.geniculata mitochondrial 16S rRNA gene	Y07551	73.81	99.39	2.17291e−18	Orthoptera	Rhaphidophoridae	Dolichopoda geniculata
Styloperla sp. mitochondrion, partial genome	KR088971	74.83	89.63	7.5842e−18	Plecoptera	Styloperlidae	Styloperla sp. YZD−2015
Allocapnia recta isolate H19 16S ribosomal RNA gen	KC881054	76.39	87.2	7.5842e−18	Plecoptera	Capniidae	Allocapnia recta
Allocapnia recta isolate H16 16S ribosomal RNA gen	KC881051	76.39	87.2	7.5842e−18	Plecoptera	Capniidae	Allocapnia recta
Allocapnia recta isolate H15 16S ribosomal RNA gen	KC881050	76.39	87.2	7.5842e−18	Plecoptera	Capniidae	Allocapnia recta
Allocapnia recta isolate H14 16S ribosomal RNA gen	KC881049	76.39	87.2	7.5842e−18	Plecoptera	Capniidae	Allocapnia recta
Allocapnia recta isolate H10 16S ribosomal RNA gen	KC881045	76.39	87.2	7.5842e−18	Plecoptera	Capniidae	Allocapnia recta
Allocapnia recta isolate H6 16S ribosomal RNA gene	KC881041	76.39	87.2	7.5842e−18	Plecoptera	Capniidae	Allocapnia recta
Dolichopoda bormansi isolate BRA3 16S ribosomal RN	AY507605	74.56	99.39	7.5842e−18	Orthoptera	Rhaphidophoridae	Dolichopoda bormansi
Doddsia occidentalis 16S ribosomal RNA gene, parti	EF623270	74.39	99.39	7.5842e−18	Plecoptera	Taeniopterygidae	Doddsia occidentalis
Skwala americana 16S ribosomal RNA gene, partial s	EF623217	76.39	87.2	7.5842e−18	Plecoptera	Perlodidae	Skwala americana
Nemurella pictetii 16S ribosomal RNA gene, partial	EF623192	81.55	62.8	7.5842e−18	Plecoptera	Nemouridae	Nemurella pictetii
Malenka californica 16S ribosomal RNA gene, partia	EF623182	81.55	62.8	7.5842e−18	Plecoptera	Nemouridae	Malenka californica
Cardioperla lobata 16S ribosomal RNA gene, partial	EF623164	75.15	99.39	7.5842e−18	Plecoptera	Gripopterygidae	Cardioperla lobata
Sasquaperla hoopa 16S ribosomal RNA gene, partial	EF623143	81.55	62.8	7.5842e−18	Plecoptera	Chloroperlidae	Sasquaperla hoopa
Tasmanoperla larvalis 16S ribosomal RNA gene, part	EF623125	76.51	90.24	7.5842e−18	Plecoptera	Austroperlidae	Tasmanoperla larvalis
Allocapnia recta haplotype 11ar: H011 16S ribosoma	EU306874	76.39	87.2	7.5842e−18	Plecoptera	Capniidae	Allocapnia recta
Allocapnia recta haplotype 7ar: H07 16S ribosomal	EU306870	76.39	87.2	7.5842e−18	Plecoptera	Capniidae	Allocapnia recta
Allocapnia recta haplotype 2ar: H02 16S ribosomal	DQ915180	76.39	87.2	7.5842e−18	Plecoptera	Capniidae	Allocapnia recta
Macroxyela ferruginea 16S ribosomal RNA gene, part	AY206773	78.69	74.39	7.5842e−18	Hymenoptera	Xyelidae	Macroxyela ferruginea
Dolichopoda bormansi isolate BRA 16S ribosomal RNA	AY793578	74.56	99.39	7.5842e−18	Orthoptera	Rhaphidophoridae	Dolichopoda bormansi
Strophopteryx appalachia 16S ribosomal RNA gene, p	EF623271	78.23	75	2.64715e−17	Plecoptera	Taeniopterygidae	Strophopteryx appalachia
Pteronarcella badia 16S ribosomal RNA gene, partia	EF623262	80.37	65.24	2.64715e−17	Plecoptera	Pteronarcyidae	Pteronarcella badia
Cerconychia sp. BYU_PL114 16S ribosomal RNA gene,	EF623260	75.17	90.85	2.64715e−17	Plecoptera	Styloperlidae	Cerconychia sp. BYU_PL114
Pictetoperla sp. BYU_PL156 16S ribosomal RNA gene,	EF623247	80.37	65.24	2.64715e−17	Plecoptera	Perlidae	Pictetoperla sp. BYU_PL156
Salmoperla sylvanica 16S ribosomal RNA gene, parti	EF623222	80.37	65.24	2.64715e−17	Plecoptera	Perlodidae	Salmoperla sylvanica
Paraleuctra vershina 16S ribosomal RNA gene, parti	EF623173	80.37	65.24	2.64715e−17	Plecoptera	Leuctridae	Paraleuctra vershina
Pelurgoperla personata 16S ribosomal RNA gene, par	EF623168	78.23	75	2.64715e−17	Plecoptera	Gripopterygidae	Pelurgoperla personata
Antarctoperla sp. BYU_PL142 16S ribosomal RNA gene	EF623163	78.23	75	2.64715e−17	Plecoptera	Gripopterygidae	Antarctoperla sp. BYU_PL142
Cosmioperla australis 16S ribosomal RNA gene, part	EF623148	74.51	89.63	2.64715e−17	Plecoptera	Eustheniidae	Cosmioperla australis
Mesocapnia arizonensis mitochondrion, partial geno	KP642637	80.58	62.8	9.23945e−17	Plecoptera	Capniidae	Mesocapnia arizonensis
Ceracris kiangsu isolate Mengla_3 16S ribosomal RN	JQ799690	75.3	99.39	9.23945e−17	Orthoptera	Acrididae	Ceracris kiangsu
Allocapnia recta isolate H13 16S ribosomal RNA gen	KC881048	80.58	62.8	9.23945e−17	Plecoptera	Capniidae	Allocapnia recta
Allocapnia recta isolate H11 16S ribosomal RNA gen	KC881046	80.58	62.8	9.23945e−17	Plecoptera	Capniidae	Allocapnia recta
Allocapnia recta isolate H7 16S ribosomal RNA gene	KC881042	80.58	62.8	9.23945e−17	Plecoptera	Capniidae	Allocapnia recta
Allocapnia recta isolate H3 16S ribosomal RNA gene	KC881038	80.58	62.8	9.23945e−17	Plecoptera	Capniidae	Allocapnia recta
Allocapnia recta isolate H1 16S ribosomal RNA gene	KC881036	80.58	62.8	9.23945e−17	Plecoptera	Capniidae	Allocapnia recta
Peltodytes quadratus voucher BMNH794142 16S riboso	HQ381466	79.13	70.12	9.23945e−17	Coleoptera	Haliplidae	Peltodytes quadratus
Beloneuria stewarti 16S ribosomal RNA gene, partia	EF623248	77.69	79.27	9.23945e−17	Plecoptera	Perlidae	Beloneuria stewarti
