# Supplementary material for: Testing the potential of a ribosomal 16S marker for DNA metabarcoding of insects
Source: PeerJ. 2016 Apr 19;4:e1966. doi: 10.7717/peerj.1966 (PMC4841222; doi:10.7717/peerj.1966)
Supplement: Supplemental Information 1 [file OTU_355.csv_plot.pdf › scripts/5 match OTUs/blast_hits/OTU_355.csv_plot.pdf]

OTU_355.csv

Leuctra inermis

Leuctridae

Plecoptera

86.79

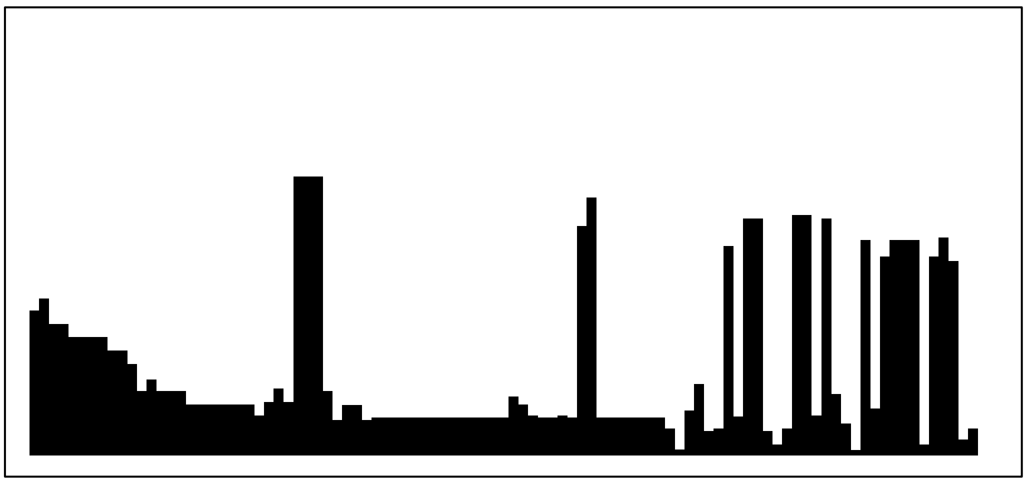

Leuctra inermis 16S ribosomal RNA gene, partial se	EF623176	86.79	100	1.45287e−45	Plecoptera	Leuctridae	Leuctra inermis
Moselia infuscata 16S ribosomal RNA gene, partial	EF623175	87.34	99.35	7.52607e−43	Plecoptera	Leuctridae	Moselia infuscata
Leuctra tenuis haplotype 8lt: HO8 16S ribosomal RN	EU327680	86.16	100	9.16864e−42	Plecoptera	Leuctridae	Leuctra tenuis
Leuctra tenuis haplotype 7lt: HO7 16S ribosomal RN	EU327679	86.16	100	9.16864e−42	Plecoptera	Leuctridae	Leuctra tenuis
Leuctra tenuis haplotype 10lt: HO10 16S ribosomal	EU327682	85.53	100	3.8986e−40	Plecoptera	Leuctridae	Leuctra tenuis
Leuctra tenuis haplotype 9lt: HO9 16S ribosomal RN	EU327681	85.53	100	3.8986e−40	Plecoptera	Leuctridae	Leuctra tenuis
Leuctra tenuis haplotype 3lt: H03 16S ribosomal RN	DQ915184	85.53	100	3.8986e−40	Plecoptera	Leuctridae	Leuctra tenuis
Leuctra tenuis haplotype 2lt: H02 16S ribosomal RN	DQ915183	85.53	100	3.8986e−40	Plecoptera	Leuctridae	Leuctra tenuis
Leuctra tenuis haplotype 6lt: HO6 16S ribosomal RN	EU327678	84.91	100	4.74947e−39	Plecoptera	Leuctridae	Leuctra tenuis
Leuctra tenuis haplotype 1lt: H01 16S ribosomal RN	DQ915182	84.91	100	4.74947e−39	Plecoptera	Leuctridae	Leuctra tenuis
Leuctra tenuis haplotype 5lt: HO5 16S ribosomal RN	EU327677	84.28	100	2.01953e−37	Plecoptera	Leuctridae	Leuctra tenuis
Leuctra tenuis haplotype 4lt: HO4 16S ribosomal RN	EU327676	83.02	100	2.46029e−36	Plecoptera	Leuctridae	Leuctra tenuis
Cascadoperla trictura 16S ribosomal RNA gene, part	EF623225	83.54	100	8.58725e−36	Plecoptera	Perlodidae	Cascadoperla trictura
Despaxia augusta 16S ribosomal RNA gene, partial s	EF623177	83.02	100	2.99724e−35	Plecoptera	Leuctridae	Despaxia augusta
Apteroperla tikumana mitochondrion, complete genom	KR604721	83.02	100	1.27446e−33	Plecoptera	Capniidae	Apteroperla tikumana
Allocapnia minima 16S ribosomal RNA gene, partial	EF623130	83.02	100	1.27446e−33	Plecoptera	Capniidae	Allocapnia minima
Allocapnia recta isolate H15 16S ribosomal RNA gen	KC881050	82.39	100	1.55261e−32	Plecoptera	Capniidae	Allocapnia recta
Allocapnia recta isolate H14 16S ribosomal RNA gen	KC881049	82.39	100	1.55261e−32	Plecoptera	Capniidae	Allocapnia recta
Allocapnia recta isolate H10 16S ribosomal RNA gen	KC881045	82.39	100	1.55261e−32	Plecoptera	Capniidae	Allocapnia recta
Allocapnia recta isolate H6 16S ribosomal RNA gene	KC881041	82.39	100	1.55261e−32	Plecoptera	Capniidae	Allocapnia recta
Isocapnia hyalita 16S ribosomal RNA gene, partial	EF623131	82.39	100	1.55261e−32	Plecoptera	Capniidae	Isocapnia hyalita
Allocapnia recta haplotype 11ar: H011 16S ribosoma	EU306874	82.39	100	1.55261e−32	Plecoptera	Capniidae	Allocapnia recta
Allocapnia recta haplotype 7ar: H07 16S ribosomal	EU306870	82.39	100	1.55261e−32	Plecoptera	Capniidae	Allocapnia recta
Oemopteryx vanduzeea 16S ribosomal RNA gene, parti	EF623266	81.88	100	5.41914e−32	Plecoptera	Taeniopterygidae	Oemopteryx vanduzeea
Nemoura cinerea 16S ribosomal RNA gene, partial se	EF623194	82.5	100	5.41914e−32	Plecoptera	Nemouridae	Nemoura cinerea
Protonemura meyeri 16S ribosomal RNA gene, partial	EF623193	83.12	100	5.41914e−32	Plecoptera	Nemouridae	Protonemura meyeri
Shipsa rotunda 16S ribosomal RNA gene, partial seq	EF623184	82.5	100	5.41914e−32	Plecoptera	Nemouridae	Shipsa rotunda
Suwallia teleckojensis 16S ribosomal RNA gene, par	EF623142	93.07	65.16	5.41914e−32	Plecoptera	Chloroperlidae	Suwallia teleckojensis
Neaviperla forcipata 16S ribosomal RNA gene, parti	EF623139	93.07	65.16	5.41914e−32	Plecoptera	Chloroperlidae	Neaviperla forcipata
Plumiperla diversa 16S ribosomal RNA gene, partial	EF623132	93.07	65.16	5.41914e−32	Plecoptera	Chloroperlidae	Plumiperla diversa
Osobenus yakimae 16S ribosomal RNA gene, partial s	EF623232	83.02	100	1.89147e−31	Plecoptera	Perlodidae	Osobenus yakimae
Soyedina producta 16S ribosomal RNA gene, partial	EF623196	81.65	100	1.89147e−31	Plecoptera	Nemouridae	Soyedina producta
Paraleuctra occidentalis 16S ribosomal RNA gene, p	EF623178	82.35	98.71	1.89147e−31	Plecoptera	Leuctridae	Paraleuctra occidentalis
Kathroperla perdita 16S ribosomal RNA gene, partia	EF623135	82.35	97.42	1.89147e−31	Plecoptera	Chloroperlidae	Kathroperla perdita
Capnura wanica 16S ribosomal RNA gene, partial seq	EF623128	81.65	100	1.89147e−31	Plecoptera	Capniidae	Capnura wanica
Mesocapnia arizonensis mitochondrion, partial geno	KP642637	81.76	100	6.60187e−31	Plecoptera	Capniidae	Mesocapnia arizonensis
Allocapnia recta isolate H19 16S ribosomal RNA gen	KC881054	81.76	100	6.60187e−31	Plecoptera	Capniidae	Allocapnia recta
Allocapnia recta isolate H17 16S ribosomal RNA gen	KC881052	81.76	100	6.60187e−31	Plecoptera	Capniidae	Allocapnia recta
Allocapnia recta isolate H16 16S ribosomal RNA gen	KC881051	81.76	100	6.60187e−31	Plecoptera	Capniidae	Allocapnia recta
Allocapnia recta isolate H13 16S ribosomal RNA gen	KC881048	81.76	100	6.60187e−31	Plecoptera	Capniidae	Allocapnia recta
Allocapnia recta isolate H12 16S ribosomal RNA gen	KC881047	81.76	100	6.60187e−31	Plecoptera	Capniidae	Allocapnia recta
Allocapnia recta isolate H11 16S ribosomal RNA gen	KC881046	81.76	100	6.60187e−31	Plecoptera	Capniidae	Allocapnia recta
Allocapnia recta isolate H9 16S ribosomal RNA gene	KC881044	81.76	100	6.60187e−31	Plecoptera	Capniidae	Allocapnia recta
Allocapnia recta isolate H8 16S ribosomal RNA gene	KC881043	81.76	100	6.60187e−31	Plecoptera	Capniidae	Allocapnia recta
Allocapnia recta isolate H7 16S ribosomal RNA gene	KC881042	81.76	100	6.60187e−31	Plecoptera	Capniidae	Allocapnia recta
Allocapnia recta isolate H5 16S ribosomal RNA gene	KC881040	81.76	100	6.60187e−31	Plecoptera	Capniidae	Allocapnia recta
Allocapnia recta isolate H4 16S ribosomal RNA gene	KC881039	81.76	100	6.60187e−31	Plecoptera	Capniidae	Allocapnia recta
Allocapnia recta isolate H3 16S ribosomal RNA gene	KC881038	81.76	100	6.60187e−31	Plecoptera	Capniidae	Allocapnia recta
Allocapnia recta isolate H1 16S ribosomal RNA gene	KC881036	81.76	100	6.60187e−31	Plecoptera	Capniidae	Allocapnia recta
Pteronarcys sachalina 16S ribosomal RNA gene, part	EF623264	82.76	93.55	6.60187e−31	Plecoptera	Pteronarcyidae	Pteronarcys sachalina
Perlinodes aurea 16S ribosomal RNA gene, partial s	EF623234	82.39	100	6.60187e−31	Plecoptera	Perlodidae	Perlinodes aurea
Yugus bulbosus 16S ribosomal RNA gene, partial seq	EF623214	81.88	100	6.60187e−31	Plecoptera	Perlodidae	Yugus bulbosus
Nemurella pictetii 16S ribosomal RNA gene, partial	EF623192	81.76	100	6.60187e−31	Plecoptera	Nemouridae	Nemurella pictetii
Ostrocerca foersteri 16S ribosomal RNA gene, parti	EF623191	81.76	100	6.60187e−31	Plecoptera	Nemouridae	Ostrocerca foersteri
Visoka cataractae 16S ribosomal RNA gene, partial	EF623189	81.88	100	6.60187e−31	Plecoptera	Nemouridae	Visoka cataractae
Nemoura trispinosa 16S ribosomal RNA gene, partial	EF623187	81.76	100	6.60187e−31	Plecoptera	Nemouridae	Nemoura trispinosa
Zapada cinctipes 16S ribosomal RNA gene, partial s	EF623183	90.74	69.03	6.60187e−31	Plecoptera	Nemouridae	Zapada cinctipes
Triznaka pintada 16S ribosomal RNA gene, partial s	EF623134	92.08	65.16	6.60187e−31	Plecoptera	Chloroperlidae	Triznaka pintada
Mesocapnia sp. BYU_PL010 16S ribosomal RNA gene, p	EF623127	81.76	100	6.60187e−31	Plecoptera	Capniidae	Mesocapnia sp. BYU_PL010
Allocapnia recta haplotype 10ar: H010 16S ribosoma	EU306873	81.76	100	6.60187e−31	Plecoptera	Capniidae	Allocapnia recta
Allocapnia recta haplotype 9ar: H09 16S ribosomal	EU306872	81.76	100	6.60187e−31	Plecoptera	Capniidae	Allocapnia recta
Allocapnia recta haplotype 8ar: H08 16S ribosomal	EU306871	81.76	100	6.60187e−31	Plecoptera	Capniidae	Allocapnia recta
Allocapnia recta haplotype 3ar: H03 16S ribosomal	DQ915181	81.76	100	6.60187e−31	Plecoptera	Capniidae	Allocapnia recta
Allocapnia recta haplotype 2ar: H02 16S ribosomal	DQ915180	81.76	100	6.60187e−31	Plecoptera	Capniidae	Allocapnia recta
Allocapnia recta haplotype 1ar: H01 16S ribosomal	DQ915179	81.76	100	6.60187e−31	Plecoptera	Capniidae	Allocapnia recta
Doddsia occidentalis 16S ribosomal RNA gene, parti	EF623270	81.25	100	2.30428e−30	Plecoptera	Taeniopterygidae	Doddsia occidentalis
Cosumnoperla hypocrena 16S ribosomal RNA gene, par	EF623226	80.26	98.06	2.30428e−30	Plecoptera	Perlodidae	Cosumnoperla hypocrena
Paranemoura claasseni 16S ribosomal RNA gene, part	EF623185	82.1	100	2.30428e−30	Plecoptera	Nemouridae	Paranemoura claasseni
Alloperla severa 16S ribosomal RNA gene, partial s	EF623141	83.33	91.61	2.30428e−30	Plecoptera	Chloroperlidae	Alloperla severa
Allocapnia recta isolate H18 16S ribosomal RNA gen	KC881053	81.13	100	8.04272e−30	Plecoptera	Capniidae	Allocapnia recta
Cultus pilatus 16S ribosomal RNA gene, partial seq	EF623212	81.25	100	8.04272e−30	Plecoptera	Perlodidae	Cultus pilatus
Udamocercia sp. BYU_PL150 16S ribosomal RNA gene,	EF623201	89.81	69.03	8.04272e−30	Plecoptera	Notonemouridae	Udamocercia sp. BYU_PL150
Nanonemoura wahkeena 16S ribosomal RNA gene, parti	EF623198	81.82	98.71	8.04272e−30	Plecoptera	Nemouridae	Nanonemoura wahkeena
Siphonoperla torrentium 16S ribosomal RNA gene, pa	EF623138	91.09	65.16	8.04272e−30	Plecoptera	Chloroperlidae	Siphonoperla torrentium
Sweltsa coloradensis 16S ribosomal RNA gene, parti	EF623133	91.09	65.16	8.04272e−30	Plecoptera	Chloroperlidae	Sweltsa coloradensis
Allocapnia recta haplotype 12ar: H12 16S ribosomal	EU306875	81.13	100	8.04272e−30	Plecoptera	Capniidae	Allocapnia recta
Bolotoperla rossi 16S ribosomal RNA gene, partial	EF623269	80.5	100	2.80719e−29	Plecoptera	Taeniopterygidae	Bolotoperla rossi
Frisonia picticeps 16S ribosomal RNA gene, partial	EF623227	81.25	100	2.80719e−29	Plecoptera	Perlodidae	Frisonia picticeps
Isoperla oxylepis 16S ribosomal RNA gene, partial	EF623221	91.26	66.45	2.80719e−29	Plecoptera	Perlodidae	Isoperla oxylepis
Isoperla davisi 16S ribosomal RNA gene, partial se	EF623206	91.26	66.45	2.80719e−29	Plecoptera	Perlodidae	Isoperla davisi
Malenka californica 16S ribosomal RNA gene, partia	EF623182	81.88	100	2.80719e−29	Plecoptera	Nemouridae	Malenka californica
Chloroperla tripunctata 16S ribosomal RNA gene, pa	EF623137	91.09	65.16	2.80719e−29	Plecoptera	Chloroperlidae	Chloroperla tripunctata
Pteronarcys princeps mitochondrion, complete genom	AY687866	82.88	93.55	2.80719e−29	Plecoptera	Pteronarcyidae	Pteronarcys princeps
Scopura montana 16S ribosomal RNA gene, partial se	EF623265	81.48	100	9.79804e−29	Plecoptera		Scopura montana
Megaleuctra kincaidi 16S ribosomal RNA gene, parti	EF623174	80.25	100	9.79804e−29	Plecoptera	Leuctridae	Megaleuctra kincaidi
Pteronarcella badia 16S ribosomal RNA gene, partia	EF623262	90.1	65.16	3.41985e−28	Plecoptera	Pteronarcyidae	Pteronarcella badia
Pteronarcys californica 16S ribosomal RNA gene, pa	EF623261	82.19	93.55	3.41985e−28	Plecoptera	Pteronarcyidae	Pteronarcys californica
Cerconychia sp. BYU_PL114 16S ribosomal RNA gene,	EF623260	89.32	66.45	3.41985e−28	Plecoptera	Styloperlidae	Cerconychia sp. BYU_PL114
Acroperla trivacauta 16S ribosomal RNA gene, parti	EF623155	90.1	65.16	3.41985e−28	Plecoptera	Gripopterygidae	Acroperla trivacauta
Sasquaperla hoopa 16S ribosomal RNA gene, partial	EF623143	90.1	65.16	3.41985e−28	Plecoptera	Chloroperlidae	Sasquaperla hoopa
Paraperla frontalis 16S ribosomal RNA gene, partia	EF623136	90.1	65.16	3.41985e−28	Plecoptera	Chloroperlidae	Paraperla frontalis
Klapopteryx armillata 16S ribosomal RNA gene, part	EF623123	80.5	100	3.41985e−28	Plecoptera	Austroperlidae	Klapopteryx armillata
Osmylus sp. EH−2004 16S ribosomal RNA gene, partia	AY620146	89.32	66.45	3.41985e−28	Neuroptera	Osmylidae	Osmylus sp. EH−2004
Prostoia besametsa 16S ribosomal RNA gene, partial	EF623188	90.2	65.16	1.19365e−27	Plecoptera	Nemouridae	Prostoia besametsa
Utaperla sopladora 16S ribosomal RNA gene, partial	EF623140	89.11	65.16	1.19365e−27	Plecoptera	Chloroperlidae	Utaperla sopladora
Isogenoides colubrinus isolate BYU_PL130 16S ribos	EF623448	80.75	100	1.19365e−27	Plecoptera	Perlodidae	Isogenoides colubrinus
Rickera sorpta 16S ribosomal RNA gene, partial seq	EF623235	81.25	100	4.16623e−27	Plecoptera	Perlodidae	Rickera sorpta
Austrocerca rieka 16S ribosomal RNA gene, partial	EF623205	79.87	100	4.16623e−27	Plecoptera	Notonemouridae	Austrocerca rieka
Austrocercoides zwicki 16S ribosomal RNA gene, par	EF623199	79.87	100	4.16623e−27	Plecoptera	Notonemouridae	Austrocercoides zwicki
Notonemouridae sp. MD−2011 voucher ZMUC:JD−1507 16	JN615257	79.38	100	1.45416e−26	Plecoptera	Notonemouridae	Notonemouridae sp. MD−2011
