# Supplementary material for: Testing the potential of a ribosomal 16S marker for DNA metabarcoding of insects
Source: PeerJ. 2016 Apr 19;4:e1966. doi: 10.7717/peerj.1966 (PMC4841222; doi:10.7717/peerj.1966)
Supplement: Supplemental Information 1 [file OTU_38.csv_plot.pdf › scripts/5 match OTUs/blast_hits/OTU_38.csv_plot.pdf]

OTU_38.csv

Drusus annulatus

Limnephilidae

Trichoptera

100

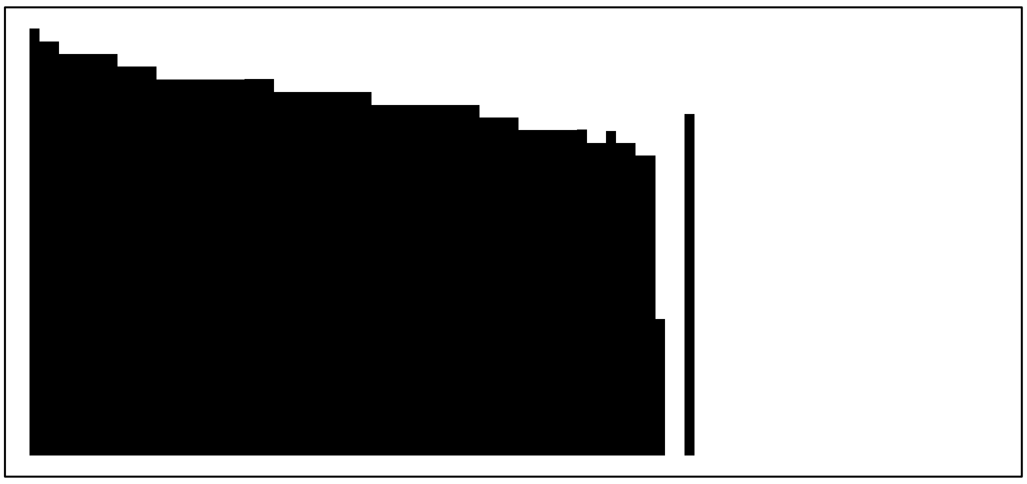

Drusus annulatus voucher DAn005 16S ribosomal RNA	EU215182	100	100	3.54059e−79	Trichoptera	Limnephilidae	Drusus annulatus
Drusus improvisus voucher RIS DImp004 16S ribosoma	EU999189	99.4	100	1.5055e−77	Trichoptera	Limnephilidae	Drusus improvisus
Drusus annulatus voucher DAn001 16S ribosomal RNA	EU215181	99.4	100	1.5055e−77	Trichoptera	Limnephilidae	Drusus annulatus
Metanoea flavipennis voucher MF001 16S ribosomal R	EU215224	98.81	100	1.83407e−76	Trichoptera	Limnephilidae	Metanoea flavipennis
Drusus rectus voucher DRec005 16S ribosomal RNA ge	EU215211	98.81	100	1.83407e−76	Trichoptera	Limnephilidae	Drusus rectus
Drusus romanicus romanicus voucher DR003 16S ribos	EU215209	98.81	100	1.83407e−76	Trichoptera	Limnephilidae	Drusus romanicus romanicus
Drusus romanicus romanicus voucher DR001 16S ribos	EU215208	98.81	100	1.83407e−76	Trichoptera	Limnephilidae	Drusus romanicus romanicus
Drusus melanchaetes voucher DMel001 16S ribosomal	EU215199	98.81	100	1.83407e−76	Trichoptera	Limnephilidae	Drusus melanchaetes
Drusus biguttatus voucher DBig003 16S ribosomal RN	EU215184	98.81	100	1.83407e−76	Trichoptera	Limnephilidae	Drusus biguttatus
Drusus improvisus voucher RIS DImp003 16S ribosoma	EU999188	98.21	100	2.23436e−75	Trichoptera	Limnephilidae	Drusus improvisus
Drusus improvisus voucher RIS DImp001 16S ribosoma	EU999187	98.21	100	7.79868e−75	Trichoptera	Limnephilidae	Drusus improvisus
Drusus mixtus voucher DMix001 16S ribosomal RNA ge	EU215200	98.21	100	7.79868e−75	Trichoptera	Limnephilidae	Drusus mixtus
Drusus brunneus voucher DBr002 16S ribosomal RNA g	EU215187	98.22	100	2.72201e−74	Trichoptera	Limnephilidae	Drusus brunneus
Drusus discolor voucher DdCAIM1 16S ribosomal RNA	KM001841	97.62	100	9.50073e−74	Trichoptera	Limnephilidae	Drusus discolor
Drusus discolor voucher DdIM2 16S ribosomal RNA ge	FJ002806	97.62	100	9.50073e−74	Trichoptera	Limnephilidae	Drusus discolor
Metanoea rhaetica voucher MR005 16S ribosomal RNA	EU215226	97.62	100	9.50073e−74	Trichoptera	Limnephilidae	Metanoea rhaetica
Metanoea rhaetica voucher MR001 16S ribosomal RNA	EU215225	97.62	100	9.50073e−74	Trichoptera	Limnephilidae	Metanoea rhaetica
Ecclisopteryx guttulata voucher EGut009 16S riboso	EU215220	97.62	100	9.50073e−74	Trichoptera	Limnephilidae	Ecclisopteryx guttulata
Drusus romanicus meridionalis voucher DR007 16S ri	EU215210	97.62	100	9.50073e−74	Trichoptera	Limnephilidae	Drusus romanicus meridionalis
Drusus discophorus pallidus voucher DDis001 16S ri	EU215195	97.62	100	9.50073e−74	Trichoptera	Limnephilidae	Drusus discophorus pallidus
Drusus destitutus voucher DDes001 16S ribosomal RN	EU215193	97.62	100	9.50073e−74	Trichoptera	Limnephilidae	Drusus destitutus
Drusus discolor voucher DD373 16S ribosomal RNA ge	EU215190	97.62	100	9.50073e−74	Trichoptera	Limnephilidae	Drusus discolor
Drusus trifidus voucher DTri007 16S ribosomal RNA	EU215215	97.63	100	3.31608e−73	Trichoptera	Limnephilidae	Drusus trifidus
Drusus trifidus voucher DTri006 16S ribosomal RNA	EU215214	97.63	100	3.31608e−73	Trichoptera	Limnephilidae	Drusus trifidus
Drusus brunneus voucher DBr001 16S ribosomal RNA g	EU215186	97.63	100	3.31608e−73	Trichoptera	Limnephilidae	Drusus brunneus
Ecclisopteryx madida voucher fEma0102M 16S ribosom	KM001738	97.02	100	4.03981e−72	Trichoptera	Limnephilidae	Ecclisopteryx madida
Ecclisopteryx madida voucher fEma0101M 16S ribosom	KM001737	97.02	100	4.03981e−72	Trichoptera	Limnephilidae	Ecclisopteryx madida
Ecclisopteryx madida voucher EM006 16S ribosomal R	KM001736	97.02	100	4.03981e−72	Trichoptera	Limnephilidae	Ecclisopteryx madida
Ecclisopteryx madida voucher EM005 16S ribosomal R	KM001735	97.02	100	4.03981e−72	Trichoptera	Limnephilidae	Ecclisopteryx madida
Ecclisopteryx madida voucher EM003 16S ribosomal R	KM001734	97.02	100	4.03981e−72	Trichoptera	Limnephilidae	Ecclisopteryx madida
Ecclisopteryx madida voucher EM001 16S ribosomal R	EU215221	97.02	100	4.03981e−72	Trichoptera	Limnephilidae	Ecclisopteryx madida
Drusus destitutus voucher DDes005 16S ribosomal RN	EU215194	97.02	100	4.03981e−72	Trichoptera	Limnephilidae	Drusus destitutus
Drusus balcanicus voucher DBal001 16S ribosomal RN	EU215183	97.02	100	4.03981e−72	Trichoptera	Limnephilidae	Drusus balcanicus
Drusus alpinus voucher DAlp003 16S ribosomal RNA g	EU215180	97.02	100	4.03981e−72	Trichoptera	Limnephilidae	Drusus alpinus
Drusus alpinus voucher DAlp002 16S ribosomal RNA g	EU215179	97.02	100	4.03981e−72	Trichoptera	Limnephilidae	Drusus alpinus
Ecclisopteryx guttulata voucher fEgu0104M 16S ribo	KM001749	96.43	100	4.9215e−71	Trichoptera	Limnephilidae	Ecclisopteryx guttulata
Ecclisopteryx guttulata voucher fEgu0103M 16S ribo	KM001748	96.43	100	4.9215e−71	Trichoptera	Limnephilidae	Ecclisopteryx guttulata
Ecclisopteryx guttulata voucher fEgu0101M 16S ribo	KM001746	96.43	100	4.9215e−71	Trichoptera	Limnephilidae	Ecclisopteryx guttulata
Ecclisopteryx madida voucher EM002 16S ribosomal R	KM001733	96.43	100	4.9215e−71	Trichoptera	Limnephilidae	Ecclisopteryx madida
Drusus vespertinus voucher DvRL1 16S ribosomal RNA	FJ002817	96.43	100	4.9215e−71	Trichoptera	Limnephilidae	Drusus vespertinus
Drusus ramae voucher DraIM1 16S ribosomal RNA gene	FJ002809	96.43	100	4.9215e−71	Trichoptera	Limnephilidae	Drusus ramae
Drusus medianus voucher DmIM1 16S ribosomal RNA ge	FJ002807	96.43	100	4.9215e−71	Trichoptera	Limnephilidae	Drusus medianus
Ecclisopteryx madida voucher EM004 16S ribosomal R	EU215222	96.41	99.4	4.9215e−71	Trichoptera	Limnephilidae	Ecclisopteryx madida
Drusus muelleri voucher DMue005 16S ribosomal RNA	EU215205	96.43	100	4.9215e−71	Trichoptera	Limnephilidae	Drusus muelleri
Drusus muelleri voucher DMue004 16S ribosomal RNA	EU215204	96.43	100	4.9215e−71	Trichoptera	Limnephilidae	Drusus muelleri
Drusus muelleri voucher DMue003 16S ribosomal RNA	EU215203	96.43	100	4.9215e−71	Trichoptera	Limnephilidae	Drusus muelleri
Ecclisopteryx guttulata voucher fEgu0102F 16S ribo	KM001747	95.83	100	2.09268e−69	Trichoptera	Limnephilidae	Ecclisopteryx guttulata
Drusus bosnicus voucher DbsVML2 16S ribosomal RNA	GQ470605	95.83	100	2.09268e−69	Trichoptera	Limnephilidae	Drusus bosnicus
Drusus radovanovici radovanovici voucher DRRIM1 16	GQ470601	95.83	100	2.09268e−69	Trichoptera	Limnephilidae	Drusus radovanovici radovanovici
Drusus bosnicus voucher DbsIM1 16S ribosomal RNA g	FJ002821	95.83	100	2.09268e−69	Trichoptera	Limnephilidae	Drusus bosnicus
Ecclisopteryx dalecarlica voucher fEda0901F 16S ri	KM001827	95.24	100	2.5494e−68	Trichoptera	Limnephilidae	Ecclisopteryx dalecarlica
Ecclisopteryx dalecarlica voucher fEda0801M 16S ri	KM001825	95.24	100	2.5494e−68	Trichoptera	Limnephilidae	Ecclisopteryx dalecarlica
Ecclisopteryx dalecarlica voucher fEda0701F 16S ri	KM001824	95.24	100	2.5494e−68	Trichoptera	Limnephilidae	Ecclisopteryx dalecarlica
Ecclisopteryx dalecarlica voucher ED002 16S riboso	EU215219	95.24	100	2.5494e−68	Trichoptera	Limnephilidae	Ecclisopteryx dalecarlica
Ecclisopteryx dalecarlica voucher ED001 16S riboso	EU215218	95.24	100	2.5494e−68	Trichoptera	Limnephilidae	Ecclisopteryx dalecarlica
Drusus franzi voucher DFrII 16S ribosomal RNA gene	EU215198	95.24	100	2.5494e−68	Trichoptera	Limnephilidae	Drusus franzi
Ecclisopteryx malickyi voucher EMal002 16S ribosom	KM001725	95.27	100	8.89828e−68	Trichoptera	Limnephilidae	Ecclisopteryx malickyi
Ecclisopteryx dalecarlica voucher fEda0501M 16S ri	KM001822	94.64	100	1.08403e−66	Trichoptera	Limnephilidae	Ecclisopteryx dalecarlica
Drusus schmidi voucher DsIM4 16S ribosomal RNA gen	FJ002816	94.64	100	1.08403e−66	Trichoptera	Limnephilidae	Drusus schmidi
Ecclisopteryx malickyi voucher EMal001 16S ribosom	EU215223	95.21	98.81	1.08403e−66	Trichoptera	Limnephilidae	Ecclisopteryx malickyi
Ecclisopteryx dalecarlica voucher Dsp023 16S ribos	EU215212	94.64	100	1.08403e−66	Trichoptera	Limnephilidae	Ecclisopteryx dalecarlica
Drusus franzi voucher DFr007 16S ribosomal RNA gen	EU215197	94.64	100	1.08403e−66	Trichoptera	Limnephilidae	Drusus franzi
Ecclisopteryx dalecarlica voucher fEda0502F 16S ri	KM001823	94.05	100	1.32062e−65	Trichoptera	Limnephilidae	Ecclisopteryx dalecarlica
Drusus schmidi voucher DsIM3 16S ribosomal RNA gen	FJ002815	94.05	100	1.32062e−65	Trichoptera	Limnephilidae	Drusus schmidi
Ecclisomyia kamtshatica mitochondrial gene for 16S	AB355444	86.39	100	1.32179e−46	Trichoptera	Limnephilidae	Ecclisomyia kamtshatica
PREDICTED: Apis florea dual serine/threonine and t	XM_012485067	72.22	53.57	6.4242	Hymenoptera	Apidae	Apis florea
Sebaldella termitidis ATCC 33386, complete genome	CP001739	76.92	30.95	6.4242	Fusobacteriales	Leptotrichiaceae	Sebaldella termitidis ATCC 33386
Homo sapiens BAC clone RP11−11P22 from 2, complete	AC092835	96	14.88	6.4242	Primates	Hominidae	Homo sapiens
