# Supplementary material for: Testing the potential of a ribosomal 16S marker for DNA metabarcoding of insects
Source: PeerJ. 2016 Apr 19;4:e1966. doi: 10.7717/peerj.1966 (PMC4841222; doi:10.7717/peerj.1966)
Supplement: Supplemental Information 1 [file OTU_399.csv_plot.pdf › scripts/5 match OTUs/blast_hits/OTU_399.csv_plot.pdf]

OTU_399.csv

Ephemera danica

Ephemeridae

Ephemeroptera

96.05

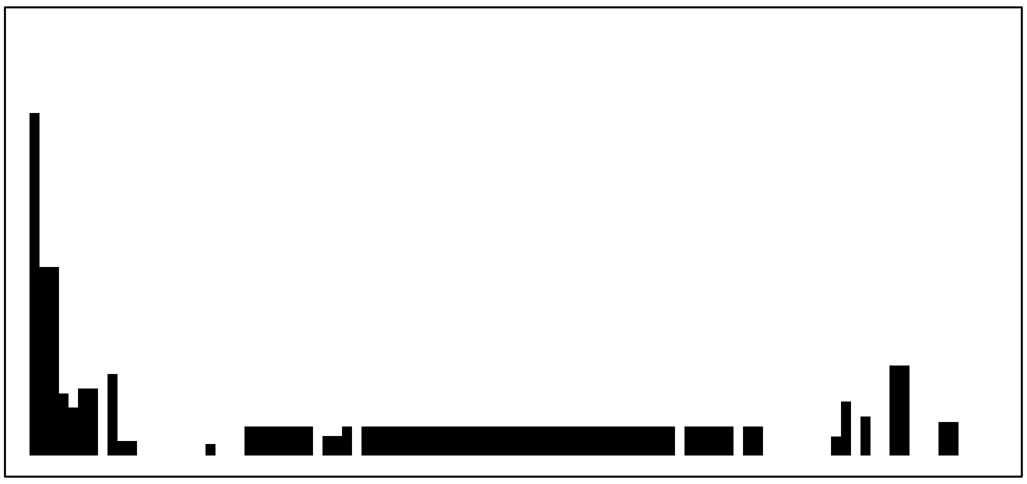

Ephemera danica 16S ribosomal RNA gene, partial se	KF855860	96.05	100	2.10049e−62	Ephemeroptera	Ephemeridae	Ephemera danica
Ephemera orientalis voucher EPH01 tRNA−Leu, 16S ri	KF256907	88.82	100	3.33141e−47	Ephemeroptera	Ephemeridae	Ephemera orientalis
Ephemera orientalis mitochondrion, complete genome	EU591678	88.82	100	3.33141e−47	Ephemeroptera	Ephemeridae	Ephemera orientalis
Ephemera simulans voucher BYU:IGCEP038 16S ribosom	GQ118285	82.89	100	2.92231e−35	Ephemeroptera	Ephemeridae	Ephemera simulans
Siphlonurus immanis voucher NKMT032 mitochondrion,	FJ606783	82.24	100	1.2426e−33	Ephemeroptera	Siphlonuridae	Siphlonurus immanis
Ephemera sp. 1 BM−2008 12S ribosomal RNA gene, par	EU477611	83.12	100	4.3371e−33	Ephemeroptera	Ephemeridae	Ephemera sp. 1 BM−2008
Ephemera sp. 'Bonn' large subunit ribosomal RNA ge	AF266047	83.12	100	4.3371e−33	Ephemeroptera	Ephemeridae	Ephemera sp. 'Bonn'
Priocera castanea voucher 7150 16S ribosomal RNA g	KC524873	79.43	90.79	1.61771e−19	Coleoptera	Cleridae	Priocera castanea
Ctenoplusia limbirena mitochondrion, complete geno	KM244665	83.81	67.76	1.97078e−18	Lepidoptera	Noctuidae	Ctenoplusia limbirena
Oxythyrea pantherina voucher 842859 16S ribosomal	JX234531	80.67	77.63	1.97078e−18	Coleoptera	Scarabaeidae	Oxythyrea pantherina
Oxythyrea funesta voucher 678443 16S ribosomal RNA	JX234432	80.67	77.63	1.97078e−18	Coleoptera	Scarabaeidae	Oxythyrea funesta
Prodasineura sita voucher RMNH.INS.229286 16S ribo	KF369864	78.79	84.21	1.97078e−18	Odonata	Protoneuridae	Prodasineura sita
Chiasmia defixaria gene for 16S rRNA, partial sequ	AB265385	79.23	82.89	1.97078e−18	Lepidoptera	Geometridae	Chiasmia defixaria
Anthopotamus sp. EP022 16S ribosomal RNA gene, par	AY749766	76.58	100	1.97078e−18	Ephemeroptera	Potamanthidae	Anthopotamus sp. EP022
Potamanthus luteus voucher BYU:IGCEP219 16S riboso	GQ118293	76.47	98.68	6.87869e−18	Ephemeroptera	Potamanthidae	Potamanthus luteus
Podallea madegassica 16S ribosomal RNA gene, parti	EU734892	78.23	92.11	6.87869e−18	Neuroptera	Berothidae	Podallea madegassica
Dichochrysa tacta isolate Dictac1 16S ribosomal RN	AY620154	76.43	99.34	6.87869e−18	Neuroptera	Chrysopidae	Pseudomallada tactus
Nipponosialis kumejimae isolate H841 16S ribosomal	KF741321	75.47	99.34	2.4009e−17	Megaloptera	Sialidae	Nipponosialis kumejimae
Ephoron album mitochondrial gene for 16S rRNA, par	AB711636	80.53	73.68	2.4009e−17	Ephemeroptera	Polymitarcyidae	Ephoron album
Osmylus fulvicephalus 16S ribosomal RNA gene, part	EU734887	79.26	85.53	2.4009e−17	Neuroptera	Osmylidae	Osmylus fulvicephalus
Comibaena procumbaria gene for 16S rRNA, partial s	AB265413	76.67	97.37	2.4009e−17	Lepidoptera	Geometridae	Comibaena procumbaria
Agabus guttatus mitochondrial 16S rRNA gene (parti	LM654777	76.22	91.45	8.37996e−17	Coleoptera	Dytiscidae	Agabus guttatus
Bombyx mori strain huayu mitochondrion, complete g	KM875545	81.36	76.32	8.37996e−17	Lepidoptera	Bombycidae	Bombyx mori
Bombyx mori strain Yu39 mitochondrion, complete ge	KP313778	81.36	76.32	8.37996e−17	Lepidoptera	Bombycidae	Bombyx mori
Bombyx mori strain Yu5 mitochondrion, complete gen	KP192479	81.36	76.32	8.37996e−17	Lepidoptera	Bombycidae	Bombyx mori
Bombyx mori strain Chunyun mitochondrion, complete	KP192478	81.36	76.32	8.37996e−17	Lepidoptera	Bombycidae	Bombyx mori
Bombyx mori strain Jin6 mitochondrion, complete ge	KP244370	81.36	76.32	8.37996e−17	Lepidoptera	Bombycidae	Bombyx mori
Bombyx mori strain Qiufeng x Baiyu mitochondrion,	KM347743	81.36	76.32	8.37996e−17	Lepidoptera	Bombycidae	Bombyx mori
Bombyx mori strain Baiyun mitochondrion, complete	KM279431	81.36	76.32	8.37996e−17	Lepidoptera	Bombycidae	Bombyx mori
Rondotia menciana voucher RM1234 mitochondrion, co	KJ647172	76.82	97.37	8.37996e−17	Lepidoptera	Bombycidae	Rondotia menciana
Ephoron leukon mitochondrial gene for 16S rRNA, pa	AB711641	80.91	71.71	8.37996e−17	Ephemeroptera	Polymitarcyidae	Ephoron leukon
Ephoron album mitochondrial gene for 16S rRNA, par	AB711635	80.91	71.71	8.37996e−17	Ephemeroptera	Polymitarcyidae	Ephoron album
Bombyx mori mRNA, clone: fprW07J07	AK406146	81.36	76.32	8.37996e−17	Lepidoptera	Bombycidae	Bombyx mori
Papilio alexanor voucher FS−b−40 16S ribosomal RNA	GQ268343	76.71	94.74	8.37996e−17	Lepidoptera	Papilionidae	Papilio alexanor
Bombyx mori isolate C108 mitochondrion, complete g	GU966630	81.36	76.32	8.37996e−17	Lepidoptera	Bombycidae	Bombyx mori
Bombyx mori isolate Handan mitochondrion, complete	GU966628	81.36	76.32	8.37996e−17	Lepidoptera	Bombycidae	Bombyx mori
Bombyx mori isolate Mutation M2 mitochondrion, com	GU966626	81.36	76.32	8.37996e−17	Lepidoptera	Bombycidae	Bombyx mori
Bombyx mori isolate Mutate15−010 mitochondrion, co	GU966625	81.36	76.32	8.37996e−17	Lepidoptera	Bombycidae	Bombyx mori
Bombyx mori isolate Xiaoshiwan mitochondrion, comp	GU966624	81.36	76.32	8.37996e−17	Lepidoptera	Bombycidae	Bombyx mori
Bombyx mori isolate Bilian mitochondrion, complete	GU966623	81.36	76.32	8.37996e−17	Lepidoptera	Bombycidae	Bombyx mori
Bombyx mori isolate J872 mitochondrion, complete g	GU966622	81.36	76.32	8.37996e−17	Lepidoptera	Bombycidae	Bombyx mori
Bombyx mori isolate J7532 mitochondrion, complete	GU966620	81.36	76.32	8.37996e−17	Lepidoptera	Bombycidae	Bombyx mori
Bombyx mori isolate Mutate02−210 mitochondrion, co	GU966618	81.36	76.32	8.37996e−17	Lepidoptera	Bombycidae	Bombyx mori
Bombyx mori isolate Sihong mitochondrion, complete	GU966617	81.36	76.32	8.37996e−17	Lepidoptera	Bombycidae	Bombyx mori
Bombyx mori isolate J106 mitochondrion, complete g	GU966615	81.36	76.32	8.37996e−17	Lepidoptera	Bombycidae	Bombyx mori
Bombyx mori isolate Ankang No. 4 mitochondrion, co	GU966614	81.36	76.32	8.37996e−17	Lepidoptera	Bombycidae	Bombyx mori
Bombyx mori isolate ZT000 mitochondrion, complete	GU966613	81.36	76.32	8.37996e−17	Lepidoptera	Bombycidae	Bombyx mori
Bombyx mori isolate J04−010 mitochondrion, complet	GU966612	81.36	76.32	8.37996e−17	Lepidoptera	Bombycidae	Bombyx mori
Bombyx mori isolate ZT500 mitochondrion, complete	GU966611	81.36	76.32	8.37996e−17	Lepidoptera	Bombycidae	Bombyx mori
Bombyx mori isolate Lao 2 mitochondrion, complete	GU966610	81.36	76.32	8.37996e−17	Lepidoptera	Bombycidae	Bombyx mori
Bombyx mori isolate Zhugui mitochondrion, complete	GU966609	81.36	76.32	8.37996e−17	Lepidoptera	Bombycidae	Bombyx mori
Bombyx mori isolate Mutate15−001 mitochondrion, co	GU966608	81.36	76.32	8.37996e−17	Lepidoptera	Bombycidae	Bombyx mori
Bombyx mori isolate Europe 18 mitochondrion, compl	GU966607	81.36	76.32	8.37996e−17	Lepidoptera	Bombycidae	Bombyx mori
Bombyx mori isolate Sichuang M3 mitochondrion, com	GU966605	81.36	76.32	8.37996e−17	Lepidoptera	Bombycidae	Bombyx mori
Bombyx mori isolate A06E mitochondrion, complete g	GU966604	81.36	76.32	8.37996e−17	Lepidoptera	Bombycidae	Bombyx mori
Bombyx mori isolate ZT100 mitochondrion, complete	GU966603	81.36	76.32	8.37996e−17	Lepidoptera	Bombycidae	Bombyx mori
Bombyx mori isolate N4 mitochondrion, complete gen	GU966602	81.36	76.32	8.37996e−17	Lepidoptera	Bombycidae	Bombyx mori
Bombyx mori isolate Cambodia mitochondrion, comple	GU966601	81.36	76.32	8.37996e−17	Lepidoptera	Bombycidae	Bombyx mori
Bombyx mori isolate ZT900 mitochondrion, complete	GU966600	81.36	76.32	8.37996e−17	Lepidoptera	Bombycidae	Bombyx mori
Bombyx mori isolate Soviet Union No. 1 mitochondri	GU966599	81.36	76.32	8.37996e−17	Lepidoptera	Bombycidae	Bombyx mori
Bombyx mori isolate Italy 16 mitochondrion, comple	GU966596	81.36	76.32	8.37996e−17	Lepidoptera	Bombycidae	Bombyx mori
Bombyx mori isolate India M3 mitochondrion, comple	GU966595	81.36	76.32	8.37996e−17	Lepidoptera	Bombycidae	Bombyx mori
Bombyx mori isolate Damao mitochondrion, complete	GU966594	81.36	76.32	8.37996e−17	Lepidoptera	Bombycidae	Bombyx mori
Bombyx mandarina from Japan mitochondrion, complet	GU966593	81.36	76.32	8.37996e−17	Lepidoptera	Bombycidae	Bombyx mandarina
Bombyx mandarina strain Qingzhou mitochondrion, co	FJ384796	81.36	76.32	8.37996e−17	Lepidoptera	Bombycidae	Bombyx mandarina
Bombyx mandarina mitochondrial DNA, complete genom	AB070263	81.36	76.32	8.37996e−17	Lepidoptera	Bombycidae	Bombyx mandarina
Agabus guttatus 16S ribosomal RNA gene, partial se	AY039262	76.22	91.45	8.37996e−17	Coleoptera	Dytiscidae	Agabus guttatus
Bombyx mori strain Xiafang mitochondrion, complete	AY048187	81.36	76.32	8.37996e−17	Lepidoptera	Bombycidae	Bombyx mori
Bombyx mori NADH dehydrogenase subunit 1 (ND1) gen	AF295563	81.36	76.32	8.37996e−17	Lepidoptera	Bombycidae	Bombyx mori
Bombyx mori mitochondrion, complete genome	AF149768	81.36	76.32	8.37996e−17	Lepidoptera	Bombycidae	Bombyx mori
Bombyx mori mitochondrial DNA, complete genome, st	AB083339	81.36	76.32	8.37996e−17	Lepidoptera	Bombycidae	Bombyx mori
Bombyx mori mitochondrial DNA, complete genome, st	AB070264	81.36	76.32	8.37996e−17	Lepidoptera	Bombycidae	Bombyx mori
Papilio alexanor mitochondrion, large subunit ribo	AJ224052	76.71	94.74	8.37996e−17	Lepidoptera	Papilionidae	Papilio alexanor
Bombyx mandarina 16S ribosomal RNA gene, mitochond	AF027951	81.36	76.32	8.37996e−17	Lepidoptera	Bombycidae	Bombyx mandarina
Bombyx mori 16S ribosomal RNA gene, mitochondrial	AF027950	81.36	76.32	8.37996e−17	Lepidoptera	Bombycidae	Bombyx mori
Kuromondokuga niphonis voucher H167 large subunit	KP081963	77.85	94.08	2.92489e−16	Lepidoptera	Lymantriidae	Kuromondokuga niphonis
Protaetia marginicollis voucher 842877 16S ribosom	JX234546	76	97.37	2.92489e−16	Coleoptera	Scarabaeidae	Protaetia marginicollis
Ephoron shigae mitochondrial gene for 16S ribosoma	AB874293	78.51	79.61	2.92489e−16	Ephemeroptera	Polymitarcyidae	Ephoron shigae
Ephoron shigae mitochondrial gene for 16S ribosoma	AB874289	78.51	79.61	2.92489e−16	Ephemeroptera	Polymitarcyidae	Ephoron shigae
Agabus caraboides mitochondrial partial 16S rRNA g	HF931451	74.52	99.34	2.92489e−16	Coleoptera	Dytiscidae	Agabus caraboides
Ephoron shigae mitochondrial gene for 16S rRNA, pa	AB711533	78.51	79.61	2.92489e−16	Ephemeroptera	Polymitarcyidae	Ephoron shigae
Ephoron shigae mitochondrial gene for 16S rRNA, pa	AB711527	78.51	79.61	2.92489e−16	Ephemeroptera	Polymitarcyidae	Ephoron shigae
Oedosmylus brevis 16S ribosomal RNA gene, partial	EU734907	80.87	73.68	2.92489e−16	Neuroptera	Osmylidae	Oedosmylus brevis
Oliarces clara 16S ribosomal RNA gene, partial seq	EU734885	82.52	65.79	2.92489e−16	Neuroptera	Ithonidae	Oliarces clara
Megalithone tillyardi 16S ribosomal RNA gene, part	EU734866	78.38	92.76	2.92489e−16	Neuroptera	Ithonidae	Megalithone tillyardi
Stylogomphus shirizui 12S ribosomal RNA gene, part	EU477679	81.82	64.47	2.92489e−16	Odonata	Gomphidae	Stylogomphus shirozui
Leucochrysa texana 16S ribosomal RNA gene, partial	DQ399279	76.25	99.34	2.92489e−16	Neuroptera	Chrysopidae	Leucochrysa texana
Agabus glacialis mitochondrial 16S rRNA gene (part	LM654776	74.36	99.34	1.02089e−15	Coleoptera	Dytiscidae	Agabus glacialis
Hygrostola dallolmoi voucher HYTAG7234 16S ribosom	KP682574	84.21	61.18	1.02089e−15	Lepidoptera	Noctuidae	Hygrostola dallolmoi
Hygrostola dallolmoi voucher HYTAG6454 16S ribosom	KP682573	84.21	61.18	1.02089e−15	Lepidoptera	Noctuidae	Hygrostola dallolmoi
Xeloma leprosa voucher 842864 16S ribosomal RNA ge	JX234536	77.4	88.82	1.02089e−15	Coleoptera	Scarabaeidae	Xeloma leprosa
Dischista cincta voucher 842787 16S ribosomal RNA	JX234481	75.86	89.47	1.02089e−15	Coleoptera	Scarabaeidae	Dischista cincta
Leptomorphus sp. DK−2013 voucher JSS1 16S ribosoma	KC435575	75.5	97.37	1.02089e−15	Diptera	Mycetophilidae	Leptomorphus sp. DK−2013
Ephoron leukon mitochondrial gene for 16S rRNA, pa	AB711638	81.55	67.11	1.02089e−15	Ephemeroptera	Polymitarcyidae	Ephoron leukon
Ephoron leukon mitochondrial gene for 16S rRNA, pa	AB711637	81.55	67.11	1.02089e−15	Ephemeroptera	Polymitarcyidae	Ephoron leukon
Phytosciara ussuriensis voucher 883 16S ribosomal	JQ613948	77.6	80.26	1.02089e−15	Diptera	Sciaridae	Phytosciara ussuriensis
Bradysia difformis voucher 122 16S ribosomal RNA g	JQ613894	75.86	91.45	1.02089e−15	Diptera	Sciaridae	Bradysia impatiens
Acraea violae isolate F472 16S ribosomal RNA gene,	JQ416072	76.35	94.74	1.02089e−15	Lepidoptera	Nymphalidae	Acraea violae
Acraea violae isolate F486 16S ribosomal RNA gene,	JQ416057	76.35	94.74	1.02089e−15	Lepidoptera	Nymphalidae	Acraea violae
Pachliopta aristolochiae isolate N296 16S ribosoma	JQ421944	76.98	88.82	1.02089e−15	Lepidoptera	Papilionidae	Pachliopta aristolochiae
