# Supplementary material for: Testing the potential of a ribosomal 16S marker for DNA metabarcoding of insects
Source: PeerJ. 2016 Apr 19;4:e1966. doi: 10.7717/peerj.1966 (PMC4841222; doi:10.7717/peerj.1966)
Supplement: Supplemental Information 1 [file OTU_411.csv_plot.pdf › scripts/5 match OTUs/blast_hits/OTU_411.csv_plot.pdf]

OTU_411.csv

Ephemera danica

Ephemeridae

Ephemeroptera

96.71

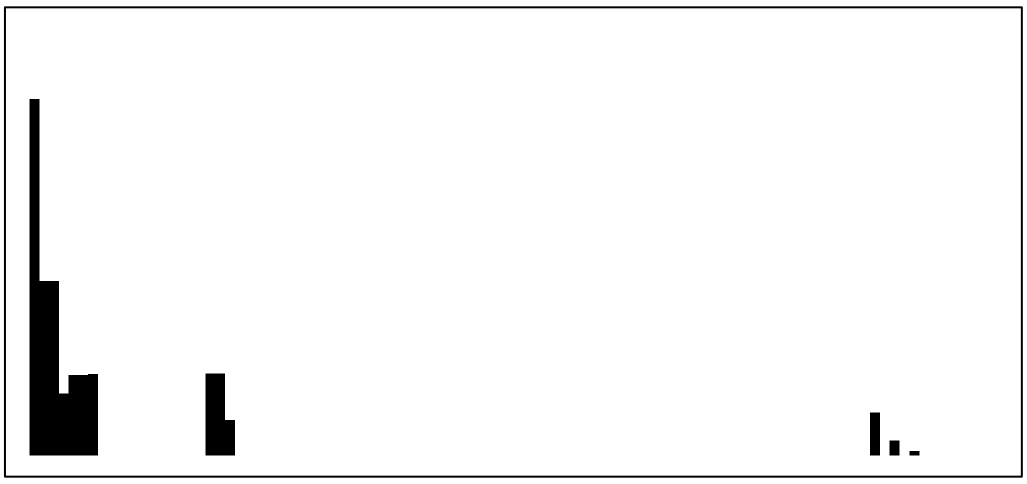

Ephemera danica 16S ribosomal RNA gene, partial se	KF855860	96.71	100	1.72419e−63	Ephemeroptera	Ephemeridae	Ephemera danica
Ephemera orientalis voucher EPH01 tRNA−Leu, 16S ri	KF256907	88.16	100	4.05849e−46	Ephemeroptera	Ephemeridae	Ephemera orientalis
Ephemera orientalis mitochondrion, complete genome	EU591678	88.16	100	4.05849e−46	Ephemeroptera	Ephemeridae	Ephemera orientalis
Ephemera simulans voucher BYU:IGCEP038 16S ribosom	GQ118285	82.89	100	2.92231e−35	Ephemeroptera	Ephemeridae	Ephemera simulans
Ephemera sp. 1 BM−2008 12S ribosomal RNA gene, par	EU477611	83.77	100	1.01999e−34	Ephemeroptera	Ephemeridae	Ephemera sp. 1 BM−2008
Ephemera sp. 'Bonn' large subunit ribosomal RNA ge	AF266047	83.77	100	1.01999e−34	Ephemeroptera	Ephemeridae	Ephemera sp. 'Bonn'
Siphlonurus immanis voucher NKMT032 mitochondrion,	FJ606783	83.8	93.42	1.2426e−33	Ephemeroptera	Siphlonuridae	Siphlonurus immanis
Anotogaster sp. RF1898 mitochondrial gene for 16S	AB707899	76.16	98.68	5.64637e−19	Odonata	Cordulegastridae	Anotogaster sp. RF1898
Anotogaster sp. RF1284 mitochondrial gene for 16S	AB707896	76.16	98.68	5.64637e−19	Odonata	Cordulegastridae	Anotogaster sp. RF1284
Anotogaster sieboldii mitochondrial gene for 16S r	AB707892	76.16	98.68	5.64637e−19	Odonata	Cordulegastridae	Anotogaster sieboldii
Anotogaster sieboldii mitochondrial gene for 16S r	AB707881	76.16	98.68	5.64637e−19	Odonata	Cordulegastridae	Anotogaster sieboldii
Anotogaster sieboldii 12S ribosomal RNA gene, part	EU477686	76.16	98.68	5.64637e−19	Odonata	Cordulegastridae	Anotogaster sieboldii
Anotogaster sieboldii mitochondrial gene for 16S r	AB127061	76.16	98.68	5.64637e−19	Odonata	Cordulegastridae	Anotogaster sieboldii
Anotogaster sieboldi large subunit ribosomal RNA g	AF266087	76.16	98.68	5.64637e−19	Odonata	Cordulegastridae	Anotogaster sieboldii
Anotogaster sp. RF1894 mitochondrial gene for 16S	AB707898	76.32	98.68	1.97078e−18	Odonata	Cordulegastridae	Anotogaster sp. RF1894
Anotogaster sieboldii mitochondrial gene for 16S r	AB707885	75.5	98.68	2.4009e−17	Odonata	Cordulegastridae	Anotogaster sieboldii
Anotogaster sieboldii mitochondrial gene for 16S r	AB707882	75.5	98.68	2.4009e−17	Odonata	Cordulegastridae	Anotogaster sieboldii
Anotogaster sieboldii mitochondrial gene for 16S r	AB707880	75.5	98.68	2.4009e−17	Odonata	Cordulegastridae	Anotogaster sieboldii
Hygrostola dallolmoi voucher HYTAG7234 16S ribosom	KP682574	83.84	63.82	8.37996e−17	Lepidoptera	Noctuidae	Hygrostola dallolmoi
Hygrostola dallolmoi voucher HYTAG6454 16S ribosom	KP682573	83.84	63.82	8.37996e−17	Lepidoptera	Noctuidae	Hygrostola dallolmoi
Ctenoplusia limbirena mitochondrion, complete geno	KM244665	81.65	70.39	8.37996e−17	Lepidoptera	Noctuidae	Ctenoplusia limbirena
Neallogaster pekinensis mitochondrial gene for 16S	AB707904	74.83	98.68	2.92489e−16	Odonata	Cordulegastridae	Neallogaster pekinensis
Cordulegaster picta mitochondrial gene for 16S rRN	AB707902	74.83	98.68	2.92489e−16	Odonata	Cordulegastridae	Cordulegaster picta
Anotogaster sieboldii mitochondrial gene for 16S r	AB707886	74.83	98.68	2.92489e−16	Odonata	Cordulegastridae	Anotogaster sieboldii
Anotogaster klossi mitochondrial gene for 16S rRNA	AB707872	74.83	98.68	2.92489e−16	Odonata	Cordulegastridae	Anotogaster klossi
Potamanthus luteus voucher BYU:IGCEP219 16S riboso	GQ118293	75.82	98.68	2.92489e−16	Ephemeroptera	Potamanthidae	Potamanthus luteus
Cordulegaster sp. 1 BM−2008 12S ribosomal RNA gene	EU477687	74.83	98.68	2.92489e−16	Odonata	Cordulegastridae	Cordulegaster sp. 1 BM−2008
Phenes raptor isolate 2 12S ribosomal RNA gene, pa	EU477632	79.2	80.26	2.92489e−16	Odonata	Petaluridae	Phenes raptor
Phenes raptor 12S ribosomal RNA gene, partial sequ	EU477622	79.2	80.26	2.92489e−16	Odonata	Petaluridae	Phenes raptor
Phenes raptor 16S ribosomal RNA gene, partial sequ	EU055038	79.2	80.26	2.92489e−16	Odonata	Petaluridae	Phenes raptor
Phenes raptor large subunit ribosomal RNA gene, pa	AF266083	79.2	80.26	2.92489e−16	Odonata	Petaluridae	Phenes raptor
Anotogaster sp. RF1283 mitochondrial gene for 16S	AB707895	78.45	75.66	1.02089e−15	Odonata	Cordulegastridae	Anotogaster sp. RF1283
Anotogaster kuchenbeiseri mitochondrial gene for 1	AB707878	78.45	75.66	1.02089e−15	Odonata	Cordulegastridae	Anotogaster kuchenbeiseri
Anotogaster chaoi mitochondrial gene for 16S rRNA,	AB707858	78.45	75.66	1.02089e−15	Odonata	Cordulegastridae	Anotogaster chaoi
Kalyptogaster erronea 12S ribosomal RNA and 16S ri	EU477690	78.45	75.66	1.02089e−15	Odonata	Cordulegastridae	Kalyptogaster erronea
Cordulegaster boltonii 12S ribosomal RNA gene, par	EU477688	78.45	75.66	1.02089e−15	Odonata	Cordulegastridae	Cordulegaster boltonii
Cordulegaster dorsalis 16S ribosomal RNA gene, par	EU055084	78.45	75.66	1.02089e−15	Odonata	Cordulegastridae	Cordulegaster dorsalis
Kalyptogaster erronea voucher JW_DNA_40 16S riboso	EF631561	78.45	75.66	1.02089e−15	Odonata	Cordulegastridae	Kalyptogaster erronea
Taeniogaster obliqua voucher JW_DNA_OTO 16S riboso	EF631533	75.32	98.68	1.02089e−15	Odonata	Cordulegastridae	Taeniogaster obliqua
Cordulegaster boltoni large subunit ribosomal RNA	AF266056	78.45	75.66	1.02089e−15	Odonata	Cordulegastridae	Cordulegaster boltonii
Indolestes peregrinus voucher ZYG08 tRNA−Leu, 16S	KF256891	74.68	98.03	3.56325e−15	Odonata	Lestidae	Indolestes peregrinus
Sympecma paedisca voucher ZYG07 tRNA−Leu, 16S ribo	KF256890	74.68	98.03	3.56325e−15	Odonata	Lestidae	Sympecma paedisca
Indolestes peregrinus mitochondrial gene for 16S r	AB707356	74.68	98.03	3.56325e−15	Odonata	Lestidae	Indolestes peregrinus
Austrogomphus amphiclitus 16S ribosomal RNA gene,	EU055074	74.52	99.34	3.56325e−15	Odonata	Gomphidae	Austrogomphus amphiclitus
Nannophlebia sp. SMB−2007 16S ribosomal RNA gene,	EU055044	75.17	94.74	3.56325e−15	Odonata	Libellulidae	Nannophlebia sp. SMB−2007
Neurocordulia obsoleta voucher JW_DNA_ONO 16S ribo	EF631509	77.17	80.26	3.56325e−15	Odonata	Corduliidae	Neurocordulia obsoleta
Indolestes gracilis 16S ribosomal RNA gene, partia	EF032700	74.68	98.03	3.56325e−15	Odonata	Lestidae	Indolestes gracilis
Chalcolestes viridis large subunit ribosomal RNA g	EF044262	74.68	98.03	3.56325e−15	Odonata	Lestidae	Chalcolestes viridis
Kuromondokuga niphonis voucher H167 large subunit	KP081963	76.47	96.71	1.2437e−14	Lepidoptera	Lymantriidae	Kuromondokuga niphonis
Epacanthaclisis banksi mitochondrion, complete gen	KF701327	77.5	76.32	1.2437e−14	Neuroptera	Myrmeleontidae	Epacanthaclisis banksi
Epiophlebia superstes voucher ANISO01 tRNA−Leu, 16	KF256906	75.48	98.03	1.2437e−14	Odonata	Epiophlebiidae	Epiophlebia superstes
Prodasineura sita voucher RMNH.INS.229286 16S ribo	KF369864	76.52	84.21	1.2437e−14	Odonata	Protoneuridae	Prodasineura sita
Ephoron album mitochondrial gene for 16S rRNA, par	AB711636	78.76	73.68	1.2437e−14	Ephemeroptera	Polymitarcyidae	Ephoron album
Semirhynchia sp. n. SV01 16S ribosomal RNA gene, p	JX294172	79.46	71.05	1.2437e−14	Neuroptera	Nemopteridae	Semirhynchia sp. n. SV01
Sicyoptera cuspidata isolate SCBB01 16S ribosomal	JX294167	79.46	71.05	1.2437e−14	Neuroptera	Nemopteridae	Sicyoptera cuspidata
Sicyoptera dilatata isolate SDGG01 16S ribosomal R	JX294163	78.99	75.66	1.2437e−14	Neuroptera	Nemopteridae	Sicyoptera dilatata
Nemeura gracilis isolate NGW01 16S ribosomal RNA g	JX294132	79.46	71.05	1.2437e−14	Neuroptera	Nemopteridae	Nemeura gracilis
Anotogaster klossi mitochondrial gene for 16S rRNA	AB711440	74.17	98.68	1.2437e−14	Odonata	Cordulegastridae	Anotogaster klossi
Somatochlora arctica mitochondrial gene for 16S rR	AB707969	77.24	80.26	1.2437e−14	Odonata	Corduliidae	Somatochlora arctica
Sonjagaster insignis mitochondrial gene for 16S rR	AB707905	77.59	75.66	1.2437e−14	Odonata	Cordulegastridae	Sonjagaster insignis
Neallogaster pekinensis mitochondrial gene for 16S	AB707903	74.17	98.68	1.2437e−14	Odonata	Cordulegastridae	Neallogaster pekinensis
Anotogaster klossi mitochondrial gene for 16S rRNA	AB707876	74.17	98.68	1.2437e−14	Odonata	Cordulegastridae	Anotogaster klossi
Anotogaster klossi mitochondrial gene for 16S rRNA	AB707866	74.17	98.68	1.2437e−14	Odonata	Cordulegastridae	Anotogaster klossi
Anotogaster klossi mitochondrial gene for 16S rRNA	AB707864	74.17	98.68	1.2437e−14	Odonata	Cordulegastridae	Anotogaster klossi
Anotogaster klossi mitochondrial gene for 16S rRNA	AB707862	74.17	98.68	1.2437e−14	Odonata	Cordulegastridae	Anotogaster klossi
Anotogaster klossi mitochondrial gene for 16S rRNA	AB707860	74.17	98.68	1.2437e−14	Odonata	Cordulegastridae	Anotogaster klossi
Tanypteryx pryeri mitochondrial gene for 16S rRNA,	AB707857	76.86	78.95	1.2437e−14	Odonata	Petaluridae	Tanypteryx pryeri
Tanypteryx pryeri mitochondrial gene for 16S rRNA,	AB707855	76.86	78.95	1.2437e−14	Odonata	Petaluridae	Tanypteryx pryeri
Tanypteryx pryeri mitochondrial gene for 16S rRNA,	AB707854	76.86	78.95	1.2437e−14	Odonata	Petaluridae	Tanypteryx pryeri
Tanypteryx pryeri mitochondrial gene for 16S rRNA,	AB707852	74.32	96.05	1.2437e−14	Odonata	Petaluridae	Tanypteryx pryeri
Asiagomphus melaenops mitochondrial gene for 16S r	AB707724	76.56	83.55	1.2437e−14	Odonata	Aeshnidae	Asiagomphus melaenops
Asiagomphus melaenops mitochondrial gene for 16S r	AB707720	76.56	83.55	1.2437e−14	Odonata	Aeshnidae	Asiagomphus melaenops
Asiagomphus melaenops mitochondrial gene for 16S r	AB707719	76.56	83.55	1.2437e−14	Odonata	Aeshnidae	Asiagomphus melaenops
Planaeschna ishigakiana mitochondrial gene for 16S	AB707685	76.98	80.26	1.2437e−14	Odonata	Aeshnidae	Planaeschna ishigakiana
Epiophlebia superstes mitochondrial gene for 16S r	AB707613	75.48	98.03	1.2437e−14	Odonata	Epiophlebiidae	Epiophlebia superstes
Epiophlebia superstes mitochondrial gene for 16S r	AB707602	75.48	98.03	1.2437e−14	Odonata	Epiophlebiidae	Epiophlebia superstes
Palingenia longicauda mitochondrial partial 16S rR	HE650662	79.8	65.13	1.2437e−14	Ephemeroptera	Palingeniidae	Palingenia longicauda
Palingenia longicauda mitochondrial partial 16S rR	HE650475	79.8	65.13	1.2437e−14	Ephemeroptera	Palingeniidae	Palingenia longicauda
Palingenia longicauda mitochondrial partial 16S rR	HE650547	79.8	65.13	1.2437e−14	Ephemeroptera	Palingeniidae	Palingenia longicauda
Palingenia longicauda mitochondrial partial 16S rR	HE650502	79.8	65.13	1.2437e−14	Ephemeroptera	Palingeniidae	Palingenia longicauda
Papilio alexanor voucher FS−b−40 16S ribosomal RNA	GQ268343	75.33	97.37	1.2437e−14	Lepidoptera	Papilionidae	Papilio alexanor
Cordulegaster maculata 12S ribosomal RNA and 16S r	EU477689	77.59	75.66	1.2437e−14	Odonata	Cordulegastridae	Cordulegaster maculata
Cordulegaster picta 12S ribosomal RNA gene, partia	EU477685	77.59	75.66	1.2437e−14	Odonata	Cordulegastridae	Cordulegaster picta
Asiagomphus melaenops 12S ribosomal RNA gene, part	EU477664	76.56	83.55	1.2437e−14	Odonata	Aeshnidae	Asiagomphus melaenops
Uropetala carovei 12S ribosomal RNA gene, partial	EU477633	77.52	83.55	1.2437e−14	Odonata	Petaluridae	Uropetala carovei
Leptoperla sp. BYU_PL107 16S ribosomal RNA gene, p	EF623161	77.44	83.55	1.2437e−14	Plecoptera	Gripopterygidae	Leptoperla sp. BYU_PL107
Grylloblatta n. sp. 'Trout Lake' voucher BYU_gb23.	DQ457256	82	64.47	1.2437e−14	Grylloblattodea	Grylloblattidae	Grylloblatta sp. n. 'Trout Lake'
Uropetala carovei large subunit ribosomal RNA gene	AY274506	77.52	83.55	1.2437e−14	Odonata	Petaluridae	Uropetala carovei
Panorpa claripennis 16S ribosomal RNA gene, partia	AF180068	80.7	73.03	1.2437e−14	Mecoptera	Panorpidae	Panorpa claripennis
Cordulegaster picta large subunit ribosomal RNA ge	AF266086	77.59	75.66	1.2437e−14	Odonata	Cordulegastridae	Cordulegaster picta
Eresia plaginota isolate 38−4 16S ribosomal RNA ge	AF186912	80.2	65.79	1.2437e−14	Lepidoptera	Nymphalidae	Eresia plaginota
Papilio alexanor mitochondrion, large subunit ribo	AJ224052	75.33	97.37	1.2437e−14	Lepidoptera	Papilionidae	Papilio alexanor
Oxythyrea pantherina voucher 842859 16S ribosomal	JX234531	78.15	77.63	4.34093e−14	Coleoptera	Scarabaeidae	Oxythyrea pantherina
Oxythyrea funesta voucher 678443 16S ribosomal RNA	JX234432	78.15	77.63	4.34093e−14	Coleoptera	Scarabaeidae	Oxythyrea funesta
Ephoron leukon mitochondrial gene for 16S rRNA, pa	AB711641	79.09	71.71	4.34093e−14	Ephemeroptera	Polymitarcyidae	Ephoron leukon
Ephoron album mitochondrial gene for 16S rRNA, par	AB711635	79.09	71.71	4.34093e−14	Ephemeroptera	Polymitarcyidae	Ephoron album
Nemopterella longicornis isolate NLTS01 16S riboso	JX294149	78.15	76.97	4.34093e−14	Neuroptera	Nemopteridae	Nemopterella longicornis
Nemia karrooa isolate NAMD01 16S ribosomal RNA gen	JX294147	77.42	80.26	4.34093e−14	Neuroptera	Nemopteridae	Nemia karrooa
Pachliopta aristolochiae isolate N296 16S ribosoma	JQ421944	75.52	91.45	4.34093e−14	Lepidoptera	Papilionidae	Pachliopta aristolochiae
Pachliopta aristolochiae isolate F566 16S ribosoma	JQ421940	75.52	91.45	4.34093e−14	Lepidoptera	Papilionidae	Pachliopta aristolochiae
