# Supplementary material for: Testing the potential of a ribosomal 16S marker for DNA metabarcoding of insects
Source: PeerJ. 2016 Apr 19;4:e1966. doi: 10.7717/peerj.1966 (PMC4841222; doi:10.7717/peerj.1966)
Supplement: Supplemental Information 1 [file OTU_469.csv_plot.pdf › scripts/5 match OTUs/blast_hits/OTU_469.csv_plot.pdf]

OTU_469.csv

Drusus annulatus

Limnephilidae

Trichoptera

98.81

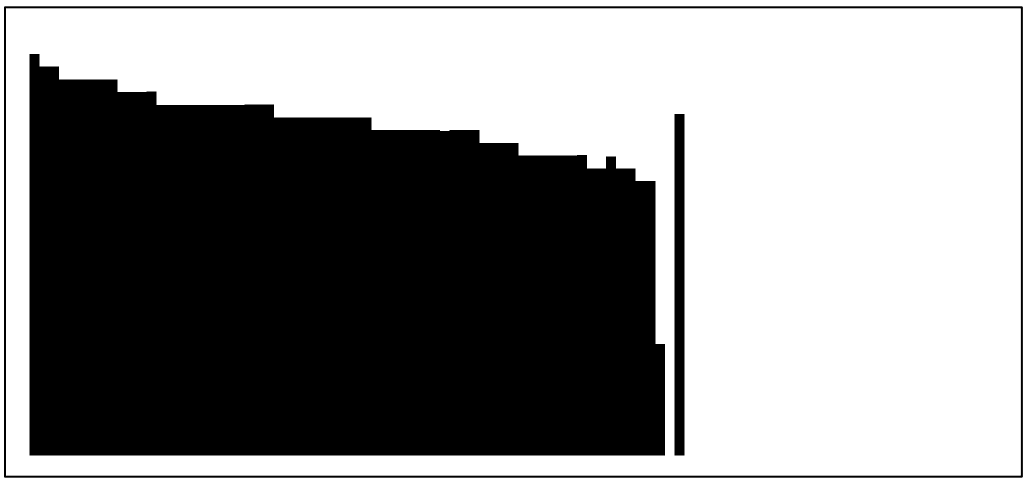

Drusus annulatus voucher DAn005 16S ribosomal RNA	EU215182	98.81	100	2.21756e−75	Trichoptera	Limnephilidae	Drusus annulatus
Drusus improvisus voucher RIS DImp004 16S ribosoma	EU999189	98.21	100	9.4293e−74	Trichoptera	Limnephilidae	Drusus improvisus
Drusus annulatus voucher DAn001 16S ribosomal RNA	EU215181	98.21	100	9.4293e−74	Trichoptera	Limnephilidae	Drusus annulatus
Metanoea flavipennis voucher MF001 16S ribosomal R	EU215224	97.62	100	1.14872e−72	Trichoptera	Limnephilidae	Metanoea flavipennis
Drusus rectus voucher DRec005 16S ribosomal RNA ge	EU215211	97.62	100	1.14872e−72	Trichoptera	Limnephilidae	Drusus rectus
Drusus romanicus romanicus voucher DR003 16S ribos	EU215209	97.62	100	1.14872e−72	Trichoptera	Limnephilidae	Drusus romanicus romanicus
Drusus romanicus romanicus voucher DR001 16S ribos	EU215208	97.62	100	1.14872e−72	Trichoptera	Limnephilidae	Drusus romanicus romanicus
Drusus melanchaetes voucher DMel001 16S ribosomal	EU215199	97.62	100	1.14872e−72	Trichoptera	Limnephilidae	Drusus melanchaetes
Drusus biguttatus voucher DBig003 16S ribosomal RN	EU215184	97.62	100	1.14872e−72	Trichoptera	Limnephilidae	Drusus biguttatus
Drusus improvisus voucher RIS DImp003 16S ribosoma	EU999188	97.02	100	1.39943e−71	Trichoptera	Limnephilidae	Drusus improvisus
Drusus improvisus voucher RIS DImp001 16S ribosoma	EU999187	97.02	100	4.8845e−71	Trichoptera	Limnephilidae	Drusus improvisus
Drusus mixtus voucher DMix001 16S ribosomal RNA ge	EU215200	97.02	100	4.8845e−71	Trichoptera	Limnephilidae	Drusus mixtus
Drusus brunneus voucher DBr002 16S ribosomal RNA g	EU215187	97.04	100	1.70486e−70	Trichoptera	Limnephilidae	Drusus brunneus
Drusus discolor voucher DdCAIM1 16S ribosomal RNA	KM001841	96.43	100	5.95054e−70	Trichoptera	Limnephilidae	Drusus discolor
Drusus discolor voucher DdIM2 16S ribosomal RNA ge	FJ002806	96.43	100	5.95054e−70	Trichoptera	Limnephilidae	Drusus discolor
Metanoea rhaetica voucher MR005 16S ribosomal RNA	EU215226	96.43	100	5.95054e−70	Trichoptera	Limnephilidae	Metanoea rhaetica
Metanoea rhaetica voucher MR001 16S ribosomal RNA	EU215225	96.43	100	5.95054e−70	Trichoptera	Limnephilidae	Metanoea rhaetica
Ecclisopteryx guttulata voucher EGut009 16S riboso	EU215220	96.43	100	5.95054e−70	Trichoptera	Limnephilidae	Ecclisopteryx guttulata
Drusus romanicus meridionalis voucher DR007 16S ri	EU215210	96.43	100	5.95054e−70	Trichoptera	Limnephilidae	Drusus romanicus meridionalis
Drusus discophorus pallidus voucher DDis001 16S ri	EU215195	96.43	100	5.95054e−70	Trichoptera	Limnephilidae	Drusus discophorus pallidus
Drusus destitutus voucher DDes001 16S ribosomal RN	EU215193	96.43	100	5.95054e−70	Trichoptera	Limnephilidae	Drusus destitutus
Drusus discolor voucher DD373 16S ribosomal RNA ge	EU215190	96.43	100	5.95054e−70	Trichoptera	Limnephilidae	Drusus discolor
Drusus trifidus voucher DTri007 16S ribosomal RNA	EU215215	96.45	100	2.07694e−69	Trichoptera	Limnephilidae	Drusus trifidus
Drusus trifidus voucher DTri006 16S ribosomal RNA	EU215214	96.45	100	2.07694e−69	Trichoptera	Limnephilidae	Drusus trifidus
Drusus brunneus voucher DBr001 16S ribosomal RNA g	EU215186	96.45	100	2.07694e−69	Trichoptera	Limnephilidae	Drusus brunneus
Ecclisopteryx madida voucher fEma0102M 16S ribosom	KM001738	95.83	100	2.53023e−68	Trichoptera	Limnephilidae	Ecclisopteryx madida
Ecclisopteryx madida voucher fEma0101M 16S ribosom	KM001737	95.83	100	2.53023e−68	Trichoptera	Limnephilidae	Ecclisopteryx madida
Ecclisopteryx madida voucher EM006 16S ribosomal R	KM001736	95.83	100	2.53023e−68	Trichoptera	Limnephilidae	Ecclisopteryx madida
Ecclisopteryx madida voucher EM005 16S ribosomal R	KM001735	95.83	100	2.53023e−68	Trichoptera	Limnephilidae	Ecclisopteryx madida
Ecclisopteryx madida voucher EM003 16S ribosomal R	KM001734	95.83	100	2.53023e−68	Trichoptera	Limnephilidae	Ecclisopteryx madida
Ecclisopteryx madida voucher EM001 16S ribosomal R	EU215221	95.83	100	2.53023e−68	Trichoptera	Limnephilidae	Ecclisopteryx madida
Drusus destitutus voucher DDes005 16S ribosomal RN	EU215194	95.83	100	2.53023e−68	Trichoptera	Limnephilidae	Drusus destitutus
Drusus balcanicus voucher DBal001 16S ribosomal RN	EU215183	95.83	100	2.53023e−68	Trichoptera	Limnephilidae	Drusus balcanicus
Drusus alpinus voucher DAlp003 16S ribosomal RNA g	EU215180	95.83	100	2.53023e−68	Trichoptera	Limnephilidae	Drusus alpinus
Drusus alpinus voucher DAlp002 16S ribosomal RNA g	EU215179	95.83	100	2.53023e−68	Trichoptera	Limnephilidae	Drusus alpinus
Ecclisopteryx guttulata voucher fEgu0104M 16S ribo	KM001749	95.24	100	3.08245e−67	Trichoptera	Limnephilidae	Ecclisopteryx guttulata
Ecclisopteryx guttulata voucher fEgu0103M 16S ribo	KM001748	95.24	100	3.08245e−67	Trichoptera	Limnephilidae	Ecclisopteryx guttulata
Ecclisopteryx guttulata voucher fEgu0101M 16S ribo	KM001746	95.24	100	3.08245e−67	Trichoptera	Limnephilidae	Ecclisopteryx guttulata
Ecclisopteryx madida voucher EM002 16S ribosomal R	KM001733	95.24	100	3.08245e−67	Trichoptera	Limnephilidae	Ecclisopteryx madida
Drusus vespertinus voucher DvRL1 16S ribosomal RNA	FJ002817	95.24	100	3.08245e−67	Trichoptera	Limnephilidae	Drusus vespertinus
Drusus ramae voucher DraIM1 16S ribosomal RNA gene	FJ002809	95.24	100	3.08245e−67	Trichoptera	Limnephilidae	Drusus ramae
Drusus medianus voucher DmIM1 16S ribosomal RNA ge	FJ002807	95.24	100	3.08245e−67	Trichoptera	Limnephilidae	Drusus medianus
Ecclisopteryx madida voucher EM004 16S ribosomal R	EU215222	95.21	99.4	3.08245e−67	Trichoptera	Limnephilidae	Ecclisopteryx madida
Drusus muelleri voucher DMue005 16S ribosomal RNA	EU215205	95.24	100	3.08245e−67	Trichoptera	Limnephilidae	Drusus muelleri
Drusus muelleri voucher DMue004 16S ribosomal RNA	EU215204	95.24	100	3.08245e−67	Trichoptera	Limnephilidae	Drusus muelleri
Drusus muelleri voucher DMue003 16S ribosomal RNA	EU215203	95.24	100	3.08245e−67	Trichoptera	Limnephilidae	Drusus muelleri
Ecclisopteryx guttulata voucher fEgu0102F 16S ribo	KM001747	94.64	100	1.31069e−65	Trichoptera	Limnephilidae	Ecclisopteryx guttulata
Drusus bosnicus voucher DbsVML2 16S ribosomal RNA	GQ470605	94.64	100	1.31069e−65	Trichoptera	Limnephilidae	Drusus bosnicus
Drusus radovanovici radovanovici voucher DRRIM1 16	GQ470601	94.64	100	1.31069e−65	Trichoptera	Limnephilidae	Drusus radovanovici radovanovici
Drusus bosnicus voucher DbsIM1 16S ribosomal RNA g	FJ002821	94.64	100	1.31069e−65	Trichoptera	Limnephilidae	Drusus bosnicus
Ecclisopteryx dalecarlica voucher fEda0901F 16S ri	KM001827	94.05	100	1.59675e−64	Trichoptera	Limnephilidae	Ecclisopteryx dalecarlica
Ecclisopteryx dalecarlica voucher fEda0801M 16S ri	KM001825	94.05	100	1.59675e−64	Trichoptera	Limnephilidae	Ecclisopteryx dalecarlica
Ecclisopteryx dalecarlica voucher fEda0701F 16S ri	KM001824	94.05	100	1.59675e−64	Trichoptera	Limnephilidae	Ecclisopteryx dalecarlica
Ecclisopteryx dalecarlica voucher ED002 16S riboso	EU215219	94.05	100	1.59675e−64	Trichoptera	Limnephilidae	Ecclisopteryx dalecarlica
Ecclisopteryx dalecarlica voucher ED001 16S riboso	EU215218	94.05	100	1.59675e−64	Trichoptera	Limnephilidae	Ecclisopteryx dalecarlica
Drusus franzi voucher DFrII 16S ribosomal RNA gene	EU215198	94.05	100	1.59675e−64	Trichoptera	Limnephilidae	Drusus franzi
Ecclisopteryx malickyi voucher EMal002 16S ribosom	KM001725	94.08	100	5.57321e−64	Trichoptera	Limnephilidae	Ecclisopteryx malickyi
Ecclisopteryx dalecarlica voucher fEda0501M 16S ri	KM001822	93.45	100	6.78956e−63	Trichoptera	Limnephilidae	Ecclisopteryx dalecarlica
Drusus schmidi voucher DsIM4 16S ribosomal RNA gen	FJ002816	93.45	100	6.78956e−63	Trichoptera	Limnephilidae	Drusus schmidi
Ecclisopteryx malickyi voucher EMal001 16S ribosom	EU215223	94.01	98.8	6.78956e−63	Trichoptera	Limnephilidae	Ecclisopteryx malickyi
Ecclisopteryx dalecarlica voucher Dsp023 16S ribos	EU215212	93.45	100	6.78956e−63	Trichoptera	Limnephilidae	Ecclisopteryx dalecarlica
Drusus franzi voucher DFr007 16S ribosomal RNA gen	EU215197	93.45	100	6.78956e−63	Trichoptera	Limnephilidae	Drusus franzi
Ecclisopteryx dalecarlica voucher fEda0502F 16S ri	KM001823	92.86	100	8.27137e−62	Trichoptera	Limnephilidae	Ecclisopteryx dalecarlica
Drusus schmidi voucher DsIM3 16S ribosomal RNA gen	FJ002815	92.86	100	8.27137e−62	Trichoptera	Limnephilidae	Drusus schmidi
Ecclisomyia kamtshatica mitochondrial gene for 16S	AB355444	85.21	100	8.27868e−43	Trichoptera	Limnephilidae	Ecclisomyia kamtshatica
Sebaldella termitidis ATCC 33386, complete genome	CP001739	76.92	31.14	6.3759	Fusobacteriales	Leptotrichiaceae	Sebaldella termitidis ATCC 33386
Homo sapiens BAC clone RP11−11P22 from 2, complete	AC092835	96	14.97	6.3759	Primates	Hominidae	Homo sapiens
