# Supplementary material for: Testing the potential of a ribosomal 16S marker for DNA metabarcoding of insects
Source: PeerJ. 2016 Apr 19;4:e1966. doi: 10.7717/peerj.1966 (PMC4841222; doi:10.7717/peerj.1966)
Supplement: Supplemental Information 1 [file OTU_49.csv_plot.pdf › scripts/5 match OTUs/blast_hits/OTU_49.csv_plot.pdf]

OTU_49.csv

Asellus aquaticus

Asellidae

Isopoda

96.32

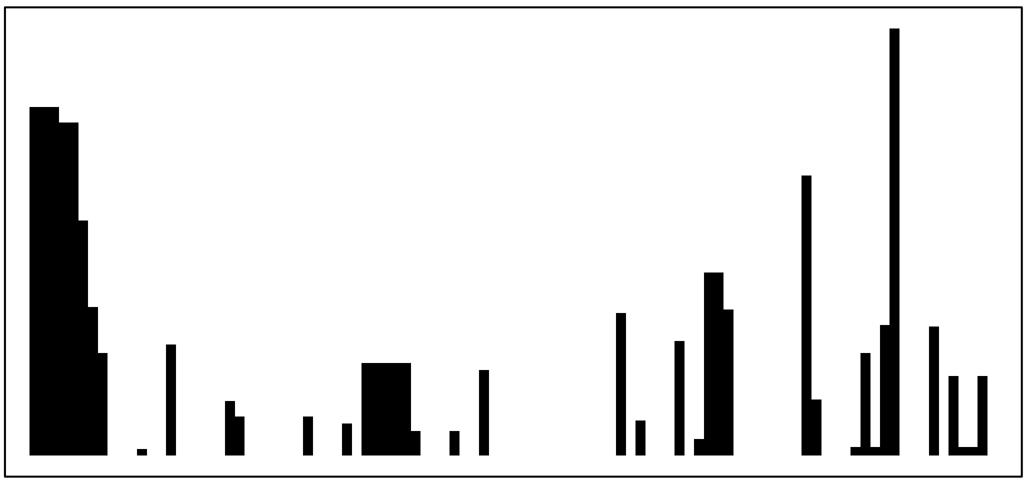

Asellus aquaticus isolate s180 16S ribosomal RNA g	KC610270	96.32	100	8.71014e−54	Isopoda	Asellidae	Asellus aquaticus
Asellus aquaticus 16S ribosomal RNA gene, partial	DQ305105	96.32	100	8.71014e−54	Isopoda	Asellidae	Asellus aquaticus
Asellus aquaticus 16S ribosomal RNA gene, partial	AF532161	96.32	100	8.71014e−54	Isopoda	Asellidae	Asellus aquaticus
Asellus aquaticus 16S ribosomal RNA gene, partial	DQ305106	95.59	100	1.06111e−52	Isopoda	Asellidae	Asellus aquaticus
Asellus aquaticus partial mitochondrial 16S rRNA g	AJ388077	95.59	100	1.06111e−52	Isopoda	Asellidae	Asellus aquaticus
Asellus levanidovorum 16S ribosomal RNA gene, part	AF532160	91.01	65.19	1.79847e−24	Isopoda	Asellidae	Asellus levanidovorum
Mesoasellus dybowskii 16S ribosomal RNA gene, part	AF532158	86.96	65.19	1.68443e−18	Isopoda	Asellidae	Mesoasellus dybowskii
Asellid sp. BH−2002 16S ribosomal RNA gene, partia	AF532159	84.78	65.19	8.72554e−16	Isopoda	Asellidae	Asellid sp. BH−2002
Bragasellus cortesi isolate POUCA_201010_1ID_YCD83	JQ921676	77.42	65.93	1.21287e−07	Isopoda	Asellidae	Bragasellus cortesi
Stenasellus virei isolate ORFES_200906_2_YLK394 16	JQ921839	77.01	63.7	4.23333e−07	Isopoda	Stenasellidae	Stenasellus virei
Proasellus beroni isolate s202 16S ribosomal RNA g	KC610292	78.21	57.04	5.15725e−06	Isopoda	Asellidae	Proasellus beroni
Proasellus spelaeus isolate ETCHEVE_200811_1ex_YAS	JQ921811	80.3	48.89	5.15725e−06	Isopoda	Asellidae	Proasellus spelaeus
Proasellus aquaecalidae isolate PEAUXCH_200811_1_Y	JQ921704	75.28	65.19	1.80006e−05	Isopoda	Asellidae	Proasellus aquaecalidae
Proasellus aff. lagari Henry & Magniez, 1982 isola	JQ921700	79.1	49.63	1.80006e−05	Isopoda	Asellidae	Proasellus aff. lagari Henry & Magniez, 1982
Julus scandinavius voucher ZMUC200018 16S ribosoma	JF320957	85.19	39.26	1.80006e−05	Julida	Julidae	Julus scandinavius
Proasellus ebrensis isolate s207 16S ribosomal RNA	KC610297	78.79	48.89	6.28282e−05	Isopoda	Asellidae	Proasellus ebrensis
Proasellus ebrensis isolate s206 16S ribosomal RNA	KC610296	78.79	48.89	6.28282e−05	Isopoda	Asellidae	Proasellus ebrensis
Proasellus sp. type locality of P. gourbaultae iso	KC610099	77.46	52.59	6.28282e−05	Isopoda	Asellidae	Proasellus sp. type locality of P. gourbaultae
Proasellus ebrensis isolate CERECEDA_200906_1_YLK1	JQ921747	78.79	48.89	6.28282e−05	Isopoda	Asellidae	Proasellus ebrensis
Proasellus beroni isolate ORTAMIN_200810_1ex_YAS42	JQ921715	78.08	53.33	6.28282e−05	Isopoda	Asellidae	Proasellus beroni
Megaphyllum montivagum voucher ZMUC:00101492 small	KF701067	82.54	44.44	0.000219292	Julida	Julidae	Megaphyllum montivagum
Proasellus meijersae isolate s223 16S ribosomal RN	KC610313	81.82	40.74	0.000219292	Isopoda	Asellidae	Proasellus meijersae
Proasellus slavus isolate s118 16S ribosomal RNA g	KC610208	76.83	57.78	0.000219292	Isopoda	Asellidae	Proasellus slavus
Proasellus slavus isolate s117 16S ribosomal RNA g	KC610207	76.83	57.78	0.000219292	Isopoda	Asellidae	Proasellus slavus
Proasellus slavus isolate s105 16S ribosomal RNA g	KC610195	76.83	57.78	0.000219292	Isopoda	Asellidae	Proasellus slavus
Proasellus slavus isolate s104 16S ribosomal RNA g	KC610194	76.83	57.78	0.000219292	Isopoda	Asellidae	Proasellus slavus
Proasellus slavus isolate s103 16S ribosomal RNA g	KC610193	76.83	57.78	0.000219292	Isopoda	Asellidae	Proasellus slavus
Proasellus slavus isolate PRVOVERZ_201009_1ID_YCD5	JQ921809	76.83	57.78	0.000219292	Isopoda	Asellidae	Proasellus slavus
Proasellus meijersae isolate MUNDO_200910_3_YDE001	JQ921777	81.82	40.74	0.000219292	Isopoda	Asellidae	Proasellus meijersae
Proasellus stocki isolate SAJA_201010_1ID_YCD838 1	JQ921730	76	55.56	0.000219292	Isopoda	Asellidae	Proasellus stocki
Proasellus karamani isolate ZALOMSKA_200305_1_YLK3	JQ921766	77.61	49.63	0.000765404	Isopoda	Asellidae	Proasellus karamani
Proasellus escolai isolate SALAR_200910_2_YCD140 1	JQ921749	79.37	46.67	0.000765404	Isopoda	Asellidae	Proasellus escolai
Proasellus coxalis isolate BUNAR_200402_1ID_YLK392	JQ921740	81.48	40	0.000765404	Isopoda	Asellidae	Proasellus coxalis
Bragasellus comasi isolate COBALLE2_200906_1_YLK19	JQ921672	73.12	65.19	0.000765404	Isopoda	Asellidae	Bragasellus comasi
Mysmeninae sp. MYSM−033−MAD 16S ribosomal RNA gene	GU456754	84.31	37.78	0.000765404	Araneae	Mysmenidae	Mysmeninae sp. MYSM−033−MAD
Mysmeninae sp. MYSM−032−MAD 16S ribosomal RNA gene	GU456753	84.31	37.78	0.000765404	Araneae	Mysmenidae	Mysmeninae sp. MYSM−032−MAD
Mysmeninae sp. MYSM−031−MAD 16S ribosomal RNA gene	GU456752	84.31	37.78	0.000765404	Araneae	Mysmenidae	Mysmeninae sp. MYSM−031−MAD
Mysmeninae sp. MYSM−026−MAD 16S ribosomal RNA gene	GU456751	84.31	37.78	0.000765404	Araneae	Mysmenidae	Mysmeninae sp. MYSM−026−MAD
Leucauge argyra voucher FAPDNA041 16S ribosomal RN	EU003264	84.31	37.78	0.000765404	Araneae	Tetragnathidae	Leucauge argyra
Proasellus chauvini isolate s204 16S ribosomal RNA	KC610294	81.13	39.26	0.00267152	Isopoda	Asellidae	Proasellus chauvini
Proasellus aquaecalidae isolate s198 16S ribosomal	KC610288	74.16	65.19	0.00267152	Isopoda	Asellidae	Proasellus aquaecalidae
Synasellus sp. n. Aboim isolate ABOIM_201010_1ID_Y	JQ921860	74.16	65.19	0.00267152	Isopoda	Asellidae	Synasellus sp. n. Aboim
Proasellus sp. n. Maglia isolate MAGLIA_200908_1ID	JQ921791	77.27	48.89	0.00267152	Isopoda	Asellidae	Proasellus sp. n. Maglia
Proasellus chauvini isolate HAYTZA_200811_2_YAS534	JQ921733	81.13	39.26	0.00267152	Isopoda	Asellidae	Proasellus chauvini
Proasellus ebrensis isolate RUDRON_201010_1IDex_YC	JQ921720	77.27	48.89	0.00267152	Isopoda	Asellidae	Proasellus ebrensis
Proasellus aquaecalidae isolate OUEILNEE_200811_2_	JQ921703	74.16	65.19	0.00267152	Isopoda	Asellidae	Proasellus aquaecalidae
Microdipoena guttata 16S ribosomal RNA gene, parti	GU456747	84	37.04	0.00267152	Araneae	Mysmenidae	Microdipoena guttata
Proasellus sp. Netherlands−BH−2002 16S ribosomal R	AF532163	73.91	65.93	0.00267152	Isopoda	Asellidae	Proasellus sp. Netherlands−BH−2002
Proasellus sp. n. Verclause isolate s230 16S ribos	KC610320	76.12	49.63	0.00932453	Isopoda	Asellidae	Proasellus sp. n. Verclause
Proasellus aff. escolai s209 16S ribosomal RNA gen	KC610299	80	40.74	0.00932453	Isopoda	Asellidae	Proasellus aff. escolai s209
Proasellus sp. n. Verclause isolate LAPENNE_201002	JQ921797	76.12	49.63	0.00932453	Isopoda	Asellidae	Proasellus sp. n. Verclause
Proasellus coxalis isolate STENSAN_200811_2ID_YAS4	JQ921743	74.47	65.93	0.00932453	Isopoda	Asellidae	Proasellus coxalis
Proasellus cantabricus isolate RESCANO_200906_1ID_	JQ921719	76.12	49.63	0.00932453	Isopoda	Asellidae	Proasellus cantabricus
Proasellus aff. escolai Henry & Magniez, 1982 isol	JQ921699	80	40.74	0.00932453	Isopoda	Asellidae	Proasellus aff. escolai Henry & Magniez, 1982
Asellus aquaticus mitochondrion, partial genome	GU130252	74.47	65.93	0.00932453	Isopoda	Asellidae	Asellus aquaticus
Stenasellus breuili isolate s237 16S ribosomal RNA	KC610327	78.57	41.48	0.0325458	Isopoda	Stenasellidae	Stenasellus breuili
Stenasellus breuili isolate ALLI_200906_1ID_YLK130	JQ921830	78.57	41.48	0.0325458	Isopoda	Stenasellidae	Stenasellus breuili
Proasellus istrianus isolate GLINSCIC_200701_1ID_Y	JQ921764	78.57	41.48	0.0325458	Isopoda	Asellidae	Proasellus istrianus
Proasellus aragonensis isolate BURGUI_200906_2_YLK	JQ921706	75.76	48.89	0.0325458	Isopoda	Asellidae	Proasellus aragonensis
Proasellus aragonensis isolate BURGUI_200906_1ID_Y	JQ921705	75.76	48.89	0.0325458	Isopoda	Asellidae	Proasellus aragonensis
Ophyiulus pilosus voucher ZMUC200176 16S ribosomal	JF320969	86.67	32.59	0.0325458	Julida	Julidae	Ophyiulus pilosus
Caecosphaeroma virei isolate FRA−Doubs 16S ribosom	FJ842022	74.16	64.44	0.0325458	Isopoda	Sphaeromatidae	Caecosphaeroma virei
Oxidus gracilis voucher MTX0216 small subunit ribo	KR135959	81.63	34.81	0.113596	Polydesmida	Paradoxosomatidae	Oxidus gracilis
Proasellus aff. escolai s208 16S ribosomal RNA gen	KC610298	78.18	40.74	0.113596	Isopoda	Asellidae	Proasellus aff. escolai s208
Proasellus ebrensis isolate MARFIL_200906_1ID_YAS8	JQ921748	72.73	48.89	0.113596	Isopoda	Asellidae	Proasellus ebrensis
Proasellus sp. n. Aquamalas Pontones isolate AQUAL	JQ921727	78.18	40.74	0.113596	Isopoda	Asellidae	Proasellus sp. n. Aquamalas Pontones
Parachtes limbarae voucher crba001231 16S ribosoma	JN705772	85.37	28.15	0.113596	Araneae	Dysderidae	Parachtes limbarae
Modisimus sp. nov. A BAH−2009 voucher DNA07−NF69 1	FJ227995	77.46	50.37	0.113596	Araneae	Pholcidae	Modisimus berac
Phonognatha graeffei voucher FAPMCZ02 16S ribosoma	EU003275	80.77	37.78	0.113596	Araneae	Tetragnathidae	Phonognatha graeffei
Betamorpha fusiformis isolate BF138 16S ribosomal	EF116535	88.57	25.93	0.113596	Isopoda	Munnopsidae	Betamorpha fusiformis
Betamorpha fusiformis isolate BF91 16S ribosomal R	EF116533	88.57	25.93	0.113596	Isopoda	Munnopsidae	Betamorpha fusiformis
Betamorpha fusiformis isolate BF57 16S ribosomal R	EF116529	86.84	28.15	0.113596	Isopoda	Munnopsidae	Betamorpha fusiformis
Synalpheus williamsi 16S ribosomal RNA gene, parti	AF230264	76.14	57.78	0.113596	Decapoda	Alpheidae	Synalpheus williamsi
Stemonyphantes abantensis 16S ribosomal RNA gene,	KT003177	79.63	40	0.396489	Araneae	Linyphiidae	Stemonyphantes abantensis
Megaphyllum bosniense voucher ZMUC:00021391 small	KF701073	72.53	63.7	0.396489	Julida	Julidae	Megaphyllum bosniense
Proasellus lescherae isolate s219 16S ribosomal RN	KC610309	77.19	42.22	0.396489	Isopoda	Asellidae	Proasellus lescherae
Proasellus slavus isolate s110 16S ribosomal RNA g	KC610200	73.33	65.19	0.396489	Isopoda	Asellidae	Proasellus slavus
Proasellus slavus isolate s108 16S ribosomal RNA g	KC610198	73.33	65.19	0.396489	Isopoda	Asellidae	Proasellus slavus
Proasellus lescherae isolate MATARRA_200910_1ID_YC	JQ921770	77.19	42.22	0.396489	Isopoda	Asellidae	Proasellus lescherae
Brachyiulus bagnalli voucher ZMUC100790 16S riboso	JF320984	93.1	21.48	0.396489	Julida	Julidae	Brachyiulus bagnalli
Brachyiulus pusillus voucher ZMUC100789 16S riboso	JF320983	82.61	34.07	0.396489	Julida	Julidae	Brachyiulus pusillus
Dolichoiulus tongiorgii voucher ZMUC200178 16S rib	JF320971	79.63	39.26	0.396489	Julida	Julidae	Dolichoiulus tongiorgii
Megaphyllum projectum voucher ZMUC200023 16S ribos	JF320963	72.73	61.48	0.396489	Julida	Julidae	Megaphyllum projectum
Processa japonica 16S ribosomal RNA gene, partial	GQ131909	79.63	40	0.396489	Decapoda	Processidae	Processa japonica
Microdipoena sp. ATOL_ARAGH000003 16S ribosomal RN	HM030404	80.39	37.78	0.396489	Araneae	Mysmenidae	Microdipoena sp. ATOL_ARAGH000003
Ariadna fidicina 16S ribosomal RNA gene, partial s	FJ607447	84.78	34.07	0.396489	Araneae	Segestriidae	Ariadna fidicina
Theridion differens 16S ribosomal RNA gene, partia	EF449630	80.39	37.78	0.396489	Araneae	Theridiidae	Theridion differens
PREDICTED: Stomoxys calcitrans zinc finger protein	XM_013262361	86.11	26.67	1.38388	Diptera	Muscidae	Stomoxys calcitrans
Strongyloides ratti genome assembly S_ratti_ED321	LN609405	100	17.04	1.38388	Rhabditida	Strongyloididae	Strongyloides ratti
Proasellus slavus isolate s123 16S ribosomal RNA g	KC610213	79.17	35.56	1.38388	Isopoda	Asellidae	Proasellus slavus
Proasellus slavus isolate s122 16S ribosomal RNA g	KC610212	79.17	35.56	1.38388	Isopoda	Asellidae	Proasellus slavus
Nephilengys papuana voucher NEP_ngpap_50 16S ribos	KC849141	80	37.04	1.38388	Araneae	Nephilidae	Nephilengys papuana
Carapoia sp. DNA100446405 bio−material ZFMK:DNA100	JX023900	86.05	31.11	1.38388	Araneae	Pholcidae	Carapoia sp. DNA100446405
Carapoia genitalis bio−material ZFMK:DNA100446401	JX023897	75.34	52.59	1.38388	Araneae	Pholcidae	Carapoia genitalis
Dysdera verneaui isolate V194LR 16S ribosomal RNA	KC785865	83.72	31.85	1.38388	Araneae	Dysderidae	Dysdera verneaui
Dysdera verneaui isolate V179ECH 16S ribosomal RNA	KC785863	80.39	36.3	1.38388	Araneae	Dysderidae	Dysdera verneaui
Dysdera verneaui isolate V172ECH 16S ribosomal RNA	KC785862	80.39	36.3	1.38388	Araneae	Dysderidae	Dysdera verneaui
Dysdera verneaui isolate V170CRC 16S ribosomal RNA	KC785860	83.72	31.85	1.38388	Araneae	Dysderidae	Dysdera verneaui
Proasellus slovenicus isolate JAMAPOD_200409_1_YLK	JQ921810	77.78	39.26	1.38388	Isopoda	Asellidae	Proasellus slovenicus
Proasellus slavus isolate KLUZA_201007_2_YCD675 16	JQ921808	79.17	35.56	1.38388	Isopoda	Asellidae	Proasellus slavus
