# Supplementary material for: Testing the potential of a ribosomal 16S marker for DNA metabarcoding of insects
Source: PeerJ. 2016 Apr 19;4:e1966. doi: 10.7717/peerj.1966 (PMC4841222; doi:10.7717/peerj.1966)
Supplement: Supplemental Information 1 [file OTU_498.csv_plot.pdf › scripts/5 match OTUs/blast_hits/OTU_498.csv_plot.pdf]

OTU_498.csv

Rhithrogena semicolorata

Heptageniidae

Ephemeroptera

97.44

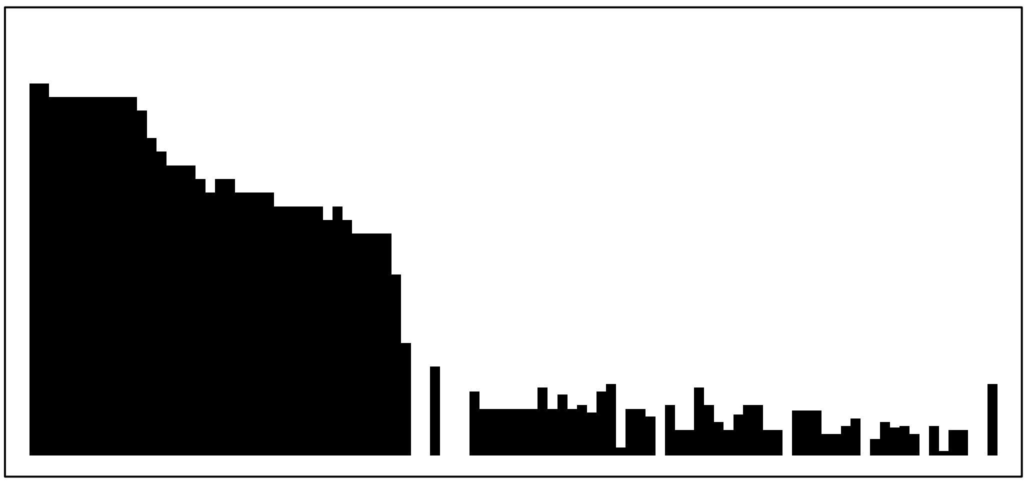

Rhithrogena semicolorata mitochondrial partial 16S	LN868673	97.44	100	2.82558e−67	Ephemeroptera	Heptageniidae	Rhithrogena semicolorata
Rhithrogena sp. 4 LV−2015 mitochondrial partial 16	LN868652	97.44	100	2.82558e−67	Ephemeroptera	Heptageniidae	Rhithrogena sp. 4 LV−2015
Rhithrogena sp. 13 LV−2015 mitochondrial partial 1	LN868672	96.79	100	1.20147e−65	Ephemeroptera	Heptageniidae	Rhithrogena sp. 13 LV−2015
Rhithrogena sp. 12 LV−2015 mitochondrial partial 1	LN868671	96.79	100	1.20147e−65	Ephemeroptera	Heptageniidae	Rhithrogena sp. 12 LV−2015
Rhithrogena picteti mitochondrial partial 16S rRNA	LN868663	96.79	100	1.20147e−65	Ephemeroptera	Heptageniidae	Rhithrogena picteti
Rhithrogena sp. 3 LV−2015 mitochondrial partial 16	LN868659	96.79	100	1.20147e−65	Ephemeroptera	Heptageniidae	Rhithrogena sp. 3 LV−2015
Rhithrogena fonticola mitochondrial partial 16S rR	LN868656	96.79	100	1.20147e−65	Ephemeroptera	Heptageniidae	Rhithrogena fonticola
Rhithrogena sp. 2 LV−2015 mitochondrial partial 16	LN868654	96.79	100	1.20147e−65	Ephemeroptera	Heptageniidae	Rhithrogena sp. 2 LV−2015
Rhithrogena puytoraci mitochondrial partial 16S rR	LN868651	96.79	100	1.20147e−65	Ephemeroptera	Heptageniidae	Rhithrogena puytoraci
Rhithrogena iridina mitochondrial partial 16S rRNA	LN868646	96.79	100	1.20147e−65	Ephemeroptera	Heptageniidae	Rhithrogena iridina
Rhithrogena taurisca mitochondrial partial 16S rRN	LN868638	96.79	100	1.20147e−65	Ephemeroptera	Heptageniidae	Rhithrogena taurisca
Rhithrogena sp. 5 LV−2015 mitochondrial partial 16	LN868653	96.15	100	1.46369e−64	Ephemeroptera	Heptageniidae	Rhithrogena sp. 5 LV−2015
Rhithrogena landai mitochondrial partial 16S rRNA	LN868644	94.87	100	9.23688e−61	Ephemeroptera	Heptageniidae	Rhithrogena landai
Rhithrogena eatoni mitochondrial partial 16S rRNA	LN868666	94.23	100	3.92762e−59	Ephemeroptera	Heptageniidae	Rhithrogena eatoni
Rhithrogena degrangei mitochondrial partial 16S rR	LN868674	93.59	100	4.78482e−58	Ephemeroptera	Heptageniidae	Rhithrogena degrangei
Rhithrogena alpestris mitochondrial partial 16S rR	LN868657	93.59	100	4.78482e−58	Ephemeroptera	Heptageniidae	Rhithrogena alpestris
Rhithrogena sp. 9 LV−2015 mitochondrial partial 16	LN868650	93.59	100	4.78482e−58	Ephemeroptera	Heptageniidae	Rhithrogena sp. 9 LV−2015
Rhithrogena sp. 7 LV−2015 mitochondrial partial 16	LN868669	92.95	100	2.03456e−56	Ephemeroptera	Heptageniidae	Rhithrogena sp. 7 LV−2015
Rhithrogena germanica mitochondrial partial 16S rR	LN868660	92.31	100	2.03456e−56	Ephemeroptera	Heptageniidae	Rhithrogena germanica
Rhithrogena gratianopolitana mitochondrial partial	LN868647	92.95	100	2.03456e−56	Ephemeroptera	Heptageniidae	Rhithrogena gratianopolitana
Rhithrogena nivata mitochondrial partial 16S rRNA	LN868643	92.95	100	2.03456e−56	Ephemeroptera	Heptageniidae	Rhithrogena nivata
Rhithrogena corcontica mitochondrial partial 16S r	LN868662	92.31	100	2.4786e−55	Ephemeroptera	Heptageniidae	Rhithrogena corcontica
Rhithrogena gratianopolitana mitochondrial partial	LN868648	92.31	100	2.4786e−55	Ephemeroptera	Heptageniidae	Rhithrogena gratianopolitana
Rhithrogena hybrida mitochondrial partial 16S rRNA	LN868642	92.31	100	2.4786e−55	Ephemeroptera	Heptageniidae	Rhithrogena hybrida
Rhithrogena circumtatrica mitochondrial partial 16	LN868641	92.31	100	2.4786e−55	Ephemeroptera	Heptageniidae	Rhithrogena circumtatrica
Rhithrogena savoiensis mitochondrial partial 16S r	LN868658	91.67	100	8.65116e−55	Ephemeroptera	Heptageniidae	Rhithrogena savoiensis
Rhithrogena sp. 11 LV−2015 mitochondrial partial 1	LN868668	91.67	100	1.05393e−53	Ephemeroptera	Heptageniidae	Rhithrogena sp. 11 LV−2015
Rhithrogena nuragica mitochondrial partial 16S rRN	LN868667	91.67	100	1.05393e−53	Ephemeroptera	Heptageniidae	Rhithrogena nuragica
Rhithrogena sp. 8 LV−2015 mitochondrial partial 16	LN868661	91.67	100	1.05393e−53	Ephemeroptera	Heptageniidae	Rhithrogena sp. 8 LV−2015
Rhithrogena sp. 6 LV−2015 mitochondrial partial 16	LN868655	91.67	100	1.05393e−53	Ephemeroptera	Heptageniidae	Rhithrogena sp. 6 LV−2015
Rhithrogena beskidensis mitochondrial partial 16S	LN868645	91.03	100	1.05393e−53	Ephemeroptera	Heptageniidae	Rhithrogena beskidensis
Rhithrogena diensis mitochondrial partial 16S rRNA	LN868637	91.67	100	1.05393e−53	Ephemeroptera	Heptageniidae	Rhithrogena diensis
Rhithrogena allobrogica mitochondrial partial 16S	LN868640	91.03	100	1.28395e−52	Ephemeroptera	Heptageniidae	Rhithrogena allobrogica
Rhithrogena sp. 10 LV−2015 mitochondrial partial 1	LN868664	90.38	100	4.48141e−52	Ephemeroptera	Heptageniidae	Rhithrogena sp. 10 LV−2015
Rhithrogena dorieri mitochondrial partial 16S rRNA	LN868649	90.38	100	4.48141e−52	Ephemeroptera	Heptageniidae	Rhithrogena dorieri
Rhithrogena insularis mitochondrial partial 16S rR	LN868665	90.38	100	5.45948e−51	Ephemeroptera	Heptageniidae	Rhithrogena insularis
Rhithrogena grischuna mitochondrial partial 16S rR	LN868639	90.38	100	5.45948e−51	Ephemeroptera	Heptageniidae	Rhithrogena grischuna
Rhithrogena sartorii mitochondrial partial 16S rRN	LN868670	88.46	100	2.82808e−48	Ephemeroptera	Heptageniidae	Rhithrogena sartorii
Rhithrogena sp. EP021 16S ribosomal RNA gene, part	AY749765	85.26	100	3.22684e−41	Ephemeroptera	Heptageniidae	Rhithrogena sp. EP021
Cinygmula sp. m185 mitochondrial partial 16S rRNA	LN868675	79.49	100	2.83058e−29	Ephemeroptera	Heptageniidae	Cinygmula sp. m185
Cinygmula sp. EP013 16S ribosomal RNA gene, partia	AY749760	79.49	100	2.83058e−29	Ephemeroptera	Heptageniidae	Cinygmula sp. EP013
Cultus pilatus 16S ribosomal RNA gene, partial seq	EF623212	84.16	64.74	4.79328e−20	Plecoptera	Perlodidae	Cultus pilatus
Heptagenia sp. EP018 16S ribosomal RNA gene, parti	AY749762	78.26	87.82	4.79328e−20	Ephemeroptera	Heptageniidae	Heptagenia sp. EP018
Paegniodes cupulatus mitochondrion, complete genom	HM004123	76.43	100	1.67302e−19	Ephemeroptera	Heptageniidae	Paegniodes cupulatus
Chiloporter sp. EP051 16S ribosomal RNA gene, part	AY749776	75.8	100	2.03816e−18	Ephemeroptera	Ameletopsidae	Chiloporter sp. EP051
Remenus bilobatus 16S ribosomal RNA gene, partial	EF623233	83	64.1	7.11386e−18	Plecoptera	Perlodidae	Remenus bilobatus
Dolichopoda noctivaga isolate Zef1 16S ribosomal R	KC783279	82.18	64.74	2.48298e−17	Orthoptera	Rhaphidophoridae	Dolichopoda noctivaga
Dolichopoda noctivaga isolate Ana1 16S ribosomal R	KC783278	82.18	64.74	2.48298e−17	Orthoptera	Rhaphidophoridae	Dolichopoda noctivaga
Dolichopoda noctivaga isolate Kel1 16S ribosomal R	KC783277	82.18	64.74	2.48298e−17	Orthoptera	Rhaphidophoridae	Dolichopoda noctivaga
Dolichopoda noctivaga isolate Sar1 16S ribosomal R	KC783276	82.18	64.74	2.48298e−17	Orthoptera	Rhaphidophoridae	Dolichopoda noctivaga
Cryptoperla japonica mitochondrial gene for 16S rR	AB711202	82.18	64.74	2.48298e−17	Plecoptera	Peltoperlidae	Cryptoperla japonica
Cryptoperla japonica mitochondrial gene for 16S rR	AB711184	82.18	64.74	2.48298e−17	Plecoptera	Peltoperlidae	Cryptoperla japonica
Notonemouridae sp. MD−2011 voucher ZMUC:JD−1507 16	JN615257	83.17	62.82	2.48298e−17	Plecoptera	Notonemouridae	Notonemouridae sp. MD−2011
Dolichopoda hyrcana voucher LEN 16S ribosomal RNA	EU887871	82.18	64.74	2.48298e−17	Orthoptera	Rhaphidophoridae	Dolichopoda hyrcana
Isoperla oxylepis 16S ribosomal RNA gene, partial	EF623221	82.86	66.67	2.48298e−17	Plecoptera	Perlodidae	Isoperla oxylepis
Dolichopoda euxina isolate GOL 16S ribosomal RNA g	AY793565	82.18	64.74	2.48298e−17	Orthoptera	Rhaphidophoridae	Dolichopoda euxina
Perlinodes aurea 16S ribosomal RNA gene, partial s	EF623234	82.35	64.74	8.66646e−17	Plecoptera	Perlodidae	Perlinodes aurea
Dipteromimus sp. Ofunato−1 mitochondrial gene for	AB110236	82	64.1	8.66646e−17	Ephemeroptera	Dipteromimidae	Dipteromimus sp. Ofunato−1
Cinygma sp. EP123 16S ribosomal RNA gene, partial	AY749804	83	64.1	8.66646e−17	Ephemeroptera	Heptageniidae	Cinygma sp. EP123
Analetris eximia 16S ribosomal RNA gene, partial s	AY749797	83.33	64.74	8.66646e−17	Ephemeroptera	Acanthametropodidae	Analetris eximia
Heptagenia sulphurea mitochondrial partial 16S rRN	HE651431	80.37	67.95	3.02489e−16	Ephemeroptera	Heptageniidae	Heptagenia sulphurea
Salmoperla sylvanica 16S ribosomal RNA gene, parti	EF623222	82.18	64.1	3.02489e−16	Plecoptera	Perlodidae	Salmoperla sylvanica
Megarcys signata 16S ribosomal RNA gene, partial s	EF623215	82.18	64.1	3.02489e−16	Plecoptera	Perlodidae	Megarcys signata
Arthroplea bipunctata 16S ribosomal RNA gene, part	AY749817	81.82	63.46	3.02489e−16	Ephemeroptera	Arthropleidae	Arthroplea bipunctata
Cloeon dipterum voucher EPH02 tRNA−Leu, 16S riboso	KF256908	76.1	99.36	1.05579e−15	Ephemeroptera	Baetidae	Cloeon dipterum
Cryptoperla sp. YK−2012−2 mitochondrial gene for 1	AB711244	82.35	64.74	1.05579e−15	Plecoptera	Peltoperlidae	Cryptoperla sp. YK−2012−2
Cryptoperla japonica mitochondrial gene for 16S rR	AB711183	81.19	64.74	1.05579e−15	Plecoptera	Peltoperlidae	Cryptoperla japonica
Cryptoperla japonica mitochondrial gene for 16S rR	AB711182	81.19	64.74	1.05579e−15	Plecoptera	Peltoperlidae	Cryptoperla japonica
Simocephalus sp. EIZ−2011 isolate Brb1−Sim1 16S ri	JN874613	83.17	63.46	1.05579e−15	Diplostraca	Daphniidae	Simocephalus sp. EIZ−2011
Pachyrhamma edwardsii voucher WEL 16S ribosomal RN	HM594509	82.35	64.74	1.05579e−15	Orthoptera	Rhaphidophoridae	Pachyrhamma edwardsii
Dolichopoda geniculata pontiana isolate PNZ1 16S r	AY507588	81.55	65.38	1.05579e−15	Orthoptera	Rhaphidophoridae	Dolichopoda geniculata pontiana
Dolichopoda noctivaga voucher COR 16S ribosomal RN	EU887872	81.19	64.74	1.05579e−15	Orthoptera	Rhaphidophoridae	Dolichopoda noctivaga
Isoperla davisi 16S ribosomal RNA gene, partial se	EF623206	81.9	66.67	1.05579e−15	Plecoptera	Perlodidae	Isoperla davisi
Neonemura sp. BYU_PL153 16S ribosomal RNA gene, pa	EF623204	82.35	64.74	1.05579e−15	Plecoptera	Notonemouridae	Neonemura sp. BYU_PL153
Kimminsoperla albomacula 16S ribosomal RNA gene, p	EF623202	82.35	64.74	1.05579e−15	Plecoptera	Notonemouridae	Kimminsoperla albomacula
Eusthenia costalis 16S ribosomal RNA gene, partial	EF623150	81.19	64.74	1.05579e−15	Plecoptera	Eustheniidae	Eusthenia costalis
Neopanorpa sp. M24899 16S ribosomal RNA gene, part	AF180059	81.19	64.74	1.05579e−15	Mecoptera	Panorpidae	Neopanorpa sp. M24899
Parameletus columbiae 16S ribosomal RNA gene, part	AY749768	74.52	100	1.05579e−15	Ephemeroptera	Siphlonuridae	Parameletus columbiae
Actias sinensis subaurea mitochondrial gene for 16	AB841441	82.11	60.9	3.68507e−15	Lepidoptera	Saturniidae	Actias sinensis subaurea
Actias sinensis subaurea mitochondrial gene for 16	AB841440	82.11	60.9	3.68507e−15	Lepidoptera	Saturniidae	Actias sinensis subaurea
Actias sinensis subaurea mitochondrial gene for 16	AB841439	82.11	60.9	3.68507e−15	Lepidoptera	Saturniidae	Actias sinensis subaurea
Cryptoperla sp. YK−2012−1 mitochondrial gene for 1	AB711243	81	64.1	3.68507e−15	Plecoptera	Peltoperlidae	Cryptoperla sp. YK−2012−1
Cryptoperla ishigakiensis mitochondrial gene for 1	AB711242	81	64.1	3.68507e−15	Plecoptera	Peltoperlidae	Cryptoperla ishigakiensis
Baumanella alameda 16S ribosomal RNA gene, partial	EF623236	81.37	64.74	3.68507e−15	Plecoptera	Perlodidae	Baumanella alameda
Neuroperla schedingi 16S ribosomal RNA gene, parti	EF623151	81.73	66.03	3.68507e−15	Plecoptera	Eustheniidae	Neuroperla schedingi
Cosmioperla australis 16S ribosomal RNA gene, part	EF623148	79.44	68.59	3.68507e−15	Plecoptera	Eustheniidae	Cosmioperla australis
Chloroperla tripunctata 16S ribosomal RNA gene, pa	EF623137	80.77	66.03	3.68507e−15	Plecoptera	Chloroperlidae	Chloroperla tripunctata
Capnura wanica 16S ribosomal RNA gene, partial seq	EF623128	81.55	64.74	3.68507e−15	Plecoptera	Capniidae	Capnura wanica
Simocephalus vetulus 16S ribosomal RNA gene, parti	DQ470576	81.31	67.31	3.68507e−15	Diplostraca	Daphniidae	Simocephalus vetulus
Dolichopoda geniculata pontiana isolate PNZ 16S ri	AY793586	81.37	64.74	3.68507e−15	Orthoptera	Rhaphidophoridae	Dolichopoda geniculata pontiana
Tasmanophlebia sp. EP158 16S ribosomal RNA gene, p	AY749825	81	64.1	3.68507e−15	Ephemeroptera	Oniscigastridae	Tasmanophlebia sp. EP158
Epeorus longimanus 16S ribosomal RNA gene, partial	AY749757	74.36	99.36	3.68507e−15	Ephemeroptera	Heptageniidae	Epeorus longimanus
D.geniculata mitochondrial 16S rRNA gene	Y07551	81.37	64.74	3.68507e−15	Orthoptera	Rhaphidophoridae	Dolichopoda geniculata
Apterodorcus bacchus voucher SK121 16S ribosomal R	KP250211	80.2	64.74	1.28622e−14	Coleoptera	Lucanidae	Apterodorcus bacchus
Semirhynchia sp. n. SV01 16S ribosomal RNA gene, p	JX294172	81.19	64.74	1.28622e−14	Neuroptera	Nemopteridae	Semirhynchia sp. n. SV01
Sicyoptera dilatata isolate SDGG01 16S ribosomal R	JX294163	81.19	64.74	1.28622e−14	Neuroptera	Nemopteridae	Sicyoptera dilatata
Cryptoperla kawasawai mitochondrial gene for 16S r	AB711236	78.76	72.44	1.28622e−14	Plecoptera	Peltoperlidae	Cryptoperla kawasawai
Hansonoperla appalachia 16S ribosomal RNA gene, pa	EF623249	80	64.1	1.28622e−14	Plecoptera	Perlidae	Hansonoperla appalachia
Rickera sorpta 16S ribosomal RNA gene, partial seq	EF623235	83.33	64.1	1.28622e−14	Plecoptera	Perlodidae	Rickera sorpta
Osobenus yakimae 16S ribosomal RNA gene, partial s	EF623232	79.65	71.79	1.28622e−14	Plecoptera	Perlodidae	Osobenus yakimae
