# Supplementary material for: Testing the potential of a ribosomal 16S marker for DNA metabarcoding of insects
Source: PeerJ. 2016 Apr 19;4:e1966. doi: 10.7717/peerj.1966 (PMC4841222; doi:10.7717/peerj.1966)
Supplement: Supplemental Information 1 [file OTU_5.csv_plot.pdf › scripts/5 match OTUs/blast_hits/OTU_5.csv_plot.pdf]

OTU_5.csv

Ecdyonurus dispar

Heptageniidae

Ephemeroptera

91.61

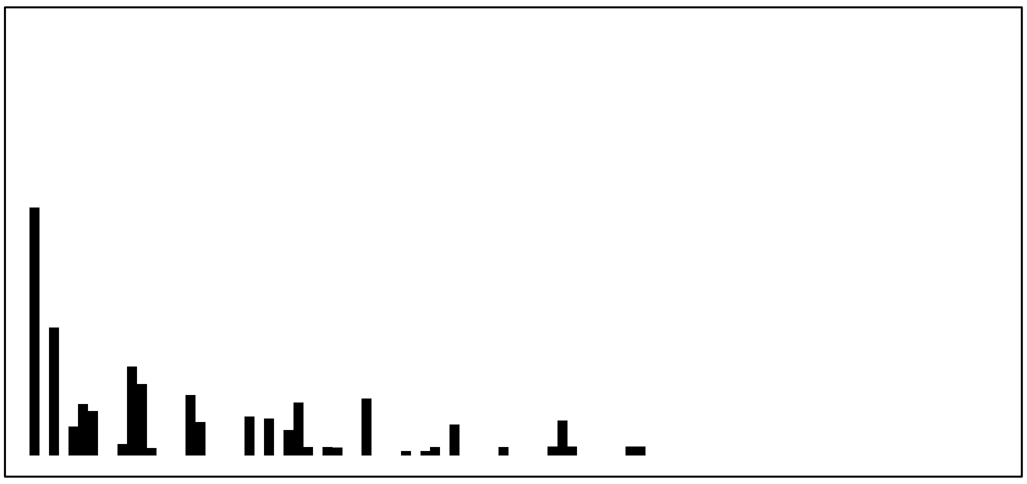

Ecdyonurus dispar 16S ribosomal RNA gene, partial	AY749775	91.61	100	2.9946e−54	Ephemeroptera	Heptageniidae	Ecdyonurus dispar
Parafronurus youi mitochondrion, complete genome	EU349015	77.92	99.35	2.15816e−24	Ephemeroptera	Heptageniidae	Parafronurus youi
Afronurus sp. 212BoAf mitochondrial partial 16S rR	HE651421	86	64.52	2.62918e−23	Ephemeroptera	Heptageniidae	Afronurus sp. 212BoAf
Clioperla clio 16S ribosomal RNA gene, partial seq	EF623237	76.28	100	3.90205e−21	Plecoptera	Perlodidae	Clioperla clio
Siphlonurus sp. EP084 16S ribosomal RNA gene, part	AY749786	81.36	75.48	4.75367e−20	Ephemeroptera	Siphlonuridae	Siphlonurus sp. EP084
Siphlonurus sp. MT−2014 isolate CL142 mitochondrio	KM244684	82.41	69.68	1.65913e−19	Ephemeroptera	Siphlonuridae	Siphlonurus sp. MT−2014
Pteronarcys sachalina 16S ribosomal RNA gene, part	EF623264	82.08	68.39	1.65919e−19	Plecoptera	Pteronarcyidae	Pteronarcys sachalina
Siphlonurus binotatus mitochondrial gene for 16S r	AB110269	76.81	89.03	1.65919e−19	Ephemeroptera	Siphlonuridae	Siphlonurus binotatus
Oniscigaster distans 16S ribosomal RNA gene, parti	AY749818	76.43	100	1.65919e−19	Ephemeroptera	Oniscigastridae	Oniscigaster distans
Pseudiron centralis 16S ribosomal RNA gene, partia	AY749799	80.53	72.9	1.65919e−19	Ephemeroptera	Pseudironidae	Pseudiron centralis
Afronurus peringueyi 16S ribosomal RNA gene, parti	AY749790	84.16	63.87	1.65919e−19	Ephemeroptera	Heptageniidae	Afronurus peringueyi
Heptagenia sulphurea mitochondrial partial 16S rRN	HE651431	83.33	65.16	5.79115e−19	Ephemeroptera	Heptageniidae	Heptagenia sulphurea
Thalerosphyrus lamuriensis mitochondrial partial 1	HE651425	80.34	75.48	5.79115e−19	Ephemeroptera	Heptageniidae	Thalerosphyrus lamuriensis
Atopopus tarsalis mitochondrial partial 16S rRNA g	HE651420	74.84	100	5.79115e−19	Ephemeroptera	Heptageniidae	Atopopus tarsalis
Atopopus tarsalis mitochondrial partial 16S rRNA g	HE651419	74.84	100	5.79115e−19	Ephemeroptera	Heptageniidae	Atopopus tarsalis
Osmylus sp. EH−2004 16S ribosomal RNA gene, partia	AY620146	75.64	100	5.79115e−19	Neuroptera	Osmylidae	Osmylus sp. EH−2004
Afronurus sp. 213BoAf mitochondrial partial 16S rR	HE651422	82.83	63.87	2.02131e−18	Ephemeroptera	Heptageniidae	Afronurus sp. 213BoAf
Paegniodes cupulatus mitochondrion, complete genom	HM004123	81.55	66.45	7.05507e−18	Ephemeroptera	Heptageniidae	Paegniodes cupulatus
Thalerosphyrus lamuriensis mitochondrial partial 1	HE651429	79.49	75.48	7.05507e−18	Ephemeroptera	Heptageniidae	Thalerosphyrus lamuriensis
Cinygmina sp. EP133 16S ribosomal RNA gene, partia	AY749811	74.84	100	7.05507e−18	Ephemeroptera	Heptageniidae	Cinygmina sp. EP133
Heteromallus cavicola voucher MIL 16S ribosomal RN	HM594499	80	72.9	2.46246e−17	Orthoptera	Rhaphidophoridae	Heteromallus cavicola
Rallidens mcfarlanei 16S ribosomal RNA gene, parti	AY749794	75.16	100	2.46246e−17	Ephemeroptera	Rallidentidae	Rallidens mcfarlanei
Stenonema sp. EP026 16S ribosomal RNA gene, partia	AY749769	81.82	63.87	2.46246e−17	Ephemeroptera	Heptageniidae	Stenonema sp. EP026
Dolichopoda sp. MR−2008 16S ribosomal RNA gene, pa	EU887863	80	72.9	8.59483e−17	Orthoptera	Rhaphidophoridae	Dolichopoda lustriae
Yugus bulbosus 16S ribosomal RNA gene, partial seq	EF623214	81.73	65.81	8.59483e−17	Plecoptera	Perlodidae	Yugus bulbosus
Ametropus neavei 16S ribosomal RNA gene, partial s	AY749800	75.47	100	8.59483e−17	Ephemeroptera	Ametropodidae	Ametropus neavei
Parameletus columbiae 16S ribosomal RNA gene, part	AY749768	81.19	65.16	8.59483e−17	Ephemeroptera	Siphlonuridae	Parameletus columbiae
Afronurus sp. 338SuAf mitochondrial partial 16S rR	HE651428	82.47	62.58	2.99989e−16	Ephemeroptera	Heptageniidae	Afronurus sp. 338SuAf
Afronurus sp. 297SAAf mitochondrial partial 16S rR	HE651424	80.39	65.81	2.99989e−16	Ephemeroptera	Heptageniidae	Afronurus sp. 297SAAf
Compsoneuria sp. 4 LV−2012 mitochondrial partial 1	HE651398	79.31	74.19	2.99989e−16	Ephemeroptera	Heptageniidae	Compsoneuria sp. 4 LV−2012
Isoperla oxylepis 16S ribosomal RNA gene, partial	EF623221	80.39	65.81	2.99989e−16	Plecoptera	Perlodidae	Isoperla oxylepis
Isoperla davisi 16S ribosomal RNA gene, partial se	EF623206	80.37	68.39	2.99989e−16	Plecoptera	Perlodidae	Isoperla davisi
Panorpa acuta 16S ribosomal RNA gene, partial sequ	AF180047	78.95	72.9	2.99989e−16	Mecoptera	Panorpidae	Panorpa acuta
Pteronarcys scotti 16S ribosomal RNA gene, partial	EF623263	79.25	68.39	1.04707e−15	Plecoptera	Pteronarcyidae	Pteronarcys scotti
Osobenus yakimae 16S ribosomal RNA gene, partial s	EF623232	82.65	62.58	1.04707e−15	Plecoptera	Perlodidae	Osobenus yakimae
Cosumnoperla hypocrena 16S ribosomal RNA gene, par	EF623226	78.45	74.19	1.04707e−15	Plecoptera	Perlodidae	Cosumnoperla hypocrena
Cascadoperla trictura 16S ribosomal RNA gene, part	EF623225	79.28	70.97	1.04707e−15	Plecoptera	Perlodidae	Cascadoperla trictura
Isoperla fulva 16S ribosomal RNA gene, partial seq	EF623207	73.72	100	1.04707e−15	Plecoptera	Perlodidae	Isoperla fulva
Amphinemura nigritta 16S ribosomal RNA gene, parti	EF623186	80.19	67.74	1.04707e−15	Plecoptera	Nemouridae	Amphinemura nigritta
Neuroperlopsis patris 16S ribosomal RNA gene, part	EF623152	74.32	94.84	1.04707e−15	Plecoptera	Eustheniidae	Neuroperlopsis patris
Isogenoides varians isolate BYU_PL079 16S ribosoma	EF623445	80.2	65.16	1.04707e−15	Plecoptera	Perlodidae	Isogenoides varians
Perlodes mortoni 16S ribosomal RNA gene, partial s	KF855870	80.39	65.16	3.65462e−15	Plecoptera	Perlodidae	Perlodes mortoni
Thalerosphyrus sinuosus mitochondrial partial 16S	HE651430	75.34	90.32	3.65462e−15	Ephemeroptera	Heptageniidae	Thalerosphyrus sinuosus
Afronurus sp. 336SuAf mitochondrial partial 16S rR	HE651427	81.44	62.58	3.65462e−15	Ephemeroptera	Heptageniidae	Afronurus sp. 336SuAf
Notonurus josettae mitochondrial partial 16S rRNA	HE651408	78.26	74.19	3.65462e−15	Ephemeroptera	Heptageniidae	Notonurus josettae
Notonurus josettae mitochondrial partial 16S rRNA	HE651399	78.26	74.19	3.65462e−15	Ephemeroptera	Heptageniidae	Notonurus josettae
Talitropsis sedilloti voucher CW160 16S ribosomal	HM594511	80	64.52	3.65462e−15	Orthoptera	Rhaphidophoridae	Talitropsis sedilloti
Parudenus falklandicus voucher PAR 16S ribosomal R	HM594502	78.95	72.26	3.65462e−15	Orthoptera	Rhaphidophoridae	Parudenus falklandicus
Isogenoides olivaceus 16S ribosomal RNA gene, part	EF623224	80.39	65.16	3.65462e−15	Plecoptera	Perlodidae	Isogenoides olivaceus
Kimminsoperla albomacula 16S ribosomal RNA gene, p	EF623202	77.59	72.9	3.65462e−15	Plecoptera	Notonemouridae	Kimminsoperla albomacula
Suwallia teleckojensis 16S ribosomal RNA gene, par	EF623142	74.07	100	3.65462e−15	Plecoptera	Chloroperlidae	Suwallia teleckojensis
Chiloporter sp. EP051 16S ribosomal RNA gene, part	AY749776	80	64.52	3.65462e−15	Ephemeroptera	Ameletopsidae	Chiloporter sp. EP051
Rhithrogena nuragica mitochondrial partial 16S rRN	LN868667	73.25	100	1.27559e−14	Ephemeroptera	Heptageniidae	Rhithrogena nuragica
Hydroperla crosbyi 16S ribosomal RNA gene, partial	EF623219	80.41	62.58	1.27559e−14	Plecoptera	Perlodidae	Hydroperla crosbyi
Kogotus nonus 16S ribosomal RNA gene, partial sequ	EF623213	81.63	62.58	1.27559e−14	Plecoptera	Perlodidae	Kogotus nonus
Despaxia augusta 16S ribosomal RNA gene, partial s	EF623177	80.41	62.58	1.27559e−14	Plecoptera	Leuctridae	Despaxia augusta
Diamphipnopsis sp. BYU_PL138 16S ribosomal RNA gen	EF623145	75.36	87.74	1.27559e−14	Plecoptera	Diamphipnoidae	Diamphipnopsis sp. BYU_PL138
Plumiperla diversa 16S ribosomal RNA gene, partial	EF623132	74.38	100	1.27559e−14	Plecoptera	Chloroperlidae	Plumiperla diversa
Neopanorpa sp. M24899 16S ribosomal RNA gene, part	AF180059	78.07	72.9	1.27559e−14	Mecoptera	Panorpidae	Neopanorpa sp. M24899
Panorpa nebulosa 16S ribosomal RNA gene, partial s	AF180058	78.07	72.9	1.27559e−14	Mecoptera	Panorpidae	Panorpa nebulosa
Panorpa alpina 16S ribosomal RNA gene, partial seq	AF180046	78.07	72.9	1.27559e−14	Mecoptera	Panorpidae	Panorpa alpina
Tasmanophlebia sp. EP158 16S ribosomal RNA gene, p	AY749825	80.41	62.58	1.27559e−14	Ephemeroptera	Oniscigastridae	Tasmanophlebia sp. EP158
Siphlonella sp. EP083 16S ribosomal RNA gene, part	AY749785	80.41	62.58	1.27559e−14	Ephemeroptera	Oniscigastridae	Siphlonella sp. EP083
Chaquihua sp. EP080 16S ribosomal RNA gene, partia	AY749784	79.41	65.81	1.27559e−14	Ephemeroptera	Ameletopsidae	Chaquihua sp. EP080
Heptagenia sp. EP018 16S ribosomal RNA gene, parti	AY749762	74.82	89.03	1.27559e−14	Ephemeroptera	Heptageniidae	Heptagenia sp. EP018
Compsoneuria sp. 10 LV−2012 mitochondrial partial	HE651412	77.39	74.19	4.45223e−14	Ephemeroptera	Heptageniidae	Compsoneuria sp. 10 LV−2012
Notonemouridae sp. MD−2011 voucher ZMUC:JD−1507 16	JN615257	79.05	67.1	4.45223e−14	Plecoptera	Notonemouridae	Notonemouridae sp. MD−2011
Hexagenia sp. BYU IGCEP003 16S ribosomal RNA gene	GQ118286	77.88	72.9	4.45223e−14	Ephemeroptera	Ephemeridae	Hexagenia sp. BYU IGCEP003
Ephemera simulans voucher BYU:IGCEP038 16S ribosom	GQ118285	77.69	74.19	4.45223e−14	Ephemeroptera	Ephemeridae	Ephemera simulans
Dolichopoda sp. VAR 16S ribosomal RNA gene, partia	EU887865	78.26	72.9	4.45223e−14	Orthoptera	Rhaphidophoridae	Dolichopoda sp. VAR
Dolichopoda sp. TAY 16S ribosomal RNA gene, partia	EU887864	78.26	72.9	4.45223e−14	Orthoptera	Rhaphidophoridae	Dolichopoda sp. TAY
Dolichopoda annae voucher OLI 16S ribosomal RNA ge	EU887860	78.26	72.9	4.45223e−14	Orthoptera	Rhaphidophoridae	Dolichopoda annae
Dolichopoda pavesii voucher SPI 16S ribosomal RNA	EF216970	79.05	67.1	4.45223e−14	Orthoptera	Rhaphidophoridae	Dolichopoda pavesii
Pteronarcys californica 16S ribosomal RNA gene, pa	EF623261	78.3	68.39	4.45223e−14	Plecoptera	Pteronarcyidae	Pteronarcys californica
Baumanella alameda 16S ribosomal RNA gene, partial	EF623236	79.61	65.81	4.45223e−14	Plecoptera	Perlodidae	Baumanella alameda
Perlinodes aurea 16S ribosomal RNA gene, partial s	EF623234	79.61	65.81	4.45223e−14	Plecoptera	Perlodidae	Perlinodes aurea
Frisonia picticeps 16S ribosomal RNA gene, partial	EF623227	78.57	70.97	4.45223e−14	Plecoptera	Perlodidae	Frisonia picticeps
Isoperla phalerata 16S ribosomal RNA gene, partial	EF623208	78.64	66.45	4.45223e−14	Plecoptera	Perlodidae	Isoperla phalerata
Udamocercia sp. BYU_PL150 16S ribosomal RNA gene,	EF623201	79.09	70.32	4.45223e−14	Plecoptera	Notonemouridae	Udamocercia sp. BYU_PL150
Nanonemoura wahkeena 16S ribosomal RNA gene, parti	EF623198	73.86	91.61	4.45223e−14	Plecoptera	Nemouridae	Nanonemoura wahkeena
Zapada cinctipes 16S ribosomal RNA gene, partial s	EF623183	79.61	65.81	4.45223e−14	Plecoptera	Nemouridae	Zapada cinctipes
Zealeuctra arnoldi 16S ribosomal RNA gene, partial	EF623180	78.5	67.74	4.45223e−14	Plecoptera	Leuctridae	Zealeuctra arnoldi
Pteronarcys princeps mitochondrion, complete genom	AY687866	78.3	68.39	4.45223e−14	Plecoptera	Pteronarcyidae	Pteronarcys princeps
Cryptoperla japonica mitochondrial gene for 16S rR	AB711202	79.81	66.45	1.55398e−13	Plecoptera	Peltoperlidae	Cryptoperla japonica
Cryptoperla japonica mitochondrial gene for 16S rR	AB711184	79.81	66.45	1.55398e−13	Plecoptera	Peltoperlidae	Cryptoperla japonica
Dolichopoda remyi voucher EDE 16S ribosomal RNA ge	EF216969	77.19	72.9	1.55398e−13	Orthoptera	Rhaphidophoridae	Dolichopoda remyi
Isogenoides hansoni 16S ribosomal RNA gene, partia	EF623223	79.41	65.16	1.55398e−13	Plecoptera	Perlodidae	Isogenoides hansoni
Skwala americana 16S ribosomal RNA gene, partial s	EF623217	79.05	65.81	1.55398e−13	Plecoptera	Perlodidae	Skwala americana
Isogenoides zionensis 16S ribosomal RNA gene, part	EF623210	79.41	65.16	1.55398e−13	Plecoptera	Perlodidae	Isogenoides zionensis
Neofulla sp. BYU_PL152 16S ribosomal RNA gene, par	EF623203	77.68	71.61	1.55398e−13	Plecoptera	Notonemouridae	Neofulla sp. BYU_PL152
Austronemoura sp. BYU_PL149 16S ribosomal RNA gene	EF623200	77.59	73.55	1.55398e−13	Plecoptera	Notonemouridae	Austronemoura sp. BYU_PL149
Soyedina producta 16S ribosomal RNA gene, partial	EF623196	79.38	62.58	1.55398e−13	Plecoptera	Nemouridae	Soyedina producta
Moselia infuscata 16S ribosomal RNA gene, partial	EF623175	79.63	69.03	1.55398e−13	Plecoptera	Leuctridae	Moselia infuscata
Neaviperla forcipata 16S ribosomal RNA gene, parti	EF623139	73.46	100	1.55398e−13	Plecoptera	Chloroperlidae	Neaviperla forcipata
Isogenoides doratus isolate BYU_PL131 16S ribosoma	EF623449	79.41	65.16	1.55398e−13	Plecoptera	Perlodidae	Isogenoides doratus
Isogenoides colubrinus isolate BYU_PL130 16S ribos	EF623448	79.41	65.16	1.55398e−13	Plecoptera	Perlodidae	Isogenoides colubrinus
Isogenoides elongatus isolate BYU_PL082 16S riboso	EF623447	79.41	65.16	1.55398e−13	Plecoptera	Perlodidae	Isogenoides elongatus
Isogenoides frontalis isolate BYU_PL080 16S riboso	EF623446	79.41	65.16	1.55398e−13	Plecoptera	Perlodidae	Isogenoides frontalis
Dolichopoda remyi isolate EDE 16S ribosomal RNA ge	AY793589	77.19	72.9	1.55398e−13	Orthoptera	Rhaphidophoridae	Dolichopoda remyi
Panorpa claripennis 16S ribosomal RNA gene, partia	AF180068	77.19	72.9	1.55398e−13	Mecoptera	Panorpidae	Panorpa claripennis
