# Supplementary material for: Testing the potential of a ribosomal 16S marker for DNA metabarcoding of insects
Source: PeerJ. 2016 Apr 19;4:e1966. doi: 10.7717/peerj.1966 (PMC4841222; doi:10.7717/peerj.1966)
Supplement: Supplemental Information 1 [file OTU_51.csv_plot.pdf › scripts/5 match OTUs/blast_hits/OTU_51.csv_plot.pdf]

OTU_51.csv

Erpobdella obscura

Erpobdellidae

Arhynchobdellida

86.3

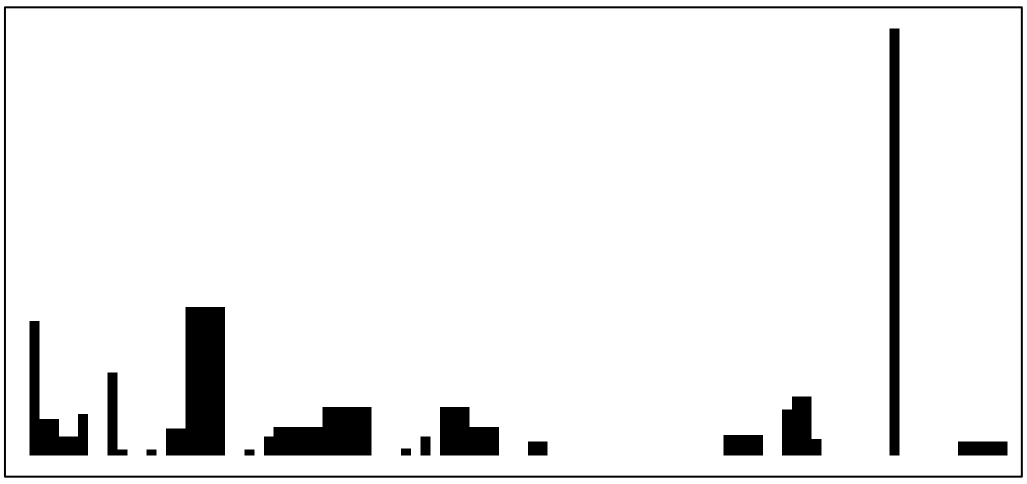

Erpobdella obscura isolate Eobs 16S ribosomal RNA	JQ821464	86.3	57.94	4.11315e−13	Arhynchobdellida	Erpobdellidae	Erpobdella obscura
Octochaetus multiporus isolate YN1 16S ribosomal R	KP780262	81.69	55.56	1.3446e−06	Haplotaxida	Octochaetidae	Octochaetus multiporus
Octochaetus kenleei 16S ribosomal RNA gene, partia	HQ529286	81.69	55.56	1.3446e−06	Haplotaxida	Octochaetidae	Octochaetus kenleei
Lumbricillus tuba isolate CE879 16S ribosomal RNA	GU901831	80.88	53.97	4.6931e−06	Haplotaxida	Enchytraeidae	Lumbricillus tuba
Parachilota sp. 570 16S ribosomal RNA gene, partia	HQ232624	80.88	53.97	4.6931e−06	Haplotaxida	Acanthodrilidae	Parachilota sp. 570
Megascolecidae sp. 1 SB−2015 isolate YN7_White 16S	KP771677	81.94	55.56	5.71736e−05	Haplotaxida	Megascolecidae	Megascolecidae sp. 1 SB−2015
Hormogaster elisae voucher SEV12 16S ribosomal RNA	JN209366	80	54.76	5.71736e−05	Haplotaxida	Hormogastridae	Hormogaster elisae
Hormogaster elisae voucher SEV11 16S ribosomal RNA	JN209365	80	54.76	5.71736e−05	Haplotaxida	Hormogastridae	Hormogaster elisae
Udeina sp. 586 16S ribosomal RNA gene, partial seq	HQ232609	83.87	48.41	5.71736e−05	Haplotaxida	Acanthodrilidae	Udeina sp. 586
Acanthodrilinae sp. 830 16S ribosomal RNA gene, pa	HQ232498	80.28	53.97	5.71736e−05	Haplotaxida	Acanthodrilidae	Acanthodrilinae sp. 830
Hormogaster elisae isolate HSEV2 16S ribosomal RNA	GQ409708	80	54.76	5.71736e−05	Haplotaxida	Hormogastridae	Hormogaster elisae
Hormogaster elisae isolate HSEV1 16S ribosomal RNA	GQ409707	80	54.76	5.71736e−05	Haplotaxida	Hormogastridae	Hormogaster elisae
Thalassodrilus prostatus isolate CE970 16S ribosom	KJ753883	80.28	55.56	0.000199556	Haplotaxida	Naididae	Thalassodrilus prostatus
Limnodrilus udekemianus isolate 95BTub 16S ribosom	HM991165	79.1	53.17	0.000199556	Haplotaxida	Naididae	Limnodrilus udekemianus
Rhynchelmis rostrata variant isolate CE555 16S rib	GU592345	81.25	50.79	0.000199556	Lumbriculida	Lumbriculidae	Rhynchelmis rostrata
Rhynchelmis rostrata isolate CE1070 16S ribosomal	GU592336	81.25	50.79	0.000199556	Lumbriculida	Lumbriculidae	Rhynchelmis rostrata
Hormogaster samnitica voucher UCMLT 00179 16S ribo	KF975153	86.96	36.51	0.000696518	Haplotaxida	Hormogastridae	Hormogaster samnitica
Hormogaster samnitica voucher UCMLT 00178 16S ribo	KF975152	86.96	36.51	0.000696518	Haplotaxida	Hormogastridae	Hormogaster samnitica
Hormogaster samnitica voucher UCMLT 00177 16S ribo	KF975151	86.96	36.51	0.000696518	Haplotaxida	Hormogastridae	Hormogaster samnitica
Hormogaster samnitica voucher UCMLT 00176 16S ribo	KF975150	86.96	36.51	0.000696518	Haplotaxida	Hormogastridae	Hormogaster samnitica
Drawida japonica isolate H7 16S ribosomal RNA gene	JF714597	73.33	77.78	0.000696518	Moniligastrida	Moniligastridae	Drawida japonica
Drawida japonica isolate H26 16S ribosomal RNA gen	JF714616	73.33	77.78	0.000696518	Moniligastrida	Moniligastridae	Drawida japonica
Plutellus sp. 5 PK−2012 16S ribosomal RNA gene, pa	JX157597	80.28	54.76	0.000696518	Haplotaxida	Megascolecidae	Plutellus sp. 5 PK−2012
Drawida sp. 1 PK−2012 16S ribosomal RNA gene, part	JX046752	77.94	53.97	0.000696518	Moniligastrida	Moniligastridae	Drawida sp. 1 PK−2012
Diplotrema sp. 904 16S ribosomal RNA gene, partial	JF267871	80.88	53.97	0.000696518	Haplotaxida	Megascolecidae	Diplotrema sp. 904
Hormogaster elisae voucher UCE9 16S ribosomal RNA	JN209412	81.33	57.14	0.000696518	Haplotaxida	Hormogastridae	Hormogaster elisae
Hormogaster elisae voucher UCE8 16S ribosomal RNA	JN209411	81.33	57.14	0.000696518	Haplotaxida	Hormogastridae	Hormogaster elisae
Hormogaster elisae voucher UCE12 16S ribosomal RNA	JN209408	81.33	57.14	0.000696518	Haplotaxida	Hormogastridae	Hormogaster elisae
Hormogaster elisae voucher UCE6 16S ribosomal RNA	JN209409	81.33	57.14	0.000696518	Haplotaxida	Hormogastridae	Hormogaster elisae
Hormogaster elisae voucher UCE10 16S ribosomal RNA	JN209407	81.33	57.14	0.000696518	Haplotaxida	Hormogastridae	Hormogaster elisae
Hormogaster elisae voucher NAV4 16S ribosomal RNA	JN209306	82.26	48.41	0.000696518	Haplotaxida	Hormogastridae	Hormogaster elisae
Hormogaster elisae voucher NAV10 16S ribosomal RNA	JN209303	82.26	48.41	0.000696518	Haplotaxida	Hormogastridae	Hormogaster elisae
Hormogaster elisae voucher NAV11 16S ribosomal RNA	JN209304	82.26	48.41	0.000696518	Haplotaxida	Hormogastridae	Hormogaster elisae
Hormogaster elisae voucher CAB9 16S ribosomal RNA	JN209226	82.26	48.41	0.000696518	Haplotaxida	Hormogastridae	Hormogaster elisae
Hormogaster elisae voucher CAB10 16S ribosomal RNA	JN209221	82.26	48.41	0.000696518	Haplotaxida	Hormogastridae	Hormogaster elisae
Drawida gracilis 16S ribosomal RNA gene, partial s	JN793520	74.42	68.25	0.000696518	Moniligastrida	Moniligastridae	Drawida gracilis
Drawida gracilis isolate SR−04−E1B 16S ribosomal R	JN887900	74.42	68.25	0.000696518	Moniligastrida	Moniligastridae	Drawida gracilis
Drawida gracilis isolate SR−04−E1A 16S ribosomal R	JN887899	74.42	68.25	0.000696518	Moniligastrida	Moniligastridae	Drawida gracilis
Toutellus adecus isolate 894 16S ribosomal RNA gen	HQ232611	80.33	48.41	0.000696518	Haplotaxida	Megascolecidae	Toutellus adecus
Octochaetus sp. WM113 16S ribosomal RNA gene, part	HQ232517	78.87	55.56	0.000696518	Haplotaxida	Octochaetidae	Octochaetus sp. WM113
Diplotrema sp. 904 16S ribosomal RNA gene, partial	HQ232497	80.88	53.97	0.000696518	Haplotaxida	Megascolecidae	Diplotrema sp. 904
Diplocardia farmvillensis isolate 420 16S ribosoma	HQ232491	77.63	57.14	0.000696518	Haplotaxida	Megascolecidae	Diplocardia farmvillensis
Hormogaster elisae isolate HNAV2 16S ribosomal RNA	GQ409731	82.26	48.41	0.000696518	Haplotaxida	Hormogastridae	Hormogaster elisae
Hormogaster elisae isolate HNAV1 16S ribosomal RNA	GQ409730	82.26	48.41	0.000696518	Haplotaxida	Hormogastridae	Hormogaster elisae
Hormogaster elisae isolate HCAB1 16S ribosomal RNA	GQ409729	82.26	48.41	0.000696518	Haplotaxida	Hormogastridae	Hormogaster elisae
Hormogaster elisae isolate HUCE2 16S ribosomal RNA	GQ409722	81.33	57.14	0.000696518	Haplotaxida	Hormogastridae	Hormogaster elisae
Hormogaster elisae isolate HUCE3 16S ribosomal RNA	GQ409721	81.33	57.14	0.000696518	Haplotaxida	Hormogastridae	Hormogaster elisae
Hormogaster elisae isolate HUCE1 16S ribosomal RNA	GQ409720	81.33	57.14	0.000696518	Haplotaxida	Hormogastridae	Hormogaster elisae
Hormogaster elisae isolate HPAR1 16S ribosomal RNA	GQ409709	79.37	50	0.000696518	Haplotaxida	Hormogastridae	Hormogaster elisae
Eunoe nodosa voucher SMNH118963 16S ribosomal RNA	JN852892	78.38	57.94	0.00243109	Phyllodocida	Polynoidae	Eunoe nodosa
Parachilota sp. 561 16S ribosomal RNA gene, partia	HQ232623	77.94	53.97	0.00243109	Haplotaxida	Acanthodrilidae	Parachilota sp. 561
Millsonia sp. 1102 isolate 1101 16S ribosomal RNA	HQ232621	80.65	49.21	0.00243109	Haplotaxida	Megascolecidae	Millsonia sp. 1102
Benhaminae sp. 1093 16S ribosomal RNA gene, partia	HQ232619	80.65	49.21	0.00243109	Haplotaxida	Acanthodrilidae	Benhaminae sp. 1093
Microscolex dubius isolate MicrclexDubius 16S rib	HQ232608	79.71	53.97	0.00243109	Haplotaxida	Megascolecidae	Microscolex dubius
Diplocardia hulberti isolate 431 16S ribosomal RNA	HQ232494	79.45	55.56	0.00243109	Haplotaxida	Megascolecidae	Diplocardia hulberti
Rhynchelmis gustafsoni isolate CE507 16S ribosomal	GU592339	79.17	56.35	0.00243109	Lumbriculida	Lumbriculidae	Rhynchelmis gustafsoni
Rhynchelmis cf. yakimorum PDW−2010 isolate CE498 1	GU592357	79.17	56.35	0.00243109	Lumbriculida	Lumbriculidae	Rhynchelmis cf. yakimorum PDW−2010
Rhynchelmis yakimorum variant 1 isolate CE1087 16S	GU592354	79.17	56.35	0.00243109	Lumbriculida	Lumbriculidae	Rhynchelmis yakimorum
Rhynchelmis gustafsoni isolate CE584 16S ribosomal	GU592344	79.17	56.35	0.00243109	Lumbriculida	Lumbriculidae	Rhynchelmis gustafsoni
Rhynchelmis gustafsoni isolate CE583 16S ribosomal	GU592343	79.17	56.35	0.00243109	Lumbriculida	Lumbriculidae	Rhynchelmis gustafsoni
Rhynchelmis gustafsoni isolate CE582 16S ribosomal	GU592342	79.17	56.35	0.00243109	Lumbriculida	Lumbriculidae	Rhynchelmis gustafsoni
Rhynchelmis gustafsoni isolate CE508 16S ribosomal	GU592340	79.17	56.35	0.00243109	Lumbriculida	Lumbriculidae	Rhynchelmis gustafsoni
Diplocardia caroliniana isolate 23.16S2 16S riboso	DQ533724	79.45	55.56	0.00243109	Haplotaxida	Megascolecidae	Diplocardia caroliniana
Rhynchelmis gustafsoni 16S ribosomal RNA gene, par	AY764153	79.17	56.35	0.00243109	Lumbriculida	Lumbriculidae	Rhynchelmis gustafsoni
Limnodrilus udekemianus 16S ribosomal RNA gene, pa	AY885612	77.61	53.17	0.00243109	Haplotaxida	Naididae	Limnodrilus udekemianus
Amynthas sp. FJ201108−06 isolate S33J 16S ribosoma	KF205700	76.32	59.52	0.00848532	Haplotaxida	Megascolecidae	Amynthas sp. FJ201108−06
Eisenia fetida isolate H12 16S ribosomal RNA gene,	JQ739913	73.53	77.78	0.00848532	Haplotaxida	Lumbricidae	Eisenia fetida
Drawida sp. 3 PK−2012 16S ribosomal RNA gene, part	JX157600	78.69	48.41	0.00848532	Moniligastrida	Moniligastridae	Drawida sp. 3 PK−2012
Drawida sp. 5 PK−2012 16S ribosomal RNA gene, part	JX157595	78.69	48.41	0.00848532	Moniligastrida	Moniligastridae	Drawida sp. 5 PK−2012
Fimoscolex sp. PG 16S ribosomal RNA gene, partial	JF267885	77.46	55.56	0.00848532	Haplotaxida	Glossoscolecidae	Fimoscolex sp. PG
Marionina communis isolate CE811 16S ribosomal RNA	GU901839	77.46	55.56	0.00848532	Haplotaxida	Enchytraeidae	Marionina communis
Rhododrilus huttoni isolate WM98 16S ribosomal RNA	HQ232599	80.95	49.21	0.00848532	Haplotaxida	Megascolecidae	Rhododrilus huttoni
Rhododrilus cockaynei isolate WM9 16S ribosomal RN	HQ232590	80.95	49.21	0.00848532	Haplotaxida	Megascolecidae	Rhododrilus cockaynei
Rhododrilus intermedius isolate WM66 16S ribosomal	HQ232568	80.95	49.21	0.00848532	Haplotaxida	Megascolecidae	Rhododrilus intermedius
Rhododrilus n. sp. 6 TRB−2010 isolate WM100 16S ri	HQ232506	80.95	49.21	0.00848532	Haplotaxida	Megascolecidae	Rhododrilus n. sp. 6 TRB−2010
Acanthodrilinae sp. 503 16S ribosomal RNA gene, pa	HQ232495	78.57	53.97	0.00848532	Haplotaxida	Acanthodrilidae	Acanthodrilinae sp. 503
Eclipidrilus frigidus isolate CE557 16S ribosomal	GU592329	78.79	52.38	0.00848532	Lumbriculida	Lumbriculidae	Eclipidrilus frigidus
Perionyx sp. 2 WL−2010 clone B55 16S ribosomal RNA	HM219177	82.14	44.44	0.00848532	Haplotaxida	Megascolecidae	Perionyx sp. 2 WL−2010
Diplocardia caroliniana isolate 21.16S2 16S riboso	DQ533731	82.76	44.44	0.00848532	Haplotaxida	Megascolecidae	Diplocardia caroliniana
Diplocardia caroliniana isolate 19.16S1 16S riboso	DQ533729	82.76	44.44	0.00848532	Haplotaxida	Megascolecidae	Diplocardia caroliniana
Oryza sativa microsatellite MRG0392 containing (AT	AY018067	80.77	41.27	0.00848532	Poales	Poaceae	Oryza sativa
Diplocardia komareki clone 8O 16S ribosomal RNA ge	DQ388662	77.94	53.97	0.00848532	Haplotaxida	Megascolecidae	Diplocardia komareki
Diplocardia komareki isolate 2O 16S ribosomal RNA	DQ257315	77.94	53.97	0.00848532	Haplotaxida	Megascolecidae	Diplocardia komareki
Diplocardia komareki isolate 6O 16S ribosomal RNA	DQ257314	77.94	53.97	0.00848532	Haplotaxida	Megascolecidae	Diplocardia komareki
Diplocardia komareki isolate 5O 16S ribosomal RNA	DQ257313	77.94	53.97	0.00848532	Haplotaxida	Megascolecidae	Diplocardia komareki
Diplocardia komareki isolate 4O 16S ribosomal RNA	DQ257312	77.94	53.97	0.00848532	Haplotaxida	Megascolecidae	Diplocardia komareki
Diplocardia komareki isolate 270404 16S ribosomal	DQ247768	77.94	53.97	0.00848532	Haplotaxida	Megascolecidae	Diplocardia komareki
Megascolecidae sp. 3 SB−2015 isolate End9_grey 16S	KP771678	79.17	55.56	0.0296167	Haplotaxida	Megascolecidae	Megascolecidae sp. 3 SB−2015
Megascolex insignis 16S ribosomal RNA gene, partia	JX157594	100	20.63	0.0296167	Haplotaxida	Megascolecidae	Megascolex insignis
Drawida sp. br 16S ribosomal RNA gene, partial seq	JF267918	78.57	55.56	0.0296167	Moniligastrida	Moniligastridae	Drawida sp. br
Neotrigaster rufa 16S ribosomal RNA gene, partial	JF267866	79.17	55.56	0.0296167	Haplotaxida	Megascolecidae	Neotrigaster rufa
Hormogaster samnitica lirapora voucher TES5 16S ri	JN209392	78.57	55.56	0.0296167	Haplotaxida	Hormogastridae	Hormogaster samnitica lirapora
Hormogaster samnitica lirapora voucher TES3 16S ri	JN209390	78.57	55.56	0.0296167	Haplotaxida	Hormogastridae	Hormogaster samnitica lirapora
Hormogaster samnitica lirapora voucher TES2 16S ri	JN209389	78.57	55.56	0.0296167	Haplotaxida	Hormogastridae	Hormogaster samnitica lirapora
Hormogaster sp. n. SAN voucher SAN3 16S ribosomal	JN209360	76	59.52	0.0296167	Haplotaxida	Hormogastridae	Hormogaster sp. n. SAN
Hormogaster elisae voucher LOZ7 16S ribosomal RNA	JN209285	80.65	48.41	0.0296167	Haplotaxida	Hormogastridae	Hormogaster elisae
Hormogaster elisae voucher LOZ24 16S ribosomal RNA	JN209283	80.65	48.41	0.0296167	Haplotaxida	Hormogastridae	Hormogaster elisae
Hormogaster elisae voucher LOZ14 16S ribosomal RNA	JN209273	80.65	48.41	0.0296167	Haplotaxida	Hormogastridae	Hormogaster elisae
Hormogaster elisae voucher LOZ17 16S ribosomal RNA	JN209276	80.65	48.41	0.0296167	Haplotaxida	Hormogastridae	Hormogaster elisae
Hormogaster elisae voucher LOZ11 16S ribosomal RNA	JN209271	80.65	48.41	0.0296167	Haplotaxida	Hormogastridae	Hormogaster elisae
