# Supplementary material for: Testing the potential of a ribosomal 16S marker for DNA metabarcoding of insects
Source: PeerJ. 2016 Apr 19;4:e1966. doi: 10.7717/peerj.1966 (PMC4841222; doi:10.7717/peerj.1966)
Supplement: Supplemental Information 1 [file OTU_529.csv_plot.pdf › scripts/5 match OTUs/blast_hits/OTU_529.csv_plot.pdf]

OTU_529.csv

Habroleptoides confusa

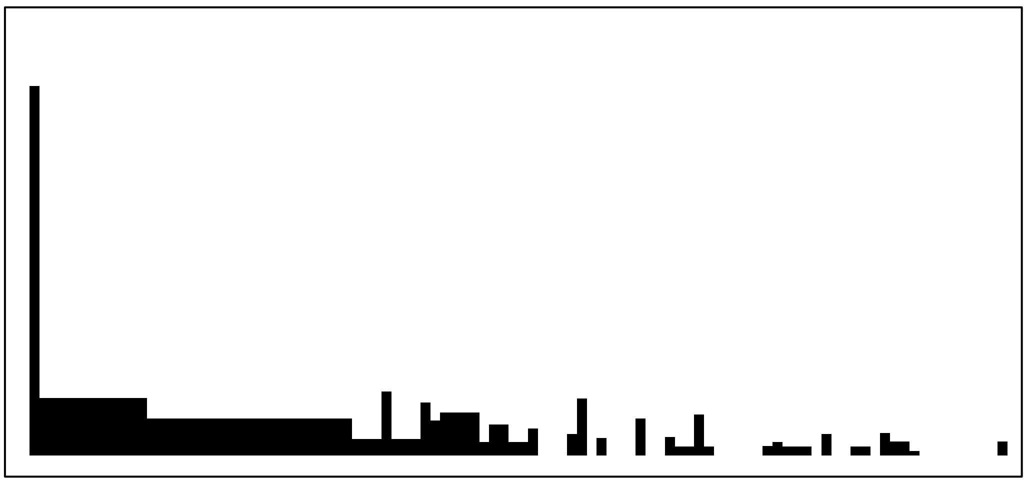

Ephemeroptera

97.32

Habroleptoides confusa voucher BYU:IGCEP153 16S ri	GQ118289	97.32	98.68	1.70945e−63	Ephemeroptera		Habroleptoides confusa
Ephoron shigae mitochondrial gene for 16S ribosoma	AB874306	82.69	68.21	5.59811e−19	Ephemeroptera	Polymitarcyidae	Ephoron shigae
Ephoron shigae mitochondrial gene for 16S ribosoma	AB874293	82.69	68.21	5.59811e−19	Ephemeroptera	Polymitarcyidae	Ephoron shigae
Ephoron shigae mitochondrial gene for 16S ribosoma	AB874289	82.69	68.21	5.59811e−19	Ephemeroptera	Polymitarcyidae	Ephoron shigae
Ephoron shigae mitochondrial gene for 16S ribosoma	AB874283	82.69	68.21	5.59811e−19	Ephemeroptera	Polymitarcyidae	Ephoron shigae
Ephoron shigae mitochondrial gene for 16S ribosoma	AB874278	82.69	68.21	5.59811e−19	Ephemeroptera	Polymitarcyidae	Ephoron shigae
Ephoron shigae mitochondrial gene for 16S rRNA, pa	AB711533	82.69	68.21	5.59811e−19	Ephemeroptera	Polymitarcyidae	Ephoron shigae
Ephoron shigae mitochondrial gene for 16S rRNA, pa	AB711527	82.69	68.21	5.59811e−19	Ephemeroptera	Polymitarcyidae	Ephoron shigae
Ephoron shigae mitochondrial gene for 16S rRNA, pa	AB711523	82.69	68.21	5.59811e−19	Ephemeroptera	Polymitarcyidae	Ephoron shigae
Ephoron shigae mitochondrial gene for 16S rRNA, pa	AB711500	82.69	68.21	5.59811e−19	Ephemeroptera	Polymitarcyidae	Ephoron shigae
Ephoron shigae mitochondrial gene for 16S rRNA, pa	AB711493	82.69	68.21	5.59811e−19	Ephemeroptera	Polymitarcyidae	Ephoron shigae
Ephoron shigae mitochondrial gene for 16S rRNA, pa	AB711471	82.69	68.21	5.59811e−19	Ephemeroptera	Polymitarcyidae	Ephoron shigae
Ephoron shigae mitochondrial gene for 16S ribosoma	AB874305	81.73	68.21	2.38038e−17	Ephemeroptera	Polymitarcyidae	Ephoron shigae
Ephoron shigae mitochondrial gene for 16S ribosoma	AB874303	81.73	68.21	2.38038e−17	Ephemeroptera	Polymitarcyidae	Ephoron shigae
Ephoron shigae mitochondrial gene for 16S ribosoma	AB874302	81.73	68.21	2.38038e−17	Ephemeroptera	Polymitarcyidae	Ephoron shigae
Ephoron shigae mitochondrial gene for 16S ribosoma	AB874301	81.73	68.21	2.38038e−17	Ephemeroptera	Polymitarcyidae	Ephoron shigae
Ephoron shigae mitochondrial gene for 16S ribosoma	AB874299	81.73	68.21	2.38038e−17	Ephemeroptera	Polymitarcyidae	Ephoron shigae
Ephoron shigae mitochondrial gene for 16S ribosoma	AB874288	81.73	68.21	2.38038e−17	Ephemeroptera	Polymitarcyidae	Ephoron shigae
Ephoron shigae mitochondrial gene for 16S ribosoma	AB874284	81.73	68.21	2.38038e−17	Ephemeroptera	Polymitarcyidae	Ephoron shigae
Ephoron shigae mitochondrial gene for 16S ribosoma	AB874279	81.73	68.21	2.38038e−17	Ephemeroptera	Polymitarcyidae	Ephoron shigae
Ephoron shigae mitochondrial gene for 16S ribosoma	AB874277	81.73	68.21	2.38038e−17	Ephemeroptera	Polymitarcyidae	Ephoron shigae
Ephoron shigae mitochondrial gene for 16S rRNA, pa	AB711590	81.73	68.21	2.38038e−17	Ephemeroptera	Polymitarcyidae	Ephoron shigae
Ephoron shigae mitochondrial gene for 16S rRNA, pa	AB711582	81.73	68.21	2.38038e−17	Ephemeroptera	Polymitarcyidae	Ephoron shigae
Ephoron shigae mitochondrial gene for 16S rRNA, pa	AB711581	81.73	68.21	2.38038e−17	Ephemeroptera	Polymitarcyidae	Ephoron shigae
Ephoron shigae mitochondrial gene for 16S rRNA, pa	AB711575	81.73	68.21	2.38038e−17	Ephemeroptera	Polymitarcyidae	Ephoron shigae
Ephoron shigae mitochondrial gene for 16S rRNA, pa	AB711531	81.73	68.21	2.38038e−17	Ephemeroptera	Polymitarcyidae	Ephoron shigae
Ephoron shigae mitochondrial gene for 16S rRNA, pa	AB711530	81.73	68.21	2.38038e−17	Ephemeroptera	Polymitarcyidae	Ephoron shigae
Ephoron shigae mitochondrial gene for 16S rRNA, pa	AB711528	81.73	68.21	2.38038e−17	Ephemeroptera	Polymitarcyidae	Ephoron shigae
Ephoron shigae mitochondrial gene for 16S rRNA, pa	AB711521	81.73	68.21	2.38038e−17	Ephemeroptera	Polymitarcyidae	Ephoron shigae
Ephoron shigae mitochondrial gene for 16S rRNA, pa	AB711514	81.73	68.21	2.38038e−17	Ephemeroptera	Polymitarcyidae	Ephoron shigae
Ephoron shigae mitochondrial gene for 16S rRNA, pa	AB711501	81.73	68.21	2.38038e−17	Ephemeroptera	Polymitarcyidae	Ephoron shigae
Ephoron shigae mitochondrial gene for 16S rRNA, pa	AB711492	81.73	68.21	2.38038e−17	Ephemeroptera	Polymitarcyidae	Ephoron shigae
Ephoron shigae mitochondrial gene for 16S rRNA, pa	AB711482	81.73	68.21	2.38038e−17	Ephemeroptera	Polymitarcyidae	Ephoron shigae
Ephoron shigae mitochondrial gene for 16S ribosoma	AB874304	80.77	68.21	2.8999e−16	Ephemeroptera	Polymitarcyidae	Ephoron shigae
Ephoron shigae mitochondrial gene for 16S ribosoma	AB874300	80.77	68.21	2.8999e−16	Ephemeroptera	Polymitarcyidae	Ephoron shigae
Ephoron shigae mitochondrial gene for 16S ribosoma	AB874297	80.77	68.21	2.8999e−16	Ephemeroptera	Polymitarcyidae	Ephoron shigae
Ephoron eophilum mitochondrial gene for 16S rRNA,	AB711631	83	64.24	2.8999e−16	Ephemeroptera	Polymitarcyidae	Ephoron eophilum
Ephoron shigae mitochondrial gene for 16S rRNA, pa	AB711598	80.77	68.21	2.8999e−16	Ephemeroptera	Polymitarcyidae	Ephoron shigae
Ephoron shigae mitochondrial gene for 16S rRNA, pa	AB711532	80.77	68.21	2.8999e−16	Ephemeroptera	Polymitarcyidae	Ephoron shigae
Ephoron shigae mitochondrial gene for 16S rRNA, pa	AB711522	80.77	68.21	2.8999e−16	Ephemeroptera	Polymitarcyidae	Ephoron shigae
Penaphlebia sp. EP076 16S ribosomal RNA gene, part	AY749782	82.47	64.24	2.8999e−16	Ephemeroptera		Penaphlebia sp. EP076
Anthopotamus sp. EP022 16S ribosomal RNA gene, par	AY749766	81.63	64.24	1.01216e−15	Ephemeroptera	Potamanthidae	Anthopotamus sp. EP022
Ephoron eophilum mitochondrial gene for 16S rRNA,	AB711630	82	64.24	3.5328e−15	Ephemeroptera	Polymitarcyidae	Ephoron eophilum
Ephoron eophilum mitochondrial gene for 16S rRNA,	AB711621	82	64.24	3.5328e−15	Ephemeroptera	Polymitarcyidae	Ephoron eophilum
Ephoron eophilum mitochondrial gene for 16S rRNA,	AB711619	82	64.24	3.5328e−15	Ephemeroptera	Polymitarcyidae	Ephoron eophilum
Ephoron eophilum mitochondrial gene for 16S rRNA,	AB711615	82	64.24	3.5328e−15	Ephemeroptera	Polymitarcyidae	Ephoron eophilum
Ephemera orientalis voucher EPH01 tRNA−Leu, 16S ri	KF256907	80.61	64.24	4.30383e−14	Ephemeroptera	Ephemeridae	Ephemera orientalis
Hypattalus sp. NLG−2013 voucher COL514 12S ribosom	KC524788	81.44	62.91	4.30383e−14	Coleoptera	Melyridae	Hypattalus sp. NLG−2013
Enochrus talamanca 16S ribosomal RNA gene, partial	KC992672	81.44	62.91	4.30383e−14	Coleoptera	Hydrophilidae	Enochrus talamanca
Ephemera orientalis mitochondrion, complete genome	EU591678	80.61	64.24	4.30383e−14	Ephemeroptera	Ephemeridae	Ephemera orientalis
Anoplotrupes stercorosus voucher SK300 16S ribosom	KP250244	80.61	62.91	1.50218e−13	Coleoptera	Geotrupidae	Anoplotrupes stercorosus
Phyllobaenus testaceus voucher 7228 16S ribosomal	KC524896	81.25	62.25	1.50218e−13	Coleoptera	Cleridae	Phyllobaenus testaceus
Ephoron leukon mitochondrial gene for 16S rRNA, pa	AB711641	79.41	66.89	1.50218e−13	Ephemeroptera	Polymitarcyidae	Ephoron leukon
Ephoron album mitochondrial gene for 16S rRNA, par	AB711636	79.41	66.89	1.50218e−13	Ephemeroptera	Polymitarcyidae	Ephoron album
Ephoron album mitochondrial gene for 16S rRNA, par	AB711635	79.41	66.89	1.50218e−13	Ephemeroptera	Polymitarcyidae	Ephoron album
Ephoron eophilum mitochondrial gene for 16S rRNA,	AB711633	81	64.24	1.50218e−13	Ephemeroptera	Polymitarcyidae	Ephoron eophilum
Afronurus sp. 213BoAf mitochondrial partial 16S rR	HE651422	82.65	62.91	1.50218e−13	Ephemeroptera	Heptageniidae	Afronurus sp. 213BoAf
Hexagenia sp. BYU IGCEP003 16S ribosomal RNA gene,	GQ118286	79.41	66.89	1.50218e−13	Ephemeroptera	Ephemeridae	Hexagenia sp. BYU IGCEP003
Hercostomus brevidigitalis voucher RMBR:103445 16S	FJ808172	80.81	64.24	1.50218e−13	Diptera	Dolichopodidae	Hercostomus brevidigitalis
Paraleptophlebia vaciva 16S ribosomal RNA gene, pa	AY749759	79.38	64.24	1.50218e−13	Ephemeroptera		Paraleptophlebia vaciva
Protohermes yangi 16S ribosomal RNA gene, partial	KF944544	79.59	64.24	5.24313e−13	Megaloptera	Corydalidae	Protohermes yangi
Protohermes hunanensis 16S ribosomal RNA gene, par	KF944532	79.59	64.24	5.24313e−13	Megaloptera	Corydalidae	Protohermes hunanensis
Neptis thisbe 16S ribosomal RNA gene, partial sequ	JX185871	81.72	60.26	5.24313e−13	Lepidoptera	Nymphalidae	Neptis thisbe
Jellisonia amadoi mitochondrion, complete genome	KF322091	79.59	64.24	5.24313e−13	Siphonaptera	Ceratophyllidae	Jellisonia amadoi
Hymenoplia fulvipennis voucher DA0116 16S ribosoma	FJ956720	79.59	64.24	5.24313e−13	Coleoptera	Scarabaeidae	Hymenoplia fulvipennis
Epilachna tumida voucher KS135 16S ribosomal RNA g	KP143571	80.85	61.59	1.83003e−12	Coleoptera	Coccinellidae	Epilachna tumida
Protaetia fusca voucher 842831 16S ribosomal RNA g	JX234512	80.41	62.91	1.83003e−12	Coleoptera	Scarabaeidae	Protaetia fusca
Atrichiana placida voucher 842794 16S ribosomal RN	JX234487	80.41	62.91	1.83003e−12	Coleoptera	Scarabaeidae	Atrichiana placida
Neptis tshetverikovi 16S ribosomal RNA gene, parti	JX185894	81.91	60.26	1.83003e−12	Lepidoptera	Nymphalidae	Neptis tshetverikovi
Trypocopris sp. BMNH 793041 16S ribosomal RNA gene	JN969126	80.41	62.91	1.83003e−12	Coleoptera	Geotrupidae	Trypocopris sp. BMNH 793041
Myrmeleon bore isolate 74−boreD1 16S ribosomal RNA	KC195845	79.8	64.24	1.83003e−12	Neuroptera	Myrmeleontidae	Myrmeleon bore
Myrmeleon bore isolate 113−bore2 16S ribosomal RNA	KC195844	79.8	64.24	1.83003e−12	Neuroptera	Myrmeleontidae	Myrmeleon bore
Ephoron leukon mitochondrial gene for 16S rRNA, pa	AB711638	78.43	66.89	1.83003e−12	Ephemeroptera	Polymitarcyidae	Ephoron leukon
Ephoron leukon mitochondrial gene for 16S rRNA, pa	AB711637	78.43	66.89	1.83003e−12	Ephemeroptera	Polymitarcyidae	Ephoron leukon
Nemopterella longicornis isolate NLTS02 16S riboso	JX294150	79.8	64.24	1.83003e−12	Neuroptera	Nemopteridae	Nemopterella longicornis
Xylotrechus rusticus 16S ribosomal RNA gene, parti	JX177496	80.43	60.26	1.83003e−12	Coleoptera	Cerambycidae	Rusticoclytus rusticus
Afronurus sp. 212BoAf mitochondrial partial 16S rR	HE651421	80.61	62.91	1.83003e−12	Ephemeroptera	Heptageniidae	Afronurus sp. 212BoAf
Trox fascicularis rowei isolate 46A 16S ribosomal	EF570396	80.41	62.91	1.83003e−12	Coleoptera	Trogidae	Trox fascicularis rowei
Trox arcuatus isolate 69 16S ribosomal RNA gene, p	EF570395	80.41	62.91	1.83003e−12	Coleoptera	Trogidae	Trox arcuatus
Trox fascicularis rowei isolate 57A 16S ribosomal	EF570361	80.41	62.91	1.83003e−12	Coleoptera	Trogidae	Trox fascicularis rowei
Tachytrechus notatus voucher Pollet 0279 (P) 16S r	EU864017	79.8	64.24	1.83003e−12	Diptera	Dolichopodidae	Tachytrechus notatus
Eatonica denysae voucher BYU:IGCEP225 16S ribosoma	GQ118284	81	64.24	1.83003e−12	Ephemeroptera	Ephemeridae	Eatonica denysae
Leucochrysa robusta voucher CASC 00213 16S ribosom	DQ399281	79.8	64.24	1.83003e−12	Neuroptera	Chrysopidae	Leucochrysa robusta
Leucochrysa texana 16S ribosomal RNA gene, partial	DQ399279	79.8	64.24	1.83003e−12	Neuroptera	Chrysopidae	Leucochrysa texana
Phelotrupes laevistriatus mitochondrial gene for 1	AB178317	80.41	62.91	1.83003e−12	Coleoptera	Geotrupidae	Phelotrupes laevistriatus
Pseudiron centralis 16S ribosomal RNA gene, partia	AY749799	80.41	62.91	1.83003e−12	Ephemeroptera	Pseudironidae	Pseudiron centralis
Chaquihua sp. EP080 16S ribosomal RNA gene, partia	AY749784	79.8	64.24	1.83003e−12	Ephemeroptera	Ameletopsidae	Chaquihua sp. EP080
Epilachna cruciata voucher KS223 16S ribosomal RNA	KP143627	81.05	61.59	6.38744e−12	Coleoptera	Coccinellidae	Epilachna cruciata
Abrota ganga isolate N793 mitochondrion, complete	KF590536	80.65	60.26	6.38744e−12	Lepidoptera	Nymphalidae	Abrota ganga
Dophla evelina isolate N529 mitochondrion, complet	KF590532	80.65	60.26	6.38744e−12	Lepidoptera	Nymphalidae	Dophla evelina
Cephaloclerus sp. 1 NLG−2013 voucher 7183 16S ribo	KC524887	80.21	62.25	6.38744e−12	Coleoptera	Cleridae	Cephaloclerus sp. 1 NLG−2013
Myrmeleon inconspicuus isolate 6−incgre1 16S ribos	KC195843	79	64.24	6.38744e−12	Neuroptera	Myrmeleontidae	Myrmeleon inconspicuus
Myrmeleon inconspicuus isolate 49−inclaz 16S ribos	KC195841	79	64.24	6.38744e−12	Neuroptera	Myrmeleontidae	Myrmeleon inconspicuus
Myrmeleon inconspicuus isolate 40−inctu1 16S ribos	KC195839	79	64.24	6.38744e−12	Neuroptera	Myrmeleontidae	Myrmeleon inconspicuus
Myrmeleon inconspicuus isolate 67−gamscur 16S ribo	KC195836	79	64.24	6.38744e−12	Neuroptera	Myrmeleontidae	Myrmeleon inconspicuus
Myrmeleon inconspicuus isolate 41−incro1 16S ribos	KC195835	79	64.24	6.38744e−12	Neuroptera	Myrmeleontidae	Myrmeleon inconspicuus
Myrmeleon inconspicuus isolate 5−incgre2 16S ribos	KC195834	79	64.24	6.38744e−12	Neuroptera	Myrmeleontidae	Myrmeleon inconspicuus
Myrmeleon inconspicuus isolate 88−bouhedma 16S rib	KC195833	79	64.24	6.38744e−12	Neuroptera	Myrmeleontidae	Myrmeleon inconspicuus
Myrmeleon inconspicuus isolate 99−incsal1 16S ribo	KC195832	79	64.24	6.38744e−12	Neuroptera	Myrmeleontidae	Myrmeleon inconspicuus
Dophla evelina isolate F90 16S ribosomal RNA gene,	JQ416135	80.65	60.26	6.38744e−12	Lepidoptera	Nymphalidae	Dophla evelina
