# Supplementary material for: Testing the potential of a ribosomal 16S marker for DNA metabarcoding of insects
Source: PeerJ. 2016 Apr 19;4:e1966. doi: 10.7717/peerj.1966 (PMC4841222; doi:10.7717/peerj.1966)
Supplement: Supplemental Information 1 [file OTU_577.csv_plot.pdf › scripts/5 match OTUs/blast_hits/OTU_577.csv_plot.pdf]

OTU_577.csv

Ecdyonurus dispar

Heptageniidae

Ephemeroptera

90.32

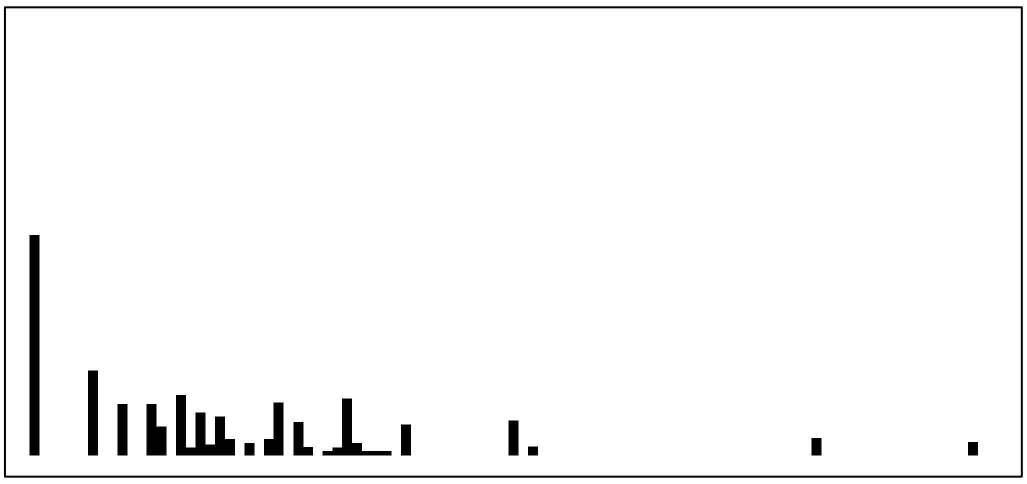

Ecdyonurus dispar 16S ribosomal RNA gene, partial	AY749775	90.32	100	5.35152e−51	Ephemeroptera	Heptageniidae	Ecdyonurus dispar
Afronurus sp. 212BoAf mitochondrial partial 16S rR	HE651421	78.06	100	6.11148e−25	Ephemeroptera	Heptageniidae	Afronurus sp. 212BoAf
Parafronurus youi mitochondrion, complete genome	EU349015	78.57	99.35	6.11148e−25	Ephemeroptera	Heptageniidae	Parafronurus youi
Pseudiron centralis 16S ribosomal RNA gene, parti	AY749799	78.26	88.89	3.85676e−21	Ephemeroptera	Pseudironidae	Pseudiron centralis
Heptagenia sulphurea mitochondrial partial 16S rRN	HE651431	78.42	88.89	1.34614e−20	Ephemeroptera	Heptageniidae	Heptagenia sulphurea
Clioperla clio 16S ribosomal RNA gene, partial seq	EF623237	75.64	100	1.34614e−20	Plecoptera	Perlodidae	Clioperla clio
Afronurus peringueyi 16S ribosomal RNA gene, parti	AY749790	83.96	67.97	1.34614e−20	Ephemeroptera	Heptageniidae	Afronurus peringueyi
Siphlonurus binotatus mitochondrial gene for 16S r	AB110269	77.54	88.89	4.6985e−20	Ephemeroptera	Siphlonuridae	Siphlonurus binotatus
Oniscigaster distans 16S ribosomal RNA gene, parti	AY749818	76.43	100	4.6985e−20	Ephemeroptera	Oniscigastridae	Oniscigaster distans
Siphlonurus sp. MT−2014 isolate CL142 mitochondrio	KM244684	82.41	70.59	1.63994e−19	Ephemeroptera	Siphlonuridae	Siphlonurus sp. MT−2014
Atopopus tarsalis mitochondrial partial 16S rRNA g	HE651420	75.48	100	1.63994e−19	Ephemeroptera	Heptageniidae	Atopopus tarsalis
Atopopus tarsalis mitochondrial partial 16S rRNA g	HE651419	75.48	100	1.63994e−19	Ephemeroptera	Heptageniidae	Atopopus tarsalis
Siphlonurus sp. EP084 16S ribosomal RNA gene, part	AY749786	82.41	70.59	1.63994e−19	Ephemeroptera	Siphlonuridae	Siphlonurus sp. EP084
Thalerosphyrus lamuriensis mitochondrial partial 1	HE651425	81.36	75.82	5.72394e−19	Ephemeroptera	Heptageniidae	Thalerosphyrus lamuriensis
Rallidens mcfarlanei 16S ribosomal RNA gene, parti	AY749794	74.68	100	5.72394e−19	Ephemeroptera	Rallidentidae	Rallidens mcfarlanei
Afronurus sp. 213BoAf mitochondrial partial 16S rR	HE651422	82.83	64.71	1.99785e−18	Ephemeroptera	Heptageniidae	Afronurus sp. 213BoAf
Pteronarcys sachalina 16S ribosomal RNA gene, part	EF623264	80.36	73.2	1.99785e−18	Plecoptera	Pteronarcyidae	Pteronarcys sachalina
Paegniodes cupulatus mitochondrion, complete genom	HM004123	82	65.36	6.97319e−18	Ephemeroptera	Heptageniidae	Paegniodes cupulatus
Thalerosphyrus lamuriensis mitochondrial partial 1	HE651429	80.51	75.82	2.43388e−17	Ephemeroptera	Heptageniidae	Thalerosphyrus lamuriensis
Stenonema sp. EP026 16S ribosomal RNA gene, partia	AY749769	81.82	64.71	2.43388e−17	Ephemeroptera	Heptageniidae	Stenonema sp. EP026
Parameletus columbiae 16S ribosomal RNA gene, part	AY749768	80.77	67.97	2.43388e−17	Ephemeroptera	Siphlonuridae	Parameletus columbiae
Heteromallus cavicola voucher MIL 16S ribosomal RN	HM594499	78.95	72.55	8.49508e−17	Orthoptera	Rhaphidophoridae	Heteromallus cavicola
Isoperla fulva 16S ribosomal RNA gene, partial seq	EF623207	80.58	67.32	8.49508e−17	Plecoptera	Perlodidae	Isoperla fulva
Osmylus sp. EH−2004 16S ribosomal RNA gene, partia	AY620146	79.46	73.2	8.49508e−17	Neuroptera	Osmylidae	Osmylus sp. EH−2004
Perlodes mortoni 16S ribosomal RNA gene, partial s	KF855870	80.77	67.32	2.96508e−16	Plecoptera	Perlodidae	Perlodes mortoni
Afronurus sp. 338SuAf mitochondrial partial 16S rR	HE651428	82.47	63.4	2.96508e−16	Ephemeroptera	Heptageniidae	Afronurus sp. 338SuAf
Parudenus falklandicus voucher PAR 16S ribosomal R	HM594502	78.76	71.9	2.96508e−16	Orthoptera	Rhaphidophoridae	Parudenus falklandicus
Yugus bulbosus 16S ribosomal RNA gene, partial seq	EF623214	81.55	66.01	2.96508e−16	Plecoptera	Perlodidae	Yugus bulbosus
Isogenoides varians isolate BYU_PL079 16S ribosoma	EF623445	80.39	66.67	2.96508e−16	Plecoptera	Perlodidae	Isogenoides varians
Ametropus neavei 16S ribosomal RNA gene, partial s	AY749800	74.52	100	2.96508e−16	Ephemeroptera	Ametropodidae	Ametropus neavei
Afronurus sp. 297SAAf mitochondrial partial 16S rR	HE651424	80.2	66.01	1.03491e−15	Ephemeroptera	Heptageniidae	Afronurus sp. 297SAAf
Compsoneuria sp. 4 LV−2012 mitochondrial partial 1	HE651398	80.37	69.28	1.03491e−15	Ephemeroptera	Heptageniidae	Compsoneuria sp. 4 LV−2012
Osobenus yakimae 16S ribosomal RNA gene, partial s	EF623232	82.65	63.4	1.03491e−15	Plecoptera	Perlodidae	Osobenus yakimae
Isogenoides olivaceus 16S ribosomal RNA gene, part	EF623224	80.58	66.67	1.03491e−15	Plecoptera	Perlodidae	Isogenoides olivaceus
Isoperla oxylepis 16S ribosomal RNA gene, partial	EF623221	80.2	66.01	1.03491e−15	Plecoptera	Perlodidae	Isoperla oxylepis
Isoperla davisi 16S ribosomal RNA gene, partial se	EF623206	80.2	66.01	1.03491e−15	Plecoptera	Perlodidae	Isoperla davisi
Amphinemura nigritta 16S ribosomal RNA gene, parti	EF623186	80.19	68.63	1.03491e−15	Plecoptera	Nemouridae	Amphinemura nigritta
Cinygmina sp. EP133 16S ribosomal RNA gene, partia	AY749811	74.84	100	1.03491e−15	Ephemeroptera	Heptageniidae	Cinygmina sp. EP133
Afronurus sp. 336SuAf mitochondrial partial 16S rR	HE651427	81.44	63.4	3.6122e−15	Ephemeroptera	Heptageniidae	Afronurus sp. 336SuAf
Talitropsis sedilloti voucher CW160 16S ribosomal	HM594511	80	65.36	3.6122e−15	Orthoptera	Rhaphidophoridae	Talitropsis sedilloti
Dolichopoda sp. MR−2008 16S ribosomal RNA gene, pa	EU887863	79.28	71.9	3.6122e−15	Orthoptera	Rhaphidophoridae	Dolichopoda lustriae
Cosumnoperla hypocrena 16S ribosomal RNA gene, par	EF623226	74.66	94.77	3.6122e−15	Plecoptera	Perlodidae	Cosumnoperla hypocrena
Hydroperla crosbyi 16S ribosomal RNA gene, partial	EF623219	79.61	67.32	3.6122e−15	Plecoptera	Perlodidae	Hydroperla crosbyi
Udamocercia sp. BYU_PL150 16S ribosomal RNA gene,	EF623201	80	67.97	3.6122e−15	Plecoptera	Notonemouridae	Udamocercia sp. BYU_PL150
Tasmanophlebia sp. EP158 16S ribosomal RNA gene, p	AY749825	73.72	100	3.6122e−15	Ephemeroptera	Oniscigastridae	Tasmanophlebia sp. EP158
Siphlonella sp. EP083 16S ribosomal RNA gene, part	AY749785	73.72	100	3.6122e−15	Ephemeroptera	Oniscigastridae	Siphlonella sp. EP083
Chiloporter sp. EP051 16S ribosomal RNA gene, part	AY749776	80	65.36	3.6122e−15	Ephemeroptera	Ameletopsidae	Chiloporter sp. EP051
Heptagenia sp. EP018 16S ribosomal RNA gene, parti	AY749762	75.54	88.89	3.6122e−15	Ephemeroptera	Heptageniidae	Heptagenia sp. EP018
Hexagenia sp. BYU IGCEP003 16S ribosomal RNA gene,	GQ118286	78.76	72.55	1.26078e−14	Ephemeroptera	Ephemeridae	Hexagenia sp. BYU IGCEP003
Kogotus nonus 16S ribosomal RNA gene, partial sequ	EF623213	81.63	63.4	1.26078e−14	Plecoptera	Perlodidae	Kogotus nonus
Zealeuctra arnoldi 16S ribosomal RNA gene, partial	EF623180	78.18	69.93	1.26078e−14	Plecoptera	Leuctridae	Zealeuctra arnoldi
Despaxia augusta 16S ribosomal RNA gene, partial s	EF623177	80.41	63.4	1.26078e−14	Plecoptera	Leuctridae	Despaxia augusta
Plumiperla diversa 16S ribosomal RNA gene, partial	EF623132	75	94.12	1.26078e−14	Plecoptera	Chloroperlidae	Plumiperla diversa
Chaquihua sp. EP080 16S ribosomal RNA gene, partia	AY749784	78.9	71.24	1.26078e−14	Ephemeroptera	Ameletopsidae	Chaquihua sp. EP080
Thalerosphyrus sinuosus mitochondrial partial 16S	HE651430	72.9	100	4.40056e−14	Ephemeroptera	Heptageniidae	Thalerosphyrus sinuosus
Udenus wnigrum voucher MAY 16S ribosomal RNA gene,	HM594504	77.19	72.55	4.40056e−14	Orthoptera	Rhaphidophoridae	Udenus wnigrum
Udenus wnigrum voucher CER 16S ribosomal RNA gene,	HM594503	77.19	72.55	4.40056e−14	Orthoptera	Rhaphidophoridae	Udenus wnigrum
Dolichopoda pavesii voucher SPI 16S ribosomal RNA	EF216951	78.38	71.9	4.40056e−14	Orthoptera	Rhaphidophoridae	Dolichopoda pavesii
Pteronarcys scotti 16S ribosomal RNA gene, partial	EF623263	79.05	67.97	4.40056e−14	Plecoptera	Pteronarcyidae	Pteronarcys scotti
Cascadoperla trictura 16S ribosomal RNA gene, part	EF623225	79.21	66.01	4.40056e−14	Plecoptera	Perlodidae	Cascadoperla trictura
Isogenoides hansoni 16S ribosomal RNA gene, partia	EF623223	79.61	66.67	4.40056e−14	Plecoptera	Perlodidae	Isogenoides hansoni
Isogenoides zionensis 16S ribosomal RNA gene, part	EF623210	79.61	66.67	4.40056e−14	Plecoptera	Perlodidae	Isogenoides zionensis
Zapada cinctipes 16S ribosomal RNA gene, partial s	EF623183	79.05	67.97	4.40056e−14	Plecoptera	Nemouridae	Zapada cinctipes
Moselia infuscata 16S ribosomal RNA gene, partial	EF623175	80	67.97	4.40056e−14	Plecoptera	Leuctridae	Moselia infuscata
Neuroperlopsis patris 16S ribosomal RNA gene, part	EF623152	74	94.77	4.40056e−14	Plecoptera	Eustheniidae	Neuroperlopsis patris
Isogenoides doratus isolate BYU_PL131 16S ribosoma	EF623449	79.61	66.67	4.40056e−14	Plecoptera	Perlodidae	Isogenoides doratus
Isogenoides colubrinus isolate BYU_PL130 16S ribos	EF623448	79.61	66.67	4.40056e−14	Plecoptera	Perlodidae	Isogenoides colubrinus
Isogenoides elongatus isolate BYU_PL082 16S riboso	EF623447	79.61	66.67	4.40056e−14	Plecoptera	Perlodidae	Isogenoides elongatus
Isogenoides frontalis isolate BYU_PL080 16S riboso	EF623446	79.61	66.67	4.40056e−14	Plecoptera	Perlodidae	Isogenoides frontalis
Neopanorpa sp. M24899 16S ribosomal RNA gene, part	AF180059	78.07	72.55	4.40056e−14	Mecoptera	Panorpidae	Neopanorpa sp. M24899
Baumanella alameda 16S ribosomal RNA gene, partial	EF623236	79.41	66.01	1.53595e−13	Plecoptera	Perlodidae	Baumanella alameda
Perlinodes aurea 16S ribosomal RNA gene, partial s	EF623234	79.41	66.01	1.53595e−13	Plecoptera	Perlodidae	Perlinodes aurea
Susulus venastus 16S ribosomal RNA gene, partial s	EF623229	78.85	67.32	1.53595e−13	Plecoptera	Perlodidae	Susulus venastus
Isoperla phalerata 16S ribosomal RNA gene, partial	EF623208	79.38	63.4	1.53595e−13	Plecoptera	Perlodidae	Isoperla phalerata
Neofulla sp. BYU_PL152 16S ribosomal RNA gene, par	EF623203	79.38	63.4	1.53595e−13	Plecoptera	Notonemouridae	Neofulla sp. BYU_PL152
Kimminsoperla albomacula 16S ribosomal RNA gene, p	EF623202	79	65.36	1.53595e−13	Plecoptera	Notonemouridae	Kimminsoperla albomacula
Nanonemoura wahkeena 16S ribosomal RNA gene, parti	EF623198	72.55	91.5	1.53595e−13	Plecoptera	Nemouridae	Nanonemoura wahkeena
Soyedina producta 16S ribosomal RNA gene, partial	EF623196	79.38	63.4	1.53595e−13	Plecoptera	Nemouridae	Soyedina producta
Suwallia teleckojensis 16S ribosomal RNA gene, par	EF623142	73.75	100	1.53595e−13	Plecoptera	Chloroperlidae	Suwallia teleckojensis
Triznaka pintada 16S ribosomal RNA gene, partial s	EF623134	78.38	71.9	1.53595e−13	Plecoptera	Chloroperlidae	Triznaka pintada
Ichthybotus hudsoni 16S ribosomal RNA gene, partia	AY749816	80.81	64.71	1.53595e−13	Ephemeroptera	Ichthybotidae	Ichthybotus hudsoni
Compsoneuria sp. 10 LV−2012 mitochondrial partial	HE651412	78.3	69.28	5.36098e−13	Ephemeroptera	Heptageniidae	Compsoneuria sp. 10 LV−2012
Notonurus josettae mitochondrial partial 16S rRNA	HE651408	79.05	67.97	5.36098e−13	Ephemeroptera	Heptageniidae	Notonurus josettae
Compsoneuria sp. 1 LV−2012 mitochondrial partial 1	HE651407	79.05	68.63	5.36098e−13	Ephemeroptera	Heptageniidae	Compsoneuria sp. 1 LV−2012
Compsoneuria sp. 1 LV−2012 mitochondrial partial 1	HE651405	79.05	68.63	5.36098e−13	Ephemeroptera	Heptageniidae	Compsoneuria sp. 1 LV−2012
Compsoneuria sp. 1 LV−2012 mitochondrial partial 1	HE651402	79.05	68.63	5.36098e−13	Ephemeroptera	Heptageniidae	Compsoneuria sp. 1 LV−2012
Compsoneuria sp. 1 LV−2012 mitochondrial partial 1	HE651401	79.05	68.63	5.36098e−13	Ephemeroptera	Heptageniidae	Compsoneuria sp. 1 LV−2012
Compsoneuria sp. 1 LV−2012 mitochondrial partial 1	HE651400	79.05	68.63	5.36098e−13	Ephemeroptera	Heptageniidae	Compsoneuria sp. 1 LV−2012
Notonurus josettae mitochondrial partial 16S rRNA	HE651399	79.05	67.97	5.36098e−13	Ephemeroptera	Heptageniidae	Notonurus josettae
Notonemouridae sp. MD−2011 voucher ZMUC:JD−1507 16	JN615257	79.05	67.32	5.36098e−13	Plecoptera	Notonemouridae	Notonemouridae sp. MD−2011
Saga ornata isolate Sorn27 16S ribosomal RNA gene,	GU206322	76.58	72.55	5.36098e−13	Orthoptera	Tettigoniidae	Saga ornata
Macropathus filifer voucher CW301 16S ribosomal RN	HM594510	76.79	71.24	5.36098e−13	Orthoptera	Rhaphidophoridae	Macropathus filifer
Ephemera simulans voucher BYU:IGCEP038 16S ribosom	GQ118285	79.8	63.4	5.36098e−13	Ephemeroptera	Ephemeridae	Ephemera simulans
Dolichopoda giachinoi voucher ORO 16S ribosomal RN	EF216952	77.27	71.9	5.36098e−13	Orthoptera	Rhaphidophoridae	Dolichopoda giachinoi
Pteronarcella badia 16S ribosomal RNA gene, partia	EF623262	79.59	63.4	5.36098e−13	Plecoptera	Pteronarcyidae	Pteronarcella badia
Pteronarcys californica 16S ribosomal RNA gene, pa	EF623261	76.79	73.2	5.36098e−13	Plecoptera	Pteronarcyidae	Pteronarcys californica
Rickera sorpta 16S ribosomal RNA gene, partial seq	EF623235	80.61	63.4	5.36098e−13	Plecoptera	Perlodidae	Rickera sorpta
Helopicus bogaloosa 16S ribosomal RNA gene, partia	EF623228	79.59	63.4	5.36098e−13	Plecoptera	Perlodidae	Helopicus bogaloosa
Skwala americana 16S ribosomal RNA gene, partial s	EF623217	78.85	66.01	5.36098e−13	Plecoptera	Perlodidae	Skwala americana
Chernokrilus misnomus 16S ribosomal RNA gene, part	EF623209	77.88	67.97	5.36098e−13	Plecoptera	Perlodidae	Chernokrilus misnomus
