# Supplementary material for: Testing the potential of a ribosomal 16S marker for DNA metabarcoding of insects
Source: PeerJ. 2016 Apr 19;4:e1966. doi: 10.7717/peerj.1966 (PMC4841222; doi:10.7717/peerj.1966)
Supplement: Supplemental Information 1 [file peerj-04-1966-s006.zip › scripts/5 match OTUs/blast_hits/OTU_62.csv_plot.pdf]

OTU_62.csv

Habroleptoides confusa

Ephemeroptera

96.69

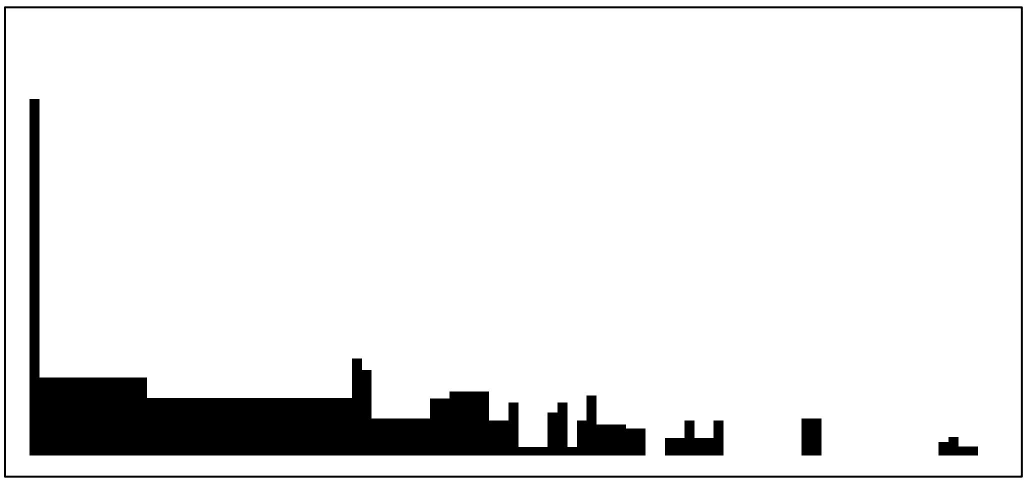

Habroleptoides confusa voucher BYU:IGCEP153 16S ri	GQ118289	96.69	100	5.96658e−63	Ephemeroptera		Habroleptoides confusa
Ephoron shigae mitochondrial gene for 16S ribosoma	AB874306	83.65	68.21	4.59521e−20	Ephemeroptera	Polymitarcyidae	Ephoron shigae
Ephoron shigae mitochondrial gene for 16S ribosoma	AB874293	83.65	68.21	4.59521e−20	Ephemeroptera	Polymitarcyidae	Ephoron shigae
Ephoron shigae mitochondrial gene for 16S ribosoma	AB874289	83.65	68.21	4.59521e−20	Ephemeroptera	Polymitarcyidae	Ephoron shigae
Ephoron shigae mitochondrial gene for 16S ribosoma	AB874283	83.65	68.21	4.59521e−20	Ephemeroptera	Polymitarcyidae	Ephoron shigae
Ephoron shigae mitochondrial gene for 16S ribosoma	AB874278	83.65	68.21	4.59521e−20	Ephemeroptera	Polymitarcyidae	Ephoron shigae
Ephoron shigae mitochondrial gene for 16S rRNA, pa	AB711533	83.65	68.21	4.59521e−20	Ephemeroptera	Polymitarcyidae	Ephoron shigae
Ephoron shigae mitochondrial gene for 16S rRNA, pa	AB711527	83.65	68.21	4.59521e−20	Ephemeroptera	Polymitarcyidae	Ephoron shigae
Ephoron shigae mitochondrial gene for 16S rRNA, pa	AB711523	83.65	68.21	4.59521e−20	Ephemeroptera	Polymitarcyidae	Ephoron shigae
Ephoron shigae mitochondrial gene for 16S rRNA, pa	AB711500	83.65	68.21	4.59521e−20	Ephemeroptera	Polymitarcyidae	Ephoron shigae
Ephoron shigae mitochondrial gene for 16S rRNA, pa	AB711493	83.65	68.21	4.59521e−20	Ephemeroptera	Polymitarcyidae	Ephoron shigae
Ephoron shigae mitochondrial gene for 16S rRNA, pa	AB711471	83.65	68.21	4.59521e−20	Ephemeroptera	Polymitarcyidae	Ephoron shigae
Ephoron shigae mitochondrial gene for 16S ribosoma	AB874305	82.69	68.21	5.59811e−19	Ephemeroptera	Polymitarcyidae	Ephoron shigae
Ephoron shigae mitochondrial gene for 16S ribosoma	AB874303	82.69	68.21	5.59811e−19	Ephemeroptera	Polymitarcyidae	Ephoron shigae
Ephoron shigae mitochondrial gene for 16S ribosoma	AB874302	82.69	68.21	5.59811e−19	Ephemeroptera	Polymitarcyidae	Ephoron shigae
Ephoron shigae mitochondrial gene for 16S ribosoma	AB874301	82.69	68.21	5.59811e−19	Ephemeroptera	Polymitarcyidae	Ephoron shigae
Ephoron shigae mitochondrial gene for 16S ribosoma	AB874299	82.69	68.21	5.59811e−19	Ephemeroptera	Polymitarcyidae	Ephoron shigae
Ephoron shigae mitochondrial gene for 16S ribosoma	AB874288	82.69	68.21	5.59811e−19	Ephemeroptera	Polymitarcyidae	Ephoron shigae
Ephoron shigae mitochondrial gene for 16S ribosoma	AB874284	82.69	68.21	5.59811e−19	Ephemeroptera	Polymitarcyidae	Ephoron shigae
Ephoron shigae mitochondrial gene for 16S ribosoma	AB874279	82.69	68.21	5.59811e−19	Ephemeroptera	Polymitarcyidae	Ephoron shigae
Ephoron shigae mitochondrial gene for 16S ribosoma	AB874277	82.69	68.21	5.59811e−19	Ephemeroptera	Polymitarcyidae	Ephoron shigae
Ephoron shigae mitochondrial gene for 16S rRNA, pa	AB711590	82.69	68.21	5.59811e−19	Ephemeroptera	Polymitarcyidae	Ephoron shigae
Ephoron shigae mitochondrial gene for 16S rRNA, pa	AB711582	82.69	68.21	5.59811e−19	Ephemeroptera	Polymitarcyidae	Ephoron shigae
Ephoron shigae mitochondrial gene for 16S rRNA, pa	AB711581	82.69	68.21	5.59811e−19	Ephemeroptera	Polymitarcyidae	Ephoron shigae
Ephoron shigae mitochondrial gene for 16S rRNA, pa	AB711575	82.69	68.21	5.59811e−19	Ephemeroptera	Polymitarcyidae	Ephoron shigae
Ephoron shigae mitochondrial gene for 16S rRNA, pa	AB711531	82.69	68.21	5.59811e−19	Ephemeroptera	Polymitarcyidae	Ephoron shigae
Ephoron shigae mitochondrial gene for 16S rRNA, pa	AB711530	82.69	68.21	5.59811e−19	Ephemeroptera	Polymitarcyidae	Ephoron shigae
Ephoron shigae mitochondrial gene for 16S rRNA, pa	AB711528	82.69	68.21	5.59811e−19	Ephemeroptera	Polymitarcyidae	Ephoron shigae
Ephoron shigae mitochondrial gene for 16S rRNA, pa	AB711521	82.69	68.21	5.59811e−19	Ephemeroptera	Polymitarcyidae	Ephoron shigae
Ephoron shigae mitochondrial gene for 16S rRNA, pa	AB711514	82.69	68.21	5.59811e−19	Ephemeroptera	Polymitarcyidae	Ephoron shigae
Ephoron shigae mitochondrial gene for 16S rRNA, pa	AB711501	82.69	68.21	5.59811e−19	Ephemeroptera	Polymitarcyidae	Ephoron shigae
Ephoron shigae mitochondrial gene for 16S rRNA, pa	AB711492	82.69	68.21	5.59811e−19	Ephemeroptera	Polymitarcyidae	Ephoron shigae
Ephoron shigae mitochondrial gene for 16S rRNA, pa	AB711482	82.69	68.21	5.59811e−19	Ephemeroptera	Polymitarcyidae	Ephoron shigae
Penaphlebia sp. EP076 16S ribosomal RNA gene, part	AY749782	84.54	64.24	5.59811e−19	Ephemeroptera		Penaphlebia sp. EP076
Ephoron eophilum mitochondrial gene for 16S rRNA,	AB711631	84	64.24	6.8199e−18	Ephemeroptera	Polymitarcyidae	Ephoron eophilum
Ephoron shigae mitochondrial gene for 16S ribosoma	AB874304	81.73	68.21	2.38038e−17	Ephemeroptera	Polymitarcyidae	Ephoron shigae
Ephoron shigae mitochondrial gene for 16S ribosoma	AB874300	81.73	68.21	2.38038e−17	Ephemeroptera	Polymitarcyidae	Ephoron shigae
Ephoron shigae mitochondrial gene for 16S ribosoma	AB874297	81.73	68.21	2.38038e−17	Ephemeroptera	Polymitarcyidae	Ephoron shigae
Ephoron shigae mitochondrial gene for 16S rRNA, pa	AB711598	81.73	68.21	2.38038e−17	Ephemeroptera	Polymitarcyidae	Ephoron shigae
Ephoron shigae mitochondrial gene for 16S rRNA, pa	AB711532	81.73	68.21	2.38038e−17	Ephemeroptera	Polymitarcyidae	Ephoron shigae
Ephoron shigae mitochondrial gene for 16S rRNA, pa	AB711522	81.73	68.21	2.38038e−17	Ephemeroptera	Polymitarcyidae	Ephoron shigae
Ephemera orientalis voucher EPH01 tRNA−Leu, 16S ri	KF256907	82.65	64.24	8.30834e−17	Ephemeroptera	Ephemeridae	Ephemera orientalis
Ephemera orientalis mitochondrion, complete genome	EU591678	82.65	64.24	8.30834e−17	Ephemeroptera	Ephemeridae	Ephemera orientalis
Ephoron eophilum mitochondrial gene for 16S rRNA,	AB711630	83	64.24	2.8999e−16	Ephemeroptera	Polymitarcyidae	Ephoron eophilum
Ephoron eophilum mitochondrial gene for 16S rRNA,	AB711621	83	64.24	2.8999e−16	Ephemeroptera	Polymitarcyidae	Ephoron eophilum
Ephoron eophilum mitochondrial gene for 16S rRNA,	AB711619	83	64.24	2.8999e−16	Ephemeroptera	Polymitarcyidae	Ephoron eophilum
Ephoron eophilum mitochondrial gene for 16S rRNA,	AB711615	83	64.24	2.8999e−16	Ephemeroptera	Polymitarcyidae	Ephoron eophilum
Jellisonia amadoi mitochondrion, complete genome	KF322091	81.63	64.24	1.01216e−15	Siphonaptera	Ceratophyllidae	Jellisonia amadoi
Anthopotamus sp. EP022 16S ribosomal RNA gene, par	AY749766	81.63	64.24	1.01216e−15	Ephemeroptera	Potamanthidae	Anthopotamus sp. EP022
Atrichiana placida voucher 842794 16S ribosomal RN	JX234487	82.47	62.91	3.5328e−15	Coleoptera	Scarabaeidae	Atrichiana placida
Ephoron leukon mitochondrial gene for 16S rRNA, pa	AB711641	80.39	66.89	3.5328e−15	Ephemeroptera	Polymitarcyidae	Ephoron leukon
Ephoron album mitochondrial gene for 16S rRNA, par	AB711636	80.39	66.89	3.5328e−15	Ephemeroptera	Polymitarcyidae	Ephoron album
Ephoron album mitochondrial gene for 16S rRNA, par	AB711635	80.39	66.89	3.5328e−15	Ephemeroptera	Polymitarcyidae	Ephoron album
Ephoron eophilum mitochondrial gene for 16S rRNA,	AB711633	82	64.24	3.5328e−15	Ephemeroptera	Polymitarcyidae	Ephoron eophilum
Trox arcuatus isolate 69 16S ribosomal RNA gene, p	EF570395	82.47	62.91	3.5328e−15	Coleoptera	Trogidae	Trox arcuatus
Hexagenia sp. BYU IGCEP003 16S ribosomal RNA gene,	GQ118286	80.39	66.89	3.5328e−15	Ephemeroptera	Ephemeridae	Hexagenia sp. BYU IGCEP003
Habrophlebiodes zijinensis mitochondrion, partial	GU936203	81.63	64.24	1.23307e−14	Ephemeroptera		Habrophlebiodes zijinensis
Eresia perilla isolate 38−9 16S ribosomal RNA gene	AF186906	82.8	60.26	1.23307e−14	Lepidoptera	Nymphalidae	Castilia perilla
Hypattalus sp. NLG−2013 voucher COL514 12S ribosom	KC524788	81.44	62.91	4.30383e−14	Coleoptera	Melyridae	Hypattalus sp. NLG−2013
Enochrus talamanca 16S ribosomal RNA gene, partial	KC992672	81.44	62.91	4.30383e−14	Coleoptera	Hydrophilidae	Enochrus talamanca
Afronurus sp. 212BoAf mitochondrial partial 16S rR	HE651421	81.44	62.91	4.30383e−14	Ephemeroptera	Heptageniidae	Afronurus sp. 212BoAf
Phyllobaenus testaceus voucher 7228 16S ribosomal	KC524896	81.25	62.25	1.50218e−13	Coleoptera	Cleridae	Phyllobaenus testaceus
Cephaloclerus sp. 1 NLG−2013 voucher 7183 16S ribo	KC524887	81.25	62.25	1.50218e−13	Coleoptera	Cleridae	Cephaloclerus sp. 1 NLG−2013
Ephoron leukon mitochondrial gene for 16S rRNA, pa	AB711638	79.41	66.89	1.50218e−13	Ephemeroptera	Polymitarcyidae	Ephoron leukon
Ephoron leukon mitochondrial gene for 16S rRNA, pa	AB711637	79.41	66.89	1.50218e−13	Ephemeroptera	Polymitarcyidae	Ephoron leukon
Nemopterella longicornis isolate NLTS02 16S riboso	JX294150	80.81	64.24	1.50218e−13	Neuroptera	Nemopteridae	Nemopterella longicornis
Tachytrechus notatus voucher Pollet 0279 (P) 16S r	EU864017	80.81	64.24	1.50218e−13	Diptera	Dolichopodidae	Tachytrechus notatus
Ephemerella atagosana 16S ribosomal RNA gene, part	FJ443039	81.63	64.24	1.50218e−13	Ephemeroptera	Ephemerellidae	Ephemerella atagosana
Hercostomus brevidigitalis voucher RMBR:103445 16S	FJ808172	80.81	64.24	1.50218e−13	Diptera	Dolichopodidae	Hercostomus brevidigitalis
Leucochrysa texana 16S ribosomal RNA gene, partial	DQ399279	80.81	64.24	1.50218e−13	Neuroptera	Chrysopidae	Leucochrysa texana
Pseudiron centralis 16S ribosomal RNA gene, partia	AY749799	81.63	62.91	1.50218e−13	Ephemeroptera	Pseudironidae	Pseudiron centralis
Paraleptophlebia vaciva 16S ribosomal RNA gene, pa	AY749759	79.38	64.24	1.50218e−13	Ephemeroptera		Paraleptophlebia vaciva
Protohermes yangi 16S ribosomal RNA gene, partial	KF944544	79.59	64.24	5.24313e−13	Megaloptera	Corydalidae	Protohermes yangi
Protohermes sinuolatus 16S ribosomal RNA gene, par	KF944540	79.59	64.24	5.24313e−13	Megaloptera	Corydalidae	Protohermes sinuolatus
Protohermes hunanensis 16S ribosomal RNA gene, par	KF944532	79.59	64.24	5.24313e−13	Megaloptera	Corydalidae	Protohermes hunanensis
Platyneuromus sorror 16S ribosomal RNA gene, parti	KF944516	79.59	64.24	5.24313e−13	Megaloptera	Corydalidae	Platyneuromus sorror
Protohermes costalis isolate CAU00586 16S ribosoma	KF741309	79.59	64.24	5.24313e−13	Megaloptera	Corydalidae	Protohermes costalis
Protohermes costalis isolate CAU00209 16S ribosoma	KF741305	79.59	64.24	5.24313e−13	Megaloptera	Corydalidae	Protohermes costalis
Ephemera danica 16S ribosomal RNA gene, partial se	KF855860	79.59	64.24	5.24313e−13	Ephemeroptera	Ephemeridae	Ephemera danica
Abrota ganga isolate N793 mitochondrion, complete	KF590536	81.72	60.26	5.24313e−13	Lepidoptera	Nymphalidae	Abrota ganga
Neptis thisbe 16S ribosomal RNA gene, partial sequ	JX185871	81.72	60.26	5.24313e−13	Lepidoptera	Nymphalidae	Neptis thisbe
Myrmeleon inconspicuus isolate 6−incgre1 16S ribos	KC195843	80	64.24	5.24313e−13	Neuroptera	Myrmeleontidae	Myrmeleon inconspicuus
Myrmeleon inconspicuus isolate 49−inclaz 16S ribos	KC195841	80	64.24	5.24313e−13	Neuroptera	Myrmeleontidae	Myrmeleon inconspicuus
Myrmeleon inconspicuus isolate 40−inctu1 16S ribos	KC195839	80	64.24	5.24313e−13	Neuroptera	Myrmeleontidae	Myrmeleon inconspicuus
Myrmeleon inconspicuus isolate 67−gamscur 16S ribo	KC195836	80	64.24	5.24313e−13	Neuroptera	Myrmeleontidae	Myrmeleon inconspicuus
Myrmeleon inconspicuus isolate 41−incro1 16S ribos	KC195835	80	64.24	5.24313e−13	Neuroptera	Myrmeleontidae	Myrmeleon inconspicuus
Myrmeleon inconspicuus isolate 5−incgre2 16S ribos	KC195834	80	64.24	5.24313e−13	Neuroptera	Myrmeleontidae	Myrmeleon inconspicuus
Myrmeleon inconspicuus isolate 88−bouhedma 16S rib	KC195833	80	64.24	5.24313e−13	Neuroptera	Myrmeleontidae	Myrmeleon inconspicuus
Myrmeleon inconspicuus isolate 99−incsal1 16S ribo	KC195832	80	64.24	5.24313e−13	Neuroptera	Myrmeleontidae	Myrmeleon inconspicuus
Omorgus asperulatus isolate 31 16S ribosomal RNA g	EF570412	79.61	66.89	5.24313e−13	Coleoptera	Trogidae	Omorgus asperulatus
Omorgus cf. asperulatus YvdM−2007 isolate 102 16S	EF570411	79.61	66.89	5.24313e−13	Coleoptera	Trogidae	Omorgus cf. asperulatus YvdM−2007
Tachytrechus transitorius voucher Pollet 0101 (P)	EU864018	80	64.24	5.24313e−13	Diptera	Dolichopodidae	Tachytrechus transitorius
Hymenoplia fulvipennis voucher DA0116 16S ribosoma	FJ956720	79.59	64.24	5.24313e−13	Coleoptera	Scarabaeidae	Hymenoplia fulvipennis
Leptophlebiidae sp. EP030 16S ribosomal RNA gene,	AY749770	80.61	64.24	5.24313e−13	Ephemeroptera		Leptophlebiidae sp. EP030
Epilachna tumida voucher KS135 16S ribosomal RNA g	KP143571	80.85	61.59	1.83003e−12	Coleoptera	Coccinellidae	Epilachna tumida
Protaetia fusca voucher 842831 16S ribosomal RNA g	JX234512	80.41	62.91	1.83003e−12	Coleoptera	Scarabaeidae	Protaetia fusca
Trox fascicularis fascicularis isolate ffSn03 16S	KC801087	80.41	62.91	1.83003e−12	Coleoptera	Trogidae	Trox fascicularis fascicularis
Ephoron leukon mitochondrial gene for 16S rRNA, pa	AB711640	78.43	66.89	1.83003e−12	Ephemeroptera	Polymitarcyidae	Ephoron leukon
Semirhynchia sp. n. SV01 16S ribosomal RNA gene, p	JX294172	79.8	64.24	1.83003e−12	Neuroptera	Nemopteridae	Semirhynchia sp. n. SV01
Sicyoptera cuspidata isolate SCBB01 16S ribosomal	JX294167	79.8	64.24	1.83003e−12	Neuroptera	Nemopteridae	Sicyoptera cuspidata
