# Supplementary material for: Testing the potential of a ribosomal 16S marker for DNA metabarcoding of insects
Source: PeerJ. 2016 Apr 19;4:e1966. doi: 10.7717/peerj.1966 (PMC4841222; doi:10.7717/peerj.1966)
Supplement: Supplemental Information 1 [file peerj-04-1966-s006.zip › scripts/5 match OTUs/blast_hits/OTU_623.csv_plot.pdf]

OTU_623.csv

Parochlus steinenii

Chironomidae

Diptera

87.82

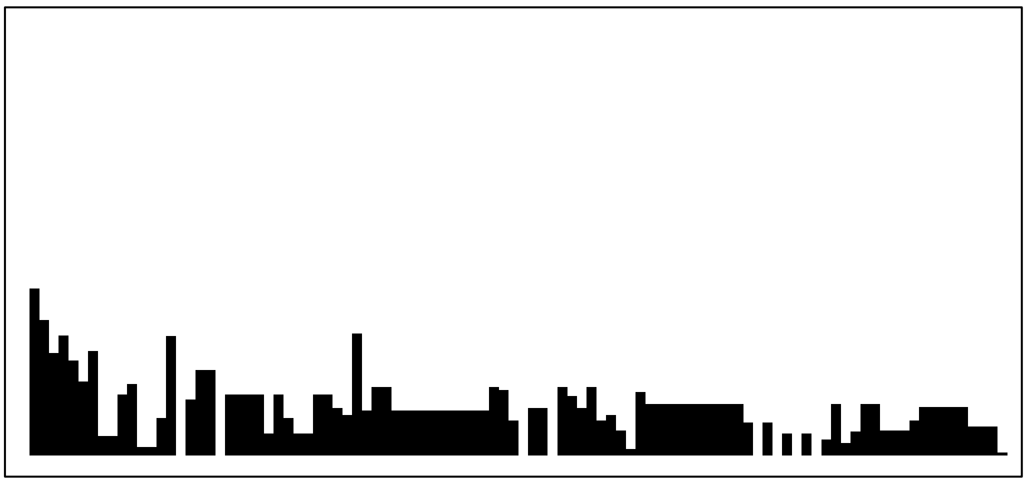

Parochlus steinenii isolate Sejong01 mitochondrion	KT003702	87.82	100	1.20872e−46	Diptera	Chironomidae	Parochlus steinenii
Bolitophila sp. ER−2009 16S ribosomal RNA gene, pa	FJ172021	86.33	87.82	3.70077e−34	Diptera	Mycetophilidae	Bolitophila sp. ER−2009
Bolitophila occlusa voucher JSB2 16S ribosomal RNA	KJ136713	84.78	87.82	4.50846e−33	Diptera	Mycetophilidae	Bolitophila occlusa
Bolitophila cinerea voucher JSB1 16S ribosomal RNA	KJ136712	85.61	87.82	4.50846e−33	Diptera	Mycetophilidae	Bolitophila cinerea
Edwardsina gigantea 16S ribosomal RNA gene, partia	KC177460	84.44	86.54	1.57361e−32	Diptera	Blephariceridae	Edwardsina gigantea
Pedicia albivitta voucher O'Grady 200012 16S ribos	EU005435	83.45	89.1	5.49242e−32	Diptera	Pediciidae	Pedicia albivitta
Bolitophila hybrida 16S ribosomal RNA gene, partia	FJ172002	84.89	87.82	1.91704e−31	Diptera	Mycetophilidae	Bolitophila hybrida
Tipula abdominalis 16S ribosomal RNA gene, partial	KC177457	80.89	100	2.33544e−30	Diptera	Tipulidae	Tipula abdominalis
Tipula abdominalis mitochondrion, partial genome	JN861743	80.89	100	2.33544e−30	Diptera	Tipulidae	Tipula abdominalis
Tipula sp. PMO 200011 16S ribosomal RNA gene, part	EU005437	82.86	89.1	2.84515e−29	Diptera	Tipulidae	Tipula sp. PMO 200011
Arachnocampa luminosa biotype 1 16S large subunit	AY576329	83.33	87.82	2.84515e−29	Diptera	Keroplatidae	Arachnocampa luminosa
Sylvicola fenestralis 16S ribosomal RNA gene, part	KC177461	80.38	100	9.93054e−29	Diptera	Anisopodidae	Sylvicola fenestralis
Sylvicola fenestralis mitochondrion, complete geno	JN861752	80.38	100	9.93054e−29	Diptera	Anisopodidae	Sylvicola fenestralis
Callomyia admirabilis isolate CA42 16S ribosomal R	KC699326	81.75	87.82	3.4661e−28	Diptera	Platypezidae	Callomyia admirabilis
Hercostomus meieri voucher RMBR:103442 16S ribosom	FJ808173	85.6	79.49	3.4661e−28	Diptera	Dolichopodidae	Hercostomus meieri
Empis producta voucher RMBR:103430 16S ribosomal R	FJ808167	79.49	99.36	3.4661e−28	Diptera	Empididae	Empis producta
Arachnocampa luminosa biotype 3 16S large subunit	AY576331	82.61	87.82	3.4661e−28	Diptera	Keroplatidae	Arachnocampa luminosa
Simulium tenederoi mitochondrial gene for 16S ribo	LC035002	84	79.49	1.20979e−27	Diptera	Simuliidae	Simulium tenederoi
Simulium tenederoi mitochondrial gene for 16S ribo	LC035001	84	79.49	1.20979e−27	Diptera	Simuliidae	Simulium tenederoi
Dichopygina aculeata voucher 947 16S ribosomal RNA	JQ613961	79.74	97.44	1.20979e−27	Diptera	Sciaridae	Dichopygina aculeata
Simulium sheilae isolate SH1B 16S ribosomal RNA ge	KM410213	82.84	85.26	4.22257e−27	Diptera	Simuliidae	Simulium sheilae
Simulium roslihashimi isolate R1B 16S ribosomal RN	KM410192	82.84	85.26	4.22257e−27	Diptera	Simuliidae	Simulium roslihashimi
Simulium izuae isolate I3B 16S ribosomal RNA gene,	KM410191	82.84	85.26	4.22257e−27	Diptera	Simuliidae	Simulium izuae
Simulium izuae isolate I2B 16S ribosomal RNA gene,	KM410190	82.84	85.26	4.22257e−27	Diptera	Simuliidae	Simulium izuae
Haematopota pluvialis 16S ribosomal RNA gene, part	KC177454	81.02	87.82	4.22257e−27	Diptera	Tabanidae	Haematopota pluvialis
Simulium tuenense mitochondrial gene for 16S ribos	AB591244	82.84	85.26	4.22257e−27	Diptera	Simuliidae	Simulium tuenense
Beris morrisii 16S ribosomal RNA gene, partial seq	GU947373	81.75	87.82	4.22257e−27	Diptera	Stratiomyidae	Beris morrisii
Chrysops viduatus 16S ribosomal RNA gene, partial	HM132115	81.02	87.82	4.22257e−27	Diptera	Tabanidae	Chrysops viduatus
Tabanus bromius 16S ribosomal RNA gene, partial se	HM132111	81.02	87.82	4.22257e−27	Diptera	Tabanidae	Tabanus bromius
Simulium inthanonense 16S ribosomal RNA gene, part	GQ865995	82.84	85.26	4.22257e−27	Diptera	Simuliidae	Simulium inthanonense
Simulium cabrerai mitochondrial gene for 16S rRNA,	AB093128	82.84	85.26	4.22257e−27	Diptera	Simuliidae	Simulium cabrerai
Tongamya stuckenbergi 16S ribosomal RNA gene, part	KM878969	82.22	85.26	1.47382e−26	Diptera	Mydidae	Tongamya stuckenbergi
Callomyia amoena isolate CA06 16S ribosomal RNA ge	KC699325	81.88	87.82	1.47382e−26	Diptera	Platypezidae	Callomyia amoena
Paracladura trichoptera mitochondrion, complete ge	JN861751	85.71	75.64	1.47382e−26	Diptera	Trichoceridae	Paracladura trichoptera
Simulium suyoense mitochondrial gene for 16S ribos	LC035000	82.09	85.26	5.14415e−26	Diptera	Simuliidae	Simulium suyoense
Simulium claveriaense mitochondrial gene for 16S r	LC034988	83.2	79.49	5.14415e−26	Diptera	Simuliidae	Simulium claveriaense
Simulium claveriaense mitochondrial gene for 16S r	LC034987	83.2	79.49	5.14415e−26	Diptera	Simuliidae	Simulium claveriaense
Simulium sheilae isolate SH2B 16S ribosomal RNA ge	KM410214	82.09	85.26	5.14415e−26	Diptera	Simuliidae	Simulium sheilae
Simulium brinchangense isolate B2B 16S ribosomal R	KM410202	82.09	85.26	5.14415e−26	Diptera	Simuliidae	Simulium brinchangense
Simulium brinchangense isolate B1B 16S ribosomal R	KM410201	82.09	85.26	5.14415e−26	Diptera	Simuliidae	Simulium brinchangense
Simulium tanahrataense isolate TR2B 16S ribosomal	KM410196	82.09	85.26	5.14415e−26	Diptera	Simuliidae	Simulium tanahrataense
Simulium tanahrataense isolate TR1B 16S ribosomal	KM410195	82.09	85.26	5.14415e−26	Diptera	Simuliidae	Simulium tanahrataense
Simulium izuae isolate I1B 16S ribosomal RNA gene,	KM410189	82.09	85.26	5.14415e−26	Diptera	Simuliidae	Simulium izuae
Cyphomyia notabilis 16S ribosomal RNA gene, partia	GU947403	82.09	85.26	5.14415e−26	Diptera	Stratiomyidae	Cyphomyia notabilis
Simulium chumpornense 16S ribosomal RNA gene, part	GQ865998	82.09	85.26	5.14415e−26	Diptera	Simuliidae	Simulium chumpornense
Simulium decuplum 16S ribosomal RNA gene, partial	GQ865986	82.09	85.26	5.14415e−26	Diptera	Simuliidae	Simulium decuplum
Simulium recurvum mitochondrial gene for 16S ribos	AB334097	82.09	85.26	5.14415e−26	Diptera	Simuliidae	Simulium recurvum
Simulium claveriaense mitochondrial gene for 16S r	AB334096	83.2	79.49	5.14415e−26	Diptera	Simuliidae	Simulium claveriaense
Simulium tuyense mitochondrial gene for 16S riboso	LC035004	83.06	78.85	1.79548e−25	Diptera	Simuliidae	Simulium tuyense
Simulium spinosibranchium mitochondrial gene for 1	LC034996	81.62	85.26	1.79548e−25	Diptera	Simuliidae	Simulium spinosibranchium
Microsania collarti isolate CAN8 16S ribosomal RNA	KC699334	78.21	99.36	1.79548e−25	Diptera	Platypezidae	Microsania collarti
Simulium khunklangense mitochondrial gene for 16S	AB820367	82.22	85.26	1.79548e−25	Diptera	Simuliidae	Simulium khunklangense
Simulium chomthongense mitochondrial gene for 16S	AB699899	82.22	85.26	1.79548e−25	Diptera	Simuliidae	Simulium chomthongense
Chorisops tunisiae 16S ribosomal RNA gene, partial	GU947379	78.34	99.36	1.79548e−25	Diptera	Stratiomyidae	Chorisops tunisiae
Poecilobothrus principalis voucher Pollet 0198 (P)	EU864010	83.2	79.49	1.79548e−25	Diptera	Dolichopodidae	Poecilobothrus principalis
Gymnopternus blankaartensis voucher Pollet 0090 (P	EU863973	82.79	78.21	1.79548e−25	Diptera	Dolichopodidae	Gymnopternus blankaartensis
Simulium caudisclerum 16S ribosomal RNA gene, part	GQ865999	82.22	85.26	1.79548e−25	Diptera	Simuliidae	Simulium caudisclerum
Leptomorphus walkeri 16S ribosomal RNA gene, parti	FJ172020	83.19	76.28	1.79548e−25	Diptera	Mycetophilidae	Leptomorphus walkeri
Simulium spinosibranchium mitochondrial gene for 1	AB334098	81.62	85.26	1.79548e−25	Diptera	Simuliidae	Simulium spinosibranchium
Somatia sp. YSUW04038 16S ribosomal RNA gene, part	AY573134	81.89	81.41	1.79548e−25	Diptera	Somatiidae	Somatia sp. YSUW04038
Arachnocampa luminosa biotype 2 16S large subunit	AY576330	81.16	87.82	1.79548e−25	Diptera	Keroplatidae	Arachnocampa luminosa
Coelopa alluaudi 16S ribosomal RNA gene, partial s	AF403455	80.29	87.82	1.79548e−25	Diptera	Coelopidae	Coelopa alluaudi
Simulium palauense mitochondrial gene for 16S ribo	AB056742	82.96	85.26	1.79548e−25	Diptera	Simuliidae	Simulium palauense
Simulium nobile isolate N5 16S ribosomal RNA gene,	KP661530	82.4	79.49	6.26685e−25	Diptera	Simuliidae	Simulium nobile
Simulium nobile isolate N4 16S ribosomal RNA gene,	KP661529	82.4	79.49	6.26685e−25	Diptera	Simuliidae	Simulium nobile
Simulium nobile isolate N3 16S ribosomal RNA gene,	KP661528	82.4	79.49	6.26685e−25	Diptera	Simuliidae	Simulium nobile
Simulium nobile isolate N2 16S ribosomal RNA gene,	KP661527	82.4	79.49	6.26685e−25	Diptera	Simuliidae	Simulium nobile
Simulium nobile isolate N1 16S ribosomal RNA gene,	KP661526	82.4	79.49	6.26685e−25	Diptera	Simuliidae	Simulium nobile
Simulium timorense isolate TM5 16S ribosomal RNA g	KP661525	82.4	79.49	6.26685e−25	Diptera	Simuliidae	Simulium timorense
Simulium timorense isolate TM4 16S ribosomal RNA g	KP661524	82.4	79.49	6.26685e−25	Diptera	Simuliidae	Simulium timorense
Simulium timorense isolate TM3 16S ribosomal RNA g	KP661523	82.4	79.49	6.26685e−25	Diptera	Simuliidae	Simulium timorense
Simulium timorense isolate TM2 16S ribosomal RNA g	KP661522	82.4	79.49	6.26685e−25	Diptera	Simuliidae	Simulium timorense
Simulium timorense isolate TM1 16S ribosomal RNA g	KP661521	82.4	79.49	6.26685e−25	Diptera	Simuliidae	Simulium timorense
Diadocidia stanfordensis voucher JSSJ14 16S riboso	KJ136742	81.54	82.69	6.26685e−25	Diptera	Diadocidiidae	Diadocidia stanfordensis
Liriomyza sativae mitochondrion, complete genome	JQ862475	77.71	99.36	6.26685e−25	Diptera	Agromyzidae	Liriomyza sativae
Keilbachia subacumina voucher 890 16S ribosomal RN	JQ613951	81.54	82.05	6.26685e−25	Diptera	Sciaridae	Keilbachia subacumina
Trichopeza longicornis voucher Pollet 0243 (P) 16S	HQ449035	78.98	99.36	6.26685e−25	Diptera	Empididae	Trichopeza longicornis
Cramptonomyia spenceri mitochondrion, complete gen	JN861747	81.02	86.54	6.26685e−25	Diptera	Pachyneuridae	Cramptonomyia spenceri
Ptychoptera sp. ATB−2011 mitochondrion, complete g	JN861744	80	89.1	6.26685e−25	Diptera	Ptychopteridae	Ptychoptera sp. ATB−2011
Musca ventrosa 16S ribosomal RNA gene, partial seq	JN226633	81.02	87.18	6.26685e−25	Diptera	Muscidae	Musca ventrosa
Liriomyza sativae mitochondrion, complete genome	HQ333260	77.71	99.36	6.26685e−25	Diptera	Agromyzidae	Liriomyza sativae
Pachygaster atra 16S ribosomal RNA gene, partial s	GU947397	80.74	86.54	6.26685e−25	Diptera	Stratiomyidae	Pachygaster atra
Simulium nobile 16S ribosomal RNA gene, partial se	GQ866008	82.4	79.49	6.26685e−25	Diptera	Simuliidae	Simulium nobile
Simulium chiharuae mitochondrial gene for 16S ribo	AB334092	80.58	87.82	6.26685e−25	Diptera	Simuliidae	Simulium chiharuae
Caenophthalmus capensis voucher MEI 120162 16S rib	DQ250838	81.12	89.1	6.26685e−25	Diptera	Therevidae	Caenophthalmus capensis
Simulium nobile mitochondrial gene for 16S rRNA, p	AB093115	82.4	79.49	6.26685e−25	Diptera	Simuliidae	Simulium nobile
Simulium baltazarae mitochondrial gene for 16S rRN	AB093114	82.4	79.49	6.26685e−25	Diptera	Simuliidae	Simulium baltazarae
Simulium mie mitochondrial gene for 16S ribosomal	AB056732	81.16	86.54	6.26685e−25	Diptera	Simuliidae	Simulium mie
Simulium mie mitochondrial gene for 16S ribosomal	AB056731	81.16	86.54	6.26685e−25	Diptera	Simuliidae	Simulium mie
Simulium mie mitochondrial gene for 16S ribosomal	AB056730	81.16	86.54	6.26685e−25	Diptera	Simuliidae	Simulium mie
Cnephia dacotensis mitochondrial 16S ribosomal RNA	L13403	81.62	85.26	6.26685e−25	Diptera	Simuliidae	Cnephia dacotensis
Simulium tuyense mitochondrial gene for 16S riboso	LC035008	82.26	78.85	2.18735e−24	Diptera	Simuliidae	Simulium tuyense
Simulium tuyense mitochondrial gene for 16S riboso	LC035007	82.26	78.85	2.18735e−24	Diptera	Simuliidae	Simulium tuyense
Simulium tuyense mitochondrial gene for 16S riboso	LC035006	82.26	78.85	2.18735e−24	Diptera	Simuliidae	Simulium tuyense
Simulium tuyense mitochondrial gene for 16S riboso	LC035005	82.26	78.85	2.18735e−24	Diptera	Simuliidae	Simulium tuyense
Simulium tuyense mitochondrial gene for 16S riboso	LC035003	82.26	78.85	2.18735e−24	Diptera	Simuliidae	Simulium tuyense
Simulium suyoense mitochondrial gene for 16S ribos	LC034999	81.34	85.26	2.18735e−24	Diptera	Simuliidae	Simulium suyoense
Simulium suyoense mitochondrial gene for 16S ribos	LC034998	81.34	85.26	2.18735e−24	Diptera	Simuliidae	Simulium suyoense
Simulium suyoense mitochondrial gene for 16S ribos	LC034997	81.34	85.26	2.18735e−24	Diptera	Simuliidae	Simulium suyoense
Corynoptera boletiphaga voucher 995 16S ribosomal	JQ613974	80.13	97.44	2.18735e−24	Diptera	Sciaridae	Corynoptera boletiphaga
