# Supplementary material for: Testing the potential of a ribosomal 16S marker for DNA metabarcoding of insects
Source: PeerJ. 2016 Apr 19;4:e1966. doi: 10.7717/peerj.1966 (PMC4841222; doi:10.7717/peerj.1966)
Supplement: Supplemental Information 1 [file peerj-04-1966-s006.zip › scripts/5 match OTUs/blast_hits/OTU_625.csv_plot.pdf]

OTU_625.csv

Pedicia albivitta

Pediciidae

Diptera

85.9

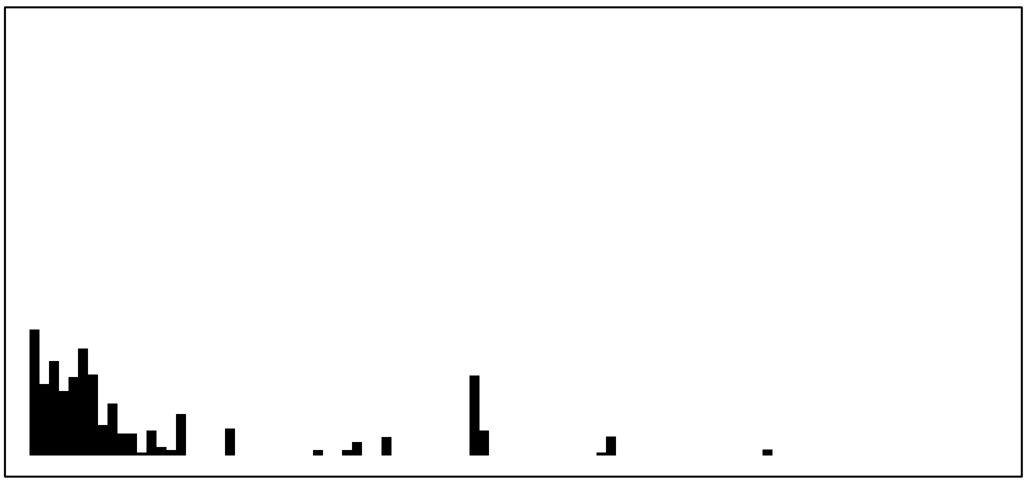

Pedicia albivitta voucher O'Grady 200012 16S ribos	EU005435	85.9	99.36	9.29262e−42	Diptera	Pediciidae	Pedicia albivitta
Edwardsina gigantea 16S ribosomal RNA gene, partia	KC177460	83.33	100	2.04684e−37	Diptera	Blephariceridae	Edwardsina gigantea
Bolitophila cinerea voucher JSB1 16S ribosomal RNA	KJ136712	84.42	97.44	7.14416e−37	Diptera	Mycetophilidae	Bolitophila cinerea
Corynoptera blanda voucher 955 16S ribosomal RNA g	JQ613965	83.01	97.44	1.06029e−34	Diptera	Sciaridae	Corynoptera blanda
Parochlus steinenii isolate Sejong01 mitochondrion	KT003702	83.67	94.23	3.70077e−34	Diptera	Chironomidae	Parochlus steinenii
Bolitophila sp. ER−2009 16S ribosomal RNA gene, pa	FJ172021	85	89.1	1.29169e−33	Diptera	Mycetophilidae	Bolitophila sp. ER−2009
Tipula sp. PMO 200011 16S ribosomal RNA gene, part	EU005437	83.78	94.23	1.29169e−33	Diptera	Tipulidae	Tipula sp. PMO 200011
Derodontus emaculatus voucher BMNH:679374 16S ribo	DQ202609	81.41	99.36	5.49242e−32	Coleoptera		Derodontus emaculatus
Eudicrana sp. DK−2013 voucher JSS12 16S ribosomal	KC435571	82.43	93.59	1.91704e−31	Diptera	Mycetophilidae	Eudicrana sp. DK−2013
Tipula abdominalis 16S ribosomal RNA gene, partial	KC177457	81.01	99.36	2.33544e−30	Diptera	Tipulidae	Tipula abdominalis
Tipula abdominalis mitochondrion, partial genome	JN861743	81.01	99.36	2.33544e−30	Diptera	Tipulidae	Tipula abdominalis
Minettia longipennis mitochondrion partial 16S rRN	AJ439184	80.13	100	2.33544e−30	Diptera	Lauxaniidae	Minettia longipennis
Phytosciara flavipes voucher JSOUT8 16S ribosomal	KJ136731	81.17	97.44	8.15148e−30	Diptera	Sciaridae	Phytosciara flavipes
Gonocephalum pusillum isolate CSU0904046030 16S ri	GQ118381	80.38	100	2.84515e−29	Coleoptera	Tenebrionidae	Gonocephalum pusillum
Culcua kolibaci 16S ribosomal RNA gene, partial se	GU947391	80.25	100	9.93054e−29	Diptera	Stratiomyidae	Culcua kolibaci
Bolitophila hybrida 16S ribosomal RNA gene, partia	FJ172016	81.94	91.67	9.93054e−29	Diptera	Mycetophilidae	Bolitophila hybrida
Drosophila funebris voucher 103952 NADH dehydrogen	EU494313	79.75	100	9.93054e−29	Diptera	Drosophilidae	Drosophila funebris
Hybos femoratus voucher Pollet 0247 (P) 16S riboso	HQ449036	79.11	100	3.4661e−28	Diptera	Hybotidae	Hybos femoratus
Cramptonomyia spenceri mitochondrion, complete gen	JN861747	79.49	99.36	3.4661e−28	Diptera	Pachyneuridae	Cramptonomyia spenceri
Ptychoptera sp. ATB−2011 mitochondrion, complete g	JN861744	79.49	99.36	3.4661e−28	Diptera	Ptychopteridae	Ptychoptera sp. ATB−2011
Bolitophila occlusa voucher JSB2 16S ribosomal RNA	KJ136713	81.25	91.67	1.20979e−27	Diptera	Mycetophilidae	Bolitophila occlusa
Lycoriella flavicornis voucher 62 16S ribosomal RN	JQ613885	79.74	97.44	1.20979e−27	Diptera	Sciaridae	Lycoriella flavicornis
Minettia flaveola 16S ribosomal RNA gene, partial	KC177448	78.85	100	1.20979e−27	Diptera	Lauxaniidae	Minettia flaveola
Hercostomus praeceps voucher Pollet 0141 (P) 16S r	EU864003	79.62	100	1.20979e−27	Diptera	Dolichopodidae	Hercostomus praeceps
Hercostomus fulvicaudis voucher Pollet 0154 (P) 16	EU863992	79.62	100	1.20979e−27	Diptera	Dolichopodidae	Hercostomus fulvicaudis
Grzegorzekia collaris 16S ribosomal RNA gene, part	FJ172016	79.74	97.44	1.20979e−27	Diptera	Mycetophilidae	Grzegorzekia collaris
Drosophila malerkotliana voucher 105504 NADH dehyd	EU494357	78.85	100	1.20979e−27	Diptera	Drosophilidae	Drosophila malerkotliana
Drosophila signata voucher 109405 NADH dehydrogena	EU494328	78.85	100	1.20979e−27	Diptera	Drosophilidae	Drosophila signata
Drosophila kepulauana voucher 109407 NADH dehydrog	EU494321	78.85	100	1.20979e−27	Diptera	Drosophilidae	Drosophila kepulauana
Panorpa takenouchii isolate 26tak 16S ribosomal RN	EF041520	80.25	99.36	1.20979e−27	Mecoptera	Panorpidae	Panorpa takenouchii
Drosophila kepulauana 16S ribosomal RNA gene, part	AF387334	78.85	100	1.20979e−27	Diptera	Drosophilidae	Drosophila kepulauana
Drosophila malerkotliana cytochrome b (cytb) gene,	AF164579	78.85	100	1.20979e−27	Diptera	Drosophilidae	Drosophila malerkotliana
Panorpa takenouchii 16S ribosomal RNA gene, partia	AF180064	80.25	99.36	1.20979e−27	Mecoptera	Panorpidae	Panorpa takenouchii
Acilius athabascae voucher 305G09/BMNH799009 16S r	KF979079	80.62	100	4.22257e−27	Coleoptera	Dytiscidae	Acilius athabascae
Phytosciara flavipes voucher 88 16S ribosomal RNA	JQ613889	79.87	97.44	4.22257e−27	Diptera	Sciaridae	Phytosciara flavipes
Ochlerotatus nipponicus isolate A75 16S ribosomal	DQ397914	79.75	100	4.22257e−27	Diptera	Culicidae	Aedes nipponicus
Arachnocampa luminosa biotype 1 16S large subunit	AY576329	80.85	90.38	4.22257e−27	Diptera	Keroplatidae	Arachnocampa luminosa
Drosophila albomicans mitochondrion, complete geno	KT119344	78.21	100	1.47382e−26	Diptera	Drosophilidae	Drosophila albomicans
Lycoriella gerbatshevskayae voucher 601 16S riboso	JQ613919	79.08	97.44	1.47382e−26	Diptera	Sciaridae	Lycoriella gerbatshevskayae
Zygoneura sciarina voucher 311 16S ribosomal RNA g	JQ613909	79.08	97.44	1.47382e−26	Diptera	Sciaridae	Zygoneura sciarina
Phytosciara semiferruginea voucher 220 16S ribosom	JQ613900	78.98	97.44	1.47382e−26	Diptera	Sciaridae	Phytosciara semiferruginea
Colobaea bifasciella voucher MU_NUS_SG3 16S riboso	JN828202	78.21	100	1.47382e−26	Diptera	Sciomyzidae	Colobaea bifasciella
Eudmeta marginata 16S ribosomal RNA gene, partial	GU947400	78.85	100	1.47382e−26	Diptera	Stratiomyidae	Eudmeta marginata
Tinda javana 16S ribosomal RNA gene, partial seque	GU947393	78.85	100	1.47382e−26	Diptera	Stratiomyidae	Tinda javana
Hercostomus verbekei voucher Pollet 0263 (P) 16S r	EU864004	78.21	100	1.47382e−26	Diptera	Dolichopodidae	Hercostomus verbekei
Leptomorphus walkeri 16S ribosomal RNA gene, parti	FJ172020	83.74	78.21	1.47382e−26	Diptera	Mycetophilidae	Leptomorphus walkeri
Pyratula zonata 16S ribosomal RNA gene, partial se	FJ172004	81.16	88.46	1.47382e−26	Diptera	Keroplatidae	Pyratula zonata
Drosophila putrida voucher 103964 NADH dehydrogena	EU494348	78.21	100	1.47382e−26	Diptera	Drosophilidae	Drosophila putrida
Drosophila rubida voucher 109400 NADH dehydrogenas	EU494327	78.21	100	1.47382e−26	Diptera	Drosophilidae	Drosophila rubida
Drosophila nasuta voucher 103957 NADH dehydrogenas	EU494323	78.21	100	1.47382e−26	Diptera	Drosophilidae	Drosophila nasuta
Drosophila hypocausta voucher 103972 NADH dehydrog	EU494319	78.21	100	1.47382e−26	Diptera	Drosophilidae	Drosophila hypocausta
Drosophila albomicans voucher 109408 NADH dehydrog	EU494318	78.21	100	1.47382e−26	Diptera	Drosophilidae	Drosophila albomicans
Drosophila albomicans 16S ribosomal RNA gene, part	AF387333	78.21	100	1.47382e−26	Diptera	Drosophilidae	Drosophila albomicans
Drosophila sulfurigaster albostrigata 16S ribosoma	AF387336	78.21	100	1.47382e−26	Diptera	Drosophilidae	Drosophila sulfurigaster albostrigata
Drosophila nasuta 16S ribosomal RNA gene, partial	AF387335	78.21	100	1.47382e−26	Diptera	Drosophilidae	Drosophila nasuta
Drosophila kohkoa 16S ribosomal RNA gene, partial	AF387332	78.21	100	1.47382e−26	Diptera	Drosophilidae	Drosophila kohkoa
Hercostomus longiventris voucher Pollet 0274 (P) 1	EU863994	78.98	100	5.14415e−26	Diptera	Dolichopodidae	Hercostomus longiventris
Drosophila acanthoptera voucher 105622 NADH dehydr	EU494332	78.48	100	5.14415e−26	Diptera	Drosophilidae	Drosophila acanthoptera
Culex mimeticus 16S ribosomal RNA gene, partial se	EF593021	80.12	100	5.14415e−26	Diptera	Culicidae	Culex mimeticus
Arachnocampa tropicus biotype 6 16S large subunit	AY576335	80.88	87.18	5.14415e−26	Diptera	Keroplatidae	Arachnocampa tropicus
Gitona bivisualis 16S ribosomal RNA gene, partial	AF479793	78.34	100	5.14415e−26	Diptera	Drosophilidae	Gitona bivisualis
Platambus princeps 16S ribosomal RNA gene, partial	AY138677	78.95	97.44	5.14415e−26	Coleoptera	Dytiscidae	Platambus princeps
Bradysiopsis vittigera voucher 948 16S ribosomal R	JQ613962	79.38	97.44	1.79548e−25	Diptera	Sciaridae	Bradysiopsis vittigera
Phytosciara ussuriensis voucher 883 16S ribosomal	JQ613948	78.29	97.44	1.79548e−25	Diptera	Sciaridae	Phytosciara ussuriensis
Pseudomigadops n. sp. MSL2007 voucher 668381 16S r	EF214073	78.48	100	1.79548e−25	Coleoptera	Carabidae	Pseudomigadops n. sp. MSL2007
Pseudomigadops n. sp. MSL2007 voucher 668379 16S r	EF214072	78.48	100	1.79548e−25	Coleoptera	Carabidae	Pseudomigadops n. sp. MSL2007
Pseudomigadops n. sp. MSL2007 voucher 668338 16S r	EF214034	78.48	100	1.79548e−25	Coleoptera	Carabidae	Pseudomigadops n. sp. MSL2007
Pseudomigadops n. sp. MSL2007 voucher 668317 16S r	EF214022	78.48	100	1.79548e−25	Coleoptera	Carabidae	Pseudomigadops n. sp. MSL2007
Pseudomigadops n. sp. MSL2007 voucher 668302 16S r	EF214012	78.48	100	1.79548e−25	Coleoptera	Carabidae	Pseudomigadops n. sp. MSL2007
Pseudomigadops n. sp. MSL2007 voucher 668300 16S r	EF214010	78.48	100	1.79548e−25	Coleoptera	Carabidae	Pseudomigadops n. sp. MSL2007
Pseudomigadops n. sp. MSL2007 voucher 668299 16S r	EF214009	78.48	100	1.79548e−25	Coleoptera	Carabidae	Pseudomigadops n. sp. MSL2007
Sphenella marginata 16S ribosomal RNA gene, partia	GQ175836	77.99	100	1.79548e−25	Diptera	Tephritidae	Sphenella marginata
Pseudomigadops sp. PSM1 mitochondrial 16S rRNA gen	FM994738	78.48	100	1.79548e−25	Coleoptera	Carabidae	Pseudomigadops sp. PSM1
Pseudomigadops n. sp. MSL2007 voucher 668369 16S r	EF214062	78.48	100	1.79548e−25	Coleoptera	Carabidae	Pseudomigadops n. sp. MSL2007
Drosophila tripunctata voucher 107541 NADH dehydro	EU494351	77.99	100	1.79548e−25	Diptera	Drosophilidae	Drosophila tripunctata
Thaumatoperla sp. BYU_PL109 16S ribosomal RNA gene	EF623149	80.27	93.59	1.79548e−25	Plecoptera	Eustheniidae	Thaumatoperla sp. BYU_PL109
Drosophila tripunctata 16S ribosomal RNA gene, par	AF479806	77.99	100	1.79548e−25	Diptera	Drosophilidae	Drosophila tripunctata
Neoconops longicornis 16S ribosomal RNA gene, part	AF154769	78.21	100	1.79548e−25	Diptera	Conopidae	Neoconops longicornis
Neoconops longicornis 16S ribosomal RNA gene, part	AF154768	78.21	100	1.79548e−25	Diptera	Conopidae	Neoconops longicornis
Neoconops longicornis 16S ribosomal RNA gene, part	AF154767	78.21	100	1.79548e−25	Diptera	Conopidae	Neoconops longicornis
Aedes w−albus 16S ribosomal RNA gene, mitochondria	AF034472	79.38	100	1.79548e−25	Diptera	Culicidae	Aedes w−albus
Aedes vigilax isolate Mi139 mitochondrion, complet	KP995260	78.26	100	6.26685e−25	Diptera	Culicidae	Ochlerotatus vigilax
Bertamyia notata isolate BN39 16S ribosomal RNA ge	KC699324	79.38	100	6.26685e−25	Diptera	Platypezidae	Bertamyia notata
Ectinorhynchus sp. 'Bribie' voucher UQIC41178 16S	KM212983	77.56	100	6.26685e−25	Diptera	Therevidae	Ectinorhynchus sp. 'Bribie'
Ectinorhynchus sp. 'Moffatt' voucher QMT183203 16S	KM212976	77.56	100	6.26685e−25	Diptera	Therevidae	Ectinorhynchus sp. 'Moffatt'
Ectinorhynchus sp. 'warrumbungles' voucher QMT1832	KM212974	77.56	100	6.26685e−25	Diptera	Therevidae	Ectinorhynchus sp. 'warrumbungles'
Wuchereria bancrofti genome assembly W_bancrofti_J	LM000940	79.5	100	6.26685e−25	Spirurida	Onchocercidae	Wuchereria bancrofti
Drosophila melanogaster 16S ribosomal RNA gene, pa	KJ919952	77.56	100	6.26685e−25	Diptera	Drosophilidae	Drosophila melanogaster
Rhantaticus congestus voucher NHRS/JLK000000091 16	KF979103	79.19	93.59	6.26685e−25	Coleoptera	Dytiscidae	Rhantaticus congestus
Rhantaticus congestus voucher 412B12_BMNH832121 16	KF979100	79.19	93.59	6.26685e−25	Coleoptera	Dytiscidae	Rhantaticus congestus
Acilius abbreviatus voucher KBMAcab72 16S ribosoma	KF979078	79.74	97.44	6.26685e−25	Coleoptera	Dytiscidae	Acilius abbreviatus
Protoplasa fitchii 16S ribosomal RNA gene, partial	KC177462	78.21	97.44	6.26685e−25	Diptera	Tanyderidae	Protoplasa fitchii
Sciadocera rufomaculata 16S ribosomal RNA gene, pa	JN196447	78.62	100	6.26685e−25	Diptera	Sciadoceridae	Sciadocera rufomaculata
Protoplasa fitchii mitochondrion, complete genome	JN861746	78.21	97.44	6.26685e−25	Diptera	Tanyderidae	Protoplasa fitchii
Rhantaticus congestus voucher BMNH829999 16S ribos	HQ381497	79.19	93.59	6.26685e−25	Coleoptera	Dytiscidae	Rhantaticus congestus
Culcua nigra 16S ribosomal RNA gene, partial seque	GU947392	78.34	100	6.26685e−25	Diptera	Stratiomyidae	Culcua nigra
Culex quinquefasciatus from USA mitochondrion, com	HQ724617	79.5	100	6.26685e−25	Diptera	Culicidae	Culex quinquefasciatus
Culex pipiens pipiens from Turkey mitochondrion, c	HQ724616	79.5	100	6.26685e−25	Diptera	Culicidae	Culex pipiens pipiens
Culex pipiens pipiens from Tunisia mitochondrion,	HQ724614	79.5	100	6.26685e−25	Diptera	Culicidae	Culex pipiens pipiens
Culex quinquefasciatus mitochondrion, complete gen	GU188856	79.5	100	6.26685e−25	Diptera	Culicidae	Culex quinquefasciatus
