# Supplementary material for: Testing the potential of a ribosomal 16S marker for DNA metabarcoding of insects
Source: PeerJ. 2016 Apr 19;4:e1966. doi: 10.7717/peerj.1966 (PMC4841222; doi:10.7717/peerj.1966)
Supplement: Supplemental Information 1 [file peerj-04-1966-s006.zip › scripts/5 match OTUs/blast_hits/OTU_636.csv_plot.pdf]

OTU_636.csv

Pedicia albivitta

Pediciidae

Diptera

85.9

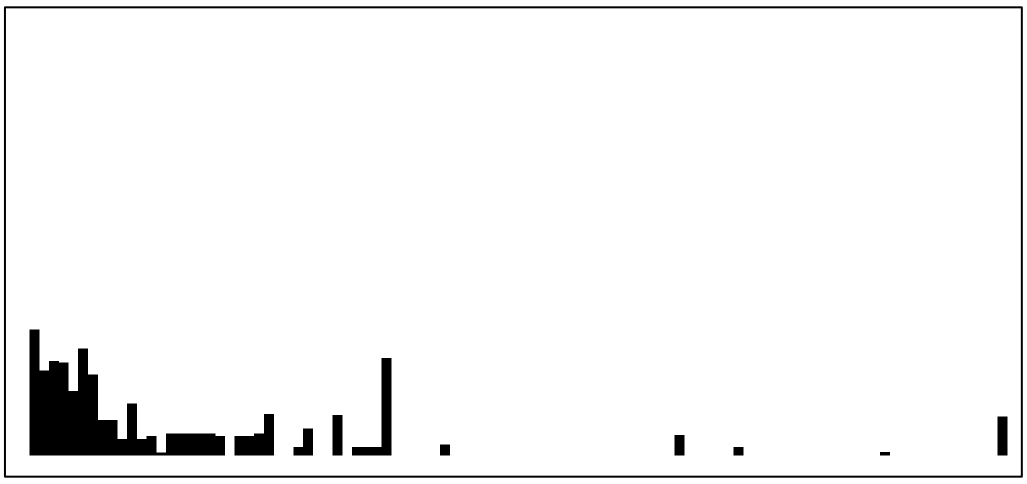

Pedicia albivitta voucher O'Grady 200012 16S ribos	EU005435	85.9	99.36	9.29262e−42	Diptera	Pediciidae	Pedicia albivitta
Edwardsina gigantea 16S ribosomal RNA gene, partia	KC177460	83.97	100	1.68015e−38	Diptera	Blephariceridae	Edwardsina gigantea
Bolitophila cinerea voucher JSB1 16S ribosomal RNA	KJ136712	84.42	97.44	7.14416e−37	Diptera	Mycetophilidae	Bolitophila cinerea
Parochlus steinenii isolate Sejong01 mitochondrion	KT003702	84.35	94.23	3.03777e−35	Diptera	Chironomidae	Parochlus steinenii
Corynoptera blanda voucher 955 16S ribosomal RNA g	JQ613965	83.01	97.44	1.06029e−34	Diptera	Sciaridae	Corynoptera blanda
Bolitophila sp. ER−2009 16S ribosomal RNA gene, pa	FJ172021	85	89.1	1.29169e−33	Diptera	Mycetophilidae	Bolitophila sp. ER−2009
Tipula sp. PMO 200011 16S ribosomal RNA gene, part	EU005437	83.78	94.23	1.29169e−33	Diptera	Tipulidae	Tipula sp. PMO 200011
Tipula abdominalis 16S ribosomal RNA gene, partial	KC177457	81.65	99.36	5.49242e−32	Diptera	Tipulidae	Tipula abdominalis
Tipula abdominalis mitochondrion, partial genome	JN861743	81.65	99.36	5.49242e−32	Diptera	Tipulidae	Tipula abdominalis
Minettia longipennis mitochondrion partial 16S rRN	AJ439184	80.77	100	5.49242e−32	Diptera	Lauxaniidae	Minettia longipennis
Eudicrana sp. DK−2013 voucher JSS12 16S ribosomal	KC435571	82.43	93.59	1.91704e−31	Diptera	Mycetophilidae	Eudicrana sp. DK−2013
Derodontus emaculatus voucher BMNH:679374 16S ribo	DQ202609	80.77	99.36	6.69114e−31	Coleoptera		Derodontus emaculatus
Culcua kolibaci 16S ribosomal RNA gene, partial se	GU947391	80.89	100	2.33544e−30	Diptera	Stratiomyidae	Culcua kolibaci
Drosophila takahashii cytochrome b (cytb) gene, pa	AF164592	80.13	100	2.33544e−30	Diptera	Drosophilidae	Drosophila takahashii
Chrysoperla nipponensis mitochondrial DNA, complet	AP011623	81.01	100	8.15148e−30	Neuroptera	Chrysopidae	Chrysoperla nipponensis
Chrysoperla agilis isolate A−083 16S large subunit	AY743751	81.01	100	8.15148e−30	Neuroptera	Chrysopidae	Chrysoperla agilis
Chrysoperla lucasina isolate L−079 16S large subun	AY743746	81.01	100	8.15148e−30	Neuroptera	Chrysopidae	Chrysoperla lucasina
Chrysoperla lucasina isolate L−029 16S large subun	AY743737	81.01	100	8.15148e−30	Neuroptera	Chrysopidae	Chrysoperla lucasina
Chrysoperla lucasina isolate L−099 16S large subun	AY743732	81.01	100	8.15148e−30	Neuroptera	Chrysopidae	Chrysoperla lucasina
Panorpa takenouchii isolate 26tak 16S ribosomal RN	EF041520	80.89	99.36	2.84515e−29	Mecoptera	Panorpidae	Panorpa takenouchii
Chrysoperla carnea isolate Chrcar3 16S ribosomal R	AY620150	80	100	2.84515e−29	Neuroptera	Chrysopidae	Chrysoperla carnea
Panorpa takenouchii 16S ribosomal RNA gene, partia	AF180064	80.89	99.36	2.84515e−29	Mecoptera	Panorpidae	Panorpa takenouchii
Chrysoperla lucasina isolate L−070 16S large subun	AY743744	80.89	99.36	2.84515e−29	Neuroptera	Chrysopidae	Chrysoperla lucasina
Cychrus caraboides caraboides voucher CC00817 16S	JQ647184	81.01	99.36	9.93054e−29	Coleoptera	Carabidae	Cychrus caraboides caraboides
Bolitophila hybrida 16S ribosomal RNA gene, partia	FJ172002	81.94	91.67	9.93054e−29	Diptera	Mycetophilidae	Bolitophila hybrida
Drosophila funebris voucher 103952 NADH dehydrogen	EU494313	79.75	100	9.93054e−29	Diptera	Drosophilidae	Drosophila funebris
Leucochrysa marquezi voucher CASC 00212 16S riboso	DQ399280	79.75	100	9.93054e−29	Neuroptera	Chrysopidae	Leucochrysa marquezi
Chrysoperla nipponensis mitochondrion, complete ge	KP027407	80.38	100	3.4661e−28	Neuroptera	Chrysopidae	Chrysoperla nipponensis
Acilius athabascae voucher 305G09/BMNH799009 16S r	KF979079	81.25	100	3.4661e−28	Coleoptera	Dytiscidae	Acilius athabascae
Ptychoptera sp. ATB−2011 mitochondrion, complete g	JN861744	79.49	99.36	3.4661e−28	Diptera	Ptychopteridae	Ptychoptera sp. ATB−2011
Tinda javana 16S ribosomal RNA gene, partial seque	GU947393	79.49	100	3.4661e−28	Diptera	Stratiomyidae	Tinda javana
Pyratula zonata 16S ribosomal RNA gene, partial se	FJ172004	81.88	88.46	3.4661e−28	Diptera	Keroplatidae	Pyratula zonata
Mallada luctuosus 16S ribosomal RNA gene, partial	DQ399288	79.14	100	3.4661e−28	Neuroptera	Chrysopidae	Pseudomallada luctuosus
Chrysoperla carnea isolate Chrcar2 16S ribosomal R	AY620152	80.38	100	3.4661e−28	Neuroptera	Chrysopidae	Chrysoperla carnea
Chrysoperla sp. isolate 82 16S large subunit ribos	AY743750	80.38	100	3.4661e−28	Neuroptera	Chrysopidae	Chrysoperla sp. isolate 82
Chrysoperla carnea isolate C−097 16S large subunit	AY743731	80.38	100	3.4661e−28	Neuroptera	Chrysopidae	Chrysoperla carnea
Leptomorphus walkeri 16S ribosomal RNA gene, parti	FJ172020	84.55	78.21	1.20979e−27	Diptera	Mycetophilidae	Leptomorphus walkeri
Drosophila malerkotliana voucher 105504 NADH dehyd	EU494357	78.85	100	1.20979e−27	Diptera	Drosophilidae	Drosophila malerkotliana
Drosophila rubida voucher 109400 NADH dehydrogenas	EU494327	78.85	100	1.20979e−27	Diptera	Drosophilidae	Drosophila rubida
Drosophila hypocausta voucher 103972 NADH dehydrog	EU494319	78.85	100	1.20979e−27	Diptera	Drosophilidae	Drosophila hypocausta
Apertochrysa edwardsi 16S ribosomal RNA gene, part	DQ399265	80	100	1.20979e−27	Neuroptera	Chrysopidae	Apertochrysa edwardsi
Drosophila malerkotliana cytochrome b (cytb) gene,	AF164579	78.85	100	1.20979e−27	Diptera	Drosophilidae	Drosophila malerkotliana
Chrysoperla lucasina isolate L−078 16S large subun	AY743776	80.5	100	1.20979e−27	Neuroptera	Chrysopidae	Chrysoperla lucasina
Calosoma sp. BYU−CO241 mitochondrion, complete gen	GU176340	79.01	99.36	4.22257e−27	Coleoptera	Carabidae	Calosoma sp. BYU−CO241
Calosoma scrutator 16S ribosomal RNA gene, partial	EU734850	79.01	99.36	4.22257e−27	Coleoptera	Carabidae	Calosoma scrutator
Leucochrysa lancala voucher CASC 00211 16S ribosom	DQ399277	79.11	100	4.22257e−27	Neuroptera	Chrysopidae	Leucochrysa lancala
Chrysoperla carnea isolate C−116 16S large subunit	AY743760	79.75	100	4.22257e−27	Neuroptera	Chrysopidae	Chrysoperla carnea
Chrysoperla carnea isolate C−102 16S large subunit	AY743755	79.75	100	4.22257e−27	Neuroptera	Chrysopidae	Chrysoperla carnea
Chrysoperla rufilabris isolate R−065 16S large sub	AY743741	79.01	100	4.22257e−27	Neuroptera	Chrysopidae	Chrysoperla rufilabris
Chrysoperla carnea isolate C−089 16S large subunit	AY743729	79.75	100	4.22257e−27	Neuroptera	Chrysopidae	Chrysoperla carnea
Dysmicohermes ingens mitochondrion, complete genom	KJ806318	79.87	100	1.47382e−26	Megaloptera	Corydalidae	Dysmicohermes ingens
Drosophila ananassae isolate Delhi1 16S ribosomal	JX896435	78.21	100	1.47382e−26	Diptera	Drosophilidae	Drosophila ananassae
Colobaea bifasciella voucher MU_NUS_SG3 16S riboso	JN828202	78.21	100	1.47382e−26	Diptera	Sciomyzidae	Colobaea bifasciella
Eudmeta marginata 16S ribosomal RNA gene, partial	GU947400	78.85	100	1.47382e−26	Diptera	Stratiomyidae	Eudmeta marginata
Pseudomigadops n. sp. MSL2007 voucher 668381 16S r	EF214073	79.11	100	1.47382e−26	Coleoptera	Carabidae	Pseudomigadops n. sp. MSL2007
Pseudomigadops n. sp. MSL2007 voucher 668379 16S r	EF214072	79.11	100	1.47382e−26	Coleoptera	Carabidae	Pseudomigadops n. sp. MSL2007
Pseudomigadops n. sp. MSL2007 voucher 668338 16S r	EF214034	79.11	100	1.47382e−26	Coleoptera	Carabidae	Pseudomigadops n. sp. MSL2007
Pseudomigadops n. sp. MSL2007 voucher 668317 16S r	EF214022	79.11	100	1.47382e−26	Coleoptera	Carabidae	Pseudomigadops n. sp. MSL2007
Pseudomigadops n. sp. MSL2007 voucher 668302 16S r	EF214012	79.11	100	1.47382e−26	Coleoptera	Carabidae	Pseudomigadops n. sp. MSL2007
Pseudomigadops n. sp. MSL2007 voucher 668300 16S r	EF214010	79.11	100	1.47382e−26	Coleoptera	Carabidae	Pseudomigadops n. sp. MSL2007
Pseudomigadops n. sp. MSL2007 voucher 668299 16S r	EF214009	79.11	100	1.47382e−26	Coleoptera	Carabidae	Pseudomigadops n. sp. MSL2007
Hercostomus verbekei voucher Pollet 0263 (P) 16S r	EU864004	78.21	100	1.47382e−26	Diptera	Dolichopodidae	Hercostomus verbekei
Grzegorzekia collaris 16S ribosomal RNA gene, part	FJ172016	79.08	97.44	1.47382e−26	Diptera	Mycetophilidae	Grzegorzekia collaris
Pseudomigadops sp. PSM1 mitochondrial 16S rRNA gen	FM994738	79.11	100	1.47382e−26	Coleoptera	Carabidae	Pseudomigadops sp. PSM1
Pseudomigadops n. sp. MSL2007 voucher 668369 16S r	EF214062	79.11	100	1.47382e−26	Coleoptera	Carabidae	Pseudomigadops n. sp. MSL2007
Drosophila putrida voucher 103964 NADH dehydrogena	EU494348	78.21	100	1.47382e−26	Diptera	Drosophilidae	Drosophila putrida
Thaumatoperla sp. BYU_PL109 16S ribosomal RNA gene	EF623149	80.95	93.59	1.47382e−26	Plecoptera	Eustheniidae	Thaumatoperla sp. BYU_PL109
Libnotes perkinsi voucher O'Grady 200981 16S ribos	EU005433	78.85	99.36	1.47382e−26	Diptera	Limoniidae	Libnotes perkinsi
Libnotes perkinsi voucher O'Grady 200947 16S ribos	EU005432	78.85	99.36	1.47382e−26	Diptera	Limoniidae	Libnotes perkinsi
Chrysoperla carnea isolate C−101 16S large subunit	AY743754	79.38	100	1.47382e−26	Neuroptera	Chrysopidae	Chrysoperla carnea
Sapromyza madeirensis mitochondrion partial 16S rR	AJ439187	78.21	100	1.47382e−26	Diptera	Lauxaniidae	Sapromyza madeirensis
Bolopus furcatus isolate 1305 16S ribosomal RNA ge	KC699315	78.34	100	5.14415e−26	Diptera	Platypezidae	Bolopus furcatus
Acilius abbreviatus voucher KBMAcab72 16S ribosoma	KF979078	80.39	97.44	5.14415e−26	Coleoptera	Dytiscidae	Acilius abbreviatus
Culcua nigra 16S ribosomal RNA gene, partial seque	GU947392	78.98	100	5.14415e−26	Diptera	Stratiomyidae	Culcua nigra
Hercostomus longiventris voucher Pollet 0274 (P) 1	EU863994	78.98	100	5.14415e−26	Diptera	Dolichopodidae	Hercostomus longiventris
Empis producta voucher RMBR:103430 16S ribosomal R	FJ808167	78.34	100	5.14415e−26	Diptera	Empididae	Empis producta
Libnotes orofenaae voucher O'Grady 202034 16S ribo	EU005431	78.48	99.36	5.14415e−26	Diptera	Limoniidae	Libnotes orofenaae
Leucochrysa texana 16S ribosomal RNA gene, partial	DQ399279	78.48	100	5.14415e−26	Neuroptera	Chrysopidae	Leucochrysa texana
Brugia timori genome assembly B_timori_Indonesia_v	LK908083	79.25	100	1.79548e−25	Spirurida	Onchocercidae	Brugia timori
Acilius sylvanus voucher KBMAcsy51 16S ribosomal R	KF979086	80	100	1.79548e−25	Coleoptera	Dytiscidae	Acilius sylvanus
Acilius semisulcatus voucher KBMAcse7 16S ribosoma	KF979085	80	100	1.79548e−25	Coleoptera	Dytiscidae	Acilius semisulcatus
Laemostenus terricola 16S ribosomal RNA (rrnl) gen	JF778796	79.25	100	1.79548e−25	Coleoptera	Carabidae	Laemostenus terricola
Sphenella marginata 16S ribosomal RNA gene, partia	GQ175836	77.99	100	1.79548e−25	Diptera	Tephritidae	Sphenella marginata
Phthinia humilis 16S ribosomal RNA gene, partial s	FJ172009	79.49	97.44	1.79548e−25	Diptera	Mycetophilidae	Phthinia humilis
Trachypachus holmbergi mitochondrion, complete gen	EU877954	79.25	99.36	1.79548e−25	Coleoptera	Trachypachidae	Trachypachus holmbergi
Drosophila tripunctata voucher 107541 NADH dehydro	EU494351	77.99	100	1.79548e−25	Diptera	Drosophilidae	Drosophila tripunctata
Notomma n. sp. 1000 16S ribosomal RNA gene, partia	AY792108	79.59	93.59	1.79548e−25	Diptera	Tephritidae	Notomma n. sp. 1000
Arachnocampa luminosa biotype 2 16S subunit riboso	AY576330	80.14	90.38	1.79548e−25	Diptera	Keroplatidae	Arachnocampa luminosa
Drosophila tripunctata 16S ribosomal RNA gene, par	AF479806	77.99	100	1.79548e−25	Diptera	Drosophilidae	Drosophila tripunctata
Neoconops longicornis 16S ribosomal RNA gene, part	AF154769	78.21	100	1.79548e−25	Diptera	Conopidae	Neoconops longicornis
Neoconops longicornis 16S ribosomal RNA gene, part	AF154768	78.21	100	1.79548e−25	Diptera	Conopidae	Neoconops longicornis
Neoconops longicornis 16S ribosomal RNA gene, part	AF154767	78.21	100	1.79548e−25	Diptera	Conopidae	Neoconops longicornis
Ectinorhynchus sp. 'Bribie' voucher UQIC41178 16S	KM212983	77.56	100	6.26685e−25	Diptera	Therevidae	Ectinorhynchus sp. 'Bribie'
Ectinorhynchus sp. 'Moffatt' voucher QMT183203 16S	KM212976	77.56	100	6.26685e−25	Diptera	Therevidae	Ectinorhynchus sp. 'Moffatt'
Ectinorhynchus sp. 'warrumbungles' voucher QMT1832	KM212974	77.56	100	6.26685e−25	Diptera	Therevidae	Ectinorhynchus sp. 'warrumbungles'
Dicranomyia iniquispina voucher 205222 16S ribosom	KC692800	78.48	99.36	6.26685e−25	Diptera	Limoniidae	Dicranomyia iniquispina
Sciadocera rufomaculata 16S ribosomal RNA gene, pa	JN196447	78.62	100	6.26685e−25	Diptera	Sciadoceridae	Sciadocera rufomaculata
Penetretus temporalis genomic DNA containing mitoc	FR729595	77.99	100	6.26685e−25	Coleoptera	Carabidae	Penetretus temporalis
Tachytrechus notatus voucher Pollet 0279 (P) 16S r	EU864017	78.34	100	6.26685e−25	Diptera	Dolichopodidae	Tachytrechus notatus
Pseudogonia rufifrons mitochondrial gene for 16S r	AB465942	81.82	83.97	6.26685e−25	Diptera	Tachinidae	Pseudogonia rufifrons
