# Supplementary material for: Testing the potential of a ribosomal 16S marker for DNA metabarcoding of insects
Source: PeerJ. 2016 Apr 19;4:e1966. doi: 10.7717/peerj.1966 (PMC4841222; doi:10.7717/peerj.1966)
Supplement: Supplemental Information 1 [file peerj-04-1966-s006.zip › scripts/5 match OTUs/blast_hits/OTU_64.csv_plot.pdf]

OTU_64.csv

Oreodytes sanmarkii

Dytiscidae

Coleoptera

96.82

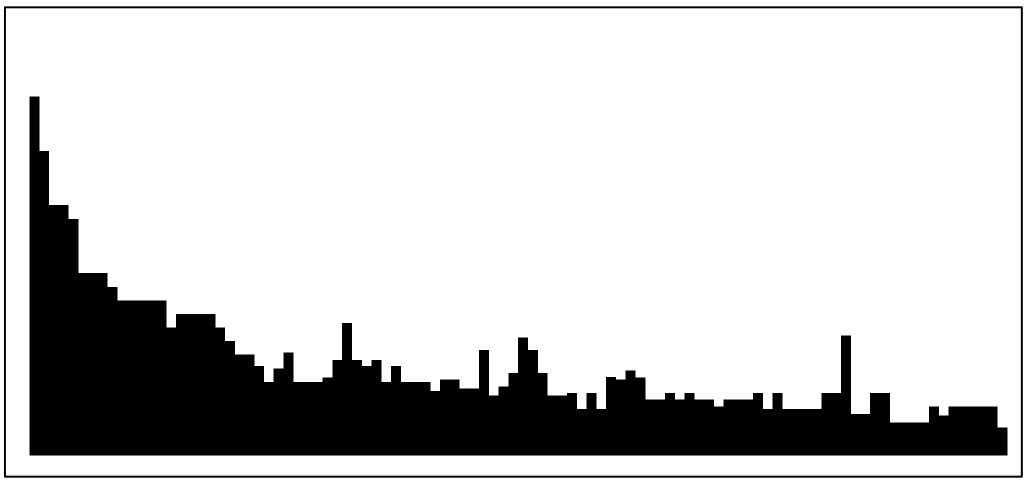

Oreodytes sanmarkii mitochondrial 16S rRNA gene (p	HF931524	96.82	100	3.47071e−66	Coleoptera	Dytiscidae	Oreodytes sanmarkii
Oreodytes obesus cordillerensis mitochondrial part	HF931516	94.27	100	9.31322e−61	Coleoptera	Dytiscidae	Oreodytes obesus cordillerensis
Oreodytes crassulus partial 16S rRNA gene	AJ850348	91.72	100	2.49908e−55	Coleoptera	Dytiscidae	Oreodytes crassulus
Oreodytes congruus partial 16S rRNA gene	AJ850347	91.72	100	2.49908e−55	Coleoptera	Dytiscidae	Oreodytes congruus
Oreodytes congruus 16S ribosomal RNA gene, partial	KF575550	91.08	100	3.04451e−54	Coleoptera	Dytiscidae	Oreodytes congruus
Oreodytes mongolicus mitochondrial 16S rRNA gene (	HF931491	88.54	100	8.16955e−49	Coleoptera	Dytiscidae	Oreodytes mongolicus
Oreodytes abbreviatus mitochondrial 16S rRNA gene	HF931477	88.54	100	8.16955e−49	Coleoptera	Dytiscidae	Oreodytes abbreviatus
Oreodytes rhyacophilus partial 16S rRNA gene	AJ850352	88.54	100	8.16955e−49	Coleoptera	Dytiscidae	Oreodytes rhyacophilus
Oreodytes snoqualmie mitochondrial partial 16S rRN	HF931519	87.9	100	3.47378e−47	Coleoptera	Dytiscidae	Oreodytes snoqualmie
Oreodytes septentrionalis mitochondrial 16S rRNA g	HF931492	87.26	100	4.23193e−46	Coleoptera	Dytiscidae	Oreodytes septentrionalis
Oreodytes shorti mitochondrial 16S rRNA gene (part	HF931488	87.26	100	4.23193e−46	Coleoptera	Dytiscidae	Oreodytes shorti
Boreonectes riberae mitochondrial 16S rRNA gene (p	HF931461	87.26	100	5.15555e−45	Coleoptera	Dytiscidae	Boreonectes riberae
Boreonectes griseostriatus mitochondrial partial 1	HF931387	87.26	100	5.15555e−45	Coleoptera	Dytiscidae	Boreonectes griseostriatus
Boreonectes multilineatus mitochondrial partial 16	HF931384	87.26	100	5.15555e−45	Coleoptera	Dytiscidae	Boreonectes multilineatus
Laccornis difformis 16S ribosomal RNA gene, partia	KF575544	85.99	100	2.19219e−43	Coleoptera	Dytiscidae	Laccornis difformis
Boreonectes alpestris mitochondrial 16S rRNA gene	HF931512	86.62	100	2.19219e−43	Coleoptera	Dytiscidae	Boreonectes alpestris
Boreonectes striatellus mitochondrial 16S rRNA gen	HF931511	86.62	100	2.19219e−43	Coleoptera	Dytiscidae	Boreonectes striatellus
Boreonectes macedonicus mitochondrial 16S rRNA gen	HF931359	86.62	100	2.19219e−43	Coleoptera	Dytiscidae	Boreonectes macedonicus
Stictotarsus ibericus 16S ribosomal RNA (rrnL) gen	EF670030	86.62	100	2.19219e−43	Coleoptera	Dytiscidae	Stictotarsus ibericus
Boreonectes griseostriatus mitochondrial 16S rRNA	HF931541	85.99	100	2.67064e−42	Coleoptera	Dytiscidae	Boreonectes griseostriatus
Deronectes parvicollis mitochondrial 16S rRNA gene	HF931454	85.35	100	9.32145e−42	Coleoptera	Dytiscidae	Deronectes parvicollis
Oreodytes scitulus 16S ribosomal RNA gene, partial	KF575551	84.71	100	1.13558e−40	Coleoptera	Dytiscidae	Oreodytes scitulus
Oreodytes scitulus mitochondrial partial 16S rRNA	HF931515	84.71	100	1.13558e−40	Coleoptera	Dytiscidae	Oreodytes scitulus
Nebrioporus formaster mitochondrial partial 16S rR	HF931377	84.18	100	1.68536e−38	Coleoptera	Dytiscidae	Nebrioporus formaster
Oreodytes quadrimaculatus 16S ribosomal RNA gene,	KF575567	83.44	100	5.88247e−38	Coleoptera	Dytiscidae	Oreodytes quadrimaculatus
Boreonectes griseostriatus mitochondrial 16S rRNA	HF931385	84.08	100	5.88247e−38	Coleoptera	Dytiscidae	Boreonectes griseostriatus
Hydroporus foveolatus mitochondrial 16S rRNA gene	HE613724	84.81	100	5.88247e−38	Coleoptera	Dytiscidae	Hydroporus foveolatus
Laccornis kocae partial 16S rRNA gene	AJ850419	83.44	100	5.88247e−38	Coleoptera	Dytiscidae	Laccornis kocae
Oreodytes quadrimaculatus partial 16S rRNA gene	AJ850351	83.44	100	5.88247e−38	Coleoptera	Dytiscidae	Oreodytes quadrimaculatus
Oreodytes natrix partial 16S rRNA gene	AJ850349	83.44	100	5.88247e−38	Coleoptera	Dytiscidae	Oreodytes natrix
Aethionectes fulvonotatus voucher KBMAeop406 16S r	KF979088	83.65	100	2.05319e−37	Coleoptera	Dytiscidae	Aethionectes fulvonotatus
Hydroporus tessellatus 16S ribosomal RNA gene, par	JX434770	84.47	100	2.05319e−37	Coleoptera	Dytiscidae	Hydroporus tessellatus
Deronectes moestus mitochondrial 16S rRNA gene (pa	HE613726	86.21	91.72	2.05319e−37	Coleoptera	Dytiscidae	Deronectes moestus
Hydroporus basinotatus mitochondrial 16S rRNA gene	HE613678	84.47	100	2.05319e−37	Coleoptera	Dytiscidae	Hydroporus basinotatus
Stictotarsus maghrebinus 16S ribosomal RNA gene, p	AY250947	84.18	100	2.05319e−37	Coleoptera	Dytiscidae	Stictotarsus maghrebinus
Hydroporus basinotatus 16S ribosomal RNA gene, par	AF518258	84.47	100	2.05319e−37	Coleoptera	Dytiscidae	Hydroporus basinotatus
Hygrotus novemlineatus mitochondrial partial 16S r	HF931523	83.44	100	7.16632e−37	Coleoptera	Dytiscidae	Hygrotus novemlineatus
Hydroporus zimmermanni mitochondrial 16S rRNA gene	HE613679	84.18	100	7.16632e−37	Coleoptera	Dytiscidae	Hydroporus zimmermanni
Hygrotus turbidus partial 16S rRNA gene	AJ850390	83.44	100	7.16632e−37	Coleoptera	Dytiscidae	Hygrotus turbidus
Hygrotus corpulentus partial 16S rRNA gene	AJ850387	83.44	100	7.16632e−37	Coleoptera	Dytiscidae	Hygrotus corpulentus
Hydroporus glabriusculus 16S ribosomal RNA gene, p	AY365270	83.44	100	7.16632e−37	Coleoptera	Dytiscidae	Hydroporus glabriusculus
Scarodytes sp. IR−2003 16S ribosomal RNA gene, par	AY250939	83.02	100	7.16632e−37	Coleoptera	Dytiscidae	Scarodytes sp. IR−2003
Stictotarsus procerus mitochondrial 16S rRNA gene	HF931379	83.54	100	2.50129e−36	Coleoptera	Dytiscidae	Stictotarsus procerus
Deronectes opatrinus mitochondrial 16S rRNA gene (	HE613701	83.54	100	2.50129e−36	Coleoptera	Dytiscidae	Deronectes opatrinus
Hydroporus marginatus mitochondrial 16S rRNA gene	HE613699	83.12	100	2.50129e−36	Coleoptera	Dytiscidae	Hydroporus marginatus
Hydroporus marginatus 16S ribosomal RNA gene, part	AF518267	83.12	100	2.50129e−36	Coleoptera	Dytiscidae	Hydroporus marginatus
Deronectes moestus 16S ribosomal RNA gene, partial	KF575561	84.93	91.72	8.73037e−36	Coleoptera	Dytiscidae	Deronectes moestus
Hygrotus patruelis mitochondrial 16S rRNA gene (pa	HF931388	82.8	100	8.73037e−36	Coleoptera	Dytiscidae	Hygrotus patruelis
Hydroporus analis mitochondrial partial 16S rRNA g	HE613731	83.23	100	8.73037e−36	Coleoptera	Dytiscidae	Hydroporus analis
Hydroporus fuscipennis mitochondrial 16S rRNA gene	HE613730	83.85	100	8.73037e−36	Coleoptera	Dytiscidae	Hydroporus fuscipennis
Deronectes brannani mitochondrial 16S rRNA gene (p	HE613695	85.52	91.72	8.73037e−36	Coleoptera	Dytiscidae	Deronectes brannanii
Deronectes moestus mitochondrial 16S rRNA gene (pa	HE613683	84.93	91.72	8.73037e−36	Coleoptera	Dytiscidae	Deronectes moestus
Hydroporus fuscipennis 16S ribosomal RNA gene, par	AF518262	83.85	100	8.73037e−36	Coleoptera	Dytiscidae	Hydroporus fuscipennis
Agabus didymus mitochondrial 16S rRNA gene (partia	LM654773	82.8	100	3.0472e−35	Coleoptera	Dytiscidae	Agabus didymus
Agabus didymus voucher UNM KBMPmsp730 16S ribosoma	KJ548392	82.8	100	3.0472e−35	Coleoptera	Dytiscidae	Agabus didymus
Suphrodytes dorsalis 16S ribosomal RNA gene, parti	KF575577	82.91	100	3.0472e−35	Coleoptera	Dytiscidae	Suphrodytes dorsalis
Agabus dichrous mitochondrial partial 16S rRNA gen	HF931378	82.17	100	3.0472e−35	Coleoptera	Dytiscidae	Agabus dichrous
Suphrodytes dorsalis voucher BMNH:725966 16S ribos	JX221574	82.91	100	3.0472e−35	Coleoptera	Dytiscidae	Suphrodytes dorsalis
Deronectes bicostatus mitochondrial 16S rRNA gene	HE613705	82.17	100	3.0472e−35	Coleoptera	Dytiscidae	Deronectes bicostatus
Hygrotus impressopunctatus partial 16S rRNA gene	AJ850405	83.66	97.45	3.0472e−35	Coleoptera	Dytiscidae	Hygrotus impressopunctatus
Hydroporus rufifrons 16S ribosomal RNA gene, parti	AF518277	83.54	100	3.0472e−35	Coleoptera	Dytiscidae	Hydroporus rufifrons
Hygrotus impressopunctatus voucher UNM KBMHtim80 1	KJ548334	83.97	97.45	1.06358e−34	Coleoptera	Dytiscidae	Hygrotus impressopunctatus
Hygrotus armeniacus mitochondrial partial 16S rRNA	HF931520	83.65	100	1.06358e−34	Coleoptera	Dytiscidae	Hygrotus armeniacus
Hydroporus discretus mitochondrial 16S rRNA gene (	HE613723	82.61	100	1.06358e−34	Coleoptera	Dytiscidae	Hydroporus discretus
Hydroporus decipiens mitochondrial 16S rRNA gene (	HE613722	82.61	100	1.06358e−34	Coleoptera	Dytiscidae	Hydroporus decipiens
Deronectes doriae mitochondrial 16S rRNA gene (par	HE613710	82.91	100	1.06358e−34	Coleoptera	Dytiscidae	Deronectes doriae
Hydroporus errans mitochondrial 16S rRNA gene (par	HE613704	82.61	100	1.06358e−34	Coleoptera	Dytiscidae	Hydroporus errans
Deronectes sahlbergi mitochondrial 16S rRNA gene (	HE613680	82.91	100	1.06358e−34	Coleoptera	Dytiscidae	Deronectes sahlbergi
Hydroporus compunctus mitochondrial 16S rRNA gene	HE599693	82.61	100	1.06358e−34	Coleoptera	Dytiscidae	Hydroporus compunctus
Hydroporus pubescens voucher NHM−IR39 16S ribosoma	EF419327	82.61	100	1.06358e−34	Coleoptera	Dytiscidae	Hydroporus pubescens
Scarodytes halensis haplotype 3 16S ribosomal RNA	AY250937	82.28	100	1.06358e−34	Coleoptera	Dytiscidae	Scarodytes halensis
Hydroporus compunctus 16S ribosomal RNA gene, part	AF518259	82.61	100	1.06358e−34	Coleoptera	Dytiscidae	Hydroporus compunctus
Hydroporus discretus 16S ribosomal RNA gene, parti	AF518260	82.61	100	1.06358e−34	Coleoptera	Dytiscidae	Hydroporus discretus
Hydroporus analis 16S ribosomal RNA gene, partial	AF518257	82.61	100	1.06358e−34	Coleoptera	Dytiscidae	Hydroporus analis
Hydroporus rufifrons 16S ribosomal RNA gene, parti	JX434758	82.91	100	3.71225e−34	Coleoptera	Dytiscidae	Hydroporus rufifrons
Hygrotus lutescens mitochondrial partial 16S rRNA	HF931518	82.17	100	3.71225e−34	Coleoptera	Dytiscidae	Hygrotus lutescens
Hydroporus foveolatus mitochondrial 16S rRNA gene	HF931502	82.91	100	3.71225e−34	Coleoptera	Dytiscidae	Hydroporus foveolatus
Hygrotus fontinalis mitochondrial 16S rRNA gene (p	HF931494	82.17	100	3.71225e−34	Coleoptera	Dytiscidae	Hygrotus fontinalis
Hygrotus marklini mitochondrial 16S rRNA gene (par	HF931467	82.17	100	3.71225e−34	Coleoptera	Dytiscidae	Hygrotus marklini
Boreonectes funereus mitochondrial 16S rRNA gene (	HF931393	82.17	100	3.71225e−34	Coleoptera	Dytiscidae	Boreonectes funereus
Suphrodytes figuratus voucher BMNH:722148 16S ribo	JX221571	82.17	100	3.71225e−34	Coleoptera	Dytiscidae	Suphrodytes figuratus
Hydroporus pubescens mitochondrial 16S rRNA gene (	HE613707	82.91	100	3.71225e−34	Coleoptera	Dytiscidae	Hydroporus pubescens
Hydroporus limbatus mitochondrial 16S rRNA gene (p	HE613706	82.91	100	3.71225e−34	Coleoptera	Dytiscidae	Hydroporus limbatus
Hovahydrus sp. BI−2011 voucher BMNH831333 16S ribo	HQ381524	85.62	91.72	3.71225e−34	Coleoptera	Dytiscidae	Hovahydrus sp. BI−2011
Antiporus hollingsworthi mitochondrial partial 16S	FR727246	81.94	98.73	3.71225e−34	Coleoptera	Dytiscidae	Antiporus hollingsworthi
Nebrioporus assimilis 16S ribosomal RNA gene, part	AY250921	81.94	98.73	3.71225e−34	Coleoptera	Dytiscidae	Nebrioporus assimilis
Hydroporus pubescens 16S ribosomal RNA gene, parti	AF518276	82.91	100	3.71225e−34	Coleoptera	Dytiscidae	Hydroporus pubescens
Hydroporus limbatus 16S ribosomal RNA gene, partia	AF518264	82.91	100	3.71225e−34	Coleoptera	Dytiscidae	Hydroporus limbatus
Ilybius dettneri mitochondrial 16S rRNA gene (part	LM654797	81.53	100	1.2957e−33	Coleoptera	Dytiscidae	Ilybius dettneri
Agabus nebulosus mitochondrial 16S rRNA gene (part	LM654779	81.53	100	1.2957e−33	Coleoptera	Dytiscidae	Agabus nebulosus
Agabus glacialis mitochondrial 16S rRNA gene (part	LM654776	81.53	100	1.2957e−33	Coleoptera	Dytiscidae	Agabus glacialis
Cybister marginicollis voucher UNM KBMCyma416 16S	KJ548319	81.53	100	1.2957e−33	Coleoptera	Dytiscidae	Cybister marginicollis
Deronectes aubei voucher UNM KBMDeau758 16S riboso	KJ548292	82.28	100	1.2957e−33	Coleoptera	Dytiscidae	Deronectes aubei
Hydroporus planus mitochondrial 16S rRNA gene (par	HE613708	81.88	100	1.2957e−33	Coleoptera	Dytiscidae	Hydroporus planus
Deronectes costipennis mitochondrial 16S rRNA gene	HE613697	82.28	100	1.2957e−33	Coleoptera	Dytiscidae	Deronectes costipennis
Deronectes platynotus mitochondrial 16S rRNA gene	HE613682	82.28	100	1.2957e−33	Coleoptera	Dytiscidae	Deronectes platynotus
Deronectes depressicollis mitochondrial 16S rRNA g	HE613681	82.28	100	1.2957e−33	Coleoptera	Dytiscidae	Deronectes depressicollis
Deronectes semirufus mitochondrial 16S rRNA gene (	HE613676	82.28	100	1.2957e−33	Coleoptera	Dytiscidae	Deronectes semirufus
Deronectes semirufus mitochondrial 16S rRNA gene (	HE613674	82.28	100	1.2957e−33	Coleoptera	Dytiscidae	Deronectes semirufus
Antiporus hollingsworthi mitochondrial partial 16S	FR727247	81.29	98.73	4.52244e−33	Coleoptera	Dytiscidae	Antiporus hollingsworthi
