# Supplementary material for: Testing the potential of a ribosomal 16S marker for DNA metabarcoding of insects
Source: PeerJ. 2016 Apr 19;4:e1966. doi: 10.7717/peerj.1966 (PMC4841222; doi:10.7717/peerj.1966)
Supplement: Supplemental Information 1 [file peerj-04-1966-s006.zip › scripts/5 match OTUs/blast_hits/OTU_640.csv_plot.pdf]

OTU_640.csv

Oreodytes sanmarkii

Dytiscidae

Coleoptera

98.09

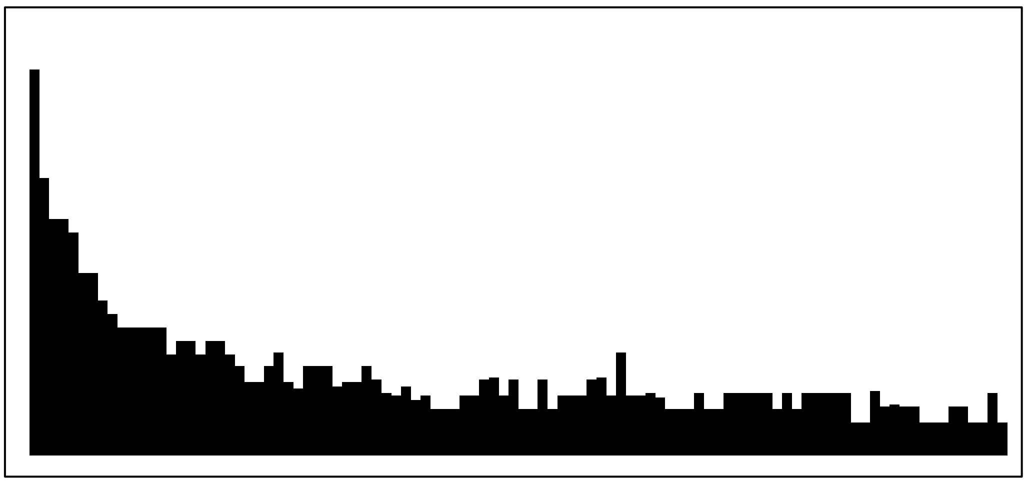

Oreodytes sanmarkii mitochondrial 16S rRNA gene (p	HF931524	98.09	100	6.73453e−69	Coleoptera	Dytiscidae	Oreodytes sanmarkii
Oreodytes obesus cordillerensis mitochondrial part	HF931516	92.99	100	4.8492e−58	Coleoptera	Dytiscidae	Oreodytes obesus cordillerensis
Oreodytes crassulus partial 16S rRNA gene	AJ850348	91.08	100	3.06018e−54	Coleoptera	Dytiscidae	Oreodytes crassulus
Oreodytes congruus partial 16S rRNA gene	AJ850347	91.08	100	3.06018e−54	Coleoptera	Dytiscidae	Oreodytes congruus
Oreodytes congruus 16S ribosomal RNA gene, partial	KF575550	90.45	100	1.30122e−52	Coleoptera	Dytiscidae	Oreodytes congruus
Oreodytes abbreviatus mitochondrial 16S rRNA gene	HF931477	88.54	100	8.21159e−49	Coleoptera	Dytiscidae	Oreodytes abbreviatus
Oreodytes rhyacophilus partial 16S rRNA gene	AJ850352	88.54	100	8.21159e−49	Coleoptera	Dytiscidae	Oreodytes rhyacophilus
Oreodytes mongolicus mitochondrial 16S rRNA gene (	HF931491	87.26	100	4.25371e−46	Coleoptera	Dytiscidae	Oreodytes mongolicus
Oreodytes snoqualmie mitochondrial partial 16S rRN	HF931519	86.62	100	1.80872e−44	Coleoptera	Dytiscidae	Oreodytes snoqualmie
Oreodytes septentrionalis mitochondrial 16S rRNA g	HF931492	85.99	100	2.20348e−43	Coleoptera	Dytiscidae	Oreodytes septentrionalis
Oreodytes shorti mitochondrial 16S rRNA gene (part	HF931488	85.99	100	2.20348e−43	Coleoptera	Dytiscidae	Oreodytes shorti
Boreonectes riberae mitochondrial 16S rRNA gene (p	HF931461	85.99	100	2.68438e−42	Coleoptera	Dytiscidae	Boreonectes riberae
Boreonectes griseostriatus mitochondrial partial 1	HF931387	85.99	100	2.68438e−42	Coleoptera	Dytiscidae	Boreonectes griseostriatus
Boreonectes multilineatus mitochondrial partial 16	HF931384	85.99	100	2.68438e−42	Coleoptera	Dytiscidae	Boreonectes multilineatus
Laccornis difformis 16S ribosomal RNA gene, partia	KF575544	84.71	100	1.14143e−40	Coleoptera	Dytiscidae	Laccornis difformis
Boreonectes alpestris mitochondrial 16S rRNA gene	HF931512	85.35	100	1.14143e−40	Coleoptera	Dytiscidae	Boreonectes alpestris
Boreonectes striatellus mitochondrial 16S rRNA gen	HF931511	85.35	100	1.14143e−40	Coleoptera	Dytiscidae	Boreonectes striatellus
Deronectes parvicollis mitochondrial 16S rRNA gene	HF931454	84.71	100	1.14143e−40	Coleoptera	Dytiscidae	Deronectes parvicollis
Boreonectes macedonicus mitochondrial 16S rRNA gen	HF931359	85.35	100	1.14143e−40	Coleoptera	Dytiscidae	Boreonectes macedonicus
Stictotarsus ibericus 16S ribosomal RNA (rrnL) gen	EF670030	85.35	100	1.14143e−40	Coleoptera	Dytiscidae	Stictotarsus ibericus
Boreonectes griseostriatus mitochondrial 16S rRNA	HF931541	84.71	100	1.39055e−39	Coleoptera	Dytiscidae	Boreonectes griseostriatus
Suphrodytes dorsalis 16S ribosomal RNA gene, parti	KF575577	84.18	100	5.91275e−38	Coleoptera	Dytiscidae	Suphrodytes dorsalis
Oreodytes scitulus 16S ribosomal RNA gene, partial	KF575551	83.44	100	5.91275e−38	Coleoptera	Dytiscidae	Oreodytes scitulus
Oreodytes scitulus mitochondrial partial 16S rRNA	HF931515	83.44	100	5.91275e−38	Coleoptera	Dytiscidae	Oreodytes scitulus
Suphrodytes dorsalis voucher BMNH:725966 16S ribos	JX221574	84.18	100	5.91275e−38	Coleoptera	Dytiscidae	Suphrodytes dorsalis
Hydroporus foveolatus mitochondrial 16S rRNA gene	HE613724	84.81	100	5.91275e−38	Coleoptera	Dytiscidae	Hydroporus foveolatus
Laccornis kocae partial 16S rRNA gene	AJ850419	83.44	100	5.91275e−38	Coleoptera	Dytiscidae	Laccornis kocae
Aethionectes fulvonotatus voucher KBMAeop406 16S r	KF979088	83.12	100	7.2032e−37	Coleoptera	Dytiscidae	Aethionectes fulvonotatus
Hydroporus tessellatus 16S ribosomal RNA gene, par	JX434770	84.18	100	7.2032e−37	Coleoptera	Dytiscidae	Hydroporus tessellatus
Hydroporus zimmermanni mitochondrial 16S rRNA gene	HE613679	84.18	100	7.2032e−37	Coleoptera	Dytiscidae	Hydroporus zimmermanni
Hydroporus basinotatus mitochondrial 16S rRNA gene	HE613678	84.18	100	7.2032e−37	Coleoptera	Dytiscidae	Hydroporus basinotatus
Antiporus hollingsworthi mitochondrial partial 16S	FR727246	83.23	98.73	7.2032e−37	Coleoptera	Dytiscidae	Antiporus hollingsworthi
Hydroporus glabriusculus 16S ribosomal RNA gene, p	AY365270	83.44	100	7.2032e−37	Coleoptera	Dytiscidae	Hydroporus glabriusculus
Hydroporus sp. IR33.4 16S ribosomal RNA gene, part	AY365261	83.44	100	7.2032e−37	Coleoptera	Dytiscidae	Hydroporus sp. IR33.4
Hydroporus basinotatus 16S ribosomal RNA gene, par	AF518258	84.18	100	7.2032e−37	Coleoptera	Dytiscidae	Hydroporus basinotatus
Nirripirti megamacrocephalus isolate ABTC78781 16S	EU616935	83.54	100	2.51416e−36	Coleoptera	Dytiscidae	Paroster megamacrocephalus
Nebrioporus formaster mitochondrial partial 16S rR	HF931377	82.91	100	8.7753e−36	Coleoptera	Dytiscidae	Nebrioporus formaster
Suphrodytes figuratus voucher BMNH:722148 16S ribo	JX221571	82.8	100	8.7753e−36	Coleoptera	Dytiscidae	Suphrodytes figuratus
Hydroporus marginatus mitochondrial 16S rRNA gene	HE613699	83.23	100	8.7753e−36	Coleoptera	Dytiscidae	Hydroporus marginatus
Antiporus hollingsworthi mitochondrial partial 16S	FR727247	82.58	98.73	8.7753e−36	Coleoptera	Dytiscidae	Antiporus hollingsworthi
Hydroporus marginatus 16S ribosomal RNA gene, part	AF518267	82.8	100	8.7753e−36	Coleoptera	Dytiscidae	Hydroporus marginatus
Lancetes varius voucher UNM KBMLcva196 16S ribosom	KJ548351	82.17	100	3.06288e−35	Coleoptera	Dytiscidae	Lancetes varius
Lancetes angusticollis voucher UNM KBMLcan301 16S	KJ548347	82.17	100	3.06288e−35	Coleoptera	Dytiscidae	Lancetes angusticollis
Hydaticus cinctipennis voucher UNM KBMHyci20 16S r	KJ548338	82.17	100	3.06288e−35	Coleoptera	Dytiscidae	Hydaticus cinctipennis
Hydroporus appalachius 16S ribosomal RNA gene, par	JX434765	82.8	100	3.06288e−35	Coleoptera	Dytiscidae	Hydroporus appalachius
Boreonectes griseostriatus mitochondrial 16S rRNA	HF931385	82.8	100	3.06288e−35	Coleoptera	Dytiscidae	Boreonectes griseostriatus
Hydroporus analis mitochondrial partial 16S rRNA g	HE613731	83.54	100	3.06288e−35	Coleoptera	Dytiscidae	Hydroporus analis
Hydroporus fuscipennis mitochondrial 16S rRNA gene	HE613730	83.65	100	3.06288e−35	Coleoptera	Dytiscidae	Hydroporus fuscipennis
Hydroporus pervicinus mitochondrial 16S rRNA gene	HE599695	82.8	100	3.06288e−35	Coleoptera	Dytiscidae	Hydroporus pervicinus
Hydroporus compunctus mitochondrial 16S rRNA gene	HE599693	83.54	100	3.06288e−35	Coleoptera	Dytiscidae	Hydroporus compunctus
Copelatus sp. BI−2011 voucher BMNH792945 16S ribos	HQ381441	82.17	100	3.06288e−35	Coleoptera	Dytiscidae	Copelatus sp. BI−2011
Oreodytes natrix partial 16S rRNA gene	AJ850349	82.17	100	3.06288e−35	Coleoptera	Dytiscidae	Oreodytes natrix
Nirripirti bulbus isolate ABTC78658−9 16S ribosoma	EU616918	83.54	100	3.06288e−35	Coleoptera	Dytiscidae	Paroster bulbus
Nirripirti byroensis isolate ABTC78677−8 16S ribos	EU616910	82.17	100	3.06288e−35	Coleoptera	Dytiscidae	Paroster byroensis
Nirripirti newhavenensis 16S ribosomal RNA gene, p	AY353844	82.8	100	3.06288e−35	Coleoptera	Dytiscidae	Paroster newhavenensis
Hydroporus occidentalis 16S ribosomal RNA gene, pa	AY365280	82.8	100	3.06288e−35	Coleoptera	Dytiscidae	Hydroporus occidentalis
Hydroporus hirtellus 16S ribosomal RNA gene, parti	AY365271	82.8	100	3.06288e−35	Coleoptera	Dytiscidae	Hydroporus hirtellus
Hydroporus compunctus 16S ribosomal RNA gene, part	AF518259	83.54	100	3.06288e−35	Coleoptera	Dytiscidae	Hydroporus compunctus
Hydroporus fuscipennis 16S ribosomal RNA gene, par	AF518262	83.65	100	3.06288e−35	Coleoptera	Dytiscidae	Hydroporus fuscipennis
Hydrocolus paugus 16S ribosomal RNA gene, partial	KF575539	82.8	100	1.06905e−34	Coleoptera	Dytiscidae	Hydrocolus paugus
Deronectes moestus mitochondrial 16S rRNA gene (pa	HE613726	84.83	91.72	1.06905e−34	Coleoptera	Dytiscidae	Deronectes moestus
Hydrocolus sahlbergi mitochondrial 16S rRNA gene (	HE599691	82.8	100	1.06905e−34	Coleoptera	Dytiscidae	Hydrocolus sahlbergi
Hydrocolus picicornis partial 16S rRNA gene	AJ850379	82.8	100	1.06905e−34	Coleoptera	Dytiscidae	Hydrocolus picicornis
Stictotarsus maghrebinus 16S ribosomal RNA gene, p	AY250947	82.91	100	1.06905e−34	Coleoptera	Dytiscidae	Stictotarsus maghrebinus
Scarodytes sp. IR−2003 16S ribosomal RNA gene, par	AY250939	82.72	100	1.06905e−34	Coleoptera	Dytiscidae	Scarodytes sp. IR−2003
Agabus didymus mitochondrial 16S rRNA gene (partia	LM654773	82.17	100	3.73135e−34	Coleoptera	Dytiscidae	Agabus didymus
Hydroporus umbrosus voucher UNM KBMHyum551 16S rib	KJ548394	82.17	100	3.73135e−34	Coleoptera	Dytiscidae	Hydroporus umbrosus
Agabus didymus voucher UNM KBMPmsp730 16S ribosoma	KJ548392	82.17	100	3.73135e−34	Coleoptera	Dytiscidae	Agabus didymus
Hydroporus foveolatus mitochondrial 16S rRNA gene	HF931502	82.91	100	3.73135e−34	Coleoptera	Dytiscidae	Hydroporus foveolatus
Hydroporus brancoi gredensis mitochondrial partial	HF931329	82.17	100	3.73135e−34	Coleoptera	Dytiscidae	Hydroporus brancoi gredensis
Suphrodytes figuratus voucher BMNH:722150 16S ribo	JX221572	82.17	100	3.73135e−34	Coleoptera	Dytiscidae	Suphrodytes figuratus
Hydroporus discretus mitochondrial 16S rRNA gene (	HE613723	82.91	100	3.73135e−34	Coleoptera	Dytiscidae	Hydroporus discretus
Hydroporus decipiens mitochondrial 16S rRNA gene (	HE613722	82.91	100	3.73135e−34	Coleoptera	Dytiscidae	Hydroporus decipiens
Hydroporus pubescens mitochondrial 16S rRNA gene (	HE613707	82.91	100	3.73135e−34	Coleoptera	Dytiscidae	Hydroporus pubescens
Hydroporus limbatus mitochondrial 16S rRNA gene (p	HE613706	82.91	100	3.73135e−34	Coleoptera	Dytiscidae	Hydroporus limbatus
Hydroporus errans mitochondrial 16S rRNA gene (par	HE613704	82.91	100	3.73135e−34	Coleoptera	Dytiscidae	Hydroporus errans
Suphrodytes dorsalis mitochondrial 16S rRNA gene (	HE599700	82.17	100	3.73135e−34	Coleoptera	Dytiscidae	Suphrodytes dorsalis
Hydroporus pubescens voucher NHM−IR39 16S ribosoma	EF419327	82.91	100	3.73135e−34	Coleoptera	Dytiscidae	Hydroporus pubescens
Hydroporus umbrosus 16S ribosomal RNA gene, partia	AF518280	82.17	100	3.73135e−34	Coleoptera	Dytiscidae	Hydroporus umbrosus
Hydroporus rufifrons 16S ribosomal RNA gene, parti	AF518277	82.91	100	3.73135e−34	Coleoptera	Dytiscidae	Hydroporus rufifrons
Hydroporus pubescens 16S ribosomal RNA gene, parti	AF518276	82.91	100	3.73135e−34	Coleoptera	Dytiscidae	Hydroporus pubescens
Hydroporus limbatus 16S ribosomal RNA gene, partia	AF518264	82.91	100	3.73135e−34	Coleoptera	Dytiscidae	Hydroporus limbatus
Hydroporus discretus 16S ribosomal RNA gene, parti	AF518260	82.91	100	3.73135e−34	Coleoptera	Dytiscidae	Hydroporus discretus
Hydroporus analis 16S ribosomal RNA gene, partial	AF518257	82.91	100	3.73135e−34	Coleoptera	Dytiscidae	Hydroporus analis
Lancetes lanceolatus voucher UNM KBMLcla91 16S rib	KJ548348	81.53	100	1.30237e−33	Coleoptera	Dytiscidae	Lancetes lanceolatus
Oreodytes quadrimaculatus 16S ribosomal RNA gene,	KF575567	81.53	100	1.30237e−33	Coleoptera	Dytiscidae	Oreodytes quadrimaculatus
Hygrotus armeniacus mitochondrial partial 16S rRNA	HF931520	83.02	100	1.30237e−33	Coleoptera	Dytiscidae	Hygrotus armeniacus
Stictotarsus procerus mitochondrial 16S rRNA gene	HF931379	82.28	100	1.30237e−33	Coleoptera	Dytiscidae	Stictotarsus procerus
Hydroporus planus mitochondrial 16S rRNA gene (par	HE613708	82.39	100	1.30237e−33	Coleoptera	Dytiscidae	Hydroporus planus
Deronectes opatrinus mitochondrial 16S rRNA gene (	HE613701	82.28	100	1.30237e−33	Coleoptera	Dytiscidae	Deronectes opatrinus
Paroster sp. R401 Newhaven voucher ABTC:78974 16S	JQ745754	82.28	100	1.30237e−33	Coleoptera	Dytiscidae	Paroster sp. R401 Newhaven
Copelatus sp. BI−2011 voucher BMNH671215 16S ribos	HQ381377	81.53	100	1.30237e−33	Coleoptera	Dytiscidae	Copelatus sp. BI−2011
Antiporus sp. MB1421 mitochondrial partial 16S rRN	FR727245	81.53	100	1.30237e−33	Coleoptera	Dytiscidae	Antiporus sp. MB1421
Oreodytes quadrimaculatus partial 16S rRNA gene	AJ850351	81.53	100	1.30237e−33	Coleoptera	Dytiscidae	Oreodytes quadrimaculatus
Paroster sp. 2 RL−2008 16S ribosomal RNA gene, par	EU616958	82.28	100	1.30237e−33	Coleoptera	Dytiscidae	Paroster sp. 2 RL−2008
Paroster sp. spnMtWedge 16S ribosomal RNA gene, pa	EU616954	82.28	100	1.30237e−33	Coleoptera	Dytiscidae	Paroster sp. spnMtWedge
Lancetes lanceolatus 16S ribosomal RNA gene, parti	AY138673	81.53	100	1.30237e−33	Coleoptera	Dytiscidae	Lancetes lanceolatus
Ilybius gagates 16S ribosomal RNA gene, partial se	AY138663	81.53	100	1.30237e−33	Coleoptera	Dytiscidae	Ilybius gagates
Ilybius erichsoni 16S ribosomal RNA gene, partial	AY138661	82.91	100	1.30237e−33	Coleoptera	Dytiscidae	Ilybius erichsoni
Nirripirti wedgeensis isolate ABTC78773−4 16S ribo	EU616938	81.53	100	4.54572e−33	Coleoptera	Dytiscidae	Paroster wedgeensis
