# Supplementary material for: Testing the potential of a ribosomal 16S marker for DNA metabarcoding of insects
Source: PeerJ. 2016 Apr 19;4:e1966. doi: 10.7717/peerj.1966 (PMC4841222; doi:10.7717/peerj.1966)
Supplement: Supplemental Information 1 [file peerj-04-1966-s006.zip › scripts/5 match OTUs/blast_hits/OTU_65.csv_plot.pdf]

OTU_65.csv

Ecdyonurus dispar

Heptageniidae

Ephemeroptera

90.54

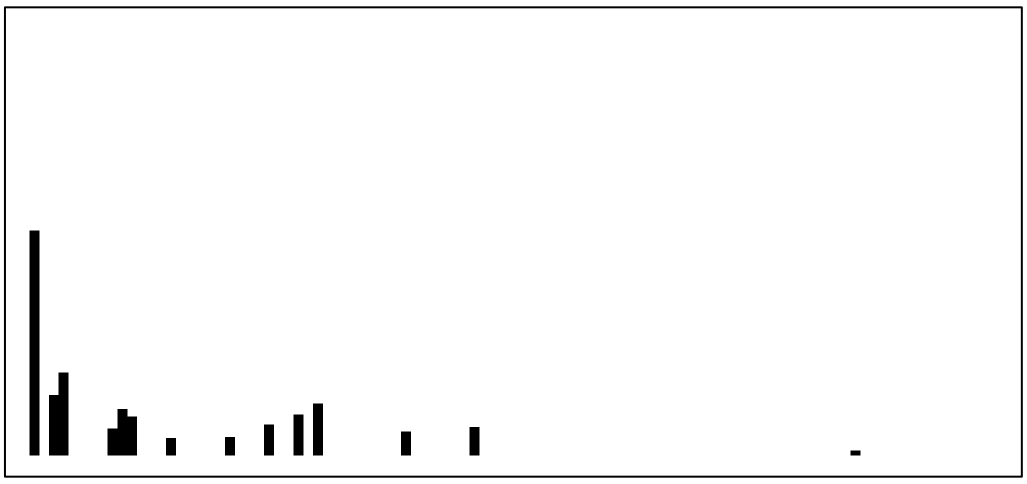

Ecdyonurus dispar 16S ribosomal RNA gene, partial	AY749775	90.54	95.48	2.30224e−49	Ephemeroptera	Heptageniidae	Ecdyonurus dispar
Parafronurus youi mitochondrion, complete genome	EU349015	77.55	94.84	1.11796e−21	Ephemeroptera	Heptageniidae	Parafronurus youi
Afronurus sp. 213BoAf mitochondrial partial 16S rR	HE651422	82.83	63.87	2.02131e−18	Ephemeroptera	Heptageniidae	Afronurus sp. 213BoAf
Afronurus sp. 212BoAf mitochondrial partial 16S rR	HE651421	83.87	60	7.05507e−18	Ephemeroptera	Heptageniidae	Afronurus sp. 212BoAf
Atopopus tarsalis mitochondrial partial 16S rRNA g	HE651420	75	95.48	7.05507e−18	Ephemeroptera	Heptageniidae	Atopopus tarsalis
Atopopus tarsalis mitochondrial partial 16S rRNA g	HE651419	75	95.48	7.05507e−18	Ephemeroptera	Heptageniidae	Atopopus tarsalis
Siphlonurus binotatus mitochondrial gene for 16S r	AB110269	76.09	89.03	7.05507e−18	Ephemeroptera	Siphlonuridae	Siphlonurus binotatus
Pseudiron centralis 16S ribosomal RNA gene, partia	AY749799	76.09	89.03	7.05507e−18	Ephemeroptera	Pseudironidae	Pseudiron centralis
Siphlonurus sp. EP084 16S ribosomal RNA gene, part	AY749786	81.25	71.61	7.05507e−18	Ephemeroptera	Siphlonuridae	Siphlonurus sp. EP084
Siphlonurus sp. MT−2014 isolate CL142 mitochondrio	KM244684	82.18	65.16	2.46246e−17	Ephemeroptera	Siphlonuridae	Siphlonurus sp. MT−2014
Pteronarcys sachalina 16S ribosomal RNA gene, part	EF623264	81.82	63.87	2.46246e−17	Plecoptera	Pteronarcyidae	Pteronarcys sachalina
Oniscigaster distans 16S ribosomal RNA gene, parti	AY749818	76	95.48	2.46246e−17	Ephemeroptera	Oniscigastridae	Oniscigaster distans
Clioperla clio 16S ribosomal RNA gene, partial seq	EF623237	74.5	95.48	2.99989e−16	Plecoptera	Perlodidae	Clioperla clio
Neuroperlopsis patris 16S ribosomal RNA gene, part	EF623152	75.18	90.32	1.04707e−15	Plecoptera	Eustheniidae	Neuroperlopsis patris
Osmylus sp. EH−2004 16S ribosomal RNA gene, partia	AY620146	80.81	63.87	1.04707e−15	Neuroptera	Osmylidae	Osmylus sp. EH−2004
Heptagenia sulphurea mitochondrial partial 16S rRN	HE651431	75.91	87.74	3.65462e−15	Ephemeroptera	Heptageniidae	Heptagenia sulphurea
Cinygmina sp. EP133 16S ribosomal RNA gene, partia	AY749811	73.55	100	3.65462e−15	Ephemeroptera	Heptageniidae	Cinygmina sp. EP133
Thalerosphyrus lamuriensis mitochondrial partial 1	HE651425	78.38	71.61	1.27559e−14	Ephemeroptera	Heptageniidae	Thalerosphyrus lamuriensis
Heteromallus cavicola voucher MIL 16S ribosomal RN	HM594499	78.26	72.9	1.27559e−14	Orthoptera	Rhaphidophoridae	Heteromallus cavicola
Rallidens mcfarlanei 16S ribosomal RNA gene, parti	AY749794	74.67	95.48	1.27559e−14	Ephemeroptera	Rallidentidae	Rallidens mcfarlanei
Parameletus columbiae 16S ribosomal RNA gene, part	AY749768	80.85	60.65	1.27559e−14	Ephemeroptera	Siphlonuridae	Parameletus columbiae
Hexagenia sp. BYU IGCEP003 16S ribosomal RNA gene,	GQ118286	77.88	72.9	4.45223e−14	Ephemeroptera	Ephemeridae	Hexagenia sp. BYU IGCEP003
Dolichopoda sp. MR−2008 16S ribosomal RNA gene, pa	EU887863	78.26	72.9	4.45223e−14	Orthoptera	Rhaphidophoridae	Dolichopoda lustriae
Cosumnoperla hypocrena 16S ribosomal RNA gene, par	EF623226	74.47	90.32	4.45223e−14	Plecoptera	Perlodidae	Cosumnoperla hypocrena
Yugus bulbosus 16S ribosomal RNA gene, partial seq	EF623214	81.44	61.29	4.45223e−14	Plecoptera	Perlodidae	Yugus bulbosus
Acruroperla atra 16S ribosomal RNA gene, partial s	EF623121	74	95.48	4.45223e−14	Plecoptera	Austroperlidae	Acruroperla atra
Ametropus neavei 16S ribosomal RNA gene, partial s	AY749800	75.51	87.74	4.45223e−14	Ephemeroptera	Ametropodidae	Ametropus neavei
Afronurus peringueyi 16S ribosomal RNA gene, parti	AY749790	81.91	59.35	4.45223e−14	Ephemeroptera	Heptageniidae	Afronurus peringueyi
Afronurus sp. 297SAAf mitochondrial partial 16S rR	HE651424	80	61.29	1.55398e−13	Ephemeroptera	Heptageniidae	Afronurus sp. 297SAAf
Osobenus yakimae 16S ribosomal RNA gene, partial s	EF623232	82.42	58.06	1.55398e−13	Plecoptera	Perlodidae	Osobenus yakimae
Isoperla davisi 16S ribosomal RNA gene, partial se	EF623206	74.29	89.68	1.55398e−13	Plecoptera	Perlodidae	Isoperla davisi
Thalerosphyrus lamuriensis mitochondrial partial 1	HE651429	77.48	71.61	5.42393e−13	Ephemeroptera	Heptageniidae	Thalerosphyrus lamuriensis
Tettigonia ussuriana isolate CLN008 16S large subu	EF198430	78.64	66.45	5.42393e−13	Orthoptera	Tettigoniidae	Tettigonia ussuriana
Zealeuctra arnoldi 16S ribosomal RNA gene, partial	EF623180	79	63.23	5.42393e−13	Plecoptera	Leuctridae	Zealeuctra arnoldi
Isogenoides varians isolate BYU_PL079 16S ribosoma	EF623445	79.79	60.65	5.42393e−13	Plecoptera	Perlodidae	Isogenoides varians
Tettigonia viridissima mitochondrial 16S rRNA gene	Z97622	78.64	66.45	5.42393e−13	Orthoptera	Tettigoniidae	Tettigonia viridissima
Perlodes mortoni 16S ribosomal RNA gene, partial s	KF855870	80	60.65	1.89314e−12	Plecoptera	Perlodidae	Perlodes mortoni
Thalerosphyrus sinuosus mitochondrial partial 16S	HE651430	74.82	85.81	1.89314e−12	Ephemeroptera	Heptageniidae	Thalerosphyrus sinuosus
Afronurus sp. 336SuAf mitochondrial partial 16S rR	HE651427	81.11	58.06	1.89314e−12	Ephemeroptera	Heptageniidae	Afronurus sp. 336SuAf
Talitropsis sedilloti voucher CW160 16S ribosomal	HM594511	78	64.52	1.89314e−12	Orthoptera	Rhaphidophoridae	Talitropsis sedilloti
Parudenus falklandicus voucher PAR 16S ribosomal R	HM594502	77.19	72.26	1.89314e−12	Orthoptera	Rhaphidophoridae	Parudenus falklandicus
Isogenoides olivaceus 16S ribosomal RNA gene, part	EF623224	80	60.65	1.89314e−12	Plecoptera	Perlodidae	Isogenoides olivaceus
Despaxia augusta 16S ribosomal RNA gene, partial s	EF623177	80	58.06	1.89314e−12	Plecoptera	Leuctridae	Despaxia augusta
Chaquihua sp. EP080 16S ribosomal RNA gene, partia	AY749784	78.95	61.29	1.89314e−12	Ephemeroptera	Ameletopsidae	Chaquihua sp. EP080
Baumanella alameda 16S ribosomal RNA gene, partial	EF623236	79.17	61.29	6.6077e−12	Plecoptera	Perlodidae	Baumanella alameda
Kogotus nonus 16S ribosomal RNA gene, partial sequ	EF623213	81.32	58.06	6.6077e−12	Plecoptera	Perlodidae	Kogotus nonus
Udamocercia sp. BYU_PL150 16S ribosomal RNA gene,	EF623201	78.64	65.81	6.6077e−12	Plecoptera	Notonemouridae	Udamocercia sp. BYU_PL150
Stenonema sp. EP026 16S ribosomal RNA gene, partia	AY749769	79.35	59.35	6.6077e−12	Ephemeroptera	Heptageniidae	Stenonema sp. EP026
Afronurus sp. 338SuAf mitochondrial partial 16S rR	HE651428	80	58.06	2.30631e−11	Ephemeroptera	Heptageniidae	Afronurus sp. 338SuAf
Notonurus josettae mitochondrial partial 16S rRNA	HE651408	72.37	95.48	2.30631e−11	Ephemeroptera	Heptageniidae	Notonurus josettae
Notonurus josettae mitochondrial partial 16S rRNA	HE651399	72.37	95.48	2.30631e−11	Ephemeroptera	Heptageniidae	Notonurus josettae
Compsoneuria sp. 4 LV−2012 mitochondrial partial 1	HE651398	77.06	69.68	2.30631e−11	Ephemeroptera	Heptageniidae	Compsoneuria sp. 4 LV−2012
Orthochtha dasycnemis 16S ribosomal RNA gene, part	JF932466	73.42	100	2.30631e−11	Orthoptera	Acrididae	Orthochtha dasycnemis
Tettigonia viridissima voucher 200 16S ribosomal R	EU203876	77.67	66.45	2.30631e−11	Orthoptera	Tettigoniidae	Tettigonia viridissima
Tettigonia viridissima voucher 122 16S ribosomal R	EU203862	77.67	66.45	2.30631e−11	Orthoptera	Tettigoniidae	Tettigonia viridissima
Dolichopoda sp. VAR 16S ribosomal RNA gene, partia	EU887865	76.52	72.9	2.30631e−11	Orthoptera	Rhaphidophoridae	Dolichopoda sp. VAR
Dolichopoda sp. TAY 16S ribosomal RNA gene, partia	EU887864	76.52	72.9	2.30631e−11	Orthoptera	Rhaphidophoridae	Dolichopoda sp. TAY
Dolichopoda annae voucher OLI 16S ribosomal RNA ge	EU887860	76.52	72.9	2.30631e−11	Orthoptera	Rhaphidophoridae	Dolichopoda annae
Tettigonia hispanica haplotype THISPA 16S ribosoma	EF515156	77.67	66.45	2.30631e−11	Orthoptera	Tettigoniidae	Tettigonia hispanica
Tettigonia viridissima haplotype TETI 16S ribosoma	EF515155	77.67	66.45	2.30631e−11	Orthoptera	Tettigoniidae	Tettigonia viridissima
Psalmatophanes barretoi haplotype MA6 16S ribosoma	EF515154	76.36	70.97	2.30631e−11	Orthoptera	Tettigoniidae	Psalmatophanes barretoi
Psalmatophanes barretoi haplotype MA5 16S ribosoma	EF515153	76.36	70.97	2.30631e−11	Orthoptera	Tettigoniidae	Psalmatophanes barretoi
Psalmatophanes barretoi haplotype MA4 16S ribosoma	EF515152	76.36	70.97	2.30631e−11	Orthoptera	Tettigoniidae	Psalmatophanes barretoi
Psalmatophanes barretoi haplotype MA3 16S ribosoma	EF515151	76.36	70.97	2.30631e−11	Orthoptera	Tettigoniidae	Psalmatophanes barretoi
Psalmatophanes barretoi haplotype MA2 16S ribosoma	EF515150	76.36	70.97	2.30631e−11	Orthoptera	Tettigoniidae	Psalmatophanes barretoi
Psalmatophanes barretoi haplotype MA1 16S ribosoma	EF515149	76.36	70.97	2.30631e−11	Orthoptera	Tettigoniidae	Psalmatophanes barretoi
Metrioptera bicolor isolate CLN011 16S large subun	EF198433	78	64.52	2.30631e−11	Orthoptera	Tettigoniidae	Bicolorana bicolor
Tettigonia cantans isolate CLN007 16S large subuni	EF198429	78	64.52	2.30631e−11	Orthoptera	Tettigoniidae	Tettigonia cantans
Tettigonia caudata isolate CLN006 16S large subuni	EF198428	78	64.52	2.30631e−11	Orthoptera	Tettigoniidae	Tettigonia caudata
Dolichopoda pavesii voucher SPI 16S ribosomal RNA	EF216951	77.14	67.1	2.30631e−11	Orthoptera	Rhaphidophoridae	Dolichopoda pavesii
Isogenoides hansoni 16S ribosomal RNA gene, partia	EF623223	78.95	60.65	2.30631e−11	Plecoptera	Perlodidae	Isogenoides hansoni
Isogenoides zionensis 16S ribosomal RNA gene, part	EF623210	78.95	60.65	2.30631e−11	Plecoptera	Perlodidae	Isogenoides zionensis
Neofulla sp. BYU_PL152 16S ribosomal RNA gene, par	EF623203	77.14	67.1	2.30631e−11	Plecoptera	Notonemouridae	Neofulla sp. BYU_PL152
Dinotoperla sp. BYU_PL105 16S ribosomal RNA gene,	EF623160	72.56	100	2.30631e−11	Plecoptera	Gripopterygidae	Dinotoperla sp. BYU_PL105
Isogenoides doratus isolate BYU_PL131 16S ribosoma	EF623449	78.95	60.65	2.30631e−11	Plecoptera	Perlodidae	Isogenoides doratus
Isogenoides colubrinus isolate BYU_PL130 16S ribos	EF623448	78.95	60.65	2.30631e−11	Plecoptera	Perlodidae	Isogenoides colubrinus
Isogenoides elongatus isolate BYU_PL082 16S riboso	EF623447	78.95	60.65	2.30631e−11	Plecoptera	Perlodidae	Isogenoides elongatus
Isogenoides frontalis isolate BYU_PL080 16S riboso	EF623446	78.95	60.65	2.30631e−11	Plecoptera	Perlodidae	Isogenoides frontalis
Tettigonia viridissima voucher 417 16S ribosomal R	EU120825	77.67	66.45	2.30631e−11	Orthoptera	Tettigoniidae	Tettigonia viridissima
Deracantha onos mitochondrion, complete genome	EU137664	78.35	62.58	8.04983e−11	Orthoptera	Tettigoniidae	Deracantha onos
Psorodonotus venosus voucher 406 16S ribosomal RNA	EU203911	78.35	62.58	8.04983e−11	Orthoptera	Tettigoniidae	Psorodonotus venosus
Psorodonotus caucasicus voucher 397 16S ribosomal	EU203908	78.35	62.58	8.04983e−11	Orthoptera	Tettigoniidae	Psorodonotus caucasicus
Psorodonotus sp. MMN−2008 voucher 393 16S ribosoma	EU203906	78.35	62.58	8.04983e−11	Orthoptera	Tettigoniidae	Psorodonotus sp. MMN−2008
Dolichopoda remyi voucher EDE 16S ribosomal RNA ge	EF216969	75.44	72.9	8.04983e−11	Orthoptera	Rhaphidophoridae	Dolichopoda remyi
Rickera sorpta 16S ribosomal RNA gene, partial seq	EF623235	80.22	58.06	8.04983e−11	Plecoptera	Perlodidae	Rickera sorpta
Isoperla oxylepis 16S ribosomal RNA gene, partial	EF623221	77.89	61.29	8.04983e−11	Plecoptera	Perlodidae	Isoperla oxylepis
Hydroperla crosbyi 16S ribosomal RNA gene, partial	EF623219	78.89	58.06	8.04983e−11	Plecoptera	Perlodidae	Hydroperla crosbyi
Moselia infuscata 16S ribosomal RNA gene, partial	EF623175	79.21	64.52	8.04983e−11	Plecoptera	Leuctridae	Moselia infuscata
Diamphipnopsis sp. BYU_PL138 16S ribosomal RNA gen	EF623145	74.05	83.23	8.04983e−11	Plecoptera	Diamphipnoidae	Diamphipnopsis sp. BYU_PL138
Triznaka pintada 16S ribosomal RNA gene, partial s	EF623134	73.43	90.97	8.04983e−11	Plecoptera	Chloroperlidae	Triznaka pintada
Psorodonotus fieberi voucher 198 16S ribosomal RNA	EU120806	78.35	62.58	8.04983e−11	Orthoptera	Tettigoniidae	Psorodonotus fieberi
Dolichopoda remyi isolate EDE 16S ribosomal RNA ge	AY793589	75.44	72.9	8.04983e−11	Orthoptera	Rhaphidophoridae	Dolichopoda remyi
Panorpa acuta 16S ribosomal RNA gene, partial sequ	AF180047	76.64	68.39	8.04983e−11	Mecoptera	Panorpidae	Panorpa acuta
Ichthybotus hudsoni 16S ribosomal RNA gene, partia	AY749816	78.79	63.87	8.04983e−11	Ephemeroptera	Ichthybotidae	Ichthybotus hudsoni
Heptagenia sp. EP018 16S ribosomal RNA gene, parti	AY749762	73.48	84.52	8.04983e−11	Ephemeroptera	Heptageniidae	Heptagenia sp. EP018
Parapholidoptera distincta haplotype 51−698 16S ri	JX024230	78.49	60	2.80967e−10	Orthoptera	Tettigoniidae	Parapholidoptera distincta
Compsoneuria sp. 9 LV−2012 mitochondrial partial 1	HE651406	72.6	92.26	2.80967e−10	Ephemeroptera	Heptageniidae	Compsoneuria sp. 9 LV−2012
Udenus wnigrum voucher MAY 16S ribosomal RNA gene,	HM594504	74.78	72.26	2.80967e−10	Orthoptera	Rhaphidophoridae	Udenus wnigrum
Udenus wnigrum voucher CER 16S ribosomal RNA gene,	HM594503	74.78	72.26	2.80967e−10	Orthoptera	Rhaphidophoridae	Udenus wnigrum
Parapholidoptera spinulosa voucher 388 16S ribosom	EU203904	78.49	60	2.80967e−10	Orthoptera	Tettigoniidae	Parapholidoptera spinulosa
