# Supplementary material for: Testing the potential of a ribosomal 16S marker for DNA metabarcoding of insects
Source: PeerJ. 2016 Apr 19;4:e1966. doi: 10.7717/peerj.1966 (PMC4841222; doi:10.7717/peerj.1966)
Supplement: Supplemental Information 1 [file peerj-04-1966-s006.zip › scripts/5 match OTUs/blast_hits/OTU_655.csv_plot.pdf]

OTU_655.csv

Parochlus steinenii

Chironomidae

Diptera

85.9

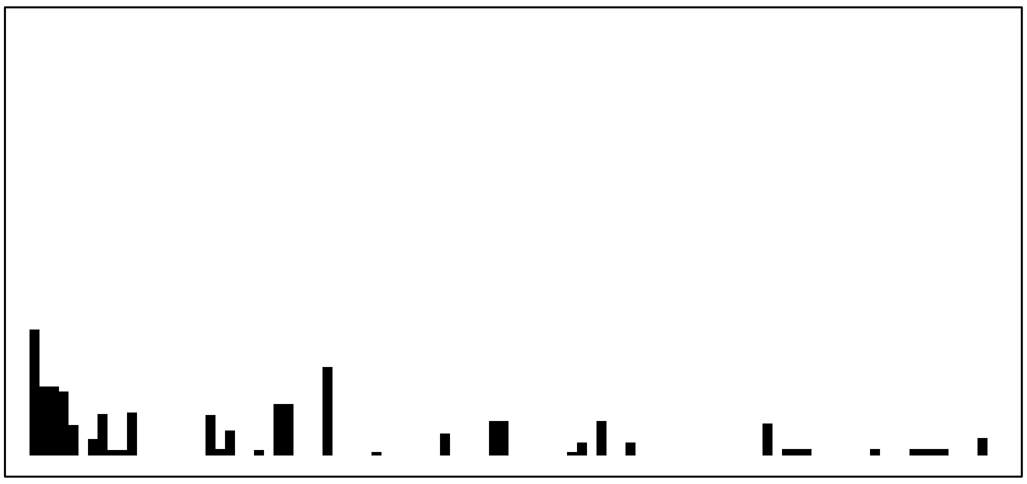

Parochlus steinenii isolate Sejong01 mitochondrion	KT003702	85.9	100	7.62785e−43	Diptera	Chironomidae	Parochlus steinenii
Bolitophila cinerea voucher JSB1 16S ribosomal RNA	KJ136712	83.23	97.44	1.29169e−33	Diptera	Mycetophilidae	Bolitophila cinerea
Bolitophila sp. ER−2009 16S ribosomal RNA gene, pa	FJ172021	83.23	97.44	1.29169e−33	Diptera	Mycetophilidae	Bolitophila sp. ER−2009
Bolitophila occlusa voucher JSB2 16S ribosomal RNA	KJ136713	82.99	93.59	1.91704e−31	Diptera	Mycetophilidae	Bolitophila occlusa
Agathomyia zetterstedti isolate 814 16S ribosomal	KC699312	81.41	99.36	6.69114e−31	Diptera	Platypezidae	Agathomyia zetterstedti
Agathomyia elegantula isolate AE04 16S ribosomal R	KC699320	80	99.36	8.15148e−30	Diptera	Platypezidae	Agathomyia elegantula
Agathomyia lundbecki isolate 233 16S ribosomal RNA	KC699306	80.77	99.36	8.15148e−30	Diptera	Platypezidae	Agathomyia lundbecki
Bolitophila hybrida 16S ribosomal RNA gene, partia	FJ172002	81.94	97.44	8.15148e−30	Diptera	Mycetophilidae	Bolitophila hybrida
Tipula sp. PMO 200011 16S ribosomal RNA gene, part	EU005437	80.25	100	8.15148e−30	Diptera	Tipulidae	Tipula sp. PMO 200011
Pedicia albivitta voucher O'Grady 200012 16S ribos	EU005435	80.25	100	8.15148e−30	Diptera	Pediciidae	Pedicia albivitta
Arachnocampa luminosa biotype 1 16S large subunit	AY576329	82	94.23	8.15148e−30	Diptera	Keroplatidae	Arachnocampa luminosa
Edwardsina gigantea 16S ribosomal RNA gene, partia	KC177460	79.35	99.36	9.93054e−29	Diptera	Blephariceridae	Edwardsina gigantea
Clinocera sp. Pollet 0249 16S ribosomal RNA gene,	HQ449032	79.75	99.36	3.4661e−28	Diptera	Empididae	Clinocera sp. Pollet 0249
Geomyza tripunctata 16S ribosomal RNA gene, partia	EU268537	78.71	99.36	4.22257e−27	Diptera	Opomyzidae	Geomyza tripunctata
Sylvicola fenestralis 16S ribosomal RNA gene, part	KC177461	78.85	100	1.47382e−26	Diptera	Anisopodidae	Sylvicola fenestralis
Tipula abdominalis 16S ribosomal RNA gene, partial	KC177457	78.21	100	1.47382e−26	Diptera	Tipulidae	Tipula abdominalis
Sylvicola fenestralis mitochondrion, complete geno	JN861752	78.85	100	1.47382e−26	Diptera	Anisopodidae	Sylvicola fenestralis
Tipula abdominalis mitochondrion, partial genome	JN861743	78.21	100	1.47382e−26	Diptera	Tipulidae	Tipula abdominalis
Arachnocampa luminosa biotype 3 16S large subunit	AY576331	81.88	87.82	1.47382e−26	Diptera	Keroplatidae	Arachnocampa luminosa
Platypezina diversa isolate PD29 16S ribosomal RNA	KC699349	80.29	87.82	1.79548e−25	Diptera	Platypezidae	Platypezina diversa
Callomyia admirabilis isolate CA42 16S ribosomal R	KC699326	81.16	87.82	1.79548e−25	Diptera	Platypezidae	Callomyia admirabilis
Agathomyia viduella isolate AV01 16S ribosomal RNA	KC699317	78.85	99.36	1.79548e−25	Diptera	Platypezidae	Agathomyia viduella
Agathomyia sexmaculata isolate 816 16S ribosomal R	KC699313	78.21	99.36	1.79548e−25	Diptera	Platypezidae	Agathomyia sexmaculata
Beris morrisii 16S ribosomal RNA gene, partial seq	GU947373	80.25	99.36	1.79548e−25	Diptera	Stratiomyidae	Beris morrisii
Somatia sp. YSUW04038 16S ribosomal RNA gene, part	AY573134	78.91	94.23	1.79548e−25	Diptera	Somatiidae	Somatia sp. YSUW04038
Simulium tenederoi mitochondrial gene for 16S ribo	LC035002	82.4	79.49	6.26685e−25	Diptera	Simuliidae	Simulium tenederoi
Simulium tenederoi mitochondrial gene for 16S ribo	LC035001	82.4	79.49	6.26685e−25	Diptera	Simuliidae	Simulium tenederoi
Simulium suyoense mitochondrial gene for 16S ribos	LC035000	79.63	100	6.26685e−25	Diptera	Simuliidae	Simulium suyoense
Agathomyia woodella isolate 448 16S ribosomal RNA	KC699307	79.59	93.59	6.26685e−25	Diptera	Platypezidae	Agathomyia woodella
Trichopeza longicornis voucher Pollet 0243 (P) 16S	HQ449035	79.75	99.36	6.26685e−25	Diptera	Empididae	Trichopeza longicornis
Hercostomus meieri voucher RMBR:103442 16S ribosom	FJ808173	84.13	79.49	6.26685e−25	Diptera	Dolichopodidae	Hercostomus meieri
Empis producta voucher RMBR:103430 16S ribosomal R	FJ808167	78.98	99.36	6.26685e−25	Diptera	Empididae	Empis producta
Chydaeopeza tibialis isolate CT44 16S ribosomal RN	KC699336	78.21	99.36	2.18735e−24	Diptera	Platypezidae	Chydaeopeza tibialis
Agathomyia falleni isolate AF03 16S ribosomal RNA	KC699319	77.42	99.36	2.18735e−24	Diptera	Platypezidae	Agathomyia falleni
Grossoseta pacifica isolate 1384 16S ribosomal RNA	KC699316	79.56	87.82	2.18735e−24	Diptera	Platypezidae	Grossoseta pacifica
Ptychoptera sp. ATB−2011 mitochondrion, complete g	JN861744	80.14	89.1	2.18735e−24	Diptera	Ptychopteridae	Ptychoptera sp. ATB−2011
Arachnocampa luminosa biotype 2 16S large subunit	AY576330	79.33	94.23	2.18735e−24	Diptera	Keroplatidae	Arachnocampa luminosa
Coelopa alluaudi 16S ribosomal RNA gene, partial s	AF403455	79.56	87.82	2.18735e−24	Diptera	Coelopidae	Coelopa alluaudi
Chaetocoelopa littoralis 16S ribosomal RNA gene, p	AF403453	78.21	99.36	2.18735e−24	Diptera	Coelopidae	Chaetocoelopa littoralis
Simulium mie mitochondrial gene for 16S ribosomal	AB056732	78.62	100	2.18735e−24	Diptera	Simuliidae	Simulium mie
Simulium mie mitochondrial gene for 16S ribosomal	AB056731	78.62	100	2.18735e−24	Diptera	Simuliidae	Simulium mie
Simulium mie mitochondrial gene for 16S ribosomal	AB056730	78.62	100	2.18735e−24	Diptera	Simuliidae	Simulium mie
Simulium izuae isolate I1B 16S ribosomal RNA gene,	KM410189	81.02	85.26	7.63459e−24	Diptera	Simuliidae	Simulium izuae
Simulium tuyense mitochondrial gene for 16S riboso	LC035004	78.43	97.44	2.66473e−23	Diptera	Simuliidae	Simulium tuyense
Simulium suyoense mitochondrial gene for 16S ribos	LC034999	79.01	100	2.66473e−23	Diptera	Simuliidae	Simulium suyoense
Simulium suyoense mitochondrial gene for 16S ribos	LC034998	79.01	100	2.66473e−23	Diptera	Simuliidae	Simulium suyoense
Simulium suyoense mitochondrial gene for 16S ribos	LC034997	79.01	100	2.66473e−23	Diptera	Simuliidae	Simulium suyoense
Simulium claveriaense mitochondrial gene for 16S r	LC034988	81.6	79.49	2.66473e−23	Diptera	Simuliidae	Simulium claveriaense
Simulium claveriaense mitochondrial gene for 16S r	LC034987	81.6	79.49	2.66473e−23	Diptera	Simuliidae	Simulium claveriaense
Simulium sheilae isolate SH2B 16S ribosomal RNA ge	KM410214	77.71	100	2.66473e−23	Diptera	Simuliidae	Simulium sheilae
Protoclythia rufa isolate PR22 16S ribosomal RNA g	KC699354	76.77	99.36	2.66473e−23	Diptera	Platypezidae	Protoclythia rufa
Protoclythia modesta isolate PM21 16S ribosomal RN	KC699350	76.77	99.36	2.66473e−23	Diptera	Platypezidae	Protoclythia modesta
Ectinorhynchus sp. 'Bribie' voucher UQIC41178 16S	KM212983	78.75	99.36	2.66473e−23	Diptera	Therevidae	Ectinorhynchus sp. 'Bribie'
Ectinorhynchus sp. 'Moffatt' voucher QMT183203 16S	KM212976	78.75	99.36	2.66473e−23	Diptera	Therevidae	Ectinorhynchus sp. 'Moffatt'
Ectinorhynchus sp. 'warrumbungles' voucher QMT1832	KM212974	78.75	99.36	2.66473e−23	Diptera	Therevidae	Ectinorhynchus sp. 'warrumbungles'
Paracladura trichoptera mitochondrion, complete ge	JN861751	80.14	86.54	2.66473e−23	Diptera	Trichoceridae	Paracladura trichoptera
Simulium decuplum 16S ribosomal RNA gene, partial	GQ865986	80.6	85.26	2.66473e−23	Diptera	Simuliidae	Simulium decuplum
Simulium suyoense mitochondrial gene for 16S ribos	AB334099	79.01	100	2.66473e−23	Diptera	Simuliidae	Simulium suyoense
Simulium claveriaense mitochondrial gene for 16S r	AB334096	81.6	79.49	2.66473e−23	Diptera	Simuliidae	Simulium claveriaense
Dialysis sp. SLW−2002 16S ribosomal RNA gene, part	AY140888	76.77	99.36	2.66473e−23	Diptera	Xylophagidae	Dialysis sp. SLW−2002
Caenophthalmus capensis voucher MEI 120162 16S rib	DQ250838	79.73	92.31	2.66473e−23	Diptera	Therevidae	Caenophthalmus capensis
Simulium cabrerai mitochondrial gene for 16S rRNA,	AB093128	80.6	85.26	2.66473e−23	Diptera	Simuliidae	Simulium cabrerai
Protoclythia modesta 16S ribosomal RNA gene, parti	AF126348	76.77	99.36	2.66473e−23	Diptera	Platypezidae	Protoclythia modesta
Belosta albipilosa 16S ribosomal RNA gene, partial	KM878903	78.62	99.36	9.30083e−23	Diptera	Scenopinidae	Belosta albipilosa
Callomyia amoena isolate CA06 16S ribosomal RNA ge	KC699325	79.71	87.82	9.30083e−23	Diptera	Platypezidae	Callomyia amoena
Agathomyia unicolor isolate AU2 16S ribosomal RNA	KC699318	77.56	99.36	9.30083e−23	Diptera	Platypezidae	Agathomyia unicolor
Simulium tribulatum isolate LAC 17−5 16S ribosomal	KP233212	77.99	100	9.30083e−23	Diptera	Simuliidae	Simulium tribulatum
Haematobia irritans mitochondrion, partial genome	KM669714	77.56	99.36	9.30083e−23	Diptera	Muscidae	Haematobia irritans
Protoplasa fitchii 16S ribosomal RNA gene, partial	KC177462	76.73	97.44	9.30083e−23	Diptera	Tanyderidae	Protoplasa fitchii
Haematobia irritans isolate D50 small subunit ribo	JQ246755	77.56	99.36	9.30083e−23	Diptera	Muscidae	Haematobia irritans
Protoplasa fitchii mitochondrion, complete genome	JN861746	76.73	97.44	9.30083e−23	Diptera	Tanyderidae	Protoplasa fitchii
Haematobia irritans 16S ribosomal RNA gene, partia	FJ025436	77.56	99.36	9.30083e−23	Diptera	Muscidae	Haematobia irritans
Limonia sp. PMO 200984 16S ribosomal RNA gene, par	EU005416	76.62	98.72	9.30083e−23	Diptera	Limoniidae	Limonia sp. PMO 200984
Haematobia irritans irritans mitochondrion, comple	DQ029097	77.56	99.36	9.30083e−23	Diptera	Muscidae	Haematobia irritans irritans
This canus 16S ribosomal RNA gene, partial sequenc	AF403473	78.42	89.1	9.30083e−23	Diptera	Coelopidae	This canus
Simulium palauense mitochondrial gene for 16S ribo	AB056742	81.48	85.26	9.30083e−23	Diptera	Simuliidae	Simulium palauense
Simulium sheilae isolate SH1B 16S ribosomal RNA ge	KM410213	77.07	100	3.24631e−22	Diptera	Simuliidae	Simulium sheilae
Simulium roslihashimi isolate R1B 16S ribosomal RN	KM410192	80.29	85.26	3.24631e−22	Diptera	Simuliidae	Simulium roslihashimi
Simulium izuae isolate I3B 16S ribosomal RNA gene,	KM410191	80.29	85.26	3.24631e−22	Diptera	Simuliidae	Simulium izuae
Simulium izuae isolate I2B 16S ribosomal RNA gene,	KM410190	80.29	85.26	3.24631e−22	Diptera	Simuliidae	Simulium izuae
Tongamya stuckenbergi 16S ribosomal RNA gene, part	KM878969	78.12	99.36	3.24631e−22	Diptera	Mydidae	Tongamya stuckenbergi
Atherigona varia voucher RMBR:116181 16S ribosomal	KJ476297	78.48	99.36	3.24631e−22	Diptera	Muscidae	Atherigona varia
Simulium khunklangense mitochondrial gene for 16S	AB820367	78.77	92.31	3.24631e−22	Diptera	Simuliidae	Simulium khunklangense
Liriomyza sativae mitochondrion, complete genome	JQ862475	76.43	99.36	3.24631e−22	Diptera	Agromyzidae	Liriomyza sativae
Simulium chomthongense mitochondrial gene for 16S	AB699899	78.77	92.31	3.24631e−22	Diptera	Simuliidae	Simulium chomthongense
Haematopota pluvialis 16S ribosomal RNA gene, part	KC177454	78	94.23	3.24631e−22	Diptera	Tabanidae	Haematopota pluvialis
Simulium tuenense mitochondrial gene for 16S ribos	AB591244	80.29	85.26	3.24631e−22	Diptera	Simuliidae	Simulium tuenense
Liriomyza sativae mitochondrion, complete genome	HQ333260	76.43	99.36	3.24631e−22	Diptera	Agromyzidae	Liriomyza sativae
Tabanus bromius 16S ribosomal RNA gene, partial se	HM132111	76.58	99.36	3.24631e−22	Diptera	Tabanidae	Tabanus bromius
Simulium caudisclerum 16S ribosomal RNA gene, part	GQ865999	78.77	92.31	3.24631e−22	Diptera	Simuliidae	Simulium caudisclerum
Simulium chumpornense 16S ribosomal RNA gene, part	GQ865998	80.29	85.26	3.24631e−22	Diptera	Simuliidae	Simulium chumpornense
Simulium inthanonense 16S ribosomal RNA gene, part	GQ865995	80.29	85.26	3.24631e−22	Diptera	Simuliidae	Simulium inthanonense
Simulium decuplum 16S ribosomal RNA gene, partial	GQ865987	80.29	85.26	3.24631e−22	Diptera	Simuliidae	Simulium decuplum
Simulium angulistylum 16S ribosomal RNA gene, part	GQ865985	80.29	85.26	3.24631e−22	Diptera	Simuliidae	Simulium angulistylum
Drosophila rubida voucher 109400 NADH dehydrogenas	EU494327	78.52	92.31	3.24631e−22	Diptera	Drosophilidae	Drosophila rubida
Drosophila hypocausta voucher 103972 NADH dehydrog	EU494319	78.52	92.31	3.24631e−22	Diptera	Drosophilidae	Drosophila hypocausta
Simulium oblongum mitochondrial gene for 16S ribos	AB334089	77.07	100	3.24631e−22	Diptera	Simuliidae	Simulium oblongum
Simulium baltazarae mitochondrial gene for 16S rRN	AB093114	80.8	79.49	3.24631e−22	Diptera	Simuliidae	Simulium baltazarae
Simulium sasai mitochondrial gene for 16S ribosoma	AB056734	78.38	91.67	3.24631e−22	Diptera	Simuliidae	Simulium sasai
Simulium feuerborni mitochondrial gene for 16S rib	AB056728	78.38	91.67	3.24631e−22	Diptera	Simuliidae	Simulium feuerborni
