# Supplementary material for: Testing the potential of a ribosomal 16S marker for DNA metabarcoding of insects
Source: PeerJ. 2016 Apr 19;4:e1966. doi: 10.7717/peerj.1966 (PMC4841222; doi:10.7717/peerj.1966)
Supplement: Supplemental Information 1 [file peerj-04-1966-s006.zip › scripts/5 match OTUs/blast_hits/OTU_657.csv_plot.pdf]

OTU_657.csv

Pedicia albivitta

Pediciidae

Diptera

87.07

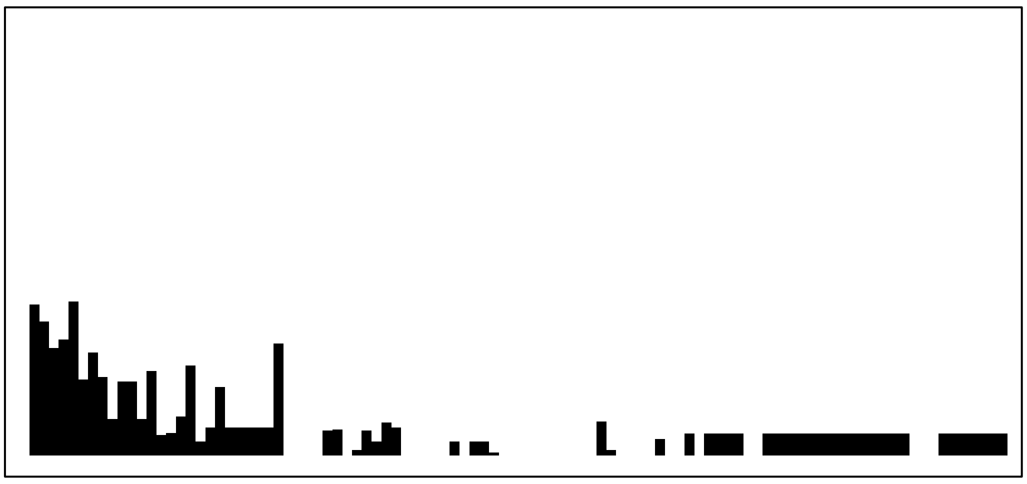

Pedicia albivitta voucher O'Grady 200012 16S ribos	EU005435	87.07	94.81	1.11336e−40	Diptera	Pediciidae	Pedicia albivitta
Bolitophila cinerea voucher JSB1 16S ribosomal RNA	KJ136712	86.27	97.4	4.73413e−39	Diptera	Mycetophilidae	Bolitophila cinerea
Edwardsina gigantea 16S ribosomal RNA gene, partia	KC177460	85.03	95.45	5.76735e−38	Diptera	Blephariceridae	Edwardsina gigantea
Parochlus steinenii isolate Sejong01 mitochondrion	KT003702	85.42	92.86	2.98756e−35	Diptera	Chironomidae	Parochlus steinenii
Bolitophila sp. ER−2009 16S ribosomal RNA gene, pa	FJ172021	87.22	85.71	2.98756e−35	Diptera	Mycetophilidae	Bolitophila sp. ER−2009
Corynoptera blanda voucher 955 16S ribosomal RNA g	JQ613965	83.55	97.4	3.6396e−34	Diptera	Sciaridae	Corynoptera blanda
Tipula sp. PMO 200011 16S ribosomal RNA gene, part	EU005437	84.83	92.86	1.27034e−33	Diptera	Tipulidae	Tipula sp. PMO 200011
Eudicrana sp. DK−2013 voucher JSS12 16S ribosomal	KC435571	83.67	93.51	1.5476e−32	Diptera	Mycetophilidae	Eudicrana sp. DK−2013
Minettia longipennis mitochondrion partial 16S rRN	AJ439184	81.7	98.7	5.40164e−32	Diptera	Lauxaniidae	Minettia longipennis
Tipula abdominalis 16S ribosomal RNA gene, partial	KC177457	83.45	92.21	1.88536e−31	Diptera	Tipulidae	Tipula abdominalis
Tipula abdominalis mitochondrion, partial genome	JN861743	83.45	92.21	1.88536e−31	Diptera	Tipulidae	Tipula abdominalis
Derodontus emaculatus voucher BMNH:679374 16S ribo	DQ202609	81.7	98.05	6.58055e−31	Coleoptera		Derodontus emaculatus
Bolitophila hybrida 16S ribosomal RNA gene, partia	FJ172002	83.94	88.31	2.29684e−30	Diptera	Mycetophilidae	Bolitophila hybrida
Drosophila takahashii cytochrome b (cytb) gene, pa	AF164592	80.95	95.45	8.01675e−30	Diptera	Drosophilidae	Drosophila takahashii
Ptychoptera sp. ATB−2011 mitochondrion, complete g	JN861744	81.05	98.05	2.79812e−29	Diptera	Ptychopteridae	Ptychoptera sp. ATB−2011
Tinda javana 16S ribosomal RNA gene, partial seque	GU947393	81.82	92.86	2.79812e−29	Diptera	Stratiomyidae	Tinda javana
Culcua kolibaci 16S ribosomal RNA gene, partial se	GU947391	84.21	85.71	2.79812e−29	Diptera	Stratiomyidae	Culcua kolibaci
Drosophila funebris voucher 103952 NADH dehydrogen	EU494313	80.65	98.7	9.7664e−29	Diptera	Drosophilidae	Drosophila funebris
Chrysoperla nipponensis mitochondrial DNA, complet	AP011623	81.29	98.7	3.40881e−28	Neuroptera	Chrysopidae	Chrysoperla nipponensis
Pyratula zonata 16S ribosomal RNA gene, partial se	FJ172004	83.21	85.06	3.40881e−28	Diptera	Keroplatidae	Pyratula zonata
Chrysoperla agilis isolate A−083 16S large subunit	AY743751	81.29	98.7	3.40881e−28	Neuroptera	Chrysopidae	Chrysoperla agilis
Chrysoperla lucasina isolate L−079 16S large subun	AY743746	81.29	98.7	3.40881e−28	Neuroptera	Chrysopidae	Chrysoperla lucasina
Chrysoperla lucasina isolate L−070 16S large subun	AY743744	81.29	98.7	3.40881e−28	Neuroptera	Chrysopidae	Chrysoperla lucasina
Chrysoperla lucasina isolate L−029 16S large subun	AY743737	81.29	98.7	3.40881e−28	Neuroptera	Chrysopidae	Chrysoperla lucasina
Chrysoperla lucasina isolate L−099 16S large subun	AY743732	81.29	98.7	3.40881e−28	Neuroptera	Chrysopidae	Chrysoperla lucasina
Leptomorphus walkeri 16S ribosomal RNA gene, parti	FJ172020	85.25	77.92	1.18979e−27	Diptera	Mycetophilidae	Leptomorphus walkeri
Drosophila signata voucher 109405 NADH dehydrogena	EU494328	79.74	98.7	1.18979e−27	Diptera	Drosophilidae	Drosophila signata
Drosophila rubida voucher 109400 NADH dehydrogenas	EU494327	79.74	98.7	1.18979e−27	Diptera	Drosophilidae	Drosophila rubida
Drosophila kepulauana voucher 109407 NADH dehydrog	EU494321	79.74	98.7	1.18979e−27	Diptera	Drosophilidae	Drosophila kepulauana
Drosophila hypocausta voucher 103972 NADH dehydrog	EU494319	79.74	98.7	1.18979e−27	Diptera	Drosophilidae	Drosophila hypocausta
Panorpa takenouchii isolate 26tak 16S ribosomal RN	EF041520	81.17	98.05	1.18979e−27	Mecoptera	Panorpidae	Panorpa takenouchii
Ochlerotatus nipponicus isolate A75 16S ribosomal	DQ397914	81.21	95.45	1.18979e−27	Diptera	Culicidae	Aedes nipponicus
Drosophila kepulauana 16S ribosomal RNA gene, part	AF387334	79.74	98.7	1.18979e−27	Diptera	Drosophilidae	Drosophila kepulauana
Chrysoperla carnea isolate Chrcar3 16S ribosomal R	AY620150	80.25	98.7	1.18979e−27	Neuroptera	Chrysopidae	Chrysoperla carnea
Panorpa takenouchii 16S ribosomal RNA gene, partia	AF180064	81.17	98.05	1.18979e−27	Mecoptera	Panorpidae	Panorpa takenouchii
Chrysoperla nipponensis mitochondrion, complete ge	KP027407	80.65	98.7	4.15278e−27	Neuroptera	Chrysopidae	Chrysoperla nipponensis
Acilius athabascae voucher 305G09/BMNH799009 16S r	KF979079	81.53	98.7	4.15278e−27	Coleoptera	Dytiscidae	Acilius athabascae
Cychrus caraboides caraboides voucher CC00817 16S	JQ647184	81.29	98.05	4.15278e−27	Coleoptera	Carabidae	Cychrus caraboides caraboides
Calosoma sp. BYU−CO241 mitochondrion, complete gen	GU176340	79.87	98.05	4.15278e−27	Coleoptera	Carabidae	Calosoma sp. BYU−CO241
Calosoma scrutator 16S ribosomal RNA gene, partial	EU734850	79.87	98.05	4.15278e−27	Coleoptera	Carabidae	Calosoma scrutator
Drosophila malerkotliana voucher 105504 NADH dehyd	EU494357	79.59	95.45	4.15278e−27	Diptera	Drosophilidae	Drosophila malerkotliana
Mallada luctuosus 16S ribosomal RNA gene, partial	DQ399288	79.38	98.7	4.15278e−27	Neuroptera	Chrysopidae	Pseudomallada luctuosus
Leucochrysa marquezi voucher CASC 00212 16S riboso	DQ399280	80	98.7	4.15278e−27	Neuroptera	Chrysopidae	Leucochrysa marquezi
Chrysoperla carnea isolate Chrcar2 16S ribosomal R	AY620152	80.65	98.7	4.15278e−27	Neuroptera	Chrysopidae	Chrysoperla carnea
Drosophila malerkotliana cytochrome b (cytb) gene,	AF164579	79.59	95.45	4.15278e−27	Diptera	Drosophilidae	Drosophila malerkotliana
Chrysoperla sp. isolate 82 16S large subunit ribos	AY743750	80.65	98.7	4.15278e−27	Neuroptera	Chrysopidae	Chrysoperla sp. isolate 82
Chrysoperla carnea isolate C−097 16S large subunit	AY743731	80.65	98.7	4.15278e−27	Neuroptera	Chrysopidae	Chrysoperla carnea
Sarcophagidae sp. BAHAR107−14 voucher BAH_201312_P	KP253964	80.14	92.86	1.44946e−26	Diptera	Sarcophagidae	Sarcophagidae sp. BAHAR107−14
Protoplasa fitchii 16S ribosomal RNA gene, partial	KC177462	79.87	94.16	1.44946e−26	Diptera	Tanyderidae	Protoplasa fitchii
Colobaea bifasciella voucher MU_NUS_SG3 16S riboso	JN828202	79.08	98.7	1.44946e−26	Diptera	Sciomyzidae	Colobaea bifasciella
Protoplasa fitchii mitochondrion, complete genome	JN861746	79.87	94.16	1.44946e−26	Diptera	Tanyderidae	Protoplasa fitchii
Eudmeta marginata 16S ribosomal RNA gene, partial	GU947400	79.74	98.7	1.44946e−26	Diptera	Stratiomyidae	Eudmeta marginata
Hercostomus verbekei voucher Pollet 0263 (P) 16S r	EU864004	79.08	98.7	1.44946e−26	Diptera	Dolichopodidae	Hercostomus verbekei
Drosophila putrida voucher 103964 NADH dehydrogena	EU494348	79.08	98.7	1.44946e−26	Diptera	Drosophilidae	Drosophila putrida
Drosophila nasuta voucher 103957 NADH dehydrogenas	EU494323	79.08	98.7	1.44946e−26	Diptera	Drosophilidae	Drosophila nasuta
Drosophila albomicans voucher 109408 NADH dehydrog	EU494318	79.08	98.7	1.44946e−26	Diptera	Drosophilidae	Drosophila albomicans
Libnotes perkinsi voucher O'Grady 200981 16S ribos	EU005433	79.74	98.05	1.44946e−26	Diptera	Limoniidae	Libnotes perkinsi
Libnotes perkinsi voucher O'Grady 200947 16S ribos	EU005432	79.74	98.05	1.44946e−26	Diptera	Limoniidae	Libnotes perkinsi
Culex mimeticus 16S ribosomal RNA gene, partial se	EF593021	81.58	95.45	1.44946e−26	Diptera	Culicidae	Culex mimeticus
Apertochrysa edwardsi 16S ribosomal RNA gene, part	DQ399265	80.25	98.7	1.44946e−26	Neuroptera	Chrysopidae	Apertochrysa edwardsi
Drosophila albomicans 16S ribosomal RNA gene, part	AF387333	79.08	98.7	1.44946e−26	Diptera	Drosophilidae	Drosophila albomicans
Drosophila sulfurigaster albostrigata 16S ribosoma	AF387336	79.08	98.7	1.44946e−26	Diptera	Drosophilidae	Drosophila sulfurigaster albostrigata
Drosophila nasuta 16S ribosomal RNA gene, partial	AF387335	79.08	98.7	1.44946e−26	Diptera	Drosophilidae	Drosophila nasuta
Drosophila kohkoa 16S ribosomal RNA gene, partial	AF387332	79.08	98.7	1.44946e−26	Diptera	Drosophilidae	Drosophila kohkoa
Chrysoperla lucasina isolate L−078 16S large subun	AY743776	80.77	98.7	1.44946e−26	Neuroptera	Chrysopidae	Chrysoperla lucasina
Sapromyza madeirensis mitochondrion partial 16S rR	AJ439187	79.08	98.7	1.44946e−26	Diptera	Lauxaniidae	Sapromyza madeirensis
Bolopus furcatus isolate 1305 16S ribosomal RNA ge	KC699315	79.22	98.7	5.05912e−26	Diptera	Platypezidae	Bolopus furcatus
Wuchereria bancrofti genome assembly W_bancrofti_J	LM000940	81.01	98.7	5.05912e−26	Spirurida	Onchocercidae	Wuchereria bancrofti
Keilbachia subacumina voucher 890 16S ribosomal RN	JQ613951	80	94.16	5.05912e−26	Diptera	Sciaridae	Keilbachia subacumina
Culex quinquefasciatus from USA mitochondrion, com	HQ724617	81.01	98.7	5.05912e−26	Diptera	Culicidae	Culex quinquefasciatus
Culex pipiens pipiens from Turkey mitochondrion, c	HQ724616	81.01	98.7	5.05912e−26	Diptera	Culicidae	Culex pipiens pipiens
Culex pipiens pipiens from Tunisia mitochondrion,	HQ724614	81.01	98.7	5.05912e−26	Diptera	Culicidae	Culex pipiens pipiens
Culex quinquefasciatus mitochondrion, complete gen	GU188856	81.01	98.7	5.05912e−26	Diptera	Culicidae	Culex quinquefasciatus
Hercostomus longiventris voucher Pollet 0274 (P) 1	EU863994	79.87	98.7	5.05912e−26	Diptera	Dolichopodidae	Hercostomus longiventris
Grzegorzekia collaris 16S ribosomal RNA gene, part	FJ172016	79.61	97.4	5.05912e−26	Diptera	Mycetophilidae	Grzegorzekia collaris
Culex quinquefasciatus isolate A−373 16S ribosomal	EU711101	81.01	98.7	5.05912e−26	Diptera	Culicidae	Culex quinquefasciatus
Culex quinquefasciatus isolate A−365 16S ribosomal	EU711099	81.01	98.7	5.05912e−26	Diptera	Culicidae	Culex quinquefasciatus
Culex quinquefasciatus isolate A−274 16S ribosomal	EU711098	81.01	98.7	5.05912e−26	Diptera	Culicidae	Culex quinquefasciatus
Culex quinquefasciatus isolate A−266 16S ribosomal	EU711095	81.01	98.7	5.05912e−26	Diptera	Culicidae	Culex quinquefasciatus
Culex quinquefasciatus isolate A−263 16S ribosomal	EU711094	81.01	98.7	5.05912e−26	Diptera	Culicidae	Culex quinquefasciatus
Culex quinquefasciatus isolate A−128 16S ribosomal	EU711088	81.01	98.7	5.05912e−26	Diptera	Culicidae	Culex quinquefasciatus
Culex quinquefasciatus isolate A−173 16S ribosomal	EU711092	81.01	98.7	5.05912e−26	Diptera	Culicidae	Culex quinquefasciatus
Culex quinquefasciatus isolate A−169 16S ribosomal	EU711091	81.01	98.7	5.05912e−26	Diptera	Culicidae	Culex quinquefasciatus
Culex quinquefasciatus isolate A−137 16S ribosomal	EU711090	81.01	98.7	5.05912e−26	Diptera	Culicidae	Culex quinquefasciatus
Culex quinquefasciatus isolate A−84 16S ribosomal	EU711087	81.01	98.7	5.05912e−26	Diptera	Culicidae	Culex quinquefasciatus
Culex quinquefasciatus isolate A−66 16S ribosomal	EU711086	81.01	98.7	5.05912e−26	Diptera	Culicidae	Culex quinquefasciatus
Culex quinquefasciatus isolate A−59 16S ribosomal	EU711085	81.01	98.7	5.05912e−26	Diptera	Culicidae	Culex quinquefasciatus
Culex quinquefasciatus isolate A−52 16S ribosomal	EU711084	81.01	98.7	5.05912e−26	Diptera	Culicidae	Culex quinquefasciatus
Culex quinquefasciatus isolate A−40 16S ribosomal	EU711083	81.01	98.7	5.05912e−26	Diptera	Culicidae	Culex quinquefasciatus
Culex quinquefasciatus isolate A−35 16S ribosomal	EU711082	81.01	98.7	5.05912e−26	Diptera	Culicidae	Culex quinquefasciatus
Thinophilus sp. 4 SNK−2009 voucher RMBR:103514 16S	FJ808217	79.38	98.7	5.05912e−26	Diptera	Dolichopodidae	Thinophilus sp. 4 SNK−2009
Hercostomus meieri voucher RMBR:103442 16S ribosom	FJ808173	79.87	98.7	5.05912e−26	Diptera	Dolichopodidae	Hercostomus meieri
Empis producta voucher RMBR:103430 16S ribosomal R	FJ808167	79.22	98.7	5.05912e−26	Diptera	Empididae	Empis producta
Culex quinquefasciatus from India: Jamnagar 16S ri	EU555416	81.01	98.7	5.05912e−26	Diptera	Culicidae	Culex quinquefasciatus
Culex quinquefasciatus from India: Jodhpur 16S rib	EU555415	81.01	98.7	5.05912e−26	Diptera	Culicidae	Culex quinquefasciatus
Culex quinquefasciatus from India: Bikaner 16S rib	EU555414	81.01	98.7	5.05912e−26	Diptera	Culicidae	Culex quinquefasciatus
Culex quinquefasciatus from India: Bhatinda 16S ri	EU555413	81.01	98.7	5.05912e−26	Diptera	Culicidae	Culex quinquefasciatus
Culex quinquefasciatus isolate A81 16S ribosomal R	EU339142	81.01	98.7	5.05912e−26	Diptera	Culicidae	Culex quinquefasciatus
Culex quinquefasciatus isolate A12 16S ribosomal R	EU339146	81.01	98.7	5.05912e−26	Diptera	Culicidae	Culex quinquefasciatus
Culex quinquefasciatus isolate A77 16S ribosomal R	EU339144	81.01	98.7	5.05912e−26	Diptera	Culicidae	Culex quinquefasciatus
