# Supplementary material for: Testing the potential of a ribosomal 16S marker for DNA metabarcoding of insects
Source: PeerJ. 2016 Apr 19;4:e1966. doi: 10.7717/peerj.1966 (PMC4841222; doi:10.7717/peerj.1966)
Supplement: Supplemental Information 1 [file peerj-04-1966-s006.zip › scripts/5 match OTUs/blast_hits/OTU_662.csv_plot.pdf]

OTU_662.csv

Oreodytes sanmarkii

Dytiscidae

Coleoptera

98.09

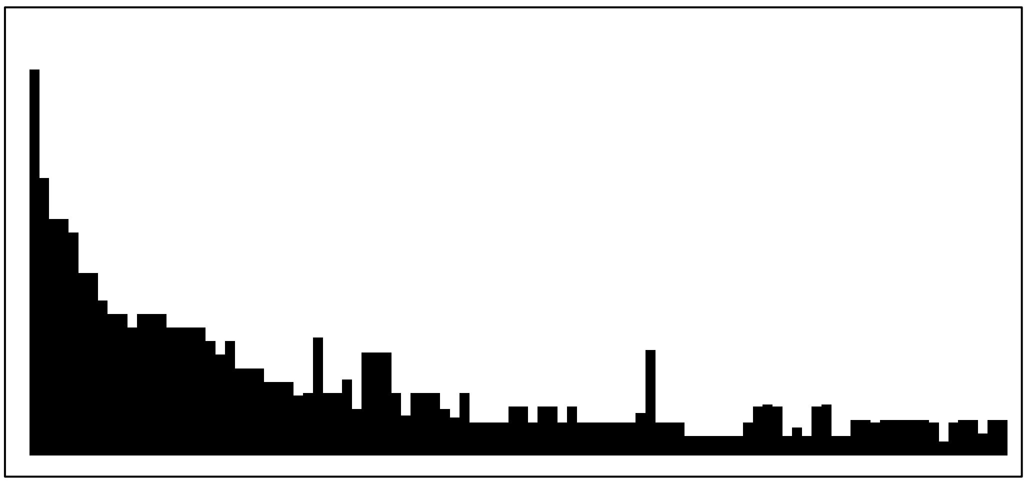

Oreodytes sanmarkii mitochondrial 16S rRNA gene (p	HF931524	98.09	100	6.73453e−69	Coleoptera	Dytiscidae	Oreodytes sanmarkii
Oreodytes obesus cordillerensis mitochondrial part	HF931516	92.99	100	4.8492e−58	Coleoptera	Dytiscidae	Oreodytes obesus cordillerensis
Oreodytes crassulus partial 16S rRNA gene	AJ850348	91.08	100	3.06018e−54	Coleoptera	Dytiscidae	Oreodytes crassulus
Oreodytes congruus partial 16S rRNA gene	AJ850347	91.08	100	3.06018e−54	Coleoptera	Dytiscidae	Oreodytes congruus
Oreodytes congruus 16S ribosomal RNA gene, partial	KF575550	90.45	100	1.30122e−52	Coleoptera	Dytiscidae	Oreodytes congruus
Oreodytes abbreviatus mitochondrial 16S rRNA gene	HF931477	88.54	100	8.21159e−49	Coleoptera	Dytiscidae	Oreodytes abbreviatus
Oreodytes rhyacophilus partial 16S rRNA gene	AJ850352	88.54	100	8.21159e−49	Coleoptera	Dytiscidae	Oreodytes rhyacophilus
Oreodytes mongolicus mitochondrial 16S rRNA gene (	HF931491	87.26	100	4.25371e−46	Coleoptera	Dytiscidae	Oreodytes mongolicus
Oreodytes snoqualmie mitochondrial partial 16S rRN	HF931519	86.62	100	1.80872e−44	Coleoptera	Dytiscidae	Oreodytes snoqualmie
Oreodytes septentrionalis mitochondrial 16S rRNA g	HF931492	86.62	100	1.80872e−44	Coleoptera	Dytiscidae	Oreodytes septentrionalis
Oreodytes shorti mitochondrial 16S rRNA gene (part	HF931488	85.99	100	2.20348e−43	Coleoptera	Dytiscidae	Oreodytes shorti
Boreonectes riberae mitochondrial 16S rRNA gene (p	HF931461	86.62	100	2.20348e−43	Coleoptera	Dytiscidae	Boreonectes riberae
Boreonectes griseostriatus mitochondrial partial 1	HF931387	86.62	100	2.20348e−43	Coleoptera	Dytiscidae	Boreonectes griseostriatus
Boreonectes multilineatus mitochondrial partial 16	HF931384	86.62	100	2.20348e−43	Coleoptera	Dytiscidae	Boreonectes multilineatus
Boreonectes alpestris mitochondrial 16S rRNA gene	HF931512	85.99	100	2.68438e−42	Coleoptera	Dytiscidae	Boreonectes alpestris
Boreonectes striatellus mitochondrial 16S rRNA gen	HF931511	85.99	100	2.68438e−42	Coleoptera	Dytiscidae	Boreonectes striatellus
Boreonectes macedonicus mitochondrial 16S rRNA gen	HF931359	85.99	100	2.68438e−42	Coleoptera	Dytiscidae	Boreonectes macedonicus
Stictotarsus ibericus 16S ribosomal RNA (rrnL) gen	EF670030	85.99	100	2.68438e−42	Coleoptera	Dytiscidae	Stictotarsus ibericus
Deronectes parvicollis mitochondrial 16S rRNA gene	HF931454	85.35	100	9.36942e−42	Coleoptera	Dytiscidae	Deronectes parvicollis
Laccornis difformis 16S ribosomal RNA gene, partia	KF575544	84.71	100	1.14143e−40	Coleoptera	Dytiscidae	Laccornis difformis
Boreonectes griseostriatus mitochondrial 16S rRNA	HF931541	85.35	100	1.14143e−40	Coleoptera	Dytiscidae	Boreonectes griseostriatus
Oreodytes scitulus 16S ribosomal RNA gene, partial	KF575551	84.08	100	4.85348e−39	Coleoptera	Dytiscidae	Oreodytes scitulus
Oreodytes scitulus mitochondrial partial 16S rRNA	HF931515	84.08	100	4.85348e−39	Coleoptera	Dytiscidae	Oreodytes scitulus
Laccornis kocae partial 16S rRNA gene	AJ850419	84.08	100	4.85348e−39	Coleoptera	Dytiscidae	Laccornis kocae
Lancetes nigriceps partial 16S rRNA gene	AJ850418	83.44	100	5.91275e−38	Coleoptera	Dytiscidae	Lancetes nigriceps
Lancetes nigriceps 16S ribosomal RNA gene, partial	AY334132	83.44	100	5.91275e−38	Coleoptera	Dytiscidae	Lancetes nigriceps
Boreonectes griseostriatus mitochondrial 16S rRNA	HF931385	83.44	100	7.2032e−37	Coleoptera	Dytiscidae	Boreonectes griseostriatus
Lancetes nigriceps voucher UNM KBMLcni390 16S ribo	KJ548350	82.8	100	2.51416e−36	Coleoptera	Dytiscidae	Lancetes nigriceps
Nebrioporus formaster mitochondrial partial 16S rR	HF931377	82.91	100	8.7753e−36	Coleoptera	Dytiscidae	Nebrioporus formaster
Deronectes moestus mitochondrial 16S rRNA gene (pa	HE613726	85.52	91.72	8.7753e−36	Coleoptera	Dytiscidae	Deronectes moestus
Suphrodytes dorsalis 16S ribosomal RNA gene, parti	KF575577	82.91	100	3.06288e−35	Coleoptera	Dytiscidae	Suphrodytes dorsalis
Suphrodytes dorsalis voucher BMNH:725966 16S ribos	JX221574	82.91	100	3.06288e−35	Coleoptera	Dytiscidae	Suphrodytes dorsalis
Hydroporus foveolatus mitochondrial 16S rRNA gene	HE613724	83.54	100	3.06288e−35	Coleoptera	Dytiscidae	Hydroporus foveolatus
Oreodytes natrix partial 16S rRNA gene	AJ850349	82.17	100	3.06288e−35	Coleoptera	Dytiscidae	Oreodytes natrix
Deronectes moestus 16S ribosomal RNA gene, partial	KF575561	84.83	91.72	1.06905e−34	Coleoptera	Dytiscidae	Deronectes moestus
Deronectes brannani mitochondrial 16S rRNA gene (p	HE613695	84.83	91.72	1.06905e−34	Coleoptera	Dytiscidae	Deronectes brannanii
Deronectes moestus mitochondrial 16S rRNA gene (pa	HE613683	84.83	91.72	1.06905e−34	Coleoptera	Dytiscidae	Deronectes moestus
Stictotarsus maghrebinus 16S ribosomal RNA gene, p	AY250947	82.91	100	1.06905e−34	Coleoptera	Dytiscidae	Stictotarsus maghrebinus
Aethionectes fulvonotatus voucher KBMAeop406 16S r	KF979088	81.88	100	3.73135e−34	Coleoptera	Dytiscidae	Aethionectes fulvonotatus
Hydroporus tessellatus 16S ribosomal RNA gene, par	JX434770	82.91	100	3.73135e−34	Coleoptera	Dytiscidae	Hydroporus tessellatus
Hydroporus zimmermanni mitochondrial 16S rRNA gene	HE613679	82.91	100	3.73135e−34	Coleoptera	Dytiscidae	Hydroporus zimmermanni
Hydroporus basinotatus mitochondrial 16S rRNA gene	HE613678	82.91	100	3.73135e−34	Coleoptera	Dytiscidae	Hydroporus basinotatus
Hydroporus glabriusculus 16S ribosomal RNA gene, p	AY365270	82.17	100	3.73135e−34	Coleoptera	Dytiscidae	Hydroporus glabriusculus
Scarodytes sp. IR−2003 16S ribosomal RNA gene, par	AY250939	81.76	100	3.73135e−34	Coleoptera	Dytiscidae	Scarodytes sp. IR−2003
Hydroporus basinotatus 16S ribosomal RNA gene, par	AF518258	82.91	100	3.73135e−34	Coleoptera	Dytiscidae	Hydroporus basinotatus
Lancetes varius voucher UNM KBMLcva196 16S ribosom	KJ548351	81.53	100	1.30237e−33	Coleoptera	Dytiscidae	Lancetes varius
Lancetes angusticollis voucher UNM KBMLcan301 16S	KJ548347	81.53	100	1.30237e−33	Coleoptera	Dytiscidae	Lancetes angusticollis
Hydaticus cinctipennis voucher UNM KBMHyci20 16S r	KJ548338	81.53	100	1.30237e−33	Coleoptera	Dytiscidae	Hydaticus cinctipennis
Oreodytes quadrimaculatus 16S ribosomal RNA gene,	KF575567	81.53	100	1.30237e−33	Coleoptera	Dytiscidae	Oreodytes quadrimaculatus
Stictotarsus procerus mitochondrial 16S rRNA gene	HF931379	82.28	100	1.30237e−33	Coleoptera	Dytiscidae	Stictotarsus procerus
Deronectes doriae mitochondrial 16S rRNA gene (par	HE613710	82.28	100	1.30237e−33	Coleoptera	Dytiscidae	Deronectes doriae
Deronectes bicostatus mitochondrial 16S rRNA gene	HE613705	81.53	100	1.30237e−33	Coleoptera	Dytiscidae	Deronectes bicostatus
Deronectes opatrinus mitochondrial 16S rRNA gene (	HE613701	82.28	100	1.30237e−33	Coleoptera	Dytiscidae	Deronectes opatrinus
Deronectes sahlbergi mitochondrial 16S rRNA gene (	HE613680	82.28	100	1.30237e−33	Coleoptera	Dytiscidae	Deronectes sahlbergi
Copelatus sp. BI−2011 voucher BMNH792945 16S ribos	HQ381441	81.53	100	1.30237e−33	Coleoptera	Dytiscidae	Copelatus sp. BI−2011
Stictotarsus falli 16S ribosomal RNA (rrnL) gene,	EF670029	82.28	100	1.30237e−33	Coleoptera	Dytiscidae	Stictotarsus falli
Oreodytes quadrimaculatus partial 16S rRNA gene	AJ850351	81.53	100	1.30237e−33	Coleoptera	Dytiscidae	Oreodytes quadrimaculatus
Ilybius gagates 16S ribosomal RNA gene, partial se	AY138663	81.53	100	1.30237e−33	Coleoptera	Dytiscidae	Ilybius gagates
Hygrotus novemlineatus mitochondrial partial 16S r	HF931523	81.53	100	4.54572e−33	Coleoptera	Dytiscidae	Hygrotus novemlineatus
Hygrotus marklini mitochondrial 16S rRNA gene (par	HF931467	81.53	100	4.54572e−33	Coleoptera	Dytiscidae	Hygrotus marklini
Hygrotus patruelis mitochondrial 16S rRNA gene (pa	HF931388	81.53	100	4.54572e−33	Coleoptera	Dytiscidae	Hygrotus patruelis
Suphrodytes figuratus voucher BMNH:722148 16S ribo	JX221571	81.53	100	4.54572e−33	Coleoptera	Dytiscidae	Suphrodytes figuratus
Hydroporus marginatus mitochondrial 16S rRNA gene	HE613699	81.99	100	4.54572e−33	Coleoptera	Dytiscidae	Hydroporus marginatus
Hovahydrus sp. BI−2011 voucher BMNH831333 16S ribo	HQ381524	84.93	91.72	4.54572e−33	Coleoptera	Dytiscidae	Hovahydrus sp. BI−2011
Hygrotus turbidus partial 16S rRNA gene	AJ850390	81.53	100	4.54572e−33	Coleoptera	Dytiscidae	Hygrotus turbidus
Hydroporus sp. IR33.4 16S ribosomal RNA gene, part	AY365261	81.53	100	4.54572e−33	Coleoptera	Dytiscidae	Hydroporus sp. IR33.4
Hydroporus marginatus 16S ribosomal RNA gene, part	AF518267	81.53	100	4.54572e−33	Coleoptera	Dytiscidae	Hydroporus marginatus
Ilybius fraterculus mitochondrial 16S rRNA gene (p	LM654800	80.89	100	1.58661e−32	Coleoptera	Dytiscidae	Ilybius fraterculus
Ilybius ater mitochondrial 16S rRNA gene (partial)	LM654794	80.89	100	1.58661e−32	Coleoptera	Dytiscidae	Ilybius ater
Deronectes latus voucher UNM KBMDnla560 16S riboso	KJ548380	80.89	100	1.58661e−32	Coleoptera	Dytiscidae	Deronectes latus
Lancetes lanceolatus voucher UNM KBMLcla91 16S rib	KJ548348	80.89	100	1.58661e−32	Coleoptera	Dytiscidae	Lancetes lanceolatus
Ilybius fraterculus voucher UNM KBMIlfr409 16S rib	KJ548342	80.89	100	1.58661e−32	Coleoptera	Dytiscidae	Ilybius fraterculus
Ilybius sp. UNM KBMAgsp411 16S ribosomal RNA gene,	KJ548304	80.89	100	1.58661e−32	Coleoptera	Dytiscidae	Ilybius sp. UNM KBMAgsp411
Boreonectes funereus mitochondrial 16S rRNA gene (	HF931393	81.53	100	1.58661e−32	Coleoptera	Dytiscidae	Boreonectes funereus
Hydroporus analis mitochondrial partial 16S rRNA g	HE613731	82.28	100	1.58661e−32	Coleoptera	Dytiscidae	Hydroporus analis
Hydroporus fuscipennis mitochondrial 16S rRNA gene	HE613730	82.39	100	1.58661e−32	Coleoptera	Dytiscidae	Hydroporus fuscipennis
Hydroporus compunctus mitochondrial 16S rRNA gene	HE599693	82.28	100	1.58661e−32	Coleoptera	Dytiscidae	Hydroporus compunctus
Copelatus sp. BI−2011 voucher BMNH671215 16S ribos	HQ381377	80.89	100	1.58661e−32	Coleoptera	Dytiscidae	Copelatus sp. BI−2011
Antiporus hollingsworthi mitochondrial partial 16S	FR727246	81.29	98.73	1.58661e−32	Coleoptera	Dytiscidae	Antiporus hollingsworthi
Lancetes varius 16S ribosomal RNA gene, partial se	AY071784	80.89	100	1.58661e−32	Coleoptera	Dytiscidae	Lancetes varius
Hydroporus compunctus 16S ribosomal RNA gene, part	AF518259	82.28	100	1.58661e−32	Coleoptera	Dytiscidae	Hydroporus compunctus
Hydroporus fuscipennis 16S ribosomal RNA gene, par	AF518262	82.39	100	1.58661e−32	Coleoptera	Dytiscidae	Hydroporus fuscipennis
Lancetes lanceolatus 16S ribosomal RNA gene, parti	AY138673	80.89	100	1.58661e−32	Coleoptera	Dytiscidae	Lancetes lanceolatus
Aglymbus gestroi 16S ribosomal RNA gene, partial s	AY138639	80.89	100	1.58661e−32	Coleoptera	Dytiscidae	Aglymbus gestroi
Deronectes aubei voucher UNM KBMDeau758 16S riboso	KJ548292	81.65	100	5.53782e−32	Coleoptera	Dytiscidae	Deronectes aubei
Sanfilippodytes sp. KBM−2014 16S ribosomal RNA gen	KF575554	81.65	100	5.53782e−32	Coleoptera	Dytiscidae	Sanfilippodytes sp. KBM−2014
Hydrocolus paugus 16S ribosomal RNA gene, partial	KF575539	81.53	100	5.53782e−32	Coleoptera	Dytiscidae	Hydrocolus paugus
Deronectes costipennis mitochondrial 16S rRNA gene	HE613697	81.65	100	5.53782e−32	Coleoptera	Dytiscidae	Deronectes costipennis
Deronectes platynotus mitochondrial 16S rRNA gene	HE613682	81.65	100	5.53782e−32	Coleoptera	Dytiscidae	Deronectes platynotus
Deronectes depressicollis mitochondrial 16S rRNA g	HE613681	81.65	100	5.53782e−32	Coleoptera	Dytiscidae	Deronectes depressicollis
Deronectes semirufus mitochondrial 16S rRNA gene (	HE613676	81.65	100	5.53782e−32	Coleoptera	Dytiscidae	Deronectes semirufus
Deronectes semirufus mitochondrial 16S rRNA gene (	HE613674	81.65	100	5.53782e−32	Coleoptera	Dytiscidae	Deronectes semirufus
Hydrocolus sahlbergi mitochondrial 16S rRNA gene (	HE599691	81.53	100	5.53782e−32	Coleoptera	Dytiscidae	Hydrocolus sahlbergi
Antiporus hollingsworthi mitochondrial partial 16S	FR727247	80.65	98.73	5.53782e−32	Coleoptera	Dytiscidae	Antiporus hollingsworthi
Hydrocolus picicornis partial 16S rRNA gene	AJ850379	81.53	100	5.53782e−32	Coleoptera	Dytiscidae	Hydrocolus picicornis
Nirripirti megamacrocephalus isolate ABTC78781 16S	EU616935	81.65	100	5.53782e−32	Coleoptera	Dytiscidae	Paroster megamacrocephalus
Deronectes costipennis 16S ribosomal RNA gene, par	AY250951	81.65	100	5.53782e−32	Coleoptera	Dytiscidae	Deronectes costipennis
Scarodytes halensis haplotype 3 16S ribosomal RNA	AY250937	81.01	100	5.53782e−32	Coleoptera	Dytiscidae	Scarodytes halensis
Deronectes aubei sanfilippoi haplotype 2 16S ribos	AY250908	81.65	100	5.53782e−32	Coleoptera	Dytiscidae	Deronectes aubei sanfilippoi
Deronectes aubei sanfilippoi haplotype 1 16S ribos	AY250907	81.65	100	5.53782e−32	Coleoptera	Dytiscidae	Deronectes aubei sanfilippoi
