# Supplementary material for: Testing the potential of a ribosomal 16S marker for DNA metabarcoding of insects
Source: PeerJ. 2016 Apr 19;4:e1966. doi: 10.7717/peerj.1966 (PMC4841222; doi:10.7717/peerj.1966)

OTU_67.csv

Comostola subtiliaria

Geometridae

Lepidoptera

68

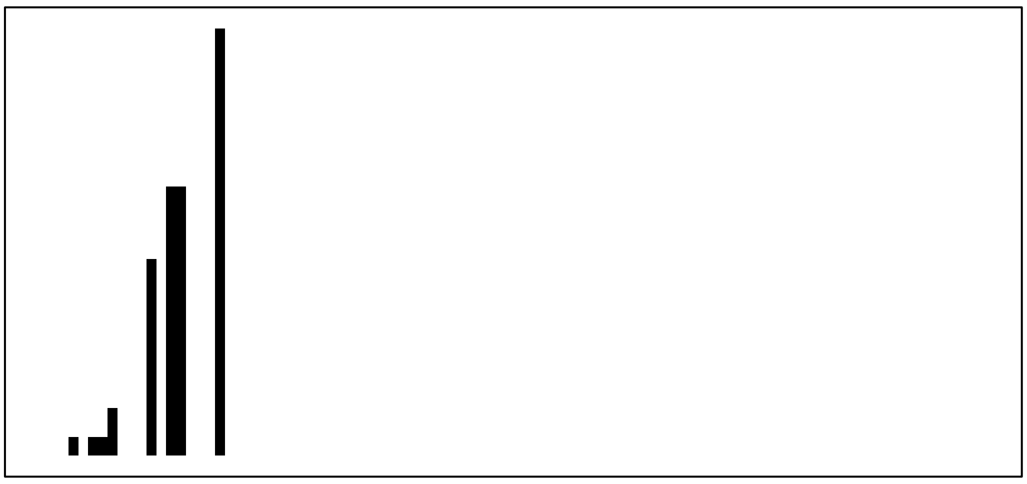

Comostola subtiliaria gene for 16S rRNA, partial s	AB265414	68	99.43	8.79594e−05	Lepidoptera	Geometridae	Comostola subtiliaria
Papuadytes munaso mitochondrial 16S rRNA gene (par	AM404033	67.63	98.29	0.000307009	Coleoptera	Dytiscidae	Papuadytes munaso
Papuadytes sp. MB262 16S ribosomal RNA gene, parti	AY334144	67.63	98.29	0.000307009	Coleoptera	Dytiscidae	Papuadytes sp. MB262
Cyprinus carpio genome assembly common carp genome	LN594162	68.98	98.29	0.00374013	Cypriniformes	Cyprinidae	Cyprinus carpio
Ovis canadensis canadensis isolate 43U chromosome	CP011886	80.85	25.14	1.93744		Bovidae	Ovis canadensis canadensis
Rhinorhynchidius australasiae isolate COL1255 16S	KF016286	67.55	98.29	1.93744	Coleoptera	Brentidae	Rhinorhynchidius australasiae
PREDICTED: Ovis aries musimon uncharacterized LOC1	XR_001036254	80.85	25.14	1.93744		Bovidae	Ovis aries musimon
PREDICTED: Ovis aries uncharacterized LOC105612514	XR_001040876	80.85	25.14	1.93744		Bovidae	Ovis aries
TPA_asm: Oryzias latipes strain Hd−rR, complete ge	HG313997	82.22	25.14	1.93744	Beloniformes	Adrianichthyidae	Oryzias latipes
Solanum pennellii chromosome ch01, complete genome	HG975440	72.16	53.14	1.93744	Solanales	Solanaceae	Solanum pennellii
Vitis vinifera contig VV78X180138.30, whole genome	AM468006	70.59	52	1.93744	Vitales	Vitaceae	Vitis vinifera
Vitis vinifera contig VV78X261535.7, whole genome	AM466085	67.63	88.57	1.93744	Vitales	Vitaceae	Vitis vinifera
Genomic sequence for Brassica oleracea, clone B21H	AC122543	89.19	21.14	1.93744	Brassicales	Brassicaceae	Brassica oleracea
Strongyloides ratti genome assembly S_ratti_ED321	LN609529	76.92	29.71	6.76232	Rhabditida	Strongyloididae	Strongyloides ratti
Solanum lycopersicum chromosome ch01, complete gen	HG975513	92.59	15.43	6.76232	Solanales	Solanaceae	Solanum lycopersicum
Zebrafish DNA sequence from clone DKEYP−93H6 in li	BX510992	92.59	15.43	6.76232	Cypriniformes	Cyprinidae	Danio rerio
Rhipicephalus sp. Morphotype 4 haplotype 2 16S rib	KC243850	80	25.71	6.76232	Ixodida	Ixodidae	Rhipicephalus sp. Morphotype 4
Hyalonthophagus alcyon 16S ribosomal RNA gene, par	AY131565	68.91	68	6.76232	Coleoptera	Scarabaeidae	Hyalonthophagus alcyon
Aleiantus viridicollis 12S ribosomal RNA gene, par	DQ369356	71	54.86	6.76232	Coleoptera	Scarabaeidae	Epactoides viridicollis
Zebrafish DNA sequence from clone DKEY−225H23 in l	BX649231	100	12.57	6.76232	Cypriniformes	Cyprinidae	Danio rerio

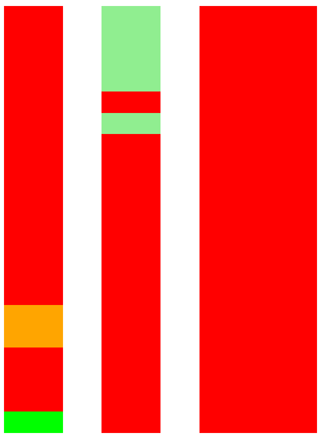

Supplement: Supplemental Information 1 [file peerj-04-1966-s006.zip › scripts/5 match OTUs/blast_hits/OTU_67.csv_plot.pdf]
