# Supplementary material for: Testing the potential of a ribosomal 16S marker for DNA metabarcoding of insects
Source: PeerJ. 2016 Apr 19;4:e1966. doi: 10.7717/peerj.1966 (PMC4841222; doi:10.7717/peerj.1966)
Supplement: Supplemental Information 1 [file peerj-04-1966-s006.zip › scripts/5 match OTUs/blast_hits/OTU_69.csv_plot.pdf]

# OTU_69.csv

## Salina sp. BXW−2014

## Paronellidae

# 90.48

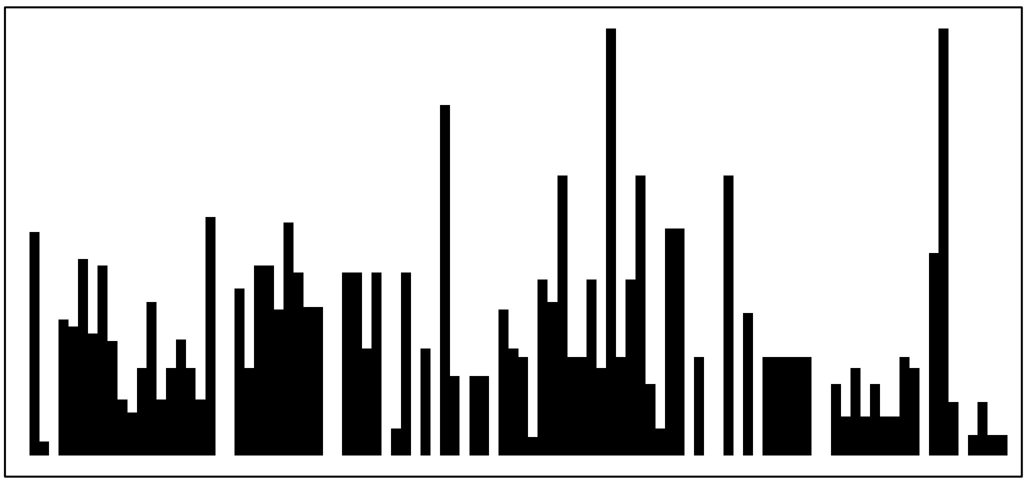

| | | | | | | | |
|---|---|---|---|---|---|---|---|
| Salina sp. BXW−2014 16S ribosomal RNA gene, partia | KM978386 | 90.48 | 28.77 | 1.99806e−05 | | Paronellidae | Salina sp. BXW−2014 |
| Orchesellides sp. 9688−2 FZ−2013 16S ribosomal RNA | KC236217 | 80.65 | 42.47 | 1.99806e−05 | | Entomobryidae | Orchesellides sp. 9688−2 FZ−2013 |
| Sinella cf. colorata FZ−2013 16S ribosomal RNA gen | KC236216 | 76.25 | 54.11 | 0.000243414 | | Entomobryidae | Sinella cf. colorata FZ−2013 |
| Angela sp. MN120 16S ribosomal RNA gene, partial s | EF383381 | 86.36 | 30.14 | 0.000849598 | Mantodea | Mantidae | Angela sp. MN120 |
| Callyntrura guangdongensis 16S ribosomal RNA gene, | KM978382 | 86.05 | 29.45 | 0.00296539 | | Paronellidae | Callyntrura guangdongensis |
| Drepanura sp. 1 FZ−2013 16S ribosomal RNA gene, pa | KC236193 | 89.19 | 25.34 | 0.0103502 | | Entomobryidae | Drepanura sp. 1 FZ−2013 |
| Milionia basalis gene for 16S rRNA, partial sequen | AB265396 | 85.71 | 28.77 | 0.0103502 | Lepidoptera | Geometridae | Milionia basalis |
| Cyphoderus javanus 16S ribosomal RNA gene, partial | KM978390 | 88.89 | 24.66 | 0.0361258 | | Cyphoderidae | Cyphoderus javanus |
| Plaxiphora biramosa 16S ribosomal RNA gene, partia | KJ574069 | 85.37 | 28.08 | 0.0361258 | Neoloricata | Mopaliidae | Plaxiphora biramosa |
| Dracunculus medinensis genome assembly D_medinensi | LK978224 | 82.61 | 31.51 | 0.0361258 | Spirurida | Dracunculidae | Dracunculus medinensis |
| Iravadiidae gen. 3 sp. 1 REG−2013 isolate IC−BOW−A | KC439885 | 82 | 34.25 | 0.0361258 | | Iravadiidae | Iravadiidae gen. 3 sp. 1 REG−2013 |
| Tomocerus similis voucher C9674_1 16S ribosomal RN | KF591903 | 84.09 | 30.14 | 0.0361258 | | Tomoceridae | Tomocerus similis |
| Folsomia quadrioculata 16S ribosomal RNA gene, par | KC236199 | 87.18 | 26.71 | 0.0361258 | | Isotomidae | Folsomia quadrioculata |
| Epitonium ancillotoi voucher RMNH88484 16S ribosom | JF750949 | 82.61 | 31.51 | 0.0361258 | | Epitoniidae | Epitonium ancillotoi |
| Decellebruchus atrolineatus voucher BRU−Aa11 16S r | HQ178239 | 84.09 | 30.14 | 0.0361258 | Coleoptera | Chrysomelidae | Decellebruchus atrolineatus |
| Crenatula avicularis voucher USNM:795306 16S ribos | HQ329399 | 85.42 | 32.19 | 0.0361258 | Pterioida | Isognomonidae | Crenatula avicularis |
| Noualhierana furtiva voucher CW 224 16S ribosomal | FJ230432 | 84.09 | 30.14 | 0.0361258 | Hemiptera | Reduviidae | Noualhierana furtiva |
| Glycine max strain Williams 82 clone GM_WBb0093C02 | AC235350 | 82.61 | 31.51 | 0.0361258 | Fabales | Fabaceae | Glycine max |
| Candidatus Azobacteroides pseudotrichonymphae geno | AP010656 | 91.18 | 23.29 | 0.0361258 | Bacteroidales | | Candidatus Azobacteroides pseudotrichonymphae genomovar. CF |
| Friesea grisea mitochondrion, complete genome | EU016196 | 73.42 | 54.11 | 0.0361258 | | Neanuridae | Friesea grisea |
| Orchesella villosa mitochondrion, complete genome | EU016195 | 75.34 | 49.32 | 0.0361258 | | Entomobryidae | Orchesella villosa |
| Neogovea sp. MCZDNA101409 16S ribosomal RNA gene, | DQ825619 | 87.8 | 27.4 | 0.0361258 | Opiliones | Neogoveidae | Neogovea sp. MCZDNA101409 |
| Archiearis parthenias gene for 16S rRNA, partial s | AB265368 | 84.09 | 30.14 | 0.0361258 | Lepidoptera | Geometridae | Archiearis parthenias |
| Eublaberus posticus voucher G49 16S ribosomal RNA | EF363268 | 88.89 | 30.14 | 0.0361258 | Blattodea | Blaberidae | Eublaberus posticus |
| Eublaberus posticus mitochondrion 16S ribosomal RN | U17785 | 88.89 | 30.14 | 0.0361258 | Blattodea | Blaberidae | Eublaberus posticus |
| Salina celebensis 16S ribosomal RNA gene, partial | KM978388 | 86.84 | 26.03 | 0.126092 | | Paronellidae | Salina celebensis |
| Ovis canadensis canadensis isolate 43U chromosome | CP011888 | 90.91 | 22.6 | 0.126092 | | Bovidae | Ovis canadensis canadensis |
| Enterobius vermicularis genome assembly E_vermicul | LM416355 | 88.57 | 23.97 | 0.126092 | Oxyurida | Oxyuridae | Enterobius vermicularis |
| Schistosoma mattheei genome assembly S_mattheei_De | LM156157 | 86.96 | 30.14 | 0.126092 | Strigeidida | Schistosomatidae | Schistosoma mattheei |
| Schistosoma curassoni genome assembly S_curassoni_ | LM077553 | 86.96 | 30.14 | 0.126092 | Strigeidida | Schistosomatidae | Schistosoma curassoni |
| Rhodinia verecunda mitochondrial gene for 16S ribo | AB841469 | 78.57 | 38.36 | 0.126092 | Lepidoptera | Saturniidae | Rhodinia verecunda |
| Rhodinia verecunda mitochondrial gene for 16S ribo | AB841468 | 78.57 | 38.36 | 0.126092 | Lepidoptera | Saturniidae | Rhodinia verecunda |
| PREDICTED: Monodelphis domestica TBC1 domain famil | XM_001369738 | 88.57 | 23.97 | 0.126092 | Didelphimorphia | Didelphidae | Monodelphis domestica |
| PREDICTED: Monodelphis domestica TBC1 domain famil | XM_007501140 | 88.57 | 23.97 | 0.126092 | Didelphimorphia | Didelphidae | Monodelphis domestica |
| Folsomia candida voucher Folsomia_Candida 16S ribo | KF591920 | 85 | 27.4 | 0.126092 | | Isotomidae | Folsomia candida |
| PREDICTED: Saccoglossus kowalevskii uncharacterize | XM_002739036 | 88.57 | 23.97 | 0.126092 | | Harrimaniidae | Saccoglossus kowalevskii |
| Peripatoides sp. DNA103691 16S ribosomal RNA gene, | KC754561 | 76.19 | 43.15 | 0.126092 | | Peripatopsidae | Peripatoides sp. DNA103691 |
| Stenygrinum quadrinotatum voucher HNAU.PP 013 16S | KF737729 | 81.25 | 32.88 | 0.126092 | Coleoptera | Cerambycidae | Stenygrinum quadrinotatum |
| Lamellidens generosus voucher UMMZ:MC:304346 16S l | KF011263 | 88.57 | 23.97 | 0.126092 | Unionoida | Unionidae | Lamellidens generosus |
| Orchesellides sinensis 16S ribosomal RNA gene, par | KC236209 | 74.07 | 54.79 | 0.126092 | | Entomobryidae | Orchesellides sinensis |
| Folsomia candida 16S ribosomal RNA gene, partial s | KC236200 | 85 | 27.4 | 0.126092 | | Isotomidae | Folsomia candida |
| Peripatoides sympatrica mitochondrion, partial gen | JF800075 | 76.19 | 43.15 | 0.126092 | | Peripatopsidae | Peripatoides sympatrica |
| Zebrafish DNA sequence from clone DKEY−202K7 in li | CU138513 | 96.43 | 19.18 | 0.126092 | Cypriniformes | Cyprinidae | Danio rerio |
| Stagmomantis sp. MN278 16S ribosomal RNA gene, par | FJ806254 | 83.72 | 29.45 | 0.126092 | Mantodea | Mantidae | Stagmomantis sp. MN278 |
| Glycine max strain Williams 82 clone GM_WBb0002A11 | AC235169 | 76.4 | 57.53 | 0.126092 | Fabales | Fabaceae | Glycine max |
| Metilia brunnerii isolate MN146 16S ribosomal RNA | EF383392 | 83.72 | 29.45 | 0.126092 | Mantodea | Acanthopidae | Metilia brunnerii |
| Lithobius variegatus rubriceps 16S ribosomal RNA g | AY084071 | 83.72 | 29.45 | 0.126092 | Lithobiomorpha | Lithobiidae | Lithobius variegatus rubriceps |
| Homo sapiens cDNA FLJ38749 fis, clone KIDNE2012453 | AK096068 | 77.78 | 41.78 | 0.126092 | Primates | Hominidae | Homo sapiens |
| Gomphiocephalus hodgsoni mitochondrion, complete g | AY191995 | 86.84 | 26.03 | 0.126092 | | Hypogastruridae | Gomphiocephalus hodgsoni |
| Mainwaringia rhizophila 16S ribosomal RNA gene, mi | U46829 | 85 | 27.4 | 0.126092 | | Littorinidae | Mainwaringia rhizophila |
| Candidatus Westeberhardia cardiocondylae genome as | LN774881 | 84.62 | 26.71 | 0.440103 | Enterobacteriales | Enterobacteriaceae | Candidatus Westeberhardia cardiocondylae |
| PREDICTED: Microcebus murinus pleiomorphic adenoma | XM_012774084 | 80.85 | 32.19 | 0.440103 | Primates | Cheirogaleidae | Microcebus murinus |
| Bryopsis plumosa chloroplast complete genome, stra | LN810504 | 88.24 | 23.29 | 0.440103 | Bryopsidales | Bryopsidaceae | Bryopsis plumosa |
| Caulibugula pearsei voucher BRBRY−Cau2 16S ribosom | KF714807 | 87.18 | 26.71 | 0.440103 | Hemiptera | Reduviidae | Caulibugula pearsei |
| Protopolystoma xenopodis genome assembly P_xenopod | LM861591 | 93.1 | 19.86 | 0.440103 | Polyopisthocotylea | Polystomatidae | Protopolystoma xenopodis |
| Schistosoma margrebowiei genome assembly S_margreb | LL888452 | 84.62 | 26.71 | 0.440103 | Strigeidida | Schistosomatidae | Schistosoma margrebowiei |
| Schistosoma margrebowiei genome assembly S_margreb | LL883872 | 84.62 | 26.71 | 0.440103 | Strigeidida | Schistosomatidae | Schistosoma margrebowiei |
| Gongylonema pulchrum genome assembly G_pulchrum_Ho | LL748524 | 88.24 | 23.29 | 0.440103 | Spirurida | Gongylonematidae | Gongylonema pulchrum |
| Brugia pahangi genome assembly B_pahangi_Glasgow , | LK964536 | 84.09 | 30.14 | 0.440103 | Spirurida | Onchocercidae | Brugia pahangi |
| Angiostrongylus costaricensis genome assembly A_co | LK939637 | 100 | 16.44 | 0.440103 | Rhabditida | Angiostrongylidae | Angiostrongylus costaricensis |
| Schistosoma mattheei genome assembly S_mattheei_De | LM180696 | 84.62 | 26.71 | 0.440103 | Strigeidida | Schistosomatidae | Schistosoma mattheei |
| Strongyloides ratti genome assembly S_ratti_ED321 | LN609528 | 88.24 | 23.29 | 0.440103 | Rhabditida | Strongyloididae | Strongyloides ratti |
| Plasmodium chabaudi chabaudi genome assembly PCHAS | LK022885 | 93.1 | 19.86 | 0.440103 | Haemosporida | Plasmodiidae | Plasmodium chabaudi chabaudi |
| Emmelina monodactyla mitochondrion, partial genome | KJ508063 | 83.33 | 28.77 | 0.440103 | Lepidoptera | Pterophoridae | Emmelina monodactyla |
| Monochamus alternatus mitochondrion, complete geno | KJ809086 | 81.25 | 43.15 | 0.440103 | Coleoptera | Cerambycidae | Monochamus alternatus |
| PREDICTED: Nasonia vitripennis uncharacterized LOC | XM_008217494 | 90.62 | 21.92 | 0.440103 | Hymenoptera | Pteromalidae | Nasonia vitripennis |
| PREDICTED: Nasonia vitripennis uncharacterized LOC | XM_003424965 | 90.62 | 21.92 | 0.440103 | Hymenoptera | Pteromalidae | Nasonia vitripennis |
| Coecobrya communis 16S ribosomal RNA gene, partial | KC236218 | 80 | 34.25 | 0.440103 | | Entomobryidae | Coecobrya communis |
| Entomobrya sp. 2 FZ−2013 16S ribosomal RNA gene, p | KC236198 | 84.62 | 26.71 | 0.440103 | | Entomobryidae | Entomobrya sp. 2 FZ−2013 |
| Coecobrya tenebricosa 16S ribosomal RNA gene, part | KC236191 | 80 | 34.25 | 0.440103 | | Entomobryidae | Coecobrya tenebricosa |
| Phaedrotoma rudis isolate CVA4259 16S ribosomal RN | JQ736266 | 71.13 | 60.27 | 0.440103 | Hymenoptera | Braconidae | Phaedrotoma rudis |
| Ciona intestinalis clone 4H2_9L22 genomic sequence | JF357717 | 93.1 | 19.86 | 0.440103 | Enterogona | Cionidae | Ciona intestinalis |
| Hypogastrura vernalis 16S ribosomal RNA gene, part | JN970945 | 73.2 | 64.38 | 0.440103 | | Hypogastruridae | Hypogastrura vernalis |
| Eublaberus posticus voucher ZMUC:JD−879 16S riboso | JN615276 | 86.67 | 30.14 | 0.440103 | Blattodea | Blaberidae | Eublaberus posticus |
| Dichromacalles diocletianus bio−material ZFMK−DNA | GU988590 | 76.19 | 43.15 | 0.440103 | Coleoptera | Curculionidae | Dichromacalles diocletianus |
| Armatoglyptes habei isolate Acro−sp−13 16S ribosom | HQ148947 | 84.62 | 26.71 | 0.440103 | Pygophora | Lithoglyptidae | Armatoglyptes habei |
| Armatoglyptes habei isolate Acro−sp−10 16S ribosom | HQ148946 | 84.62 | 26.71 | 0.440103 | Pygophora | Lithoglyptidae | Armatoglyptes habei |
| Armatoglyptes habei isolate Acro−sp−8 16S ribosom | HQ148945 | 84.62 | 26.71 | 0.440103 | Pygophora | Lithoglyptidae | Armatoglyptes habei |
| Armatoglyptes habei isolate Acro−sp−6 16S ribosoma | HQ148944 | 84.62 | 26.71 | 0.440103 | Pygophora | Lithoglyptidae | Armatoglyptes habei |
| Armatoglyptes habei isolate Acro−sp−3 16S ribosoma | HQ148943 | 84.62 | 26.71 | 0.440103 | Pygophora | Lithoglyptidae | Armatoglyptes habei |
| Aphengoecus multiserratus isolate AMDAR03 16S ribo | GQ289726 | 79.37 | 42.47 | 0.440103 | Coleoptera | Scarabaeidae | Aphengoecus multiserratus |
| Aphengoecus multiserratus isolate AMDAR02 16S ribo | GQ289725 | 79.37 | 42.47 | 0.440103 | Coleoptera | Scarabaeidae | Aphengoecus multiserratus |
| Xanthomantis mantispoides isolate MN335 16S riboso | FJ806306 | 83.33 | 28.77 | 0.440103 | Mantodea | Iridopterygidae | Xanthomantis mantispoides |
| Empusa sp. MN131 16S ribosomal RNA gene, partial s | FJ806202 | 81.82 | 30.14 | 0.440103 | Mantodea | Empusidae | Empusa sp. MN131 |
| Parastagmatoptera sp. MN028 16S ribosomal RNA gene | FJ806159 | 84.09 | 30.14 | 0.440103 | Mantodea | Mantidae | Parastagmatoptera sp. MN028 |
| Psychobiella sordida 16S ribosomal RNA gene, parti | EU734896 | 81.82 | 30.14 | 0.440103 | Neuroptera | Hemerobiidae | Psychobiella sordida |
| Notocrypta curvifascia 16S ribosomal RNA gene, par | DQ445109 | 83.33 | 28.77 | 0.440103 | Lepidoptera | Hesperiidae | Notocrypta curvifascia |
| Brotia verbecki voucher ZMB 200.118b 16S ribosomal | AY330780 | 81.82 | 30.14 | 0.440103 | | Pachychilidae | Brotia verbecki |
| Brotia verbecki voucher ZMB 200.118 16S ribosomal | AY330779 | 81.82 | 30.14 | 0.440103 | | Pachychilidae | Brotia verbecki |
| Podura aquatica mitochondrion, partial genome | AY639939 | 84.62 | 26.71 | 0.440103 | | Poduridae | Podura aquatica |
| Parastagmatoptera sp. MN028 16S ribosomal RNA gene | AY491117 | 84.09 | 30.14 | 0.440103 | Mantodea | Mantidae | Parastagmatoptera sp. MN028 |
| Pachysoma bennigseni 16S ribosomal RNA gene, parti | AF499698 | 77.78 | 43.15 | 0.440103 | Coleoptera | Scarabaeidae | Pachysoma bennigseni |
| Zebrafish DNA sequence from clone RP71−4M17 in lin | BX296520 | 89.47 | 24.66 | 0.440103 | Cypriniformes | Cyprinidae | Danio rerio |
| Zebrafish DNA sequence from clone CH211−132F13 in | BX005096 | 100 | 16.44 | 0.440103 | Cypriniformes | Cyprinidae | Danio rerio |
| Folsomia candida 16S ribosomal RNA gene, partial s | JN970944 | 82.5 | 27.4 | 1.53611 | | Isotomidae | Folsomia candida |
| Monochamus alternatus endai mitochondrial gene for | AB533599 | 79.69 | 43.15 | 1.53611 | Coleoptera | Cerambycidae | Monochamus alternatus endai |
| Epactoides mangabeensis voucher Ale.AND24.32.4 16S | EU030499 | 80.95 | 28.77 | 1.53611 | Coleoptera | Scarabaeidae | Epactoides mangabeensis |
| Folsomia candida 16S ribosomal RNA gene, partial s | AY555551 | 82.5 | 27.4 | 1.53611 | | Isotomidae | Folsomia candida |
| Epactoides mangabeensis voucher Ale.AND24.32.2 16S | EU030498 | 80.95 | 28.77 | 5.36155 | Coleoptera | Scarabaeidae | Epactoides mangabeensis |
| Epactoides mangabeensis voucher Ale.AND24.32.1 16S | EU030497 | 80.95 | 28.77 | 5.36155 | Coleoptera | Scarabaeidae | Epactoides mangabeensis |
