# Supplementary material for: Testing the potential of a ribosomal 16S marker for DNA metabarcoding of insects
Source: PeerJ. 2016 Apr 19;4:e1966. doi: 10.7717/peerj.1966 (PMC4841222; doi:10.7717/peerj.1966)
Supplement: Supplemental Information 1 [file peerj-04-1966-s006.zip › scripts/5 match OTUs/blast_hits/OTU_7.csv_plot.pdf]

OTU_7.csv

Epeorus longimanus

Heptageniidae

Ephemeroptera

87.1

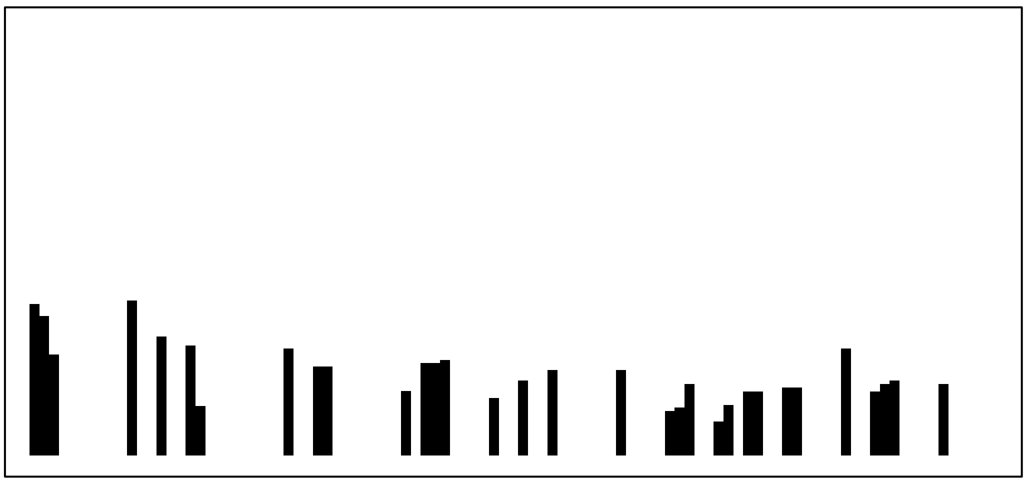

Epeorus longimanus 16S ribosomal RNA gene, partial	AY749757	87.1	100	5.07103e−45	Ephemeroptera	Heptageniidae	Epeorus longimanus
Epeorus sp. MT−2014 mitochondrion, complete genome	KM244708	86.54	100	9.16864e−42	Ephemeroptera	Heptageniidae	Epeorus sp. MT−2014
Cloeon dipterum voucher EPH02 tRNA−Leu, 16S ribosо	KF256908	84.71	100	4.74947e−39	Ephemeroptera	Baetidae	Cloeon dipterum
Cryptoperla japonica mitochondrial gene for 16S rR	AB711184	79.75	99.35	6.18324e−25	Plecoptera	Peltoperlidae	Cryptoperla japonica
Cryptoperla japonica mitochondrial gene for 16S rR	AB711183	79.75	99.35	6.18324e−25	Plecoptera	Peltoperlidae	Cryptoperla japonica
Cryptoperla japonica mitochondrial gene for 16S rR	AB711182	79.75	99.35	6.18324e−25	Plecoptera	Peltoperlidae	Cryptoperla japonica
Siphlonurus sp. EP084 16S ribosomal RNA gene, part	AY749786	78.71	99.35	6.18324e−25	Ephemeroptera	Siphlonuridae	Siphlonurus sp. EP084
Parameletus columbiae 16S ribosomal RNA gene, part	AY749768	79.49	99.35	6.18324e−25	Ephemeroptera	Siphlonuridae	Parameletus columbiae
Siphlonella sp. EP083 16S ribosomal RNA gene, part	AY749785	78.98	99.35	2.15816e−24	Ephemeroptera	Oniscigastridae	Siphlonella sp. EP083
Cryptoperla japonica mitochondrial gene for 16S rR	AB711202	78.48	99.35	2.62918e−23	Plecoptera	Peltoperlidae	Cryptoperla japonica
Cultus pilatus 16S ribosomal RNA gene, partial seq	EF623212	87.25	65.16	2.62918e−23	Plecoptera	Perlodidae	Cultus pilatus
Cryptoperla japonica mitochondrial gene for 16S rR	AB711189	78.48	99.35	3.203e−22	Plecoptera	Peltoperlidae	Cryptoperla japonica
Perlodes mortoni 16S ribosomal RNA gene, partial s	KF855870	77.36	99.35	1.11796e−21	Plecoptera	Perlodidae	Perlodes mortoni
Despaxia augusta 16S ribosomal RNA gene, partial s	EF623177	85.58	66.45	1.11796e−21	Plecoptera	Leuctridae	Despaxia augusta
Tasmanophlebia sp. EP158 16S ribosomal RNA gene, p	AY749825	77.71	99.35	1.11796e−21	Ephemeroptera	Oniscigastridae	Tasmanophlebia sp. EP158
Cryptoperla ishigakiensis mitochondrial gene for 1	AB711242	77.85	99.35	1.36195e−20	Plecoptera	Peltoperlidae	Cryptoperla ishigakiensis
Rhithrogena sp. 8 LV−2015 mitochondrial partial 16	LN868661	85.15	64.52	4.75367e−20	Ephemeroptera	Heptageniidae	Rhithrogena sp. 8 LV−2015
Rhithrogena nivata mitochondrial partial 16S rRNA	LN868643	82.3	72.26	4.75367e−20	Ephemeroptera	Heptageniidae	Rhithrogena nivata
Oyamia seminigra mitochondrial gene for 16S riboso	AB771688	79.26	83.23	4.75367e−20	Plecoptera	Perlidae	Oyamia seminigra
Scopura montana 16S ribosomal RNA gene, partial se	EF623265	76.88	99.35	4.75367e−20	Plecoptera		Scopura montana
Neuroperlopsis patris 16S ribosomal RNA gene, part	EF623152	77.07	99.35	4.75367e−20	Plecoptera	Eustheniidae	Neuroperlopsis patris
Paraperla frontalis 16S ribosomal RNA gene, partia	EF623136	77.07	99.35	4.75367e−20	Plecoptera	Chloroperlidae	Paraperla frontalis
Allocapnia recta haplotype 12ar: H12 16S ribosomal	EU306875	77.07	99.35	4.75367e−20	Plecoptera	Capniidae	Allocapnia recta
Rhithrogena insularis mitochondrial partial 16S rR	LN868665	76.13	99.35	1.65919e−19	Ephemeroptera	Heptageniidae	Rhithrogena insularis
Cryptoperla sp. YK−2012−2 mitochondrial gene for 1	AB711244	76.58	99.35	1.65919e−19	Plecoptera	Peltoperlidae	Cryptoperla sp. YK−2012−2
Cryptoperla sp. YK−2012−1 mitochondrial gene for 1	AB711243	77.22	99.35	1.65919e−19	Plecoptera	Peltoperlidae	Cryptoperla sp. YK−2012−1
Neofulla sp. BYU_PL152 16S ribosomal RNA gene, par	EF623203	85	63.87	1.65919e−19	Plecoptera	Notonemouridae	Neofulla sp. BYU_PL152
Pelurgoperla personata 16S ribosomal RNA gene, par	EF623168	76.43	99.35	1.65919e−19	Plecoptera	Gripopterygidae	Pelurgoperla personata
Klapopteryx armillata 16S ribosomal RNA gene, part	EF623123	76.43	99.35	1.65919e−19	Plecoptera	Austroperlidae	Klapopteryx armillata
Rhithrogena sp. 7 LV−2015 mitochondrial partial 16	LN868669	84.16	64.52	5.79115e−19	Ephemeroptera	Heptageniidae	Rhithrogena sp. 7 LV−2015
Rhithrogena sp. 9 LV−2015 mitochondrial partial 16	LN868650	84.16	64.52	5.79115e−19	Ephemeroptera	Heptageniidae	Rhithrogena sp. 9 LV−2015
Apteroperla tikumana mitochondrion, complete genom	KR604721	75.64	99.35	5.79115e−19	Plecoptera	Capniidae	Apteroperla tikumana
Mesocapnia arizonensis mitochondrion, partial geno	KP642637	75.64	99.35	5.79115e−19	Plecoptera	Capniidae	Mesocapnia arizonensis
Allocapnia recta isolate H13 16S ribosomal RNA gen	KC881048	75.64	99.35	5.79115e−19	Plecoptera	Capniidae	Allocapnia recta
Allocapnia recta isolate H11 16S ribosomal RNA gen	KC881046	75.64	99.35	5.79115e−19	Plecoptera	Capniidae	Allocapnia recta
Allocapnia recta isolate H7 16S ribosomal RNA gene	KC881042	75.64	99.35	5.79115e−19	Plecoptera	Capniidae	Allocapnia recta
Allocapnia recta isolate H3 16S ribosomal RNA gene	KC881038	75.64	99.35	5.79115e−19	Plecoptera	Capniidae	Allocapnia recta
Allocapnia recta isolate H1 16S ribosomal RNA gene	KC881036	75.64	99.35	5.79115e−19	Plecoptera	Capniidae	Allocapnia recta
Oyamia seminigra mitochondrial gene for 16S riboso	AB771693	83.02	66.45	5.79115e−19	Plecoptera	Perlidae	Oyamia seminigra
Oyamia seminigra mitochondrial gene for 16S riboso	AB771690	78.95	83.23	5.79115e−19	Plecoptera	Perlidae	Oyamia seminigra
Anterastes serbicus haplotype 38BUL 16S ribosomal	HM856177	84.31	64.52	5.79115e−19	Orthoptera	Tettigoniidae	Anterastes serbicus
Anterastes serbicus haplotype 37BUL 16S ribosomal	HM856173	84.31	64.52	5.79115e−19	Orthoptera	Tettigoniidae	Anterastes serbicus
Isoperla oxylepis 16S ribosomal RNA gene, partial	EF623221	84.47	65.16	5.79115e−19	Plecoptera	Perlodidae	Isoperla oxylepis
Pictetiella expansa 16S ribosomal RNA gene, partia	EF623216	76.28	99.35	5.79115e−19	Plecoptera	Perlodidae	Pictetiella expansa
Nemoura cinerea 16S ribosomal RNA gene, partial se	EF623194	76.73	99.35	5.79115e−19	Plecoptera	Nemouridae	Nemoura cinerea
Moselia infuscata 16S ribosomal RNA gene, partial	EF623175	76.73	100	5.79115e−19	Plecoptera	Leuctridae	Moselia infuscata
Mesocapnia sp. BYU_PL010 16S ribosomal RNA gene, p	EF623127	75.64	99.35	5.79115e−19	Plecoptera	Capniidae	Mesocapnia sp. BYU_PL010
Tasmanoperla larvalis 16S ribosomal RNA gene, part	EF623125	82.69	66.45	5.79115e−19	Plecoptera	Austroperlidae	Tasmanoperla larvalis
Allocapnia recta haplotype 3ar: H03 16S ribosomal	DQ915181	75.64	99.35	5.79115e−19	Plecoptera	Capniidae	Allocapnia recta
Rhithrogena nuragica mitochondrial partial 16S rRN	LN868667	75.48	99.35	2.02131e−18	Ephemeroptera	Heptageniidae	Rhithrogena nuragica
Rhithrogena grischuna mitochondrial partial 16S rR	LN868639	83.5	64.52	2.02131e−18	Ephemeroptera	Heptageniidae	Rhithrogena grischuna
Siphlonurus sp. MT−2014 isolate CL142 mitochondrio	KM244684	75.48	99.35	2.02131e−18	Ephemeroptera	Siphlonuridae	Siphlonurus sp. MT−2014
Epitheca bimaculata mitochondrial gene for 16S rRN	AB707954	76.92	99.35	2.02131e−18	Odonata	Corduliidae	Epitheca bimaculata
Anterastes disparalatus haplotype 46BO 16S ribosom	HQ850975	84	63.87	2.02131e−18	Orthoptera	Tettigoniidae	Anterastes disparalatus
Epitheca bimaculata 12S ribosomal RNA gene, partia	EU477708	76.92	99.35	2.02131e−18	Odonata	Corduliidae	Epitheca bimaculata
Brachyptera seticornis 16S ribosomal RNA gene, par	EF623268	77.02	99.35	2.02131e−18	Plecoptera	Taeniopterygidae	Brachyptera seticornis
Pteronarcys californica 16S ribosomal RNA gene, pa	EF623261	76.1	99.35	2.02131e−18	Plecoptera	Pteronarcyidae	Pteronarcys californica
Lednia tumana 16S ribosomal RNA gene, partial sequ	EF623197	76.43	99.35	2.02131e−18	Plecoptera	Nemouridae	Lednia tumana
Claudioperla sp. BYU_PL158 16S ribosomal RNA gene,	EF623169	76.43	99.35	2.02131e−18	Plecoptera	Gripopterygidae	Claudioperla sp. BYU_PL158
Antarctoperla sp. BYU_PL142 16S ribosomal RNA gene	EF623163	75.8	99.35	2.02131e−18	Plecoptera	Gripopterygidae	Antarctoperla sp. BYU_PL142
Koroglus disparalatus voucher 156 16S ribosomal RN	EU120777	84	63.87	2.02131e−18	Orthoptera	Tettigoniidae	Anterastes disparalatus
Rallidens mcfarlanei 16S ribosomal RNA gene, parti	AY749794	75.48	99.35	2.02131e−18	Ephemeroptera	Rallidentidae	Rallidens mcfarlanei
Chaquihua sp. EP080 16S ribosomal RNA gene, partia	AY749784	75.48	99.35	2.02131e−18	Ephemeroptera	Ameletopsidae	Chaquihua sp. EP080
Chiloporter sp. EP051 16S ribosomal RNA gene, part	AY749776	75.47	99.35	2.02131e−18	Ephemeroptera	Ameletopsidae	Chiloporter sp. EP051
Jellisonia amadoi mitochondrion, complete genome	KF322091	75.64	99.35	7.05507e−18	Siphonaptera	Ceratophyllidae	Jellisonia amadoi
Oyamia seminigra mitochondrial gene for 16S riboso	AB771692	82.08	66.45	7.05507e−18	Plecoptera	Perlidae	Oyamia seminigra
Taenionema palladium 16S ribosomal RNA gene, parti	EF623267	82.24	66.45	7.05507e−18	Plecoptera	Taeniopterygidae	Taenionema palladium
Pteronarcys sachalina 16S ribosomal RNA gene, part	EF623264	83.33	65.16	7.05507e−18	Plecoptera	Pteronarcyidae	Pteronarcys sachalina
Frisonia picticeps 16S ribosomal RNA gene, partial	EF623227	76.88	99.35	7.05507e−18	Plecoptera	Perlodidae	Frisonia picticeps
Perlodes dispar 16S ribosomal RNA gene, partial se	EF623220	75.47	99.35	7.05507e−18	Plecoptera	Perlodidae	Perlodes dispar
Isoperla davisi 16S ribosomal RNA gene, partial se	EF623206	81.58	72.26	7.05507e−18	Plecoptera	Perlodidae	Isoperla davisi
Nemurella pictetii 16S ribosomal RNA gene, partial	EF623192	82.35	65.16	7.05507e−18	Plecoptera	Nemouridae	Nemurella pictetii
Malenka californica 16S ribosomal RNA gene, partia	EF623182	76.1	99.35	7.05507e−18	Plecoptera	Nemouridae	Malenka californica
Leuctra tenuis haplotype 8lt: HO8 16S ribosomal RN	EU327680	83	63.87	7.05507e−18	Plecoptera	Leuctridae	Leuctra tenuis
Leuctra tenuis haplotype 7lt: HO7 16S ribosomal RN	EU327679	83	63.87	7.05507e−18	Plecoptera	Leuctridae	Leuctra tenuis
Cinygma sp. EP123 16S ribosomal RNA gene, partial	AY749804	75.64	100	7.05507e−18	Ephemeroptera	Heptageniidae	Cinygma sp. EP123
Cinygmula sp. m185 mitochondrial partial 16S rRNA	LN868675	76.43	99.35	2.46246e−17	Ephemeroptera	Heptageniidae	Cinygmula sp. m185
Rhithrogena degrangei mitochondrial partial 16S rR	LN868674	83.17	64.52	2.46246e−17	Ephemeroptera	Heptageniidae	Rhithrogena degrangei
Rhithrogena alpestris mitochondrial partial 16S rR	LN868657	83.17	64.52	2.46246e−17	Ephemeroptera	Heptageniidae	Rhithrogena alpestris
Allocapnia recta isolate H15 16S ribosomal RNA gen	KC881050	75	99.35	2.46246e−17	Plecoptera	Capniidae	Allocapnia recta
Allocapnia recta isolate H14 16S ribosomal RNA gen	KC881049	75	99.35	2.46246e−17	Plecoptera	Capniidae	Allocapnia recta
Allocapnia recta isolate H10 16S ribosomal RNA gen	KC881045	75	99.35	2.46246e−17	Plecoptera	Capniidae	Allocapnia recta
Allocapnia recta isolate H6 16S ribosomal RNA gene	KC881041	75	99.35	2.46246e−17	Plecoptera	Capniidae	Allocapnia recta
Isonychia ignota mitochondrion, complete genome	HM143892	85	63.87	2.46246e−17	Ephemeroptera	Isonychiidae	Isonychia ignota
Anterastes davrazensis haplotype 52−972 16S riboso	JX024231	75.16	100	2.46246e−17	Orthoptera	Tettigoniidae	Anterastes davrazensis
Notonemouridae sp. MD−2011 voucher ZMUC:JD−1507 16	JN615257	75	99.35	2.46246e−17	Plecoptera	Notonemouridae	Notonemouridae sp. MD−2011
Anterastes disparalatus haplotype 45BO 16S ribosom	HQ850976	83	63.87	2.46246e−17	Orthoptera	Tettigoniidae	Anterastes disparalatus
Anterastes serbicus haplotype 32BUL 16S ribosomal	HM856167	83.33	64.52	2.46246e−17	Orthoptera	Tettigoniidae	Anterastes serbicus
Baumanella alameda 16S ribosomal RNA gene, partial	EF623236	83.5	65.16	2.46246e−17	Plecoptera	Perlodidae	Baumanella alameda
Austronemoura sp. BYU_PL149 16S ribosomal RNA gene	EF623200	75	99.35	2.46246e−17	Plecoptera	Notonemouridae	Austronemoura sp. BYU_PL149
Nanonemoura wahkeena 16S ribosomal RNA gene, parti	EF623198	75	99.35	2.46246e−17	Plecoptera	Nemouridae	Nanonemoura wahkeena
Paranemoura claasseni 16S ribosomal RNA gene, part	EF623185	75	99.35	2.46246e−17	Plecoptera	Nemouridae	Paranemoura claasseni
Shipsa rotunda 16S ribosomal RNA gene, partial seq	EF623184	75	99.35	2.46246e−17	Plecoptera	Nemouridae	Shipsa rotunda
Leuctra inermis 16S ribosomal RNA gene, partial se	EF623176	83.33	63.87	2.46246e−17	Plecoptera	Leuctridae	Leuctra inermis
Allocapnia minima 16S ribosomal RNA gene, partial	EF623130	75	99.35	2.46246e−17	Plecoptera	Capniidae	Allocapnia minima
Capnura wanica 16S ribosomal RNA gene, partial seq	EF623128	75.32	99.35	2.46246e−17	Plecoptera	Capniidae	Capnura wanica
Isogenoides varians isolate BYU_PL079 16S ribosoma	EF623445	75.95	99.35	2.46246e−17	Plecoptera	Perlodidae	Isogenoides varians
Allocapnia recta haplotype 11ar: H011 16S ribosoma	EU306874	75	99.35	2.46246e−17	Plecoptera	Capniidae	Allocapnia recta
Allocapnia recta haplotype 7ar: H07 16S ribosomal	EU306870	75	99.35	2.46246e−17	Plecoptera	Capniidae	Allocapnia recta
Cinygmula sp. EP013 16S ribosomal RNA gene, partia	AY749760	76.43	99.35	2.46246e−17	Ephemeroptera	Heptageniidae	Cinygmula sp. EP013
