# Supplementary material for: Testing the potential of a ribosomal 16S marker for DNA metabarcoding of insects
Source: PeerJ. 2016 Apr 19;4:e1966. doi: 10.7717/peerj.1966 (PMC4841222; doi:10.7717/peerj.1966)
Supplement: Supplemental Information 1 [file peerj-04-1966-s006.zip › scripts/5 match OTUs/blast_hits/OTU_71.csv_plot.pdf]

OTU_71.csv

Isoperla oxylepis

Perlodidae

Plecoptera

93.04

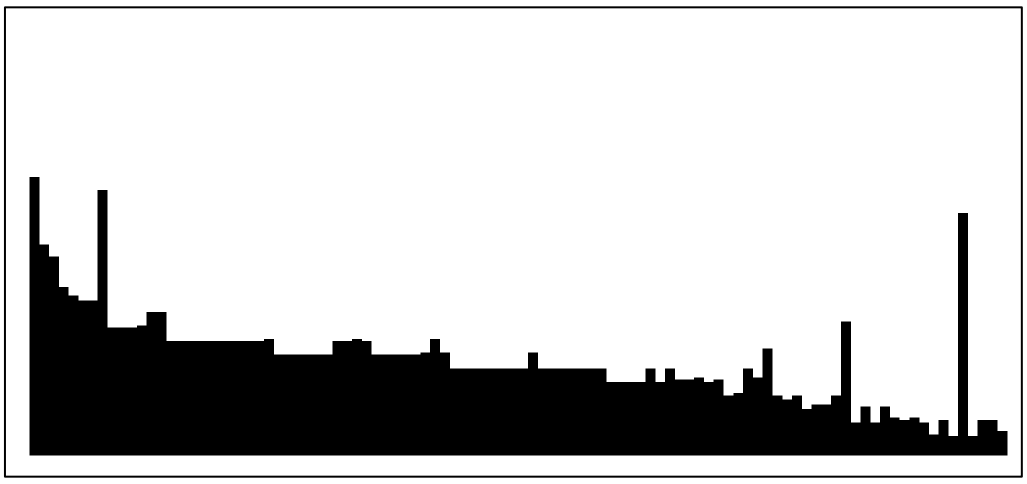

Isoperla oxylepis 16S ribosomal RNA gene, partial	EF623221	93.04	100	5.87728e−57	Plecoptera	Perlodidae	Isoperla oxylepis
Brachyptera seticornis 16S ribosomal RNA gene, par	EF623268	89.87	100	5.5046e−51	Plecoptera	Taeniopterygidae	Brachyptera seticornis
Nemoura cinerea 16S ribosomal RNA gene, partial se	EF623194	89.31	100	9.95255e−48	Plecoptera	Nemouridae	Nemoura cinerea
Zapada cinctipes 16S ribosomal RNA gene, partial s	EF623183	87.9	100	3.47378e−47	Plecoptera	Nemouridae	Zapada cinctipes
Malenka californica 16S ribosomal RNA gene, partia	EF623182	87.5	100	1.21247e−46	Plecoptera	Nemouridae	Malenka californica
Nemoura trispinosa 16S ribosomal RNA gene, partial	EF623187	87.26	100	4.23193e−46	Plecoptera	Nemouridae	Nemoura trispinosa
Isoperla fulva 16S ribosomal RNA gene, partial seq	EF623207	87.26	100	5.15555e−45	Plecoptera	Perlodidae	Isoperla fulva
Protonemura meyeri 16S ribosomal RNA gene, partial	EF623193	92.42	83.44	6.28074e−44	Plecoptera	Nemouridae	Protonemura meyeri
Nanonemoura wahkeena 16S ribosomal RNA gene, parti	EF623198	85.99	100	2.19219e−43	Plecoptera	Nemouridae	Nanonemoura wahkeena
Ostrocerca foersteri 16S ribosomal RNA gene, parti	EF623191	85.99	100	2.19219e−43	Plecoptera	Nemouridae	Ostrocerca foersteri
Visoka cataractae 16S ribosomal RNA gene, partial	EF623189	85.99	100	2.19219e−43	Plecoptera	Nemouridae	Visoka cataractae
Doddsia occidentalis 16S ribosomal RNA gene, parti	EF623270	86.08	100	7.65151e−43	Plecoptera	Taeniopterygidae	Doddsia occidentalis
Lednia tumana 16S ribosomal RNA gene, partial sequ	EF623197	86.71	100	7.65151e−43	Plecoptera	Nemouridae	Lednia tumana
Shipsa rotunda 16S ribosomal RNA gene, partial seq	EF623184	86.71	100	7.65151e−43	Plecoptera	Nemouridae	Shipsa rotunda
Mesocapnia arizonensis mitochondrion, partial geno	KP642637	85.35	100	9.32145e−42	Plecoptera	Capniidae	Mesocapnia arizonensis
Allocapnia recta isolate H13 16S ribosomal RNA gen	KC881048	85.35	100	9.32145e−42	Plecoptera	Capniidae	Allocapnia recta
Allocapnia recta isolate H11 16S ribosomal RNA gen	KC881046	85.35	100	9.32145e−42	Plecoptera	Capniidae	Allocapnia recta
Allocapnia recta isolate H7 16S ribosomal RNA gene	KC881042	85.35	100	9.32145e−42	Plecoptera	Capniidae	Allocapnia recta
Allocapnia recta isolate H3 16S ribosomal RNA gene	KC881038	85.35	100	9.32145e−42	Plecoptera	Capniidae	Allocapnia recta
Allocapnia recta isolate H1 16S ribosomal RNA gene	KC881036	85.35	100	9.32145e−42	Plecoptera	Capniidae	Allocapnia recta
Paranemoura claasseni 16S ribosomal RNA gene, part	EF623185	85.35	100	9.32145e−42	Plecoptera	Nemouridae	Paranemoura claasseni
Mesocapnia sp. BYU_PL010 16S ribosomal RNA gene, p	EF623127	85.35	100	9.32145e−42	Plecoptera	Capniidae	Mesocapnia sp. BYU_PL010
Allocapnia recta haplotype 12ar: H12 16S ribosomal	EU306875	85.35	100	9.32145e−42	Plecoptera	Capniidae	Allocapnia recta
Allocapnia recta haplotype 3ar: H03 16S ribosomal	DQ915181	85.35	100	9.32145e−42	Plecoptera	Capniidae	Allocapnia recta
Oemopteryx vanduzeea 16S ribosomal RNA gene, parti	EF623266	85.44	100	3.2535e−41	Plecoptera	Taeniopterygidae	Oemopteryx vanduzeea
Allocapnia recta isolate H19 16S ribosomal RNA gen	KC881054	84.71	100	1.13558e−40	Plecoptera	Capniidae	Allocapnia recta
Allocapnia recta isolate H16 16S ribosomal RNA gen	KC881051	84.71	100	1.13558e−40	Plecoptera	Capniidae	Allocapnia recta
Allocapnia recta isolate H15 16S ribosomal RNA gen	KC881050	84.71	100	1.13558e−40	Plecoptera	Capniidae	Allocapnia recta
Allocapnia recta isolate H14 16S ribosomal RNA gen	KC881049	84.71	100	1.13558e−40	Plecoptera	Capniidae	Allocapnia recta
Allocapnia recta isolate H10 16S ribosomal RNA gen	KC881045	84.71	100	1.13558e−40	Plecoptera	Capniidae	Allocapnia recta
Allocapnia recta isolate H6 16S ribosomal RNA gene	KC881041	84.71	100	1.13558e−40	Plecoptera	Capniidae	Allocapnia recta
Clioperla clio 16S ribosomal RNA gene, partial seq	EF623237	85.35	100	1.13558e−40	Plecoptera	Perlodidae	Clioperla clio
Isoperla phalerata 16S ribosomal RNA gene, partial	EF623208	85.35	100	1.13558e−40	Plecoptera	Perlodidae	Isoperla phalerata
Isoperla davisi 16S ribosomal RNA gene, partial se	EF623206	85.44	100	1.13558e−40	Plecoptera	Perlodidae	Isoperla davisi
Soyedina producta 16S ribosomal RNA gene, partial	EF623196	85.35	100	1.13558e−40	Plecoptera	Nemouridae	Soyedina producta
Nemurella pictetii 16S ribosomal RNA gene, partial	EF623192	84.71	100	1.13558e−40	Plecoptera	Nemouridae	Nemurella pictetii
Triznaka pintada 16S ribosomal RNA gene, partial s	EF623134	84.71	100	1.13558e−40	Plecoptera	Chloroperlidae	Triznaka pintada
Allocapnia recta haplotype 11ar: H011 16S ribosoma	EU306874	84.71	100	1.13558e−40	Plecoptera	Capniidae	Allocapnia recta
Allocapnia recta haplotype 7ar: H07 16S ribosomal	EU306870	84.71	100	1.13558e−40	Plecoptera	Capniidae	Allocapnia recta
Allocapnia recta haplotype 2ar: H02 16S ribosomal	DQ915180	84.71	100	1.13558e−40	Plecoptera	Capniidae	Allocapnia recta
Scopura montana 16S ribosomal RNA gene, partial se	EF623265	84.81	100	3.96358e−40	Plecoptera		Scopura montana
Prostoia besametsa 16S ribosomal RNA gene, partial	EF623188	85.44	100	3.96358e−40	Plecoptera	Nemouridae	Prostoia besametsa
Suwallia teleckojensis 16S ribosomal RNA gene, par	EF623142	84.81	100	3.96358e−40	Plecoptera	Chloroperlidae	Suwallia teleckojensis
Apteroperla tikumana mitochondrion, complete genom	KR604721	84.08	100	4.82863e−39	Plecoptera	Capniidae	Apteroperla tikumana
Allocapnia recta isolate H18 16S ribosomal RNA gen	KC881053	84.08	100	4.82863e−39	Plecoptera	Capniidae	Allocapnia recta
Allocapnia recta isolate H17 16S ribosomal RNA gen	KC881052	84.08	100	4.82863e−39	Plecoptera	Capniidae	Allocapnia recta
Allocapnia recta isolate H12 16S ribosomal RNA gen	KC881047	84.08	100	4.82863e−39	Plecoptera	Capniidae	Allocapnia recta
Allocapnia recta isolate H9 16S ribosomal RNA gene	KC881044	84.08	100	4.82863e−39	Plecoptera	Capniidae	Allocapnia recta
Allocapnia recta isolate H8 16S ribosomal RNA gene	KC881043	84.08	100	4.82863e−39	Plecoptera	Capniidae	Allocapnia recta
Allocapnia recta isolate H5 16S ribosomal RNA gene	KC881040	84.08	100	4.82863e−39	Plecoptera	Capniidae	Allocapnia recta
Allocapnia recta isolate H4 16S ribosomal RNA gene	KC881039	84.08	100	4.82863e−39	Plecoptera	Capniidae	Allocapnia recta
Cosumnoperla hypocrena 16S ribosomal RNA gene, par	EF623226	84.81	100	4.82863e−39	Plecoptera	Perlodidae	Cosumnoperla hypocrena
Allocapnia minima 16S ribosomal RNA gene, partial	EF623130	84.08	100	4.82863e−39	Plecoptera	Capniidae	Allocapnia minima
Allocapnia recta haplotype 10ar: H010 16S ribosoma	EU306873	84.08	100	4.82863e−39	Plecoptera	Capniidae	Allocapnia recta
Allocapnia recta haplotype 9ar: H09 16S ribosomal	EU306872	84.08	100	4.82863e−39	Plecoptera	Capniidae	Allocapnia recta
Allocapnia recta haplotype 8ar: H08 16S ribosomal	EU306871	84.08	100	4.82863e−39	Plecoptera	Capniidae	Allocapnia recta
Allocapnia recta haplotype 1ar: H01 16S ribosomal	DQ915179	84.08	100	4.82863e−39	Plecoptera	Capniidae	Allocapnia recta
Pteronarcys sachalina 16S ribosomal RNA gene, part	EF623264	84.08	100	5.88247e−38	Plecoptera	Pteronarcyidae	Pteronarcys sachalina
Pteronarcys californica 16S ribosomal RNA gene, pa	EF623261	84.08	100	5.88247e−38	Plecoptera	Pteronarcyidae	Pteronarcys californica
Frisonia picticeps 16S ribosomal RNA gene, partial	EF623227	83.44	100	5.88247e−38	Plecoptera	Perlodidae	Frisonia picticeps
Austrocerca rieka 16S ribosomal RNA gene, partial	EF623205	83.44	100	5.88247e−38	Plecoptera	Notonemouridae	Austrocerca rieka
Austrocercoides zwicki 16S ribosomal RNA gene, par	EF623199	83.44	100	5.88247e−38	Plecoptera	Notonemouridae	Austrocercoides zwicki
Paraperla frontalis 16S ribosomal RNA gene, partia	EF623136	83.44	100	5.88247e−38	Plecoptera	Chloroperlidae	Paraperla frontalis
Sweltsa coloradensis 16S ribosomal RNA gene, parti	EF623133	84.08	100	5.88247e−38	Plecoptera	Chloroperlidae	Sweltsa coloradensis
Plumiperla diversa 16S ribosomal RNA gene, partial	EF623132	83.44	100	5.88247e−38	Plecoptera	Chloroperlidae	Plumiperla diversa
Capnura wanica 16S ribosomal RNA gene, partial seq	EF623128	84.08	100	5.88247e−38	Plecoptera	Capniidae	Capnura wanica
Bolotoperla rossi 16S ribosomal RNA gene, partial	EF623269	83.54	100	2.05319e−37	Plecoptera	Taeniopterygidae	Bolotoperla rossi
Neaviperla forcipata 16S ribosomal RNA gene, parti	EF623139	83.54	100	2.05319e−37	Plecoptera	Chloroperlidae	Neaviperla forcipata
Taenionema palladium 16S ribosomal RNA gene, parti	EF623267	83.65	100	7.16632e−37	Plecoptera	Taeniopterygidae	Taenionema palladium
Pteronarcys princeps mitochondrion, complete genom	AY687866	83.44	100	7.16632e−37	Plecoptera	Pteronarcyidae	Pteronarcys princeps
Notonemouridae sp. MD−2011 voucher ZMUC:JD−1507 16	JN615257	83.54	100	2.50129e−36	Plecoptera	Notonemouridae	Notonemouridae sp. MD−2011
Perlinodes aurea 16S ribosomal RNA gene, partial s	EF623234	82.8	100	2.50129e−36	Plecoptera	Perlodidae	Perlinodes aurea
Setvena wahkeena 16S ribosomal RNA gene, partial s	EF623211	82.91	100	8.73037e−36	Plecoptera	Perlodidae	Setvena wahkeena
Moselia infuscata 16S ribosomal RNA gene, partial	EF623175	84.08	99.36	8.73037e−36	Plecoptera	Leuctridae	Moselia infuscata
Megaleuctra kincaidi 16S ribosomal RNA gene, parti	EF623174	83.65	100	8.73037e−36	Plecoptera	Leuctridae	Megaleuctra kincaidi
Strophopteryx appalachia 16S ribosomal RNA gene, p	EF623271	85	89.17	3.0472e−35	Plecoptera	Taeniopterygidae	Strophopteryx appalachia
Cascadoperla trictura 16S ribosomal RNA gene, part	EF623225	82.8	100	3.0472e−35	Plecoptera	Perlodidae	Cascadoperla trictura
Thaumatoperla sp. BYU_PL109 16S ribosomal RNA gene	EF623149	82.61	100	3.0472e−35	Plecoptera	Eustheniidae	Thaumatoperla sp. BYU_PL109
Siphonoperla torrentium 16S ribosomal RNA gene, pa	EF623138	82.8	100	3.0472e−35	Plecoptera	Chloroperlidae	Siphonoperla torrentium
Chloroperla tripunctata 16S ribosomal RNA gene, pa	EF623137	82.17	100	3.0472e−35	Plecoptera	Chloroperlidae	Chloroperla tripunctata
Megarcys signata 16S ribosomal RNA gene, partial s	EF623215	82.39	100	1.06358e−34	Plecoptera	Perlodidae	Megarcys signata
Utaperla sopladora 16S ribosomal RNA gene, partial	EF623140	82.39	100	3.71225e−34	Plecoptera	Chloroperlidae	Utaperla sopladora
Osmylus sp. EH−2004 16S ribosomal RNA gene, partia	AY620146	82.8	100	3.71225e−34	Neuroptera	Osmylidae	Osmylus sp. EH−2004
Peltoperla arcuata 16S ribosomal RNA gene, partial	EF623258	86.26	83.44	1.2957e−33	Plecoptera	Peltoperlidae	Peltoperla arcuata
Baumanella alameda 16S ribosomal RNA gene, partial	EF623236	81.53	100	1.2957e−33	Plecoptera	Perlodidae	Baumanella alameda
Oconoperla innubila 16S ribosomal RNA gene, partia	EF623230	82.28	100	1.2957e−33	Plecoptera	Perlodidae	Oconoperla innubila
Salmoperla sylvanica 16S ribosomal RNA gene, parti	EF623222	81.53	100	1.2957e−33	Plecoptera	Perlodidae	Salmoperla sylvanica
Pictetiella expansa 16S ribosomal RNA gene, partia	EF623216	82.28	100	1.2957e−33	Plecoptera	Perlodidae	Pictetiella expansa
Skwala americana 16S ribosomal RNA gene, partial s	EF623217	81.76	100	4.52244e−33	Plecoptera	Perlodidae	Skwala americana
Alloperla severa 16S ribosomal RNA gene, partial s	EF623141	81.65	100	4.52244e−33	Plecoptera	Chloroperlidae	Alloperla severa
Isogenoides frontalis isolate BYU_PL080 16S riboso	EF623446	81.76	100	4.52244e−33	Plecoptera	Perlodidae	Isogenoides frontalis
Pteronarcys scotti 16S ribosomal RNA gene, partial	EF623263	81.53	100	1.57849e−32	Plecoptera	Pteronarcyidae	Pteronarcys scotti
Pteronarcella badia 16S ribosomal RNA gene, partia	EF623262	80.98	100	1.57849e−32	Plecoptera	Pteronarcyidae	Pteronarcella badia
Rickera sorpta 16S ribosomal RNA gene, partial seq	EF623235	81.65	100	1.57849e−32	Plecoptera	Perlodidae	Rickera sorpta
Helopicus bogaloosa 16S ribosomal RNA gene, partia	EF623228	80.89	100	1.57849e−32	Plecoptera	Perlodidae	Helopicus bogaloosa
Amphinemura sulcicollis 16S ribosomal RNA gene, pa	EF623195	91.35	66.24	5.50946e−32	Plecoptera	Nemouridae	Amphinemura sulcicollis
Sasquaperla hoopa 16S ribosomal RNA gene, partial	EF623143	80.89	100	5.50946e−32	Plecoptera	Chloroperlidae	Sasquaperla hoopa
Kathroperla perdita 16S ribosomal RNA gene, partia	EF623135	81.65	100	5.50946e−32	Plecoptera	Chloroperlidae	Kathroperla perdita
Isocapnia hyalita 16S ribosomal RNA gene, partial	EF623131	81.65	100	5.50946e−32	Plecoptera	Capniidae	Isocapnia hyalita
Isogenoides colubrinus isolate BYU_PL130 16S ribos	EF623448	81.13	100	5.50946e−32	Plecoptera	Perlodidae	Isogenoides colubrinus
