# Supplementary material for: Testing the potential of a ribosomal 16S marker for DNA metabarcoding of insects
Source: PeerJ. 2016 Apr 19;4:e1966. doi: 10.7717/peerj.1966 (PMC4841222; doi:10.7717/peerj.1966)
Supplement: Supplemental Information 1 [file peerj-04-1966-s006.zip › scripts/5 match OTUs/blast_hits/OTU_72.csv_plot.pdf]

OTU_72.csv

Dinocras cephalotes

Perlidae

Plecoptera

95.78

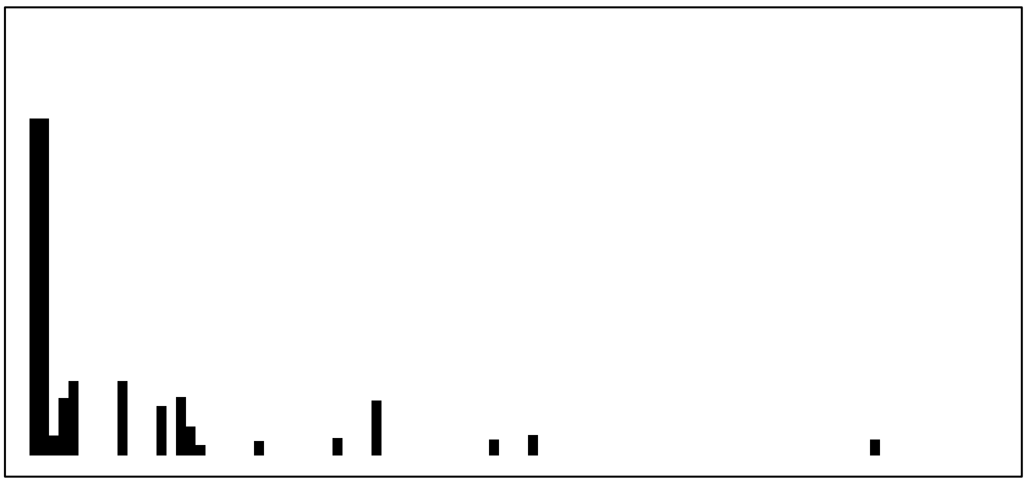

Dinocras cephalotes mitochondrion, complete genome	KF484757	95.78	100	8.76447e−68	Plecoptera	Perlidae	Dinocras cephalotes
Dinocras cephalotes 16S ribosomal RNA gene, partia	EF623244	95.78	100	8.76447e−68	Plecoptera	Perlidae	Dinocras cephalotes
Calineuria californica 16S ribosomal RNA gene, par	EF623239	80.92	90.36	1.06962e−28	Plecoptera	Perlidae	Calineuria californica
Perlesta decipines 16S ribosomal RNA gene, partial	EF623242	82.68	76.51	6.75003e−25	Plecoptera	Perlidae	Perlesta decipines
Agnetina capitata 16S ribosomal RNA gene, partial	EF623241	83.47	72.89	6.75003e−25	Plecoptera	Perlidae	Agnetina capitata
Perlinella drymo 16S ribosomal RNA gene, partial s	EF623240	80	87.35	6.75003e−25	Plecoptera	Perlidae	Perlinella drymo
Pteronarcys princeps mitochondrion, complete genom	AY687866	78.77	87.35	1.00179e−22	Plecoptera	Pteronarcyidae	Pteronarcys princeps
Claassenia sabulosa 16S ribosomal RNA gene, partia	EF623250	76.47	92.17	3.4966e−22	Plecoptera	Perlidae	Claassenia sabulosa
Moselia infuscata 16S ribosomal RNA gene, partial	EF623175	76.05	100	3.4966e−22	Plecoptera	Leuctridae	Moselia infuscata
Kathroperla perdita 16S ribosomal RNA gene, partia	EF623135	83.49	65.66	1.22043e−21	Plecoptera	Chloroperlidae	Kathroperla perdita
Styloperla sp. mitochondrion, partial genome	KR088971	75.95	93.37	4.25974e−21	Plecoptera	Styloperlidae	Styloperla sp. YZD−2015
Pteronarcys sachalina 16S ribosomal RNA gene, part	EF623264	78.23	87.35	4.25974e−21	Plecoptera	Pteronarcyidae	Pteronarcys sachalina
Pteronarcys californica 16S ribosomal RNA gene, pa	EF623261	78.08	87.35	4.25974e−21	Plecoptera	Pteronarcyidae	Pteronarcys californica
Acroneuria lycorias 16S ribosomal RNA gene, partial sa	EF623251	82.3	67.47	4.25974e−21	Plecoptera	Perlidae	Acroneuria lycorias
Taenionema palladium 16S ribosomal RNA gene, parti	EF623267	75.9	99.4	1.48679e−20	Plecoptera	Taeniopterygidae	Taenionema palladium
Hansonoperla appalachia 16S ribosomal RNA gene, pa	EF623249	82.73	66.27	1.48679e−20	Plecoptera	Perlidae	Hansonoperla appalachia
Udamocercia sp. BYU_PL150 16S ribosomal RNA gene,	EF623201	81.36	71.08	1.48679e−20	Plecoptera	Notonemouridae	Udamocercia sp. BYU_PL150
Despaxia augusta 16S ribosomal RNA gene, partial s	EF623177	80.49	73.49	1.48679e−20	Plecoptera	Leuctridae	Despaxia augusta
Leuctra tenuis haplotype 10lt: HO10 16S ribosomal	EU327682	76.05	99.4	1.48679e−20	Plecoptera	Leuctridae	Leuctra tenuis
Leuctra tenuis haplotype 9lt: HO9 16S ribosomal RN	EU327681	76.05	99.4	1.48679e−20	Plecoptera	Leuctridae	Leuctra tenuis
Leuctra tenuis haplotype 8lt: HO8 16S ribosomal RN	EU327680	76.05	99.4	1.48679e−20	Plecoptera	Leuctridae	Leuctra tenuis
Leuctra tenuis haplotype 7lt: HO7 16S ribosomal RN	EU327679	76.05	99.4	1.48679e−20	Plecoptera	Leuctridae	Leuctra tenuis
Leuctra tenuis haplotype 6lt: HO6 16S ribosomal RN	EU327678	76.05	99.4	1.48679e−20	Plecoptera	Leuctridae	Leuctra tenuis
Leuctra tenuis haplotype 4lt: HO4 16S ribosomal RN	EU327676	80.67	71.69	1.48679e−20	Plecoptera	Leuctridae	Leuctra tenuis
Leuctra tenuis haplotype 3lt: H03 16S ribosomal RN	DQ915184	76.05	99.4	1.48679e−20	Plecoptera	Leuctridae	Leuctra tenuis
Leuctra tenuis haplotype 2lt: H02 16S ribosomal RN	DQ915183	76.05	99.4	1.48679e−20	Plecoptera	Leuctridae	Leuctra tenuis
Leuctra tenuis haplotype 1lt: H01 16S ribosomal RN	DQ915182	76.05	99.4	1.48679e−20	Plecoptera	Leuctridae	Leuctra tenuis
Pteronarcys scotti 16S ribosomal RNA gene, partial	EF623263	77.24	87.35	5.18942e−20	Plecoptera	Pteronarcyidae	Pteronarcys scotti
Leuctra inermis 16S ribosomal RNA gene, partial se	EF623176	79.67	73.49	5.18942e−20	Plecoptera	Leuctridae	Leuctra inermis
Dolichopoda bormansi isolate BRA3 16S ribosomal RN	AY507605	74.85	99.4	1.81129e−19	Orthoptera	Rhaphidophoridae	Dolichopoda bormansi
Scopura montana 16S ribosomal RNA gene, partial se	EF623265	79.37	74.7	1.81129e−19	Plecoptera		Scopura montana
Chloroperla tripunctata 16S ribosomal RNA gene, pa	EF623137	80.8	74.7	1.81129e−19	Plecoptera	Chloroperlidae	Chloroperla tripunctata
Plumiperla diversa 16S ribosomal RNA gene, partial	EF623132	77.33	89.16	1.81129e−19	Plecoptera	Chloroperlidae	Plumiperla diversa
Leuctra tenuis haplotype 5lt: HO5 16S ribosomal RN	EU327677	75.45	99.4	1.81129e−19	Plecoptera	Leuctridae	Leuctra tenuis
Dolichopoda bormansi isolate BRA 16S ribosomal RNA	AY793578	74.85	99.4	1.81129e−19	Orthoptera	Rhaphidophoridae	Dolichopoda bormansi
Pteronarcella badia 16S ribosomal RNA gene, partial	EF623262	82.57	65.66	6.32201e−19	Plecoptera	Pteronarcyidae	Pteronarcella badia
Cardioperla lobata 16S ribosomal RNA gene, partial	EF623164	76.62	91.57	6.32201e−19	Plecoptera	Gripopterygidae	Cardioperla lobata
Neaviperla forcipata 16S ribosomal RNA gene, parti	EF623139	75.64	93.98	6.32201e−19	Plecoptera	Chloroperlidae	Neaviperla forcipata
Notonemouridae sp. MD−2011 voucher ZMUC:JD−1507 16	JN615257	78.74	75.9	2.2066e−18	Plecoptera	Notonemouridae	Notonemouridae sp. MD−2011
Doddsia occidentalis 16S ribosomal RNA gene, parti	EF623270	74.7	99.4	2.2066e−18	Plecoptera	Taeniopterygidae	Doddsia occidentalis
Cosumnoperla hypocrena 16S ribosomal RNA gene, par	EF623226	75.86	87.35	2.2066e−18	Plecoptera	Perlodidae	Cosumnoperla hypocrena
Prostoia besametsa 16S ribosomal RNA gene, partial	EF623188	75.31	96.99	2.2066e−18	Plecoptera	Nemouridae	Prostoia besametsa
Cryptoperla sp. YK−2012−1 mitochondrial gene for 1	AB711243	76.92	84.94	7.70178e−18	Plecoptera	Peltoperlidae	Cryptoperla sp. YK−2012−1
Dolichopoda bormansi isolate BRA5 16S ribosomal RN	AY507606	74.27	99.4	7.70178e−18	Orthoptera	Rhaphidophoridae	Dolichopoda bormansi
Eccoptura xanthenes 16S ribosomal RNA gene, partia	EF623252	78.57	75.9	7.70178e−18	Plecoptera	Perlidae	Eccoptura xanthenes
Salmoperla sylvanica 16S ribosomal RNA gene, parti	EF623222	75	93.37	7.70178e−18	Plecoptera	Perlodidae	Salmoperla sylvanica
Lednia tumana 16S ribosomal RNA gene, partial sequ	EF623197	78.12	75.9	7.70178e−18	Plecoptera	Nemouridae	Lednia tumana
Nemoura cinerea 16S ribosomal RNA gene, partial se	EF623194	80.73	65.66	7.70178e−18	Plecoptera	Nemouridae	Nemoura cinerea
Claudioperla sp. BYU_PL158 16S ribosomal RNA gene,	EF623169	75.97	91.57	7.70178e−18	Plecoptera	Gripopterygidae	Claudioperla sp. BYU_PL158
Triznaka pintada 16S ribosomal RNA gene, partial s	EF623134	80	74.7	7.70178e−18	Plecoptera	Chloroperlidae	Triznaka pintada
Peltodytes quadratus voucher BMNH794142 16S riboso	HQ381466	78.57	75.9	2.68819e−17	Coleoptera	Haliplidae	Peltodytes quadratus
Cultus pilatus 16S ribosomal RNA gene, partial seq	EF623212	80.95	63.25	2.68819e−17	Plecoptera	Perlodidae	Cultus pilatus
Suwallia teleckojensis 16S ribosomal RNA gene, par	EF623142	79.31	69.88	2.68819e−17	Plecoptera	Chloroperlidae	Suwallia teleckojensis
Penturoperla barbata 16S ribosomal RNA gene, parti	EF623124	80	69.28	2.68819e−17	Plecoptera	Austroperlidae	Penturoperla barbata
Osmylus sp. EH−2004 16S ribosomal RNA gene, partia	AY620146	76.19	87.35	2.68819e−17	Neuroptera	Osmylidae	Osmylus sp. EH−2004
D.geniculata mitochondrial 16S rRNA gene	Y07551	73.53	99.4	2.68819e−17	Orthoptera	Rhaphidophoridae	Dolichopoda geniculata
Allocapnia recta isolate H18 16S ribosomal RNA gen	KC881053	76.03	87.35	9.38269e−17	Plecoptera	Capniidae	Allocapnia recta
Allocapnia recta isolate H17 16S ribosomal RNA gen	KC881052	76.03	87.35	9.38269e−17	Plecoptera	Capniidae	Allocapnia recta
Allocapnia recta isolate H12 16S ribosomal RNA gen	KC881047	76.03	87.35	9.38269e−17	Plecoptera	Capniidae	Allocapnia recta
Allocapnia recta isolate H9 16S ribosomal RNA gene	KC881044	76.03	87.35	9.38269e−17	Plecoptera	Capniidae	Allocapnia recta
Allocapnia recta isolate H8 16S ribosomal RNA gene	KC881043	76.03	87.35	9.38269e−17	Plecoptera	Capniidae	Allocapnia recta
Allocapnia recta isolate H5 16S ribosomal RNA gene	KC881040	76.03	87.35	9.38269e−17	Plecoptera	Capniidae	Allocapnia recta
Allocapnia recta isolate H4 16S ribosomal RNA gene	KC881039	76.03	87.35	9.38269e−17	Plecoptera	Capniidae	Allocapnia recta
Cascadoperla trictura 16S ribosomal RNA gene, part	EF623225	76.03	86.14	9.38269e−17	Plecoptera	Perlodidae	Cascadoperla trictura
Skwala americana 16S ribosomal RNA gene, partial s	EF623217	76.03	87.35	9.38269e−17	Plecoptera	Perlodidae	Skwala americana
Nemoura trispinosa 16S ribosomal RNA gene, partial	EF623187	77.95	75.9	9.38269e−17	Plecoptera	Nemouridae	Nemoura trispinosa
Paranemoura claasseni 16S ribosomal RNA gene, part	EF623185	77.95	75.9	9.38269e−17	Plecoptera	Nemouridae	Paranemoura claasseni
Shipsa rotunda 16S ribosomal RNA gene, partial seq	EF623184	77.95	75.9	9.38269e−17	Plecoptera	Nemouridae	Shipsa rotunda
Riekoperla triloba 16S ribosomal RNA gene, partial	EF623171	74.1	99.4	9.38269e−17	Plecoptera	Gripopterygidae	Riekoperla triloba
Dinotoperla sp. BYU_PL105 16S ribosomal RNA gene,	EF623160	74.1	99.4	9.38269e−17	Plecoptera	Gripopterygidae	Dinotoperla sp. BYU_PL105
Acroperla trivacauta 16S ribosomal RNA gene, partial	EF623155	75	95.78	9.38269e−17	Plecoptera	Gripopterygidae	Acroperla trivacauta
Cosmioperla australis 16S ribosomal RNA gene, part	EF623148	74.05	91.57	9.38269e−17	Plecoptera	Eustheniidae	Cosmioperla australis
Alloperla severa 16S ribosomal RNA gene, partial s	EF623141	75.82	89.16	9.38269e−17	Plecoptera	Chloroperlidae	Alloperla severa
Utaperla sopladora 16S ribosomal RNA gene, partial	EF623140	74.85	95.78	9.38269e−17	Plecoptera	Chloroperlidae	Utaperla sopladora
Acruroperla atra 16S ribosomal RNA gene, partial s	EF623121	75	95.78	9.38269e−17	Plecoptera	Austroperlidae	Acruroperla atra
Allocapnia recta haplotype 10ar: H010 16S ribosoma	EU306873	76.03	87.35	9.38269e−17	Plecoptera	Capniidae	Allocapnia recta
Allocapnia recta haplotype 9ar: H09 16S ribosomal	EU306872	76.03	87.35	9.38269e−17	Plecoptera	Capniidae	Allocapnia recta
Allocapnia recta haplotype 8ar: H08 16S ribosomal	EU306871	76.03	87.35	9.38269e−17	Plecoptera	Capniidae	Allocapnia recta
Allocapnia recta haplotype 1ar: H01 16S ribosomal	DQ915179	76.03	87.35	9.38269e−17	Plecoptera	Capniidae	Allocapnia recta
Cryptoperla ishigakiensis mitochondrial gene for 1	AB711242	77.97	71.08	3.27488e−16	Plecoptera	Peltoperlidae	Cryptoperla ishigakiensis
Eupholidoptera krueperi voucher 408 16S ribosomal	EU203912	74.17	88.55	3.27488e−16	Orthoptera	Tettigoniidae	Eupholidoptera krueperi
Paragnetina media 16S ribosomal RNA gene, partial	EF623253	73.05	100	3.27488e−16	Plecoptera	Perlidae	Paragnetina media
Pictetoperla sp. BYU_PL156 16S ribosomal RNA gene,	EF623247	79.82	65.66	3.27488e−16	Plecoptera	Perlidae	Pictetoperla sp. BYU_PL156
Kempnyella sp. BYU_PL155 16S ribosomal RNA gene, p	EF623246	78.38	66.87	3.27488e−16	Plecoptera	Perlidae	Kempnyella sp. BYU_PL155
Osobenus yakimae 16S ribosomal RNA gene, partial s	EF623232	75	90.96	3.27488e−16	Plecoptera	Perlodidae	Osobenus yakimae
Isogenoides olivaceus 16S ribosomal RNA gene, part	EF623224	78.95	68.67	3.27488e−16	Plecoptera	Perlodidae	Isogenoides olivaceus
Paraleuctra vershina 16S ribosomal RNA gene, parti	EF623173	80.73	65.66	3.27488e−16	Plecoptera	Leuctridae	Paraleuctra vershina
Pelurgoperla personata 16S ribosomal RNA gene, par	EF623168	75.32	91.57	3.27488e−16	Plecoptera	Gripopterygidae	Pelurgoperla personata
Antarctoperla sp. BYU_PL142 16S ribosomal RNA gene	EF623163	75.32	91.57	3.27488e−16	Plecoptera	Gripopterygidae	Antarctoperla sp. BYU_PL142
Tasmanoperla larvalis 16S ribosomal RNA gene, part	EF623125	75.16	93.98	3.27488e−16	Plecoptera	Austroperlidae	Tasmanoperla larvalis
Pholidoptera fallax voucher 160 16S ribosomal RNA	EU120781	74.56	100	3.27488e−16	Orthoptera	Tettigoniidae	Pholidoptera fallax
Namaquaphasma ookiepense isolate 821 16S large sub	DQ890293	74.4	100	3.27488e−16	Mantophasmatodea	Austrophasmatidae	Namaquaphasma ookiepense
Namaquaphasma ookiepense isolate 813 16S large sub	DQ890292	74.4	100	3.27488e−16	Mantophasmatodea	Austrophasmatidae	Namaquaphasma ookiepense
Graphelmis picea 16S ribosomal RNA gene, partial s	DQ266472	73.42	92.17	3.27488e−16	Coleoptera	Elmidae	Graphelmis picea
Ceuthophilus latens 16S ribosomal RNA gene, partia	AF212055	78.95	68.67	3.27488e−16	Orthoptera	Rhaphidophoridae	Ceuthophilus latens
Allocapnia recta isolate H19 16S ribosomal RNA gen	KC881054	75.34	87.35	1.14305e−15	Plecoptera	Capniidae	Allocapnia recta
Allocapnia recta isolate H16 16S ribosomal RNA gen	KC881051	75.34	87.35	1.14305e−15	Plecoptera	Capniidae	Allocapnia recta
Allocapnia recta isolate H15 16S ribosomal RNA gen	KC881050	75.34	87.35	1.14305e−15	Plecoptera	Capniidae	Allocapnia recta
Allocapnia recta isolate H14 16S ribosomal RNA gen	KC881049	75.34	87.35	1.14305e−15	Plecoptera	Capniidae	Allocapnia recta
Allocapnia recta isolate H10 16S ribosomal RNA gen	KC881045	75.34	87.35	1.14305e−15	Plecoptera	Capniidae	Allocapnia recta
