# Supplementary material for: Testing the potential of a ribosomal 16S marker for DNA metabarcoding of insects
Source: PeerJ. 2016 Apr 19;4:e1966. doi: 10.7717/peerj.1966 (PMC4841222; doi:10.7717/peerj.1966)
Supplement: Supplemental Information 1 [file peerj-04-1966-s006.zip › scripts/5 match OTUs/blast_hits/OTU_74.csv_plot.pdf]

OTU_74.csv

Isoperla oxylepis

Perlodidae

Plecoptera

87.34

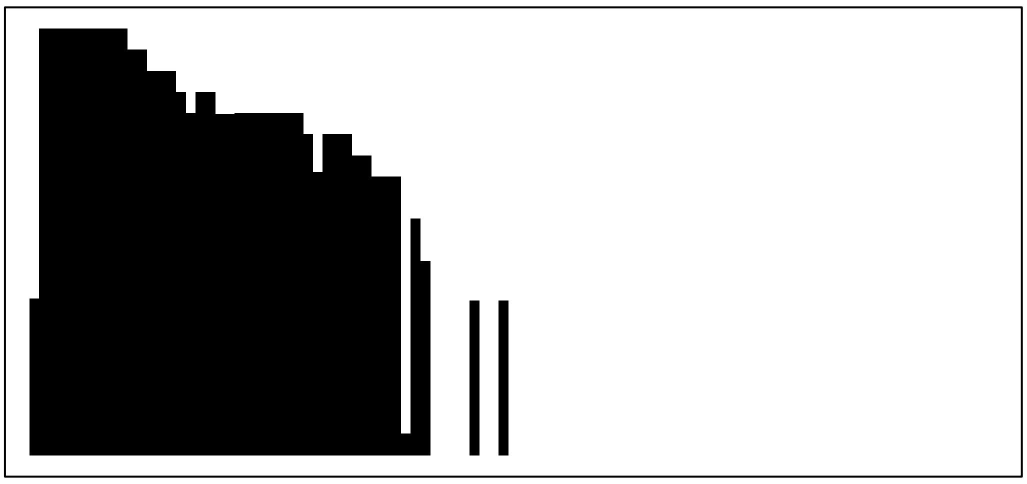

Isoperla oxylepis 16S ribosomal RNA gene, partial	EF623221	87.34	100	2.19219e−43	Plecoptera	Perlodidae	Isoperla oxylepis
Rhithrogena sp. 13 LV−2015 mitochondrial partial 1	LN868672	100	64.33	7.65151e−43	Ephemeroptera	Heptageniidae	Rhithrogena sp. 13 LV−2015
Rhithrogena sp. 12 LV−2015 mitochondrial partial 1	LN868671	100	64.33	7.65151e−43	Ephemeroptera	Heptageniidae	Rhithrogena sp. 12 LV−2015
Rhithrogena picteti mitochondrial partial 16S rRNA	LN868663	100	64.33	7.65151e−43	Ephemeroptera	Heptageniidae	Rhithrogena picteti
Rhithrogena sp. 3 LV−2015 mitochondrial partial 16	LN868659	100	64.33	7.65151e−43	Ephemeroptera	Heptageniidae	Rhithrogena sp. 3 LV−2015
Rhithrogena sp. 2 LV−2015 mitochondrial partial 16	LN868654	100	64.33	7.65151e−43	Ephemeroptera	Heptageniidae	Rhithrogena sp. 2 LV−2015
Rhithrogena puytoraci mitochondrial partial 16S rR	LN868651	100	64.33	7.65151e−43	Ephemeroptera	Heptageniidae	Rhithrogena puytoraci
Rhithrogena iridina mitochondrial partial 16S rRNA	LN868646	100	64.33	7.65151e−43	Ephemeroptera	Heptageniidae	Rhithrogena iridina
Rhithrogena taurisca mitochondrial partial 16S rRN	LN868638	100	64.33	7.65151e−43	Ephemeroptera	Heptageniidae	Rhithrogena taurisca
Rhithrogena sp. 5 LV−2015 mitochondrial partial 16	LN868653	100	63.69	2.67064e−42	Ephemeroptera	Heptageniidae	Rhithrogena sp. 5 LV−2015
Rhithrogena semicolorata mitochondrial partial 16S	LN868673	99.01	64.33	3.2535e−41	Ephemeroptera	Heptageniidae	Rhithrogena semicolorata
Rhithrogena sp. 4 LV−2015 mitochondrial partial 16	LN868652	99.01	64.33	3.2535e−41	Ephemeroptera	Heptageniidae	Rhithrogena sp. 4 LV−2015
Rhithrogena fonticola mitochondrial partial 16S rR	LN868656	98.02	64.33	3.96358e−40	Ephemeroptera	Heptageniidae	Rhithrogena fonticola
Rhithrogena sp. 7 LV−2015 mitochondrial partial 16	LN868669	98.02	64.33	4.82863e−39	Ephemeroptera	Heptageniidae	Rhithrogena sp. 7 LV−2015
Rhithrogena nivata mitochondrial partial 16S rRNA	LN868643	98.02	64.33	4.82863e−39	Ephemeroptera	Heptageniidae	Rhithrogena nivata
Rhithrogena sp. 8 LV−2015 mitochondrial partial 16	LN868661	97.03	64.33	2.05319e−37	Ephemeroptera	Heptageniidae	Rhithrogena sp. 8 LV−2015
Rhithrogena savoiensis mitochondrial partial 16S r	LN868658	96.04	64.33	2.05319e−37	Ephemeroptera	Heptageniidae	Rhithrogena savoiensis
Rhithrogena sp. 9 LV−2015 mitochondrial partial 16	LN868650	97.03	64.33	2.05319e−37	Ephemeroptera	Heptageniidae	Rhithrogena sp. 9 LV−2015
Rhithrogena grischuna mitochondrial partial 16S rR	LN868639	97.03	64.33	2.05319e−37	Ephemeroptera	Heptageniidae	Rhithrogena grischuna
Rhithrogena sp. 10 LV−2015 mitochondrial partial 1	LN868664	96	63.69	7.16632e−37	Ephemeroptera	Heptageniidae	Rhithrogena sp. 10 LV−2015
Rhithrogena beskidensis mitochondrial partial 16S	LN868645	96	63.69	7.16632e−37	Ephemeroptera	Heptageniidae	Rhithrogena beskidensis
Rhithrogena degrangei mitochondrial partial 16S rR	LN868674	96.04	64.33	2.50129e−36	Ephemeroptera	Heptageniidae	Rhithrogena degrangei
Rhithrogena sp. 11 LV−2015 mitochondrial partial 1	LN868668	96.04	64.33	2.50129e−36	Ephemeroptera	Heptageniidae	Rhithrogena sp. 11 LV−2015
Rhithrogena alpestris mitochondrial partial 16S rR	LN868657	96.04	64.33	2.50129e−36	Ephemeroptera	Heptageniidae	Rhithrogena alpestris
Rhithrogena sp. 6 LV−2015 mitochondrial partial 16	LN868655	96.04	64.33	2.50129e−36	Ephemeroptera	Heptageniidae	Rhithrogena sp. 6 LV−2015
Rhithrogena hybrida mitochondrial partial 16S rRNA	LN868642	96.04	64.33	2.50129e−36	Ephemeroptera	Heptageniidae	Rhithrogena hybrida
Rhithrogena circumtatrica mitochondrial partial 16	LN868641	96.04	64.33	2.50129e−36	Ephemeroptera	Heptageniidae	Rhithrogena circumtatrica
Rhithrogena diensis mitochondrial partial 16S rRNA	LN868637	96.04	64.33	2.50129e−36	Ephemeroptera	Heptageniidae	Rhithrogena diensis
Rhithrogena germanica mitochondrial partial 16S rR	LN868660	95.05	64.33	8.73037e−36	Ephemeroptera	Heptageniidae	Rhithrogena germanica
Rhithrogena sartorii mitochondrial partial 16S rRN	LN868670	93.27	66.24	1.06358e−34	Ephemeroptera	Heptageniidae	Rhithrogena sartorii
Rhithrogena insularis mitochondrial partial 16S rR	LN868665	95.05	64.33	1.06358e−34	Ephemeroptera	Heptageniidae	Rhithrogena insularis
Rhithrogena gratianopolitana mitochondrial partial	LN868648	95.05	64.33	1.06358e−34	Ephemeroptera	Heptageniidae	Rhithrogena gratianopolitana
Rhithrogena gratianopolitana mitochondrial partial	LN868647	95.05	64.33	1.06358e−34	Ephemeroptera	Heptageniidae	Rhithrogena gratianopolitana
Rhithrogena nuragica mitochondrial partial 16S rRN	LN868667	94.06	64.33	1.2957e−33	Ephemeroptera	Heptageniidae	Rhithrogena nuragica
Rhithrogena eatoni mitochondrial partial 16S rRNA	LN868666	94.06	64.33	1.2957e−33	Ephemeroptera	Heptageniidae	Rhithrogena eatoni
Rhithrogena dorieri mitochondrial partial 16S rRNA	LN868649	93.07	64.33	4.52244e−33	Ephemeroptera	Heptageniidae	Rhithrogena dorieri
Rhithrogena corcontica mitochondrial partial 16S r	LN868662	93.07	64.33	5.50946e−32	Ephemeroptera	Heptageniidae	Rhithrogena corcontica
Rhithrogena landai mitochondrial partial 16S rRNA	LN868644	93.07	64.33	5.50946e−32	Ephemeroptera	Heptageniidae	Rhithrogena landai
Isoperla davisi 16S ribosomal RNA gene, partial se	EF623206	81.01	100	8.17677e−30	Plecoptera	Perlodidae	Isoperla davisi
Rhithrogena allobrogica mitochondrial partial 16S	LN868640	91.09	64.33	2.85397e−29	Ephemeroptera	Heptageniidae	Rhithrogena allobrogica
Rhithrogena sp. EP021 16S ribosomal RNA gene, part	AY749765	89.11	64.33	1.21354e−27	Ephemeroptera	Heptageniidae	Rhithrogena sp. EP021
Brachyptera seticornis 16S ribosomal RNA gene, par	EF623268	79.5	100	4.23567e−27	Plecoptera	Taeniopterygidae	Brachyptera seticornis
Isoperla fulva 16S ribosomal RNA gene, partial seq	EF623207	78.75	100	1.47839e−26	Plecoptera	Perlodidae	Isoperla fulva
Neopanorpa pulchra mitochondrion, complete genome	FJ169955	78.88	100	5.1601e−26	Mecoptera	Panorpidae	Neopanorpa pulchra
Isoperla phalerata 16S ribosomal RNA gene, partial	EF623208	78.34	100	5.1601e−26	Plecoptera	Perlodidae	Isoperla phalerata
Cinygmula sp. m185 mitochondrial partial 16S rRNA	LN868675	87.25	64.97	1.80105e−25	Ephemeroptera	Heptageniidae	Cinygmula sp. m185
Perlinodes aurea 16S ribosomal RNA gene, partial s	EF623234	78.48	100	1.80105e−25	Plecoptera	Perlodidae	Perlinodes aurea
Capnura wanica 16S ribosomal RNA gene, partial seq	EF623128	78.62	100	1.80105e−25	Plecoptera	Capniidae	Capnura wanica
Cinygmula sp. EP013 16S ribosomal RNA gene, partia	AY749760	87.25	64.97	1.80105e−25	Ephemeroptera	Heptageniidae	Cinygmula sp. EP013
Neopanorpa pulchra mitochondrion, complete genome	JX569848	78.26	100	6.28629e−25	Mecoptera	Panorpidae	Neopanorpa pulchra
Clioperla clio 16S ribosomal RNA gene, partial seq	EF623237	78.12	100	6.28629e−25	Plecoptera	Perlodidae	Clioperla clio
Cultus pilatus 16S ribosomal RNA gene, partial seq	EF623212	77.71	100	2.19413e−24	Plecoptera	Perlodidae	Cultus pilatus
Thaumatoperla sp. BYU_PL109 16S ribosomal RNA gene	EF623149	77.64	100	2.19413e−24	Plecoptera	Eustheniidae	Thaumatoperla sp. BYU_PL109
Megarcys signata 16S ribosomal RNA gene, partial s	EF623215	77.85	100	7.65827e−24	Plecoptera	Perlodidae	Megarcys signata
Soyedina producta 16S ribosomal RNA gene, partial	EF623196	77.99	100	7.65827e−24	Plecoptera	Nemouridae	Soyedina producta
Mesocapnia arizonensis mitochondrion, partial geno	KP642637	77.5	100	2.673e−23	Plecoptera	Capniidae	Mesocapnia arizonensis
Allocapnia recta isolate H13 16S ribosomal RNA gen	KC881048	77.5	100	2.673e−23	Plecoptera	Capniidae	Allocapnia recta
Allocapnia recta isolate H11 16S ribosomal RNA gen	KC881046	77.5	100	2.673e−23	Plecoptera	Capniidae	Allocapnia recta
Allocapnia recta isolate H7 16S ribosomal RNA gene	KC881042	77.5	100	2.673e−23	Plecoptera	Capniidae	Allocapnia recta
Allocapnia recta isolate H3 16S ribosomal RNA gene	KC881038	77.5	100	2.673e−23	Plecoptera	Capniidae	Allocapnia recta
Mesocapnia sp. BYU_PL010 16S ribosomal RNA gene, p	EF623127	77.5	100	2.673e−23	Plecoptera	Capniidae	Mesocapnia sp. BYU_PL010
Allocapnia recta haplotype 12ar: H12 16S ribosomal	EU306875	77.5	100	2.673e−23	Plecoptera	Capniidae	Allocapnia recta
Allocapnia recta haplotype 3ar: H03 16S ribosomal	DQ915181	77.5	100	2.673e−23	Plecoptera	Capniidae	Allocapnia recta
Frisonia picticeps 16S ribosomal RNA gene, partial	EF623227	77.22	100	9.32968e−23	Plecoptera	Perlodidae	Frisonia picticeps
Apteroperla tikumana mitochondrion, complete genom	KR604721	76.88	100	3.25638e−22	Plecoptera	Capniidae	Apteroperla tikumana
Allocapnia recta isolate H19 16S ribosomal RNA gen	KC881054	76.88	100	3.25638e−22	Plecoptera	Capniidae	Allocapnia recta
Allocapnia recta isolate H16 16S ribosomal RNA gen	KC881051	76.88	100	3.25638e−22	Plecoptera	Capniidae	Allocapnia recta
Allocapnia recta isolate H15 16S ribosomal RNA gen	KC881050	76.88	100	3.25638e−22	Plecoptera	Capniidae	Allocapnia recta
Allocapnia recta isolate H14 16S ribosomal RNA gen	KC881049	76.88	100	3.25638e−22	Plecoptera	Capniidae	Allocapnia recta
Allocapnia recta isolate H10 16S ribosomal RNA gen	KC881045	76.88	100	3.25638e−22	Plecoptera	Capniidae	Allocapnia recta
Allocapnia recta isolate H6 16S ribosomal RNA gene	KC881041	76.88	100	3.25638e−22	Plecoptera	Capniidae	Allocapnia recta
Allocapnia recta isolate H1 16S ribosomal RNA gene	KC881036	76.88	100	3.25638e−22	Plecoptera	Capniidae	Allocapnia recta
Paraperla frontalis 16S ribosomal RNA gene, partia	EF623136	77.36	100	3.25638e−22	Plecoptera	Chloroperlidae	Paraperla frontalis
Allocapnia recta haplotype 11ar: H011 16S ribosoma	EU306874	76.88	100	3.25638e−22	Plecoptera	Capniidae	Allocapnia recta
Allocapnia recta haplotype 7ar: H07 16S ribosomal	EU306870	76.88	100	3.25638e−22	Plecoptera	Capniidae	Allocapnia recta
Allocapnia recta haplotype 2ar: H02 16S ribosomal	DQ915180	76.88	100	3.25638e−22	Plecoptera	Capniidae	Allocapnia recta
Pteronarcys californica 16S ribosomal RNA gene, pa	EF623261	76.43	100	1.13659e−21	Plecoptera	Pteronarcyidae	Pteronarcys californica
Rickera sorpta 16S ribosomal RNA gene, partial seq	EF623235	77.22	100	1.13659e−21	Plecoptera	Perlodidae	Rickera sorpta
Neopanorpa sp. M24899 16S ribosomal RNA gene, part	AF180059	77.16	100	1.13659e−21	Mecoptera	Panorpidae	Neopanorpa sp. M24899
Salmoperla sylvanica 16S ribosomal RNA gene, parti	EF623222	76.58	100	3.96708e−21	Plecoptera	Perlodidae	Salmoperla sylvanica
Nemoura cinerea 16S ribosomal RNA gene, partial se	EF623194	77.78	100	3.96708e−21	Plecoptera	Nemouridae	Nemoura cinerea
Chloroperla tripunctata 16S ribosomal RNA gene, pa	EF623137	76.58	100	3.96708e−21	Plecoptera	Chloroperlidae	Chloroperla tripunctata
Allocapnia recta isolate H18 16S ribosomal RNA gen	KC881053	76.25	100	1.38465e−20	Plecoptera	Capniidae	Allocapnia recta
Allocapnia recta isolate H17 16S ribosomal RNA gen	KC881052	76.25	100	1.38465e−20	Plecoptera	Capniidae	Allocapnia recta
Allocapnia recta isolate H12 16S ribosomal RNA gen	KC881047	76.25	100	1.38465e−20	Plecoptera	Capniidae	Allocapnia recta
Allocapnia recta isolate H9 16S ribosomal RNA gene	KC881044	76.25	100	1.38465e−20	Plecoptera	Capniidae	Allocapnia recta
Allocapnia recta isolate H8 16S ribosomal RNA gene	KC881043	76.25	100	1.38465e−20	Plecoptera	Capniidae	Allocapnia recta
Allocapnia recta isolate H5 16S ribosomal RNA gene	KC881040	76.25	100	1.38465e−20	Plecoptera	Capniidae	Allocapnia recta
Allocapnia recta isolate H4 16S ribosomal RNA gene	KC881039	76.25	100	1.38465e−20	Plecoptera	Capniidae	Allocapnia recta
Cascadoperla trictura 16S ribosomal RNA gene, part	EF623225	75.8	100	1.38465e−20	Plecoptera	Perlodidae	Cascadoperla trictura
Setvena wahkeena 16S ribosomal RNA gene, partial s	EF623211	76.1	100	1.38465e−20	Plecoptera	Perlodidae	Setvena wahkeena
Austrocerca rieka 16S ribosomal RNA gene, partial	EF623205	76.88	100	1.38465e−20	Plecoptera	Notonemouridae	Austrocerca rieka
Austrocercoides zwicki 16S ribosomal RNA gene, par	EF623199	76.88	100	1.38465e−20	Plecoptera	Notonemouridae	Austrocercoides zwicki
Nemoura trispinosa 16S ribosomal RNA gene, partial	EF623187	76.25	100	1.38465e−20	Plecoptera	Nemouridae	Nemoura trispinosa
Zapada cinctipes 16S ribosomal RNA gene, partial s	EF623183	76.25	100	1.38465e−20	Plecoptera	Nemouridae	Zapada cinctipes
Allocapnia minima 16S ribosomal RNA gene, partial	EF623130	76.25	100	1.38465e−20	Plecoptera	Capniidae	Allocapnia minima
Allocapnia recta haplotype 10ar: H010 16S ribosoma	EU306873	76.25	100	1.38465e−20	Plecoptera	Capniidae	Allocapnia recta
Allocapnia recta haplotype 9ar: H09 16S ribosomal	EU306872	76.25	100	1.38465e−20	Plecoptera	Capniidae	Allocapnia recta
Allocapnia recta haplotype 8ar: H08 16S ribosomal	EU306871	76.25	100	1.38465e−20	Plecoptera	Capniidae	Allocapnia recta
Allocapnia recta haplotype 1ar: H01 16S ribosomal	DQ915179	76.25	100	1.38465e−20	Plecoptera	Capniidae	Allocapnia recta
