# Supplementary material for: Testing the potential of a ribosomal 16S marker for DNA metabarcoding of insects
Source: PeerJ. 2016 Apr 19;4:e1966. doi: 10.7717/peerj.1966 (PMC4841222; doi:10.7717/peerj.1966)
Supplement: Supplemental Information 1 [file peerj-04-1966-s006.zip › scripts/5 match OTUs/blast_hits/OTU_75.csv_plot.pdf]

OTU_75.csv

Chorisops tunisiae

Stratiomyidae

Diptera

85.35

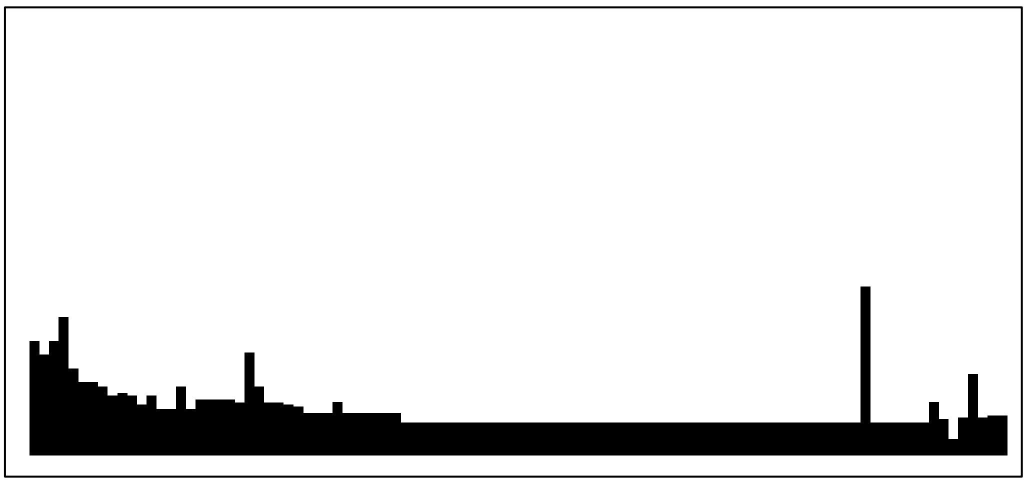

Chorisops tunisiae 16S ribosomal RNA gene, partial	GU947379	85.35	100	1.13558e−40	Diptera	Stratiomyidae	Chorisops tunisiae
Actina chalybea 16S ribosomal RNA gene, partial se	GU947372	84.71	100	1.38343e−39	Diptera	Acroceridae	Actina chalybea
Pedicia albivitta voucher O'Grady 200012 16S ribos	EU005435	85.35	99.36	1.38343e−39	Diptera	Pediciidae	Pedicia albivitta
Parochlus steinenii isolate Sejong01 mitochondrion	KT003702	86.49	94.27	4.82863e−39	Diptera	Chironomidae	Parochlus steinenii
Edwardsina gigantea 16S ribosomal RNA gene, partia	KC177460	84.08	100	5.88247e−38	Diptera	Blephariceridae	Edwardsina gigantea
Hermetia illucens 16S ribosomal RNA gene, partial	GU947384	83.44	100	7.16632e−37	Diptera	Stratiomyidae	Hermetia illucens
Ptychoptera sp. ATB−2011 mitochondrion, complete g	JN861744	83.44	99.36	8.73037e−36	Diptera	Ptychopteridae	Ptychoptera sp. ATB−2011
Corynoptera subtilis voucher T34 16S ribosomal RNA	JQ613978	83.23	97.45	3.0472e−35	Diptera	Sciaridae	Corynoptera subtilis
Hermetia illucens 16S ribosomal RNA gene, partial	KC177455	82.8	100	3.0472e−35	Diptera	Stratiomyidae	Hermetia illucens
Minettia flaveola 16S ribosomal RNA gene, partial	KC177448	82.91	100	3.0472e−35	Diptera	Lauxaniidae	Minettia flaveola
Lasiopa balius 16S ribosomal RNA gene, partial seq	GU947382	82.8	100	3.0472e−35	Diptera	Stratiomyidae	Lasiopa balius
Minettia longipennis mitochondrion partial 16S rRN	AJ439184	82.39	100	1.06358e−34	Diptera	Lauxaniidae	Minettia longipennis
Clitellaria ephippium 16S ribosomal RNA gene, part	GU947399	82.8	100	3.71225e−34	Diptera	Stratiomyidae	Clitellaria ephippium
Hercostomus verbekei voucher Pollet 0263 (P) 16S r	EU864004	82.17	100	3.71225e−34	Diptera	Dolichopodidae	Hercostomus verbekei
Drosophila acanthoptera voucher 105622 NADH dehydr	EU494332	82.17	100	3.71225e−34	Diptera	Drosophilidae	Drosophila acanthoptera
Armigeres subalbatus ASAP ID: 43761 large ribosoma	AY439688	83.23	100	3.71225e−34	Diptera	Culicidae	Armigeres subalbatus
Chrysops carbonarius 16S ribosomal RNA gene, parti	AY123341	82.17	100	3.71225e−34	Diptera	Tabanidae	Chrysops carbonarius
Drosophila arawakana dominicensis NADH dehydrogena	AF246516	82.61	100	3.71225e−34	Diptera	Drosophilidae	Drosophila arawakana dominicensis
Drosophila arawakana arawakana strain 15182−2261.0	AF246499	82.61	100	3.71225e−34	Diptera	Drosophilidae	Drosophila arawakana arawakana
Drosophila arawakana arawakana strain 15182−2261.1	AF246500	82.61	100	3.71225e−34	Diptera	Drosophilidae	Drosophila arawakana arawakana
Drosophila nigrodunni NADH dehydrogenase subunit 1	AF246494	82.61	100	3.71225e−34	Diptera	Drosophilidae	Drosophila nigrodunni
Phytosciara flavipes voucher JSOUT8 16S ribosomal	KJ136731	82.47	97.45	1.2957e−33	Diptera	Sciaridae	Phytosciara flavipes
Bolitophila occlusa voucher JSB2 16S ribosomal RNA	KJ136713	84.83	91.72	1.2957e−33	Diptera	Mycetophilidae	Bolitophila occlusa
Bolitophila cinerea voucher JSB1 16S ribosomal RNA	KJ136712	83.23	97.45	1.2957e−33	Diptera	Mycetophilidae	Bolitophila cinerea
Camptochaeta camptochaeta voucher 972 16S ribosoma	JQ613970	82.47	97.45	1.2957e−33	Diptera	Sciaridae	Camptochaeta camptochaeta
Phytosciara flavipes voucher 88 16S ribosomal RNA	JQ613889	82.47	97.45	1.2957e−33	Diptera	Sciaridae	Phytosciara flavipes
Libnotes orofenaae voucher O'Grady 202034 16S ribo	EU005431	82.39	99.36	1.2957e−33	Diptera	Limoniidae	Libnotes orofenaae
Limonia tahitiensis voucher O'Grady 202004 16S rib	EU005425	82.28	99.36	1.2957e−33	Diptera	Limoniidae	Dicranomyia tahitiensis
Drosophila dunni voucher 103969 NADH dehydrogenase	EU494311	81.99	100	4.52244e−33	Diptera	Drosophilidae	Drosophila dunni
Drosophila similis grenadensis NADH dehydrogenase	AF246519	81.99	100	4.52244e−33	Diptera	Drosophilidae	Drosophila similis grenadensis
Drosophila dunni dunni strain Barranquitas, line 1	AF246512	81.99	100	4.52244e−33	Diptera	Drosophilidae	Drosophila dunni dunni
Drosophila procardinoides NADH dehydrogenase subun	AF246508	82.5	100	4.52244e−33	Diptera	Drosophilidae	Drosophila procardinoides
Drosophila acutilabella strain 15181−2171.9 NADH d	AF246509	81.99	100	4.52244e−33	Diptera	Drosophilidae	Drosophila acutilabella
Drosophila dunni thomasensis NADH dehydrogenase su	AF246501	81.99	100	4.52244e−33	Diptera	Drosophilidae	Drosophila dunni thomasensis
Drosophila dunni dunni strain 15182−2291.0 NADH de	AF246497	81.99	100	4.52244e−33	Diptera	Drosophilidae	Drosophila dunni dunni
Drosophila acutilabella strain 15181−2171.2 NADH d	AF246496	81.99	100	4.52244e−33	Diptera	Drosophilidae	Drosophila acutilabella
Drosophila similis similis NADH dehydrogenase subu	AF246495	81.99	100	4.52244e−33	Diptera	Drosophilidae	Drosophila similis similis
Drosophila caribiana NADH dehydrogenase subunit 1	AF246493	81.99	100	4.52244e−33	Diptera	Drosophilidae	Drosophila caribiana
Drosophila yakuba strain yak_GN110 mitochondrion,	KF824901	81.53	100	1.57849e−32	Diptera	Drosophilidae	Drosophila yakuba
Drosophila yakuba strain yak_ZW162 mitochondrion,	KF824900	81.53	100	1.57849e−32	Diptera	Drosophilidae	Drosophila yakuba
Drosophila yakuba strain yak_ZW121 mitochondrion,	KF824899	81.53	100	1.57849e−32	Diptera	Drosophilidae	Drosophila yakuba
Drosophila yakuba strain yak_ZW002A mitochondrion,	KF824898	81.53	100	1.57849e−32	Diptera	Drosophilidae	Drosophila yakuba
Drosophila yakuba strain yak_TZ32 mitochondrion, c	KF824897	81.53	100	1.57849e−32	Diptera	Drosophilidae	Drosophila yakuba
Drosophila yakuba strain yak_TZ025 mitochondrion,	KF824896	81.53	100	1.57849e−32	Diptera	Drosophilidae	Drosophila yakuba
Drosophila yakuba strain yak_ST mitochondrion, com	KF824895	81.53	100	1.57849e−32	Diptera	Drosophilidae	Drosophila yakuba
Drosophila yakuba strain yak_SN2 mitochondrion, co	KF824894	81.53	100	1.57849e−32	Diptera	Drosophilidae	Drosophila yakuba
Drosophila yakuba strain yak_Rain6 mitochondrion,	KF824893	81.53	100	1.57849e−32	Diptera	Drosophilidae	Drosophila yakuba
Drosophila yakuba strain yak_Rain5 mitochondrion,	KF824892	81.53	100	1.57849e−32	Diptera	Drosophilidae	Drosophila yakuba
Drosophila yakuba strain yak_Rain4 mitochondrion,	KF824891	81.53	100	1.57849e−32	Diptera	Drosophilidae	Drosophila yakuba
Drosophila yakuba strain yak_Rain3 mitochondrion,	KF824890	81.53	100	1.57849e−32	Diptera	Drosophilidae	Drosophila yakuba
Drosophila yakuba strain yak_Rain2 mitochondrion,	KF824889	81.53	100	1.57849e−32	Diptera	Drosophilidae	Drosophila yakuba
Drosophila yakuba strain yak_Rain1 mitochondrion,	KF824888	81.53	100	1.57849e−32	Diptera	Drosophilidae	Drosophila yakuba
Drosophila yakuba strain yak_Obat mitochondrion, c	KF824887	81.53	100	1.57849e−32	Diptera	Drosophilidae	Drosophila yakuba
Drosophila yakuba strain yak_LP6 mitochondrion, co	KF824886	81.53	100	1.57849e−32	Diptera	Drosophilidae	Drosophila yakuba
Drosophila yakuba strain yak_KY221 mitochondrion,	KF824885	81.53	100	1.57849e−32	Diptera	Drosophilidae	Drosophila yakuba
Drosophila yakuba strain yak_G46 mitochondrion, co	KF824884	81.53	100	1.57849e−32	Diptera	Drosophilidae	Drosophila yakuba
Drosophila yakuba strain yak_field3 mitochondrion,	KF824883	81.53	100	1.57849e−32	Diptera	Drosophilidae	Drosophila yakuba
Drosophila yakuba strain yak_field2 mitochondrion,	KF824882	81.53	100	1.57849e−32	Diptera	Drosophilidae	Drosophila yakuba
Drosophila yakuba strain yak_field1 mitochondrion,	KF824881	81.53	100	1.57849e−32	Diptera	Drosophilidae	Drosophila yakuba
Drosophila yakuba strain yak_Cost mitochondrion, c	KF824880	81.53	100	1.57849e−32	Diptera	Drosophilidae	Drosophila yakuba
Drosophila yakuba strain yak_cam115 mitochondrion,	KF824879	81.53	100	1.57849e−32	Diptera	Drosophilidae	Drosophila yakuba
Drosophila yakuba strain yak_BS4 mitochondrion, co	KF824878	81.53	100	1.57849e−32	Diptera	Drosophilidae	Drosophila yakuba
Drosophila yakuba strain yak_BS3 mitochondrion, co	KF824877	81.53	100	1.57849e−32	Diptera	Drosophilidae	Drosophila yakuba
Drosophila yakuba strain yak_bosu mitochondrion, c	KF824876	81.53	100	1.57849e−32	Diptera	Drosophilidae	Drosophila yakuba
Drosophila yakuba strain yak_42Y mitochondrion, co	KF824875	81.53	100	1.57849e−32	Diptera	Drosophilidae	Drosophila yakuba
Drosophila yakuba strain yak_21Y mitochondrion, co	KF824874	81.53	100	1.57849e−32	Diptera	Drosophilidae	Drosophila yakuba
Drosophila yakuba strain yak_12Y mitochondrion, co	KF824873	81.53	100	1.57849e−32	Diptera	Drosophilidae	Drosophila yakuba
Drosophila santomea strain san_Thena5 mitochondrio	KF824872	81.53	100	1.57849e−32	Diptera	Drosophilidae	Drosophila santomea
Drosophila santomea strain san_thena2 mitochondrio	KF824871	81.53	100	1.57849e−32	Diptera	Drosophilidae	Drosophila santomea
Drosophila santomea strain san_sto7 mitochondrion,	KF824870	81.53	100	1.57849e−32	Diptera	Drosophilidae	Drosophila santomea
Drosophila santomea strain san_sto4 mitochondrion,	KF824869	81.53	100	1.57849e−32	Diptera	Drosophilidae	Drosophila santomea
Drosophila santomea strain san_sto18 mitochondrion	KF824868	81.53	100	1.57849e−32	Diptera	Drosophilidae	Drosophila santomea
Drosophila santomea strain san_Rain40 mitochondrio	KF824867	81.53	100	1.57849e−32	Diptera	Drosophilidae	Drosophila santomea
Drosophila santomea strain san_Rain36 mitochondrio	KF824866	81.53	100	1.57849e−32	Diptera	Drosophilidae	Drosophila santomea
Drosophila santomea strain san_Quija24 mitochondri	KF824865	81.53	100	1.57849e−32	Diptera	Drosophilidae	Drosophila santomea
Drosophila santomea strain san_Quija12 mitochondri	KF824864	81.53	100	1.57849e−32	Diptera	Drosophilidae	Drosophila santomea
Drosophila santomea strain san_Quija1 mitochondrio	KF824863	81.53	100	1.57849e−32	Diptera	Drosophilidae	Drosophila santomea
Drosophila santomea strain san_field6 mitochondrio	KF824862	81.53	100	1.57849e−32	Diptera	Drosophilidae	Drosophila santomea
Drosophila santomea strain san_field4 mitochondrio	KF824861	81.53	100	1.57849e−32	Diptera	Drosophilidae	Drosophila santomea
Drosophila santomea strain san_Cost mitochondrion,	KF824860	81.53	100	1.57849e−32	Diptera	Drosophilidae	Drosophila santomea
Drosophila santomea strain san_Car mitochondrion,	KF824859	81.53	100	1.57849e−32	Diptera	Drosophilidae	Drosophila santomea
Drosophila santomea strain san_cabumbe mitochondri	KF824858	81.53	100	1.57849e−32	Diptera	Drosophilidae	Drosophila santomea
Drosophila santomea strain san_BS13 mitochondrion,	KF824857	81.53	100	1.57849e−32	Diptera	Drosophilidae	Drosophila santomea
Drosophila santomea strain san_BS11 mitochondrion,	KF824856	81.53	100	1.57849e−32	Diptera	Drosophilidae	Drosophila santomea
Tabanus maculicornis 16S ribosomal RNA gene, parti	HM132112	81.53	100	1.57849e−32	Diptera	Tabanidae	Tabanus maculicornis
Leptomorphus walkeri 16S ribosomal RNA gene, parti	FJ172020	87.9	78.34	1.57849e−32	Diptera	Mycetophilidae	Leptomorphus walkeri
Drosophila rubida voucher 109400 NADH dehydrogenas	EU494327	81.53	100	1.57849e−32	Diptera	Drosophilidae	Drosophila rubida
Drosophila hypocausta voucher 103972 NADH dehydrog	EU494319	81.53	100	1.57849e−32	Diptera	Drosophilidae	Drosophila hypocausta
Drosophila takahashii cytochrome b (cytb) gene, pa	AF164592	81.53	100	1.57849e−32	Diptera	Drosophilidae	Drosophila takahashii
Drosophila teissieri cytochrome b (cytb) gene, par	AF164586	81.53	100	1.57849e−32	Diptera	Drosophilidae	Drosophila teissieri
Drosophila orena cytochrome b (cytb) gene, partial	AF164584	81.53	100	1.57849e−32	Diptera	Drosophilidae	Drosophila orena
Drosophila yakuba complete mitochondrial sequence	X03240	81.53	100	1.57849e−32	Diptera	Drosophilidae	Drosophila yakuba
Bradysiopsis vittigera voucher 948 16S ribosomal R	JQ613962	82.5	97.45	5.50946e−32	Diptera	Sciaridae	Bradysiopsis vittigera
Bradysia lapponica voucher 607 16S ribosomal RNA g	JQ613920	81.7	97.45	5.50946e−32	Diptera	Sciaridae	Bradysia lapponica
Cramptonomyia spenceri mitochondrion, complete gen	JN861747	80.77	99.36	5.50946e−32	Diptera	Pachyneuridae	Cramptonomyia spenceri
Drosophila ornatipennis voucher 103965 NADH dehydr	EU494307	81.76	100	5.50946e−32	Diptera	Drosophilidae	Drosophila ornatipennis
Arachnocampa luminosa biotype 1 16S large subunit	AY576329	83.8	90.45	5.50946e−32	Diptera	Keroplatidae	Arachnocampa luminosa
Drosophila ornatipennis 16S ribosomal RNA gene, pa	AF479799	81.76	100	5.50946e−32	Diptera	Drosophilidae	Drosophila ornatipennis
Drosophila neocardini NADH dehydrogenase subunit 1	AF246505	81.88	100	5.50946e−32	Diptera	Drosophilidae	Drosophila neocardini
Drosophila parthenogenetica strain 15181−2221.0 NA	AF246503	81.88	100	5.50946e−32	Diptera	Drosophilidae	Drosophila parthenogenetica
