# Supplementary material for: Testing the potential of a ribosomal 16S marker for DNA metabarcoding of insects
Source: PeerJ. 2016 Apr 19;4:e1966. doi: 10.7717/peerj.1966 (PMC4841222; doi:10.7717/peerj.1966)
Supplement: Supplemental Information 1 [file peerj-04-1966-s006.zip › scripts/5 match OTUs/blast_hits/OTU_76.csv_plot.pdf]

# OTU_76.csv

## Cascadoperla trictura

## Perlodidae

## Plecoptera

## 85.35

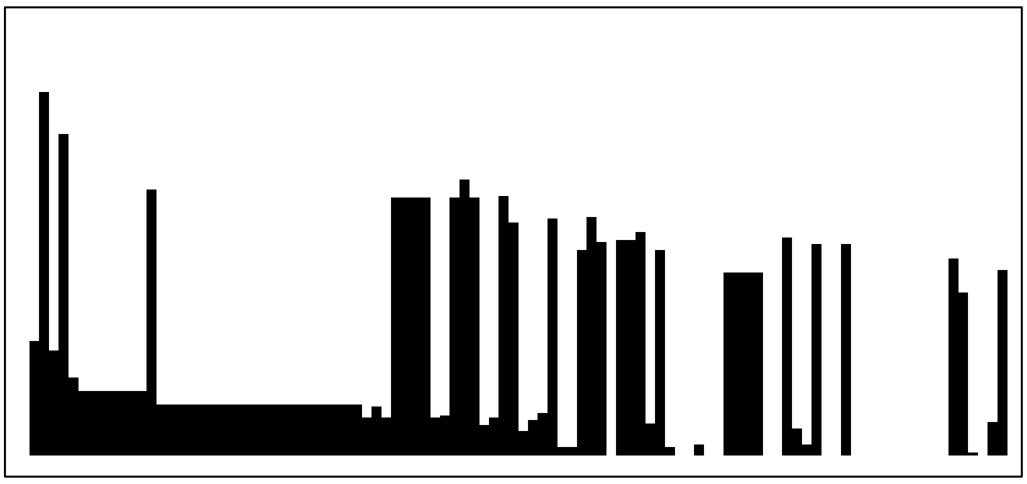

| | | | | | | | |
|---|---|---|---|---|---|---|---|
| Cascadoperla trictura 16S ribosomal RNA gene, part | EF623225 | 85.35 | 100 | 1.13558e−40 | Plecoptera | Perlodidae | Cascadoperla trictura |
| Isoperla oxylepis 16S ribosomal RNA gene, partial | EF623221 | 97.03 | 64.33 | 1.68536e−38 | Plecoptera | Perlodidae | Isoperla oxylepis |
| Perlinodes aurea 16S ribosomal RNA gene, partial s | EF623234 | 84.91 | 100 | 2.05319e−37 | Plecoptera | Perlodidae | Perlinodes aurea |
| Isoperla davisi 16S ribosomal RNA gene, partial se | EF623206 | 95.05 | 64.33 | 8.73037e−36 | Plecoptera | Perlodidae | Isoperla davisi |
| Allocapnia minima 16S ribosomal RNA gene, partial | EF623130 | 83.65 | 100 | 8.73037e−36 | Plecoptera | Capniidae | Allocapnia minima |
| Apteroperla tikumana mitochondrion, complete genom | KR604721 | 83.02 | 100 | 1.06358e−34 | Plecoptera | Capniidae | Apteroperla tikumana |
| Allocapnia recta isolate H15 16S ribosomal RNA gen | KC881050 | 83.02 | 100 | 1.06358e−34 | Plecoptera | Capniidae | Allocapnia recta |
| Allocapnia recta isolate H14 16S ribosomal RNA gen | KC881049 | 83.02 | 100 | 1.06358e−34 | Plecoptera | Capniidae | Allocapnia recta |
| Allocapnia recta isolate H10 16S ribosomal RNA gen | KC881045 | 83.02 | 100 | 1.06358e−34 | Plecoptera | Capniidae | Allocapnia recta |
| Allocapnia recta isolate H6 16S ribosomal RNA gene | KC881041 | 83.02 | 100 | 1.06358e−34 | Plecoptera | Capniidae | Allocapnia recta |
| Allocapnia recta haplotype 11ar: H011 16S ribosoma | EU306874 | 83.02 | 100 | 1.06358e−34 | Plecoptera | Capniidae | Allocapnia recta |
| Allocapnia recta haplotype 7ar: H07 16S ribosomal | EU306870 | 83.02 | 100 | 1.06358e−34 | Plecoptera | Capniidae | Allocapnia recta |
| Cryptoperla sp. YK−2012−1 mitochondrial gene for 1 | AB711243 | 92.45 | 67.52 | 1.2957e−33 | Plecoptera | Peltoperlidae | Cryptoperla sp. YK−2012−1 |
| Mesocapnia arizonensis mitochondrion, partial geno | KP642637 | 82.39 | 100 | 4.52244e−33 | Plecoptera | Capniidae | Mesocapnia arizonensis |
| Allocapnia recta isolate H19 16S ribosomal RNA gen | KC881054 | 82.39 | 100 | 4.52244e−33 | Plecoptera | Capniidae | Allocapnia recta |
| Allocapnia recta isolate H17 16S ribosomal RNA gen | KC881052 | 82.39 | 100 | 4.52244e−33 | Plecoptera | Capniidae | Allocapnia recta |
| Allocapnia recta isolate H16 16S ribosomal RNA gen | KC881051 | 82.39 | 100 | 4.52244e−33 | Plecoptera | Capniidae | Allocapnia recta |
| Allocapnia recta isolate H13 16S ribosomal RNA gen | KC881048 | 82.39 | 100 | 4.52244e−33 | Plecoptera | Capniidae | Allocapnia recta |
| Allocapnia recta isolate H12 16S ribosomal RNA gen | KC881047 | 82.39 | 100 | 4.52244e−33 | Plecoptera | Capniidae | Allocapnia recta |
| Allocapnia recta isolate H11 16S ribosomal RNA gen | KC881046 | 82.39 | 100 | 4.52244e−33 | Plecoptera | Capniidae | Allocapnia recta |
| Allocapnia recta isolate H9 16S ribosomal RNA gene | KC881044 | 82.39 | 100 | 4.52244e−33 | Plecoptera | Capniidae | Allocapnia recta |
| Allocapnia recta isolate H8 16S ribosomal RNA gene | KC881043 | 82.39 | 100 | 4.52244e−33 | Plecoptera | Capniidae | Allocapnia recta |
| Allocapnia recta isolate H7 16S ribosomal RNA gene | KC881042 | 82.39 | 100 | 4.52244e−33 | Plecoptera | Capniidae | Allocapnia recta |
| Allocapnia recta isolate H5 16S ribosomal RNA gene | KC881040 | 82.39 | 100 | 4.52244e−33 | Plecoptera | Capniidae | Allocapnia recta |
| Allocapnia recta isolate H4 16S ribosomal RNA gene | KC881039 | 82.39 | 100 | 4.52244e−33 | Plecoptera | Capniidae | Allocapnia recta |
| Allocapnia recta isolate H3 16S ribosomal RNA gene | KC881038 | 82.39 | 100 | 4.52244e−33 | Plecoptera | Capniidae | Allocapnia recta |
| Allocapnia recta isolate H1 16S ribosomal RNA gene | KC881036 | 82.39 | 100 | 4.52244e−33 | Plecoptera | Capniidae | Allocapnia recta |
| Mesocapnia sp. BYU_PL010 16S ribosomal RNA gene, p | EF623127 | 82.39 | 100 | 4.52244e−33 | Plecoptera | Capniidae | Mesocapnia sp. BYU_PL010 |
| Allocapnia recta haplotype 10ar: H010 16S ribosoma | EU306873 | 82.39 | 100 | 4.52244e−33 | Plecoptera | Capniidae | Allocapnia recta |
| Allocapnia recta haplotype 9ar: H09 16S ribosomal | EU306872 | 82.39 | 100 | 4.52244e−33 | Plecoptera | Capniidae | Allocapnia recta |
| Allocapnia recta haplotype 8ar: H08 16S ribosomal | EU306871 | 82.39 | 100 | 4.52244e−33 | Plecoptera | Capniidae | Allocapnia recta |
| Allocapnia recta haplotype 3ar: H03 16S ribosomal | DQ915181 | 82.39 | 100 | 4.52244e−33 | Plecoptera | Capniidae | Allocapnia recta |
| Allocapnia recta haplotype 2ar: H02 16S ribosomal | DQ915180 | 82.39 | 100 | 4.52244e−33 | Plecoptera | Capniidae | Allocapnia recta |
| Allocapnia recta haplotype 1ar: H01 16S ribosomal | DQ915179 | 82.39 | 100 | 4.52244e−33 | Plecoptera | Capniidae | Allocapnia recta |
| Leuctra inermis 16S ribosomal RNA gene, partial se | EF623176 | 81.76 | 100 | 1.57849e−32 | Plecoptera | Leuctridae | Leuctra inermis |
| Capnura wanica 16S ribosomal RNA gene, partial seq | EF623128 | 82.28 | 100 | 1.57849e−32 | Plecoptera | Capniidae | Capnura wanica |
| Allocapnia recta isolate H18 16S ribosomal RNA gen | KC881053 | 81.76 | 100 | 5.50946e−32 | Plecoptera | Capniidae | Allocapnia recta |
| Cryptoperla japonica mitochondrial gene for 16S rR | AB711202 | 92.08 | 64.33 | 5.50946e−32 | Plecoptera | Peltoperlidae | Cryptoperla japonica |
| Cryptoperla japonica mitochondrial gene for 16S rR | AB711184 | 92.08 | 64.33 | 5.50946e−32 | Plecoptera | Peltoperlidae | Cryptoperla japonica |
| Cryptoperla japonica mitochondrial gene for 16S rR | AB711183 | 92.08 | 64.33 | 5.50946e−32 | Plecoptera | Peltoperlidae | Cryptoperla japonica |
| Cryptoperla japonica mitochondrial gene for 16S rR | AB711182 | 92.08 | 64.33 | 5.50946e−32 | Plecoptera | Peltoperlidae | Cryptoperla japonica |
| Notonemouridae sp. MD−2011 voucher ZMUC:JD−1507 16 | JN615257 | 81.76 | 100 | 5.50946e−32 | Plecoptera | Notonemouridae | Notonemouridae sp. MD−2011 |
| Osobenus yakimae 16S ribosomal RNA gene, partial s | EF623232 | 81.88 | 100 | 5.50946e−32 | Plecoptera | Perlodidae | Osobenus yakimae |
| Cosumnoperla hypocrena 16S ribosomal RNA gene, par | EF623226 | 92.08 | 64.33 | 5.50946e−32 | Plecoptera | Perlodidae | Cosumnoperla hypocrena |
| Chernokrilus misnomus 16S ribosomal RNA gene, part | EF623209 | 92.93 | 63.06 | 5.50946e−32 | Plecoptera | Perlodidae | Chernokrilus misnomus |
| Isoperla fulva 16S ribosomal RNA gene, partial seq | EF623207 | 92.08 | 64.33 | 5.50946e−32 | Plecoptera | Perlodidae | Isoperla fulva |
| Moselia infuscata 16S ribosomal RNA gene, partial | EF623175 | 81.41 | 99.36 | 5.50946e−32 | Plecoptera | Leuctridae | Mosielia infuscata |
| Allocapnia recta haplotype 12ar: H12 16S ribosomal | EU306875 | 81.76 | 100 | 5.50946e−32 | Plecoptera | Capniidae | Allocapnia recta |
| Frisonia picticeps 16S ribosomal RNA gene, partial | EF623227 | 92.16 | 64.33 | 1.92299e−31 | Plecoptera | Perlodidae | Frisonia picticeps |
| Rickera sorpta 16S ribosomal RNA gene, partial seq | EF623235 | 90.91 | 68.15 | 6.7119e−31 | Plecoptera | Perlodidae | Rickera sorpta |
| Cultus pilatus 16S ribosomal RNA gene, partial seq | EF623212 | 81.13 | 100 | 6.7119e−31 | Plecoptera | Perlodidae | Cultus pilatus |
| Neonemura sp. BYU_PL153 16S ribosomal RNA gene, pa | EF623204 | 81.65 | 100 | 6.7119e−31 | Plecoptera | Notonemouridae | Neonemura sp. BYU_PL153 |
| Megaleuctra kincaidi 16S ribosomal RNA gene, parti | EF623174 | 81.99 | 100 | 6.7119e−31 | Plecoptera | Leuctridae | Megaleuctra kincaidi |
| Isoperla phalerata 16S ribosomal RNA gene, partial | EF623208 | 91.09 | 64.33 | 2.34268e−30 | Plecoptera | Perlodidae | Isoperla phalerata |
| Leuctra tenuis haplotype 8lt: HO8 16S ribosomal RN | EU327680 | 80.38 | 100 | 2.34268e−30 | Plecoptera | Leuctridae | Leuctra tenuis |
| Leuctra tenuis haplotype 7lt: HO7 16S ribosomal RN | EU327679 | 80.38 | 100 | 2.34268e−30 | Plecoptera | Leuctridae | Leuctra tenuis |
| Cryptoperla ishigakiensis mitochondrial gene for 1 | AB711242 | 89.62 | 67.52 | 8.17677e−30 | Plecoptera | Peltoperlidae | Cryptoperla ishigakiensis |
| Perlodes dispar 16S ribosomal RNA gene, partial se | EF623220 | 91.18 | 64.33 | 8.17677e−30 | Plecoptera | Perlodidae | Perlodes dispar |
| Kogotus nonus 16S ribosomal RNA gene, partial sequ | EF623213 | 90 | 68.15 | 8.17677e−30 | Plecoptera | Perlodidae | Kogotus nonus |
| Despaxia augusta 16S ribosomal RNA gene, partial s | EF623177 | 79.62 | 100 | 8.17677e−30 | Plecoptera | Leuctridae | Despaxia augusta |
| Cryptoperla sp. YK−2012−2 mitochondrial gene for 1 | AB711244 | 90.1 | 64.33 | 2.85397e−29 | Plecoptera | Peltoperlidae | Cryptoperla sp. YK−2012−2 |
| Cryptoperla japonica mitochondrial gene for 16S rR | AB711189 | 90.1 | 64.33 | 2.85397e−29 | Plecoptera | Peltoperlidae | Cryptoperla japonica |
| Dolichopoda geniculata geniculata isolate PRA20 16 | AY507590 | 90.48 | 65.61 | 2.85397e−29 | Orthoptera | Rhaphidophoridae | Dolichopoda geniculata geniculata |
| Scopura montana 16S ribosomal RNA gene, partial se | EF623265 | 81.48 | 100 | 2.85397e−29 | Plecoptera | | Scopura montana |
| Pictetiella expansa 16S ribosomal RNA gene, partia | EF623216 | 89.62 | 67.52 | 2.85397e−29 | Plecoptera | Perlodidae | Pictetiella expansa |
| Megarcys signata 16S ribosomal RNA gene, partial s | EF623215 | 80.38 | 100 | 2.85397e−29 | Plecoptera | Perlodidae | Megarcys signata |
| Leuctra tenuis haplotype 10lt: HO10 16S ribosomal | EU327682 | 79.75 | 100 | 2.85397e−29 | Plecoptera | Leuctridae | Leuctra tenuis |
| Leuctra tenuis haplotype 9lt: HO9 16S ribosomal RN | EU327681 | 79.75 | 100 | 2.85397e−29 | Plecoptera | Leuctridae | Leuctra tenuis |
| Brachypanorpa carolinensis isolate 52Bcar 16S ribo | EF041526 | 80.5 | 100 | 2.85397e−29 | Mecoptera | Panorpodidae | Brachypanorpa carolinensis |
| Leuctra tenuis haplotype 3lt: H03 16S ribosomal RN | DQ915184 | 79.75 | 100 | 2.85397e−29 | Plecoptera | Leuctridae | Leuctra tenuis |
| Leuctra tenuis haplotype 2lt: H02 16S ribosomal RN | DQ915183 | 79.75 | 100 | 2.85397e−29 | Plecoptera | Leuctridae | Leuctra tenuis |
| Dolichopoda naxia voucher ZEU 16S ribosomal RNA ge | EU887868 | 88.57 | 66.88 | 9.96134e−29 | Orthoptera | Rhaphidophoridae | Dolichopoda naxia |
| Dolichopoda naxia voucher STA 16S ribosomal RNA ge | EU887867 | 88.57 | 66.88 | 9.96134e−29 | Orthoptera | Rhaphidophoridae | Dolichopoda naxia |
| Dolichopoda sp. SKA 16S ribosomal RNA gene, partia | EF216964 | 88.57 | 66.88 | 9.96134e−29 | Orthoptera | Rhaphidophoridae | Dolichopoda sp. SKA |
| Dolichopoda sp. EPT 16S ribosomal RNA gene, partia | EF216963 | 88.57 | 66.88 | 9.96134e−29 | Orthoptera | Rhaphidophoridae | Dolichopoda sp. EPT |
| Doddsia occidentalis 16S ribosomal RNA gene, parti | EF623270 | 79.87 | 100 | 9.96134e−29 | Plecoptera | Taeniopterygidae | Doddsia occidentalis |
| Oemopteryx vanduzeea 16S ribosomal RNA gene, parti | EF623266 | 79.87 | 100 | 9.96134e−29 | Plecoptera | Taeniopterygidae | Oemopteryx vanduzeea |
| Austronemoura sp. BYU_PL149 16S ribosomal RNA gene | EF623200 | 90.2 | 64.33 | 9.96134e−29 | Plecoptera | Notonemouridae | Austronemoura sp. BYU_PL149 |
| Protonemura meyeri 16S ribosomal RNA gene, partial | EF623193 | 81.25 | 100 | 9.96134e−29 | Plecoptera | Nemouridae | Protonemura meyeri |
| Cosmioperla australis 16S ribosomal RNA gene, part | EF623148 | 80.5 | 100 | 9.96134e−29 | Plecoptera | Eustheniidae | Cosmioperla australis |
| Peltoperla arcuata 16S ribosomal RNA gene, partial | EF623258 | 89.9 | 63.06 | 3.47685e−28 | Plecoptera | Peltoperlidae | Peltoperla arcuata |
| Skwala americana 16S ribosomal RNA gene, partial s | EF623217 | 79.75 | 100 | 3.47685e−28 | Plecoptera | Perlodidae | Skwala americana |
| Thaumatoperla sp. BYU_PL109 16S ribosomal RNA gene | EF623149 | 79.75 | 100 | 3.47685e−28 | Plecoptera | Eustheniidae | Thaumatoperla sp. BYU_PL109 |
| Sweltsa coloradensis 16S ribosomal RNA gene, parti | EF623133 | 89.9 | 63.06 | 3.47685e−28 | Plecoptera | Chloroperlidae | Sweltsa coloradensis |
| Dolichopoda sutini isolate Sut1 16S ribosomal RNA | KC783268 | 79.35 | 98.73 | 1.21354e−27 | Orthoptera | Rhaphidophoridae | Dolichopoda sutini |
| Dolichopoda petrochilosi voucher JOA 16S ribosomal | EF216967 | 80 | 98.73 | 1.21354e−27 | Orthoptera | Rhaphidophoridae | Dolichopoda petrochilosi |
| Salmoperla sylvanica 16S ribosomal RNA gene, parti | EF623222 | 79.87 | 100 | 1.21354e−27 | Plecoptera | Perlodidae | Salmoperla sylvanica |
| Nanonemoura wahkeena 16S ribosomal RNA gene, parti | EF623198 | 79.87 | 100 | 1.21354e−27 | Plecoptera | Nemouridae | Nanonemoura wahkeena |
| Neuroperla schedingi 16S ribosomal RNA gene, parti | EF623151 | 80 | 100 | 1.21354e−27 | Plecoptera | Eustheniidae | Neuroperla schedingi |
| Kathroperla perdita 16S ribosomal RNA gene, partia | EF623135 | 79.87 | 100 | 1.21354e−27 | Plecoptera | Chloroperlidae | Kathroperla perdita |
| Isocapnia hyalita 16S ribosomal RNA gene, partial | EF623131 | 79.87 | 100 | 1.21354e−27 | Plecoptera | Capniidae | Isocapnia hyalita |
| Leuctra tenuis haplotype 6lt: HO6 16S ribosomal RN | EU327678 | 79.11 | 100 | 1.21354e−27 | Plecoptera | Leuctridae | Leuctra tenuis |
| Leuctra tenuis haplotype 4lt: HO4 16S ribosomal RN | EU327676 | 79.11 | 100 | 1.21354e−27 | Plecoptera | Leuctridae | Leuctra tenuis |
| Leuctra tenuis haplotype 1lt: H01 16S ribosomal RN | DQ915182 | 79.11 | 100 | 1.21354e−27 | Plecoptera | Leuctridae | Leuctra tenuis |
| Perlodes mortoni 16S ribosomal RNA gene, partial s | KF855870 | 89.22 | 64.33 | 4.23567e−27 | Plecoptera | Perlodidae | Perlodes mortoni |
| Dolichopoda sutini isolate Ask1 16S ribosomal RNA | KC783269 | 87.62 | 66.88 | 4.23567e−27 | Orthoptera | Rhaphidophoridae | Dolichopoda sutini |
| Yugus bulbosus 16S ribosomal RNA gene, partial seq | EF623214 | 80.12 | 100 | 4.23567e−27 | Plecoptera | Perlodidae | Yugus bulbosus |
| Panorpodes colei isolate 117Pcol 16S ribosomal RNA | EF041524 | 79.11 | 100 | 4.23567e−27 | Mecoptera | Panorpodidae | Panorpodes colei |
| Pteronarcys princeps mitochondrion, complete genom | AY687866 | 81.56 | 89.17 | 4.23567e−27 | Plecoptera | Pteronarcyidae | Pteronarcys princeps |
| Parameletus columbiae 16S ribosomal RNA gene, part | AY749768 | 88.68 | 66.88 | 4.23567e−27 | Ephemeroptera | Siphlonuridae | Parameletus columbiae |
