# Supplementary material for: Testing the potential of a ribosomal 16S marker for DNA metabarcoding of insects
Source: PeerJ. 2016 Apr 19;4:e1966. doi: 10.7717/peerj.1966 (PMC4841222; doi:10.7717/peerj.1966)
Supplement: Supplemental Information 1 [file peerj-04-1966-s006.zip › scripts/5 match OTUs/blast_hits/OTU_78.csv_plot.pdf]

OTU_78.csv

Rhithrogena alpestris

Heptageniidae

Ephemeroptera

87.82

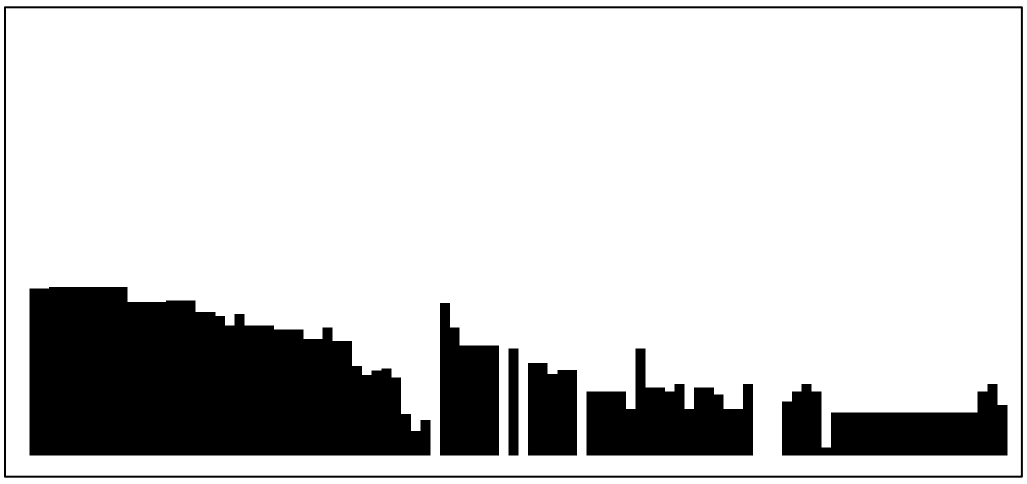

Rhithrogena alpestris mitochondrial partial 16S rR	LN868657	87.82	100	1.76996e−44	Ephemeroptera	Heptageniidae	Rhithrogena alpestris
Rhithrogena nivata mitochondrial partial 16S rRNA	LN868643	87.82	100	1.76996e−44	Ephemeroptera	Heptageniidae	Rhithrogena nivata
Rhithrogena sp. 13 LV−2015 mitochondrial partial 1	LN868672	87.9	100	6.17778e−44	Ephemeroptera	Heptageniidae	Rhithrogena sp. 13 LV−2015
Rhithrogena sp. 12 LV−2015 mitochondrial partial 1	LN868671	87.9	100	6.17778e−44	Ephemeroptera	Heptageniidae	Rhithrogena sp. 12 LV−2015
Rhithrogena picteti mitochondrial partial 16S rRNA	LN868663	87.9	100	6.17778e−44	Ephemeroptera	Heptageniidae	Rhithrogena picteti
Rhithrogena sp. 3 LV−2015 mitochondrial partial 16	LN868659	87.9	100	6.17778e−44	Ephemeroptera	Heptageniidae	Rhithrogena sp. 3 LV−2015
Rhithrogena sp. 2 LV−2015 mitochondrial partial 16	LN868654	87.9	100	6.17778e−44	Ephemeroptera	Heptageniidae	Rhithrogena sp. 2 LV−2015
Rhithrogena puytoraci mitochondrial partial 16S rR	LN868651	87.9	100	6.17778e−44	Ephemeroptera	Heptageniidae	Rhithrogena puytoraci
Rhithrogena iridina mitochondrial partial 16S rRNA	LN868646	87.9	100	6.17778e−44	Ephemeroptera	Heptageniidae	Rhithrogena iridina
Rhithrogena taurisca mitochondrial partial 16S rRN	LN868638	87.9	100	6.17778e−44	Ephemeroptera	Heptageniidae	Rhithrogena taurisca
Rhithrogena degrangei mitochondrial partial 16S rR	LN868674	87.18	100	2.15626e−43	Ephemeroptera	Heptageniidae	Rhithrogena degrangei
Rhithrogena sp. 7 LV−2015 mitochondrial partial 16	LN868669	87.18	100	2.15626e−43	Ephemeroptera	Heptageniidae	Rhithrogena sp. 7 LV−2015
Rhithrogena sp. 8 LV−2015 mitochondrial partial 16	LN868661	87.18	100	2.15626e−43	Ephemeroptera	Heptageniidae	Rhithrogena sp. 8 LV−2015
Rhithrogena sp. 9 LV−2015 mitochondrial partial 16	LN868650	87.18	100	2.15626e−43	Ephemeroptera	Heptageniidae	Rhithrogena sp. 9 LV−2015
Rhithrogena semicolorata mitochondrial partial 16S	LN868673	87.26	100	7.52607e−43	Ephemeroptera	Heptageniidae	Rhithrogena semicolorata
Rhithrogena sp. 5 LV−2015 mitochondrial partial 16	LN868653	87.26	100	7.52607e−43	Ephemeroptera	Heptageniidae	Rhithrogena sp. 5 LV−2015
Rhithrogena sp. 4 LV−2015 mitochondrial partial 16	LN868652	87.26	100	7.52607e−43	Ephemeroptera	Heptageniidae	Rhithrogena sp. 4 LV−2015
Rhithrogena hybrida mitochondrial partial 16S rRNA	LN868642	86.71	100	7.52607e−43	Ephemeroptera	Heptageniidae	Rhithrogena hybrida
Rhithrogena circumtatrica mitochondrial partial 16	LN868641	86.71	100	7.52607e−43	Ephemeroptera	Heptageniidae	Rhithrogena circumtatrica
Rhithrogena landai mitochondrial partial 16S rRNA	LN868644	86.54	100	9.16864e−42	Ephemeroptera	Heptageniidae	Rhithrogena landai
Rhithrogena sp. 11 LV−2015 mitochondrial partial 1	LN868668	86.08	100	3.20017e−41	Ephemeroptera	Heptageniidae	Rhithrogena sp. 11 LV−2015
Rhithrogena fonticola mitochondrial partial 16S rR	LN868656	86.62	100	3.20017e−41	Ephemeroptera	Heptageniidae	Rhithrogena fonticola
Rhithrogena sp. 6 LV−2015 mitochondrial partial 16	LN868655	86.08	100	3.20017e−41	Ephemeroptera	Heptageniidae	Rhithrogena sp. 6 LV−2015
Rhithrogena gratianopolitana mitochondrial partial	LN868647	86.08	100	3.20017e−41	Ephemeroptera	Heptageniidae	Rhithrogena gratianopolitana
Rhithrogena diensis mitochondrial partial 16S rRNA	LN868637	86.08	100	3.20017e−41	Ephemeroptera	Heptageniidae	Rhithrogena diensis
Rhithrogena nuragica mitochondrial partial 16S rRN	LN868667	85.9	100	1.11697e−40	Ephemeroptera	Heptageniidae	Rhithrogena nuragica
Rhithrogena eatoni mitochondrial partial 16S rRNA	LN868666	85.9	100	1.11697e−40	Ephemeroptera	Heptageniidae	Rhithrogena eatoni
Rhithrogena insularis mitochondrial partial 16S rR	LN868665	85.9	100	1.11697e−40	Ephemeroptera	Heptageniidae	Rhithrogena insularis
Rhithrogena corcontica mitochondrial partial 16S r	LN868662	85.44	100	3.8986e−40	Ephemeroptera	Heptageniidae	Rhithrogena corcontica
Rhithrogena gratianopolitana mitochondrial partial	LN868648	85.44	100	3.8986e−40	Ephemeroptera	Heptageniidae	Rhithrogena gratianopolitana
Rhithrogena beskidensis mitochondrial partial 16S	LN868645	85.99	100	3.8986e−40	Ephemeroptera	Heptageniidae	Rhithrogena beskidensis
Rhithrogena sp. 10 LV−2015 mitochondrial partial 1	LN868664	85.35	100	1.65773e−38	Ephemeroptera	Heptageniidae	Rhithrogena sp. 10 LV−2015
Rhithrogena germanica mitochondrial partial 16S rR	LN868660	85.35	100	1.65773e−38	Ephemeroptera	Heptageniidae	Rhithrogena germanica
Rhithrogena grischuna mitochondrial partial 16S rR	LN868639	84.18	100	2.01953e−37	Ephemeroptera	Heptageniidae	Rhithrogena grischuna
Epeorus longimanus 16S ribosomal RNA gene, partial	AY749757	83.77	99.35	2.01953e−37	Ephemeroptera	Heptageniidae	Epeorus longimanus
Rhithrogena allobrogica mitochondrial partial 16S	LN868640	83.97	100	2.46029e−36	Ephemeroptera	Heptageniidae	Rhithrogena allobrogica
Rhithrogena dorieri mitochondrial partial 16S rRNA	LN868649	84.08	100	8.58725e−36	Ephemeroptera	Heptageniidae	Rhithrogena dorieri
Rhithrogena savoiensis mitochondrial partial 16S r	LN868658	83.65	100	2.99724e−35	Ephemeroptera	Heptageniidae	Rhithrogena savoiensis
Epeorus sp. MT−2014 mitochondrion, complete genome	KM244708	81.94	99.35	5.41914e−32	Ephemeroptera	Heptageniidae	Epeorus sp. MT−2014
Rhithrogena sartorii mitochondrial partial 16S rRN	LN868670	81.13	100	8.04272e−30	Ephemeroptera	Heptageniidae	Rhithrogena sartorii
Cloeon dipterum voucher EPH02 tRNA−Leu, 16S riboso	KF256908	81.65	99.35	8.04272e−30	Ephemeroptera	Baetidae	Cloeon dipterum
Rhithrogena sp. EP021 16S ribosomal RNA gene, part	AY749765	78.34	100	7.53272e−24	Ephemeroptera	Heptageniidae	Rhithrogena sp. EP021
Cultus pilatus 16S ribosomal RNA gene, partial seq	EF623212	87.13	64.52	9.17674e−23	Plecoptera	Perlodidae	Cultus pilatus
Despaxia augusta 16S ribosomal RNA gene, partial s	EF623177	86	63.87	3.90205e−21	Plecoptera	Leuctridae	Despaxia augusta
Cryptoperla japonica mitochondrial gene for 16S rR	AB711202	85.15	64.52	4.75367e−20	Plecoptera	Peltoperlidae	Cryptoperla japonica
Cryptoperla japonica mitochondrial gene for 16S rR	AB711184	85.15	64.52	4.75367e−20	Plecoptera	Peltoperlidae	Cryptoperla japonica
Cryptoperla japonica mitochondrial gene for 16S rR	AB711183	85.15	64.52	4.75367e−20	Plecoptera	Peltoperlidae	Cryptoperla japonica
Cryptoperla japonica mitochondrial gene for 16S rR	AB711182	85.15	64.52	4.75367e−20	Plecoptera	Peltoperlidae	Cryptoperla japonica
Cinygmula sp. m185 mitochondrial partial 16S rRNA	LN868675	76.43	100	1.65919e−19	Ephemeroptera	Heptageniidae	Cinygmula sp. m185
Neofulla sp. BYU_PL152 16S ribosomal RNA gene, par	EF623203	85	63.87	1.65919e−19	Plecoptera	Notonemouridae	Neofulla sp. BYU_PL152
Cinygmula sp. EP013 16S ribosomal RNA gene, partia	AY749760	76.43	100	1.65919e−19	Ephemeroptera	Heptageniidae	Cinygmula sp. EP013
Anterastes serbicus haplotype 38BUL 16S ribosomal	HM856177	84.31	64.52	5.79115e−19	Orthoptera	Tettigoniidae	Anterastes serbicus
Anterastes serbicus haplotype 37BUL 16S ribosomal	HM856173	84.31	64.52	5.79115e−19	Orthoptera	Tettigoniidae	Anterastes serbicus
Isoperla oxylepis 16S ribosomal RNA gene, partial	EF623221	83.81	66.45	5.79115e−19	Plecoptera	Perlodidae	Isoperla oxylepis
Anterastes disparalatus haplotype 46BO 16S ribosom	HQ850975	84	63.87	2.02131e−18	Orthoptera	Tettigoniidae	Anterastes disparalatus
Koroglus disparalatus voucher 156 16S ribosomal RN	EU120777	84	63.87	2.02131e−18	Orthoptera	Tettigoniidae	Anterastes disparalatus
Parameletus columbiae 16S ribosomal RNA gene, part	AY749768	76.43	100	2.02131e−18	Ephemeroptera	Siphlonuridae	Parameletus columbiae
Nemoura cinerea 16S ribosomal RNA gene, partial se	EF623194	83	63.87	7.05507e−18	Plecoptera	Nemouridae	Nemoura cinerea
Tasmanoperla larvalis 16S ribosomal RNA gene, part	EF623125	83	63.87	7.05507e−18	Plecoptera	Austroperlidae	Tasmanoperla larvalis
Leuctra tenuis haplotype 8lt: HO8 16S ribosomal RN	EU327680	83	63.87	7.05507e−18	Plecoptera	Leuctridae	Leuctra tenuis
Leuctra tenuis haplotype 7lt: HO7 16S ribosomal RN	EU327679	83	63.87	7.05507e−18	Plecoptera	Leuctridae	Leuctra tenuis
Perlodes mortoni 16S ribosomal RNA gene, partial s	KF855870	82.18	64.52	2.46246e−17	Plecoptera	Perlodidae	Perlodes mortoni
Isonychia ignota mitochondrion, complete genome	HM143892	85	63.87	2.46246e−17	Ephemeroptera	Isonychiidae	Isonychia ignota
Cryptoperla sp. YK−2012−2 mitochondrial gene for 1	AB711244	83.17	64.52	2.46246e−17	Plecoptera	Peltoperlidae	Cryptoperla sp. YK−2012−2
Cryptoperla japonica mitochondrial gene for 16S rR	AB711189	83.17	64.52	2.46246e−17	Plecoptera	Peltoperlidae	Cryptoperla japonica
Anterastes disparalatus haplotype 45BO 16S ribosom	HQ850976	83	63.87	2.46246e−17	Orthoptera	Tettigoniidae	Anterastes disparalatus
Anterastes serbicus haplotype 32BUL 16S ribosomal	HM856167	83.33	64.52	2.46246e−17	Orthoptera	Tettigoniidae	Anterastes serbicus
Scopura montana 16S ribosomal RNA gene, partial se	EF623265	82.18	64.52	2.46246e−17	Plecoptera		Scopura montana
Pteronarcys sachalina 16S ribosomal RNA gene, part	EF623264	83.17	64.52	2.46246e−17	Plecoptera	Pteronarcyidae	Pteronarcys sachalina
Pictetiella expansa 16S ribosomal RNA gene, partia	EF623216	83.17	64.52	2.46246e−17	Plecoptera	Perlodidae	Pictetiella expansa
Isoperla davisi 16S ribosomal RNA gene, partial se	EF623206	82.86	66.45	2.46246e−17	Plecoptera	Perlodidae	Isoperla davisi
Nemurella pictetii 16S ribosomal RNA gene, partial	EF623192	82.18	64.52	2.46246e−17	Plecoptera	Nemouridae	Nemurella pictetii
Malenka californica 16S ribosomal RNA gene, partia	EF623182	82.18	64.52	2.46246e−17	Plecoptera	Nemouridae	Malenka californica
Leuctra inermis 16S ribosomal RNA gene, partial se	EF623176	83.33	63.87	2.46246e−17	Plecoptera	Leuctridae	Leuctra inermis
Klapopteryx armillata 16S ribosomal RNA gene, part	EF623123	76.81	87.74	2.46246e−17	Plecoptera	Austroperlidae	Klapopteryx armillata
Chiloporter sp. EP051 16S ribosomal RNA gene, part	AY749776	75	100	2.46246e−17	Ephemeroptera	Ameletopsidae	Chiloporter sp. EP051
Heptagenia sp. EP018 16S ribosomal RNA gene, parti	AY749762	77.54	87.74	2.46246e−17	Ephemeroptera	Heptageniidae	Heptagenia sp. EP018
Taenionema palladium 16S ribosomal RNA gene, parti	EF623267	82.52	63.87	8.59483e−17	Plecoptera	Taeniopterygidae	Taenionema palladium
Pteronarcys californica 16S ribosomal RNA gene, pa	EF623261	83	63.87	8.59483e−17	Plecoptera	Pteronarcyidae	Pteronarcys californica
Baumanella alameda 16S ribosomal RNA gene, partial	EF623236	83.33	64.52	8.59483e−17	Plecoptera	Perlodidae	Baumanella alameda
Remenus bilobatus 16S ribosomal RNA gene, partial	EF623233	83	63.87	8.59483e−17	Plecoptera	Perlodidae	Remenus bilobatus
Osobenus yakimae 16S ribosomal RNA gene, partial s	EF623232	80.36	71.61	8.59483e−17	Plecoptera	Perlodidae	Osobenus yakimae
Diura knowltoni 16S ribosomal RNA gene, partial se	EF623218	82	63.87	8.59483e−17	Plecoptera	Perlodidae	Diura knowltoni
Prostoia besametsa 16S ribosomal RNA gene, partial	EF623188	82	63.87	8.59483e−17	Plecoptera	Nemouridae	Prostoia besametsa
Nemoura trispinosa 16S ribosomal RNA gene, partial	EF623187	82	63.87	8.59483e−17	Plecoptera	Nemouridae	Nemoura trispinosa
Paranemoura claasseni 16S ribosomal RNA gene, part	EF623185	82	63.87	8.59483e−17	Plecoptera	Nemouridae	Paranemoura claasseni
Shipsa rotunda 16S ribosomal RNA gene, partial seq	EF623184	82	63.87	8.59483e−17	Plecoptera	Nemouridae	Shipsa rotunda
Claudioperla sp. BYU_PL158 16S ribosomal RNA gene,	EF623169	82	63.87	8.59483e−17	Plecoptera	Gripopterygidae	Claudioperla sp. BYU_PL158
Pelurgoperla personata 16S ribosomal RNA gene, par	EF623168	82	63.87	8.59483e−17	Plecoptera	Gripopterygidae	Pelurgoperla personata
Antarctoperla sp. BYU_PL142 16S ribosomal RNA gene	EF623163	82	63.87	8.59483e−17	Plecoptera	Gripopterygidae	Antarctoperla sp. BYU_PL142
Leuctra tenuis haplotype 10lt: HO10 16S ribosomal	EU327682	82	63.87	8.59483e−17	Plecoptera	Leuctridae	Leuctra tenuis
Leuctra tenuis haplotype 9lt: HO9 16S ribosomal RN	EU327681	82	63.87	8.59483e−17	Plecoptera	Leuctridae	Leuctra tenuis
Leuctra tenuis haplotype 6lt: HO6 16S ribosomal RN	EU327678	82	63.87	8.59483e−17	Plecoptera	Leuctridae	Leuctra tenuis
Leuctra tenuis haplotype 5lt: HO5 16S ribosomal RN	EU327677	82	63.87	8.59483e−17	Plecoptera	Leuctridae	Leuctra tenuis
Leuctra tenuis haplotype 3lt: H03 16S ribosomal RN	DQ915184	82	63.87	8.59483e−17	Plecoptera	Leuctridae	Leuctra tenuis
Leuctra tenuis haplotype 2lt: H02 16S ribosomal RN	DQ915183	82	63.87	8.59483e−17	Plecoptera	Leuctridae	Leuctra tenuis
Leuctra tenuis haplotype 1lt: H01 16S ribosomal RN	DQ915182	82	63.87	8.59483e−17	Plecoptera	Leuctridae	Leuctra tenuis
Pteronarcys princeps mitochondrion, complete genom	AY687866	83	63.87	8.59483e−17	Plecoptera	Pteronarcyidae	Pteronarcys princeps
Polyphylla decemlineata 16S ribosomal RNA gene, pa	JX994048	83.33	61.29	2.99989e−16	Coleoptera	Scarabaeidae	Polyphylla decemlineata
Hesperoperla pacifica strain PL008 16S ribosomal R	JN800541	82.35	63.87	2.99989e−16	Plecoptera	Perlidae	Hesperoperla pacifica
