# Supplementary material for: Testing the potential of a ribosomal 16S marker for DNA metabarcoding of insects
Source: PeerJ. 2016 Apr 19;4:e1966. doi: 10.7717/peerj.1966 (PMC4841222; doi:10.7717/peerj.1966)
Supplement: Supplemental Information 1 [file peerj-04-1966-s006.zip › scripts/5 match OTUs/blast_hits/OTU_79.csv_plot.pdf]

OTU_79.csv

Stylodrilus heringianus

Lumbriculidae

Lumbriculida

95.16

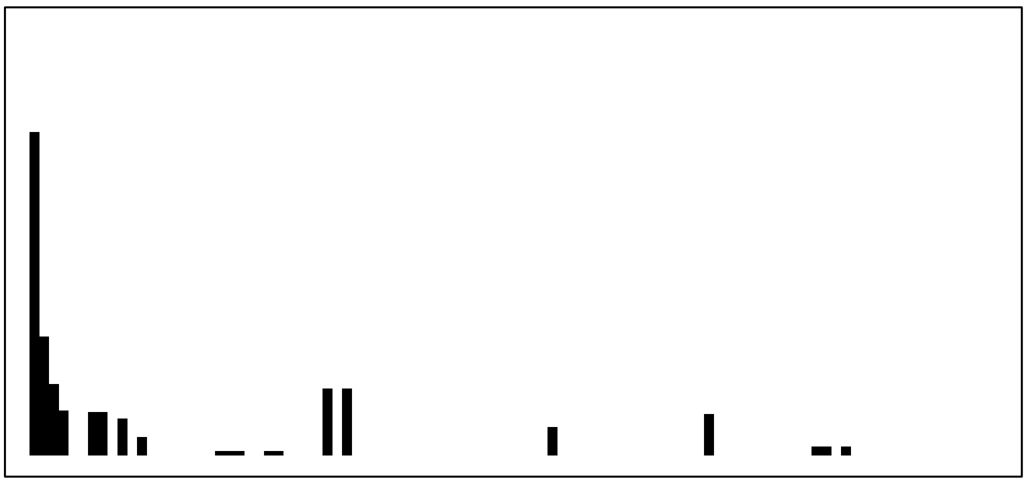

Stylodrilus heringianus 16S ribosomal RNA gene, pa	AY885577	95.16	100	3.05254e−46	Lumbriculida	Lumbriculidae	Stylodrilus heringianus
Marionina southerni clone CE674 16S ribosomal RNA	JN799953	85.56	72.36	7.67848e−16	Haplotaxida	Enchytraeidae	Marionina southerni
Rhyacodrilus coccineus 16S ribosomal RNA gene, par	DQ459931	83.33	71.54	9.3543e−15	Haplotaxida	Naididae	Rhyacodrilus coccineus
Eremidrilus coyote isolate Ecoy 16S ribosomal RNA	JQ821462	82.11	74.8	1.13959e−13	Lumbriculida	Lumbriculidae	Eremidrilus coyote
Kincaidiana hexatheca isolate CE861 16S ribosomal	GU592335	76.34	100	1.13959e−13	Lumbriculida	Lumbriculidae	Kincaidiana hexatheca
Guestphalinus sp. PDW−2010 isolate CE865 16S ribos	GU592334	76.15	100	3.97755e−13	Lumbriculida	Lumbriculidae	Guestphalinus sp. PDW−2010
Aeolosoma viride 16S ribosomal RNA gene, partial s	DQ779600	82.02	69.92	3.97755e−13	Haplotaxida	Aeolosomatidae	Aeolosoma viride
Epirodrilus pygmaeus 16S ribosomal RNA gene, parti	DQ459936	82.02	69.92	3.97755e−13	Haplotaxida	Naididae	Epirodrilus pygmaeus
Limnodrilus hoffmeisteri haplotype 13_I 16S riboso	EU160471	75.57	100	1.3883e−12	Haplotaxida	Naididae	Limnodrilus hoffmeisteri
Diporochaeta sp. Cradle Mt 16S ribosomal RNA gene,	AF406574	81.72	72.36	1.3883e−12	Haplotaxida	Megascolecidae	Diporochaeta sp. Cradle Mt
Rhynchelmis yakimorum variant 1 isolate CE860 16S	GU592355	75.76	100	4.84565e−12	Lumbriculida	Lumbriculidae	Rhynchelmis yakimorum
Eremidrilus coyote isolate CE702 16S ribosomal RNA	GU592328	80.85	73.98	4.84565e−12	Lumbriculida	Lumbriculidae	Eremidrilus coyote
Limnodrilus hoffmeisteri haplotype 19_II 16S ribos	EU160487	76.15	100	4.84565e−12	Haplotaxida	Naididae	Limnodrilus hoffmeisteri
Limnodrilus hoffmeisteri haplotype 16_II 16S ribos	EU160484	76.15	100	4.84565e−12	Haplotaxida	Naididae	Limnodrilus hoffmeisteri
Limnodrilus hoffmeisteri haplotype 21_II 16S ribos	EU160482	76.15	100	4.84565e−12	Haplotaxida	Naididae	Limnodrilus hoffmeisteri
Limnodrilus hoffmeisteri haplotype 15_II 16S ribos	EU160478	76.15	100	4.84565e−12	Haplotaxida	Naididae	Limnodrilus hoffmeisteri
Limnodrilus hoffmeisteri haplotype 20_II 16S ribos	EU160463	76.15	100	4.84565e−12	Haplotaxida	Naididae	Limnodrilus hoffmeisteri
Limnodrilus hoffmeisteri haplotype 14_II 16S ribos	EU160462	76.15	100	4.84565e−12	Haplotaxida	Naididae	Limnodrilus hoffmeisteri
Sparganophilus sp. G1 16S ribosomal RNA gene, part	JF267907	80	73.17	1.6913e−11	Lumbriculida	Sparganophilidae	Sparganophilus sp. G1
Rhynchelmis yakimorum variant 1 isolate CE1087 16S	GU592354	80.21	73.98	1.6913e−11	Lumbriculida	Lumbriculidae	Rhynchelmis yakimorum
Rhynchelmis glandula isolate CE864 16S ribosomal R	GU592338	80.21	73.98	1.6913e−11	Lumbriculida	Lumbriculidae	Rhynchelmis glandula
Rhynchelmis elrodi isolate CE580 16S ribosomal RNA	GU592337	80.21	73.98	1.6913e−11	Lumbriculida	Lumbriculidae	Rhynchelmis elrodi
Rhyacodrilus subterraneus 16S ribosomal RNA gene,	DQ459937	76.98	100	1.6913e−11	Haplotaxida	Naididae	Rhyacodrilus subterraneus
Diplocardia komareki isolate 7.16S2 16S ribosomal	DQ533732	78.7	84.55	1.6913e−11	Haplotaxida	Megascolecidae	Diplocardia komareki
Rhynchelmis gustafsoni 16S ribosomal RNA gene, par	AY764153	80.21	73.98	1.6913e−11	Lumbriculida	Lumbriculidae	Rhynchelmis gustafsoni
Rhynchelmis elrodi 16S ribosomal RNA gene, partial	AY764152	80.21	73.98	1.6913e−11	Lumbriculida	Lumbriculidae	Rhynchelmis elrodi
Drawida ghilarovi mitochondrial partial 16S rRNA g	HG970208	79.17	73.17	5.90321e−11	Moniligastrida	Moniligastridae	Drawida ghilarovi
Drawida ghilarovi mitochondrial partial 16S rRNA g	HG970207	79.17	73.17	5.90321e−11	Moniligastrida	Moniligastridae	Drawida ghilarovi
Drawida ghilarovi mitochondrial partial 16S rRNA g	HG970206	79.17	73.17	5.90321e−11	Moniligastrida	Moniligastridae	Drawida ghilarovi
Chamaedrilus aff. glandulosus B SM−2014 isolate CE	KF672439	77.78	100	5.90321e−11	Haplotaxida	Enchytraeidae	Chamaedrilus aff. glandulosus B SM−2014
Nematogenia sp. 341 16S ribosomal RNA gene, partia	JF267903	83.13	65.04	5.90321e−11	Haplotaxida	Ocnerodrilidae	Nematogenia sp. 341
Limnodrilus hoffmeisteri isolate 233BTub 16S ribos	JF783983	74.42	100	5.90321e−11	Haplotaxida	Naididae	Limnodrilus hoffmeisteri
Nematogenia sp. 341 16S ribosomal RNA gene, partia	HQ232501	83.13	65.04	5.90321e−11	Haplotaxida	Ocnerodrilidae	Nematogenia sp. 341
Eclipidrilus frigidus isolate CE557 16S ribosomal	GU592329	75.59	100	5.90321e−11	Lumbriculida	Lumbriculidae	Eclipidrilus frigidus
Limnodrilus hoffmeisteri haplotype 33_VII 16S ribo	EU160485	74.42	100	5.90321e−11	Haplotaxida	Naididae	Limnodrilus hoffmeisteri
Limnodrilus hoffmeisteri haplotype 07_I 16S riboso	EU160483	74.81	100	5.90321e−11	Haplotaxida	Naididae	Limnodrilus hoffmeisteri
Limnodrilus hoffmeisteri haplotype 06_I 16S riboso	EU160481	74.81	100	5.90321e−11	Haplotaxida	Naididae	Limnodrilus hoffmeisteri
Limnodrilus hoffmeisteri haplotype 11_I 16S riboso	EU160480	74.81	100	5.90321e−11	Haplotaxida	Naididae	Limnodrilus hoffmeisteri
Limnodrilus hoffmeisteri haplotype 08_I 16S riboso	EU160476	74.81	100	5.90321e−11	Haplotaxida	Naididae	Limnodrilus hoffmeisteri
Limnodrilus hoffmeisteri haplotype 09_I 16S riboso	EU160475	74.81	100	5.90321e−11	Haplotaxida	Naididae	Limnodrilus hoffmeisteri
Limnodrilus hoffmeisteri haplotype 04_I 16S riboso	EU160474	74.81	100	5.90321e−11	Haplotaxida	Naididae	Limnodrilus hoffmeisteri
Limnodrilus hoffmeisteri haplotype 05_I 16S riboso	EU160473	74.81	100	5.90321e−11	Haplotaxida	Naididae	Limnodrilus hoffmeisteri
Limnodrilus hoffmeisteri haplotype 10_I 16S riboso	EU160472	74.81	100	5.90321e−11	Haplotaxida	Naididae	Limnodrilus hoffmeisteri
Limnodrilus hoffmeisteri haplotype 12_I 16S riboso	EU160469	74.81	100	5.90321e−11	Haplotaxida	Naididae	Limnodrilus hoffmeisteri
Limnodrilus hoffmeisteri haplotype 02_I 16S riboso	EU160468	74.81	100	5.90321e−11	Haplotaxida	Naididae	Limnodrilus hoffmeisteri
Limnodrilus hoffmeisteri haplotype 03_I 16S riboso	EU160467	74.81	100	5.90321e−11	Haplotaxida	Naididae	Limnodrilus hoffmeisteri
Limnodrilus hoffmeisteri haplotype 01_I 16S riboso	EU160464	74.81	100	5.90321e−11	Haplotaxida	Naididae	Limnodrilus hoffmeisteri
Limnodrilus hoffmeisteri Mitochondrion 16S Ribosom	AJ225912	74.81	100	5.90321e−11	Haplotaxida	Naididae	Limnodrilus hoffmeisteri
Drawida ghilarovi mitochondrial partial 16S rRNA g	HG970219	78.35	73.17	2.06042e−10	Moniligastrida	Moniligastridae	Drawida ghilarovi
Chamaedrilus cognettii isolate CE1043 16S ribosoma	KF672430	79.35	73.17	2.06042e−10	Haplotaxida	Enchytraeidae	Chamaedrilus cognettii
Sparganophilus sp. G2 16S ribosomal RNA gene, part	JF267914	79.79	73.17	2.06042e−10	Lumbriculida	Sparganophilidae	Sparganophilus sp. G2
Ocnerodrilidae sp. PR 16S ribosomal RNA gene, part	JF267905	77.66	72.36	2.06042e−10	Haplotaxida	Ocnerodrilidae	Ocnerodrilidae sp. PR
Fimoscolex sp. PG 16S ribosomal RNA gene, partial	JF267885	79.17	73.17	2.06042e−10	Haplotaxida	Glossoscolecidae	Fimoscolex sp. PG
Rhyacodriloides sp. 2 PM−2010 partial 16S rRNA gen	FN796450	81.32	69.92	2.06042e−10	Haplotaxida	Naididae	Rhyacodriloides sp. 2 PM−2010
Cognettia cognetti isolate CE1042 16S ribosomal RN	GU901781	79.35	73.17	2.06042e−10	Haplotaxida	Enchytraeidae	Chamaedrilus cognettii
Rhynchelmis gustafsoni isolate CE507 16S ribosomal	GU592339	79.17	73.98	2.06042e−10	Lumbriculida	Lumbriculidae	Rhynchelmis gustafsoni
Rhynchelmis cf. yakimorum PDW−2010 isolate CE498 1	GU592357	79.17	73.98	2.06042e−10	Lumbriculida	Lumbriculidae	Rhynchelmis cf. yakimorum PDW−2010
Rhynchelmis yakimorum variant 1 isolate CE867 16S	GU592356	75	100	2.06042e−10	Lumbriculida	Lumbriculidae	Rhynchelmis yakimorum
Rhynchelmis gustafsoni isolate CE584 16S ribosomal	GU592344	79.17	73.98	2.06042e−10	Lumbriculida	Lumbriculidae	Rhynchelmis gustafsoni
Rhynchelmis gustafsoni isolate CE582 16S ribosomal	GU592342	79.17	73.98	2.06042e−10	Lumbriculida	Lumbriculidae	Rhynchelmis gustafsoni
Rhynchelmis gustafsoni isolate CE508 16S ribosomal	GU592340	79.17	73.98	2.06042e−10	Lumbriculida	Lumbriculidae	Rhynchelmis gustafsoni
Eclipidrilus palustris isolate CE868 16S ribosomal	GU592333	75.65	91.06	2.06042e−10	Lumbriculida	Lumbriculidae	Eclipidrilus palustris
Limnodrilus hoffmeisteri haplotype 17_II 16S ribos	EU160477	75.38	100	2.06042e−10	Haplotaxida	Naididae	Limnodrilus hoffmeisteri
Drawida ghilarovi mitochondrial partial 16S rRNA g	HG970211	77.55	73.17	7.19158e−10	Moniligastrida	Moniligastridae	Drawida ghilarovi
Drawida ghilarovi mitochondrial partial 16S rRNA g	HG970210	77.55	73.17	7.19158e−10	Moniligastrida	Moniligastridae	Drawida ghilarovi
Glycera southeastatlantica isolate st. 532b 16S ri	KJ847300	79.55	68.29	7.19158e−10	Phyllodocida	Glyceridae	Glycera southeastatlantica
Marionina tumulicola clone CE571 16S ribosomal RNA	JN799952	78.89	73.17	7.19158e−10	Haplotaxida	Enchytraeidae	Marionina tumulicola
Rhynchelmis saxosa isolate CE706 16S ribosomal RNA	GU592346	78.72	73.98	7.19158e−10	Lumbriculida	Lumbriculidae	Rhynchelmis saxosa
Glycera southeastatlantica 16S ribosomal RNA gene,	GQ426609	79.55	68.29	7.19158e−10	Phyllodocida	Glyceridae	Glycera southeastatlantica
Chaetogaster limnaei 16S ribosomal RNA gene, parti	GQ355405	81.93	65.04	7.19158e−10	Haplotaxida	Naididae	Chaetogaster limnaei
Limnodrilus hoffmeisteri voucher Lh1 16S ribosomal	EU117546	74.05	100	7.19158e−10	Haplotaxida	Naididae	Limnodrilus hoffmeisteri
Limnodrilus hoffmeisteri haplotype 24_III 16S ribo	EU160492	75.76	100	7.19158e−10	Haplotaxida	Naididae	Limnodrilus hoffmeisteri
Diplocardia komareki isolate 6.16S1 16S ribosomal	DQ533722	77.78	84.55	7.19158e−10	Haplotaxida	Megascolecidae	Diplocardia komareki
Drawida ghilarovi mitochondrial partial 16S rRNA g	HG970218	78.12	73.17	2.51011e−09	Moniligastrida	Moniligastridae	Drawida ghilarovi
Drawida ghilarovi mitochondrial partial 16S rRNA g	HG970214	78.12	73.17	2.51011e−09	Moniligastrida	Moniligastridae	Drawida ghilarovi
Drawida ghilarovi mitochondrial partial 16S rRNA g	HG970213	78.12	73.17	2.51011e−09	Moniligastrida	Moniligastridae	Drawida ghilarovi
Drawida ghilarovi mitochondrial partial 16S rRNA g	HG970212	78.12	73.17	2.51011e−09	Moniligastrida	Moniligastridae	Drawida ghilarovi
Annelida environmental sample clone Net14 large su	KM200891	77.32	74.8	2.51011e−09			Annelida environmental sample
Benhamiona sp. Gh 16S ribosomal RNA gene, partial	JF267872	79.79	73.17	2.51011e−09	Haplotaxida	Acanthodrilidae	Benhamiona sp. Gh
Benhamiona sp. 1042 16S ribosomal RNA gene, partia	HQ232615	79.79	73.17	2.51011e−09	Haplotaxida	Acanthodrilidae	Benhamiona sp. 1042
Rhynchelmis tetratheca isolate CE641 16S ribosomal	GU592348	80.41	73.17	2.51011e−09	Lumbriculida	Lumbriculidae	Rhynchelmis tetratheca
Rhynchelmis tetratheca isolate CE586 16S ribosomal	GU592347	80.41	73.17	2.51011e−09	Lumbriculida	Lumbriculidae	Rhynchelmis tetratheca
Chaetogaster diaphanus 16S ribosomal RNA gene, par	GQ355403	79.55	69.92	2.51011e−09	Haplotaxida	Naididae	Chaetogaster diaphanus
Rhynchelmis tetratheca 16S ribosomal RNA gene, par	AY340477	80.41	73.17	2.51011e−09	Lumbriculida	Lumbriculidae	Rhynchelmis tetratheca
Glycera southeastatlantica isolate st. 534b 16S ri	KJ847301	78.41	68.29	8.76114e−09	Phyllodocida	Glyceridae	Glycera southeastatlantica
Chamaedrilus chalupskyi isolate CE823 16S ribosoma	KF672456	79.57	73.17	8.76114e−09	Haplotaxida	Enchytraeidae	Chamaedrilus chalupskyi
Chamaedrilus chalupskyi isolate CE722 16S ribosoma	KF672455	79.57	73.17	8.76114e−09	Haplotaxida	Enchytraeidae	Chamaedrilus chalupskyi
Chamaedrilus chalupskyi isolate CE2055 16S ribosom	KF672452	79.57	73.17	8.76114e−09	Haplotaxida	Enchytraeidae	Chamaedrilus chalupskyi
Chamaedrilus chalupskyi isolate CE1719 16S ribosom	KF672451	79.57	73.17	8.76114e−09	Haplotaxida	Enchytraeidae	Chamaedrilus chalupskyi
Chamaedrilus chalupskyi isolate CE11325 16S riboso	KF672450	79.57	73.17	8.76114e−09	Haplotaxida	Enchytraeidae	Chamaedrilus chalupskyi
Lumbricillus kaloensis isolate CE977 16S ribosomal	GU901828	79.35	73.17	8.76114e−09	Haplotaxida	Enchytraeidae	Lumbricillus kaloensis
Enchytraeus lacteus isolate CE813 16S ribosomal RN	GU901791	74.19	100	8.76114e−09	Haplotaxida	Enchytraeidae	Enchytraeus lacteus
Rhynchelmis rostrata variant isolate CE555 16S rib	GU592345	78.12	73.98	8.76114e−09	Lumbriculida	Lumbriculidae	Rhynchelmis rostrata
Rhynchelmis gustafsoni isolate CE583 16S ribosomal	GU592343	78.12	73.98	8.76114e−09	Lumbriculida	Lumbriculidae	Rhynchelmis gustafsoni
Rhynchelmis rostrata isolate CE1070 16S ribosomal	GU592336	78.12	73.98	8.76114e−09	Lumbriculida	Lumbriculidae	Rhynchelmis rostrata
Eclipidrilus pacificus variant isolate CE863 16S r	GU592331	78.12	73.98	8.76114e−09	Lumbriculida	Lumbriculidae	Eclipidrilus pacificus
Dichogaster annae 16S ribosomal RNA gene, partial	FJ986145	77.66	73.17	8.76114e−09	Haplotaxida	Megascolecidae	Dichogaster annae
Chaetogaster diaphanus 16S ribosomal RNA gene, par	DQ459956	78.65	69.92	8.76114e−09	Haplotaxida	Naididae	Chaetogaster diaphanus
Rhyacodrilus hiemalis 16S ribosomal RNA gene, part	DQ459932	78.26	73.17	8.76114e−09	Haplotaxida	Naididae	Rhyacodrilus hiemalis
Drawida ghilarovi mitochondrial partial 16S rRNA g	HG970209	76.53	73.17	3.05794e−08	Moniligastrida	Moniligastridae	Drawida ghilarovi
