# Supplementary material for: Testing the potential of a ribosomal 16S marker for DNA metabarcoding of insects
Source: PeerJ. 2016 Apr 19;4:e1966. doi: 10.7717/peerj.1966 (PMC4841222; doi:10.7717/peerj.1966)
Supplement: Supplemental Information 1 [file peerj-04-1966-s006.zip › scripts/5 match OTUs/blast_hits/OTU_8.csv_plot.pdf]

OTU_8.csv

Oreodytes sanmarkii

Dytiscidae

Coleoptera

98.73

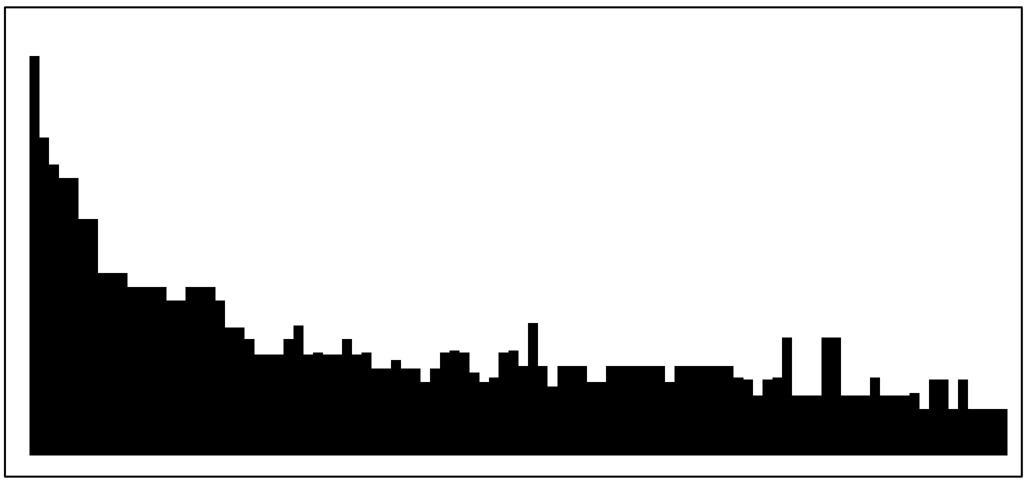

Oreodytes sanmarkii mitochondrial 16S rRNA gene (p	HF931524	98.73	100	1.5757e−70	Coleoptera	Dytiscidae	Oreodytes sanmarkii
Oreodytes obesus cordillerensis mitochondrial part	HF931516	94.9	100	2.19026e−62	Coleoptera	Dytiscidae	Oreodytes obesus cordillerensis
Oreodytes congruus 16S ribosomal RNA gene, partial	KF575550	93.63	100	1.13458e−59	Coleoptera	Dytiscidae	Oreodytes congruus
Oreodytes crassulus partial 16S rRNA gene	AJ850348	92.99	100	4.82437e−58	Coleoptera	Dytiscidae	Oreodytes crassulus
Oreodytes congruus partial 16S rRNA gene	AJ850347	92.99	100	4.82437e−58	Coleoptera	Dytiscidae	Oreodytes congruus
Oreodytes abbreviatus mitochondrial 16S rRNA gene	HF931477	91.08	100	3.04451e−54	Coleoptera	Dytiscidae	Oreodytes abbreviatus
Oreodytes rhyacophilus partial 16S rRNA gene	AJ850352	91.08	100	3.04451e−54	Coleoptera	Dytiscidae	Oreodytes rhyacophilus
Oreodytes mongolicus mitochondrial 16S rRNA gene (	HF931491	88.54	100	8.16955e−49	Coleoptera	Dytiscidae	Oreodytes mongolicus
Boreonectes riberae mitochondrial 16S rRNA gene (p	HF931461	88.54	100	9.95255e−48	Coleoptera	Dytiscidae	Boreonectes riberae
Boreonectes multilineatus mitochondrial partial 16	HF931384	88.54	100	9.95255e−48	Coleoptera	Dytiscidae	Boreonectes multilineatus
Oreodytes snoqualmie mitochondrial partial 16S rRN	HF931519	87.9	100	3.47378e−47	Coleoptera	Dytiscidae	Oreodytes snoqualmie
Deronectes parvicollis mitochondrial 16S rRNA gene	HF931454	87.9	100	3.47378e−47	Coleoptera	Dytiscidae	Deronectes parvicollis
Boreonectes alpestris mitochondrial 16S rRNA gene	HF931512	87.9	100	4.23193e−46	Coleoptera	Dytiscidae	Boreonectes alpestris
Boreonectes striatellus mitochondrial 16S rRNA gen	HF931511	87.9	100	4.23193e−46	Coleoptera	Dytiscidae	Boreonectes striatellus
Oreodytes septentrionalis mitochondrial 16S rRNA g	HF931492	87.26	100	4.23193e−46	Coleoptera	Dytiscidae	Oreodytes septentrionalis
Oreodytes shorti mitochondrial 16S rRNA gene (part	HF931488	87.26	100	4.23193e−46	Coleoptera	Dytiscidae	Oreodytes shorti
Boreonectes griseostriatus mitochondrial partial 1	HF931387	87.9	100	4.23193e−46	Coleoptera	Dytiscidae	Boreonectes griseostriatus
Boreonectes macedonicus mitochondrial 16S rRNA gen	HF931359	87.9	100	4.23193e−46	Coleoptera	Dytiscidae	Boreonectes macedonicus
Stictotarsus ibericus 16S ribosomal RNA (rrnL) gen	EF670030	87.9	100	4.23193e−46	Coleoptera	Dytiscidae	Stictotarsus ibericus
Boreonectes griseostriatus mitochondrial 16S rRNA	HF931541	87.26	100	5.15555e−45	Coleoptera	Dytiscidae	Boreonectes griseostriatus
Laccornis difformis 16S ribosomal RNA gene, partia	KF575544	85.99	100	2.19219e−43	Coleoptera	Dytiscidae	Laccornis difformis
Laccornis kocae partial 16S rRNA gene	AJ850419	85.99	100	2.19219e−43	Coleoptera	Dytiscidae	Laccornis kocae
Suphrodytes dorsalis 16S ribosomal RNA gene, parti	KF575577	85.44	100	1.13558e−40	Coleoptera	Dytiscidae	Suphrodytes dorsalis
Oreodytes quadrimaculatus 16S ribosomal RNA gene,	KF575567	84.71	100	1.13558e−40	Coleoptera	Dytiscidae	Oreodytes quadrimaculatus
Oreodytes scitulus 16S ribosomal RNA gene, partial	KF575551	84.71	100	1.13558e−40	Coleoptera	Dytiscidae	Oreodytes scitulus
Oreodytes scitulus mitochondrial partial 16S rRNA	HF931515	84.71	100	1.13558e−40	Coleoptera	Dytiscidae	Oreodytes scitulus
Suphrodytes dorsalis voucher BMNH:725966 16S ribos	JX221574	85.44	100	1.13558e−40	Coleoptera	Dytiscidae	Suphrodytes dorsalis
Hydroporus foveolatus mitochondrial 16S rRNA gene	HE613724	86.08	100	1.13558e−40	Coleoptera	Dytiscidae	Hydroporus foveolatus
Oreodytes quadrimaculatus partial 16S rRNA gene	AJ850351	84.71	100	1.13558e−40	Coleoptera	Dytiscidae	Oreodytes quadrimaculatus
Nebrioporus formaster mitochondrial partial 16S rR	HF931377	84.81	100	3.96358e−40	Coleoptera	Dytiscidae	Nebrioporus formaster
Hygrotus novemlineatus mitochondrial partial 16S r	HF931523	84.71	100	1.38343e−39	Coleoptera	Dytiscidae	Hygrotus novemlineatus
Hygrotus marklini mitochondrial 16S rRNA gene (par	HF931467	84.71	100	1.38343e−39	Coleoptera	Dytiscidae	Hygrotus marklini
Hydroporus zimmermanni mitochondrial 16S rRNA gene	HE613679	85.44	100	1.38343e−39	Coleoptera	Dytiscidae	Hydroporus zimmermanni
Hygrotus turbidus partial 16S rRNA gene	AJ850390	84.71	100	1.38343e−39	Coleoptera	Dytiscidae	Hygrotus turbidus
Stictotarsus maghrebinus 16S ribosomal RNA gene, p	AY250947	84.81	100	4.82863e−39	Coleoptera	Dytiscidae	Stictotarsus maghrebinus
Hygrotus patruelis mitochondrial 16S rRNA gene (pa	HF931388	84.08	100	1.68536e−38	Coleoptera	Dytiscidae	Hygrotus patruelis
Suphrodytes figuratus voucher BMNH:722148 16S ribo	JX221571	84.08	100	1.68536e−38	Coleoptera	Dytiscidae	Suphrodytes figuratus
Hydroporus marginatus mitochondrial 16S rRNA gene	HE613699	84.47	100	1.68536e−38	Coleoptera	Dytiscidae	Hydroporus marginatus
Hydroporus glabriusculus 16S ribosomal RNA gene, p	AY365270	84.08	100	1.68536e−38	Coleoptera	Dytiscidae	Hydroporus glabriusculus
Hydroporus marginatus 16S ribosomal RNA gene, part	AF518267	84.08	100	1.68536e−38	Coleoptera	Dytiscidae	Hydroporus marginatus
Aethionectes fulvonotatus voucher KBMAeop406 16S r	KF979088	83.44	100	5.88247e−38	Coleoptera	Dytiscidae	Aethionectes fulvonotatus
Boreonectes griseostriatus mitochondrial 16S rRNA	HF931385	84.08	100	5.88247e−38	Coleoptera	Dytiscidae	Boreonectes griseostriatus
Hydroporus analis mitochondrial partial 16S rRNA g	HE613731	84.81	100	5.88247e−38	Coleoptera	Dytiscidae	Hydroporus analis
Hydroporus fuscipennis mitochondrial 16S rRNA gene	HE613730	84.91	100	5.88247e−38	Coleoptera	Dytiscidae	Hydroporus fuscipennis
Hydroporus compunctus mitochondrial 16S rRNA gene	HE599693	84.81	100	5.88247e−38	Coleoptera	Dytiscidae	Hydroporus compunctus
Antiporus hollingsworthi mitochondrial partial 16S	FR727246	83.87	98.73	5.88247e−38	Coleoptera	Dytiscidae	Antiporus hollingsworthi
Oreodytes natrix partial 16S rRNA gene	AJ850349	83.44	100	5.88247e−38	Coleoptera	Dytiscidae	Oreodytes natrix
Scarodytes sp. IR−2003 16S ribosomal RNA gene, par	AY250939	83.65	100	5.88247e−38	Coleoptera	Dytiscidae	Scarodytes sp. IR−2003
Hydroporus compunctus 16S ribosomal RNA gene, part	AF518259	84.81	100	5.88247e−38	Coleoptera	Dytiscidae	Hydroporus compunctus
Hydroporus fuscipennis 16S ribosomal RNA gene, par	AF518262	84.91	100	5.88247e−38	Coleoptera	Dytiscidae	Hydroporus fuscipennis
Stictotarsus procerus mitochondrial 16S rRNA gene	HF931379	84.18	100	2.05319e−37	Coleoptera	Dytiscidae	Stictotarsus procerus
Deronectes moestus mitochondrial 16S rRNA gene (pa	HE613726	86.21	91.72	2.05319e−37	Coleoptera	Dytiscidae	Deronectes moestus
Deronectes doriae mitochondrial 16S rRNA gene (par	HE613710	84.18	100	2.05319e−37	Coleoptera	Dytiscidae	Deronectes doriae
Antiporus hollingsworthi mitochondrial partial 16S	FR727247	83.23	98.73	2.05319e−37	Coleoptera	Dytiscidae	Antiporus hollingsworthi
Nirripirti megamacrocephalus isolate ABTC78781 16S	EU616935	84.18	100	2.05319e−37	Coleoptera	Dytiscidae	Paroster megamacrocephalus
Hydroporus tessellatus 16S ribosomal RNA gene, par	JX434770	84.18	100	7.16632e−37	Coleoptera	Dytiscidae	Hydroporus tessellatus
Hydroporus foveolatus mitochondrial 16S rRNA gene	HF931502	84.18	100	7.16632e−37	Coleoptera	Dytiscidae	Hydroporus foveolatus
Hydroporus brancoi gredensis mitochondrial partial	HF931329	83.44	100	7.16632e−37	Coleoptera	Dytiscidae	Hydroporus brancoi gredensis
Suphrodytes figuratus voucher BMNH:722150 16S ribo	JX221572	83.44	100	7.16632e−37	Coleoptera	Dytiscidae	Suphrodytes figuratus
Hydroporus discretus mitochondrial 16S rRNA gene (	HE613723	84.18	100	7.16632e−37	Coleoptera	Dytiscidae	Hydroporus discretus
Hydroporus decipiens mitochondrial 16S rRNA gene (	HE613722	84.18	100	7.16632e−37	Coleoptera	Dytiscidae	Hydroporus decipiens
Hydroporus pubescens mitochondrial 16S rRNA gene (	HE613707	84.18	100	7.16632e−37	Coleoptera	Dytiscidae	Hydroporus pubescens
Hydroporus limbatus mitochondrial 16S rRNA gene (p	HE613706	84.18	100	7.16632e−37	Coleoptera	Dytiscidae	Hydroporus limbatus
Hydroporus errans mitochondrial 16S rRNA gene (par	HE613704	84.18	100	7.16632e−37	Coleoptera	Dytiscidae	Hydroporus errans
Hydroporus basinotatus mitochondrial 16S rRNA gene	HE613678	84.18	100	7.16632e−37	Coleoptera	Dytiscidae	Hydroporus basinotatus
Suphrodytes dorsalis mitochondrial 16S rRNA gene (	HE599700	83.44	100	7.16632e−37	Coleoptera	Dytiscidae	Suphrodytes dorsalis
Hydroporus pubescens voucher NHM−IR39 16S ribosoma	EF419327	84.18	100	7.16632e−37	Coleoptera	Dytiscidae	Hydroporus pubescens
Hydroporus basinotatus 16S ribosomal RNA gene, par	AF518258	84.18	100	7.16632e−37	Coleoptera	Dytiscidae	Hydroporus basinotatus
Hydroporus pubescens 16S ribosomal RNA gene, parti	AF518276	84.18	100	7.16632e−37	Coleoptera	Dytiscidae	Hydroporus pubescens
Hydroporus limbatus 16S ribosomal RNA gene, partia	AF518264	84.18	100	7.16632e−37	Coleoptera	Dytiscidae	Hydroporus limbatus
Hydroporus discretus 16S ribosomal RNA gene, parti	AF518260	84.18	100	7.16632e−37	Coleoptera	Dytiscidae	Hydroporus discretus
Hydroporus analis 16S ribosomal RNA gene, partial	AF518257	84.18	100	7.16632e−37	Coleoptera	Dytiscidae	Hydroporus analis
Hydroporus planus mitochondrial 16S rRNA gene (par	HE613708	83.65	100	2.50129e−36	Coleoptera	Dytiscidae	Hydroporus planus
Deronectes opatrinus mitochondrial 16S rRNA gene (	HE613701	83.54	100	2.50129e−36	Coleoptera	Dytiscidae	Deronectes opatrinus
Copelatus sp. BI−2011 voucher BMNH792945 16S ribos	HQ381441	82.8	100	2.50129e−36	Coleoptera	Dytiscidae	Copelatus sp. BI−2011
Stictotarsus falli 16S ribosomal RNA (rrnL) gene,	EF670029	83.54	100	2.50129e−36	Coleoptera	Dytiscidae	Stictotarsus falli
Hydroporus planus 16S ribosomal RNA gene, partial	AF518275	83.65	100	2.50129e−36	Coleoptera	Dytiscidae	Hydroporus planus
Deronectes moestus 16S ribosomal RNA gene, partial	KF575561	85.52	91.72	8.73037e−36	Coleoptera	Dytiscidae	Deronectes moestus
Hydroporus semenowi mitochondrial partial 16S rRNA	HF931509	82.8	100	8.73037e−36	Coleoptera	Dytiscidae	Hydroporus semenowi
Hygrotus tumidiventris mitochondrial 16S rRNA gene	HF931496	82.8	100	8.73037e−36	Coleoptera	Dytiscidae	Hygrotus tumidiventris
Hygrotus unguicularis mitochondrial partial 16S rR	HF931489	82.8	100	8.73037e−36	Coleoptera	Dytiscidae	Hygrotus unguicularis
Deronectes brannani mitochondrial 16S rRNA gene (p	HE613695	85.52	91.72	8.73037e−36	Coleoptera	Dytiscidae	Deronectes brannanii
Deronectes moestus mitochondrial 16S rRNA gene (pa	HE613683	85.52	91.72	8.73037e−36	Coleoptera	Dytiscidae	Deronectes moestus
Hydroporus necopinatus mitochondrial partial 16S r	HE599692	82.8	100	8.73037e−36	Coleoptera	Dytiscidae	Hydroporus necopinatus
Hydroporus brancoi mitochondrial 16S rRNA gene (pa	HE599678	82.8	100	8.73037e−36	Coleoptera	Dytiscidae	Hydroporus brancoi
Hydroporus cantabricus mitochondrial partial 16S r	HE599676	82.8	100	8.73037e−36	Coleoptera	Dytiscidae	Hydroporus cantabricus
Nirripirti napperbyensis 16S ribosomal RNA gene, p	AY353843	83.65	100	8.73037e−36	Coleoptera	Dytiscidae	Paroster napperbyensis
Hydroporus necopinatus 16S ribosomal RNA gene, par	AY365275	82.8	100	8.73037e−36	Coleoptera	Dytiscidae	Hydroporus necopinatus
Hydroporus morio 16S ribosomal RNA gene, partial s	AY365274	82.8	100	8.73037e−36	Coleoptera	Dytiscidae	Hydroporus morio
Hydroporus sp. IR33.4 16S ribosomal RNA gene, part	AY365261	82.8	100	8.73037e−36	Coleoptera	Dytiscidae	Hydroporus sp. IR33.4
Scarodytes halensis haplotype 3 16S ribosomal RNA	AY250937	82.91	100	8.73037e−36	Coleoptera	Dytiscidae	Scarodytes halensis
Hydaticus cinctipennis voucher UNM KBMHyci20 16S r	KJ548338	82.17	100	3.0472e−35	Coleoptera	Dytiscidae	Hydaticus cinctipennis
Hydroporus discretus mitochondrial 16S rRNA gene (	HF931449	83.54	100	3.0472e−35	Coleoptera	Dytiscidae	Hydroporus discretus
Hydroporus thracicus mitochondrial 16S rRNA gene (	HF931413	83.54	100	3.0472e−35	Coleoptera	Dytiscidae	Hydroporus thracicus
Deronectes bicostatus mitochondrial 16S rRNA gene	HE613705	82.17	100	3.0472e−35	Coleoptera	Dytiscidae	Deronectes bicostatus
Hydroporus macedonicus mitochondrial 16S rRNA gene	HE613690	83.54	100	3.0472e−35	Coleoptera	Dytiscidae	Hydroporus macedonicus
Copelatus sp. BI−2011 voucher BMNH671215 16S ribos	HQ381377	82.17	100	3.0472e−35	Coleoptera	Dytiscidae	Copelatus sp. BI−2011
Antiporus sp. MB1421 mitochondrial partial 16S rRN	FR727245	82.17	100	3.0472e−35	Coleoptera	Dytiscidae	Antiporus sp. MB1421
Oreodytes picturatus partial 16S rRNA gene	AJ850350	82.17	100	3.0472e−35	Coleoptera	Dytiscidae	Oreodytes picturatus
Nirripirti wedgeensis isolate ABTC78773−4 16S ribo	EU616938	82.17	100	1.06358e−34	Coleoptera	Dytiscidae	Paroster wedgeensis
