# Supplementary material for: Testing the potential of a ribosomal 16S marker for DNA metabarcoding of insects
Source: PeerJ. 2016 Apr 19;4:e1966. doi: 10.7717/peerj.1966 (PMC4841222; doi:10.7717/peerj.1966)
Supplement: Supplemental Information 1 [file peerj-04-1966-s006.zip › scripts/5 match OTUs/blast_hits/OTU_80.csv_plot.pdf]

OTU_80.csv

Dinocras cephalotes

Perlidae

Plecoptera

85.63

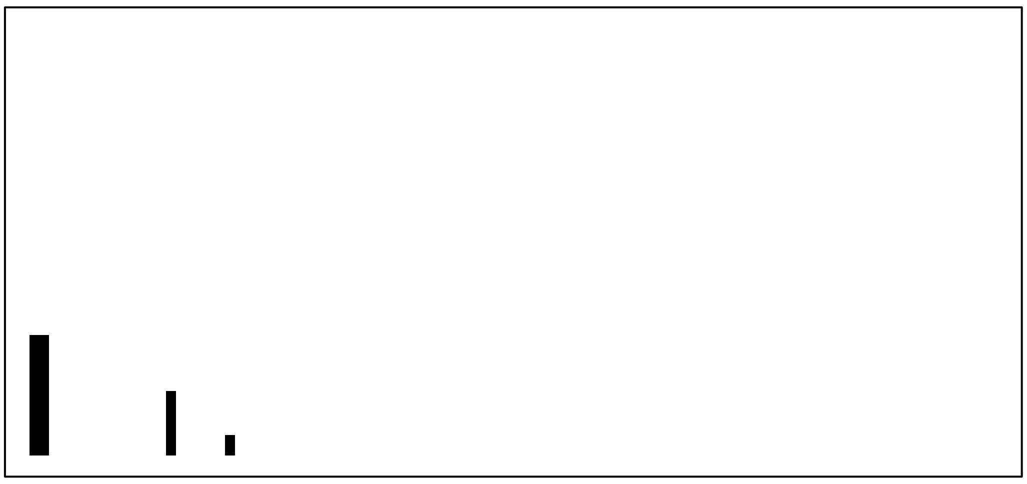

Dinocras cephalotes mitochondrion, complete genome	KF484757	85.63	100	2.80198e−42	Plecoptera	Perlidae	Dinocras cephalotes
Dinocras cephalotes 16S ribosomal RNA gene, partia	EF623244	85.63	100	2.80198e−42	Plecoptera	Perlidae	Dinocras cephalotes
Claassenia sabulosa 16S ribosomal RNA gene, partia	EF623250	77.91	100	2.30204e−24	Plecoptera	Perlidae	Claassenia sabulosa
Pteronarcys sachalina 16S ribosomal RNA gene, part	EF623264	75.93	99.39	1.45275e−20	Plecoptera	Pteronarcyidae	Pteronarcys sachalina
Calineuria californica 16S ribosomal RNA gene, par	EF623239	76.36	99.39	5.07058e−20	Plecoptera	Perlidae	Calineuria californica
Agnetina capitata 16S ribosomal RNA gene, partial	EF623241	76.79	100	1.76981e−19	Plecoptera	Perlidae	Agnetina capitata
Pteronarcys princeps mitochondrion, complete genom	AY687866	75.31	99.39	1.76981e−19	Plecoptera	Pteronarcyidae	Pteronarcys princeps
Cosumnoperla hypocrena 16S ribosomal RNA gene, par	EF623226	75.78	98.77	6.17723e−19	Plecoptera	Perlodidae	Cosumnoperla hypocrena
Pteronarcys californica 16S ribosomal RNA gene, pa	EF623261	74.69	99.39	7.52541e−18	Plecoptera	Pteronarcyidae	Pteronarcys californica
Austrocerca rieka 16S ribosomal RNA gene, partial	EF623205	74.69	99.39	7.52541e−18	Plecoptera	Notonemouridae	Austrocerca rieka
Austrocercoides zwicki 16S ribosomal RNA gene, par	EF623199	74.69	99.39	7.52541e−18	Plecoptera	Notonemouridae	Austrocercoides zwicki
Moselia infuscata 16S ribosomal RNA gene, partial	EF623175	74.7	100	7.52541e−18	Plecoptera	Leuctridae	Moselia infuscata
Eupholidoptera krueperi voucher 408 16S ribosomal	EU203912	75.84	88.96	2.62662e−17	Orthoptera	Tettigoniidae	Eupholidoptera krueperi
Pteronarcys scotti 16S ribosomal RNA gene, partial	EF623263	74.07	99.39	9.16782e−17	Plecoptera	Pteronarcyidae	Pteronarcys scotti
Hansonoperla appalachia 16S ribosomal RNA gene, pa	EF623249	83.02	64.42	9.16782e−17	Plecoptera	Perlidae	Hansonoperla appalachia
Grylloblatta gurneyi voucher BYU_gb10.2 16S riboso	DQ457240	78.1	84.05	9.16782e−17	Grylloblattodea	Grylloblattidae	Grylloblatta gurneyi
Grylloblatta gurneyi voucher BYU_gb10.1 16S riboso	DQ457239	78.1	84.05	9.16782e−17	Grylloblattodea	Grylloblattidae	Grylloblatta gurneyi
Osmylus sp. EH−2004 16S ribosomal RNA gene, partia	AY620146	74.69	99.39	9.16782e−17	Neuroptera	Osmylidae	Osmylus sp. EH−2004
Taenionema palladium 16S ribosomal RNA gene, parti	EF623267	74.7	99.39	3.19988e−16	Plecoptera	Taeniopterygidae	Taenionema palladium
Styloperla wui 16S ribosomal RNA gene, partial seq	EF623259	73.46	99.39	3.19988e−16	Plecoptera	Styloperlidae	Styloperla wui
Perlinella drymo 16S ribosomal RNA gene, partial	EF623240	80.95	64.42	3.19988e−16	Plecoptera	Perlidae	Perlinella drymo
Triznaka pintada 16S ribosomal RNA gene, partial s	EF623134	75.78	95.71	3.19988e−16	Plecoptera	Chloroperlidae	Triznaka pintada
Grylloblatta gurneyi voucher BYU_gb26.1 16S riboso	DQ457259	77.37	84.05	3.19988e−16	Grylloblattodea	Grylloblattidae	Grylloblatta gurneyi
Anterastes burri haplotype 16BO 16S ribosomal RNA	HM856171	76.39	86.5	1.11687e−15	Orthoptera	Tettigoniidae	Anterastes burri
Anterastes burri haplotype 17BO 16S ribosomal RNA	HM856169	76.92	86.5	1.11687e−15	Orthoptera	Tettigoniidae	Anterastes burri
Cleptelmis sp. FC_E13 16S ribosomal RNA (rrnL) gen	EF209444	73.75	93.25	1.11687e−15	Coleoptera	Elmidae	Cleptelmis sp. FC_E13
Suwallia teleckojensis 16S ribosomal RNA gene, par	EF623142	75	99.39	1.11687e−15	Plecoptera	Chloroperlidae	Suwallia teleckojensis
Neaviperla forcipata 16S ribosomal RNA gene, parti	EF623139	76.51	90.18	1.11687e−15	Plecoptera	Chloroperlidae	Neaviperla forcipata
Styloperla sp. mitochondrion, partial genome	KR088971	74.83	90.18	3.89826e−15	Plecoptera	Styloperlidae	Styloperla sp. YZD−2015
Cryptoperla sp. YK−2012−1 mitochondrial gene for 1	AB711243	75.15	99.39	3.89826e−15	Plecoptera	Peltoperlidae	Cryptoperla sp. YK−2012−1
Dolichopoda geniculata geniculata isolate PRA20 16	AY507590	73.49	99.39	3.89826e−15	Orthoptera	Rhaphidophoridae	Dolichopoda geniculata geniculata
Doddsia occidentalis 16S ribosomal RNA gene, parti	EF623270	75.76	99.39	3.89826e−15	Plecoptera	Taeniopterygidae	Doddsia occidentalis
Scopura montana 16S ribosomal RNA gene, partial se	EF623265	76.8	76.69	3.89826e−15	Plecoptera		Scopura montana
Neonemura sp. BYU_PL153 16S ribosomal RNA gene, pa	EF623204	74.23	99.39	3.89826e−15	Plecoptera	Notonemouridae	Neonemura sp. BYU_PL153
Cosmioperla australis 16S ribosomal RNA gene, part	EF623148	75.15	99.39	3.89826e−15	Plecoptera	Eustheniidae	Cosmioperla australis
Xestoblatta cavicola voucher MNHN G21 16S ribosoma	KF372496	78.05	75.46	1.36063e−14	Blattodea	Ectobiidae	Xestoblatta cavicola
Periplaneta americana mitochondrion, complete geno	GU947663	75.66	90.18	1.36063e−14	Blattodea	Blattidae	Periplaneta americana
Brachyptera seticornis 16S ribosomal RNA gene, par	EF623268	74.23	99.39	1.36063e−14	Plecoptera	Taeniopterygidae	Brachyptera seticornis
Oemopteryx vanduzeea 16S ribosomal RNA gene, parti	EF623266	74.25	99.39	1.36063e−14	Plecoptera	Taeniopterygidae	Oemopteryx vanduzeea
Perlesta decipines 16S ribosomal RNA gene, partial	EF623242	77.42	76.07	1.36063e−14	Plecoptera	Perlidae	Perlesta decipines
Megaleuctra kincaidi 16S ribosomal RNA gene, parti	EF623174	73.62	99.39	1.36063e−14	Plecoptera	Leuctridae	Megaleuctra kincaidi
Anonconotus baracunensis voucher 194 16S ribosomal	EU120803	75.17	88.96	1.36063e−14	Orthoptera	Tettigoniidae	Anonconotus baracunensis
Metrioptera roeselii voucher 113 16S ribosomal RNA	EU120763	75	87.73	1.36063e−14	Orthoptera	Tettigoniidae	Bicolorana roeselii
Grylloblatta gurneyi voucher BYU_gb05.2 16S riboso	DQ457232	75	99.39	1.36063e−14	Grylloblattodea	Grylloblattidae	Grylloblatta gurneyi
Grylloblatta gurneyi voucher BYU_gb05.1 16S riboso	DQ457231	75	99.39	1.36063e−14	Grylloblattodea	Grylloblattidae	Grylloblatta gurneyi
D.geniculata mitochondrial 16S rRNA gene	Y07551	73.21	99.39	1.36063e−14	Orthoptera	Rhaphidophoridae	Dolichopoda geniculata
Periplaneta americana mitochondrion 16S ribosomal	U17806	75.66	90.18	1.36063e−14	Blattodea	Blattidae	Periplaneta americana
Acroneuria hainana mitochondrion, complete genome	KM199685	73.81	99.39	4.74905e−14	Plecoptera	Perlidae	Acroneuria hainana
Myrmeleon fasciatus isolate 87−fasmar 16S ribosoma	KC195819	77.6	76.07	4.74905e−14	Neuroptera	Myrmeleontidae	Myrmeleon fasciatus
Myrmeleon fasciatus isolate 68−fasmar11 16S riboso	KC195818	77.6	76.07	4.74905e−14	Neuroptera	Myrmeleontidae	Myrmeleon fasciatus
Anterastes burri haplotype 18BO 16S ribosomal RNA	HM856174	75.69	86.5	4.74905e−14	Orthoptera	Tettigoniidae	Anterastes burri
Anterastes burri haplotype 19BO 16S ribosomal RNA	HM856168	75.69	86.5	4.74905e−14	Orthoptera	Tettigoniidae	Anterastes burri
Anterastes burri haplotype 23CA 16S ribosomal RNA	HM856165	75.69	86.5	4.74905e−14	Orthoptera	Tettigoniidae	Anterastes burri
Anterastes serbicus haplotype 29KS 16S ribosomal R	HM856149	75.69	86.5	4.74905e−14	Orthoptera	Tettigoniidae	Anterastes serbicus
Anterastes serbicus haplotype 31KS 16S ribosomal R	HM856148	75.69	86.5	4.74905e−14	Orthoptera	Tettigoniidae	Anterastes serbicus
Anterastes serbicus haplotype 30KS 16S ribosomal R	HM856146	75.69	86.5	4.74905e−14	Orthoptera	Tettigoniidae	Anterastes serbicus
Anterastes serbicus haplotype 28SI 16S ribosomal R	HM856145	75.69	86.5	4.74905e−14	Orthoptera	Tettigoniidae	Anterastes serbicus
Anterastes antitauricus haplotype 13KO 16S ribosom	HM856142	75.69	86.5	4.74905e−14	Orthoptera	Tettigoniidae	Anterastes antitauricus
Austrolimnius sp. FC_E14 16S ribosomal RNA (rrnL)	EF209443	78.1	64.42	4.74905e−14	Coleoptera	Elmidae	Austrolimnius sp. FC_E14
Parapholidoptera spinulosa voucher 388 16S ribosom	EU203904	74.83	88.96	4.74905e−14	Orthoptera	Tettigoniidae	Parapholidoptera spinulosa
Isoperla fulva 16S ribosomal RNA gene, partial seq	EF623207	75.97	77.91	4.74905e−14	Plecoptera	Perlodidae	Isoperla fulva
Neuroperlopsis patris 16S ribosomal RNA gene, part	EF623152	73.94	99.39	4.74905e−14	Plecoptera	Eustheniidae	Neuroperlopsis patris
Chloroperla tripunctata 16S ribosomal RNA gene, pa	EF623137	75.66	89.57	4.74905e−14	Plecoptera	Chloroperlidae	Chloroperla tripunctata
Parapholidoptera cf. noxia 422 voucher 422 16S rib	EU120827	74.69	96.93	4.74905e−14	Orthoptera	Tettigoniidae	Parapholidoptera cf. noxia 422
Anterastes serbicus voucher 161 16S ribosomal RNA	EU120782	75.69	86.5	4.74905e−14	Orthoptera	Tettigoniidae	Anterastes serbicus
Xyela sp. Sus1 16S ribosomal RNA gene, partial seq	AY206772	73.49	99.39	4.74905e−14	Hymenoptera	Xyelidae	Xyela sp. Sus1
Yoraperla uenoi mitochondrial gene for 16S rRNA, p	AB712375	73.83	90.18	1.65758e−13	Plecoptera	Peltoperlidae	Yoraperla uenoi
Thoracites sp. MATM−2012 small subunit ribosomal R	JQ246745	73.65	100	1.65758e−13	Diptera	Calliphoridae	Thoracites sp. MATM−2012
Galloisiana yuasai voucher ZMUC:JD−650 16S ribosom	JN615261	78.05	75.46	1.65758e−13	Grylloblattodea	Grylloblattidae	Galloisiana yuasai
Anterastes burri haplotype 3BU 16S ribosomal RNA g	HM856156	73.65	100	1.65758e−13	Orthoptera	Tettigoniidae	Anterastes burri
Dolichopoda geniculata geniculata isolate VAL1 16S	AY507600	72.89	99.39	1.65758e−13	Orthoptera	Rhaphidophoridae	Dolichopoda geniculata geniculata
Dolichopoda laetitiae isolate DIA18 16S ribosomal	AY507578	72.89	99.39	1.65758e−13	Orthoptera	Rhaphidophoridae	Dolichopoda laetitiae
Dolichopoda geniculata geniculata isolate AUS1 16S	AY507566	72.89	99.39	1.65758e−13	Orthoptera	Rhaphidophoridae	Dolichopoda geniculata geniculata
Metrioptera bicolor isolate CLN011 16S large subun	EF198433	74.64	84.66	1.65758e−13	Orthoptera	Tettigoniidae	Bicolorana bicolor
Dolichopoda pavesii voucher SPI 16S ribosomal RNA	EF216951	73.94	99.39	1.65758e−13	Orthoptera	Rhaphidophoridae	Dolichopoda pavesii
Salmoperla sylvanica 16S ribosomal RNA gene, parti	EF623222	73.62	99.39	1.65758e−13	Plecoptera	Perlodidae	Salmoperla sylvanica
Eupholidoptera mersinensis voucher 415 16S ribosom	EU120823	75.35	86.5	1.65758e−13	Orthoptera	Tettigoniidae	Eupholidoptera mersinensis
Metrioptera bicolor voucher 178 16S ribosomal RNA	EU120795	76.42	74.85	1.65758e−13	Orthoptera	Tettigoniidae	Bicolorana bicolor
Eupholidoptera prasina voucher 176 16S ribosomal R	EU120793	76.61	76.07	1.65758e−13	Orthoptera	Tettigoniidae	Eupholidoptera prasina
Galloisiana yuasai 16S large subunit ribosomal RNA	DQ890284	78.05	75.46	1.65758e−13	Grylloblattodea	Grylloblattidae	Galloisiana yuasai
Ceraeochrysa cincta voucher CASC 00217 16S ribosom	DQ399268	78.26	70.55	1.65758e−13	Neuroptera	Chrysopidae	Ceraeochrysa cincta
Dolichopoda geniculata isolate VAL 16S ribosomal R	AY793583	72.89	99.39	1.65758e−13	Orthoptera	Rhaphidophoridae	Dolichopoda geniculata
Dolichopoda laetitiae etrusca isolate DIA 16S ribo	AY793580	72.89	99.39	1.65758e−13	Orthoptera	Rhaphidophoridae	Dolichopoda laetitiae etrusca
Nebrioporus lanceolatus 16S ribosomal RNA gene, pa	AY250927	73.49	100	1.65758e−13	Coleoptera	Dytiscidae	Nebrioporus lanceolatus
Methana athertonensis 16S ribosomal RNA gene, part	KP986308	73.72	93.87	5.78553e−13	Blattodea	Blattidae	Methana athertonensis
Eupholidoptera magnifica isolate SIL1 16S ribosoma	KC852281	74.32	90.18	5.78553e−13	Orthoptera	Tettigoniidae	Eupholidoptera magnifica
Myrmeleon fasciatus group sp. 17−pfro1 16S ribosom	KF321969	76.8	76.07	5.78553e−13	Neuroptera	Myrmeleontidae	Myrmeleon fasciatus group sp. 17−pfro1
Tricyclea sp. MATM−2012 small subunit ribosomal RN	JQ246733	73.65	100	5.78553e−13	Diptera	Calliphoridae	Tricyclea sp. MATM−2012
Hemigymnochaeta unicolor isolate D26 small subunit	JQ246731	73.65	100	5.78553e−13	Diptera	Calliphoridae	Hemigymnochaeta unicolor
Madaglymbus sp. BI−2011 voucher BMNH798495 16S rib	HQ381489	72.12	100	5.78553e−13	Coleoptera	Dytiscidae	Madaglymbus sp. BI−2011
Bruchus pisorum voucher BRU−Zp11 16S ribosomal RNA	HQ178237	74.36	95.09	5.78553e−13	Coleoptera	Chrysomelidae	Bruchus pisorum
Anterastes antitauricus haplotype 15KA 16S ribosom	HM856170	75	86.5	5.78553e−13	Orthoptera	Tettigoniidae	Anterastes antitauricus
Anterastes burri haplotype 25CA 16S ribosomal RNA	HM856163	75	86.5	5.78553e−13	Orthoptera	Tettigoniidae	Anterastes burri
Anterastes antitauricus haplotype 22AF 16S ribosom	HM856161	75	86.5	5.78553e−13	Orthoptera	Tettigoniidae	Anterastes antitauricus
Anterastes antitauricus haplotype 21AF 16S ribosom	HM856160	75	86.5	5.78553e−13	Orthoptera	Tettigoniidae	Anterastes antitauricus
Anterastes burri haplotype 24CA 16S ribosomal RNA	HM856155	75	86.5	5.78553e−13	Orthoptera	Tettigoniidae	Anterastes burri
Anterastes serbicus haplotype 27GI 16S ribosomal R	HM856147	75	86.5	5.78553e−13	Orthoptera	Tettigoniidae	Anterastes serbicus
Anterastes antitauricus haplotype 12KO 16S ribosom	HM856143	75	86.5	5.78553e−13	Orthoptera	Tettigoniidae	Anterastes antitauricus
Anterastes antitauricus haplotype 14KO 16S ribosom	HM856140	75	86.5	5.78553e−13	Orthoptera	Tettigoniidae	Anterastes antitauricus
Eupholidoptera mersinensis voucher 414 16S ribosom	EU203914	75.81	76.07	2.01935e−12	Orthoptera	Tettigoniidae	Eupholidoptera mersinensis
