# Supplementary material for: Testing the potential of a ribosomal 16S marker for DNA metabarcoding of insects
Source: PeerJ. 2016 Apr 19;4:e1966. doi: 10.7717/peerj.1966 (PMC4841222; doi:10.7717/peerj.1966)
Supplement: Supplemental Information 1 [file peerj-04-1966-s006.zip › scripts/5 match OTUs/blast_hits/OTU_81.csv_plot.pdf]

OTU_81.csv

Velia caprai

Veliidae

Hemiptera

96.18

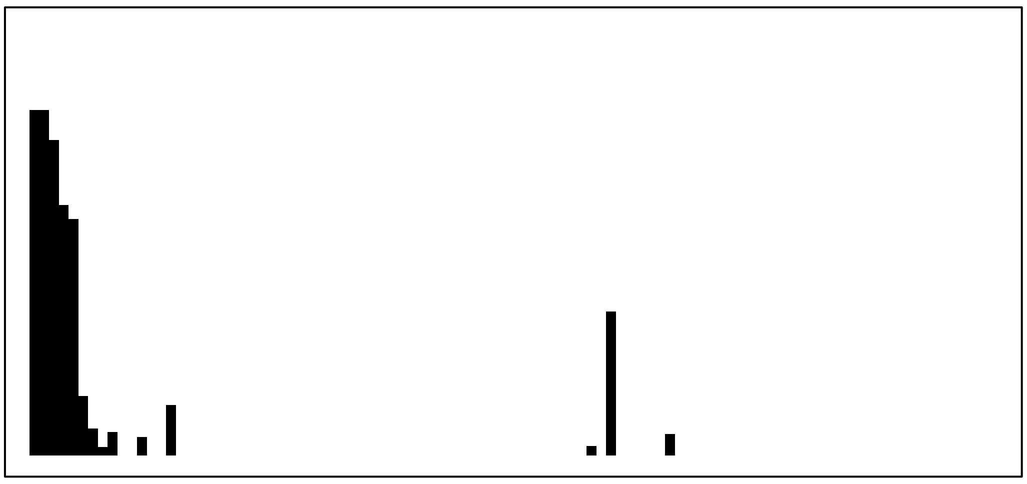

Velia caprai isolate 943 16S ribosomal RNA gene, p	EU871185	96.18	100	5.15099e−64	Hemiptera	Veliidae	Velia caprai
Velia caprai 16S ribosomal RNA gene, partial seque	AY648139	96.18	100	5.15099e−64	Hemiptera	Veliidae	Velia caprai
Velia hoberlandti isolate 941 16S ribosomal RNA ge	EU871188	94.77	97.45	3.96008e−59	Hemiptera	Veliidae	Velia hoberlandti
Velia maderensis isolate 1862 16S ribosomal RNA ge	EU871187	91.72	100	8.72266e−55	Hemiptera	Veliidae	Velia maderensis
Velia affinis isolate 373 16S ribosomal RNA gene,	EU871189	91.08	100	3.70897e−53	Hemiptera	Veliidae	Velia affinis
Rhagovelia tenuipes voucher MC36a 16S ribosomal RN	KC594837	82.78	94.27	6.7119e−31	Hemiptera	Veliidae	Rhagovelia tenuipes
Rhagovelia robusta voucher MC35a 16S ribosomal RNA	KC594836	81.25	99.36	1.21354e−27	Hemiptera	Veliidae	Rhagovelia robusta
Ventidius usingeri isolate 1059 16S ribosomal RNA	EU871207	80.38	97.45	1.21354e−27	Hemiptera	Gerridae	Ventidius usingeri
Perittopus asiaticus isolate 846 16S ribosomal RNA	EU871165	81.1	100	1.21354e−27	Hemiptera	Veliidae	Perittopus asiaticus
Limnoporus genitalis 16S ribosomal RNA gene, parti	AY425217	78.85	97.45	1.47839e−26	Hemiptera	Gerridae	Limnoporus genitalis
Limnoporus canaliculatus 16S ribosomal RNA gene, p	AY425208	78.85	97.45	1.47839e−26	Hemiptera	Gerridae	Limnoporus canaliculatus
Xenobates argentatus isolate 973 16S ribosomal RNA	EU871177	80.85	88.54	5.1601e−26	Hemiptera	Veliidae	Xenobates argentatus
Gerris argenticollis 16S large subunit ribosomal R	AY951329	78.85	97.45	1.80105e−25	Hemiptera	Gerridae	Gerris argenticollis
Limnogonus hungerfordi isolate 1431 16S large subu	GU645089	78.98	97.45	6.28629e−25	Hemiptera	Gerridae	Limnogonus hungerfordi
Halovelia sp. JD−2008 isolate 1950 16S ribosomal R	EU871176	82.35	85.35	6.28629e−25	Hemiptera	Veliidae	Halovelia sp. JD−2008
Limnoporus esakii 16S ribosomal RNA gene, partial	AY425216	78.21	97.45	6.28629e−25	Hemiptera	Gerridae	Limnoporus esakii
Limnoporus esakii mitochondrial gene for 16S ribos	AB026592	78.21	97.45	6.28629e−25	Hemiptera	Gerridae	Limnoporus esakii
Limnogonus luctuosus isolate 2294 16S large subuni	GU645092	78.48	97.45	2.19413e−24	Hemiptera	Gerridae	Limnogonus luctuosus
Microvelia reticulata isolate 292 16S ribosomal RN	EU871172	79.11	97.45	2.19413e−24	Hemiptera	Veliidae	Microvelia reticulata
Halobates mjobergi 16S ribosomal RNA gene, partial	AY648134	77.78	98.09	2.19413e−24	Hemiptera	Gerridae	Halobates mjobergi
Amemboa sp. JD−2014 isolate 2440 16S large subunit	KC880875	78.75	97.45	7.65827e−24	Hemiptera	Gerridae	Amemboa sp. JD−2014
Gerris gracilicornis mitochondrial genes for 12S r	AB679162	78.21	97.45	7.65827e−24	Hemiptera	Gerridae	Gerris gracilicornis
Gerris gracilicornis mitochondrial genes for 12S r	AB679161	78.21	97.45	7.65827e−24	Hemiptera	Gerridae	Gerris gracilicornis
Gerris gracilicornis mitochondrial genes for 12S r	AB679160	78.21	97.45	7.65827e−24	Hemiptera	Gerridae	Gerris gracilicornis
Gerris gracilicornis mitochondrial genes for 12S r	AB679153	77.56	97.45	7.65827e−24	Hemiptera	Gerridae	Gerris gracilicornis
Limnogonus intermedius isolate 140 16S large subun	GU645053	78.34	97.45	2.673e−23	Hemiptera	Gerridae	Limnogonus intermedius
Gerris sphagnetorum 16S ribosomal RNA gene, partia	AY425206	78.4	97.45	2.673e−23	Hemiptera	Gerridae	Gerris sphagnetorum
Aquarius ventralis 16S ribosomal RNA gene, partial	AY425196	78.06	97.45	2.673e−23	Hemiptera	Gerridae	Aquarius ventralis
Gerris gracilicornis mitochondrial genes for 12S r	AB679157	77.56	97.45	9.32968e−23	Hemiptera	Gerridae	Gerris gracilicornis
Gerris gracilicornis mitochondrial genes for 12S r	AB679158	77.56	97.45	9.32968e−23	Hemiptera	Gerridae	Gerris gracilicornis
Gerris gracilicornis mitochondrial genes for 12S r	AB679152	77.56	97.45	9.32968e−23	Hemiptera	Gerridae	Gerris gracilicornis
Gerris gracilicornis mitochondrial genes for 12S r	AB679151	77.56	97.45	9.32968e−23	Hemiptera	Gerridae	Gerris gracilicornis
Gerris gracilicornis mitochondrial genes for 12S r	AB679150	77.56	97.45	9.32968e−23	Hemiptera	Gerridae	Gerris gracilicornis
Gerris gracilicornis mitochondrial genes for 12S r	AB679149	77.56	97.45	9.32968e−23	Hemiptera	Gerridae	Gerris gracilicornis
Potamometropsis sumaldei 16S ribosomal RNA gene, p	AY743237	77.85	97.45	9.32968e−23	Hemiptera	Gerridae	Potamometropsis sumaldei
Amemboa sexualis 16S ribosomal RNA gene, partial s	AY648138	77.99	97.45	9.32968e−23	Hemiptera	Gerridae	Amemboa sexualis
Gerris caucasicus 16S large subunit ribosomal RNA	AY951328	77.56	97.45	9.32968e−23	Hemiptera	Gerridae	Gerris caucasicus
Gerris odontogaster 16S ribosomal RNA gene, partia	AY425219	77.56	97.45	9.32968e−23	Hemiptera	Gerridae	Gerris odontogaster
Gerris yezoensis 16S ribosomal RNA gene, partial s	AY425203	77.56	97.45	9.32968e−23	Hemiptera	Gerridae	Gerris yezoensis
Gerris gracilicornis 16S ribosomal RNA gene, parti	AY425201	77.56	97.45	9.32968e−23	Hemiptera	Gerridae	Gerris gracilicornis
Gerris argentatus 16S ribosomal RNA gene, partial	AY425194	77.56	97.45	9.32968e−23	Hemiptera	Gerridae	Gerris argentatus
Gerris buenoi 16S ribosomal RNA gene, partial sequ	AY425191	77.56	97.45	9.32968e−23	Hemiptera	Gerridae	Gerris buenoi
Microvelia horvathi mitochondrial gene for 16S rib	AB026612	77.36	97.45	9.32968e−23	Hemiptera	Veliidae	Microvelia horvathi
Rheumatogonus luzonicus mitochondrial gene for 16S	AB026609	78.62	97.45	9.32968e−23	Hemiptera	Gerridae	Rheumatogonus luzonicus
Gerris babai mitochondrial gene for 16S ribosomal	AB026585	77.56	97.45	9.32968e−23	Hemiptera	Gerridae	Gerris babai
Hypselonotus fulvus 16S ribosomal RNA gene, partia	KC537015	76.73	97.45	3.25638e−22	Hemiptera	Coreidae	Hypselonotus fulvus
Gerris gracilicornis mitochondrial genes for 12S r	AB679164	76.92	97.45	3.25638e−22	Hemiptera	Gerridae	Gerris gracilicornis
Gerris gracilicornis mitochondrial genes for 12S r	AB679163	76.92	97.45	3.25638e−22	Hemiptera	Gerridae	Gerris gracilicornis
Gerris gracilicornis mitochondrial genes for 12S r	AB679156	76.92	97.45	3.25638e−22	Hemiptera	Gerridae	Gerris gracilicornis
Gerris gracilicornis mitochondrial genes for 12S r	AB679154	76.92	97.45	3.25638e−22	Hemiptera	Gerridae	Gerris gracilicornis
Gerris sp. YT−2009 16S ribosomal RNA gene, partial	FJ372633	76.92	97.45	3.25638e−22	Hemiptera	Gerridae	Gerris sp. YT−2009
Limnogonus hungerfordi isolate 207 16S large subun	GU645085	77.71	97.45	3.25638e−22	Hemiptera	Gerridae	Limnogonus hungerfordi
Halobates micans isolate H12 16S ribosomal RNA gen	EU871204	77.02	97.45	3.25638e−22	Hemiptera	Gerridae	Halobates micans
Rheumatogonus luzonicus isolate 1062 16S ribosomal	EU871202	77.85	97.45	3.25638e−22	Hemiptera	Gerridae	Rheumatogonus luzonicus
Gerris lateralis 16S ribosomal RNA gene, partial s	AY425231	76.92	97.45	3.25638e−22	Hemiptera	Gerridae	Gerris lateralis
Gerris asper 16S ribosomal RNA gene, partial seque	AY425184	76.92	97.45	3.25638e−22	Hemiptera	Gerridae	Gerris asper
Leptinopterus femoratus voucher SK231 16S ribosoma	KP250254	77.64	98.73	1.13659e−21	Coleoptera	Lucanidae	Leptinopterus femoratus
Tenagogonus australiensis isolate 1482 16S large s	KC880888	80.43	85.35	1.13659e−21	Hemiptera	Gerridae	Tenagogonus australiensis
Cryphocricos hungerfordi isolate 544 16S ribosomal	AY585771	79.87	97.45	1.13659e−21	Hemiptera	Naucoridae	Cryphocricos hungerfordi
Rhagovelia sp. Los Banos mitochondrial gene for 16	AB026610	86.73	61.15	1.13659e−21	Hemiptera	Veliidae	Rhagovelia sp. Los Banos
Metrocoris philippinensis mitochondrial gene for 1	AB026605	78.62	97.45	1.13659e−21	Hemiptera	Gerridae	Metrocoris philippinensis
Tenagogonus australiensis isolate 1479 16S large s	KC880887	79.86	85.35	3.96708e−21	Hemiptera	Gerridae	Tenagogonus australiensis
Gerris insularis mitochondrial genes for 12S rRNA,	AB679166	76.28	97.45	3.96708e−21	Hemiptera	Gerridae	Gerris insularis
Gerris cf. insularis 'type B' mitochondrial genes	AB679165	76.28	97.45	3.96708e−21	Hemiptera	Gerridae	Gerris cf. insularis 'type B'
Grylloblattina djakonovi djakonovi voucher BYU_gb1	DQ457247	78.48	97.45	3.96708e−21	Grylloblattodea	Grylloblattidae	Grylloblattina djakonovi djakonovi
Paraplea sp. MBH−2004 16S ribosomal RNA gene, part	AY585786	80.99	85.35	3.96708e−21	Hemiptera	Pleidae	Paraplea sp. MBH−2004
Gerris insularis 16S large subunit ribosomal RNA g	AY951327	76.28	97.45	3.96708e−21	Hemiptera	Gerridae	Gerris insularis
Aquarius philippinensis 16S large subunit ribosoma	AY951324	77.71	97.45	3.96708e−21	Hemiptera	Gerridae	Aquarius philippinensis
Aquarius cinereus 16S ribosomal RNA gene, partial	AY425234	76.92	97.45	3.96708e−21	Hemiptera	Gerridae	Aquarius cinereus
Gerris brasili 16S ribosomal RNA gene, partial seq	AY425229	76.92	97.45	3.96708e−21	Hemiptera	Gerridae	Gerris brasili
Gerris swakopensis 16S ribosomal RNA gene, partial	AY425223	76.92	97.45	3.96708e−21	Hemiptera	Gerridae	Gerris swakopensis
Aquarius elongatus 16S ribosomal RNA gene, partial	AY425200	76.92	97.45	3.96708e−21	Hemiptera	Gerridae	Aquarius elongatus
Aquarius najas 16S ribosomal RNA gene, partial seq	AY425199	76.92	97.45	3.96708e−21	Hemiptera	Gerridae	Aquarius najas
Aquarius chilensis 16S ribosomal RNA gene, partial	AY425189	76.92	97.45	3.96708e−21	Hemiptera	Gerridae	Aquarius chilensis
Aquarius elongatus mitochondrial gene for 16S ribo	AB026593	76.92	97.45	3.96708e−21	Hemiptera	Gerridae	Aquarius elongatus
Limnogonus skusei isolate 149 16S large subunit ri	GU645094	77.07	97.45	1.38465e−20	Hemiptera	Gerridae	Limnogonus luctuosus
Limnogonus hungerfordi isolate 1014 16S large subu	GU645086	76.43	97.45	1.38465e−20	Hemiptera	Gerridae	Limnogonus hungerfordi
Limnogonus hungerfordi isolate 148 16S large subun	GU645084	77.07	97.45	1.38465e−20	Hemiptera	Gerridae	Limnogonus hungerfordi
Eurygerris fuscinervis 16S ribosomal RNA gene, par	AY648137	76.58	97.45	1.38465e−20	Hemiptera	Gerridae	Eurygerris fuscinervis
Aquarius lili 16S ribosomal RNA gene, partial sequ	AY425232	77.22	97.45	1.38465e−20	Hemiptera	Gerridae	Aquarius lili
Aquarius adelaidis 16S ribosomal RNA gene, partial	AY425202	77.22	97.45	1.38465e−20	Hemiptera	Gerridae	Aquarius adelaidis
Aquarius distanti 16S ribosomal RNA gene, partial	AY425198	76.43	97.45	1.38465e−20	Hemiptera	Gerridae	Aquarius distanti
Aquarius remigis 16S ribosomal RNA gene, partial s	AY425190	76.43	97.45	1.38465e−20	Hemiptera	Gerridae	Aquarius remigis
Gerris gracilicornis mitochondrial gene for 16S ri	AB026590	76.43	97.45	1.38465e−20	Hemiptera	Gerridae	Gerris gracilicornis
Limnometra lipovskyi isolate 1071 16S large subuni	KC880898	78.68	84.71	4.8329e−20	Hemiptera	Gerridae	Limnometra lipovskyi
Palaeodocosia vittata voucher JSM6 16S ribosomal R	KC435585	77.07	92.36	4.8329e−20	Diptera	Mycetophilidae	Palaeodocosia vittata
Amphiareus morimotoi 16S ribosomal RNA gene, parti	GQ258385	78.85	94.27	4.8329e−20	Hemiptera	Anthocoridae	Amphiareus morimotoi
Onychotrechus esakii isolate 259 16S ribosomal RNA	EU871191	76.88	97.45	4.8329e−20	Hemiptera	Gerridae	Onychotrechus esakii
Gerris sahlbergi isolate 109 16S ribosomal RNA gen	DQ988688	76.28	97.45	4.8329e−20	Hemiptera	Gerridae	Gerris sahlbergi
Aquarius nyctalis 16S ribosomal RNA gene, partial	AY425233	76.28	97.45	4.8329e−20	Hemiptera	Gerridae	Aquarius nyctalis
Aquarius remigoides 16S ribosomal RNA gene, partia	AY425230	76.28	97.45	4.8329e−20	Hemiptera	Gerridae	Aquarius remigoides
Gerris lacustris 16S ribosomal RNA gene, partial s	AY425227	76.28	97.45	4.8329e−20	Hemiptera	Gerridae	Gerris lacustris
Gerris costae costae 16S ribosomal RNA gene, parti	AY425226	76.28	97.45	4.8329e−20	Hemiptera	Gerridae	Gerris costae costae
Gerris latiabdominis 16S ribosomal RNA gene, parti	AY425215	76.28	97.45	4.8329e−20	Hemiptera	Gerridae	Gerris latiabdominis
Gerris gibbifer 16S ribosomal RNA gene, partial se	AY425195	76.28	97.45	4.8329e−20	Hemiptera	Gerridae	Gerris gibbifer
Gerris argenticollis 16S ribosomal RNA gene, parti	AY425193	76.28	97.45	4.8329e−20	Hemiptera	Gerridae	Gerris argenticollis
Limnogonus fossarum fossarum mitochondrial gene fo	AB026601	76.58	97.45	4.8329e−20	Hemiptera	Gerridae	Limnogonus fossarum fossarum
Limnogonus hungerfordi mitochondrial gene for 16S	AB026600	75.95	97.45	4.8329e−20	Hemiptera	Gerridae	Limnogonus hungerfordi
Gerris latiabdominis mitochondrial gene for 16S ri	AB026586	76.28	97.45	4.8329e−20	Hemiptera	Gerridae	Gerris latiabdominis
Hydrobius convexus voucher IBE<ESP>:AB31 large sub	KF755287	77.07	97.45	1.68585e−19	Coleoptera	Hydrophilidae	Hydrobius convexus
