# Supplementary material for: Testing the potential of a ribosomal 16S marker for DNA metabarcoding of insects
Source: PeerJ. 2016 Apr 19;4:e1966. doi: 10.7717/peerj.1966 (PMC4841222; doi:10.7717/peerj.1966)
Supplement: Supplemental Information 1 [file peerj-04-1966-s006.zip › scripts/5 match OTUs/blast_hits/OTU_82.csv_plot.pdf]

OTU_82.csv

Sericostoma personatum

Sericostomatidae

Trichoptera

77.88

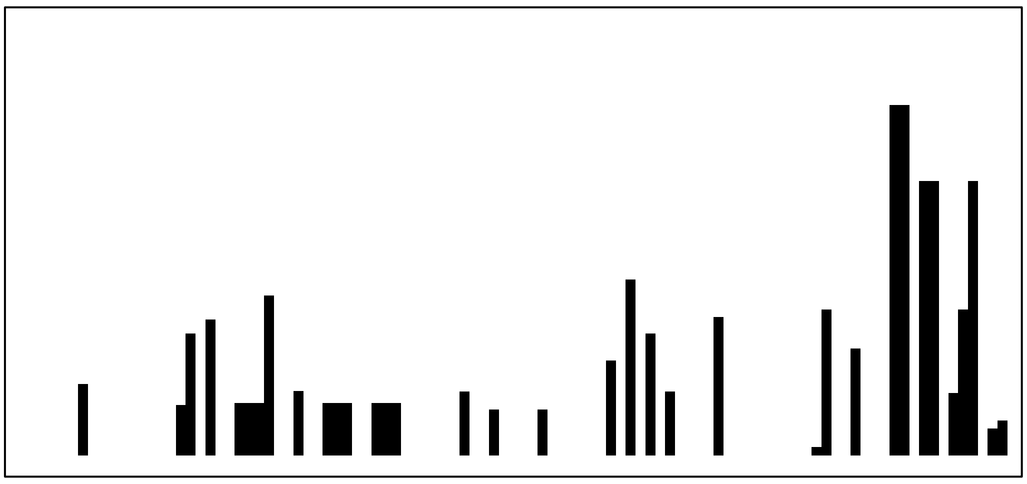

Sericostoma personatum mitochondrion, partial geno	KP455290	77.88	71.23	7.44609e−11	Trichoptera	Sericostomatidae	Sericostoma personatum
Bradysiopsis vittigera voucher 948 16S ribosomal R	JQ613962	74.04	66.44	6.97393e−05	Diptera	Sciaridae	Bradysiopsis vittigera
Bactrocera minax mitochondrion, complete genome	HM776033	76.34	60.27	6.97393e−05	Diptera	Tephritidae	Bactrocera minax
Bactrocera minax 16S ribosomal RNA gene, partial s	FJ866821	76.34	60.27	6.97393e−05	Diptera	Tephritidae	Bactrocera minax
Trifolium pratense genome assembly redclover, chro	LN846354	80	50	0.000243414	Fabales	Fabaceae	Trifolium pratense
Hamadryas epinome voucher epinome cracker mitochon	KM378244	83.33	35.62	0.000243414	Lepidoptera	Nymphalidae	Hamadryas epinome
Neptis rivularis 16S ribosomal RNA gene, partial s	JX185895	72.73	63.7	0.000243414	Lepidoptera	Nymphalidae	Neptis rivularis
Ochthebius rugulosus mitochondrial 16S rRNA gene (	HF931447	74.53	69.18	0.000243414	Coleoptera	Hydraenidae	Ochthebius rugulosus
Hydraena dentipes isolate 123_1 large subunit ribo	HQ425847	74.74	60.96	0.000849598	Coleoptera	Hydraenidae	Hydraena dentipes
Hydraena dentipes voucher MNCN−AI361 large subunit	HM588458	74.74	60.96	0.000849598	Coleoptera	Hydraenidae	Hydraena dentipes
Zebrafish DNA sequence from clone CH211−4C18 in li	CR847949	74.19	63.01	0.000849598	Cypriniformes	Cyprinidae	Danio rerio
Zebrafish DNA sequence from clone DKEY−78N12 in li	CR388371	74.19	63.01	0.000849598	Cypriniformes	Cyprinidae	Danio rerio
Neoneuromus orientalis 16S ribosomal RNA gene, par	KF944509	74.19	60.96	0.00296539	Megaloptera	Corydalidae	Neoneuromus orientalis
Strongyloides venezuelensis genome assembly S_vene	LM524970	72.32	74.66	0.0103502	Rhabditida	Strongyloididae	Strongyloides venezuelensis
Limnebius simplex mitochondrial 16S rRNA gene (par	HF931327	75.68	50.68	0.0103502	Coleoptera	Hydraenidae	Limnebius simplex
Plasmodium falciparum 3D7 chromosome 9	AL844508	82.35	34.93	0.0103502	Haemosporida	Plasmodiidae	Plasmodium falciparum 3D7
Vitis vinifera contig VV78X256038.6, whole genome	AM478677	85.71	28.77	0.0103502	Vitales	Vitaceae	Vitis vinifera
Zebrafish DNA sequence from clone CH73−316N6 in li	CT826381	75	59.59	0.0103502	Cypriniformes	Cyprinidae	Danio rerio
Lotharella vacuolata nucleomorph DNA, chromosome:	AB996601	86.36	28.08	0.0361258			Lotharella vacuolata
Tetrahymena thermophila SB210 Myb−like DNA−binding	XM_012801001	75.61	52.74	0.0361258	Hymenostomatida	Tetrahymenidae	Tetrahymena thermophila SB210
Strongyloides venezuelensis genome assembly S_vene	LM524980	76.47	42.47	0.0361258	Rhabditida	Strongyloididae	Strongyloides venezuelensis
Clostridium botulinum CDC_1436, complete genome	CP006908	82.46	39.04	0.0361258	Clostridiales	Clostridiaceae	Clostridium botulinum CDC_1436
Clostridium botulinum CDC_297, complete genome	CP006907	82.46	39.04	0.0361258	Clostridiales	Clostridiaceae	Clostridium botulinum CDC_297
Clostridium botulinum DNA, complete genome, strain	AP014696	82.46	39.04	0.0361258	Clostridiales	Clostridiaceae	Clostridium botulinum
Aporia intercostata mitochondrion, complete genome	KC461928	87.5	26.03	0.0361258	Lepidoptera	Pieridae	Aporia intercostata
Strongyloides stercoralis genome assembly S_sterco	LL999087	72.34	63.7	0.0361258	Rhabditida	Strongyloididae	Strongyloides stercoralis
Strongyloides stercoralis genome assembly S_sterco	LL999054	71.96	73.29	0.0361258	Rhabditida	Strongyloididae	Strongyloides stercoralis
Cyprinus carpio genome assembly common carp genome	LN595520	83.02	34.93	0.0361258	Cypriniformes	Cyprinidae	Cyprinus carpio
Bactrocera minax voucher Bminax446 16S ribosomal R	JQ671091	73.12	60.27	0.0361258	Diptera	Tephritidae	Bactrocera minax
Hydraena producta voucher MNCN−AI402 large subunit	HM588466	73.68	60.96	0.0361258	Coleoptera	Hydraenidae	Hydraena producta
Clostridium botulinum H04402 065, complete genome	FR773526	82.46	39.04	0.0361258	Clostridiales	Clostridiaceae	Clostridium botulinum H04402 065
Clostridium botulinum Ba4 str. 657, complete genom	CP001083	82.46	39.04	0.0361258	Clostridiales	Clostridiaceae	Clostridium botulinum Ba4 str. 657
Clostridium botulinum A2 str. Kyoto, complete geno	CP001581	82.46	39.04	0.0361258	Clostridiales	Clostridiaceae	Clostridium botulinum A2 str. Kyoto
Amygdalops thomasseti 16S ribosomal RNA gene, part	EU268522	73.47	63.7	0.0361258	Diptera	Anthomyzidae	Amygdalops thomasseti
Drosophila equinoxialis voucher 107548 NADH dehydr	EU494368	72.73	63.7	0.0361258	Diptera	Drosophilidae	Drosophila equinoxialis
Clostridium botulinum A3 str. Loch Maree, complete	CP000962	82.46	39.04	0.0361258	Clostridiales	Clostridiaceae	Clostridium botulinum A3 str. Loch Maree
Clostridium botulinum B1 str. Okra, complete genom	CP000939	82.46	39.04	0.0361258	Clostridiales	Clostridiaceae	Clostridium botulinum B1 str. Okra
Clostridium botulinum A str. ATCC 3502 complete ge	AM412317	82.46	39.04	0.0361258	Clostridiales	Clostridiaceae	Clostridium botulinum A str. ATCC 3502
Zebrafish DNA sequence from clone CH211−225M7 in l	BX510332	73.49	55.48	0.0361258	Cypriniformes	Cyprinidae	Danio rerio
Zebrafish DNA sequence from clone DKEY−82F1 in lin	BX276120	72.38	71.92	0.0361258	Cypriniformes	Cyprinidae	Danio rerio
Zebrafish DNA sequence from clone CH211−120C8 in l	BX005357	73.49	55.48	0.0361258	Cypriniformes	Cyprinidae	Danio rerio
Strongyloides venezuelensis genome assembly S_vene	LM524968	75	43.84	0.126092	Rhabditida	Strongyloididae	Strongyloides venezuelensis
Schistosoma margrebowiei genome assembly S_margreb	LL880854	73.68	46.58	0.126092	Strigeidida	Schistosomatidae	Schistosoma margrebowiei
Diphyllobothrium latum genome assembly D_latum_Gen	LL593258	79.63	35.62	0.126092	Diphyllobothriidea	Diphyllobothriidae	Diphyllobothrium latum
Thelazia callipaeda genome assembly T_callipaeda_T	LK981667	82.98	31.51	0.126092	Spirurida	Thelaziidae	Thelazia callipaeda
Solanum pennellii chromosome ch01, complete genome	HG975440	78.33	40.41	0.126092	Solanales	Solanaceae	Solanum pennellii
Muscina levida 16S ribosomal RNA gene, partial seq	JN226648	76.32	50.68	0.126092	Diptera	Muscidae	Muscina levida
Clostridium botulinum F str. 230613, complete geno	CP002011	82.14	38.36	0.126092	Clostridiales	Clostridiaceae	Clostridium botulinum F str. 230613
Plasmodium falciparum 3D7 chromosome 3	AL844502	70.34	78.08	0.126092	Haemosporida	Plasmodiidae	Plasmodium falciparum 3D7
Muscina levida 16S ribosomal RNA gene, partial seq	FJ025458	76.32	50.68	0.126092	Diptera	Muscidae	Muscina levida
Populus trichocarpa clone POP009−F11, complete seq	AC210129	71.79	76.03	0.126092	Malpighiales	Salicaceae	Populus trichocarpa
Zebrafish DNA sequence from clone CH73−171F19 in l	CU075695	72.73	67.81	0.126092	Cypriniformes	Cyprinidae	Danio rerio
Clostridium botulinum F str. Langeland, complete g	CP000728	82.14	38.36	0.126092	Clostridiales	Clostridiaceae	Clostridium botulinum F str. Langeland
Vitis vinifera contig VV78X221502.6, whole genome	AM426853	72.83	61.64	0.126092	Vitales	Vitaceae	Vitis vinifera
Vitis vinifera, whole genome shotgun sequence, con	AM446276	71.72	67.12	0.126092	Vitales	Vitaceae	Vitis vinifera
Vitis vinifera, whole genome shotgun sequence, con	AM485573	76.62	51.37	0.126092	Vitales	Vitaceae	Vitis vinifera
Populus trichocarpa clone Pop1−57B11, complete seq	AC184084	71.79	76.03	0.126092	Malpighiales	Salicaceae	Populus trichocarpa
Drosophila busckii chromosome 3R sequence	CP012526	71.57	63.7	0.440103	Diptera	Drosophilidae	Drosophila busckii
Neoneuromus tonkinensis mitochondrion, complete ge	KP126231	73.97	47.95	0.440103	Megaloptera	Corydalidae	Neoneuromus tonkinensis
Apteryx australis mantelli genome assembly AptMant	LK391417	84.44	30.14	0.440103	Apterygiformes	Apterygidae	Apteryx australis mantelli
Wuchereria bancrofti genome assembly W_bancrofti_J	LM002417	72.18	86.3	0.440103	Spirurida	Onchocercidae	Wuchereria bancrofti
Strongyloides ratti genome assembly S_ratti_ED321	LN609528	88.24	23.29	0.440103	Rhabditida	Strongyloididae	Strongyloides ratti
Chilo auricilius mitochondrion, complete genome	KJ174087	78.85	35.62	0.440103	Lepidoptera	Crambidae	Chilo auricilius
Solanum lycopersicum chromosome ch09, complete gen	HG975521	85.71	28.08	0.440103	Solanales	Solanaceae	Solanum lycopersicum
Solanum pennellii chromosome ch10, complete genome	HG975449	80	34.25	0.440103	Solanales	Solanaceae	Solanum pennellii
Lotharella sp. CCMP622 chromosome 1 nucleomorph se	CP006627	82.98	30.14	0.440103			Lotharella oceanica
Clostridium saccharoperbutylacetonicum N1−4(HMT) p	CP004122	78.46	42.47	0.440103	Clostridiales	Clostridiaceae	Clostridium saccharoperbutylacetonicum N1−4(HMT)
Limnia boscii voucher F−121 16S ribosomal RNA gene	JN816287	72.28	64.38	0.440103	Diptera	Sciomyzidae	Limnia boscii
Ochthebius granulatus voucher MNCN−AI291/23637 16S	GU143731	72.92	63.7	0.440103	Coleoptera	Hydraenidae	Ochthebius granulatus
Zebrafish DNA sequence from clone CH73−306G17 in l	CU856364	75	49.32	0.440103	Cypriniformes	Cyprinidae	Danio rerio
Brassica rapa subsp. pekinensis clone KBrB057E05,	AC189401	86.49	25.34	0.440103	Brassicales	Brassicaceae	Brassica rapa subsp. pekinensis
M.truncatula DNA sequence from clone MTH2−59E13 on	CU468266	71.72	63.7	0.440103	Fabales	Fabaceae	Medicago truncatula
Ostertagia kolchida partial nd4 gene for NADH dehy	AM261319	73.96	58.22	0.440103	Rhabditida	Trichostrongylidae	Ostertagia kolchida
Rivellia cestoventris 16S ribosomal RNA gene, part	DQ534711	72.22	58.22	0.440103	Diptera	Platystomatidae	Rivellia cestoventris
Limnia boscii 16S ribosomal RNA gene, partial sequ	AY875094	72.28	64.38	0.440103	Diptera	Sciomyzidae	Limnia boscii
Populus trichocarpa clone Pop1−53P10, complete seq	AC184079	67.91	84.25	0.440103	Malpighiales	Salicaceae	Populus trichocarpa
Diasemopsis obstans 16S ribosomal RNA gene, partia	AF304718	75.32	50.68	0.440103	Diptera	Diopsidae	Diasemopsis obstans
Neoconops longicornis 16S ribosomal RNA gene, part	AF154769	72.45	63.01	0.440103	Diptera	Conopidae	Neoconops longicornis
Neoconops longicornis 16S ribosomal RNA gene, part	AF154768	72.45	63.01	0.440103	Diptera	Conopidae	Neoconops longicornis
Neoconops longicornis 16S ribosomal RNA gene, part	AF154767	72.45	63.01	0.440103	Diptera	Conopidae	Neoconops longicornis
Zebrafish DNA sequence from clone RP71−76A6 in lin	AL606972	80.39	34.93	0.440103	Cypriniformes	Cyprinidae	Danio rerio
Quercusia quercus mitochondrion, partial genome	KM592971	86.84	26.03	1.53611	Lepidoptera	Lycaenidae	Quercusia quercus
Kirinia epaminondas 16S ribosomal RNA gene, partia	KM200153	71.72	61.64	1.53611	Lepidoptera	Nymphalidae	Kirinia epaminondas
Ovis canadensis canadensis isolate 43U chromosome	CP011895	73.49	56.85	1.53611		Bovidae	Ovis canadensis canadensis
Strongyloides venezuelensis genome assembly S_vene	LM524999	85	26.71	1.53611	Rhabditida	Strongyloididae	Strongyloides venezuelensis
Ceratophyllus rusticus isolate F300 16S ribosomal	KM891342	77.78	40.41	1.53611	Siphonaptera	Ceratophyllidae	Ceratophyllus rusticus
Peromyscopsylla selenis isolate F038 16S ribosomal	KM891269	77.78	40.41	1.53611	Siphonaptera	Leptopsyllidae	Peromyscopsylla selenis
Opomyza florum isolate A62 16S ribosomal RNA gene,	KJ418480	69.61	63.7	1.53611	Diptera	Opomyzidae	Opomyza florum
Cucumis melo genomic chromosome, chr_12	LN713266	96.43	19.18	1.53611	Cucurbitales	Cucurbitaceae	Cucumis melo
Cucumis melo genomic scaffold, anchoredscaffold000	LN681926	96.43	19.18	1.53611	Cucurbitales	Cucurbitaceae	Cucumis melo
Ailuropoda melanoleuca microsatellite GPL−80 seque	KF907155	71.95	54.79	1.53611	Carnivora	Ursidae	Ailuropoda melanoleuca
Haemonchus placei genome assembly H_placei_MHpl1 ,	LM586531	92.86	19.18	1.53611	Rhabditida	Haemonchidae	Haemonchus placei
Rhabditophanes sp. KR3021 genome assembly Rhabdito	LK995558	92.86	19.18	1.53611	Rhabditida	Alloionematidae	Rhabditophanes sp. KR3021
Brugia pahangi genome assembly B_pahangi_Glasgow ,	LK973184	76.47	45.89	1.53611	Spirurida	Onchocercidae	Brugia pahangi
Parastrongyloides trichosuri genome assembly P_tri	LM523165	82.93	28.08	1.53611	Rhabditida	Strongyloididae	Parastrongyloides trichosuri
Cyprinus carpio genome assembly common carp genome	LN593337	86.84	26.03	1.53611	Cypriniformes	Cyprinidae	Cyprinus carpio
Solanum lycopersicum chromosome ch02, complete gen	HG975514	92.86	19.18	1.53611	Solanales	Solanaceae	Solanum lycopersicum
Solanum lycopersicum chromosome ch01, complete gen	HG975513	79.31	36.3	1.53611	Solanales	Solanaceae	Solanum lycopersicum
Solanum pennellii chromosome ch11, complete genome	HG975450	81.25	32.88	1.53611	Solanales	Solanaceae	Solanum pennellii
Solanum pennellii chromosome ch07, complete genome	HG975446	81.63	32.88	1.53611	Solanales	Solanaceae	Solanum pennellii
