# Supplementary material for: Testing the potential of a ribosomal 16S marker for DNA metabarcoding of insects
Source: PeerJ. 2016 Apr 19;4:e1966. doi: 10.7717/peerj.1966 (PMC4841222; doi:10.7717/peerj.1966)
Supplement: Supplemental Information 1 [file peerj-04-1966-s006.zip › scripts/5 match OTUs/blast_hits/OTU_84.csv_plot.pdf]

OTU_84.csv

Leuctra inermis

Leuctridae

Plecoptera

87.5

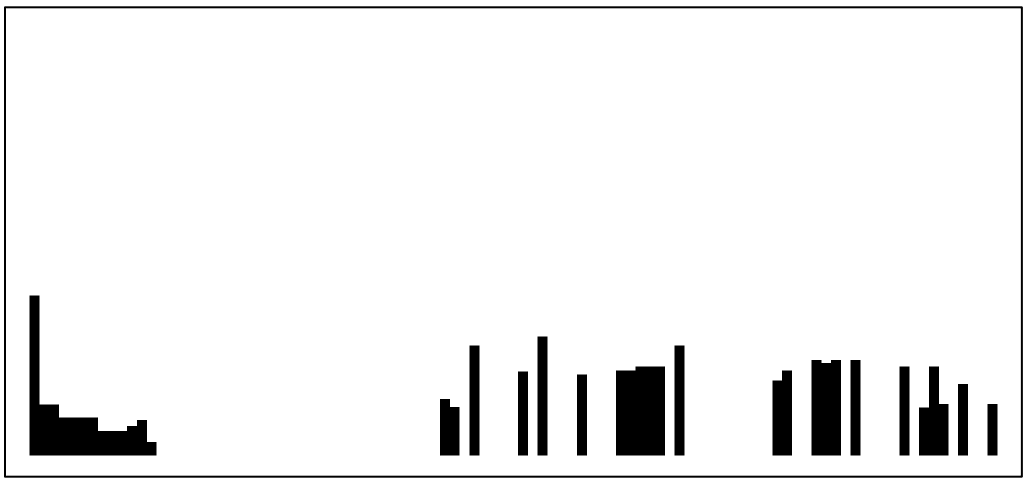

Leuctra inermis 16S ribosomal RNA gene, partial se	EF623176	87.5	100	1.5013e−45	Plecoptera	Leuctridae	Leuctra inermis
Leuctra tenuis haplotype 8lt: HO8 16S ribosomal RN	EU327680	82.39	100	3.09715e−35	Plecoptera	Leuctridae	Leuctra tenuis
Leuctra tenuis haplotype 7lt: HO7 16S ribosomal RN	EU327679	82.39	100	3.09715e−35	Plecoptera	Leuctridae	Leuctra tenuis
Leuctra tenuis haplotype 10lt: HO10 16S ribosomal	EU327682	81.76	100	1.31694e−33	Plecoptera	Leuctridae	Leuctra tenuis
Leuctra tenuis haplotype 9lt: HO9 16S ribosomal RN	EU327681	81.76	100	1.31694e−33	Plecoptera	Leuctridae	Leuctra tenuis
Leuctra tenuis haplotype 3lt: H03 16S ribosomal RN	DQ915184	81.76	100	1.31694e−33	Plecoptera	Leuctridae	Leuctra tenuis
Leuctra tenuis haplotype 2lt: H02 16S ribosomal RN	DQ915183	81.76	100	1.31694e−33	Plecoptera	Leuctridae	Leuctra tenuis
Leuctra tenuis haplotype 6lt: HO6 16S ribosomal RN	EU327678	81.13	100	1.60436e−32	Plecoptera	Leuctridae	Leuctra tenuis
Leuctra tenuis haplotype 5lt: HO5 16S ribosomal RN	EU327677	81.13	100	1.60436e−32	Plecoptera	Leuctridae	Leuctra tenuis
Leuctra tenuis haplotype 1lt: H01 16S ribosomal RN	DQ915182	81.13	100	1.60436e−32	Plecoptera	Leuctridae	Leuctra tenuis
Despaxia augusta 16S ribosomal RNA gene, partial s	EF623177	81.37	100	6.82193e−31	Plecoptera	Leuctridae	Despaxia augusta
Moselia infuscata 16S ribosomal RNA gene, partial	EF623175	81.65	99.37	6.82193e−31	Plecoptera	Leuctridae	Moselia infuscata
Leuctra tenuis haplotype 4lt: HO4 16S ribosomal RN	EU327676	80.62	100	2.90076e−29	Plecoptera	Leuctridae	Leuctra tenuis
Soyedina producta 16S ribosomal RNA gene, partial	EF623196	79.88	100	6.38935e−25	Plecoptera	Nemouridae	Soyedina producta
Nemoura trispinosa 16S ribosomal RNA gene, partial	EF623187	79.27	100	2.2301e−24	Plecoptera	Nemouridae	Nemoura trispinosa
Pteronarcys princeps mitochondrion, complete genom	AY687866	78.26	100	2.2301e−24	Plecoptera	Pteronarcyidae	Pteronarcys princeps
Nemurella pictetii 16S ribosomal RNA gene, partial	EF623192	79.04	100	7.78382e−24	Plecoptera	Nemouridae	Nemurella pictetii
Kathroperla perdita 16S ribosomal RNA gene, partia	EF623135	77.36	100	2.71682e−23	Plecoptera	Chloroperlidae	Kathroperla perdita
Pteronarcys sachalina 16S ribosomal RNA gene, part	EF623264	77.64	100	9.48263e−23	Plecoptera	Pteronarcyidae	Pteronarcys sachalina
Pteronarcys californica 16S ribosomal RNA gene, pa	EF623261	77.64	100	9.48263e−23	Plecoptera	Pteronarcyidae	Pteronarcys californica
Nemoura cinerea 16S ribosomal RNA gene, partial se	EF623194	78.05	100	9.48263e−23	Plecoptera	Nemouridae	Nemoura cinerea
Protonemura meyeri 16S ribosomal RNA gene, partial	EF623193	78.88	100	9.48263e−23	Plecoptera	Nemouridae	Protonemura meyeri
Shipsa rotunda 16S ribosomal RNA gene, partial seq	EF623184	78.31	100	9.48263e−23	Plecoptera	Nemouridae	Shipsa rotunda
Allocapnia recta isolate H17 16S ribosomal RNA gen	KC881052	77.78	100	3.30976e−22	Plecoptera	Capniidae	Allocapnia recta
Allocapnia recta isolate H12 16S ribosomal RNA gen	KC881047	77.78	100	3.30976e−22	Plecoptera	Capniidae	Allocapnia recta
Allocapnia recta isolate H9 16S ribosomal RNA gene	KC881044	77.78	100	3.30976e−22	Plecoptera	Capniidae	Allocapnia recta
Allocapnia recta isolate H8 16S ribosomal RNA gene	KC881043	77.78	100	3.30976e−22	Plecoptera	Capniidae	Allocapnia recta
Allocapnia recta isolate H5 16S ribosomal RNA gene	KC881040	77.78	100	3.30976e−22	Plecoptera	Capniidae	Allocapnia recta
Allocapnia recta isolate H4 16S ribosomal RNA gene	KC881039	77.78	100	3.30976e−22	Plecoptera	Capniidae	Allocapnia recta
Paraperla frontalis 16S ribosomal RNA gene, partia	EF623136	79.01	100	3.30976e−22	Plecoptera	Chloroperlidae	Paraperla frontalis
Allocapnia recta haplotype 10ar: H010 16S ribosoma	EU306873	77.78	100	3.30976e−22	Plecoptera	Capniidae	Allocapnia recta
Allocapnia recta haplotype 9ar: H09 16S ribosomal	EU306872	77.78	100	3.30976e−22	Plecoptera	Capniidae	Allocapnia recta
Allocapnia recta haplotype 8ar: H08 16S ribosomal	EU306871	77.78	100	3.30976e−22	Plecoptera	Capniidae	Allocapnia recta
Allocapnia recta haplotype 1ar: H01 16S ribosomal	DQ915179	77.78	100	3.30976e−22	Plecoptera	Capniidae	Allocapnia recta
Anterastes serbicus haplotype 38BUL 16S ribosomal	HM856177	78.53	99.37	1.15522e−21	Orthoptera	Tettigoniidae	Anterastes serbicus
Apteroperla tikumana mitochondrion, complete genom	KR604721	77.16	100	4.03212e−21	Plecoptera	Capniidae	Apteroperla tikumana
Allocapnia recta isolate H18 16S ribosomal RNA gen	KC881053	77.16	100	4.03212e−21	Plecoptera	Capniidae	Allocapnia recta
Allocapnia recta isolate H15 16S ribosomal RNA gen	KC881050	77.16	100	4.03212e−21	Plecoptera	Capniidae	Allocapnia recta
Allocapnia recta isolate H14 16S ribosomal RNA gen	KC881049	77.16	100	4.03212e−21	Plecoptera	Capniidae	Allocapnia recta
Allocapnia recta isolate H10 16S ribosomal RNA gen	KC881045	77.16	100	4.03212e−21	Plecoptera	Capniidae	Allocapnia recta
Allocapnia recta isolate H6 16S ribosomal RNA gene	KC881041	77.16	100	4.03212e−21	Plecoptera	Capniidae	Allocapnia recta
Doddsia occidentalis 16S ribosomal RNA gene, parti	EF623270	76.4	100	4.03212e−21	Plecoptera	Taeniopterygidae	Doddsia occidentalis
Ostrocerca foersteri 16S ribosomal RNA gene, parti	EF623191	82.64	74.21	4.03212e−21	Plecoptera	Nemouridae	Ostrocerca foersteri
Visoka cataractae 16S ribosomal RNA gene, partial	EF623189	82.26	76.1	4.03212e−21	Plecoptera	Nemouridae	Visoka cataractae
Allocapnia minima 16S ribosomal RNA gene, partial	EF623130	77.16	100	4.03212e−21	Plecoptera	Capniidae	Allocapnia minima
Tasmanoperla larvalis 16S ribosomal RNA gene, part	EF623125	85.15	63.52	4.03212e−21	Plecoptera	Austroperlidae	Tasmanoperla larvalis
Klapopteryx armillata 16S ribosomal RNA gene, part	EF623123	76.88	100	4.03212e−21	Plecoptera	Austroperlidae	Klapopteryx armillata
Allocapnia recta haplotype 11ar: H011 16S ribosoma	EU306874	77.16	100	4.03212e−21	Plecoptera	Capniidae	Allocapnia recta
Allocapnia recta haplotype 7ar: H07 16S ribosomal	EU306870	77.16	100	4.03212e−21	Plecoptera	Capniidae	Allocapnia recta
Ameletoides sp. EP159 16S ribosomal RNA gene, part	AY749826	78.87	89.31	4.03212e−21	Ephemeroptera	Nesameletidae	Ameletoides sp. EP159
Rallidens mcfarlanei 16S ribosomal RNA gene, partial	AY749794	83.93	70.44	4.03212e−21	Ephemeroptera	Rallidentidae	Rallidens mcfarlanei
Yugus bulbosus 16S ribosomal RNA gene, partial seq	EF623214	78.53	100	1.40735e−20	Plecoptera	Perlodidae	Yugus bulbosus
Lednia tumana 16S ribosomal RNA gene, partial sequ	EF623197	85.58	64.78	1.40735e−20	Plecoptera	Nemouridae	Lednia tumana
Malenka californica 16S ribosomal RNA gene, partia	EF623182	77.3	100	1.40735e−20	Plecoptera	Nemouridae	Malenka californica
Triznaka pintada 16S ribosomal RNA gene, partial s	EF623134	78.53	100	1.40735e−20	Plecoptera	Chloroperlidae	Triznaka pintada
Isocapnia hyalita 16S ribosomal RNA gene, partial	EF623131	76.69	100	1.40735e−20	Plecoptera	Capniidae	Isocapnia hyalita
Siphlonurus binotatus mitochondrial gene for 16S r	AB110269	83.78	69.81	1.40735e−20	Ephemeroptera	Siphlonuridae	Siphlonurus binotatus
Anterastes serbicus haplotype 37BUL 16S ribosomal	HM856173	77.91	99.37	4.91212e−20	Orthoptera	Tettigoniidae	Anterastes serbicus
Pteronarcys scotti 16S ribosomal RNA gene, partial	EF623263	79.58	88.05	4.91212e−20	Plecoptera	Pteronarcyidae	Pteronarcys scotti
Cultus pilatus 16S ribosomal RNA gene, partial seq	EF623212	77.02	100	4.91212e−20	Plecoptera	Perlodidae	Cultus pilatus
Prostoia besametsa 16S ribosomal RNA gene, partial	EF623188	83.96	64.78	4.91212e−20	Plecoptera	Nemouridae	Prostoia besametsa
Paranemoura claasseni 16S ribosomal RNA gene, part	EF623185	83.96	64.78	4.91212e−20	Plecoptera	Nemouridae	Paranemoura claasseni
Acroperla trivacauta 16S ribosomal RNA gene, parti	EF623155	84.16	63.52	4.91212e−20	Plecoptera	Gripopterygidae	Acroperla trivacauta
Sasquaperla hoopa 16S ribosomal RNA gene, partial	EF623143	84.16	63.52	4.91212e−20	Plecoptera	Chloroperlidae	Sasquaperla hoopa
Chloroperla tripunctata 16S ribosomal RNA gene, pa	EF623137	84.16	63.52	4.91212e−20	Plecoptera	Chloroperlidae	Chloroperla tripunctata
Capnura wanica 16S ribosomal RNA gene, partial seq	EF623128	77.02	100	4.91212e−20	Plecoptera	Capniidae	Capnura wanica
Penturoperla barbata 16S ribosomal RNA gene, parti	EF623124	85.15	63.52	4.91212e−20	Plecoptera	Austroperlidae	Penturoperla barbata
Mesocapnia arizonensis mitochondrion, partial geno	KP642637	76.54	100	1.7145e−19	Plecoptera	Capniidae	Mesocapnia arizonensis
Allocapnia recta isolate H19 16S ribosomal RNA gen	KC881054	76.54	100	1.7145e−19	Plecoptera	Capniidae	Allocapnia recta
Allocapnia recta isolate H16 16S ribosomal RNA gen	KC881051	76.54	100	1.7145e−19	Plecoptera	Capniidae	Allocapnia recta
Allocapnia recta isolate H13 16S ribosomal RNA gen	KC881048	76.54	100	1.7145e−19	Plecoptera	Capniidae	Allocapnia recta
Allocapnia recta isolate H11 16S ribosomal RNA gen	KC881046	76.54	100	1.7145e−19	Plecoptera	Capniidae	Allocapnia recta
Allocapnia recta isolate H7 16S ribosomal RNA gene	KC881042	76.54	100	1.7145e−19	Plecoptera	Capniidae	Allocapnia recta
Allocapnia recta isolate H3 16S ribosomal RNA gene	KC881038	76.54	100	1.7145e−19	Plecoptera	Capniidae	Allocapnia recta
Anterastes serbicus haplotype 32BUL 16S ribosomal	HM856167	77.02	99.37	1.7145e−19	Orthoptera	Tettigoniidae	Anterastes serbicus
Oemopteryx vanduzeea 16S ribosomal RNA gene, parti	EF623266	76.54	100	1.7145e−19	Plecoptera	Taeniopterygidae	Oemopteryx vanduzeea
Pteronarcella badia 16S ribosomal RNA gene, partia	EF623262	83.5	64.78	1.7145e−19	Plecoptera	Pteronarcyidae	Pteronarcella badia
Acroneuria lycorias 16S ribosomal RNA gene, partia	EF623251	83.96	64.78	1.7145e−19	Plecoptera	Perlidae	Acroneuria lycorias
Susulus venastus 16S ribosomal RNA gene, partial s	EF623229	77.16	100	1.7145e−19	Plecoptera	Perlodidae	Susulus venastus
Amphinemura nigritta 16S ribosomal RNA gene, parti	EF623186	76.54	100	1.7145e−19	Plecoptera	Nemouridae	Amphinemura nigritta
Suwallia teleckojensis 16S ribosomal RNA gene, par	EF623142	84.47	63.52	1.7145e−19	Plecoptera	Chloroperlidae	Suwallia teleckojensis
Utaperla sopladora 16S ribosomal RNA gene, partial	EF623140	84.31	63.52	1.7145e−19	Plecoptera	Chloroperlidae	Utaperla sopladora
Neaviperla forcipata 16S ribosomal RNA gene, parti	EF623139	84.47	63.52	1.7145e−19	Plecoptera	Chloroperlidae	Neaviperla forcipata
Sweltsa coloradensis 16S ribosomal RNA gene, parti	EF623133	77.91	100	1.7145e−19	Plecoptera	Chloroperlidae	Sweltsa coloradensis
Plumiperla diversa 16S ribosomal RNA gene, partial	EF623132	84.47	63.52	1.7145e−19	Plecoptera	Chloroperlidae	Plumiperla diversa
Mesocapnia sp. BYU_PL010 16S ribosomal RNA gene, p	EF623127	76.54	100	1.7145e−19	Plecoptera	Capniidae	Mesocapnia sp. BYU_PL010
Allocapnia recta haplotype 3ar: H03 16S ribosomal	DQ915181	76.54	100	1.7145e−19	Plecoptera	Capniidae	Allocapnia recta
Allocapnia recta haplotype 2ar: H02 16S ribosomal	DQ915180	76.54	100	1.7145e−19	Plecoptera	Capniidae	Allocapnia recta
Brachyptera seticornis 16S ribosomal RNA gene, par	EF623268	77.44	100	5.98419e−19	Plecoptera	Taeniopterygidae	Brachyptera seticornis
Cerconychia sp. BYU_PL114 16S ribosomal RNA gene,	EF623260	84.16	63.52	5.98419e−19	Plecoptera	Styloperlidae	Cerconychia sp. BYU_PL114
Cascadoperla trictura 16S ribosomal RNA gene, part	EF623225	76.25	100	5.98419e−19	Plecoptera	Perlodidae	Cascadoperla trictura
Amphinemura sulcicollis 16S ribosomal RNA gene, pa	EF623195	82.24	67.3	5.98419e−19	Plecoptera	Nemouridae	Amphinemura sulcicollis
Epeorus longimanus 16S ribosomal RNA gene, partial	AY749757	84.16	63.52	5.98419e−19	Ephemeroptera	Heptageniidae	Epeorus longimanus
Dinocras cephalotes mitochondrion, complete genome	KF484757	82.41	64.78	2.08869e−18	Plecoptera	Perlidae	Dinocras cephalotes
Allocapnia recta isolate H1 16S ribosomal RNA gene	KC881036	75.93	100	2.08869e−18	Plecoptera	Capniidae	Allocapnia recta
Anterastes disparalatus haplotype 46BO 16S ribosom	HQ850975	83.33	64.15	2.08869e−18	Orthoptera	Tettigoniidae	Anterastes disparalatus
Anterastes serbicus haplotype 35BUL 16S ribosomal	HM856162	76.25	99.37	2.08869e−18	Orthoptera	Tettigoniidae	Anterastes serbicus
Metrioptera engelhardti isolate CLN012 16S large s	EF198434	75.47	99.37	2.08869e−18	Orthoptera	Tettigoniidae	Metrioptera engelhardti
Dinocras cephalotes 16S ribosomal RNA gene, partia	EF623244	82.41	64.78	2.08869e−18	Plecoptera	Perlidae	Dinocras cephalotes
Osobenus yakimae 16S ribosomal RNA gene, partial s	EF623232	77.3	100	2.08869e−18	Plecoptera	Perlodidae	Osobenus yakimae
