# Supplementary material for: Testing the potential of a ribosomal 16S marker for DNA metabarcoding of insects
Source: PeerJ. 2016 Apr 19;4:e1966. doi: 10.7717/peerj.1966 (PMC4841222; doi:10.7717/peerj.1966)
Supplement: Supplemental Information 1 [file peerj-04-1966-s006.zip › scripts/5 match OTUs/blast_hits/OTU_85.csv_plot.pdf]

OTU_85.csv

Xenorhabdus bovienii

Enterobacteriaceae

Enterobacteriales

76.39

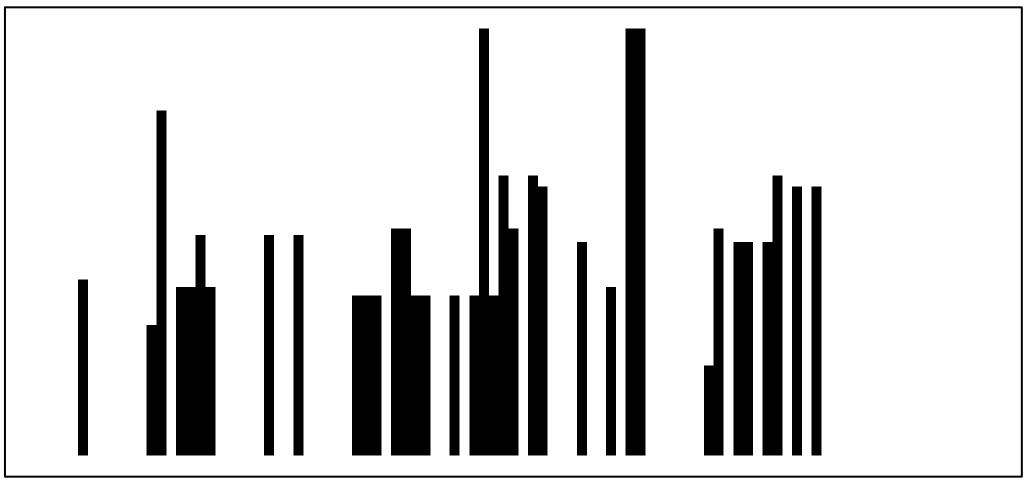

Xenorhabdus bovienii str. CS03 chromosome, complet	FO818637	76.39	51.8	0.00969751	Enterobacteriales	Enterobacteriaceae	Xenorhabdus bovienii
Pyramidula tetragona voucher Ros et al. 15/3/1997	EF173126	73.33	73.38	0.00969751	Funariales	Funariaceae	Pyramidula tetragona
Strongyloides ratti genome assembly S_ratti_ED321	LN609529	71.43	76.98	0.11814	Rhabditida	Strongyloididae	Strongyloides ratti
Solanum lycopersicum chromosome ch02, complete gen	HG975514	70.3	70.5	0.11814	Solanales	Solanaceae	Solanum lycopersicum
Solanum lycopersicum chromosome 2 clone C02HBa0167	AC215407	70.3	70.5	0.11814	Solanales	Solanaceae	Solanum lycopersicum
Enterobius vermicularis genome assembly E_vermicul	LM413771	88.24	24.46	0.412349	Oxyurida	Oxyuridae	Enterobius vermicularis
Parastrongyloides trichosuri genome assembly P_tri	LM523176	71.43	70.5	0.412349	Rhabditida	Strongyloididae	Parastrongyloides trichosuri
Zebrafish DNA sequence from clone CH211−87J1 in li	FP102071	69.64	78.42	0.412349	Cypriniformes	Cyprinidae	Danio rerio
Zebrafish DNA sequence from clone CH1073−416M20 in	CU651673	69.64	78.42	0.412349	Cypriniformes	Cyprinidae	Danio rerio
Oryza sativa (japonica cultivar−group) chromosome	AC129224	75.64	56.12	0.412349	Poales	Poaceae	Oryza sativa Japonica Group
Oryza sativa (japonica cultivar−group) chromosome	AC129225	75.64	56.12	0.412349	Poales	Poaceae	Oryza sativa Japonica Group
Trifolium pratense genome assembly redclover, chro	LN846349	75.76	47.48	1.43924	Fabales	Fabaceae	Trifolium pratense
PREDICTED: Strongylocentrotus purpuratus uncharact	XR_972630	86.11	22.3	1.43924	Echinoida	Strongylocentrotidae	Strongylocentrotus purpuratus
Protopolystoma xenopodis genome assembly P_xenopod	LM911455	96.15	18.71	1.43924	Polyopisthocotylea	Polystomatidae	Protopolystoma xenopodis
Strongyloides papillosus genome assembly S_papillo	LM525843	73.24	51.08	1.43924	Rhabditida	Strongyloididae	Strongyloides papillosus
Hymenolepis nana genome assembly H_nana_Japan ,sca	LM402034	87.88	23.74	1.43924	Cyclophyllidea	Hymenolepididae	Hymenolepis nana
Wuchereria bancrofti genome assembly W_bancrofti_J	LM000918	87.88	23.74	1.43924	Spirurida	Onchocercidae	Wuchereria bancrofti
Schistosoma rodhaini genome assembly S_rodhaini_Bu	LL959551	90.32	22.3	1.43924	Strigeidida	Schistosomatidae	Schistosoma rodhaini
Cyprinus carpio genome assembly common carp genome	LN591209	87.88	23.74	1.43924	Cypriniformes	Cyprinidae	Cyprinus carpio
PREDICTED: Apis dorsata uncharacterized LOC1026712	XM_006616221	75.29	57.55	1.43924	Hymenoptera	Apidae	Apis dorsata
PREDICTED: Apis dorsata uncharacterized LOC1026712	XM_006616220	75.29	57.55	1.43924	Hymenoptera	Apidae	Apis dorsata
PREDICTED: Apis dorsata uncharacterized LOC1026712	XM_006616219	75.29	57.55	1.43924	Hymenoptera	Apidae	Apis dorsata
PREDICTED: Apis dorsata uncharacterized LOC1026712	XM_006616218	75.29	57.55	1.43924	Hymenoptera	Apidae	Apis dorsata
PREDICTED: Apis dorsata uncharacterized LOC1026712	XM_006616217	75.29	57.55	1.43924	Hymenoptera	Apidae	Apis dorsata
Schistosoma mansoni strain Puerto Rico chromosome	HE601630	90.32	22.3	1.43924	Strigeidida	Schistosomatidae	Schistosoma mansoni
Dictyostelium purpureum hypothetical protein, mRNA	XM_003284470	73.68	53.96	1.43924	Dictyosteliida		Dictyostelium purpureum
Solanum lycopersicum clone BAC CaCM511O09−Tomato,	GU048895	74.19	44.6	1.43924	Solanales	Solanaceae	Solanum lycopersicum
Plasmodium falciparum 3D7 chromosome 4	AL844503	90.32	22.3	1.43924	Haemosporida	Plasmodiidae	Plasmodium falciparum 3D7
Solanum lycopersicum chromosome 2 clone C02SLm0049	AC215479	74.19	44.6	1.43924	Solanales	Solanaceae	Solanum lycopersicum
Solanum lycopersicum chromosome 2 clone C02HBa0165	AC215406	74.19	44.6	1.43924	Solanales	Solanaceae	Solanum lycopersicum
Strongyloides venezuelensis genome assembly S_vene	LM524970	75.36	49.64	5.02343	Rhabditida	Strongyloididae	Strongyloides venezuelensis
Trifolium pratense genome assembly redclover, chro	LN846354	73.91	49.64	5.02343	Fabales	Fabaceae	Trifolium pratense
Campylobacter jejuni subsp. jejuni strain 00−6200,	CP010307	74.24	46.04	5.02343	Campylobacterales	Campylobacteraceae	Campylobacter jejuni subsp. jejuni
PREDICTED: Musca domestica repetitive proline−rich	XM_005188683	87.5	23.02	5.02343	Diptera	Muscidae	Musca domestica
PREDICTED: Musca domestica repetitive proline−rich	XM_011296334	87.5	23.02	5.02343	Diptera	Muscidae	Musca domestica
PREDICTED: Musca domestica repetitive proline−rich	XM_011296332	87.5	23.02	5.02343	Diptera	Muscidae	Musca domestica
Campylobacter jejuni subsp. jejuni strain YH001, c	CP010058	74.24	46.04	5.02343	Campylobacterales	Campylobacteraceae	Campylobacter jejuni subsp. jejuni
Cucumis melo genomic chromosome, chr_3	LN713257	90.62	23.02	5.02343	Cucurbitales	Cucurbitaceae	Cucumis melo
Cucumis melo genomic scaffold, anchoredscaffold000	LN681823	90.62	23.02	5.02343	Cucurbitales	Cucurbitaceae	Cucumis melo
[Clostridium] sordellii genome assembly JGS6382, c	LN681234	87.5	23.02	5.02343	Clostridiales	Peptostreptococcaceae	[Clostridium] sordellii
[Clostridium] sordellii genome assembly ATCC9714_	LN679998	87.5	23.02	5.02343	Clostridiales	Peptostreptococcaceae	[Clostridium] sordellii
Campylobacter jejuni subsp. jejuni F38011, complet	CP006851	74.24	46.04	5.02343	Campylobacterales	Campylobacteraceae	Campylobacter jejuni subsp. jejuni F38011
Protopolystoma xenopodis genome assembly P_xenopod	LM803609	75	43.17	5.02343	Polyopisthocotylea	Polystomatidae	Protopolystoma xenopodis
Strongyloides stercoralis genome assembly S_sterco	LL999123	87.5	23.02	5.02343	Rhabditida	Strongyloididae	Strongyloides stercoralis
Strongyloides stercoralis genome assembly S_sterco	LL999063	75.81	44.6	5.02343	Rhabditida	Strongyloididae	Strongyloides stercoralis
Schistosoma rodhaini genome assembly S_rodhaini_Bu	LL970877	87.5	23.02	5.02343	Strigeidida	Schistosomatidae	Schistosoma rodhaini
Schistosoma rodhaini genome assembly S_rodhaini_Bu	LL959020	100	15.83	5.02343	Strigeidida	Schistosomatidae	Schistosoma rodhaini
Schistosoma rodhaini genome assembly S_rodhaini_Bu	LL957480	87.5	23.02	5.02343	Strigeidida	Schistosomatidae	Schistosoma rodhaini
Schistosoma margrebowiei genome assembly S_margreb	LL878558	93.1	20.86	5.02343	Strigeidida	Schistosomatidae	Schistosoma margrebowiei
Schistosoma margrebowiei genome assembly S_margreb	LL877077	90.62	22.3	5.02343	Strigeidida	Schistosomatidae	Schistosoma margrebowiei
Caenorhabditis elegans genome assembly C_elegans_B	LK927976	70.54	76.98	5.02343	Rhabditida	Rhabditidae	Caenorhabditis elegans
Onchocerca flexuosa genome assembly O_flexuosa_Cor	LM541621	93.1	20.86	5.02343	Spirurida	Onchocercidae	Onchocerca flexuosa
Strongyloides papillosus genome assembly S_papillo	LM525557	92.59	19.42	5.02343	Rhabditida	Strongyloididae	Strongyloides papillosus
Cyprinus carpio genome assembly common carp genome	LN590688	73.61	51.8	5.02343	Cypriniformes	Cyprinidae	Cyprinus carpio
Plasmodium berghei ANKA genome assembly PBANKA01,	LK023128	74.03	52.52	5.02343	Haemosporida	Plasmodiidae	Plasmodium berghei ANKA
Campylobacter jejuni subsp. jejuni strain MTVDSCj2	CP008787	74.24	46.04	5.02343	Campylobacterales	Campylobacteraceae	Campylobacter jejuni subsp. jejuni
Solanum lycopersicum chromosome ch10, complete gen	HG975522	90	21.58	5.02343	Solanales	Solanaceae	Solanum lycopersicum
Campylobacter jejuni subsp. jejuni R14, complete g	CP005081	74.24	46.04	5.02343	Campylobacterales	Campylobacteraceae	Campylobacter jejuni subsp. jejuni R14
Campylobacter jejuni 32488, complete sequence	CP006006	74.24	46.04	5.02343	Campylobacterales	Campylobacteraceae	Campylobacter jejuni 32488
Vibrio parahaemolyticus O1:Kuk str. FDA_R31 chromo	CP006005	87.88	23.74	5.02343	Vibrionales	Vibrionaceae	Vibrio parahaemolyticus O1:Kuk str. FDA_R31
Cyprinus carpio clone 430105 microsatellite sequen	JN741051	74.65	51.08	5.02343	Cypriniformes	Cyprinidae	Cyprinus carpio
Schistosoma mansoni strain Puerto Rico chromosome	HE601626	100	15.83	5.02343	Strigeidida	Schistosomatidae	Schistosoma mansoni
Schistosoma mansoni strain Puerto Rico chromosome	HE601625	100	15.83	5.02343	Strigeidida	Schistosomatidae	Schistosoma mansoni
Dictyostelium purpureum hypothetical protein, mRNA	XM_003283811	75.76	46.76	5.02343	Dictyosteliida		Dictyostelium purpureum
Campylobacter jejuni subsp. jejuni S3, complete ge	CP001960	74.24	46.04	5.02343	Campylobacterales	Campylobacteraceae	Campylobacter jejuni subsp. jejuni S3
Campylobacter jejuni subsp. jejuni ICDCCJ07001, co	CP002029	74.24	46.04	5.02343	Campylobacterales	Campylobacteraceae	Campylobacter jejuni subsp. jejuni ICDCCJ07001
Campylobacter jejuni subsp. jejuni M1, complete ge	CP001900	74.24	46.04	5.02343	Campylobacterales	Campylobacteraceae	Campylobacter jejuni subsp. jejuni M1
Campylobacter jejuni subsp. jejuni IA3902, complet	CP001876	74.24	46.04	5.02343	Campylobacterales	Campylobacteraceae	Campylobacter jejuni subsp. jejuni IA3902
Plasmodium falciparum 3D7 chromosome 11, complete	AE014186	72.03	80.58	5.02343	Haemosporida	Plasmodiidae	Plasmodium falciparum 3D7
Pig DNA sequence from clone CH242−207C4 on chromos	CU618312	84.21	25.18	5.02343		Suidae	Sus scrofa
Populus trichocarpa clone POP016−O23, complete seq	AC213416	90.62	22.3	5.02343	Malpighiales	Salicaceae	Populus trichocarpa
Campylobacter jejuni subsp. jejuni 81116, complete	CP000814	74.24	46.04	5.02343	Campylobacterales	Campylobacteraceae	Campylobacter jejuni subsp. jejuni 81116
Vitis vinifera contig VV78X221063.5, whole genome	AM442003	90	21.58	5.02343	Vitales	Vitaceae	Vitis vinifera
Vitis vinifera, whole genome shotgun sequence, con	AM452356	90	21.58	5.02343	Vitales	Vitaceae	Vitis vinifera
Campylobacter jejuni subsp. jejuni 81−176, complet	CP000538	74.24	46.04	5.02343	Campylobacterales	Campylobacteraceae	Campylobacter jejuni subsp. jejuni 81−176
Solanum lycopersicum chromosome 10 clone C10SLe004	AC193781	90	21.58	5.02343	Solanales	Solanaceae	Solanum lycopersicum
Zebrafish DNA sequence from clone DKEY−193L8 in li	CR356247	93.1	20.86	5.02343	Cypriniformes	Cyprinidae	Danio rerio
Campylobacter jejuni RM1221, complete genome	CP000025	74.24	46.04	5.02343	Campylobacterales	Campylobacteraceae	Campylobacter jejuni RM1221
Homo sapiens chromosome 11, clone RP11−283H3, comp	AC036114	92.59	19.42	5.02343	Primates	Hominidae	Homo sapiens
Campylobacter jejuni isolate Contig_139 Cj81−094 g	AY681273	74.24	46.04	5.02343	Campylobacterales	Campylobacteraceae	Campylobacter jejuni subsp. jejuni 81−176
Zebrafish DNA sequence from clone DKEY−227H16 in l	BX248324	92.59	19.42	5.02343	Cypriniformes	Cyprinidae	Danio rerio
Caenorhabditis elegans YAC Y38H8A, complete sequen	AL021483	70.54	76.98	5.02343	Rhabditida	Rhabditidae	Caenorhabditis elegans
