# Supplementary material for: Testing the potential of a ribosomal 16S marker for DNA metabarcoding of insects
Source: PeerJ. 2016 Apr 19;4:e1966. doi: 10.7717/peerj.1966 (PMC4841222; doi:10.7717/peerj.1966)
Supplement: Supplemental Information 1 [file peerj-04-1966-s006.zip › scripts/5 match OTUs/blast_hits/OTU_86.csv_plot.pdf]

OTU_86.csv

Hansonoperla appalachia

Perlidae

Plecoptera

88.35

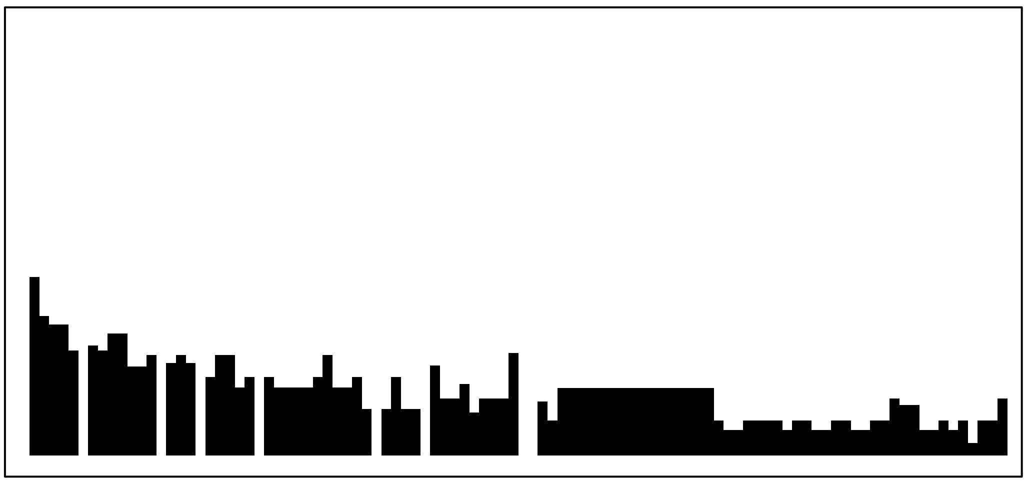

Hansonoperla appalachia 16S ribosomal RNA gene, pa	EF623249	88.35	62.73	1.8601e−25	Plecoptera	Perlidae	Hansonoperla appalachia
Acroneuria lycorias 16S ribosomal RNA gene, partia	EF623251	86.54	62.73	6.4924e−25	Plecoptera	Perlidae	Acroneuria lycorias
Megaleuctra kincaidi 16S ribosomal RNA gene, parti	EF623174	86.14	62.73	7.90936e−24	Plecoptera	Leuctridae	Megaleuctra kincaidi
Calineuria californica 16S ribosomal RNA gene, par	EF623239	86.14	62.73	9.63557e−23	Plecoptera	Perlidae	Calineuria californica
Dinocras cephalotes mitochondrion, complete genome	KF484757	84.91	62.73	3.36315e−22	Plecoptera	Perlidae	Dinocras cephalotes
Pteronarcys sachalina 16S ribosomal RNA gene, part	EF623264	79.56	84.47	3.36315e−22	Plecoptera	Pteronarcyidae	Pteronarcys sachalina
Pictetoperla sp. BYU_PL156 16S ribosomal RNA gene,	EF623247	85.15	62.73	3.36315e−22	Plecoptera	Perlidae	Pictetoperla sp. BYU_PL156
Dinocras cephalotes 16S ribosomal RNA gene, partia	EF623244	84.91	62.73	3.36315e−22	Plecoptera	Perlidae	Dinocras cephalotes
Cryptoperla japonica mitochondrial gene for 16S rR	AB711183	85.71	60.87	4.09715e−21	Plecoptera	Peltoperlidae	Cryptoperla japonica
Cryptoperla japonica mitochondrial gene for 16S rR	AB711182	85.71	60.87	4.09715e−21	Plecoptera	Peltoperlidae	Cryptoperla japonica
Dolichopoda pavesii voucher SPI 16S ribosomal RNA	EF216951	84.16	62.73	4.09715e−21	Orthoptera	Rhaphidophoridae	Dolichopoda pavesii
Nemoura cinerea 16S ribosomal RNA gene, partial se	EF623194	84.16	62.73	4.09715e−21	Plecoptera	Nemouridae	Nemoura cinerea
Scopura montana 16S ribosomal RNA gene, partial se	EF623265	84.69	60.87	1.43005e−20	Plecoptera		Scopura montana
Pteronarcys scotti 16S ribosomal RNA gene, partial	EF623263	78.83	84.47	1.43005e−20	Plecoptera	Pteronarcyidae	Pteronarcys scotti
Eccoptura xanthenes 16S ribosomal RNA gene, partia	EF623252	84.31	62.73	1.43005e−20	Plecoptera	Perlidae	Eccoptura xanthenes
Agnetina capitata 16S ribosomal RNA gene, partial	EF623241	84.69	60.87	1.43005e−20	Plecoptera	Perlidae	Agnetina capitata
Perlinella drymo 16S ribosomal RNA gene, partial s	EF623240	84.31	62.73	1.43005e−20	Plecoptera	Perlidae	Perlinella drymo
Pteronarcys princeps mitochondrion, complete genom	AY687866	78.83	84.47	1.43005e−20	Plecoptera	Pteronarcyidae	Pteronarcys princeps
Apteroperla tikumana mitochondrion, complete genom	KR604721	83.67	60.87	1.74215e−19	Plecoptera	Capniidae	Apteroperla tikumana
Cryptoperla japonica mitochondrial gene for 16S rR	AB711202	84.69	60.87	1.74215e−19	Plecoptera	Peltoperlidae	Cryptoperla japonica
Cryptoperla japonica mitochondrial gene for 16S rR	AB711184	84.69	60.87	1.74215e−19	Plecoptera	Peltoperlidae	Cryptoperla japonica
Notonemouridae sp. MD−2011 voucher ZMUC:JD−1507 16	JN615257	83.17	62.73	1.74215e−19	Plecoptera	Notonemouridae	Notonemouridae sp. MD−2011
Dolichopoda sp. MR−2008 16S ribosomal RNA gene, pa	EU887863	83.67	60.87	1.74215e−19	Orthoptera	Rhaphidophoridae	Dolichopoda lustriae
Pteronarcys californica 16S ribosomal RNA gene, pa	EF623261	78.1	84.47	1.74215e−19	Plecoptera	Pteronarcyidae	Pteronarcys californica
Nemurella pictetii 16S ribosomal RNA gene, partial	EF623192	83.67	60.87	1.74215e−19	Plecoptera	Nemouridae	Nemurella pictetii
Prostoia besametsa 16S ribosomal RNA gene, partial	EF623188	83.17	62.73	1.74215e−19	Plecoptera	Nemouridae	Prostoia besametsa
Nemoura trispinosa 16S ribosomal RNA gene, partial	EF623187	83.17	62.73	1.74215e−19	Plecoptera	Nemouridae	Nemoura trispinosa
Paranemoura claasseni 16S ribosomal RNA gene, part	EF623185	83.17	62.73	1.74215e−19	Plecoptera	Nemouridae	Paranemoura claasseni
Shipsa rotunda 16S ribosomal RNA gene, partial seq	EF623184	83.17	62.73	1.74215e−19	Plecoptera	Nemouridae	Shipsa rotunda
Malenka californica 16S ribosomal RNA gene, partia	EF623182	83.67	60.87	1.74215e−19	Plecoptera	Nemouridae	Malenka californica
Osmylus sp. EH−2004 16S ribosomal RNA gene, partia	AY620146	84.69	60.87	1.74215e−19	Neuroptera	Osmylidae	Osmylus sp. EH−2004
Cryptoperla sp. YK−2012−1 mitochondrial gene for 1	AB711243	83.17	62.73	2.12238e−18	Plecoptera	Peltoperlidae	Cryptoperla sp. YK−2012−1
Cryptoperla ishigakiensis mitochondrial gene for 1	AB711242	83.17	62.73	2.12238e−18	Plecoptera	Peltoperlidae	Cryptoperla ishigakiensis
Cryptoperla japonica mitochondrial gene for 16S rR	AB711189	83.67	60.87	2.12238e−18	Plecoptera	Peltoperlidae	Cryptoperla japonica
Doddsia occidentalis 16S ribosomal RNA gene, parti	EF623270	82.18	62.73	2.12238e−18	Plecoptera	Taeniopterygidae	Doddsia occidentalis
Brachyptera seticornis 16S ribosomal RNA gene, par	EF623268	79.83	73.91	2.12238e−18	Plecoptera	Taeniopterygidae	Brachyptera seticornis
Pteronarcella badia 16S ribosomal RNA gene, partia	EF623262	82.18	62.73	2.12238e−18	Plecoptera	Pteronarcyidae	Pteronarcella badia
Cosumnoperla hypocrena 16S ribosomal RNA gene, par	EF623226	83.67	60.87	2.12238e−18	Plecoptera	Perlodidae	Cosumnoperla hypocrena
Lednia tumana 16S ribosomal RNA gene, partial sequ	EF623197	82.18	62.73	2.12238e−18	Plecoptera	Nemouridae	Lednia tumana
Protonemura meyeri 16S ribosomal RNA gene, partial	EF623193	82.18	62.73	2.12238e−18	Plecoptera	Nemouridae	Protonemura meyeri
Cosmioperla australis 16S ribosomal RNA gene, part	EF623148	76.67	92.55	2.12238e−18	Plecoptera	Eustheniidae	Cosmioperla australis
Eupholidoptera chabrieri voucher 173 16S ribosomal	EU120792	84.21	59.01	2.12238e−18	Orthoptera	Tettigoniidae	Eupholidoptera chabrieri
Dolichopoda steriotisi voucher ANT 16S ribosomal R	EF216955	82.65	60.87	7.40782e−18	Orthoptera	Rhaphidophoridae	Dolichopoda steriotisi
Bolotoperla rossi 16S ribosomal RNA gene, partial	EF623269	82.65	60.87	7.40782e−18	Plecoptera	Taeniopterygidae	Bolotoperla rossi
Kempnyella sp. BYU_PL155 16S ribosomal RNA gene, p	EF623246	83.33	62.73	7.40782e−18	Plecoptera	Perlidae	Kempnyella sp. BYU_PL155
Udamocercia sp. BYU_PL150 16S ribosomal RNA gene,	EF623201	82	62.11	7.40782e−18	Plecoptera	Notonemouridae	Udamocercia sp. BYU_PL150
Nanonemoura wahkeena 16S ribosomal RNA gene, parti	EF623198	82.65	60.87	7.40782e−18	Plecoptera	Nemouridae	Nanonemoura wahkeena
Ostrocerca foersteri 16S ribosomal RNA gene, parti	EF623191	82.65	60.87	7.40782e−18	Plecoptera	Nemouridae	Ostrocerca foersteri
Visoka cataractae 16S ribosomal RNA gene, partial	EF623189	82.65	60.87	7.40782e−18	Plecoptera	Nemouridae	Visoka cataractae
Grylloblatta sp. BYU_gb29.1 16S ribosomal RNA gene	DQ457261	84.78	57.14	7.40782e−18	Grylloblattodea	Grylloblattidae	Grylloblatta sp. BYU_gb29.1
Dolichopoda sp. VAR 16S ribosomal RNA gene, partia	EU887865	76.81	84.47	2.58558e−17	Orthoptera	Rhaphidophoridae	Dolichopoda sp. VAR
Dolichopoda sp. TAY 16S ribosomal RNA gene, partia	EU887864	76.81	84.47	2.58558e−17	Orthoptera	Rhaphidophoridae	Dolichopoda sp. TAY
Perlesta decipines 16S ribosomal RNA gene, partial	EF623242	82.52	62.73	2.58558e−17	Plecoptera	Perlidae	Perlesta decipines
Mesocapnia arizonensis mitochondrion, partial geno	KP642637	81.63	60.87	9.02457e−17	Plecoptera	Capniidae	Mesocapnia arizonensis
Eupholidoptera uvarovi isolate KAS1 16S ribosomal	KC852312	83.16	59.01	9.02457e−17	Orthoptera	Tettigoniidae	Eupholidoptera uvarovi
Eupholidoptera smyrnensis isolate KOY1 16S ribosom	KC852309	83.16	59.01	9.02457e−17	Orthoptera	Tettigoniidae	Eupholidoptera smyrnensis
Eupholidoptera megastyla isolate ALE1 16S ribosoma	KC852308	83.16	59.01	9.02457e−17	Orthoptera	Tettigoniidae	Eupholidoptera megastyla
Eupholidoptera hesperica isolate MAR3 16S ribosoma	KC852304	83.16	59.01	9.02457e−17	Orthoptera	Tettigoniidae	Eupholidoptera hesperica
Eupholidoptera hesperica isolate MAR1 16S ribosoma	KC852302	83.16	59.01	9.02457e−17	Orthoptera	Tettigoniidae	Eupholidoptera hesperica
Eupholidoptera danconai isolate ROG1 16S ribosomal	KC852297	83.16	59.01	9.02457e−17	Orthoptera	Tettigoniidae	Eupholidoptera danconai
Eupholidoptera danconai isolate TRL2 16S ribosomal	KC852296	83.16	59.01	9.02457e−17	Orthoptera	Tettigoniidae	Eupholidoptera danconai
Eupholidoptera danconai isolate JEN1 16S ribosomal	KC852294	83.16	59.01	9.02457e−17	Orthoptera	Tettigoniidae	Eupholidoptera danconai
Eupholidoptera danconai isolate PIE1 16S ribosomal	KC852292	83.16	59.01	9.02457e−17	Orthoptera	Tettigoniidae	Eupholidoptera danconai
Eupholidoptera bimucronata isolate MAD1 16S riboso	KC852289	83.16	59.01	9.02457e−17	Orthoptera	Tettigoniidae	Eupholidoptera bimucronata
Eupholidoptera bimucronata isolate PTR1 16S riboso	KC852287	83.16	59.01	9.02457e−17	Orthoptera	Tettigoniidae	Eupholidoptera bimucronata
Eupholidoptera bimucronata isolate GIB3 16S riboso	KC852285	83.16	59.01	9.02457e−17	Orthoptera	Tettigoniidae	Eupholidoptera bimucronata
Eupholidoptera bimucronata isolate GIB2 16S riboso	KC852284	83.16	59.01	9.02457e−17	Orthoptera	Tettigoniidae	Eupholidoptera bimucronata
Eupholidoptera bimucronata isolate GIB1 16S riboso	KC852283	83.16	59.01	9.02457e−17	Orthoptera	Tettigoniidae	Eupholidoptera bimucronata
Eupholidoptera magnifica isolate ARB1 16S ribosoma	KC852277	83.16	59.01	9.02457e−17	Orthoptera	Tettigoniidae	Eupholidoptera magnifica
Eupholidoptera magnifica isolate BIG1 16S ribosoma	KC852275	83.16	59.01	9.02457e−17	Orthoptera	Tettigoniidae	Eupholidoptera magnifica
Allocapnia recta isolate H19 16S ribosomal RNA gen	KC881054	81.63	60.87	9.02457e−17	Plecoptera	Capniidae	Allocapnia recta
Allocapnia recta isolate H18 16S ribosomal RNA gen	KC881053	81.19	62.73	9.02457e−17	Plecoptera	Capniidae	Allocapnia recta
Allocapnia recta isolate H17 16S ribosomal RNA gen	KC881052	81.19	62.73	9.02457e−17	Plecoptera	Capniidae	Allocapnia recta
Allocapnia recta isolate H16 16S ribosomal RNA gen	KC881051	81.63	60.87	9.02457e−17	Plecoptera	Capniidae	Allocapnia recta
Allocapnia recta isolate H15 16S ribosomal RNA gen	KC881050	81.63	60.87	9.02457e−17	Plecoptera	Capniidae	Allocapnia recta
Allocapnia recta isolate H14 16S ribosomal RNA gen	KC881049	81.63	60.87	9.02457e−17	Plecoptera	Capniidae	Allocapnia recta
Allocapnia recta isolate H13 16S ribosomal RNA gen	KC881048	81.63	60.87	9.02457e−17	Plecoptera	Capniidae	Allocapnia recta
Allocapnia recta isolate H12 16S ribosomal RNA gen	KC881047	81.19	62.73	9.02457e−17	Plecoptera	Capniidae	Allocapnia recta
Allocapnia recta isolate H11 16S ribosomal RNA gen	KC881046	81.63	60.87	9.02457e−17	Plecoptera	Capniidae	Allocapnia recta
Allocapnia recta isolate H10 16S ribosomal RNA gen	KC881045	81.63	60.87	9.02457e−17	Plecoptera	Capniidae	Allocapnia recta
Allocapnia recta isolate H9 16S ribosomal RNA gene	KC881044	81.19	62.73	9.02457e−17	Plecoptera	Capniidae	Allocapnia recta
Allocapnia recta isolate H8 16S ribosomal RNA gene	KC881043	81.19	62.73	9.02457e−17	Plecoptera	Capniidae	Allocapnia recta
Allocapnia recta isolate H7 16S ribosomal RNA gene	KC881042	81.63	60.87	9.02457e−17	Plecoptera	Capniidae	Allocapnia recta
Allocapnia recta isolate H6 16S ribosomal RNA gene	KC881041	81.63	60.87	9.02457e−17	Plecoptera	Capniidae	Allocapnia recta
Allocapnia recta isolate H5 16S ribosomal RNA gene	KC881040	81.19	62.73	9.02457e−17	Plecoptera	Capniidae	Allocapnia recta
Allocapnia recta isolate H4 16S ribosomal RNA gene	KC881039	81.19	62.73	9.02457e−17	Plecoptera	Capniidae	Allocapnia recta
Allocapnia recta isolate H3 16S ribosomal RNA gene	KC881038	81.63	60.87	9.02457e−17	Plecoptera	Capniidae	Allocapnia recta
Allocapnia recta isolate H1 16S ribosomal RNA gene	KC881036	81.63	60.87	9.02457e−17	Plecoptera	Capniidae	Allocapnia recta
Cryptoperla sp. YK−2012−2 mitochondrial gene for 1	AB711244	82.65	60.87	9.02457e−17	Plecoptera	Peltoperlidae	Cryptoperla sp. YK−2012−2
Micropathus tasmaniensis voucher JNC 16S ribosomal	HM594507	82.35	62.73	9.02457e−17	Orthoptera	Rhaphidophoridae	Micropathus tasmaniensis
Micropathus tasmaniensis voucher IDB 16S ribosomal	HM594505	82.35	62.73	9.02457e−17	Orthoptera	Rhaphidophoridae	Micropathus tasmaniensis
Dolichopoda hussoni voucher IZB 16S ribosomal RNA	EF216973	81.19	62.73	9.02457e−17	Orthoptera	Rhaphidophoridae	Dolichopoda hussoni
Dolichopoda thasosensis voucher DRA 16S ribosomal	EF216956	81.19	62.73	9.02457e−17	Orthoptera	Rhaphidophoridae	Dolichopoda thasosensis
Dolichopoda kiriakii voucher AGH 16S ribosomal RNA	EF216954	81.63	60.87	9.02457e−17	Orthoptera	Rhaphidophoridae	Dolichopoda kiriakii
Strophopteryx appalachia 16S ribosomal RNA gene, p	EF623271	81.19	62.73	9.02457e−17	Plecoptera	Taeniopterygidae	Strophopteryx appalachia
Oemopteryx vanduzeea 16S ribosomal RNA gene, parti	EF623266	81.63	60.87	9.02457e−17	Plecoptera	Taeniopterygidae	Oemopteryx vanduzeea
Beloneuria stewarti 16S ribosomal RNA gene, partia	EF623248	80.58	63.98	9.02457e−17	Plecoptera	Perlidae	Beloneuria stewarti
Hesperoperla pacifica 16S ribosomal RNA gene, part	EF623238	81.63	60.87	9.02457e−17	Plecoptera	Perlidae	Hesperoperla pacifica
Zapada cinctipes 16S ribosomal RNA gene, partial s	EF623183	81.63	60.87	9.02457e−17	Plecoptera	Nemouridae	Zapada cinctipes
Moselia infuscata 16S ribosomal RNA gene, partial	EF623175	82.65	60.87	9.02457e−17	Plecoptera	Leuctridae	Moselia infuscata
