# Supplementary material for: Testing the potential of a ribosomal 16S marker for DNA metabarcoding of insects
Source: PeerJ. 2016 Apr 19;4:e1966. doi: 10.7717/peerj.1966 (PMC4841222; doi:10.7717/peerj.1966)
Supplement: Supplemental Information 1 [file peerj-04-1966-s006.zip › scripts/5 match OTUs/blast_hits/OTU_87.csv_plot.pdf]

OTU_87.csv

Apis andreniformis

Apidae

Hymenoptera

72.19

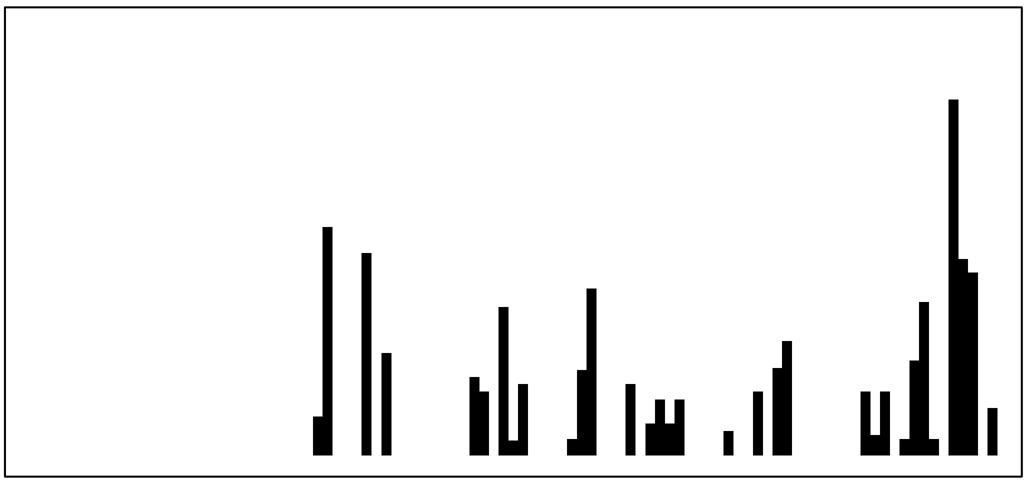

Apis andreniformis mitochondrion, partial genome	KF736157	72.19	84.83	7.87418e−12	Hymenoptera	Apidae	Apis andreniformis
Apis andreniformis isolate HX1 16S large subunit r	JQ317321	72.19	84.83	7.87418e−12	Hymenoptera	Apidae	Apis andreniformis
Apis andreniformis voucher MP145 16S ribosomal RNA	EU162957	72.19	84.83	7.87418e−12	Hymenoptera	Apidae	Apis andreniformis
Apis andreniformis isolate Thailand5 large subunit	FJ348343	72.19	84.83	7.87418e−12	Hymenoptera	Apidae	Apis andreniformis
Apis andreniformis large subunit ribosomal RNA (rr	AY588425	72.19	84.83	7.87418e−12	Hymenoptera	Apidae	Apis andreniformis
Bombus pullatus 16S ribosomal RNA gene, partial se	AY268411	75.63	64.04	4.07892e−09	Hymenoptera	Apidae	Bombus pullatus
Laryngodus australiae voucher AMNH_PBI 00088391 16	FJ824817	70	99.44	4.96915e−08	Hemiptera	Rhyparochromidae	Laryngodus australiae
Selenopsocus sp. KY198 mitochondrial gene for 16S	AB918939	73.73	64.04	1.7344e−07	Psocoptera	Troctopsocidae	Selenopsocus sp. KY198
Bombus hortulanus voucher SC200 16S ribosomal RNA	DQ788025	72.5	66.85	1.7344e−07	Hymenoptera	Apidae	Bombus hortulanus
Bombus hortulanus voucher SC200 16S ribosomal RNA	AY737342	72.5	66.85	1.7344e−07	Hymenoptera	Apidae	Bombus hortulanus
Selenopsocus sp. KY−2004 16S ribosomal RNA gene, p	DQ104761	73.73	64.04	1.7344e−07	Psocoptera	Troctopsocidae	Selenopsocus sp. KY−2004
Bombus eximius voucher SC380 16S ribosomal RNA gen	FJ159115	71.43	70.79	6.05366e−07	Hymenoptera	Apidae	Bombus eximius
Laryngodus luteomaculatus voucher AMNH_ENT 0002413	FJ824820	69.27	99.44	6.05366e−07	Hemiptera	Rhyparochromidae	Laryngodus luteomaculatus
Bombus eximius voucher SC049 16S ribosomal RNA gen	DQ788005	71.43	70.79	6.05366e−07	Hymenoptera	Apidae	Bombus eximius
Bombus eximius voucher SC049 16S ribosomal RNA gen	AY737335	71.43	70.79	6.05366e−07	Hymenoptera	Apidae	Bombus eximius
Bombus bellicosus 16S ribosomal RNA gene, partial	AY268399	76.24	55.06	6.05366e−07	Hymenoptera	Apidae	Bombus bellicosus
Apis koschevnikovi haplotype 2 16S ribosomal RNA g	AF153097	72.66	71.91	6.05366e−07	Hymenoptera	Apidae	Apis koschevnikovi
Bombus nr. avanus HMH−2009 voucher SC306 16S ribos	FJ159117	74.17	63.48	2.11294e−06	Hymenoptera	Apidae	Bombus nr. avanus HMH−2009
Bombus ecuadorius voucher SC135 16S ribosomal RNA	DQ788001	71.67	66.85	2.11294e−06	Hymenoptera	Apidae	Bombus ecuadorius
Theobroma cacao Phosphatase 2c, putative isoform 1	XM_007013023	72.11	76.4	7.37487e−06	Malvales	Malvaceae	Theobroma cacao
Zebrafish DNA sequence from clone CH1073−102D20 in	FP017190	68.62	98.88	7.37487e−06	Cypriniformes	Cyprinidae	Danio rerio
Laryngodus cervantes voucher AMNH_PBI 00140618 16S	FJ824819	68.72	99.44	2.57408e−05	Hemiptera	Rhyparochromidae	Laryngodus cervantes
Laryngodus cervantes voucher AMNH_ENT 00024139 16S	FJ824818	68.72	99.44	2.57408e−05	Hemiptera	Rhyparochromidae	Laryngodus cervantes
Bombus avanus voucher SC272 16S ribosomal RNA gene	EF032344	73.33	63.48	2.57408e−05	Hymenoptera	Apidae	Bombus avanus
Bombus vogti voucher SC172 16S ribosomal RNA gene,	DQ788130	71.43	66.85	2.57408e−05	Hymenoptera	Apidae	Bombus vogti
Laryngodus sp. WCW−2003 16S ribosomal RNA gene, pa	AY252680	68.72	99.44	2.57408e−05	Hemiptera	Rhyparochromidae	Laryngodus sp. WCW−2003
Arabidopsis thaliana chromosome 3, complete sequen	CP002686	75	52.81	8.98443e−05	Brassicales	Brassicaceae	Arabidopsis thaliana
Cotesia anisotae partial mitochondrial 16S rRNA ge	AJ535915	73.17	67.98	8.98443e−05	Hymenoptera	Braconidae	Cotesia anisotae
Arabidopsis thaliana genomic DNA, chromosome 3, TA	AB025608	75	52.81	8.98443e−05	Brassicales	Brassicaceae	Arabidopsis thaliana
Typhlatya miravetensis mitochondrial partial 16S r	HE801012	81.82	30.9	0.000313587	Decapoda	Atyidae	Typhlatya miravetensis
Cyanophora paradoxa strain NIES−763 cyanelle, part	KM198929	90.7	24.16	0.00109453		Cyanophoraceae	Cyanophora paradoxa
Dracunculus medinensis genome assembly D_medinensi	LK978299	72.97	61.24	0.00109453	Spirurida	Dracunculidae	Dracunculus medinensis
Leiodytes sp. BMNH 681747 partial 16S rRNA gene	AJ850327	75.29	47.75	0.00109453	Coleoptera	Dytiscidae	Leiodytes sp. BMNH 681747
Trifolium pratense genome assembly redclover, chro	LN846352	72.55	56.74	0.00382028	Fabales	Fabaceae	Trifolium pratense
Dracunculus medinensis genome assembly D_medinensi	LK978199	89.47	21.35	0.00382028	Spirurida	Dracunculidae	Dracunculus medinensis
Strongyloides ratti genome assembly S_ratti_ED321	LN609530	69.38	79.78	0.00382028	Rhabditida	Strongyloididae	Strongyloides ratti
Solanum lycopersicum chromosome ch06, complete gen	HG975518	84.78	25.84	0.00382028	Solanales	Solanaceae	Solanum lycopersicum
Solanum lycopersicum chromosome ch05, complete gen	HG975517	75.53	51.12	0.00382028	Solanales	Solanaceae	Solanum lycopersicum
Sulabanus sp. UPOL A00075 voucher UPOLA00075 16S r	KC538751	78.67	39.89	0.00382028	Coleoptera	Lycidae	Sulabanus sp. UPOL A00075
Sulabanus sp. UPOL A00066 voucher UPOLA00066 16S r	KC538743	78.67	39.89	0.00382028	Coleoptera	Lycidae	Sulabanus sp. UPOL A00066
Hipparchia autonoe mitochondrion, complete genome	GQ868707	70.5	73.6	0.00382028	Lepidoptera	Nymphalidae	Hipparchia autonoe
Cymus sp. WCW−2003e 16S ribosomal RNA gene, partia	AY252679	68.16	100	0.00382028	Hemiptera	Lygaeidae	Cymus sp. WCW−2003e
Clostridium perfringens plasmid pJIR4150, complete	LN835295	70	79.78	0.0133341	Clostridiales	Clostridiaceae	Clostridium perfringens
Protopolystoma xenopodis genome assembly P_xenopod	LM751559	75.32	37.64	0.0133341	Polyopisthocotylea	Polystomatidae	Protopolystoma xenopodis
Carposina sasakii mitochondrion, complete genome	HQ840719	71.55	62.92	0.0133341	Lepidoptera	Carposinidae	Carposina sasakii
Apostasia sp. G244 hypothetical protein Ycf1 (ycf1	JN181532	83.67	26.97	0.0133341	Asparagales	Orchidaceae	Apostasia sp. G244
Thermoanaerobacterium thermosaccharolyticum DSM 57	CP002171	82.98	26.4	0.0133341	Thermoanaerobacterales	Thermoanaerobacterales Family III. Incertae Sedis	Thermoanaerobacterium thermosaccharolyticum DSM 571
Rivellia flaviventris 16S ribosomal RNA gene, part	DQ534713	67.65	76.4	0.0133341	Diptera	Platystomatidae	Rivellia flaviventris
Genomic sequence for Brassica oleracea, clone B21H	AC122543	86.96	25.84	0.0133341	Brassicales	Brassicaceae	Brassica oleracea
Human DNA sequence from clone RP11−412H14 on chrom	AL135789	80.7	32.02	0.0133341	Primates	Hominidae	Homo sapiens
Ovis canadensis canadensis isolate 43U chromosome	CP011891	83.33	26.4	0.0465405		Bovidae	Ovis canadensis canadensis
Trifolium pratense genome assembly redclover, chro	LN846355	72.55	56.74	0.0465405	Fabales	Fabaceae	Trifolium pratense
Trifolium pratense genome assembly redclover, chro	LN846354	72.55	56.74	0.0465405	Fabales	Fabaceae	Trifolium pratense
Strongyloides stercoralis genome assembly S_sterco	LL999054	79.71	38.2	0.0465405	Rhabditida	Strongyloididae	Strongyloides stercoralis
Syphacia muris genome assembly S_muris_Valencia ,s	LK996816	78.57	31.46	0.0465405	Oxyurida	Oxyuridae	Syphacia muris
Dracunculus medinensis genome assembly D_medinensi	LK978425	80.77	27.53	0.0465405	Spirurida	Dracunculidae	Dracunculus medinensis
Dracunculus medinensis genome assembly D_medinensi	LK978201	84	28.09	0.0465405	Spirurida	Dracunculidae	Dracunculus medinensis
Dracunculus medinensis genome assembly D_medinensi	LK978249	87.8	23.03	0.0465405	Spirurida	Dracunculidae	Dracunculus medinensis
Dracunculus medinensis genome assembly D_medinensi	LK978345	73.79	53.37	0.0465405	Spirurida	Dracunculidae	Dracunculus medinensis
Brugia pahangi genome assembly B_pahangi_Glasgow ,	LK974278	77.22	44.38	0.0465405	Spirurida	Onchocercidae	Brugia pahangi
Wuchereria bancrofti genome assembly W_bancrofti_J	LM004263	77.14	39.33	0.0465405	Spirurida	Onchocercidae	Wuchereria bancrofti
Cyprinus carpio genome assembly common carp genome	LN591153	83.33	28.09	0.0465405	Cypriniformes	Cyprinidae	Cyprinus carpio
Cyprinus carpio genome assembly common carp genome	LN590702	69.03	81.46	0.0465405	Cypriniformes	Cyprinidae	Cyprinus carpio
Solanum lycopersicum chromosome ch09, complete gen	HG975521	81.48	28.65	0.0465405	Solanales	Solanaceae	Solanum lycopersicum
Solanum pennellii chromosome ch10, complete genome	HG975449	82.61	25.84	0.0465405	Solanales	Solanaceae	Solanum pennellii
Solanum pennellii chromosome ch09, complete genome	HG975448	81.48	28.65	0.0465405	Solanales	Solanaceae	Solanum pennellii
Solanum pennellii chromosome ch06, complete genome	HG975445	82.61	25.84	0.0465405	Solanales	Solanaceae	Solanum pennellii
Solanum pennellii chromosome ch05, complete genome	HG975444	77.03	39.89	0.0465405	Solanales	Solanaceae	Solanum pennellii
Solanum pennellii chromosome ch03, complete genome	HG975442	75.49	54.49	0.0465405	Solanales	Solanaceae	Solanum pennellii
Theobroma cacao Uncharacterized protein (TCM_00992	XM_007044293	69.18	76.4	0.0465405	Malvales	Malvaceae	Theobroma cacao
Theobroma cacao SAC domain−containing protein 8 is	XM_007026601	69.01	92.7	0.0465405	Malvales	Malvaceae	Theobroma cacao
Citrus clementina hypothetical protein (CICLE_v100	XM_006441473	81.13	29.78	0.0465405	Sapindales	Rutaceae	Citrus clementina
Sulabanus lineatus voucher UPOLMD0034 16S ribosoma	KC538784	73.42	44.38	0.0465405	Coleoptera	Lycidae	Sulabanus lineatus
Candidatus Carsonella ruddii HT isolate Thao2000,	CP003544	72.12	57.87	0.0465405	Oceanospirillales	Halomonadaceae	Candidatus Carsonella ruddii HT isolate Thao2000
Mus musculus targeted deletion, lacZ−tagged mutant	JN958043	82.98	26.4	0.0465405	Rodentia	Muridae	Mus musculus
Glycine max strain Williams 82 clone GM_WBb0027P13	AC235233	67.72	82.02	0.0465405	Fabales	Fabaceae	Glycine max
Populus trichocarpa clone POP037−E08, complete seq	AC214417	84.09	24.72	0.0465405	Malpighiales	Salicaceae	Populus trichocarpa
Vitis vinifera contig VV78X123196.5, whole genome	AM486567	85.37	23.03	0.0465405	Vitales	Vitaceae	Vitis vinifera
Vitis vinifera contig VV78X083924.7, whole genome	AM424826	79.03	32.58	0.0465405	Vitales	Vitaceae	Vitis vinifera
Vitis vinifera contig VV78X013765.8, whole genome	AM457032	77.46	38.2	0.0465405	Vitales	Vitaceae	Vitis vinifera
Vitis vinifera, whole genome shotgun sequence, con	AM455437	78.12	33.71	0.0465405	Vitales	Vitaceae	Vitis vinifera
Bigelowiella natans nucleomorph chromosome 2, comp	DQ158857	79.69	35.39	0.0465405			Bigelowiella natans
Mus musculus BAC clone RP23−425F8 from chromosome	AC147376	77.61	37.64	0.0465405	Rodentia	Muridae	Mus musculus
Mus musculus BAC clone RP24−449H18 from 5, complet	AC122045	77.61	37.64	0.0465405	Rodentia	Muridae	Mus musculus
Lotus japonicus genomic DNA, chromosome 5, clone:	AP004969	79.1	37.64	0.0465405	Fabales	Fabaceae	Lotus japonicus
Mouse DNA sequence from clone RP23−400P17 on chrom	AL662887	82.98	26.4	0.0465405	Rodentia	Muridae	Mus musculus
PREDICTED: Zonotrichia albicollis F−box and leucin	XM_005487504	80.95	32.58	0.162442	Passeriformes	Passerellidae	Zonotrichia albicollis
PREDICTED: Bactrocera oleae trimethylguanosine syn	XM_014242963	82.98	26.4	0.162442	Diptera	Tephritidae	Bactrocera oleae
Drosophila busckii chromosome X sequence	CP012528	76.71	37.64	0.162442	Diptera	Drosophilidae	Drosophila busckii
Ovis canadensis canadensis isolate 43U chromosome	CP011889	80.77	28.09	0.162442		Bovidae	Ovis canadensis canadensis
Strongyloides venezuelensis genome assembly S_vene	LM524987	84.44	25.28	0.162442	Rhabditida	Strongyloididae	Strongyloides venezuelensis
Strongylus vulgaris genome assembly S_vulgaris_Ken	LM216158	87.18	21.91	0.162442	Rhabditida	Strongylidae	Strongylus vulgaris
Strongylus vulgaris genome assembly S_vulgaris_Ken	LM211431	80.77	28.65	0.162442	Rhabditida	Strongylidae	Strongylus vulgaris
Toxocara canis genome assembly T_canis_Equador ,sc	LM047765	72.13	66.85	0.162442	Ascaridida	Toxocaridae	Toxocara canis
Wuchereria bancrofti genome assembly W_bancrofti_J	LM000784	96.67	16.29	0.162442	Spirurida	Onchocercidae	Wuchereria bancrofti
Trichobilharzia regenti genome assembly T_regenti_	LL058671	89.19	20.79	0.162442	Strigeidida	Schistosomatidae	Trichobilharzia regenti
Syphacia muris genome assembly S_muris_Valencia ,s	LK996104	88.57	19.66	0.162442	Oxyurida	Oxyuridae	Syphacia muris
Rhabditophanes sp. KR3021 genome assembly Rhabdito	LK995736	70.69	62.92	0.162442	Rhabditida	Alloionematidae	Rhabditophanes sp. KR3021
Thelazia callipaeda genome assembly T_callipaeda_T	LK979729	82.22	22.47	0.162442	Spirurida	Thelaziidae	Thelazia callipaeda
Dracunculus medinensis genome assembly D_medinensi	LK978668	79.69	34.83	0.162442	Spirurida	Dracunculidae	Dracunculus medinensis
