# Supplementary material for: Testing the potential of a ribosomal 16S marker for DNA metabarcoding of insects
Source: PeerJ. 2016 Apr 19;4:e1966. doi: 10.7717/peerj.1966 (PMC4841222; doi:10.7717/peerj.1966)
Supplement: Supplemental Information 1 [file peerj-04-1966-s006.zip › scripts/5 match OTUs/blast_hits/OTU_88.csv_plot.pdf]

OTU_88.csv

Ptychoptera sp. ATB−2011

Ptychopteridae

Diptera

91.14

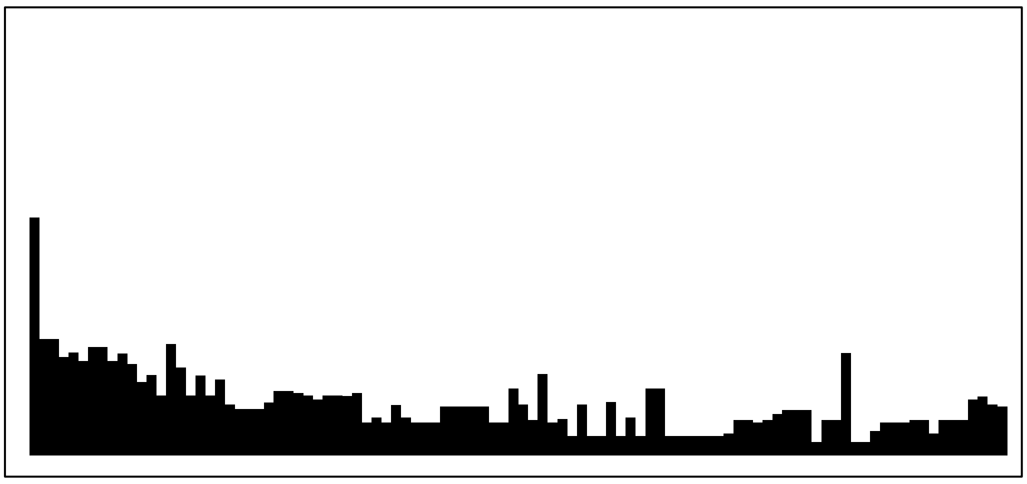

Ptychoptera sp. ATB−2011 mitochondrion, complete g	JN861744	91.14	100	1.29456e−52	Diptera	Ptychopteridae	Ptychoptera sp. ATB−2011
Trichocera brevicornis 16S ribosomal RNA gene, par	KC177458	85.44	100	1.13558e−40	Diptera	Trichoceridae	Trichocera brevicornis
Trichocera bimacula mitochondrion, complete genome	JN861750	85.44	100	1.13558e−40	Diptera	Trichoceridae	Trichocera bimacula
Edwardsina gigantea 16S ribosomal RNA gene, partia	KC177460	84.62	99.36	4.82863e−39	Diptera	Blephariceridae	Edwardsina gigantea
Pedicia albivitta voucher O'Grady 200012 16S ribos	EU005435	84.81	100	4.82863e−39	Diptera	Pediciidae	Pedicia albivitta
Bolitophila cinerea voucher JSB1 16S ribosomal RNA	KJ136712	84.42	97.45	5.88247e−38	Diptera	Mycetophilidae	Bolitophila cinerea
Eudicrana sp. DK−2013 voucher JSS12 16S ribosomal	KC435571	85.06	97.45	5.88247e−38	Diptera	Mycetophilidae	Eudicrana sp. DK−2013
Urytalpa macrocera 16S ribosomal RNA gene, partial	FJ172025	85.06	97.45	5.88247e−38	Diptera	Keroplatidae	Urytalpa macrocera
Bolitophila sp. ER−2009 16S ribosomal RNA gene, pa	FJ172021	84.42	97.45	5.88247e−38	Diptera	Mycetophilidae	Bolitophila sp. ER−2009
Speolepta leptogaster 16S ribosomal RNA gene, part	FJ172033	84.77	96.18	2.05319e−37	Diptera	Mycetophilidae	Speolepta leptogaster
Tipula sp. PMO 200011 16S ribosomal RNA gene, part	EU005437	84.28	100	2.05319e−37	Diptera	Tipulidae	Tipula sp. PMO 200011
Derodontus emaculatus voucher BMNH:679374 16S ribo	DQ202609	83.44	100	2.05319e−37	Coleoptera		Derodontus emaculatus
Bolitophila hybrida 16S ribosomal RNA gene, partia	FJ172002	83.77	97.45	2.50129e−36	Diptera	Mycetophilidae	Bolitophila hybrida
Parochlus steinenii isolate Sejong01 mitochondrion	KT003702	82.8	100	3.0472e−35	Diptera	Chironomidae	Parochlus steinenii
Boletina gripha isolate 057362 16S ribosomal RNA g	HQ230382	85.21	90.45	3.0472e−35	Diptera	Mycetophilidae	Boletina gripha
Gnoriste longirostris isolate 014972 16S ribosomal	HQ230376	84.11	96.18	3.0472e−35	Diptera	Mycetophilidae	Gnoriste longirostris
Simulium rufibasis 16S ribosomal RNA gene, partial	GQ866016	82.8	100	3.0472e−35	Diptera	Simuliidae	Simulium rufibasis
Clusiodes ruficollis 16S ribosomal RNA gene, parti	EU268538	83.75	99.36	3.0472e−35	Diptera	Clusiidae	Clusiodes ruficollis
Simulium rufibasis mitochondrial gene for 16S rRNA	AB093121	82.8	100	3.0472e−35	Diptera	Simuliidae	Simulium rufibasis
Limnophora riparia 16S ribosomal RNA gene, partial	FJ025449	83.54	99.36	3.71225e−34	Diptera	Muscidae	Limnophora riparia
Limonia tahitiensis voucher O'Grady 202004 16S rib	EU005425	82.39	100	3.71225e−34	Diptera	Limoniidae	Dicranomyia tahitiensis
Simulium nikkoense mitochondrial gene for 16S rRNA	AB093116	82.17	100	3.71225e−34	Diptera	Simuliidae	Simulium nikkoense
Simulium dumogaense mitochondrial gene for 16S rRN	AB093106	82.17	100	3.71225e−34	Diptera	Simuliidae	Simulium dumogaense
Simulium konoi mitochondrial gene for 16S ribosoma	AB056741	82.17	100	3.71225e−34	Diptera	Simuliidae	Simulium konoi
Bolitophila occlusa voucher JSB2 16S ribosomal RNA	KJ136713	82.47	97.45	1.2957e−33	Diptera	Mycetophilidae	Bolitophila occlusa
Liriomyza huidobrensis mitochondrion, complete gen	JQ862474	83.02	99.36	1.2957e−33	Diptera	Agromyzidae	Liriomyza huidobrensis
Liriomyza huidobrensis mitochondrion, complete gen	JN570505	83.02	99.36	1.2957e−33	Diptera	Agromyzidae	Liriomyza huidobrensis
Simulium siripoomense 16S ribosomal RNA gene, part	GQ866003	82.91	100	1.2957e−33	Diptera	Simuliidae	Simulium siripoomense
Lispe tentaculata 16S ribosomal RNA gene, partial	FJ025450	82.8	99.36	1.2957e−33	Diptera	Muscidae	Lispe tentaculata
Thaumatomyia notata 16S ribosomal RNA gene, partia	KC177449	82.61	99.36	4.52244e−33	Diptera	Chloropidae	Thaumatomyia notata
Hercostomus praeceps voucher Pollet 0141 (P) 16S r	EU864003	82.8	99.36	4.52244e−33	Diptera	Dolichopodidae	Hercostomus praeceps
Hercostomus fulvicaudis voucher Pollet 0154 (P) 16	EU863992	82.8	99.36	4.52244e−33	Diptera	Dolichopodidae	Hercostomus fulvicaudis
Leptomorphus walkeri 16S ribosomal RNA gene, parti	FJ172020	82.78	93.63	4.52244e−33	Diptera	Mycetophilidae	Leptomorphus walkeri
Hercostomus meieri voucher RMBR:103442 16S ribosom	FJ808173	82.91	99.36	4.52244e−33	Diptera	Dolichopodidae	Hercostomus meieri
Tipula abdominalis 16S ribosomal RNA gene, partial	KC177457	81.53	100	1.57849e−32	Diptera	Tipulidae	Tipula abdominalis
Liriomyza trifolii mitochondrion, complete genome	JN570506	81.76	99.36	1.57849e−32	Diptera	Agromyzidae	Liriomyza trifolii
Tipula abdominalis mitochondrion, partial genome	JN861743	81.53	100	1.57849e−32	Diptera	Tipulidae	Tipula abdominalis
Boletina sp. 014967 16S ribosomal RNA gene, partia	HQ230375	82.35	97.45	1.57849e−32	Diptera	Mycetophilidae	Boletina sp. 014967
Liriomyza trifolii mitochondrion, complete genome	GU327644	81.76	99.36	1.57849e−32	Diptera	Agromyzidae	Liriomyza trifolii
Simulium iridescens mitochondrial gene for 16S rRN	AB093127	81.53	100	1.57849e−32	Diptera	Simuliidae	Simulium iridescens
Simulium taalense mitochondrial gene for 16S rRNA,	AB093109	81.53	100	1.57849e−32	Diptera	Simuliidae	Simulium taalense
Simulium melanopus mitochondrial gene for 16S rRNA	AB093108	81.53	100	1.57849e−32	Diptera	Simuliidae	Simulium melanopus
Simulium laterale mitochondrial gene for 16S rRNA,	AB093107	82.28	100	1.57849e−32	Diptera	Simuliidae	Simulium laterale
Simulium bishopi mitochondrial gene for 16S rRNA,	AB093105	82.28	100	1.57849e−32	Diptera	Simuliidae	Simulium bishopi
Simulium maenoi mitochondrial gene for 16S rRNA, p	AB093104	82.28	100	1.57849e−32	Diptera	Simuliidae	Simulium maenoi
Simulium japonicum mitochondrial gene for 16S rRNA	AB093103	82.28	100	1.57849e−32	Diptera	Simuliidae	Simulium japonicum
Simulium nigrogilvum mitochondrial gene for 16S rR	AB093099	82.28	100	1.57849e−32	Diptera	Simuliidae	Simulium nigrogilvum
Simulium aokii mitochondrial gene for 16S ribosoma	AB056745	81.53	100	1.57849e−32	Diptera	Simuliidae	Simulium aokii
Simulium bivittatum mitochondrion 16S ribosomal RN	U17727	81.53	100	1.57849e−32	Diptera	Simuliidae	Simulium bivittatum
Limnophora olympiae 16S ribosomal RNA gene, partia	FJ025448	83.12	99.36	5.50946e−32	Diptera	Muscidae	Limnophora olympiae
Thraxan ebenus mitochondrial NADH1 gene (partial),	AJ391517	82.39	99.36	5.50946e−32	Diptera	Bombyliidae	Thraxan ebenus
Stegopterna mutata mitochondrial 16S ribosomal RNA	L13404	81.65	100	5.50946e−32	Diptera	Simuliidae	Stegopterna mutata
Diadocidia bruneicola voucher JSSJ16 16S ribosomal	KJ136744	83.8	90.45	1.92299e−31	Diptera	Diadocidiidae	Diadocidia bruneicola
Cramptonomyia spenceri mitochondrion, complete gen	JN861747	81.53	99.36	1.92299e−31	Diptera	Pachyneuridae	Cramptonomyia spenceri
Boletina villosa isolate 014966 16S ribosomal RNA	HQ230374	81.7	97.45	1.92299e−31	Diptera	Mycetophilidae	Boletina villosa
Cyphomyia notabilis 16S ribosomal RNA gene, partia	GU947450	80.89	100	1.92299e−31	Diptera	Stratiomyidae	Cyphomyia notabilis
Dohrniphora cornuta isolate 200862 16S ribosomal R	GU361998	82.39	99.36	1.92299e−31	Diptera	Phoridae	Dohrniphora cornuta
Simulium chamlongi 16S ribosomal RNA gene, partial	GQ866020	80.89	100	1.92299e−31	Diptera	Simuliidae	Simulium chamlongi
Simulium yuphae 16S ribosomal RNA gene, partial se	GQ866015	80.89	100	1.92299e−31	Diptera	Simuliidae	Simulium yuphae
Blondelia nigripes mitochondrial gene for 16S rRNA	AB465902	82.5	99.36	1.92299e−31	Diptera	Tachinidae	Blondelia nigripes
Simulium nili 16S ribosomal RNA gene, partial sequ	AY944188	80.89	100	1.92299e−31	Diptera	Simuliidae	Simulium nili
Pelurgoperla personata 16S ribosomal RNA gene, par	EF623168	81.76	100	1.92299e−31	Plecoptera	Gripopterygidae	Pelurgoperla personata
Limonia hawaiiensis voucher O'Grady 200106 16S rib	EU005403	80.89	100	1.92299e−31	Diptera	Limoniidae	Dicranomyia hawaiiensis
Panorpa takenouchii isolate 26tak 16S ribosomal RN	EF041520	83.12	100	1.92299e−31	Mecoptera	Panorpidae	Panorpa takenouchii
Panorpa takenouchii 16S ribosomal RNA gene, partia	AF180064	83.12	100	1.92299e−31	Mecoptera	Panorpidae	Panorpa takenouchii
Simulium chamlongi mitochondrial gene for 16S rRNA	AB093124	80.89	100	1.92299e−31	Diptera	Simuliidae	Simulium chamlongi
Simulium saitoi mitochondrial gene for 16S ribosom	AB056733	80.89	100	1.92299e−31	Diptera	Simuliidae	Simulium saitoi
Simulium feuerborni mitochondrial gene for 16S rib	AB056729	80.89	100	1.92299e−31	Diptera	Simuliidae	Simulium feuerborni
Simulium venustum (individual AC(gB)) mitochondria	L13412	80.89	100	1.92299e−31	Diptera	Simuliidae	Simulium venustum
Simulium venustum (individual CC3) mitochondrial 1	L13410	80.89	100	1.92299e−31	Diptera	Simuliidae	Simulium venustum
Simulium venustum (individual CC) mitochondrial 16	L13409	80.89	100	1.92299e−31	Diptera	Simuliidae	Simulium venustum
Evocoa chilensis 16S ribosomal RNA gene, partial s	KM878914	81.01	99.36	6.7119e−31	Diptera	Evocoidae	Evocoa chilensis
Simulium tribulatum isolate LAC 17−5 16S ribosomal	KP233212	81.65	100	6.7119e−31	Diptera	Simuliidae	Simulium tribulatum
Simulium tribulatum isolate LAC 3−2 16S ribosomal	KP233211	81.65	100	6.7119e−31	Diptera	Simuliidae	Simulium tribulatum
Lispe nana voucher RMBR:116206 16S ribosomal RNA g	KJ476314	81.53	99.36	6.7119e−31	Diptera	Muscidae	Lispe nana
Simulium atipornae mitochondrial gene for 16S ribo	AB893948	81.65	100	6.7119e−31	Diptera	Simuliidae	Simulium atipornae
Phytosciara semiferruginea voucher 220 16S ribosom	JQ613900	81.94	97.45	6.7119e−31	Diptera	Sciaridae	Phytosciara semiferruginea
Boletina nigricans isolate 057488 16S ribosomal RN	HQ230387	82.12	96.18	6.7119e−31	Diptera	Mycetophilidae	Boletina nigricans
Boletina trispinosa isolate 012711 16S ribosomal R	HQ230373	82.12	96.18	6.7119e−31	Diptera	Mycetophilidae	Boletina trispinosa
Boletina nigricans isolate 008071 16S ribosomal RN	HQ230368	82.12	96.18	6.7119e−31	Diptera	Mycetophilidae	Boletina nigricans
Calosoma sp. BYU−CO241 mitochondrion, complete gen	GU176340	80.62	100	6.7119e−31	Coleoptera	Carabidae	Calosoma sp. BYU−CO241
Simulium chiangmaiense 16S ribosomal RNA gene, par	GQ866009	81.65	100	6.7119e−31	Diptera	Simuliidae	Simulium chiangmaiense
Simulium fenestratum 16S ribosomal RNA gene, parti	GQ866006	81.65	100	6.7119e−31	Diptera	Simuliidae	Simulium fenestratum
Conops sp. JFC−2009 isolate CSU0904046119 16S ribo	GQ118390	84.78	87.26	6.7119e−31	Diptera	Conopidae	Conops sp. JFC−2009
Calosoma scrutator 16S ribosomal RNA gene, partial	EU734850	80.62	100	6.7119e−31	Coleoptera	Carabidae	Calosoma scrutator
Leucochrysa marquezi voucher CASC 00212 16S riboso	DQ399280	80.62	99.36	6.7119e−31	Neuroptera	Chrysopidae	Leucochrysa marquezi
Papuadytes cf. ferrugineus MB104 16S ribosomal RNA	AY334152	81.13	99.36	6.7119e−31	Coleoptera	Dytiscidae	Papuadytes cf. ferrugineus MB104
Neoconops longicornis 16S ribosomal RNA gene, part	AF154769	81.53	99.36	6.7119e−31	Diptera	Conopidae	Neoconops longicornis
Neoconops longicornis 16S ribosomal RNA gene, part	AF154768	81.53	99.36	6.7119e−31	Diptera	Conopidae	Neoconops longicornis
Neoconops longicornis 16S ribosomal RNA gene, part	AF154767	81.53	99.36	6.7119e−31	Diptera	Conopidae	Neoconops longicornis
Simulium arakawae mitochondrial gene for 16S rRNA,	AB093125	81.65	100	6.7119e−31	Diptera	Simuliidae	Simulium arakawae
Simulium tumpaense mitochondrial gene for 16S rRNA	AB093110	81.65	100	6.7119e−31	Diptera	Simuliidae	Simulium tumpaense
Simulium grossifilum mitochondrial gene for 16S rR	AB093102	81.01	100	6.7119e−31	Diptera	Simuliidae	Simulium grossifilum
Simulium upikae mitochondrial gene for 16S rRNA, p	AB093101	81.65	100	6.7119e−31	Diptera	Simuliidae	Simulium upikae
Simulium eximium mitochondrial gene for 16S rRNA,	AB093100	81.65	100	6.7119e−31	Diptera	Simuliidae	Simulium eximium
Simulium vittatum mitochondrial 16S ribosomal RNA	L13400	81.65	100	6.7119e−31	Diptera	Simuliidae	Simulium vittatum
Heliographa ceylanica voucher RMBR:116201 16S ribo	KJ476310	82.61	99.36	2.34268e−30	Diptera	Muscidae	Heliographa ceylanica
Diadocidia sp. Thailand DK−2014 voucher JSSJ20 16S	KJ136748	82.76	89.17	2.34268e−30	Diptera	Diadocidiidae	Diadocidia sp. Thailand DK−2014
Acilius athabascae voucher 305G09/BMNH799009 16S r	KF979079	82.39	99.36	2.34268e−30	Coleoptera	Dytiscidae	Acilius athabascae
Acilius abbreviatus voucher KBMAcab72 16S ribosoma	KF979078	82.28	99.36	2.34268e−30	Coleoptera	Dytiscidae	Acilius abbreviatus
