# Supplementary material for: Testing the potential of a ribosomal 16S marker for DNA metabarcoding of insects
Source: PeerJ. 2016 Apr 19;4:e1966. doi: 10.7717/peerj.1966 (PMC4841222; doi:10.7717/peerj.1966)
Supplement: Supplemental Information 1 [file peerj-04-1966-s006.zip › scripts/5 match OTUs/blast_hits/OTU_89.csv_plot.pdf]

OTU_89.csv

Zygoneura sciarina

Sciaridae

Diptera

97.45

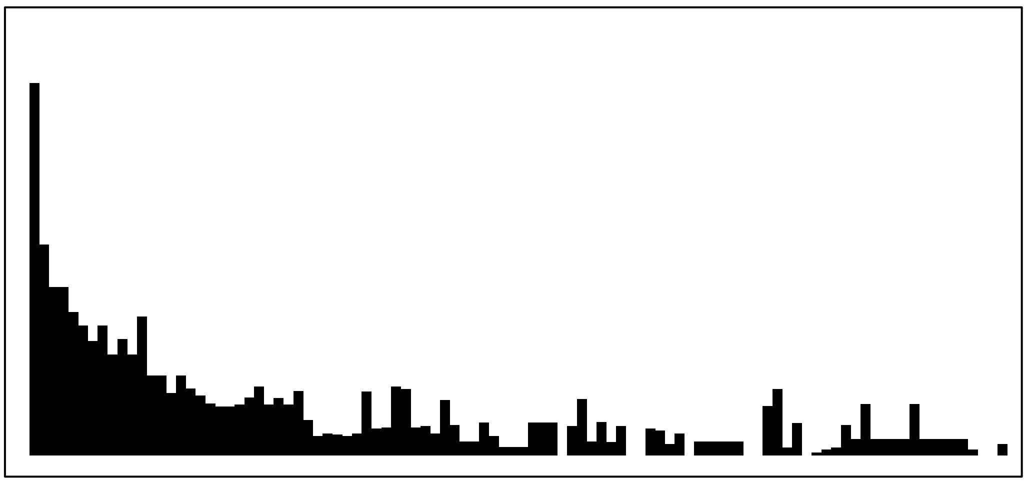

Zygoneura sciarina voucher 311 16S ribosomal RNA g	JQ613909	97.45	100	8.16233e−68	Diptera	Sciaridae	Zygoneura sciarina
Zygoneura bidens voucher 312 16S ribosomal RNA gen	JQ613910	89.87	100	6.70597e−50	Diptera	Sciaridae	Zygoneura bidens
Scatopsciara atomaria gr. sp. SS−2012 voucher 978	JQ613972	87.9	100	3.47378e−47	Diptera	Sciaridae	Scatopsciara atomaria gr. sp. SS−2012
Zygoneura flavicornis voucher 300 16S ribosomal RN	JQ613907	87.9	100	1.21247e−46	Diptera	Sciaridae	Zygoneura flavicornis
Ctenosciara insolita voucher 803 16S ribosomal RNA	JQ613935	86.71	100	7.65151e−43	Diptera	Sciaridae	Ctenosciara insolita
Scatopsciara necopinata voucher 623 16S ribosomal	JQ613921	86.08	99.36	2.67064e−42	Diptera	Sciaridae	Scatopsciara necopinata
Scatopsciara atomaria voucher 981 16S ribosomal RN	JQ613973	85.35	100	9.32145e−42	Diptera	Sciaridae	Scatopsciara atomaria
Camptochaeta tenuipalpalis voucher 566 16S ribosom	JQ613915	86.08	100	1.13558e−40	Diptera	Sciaridae	Camptochaeta tenuipalpalis
Scatopsciara atomaria voucher 90 16S ribosomal RNA	JQ613890	84.71	100	1.13558e−40	Diptera	Sciaridae	Scatopsciara atomaria
Bradysia cinerascens voucher T36 16S ribosomal RNA	JQ613979	85.44	100	3.96358e−40	Diptera	Sciaridae	Bradysia cinerascens
Ctenosciara hyalipennis voucher T08 16S ribosomal	JQ613976	84.71	100	1.38343e−39	Diptera	Sciaridae	Ctenosciara hyalipennis
Pseudolycoriella horribilis voucher 74 16S ribosom	JQ613887	86.5	100	1.38343e−39	Diptera	Sciaridae	Pseudolycoriella horribilis
Trichosia sp. 3 nr. morio SS−2012 voucher 805 16S	JQ613936	83.75	100	4.82863e−39	Diptera	Sciaridae	Trichosia sp. 3 nr. morio SS−2012
Phytosciara flavipes voucher 88 16S ribosomal RNA	JQ613889	83.75	100	7.16632e−37	Diptera	Sciaridae	Phytosciara flavipes
Trichosia edwardsi voucher T39 16S ribosomal RNA g	JQ613980	82.91	98.73	2.50129e−36	Diptera	Sciaridae	Trichosia edwardsi
Phytosciara ussuriensis voucher 883 16S ribosomal	JQ613948	83.75	100	8.73037e−36	Diptera	Sciaridae	Phytosciara ussuriensis
Phytosciara flavipes voucher JSOUT8 16S ribosomal	KJ136731	83.12	100	3.0472e−35	Diptera	Sciaridae	Phytosciara flavipes
Corynoptera vagula voucher 959 16S ribosomal RNA g	JQ613967	82.8	100	3.0472e−35	Diptera	Sciaridae	Corynoptera vagula
Bradysiopsis vittigera voucher 948 16S ribosomal R	JQ613962	82.42	100	3.0472e−35	Diptera	Sciaridae	Bradysiopsis vittigera
Bolitophila occlusa voucher JSB2 16S ribosomal RNA	KJ136713	82.28	100	1.06358e−34	Diptera	Mycetophilidae	Bolitophila occlusa
Camptochaeta camptochaeta voucher 972 16S ribosoma	JQ613970	82.28	100	1.06358e−34	Diptera	Sciaridae	Camptochaeta camptochaeta
Trichosia confusa voucher 838 16S ribosomal RNA ge	JQ613942	82.39	98.73	1.06358e−34	Diptera	Sciaridae	Trichosia confusa
Pseudolycoriella sp. 1 SS−2012 voucher 586 16S rib	JQ613916	82.72	100	1.06358e−34	Diptera	Sciaridae	Pseudolycoriella sp. 1 SS−2012
Xylosciara inornata voucher 230 16S ribosomal RNA	JQ613902	83.23	100	1.06358e−34	Diptera	Sciaridae	Xylosciara inornata
Phytosciara semiferruginea voucher 220 16S ribosom	JQ613900	82.39	100	1.06358e−34	Diptera	Sciaridae	Phytosciara semiferruginea
Scatopsciara nr. amplituda SS−2012 voucher 902 16S	JQ613953	82.69	97.45	3.71225e−34	Diptera	Sciaridae	Scatopsciara nr. amplituda SS−2012
Scatopsciara vitripennis voucher 112 16S ribosomal	JQ613892	82.39	100	3.71225e−34	Diptera	Sciaridae	Scatopsciara vitripennis
Thaumatomyia notata 16S ribosomal RNA gene, partia	KC177449	83.02	98.09	1.2957e−33	Diptera	Chloropidae	Thaumatomyia notata
Bradysia atracornea voucher 133 16S ribosomal RNA	JQ613895	81.65	100	1.57849e−32	Diptera	Sciaridae	Bradysia atracornea
Lycoriella flavicornis voucher 62 16S ribosomal RN	JQ613885	80.89	100	1.57849e−32	Diptera	Sciaridae	Lycoriella flavicornis
Bolitophila cinerea voucher JSB1 16S ribosomal RNA	KJ136712	81.01	100	5.50946e−32	Diptera	Mycetophilidae	Bolitophila cinerea
Lycoriella ingenua voucher 229 16S ribosomal RNA g	JQ613901	80.98	100	5.50946e−32	Diptera	Sciaridae	Lycoriella ingenua
Bradysia lapponica voucher 607 16S ribosomal RNA g	JQ613920	80.89	100	1.92299e−31	Diptera	Sciaridae	Bradysia lapponica
Phytosciara orcina voucher 595 16S ribosomal RNA g	JQ613918	81.01	100	6.7119e−31	Diptera	Sciaridae	Phytosciara orcina
Lipoptena cervi 16S ribosomal RNA gene, partial se	AF322437	82.99	91.72	6.7119e−31	Diptera	Hippoboscidae	Lipoptena cervi
Corynoptera blanda voucher 955 16S ribosomal RNA g	JQ613965	81.25	100	2.34268e−30	Diptera	Sciaridae	Corynoptera blanda
Drosophila sordidula voucher 103954 NADH dehydroge	EU494314	81.29	97.45	2.34268e−30	Diptera	Drosophilidae	Drosophila sordidula
Lipoptena cervi 16S ribosomal RNA gene, partial se	EF531114	83.22	91.72	2.34268e−30	Diptera	Hippoboscidae	Lipoptena cervi
Aedes maehleri isolate A31 16S ribosomal RNA gene,	DQ397930	83.11	92.99	2.34268e−30	Diptera	Culicidae	Aedes maehleri
Drosophila albomicans mitochondrion, complete geno	KT119344	81.29	97.45	8.17677e−30	Diptera	Drosophilidae	Drosophila albomicans
Trichosia splendens voucher 970 16S ribosomal RNA	JQ613969	81.37	98.73	8.17677e−30	Diptera	Sciaridae	Trichosia splendens
Ctenosciara angustistylata voucher 255 16S ribosom	JQ613904	81.01	100	8.17677e−30	Diptera	Sciaridae	Ctenosciara angustistylata
Bradysia procera voucher 147 16S ribosomal RNA gen	JQ613896	82.58	92.99	8.17677e−30	Diptera	Sciaridae	Bradysia procera
Lonchoptera uniseta 16S ribosomal RNA gene, partia	KC177456	81.41	97.45	8.17677e−30	Diptera	Lonchopteridae	Lonchoptera uniseta
Drosophila robusta voucher 103967 NADH dehydrogena	EU494347	80.65	97.45	8.17677e−30	Diptera	Drosophilidae	Drosophila robusta
Drosophila parthenogenetica strain 15181−2221.0 NA	AF246503	80.65	97.45	8.17677e−30	Diptera	Drosophilidae	Drosophila parthenogenetica
Bolopus furcatus isolate 1305 16S ribosomal RNA ge	KC699315	81.53	97.45	2.85397e−29	Diptera	Platypezidae	Bolopus furcatus
Nolima pinal 16S ribosomal RNA gene, partial seque	KM051514	80.89	97.45	2.85397e−29	Neuroptera	Mantispidae	Nolima pinal
Corynoptera subtilis voucher T34 16S ribosomal RNA	JQ613978	80.38	100	2.85397e−29	Diptera	Sciaridae	Corynoptera subtilis
Lycoriella gerbatshevskayae voucher 601 16S riboso	JQ613919	80.38	100	2.85397e−29	Diptera	Sciaridae	Lycoriella gerbatshevskayae
Elgiva solicita voucher F−6 16S ribosomal RNA gene	JN816284	80.39	97.45	2.85397e−29	Diptera	Sciomyzidae	Elgiva solicita
Gymnopternus celer voucher Pollet 0018 (P) 16S rib	EU863977	81.53	97.45	2.85397e−29	Diptera	Dolichopodidae	Gymnopternus celer
Gymnopternus celer voucher Pollet 0051 (P) 16S rib	EU863978	81.53	97.45	2.85397e−29	Diptera	Dolichopodidae	Gymnopternus celer
Gymnopternus brevicornis voucher Pollet 0036 (P) 1	EU863974	81.53	97.45	2.85397e−29	Diptera	Dolichopodidae	Gymnopternus brevicornis
Pyratula zonata 16S ribosomal RNA gene, partial se	FJ172004	79.87	100	2.85397e−29	Diptera	Keroplatidae	Pyratula zonata
Melanostoma scalare 16S ribosomal RNA gene, partia	AF126336	81.37	97.45	2.85397e−29	Diptera	Syrphidae	Melanostoma scalare
Bertamyia notata isolate 1304 16S ribosomal RNA ge	KC699314	82.64	87.9	9.96134e−29	Diptera	Platypezidae	Bertamyia notata
Drosophila melanogaster 16S ribosomal RNA gene, pa	KJ919952	80.65	97.45	9.96134e−29	Diptera	Drosophilidae	Drosophila melanogaster
Scaptomyza nigrita voucher 10004 16S ribosomal RNA	KC609624	81.56	87.9	9.96134e−29	Diptera	Drosophilidae	Scaptomyza nigrita
Dichopygina aculeata voucher 947 16S ribosomal RNA	JQ613961	80.62	100	9.96134e−29	Diptera	Sciaridae	Dichopygina aculeata
Pseudolycoriella sp. SS−2012 voucher 84 16S riboso	JQ613888	81.37	100	9.96134e−29	Diptera	Sciaridae	Pseudolycoriella sp. SS−2012
Scaptomyza magnipalpa voucher O201056 16S ribosoma	HQ171048	80	97.45	9.96134e−29	Diptera	Drosophilidae	Scaptomyza magnipalpa
Urytalpa macrocera 16S ribosomal RNA gene, partial	FJ172025	80	100	9.96134e−29	Diptera	Keroplatidae	Urytalpa macrocera
Hemerodromia sp. SNK−2009 voucher RMBR:103443 16S	FJ808170	81.25	97.45	9.96134e−29	Diptera	Empididae	Hemerodromia sp. SNK−2009
Thricops genarum 16S ribosomal RNA gene, partial s	FJ025479	81.17	97.45	9.96134e−29	Diptera	Muscidae	Thricops genarum
Thricops aculeipes 16S ribosomal RNA gene, partial	FJ025477	80.52	97.45	9.96134e−29	Diptera	Muscidae	Thricops aculeipes
Scaptodrosophila lebanonensis voucher 105639 NADH	EU494411	81.01	97.45	9.96134e−29	Diptera	Drosophilidae	Scaptodrosophila lebanonensis
Drosophila acanthoptera voucher 105622 NADH dehydr	EU494332	80	97.45	9.96134e−29	Diptera	Drosophilidae	Drosophila acanthoptera
Drosophila signata voucher 109405 NADH dehydrogena	EU494328	80.65	97.45	9.96134e−29	Diptera	Drosophilidae	Drosophila signata
Drosophila kepulauana voucher 109407 NADH dehydrog	EU494321	80.65	97.45	9.96134e−29	Diptera	Drosophilidae	Drosophila kepulauana
Drosophila immigrans voucher 103956 NADH dehydroge	EU494320	80.65	97.45	9.96134e−29	Diptera	Drosophilidae	Drosophila immigrans
Drosophila kepulauana 16S ribosomal RNA gene, part	AF387334	80.65	97.45	9.96134e−29	Diptera	Drosophilidae	Drosophila kepulauana
Drosophila kohkoa 16S ribosomal RNA gene, partial	AF387332	80.65	97.45	9.96134e−29	Diptera	Drosophilidae	Drosophila kohkoa
Drosophila procardinoides NADH dehydrogenase subun	AF246508	80	97.45	9.96134e−29	Diptera	Drosophilidae	Drosophila procardinoides
Drosophila neocardini NADH dehydrogenase subunit 1	AF246505	80	97.45	9.96134e−29	Diptera	Drosophilidae	Drosophila neocardini
Lipoptena cervi 16S ribosomal RNA gene, partial se	DQ133043	82.31	91.72	9.96134e−29	Diptera	Hippoboscidae	Lipoptena cervi
Aedes notoscriptus isolate MC04 mitochondrion, com	KM676218	83.11	92.36	3.47685e−28	Diptera	Culicidae	Aedes notoscriptus
Diadocidia valida voucher JSSJ4 16S ribosomal RNA	KJ136735	80.37	100	3.47685e−28	Diptera	Diadocidiidae	Diadocidia valida
Trichosia sp. 1 nr. borealis SS−2012 voucher 858 1	JQ613945	81.51	91.72	3.47685e−28	Diptera	Sciaridae	Trichosia sp. 1 nr. borealis SS−2012
Leptosciarella dentata voucher 841 16S ribosomal R	JQ613943	79.5	100	3.47685e−28	Diptera	Sciaridae	Leptosciarella dentata
Pherbellia albocostata voucher MU_NUS_SG56 16S rib	JN828219	80.13	97.45	3.47685e−28	Diptera	Sciomyzidae	Pherbellia albocostata
Papilio alexanor voucher FS−b−40 16S ribosomal RNA	GQ268343	80.26	94.9	3.47685e−28	Lepidoptera	Papilionidae	Papilio alexanor
Diadocidia valida 16S ribosomal RNA gene, partial	FJ172022	80.37	100	3.47685e−28	Diptera	Diadocidiidae	Diadocidia valida
Empis producta voucher RMBR:103430 16S ribosomal R	FJ808167	81.41	97.45	3.47685e−28	Diptera	Empididae	Empis producta
Drosophila tripunctata voucher 107541 NADH dehydro	EU494351	80.77	97.45	3.47685e−28	Diptera	Drosophilidae	Drosophila tripunctata
Drosophila quinaria voucher 107542 NADH dehydrogen	EU494338	82.39	89.81	3.47685e−28	Diptera	Drosophilidae	Drosophila quinaria
Drosophila griseolineata voucher 103986 NADH dehyd	EU494315	80.77	97.45	3.47685e−28	Diptera	Drosophilidae	Drosophila griseolineata
Drosophila funebris voucher 103952 NADH dehydrogen	EU494313	80.77	97.45	3.47685e−28	Diptera	Drosophilidae	Drosophila funebris
Drosophila ornatipennis voucher 103965 NADH dehydr	EU494307	80.77	97.45	3.47685e−28	Diptera	Drosophilidae	Drosophila ornatipennis
Drosophila tripunctata 16S ribosomal RNA gene, par	AF479806	80.77	97.45	3.47685e−28	Diptera	Drosophilidae	Drosophila tripunctata
Drosophila falleni 16S ribosomal RNA gene, partial	AF479802	82.39	89.81	3.47685e−28	Diptera	Drosophilidae	Drosophila falleni
Drosophila ornatipennis 16S ribosomal RNA gene, pa	AF479799	80.77	97.45	3.47685e−28	Diptera	Drosophilidae	Drosophila ornatipennis
Drosophila arawakana dominicensis NADH dehydrogena	AF246516	80.77	97.45	3.47685e−28	Diptera	Drosophilidae	Drosophila arawakana dominicensis
Drosophila arawakana arawakana strain 15182−2261.0	AF246499	80.77	97.45	3.47685e−28	Diptera	Drosophilidae	Drosophila arawakana arawakana
Drosophila arawakana arawakana strain 15182−2261.1	AF246500	80.77	97.45	3.47685e−28	Diptera	Drosophilidae	Drosophila arawakana arawakana
Drosophila nigrodunni NADH dehydrogenase subunit 1	AF246494	80.77	97.45	3.47685e−28	Diptera	Drosophilidae	Drosophila nigrodunni
Papilio alexanor mitochondrion, large subunit ribo	AJ224052	80.26	94.9	3.47685e−28	Lepidoptera	Papilionidae	Papilio alexanor
Diadocidia stanfordensis voucher JSSJ14 16S riboso	KJ136742	79.75	100	1.21354e−27	Diptera	Diadocidiidae	Diadocidia stanfordensis
Edwardsina gigantea 16S ribosomal RNA gene, partia	KC177460	79.74	97.45	1.21354e−27	Diptera	Blephariceridae	Edwardsina gigantea
Pharmacophagus antenor mitochondrial partial 16S r	AM283054	80.52	94.9	1.21354e−27	Lepidoptera	Papilionidae	Pharmacophagus antenor
