# Supplementary material for: Testing the potential of a ribosomal 16S marker for DNA metabarcoding of insects
Source: PeerJ. 2016 Apr 19;4:e1966. doi: 10.7717/peerj.1966 (PMC4841222; doi:10.7717/peerj.1966)
Supplement: Supplemental Information 1 [file peerj-04-1966-s006.zip › scripts/5 match OTUs/blast_hits/OTU_9.csv_plot.pdf]

OTU_9.csv

Pedicia albivitta

Pediciidae

Diptera

87.82

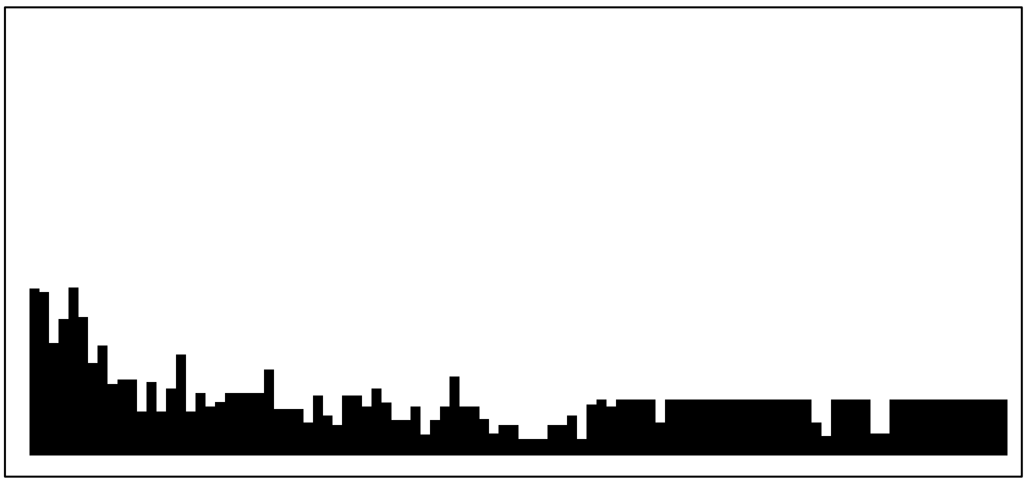

Pedicia albivitta voucher O'Grady 200012 16S ribos	EU005435	87.82	99.36	1.46498e−45	Diptera	Pediciidae	Pedicia albivitta
Bolitophila cinerea voucher JSB1 16S ribosomal RNA	KJ136712	87.66	97.44	2.17423e−43	Diptera	Mycetophilidae	Bolitophila cinerea
Edwardsina gigantea 16S ribosomal RNA gene, partia	KC177460	85.26	100	3.22684e−41	Diptera	Blephariceridae	Edwardsina gigantea
Parochlus steinenii isolate Sejong01 mitochondrion	KT003702	86.39	94.23	1.37209e−39	Diptera	Chironomidae	Parochlus steinenii
Bolitophila sp. ER−2009 16S ribosomal RNA gene, pa	FJ172021	87.86	89.1	4.78905e−39	Diptera	Mycetophilidae	Bolitophila sp. ER−2009
Tipula sp. PMO 200011 16S ribosomal RNA gene, part	EU005437	86.49	94.23	4.78905e−39	Diptera	Tipulidae	Tipula sp. PMO 200011
Corynoptera blanda voucher 955 16S ribosomal RNA g	JQ613965	84.31	97.44	2.03636e−37	Diptera	Sciaridae	Corynoptera blanda
Eudicrana sp. DK−2013 voucher JSS12 16S ribosomal	KC435571	85.14	93.59	7.10758e−37	Diptera	Mycetophilidae	Eudicrana sp. DK−2013
Derodontus emaculatus voucher BMNH:679374 16S ribo	DQ202609	83.33	99.36	2.48079e−36	Coleoptera		Derodontus emaculatus
Tipula abdominalis 16S ribosomal RNA gene, partial	KC177457	83.54	99.36	8.65881e−36	Diptera	Tipulidae	Tipula abdominalis
Tipula abdominalis mitochondrion, partial genome	JN861743	83.54	99.36	8.65881e−36	Diptera	Tipulidae	Tipula abdominalis
Minettia flaveola 16S ribosomal RNA gene, partial	KC177448	82.05	100	1.05486e−34	Diptera	Lauxaniidae	Minettia flaveola
Ptychoptera sp. ATB−2011 mitochondrion, complete g	JN861744	83.44	99.36	1.05486e−34	Diptera	Ptychopteridae	Ptychoptera sp. ATB−2011
Minettia longipennis mitochondrion partial 16S rRN	AJ439184	82.05	100	1.05486e−34	Diptera	Lauxaniidae	Minettia longipennis
Phytosciara flavipes voucher JSOUT8 16S ribosomal	KJ136731	83.12	97.44	3.68182e−34	Diptera	Sciaridae	Phytosciara flavipes
Bolitophila hybrida 16S ribosomal RNA gene, partia	FJ172002	84.72	91.67	3.68182e−34	Diptera	Mycetophilidae	Bolitophila hybrida
Cramptonomyia spenceri mitochondrion, complete gen	JN861747	82.05	99.36	1.28508e−33	Diptera	Pachyneuridae	Cramptonomyia spenceri
Chrysoperla nipponensis mitochondrial DNA, complet	AP011623	82.91	100	1.28508e−33	Neuroptera	Chrysopidae	Chrysoperla nipponensis
Gonocephalum pusillum isolate CSU0904046030 16S ri	GQ118381	82.28	100	1.28508e−33	Coleoptera	Tenebrionidae	Gonocephalum pusillum
Drosophila funebris voucher 103952 NADH dehydrogen	EU494313	82.5	100	1.28508e−33	Diptera	Drosophilidae	Drosophila funebris
Chrysoperla agilis isolate A−083 16S large subunit	AY743751	82.91	100	1.28508e−33	Neuroptera	Chrysopidae	Chrysoperla agilis
Chrysoperla lucasina isolate L−079 16S large subun	AY743746	82.91	100	1.28508e−33	Neuroptera	Chrysopidae	Chrysoperla lucasina
Chrysoperla lucasina isolate L−029 16S large subun	AY743737	82.91	100	1.28508e−33	Neuroptera	Chrysopidae	Chrysoperla lucasina
Chrysoperla lucasina isolate L−099 16S large subun	AY743732	82.91	100	1.28508e−33	Neuroptera	Chrysopidae	Chrysoperla lucasina
Bolitophila occlusa voucher JSB2 16S ribosomal RNA	KJ136713	84.03	91.67	4.48537e−33	Diptera	Mycetophilidae	Bolitophila occlusa
Culcua kolibaci 16S ribosomal RNA gene, partial se	GU947391	82.17	100	4.48537e−33	Diptera	Stratiomyidae	Culcua kolibaci
Hercostomus praeceps voucher Pollet 0141 (P) 16S r	EU864003	82.17	100	4.48537e−33	Diptera	Dolichopodidae	Hercostomus praeceps
Hercostomus fulvicaudis voucher Pollet 0154 (P) 16	EU863992	82.17	100	4.48537e−33	Diptera	Dolichopodidae	Hercostomus fulvicaudis
Limonia tahitiensis voucher O'Grady 202004 16S rib	EU005425	81.53	99.36	4.48537e−33	Diptera	Limoniidae	Dicranomyia tahitiensis
Panorpa takenouchii isolate 26tak 16S ribosomal RN	EF041520	82.8	99.36	4.48537e−33	Mecoptera	Panorpidae	Panorpa takenouchii
Chrysoperla carnea isolate Chrcar3 16S ribosomal R	AY620150	81.88	100	4.48537e−33	Neuroptera	Chrysopidae	Chrysoperla carnea
Drosophila takahashii cytochrome b (cytb) gene, pa	AF164592	81.41	100	4.48537e−33	Diptera	Drosophilidae	Drosophila takahashii
Panorpa takenouchii 16S ribosomal RNA gene, partia	AF180064	82.8	99.36	4.48537e−33	Mecoptera	Panorpidae	Panorpa takenouchii
Chrysoperla lucasina isolate L−070 16S large subun	AY743744	82.8	99.36	4.48537e−33	Neuroptera	Chrysopidae	Chrysoperla lucasina
Chrysoperla nipponensis mitochondrion, complete ge	KP027407	82.28	100	1.56555e−32	Neuroptera	Chrysopidae	Chrysoperla nipponensis
Acilius athabascae voucher 305G09/BMNH799009 16S r	KF979079	83.12	100	1.56555e−32	Coleoptera	Dytiscidae	Acilius athabascae
Phytosciara flavipes voucher 88 16S ribosomal RNA	JQ613889	82.47	97.44	1.56555e−32	Diptera	Sciaridae	Phytosciara flavipes
Drosophila rubida voucher 109400 NADH dehydrogenas	EU494327	81.65	100	1.56555e−32	Diptera	Drosophilidae	Drosophila rubida
Drosophila hypocausta voucher 103972 NADH dehydrog	EU494319	81.65	100	1.56555e−32	Diptera	Drosophilidae	Drosophila hypocausta
Ochlerotatus nipponicus isolate A75 16S ribosomal	DQ397914	82.28	100	1.56555e−32	Diptera	Culicidae	Aedes nipponicus
Mallada luctuosus 16S ribosomal RNA gene, partial	DQ399288	80.98	100	1.56555e−32	Neuroptera	Chrysopidae	Pseudomallada luctuosus
Leucochrysa marquezi voucher CASC 00212 16S riboso	DQ399280	81.65	100	1.56555e−32	Neuroptera	Chrysopidae	Leucochrysa marquezi
Chrysoperla carnea isolate Chrcar2 16S ribosomal R	AY620152	82.28	100	1.56555e−32	Neuroptera	Chrysopidae	Chrysoperla carnea
Arachnocampa luminosa biotype 1 16S large subunit	AY576329	83.69	90.38	1.56555e−32	Diptera	Keroplatidae	Arachnocampa luminosa
Chrysoperla sp. isolate 82 16S large subunit ribos	AY743750	82.28	100	1.56555e−32	Neuroptera	Chrysopidae	Chrysoperla sp. isolate 82
Chrysoperla carnea isolate C−097 16S large subunit	AY743731	82.28	100	1.56555e−32	Neuroptera	Chrysopidae	Chrysoperla carnea
Lycoriella flavicornis voucher 62 16S ribosomal RN	JQ613885	81.7	97.44	5.4643e−32	Diptera	Sciaridae	Lycoriella flavicornis
Hybos femoratus voucher Pollet 0247 (P) 16S riboso	HQ449036	81.01	100	5.4643e−32	Diptera	Hybotidae	Hybos femoratus
Eudmeta marginata 16S ribosomal RNA gene, partial	GU947400	81.41	100	5.4643e−32	Diptera	Stratiomyidae	Eudmeta marginata
Tinda javana 16S ribosomal RNA gene, partial seque	GU947393	81.41	100	5.4643e−32	Diptera	Stratiomyidae	Tinda javana
Hercostomus verbekei voucher Pollet 0263 (P) 16S r	EU864004	80.77	100	5.4643e−32	Diptera	Dolichopodidae	Hercostomus verbekei
Drosophila signata voucher 109405 NADH dehydrogena	EU494328	80.77	100	5.4643e−32	Diptera	Drosophilidae	Drosophila signata
Drosophila kepulauana voucher 109407 NADH dehydrog	EU494321	80.77	100	5.4643e−32	Diptera	Drosophilidae	Drosophila kepulauana
Libnotes perkinsi voucher O'Grady 200981 16S ribos	EU005433	81.41	99.36	5.4643e−32	Diptera	Limoniidae	Libnotes perkinsi
Libnotes perkinsi voucher O'Grady 200947 16S ribos	EU005432	81.41	99.36	5.4643e−32	Diptera	Limoniidae	Libnotes perkinsi
Apertochrysa edwardsi 16S ribosomal RNA gene, part	DQ399265	81.88	100	5.4643e−32	Neuroptera	Chrysopidae	Apertochrysa edwardsi
Drosophila kepulauana 16S ribosomal RNA gene, part	AF387334	80.77	100	5.4643e−32	Diptera	Drosophilidae	Drosophila kepulauana
Chrysoperla lucasina isolate L−078 16S large subun	AY743776	82.39	100	5.4643e−32	Neuroptera	Chrysopidae	Chrysoperla lucasina
Wuchereria bancrofti genome assembly W_bancrofti_J	LM000940	82.61	100	1.90723e−31	Spirurida	Onchocercidae	Wuchereria bancrofti
Cychrus caraboides caraboides voucher CC00817 16S	JQ647184	82.28	99.36	1.90723e−31	Coleoptera	Carabidae	Cychrus caraboides caraboides
Culex quinquefasciatus from USA mitochondrion, com	HQ724617	82.61	100	1.90723e−31	Diptera	Culicidae	Culex quinquefasciatus
Culex pipiens pipiens from Turkey mitochondrion, c	HQ724616	82.61	100	1.90723e−31	Diptera	Culicidae	Culex pipiens pipiens
Culex pipiens pipiens from Tunisia mitochondrion,	HQ724614	82.61	100	1.90723e−31	Diptera	Culicidae	Culex pipiens pipiens
Culex quinquefasciatus mitochondrion, complete gen	GU188856	82.61	100	1.90723e−31	Diptera	Culicidae	Culex quinquefasciatus
Hercostomus longiventris voucher Pollet 0274 (P) 1	EU863994	81.53	100	1.90723e−31	Diptera	Dolichopodidae	Hercostomus longiventris
Culex quinquefasciatus isolate A−373 16S ribosomal	EU711101	82.61	100	1.90723e−31	Diptera	Culicidae	Culex quinquefasciatus
Culex quinquefasciatus isolate A−365 16S ribosomal	EU711099	82.61	100	1.90723e−31	Diptera	Culicidae	Culex quinquefasciatus
Culex quinquefasciatus isolate A−274 16S ribosomal	EU711098	82.61	100	1.90723e−31	Diptera	Culicidae	Culex quinquefasciatus
Culex quinquefasciatus isolate A−266 16S ribosomal	EU711095	82.61	100	1.90723e−31	Diptera	Culicidae	Culex quinquefasciatus
Culex quinquefasciatus isolate A−263 16S ribosomal	EU711094	82.61	100	1.90723e−31	Diptera	Culicidae	Culex quinquefasciatus
Culex quinquefasciatus isolate A−128 16S ribosomal	EU711088	82.61	100	1.90723e−31	Diptera	Culicidae	Culex quinquefasciatus
Culex quinquefasciatus isolate A−173 16S ribosomal	EU711092	82.61	100	1.90723e−31	Diptera	Culicidae	Culex quinquefasciatus
Culex quinquefasciatus isolate A−169 16S ribosomal	EU711091	82.61	100	1.90723e−31	Diptera	Culicidae	Culex quinquefasciatus
Culex quinquefasciatus isolate A−137 16S ribosomal	EU711090	82.61	100	1.90723e−31	Diptera	Culicidae	Culex quinquefasciatus
Culex quinquefasciatus isolate A−84 16S ribosomal	EU711087	82.61	100	1.90723e−31	Diptera	Culicidae	Culex quinquefasciatus
Culex quinquefasciatus isolate A−66 16S ribosomal	EU711086	82.61	100	1.90723e−31	Diptera	Culicidae	Culex quinquefasciatus
Culex quinquefasciatus isolate A−59 16S ribosomal	EU711085	82.61	100	1.90723e−31	Diptera	Culicidae	Culex quinquefasciatus
Culex quinquefasciatus isolate A−52 16S ribosomal	EU711084	82.61	100	1.90723e−31	Diptera	Culicidae	Culex quinquefasciatus
Culex quinquefasciatus isolate A−40 16S ribosomal	EU711083	82.61	100	1.90723e−31	Diptera	Culicidae	Culex quinquefasciatus
Culex quinquefasciatus isolate A−35 16S ribosomal	EU711082	82.61	100	1.90723e−31	Diptera	Culicidae	Culex quinquefasciatus
Hercostomus meieri voucher RMBR:103442 16S ribosom	FJ808173	81.53	100	1.90723e−31	Diptera	Dolichopodidae	Hercostomus meieri
Empis producta voucher RMBR:103430 16S ribosomal R	FJ808167	80.89	100	1.90723e−31	Diptera	Empididae	Empis producta
Culex quinquefasciatus from India: Jamnagar 16S ri	EU555416	82.61	100	1.90723e−31	Diptera	Culicidae	Culex quinquefasciatus
Culex quinquefasciatus from India: Jodhpur 16S rib	EU555415	82.61	100	1.90723e−31	Diptera	Culicidae	Culex quinquefasciatus
Culex quinquefasciatus from India: Bikaner 16S rib	EU555414	82.61	100	1.90723e−31	Diptera	Culicidae	Culex quinquefasciatus
Culex quinquefasciatus from India: Bhatinda 16S ri	EU555413	82.61	100	1.90723e−31	Diptera	Culicidae	Culex quinquefasciatus
Drosophila putrida voucher 103964 NADH dehydrogena	EU494348	81.01	100	1.90723e−31	Diptera	Drosophilidae	Drosophila putrida
Drosophila acanthoptera voucher 105622 NADH dehydr	EU494332	81.01	100	1.90723e−31	Diptera	Drosophilidae	Drosophila acanthoptera
Culex quinquefasciatus isolate A81 16S ribosomal R	EU339142	82.61	100	1.90723e−31	Diptera	Culicidae	Culex quinquefasciatus
Culex quinquefasciatus isolate A12 16S ribosomal R	EU339146	82.61	100	1.90723e−31	Diptera	Culicidae	Culex quinquefasciatus
Culex quinquefasciatus isolate A77 16S ribosomal R	EU339144	82.61	100	1.90723e−31	Diptera	Culicidae	Culex quinquefasciatus
Culex quinquefasciatus isolate A16 16S ribosomal R	EU339143	82.61	100	1.90723e−31	Diptera	Culicidae	Culex quinquefasciatus
Culex quinquefasciatus isolate A160 16S ribosomal	EU339141	82.61	100	1.90723e−31	Diptera	Culicidae	Culex quinquefasciatus
Culex quinquefasciatus isolate A181 16S ribosomal	EU339140	82.61	100	1.90723e−31	Diptera	Culicidae	Culex quinquefasciatus
Culex quinquefasciatus isolate A4 16S ribosomal RN	EU339139	82.61	100	1.90723e−31	Diptera	Culicidae	Culex quinquefasciatus
Culex quinquefasciatus isolate A22 16S ribosomal R	EU339138	82.61	100	1.90723e−31	Diptera	Culicidae	Culex quinquefasciatus
Culex quinquefasciatus isolate A7 16S ribosomal RN	EU339137	82.61	100	1.90723e−31	Diptera	Culicidae	Culex quinquefasciatus
Culex quinquefasciatus isolate A141 16S ribosomal	EU339136	82.61	100	1.90723e−31	Diptera	Culicidae	Culex quinquefasciatus
Culex quinquefasciatus isolate A190 16S ribosomal	EU339135	82.61	100	1.90723e−31	Diptera	Culicidae	Culex quinquefasciatus
Culex quinquefasciatus isolate A144 16S ribosomal	EU339134	82.61	100	1.90723e−31	Diptera	Culicidae	Culex quinquefasciatus
