# Supplementary material for: Testing the potential of a ribosomal 16S marker for DNA metabarcoding of insects
Source: PeerJ. 2016 Apr 19;4:e1966. doi: 10.7717/peerj.1966 (PMC4841222; doi:10.7717/peerj.1966)
Supplement: Supplemental Information 1 [file peerj-04-1966-s006.zip › scripts/5 match OTUs/blast_hits/OTU_90.csv_plot.pdf]

OTU_90.csv

Nanonemoura wahkeena

Nemouridae

Plecoptera

89.87

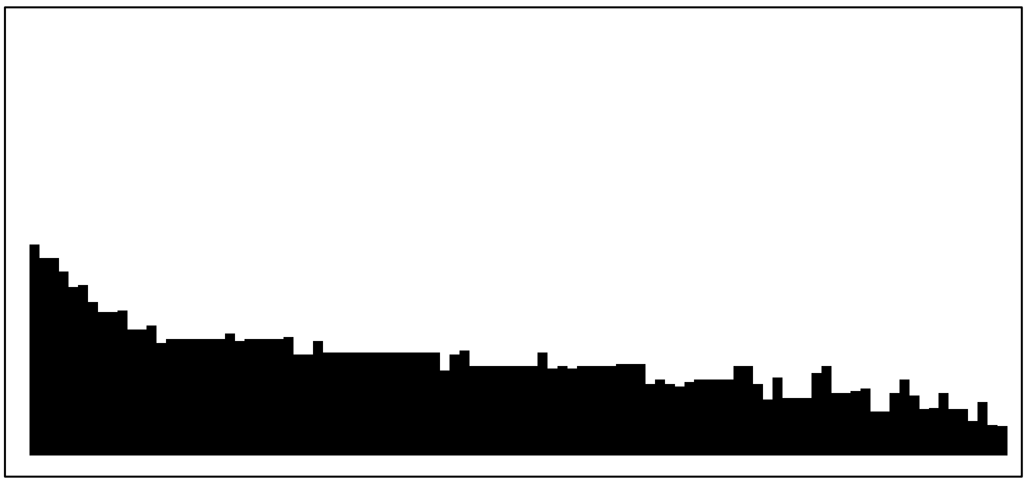

Nanonemoura wahkeena 16S ribosomal RNA gene, parti	EF623198	89.87	100	6.65101e−50	Plecoptera	Nemouridae	Nanonemoura wahkeena
Nemurella pictetii 16S ribosomal RNA gene, partial	EF623192	89.24	100	8.10258e−49	Plecoptera	Nemouridae	Nemurella pictetii
Ostrocerca foersteri 16S ribosomal RNA gene, parti	EF623191	89.24	100	8.10258e−49	Plecoptera	Nemouridae	Ostrocerca foersteri
Visoka cataractae 16S ribosomal RNA gene, partial	EF623189	88.61	100	3.44531e−47	Plecoptera	Nemouridae	Visoka cataractae
Perlinodes aurea 16S ribosomal RNA gene, partial s	EF623234	87.9	100	4.19724e−46	Plecoptera	Perlodidae	Perlinodes aurea
Shipsa rotunda 16S ribosomal RNA gene, partial seq	EF623184	87.97	100	4.19724e−46	Plecoptera	Nemouridae	Shipsa rotunda
Isoperla oxylepis 16S ribosomal RNA gene, partial	EF623221	87.18	100	1.46498e−45	Plecoptera	Perlodidae	Isoperla oxylepis
Paranemoura claasseni 16S ribosomal RNA gene, part	EF623185	86.71	100	2.17423e−43	Plecoptera	Nemouridae	Paranemoura claasseni
Zapada cinctipes 16S ribosomal RNA gene, partial s	EF623183	86.71	100	2.17423e−43	Plecoptera	Nemouridae	Zapada cinctipes
Scopura montana 16S ribosomal RNA gene, partial se	EF623265	86.79	100	7.58879e−43	Plecoptera		Scopura montana
Neonemura sp. BYU_PL153 16S ribosomal RNA gene, pa	EF623204	85.9	100	7.58879e−43	Plecoptera	Notonemouridae	Neonemura sp. BYU_PL153
Soyedina producta 16S ribosomal RNA gene, partial	EF623196	85.9	100	7.58879e−43	Plecoptera	Nemouridae	Soyedina producta
Protonemura meyeri 16S ribosomal RNA gene, partial	EF623193	86.08	100	9.24504e−42	Plecoptera	Nemouridae	Protonemura meyeri
Cascadoperla trictura 16S ribosomal RNA gene, part	EF623225	85.26	100	3.22684e−41	Plecoptera	Perlodidae	Cascadoperla trictura
Mesocapnia arizonensis mitochondrion, partial geno	KP642637	85.44	100	1.12628e−40	Plecoptera	Capniidae	Mesocapnia arizonensis
Allocapnia recta isolate H13 16S ribosomal RNA gen	KC881048	85.44	100	1.12628e−40	Plecoptera	Capniidae	Allocapnia recta
Allocapnia recta isolate H11 16S ribosomal RNA gen	KC881046	85.44	100	1.12628e−40	Plecoptera	Capniidae	Allocapnia recta
Allocapnia recta isolate H7 16S ribosomal RNA gene	KC881042	85.44	100	1.12628e−40	Plecoptera	Capniidae	Allocapnia recta
Allocapnia recta isolate H3 16S ribosomal RNA gene	KC881038	85.44	100	1.12628e−40	Plecoptera	Capniidae	Allocapnia recta
Allocapnia recta isolate H1 16S ribosomal RNA gene	KC881036	85.44	100	1.12628e−40	Plecoptera	Capniidae	Allocapnia recta
Oemopteryx vanduzeea 16S ribosomal RNA gene, parti	EF623266	85.71	100	1.12628e−40	Plecoptera	Taeniopterygidae	Oemopteryx vanduzeea
Megarcys signata 16S ribosomal RNA gene, partial s	EF623215	85.35	100	1.12628e−40	Plecoptera	Perlodidae	Megarcys signata
Nemoura trispinosa 16S ribosomal RNA gene, partial	EF623187	85.44	100	1.12628e−40	Plecoptera	Nemouridae	Nemoura trispinosa
Allocapnia minima 16S ribosomal RNA gene, partial	EF623130	85.44	100	1.12628e−40	Plecoptera	Capniidae	Allocapnia minima
Mesocapnia sp. BYU_PL010 16S ribosomal RNA gene, p	EF623127	85.44	100	1.12628e−40	Plecoptera	Capniidae	Mesocapnia sp. BYU_PL010
Allocapnia recta haplotype 3ar: H03 16S ribosomal	DQ915181	85.44	100	1.12628e−40	Plecoptera	Capniidae	Allocapnia recta
Megaleuctra kincaidi 16S ribosomal RNA gene, parti	EF623174	85.53	100	3.93109e−40	Plecoptera	Leuctridae	Megaleuctra kincaidi
Salmoperla sylvanica 16S ribosomal RNA gene, parti	EF623222	84.71	100	1.37209e−39	Plecoptera	Perlodidae	Salmoperla sylvanica
Lednia tumana 16S ribosomal RNA gene, partial sequ	EF623197	84.71	100	1.37209e−39	Plecoptera	Nemouridae	Lednia tumana
Capnura wanica 16S ribosomal RNA gene, partial seq	EF623128	85.35	100	1.37209e−39	Plecoptera	Capniidae	Capnura wanica
Apteroperla tikumana mitochondrion, complete genom	KR604721	84.81	100	4.78905e−39	Plecoptera	Capniidae	Apteroperla tikumana
Allocapnia recta isolate H19 16S ribosomal RNA gen	KC881054	84.81	100	4.78905e−39	Plecoptera	Capniidae	Allocapnia recta
Allocapnia recta isolate H16 16S ribosomal RNA gen	KC881051	84.81	100	4.78905e−39	Plecoptera	Capniidae	Allocapnia recta
Allocapnia recta isolate H15 16S ribosomal RNA gen	KC881050	84.81	100	4.78905e−39	Plecoptera	Capniidae	Allocapnia recta
Allocapnia recta isolate H14 16S ribosomal RNA gen	KC881049	84.81	100	4.78905e−39	Plecoptera	Capniidae	Allocapnia recta
Allocapnia recta isolate H10 16S ribosomal RNA gen	KC881045	84.81	100	4.78905e−39	Plecoptera	Capniidae	Allocapnia recta
Allocapnia recta isolate H6 16S ribosomal RNA gene	KC881041	84.81	100	4.78905e−39	Plecoptera	Capniidae	Allocapnia recta
Cryptoperla sp. YK−2012−1 mitochondrial gene for 1	AB711243	84.81	100	4.78905e−39	Plecoptera	Peltoperlidae	Cryptoperla sp. YK−2012−1
Allocapnia recta haplotype 12ar: H12 16S ribosomal	EU306875	84.81	100	4.78905e−39	Plecoptera	Capniidae	Allocapnia recta
Allocapnia recta haplotype 11ar: H011 16S ribosoma	EU306874	84.81	100	4.78905e−39	Plecoptera	Capniidae	Allocapnia recta
Allocapnia recta haplotype 7ar: H07 16S ribosomal	EU306870	84.81	100	4.78905e−39	Plecoptera	Capniidae	Allocapnia recta
Allocapnia recta haplotype 2ar: H02 16S ribosomal	DQ915180	84.81	100	4.78905e−39	Plecoptera	Capniidae	Allocapnia recta
Pteronarcys californica 16S ribosomal RNA gene, pa	EF623261	83.97	100	1.67154e−38	Plecoptera	Pteronarcyidae	Pteronarcys californica
Isoperla davisi 16S ribosomal RNA gene, partial se	EF623206	84.71	100	1.67154e−38	Plecoptera	Perlodidae	Isoperla davisi
Nemoura cinerea 16S ribosomal RNA gene, partial se	EF623194	84.91	100	1.67154e−38	Plecoptera	Nemouridae	Nemoura cinerea
Allocapnia recta isolate H18 16S ribosomal RNA gen	KC881053	84.18	100	5.83426e−38	Plecoptera	Capniidae	Allocapnia recta
Allocapnia recta isolate H17 16S ribosomal RNA gen	KC881052	84.18	100	5.83426e−38	Plecoptera	Capniidae	Allocapnia recta
Allocapnia recta isolate H12 16S ribosomal RNA gen	KC881047	84.18	100	5.83426e−38	Plecoptera	Capniidae	Allocapnia recta
Allocapnia recta isolate H9 16S ribosomal RNA gene	KC881044	84.18	100	5.83426e−38	Plecoptera	Capniidae	Allocapnia recta
Allocapnia recta isolate H8 16S ribosomal RNA gene	KC881043	84.18	100	5.83426e−38	Plecoptera	Capniidae	Allocapnia recta
Allocapnia recta isolate H5 16S ribosomal RNA gene	KC881040	84.18	100	5.83426e−38	Plecoptera	Capniidae	Allocapnia recta
Allocapnia recta isolate H4 16S ribosomal RNA gene	KC881039	84.18	100	5.83426e−38	Plecoptera	Capniidae	Allocapnia recta
Frisonia picticeps 16S ribosomal RNA gene, partial	EF623227	84.81	100	5.83426e−38	Plecoptera	Perlodidae	Frisonia picticeps
Austronemoura sp. BYU_PL149 16S ribosomal RNA gene	EF623200	84.08	100	5.83426e−38	Plecoptera	Notonemouridae	Austronemoura sp. BYU_PL149
Malenka californica 16S ribosomal RNA gene, partia	EF623182	84.18	100	5.83426e−38	Plecoptera	Nemouridae	Malenka californica
Thaumatoperla sp. BYU_PL109 16S ribosomal RNA gene	EF623149	84.08	100	5.83426e−38	Plecoptera	Eustheniidae	Thaumatoperla sp. BYU_PL109
Allocapnia recta haplotype 10ar: H010 16S ribosoma	EU306873	84.18	100	5.83426e−38	Plecoptera	Capniidae	Allocapnia recta
Allocapnia recta haplotype 9ar: H09 16S ribosomal	EU306872	84.18	100	5.83426e−38	Plecoptera	Capniidae	Allocapnia recta
Allocapnia recta haplotype 8ar: H08 16S ribosomal	EU306871	84.18	100	5.83426e−38	Plecoptera	Capniidae	Allocapnia recta
Allocapnia recta haplotype 1ar: H01 16S ribosomal	DQ915179	84.18	100	5.83426e−38	Plecoptera	Capniidae	Allocapnia recta
Cryptoperla japonica mitochondrial gene for 16S rR	AB711184	84.28	100	2.03636e−37	Plecoptera	Peltoperlidae	Cryptoperla japonica
Cryptoperla japonica mitochondrial gene for 16S rR	AB711183	84.28	100	2.03636e−37	Plecoptera	Peltoperlidae	Cryptoperla japonica
Cryptoperla japonica mitochondrial gene for 16S rR	AB711182	84.28	100	2.03636e−37	Plecoptera	Peltoperlidae	Cryptoperla japonica
Pteronarcys sachalina 16S ribosomal RNA gene, part	EF623264	83.33	100	2.03636e−37	Plecoptera	Pteronarcyidae	Pteronarcys sachalina
Peltoperla arcuata 16S ribosomal RNA gene, partial	EF623258	83.54	100	2.03636e−37	Plecoptera	Peltoperlidae	Peltoperla arcuata
Pteronarcys princeps mitochondrion, complete genom	AY687866	83.33	100	2.03636e−37	Plecoptera	Pteronarcyidae	Pteronarcys princeps
Dolichopoda sp. SKA 16S ribosomal RNA gene, partia	EF216964	83.23	100	7.10758e−37	Orthoptera	Rhaphidophoridae	Dolichopoda sp. SKA
Skwala americana 16S ribosomal RNA gene, partial s	EF623217	83.44	100	7.10758e−37	Plecoptera	Perlodidae	Skwala americana
Cryptoperla japonica mitochondrial gene for 16S rR	AB711202	83.54	100	2.48079e−36	Plecoptera	Peltoperlidae	Cryptoperla japonica
Austrocerca rieka 16S ribosomal RNA gene, partial	EF623205	83.54	100	2.48079e−36	Plecoptera	Notonemouridae	Austrocerca rieka
Austrocercoides zwicki 16S ribosomal RNA gene, par	EF623199	83.54	100	2.48079e−36	Plecoptera	Notonemouridae	Austrocercoides zwicki
Prostoia besametsa 16S ribosomal RNA gene, partial	EF623188	83.54	100	2.48079e−36	Plecoptera	Nemouridae	Prostoia besametsa
Eusthenia costalis 16S ribosomal RNA gene, partial	EF623150	84.18	100	2.48079e−36	Plecoptera	Eustheniidae	Eusthenia costalis
Triznaka pintada 16S ribosomal RNA gene, partial s	EF623134	84.18	100	2.48079e−36	Plecoptera	Chloroperlidae	Triznaka pintada
Osmylus sp. EH−2004 16S ribosomal RNA gene, partia	AY620146	83.33	100	2.48079e−36	Neuroptera	Osmylidae	Osmylus sp. EH−2004
Dolichopoda sp. EPT 16S ribosomal RNA gene, partia	EF216963	82.61	100	8.65881e−36	Orthoptera	Rhaphidophoridae	Dolichopoda sp. EPT
Bolotoperla rossi 16S ribosomal RNA gene, partial	EF623269	83.65	100	8.65881e−36	Plecoptera	Taeniopterygidae	Bolotoperla rossi
Osobenus yakimae 16S ribosomal RNA gene, partial s	EF623232	82.69	100	8.65881e−36	Plecoptera	Perlodidae	Osobenus yakimae
Isoperla phalerata 16S ribosomal RNA gene, partial	EF623208	82.69	100	8.65881e−36	Plecoptera	Perlodidae	Isoperla phalerata
Limnoperla sp. BYU_PL145 16S ribosomal RNA gene, p	EF623166	82.69	100	8.65881e−36	Plecoptera	Gripopterygidae	Limnoperla sp. BYU_PL145
Doddsia occidentalis 16S ribosomal RNA gene, parti	EF623270	83.85	100	3.02222e−35	Plecoptera	Taeniopterygidae	Doddsia occidentalis
Rickera sorpta 16S ribosomal RNA gene, partial seq	EF623235	84.18	100	3.02222e−35	Plecoptera	Perlodidae	Rickera sorpta
Cosumnoperla hypocrena 16S ribosomal RNA gene, par	EF623226	82.91	100	3.02222e−35	Plecoptera	Perlodidae	Cosumnoperla hypocrena
Cryptoperla ishigakiensis mitochondrial gene for 1	AB711242	82.91	100	1.05486e−34	Plecoptera	Peltoperlidae	Cryptoperla ishigakiensis
Cryptoperla japonica mitochondrial gene for 16S rR	AB711189	83.02	100	1.05486e−34	Plecoptera	Peltoperlidae	Cryptoperla japonica
Notonemouridae sp. MD−2011 voucher ZMUC:JD−1507 16	JN615257	83.12	100	1.05486e−34	Plecoptera	Notonemouridae	Notonemouridae sp. MD−2011
Chernokrilus misnomus 16S ribosomal RNA gene, part	EF623209	82.05	100	1.05486e−34	Plecoptera	Perlodidae	Chernokrilus misnomus
Isoperla fulva 16S ribosomal RNA gene, partial seq	EF623207	82.05	100	1.05486e−34	Plecoptera	Perlodidae	Isoperla fulva
Soliperla campanula 16S ribosomal RNA gene, partia	EF623256	82.91	99.36	3.68182e−34	Plecoptera	Peltoperlidae	Soliperla campanula
Kogotus nonus 16S ribosomal RNA gene, partial sequ	EF623213	83.54	100	3.68182e−34	Plecoptera	Perlodidae	Kogotus nonus
Kimminsoperla albomacula 16S ribosomal RNA gene, p	EF623202	82.8	100	3.68182e−34	Plecoptera	Notonemouridae	Kimminsoperla albomacula
Paraperla frontalis 16S ribosomal RNA gene, partia	EF623136	82.17	100	3.68182e−34	Plecoptera	Chloroperlidae	Paraperla frontalis
Dolichopoda geniculata geniculata isolate PRA20 16	AY507590	82.21	100	1.28508e−33	Orthoptera	Rhaphidophoridae	Dolichopoda geniculata geniculata
Plumiperla diversa 16S ribosomal RNA gene, partial	EF623132	82.91	100	1.28508e−33	Plecoptera	Chloroperlidae	Plumiperla diversa
Microchorista philpotti 16S ribosomal RNA gene, pa	KC177459	82.17	100	4.48537e−33	Mecoptera	Nannochoristidae	Nannochorista philpotti
Microchorista philpotti mitochondrion, partial gen	HQ696580	82.17	100	4.48537e−33	Mecoptera	Nannochoristidae	Nannochorista philpotti
Dolichopoda graeca voucher PER 16S ribosomal RNA g	EF216953	81.6	100	4.48537e−33	Orthoptera	Rhaphidophoridae	Dolichopoda graeca
Brachyptera seticornis 16S ribosomal RNA gene, par	EF623268	82.5	100	4.48537e−33	Plecoptera	Taeniopterygidae	Brachyptera seticornis
Pteronarcys scotti 16S ribosomal RNA gene, partial	EF623263	81.41	100	4.48537e−33	Plecoptera	Pteronarcyidae	Pteronarcys scotti
Pteronarcella badia 16S ribosomal RNA gene, partia	EF623262	81.37	100	4.48537e−33	Plecoptera	Pteronarcyidae	Pteronarcella badia
