# Supplementary material for: Testing the potential of a ribosomal 16S marker for DNA metabarcoding of insects
Source: PeerJ. 2016 Apr 19;4:e1966. doi: 10.7717/peerj.1966 (PMC4841222; doi:10.7717/peerj.1966)
Supplement: Supplemental Information 1 [file peerj-04-1966-s006.zip › scripts/5 match OTUs/blast_hits/OTU_91.csv_plot.pdf]

OTU_91.csv

Ephemera danica

Ephemeridae

Ephemeroptera

100

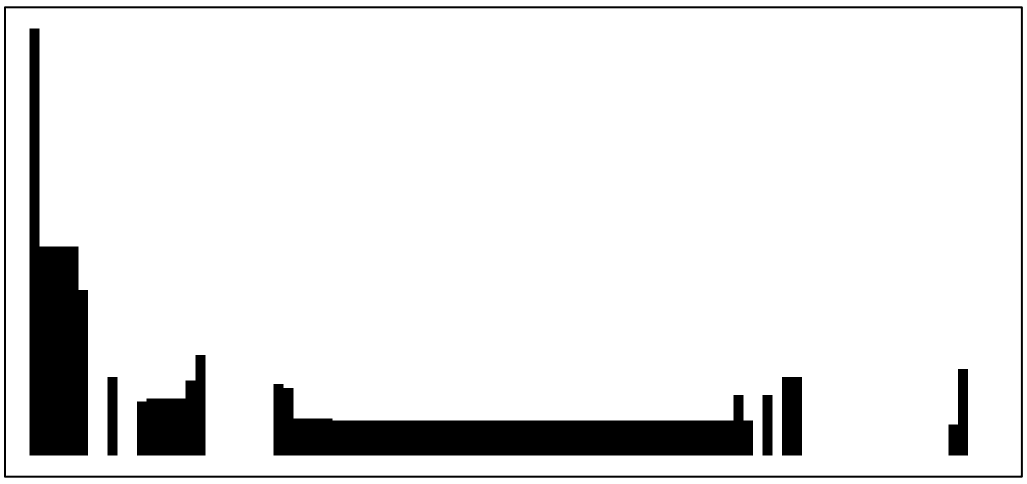

Ephemera danica 16S ribosomal RNA gene, partial se	KF855860	100	62.82	3.22684e−41	Ephemeroptera	Ephemeridae	Ephemera danica
Ephemera orientalis voucher EPH01 tRNA−Leu, 16S ri	KF256907	89.8	62.82	1.20359e−27	Ephemeroptera	Ephemeridae	Ephemera orientalis
Ephemera orientalis mitochondrion, complete genome	EU591678	89.8	62.82	1.20359e−27	Ephemeroptera	Ephemeridae	Ephemera orientalis
Ephemera sp. 1 BM−2008 12S ribosomal RNA gene, par	EU477611	89.8	62.82	1.46628e−26	Ephemeroptera	Ephemeridae	Ephemera sp. 1 BM−2008
Ephemera sp. 'Bonn' large subunit ribosomal RNA ge	AF266047	89.8	62.82	1.46628e−26	Ephemeroptera	Ephemeridae	Ephemera sp. 'Bonn'
Ephemera simulans voucher BYU:IGCEP038 16S ribosom	GQ118285	87.76	62.82	6.23476e−25	Ephemeroptera	Ephemeridae	Ephemera simulans
Apteroperla tikumana mitochondrion, complete genom	KR604721	76.58	100	4.79328e−20	Plecoptera	Capniidae	Apteroperla tikumana
Moselia infuscata 16S ribosomal RNA gene, partial	EF623175	76.28	99.36	4.79328e−20	Plecoptera	Leuctridae	Moselia infuscata
Siphlonurus immanis voucher NKMT032 mitochondrion,	FJ606783	83.67	62.82	1.67302e−19	Ephemeroptera	Siphlonuridae	Siphlonurus immanis
Leuctra inermis 16S ribosomal RNA gene, partial se	EF623176	76.4	100	5.83941e−19	Plecoptera	Leuctridae	Leuctra inermis
Allocapnia minima 16S ribosomal RNA gene, partial	EF623130	75.95	100	5.83941e−19	Plecoptera	Capniidae	Allocapnia minima
Atrophaneura dasarada 16S ribosomal RNA gene, part	DQ673329	82.52	65.38	2.03816e−18	Lepidoptera	Papilionidae	Atrophaneura dasarada
Palingenia longicauda mitochondrial partial 16S rR	HE650662	82.65	62.82	7.11386e−18	Ephemeroptera	Palingeniidae	Palingenia longicauda
Palingenia longicauda mitochondrial partial 16S rR	HE650475	82.65	62.82	7.11386e−18	Ephemeroptera	Palingeniidae	Palingenia longicauda
Palingenia longicauda mitochondrial partial 16S rR	HE650547	82.65	62.82	7.11386e−18	Ephemeroptera	Palingeniidae	Palingenia longicauda
Palingenia longicauda mitochondrial partial 16S rR	HE650502	82.65	62.82	7.11386e−18	Ephemeroptera	Palingeniidae	Palingenia longicauda
Eresia plaginota isolate 38−4 16S ribosomal RNA ge	AF186912	83.51	61.54	7.11386e−18	Lepidoptera	Nymphalidae	Eresia plaginota
Ctenoplusia limbirena mitochondrion, complete geno	KM244665	84.69	61.54	2.48298e−17	Lepidoptera	Noctuidae	Ctenoplusia limbirena
Allocapnia recta isolate H15 16S ribosomal RNA gen	KC881050	75.32	100	2.48298e−17	Plecoptera	Capniidae	Allocapnia recta
Allocapnia recta isolate H14 16S ribosomal RNA gen	KC881049	75.32	100	2.48298e−17	Plecoptera	Capniidae	Allocapnia recta
Allocapnia recta isolate H10 16S ribosomal RNA gen	KC881045	75.32	100	2.48298e−17	Plecoptera	Capniidae	Allocapnia recta
Allocapnia recta isolate H6 16S ribosomal RNA gene	KC881041	75.32	100	2.48298e−17	Plecoptera	Capniidae	Allocapnia recta
Isocapnia hyalita 16S ribosomal RNA gene, partial	EF623131	75.32	100	2.48298e−17	Plecoptera	Capniidae	Isocapnia hyalita
Allocapnia recta haplotype 11ar: H011 16S ribosoma	EU306874	75.32	100	2.48298e−17	Plecoptera	Capniidae	Allocapnia recta
Allocapnia recta haplotype 7ar: H07 16S ribosomal	EU306870	75.32	100	2.48298e−17	Plecoptera	Capniidae	Allocapnia recta
Ichthybotus hudsoni 16S ribosomal RNA gene, partia	AY749816	83.33	61.54	2.48298e−17	Ephemeroptera	Ichthybotidae	Ichthybotus hudsoni
Leucocelis rubra voucher 842824 16S ribosomal RNA	JX234507	83.16	60.26	8.66646e−17	Coleoptera	Scarabaeidae	Leucocelis rubra
Semirhynchia sp. n. SV01 16S ribosomal RNA gene, p	JX294172	81.73	64.1	8.66646e−17	Neuroptera	Nemopteridae	Semirhynchia sp. n. SV01
Sicyoptera cuspidata isolate SCBB01 16S ribosomal	JX294167	81.73	64.1	8.66646e−17	Neuroptera	Nemopteridae	Sicyoptera cuspidata
Sicyoptera dilatata isolate SDGG01 16S ribosomal R	JX294163	81.73	64.1	8.66646e−17	Neuroptera	Nemopteridae	Sicyoptera dilatata
Nemeura gracilis isolate NGW01 16S ribosomal RNA g	JX294132	81.73	64.1	8.66646e−17	Neuroptera	Nemopteridae	Nemeura gracilis
Palingenia longicauda mitochondrial partial 16S rR	HE650589	81.63	62.82	8.66646e−17	Ephemeroptera	Palingeniidae	Palingenia longicauda
Palingenia longicauda mitochondrial partial 16S rR	HE650657	81.63	62.82	8.66646e−17	Ephemeroptera	Palingeniidae	Palingenia longicauda
Palingenia longicauda mitochondrial partial 16S rR	HE650650	81.63	62.82	8.66646e−17	Ephemeroptera	Palingeniidae	Palingenia longicauda
Palingenia longicauda mitochondrial partial 16S rR	HE650647	81.63	62.82	8.66646e−17	Ephemeroptera	Palingeniidae	Palingenia longicauda
Palingenia longicauda mitochondrial partial 16S rR	HE650637	81.63	62.82	8.66646e−17	Ephemeroptera	Palingeniidae	Palingenia longicauda
Palingenia longicauda mitochondrial partial 16S rR	HE650635	81.63	62.82	8.66646e−17	Ephemeroptera	Palingeniidae	Palingenia longicauda
Palingenia longicauda mitochondrial partial 16S rR	HE650631	81.63	62.82	8.66646e−17	Ephemeroptera	Palingeniidae	Palingenia longicauda
Palingenia longicauda mitochondrial partial 16S rR	HE650625	81.63	62.82	8.66646e−17	Ephemeroptera	Palingeniidae	Palingenia longicauda
Palingenia longicauda mitochondrial partial 16S rR	HE650623	81.63	62.82	8.66646e−17	Ephemeroptera	Palingeniidae	Palingenia longicauda
Palingenia longicauda mitochondrial partial 16S rR	HE650622	81.63	62.82	8.66646e−17	Ephemeroptera	Palingeniidae	Palingenia longicauda
Palingenia longicauda mitochondrial partial 16S rR	HE650618	81.63	62.82	8.66646e−17	Ephemeroptera	Palingeniidae	Palingenia longicauda
Palingenia longicauda mitochondrial partial 16S rR	HE650617	81.63	62.82	8.66646e−17	Ephemeroptera	Palingeniidae	Palingenia longicauda
Palingenia longicauda mitochondrial partial 16S rR	HE650616	81.63	62.82	8.66646e−17	Ephemeroptera	Palingeniidae	Palingenia longicauda
Palingenia longicauda mitochondrial partial 16S rR	HE650604	81.63	62.82	8.66646e−17	Ephemeroptera	Palingeniidae	Palingenia longicauda
Palingenia longicauda mitochondrial partial 16S rR	HE650594	81.63	62.82	8.66646e−17	Ephemeroptera	Palingeniidae	Palingenia longicauda
Palingenia longicauda mitochondrial partial 16S rR	HE650588	81.63	62.82	8.66646e−17	Ephemeroptera	Palingeniidae	Palingenia longicauda
Palingenia longicauda mitochondrial partial 16S rR	HE650575	81.63	62.82	8.66646e−17	Ephemeroptera	Palingeniidae	Palingenia longicauda
Palingenia longicauda mitochondrial partial 16S rR	HE650569	81.63	62.82	8.66646e−17	Ephemeroptera	Palingeniidae	Palingenia longicauda
Palingenia longicauda mitochondrial partial 16S rR	HE650550	81.63	62.82	8.66646e−17	Ephemeroptera	Palingeniidae	Palingenia longicauda
Palingenia longicauda mitochondrial partial 16S rR	HE650542	81.63	62.82	8.66646e−17	Ephemeroptera	Palingeniidae	Palingenia longicauda
Palingenia longicauda mitochondrial partial 16S rR	HE650533	81.63	62.82	8.66646e−17	Ephemeroptera	Palingeniidae	Palingenia longicauda
Palingenia longicauda mitochondrial partial 16S rR	HE650503	81.63	62.82	8.66646e−17	Ephemeroptera	Palingeniidae	Palingenia longicauda
Palingenia longicauda mitochondrial partial 16S rR	HE650520	81.63	62.82	8.66646e−17	Ephemeroptera	Palingeniidae	Palingenia longicauda
Palingenia longicauda mitochondrial partial 16S rR	HE650519	81.63	62.82	8.66646e−17	Ephemeroptera	Palingeniidae	Palingenia longicauda
Palingenia longicauda mitochondrial partial 16S rR	HE650515	81.63	62.82	8.66646e−17	Ephemeroptera	Palingeniidae	Palingenia longicauda
Palingenia longicauda mitochondrial partial 16S rR	HE650514	81.63	62.82	8.66646e−17	Ephemeroptera	Palingeniidae	Palingenia longicauda
Palingenia longicauda mitochondrial partial 16S rR	HE650509	81.63	62.82	8.66646e−17	Ephemeroptera	Palingeniidae	Palingenia longicauda
Palingenia longicauda mitochondrial partial 16S rR	HE650507	81.63	62.82	8.66646e−17	Ephemeroptera	Palingeniidae	Palingenia longicauda
Palingenia longicauda mitochondrial partial 16S rR	HE650505	81.63	62.82	8.66646e−17	Ephemeroptera	Palingeniidae	Palingenia longicauda
Palingenia longicauda mitochondrial partial 16S rR	HE650499	81.63	62.82	8.66646e−17	Ephemeroptera	Palingeniidae	Palingenia longicauda
Palingenia longicauda mitochondrial partial 16S rR	HE650446	81.63	62.82	8.66646e−17	Ephemeroptera	Palingeniidae	Palingenia longicauda
Palingenia longicauda mitochondrial partial 16S rR	HE650435	81.63	62.82	8.66646e−17	Ephemeroptera	Palingeniidae	Palingenia longicauda
Palingenia longicauda mitochondrial partial 16S rR	HE650461	81.63	62.82	8.66646e−17	Ephemeroptera	Palingeniidae	Palingenia longicauda
Palingenia longicauda mitochondrial partial 16S rR	HE650449	81.63	62.82	8.66646e−17	Ephemeroptera	Palingeniidae	Palingenia longicauda
Palingenia longicauda mitochondrial partial 16S rR	HE650420	81.63	62.82	8.66646e−17	Ephemeroptera	Palingeniidae	Palingenia longicauda
Palingenia longicauda mitochondrial partial 16S rR	HE650440	81.63	62.82	8.66646e−17	Ephemeroptera	Palingeniidae	Palingenia longicauda
Palingenia longicauda mitochondrial partial 16S rR	HE650434	81.63	62.82	8.66646e−17	Ephemeroptera	Palingeniidae	Palingenia longicauda
Palingenia longicauda mitochondrial partial 16S rR	HE650430	81.63	62.82	8.66646e−17	Ephemeroptera	Palingeniidae	Palingenia longicauda
Palingenia longicauda mitochondrial partial 16S rR	HE650421	81.63	62.82	8.66646e−17	Ephemeroptera	Palingeniidae	Palingenia longicauda
Palingenia longicauda mitochondrial partial 16S rR	HE650424	81.63	62.82	8.66646e−17	Ephemeroptera	Palingeniidae	Palingenia longicauda
Palingenia longicauda mitochondrial partial 16S rR	HE650422	81.63	62.82	8.66646e−17	Ephemeroptera	Palingeniidae	Palingenia longicauda
Euryades corethrus voucher FS−b−3716 16S ribosomal	GQ268348	82.83	61.54	8.66646e−17	Lepidoptera	Papilionidae	Euryades corethrus
Palingenia longicauda voucher BYU:IGCEP216 16S rib	GQ118292	81.63	62.82	8.66646e−17	Ephemeroptera	Palingeniidae	Palingenia longicauda
Lertha barbara 16S ribosomal RNA gene, partial seq	EU734879	75.16	99.36	8.66646e−17	Neuroptera	Nemopteridae	Lertha barbara
Euryades corethrus mitochondrial partial 16S rRNA	AM283053	82.83	61.54	8.66646e−17	Lepidoptera	Papilionidae	Euryades corethrus
Papilio delalandei mitochondrion, large subunit ri	AJ224060	76.43	97.44	8.66646e−17	Lepidoptera	Papilionidae	Papilio delalandei
Hygrostola dallolmoi voucher HYTAG7234 16S ribosom	KP682574	83.67	61.54	3.02489e−16	Lepidoptera	Noctuidae	Hygrostola dallolmoi
Hygrostola dallolmoi voucher HYTAG6454 16S ribosom	KP682573	83.67	61.54	3.02489e−16	Lepidoptera	Noctuidae	Hygrostola dallolmoi
Mesocapnia arizonensis mitochondrion, partial geno	KP642637	74.68	100	3.02489e−16	Plecoptera	Capniidae	Mesocapnia arizonensis
Neptis clinia mitochondrion, complete genome	KM244664	75.48	97.44	3.02489e−16	Lepidoptera	Nymphalidae	Neptis clinia
Allocapnia recta isolate H19 16S ribosomal RNA gen	KC881054	74.68	100	3.02489e−16	Plecoptera	Capniidae	Allocapnia recta
Allocapnia recta isolate H17 16S ribosomal RNA gen	KC881052	74.68	100	3.02489e−16	Plecoptera	Capniidae	Allocapnia recta
Allocapnia recta isolate H16 16S ribosomal RNA gen	KC881051	74.68	100	3.02489e−16	Plecoptera	Capniidae	Allocapnia recta
Allocapnia recta isolate H13 16S ribosomal RNA gen	KC881048	74.68	100	3.02489e−16	Plecoptera	Capniidae	Allocapnia recta
Allocapnia recta isolate H12 16S ribosomal RNA gen	KC881047	74.68	100	3.02489e−16	Plecoptera	Capniidae	Allocapnia recta
Allocapnia recta isolate H11 16S ribosomal RNA gen	KC881046	74.68	100	3.02489e−16	Plecoptera	Capniidae	Allocapnia recta
Allocapnia recta isolate H9 16S ribosomal RNA gene	KC881044	74.68	100	3.02489e−16	Plecoptera	Capniidae	Allocapnia recta
Allocapnia recta isolate H8 16S ribosomal RNA gene	KC881043	74.68	100	3.02489e−16	Plecoptera	Capniidae	Allocapnia recta
Allocapnia recta isolate H7 16S ribosomal RNA gene	KC881042	74.68	100	3.02489e−16	Plecoptera	Capniidae	Allocapnia recta
Allocapnia recta isolate H5 16S ribosomal RNA gene	KC881040	74.68	100	3.02489e−16	Plecoptera	Capniidae	Allocapnia recta
Allocapnia recta isolate H4 16S ribosomal RNA gene	KC881039	74.68	100	3.02489e−16	Plecoptera	Capniidae	Allocapnia recta
Allocapnia recta isolate H3 16S ribosomal RNA gene	KC881038	74.68	100	3.02489e−16	Plecoptera	Capniidae	Allocapnia recta
Allocapnia recta isolate H1 16S ribosomal RNA gene	KC881036	74.68	100	3.02489e−16	Plecoptera	Capniidae	Allocapnia recta
Palingenia longicauda mitochondrial partial 16S rR	HE650510	81.44	62.18	3.02489e−16	Ephemeroptera	Palingeniidae	Palingenia longicauda
Trox niponensis isolate 100 16S ribosomal RNA gene	EF570413	84.04	58.97	3.02489e−16	Coleoptera	Trogidae	Trox niponensis
Mesocapnia sp. BYU_PL010 16S ribosomal RNA gene, p	EF623127	74.68	100	3.02489e−16	Plecoptera	Capniidae	Mesocapnia sp. BYU_PL010
Allocapnia recta haplotype 10ar: H010 16S ribosoma	EU306873	74.68	100	3.02489e−16	Plecoptera	Capniidae	Allocapnia recta
Allocapnia recta haplotype 9ar: H09 16S ribosomal	EU306872	74.68	100	3.02489e−16	Plecoptera	Capniidae	Allocapnia recta
Allocapnia recta haplotype 8ar: H08 16S ribosomal	EU306871	74.68	100	3.02489e−16	Plecoptera	Capniidae	Allocapnia recta
