# Supplementary material for: Testing the potential of a ribosomal 16S marker for DNA metabarcoding of insects
Source: PeerJ. 2016 Apr 19;4:e1966. doi: 10.7717/peerj.1966 (PMC4841222; doi:10.7717/peerj.1966)
Supplement: Supplemental Information 1 [file peerj-04-1966-s006.zip › scripts/5 match OTUs/blast_hits/OTU_93.csv_plot.pdf]

OTU_93.csv

Ephemera danica

Ephemeridae

Ephemeroptera

84.62

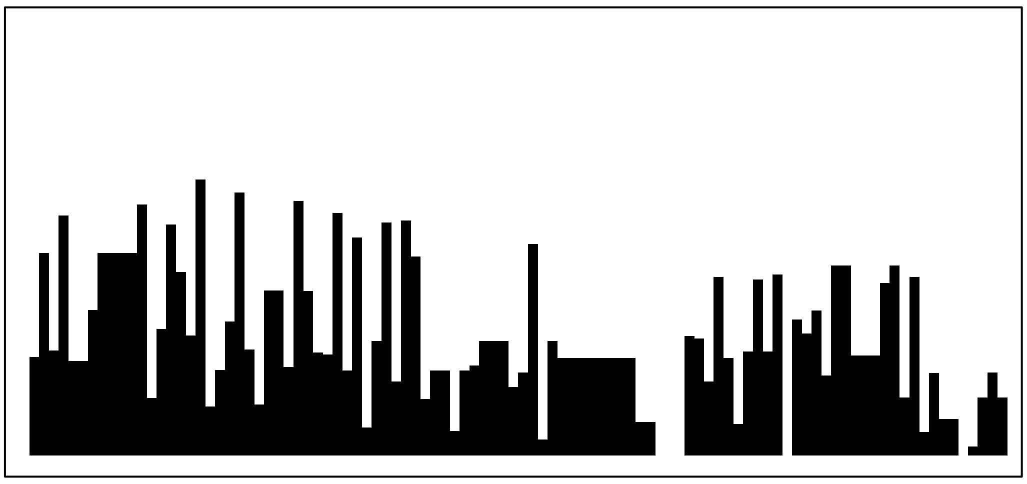

Ephemera danica 16S ribosomal RNA gene, partial se	KF855860	84.62	100	5.68961e−38	Ephemeroptera	Ephemeridae	Ephemera danica
Isoperla oxylepis 16S ribosomal RNA gene, partial	EF623221	89.47	84.97	5.68961e−38	Plecoptera	Perlodidae	Isoperla oxylepis
Isoperla davisi 16S ribosomal RNA gene, partial se	EF623206	84.91	100	1.98587e−37	Plecoptera	Perlodidae	Isoperla davisi
Cryptoperla sp. YK−2012−1 mitochondrial gene for 1	AB711243	91.23	74.51	2.41928e−36	Plecoptera	Peltoperlidae	Cryptoperla sp. YK−2012−1
Ephemera orientalis voucher EPH01 tRNA−Leu, 16S ri	KF256907	84.42	100	8.44412e−36	Ephemeroptera	Ephemeridae	Ephemera orientalis
Ephemera orientalis mitochondrion, complete genome	EU591678	84.42	100	8.44412e−36	Ephemeroptera	Ephemeridae	Ephemera orientalis
Perlinodes aurea 16S ribosomal RNA gene, partial s	EF623234	86.81	92.16	8.44412e−36	Plecoptera	Perlodidae	Perlinodes aurea
Cryptoperla japonica mitochondrial gene for 16S rR	AB711202	89.47	74.51	1.25322e−33	Plecoptera	Peltoperlidae	Cryptoperla japonica
Cryptoperla japonica mitochondrial gene for 16S rR	AB711184	89.47	74.51	1.25322e−33	Plecoptera	Peltoperlidae	Cryptoperla japonica
Cryptoperla japonica mitochondrial gene for 16S rR	AB711183	89.47	74.51	1.25322e−33	Plecoptera	Peltoperlidae	Cryptoperla japonica
Cryptoperla japonica mitochondrial gene for 16S rR	AB711182	89.47	74.51	1.25322e−33	Plecoptera	Peltoperlidae	Cryptoperla japonica
Cascadoperla trictura 16S ribosomal RNA gene, part	EF623225	91.74	71.24	1.25322e−33	Plecoptera	Perlodidae	Cascadoperla trictura
Leucochrysa texana 16S ribosomal RNA gene, partial	DQ399279	82.69	99.35	4.37416e−33	Neuroptera	Chrysopidae	Leucochrysa texana
Notonemouridae sp. MD−2011 voucher ZMUC:JD−1507 16	JN615257	85.93	85.62	1.52673e−32	Plecoptera	Notonemouridae	Notonemouridae sp. MD−2011
Cosumnoperla hypocrena 16S ribosomal RNA gene, par	EF623226	90.83	71.24	1.52673e−32	Plecoptera	Perlodidae	Cosumnoperla hypocrena
Cryptoperla ishigakiensis mitochondrial gene for 1	AB711242	88.6	74.51	5.32882e−32	Plecoptera	Peltoperlidae	Cryptoperla ishigakiensis
Frisonia picticeps 16S ribosomal RNA gene, partial	EF623227	85.61	84.31	5.32882e−32	Plecoptera	Perlodidae	Frisonia picticeps
Chernokrilus misnomus 16S ribosomal RNA gene, part	EF623209	92.93	64.71	5.32882e−32	Plecoptera	Perlodidae	Chernokrilus misnomus
Plumiperla diversa 16S ribosomal RNA gene, partial	EF623132	82.28	100	5.32882e−32	Plecoptera	Chloroperlidae	Plumiperla diversa
Neopanorpa sp. M24899 16S ribosomal RNA gene, part	AF180059	84	98.04	5.32882e−32	Mecoptera	Panorpidae	Neopanorpa sp. M24899
Peltoperla arcuata 16S ribosomal RNA gene, partial	EF623258	86.26	83.66	1.85994e−31	Plecoptera	Peltoperlidae	Peltoperla arcuata
Rickera sorpta 16S ribosomal RNA gene, partial seq	EF623235	92.31	67.32	1.85994e−31	Plecoptera	Perlodidae	Rickera sorpta
Isoperla phalerata 16S ribosomal RNA gene, partial	EF623208	84.96	84.97	1.85994e−31	Plecoptera	Perlodidae	Isoperla phalerata
Triznaka pintada 16S ribosomal RNA gene, partial s	EF623134	82.39	100	1.85994e−31	Plecoptera	Chloroperlidae	Triznaka pintada
Cryptoperla sp. YK−2012−2 mitochondrial gene for 1	AB711244	87.72	74.51	6.49184e−31	Plecoptera	Peltoperlidae	Cryptoperla sp. YK−2012−2
Cryptoperla japonica mitochondrial gene for 16S rR	AB711189	87.72	74.51	6.49184e−31	Plecoptera	Peltoperlidae	Cryptoperla japonica
Salmoperla sylvanica 16S ribosomal RNA gene, parti	EF623222	84.14	92.81	6.49184e−31	Plecoptera	Perlodidae	Salmoperla sylvanica
Isoperla fulva 16S ribosomal RNA gene, partial seq	EF623207	91.92	64.71	6.49184e−31	Plecoptera	Perlodidae	Isoperla fulva
Megaleuctra kincaidi 16S ribosomal RNA gene, parti	EF623174	87.7	77.12	6.49184e−31	Plecoptera	Leuctridae	Megaleuctra kincaidi
Megarcys signata 16S ribosomal RNA gene, partial s	EF623215	84.83	92.16	2.26587e−30	Plecoptera	Perlodidae	Megarcys signata
Thaumatoperla sp. BYU_PL109 16S ribosomal RNA gene	EF623149	84.73	83.66	2.26587e−30	Plecoptera	Eustheniidae	Thaumatoperla sp. BYU_PL109
Kogotus nonus 16S ribosomal RNA gene, partial sequ	EF623213	91.35	67.32	7.90868e−30	Plecoptera	Perlodidae	Kogotus nonus
Limnoperla sp. BYU_PL145 16S ribosomal RNA gene, p	EF623166	83.97	84.31	7.90868e−30	Plecoptera	Gripopterygidae	Limnoperla sp. BYU_PL145
Sweltsa coloradensis 16S ribosomal RNA gene, parti	EF623133	90.2	66.67	7.90868e−30	Plecoptera	Chloroperlidae	Sweltsa coloradensis
Nannoleon michaelseni isolate Nanmic1 16S ribosoma	AY620177	81.29	98.69	7.90868e−30	Neuroptera	Myrmeleontidae	Nannoleon michaelseni
Myrmeleon hyalinus isolate 58−hysic2 16S ribosomal	KC195806	85.37	79.74	2.7604e−29	Neuroptera	Myrmeleontidae	Myrmeleon hyalinus
Osobenus yakimae 16S ribosomal RNA gene, partial s	EF623232	90.91	64.71	2.7604e−29	Plecoptera	Perlodidae	Osobenus yakimae
Dolichopoda sp. SPS 16S ribosomal RNA gene, partia	EF216965	83.46	84.31	9.63474e−29	Orthoptera	Rhaphidophoridae	Dolichopoda sp. SPS
Perlodes dispar 16S ribosomal RNA gene, partial se	EF623220	91	64.71	9.63474e−29	Plecoptera	Perlodidae	Perlodes dispar
Pictetiella expansa 16S ribosomal RNA gene, partia	EF623216	89.32	67.32	9.63474e−29	Plecoptera	Perlodidae	Pictetiella expansa
Austronemoura sp. BYU_PL149 16S ribosomal RNA gene	EF623200	82.64	90.85	9.63474e−29	Plecoptera	Notonemouridae	Austronemoura sp. BYU_PL149
Eusthenia costalis 16S ribosomal RNA gene, partial	EF623150	83.97	83.66	9.63474e−29	Plecoptera	Eustheniidae	Eusthenia costalis
Diamphipnopsis sp. BYU_PL138 16S ribosomal RNA gen	EF623145	83.97	83.66	9.63474e−29	Plecoptera	Diamphipnoidae	Diamphipnopsis sp. BYU_PL138
Paraperla frontalis 16S ribosomal RNA gene, partia	EF623136	81.13	100	9.63474e−29	Plecoptera	Chloroperlidae	Paraperla frontalis
Klapopteryx armillata 16S ribosomal RNA gene, part	EF623123	83.97	83.66	9.63474e−29	Plecoptera	Austroperlidae	Klapopteryx armillata
Perlodes mortoni 16S ribosomal RNA gene, partial s	KF855870	84.21	83.66	3.36285e−28	Plecoptera	Perlodidae	Perlodes mortoni
Dolichopoda remyi voucher EDE 16S ribosomal RNA ge	EF216969	85.37	80.39	3.36285e−28	Orthoptera	Rhaphidophoridae	Dolichopoda remyi
Dolichopoda sp. SKA 16S ribosomal RNA gene, partia	EF216964	85.37	80.39	3.36285e−28	Orthoptera	Rhaphidophoridae	Dolichopoda sp. SKA
Dolichopoda sp. EPT 16S ribosomal RNA gene, partia	EF216963	85.37	80.39	3.36285e−28	Orthoptera	Rhaphidophoridae	Dolichopoda sp. EPT
Neonemura sp. BYU_PL153 16S ribosomal RNA gene, pa	EF623204	83.21	84.31	3.36285e−28	Plecoptera	Notonemouridae	Neonemura sp. BYU_PL153
Notoperla sp. BYU_PL146 16S ribosomal RNA gene, pa	EF623167	83.87	81.05	3.36285e−28	Plecoptera	Gripopterygidae	Notoperla sp. BYU_PL146
Cosmioperla australis 16S ribosomal RNA gene, part	EF623148	89.9	64.71	3.36285e−28	Plecoptera	Eustheniidae	Cosmioperla australis
Suwallia teleckojensis 16S ribosomal RNA gene, par	EF623142	80.75	100	3.36285e−28	Plecoptera	Chloroperlidae	Suwallia teleckojensis
Dolichopoda remyi isolate EDE 16S ribosomal RNA ge	AY793589	85.37	80.39	3.36285e−28	Orthoptera	Rhaphidophoridae	Dolichopoda remyi
Myrmeleon hyalinus isolate 38−hyalalg1 16S ribosom	KC195816	84.55	79.74	1.17375e−27	Neuroptera	Myrmeleontidae	Myrmeleon hyalinus
Myrmeleon hyalinus isolate 55−hyalg2 16S ribosomal	KC195815	84.55	79.74	1.17375e−27	Neuroptera	Myrmeleontidae	Myrmeleon hyalinus
Myrmeleon hyalinus isolate 85−hyalnero 16S ribosom	KC195814	84.55	79.74	1.17375e−27	Neuroptera	Myrmeleontidae	Myrmeleon hyalinus
Myrmeleon hyalinus isolate 50−hysic1 16S ribosomal	KC195813	84.55	79.74	1.17375e−27	Neuroptera	Myrmeleontidae	Myrmeleon hyalinus
Myrmeleon hyalinus isolate 21−hyalcre2 16S ribosom	KC195811	84.55	79.74	1.17375e−27	Neuroptera	Myrmeleontidae	Myrmeleon hyalinus
Myrmeleon hyalinus isolate 57−hyaro1 16S ribosomal	KC195809	84.55	79.74	1.17375e−27	Neuroptera	Myrmeleontidae	Myrmeleon hyalinus
Myrmeleon hyalinus isolate 53−hyeg1 16S ribosomal	KC195808	84.55	79.74	1.17375e−27	Neuroptera	Myrmeleontidae	Myrmeleon hyalinus
Myrmeleon hyalinus isolate 64−hyisr 16S ribosomal	KC195807	84.55	79.74	1.17375e−27	Neuroptera	Myrmeleontidae	Myrmeleon hyalinus
Microchorista philpotti 16S ribosomal RNA gene, pa	KC177459	81.56	92.16	1.17375e−27	Mecoptera	Nannochoristidae	Nannochorista philpotti
Microchorista philpotti mitochondrion, partial gen	HQ696580	81.56	92.16	1.17375e−27	Mecoptera	Nannochoristidae	Nannochorista philpotti
Pterostichus shibatai mitochondrial gene for 16S r	AB253863	79.75	100	1.17375e−27	Coleoptera	Carabidae	Pterostichus shibatai
Siphlonurus immanis voucher NKMT032 mitochondrion,	FJ606783	79.74	100	1.17375e−27	Ephemeroptera	Siphlonuridae	Siphlonurus immanis
Ephemera simulans voucher BYU:IGCEP038 16S ribosom	GQ118285	79.74	100	1.17375e−27	Ephemeroptera	Ephemeridae	Ephemera simulans
Dolichopoda geniculata geniculata isolate PRA20 16	AY507590	85.6	80.39	1.17375e−27	Orthoptera	Rhaphidophoridae	Dolichopoda geniculata geniculata
Dolichopoda sp. MR−2008 16S ribosomal RNA gene, pa	EU887863	85.48	80.39	1.17375e−27	Orthoptera	Rhaphidophoridae	Dolichopoda lustriae
Doddsia occidentalis 16S ribosomal RNA gene, parti	EF623270	83.46	84.31	1.17375e−27	Plecoptera	Taeniopterygidae	Doddsia occidentalis
Cultus pilatus 16S ribosomal RNA gene, partial seq	EF623212	88.35	67.32	1.17375e−27	Plecoptera	Perlodidae	Cultus pilatus
Dolichopoda sutini isolate Ask1 16S ribosomal RNA	KC783269	84.55	80.39	4.0968e−27	Orthoptera	Rhaphidophoridae	Dolichopoda sutini
Neopanorpa pulchra mitochondrion, complete genome	JX569848	81.46	98.69	4.0968e−27	Mecoptera	Panorpidae	Neopanorpa pulchra
Myrmeleon hyalinus isolate 20−hyalcre1 16S ribosom	KC195817	84.87	77.12	4.0968e−27	Neuroptera	Myrmeleontidae	Myrmeleon hyalinus
Cryptoperla kawasawai mitochondrial gene for 16S r	AB711236	88.24	66.67	4.0968e−27	Plecoptera	Peltoperlidae	Cryptoperla kawasawai
Soliperla campanula 16S ribosomal RNA gene, partia	EF623256	84.87	76.47	4.0968e−27	Plecoptera	Peltoperlidae	Soliperla campanula
Skwala americana 16S ribosomal RNA gene, partial s	EF623217	88.46	67.32	4.0968e−27	Plecoptera	Perlodidae	Skwala americana
Nemoura cinerea 16S ribosomal RNA gene, partial se	EF623194	80	100	4.0968e−27	Plecoptera	Nemouridae	Nemoura cinerea
Despaxia augusta 16S ribosomal RNA gene, partial s	EF623177	86.36	71.9	4.0968e−27	Plecoptera	Leuctridae	Despaxia augusta
Coptaspis sp. MCJ−2002 16S ribosomal RNA gene, par	AF514494	85.71	77.12	4.0968e−27	Orthoptera	Tettigoniidae	Coptaspis sp. MCJ−2002
Dolichopoda sutini isolate Sut1 16S ribosomal RNA	KC783268	86.79	69.28	1.42992e−26	Orthoptera	Rhaphidophoridae	Dolichopoda sutini
Myrmeleon hyalinus isolate 45−hyalro1 16S ribosoma	KC195810	83.74	79.74	1.42992e−26	Neuroptera	Myrmeleontidae	Myrmeleon hyalinus
Dolichopoda naxia voucher ZEU 16S ribosomal RNA ge	EU887868	88.89	64.71	1.42992e−26	Orthoptera	Rhaphidophoridae	Dolichopoda naxia
Dolichopoda naxia voucher STA 16S ribosomal RNA ge	EU887867	88.89	64.71	1.42992e−26	Orthoptera	Rhaphidophoridae	Dolichopoda naxia
Dolichopoda annae voucher OLI 16S ribosomal RNA ge	EU887860	84.68	80.39	1.42992e−26	Orthoptera	Rhaphidophoridae	Dolichopoda annae
Dolichopoda hussoni voucher IZB 16S ribosomal RNA	EF216973	84.68	80.39	1.42992e−26	Orthoptera	Rhaphidophoridae	Dolichopoda hussoni
Dolichopoda thasosensis voucher DRA 16S ribosomal	EF216956	84.68	80.39	1.42992e−26	Orthoptera	Rhaphidophoridae	Dolichopoda thasosensis
Scopura montana 16S ribosomal RNA gene, partial se	EF623265	88.07	69.93	1.42992e−26	Plecoptera		Scopura montana
Malirekus iroquois 16S ribosomal RNA gene, partial	EF623231	88.89	64.71	1.42992e−26	Plecoptera	Perlodidae	Malirekus iroquois
Neofulla sp. BYU_PL152 16S ribosomal RNA gene, par	EF623203	82.71	85.62	1.42992e−26	Plecoptera	Notonemouridae	Neofulla sp. BYU_PL152
Neaviperla forcipata 16S ribosomal RNA gene, parti	EF623139	88.35	66.67	1.42992e−26	Plecoptera	Chloroperlidae	Neaviperla forcipata
Kathroperla perdita 16S ribosomal RNA gene, partia	EF623135	81.08	94.12	1.42992e−26	Plecoptera	Chloroperlidae	Kathroperla perdita
Psorodonotus fieberi voucher 198 16S ribosomal RNA	EU120806	83.85	83.66	1.42992e−26	Orthoptera	Tettigoniidae	Psorodonotus fieberi
Tyrannophasma gladiator 16S large subunit ribosoma	DQ890285	81.69	90.85	1.42992e−26	Mantophasmatodea		Tyrannophasma gladiator
Tyrannophasma gladiator voucher BYU_mp02 16S ribos	DQ457266	81.69	90.85	1.42992e−26	Mantophasmatodea		Tyrannophasma gladiator
Leucochrysa marquezi voucher CASC 00212 16S riboso	DQ399280	79.49	99.35	1.42992e−26	Neuroptera	Chrysopidae	Leucochrysa marquezi
Chrysopodes polygonica voucher CASC 00222 16S ribo	DQ399273	80.42	92.81	1.42992e−26	Neuroptera	Chrysopidae	Chrysopodes polygonica
Mesocapnia arizonensis mitochondrion, partial geno	KP642637	82.71	84.31	4.99092e−26	Plecoptera	Capniidae	Mesocapnia arizonensis
Carphurus sp. 2 NLG−2013 voucher COL358 12S riboso	KC524784	83.87	79.74	4.99092e−26	Coleoptera	Melyridae	Carphurus sp. 2 NLG−2013
Allocapnia recta isolate H13 16S ribosomal RNA gen	KC881048	82.71	84.31	4.99092e−26	Plecoptera	Capniidae	Allocapnia recta
