# Supplementary material for: Testing the potential of a ribosomal 16S marker for DNA metabarcoding of insects
Source: PeerJ. 2016 Apr 19;4:e1966. doi: 10.7717/peerj.1966 (PMC4841222; doi:10.7717/peerj.1966)
Supplement: Supplemental Information 1 [file peerj-04-1966-s006.zip › scripts/5 match OTUs/blast_hits/OTU_94.csv_plot.pdf]

OTU_94.csv

Hansonoperla appalachia

Perlidae

Plecoptera

87.38

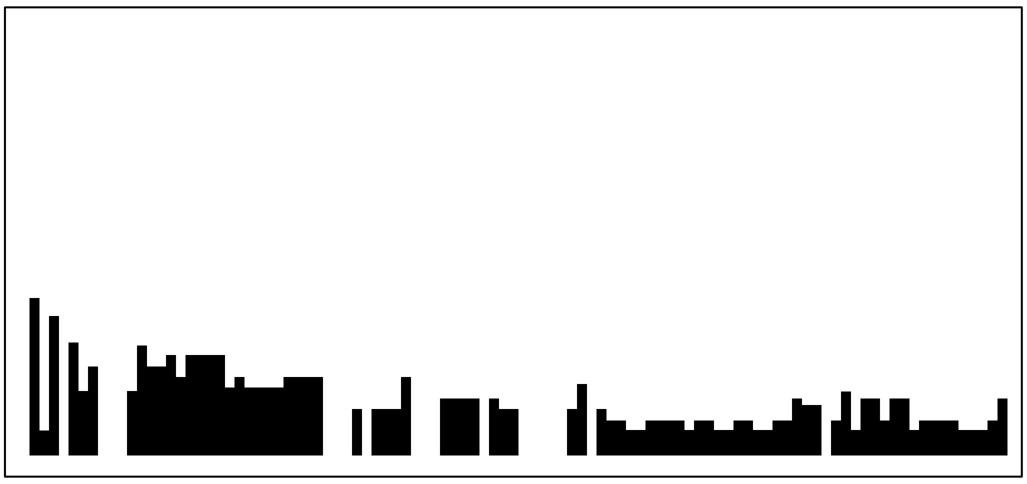

Hansonoperla appalachia 16S ribosomal RNA gene, pa	EF623249	87.38	62.35	1.87487e−25	Plecoptera	Perlidae	Hansonoperla appalachia
Eccoptura xanthenes 16S ribosomal RNA gene, partia	EF623252	81.16	84.57	6.54393e−25	Plecoptera	Perlidae	Eccoptura xanthenes
Acroneuria lycorias 16S ribosomal RNA gene, partia	EF623251	86.54	62.35	6.54393e−25	Plecoptera	Perlidae	Acroneuria lycorias
Agnetina capitata 16S ribosomal RNA gene, partial	EF623241	77.7	91.36	2.78255e−23	Plecoptera	Perlidae	Agnetina capitata
Perlinella drymo 16S ribosomal RNA gene, partial s	EF623240	85.29	62.35	1.18317e−21	Plecoptera	Perlidae	Perlinella drymo
Dinocras cephalotes mitochondrion, complete genome	KF484757	83.02	62.35	4.12967e−21	Plecoptera	Perlidae	Dinocras cephalotes
Notonemouridae sp. MD−2011 voucher ZMUC:JD−1507 16	JN615257	84.16	62.35	4.12967e−21	Plecoptera	Notonemouridae	Notonemouridae sp. MD−2011
Dolichopoda sp. MR−2008 16S ribosomal RNA gene, pa	EU887863	77.94	83.95	4.12967e−21	Orthoptera	Rhaphidophoridae	Dolichopoda lustriae
Dolichopoda pavesii voucher SPI 16S ribosomal RNA	EF216951	78.36	82.72	4.12967e−21	Orthoptera	Rhaphidophoridae	Dolichopoda pavesii
Brachyptera seticornis 16S ribosomal RNA gene, par	EF623268	78.01	87.04	4.12967e−21	Plecoptera	Taeniopterygidae	Brachyptera seticornis
Dinocras cephalotes 16S ribosomal RNA gene, partia	EF623244	83.02	62.35	4.12967e−21	Plecoptera	Perlidae	Dinocras cephalotes
Calineuria californica 16S ribosomal RNA gene, par	EF623239	85.15	62.35	4.12967e−21	Plecoptera	Perlidae	Calineuria californica
Nemoura cinerea 16S ribosomal RNA gene, partial se	EF623194	84.16	62.35	4.12967e−21	Plecoptera	Nemouridae	Nemoura cinerea
Megaleuctra kincaidi 16S ribosomal RNA gene, parti	EF623174	84.16	62.35	4.12967e−21	Plecoptera	Leuctridae	Megaleuctra kincaidi
Scopura montana 16S ribosomal RNA gene, partial se	EF623265	84.69	60.49	1.4414e−20	Plecoptera		Scopura montana
Apteroperla tikumana mitochondrion, complete genom	KR604721	83.67	60.49	1.75598e−19	Plecoptera	Capniidae	Apteroperla tikumana
Cryptoperla japonica mitochondrial gene for 16S rR	AB711202	84.69	60.49	1.75598e−19	Plecoptera	Peltoperlidae	Cryptoperla japonica
Cryptoperla japonica mitochondrial gene for 16S rR	AB711184	84.69	60.49	1.75598e−19	Plecoptera	Peltoperlidae	Cryptoperla japonica
Pleioplectron simplex voucher CW306 16S ribosomal	HM594512	84.69	60.49	1.75598e−19	Orthoptera	Rhaphidophoridae	Pleioplectron simplex
Pteronarcys sachalina 16S ribosomal RNA gene, part	EF623264	84.69	60.49	1.75598e−19	Plecoptera	Pteronarcyidae	Pteronarcys sachalina
Pictetoperla sp. BYU_PL156 16S ribosomal RNA gene,	EF623247	83.17	62.35	1.75598e−19	Plecoptera	Perlidae	Pictetoperla sp. BYU_PL156
Nemurella pictetii 16S ribosomal RNA gene, partial	EF623192	83.67	60.49	1.75598e−19	Plecoptera	Nemouridae	Nemurella pictetii
Prostoia besametsa 16S ribosomal RNA gene, partial	EF623188	83.17	62.35	1.75598e−19	Plecoptera	Nemouridae	Prostoia besametsa
Nemoura trispinosa 16S ribosomal RNA gene, partial	EF623187	83.17	62.35	1.75598e−19	Plecoptera	Nemouridae	Nemoura trispinosa
Paranemoura claasseni 16S ribosomal RNA gene, part	EF623185	83.17	62.35	1.75598e−19	Plecoptera	Nemouridae	Paranemoura claasseni
Shipsa rotunda 16S ribosomal RNA gene, partial seq	EF623184	83.17	62.35	1.75598e−19	Plecoptera	Nemouridae	Shipsa rotunda
Malenka californica 16S ribosomal RNA gene, partia	EF623182	83.67	60.49	1.75598e−19	Plecoptera	Nemouridae	Malenka californica
Cryptoperla japonica mitochondrial gene for 16S rR	AB711183	83.67	60.49	2.13922e−18	Plecoptera	Peltoperlidae	Cryptoperla japonica
Cryptoperla japonica mitochondrial gene for 16S rR	AB711182	83.67	60.49	2.13922e−18	Plecoptera	Peltoperlidae	Cryptoperla japonica
Pachyrhamma edwardsii voucher CW122 16S ribosomal	HM594508	83.67	60.49	2.13922e−18	Orthoptera	Rhaphidophoridae	Pachyrhamma edwardsii
Dolichopoda hussoni voucher IZB 16S ribosomal RNA	EF216973	76.47	83.95	2.13922e−18	Orthoptera	Rhaphidophoridae	Dolichopoda hussoni
Dolichopoda thasosensis voucher DRA 16S ribosomal	EF216956	76.47	83.95	2.13922e−18	Orthoptera	Rhaphidophoridae	Dolichopoda thasosensis
Dolichopoda steriotisi voucher ANT 16S ribosomal R	EF216955	76.87	82.72	2.13922e−18	Orthoptera	Rhaphidophoridae	Dolichopoda steriotisi
Doddsia occidentalis 16S ribosomal RNA gene, parti	EF623270	82.18	62.35	2.13922e−18	Plecoptera	Taeniopterygidae	Doddsia occidentalis
Pteronarcys scotti 16S ribosomal RNA gene, partial	EF623263	76.47	93.83	2.13922e−18	Plecoptera	Pteronarcyidae	Pteronarcys scotti
Pteronarcella badia 16S ribosomal RNA gene, parti	EF623262	82.18	62.35	2.13922e−18	Plecoptera	Pteronarcyidae	Pteronarcella badia
Lednia tumana 16S ribosomal RNA gene, partial sequ	EF623197	82.18	62.35	2.13922e−18	Plecoptera	Nemouridae	Lednia tumana
Protonemura meyeri 16S ribosomal RNA gene, partial	EF623193	82.18	62.35	2.13922e−18	Plecoptera	Nemouridae	Protonemura meyeri
Osmylus sp. EH−2004 16S ribosomal RNA gene, partia	AY620146	83.67	60.49	2.13922e−18	Neuroptera	Osmylidae	Osmylus sp. EH−2004
Eupholidoptera smyrnensis isolate KOY1 16S ribosom	KC852309	77.34	79.01	7.46661e−18	Orthoptera	Tettigoniidae	Eupholidoptera smyrnensis
Eupholidoptera magnifica isolate ARB1 16S ribosoma	KC852277	76.06	86.42	7.46661e−18	Orthoptera	Tettigoniidae	Eupholidoptera magnifica
Dolichopoda ithakii voucher ITA 16S ribosomal RNA	EF216949	76.76	87.04	7.46661e−18	Orthoptera	Rhaphidophoridae	Dolichopoda ithakii
Hesperoperla pacifica 16S ribosomal RNA gene, part	EF623238	82.65	60.49	7.46661e−18	Plecoptera	Perlidae	Hesperoperla pacifica
Nanonemoura wahkeena 16S ribosomal RNA gene, parti	EF623198	82.65	60.49	7.46661e−18	Plecoptera	Nemouridae	Nanonemoura wahkeena
Ostrocerca foersteri 16S ribosomal RNA gene, parti	EF623191	82.65	60.49	7.46661e−18	Plecoptera	Nemouridae	Ostrocerca foersteri
Visoka cataractae 16S ribosomal RNA gene, partial	EF623189	82.65	60.49	7.46661e−18	Plecoptera	Nemouridae	Visoka cataractae
Eupholidoptera chabrieri voucher 173 16S ribosomal	EU120792	76.06	86.42	7.46661e−18	Orthoptera	Tettigoniidae	Eupholidoptera chabrieri
Dolichopoda muceddai isolate SAR1 16S ribosomal RN	AY793575	82.65	60.49	7.46661e−18	Orthoptera	Rhaphidophoridae	Dolichopoda muceddai
Cryptoperla sp. YK−2012−1 mitochondrial gene for 1	AB711243	82.18	62.35	2.6061e−17	Plecoptera	Peltoperlidae	Cryptoperla sp. YK−2012−1
Cryptoperla ishigakiensis mitochondrial gene for 1	AB711242	82.18	62.35	2.6061e−17	Plecoptera	Peltoperlidae	Cryptoperla ishigakiensis
Dolichopoda sp. KOR 16S ribosomal RNA gene, partia	EU887866	76.47	83.95	2.6061e−17	Orthoptera	Rhaphidophoridae	Dolichopoda sp. KOR
Dolichopoda remyi voucher EDE 16S ribosomal RNA ge	EF216969	76.47	83.95	2.6061e−17	Orthoptera	Rhaphidophoridae	Dolichopoda remyi
Dolichopoda dalensi voucher KEF 16S ribosomal RNA	EF216959	75.89	87.04	2.6061e−17	Orthoptera	Rhaphidophoridae	Dolichopoda dalensi
Dolichopoda kiriakii voucher AGH 16S ribosomal RNA	EF216954	76.12	82.72	2.6061e−17	Orthoptera	Rhaphidophoridae	Dolichopoda kiriakii
Dolichopoda giachinoi voucher ORO 16S ribosomal RN	EF216952	76.6	87.04	2.6061e−17	Orthoptera	Rhaphidophoridae	Dolichopoda giachinoi
Pteronarcys californica 16S ribosomal RNA gene, pa	EF623261	82.18	62.35	2.6061e−17	Plecoptera	Pteronarcyidae	Pteronarcys californica
Grylloblatta sp. BYU_gb29.1 16S ribosomal RNA gene	DQ457261	83.33	59.26	2.6061e−17	Grylloblattodea	Grylloblattidae	Grylloblatta sp. BYU_gb29.1
Dolichopoda remyi isolate EDE 16S ribosomal RNA ge	AY793589	76.47	83.95	2.6061e−17	Orthoptera	Rhaphidophoridae	Dolichopoda remyi
Pteronarcys princeps mitochondrion, complete genom	AY687866	82.18	62.35	2.6061e−17	Plecoptera	Pteronarcyidae	Pteronarcys princeps
Mesocapnia arizonensis mitochondrion, partial geno	KP642637	81.63	60.49	9.0962e−17	Plecoptera	Capniidae	Mesocapnia arizonensis
Allocapnia recta isolate H19 16S ribosomal RNA gen	KC881054	81.63	60.49	9.0962e−17	Plecoptera	Capniidae	Allocapnia recta
Allocapnia recta isolate H18 16S ribosomal RNA gen	KC881053	81.19	62.35	9.0962e−17	Plecoptera	Capniidae	Allocapnia recta
Allocapnia recta isolate H17 16S ribosomal RNA gen	KC881052	81.19	62.35	9.0962e−17	Plecoptera	Capniidae	Allocapnia recta
Allocapnia recta isolate H16 16S ribosomal RNA gen	KC881051	81.63	60.49	9.0962e−17	Plecoptera	Capniidae	Allocapnia recta
Allocapnia recta isolate H15 16S ribosomal RNA gen	KC881050	81.63	60.49	9.0962e−17	Plecoptera	Capniidae	Allocapnia recta
Allocapnia recta isolate H14 16S ribosomal RNA gen	KC881049	81.63	60.49	9.0962e−17	Plecoptera	Capniidae	Allocapnia recta
Allocapnia recta isolate H13 16S ribosomal RNA gen	KC881048	81.63	60.49	9.0962e−17	Plecoptera	Capniidae	Allocapnia recta
Allocapnia recta isolate H12 16S ribosomal RNA gen	KC881047	81.19	62.35	9.0962e−17	Plecoptera	Capniidae	Allocapnia recta
Allocapnia recta isolate H11 16S ribosomal RNA gen	KC881046	81.63	60.49	9.0962e−17	Plecoptera	Capniidae	Allocapnia recta
Allocapnia recta isolate H10 16S ribosomal RNA gen	KC881045	81.63	60.49	9.0962e−17	Plecoptera	Capniidae	Allocapnia recta
Allocapnia recta isolate H9 16S ribosomal RNA gene	KC881044	81.19	62.35	9.0962e−17	Plecoptera	Capniidae	Allocapnia recta
Allocapnia recta isolate H8 16S ribosomal RNA gene	KC881043	81.19	62.35	9.0962e−17	Plecoptera	Capniidae	Allocapnia recta
Allocapnia recta isolate H7 16S ribosomal RNA gene	KC881042	81.63	60.49	9.0962e−17	Plecoptera	Capniidae	Allocapnia recta
Allocapnia recta isolate H6 16S ribosomal RNA gene	KC881041	81.63	60.49	9.0962e−17	Plecoptera	Capniidae	Allocapnia recta
Allocapnia recta isolate H5 16S ribosomal RNA gene	KC881040	81.19	62.35	9.0962e−17	Plecoptera	Capniidae	Allocapnia recta
Allocapnia recta isolate H4 16S ribosomal RNA gene	KC881039	81.19	62.35	9.0962e−17	Plecoptera	Capniidae	Allocapnia recta
Allocapnia recta isolate H3 16S ribosomal RNA gene	KC881038	81.63	60.49	9.0962e−17	Plecoptera	Capniidae	Allocapnia recta
Allocapnia recta isolate H1 16S ribosomal RNA gene	KC881036	81.63	60.49	9.0962e−17	Plecoptera	Capniidae	Allocapnia recta
Pachyrhamma edwardsii voucher WEL 16S ribosomal RN	HM594509	82.65	60.49	9.0962e−17	Orthoptera	Rhaphidophoridae	Pachyrhamma edwardsii
Micropathus tasmaniensis voucher JNC 16S ribosomal	HM594507	82.35	62.35	9.0962e−17	Orthoptera	Rhaphidophoridae	Micropathus tasmaniensis
Micropathus tasmaniensis voucher IDB 16S ribosomal	HM594505	82.35	62.35	9.0962e−17	Orthoptera	Rhaphidophoridae	Micropathus tasmaniensis
Dolichopoda graeca voucher PER 16S ribosomal RNA g	EF216953	76.3	82.72	9.0962e−17	Orthoptera	Rhaphidophoridae	Dolichopoda graeca
Oemopteryx vanduzeea 16S ribosomal RNA gene, parti	EF623266	81.63	60.49	9.0962e−17	Plecoptera	Taeniopterygidae	Oemopteryx vanduzeea
Claassenia sabulosa 16S ribosomal RNA gene, partia	EF623250	83	60.49	9.0962e−17	Plecoptera	Perlidae	Claassenia sabulosa
Kempnyella sp. BYU_PL155 16S ribosomal RNA gene, p	EF623246	81.19	62.35	9.0962e−17	Plecoptera	Perlidae	Kempnyella sp. BYU_PL155
Cosumnoperla hypocrena 16S ribosomal RNA gene, par	EF623226	82.65	60.49	9.0962e−17	Plecoptera	Perlodidae	Cosumnoperla hypocrena
Neonemura sp. BYU_PL153 16S ribosomal RNA gene, pa	EF623204	82.65	60.49	9.0962e−17	Plecoptera	Notonemouridae	Neonemura sp. BYU_PL153
Zapada cinctipes 16S ribosomal RNA gene, partial s	EF623183	81.63	60.49	9.0962e−17	Plecoptera	Nemouridae	Zapada cinctipes
Moselia infuscata 16S ribosomal RNA gene, partial	EF623175	82.65	60.49	9.0962e−17	Plecoptera	Leuctridae	Moselia infuscata
Galloisiana nipponensis 16S ribosomal RNA gene, pa	EF623153	82.65	60.49	9.0962e−17	Grylloblattodea	Grylloblattidae	Galloisiana nipponensis
Kathroperla perdita 16S ribosomal RNA gene, partia	EF623135	81.19	62.35	9.0962e−17	Plecoptera	Chloroperlidae	Kathroperla perdita
Allocapnia minima 16S ribosomal RNA gene, partial	EF623130	81.63	60.49	9.0962e−17	Plecoptera	Capniidae	Allocapnia minima
Mesocapnia sp. BYU_PL010 16S ribosomal RNA gene, p	EF623127	81.63	60.49	9.0962e−17	Plecoptera	Capniidae	Mesocapnia sp. BYU_PL010
Allocapnia recta haplotype 12ar: H12 16S ribosomal	EU306875	81.63	60.49	9.0962e−17	Plecoptera	Capniidae	Allocapnia recta
Allocapnia recta haplotype 11ar: H011 16S ribosoma	EU306874	81.63	60.49	9.0962e−17	Plecoptera	Capniidae	Allocapnia recta
Allocapnia recta haplotype 10ar: H010 16S ribosoma	EU306873	81.19	62.35	9.0962e−17	Plecoptera	Capniidae	Allocapnia recta
Allocapnia recta haplotype 9ar: H09 16S ribosomal	EU306872	81.19	62.35	9.0962e−17	Plecoptera	Capniidae	Allocapnia recta
Allocapnia recta haplotype 8ar: H08 16S ribosomal	EU306871	81.19	62.35	9.0962e−17	Plecoptera	Capniidae	Allocapnia recta
Allocapnia recta haplotype 7ar: H07 16S ribosomal	EU306870	81.63	60.49	9.0962e−17	Plecoptera	Capniidae	Allocapnia recta
Galloisiana yuasai voucher BYU_gb13.2 16S ribosoma	DQ457244	82.65	60.49	9.0962e−17	Grylloblattodea	Grylloblattidae	Galloisiana yuasai
