# Supplementary material for: Testing the potential of a ribosomal 16S marker for DNA metabarcoding of insects
Source: PeerJ. 2016 Apr 19;4:e1966. doi: 10.7717/peerj.1966 (PMC4841222; doi:10.7717/peerj.1966)
Supplement: Supplemental Information 1 [file peerj-04-1966-s006.zip › scripts/5 match OTUs/blast_hits/OTU_95.csv_plot.pdf]

OTU_95.csv

Calliteara wolongensis

Lymantriidae

Lepidoptera

81.37

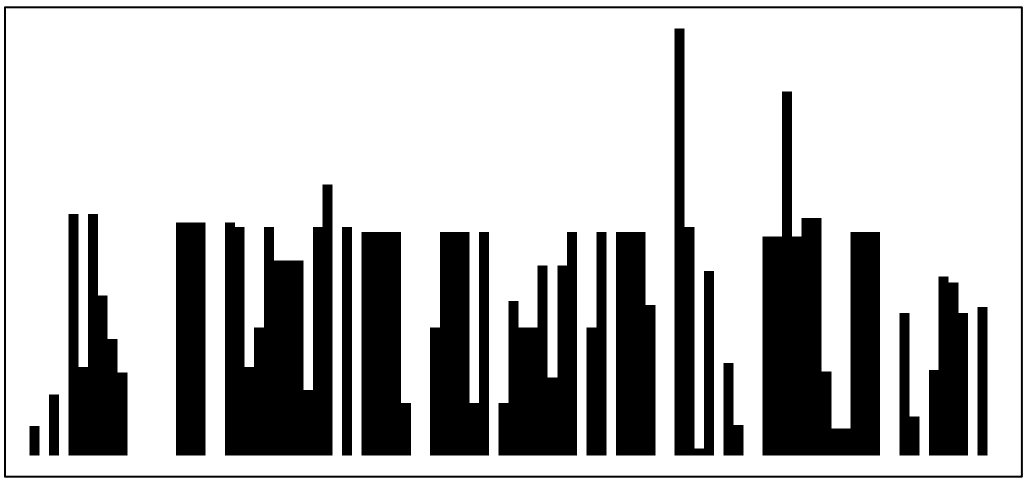

Calliteara wolongensis voucher H248 large subunit	KP082008	81.37	48.33	5.29919e−15	Lepidoptera	Lymantriidae	Calliteara wolongensis
Ditomyia fasciata voucher JSSJ3 16S ribosomal RNA	KJ136734	75.42	56.46	4.07402e−10	Diptera	Ditomyiidae	Ditomyia fasciata
Vitis vinifera contig VV78X187583.5, whole genome	AM483039	82.86	33.01	1.73232e−08	Vitales	Vitaceae	Vitis vinifera
Zebrafish DNA sequence from clone CH211−272F15 in	BX072559	71.69	76.56	6.04637e−08	Cypriniformes	Cyprinidae	Danio rerio
TPA_asm: Neospora caninum Liverpool, chromosome ch	LN714482	91.3	22.01	2.11039e−07	Eucoccidiorida	Sarcocystidae	Neospora caninum Liverpool
Schistosoma mansoni strain Puerto Rico chromosome	HE601627	84.13	30.14	2.11039e−07	Strigeidida	Schistosomatidae	Schistosoma mansoni
Neospora caninum Liverpool complete genome, chromo	FR823389	91.3	22.01	2.11039e−07	Eucoccidiorida	Sarcocystidae	Neospora caninum Liverpool
Vitis vinifera contig VV78X188988.15, whole genome	AM476185	87.5	26.79	2.11039e−07	Vitales	Vitaceae	Vitis vinifera
Solanum lycopersicum chromosome ch12, complete gen	HG975524	85.45	26.32	7.36599e−07	Solanales	Solanaceae	Solanum lycopersicum
Solanum pennellii chromosome ch01, complete genome	HG975440	83.87	29.67	7.36599e−07	Solanales	Solanaceae	Solanum pennellii
Kallima inachus mitochondrion, complete genome	HM243591	75	51.67	7.36599e−07	Lepidoptera	Nymphalidae	Kallima inachus
Kallima inachus mitochondrion, complete genome	JN857943	75	51.67	7.36599e−07	Lepidoptera	Nymphalidae	Kallima inachus
Kallima inachus isolate KYI−Rrna369 16S ribosomal	HM368519	75	51.67	7.36599e−07	Lepidoptera	Nymphalidae	Kallima inachus
Kallima inachus voucher jshao2007004 16S ribosomal	EU334569	75	51.67	7.36599e−07	Lepidoptera	Nymphalidae	Kallima inachus
Zebrafish DNA sequence from clone DKEY−155O15 in l	AL844521	70.22	81.82	7.36599e−07	Cypriniformes	Cyprinidae	Danio rerio
Cucumis melo genomic chromosome, chr_12	LN713266	90.91	21.05	2.57098e−06	Cucurbitales	Cucurbitaceae	Cucumis melo
Cucumis melo genomic scaffold, anchoredscaffold000	LN681932	90.91	21.05	2.57098e−06	Cucurbitales	Cucurbitaceae	Cucumis melo
Cyprinus carpio genome assembly common carp genome	LN594840	90.91	21.05	2.57098e−06	Cypriniformes	Cyprinidae	Cyprinus carpio
Helobdella robusta hypothetical protein mRNA	XM_009029618	80	35.89	2.57098e−06	Rhynchobdellida	Glossiphoniidae	Helobdella robusta
Glycine max clone GM_WBb0105E20, complete sequence	AC235863	79.73	35.41	2.57098e−06	Fabales	Fabaceae	Glycine max
Zebrafish DNA sequence from clone CH211−187D7 in l	CU571062	90.91	21.05	2.57098e−06	Cypriniformes	Cyprinidae	Danio rerio
Protopolystoma xenopodis genome assembly P_xenopod	LM937114	90.7	20.57	8.97362e−06	Polyopisthocotylea	Polystomatidae	Protopolystoma xenopodis
Solanum lycopersicum chromosome ch11, complete gen	HG975523	84.13	29.19	8.97362e−06	Solanales	Solanaceae	Solanum lycopersicum
Solanum pennellii chromosome ch12, complete genome	HG975451	86	23.92	8.97362e−06	Solanales	Solanaceae	Solanum pennellii
Homo sapiens coiled−coil domain containing 88A (CC	NG_031944	90.7	20.57	8.97362e−06	Primates	Hominidae	Homo sapiens
Zebrafish DNA sequence from clone CH73−170L9 in li	FP236529	89.13	22.01	8.97362e−06	Cypriniformes	Cyprinidae	Danio rerio
Mus musculus chromosome 7, clone RP23−325M1, compl	AC091333	89.13	22.01	8.97362e−06	Rodentia	Muridae	Mus musculus
Mus musculus BAC clone RP23−173C1 from chromosome	AC138549	89.13	22.01	8.97362e−06	Rodentia	Muridae	Mus musculus
Zebrafish DNA sequence from clone DKEY−60B23 in li	BX649558	83.05	28.23	8.97362e−06	Cypriniformes	Cyprinidae	Danio rerio
Homo sapiens BAC clone RP11−369J6 from 2, complete	AC019198	90.7	20.57	8.97362e−06	Primates	Hominidae	Homo sapiens
Oryza sativa Japonica Group genomic DNA, chromosom	AP003048	92.68	19.62	8.97362e−06	Poales	Poaceae	Oryza sativa Japonica Group
Mouse DNA sequence from clone RP24−298F18 on chrom	CT033786	78.21	37.32	8.97362e−06	Rodentia	Muridae	Mus musculus
Mouse DNA sequence from clone RP23−39I7 on chromos	AL672284	90.7	20.57	8.97362e−06	Rodentia	Muridae	Mus musculus
Homo sapiens myocardial infarction associated tran	NG_016621	76.19	36.84	3.1321e−05	Primates	Hominidae	Homo sapiens
Schistosoma curassoni genome assembly S_curassoni_	LM112551	90.48	20.1	3.1321e−05	Strigeidida	Schistosomatidae	Schistosoma curassoni
Trichobilharzia regenti genome assembly T_regenti_	LL013282	90.48	20.1	3.1321e−05	Strigeidida	Schistosomatidae	Trichobilharzia regenti
Trichobilharzia regenti genome assembly T_regenti_	LL006296	90.48	20.1	3.1321e−05	Strigeidida	Schistosomatidae	Trichobilharzia regenti
Ascaris lumbricoides genome assembly A_lumbricoide	LK873347	90.48	20.1	3.1321e−05	Ascaridida	Ascarididae	Ascaris lumbricoides
Cyprinus carpio genome assembly common carp genome	LN590753	82.46	27.27	3.1321e−05	Cypriniformes	Cyprinidae	Cyprinus carpio
Plasmodium yoelii genome assembly PY17X01, chromos	LM993663	69.04	90.91	3.1321e−05	Haemosporida	Plasmodiidae	Plasmodium yoelii
Plasmodium yoelii genome assembly PYYM01, chromoso	LK934637	69.04	90.91	3.1321e−05	Haemosporida	Plasmodiidae	Plasmodium yoelii
Solanum pennellii chromosome ch07, complete genome	HG975446	86	23.92	3.1321e−05	Solanales	Solanaceae	Solanum pennellii
Rattus norvegicus Y Chr BAC RNECO−185E18 (Amplicon	AC242886	90.48	20.1	3.1321e−05	Rodentia	Muridae	Rattus norvegicus
Rattus norvegicus Y Chr BAC RNAEX−124D15 (Amplicon	AC241595	90.48	20.1	3.1321e−05	Rodentia	Muridae	Rattus norvegicus
Rattus norvegicus Y Chr BAC RNAEX−23J03 (Amplicon	AC241446	90.48	20.1	3.1321e−05	Rodentia	Muridae	Rattus norvegicus
Medicago truncatula clone mth2−129b5, complete seq	AC225529	82.46	27.27	3.1321e−05	Fabales	Fabaceae	Medicago truncatula
Mouse DNA sequence from clone RP23−150H18 on chrom	CT010588	90.48	20.1	3.1321e−05	Rodentia	Muridae	Mus musculus
Homo sapiens non−coding RNA, MIAT, exon 1, 2, 3, 4	AB266844	76.19	36.84	3.1321e−05	Primates	Hominidae	Homo sapiens
Medicago truncatula chromosome 7 BAC clone mth2−81	AC169182	82.46	27.27	3.1321e−05	Fabales	Fabaceae	Medicago truncatula
Zebrafish DNA sequence from clone CH211−167J9 in l	CR848814	87.23	22.49	3.1321e−05	Cypriniformes	Cyprinidae	Danio rerio
Mus musculus BAC clone RP23−330A5 from chromosome	AC127592	86	23.92	3.1321e−05	Rodentia	Muridae	Mus musculus
Mus musculus chromosome 16, clone RP24−383M8, comp	AC102503	86	23.92	3.1321e−05	Rodentia	Muridae	Mus musculus
Oryza sativa chromosome 3 BAC OSJNBa0033P04 genomi	AC092263	88.89	21.53	3.1321e−05	Poales	Poaceae	Oryza sativa Japonica Group
Zebrafish DNA sequence from clone DKEY−15F11 in li	BX465201	83.64	26.32	3.1321e−05	Cypriniformes	Cyprinidae	Danio rerio
Oryza sativa chromosome 3 BAC OSJNBa0059G06 genomi	AC096690	88.89	21.53	3.1321e−05	Poales	Poaceae	Oryza sativa Japonica Group
Human DNA sequence from clone RP11−114A21 on chrom	AL357129	90.48	20.1	3.1321e−05	Primates	Hominidae	Homo sapiens
Human DNA sequence from clone CTA−373H7 on chromos	Z99774	76.19	36.84	3.1321e−05	Primates	Hominidae	Homo sapiens
Oryza sativa Japonica Group genomic DNA, chromosom	AP005450	86	23.92	3.1321e−05	Poales	Poaceae	Oryza sativa Japonica Group
Zebrafish DNA sequence from clone CH211−190F24 in	BX548035	90.48	20.1	3.1321e−05	Cypriniformes	Cyprinidae	Danio rerio
Zebrafish DNA sequence from clone DKEY−38L7 in lin	BX255933	69.88	78.47	3.1321e−05	Cypriniformes	Cyprinidae	Danio rerio
Mouse DNA sequence from clone RP23−34B24 on chromo	AL772310	90.48	20.1	3.1321e−05	Rodentia	Muridae	Mus musculus
Mouse DNA sequence from clone RP23−134A17 on chrom	AL732521	90.48	20.1	3.1321e−05	Rodentia	Muridae	Mus musculus
Human DNA sequence from clone RP11−562E17 on chrom	AL157764	90.48	20.1	3.1321e−05	Primates	Hominidae	Homo sapiens
Strongyloides venezuelensis genome assembly S_vene	LM524983	87.04	25.36	0.000109321	Rhabditida	Strongyloididae	Strongyloides venezuelensis
Cucumis melo genomic chromosome, chr_7	LN713261	74.29	50.24	0.000109321	Cucurbitales	Cucurbitaceae	Cucumis melo
Cucumis melo genomic scaffold, anchoredscaffold000	LN681861	74.29	50.24	0.000109321	Cucurbitales	Cucurbitaceae	Cucumis melo
Trichobilharzia regenti genome assembly T_regenti_	LL033098	100	14.83	0.000109321	Strigeidida	Schistosomatidae	Trichobilharzia regenti
Trichobilharzia regenti genome assembly T_regenti_	LL007341	90.7	20.57	0.000109321	Strigeidida	Schistosomatidae	Trichobilharzia regenti
Ascaris lumbricoides genome assembly A_lumbricoide	LK875739	80.33	29.19	0.000109321	Ascaridida	Ascarididae	Ascaris lumbricoides
Schistosoma mattheei genome assembly S_mattheei_De	LM151207	88.64	21.05	0.000109321	Strigeidida	Schistosomatidae	Schistosoma mattheei
Cyprinus carpio genome assembly common carp genome	LN590718	71.27	79.43	0.000109321	Cypriniformes	Cyprinidae	Cyprinus carpio
Solanum lycopersicum chromosome ch09, complete gen	HG975521	84.31	24.4	0.000109321	Solanales	Solanaceae	Solanum lycopersicum
Solanum lycopersicum chromosome ch06, complete gen	HG975518	81.43	30.62	0.000109321	Solanales	Solanaceae	Solanum lycopersicum
Plasmodium falciparum 3D7 chromosome 12, complete	AE014188	75	43.54	0.000109321	Haemosporida	Plasmodiidae	Plasmodium falciparum 3D7
Zebrafish DNA sequence from clone DKEY−194E13 in l	CU570896	73.55	57.89	0.000109321	Cypriniformes	Cyprinidae	Danio rerio
Vitis vinifera contig VV78X277513.23, whole genome	AM450518	90.24	19.62	0.000109321	Vitales	Vitaceae	Vitis vinifera
Mus musculus chromosome 3, clone RP23−290N20, comp	AC161931	90.24	19.62	0.000109321	Rodentia	Muridae	Mus musculus
Mus musculus BAC clone RP24−218F22 from chromosome	AC126255	97.06	16.27	0.000109321	Rodentia	Muridae	Mus musculus
Mus musculus chromosome 3, clone RP23−468J10, comp	AC101891	90.24	19.62	0.000109321	Rodentia	Muridae	Mus musculus
Zebrafish DNA sequence from clone CH211−66B9 in li	BX511268	91.11	21.05	0.000109321	Cypriniformes	Cyprinidae	Danio rerio
Zebrafish DNA sequence from clone CH211−119H23 in	BX005444	91.11	21.05	0.000109321	Cypriniformes	Cyprinidae	Danio rerio
Mouse DNA sequence from clone RP23−318H22 on chrom	BX119995	83.93	26.32	0.000109321	Rodentia	Muridae	Mus musculus
Cucumis melo genomic chromosome, chr_10	LN713264	81.25	30.62	0.000381568	Cucurbitales	Cucurbitaceae	Cucumis melo
Cucumis melo genomic scaffold, anchoredscaffold000	LN681905	81.25	30.62	0.000381568	Cucurbitales	Cucurbitaceae	Cucumis melo
Echinostoma caproni genome assembly E_caproni_Egyp	LL243333	90.48	20.1	0.000381568	Echinostomida	Echinostomatidae	Echinostoma caproni
Trichobilharzia regenti genome assembly T_regenti_	LL040982	90.48	20.1	0.000381568	Strigeidida	Schistosomatidae	Trichobilharzia regenti
Trichobilharzia regenti genome assembly T_regenti_	LL029588	90.48	20.1	0.000381568	Strigeidida	Schistosomatidae	Trichobilharzia regenti
Cyprinus carpio genome assembly common carp genome	LN593527	76.25	37.32	0.000381568	Cypriniformes	Cyprinidae	Cyprinus carpio
Cyprinus carpio genome assembly common carp genome	LN590688	76.32	36.36	0.000381568	Cypriniformes	Cyprinidae	Cyprinus carpio
Elaeophora elaphi genome assembly E_elaphi ,scaffo	LL710598	86.67	21.53	0.000381568	Spirurida	Onchocercidae	Elaeophora elaphi
Solanum lycopersicum chromosome ch04, complete gen	HG975516	81.82	26.32	0.000381568	Solanales	Solanaceae	Solanum lycopersicum
Arabidopsis lyrata subsp. lyrata predicted protein	XM_002888930	68.33	84.69	0.000381568	Brassicales	Brassicaceae	Arabidopsis lyrata subsp. lyrata
Pongo abelii BAC clone CH276−259P23 from chromosom	AC205929	84	23.92	0.000381568	Primates	Hominidae	Pongo abelii
Angiostrongylus cantonensis genome assembly A_cant	LK946731	88.37	20.57	0.0013318	Rhabditida	Angiostrongylidae	Angiostrongylus cantonensis
Angiostrongylus cantonensis genome assembly A_cant	LK946422	88.1	20.1	0.0013318	Rhabditida	Angiostrongylidae	Angiostrongylus cantonensis
Strongyloides ratti genome assembly S_ratti_ED321	LN609528	86.67	21.53	0.00464845	Rhabditida	Strongyloididae	Strongyloides ratti
Cyprinus carpio genome assembly common carp genome	LN590925	67.44	80.86	0.00464845	Cypriniformes	Cyprinidae	Cyprinus carpio
Onchocerca ochengi genome assembly O_ochengi_Ngaou	LL519922	86.96	21.53	0.0162247	Spirurida	Onchocercidae	Onchocerca ochengi
Cyprinus carpio genome assembly common carp genome	LN598389	80	26.32	0.0162247	Cypriniformes	Cyprinidae	Cyprinus carpio
