# Supplementary material for: Testing the potential of a ribosomal 16S marker for DNA metabarcoding of insects
Source: PeerJ. 2016 Apr 19;4:e1966. doi: 10.7717/peerj.1966 (PMC4841222; doi:10.7717/peerj.1966)
Supplement: Supplemental Information 1 [file peerj-04-1966-s006.zip › scripts/5 match OTUs/blast_hits/OTU_96.csv_plot.pdf]

OTU_96.csv

Hansonoperla appalachia

Perlidae

Plecoptera

86

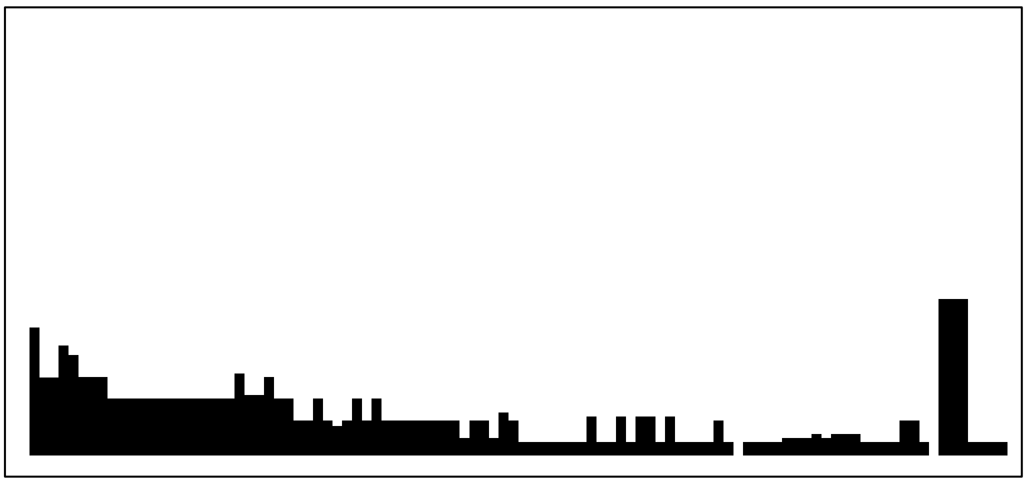

Hansonoperla appalachia 16S ribosomal RNA gene, pa	EF623249	86	60.87	3.36315e−22	Plecoptera	Perlidae	Hansonoperla appalachia
Dinocras cephalotes mitochondrion, complete genome	KF484757	83.65	61.49	4.09715e−21	Plecoptera	Perlidae	Dinocras cephalotes
Dinocras cephalotes 16S ribosomal RNA gene, partia	EF623244	83.65	61.49	4.09715e−21	Plecoptera	Perlidae	Dinocras cephalotes
Acroneuria lycorias 16S ribosomal RNA gene, partia	EF623251	85.15	60.87	1.43005e−20	Plecoptera	Perlidae	Acroneuria lycorias
Agnetina capitata 16S ribosomal RNA gene, partial	EF623241	84.69	60.87	1.43005e−20	Plecoptera	Perlidae	Agnetina capitata
Lednia tumana 16S ribosomal RNA gene, partial sequ	EF623197	83.67	60.87	1.74215e−19	Plecoptera	Nemouridae	Lednia tumana
Nemoura cinerea 16S ribosomal RNA gene, partial se	EF623194	83.67	60.87	1.74215e−19	Plecoptera	Nemouridae	Nemoura cinerea
Calineuria californica 16S ribosomal RNA gene, par	EF623239	83.67	60.87	2.12238e−18	Plecoptera	Perlidae	Calineuria californica
Dolichopoda geniculata geniculata isolate PRA20 16	AY507590	82.65	60.87	7.40782e−18	Orthoptera	Rhaphidophoridae	Dolichopoda geniculata geniculata
Dolichopoda sp. VAR 16S ribosomal RNA gene, partia	EU887865	82.65	60.87	7.40782e−18	Orthoptera	Rhaphidophoridae	Dolichopoda sp. VAR
Dolichopoda sp. TAY 16S ribosomal RNA gene, partia	EU887864	82.65	60.87	7.40782e−18	Orthoptera	Rhaphidophoridae	Dolichopoda sp. TAY
Dolichopoda sp. MR−2008 16S ribosomal RNA gene, pa	EU887863	82.65	60.87	7.40782e−18	Orthoptera	Rhaphidophoridae	Dolichopoda lustriae
Dolichopoda annae voucher OLI 16S ribosomal RNA ge	EU887860	82.65	60.87	7.40782e−18	Orthoptera	Rhaphidophoridae	Dolichopoda annae
Dolichopoda hussoni voucher IZB 16S ribosomal RNA	EF216973	82.65	60.87	7.40782e−18	Orthoptera	Rhaphidophoridae	Dolichopoda hussoni
Dolichopoda thasosensis voucher DRA 16S ribosomal	EF216956	82.65	60.87	7.40782e−18	Orthoptera	Rhaphidophoridae	Dolichopoda thasosensis
Dolichopoda kiriakii voucher AGH 16S ribosomal RNA	EF216954	82.65	60.87	7.40782e−18	Orthoptera	Rhaphidophoridae	Dolichopoda kiriakii
Dolichopoda pavesii voucher SPI 16S ribosomal RNA	EF216951	82.65	60.87	7.40782e−18	Orthoptera	Rhaphidophoridae	Dolichopoda pavesii
Prostoia besametsa 16S ribosomal RNA gene, partial	EF623188	82.65	60.87	7.40782e−18	Plecoptera	Nemouridae	Prostoia besametsa
Paranemoura claasseni 16S ribosomal RNA gene, part	EF623185	82.65	60.87	7.40782e−18	Plecoptera	Nemouridae	Paranemoura claasseni
Shipsa rotunda 16S ribosomal RNA gene, partial seq	EF623184	82.65	60.87	7.40782e−18	Plecoptera	Nemouridae	Shipsa rotunda
Malenka californica 16S ribosomal RNA gene, partia	EF623182	82.65	60.87	7.40782e−18	Plecoptera	Nemouridae	Malenka californica
Sasquaperla hoopa 16S ribosomal RNA gene, partial	EF623143	83.84	60.87	7.40782e−18	Plecoptera	Chloroperlidae	Sasquaperla hoopa
Dolichopoda graeca voucher PER 16S ribosomal RNA g	EF216953	82.83	60.87	2.58558e−17	Orthoptera	Rhaphidophoridae	Dolichopoda graeca
Eccoptura xanthenes 16S ribosomal RNA gene, partia	EF623252	82.83	60.87	2.58558e−17	Plecoptera	Perlidae	Eccoptura xanthenes
Macroxyela ferruginea 16S ribosomal RNA gene, part	AY206773	83.67	60.87	2.58558e−17	Hymenoptera	Xyelidae	Macroxyela ferruginea
Peltodytes quadratus voucher BMNH794142 16S riboso	HQ381466	82.65	60.87	9.02457e−17	Coleoptera	Haliplidae	Peltodytes quadratus
Dolichopoda remyi voucher EDE 16S ribosomal RNA ge	EF216969	82.65	60.87	9.02457e−17	Orthoptera	Rhaphidophoridae	Dolichopoda remyi
Dolichopoda dalensi voucher KEF 16S ribosomal RNA	EF216959	81.63	60.87	9.02457e−17	Orthoptera	Rhaphidophoridae	Dolichopoda dalensi
Dolichopoda steriotisi voucher ANT 16S ribosomal R	EF216955	81.63	60.87	9.02457e−17	Orthoptera	Rhaphidophoridae	Dolichopoda steriotisi
Dolichopoda giachinoi voucher ORO 16S ribosomal RN	EF216952	82.65	60.87	9.02457e−17	Orthoptera	Rhaphidophoridae	Dolichopoda giachinoi
Scopura montana 16S ribosomal RNA gene, partial se	EF623265	81.63	60.87	9.02457e−17	Plecoptera		Scopura montana
Perlinella drymo 16S ribosomal RNA gene, partial s	EF623240	81.37	60.87	9.02457e−17	Plecoptera	Perlidae	Perlinella drymo
Nanonemoura wahkeena 16S ribosomal RNA gene, parti	EF623198	81.63	60.87	9.02457e−17	Plecoptera	Nemouridae	Nanonemoura wahkeena
Moselia infuscata 16S ribosomal RNA gene, partial	EF623175	82.65	60.87	9.02457e−17	Plecoptera	Leuctridae	Moselia infuscata
Megaleuctra kincaidi 16S ribosomal RNA gene, parti	EF623174	81.63	60.87	9.02457e−17	Plecoptera	Leuctridae	Megaleuctra kincaidi
Dolichopoda remyi isolate EDE 16S ribosomal RNA ge	AY793589	82.65	60.87	9.02457e−17	Orthoptera	Rhaphidophoridae	Dolichopoda remyi
Dolichopoda cyrnensis isolate SAB 16S ribosomal RN	AY793576	81.63	60.87	9.02457e−17	Orthoptera	Rhaphidophoridae	Dolichopoda cyrnensis
Dolichopoda muceddai isolate SAR1 16S ribosomal RN	AY793575	81.63	60.87	9.02457e−17	Orthoptera	Rhaphidophoridae	Dolichopoda muceddai
D.geniculata mitochondrial 16S rRNA gene	Y07551	81.63	60.87	9.02457e−17	Orthoptera	Rhaphidophoridae	Dolichopoda geniculata
Cryptoperla sp. YK−2012−1 mitochondrial gene for 1	AB711243	81.63	60.87	1.09942e−15	Plecoptera	Peltoperlidae	Cryptoperla sp. YK−2012−1
Cryptoperla japonica mitochondrial gene for 16S rR	AB711202	81.63	60.87	1.09942e−15	Plecoptera	Peltoperlidae	Cryptoperla japonica
Cryptoperla japonica mitochondrial gene for 16S rR	AB711184	81.63	60.87	1.09942e−15	Plecoptera	Peltoperlidae	Cryptoperla japonica
Anterastes davrazensis haplotype 53−973 16S riboso	JX024232	81.63	60.87	1.09942e−15	Orthoptera	Tettigoniidae	Anterastes davrazensis
Anterastes davrazensis haplotype 52−972 16S riboso	JX024231	81.63	60.87	1.09942e−15	Orthoptera	Tettigoniidae	Anterastes davrazensis
Notonemouridae sp. MD−2011 voucher ZMUC:JD−1507 16	JN615257	80.81	61.49	1.09942e−15	Plecoptera	Notonemouridae	Notonemouridae sp. MD−2011
Dolichopoda cassagnaui voucher TRI 16S ribosomal R	EF216961	81.63	60.87	1.09942e−15	Orthoptera	Rhaphidophoridae	Dolichopoda cassagnaui
Pteronarcys sachalina 16S ribosomal RNA gene, part	EF623264	81.63	60.87	1.09942e−15	Plecoptera	Pteronarcyidae	Pteronarcys sachalina
Pictetoperla sp. BYU_PL156 16S ribosomal RNA gene,	EF623247	80.81	61.49	1.09942e−15	Plecoptera	Perlidae	Pictetoperla sp. BYU_PL156
Perlesta decipines 16S ribosomal RNA gene, partial	EF623242	82	60.87	1.09942e−15	Plecoptera	Perlidae	Perlesta decipines
Despaxia augusta 16S ribosomal RNA gene, partial s	EF623177	81.63	60.87	1.09942e−15	Plecoptera	Leuctridae	Despaxia augusta
Dolichopoda bormansi isolate BRA5 16S ribosomal RN	AY507606	80.61	60.87	3.83735e−15	Orthoptera	Rhaphidophoridae	Dolichopoda bormansi
Dolichopoda bormansi isolate BRA3 16S ribosomal RN	AY507605	80.61	60.87	3.83735e−15	Orthoptera	Rhaphidophoridae	Dolichopoda bormansi
Dolichopoda geniculata geniculata isolate VAL1 16S	AY507600	80.61	60.87	3.83735e−15	Orthoptera	Rhaphidophoridae	Dolichopoda geniculata geniculata
Dolichopoda baccettii isolate PST17 16S ribosomal	AY507593	80.61	60.87	3.83735e−15	Orthoptera	Rhaphidophoridae	Dolichopoda baccettii
Dolichopoda bolivari isolate FRN2 16S ribosomal RN	AY507579	80.61	60.87	3.83735e−15	Orthoptera	Rhaphidophoridae	Dolichopoda bolivari
Dolichopoda laetitiae isolate DIA18 16S ribosomal	AY507578	80.61	60.87	3.83735e−15	Orthoptera	Rhaphidophoridae	Dolichopoda laetitiae
Dolichopoda geniculata geniculata isolate AUS1 16S	AY507566	80.61	60.87	3.83735e−15	Orthoptera	Rhaphidophoridae	Dolichopoda geniculata geniculata
Dolichopoda unicolor voucher DIR 16S ribosomal RNA	EF216970	81.82	60.87	3.83735e−15	Orthoptera	Rhaphidophoridae	Dolichopoda unicolor
Doddsia occidentalis 16S ribosomal RNA gene, parti	EF623270	80.61	60.87	3.83735e−15	Plecoptera	Taeniopterygidae	Doddsia occidentalis
Brachyptera seticornis 16S ribosomal RNA gene, par	EF623268	80.61	60.87	3.83735e−15	Plecoptera	Taeniopterygidae	Brachyptera seticornis
Cerconychia sp. BYU_PL114 16S ribosomal RNA gene,	EF623260	81.82	60.87	3.83735e−15	Plecoptera	Styloperlidae	Cerconychia sp. BYU_PL114
Zapada cinctipes 16S ribosomal RNA gene, partial s	EF623183	80.61	60.87	3.83735e−15	Plecoptera	Nemouridae	Zapada cinctipes
Cosmioperla australis 16S ribosomal RNA gene, part	EF623148	81.82	60.87	3.83735e−15	Plecoptera	Eustheniidae	Cosmioperla australis
Chloroperla tripunctata 16S ribosomal RNA gene, pa	EF623137	81.82	60.87	3.83735e−15	Plecoptera	Chloroperlidae	Chloroperla tripunctata
Kathroperla perdita 16S ribosomal RNA gene, partia	EF623135	80.61	60.87	3.83735e−15	Plecoptera	Chloroperlidae	Kathroperla perdita
Sweltsa coloradensis 16S ribosomal RNA gene, parti	EF623133	81.82	60.87	3.83735e−15	Plecoptera	Chloroperlidae	Sweltsa coloradensis
Dolichopoda geniculata isolate VAL 16S ribosomal R	AY793583	80.61	60.87	3.83735e−15	Orthoptera	Rhaphidophoridae	Dolichopoda geniculata
Dolichopoda laetitiae etrusca isolate DIA 16S ribo	AY793580	80.61	60.87	3.83735e−15	Orthoptera	Rhaphidophoridae	Dolichopoda laetitiae etrusca
Dolichopoda bormansi isolate BRA 16S ribosomal RNA	AY793578	80.61	60.87	3.83735e−15	Orthoptera	Rhaphidophoridae	Dolichopoda bormansi
Dolichopoda baccettii isolate PST 16S ribosomal RN	AY793571	80.61	60.87	3.83735e−15	Orthoptera	Rhaphidophoridae	Dolichopoda baccettii
Eupholidoptera uvarovi isolate KAS1 16S ribosomal	KC852312	81.63	60.87	1.33937e−14	Orthoptera	Tettigoniidae	Eupholidoptera uvarovi
Cylindera belli 16S ribosomal RNA gene, partial se	KC963544	80.61	60.87	1.33937e−14	Coleoptera	Carabidae	Cylindera belli
Yoraperla uenoi mitochondrial gene for 16S rRNA, p	AB712375	80	63.98	1.33937e−14	Plecoptera	Peltoperlidae	Yoraperla uenoi
Anterastes disparalatus haplotype 46BO 16S ribosom	HQ850975	80.61	60.87	1.33937e−14	Orthoptera	Tettigoniidae	Anterastes disparalatus
Anterastes burri haplotype 18BO 16S ribosomal RNA	HM856174	80.61	60.87	1.33937e−14	Orthoptera	Tettigoniidae	Anterastes burri
Anterastes burri haplotype 19BO 16S ribosomal RNA	HM856168	80.61	60.87	1.33937e−14	Orthoptera	Tettigoniidae	Anterastes burri
Anterastes uludaghensis haplotype 39BU 16S ribosom	HM856139	80.61	60.87	1.33937e−14	Orthoptera	Tettigoniidae	Anterastes uludaghensis
Dolichopoda patrizii voucher PET 16S ribosomal RNA	EU887862	80.81	60.87	1.33937e−14	Orthoptera	Rhaphidophoridae	Dolichopoda patrizii
Dolichopoda ithakii voucher ITA 16S ribosomal RNA	EF216949	80.81	60.87	1.33937e−14	Orthoptera	Rhaphidophoridae	Dolichopoda ithakii
Taenionema palladium 16S ribosomal RNA gene, parti	EF623267	80.81	60.87	1.33937e−14	Plecoptera	Taeniopterygidae	Taenionema palladium
Suwallia teleckojensis 16S ribosomal RNA gene, par	EF623142	81	60.87	1.33937e−14	Plecoptera	Chloroperlidae	Suwallia teleckojensis
Alloperla severa 16S ribosomal RNA gene, partial s	EF623141	80.81	60.87	1.33937e−14	Plecoptera	Chloroperlidae	Alloperla severa
Neaviperla forcipata 16S ribosomal RNA gene, parti	EF623139	81	60.87	1.33937e−14	Plecoptera	Chloroperlidae	Neaviperla forcipata
Triznaka pintada 16S ribosomal RNA gene, partial s	EF623134	81	60.87	1.33937e−14	Plecoptera	Chloroperlidae	Triznaka pintada
Plumiperla diversa 16S ribosomal RNA gene, partial	EF623132	81	60.87	1.33937e−14	Plecoptera	Chloroperlidae	Plumiperla diversa
Tessellana veyseli voucher 260 16S ribosomal RNA g	EU120811	80.61	60.87	1.33937e−14	Orthoptera	Tettigoniidae	Tessellana veyseli
Anterastes serbicus voucher 161 16S ribosomal RNA	EU120782	80.61	60.87	1.33937e−14	Orthoptera	Tettigoniidae	Anterastes serbicus
Koroglus disparalatus voucher 156 16S ribosomal RN	EU120777	80.61	60.87	1.33937e−14	Orthoptera	Tettigoniidae	Anterastes disparalatus
Xyela sp. Sus1 16S ribosomal RNA gene, partial seq	AY206772	80.61	60.87	1.33937e−14	Hymenoptera	Xyelidae	Xyela sp. Sus1
Cicindela (Rivacindela) sp. JP−212 mitochondrial p	AJ619543	81.63	60.87	1.33937e−14	Coleoptera	Carabidae	Cicindela (Rivacindela) sp. JP−212
Cicindela (Rivacindela) sp. JP−59b mitochondrial p	AJ619322	81.63	60.87	1.33937e−14	Coleoptera	Carabidae	Cicindela (Rivacindela) sp. JP−59b
Ifasina belli mitochondrial 16S rRNA (partial), tR	AJ515112	80.61	60.87	1.33937e−14	Coleoptera	Carabidae	Cylindera belli
Apteroperla tikumana mitochondrion, complete genom	KR604721	79.59	60.87	4.67485e−14	Plecoptera	Capniidae	Apteroperla tikumana
Oyamia cryptomeria mitochondrial gene for 16S ribo	AB771687	87.32	44.1	4.67485e−14	Plecoptera	Perlidae	Oyamia cryptomeria
Oyamia cryptomeria mitochondrial gene for 16S ribo	AB771686	87.32	44.1	4.67485e−14	Plecoptera	Perlidae	Oyamia cryptomeria
Oyamia cryptomeria mitochondrial gene for 16S ribo	AB771685	87.32	44.1	4.67485e−14	Plecoptera	Perlidae	Oyamia cryptomeria
Cryptoperla ishigakiensis mitochondrial gene for 1	AB711242	80.61	60.87	4.67485e−14	Plecoptera	Peltoperlidae	Cryptoperla ishigakiensis
Cryptoperla japonica mitochondrial gene for 16S rR	AB711183	80.61	60.87	4.67485e−14	Plecoptera	Peltoperlidae	Cryptoperla japonica
Cryptoperla japonica mitochondrial gene for 16S rR	AB711182	80.61	60.87	4.67485e−14	Plecoptera	Peltoperlidae	Cryptoperla japonica
Spelaeiacris tabulae voucher KAL 16S ribosomal RNA	HM594496	80.61	60.87	4.67485e−14	Orthoptera	Rhaphidophoridae	Spelaeiacris tabulae
