# Supplementary material for: Testing the potential of a ribosomal 16S marker for DNA metabarcoding of insects
Source: PeerJ. 2016 Apr 19;4:e1966. doi: 10.7717/peerj.1966 (PMC4841222; doi:10.7717/peerj.1966)
Supplement: Supplemental Information 1 [file peerj-04-1966-s006.zip › scripts/5 match OTUs/blast_hits/OTU_97.csv_plot.pdf]

OTU_97.csv

Acroneuria lycorias

Perlidae

Plecoptera

87.5

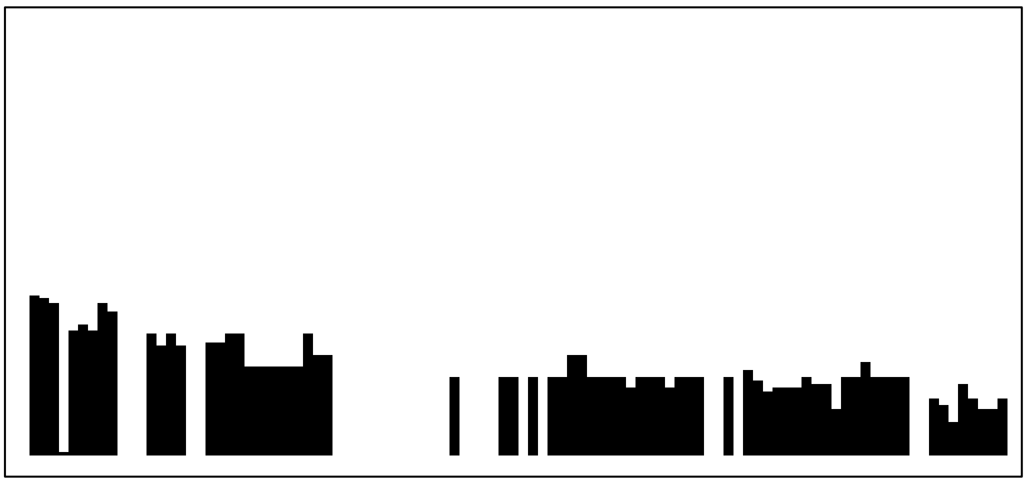

Acroneuria lycorias 16S ribosomal RNA gene, partia	EF623251	87.5	62.73	5.32929e−26	Plecoptera	Perlidae	Acroneuria lycorias
Hansonoperla appalachia 16S ribosomal RNA gene, pa	EF623249	87.38	62.73	1.8601e−25	Plecoptera	Perlidae	Hansonoperla appalachia
Megaleuctra kincaidi 16S ribosomal RNA gene, parti	EF623174	87.13	62.73	6.4924e−25	Plecoptera	Leuctridae	Megaleuctra kincaidi
Pteronarcys sachalina 16S ribosomal RNA gene, part	EF623264	80.14	86.96	2.26607e−24	Plecoptera	Pteronarcyidae	Pteronarcys sachalina
Dinocras cephalotes mitochondrion, complete genome	KF484757	85.85	62.73	7.90936e−24	Plecoptera	Perlidae	Dinocras cephalotes
Pictetoperla sp. BYU_PL156 16S ribosomal RNA gene,	EF623247	86.14	62.73	7.90936e−24	Plecoptera	Perlidae	Pictetoperla sp. BYU_PL156
Dinocras cephalotes 16S ribosomal RNA gene, partia	EF623244	85.85	62.73	7.90936e−24	Plecoptera	Perlidae	Dinocras cephalotes
Calineuria californica 16S ribosomal RNA gene, par	EF623239	87.13	62.73	7.90936e−24	Plecoptera	Perlidae	Calineuria californica
Scopura montana 16S ribosomal RNA gene, partial se	EF623265	86.73	60.87	2.76064e−23	Plecoptera		Scopura montana
Brachyptera seticornis 16S ribosomal RNA gene, par	EF623268	79.43	86.96	9.63557e−23	Plecoptera	Taeniopterygidae	Brachyptera seticornis
Pteronarcys scotti 16S ribosomal RNA gene, partial	EF623263	79.43	86.96	9.63557e−23	Plecoptera	Pteronarcyidae	Pteronarcys scotti
Pteronarcys princeps mitochondrion, complete genom	AY687866	79.43	86.96	9.63557e−23	Plecoptera	Pteronarcyidae	Pteronarcys princeps
Apteroperla tikumana mitochondrion, complete genom	KR604721	85.71	60.87	3.36315e−22	Plecoptera	Capniidae	Apteroperla tikumana
Notonemouridae sp. MD−2011 voucher ZMUC:JD−1507 16	JN615257	85.15	62.73	3.36315e−22	Plecoptera	Notonemouridae	Notonemouridae sp. MD−2011
Agnetina capitata 16S ribosomal RNA gene, partial	EF623241	85.71	60.87	3.36315e−22	Plecoptera	Perlidae	Agnetina capitata
Nemoura cinerea 16S ribosomal RNA gene, partial se	EF623194	85.15	62.73	3.36315e−22	Plecoptera	Nemouridae	Nemoura cinerea
Doddsia occidentalis 16S ribosomal RNA gene, parti	EF623270	77.85	91.93	1.17385e−21	Plecoptera	Taeniopterygidae	Doddsia occidentalis
Pteronarcys californica 16S ribosomal RNA gene, pa	EF623261	78.72	86.96	1.17385e−21	Plecoptera	Pteronarcyidae	Pteronarcys californica
Eccoptura xanthenes 16S ribosomal RNA gene, partia	EF623252	85.29	62.73	1.17385e−21	Plecoptera	Perlidae	Eccoptura xanthenes
Perlinella drymo 16S ribosomal RNA gene, partial s	EF623240	85.29	62.73	1.17385e−21	Plecoptera	Perlidae	Perlinella drymo
Cryptoperla japonica mitochondrial gene for 16S rR	AB711183	85.71	60.87	4.09715e−21	Plecoptera	Peltoperlidae	Cryptoperla japonica
Cryptoperla japonica mitochondrial gene for 16S rR	AB711182	85.71	60.87	4.09715e−21	Plecoptera	Peltoperlidae	Cryptoperla japonica
Dolichopoda pavesii voucher SPI 16S ribosomal RNA	EF216951	84.16	62.73	4.09715e−21	Orthoptera	Rhaphidophoridae	Dolichopoda pavesii
Protonemura meyeri 16S ribosomal RNA gene, partial	EF623193	84.16	62.73	4.09715e−21	Plecoptera	Nemouridae	Protonemura meyeri
Prostoia besametsa 16S ribosomal RNA gene, partial	EF623188	84.16	62.73	4.09715e−21	Plecoptera	Nemouridae	Prostoia besametsa
Nemoura trispinosa 16S ribosomal RNA gene, partial	EF623187	84.16	62.73	4.09715e−21	Plecoptera	Nemouridae	Nemoura trispinosa
Paranemoura claasseni 16S ribosomal RNA gene, part	EF623185	84.16	62.73	4.09715e−21	Plecoptera	Nemouridae	Paranemoura claasseni
Shipsa rotunda 16S ribosomal RNA gene, partial seq	EF623184	84.16	62.73	4.09715e−21	Plecoptera	Nemouridae	Shipsa rotunda
Osmylus sp. EH−2004 16S ribosomal RNA gene, partia	AY620146	85.71	60.87	4.09715e−21	Neuroptera	Osmylidae	Osmylus sp. EH−2004
Nemurella pictetii 16S ribosomal RNA gene, partial	EF623192	84.69	60.87	1.43005e−20	Plecoptera	Nemouridae	Nemurella pictetii
Malenka californica 16S ribosomal RNA gene, partia	EF623182	84.69	60.87	1.43005e−20	Plecoptera	Nemouridae	Malenka californica
Allocapnia recta isolate H18 16S ribosomal RNA gen	KC881053	77.14	86.96	4.99135e−20	Plecoptera	Capniidae	Allocapnia recta
Allocapnia recta isolate H17 16S ribosomal RNA gen	KC881052	77.14	86.96	4.99135e−20	Plecoptera	Capniidae	Allocapnia recta
Allocapnia recta isolate H12 16S ribosomal RNA gen	KC881047	77.14	86.96	4.99135e−20	Plecoptera	Capniidae	Allocapnia recta
Allocapnia recta isolate H9 16S ribosomal RNA gene	KC881044	77.14	86.96	4.99135e−20	Plecoptera	Capniidae	Allocapnia recta
Allocapnia recta isolate H8 16S ribosomal RNA gene	KC881043	77.14	86.96	4.99135e−20	Plecoptera	Capniidae	Allocapnia recta
Allocapnia recta isolate H5 16S ribosomal RNA gene	KC881040	77.14	86.96	4.99135e−20	Plecoptera	Capniidae	Allocapnia recta
Allocapnia recta isolate H4 16S ribosomal RNA gene	KC881039	77.14	86.96	4.99135e−20	Plecoptera	Capniidae	Allocapnia recta
Cosmioperla australis 16S ribosomal RNA gene, part	EF623148	78	91.93	4.99135e−20	Plecoptera	Eustheniidae	Cosmioperla australis
Allocapnia recta haplotype 10ar: H010 16S ribosoma	EU306873	77.14	86.96	4.99135e−20	Plecoptera	Capniidae	Allocapnia recta
Allocapnia recta haplotype 9ar: H09 16S ribosomal	EU306872	77.14	86.96	4.99135e−20	Plecoptera	Capniidae	Allocapnia recta
Allocapnia recta haplotype 8ar: H08 16S ribosomal	EU306871	77.14	86.96	4.99135e−20	Plecoptera	Capniidae	Allocapnia recta
Allocapnia recta haplotype 1ar: H01 16S ribosomal	DQ915179	77.14	86.96	4.99135e−20	Plecoptera	Capniidae	Allocapnia recta
Mesocapnia arizonensis mitochondrion, partial geno	KP642637	83.67	60.87	1.74215e−19	Plecoptera	Capniidae	Mesocapnia arizonensis
Allocapnia recta isolate H19 16S ribosomal RNA gen	KC881054	78.17	86.96	1.74215e−19	Plecoptera	Capniidae	Allocapnia recta
Allocapnia recta isolate H16 16S ribosomal RNA gen	KC881051	78.17	86.96	1.74215e−19	Plecoptera	Capniidae	Allocapnia recta
Allocapnia recta isolate H15 16S ribosomal RNA gen	KC881050	78.17	86.96	1.74215e−19	Plecoptera	Capniidae	Allocapnia recta
Allocapnia recta isolate H14 16S ribosomal RNA gen	KC881049	78.17	86.96	1.74215e−19	Plecoptera	Capniidae	Allocapnia recta
Allocapnia recta isolate H13 16S ribosomal RNA gen	KC881048	83.67	60.87	1.74215e−19	Plecoptera	Capniidae	Allocapnia recta
Allocapnia recta isolate H11 16S ribosomal RNA gen	KC881046	83.67	60.87	1.74215e−19	Plecoptera	Capniidae	Allocapnia recta
Allocapnia recta isolate H10 16S ribosomal RNA gen	KC881045	78.17	86.96	1.74215e−19	Plecoptera	Capniidae	Allocapnia recta
Allocapnia recta isolate H7 16S ribosomal RNA gene	KC881042	83.67	60.87	1.74215e−19	Plecoptera	Capniidae	Allocapnia recta
Allocapnia recta isolate H6 16S ribosomal RNA gene	KC881041	78.17	86.96	1.74215e−19	Plecoptera	Capniidae	Allocapnia recta
Allocapnia recta isolate H3 16S ribosomal RNA gene	KC881038	83.67	60.87	1.74215e−19	Plecoptera	Capniidae	Allocapnia recta
Allocapnia recta isolate H1 16S ribosomal RNA gene	KC881036	83.67	60.87	1.74215e−19	Plecoptera	Capniidae	Allocapnia recta
Cryptoperla japonica mitochondrial gene for 16S rR	AB711202	84.69	60.87	1.74215e−19	Plecoptera	Peltoperlidae	Cryptoperla japonica
Cryptoperla japonica mitochondrial gene for 16S rR	AB711184	84.69	60.87	1.74215e−19	Plecoptera	Peltoperlidae	Cryptoperla japonica
Dolichopoda sp. MR−2008 16S ribosomal RNA gene, pa	EU887863	83.67	60.87	1.74215e−19	Orthoptera	Rhaphidophoridae	Dolichopoda lustriae
Bolotoperla rossi 16S ribosomal RNA gene, partial	EF623269	83.67	60.87	1.74215e−19	Plecoptera	Taeniopterygidae	Bolotoperla rossi
Oemopteryx vanduzeea 16S ribosomal RNA gene, parti	EF623266	83.67	60.87	1.74215e−19	Plecoptera	Taeniopterygidae	Oemopteryx vanduzeea
Nanonemoura wahkeena 16S ribosomal RNA gene, parti	EF623198	83.67	60.87	1.74215e−19	Plecoptera	Nemouridae	Nanonemoura wahkeena
Lednia tumana 16S ribosomal RNA gene, partial sequ	EF623197	83.17	62.73	1.74215e−19	Plecoptera	Nemouridae	Lednia tumana
Ostrocerca foersteri 16S ribosomal RNA gene, partia	EF623191	83.67	60.87	1.74215e−19	Plecoptera	Nemouridae	Ostrocerca foersteri
Visoka cataractae 16S ribosomal RNA gene, partial	EF623189	83.67	60.87	1.74215e−19	Plecoptera	Nemouridae	Visoka cataractae
Zapada cinctipes 16S ribosomal RNA gene, partial s	EF623183	83.67	60.87	1.74215e−19	Plecoptera	Nemouridae	Zapada cinctipes
Kathroperla perdita 16S ribosomal RNA gene, partia	EF623135	83.17	62.73	1.74215e−19	Plecoptera	Chloroperlidae	Kathroperla perdita
Allocapnia minima 16S ribosomal RNA gene, partial	EF623130	83.67	60.87	1.74215e−19	Plecoptera	Capniidae	Allocapnia minima
Mesocapnia sp. BYU_PL010 16S ribosomal RNA gene, p	EF623127	83.67	60.87	1.74215e−19	Plecoptera	Capniidae	Mesocapnia sp. BYU_PL010
Allocapnia recta haplotype 12ar: H12 16S ribosomal	EU306875	83.67	60.87	1.74215e−19	Plecoptera	Capniidae	Allocapnia recta
Allocapnia recta haplotype 11ar: H011 16S ribosoma	EU306874	78.17	86.96	1.74215e−19	Plecoptera	Capniidae	Allocapnia recta
Allocapnia recta haplotype 7ar: H07 16S ribosomal	EU306870	78.17	86.96	1.74215e−19	Plecoptera	Capniidae	Allocapnia recta
Allocapnia recta haplotype 3ar: H03 16S ribosomal	DQ915181	83.67	60.87	1.74215e−19	Plecoptera	Capniidae	Allocapnia recta
Allocapnia recta haplotype 2ar: H02 16S ribosomal	DQ915180	78.17	86.96	1.74215e−19	Plecoptera	Capniidae	Allocapnia recta
Cryptoperla sp. YK−2012−2 mitochondrial gene for 1	AB711244	84	60.87	6.08071e−19	Plecoptera	Peltoperlidae	Cryptoperla sp. YK−2012−2
Pteronarcella badia 16S ribosomal RNA gene, partia	EF623262	83.5	62.73	6.08071e−19	Plecoptera	Pteronarcyidae	Pteronarcella badia
Udamocercia sp. BYU_PL150 16S ribosomal RNA gene,	EF623201	83	62.11	6.08071e−19	Plecoptera	Notonemouridae	Udamocercia sp. BYU_PL150
Eupholidoptera chabrieri voucher 173 16S ribosomal	EU120792	83.17	62.73	6.08071e−19	Orthoptera	Tettigoniidae	Eupholidoptera chabrieri
Cryptoperla sp. YK−2012−1 mitochondrial gene for 1	AB711243	83.17	62.73	2.12238e−18	Plecoptera	Peltoperlidae	Cryptoperla sp. YK−2012−1
Cryptoperla ishigakiensis mitochondrial gene for 1	AB711242	83.17	62.73	2.12238e−18	Plecoptera	Peltoperlidae	Cryptoperla ishigakiensis
Cryptoperla japonica mitochondrial gene for 16S rR	AB711189	83.67	60.87	2.12238e−18	Plecoptera	Peltoperlidae	Cryptoperla japonica
Micropathus tasmaniensis voucher JNC 16S ribosomal	HM594507	83.33	62.73	2.12238e−18	Orthoptera	Rhaphidophoridae	Micropathus tasmaniensis
Micropathus tasmaniensis voucher IDB 16S ribosomal	HM594505	83.33	62.73	2.12238e−18	Orthoptera	Rhaphidophoridae	Micropathus tasmaniensis
Strophopteryx appalachia 16S ribosomal RNA gene, p	EF623271	82.18	62.73	2.12238e−18	Plecoptera	Taeniopterygidae	Strophopteryx appalachia
Cosumnoperla hypocrena 16S ribosomal RNA gene, par	EF623226	83.67	60.87	2.12238e−18	Plecoptera	Perlodidae	Cosumnoperla hypocrena
Moselia infuscata 16S ribosomal RNA gene, partial	EF623175	83.67	60.87	2.12238e−18	Plecoptera	Leuctridae	Moselia infuscata
Grylloblatta sp. BYU_gb29.1 16S ribosomal RNA gene	DQ457261	84.38	59.63	2.12238e−18	Grylloblattodea	Grylloblattidae	Grylloblatta sp. BYU_gb29.1
Galloisiana yuasai voucher BYU_gb12.1 16S ribosoma	DQ457242	83.67	60.87	2.12238e−18	Grylloblattodea	Grylloblattidae	Galloisiana yuasai
Grylloblatta gurneyi voucher BYU_gb10.2 16S riboso	DQ457240	83.67	60.87	2.12238e−18	Grylloblattodea	Grylloblattidae	Grylloblatta gurneyi
Grylloblatta gurneyi voucher BYU_gb10.1 16S riboso	DQ457239	83.67	60.87	2.12238e−18	Grylloblattodea	Grylloblattidae	Grylloblatta gurneyi
Macroxyela ferruginea 16S ribosomal RNA gene, part	AY206773	83.67	60.87	2.12238e−18	Hymenoptera	Xyelidae	Macroxyela ferruginea
Dolichopoda sp. VAR 16S ribosomal RNA gene, partia	EU887865	76.6	86.34	7.40782e−18	Orthoptera	Rhaphidophoridae	Dolichopoda sp. VAR
Dolichopoda sp. TAY 16S ribosomal RNA gene, partia	EU887864	76.6	86.34	7.40782e−18	Orthoptera	Rhaphidophoridae	Dolichopoda sp. TAY
Dolichopoda steriotisi voucher ANT 16S ribosomal R	EF216955	82.65	60.87	7.40782e−18	Orthoptera	Rhaphidophoridae	Dolichopoda steriotisi
Taenionema palladium 16S ribosomal RNA gene, parti	EF623267	82.35	62.73	7.40782e−18	Plecoptera	Taeniopterygidae	Taenionema palladium
Beloneuria stewarti 16S ribosomal RNA gene, partia	EF623248	81.55	63.98	7.40782e−18	Plecoptera	Perlidae	Beloneuria stewarti
Kempnyella sp. BYU_PL155 16S ribosomal RNA gene, p	EF623246	83.33	62.73	7.40782e−18	Plecoptera	Perlidae	Kempnyella sp. BYU_PL155
Hesperoperla pacifica 16S ribosomal RNA gene, part	EF623238	82.65	60.87	7.40782e−18	Plecoptera	Perlidae	Hesperoperla pacifica
Eupholidoptera uvarovi isolate KAS1 16S ribosomal	KC852312	82.18	62.73	2.58558e−17	Orthoptera	Tettigoniidae	Eupholidoptera uvarovi
Eupholidoptera smyrnensis isolate KOY1 16S ribosom	KC852309	82.18	62.73	2.58558e−17	Orthoptera	Tettigoniidae	Eupholidoptera smyrnensis
Grylloblatta gurneyi voucher BYU_gb26.1 16S riboso	DQ457259	82.65	60.87	2.58558e−17	Grylloblattodea	Grylloblattidae	Grylloblatta gurneyi
