# Supplementary material for: Testing the potential of a ribosomal 16S marker for DNA metabarcoding of insects
Source: PeerJ. 2016 Apr 19;4:e1966. doi: 10.7717/peerj.1966 (PMC4841222; doi:10.7717/peerj.1966)
Supplement: Supplemental Information 1 [file peerj-04-1966-s006.zip › scripts/5 match OTUs/blast_hits/OTU_98.csv_plot.pdf]

OTU_98.csv

Sericostoma clypeatum

Sericostomatidae

Trichoptera

87.77

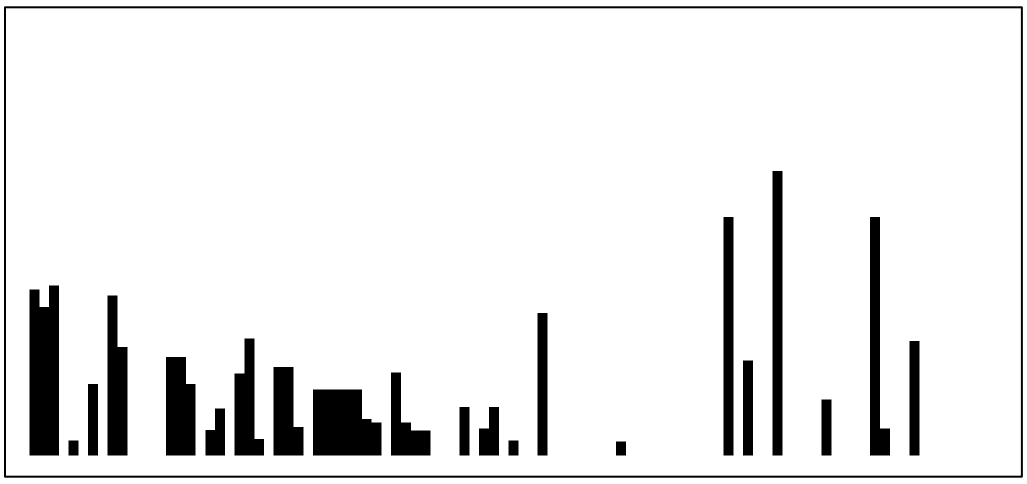

Sericostoma clypeatum voucher NHRS−EN CC4 16S ribo	FJ263207	87.77	94.44	1.23601e−39	Trichoptera	Sericostomatidae	Sericostoma clypeatum
Sericostoma personatum mitochondrion, partial geno	KP455290	86.96	94.44	1.8344e−37	Trichoptera	Sericostomatidae	Sericostoma personatum
Notidobiella chacayana isolate CL5 16S ribosomal R	EF394993	87.96	73.61	1.60914e−25	Trichoptera	Sericostomatidae	Notidobiella chacayana
Helicopsyche albescens isolate BR5 16S ribosomal R	EF394986	77.44	89.58	3.31961e−15	Trichoptera	Helicopsychidae	Helicopsyche albescens
Leptinotarsa juncta mitochondrial partial 16S rRNA	AJ841314	80.68	60.42	7.31193e−11	Coleoptera	Chrysomelidae	Leptinotarsa juncta
Notidobiella sp. CM6 16S ribosomal RNA gene, parti	EF394994	73.91	94.44	7.31193e−11	Trichoptera	Sericostomatidae	Notidobiella sp. CM6
Drepanepteryx phalaenoides 16S ribosomal RNA gene,	EU734861	83.33	52.78	2.55211e−10	Neuroptera	Hemerobiidae	Drepanepteryx phalaenoides
Helicopsyche cochleaetesta isolate CP5 16S ribosom	EF394996	74.1	95.83	2.55211e−10	Trichoptera	Helicopsychidae	Helicopsyche cochleaetesta
Stromboceros koebelei mitochondrial gene for 16S r	AB859180	87.5	43.75	8.90775e−10	Hymenoptera	Tenthredinidae	Stromboceros koebelei
Mordellista brevicauda voucher UPOL ZL0070 16S rib	FJ903717	85.07	45.83	8.90775e−10	Coleoptera	Mordellidae	Mordellista brevicauda
Caloca saneva mitochondrial 16S rRNA gene, specime	FN257670	78.3	72.92	8.90775e−10	Trichoptera	Calocidae	Caloca saneva
Tamasia variegata mitochondrial 16S rRNA gene, spe	FN257669	78.5	72.92	8.90775e−10	Trichoptera	Calocidae	Tamasia variegata
Orfelia fasciata 16S ribosomal RNA gene, partial s	FJ172008	76.47	70.14	8.90775e−10	Diptera	Keroplatidae	Orfelia fasciata
Caenota plicata isolate CR9 16S ribosomal RNA gene	EF395003	77.14	70.83	3.10911e−09	Trichoptera	Calocidae	Caenota plicata
Colophon haughtoni isolate C14 16S ribosomal RNA g	KC763258	84.62	44.44	1.08519e−08	Coleoptera	Lucanidae	Colophon haughtoni
Colophon haughtoni isolate C121 16S ribosomal RNA	KC763256	84.62	44.44	1.08519e−08	Coleoptera	Lucanidae	Colophon haughtoni
Jellisonia amadoi mitochondrion, complete genome	KF322091	83.33	45.83	1.08519e−08	Siphonaptera	Ceratophyllidae	Jellisonia amadoi
Renocera johnsoni voucher F−92 16S ribosomal RNA g	JN816289	75.45	75.69	1.08519e−08	Diptera	Sciomyzidae	Renocera johnsoni
Liodessus luteopictus voucher BMNH674989 16S ribos	HQ381386	81.18	58.33	1.08519e−08	Coleoptera	Dytiscidae	Liodessus luteopictus
Catops picipes mitochondrial partial 16S rRNA gene	FM209287	82.19	50	1.08519e−08	Coleoptera	Leiodidae	Catops picipes
Pliocaloca sp. CQ3 16S ribosomal RNA gene, partial	EF394999	76	85.42	1.08519e−08	Trichoptera	Calocidae	Pliocaloca sp. CQ3
Eutrichapion ervi partial mitochondrial 16S rRNA g	AJ495468	83.82	46.53	1.08519e−08	Coleoptera	Brentidae	Eutrichapion ervi
Colophon haughtoni isolate C13 16S ribosomal RNA g	KC763257	85.48	42.36	3.78767e−08	Coleoptera	Lucanidae	Colophon haughtoni
Stenocrates laevicollis 16S ribosomal RNA gene, pa	JX982897	80.77	53.47	3.78767e−08	Coleoptera	Scarabaeidae	Stenocrates laevicollis
Euparagia desertorum 16S ribosomal RNA gene, parti	GU596598	76.77	68.06	3.78767e−08	Hymenoptera	Vespidae	Euparagia desertorum
Trox caffer caffer isolate Tcaf02 16S ribosomal RN	KM371688	84.13	43.06	1.32203e−07	Coleoptera	Trogidae	Trox caffer caffer
Trox caffer caffer isolate Tcaf02 16S ribosomal RN	KC801076	84.13	43.06	1.32203e−07	Coleoptera	Trogidae	Trox caffer caffer
Dohrniphora porrasae isolate 6.iii.08−450 16S ribo	HM366948	81.33	50	1.32203e−07	Diptera	Phoridae	Dohrniphora porrasae
Alloecentrella sp. BP8 16S ribosomal RNA gene, par	EF394983	75.24	72.92	1.32203e−07	Trichoptera	Calocidae	Alloecentrella sp. BP8
Colophon stokoei voucher C131 16S ribosomal RNA ge	KP250230	83.08	44.44	4.61433e−07	Coleoptera	Lucanidae	Colophon stokoei
Colophon eastmani eastmani isolate C83 16S ribosom	KC763279	83.08	44.44	4.61433e−07	Coleoptera	Lucanidae	Colophon eastmani eastmani
Colophon stokoei isolate C132 16S ribosomal RNA ge	KC763244	83.08	44.44	4.61433e−07	Coleoptera	Lucanidae	Colophon stokoei
Colophon stokoei isolate C07 16S ribosomal RNA gen	KC763242	83.08	44.44	4.61433e−07	Coleoptera	Lucanidae	Colophon stokoei
Colophon stokoei isolate C01 16S ribosomal RNA gen	KC763240	83.08	44.44	4.61433e−07	Coleoptera	Lucanidae	Colophon stokoei
Yola bicarinata voucher NHM−IR16 16S ribosomal RNA	EF056700	81.69	47.92	4.61433e−07	Coleoptera	Dytiscidae	Yola bicarinata
Papuadytes sp. MB657 mitochondrial 16S rRNA gene (	AM404056	81.54	45.14	4.61433e−07	Coleoptera	Dytiscidae	Papuadytes sp. MB657
Pycnocentrella eruensis isolate BR4 16S ribosomal	EF394985	75.93	72.92	1.61056e−06	Trichoptera	Calocidae	Pycnocentrella eruensis
Gonocephalum adpressiforme 16S ribosomal RNA gene,	KJ003046	83.87	42.36	5.6214e−06	Coleoptera	Tenebrionidae	Gonocephalum adpressiforme
Colophon kawaii isolate C37 16S ribosomal RNA gene	KC763270	81.54	44.44	5.6214e−06	Coleoptera	Lucanidae	Colophon kawaii
Acalles lemur cisalpinus bio−material ZFMK−DNA JJ0	GU988426	81.16	46.53	5.6214e−06	Coleoptera	Curculionidae	Acalles lemur cisalpinus
Acalles lemur cisalpinus bio−material ZFMK−DNA JJ0	GU988416	81.16	46.53	5.6214e−06	Coleoptera	Curculionidae	Acalles lemur cisalpinus
Malaxella flava isolate 91 16S ribosomal RNA gene,	HM233772	72.34	95.83	5.6214e−06	Hemiptera	Delphacidae	Malaxella flava
Denticollis sp. UPOL RK0006 16S ribosomal RNA gene	KF626041	74.47	64.58	1.96206e−05	Coleoptera	Elateridae	Denticollis sp. UPOL RK0006
Strongylogaster moiwana mitochondrial gene for 16S	AB859189	72.92	66.67	6.84827e−05	Hymenoptera	Tenthredinidae	Strongylogaster moiwana
Chariotheca sp. 1 GJK−2014 16S ribosomal RNA gene,	KJ003016	82.26	42.36	0.000239028	Coleoptera	Tenebrionidae	Chariotheca sp. 1 GJK−2014
Myrmeleon quinquemaculatus isolate 92−quinq 16S ri	KF321974	79.37	43.75	0.000239028	Neuroptera	Myrmeleontidae	Myrmeleon quinquemaculatus
Lytta vesicatoria 16S ribosomal RNA gene, partial	EF490140	81.25	43.75	0.000239028	Coleoptera	Meloidae	Lytta vesicatoria
Gonocephalum subspinosum 16S ribosomal RNA gene, p	AY663870	82.26	42.36	0.000239028	Coleoptera	Tenebrionidae	Gonocephalum subspinosum
Laccodytes sp. UNM KBMLmsp764 16S ribosomal RNA ge	KJ548297	78.12	44.44	0.00083429	Coleoptera	Dytiscidae	Laccodytes sp. UNM KBMLmsp764
Hydrophilomima sp. SLE0067 16S ribosomal RNA gene,	KC992686	80.7	39.58	0.00083429	Coleoptera	Hydrophilidae	Hydrophilomima sp. SLE0067
Acalles lemur cisalpinus bio−material ZFMK−DNA JJ0	GU988410	76.32	52.08	0.00083429	Coleoptera	Curculionidae	Acalles lemur cisalpinus
Calocoides aquilonia isolate CR1 16S ribosomal RNA	EF395000	73.33	70.83	0.00083429	Trichoptera	Calocidae	Calocoides aquilonia
Gongylonema pulchrum genome assembly G_pulchrum_Ho	LL750841	86.67	31.25	0.00291196	Spirurida	Gongylonematidae	Gongylonema pulchrum
Rhagophthalmidae sp. UPOL 001363 16S ribosomal RNA	KF626006	73.47	67.36	0.00291196	Coleoptera	Phengodidae	Rhagophthalmidae sp. UPOL 001363
Callytron inspeculare 16S ribosomal RNA gene, part	KC963553	72.82	70.14	0.00291196	Coleoptera	Carabidae	Callytron inspeculare
Callytron yuasai 16S ribosomal RNA gene, partial s	KC963552	72.82	70.14	0.00291196	Coleoptera	Carabidae	Callytron yuasai
Euparagia scutellaris 16S ribosomal RNA gene, part	GU596692	78.46	43.75	0.00291196	Hymenoptera	Vespidae	Euparagia scutellaris
Polistes occipitalis 16S ribosomal RNA gene, parti	GU596664	77.33	52.08	0.00291196	Hymenoptera	Vespidae	Polistes occipitalis
Helicopsyche murrumba isolate CP6 16S ribosomal RN	EF394997	70.68	90.28	0.00291196	Trichoptera	Helicopsychidae	Helicopsyche murrumba
Paramonoctonus angustivalvus 16S ribosomal RNA gen	AF174342	77.27	45.83	0.00291196	Hymenoptera	Braconidae	Paramonoctonus angustivalvus
Gonocephalum reticulatum 16S ribosomal RNA gene, p	AY663879	80.65	42.36	0.00291196	Coleoptera	Tenebrionidae	Gonocephalum reticulatum
PREDICTED: Mus musculus interleukin 17F (Il17f), t	XM_006495523	78.57	47.22	0.0101637	Rodentia	Muridae	Mus musculus
PREDICTED: Mus musculus interleukin 17F (Il17f), t	XM_006495522	78.57	47.22	0.0101637	Rodentia	Muridae	Mus musculus
Berberomeloe majalis voucher MAB107 16S ribosomal	KC853076	77.42	43.06	0.0101637	Coleoptera	Meloidae	Berberomeloe majalis
Mus musculus interleukin 17F (Il17f), mRNA	NM_145856	78.57	47.22	0.0101637	Rodentia	Muridae	Mus musculus
Mus musculus chromosome 1, clone RP24−305O23, comp	AC159614	78.57	47.22	0.0101637	Rodentia	Muridae	Mus musculus
Mus musculus interleukin 17F mRNA, complete cds	AY380822	78.57	47.22	0.0101637	Rodentia	Muridae	Mus musculus
Mus musculus activated spleen cDNA, RIKEN full−len	AK157180	78.57	47.22	0.0101637	Rodentia	Muridae	Mus musculus
Mus musculus chromosome 1, clone RP23−191A12, comp	AC156980	78.57	47.22	0.0101637	Rodentia	Muridae	Mus musculus
Polyzonus fasciatus voucher HNAU.PP 012 16S riboso	KF737728	76.47	46.53	0.0354749	Coleoptera	Cerambycidae	Polyzonus fasciatus
Plutella xylostella mitochondrion, partial genome	JF911819	75.61	54.86	0.0354749	Lepidoptera	Plutellidae	Plutella xylostella
Rattus norvegicus interleukin 17F (Il17f), mRNA	NM_001015011	91.18	22.22	0.12382	Rodentia	Muridae	Rattus norvegicus
Strongyloides stercoralis genome assembly S_sterco	LL999056	71.56	69.44	0.12382	Rhabditida	Strongyloididae	Strongyloides stercoralis
Gongylonema pulchrum genome assembly G_pulchrum_Ho	LL748765	84.44	31.25	0.12382	Spirurida	Gongylonematidae	Gongylonema pulchrum
Strongyloides ratti genome assembly S_ratti_ED321	LN609529	75.9	55.56	0.12382	Rhabditida	Strongyloididae	Strongyloides ratti
Cyprinus carpio genome assembly common carp genome	LN594863	70.71	66.67	0.12382	Cypriniformes	Cyprinidae	Cyprinus carpio
Cyprinus carpio genome assembly common carp genome	LN590706	93.33	20.83	0.12382	Cypriniformes	Cyprinidae	Cyprinus carpio
Ochthebius depressionis mitochondrial 16S rRNA gen	HF931340	72.5	55.56	0.12382	Coleoptera	Hydraenidae	Ochthebius depressionis
Cyprinus carpio clone 888268 microsatellite sequen	JN777282	70.71	66.67	0.12382	Cypriniformes	Cyprinidae	Cyprinus carpio
Aphanocephalus sp. 1 NPL−2007 16S ribosomal RNA ge	EU164591	68.63	70.83	0.12382	Coleoptera	Discolomatidae	Aphanocephalus sp. 1 NPL−2007
Elasmostethus scotti 16S ribosomal RNA gene, parti	AY986804	69.49	81.25	0.12382	Hemiptera	Acanthosomatidae	Elasmostethus scotti
Bacillus cytotoxicus NVH 391−98, complete genome	CP000764	82.61	31.94	0.12382	Bacillales	Bacillaceae	Bacillus cytotoxicus NVH 391−98
Vitis vinifera contig VV78X035146.8, whole genome	AM481609	72.27	79.17	0.12382	Vitales	Vitaceae	Vitis vinifera
Bombus sibiricus voucher SC274 16S ribosomal RNA g	EF032348	70.37	73.61	0.12382	Hymenoptera	Apidae	Bombus sibiricus
Stauralia compuncta 16S ribosomal RNA gene, partia	AY252741	69.34	93.06	0.12382	Hemiptera	Acanthosomatidae	Stauralia compuncta
Mylabris obsoleta mitochondrial partial 16S rRNA g	AJ633656	76.19	43.75	0.12382	Coleoptera	Meloidae	Mylabris obsoleta
Rattus norvegicus interleukin 17F, mRNA (cDNA clon	BC091568	91.18	22.22	0.12382	Rodentia	Muridae	Rattus norvegicus
PREDICTED: Megachile rotundata SLIT and NTRK−like	XM_003703951	81.25	31.94	0.432173	Hymenoptera	Megachilidae	Megachile rotundata
Cucumis melo genomic chromosome, chr_12	LN713266	73.4	63.19	0.432173	Cucurbitales	Cucurbitaceae	Cucumis melo
Cucumis melo genomic scaffold, anchoredscaffold000	LN681928	73.4	63.19	0.432173	Cucurbitales	Cucurbitaceae	Cucumis melo
Schistosoma curassoni genome assembly S_curassoni_	LM065488	85.37	28.47	0.432173	Strigeidida	Schistosomatidae	Schistosoma curassoni
Plasmodium yoelii genome assembly PY17X01, chromos	LM993666	72.53	61.81	0.432173	Haemosporida	Plasmodiidae	Plasmodium yoelii
Plasmodium yoelii genome assembly PYYM01, chromoso	LK934640	72.53	61.81	0.432173	Haemosporida	Plasmodiidae	Plasmodium yoelii
Lamellipalpus pacholatkoi voucher UPOL RK0379 16S	KF625955	71.74	61.11	0.432173	Coleoptera	Lampyridae	Lamellipalpus pacholatkoi
PREDICTED: Macaca fascicularis ATP−binding cassett	XM_005550330	79.63	36.81	0.432173	Primates	Cercopithecidae	Macaca fascicularis
Apimela sp. ZMUN 10030869 16S ribosomal RNA gene,	JX536421	67.91	87.5	0.432173	Coleoptera	Staphylinidae	Apimela sp. ZMUN 10030869
Berberomeloe majalis voucher MAB122 16S ribosomal	KC853081	75.81	43.06	0.432173	Coleoptera	Meloidae	Berberomeloe majalis
Berberomeloe majalis voucher MAB500 16S ribosomal	KC853077	75.81	43.06	0.432173	Coleoptera	Meloidae	Berberomeloe majalis
Berberomeloe majalis voucher MAB169 16S ribosomal	KC853068	75.81	43.06	0.432173	Coleoptera	Meloidae	Berberomeloe majalis
Vitis vinifera contig VV78X051662.5, whole genome	AM449991	68.47	75	1.50843	Vitales	Vitaceae	Vitis vinifera
