# Supplementary material for: Testing the potential of a ribosomal 16S marker for DNA metabarcoding of insects
Source: PeerJ. 2016 Apr 19;4:e1966. doi: 10.7717/peerj.1966 (PMC4841222; doi:10.7717/peerj.1966)
Supplement: Supplemental Information 1 [file peerj-04-1966-s006.zip › scripts/5 match OTUs/blast_hits/OTU_99.csv_plot.pdf]

OTU_99.csv

Isoperla oxylepis

Perlodidae

Plecoptera

88.61

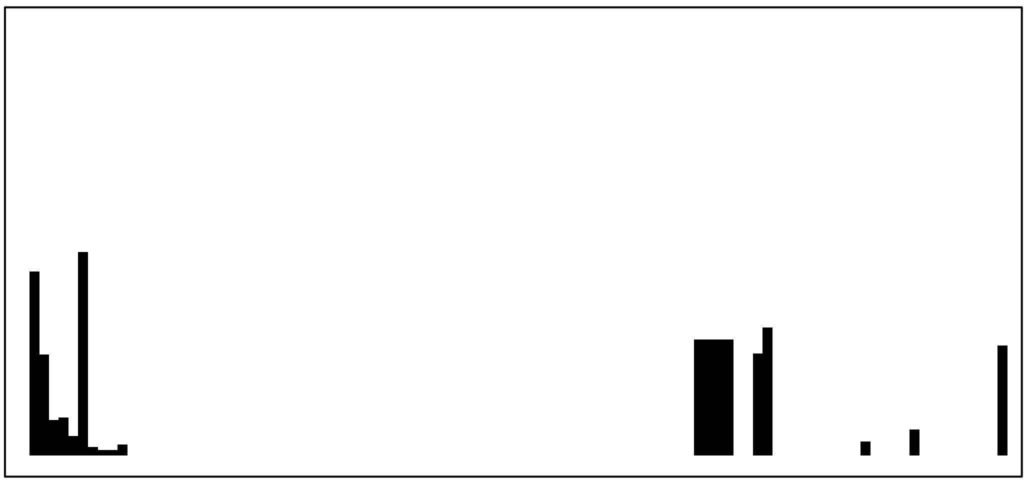

Isoperla oxylepis 16S ribosomal RNA gene, partial	EF623221	88.61	100	4.19724e−46	Plecoptera	Perlodidae	Isoperla oxylepis
Epeorus longimanus 16S ribosomal RNA gene, partial	AY749757	84.71	99.36	5.83426e−38	Ephemeroptera	Heptageniidae	Epeorus longimanus
Isoperla davisi 16S ribosomal RNA gene, partial se	EF623206	81.65	100	1.90723e−31	Plecoptera	Perlodidae	Isoperla davisi
Cloeon dipterum voucher EPH02 tRNA−Leu, 16S riboso	KF256908	81.76	99.36	2.32348e−30	Ephemeroptera	Baetidae	Cloeon dipterum
Isoperla fulva 16S ribosomal RNA gene, partial seq	EF623207	80.89	100	2.32348e−30	Plecoptera	Perlodidae	Isoperla fulva
Epeorus sp. MT−2014 mitochondrion, complete genome	KM244708	89.52	67.31	8.10974e−30	Ephemeroptera	Heptageniidae	Epeorus sp. MT−2014
Brachyptera seticornis 16S ribosomal RNA gene, par	EF623268	80.38	100	2.83058e−29	Plecoptera	Taeniopterygidae	Brachyptera seticornis
Cultus pilatus 16S ribosomal RNA gene, partial seq	EF623212	80.25	100	9.87969e−29	Plecoptera	Perlodidae	Cultus pilatus
Isoperla phalerata 16S ribosomal RNA gene, partial	EF623208	80.25	100	9.87969e−29	Plecoptera	Perlodidae	Isoperla phalerata
Nemoura cinerea 16S ribosomal RNA gene, partial se	EF623194	80.5	100	1.20359e−27	Plecoptera	Nemouridae	Nemoura cinerea
Malenka californica 16S ribosomal RNA gene, partia	EF623182	78.75	100	1.46628e−26	Plecoptera	Nemouridae	Malenka californica
Clioperla clio 16S ribosomal RNA gene, partial seq	EF623237	78.98	100	5.11781e−26	Plecoptera	Perlodidae	Clioperla clio
Nemoura trispinosa 16S ribosomal RNA gene, partial	EF623187	78.34	100	5.11781e−26	Plecoptera	Nemouridae	Nemoura trispinosa
Perlinodes aurea 16S ribosomal RNA gene, partial s	EF623234	78.62	100	1.78629e−25	Plecoptera	Perlodidae	Perlinodes aurea
Paraperla frontalis 16S ribosomal RNA gene, partia	EF623136	79.11	100	1.78629e−25	Plecoptera	Chloroperlidae	Paraperla frontalis
Pictetiella expansa 16S ribosomal RNA gene, partia	EF623216	78.34	100	6.23476e−25	Plecoptera	Perlodidae	Pictetiella expansa
Soyedina producta 16S ribosomal RNA gene, partial	EF623196	78.34	100	6.23476e−25	Plecoptera	Nemouridae	Soyedina producta
Baumanella alameda 16S ribosomal RNA gene, partial	EF623236	78.48	100	2.17615e−24	Plecoptera	Perlodidae	Baumanella alameda
Megarcys signata 16S ribosomal RNA gene, partial s	EF623215	77.71	100	2.17615e−24	Plecoptera	Perlodidae	Megarcys signata
Nemurella pictetii 16S ribosomal RNA gene, partial	EF623192	77.71	100	2.17615e−24	Plecoptera	Nemouridae	Nemurella pictetii
Zapada cinctipes 16S ribosomal RNA gene, partial s	EF623183	77.71	100	2.17615e−24	Plecoptera	Nemouridae	Zapada cinctipes
Frisonia picticeps 16S ribosomal RNA gene, partial	EF623227	77.99	100	7.5955e−24	Plecoptera	Perlodidae	Frisonia picticeps
Setvena wahkeena 16S ribosomal RNA gene, partial s	EF623211	77.99	100	7.5955e−24	Plecoptera	Perlodidae	Setvena wahkeena
Mesocapnia arizonensis mitochondrion, partial geno	KP642637	77.07	100	2.65109e−23	Plecoptera	Capniidae	Mesocapnia arizonensis
Allocapnia recta isolate H13 16S ribosomal RNA gen	KC881048	77.07	100	2.65109e−23	Plecoptera	Capniidae	Allocapnia recta
Allocapnia recta isolate H11 16S ribosomal RNA gen	KC881046	77.07	100	2.65109e−23	Plecoptera	Capniidae	Allocapnia recta
Allocapnia recta isolate H7 16S ribosomal RNA gene	KC881042	77.07	100	2.65109e−23	Plecoptera	Capniidae	Allocapnia recta
Allocapnia recta isolate H3 16S ribosomal RNA gene	KC881038	77.07	100	2.65109e−23	Plecoptera	Capniidae	Allocapnia recta
Allocapnia recta isolate H1 16S ribosomal RNA gene	KC881036	77.07	100	2.65109e−23	Plecoptera	Capniidae	Allocapnia recta
Pteronarcys californica 16S ribosomal RNA gene, pa	EF623261	77.71	100	2.65109e−23	Plecoptera	Pteronarcyidae	Pteronarcys californica
Rickera sorpta 16S ribosomal RNA gene, partial seq	EF623235	77.71	100	2.65109e−23	Plecoptera	Perlodidae	Rickera sorpta
Cascadoperla trictura 16S ribosomal RNA gene, part	EF623225	77.71	100	2.65109e−23	Plecoptera	Perlodidae	Cascadoperla trictura
Nanonemoura wahkeena 16S ribosomal RNA gene, parti	EF623198	77.07	100	2.65109e−23	Plecoptera	Nemouridae	Nanonemoura wahkeena
Ostrocerca foersteri 16S ribosomal RNA gene, parti	EF623191	77.07	100	2.65109e−23	Plecoptera	Nemouridae	Ostrocerca foersteri
Visoka cataractae 16S ribosomal RNA gene, partial	EF623189	77.07	100	2.65109e−23	Plecoptera	Nemouridae	Visoka cataractae
Sweltsa coloradensis 16S ribosomal RNA gene, parti	EF623133	77.85	100	2.65109e−23	Plecoptera	Chloroperlidae	Sweltsa coloradensis
Mesocapnia sp. BYU_PL010 16S ribosomal RNA gene, p	EF623127	77.07	100	2.65109e−23	Plecoptera	Capniidae	Mesocapnia sp. BYU_PL010
Allocapnia recta haplotype 12ar: H12 16S ribosomal	EU306875	77.07	100	2.65109e−23	Plecoptera	Capniidae	Allocapnia recta
Allocapnia recta haplotype 3ar: H03 16S ribosomal	DQ915181	77.07	100	2.65109e−23	Plecoptera	Capniidae	Allocapnia recta
Perlodes mortoni 16S ribosomal RNA gene, partial s	KF855870	77.22	100	9.25321e−23	Plecoptera	Perlodidae	Perlodes mortoni
Taenionema palladium 16S ribosomal RNA gene, parti	EF623267	77.64	100	9.25321e−23	Plecoptera	Taeniopterygidae	Taenionema palladium
Cosumnoperla hypocrena 16S ribosomal RNA gene, par	EF623226	77.99	100	9.25321e−23	Plecoptera	Perlodidae	Cosumnoperla hypocrena
Perlodes dispar 16S ribosomal RNA gene, partial se	EF623220	77.22	100	9.25321e−23	Plecoptera	Perlodidae	Perlodes dispar
Lednia tumana 16S ribosomal RNA gene, partial sequ	EF623197	77.85	100	9.25321e−23	Plecoptera	Nemouridae	Lednia tumana
Shipsa rotunda 16S ribosomal RNA gene, partial seq	EF623184	77.85	100	9.25321e−23	Plecoptera	Nemouridae	Shipsa rotunda
Neopanorpa pulchra mitochondrion, complete genome	FJ169955	77.64	100	3.22969e−22	Mecoptera	Panorpidae	Neopanorpa pulchra
Pteronarcys sachalina 16S ribosomal RNA gene, part	EF623264	77.07	100	3.22969e−22	Plecoptera	Pteronarcyidae	Pteronarcys sachalina
Pteronarcella badia 16S ribosomal RNA gene, partia	EF623262	77.16	100	3.22969e−22	Plecoptera	Pteronarcyidae	Pteronarcella badia
Kogotus nonus 16S ribosomal RNA gene, partial sequ	EF623213	77.07	100	3.22969e−22	Plecoptera	Perlodidae	Kogotus nonus
Thaumatoperla sp. BYU_PL109 16S ribosomal RNA gene	EF623149	77.02	100	3.22969e−22	Plecoptera	Eustheniidae	Thaumatoperla sp. BYU_PL109
Suwallia teleckojensis 16S ribosomal RNA gene, par	EF623142	77.5	100	3.22969e−22	Plecoptera	Chloroperlidae	Suwallia teleckojensis
Pteronarcys princeps mitochondrion, complete genom	AY687866	77.07	100	3.22969e−22	Plecoptera	Pteronarcyidae	Pteronarcys princeps
Apteroperla tikumana mitochondrion, complete genom	KR604721	76.43	100	1.12727e−21	Plecoptera	Capniidae	Apteroperla tikumana
Allocapnia recta isolate H19 16S ribosomal RNA gen	KC881054	76.43	100	1.12727e−21	Plecoptera	Capniidae	Allocapnia recta
Allocapnia recta isolate H16 16S ribosomal RNA gen	KC881051	76.43	100	1.12727e−21	Plecoptera	Capniidae	Allocapnia recta
Allocapnia recta isolate H15 16S ribosomal RNA gen	KC881050	76.43	100	1.12727e−21	Plecoptera	Capniidae	Allocapnia recta
Allocapnia recta isolate H14 16S ribosomal RNA gen	KC881049	76.43	100	1.12727e−21	Plecoptera	Capniidae	Allocapnia recta
Allocapnia recta isolate H10 16S ribosomal RNA gen	KC881045	76.43	100	1.12727e−21	Plecoptera	Capniidae	Allocapnia recta
Allocapnia recta isolate H6 16S ribosomal RNA gene	KC881041	76.43	100	1.12727e−21	Plecoptera	Capniidae	Allocapnia recta
Salmoperla sylvanica 16S ribosomal RNA gene, parti	EF623222	76.43	100	1.12727e−21	Plecoptera	Perlodidae	Salmoperla sylvanica
Yugus bulbosus 16S ribosomal RNA gene, partial seq	EF623214	77.22	100	1.12727e−21	Plecoptera	Perlodidae	Yugus bulbosus
Paranemoura claasseni 16S ribosomal RNA gene, part	EF623185	76.43	100	1.12727e−21	Plecoptera	Nemouridae	Paranemoura claasseni
Chloroperla tripunctata 16S ribosomal RNA gene, pa	EF623137	76.43	100	1.12727e−21	Plecoptera	Chloroperlidae	Chloroperla tripunctata
Capnura wanica 16S ribosomal RNA gene, partial seq	EF623128	76.73	100	1.12727e−21	Plecoptera	Capniidae	Capnura wanica
Allocapnia recta haplotype 11ar: H011 16S ribosoma	EU306874	76.43	100	1.12727e−21	Plecoptera	Capniidae	Allocapnia recta
Allocapnia recta haplotype 7ar: H07 16S ribosomal	EU306870	76.43	100	1.12727e−21	Plecoptera	Capniidae	Allocapnia recta
Allocapnia recta haplotype 2ar: H02 16S ribosomal	DQ915180	76.43	100	1.12727e−21	Plecoptera	Capniidae	Allocapnia recta
Neopanorpa pulchra mitochondrion, complete genome	JX569848	77.02	100	3.93457e−21	Mecoptera	Panorpidae	Neopanorpa pulchra
Cryptoperla japonica mitochondrial gene for 16S rR	AB711202	85.44	65.38	3.93457e−21	Plecoptera	Peltoperlidae	Cryptoperla japonica
Cryptoperla japonica mitochondrial gene for 16S rR	AB711184	85.44	65.38	3.93457e−21	Plecoptera	Peltoperlidae	Cryptoperla japonica
Cryptoperla japonica mitochondrial gene for 16S rR	AB711183	85.44	65.38	3.93457e−21	Plecoptera	Peltoperlidae	Cryptoperla japonica
Cryptoperla japonica mitochondrial gene for 16S rR	AB711182	85.44	65.38	3.93457e−21	Plecoptera	Peltoperlidae	Cryptoperla japonica
Doddsia occidentalis 16S ribosomal RNA gene, parti	EF623270	76.58	100	3.93457e−21	Plecoptera	Taeniopterygidae	Doddsia occidentalis
Scopura montana 16S ribosomal RNA gene, partial se	EF623265	76.58	100	3.93457e−21	Plecoptera		Scopura montana
Neofulla sp. BYU_PL152 16S ribosomal RNA gene, par	EF623203	84.76	66.67	3.93457e−21	Plecoptera	Notonemouridae	Neofulla sp. BYU_PL152
Despaxia augusta 16S ribosomal RNA gene, partial s	EF623177	86	63.46	3.93457e−21	Plecoptera	Leuctridae	Despaxia augusta
Triznaka pintada 16S ribosomal RNA gene, partial s	EF623134	76.73	100	3.93457e−21	Plecoptera	Chloroperlidae	Triznaka pintada
Jellisonia amadoi mitochondrion, complete genome	KF322091	77.36	100	1.3733e−20	Siphonaptera	Ceratophyllidae	Jellisonia amadoi
Allocapnia recta isolate H18 16S ribosomal RNA gen	KC881053	75.8	100	1.3733e−20	Plecoptera	Capniidae	Allocapnia recta
Allocapnia recta isolate H17 16S ribosomal RNA gen	KC881052	75.8	100	1.3733e−20	Plecoptera	Capniidae	Allocapnia recta
Allocapnia recta isolate H12 16S ribosomal RNA gen	KC881047	75.8	100	1.3733e−20	Plecoptera	Capniidae	Allocapnia recta
Allocapnia recta isolate H9 16S ribosomal RNA gene	KC881044	75.8	100	1.3733e−20	Plecoptera	Capniidae	Allocapnia recta
Allocapnia recta isolate H8 16S ribosomal RNA gene	KC881043	75.8	100	1.3733e−20	Plecoptera	Capniidae	Allocapnia recta
Allocapnia recta isolate H5 16S ribosomal RNA gene	KC881040	75.8	100	1.3733e−20	Plecoptera	Capniidae	Allocapnia recta
Allocapnia recta isolate H4 16S ribosomal RNA gene	KC881039	75.8	100	1.3733e−20	Plecoptera	Capniidae	Allocapnia recta
Cryptoperla sp. YK−2012−2 mitochondrial gene for 1	AB711244	80.65	78.85	1.3733e−20	Plecoptera	Peltoperlidae	Cryptoperla sp. YK−2012−2
Hydroperla crosbyi 16S ribosomal RNA gene, partial	EF623219	76.43	100	1.3733e−20	Plecoptera	Perlodidae	Hydroperla crosbyi
Diura knowltoni 16S ribosomal RNA gene, partial se	EF623218	75.8	100	1.3733e−20	Plecoptera	Perlodidae	Diura knowltoni
Austrocerca rieka 16S ribosomal RNA gene, partial	EF623205	75.8	100	1.3733e−20	Plecoptera	Notonemouridae	Austrocerca rieka
Austrocercoides zwicki 16S ribosomal RNA gene, par	EF623199	75.8	100	1.3733e−20	Plecoptera	Notonemouridae	Austrocercoides zwicki
Protonemura meyeri 16S ribosomal RNA gene, partial	EF623193	81.2	83.33	1.3733e−20	Plecoptera	Nemouridae	Protonemura meyeri
Neuroperlopsis patris 16S ribosomal RNA gene, part	EF623152	77.16	100	1.3733e−20	Plecoptera	Eustheniidae	Neuroperlopsis patris
Allocapnia minima 16S ribosomal RNA gene, partial	EF623130	75.8	100	1.3733e−20	Plecoptera	Capniidae	Allocapnia minima
Isogenoides frontalis isolate BYU_PL080 16S riboso	EF623446	75.8	100	1.3733e−20	Plecoptera	Perlodidae	Isogenoides frontalis
Allocapnia recta haplotype 10ar: H010 16S ribosoma	EU306873	75.8	100	1.3733e−20	Plecoptera	Capniidae	Allocapnia recta
Allocapnia recta haplotype 9ar: H09 16S ribosomal	EU306872	75.8	100	1.3733e−20	Plecoptera	Capniidae	Allocapnia recta
Allocapnia recta haplotype 8ar: H08 16S ribosomal	EU306871	75.8	100	1.3733e−20	Plecoptera	Capniidae	Allocapnia recta
Allocapnia recta haplotype 1ar: H01 16S ribosomal	DQ915179	75.8	100	1.3733e−20	Plecoptera	Capniidae	Allocapnia recta
Neopanorpa sp. M24899 16S ribosomal RNA gene, part	AF180059	77.16	100	1.3733e−20	Mecoptera	Panorpidae	Neopanorpa sp. M24899
Rhithrogena sp. 8 LV−2015 mitochondrial partial 16	LN868661	85.15	64.1	4.79328e−20	Ephemeroptera	Heptageniidae	Rhithrogena sp. 8 LV−2015
