# Supplementary material for: Testing the potential of a ribosomal 16S marker for DNA metabarcoding of insects
Source: PeerJ. 2016 Apr 19;4:e1966. doi: 10.7717/peerj.1966 (PMC4841222; doi:10.7717/peerj.1966)
Supplement: Supplemental Information 1 [file peerj-04-1966-s006.zip › scripts/6 statistics/new figure 1 scripts/1 PLOTS + tables/151009_hit_incsequ_SUB_v11_16S_plot.pdf]

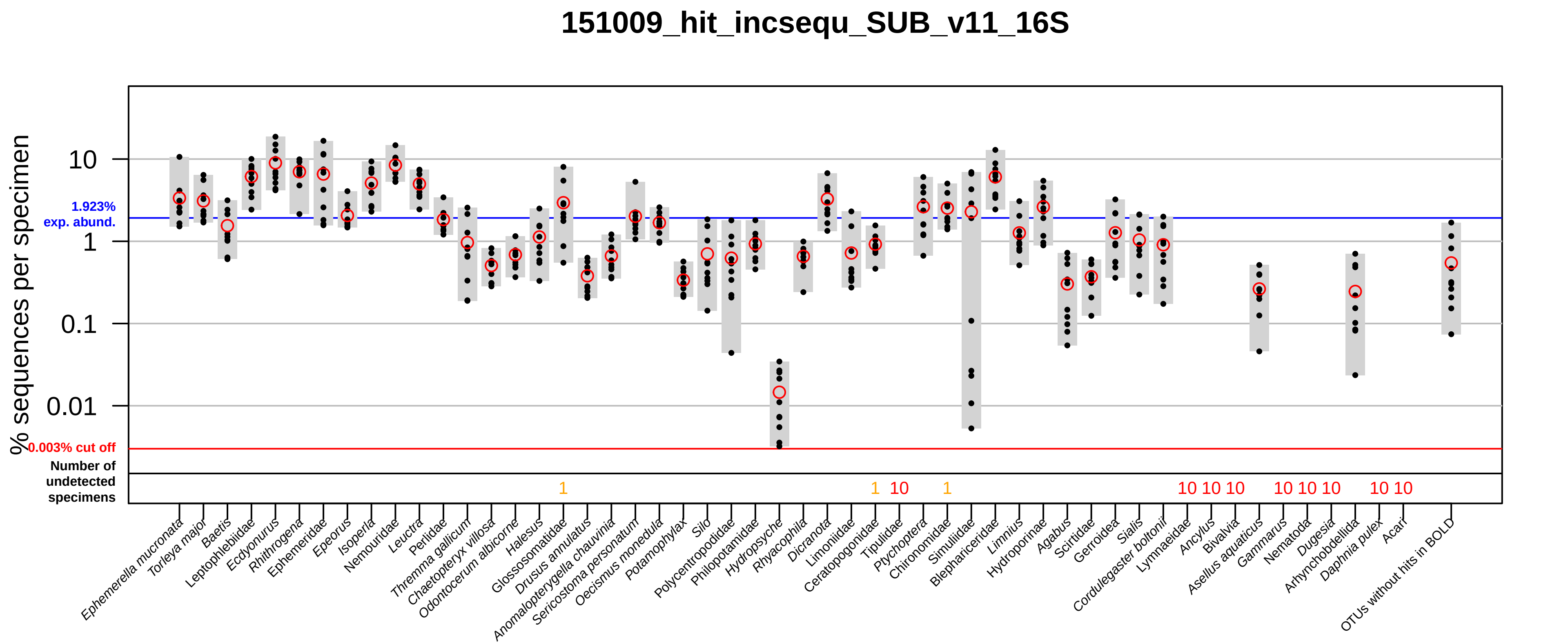
151009_hit_incsequ_SUB_v11_16S
% sequences per specimen
10
1
0.1
0.01
1.923%
exp. abund.
0.003% cut off
Number of undetected specimens
1
1 10
1
10 10 10
10 10 10
10 10
Ephemerella mucronata
Torleya major
Baetis
Leptophlebiidae
Ecdyonurus
Rhithrogena
Ephemeridae
Epeorus
Isoperla
Nemouridae
Leuctra
Perlidae
Thremma gallicum
Chaetopteryx villosa
Odontocerum albicorne
Halesus
Glossosomatidae
Drusus annulatus
Anomalopterygella chauvinia
Sericostoma personatum
Oecismus monedula
Potamophylax
Silo
Polycentropodidae
Philopotamidae
Hydropsyche
Rhyacophila
Dicranota
Limoniidae
Ceratopogonidae
Tipulidae
Ptychoptera
Chironomidae
Simuliidae
Blephariceridae
Limnius
Hydroporinae
Agabus
Scirtidae
Gerroidea
Sialis
Cordulegaster boltonii
Lymnaeidae
Ancylus
Bivalvia
Asellus aquaticus
Gammarus
Nematoda
Dugesia
Arhynchobdellida
Daphnia pulex
Acari
OTUs without hits in BOLD
