# Supplementary material for: Testing the potential of a ribosomal 16S marker for DNA metabarcoding of insects
Source: PeerJ. 2016 Apr 19;4:e1966. doi: 10.7717/peerj.1966 (PMC4841222; doi:10.7717/peerj.1966)
Supplement: Supplemental Information 1 [file peerj-04-1966-s006.zip › scripts/manual_16S_v1.doc]

NextSeq 16S data 2015 - Manual

**1) Check md5 checksums to verify that files were downloaded correctly.**

cd "/Users/luckylion/Documents/UNI_und_VORLESUNGEN/11 phd projects/1 Meta Taberlet 16S/150610_NS500639_0005_AH2WMCAFXX_fq/"

md5 Undetermined_S0_L001_R1_001.fastq.gz

md5 Undetermined_S0_L001_R2_001.fastq.gz

md5 Undetermined_S0_L002_R1_001.fastq.gz

md5 Undetermined_S0_L002_R2_001.fastq.gz

md5 Undetermined_S0_L003_R1_001.fastq.gz

md5 Undetermined_S0_L003_R2_001.fastq.gz

md5 Undetermined_S0_L004_R1_001.fastq.gz

md5 Undetermined_S0_L004_R2_001.fastq.gz

**2) Load raw data into fastQC (unzip first, using finder is ok)**

(ggf save FastQC reports),

write down total number of reads!

**3) Split ready by tag using R script** **“splitreads_ins_v11.R”**

- load individual fastq files into FastQC to check they can be read correctly.

Add up number of reads and compare sum to sum of reads in raw files. They should match. Save number of read per tag, for statistics later on (Plot library distribution, loss of sequences in sequence processing down the road).

- You can count the number of reads with

fgrep -o @ N5_F0+N7_R4_r1.txt | wc -l

**4) merge PE reads with USEARCH: -fastq_mergepairs**

more info: http://www.drive5.com/usearch/manual/cmd_fastq_mergepairs.html

usearch -fastq_mergepairs N5_R0+N7_F4_r1.txt -reverse N5_R0+N7_F4_r2.txt -fastaout "../../2 Build PE reads/N5_R0+N7_F4_merged_e1.txt" -fastq_allowmergestagger -fastq_merge_maxee 1.0

-fastq_merge_maxee 1.0 allows for a total of 1 error per merged sequence pair. The error rate is calculated after merging for the complete read! Is the same as “fastq_filter” (i have tested that)

fastq_allowmergestagger allows for overlaps which are illumina reads, but the overlaps are automatically discarded. This is the case for very few reads in the dataset as the overlap is typically not high enough for creating overlaps into the other primer / illumina adapter. However, we need to add the fastq_allowmergestagger option to keep these reads!

USEARCH 32 bit cant load documents bigger than 4 GB, so you need to divide files > 4 GB in size, process and merge the results using:

\# Split reads into smaller files an rename, number of lines to split hast to be dividable by 4 and varies on the sequencing length (longer reads take up more space, so you might need less reads per file!)

split -l 30000000 N5_F2+N7_R2_r1.txt

mv xaa xaa_r1

mv xab xab_r1

split -l 30000000 N5_F2+N7_R2_r2.txt

mv xaa xaa_r2

mv xab xab_r2

\# merge splitted reads

usearch -fastq_mergepairs xaa_r1 -reverse xaa_r2 -fastaout xaa_merged_e1.txt -fastq_allowmergestagger -fastq_merge_maxee 1.0

usearch -fastq_mergepairs xab_r1 -reverse xab_r2 -fastaout xab_merged_e1.txt -fastq_allowmergestagger -fastq_merge_maxee 1.0

#combine merged reads into one file

cat xaa_merged_e1.txt xab_merged_e1.txt > "../../2 Build PE reads/N5_F2+N7_R2_merged_e1.txt"

Read the number of PE reads merged with the following command to calculate the number of reads discarded in each replica (for statistic reasons). Also notice that we are now saving the files without quality scores as fasta files (Vsearch can't handle fastq files)!

fgrep -o ">" "../../2 Build PE reads/N5_R1+N7_F3_merged_e1.txt" | wc -l

make sure to save counts and console outputs of PE merging in a log txt file (copy and paste).

**5) remove complete adaptor sequences from the PE reads with Cutadapt**

cutadapt can discard sequences in which one adapter is not removed (due to high error rates or mismatches) using the “--discard-untrimmed” option. However when matching both adapters in the same step, if only one adapter is matched, the read is till kept and the other adapter is not removed. Thus, you have to remove the adapter one at a time, instead of removing both in the same run!

Minimum overlap is not needed (as used in the paper before, --overlap 10), as we used exact matches for both PE reads when splitting. So the adapters do match at least 10 bases (combined) and are quality filtered after PE merging.

#N5_F0+N7_R4

cutadapt -g ^RGACGAGAAGACCCTATARA -o temp.txt "../2 Build PE reads/N5_F0+N7_R4_merged_e1.txt" -f fasta --discard-untrimmed

\# make sure to build the reverse complement of the primer sequence + adaptor you are looking for, as we are dealing with PE reads!

cutadapt -a TACYTTAGGGATAACAGCGTAACG$ -o N5_F0+N7_R4_merged_e1_cut.txt temp.txt -f fasta --discard-untrimmed

make sure to save counts and console outputs from primer cuting in a log txt file (copy and paste).

**6) Remove singleton sequences and sort sequences by abundance (not sequence length!), use Vsearch if no 64 bit version of Usearch is available**

One could merge all files direclty, and than dereplicate, but you might run into processing power issues!

A) Dereplicate each individual file incl. Sizeout option (contains the read counts!)

Vsearch -derep_fulllength "../3 cut adapt/N5_F0+N7_R4_merged_e1_cut.txt" -output N5_F0+N7_R4_merged_e1_cut_derep.txt -sizeout

B) Merge all files using cat!

cat N5_F0+N7_R4_merged_e1_cut_derep.txt N5_F1+N7_R3_merged_e1_cut_derep.txt N5_F2+N7_R2_merged_e1_cut_derep.txt N5_F3+N7_R1_merged_e1_cut_derep.txt N5_F4+N7_R0_merged_e1_cut_derep.txt N5_R0+N7_F4_merged_e1_cut_derep.txt N5_R1+N7_F3_merged_e1_cut_derep.txt N5_R2+N7_F2_merged_e1_cut_derep.txt N5_R3+N7_F1_merged_e1_cut_derep.txt N5_R4+N7_F0_merged_e1_cut_derep.txt > 150724_16s_derep.txt

C) dereplicate the pooled files with sizeout + sizein to keep read abundances! Use minuniquesize 2 to remove singletons!

Vsearch -derep_fulllength 150724_16s_derep.txt -output 150724_16s_derep_min2.txt -sizein -sizeout -minuniquesize 2

**7) clustering with USEARCH -**

Cluster with 3% similarity on both strands!

usearch -cluster_otus 150724_16s_derep_min2.txt -otus 150729_otus.txt -uparseout 150729_out_table.txt -relabel OTU_ -sizein -sizeout -otu_radius_pct 3 -strand both

Rename OTUs, actually keep chimeras to see it they are really OK to exclude or if taxa get lost by this (check both!)

**8) remove chimeras denovo**

usearch -uchime_denovo 150729_otus.txt -nonchimeras 150729_otus_nochim.txt -chimeras 150726_otus_chimeras.txt

keep chimera / non chimera files, but make sure to blast the complete OTU table.

In the 16S dataset: 192/855 chimeras found (22.5%)

- Thats quite a lot of OTUs for 52 taxa, but probably chimeras, Read and PCR errors and the difficulty of aligning reads in the 16S region might contribute!

Bug in USEARCH - not all non chimeric sequences are saved! Take OTU list and annotate chimeras out in that file and blast all sequences against it.

~~Actually! Rename OTUs and chimeric sequences but keep them in the same file! Then compare sequences agains the whole sequence pool! Other wise sequences which are derived from chimeric sequences could be matched to “sort of matching OTUs”. So compare the sequences against all, and exclude chimeric sequences in the later analysis.~~

~~(potential inconsistencies with chimeras removed in OTU clustering?)~~

actually, not many hits for chimeras, lets just remove them!

150729_OTU_renamed.txt renamed with rename_OTU_v3.R

keep only OTUs without chimeras!

**9) compare dereplicated reads of each replica against the OTUs without chimeras**

usearch -usearch_global N5_F0+N7_R4_merged_e1_cut_derep.txt -db 150729_OTU_nonchim_renamed.txt -strand both -id 0.97 -blast6out Usearch_global/N5_F0+N7_R4.txt -maxhits 1

**10) convert usearch raw hit tables to OTU table, for plotting etc (R script)**

Blast_16S_v2_hittable.R
